# Genomic Insights into *Fusarium verticillioides* Diversity: The Genome of Two Clinical Isolates and Their Demethylase Inhibitor Fungicides Susceptibility

**DOI:** 10.3390/pathogens13121062

**Published:** 2024-12-03

**Authors:** Luca Degradi, Valeria Tava, Maria Carmela Esposto, Anna Prigitano, Daniela Bulgari, Andrea Kunova, Marco Saracchi, Paolo Cortesi, Matias Pasquali

**Affiliations:** 1 Department of Food, Environmental and Nutritional Sciences (DeFENS), University of Milan, Via Celoria 2, 20133 Milan, Italy; luca.degradi@unimi.it (L.D.); valeria.tava@unimi.it (V.T.); daniela.bulgari@unimi.it (D.B.); andrea.kunova@unimi.it (A.K.); marco.saracchi@unimi.it (M.S.); paolo.cortesi@unimi.it (P.C.); 2 Department of Imaging & Pathology, KU Leuven, RK-Herestraat 49, 3000 Leuven, Belgium; 3 Department of Biomedical Sciences for Health (SCIBIS), University of Milan, Via Pascal 36, 20133 Milan, Italy; maria.esposto@unimi.it (M.C.E.); anna.prigitano@unimi.it (A.P.)

**Keywords:** *Fusarium fujikuroii* species complex, human infection, transkingdom pathogen, comparative genomics, fungicide susceptibility, docking

## Abstract

*Fusarium verticillioides* is an important plant pathogen in maize and other cereals that is seldom detected as the cause of human fusariosis. Here, we provide the analysis of the available diversity of *F. verticillioides* sequenced worldwide and report the first two genome assemblies and annotations (including mitochondrial DNA) of *Fusarium verticillioides* from clinical settings. *Fusarium verticillioides* 05-0160 (IUM05-0160) and *Fusarium verticillioides* 09-1037 (IUM09-1037) strains were obtained from the bone marrow and blood of two immunocompromised patients, respectively. The phylogenomic analysis confirmed the species identity of our two strains. Comparative genomic analyses among the reannotated *F. verticillioides* genomes (*n* = 46) did not lead to the identification of unique genes specific to the clinical samples. Two subgroups in the *F. verticillioides* clade were also identified and confirmed by a mitochondrial diversity study. Clinical strains (*n* = 4) were positioned in the multigene phylogenetic tree without any correlation between the host and the tree branches, grouping with plant-derived strains. To investigate the existence of a potential fitness advantage of our two clinical strains, we compared demethylase inhibitor fungicides susceptibility against the reference *Fusarium verticillioides* 7600, showing, on average, lower susceptibility to agricultural and medical-used antifungals. A significant reduction in susceptibility was observed for itraconazole and tetraconazole, which might be explained by structural changes in CYP51A and CYP51C sequences. By providing the first two annotated genomes of *F. verticillioides* from clinical settings comprehensive of their mitogenomes, this study can serve as a base for exploring the fitness and adaptation capacities of *Fusarium verticillioides* infecting different kingdoms.

## 1. Introduction

Fungal infections represent a threat to humans, animals, and plants [1]. The genus *Fusarium* is included by WHO in the “high priority group” of pathogens with important public health impacts within the fungal priority pathogens list (FPPL) [2]. *Fusarium* spp. are known for their inherent resistance to many antifungals and for their ability to cause invasive fungal diseases globally in patients with compromised immune systems [3].

**Figure 1:**
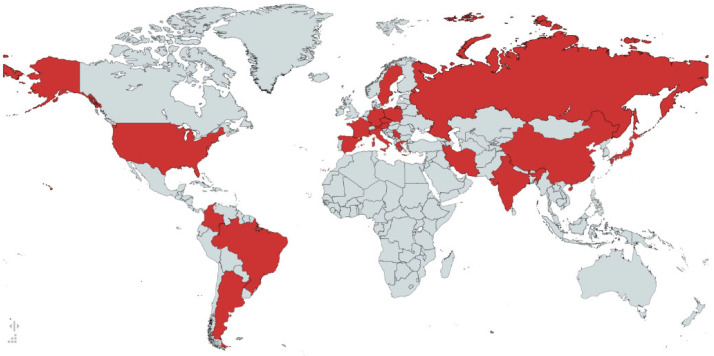
Distribution of clinical cases of *Fusarium verticillioides*. In red countries with at least 1 case of fusariosis caused by *Fusarium verticillioides* is reported.

*Fusarium verticillioides* Sacc. Nirenberg (=*Fusarium moniliforme* Sheldon) is a necrotrophic pathogen, which infects important crops such as sorghum, sugarcane, and maize. It occurs worldwide, both in the soils of tropical and subtropical regions, as well as humid and sub-humid temperate zones. *F. verticillioides* is probably the most common pathogen of maize crops throughout the world—it causes stalk, root, ear, kernel rot, and seedling blight. This pathogen may cause damage during all stages of the plant growth, although in most cases, the infection is asymptomatic and can be characterized as an endophytic relationship. Like other *Fusaria*, *F. verticillioides* is considered non-pathogenic to healthy human beings, but it can become a serious threat to individuals with compromised immune systems. *F. verticillioides* has also been reported in different regions of the world to be the etiological agent of superficial and disseminated infections in humans (Figure 1 [4,5,6,7,8,9,10,11,12,13,14,15,16,17,18,19]). *Fusarium* spp. infections are considered globally relevant, especially in immunosuppressed patients, and are frequently associated with high mortality due to limited therapeutic options and diagnostic challenges. Therefore, elucidating molecular mechanisms underlying fungal pathogenesis and fungicide resistance in *F. verticillioides* is crucial to both agriculture and public health.

Given the association of *F. verticilliodes* to clinical cases, it is important to understand the ability of the fungus to adapt to hosts with complex immune systems. One of the major threats of fungal human infection is associated with the limited availability of effective molecules able to control the infection [20]. Among the currently available treatments, demethylase inhibitors (DMIs) represent an important class of molecules used in clinical settings, so reduced susceptibility to this class of antifungal drugs must be monitored constantly [21]. Reduced DMI susceptibility may represent a factor favouring the fitness of a strain in clinical settings as observed in agricultural settings for other *Fusarium* species [22,23]. Mutations of CYP51A, B, and C observed in other fungal species seem to explain the decreased effectiveness of specific DMI molecules [24].

Understanding what allows *F. verticillioides* to cause diseases in hosts from different kingdoms is a crucial question to comprehensively address the challenges that emerging fungal species pose to the traditional separation of clinical and agricultural pathogens [25].

In the literature, only one study compared strains from human patients with strains of plant origin in Brazil, exploring their diversity with molecular markers [7]. In this work, the authors observed a high degree of homogeneity among clinical isolates in contrast to the high variability observed in phytopathogenic isolates of *F. verticillioides* for most of the molecular markers tested. Moreover, the grouping of some clinical and phytopathogenic strains in the same clades, according to the authors, suggests that mycoses, caused by *F. verticillioides*, may be acquired in the field after traumatic exposure to plants [7].

The availability of full genome sequences enables a precise classification of fungal strains [25] as well as exploring the mechanisms of fungal pathogenesis and host adaptation effectively [26,27,28,29,30]. Therefore, *F. verticillioides’* capability to adapt to human hosts can be explored using genomic data. Here, we sequenced and annotated two *F. verticillioides* genomes obtained from human patients and compared them to the genomes of *F. verticillioides* deposited in NCBI. Moreover, we define the susceptibility to DMIs of these two strains, evidencing a lower susceptibility to most of the tested molecules.

## 2. Materials and Methods

### 2.1. DNA Extraction and Sequencing

High-molecular-weight DNA was extracted from lyophilized mycelia of *Fusarium verticillioides* IUM05-0160 and IUM09-1037 strains. Samples are stored on potato agar discs in distilled water at 4 °C in the Defens Collection (University of Milan). They were obtained from immunocompromised patients after bone-marrow transplantation and leukaemia disease, respectively [13,16]. CTAB extraction followed by Qiagen (Hilden, Germany) genomic tip procedure was used for DNA extraction according to the manufacturer’s instructions [31]. The obtained DNA was processed with MiSeq Reagent Kit v2 and sequenced using the MiSeq Illumina sequencer (Illumina Inc., San Diego, CA, USA) by the service provider (Eurofins Genomics, Ebersberg, Germany).

### 2.2. Genome Assembly and Annotations

Nuclear genome assembly was performed on FASTP (Galaxy version 0.20.1+galaxy0; https://github.com/galaxyproject/tools-iuc/tree/master/tools/fastp, accessed on 2 February 2022) processed reads (filtering reads when Q score was below 30) using the Shovill tool (Galaxy version 1.1.0+galaxy0; https://github.com/galaxyproject/tools-iuc/tree/master/tools/shovill, accessed on 2 February 2022) with default setting as well as a SPAdes assembler. The completeness of the genome assemblies was performed with Benchmarking Universal Single-Copy Orthologs (BUSCO) implemented in the Galaxy platform (Galaxy Version 5.4.6+galaxy0) using the *hypocreales_odb10* lineage dataset. Genome assembly quality was determined on QUAST (Galaxy Version 5.2.0+galaxy1), which provided detailed information about the contigs, such as the N50 length.

Mitochondrial DNA assembly was carried out with NOVOplasty (Galaxy version 4.3.1+galaxy0) using the strain 7600 as a reference and the first 715 bp from the *cox1* gene from the same strain as a seed sequence. The obtained mitogenomes were then annotated by integrating results from Mfannot [32] and RNAWeasel [33].

Annotations were made using the *F. verticillioides* strain 7600, which is the RefSeq strain for *F. verticillioides*. Gene prediction was performed using Augustus implemented in the Galaxy platform (Galaxy Version 3.4.0+galaxy1), and functional annotation and Gene Ontology (GO) analyses were carried out using Omicsbox (formerly known as Blast2GO) (v2.1.14, Biobam, Valencia, Spain). The list of annotated protein sequences was imported into OmicsBox to perform Gene Ontology (GO) annotation. The results from this search were used for downstream analyses including mapping, annotation, gene ontology, and pathway analysis, using default values. Subsequently, the GO analysis was classified into three categories, namely, cellular components, molecular function, and biological process [34].

Antismash [35] tool (Fungal version v. 7.0) was used for secondary metabolite cluster prediction. EffectorP 3.0 [36] on annotated protein sequences was used to analyse effectors. Sequences with a threshold >90% and >50% in EffectorP genes prediction analysis were retained.

### 2.3. Intraspecies Analysis

All the available sequences of the strains of *F. verticillioides* were downloaded from the NCBI database (Table S1). The BUSCO tool, used to measure completeness, defined the strains used for further analysis with a threshold of 98%. Filtered genomes were first analysed using QUAST to have an overview of the assemblies, then annotated following the same workflow described for our two strains, processed with Antismash 7.0 and EffectorP 3.0 to predict secondary metabolite clusters and putative effectors genes, respectively.

To position and analyse our strains among the 46 comparable genomes of *F. verticillioides* (Table 1), multiple approaches were used.

Phylogenetic tree was built using 18 conserved genes [37] obtained from assembled genomes on NCBI: ((*Actin* (1419 bp), *ATP citrate lyase subunit 2* (1470 bp), *Atp dependent DNA helicase II* (1935 bp), *Calmodulin* (450 bp), *cytochrome p450 reductase* (2062 bp), *DNA polymerase alpha subunit* (4356 bp), *DNA polymerase epsilon subunit* (6636 bp), *Translation elongation factor 1-alpha* (1383 bp), *fatty acid alpha* (5568 bp), *fatty acid synthase beta subunit* (6312 bp), *phosphoglycerate kinase* (1257 bp), *ribosomal biogenesis protein* (2451 bp), *RNA polymerase largest subunit* (5223 bp), *RNA polymerase second largest subunit* (3813 bp), *sphinganine palmitoyl transferase subunit* (1953 bp), *topoisomerase* (2730 bp), *tubulin alpha* (1350 bp), *tubulin beta* (1341 bp)). After concatenation and alignment using MAFFT, the result was then processed on IQTREE [38] for training models, tree construction, and visualization.

To better understand their similarity within the species at the genomic level, available assembled genomes were aligned using FastANI tool (Galaxy Version 1.3) using the function “all against all”.

Mitogenomes of available sequences were processed and annotated as described above for our two strains. All alignments were performed using the Geneious platform and manually checked.

Finally, the search for putatively unique genes among clinical strains and non-clinical strains was done using the following protocol. Reciprocal Blast (RBH) Galaxy Version 0.3.0 (threshold for identity and coverage of 95%) was used to define the shared genes among the four human isolates. Once the pool of shared genes among human strains was obtained, RBH against non-clinical strains was used to find unique genes shared only by the human-derived strains.

### 2.4. Susceptibility of F. verticillioides to Medical and Agricultural DMI Fungicides

Three *F. verticillioides* strains (7600 reference from maize, IUM05-0160 and IUM09-1037 from human patients) were tested for in vitro susceptibility to four medical DMIs—itraconazole, voriconazole, posaconazole, and isavuconazole (Sigma-Aldrich, St. Louis, MO, USA)—and to eight DMIs used in crop protection—prochloraz, tebuconazole, epoxiconazole, difenoconazole, propiconazole, tetraconazole, flusilazole, and fenbuconazole (Sigma-Aldrich, St. Louis, MO, USA). Susceptibility assays were performed with the broth microdilution method according to the Clinical and Laboratory Standards Institute (CLSI) guidelines for filamentous fungi (Reference CLSI M38-A2). All molecules were prepared at final concentrations ranging from 0.03 to 16 mg/L. Broth microdilution assay was performed in RPMI-1640 medium with glutamine, without bicarbonate (Sigma-Aldrich, St. Louis, MO, USA). Conidia suspensions were collected from 2- to 5-day-old colonies grown in potato dextrose agar (Sigma-Aldrich, St. Louis, MO, USA). Using a haemocytometer, the conidia concentration was adjusted to the final working inoculum of 0.5–5 × 10^4^ CFU/mL. The conidial suspensions were inoculated to the RPMI-1640 medium containing increasing concentrations of the tested fungicides in 96-well microplates, which were incubated at 28 °C for 48 h. The minimum inhibitory concentration (MIC) value was the concentration of fungicide yielding no fungal growth at visual reading: no mycelium was visible, and the medium appeared crystal-clear by looking with the naked eye. Tests were performed in duplicate. Reference strains *Candida parapsilosis* ATCC 22019 and *Candida krusei* ATCC 6258 were used as controls [39].

To correlate the DMI’s susceptibility to mutations in the CYP51 genes, the sequences were retrieved from the assemblies of the two human strains and the reference *F. verticillioides* 7600.

CYP51A (XP_018757407), CYP51B (XM_018743733.1), and CYP51C (XP_018760287.1) sequences were used to retrieve the three CYP51 genes in all the strains and aligned and analysed using Geneious prime software (v. 2024.0.5, Biomatters Ltd., Auckland, New Zealand) and Alphafold 2 software Galaxy Version 2.3.1+galaxy5 (https://radegast.galaxyproject.org/repos/galaxy-australia/alphafold2, accessed on 1 October 2024).

Docking of the proteins with itraconazole and tetraconazole was performed on the neurosnap platform (www.neurosnap.ai, accessed on 1 October 2024) using DiffDock-L software (web version on Neurosnap, Wilmington, DE, USA), which requires a .pdb file of the protein and SMILE sequence of the chemical compound. The prediction of interaction was performed both on CYP51A and CYP51C sequences of strains 7600, IUM05-0160, and IUM09-1037 for tetraconazole and itraconazole.

## 3. Results

### 3.1. Genome Assemblies and Comparative Analysis

Illumina sequencing produced 13,119,244 paired-end reads for IUM05-0160 and 16,799,831 reads for IUM09-1037. The genome sizes of IUM05-0160 and IUM09-1037 were 43.6 Mb and 43.27 Mb (1.8 Mb and 1.4 Mb more than the reference 7600), respectively, divided into 102 and 101 contigs plus circular mitochondrial DNA (mtDNA). N50 of the final assemblies were 13 for IUM05-0160 and 12 for IUM09-1037. BUSCO results show more than 99% of genes present (using hypocreales_odb10) (Table 2). The final functional annotation includes 14,397 genes for IUM05-0160 and 13,860 for IUM09-1037 (Tables S2 and S3). Among the annotated genes, for IUM05-0160, 75.66% are associated with at least one gene ontology term while IUM09-1037 has 73.21% of genes with at least one associated GO term.

Secondary metabolites were 45 and 48 for IUM05-0160 and IUM09-1037, respectively, according to Antismash annotation (Tables S4 and S5). Overall, IUM05-0160 has a reduced number of NRPS (*n* = 12) compared to IUM09-1037 (*n* = 15). IUM05-0160 has one more cluster associated with terpene synthesis compared to the other human strain. The results indicate that the profile of the two human strains appears very similar. For what concerns “molecular function” analysis, IUM09-1037 showed a higher percentage of GO related to “cellular process” compared to IUM05-0160 (33% vs. 25%), while IUM05-0160 showed a higher percentage for “developmental and multicellular organismal processes”. Analyzing “biological activity”, IUM09-1037 shows a higher percentage of genes classified as “catalytic activity” (Figure S3) compared to IUM05-0160, which presented a higher value for “molecular function regulator activity”.

In order to position our strains in *F. verticillioides* diversity, we explored the NCBI database, finding 58 assemblies of *F. verticillioides*. BUSCO threshold selection (>98%) reduced our collection to 46 comparable strains for further analysis (Table 2). Overall diversity of the 46 *F. verticillioides* genomes showed that genome sizes ranged from 41.02 Mb to 44.65 Mb, and gene numbers from 13,860 to 14,610. Effectors identified with effector P ranged from 4651 to 4943 and secondary metabolites clusters ranged from 43 to 51 (Table 2).

Our dataset included four clinical samples, which we used to test the hypothesis of diversity between human and plant/environmental strains. Specifically, we evaluated the presence of significant differences in the genome size, gene number, effector number, or secondary metabolites cluster number. T-test (Table S6) showed no significant differences between the human-derived strains group and other environmental samples. We further explored the number of shared genes among the four human isolates, finding 11,697 common genes (Table S7). Moreover, we checked the presence of unique genes belonging only to the clinical strains, but no genes were found to be unique for this group of strains.

### 3.2. Fusarium verticillioides Diversity

**Figure 2:**
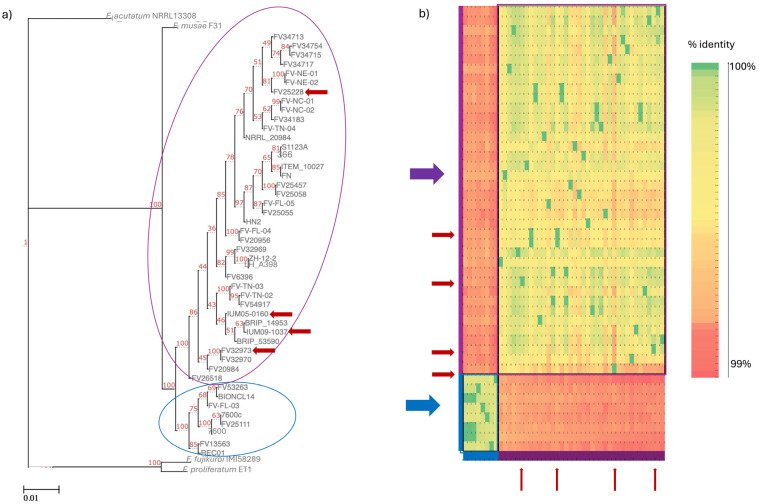
(**a**) Phylogenetic three based on the analysis of 18 conserved genes. IQ-Tree was used for model construction, tree construction and visualization. Numbers at nodes refer to the Bootstrap value. (**b**) FastANI results of *F. verticilliodes* whole genome alignment. Red arrows refer to human-derived strains; blue and purple colours highlight two distinct groups present in the tree.

MAFFT alignment of the 18 concatenated genes gave 99.3% pairwise identity and 95.8% identical sites among all the strains. TIM2 + F2 + R was the model obtained from Web-IQ-Tree used to produce the phylogenetic tree (Figure 2) (Bootstrap threshold 50%), which grouped human-derived strains with non-clinical ones. The phylogenetic analysis showed that all the clinical strains were interspersed in the *F. verticilliodies* clade. Moreover, two groups within *F. verticillioides* could be identified: one group included clinical samples and environmental samples, and the other group comprised the reference strain 7600 and a few other environmental samples (Figure 2a). FastANI alignment confirmed the presence of the same two groups within the *F. verticillioides* clade (Figure 2b).

### 3.3. Mitogenomes

**Figure 3:**
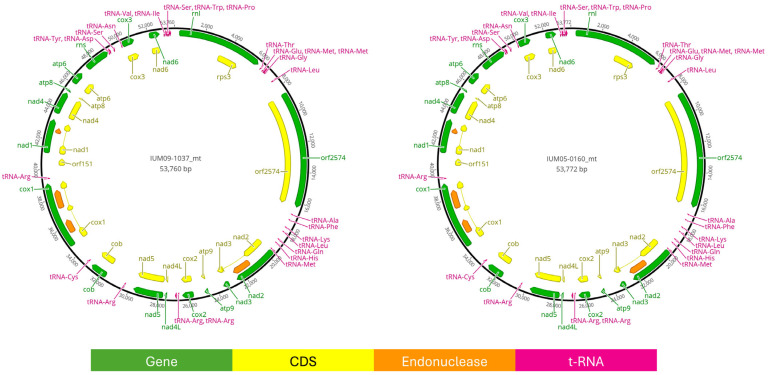
Graphical representation of assembled and annotated mitochondrial genomes of IUM05-0160 and IUM09-1037.

Assembled mitochondrial DNAs resulted in 53,772 bp long for IUM05-0160 and 53,760 bp for IUM09-1037. The annotation includes 15 genes, a small and large ribosomal subunit and 4 endonucleases belonging to the GIY-YIG and LAGLIDADG family (Figure 3). Six other mitogenomes were found in the assemblies of the 46 analysed *F. verticillioides* strains (Table 2). Mitogenomes had sizes ranging from 53,536 bp to 53,870 bp. Aligning aminoacidic sequences of all the 15 genes of the eight strains, we observed that seven strains, including our two clinical samples, showed four mutations compared to strain 7600 in the *Nad5* gene (Figure S1). Despite the analysis being performed on a smaller number of samples, the same groupings of *nad5* diversity mimic multigene phylogeny and FastANI grouping.

### 3.4. DMI Susceptibility and CYP51 Analysis

The antifungal susceptibility assay highlighted, on average, higher resistance to DMIs for our two clinical strains compared to the environmental one (Table 3). Highest significant differences in susceptibility could be observed for itraconazole and tetraconazole.

**Figure 4:**
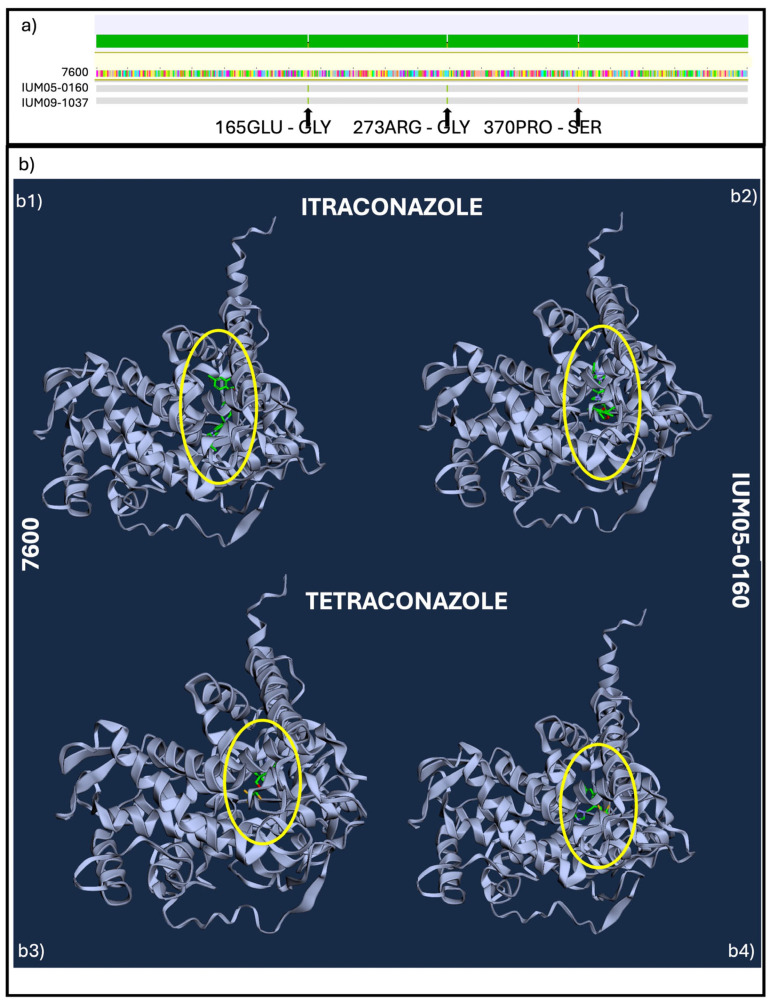
(**a**) Alignment of CYP51A aminoacidic sequences of 7600 (reference), IUM05-0160 and IUM09-1037, arrows indicate mutations compared with the reference. (**b**) Diffdock L output representing the point of interaction between (**b1**) itraconazole 7600 (MIC = 0.5); (**b2**) itraconazole IUM05-0160 (MIC = 4 for IUM05-160/MIC > 16 for IUM09-1037); (**b3**) tetraconazole 7600 (MIC = 1) and (**b4**) tetraconazole and IUM05-0160 (MIC > 16). Yellow circles indicate the position of potential interactions between the protein and fungicides.

Alignment of the CYP51A proteins highlighted 100% of identical sites for the two human strains (Figure 4), while three substitutions were found compared with the reference (7600): two related to a charge difference in the final protein due to aminoacidic substitution of *Glu* to *Gly* and of *Gly* to *Arg* (highlighted in Figure 4a), while the third one is a substitution that changes the polarity moving from polar AA (*Ser*) to a non-polar AA (*Pro*). The difference in the amino acid sequences may introduce structural changes in the folded protein as evidenced by a docking study (Figure 4b) with itraconazole and tetraconazole (Video S1a–d, Tables S8 and S9a–d), which can determine the diverse position and binding with the molecules.

The alignment of CYP51A in all the available strains showed a total of nine different isoforms of the protein. All the clinical strains shared the same sequence (Figure S2).

CYP51B was identical for all the strains (Figure S3), while CYP51C showed a higher level of polymorphism both within our three strains and within the overall collection.

**Figure 5:**
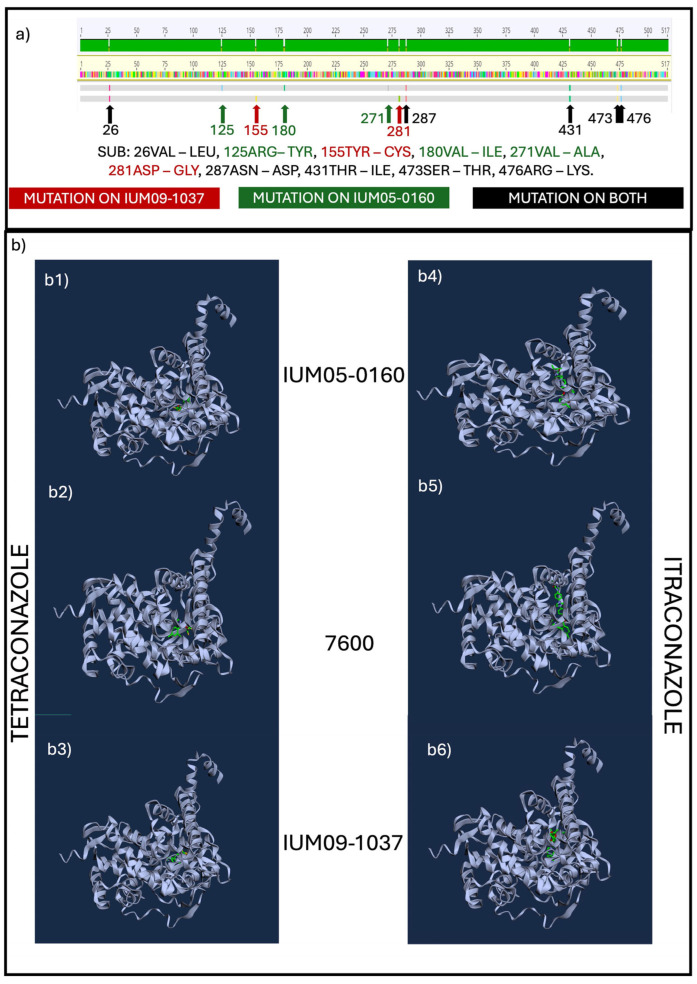
(**a**) Alignment of CYP51C aminoacidic sequences of 7600 (reference), IUM05-0160, and IUM09-1037; arrows indicate mutations compared with the reference. In red mutation only on IUM09-1037, in green on IUM05-0160, and black for both IUM05-160 and IUM09-1037, numbers are referred to the aminoacidic position of mutation of the 7600 sequence. (**b**) Diffdock-L output representing the point of interaction between (**b1**) tetraconazole and IUM05-0160 (MIC > 16 mg/L); (**b2**) tetraconazole and 7600 (MIC = 1 mg/L); (**b3**) tetraconazole and IUM09-1037 (MIC > 16 mg/L); (**b4**) itraconazole and IUM05-0160 (MIC = 4 mg/L); (**b5**) itraconazole and 7600 (MIC = 0.5 mg/L); (**b6**) itraconazole and IUM09-1037 (MIC > 16 mg/L). Yellow circles indicate the position of potential interactions between the protein and the fungicides.

CYP51C alignment of all 46 strains (Figure S4) showed 29 groups of protein isoforms. As for CYP51A changes in our three reference strains, we observed mutations leading to a shift in charge and polarity (Figure 5a) that have a significant impact on the protein structure and interaction with the molecules (Figure 5b, VideoS1e–l, Tables S8 and S9e–l). The diverse structure of CYP51C between the two human strains IUM05-160 and IUM09-1037 might explain the diverse susceptibility to itraconazole (4 mg/L and >16 mg/L, respectively).

## 4. Discussion

To our knowledge, this work analyzed and annotated the first two genomes and mitochondrial genomes of *F. verticillioides* strains from human patients. We also compared a large dataset of *F. verticillioides* genomes available from diverse origins, providing a diversity of the species from a genomic perspective. Mitogenome diversity, multigene phylogeny, and overall genome identity seem to suggest the existence of two subgroups within *F. verticillioides* strains. Further verification is required to understand whether the two groups represent an ongoing process of speciation or two separate subspecies.

The clinical strains, whose genomes are available, are interspersed in the phylogeny of *F. verticillioides* isolated from plants, confirming previous results. Indeed, Chang and co-authors [7] already demonstrated the presence of *F. verticillioides* in hosts belonging to different kingdoms with the use of molecular markers. No specific genomic features could be associated with clinical strains such as genome size, secondary metabolite clusters, or number of effectors. Indeed, no unique genes could differentiate clinical strains from other environmental strains of *F. verticillioides*. Observed in other *Fusarium* species [27], some determinants may be acquired and maintained in the genome favouring the fitness in colonizing different hosts such as supplementary chromosomes in *F. oxysporum*. *F. verticilliodes*, on the contrary, seem to be able to infect diverse hosts without unique genomic acquisitions, suggesting that modulation of the infecting arsenal, and not the acquisition of specific novel host-specific genes, may play a role in successful virulence in patients. A possible explanation of effective infections in clinical settings can be associated with the ability to adapt to antifungal drugs; therefore, diverse sensitivity to drugs might partially explain the clinical success of fungal infections.

Interestingly, on average, the level of susceptibility to DMIs of human strains was reduced compared to the strain 7600 used as reference, possibly suggesting some additional advantage of these strains in clinical settings. One explanation for the diversity in susceptibility was associated with the modification of the *CYP51* genes in fungal species [22,40]. In *F. verticillioides*, laboratory mutants showed resistance to prochloraz by showing a single AA mutation in CYP51B [41]. Our docking studies are compatible with other observations that link mutations in CYP51A in different *Fusarium* species with clinical and environmental origin to a certain degree of resistance against different azoles [23]. In other fungal species, hot spots for mutations in Cyp51 proteins (including CYP51C) play an important role in interaction with azole drugs [42]. Also in *F. fujikuroi*, CYP51B and partially also CYP51A and CYP51C were shown to affect susceptibility to some agricultural DMIs [43]. As observed for other species, the set of mutations in the target enzymes may explain the susceptibility to fungicides and inform on the best therapy for fungal infections, especially in clinical cases [22,39]. For this reason, our comparative analysis of CYP51 genes in *F. verticillioides* may also guide the potential prediction of diverse levels of susceptibility to DMIs, as suggested in *Fusarium solani* [23].

Our results evidence that CYP51C is highly polymorphic compared to the other CYP51s in *F. verticillioides*. As our docking analysis showed, structural changes in CYP51C might represent a way *F. verticillioides* adapt to DMI fungicides. This will require future functional validations of the role of CYP51C in determining the susceptibility to clinical DMIs. Nonetheless other mechanisms apart from mutations in the CYP51 protein [44,45] can lead to decreased susceptibility, as shown for transcriptional regulators [42] as well as post-transcriptional events on CYP51 [46]. Future studies coupled with experimental susceptibility measures will have to functionally determine which structural changes play an effective role in strain fitness against DMIs in *F. verticillioides*.

Overall, our work provides information on genomic diversity among strains infecting plants and animals (humans), showing that mechanisms leading to trans-kingdom infections [13,18,47,48] are intrinsic to the genomic structure of *F. verticillioides* and generate an interesting future hypothesis to be tested that shall evaluate whether successful infection might be associated with lower susceptibility to DMIs. Here, we showed that comparative genomics is also useful for correlating diversity data with the fitness characteristics of the fungi.

Our work sets the foundations for exploring genomic data in *F. verticilliodes,* with the aim to better define overall diversity within the species, a fundamental step to understanding their role in human infections. Moreover, we provide the fully annotated versions of two *F. verticillioides* human pathogens to the community.

## Figures and Tables

**Table 1:** Strain ID, NCBI assembly Accession number, year of isolation, host and country of isolation of the strains used in this study. The two strains sequenced in this study are in bold.

Strain ID	Accession Number	Isolation Year	Isolation Host	Isolation Country
FV54917	GCA_040113465.1	2022	MAIZE ROOT	USA
FV-FL-03	GCA_040113355.1	2022	MAIZE KERNEL	USA
FV-FL-04	GCA_040113345.1	2022	MAIZE KERNEL	USA
FV-FL-05	GCA_040113015.1	2022	ND	USA
FV-NC-01	GCA_040112945.1	2022	MAIZE KERNEL	USA
FV-NC-02	GCA_040112935.1	2022	MAIZE KERNEL	USA
FV-NE-01	GCA_040112915.1	2022	MAIZE STALK	USA
FV-NE-02	GCA_040112925.1	2022	MAIZE STALK	USA
FV-TN-02	GCA_040112855.1	2022	MAIZE KERNEL	USA
FV-TN-03	GCA_040112835.1	2022	MAIZE KERNEL	USA
FV-TN-04	GCA_040112765.1	2022	MAIZE KERNEL	USA
HN2	GCA_026119585.1	ND	MAIZE	CHINA
LH-A398	GCA_037043915.1	2021	HENS FECES	ND
NRRL_20984	GCA_013759275.1	ND	MAIZE	USA
REC01	GCA_033807555.1	2022	MAIZE	PERU
S1123A	GCA_025503005.1	2019	HUMAN FECES	ND
ZH12-2	GCA_037214365.1	ND	MAIZE	ND
**IUM05-0160**	**This study**	**2005**	**HUMAN BLOOD**	**ITALY**
366	GCA_037954515.1	ND	ND	ND
**IUM09-1037**	**This study**	**2009**	**HUMAN BLOOD**	**ITALY**
7600c	GCA_027571605.1	ND	MAIZE	ND
7600	GCA_000149555.1	ND	MAIZE	ND
ITEM 10027	GCA_031360185.1	2000	MAIZE	ITALY
BRIP_14953	GCA_003316975.2	1977	MAIZE	AUSTRALIA
BRIP_53263	GCA_003317015.2	2009	SORGHUM	AUSTRALIA
BRIP_53590	GCA_003316995.2	2010	MAIZE	AUSTRALIA
BIONCL14	GCA_033110985.1	ND	ND	ND
FN	GCA_031841155.1	2015	SUGARCANE LEAVES	CHINA
FV6396	GCA_040113455.1	2022	CHICKEN FEED	USA
FV13563	GCA_040113975.1	2021	PINUS TAEDA	USA
FV20956	GCA_040113985.1	2021	MAIZE	USA
FV20984	GCA_040113995.1	2021	MAIZE	USA
FV25055	GCA_040113965.1	2021	CLINICAL ISOLATE	ND
FV25058	GCA_040113955.1	2021	PINUS SEED	USA
FV25111	GCA_040113895.1	2021	LEMON TREE	USA
FV25228	GCA_040113885.1	2021	HUMAN HAND	ND
FV25457	GCA_040113875.1	2021	MAIZE KERNEL	GEORGIA (USA)
FV26518	GCA_040113865.1	2021	GARDEN SOIL	USA
FV32969	GCA_040113675.1	2021	ANIMAL FEED	GEORGIA (USA)
FV32970	GCA_040113665.1	2021	ND	ND
FV32973	GCA_040113655.1	2021	HUMAN SKIN	USA
FV34183	GCA_040113695.1	2021	ND	ND
FV34713	GCA_040113565.1	2021	MAIZE KERNEL	GUATEMALA
FV34715	GCA_040113575.1	2021	MAIZE KERNEL	GUATEMALA
FV34717	GCA_040113545.1	2021	MAIZE KERNEL	GUATEMALA
FV34754	GCA_040113585.1	2021	MAIZE KERNEL	GUATEMALA

**Table 2:** Strain ID, BUSCO value, genome size, number of genes, number of effectors with 90% confidence, number of effectors with 50% confidence, number of secondary metabolites clusters, and mitogenome size (if available) of the *Fusarium verticillioides* strains obtained from NCBI. The two clinical strains sequenced and annotated in this study are in bold.

Strain ID	Busco %	Genome Size (Mb)	Genes n°	Effectors > 90%	Effectors > 50%	Secondary Metabolite Clusters	mtDNA
FV54917	99.8	41.95	14,251	349	4764	44	NA
FV-FL-03	99.8	42.02	14,279	337	4784	48	NA
FV-FL-04	99.8	42.02	14,279	349	4749	50	NA
FV-FL-05	99.8	42.15	14,336	355	4804	45	NA
FV-NC-01	99.8	43.07	14,450	373	4856	49	NA
FV-NC-02	99.7	42.46	14,365	373	4825	47	NA
FV-NE-01	99.8	42.54	14,426	358	4822	46	NA
FV-NE-02	99.8	42.82	14,424	354	4820	45	NA
FV-TN-02	99.8	42.48	14,336	355	4746	48	NA
FV-TN-03	99.5	41.31	14,115	350	4684	45	NA
FV-TN-04	99.7	42.30	14,294	355	4747	46	NA
HN2	99.8	42.81	14,103	342	4713	46	53,764 bp
LH-A398	99.7	43.18	14,270	347	4772	47	NA
NRRL_20984	99.5	41.92	14,343	351	4787	46	NA
REC01	99.6	42.82	14,396	345	4833	48	NA
S1123A	99.8	43.18	14,299	349	4754	46	58,870 bp
ZH12-2	99.8	43.12	14,267	350	4742	45	53,773 bp
**IUM05-0160**	**99.8**	**43.61**	**14,397**	**351**	**4814**	**45**	**53,772 bp**
366	99.9	44.03	14,233	348	4723	46	NA
**IUM09-1037**	**99.8**	**43.43**	**13,860**	**343**	**4712**	**48**	**53,760 bp**
7600c	99.8	41.99	14,185	338	4728	48	NA
7600	99.6	41.88	16,290	--	--	47	53,753 bp
ITEM 10027	99.8	43.50	14,329	300	4754	47	53,763 bp
BRIP_14953	99.7	42.54	14,185	336	4722	43	NA
BRIP_53263	99.7	42.40	14,289	363	4773	46	NA
BRIP_53590	99.8	42.29	14,193	350	4731	44	NA
BIONCL14	98.5	41.39	14,031	360	4718	46	NA
FN	99.3	44.65	14,610	352	4943	46	53,536 bp
FV6396	99.6	42.59	14,358	363	4823	45	NA
FV13563	99.6	42.02	14,314	360	4789	48	NA
FV20956	99.6	42.10	14,251	354	4759	48	NA
FV20984	99.7	42.11	14,299	347	4750	46	NA
FV25055	99.7	42.70	14,420	357	4800	48	NA
FV25058	99.8	41.72	14,131	347	4707	45	NA
FV25111	99.8	41.81	14,260	339	4759	48	NA
FV25228	99.8	41.79	14,214	349	4741	47	NA
FV25457	99.8	42.06	14,264	353	4746	47	NA
FV26518	99.7	41.25	14,061	350	4713	43	NA
FV32969	99.4	41.02	14,014	343	4688	44	NA
FV32970	99.7	41.47	14,139	357	4730	45	NA
FV32973	99.5	41.47	14,128	353	4730	44	NA
FV34183	99.8	42.25	14,326	354	4759	45	NA
FV34713	99.8	42.26	14,293	343	4772	48	NA
FV34715	99.7	41.96	14,263	343	4763	48	NA
FV34717	99.7	41.16	13,896	345	4683	43	NA
FV34754	99.3	41.13	13,920	341	4651	45	NA

**Table 3:** DMIs analysis results: MIC100 evaluation for 12 different compounds (mg/L): ITRA: itraconazole; VORI: voriconazole; POSA: posaconazole; ISAV: isavuconazole; PROCH: prochloraz; TEBU: tebuconazole; EPOXI: epoxiconazole; DIFENO: difenoconazole; PROPI: propiconazole; TETRA: tetraconazole; FLUSI: flusiconazole; FENBU: fembuconazole.

Isolation Source/Strain	ITRA	VORI	POSA	ISAV	PROCH	TEBU	EPOXI	DIFENO	PROPI	TETRA	FLUSI	FENBU
Human/IUM09-1037	>16	1	0.5	2	0.12	1	0.5	2	0.5	>16	0.5	2
Human/IUM05-0160	4	1	0.5	1	0.12	1	0.5	2	1	>16	1	2
Maize/7600	0.5	1	0.5	1	0.25	0.5	0.12	2	0.25	1	0.25	0.5

## Data Availability

The IUM09-1037 and IUM05-0160 genome projects can be found in the ENA database under BioProject accession number PRJEB74360. Genome assemblies accession numbers are ERZ23880143 and ERZ23878298, respectively, while Illumina paired-end reads are submitted under the same project under the accession numbers ERR13431485 and ERR13431426, respectively, for IUM09-1037 and IUM05-0160.

## Supplementary Materials

The following supporting information can be downloaded at: https://www.mdpi.com/article/10.3390/pathogens13121062/s1, https://figshare.com/articles/media/Genomic_Insights_into_i_Fusarium_verticillioides_i_diversity_the_genome_of_two_Clinical_Isolates_and_their_demethylase_inhibitor_fungicides_susceptibility_-_Supplementary_materialsGenomic_Insights_into_Fusarium_verticillioides_diversity_the/27689310?file=50425581, Figure S1: Alignment of the mitochondrial *nad5* gene highlights mutations of the strains compared to the strain 7600 used as reference, and confirms groups obtained with multigene phylogeny and fastANI comparison; Figure S2: Aminoacidic alignment of CYP51A gene extracted from all the strains present in the collection; red arrow indicates strains isolated from humans; Figure S3: Aminoacidic alignment of CYP51B gene extracted from all the strains present in the collection; red arrow indicates strains isolated from humans; Figure S4: Aminoacidic alignment of CYP51C gene extracted from all the strains present in the collection; red arrow indicates strains isolated from humans; Table S1: List with all the downloaded strains from NCBI with their year, host, and country of isolation. Last column reports the BUSCO percentage, calculated using Augustus, and Hypocreales_odb10. The two human strains sequenced and anotated in this study are highlighted in bold; Table S2: List of annotated genes of *Fusarium verticilliodies* IUM05-0160; Table S3: List of annotated genes of *Fusarium verticilliodies* IUM09-1037; Table S4: Antismash output of *Fusarium verticilliodies* IUM05-0160; Table S5: Antismash output of *Fusarium verticilliodies* IUM09-1037; Table S6: T-test data and output measured on: genome size, total gene numbers, effectors number, secondary metabolite clusters number; Table S7: List of *Fusarium verticillioides* genes shared with all the other human derived strains (IUM09-1037, FV25228 and FV32973); Table S8a: Rank_1 confidence IUM05-0160—CYP51A—itraconazole; Table S9a: Diffdock output IUM05-0160—CYP51A—itraconazole; Table S8b: Rank_1 confidence IUM05-0160—CYP51A—tetraconazole; Table S9b: Diffdock output IUM05-0160—CYP51A—tetraconazole; Table S8c: Rank_1 confidence 7600—CYP51A—itraconazole; Table S9c: Diffdock output 7600—CYP51A—itraconazole; Table S8d: Rank_1 confidence 7600—CYP51A—tetraconazole; Table S9d: Diffdock output 7600—CYP51A—tetraconazole; Table S8e: Rank_1 confidence IUM05-0160—CYP51C—itraconazole; Table S9e: Diffdock output IUM05-0160—CYP51C—itraconazole; Table S8f: Rank_1 confidence IUM05-0160—CYP51C—tetraconazole; Table S9f: Diffdock output IUM05-0160—CYP51C—tetraconazole; Table S8g: Rank_1 confidence IUM09-1037—CYP51C—itraconazole; Table S9g: Diffdock output IUM09-1037—CYP51C—itraconazole; Table S8h: Rank_1 confidence IUM09-1037—CYP51C—tetraconazole; Table S9h: Diffdock output IUM09-1037—CYP51C—tetraconazole; Table S8i: Rank_1 confidence 7600—CYP51C—itraconazole; Table S9i: Diffdock output 7600—CYP51C—itraconazole; Video S1l: Table S8l: Rank_1 confidence 7600—CYP51C—tetraconazole; Table S9l: Diffdock output 7600—CYP51C—tetraconazole; Video S1a: Video of docking between Cyp51a gene from IUM05-0160 and Itraconazole; Video S1b: Video of docking between Cyp51a gene from IUM05-0160 and Tetraconazole; Video S1c: Video of docking between CYP51A gene from 7600 and Itraconazole; Video S1d: Video of docking between CYP51A gene from 7600 and Tetraconazole; Video S1e: Video of docking between CYP51C gene from IUM05-0160 and Itraconazole; Video S1f: Video of docking between CYP51C gene from IUM05-0160 and Tetraconazole; Video S1g: Video of docking between CYP51C gene from IUM09-1037 and Itraconazole; Video S1h: Video of docking between CYP51C gene from IUM09-1037 and Tetraconazole; Video S1i: Video of docking between CYP51C gene from 7600 and Itraconazole; Video S1l: Video of docking between CYP51C gene from IUM05-0160 and Tetraconazole.
