# Supplementary material for: Genomic Insights into Fusarium verticillioides Diversity: The Genome of Two Clinical Isolates and Their Demethylase Inhibitor Fungicides Susceptibility
Source: Pathogens. 2024 Dec 3;13(12):1062. doi: 10.3390/pathogens13121062 (PMC11728828; doi:10.3390/pathogens13121062)
Supplement: Supplementary file 1 [file pathogens-13-01062-s001.zip › Table S1.pdf]

Origin	Reference(s)
Greece	(Georgiadou et al., 2014)
Italy	(Cocchi et al., 2011; Fabris et al., 1983; On the behalf of the ECMM Working Group et al., 2014; Tortorano et al., 2008)
China	(Sun et al., 2015)
Germany	(Azor et al., 2008)
Sweden	(Azor et al., 2008)
Spain	7/30/2024 6:49:00 PM
URSS	(Azor et al., 2008)
Colombia	(Castro López et al., 2009)
France	(Fabris et al., 1983)
Austria	(Dornbusch et al., 2005)
Japan	(Mochizuki et al., 2012)
USA	(Siegel et al., 2010)
Poland	(Twarużek et al., 2014)
Brazil	(Chang et al., 2013; Macêdo et al., 2008)
Iran	(Yassin et al., 2022)
India	(Tupaki-Sreepurna et al., 2018)
Serbia	(On the behalf of the ECMM Working Group et al., 2014)
Czech Republic	(On the behalf of the ECMM Working Group et al., 2014)
Argentina	(Barberis et al., 2021)

Azor, M., Gené, J., Cano, J., Sutton, D.A., Fothergill, A.W., Rinaldi, M.G., Guarro, J., 2008. In Vitro Antifungal Susceptibility and Molecular Characterization of Clinical Isolates of *Fusarium verticillioides* (*F. moniliforme*) and *Fusarium thapsinum*. Antimicrob Agents Chemother 52, 2228–2231. https://doi.org/10.1128/AAC.00176-08

Barberis, F., Benedetti, M.F., de Abreu, M.S., Pola, S.J., Posse, G., Capece, P., Lausi, A.F., Nusblat, A., Cuestas, M.L., 2021. Invasive fusariosis in a critically ill patient with severe COVID-19 pneumonia: A case report. Med Mycol Case Rep 35, 5–8. https://doi.org/10.1016/j.mmcr.2021.12.003

Castro López, N., Casas, C., Sopo, L., Rojas, A., Del Portillo, P., Cepero de García, M.C., Restrepo, S., 2009. Fusarium species detected in onychomycosis in Colombia. Mycoses 52, 350–356. https://doi.org/10.1111/j.1439-0507.2008.01619.x

Chang, S.C., Macêdo, D.P.C., Souza-Motta, C.M., Oliveira, N.T., 2013. Use of molecular markers to compare Fusarium verticillioides pathogenic strains isolated from plants and humans. Genet. Mol. Res. 12, 2863–2875. https://doi.org/10.4238/2013.August.12.2

Cocchi, S., Codeluppi, M., Venturelli, C., Bedini, A., Grottola, A., Gennari, W., Cavrini, F., Di Benedetto, F., De Ruvo, N., Rumpianesi, F., Gerunda, G.E., Guaraldi, G., 2011. Fusarium verticillioides fungemia in a liver transplantation patient: successful treatment with voriconazole. Diagnostic Microbiology and Infectious Disease 71, 438–441. https://doi.org/10.1016/j.diagmicrobio.2011.08.024

Dornbusch, H.J., Buzina, W., Summerbell, R.C., Lass-Flörl, C., Lackner, H., Schwinger, W., Sovinz, P., Urban, C., 2005. Fusarium verticillioides Abscess of the Nasal Septum in an Immunosuppressed Child: Case Report and Identification of the Morphologically Atypical Fungal Strain. Journal of Clinical Microbiology 43, 1998–2001. https://doi.org/10.1128/jcm.43.4.1998-2001.2004

Fabris, A., Biasioli, S., Chiaramonte, S., Feriani, M., Piacentini, I., Pisani, E., Ronco, C., Viviani, A., Lagreca, G., 1983. AN UNUSUAL FORM OF FUSARIUM-VERTICILLIOIDES PERITONITIS IN A PATIENT ON CHRONIC PERITONEAL-DIALYSIS.

Georgiadou, S.P., Velegraki, A., Arabatzis, M., Neonakis, I., Chatzipanagiotou, S., Dalekos, G.N., Petinaki, E., 2014. Cluster of Fusarium verticillioides bloodstream infections among immunocompetent patients in an internal medicine department after reconstruction works in Larissa, Central Greece. Journal of Hospital Infection 86, 267–271. https://doi.org/10.1016/j.jhin.2014.01.011

Macêdo, D.P.C., Neves, R.P., Fontan, J., Souza-Motta, C.M., Lima, D., 2008. A case of invasive rhinosinusitis by Fusarium verticillioides (Saccardo) Nirenberg in an apparently immunocompetent patient. Medical Mycology 46, 499–503. https://doi.org/10.1080/13693780701861462

Mochizuki, K., Shiraki, I., Murase, H., Ohkusu, K., Nishimura, K., 2012. Identification and sensitivity of two rare fungal species isolated from two patients with *Fusarium* keratomycosis. Journal of Infection and Chemotherapy 18, 939–944. https://doi.org/10.1007/s10156-012-0389-4

On the behalf of the ECMM Working Group, Tortorano, A.M., Prigitano, A., Esposto, M.C., Arsic Arsenijevic, V., Kolarovic, J., Ivanovic, D., Paripovic, L., Klingspor, L., Nordøy, I., Hamal, P., Arikan Akdagli, S., Ossi, C., Grancini, A., Cavanna, C., Lo Cascio, G., Scarparo, C., Candoni, A., Caira, M., Drogari Apiranthitou, M., 2014. European Confederation of Medical Mycology (ECMM) epidemiological survey on invasive infections due to Fusarium species in Europe. Eur J Clin Microbiol Infect Dis 33, 1623–1630. https://doi.org/10.1007/s10096-014-2111-1

Siegel, M., Kan, V., Varma, A., Benator, D., 2010. Successful Treatment of Invasive Fusarium verticillioides Infection With Posaconazole in a Man With Acute Myelogenous Leukemia. Infectious Diseases in Clinical Practice 18, 71–74. https://doi.org/10.1097/IPC.0b013e3181b21a01

Sun, S., Lyu, Q., Han, L., Ma, Q., Hu, H., He, S., Tao, S., Zhang, J., Zhang, H., Wang, L., 2015. Molecular identification and in vitro susceptibility of Fusarium from fungal keratitis in central China. Chinese Journal of Ophthalmology 51, 660–667. https://doi.org/10.3760/cma.j.issn.0412-4081.2015.09.005

Tortorano, A.M., Prigitano, A., Dho, G., Esposto, M.C., Gianni, C., Grancini, A., Ossi, C., Viviani, M.A., 2008. Species Distribution and In Vitro Antifungal Susceptibility Patterns of 75 Clinical Isolates of *Fusarium* spp. from Northern Italy. Antimicrob Agents Chemother 52, 2683–2685. https://doi.org/10.1128/AAC.00272-08

Tupaki-Sreepurna, A., Thanneru, V., Natarajan, S., Sharma, S., Gopi, A., Sundaram, M., Kindo, A.J., 2018. Phylogenetic Diversity and In Vitro Susceptibility Profiles of Human Pathogenic Members of the Fusarium fujikuroi Species Complex Isolated from South India. Mycopathologia 183, 529–540. https://doi.org/10.1007/s11046-018-0248-7

Twarużek, M., Soszczyńska, E., Winiarski, P., Zwierz, A., Grajewski, J., 2014. The occurrence of molds in patients with chronic sinusitis. Eur Arch Otorhinolaryngol 271, 1143–1148. https://doi.org/10.1007/s00405-013-2737-0

Yassin, Z., Salehi, Z., Soleimani, M., Lotfali, E., Fattahi, M., Sharifynia, S., 2022. Phylogenetic relationship of Fusarium species isolated from keratitis using TEF1 and RPB2 gene sequences. Iran J Microbiol 14, 417–422. https://doi.org/10.18502/ijm.v14i3.9794
