# Supplementary material for: Genomic Insights into Fusarium verticillioides Diversity: The Genome of Two Clinical Isolates and Their Demethylase Inhibitor Fungicides Susceptibility
Source: Pathogens. 2024 Dec 3;13(12):1062. doi: 10.3390/pathogens13121062 (PMC11728828; doi:10.3390/pathogens13121062)
Supplement: Supplementary file 1 [file pathogens-13-01062-s001.zip › Table S2.pdf]

ID	Length	Description, GO
Fv_160_1.g1	762	ID=Fv_160_1.g1;Description=hypothetical protein FVEG_14759 [Fusarium verticillioides 7600]
Fv_160_1.g2	491	ID=Fv_160_1.g2;Description=hypothetical protein FVEG_00982 [Fusarium verticillioides 7600];Gene=FOXB_10650;Ontology_term=membrane,hydrolase activity, hydrolyzing O-glycosyl compounds,polysaccharide catabolic process;Ontology_id=GO:0016020,GO:0004553,GO:0000272;Enzyme_code=EC:3.2.1;Enzyme_name=Glycosylases
Fv_160_1.g3	489	ID=Fv_160_1.g3;Description=hypothetical protein FVEG_00981 [Fusarium verticillioides 7600];Gene=HZS61_000919;Ontology_term=membrane,G protein-coupled receptor activity,cell surface receptor signaling pathway,G protein-coupled receptor signaling pathway;Ontology_id=GO:0016020,GO:0004930,GO:0007166,GO:0007186
Fv_160_1.g4	313	ID=Fv_160_1.g4;Description=hypothetical protein FVEG_00980 [Fusarium verticillioides 7600];Gene=FDENT_3736;Ontology_term=dihydroneopterin aldolase activity,folic acid biosynthetic process;Ontology_id=GO:0004150,GO:0046656;Enzyme_code=EC:4.1.2.25;Enzyme_name=dihydroneopterin aldolase
Fv_160_1.g5	224	ID=Fv_160_1.g5;Description=hypothetical protein FVEG_00979 [Fusarium verticillioides 7600]
Fv_160_1.g6	74	ID=Fv_160_1.g6;Description=utp-glucose-1-phosphate uridylyltransferase [Fusarium napiforme];Gene=FOXB_13474;Ontology_term=UTP:glucose-1-phosphate uridylyltransferase activity,glycogen biosynthetic process,trehalose biosynthetic process,UDP-glucose metabolic process,(1->6)-beta-D-glucan biosynthetic process;Ontology_id=GO:0003983,GO:0005978,GO:0005992,GO:0006011,GO:0006078;Enzyme_code=EC:2.7.7.64,EC:2.7.7.9;Enzym e_name=UTP-monosaccharide-1-phosphate uridylyltransferase,UTP--glucose-1-phosphate uridylyltransferase
Fv_160_1.g7	450	ID=Fv_160_1.g7;Description=UTP--glucose-1-phosphate uridylyltransferase-domain-containing protein [Fusarium redolens];Gene=FOXB_13474;Ontology_term=UTP:glucose-1-phosphate uridylyltransferase activity,glycogen biosynthetic process,trehalose biosynthetic process,UDP-glucose metabolic process,(1->6)-beta-D-glucan biosynthetic process;Ontology_id=GO:0003983,GO:0005978,GO:0005992,GO:0006011,GO:0006078;Enzyme_code=EC:2.7.7.64,EC:2.7.7.9;Enzym e_name=UTP-monosaccharide-1-phosphate uridylyltransferase,UTP--glucose-1-phosphate uridylyltransferase
Fv_160_1.g8	211	ID=Fv_160_1.g8;Description=hypothetical protein FVEG_00977 [Fusarium verticillioides 7600]
Fv_160_1.g9	194	ID=Fv_160_1.g9;Description=hypothetical protein FVEG_00976 [Fusarium verticillioides 7600]

Fv_160_1.g10	567	ID=Fv_160_1.g10;Description=hypothetical protein FVER53263_00975 [Fusarium verticillioides];Gene=FOYG_06805;Ontology_term=triglyceride lipase activity,triglyceride metabolic process,lipid catabolic process;Ontology_id=GO:0004806,GO:0006641,GO:0016042;Enzyme_code=EC:3.1.1.3;Enzyme_name=triacylglycerol lipase
Fv_160_1.g11	406	ID=Fv_160_1.g11;Description=hypothetical protein FVEG_00974 [Fusarium verticillioides 7600];Gene=FDENT_3729;Ontology_term=Cdc73/Paf1 complex,alcohol dehydrogenase (NAD+) activity,alcohol metabolic process,transcription elongation by RNA polymerase
Fv_160_1.g12	348	ID=Fv_160_1.g12;Description=maintenance of ploidy mob2 [Fusarium pseudoanthophilum]
Fv_160_1.g13	174	ID=Fv_160_1.g13;Description=hypothetical protein FVEG_00972 [Fusarium verticillioides 7600];Gene=Adh;Ontology_term=alcohol dehydrogenase (NAD+) activity,alcohol metabolic
Fv_160_1.g14	612	ID=Fv_160_1.g14;Description=hypothetical protein FVER14953_00971 [Fusarium verticillioides];Gene=FPRO05_11637;Ontology_term=nucleosome,nucleus,DNA binding,DNA-binding transcription factor activity,structural constituent of chromatin,protein heterodimerization activity,regulation of DNA-templated
Fv_160_1.g15	354	ID=Fv_160_1.g15;Description=hypothetical protein FVEG_00970 [Fusarium verticillioides 7600]
Fv_160_1.g16	466	ID=Fv_160_1.g16;Description=hypothetical protein FVEG_00969 [Fusarium verticillioides
Fv_160_1.g17	354	ID=Fv_160_1.g17;Description=hypothetical protein FVEG_00968 [Fusarium verticillioides 7600];Gene=FOXB_13485;Ontology_term=THO complex part of transcription export complex,mRNA
Fv_160_1.g18	118	ID=Fv_160_1.g18;Description=tubulin binding cofactor A [Fusarium verticillioides 7600];Gene=FTJAE_10365;Ontology_term=cytoplasm,microtubule,beta-tubulin binding,tubulin complex assembly,post-chaperonin
Fv_160_1.g19	617	ID=Fv_160_1.g19;Description=cohesin complex subunit SCC1 [Fusarium verticillioides 7600];Gene=FTJAE_10366;Ontology_term=nucleus,cohesin complex,sister chromatid
Fv_160_1.g20	1841	ID=Fv_160_1.g20;Description=calcium-transporting ATPase 1 [Fusarium subglutinans];Gene=FCIRC_10305;Ontology_term=Golgi apparatus,membrane,ATP binding,ATP hydrolysis activity,protein
Fv_160_1.g21	431	ID=Fv_160_1.g21;Description=tRNA threonylcarbamoyladenosine biosynthesis protein [Fusarium verticillioides 7600];Gene=FDENT_3719;Ontology_term=Golgi apparatus,membrane,double-stranded RNA binding,ATP binding,ATP hydrolysis activity,L-threonylcarbamoyladenylate synthase,tRNA processing,protein transport;Ontology_id=GO:0005794,GO:0016020,GO:0003725,GO:0005524,GO:0016887,GO:0061710,GO:0008033,GO:0015031;En
Fv_160_1.g22	197	ID=Fv_160_1.g22;Description=hypothetical protein FVEG_00962 [Fusarium verticillioides 7600];Gene=FDENT_3718;Ontology_term=Golgi membrane,COPI vesicle coat,alcohol dehydrogenase (NAD+) activity,alcohol metabolic process,retrograde vesicle-mediated transport, Golgi to endoplasmic reticulum,protein transport;Ontology_id=GO:0000139,GO:0030126,GO:0004022,GO:0006066,GO:0006890,GO:0015031;Enzyme_code=EC:1.1.1.1,EC:

Fv_160_1.g23	1006	ID=Fv_160_1.g23;Description=hypothetical protein FVER53590_00961 [Fusarium verticillioides];Gene=FANTH_970;Ontology_term=cell division,nuclear DNA replication,ATP binding,nuclear pre-replicative complex,DNA binding,DNA helicase activity,regulation of DNA-templated DNA replication initiation,DNA duplex unwinding,DNA replication preinitiation complex,nuclear replication fork,MCM complex,hydrolase activity;Ontology_id=GO:0051301,GO:0033260,GO:0005524,GO:0005656,GO:0003677,GO:0003678,GO:0030174,GO:0032508,GO:0
Fv_160_1.g24	566	ID=Fv_160_1.g24;Description=hypothetical protein FVEG_00960 [Fusarium verticillioides 7600];Gene=FPRO05_11648;Ontology_term=vacuolar membrane,alcohol dehydrogenase (NAD+) activity,lyase activity,pyridoxal phosphate binding,alcohol metabolic process,transsulfuration;Ontology_id=GO:0005774,GO:0004022,GO:0016829,GO:0030170,GO:0006066,GO:0019346;Enzyme_code
Fv_160_1.g25	806	ID=Fv_160_1.g25;Description=hypothetical protein FVER53590_00959 [Fusarium verticillioides];Gene=FGADI_4670;Ontology_term=vacuolar membrane,endoplasmic reticulum,vacuolar transporter chaperone complex,inositol hexakisphosphate binding,calmodulin binding,polyphosphate kinase activity,pyridoxal phosphate binding,polyphosphate biosynthetic process,vacuolar transport,microautophagy,transsulfuration,vacuole fusion, non-autophagic;Ontology_id=GO:0005774,GO:0005783,GO:0033254,GO:0000822,GO:0005516,GO:0008976,GO:0030170,GO:0006799,
Fv_160_1.g26	427	ID=Fv_160_1.g26;Description=arginine N-methyltransferase 2 [Fusarium verticillioides 7600];Gene=RMT2;Ontology_term=nucleus,cytoplasm,protein arginine N5-methyltransferase
Fv_160_1.g27	492	ID=Fv_160_1.g27;Description=hexokinase [Fusarium verticillioides 7600];Gene=FGADI_4672;Ontology_term=hexokinase activity,ATP binding,glucose binding,intracellular glucose homeostasis,glycolytic process,carbohydrate phosphorylation;Ontology_id=GO:0004396,GO:0005524,GO:0005536,GO:0001678,GO:0006096,GO:0046835;Enzyme_code=EC:2.7.
Fv_160_1.g28	499	ID=Fv_160_1.g28;Description=oligosaccharyl transferase complex subunit OST4 [Fusarium verticillioides 7600];Gene=FVEG_00956;Ontology_term=oligosaccharyltransferase complex,transferase
Fv_160_1.g29	340	ID=Fv_160_1.g29;Description=hypothetical protein FVEG_00954 [Fusarium verticillioides 7600];Gene=FVEG_00954;Ontology_term=oligosaccharyltransferase complex,transferase
Fv_160_1.g30	414	ID=Fv_160_1.g30;Description=inorganic pyrophosphatase [Fusarium verticillioides 7600];Gene=FVEG_00953;Ontology_term=cytoplasm,magnesium ion binding,alcohol dehydrogenase (NAD+) activity,inorganic diphosphate phosphatase activity,alcohol metabolic process,phosphate-containing compound metabolic process;Ontology_id=GO:0005737,GO:0000287,GO:0004022,GO:0004427,GO:0006066,GO:0006796;Enzyme_code=EC:3.6.1.1,EC:1.
Fv_160_1.g31	97	ID=Fv_160_1.g31;Description=hypothetical protein FOXG_00561 [Fusarium oxysporum f. sp. lycopersici 4287];Gene=FOC1_g10015857;Ontology_term=mitochondrion,ribosome,ribonucleoprotein
Fv_160_1.g32	385	ID=Fv_160_1.g32;Description=hypothetical protein FVEG_00952 [Fusarium verticillioides 7600];Gene=FTJAE_10379;Ontology_term=membrane,SNAP receptor activity,retrograde vesicle-mediated transport, Golgi to

Fv_160_1.g33	255	ID=Fv_160_1.g33;Description=hypothetical protein FVEG_00951 [Fusarium verticillioides 7600];Gene=28942866;Ontology_term=cytoplasm,zinc ion binding,methylthioribulose 1-phosphate dehydratase activity,L-methionine salvage from S-adenosylmethionine,L-methionine salvage from methylthioadenosine;Ontology_id=GO:0005737,GO:0008270,GO:0046570,GO:0019284,GO:0019509;Enzyme_code=EC:4.2.1.109;En
Fv_160_1.g34	200	ID=Fv_160_1.g34;Description=hypothetical protein FVER14953_00950 [Fusarium verticillioides];Gene=Adh;Ontology_term=alcohol dehydrogenase (NAD+) activity,alcohol metabolic
Fv_160_1.g35	561	ID=Fv_160_1.g35;Description=calnexin [Fusarium verticillioides 7600];Gene=FDENT_3706;Ontology_term=endoplasmic reticulum,membrane,calcium ion binding,unfolded protein binding,protein
Fv_160_1.g36	570	ID=Fv_160_1.g36;Description=methylmalonate-semialdehyde dehydrogenase (acylating) [Fusarium verticillioides 7600];Gene=F25303_1564;Ontology_term=alcohol dehydrogenase (NAD+) activity,methylmalonate-semialdehyde dehydrogenase (acylating, NAD) activity,alcohol metabolic process;Ontology_id=GO:0004022,GO:0004491,GO:0006066;Enzyme_code=EC:1.2.1.27,EC:1.1.1.1,EC:1.1.1.71;Enzyme_name=meth
Fv_160_1.g37	658	ID=Fv_160_1.g37;Description=hypothetical protein FVEG_00947 [Fusarium verticillioides 7600];Gene=F25303_1565;Ontology_term=monooxygenase activity,FAD binding,biosynthetic
Fv_160_1.g38	378	ID=Fv_160_1.g38;Description=hypothetical protein FVEG_00946 [Fusarium verticillioides 7600]
Fv_160_1.g39	258	ID=Fv_160_1.g39;Description=wd40 repeat [Fusarium agapanthi];Gene=Tip60
Fv_160_1.g40	783	ID=Fv_160_1.g40;Description=hypothetical protein FVER53263_00944 [Fusarium verticillioides];Gene=FDENT_3702;Ontology_term=pseudouridylate synthase activity,kinase activity,hydrolase activity, acting on glycosyl bonds,metal ion binding,pseudouridine synthesis,phosphorylation;Ontology_id=GO:0004730,GO:0016301,GO:0016798,GO:0046872,GO:0001522,GO:0016310;Enzyme_cod
Fv_160_1.g41	581	ID=Fv_160_1.g41;Description=hypothetical protein FVER53590_00943 [Fusarium verticillioides];Gene=FVEG_00943;Ontology_term=nucleolus,rRNA processing;Ontology_id=GO:0005730,GO:0006364
Fv_160_1.g42	136	ID=Fv_160_1.g42;Description=hypothetical protein FVEG_14754 [Fusarium verticillioides 7600];Gene=Adh;Ontology_term=alcohol dehydrogenase (NAD+) activity,alcohol metabolic
Fv_160_1.g43	139	ID=Fv_160_1.g43;Description=hypothetical protein FVER53590_00942 [Fusarium verticillioides]
Fv_160_1.g44	1248	ID=Fv_160_1.g44;Description=hypothetical protein FVER14953_00941 [Fusarium verticillioides]
Fv_160_1.g45	491	ID=Fv_160_1.g45;Description=DNA replication complex gins sld5 [Fusarium albosuccineum];Gene=FALBO_3348;Ontology_term=nucleus,DNA-templated DNA
Fv_160_1.g46	235	ID=Fv_160_1.g46;Description=hypothetical protein FVER53263_00938 [Fusarium
Fv_160_1.g47	621	ID=Fv_160_1.g47;Description=peptidylprolyl isomerase [Fusarium verticillioides 7600];Gene=BFJ69_g12640;Ontology_term=membrane,peptidyl-prolyl cis-trans isomerase activity,protein

Fv_160_1.g48	372	ID=Fv_160_1.g48;Description=Phospho-2-dehydro-3-deoxyheptonate aldolase amt16 [Fusarium musae];Gene=F25303_1576;Ontology_term=3-deoxy-7-phosphoheptulonate synthase activity,amino acid biosynthetic process,aromatic amino acid family biosynthetic
Fv_160_1.g49	367	ID=Fv_160_1.g49;Description=hypothetical protein FVEG_00935 [Fusarium verticillioides
Fv_160_1.g50	172	ID=Fv_160_1.g50;Description=hypothetical protein FVEG_00934 [Fusarium verticillioides
Fv_160_1.g51	574	ID=Fv_160_1.g51;Description=cytosolic Fe-S cluster assembly factor NAR1 [Fusarium verticillioides 7600];Gene=FVEG_00933;Ontology_term=alcohol dehydrogenase (NAD+) activity,4 iron, 4 sulfur cluster binding,alcohol metabolic process;Ontology_id=GO:0004022,GO:0051539,GO:0006066;Enzyme_code=EC:1.1.1.1,EC:1.1.1.71;Enzyme_name=alcohol
Fv_160_1.g52	280	ID=Fv_160_1.g52;Description=hypothetical protein FVER53590_00932 [Fusarium verticillioides]
Fv_160_1.g53	893	ID=Fv_160_1.g53;Description=AGC/AKT protein kinase [Fusarium verticillioides 7600];Gene=FVEG_00931;Ontology_term=protein serine/threonine kinase activity,ATP
Fv_160_1.g54	128	ID=Fv_160_1.g54;Description=F-type H+-transporting ATPase subunit H [Fusarium verticillioides 7600];Gene=FNAPI_13229;Ontology_term=mitochondrial proton-transporting ATP synthase complex, coupling factor F(o),proton
Fv_160_1.g55	392	ID=Fv_160_1.g55;Description=hypothetical protein FVEG_00929 [Fusarium verticillioides
Fv_160_1.g56	515	ID=Fv_160_1.g56;Description=hypothetical protein FVER53263_00928 [Fusarium verticillioides];Gene=FFUJ_00987;Ontology_term=cytoplasm,ribosomal protein S6 kinase activity,ATP binding,phosphorylation,TORC1
Fv_160_1.g57	519	ID=Fv_160_1.g57;Description=serine/threonine-protein kinase PRP4 [Fusarium verticillioides 7600];Gene=C2S_12462;Ontology_term=U4/U6 x U5 tri-snRNP complex,alcohol dehydrogenase (NAD+) activity,kinase activity,spliceosomal conformational changes to generate catalytic conformation,alcohol metabolic process,phosphorylation,snoRNA splicing;Ontology_id=GO:0046540,GO:0004022,GO:0016301,GO:0000393,GO:0006066,GO:0016310,GO:0034247;Enzyme_code=EC:
Fv_160_1.g58	443	ID=Fv_160_1.g58;Description=hypothetical protein FVEG_00926 [Fusarium verticillioides 7600];Gene=FVER53263_00926;Ontology_term=membrane,kinase activity,cyclin-dependent protein serine/threonine kinase regulator activity,regulation of transcription by RNA polymerase
Fv_160_1.g59	99	ID=Fv_160_1.g59;Description=hypothetical protein FVEG_00925 [Fusarium verticillioides 7600];Gene=LSM8;Ontology_term=spliceosomal complex,U6 snRNP,U4/U6 x U5 tri-snRNP complex,RNA binding,mRNA splicing, via
Fv_160_1.g60	432	ID=Fv_160_1.g60;Description=hypothetical protein J7337_001072 [Fusarium musae];Gene=FMAN_01116;Ontology_term=hydrolase activity, hydrolyzing N-glycosyl compounds,nucleobase-containing compound metabolic process,organonitrogen compound metabolic process;Ontology_id=GO:0016799,GO:0006139,GO:1901564;Enzyme_code=EC:3.2.2;Enzyme_name=Glycosylases
Fv_160_1.g61	400	ID=Fv_160_1.g61;Description=hypothetical protein FVEG_00923 [Fusarium verticillioides
Fv_160_1.g62	408	ID=Fv_160_1.g62;Description=hypothetical protein FVEG_00922 [Fusarium verticillioides 7600]

Fv_160_1.g63	201	ID=Fv_160_1.g63;Description=hypothetical protein FVEG_00921 [Fusarium verticillioides 7600];Gene=F25303_3774;Ontology_term=alcohol dehydrogenase (NAD+) activity,alcohol metabolic process,proteasome regulatory particle assembly;Ontology_id=GO:0004022,GO:0006066,GO:0070682;Enzyme_code=EC:1.1.1.1,EC:1.1.1.71;Enzyme_name=alcohol
Fv_160_1.g64	561	ID=Fv_160_1.g64;Description=hypothetical protein FVER53263_00920 [Fusarium verticillioides];Gene=FNAPI_4565;Ontology_term=membrane,serine-type endopeptidase
Fv_160_1.g65	623	ID=Fv_160_1.g65;Description=hypothetical protein FVER53590_00919 [Fusarium verticillioides];Gene=FSUBG_14069;Ontology_term=transferase activity,metal ion binding,NAD+
Fv_160_1.g66	484	ID=Fv_160_1.g66;Description=hypothetical protein FVEG_00918 [Fusarium verticillioides 7600]
Fv_160_1.g67	114	ID=Fv_160_1.g67;Description=U4/U6-U5 snRNP complex subunit lsm6 [Fusarium musae];Gene=FOPG_01933;Ontology_term=spliceosomal complex,intracellular non-membrane-bounded organelle,spliceosomal
Fv_160_1.g68	282	ID=Fv_160_1.g68;Description=hypothetical protein FVEG_00916 [Fusarium verticillioides 7600];Gene=FCIRC_9868;Ontology_term=membrane,S-adenosylmethionine-dependent methyltransferase
Fv_160_1.g69	273	ID=Fv_160_1.g69;Description=hypothetical protein FNAPI_12394 [Fusarium napiforme];Gene=FFUJ_00974;Ontology_term=membrane,S-adenosylmethionine-dependent methyltransferase
Fv_160_1.g70	241	ID=Fv_160_1.g70;Description=hypothetical protein FVEG_00914 [Fusarium verticillioides 7600];Gene=Adh;Ontology_term=alcohol dehydrogenase (NAD+) activity,alcohol metabolic
Fv_160_1.g71	428	ID=Fv_160_1.g71;Description=cystathionine beta-lyase [Fusarium verticillioides 7600];Gene=FMAN_01126;Ontology_term=lyase activity,pyridoxal phosphate
Fv_160_1.g72	214	ID=Fv_160_1.g72;Description=hypothetical protein FVEG_00912 [Fusarium verticillioides 7600]
Fv_160_1.g73	332	ID=Fv_160_1.g73;Description=hypothetical protein FVER14953_00911 [Fusarium verticillioides];Gene=FTJAE_5394;Ontology_term=membrane,hydrolase activity, acting on carbon-nitrogen (but not peptide) bonds,carbohydrate metabolic
Fv_160_1.g74	358	ID=Fv_160_1.g74;Description=hypothetical protein FVER14953_00910 [Fusarium verticillioides];Gene=FFC1_01042;Ontology_term=lyase activity,metal ion
Fv_160_1.g75	300	ID=Fv_160_1.g75;Description=hypothetical protein FVEG_00909 [Fusarium verticillioides 7600]
Fv_160_1.g76	540	ID=Fv_160_1.g76;Description=hypothetical protein FVEG_00908 [Fusarium verticillioides 7600];Gene=FMUND_10233;Ontology_term=nucleus,ribonucleoprotein complex,DNA binding,DNA-binding transcription factor activity,alcohol dehydrogenase (NAD+) activity,helicase activity,ATP binding,hydrolase activity,alcohol metabolic process,regulation of DNA-templated transcription,rRNA processing;Ontology_id=GO:0005634,GO:1990904,GO:0003677,GO:0003700,GO:0004022,GO:0004386,GO:0005524,GO:0016787,G
Fv_160_1.g77	974	ID=Fv_160_1.g77;Description=adenosinetriphosphatase [Fusarium verticillioides 7600];Gene=FPRO05_11699;Ontology_term=ribonucleoprotein complex,nucleic acid binding,helicase activity,ATP binding,hydrolase

Fv_160_1.g78	91	ID=Fv_160_1.g78;Description=acylphosphatase [Fusarium verticillioides 7600];Gene=BFJ68_g12320;Ontology_term=acylphosphatase
Fv_160_1.g79	347	ID=Fv_160_1.g79;Description=hypothetical protein FVEG_00906 [Fusarium verticillioides
Fv_160_1.g80	463	ID=Fv_160_1.g80;Description=hypothetical protein FVEG_00905 [Fusarium verticillioides 7600];Gene=Adh;Ontology_term=alcohol dehydrogenase (NAD+) activity,alcohol metabolic
Fv_160_1.g81	284	ID=Fv_160_1.g81;Description=hypothetical protein FVEG_00904 [Fusarium verticillioides
Fv_160_1.g82	202	ID=Fv_160_1.g82;Description=hypothetical protein J7337_001094 [Fusarium musae];Gene=FMUND_10228;Ontology_term=gamma-glutamylcyclotransferase activity,glutathione specific gamma-glutamylcyclotransferase activity,glutathione catabolic process;Ontology_id=GO:0003839,GO:0061928,GO:0006751;Enzyme_code=EC:4.3.2.7,EC:4.3.2.9;Enzyme_name=glutathione-specific gamma-glutamylcyclotransferase,gamma-glutamylcyclotransferase
Fv_160_1.g83	195	ID=Fv_160_1.g83;Description=hypothetical protein FVER53590_00902 [Fusarium verticillioides];Gene=FCIRC_11247;Ontology_term=RNA binding,alcohol dehydrogenase (NAD+) activity,alcohol metabolic process;Ontology_id=GO:0003723,GO:0004022,GO:0006066;Enzyme_code=EC:1.1.1.1,EC:1.1.1.71;Enzyme_name=alcohol
Fv_160_1.g84	540	ID=Fv_160_1.g84;Description=hypothetical protein FVEG_00901 [Fusarium verticillioides 7600]
Fv_160_1.g85	317	ID=Fv_160_1.g85;Description=citrate lyase subunit beta-like protein [Fusarium verticillioides 7600];Gene=FOYG_06723;Ontology_term=lyase activity,metal ion
Fv_160_1.g86	194	ID=Fv_160_1.g86;Description=golgi apparatus membrane protein TVP23 [Fusarium oxysporum f. sp. lycopersici 4287];Gene=BFJ68_g4840;Ontology_term=Golgi membrane,vesicle-mediated transport;Ontology_id=GO:0000139,GO:0016192
Fv_160_1.g87	389	ID=Fv_160_1.g87;Description=hypothetical protein FVEG_00898 [Fusarium verticillioides
Fv_160_1.g88	1166	ID=Fv_160_1.g88;Description=hypothetical protein FVER53590_00897 [Fusarium
Fv_160_1.g89	287	ID=Fv_160_1.g89;Description=hypothetical protein FVER14953_00896 [Fusarium verticillioides];Gene=FVEG_00896;Ontology_term=nucleolus,small-subunit processome,rRNA
Fv_160_1.g90	381	ID=Fv_160_1.g90;Description=hypothetical protein FVEG_00895 [Fusarium verticillioides 7600];Gene=FOBC_01329;Ontology_term=myosin phosphatase activity,metal ion
Fv_160_1.g91	1383	ID=Fv_160_1.g91;Description=hypothetical protein FVER14953_00894 [Fusarium verticillioides]
Fv_160_1.g92	1194	ID=Fv_160_1.g92;Description=serine/threonine protein kinase [Fusarium verticillioides 7600];Gene=FMUND_10218;Ontology_term=phagophore assembly site membrane,protein serine/threonine kinase activity,ATP binding,autophagy,phosphorylation;Ontology_id=GO:0034045,GO:0004674,GO:0005524,GO:0006914,GO:0016310;Enzyme_code=EC:2.7.11.1;Enzyme_name=non-specific serine/threonine protein kinase
Fv_160_1.g93	977	ID=Fv_160_1.g93;Description=uncharacterized protein FTJAE_5376 [Fusarium tjaetaba];Gene=Adh;Ontology_term=membrane,alcohol dehydrogenase (NAD+) activity,oligosaccharyl transferase activity,metal ion binding,alcohol metabolic process;Ontology_id=GO:0016020,GO:0004022,GO:0004576,GO:0046872,GO:0006066;Enzyme_code=EC:2.4.1,EC:1.1.1.1,EC:1.1.1.7

Fv_160_1.g94	749	ID=Fv_160_1.g94;Description=dolichyl-diphosphooligosaccharide-protein glycosyltransferase [Fusarium verticillioides 7600];Gene=FPANT_213;Ontology_term=membrane,dolichyl-diphosphooligosaccharide-protein glycotransferase activity,metal ion binding;Ontology_id=GO:0016020,GO:0004579,GO:0046872;Enzyme_code=EC:2.4.99.18,EC:2.4.1;Enzyme_name=dolichyl-diphosphooligosaccharide--protein glycotransferase,Glycosyltransferases
Fv_160_1.g95	212	ID=Fv_160_1.g95;Description=hypothetical protein J7337_001106 [Fusarium musae];Gene=FPANT_212;Ontology_term=mitochondrial proton-transporting ATP synthase complex, coupling factor F(o),oligosaccharyl transferase activity,proton transmembrane transporter activity,proton motive force-driven ATP synthesis,proton
Fv_160_1.g96	692	ID=Fv_160_1.g96;Description=hypothetical protein FVER53590_00889 [Fusarium verticillioides]
Fv_160_1.g97	329	ID=Fv_160_1.g97;Description=hypothetical protein FVEG_00888 [Fusarium verticillioides 7600]
Fv_160_1.g98	314	ID=Fv_160_1.g98;Description=hypothetical protein FVEG_00887 [Fusarium verticillioides
Fv_160_1.g99	579	ID=Fv_160_1.g99;Description=hypothetical protein FVEG_00886 [Fusarium verticillioides 7600]
Fv_160_1.g100	441	ID=Fv_160_1.g100;Description=hypothetical protein FVEG_00885 [Fusarium verticillioides
Fv_160_1.g101	471	ID=Fv_160_1.g101;Description=hypothetical protein FVER14953_00884 [Fusarium verticillioides];Gene=FACUT_11460;Ontology_term=phosphoric diester hydrolase activity,lipid metabolic
Fv_160_1.g102	206	ID=Fv_160_1.g102;Description=hypothetical protein FVER14953_00883 [Fusarium verticillioides];Gene=Adh;Ontology_term=alcohol dehydrogenase (NAD+) activity,alcohol metabolic
Fv_160_1.g103	403	ID=Fv_160_1.g103;Description=S-adenosylmethionine synthase [Fusarium oxysporum f. sp. lycopersici 4287];Gene=FCIRC_11266;Ontology_term=methionine adenosyltransferase activity,ATP binding,metal ion binding,methionine metabolic process,S-adenosylmethionine biosynthetic process,one-carbon metabolic
Fv_160_1.g104	300	ID=Fv_160_1.g104;Description=GPN-loop GTPase 3 like [Fusarium verticillioides 7600];Gene=FCIRC_11267;Ontology_term=GTP binding,hydrolase activity,protein import into nucleus,mitotic sister chromatid
Fv_160_1.g105	179	ID=Fv_160_1.g105;Description=hypothetical protein FCOIX_13170 [Fusarium
Fv_160_1.g106	899	ID=Fv_160_1.g106;Description=hypothetical protein FVER14953_00879 [Fusarium verticillioides]
Fv_160_1.g107	338	ID=Fv_160_1.g107;Description=hypothetical protein LB506_000603 [Fusarium annulatum];Gene=Focb16_v001182;Ontology_term=cytosol,methylenetetrahydrofolate dehydrogenase (NAD+) activity,methylenetetrahydrofolate dehydrogenase (NADP+) activity,one-carbon metabolic process,purine nucleobase biosynthetic process,folic acid-containing compound biosynthetic process;Ontology_id=GO:0005829,GO:0004487,GO:0004488,GO:0006730,GO:0009113,GO:0009396;Enzyme_code=EC:1.5.1.15,EC:1
Fv_160_1.g108	513	ID=Fv_160_1.g108;Description=hypothetical protein FVER14953_00877 [Fusarium verticillioides];Gene=FVEG_00877;Ontology_term=plasma membrane,nitrate transmembrane transporter activity,nitrite transmembrane transporter activity,nitrate transmembrane transport,nitrite transport,nitrate
Fv_160_1.g109	262	ID=Fv_160_1.g109;Description=hypothetical protein J7337_001120 [Fusarium musae]
Fv_160_1.g110	421	ID=Fv_160_1.g110;Description=hypothetical protein FVER53590_00875 [Fusarium verticillioides]

Fv_160_1.g111	594	ID=Fv_160_1.g111;Description=hypothetical protein FVER14953_00874 [Fusarium verticillioides];Gene=FTJAE_5358;Ontology_term=ATP binding,ATP-dependent protein folding chaperone,protein
Fv_160_1.g112	489	ID=Fv_160_1.g112;Description=hypothetical protein FVEG_00873 [Fusarium verticillioides 7600]
Fv_160_1.g113	433	ID=Fv_160_1.g113;Description=hypothetical protein FVEG_00872 [Fusarium verticillioides 7600]
Fv_160_1.g114	346	ID=Fv_160_1.g114;Description=hypothetical protein FVEG_00871 [Fusarium verticillioides 7600]
Fv_160_1.g115	119	ID=Fv_160_1.g115;Description=hypothetical protein FNYG_09318 [Fusarium nygamai];Gene=FPRO05_11736;Ontology_term=nucleus,cytoplasm,cytidine deaminase activity,zinc ion binding,identical protein binding,deoxycytidine deaminase activity,deoxycytidine catabolic process,pyrimidine-containing compound salvage,cytidine deamination;Ontology_id=GO:0005634,GO:0005737,GO:0004126,GO:0008270,GO:0042802,GO:0047844,GO:0006217,GO:0008655,
Fv_160_1.g116	1340	ID=Fv_160_1.g116;Description=STE/STE11/SSK protein kinase [Fusarium verticillioides 7600];Gene=FOVG_00824;Ontology_term=cytoplasm,MAP kinase kinase kinase activity,ATP binding,nucleobase-containing compound metabolic process,phosphorylation,stress-activated MAPK cascade;Ontology_id=GO:0005737,GO:0004709,GO:0005524,GO:0006139,GO:0016310,GO:0051403;Enzyme_code=EC:2.7.11.25,EC
Fv_160_1.g117	307	ID=Fv_160_1.g117;Description=2,4-dienoyl-CoA reductase (NADPH2) [Fusarium verticillioides 7600];Gene=FVEG_00866;Ontology_term=2,4-dienoyl-CoA reductase (NADPH) activity,fatty acid catabolic
Fv_160_1.g118	470	ID=Fv_160_1.g118;Description=hypothetical protein FVER53590_00865 [Fusarium
Fv_160_1.g119	572	ID=Fv_160_1.g119;Description=hypothetical protein FVEG_00864 [Fusarium verticillioides 7600];Gene=FTJAE_5351;Ontology_term=membrane,transmembrane transporter activity,transmembrane
Fv_160_1.g120	704	ID=Fv_160_1.g120;Description=hypothetical protein FVEG_14747 [Fusarium verticillioides 7600];Gene=FTJAE_5350;Ontology_term=nucleus,DNA binding,zinc ion binding,DNA-binding transcription factor activity, RNA
Fv_160_1.g121	984	ID=Fv_160_1.g121;Description=hypothetical protein FVER53263_00860 [Fusarium verticillioides]
Fv_160_1.g122	419	ID=Fv_160_1.g122;Description=chromosome segregation smc [Fusarium pseudocircinatum];Gene=Adh;Ontology_term=plasma membrane,alcohol dehydrogenase (NAD+) activity,alcohol metabolic process;Ontology_id=GO:0005886,GO:0004022,GO:0006066;Enzyme_code=EC:1.1.1.1,EC:1.1.1.71;Enzyme_name=alcohol
Fv_160_1.g123	715	ID=Fv_160_1.g123;Description=hypothetical protein FVEG_00858 [Fusarium verticillioides 7600];Gene=FNAPI_2344;Ontology_term=nucleus,mRNA processing;Ontology_id=GO:0005634,GO:0006397
Fv_160_1.g124	74	ID=Fv_160_1.g124;Description=hypothetical protein FVEG_14746 [Fusarium verticillioides 7600];Gene=NECHADRAFT_73390;Ontology_term=mitochondrial inner membrane,mitochondrial cytochrome c oxidase
Fv_160_1.g125	634	ID=Fv_160_1.g125;Description=hypothetical protein FVEG_00857 [Fusarium verticillioides
Fv_160_1.g126	570	ID=Fv_160_1.g126;Description=hypothetical protein FVER53590_00856 [Fusarium
Fv_160_1.g127	453	ID=Fv_160_1.g127;Description=tubulin alpha chain [Fusarium oxysporum f. sp. lycopersici 4287];Gene=CEP52_001593;Ontology_term=microtubule,structural constituent of cytoskeleton,GTP binding,hydrolase activity,metal ion binding,cytoskeleton organization,microtubule-based

Fv_160_1.g128	359	ID=Fv_160_1.g128;Description=hypothetical protein FVEG_14745 [Fusarium verticillioides 7600];Gene=FOVG_00810;Ontology_term=membrane,transmembrane transporter activity,transmembrane
Fv_160_1.g129	463	ID=Fv_160_1.g129;Description=hypothetical protein FVER14953_00854 [Fusarium verticillioides];Gene=BFJ70_g15781;Ontology_term=oxidoreductase
Fv_160_1.g130	446	ID=Fv_160_1.g130;Description=hypothetical protein FVEG_00853 [Fusarium verticillioides 7600];Gene=F25303_1487;Ontology_term=oxidoreductase activity, acting on the aldehyde or oxo group of donors, NAD or NADP as
Fv_160_1.g131	373	ID=Fv_160_1.g131;Description=hypothetical protein FNYG_09338 [Fusarium
Fv_160_1.g132	152	ID=Fv_160_1.g132;Description=hypothetical protein FPSE_00057 [Fusarium pseudograminearum CS3096];Gene=CEP52_001591;Ontology_term=small ribosomal subunit,RNA binding,structural constituent of
Fv_160_1.g133	85	ID=Fv_160_1.g133;Description=hypothetical protein FVER53590_30382 [Fusarium verticillioides];Gene=F52700_12624;Ontology_term=methyltransferase
Fv_160_1.g134	165	ID=Fv_160_1.g134;Description=hypothetical protein FVEG_00850 [Fusarium verticillioides
Fv_160_1.g135	105	ID=Fv_160_1.g135;Description=hypothetical protein FVEG_00849 [Fusarium verticillioides 7600];Gene=Adh;Ontology_term=alcohol dehydrogenase (NAD+) activity,alcohol metabolic
Fv_160_1.g136	422	ID=Fv_160_1.g136;Description=hypothetical protein FVEG_00848 [Fusarium verticillioides 7600];Gene=FNAPI_2333;Ontology_term=hydrolase
Fv_160_1.g137	103	ID=Fv_160_1.g137;Description=hypothetical protein LB503_000708 [Fusarium chuoi];Gene=Adh;Ontology_term=alcohol dehydrogenase (NAD+) activity,alcohol metabolic
Fv_160_1.g138	121	ID=Fv_160_1.g138;Description=hypothetical protein FVER53590_00846 [Fusarium verticillioides]
Fv_160_1.g139	416	ID=Fv_160_1.g139;Description=hypothetical protein FVEG_00845 [Fusarium verticillioides
Fv_160_1.g140	537	ID=Fv_160_1.g140;Description=hypothetical protein FVER14953_00844 [Fusarium
Fv_160_1.g141	213	ID=Fv_160_1.g141;Description=hypothetical protein FVER14953_00843 [Fusarium verticillioides];Gene=FCIRC_9658;Ontology_term=3'-5'-RNA exonuclease activity,nucleic acid binding,RNA metabolic
Fv_160_1.g142	553	ID=Fv_160_1.g142;Description=phosphoglucomutase [Fusarium verticillioides 7600];Gene=FANTH_9683;Ontology_term=magnesium ion binding,phosphoglucomutase activity,carbohydrate metabolic process;Ontology_id=GO:0000287,GO:0004614,GO:0005975;Enzyme_code=EC:5.4.2.2;Enzyme_name=phosphoglucomutase (alpha-
Fv_160_1.g143	430	ID=Fv_160_1.g143;Description=hypothetical protein FVEG_00841 [Fusarium verticillioides 7600]
Fv_160_1.g144	102	ID=Fv_160_1.g144;Description=Non-histone chromosomal protein 6 [Fusarium oxysporum f. sp. cubense];Gene=F25303_1500;Ontology_term=nucleus,DNA binding;Ontology_id=GO:0005634,GO:0003677
Fv_160_1.g145	232	ID=Fv_160_1.g145;Description=hypothetical protein FVEG_00839 [Fusarium verticillioides 7600];Gene=FOXB_13089;Ontology_term=kinetochore,nucleus,kinetochore
Fv_160_1.g146	1404	ID=Fv_160_1.g146;Description=hypothetical protein FVER53263_00838 [Fusarium verticillioides];Gene=FPANT_2717;Ontology_term=vacuolar membrane,phagophore assembly site membrane,autophagosome

Fv_160_1.g147	362	ID=Fv_160_1.g147;Description=hypothetical protein FVEG_00837 [Fusarium verticillioides 7600]
Fv_160_1.g148	331	ID=Fv_160_1.g148;Description=hypothetical protein FVEG_00836 [Fusarium verticillioides 7600]
Fv_160_1.g149	621	ID=Fv_160_1.g149;Description=hypothetical protein FVEG_00835 [Fusarium verticillioides 7600];Gene=FGLOB1_8093;Ontology_term=membrane,cysteine-type deubiquitinase
Fv_160_1.g150	454	ID=Fv_160_1.g150;Description=hypothetical protein FVEG_00834 [Fusarium verticillioides 7600];Gene=FocTR4_00000941;Ontology_term=membrane,transferase
Fv_160_1.g151	724	ID=Fv_160_1.g151;Description=hypothetical protein FPRO03_00805 [Fusarium proliferatum];Gene=FocTR4_00000940;Ontology_term=nucleus,DNA-binding transcription factor activity, RNA polymerase II-specific,alcohol dehydrogenase (NAD+) activity,zinc ion binding,alcohol metabolic process,regulation of transcription by RNA polymerase
Fv_160_1.g152	209	ID=Fv_160_1.g152;Description=hypothetical protein FVEG_00832 [Fusarium verticillioides 7600];Gene=FVEG_00832;Ontology_term=acyltransferase activity, transferring groups other than amino-acyl
Fv_160_1.g153	468	ID=Fv_160_1.g153;Description=NADH-ubiquinone oxidoreductase 49 kDa subunit, mitochondrial [Fusarium verticillioides 7600];Gene=FVEG_00831;Ontology_term=oxidoreductase activity, acting on NAD(P)H,acyltransferase activity, transferring groups other than amino-acyl groups,quinone binding,NAD
Fv_160_1.g154	487	ID=Fv_160_1.g154;Description=ubiquinone biosynthesis monooxygenase Coq6 [Fusarium verticillioides 7600];Gene=COQ6;Ontology_term=extrinsic component of mitochondrial inner membrane,2-octaprenyl-6-methoxyphenol hydroxylase activity,oxidoreductase activity, acting on paired donors, with incorporation or reduction of molecular oxygen, NAD(P)H as one donor, and incorporation of one atom of oxygen,FAD binding,4-hydroxy-3-all-trans-hexaprenylbenzoate oxygenase activity,ubiquinone biosynthetic process;Ontology_id=GO:0031314,GO:0008681,GO:0016709,GO:0071949,GO:0106364,GO:0006744;Enzyme_code=EC:1.14.14,EC:1.
Fv_160_1.g155	671	ID=Fv_160_1.g155;Description=hypothetical protein FVER53590_00829 [Fusarium verticillioides];Gene=FACUT_11450;Ontology_term=DNA polymerase processivity factor activity,DNA binding,nuclear inner membrane,cytoplasm,positive regulation of DNA-directed DNA polymerase activity,chromosome,chromatin
Fv_160_1.g156	242	ID=Fv_160_1.g156;Description=atp3 gamma subunit of the F1 sector of mitochondrial F1F0 ATP synthase [Fusarium musae];Gene=Forpe1208_v000797;Ontology_term=mitochondrial proton-transporting ATP synthase, stator stalk,proton-transporting ATP synthase activity, rotational mechanism,proton-transporting ATPase activity, rotational mechanism,proton motive force-driven ATP synthesis,protein-containing complex assembly,proton transmembrane transport;Ontology_id=GO:0000274,GO:0046933,GO:0046961,GO:0015986,GO:0065003,GO:1902600;Enzyme_code=EC:7.1.2.2,EC:
Fv_160_1.g157	549	ID=Fv_160_1.g157;Description=hypothetical protein FVER14953_00827 [Fusarium verticillioides];Gene=FOC1_g10015989;Ontology_term=intracellular membrane-bounded organelle,ribosome
Fv_160_1.g158	833	ID=Fv_160_1.g158;Description=hypothetical protein FVER53590_00826 [Fusarium verticillioides];Gene=FNAPI_2311;Ontology_term=membrane,magnesium ion transmembrane transporter activity,magnesium ion

Fv_160_1.g159	293	ID=Fv_160_1.g159;Description=hypothetical protein FVEG_00825 [Fusarium verticillioides 7600];Gene=FMAN_01216;Ontology_term=nucleus,RNA binding,mRNA 3'-end
Fv_160_1.g160	439	ID=Fv_160_1.g160;Description=hypothetical protein FVEG_00824 [Fusarium verticillioides 7600];Gene=FCIRC_9637;Ontology_term=Golgi membrane,endoplasmic reticulum membrane,COPII-coated ER to Golgi transport vesicle,endoplasmic reticulum-Golgi intermediate compartment membrane,retrograde transporter complex, Golgi to ER,endoplasmic reticulum to Golgi vesicle-mediated transport,retrograde vesicle-mediated transport, Golgi to endoplasmic
Fv_160_1.g161	735	ID=Fv_160_1.g161;Description=hypothetical protein FVEG_00823 [Fusarium verticillioides 7600]
Fv_160_1.g162	270	ID=Fv_160_1.g162;Description=probable proteasome endopeptidase complex chain PUP1 [Fusarium fujikuroi IMI 58289];Gene=FCIRC_9635;Ontology_term=nucleus,proteasome core complex, beta-subunit complex,proteasome storage granule,threonine-type endopeptidase activity,proteasomal ubiquitin-independent protein catabolic process,proteasome-mediated ubiquitin-dependent protein catabolic
Fv_160_1.g163	1501	ID=Fv_160_1.g163;Description=hypothetical protein FVER14953_00821 [Fusarium
Fv_160_1.g164	381	ID=Fv_160_1.g164;Description=DNA-directed RNA polymerase I and III subunit RPAC1 [Fusarium verticillioides 7600];Gene=FOPG_01815;Ontology_term=RNA polymerase III complex,RNA polymerase I complex,RNA polymerase I activity,RNA polymerase III activity,DNA binding,protein dimerization activity,transcription initiation at RNA polymerase I promoter,transcription elongation by RNA polymerase I,termination of RNA polymerase I transcription,transcription initiation at RNA polymerase III promoter,termination of RNA polymerase III transcription,tRNA transcription by RNA polymerase III;Ontology_id=GO:0005666,GO:0005736,GO:0001054,GO:0001056,GO:0003677,GO:0046983,GO:0006361,GO:0006362,GO:00063
Fv_160_1.g165	433	ID=Fv_160_1.g165;Description=ubiquitin-activating enzyme E1 C [Fusarium verticillioides 7600];Gene=F25303_1522;Ontology_term=DNA-directed RNA polymerase complex,nucleus,DNA binding,DNA-directed 5'-3' RNA polymerase activity,alcohol dehydrogenase (NAD+) activity,ATP binding,NEDD8 activating enzyme activity,metal ion binding,alcohol metabolic process,transcription by RNA polymerase II,protein neddylation;Ontology_id=GO:0000428,GO:0005634,GO:0003677,GO:0003899,GO:0004022,GO:0005524,GO:0019781,GO:0046872,
Fv_160_1.g166	988	ID=Fv_160_1.g166;Description=hypothetical protein FVEG_00817 [Fusarium verticillioides 7600]
Fv_160_1.g167	838	ID=Fv_160_1.g167;Description=STE/STE20/YSK protein kinase [Fusarium verticillioides 7600];Gene=FPANT_2739;Ontology_term=alcohol dehydrogenase (NAD+) activity,protein kinase activity,ATP binding,alcohol metabolic process,phosphorylation;Ontology_id=GO:0004022,GO:0004672,GO:0005524,GO:0006066,GO:0016310;Enzyme_code=EC:2.7.1,EC:
Fv_160_1.g168	169	ID=Fv_160_1.g168;Description=hypothetical protein FVEG_14739 [Fusarium verticillioides 7600]
Fv_160_1.g169	849	ID=Fv_160_1.g169;Description=hypothetical protein FVER53590_00815 [Fusarium verticillioides];Gene=FCIRC_8668;Ontology_term=nucleus,DNA damage response;Ontology_id=GO:0005634,GO:0006974

Fv_160_1.g170	98	ID=Fv_160_1.g170;Description=hypothetical protein FPSE_00018 [Fusarium pseudograminearum CS3096];Gene=CDV36_008086;Ontology_term=P-body,spliceosomal complex,U5 snRNP,U6 snRNP,nucleolus,sno(s)RNA-containing ribonucleoprotein complex,U4/U6 x U5 tri-snRNP complex,Lsm1-7-Pat1 complex,poly(U) RNA binding,U2 snRNA binding,deadenylation-dependent decapping of nuclear-transcribed mRNA,mRNA splicing, via
Fv_160_1.g171	839	ID=Fv_160_1.g171;Description=Cullin 3 [Fusarium verticillioides 7600];Gene=FGLOB1_8070;Ontology_term=alcohol dehydrogenase (NAD+) activity,ubiquitin protein ligase binding,alcohol metabolic process,ubiquitin-dependent protein catabolic process;Ontology_id=GO:0004022,GO:0031625,GO:0006066,GO:0006511;Enzyme_code=EC:1.1.1.1,EC:1.1.1.71;Enzyme_name=alco
Fv_160_1.g172	123	ID=Fv_160_1.g172;Description=small nuclear ribonucleoprotein D1 [Fusarium oxysporum f. sp. lycopersici 4287];Gene=FNAPI_2298;Ontology_term=spliceosomal snRNP complex,RNA binding,spliceosomal snRNP
Fv_160_1.g173	254	ID=Fv_160_1.g173;Description=hypothetical protein FVEG_00811 [Fusarium verticillioides 7600];Gene=FVEG_00811;Ontology_term=nucleic acid binding,S-adenosylmethionine-dependent methyltransferase activity,nitrogen compound metabolic process,macromolecule methylation,primary metabolic
Fv_160_1.g174	737	ID=Fv_160_1.g174;Description=YVC1 vacuolar cation channel [Fusarium subglutinans];Gene=FVEG_00810;Ontology_term=membrane,protein kinase
Fv_160_1.g175	367	ID=Fv_160_1.g175;Description=hypothetical protein FVER53590_30002 [Fusarium verticillioides];Gene=FDENT_5262;Ontology_term=membrane,protein kinase
Fv_160_1.g176	752	ID=Fv_160_1.g176;Description=IQ domain-containing protein containing GTPase activating protein [Fusarium oxysporum f. sp. lycopersici 4287];Gene=FOXG_00703;Ontology_term=membrane,protein kinase activity;Ontology_id=GO:0016020,GO:0004672;Enzyme_code=EC:2.7.1;Enzyme_name=Transferring phosphorus-containing groups
Fv_160_1.g177	187	ID=Fv_160_1.g177;Description=hypothetical protein FVEG_00809 [Fusarium verticillioides 7600]
Fv_160_1.g178	184	ID=Fv_160_1.g178;Description=peroxiredoxin (alkyl hydroperoxide reductase subunit C) [Fusarium oxysporum f. sp. lycopersici 4287];Gene=FOVG_00748;Ontology_term=thioredoxin peroxidase activity,cellular response to oxidative stress,cellular oxidant detoxification;Ontology_id=GO:0008379,GO:0034599,GO:0098869;Enzyme_code=EC:1.11.1.24;Enzyme_name=thioredoxin-
Fv_160_1.g179	302	ID=Fv_160_1.g179;Description=nuclear transcription factor Y, alpha [Fusarium verticillioides 7600];Gene=FOVG_00746;Ontology_term=nucleus,DNA binding,DNA-binding transcription factor activity,regulation of DNA-
Fv_160_1.g180	771	ID=Fv_160_1.g180;Description=hypothetical protein FVEG_00806 [Fusarium verticillioides 7600];Gene=FPANT_12976;Ontology_term=nucleus,membrane,metal ion transmembrane transporter activity,chromatin

Fv_160_1.g181	282	ID=Fv_160_1.g181;Description=histone chaperone ASF1 [Fusarium verticillioides 7600];Gene=FCIRC_9956;Ontology_term=chromosome, telomeric region,nucleus,cytosol,membrane,H3 histone acetyltransferase complex,acetyltransferase activator activity,histone binding,metal ion transmembrane transporter activity,regulation of protein phosphorylation,nucleosome assembly,DNA replication-dependent chromatin assembly,nucleosome disassembly,magnesium ion transport,silent mating-type cassette heterochromatin formation,subtelomeric heterochromatin formation,positive regulation of transcription elongation by RNA polymerase II,positive regulation of histone acetylation,transmembrane transport;Ontology_id=GO:0000781,GO:0005634,GO:0005829,GO:0016020,GO:0070775,GO:0010698,GO:0042393,GO:0046873,GO
Fv_160_1.g182	992	ID=Fv_160_1.g182;Description=hypothetical protein FVER14953_00804 [Fusarium verticillioides];Gene=BFJ68_g4887;Ontology_term=nucleus,cytoplasm,mRNA
Fv_160_1.g183	988	ID=Fv_160_1.g183;Description=hypothetical protein FVEG_00803 [Fusarium verticillioides
Fv_160_1.g184	339	ID=Fv_160_1.g184;Description=hypothetical protein FVER53263_00802 [Fusarium verticillioides]
Fv_160_1.g185	450	ID=Fv_160_1.g185;Description=saccharopine dehydrogenase [NADP+, L-glutamate-forming] [Fusarium oxysporum f. sp. conglutinans race 2 54008];Gene=F25303_1541;Ontology_term=oxidoreductase
Fv_160_1.g186	531	ID=Fv_160_1.g186;Description=hypothetical protein FVEG_00799 [Fusarium verticillioides 7600];Gene=FVEG_00799;Ontology_term=membrane,transmembrane transporter activity,transmembrane
Fv_160_1.g187	186	ID=Fv_160_1.g187;Description=hypothetical protein FVEG_14736 [Fusarium verticillioides 7600];Gene=FTJAE_4151;Ontology_term=membrane,transmembrane transporter activity,transmembrane
Fv_160_1.g188	984	ID=Fv_160_1.g188;Description=hypothetical protein J7337_001196 [Fusarium musae];Gene=FOVG_00736;Ontology_term=cellular
Fv_160_1.g189	299	ID=Fv_160_1.g189;Description=hypothetical protein FVEG_00797 [Fusarium verticillioides 7600]
Fv_160_1.g190	92	ID=Fv_160_1.g190;Description=hypothetical protein FCOIX_4630 [Fusarium coicis]
Fv_160_1.g191	77	ID=Fv_160_1.g191;Description=---NA---
Fv_160_1.g192	95	ID=Fv_160_1.g192;Description=hypothetical protein FVEG_00795 [Fusarium verticillioides 7600]
Fv_160_1.g193	683	ID=Fv_160_1.g193;Description=hypothetical protein FVEG_00794 [Fusarium verticillioides 7600];Gene=FPCIR_7072;Ontology_term=nucleus,DNA binding,zinc ion binding,DNA-binding transcription factor activity, RNA
Fv_160_1.g194	736	ID=Fv_160_1.g194;Description=polynucleotide 5'-hydroxyl-kinase GRC3 [Fusarium verticillioides 7600];Gene=FOXG_00722;Ontology_term=ATP binding,polynucleotide 5'-hydroxyl-kinase activity,RNA processing,phosphorylation;Ontology_id=GO:0005524,GO:0051731,GO:0006396,GO:0016310;Enzyme_code=EC:2.7.1;Enzyme_nam
Fv_160_1.g195	658	ID=Fv_160_1.g195;Description=hypothetical protein FVER14953_00792 [Fusarium verticillioides];Gene=FCIRC_1786;Ontology_term=transcription by RNA polymerase I;Ontology_id=GO:0006360
Fv_160_1.g196	454	ID=Fv_160_1.g196;Description=hypothetical protein FVEG_00790 [Fusarium verticillioides 7600];Gene=FGADI_5100;Ontology_term=membrane,lipid metabolic process;Ontology_id=GO:0016020,GO:0006629
Fv_160_1.g197	410	ID=Fv_160_1.g197;Description=CAMK/CAMK1 protein kinase [Fusarium verticillioides 7600];Gene=FGADI_5101;Ontology_term=protein serine/threonine kinase activity,ATP

Fv_160_1.g198	192	ID=Fv_160_1.g198;Description=hypothetical protein FVEG_14731 [Fusarium verticillioides 7600];Gene=FOVG_00725;Ontology_term=translation release factor activity,translational
Fv_160_1.g199	861	ID=Fv_160_1.g199;Description=hypothetical protein FVER14953_00788 [Fusarium verticillioides];Gene=FVEG_00788;Ontology_term=plasma membrane,RNA binding,heparin binding,cell
Fv_160_1.g200	570	ID=Fv_160_1.g200;Description=hypothetical protein FVER14953_00787 [Fusarium verticillioides];Gene=FocTR4_00000885;Ontology_term=membrane,ferric-chelate reductase activity,iron ion
Fv_160_1.g201	359	ID=Fv_160_1.g201;Description=hypothetical protein FVEG_00786 [Fusarium verticillioides 7600];Gene=FPANT_11922;Ontology_term=membrane,retrograde vesicle-mediated transport, Golgi to endoplasmic
Fv_160_1.g202	642	ID=Fv_160_1.g202;Description=hypothetical protein FVER14953_00784 [Fusarium verticillioides];Gene=FPANT_11921;Ontology_term=nucleus,transcription corepressor activity,negative regulation of DNA-templated
Fv_160_1.g203	139	ID=Fv_160_1.g203;Description=hypothetical protein FVEG_00783 [Fusarium verticillioides 7600]
Fv_160_1.g204	773	ID=Fv_160_1.g204;Description=hypothetical protein FVER14953_00782 [Fusarium verticillioides];Gene=FPCIR_7082;Ontology_term=membrane,metalloendopeptidase activity,metal ion binding,proteolysis,integrin-mediated signaling
Fv_160_1.g205	710	ID=Fv_160_1.g205;Description=acetyl-coenzyme A synthetase [Fusarium tjaetaba];Gene=FACUT_8468;Ontology_term=acetate-CoA ligase activity,ATP binding,AMP binding,acetyl-CoA biosynthetic process from
Fv_160_1.g206	352	ID=Fv_160_1.g206;Description=hypothetical protein FVER14953_00780 [Fusarium verticillioides];Gene=FCIRC_1776;Ontology_term=DNA replication factor C complex,ribosome,Rad17 RFC-like complex,Ctf18 RFC-like complex,Elg1 RFC-like complex,ribonucleoprotein complex,DNA binding,DNA clamp loader activity,structural constituent of ribosome,ATP hydrolysis activity,rRNA binding,leading strand elongation,translation,sister chromatid cohesion,UV-damage excision repair,DNA clamp
Fv_160_1.g207	252	ID=Fv_160_1.g207;Description=Replication factor C subunit 5 [Fusarium napiforme];Gene=BFJ72_g9808;Ontology_term=mitochondrial large ribosomal subunit,DNA binding,structural constituent of ribosome,ATP hydrolysis activity,rRNA binding,DNA strand elongation involved in DNA replication,mitochondrial translation;Ontology_id=GO:0005762,GO:0003677,GO:0003735,GO:0016887,GO:0019843,GO:0006271,GO:0032543;Enzyme_code=
Fv_160_1.g208	1046	ID=Fv_160_1.g208;Description=hypothetical protein FVER14953_00778 [Fusarium verticillioides];Gene=per;Ontology_term=nucleus,DNA replication factor C complex,perinuclear region of cytoplasm,DNA binding,chromatin binding,DNA clamp loader activity,ATP binding,ATP hydrolysis activity,mitotic cell cycle,leading strand elongation,mismatch repair,regulation of transcription by RNA polymerase II,rhythmic process,UV-damage excision repair;Ontology_id=GO:0005634,GO:0005663,GO:0048471,GO:0003677,GO:0003682,GO:0003689,GO:0005524,GO:0016887,GO:00
Fv_160_1.g209	1028	ID=Fv_160_1.g209;Description=hypothetical protein FVER53590_00776 [Fusarium verticillioides]
Fv_160_1.g210	125	ID=Fv_160_1.g210;Description=hypothetical protein J7337_001218 [Fusarium musae];Gene=FVEG_00775;Ontology_term=cytoplasm,microtubule,DASH complex,mitotic spindle,DNA binding,cell cycle,cell

Fv_160_1.g211	2232	ID=Fv_160_1.g211;Description=hypothetical protein FVEG_14727 [Fusarium verticillioides 7600];Gene=SNT1;Ontology_term=cytoplasm,microtubule,DASH complex,mitotic spindle,DNA binding,cell cycle,cell
Fv_160_1.g212	357	ID=Fv_160_1.g212;Description=deoxyhypusine synthase [Fusarium verticillioides 7600];Gene=Rh2;Ontology_term=membrane,G protein-coupled photoreceptor activity,deoxyhypusine synthase activity,G protein-coupled receptor signaling pathway,visual perception,phototransduction,detection of visible
Fv_160_1.g213	279	ID=Fv_160_1.g213;Description=hypothetical protein FVER14953_20321 [Fusarium verticillioides]
Fv_160_1.g214	300	ID=Fv_160_1.g214;Description=hypothetical protein FVEG_14725 [Fusarium verticillioides 7600]
Fv_160_1.g215	391	ID=Fv_160_1.g215;Description=oxidoreductase [Fusarium verticillioides 7600];Gene=FOVG_00703;Ontology_term=nucleotide
Fv_160_1.g216	280	ID=Fv_160_1.g216;Description=hypothetical protein FVEG_00768 [Fusarium verticillioides 7600];Gene=FOVG_00702;Ontology_term=S-adenosylmethionine-dependent methyltransferase
Fv_160_1.g217	572	ID=Fv_160_1.g217;Description=hypothetical protein FVER53263_00767 [Fusarium verticillioides]
Fv_160_1.g218	265	ID=Fv_160_1.g218;Description=hypothetical protein J7337_001226 [Fusarium musae];Gene=FNAPI_5843;Ontology_term=cytoplasm,alpha-tubulin binding,tubulin complex assembly,post-chaperonin tubulin
Fv_160_1.g219	517	ID=Fv_160_1.g219;Description=hypothetical protein FVEG_00763 [Fusarium verticillioides
Fv_160_1.g220	422	ID=Fv_160_1.g220;Description=hypothetical protein FVER53590_00762 [Fusarium
Fv_160_1.g221	391	ID=Fv_160_1.g221;Description=hypothetical protein FVER14953_00761 [Fusarium verticillioides];Gene=FNAPI_5840;Ontology_term=nucleus,DNA-binding transcription factor activity, RNA polymerase II-specific,DNA binding,hydrolase activity,regulation of transcription by RNA polymerase
Fv_160_1.g222	149	ID=Fv_160_1.g222;Description=---NA---
Fv_160_1.g223	450	ID=Fv_160_1.g223;Description=hypothetical protein FVER53263_00760 [Fusarium verticillioides];Gene=FOXG_00755;Ontology_term=nucleus,DNA-binding transcription factor activity, RNA polymerase II-specific,zinc
Fv_160_1.g224	309	ID=Fv_160_1.g224;Description=hypothetical protein FVEG_00759 [Fusarium verticillioides 7600]
Fv_160_1.g225	652	ID=Fv_160_1.g225;Description=hypothetical protein FVER14953_00758 [Fusarium
Fv_160_1.g226	72	ID=Fv_160_1.g226;Description=F-type H+-transporting ATPase subunit epsilon [Fusarium verticillioides 7600];Gene=BKA59DRAFT_507637;Ontology_term=mitochondrial proton-transporting ATP synthase complex, catalytic sector F(1),proton-transporting ATP synthase activity, rotational mechanism,proton motive force-driven ATP
Fv_160_1.g227	757	ID=Fv_160_1.g227;Description=hypothetical protein FVER14953_00756 [Fusarium verticillioides];Gene=BFJ69_g897;Ontology_term=NatC complex,transferase
Fv_160_1.g228	346	ID=Fv_160_1.g228;Description=hypothetical protein FVEG_00755 [Fusarium verticillioides 7600];Gene=FVEG_00755;Ontology_term=mitochondrial inner membrane;Ontology_id=GO:0005743

Fv_160_1.g229	1550	ID=Fv_160_1.g229;Description=hypothetical protein FVER53263_00754 [Fusarium verticillioides];Gene=Lcp4;Ontology_term=nucleus,transcription regulator complex,sequence-specific DNA binding,protein heterodimerization activity,positive regulation of antibacterial peptide biosynthetic process,peripheral nervous system development,chaeta morphogenesis,epithelial cell proliferation involved in Malpighian tubule morphogenesis,Malpighian tubule tip
Fv_160_1.g230	423	ID=Fv_160_1.g230;Description=26S proteasome regulatory subunit N6 [Fusarium verticillioides 7600];Gene=FMEXI_7907;Ontology_term=proteasome regulatory particle, lid subcomplex,proteasome storage granule,structural molecule activity,proteasome-mediated ubiquitin-dependent protein catabolic process,proteasome
Fv_160_1.g231	244	ID=Fv_160_1.g231;Description=molecular chaperone GrpE [Fusarium verticillioides 7600];Gene=FOPG_01737;Ontology_term=PAM complex, Tim23 associated import motor,adenyl-nucleotide exchange factor activity,protein homodimerization activity,protein-folding chaperone binding,protein import into mitochondrial matrix,protein
Fv_160_1.g232	269	ID=Fv_160_1.g232;Description=rho family, other [Fusarium verticillioides 7600];Gene=FMEXI_7909;Ontology_term=division septum,cell cortex,membrane,GTPase activity,GTP binding,division septum assembly,small GTPase mediated signal transduction,protein localization;Ontology_id=GO:0000935,GO:0005938,GO:0016020,GO:0003924,GO:0005525,GO:0000917,GO:0007264,GO:0008104;E
Fv_160_1.g233	145	ID=Fv_160_1.g233;Description=transcription elongation factor B, polypeptide 1 [Fusarium verticillioides 7600];Gene=C2S_5907;Ontology_term=cytosol,protein-containing complex,translation elongation factor activity,alcohol dehydrogenase (NAD+) activity,acetaldehyde dehydrogenase (acetylating) activity,protein homodimerization activity,ethanol oxidation,acetaldehyde metabolic process,translational elongation,ubiquitin-dependent protein catabolic process,NADH metabolic process,alcohol catabolic process,behavioral response to ethanol;Ontology_id=GO:0005829,GO:0032991,GO:0003746,GO:0004022,GO:0008774,GO:0042803,GO:0006069,GO:0006117,GO:
Fv_160_1.g234	542	ID=Fv_160_1.g234;Description=hypothetical protein FVEG_00749 [Fusarium verticillioides 7600];Gene=FTJAE_12885;Ontology_term=nucleus,RNA lariat debranching enzyme activity,metal ion binding,mRNA processing;Ontology_id=GO:0005634,GO:0008419,GO:0046872,GO:0006397;Enzyme_code=EC:3.1.30,EC:3.1.26;Enzyme_name=Acti
Fv_160_1.g235	257	ID=Fv_160_1.g235;Description=maintenance-ploidy mob1 [Fusarium napiforme];Gene=9.0;Ontology_term=nucleus,membrane,hydrolase activity, acting on ester bonds,metal ion binding,proton-transporting ATP synthase activity, rotational mechanism,mRNA processing,proton motive force-driven ATP synthesis,proton transmembrane
Fv_160_1.g236	226	ID=Fv_160_1.g236;Description=ATP synthase F1, delta subunit [Fusarium verticillioides 7600];Gene=FOXG_00768;Ontology_term=mitochondrial proton-transporting ATP synthase, stator stalk,proton-transporting ATP synthase activity, rotational mechanism,proton motive force-driven ATP synthesis,proton transmembrane
Fv_160_1.g237	1026	ID=Fv_160_1.g237;Description=Six-hairpin glycosidase-like protein [Fusarium oxysporum f. sp. albedinis];Gene=FOXG_00769;Ontology_term=Glc3Man9GlcNAc2 oligosaccharide glucosidase activity,oligosaccharide metabolic

Fv_160_1.g238	363	ID=Fv_160_1.g238;Description=undecaprenyl diphosphate synthase [Fusarium verticillioides 7600];Gene=C2S_5904;Ontology_term=dehydrodolichyl diphosphate synthase activity,dolichol biosynthetic process;Ontology_id=GO:0045547,GO:0019408;Enzyme_code=EC:2.5.1.87;Enzyme_name=ditrans,polycis-polyprenyl diphosphate
Fv_160_1.g239	728	ID=Fv_160_1.g239;Description=hypothetical protein J7337_001247 [Fusarium musae];Gene=FOPG_01729;Ontology_term=chromatin,nucleus,metal ion binding,chromatin
Fv_160_1.g240	614	ID=Fv_160_1.g240;Description=hypothetical protein FVEG_00743 [Fusarium verticillioides 7600]
Fv_160_1.g241	860	ID=Fv_160_1.g241;Description=Leucine Rich Repeat domain protein [Fusarium fujikuroi];Gene=FOMG_01519;Ontology_term=vacuolar proton-transporting V-type ATPase, V0 domain,proton-transporting ATPase activity, rotational mechanism,proton transmembrane
Fv_160_1.g242	832	ID=Fv_160_1.g242;Description=endoribonuclease ysh1 [Fusarium musae];Gene=gC;Ontology_term=nucleus,cytosol,protein-containing complex,alcohol dehydrogenase (NAD+) activity,acetaldehyde dehydrogenase (acetylating) activity,protein homodimerization activity,ethanol oxidation,acetaldehyde metabolic process,mRNA processing,NADH metabolic process,alcohol catabolic process,behavioral response to ethanol;Ontology_id=GO:0005634,GO:0005829,GO:0032991,GO:0004022,GO:0008774,GO:0042803,GO:0006069,GO:0006117,GO:
Fv_160_1.g243	643	ID=Fv_160_1.g243;Description=hypothetical protein FVEG_00739 [Fusarium verticillioides 7600];Gene=FPCIR_8493;Ontology_term=transcription factor TFIID complex,hydrolase activity, acting on carbon-nitrogen (but not peptide) bonds, in linear amides,DNA-templated transcription
Fv_160_1.g244	295	ID=Fv_160_1.g244;Description=hypothetical protein FVER53590_00739 [Fusarium verticillioides];Gene=FMAN_01301;Ontology_term=transcription factor TFIID complex,hydrolase activity, acting on carbon-nitrogen (but not peptide) bonds, in linear amides,DNA-templated transcription
Fv_160_1.g245	201	ID=Fv_160_1.g245;Description=actin like protein 2/3 complex, subunit 5 [Fusarium verticillioides 7600];Gene=FMEXI_10181;Ontology_term=cytoplasm,Arp2/3 protein complex,regulation of actin filament polymerization,Arp2/3
Fv_160_1.g246	348	ID=Fv_160_1.g246;Description=hypothetical protein FVER53590_00736 [Fusarium verticillioides];Gene=FPANT_1836;Ontology_term=S-adenosylmethionine-dependent methyltransferase
Fv_160_1.g247	985	ID=Fv_160_1.g247;Description=hypothetical protein J7337_001255 [Fusarium musae];Gene=FPANT_1837;Ontology_term=AP-2 adaptor complex,clathrin adaptor activity,intracellular protein transport,clathrin-dependent
Fv_160_1.g248	263	ID=Fv_160_1.g248;Description=YggS family pyridoxal phosphate enzyme [Fusarium verticillioides 7600];Gene=FVEG_00734;Ontology_term=pyridoxal phosphate binding;Ontology_id=GO:0030170
Fv_160_1.g249	374	ID=Fv_160_1.g249;Description=hypothetical protein FVER14953_00733 [Fusarium verticillioides]
Fv_160_1.g250	542	ID=Fv_160_1.g250;Description=hypothetical protein J7337_001258 [Fusarium
Fv_160_1.g251	282	ID=Fv_160_1.g251;Description=hypothetical protein FVEG_00731 [Fusarium verticillioides 7600];Gene=FVEG_00731;Ontology_term=oxidoreductase
Fv_160_1.g252	650	ID=Fv_160_1.g252;Description=hypothetical protein FVER14953_00730 [Fusarium verticillioides]

Fv_160_1.g253	653	ID=Fv_160_1.g253;Description=hypothetical protein FVEG_00729 [Fusarium verticillioides 7600];Gene=CEK26_001240;Ontology_term=extracellular region,membrane,egg chorion,transmembrane transporter
Fv_160_1.g254	870	ID=Fv_160_1.g254;Description=hypothetical protein FVER14953_00727 [Fusarium verticillioides];Gene=TRX2;Ontology_term=extracellular region,membrane,egg chorion,calcium ion binding,transmembrane
Fv_160_1.g255	275	ID=Fv_160_1.g255;Description=hypothetical protein FVER53263_00726 [Fusarium verticillioides];Gene=FOXG_00789;Ontology_term=membrane,hydrolase
Fv_160_1.g256	860	ID=Fv_160_1.g256;Description=protein SEY1 [Fusarium verticillioides 7600];Gene=SEY1;Ontology_term=endoplasmic reticulum membrane,GTPase activity,GTP binding,carbohydrate binding,endoplasmic reticulum organization;Ontology_id=GO:0005789,GO:0003924,GO:0005525,GO:0030246,GO:0007029;Enzyme_code=EC:3.6.1.15;Enzyme_na
Fv_160_1.g257	339	ID=Fv_160_1.g257;Description=hypothetical protein FVER14953_00724 [Fusarium verticillioides];Gene=FOYG_06528;Ontology_term=dorsal closure,wing disc development,peptidoglycan recognition protein signaling pathway,imaginal disc fusion, thorax closure,DNA-binding transcription activator activity, RNA polymerase II-specific,positive regulation of border follicle cell migration,R3/R4 cell fate commitment,positive regulation of transcription by RNA polymerase II,dendrite morphogenesis,RNA catabolic process,DNA binding,RNA-DNA hybrid ribonuclease activity,wound healing,RNA binding,synaptic assembly at neuromuscular junction,protein heterodimerization activity,metal ion binding,locomotor rhythm,transcription factor AP-1 complex,second mitotic wave involved in compound eye morphogenesis,DNA-binding transcription factor
Fv_160_1.g258	649	ID=Fv_160_1.g258;Description=hypothetical protein FVER14953_00723 [Fusarium verticillioides];Gene=ORF30;Ontology_term=kinetochore,nucleosome,nucleus,centromeric DNA binding,structural constituent of chromatin,nucleosome assembly,kinetochore
Fv_160_1.g259	915	ID=Fv_160_1.g259;Description=hypothetical protein FVEG_00722 [Fusarium verticillioides 7600];Gene=FVEG_00722;Ontology_term=mitochondrial inner membrane,RNA binding,mitochondrial genome maintenance,mRNA
Fv_160_1.g260	220	ID=Fv_160_1.g260;Description=hypothetical protein FVER53263_00721 [Fusarium verticillioides]
Fv_160_1.g261	652	ID=Fv_160_1.g261;Description=hsp70-like protein [Fusarium verticillioides 7600];Gene=FNAPI_8411;Ontology_term=ATP binding,ATP-dependent protein folding chaperone,protein folding;Ontology_id=GO:0005524,GO:0140662,GO:0006457
Fv_160_1.g262	271	ID=Fv_160_1.g262;Description=hypothetical protein FVEG_00719 [Fusarium verticillioides 7600]
Fv_160_1.g263	642	ID=Fv_160_1.g263;Description=hypothetical protein FVEG_00718 [Fusarium verticillioides 7600];Gene=FCIRC_12510;Ontology_term=membrane,transmembrane transporter activity,transmembrane
Fv_160_1.g264	859	ID=Fv_160_1.g264;Description=carnitine O-acetyltransferase [Fusarium verticillioides 7600];Gene=FOVG_00649;Ontology_term=mitochondrion,acyltransferase activity,fatty acid metabolic process,carnitine metabolic
Fv_160_1.g265	1080	ID=Fv_160_1.g265;Description=hypothetical protein FVER53263_00716 [Fusarium verticillioides];Gene=FOVG_00648;Ontology_term=nucleolus,RNA binding,rRNA

Fv_160_1.g266	1157	ID=Fv_160_1.g266;Description=DNA-directed RNA polymerase III subunit RPC2 [Fusarium verticillioides 7600];Gene=BFJ68_g14322;Ontology_term=chromatin,RNA polymerase III complex,RNA polymerase III activity,DNA binding,ribonucleoside binding,metal ion binding,transcription initiation at RNA polymerase III promoter,termination of RNA polymerase III transcription,tRNA transcription by RNA polymerase III;Ontology_id=GO:0000785,GO:0005666,GO:0001056,GO:0003677,GO:0032549,GO:0046872,GO:0006384,GO:0006386,GO:00427
Fv_160_1.g267	439	ID=Fv_160_1.g267;Description=DNA repair protein RAD57 [Fusarium verticillioides 7600];Gene=FMAN_01325;Ontology_term=ribosome,site of double-strand break,ribonucleoprotein complex,DNA binding,structural constituent of ribosome,ATP binding,ATP hydrolysis activity,ATP-dependent DNA damage sensor activity,DNA recombinase assembly,translation,reciprocal meiotic recombination;Ontology_id=GO:0005840,GO:0035861,GO:1990904,GO:0003677,GO:0003735,GO:0005524,GO:0016887,GO:01406
Fv_160_1.g268	203	ID=Fv_160_1.g268;Description=Ff.00g091400.m01.CDS01 [Fusarium sp. VM40];Gene=FOXG_00802;Ontology_term=ribosome,ribonucleoprotein complex,structural constituent of
Fv_160_1.g269	247	ID=Fv_160_1.g269;Description=hypothetical protein FVEG_00711 [Fusarium verticillioides 7600];Gene=FPANT_11459;Ontology_term=ribosome,ribonucleoprotein complex,structural constituent of
Fv_160_1.g270	896	ID=Fv_160_1.g270;Description=hypothetical protein FVER53263_00710 [Fusarium verticillioides]
Fv_160_1.g271	1077	ID=Fv_160_1.g271;Description=hypothetical protein FVEG_00707 [Fusarium verticillioides 7600]
Fv_160_1.g272	339	ID=Fv_160_1.g272;Description=hypothetical protein FVEG_00706 [Fusarium verticillioides
Fv_160_1.g273	383	ID=Fv_160_1.g273;Description=hypothetical protein FNAPI_13120 [Fusarium napiforme];Gene=ORF45;Ontology_term=extracellular space,mating behavior,sexual reproduction;Ontology_id=GO:0005615,GO:0007617,GO:0019953
Fv_160_1.g274	532	ID=Fv_160_1.g274;Description=protein phosphatase [Fusarium verticillioides 7600];Gene=BFJ63_vAg12752;Ontology_term=fungal-type vacuole,myosin phosphatase activity,metal ion
Fv_160_1.g275	348	ID=Fv_160_1.g275;Description=hypothetical protein FVER14953_00703 [Fusarium verticillioides]
Fv_160_1.g276	272	ID=Fv_160_1.g276;Description=hypothetical protein FVEG_00702 [Fusarium verticillioides
Fv_160_1.g277	552	ID=Fv_160_1.g277;Description=hypothetical protein FVER53263_00701 [Fusarium verticillioides];Gene=FMAN_01336;Ontology_term=membrane,UDP-glycosyltransferase
Fv_160_1.g278	922	ID=Fv_160_1.g278;Description=hypothetical protein FVER14953_00700 [Fusarium
Fv_160_1.g279	504	ID=Fv_160_1.g279;Description=hypothetical protein FVEG_00699 [Fusarium verticillioides 7600];Gene=ORF52

Fv_160_1.g280	255	ID=Fv_160_1.g280;Description=RNA polymerase II subunit A domain phosphatase SSU72 [Fusarium verticillioides 7600];Gene=FOVG_00526;Ontology_term=chromatin,mRNA cleavage and polyadenylation specificity factor complex,protein tyrosine phosphatase activity,RNA polymerase II CTD heptapeptide repeat phosphatase activity,myosin phosphatase activity,transcriptional start site selection at RNA polymerase II promoter,regulation of transcription by RNA polymerase II,transcription elongation by RNA polymerase II,mRNA polyadenylation,sno(s)RNA transcription,termination of RNA polymerase II transcription, poly(A)-coupled,termination of RNA polymerase II transcription, exosome-dependent,transcription antitermination,positive regulation of telomere maintenance via semi-conservative replication,regulation of siRNA-independent facultative heterochromatin
Fv_160_1.g281	431	ID=Fv_160_1.g281;Description=hypothetical protein FVEG_00697 [Fusarium verticillioides 7600]
Fv_160_1.g282	332	ID=Fv_160_1.g282;Description=NADH-cytochrome b5 reductase 2 [Fusarium verticillioides 7600];Gene=FOXG_00901;Ontology_term=mitochondrial outer membrane,cytochrome-b5 reductase activity, acting on
Fv_160_1.g283	800	ID=Fv_160_1.g283;Description=hypothetical protein FVER14953_00695 [Fusarium verticillioides];Gene=FACUT_11483;Ontology_term=membrane,carboxypeptidase activity,metallopeptidase
Fv_160_1.g284	559	ID=Fv_160_1.g284;Description=hypothetical protein FVER14953_00694 [Fusarium verticillioides];Gene=FOXG_00898;Ontology_term=membrane,transmembrane transporter activity,transmembrane
Fv_160_1.g285	449	ID=Fv_160_1.g285;Description=hypothetical protein FVER14953_00693 [Fusarium verticillioides]
Fv_160_1.g286	500	ID=Fv_160_1.g286;Description=hypothetical protein FVEG_14720 [Fusarium verticillioides 7600]
Fv_160_1.g287	617	ID=Fv_160_1.g287;Description=hypothetical protein FVEG_00691 [Fusarium verticillioides 7600]
Fv_160_1.g288	816	ID=Fv_160_1.g288;Description=hypothetical protein FVEG_00690 [Fusarium verticillioides
Fv_160_1.g289	78	ID=Fv_160_1.g289;Description=hypothetical protein FVER14953_20722 [Fusarium verticillioides]
Fv_160_1.g290	350	ID=Fv_160_1.g290;Description=hypothetical protein FVER14953_00689 [Fusarium verticillioides]
Fv_160_1.g291	614	ID=Fv_160_1.g291;Description=heat shock protein SSB1 [Fusarium verticillioides 7600];Gene=FOMG_01658;Ontology_term=cytosol,ATP binding,ATP-dependent protein folding chaperone,protein
Fv_160_1.g292	112	ID=Fv_160_1.g292;Description=hypothetical protein FVEG_14718 [Fusarium verticillioides 7600]
Fv_160_1.g293	410	ID=Fv_160_1.g293;Description=hypothetical protein FVEG_00686 [Fusarium verticillioides
Fv_160_1.g294	257	ID=Fv_160_1.g294;Description=hypothetical protein FVER14953_00685 [Fusarium verticillioides]
Fv_160_1.g295	91	ID=Fv_160_1.g295;Description=hypothetical protein FVEG_00684 [Fusarium verticillioides 7600];Gene=gp64
Fv_160_1.g296	1750	ID=Fv_160_1.g296;Description=DNA-directed RNA polymerase II subunit RPB1 [Fusarium verticillioides 7600];Gene=A0A4D6RH46;Ontology_term=DNA-directed RNA polymerase complex,nucleus,DNA binding,DNA-directed 5'-3' RNA polymerase activity,metal ion binding,transcription by RNA polymerase
Fv_160_1.g297	463	ID=Fv_160_1.g297;Description=hypothetical protein FVEG_00682 [Fusarium verticillioides

Fv_160_1.g298	288	ID=Fv_160_1.g298;Description=tRNA(His) guanylyltransferase [Fusarium verticillioides 7600];Gene=FOVG_00549;Ontology_term=magnesium ion binding,GTP binding,tRNA guanylyltransferase activity,tRNA modification,tRNA 5'-end
Fv_160_1.g299	219	ID=Fv_160_1.g299;Description=hypothetical protein FVER14953_00680 [Fusarium verticillioides];Gene=FNAPI_4653;Ontology_term=DNA-directed RNA polymerase complex;Ontology_id=GO:0000428
Fv_160_1.g300	308	ID=Fv_160_1.g300;Description=transcription elongation factor S-II [Fusarium verticillioides 7600];Gene=FMAN_01359;Ontology_term=nucleus,cytoplasm,DNA binding,translation elongation factor activity,zinc ion binding,transcription elongation by RNA polymerase II,translational
Fv_160_1.g301	814	ID=Fv_160_1.g301;Description=hypothetical protein FVER14953_00678 [Fusarium
Fv_160_1.g302	445	ID=Fv_160_1.g302;Description=cysteine synthase A [Fusarium verticillioides 7600];Gene=FCIRC_3677;Ontology_term=membrane,cysteine biosynthetic process from
Fv_160_1.g303	507	ID=Fv_160_1.g303;Description=hypothetical protein J7337_001310 [Fusarium musae];Gene=FocTR4_00000726;Ontology_term=mitochondrion,protein-arginine omega-N symmetric methyltransferase
Fv_160_1.g304	959	ID=Fv_160_1.g304;Description=Alanine--tRNA ligase [Fusarium musae];Gene=ala1;Ontology_term=mitochondrion,tRNA binding,alanine-tRNA ligase activity,ATP binding,zinc ion binding,mitochondrial alanyl-tRNA aminoacylation;Ontology_id=GO:0005739,GO:0000049,GO:0004813,GO:0005524,GO:0008270,GO:0070143;Enzyme_code=EC:6.1.1
Fv_160_1.g305	220	ID=Fv_160_1.g305;Description=hypothetical protein FVER14953_00674 [Fusarium verticillioides]
Fv_160_1.g306	166	ID=Fv_160_1.g306;Description=hypothetical protein FVEG_00673 [Fusarium verticillioides 7600];Gene=FANTH_9025;Ontology_term=membrane,ligase
Fv_160_1.g307	824	ID=Fv_160_1.g307;Description=hypothetical protein J7337_001314 [Fusarium
Fv_160_1.g308	368	ID=Fv_160_1.g308;Description=uracil-DNA glycosylase [Fusarium verticillioides 7600];Gene=ung1;Ontology_term=nucleus,mitochondrion,uracil DNA N-glycosylase activity,base-excision
Fv_160_1.g309	1046	ID=Fv_160_1.g309;Description=hypothetical protein FVER14953_00670 [Fusarium verticillioides]
Fv_160_1.g310	1231	ID=Fv_160_1.g310;Description=hypothetical protein FVER14953_00669 [Fusarium verticillioides];Gene=FMUND_3678;Ontology_term=nucleus,single-stranded DNA binding,endonuclease activity,metal ion binding,nucleotide-excision repair involved in interstrand cross-link
Fv_160_1.g311	106	ID=Fv_160_1.g311;Description=hypothetical protein FVEG_00668 [Fusarium verticillioides 7600];Gene=C2S_5827;Ontology_term=NatC complex,transferase
Fv_160_1.g312	352	ID=Fv_160_1.g312;Description=hypothetical protein FOXG_00871 [Fusarium oxysporum f. sp. lycopersici 4287];Gene=C2S_5826;Ontology_term=cellular anatomical entity;Ontology_id=GO:0110165
Fv_160_1.g313	1142	ID=Fv_160_1.g313;Description=hypothetical protein FVER14953_00666 [Fusarium verticillioides];Gene=BFJ69_g852;Ontology_term=histone H3K27 trimethyltransferase activity,chromatin remodeling,methylation;Ontology_id=GO:0140951,GO:0006338,GO:0032259;Enzyme_code=EC:2.1.1.356;Enzyme_name=[histone

Fv_160_1.g314	213	ID=Fv_160_1.g314;Description=hypothetical protein FVEG_00665 [Fusarium verticillioides 7600];Gene=F25303_5269;Ontology_term=endoplasmic reticulum membrane,intracellular protein transport,endoplasmic reticulum to Golgi vesicle-mediated transport,protein localization to endoplasmic reticulum exit
Fv_160_1.g315	478	ID=Fv_160_1.g315;Description=hypothetical protein FVEG_00664 [Fusarium verticillioides 7600]
Fv_160_1.g316	266	ID=Fv_160_1.g316;Description=hypothetical protein FVEG_00663 [Fusarium verticillioides 7600];Gene=F52700_7530;Ontology_term=catalytic activity,DNA repair;Ontology_id=GO:0003824,GO:0006281
Fv_160_1.g317	481	ID=Fv_160_1.g317;Description=hypothetical protein FVEG_00662 [Fusarium verticillioides 7600];Gene=FOVG_00569;Ontology_term=oxidoreductase activity,FAD
Fv_160_1.g318	74	ID=Fv_160_1.g318;Description=hypothetical protein FOXG_17964 [Fusarium oxysporum f. sp. lycopersici 4287]
Fv_160_1.g319	389	ID=Fv_160_1.g319;Description=hypothetical protein FVER14953_00660 [Fusarium verticillioides];Gene=FTJAE_13126;Ontology_term=oxidoreductase activity,metal ion binding,small molecule biosynthetic process,organic cyclic compound biosynthetic
Fv_160_1.g320	962	ID=Fv_160_1.g320;Description=hypothetical protein FVER14953_00659 [Fusarium verticillioides];Gene=BFJ69_g941;Ontology_term=ribonucleoprotein complex;Ontology_id=GO:1990904
Fv_160_1.g321	80	ID=Fv_160_1.g321;Description=DNA-directed RNA polymerase I, II, and III subunit RPABC5 [Fusarium oxysporum f. sp. lycopersici 4287];Gene=F66182_11002;Ontology_term=DNA-directed RNA polymerase complex,DNA binding,DNA-directed 5'-3' RNA polymerase activity,zinc ion binding,DNA-templated
Fv_160_1.g322	708	ID=Fv_160_1.g322;Description=hypothetical protein FVER14953_00655 [Fusarium verticillioides];Gene=94
Fv_160_1.g323	108	ID=Fv_160_1.g323;Description=hypothetical protein FVER14953_00654 [Fusarium verticillioides]
Fv_160_1.g324	262	ID=Fv_160_1.g324;Description=hypothetical protein FVEG_14713 [Fusarium verticillioides 7600];Gene=FVER53263_00653;Ontology_term=S-adenosylmethionine-dependent methyltransferase
Fv_160_1.g325	353	ID=Fv_160_1.g325;Description=hypothetical protein FVEG_00653 [Fusarium verticillioides 7600];Gene=F25303_5259;Ontology_term=S-adenosylmethionine-dependent methyltransferase
Fv_160_1.g326	260	ID=Fv_160_1.g326;Description=hypothetical protein FVER14953_00652 [Fusarium verticillioides]
Fv_160_1.g327	661	ID=Fv_160_1.g327;Description=hypothetical protein FVER14953_00651 [Fusarium
Fv_160_1.g328	103	ID=Fv_160_1.g328;Description=dynein light chain LC8-type [Fusarium oxysporum f. sp. lycopersici 4287];Gene=FGRA07_03726;Ontology_term=nuclear pore,peroxisome,cytoplasmic dynein complex,cytoplasmic microtubule,nuclear periphery,meiotic spindle pole body,peroxisomal importomer complex,plus-end-directed microtubule motor activity,protein-containing complex binding,nuclear migration along microtubule,establishment of mitotic spindle localization,nuclear pore complex assembly;Ontology_id=GO:0005643,GO:0005777,GO:0005868,GO:0005881,GO:0034399,GO:0035974,GO:1990429,GO:0008574,GO

Fv_160_1.g329	857	ID=Fv_160_1.g329;Description=hypothetical protein FVEG_00647 [Fusarium verticillioides 7600];Gene=FTJAE_13116;Ontology_term=nucleolus,90S preribosome,small-subunit processome,rRNA primary transcript binding,endonucleolytic cleavage in ITS1 to separate SSU-rRNA from 5.8S rRNA and LSU-rRNA from tricistronic rRNA transcript (SSU-rRNA, 5.8S rRNA, LSU-rRNA),endonucleolytic cleavage to generate mature 5'-end of SSU-rRNA from (SSU-rRNA, 5.8S rRNA, LSU-rRNA),endonucleolytic cleavage in 5'-ETS of tricistronic rRNA transcript (SSU-rRNA, 5.8S rRNA, LSU-rRNA),small-subunit processome
Fv_160_1.g330	863	ID=Fv_160_1.g330;Description=hypothetical protein FVER53590_00646 [Fusarium verticillioides];Gene=FVEG_00646;Ontology_term=nucleus,DNA-binding transcription factor activity,RNA binding,regulation of DNA-
Fv_160_1.g331	519	ID=Fv_160_1.g331;Description=hypothetical protein FVEG_00645 [Fusarium verticillioides 7600]
Fv_160_1.g332	182	ID=Fv_160_1.g332;Description=peptidyl-prolyl cis-trans isomerase H [Fusarium napiforme];Gene=FDENT_11091;Ontology_term=peptidyl-prolyl cis-trans isomerase activity,protein
Fv_160_1.g333	448	ID=Fv_160_1.g333;Description=hypothetical protein FVEG_00643 [Fusarium verticillioides
Fv_160_1.g334	310	ID=Fv_160_1.g334;Description=hypothetical protein FVEG_00642 [Fusarium verticillioides 7600]
Fv_160_1.g335	1592	ID=Fv_160_1.g335;Description=hypothetical protein FVER53590_00640 [Fusarium verticillioides];Gene=FRV6_03502;Ontology_term=membrane,protein targeting to vacuole;Ontology_id=GO:0016020,GO:0006623
Fv_160_1.g336	241	ID=Fv_160_1.g336;Description=hypothetical protein J7337_001343 [Fusarium musae];Gene=FOXG_00844;Ontology_term=membrane,protein targeting to vacuole;Ontology_id=GO:0016020,GO:0006623
Fv_160_1.g337	453	ID=Fv_160_1.g337;Description=unnamed protein product [Fusarium graminearum];Gene=C2S_5803;Ontology_term=membrane,metal ion transmembrane transporter activity,magnesium ion
Fv_160_1.g338	627	ID=Fv_160_1.g338;Description=hypothetical protein FVEG_00638 [Fusarium verticillioides 7600];Gene=107
Fv_160_1.g339	539	ID=Fv_160_1.g339;Description=hypothetical protein FVEG_14705 [Fusarium verticillioides 7600];Gene=Rh3;Ontology_term=membrane,G protein-coupled receptor activity,photoreceptor activity,G protein-coupled receptor signaling pathway,visual
Fv_160_1.g340	308	ID=Fv_160_1.g340;Description=hypothetical protein FVER53263_20989 [Fusarium verticillioides]
Fv_160_1.g341	208	ID=Fv_160_1.g341;Description=hypothetical protein FVEG_00634 [Fusarium verticillioides 7600]
Fv_160_1.g342	692	ID=Fv_160_1.g342;Description=hypothetical protein FVER14953_00633 [Fusarium verticillioides];Gene=BFJ68_g7104;Ontology_term=RNA binding,RNA helicase activity,ATP binding,hydrolase activity,maturation of
Fv_160_1.g343	462	ID=Fv_160_1.g343;Description=hypothetical protein FVEG_00632 [Fusarium verticillioides 7600]
Fv_160_1.g344	619	ID=Fv_160_1.g344;Description=hypothetical protein FVER53590_00631 [Fusarium verticillioides];Gene=FFUJ_00688;Ontology_term=cytoplasm,nucleic acid binding,lysine-tRNA ligase activity,ATP binding,lysyl-tRNA
Fv_160_1.g345	472	ID=Fv_160_1.g345;Description=hypothetical protein FVER14953_00630 [Fusarium verticillioides]
Fv_160_1.g346	319	ID=Fv_160_1.g346;Description=TDG/mug DNA glycosylase [Fusarium verticillioides 7600];Gene=FACUT_2846;Ontology_term=mismatch base pair DNA N-glycosylase activity,base-excision repair, AP site

Fv_160_1.g347	502	ID=Fv_160_1.g347;Description=UDP-N-acetylglucosamine pyrophosphorylase [Fusarium verticillioides 7600];Gene=FNAPI_8630;Ontology_term=uridylyltransferase
Fv_160_1.g348	330	ID=Fv_160_1.g348;Description=thioredoxin reductase [Fusarium verticillioides 7600];Gene=FOVG_00605;Ontology_term=GARP complex,cytosol,endosome membrane,thioredoxin-disulfide reductase (NADP) activity,removal of superoxide radicals,retrograde transport, endosome to
Fv_160_1.g349	826	ID=Fv_160_1.g349;Description=hypothetical protein FVEG_00626 [Fusarium verticillioides 7600];Gene=FocTR4_00000775;Ontology_term=GARP complex,cytosol,endosome membrane,retrograde transport, endosome to
Fv_160_1.g350	1141	ID=Fv_160_1.g350;Description=hypothetical protein FVER14953_00625 [Fusarium
Fv_160_1.g351	780	ID=Fv_160_1.g351;Description=hypothetical protein FVEG_00623 [Fusarium verticillioides 7600];Gene=FFB14_04053;Ontology_term=endoplasmic reticulum organization,lipid storage,nuclear membrane
Fv_160_1.g352	854	ID=Fv_160_1.g352;Description=histidine biosynthesis trifunctional protein [Fusarium verticillioides 7600];Gene=FTJAE_3021;Ontology_term=histidinol dehydrogenase activity,phosphoribosyl-AMP cyclohydrolase activity,phosphoribosyl-ATP diphosphatase activity,ATP binding,metal ion binding,NAD binding,histidine biosynthetic process;Ontology_id=GO:0004399,GO:0004635,GO:0004636,GO:0005524,GO:0046872,GO:0051287,GO:0000105;Enzyme_code=EC
Fv_160_1.g353	253	ID=Fv_160_1.g353;Description=transcription initiation factor TFIID subunit 9B [Fusarium verticillioides 7600];Gene=FCIRC_8943;Ontology_term=nucleus,translation initiation factor activity,protein heterodimerization activity,DNA-
Fv_160_1.g354	474	ID=Fv_160_1.g354;Description=mitochondrial-processing peptidase subunit beta [Fusarium oxysporum f. sp. lycopersici 4287];Gene=FCIRC_8944;Ontology_term=mitochondrial processing peptidase complex,metalloendopeptidase activity,metal ion binding,protein processing involved in protein targeting to
Fv_160_1.g355	891	ID=Fv_160_1.g355;Description=hypothetical protein FVER53263_00619 [Fusarium verticillioides];Gene=BFJ65_g1072;Ontology_term=cytoplasm,nucleotide binding,IMP dehydrogenase activity,alpha-galactosidase activity,glycosyltransferase activity,metal ion binding,carbohydrate metabolic process,GMP biosynthetic process;Ontology_id=GO:0005737,GO:0000166,GO:0003938,GO:0004557,GO:0016757,GO:0046872,GO:0005975,GO:0006177;Enzy
Fv_160_1.g356	532	ID=Fv_160_1.g356;Description=inosine-5'-monophosphate dehydrogenase IMD4 [Fusarium oxysporum f. sp. lycopersici 4287];Gene=FNAPI_12812;Ontology_term=cytoplasm,nucleotide binding,IMP dehydrogenase activity,metal ion binding,carbohydrate metabolic process,GMP biosynthetic
Fv_160_1.g357	199	ID=Fv_160_1.g357;Description=hypothetical protein FVEG_00617 [Fusarium verticillioides 7600];Gene=Adh;Ontology_term=cytosol,protein-containing complex,alcohol dehydrogenase (NAD+) activity,acetaldehyde dehydrogenase (acetylating) activity,protein homodimerization activity,ethanol oxidation,acetaldehyde metabolic process,NADH metabolic process,alcohol catabolic process,behavioral response to ethanol;Ontology_id=GO:0005829,GO:0032991,GO:0004022,GO:0008774,GO:0042803,GO:0006069,GO:0006117,GO:0006734,GO:

Fv_160_1.g358	243	ID=Fv_160_1.g358;Description=hypothetical protein FVEG_00616 [Fusarium verticillioides 7600];Gene=FVER53590_00616;Ontology_term=nucleus,cytoplasm,histone H4 acetyltransferase activity,histone H2A acetyltransferase activity,chromatin
Fv_160_1.g359	102	ID=Fv_160_1.g359;Description=hypothetical protein FVER14953_20830 [Fusarium verticillioides]
Fv_160_1.g360	99	ID=Fv_160_1.g360;Description=hypothetical protein FVEG_00615 [Fusarium verticillioides 7600];Gene=FocTR4_00000787;Ontology_term=cytosol,ribosome,ribonucleoprotein complex,structural constituent of ribosome,alcohol dehydrogenase (NAD+) activity,acetaldehyde dehydrogenase (acetylating) activity,protein homodimerization activity,ethanol oxidation,acetaldehyde metabolic process,translation,NADH metabolic process,alcohol catabolic process,behavioral response to
Fv_160_1.g361	805	ID=Fv_160_1.g361;Description=hypothetical protein FVER53590_00614 [Fusarium verticillioides]
Fv_160_1.g362	565	ID=Fv_160_1.g362;Description=hypothetical protein FVEG_00613 [Fusarium verticillioides 7600]
Fv_160_1.g363	527	ID=Fv_160_1.g363;Description=hypothetical protein FVER14953_00612 [Fusarium verticillioides];Gene=FVEG_00612;Ontology_term=succinate-semialdehyde dehydrogenase (NAD+) activity,succinate-semialdehyde dehydrogenase (NADP+) activity,gamma-aminobutyric acid catabolic process;Ontology_id=GO:0004777,GO:0036243,GO:0009450;Enzyme_code=EC:1.2.1.16,EC:1.2.1.24,EC:1.2.1.79;Enzyme_name=succ
Fv_160_1.g364	287	ID=Fv_160_1.g364;Description=hypothetical protein FVEG_00611 [Fusarium verticillioides
Fv_160_1.g365	1511	ID=Fv_160_1.g365;Description=hypothetical protein FVER53263_00610 [Fusarium verticillioides];Gene=FFUJ_00666;Ontology_term=phosphorelay sensor kinase activity,phosphorelay signal transduction system,phosphorylation;Ontology_id=GO:0000155,GO:0000160,GO:0016310;Enzyme_code=EC:2.7.3,EC:2.7.13.3;Enzyme_name=Tra
Fv_160_1.g366	480	ID=Fv_160_1.g366;Description=hypothetical protein FVEG_00609 [Fusarium verticillioides 7600];Gene=FACUT_2825;Ontology_term=methyltransferase
Fv_160_1.g367	373	ID=Fv_160_1.g367;Description=hypothetical protein FVEG_00608 [Fusarium verticillioides
Fv_160_1.g368	228	ID=Fv_160_1.g368;Description=hypothetical protein FPANT_12904 [Fusarium pseudoanthophilum];Gene=LSDV139;Ontology_term=oxidoreductase activity,ethanol metabolic process,cellular metabolic
Fv_160_1.g369	167	ID=Fv_160_1.g369;Description=peptidyl-prolyl cis-trans isomerase-like 3 [Fusarium verticillioides 7600];Gene=FFUJ_00662;Ontology_term=peptidyl-prolyl cis-trans isomerase activity,protein
Fv_160_1.g370	302	ID=Fv_160_1.g370;Description=hypothetical protein FVER53590_00605 [Fusarium verticillioides]
Fv_160_1.g371	550	ID=Fv_160_1.g371;Description=Peptidyl-prolyl isomerase cwc27 [Fusarium musae];Gene=FNAPI_2388;Ontology_term=peptidyl-prolyl cis-trans isomerase activity,protein
Fv_160_1.g372	764	ID=Fv_160_1.g372;Description=hypothetical protein FVER53590_00603 [Fusarium verticillioides];Gene=FNAPI_2389;Ontology_term=nucleus,DNA-binding transcription factor activity,sequence-specific DNA
Fv_160_1.g373	394	ID=Fv_160_1.g373;Description=hypothetical protein FVEG_00602 [Fusarium verticillioides 7600]
Fv_160_1.g374	813	ID=Fv_160_1.g374;Description=hypothetical protein FVER14953_00598 [Fusarium

Fv_160_1.g375	577	ID=Fv_160_1.g375;Description=hypothetical protein FVER14953_00597 [Fusarium verticillioides];Gene=FPANT_11068;Ontology_term=membrane,monoatomic cation transmembrane transporter activity,intracellular monoatomic cation homeostasis,monoatomic cation transmembrane transport,inorganic ion
Fv_160_1.g376	455	ID=Fv_160_1.g376;Description=hypothetical protein FVEG_00596 [Fusarium verticillioides 7600];Gene=FOXG_00917
Fv_160_1.g377	324	ID=Fv_160_1.g377;Description=hypothetical protein FVEG_00595 [Fusarium verticillioides 7600]
Fv_160_1.g378	451	ID=Fv_160_1.g378;Description=syntaxin 1B 2 3 [Fusarium tjaetaba];Gene=FGADI_1494;Ontology_term=membrane,vesicle-mediated
Fv_160_1.g379	237	ID=Fv_160_1.g379;Description=hypothetical protein FVEG_00593 [Fusarium verticillioides 7600];Gene=Adh;Ontology_term=cytosol,protein-containing complex,alcohol dehydrogenase (NAD+) activity,acetaldehyde dehydrogenase (acetylating) activity,protein homodimerization activity,ethanol oxidation,acetaldehyde metabolic process,NADH metabolic process,alcohol catabolic process,behavioral response to ethanol;Ontology_id=GO:0005829,GO:0032991,GO:0004022,GO:0008774,GO:0042803,GO:0006069,GO:0006117,GO:0006734,GO:
Fv_160_1.g380	357	ID=Fv_160_1.g380;Description=hypothetical protein FVER14953_00592 [Fusarium verticillioides];Gene=C2S_13470;Ontology_term=chitinase activity,chitin binding,carbohydrate metabolic
Fv_160_1.g381	359	ID=Fv_160_1.g381;Description=hypothetical protein FVER53590_00591 [Fusarium verticillioides];Gene=FNAPI_2398;Ontology_term=S-adenosylmethionine-dependent methyltransferase
Fv_160_1.g382	252	ID=Fv_160_1.g382;Description=hypothetical protein FVEG_00590 [Fusarium verticillioides 7600];Gene=FNAPI_2399;Ontology_term=cytosol,mRNA cleavage and polyadenylation specificity factor complex,RNA binding,S-adenosylmethionine-dependent methyltransferase activity,metal ion binding,mRNA polyadenylation,methylation;Ontology_id=GO:0005829,GO:0005847,GO:0003723,GO:0008757,GO:0046872,GO:0006378,GO:00322
Fv_160_1.g383	495	ID=Fv_160_1.g383;Description=hypothetical protein FVEG_00589 [Fusarium verticillioides 7600];Gene=148
Fv_160_1.g384	161	ID=Fv_160_1.g384;Description=hypothetical protein FVEG_14694 [Fusarium verticillioides 7600]
Fv_160_1.g385	68	ID=Fv_160_1.g385;Description=hypothetical protein FVER14953_20834 [Fusarium verticillioides]
Fv_160_1.g386	422	ID=Fv_160_1.g386;Description=hypothetical protein FVEG_00588 [Fusarium verticillioides 7600]
Fv_160_1.g387	173	ID=Fv_160_1.g387;Description=hypothetical protein FVER14953_20835 [Fusarium verticillioides]
Fv_160_1.g388	77	ID=Fv_160_1.g388;Description=hypothetical protein FVER14953_20836 [Fusarium
Fv_160_1.g389	493	ID=Fv_160_1.g389;Description=aldehyde dehydrogenase [Fusarium verticillioides 7600];Gene=FACUT_2803;Ontology_term=oxidoreductase activity, acting on the aldehyde or oxo group of donors, NAD or NADP as
Fv_160_1.g390	169	ID=Fv_160_1.g390;Description=hypothetical protein FVEG_14690 [Fusarium verticillioides 7600]
Fv_160_1.g391	289	ID=Fv_160_1.g391;Description=hypothetical protein FVER53590_00584 [Fusarium verticillioides]
Fv_160_1.g392	821	ID=Fv_160_1.g392;Description=hypothetical protein FVEG_00583 [Fusarium verticillioides 7600];Gene=157
Fv_160_1.g393	1019	ID=Fv_160_1.g393;Description=hypothetical protein FVEG_00582 [Fusarium verticillioides 7600]
Fv_160_1.g394	1015	ID=Fv_160_1.g394;Description=hypothetical protein FVER53263_00581 [Fusarium verticillioides]

Fv_160_1.g395	501	ID=Fv_160_1.g395;Description=phosphatidylinositol N-acetylglucosaminyltransferase gpi3 subunit [Fusarium verticillioides 7600];Gene=FPANT_11026;Ontology_term=glycosylphosphatidylinositol-N-acetylglucosaminyltransferase (GPI-GnT) complex,phosphatidylinositol N-acetylglucosaminyltransferase
Fv_160_1.g396	900	ID=Fv_160_1.g396;Description=hypothetical protein FVER14953_00579 [Fusarium verticillioides];Gene=FVEG_00579;Ontology_term=RNA binding,helicase activity,ATP binding,hydrolase
Fv_160_1.g397	592	ID=Fv_160_1.g397;Description=hypothetical protein FVEG_00577 [Fusarium verticillioides 7600]
Fv_160_1.g398	220	ID=Fv_160_1.g398;Description=hypothetical protein FVEG_00576 [Fusarium verticillioides
Fv_160_1.g399	488	ID=Fv_160_1.g399;Description=hypothetical protein FVEG_00575 [Fusarium verticillioides
Fv_160_1.g400	816	ID=Fv_160_1.g400;Description=hypothetical protein FVEG_00574 [Fusarium verticillioides 7600];Gene=FMAN_01456;Ontology_term=nucleus,DNA binding,zinc ion binding,DNA-binding transcription factor activity, RNA
Fv_160_1.g401	757	ID=Fv_160_1.g401;Description=hypothetical protein FVEG_00572 [Fusarium verticillioides 7600];Gene=BFJ72_g2948;Ontology_term=nucleus,DNA binding,zinc ion binding,DNA-binding transcription factor activity, RNA
Fv_160_1.g402	481	ID=Fv_160_1.g402;Description=hypothetical protein FVER53263_21151 [Fusarium verticillioides];Gene=FocTR4_00000654;Ontology_term=nucleus,nucleic acid binding,helicase activity,ATP binding,ATP hydrolysis activity,DNA
Fv_160_1.g403	982	ID=Fv_160_1.g403;Description=hypothetical protein FVEG_00570 [Fusarium verticillioides 7600];Gene=165
Fv_160_1.g404	271	ID=Fv_160_1.g404;Description=hypothetical protein FVEG_00569 [Fusarium verticillioides 7600];Gene=FVEG_00569;Ontology_term=BLOC-1 complex,localization;Ontology_id=GO:0031083,GO:0051179
Fv_160_1.g405	308	ID=Fv_160_1.g405;Description=enoyl reductase [Fusarium verticillioides 7600];Gene=FOXG_00946;Ontology_term=membrane,oxidoreductase activity, acting on the CH-CH group of donors,lipid metabolic
Fv_160_1.g406	783	ID=Fv_160_1.g406;Description=hypothetical protein FPCIR_13587 [Fusarium pseudocircinatum];Gene=FOXB_09841;Ontology_term=ribosome,ribonucleoprotein complex,structural constituent of
Fv_160_1.g407	206	ID=Fv_160_1.g407;Description=40S ribosomal protein S8 [Fusarium oxysporum f. sp. lycopersici 4287];Gene=FGADI_1461;Ontology_term=ribosome,ribonucleoprotein complex,structural constituent of
Fv_160_1.g408	968	ID=Fv_160_1.g408;Description=hypothetical protein FVER14953_00565 [Fusarium verticillioides]
Fv_160_1.g409	3993	ID=Fv_160_1.g409;Description=hypothetical protein FVER53263_00563 [Fusarium verticillioides];Gene=FMUND_4874;Ontology_term=ubiquitin-protein transferase activity,ligase activity,protein
Fv_160_1.g410	239	ID=Fv_160_1.g410;Description=probable 40s ribosomal protein S6.e, cytosolic [Fusarium proliferatum ET1];Gene=FOYG_06348;Ontology_term=ribosome,ribonucleoprotein complex,structural constituent of
Fv_160_1.g411	146	ID=Fv_160_1.g411;Description=chromosome transmission fidelity protein 8 [Fusarium verticillioides 7600];Gene=FPANT_11009;Ontology_term=Ctf18 RFC-like complex,mitotic sister chromatid

Fv_160_1.g412	454	ID=Fv_160_1.g412;Description=diphthamide biosynthesis protein 1 [Fusarium verticillioides 7600];Gene=FPANT_11008;Ontology_term=metal ion binding,iron-sulfur cluster binding,2-(3-amino-3-carboxypropyl)histidine synthase activity;Ontology_id=GO:0046872,GO:0051536,GO:0090560;Enzyme_code=EC:2.5.1.108;Enzyme_name=2-(3-amino-3-
Fv_160_1.g413	511	ID=Fv_160_1.g413;Description=P-loop containing nucleoside triphosphate hydrolase protein [Fusarium redolens];Gene=FNAPI_2059;Ontology_term=proton-transporting V-type ATPase, V1 domain,catalytic activity,ATP binding,proton-transporting ATPase activity, rotational mechanism,small molecule biosynthetic process,ATP metabolic process,proton transmembrane
Fv_160_1.g414	369	ID=Fv_160_1.g414;Description=hydroxymethylglutaryl-CoA lyase [Fusarium verticillioides 7600];Gene=FOMG_01727;Ontology_term=microtubule,hydroxymethylglutaryl-CoA lyase activity,structural constituent of cytoskeleton,GTP binding,hydrolase activity,metal ion binding,cytoskeleton organization,microtubule-based process,small molecule biosynthetic
Fv_160_1.g415	449	ID=Fv_160_1.g415;Description=hypothetical protein LB505_000615 [Fusarium chuoi];Gene=FNAPI_2058;Ontology_term=microtubule,structural constituent of cytoskeleton,GTP binding,hydrolase activity,metal ion binding,cytoskeleton organization,microtubule-based process,small molecule biosynthetic
Fv_160_1.g416	243	ID=Fv_160_1.g416;Description=hypothetical protein FVEG_00556 [Fusarium verticillioides
Fv_160_1.g417	517	ID=Fv_160_1.g417;Description=hypothetical protein FVEG_00555 [Fusarium verticillioides 7600]
Fv_160_1.g418	432	ID=Fv_160_1.g418;Description=spore coat SP96 precursor [Fusarium coicis];Gene=speE
Fv_160_1.g419	913	ID=Fv_160_1.g419;Description=hypothetical protein J7337_001415 [Fusarium musae];Gene=FDENT_13191;Ontology_term=RNA
Fv_160_1.g420	454	ID=Fv_160_1.g420;Description=ubiquinol-cytochrome c reductase core subunit 2 [Fusarium verticillioides 7600];Gene=FPHYL_5380;Ontology_term=metal ion binding;Ontology_id=GO:0046872
Fv_160_1.g421	633	ID=Fv_160_1.g421;Description=hypothetical protein FVER14953_00551 [Fusarium verticillioides];Gene=FDENT_13193;Ontology_term=RNA polymerase III complex,single-stranded DNA binding,DNA-templated
Fv_160_1.g422	303	ID=Fv_160_1.g422;Description=transport uso1 [Fusarium longipes];Gene=FANTH_8850;Ontology_term=mitochondrial inner membrane,guanine nucleotide transmembrane transporter activity,mitochondrial genome maintenance,intracellular iron ion homeostasis,guanine nucleotide transmembrane
Fv_160_1.g423	2368	ID=Fv_160_1.g423;Description=hypothetical protein FVER14953_00549 [Fusarium verticillioides]
Fv_160_1.g424	91	ID=Fv_160_1.g424;Description=uncharacterized protein FFUJ_00604 [Fusarium fujikuroi IMI 58289];Gene=FGADI_7266;Ontology_term=cytoplasm,microtubule,DASH complex,mitotic spindle,attachment of spindle
Fv_160_1.g425	502	ID=Fv_160_1.g425;Description=hypothetical protein J7337_001421 [Fusarium musae];Gene=F25303_5742;Ontology_term=ribosome,ribonucleoprotein complex,RNA binding,structural constituent of
Fv_160_1.g426	77	ID=Fv_160_1.g426;Description=hypothetical protein FOXG_17986 [Fusarium oxysporum f. sp. lycopersici
Fv_160_1.g427	526	ID=Fv_160_1.g427;Description=hypothetical protein FVEG_00546 [Fusarium verticillioides 7600];Gene=FNAPI_2046;Ontology_term=guanyl-nucleotide exchange factor activity,small GTPase mediated signal

Fv_160_1.g428	910	ID=Fv_160_1.g428;Description=hypothetical protein FVEG_00545 [Fusarium verticillioides 7600];Gene=FOXG_00967;Ontology_term=guanyl-nucleotide exchange factor activity,small GTPase mediated signal
Fv_160_1.g429	432	ID=Fv_160_1.g429;Description=hypothetical protein FVER53590_00544 [Fusarium verticillioides];Gene=191;Ontology_term=external side of plasma membrane,T cell mediated immunity,cell surface receptor
Fv_160_1.g430	233	ID=Fv_160_1.g430;Description=hypothetical protein FVEG_00543 [Fusarium verticillioides 7600]
Fv_160_1.g431	303	ID=Fv_160_1.g431;Description=Fe/S biogenesis protein NfuA [Fusarium verticillioides 7600];Gene=Forpe1208_v000521;Ontology_term=mitochondrial matrix,iron ion binding,iron-sulfur cluster binding,iron-sulfur cluster
Fv_160_1.g432	375	ID=Fv_160_1.g432;Description=L-iditol 2-dehydrogenase [Fusarium verticillioides 7600];Gene=FOVG_00449;Ontology_term=oxidoreductase activity, acting on the CH-OH group of donors, NAD or NADP as
Fv_160_1.g433	197	ID=Fv_160_1.g433;Description=F-type H+-transporting ATPase subunit G [Fusarium verticillioides 7600];Gene=FocTR4_00000622;Ontology_term=mitochondrial proton-transporting ATP synthase complex, coupling factor F(o),proton transmembrane transporter activity,proton motive force-driven ATP synthesis,proton transmembrane
Fv_160_1.g434	299	ID=Fv_160_1.g434;Description=methyl transferase [Fusarium sp. NRRL 52700];Gene=FCIRC_6444;Ontology_term=nucleus,membrane,S-adenosylmethionine-dependent methyltransferase activity,sporulation resulting in formation of a cellular
Fv_160_1.g435	397	ID=Fv_160_1.g435;Description=hypothetical protein FVEG_00538 [Fusarium verticillioides 7600];Gene=FNYG_13002;Ontology_term=transcription factor TFIIA complex,translation initiation factor activity,transcription
Fv_160_1.g436	584	ID=Fv_160_1.g436;Description=hypothetical protein FVER14953_00537 [Fusarium verticillioides];Gene=FOXG_00977;Ontology_term=membrane,mannosyl-oligosaccharide 1,2-alpha-mannosidase activity,calcium ion binding,carbohydrate metabolic process,ubiquitin-dependent ERAD pathway;Ontology_id=GO:0016020,GO:0004571,GO:0005509,GO:0005975,GO:0030433;Enzyme_code=EC:3.2.1.113,EC:3.2.1.24;Enz
Fv_160_1.g437	602	ID=Fv_160_1.g437;Description=related to cell cycle protein p55cdc [Fusarium fujikuroi IMI 58289];Gene=FVEG_00536;Ontology_term=anaphase-promoting complex binding,ubiquitin-protein transferase activator activity,cell
Fv_160_1.g438	757	ID=Fv_160_1.g438;Description=hypothetical protein FVEG_00535 [Fusarium verticillioides 7600]
Fv_160_1.g439	689	ID=Fv_160_1.g439;Description=hypothetical protein FVEG_00534 [Fusarium verticillioides 7600]
Fv_160_1.g440	231	ID=Fv_160_1.g440;Description=hypothetical protein FVEG_00532 [Fusarium verticillioides
Fv_160_1.g441	505	ID=Fv_160_1.g441;Description=hypothetical protein FVER14953_00531 [Fusarium verticillioides]
Fv_160_1.g442	765	ID=Fv_160_1.g442;Description=tRNA-dihydrouridine synthase 3 [Fusarium verticillioides 7600];Gene=FOVG_00438;Ontology_term=flavin adenine dinucleotide binding,tRNA-dihydrouridine47 synthase activity,tRNA dihydrouridine synthesis,mRNA
Fv_160_1.g443	413	ID=Fv_160_1.g443;Description=hypothetical protein FVEG_00529 [Fusarium verticillioides 7600];Gene=FOXB_11099;Ontology_term=peroxisomal membrane,peroxisomal importomer complex,protein transmembrane transporter activity,protein-macromolecule adaptor activity,protein import into peroxisome matrix,

Fv_160_1.g444	407	ID=Fv_160_1.g444;Description=hypothetical protein FVEG_00528 [Fusarium verticillioides 7600];Gene=206;Ontology_term=zinc ion binding,positive regulation of epidermal growth factor receptor signaling pathway,3',5'-cyclic-GMP phosphodiesterase activity,positive regulation of MAPK cascade,visual perception,photoreceptor disc membrane,cGMP binding,photoreceptor outer segment membrane,molecular adaptor activity,positive regulation of G protein-coupled receptor signaling pathway;Ontology_id=GO:0008270,GO:0045742,GO:0047555,GO:0043410,GO:0007601,GO:0097381,GO:0030553,GO:0042622,GO:
Fv_160_1.g445	715	ID=Fv_160_1.g445;Description=chromatin assembly factor 1 subunit B [Fusarium coicis];Gene=FMAN_01501;Ontology_term=extracellular region,collagen trimer,nucleus,DNA replication-dependent chromatin
Fv_160_1.g446	515	ID=Fv_160_1.g446;Description=hypothetical protein FVEG_00526 [Fusarium verticillioides 7600];Gene=ecdD-3;Ontology_term=membrane,transmembrane transporter activity,carbohydrate transport,transmembrane
Fv_160_1.g447	556	ID=Fv_160_1.g447;Description=hypothetical protein FVER14953_00525 [Fusarium verticillioides];Gene=FVEG_00525;Ontology_term=sulfuric ester hydrolase
Fv_160_1.g448	1550	ID=Fv_160_1.g448;Description=hypothetical protein FVER14953_00524 [Fusarium verticillioides];Gene=FVEG_00524;Ontology_term=membrane,ATP binding,ATP hydrolysis activity,ABC-type transporter activity,transmembrane transport;Ontology_id=GO:0016020,GO:0005524,GO:0016887,GO:0140359,GO:0055085;Enzyme_code=EC:7.2.2,EC:3.6.1.15;Enzym
Fv_160_1.g449	687	ID=Fv_160_1.g449;Description=hypothetical protein FVEG_00523 [Fusarium verticillioides 7600];Gene=FNYG_13551;Ontology_term=nucleus,DNA-binding transcription factor activity, RNA polymerase II-specific,zinc ion
Fv_160_1.g450	822	ID=Fv_160_1.g450;Description=hypothetical protein FVEG_00522 [Fusarium verticillioides 7600];Gene=FNYG_13550;Ontology_term=ATP binding,ATP hydrolysis
Fv_160_1.g451	322	ID=Fv_160_1.g451;Description=hypothetical protein FVEG_00521 [Fusarium verticillioides 7600];Gene=COLNF6;Ontology_term=collagen trimer,autophagy;Ontology_id=GO:0005581,GO:0006914
Fv_160_1.g452	486	ID=Fv_160_1.g452;Description=Vacuolar protein-sorting-associated protein 30 [Fusarium
Fv_160_1.g453	925	ID=Fv_160_1.g453;Description=hypothetical protein FVER53590_00519 [Fusarium verticillioides];Gene=FTJAE_7706;Ontology_term=nucleus,histone binding,protein heterodimerization
Fv_160_1.g454	340	ID=Fv_160_1.g454;Description=CMGC CK2 kinase [Fusarium tjaetaba];Gene=F25303_3343;Ontology_term=protein serine/threonine kinase activity,ATP
Fv_160_1.g455	347	ID=Fv_160_1.g455;Description=hypothetical protein FVER53590_00517 [Fusarium verticillioides]
Fv_160_1.g456	494	ID=Fv_160_1.g456;Description=hypothetical protein FVER53263_00516 [Fusarium verticillioides];Gene=FVEG_00516;Ontology_term=protein-containing complex,cellular anatomical
Fv_160_1.g457	306	ID=Fv_160_1.g457;Description=hypothetical protein FVER14953_00515 [Fusarium verticillioides]
Fv_160_1.g458	173	ID=Fv_160_1.g458;Description=ribosomal 40S subunit protein S11A [Fusarium musae];Gene=FOC1_g10016308;Ontology_term=ribosome,ribonucleoprotein complex,structural constituent of

Fv_160_1.g459	1484	ID=Fv_160_1.g459;Description=hypothetical protein FVEG_00513 [Fusarium verticillioides 7600];Gene=FVER53263_00513;Ontology_term=nuclear pore,membrane;Ontology_id=GO:0005643,GO:0016020
Fv_160_1.g460	1385	ID=Fv_160_1.g460;Description=hypothetical protein FVER14953_00511 [Fusarium verticillioides];Gene=FVEG_00512;Ontology_term=nuclear pore,membrane;Ontology_id=GO:0005643,GO:0016020
Fv_160_1.g461	578	ID=Fv_160_1.g461;Description=hypothetical protein FVER14953_00509 [Fusarium verticillioides];Gene=sinu;Ontology_term=mitochondrial respiratory chain complex I,mitochondrial intermembrane
Fv_160_1.g462	357	ID=Fv_160_1.g462;Description=hypothetical protein FVEG_00508 [Fusarium verticillioides 7600];Gene=FGADI_7145;Ontology_term=carbon-sulfur lyase activity,metal ion
Fv_160_1.g463	490	ID=Fv_160_1.g463;Description=hypothetical protein FVER14953_00506 [Fusarium verticillioides];Gene=FNAPI_10745;Ontology_term=mitochondrial inner membrane,tetrahydrofolylpolyglutamate synthase activity,ATP binding,metal ion binding,one-carbon metabolic process,tetrahydrofolylpolyglutamate biosynthetic
Fv_160_1.g464	492	ID=Fv_160_1.g464;Description=hypothetical protein FVEG_00507 [Fusarium verticillioides 7600];Gene=FPANT_1;Ontology_term=mitochondrial inner membrane,tetrahydrofolylpolyglutamate synthase activity,ATP binding,metal ion binding,one-carbon metabolic process,tetrahydrofolylpolyglutamate biosynthetic
Fv_160_1.g465	636	ID=Fv_160_1.g465;Description=hypothetical protein FVEG_00505 [Fusarium verticillioides 7600];Gene=FGLOB1_10713;Ontology_term=mitochondrial inner membrane;Ontology_id=GO:0005743
Fv_160_1.g466	220	ID=Fv_160_1.g466;Description=GTP-binding protein ypt5 [Fusarium oxysporum f. sp. lycopersici 4287];Gene=BFJ68_g12203;Ontology_term=early endosome membrane,GTPase activity,GTP binding,endosomal vesicle fusion;Ontology_id=GO:0031901,GO:0003924,GO:0005525,GO:0034058;Enzyme_code=EC:3.6.1.15;Enzyme_name=nucleoside-
Fv_160_1.g467	709	ID=Fv_160_1.g467;Description=hypothetical protein FVEG_00503 [Fusarium verticillioides 7600];Gene=FOBC_00914;Ontology_term=Golgi membrane,endoplasmic reticulum membrane,Golgi medial cisterna,cortical endoplasmic reticulum,SPOTS complex,host cell viral assembly compartment,phosphatidylinositol-3-phosphate phosphatase activity,phosphatidylinositol-4-phosphate phosphatase activity,phosphatidylinositol-3,5-bisphosphate 3-phosphatase activity,phosphatidylinositol
Fv_160_1.g468	1216	ID=Fv_160_1.g468;Description=hypothetical protein FVEG_00502 [Fusarium verticillioides
Fv_160_1.g469	941	ID=Fv_160_1.g469;Description=hypothetical protein FVER53590_00501 [Fusarium verticillioides];Gene=FVEG_00501;Ontology_term=tRNA binding,aminoacyl-tRNA editing activity,isoleucine-tRNA ligase activity,ATP binding,isoleucyl-tRNA aminoacylation,aminoacyl-tRNA metabolism involved in translational fidelity;Ontology_id=GO:0000049,GO:0002161,GO:0004822,GO:0005524,GO:0006428,GO:0106074;Enzyme_code=EC:6.1.1.5,EC:3.1
Fv_160_1.g470	278	ID=Fv_160_1.g470;Description=hypothetical protein FVEG_00500 [Fusarium verticillioides 7600]
Fv_160_1.g471	427	ID=Fv_160_1.g471;Description=hypothetical protein FVER14953_00498 [Fusarium verticillioides];Gene=FOC1_g10016326;Ontology_term=SAM complex,protein transport;Ontology_id=GO:0001401,GO:0015031

Fv_160_1.g472	425	ID=Fv_160_1.g472;Description=phosphatidylinositol glycan, class U [Fusarium verticillioides 7600];Gene=FOMG_01798;Ontology_term=GPI-anchor transamidase complex,attachment of GPI anchor to protein,cell
Fv_160_1.g473	657	ID=Fv_160_1.g473;Description=hypothetical protein FVEG_00496 [Fusarium verticillioides 7600];Gene=NOP7;Ontology_term=nucleoplasm,nucleolus,preribosome, large subunit precursor,ribonucleoprotein complex binding,maturation of LSU-rRNA from tricistronic rRNA transcript (SSU-rRNA, 5.8S rRNA, LSU-rRNA),maturation of 5.8S rRNA from
Fv_160_1.g474	636	ID=Fv_160_1.g474;Description=hypothetical protein FVER53590_00495 [Fusarium
Fv_160_1.g475	1163	ID=Fv_160_1.g475;Description=hypothetical protein FVER14953_00494 [Fusarium verticillioides];Gene=FFUJ_00546;Ontology_term=mitochondrial respiratory chain complex I,microtubule motor activity,ATP binding,microtubule binding,response to oxidative stress,microtubule-based movement,mitochondrial respiratory chain complex I
Fv_160_1.g476	460	ID=Fv_160_1.g476;Description=hypothetical protein FVER14953_00493 [Fusarium verticillioides];Gene=F25303_3365;Ontology_term=protein phosphatase 4 complex,protein phosphatase regulator
Fv_160_1.g477	395	ID=Fv_160_1.g477;Description=hypothetical protein FVEG_00492 [Fusarium verticillioides 7600];Gene=242
Fv_160_1.g478	713	ID=Fv_160_1.g478;Description=hypothetical protein J7337_001476 [Fusarium musae];Gene=FVER53590_00491;Ontology_term=vacuolar membrane,calcium:proton antiporter activity,calcium ion
Fv_160_1.g479	1103	ID=Fv_160_1.g479;Description=hypothetical protein FVER53590_00490 [Fusarium verticillioides];Gene=BFJ68_g14686;Ontology_term=protein serine/threonine kinase activity,ATP
Fv_160_1.g480	643	ID=Fv_160_1.g480;Description=hypothetical protein FVER53590_25962 [Fusarium verticillioides];Gene=FOBC_00898;Ontology_term=endoplasmic reticulum membrane,O-acyltransferase
Fv_160_1.g481	392	ID=Fv_160_1.g481;Description=hypothetical protein FVER53263_20853 [Fusarium verticillioides];Gene=prfA;Ontology_term=mitochondrial respiratory chain complex I,mitochondrial respiratory chain complex I
Fv_160_1.g482	529	ID=Fv_160_1.g482;Description=hypothetical protein FVEG_00488 [Fusarium verticillioides 7600];Gene=FocTR4_00000568;Ontology_term=membrane,carbohydrate binding;Ontology_id=GO:0016020,GO:0030246
Fv_160_1.g483	470	ID=Fv_160_1.g483;Description=hypothetical protein J7337_001481 [Fusarium musae]
Fv_160_1.g484	302	ID=Fv_160_1.g484;Description=enoyl-CoA hydratase [Fusarium verticillioides 7600]
Fv_160_1.g485	677	ID=Fv_160_1.g485;Description=hypothetical protein FVEG_00483 [Fusarium verticillioides 7600]
Fv_160_1.g486	616	ID=Fv_160_1.g486;Description=D-lactate dehydrogenase (cytochrome) [Fusarium verticillioides 7600];Gene=FOTG_04429;Ontology_term=membrane,catalytic activity,FAD
Fv_160_1.g487	127	ID=Fv_160_1.g487;Description=hypothetical protein FVEG_14671 [Fusarium verticillioides 7600]
Fv_160_1.g488	512	ID=Fv_160_1.g488;Description=hypothetical protein FVEG_14670 [Fusarium verticillioides 7600]
Fv_160_1.g489	592	ID=Fv_160_1.g489;Description=hypothetical protein FVER53263_00480 [Fusarium
Fv_160_1.g490	243	ID=Fv_160_1.g490;Description=hypothetical protein FVEG_00479 [Fusarium verticillioides 7600]
Fv_160_1.g491	513	ID=Fv_160_1.g491;Description=cutinase palindrome-binding protein [Fusarium verticillioides 7600];Gene=BFJ68_g14674;Ontology_term=zinc ion binding,sequence-specific DNA binding,regulation of DNA-templated

Fv_160_1.g492	1307	ID=Fv_160_1.g492;Description=hypothetical protein FVER14953_00477 [Fusarium verticillioides];Gene=FocTR4_00000556;Ontology_term=Mre11 complex,ATP hydrolysis activity,telomere maintenance,double-strand break
Fv_160_1.g493	606	ID=Fv_160_1.g493;Description=DNA repair protein RAD50 [Fusarium verticillioides 7600];Gene=FNAPI_4468;Ontology_term=membrane,transmembrane transporter activity,transmembrane
Fv_160_1.g494	491	ID=Fv_160_1.g494;Description=hypothetical protein FVEG_00475 [Fusarium verticillioides 7600];Gene=FMAN_01554;Ontology_term=N,N-dimethylaniline monooxygenase activity,flavin adenine dinucleotide binding,NADP binding;Ontology_id=GO:0004499,GO:0050660,GO:0050661;Enzyme_code=EC:1.14.13.8;Enzyme_name=flavin-containing
Fv_160_1.g495	746	ID=Fv_160_1.g495;Description=hypothetical protein FVER53590_00473 [Fusarium verticillioides];Gene=FNAPI_4466;Ontology_term=nucleus,DNA binding,zinc ion binding,DNA-binding transcription factor activity,
Fv_160_1.g496	438	ID=Fv_160_1.g496;Description=hypothetical protein FVEG_00472 [Fusarium verticillioides
Fv_160_1.g497	431	ID=Fv_160_1.g497;Description=hypothetical protein FVEG_00471 [Fusarium verticillioides 7600];Gene=FMAN_01557;Ontology_term=cytoplasm,metal ion binding,ubiquitin protein ligase activity,proteasome-mediated ubiquitin-dependent protein catabolic process,negative regulation of
Fv_160_1.g498	898	ID=Fv_160_1.g498;Description=hypothetical protein FVEG_00470 [Fusarium verticillioides 7600]
Fv_160_1.g499	95	ID=Fv_160_1.g499;Description=hypothetical protein FVEG_00469 [Fusarium verticillioides 7600];Gene=FAUST_3;Ontology_term=microtubule,mitochondrial intermembrane space protein transporter complex,TIM22 mitochondrial import inner membrane insertion complex,calcium ion binding,calcium-dependent phospholipid binding,unfolded protein binding,protein transporter activity,cytoskeleton organization,protein insertion into mitochondrial inner
Fv_160_1.g500	477	ID=Fv_160_1.g500;Description=hypothetical protein FVEG_00468 [Fusarium verticillioides 7600];Gene=FVEG_00468;Ontology_term=extracellular region,collagen trimer,oxidoreductase activity, acting on the aldehyde or oxo group of donors, NAD or NADP as acceptor,metal ion
Fv_160_1.g501	69	ID=Fv_160_1.g501;Description=hypothetical protein FNAPI_4460 [Fusarium napiforme]
Fv_160_1.g502	748	ID=Fv_160_1.g502;Description=hypothetical protein FVER14953_00466 [Fusarium verticillioides];Gene=FNYG_13497;Ontology_term=nucleus,DNA-binding transcription factor activity, RNA polymerase II-specific,zinc
Fv_160_1.g503	518	ID=Fv_160_1.g503;Description=hypothetical protein FVEG_00464 [Fusarium verticillioides 7600]
Fv_160_1.g504	1018	ID=Fv_160_1.g504;Description=hypothetical protein FVER53263_00463 [Fusarium verticillioides];Gene=FNYG_13495;Ontology_term=endoplasmic reticulum,membrane,mannosyl-oligosaccharide 1,2-alpha-mannosidase activity,calcium ion binding,carbohydrate metabolic process,ubiquitin-dependent ERAD pathway;Ontology_id=GO:0005783,GO:0016020,GO:0004571,GO:0005509,GO:0005975,GO:0030433;Enzyme_code=EC:3.2.1.113,E
Fv_160_1.g505	223	ID=Fv_160_1.g505;Description=adenine phosphoribosyltransferase [Fusarium verticillioides 7600];Gene=BFJ70_g8483;Ontology_term=cytoplasm,adenine phosphoribosyltransferase activity,purine ribonucleoside salvage,adenine salvage,AMP

Fv_160_1.g506	601	ID=Fv_160_1.g506;Description=hypothetical protein FVEG_00461 [Fusarium verticillioides 7600];Gene=FNAPI_1344;Ontology_term=peroxisomal membrane,metal ion binding,protein import into peroxisome matrix,
Fv_160_1.g507	437	ID=Fv_160_1.g507;Description=hypothetical protein FVEG_00460 [Fusarium verticillioides 7600];Gene=FNYG_13492;Ontology_term=peroxisomal membrane,zinc ion binding,transferase activity,protein import into peroxisome matrix, receptor recycling,protein
Fv_160_1.g508	540	ID=Fv_160_1.g508;Description=hypothetical protein FVEG_00459 [Fusarium verticillioides 7600];Gene=FVEG_00459;Ontology_term=nucleus,DNA binding,zinc ion binding,DNA-binding transcription factor activity, RNA
Fv_160_1.g509	600	ID=Fv_160_1.g509;Description=hypothetical protein FVEG_00458 [Fusarium verticillioides
Fv_160_1.g510	570	ID=Fv_160_1.g510;Description=hypothetical protein J7337_001506 [Fusarium
Fv_160_1.g511	923	ID=Fv_160_1.g511;Description=hypothetical protein FVEG_00456 [Fusarium verticillioides 7600]
Fv_160_1.g512	354	ID=Fv_160_1.g512;Description=hypothetical protein FVEG_00454 [Fusarium verticillioides 7600];Gene=FGADI_6035;Ontology_term=nucleus,DNA binding;Ontology_id=GO:0005634,GO:0003677
Fv_160_1.g513	257	ID=Fv_160_1.g513;Description=hypothetical protein FVEG_14663 [Fusarium verticillioides
Fv_160_1.g514	175	ID=Fv_160_1.g514;Description=protein SYM1 [Fusarium verticillioides 7600];Gene=FOIG_13152;Ontology_term=mitochondrial inner membrane,ethanol metabolic process;Ontology_id=GO:0005743,GO:0006067
Fv_160_1.g515	335	ID=Fv_160_1.g515;Description=hypothetical protein FVEG_00451 [Fusarium verticillioides 7600]
Fv_160_1.g516	1066	ID=Fv_160_1.g516;Description=tRNA(Met) cytidine acetyltransferase [Fusarium verticillioides 7600];Gene=NAT10;Ontology_term=nucleolus,ATP binding,N-acetyltransferase activity,rRNA modification,ribosomal small subunit biogenesis,tRNA
Fv_160_1.g517	236	ID=Fv_160_1.g517;Description=hypothetical protein FVEG_00449 [Fusarium verticillioides 7600]
Fv_160_1.g518	274	ID=Fv_160_1.g518;Description=hypothetical protein FVER14953_00448 [Fusarium verticillioides];Gene=FNAPI_1332;Ontology_term=S-adenosylmethionine-dependent methyltransferase
Fv_160_1.g519	408	ID=Fv_160_1.g519;Description=hypothetical protein FVEG_00447 [Fusarium verticillioides 7600];Gene=FANTH_2064;Ontology_term=metallopeptidase activity,S-adenosylmethionine-dependent methyltransferase activity,metal ion binding,methylation;Ontology_id=GO:0008237,GO:0008757,GO:0046872,GO:0032259;Enzyme_code=EC:3.4,EC:2.1.1;Enzyme_name
Fv_160_1.g520	66	ID=Fv_160_1.g520;Description=hypothetical protein FVER14953_20074 [Fusarium verticillioides]
Fv_160_1.g521	1369	ID=Fv_160_1.g521;Description=hypothetical protein FVEG_00446 [Fusarium verticillioides 7600];Gene=FPANT_1858;Ontology_term=phosphorelay sensor kinase activity,phosphorelay signal transduction system,phosphorylation;Ontology_id=GO:0000155,GO:0000160,GO:0016310;Enzyme_code=EC:2.7.3,EC:2.7.13.3;Enzyme_name=Tra
Fv_160_1.g522	1224	ID=Fv_160_1.g522;Description=hypothetical protein FVER14953_00445 [Fusarium verticillioides];Gene=FVER53263_00445;Ontology_term=membrane,lipid binding,lipid transport,endoplasmic reticulum-plasma

Fv_160_1.g523	557	ID=Fv_160_1.g523;Description=NADPH oxidase [Fusarium verticillioides 7600];Gene=FOMG_01857;Ontology_term=membrane,ferric-chelate reductase activity,iron ion
Fv_160_1.g524	836	ID=Fv_160_1.g524;Description=urease [Fusarium verticillioides 7600];Gene=FOMG_01858;Ontology_term=urease complex,urease activity,nickel cation binding,urea catabolic
Fv_160_1.g525	636	ID=Fv_160_1.g525;Description=hypothetical protein FVEG_00442 [Fusarium verticillioides
Fv_160_1.g526	840	ID=Fv_160_1.g526;Description=hypothetical protein FVEG_00441 [Fusarium verticillioides 7600];Gene=FMEXI_300;Ontology_term=S-adenosylmethionine-dependent methyltransferase
Fv_160_1.g527	182	ID=Fv_160_1.g527;Description=succinate dehydrogenase (ubiquinone) membrane anchor subunit [Fusarium verticillioides 7600];Gene=FNYG_13473;Ontology_term=mitochondrial inner membrane;Ontology_id=GO:0005743
Fv_160_1.g528	615	ID=Fv_160_1.g528;Description=tyrosyl-tRNA synthetase [Fusarium verticillioides 7600];Gene=FNYG_13472;Ontology_term=RNA binding,tyrosine-tRNA ligase activity,ATP binding,tyrosyl-tRNA
Fv_160_1.g529	411	ID=Fv_160_1.g529;Description=hypothetical protein FVEG_00438 [Fusarium verticillioides 7600];Gene=28943350;Ontology_term=GATOR1 complex,GTPase activator activity,negative regulation of TORC1
Fv_160_1.g530	443	ID=Fv_160_1.g530;Description=hypothetical protein FVEG_00437 [Fusarium verticillioides 7600];Gene=FVEG_00437;Ontology_term=protein-macromolecule adaptor activity,polyubiquitin modification-dependent protein binding,ubiquitin-dependent ERAD pathway,spindle pole body duplication,protein localization to
Fv_160_1.g531	335	ID=Fv_160_1.g531;Description=NADPH2:quinone reductase [Fusarium verticillioides 7600];Gene=FANTH_2075;Ontology_term=zinc ion binding,oxidoreductase activity;Ontology_id=GO:0008270,GO:0016491;Enzyme_code=EC:1;Enzyme_name=Oxidoreductases
Fv_160_1.g532	1049	ID=Fv_160_1.g532;Description=hypothetical protein FVEG_00435 [Fusarium verticillioides 7600];Gene=FANTH_2076;Ontology_term=nucleus,cytoplasm,microtubule,structural constituent of cytoskeleton,small GTPase binding,intracellular protein transport,cytoskeleton
Fv_160_1.g533	498	ID=Fv_160_1.g533;Description=hypothetical protein FVER14953_00434 [Fusarium verticillioides];Gene=Forpe1208_v000423;Ontology_term=mitochondrion,threonine-tRNA ligase activity,ATP binding,mitochondrial threonyl-tRNA
Fv_160_1.g534	116	ID=Fv_160_1.g534;Description=hypothetical protein FVEG_00433 [Fusarium verticillioides 7600];Gene=FGADI_6055;Ontology_term=protein dimerization activity;Ontology_id=GO:0046983
Fv_160_1.g535	269	ID=Fv_160_1.g535;Description=hypothetical protein FVER14953_00432 [Fusarium verticillioides];Gene=FOVG_00329;Ontology_term=nucleoside triphosphate diphosphatase
Fv_160_1.g536	678	ID=Fv_160_1.g536;Description=hypothetical protein FVER14953_00431 [Fusarium verticillioides];Gene=FNYG_13464;Ontology_term=mitochondrial outer membrane,endoplasmic reticulum membrane,cytosol,RSC-type complex,enzyme binding,heme binding,metal ion binding,chromatin remodeling,transcription by RNA polymerase
Fv_160_1.g537	438	ID=Fv_160_1.g537;Description=hypothetical protein FVEG_00430 [Fusarium verticillioides
Fv_160_1.g538	368	ID=Fv_160_1.g538;Description=hypothetical protein FVEG_00429 [Fusarium verticillioides

Fv_160_1.g539	699	ID=Fv_160_1.g539;Description=hypothetical protein FVER53590_00428 [Fusarium verticillioides];Gene=FVER53590_00428;Ontology_term=nucleus,DNA-binding transcription factor activity, RNA polymerase II-
Fv_160_1.g540	581	ID=Fv_160_1.g540;Description=hypothetical protein FVER14953_00425 [Fusarium
Fv_160_1.g541	213	ID=Fv_160_1.g541;Description=hypothetical protein F25303_10891 [Fusarium sp. NRRL 25303];Gene=FOC4_g10014348;Ontology_term=cytoplasm,arginine-tRNA ligase activity,ATP binding,arginyl-tRNA
Fv_160_1.g542	635	ID=Fv_160_1.g542;Description=hypothetical protein FVER53590_00424 [Fusarium verticillioides];Gene=Forpi1262_v000443;Ontology_term=cytoplasm,arginine-tRNA ligase activity,ATP binding,arginyl-tRNA
Fv_160_1.g543	595	ID=Fv_160_1.g543;Description=hypothetical protein J7337_001538 [Fusarium musae];Gene=FFUJ_00479;Ontology_term=cytoplasm,mRNA binding,Hsp70 protein binding,ATPase inhibitor activity,Hsp90 protein binding,protein folding,protein targeting to
Fv_160_1.g544	455	ID=Fv_160_1.g544;Description=hypothetical protein FVER14953_20081 [Fusarium verticillioides];Gene=FTJAE_6373;Ontology_term=Golgi membrane,plasma membrane,glutamatergic synapse,dopamine neurotransmitter receptor activity, coupled via Gi/Go,G protein-coupled receptor activity,nitric oxide dioxygenase activity,oxygen binding,heme binding,synaptic transmission, dopaminergic,adenylate cyclase-inhibiting dopamine receptor signaling pathway,regulation of dopamine secretion,regulation of potassium ion transport,negative regulation of cytosolic calcium ion concentration,negative regulation of synaptic transmission, glutamatergic,phospholipase C-activating dopamine receptor signaling pathway,adenylate cyclase-activating adrenergic receptor signaling pathway,negative regulation of prolactin secretion,negative regulation of lactation,positive regulation of mammary gland involution,hyaloid vascular plexus
Fv_160_1.g545	220	ID=Fv_160_1.g545;Description=hypothetical protein FNAPI_7396 [Fusarium napiforme];Gene=FANTH_2089;Ontology_term=metal
Fv_160_1.g546	518	ID=Fv_160_1.g546;Description=hypothetical protein FVEG_14659 [Fusarium verticillioides 7600]
Fv_160_1.g547	241	ID=Fv_160_1.g547;Description=hypothetical protein FVEG_00418 [Fusarium verticillioides 7600];Gene=FTJAE_6376;Ontology_term=transferase
Fv_160_1.g548	247	ID=Fv_160_1.g548;Description=hypothetical protein FVEG_00417 [Fusarium verticillioides 7600]
Fv_160_1.g549	492	ID=Fv_160_1.g549;Description=hypothetical protein FVER53263_00416 [Fusarium verticillioides];Gene=FANTH_2093;Ontology_term=N,N-dimethylaniline monooxygenase activity,flavin adenine dinucleotide binding,NADP binding;Ontology_id=GO:0004499,GO:0050660,GO:0050661;Enzyme_code=EC:1.14.13.8;Enzyme_name=flavin-
Fv_160_1.g550	462	ID=Fv_160_1.g550;Description=hypothetical protein FVEG_00415 [Fusarium verticillioides 7600];Gene=FVEG_00415;Ontology_term=monooxygenase activity,iron ion binding,methyltransferase activity,oxidoreductase activity, acting on paired donors, with incorporation or reduction of molecular oxygen,heme binding,methylation;Ontology_id=GO:0004497,GO:0005506,GO:0008168,GO:0016705,GO:0020037,GO:0032259;Enzyme_code=EC:
Fv_160_1.g551	484	ID=Fv_160_1.g551;Description=hypothetical protein FVER53263_00414 [Fusarium verticillioides]

Fv_160_1.g552	91	ID=Fv_160_1.g552;Description=hypothetical protein FVER53263_20561 [Fusarium verticillioides];Gene=FOC1_g10016409;Ontology_term=U5 snRNP,U1 snRNP,U2 snRNP,U4/U6 x U5 tri-snRNP complex,U2-type prespliceosome,post-mRNA release spliceosomal complex,RNA binding,spliceosomal snRNP
Fv_160_1.g553	159	ID=Fv_160_1.g553;Description=hypothetical protein FVEG_00413 [Fusarium verticillioides 7600];Gene=P01512;Ontology_term=extracellular region,defense response to bacterium,innate immune
Fv_160_1.g554	836	ID=Fv_160_1.g554;Description=hypothetical protein FVER53263_00412 [Fusarium verticillioides];Gene=FACUT_3759;Ontology_term=beta-glucosidase activity,scopolin beta-glucosidase activity,cellulose catabolic
Fv_160_1.g555	449	ID=Fv_160_1.g555;Description=hypothetical protein FVEG_00411 [Fusarium verticillioides 7600];Gene=FocTR4_00000489;Ontology_term=membrane,metal ion transmembrane transporter activity,metal ion
Fv_160_1.g556	296	ID=Fv_160_1.g556;Description=hypothetical protein FVEG_00410 [Fusarium verticillioides 7600];Gene=FOVG_00307;Ontology_term=monoatomic cation homeostasis,inorganic ion
Fv_160_1.g557	100	ID=Fv_160_1.g557;Description=hypothetical protein FVEG_14657 [Fusarium verticillioides
Fv_160_1.g558	263	ID=Fv_160_1.g558;Description=hypothetical protein FVEG_00409 [Fusarium verticillioides
Fv_160_1.g559	121	ID=Fv_160_1.g559;Description=hypothetical protein FVER14953_00408 [Fusarium verticillioides]
Fv_160_1.g560	227	ID=Fv_160_1.g560;Description=hypothetical protein J7337_001554 [Fusarium musae];Gene=FPANT_1896;Ontology_term=membrane,copper ion transmembrane transporter activity,copper ion transmembrane
Fv_160_1.g561	611	ID=Fv_160_1.g561;Description=hypothetical protein FVEG_00406 [Fusarium verticillioides 7600];Gene=FOXG_01108;Ontology_term=nucleus,DNA-binding transcription factor activity, RNA polymerase II-specific,zinc ion
Fv_160_1.g562	307	ID=Fv_160_1.g562;Description=uncharacterized protein FOBCDRAFT_194447 [Fusarium oxysporum Fo47];Gene=FOBC_00814;Ontology_term=growth factor activity,branch elongation involved in ureteric bud branching,fibroblast growth factor receptor signaling pathway,integrin binding,heparin binding,positive regulation of cholesterol biosynthetic process,positive regulation of transcription by RNA polymerase II,nucleoplasm,wound healing,cellular response to heat,positive regulation of cell division,cytosol,extracellular matrix,positive regulation of epithelial cell proliferation,positive regulation of endothelial cell migration,lung development,positive regulation of sprouting angiogenesis,S100 protein binding,fibroblast growth factor receptor binding,extracellular space,regulation of endothelial tube morphogenesis,cell cortex,positive regulation of protein phosphorylation,positive regulation of ERK1 and ERK2 cascade,membrane,organ induction,cell differentiation,regulation of
Fv_160_1.g563	538	ID=Fv_160_1.g563;Description=hypothetical protein FVEG_00404 [Fusarium verticillioides 7600];Gene=rplQ
Fv_160_1.g564	124	ID=Fv_160_1.g564;Description=hypothetical protein FVEG_14653 [Fusarium verticillioides 7600]
Fv_160_1.g565	224	ID=Fv_160_1.g565;Description=peptidyl-prolyl cis-trans isomerase, mitochondrial [Fusarium verticillioides 7600];Gene=Forpi1262_v000421;Ontology_term=peptidyl-prolyl cis-trans isomerase activity,protein
Fv_160_1.g566	383	ID=Fv_160_1.g566;Description=DNA-directed RNA polymerase III subunit RPC6 [Fusarium verticillioides 7600];Gene=FMEXI_632;Ontology_term=RNA polymerase III complex,transcription by RNA polymerase

Fv_160_1.g567	158	ID=Fv_160_1.g567;Description=hypothetical protein FVEG_00401 [Fusarium verticillioides 7600];Gene=act-2;Ontology_term=actin filament,cell cortex,ATP binding,hydrolase activity,mitotic cytokinesis,meiosis II cytokinesis,embryo development ending in birth or egg hatching,cortical actin cytoskeleton organization,locomotion;Ontology_id=GO:0005884,GO:0005938,GO:0005524,GO:0016787,GO:0000281,GO:0007111,GO:0009792,
Fv_160_1.g568	650	ID=Fv_160_1.g568;Description=hypothetical protein FVER53590_00400 [Fusarium verticillioides];Gene=FOMG_01903;Ontology_term=histone deacetylase activity,chromatin remodeling,regulation of DNA-templated
Fv_160_1.g569	337	ID=Fv_160_1.g569;Description=26S proteasome regulatory subunit rpn11 [Fusarium verticillioides 7600];Gene=FOC1_g10016425;Ontology_term=mitochondrion,cytosol,proteasome regulatory particle, lid subcomplex,nuclear periphery,proteasome storage granule,metallopeptidase activity,mitochondrial fission,peroxisome fission,proteasome-mediated ubiquitin-dependent protein catabolic process,proteasome assembly,proteasome storage granule assembly;Ontology_id=GO:0005739,GO:0005829,GO:0008541,GO:0034399,GO:0034515,GO:0008237,GO:0000266,GO:0016559,GO
Fv_160_1.g570	400	ID=Fv_160_1.g570;Description=hypothetical protein FVEG_00398 [Fusarium verticillioides 7600];Gene=330
Fv_160_1.g571	227	ID=Fv_160_1.g571;Description=hypothetical protein FVEG_00397 [Fusarium verticillioides 7600];Gene=FNAPI_7369;Ontology_term=membrane,carboxylic ester hydrolase
Fv_160_1.g572	228	ID=Fv_160_1.g572;Description=hypothetical protein FVEG_00396 [Fusarium verticillioides 7600];Gene=FPRO_01705;Ontology_term=carboxylic ester hydrolase
Fv_160_1.g573	164	ID=Fv_160_1.g573;Description=hypothetical protein FVEG_00395 [Fusarium verticillioides 7600]
Fv_160_1.g574	183	ID=Fv_160_1.g574;Description=hypothetical protein FNYG_06789 [Fusarium nygamai]
Fv_160_1.g575	575	ID=Fv_160_1.g575;Description=CAMK/CAMK1/CAMK1-RCK protein kinase [Fusarium verticillioides 7600];Gene=FACUT_3739;Ontology_term=lysozyme activity,protein serine/threonine kinase activity,ATP binding,phosphorylation,killing of cells of another organism,defense response to bacterium;Ontology_id=GO:0003796,GO:0004674,GO:0005524,GO:0016310,GO:0031640,GO:0042742;Enzyme_code=EC:2.7.11.1,E
Fv_160_1.g576	496	ID=Fv_160_1.g576;Description=hypothetical protein FVEG_00393 [Fusarium verticillioides 7600];Gene=BFJ72_g2989;Ontology_term=membrane,transmembrane transporter activity,transmembrane
Fv_160_1.g577	274	ID=Fv_160_1.g577;Description=Protein of unknown function DUF1275 [Fusarium oxysporum f. sp.
Fv_160_1.g578	670	ID=Fv_160_1.g578;Description=hypothetical protein FVER14953_00392 [Fusarium verticillioides]
Fv_160_1.g579	331	ID=Fv_160_1.g579;Description=hypothetical protein FVER53590_00391 [Fusarium
Fv_160_1.g580	340	ID=Fv_160_1.g580;Description=hypothetical protein FVER14953_00390 [Fusarium verticillioides]
Fv_160_1.g581	648	ID=Fv_160_1.g581;Description=hypothetical protein FVEG_00389 [Fusarium verticillioides 7600];Gene=FPRO_01714;Ontology_term=aminopeptidase activity,serine-type peptidase
Fv_160_1.g582	400	ID=Fv_160_1.g582;Description=hypothetical protein FVER14953_00388 [Fusarium verticillioides]
Fv_160_1.g583	624	ID=Fv_160_1.g583;Description=hypothetical protein FVER14953_00387 [Fusarium verticillioides];Gene=sid1-0;Ontology_term=protein kinase activity,ATP

Fv_160_1.g584	221	ID=Fv_160_1.g584;Description=hypothetical protein J7337_001574 [Fusarium
Fv_160_1.g585	484	ID=Fv_160_1.g585;Description=hypothetical protein FVEG_00385 [Fusarium verticillioides 7600];Gene=FVEG_00385;Ontology_term=mRNA cis splicing, via spliceosome;Ontology_id=GO:0045292
Fv_160_1.g586	617	ID=Fv_160_1.g586;Description=hypothetical protein FVER14953_00384 [Fusarium verticillioides];Gene=FFUJ_00438;Ontology_term=nucleus,DNA-binding transcription factor activity,sequence-specific DNA
Fv_160_1.g587	542	ID=Fv_160_1.g587;Description=hypothetical protein FVEG_00383 [Fusarium verticillioides 7600];Gene=FOMG_01924;Ontology_term=nucleus,double-strand break repair via nonhomologous end
Fv_160_1.g588	589	ID=Fv_160_1.g588;Description=deoxyribodipyrimidine photo-lyase [Fusarium verticillioides 7600];Gene=FOZG_01912;Ontology_term=lyase activity,organic cyclic compound binding,heterocyclic compound binding,nucleobase-containing compound metabolic process,response to stress,cellular response to
Fv_160_1.g589	87	ID=Fv_160_1.g589;Description=hypothetical protein FPSE_12018 [Fusarium pseudograminearum CS3096];Gene=BHE90_003411;Ontology_term=cytoplasm,ribosome,ribonucleoprotein complex,structural constituent of
Fv_160_1.g590	889	ID=Fv_160_1.g590;Description=hypothetical protein FVER53590_00380 [Fusarium verticillioides];Gene=SLX4;Ontology_term=Slx1-Slx4 complex,DNA binding,5'-flap endonuclease activity,DNA replication,DNA repair,DNA recombination,organelle organization;Ontology_id=GO:0033557,GO:0003677,GO:0017108,GO:0006260,GO:0006281,GO:0006310,GO:0006996;Enzyme_code=EC:3.1.30,EC:3.1.21;Enzyme_name=Acting on ester bonds,Acting on ester bonds
Fv_160_1.g591	947	ID=Fv_160_1.g591;Description=hypothetical protein FVEG_00379 [Fusarium verticillioides 7600];Gene=FVEG_00379;Ontology_term=nucleus,DNA binding,zinc ion binding,DNA-binding transcription factor activity, RNA
Fv_160_1.g592	350	ID=Fv_160_1.g592;Description=hypothetical protein FVEG_00378 [Fusarium verticillioides 7600];Gene=F25303_6169;Ontology_term=membrane,ATP binding;Ontology_id=GO:0016020,GO:0005524
Fv_160_1.g593	348	ID=Fv_160_1.g593;Description=hypothetical protein FVEG_00377 [Fusarium verticillioides 7600];Gene=FPRO_01726;Ontology_term=hydrolase activity, acting on acid anhydrides, in phosphorus-containing anhydrides,metal
Fv_160_1.g594	139	ID=Fv_160_1.g594;Description=hypothetical protein FPSE_09944 [Fusarium pseudograminearum CS3096];Gene=FSPOR_2286;Ontology_term=ribosome,ribonucleoprotein complex,structural constituent of
Fv_160_1.g595	1467	ID=Fv_160_1.g595;Description=hypothetical protein FVER53590_00375 [Fusarium verticillioides]
Fv_160_1.g596	503	ID=Fv_160_1.g596;Description=hypothetical protein FVEG_00374 [Fusarium verticillioides 7600]
Fv_160_1.g597	617	ID=Fv_160_1.g597;Description=malate dehydrogenase (oxaloacetate-decarboxylating) [Fusarium verticillioides 7600];Gene=FOMG_01936;Ontology_term=malate dehydrogenase (decarboxylating) (NAD+) activity,metal ion binding,NAD binding;Ontology_id=GO:0004471,GO:0046872,GO:0051287;Enzyme_code=EC:1.1.1.38,EC:1.1.1.39;Enzyme_name=malate
Fv_160_1.g598	559	ID=Fv_160_1.g598;Description=hypothetical protein J7337_001589 [Fusarium musae];Gene=FFUJ_00426;Ontology_term=collagen trimer,membrane,transmembrane transporter activity,transmembrane
Fv_160_1.g599	383	ID=Fv_160_1.g599;Description=hypothetical protein FVER14953_00371 [Fusarium

Fv_160_1.g600	416	ID=Fv_160_1.g600;Description=hypothetical protein FVEG_00370 [Fusarium verticillioides 7600];Gene=FFUJ_00424;Ontology_term=collagen trimer,cellular anatomical entity,serine-type endopeptidase
Fv_160_1.g601	855	ID=Fv_160_1.g601;Description=beta-mannosidase [Fusarium verticillioides 7600];Gene=BFJ68_g6774;Ontology_term=GTPase activity,hydrolase activity, hydrolyzing O-glycosyl compounds,GTP binding,carbohydrate metabolic process;Ontology_id=GO:0003924,GO:0004553,GO:0005525,GO:0005975;Enzyme_code=EC:3.6.1.15,EC:3.2.1;Enzyme_name=nucle
Fv_160_1.g602	339	ID=Fv_160_1.g602;Description=hypothetical protein FVEG_00367 [Fusarium verticillioides 7600];Gene=FPRO_01736;Ontology_term=GTPase activity,GTP
Fv_160_1.g603	399	ID=Fv_160_1.g603;Description=homocitrate synthase [Fusarium verticillioides 7600];Gene=FNAPI_7338;Ontology_term=acyltransferase activity, acyl groups converted into alkyl on transfer,carboxylic acid metabolic process,cellular biosynthetic process,small molecule biosynthetic process,organonitrogen compound biosynthetic
Fv_160_1.g604	262	ID=Fv_160_1.g604;Description=hypothetical protein FVEG_00365 [Fusarium verticillioides 7600];Gene=BFJ69_g11584;Ontology_term=oxidoreductase activity, acting on the CH-OH group of donors, NAD or NADP as acceptor,NAD binding,cellular biosynthetic
Fv_160_1.g605	361	ID=Fv_160_1.g605;Description=glycerate dehydrogenase [Fusarium tjaetaba];Gene=FNAPI_7336;Ontology_term=oxidoreductase activity, acting on the CH-OH group of donors, NAD or NADP as acceptor,NAD binding,cellular biosynthetic process;Ontology_id=GO:0016616,GO:0051287,GO:0044249;Enzyme_code=EC:1.1.1;Enzyme_name=Acting on the CH-OH group of
Fv_160_1.g606	320	ID=Fv_160_1.g606;Description=hypothetical protein J7337_001598 [Fusarium musae];Gene=FMAN_01666;Ontology_term=membrane,channel activity,transmembrane
Fv_160_1.g607	191	ID=Fv_160_1.g607;Description=hypothetical protein FVEG_14645 [Fusarium verticillioides
Fv_160_1.g608	684	ID=Fv_160_1.g608;Description=hypothetical protein FVEG_00362 [Fusarium verticillioides 7600];Gene=FOPG_04649;Ontology_term=nucleus,DNA binding,zinc ion binding,DNA-binding transcription factor activity, RNA
Fv_160_1.g609	296	ID=Fv_160_1.g609;Description=hypothetical protein FVER14953_00361 [Fusarium verticillioides];Gene=FDENT_4989;Ontology_term=cytoplasmic vesicle membrane,dendrite membrane,chloride channel complex,postsynaptic membrane,GABA-A receptor complex,GABA-A receptor activity,inhibitory extracellular ligand-gated monoatomic ion channel activity,benzodiazepine receptor activity,hydrolase activity,GABA-gated chloride ion channel activity,gamma-aminobutyric acid signaling pathway,synaptic transmission, GABAergic,regulation of postsynaptic membrane potential,chloride transmembrane transport,inhibitory synapse
Fv_160_1.g610	205	ID=Fv_160_1.g610;Description=MXR1-responsible for the reduction of methionine sulfoxide [Fusarium subglutinans];Gene=FOVG_00247;Ontology_term=cytoplasm,peptide-methionine (S)-S-oxide reductase activity,cellular response to oxidative stress;Ontology_id=GO:0005737,GO:0008113,GO:0034599;Enzyme_code=EC:1.8.4.11;Enzyme_name=peptide-methionine
Fv_160_1.g611	345	ID=Fv_160_1.g611;Description=hypothetical protein QL093DRAFT_2559366 [Fusarium
Fv_160_1.g612	992	ID=Fv_160_1.g612;Description=hypothetical protein FVER53590_00358 [Fusarium verticillioides]
Fv_160_1.g613	139	ID=Fv_160_1.g613;Description=hypothetical protein FVER14953_00356 [Fusarium verticillioides]

Fv_160_1.g614	364	ID=Fv_160_1.g614;Description=hypothetical protein FVEG_14644 [Fusarium verticillioides 7600];Gene=374
Fv_160_1.g615	525	ID=Fv_160_1.g615;Description=hypothetical protein FVEG_14643 [Fusarium verticillioides 7600];Gene=1015
Fv_160_1.g616	406	ID=Fv_160_1.g616;Description=hypothetical protein FVEG_14642 [Fusarium verticillioides 7600]
Fv_160_1.g617	584	ID=Fv_160_1.g617;Description=threonine ammonia-lyase, biosynthetic [Fusarium verticillioides 7600];Gene=FOMG_01958;Ontology_term=L-threonine ammonia-lyase activity,pyridoxal phosphate binding,isoleucine biosynthetic process;Ontology_id=GO:0004794,GO:0030170,GO:0009097;Enzyme_code=EC:4.3.1.19;Enzyme_name=threonine ammonia-lyase
Fv_160_1.g618	161	ID=Fv_160_1.g618;Description=hypothetical protein FVEG_00353 [Fusarium verticillioides
Fv_160_1.g619	616	ID=Fv_160_1.g619;Description=hypothetical protein FVEG_00352 [Fusarium verticillioides 7600];Gene=FOVG_00237;Ontology_term=hydrolase activity,carbohydrate metabolic
Fv_160_1.g620	1057	ID=Fv_160_1.g620;Description=hypothetical protein FVEG_00351 [Fusarium verticillioides 7600];Gene=FNAPI_7322;Ontology_term=cytoplasm,microtubule binding,kinase
Fv_160_1.g621	778	ID=Fv_160_1.g621;Description=hypothetical protein FVEG_00350 [Fusarium verticillioides
Fv_160_1.g622	237	ID=Fv_160_1.g622;Description=hypothetical protein FVEG_00349 [Fusarium verticillioides 7600];Gene=FMEXI_578;Ontology_term=ESCRT I complex,protein-containing complex binding,protein targeting to membrane,protein targeting to vacuole,protein transport to vacuole involved in ubiquitin-dependent protein catabolic process via
Fv_160_1.g623	422	ID=Fv_160_1.g623;Description=hypothetical protein LB503_000218 [Fusarium chuoi];Gene=MDM12;Ontology_term=endoplasmic reticulum membrane,ERMES complex,lipid binding,kinase activity,mitochondrial genome maintenance,lipid transport,phosphorylation,protein insertion into mitochondrial outer membrane;Ontology_id=GO:0005789,GO:0032865,GO:0008289,GO:0016301,GO:0000002,GO:0006869,GO:0016310,GO:0045040;E
Fv_160_1.g624	2517	ID=Fv_160_1.g624;Description=hypothetical protein FVER14953_00347 [Fusarium verticillioides];Gene=FMEXI_576;Ontology_term=1-phosphatidylinositol-3-phosphate 5-kinase activity,ATP binding,metal ion binding,phosphorylation,phosphatidylinositol metabolic
Fv_160_1.g625	742	ID=Fv_160_1.g625;Description=hypothetical protein FVEG_00346 [Fusarium verticillioides 7600]
Fv_160_1.g626	121	ID=Fv_160_1.g626;Description=BolA protein [Fusarium verticillioides 7600]
Fv_160_1.g627	596	ID=Fv_160_1.g627;Description=hypothetical protein FVEG_00344 [Fusarium verticillioides 7600]
Fv_160_1.g628	256	ID=Fv_160_1.g628;Description=translation initiation factor 4E [Fusarium verticillioides 7600];Gene=FOYG_06109;Ontology_term=cytoplasm,RNA binding,translation initiation factor activity,translational
Fv_160_1.g629	396	ID=Fv_160_1.g629;Description=hypothetical protein FVEG_00342 [Fusarium verticillioides
Fv_160_1.g630	2412	ID=Fv_160_1.g630;Description=hypothetical protein FAUST_8208 [Fusarium austroamericanum];Gene=MDCFG851_LOCUS569271;Ontology_term=hydrolase
Fv_160_1.g631	80	ID=Fv_160_1.g631;Description=hypothetical protein FVER14953_00339 [Fusarium verticillioides];Gene=FOXG_18021;Ontology_term=anaphase-promoting complex,anaphase-promoting complex-dependent
Fv_160_1.g632	720	ID=Fv_160_1.g632;Description=hypothetical protein FVEG_00338 [Fusarium verticillioides 7600]

Fv_160_1.g633	324	ID=Fv_160_1.g633;Description=cell cycle checkpoint protein [Fusarium verticillioides 7600];Gene=FVEG_00337;Ontology_term=nucleus,membrane,exonuclease activity,DNA damage checkpoint signaling,DNA
Fv_160_1.g634	79	ID=Fv_160_1.g634;Description=cell cycle checkpoint protein [Fusarium verticillioides 7600];Gene=CDV36_009254;Ontology_term=nucleus,oligosaccharyltransferase I complex,DNA damage checkpoint signaling,DNA
Fv_160_1.g635	396	ID=Fv_160_1.g635;Description=histone acetyltransferase [Fusarium musae];Gene=BFJ63_vAg8709;Ontology_term=SAGA complex,chromosome, centromeric region,nucleus,cytosol,SLIK (SAGA-like) complex,ADA complex,transcription coregulator activity,histone H3K14 acetyltransferase activity,histone H3K9 acetyltransferase activity,histone H3K18 acetyltransferase activity,lysine-acetylated histone binding,histone crotonyltransferase activity,negative regulation of induction of conjugation with cellular fusion,positive regulation of transcription elongation by RNA polymerase II,transcription initiation-coupled chromatin remodeling;Ontology_id=GO:0000124,GO:0000775,GO:0005634,GO:0005829,GO:0046695,GO:0140671,GO:0003712,GO:0036408,
Fv_160_1.g636	332	ID=Fv_160_1.g636;Description=hypothetical protein FVEG_00335 [Fusarium verticillioides 7600]
Fv_160_1.g637	410	ID=Fv_160_1.g637;Description=hypothetical protein FVER14953_00334 [Fusarium verticillioides];Gene=FMAN_01698;Ontology_term=biotin synthase activity,metal ion binding,2 iron, 2 sulfur cluster binding,4 iron, 4 sulfur cluster binding,biotin biosynthetic
Fv_160_1.g638	563	ID=Fv_160_1.g638;Description=hypothetical protein FVEG_00333 [Fusarium verticillioides 7600];Gene=crt-1;Ontology_term=endoplasmic reticulum lumen,endoplasmic reticulum membrane,calcium ion binding,carbohydrate binding,unfolded protein binding,protein folding,response to heat,regulation of gene expression,programmed cell death,defecation,ubiquitin-dependent ERAD pathway,endoplasmic reticulum unfolded protein response,hemidesmosome assembly,response to ethanol,multicellular organismal reproductive process;Ontology_id=GO:0005788,GO:0005789,GO:0005509,GO:0030246,GO:0051082,GO:0006457,GO:0009408,GO:0010468,GO:
Fv_160_1.g639	288	ID=Fv_160_1.g639;Description=hypothetical protein FVER53590_00332 [Fusarium
Fv_160_1.g640	203	ID=Fv_160_1.g640;Description=hypothetical protein FVEG_00331 [Fusarium verticillioides 7600]
Fv_160_1.g641	440	ID=Fv_160_1.g641;Description=hypothetical protein FVER53590_00330 [Fusarium verticillioides]
Fv_160_1.g642	1433	ID=Fv_160_1.g642;Description=RecName: Full=ABC transporter FUM19;AltNameFull=Fumonisin biosynthesis cluster protein 19 [Fusarium verticillioides 7600];Gene=FOXYS1_39;Ontology_term=plasma membrane,ATP binding,ATP hydrolysis activity,ABC-type transporter activity,transmembrane transport;Ontology_id=GO:0005886,GO:0005524,GO:0016887,GO:0140359,GO:0055085;Enzyme_code=EC:7.2.2,EC:3.6.1.15;Enzym
Fv_160_1.g643	429	ID=Fv_160_1.g643;Description=RecName: Full=Sphingosine N-acyltransferase-like protein FUM18;AltNameFull=Fumonisin biosynthesis cluster protein 18 [Fusarium verticillioides 7600];Gene=FPRO_13581;Ontology_term=membrane,sphingosine N-acyltransferase activity,ceramide biosynthetic
Fv_160_1.g644	255	ID=Fv_160_1.g644;Description=hypothetical protein FVER53590_00327 [Fusarium verticillioides];Gene=FALBO_13101;Ontology_term=membrane,sphingosine N-acyltransferase activity,ceramide biosynthetic

Fv_160_1.g645	676	ID=Fv_160_1.g645;Description=hypothetical protein FVER53263_00326 [Fusarium verticillioides];Gene=Prx1;Ontology_term=nucleus,DNA binding,endonuclease activity,ATP binding,ligase activity,DNA transposition,DNA integration;Ontology_id=GO:0005634,GO:0003677,GO:0004519,GO:0005524,GO:0016874,GO:0006313,GO:0015074;Enzyme_code
Fv_160_1.g646	596	ID=Fv_160_1.g646;Description=hypothetical protein FVER53590_25705 [Fusarium verticillioides];Gene=FPRO_13583;Ontology_term=membrane,monooxygenase activity,iron ion binding,oxidoreductase activity, acting on paired donors, with incorporation or reduction of molecular oxygen,heme binding,fumonisin biosynthetic process;Ontology_id=GO:0016020,GO:0004497,GO:0005506,GO:0016705,GO:0020037,GO:1900541;Enzyme_code=EC:1.14;Enzyme
Fv_160_1.g647	589	ID=Fv_160_1.g647;Description=hypothetical protein FVER53590_00325 [Fusarium verticillioides];Gene=FVER53263_00325;Ontology_term=ligase activity,fumonisin biosynthetic
Fv_160_1.g648	369	ID=Fv_160_1.g648;Description=hypothetical protein FVER53590_00324 [Fusarium verticillioides];Gene=FUM13;Ontology_term=oxidoreductase activity,fumonisin biosynthetic
Fv_160_1.g649	473	ID=Fv_160_1.g649;Description=hypothetical protein FVER53590_00323 [Fusarium verticillioides];Gene=FVER53263_00323;Ontology_term=membrane,monooxygenase activity,iron ion binding,oxidoreductase activity, acting on paired donors, with incorporation or reduction of molecular oxygen,heme binding,fumonisin biosynthetic process;Ontology_id=GO:0016020,GO:0004497,GO:0005506,GO:0016705,GO:0020037,GO:1900541;Enzyme_code=EC:1.14;Enzyme
Fv_160_1.g650	493	ID=Fv_160_1.g650;Description=RecName: Full=Acyl-CoA synthetase FUM10;AltNameFull=Fumonisin biosynthesis cluster protein 10 [Fusarium verticillioides 7600];Gene=FUM10;Ontology_term=ATP binding,ligase activity,fumonisin biosynthetic process;Ontology_id=GO:0005524,GO:0016874,GO:1900541;Enzyme_code=EC:6;Enzyme_name=Ligases
Fv_160_1.g651	300	ID=Fv_160_1.g651;Description=hypothetical protein FVEG_00320 [Fusarium verticillioides 7600];Gene=FMUND_4256;Ontology_term=metal ion binding,dioxygenase activity,fumonisin biosynthetic
Fv_160_1.g652	289	ID=Fv_160_1.g652;Description=hypothetical protein FVEG_14635 [Fusarium verticillioides 7600];Gene=BFJ72_g3198;Ontology_term=transaminase activity,pyridoxal phosphate binding,biosynthetic
Fv_160_1.g653	553	ID=Fv_160_1.g653;Description=FUM8p [[Gibberella] fujikuroi var. moniliformis];Gene=FANTH_8694;Ontology_term=transaminase activity,pyridoxal phosphate binding,fumonisin biosynthetic
Fv_160_1.g654	424	ID=Fv_160_1.g654;Description=hypothetical protein FVER53590_00319 [Fusarium verticillioides];Gene=FANTH_8693;Ontology_term=myosin complex,A band,myosin filament,cytoskeletal protein binding,oxidoreductase activity,SH3 domain binding,protein kinase binding,metal ion binding,skeletal muscle myosin thick filament assembly,locomotion,positive regulation of ovulation,positive regulation of sarcomere organization,fumonisin biosynthetic process;Ontology_id=GO:0016459,GO:0031672,GO:0032982,GO:0008092,GO:0016491,GO:0017124,GO:0019901,GO:0046872,GO:

Fv_160_1.g655	1132	ID=Fv_160_1.g655;Description=FUM6p [[Gibberella] fujikuroi var. moniliformis];Gene=FPHYL_321;Ontology_term=NADPH-hemoprotein reductase activity,iron ion binding,FMN binding,heme binding,aromatase activity,fumonisin biosynthetic process;Ontology_id=GO:0003958,GO:0005506,GO:0010181,GO:0020037,GO:0070330,GO:1900541;Enzyme_code=EC:1.6.2.4,EC:1.14.14.1;Enzyme_name=NADPH--hemoprotein reductase,unspecific monooxygenase
Fv_160_1.g656	2597	ID=Fv_160_1.g656;Description=acetyltransferase [Fusarium verticillioides 7600];Gene=FPRO_13593;Ontology_term=3-oxoacyl-[acyl-carrier-protein] synthase activity,S-adenosylmethionine-dependent methyltransferase activity,oxidoreductase activity,phosphopantetheine binding,fatty acid biosynthetic process,methylation,fumonisin biosynthetic process;Ontology_id=GO:0004315,GO:0008757,GO:0016491,GO:0031177,GO:0006633,GO:0032259,GO:1900541;Enzyme_code=EC
Fv_160_1.g657	512	ID=Fv_160_1.g657;Description=hypothetical protein FVER53590_00314 [Fusarium verticillioides];Gene=FUM21;Ontology_term=nucleus,DNA binding,zinc ion binding,oxidoreductase activity,positive regulation of fumonisin biosynthetic process,DNA-binding transcription factor activity, RNA polymerase II-specific,regulation of transcription by
Fv_160_1.g658	364	ID=Fv_160_1.g658;Description=hypothetical protein FVER14953_00314 [Fusarium verticillioides];Gene=FPANT_3839;Ontology_term=nucleus,cytoplasmic stress granule,P granule,DNA-binding transcription factor activity, RNA polymerase II-specific,RNA binding,RNA helicase activity,translation initiation factor activity,ATP binding,zinc ion binding,oxidoreductase activity,ATP hydrolysis activity,cytoplasmic translational initiation,regulation of transcription by RNA polymerase
Fv_160_1.g659	336	ID=Fv_160_1.g659;Description=hypothetical protein FVER53590_00313 [Fusarium verticillioides];Gene=FNAPI_4730;Ontology_term=zinc ion binding,ubiquitin protein ligase
Fv_160_1.g660	450	ID=Fv_160_1.g660;Description=protein PNG1 [Fusarium verticillioides 7600];Gene=FOC1_g10016503;Ontology_term=metal ion binding,response to stress;Ontology_id=GO:0046872,GO:0006950
Fv_160_1.g661	879	ID=Fv_160_1.g661;Description=hypothetical protein FVEG_00311 [Fusarium verticillioides 7600];Gene=FMEXI_554;Ontology_term=90S preribosome,maturation of SSU-rRNA from tricistronic rRNA transcript (SSU-rRNA, 5.8S
Fv_160_1.g662	459	ID=Fv_160_1.g662;Description=nicotinate phosphoribosyltransferase [Fusarium coicis];Gene=BFJ68_g6797;Ontology_term=chromosome, telomeric region,nucleus,nicotinate phosphoribosyltransferase activity,glycosyltransferase activity,rDNA heterochromatin formation,NAD biosynthetic process,nicotinate nucleotide salvage,subtelomeric heterochromatin formation;Ontology_id=GO:0000781,GO:0005634,GO:0004516,GO:0016757,GO:0000183,GO:0009435,GO:0019358,GO:0031509;En
Fv_160_1.g663	396	ID=Fv_160_1.g663;Description=hypothetical protein FVEG_00309 [Fusarium verticillioides 7600];Gene=FOC1_g10016506;Ontology_term=nucleosome,nucleus,cytoplasm,plasma membrane,DNA-binding transcription factor activity, RNA polymerase II-specific,DNA binding,zinc ion binding,glycosyltransferase activity,structural constituent of chromatin,protein heterodimerization activity,carboxylesterase activity,regulation of transcription by RNA polymerase II;Ontology_id=GO:0000786,GO:0005634,GO:0005737,GO:0005886,GO:0000981,GO:0003677,GO:0008270,GO:0016757,GO:003052

Fv_160_1.g664	376	ID=Fv_160_1.g664;Description=hypothetical protein FVER53590_00308 [Fusarium verticillioides];Gene=FPCIR_687;Ontology_term=nucleus,vacuolar membrane,DNA-binding transcription factor activity, RNA polymerase II-specific,zinc ion binding,glycosyltransferase activity,regulation of transcription by RNA polymerase
Fv_160_1.g665	581	ID=Fv_160_1.g665;Description=hypothetical protein FVEG_14631 [Fusarium verticillioides
Fv_160_1.g666	695	ID=Fv_160_1.g666;Description=hypothetical protein FVEG_00306 [Fusarium verticillioides 7600]
Fv_160_1.g667	440	ID=Fv_160_1.g667;Description=hypothetical protein FVER53263_00303 [Fusarium verticillioides];Gene=FVEG_00303;Ontology_term=nucleus,Rix1 complex,rRNA
Fv_160_1.g668	113	ID=Fv_160_1.g668;Description=hypothetical protein FOXG_01197 [Fusarium oxysporum f. sp. lycopersici 4287];Gene=F25303_4751;Ontology_term=iron-sulfur cluster assembly;Ontology_id=GO:0016226
Fv_160_1.g669	854	ID=Fv_160_1.g669;Description=hypothetical protein FVER14953_00301 [Fusarium verticillioides]
Fv_160_1.g670	369	ID=Fv_160_1.g670;Description=hypothetical protein FVEG_00300 [Fusarium verticillioides
Fv_160_1.g671	966	ID=Fv_160_1.g671;Description=hypothetical protein FVEG_00299 [Fusarium verticillioides
Fv_160_1.g672	82	ID=Fv_160_1.g672;Description=hypothetical protein FVEG_00298 [Fusarium verticillioides 7600];Gene=Q01821;Ontology_term=plasma membrane,signal transduction,visual
Fv_160_1.g673	608	ID=Fv_160_1.g673;Description=hypothetical protein FVER14953_00297 [Fusarium verticillioides];Gene=FFUJ_00359;Ontology_term=cytosol,hydrolase activity,nucleotide catabolic
Fv_160_1.g674	409	ID=Fv_160_1.g674;Description=solute carrier family 35 member F2 [Fusarium pseudoanthophilum];Gene=FMAN_01721;Ontology_term=membrane,transmembrane transporter activity,transmembrane
Fv_160_1.g675	365	ID=Fv_160_1.g675;Description=hypothetical protein FVER14953_00295 [Fusarium verticillioides]
Fv_160_1.g676	396	ID=Fv_160_1.g676;Description=hypothetical protein FVER53263_00294 [Fusarium verticillioides]
Fv_160_1.g677	388	ID=Fv_160_1.g677;Description=hypothetical protein FVEG_00293 [Fusarium verticillioides 7600]
Fv_160_1.g678	358	ID=Fv_160_1.g678;Description=hypothetical protein FVER53590_00292 [Fusarium verticillioides]
Fv_160_1.g679	1057	ID=Fv_160_1.g679;Description=hypothetical protein FVER53590_00291 [Fusarium verticillioides];Gene=gaoC;Ontology_term=galactose oxidase
Fv_160_1.g680	1307	ID=Fv_160_1.g680;Description=uncharacterized protein FTJAE_1672 [Fusarium tjaetaba]
Fv_160_1.g681	318	ID=Fv_160_1.g681;Description=hypothetical protein FVEG_00289 [Fusarium verticillioides 7600]
Fv_160_1.g682	1280	ID=Fv_160_1.g682;Description=hypothetical protein FVER14953_00288 [Fusarium verticillioides];Gene=FFUJ_00347;Ontology_term=poly(A) RNA polymerase
Fv_160_1.g683	628	ID=Fv_160_1.g683;Description=hypothetical protein FMUND_4290 [Fusarium mundagurra]
Fv_160_1.g684	276	ID=Fv_160_1.g684;Description=hypothetical protein FVEG_14626 [Fusarium verticillioides 7600];Gene=LIP;Ontology_term=cytosolic large ribosomal subunit,structural constituent of ribosome,cytoplasmic translational
Fv_160_1.g685	388	ID=Fv_160_1.g685;Description=hypothetical protein FVER14953_20306 [Fusarium verticillioides]
Fv_160_1.g686	707	ID=Fv_160_1.g686;Description=hypothetical protein FVER14953_00286 [Fusarium verticillioides];Gene=431

Fv_160_1.g687	670	ID=Fv_160_1.g687;Description=hypothetical protein FVER14953_00285 [Fusarium verticillioides]
Fv_160_1.g688	191	ID=Fv_160_1.g688;Description=hypothetical protein FVER53263_00284 [Fusarium verticillioides]
Fv_160_1.g689	679	ID=Fv_160_1.g689;Description=hypothetical protein FVER14953_00283 [Fusarium verticillioides]
Fv_160_1.g690	280	ID=Fv_160_1.g690;Description=hypothetical protein FVER14953_00282 [Fusarium verticillioides];Gene=FFUJ_00337;Ontology_term=O-methyltransferase activity,S-adenosylmethionine-dependent methyltransferase
Fv_160_1.g691	354	ID=Fv_160_1.g691;Description=hypothetical protein FVEG_00281 [Fusarium verticillioides 7600];Gene=LIP';Ontology_term=cytosolic large ribosomal subunit,structural constituent of ribosome,cytoplasmic translational
Fv_160_1.g692	815	ID=Fv_160_1.g692;Description=hypothetical protein FVEG_00280 [Fusarium verticillioides 7600]
Fv_160_1.g693	735	ID=Fv_160_1.g693;Description=hypothetical protein J7337_001674 [Fusarium musae]
Fv_160_1.g694	504	ID=Fv_160_1.g694;Description=hypothetical protein FVER53263_00277 [Fusarium verticillioides]
Fv_160_1.g695	189	ID=Fv_160_1.g695;Description=hypothetical protein FVER14953_20090 [Fusarium verticillioides];Gene=438;Ontology_term=hydrolase
Fv_160_1.g696	276	ID=Fv_160_1.g696;Description=hypothetical protein FVER53263_00276 [Fusarium verticillioides];Gene=FNAPI_2432;Ontology_term=guanosine tetraphosphate metabolic process;Ontology_id=GO:0015969
Fv_160_1.g697	392	ID=Fv_160_1.g697;Description=hypothetical protein FVER53590_00275 [Fusarium verticillioides];Gene=FPHYL_277;Ontology_term=extracellular region,hormone activity,hydrolase activity,signal
Fv_160_1.g698	714	ID=Fv_160_1.g698;Description=zinc finger BTB domain-containing protein [Fusarium circinatum];Gene=FPHYL_276;Ontology_term=transferase
Fv_160_1.g699	675	ID=Fv_160_1.g699;Description=unnamed protein product [Fusarium
Fv_160_1.g700	736	ID=Fv_160_1.g700;Description=hypothetical protein FVEG_00270 [Fusarium verticillioides 7600];Gene=443
Fv_160_1.g701	213	ID=Fv_160_1.g701;Description=hypothetical protein FVER53263_00269 [Fusarium verticillioides];Gene=444
Fv_160_1.g702	305	ID=Fv_160_1.g702;Description=hypothetical protein FVER14953_00268 [Fusarium verticillioides];Gene=445;Ontology_term=extracellular space,nucleus,cytosol,glyceraldehyde-3-phosphate dehydrogenase (NAD+) (phosphorylating) activity,cytokine activity,interleukin-1 receptor binding,copper ion binding,NADP binding,NAD binding,fever generation,connective tissue replacement involved in inflammatory response wound healing,glucose metabolic process,glycolytic process,intracellular sodium ion homeostasis,immune response,negative regulation of cell population proliferation,positive regulation of vascular endothelial growth factor production,cytokine-mediated signaling pathway,positive regulation of interleukin-2 production,positive regulation of immature T cell proliferation in thymus,cellular response to heat,ectopic germ cell programmed cell death,positive regulation of angiogenesis,positive regulation of mitotic nuclear division,positive regulation of transcription by RNA polymerase II,response to copper ion,positive regulation of protein secretion,positive regulation of cell division,cellular response to lipopolysaccharide,extrinsic apoptotic signaling pathway in absence of ligand;Ontology_id=GO:0005615,GO:0005634,GO:0005829,GO:0004365,GO:0005125,GO:0005149,GO:0005507,GO:0050661,GO:00
Fv_160_1.g703	267	ID=Fv_160_1.g703;Description=hypothetical protein FVER14953_20091 [Fusarium verticillioides];Gene=446

Fv_160_1.g704	206	ID=Fv_160_1.g704;Description=hypothetical protein FVEG_00266 [Fusarium verticillioides 7600]
Fv_160_1.g705	125	ID=Fv_160_1.g705;Description=hypothetical protein FVEG_00265 [Fusarium verticillioides 7600];Gene=448
Fv_160_1.g706	166	ID=Fv_160_1.g706;Description=hypothetical protein FVER53590_00264 [Fusarium verticillioides]
Fv_160_1.g707	185	ID=Fv_160_1.g707;Description=hypothetical protein FVEG_00263 [Fusarium verticillioides 7600]
Fv_160_1.g708	638	ID=Fv_160_1.g708;Description=hypothetical protein FVER53590_00262 [Fusarium verticillioides];Gene=FPANT_3896;Ontology_term=oxidoreductase activity, acting on CH-OH group of donors,flavin adenine
Fv_160_1.g709	199	ID=Fv_160_1.g709;Description=hypothetical protein FVER53263_00261 [Fusarium verticillioides];Gene=FGADI_4188;Ontology_term=membrane,oxidoreductase activity, acting on CH-OH group of donors,flavin
Fv_160_1.g710	388	ID=Fv_160_1.g710;Description=hypothetical protein FVEG_00260 [Fusarium verticillioides 7600];Gene=FMUND_7305;Ontology_term=nucleus,DNA-binding transcription factor activity, RNA polymerase II-specific,zinc ion
Fv_160_1.g711	1025	ID=Fv_160_1.g711;Description=hypothetical protein FVER53590_00259 [Fusarium verticillioides];Gene=FPHYL_262;Ontology_term=membrane,chitin synthase
Fv_160_1.g712	515	ID=Fv_160_1.g712;Description=hypothetical protein FVER53590_00258 [Fusarium verticillioides];Gene=FMAN_01766;Ontology_term=membrane,monooxygenase activity,iron ion binding,oxidoreductase activity, acting on paired donors, with incorporation or reduction of molecular oxygen,heme binding;Ontology_id=GO:0016020,GO:0004497,GO:0005506,GO:0016705,GO:0020037;Enzyme_code=EC:1.14;Enzyme_name=Actin
Fv_160_1.g713	237	ID=Fv_160_1.g713;Description=hypothetical protein FVER14953_00255 [Fusarium verticillioides]
Fv_160_1.g714	207	ID=Fv_160_1.g714;Description=GTPase-activating of the rho rac family (LRG1) [Fusarium pseudoanthophilum];Gene=FPANT_3905;Ontology_term=cellular anatomical entity,GTPase regulator activity,metal ion
Fv_160_1.g715	302	ID=Fv_160_1.g715;Description=hypothetical protein FVEG_00254 [Fusarium verticillioides 7600];Gene=461;Ontology_term=glycosome,glyceraldehyde-3-phosphate dehydrogenase (NAD+) (phosphorylating) activity,NADP binding,NAD binding,glucose metabolic process,glycolytic process;Ontology_id=GO:0020015,GO:0004365,GO:0050661,GO:0051287,GO:0006006,GO:0006096;Enzyme_code=EC:1.2.1.59,EC:1
Fv_160_1.g716	812	ID=Fv_160_1.g716;Description=hypothetical protein FVER53590_00253 [Fusarium
Fv_160_1.g717	657	ID=Fv_160_1.g717;Description=hypothetical protein FVEG_00252 [Fusarium verticillioides 7600];Gene=FACUT_4165;Ontology_term=membrane,acyltransferase activity, transferring groups other than amino-acyl
Fv_160_1.g718	191	ID=Fv_160_1.g718;Description=hypothetical protein FVER14953_00251 [Fusarium verticillioides];Gene=BFJ68_g11397;Ontology_term=acyltransferase activity, transferring groups other than amino-acyl
Fv_160_1.g719	434	ID=Fv_160_1.g719;Description=alpha-glucosidase (maltase) [Fusarium subglutinans];Gene=FANTH_676;Ontology_term=maltose metabolic process,nucleus,DNA binding,zinc ion binding,alpha-glucosidase activity,carbohydrate catabolic process,lyase activity,DNA-binding transcription factor activity, RNA polymerase II-specific,regulation of transcription by RNA polymerase II;Ontology_id=GO:0000023,GO:0005634,GO:0003677,GO:0008270,GO:0090599,GO:0016052,GO:0016829,GO:0000981,GO:0006357;Enzyme_code=EC:3.2.1.20,EC:4;Enzyme_name=alpha-glucosidase,Lyases

Fv_160_1.g720	613	ID=Fv_160_1.g720;Description=trehalose-6-phosphate hydrolase [Fusarium verticillioides 7600];Gene=FVEG_00249;Ontology_term=nucleus,DNA-binding transcription factor activity, RNA polymerase II-specific,zinc ion binding,alpha-glucosidase activity,maltose metabolic process,regulation of transcription by RNA polymerase II,carbohydrate
Fv_160_1.g721	507	ID=Fv_160_1.g721;Description=hypothetical protein FVEG_00248 [Fusarium verticillioides 7600];Gene=FANTH_675;Ontology_term=membrane,transmembrane transporter activity,carbohydrate transport,transmembrane
Fv_160_1.g722	154	ID=Fv_160_1.g722;Description=hypothetical protein FVER14953_00247 [Fusarium verticillioides];Gene=FMAN_01777;Ontology_term=growth factor activity,interleukin-2-mediated signaling pathway,positive regulation of plasma cell differentiation,adaptive immune response,interleukin-2 receptor binding,positive regulation of tyrosine phosphorylation of STAT protein,cytokine activity,positive regulation of activated T cell proliferation,extracellular space,phosphorelay signal transduction system,negative regulation of B cell apoptotic process,positive regulation of immunoglobulin production,positive regulation of interleukin-17 production,positive regulation of B cell
Fv_160_1.g723	302	ID=Fv_160_1.g723;Description=hypothetical protein FVER53263_00246 [Fusarium verticillioides]
Fv_160_1.g724	502	ID=Fv_160_1.g724;Description=hypothetical protein FVER14953_00245 [Fusarium verticillioides]
Fv_160_1.g725	299	ID=Fv_160_1.g725;Description=hypothetical protein FVER53263_00244 [Fusarium verticillioides]
Fv_160_1.g726	1155	ID=Fv_160_1.g726;Description=heterokaryon incompatibility 6 OR allele [Fusarium tjaetaba]
Fv_160_1.g727	136	ID=Fv_160_1.g727;Description=hypothetical protein FVER14953_00241 [Fusarium verticillioides];Gene=Forpe1208_v000257;Ontology_term=carbon-sulfur lyase activity,metal ion
Fv_160_1.g728	690	ID=Fv_160_1.g728;Description=hypothetical protein FVER14953_00240 [Fusarium verticillioides];Gene=FOXB_14750;Ontology_term=nucleus,DNA binding,zinc ion binding,DNA-binding transcription factor activity,
Fv_160_1.g729	714	ID=Fv_160_1.g729;Description=hypothetical protein FVER53263_00239 [Fusarium
Fv_160_1.g730	1075	ID=Fv_160_1.g730;Description=hypothetical protein FVER53590_00238 [Fusarium verticillioides];Gene=FVEG_00238;Ontology_term=isomerase activity,phosphopantetheine binding,secondary metabolite biosynthetic process,organic substance biosynthetic
Fv_160_1.g731	303	ID=Fv_160_1.g731;Description=hypothetical protein FVEG_00237 [Fusarium verticillioides 7600];Gene=477
Fv_160_1.g732	124	ID=Fv_160_1.g732;Description=unnamed protein product [Fusarium fujikuroi]
Fv_160_1.g733	696	ID=Fv_160_1.g733;Description=hypothetical protein FVER14953_00236 [Fusarium verticillioides];Gene=Slc6a4;Ontology_term=membrane,membrane-bounded organelle,ATP binding,ATP-dependent protein folding
Fv_160_1.g734	414	ID=Fv_160_1.g734;Description=hypothetical protein FVER14953_00235 [Fusarium verticillioides];Gene=FPCIR_4239;Ontology_term=metal ion binding,metallodipeptidase
Fv_160_1.g735	426	ID=Fv_160_1.g735;Description=hypothetical protein FVER14953_00233 [Fusarium verticillioides];Gene=FDENT_4059;Ontology_term=membrane,transmembrane transporter activity,transmembrane
Fv_160_1.g736	501	ID=Fv_160_1.g736;Description=hypothetical protein FVER53590_00232 [Fusarium verticillioides];Gene=FPANT_3926;Ontology_term=membrane,transmembrane transporter activity,transmembrane

Fv_160_1.g737	354	ID=Fv_160_1.g737;Description=hypothetical protein FVER14953_00231 [Fusarium verticillioides];Gene=FDENT_4055;Ontology_term=3-beta-hydroxy-delta5-steroid dehydrogenase activity,steroid biosynthetic
Fv_160_1.g738	818	ID=Fv_160_1.g738;Description=pyrimidine 5 -nucleotidase [Fusarium agapanthi];Gene=FPANT_3927;Ontology_term=3-beta-hydroxy-delta5-steroid dehydrogenase activity,steroid biosynthetic
Fv_160_1.g739	528	ID=Fv_160_1.g739;Description=hypothetical protein FVEG_00229 [Fusarium verticillioides 7600];Gene=FNAPI_2476;Ontology_term=membrane,transmembrane transporter activity,transmembrane
Fv_160_1.g740	582	ID=Fv_160_1.g740;Description=hypothetical protein FVEG_00228 [Fusarium verticillioides 7600];Gene=FVEG_00228;Ontology_term=nucleus,DNA binding,zinc ion binding,DNA-binding transcription factor activity, RNA
Fv_160_1.g741	77	ID=Fv_160_1.g741;Description=conidiation-specific protein 10 [Fusarium verticillioides 7600]
Fv_160_1.g742	85	ID=Fv_160_1.g742;Description=hypothetical protein FVEG_00226 [Fusarium verticillioides 7600];Gene=KNG2;Ontology_term=maturation of SSU-rRNA from tricistronic rRNA transcript (SSU-rRNA, 5.8S rRNA, LSU-rRNA),inflammatory response,extracellular space,structural constituent of ribosome,cysteine-type endopeptidase inhibitor activity,cytosolic small ribosomal subunit,negative regulation of blood coagulation,vasodilation,nucleolus,translation,positive
Fv_160_1.g743	327	ID=Fv_160_1.g743;Description=hypothetical protein FVEG_00225 [Fusarium verticillioides 7600]
Fv_160_1.g744	569	ID=Fv_160_1.g744;Description=hypothetical protein FVER14953_00224 [Fusarium
Fv_160_1.g745	446	ID=Fv_160_1.g745;Description=hypothetical protein FVEG_00223 [Fusarium verticillioides 7600];Gene=FOBC_00647;Ontology_term=muscle myosin complex,striated muscle myosin thick filament,membrane,A band,microfilament motor activity,ATP binding,structural constituent of muscle,transmembrane transporter activity,actin filament binding,muscle contraction,egg-laying behavior,skeletal muscle myosin thick filament assembly,locomotion,sarcomere organization,transmembrane
Fv_160_1.g746	462	ID=Fv_160_1.g746;Description=hypothetical protein FVER53263_00222 [Fusarium verticillioides];Gene=FPRO_01879;Ontology_term=nucleus,inflammatory response,extracellular space,zinc ion binding,cysteine-type endopeptidase inhibitor activity,negative regulation of blood coagulation,membrane,cilium,vasodilation,DNA-binding transcription factor activity, RNA polymerase II-specific,regulation of transcription by RNA polymerase II,positive regulation of cytosolic calcium
Fv_160_1.g747	526	ID=Fv_160_1.g747;Description=hypothetical protein J7337_001728 [Fusarium musae];Gene=FPANT_12114;Ontology_term=membrane,transmembrane transporter activity,transmembrane
Fv_160_1.g748	380	ID=Fv_160_1.g748;Description=hypothetical protein FVER14953_00220 [Fusarium
Fv_160_1.g749	236	ID=Fv_160_1.g749;Description=hypothetical protein FVEG_00219 [Fusarium verticillioides 7600];Gene=FLAG1_11336;Ontology_term=acyltransferase activity, transferring groups other than amino-acyl
Fv_160_1.g750	302	ID=Fv_160_1.g750;Description=hypothetical protein FVER14953_00218 [Fusarium verticillioides];Gene=FGLOB1_2548;Ontology_term=acyltransferase activity, transferring groups other than amino-acyl
Fv_160_1.g751	564	ID=Fv_160_1.g751;Description=hypothetical protein FVER14953_00217 [Fusarium verticillioides];Gene=FVEG_00217;Ontology_term=oxidoreductase activity, acting on CH-OH group of donors,flavin adenine

Fv_160_1.g752	387	ID=Fv_160_1.g752;Description=hypothetical protein FVEG_00216 [Fusarium verticillioides
Fv_160_1.g753	644	ID=Fv_160_1.g753;Description=hypothetical protein FVEG_00215 [Fusarium verticillioides 7600];Gene=FFUJ_00259;Ontology_term=nucleus,DNA binding,zinc ion binding,membrane,DNA-binding transcription factor activity, RNA polymerase II-specific,regulation of transcription by RNA polymerase
Fv_160_1.g754	451	ID=Fv_160_1.g754;Description=hypothetical protein FVEG_00214 [Fusarium verticillioides 7600];Gene=FVEG_00214;Ontology_term=cilium,membrane,transmembrane transporter activity,transmembrane
Fv_160_1.g755	541	ID=Fv_160_1.g755;Description=hypothetical protein FVER14953_00213 [Fusarium verticillioides];Gene=FPCIR_12873;Ontology_term=membrane,1-alkyl-2-acetylglycerophosphocholine esterase activity,lipid catabolic
Fv_160_1.g756	1030	ID=Fv_160_1.g756;Description=hypothetical protein FVER53590_25989 [Fusarium verticillioides];Gene=FPANT_13206;Ontology_term=serine-type endopeptidase
Fv_160_1.g757	531	ID=Fv_160_1.g757;Description=subtilisin-like serine protease [Fusarium pseudoanthophilum];Gene=FPANT_13206;Ontology_term=vacuole,serine-type endopeptidase activity,pexophagy,proteolysis,protein catabolic process in the vacuole,sporulation resulting in formation of a cellular
Fv_160_1.g758	2609	ID=Fv_160_1.g758;Description=hypothetical protein FVER14953_00209 [Fusarium verticillioides]
Fv_160_1.g759	76	ID=Fv_160_1.g759;Description=hypothetical protein HG530_008237 [Fusarium
Fv_160_1.g760	458	ID=Fv_160_1.g760;Description=hypothetical protein FVER14953_00207 [Fusarium verticillioides];Gene=FPCIR_12869;Ontology_term=endoplasmic reticulum membrane,transferase
Fv_160_1.g761	243	ID=Fv_160_1.g761;Description=pre-mRNA-splicing factor ISY1 [Fusarium verticillioides 7600];Gene=FPCIR_4181;Ontology_term=nucleus,generation of catalytic spliceosome for second transesterification
Fv_160_1.g762	863	ID=Fv_160_1.g762;Description=hypothetical protein FVER14953_00205 [Fusarium verticillioides];Gene=508;Ontology_term=spindle,clathrin coat of trans-Golgi network vesicle,clathrin coat of coated pit,synaptic vesicle membrane,clathrin complex,postsynaptic endocytic zone cytoplasmic component,structural molecule activity,clathrin heavy chain binding,intracellular protein transport,cell cycle,clathrin coat assembly,cell division,clathrin-dependent endocytosis;Ontology_id=GO:0005819,GO:0030130,GO:0030132,GO:0030672,GO:0071439,GO:0099631,GO:0005198,GO:0032050,
Fv_160_1.g763	497	ID=Fv_160_1.g763;Description=homoserine O-acetyltransferase [Fusarium verticillioides 7600];Gene=F25303_5336;Ontology_term=serine O-acetyltransferase activity,cysteine biosynthetic process from
Fv_160_1.g764	338	ID=Fv_160_1.g764;Description=hypothetical protein FVER14953_00203 [Fusarium verticillioides]
Fv_160_1.g765	268	ID=Fv_160_1.g765;Description=hypothetical protein FVER14953_00202 [Fusarium verticillioides];Gene=FMUND_13639;Ontology_term=hydrolase
Fv_160_1.g766	204	ID=Fv_160_1.g766;Description=hypothetical protein FVER14953_00201 [Fusarium verticillioides];Gene=F25303_5333;Ontology_term=hydrolase

Fv_160_1.g767	318	ID=Fv_160_1.g767;Description=hypothetical protein FVER53590_00199 [Fusarium verticillioides];Gene=FPRO_01900;Ontology_term=membrane,hydrolase activity, hydrolyzing O-glycosyl compounds,metal ion transmembrane transporter activity,magnesium ion transport,xylan catabolic process,transmembrane
Fv_160_1.g768	888	ID=Fv_160_1.g768;Description=uncharacterized protein FFB14_04498 [Fusarium fujikuroi];Gene=FPRO_01901;Ontology_term=clathrin coat of trans-Golgi network vesicle,clathrin coat of coated pit,synaptic vesicle membrane,postsynaptic endocytic zone cytoplasmic component,structural molecule activity,clathrin heavy chain binding,metal ion transmembrane transporter activity,intracellular protein transport,magnesium ion transport,transmembrane transport,clathrin-
Fv_160_1.g769	522	ID=Fv_160_1.g769;Description=hypothetical protein FVER14953_00198 [Fusarium verticillioides];Gene=FNAPI_12999;Ontology_term=membrane,transmembrane transporter activity,transmembrane
Fv_160_1.g770	490	ID=Fv_160_1.g770;Description=COP9 signalosome complex subunit 2 [Fusarium verticillioides 7600];Gene=ceh-19;Ontology_term=nucleus,DNA-binding transcription factor activity, RNA polymerase II-specific,DNA binding,regulation of
Fv_160_1.g771	628	ID=Fv_160_1.g771;Description=hypothetical protein FVER14953_00195 [Fusarium verticillioides];Gene=FPCIR_4192;Ontology_term=cysteine-type deubiquitinase
Fv_160_1.g772	363	ID=Fv_160_1.g772;Description=hypothetical protein FVER53263_00193 [Fusarium verticillioides];Gene=FDENT_4020;Ontology_term=S-adenosylmethionine-dependent methyltransferase
Fv_160_1.g773	478	ID=Fv_160_1.g773;Description=hypothetical protein FVER14953_00192 [Fusarium verticillioides];Gene=FVER53590_00192;Ontology_term=glutamine synthetase activity,glutamine biosynthetic
Fv_160_1.g774	279	ID=Fv_160_1.g774;Description=glucose 1-dehydrogenase [Fusarium verticillioides 7600];Gene=FMUND_13648;Ontology_term=apical plasma membrane,ATP binding,ABC-type xenobiotic transporter activity,efflux transmembrane transporter activity,oxidoreductase activity,ATP hydrolysis activity,xenobiotic transport,cellular biosynthetic process,innate immune response,defense response to Gram-negative bacterium,transmembrane transport,stress response to cadmium ion;Ontology_id=GO:0016324,GO:0005524,GO:0008559,GO:0015562,GO:0016491,GO:0016887,GO:0042908,GO:0044249,GO:0045
Fv_160_1.g775	598	ID=Fv_160_1.g775;Description=hypothetical protein FVER14953_00190 [Fusarium verticillioides];Gene=FMUND_13649;Ontology_term=isocitrate lyase activity,methylisocitrate lyase activity,metal ion binding,carboxylic acid metabolic
Fv_160_1.g776	457	ID=Fv_160_1.g776;Description=2-methylcitrate synthase, mitochondrial [Fusarium verticillioides 7600];Gene=Forpe1208_v000211;Ontology_term=mitochondrial matrix,citrate (Si)-synthase activity,2-methylcitrate synthase activity,tricarboxylic acid cycle,citrate metabolic process,propionate catabolic process, 2-methylcitrate cycle;Ontology_id=GO:0005759,GO:0004108,GO:0050440,GO:0006099,GO:0006101,GO:0019629;Enzyme_code=EC:2.3.3.5,EC:2.3.3
Fv_160_1.g777	190	ID=Fv_160_1.g777;Description=hypothetical protein FVER14953_00188 [Fusarium verticillioides];Gene=FFUJ_00235;Ontology_term=acyltransferase activity, transferring groups other than amino-acyl

Fv_160_1.g778	371	ID=Fv_160_1.g778;Description=hypothetical protein FVER14953_00187 [Fusarium verticillioides];Gene=FCIRC_12454;Ontology_term=membrane,hydrolase activity, hydrolyzing O-glycosyl compounds,carbohydrate
Fv_160_1.g779	237	ID=Fv_160_1.g779;Description=glutathione S-transferase [Fusarium verticillioides 7600];Gene=FPANT_7948;Ontology_term=nucleosome,nucleus,DNA binding,transferase activity,structural constituent of chromatin,protein heterodimerization
Fv_160_1.g780	1753	ID=Fv_160_1.g780;Description=hypothetical protein FVER53263_00185 [Fusarium verticillioides];Gene=FCIRC_12456;Ontology_term=nucleus,cytoplasm,helicase activity,zinc ion
Fv_160_1.g781	162	ID=Fv_160_1.g781;Description=hypothetical protein FVEG_00184 [Fusarium verticillioides 7600]
Fv_160_1.g782	203	ID=Fv_160_1.g782;Description=hypothetical protein FVER14953_00183 [Fusarium verticillioides];Gene=FVER53590_00183;Ontology_term=cytosol,plasma membrane,cellular bud,cell division site,GTPase activity,GTP binding,microtubule cytoskeleton organization,small GTPase mediated signal transduction,regulation of septum digestion after cytokinesis,establishment or maintenance of actin cytoskeleton polarity,positive regulation of exocytosis,positive regulation of formin-nucleated actin cable
Fv_160_1.g783	377	ID=Fv_160_1.g783;Description=hypothetical protein FVER14953_00182 [Fusarium verticillioides];Gene=FACUT_3925;Ontology_term=U2-type spliceosomal complex,mRNA processing,RNA
Fv_160_1.g784	113	ID=Fv_160_1.g784;Description=hypothetical protein FOZG_02122 [Fusarium oxysporum Fo47];Gene=CEP51_005295;Ontology_term=ribosome,ribonucleoprotein complex,structural constituent of
Fv_160_1.g785	945	ID=Fv_160_1.g785;Description=hypothetical protein FVER14953_00180 [Fusarium verticillioides];Gene=BFJ72_g11372;Ontology_term=membrane,beta-glucosidase activity,scopolin beta-glucosidase activity,cellulose
Fv_160_1.g786	372	ID=Fv_160_1.g786;Description=hypothetical protein FVER14953_00179 [Fusarium verticillioides];Gene=FOYG_05937;Ontology_term=hydrolase activity,carbohydrate metabolic
Fv_160_1.g787	515	ID=Fv_160_1.g787;Description=hypothetical protein FVEG_00178 [Fusarium verticillioides 7600];Gene=FocTR4_00000248;Ontology_term=membrane,transmembrane transporter activity,transmembrane
Fv_160_1.g788	240	ID=Fv_160_1.g788;Description=hypothetical protein FVEG_00177 [Fusarium verticillioides 7600]
Fv_160_1.g789	110	ID=Fv_160_1.g789;Description=hypothetical protein FVEG_00176 [Fusarium verticillioides 7600]
Fv_160_1.g790	104	ID=Fv_160_1.g790;Description=hypothetical protein FPANT_12818 [Fusarium pseudoanthophilum]
Fv_160_1.g791	90	ID=Fv_160_1.g791;Description=hypothetical protein FVEG_14603 [Fusarium verticillioides 7600]
Fv_160_1.g792	120	ID=Fv_160_1.g792;Description=hypothetical protein FVER14953_00175 [Fusarium verticillioides]
Fv_160_1.g793	125	ID=Fv_160_1.g793;Description=hypothetical protein FVER14953_20503 [Fusarium
Fv_160_1.g794	106	ID=Fv_160_1.g794;Description=hypothetical protein FVEG_14601 [Fusarium verticillioides
Fv_160_1.g795	103	ID=Fv_160_1.g795;Description=hypothetical protein FVER14953_00174 [Fusarium verticillioides];Gene=533
Fv_160_1.g796	117	ID=Fv_160_1.g796;Description=hypothetical protein FVEG_00173 [Fusarium verticillioides 7600];Gene=534
Fv_160_1.g797	99	ID=Fv_160_1.g797;Description=hypothetical protein FVEG_00172 [Fusarium verticillioides 7600];Gene=FPCIR_4216

Fv_160_1.g798	130	ID=Fv_160_1.g798;Description=hypothetical protein FNYG_06574 [Fusarium nygamai];Gene=FPCIR_4216;Ontology_term=metal ion
Fv_160_1.g799	214	ID=Fv_160_1.g799;Description=hypothetical protein FVEG_00171 [Fusarium verticillioides 7600];Gene=FSUBG_8125;Ontology_term=oxidoreductase activity, acting on the aldehyde or oxo group of donors, NAD or NADP as
Fv_160_1.g800	498	ID=Fv_160_1.g800;Description=hypothetical protein FVER14953_00170 [Fusarium verticillioides];Gene=BFJ69_g8660;Ontology_term=oxidoreductase activity, acting on the aldehyde or oxo group of donors, NAD or
Fv_160_1.g801	194	ID=Fv_160_1.g801;Description=hypothetical protein FVER14953_20504 [Fusarium verticillioides];Gene=Forpe1208_v000190
Fv_160_1.g802	458	ID=Fv_160_1.g802;Description=hypothetical protein FVER14953_00169 [Fusarium verticillioides];Gene=FNAPI_3795;Ontology_term=oxidoreductase
Fv_160_1.g803	782	ID=Fv_160_1.g803;Description=C6 transcription factor [Fusarium coicis];Gene=FPRO_01934;Ontology_term=nucleus,DNA binding,zinc ion binding,DNA-binding transcription factor activity, RNA polymerase II-specific,regulation of transcription by RNA
Fv_160_1.g804	278	ID=Fv_160_1.g804;Description=hypothetical protein FANTH_7201 [Fusarium anthophilum];Gene=FCIRC_2961;Ontology_term=regulation of cell shape,motor neuron axon guidance,actin filament organization,neuronal cell body,cell projection,cytoplasmic side of plasma membrane,cell chemotaxis,GTP binding,cortical cytoskeleton organization,neuron migration,nematode larval development,GTPase activity,apoptotic process,cytosol,cytoplasmic vesicle,regulation of synaptic transmission, GABAergic,netrin-activated signaling pathway,left/right axis specification,negative regulation of microtubule polymerization,protein kinase binding,small GTPase mediated signal transduction,positive regulation of engulfment of apoptotic cell,positive regulation of distal tip cell migration,embryonic body morphogenesis,nematode male tail tip morphogenesis,chemorepulsion of axon,S-adenosylmethionine-dependent methyltransferase activity,regulation of dorsal/ventral axon guidance,gastrulation,axon extension involved in axon guidance,establishment of mitotic spindle orientation,cytoskeleton,regulation of actin cytoskeleton organization,establishment or maintenance of actin cytoskeleton polarity,axon
Fv_160_1.g805	1074	ID=Fv_160_1.g805;Description=hypothetical protein FVEG_00165 [Fusarium verticillioides 7600];Gene=540
Fv_160_1.g806	584	ID=Fv_160_1.g806;Description=hypothetical protein FVER53263_00160 [Fusarium verticillioides];Gene=FVER53590_00160;Ontology_term=membrane,transmembrane transporter activity,transmembrane
Fv_160_1.g807	290	ID=Fv_160_1.g807;Description=hypothetical protein FVEG_00159 [Fusarium verticillioides 7600];Gene=FVEG_00159;Ontology_term=oxidoreductase
Fv_160_1.g808	592	ID=Fv_160_1.g808;Description=heterokaryon incompatibility 6 OR allele [Fusarium tjaetaba]
Fv_160_1.g809	682	ID=Fv_160_1.g809;Description=hypothetical protein FVER14953_00157 [Fusarium verticillioides];Gene=FMAN_01865;Ontology_term=nucleus,membrane,DNA-binding transcription factor activity, RNA polymerase II-
Fv_160_1.g810	348	ID=Fv_160_1.g810;Description=hypothetical protein J7337_001792 [Fusarium musae];Gene=FocTR4_00000225;Ontology_term=oxidoreductase activity, acting on the CH-CH group of donors, NAD or NADP as
Fv_160_1.g811	410	ID=Fv_160_1.g811;Description=hypothetical protein FVEG_14594 [Fusarium verticillioides 7600];Gene=FVEG_14594;Ontology_term=cellular anatomical entity,hydrolase activity, acting on glycosyl

Fv_160_1.g812	474	ID=Fv_160_1.g812;Description=hypothetical protein FVEG_00155 [Fusarium verticillioides 7600];Gene=mtr-
Fv_160_1.g813	297	ID=Fv_160_1.g813;Description=dihydrodipicolinate synthase [Fusarium oxysporum f. sp. lycopersici 4287];Gene=FCIRC_2986;Ontology_term=lyase activity;Ontology_id=GO:0016829;Enzyme_code=EC:4;Enzyme_name=Lyases
Fv_160_1.g814	711	ID=Fv_160_1.g814;Description=hypothetical protein FVER53590_00153 [Fusarium verticillioides];Gene=FANTH_787;Ontology_term=nucleus,DNA-binding transcription factor activity, RNA polymerase II-specific,zinc
Fv_160_1.g815	314	ID=Fv_160_1.g815;Description=hypothetical protein J7337_001797 [Fusarium musae]
Fv_160_1.g816	501	ID=Fv_160_1.g816;Description=hypothetical protein J7337_001798 [Fusarium musae];Gene=FANTH_785;Ontology_term=oxidoreductase activity, acting on the aldehyde or oxo group of donors, NAD or NADP as
Fv_160_1.g817	1035	ID=Fv_160_1.g817;Description=hypothetical protein FVEG_00150 [Fusarium verticillioides 7600]
Fv_160_1.g818	821	ID=Fv_160_1.g818;Description=hypothetical protein FVER14953_00149 [Fusarium verticillioides];Gene=FPHYL_807;Ontology_term=membrane,transferase
Fv_160_1.g819	472	ID=Fv_160_1.g819;Description=hypothetical protein FVEG_00147 [Fusarium verticillioides 7600];Gene=F25303_2364;Ontology_term=nucleus,DNA-binding transcription factor activity, RNA polymerase II-specific,zinc ion
Fv_160_1.g820	258	ID=Fv_160_1.g820;Description=hypothetical protein FVEG_00146 [Fusarium verticillioides 7600];Gene=558
Fv_160_1.g821	482	ID=Fv_160_1.g821;Description=hypothetical protein FVEG_00145 [Fusarium verticillioides
Fv_160_1.g822	148	ID=Fv_160_1.g822;Description=hypothetical protein FNAPI_6334 [Fusarium napiforme]
Fv_160_1.g823	1293	ID=Fv_160_1.g823;Description=related to vegetatible incompatibility protein HET-E-1 [Fusarium fujikuroi IMI
Fv_160_1.g824	205	ID=Fv_160_1.g824;Description=phosphotransferase enzyme family [Fusarium pseudocircinatum];Gene=FPRO05_04051;Ontology_term=protein kinase activity,ATP
Fv_160_1.g825	486	ID=Fv_160_1.g825;Description=hypothetical protein FVER14953_00137 [Fusarium verticillioides];Gene=FVEG_00137;Ontology_term=extracellular region,membrane,hormone activity,transmembrane transporter activity,metal ion binding,signal transduction,positive regulation of metabolic process,transmembrane
Fv_160_1.g826	258	ID=Fv_160_1.g826;Description=hypothetical protein FVEG_00136 [Fusarium verticillioides 7600];Gene=BFJ69_g10397;Ontology_term=hydrolase
Fv_160_1.g827	240	ID=Fv_160_1.g827;Description=hypothetical protein FVER53590_00135 [Fusarium verticillioides];Gene=565
Fv_160_1.g828	253	ID=Fv_160_1.g828;Description=hypothetical protein FVER53590_00134 [Fusarium verticillioides]
Fv_160_1.g829	593	ID=Fv_160_1.g829;Description=hypothetical protein FVER53590_00133 [Fusarium verticillioides];Gene=FNYG_10202;Ontology_term=ATP binding,ATP-dependent protein folding chaperone,protein
Fv_160_1.g830	189	ID=Fv_160_1.g830;Description=hypothetical protein FVER14953_00132 [Fusarium verticillioides]
Fv_160_1.g831	237	ID=Fv_160_1.g831;Description=hypothetical protein FOXG_18075 [Fusarium oxysporum f. sp. lycopersici
Fv_160_1.g832	340	ID=Fv_160_1.g832;Description=hypothetical protein FVEG_00131 [Fusarium verticillioides 7600];Gene=FOIG_11131;Ontology_term=protein kinase activity,ATP

Fv_160_1.g833	207	ID=Fv_160_1.g833;Description=hypothetical protein FVER53590_00130 [Fusarium verticillioides];Gene=FPRO05_04064;Ontology_term=nucleus,nucleic acid binding;Ontology_id=GO:0005634,GO:0003676
Fv_160_1.g834	1032	ID=Fv_160_1.g834;Description=flavin-containing protein [Fusarium napiforme];Gene=FTJAE_7006;Ontology_term=nucleus,DNA-binding transcription factor activity, RNA polymerase II-specific,monooxygenase activity,zinc ion binding,regulation of transcription by RNA polymerase
Fv_160_1.g835	356	ID=Fv_160_1.g835;Description=hypothetical protein FVER53263_00128 [Fusarium
Fv_160_1.g836	697	ID=Fv_160_1.g836;Description=hypothetical protein FVEG_00127 [Fusarium verticillioides 7600]
Fv_160_1.g837	263	ID=Fv_160_1.g837;Description=heterokaryon incompatibility (HET) domain-containing protein [Fusarium denticulatum]
Fv_160_1.g838	833	ID=Fv_160_1.g838;Description=hypothetical protein FVER53263_00125 [Fusarium verticillioides];Gene=FCIRC_12346;Ontology_term=cytosol,brush border,membrane,myosin II complex,microfilament motor activity,protein serine/threonine kinase activity,calcium ion binding,structural constituent of muscle,transmembrane transporter activity,transmembrane transport;Ontology_id=GO:0005829,GO:0005903,GO:0016020,GO:0016460,GO:0000146,GO:0004674,GO:0005509,GO:0008307,GO
Fv_160_1.g839	136	ID=Fv_160_1.g839;Description=hypothetical protein FVEG_00123 [Fusarium verticillioides 7600];Gene=578
Fv_160_1.g840	746	ID=Fv_160_1.g840;Description=hypothetical protein FVER53263_00122 [Fusarium verticillioides];Gene=FPRO_01979;Ontology_term=vacuolar membrane,endosome
Fv_160_1.g841	874	ID=Fv_160_1.g841;Description=hypothetical protein FVER53590_27550 [Fusarium verticillioides];Gene=FANTH_710;Ontology_term=vacuolar membrane,endosome
Fv_160_1.g842	346	ID=Fv_160_1.g842;Description=hypothetical protein FVER14953_00121 [Fusarium verticillioides];Gene=FMUND_10823;Ontology_term=metallopeptidase
Fv_160_1.g843	266	ID=Fv_160_1.g843;Description=hypothetical protein FVER53590_27551 [Fusarium verticillioides]
Fv_160_1.g844	130	ID=Fv_160_1.g844;Description=hypothetical protein FVER14953_00120 [Fusarium verticillioides];Gene=FNAPI_12019;Ontology_term=hydrolase activity, hydrolyzing O-glycosyl compounds,cellulose catabolic
Fv_160_1.g845	630	ID=Fv_160_1.g845;Description=hypothetical protein FVEG_00119 [Fusarium verticillioides 7600];Gene=FOZG_02193;Ontology_term=nucleus,DNA-binding transcription factor activity, RNA polymerase II-specific,zinc ion
Fv_160_1.g846	846	ID=Fv_160_1.g846;Description=hypothetical protein FVER14953_00118 [Fusarium verticillioides];Gene=FVEG_00118;Ontology_term=beta-glucosidase activity,scopolin beta-glucosidase activity,cellulose catabolic
Fv_160_1.g847	498	ID=Fv_160_1.g847;Description=hypothetical protein FVEG_00117 [Fusarium verticillioides 7600];Gene=FVEG_00117;Ontology_term=membrane,transmembrane transporter activity,carbohydrate transport,transmembrane
Fv_160_1.g848	106	ID=Fv_160_1.g848;Description=hypothetical protein FVEG_00116 [Fusarium verticillioides 7600];Gene=FOVG_17473;Ontology_term=plasma membrane,transmembrane transporter activity,transmembrane
Fv_160_1.g849	959	ID=Fv_160_1.g849;Description=hypothetical protein FOQG_10747 [Fusarium oxysporum f. sp. raphani 54005]

Fv_160_1.g850	359	ID=Fv_160_1.g850;Description=hypothetical protein J7337_001831 [Fusarium musae];Gene=BFJ63_vAg16687;Ontology_term=maleylacetate reductase activity,metal ion
Fv_160_1.g851	284	ID=Fv_160_1.g851;Description=uncharacterized protein FRV6_10438 [Fusarium oxysporum]
Fv_160_1.g852	309	ID=Fv_160_1.g852;Description=haloacetate dehalogenase H-1 [Fusarium
Fv_160_1.g853	450	ID=Fv_160_1.g853;Description=hypothetical protein FVEG_00112 [Fusarium verticillioides 7600];Gene=FMUND_10831;Ontology_term=membrane,transmembrane transporter activity,transmembrane
Fv_160_1.g854	525	ID=Fv_160_1.g854;Description=hypothetical protein FVER53590_27554 [Fusarium verticillioides]
Fv_160_1.g855	373	ID=Fv_160_1.g855;Description=hypothetical protein FVEG_00110 [Fusarium verticillioides 7600]
Fv_160_1.g856	1174	ID=Fv_160_1.g856;Description=hypothetical protein FPANT_13589 [Fusarium pseudoanthophilum];Gene=FGLOB1_10117;Ontology_term=extracellular space,membrane,secretory granule,neurohypophyseal hormone activity,V1A vasopressin receptor binding,signal
Fv_160_1.g857	444	ID=Fv_160_1.g857;Description=hypothetical protein FVER14953_00107 [Fusarium
Fv_160_1.g858	600	ID=Fv_160_1.g858;Description=hypothetical protein FVER53590_27555 [Fusarium verticillioides]
Fv_160_1.g859	1007	ID=Fv_160_1.g859;Description=hypothetical protein FANTH_742 [Fusarium anthophilum];Gene=F25303_3169;Ontology_term=DNA binding,catalytic activity,nucleoside metabolic process;Ontology_id=GO:0003677,GO:0003824,GO:0009116
Fv_160_1.g860	697	ID=Fv_160_1.g860;Description=DNA repair and recombination RAD5C [Fusarium subglutinans];Gene=F25303_3168;Ontology_term=nucleus,cytoplasm,membrane,deoxyribose-phosphate aldolase activity,ATP binding,hydrolase activity,metal ion binding,ATP-dependent chromatin remodeler activity,chromatin remodeling,deoxyribonucleotide catabolic process,deoxyribose phosphate catabolic process;Ontology_id=GO:0005634,GO:0005737,GO:0016020,GO:0004139,GO:0005524,GO:0016787,GO:0046872,GO:0140658,GO:
Fv_160_1.g861	263	ID=Fv_160_1.g861;Description=deoxyribose-phosphate aldolase [Fusarium coicis];Gene=FNAPI_7894;Ontology_term=cytoplasm,deoxyribose-phosphate aldolase activity,deoxyribonucleotide catabolic process,deoxyribose phosphate catabolic
Fv_160_1.g862	245	ID=Fv_160_1.g862;Description=hypothetical protein FVER53263_00102 [Fusarium verticillioides];Gene=MDCFG593_LOCUS159009;Ontology_term=arylsulfatase activity,phenol-containing compound metabolic
Fv_160_1.g863	394	ID=Fv_160_1.g863;Description=hypothetical protein FVER14953_00101 [Fusarium verticillioides];Gene=FCULG_00009041;Ontology_term=membrane,transmembrane transporter activity,transmembrane
Fv_160_1.g864	368	ID=Fv_160_1.g864;Description=hypothetical protein FVER14953_00100 [Fusarium verticillioides];Gene=FOIG_11150;Ontology_term=FMN binding,oxidoreductase
Fv_160_1.g865	252	ID=Fv_160_1.g865;Description=hypothetical protein FVEG_00099 [Fusarium verticillioides 7600]
Fv_160_1.g866	350	ID=Fv_160_1.g866;Description=hypothetical protein FVEG_00098 [Fusarium verticillioides 7600]
Fv_160_1.g867	437	ID=Fv_160_1.g867;Description=hypothetical protein FVER14953_00097 [Fusarium verticillioides];Gene=FPANT_10601;Ontology_term=metallopeptidase activity,oxidoreductase

Fv_160_1.g868	391	ID=Fv_160_1.g868;Description=hypothetical protein FVER14953_00096 [Fusarium verticillioides];Gene=FPANT_10602;Ontology_term=oxidoreductase
Fv_160_1.g869	521	ID=Fv_160_1.g869;Description=hypothetical protein FVEG_00095 [Fusarium verticillioides 7600];Gene=Forpi1262_v000124;Ontology_term=membrane,transmembrane transporter activity,transmembrane
Fv_160_1.g870	3721	ID=Fv_160_1.g870;Description=hypothetical protein J7337_001850 [Fusarium musae];Gene=SERPINE1;Ontology_term=extracellular space,serine-type endopeptidase inhibitor activity;Ontology_id=GO:0005615,GO:0004867
Fv_160_1.g871	361	ID=Fv_160_1.g871;Description=hypothetical protein FVER53263_00093 [Fusarium verticillioides]
Fv_160_1.g872	660	ID=Fv_160_1.g872;Description=hypothetical protein FVEG_00092 [Fusarium verticillioides 7600];Gene=FOBC_00516;Ontology_term=nucleus,DNA binding,zinc ion binding,DNA-binding transcription factor activity, RNA
Fv_160_1.g873	494	ID=Fv_160_1.g873;Description=hypothetical protein FVEG_00091 [Fusarium verticillioides 7600];Gene=FNAPI_7884;Ontology_term=positive regulation of mechanosensory behavior,transmembrane transport,transmembrane transporter activity,positive regulation of detection of mechanical stimulus involved in sensory perception of touch,structural constituent of cytoskeleton,neuronal cell body,neuron projection,mitotic cell cycle,microtubule,GTP binding,metal ion binding,cytoplasm,GTPase activity,membrane,cytoplasmic microtubule organization;Ontology_id=GO:1905792,GO:0055085,GO:0022857,GO:1905789,GO:0005200,GO:0043025,GO:0043005,GO:0000278,
Fv_160_1.g874	342	ID=Fv_160_1.g874;Description=hypothetical protein FVER53590_00090 [Fusarium verticillioides];Gene=BFJ68_g9897;Ontology_term=nucleus,magnesium ion binding,RNA binding,ATP binding,transaminase activity,manganese ion binding,poly(A) RNA polymerase activity,mRNA polyadenylation,regulation of mRNA 3'-end processing;Ontology_id=GO:0005634,GO:0000287,GO:0003723,GO:0005524,GO:0008483,GO:0030145,GO:1990817,GO:0006378,G
Fv_160_1.g875	360	ID=Fv_160_1.g875;Description=hypothetical protein FVEG_00089 [Fusarium verticillioides 7600];Gene=FMUND_10855;Ontology_term=membrane,protein kinase activity,ATP binding,oxidoreductase activity;Ontology_id=GO:0016020,GO:0004672,GO:0005524,GO:0016491;Enzyme_code=EC:2.7.1,EC:1;Enzyme_name=Transferring
Fv_160_1.g876	1380	ID=Fv_160_1.g876;Description=hypothetical protein FVER53590_00088 [Fusarium verticillioides];Gene=FFUJ_00135;Ontology_term=A band,protein serine/threonine kinase activity,calmodulin binding,ATP binding,protein kinase binding,metal ion binding,protein serine kinase activity,adult locomotory behavior,peptidyl-serine phosphorylation,peptidyl-threonine phosphorylation,behavioral response to nicotine,positive regulation of locomotion,positive regulation of striated muscle contraction,negative regulation of cell division,positive regulation of sarcomere organization;Ontology_id=GO:0031672,GO:0004674,GO:0005516,GO:0005524,GO:0019901,GO:0046872,GO:0106310,GO:0008344,
Fv_160_1.g877	227	ID=Fv_160_1.g877;Description=serine threonine kinase [Fusarium napiforme];Gene=FMAN_01961;Ontology_term=membrane,kinase

Fv_160_1.g878	1191	ID=Fv_160_1.g878;Description=CHS-3 chitin synthase 3 [Fusarium pseudocircinatum];Gene=FVER53590_00084;Ontology_term=nucleus,plasma membrane,DNA-binding transcription factor activity, RNA polymerase II-specific,chitin synthase activity,zinc ion binding,chitin biosynthetic process,regulation of transcription by RNA polymerase II,conidium formation,cell wall organization;Ontology_id=GO:0005634,GO:0005886,GO:0000981,GO:0004100,GO:0008270,GO:0006031,GO:0006357,GO:0048315,
Fv_160_1.g879	230	ID=Fv_160_1.g879;Description=hypothetical protein FVER53263_00081 [Fusarium verticillioides]
Fv_160_1.g880	213	ID=Fv_160_1.g880;Description=hypothetical protein FVER14953_00080 [Fusarium verticillioides]
Fv_160_1.g881	2536	ID=Fv_160_1.g881;Description=hypothetical protein FVER53590_00079 [Fusarium verticillioides];Gene=PKS2;Ontology_term=3-oxoacyl-[acyl-carrier-protein] synthase activity,S-adenosylmethionine-dependent methyltransferase activity,oxidoreductase activity,phosphopantetheine binding,fatty acid biosynthetic process,secondary metabolite biosynthetic process;Ontology_id=GO:0004315,GO:0008757,GO:0016491,GO:0031177,GO:0006633,GO:0044550;Enzyme_code=EC:2.3.1.41,EC:1
Fv_160_1.g882	402	ID=Fv_160_1.g882;Description=hypothetical protein FVEG_00078 [Fusarium verticillioides
Fv_160_1.g883	500	ID=Fv_160_1.g883;Description=hypothetical protein FVEG_00077 [Fusarium verticillioides 7600];Gene=FGLOB1_10845;Ontology_term=extracellular region,cytoplasmic vesicle,vesicle lumen,yolk granule,lipid transporter activity,transferase activity,nutrient reservoir activity,lipid
Fv_160_1.g884	150	ID=Fv_160_1.g884;Description=hypothetical protein FVEG_00076 [Fusarium verticillioides 7600]
Fv_160_1.g885	473	ID=Fv_160_1.g885;Description=hypothetical protein FVER53263_00075 [Fusarium verticillioides];Gene=vit-4;Ontology_term=mesoderm development,synapse,protein phosphatase type 2A complex,chromosome, centromeric region,extracellular region,proton-transporting V-type ATPase, V1 domain,negative regulation of glycolytic process through fructose-6-phosphate,T cell homeostasis,ATP binding,meiotic cell cycle,negative regulation of epithelial to mesenchymal transition,protein heterodimerization activity,cytosol,GABA receptor binding,protein dephosphorylation,proton transmembrane transport,spindle pole,proton-transporting ATPase activity, rotational mechanism,nutrient reservoir activity,positive regulation of NLRP3 inflammasome complex assembly,plasma membrane,nucleus,lipid transport,metal ion binding,lipid transporter activity,protein tyrosine phosphatase activity,ATP metabolic process,membrane raft,regulation of G1/S transition of mitotic cell cycle,negative regulation of phosphatidylinositol 3-kinase/protein kinase B signal transduction,myosin phosphatase activity;Ontology_id=GO:0007498,GO:0045202,GO:0000159,GO:0000775,GO:0005576,GO:0033180,GO:1904539,GO:0043029,GO:0005524,GO:0051321,GO:0010719,GO:0046982,GO:0005829,GO:0050811,GO:0006470,GO:1902600,GO:0000922,GO:0046961,GO:0

Fv_160_1.g886	139	ID=Fv_160_1.g886;Description=hypothetical protein FVER53263_00074 [Fusarium verticillioides];Gene=639;Ontology_term=ruffle,cytoplasm,plasma membrane,COP9 signalosome,lamellipodium,phosphatidylinositol phospholipase C activity,lipid transporter activity,protein binding,metal ion binding,calcium-dependent phospholipase C activity,in utero embryonic development,epidermal growth factor receptor signaling pathway,positive regulation of epithelial cell migration,lipid catabolic process,cellular response to vascular endothelial growth factor stimulus,phosphatidylinositol metabolic process,phosphatidylinositol-mediated signaling,release of sequestered calcium ion into cytosol,cellular response to epidermal growth factor stimulus,negative regulation of reactive oxygen species biosynthetic process,positive regulation of cholesterol import,negative regulation of protein
Fv_160_1.g887	849	ID=Fv_160_1.g887;Description=hypothetical protein FVER14953_00073 [Fusarium verticillioides];Gene=FVER53590_00073;Ontology_term=ribosome,membrane,ribonucleoprotein complex,structural constituent of ribosome,sterol 3-beta-glucosyltransferase activity,carbohydrate metabolic process,translation,lipid glycosylation;Ontology_id=GO:0005840,GO:0016020,GO:1990904,GO:0003735,GO:0016906,GO:0005975,GO:0006412,GO:0030259
Fv_160_1.g888	161	ID=Fv_160_1.g888;Description=hypothetical protein FVEG_00072 [Fusarium verticillioides 7600];Gene=BFJ69_g16772;Ontology_term=plasma membrane,myelin sheath,structural constituent of myelin sheath,inflammatory response,positive regulation of gene expression,astrocyte development,central nervous system myelination,long-chain fatty acid biosynthetic process,cell maturation,axon
Fv_160_1.g889	211	ID=Fv_160_1.g889;Description=hypothetical protein FVEG_00071 [Fusarium verticillioides 7600]
Fv_160_1.g890	573	ID=Fv_160_1.g890;Description=hypothetical protein FVER14953_21452 [Fusarium verticillioides];Gene=FVER53590_28396;Ontology_term=protein kinase activity,ATP
Fv_160_1.g891	2241	ID=Fv_160_1.g891;Description=hypothetical protein FVER53263_00069 [Fusarium verticillioides];Gene=BFJ69_g16774;Ontology_term=protein serine/threonine kinase activity,ATP
Fv_160_1.g892	510	ID=Fv_160_1.g892;Description=hypothetical protein FVER53590_28397 [Fusarium verticillioides];Gene=F25303_1454;Ontology_term=membrane,transmembrane transporter activity,transmembrane
Fv_160_1.g893	461	ID=Fv_160_1.g893;Description=hypothetical protein FVEG_00066 [Fusarium verticillioides 7600];Gene=F25303_1455;Ontology_term=nucleus,voltage-gated potassium channel complex,axon,dendritic spine,DNA-binding transcription factor activity, RNA polymerase II-specific,delayed rectifier potassium channel activity,zinc ion binding,potassium ion binding,regulation of transcription by RNA polymerase II,regulation of monoatomic ion transmembrane transport,protein
Fv_160_1.g894	318	ID=Fv_160_1.g894;Description=hypothetical protein FVER14953_00065 [Fusarium verticillioides];Gene=FMAN_01985;Ontology_term=nitrilase activity,nitrogen compound metabolic
Fv_160_1.g895	364	ID=Fv_160_1.g895;Description=hypothetical protein FVER14953_21449 [Fusarium verticillioides];Gene=FNYG_01592;Ontology_term=lyase activity,metal ion
Fv_160_1.g896	1219	ID=Fv_160_1.g896;Description=hypothetical protein FACUT_8323 [Fusarium acutatum];Gene=FGADI_11113;Ontology_term=lipid

Fv_160_1.g897	485	ID=Fv_160_1.g897;Description=hypothetical protein F52700_4049 [Fusarium sp. NRRL 52700];Gene=FNAPI_11707;Ontology_term=hydrolase activity,lipid catabolic process,glycerolipid metabolic
Fv_160_1.g898	1485	ID=Fv_160_1.g898;Description=uncharacterized protein BKA55DRAFT_556719 [Fusarium redolens]
Fv_160_1.g899	210	ID=Fv_160_1.g899;Description=hypothetical protein FVER53263_00063 [Fusarium verticillioides]
Fv_160_1.g900	199	ID=Fv_160_1.g900;Description=hypothetical protein FVER53263_20043 [Fusarium
Fv_160_1.g901	585	ID=Fv_160_1.g901;Description=hypothetical protein FVER53590_00061 [Fusarium verticillioides];Gene=FANTH_687;Ontology_term=methyltransferase
Fv_160_1.g902	594	ID=Fv_160_1.g902;Description=hypothetical protein FVER14953_00060 [Fusarium verticillioides];Gene=FVER53590_00060;Ontology_term=membrane,solute:inorganic anion antiporter activity,monoatomic anion
Fv_160_1.g903	359	ID=Fv_160_1.g903;Description=hypothetical protein FVER14953_00058 [Fusarium verticillioides];Gene=NAEGRDRAFT_39221;Ontology_term=microtubule,structural constituent of cytoskeleton,GTP binding,hydrolase activity,metal ion binding,cytoskeleton organization,microtubule-based
Fv_160_1.g904	1004	ID=Fv_160_1.g904;Description=hypothetical protein FVER53263_00057 [Fusarium verticillioides];Gene=FTJAE_7030;Ontology_term=beta-galactosidase complex,beta-galactosidase activity,carbohydrate binding,carbohydrate metabolic process,cell wall organization,organic substance catabolic
Fv_160_1.g905	454	ID=Fv_160_1.g905;Description=hypothetical protein FVEG_00056 [Fusarium verticillioides 7600];Gene=FNAPI_11701;Ontology_term=transferase
Fv_160_1.g906	603	ID=Fv_160_1.g906;Description=hypothetical protein FVER14953_00055 [Fusarium verticillioides]
Fv_160_1.g907	275	ID=Fv_160_1.g907;Description=carbonic anhydrase [Fusarium pseudoanthophilum];Gene=FocTR4_00000105;Ontology_term=membrane,carbonate dehydratase activity,zinc ion binding,transmembrane transporter activity,transmembrane
Fv_160_1.g908	262	ID=Fv_160_1.g908;Description=hypothetical protein FOMA001_g2060 [Fusarium oxysporum f. sp. matthiolae];Gene=FOXB_13711
Fv_160_1.g909	839	ID=Fv_160_1.g909;Description=hypothetical protein FVER53590_00053 [Fusarium verticillioides]
Fv_160_1.g910	423	ID=Fv_160_1.g910;Description=hypothetical protein DER46DRAFT_693395 [Fusarium sp. MPI-SDFR-AT-0072];Gene=FPHYL_12120;Ontology_term=oxidoreductase activity,cellular biosynthetic
Fv_160_1.g911	387	ID=Fv_160_1.g911;Description=hypothetical protein FVER14953_00045 [Fusarium verticillioides];Gene=FCIRC_6842;Ontology_term=aspartic-type endopeptidase activity,beta-glucosidase activity,glucan endo-1,3-beta-glucanase activity, C-3 substituted reducing group,glucan endo-1,4-beta-glucanase activity, C-3 substituted reducing group,polysaccharide catabolic process,proteolysis,cell wall organization;Ontology_id=GO:0004190,GO:0008422,GO:0052861,GO:0052862,GO:0000272,GO:0006508,GO:0071555;Enzyme_cod

Fv_160_1.g912	783	ID=Fv_160_1.g912;Description=hypothetical protein FVER53263_00044 [Fusarium verticillioides];Gene=FNAPI_9966;Ontology_term=aspartic-type endopeptidase activity,beta-glucosidase activity,glucan endo-1,3-beta-glucanase activity, C-3 substituted reducing group,glucan endo-1,4-beta-glucanase activity, C-3 substituted reducing group,polysaccharide catabolic process,proteolysis,cell wall organization;Ontology_id=GO:0004190,GO:0008422,GO:0052861,GO:0052862,GO:0000272,GO:0006508,GO:0071555;Enzyme_cod
Fv_160_1.g913	248	ID=Fv_160_1.g913;Description=hypothetical protein H9Q70_014108 [Fusarium xylarioides]
Fv_160_1.g914	200	ID=Fv_160_1.g914;Description=hypothetical protein FVER14953_00043 [Fusarium verticillioides]
Fv_160_1.g915	569	ID=Fv_160_1.g915;Description=hypothetical protein FVEG_00042 [Fusarium verticillioides 7600];Gene=FMAN_02007;Ontology_term=oxidoreductase activity, acting on single donors with incorporation of molecular oxygen, incorporation of two atoms of oxygen,metal ion
Fv_160_1.g916	483	ID=Fv_160_1.g916;Description=hypothetical protein FVER53263_00041 [Fusarium verticillioides];Gene=FOIG_13572;Ontology_term=membrane,transmembrane transporter activity,transmembrane
Fv_160_1.g917	537	ID=Fv_160_1.g917;Description=hypothetical protein FVEG_14571 [Fusarium verticillioides
Fv_160_1.g918	333	ID=Fv_160_1.g918;Description=hypothetical protein FVEG_00040 [Fusarium verticillioides
Fv_160_1.g919	282	ID=Fv_160_1.g919;Description=hypothetical protein FVEG_00039 [Fusarium verticillioides
Fv_160_1.g920	366	ID=Fv_160_1.g920;Description=hypothetical protein FVEG_14570 [Fusarium verticillioides 7600];Gene=FVEG_14570;Ontology_term=lyase activity;Ontology_id=GO:0016829;Enzyme_code=EC:4;Enzyme_name=Lyases
Fv_160_1.g921	490	ID=Fv_160_1.g921;Description=hypothetical protein FVER53590_00038 [Fusarium verticillioides];Gene=FPANT_13944;Ontology_term=membrane,lyase activity,transmembrane transporter activity,transmembrane
Fv_160_1.g922	335	ID=Fv_160_1.g922;Description=hypothetical protein FVER14953_00037 [Fusarium verticillioides];Gene=FNAPI_9974;Ontology_term=hydrolase
Fv_160_1.g923	665	ID=Fv_160_1.g923;Description=hypothetical protein FVER53590_00036 [Fusarium
Fv_160_1.g924	532	ID=Fv_160_1.g924;Description=hypothetical protein FVER53590_00035 [Fusarium verticillioides];Gene=F52700_4008;Ontology_term=membrane,catalytic activity,GTP binding,nucleoside metabolic
Fv_160_1.g925	271	ID=Fv_160_1.g925;Description=hypothetical protein FVEG_00034 [Fusarium verticillioides 7600];Gene=FFUJ_00017;Ontology_term=extracellular region,cell surface,membrane,neuropeptide hormone activity,oxidoreductase activity,glucose metabolic process,neuropeptide signaling pathway,cellular biosynthetic
Fv_160_1.g926	458	ID=Fv_160_1.g926;Description=hypothetical protein FVEG_00033 [Fusarium verticillioides 7600]
Fv_160_1.g927	199	ID=Fv_160_1.g927;Description=hypothetical protein FVER53263_00032 [Fusarium verticillioides];Gene=FPANT_277;Ontology_term=membrane,oxidoreductase
Fv_160_1.g928	406	ID=Fv_160_1.g928;Description=hypothetical protein FVEG_00031 [Fusarium verticillioides 7600];Gene=FPANT_277;Ontology_term=membrane,oxidoreductase activity,cellular biosynthetic

Fv_160_1.g929	727	ID=Fv_160_1.g929;Description=ferric reductase Fre2p [Fusarium tjaetaba];Gene=FOVG_16173;Ontology_term=membrane,metallopeptidase activity,oxidoreductase activity,transmembrane transporter activity,glucan endo-1,3-alpha-glucosidase activity,monoatomic ion transport,transmembrane transport;Ontology_id=GO:0016020,GO:0008237,GO:0016491,GO:0022857,GO:0051118,GO:0006811,GO:0055085;Enzyme_code=E
Fv_160_1.g930	896	ID=Fv_160_1.g930;Description=glycoside hydrolase family 71 [Fusarium tjaetaba];Gene=FVEG_00029;Ontology_term=membrane,metallopeptidase activity,oxidoreductase activity,transmembrane transporter activity,glucan endo-1,3-alpha-glucosidase activity,monoatomic ion transport,transmembrane transport;Ontology_id=GO:0016020,GO:0008237,GO:0016491,GO:0022857,GO:0051118,GO:0006811,GO:0055085;Enzyme_code=E
Fv_160_1.g931	591	ID=Fv_160_1.g931;Description=hypothetical protein FVER53590_00028 [Fusarium verticillioides];Gene=BFJ68_g15367;Ontology_term=membrane,metallopeptidase activity,oxidoreductase activity,transmembrane transporter activity,glucan endo-1,3-alpha-glucosidase activity,monoatomic ion transport,transmembrane transport;Ontology_id=GO:0016020,GO:0008237,GO:0016491,GO:0022857,GO:0051118,GO:0006811,GO:0055085;Enzyme_code=E
Fv_160_1.g932	413	ID=Fv_160_1.g932;Description=subtilisin [Fusarium verticillioides 7600];Gene=FVEG_00027;Ontology_term=cellular anatomical entity,serine-type endopeptidase activity,oxidoreductase activity,proteolysis,cellular biosynthetic process;Ontology_id=GO:0110165,GO:0004252,GO:0016491,GO:0006508,GO:0044249;Enzyme_code=EC:3.4.21,EC:1;Enzyme_name=Acting on peptide bonds (peptidases),Oxidoreductases
Fv_160_1.g933	119	ID=Fv_160_1.g933;Description=hypothetical protein FVEG_00026 [Fusarium verticillioides
Fv_160_1.g934	343	ID=Fv_160_1.g934;Description=hypothetical protein FVER53263_00025 [Fusarium verticillioides];Gene=FCIRC_2840;Ontology_term=3-beta-hydroxy-delta5-steroid dehydrogenase activity,steroid biosynthetic
Fv_160_1.g935	226	ID=Fv_160_1.g935;Description=hypothetical protein FVEG_00024 [Fusarium verticillioides
Fv_160_1.g936	504	ID=Fv_160_1.g936;Description=hypothetical protein FVER53263_00023 [Fusarium verticillioides];Gene=FANTH_3120;Ontology_term=membrane,monooxygenase activity,iron ion binding,oxidoreductase activity, acting on paired donors, with incorporation or reduction of molecular oxygen,heme binding;Ontology_id=GO:0016020,GO:0004497,GO:0005506,GO:0016705,GO:0020037;Enzyme_code=EC:1.14;Enzyme_name=Actin
Fv_160_1.g937	371	ID=Fv_160_1.g937;Description=hypothetical protein FVER53590_00022 [Fusarium verticillioides];Gene=FANTH_3121;Ontology_term=membrane,oxidoreductase activity,transmembrane transporter activity,transmembrane
Fv_160_1.g938	110	ID=Fv_160_1.g938;Description=hypothetical protein FVEG_14564 [Fusarium verticillioides 7600];Gene=FGLOB1_2019;Ontology_term=transferase
Fv_160_1.g939	446	ID=Fv_160_1.g939;Description=hypothetical protein FVEG_00021 [Fusarium verticillioides 7600];Gene=F25303_5311;Ontology_term=transferase

Fv_160_1.g940	146	ID=Fv_160_1.g940;Description=hypothetical protein FVEG_00020 [Fusarium verticillioides 7600];Gene=PGKA;Ontology_term=glycosome,phosphoglycerate kinase activity,ATP binding,glycolytic process,phosphorylation;Ontology_id=GO:0020015,GO:0004618,GO:0005524,GO:0006096,GO:0016310;Enzyme_code=EC:2.7.2.3;E
Fv_160_1.g941	297	ID=Fv_160_1.g941;Description=hypothetical protein FVEG_14563 [Fusarium verticillioides 7600];Gene=FFC1_00064;Ontology_term=nucleus,DNA binding,zinc ion binding,DNA-binding transcription factor activity, RNA
Fv_160_1.g942	372	ID=Fv_160_1.g942;Description=hypothetical protein H9Q71_012271 [Fusarium xylarioides];Gene=FPANT_288;Ontology_term=oxidoreductase activity, acting on the CH-OH group of donors, NAD or NADP as acceptor,NADP binding,NAD
Fv_160_1.g943	325	ID=Fv_160_1.g943;Description=hypothetical protein FVER53263_00018 [Fusarium verticillioides];Gene=FPHYL_13222;Ontology_term=membrane,glycosyltransferase
Fv_160_1.g944	476	ID=Fv_160_1.g944;Description=hypothetical protein FVER53263_00017 [Fusarium verticillioides];Gene=FNAPI_9996;Ontology_term=nucleus,DNA binding,zinc ion binding,DNA-templated
Fv_160_1.g945	263	ID=Fv_160_1.g945;Description=hypothetical protein FVER53590_00016 [Fusarium verticillioides];Gene=FDENT_3131;Ontology_term=nucleus,DNA binding,zinc ion binding,lyase activity,DNA-templated
Fv_160_1.g946	467	ID=Fv_160_1.g946;Description=hypothetical protein FVER53263_00015 [Fusarium verticillioides];Gene=FPANT_553;Ontology_term=membrane,oxidoreductase
Fv_160_1.g947	273	ID=Fv_160_1.g947;Description=hypothetical protein FVER53263_00014 [Fusarium verticillioides]
Fv_160_1.g948	269	ID=Fv_160_1.g948;Description=hypothetical protein FVER14953_00013 [Fusarium
Fv_160_1.g949	499	ID=Fv_160_1.g949;Description=hypothetical protein FVEG_00012 [Fusarium verticillioides 7600];Gene=FANTH_3132;Ontology_term=membrane,transmembrane transporter activity,transmembrane
Fv_160_1.g950	658	ID=Fv_160_1.g950;Description=hypothetical protein FVER53590_00011 [Fusarium verticillioides];Gene=FPRO05_04194;Ontology_term=nucleus,DNA binding,zinc ion binding,DNA-templated
Fv_160_1.g951	116	ID=Fv_160_1.g951;Description=TdcF protein [Fusarium verticillioides 7600]
Fv_160_1.g952	401	ID=Fv_160_1.g952;Description=hypothetical protein FVEG_00009 [Fusarium verticillioides 7600];Gene=FOXB_12240;Ontology_term=metal ion binding,dioxygenase activity,small molecule biosynthetic process,organic cyclic compound biosynthetic
Fv_160_1.g953	357	ID=Fv_160_1.g953;Description=hypothetical protein FVEG_00008 [Fusarium verticillioides
Fv_160_1.g954	510	ID=Fv_160_1.g954;Description=hypothetical protein FVEG_00007 [Fusarium verticillioides 7600];Gene=FVEG_00007;Ontology_term=membrane,structural constituent of eye lens,transmembrane transporter activity,amino
Fv_160_1.g955	504	ID=Fv_160_1.g955;Description=hypothetical protein FVEG_00006 [Fusarium verticillioides 7600];Gene=FOVG_16153;Ontology_term=membrane,transmembrane transporter activity,transmembrane
Fv_160_1.g956	604	ID=Fv_160_1.g956;Description=hypothetical protein FVEG_00005 [Fusarium verticillioides 7600];Gene=FNAPI_9984;Ontology_term=nucleus,DNA binding,zinc ion binding,DNA-binding transcription factor activity, RNA

Fv_160_1.g957	729	ID=Fv_160_1.g957;Description=alpha-galactosidase 2 [Fusarium verticillioides 7600];Gene=FOVG_16155;Ontology_term=membrane,monooxygenase activity,iron ion binding,oxidoreductase activity, acting on paired donors, with incorporation or reduction of molecular oxygen,heme binding,raffinose alpha-galactosidase activity,carbohydrate catabolic
Fv_160_1.g958	159	ID=Fv_160_1.g958;Description=pisatin demethylase cytochrome P450 [Fusarium pseudoanthophilum];Gene=FACUT_4705;Ontology_term=membrane,monooxygenase activity,iron ion binding,methyltransferase activity,oxidoreductase activity, acting on paired donors, with incorporation or reduction of molecular oxygen,heme binding,methylation;Ontology_id=GO:0016020,GO:0004497,GO:0005506,GO:0008168,GO:0016705,GO:0020037,GO:0032259;Enzy
Fv_160_1.g959	133	ID=Fv_160_1.g959;Description=hypothetical protein FVER53263_00003 [Fusarium verticillioides]
Fv_160_1.g960	512	ID=Fv_160_1.g960;Description=hypothetical protein LB504_000036 [Fusarium proliferatum];Gene=679;Ontology_term=nucleus,DNA-binding transcription factor activity, RNA polymerase II-specific,zinc ion
Fv_160_2.g961	141	ID=Fv_160_2.g961;Description=hypothetical protein J7337_001939 [Fusarium musae]
Fv_160_2.g962	394	ID=Fv_160_2.g962;Description=hypothetical protein FPRO05_13153 [Fusarium proliferatum];Gene=FTJAE_13668;Ontology_term=nucleus,membrane,DNA binding,zinc ion binding,transmembrane transporter activity,DNA-templated transcription,carbohydrate transport,transmembrane
Fv_160_2.g963	505	ID=Fv_160_2.g963;Description=hypothetical protein FVEG_05800 [Fusarium verticillioides 7600];Gene=FGADI_3484;Ontology_term=mitochondrial inner membrane,inorganic phosphate transmembrane transporter
Fv_160_2.g964	525	ID=Fv_160_2.g964;Description=hypothetical protein FVEG_05799 [Fusarium verticillioides 7600];Gene=FANTH_9446;Ontology_term=membrane,transmembrane transporter activity,transmembrane
Fv_160_2.g965	411	ID=Fv_160_2.g965;Description=carboxymethylenebutenolidase [Fusarium verticillioides 7600];Gene=FSUBG_12454;Ontology_term=nucleus,cytoplasm,transcription repressor complex,nucleotide binding,DNA binding,chitinase activity,chitin binding,kinase inhibitor activity,identical protein binding,RNA polymerase II-specific DNA-binding transcription factor binding,toxin activity,negative regulation of transcription by RNA polymerase II,polysaccharide catabolic process,positive regulation of transcription from RNA polymerase II promoter by galactose,galactose metabolic process,chitin catabolic process,polyketide metabolic process,modulation of process of another organism,negative regulation of
Fv_160_2.g966	275	ID=Fv_160_2.g966;Description=hypothetical protein FVER14953_05797 [Fusarium verticillioides];Gene=RF1;Ontology_term=cellular anatomical entity,nucleotide binding,DNA binding,DNA-directed DNA polymerase activity,DNA replication,DNA biosynthetic process;Ontology_id=GO:0110165,GO:0000166,GO:0003677,GO:0003887,GO:0006260,GO:0071897;Enzyme_code=EC:2.7.7.7;Enzy
Fv_160_2.g967	559	ID=Fv_160_2.g967;Description=hypothetical protein FVER14953_05796 [Fusarium verticillioides];Gene=FPCIR_13141;Ontology_term=monooxygenase
Fv_160_2.g968	618	ID=Fv_160_2.g968;Description=hypothetical protein FVER53263_05795 [Fusarium verticillioides];Gene=FNAPI_8732;Ontology_term=magnesium ion binding,pyruvate decarboxylase activity,thiamine pyrophosphate

Fv_160_2.g969	500	ID=Fv_160_2.g969;Description=NAD-dependent aldehyde dehydrogenase [Fusarium verticillioides 7600];Gene=FSUBG_12458;Ontology_term=oxidoreductase activity, acting on the aldehyde or oxo group of donors, NAD or NADP as
Fv_160_2.g970	764	ID=Fv_160_2.g970;Description=hypothetical protein FVER53590_05793 [Fusarium verticillioides];Gene=FANTH_9452;Ontology_term=nucleus,DNA-binding transcription factor activity, RNA polymerase II-specific,zinc
Fv_160_2.g971	270	ID=Fv_160_2.g971;Description=3-octaprenyl-4-hydroxybenzoate carboxy-lyase UbiX [Fusarium verticillioides 7600];Gene=PAD1;Ontology_term=mitochondrion,lyase activity,flavin prenyltransferase
Fv_160_2.g972	514	ID=Fv_160_2.g972;Description=hypothetical protein FVEG_05792 [Fusarium verticillioides 7600];Gene=P18258;Ontology_term=cytosol,microtubule,cytoplasmic stress granule,translation release factor complex,translation release factor activity,GTPase activity,structural constituent of cytoskeleton,GTP binding,carboxy-lyase activity,identical protein binding,metal ion binding,nuclear-transcribed mRNA catabolic process, deadenylation-dependent decay,translational termination,cytoskeleton organization,microtubule-based process,ferulate metabolic process,cinnamic acid catabolic process;Ontology_id=GO:0005829,GO:0005874,GO:0010494,GO:0018444,GO:0003747,GO:0003924,GO:0005200,GO:0005525,GO:
Fv_160_2.g973	489	ID=Fv_160_2.g973;Description=hypothetical protein FVEG_05791 [Fusarium verticillioides 7600];Gene=FFUJ_03689;Ontology_term=DNA binding,oxidoreductase activity,FAD binding,galactose metabolic
Fv_160_2.g974	489	ID=Fv_160_2.g974;Description=hypothetical protein FVEG_05790 [Fusarium verticillioides 7600];Gene=FVER53590_05790;Ontology_term=nucleus,cytosol,mitochondrial membrane,glucokinase activity,ATP binding,glucose binding,fructokinase activity,oxidoreductase activity,mannokinase activity,intracellular glucose homeostasis,fructose metabolic process,glucose metabolic process,mannose metabolic process,glycolytic process,regulation of transcription by glucose,glucose import,carbohydrate phosphorylation,glucose 6-phosphate metabolic process,fructose import across plasma membrane,negative regulation of apoptotic signaling pathway;Ontology_id=GO:0005634,GO:0005829,GO:0031966,GO:0004340,GO:0005524,GO:0005536,GO:0008865,GO:0016491,GO:
Fv_160_2.g975	453	ID=Fv_160_2.g975;Description=hypothetical protein FVER14953_05789 [Fusarium verticillioides];Gene=FDENT_7227;Ontology_term=membrane,monooxygenase activity,transmembrane transporter activity,FAD binding,biosynthetic process,transmembrane
Fv_160_2.g976	347	ID=Fv_160_2.g976;Description=GMP synthase [Fusarium pseudoanthophilum];Gene=FSUBG_12465;Ontology_term=membrane,glutamine metabolic
Fv_160_2.g977	347	ID=Fv_160_2.g977;Description=hypothetical protein FVER53590_05787 [Fusarium verticillioides];Gene=FFC1_08127;Ontology_term=plasma membrane,eisosome,DNA-binding transcription factor activity,ATP binding,uracil:monoatomic cation symporter activity,metal ion binding,regulation of DNA-templated transcription,uracil
Fv_160_2.g978	677	ID=Fv_160_2.g978;Description=hypothetical protein FVER14953_05786 [Fusarium verticillioides];Gene=BFJ69_g11441;Ontology_term=ATP binding,metal ion binding;Ontology_id=GO:0005524,GO:0046872
Fv_160_2.g979	410	ID=Fv_160_2.g979;Description=hypothetical protein J7337_001997 [Fusarium musae];Gene=BFJ68_g9720;Ontology_term=GDP-mannose 4,6-dehydratase activity,GDP-mannose metabolic process,GDP-L-fucose biosynthetic

Fv_160_2.g980	494	ID=Fv_160_2.g980;Description=hypothetical protein BFJ69_g11423 [Fusarium oxysporum];Gene=FSUBG_12468;Ontology_term=endoplasmic reticulum,membrane,ATP binding,GDP-mannose 4,6-dehydratase activity,phosphotransferase activity, for other substituted phosphate groups,ATP-dependent protein folding chaperone,protein folding,phospholipid biosynthetic process,GDP-mannose metabolic process,GDP-L-fucose biosynthetic process;Ontology_id=GO:0005783,GO:0016020,GO:0005524,GO:0008446,GO:0016780,GO:0140662,GO:0006457,GO:0008654,GO:
Fv_160_2.g981	533	ID=Fv_160_2.g981;Description=POT family-domain-containing protein [Fusarium oxysporum f. sp. albedinis];Gene=FOC4_g10001699;Ontology_term=membrane,transmembrane transporter activity,transmembrane
Fv_160_2.g982	337	ID=Fv_160_2.g982;Description=hypothetical protein FVER14953_05782 [Fusarium verticillioides];Gene=BFJ68_g9666;Ontology_term=ATP adenylyltransferase activity,ATP binding,nucleotide metabolic
Fv_160_2.g983	228	ID=Fv_160_2.g983;Description=hypothetical protein FVER14953_05781 [Fusarium verticillioides];Gene=BFJ69_g11442;Ontology_term=2,3-bisphosphoglycerate-dependent phosphoglycerate mutase activity,glycolytic process;Ontology_id=GO:0046538,GO:0006096;Enzyme_code=EC:5.4.2.11;Enzyme_name=phosphoglycerate
Fv_160_2.g984	1022	ID=Fv_160_2.g984;Description=hypothetical protein FVER14953_05780 [Fusarium verticillioides];Gene=FGADI_11548;Ontology_term=beta-galactosidase complex,beta-galactosidase activity,carbohydrate binding,carbohydrate metabolic process,cell wall organization,organic substance catabolic
Fv_160_2.g985	523	ID=Fv_160_2.g985;Description=hypothetical protein FVEG_05779 [Fusarium verticillioides 7600];Gene=Forpe1208_v003447;Ontology_term=plasma membrane,protein binding,nucleobase transmembrane transporter activity,cytidine transmembrane transporter activity,cytosine transport,cytidine transport,purine-containing compound
Fv_160_2.g986	554	ID=Fv_160_2.g986;Description=hypothetical protein FVEG_05778 [Fusarium verticillioides 7600]
Fv_160_2.g987	311	ID=Fv_160_2.g987;Description=hypothetical protein FVER14953_05777 [Fusarium verticillioides];Gene=FNAPI_8753;Ontology_term=cytoplasm,microtubule,glucosamine-6-phosphate deaminase activity,structural constituent of cytoskeleton,GTP binding,isomerase activity,fructose 1,6-bisphosphate 1-phosphatase activity,metal ion binding,carbohydrate metabolic process,N-acetylglucosamine metabolic process,cytoskeleton organization,microtubule-based process;Ontology_id=GO:0005737,GO:0005874,GO:0004342,GO:0005200,GO:0005525,GO:0016853,GO:0042132,GO:0046872,GO:
Fv_160_2.g988	556	ID=Fv_160_2.g988;Description=hypothetical protein FVEG_05776 [Fusarium verticillioides 7600];Gene=FNAPI_8754;Ontology_term=nucleus,DNA-binding transcription factor activity, RNA polymerase II-specific,zinc ion
Fv_160_2.g989	540	ID=Fv_160_2.g989;Description=hypothetical protein FVEG_05775 [Fusarium verticillioides 7600];Gene=HZS61_010466;Ontology_term=hexokinase activity,ATP binding,glucose binding,intracellular glucose homeostasis,glycolytic process,carbohydrate
Fv_160_2.g990	223	ID=Fv_160_2.g990;Description=hypothetical protein FVER53590_05774 [Fusarium verticillioides]
Fv_160_2.g991	477	ID=Fv_160_2.g991;Description=hypothetical protein FVER14953_21632 [Fusarium verticillioides];Gene=FOPG_11629;Ontology_term=oxidoreductase

Fv_160_2.g992	255	ID=Fv_160_2.g992;Description=hypothetical protein FVEG_05772 [Fusarium verticillioides 7600];Gene=FSUBG_12322;Ontology_term=vacuole,membrane,symbiont-containing vacuole membrane,hydrolase activity, acting on glycosyl bonds,hydrolase activity, acting on carbon-nitrogen (but not peptide) bonds,xylan catabolic process;Ontology_id=GO:0005773,GO:0016020,GO:0020005,GO:0016798,GO:0016810,GO:0045493;Enzyme_code=EC:3.2,EC:3.5;En
Fv_160_2.g993	254	ID=Fv_160_2.g993;Description=hypothetical protein FVEG_05771 [Fusarium verticillioides 7600];Gene=FFUJ_03667;Ontology_term=membrane,hydrolase activity, acting on glycosyl bonds,hydrolase activity, acting on carbon-nitrogen (but not peptide) bonds,plasmid partitioning,small molecule metabolic process,xylan catabolic process;Ontology_id=GO:0016020,GO:0016798,GO:0016810,GO:0030541,GO:0044281,GO:0045493;Enzyme_code=EC:3.2,EC:3.5;En
Fv_160_2.g994	429	ID=Fv_160_2.g994;Description=hypothetical protein FVER14953_05770 [Fusarium verticillioides];Gene=2802;Ontology_term=membrane,acyltransferase activity, transferring groups other than amino-acyl
Fv_160_2.g995	492	ID=Fv_160_2.g995;Description=hypothetical protein FVEG_05769 [Fusarium verticillioides 7600];Gene=BFJ71_g13141;Ontology_term=membrane,glycosyltransferase
Fv_160_2.g996	414	ID=Fv_160_2.g996;Description=hypothetical protein FVEG_15721 [Fusarium verticillioides 7600];Gene=FSUBG_12325;Ontology_term=nucleus,DNA-binding transcription factor activity, RNA polymerase II-specific,zinc ion
Fv_160_2.g997	577	ID=Fv_160_2.g997;Description=hypothetical protein FVEG_05767 [Fusarium verticillioides 7600];Gene=GAMP;Ontology_term=extracellular region,plasma membrane,glucan 1,4-alpha-glucosidase activity,starch binding,polysaccharide catabolic
Fv_160_2.g998	371	ID=Fv_160_2.g998;Description=hypothetical protein FVER14953_05766 [Fusarium
Fv_160_2.g999	624	ID=Fv_160_2.g999;Description=hypothetical protein FVER14953_05765 [Fusarium verticillioides];Gene=FMAN_05199;Ontology_term=monooxygenase
Fv_160_2.g1000	649	ID=Fv_160_2.g1000;Description=hypothetical protein FVER53590_05764 [Fusarium verticillioides];Gene=FPANT_8483;Ontology_term=hydrolase
Fv_160_2.g1001	402	ID=Fv_160_2.g1001;Description=hypothetical protein FVER53590_05763 [Fusarium verticillioides];Gene=FPCIR_8220;Ontology_term=aspartic-type endopeptidase
Fv_160_2.g1002	451	ID=Fv_160_2.g1002;Description=hypothetical protein FVER53590_05762 [Fusarium verticillioides]
Fv_160_2.g1003	293	ID=Fv_160_2.g1003;Description=hypothetical protein FVER53590_05761 [Fusarium verticillioides]
Fv_160_2.g1004	172	ID=Fv_160_2.g1004;Description=hypothetical protein FVEG_05760 [Fusarium verticillioides 7600]
Fv_160_2.g1005	338	ID=Fv_160_2.g1005;Description=hypothetical protein FVER53590_05759 [Fusarium verticillioides];Gene=FTJAE_7296;Ontology_term=nucleus,plasma membrane,cell surface,side of membrane,nitrilase activity,DNA binding,zinc ion binding,DNA-templated

Fv_160_2.g1006	631	ID=Fv_160_2.g1006;Description=hypothetical protein FVEG_15720 [Fusarium verticillioides 7600];Gene=FFC1_08101;Ontology_term=nucleus,DNA binding,zinc ion binding,catalytic activity,ergosterol biosynthetic process,endoplasmic reticulum,isoprenoid biosynthetic process,DNA-binding transcription factor activity, RNA polymerase II-specific,farnesyl diphosphate metabolic process,regulation of transcription by RNA polymerase
Fv_160_2.g1007	496	ID=Fv_160_2.g1007;Description=hypothetical protein FVER14953_05756 [Fusarium verticillioides];Gene=FNAPI_8764;Ontology_term=membrane,transmembrane transporter activity,carbohydrate
Fv_160_2.g1008	864	ID=Fv_160_2.g1008;Description=hypothetical protein FVER53590_05755 [Fusarium verticillioides];Gene=FMUND_8835;Ontology_term=extracellular region,beta-mannosidase activity,carbohydrate metabolic
Fv_160_2.g1009	317	ID=Fv_160_2.g1009;Description=hypothetical protein H9Q69_006647 [Fusarium xylarioides];Gene=2787
Fv_160_2.g1010	390	ID=Fv_160_2.g1010;Description=hypothetical protein FVER53590_05753 [Fusarium verticillioides];Gene=FOC4_g10001741;Ontology_term=extracellular region,glucan 1,4-alpha-glucosidase activity,ferric iron binding,oxidoreductase activity, acting on single donors with incorporation of molecular oxygen, incorporation of two atoms of oxygen,starch binding,polysaccharide catabolic process,cellular aromatic compound metabolic process;Ontology_id=GO:0005576,GO:0004339,GO:0008199,GO:0016702,GO:2001070,GO:0000272,GO:0006725;Enzyme_code=EC
Fv_160_2.g1011	415	ID=Fv_160_2.g1011;Description=hypothetical protein FVER14953_05752 [Fusarium verticillioides];Gene=FNAPI_11767;Ontology_term=oxidoreductase activity,FAD binding,biosynthetic
Fv_160_2.g1012	346	ID=Fv_160_2.g1012;Description=hypothetical protein FVEG_05751 [Fusarium verticillioides 7600]
Fv_160_2.g1013	274	ID=Fv_160_2.g1013;Description=hypothetical protein FVEG_05750 [Fusarium verticillioides 7600];Gene=BFJ68_g9711;Ontology_term=membrane,orotidine-5'-phosphate decarboxylase activity,'de novo' pyrimidine nucleobase biosynthetic process,'de novo' UMP biosynthetic
Fv_160_2.g1014	351	ID=Fv_160_2.g1014;Description=hypothetical protein FVEG_05749 [Fusarium verticillioides
Fv_160_2.g1015	157	ID=Fv_160_2.g1015;Description=hypothetical protein FVEG_05748 [Fusarium verticillioides 7600]
Fv_160_2.g1016	184	ID=Fv_160_2.g1016;Description=hypothetical protein FVER14953_21634 [Fusarium
Fv_160_2.g1017	216	ID=Fv_160_2.g1017;Description=hypothetical protein FVER14953_05747 [Fusarium
Fv_160_2.g1018	1719	ID=Fv_160_2.g1018;Description=hypothetical protein FVER53590_05746 [Fusarium verticillioides];Gene=FANTH_5163;Ontology_term=RNA binding,RNA-dependent RNA polymerase activity,helicase activity,ATP hydrolysis activity,RNA-templated transcription,regulatory ncRNA-mediated gene silencing;Ontology_id=GO:0003723,GO:0003968,GO:0004386,GO:0016887,GO:0001172,GO:0031047;Enzyme_code=EC:2.7.7.48,EC:
Fv_160_2.g1019	96	ID=Fv_160_2.g1019;Description=hypothetical protein FVEG_05745 [Fusarium verticillioides 7600];Gene=DAL82;Ontology_term=nucleus,sequence-specific DNA binding,allantoin catabolic process,nitrogen catabolite activation

Fv_160_2.g1020	522	ID=Fv_160_2.g1020;Description=hypothetical protein FVEG_05744 [Fusarium verticillioides 7600];Gene=FANTH_5165;Ontology_term=peroxisome,catalase activity,oxidoreductase activity, acting on single donors with incorporation of molecular oxygen, incorporation of two atoms of oxygen,heme binding,metal ion binding,response to oxidative stress,hydrogen peroxide catabolic process,cellular oxidant detoxification;Ontology_id=GO:0005777,GO:0004096,GO:0016702,GO:0020037,GO:0046872,GO:0006979,GO:0042744,GO:009886
Fv_160_2.g1021	688	ID=Fv_160_2.g1021;Description=hypothetical protein FOTG_13907 [Fusarium oxysporum f. sp. vasinfectum 25433];Gene=FOQG_12849;Ontology_term=nucleus,DNA binding,zinc ion binding,DNA-binding transcription factor activity, RNA polymerase II-specific,regulation of transcription by RNA polymerase
Fv_160_2.g1022	574	ID=Fv_160_2.g1022;Description=transcriptional regulatory moc3 [Fusarium coicis];Gene=FMAN_05184;Ontology_term=zinc ion binding,DNA-directed RNA polymerase complex,extracellular region,DNA-binding transcription factor activity, RNA polymerase II-specific,regulation of transcription by RNA polymerase II,nucleus,DNA binding,peroxisome,amine metabolic process,DNA-directed 5'-3' RNA polymerase activity,copper ion binding,quinone binding,phenethylamine:oxygen oxidoreductase (deaminating) activity,tryptamine:oxygen oxidoreductase (deaminating) activity,aminoacetone:oxygen oxidoreductase(deaminating) activity,membrane,toxin activity,carbohydrate binding,host cell membrane,modulation of process of another organism;Ontology_id=GO:0008270,GO:0000428,GO:0005576,GO:0000981,GO:0006357,GO:0005634,GO:0003677,GO:0005777,GO:0009308,GO:0003899,GO:0005507,GO:0048038,GO:0052596,GO:0052593,GO:0052594,GO:0016020,GO:0090729,GO:0030246,GO
Fv_160_2.g1023	449	ID=Fv_160_2.g1023;Description=hypothetical protein FVEG_15715 [Fusarium verticillioides 7600];Gene=FMUND_12405;Ontology_term=eukaryotic translation initiation factor 3 complex,membrane,RNA binding,translation initiation factor activity,transmembrane transporter activity,translation initiation factor binding,translational
Fv_160_2.g1024	372	ID=Fv_160_2.g1024;Description=hypothetical protein FVER14953_05741 [Fusarium verticillioides];Gene=fgaOx3-2;Ontology_term=nucleus,DNA-binding transcription factor activity, RNA polymerase II-specific,zinc ion binding,FMN binding,oxidoreductase activity,regulation of transcription by RNA polymerase
Fv_160_2.g1025	230	ID=Fv_160_2.g1025;Description=hypothetical protein FVER53263_05740 [Fusarium verticillioides]
Fv_160_2.g1026	115	ID=Fv_160_2.g1026;Description=hypothetical protein FVER14953_05739 [Fusarium verticillioides]
Fv_160_2.g1027	266	ID=Fv_160_2.g1027;Description=hypothetical protein FVEG_05738 [Fusarium verticillioides 7600]
Fv_160_2.g1028	439	ID=Fv_160_2.g1028;Description=uncharacterized protein FFE2_04477 [Fusarium fujikuroi];Gene=flhA;Ontology_term=FAD binding,zinc ion binding,RNA polymerase II CTD heptapeptide repeat kinase activity,cyclin-dependent protein serine/threonine kinase activity,DNA-binding transcription factor activity, RNA polymerase II-specific,regulation of transcription by RNA polymerase II,cell division,nucleus,DNA binding,ATP binding,oxidoreductase activity,cytoplasm,membrane,regulation of cell cycle,phosphorylation,protein serine kinase activity;Ontology_id=GO:0071949,GO:0008270,GO:0008353,GO:0004693,GO:0000981,GO:0006357,GO:0051301,GO:0005634,GO:0

Fv_160_2.g1029	687	ID=Fv_160_2.g1029;Description=hypothetical protein FVEG_05735 [Fusarium verticillioides 7600];Gene=FOMG_16132;Ontology_term=nucleus,DNA binding,zinc ion binding,DNA-binding transcription factor activity, RNA polymerase II-specific,regulation of transcription by RNA polymerase
Fv_160_2.g1030	345	ID=Fv_160_2.g1030;Description=hypothetical protein FVEG_05734 [Fusarium verticillioides 7600];Gene=Forpi1262_v003604;Ontology_term=hydrolase
Fv_160_2.g1031	457	ID=Fv_160_2.g1031;Description=hypothetical protein FVEG_05733 [Fusarium verticillioides 7600];Gene=FANTH_10809;Ontology_term=N,N-dimethylaniline monooxygenase activity,flavin adenine dinucleotide binding,NADP binding;Ontology_id=GO:0004499,GO:0050660,GO:0050661;Enzyme_code=EC:1.14.13.8;Enzyme_name=flavin-containing
Fv_160_2.g1032	300	ID=Fv_160_2.g1032;Description=hypothetical protein FVEG_05732 [Fusarium verticillioides 7600];Gene=FPRO_05404;Ontology_term=transferase activity,hydrolase
Fv_160_2.g1033	73	ID=Fv_160_2.g1033;Description=hypothetical protein FVEG_15714 [Fusarium verticillioides 7600];Gene=DFR1;Ontology_term=extracellular region,mitochondrion,mRNA binding,dihydrofolate reductase activity,cellulose 1,4-beta-cellobiosidase activity,cellulose binding,NADP binding,glycine biosynthetic process,one-carbon metabolic process,cellulose catabolic process,dihydrofolate metabolic process,tetrahydrofolate biosynthetic process,folic acid metabolic process;Ontology_id=GO:0005576,GO:0005739,GO:0003729,GO:0004146,GO:0016162,GO:0030248,GO:0050661,GO:0006545,GO:
Fv_160_2.g1034	146	ID=Fv_160_2.g1034;Description=hypothetical protein FVEG_15712 [Fusarium verticillioides 7600];Gene=FTJAE_10841;Ontology_term=beta-glucosidase activity,scopolin beta-glucosidase activity,polysaccharide catabolic
Fv_160_2.g1035	671	ID=Fv_160_2.g1035;Description=hypothetical protein FPCIR_2211 [Fusarium pseudocircinatum];Gene=F52700_3564;Ontology_term=membrane,oxidoreductase activity,NADP binding,NAD
Fv_160_2.g1036	934	ID=Fv_160_2.g1036;Description=hypothetical protein FVER53263_05729 [Fusarium verticillioides]
Fv_160_2.g1037	197	ID=Fv_160_2.g1037;Description=uncharacterized protein LW93_1468 [Fusarium fujikuroi];Gene=3641;Ontology_term=cytoplasm,nucleotide binding,RNA helicase activity,single-stranded RNA binding,anion binding,translation;Ontology_id=GO:0005737,GO:0000166,GO:0003724,GO:0003727,GO:0043168,GO:0006412;Enzyme_code=EC:3.
Fv_160_2.g1038	421	ID=Fv_160_2.g1038;Description=hypothetical protein FVER14953_05728 [Fusarium verticillioides];Gene=FOIG_15583;Ontology_term=membrane,monooxygenase activity,FAD binding,biosynthetic
Fv_160_2.g1039	359	ID=Fv_160_2.g1039;Description=hypothetical protein FVER53263_05727 [Fusarium verticillioides];Gene=Forpi1262_v003595;Ontology_term=membrane,oxidoreductase activity,FAD binding,biosynthetic
Fv_160_2.g1040	583	ID=Fv_160_2.g1040;Description=hypothetical protein FVER53590_05726 [Fusarium verticillioides];Gene=FVEG_05726;Ontology_term=metal ion binding,linoleate 11-lipoxygenase activity,lipid oxidation,linoleic acid metabolic
Fv_160_2.g1041	266	ID=Fv_160_2.g1041;Description=hypothetical protein FVER53263_05725 [Fusarium verticillioides];Gene=FFC1_08046;Ontology_term=membrane,hydrolase activity, acting on carbon-nitrogen (but not peptide) bonds, in linear amides,ceramide metabolic

Fv_160_2.g1042	470	ID=Fv_160_2.g1042;Description=hypothetical protein FVEG_05724 [Fusarium verticillioides 7600];Gene=FSUBG_12545;Ontology_term=membrane,transferase activity,phosphopantetheine binding,organic substance biosynthetic
Fv_160_2.g1043	1638	ID=Fv_160_2.g1043;Description=hypothetical protein FVER53263_05723 [Fusarium verticillioides];Gene=FFUJ_03602;Ontology_term=membrane,phosphopantetheine binding,cellular biosynthetic process,organic
Fv_160_2.g1044	311	ID=Fv_160_2.g1044;Description=hypothetical protein FVEG_05722 [Fusarium verticillioides 7600]
Fv_160_2.g1045	705	ID=Fv_160_2.g1045;Description=hypothetical protein FVER14953_21639 [Fusarium verticillioides];Gene=FSUBG_12543;Ontology_term=nucleus,DNA binding,zinc ion binding,DNA-binding transcription factor activity, RNA polymerase II-specific,regulation of transcription by RNA polymerase
Fv_160_2.g1046	439	ID=Fv_160_2.g1046;Description=hypothetical protein FVEG_05719 [Fusarium verticillioides 7600];Gene=FTJAE_10830;Ontology_term=hydrolase activity, acting on carbon-nitrogen (but not peptide) bonds, in linear
Fv_160_2.g1047	481	ID=Fv_160_2.g1047;Description=hypothetical protein FNYG_04358 [Fusarium nygamai];Gene=FVEG_05718;Ontology_term=membrane,transmembrane transporter activity,transmembrane
Fv_160_2.g1048	365	ID=Fv_160_2.g1048;Description=hypothetical protein FVER53263_05717 [Fusarium
Fv_160_2.g1049	324	ID=Fv_160_2.g1049;Description=hypothetical protein FVER53590_05716 [Fusarium verticillioides];Gene=FPANT_14170;Ontology_term=hydrolase activity, acting on glycosyl bonds,carbohydrate metabolic
Fv_160_2.g1050	87	ID=Fv_160_2.g1050;Description=hypothetical protein FVER53590_05715 [Fusarium verticillioides];Gene=3627;Ontology_term=mitochondrial intermembrane space,respirasome,electron transfer activity,heme binding,metal ion binding,electron transport
Fv_160_2.g1051	623	ID=Fv_160_2.g1051;Description=hypothetical protein FVER53590_28837 [Fusarium verticillioides];Gene=FDENT_10778;Ontology_term=phosphoric diester hydrolase activity,lipid metabolic
Fv_160_2.g1052	503	ID=Fv_160_2.g1052;Description=D-lactate dehydrogenase (cytochrome) [Fusarium verticillioides 7600];Gene=BFJ70_g15087;Ontology_term=membrane,catalytic activity,FAD
Fv_160_2.g1053	697	ID=Fv_160_2.g1053;Description=hypothetical protein FVER53590_05712 [Fusarium verticillioides];Gene=FOMG_16088;Ontology_term=nucleus,DNA binding,zinc ion binding,DNA-binding transcription factor activity, RNA polymerase II-specific,regulation of transcription by RNA polymerase
Fv_160_2.g1054	483	ID=Fv_160_2.g1054;Description=hypothetical protein J7337_002067 [Fusarium musae];Gene=BFJ68_g10397;Ontology_term=membrane,transmembrane transporter activity,transmembrane
Fv_160_2.g1055	363	ID=Fv_160_2.g1055;Description=hypothetical protein FVER14953_05709 [Fusarium verticillioides];Gene=HAP1;Ontology_term=DNA-binding transcription factor activity,protein binding,metal ion binding,regulation of DNA-templated
Fv_160_2.g1056	236	ID=Fv_160_2.g1056;Description=hypothetical protein FVEG_05708 [Fusarium verticillioides 7600];Gene=RPL28;Ontology_term=nucleus,plasma membrane,cytosolic large ribosomal subunit,RNA binding,structural constituent of ribosome,acyltransferase activity, transferring groups other than amino-acyl groups,cytoplasmic

Fv_160_2.g1057	777	ID=Fv_160_2.g1057;Description=hypothetical protein FCOIX_8468 [Fusarium
Fv_160_2.g1058	1174	ID=Fv_160_2.g1058;Description=aminopeptidase 2 [Fusarium pseudocircinatum];Gene=FNYG_04365;Ontology_term=aminopeptidase activity,metallopeptidase activity,zinc ion
Fv_160_2.g1059	1091	ID=Fv_160_2.g1059;Description=hypothetical protein FVEG_05703 [Fusarium verticillioides 7600];Gene=FAUST_7711
Fv_160_2.g1060	261	ID=Fv_160_2.g1060;Description=hypothetical protein FVEG_05701 [Fusarium verticillioides 7600]
Fv_160_2.g1061	375	ID=Fv_160_2.g1061;Description=hypothetical protein FVER14953_05700 [Fusarium verticillioides];Gene=HZS61_010389;Ontology_term=extracellular region,pectate lyase activity,polysaccharide catabolic
Fv_160_2.g1062	666	ID=Fv_160_2.g1062;Description=amidohydrolase ytcJ-like [Fusarium verticillioides 7600];Gene=BFJ69_g10498;Ontology_term=hydrolase activity, acting on carbon-nitrogen (but not peptide)
Fv_160_2.g1063	499	ID=Fv_160_2.g1063;Description=hypothetical protein FVER53590_05698 [Fusarium verticillioides];Gene=FOPG_13372;Ontology_term=membrane,transmembrane transporter activity,transmembrane
Fv_160_2.g1064	488	ID=Fv_160_2.g1064;Description=hypothetical protein FVER53590_05697 [Fusarium verticillioides];Gene=FPHYL_3700;Ontology_term=polygalacturonase activity,carbohydrate metabolic process,cell wall
Fv_160_2.g1065	490	ID=Fv_160_2.g1065;Description=hypothetical protein FVER53590_05696 [Fusarium verticillioides];Gene=FNAPI_11611;Ontology_term=sulfuric ester hydrolase
Fv_160_2.g1066	565	ID=Fv_160_2.g1066;Description=hypothetical protein FVER53590_05695 [Fusarium verticillioides];Gene=FNAPI_11612;Ontology_term=membrane,transmembrane transporter activity,transmembrane
Fv_160_2.g1067	486	ID=Fv_160_2.g1067;Description=hypothetical protein FVER14953_05694 [Fusarium verticillioides]
Fv_160_2.g1068	440	ID=Fv_160_2.g1068;Description=hypothetical protein FVER53263_05693 [Fusarium verticillioides];Gene=FOTG_08395;Ontology_term=protein kinase activity,ATP
Fv_160_2.g1069	97	ID=Fv_160_2.g1069;Description=hypothetical protein FVEG_15707 [Fusarium verticillioides 7600]
Fv_160_2.g1070	240	ID=Fv_160_2.g1070;Description=hypothetical protein FVER14953_05692 [Fusarium verticillioides];Gene=FGADI_4374;Ontology_term=DNA-directed RNA polymerase complex,membrane,apicoplast,DNA binding,DNA-directed 5'-3' RNA polymerase activity,ribonucleoside binding,DNA-templated transcription;Ontology_id=GO:0000428,GO:0016020,GO:0020011,GO:0003677,GO:0003899,GO:0032549,GO:0006351;Enzyme_cod
Fv_160_2.g1071	346	ID=Fv_160_2.g1071;Description=hypothetical protein FVER53590_05691 [Fusarium verticillioides];Gene=HZS61_010378;Ontology_term=hydrolase
Fv_160_2.g1072	318	ID=Fv_160_2.g1072;Description=hypothetical protein FVER53263_20028 [Fusarium verticillioides];Gene=FNAPI_11617;Ontology_term=hydrolase
Fv_160_2.g1073	517	ID=Fv_160_2.g1073;Description=hypothetical protein FVEG_05690 [Fusarium verticillioides 7600];Gene=BFJ65_g5194;Ontology_term=membrane,transmembrane transporter activity,carbohydrate transport,transmembrane
Fv_160_2.g1074	660	ID=Fv_160_2.g1074;Description=hypothetical protein J7337_002086 [Fusarium musae];Gene=FFC1_07992;Ontology_term=alpha-L-arabinofuranosidase activity,arabinan catabolic process,L-arabinose metabolic

Fv_160_2.g1075	247	ID=Fv_160_2.g1075;Description=hypothetical protein FVER53590_05688 [Fusarium verticillioides];Gene=FMUND_2159;Ontology_term=extracellular matrix,metalloendopeptidase activity,zinc ion
Fv_160_2.g1076	106	ID=Fv_160_2.g1076;Description=hypothetical protein FVER53590_28833 [Fusarium verticillioides];Gene=QCR7;Ontology_term=mitochondrial respiratory chain complex III,matrix side of mitochondrial inner membrane,ubiquinol-cytochrome-c reductase activity,mitochondrial electron transport, ubiquinol to cytochrome c,mitochondrial respiratory chain complex III
Fv_160_2.g1077	107	ID=Fv_160_2.g1077;Description=hypothetical protein FVEG_15702 [Fusarium verticillioides 7600];Gene=sorD-4;Ontology_term=oxidoreductase activity,FAD
Fv_160_2.g1078	334	ID=Fv_160_2.g1078;Description=cytokinin dehydrogenase 3 [Fusarium sp. NRRL 52700];Gene=FOXB_10672;Ontology_term=oxidoreductase activity,FAD
Fv_160_2.g1079	404	ID=Fv_160_2.g1079;Description=hypothetical protein J7337_002091 [Fusarium musae]
Fv_160_2.g1080	577	ID=Fv_160_2.g1080;Description=hypothetical protein FVEG_05685 [Fusarium verticillioides 7600]
Fv_160_2.g1081	228	ID=Fv_160_2.g1081;Description=hypothetical protein FVEG_05684 [Fusarium verticillioides 7600];Gene=FPANT_8762;Ontology_term=cellular anatomical entity,hydrolase activity, acting on glycosyl bonds,inorganic cation transmembrane transporter activity,metal ion
Fv_160_2.g1082	306	ID=Fv_160_2.g1082;Description=hypothetical protein FVER14953_05683 [Fusarium verticillioides];Gene=TUB2;Ontology_term=cytoplasm,microtubule,GTPase activity,carbamoyl-phosphate synthase (glutamine-hydrolyzing) activity,structural constituent of cytoskeleton,GTP binding,metal ion binding,'de novo' pyrimidine nucleobase biosynthetic process,glutamine metabolic process,cytoskeleton organization,microtubule-based process;Ontology_id=GO:0005737,GO:0005874,GO:0003924,GO:0004088,GO:0005200,GO:0005525,GO:0046872,GO:0006207,GO:
Fv_160_2.g1083	498	ID=Fv_160_2.g1083;Description=hypothetical protein FVER53590_05682 [Fusarium verticillioides];Gene=FGADI_2158;Ontology_term=membrane,transmembrane transporter activity,transmembrane
Fv_160_2.g1084	538	ID=Fv_160_2.g1084;Description=hypothetical protein FVER53263_05680 [Fusarium verticillioides];Gene=FGADI_2157;Ontology_term=extracellular region,nucleus,membrane,DNA-binding transcription factor activity, RNA polymerase II-specific,peptidase activity,zinc ion binding,regulation of transcription by RNA polymerase II,proteolysis;Ontology_id=GO:0005576,GO:0005634,GO:0016020,GO:0000981,GO:0008233,GO:0008270,GO:0006357,GO:0006508
Fv_160_2.g1085	429	ID=Fv_160_2.g1085;Description=hypothetical protein FVER53263_05680 [Fusarium verticillioides];Gene=FVER53590_05680;Ontology_term=extracellular region,nucleus,membrane,DNA-binding transcription factor activity, RNA polymerase II-specific,zinc ion binding,dipeptidase activity,regulation of transcription by RNA polymerase II,proteolysis;Ontology_id=GO:0005576,GO:0005634,GO:0016020,GO:0000981,GO:0008270,GO:0016805,GO:0006357,GO:0006508
Fv_160_2.g1086	236	ID=Fv_160_2.g1086;Description=hypothetical protein FVER53590_05679 [Fusarium verticillioides];Gene=FNAPI_12331;Ontology_term=plasma membrane,anatomical structure

Fv_160_2.g1087	532	ID=Fv_160_2.g1087;Description=hexose transporter [Fusarium napiforme];Gene=FGADI_4386;Ontology_term=cytoplasm,membrane,magnesium ion binding,3-isopropylmalate dehydrogenase activity,hydrolase activity, hydrolyzing O-glycosyl compounds,transmembrane transporter activity,NAD binding,carbohydrate metabolic process,leucine biosynthetic process,transmembrane transport;Ontology_id=GO:0005737,GO:0016020,GO:0000287,GO:0003862,GO:0004553,GO:0022857,GO:0051287,GO:0005975,GO
Fv_160_2.g1088	505	ID=Fv_160_2.g1088;Description=hypothetical protein FVER14953_05677 [Fusarium verticillioides];Gene=FFUJ_03545;Ontology_term=membrane,hydrolase activity, hydrolyzing O-glycosyl compounds,transmembrane transporter activity,carbohydrate metabolic process,transmembrane
Fv_160_2.g1089	215	ID=Fv_160_2.g1089;Description=hypothetical protein FVER53590_28831 [Fusarium verticillioides];Gene=CPS1;Ontology_term=astral microtubule,kinetochore,nucleus,microtubule organizing center,minus-end kinesin complex,membrane,microtubule plus-end,microtubule minus-end,cortical microtubule,meiotic spindle pole,mitotic spindle polar microtubule,metallocarboxypeptidase activity,ATP binding,microtubule binding,minus-end-directed microtubule motor activity,ATP hydrolysis activity,metal ion binding,karyogamy involved in conjugation with cellular fusion,nuclear migration involved in conjugation with cellular fusion,microtubule bundle formation,nitrogen compound metabolic process,establishment or maintenance of microtubule cytoskeleton polarity,cytoplasmic microtubule organization,minus-end directed microtubule sliding,plus-end directed microtubule sliding,mitotic spindle midzone assembly,nuclear migration during mitotic telophase,mitotic spindle formation (spindle phase two),meiotic spindle formation (spindle phase two),meiotic centromere clustering,microtubule anchoring at mitotic spindle pole body,mitotic metaphase chromosome
Fv_160_2.g1090	158	ID=Fv_160_2.g1090;Description=hypothetical protein FVER53590_28830 [Fusarium verticillioides]
Fv_160_2.g1091	174	ID=Fv_160_2.g1091;Description=hypothetical protein J7337_002105 [Fusarium musae]
Fv_160_2.g1092	105	ID=Fv_160_2.g1092;Description=hypothetical protein FVER53590_25533 [Fusarium verticillioides]
Fv_160_2.g1093	538	ID=Fv_160_2.g1093;Description=hypothetical protein J7337_002106 [Fusarium musae]
Fv_160_2.g1094	371	ID=Fv_160_2.g1094;Description=hypothetical protein FVER53263_05673 [Fusarium verticillioides];Gene=FNAPI_2001;Ontology_term=glycosyltransferase
Fv_160_2.g1095	468	ID=Fv_160_2.g1095;Description=hypothetical protein FVEG_15698 [Fusarium verticillioides 7600];Gene=FFUJ_03535;Ontology_term=membrane,monooxygenase activity,iron ion binding,methyltransferase activity,oxidoreductase activity, acting on paired donors, with incorporation or reduction of molecular oxygen,hydrolase activity,heme binding,cytokinin biosynthetic process,methylation;Ontology_id=GO:0016020,GO:0004497,GO:0005506,GO:0008168,GO:0016705,GO:0016787,GO:0020037,GO:0009691,GO:0032259;Enzyme_code=EC:1.14,EC:3,EC:2.1.1;Enzyme_name=Acting on paired donors, with incorporation or reduction

Fv_160_2.g1096	469	ID=Fv_160_2.g1096;Description=hypothetical protein FVEG_15697 [Fusarium verticillioides 7600];Gene=FFUJ_03536;Ontology_term=mitochondrial respiratory chain complex IV,monooxygenase activity,alpha-amylase activity,iron ion binding,methyltransferase activity,AMP dimethylallyltransferase activity,oxidoreductase activity, acting on paired donors, with incorporation or reduction of molecular oxygen,heme binding,N6-(Delta2-isopentenyl)-adenosine 5'-monophosphate phosphoribohydrolase activity,carbohydrate metabolic process,mitochondrial electron transport, cytochrome c to oxygen,cytokinin biosynthetic process,methylation;Ontology_id=GO:0005751,GO:0004497,GO:0004556,GO:0005506,GO:0008168,GO:0009824,GO:0016705,GO:0
Fv_160_2.g1097	485	ID=Fv_160_2.g1097;Description=hypothetical protein FVEG_15696 [Fusarium verticillioides 7600];Gene=2691;Ontology_term=cytoplasm,alpha-amylase activity,transferase activity,metal ion binding,carbohydrate metabolic process;Ontology_id=GO:0005737,GO:0004556,GO:0016740,GO:0046872,GO:0005975;Enzyme_code=EC:2,EC:3.2.1.1;Enzyme_nam
Fv_160_2.g1098	495	ID=Fv_160_2.g1098;Description=hypothetical protein FVER14953_05669 [Fusarium
Fv_160_2.g1099	407	ID=Fv_160_2.g1099;Description=hypothetical protein FVEG_15695 [Fusarium verticillioides 7600];Gene=Focb16_v008380;Ontology_term=membrane,oxidoreductase activity,deaminase
Fv_160_2.g1100	389	ID=Fv_160_2.g1100;Description=hypothetical protein FVER53263_05668 [Fusarium verticillioides];Gene=FMEXI_13868;Ontology_term=membrane,oxidoreductase activity,deaminase
Fv_160_2.g1101	425	ID=Fv_160_2.g1101;Description=hypothetical protein FVER53263_05667 [Fusarium verticillioides];Gene=FPRO05_13194;Ontology_term=UDP-glucose 4-epimerase activity,galactose metabolic
Fv_160_2.g1102	655	ID=Fv_160_2.g1102;Description=hypothetical protein FVEG_05666 [Fusarium verticillioides
Fv_160_2.g1103	183	ID=Fv_160_2.g1103;Description=hypothetical protein FVEG_05665 [Fusarium verticillioides 7600]
Fv_160_2.g1104	718	ID=Fv_160_2.g1104;Description=hypothetical protein FVER14953_05664 [Fusarium verticillioides]
Fv_160_2.g1105	69	ID=Fv_160_2.g1105;Description=hypothetical protein FVER14953_21652 [Fusarium verticillioides]
Fv_160_2.g1106	523	ID=Fv_160_2.g1106;Description=hypothetical protein FVER14953_05663 [Fusarium verticillioides];Gene=FMEXI_11328;Ontology_term=membrane,transmembrane transporter activity,transmembrane
Fv_160_2.g1107	146	ID=Fv_160_2.g1107;Description=hypothetical protein FVEG_05662 [Fusarium verticillioides
Fv_160_2.g1108	525	ID=Fv_160_2.g1108;Description=hypothetical protein FVEG_05661 [Fusarium verticillioides 7600];Gene=FOMG_04529;Ontology_term=membrane,transmembrane transporter activity,amino acid transport,transmembrane
Fv_160_2.g1109	790	ID=Fv_160_2.g1109;Description=GIP2 Glc7p-interacting [Fusarium pseudoanthophilum]
Fv_160_2.g1110	619	ID=Fv_160_2.g1110;Description=hypothetical protein FVER14953_05658 [Fusarium verticillioides];Gene=FSUBG_357;Ontology_term=serine-type carboxypeptidase
Fv_160_2.g1111	658	ID=Fv_160_2.g1111;Description=hypothetical protein FVER14953_05657 [Fusarium verticillioides];Gene=infB-0;Ontology_term=translation initiation factor activity,translational initiation;Ontology_id=GO:0003743,GO:0006413

Fv_160_2.g1112	623	ID=Fv_160_2.g1112;Description=hypothetical protein FVEG_05656 [Fusarium verticillioides 7600];Gene=FMUND_9857;Ontology_term=mitochondrial respiratory chain complex IV,mitochondrial matrix,peroxisomal matrix,catalase activity,ATP binding,riboflavin kinase activity,heme binding,manganese ion binding,metalloaminopeptidase activity,response to reactive oxygen species,mitochondrial electron transport, cytochrome c to oxygen,riboflavin biosynthetic process,FMN biosynthetic process,phosphorylation,hydrogen peroxide catabolic process,cellular oxidant detoxification;Ontology_id=GO:0005751,GO:0005759,GO:0005782,GO:0004096,GO:0005524,GO:0008531,GO:0020037,GO:003014
Fv_160_2.g1113	463	ID=Fv_160_2.g1113;Description=xaa-Pro dipeptidase [Fusarium acutatum];Gene=FOZG_04713;Ontology_term=ATP binding,riboflavin kinase activity,manganese ion binding,metalloaminopeptidase activity,riboflavin biosynthetic process,FMN biosynthetic process;Ontology_id=GO:0005524,GO:0008531,GO:0030145,GO:0070006,GO:0009231,GO:0009398;Enzyme_code=EC:3.4.11,EC:2.7
Fv_160_2.g1114	375	ID=Fv_160_2.g1114;Description=hypothetical protein FVEG_05654 [Fusarium verticillioides 7600]
Fv_160_2.g1115	317	ID=Fv_160_2.g1115;Description=hypothetical protein FVER53590_05653 [Fusarium verticillioides];Gene=FNAPI_6214;Ontology_term=peroxisome,acyl-CoA oxidase activity,FAD binding,fatty acid beta-oxidation using
Fv_160_2.g1116	347	ID=Fv_160_2.g1116;Description=hypothetical protein FVEG_05652 [Fusarium verticillioides 7600];Gene=POX4;Ontology_term=peroxisome,acyl-CoA oxidase activity,FAD binding,fatty acid beta-oxidation using acyl-CoA
Fv_160_2.g1117	333	ID=Fv_160_2.g1117;Description=hypothetical protein FVEG_05651 [Fusarium verticillioides 7600];Gene=FFUJ_03513;Ontology_term=nucleus,DNA-binding transcription factor activity, RNA polymerase II-specific,DNA binding,acyl-CoA oxidase activity,zinc ion binding,positive regulation of transcription from RNA polymerase II promoter by a
Fv_160_2.g1118	560	ID=Fv_160_2.g1118;Description=hypothetical protein FVER53590_05650 [Fusarium verticillioides];Gene=FFUJ_03512;Ontology_term=aspartic-type endopeptidase
Fv_160_2.g1119	546	ID=Fv_160_2.g1119;Description=hypothetical protein FVER53590_05648 [Fusarium verticillioides];Gene=Forpe1208_v003304;Ontology_term=cytoplasm,protein serine/threonine kinase activity,ATP binding,hydrolase activity,metal ion binding,protein serine kinase activity,phosphorylation,ribosome biogenesis;Ontology_id=GO:0005737,GO:0004674,GO:0005524,GO:0016787,GO:0046872,GO:0106310,GO:0016310,GO:0042254;E
Fv_160_2.g1120	750	ID=Fv_160_2.g1120;Description=hypothetical protein FVER53590_05647 [Fusarium verticillioides];Gene=FOMG_04543;Ontology_term=nucleus,DNA binding,zinc ion binding,DNA-binding transcription factor activity, RNA polymerase II-specific,regulation of transcription by RNA polymerase
Fv_160_2.g1121	1391	ID=Fv_160_2.g1121;Description=hypothetical protein FVER14953_05646 [Fusarium verticillioides];Gene=FSUBG_367;Ontology_term=RNA binding,RNA-dependent RNA polymerase activity,RNA-templated transcription,regulatory ncRNA-mediated gene

Fv_160_2.g1122	537	ID=Fv_160_2.g1122;Description=protein PNS1 [Fusarium verticillioides 7600];Gene=FNAPI_10973;Ontology_term=plasma membrane,iron ion binding,oxidoreductase activity, acting on paired donors, with incorporation or reduction of molecular oxygen, reduced flavin or flavoprotein as one donor, and incorporation of one atom of oxygen,heme binding,transmembrane transporter activity,transmembrane transport;Ontology_id=GO:0005886,GO:0005506,GO:0016712,GO:0020037,GO:0022857,GO:0055085;Enzyme_code=EC:1.14.14;En
Fv_160_2.g1123	515	ID=Fv_160_2.g1123;Description=AAT family amino acid transporter [Fusarium verticillioides 7600];Gene=BFJ68_g4202;Ontology_term=membrane,amino acid transport,transmembrane
Fv_160_2.g1124	1284	ID=Fv_160_2.g1124;Description=hypothetical protein FVER14953_05643 [Fusarium verticillioides];Gene=FNAPI_10975;Ontology_term=iron ion binding,oxidoreductase activity, acting on paired donors, with incorporation or reduction of molecular oxygen, reduced flavin or flavoprotein as one donor, and incorporation of one atom of oxygen,isomerase activity,heme binding,secondary metabolite biosynthetic
Fv_160_2.g1125	407	ID=Fv_160_2.g1125;Description=hypothetical protein FVER53263_05642 [Fusarium verticillioides];Gene=CNA1;Ontology_term=calmodulin binding,calmodulin-dependent protein phosphatase activity,calcineurin-mediated signaling;Ontology_id=GO:0005516,GO:0033192,GO:0097720;Enzyme_code=EC:3.1.3.16;Enzyme_name=protein-
Fv_160_2.g1126	344	ID=Fv_160_2.g1126;Description=hypothetical protein FVEG_05641 [Fusarium verticillioides 7600];Gene=FNAPI_10977;Ontology_term=iron ion binding,S-adenosylmethionine-dependent methyltransferase activity,oxidoreductase activity, acting on paired donors, with incorporation or reduction of molecular oxygen, reduced flavin or flavoprotein as one donor, and incorporation of one atom of oxygen,heme binding,methylation;Ontology_id=GO:0005506,GO:0008757,GO:0016712,GO:0020037,GO:0032259;Enzyme_code=EC:1.14.14,EC:2.
Fv_160_2.g1127	257	ID=Fv_160_2.g1127;Description=hypothetical protein FVEG_05640 [Fusarium verticillioides
Fv_160_2.g1128	514	ID=Fv_160_2.g1128;Description=hypothetical protein FVER14953_05638 [Fusarium verticillioides];Gene=FGLOB1_1500;Ontology_term=phagophore assembly site membrane,autophagy of
Fv_160_2.g1129	966	ID=Fv_160_2.g1129;Description=hypothetical protein J7337_002145 [Fusarium musae];Gene=FPHYL_681;Ontology_term=DNA binding,calcium-dependent cysteine-type endopeptidase
Fv_160_2.g1130	725	ID=Fv_160_2.g1130;Description=high-affinity nickel-transporter [Fusarium denticulatum];Gene=FTJAE_2574;Ontology_term=cytoplasm,plasma membrane,calmodulin-dependent protein kinase activity,binding,nickel cation transmembrane transporter activity,signal transduction,cellular response to oxidative stress,nickel cation transmembrane
Fv_160_2.g1131	120	ID=Fv_160_2.g1131;Description=hypothetical protein FVEG_05633 [Fusarium verticillioides 7600];Gene=FVER53590_05632;Ontology_term=oxidoreductase activity,cellular biosynthetic
Fv_160_2.g1132	266	ID=Fv_160_2.g1132;Description=hypothetical protein FVER14953_05632 [Fusarium verticillioides];Gene=FTJAE_2572;Ontology_term=oxidoreductase activity,cellular biosynthetic

Fv_160_2.g1133	611	ID=Fv_160_2.g1133;Description=dihydroxy-acid dehydratase [Fusarium verticillioides 7600];Gene=ilvD1;Ontology_term=hydro-lyase activity,carboxylic acid metabolic process,primary metabolic
Fv_160_2.g1134	718	ID=Fv_160_2.g1134;Description=hypothetical protein FVEG_15686 [Fusarium verticillioides 7600];Gene=FRV6_07091;Ontology_term=nucleus,DNA binding,zinc ion binding,DNA-binding transcription factor activity, RNA
Fv_160_2.g1135	356	ID=Fv_160_2.g1135;Description=hypothetical protein FVER14953_05630 [Fusarium verticillioides];Gene=yjmC;Ontology_term=oxidoreductase
Fv_160_2.g1136	168	ID=Fv_160_2.g1136;Description=hypothetical protein FVER14953_05629 [Fusarium verticillioides];Gene=F25303_3069;Ontology_term=acyl-CoA hydrolase
Fv_160_2.g1137	171	ID=Fv_160_2.g1137;Description=hypothetical protein FVER14953_05628 [Fusarium verticillioides]
Fv_160_2.g1138	277	ID=Fv_160_2.g1138;Description=hypothetical protein FVER14953_05627 [Fusarium verticillioides]
Fv_160_2.g1139	298	ID=Fv_160_2.g1139;Description=hypothetical protein FVER14953_05626 [Fusarium verticillioides]
Fv_160_2.g1140	293	ID=Fv_160_2.g1140;Description=hypothetical protein FVEG_05625 [Fusarium verticillioides
Fv_160_2.g1141	318	ID=Fv_160_2.g1141;Description=hypothetical protein FVEG_05624 [Fusarium verticillioides 7600]
Fv_160_2.g1142	287	ID=Fv_160_2.g1142;Description=hypothetical protein FVER14953_05623 [Fusarium verticillioides]
Fv_160_2.g1143	576	ID=Fv_160_2.g1143;Description=hypothetical protein FVER14953_05622 [Fusarium verticillioides];Gene=FNAPI_10994;Ontology_term=fungal-type vacuole lumen,extracellular space,anthranilate synthase complex,anthranilate synthase activity,serine-type endopeptidase activity,tryptophan biosynthetic process,pexophagy,proteolysis,glutamine metabolic process,protein catabolic process in the vacuole,sporulation resulting in formation of a cellular
Fv_160_2.g1144	517	ID=Fv_160_2.g1144;Description=hypothetical protein FVER53590_05621 [Fusarium
Fv_160_2.g1145	289	ID=Fv_160_2.g1145;Description=hypothetical protein FVEG_05620 [Fusarium verticillioides 7600]
Fv_160_2.g1146	508	ID=Fv_160_2.g1146;Description=aminopeptidase Y [Fusarium verticillioides 7600];Gene=FNAPI_10997;Ontology_term=aminopeptidase activity,metalloexopeptidase activity,metal ion
Fv_160_2.g1147	451	ID=Fv_160_2.g1147;Description=hypothetical protein J7337_002162 [Fusarium musae];Gene=FOC1_g10009380;Ontology_term=NADPH dehydrogenase activity,FMN binding,histone H4K20 methyltransferase activity,NADP binding,chromatin remodeling,methylation;Ontology_id=GO:0003959,GO:0010181,GO:0042799,GO:0050661,GO:0006338,GO:0032259;Enzyme_code
Fv_160_2.g1148	668	ID=Fv_160_2.g1148;Description=hypothetical protein FVER14953_05617 [Fusarium verticillioides];Gene=FOC1_g10009381;Ontology_term=nucleus,histone H4K20 trimethyltransferase activity,chromatin remodeling,methylation;Ontology_id=GO:0005634,GO:0140943,GO:0006338,GO:0032259;Enzyme_code=EC:2.1.1.372;Enzyme_na
Fv_160_2.g1149	131	ID=Fv_160_2.g1149;Description=50S ribosomal protein L14 [Fusarium verticillioides 7600];Gene=FHETE_11164;Ontology_term=mitochondrial envelope,mitochondrial large ribosomal subunit,structural constituent of

Fv_160_2.g1150	186	ID=Fv_160_2.g1150;Description=DNA-directed RNA polymerase II subunit RPB9 [Fusarium verticillioides 7600];Gene=FGADI_7986;Ontology_term=RNA polymerase II, core complex,nucleic acid binding,DNA-directed 5'-3' RNA polymerase activity,zinc ion binding,DNA-templated
Fv_160_2.g1151	177	ID=Fv_160_2.g1151;Description=peptidyl-prolyl cis-trans isomerase ssp-1 [Fusarium oxysporum f. sp. lycopersici 4287];Gene=ssp-1;Ontology_term=peptidyl-prolyl cis-trans isomerase activity,regulation of nitrogen compound metabolic process,regulation of primary metabolic
Fv_160_2.g1152	621	ID=Fv_160_2.g1152;Description=hypothetical protein FVER53590_05613 [Fusarium
Fv_160_2.g1153	535	ID=Fv_160_2.g1153;Description=hypothetical protein FVER14953_05612 [Fusarium verticillioides]
Fv_160_2.g1154	674	ID=Fv_160_2.g1154;Description=hypothetical protein FVEG_15683 [Fusarium verticillioides 7600];Gene=FOZG_04757;Ontology_term=nucleus,DNA-binding transcription factor activity, RNA polymerase II-specific,zinc ion
Fv_160_2.g1155	432	ID=Fv_160_2.g1155;Description=hypothetical protein FVER14953_05609 [Fusarium verticillioides];Gene=FNAPI_11006;Ontology_term=oxidoreductase activity,flavin adenine dinucleotide
Fv_160_2.g1156	474	ID=Fv_160_2.g1156;Description=hypothetical protein FVER53590_05608 [Fusarium
Fv_160_2.g1157	515	ID=Fv_160_2.g1157;Description=hypothetical protein FVER14953_05607 [Fusarium verticillioides];Gene=FNAPI_11008;Ontology_term=membrane,N,N-dimethylaniline monooxygenase activity,flavin adenine dinucleotide binding,NADP
Fv_160_2.g1158	214	ID=Fv_160_2.g1158;Description=hypothetical protein FVEG_05606 [Fusarium verticillioides 7600];Gene=FocTR4_00005854;Ontology_term=membrane,monooxygenase
Fv_160_2.g1159	415	ID=Fv_160_2.g1159;Description=hypothetical protein FVER53590_05605 [Fusarium
Fv_160_2.g1160	718	ID=Fv_160_2.g1160;Description=palmitoyltransferase AKR1 [Fusarium verticillioides 7600];Gene=FVEG_05604;Ontology_term=early endosome membrane,protein-cysteine S-palmitoyltransferase
Fv_160_2.g1161	284	ID=Fv_160_2.g1161;Description=hypothetical protein FVEG_05603 [Fusarium verticillioides 7600];Gene=FOYG_02429;Ontology_term=endoplasmic reticulum membrane,GTP binding;Ontology_id=GO:0005789,GO:0005525
Fv_160_2.g1162	517	ID=Fv_160_2.g1162;Description=cystathionine beta-synthase [Fusarium verticillioides 7600];Gene=LW93_1609;Ontology_term=cytoplasm,cystathionine beta-synthase activity,aspartic-type endopeptidase activity,proteolysis,cysteine biosynthetic process from serine,traversing start control point of mitotic cell cycle,cysteine biosynthetic process via cystathionine,transsulfuration,hydrogen sulfide biosynthetic process;Ontology_id=GO:0005737,GO:0004122,GO:0004190,GO:0006508,GO:0006535,GO:0007089,GO:0019343,GO:0019346,GO:
Fv_160_2.g1163	491	ID=Fv_160_2.g1163;Description=hypothetical protein FVEG_05601 [Fusarium verticillioides 7600];Gene=FSUBG_411;Ontology_term=aminopeptidase activity,metalloexopeptidase activity,metal ion
Fv_160_2.g1164	294	ID=Fv_160_2.g1164;Description=hypothetical protein FVER14953_20928 [Fusarium verticillioides]
Fv_160_2.g1165	562	ID=Fv_160_2.g1165;Description=hypothetical protein FVEG_15679 [Fusarium verticillioides 7600]
Fv_160_2.g1166	432	ID=Fv_160_2.g1166;Description=hypothetical protein FVEG_05599 [Fusarium verticillioides

Fv_160_2.g1167	521	ID=Fv_160_2.g1167;Description=hypothetical protein FVER53263_05597 [Fusarium verticillioides];Gene=P62777;Ontology_term=nucleosome,nucleus,membrane,DNA-binding transcription factor activity, RNA polymerase II-specific,DNA binding,cysteine-type endopeptidase activity,zinc ion binding,transmembrane transporter activity,structural constituent of chromatin,protein heterodimerization activity,rDNA heterochromatin formation,regulation of transcription by RNA polymerase II,proteolysis,transmembrane
Fv_160_2.g1168	648	ID=Fv_160_2.g1168;Description=hypothetical protein FVER53590_29030 [Fusarium verticillioides];Gene=BFJ70_g8691;Ontology_term=nucleus,DNA-binding transcription factor activity, RNA polymerase II-specific,zinc
Fv_160_2.g1169	663	ID=Fv_160_2.g1169;Description=hypothetical protein FVER14953_05597 [Fusarium verticillioides];Gene=FPANT_3405;Ontology_term=nucleus,plasma membrane,DNA-binding transcription factor activity, RNA polymerase II-specific,chitin synthase activity,zinc ion binding,transmembrane transporter activity,regulation of transcription by RNA polymerase II,conidium formation,transmembrane transport;Ontology_id=GO:0005634,GO:0005886,GO:0000981,GO:0004100,GO:0008270,GO:0022857,GO:0006357,GO:0048315,GO
Fv_160_2.g1170	475	ID=Fv_160_2.g1170;Description=hypothetical protein FVER14953_05596 [Fusarium verticillioides];Gene=FVER53590_05596;Ontology_term=chitin binding,hydrolase activity, acting on carbon-nitrogen (but not peptide) bonds,carbohydrate metabolic
Fv_160_2.g1171	449	ID=Fv_160_2.g1171;Description=hypothetical protein FVER14953_05595 [Fusarium verticillioides];Gene=FVER53590_05595;Ontology_term=oxidoreductase activity, acting on the CH-OH group of donors, NAD or NADP as acceptor,oxidoreductase activity, acting on the CH-CH group of donors, NAD or NADP as acceptor,NAD binding,polysaccharide biosynthetic
Fv_160_2.g1172	193	ID=Fv_160_2.g1172;Description=hypothetical protein FVEG_05594 [Fusarium verticillioides 7600]
Fv_160_2.g1173	790	ID=Fv_160_2.g1173;Description=hypothetical protein J7337_002189 [Fusarium musae];Gene=FMAN_05009;Ontology_term=aldehyde-lyase activity,carbohydrate metabolic
Fv_160_2.g1174	738	ID=Fv_160_2.g1174;Description=hypothetical protein FVER53590_05591 [Fusarium verticillioides];Gene=F25303_9678;Ontology_term=catalase activity,heme binding,metal ion binding,response to oxidative stress,hydrogen peroxide catabolic process,cellular oxidant
Fv_160_2.g1175	335	ID=Fv_160_2.g1175;Description=hypothetical protein FVEG_05590 [Fusarium verticillioides 7600];Gene=ribBA;Ontology_term=ATP
Fv_160_2.g1176	453	ID=Fv_160_2.g1176;Description=hypothetical protein FVER53590_05589 [Fusarium verticillioides];Gene=FNAPI_760;Ontology_term=oxidoreductase activity,FAD
Fv_160_2.g1177	583	ID=Fv_160_2.g1177;Description=hypothetical protein FVER53263_05588 [Fusarium verticillioides];Gene=FANTH_9078;Ontology_term=nucleus,DNA binding,zinc ion binding,DNA-binding transcription factor activity, RNA polymerase II-specific,regulation of transcription by RNA polymerase
Fv_160_2.g1178	283	ID=Fv_160_2.g1178;Description=hypothetical protein FVEG_05587 [Fusarium verticillioides 7600];Gene=CEP53_001546

Fv_160_2.g1179	147	ID=Fv_160_2.g1179;Description=hypothetical protein FVEG_05586 [Fusarium verticillioides 7600];Gene=FACUT_2102;Ontology_term=carbon-sulfur lyase activity,metal ion
Fv_160_2.g1180	515	ID=Fv_160_2.g1180;Description=hypothetical protein J7337_002196 [Fusarium musae];Gene=FPCIR_2797;Ontology_term=membrane,monooxygenase activity,iron ion binding,oxidoreductase activity, acting on paired donors, with incorporation or reduction of molecular oxygen,heme binding;Ontology_id=GO:0016020,GO:0004497,GO:0005506,GO:0016705,GO:0020037;Enzyme_code=EC:1.14;Enzyme_name=Actin
Fv_160_2.g1181	321	ID=Fv_160_2.g1181;Description=hypothetical protein FVER14953_05583 [Fusarium verticillioides];Gene=FNAPI_761;Ontology_term=mitochondrial outer membrane,cytochrome-b5 reductase activity, acting on
Fv_160_2.g1182	596	ID=Fv_160_2.g1182;Description=hypothetical protein FVEG_05582 [Fusarium verticillioides 7600];Gene=FFUJ_03448;Ontology_term=magnesium ion binding,pyruvate decarboxylase activity,ATP hydrolysis activity,thiamine pyrophosphate binding,DNA replication initiation,cell division;Ontology_id=GO:0000287,GO:0004737,GO:0016887,GO:0030976,GO:0006270,GO:0051301;Enzyme_code=EC:4.1.1.1,EC:3.
Fv_160_2.g1183	339	ID=Fv_160_2.g1183;Description=hypothetical protein FVER53590_05581 [Fusarium
Fv_160_2.g1184	454	ID=Fv_160_2.g1184;Description=hypothetical protein FVER14953_05580 [Fusarium verticillioides];Gene=FVER53590_05580;Ontology_term=cytoplasm,aminoacyl-tRNA editing activity,initiator methionyl aminopeptidase activity,leucine-tRNA ligase activity,ATP binding,metal ion binding,metalloaminopeptidase activity,leucyl-tRNA aminoacylation,proteolysis,aminoacyl-tRNA metabolism involved in translational fidelity;Ontology_id=GO:0005737,GO:0002161,GO:0004239,GO:0004823,GO:0005524,GO:0046872,GO:0070006,GO:0006429,GO:0
Fv_160_2.g1185	344	ID=Fv_160_2.g1185;Description=hypothetical protein FVEG_05579 [Fusarium verticillioides 7600];Gene=recG
Fv_160_2.g1186	850	ID=Fv_160_2.g1186;Description=hypothetical protein FVER14953_05578 [Fusarium verticillioides];Gene=FDENT_10115;Ontology_term=membrane,oligopeptide transmembrane transporter activity,protein
Fv_160_2.g1187	183	ID=Fv_160_2.g1187;Description=hypothetical protein FVEG_05577 [Fusarium verticillioides 7600];Gene=FCIRC_4801;Ontology_term=membrane,kinase
Fv_160_2.g1188	618	ID=Fv_160_2.g1188;Description=hypothetical protein FVER53590_05576 [Fusarium verticillioides];Gene=FPANT_3753;Ontology_term=membrane,ferric-chelate reductase activity,iron ion
Fv_160_2.g1189	232	ID=Fv_160_2.g1189;Description=hypothetical protein FVEG_05574 [Fusarium verticillioides
Fv_160_2.g1190	1462	ID=Fv_160_2.g1190;Description=hypothetical protein FVEG_05573 [Fusarium verticillioides 7600];Gene=FNAPI_771;Ontology_term=membrane,ATP binding,ATP hydrolysis activity,ABC-type transporter activity,transmembrane transport;Ontology_id=GO:0016020,GO:0005524,GO:0016887,GO:0140359,GO:0055085;Enzyme_code=EC:7.2.2,EC:3.6.1.15;Enzym
Fv_160_2.g1191	323	ID=Fv_160_2.g1191;Description=hypothetical protein FVEG_05572 [Fusarium verticillioides 7600];Gene=FNAPI_772;Ontology_term=mitochondrial inner membrane,transmembrane

Fv_160_2.g1192	373	ID=Fv_160_2.g1192;Description=PTK9 protein tyrosine kinase 9 [Fusarium oxysporum f. sp. pisi HDV247];Gene=FOMG_04619;Ontology_term=cytoskeleton,actin binding,kinase activity,phosphorylation,negative regulation of actin filament
Fv_160_2.g1193	313	ID=Fv_160_2.g1193;Description=hypothetical protein FVER53590_05569 [Fusarium
Fv_160_2.g1194	159	ID=Fv_160_2.g1194;Description=hypothetical protein FVER14953_05568 [Fusarium verticillioides];Gene=FFC1_07872;Ontology_term=mediator complex,transcription coregulator activity,regulation of transcription by
Fv_160_2.g1195	325	ID=Fv_160_2.g1195;Description=hypothetical protein FPCIR_2812 [Fusarium
Fv_160_2.g1196	510	ID=Fv_160_2.g1196;Description=hypothetical protein FVEG_05565 [Fusarium verticillioides 7600];Gene=glmU;Ontology_term=membrane,chloride channel activity,chloride transmembrane
Fv_160_2.g1197	633	ID=Fv_160_2.g1197;Description=arginyl-tRNA synthetase [Fusarium verticillioides 7600];Gene=FOZG_04801;Ontology_term=P-body,nucleus,CCR4-NOT core complex,arginine-tRNA ligase activity,protein binding,ATP binding,nuclear-transcribed mRNA poly(A) tail shortening,deadenylation-dependent decapping of nuclear-transcribed mRNA,arginyl-tRNA aminoacylation,protein ubiquitination,positive regulation of transcription elongation by RNA polymerase II;Ontology_id=GO:0000932,GO:0005634,GO:0030015,GO:0004814,GO:0005515,GO:0005524,GO:0000289,GO:0000290,GO:000642
Fv_160_2.g1198	141	ID=Fv_160_2.g1198;Description=hypothetical protein FVEG_05563 [Fusarium verticillioides 7600];Gene=3494;Ontology_term=mitochondrial matrix,glyceraldehyde-3-phosphate dehydrogenase (NAD+) (phosphorylating) activity,NADP binding,NAD binding,glucose metabolic process,glycolytic process,protein maturation by [4Fe-4S] cluster transfer;Ontology_id=GO:0005759,GO:0004365,GO:0050661,GO:0051287,GO:0006006,GO:0006096,GO:0106035;Enzyme_code=EC
Fv_160_2.g1199	237	ID=Fv_160_2.g1199;Description=hypothetical protein FVEG_15671 [Fusarium verticillioides 7600]
Fv_160_2.g1200	378	ID=Fv_160_2.g1200;Description=arsenical-resistance protein [Fusarium verticillioides 7600];Gene=FOBC_04812;Ontology_term=plasma membrane,inorganic anion transmembrane transporter activity,antiporter
Fv_160_2.g1201	882	ID=Fv_160_2.g1201;Description=hypothetical protein FVER53590_05561 [Fusarium verticillioides];Gene=FMAN_04987;Ontology_term=membrane,serine-type endopeptidase activity,metallopeptidase activity,proteolysis;Ontology_id=GO:0016020,GO:0004252,GO:0008237,GO:0006508;Enzyme_code=EC:3.4.21;Enzyme_name=Actin
Fv_160_2.g1202	1196	ID=Fv_160_2.g1202;Description=hypothetical protein FVEG_05560 [Fusarium verticillioides 7600]
Fv_160_2.g1203	205	ID=Fv_160_2.g1203;Description=hypothetical protein J7337_002219 [Fusarium musae]
Fv_160_2.g1204	152	ID=Fv_160_2.g1204;Description=hypothetical protein FVEG_05558 [Fusarium verticillioides 7600];Gene=2596
Fv_160_2.g1205	530	ID=Fv_160_2.g1205;Description=hypothetical protein FVEG_05557 [Fusarium verticillioides 7600];Gene=FPANT_3739;Ontology_term=membrane,transmembrane transporter activity,transmembrane
Fv_160_2.g1206	2271	ID=Fv_160_2.g1206;Description=hypothetical protein FVEG_05556 [Fusarium verticillioides 7600];Gene=FOC1_g10009440;Ontology_term=acetyl-CoA carboxylase activity,biotin carboxylase activity,ATP binding,metal ion binding,fatty acid biosynthetic process,malonyl-CoA biosynthetic process;Ontology_id=GO:0003989,GO:0004075,GO:0005524,GO:0046872,GO:0006633,GO:2001295;Enzyme_code=EC:6.4.1.2,EC:6.

Fv_160_2.g1207	573	ID=Fv_160_2.g1207;Description=nuclear distribution protein nudE like 1 [Fusarium verticillioides 7600];Gene=FTJAE_11220;Ontology_term=cytoplasm,cytoskeleton,microtubule
Fv_160_2.g1208	1666	ID=Fv_160_2.g1208;Description=hypothetical protein FVER53263_05554 [Fusarium verticillioides];Gene=FTJAE_11221;Ontology_term=membrane,zinc ion binding;Ontology_id=GO:0016020,GO:0008270
Fv_160_2.g1209	1218	ID=Fv_160_2.g1209;Description=hypothetical protein FVER14953_05553 [Fusarium
Fv_160_2.g1210	252	ID=Fv_160_2.g1210;Description=hypothetical protein FVER14953_05552 [Fusarium verticillioides];Gene=2590;Ontology_term=extracellular region,membrane,serine-type peptidase
Fv_160_2.g1211	752	ID=Fv_160_2.g1211;Description=F-box and WD-40 domain-containing protein MET30 [Fusarium verticillioides 7600];Gene=BFJ71_g3208;Ontology_term=ligase activity;Ontology_id=GO:0016874;Enzyme_code=EC:6;Enzyme_name=Ligases
Fv_160_2.g1212	101	ID=Fv_160_2.g1212;Description=hypothetical protein LB504_007883 [Fusarium proliferatum]
Fv_160_2.g1213	604	ID=Fv_160_2.g1213;Description=hypothetical protein FVER53263_05547 [Fusarium verticillioides];Gene=HZS61_010257;Ontology_term=nucleus,ribosome,RNA binding,structural constituent of ribosome,pseudouridine synthase activity,translation,tRNA pseudouridine synthesis,snRNA pseudouridine synthesis,mRNA pseudouridine
Fv_160_2.g1214	203	ID=Fv_160_2.g1214;Description=40S ribosomal protein S7 [Fusarium verticillioides 7600];Gene=FNAPI_795;Ontology_term=ribosome,ribonucleoprotein complex,structural constituent of
Fv_160_2.g1215	551	ID=Fv_160_2.g1215;Description=hypothetical protein FVER53590_05545 [Fusarium verticillioides];Gene=FTJAE_6024;Ontology_term=microtubule organizing center,dynactin complex,membrane,sarcomere,monooxygenase activity,iron ion binding,oxidoreductase activity, acting on paired donors, with incorporation or reduction of molecular oxygen,heme
Fv_160_2.g1216	517	ID=Fv_160_2.g1216;Description=hypothetical protein J7337_002233 [Fusarium musae];Gene=FPCIR_2833;Ontology_term=microtubule organizing center,dynactin complex,membrane,sarcomere,monooxygenase activity,iron ion binding,oxidoreductase activity, acting on paired donors, with incorporation or reduction of molecular oxygen,heme binding;Ontology_id=GO:0005815,GO:0005869,GO:0016020,GO:0030017,GO:0004497,GO:0005506,GO:0016705,GO:0020037;Enzy
Fv_160_2.g1217	413	ID=Fv_160_2.g1217;Description=hypothetical protein FVEG_05543 [Fusarium verticillioides 7600];Gene=FMUND_5190;Ontology_term=microtubule organizing center,dynactin complex,membrane,sarcomere,monooxygenase activity,iron ion binding,O-acyltransferase activity,oxidoreductase activity, acting on paired donors, with incorporation or reduction of molecular oxygen,heme binding,lipid metabolic process;Ontology_id=GO:0005815,GO:0005869,GO:0016020,GO:0030017,GO:0004497,GO:0005506,GO:0008374,GO:0016705,GO:
Fv_160_2.g1218	525	ID=Fv_160_2.g1218;Description=hypothetical protein FVEG_05542 [Fusarium verticillioides 7600];Gene=FMUND_5191;Ontology_term=membrane,monooxygenase activity,iron ion binding,oxidoreductase activity, acting on paired donors, with incorporation or reduction of molecular oxygen,heme binding;Ontology_id=GO:0016020,GO:0004497,GO:0005506,GO:0016705,GO:0020037;Enzyme_code=EC:1.14;Enzyme_name=Actin

Fv_160_2.g1219	333	ID=Fv_160_2.g1219;Description=hypothetical protein FVEG_05541 [Fusarium verticillioides 7600];Gene=FPCIR_2836;Ontology_term=acyl-CoA hydrolase activity,acyl-CoA metabolic
Fv_160_2.g1220	585	ID=Fv_160_2.g1220;Description=hypothetical protein FVER53590_05540 [Fusarium verticillioides];Gene=FPCIR_2837;Ontology_term=hydrolase
Fv_160_2.g1221	239	ID=Fv_160_2.g1221;Description=hypothetical protein FVEG_05539 [Fusarium verticillioides 7600];Gene=FVEG_05539;Ontology_term=S-adenosylmethionine-dependent methyltransferase
Fv_160_2.g1222	526	ID=Fv_160_2.g1222;Description=hypothetical protein FVER14953_05538 [Fusarium verticillioides];Gene=FDENT_8792;Ontology_term=membrane,monooxygenase activity,iron ion binding,oxidoreductase activity, acting on paired donors, with incorporation or reduction of molecular oxygen,heme binding;Ontology_id=GO:0016020,GO:0004497,GO:0005506,GO:0016705,GO:0020037;Enzyme_code=EC:1.14;Enzyme_name=Actin
Fv_160_2.g1223	2501	ID=Fv_160_2.g1223;Description=hypothetical protein FVEG_05537 [Fusarium verticillioides 7600];Gene=FPANT_8593;Ontology_term=3-oxoacyl-[acyl-carrier-protein] synthase activity,S-adenosylmethionine-dependent methyltransferase activity,oxidoreductase activity,fatty acid biosynthetic process,secondary metabolite biosynthetic process;Ontology_id=GO:0004315,GO:0008757,GO:0016491,GO:0006633,GO:0044550;Enzyme_code=EC:2.3.1.41,EC:1,EC:2.1.1;Enz
Fv_160_2.g1224	798	ID=Fv_160_2.g1224;Description=hypothetical protein FVER53590_05536 [Fusarium verticillioides];Gene=2584;Ontology_term=nucleus,DNA binding, bending,single fertilization,positive regulation of mating-type
Fv_160_2.g1225	247	ID=Fv_160_2.g1225;Description=hypothetical protein FVEG_05535 [Fusarium verticillioides
Fv_160_2.g1226	344	ID=Fv_160_2.g1226;Description=hypothetical protein FVEG_05534 [Fusarium verticillioides 7600]
Fv_160_2.g1227	431	ID=Fv_160_2.g1227;Description=hypothetical protein FVEG_05533 [Fusarium verticillioides 7600]
Fv_160_2.g1228	555	ID=Fv_160_2.g1228;Description=hypothetical protein FVER14953_05532 [Fusarium verticillioides];Gene=FMAN_04968;Ontology_term=nucleus,DNA-binding transcription factor activity, RNA polymerase II-
Fv_160_2.g1229	357	ID=Fv_160_2.g1229;Description=hypothetical protein FVER14953_05531 [Fusarium verticillioides];Gene=F25303_2057;Ontology_term=mitochondrial intermembrane space,respirasome,peroxidase activity,electron transfer activity,heme binding,metal ion binding,electron transport chain,cellular response to oxidative stress,cellular oxidant detoxification;Ontology_id=GO:0005758,GO:0070469,GO:0004601,GO:0009055,GO:0020037,GO:0046872,GO:0022900,GO:003459
Fv_160_2.g1230	305	ID=Fv_160_2.g1230;Description=hypothetical protein FVEG_05530 [Fusarium verticillioides 7600];Gene=3471;Ontology_term=catalase activity,heme binding,response to oxidative stress,cellular oxidant
Fv_160_2.g1231	412	ID=Fv_160_2.g1231;Description=Catalase [Fusarium oxysporum f. sp. matthiolae];Gene=HZS61_010247;Ontology_term=catalase activity,heme binding,metal ion binding,response to oxidative stress,hydrogen peroxide catabolic process,cellular oxidant detoxification;Ontology_id=GO:0004096,GO:0020037,GO:0046872,GO:0006979,GO:0042744,GO:0098869;Enzyme_code=EC:1.11.1.
Fv_160_2.g1232	317	ID=Fv_160_2.g1232;Description=hypothetical protein FVER14953_05528 [Fusarium verticillioides]
Fv_160_2.g1233	181	ID=Fv_160_2.g1233;Description=hypothetical protein FVEG_05527 [Fusarium verticillioides 7600]

Fv_160_2.g1234	353	ID=Fv_160_2.g1234;Description=hypothetical protein FVER53263_05526 [Fusarium verticillioides];Gene=FFUJ_03404;Ontology_term=oxidoreductase
Fv_160_2.g1235	499	ID=Fv_160_2.g1235;Description=hypothetical protein FVEG_05525 [Fusarium verticillioides 7600];Gene=FTJAE_6043;Ontology_term=mitochondrial inner membrane,mitochondrial ribosome,LPPG:FO 2-phospho-L-lactate transferase activity,translation regulator activity,positive regulation of mitochondrial translation;Ontology_id=GO:0005743,GO:0005761,GO:0043743,GO:0045182,GO:0070131;Enzyme_code=EC:2.7.1,EC:2.7.8.28;Enzy
Fv_160_2.g1236	91	ID=Fv_160_2.g1236;Description=hypothetical protein FVEG_05524 [Fusarium verticillioides
Fv_160_2.g1237	401	ID=Fv_160_2.g1237;Description=hypothetical protein FVEG_05522 [Fusarium verticillioides 7600]
Fv_160_2.g1238	893	ID=Fv_160_2.g1238;Description=hypothetical protein J7337_002255 [Fusarium musae];Gene=FMAN_04956;Ontology_term=beta-glucosidase activity,scopolin beta-glucosidase activity,cellulose catabolic
Fv_160_2.g1239	485	ID=Fv_160_2.g1239;Description=Delta(14)-sterol reductase [Fusarium musae];Gene=FocTR4_00005777;Ontology_term=membrane,oxidoreductase activity, acting on the CH-CH group of donors, NAD or NADP as acceptor,sterol biosynthetic
Fv_160_2.g1240	313	ID=Fv_160_2.g1240;Description=hypothetical protein FVEG_05519 [Fusarium verticillioides 7600];Gene=FocTR4_00005776;Ontology_term=proton-transporting two-sector ATPase complex, catalytic domain,proton-transporting ATPase activity, rotational mechanism,proton transmembrane
Fv_160_2.g1241	377	ID=Fv_160_2.g1241;Description=hypothetical protein FVER53590_05518 [Fusarium verticillioides]
Fv_160_2.g1242	96	ID=Fv_160_2.g1242;Description=hypothetical protein FVEG_15665 [Fusarium verticillioides 7600];Gene=FCIRC_429
Fv_160_2.g1243	243	ID=Fv_160_2.g1243;Description=hypothetical protein J7337_002259 [Fusarium musae]
Fv_160_2.g1244	90	ID=Fv_160_2.g1244;Description=5 5 P-1 P-4-tetraphosphate phosphorylase 2 [Fusarium pseudoanthophilum];Gene=FPANT_12302;Ontology_term=ATP adenylyltransferase activity,ATP binding,nucleotide metabolic
Fv_160_2.g1245	342	ID=Fv_160_2.g1245;Description=hypothetical protein FVER53590_05517 [Fusarium verticillioides];Gene=FVEG_05517;Ontology_term=ATP adenylyltransferase activity,ATP binding,nucleotide metabolic
Fv_160_2.g1246	83	ID=Fv_160_2.g1246;Description=hypothetical protein FVEG_15663 [Fusarium verticillioides 7600]
Fv_160_2.g1247	238	ID=Fv_160_2.g1247;Description=hypothetical protein FVER14953_05516 [Fusarium verticillioides];Gene=FVEG_05516;Ontology_term=acyltransferase activity, transferring groups other than amino-acyl
Fv_160_2.g1248	438	ID=Fv_160_2.g1248;Description=hypothetical protein FVEG_15662 [Fusarium verticillioides 7600];Gene=FNYG_12496;Ontology_term=proton-transporting V-type ATPase, V1 domain,magnesium ion binding,RNA-directed DNA polymerase activity,pyruvate decarboxylase activity,thiamine pyrophosphate binding,proton-transporting ATPase activity, rotational mechanism,RNA-templated DNA biosynthetic process,proton transmembrane transport;Ontology_id=GO:0033180,GO:0000287,GO:0003964,GO:0004737,GO:0030976,GO:0046961,GO:0006278,GO:1902600;En
Fv_160_2.g1249	671	ID=Fv_160_2.g1249;Description=hypothetical protein FVEG_05515 [Fusarium verticillioides 7600];Gene=FACUT_5878;Ontology_term=ATP binding,ATP hydrolysis

Fv_160_2.g1250	127	ID=Fv_160_2.g1250;Description=hypothetical protein FVEG_05514 [Fusarium verticillioides 7600];Gene=FACUT_5879;Ontology_term=kinase
Fv_160_2.g1251	626	ID=Fv_160_2.g1251;Description=hypothetical protein FVER53590_05513 [Fusarium verticillioides]
Fv_160_2.g1252	435	ID=Fv_160_2.g1252;Description=beta-tubulin 2 [Fusarium tjaetaba];Gene=FMUND_13944;Ontology_term=microtubule,GTPase activity,structural constituent of cytoskeleton,GTP binding,metal ion binding,cytoskeleton organization,microtubule-based process;Ontology_id=GO:0005874,GO:0003924,GO:0005200,GO:0005525,GO:0046872,GO:0007010,GO:0007017;Enzyme_code=EC :3.6.1.15;Enzyme_name=nucleoside-triphosphate phosphatase
Fv_160_2.g1253	377	ID=Fv_160_2.g1253;Description=hypothetical protein FVEG_05511 [Fusarium verticillioides 7600];Gene=FSUBG_488;Ontology_term=zinc ion binding,oxidoreductase
Fv_160_2.g1254	177	ID=Fv_160_2.g1254;Description=hypothetical protein FVEG_15661 [Fusarium verticillioides 7600];Gene=O3G_MSEX001397;Ontology_term=zinc ion binding,oxidoreductase activity,structural constituent of
Fv_160_2.g1255	450	ID=Fv_160_2.g1255;Description=hypothetical protein FVER53590_05510 [Fusarium verticillioides];Gene=FPRO_05165;Ontology_term=extracellular region,dipeptidase
Fv_160_2.g1256	326	ID=Fv_160_2.g1256;Description=hypothetical protein FVEG_05509 [Fusarium verticillioides 7600];Gene=SCS3;Ontology_term=endoplasmic reticulum membrane,acyl-coenzyme A diphosphatase activity,phospholipid biosynthetic process,lipid droplet
Fv_160_2.g1257	812	ID=Fv_160_2.g1257;Description=hypothetical protein FVER14953_05507 [Fusarium verticillioides];Gene=BFJ72_g6065;Ontology_term=membrane,polygalacturonase
Fv_160_2.g1258	726	ID=Fv_160_2.g1258;Description=hypothetical protein FVEG_05506 [Fusarium verticillioides 7600]
Fv_160_2.g1259	166	ID=Fv_160_2.g1259;Description=hypothetical protein FVEG_05505 [Fusarium verticillioides 7600];Gene=FVEG_05505;Ontology_term=nucleus,cytoplasm,membrane,pigment
Fv_160_2.g1260	360	ID=Fv_160_2.g1260;Description=Uncharacterized protein Y057_7139 [Fusarium fujikuroi];Gene=ADH6-1;Ontology_term=F-actin capping protein complex,actin binding,zinc ion binding,oxidoreductase activity, acting on the CH-OH group of donors, NAD or NADP as acceptor,barbed-end actin filament
Fv_160_2.g1261	308	ID=Fv_160_2.g1261;Description=hypothetical protein FVEG_05503 [Fusarium verticillioides 7600]
Fv_160_2.g1262	477	ID=Fv_160_2.g1262;Description=hypothetical protein FVEG_05502 [Fusarium verticillioides 7600]
Fv_160_2.g1263	362	ID=Fv_160_2.g1263;Description=hypothetical protein FVEG_15658 [Fusarium verticillioides 7600];Gene=CAN1;Ontology_term=plasma membrane,endosome membrane,eisosome,L-arginine transmembrane transporter
Fv_160_2.g1264	287	ID=Fv_160_2.g1264;Description=hypothetical protein FVEG_05501 [Fusarium verticillioides
Fv_160_2.g1265	314	ID=Fv_160_2.g1265;Description=hypothetical protein FVEG_15655 [Fusarium verticillioides 7600];Gene=FOXG_18289;Ontology_term=membrane,transmembrane transporter activity,transmembrane

Fv_160_2.g1266	272	ID=Fv_160_2.g1266;Description=hypothetical protein FVEG_15654 [Fusarium verticillioides 7600];Gene=FNAPI_13570;Ontology_term=nucleus,viral occlusion body,DNA-binding transcription factor activity, RNA polymerase II-specific,structural molecule activity,zinc ion binding,regulation of transcription by RNA polymerase
Fv_160_2.g1267	267	ID=Fv_160_2.g1267;Description=hypothetical protein FVEG_05500 [Fusarium verticillioides 7600];Gene=FTJAE_6791;Ontology_term=membrane,cycloeucalenol cycloisomerase
Fv_160_2.g1268	82	ID=Fv_160_2.g1268;Description=hypothetical protein FVER14953_20632 [Fusarium verticillioides];Gene=FOYG_02538;Ontology_term=membrane,transmembrane transporter activity,transmembrane
Fv_160_2.g1269	273	ID=Fv_160_2.g1269;Description=hypothetical protein FVER53590_25972 [Fusarium verticillioides];Gene=FOC4_g10010688;Ontology_term=monooxygenase activity,iron ion binding,oxidoreductase activity, acting on paired donors, with incorporation or reduction of molecular oxygen,heme binding,2 iron, 2 sulfur cluster binding;Ontology_id=GO:0004497,GO:0005506,GO:0016705,GO:0020037,GO:0051537;Enzyme_code=EC:1.14;Enzyme_name=Actin
Fv_160_2.g1270	178	ID=Fv_160_2.g1270;Description=hypothetical protein FVEG_05499 [Fusarium verticillioides 7600];Gene=FOYG_02539;Ontology_term=nuclear envelope lumen,heterotrimeric G-protein complex,RNA polymerase II cis-regulatory region sequence-specific DNA binding,DNA-binding transcription repressor activity, RNA polymerase II-specific,monooxygenase activity,iron ion binding,protein binding,GTP binding,oxidoreductase activity, acting on paired donors, with incorporation or reduction of molecular oxygen,heme binding,G-protein beta/gamma-subunit complex binding,2 iron, 2 sulfur cluster binding,negative regulation of transcription by RNA polymerase II,carbon catabolite repression of transcription from RNA polymerase II promoter by glucose,G protein-coupled receptor signaling pathway,positive regulation of filamentous growth of a population of unicellular organisms in response to
Fv_160_2.g1271	338	ID=Fv_160_2.g1271;Description=hypothetical protein FVER53590_05498 [Fusarium verticillioides];Gene=FPANT_9808;Ontology_term=nucleotide binding,oxidoreductase activity, acting on
Fv_160_2.g1272	161	ID=Fv_160_2.g1272;Description=thioredoxin 1 [Fusarium verticillioides 7600];Gene=CIP1;Ontology_term=extracellular region,heme binding,metal ion binding,lactoperoxidase activity,cellular response to oxidative stress,hydrogen peroxide catabolic process,cellular oxidant
Fv_160_2.g1273	836	ID=Fv_160_2.g1273;Description=hypothetical protein FVEG_05496 [Fusarium verticillioides
Fv_160_2.g1274	361	ID=Fv_160_2.g1274;Description=hypothetical protein FVEG_05495 [Fusarium verticillioides 7600];Gene=FOMG_04708;Ontology_term=phosphatidylinositol binding;Ontology_id=GO:0035091
Fv_160_2.g1275	242	ID=Fv_160_2.g1275;Description=RP/EB family microtubule-associated protein [Fusarium oxysporum f. sp. lycopersici 4287];Gene=FDENT_6906;Ontology_term=nuclear microtubule,microtubule plus-end,mitotic spindle pole body,cell cortex of cell tip,cortical microtubule,mitotic spindle astral microtubule,mitotic spindle midzone,ATPase activator activity,cytoskeletal anchor activity,microtubule plus-end binding,microtubule lateral binding,karyogamy involved in conjugation with cellular fusion,nuclear migration involved in conjugation with cellular fusion,dynein-driven meiotic oscillatory nuclear movement,protein localization to microtubule,cell division,attachment of mitotic spindle microtubules to

Fv_160_2.g1276	790	ID=Fv_160_2.g1276;Description=hypothetical protein FVER14953_05493 [Fusarium verticillioides];Gene=FDENT_6905;Ontology_term=preribosome, large subunit precursor,nucleic acid binding,RNA helicase activity,ATP binding,hydrolase activity,ribosomal large subunit assembly,rRNA
Fv_160_2.g1277	210	ID=Fv_160_2.g1277;Description=hypothetical protein FVEG_05492 [Fusarium verticillioides 7600]
Fv_160_2.g1278	616	ID=Fv_160_2.g1278;Description=unnamed protein product [Fusarium fujikuroi];Gene=FDENT_6903;Ontology_term=2-isopropylmalate synthase activity,metal ion binding,leucine biosynthetic
Fv_160_2.g1279	1004	ID=Fv_160_2.g1279;Description=hypothetical protein FVER53263_05490 [Fusarium verticillioides];Gene=FTJAE_6780;Ontology_term=phosphatidate phosphatase
Fv_160_2.g1280	1829	ID=Fv_160_2.g1280;Description=hypothetical protein FVER53590_05489 [Fusarium verticillioides];Gene=FPANT_9907;Ontology_term=ligase
Fv_160_2.g1281	799	ID=Fv_160_2.g1281;Description=hypothetical protein FVER53590_05488 [Fusarium verticillioides]
Fv_160_2.g1282	504	ID=Fv_160_2.g1282;Description=hypothetical protein FVEG_05487 [Fusarium verticillioides 7600];Gene=FPANT_9909;Ontology_term=3',5'-cyclic-AMP phosphodiesterase activity,cAMP catabolic process;Ontology_id=GO:0004115,GO:0006198;Enzyme_code=EC:3.1.4.17,EC:3.1.4.53;Enzyme_name=3',5'-cyclic-nucleotide
Fv_160_2.g1283	287	ID=Fv_160_2.g1283;Description=hypothetical protein FVEG_05486 [Fusarium verticillioides 7600];Gene=FMAN_04910;Ontology_term=FMN binding,oxidoreductase
Fv_160_2.g1284	839	ID=Fv_160_2.g1284;Description=hypothetical protein FVER14953_05485 [Fusarium verticillioides];Gene=BFJ72_g5906;Ontology_term=3'-tRNA processing endoribonuclease activity,metal ion binding,tRNA 3'-end processing;Ontology_id=GO:0042781,GO:0046872,GO:0042780;Enzyme_code=EC:3.1.30,EC:3.1.26.11;Enzyme_name=Acting on
Fv_160_2.g1285	353	ID=Fv_160_2.g1285;Description=hypothetical protein FVEG_05484 [Fusarium verticillioides 7600];Gene=FOXG_02308;Ontology_term=mitochondrial membrane,monoatomic ion transmembrane transporter activity,serine transmembrane transporter activity,one-carbon metabolic process,monoatomic ion transmembrane transport,serine import into
Fv_160_2.g1286	704	ID=Fv_160_2.g1286;Description=CMGC/CLK protein kinase [Fusarium verticillioides 7600];Gene=BFJ72_g5904;Ontology_term=protein kinase activity,ATP
Fv_160_2.g1287	619	ID=Fv_160_2.g1287;Description=hypothetical protein FVEG_05482 [Fusarium verticillioides 7600];Gene=3419;Ontology_term=ribosome,ribonucleoprotein complex,structural constituent of
Fv_160_2.g1288	590	ID=Fv_160_2.g1288;Description=hypothetical protein FVER14953_05481 [Fusarium verticillioides];Gene=3418;Ontology_term=acyltransferase
Fv_160_2.g1289	181	ID=Fv_160_2.g1289;Description=ADP-ribosylation factor-like 2 [Fusarium verticillioides 7600];Gene=BFJ68_g4225;Ontology_term=GTPase activity,GTP
Fv_160_2.g1290	1633	ID=Fv_160_2.g1290;Description=hypothetical protein FPANT_9916 [Fusarium pseudoanthophilum];Gene=vilA;Ontology_term=GTPase activity,GTP binding,actin filament

Fv_160_2.g1291	303	ID=Fv_160_2.g1291;Description=hypothetical protein FVER53590_05478 [Fusarium verticillioides];Gene=FPANT_9917;Ontology_term=cytoplasm,lipid droplet,membrane,1-acylglycerol-3-phosphate O-acyltransferase activity,glycerophospholipid biosynthetic
Fv_160_2.g1292	618	ID=Fv_160_2.g1292;Description=hypothetical protein FVEG_05477 [Fusarium verticillioides 7600]
Fv_160_2.g1293	697	ID=Fv_160_2.g1293;Description=hypothetical protein FVER53590_05476 [Fusarium verticillioides];Gene=F25303_5201;Ontology_term=endoplasmic reticulum,membrane,protein
Fv_160_2.g1294	770	ID=Fv_160_2.g1294;Description=patatin-like phospholipase domain-containing protein [Fusarium verticillioides 7600];Gene=FPHYL_11071;Ontology_term=membrane,triglyceride lipase activity,triglyceride metabolic process,lipid catabolic process;Ontology_id=GO:0016020,GO:0004806,GO:0006641,GO:0016042;Enzyme_code=EC:3.1.1.3;Enzyme_name=triacylglycerol
Fv_160_2.g1295	189	ID=Fv_160_2.g1295;Description=small COPII coat GTPase [Fusarium oxysporum f. sp. lycopersici 4287];Gene=FPHYL_11072;Ontology_term=positive regulation of ER to Golgi vesicle-mediated transport,mitochondria-associated endoplasmic reticulum membrane,Golgi membrane,mitochondrial membrane organization,mitochondrial fission,regulation of COPII vesicle coating,endoplasmic reticulum membrane,GTP binding,nuclear envelope organization,GTPase activity,mitochondrion,positive regulation of protein exit from endoplasmic reticulum,endoplasmic reticulum exit site,COPII vesicle coat;Ontology_id=GO:1902953,GO:0044233,GO:0000139,GO:0007006,GO:0000266,GO:0003400,GO:0005789,GO:0005525,GO:000
Fv_160_2.g1296	717	ID=Fv_160_2.g1296;Description=hypothetical protein FVEG_05472 [Fusarium verticillioides 7600]
Fv_160_2.g1297	369	ID=Fv_160_2.g1297;Description=alpha-latrocrustotoxin-Lt1a [Fusarium coicis]
Fv_160_2.g1298	312	ID=Fv_160_2.g1298;Description=hypothetical protein FVER53590_05470 [Fusarium verticillioides]
Fv_160_2.g1299	168	ID=Fv_160_2.g1299;Description=ATP synthase subunit delta, mitochondrial [Fusarium odoratissimum];Gene=FSUBG_532;Ontology_term=mitochondrial proton-transporting ATP synthase complex, catalytic sector F(1),proton-transporting ATP synthase activity, rotational mechanism,monoatomic ion transport,proton motive force-driven ATP
Fv_160_2.g1300	711	ID=Fv_160_2.g1300;Description=hypothetical protein FVEG_05468 [Fusarium verticillioides 7600];Gene=FPHYL_11075;Ontology_term=proton-transporting ATP synthase complex, catalytic core F(1),proton-transporting ATP synthase activity, rotational mechanism,monoatomic ion transport,proton motive force-driven ATP
Fv_160_2.g1301	301	ID=Fv_160_2.g1301;Description=hypothetical protein FVEG_05467 [Fusarium verticillioides 7600]
Fv_160_2.g1302	558	ID=Fv_160_2.g1302;Description=hypothetical protein FVEG_05466 [Fusarium verticillioides 7600];Gene=FFUJ_03337;Ontology_term=DNA-binding transcription factor activity,regulation of DNA-templated
Fv_160_2.g1303	595	ID=Fv_160_2.g1303;Description=hypothetical protein FVER53590_05465 [Fusarium verticillioides];Gene=FPANT_10313;Ontology_term=aspartic-type endopeptidase activity,guanyl-nucleotide exchange factor activity,GTPase activator activity,Golgi membrane,pexophagy,extracellular region,fungal-type vacuole,protein secretion,regulation of COPII vesicle coating,endoplasmic reticulum membrane,oligosaccharide binding,protein-containing complex,proteolysis involved in protein catabolic process,cytoplasm to vacuole transport by the Cvt pathway,microautophagy,disordered domain specific binding;Ontology_id=GO:0004190,GO:0005085,GO:0005096,GO:0000139,GO:0000425,GO:0005576,GO:0000324,GO:0009306,GO:0

Fv_160_2.g1304	1491	ID=Fv_160_2.g1304;Description=hypothetical protein FVEG_05464 [Fusarium verticillioides 7600]
Fv_160_2.g1305	211	ID=Fv_160_2.g1305;Description=hypothetical protein FVEG_05463 [Fusarium verticillioides 7600];Gene=FMAN_04888;Ontology_term=nucleus,mitochondrial proton-transporting ATP synthase complex,proton-transporting
Fv_160_2.g1306	493	ID=Fv_160_2.g1306;Description=aspartyl aminopeptidase [Fusarium verticillioides 7600];Gene=FOPG_11130;Ontology_term=aminopeptidase activity,metallopeptidase activity,zinc ion
Fv_160_2.g1307	298	ID=Fv_160_2.g1307;Description=hypothetical protein FVEG_05461 [Fusarium verticillioides
Fv_160_2.g1308	495	ID=Fv_160_2.g1308;Description=hypothetical protein FVER14953_05460 [Fusarium
Fv_160_2.g1309	342	ID=Fv_160_2.g1309;Description=hypothetical protein FVEG_05459 [Fusarium verticillioides
Fv_160_2.g1310	1022	ID=Fv_160_2.g1310;Description=pre-tRNA nuclear export protein [Fusarium musae];Gene=FPHYL_11181;Ontology_term=nucleus,cytoplasm,tRNA binding,small GTPase binding,tRNA processing,tRNA re-export
Fv_160_2.g1311	1190	ID=Fv_160_2.g1311;Description=hypothetical protein FVER14953_05456 [Fusarium verticillioides]
Fv_160_2.g1312	766	ID=Fv_160_2.g1312;Description=hypothetical protein FVER14953_05455 [Fusarium
Fv_160_2.g1313	279	ID=Fv_160_2.g1313;Description=hypothetical protein FVER53590_05454 [Fusarium verticillioides];Gene=3394
Fv_160_2.g1314	138	ID=Fv_160_2.g1314;Description=hypothetical protein FVER53590_05453 [Fusarium verticillioides];Gene=FPRO05_06726;Ontology_term=hydrolase activity, hydrolyzing O-glycosyl compounds,carbohydrate metabolic
Fv_160_2.g1315	406	ID=Fv_160_2.g1315;Description=hypothetical protein FVER53263_05452 [Fusarium verticillioides];Gene=FNAPI_1662;Ontology_term=DNA-binding transcription factor activity,regulation of DNA-templated
Fv_160_2.g1316	723	ID=Fv_160_2.g1316;Description=hypothetical protein FVER14953_05451 [Fusarium verticillioides]
Fv_160_2.g1317	467	ID=Fv_160_2.g1317;Description=hypothetical protein FVEG_05450 [Fusarium verticillioides 7600]
Fv_160_2.g1318	316	ID=Fv_160_2.g1318;Description=hypothetical protein FVER53263_05449 [Fusarium verticillioides];Gene=FPHYL_11172;Ontology_term=cytoplasm,RNA binding;Ontology_id=GO:0005737,GO:0003723
Fv_160_2.g1319	72	ID=Fv_160_2.g1319;Description=hypothetical protein FVEG_15638 [Fusarium verticillioides 7600];Gene=RPL9A;Ontology_term=ribosome,ribonucleoprotein complex,structural constituent of ribosome,rRNA
Fv_160_2.g1320	571	ID=Fv_160_2.g1320;Description=hypothetical protein FVEG_05448 [Fusarium verticillioides 7600];Gene=FCIRC_11500;Ontology_term=membrane,ATP binding,ABC-type xenobiotic transporter activity,ATP hydrolysis activity,xenobiotic transport,response to antibiotic,transmembrane transport;Ontology_id=GO:0016020,GO:0005524,GO:0008559,GO:0016887,GO:0042908,GO:0046677,GO:0055085;Enzyme_code=E
Fv_160_2.g1321	741	ID=Fv_160_2.g1321;Description=uncharacterized protein BKA55DRAFT_534315 [Fusarium redolens];Gene=FSUBG_555;Ontology_term=Ino80 complex,ATP binding,CENP-A containing chromatin
Fv_160_2.g1322	431	ID=Fv_160_2.g1322;Description=hypothetical protein FVER53263_05446 [Fusarium verticillioides];Gene=FOYG_02606;Ontology_term=membrane,kinase activity,oxidoreductase activity,phosphorylation;Ontology_id=GO:0016020,GO:0016301,GO:0016491,GO:0016310;Enzyme_code=EC:1,EC:2.7;Enzyme_nam
Fv_160_2.g1323	242	ID=Fv_160_2.g1323;Description=hypothetical protein FVER14953_05445 [Fusarium verticillioides]

Fv_160_2.g1324	609	ID=Fv_160_2.g1324;Description=hypothetical protein FVER14953_05444 [Fusarium verticillioides]
Fv_160_2.g1325	716	ID=Fv_160_2.g1325;Description=hypothetical protein FVER14953_05443 [Fusarium verticillioides];Gene=FPHYL_1549;Ontology_term=3-isopropylmalate dehydrogenase activity,metal ion binding,leucine biosynthetic
Fv_160_2.g1326	116	ID=Fv_160_2.g1326;Description=hypothetical protein FVEG_15636 [Fusarium verticillioides 7600]
Fv_160_2.g1327	478	ID=Fv_160_2.g1327;Description=hypothetical protein FVEG_05442 [Fusarium verticillioides 7600];Gene=ARO1;Ontology_term=cytoplasm,3-dehydroquinate dehydratase activity,3-dehydroquinate synthase activity,3-phosphoshikimate 1-carboxyvinyltransferase activity,shikimate 3-dehydrogenase (NADP+) activity,shikimate kinase activity,ATP binding,metal ion binding,amino acid biosynthetic process,aromatic amino acid family biosynthetic process,chorismate biosynthetic process,phosphorylation;Ontology_id=GO:0005737,GO:0003855,GO:0003856,GO:0003866,GO:0004764,GO:0004765,GO:0005524,GO:0046872,GO:0008652,GO:0009073,GO:0009423,GO:0016310;Enzyme_code=EC:2.5.1.19,EC:1.1.1.25,EC:4.2.1.10,EC:4.2.3.4,EC:2
Fv_160_2.g1328	724	ID=Fv_160_2.g1328;Description=hypothetical protein FVER53590_05440 [Fusarium verticillioides];Gene=3380;Ontology_term=cytoplasm,microtubule,GTP binding,hydrolase
Fv_160_2.g1329	223	ID=Fv_160_2.g1329;Description=hypothetical protein FVEG_05439 [Fusarium verticillioides 7600]
Fv_160_2.g1330	167	ID=Fv_160_2.g1330;Description=negative transcription regulator [Fusarium tjaetaba];Gene=2484;Ontology_term=mediator complex,transcription coregulator activity,regulation of transcription by RNA polymerase
Fv_160_2.g1331	801	ID=Fv_160_2.g1331;Description=hypothetical protein FVER53590_05437 [Fusarium verticillioides];Gene=P42577;Ontology_term=cytoplasm,membrane,ferroxidase activity,ferric iron binding,iron ion transport,intracellular iron ion
Fv_160_2.g1332	291	ID=Fv_160_2.g1332;Description=hypothetical protein FVEG_05436 [Fusarium verticillioides
Fv_160_2.g1333	810	ID=Fv_160_2.g1333;Description=hypothetical protein FVER14953_05435 [Fusarium verticillioides]
Fv_160_2.g1334	285	ID=Fv_160_2.g1334;Description=hypothetical protein FVER53590_05434 [Fusarium verticillioides];Gene=FPCIR_13504;Ontology_term=cytosol,S-formylglutathione hydrolase activity,carboxylic ester hydrolase activity,formaldehyde catabolic
Fv_160_2.g1335	134	ID=Fv_160_2.g1335;Description=hypothetical protein FVEG_05433 [Fusarium verticillioides 7600]
Fv_160_2.g1336	154	ID=Fv_160_2.g1336;Description=DNA-directed RNA polymerase I, II, and III subunit RPABC2 [Fusarium oxysporum f. sp. lycopersici 4287];Gene=FOBC_04955;Ontology_term=RNA polymerase II, core complex,RNA polymerase III complex,RNA polymerase I complex,RNA polymerase I activity,RNA polymerase II activity,RNA polymerase III activity,DNA binding,RNA-dependent RNA polymerase activity,RNA-templated transcription,transcription initiation at RNA polymerase I promoter,transcription elongation by RNA polymerase I,termination of RNA polymerase I transcription,transcription initiation at RNA polymerase II promoter,transcription elongation by RNA polymerase II,transcription initiation at RNA polymerase III promoter,termination of RNA polymerase III transcription,tRNA transcription by RNA polymerase
Fv_160_2.g1337	500	ID=Fv_160_2.g1337;Description=hypothetical protein FVER14953_05430 [Fusarium verticillioides]

Fv_160_2.g1338	537	ID=Fv_160_2.g1338;Description=hypothetical protein FVEG_05429 [Fusarium verticillioides 7600];Gene=FMAN_04855;Ontology_term=membrane,metal ion transmembrane transporter activity,metal ion
Fv_160_2.g1339	346	ID=Fv_160_2.g1339;Description=hypothetical protein FVER14953_05428 [Fusarium verticillioides];Gene=FVEG_05428;Ontology_term=nucleic acid binding,endonuclease activity,metal ion
Fv_160_2.g1340	617	ID=Fv_160_2.g1340;Description=polyadenylation factor subunit 2 [Fusarium verticillioides 7600];Gene=FOYG_02627;Ontology_term=chromatin,mRNA cleavage and polyadenylation specificity factor complex,co-
Fv_160_2.g1341	514	ID=Fv_160_2.g1341;Description=diacylglycerol O-acyltransferase [Fusarium verticillioides 7600];Gene=HZS61_010148;Ontology_term=extracellular region,endoplasmic reticulum membrane,transport vesicle,hormone activity,O-acyltransferase activity,signal
Fv_160_2.g1342	282	ID=Fv_160_2.g1342;Description=hypothetical protein FVER53590_05425 [Fusarium verticillioides];Gene=HZS61_010147;Ontology_term=endoplasmic reticulum membrane,protein C-terminal S-isoprenylcysteine carboxyl O-methyltransferase
Fv_160_2.g1343	142	ID=Fv_160_2.g1343;Description=hypothetical protein FVEG_05424 [Fusarium verticillioides
Fv_160_2.g1344	685	ID=Fv_160_2.g1344;Description=hypothetical protein FVEG_05423 [Fusarium verticillioides 7600]
Fv_160_2.g1345	264	ID=Fv_160_2.g1345;Description=hypothetical protein FVER53590_05420 [Fusarium verticillioides]
Fv_160_2.g1346	633	ID=Fv_160_2.g1346;Description=hypothetical protein FVEG_05419 [Fusarium verticillioides 7600];Gene=FCIRC_9406;Ontology_term=phosphatidylinositol phospholipase C activity,lipid catabolic process,intracellular signal
Fv_160_2.g1347	1121	ID=Fv_160_2.g1347;Description=hypothetical protein FVEG_15635 [Fusarium verticillioides
Fv_160_2.g1348	346	ID=Fv_160_2.g1348;Description=hypothetical protein FVER53590_05415 [Fusarium verticillioides];Gene=FMAN_04844;Ontology_term=membrane,3-dehydrosphinganine reductase activity,3-keto-sphinganine metabolic process,sphingolipid biosynthetic
Fv_160_2.g1349	1361	ID=Fv_160_2.g1349;Description=hypothetical protein FVEG_05414 [Fusarium verticillioides 7600]
Fv_160_2.g1350	91	ID=Fv_160_2.g1350;Description=hypothetical protein FVER53590_05413 [Fusarium verticillioides];Gene=FOC1_g10013515;Ontology_term=mitochondrial crista junction,MICOS complex,cristae
Fv_160_2.g1351	186	ID=Fv_160_2.g1351;Description=hypothetical protein FVEG_15634 [Fusarium verticillioides
Fv_160_2.g1352	797	ID=Fv_160_2.g1352;Description=hypothetical protein FVEG_05410 [Fusarium verticillioides 7600];Gene=hpf;Ontology_term=nucleus,DNA-binding transcription factor activity, RNA polymerase II-specific,GTPase activity,GTP binding,zinc ion binding,G-protein beta/gamma-subunit complex binding,regulation of transcription by RNA polymerase II,adenylate cyclase-modulating G protein-coupled receptor signaling pathway;Ontology_id=GO:0005634,GO:0000981,GO:0003924,GO:0005525,GO:0008270,GO:0031683,GO:0006357,GO:0007188;Enz
Fv_160_2.g1353	364	ID=Fv_160_2.g1353;Description=hypothetical protein FVER14953_21024 [Fusarium verticillioides]
Fv_160_2.g1354	417	ID=Fv_160_2.g1354;Description=hypothetical protein FVEG_15632 [Fusarium verticillioides 7600];Gene=FOQG_11691;Ontology_term=nucleus,DNA-binding transcription factor activity, RNA polymerase II-specific,zinc ion

Fv_160_2.g1355	148	ID=Fv_160_2.g1355;Description=hypothetical protein FMUND_3033 [Fusarium mundagurra]
Fv_160_2.g1356	422	ID=Fv_160_2.g1356;Description=hypothetical protein FVEG_05406 [Fusarium verticillioides 7600]
Fv_160_2.g1357	247	ID=Fv_160_2.g1357;Description=triosephosphate isomerase [Fusarium verticillioides 7600];Gene=FCIRC_9417;Ontology_term=chloride channel complex,postsynaptic membrane,triose-phosphate isomerase activity,GABA-A receptor activity,extracellular ligand-gated monoatomic ion channel activity,chloride channel activity,gluconeogenesis,glycolytic process,chloride transmembrane transport;Ontology_id=GO:0034707,GO:0045211,GO:0004807,GO:0004890,GO:0005230,GO:0005254,GO:0006094,GO:0006096,GO
Fv_160_2.g1358	137	ID=Fv_160_2.g1358;Description=hypothetical protein FOXG_02232 [Fusarium oxysporum f. sp. lycopersici 4287];Gene=FOYG_02648;Ontology_term=extracellular region,nucleolus,mitochondrion,ribosome,microtubule,side of membrane,ribonucleoprotein complex,structural constituent of ribosome,GTPase activity,structural constituent of cytoskeleton,GTP binding,ribosomal large subunit assembly,cytoskeleton organization,microtubule-based process,cell adhesion,mitochondrial translation;Ontology_id=GO:0005576,GO:0005730,GO:0005739,GO:0005840,GO:0005874,GO:0098552,GO:1990904,GO:0003735,G
Fv_160_2.g1359	391	ID=Fv_160_2.g1359;Description=U1 small nuclear ribonucleoprotein 70kDa [Fusarium verticillioides 7600];Gene=FOYG_02649;Ontology_term=nucleus,ribonucleoprotein complex,U1 snRNA
Fv_160_2.g1360	244	ID=Fv_160_2.g1360;Description=mRNA turnover protein 4 [Fusarium verticillioides 7600];Gene=FOPG_02802;Ontology_term=nucleoplasm,nucleolus,cytoplasm,preribosome, large subunit precursor,small-subunit processome,ribosomal large subunit assembly,ribosomal large subunit export from nucleus,nuclear-transcribed mRNA catabolic
Fv_160_2.g1361	514	ID=Fv_160_2.g1361;Description=hypothetical protein FVEG_05401 [Fusarium verticillioides 7600];Gene=FPCIR_10606;Ontology_term=cytoplasm,intracellular organelle,RNA
Fv_160_2.g1362	420	ID=Fv_160_2.g1362;Description=hypothetical protein J7337_002372 [Fusarium musae];Gene=AGA2;Ontology_term=fungal-type cell wall,cell adhesion molecule binding,agglutination involved in conjugation with cellular
Fv_160_2.g1363	205	ID=Fv_160_2.g1363;Description=hypothetical protein FVER53263_05398 [Fusarium verticillioides];Gene=FTJAE_9253;Ontology_term=MIS12/MIND type complex,nucleus,cell cycle,cell
Fv_160_2.g1364	380	ID=Fv_160_2.g1364;Description=hypothetical protein FVEG_05397 [Fusarium verticillioides 7600];Gene=Forpi1262_v003278;Ontology_term=mitochondrion,membrane,transferase
Fv_160_2.g1365	757	ID=Fv_160_2.g1365;Description=hypothetical protein FVEG_05396 [Fusarium verticillioides 7600];Gene=HZS61_010125;Ontology_term=nucleolus,RNA binding,rRNA
Fv_160_2.g1366	193	ID=Fv_160_2.g1366;Description=hypothetical protein FVEG_05395 [Fusarium verticillioides
Fv_160_2.g1367	140	ID=Fv_160_2.g1367;Description=hypothetical protein FVEG_05394 [Fusarium verticillioides 7600];Gene=P06146;Ontology_term=nucleosome,nucleus,DNA binding,structural constituent of chromatin,protein
Fv_160_2.g1368	637	ID=Fv_160_2.g1368;Description=pH-response transcription factor pacC RIM101 [Fusarium mundagurra];Gene=thiC
Fv_160_2.g1369	351	ID=Fv_160_2.g1369;Description=hypothetical protein FVEG_05392 [Fusarium verticillioides 7600]

Fv_160_2.g1370	656	ID=Fv_160_2.g1370;Description=DNA topoisomerase III [Fusarium verticillioides 7600];Gene=FNAPI_8895;Ontology_term=DNA binding,DNA topoisomerase type I (single strand cut, ATP-independent) activity,DNA topological
Fv_160_2.g1371	356	ID=Fv_160_2.g1371;Description=hypothetical protein FVEG_05390 [Fusarium verticillioides
Fv_160_2.g1372	308	ID=Fv_160_2.g1372;Description=hypothetical protein FVER14953_05389 [Fusarium verticillioides];Gene=FOC1_g10013536;Ontology_term=alkaline phosphatase activity,phosphoprotein phosphatase activity,phosphoglycolate phosphatase activity,metal ion binding,carbohydrate metabolic
Fv_160_2.g1373	120	ID=Fv_160_2.g1373;Description=uncharacterized protein FTJAE_9263 [Fusarium tjaetaba]
Fv_160_2.g1374	190	ID=Fv_160_2.g1374;Description=hypothetical protein FVEG_05387 [Fusarium verticillioides
Fv_160_2.g1375	716	ID=Fv_160_2.g1375;Description=hypothetical protein FVER14953_05386 [Fusarium verticillioides];Gene=FTJAE_9265;Ontology_term=chromosome, telomeric region,Ku70:Ku80 complex,damaged DNA binding,helicase activity,ATP binding,ATP hydrolysis activity,telomeric DNA binding,telomere maintenance,double-strand break repair via nonhomologous end joining,DNA recombination;Ontology_id=GO:0000781,GO:0043564,GO:0003684,GO:0004386,GO:0005524,GO:0016887,GO:0042162,GO:00007
Fv_160_2.g1376	497	ID=Fv_160_2.g1376;Description=hypothetical protein FVER53590_05385 [Fusarium
Fv_160_2.g1377	105	ID=Fv_160_2.g1377;Description=mitochondrial import inner membrane translocase subunit TIM14 [Fusarium oxysporum f. sp. lycopersici 4287];Gene=FEQUK3_LOCUS6547;Ontology_term=mitochondrial inner membrane,protein
Fv_160_2.g1378	220	ID=Fv_160_2.g1378;Description=50S ribosomal protein L24e [Fusarium verticillioides 7600];Gene=FOMG_04839;Ontology_term=nucleolus,ribosome,preribosome, large subunit precursor,ATPase activator activity,phosphoribosylamine-glycine ligase activity,phosphoribosylformylglycinamidine cyclo-ligase activity,ATP binding,metal ion binding,ATPase binding,'de novo' IMP biosynthetic process,purine nucleobase biosynthetic process,ribosomal large subunit biogenesis,assembly of large subunit precursor of
Fv_160_2.g1379	600	ID=Fv_160_2.g1379;Description=hypothetical protein FVER53590_05382 [Fusarium verticillioides];Gene=FVER53590_05382;Ontology_term=membrane,hydrolase
Fv_160_2.g1380	157	ID=Fv_160_2.g1380;Description=hypothetical protein FVER14953_05381 [Fusarium verticillioides];Gene=dnaJ
Fv_160_2.g1381	430	ID=Fv_160_2.g1381;Description=hypothetical protein FVEG_05380 [Fusarium verticillioides 7600]
Fv_160_2.g1382	340	ID=Fv_160_2.g1382;Description=hypothetical protein FVER53590_05379 [Fusarium
Fv_160_2.g1383	167	ID=Fv_160_2.g1383;Description=hypothetical protein FVER53590_05378 [Fusarium verticillioides]
Fv_160_2.g1384	911	ID=Fv_160_2.g1384;Description=hypothetical protein FVEG_05377 [Fusarium verticillioides 7600];Gene=HZS61_010105;Ontology_term=chromatin,nucleus,metal ion
Fv_160_2.g1385	148	ID=Fv_160_2.g1385;Description=hypothetical protein FVEG_05376 [Fusarium verticillioides 7600]
Fv_160_2.g1386	1134	ID=Fv_160_2.g1386;Description=hypothetical protein FVER14953_05375 [Fusarium

Fv_160_2.g1387	89	ID=Fv_160_2.g1387;Description=dolichyl-phosphate mannosyltransferase polypeptide 2, regulatory subunit [Fusarium oxysporum f. sp. lycopersici 4287];Gene=FANTH_9842;Ontology_term=endoplasmic reticulum membrane,glycosyltransferase activity,enzyme regulator activity,dolichol metabolic
Fv_160_2.g1388	868	ID=Fv_160_2.g1388;Description=hypothetical protein FVER53590_05373 [Fusarium
Fv_160_2.g1389	72	ID=Fv_160_2.g1389;Description=hypothetical protein FVEG_15626 [Fusarium verticillioides
Fv_160_2.g1390	131	ID=Fv_160_2.g1390;Description=hypothetical protein FVER14953_21051 [Fusarium verticillioides];Gene=FocTR4_00005612;Ontology_term=membrane,ribonucleoprotein complex,nucleic acid binding,helicase activity,ATP binding,hydrolase
Fv_160_2.g1391	508	ID=Fv_160_2.g1391;Description=hypothetical protein FVER53590_05370 [Fusarium verticillioides];Gene=FVEG_05370;Ontology_term=flavin adenine dinucleotide binding,tRNA-dihydrouridine47 synthase activity,tRNA dihydrouridine synthesis,mRNA
Fv_160_2.g1392	717	ID=Fv_160_2.g1392;Description=hypothetical protein J7337_002403 [Fusarium musae]
Fv_160_2.g1393	421	ID=Fv_160_2.g1393;Description=hypothetical protein F25303_11385 [Fusarium sp. NRRL 25303];Gene=FANTH_9850;Ontology_term=nucleus,mitochondrial matrix,DNA-binding transcription factor activity, RNA polymerase II-specific,anthranilate synthase activity,L-aspartate:2-oxoglutarate aminotransferase activity,indole-3-glycerol-phosphate synthase activity,phosphoribosylanthranilate isomerase activity,zinc ion binding,pyridoxal phosphate binding,tryptophan biosynthetic process,2-oxoglutarate metabolic process,regulation of transcription by RNA polymerase II,aspartate catabolic process,glutamate metabolic process,glutamine metabolic process;Ontology_id=GO:0005634,GO:0005759,GO:0000981,GO:0004049,GO:0004069,GO:0004425,GO:0004640,GO:0008270,GO:
Fv_160_2.g1394	94	ID=Fv_160_2.g1394;Description=hypothetical protein FVEG_05366 [Fusarium verticillioides 7600]
Fv_160_2.g1395	1601	ID=Fv_160_2.g1395;Description=hypothetical protein FVER14953_05364 [Fusarium verticillioides];Gene=FNYG_08323;Ontology_term=proteasome complex,ATP binding,ATP hydrolysis
Fv_160_2.g1396	310	ID=Fv_160_2.g1396;Description=oxidoreductase [Fusarium verticillioides 7600];Gene=FPHYL_3070;Ontology_term=oxidoreductase activity,cellular biosynthetic process;Ontology_id=GO:0016491,GO:0044249;Enzyme_code=EC:1;Enzyme_name=Oxidoreductases
Fv_160_2.g1397	235	ID=Fv_160_2.g1397;Description=hypothetical protein FVER14953_05362 [Fusarium verticillioides];Gene=FPCIR_12659;Ontology_term=oxidoreductase activity,transferase
Fv_160_2.g1398	201	ID=Fv_160_2.g1398;Description=hypothetical protein FVER53590_30417 [Fusarium verticillioides];Gene=FPANT_1815;Ontology_term=dihydrofolate reductase activity,NADP binding,glycine biosynthetic process,one-carbon metabolic process,tetrahydrofolate biosynthetic
Fv_160_2.g1399	703	ID=Fv_160_2.g1399;Description=putative 3-oxoacyl-[acyl-carrier-protein] synthase [Fusarium proliferatum];Gene=F52700_3898;Ontology_term=3-oxoacyl-[acyl-carrier-protein] synthase activity,fatty acid biosynthetic process,signal transduction;Ontology_id=GO:0004315,GO:0006633,GO:0007165;Enzyme_code=EC:2.3.1.41;Enzyme_name=beta-

Fv_160_2.g1400	820	ID=Fv_160_2.g1400;Description=hypothetical protein FVER53590_30416 [Fusarium verticillioides];Gene=BFJ70_g8154;Ontology_term=membrane,oligopeptide transmembrane transporter activity,protein
Fv_160_2.g1401	628	ID=Fv_160_2.g1401;Description=hypothetical protein FVEG_05357 [Fusarium verticillioides 7600];Gene=BFJ68_g5695;Ontology_term=nucleus,DNA binding,zinc ion binding,DNA-binding transcription factor activity, RNA polymerase II-specific,regulation of transcription by RNA polymerase
Fv_160_2.g1402	389	ID=Fv_160_2.g1402;Description=hypothetical protein FVER14953_05355 [Fusarium verticillioides];Gene=Forpi1262_v003240;Ontology_term=pyruvate dehydrogenase complex,acyltransferase activity,acetyl-CoA biosynthetic process from
Fv_160_2.g1403	297	ID=Fv_160_2.g1403;Description=hypothetical protein FVER53590_05354 [Fusarium
Fv_160_2.g1404	847	ID=Fv_160_2.g1404;Description=hypothetical protein FVER53590_05353 [Fusarium verticillioides]
Fv_160_2.g1405	1158	ID=Fv_160_2.g1405;Description=hypothetical protein FVER14953_05352 [Fusarium
Fv_160_2.g1406	325	ID=Fv_160_2.g1406;Description=serine/threonine-protein phosphatase PP1 [Fusarium verticillioides 7600];Gene=FOIG_05619;Ontology_term=myosin phosphatase
Fv_160_2.g1407	132	ID=Fv_160_2.g1407;Description=selenoprotein W-like protein [Fusarium verticillioides 7600];Gene=2408
Fv_160_2.g1408	93	ID=Fv_160_2.g1408;Description=guanine nucleotide-binding subunit gamma [Fusarium albosuccineum];Gene=FGADI_11674;Ontology_term=G-protein beta-subunit binding,pheromone-dependent signal transduction involved in conjugation with cellular fusion,G protein-coupled receptor signaling
Fv_160_2.g1409	1055	ID=Fv_160_2.g1409;Description=translation initiation factor 3 subunit A [Fusarium verticillioides 7600];Gene=TIF32;Ontology_term=cytoplasmic stress granule,eukaryotic 43S preinitiation complex,eukaryotic 48S preinitiation complex,eukaryotic translation initiation factor 3 complex, eIF3e,eukaryotic translation initiation factor 3 complex, eIF3m,RNA binding,translation initiation factor activity,formation of cytoplasmic translation initiation
Fv_160_2.g1410	680	ID=Fv_160_2.g1410;Description=hypothetical protein FVEG_05347 [Fusarium verticillioides 7600];Gene=FVER53590_05347;Ontology_term=membrane,3-oxo-5-alpha-steroid 4-dehydrogenase activity,dolichol-linked oligosaccharide biosynthetic
Fv_160_2.g1411	327	ID=Fv_160_2.g1411;Description=hypothetical protein FVER53590_05347 [Fusarium verticillioides];Gene=C2S_13205;Ontology_term=endoplasmic reticulum membrane,3-oxo-5-alpha-steroid 4-dehydrogenase activity,NAD(P)H dehydrogenase (quinone) activity,FMN binding,polyprenol reductase activity,dolichol-linked oligosaccharide biosynthetic process,polyprenol catabolic process;Ontology_id=GO:0005789,GO:0003865,GO:0003955,GO:0010181,GO:0102389,GO:0006488,GO:0016095;Enzyme_code=EC
Fv_160_2.g1412	252	ID=Fv_160_2.g1412;Description=alkylated DNA repair protein alkB like 6 [Fusarium verticillioides 7600];Gene=BFJ69_g10289;Ontology_term=nucleus,3-dehydroquinate dehydratase activity,cysteine-type peptidase activity,metal ion binding,dioxygenase activity,proteolysis,quinate catabolic process,3,4-dihydroxybenzoate biosynthetic process;Ontology_id=GO:0005634,GO:0003855,GO:0008234,GO:0046872,GO:0051213,GO:0006508,GO:0019631,GO:0046279;Enzy

Fv_160_2.g1413	1525	ID=Fv_160_2.g1413;Description=hypothetical protein FVER14953_05344 [Fusarium verticillioides]
Fv_160_2.g1414	382	ID=Fv_160_2.g1414;Description=ribosome-interacting GTPase 1 [Fusarium oxysporum f. sp. pisi HDV247];Gene=FOYG_02717;Ontology_term=polysome,3-dehydroquinate dehydratase activity,GTPase activity,GTP binding,cytoplasmic translation,quinate catabolic process,3,4-dihydroxybenzoate biosynthetic process,positive regulation of cellular response to amino acid
Fv_160_2.g1415	547	ID=Fv_160_2.g1415;Description=hypothetical protein FVEG_05341 [Fusarium verticillioides
Fv_160_2.g1416	544	ID=Fv_160_2.g1416;Description=hypothetical protein FVER53590_05340 [Fusarium
Fv_160_2.g1417	503	ID=Fv_160_2.g1417;Description=hypothetical protein FVEG_05339 [Fusarium verticillioides 7600];Gene=P23631;Ontology_term=extracellular region,membrane,other organism cell membrane,host cell presynaptic membrane,inositol monophosphate 1-phosphatase activity,metal ion binding,toxin activity,inositol metabolic process,exocytosis,signal transduction,quinate metabolic process,modulation of process of another organism,phosphatidylinositol phosphate biosynthetic
Fv_160_2.g1418	1212	ID=Fv_160_2.g1418;Description=hypothetical protein FVER14953_05338 [Fusarium verticillioides];Gene=FSUBG_6529;Ontology_term=nucleus,nucleic acid binding;Ontology_id=GO:0005634,GO:0003676
Fv_160_2.g1419	260	ID=Fv_160_2.g1419;Description=hypothetical protein FVEG_05336 [Fusarium verticillioides 7600]
Fv_160_2.g1420	400	ID=Fv_160_2.g1420;Description=hypothetical protein FVER14953_05335 [Fusarium verticillioides];Gene=FOTG_00968;Ontology_term=regulation of alternative mRNA splicing, via
Fv_160_2.g1421	85	ID=Fv_160_2.g1421;Description=uncharacterized protein FOIG_05634 [Fusarium odoratissimum NRRL 54006];Gene=FOQG_00989;Ontology_term=mitochondrial inner membrane,mitochondrial intermembrane space protein transporter complex,metal ion binding,protein transporter activity,protein insertion into mitochondrial inner
Fv_160_2.g1422	322	ID=Fv_160_2.g1422;Description=lectin, mannose-binding 2 [Fusarium verticillioides 7600];Gene=FACUT_529;Ontology_term=membrane,carbohydrate binding;Ontology_id=GO:0016020,GO:0030246
Fv_160_2.g1423	569	ID=Fv_160_2.g1423;Description=hypothetical protein FVER14953_05331 [Fusarium verticillioides];Gene=FOYG_02727;Ontology_term=fungal-type vacuole,extracellular region,nucleus,cytosol,cAMP-dependent protein kinase complex,cAMP-dependent protein kinase activity,ATP binding,3-phytase activity,negative regulation of transcription by RNA polymerase II,negative regulation of induction of conjugation with cellular fusion,adenylate cyclase-activating glucose-activated G protein-coupled receptor signaling pathway,phosphorylation,negative regulation of gluconeogenesis,positive regulation of protein export from
Fv_160_2.g1424	487	ID=Fv_160_2.g1424;Description=hypothetical protein FVER53263_05330 [Fusarium
Fv_160_2.g1425	700	ID=Fv_160_2.g1425;Description=hypothetical protein FVEG_05329 [Fusarium verticillioides 7600];Gene=FVEG_05329;Ontology_term=membrane,cobalamin binding;Ontology_id=GO:0016020,GO:0031419

Fv_160_2.g1426	479	ID=Fv_160_2.g1426;Description=histone acetyltransferase type B catalytic subunit [Fusarium verticillioides 7600];Gene=FNAPI_4169;Ontology_term=chromosome, telomeric region,nucleus,cytoplasm,histone acetyltransferase activity,histone binding,subtelomeric heterochromatin
Fv_160_2.g1427	343	ID=Fv_160_2.g1427;Description=pyrroline-5-carboxylate reductase [Fusarium verticillioides 7600];Gene=P5CR-0;Ontology_term=membrane,pyrroline-5-carboxylate reductase activity,L-proline biosynthetic
Fv_160_2.g1428	257	ID=Fv_160_2.g1428;Description=hypothetical protein FVEG_05325 [Fusarium verticillioides 7600];Gene=FPANT_4935;Ontology_term=CTDK-1 complex,kinase activity,phosphorylation,positive regulation of DNA-templated transcription,
Fv_160_2.g1429	179	ID=Fv_160_2.g1429;Description=hypothetical protein FVEG_05324 [Fusarium verticillioides 7600];Gene=Forpe1208_v003007;Ontology_term=extracellular region,mitochondrial large ribosomal subunit,mitochondrial nucleoid,structural constituent of ribosome,polygalacturonase activity,translation,pectin catabolic process,cell wall organization;Ontology_id=GO:0005576,GO:0005762,GO:0042645,GO:0003735,GO:0004650,GO:0006412,GO:0045490,GO:0071555;
Fv_160_2.g1430	4924	ID=Fv_160_2.g1430;Description=hypothetical protein FVER53590_05323 [Fusarium verticillioides];Gene=FPANT_4937;Ontology_term=nucleoplasm,nucleolus,ATP binding,ATP hydrolysis activity,ribosomal large subunit
Fv_160_2.g1431	183	ID=Fv_160_2.g1431;Description=hypothetical protein J7337_002442 [Fusarium musae];Gene=CEK26_006349;Ontology_term=extracellular region,membrane,metalloendopeptidase activity,polygalacturonase activity,metal ion binding,proteolysis,cell adhesion,pectin catabolic process,cell wall organization;Ontology_id=GO:0005576,GO:0016020,GO:0004222,GO:0004650,GO:0046872,GO:0006508,GO:0007155,GO:0045490,
Fv_160_2.g1432	619	ID=Fv_160_2.g1432;Description=hypothetical protein FVEG_05321 [Fusarium verticillioides 7600];Gene=FGLOB1_723;Ontology_term=hydrolase
Fv_160_2.g1433	269	ID=Fv_160_2.g1433;Description=hypothetical protein FVEG_05319 [Fusarium verticillioides 7600];Gene=GAPC;Ontology_term=extracellular region,cytoplasm,glyceraldehyde-3-phosphate dehydrogenase (NAD+) (phosphorylating) activity,polygalacturonase activity,NADP binding,NAD binding,glucose metabolic process,glycolytic process,pectin catabolic process,cell wall organization;Ontology_id=GO:0005576,GO:0005737,GO:0004365,GO:0004650,GO:0050661,GO:0051287,GO:0006006,GO:0006096,GO:0045490,GO:0071555;Enzyme_code=EC:1.2.1.59,EC:1.2.1.12,EC:3.2.1.15;Enzyme_name=glyceraldehyde-3-phosphate
Fv_160_2.g1434	1807	ID=Fv_160_2.g1434;Description=hypothetical protein FVEG_05318 [Fusarium verticillioides 7600];Gene=FPHYL_9284;Ontology_term=nucleolus,ribonucleoprotein complex,rRNA
Fv_160_2.g1435	456	ID=Fv_160_2.g1435;Description=hypothetical protein FVER14953_05317 [Fusarium verticillioides];Gene=FPCIR_7329;Ontology_term=nucleolus,ribonucleoprotein complex,NADH dehydrogenase activity,NADH oxidation,rRNA

Fv_160_2.g1436	508	ID=Fv_160_2.g1436;Description=hypothetical protein FVEG_05316 [Fusarium verticillioides 7600];Gene=FPHYL_9282;Ontology_term=magnesium ion binding,hydroxyethylthiazole kinase activity,thiamine-phosphate diphosphorylase activity,ATP binding,thiamine biosynthetic process,thiamine diphosphate biosynthetic process,phosphorylation;Ontology_id=GO:0000287,GO:0004417,GO:0004789,GO:0005524,GO:0009228,GO:0009229,GO:0016310;E
Fv_160_2.g1437	600	ID=Fv_160_2.g1437;Description=hypothetical protein FVEG_05315 [Fusarium verticillioides 7600];Gene=BFJ70_g8242;Ontology_term=nucleus,peroxisomal matrix,DNA-binding transcription factor activity, RNA polymerase II-specific,zinc ion binding,isopenicillin-N N-acyltransferase activity,acyl coenzyme A: isopenicillin N acyltransferase activity,regulation of transcription by RNA polymerase II,penicillin biosynthetic process;Ontology_id=GO:0005634,GO:0005782,GO:0000981,GO:0008270,GO:0050640,GO:0102920,GO:0006357,GO:0042318;Enzy
Fv_160_2.g1438	619	ID=Fv_160_2.g1438;Description=hypothetical protein FVER14953_05314 [Fusarium verticillioides];Gene=FOTG_00948;Ontology_term=mitochondrial matrix,5-aminolevulinate synthase activity,pyridoxal phosphate binding,protoporphyrinogen IX biosynthetic process,positive regulation of organelle
Fv_160_2.g1439	1880	ID=Fv_160_2.g1439;Description=hypothetical protein FVER53590_05313 [Fusarium verticillioides];Gene=FMUND_6245;Ontology_term=nucleus,DNA binding,ATP binding,ATP hydrolysis activity,ATP-dependent chromatin remodeler activity,chromatin
Fv_160_2.g1440	513	ID=Fv_160_2.g1440;Description=glutathione synthetase [Fusarium verticillioides 7600];Gene=FCIRC_1059;Ontology_term=magnesium ion binding,glutathione synthase activity,ATP binding,glutathione binding,glutathione biosynthetic
Fv_160_2.g1441	138	ID=Fv_160_2.g1441;Description=hypothetical protein FVEG_05311 [Fusarium verticillioides 7600];Gene=pelA;Ontology_term=extracellular region,single-stranded DNA binding,pectate lyase activity,pectin lyase activity,polysaccharide catabolic process,DNA replication,cell wall organization;Ontology_id=GO:0005576,GO:0003697,GO:0030570,GO:0047490,GO:0000272,GO:0006260,GO:0071555;Enzyme_cod
Fv_160_2.g1442	560	ID=Fv_160_2.g1442;Description=pheromone a factor receptor [Fusarium verticillioides 7600];Gene=FACUT_509;Ontology_term=membrane,mating-type factor pheromone receptor activity,G protein-coupled receptor
Fv_160_2.g1443	1115	ID=Fv_160_2.g1443;Description=hypothetical protein FVER53590_05309 [Fusarium verticillioides];Gene=FACUT_508;Ontology_term=extracellular region,membrane,pectinesterase activity,aspartyl esterase activity,cell wall modification,pectin catabolic
Fv_160_2.g1444	487	ID=Fv_160_2.g1444;Description=hypothetical protein FVER53590_05307 [Fusarium
Fv_160_2.g1445	648	ID=Fv_160_2.g1445;Description=hypothetical protein FVEG_05306 [Fusarium verticillioides 7600];Gene=FOMG_04917;Ontology_term=endoplasmic reticulum membrane;Ontology_id=GO:0005789
Fv_160_2.g1446	451	ID=Fv_160_2.g1446;Description=hypothetical protein FVEG_05305 [Fusarium verticillioides 7600];Gene=BFJ69_g11232;Ontology_term=endoplasmic reticulum membrane;Ontology_id=GO:0005789

Fv_160_2.g1447	490	ID=Fv_160_2.g1447;Description=beta-glucosidase [Fusarium verticillioides 7600];Gene=FACUT_504;Ontology_term=beta-glucosidase activity,scopolin beta-glucosidase activity,carbohydrate metabolic
Fv_160_2.g1448	240	ID=Fv_160_2.g1448;Description=hypothetical protein FVER14953_05303 [Fusarium verticillioides];Gene=pmeA;Ontology_term=extracellular region,pectinesterase activity,aspartyl esterase activity,cell wall modification,pectin catabolic
Fv_160_2.g1449	295	ID=Fv_160_2.g1449;Description=hypothetical protein FVEG_05302 [Fusarium verticillioides 7600];Gene=FACUT_11791;Ontology_term=zinc ion binding,D-xylulose reductase
Fv_160_2.g1450	588	ID=Fv_160_2.g1450;Description=hypothetical protein FVEG_05301 [Fusarium verticillioides 7600];Gene=3262;Ontology_term=extracellular region,cytoplasm,spindle pole body,microtubule,membrane,other organism cell membrane,host cell presynaptic membrane,unfolded protein binding,toxin activity,karyogamy,protein folding,exocytosis,cell cycle,nuclear migration along microtubule,fungal-type cell wall organization,modulation of process of another organism,cell division;Ontology_id=GO:0005576,GO:0005737,GO:0005816,GO:0005874,GO:0016020,GO:0044218,GO:0044231,GO:0051082,GO:
Fv_160_2.g1451	708	ID=Fv_160_2.g1451;Description=hypothetical protein FVER53590_05300 [Fusarium verticillioides];Gene=BFJ69_g11208;Ontology_term=guanyl-nucleotide exchange factor activity,vesicle-mediated
Fv_160_2.g1452	937	ID=Fv_160_2.g1452;Description=hypothetical protein FVEG_05299 [Fusarium verticillioides 7600];Gene=HZS61_010037;Ontology_term=plasma membrane,potassium ion transmembrane transporter activity,intracellular
Fv_160_2.g1453	825	ID=Fv_160_2.g1453;Description=DNA excision repair protein ERCC-3 [Fusarium verticillioides 7600];Gene=FocTR4_00005535;Ontology_term=nucleotide-excision repair factor 3 complex,transcription factor TFIIH core complex,transcription factor TFIIH holo complex,cytosol,transcription preinitiation complex,DNA binding,DNA helicase activity,ATP binding,DNA translocase activity,hydrolase activity,regulation of mitotic recombination,RNA polymerase II promoter clearance,transcription open complex formation at RNA polymerase II promoter,nucleotide-excision repair,poly(A)+ mRNA export from nucleus,DNA duplex
Fv_160_2.g1454	122	ID=Fv_160_2.g1454;Description=hypothetical protein FOXG_02135 [Fusarium oxysporum f. sp. lycopersici 4287];Gene=BFJ68_g5752;Ontology_term=zinc ion binding;Ontology_id=GO:0008270
Fv_160_2.g1455	730	ID=Fv_160_2.g1455;Description=hypothetical protein KAF25_007314 [Fusarium avenaceum];Gene=FOMG_04928;Ontology_term=nucleus,mitochondrial matrix,DNA-binding transcription factor activity, RNA polymerase II-specific,5-aminolevulinate synthase activity,zinc ion binding,pyridoxal phosphate binding,FAD binding,regulation of transcription by RNA polymerase II,protoporphyrinogen IX biosynthetic process,positive regulation of organelle assembly;Ontology_id=GO:0005634,GO:0005759,GO:0000981,GO:0003870,GO:0008270,GO:0030170,GO:0071949,GO:0006357,GO
Fv_160_2.g1456	593	ID=Fv_160_2.g1456;Description=hypothetical protein J7337_002466 [Fusarium musae];Gene=FOC1_g10013626;Ontology_term=extracellular region,nucleus,DNA-binding transcription factor activity, RNA polymerase II-specific,catalytic activity,hormone activity,zinc ion binding,FAD binding,regulation of transcription by RNA polymerase

Fv_160_2.g1457	655	ID=Fv_160_2.g1457;Description=hypothetical protein FVER53263_05294 [Fusarium verticillioides];Gene=FOTG_00926;Ontology_term=actin binding,calcium ion binding,cellular component
Fv_160_2.g1458	229	ID=Fv_160_2.g1458;Description=hypothetical protein FVEG_05293 [Fusarium verticillioides 7600];Gene=FVER53263_05293;Ontology_term=ribosome,structural constituent of
Fv_160_2.g1459	406	ID=Fv_160_2.g1459;Description=hypothetical protein FVER14953_05292 [Fusarium verticillioides]
Fv_160_2.g1460	371	ID=Fv_160_2.g1460;Description=cysteine synthase [Fusarium verticillioides 7600];Gene=FOQG_00946;Ontology_term=cysteine biosynthetic process from serine;Ontology_id=GO:0006535
Fv_160_2.g1461	937	ID=Fv_160_2.g1461;Description=hypothetical protein FVEG_05290 [Fusarium verticillioides 7600];Gene=FOQG_00945;Ontology_term=metal ion binding,cysteine biosynthetic process from serine,regulation of signal
Fv_160_2.g1462	290	ID=Fv_160_2.g1462;Description=enoyl-CoA hydratase [Fusarium verticillioides 7600];Gene=Forpe1208_v002974;Ontology_term=extracellular region,glucose oxidase activity,flavin adenine dinucleotide
Fv_160_2.g1463	698	ID=Fv_160_2.g1463;Description=DNA-directed RNA polymerase II regulator [Fusarium mexicanum];Gene=FMEXI_1717;Ontology_term=membrane,Cdc73/Paf1 complex,cytosolic large ribosomal subunit,structural constituent of ribosome,cytoplasmic translational elongation,transcription elongation by RNA polymerase
Fv_160_2.g1464	626	ID=Fv_160_2.g1464;Description=hypothetical protein FVER14953_05286 [Fusarium verticillioides];Gene=FMEXI_1718;Ontology_term=transferase
Fv_160_2.g1465	130	ID=Fv_160_2.g1465;Description=40S ribosomal protein S22 [Fusarium oxysporum f. sp. lycopersici 4287];Gene=FOC1_g10013636;Ontology_term=ribosome,ribonucleoprotein complex,structural constituent of
Fv_160_2.g1466	154	ID=Fv_160_2.g1466;Description=40S ribosomal protein S12 [Fusarium musae];Gene=CDV36_008481;Ontology_term=cytosolic small ribosomal subunit,structural constituent of ribosome,translation;Ontology_id=GO:0022627,GO:0003735,GO:0006412
Fv_160_2.g1467	127	ID=Fv_160_2.g1467;Description=peptidyl-prolyl cis-trans isomerase PIN4 [Fusarium verticillioides 7600];Gene=FOC1_g10013638;Ontology_term=DNA binding,peptidyl-prolyl cis-trans isomerase activity,rRNA
Fv_160_2.g1468	385	ID=Fv_160_2.g1468;Description=hypothetical protein FVEG_05282 [Fusarium verticillioides 7600]
Fv_160_2.g1469	744	ID=Fv_160_2.g1469;Description=hypothetical protein FVER53590_05281 [Fusarium verticillioides];Gene=BFJ69_g11199;Ontology_term=RNA binding,pseudouridine synthase activity,pseudouridine
Fv_160_2.g1470	565	ID=Fv_160_2.g1470;Description=STE STE7 MKK kinase [Fusarium napiforme];Gene=FOMG_04945;Ontology_term=division septum,cytoplasm,MAP kinase kinase activity,ATP binding,cell wall integrity MAPK cascade,phosphorylation,positive regulation of calcium-mediated signaling;Ontology_id=GO:0000935,GO:0005737,GO:0004708,GO:0005524,GO:0000196,GO:0016310,GO:0050850;Enzyme_code=E
Fv_160_2.g1471	539	ID=Fv_160_2.g1471;Description=ATP-dependent RNA helicase DBP8 [Fusarium verticillioides 7600];Gene=FSUBG_12663;Ontology_term=RNA binding,RNA helicase activity,ATP binding,hydrolase

Fv_160_2.g1472	250	ID=Fv_160_2.g1472;Description=hypothetical protein FVEG_05278 [Fusarium verticillioides 7600];Gene=GLAA;Ontology_term=endoplasmic reticulum,glucan 1,4-alpha-glucosidase activity,starch binding,polysaccharide catabolic process;Ontology_id=GO:0005783,GO:0004339,GO:2001070,GO:0000272;Enzyme_code=EC:3.2.1.3;Enzyme_name=glucan
Fv_160_2.g1473	395	ID=Fv_160_2.g1473;Description=hypothetical protein FVEG_05277 [Fusarium verticillioides 7600];Gene=FMAN_04712;Ontology_term=mitochondrion,glucan 1,4-alpha-glucosidase activity,transferase activity,starch binding,polysaccharide catabolic
Fv_160_2.g1474	511	ID=Fv_160_2.g1474;Description=hypothetical protein FVEG_05276 [Fusarium verticillioides 7600]
Fv_160_2.g1475	282	ID=Fv_160_2.g1475;Description=hypothetical protein FVEG_05275 [Fusarium verticillioides 7600];Gene=2341;Ontology_term=extracellular region,nucleus,cytosol,plasma membrane,translation release factor activity,GTPase activity,acetate-CoA ligase activity,ATP binding,GTP binding,AMP binding,nuclear-transcribed mRNA catabolic process, deadenylation-dependent decay,translational termination,signal transduction,visual perception,carbon utilization,acetyl-CoA biosynthetic process from acetate,acetate catabolic
Fv_160_2.g1476	378	ID=Fv_160_2.g1476;Description=hypothetical protein FVEG_15613 [Fusarium verticillioides 7600];Gene=FNAPI_8215;Ontology_term=hydrolase activity,carboxy-lyase
Fv_160_2.g1477	335	ID=Fv_160_2.g1477;Description=hypothetical protein FVEG_05274 [Fusarium verticillioides 7600];Gene=FPRO_04927;Ontology_term=endoplasmic reticulum membrane,lyase activity,metal ion binding,dioxygenase activity,fatty acid biosynthetic process,organic cyclic compound biosynthetic
Fv_160_2.g1478	223	ID=Fv_160_2.g1478;Description=3-hydroxy acyl-CoA dehydratase [Fusarium verticillioides 7600];Gene=FDENT_9928;Ontology_term=endoplasmic reticulum membrane,monooxygenase activity,lyase activity,fatty acid biosynthetic process,organic cyclic compound biosynthetic
Fv_160_2.g1479	408	ID=Fv_160_2.g1479;Description=hypothetical protein FVEG_05272 [Fusarium verticillioides 7600]
Fv_160_2.g1480	1421	ID=Fv_160_2.g1480;Description=hypothetical protein FVEG_05271 [Fusarium verticillioides 7600];Gene=FACUT_12051;Ontology_term=SWI/SNF complex,rDNA binding,helicase activity,ATP binding,hydrolase activity,nucleosomal DNA binding,RNA polymerase II-specific DNA-binding transcription factor binding,lysine-acetylated histone binding,ATP-dependent chromatin remodeler activity,DNA-templated DNA replication,double-strand break repair,chromatin remodeling,positive regulation of mating type switching,aggrephagy,DNA strand invasion,positive regulation of transcription by RNA polymerase II,positive regulation of cell adhesion involved in single-species biofilm formation,positive regulation of invasive growth in response to glucose
Fv_160_2.g1481	568	ID=Fv_160_2.g1481;Description=hypothetical protein FVER14953_05270 [Fusarium verticillioides];Gene=FVEG_05270;Ontology_term=nucleus,site of double-strand break,Cul4-RING E3 ubiquitin ligase complex,double-
Fv_160_2.g1482	320	ID=Fv_160_2.g1482;Description=SWR1-complex protein 5 [Fusarium verticillioides 7600];Gene=FPANT_7938;Ontology_term=nucleus,chromatin organization;Ontology_id=GO:0005634,GO:0006325
Fv_160_2.g1483	297	ID=Fv_160_2.g1483;Description=hypothetical protein FVEG_05268 [Fusarium verticillioides 7600];Gene=2333

Fv_160_2.g1484	701	ID=Fv_160_2.g1484;Description=transcription factor STE12 [Fusarium verticillioides 7600];Gene=HZS61_010004;Ontology_term=nucleus,DNA-binding transcription factor activity,regulation of DNA-templated
Fv_160_2.g1485	602	ID=Fv_160_2.g1485;Description=hypothetical protein FVEG_05265 [Fusarium verticillioides 7600];Gene=FTJAE_8583;Ontology_term=actin filament binding,endocytic vesicle,mitotic actomyosin contractile ring,positive regulation of Arp2/3 complex-mediated actin nucleation,protein-macromolecule adaptor activity,actin cortical patch localization,actin cortical patch,negative regulation of Arp2/3 complex-mediated actin nucleation,microtubule-based process,Arp2/3
Fv_160_2.g1486	1429	ID=Fv_160_2.g1486;Description=hypothetical protein FVEG_15612 [Fusarium verticillioides 7600]
Fv_160_2.g1487	259	ID=Fv_160_2.g1487;Description=hypothetical protein FVER14953_05262 [Fusarium verticillioides];Gene=2329;Ontology_term=nucleosome,nucleus,cytoplasm,DNA binding,1-(5-phosphoribosyl)-5-[(5-phosphoribosylamino)methylideneamino]imidazole-4-carboxamide isomerase activity,structural constituent of chromatin,protein heterodimerization activity,histidine biosynthetic process;Ontology_id=GO:0000786,GO:0005634,GO:0005737,GO:0003677,GO:0003949,GO:0030527,GO:0046982,GO:0000105;Enzy
Fv_160_2.g1488	280	ID=Fv_160_2.g1488;Description=hypothetical protein J7337_002500 [Fusarium musae];Gene=FNAPI_8226;Ontology_term=hydrolase
Fv_160_2.g1489	821	ID=Fv_160_2.g1489;Description=hypothetical protein FVER14953_05260 [Fusarium verticillioides]
Fv_160_2.g1490	460	ID=Fv_160_2.g1490;Description=hypothetical protein FVEG_05259 [Fusarium verticillioides 7600];Gene=FACUT_12061;Ontology_term=membrane,alpha-amylase activity,nucleoside transmembrane transporter activity,calcium ion binding,carbohydrate catabolic process,nucleoside transmembrane
Fv_160_2.g1491	476	ID=Fv_160_2.g1491;Description=hypothetical protein FVEG_05258 [Fusarium verticillioides 7600];Gene=FACUT_12062;Ontology_term=ATP binding,inositol pentakisphosphate 2-kinase
Fv_160_2.g1492	541	ID=Fv_160_2.g1492;Description=mitochondrial distribution and morphology protein 34 [Fusarium verticillioides 7600];Gene=MDM34;Ontology_term=ERMES complex,lipid binding,mitochondrial genome maintenance,lipid
Fv_160_2.g1493	268	ID=Fv_160_2.g1493;Description=protein AF-9 [Fusarium verticillioides 7600];Gene=FOPG_02647;Ontology_term=Swr1 complex,NuA4 histone acetyltransferase complex,regulation of DNA-templated
Fv_160_2.g1494	289	ID=Fv_160_2.g1494;Description=hypothetical protein FVEG_05254 [Fusarium verticillioides 7600]
Fv_160_2.g1495	264	ID=Fv_160_2.g1495;Description=pre-mRNA-splicing factor SYF2 [Fusarium verticillioides 7600];Gene=FSUBG_12124;Ontology_term=Prp19 complex,post-mRNA release spliceosomal complex,mRNA splicing, via
Fv_160_2.g1496	1234	ID=Fv_160_2.g1496;Description=hypothetical protein FDENT_9910 [Fusarium denticulatum];Gene=FPRO_04905;Ontology_term=vacuolar membrane,cytosol,ATP binding,phosphopantetheine binding,N-(5-amino-5-carboxypentanoyl)-L-cysteinyl-D-valine synthase activity,penicillin biosynthetic process;Ontology_id=GO:0005774,GO:0005829,GO:0005524,GO:0031177,GO:0050564,GO:0042318;Enzyme_code=EC:6.3.2.26;Enz
Fv_160_2.g1497	261	ID=Fv_160_2.g1497;Description=hypothetical protein FVEG_05249 [Fusarium verticillioides 7600]

Fv_160_2.g1498	1364	ID=Fv_160_2.g1498;Description=phospholipid-translocating ATPase [Fusarium verticillioides 7600];Gene=FNAPI_8237;Ontology_term=peroxisomal matrix,glyoxysome,membrane,magnesium ion binding,isocitrate lyase activity,ATP binding,ATP hydrolysis activity,methylisocitrate lyase activity,phosphatidylethanolamine flippase activity,glyoxylate cycle,tricarboxylic acid cycle,fatty acid catabolic process,phospholipid transport,carbon utilization,lipid translocation,acetate catabolic process;Ontology_id=GO:0005782,GO:0009514,GO:0016020,GO:0000287,GO:0004451,GO:0005524,GO:0016887,GO:0046421,GO:
Fv_160_2.g1499	546	ID=Fv_160_2.g1499;Description=HASPIN protein kinase [Fusarium verticillioides 7600];Gene=FDENT_9907;Ontology_term=chromosome,protein serine/threonine kinase activity,ATP binding,histone kinase activity,chromatin remodeling,phosphorylation;Ontology_id=GO:0005694,GO:0004674,GO:0005524,GO:0035173,GO:0006338,GO:0016310;Enzyme_c
Fv_160_2.g1500	876	ID=Fv_160_2.g1500;Description=hypothetical protein J7337_002512 [Fusarium musae];Gene=FACUT_13849;Ontology_term=nucleus,hydrolase activity,mRNA processing,RNA
Fv_160_2.g1501	344	ID=Fv_160_2.g1501;Description=uncharacterized protein BKA55DRAFT_501511 [Fusarium redolens];Gene=FACUT_13849;Ontology_term=hydrolase
Fv_160_2.g1502	434	ID=Fv_160_2.g1502;Description=hypothetical protein FVER14953_05242 [Fusarium verticillioides];Gene=Forpe1208_v002932;Ontology_term=extracellular region,endoplasmic reticulum,membrane,RNA binding,RNA endonuclease activity,calcium ion binding,lyase activity,rRNA endonuclease activity,endoplasmic reticulum calcium ion homeostasis,negative regulation of cytoplasmic translation;Ontology_id=GO:0005576,GO:0005783,GO:0016020,GO:0003723,GO:0004521,GO:0005509,GO:0016829,GO:0033902,G
Fv_160_2.g1503	762	ID=Fv_160_2.g1503;Description=hypothetical protein FVEG_05240 [Fusarium verticillioides 7600];Gene=Forpi1262_v003135;Ontology_term=nucleus,DNA binding,zinc ion binding,DNA-binding transcription factor activity, RNA polymerase II-specific,regulation of transcription by RNA polymerase
Fv_160_2.g1504	459	ID=Fv_160_2.g1504;Description=4-aminobutyrate aminotransferase [Fusarium verticillioides 7600];Gene=FOTG_00869;Ontology_term=pyridoxal phosphate binding,4-aminobutyrate:2-oxoglutarate transaminase activity,gamma-aminobutyric acid metabolic
Fv_160_2.g1505	490	ID=Fv_160_2.g1505;Description=succinate-semialdehyde dehydrogenase [Fusarium verticillioides 7600];Gene=FOQG_00889;Ontology_term=extracellular region,host cell cytoplasm,succinate-semialdehyde dehydrogenase (NAD+) activity,succinate-semialdehyde dehydrogenase (NADP+) activity,gamma-aminobutyric acid catabolic process,killing of cells of another organism,defense response to fungus;Ontology_id=GO:0005576,GO:0030430,GO:0004777,GO:0036243,GO:0009450,GO:0031640,GO:0050832;Enzyme_code=EC:
Fv_160_2.g1506	125	ID=Fv_160_2.g1506;Description=hypothetical protein FVEG_05237 [Fusarium verticillioides 7600];Gene=FOXB_10414;Ontology_term=phosphoric diester hydrolase activity,lipid metabolic

Fv_160_2.g1507	362	ID=Fv_160_2.g1507;Description=hypothetical protein FVER14953_05236 [Fusarium verticillioides];Gene=Forpi1262_v003131;Ontology_term=phosphoric diester hydrolase activity,lipid metabolic
Fv_160_2.g1508	480	ID=Fv_160_2.g1508;Description=cell division cycle protein 37 [Fusarium verticillioides 7600];Gene=FPANT_11349;Ontology_term=nucleus,cytoplasm,protein kinase binding,heat shock protein binding,unfolded protein binding,osmosensory signaling MAPK cascade,protein folding,spindle pole body duplication,positive regulation of MAPK cascade,protein stabilization,cell division,regulation of cell
Fv_160_2.g1509	1895	ID=Fv_160_2.g1509;Description=hypothetical protein FVER14953_05234 [Fusarium verticillioides];Gene=FFUJ_03118;Ontology_term=nuclear chromosome,transcription regulator complex,DNA binding,helicase activity,ATP binding,ATP hydrolysis activity,TBP-class protein binding,ATP-dependent chromatin remodeler activity,chromatin remodeling,rRNA processing,nucleolar large rRNA transcription by RNA polymerase I,negative regulation of DNA-templated transcription,regulation of RNA polymerase II transcription preinitiation complex assembly;Ontology_id=GO:0000228,GO:0005667,GO:0003677,GO:0004386,GO:0005524,GO:0016887,GO:0017025,GO:0140658,GO
Fv_160_2.g1510	1198	ID=Fv_160_2.g1510;Description=structural maintenance-chromosome 3 (chondroitin sulfate proteoglycan 6) [Fusarium verticillioides 7600];Gene=FPHYL_13082;Ontology_term=nucleus,chromosome,ATP binding,ATP hydrolysis activity,chromosome organization,cell
Fv_160_2.g1511	339	ID=Fv_160_2.g1511;Description=hypothetical protein FVEG_05232 [Fusarium verticillioides 7600];Gene=FFUJ_03116;Ontology_term=mitochondrion,ribosome,ribonucleoprotein complex,structural constituent of
Fv_160_2.g1512	673	ID=Fv_160_2.g1512;Description=hypothetical protein FVEG_05231 [Fusarium verticillioides
Fv_160_2.g1513	92	ID=Fv_160_2.g1513;Description=ribosomal protein L37ae [Fusarium oxysporum f. sp. lycopersici 4287];Gene=NECHADRAFT_102575;Ontology_term=ribosome,structural constituent of
Fv_160_2.g1514	422	ID=Fv_160_2.g1514;Description=hypothetical protein FVEG_05229 [Fusarium verticillioides 7600];Gene=FTJAE_12577;Ontology_term=membrane,oxidoreductase
Fv_160_2.g1515	527	ID=Fv_160_2.g1515;Description=hypothetical protein FVEG_05228 [Fusarium verticillioides 7600];Gene=FTJAE_12576;Ontology_term=mitochondrion,membrane;Ontology_id=GO:0005739,GO:0016020
Fv_160_2.g1516	270	ID=Fv_160_2.g1516;Description=hypothetical protein FNYG_00328 [Fusarium
Fv_160_2.g1517	547	ID=Fv_160_2.g1517;Description=T-complex protein 1 subunit theta [Fusarium verticillioides 7600];Gene=FPHYL_13075;Ontology_term=chaperonin-containing T-complex,ATP binding,ATP hydrolysis activity,unfolded protein binding,ATP-dependent protein folding chaperone,chaperone mediated protein folding independent of
Fv_160_2.g1518	1039	ID=Fv_160_2.g1518;Description=hypothetical protein FVEG_05225 [Fusarium verticillioides 7600]
Fv_160_2.g1519	273	ID=Fv_160_2.g1519;Description=hypothetical protein FVEG_05224 [Fusarium verticillioides 7600];Gene=FPANT_3729;Ontology_term=snoRNA binding,rRNA processing;Ontology_id=GO:0030515,GO:0006364
Fv_160_2.g1520	193	ID=Fv_160_2.g1520;Description=hypothetical protein FVER14953_05223 [Fusarium verticillioides];Gene=3188

Fv_160_2.g1521	637	ID=Fv_160_2.g1521;Description=hypothetical protein FVER14953_05222 [Fusarium verticillioides];Gene=FRV6_07488;Ontology_term=lyase
Fv_160_2.g1522	471	ID=Fv_160_2.g1522;Description=hypothetical protein FVER14953_05221 [Fusarium
Fv_160_2.g1523	390	ID=Fv_160_2.g1523;Description=UV excision repair protein Rad23 [Fusarium verticillioides 7600];Gene=FOPG_08448;Ontology_term=nucleotide-excision repair factor 2 complex,nucleoplasm,cytosol,PNGase complex,peptide-N4-(N-acetyl-beta-glucosaminyl)asparagine amidase activity,damaged DNA binding,protein-macromolecule adaptor activity,polyubiquitin modification-dependent protein binding,ubiquitin binding,proteasome binding,negative regulation of transcription by RNA polymerase II,nucleotide-excision repair,ubiquitin-dependent glycoprotein ERAD pathway;Ontology_id=GO:0000111,GO:0005654,GO:0005829,GO:0120125,GO:0000224,GO:0003684,GO:0030674,GO:0031593,GO:
Fv_160_2.g1524	577	ID=Fv_160_2.g1524;Description=hypothetical protein FVEG_05219 [Fusarium verticillioides 7600]
Fv_160_2.g1525	702	ID=Fv_160_2.g1525;Description=E3 ubiquitin-protein ligase bre1 [Fusarium musae];Gene=FOQG_00869;Ontology_term=nucleus,ubiquitin-protein transferase activity,metal ion binding,chromatin organization,protein
Fv_160_2.g1526	381	ID=Fv_160_2.g1526;Description=hypothetical protein FVER53263_05217 [Fusarium verticillioides];Gene=FNAPI_11800;Ontology_term=endoplasmic reticulum membrane,regulation of store-operated calcium
Fv_160_2.g1527	1349	ID=Fv_160_2.g1527;Description=hypothetical protein FVER14953_05216 [Fusarium verticillioides];Gene=FPCIR_11825;Ontology_term=membrane,ATP binding,ATP hydrolysis activity,ABC-type transporter activity,transmembrane transport;Ontology_id=GO:0016020,GO:0005524,GO:0016887,GO:0140359,GO:0055085;Enzyme_code=EC:7.2.2,EC:3.6.1.15;Enzym
Fv_160_2.g1528	357	ID=Fv_160_2.g1528;Description=hypothetical protein FVEG_05215 [Fusarium verticillioides 7600];Gene=cbh1;Ontology_term=extracellular region,collagen trimer,cellulose 1,4-beta-cellobiosidase activity,cellulose binding,cellulose catabolic
Fv_160_2.g1529	425	ID=Fv_160_2.g1529;Description=hypothetical protein J7337_002541 [Fusarium musae];Gene=FMEXI_3608;Ontology_term=nucleus,sporulation resulting in formation of a cellular
Fv_160_2.g1530	522	ID=Fv_160_2.g1530;Description=hypothetical protein FVER14953_05213 [Fusarium verticillioides]
Fv_160_2.g1531	611	ID=Fv_160_2.g1531;Description=hypothetical protein FVER53590_05212 [Fusarium verticillioides];Gene=BFJ70_g16267;Ontology_term=RNA binding,RNA methyltransferase activity,S-adenosylmethionine-dependent methyltransferase activity,RNA
Fv_160_2.g1532	721	ID=Fv_160_2.g1532;Description=minichromosome maintenance protein 5 (cell division control protein 46) [Fusarium verticillioides 7600];Gene=FOC1_g10013713;Ontology_term=ATP hydrolysis activity,cell division,nuclear DNA replication,DNA replication origin binding,ATP binding,nuclear pre-replicative complex,regulation of DNA-templated DNA replication initiation,DNA duplex unwinding,DNA replication preinitiation complex,nuclear replication fork,helicase activity,MCM complex;Ontology_id=GO:0016887,GO:0051301,GO:0033260,GO:0003688,GO:0005524,GO:0005656,GO:0030174,GO:0032508,GO:

Fv_160_2.g1533	1106	ID=Fv_160_2.g1533;Description=hypothetical protein FVER14953_05210 [Fusarium verticillioides];Gene=FMAN_04646;Ontology_term=GARP complex,cytosol,protein transport,retrograde transport, endosome to
Fv_160_2.g1534	166	ID=Fv_160_2.g1534;Description=hypothetical protein J7337_002546 [Fusarium musae];Gene=2276
Fv_160_2.g1535	253	ID=Fv_160_2.g1535;Description=hypothetical protein FVEG_05208 [Fusarium verticillioides 7600];Gene=LOX2;Ontology_term=nucleus,RNA polymerase II cis-regulatory region sequence-specific DNA binding,DNA-binding transcription factor activity, RNA polymerase II-specific,regulation of transcription by RNA polymerase II,anterior/posterior pattern
Fv_160_2.g1536	665	ID=Fv_160_2.g1536;Description=anaphase-promoting complex subunit 8 [Fusarium verticillioides 7600];Gene=FOVG_02726;Ontology_term=anaphase-promoting complex,cyclin binding,ubiquitin protein ligase activity,protein ubiquitination,anaphase-promoting complex-dependent catabolic process,cell division,mitotic sister chromatid
Fv_160_2.g1537	114	ID=Fv_160_2.g1537;Description=hypothetical protein FVEG_05206 [Fusarium verticillioides 7600];Gene=3169
Fv_160_2.g1538	347	ID=Fv_160_2.g1538;Description=farnesyl pyrophosphate synthase [Fusarium verticillioides 7600];Gene=FACUT_9519;Ontology_term=dimethylallyltranstransferase activity,geranyltranstransferase activity,isoprenoid biosynthetic process,ketone biosynthetic process,mycotoxin biosynthetic process,alcohol biosynthetic process,organic cyclic compound biosynthetic
Fv_160_2.g1539	1015	ID=Fv_160_2.g1539;Description=hypothetical protein FVEG_05203 [Fusarium verticillioides 7600];Gene=FPRO05_06505;Ontology_term=DNA ligase
Fv_160_2.g1540	361	ID=Fv_160_2.g1540;Description=hypothetical protein J7337_002551 [Fusarium musae];Gene=FOXYS1_485;Ontology_term=DNA ligase activity,transferase activity,S-adenosylmethionine biosynthetic
Fv_160_2.g1541	523	ID=Fv_160_2.g1541;Description=hypothetical protein FVEG_05201 [Fusarium verticillioides 7600]
Fv_160_2.g1542	908	ID=Fv_160_2.g1542;Description=hypothetical protein FVER53590_05200 [Fusarium verticillioides];Gene=FMAN_04638;Ontology_term=nucleolus,small-subunit processome,rRNA
Fv_160_2.g1543	538	ID=Fv_160_2.g1543;Description=ubiquitin thiolesterase [Fusarium verticillioides 7600];Gene=ubp10;Ontology_term=nucleus,cysteine-type deubiquitinase activity,zinc ion binding,spliceosomal complex
Fv_160_2.g1544	341	ID=Fv_160_2.g1544;Description=translation initiation factor 4E [Fusarium verticillioides 7600];Gene=FANTH_8056;Ontology_term=cytoplasm,RNA binding,translation initiation factor activity,translational
Fv_160_2.g1545	195	ID=Fv_160_2.g1545;Description=hypothetical protein FVEG_05197 [Fusarium verticillioides 7600];Gene=FMAN_04635;Ontology_term=ribosome,mRNA binding,ribosomal large subunit biogenesis,mRNA
Fv_160_2.g1546	400	ID=Fv_160_2.g1546;Description=hypothetical protein J7337_002557 [Fusarium musae];Gene=FVER53590_05196;Ontology_term=cytoplasm,cysteine-type endopeptidase activity,phosphoglycerate kinase activity,ATP binding,glycolytic process,proteolysis,phosphorylation;Ontology_id=GO:0005737,GO:0004197,GO:0004618,GO:0005524,GO:0006096,GO:0006508,G
Fv_160_2.g1547	978	ID=Fv_160_2.g1547;Description=CMGC CDK CRK7 kinase [Fusarium tjaetaba];Gene=FOVG_02737;Ontology_term=protein kinase activity,ATP

Fv_160_2.g1548	504	ID=Fv_160_2.g1548;Description=hypothetical protein FVEG_05194 [Fusarium verticillioides 7600]
Fv_160_2.g1549	95	ID=Fv_160_2.g1549;Description=hypothetical protein FVER53590_05193 [Fusarium
Fv_160_2.g1550	633	ID=Fv_160_2.g1550;Description=hypothetical protein FVER14953_05191 [Fusarium verticillioides];Gene=FOC1_g10013732;Ontology_term=nucleus,protein aggregate center,3'-5'-RNA exonuclease activity,RNA binding,metal ion binding,RNA metabolic process,regulation of heterochromatin formation,cellular response to temperature stimulus;Ontology_id=GO:0005634,GO:0140453,GO:0000175,GO:0003723,GO:0046872,GO:0016070,GO:0031445,GO:0071502;Enz
Fv_160_2.g1551	698	ID=Fv_160_2.g1551;Description=hypothetical protein FVEG_05190 [Fusarium verticillioides 7600]
Fv_160_2.g1552	433	ID=Fv_160_2.g1552;Description=hypothetical protein FVER14953_05189 [Fusarium verticillioides];Gene=FOC1_g10013734;Ontology_term=nucleus,transferase
Fv_160_2.g1553	1415	ID=Fv_160_2.g1553;Description=hypothetical protein FVER53590_05188 [Fusarium verticillioides];Gene=FOC1_g10013735;Ontology_term=cytoplasm,RNA binding,5'-3' RNA exonuclease activity,nuclear-transcribed mRNA catabolic process, nonsense-mediated
Fv_160_2.g1554	218	ID=Fv_160_2.g1554;Description=hypothetical protein J7337_002565 [Fusarium musae];Gene=FMAN_04626;Ontology_term=signal peptidase complex,signal peptide processing;Ontology_id=GO:0005787,GO:0006465
Fv_160_2.g1555	1095	ID=Fv_160_2.g1555;Description=hypothetical protein FVEG_15605 [Fusarium verticillioides 7600];Gene=FVEG_15605;Ontology_term=cysteine-type peptidase activity,ubiquitin-like protein peptidase
Fv_160_2.g1556	582	ID=Fv_160_2.g1556;Description=hypothetical protein FVEG_05184 [Fusarium verticillioides 7600]
Fv_160_2.g1557	182	ID=Fv_160_2.g1557;Description=hypothetical protein FVER14953_05183 [Fusarium verticillioides];Gene=FMAN_04623;Ontology_term=nucleus,endoplasmic reticulum membrane,proteasome core complex, beta-subunit complex,endopeptidase activator activity,proteasomal ubiquitin-independent protein catabolic process,proteasome-
Fv_160_2.g1558	670	ID=Fv_160_2.g1558;Description=ATP-dependent RNA helicase DED1 [Fusarium verticillioides 7600];Gene=FOVG_02748;Ontology_term=positive regulation of formation of translation preinitiation complex,cytoplasmic stress granule,eukaryotic initiation factor 4G binding,RNA strand annealing activity,nucleus,translation initiation factor activity,ATP binding,RNA helicase activity,spliceosomal complex disassembly,negative regulation of cytoplasmic translational initiation in response to stress,mRNA binding,regulatory ncRNA-mediated gene silencing,hydrolase activity;Ontology_id=GO:1901195,GO:0010494,GO:0031370,GO:0033592,GO:0005634,GO:0003743,GO:0005524,GO:0003724,GO:0
Fv_160_2.g1559	561	ID=Fv_160_2.g1559;Description=hypothetical protein FVER14953_05181 [Fusarium verticillioides];Gene=2250
Fv_160_2.g1560	775	ID=Fv_160_2.g1560;Description=hypothetical protein FVEG_05180 [Fusarium verticillioides 7600]
Fv_160_2.g1561	789	ID=Fv_160_2.g1561;Description=hypothetical protein FVER14953_05179 [Fusarium verticillioides]
Fv_160_2.g1562	715	ID=Fv_160_2.g1562;Description=hypothetical protein FVER14953_05177 [Fusarium
Fv_160_2.g1563	892	ID=Fv_160_2.g1563;Description=hypothetical protein FVER53590_05176 [Fusarium verticillioides];Gene=rplW;Ontology_term=nucleus,DNA binding,zinc ion binding,DNA-binding transcription factor activity, RNA

Fv_160_2.g1564	344	ID=Fv_160_2.g1564;Description=hypothetical protein FVEG_05174 [Fusarium verticillioides 7600];Gene=ROX3;Ontology_term=mediator complex,transcription coregulator activity,regulation of transcription by RNA
Fv_160_2.g1565	507	ID=Fv_160_2.g1565;Description=hypothetical protein FVEG_05173 [Fusarium verticillioides 7600];Gene=FOTG_00803;Ontology_term=nucleus,DNA binding;Ontology_id=GO:0005634,GO:0003677
Fv_160_2.g1566	319	ID=Fv_160_2.g1566;Description=hypothetical protein FVER53263_05172 [Fusarium verticillioides];Gene=FGLOB1_11668;Ontology_term=oxidoreductase activity, acting on the CH-OH group of donors, NAD or NADP as acceptor,NAD+ binding,fatty acid metabolic
Fv_160_2.g1567	1147	ID=Fv_160_2.g1567;Description=replication fork protection complex subunit Tof1/Swi1 [Fusarium verticillioides 7600];Gene=FOVG_02759;Ontology_term=nucleus,isomerase activity,negative regulation of DNA replication,meiotic cell
Fv_160_2.g1568	609	ID=Fv_160_2.g1568;Description=hypothetical protein FVEG_05170 [Fusarium verticillioides 7600];Gene=BFJ68_g11672;Ontology_term=membrane,solute:inorganic anion antiporter activity,monoatomic anion
Fv_160_2.g1569	890	ID=Fv_160_2.g1569;Description=AFG3 family protein [Fusarium verticillioides 7600];Gene=BFJ70_g9175;Ontology_term=fungal-type vacuole membrane,extracellular region,m-AAA complex,mitochondrial inner boundary membrane,ATP-dependent peptidase activity,metalloendopeptidase activity,ATP binding,zinc ion binding,ATP hydrolysis activity,signal peptide processing,protein import into mitochondrial matrix,protein catabolic process,defense response to bacterium,innate immune response,protein-containing complex
Fv_160_2.g1570	238	ID=Fv_160_2.g1570;Description=hypothetical protein FVER53590_25034 [Fusarium verticillioides];Gene=FNAPI_6126;Ontology_term=DNA binding,N-methyltransferase activity,protein methyltransferase activity,site-specific DNA-methyltransferase (adenine-specific) activity,protein metabolic process,DNA methylation on adenine;Ontology_id=GO:0003677,GO:0008170,GO:0008276,GO:0009007,GO:0019538,GO:0032775;Enzyme_code=EC:2.1.1.72;Enz
Fv_160_2.g1571	136	ID=Fv_160_2.g1571;Description=related to DNA-directed RNA polymerase 13.3K chain [Fusarium fujikuroi IMI 58289];Gene=FOC1_g10013754;Ontology_term=RNA polymerase II, core complex,RNA polymerase II activity,DNA binding,protein dimerization activity,transcription by RNA polymerase
Fv_160_2.g1572	83	ID=Fv_160_2.g1572;Description=hypothetical protein HZS61_009913 [Fusarium oxysporum f. sp. conglutinans];Gene=LSM5;Ontology_term=spliceosomal complex,RNA binding,mRNA processing,RNA
Fv_160_2.g1573	552	ID=Fv_160_2.g1573;Description=hypothetical protein FVER53590_05166 [Fusarium verticillioides];Gene=BFJ68_g11676;Ontology_term=nucleic acid binding,helicase activity,3'-5' exonuclease activity,nucleobase-containing compound metabolic
Fv_160_2.g1574	475	ID=Fv_160_2.g1574;Description=ribosome biogenesis protein ytm-1 [Fusarium verticillioides 7600];Gene=YTM1;Ontology_term=nucleoplasm,preribosome, large subunit precursor,PeBoW complex,ribonucleoprotein complex binding,maturation of LSU-rRNA from tricistronic rRNA transcript (SSU-rRNA, 5.8S rRNA, LSU-rRNA),maturation of 5.8S rRNA from tricistronic rRNA transcript (SSU-rRNA, 5.8S rRNA, LSU-rRNA),chromosome organization,protein-RNA complex

Fv_160_2.g1575	705	ID=Fv_160_2.g1575;Description=glycogen [starch] synthase [Fusarium verticillioides 7600];Gene=FOYG_02901;Ontology_term=glycogen (starch) synthase activity,glycogen biosynthetic
Fv_160_2.g1576	809	ID=Fv_160_2.g1576;Description=hypothetical protein FVER14953_05163 [Fusarium
Fv_160_2.g1577	457	ID=Fv_160_2.g1577;Description=hypothetical protein FVEG_05162 [Fusarium verticillioides 7600];Gene=FMAN_04603;Ontology_term=DNA-binding transcription factor activity,regulation of DNA-templated
Fv_160_2.g1578	416	ID=Fv_160_2.g1578;Description=DnaJ like subfamily A member 2 [Fusarium verticillioides 7600];Gene=FOYG_02904;Ontology_term=cytosol,protein aggregate center,ATP binding,Hsp70 protein binding,metal ion binding,unfolded protein binding,regulatory ncRNA-mediated heterochromatin formation,cellular response to heat,protein refolding,protein aggregate center assembly,cytoplasm protein quality
Fv_160_2.g1579	547	ID=Fv_160_2.g1579;Description=hypothetical protein FVEG_05160 [Fusarium verticillioides 7600];Gene=FVER53590_05160;Ontology_term=nucleus,methyltransferase activity,tRNA methylation,RNA (guanine-N7)-
Fv_160_2.g1580	313	ID=Fv_160_2.g1580;Description=60S acidic ribosomal protein P0 [Fusarium verticillioides 7600];Gene=BFJ68_g11694;Ontology_term=cytosolic large ribosomal subunit,structural constituent of ribosome,large ribosomal subunit rRNA binding,ribosomal large subunit assembly,cytoplasmic
Fv_160_2.g1581	1105	ID=Fv_160_2.g1581;Description=hypothetical protein FVER53263_05158 [Fusarium verticillioides];Gene=FTJAE_4690;Ontology_term=ATP binding,mismatched DNA binding,ATP-dependent DNA damage sensor
Fv_160_2.g1582	342	ID=Fv_160_2.g1582;Description=hypothetical protein FVEG_05156 [Fusarium verticillioides 7600]
Fv_160_2.g1583	1080	ID=Fv_160_2.g1583;Description=alpha-mannosidase [Fusarium verticillioides 7600];Gene=FNAPI_12603;Ontology_term=alpha-mannosidase activity,carbohydrate binding,metal ion binding,mannose metabolic
Fv_160_2.g1584	402	ID=Fv_160_2.g1584;Description=NAK protein kinase [Fusarium verticillioides 7600];Gene=CEK26_006190;Ontology_term=protein serine/threonine kinase activity,ATP
Fv_160_2.g1585	1530	ID=Fv_160_2.g1585;Description=hypothetical protein FVER53263_05152 [Fusarium verticillioides];Gene=FANTH_8621;Ontology_term=nucleus,chromatin binding,metal ion
Fv_160_2.g1586	907	ID=Fv_160_2.g1586;Description=DNA polymerase theta subunit [Fusarium verticillioides 7600];Gene=FOYG_02912;Ontology_term=nucleic acid binding,helicase activity,ATP binding,hydrolase
Fv_160_2.g1587	429	ID=Fv_160_2.g1587;Description=DNA polymerase theta subunit [Fusarium coicis];Gene=FPHYL_10889;Ontology_term=nucleic acid binding,helicase activity,ATP binding,hydrolase
Fv_160_2.g1588	481	ID=Fv_160_2.g1588;Description=hypothetical protein FVEG_05150 [Fusarium verticillioides 7600];Gene=FNYG_12067;Ontology_term=aminopeptidase
Fv_160_2.g1589	501	ID=Fv_160_2.g1589;Description=hypothetical protein FVEG_05149 [Fusarium verticillioides 7600]
Fv_160_2.g1590	385	ID=Fv_160_2.g1590;Description=hypothetical protein J7337_002601 [Fusarium musae];Gene=FMUND_11549;Ontology_term=Golgi membrane,RNA binding,metal ion binding,negative regulation of DNA-templated

Fv_160_2.g1591	723	ID=Fv_160_2.g1591;Description=hypothetical protein FVEG_05147 [Fusarium verticillioides 7600];Gene=F25303_7027;Ontology_term=RNA binding,negative regulation of DNA-templated transcription,metal ion
Fv_160_2.g1592	499	ID=Fv_160_2.g1592;Description=hypothetical protein FVEG_05146 [Fusarium verticillioides 7600]
Fv_160_2.g1593	396	ID=Fv_160_2.g1593;Description=ATP-dependent RNA helicase eIF4A [Fusarium oxysporum f. sp. lycopersici 4287];Gene=FGRA07_05449;Ontology_term=RNA helicase activity,translation initiation factor activity,ATP binding,hydrolase activity,cytoplasmic translational
Fv_160_2.g1594	485	ID=Fv_160_2.g1594;Description=hypothetical protein FVEG_05144 [Fusarium verticillioides 7600];Gene=FACUT_6946;Ontology_term=3-isopropylmalate dehydratase complex,membrane,3-isopropylmalate dehydratase activity,metal ion binding,4 iron, 4 sulfur cluster binding,leucine biosynthetic
Fv_160_2.g1595	509	ID=Fv_160_2.g1595;Description=eukaryotic translation initiation factor 2 subunit gamma [Fusarium tjaetaba];Gene=FTJAE_12114;Ontology_term=ribosome,eukaryotic translation initiation factor 2 complex,eukaryotic 43S preinitiation complex,eukaryotic 48S preinitiation complex,multi-eIF complex,translation initiation factor activity,GTPase activity,GTP binding,translation initiation factor binding,methionyl-initiator methionine tRNA binding,formation of translation preinitiation complex,positive regulation of translational
Fv_160_2.g1596	485	ID=Fv_160_2.g1596;Description=hypothetical protein FVEG_05142 [Fusarium verticillioides 7600];Gene=hslU
Fv_160_2.g1597	714	ID=Fv_160_2.g1597;Description=hypothetical protein FVEG_05141 [Fusarium verticillioides 7600];Gene=FVER53590_05141;Ontology_term=nuclear pore,cytoplasm,microtubule,structural constituent of cytoskeleton,structural constituent of nuclear pore,nucleocytoplasmic transport,cytoskeleton organization,protein
Fv_160_2.g1598	96	ID=Fv_160_2.g1598;Description=hypothetical protein FVEG_05140 [Fusarium verticillioides 7600];Gene=FOYG_02924;Ontology_term=mitochondrion,ATPase inhibitor activity;Ontology_id=GO:0005739,GO:0042030
Fv_160_2.g1599	268	ID=Fv_160_2.g1599;Description=14-3-3 family protein [Fusarium verticillioides 7600];Gene=FMUND_11558;Ontology_term=monooxygenase
Fv_160_2.g1600	340	ID=Fv_160_2.g1600;Description=hypothetical protein FOFC_04799, partial [Fusarium oxysporum];Gene=F52700_11678;Ontology_term=mitochondrial large ribosomal subunit,structural constituent of
Fv_160_2.g1601	723	ID=Fv_160_2.g1601;Description=pre-mRNA-splicing ATP-dependent RNA helicase PRP28 [Fusarium verticillioides 7600];Gene=BFJ70_g9122;Ontology_term=U5 snRNP,first spliceosomal transesterification activity,RNA binding,RNA helicase activity,ATP binding,hydrolase activity,mRNA 5'-splice site
Fv_160_2.g1602	1086	ID=Fv_160_2.g1602;Description=valyl-tRNA synthetase [Fusarium verticillioides 7600];Gene=Forpe1208_v002832;Ontology_term=cytoplasm,aminoacyl-tRNA editing activity,valine-tRNA ligase activity,ATP binding,valyl-tRNA aminoacylation,aminoacyl-tRNA metabolism involved in translational fidelity;Ontology_id=GO:0005737,GO:0002161,GO:0004832,GO:0005524,GO:0006438,GO:0106074;Enzyme_code=EC:6.1.1.9,EC:3.1
Fv_160_2.g1603	494	ID=Fv_160_2.g1603;Description=v-type c subunit family [Fusarium napiforme]

Fv_160_2.g1604	329	ID=Fv_160_2.g1604;Description=hypothetical protein FVER53263_05134 [Fusarium verticillioides];Gene=FNYG_15685;Ontology_term=oxidoreductase activity,NADP
Fv_160_2.g1605	225	ID=Fv_160_2.g1605;Description=hypothetical protein FVER53590_05133 [Fusarium verticillioides]
Fv_160_2.g1606	317	ID=Fv_160_2.g1606;Description=hypothetical protein FVEG_05132 [Fusarium verticillioides
Fv_160_2.g1607	375	ID=Fv_160_2.g1607;Description=mannan polymerase complexes MNN9 subunit [Fusarium verticillioides 7600];Gene=FFUJ_03016;Ontology_term=glycosyltransferase
Fv_160_2.g1608	172	ID=Fv_160_2.g1608;Description=peroxin-4 [Fusarium verticillioides 7600]
Fv_160_2.g1609	543	ID=Fv_160_2.g1609;Description=hypothetical protein J7337_002620 [Fusarium musae];Gene=FFC1_07431;Ontology_term=nucleus,membrane,DNA-binding transcription factor activity, RNA polymerase II-
Fv_160_2.g1610	715	ID=Fv_160_2.g1610;Description=hypothetical protein FVEG_05127 [Fusarium verticillioides 7600];Gene=BFJ69_g9480;Ontology_term=cytoplasm,SH3 domain binding,apoptotic
Fv_160_2.g1611	209	ID=Fv_160_2.g1611;Description=splicing factor U2AF 23 kDa subunit [Fusarium verticillioides 7600];Gene=FOC1_g10013797;Ontology_term=commitment complex,U2-type prespliceosome,U2AF complex,RNA binding,metal ion binding,mRNA cis splicing, via
Fv_160_2.g1612	507	ID=Fv_160_2.g1612;Description=hypothetical protein FVEG_05125 [Fusarium verticillioides
Fv_160_2.g1613	1166	ID=Fv_160_2.g1613;Description=hypothetical protein FVEG_05124 [Fusarium verticillioides 7600]
Fv_160_2.g1614	420	ID=Fv_160_2.g1614;Description=hypothetical protein FVER14953_05123 [Fusarium verticillioides];Gene=FNYG_15696;Ontology_term=flavin adenine dinucleotide binding,tRNA-dihydrouridine47 synthase activity,tRNA dihydrouridine synthesis,mRNA
Fv_160_2.g1615	249	ID=Fv_160_2.g1615;Description=hypothetical protein FVEG_05122 [Fusarium verticillioides 7600];Gene=FOTG_00748;Ontology_term=MICOS complex,cristae formation;Ontology_id=GO:0061617,GO:0042407
Fv_160_2.g1616	787	ID=Fv_160_2.g1616;Description=hypothetical protein FVEG_05121 [Fusarium verticillioides 7600];Gene=FOIG_12220;Ontology_term=mitochondrial matrix,metalloendopeptidase activity,metal ion
Fv_160_2.g1617	689	ID=Fv_160_2.g1617;Description=hypothetical protein FVEG_05120 [Fusarium verticillioides 7600]
Fv_160_2.g1618	689	ID=Fv_160_2.g1618;Description=hypothetical protein FVEG_05119 [Fusarium verticillioides 7600];Gene=FOYG_02946;Ontology_term=vacuolar membrane,helicase activity,ATP hydrolysis
Fv_160_2.g1619	247	ID=Fv_160_2.g1619;Description=hypothetical protein FVEG_05118 [Fusarium verticillioides 7600];Gene=FCIRC_2884;Ontology_term=vacuolar membrane,DNA binding;Ontology_id=GO:0005774,GO:0003677
Fv_160_2.g1620	330	ID=Fv_160_2.g1620;Description=hypothetical protein FVEG_05117 [Fusarium verticillioides 7600]
Fv_160_2.g1621	841	ID=Fv_160_2.g1621;Description=hypothetical protein FVER53590_05116 [Fusarium verticillioides];Gene=FOQG_00758;Ontology_term=diphthine-ammonia ligase
Fv_160_2.g1622	547	ID=Fv_160_2.g1622;Description=hypothetical protein FVER53263_05115 [Fusarium verticillioides]

Fv_160_2.g1623	375	ID=Fv_160_2.g1623;Description=licheninase [Fusarium verticillioides 7600];Gene=FOVG_02825;Ontology_term=incipient cellular bud site,fungal-type cell wall,membrane,hydrolase activity, hydrolyzing O-glycosyl compounds,glycosyltransferase activity,carbohydrate metabolic process,cell wall chitin metabolic process,fungal-type cell wall organization;Ontology_id=GO:0000131,GO:0009277,GO:0016020,GO:0004553,GO:0016757,GO:0005975,GO:0006037,GO:0031505;
Fv_160_2.g1624	909	ID=Fv_160_2.g1624;Description=hypothetical protein FVER14953_05113 [Fusarium verticillioides];Gene=FDENT_5308;Ontology_term=nucleolus,cytosol,replication fork protection complex,DNA binding,DNA topoisomerase type I (single strand cut, ATP-independent) activity,regulation of mitotic recombination,rDNA heterochromatin formation,DNA topological change,DNA strand elongation involved in DNA replication,regulation of transcription by RNA polymerase II,transcription elongation by RNA polymerase II,mitotic chromosome condensation,nuclear migration,rRNA transcription;Ontology_id=GO:0005730,GO:0005829,GO:0031298,GO:0003677,GO:0003917,GO:0000019,GO:0000183,GO:0006265
Fv_160_2.g1625	301	ID=Fv_160_2.g1625;Description=F-type H+-transporting ATPase subunit gamma [Fusarium oxysporum f. sp. lycopersici 4287];Gene=FTJAE_10951;Ontology_term=mitochondrial proton-transporting ATP synthase, central stalk,proton-transporting ATP synthase activity, rotational mechanism,proton-transporting ATPase activity, rotational mechanism,proton motive force-driven ATP synthesis,proton transmembrane transport;Ontology_id=GO:0005756,GO:0046933,GO:0046961,GO:0015986,GO:1902600;Enzyme_code=EC:7.1.2.2,EC:6,EC:7.2.2;Enz
Fv_160_2.g1626	1090	ID=Fv_160_2.g1626;Description=hypothetical protein FVER53263_05111 [Fusarium verticillioides];Gene=FCIRC_2876;Ontology_term=nucleus,DNA binding,methyltransferase activity,oxidoreductase activity,vesicle-mediated transport,methylation;Ontology_id=GO:0005634,GO:0003677,GO:0008168,GO:0016491,GO:0016192,GO:0032259;Enzyme_code=E
Fv_160_2.g1627	660	ID=Fv_160_2.g1627;Description=lysine-specific histone demethylase 1 [Fusarium verticillioides 7600];Gene=FVEG_05109;Ontology_term=membrane,methyltransferase activity,vesicle-mediated
Fv_160_2.g1628	158	ID=Fv_160_2.g1628;Description=ubiquitin-conjugating enzyme E2 W [Fusarium verticillioides 7600]
Fv_160_2.g1629	363	ID=Fv_160_2.g1629;Description=CAMK/CAMK1/CAMK1-CMK protein kinase [Fusarium verticillioides 7600];Gene=FOQG_00748;Ontology_term=protein serine/threonine kinase activity,ATP
Fv_160_2.g1630	398	ID=Fv_160_2.g1630;Description=hypothetical protein FVER14953_05105 [Fusarium verticillioides]
Fv_160_2.g1631	551	ID=Fv_160_2.g1631;Description=histidyl-tRNA synthetase [Fusarium verticillioides 7600];Gene=FNYG_14046;Ontology_term=cytoplasm,histidine-tRNA ligase activity,ATP binding,histidyl-tRNA
Fv_160_2.g1632	1318	ID=Fv_160_2.g1632;Description=hypothetical protein FVER14953_20066 [Fusarium verticillioides];Gene=FNAPI_6468;Ontology_term=plasma membrane,GTPase activity,ATP binding,GTP binding,ATP hydrolysis activity,ABC-type transporter activity,signal transduction,transmembrane transport;Ontology_id=GO:0005886,GO:0003924,GO:0005524,GO:0005525,GO:0016887,GO:0140359,GO:0007165,GO:0055085;En

Fv_160_2.g1633	382	ID=Fv_160_2.g1633;Description=hypothetical protein FVER53590_05102 [Fusarium verticillioides];Gene=FNAPI_6467;Ontology_term=nucleus,DNA binding,zinc ion binding,DNA-binding transcription factor activity, RNA polymerase II-specific,regulation of transcription by RNA polymerase II,hydrolase
Fv_160_2.g1634	923	ID=Fv_160_2.g1634;Description=hypothetical protein FVER53590_14062 [Fusarium verticillioides];Gene=FPANT_11777;Ontology_term=nucleus,DNA binding,zinc ion binding,DNA-binding transcription factor activity, RNA polymerase II-specific,regulation of transcription by RNA polymerase
Fv_160_2.g1635	604	ID=Fv_160_2.g1635;Description=hypothetical protein FVEG_14060 [Fusarium verticillioides
Fv_160_2.g1636	487	ID=Fv_160_2.g1636;Description=P-loop containing nucleoside triphosphate hydrolase protein [Fusarium oxysporum];Gene=FDENT_5295;Ontology_term=DNA binding,DNA helicase activity,ATP binding,hydrolase activity,DNA duplex unwinding;Ontology_id=GO:0003677,GO:0003678,GO:0005524,GO:0016787,GO:0032508;Enzyme_code=EC:3;Enzyme_name=Hydr
Fv_160_2.g1637	1486	ID=Fv_160_2.g1637;Description=hypothetical protein FVER14953_14058 [Fusarium verticillioides];Gene=FPCIR_9191;Ontology_term=membrane,lipid binding,lipid transport,endoplasmic reticulum-plasma membrane
Fv_160_2.g1638	282	ID=Fv_160_2.g1638;Description=hypothetical protein FOXB_00423 [Fusarium oxysporum f. sp. conglutinans Fo5176];Gene=FPCIR_9192;Ontology_term=nucleus,cytoplasm,membrane,proteasome core complex, beta-subunit complex,threonine-type endopeptidase activity,lipid binding,lipid transport,proteasomal ubiquitin-independent protein catabolic process,proteasome-mediated ubiquitin-dependent protein catabolic process,endoplasmic reticulum-plasma membrane tethering,proteasome core complex assembly;Ontology_id=GO:0005634,GO:0005737,GO:0016020,GO:0019774,GO:0004298,GO:0008289,GO:0006869,GO:0010499,GO
Fv_160_2.g1639	455	ID=Fv_160_2.g1639;Description=hypothetical protein FVEG_14056 [Fusarium verticillioides 7600];Gene=FPCIR_9193;Ontology_term=mitochondrion,membrane,peptidase activity,metal ion binding,N(6)-L-threonylcarbamoyladenine synthase activity,tRNA threonylcarbamoyladenosine modification,proteolysis;Ontology_id=GO:0005739,GO:0016020,GO:0008233,GO:0046872,GO:0061711,GO:0002949,GO:0006508;E
Fv_160_2.g1640	1007	ID=Fv_160_2.g1640;Description=hypothetical protein FVER14953_14055 [Fusarium verticillioides];Gene=FOQG_00736;Ontology_term=mitochondrion,membrane,metal ion binding,N(6)-L-threonylcarbamoyladenine synthase activity,tRNA threonylcarbamoyladenosine
Fv_160_2.g1641	452	ID=Fv_160_2.g1641;Description=mannan polymerase II complex MNN10 subunit [Fusarium verticillioides 7600];Gene=FOC1_g10013834;Ontology_term=mannan polymerase complex,alpha-1,6-mannosyltransferase activity,cell wall mannoprotein biosynthetic process,division septum assembly,cell
Fv_160_2.g1642	511	ID=Fv_160_2.g1642;Description=hypothetical protein FVER53590_14052 [Fusarium verticillioides];Gene=FNYG_14058;Ontology_term=hydrolase activity, hydrolyzing O-glycosyl compounds,carbohydrate metabolic
Fv_160_2.g1643	266	ID=Fv_160_2.g1643;Description=hypothetical protein FAGAP_13020 [Fusarium
Fv_160_2.g1644	643	ID=Fv_160_2.g1644;Description=hypothetical protein FVEG_14050 [Fusarium verticillioides 7600];Gene=FNAPI_13625;Ontology_term=membrane,lyase activity,quinate metabolic process,3,4-dihydroxybenzoate biosynthetic

Fv_160_2.g1645	159	ID=Fv_160_2.g1645;Description=hypothetical protein FVEG_14049 [Fusarium verticillioides 7600];Gene=2166;Ontology_term=extracellular region,nucleus,early endosome,Golgi-associated vesicle,clathrin-coated pit,cell surface,growth cone,perikaryon,recycling endosome,metal ion
Fv_160_2.g1646	321	ID=Fv_160_2.g1646;Description=hypothetical protein FVEG_14048 [Fusarium verticillioides 7600];Gene=3061
Fv_160_2.g1647	156	ID=Fv_160_2.g1647;Description=hypothetical protein FPSE_11766 [Fusarium pseudograminearum CS3096];Gene=F53441_4880;Ontology_term=ribosome,ribonucleoprotein complex,RNA binding,structural constituent of
Fv_160_2.g1648	308	ID=Fv_160_2.g1648;Description=hypothetical protein FVEG_14046 [Fusarium verticillioides 7600];Gene=FGLOB1_9370;Ontology_term=guanyl-nucleotide exchange factor activity,cytosolic ribosome
Fv_160_2.g1649	541	ID=Fv_160_2.g1649;Description=hypothetical protein FVER14953_14045 [Fusarium verticillioides];Gene=FTJAE_9760;Ontology_term=vacuole,serine-type carboxypeptidase
Fv_160_2.g1650	461	ID=Fv_160_2.g1650;Description=hypothetical protein FVER14953_14044 [Fusarium verticillioides];Gene=FMAN_04527;Ontology_term=membrane,fatty acid elongase activity,fatty acid biosynthetic
Fv_160_2.g1651	356	ID=Fv_160_2.g1651;Description=VPS27-vacuolar sorting-associated [Fusarium
Fv_160_2.g1652	654	ID=Fv_160_2.g1652;Description=hypothetical protein FVEG_17707 [Fusarium verticillioides 7600];Gene=FOTG_00704;Ontology_term=molybdopterin synthase complex,GTP binding,metal ion binding,4 iron, 4 sulfur cluster binding,GTP 3',8'-cyclase activity,cyclic pyranopterin monophosphate synthase activity,Mo-molybdopterin cofactor biosynthetic process;Ontology_id=GO:0019008,GO:0005525,GO:0046872,GO:0051539,GO:0061798,GO:0061799,GO:0006777;Enzyme_code=EC
Fv_160_2.g1653	511	ID=Fv_160_2.g1653;Description=hypothetical protein FVEG_14040 [Fusarium verticillioides
Fv_160_2.g1654	433	ID=Fv_160_2.g1654;Description=hypothetical protein FVER53590_14038 [Fusarium
Fv_160_2.g1655	2058	ID=Fv_160_2.g1655;Description=hypothetical protein FVER14953_14037 [Fusarium verticillioides];Gene=HNF1B;Ontology_term=nucleus,RNA polymerase II cis-regulatory region sequence-specific DNA binding,DNA-binding transcription factor activity, RNA polymerase II-specific,protein homodimerization activity,kidney development,liver development,regulation of transcription by RNA polymerase II,insulin secretion,endocrine pancreas development,positive regulation
Fv_160_2.g1656	297	ID=Fv_160_2.g1656;Description=hypothetical protein FVEG_17705 [Fusarium verticillioides 7600]
Fv_160_2.g1657	92	ID=Fv_160_2.g1657;Description=hypothetical protein FVEG_14036 [Fusarium verticillioides 7600];Gene=FNAPI_4158;Ontology_term=mitochondrion,membrane,mitochondrial cytochrome c oxidase
Fv_160_2.g1658	608	ID=Fv_160_2.g1658;Description=hypothetical protein FVER14953_14034 [Fusarium verticillioides]
Fv_160_2.g1659	264	ID=Fv_160_2.g1659;Description=hypothetical protein FVEG_14033 [Fusarium verticillioides 7600];Gene=FOXG_01912;Ontology_term=cytoplasm,mRNA cleavage factor complex,mRNA binding,mRNA
Fv_160_2.g1660	211	ID=Fv_160_2.g1660;Description=hypothetical protein FVEG_17704 [Fusarium verticillioides
Fv_160_2.g1661	164	ID=Fv_160_2.g1661;Description=hypothetical protein FVER14953_14032 [Fusarium verticillioides]

Fv_160_2.g1662	271	ID=Fv_160_2.g1662;Description=hypothetical protein FVER14953_14031 [Fusarium verticillioides];Gene=FTJAE_13019;Ontology_term=extracellular region,mitochondrial matrix,respirasome,nucleic acid binding,pheromone activity,zinc ion binding,protein-containing complex binding,signal transduction,ubiquinone-6 biosynthetic
Fv_160_2.g1663	1023	ID=Fv_160_2.g1663;Description=insulysin [Fusarium verticillioides 7600];Gene=BFJ70_g17464;Ontology_term=metallopeptidase activity,metal ion
Fv_160_2.g1664	1635	ID=Fv_160_2.g1664;Description=hypothetical protein FNYG_06420 [Fusarium nygamai];Gene=FMUND_72;Ontology_term=cysteine-type deubiquitinase activity,metallopeptidase activity,metal ion
Fv_160_2.g1665	876	ID=Fv_160_2.g1665;Description=hypothetical protein FVER14953_08285 [Fusarium verticillioides];Gene=FTJAE_13016;Ontology_term=3',5'-cyclic-nucleotide phosphodiesterase activity,metal ion binding,signal
Fv_160_2.g1666	640	ID=Fv_160_2.g1666;Description=hypothetical protein FVER53263_08284 [Fusarium verticillioides];Gene=FVER53590_08284;Ontology_term=nucleus,DNA binding,zinc ion binding,DNA-binding transcription factor activity, RNA polymerase II-specific,regulation of transcription by RNA polymerase
Fv_160_2.g1667	503	ID=Fv_160_2.g1667;Description=hypothetical protein FVEG_08283 [Fusarium verticillioides 7600];Gene=FPHYL_9691;Ontology_term=membrane,transmembrane transporter activity,transmembrane
Fv_160_2.g1668	352	ID=Fv_160_2.g1668;Description=hypothetical protein FVEG_08282 [Fusarium verticillioides 7600]
Fv_160_2.g1669	751	ID=Fv_160_2.g1669;Description=hypothetical protein FVER53590_08281 [Fusarium verticillioides];Gene=FPANT_1638;Ontology_term=membrane,long-chain-alcohol oxidase activity,flavin adenine dinucleotide
Fv_160_3.g1670	443	ID=Fv_160_3.g1670;Description=choline-phosphate cytidylyltransferase [Fusarium verticillioides 7600];Gene=FOC1_g10012845;Ontology_term=extracellular space,nuclear envelope,NLS-dependent protein nuclear import complex,choline-phosphate cytidylyltransferase activity,hormone activity,CDP-choline pathway,G protein-coupled receptor signaling pathway,response to food;Ontology_id=GO:0005615,GO:0005635,GO:0042564,GO:0004105,GO:0005179,GO:0006657,GO:0007186,GO:0032094;Enzyme
Fv_160_3.g1671	956	ID=Fv_160_3.g1671;Description=hypothetical protein FVEG_02726 [Fusarium verticillioides 7600];Gene=FOTG_02879;Ontology_term=nucleus,DNA binding,zinc ion binding,DNA-templated
Fv_160_3.g1672	1133	ID=Fv_160_3.g1672;Description=hypothetical protein FVER53590_02727 [Fusarium verticillioides];Gene=psd3;Ontology_term=Golgi membrane,Golgi stack,endosome membrane,phosphatidylserine decarboxylase activity,calcium ion binding,phosphatidylethanolamine biosynthetic process,protein autoprocessing;Ontology_id=GO:0000139,GO:0005795,GO:0010008,GO:0004609,GO:0005509,GO:0006646,GO:0016540;Enzyme_c
Fv_160_3.g1673	71	ID=Fv_160_3.g1673;Description=hypothetical protein FVEG_02728 [Fusarium verticillioides
Fv_160_3.g1674	690	ID=Fv_160_3.g1674;Description=hypothetical protein FVEG_02729 [Fusarium verticillioides
Fv_160_3.g1675	1182	ID=Fv_160_3.g1675;Description=hypothetical protein FocTR4_00008650 [Fusarium oxysporum f. sp. cubense]

Fv_160_3.g1676	302	ID=Fv_160_3.g1676;Description=60S ribosomal protein L5 [Fusarium oxysporum f. sp. lycopersici 4287];Gene=FOMG_06802;Ontology_term=cytosolic large ribosomal subunit,structural constituent of ribosome,beta-fructofuranosidase activity,5S rRNA binding,ribosomal large subunit assembly,carbohydrate metabolic process,translation;Ontology_id=GO:0022625,GO:0003735,GO:0004564,GO:0008097,GO:0000027,GO:0005975,GO:0006412;Enzym
Fv_160_3.g1677	284	ID=Fv_160_3.g1677;Description=hypothetical protein FVER53590_02732 [Fusarium verticillioides];Gene=ACAD10;Ontology_term=phosphatase
Fv_160_3.g1678	105	ID=Fv_160_3.g1678;Description=hypothetical protein FVER53590_25966 [Fusarium verticillioides];Gene=FMAN_07615;Ontology_term=S-adenosylmethionine-dependent methyltransferase
Fv_160_3.g1679	89	ID=Fv_160_3.g1679;Description=hypothetical protein FVER53263_20248 [Fusarium verticillioides];Gene=FMAN_07615;Ontology_term=S-adenosylmethionine-dependent methyltransferase
Fv_160_3.g1680	421	ID=Fv_160_3.g1680;Description=hypothetical protein FVEG_02733 [Fusarium verticillioides 7600]
Fv_160_3.g1681	521	ID=Fv_160_3.g1681;Description=hypothetical protein FVEG_02734 [Fusarium verticillioides 7600]
Fv_160_3.g1682	1153	ID=Fv_160_3.g1682;Description=hypothetical protein FVER53590_02735 [Fusarium
Fv_160_3.g1683	935	ID=Fv_160_3.g1683;Description=hypothetical protein FVEG_02736 [Fusarium verticillioides 7600]
Fv_160_3.g1684	1298	ID=Fv_160_3.g1684;Description=hypothetical protein FVER53263_02737 [Fusarium verticillioides];Gene=FTJAE_8176;Ontology_term=nucleus,DNA-binding transcription factor activity,sequence-specific DNA
Fv_160_3.g1685	440	ID=Fv_160_3.g1685;Description=hypothetical protein FVEG_02739 [Fusarium verticillioides 7600];Gene=FTJAE_8175;Ontology_term=membrane,molybdate ion transmembrane transporter activity,molybdate ion
Fv_160_3.g1686	330	ID=Fv_160_3.g1686;Description=hypothetical protein FVER14953_02741 [Fusarium verticillioides];Gene=FPCIR_13262;Ontology_term=endoplasmic reticulum membrane,hydrolase activity, acting on ester
Fv_160_3.g1687	552	ID=Fv_160_3.g1687;Description=hypothetical protein FVER14953_02742 [Fusarium verticillioides]
Fv_160_3.g1688	394	ID=Fv_160_3.g1688;Description=hypothetical protein FVEG_02743 [Fusarium verticillioides
Fv_160_3.g1689	381	ID=Fv_160_3.g1689;Description=hypothetical protein FVEG_02744 [Fusarium verticillioides
Fv_160_3.g1690	604	ID=Fv_160_3.g1690;Description=hypothetical protein FVER14953_02745 [Fusarium verticillioides]
Fv_160_3.g1691	294	ID=Fv_160_3.g1691;Description=hypothetical protein J7337_005660 [Fusarium musae];Gene=FOC1_g10012823;Ontology_term=oxidoreductase activity,cellular biosynthetic
Fv_160_3.g1692	310	ID=Fv_160_3.g1692;Description=hypothetical protein FVEG_02747 [Fusarium verticillioides 7600];Gene=FOPG_04905;Ontology_term=oxidoreductase activity,cellular biosynthetic
Fv_160_3.g1693	514	ID=Fv_160_3.g1693;Description=hypothetical protein FVEG_02748 [Fusarium verticillioides 7600];Gene=1824;Ontology_term=membrane,ferric-chelate reductase activity,mating-type factor pheromone receptor activity,transition metal ion transport,G protein-coupled receptor signaling
Fv_160_3.g1694	953	ID=Fv_160_3.g1694;Description=hypothetical protein FVEG_02749 [Fusarium verticillioides

Fv_160_3.g1695	598	ID=Fv_160_3.g1695;Description=hypothetical protein FVEG_02750 [Fusarium verticillioides 7600];Gene=FTJAE_8165;Ontology_term=membrane,transmembrane transporter activity,transmembrane
Fv_160_3.g1696	920	ID=Fv_160_3.g1696;Description=nitrate assimilation regulatory nirA [Fusarium pseudoanthophilum];Gene=FPCIR_10716;Ontology_term=nucleus,DNA binding,zinc ion binding,DNA-templated
Fv_160_3.g1697	759	ID=Fv_160_3.g1697;Description=3-dehydroquinate dehydratase I [Fusarium verticillioides 7600];Gene=FNAPI_9127;Ontology_term=3-dehydroquinate dehydratase
Fv_160_3.g1698	116	ID=Fv_160_3.g1698;Description=hypothetical protein FVEG_02754 [Fusarium verticillioides 7600]
Fv_160_3.g1699	522	ID=Fv_160_3.g1699;Description=HAT1-interacting factor 1 [Fusarium verticillioides 7600];Gene=1817
Fv_160_3.g1700	709	ID=Fv_160_3.g1700;Description=hypothetical protein FVER14953_02756 [Fusarium verticillioides];Gene=FVEG_02756;Ontology_term=sno(s)RNA-containing ribonucleoprotein complex,Mpp10 complex,rRNA
Fv_160_3.g1701	113	ID=Fv_160_3.g1701;Description=hypothetical protein FVEG_15126 [Fusarium verticillioides 7600];Gene=dddP-0;Ontology_term=aminopeptidase activity,lyase
Fv_160_3.g1702	332	ID=Fv_160_3.g1702;Description=AP complex, mu/sigma subunit [Fusarium oxysporum f. sp. vasinfectum];Gene=FVEG_02757;Ontology_term=nucleus,AP-1 adaptor complex,clathrin adaptor activity,vesicle-mediated
Fv_160_3.g1703	197	ID=Fv_160_3.g1703;Description=clathrin coat assembly AP19 [Fusarium sp. NRRL 52700];Gene=FGADI_8676;Ontology_term=AP-1 adaptor complex,clathrin adaptor activity,intracellular protein transport,vesicle-mediated
Fv_160_3.g1704	312	ID=Fv_160_3.g1704;Description=hypothetical protein FVEG_02759 [Fusarium verticillioides 7600];Gene=FTJAE_10742;Ontology_term=ligase activity,glutamate-cysteine ligase catalytic subunit binding,glutathione biosynthetic
Fv_160_3.g1705	262	ID=Fv_160_3.g1705;Description=BUD32 protein kinase [Fusarium verticillioides 7600];Gene=FMEXI_4717;Ontology_term=chromosome, telomeric region,protein serine/threonine kinase activity,ATP binding,tRNA processing,phosphorylation;Ontology_id=GO:0000781,GO:0004674,GO:0005524,GO:0008033,GO:0016310;Enzyme_code=EC:2.7.11
Fv_160_3.g1706	252	ID=Fv_160_3.g1706;Description=hypothetical protein FVEG_02761 [Fusarium verticillioides
Fv_160_3.g1707	190	ID=Fv_160_3.g1707;Description=hypothetical protein FVEG_02762 [Fusarium verticillioides 7600];Gene=FMEXI_4719;Ontology_term=peroxiredoxin activity,cellular oxidant
Fv_160_3.g1708	1032	ID=Fv_160_3.g1708;Description=FACT complex subunit SPT16 [Fusarium verticillioides 7600];Gene=FACUT_3958;Ontology_term=DNA repair,FACT complex,DNA-templated DNA replication,integrin binding,constitutive heterochromatin formation,positive regulation of cell division,histone chaperone activity,regulation of defense response to virus by host,positive regulation of type II interferon production,cytosol,positive regulation of T-helper 1 cell cytokine production,replication fork protection complex,vascular endothelial growth factor production,signal transduction,regulation of sister chromatid cohesion,nucleosome binding,cytokine activity,fever generation,defense response to bacterium,nucleosome assembly,interleukin-1 receptor binding,lysosome,positive regulation of RNA polymerase II transcription preinitiation complex assembly,extracellular space,histone

Fv_160_3.g1709	426	ID=Fv_160_3.g1709;Description=hypothetical protein FVEG_02764 [Fusarium verticillioides 7600];Gene=FACUT_3959;Ontology_term=membrane,phospholipid translocation,cell
Fv_160_3.g1710	1150	ID=Fv_160_3.g1710;Description=hypothetical protein FVER14953_02765 [Fusarium verticillioides];Gene=FVER53263_02765;Ontology_term=membrane,phosphorelay sensor kinase activity,phosphorelay signal transduction system,phosphorylation;Ontology_id=GO:0016020,GO:0000155,GO:0000160,GO:0016310;Enzyme_code=EC:2.7.3,EC:2.7.13.3;Enzy
Fv_160_3.g1711	862	ID=Fv_160_3.g1711;Description=hypothetical protein FVEG_02766 [Fusarium verticillioides 7600];Gene=FVEG_02766;Ontology_term=glutamine synthetase activity,hydrolase activity,glutamine biosynthetic
Fv_160_3.g1712	358	ID=Fv_160_3.g1712;Description=hypothetical protein FVEG_02768 [Fusarium verticillioides
Fv_160_3.g1713	1265	ID=Fv_160_3.g1713;Description=hypothetical protein FVEG_02770 [Fusarium verticillioides 7600];Gene=F25303_12900;Ontology_term=protein heterodimerization activity;Ontology_id=GO:0046982
Fv_160_3.g1714	615	ID=Fv_160_3.g1714;Description=hypothetical protein FVER14953_02771 [Fusarium verticillioides]
Fv_160_3.g1715	434	ID=Fv_160_3.g1715;Description=hypothetical protein FVEG_02772 [Fusarium verticillioides 7600];Gene=FSUBG_13311;Ontology_term=cytoplasm,endocytosis,actin cortical patch
Fv_160_3.g1716	502	ID=Fv_160_3.g1716;Description=cysteine desulfurase [Fusarium verticillioides 7600];Gene=FMUND_15869;Ontology_term=nucleus,mitochondrion,L-cysteine desulfurase complex,pyridoxal phosphate binding,cysteine desulfurase activity,metal ion binding,iron-sulfur cluster binding,tRNA wobble position uridine thiolation,intracellular iron ion homeostasis,[2Fe-2S] cluster assembly,mitochondrial tRNA thio-modification;Ontology_id=GO:0005634,GO:0005739,GO:1990221,GO:0030170,GO:0031071,GO:0046872,GO:0051536,GO:0002143,
Fv_160_3.g1717	181	ID=Fv_160_3.g1717;Description=hypothetical protein FVEG_02774 [Fusarium verticillioides 7600]

Fv_160_3.g1718	576	ID=Fv_160_3.g1718;Description=hypothetical protein CEK27_008507 [Fusarium fujikuroi];Gene=CEK26_008490;Ontology_term=extracellular space,nucleolus,endoplasmic reticulum membrane,ribonucleoprotein complex,delta24(24-1) sterol reductase activity,RNA binding,cytokine activity,type II interferon receptor binding,pyridoxal phosphate binding,cysteine desulfurase activity,metal ion binding,protein heterodimerization activity,delta24-sterol reductase activity,iron-sulfur cluster binding,microglial cell activation,macrophage activation involved in immune response,transcription by RNA polymerase II,ergosterol biosynthetic process,positive regulation of autophagy,positive regulation of epithelial cell migration,macrophage differentiation,positive regulation of protein-containing complex assembly,negative regulation of interleukin-17 production,positive regulation of chemokine production,positive regulation of interleukin-12 production,positive regulation of interleukin-23 production,positive regulation of interleukin-6 production,positive regulation of CD4-positive, CD25-positive, alpha-beta regulatory T cell differentiation involved in immune response,positive regulation of peptidyl-serine phosphorylation of STAT protein,positive regulation of smooth muscle cell apoptotic process,type III interferon-mediated signaling pathway,ribosome biogenesis,positive regulation of protein import into nucleus,positive regulation of tyrosine phosphorylation of STAT protein,[2Fe-2S] cluster assembly,positive regulation of nitric oxide biosynthetic process,positive regulation of osteoclast differentiation,negative regulation of DNA-templated transcription,astrocyte activation,negative regulation of smooth muscle cell proliferation,positive regulation of inflammatory response,positive regulation of neurogenesis,regulation of insulin secretion,positive regulation of membrane protein ectodomain proteolysis,defense response to virus,positive regulation of killing of cells of another organism,type II interferon-mediated signaling pathway,positive regulation of fructose 1,6-bisphosphate metabolic process,positive regulation of vitamin D biosynthetic process,positive regulation of protein deacetylation,extrinsic apoptotic signaling pathway,positive regulation of amyloid-beta formation,positive regulation of tumor necrosis factor (ligand) superfamily member 11
Fv_160_3.g1719	161	ID=Fv_160_3.g1719;Description=V-type proton ATPase proteolipid subunit [Fusarium oxysporum f. sp. lycopersici 4287];Gene=BFJ65_g7292;Ontology_term=vacuolar membrane,proton-transporting V-type ATPase, V0 domain,proton-transporting ATPase activity, rotational mechanism,proton transmembrane
Fv_160_3.g1720	124	ID=Fv_160_3.g1720;Description=ribonucleoprotein-associated protein [Fusarium oxysporum f. sp. lycopersici 4287];Gene=FNAPI_12537;Ontology_term=nucleolus,ribosome,membrane,box C/D RNP complex,small-subunit processome,U4/U6 x U5 tri-snRNP complex,oxidoreductase activity, acting on the CH-CH group of donors, NAD or NADP as acceptor,transmembrane transporter activity,pyridoxal phosphate binding,U4 snRNA binding,cysteine desulfurase activity,U3 snoRNA binding,metal ion binding,protein heterodimerization activity,iron-sulfur cluster binding,mRNA splicing, via spliceosome,snoRNA guided rRNA 2'-O-methylation,maturation of SSU-rRNA from tricistronic rRNA transcript (SSU-rRNA, 5.8S rRNA, LSU-rRNA),box C/D RNA 3'-end processing,transcription by RNA polymerase II,sterol biosynthetic process,[2Fe-2S] cluster assembly,transmembrane transport;Ontology_id=GO:0005730,GO:0005840,GO:0016020,GO:0031428,GO:0032040,GO:0046540,GO:0016628,GO:0022857,GO:0030170,GO:0030621,GO:0031071,GO:0034511,GO:0046872,GO:0046982,GO:0051536,GO:0000398,GO:0000452,GO:0000462,GO
Fv_160_3.g1721	944	ID=Fv_160_3.g1721;Description=hypothetical protein FVER14953_02778 [Fusarium verticillioides];Gene=FOTG_02936;Ontology_term=cellular anatomical entity,phosphatase

Fv_160_3.g1722	397	ID=Fv_160_3.g1722;Description=hypothetical protein FVEG_02779 [Fusarium verticillioides 7600];Gene=FMEXI_11638;Ontology_term=methyltransferase
Fv_160_3.g1723	682	ID=Fv_160_3.g1723;Description=transketolase [Fusarium acutatum];Gene=FGLOB1_11516;Ontology_term=transketolase activity,metal ion binding,purine nucleotide metabolic process,carbohydrate derivative metabolic
Fv_160_3.g1724	181	ID=Fv_160_3.g1724;Description=hypothetical protein FVEG_02781 [Fusarium verticillioides 7600]
Fv_160_3.g1725	722	ID=Fv_160_3.g1725;Description=hypothetical protein FVER14953_02782 [Fusarium verticillioides];Gene=FPCIR_12645;Ontology_term=cytoplasm,exon-exon junction complex,mRNA binding,nuclear-transcribed mRNA catabolic process, nonsense-mediated decay,mRNA processing,regulation of translation,RNA splicing,mRNA
Fv_160_3.g1726	878	ID=Fv_160_3.g1726;Description=hypothetical protein FVER14953_02783 [Fusarium
Fv_160_3.g1727	1203	ID=Fv_160_3.g1727;Description=hypothetical protein FVEG_02784 [Fusarium verticillioides 7600];Gene=FNAPI_9500;Ontology_term=gamma-tubulin complex,nuclear pore,microtubule,GTP binding,metal ion binding,microtubule nucleation,cytoplasmic microtubule organization,intracellular
Fv_160_3.g1728	462	ID=Fv_160_3.g1728;Description=hypothetical protein FOXB_07321 [Fusarium oxysporum f. sp. conglutinans Fo5176];Gene=FOPG_04948;Ontology_term=fungal-type vacuole membrane,gamma-tubulin complex,nuclear pore,late endosome,peroxisome,microtubule,actin cortical patch,GTPase activity,GTP binding,microtubule binding,metal ion binding,protein targeting to vacuole,endocytosis,microtubule nucleation,peroxisome fission,actin cytoskeleton organization,cytoplasmic microtubule organization,protein retention in Golgi apparatus,cell division,meiotic cell cycle,lipid tube assembly;Ontology_id=GO:0000329,GO:0000930,GO:0005643,GO:0005770,GO:0005777,GO:0005874,GO:0030479,GO:0003924,GO
Fv_160_3.g1729	82	ID=Fv_160_3.g1729;Description=Transposable element Tc3 transposase [Fusarium oxysporum f. sp. albedinis]
Fv_160_3.g1730	419	ID=Fv_160_3.g1730;Description=hypothetical protein FVEG_02788 [Fusarium verticillioides 7600];Gene=FVEG_02788;Ontology_term=zinc ion binding,sequence-specific DNA binding,regulation of DNA-templated
Fv_160_3.g1731	716	ID=Fv_160_3.g1731;Description=glutamate-cysteine ligase [Fusarium verticillioides 7600];Gene=FOVG_06209;Ontology_term=glutamate-cysteine ligase activity,ATP binding,glutathione biosynthetic
Fv_160_3.g1732	153	ID=Fv_160_3.g1732;Description=hypothetical protein FVEG_02790 [Fusarium verticillioides 7600]
Fv_160_3.g1733	357	ID=Fv_160_3.g1733;Description=hypothetical protein FVEG_02791 [Fusarium verticillioides 7600]
Fv_160_3.g1734	354	ID=Fv_160_3.g1734;Description=guanine nucleotide-binding alpha-2 subunit [Fusarium denticulatum];Gene=FGADI_12544;Ontology_term=heterotrimeric G-protein complex,cortical dynamic polarity patch,G protein-coupled receptor binding,GTPase activity,GTP binding,G-protein beta/gamma-subunit complex binding,G protein-coupled receptor signaling pathway,negative regulation of MAPK cascade,pheromone response MAPK cascade;Ontology_id=GO:0005834,GO:0090726,GO:0001664,GO:0003924,GO:0005525,GO:0031683,GO:0007186,GO:0043409,GO:
Fv_160_3.g1735	502	ID=Fv_160_3.g1735;Description=hypothetical protein FVEG_02793 [Fusarium verticillioides 7600]
Fv_160_3.g1736	396	ID=Fv_160_3.g1736;Description=hypothetical protein FVEG_02794 [Fusarium verticillioides 7600]

Fv_160_3.g1737	547	ID=Fv_160_3.g1737;Description=imidazole glycerol phosphate synthase hisHF [Fusarium verticillioides 7600];Gene=FOMG_06729;Ontology_term=imidazoleglycerol-phosphate synthase activity,glutaminase activity,oxo-acid-lyase activity,histidine biosynthetic process,glutamine metabolic process;Ontology_id=GO:0000107,GO:0004359,GO:0016833,GO:0000105,GO:0006541;Enzyme_code=EC:3.5.1.2,EC:2.4.2,EC:4.1.3;E
Fv_160_3.g1738	277	ID=Fv_160_3.g1738;Description=hypothetical protein FVEG_02796 [Fusarium verticillioides 7600];Gene=C2S_1358;Ontology_term=membrane,ornithine decarboxylase inhibitor activity;Ontology_id=GO:0016020,GO:0008073
Fv_160_3.g1739	726	ID=Fv_160_3.g1739;Description=GTS1-transcription factor of the Gcs1p Glo3p Sps18p family [Fusarium coicis];Gene=FPANT_8729;Ontology_term=GTPase activator activity;Ontology_id=GO:0005096
Fv_160_3.g1740	981	ID=Fv_160_3.g1740;Description=hypothetical protein FVER14953_02798 [Fusarium verticillioides];Gene=FNAPI_9941;Ontology_term=plasma membrane,cortical endoplasmic reticulum,lipid binding,sterol transfer activity,karyogamy involved in conjugation with cellular fusion,invasive growth in response to glucose limitation,exocytosis,endocytosis,pseudohyphal growth,sterol transport,maintenance of cell polarity,piecemeal microautophagy of the nucleus,ER to Golgi ceramide transport,intermembrane lipid transfer;Ontology_id=GO:0005886,GO:0032541,GO:0008289,GO:0120015,GO:0000742,GO:0001403,GO:0006887,GO:0006897,GO:
Fv_160_3.g1741	447	ID=Fv_160_3.g1741;Description=hypothetical protein FVEG_02799 [Fusarium verticillioides 7600];Gene=FGADI_9487;Ontology_term=plasma membrane,side of membrane,superoxide dismutase activity,transferase activity,metal ion binding,removal of superoxide
Fv_160_3.g1742	285	ID=Fv_160_3.g1742;Description=hypothetical protein FVEG_02800 [Fusarium verticillioides 7600];Gene=FocTR4_00008568;Ontology_term=isomerase activity,fatty acid beta-
Fv_160_3.g1743	71	ID=Fv_160_3.g1743;Description=RNA polymerase II transcription factor B subunit 5 [Fusarium oxysporum f. sp. lycopersici 4287];Gene=CEP54_009034;Ontology_term=transcription factor TFIIH core complex,transcription factor TFIIH holo complex,nucleotide-excision repair,transcription initiation at RNA polymerase II
Fv_160_3.g1744	137	ID=Fv_160_3.g1744;Description=hypothetical protein FVER14953_02802 [Fusarium
Fv_160_3.g1745	1701	ID=Fv_160_3.g1745;Description=hypothetical protein FVER14953_02803 [Fusarium verticillioides]
Fv_160_3.g1746	340	ID=Fv_160_3.g1746;Description=hypothetical protein FVER14953_02804 [Fusarium verticillioides];Gene=F25303_2024;Ontology_term=mitochondrial inner membrane,transmembrane
Fv_160_3.g1747	131	ID=Fv_160_3.g1747;Description=60S ribosomal protein L22 [Fusarium tjaetaba];Gene=C2S_1348;Ontology_term=ribosome,ribonucleoprotein complex,structural constituent of
Fv_160_3.g1748	4660	ID=Fv_160_3.g1748;Description=hypothetical protein FVER14953_02808 [Fusarium verticillioides]

Fv_160_3.g1749	484	ID=Fv_160_3.g1749;Description=ATP-dependent RNA helicase DBP5 [Fusarium verticillioides 7600];Gene=BFJ69_g4072;Ontology_term=polysome,cellular bud tip,cytoplasmic stress granule,nuclear pore cytoplasmic filaments,inositol hexakisphosphate binding,nucleic acid binding,RNA helicase activity,ATP binding,hydrolase activity,tRNA export from nucleus,translational termination,poly(A)+ mRNA export from nucleus;Ontology_id=GO:0005844,GO:0005934,GO:0010494,GO:0044614,GO:0000822,GO:0003676,GO:0003724,GO:0005524,GO:
Fv_160_3.g1750	481	ID=Fv_160_3.g1750;Description=hypothetical protein FVEG_02810 [Fusarium verticillioides 7600];Gene=FVEG_02810;Ontology_term=nucleus,ribonucleoprotein complex,RNA binding,mRNA cis splicing, via
Fv_160_3.g1751	398	ID=Fv_160_3.g1751;Description=hypothetical protein FVER53590_02811 [Fusarium
Fv_160_3.g1752	361	ID=Fv_160_3.g1752;Description=DNA-3-methyladenine glycosylase II [Fusarium verticillioides 7600];Gene=FTJAE_2075;Ontology_term=catalytic activity,base-excision repair, AP site
Fv_160_3.g1753	404	ID=Fv_160_3.g1753;Description=hypothetical protein FVER53590_02813 [Fusarium verticillioides]
Fv_160_3.g1754	223	ID=Fv_160_3.g1754;Description=vacuolar-sorting protein SNF7 [Fusarium oxysporum f. sp. lycopersici 4287];Gene=FMUND_9060;Ontology_term=ESCRT III complex,plasma membrane,SWI/SNF superfamily-type complex,DNA binding,helicase activity,ATP binding,hydrolase activity,histone binding,identical protein binding,ATP-dependent chromatin remodeler activity,chromatin remodeling,regulation of DNA-templated transcription,protein transport to vacuole involved in ubiquitin-dependent protein catabolic process via the multivesicular body sorting pathway,reticulophagy,intralumenal vesicle formation,ATP export,ESCRT III complex
Fv_160_3.g1755	936	ID=Fv_160_3.g1755;Description=hypothetical protein FVER14953_21079 [Fusarium
Fv_160_3.g1756	990	ID=Fv_160_3.g1756;Description=hypothetical protein LB505_005127 [Fusarium chuoi];Gene=1757;Ontology_term=endoplasmic reticulum membrane,alpha-1,2-mannosyltransferase activity,plasmid maintenance,GPI anchor biosynthetic process;Ontology_id=GO:0005789,GO:0000026,GO:0006276,GO:0006506;Enzyme_code=EC:2.4.1;Enzyme_name=Glycosyltransfera
Fv_160_3.g1757	1396	ID=Fv_160_3.g1757;Description=hypothetical protein FVER14953_02817 [Fusarium verticillioides];Gene=BFJ70_g13372;Ontology_term=protein kinase activity,ATP
Fv_160_3.g1758	491	ID=Fv_160_3.g1758;Description=hypothetical protein FVER14953_02818 [Fusarium verticillioides];Gene=F25303_917;Ontology_term=peroxisomal membrane,protein kinase activity,calcium ion binding,ATP binding,troponin T binding,peroxisome
Fv_160_3.g1759	418	ID=Fv_160_3.g1759;Description=hypothetical protein FVER53590_02819 [Fusarium verticillioides];Gene=FTJAE_2068;Ontology_term=dimethylallyltranstransferase activity,farnesyltranstransferase activity,geranyltranstransferase activity,geranylgeranyl diphosphate biosynthetic process,ketone biosynthetic process,mycotoxin biosynthetic process,alcohol biosynthetic process,organic cyclic compound biosynthetic process;Ontology_id=GO:0004161,GO:0004311,GO:0004337,GO:0033386,GO:0042181,GO:0043386,GO:0046165,GO:1901362;Enzy

Fv_160_3.g1760	524	ID=Fv_160_3.g1760;Description=hypothetical protein FVER53263_02820 [Fusarium verticillioides];Gene=FGLOB1_11966;Ontology_term=incipient cellular bud site,plasma membrane,cellular bud neck,cell cortex,guanyl-nucleotide exchange factor activity,protein binding,transmembrane transporter activity,axial cellular bud site selection,bipolar cellular bud site selection,Ras protein signal transduction,transmembrane
Fv_160_3.g1761	244	ID=Fv_160_3.g1761;Description=haloacid dehalogenase, type II [Fusarium verticillioides 7600];Gene=FOC1_g10012744;Ontology_term=phosphatase activity,hydrolase activity, acting on acid halide bonds, in C-halide
Fv_160_3.g1762	296	ID=Fv_160_3.g1762;Description=hypothetical protein FVEG_02822 [Fusarium verticillioides 7600];Gene=FPRO_07919;Ontology_term=extracellular space,nucleus,ribokinase activity,ATP binding,ferric iron binding,intracellular iron ion homeostasis,D-ribose catabolic process,iron ion transmembrane transport,carbohydrate phosphorylation,cell division;Ontology_id=GO:0005615,GO:0005634,GO:0004747,GO:0005524,GO:0008199,GO:0006879,GO:0019303,GO:0034755,GO:
Fv_160_3.g1763	405	ID=Fv_160_3.g1763;Description=hypothetical protein FVEG_02823 [Fusarium verticillioides
Fv_160_3.g1764	619	ID=Fv_160_3.g1764;Description=hypothetical protein FVER53263_02824 [Fusarium
Fv_160_3.g1765	274	ID=Fv_160_3.g1765;Description=hypothetical protein FVEG_02825 [Fusarium verticillioides 7600];Gene=FOXB_10087;Ontology_term=Ino80 complex,chromatin remodeling;Ontology_id=GO:0031011,GO:0006338
Fv_160_3.g1766	663	ID=Fv_160_3.g1766;Description=hypothetical protein FVEG_15136 [Fusarium verticillioides 7600];Gene=BFJ69_g4086;Ontology_term=vacuolar membrane,endosome membrane,negative regulation of
Fv_160_3.g1767	289	ID=Fv_160_3.g1767;Description=hypothetical protein FVEG_02828 [Fusarium verticillioides 7600]
Fv_160_3.g1768	66	ID=Fv_160_3.g1768;Description=hypothetical protein FVEG_02829 [Fusarium verticillioides 7600];Gene=FNAPI_2586;Ontology_term=endoplasmic reticulum membrane,SNARE complex,SNAP receptor activity,methyltransferase activity,endoplasmic reticulum to Golgi vesicle-mediated transport,retrograde vesicle-mediated transport, Golgi to endoplasmic reticulum,vesicle fusion,methylation;Ontology_id=GO:0005789,GO:0031201,GO:0005484,GO:0008168,GO:0006888,GO:0006890,GO:0006906,GO:00
Fv_160_3.g1769	579	ID=Fv_160_3.g1769;Description=hypothetical protein FVER14953_02830 [Fusarium verticillioides]
Fv_160_3.g1770	281	ID=Fv_160_3.g1770;Description=thiol methyltransferase [Fusarium coicis];Gene=F25303_905;Ontology_term=S-adenosylmethionine-dependent methyltransferase
Fv_160_3.g1771	303	ID=Fv_160_3.g1771;Description=hypothetical protein FVEG_02832 [Fusarium verticillioides 7600]
Fv_160_3.g1772	1375	ID=Fv_160_3.g1772;Description=hypothetical protein FVER14953_02834 [Fusarium verticillioides];Gene=FMAN_07516;Ontology_term=cell cortex,phospholipid binding,maintenance of protein location in cell
Fv_160_3.g1773	267	ID=Fv_160_3.g1773;Description=hypothetical protein FVER14953_02836 [Fusarium verticillioides];Gene=FPRO_07908;Ontology_term=phosphatase activity,hydrolase activity, acting on acid halide bonds, in C-halide compounds,vesicle-mediated
Fv_160_3.g1774	235	ID=Fv_160_3.g1774;Description=protein Ras-2 [Fusarium verticillioides 7600];Gene=CEP53_014997;Ontology_term=plasma membrane,GTPase activity,GTP binding,signal

Fv_160_3.g1775	430	ID=Fv_160_3.g1775;Description=hypothetical protein FVEG_02838 [Fusarium verticillioides 7600];Gene=FTJAE_2053;Ontology_term=spliceosomal complex,membrane,zinc ion binding,pre-mRNA 3'-splice site binding,mRNA
Fv_160_3.g1776	901	ID=Fv_160_3.g1776;Description=chitin synthase 1 [Fusarium verticillioides 7600];Gene=FGADI_12027;Ontology_term=plasma membrane,chitin synthase activity,ATP binding,chitin biosynthetic process,conidium formation,cell wall organization;Ontology_id=GO:0005886,GO:0004100,GO:0005524,GO:0006031,GO:0048315,GO:0071555;Enzyme_code=EC:2.4.1.16
Fv_160_3.g1777	210	ID=Fv_160_3.g1777;Description=NEDD8-conjugating enzyme UBC12 [Fusarium denticulatum];Gene=UBC12;Ontology_term=plasma membrane,chitin synthase activity,ATP binding,chitin biosynthetic process,conidium formation;Ontology_id=GO:0005886,GO:0004100,GO:0005524,GO:0006031,GO:0048315;Enzyme_code=EC:2.4.1.16;Enzyme_name
Fv_160_3.g1778	405	ID=Fv_160_3.g1778;Description=Acetolactate synthase, mitochondrial [Fusarium musae];Gene=FGADI_12026;Ontology_term=mitochondrial nucleoid,double-stranded DNA binding,ketol-acid reductoisomerase activity,isomerase activity,metal ion binding,mitochondrial genome maintenance,isoleucine biosynthetic process,valine biosynthetic process;Ontology_id=GO:0042645,GO:0003690,GO:0004455,GO:0016853,GO:0046872,GO:0000002,GO:0009097,GO:0009099;Enzy
Fv_160_3.g1779	405	ID=Fv_160_3.g1779;Description=hypothetical protein FVER14953_02842 [Fusarium verticillioides]
Fv_160_3.g1780	565	ID=Fv_160_3.g1780;Description=hypothetical protein FVEG_02843 [Fusarium verticillioides 7600];Gene=OpS2-0;Ontology_term=plasma membrane,polyamine transmembrane transporter activity,efflux transmembrane transporter activity,intracellular polyamine homeostasis,ascospore wall assembly,polyamine transmembrane
Fv_160_3.g1781	445	ID=Fv_160_3.g1781;Description=hypothetical protein FVER14953_02844 [Fusarium verticillioides];Gene=BFJ65_g7495;Ontology_term=mitochondrion,glutamate-5-semialdehyde dehydrogenase activity,NADP binding,unfolded protein binding,protein folding,L-proline biosynthetic
Fv_160_3.g1782	608	ID=Fv_160_3.g1782;Description=hypothetical protein J7337_005756 [Fusarium musae];Gene=F25303_9469;Ontology_term=chromatin,condensed nuclear chromosome,DNA replication factor A complex,cytosol,site of double-strand break,chromosome, telomeric repeat region,double-stranded DNA binding,single-stranded DNA binding,sequence-specific DNA binding,metal ion binding,telomere maintenance via recombination,double-strand break repair via homologous recombination,DNA topological change,DNA unwinding involved in DNA replication,premeiotic DNA replication,nucleotide-excision repair,telomere maintenance via telomerase,reciprocal meiotic recombination,protein ubiquitination,heteroduplex formation,sporulation,establishment of protein localization,mitotic DNA
Fv_160_3.g1783	318	ID=Fv_160_3.g1783;Description=hypothetical protein FVEG_02846 [Fusarium verticillioides 7600];Gene=FMAN_07505;Ontology_term=hydrolase
Fv_160_3.g1784	335	ID=Fv_160_3.g1784;Description=NADPH2:quinone reductase [Fusarium verticillioides 7600];Gene=FocTR4_00008517;Ontology_term=oxidoreductase
Fv_160_3.g1785	148	ID=Fv_160_3.g1785;Description=hypothetical protein FVEG_02849 [Fusarium verticillioides 7600];Gene=BFJ63_vAg10256;Ontology_term=structural constituent of cell wall,fungal-type cell wall
Fv_160_3.g1786	700	ID=Fv_160_3.g1786;Description=hypothetical protein FVER14953_02850 [Fusarium verticillioides]

Fv_160_3.g1787	267	ID=Fv_160_3.g1787;Description=hypothetical protein FVEG_02851 [Fusarium verticillioides 7600]
Fv_160_3.g1788	541	ID=Fv_160_3.g1788;Description=hypothetical protein FVEG_02853 [Fusarium verticillioides 7600];Gene=FTJAE_11740;Ontology_term=nucleus,DNA binding,sporulation resulting in formation of a cellular spore,conidium
Fv_160_3.g1789	709	ID=Fv_160_3.g1789;Description=hypothetical protein FVER14953_02854 [Fusarium verticillioides];Gene=FTJAE_11741;Ontology_term=mitotic spindle organization;Ontology_id=GO:0007052
Fv_160_3.g1790	346	ID=Fv_160_3.g1790;Description=hypothetical protein FVEG_02855 [Fusarium verticillioides 7600];Gene=FCIRC_9219;Ontology_term=hydrolase
Fv_160_3.g1791	322	ID=Fv_160_3.g1791;Description=hypothetical protein FVEG_02856 [Fusarium verticillioides 7600]
Fv_160_3.g1792	207	ID=Fv_160_3.g1792;Description=hypothetical protein FVEG_02857 [Fusarium verticillioides 7600];Gene=FMAN_07496;Ontology_term=hydrolase
Fv_160_3.g1793	175	ID=Fv_160_3.g1793;Description=hypothetical protein FVEG_02858 [Fusarium verticillioides 7600]
Fv_160_3.g1794	266	ID=Fv_160_3.g1794;Description=hypothetical protein FVER14953_02859 [Fusarium verticillioides]
Fv_160_3.g1795	1017	ID=Fv_160_3.g1795;Description=hypothetical protein FVER14953_02860 [Fusarium verticillioides]
Fv_160_3.g1796	202	ID=Fv_160_3.g1796;Description=U1 small nuclear ribonucleoprotein C [Fusarium napiforme];Gene=FCIRC_11422;Ontology_term=commitment complex,U1 snRNP,U2-type prespliceosome,mRNA binding,zinc ion binding,U1 snRNA binding,spliceosomal snRNP assembly,mRNA 5'-splice site
Fv_160_3.g1797	218	ID=Fv_160_3.g1797;Description=endoplasmic reticulum vesicle protein 25 [Fusarium verticillioides 7600];Gene=ERV25;Ontology_term=membrane,COPII-coated ER to Golgi transport vesicle,endoplasmic reticulum to Golgi vesicle-
Fv_160_3.g1798	451	ID=Fv_160_3.g1798;Description=tryptophanyl-tRNA synthetase [Fusarium verticillioides 7600];Gene=FACUT_3418;Ontology_term=tryptophan-tRNA ligase activity,ATP binding,tryptophanyl-tRNA
Fv_160_3.g1799	217	ID=Fv_160_3.g1799;Description=hypothetical protein FVEG_02864 [Fusarium verticillioides 7600];Gene=FACUT_3419;Ontology_term=mitochondrial matrix,mitochondrial electron transport, succinate to
Fv_160_3.g1800	525	ID=Fv_160_3.g1800;Description=hypothetical protein FVER14953_02865 [Fusarium verticillioides];Gene=FTJAE_11752;Ontology_term=MIS12/MIND type complex,chromosome segregation,cell
Fv_160_3.g1801	526	ID=Fv_160_3.g1801;Description=hypothetical protein FVER14953_02866 [Fusarium verticillioides];Gene=FOQG_06927;Ontology_term=DNA binding,DNA-binding transcription factor activity,regulation of DNA-
Fv_160_3.g1802	453	ID=Fv_160_3.g1802;Description=hypothetical protein FVEG_02867 [Fusarium verticillioides 7600];Gene=FTJAE_11754;Ontology_term=nucleic acid binding,zinc ion binding;Ontology_id=GO:0003676,GO:0008270
Fv_160_3.g1803	331	ID=Fv_160_3.g1803;Description=Fe-S cluster assembly protein DRE2 [Fusarium verticillioides 7600];Gene=FVEG_02868;Ontology_term=mitochondrial intermembrane space,electron transfer activity,metal ion binding,2 iron, 2 sulfur cluster binding,4 iron, 4 sulfur cluster binding,iron-sulfur cluster assembly,electron transport
Fv_160_3.g1804	618	ID=Fv_160_3.g1804;Description=hypothetical protein FVER14953_02869 [Fusarium verticillioides];Gene=FNAPI_4851;Ontology_term=membrane,serine-type carboxypeptidase

Fv_160_3.g1805	207	ID=Fv_160_3.g1805;Description=carboxypeptidase D [Fusarium verticillioides 7600];Gene=FGADI_7441;Ontology_term=exocyst,RNA-directed DNA polymerase activity,carboxypeptidase activity,methyltransferase activity,RNA-templated DNA biosynthetic process,protein targeting to membrane,Golgi to plasma membrane transport,vesicle docking involved in exocytosis,methylation,vesicle tethering involved in exocytosis;Ontology_id=GO:0000145,GO:0003964,GO:0004180,GO:0008168,GO:0006278,GO:0006612,GO:0006893,GO:0006904,G
Fv_160_3.g1806	356	ID=Fv_160_3.g1806;Description=hypothetical protein FVER53590_02871 [Fusarium verticillioides];Gene=FCIRC_11412;Ontology_term=methionyl-tRNA formyltransferase activity,translational initiation,conversion of methionyl-tRNA to N-formyl-methionyl-
Fv_160_3.g1807	674	ID=Fv_160_3.g1807;Description=hypothetical protein FVER14953_02872 [Fusarium verticillioides];Gene=FCIRC_11411;Ontology_term=transcription coregulator activity,mediator complex,positive regulation of
Fv_160_3.g1808	258	ID=Fv_160_3.g1808;Description=hypothetical protein FVEG_02873 [Fusarium verticillioides 7600]
Fv_160_3.g1809	219	ID=Fv_160_3.g1809;Description=hypothetical protein FVEG_02874 [Fusarium verticillioides
Fv_160_3.g1810	242	ID=Fv_160_3.g1810;Description=hypothetical protein FVER14953_02875 [Fusarium verticillioides];Gene=FTJAE_11762;Ontology_term=O-methyltransferase activity,S-adenosylmethionine-dependent methyltransferase
Fv_160_3.g1811	493	ID=Fv_160_3.g1811;Description=hypothetical protein J7337_005783 [Fusarium musae];Gene=FPRO_07866;Ontology_term=kinase activity,phosphorylation;Ontology_id=GO:0016301,GO:0016310;Enzyme_code=EC:2.7;Enzyme_name=Transferring phosphorus-
Fv_160_3.g1812	186	ID=Fv_160_3.g1812;Description=hypothetical protein FVEG_02877 [Fusarium verticillioides 7600];Gene=FVEG_02877;Ontology_term=endoplasmic reticulum membrane;Ontology_id=GO:0005789
Fv_160_3.g1813	631	ID=Fv_160_3.g1813;Description=hypothetical protein FVER14953_02878 [Fusarium verticillioides];Gene=FNAPI_4842;Ontology_term=nucleic acid binding,zinc ion binding;Ontology_id=GO:0003676,GO:0008270
Fv_160_3.g1814	659	ID=Fv_160_3.g1814;Description=hypothetical protein FVEG_02879 [Fusarium verticillioides 7600]
Fv_160_3.g1815	225	ID=Fv_160_3.g1815;Description=hypothetical protein FVER14953_20627 [Fusarium verticillioides];Gene=BFJ68_g1551;Ontology_term=oxidoreductase
Fv_160_3.g1816	422	ID=Fv_160_3.g1816;Description=hypothetical protein FVER14953_02881 [Fusarium
Fv_160_3.g1817	302	ID=Fv_160_3.g1817;Description=hypothetical protein FVER14953_02882 [Fusarium verticillioides];Gene=F25303_3103;Ontology_term=proteasome complex,nucleus,cytoplasm,protein transport,proteasome localization,nuclear protein quality control by the ubiquitin-proteasome
Fv_160_3.g1818	519	ID=Fv_160_3.g1818;Description=hypothetical protein FVER53263_02883 [Fusarium verticillioides];Gene=F25303_3104;Ontology_term=nucleus,vacuolar membrane,DNA recombinase assembly,autophagy,amino acid transmembrane export from vacuole,double-strand break repair via single-strand
Fv_160_3.g1819	521	ID=Fv_160_3.g1819;Description=hypothetical protein FVER53590_02884 [Fusarium
Fv_160_3.g1820	130	ID=Fv_160_3.g1820;Description=hypothetical protein FVEG_02885 [Fusarium verticillioides 7600]

Fv_160_3.g1821	768	ID=Fv_160_3.g1821;Description=hypothetical protein FVEG_02886 [Fusarium verticillioides 7600]
Fv_160_3.g1822	571	ID=Fv_160_3.g1822;Description=hypothetical protein FVEG_02887 [Fusarium verticillioides 7600];Gene=FVEG_02887;Ontology_term=DNA-binding transcription factor activity,regulation of DNA-templated
Fv_160_3.g1823	624	ID=Fv_160_3.g1823;Description=hypothetical protein FVEG_02888 [Fusarium verticillioides 7600]
Fv_160_3.g1824	444	ID=Fv_160_3.g1824;Description=hypothetical protein FVEG_02889 [Fusarium verticillioides
Fv_160_3.g1825	618	ID=Fv_160_3.g1825;Description=hypothetical protein FVER14953_02891 [Fusarium verticillioides];Gene=MGR1;Ontology_term=mitochondrial inner membrane;Ontology_id=GO:0005743
Fv_160_3.g1826	756	ID=Fv_160_3.g1826;Description=hypothetical protein FVEG_02892 [Fusarium verticillioides
Fv_160_3.g1827	867	ID=Fv_160_3.g1827;Description=hypothetical protein FVER14953_02893 [Fusarium
Fv_160_3.g1828	444	ID=Fv_160_3.g1828;Description=hypothetical protein FVEG_02894 [Fusarium verticillioides 7600];Gene=FANTH_7044;Ontology_term=phosphoric diester hydrolase activity,lipid metabolic
Fv_160_3.g1829	274	ID=Fv_160_3.g1829;Description=hypothetical protein FVEG_02895 [Fusarium verticillioides 7600];Gene=FANTH_7043;Ontology_term=apical plasma membrane,lysophospholipase activity,triglyceride lipase activity,phospholipase A1 activity,acylglycerol lipase activity,calcium-independent phospholipase A2 activity,adenylyltransferase activity,phosphatidyl phospholipase B activity,NAD biosynthetic process,phosphatidylcholine catabolic process,diacylglycerol catabolic process,monoacylglycerol catabolic process;Ontology_id=GO:0016324,GO:0004622,GO:0004806,GO:0008970,GO:0047372,GO:0047499,GO:0070566,GO:0102545,GO:
Fv_160_3.g1830	490	ID=Fv_160_3.g1830;Description=hypothetical protein FVER14953_02896 [Fusarium verticillioides]
Fv_160_3.g1831	349	ID=Fv_160_3.g1831;Description=hypothetical protein FVER53263_02898 [Fusarium verticillioides];Gene=FTJAE_6587;Ontology_term=nucleus,cytoplasm,plasma membrane,calcineurin complex,side of membrane,helicase activity,calmodulin binding,ATP binding,myosin phosphatase activity,calmodulin-dependent protein phosphatase activity,metal ion binding,ATP-dependent chromatin remodeler activity,adaptation of signaling pathway by response to pheromone involved in conjugation with cellular fusion,chromatin remodeling,intracellular monoatomic ion homeostasis,regulation of cell morphogenesis,fungal-type cell wall organization,positive regulation of DNA-templated transcription,calcineurin-mediated
Fv_160_3.g1832	1595	ID=Fv_160_3.g1832;Description=hypothetical protein FVEG_15142 [Fusarium verticillioides 7600];Gene=FPCIR_9072;Ontology_term=nucleus,helicase activity,ATP binding,hydrolase activity,metal ion binding,ATP-dependent chromatin remodeler activity,chromatin
Fv_160_3.g1833	690	ID=Fv_160_3.g1833;Description=hypothetical protein FVEG_02900 [Fusarium verticillioides 7600];Gene=FPCIR_9071;Ontology_term=mitochondrion,RNA binding,RNA methyltransferase activity,rRNA
Fv_160_3.g1834	429	ID=Fv_160_3.g1834;Description=hypothetical protein FVEG_02901 [Fusarium verticillioides 7600]
Fv_160_3.g1835	136	ID=Fv_160_3.g1835;Description=hypothetical protein FVEG_02902 [Fusarium verticillioides 7600];Gene=FOC1_g10012664;Ontology_term=carbon-sulfur lyase activity,metal ion
Fv_160_3.g1836	264	ID=Fv_160_3.g1836;Description=hypothetical protein FVEG_15143 [Fusarium verticillioides

Fv_160_3.g1837	152	ID=Fv_160_3.g1837;Description=hypothetical protein FVER14953_02904 [Fusarium
Fv_160_3.g1838	294	ID=Fv_160_3.g1838;Description=hypothetical protein FVER14953_02905 [Fusarium verticillioides];Gene=FPANT_2381;Ontology_term=Golgi membrane,cytoplasmic vesicle,structural molecule activity,retrograde
Fv_160_3.g1839	173	ID=Fv_160_3.g1839;Description=40s ribosomal protein L44e [Fusarium musae];Gene=FocTR4_00008449;Ontology_term=ribosome,ribonucleoprotein complex,structural constituent of
Fv_160_3.g1840	324	ID=Fv_160_3.g1840;Description=hypothetical protein FVEG_02907 [Fusarium verticillioides 7600];Gene=F25303_3125;Ontology_term=hydrolase
Fv_160_3.g1841	247	ID=Fv_160_3.g1841;Description=ureidoglycolate hydrolase [Fusarium verticillioides 7600];Gene=FCIRC_4601;Ontology_term=medial cortex,conjugation with cellular fusion,response to osmotic stress,response to starvation,mating projection tip,actin filament organization,lipid tube assembly,lipid binding,purine nucleobase metabolic process,allantoin catabolic process,plasma membrane tubulation,ureidoglycolate lyase activity,membrane raft,ureidoglycolate hydrolase activity,Rvs161p-Rvs167p complex,regulation of endocytosis,cytoskeletal protein binding,actin cortical patch localization,actin cortical patch;Ontology_id=GO:0031097,GO:0000747,GO:0006970,GO:0042594,GO:0043332,GO:0007015,GO:0060988,GO:0008289,GO:0006144,GO:0000256,GO:0097320,GO:0050385,GO:0045121,GO:0004848,GO:1990528,GO:0030100,GO:0008092,GO:0051666,GO:00
Fv_160_3.g1842	467	ID=Fv_160_3.g1842;Description=hypothetical protein FVEG_02909 [Fusarium verticillioides
Fv_160_3.g1843	289	ID=Fv_160_3.g1843;Description=hypothetical protein FVEG_15144 [Fusarium verticillioides 7600];Gene=FPCIR_9061;Ontology_term=phosphoric diester hydrolase activity,lipid metabolic
Fv_160_3.g1844	359	ID=Fv_160_3.g1844;Description=hypothetical protein FVEG_02910 [Fusarium verticillioides
Fv_160_3.g1845	582	ID=Fv_160_3.g1845;Description=hypothetical protein FVER14953_02911 [Fusarium verticillioides];Gene=FTJAE_6573;Ontology_term=membrane,transmembrane transporter activity,transmembrane
Fv_160_3.g1846	148	ID=Fv_160_3.g1846;Description=hypothetical protein FVEG_02913 [Fusarium verticillioides 7600];Gene=FTJAE_6572;Ontology_term=nucleus,DNA binding,transcription coactivator activity,positive regulation of transcription
Fv_160_3.g1847	1090	ID=Fv_160_3.g1847;Description=hypothetical protein FVER14953_02914 [Fusarium verticillioides];Gene=Forpe1208_v007812;Ontology_term=phosphatidylinositol binding;Ontology_id=GO:0035091
Fv_160_3.g1848	678	ID=Fv_160_3.g1848;Description=hypothetical protein FVEG_02915 [Fusarium verticillioides 7600]
Fv_160_3.g1849	605	ID=Fv_160_3.g1849;Description=leukotriene A-4 hydrolase [Fusarium verticillioides 7600];Gene=C2S_14470;Ontology_term=fungal-type vacuole lumen,multivesicular body,NVT complex,aminopeptidase activity,epoxide hydrolase activity,metallopeptidase activity,zinc ion binding,proteolysis,protein catabolic process,cellular lipid metabolic process,cytoplasm to vacuole transport by the NVT pathway;Ontology_id=GO:0000328,GO:0005771,GO:0061957,GO:0004177,GO:0004301,GO:0008237,GO:0008270,GO:0006508,GO:

Fv_160_3.g1850	117	ID=Fv_160_3.g1850;Description=hypothetical protein FVEG_15147 [Fusarium verticillioides 7600];Gene=RPS25A_1;Ontology_term=positive regulation of canonical NF-kappaB signal transduction,B cell activation,positive regulation of NF-kappaB transcription factor activity,post-translational protein modification,positive regulation of angiogenesis,ATP binding,chromatin remodeling,positive regulation of DNA-templated transcription,positive regulation of vascular endothelial growth factor receptor signaling pathway,apoptotic process,phosphorylation,ribonucleoprotein complex,protein serine kinase activity,zinc ion binding,adaptive immune response,chromatin binding,ribosome,negative regulation of insulin receptor signaling pathway,calcium,diacylglycerol-dependent serine/threonine kinase activity,regulation of transcription by RNA polymerase II,positive regulation of B cell receptor signaling pathway,nucleus,negative regulation of glucose transmembrane transport,nuclear androgen receptor binding,histone binding,cytoplasm,membrane,nuclear receptor coactivator activity,histone H3T6 kinase activity;Ontology_id=GO:0043123,GO:0042113,GO:0051092,GO:0043687,GO:0045766,GO:0005524,GO:0006338,GO:0045893,GO:0
Fv_160_3.g1851	127	ID=Fv_160_3.g1851;Description=hypothetical protein FVEG_15148 [Fusarium verticillioides 7600];Gene=FVER53590_29078;Ontology_term=CST complex,single-stranded telomeric DNA binding,telomere
Fv_160_3.g1852	1767	ID=Fv_160_3.g1852;Description=hypothetical protein FVER53590_02918 [Fusarium verticillioides];Gene=FCIRC_4611;Ontology_term=nucleoplasm,cytoplasm,ribosome,cytoskeleton,basolateral plasma membrane,apical plasma membrane,membrane raft,cation-transporting ATPase complex,ribonucleoprotein complex,DNA binding,structural constituent of ribosome,microtubule motor activity,calmodulin binding,ATP binding,phospholipid binding,microtubule binding,sodium:proton antiporter activity,protein phosphatase 2B binding,identical protein binding,calcium-dependent protein binding,translation,intracellular sodium ion homeostasis,microtubule-based movement,positive regulation of cardiac muscle hypertrophy,regulation of cardiac muscle contraction by calcium ion signaling,response to muscle stretch,sodium ion export across plasma membrane,positive regulation of transcription by RNA polymerase II,stem cell differentiation,protein complex oligomerization,regulation of intracellular pH,cardiac muscle cell differentiation,positive regulation of calcineurin-NFAT signaling cascade,cellular response to acidic pH,cellular response to epinephrine stimulus,regulation of the force of heart contraction by cardiac conduction,sodium ion import across plasma membrane,positive regulation of the force of heart contraction,proton transmembrane
Fv_160_3.g1853	345	ID=Fv_160_3.g1853;Description=hypothetical protein FVER14953_02919 [Fusarium verticillioides];Gene=BFJ70_g1094;Ontology_term=S-adenosylmethionine-dependent methyltransferase
Fv_160_3.g1854	223	ID=Fv_160_3.g1854;Description=hypothetical protein FVEG_02920 [Fusarium verticillioides 7600]
Fv_160_3.g1855	371	ID=Fv_160_3.g1855;Description=hypothetical protein FVEG_02921 [Fusarium verticillioides 7600];Gene=oma1;Ontology_term=membrane,metalloendopeptidase activity,metal ion
Fv_160_3.g1856	84	ID=Fv_160_3.g1856;Description=acyl-CoA dehydrogenase [Fusarium verticillioides 7600];Gene=FGLOB1_835;Ontology_term=heme binding,metal ion binding;Ontology_id=GO:0020037,GO:0046872

Fv_160_3.g1857	500	ID=Fv_160_3.g1857;Description=hypothetical protein FVEG_02923 [Fusarium verticillioides 7600];Gene=FVEG_02923;Ontology_term=carboxy-lyase activity,heme binding,pyridoxal phosphate binding,metal ion binding,carboxylic acid metabolic
Fv_160_3.g1858	257	ID=Fv_160_3.g1858;Description=hypothetical protein FVER53590_02924 [Fusarium verticillioides];Gene=RPS6A_1;Ontology_term=carboxy-lyase activity,pyridoxal phosphate binding,carboxylic acid metabolic
Fv_160_3.g1859	87	ID=Fv_160_3.g1859;Description=Uncharacterized protein family UPF0390 [Fusarium oxysporum f. sp. vasinfectum];Gene=FSUBG_2794;Ontology_term=skeletal muscle myofibril,protein kinase activity,ATP binding,glycogen phosphorylase activity,pyridoxal phosphate binding,linear malto-oligosaccharide phosphorylase activity,SHG alpha-glucan phosphorylase activity,glycogen catabolic process,phosphorylation;Ontology_id=GO:0098723,GO:0004672,GO:0005524,GO:0008184,GO:0030170,GO:0102250,GO:0102499,
Fv_160_3.g1860	753	ID=Fv_160_3.g1860;Description=hypothetical protein FVER14953_02926 [Fusarium verticillioides];Gene=FMAN_07429;Ontology_term=protein kinase activity,ATP
Fv_160_3.g1861	570	ID=Fv_160_3.g1861;Description=hypothetical protein FVEG_02927 [Fusarium verticillioides 7600]
Fv_160_3.g1862	809	ID=Fv_160_3.g1862;Description=aconitate hydratase, mitochondrial [Fusarium verticillioides 7600];Gene=F25303_3144;Ontology_term=mitochondrion,aconitate hydratase activity,metal ion binding,4 iron, 4 sulfur cluster binding,tricarboxylic acid
Fv_160_3.g1863	1092	ID=Fv_160_3.g1863;Description=hypothetical protein FVEG_02929 [Fusarium verticillioides 7600];Gene=FPRO_07807;Ontology_term=phosphorylase kinase complex,nuclear pore outer ring,nuclear membrane,skeletal muscle myofibril,phosphorylase kinase activity,calmodulin binding,ATP binding,structural constituent of nuclear pore,tau-protein kinase activity,protein serine kinase activity,glycogen biosynthetic process,nucleocytoplasmic transport,protein transport,phosphorylation,mRNA
Fv_160_3.g1864	384	ID=Fv_160_3.g1864;Description=S-(hydroxymethyl)glutathione dehydrogenase [Fusarium verticillioides 7600];Gene=FCIRC_4622;Ontology_term=alcohol dehydrogenase (NAD+) activity,zinc ion binding,hydroxymethylfurfural reductase (NADH) activity,S-(hydroxymethyl)glutathione dehydrogenase NADP activity,S-(hydroxymethyl)glutathione dehydrogenase NAD activity,amino acid catabolic process to alcohol via Ehrlich pathway,ethanol oxidation,furaldehyde metabolic process,formaldehyde catabolic
Fv_160_3.g1865	395	ID=Fv_160_3.g1865;Description=endonuclease III [Fusarium verticillioides 7600];Gene=NTH1;Ontology_term=nucleus,mitochondrion,oxidized pyrimidine nucleobase lesion DNA N-glycosylase activity,DNA binding,endonuclease activity,class I DNA-(apurinic or apyrimidinic site) endonuclease activity,base-excision repair, AP site formation;Ontology_id=GO:0005634,GO:0005739,GO:0000703,GO:0003677,GO:0004519,GO:0140078,GO:0006285;Enzyme_code=
Fv_160_3.g1866	72	ID=Fv_160_3.g1866;Description=hypothetical protein FVEG_02932 [Fusarium verticillioides
Fv_160_3.g1867	1359	ID=Fv_160_3.g1867;Description=hypothetical protein FVER14953_02933 [Fusarium verticillioides];Gene=FTJAE_6552;Ontology_term=hydrolase

Fv_160_3.g1868	310	ID=Fv_160_3.g1868;Description=hypothetical protein FVEG_02934 [Fusarium verticillioides 7600];Gene=SLC9A1;Ontology_term=nucleoplasm,cytoplasm,basolateral plasma membrane,apical plasma membrane,membrane raft,cation-transporting ATPase complex,calmodulin binding,phospholipid binding,sodium:proton antiporter activity,protein phosphatase 2B binding,identical protein binding,calcium-dependent protein binding,intracellular sodium ion homeostasis,positive regulation of cardiac muscle hypertrophy,regulation of cardiac muscle contraction by calcium ion signaling,response to muscle stretch,sodium ion export across plasma membrane,positive regulation of transcription by RNA polymerase II,stem cell differentiation,protein complex oligomerization,regulation of intracellular pH,cardiac muscle cell differentiation,positive regulation of calcineurin-NFAT signaling cascade,cellular response to acidic pH,cellular response to epinephrine stimulus,regulation of the force of heart contraction by cardiac conduction,sodium ion import across plasma membrane,positive regulation of the force of heart contraction,proton transmembrane
Fv_160_3.g1869	638	ID=Fv_160_3.g1869;Description=hypothetical protein FVEG_02935 [Fusarium verticillioides 7600];Gene=FOPG_02924;Ontology_term=oxidoreductase activity, acting on the CH-CH group of donors,flavin adenine dinucleotide
Fv_160_3.g1870	162	ID=Fv_160_3.g1870;Description=hypothetical protein FVEG_15153 [Fusarium verticillioides 7600]
Fv_160_3.g1871	584	ID=Fv_160_3.g1871;Description=hypothetical protein FVER14953_02936 [Fusarium verticillioides];Gene=FMAN_07418;Ontology_term=ribosome,ribonucleoprotein complex,structural constituent of ribosome,deaminase
Fv_160_3.g1872	190	ID=Fv_160_3.g1872;Description=hypothetical protein FVER14953_02937 [Fusarium verticillioides];Gene=FDENT_1804;Ontology_term=NADH dehydrogenase (ubiquinone) activity,metal ion binding,quinone binding,4 iron, 4 sulfur cluster binding,electron transport chain;Ontology_id=GO:0008137,GO:0046872,GO:0048038,GO:0051539,GO:0022900;Enzyme_code=EC:7.1.1.2,EC:1.6.5.11,EC:1.6.5.
Fv_160_3.g1873	614	ID=Fv_160_3.g1873;Description=hypothetical protein FVEG_02938 [Fusarium verticillioides 7600];Gene=FANTH_7002;Ontology_term=membrane,transferase
Fv_160_3.g1874	594	ID=Fv_160_3.g1874;Description=GYP1 [Fusarium tjaetaba];Gene=1647;Ontology_term=nucleoplasm,RNA polymerase III complex,Golgi apparatus,RNA polymerase III activity,single-stranded DNA binding,GTPase activator activity,protein binding,transcription initiation at RNA polymerase III promoter,termination of RNA polymerase III transcription,vesicle-mediated transport,tRNA transcription by RNA polymerase III;Ontology_id=GO:0005654,GO:0005666,GO:0005794,GO:0001056,GO:0003697,GO:0005096,GO:0005515,GO:0006384,GO:00063
Fv_160_3.g1875	541	ID=Fv_160_3.g1875;Description=hypothetical protein FVEG_15157 [Fusarium verticillioides 7600];Gene=FCIRC_4633;Ontology_term=ribonucleoprotein complex,RNA binding;Ontology_id=GO:1990904,GO:0003723

Fv_160_3.g1876	809	ID=Fv_160_3.g1876;Description=uncharacterized protein FOBCDRAFT_34396 [Fusarium oxysporum Fo47];Gene=FocTR4_00008406;Ontology_term=ubiquitin ligase complex,nucleus,Golgi apparatus,cellular bud tip,endosome membrane,cytosolic ribosome,RSP5-BUL ubiquitin ligase complex,arylamine N-acetyltransferase activity,ligase activity,phosphatidylinositol binding,ubiquitin binding,ubiquitin protein ligase activity,chromatin organization,regulation of nitrogen utilization,mitochondrion organization,response to xenobiotic stimulus,regulation of mRNA export from nucleus,regulation of dolichol biosynthetic process,regulation of ubiquinone biosynthetic process,regulation of multivesicular body size,protein ubiquitination,poly(A)+ mRNA export from nucleus,regulation of phosphate metabolic process,positive regulation of proteasomal ubiquitin-dependent protein catabolic process,regulation of ergosterol biosynthetic process,late endosome to vacuole transport via multivesicular body sorting pathway,regulation of protein localization,regulation of actin cytoskeleton organization,ribophagy,cellular response to UV,ubiquitin-dependent protein catabolic process via the multivesicular body sorting pathway,positive regulation of fatty acid biosynthetic process,positive regulation of transcription by RNA polymerase II,positive regulation of receptor-mediated endocytosis,ubiquitin-dependent endocytosis,nonfunctional rRNA decay,mitochondria-associated ubiquitin-dependent protein catabolic process,regulation of ribosomal large subunit export from nucleus,regulation of rRNA processing,regulation of tRNA processing,regulation of tRNA export from
Fv_160_3.g1877	205	ID=Fv_160_3.g1877;Description=hypothetical protein FVEG_02944 [Fusarium verticillioides 7600];Gene=C2S_13670;Ontology_term=chromatin,nucleolus,cytoplasm,polysome,Mei2 nuclear dot complex,nucleolar peripheral inclusion body,nuclear exosome focus,mRNA binding,poly(A) binding,lncRNA binding,nuclear-transcribed mRNA catabolic process,
Fv_160_3.g1878	243	ID=Fv_160_3.g1878;Description=hypothetical protein FOXG_05188 [Fusarium oxysporum f. sp. lycopersici 4287];Gene=CDC19;Ontology_term=membrane,magnesium ion binding,pyruvate kinase activity,ATP binding,kinase activity,transmembrane transporter activity,potassium ion binding,glycolytic process,phosphorylation,transmembrane transport,nitrogen compound transport;Ontology_id=GO:0016020,GO:0000287,GO:0004743,GO:0005524,GO:0016301,GO:0022857,GO:0030955,GO:0006096,GO
Fv_160_3.g1879	1116	ID=Fv_160_3.g1879;Description=adenosinetriphosphatase [Fusarium verticillioides 7600];Gene=BFJ68_g1655;Ontology_term=RNA polymerase III complex,DNA binding,ATP binding,hydrolase activity,nucleosome binding,ATP-dependent chromatin remodeler activity,chromatin remodeling,transcription by RNA polymerase
Fv_160_3.g1880	337	ID=Fv_160_3.g1880;Description=eukaryotic translation initiation factor 3 subunit I [Fusarium verticillioides 7600];Gene=FG10268.1;Ontology_term=eukaryotic 43S preinitiation complex,eukaryotic 48S preinitiation complex,nuclear periphery,eukaryotic translation initiation factor 3 complex, eIF3e,eukaryotic translation initiation factor 3 complex, eIF3m,translation initiation factor activity,formation of cytoplasmic translation initiation

Fv_160_3.g1881	939	ID=Fv_160_3.g1881;Description=formate-tetrahydrofolate ligase [Fusarium verticillioides 7600];Gene=FOMG_06549;Ontology_term=RNA polymerase III complex,mitochondrion,formate-tetrahydrofolate ligase activity,methenyltetrahydrofolate cyclohydrolase activity,methylenetetrahydrofolate dehydrogenase (NADP+) activity,ATP binding,transcription by RNA polymerase III,tetrahydrofolate interconversion,folic acid biosynthetic process;Ontology_id=GO:0005666,GO:0005739,GO:0004329,GO:0004477,GO:0004488,GO:0005524,GO:0006383,GO:0035999,GO:
Fv_160_3.g1882	782	ID=Fv_160_3.g1882;Description=hypothetical protein FVEG_02949 [Fusarium verticillioides 7600];Gene=FMAN_07407;Ontology_term=nucleus,myosin complex,DNA binding,zinc ion binding,structural constituent of muscle,calcium ion binding,DNA-binding transcription factor activity, RNA polymerase II-specific,regulation of transcription by RNA
Fv_160_3.g1883	380	ID=Fv_160_3.g1883;Description=hypothetical protein FVEG_02950 [Fusarium verticillioides
Fv_160_3.g1884	339	ID=Fv_160_3.g1884;Description=glutamine synthetase [Fusarium mundagurra];Gene=FOPG_02946;Ontology_term=cytoplasm,glutamine synthetase activity,ATP binding,glutamine biosynthetic
Fv_160_3.g1885	74	ID=Fv_160_3.g1885;Description=hypothetical protein FVER14953_20124 [Fusarium verticillioides]
Fv_160_3.g1886	363	ID=Fv_160_3.g1886;Description=hypothetical protein FVEG_15158 [Fusarium verticillioides 7600];Gene=FVER53263_02952;Ontology_term=nucleic acid binding,zinc ion binding;Ontology_id=GO:0003676,GO:0008270
Fv_160_3.g1887	313	ID=Fv_160_3.g1887;Description=hypothetical protein FVEG_02953 [Fusarium verticillioides 7600]
Fv_160_3.g1888	692	ID=Fv_160_3.g1888;Description=hypothetical protein FVER14953_02954 [Fusarium verticillioides];Gene=FVEG_02954;Ontology_term=cellular anatomical entity;Ontology_id=GO:0110165
Fv_160_3.g1889	674	ID=Fv_160_3.g1889;Description=hypothetical protein FVEG_02955 [Fusarium verticillioides 7600]
Fv_160_3.g1890	452	ID=Fv_160_3.g1890;Description=hypothetical protein FVEG_02956 [Fusarium verticillioides 7600];Gene=Prx2;Ontology_term=myosin complex,calcium ion binding,structural constituent of muscle,plasmid
Fv_160_3.g1891	198	ID=Fv_160_3.g1891;Description=hypothetical protein FVER53263_02957 [Fusarium verticillioides];Gene=FOC1_g10011017;Ontology_term=membrane,cytosolic small ribosomal subunit,RNA binding,structural constituent of ribosome,ribosomal small subunit assembly,rRNA export from
Fv_160_3.g1892	306	ID=Fv_160_3.g1892;Description=hypothetical protein FVER14953_02958 [Fusarium verticillioides];Gene=FNAPI_3311;Ontology_term=dioxygenase
Fv_160_3.g1893	394	ID=Fv_160_3.g1893;Description=hypothetical protein FVER14953_02959 [Fusarium
Fv_160_3.g1894	253	ID=Fv_160_3.g1894;Description=20S proteasome subunit alpha 1 [Fusarium oxysporum f. sp. lycopersici 4287];Gene=FCIRC_12710;Ontology_term=nucleus,cytoplasm,proteasome core complex, alpha-subunit complex,proteasomal ubiquitin-independent protein catabolic process,proteasome-mediated ubiquitin-dependent protein catabolic
Fv_160_3.g1895	171	ID=Fv_160_3.g1895;Description=hypothetical protein FVEG_02961 [Fusarium verticillioides 7600]
Fv_160_3.g1896	149	ID=Fv_160_3.g1896;Description=hypothetical protein FVEG_02962 [Fusarium verticillioides
Fv_160_3.g1897	84	ID=Fv_160_3.g1897;Description=hypothetical protein FVEG_02963 [Fusarium verticillioides 7600]

Fv_160_3.g1898	317	ID=Fv_160_3.g1898;Description=target-rapamycin complex subunit LST8 [Fusarium oxysporum f. sp. lycopersici 4287];Gene=FDENT_1778;Ontology_term=Golgi membrane,endosome membrane,TORC1 complex,TORC2 complex,nuclear periphery,regulation of cell growth,establishment or maintenance of actin cytoskeleton polarity,fungal-type cell wall organization,TOR signaling,mitochondria-nucleus signaling
Fv_160_3.g1899	110	ID=Fv_160_3.g1899;Description=hypothetical protein FNAPI_3304 [Fusarium napiforme]
Fv_160_3.g1900	301	ID=Fv_160_3.g1900;Description=nitrilase [Fusarium verticillioides 7600];Gene=NIT3;Ontology_term=hydrolase activity, acting on carbon-nitrogen (but not peptide) bonds, in linear amides,nitrogen compound metabolic process;Ontology_id=GO:0016811,GO:0006807;Enzyme_code=EC:3.5.1;Enzyme_name=Acting on carbon-nitrogen bonds, other than
Fv_160_3.g1901	294	ID=Fv_160_3.g1901;Description=spermidine synthase [Fusarium oxysporum f. sp. lycopersici 4287];Gene=FVEG_15160;Ontology_term=spermidine synthase activity,S-adenosylmethionine-dependent methyltransferase activity,spermidine biosynthetic process,pantothenate biosynthetic
Fv_160_3.g1902	933	ID=Fv_160_3.g1902;Description=hypothetical protein FVER53263_02969 [Fusarium verticillioides]
Fv_160_3.g1903	567	ID=Fv_160_3.g1903;Description=hypothetical protein FVER14953_02970 [Fusarium verticillioides]
Fv_160_3.g1904	221	ID=Fv_160_3.g1904;Description=hypothetical protein FPSE_03626 [Fusarium pseudograminearum CS3096];Gene=FOMG_06524;Ontology_term=ribosome,ribonucleoprotein complex,structural constituent of
Fv_160_3.g1905	110	ID=Fv_160_3.g1905;Description=60S ribosomal protein L30 [Fusarium vanettenii 77-13-4];Gene=rpl-30;Ontology_term=cytosolic large ribosomal subunit,structural constituent of ribosome,pre-mRNA 5'-splice site binding,rRNA processing,negative regulation of mRNA splicing, via spliceosome;Ontology_id=GO:0022625,GO:0003735,GO:0030627,GO:0006364,GO:0048025
Fv_160_3.g1906	622	ID=Fv_160_3.g1906;Description=hypothetical protein FVEG_02973 [Fusarium verticillioides 7600]
Fv_160_3.g1907	838	ID=Fv_160_3.g1907;Description=hypothetical protein FVER53590_02974 [Fusarium
Fv_160_3.g1908	930	ID=Fv_160_3.g1908;Description=hypothetical protein FVEG_02975 [Fusarium verticillioides 7600];Gene=FVEG_02975;Ontology_term=U4/U6 x U5 tri-snRNP complex,mRNA cis splicing, via
Fv_160_3.g1909	132	ID=Fv_160_3.g1909;Description=calcofluor white hypersensitive [Fusarium
Fv_160_3.g1910	462	ID=Fv_160_3.g1910;Description=hypothetical protein FVEG_02977 [Fusarium verticillioides 7600]
Fv_160_3.g1911	845	ID=Fv_160_3.g1911;Description=hypothetical protein FVER14953_02978 [Fusarium verticillioides]
Fv_160_3.g1912	446	ID=Fv_160_3.g1912;Description=protein phosphatase [Fusarium verticillioides 7600];Gene=FVEG_02979;Ontology_term=protein serine/threonine phosphatase activity,metal ion
Fv_160_3.g1913	904	ID=Fv_160_3.g1913;Description=hypothetical protein FVER14953_02980 [Fusarium verticillioides];Gene=FPANT_13533;Ontology_term=membrane,calcium ion binding;Ontology_id=GO:0016020,GO:0005509
Fv_160_3.g1914	251	ID=Fv_160_3.g1914;Description=hypothetical protein FVEG_02981 [Fusarium verticillioides 7600];Gene=EFM6;Ontology_term=cytoplasm,protein-lysine N-methyltransferase
Fv_160_3.g1915	289	ID=Fv_160_3.g1915;Description=hypothetical protein FVER14953_02982 [Fusarium verticillioides]

Fv_160_3.g1916	780	ID=Fv_160_3.g1916;Description=hypothetical protein FVER53590_02983 [Fusarium verticillioides];Gene=FNAPI_9731;Ontology_term=membrane,Mon1-Ccz1 complex,vesicle-mediated
Fv_160_3.g1917	486	ID=Fv_160_3.g1917;Description=hypothetical protein FVEG_02984 [Fusarium verticillioides 7600];Gene=BFJ69_g6495;Ontology_term=membrane,Mon1-Ccz1 complex,vesicle-mediated
Fv_160_3.g1918	572	ID=Fv_160_3.g1918;Description=hypothetical protein FVER14953_02985 [Fusarium verticillioides]
Fv_160_3.g1919	260	ID=Fv_160_3.g1919;Description=hypothetical protein FVER53590_02986 [Fusarium verticillioides];Gene=FDENT_1758;Ontology_term=oxidoreductase activity,metal ion binding,2 iron, 2 sulfur cluster
Fv_160_3.g1920	576	ID=Fv_160_3.g1920;Description=hypothetical protein FVER53590_02988 [Fusarium verticillioides];Gene=FOC1_g10010987;Ontology_term=membrane,ligase
Fv_160_3.g1921	460	ID=Fv_160_3.g1921;Description=hypothetical protein FVEG_02989 [Fusarium verticillioides 7600];Gene=FVEG_02989;Ontology_term=extracellular space,nucleus,cytosol,cytokine activity,interleukin-1 receptor binding,copper ion binding,oxidoreductase activity,fever generation,connective tissue replacement involved in inflammatory response wound healing,intracellular sodium ion homeostasis,immune response,negative regulation of cell population proliferation,positive regulation of vascular endothelial growth factor production,cytokine-mediated signaling pathway,positive regulation of interleukin-2 production,cellular response to heat,ectopic germ cell programmed cell death,positive regulation of angiogenesis,positive regulation of mitotic nuclear division,positive regulation of transcription by RNA polymerase II,response to copper ion,positive regulation of protein secretion,positive regulation of cell division,extrinsic apoptotic signaling pathway in absence of ligand;Ontology_id=GO:0005615,GO:0005634,GO:0005829,GO:0005125,GO:0005149,GO:0005507,GO:0016491,GO:0001660,GO:00
Fv_160_3.g1922	1148	ID=Fv_160_3.g1922;Description=WEE/WEE-UNCLASSIFIED protein kinase [Fusarium verticillioides 7600];Gene=FDENT_7938;Ontology_term=nucleus,DNA binding,protein kinase activity,ATP binding,regulation of DNA-templated transcription,phosphorylation;Ontology_id=GO:0005634,GO:0003677,GO:0004672,GO:0005524,GO:0006355,GO:0016310;Enzyme_code=EC:2.7.1;Enzyme_name=Transferring phosphorus-containing groups
Fv_160_3.g1923	385	ID=Fv_160_3.g1923;Description=hypothetical protein FVER53590_02992 [Fusarium verticillioides];Gene=C2S_2439;Ontology_term=phagophore assembly site,dCMP deaminase activity,zinc ion binding,ubiquitin-like modifier activating enzyme activity,dUMP biosynthetic process,dTMP biosynthetic process,autophagy,protein transport;Ontology_id=GO:0000407,GO:0004132,GO:0008270,GO:0008641,GO:0006226,GO:0006231,GO:0006914,GO:0015031;En
Fv_160_3.g1924	695	ID=Fv_160_3.g1924;Description=Autophagy protein 7 [Fusarium musae];Gene=FOYG_07937;Ontology_term=phagophore assembly site,zinc ion binding,ubiquitin-like modifier activating enzyme activity,deaminase activity,nucleobase-containing compound metabolic process,autophagy,protein transport;Ontology_id=GO:0000407,GO:0008270,GO:0008641,GO:0019239,GO:0006139,GO:0006914,GO:0015031;Enzyme_code=E
Fv_160_3.g1925	86	ID=Fv_160_3.g1925;Description=hypothetical protein FOXG_05140 [Fusarium oxysporum f. sp. lycopersici 4287];Gene=FOPG_02989;Ontology_term=membrane;Ontology_id=GO:0016020

Fv_160_3.g1926	257	ID=Fv_160_3.g1926;Description=glycosylphosphatidylinositol anchor biosynthesis protein 11 [Fusarium verticillioides 7600];Gene=FNAPI_9555;Ontology_term=endoplasmic reticulum membrane,cytosol,glyceraldehyde-3-phosphate dehydrogenase (NAD+) (phosphorylating) activity,NADP binding,NAD binding,glucose metabolic process,glycolytic process;Ontology_id=GO:0005789,GO:0005829,GO:0004365,GO:0050661,GO:0051287,GO:0006006,GO:0006096;Enzyme_code=EC
Fv_160_3.g1927	1030	ID=Fv_160_3.g1927;Description=hypothetical protein FVER14953_02996 [Fusarium verticillioides];Gene=FNAPI_9556;Ontology_term=fungal-type vacuole membrane,nucleus,mitochondrial respiratory chain complex II, succinate dehydrogenase complex (ubiquinone),ATP binding,succinate dehydrogenase (ubiquinone) activity,electron transfer activity,hydrolase activity,metal ion binding,2 iron, 2 sulfur cluster binding,3 iron, 4 sulfur cluster binding,4 iron, 4 sulfur cluster binding,fumarate reductase (menaquinone),ATP-dependent chromatin remodeler activity,tricarboxylic acid cycle,mitochondrial electron transport, succinate to ubiquinone,chromatin remodeling,proton motive force-driven mitochondrial ATP synthesis;Ontology_id=GO:0000329,GO:0005634,GO:0005749,GO:0005524,GO:0008177,GO:0009055,GO:0016787,GO:0046872,GO
Fv_160_3.g1928	302	ID=Fv_160_3.g1928;Description=hypothetical protein FVEG_02997 [Fusarium verticillioides 7600];Gene=FVEG_02997;Ontology_term=S-adenosylmethionine-dependent methyltransferase
Fv_160_3.g1929	183	ID=Fv_160_3.g1929;Description=U3 small nucleolar ribonucleoprotein IMP3 [Fusarium oxysporum f. sp. lycopersici 4287];Gene=FVEG_02998;Ontology_term=small ribosomal subunit,small-subunit processome,Mpp10 complex,structural constituent of ribosome,rRNA binding,snoRNA binding,rRNA processing,ribosomal small subunit
Fv_160_3.g1930	212	ID=Fv_160_3.g1930;Description=hypothetical protein FVEG_02999 [Fusarium verticillioides 7600];Gene=FVEG_02999;Ontology_term=ribosome,ribonucleoprotein complex,structural constituent of
Fv_160_3.g1931	791	ID=Fv_160_3.g1931;Description=hypothetical protein FVER14953_03000 [Fusarium verticillioides]
Fv_160_3.g1932	353	ID=Fv_160_3.g1932;Description=oxidoreductase [Fusarium verticillioides 7600];Gene=F52700_13160;Ontology_term=oxidoreductase
Fv_160_3.g1933	555	ID=Fv_160_3.g1933;Description=hypothetical protein FVEG_03002 [Fusarium verticillioides 7600];Gene=FDENT_7949;Ontology_term=nucleus,membrane,cytosolic large ribosomal subunit,preribosome, large subunit precursor,DNA binding,structural constituent of ribosome,protein binding,rRNA binding,ribosomal large subunit assembly,cytoplasmic translation,regulation of DNA-templated
Fv_160_3.g1934	440	ID=Fv_160_3.g1934;Description=hypothetical protein FVER14953_03003 [Fusarium verticillioides]
Fv_160_3.g1935	290	ID=Fv_160_3.g1935;Description=hypothetical protein FVER14953_03004 [Fusarium verticillioides]
Fv_160_3.g1936	139	ID=Fv_160_3.g1936;Description=hypothetical protein FVEG_03005 [Fusarium verticillioides
Fv_160_3.g1937	356	ID=Fv_160_3.g1937;Description=homoserine kinase [Fusarium bulbicola];Gene=FPANT_4247;Ontology_term=nucleus,cytosolic large ribosomal subunit,structural constituent of ribosome,homoserine kinase activity,ATP binding,rRNA binding,ribosomal large subunit assembly,cytoplasmic translation,threonine biosynthetic process,sterol biosynthetic process,phosphorylation;Ontology_id=GO:0005634,GO:0022625,GO:0003735,GO:0004413,GO:0005524,GO:0019843,GO:0000027,

Fv_160_3.g1938	327	ID=Fv_160_3.g1938;Description=hypothetical protein FVER14953_03007 [Fusarium verticillioides];Gene=FTJAE_1390;Ontology_term=lyase
Fv_160_3.g1939	345	ID=Fv_160_3.g1939;Description=hypothetical protein FVEG_03008 [Fusarium verticillioides 7600];Gene=FVEG_03008;Ontology_term=Golgi membrane,cytosol,Ric1-Rgp1 guanyl-nucleotide exchange factor complex,guanyl-nucleotide exchange factor activity,protein binding,cell cycle,retrograde transport, endosome to Golgi,cell
Fv_160_3.g1940	358	ID=Fv_160_3.g1940;Description=hypothetical protein FVEG_03009 [Fusarium verticillioides 7600];Gene=1582
Fv_160_3.g1941	202	ID=Fv_160_3.g1941;Description=hypothetical protein FVEG_03010 [Fusarium verticillioides 7600];Gene=RGM1;Ontology_term=chromatin,nucleus,cytosol,DNA-binding transcription factor activity, RNA polymerase II-specific,DNA binding,protein binding,zinc ion binding,positive regulation of transcription by RNA polymerase
Fv_160_3.g1942	322	ID=Fv_160_3.g1942;Description=hypothetical protein FVEG_03011 [Fusarium verticillioides 7600];Gene=FOC1_g10010964;Ontology_term=glycosyltransferase
Fv_160_3.g1943	2210	ID=Fv_160_3.g1943;Description=hypothetical protein FVER53590_03012 [Fusarium verticillioides];Gene=FOTG_03206;Ontology_term=endoplasmic reticulum membrane,phagophore assembly site
Fv_160_3.g1944	539	ID=Fv_160_3.g1944;Description=hypothetical protein FVEG_03013 [Fusarium verticillioides 7600];Gene=FOYG_07915;Ontology_term=oxidoreductase
Fv_160_3.g1945	514	ID=Fv_160_3.g1945;Description=hypothetical protein FVER14953_03014 [Fusarium verticillioides];Gene=FOXYS1_12175;Ontology_term=chromosome, telomeric region,condensed nuclear chromosome,DNA replication factor A complex,site of double-strand break,double-stranded DNA binding,single-stranded DNA binding,acyl-CoA dehydrogenase activity,protein binding,heme binding,telomeric DNA binding,metal ion binding,flavin adenine dinucleotide binding,telomere maintenance via recombination,double-strand break repair via homologous recombination,DNA topological change,DNA unwinding involved in DNA replication,nucleotide-excision repair,telomere maintenance via telomerase,reciprocal meiotic recombination,protein ubiquitination,heteroduplex formation,establishment of protein localization;Ontology_id=GO:0000781,GO:0000794,GO:0005662,GO:0035861,GO:0003690,GO:0003697,GO:0003995,GO:0005515,
Fv_160_3.g1946	178	ID=Fv_160_3.g1946;Description=hypothetical protein H9Q70_006955 [Fusarium xylarioides];Gene=FOYG_07913;Ontology_term=nucleic acid binding,zinc ion binding;Ontology_id=GO:0003676,GO:0008270
Fv_160_3.g1947	158	ID=Fv_160_3.g1947;Description=hypothetical protein FVER53263_03017 [Fusarium verticillioides];Gene=1574
Fv_160_3.g1948	409	ID=Fv_160_3.g1948;Description=hypothetical protein FVEG_03018 [Fusarium verticillioides 7600];Gene=FTJAE_1381;Ontology_term=membrane,malate transmembrane transporter activity,malate transmembrane
Fv_160_3.g1949	767	ID=Fv_160_3.g1949;Description=tol [Fusarium pseudoanthophilum]
Fv_160_3.g1950	622	ID=Fv_160_3.g1950;Description=hypothetical protein FVER53590_03020 [Fusarium verticillioides];Gene=BFJ72_g1519;Ontology_term=purine-specific mismatch base pair DNA N-glycosylase activity,oxidized base lesion DNA N-glycosylase activity,metal ion binding,4 iron, 4 sulfur cluster binding,base-excision repair, AP site

Fv_160_3.g1951	692	ID=Fv_160_3.g1951;Description=hypothetical protein FVEG_15168 [Fusarium verticillioides 7600];Gene=FocTR4_00008328;Ontology_term=nucleus,DNA repair;Ontology_id=GO:0005634,GO:0006281
Fv_160_3.g1952	919	ID=Fv_160_3.g1952;Description=ubiquitin thiolesterase [Fusarium verticillioides 7600];Gene=FMUND_1172;Ontology_term=cysteine-type deubiquitinase
Fv_160_3.g1953	123	ID=Fv_160_3.g1953;Description=ubiquinol-cytochrome c reductase subunit 7 [Fusarium verticillioides 7600];Gene=BFJ68_g1710;Ontology_term=mitochondrial respiratory chain complex III,matrix side of mitochondrial inner membrane,ubiquinol-cytochrome-c reductase activity,mitochondrial electron transport, ubiquinol to cytochrome c,mitochondrial respiratory chain complex III assembly,proton transmembrane transport;Ontology_id=GO:0005750,GO:0099617,GO:0008121,GO:0006122,GO:0034551,GO:1902600;Enzyme_code=EC:1.10,EC:7.1
Fv_160_3.g1954	407	ID=Fv_160_3.g1954;Description=hypothetical protein FVEG_03025 [Fusarium verticillioides 7600];Gene=H3-3A;Ontology_term=nucleosome,nucleus,mitochondrial inner membrane,DNA binding,structural constituent of chromatin,protein heterodimerization activity,transmembrane
Fv_160_3.g1955	234	ID=Fv_160_3.g1955;Description=hypothetical protein J7337_005928 [Fusarium musae];Gene=FPRO_07708;Ontology_term=antioxidant activity,oxidoreductase activity,cellular oxidant
Fv_160_3.g1956	190	ID=Fv_160_3.g1956;Description=NADH dehydrogenase [Fusarium verticillioides 7600]
Fv_160_3.g1957	154	ID=Fv_160_3.g1957;Description=hypothetical protein FVEG_15170 [Fusarium verticillioides 7600];Gene=FPHYL_8195;Ontology_term=oxidoreductase activity,cellular biosynthetic
Fv_160_3.g1958	279	ID=Fv_160_3.g1958;Description=hypothetical protein FOXG_05107 [Fusarium oxysporum f. sp. lycopersici 4287];Gene=FOQG_15177;Ontology_term=oxidoreductase activity,cellular biosynthetic
Fv_160_3.g1959	226	ID=Fv_160_3.g1959;Description=acetyltransferase [Fusarium verticillioides 7600];Gene=FCIRC_12874;Ontology_term=NatA complex,peptide alpha-N-acetyltransferase
Fv_160_3.g1960	738	ID=Fv_160_3.g1960;Description=hypothetical protein FVER14953_03030 [Fusarium verticillioides]
Fv_160_3.g1961	457	ID=Fv_160_3.g1961;Description=hypothetical protein FVER53263_03031 [Fusarium verticillioides];Gene=FOQG_15174;Ontology_term=translation initiation factor activity,translational
Fv_160_3.g1962	515	ID=Fv_160_3.g1962;Description=hypothetical protein FVER53263_03032 [Fusarium verticillioides]
Fv_160_3.g1963	511	ID=Fv_160_3.g1963;Description=hypothetical protein FVER14953_03033 [Fusarium verticillioides];Gene=FNAPI_8069;Ontology_term=phosphatase
Fv_160_3.g1964	150	ID=Fv_160_3.g1964;Description=hypothetical protein FOXG_05101 [Fusarium oxysporum f. sp. lycopersici 4287];Gene=FOC1_g10010942;Ontology_term=membrane;Ontology_id=GO:0016020
Fv_160_3.g1965	241	ID=Fv_160_3.g1965;Description=H/ACA ribonucleoprotein complex subunit 2 [Fusarium verticillioides 7600];Gene=FOC1_g10010941;Ontology_term=ribonucleoprotein complex,ribosome
Fv_160_3.g1966	104	ID=Fv_160_3.g1966;Description=cytochrome b-c1 complex subunit 8 [Fusarium verticillioides 7600];Gene=FGADI_13039;Ontology_term=mitochondrial respiratory chain complex III,mitochondrial electron transport, ubiquinol

Fv_160_3.g1967	280	ID=Fv_160_3.g1967;Description=prohibitin-1 [Fusarium oxysporum f. sp. lycopersici 4287];Gene=FPSE_12369;Ontology_term=peroxisome,mitochondrial prohibitin complex,mitochondrion inheritance,protein folding,inner mitochondrial membrane organization,negative regulation of proteolysis,mitochondrion
Fv_160_3.g1968	652	ID=Fv_160_3.g1968;Description=hypothetical protein FVER14953_03038 [Fusarium verticillioides];Gene=rex3;Ontology_term=nucleic acid binding,exonuclease activity,RNA metabolic
Fv_160_3.g1969	444	ID=Fv_160_3.g1969;Description=hypothetical protein FVEG_03039 [Fusarium verticillioides 7600];Gene=Forpe1208_v007934;Ontology_term=membrane,lipid catabolic process;Ontology_id=GO:0016020,GO:0016042
Fv_160_3.g1970	232	ID=Fv_160_3.g1970;Description=hypothetical protein FVEG_03040 [Fusarium verticillioides 7600];Gene=FocTR4_00008306;Ontology_term=mitochondrial inner membrane,mitochondrial intermembrane
Fv_160_3.g1971	133	ID=Fv_160_3.g1971;Description=hypothetical protein FVEG_03041 [Fusarium verticillioides 7600]
Fv_160_3.g1972	162	ID=Fv_160_3.g1972;Description=hypothetical protein FVEG_03042 [Fusarium verticillioides 7600];Gene=FOPG_03037;Ontology_term=cytoplasm,acid phosphatase activity,non-membrane spanning protein tyrosine phosphatase
Fv_160_3.g1973	418	ID=Fv_160_3.g1973;Description=CMGC/MAPK/ERK1 protein kinase [Fusarium verticillioides 7600];Gene=FNAPI_8059;Ontology_term=nucleus,cytoplasm,MAP kinase activity,ATP binding,cell wall integrity MAPK cascade,phosphorylation,positive regulation of calcium-mediated signaling,negative regulation of mitotic cytokinesis,negative regulation of glucose mediated signaling pathway,positive regulation of calcium ion import across plasma membrane;Ontology_id=GO:0005634,GO:0005737,GO:0004707,GO:0005524,GO:0000196,GO:0016310,GO:0050850,GO:1902413,G
Fv_160_3.g1974	546	ID=Fv_160_3.g1974;Description=hypothetical protein FVEG_03044 [Fusarium verticillioides 7600];Gene=FNYG_06258;Ontology_term=amidase activity,indoleacetamide hydrolase
Fv_160_3.g1975	476	ID=Fv_160_3.g1975;Description=hypothetical protein FVEG_03045 [Fusarium verticillioides 7600];Gene=FVEG_03045;Ontology_term=membrane,transmembrane transporter activity,transmembrane
Fv_160_3.g1976	248	ID=Fv_160_3.g1976;Description=hypothetical protein J7337_005948 [Fusarium musae];Gene=1548;Ontology_term=calmodulin binding,L-type voltage-gated calcium channel complex,metal ion binding,cellular response to caffeine,positive regulation of muscle contraction,voltage-gated calcium channel activity,transmembrane transporter binding,regulation of ryanodine-sensitive calcium-release channel
Fv_160_3.g1977	196	ID=Fv_160_3.g1977;Description=dynactin 5 [Fusarium verticillioides 7600];Gene=BFJ68_g1776;Ontology_term=nucleus,cytoplasm,cytoskeleton,mannose-1-phosphate guanylyltransferase (GTP) activity,zinc ion
Fv_160_3.g1978	350	ID=Fv_160_3.g1978;Description=hypothetical protein FVER53263_03048 [Fusarium
Fv_160_3.g1979	184	ID=Fv_160_3.g1979;Description=hypothetical protein FVEG_03049 [Fusarium verticillioides 7600];Gene=Forpe1208_v007944;Ontology_term=mitochondrial inner membrane,mitochondrial intermembrane

Fv_160_3.g1980	441	ID=Fv_160_3.g1980;Description=methionyl aminopeptidase [Fusarium oxysporum f. sp. lycopersici 4287];Gene=FCIRC_12853;Ontology_term=endoplasmic reticulum membrane,NADPH-hemoprotein reductase activity,initiator methionyl aminopeptidase activity,FMN binding,metal ion binding,flavin adenine dinucleotide binding,NADP binding,metalloaminopeptidase activity,proteolysis;Ontology_id=GO:0005789,GO:0003958,GO:0004239,GO:0010181,GO:0046872,GO:0050660,GO:0050661,GO:00
Fv_160_3.g1981	372	ID=Fv_160_3.g1981;Description=hypothetical protein FVEG_03051 [Fusarium verticillioides 7600];Gene=1543;Ontology_term=membrane,transmembrane transporter activity,transmembrane
Fv_160_3.g1982	236	ID=Fv_160_3.g1982;Description=hypothetical protein FVEG_03052 [Fusarium verticillioides 7600];Gene=FCIRC_12851;Ontology_term=ATP binding,kinase
Fv_160_3.g1983	126	ID=Fv_160_3.g1983;Description=hypothetical protein FVEG_03053 [Fusarium verticillioides 7600]
Fv_160_3.g1984	494	ID=Fv_160_3.g1984;Description=hypothetical protein FVEG_03054 [Fusarium verticillioides 7600]
Fv_160_3.g1985	331	ID=Fv_160_3.g1985;Description=hypothetical protein FVEG_03055 [Fusarium verticillioides 7600]
Fv_160_3.g1986	385	ID=Fv_160_3.g1986;Description=hypothetical protein FVER14953_03056 [Fusarium
Fv_160_3.g1987	266	ID=Fv_160_3.g1987;Description=TKL protein kinase [Fusarium verticillioides 7600];Gene=BFJ69_g6487;Ontology_term=protein kinase activity,ATP
Fv_160_3.g1988	903	ID=Fv_160_3.g1988;Description=chitin synthase 3 [Fusarium verticillioides 7600];Gene=FSUBG_12863;Ontology_term=plasma membrane,chitosome,chitin synthase activity,septum digestion after cytokinesis,ascospore wall chitin biosynthetic process,cell wall organization;Ontology_id=GO:0005886,GO:0045009,GO:0004100,GO:0000920,GO:0034217,GO:0071555;Enzyme_code=EC:2.4.1.16
Fv_160_3.g1989	123	ID=Fv_160_3.g1989;Description=hypothetical protein FVEG_03059 [Fusarium verticillioides 7600]
Fv_160_3.g1990	534	ID=Fv_160_3.g1990;Description=hypothetical protein FVEG_03060 [Fusarium verticillioides 7600]
Fv_160_3.g1991	219	ID=Fv_160_3.g1991;Description=hypothetical protein FVEG_03061 [Fusarium verticillioides
Fv_160_3.g1992	82	ID=Fv_160_3.g1992;Description=hypothetical protein FVER14953_21276 [Fusarium verticillioides]
Fv_160_3.g1993	672	ID=Fv_160_3.g1993;Description=hypothetical protein FVER53590_03062 [Fusarium verticillioides]
Fv_160_3.g1994	328	ID=Fv_160_3.g1994;Description=hypothetical protein FVER14953_03063 [Fusarium verticillioides];Gene=FRV6_02213;Ontology_term=hydrolase
Fv_160_3.g1995	492	ID=Fv_160_3.g1995;Description=hypothetical protein FVEG_03064 [Fusarium verticillioides 7600];Gene=Forpe1208_v007957;Ontology_term=membrane,transmembrane transporter activity,carbohydrate
Fv_160_3.g1996	278	ID=Fv_160_3.g1996;Description=hypothetical protein FVEG_03065 [Fusarium verticillioides 7600];Gene=FGLOB1_911;Ontology_term=zinc ion binding,UV-damage excision repair,DNA-templated transcription,nucleotide-excision repair, DNA damage recognition,voltage-gated calcium channel complex,intracellular protein transport,voltage-gated calcium channel activity,damaged DNA binding,protein secretion,positive regulation of cytosolic calcium ion concentration,nucleotide binding,nucleotide-excision repair factor 1 complex,protein binding,prospore membrane,oxidoreductase activity,signaling,regulation of monoatomic ion transmembrane transport,regulation of

Fv_160_3.g1997	713	ID=Fv_160_3.g1997;Description=hypothetical protein FVEG_03066 [Fusarium verticillioides 7600];Gene=BFJ69_g6570;Ontology_term=nucleus,DNA binding,zinc ion binding,membrane,S-adenosylmethionine-dependent methyltransferase activity,DNA-binding transcription factor activity, RNA polymerase II-specific,regulation of transcription by RNA
Fv_160_3.g1998	278	ID=Fv_160_3.g1998;Description=hypothetical protein FVER14953_03067 [Fusarium verticillioides];Gene=FACUT_4717;Ontology_term=S-adenosylmethionine-dependent methyltransferase
Fv_160_3.g1999	543	ID=Fv_160_3.g1999;Description=hypothetical protein FVER53590_03068 [Fusarium verticillioides]
Fv_160_3.g2000	532	ID=Fv_160_3.g2000;Description=hypothetical protein FVER14953_21272 [Fusarium verticillioides];Gene=FVER53590_29970;Ontology_term=hydrolase activity, hydrolyzing O-glycosyl compounds,carbohydrate
Fv_160_3.g2001	512	ID=Fv_160_3.g2001;Description=hypothetical protein FVEG_03070 [Fusarium verticillioides 7600];Gene=FocTR4_00008269;Ontology_term=Golgi membrane,endoplasmic reticulum membrane,voltage-gated calcium channel complex,phagophore assembly site membrane,GTPase activity,protein binding,GTP binding,high voltage-gated calcium channel activity,metal ion binding,autophagosome assembly,intracellular protein transport,endoplasmic reticulum to Golgi vesicle-mediated transport,positive regulation of cytosolic calcium ion concentration,regulation of monoatomic ion transmembrane transport,calcium ion transmembrane
Fv_160_3.g2002	86	ID=Fv_160_3.g2002;Description=hypothetical protein FVER14953_21273 [Fusarium verticillioides]
Fv_160_3.g2003	76	ID=Fv_160_3.g2003;Description=hypothetical protein FVEG_15177 [Fusarium verticillioides 7600]
Fv_160_3.g2004	331	ID=Fv_160_3.g2004;Description=hypothetical protein FVER14953_03071 [Fusarium verticillioides]
Fv_160_3.g2005	618	ID=Fv_160_3.g2005;Description=hypothetical protein FVER14953_03072 [Fusarium verticillioides];Gene=FPANT_114;Ontology_term=extracellular space,serine-type endopeptidase activity,metal ion
Fv_160_3.g2006	510	ID=Fv_160_3.g2006;Description=hypothetical protein FVEG_15178 [Fusarium verticillioides 7600]
Fv_160_3.g2007	249	ID=Fv_160_3.g2007;Description=hypothetical protein FVER14953_03074 [Fusarium verticillioides];Gene=FGADI_10029;Ontology_term=nucleus,microsatellite binding,single-stranded telomeric DNA binding,GU repeat
Fv_160_3.g2008	1450	ID=Fv_160_3.g2008;Description=hypothetical protein FVER14953_03075 [Fusarium verticillioides];Gene=Forpe1208_v007968;Ontology_term=metal ion binding;Ontology_id=GO:0046872
Fv_160_3.g2009	705	ID=Fv_160_3.g2009;Description=hypothetical protein FVEG_03076 [Fusarium verticillioides 7600];Gene=vma2;Ontology_term=Golgi membrane,vacuolar proton-transporting V-type ATPase, V1 domain,endoplasmic reticulum,cytosol,COPII-coated ER to Golgi transport vesicle,pyruvate carboxylase activity,ATP binding,zinc ion binding,ATP hydrolysis activity,syntaxin binding,proton-transporting ATPase activity, rotational mechanism,pyruvate metabolic process,gluconeogenesis,endoplasmic reticulum to Golgi vesicle-mediated transport,retrograde vesicle-mediated transport, Golgi to endoplasmic reticulum,vacuolar acidification,positive regulation of SNARE complex assembly,ATP metabolic process,regulation of Ras protein signal transduction,vesicle fusion with Golgi apparatus,proton transmembrane transport;Ontology_id=GO:0000139,GO:0000221,GO:0005783,GO:0005829,GO:0030134,GO:0004736,GO:0005524,GO:0008270,GO

Fv_160_3.g2010	337	ID=Fv_160_3.g2010;Description=hypothetical protein FVER14953_03077 [Fusarium verticillioides];Gene=FGLOB1_899;Ontology_term=Ino80 complex,chromatin remodeling;Ontology_id=GO:0031011,GO:0006338
Fv_160_3.g2011	462	ID=Fv_160_3.g2011;Description=isocitrate dehydrogenase mitochondrial [Fusarium globosum];Gene=FACUT_4728;Ontology_term=magnesium ion binding,isocitrate dehydrogenase (NADP+) activity,NAD binding,tricarboxylic acid cycle,isocitrate metabolic
Fv_160_3.g2012	585	ID=Fv_160_3.g2012;Description=hypothetical protein FVER14953_03079 [Fusarium verticillioides]
Fv_160_3.g2013	728	ID=Fv_160_3.g2013;Description=hypothetical protein FCOIX_9665 [Fusarium coicis];Gene=vma3;Ontology_term=vacuolar proton-transporting V-type ATPase, V0 domain,fungal-type vacuole membrane,ATP hydrolysis activity,proton-transporting ATPase activity, rotational mechanism,proton transmembrane transport;Ontology_id=GO:0000220,GO:0000329,GO:0016887,GO:0046961,GO:1902600;Enzyme_code=EC:7.2.2,EC:3.6.1.15;Enzym
Fv_160_3.g2014	233	ID=Fv_160_3.g2014;Description=hypothetical protein FVEG_03081 [Fusarium verticillioides 7600]
Fv_160_3.g2015	625	ID=Fv_160_3.g2015;Description=hypothetical protein FVEG_03082 [Fusarium verticillioides 7600];Gene=BFJ68_g1595;Ontology_term=P-body,nucleus,CCR4-NOT core complex,nuclear-transcribed mRNA poly(A) tail
Fv_160_3.g2016	153	ID=Fv_160_3.g2016;Description=D-tyrosyl-tRNA(Tyr) deacylase [Fusarium verticillioides 7600];Gene=F53441_2021;Ontology_term=terminal cisterna,membrane,sarcoplasmic reticulum lumen,tRNA binding,CDP-diacylglycerol-serine O-phosphatidyltransferase activity,calcium ion binding,D-aminoacyl-tRNA deacylase activity,phosphatidylethanolamine biosynthetic process,phosphatidylserine biosynthetic process,aminoacyl-tRNA metabolism involved in translational
Fv_160_3.g2017	372	ID=Fv_160_3.g2017;Description=peptidyl-prolyl cis-trans isomerase D [Fusarium verticillioides 7600];Gene=FOC1_g10010888;Ontology_term=mitochondrial inner membrane,peptidyl-prolyl cis-trans isomerase activity,ribosome binding,unfolded protein binding,dihydroorotate dehydrogenase (quinone) activity,'de novo' pyrimidine nucleobase biosynthetic process,protein refolding,'de novo' UMP biosynthetic process;Ontology_id=GO:0005743,GO:0003755,GO:0043022,GO:0051082,GO:0106430,GO:0006207,GO:0042026,GO:0044205;Enzy
Fv_160_3.g2018	1237	ID=Fv_160_3.g2018;Description=hypothetical protein FVER14953_03085 [Fusarium verticillioides];Gene=FNAPI_5115;Ontology_term=nucleus,cytoplasm,GTPase activator activity,nuclear localization sequence binding,nuclear import signal receptor activity,mRNA export from nucleus,protein import into nucleus,regulation of mitotic nuclear
Fv_160_3.g2019	396	ID=Fv_160_3.g2019;Description=hypothetical protein FVER14953_03086 [Fusarium verticillioides]
Fv_160_3.g2020	206	ID=Fv_160_3.g2020;Description=hypothetical protein J7337_005990 [Fusarium musae];Gene=FOC1_g10010885;Ontology_term=ribose phosphate diphosphokinase complex,ribosome,magnesium ion binding,ribose phosphate diphosphokinase activity,ATP binding,kinase activity,5-phosphoribose 1-diphosphate biosynthetic process,ribonucleoside monophosphate biosynthetic process,nucleotide biosynthetic process,phosphorylation,fungal-type cell wall organization;Ontology_id=GO:0002189,GO:0005840,GO:0000287,GO:0004749,GO:0005524,GO:0016301,GO:0006015,GO:0009156,

Fv_160_3.g2021	471	ID=Fv_160_3.g2021;Description=hypothetical protein FVER14953_03088 [Fusarium verticillioides];Gene=CACNA1C;Ontology_term=lipid droplet,cytosol,fatty acid synthase complex,dendrite,perikaryon,postsynaptic density membrane,L-type voltage-gated calcium channel complex,fatty acid synthase activity,[acyl-carrier-protein] S-acetyltransferase activity,[acyl-carrier-protein] S-malonyltransferase activity,(3R)-hydroxypalmitoyl-[acyl-carrier-protein] dehydratase activity,enoyl-[acyl-carrier-protein] reductase (NADH) activity,enoyl-[acyl-carrier-protein] reductase (NADPH, B-specific) activity,oleoyl-[acyl-carrier-protein] hydrolase activity,fatty-acyl-CoA synthase activity,calmodulin binding,high voltage-gated calcium channel activity,(3R)-hydroxymyristoyl-[acyl-carrier-protein] dehydratase activity,myristoyl-[acyl-carrier-protein] hydrolase activity,palmitoyl-[acyl-carrier-protein] hydrolase activity,palmitoyltransferase activity,metal ion binding,regulation of cardiac muscle contraction by regulation of the release of sequestered calcium ion,long-chain fatty acid biosynthetic process,positive regulation of adenylate cyclase activity,calcium ion transport into cytosol,cardiac conduction,calcium ion transmembrane transport via high voltage-gated calcium channel,regulation of membrane repolarization during action potential;Ontology_id=GO:0005811,GO:0005829,GO:0005835,GO:0030425,GO:0043204,GO:0098839,GO:1990454,GO:0004312,GO:0004313,GO:0004314,GO:0004317,GO:0004318,GO:0004319,GO:0004320,GO:0004321,GO:0005516,GO:0008331,GO:0008659,GO
Fv_160_3.g2022	98	ID=Fv_160_3.g2022;Description=hypothetical protein FVEG_03089 [Fusarium verticillioides 7600]
Fv_160_3.g2023	853	ID=Fv_160_3.g2023;Description=hypothetical protein FVER14953_03090 [Fusarium verticillioides];Gene=1507
Fv_160_3.g2024	542	ID=Fv_160_3.g2024;Description=hypothetical protein FVEG_03091 [Fusarium verticillioides 7600];Gene=FVER53263_03091;Ontology_term=spliceosomal complex,membrane,RNA binding,zinc ion binding,transmembrane transporter activity,mRNA splicing, via spliceosome,ribonucleoprotein complex biogenesis,transmembrane
Fv_160_3.g2025	166	ID=Fv_160_3.g2025;Description=methyltransferase [Fusarium verticillioides 7600];Gene=FOVG_05869;Ontology_term=methyltransferase
Fv_160_3.g2026	332	ID=Fv_160_3.g2026;Description=hypothetical protein FVEG_03094 [Fusarium verticillioides 7600]
Fv_160_3.g2027	734	ID=Fv_160_3.g2027;Description=hypothetical protein FVER14953_03095 [Fusarium verticillioides];Gene=FVER53263_03095;Ontology_term=actin cortical patch,metal ion binding,cytoskeletal protein-membrane anchor activity,Arp2/3 complex-mediated actin nucleation,clathrin-dependent
Fv_160_3.g2028	515	ID=Fv_160_3.g2028;Description=hypothetical protein FVER14953_03096 [Fusarium verticillioides];Gene=Forpe1208_v007988;Ontology_term=ligase
Fv_160_3.g2029	121	ID=Fv_160_3.g2029;Description=hypothetical protein FVER14953_03097 [Fusarium verticillioides]

Fv_160_3.g2030	263	ID=Fv_160_3.g2030;Description=hypothetical protein FVEG_03098 [Fusarium verticillioides 7600];Gene=FNAPI_5104;Ontology_term=photoreceptor ribbon synapse,presynapse,L-type voltage-gated calcium channel complex,high voltage-gated calcium channel activity,actin filament binding,voltage-gated calcium channel activity involved in AV node cell action potential,voltage-gated calcium channel activity involved in regulation of presynaptic cytosolic calcium levels,chemical synaptic transmission,visual perception,regulation of monoatomic ion transmembrane transport,positive regulation of calcium ion transport,calcium ion import,calcium ion transmembrane transport,membrane depolarization during AV node cell action potential,regulation of heart rate by cardiac conduction,membrane depolarization during atrial cardiac muscle cell action potential,regulation of presynaptic cytosolic calcium ion
Fv_160_3.g2031	117	ID=Fv_160_3.g2031;Description=hypothetical protein FVEG_03099 [Fusarium verticillioides 7600];Gene=top1;Ontology_term=nucleus,ATP-dependent activity, acting on RNA,mitotic chromosome
Fv_160_3.g2032	453	ID=Fv_160_3.g2032;Description=hypothetical protein FVEG_03100 [Fusarium verticillioides 7600]
Fv_160_3.g2033	109	ID=Fv_160_3.g2033;Description=60S acidic ribosomal protein P1 [Fusarium verticillioides 7600];Gene=BFJ68_g1771;Ontology_term=ribosome,ribonucleoprotein complex,nucleic acid binding,structural constituent of ribosome,helicase activity,ATP binding,ATP-dependent activity, acting on RNA,hydrolase activity,RNA processing,translational elongation,ribonucleoprotein complex biogenesis;Ontology_id=GO:0005840,GO:1990904,GO:0003676,GO:0003735,GO:0004386,GO:0005524,GO:0008186,GO:0016787,G
Fv_160_3.g2034	317	ID=Fv_160_3.g2034;Description=hypothetical protein FVEG_03102 [Fusarium verticillioides 7600]
Fv_160_3.g2035	249	ID=Fv_160_3.g2035;Description=hypothetical protein FVEG_03103 [Fusarium verticillioides 7600]
Fv_160_3.g2036	290	ID=Fv_160_3.g2036;Description=hypothetical protein FVEG_03104 [Fusarium verticillioides 7600];Gene=FOVG_05857;Ontology_term=endoplasmic reticulum membrane;Ontology_id=GO:0005789
Fv_160_3.g2037	374	ID=Fv_160_3.g2037;Description=sphingolipid delta-4 desaturase [Fusarium verticillioides 7600];Gene=FDENT_3259;Ontology_term=nucleus,membrane,double-stranded DNA binding,sphingolipid delta-4 desaturase activity,gene conversion at mating-type locus,sphingosine biosynthetic
Fv_160_3.g2038	515	ID=Fv_160_3.g2038;Description=hypothetical protein FVEG_03106 [Fusarium verticillioides 7600];Gene=BFJ63_vAg1519;Ontology_term=cytosol,cytoskeleton,cell cortex,membrane,actin monomer binding,phosphatidylinositol-4,5-bisphosphate binding,proline-rich region binding,sequestering of actin monomers,intracellular transport,positive regulation of formin-nucleated actin cable assembly,mitotic actomyosin contractile ring
Fv_160_3.g2039	432	ID=Fv_160_3.g2039;Description=hypothetical protein FVER14953_03107 [Fusarium
Fv_160_3.g2040	337	ID=Fv_160_3.g2040;Description=hypothetical protein FVER14953_03108 [Fusarium verticillioides]
Fv_160_3.g2041	250	ID=Fv_160_3.g2041;Description=hypothetical protein FVEG_15182 [Fusarium verticillioides 7600]
Fv_160_3.g2042	675	ID=Fv_160_3.g2042;Description=hypothetical protein FVER14953_03109 [Fusarium verticillioides];Gene=FVEG_03109;Ontology_term=tetrahydrofolylpolyglutamate synthase activity,ATP binding,metal ion binding,one-carbon metabolic process,tetrahydrofolylpolyglutamate biosynthetic

Fv_160_3.g2043	465	ID=Fv_160_3.g2043;Description=hypothetical protein FVEG_03110 [Fusarium verticillioides 7600]
Fv_160_3.g2044	1420	ID=Fv_160_3.g2044;Description=hypothetical protein J7337_006013 [Fusarium musae];Gene=FVEG_15183;Ontology_term=protein kinase activity,ATP
Fv_160_3.g2045	552	ID=Fv_160_3.g2045;Description=hypothetical protein FVER14953_03113 [Fusarium verticillioides];Gene=PRE2;Ontology_term=cytoplasm,proteasome core complex, beta-subunit complex,threonine-type endopeptidase activity,proteasomal ubiquitin-independent protein catabolic process,proteasome-mediated ubiquitin-dependent
Fv_160_3.g2046	304	ID=Fv_160_3.g2046;Description=hypothetical protein FVEG_03114 [Fusarium verticillioides
Fv_160_3.g2047	676	ID=Fv_160_3.g2047;Description=hypothetical protein FVEG_03115 [Fusarium verticillioides 7600];Gene=FPCIR_11902;Ontology_term=RNA polymerase II transcription regulator complex,DNA-binding transcription activator activity, RNA polymerase II-specific,DNA binding,sporulation resulting in formation of a cellular spore,positive regulation of
Fv_160_3.g2048	380	ID=Fv_160_3.g2048;Description=hypothetical protein FANTH_11789 [Fusarium anthophilum];Gene=ste11;Ontology_term=nucleus,hemoglobin complex,RNA polymerase II cis-regulatory region sequence-specific
Fv_160_3.g2049	139	ID=Fv_160_3.g2049;Description=hypothetical protein FVER14953_03117 [Fusarium verticillioides];Gene=da;Ontology_term=positive regulation of transcription elongation by RNA polymerase II,nucleus,zinc ion binding,transcription elongation by RNA polymerase I,maintenance of transcriptional fidelity during transcription elongation by RNA polymerase II,RNA polymerase II transcription regulatory region sequence-specific DNA binding,transcription antitermination,regulation of mRNA 3'-end processing,RNA polymerase II complex recruiting activity,tRNA transcription by RNA polymerase III,positive regulation of RNA polymerase II
Fv_160_3.g2050	462	ID=Fv_160_3.g2050;Description=hypothetical protein FVEG_03118 [Fusarium verticillioides 7600];Gene=FVEG_03118;Ontology_term=membrane,mannan endo-1,6-alpha-mannosidase activity,carbohydrate catabolic process;Ontology_id=GO:0016020,GO:0008496,GO:0016052;Enzyme_code=EC:3.2.1.101,EC:3.2.1.24;Enzyme_name=mannan endo-
Fv_160_3.g2051	376	ID=Fv_160_3.g2051;Description=hypothetical protein FVEG_03119 [Fusarium verticillioides 7600];Gene=FOTG_15350;Ontology_term=Golgi membrane,endoplasmic reticulum membrane,COPII-coated ER to Golgi transport vesicle,endoplasmic reticulum-Golgi intermediate compartment membrane,endoplasmic reticulum to Golgi vesicle-mediated
Fv_160_3.g2052	1086	ID=Fv_160_3.g2052;Description=hypothetical protein FVEG_03120 [Fusarium verticillioides 7600]
Fv_160_3.g2053	75	ID=Fv_160_3.g2053;Description=hypothetical protein FVEG_15191 [Fusarium verticillioides 7600]
Fv_160_3.g2054	415	ID=Fv_160_3.g2054;Description=hypothetical protein FVEG_03121 [Fusarium verticillioides 7600];Gene=FFC1_02594;Ontology_term=Hsp70 protein binding,metal ion binding,unfolded protein binding,protein
Fv_160_3.g2055	876	ID=Fv_160_3.g2055;Description=hypothetical protein FVEG_03122 [Fusarium verticillioides 7600];Gene=FCIRC_13368;Ontology_term=ribonucleoprotein complex,nucleic acid binding,helicase activity,ATP binding,hydrolase
Fv_160_3.g2056	298	ID=Fv_160_3.g2056;Description=hypothetical protein FVEG_15192 [Fusarium verticillioides 7600];Gene=FocTR4_00008207;Ontology_term=iron ion binding,oxidoreductase activity, acting on paired donors, with incorporation or reduction of molecular oxygen,L-ascorbic acid binding,dioxygenase activity;Ontology_id=GO:0005506,GO:0016705,GO:0031418,GO:0051213;Enzyme_code=EC:1.14;Enzyme_name=Acting on paired

Fv_160_3.g2057	528	ID=Fv_160_3.g2057;Description=AAT family amino acid transporter [Fusarium verticillioides 7600];Gene=FOPG_12195;Ontology_term=membrane,amino acid transport,transmembrane
Fv_160_3.g2058	607	ID=Fv_160_3.g2058;Description=hypothetical protein FVER14953_03124 [Fusarium verticillioides];Gene=FPHYL_12073;Ontology_term=copper ion binding,oxidoreductase
Fv_160_3.g2059	137	ID=Fv_160_3.g2059;Description=hypothetical protein FVEG_03125 [Fusarium verticillioides 7600];Gene=FMUND_9082;Ontology_term=voltage-gated calcium channel complex,voltage-gated calcium channel activity,calcium ion binding,myosin phosphatase activity,dioxygenase activity,regulation of monoatomic ion transmembrane transport,calcium ion transmembrane
Fv_160_3.g2060	301	ID=Fv_160_3.g2060;Description=hypothetical protein FVER14953_03126 [Fusarium verticillioides]
Fv_160_3.g2061	386	ID=Fv_160_3.g2061;Description=hypothetical protein FVER14953_03127 [Fusarium
Fv_160_3.g2062	74	ID=Fv_160_3.g2062;Description=hypothetical protein FVEG_03128 [Fusarium verticillioides 7600];Gene=VMA1;Ontology_term=fungal-type vacuole membrane,proton transmembrane
Fv_160_3.g2063	325	ID=Fv_160_3.g2063;Description=hypothetical protein FVER53263_03129 [Fusarium verticillioides];Gene=FACUT_12381;Ontology_term=catalytic activity,biosynthetic process;Ontology_id=GO:0003824,GO:0009058
Fv_160_3.g2064	1154	ID=Fv_160_3.g2064;Description=hypothetical protein FVEG_15193 [Fusarium verticillioides 7600]
Fv_160_3.g2065	130	ID=Fv_160_3.g2065;Description=FMP21 [Fusarium mundagurra];Gene=PPH21;Ontology_term=fungal-type vacuole,plasma membrane,thiamine transmembrane transporter activity,myosin phosphatase activity,pyridoxal transmembrane transporter activity,pyridoxamine transmembrane transporter activity,pyridoxine transmembrane transporter activity,metal ion binding,thiamine transmembrane transport,pyridoxal transmembrane transport,pyridoxamine transmembrane transport,pyridoxine transmembrane transport;Ontology_id=GO:0000324,GO:0005886,GO:0015234,GO:0017018,GO:0031925,GO:0031927,GO:0031928,GO:0046872,GO
Fv_160_3.g2066	575	ID=Fv_160_3.g2066;Description=PiT family inorganic phosphate transporter [Fusarium verticillioides 7600];Gene=FFUJ_07046;Ontology_term=membrane,inorganic phosphate transmembrane transporter activity,symporter
Fv_160_3.g2067	431	ID=Fv_160_3.g2067;Description=hypothetical protein FVEG_15194 [Fusarium verticillioides 7600];Gene=IPP1;Ontology_term=cytoplasm,magnesium ion binding,inorganic diphosphate phosphatase activity,phosphate-containing compound metabolic
Fv_160_3.g2068	193	ID=Fv_160_3.g2068;Description=hypothetical protein FVER53590_03135 [Fusarium verticillioides]
Fv_160_3.g2069	107	ID=Fv_160_3.g2069;Description=hypothetical protein FVER14953_03136 [Fusarium verticillioides]
Fv_160_3.g2070	75	ID=Fv_160_3.g2070;Description=hypothetical protein FVER53263_03137 [Fusarium verticillioides]
Fv_160_3.g2071	305	ID=Fv_160_3.g2071;Description=hypothetical protein FVER53590_29316 [Fusarium verticillioides];Gene=POT1;Ontology_term=acyltransferase
Fv_160_3.g2072	260	ID=Fv_160_3.g2072;Description=hypothetical protein FVER53263_03138 [Fusarium
Fv_160_3.g2073	498	ID=Fv_160_3.g2073;Description=hypothetical protein FVEG_03139 [Fusarium verticillioides 7600]

Fv_160_3.g2074	580	ID=Fv_160_3.g2074;Description=hypothetical protein FVER14953_03140 [Fusarium verticillioides];Gene=FTJAE_5693;Ontology_term=extracellular region,cellulose binding,endo-1,4-beta-xylanase activity,xylan
Fv_160_3.g2075	172	ID=Fv_160_3.g2075;Description=hypothetical protein FPANT_13333 [Fusarium pseudoanthophilum];Gene=1460;Ontology_term=cellular anatomical entity,amyloid-beta binding,protein homodimerization activity,lipid metabolic process,positive regulation of cholesterol efflux,protein oxidation,peptidyl-methionine modification,positive regulation of phagocytosis,protein stabilization,organic cyclic compound metabolic process,positive regulation of phospholipid
Fv_160_3.g2076	121	ID=Fv_160_3.g2076;Description=hypothetical protein FVEG_03142 [Fusarium verticillioides 7600]
Fv_160_3.g2077	139	ID=Fv_160_3.g2077;Description=hypothetical protein FVEG_03143 [Fusarium verticillioides 7600];Gene=APOA1;Ontology_term=protein homodimerization activity,high-density lipoprotein particle,DNA replication,cholesterol metabolic process,lipid binding,lipoprotein metabolic process,positive regulation of phospholipid efflux,amyloid-beta binding,nucleotide binding,nucleus,DNA binding,DNA-directed DNA polymerase activity,metal ion binding,DNA biosynthetic process,positive regulation of cholesterol efflux,protein oxidation,4 iron, 4 sulfur cluster binding,positive regulation of phagocytosis,protein stabilization,peptidyl-methionine
Fv_160_3.g2078	330	ID=Fv_160_3.g2078;Description=inorganic pyrophosphatase [Fusarium verticillioides 7600];Gene=FNAPI_9301;Ontology_term=cytoplasm,magnesium ion binding,inorganic diphosphate phosphatase activity,phosphate-containing compound metabolic
Fv_160_3.g2079	495	ID=Fv_160_3.g2079;Description=hypothetical protein FVER14953_03145 [Fusarium verticillioides];Gene=FNAPI_9302;Ontology_term=membrane,transmembrane transporter activity,transmembrane
Fv_160_3.g2080	398	ID=Fv_160_3.g2080;Description=hypothetical protein FVEG_03146 [Fusarium verticillioides 7600];Gene=FNAPI_9303;Ontology_term=membrane,glycosyltransferase
Fv_160_3.g2081	456	ID=Fv_160_3.g2081;Description=protein BTN1 [Fusarium verticillioides 7600];Gene=FCIRC_2534;Ontology_term=fungal-type vacuole,vacuolar membrane,lysine transport,regulation of intracellular pH,L-arginine transmembrane
Fv_160_3.g2082	186	ID=Fv_160_3.g2082;Description=hypothetical protein FVEG_03148 [Fusarium verticillioides 7600]
Fv_160_3.g2083	1359	ID=Fv_160_3.g2083;Description=hypothetical protein J7337_006050 [Fusarium musae];Gene=F25303_9759;Ontology_term=membrane,ATP binding,ATP hydrolysis activity,ABC-type transporter activity,transmembrane
Fv_160_3.g2084	415	ID=Fv_160_3.g2084;Description=hypothetical protein FVER53590_03150 [Fusarium verticillioides];Gene=FVER53590_03150;Ontology_term=acyltransferase activity, transferring groups other than amino-acyl
Fv_160_3.g2085	514	ID=Fv_160_3.g2085;Description=hypothetical protein FVEG_03151 [Fusarium verticillioides 7600];Gene=FOVG_05802;Ontology_term=membrane,high-density lipoprotein particle,S-adenosylmethionine-dependent methyltransferase activity,transmembrane transporter activity,nicotianamine synthase activity,acute-phase response,nicotianamine biosynthetic process,transmembrane transport;Ontology_id=GO:0016020,GO:0034364,GO:0008757,GO:0022857,GO:0030410,GO:0006953,GO:0030418,GO:0055085;En

Fv_160_3.g2086	303	ID=Fv_160_3.g2086;Description=hypothetical protein FVEG_03152 [Fusarium verticillioides 7600];Gene=FSUBG_12410;Ontology_term=S-adenosylmethionine-dependent methyltransferase activity,nicotianamine synthase activity,nicotianamine biosynthetic
Fv_160_3.g2087	473	ID=Fv_160_3.g2087;Description=hypothetical protein FVEG_03153 [Fusarium verticillioides 7600];Gene=FANTH_1482;Ontology_term=transferase activity, transferring alkyl or aryl (other than methyl) groups,lyase activity,pyridoxal phosphate binding,transsulfuration;Ontology_id=GO:0016765,GO:0016829,GO:0030170,GO:0019346;Enzyme_code=EC:2.5.1,EC:4;Enzyme_na
Fv_160_3.g2088	676	ID=Fv_160_3.g2088;Description=hypothetical protein FVEG_03154 [Fusarium verticillioides 7600];Gene=1449;Ontology_term=RNA polymerase I complex,cytosolic large ribosomal subunit,magnesium ion binding,structural constituent of ribosome,pyruvate kinase activity,ATP binding,kinase activity,transferase activity, transferring alkyl or aryl (other than methyl) groups,pyridoxal phosphate binding,potassium ion binding,cytoplasmic translational elongation,glycolytic process,transcription elongation by RNA polymerase I,phosphorylation,transsulfuration;Ontology_id=GO:0005736,GO:0022625,GO:0000287,GO:0003735,GO:0004743,GO:0005524,GO:
Fv_160_3.g2089	379	ID=Fv_160_3.g2089;Description=hypothetical protein FVEG_03155 [Fusarium verticillioides 7600];Gene=FVER53263_03154;Ontology_term=ATP binding,metal ion binding;Ontology_id=GO:0005524,GO:0046872
Fv_160_3.g2090	278	ID=Fv_160_3.g2090;Description=hypothetical protein FVEG_03156 [Fusarium verticillioides 7600];Gene=SAA2;Ontology_term=high-density lipoprotein particle,acute-phase response;Ontology_id=GO:0034364,GO:0006953
Fv_160_3.g2091	406	ID=Fv_160_3.g2091;Description=hypothetical protein FVEG_03157 [Fusarium verticillioides 7600];Gene=FPCIR_11605;Ontology_term=oxidoreductase activity,transferase activity, transferring alkyl or aryl (other than methyl) groups,lyase activity,pyridoxal phosphate binding,transsulfuration;Ontology_id=GO:0016491,GO:0016765,GO:0016829,GO:0030170,GO:0019346;Enzyme_code=EC:2.5.1,EC:1
Fv_160_3.g2092	466	ID=Fv_160_3.g2092;Description=hypothetical protein FVEG_03158 [Fusarium verticillioides 7600];Gene=FPCIR_11606;Ontology_term=transferase activity, transferring alkyl or aryl (other than methyl) groups,lyase activity,pyridoxal phosphate binding,transsulfuration;Ontology_id=GO:0016765,GO:0016829,GO:0030170,GO:0019346;Enzyme_code=EC:2.5.1,EC:4;Enzyme_na
Fv_160_3.g2093	464	ID=Fv_160_3.g2093;Description=hypothetical protein FVER14953_03159 [Fusarium verticillioides];Gene=FCIRC_2545;Ontology_term=oxidoreductase
Fv_160_3.g2094	329	ID=Fv_160_3.g2094;Description=hypothetical protein FVEG_03160 [Fusarium verticillioides 7600];Gene=FPANT_11106;Ontology_term=metal ion binding,dioxygenase activity,small molecule biosynthetic process,organic cyclic compound biosynthetic

Fv_160_3.g2095	264	ID=Fv_160_3.g2095;Description=cytochrome c heme-lyase [Fusarium verticillioides 7600];Gene=FOTG_14562;Ontology_term=mitochondrial inner membrane,mitochondrial intermembrane space,holocytochrome-c synthase activity,metal ion binding,organic cyclic compound binding,heterocyclic compound binding,nucleobase-containing compound metabolic process,response to stress,cellular response to stimulus;Ontology_id=GO:0005743,GO:0005758,GO:0004408,GO:0046872,GO:0097159,GO:1901363,GO:0006139,GO:0006950,GO:
Fv_160_3.g2096	448	ID=Fv_160_3.g2096;Description=hypothetical protein FVEG_03162 [Fusarium verticillioides 7600]
Fv_160_3.g2097	391	ID=Fv_160_3.g2097;Description=diphosphomevalonate decarboxylase [Fusarium coicis];Gene=BFJ65_g6060;Ontology_term=cytosol,diphosphomevalonate decarboxylase activity,ATP binding,ergosterol biosynthetic process,isopentenyl diphosphate biosynthetic process, mevalonate
Fv_160_3.g2098	476	ID=Fv_160_3.g2098;Description=hypothetical protein FVEG_03164 [Fusarium verticillioides];Gene=FVER53590_03164;Ontology_term=mitochondrial inner membrane,calcium channel activity,uniporter activity,transferase activity,mitochondrial calcium ion homeostasis,calcium ion transmembrane
Fv_160_3.g2099	70	ID=Fv_160_3.g2099;Description=spore killer candidate 1 [Fusarium verticillioides]
Fv_160_3.g2100	532	ID=Fv_160_3.g2100;Description=hypothetical protein FVER14953_03165 [Fusarium verticillioides];Gene=FOTG_14566;Ontology_term=cytosolic large ribosomal subunit,structural constituent of ribosome,acyltransferase activity,cytoplasmic translation,siderophore biosynthetic
Fv_160_3.g2101	466	ID=Fv_160_3.g2101;Description=hypothetical protein J7337_006068 [Fusarium musae];Gene=FNAPI_1174;Ontology_term=oxidoreductase activity,FAD
Fv_160_3.g2102	221	ID=Fv_160_3.g2102;Description=hypothetical protein FVEG_15198 [Fusarium verticillioides];Gene=FPCIR_11615;Ontology_term=extracellular space,oxidoreductase activity,acyltransferase activity,hydrolase activity,FAD binding,regulation of immune system process,acute-phase response;Ontology_id=GO:0005615,GO:0016491,GO:0016746,GO:0016787,GO:0071949,GO:0002682,GO:0006953;Enzyme_code=E
Fv_160_3.g2103	133	ID=Fv_160_3.g2103;Description=hypothetical protein FVEG_15199 [Fusarium verticillioides]
Fv_160_3.g2104	460	ID=Fv_160_3.g2104;Description=hypothetical protein FVEG_03167 [Fusarium verticillioides];Gene=FOTG_14571;Ontology_term=oxidoreductase activity, acting on
Fv_160_3.g2105	299	ID=Fv_160_3.g2105;Description=hypothetical protein FVEG_03168 [Fusarium verticillioides 7600];Gene=BFJ68_g1534;Ontology_term=oxidoreductase
Fv_160_3.g2106	643	ID=Fv_160_3.g2106;Description=hypothetical protein FVEG_03169 [Fusarium verticillioides];Gene=BFJ65_g6062;Ontology_term=nucleus,DNA binding,zinc ion binding,DNA-binding transcription factor activity,
Fv_160_3.g2107	230	ID=Fv_160_3.g2107;Description=hypothetical protein FVEG_03170 [Fusarium verticillioides];Gene=FPRO_07553;Ontology_term=nucleus,DNA binding,zinc ion binding,transferase activity,DNA-binding transcription factor activity, RNA polymerase II-specific,regulation of transcription by RNA polymerase
Fv_160_3.g2108	373	ID=Fv_160_3.g2108;Description=hypothetical protein FVER53263_03170 [Fusarium verticillioides]

Fv_160_3.g2109	1132	ID=Fv_160_3.g2109;Description=hypothetical protein FVEG_03171 [Fusarium verticillioides]
Fv_160_3.g2110	1018	ID=Fv_160_3.g2110;Description=hypothetical protein FVEG_03172 [Fusarium verticillioides];Gene=FOXG_04959;Ontology_term=nucleus,helicase activity,ATP binding,hydrolase activity,metal ion binding,ATP-dependent chromatin remodeler activity,chromatin
Fv_160_3.g2111	976	ID=Fv_160_3.g2111;Description=hypothetical protein FVEG_03173 [Fusarium verticillioides]
Fv_160_3.g2112	620	ID=Fv_160_3.g2112;Description=hypothetical protein FVEG_03174 [Fusarium verticillioides]
Fv_160_3.g2113	484	ID=Fv_160_3.g2113;Description=hypothetical protein FVEG_03175 [Fusarium verticillioides];Gene=FPCIR_11626;Ontology_term=nucleus,DNA-binding transcription factor activity, RNA polymerase II-specific,ATP binding,zinc ion binding,hydrolase activity,ATP-dependent chromatin remodeler activity,chromatin remodeling,regulation of
Fv_160_3.g2114	139	ID=Fv_160_3.g2114;Description=hypothetical protein FVEG_03176 [Fusarium verticillioides 7600];Gene=mutS2;Ontology_term=nucleus,ATP binding,hydrolase activity,ubiquitin conjugating enzyme activity,protein ubiquitination,epigenetic regulation of gene expression;Ontology_id=GO:0005634,GO:0005524,GO:0016787,GO:0061631,GO:0016567,GO:0040029;Enzyme_code=EC:3,EC:2.3.2
Fv_160_3.g2115	288	ID=Fv_160_3.g2115;Description=hypothetical protein FVEG_15200 [Fusarium verticillioides]
Fv_160_3.g2116	1031	ID=Fv_160_3.g2116;Description=RNA-binding protein [Fusarium
Fv_160_3.g2117	359	ID=Fv_160_3.g2117;Description=hypothetical protein FVEG_03180 [Fusarium
Fv_160_3.g2118	194	ID=Fv_160_3.g2118;Description=carbonic anhydrase [Fusarium verticillioides 7600];Gene=1427;Ontology_term=extracellular region,carbonate dehydratase activity,steroid binding,zinc ion
Fv_160_3.g2119	1029	ID=Fv_160_3.g2119;Description=hypothetical protein FVEG_15203 [Fusarium verticillioides];Gene=FVER53590_29312;Ontology_term=carbonate dehydratase activity,zinc ion
Fv_160_3.g2120	667	ID=Fv_160_3.g2120;Description=hypothetical protein FVEG_03181 [Fusarium verticillioides];Gene=FOTG_14586;Ontology_term=cysteine-type endopeptidase
Fv_160_3.g2121	88	ID=Fv_160_3.g2121;Description=hypothetical protein FVEG_15204 [Fusarium verticillioides 7600]
Fv_160_3.g2122	379	ID=Fv_160_3.g2122;Description=hypothetical protein FVEG_03182 [Fusarium
Fv_160_3.g2123	179	ID=Fv_160_3.g2123;Description=hypothetical protein H9Q70_002733 [Fusarium
Fv_160_3.g2124	513	ID=Fv_160_3.g2124;Description=hypothetical protein FVER53590_03183 [Fusarium verticillioides];Gene=FVER53590_03183;Ontology_term=membrane,monooxygenase activity,iron ion binding,oxidoreductase activity, acting on paired donors, with incorporation or reduction of molecular oxygen,heme binding;Ontology_id=GO:0016020,GO:0004497,GO:0005506,GO:0016705,GO:0020037;Enzyme_code=EC:1.14;Enzyme_name=Actin
Fv_160_3.g2125	409	ID=Fv_160_3.g2125;Description=hypothetical protein FVEG_03185 [Fusarium verticillioides];Gene=FNAPI_1200;Ontology_term=FMN binding,oxidoreductase
Fv_160_3.g2126	234	ID=Fv_160_3.g2126;Description=hypothetical protein FVEG_03186 [Fusarium verticillioides]
Fv_160_3.g2127	327	ID=Fv_160_3.g2127;Description=hypothetical protein FVEG_03187 [Fusarium verticillioides]

Fv_160_3.g2128	599	ID=Fv_160_3.g2128;Description=hypothetical protein FVEG_03188 [Fusarium verticillioides];Gene=BFJ63_vAg1559;Ontology_term=membrane,transmembrane transporter activity,alpha-L-rhamnosidase activity,carbohydrate metabolic process,transmembrane
Fv_160_3.g2129	314	ID=Fv_160_3.g2129;Description=hypothetical protein FVEG_03189 [Fusarium verticillioides 7600];Gene=F25303_9327;Ontology_term=membrane,alpha-L-rhamnosidase activity,carbohydrate metabolic
Fv_160_3.g2130	207	ID=Fv_160_3.g2130;Description=hypothetical protein FVER53590_03190 [Fusarium verticillioides];Gene=F25303_9328;Ontology_term=membrane,transmembrane transporter activity,transmembrane
Fv_160_3.g2131	590	ID=Fv_160_3.g2131;Description=hypothetical protein FVEG_15207 [Fusarium
Fv_160_3.g2132	655	ID=Fv_160_3.g2132;Description=hypothetical protein FVEG_03193 [Fusarium verticillioides 7600];Gene=1416;Ontology_term=extracellular space,membrane,cytokine activity,cytokine receptor binding,hormone activity,transmembrane transporter activity,signal transduction,female pregnancy,defense response to virus,transmembrane
Fv_160_3.g2133	359	ID=Fv_160_3.g2133;Description=hypothetical protein FVEG_03194 [Fusarium verticillioides 7600];Gene=FPANT_5204;Ontology_term=monooxygenase
Fv_160_3.g2134	1781	ID=Fv_160_3.g2134;Description=hypothetical protein FVEG_03195 [Fusarium verticillioides];Gene=GRF10;Ontology_term=chromatin,cytoplasm,checkpoint clamp complex,site of double-strand break,chromosome, telomeric repeat region,DNA-binding transcription factor activity, RNA polymerase II-specific,DNA binding,protein binding,protein kinase activator activity,signaling adaptor activity,telomere maintenance,DNA repair,regulation of transcription by RNA polymerase II,mitotic intra-S DNA damage checkpoint signaling,mitotic DNA replication checkpoint signaling,cellular response to ionizing
Fv_160_3.g2135	462	ID=Fv_160_3.g2135;Description=hypothetical protein FVEG_03196 [Fusarium verticillioides]
Fv_160_3.g2136	224	ID=Fv_160_3.g2136;Description=hypothetical protein FVEG_03197 [Fusarium verticillioides]
Fv_160_3.g2137	279	ID=Fv_160_3.g2137;Description=hypothetical protein FVEG_03198 [Fusarium verticillioides];Gene=MED18;Ontology_term=mediator complex,transcription coregulator activity,regulation of transcription by RNA polymerase
Fv_160_3.g2138	459	ID=Fv_160_3.g2138;Description=hypothetical protein FVER14953_03199 [Fusarium verticillioides];Gene=F25303_11838;Ontology_term=extracellular space,cytokine activity,cytokine receptor binding,hormone activity,kinase activity,signal transduction,female pregnancy,phosphorylation,defense response to virus;Ontology_id=GO:0005615,GO:0005125,GO:0005126,GO:0005179,GO:0016301,GO:0007165,GO:0007565,GO:0016310,GO:005
Fv_160_3.g2139	559	ID=Fv_160_3.g2139;Description=hypothetical protein FVER53263_03200 [Fusarium verticillioides];Gene=FOC1_g10010765;Ontology_term=membrane,transmembrane transporter activity,transmembrane
Fv_160_3.g2140	390	ID=Fv_160_3.g2140;Description=hypothetical protein FVEG_03201 [Fusarium verticillioides 7600];Gene=FNAPI_2078;Ontology_term=catalytic activity,nucleoside metabolic process;Ontology_id=GO:0003824,GO:0009116

Fv_160_3.g2141	463	ID=Fv_160_3.g2141;Description=hypothetical protein FVEG_15208 [Fusarium verticillioides 7600];Gene=FVEG_15208;Ontology_term=nucleus,cyclin-dependent protein serine/threonine kinase inhibitor activity,protein binding,phosphoric diester hydrolase activity,ATP hydrolysis activity,ADP binding;Ontology_id=GO:0005634,GO:0004861,GO:0005515,GO:0008081,GO:0016887,GO:0043531;Enzyme_code=EC:3.1.4,EC:3.6.
Fv_160_3.g2142	92	ID=Fv_160_3.g2142;Description=hypothetical protein FVEG_03202 [Fusarium verticillioides 7600]
Fv_160_3.g2143	66	ID=Fv_160_3.g2143;Description=hypothetical protein FNAPI_2075 [Fusarium napiforme]
Fv_160_3.g2144	1460	ID=Fv_160_3.g2144;Description=hypothetical protein FVER14953_03204 [Fusarium verticillioides]
Fv_160_3.g2145	980	ID=Fv_160_3.g2145;Description=hypothetical protein FVEG_03205 [Fusarium verticillioides 7600];Gene=FVER53590_03205;Ontology_term=mRNA cis splicing, via spliceosome;Ontology_id=GO:0045292
Fv_160_3.g2146	423	ID=Fv_160_3.g2146;Description=hypothetical protein FVEG_03206 [Fusarium verticillioides
Fv_160_3.g2147	89	ID=Fv_160_3.g2147;Description=hypothetical protein FVEG_03207 [Fusarium verticillioides 7600]
Fv_160_3.g2148	433	ID=Fv_160_3.g2148;Description=hypothetical protein FVER14953_03209 [Fusarium verticillioides];Gene=FOVG_05738;Ontology_term=nucleus,DNA-binding transcription factor activity, RNA polymerase II-specific,zinc
Fv_160_3.g2149	248	ID=Fv_160_3.g2149;Description=hypothetical protein FVEG_03210 [Fusarium verticillioides 7600]
Fv_160_3.g2150	136	ID=Fv_160_3.g2150;Description=hypothetical protein J7337_006111 [Fusarium musae]
Fv_160_3.g2151	326	ID=Fv_160_3.g2151;Description=hypothetical protein FVER53590_03213 [Fusarium verticillioides]
Fv_160_3.g2152	654	ID=Fv_160_3.g2152;Description=hypothetical protein FVEG_03214 [Fusarium verticillioides
Fv_160_3.g2153	613	ID=Fv_160_3.g2153;Description=hypothetical protein FVEG_03217 [Fusarium verticillioides 7600];Gene=PCK1;Ontology_term=chromatin,alpha DNA polymerase:primase complex,site of double-strand break,chromosome, telomeric repeat region,chromatin binding,DNA replication origin binding,single-stranded DNA binding,DNA-directed DNA polymerase activity,phosphoenolpyruvate carboxykinase (ATP) activity,protein binding,ATP binding,metal ion binding,gluconeogenesis,leading strand elongation,lagging strand elongation,premeiotic DNA replication,DNA repair,gene conversion at mating-type locus,regulation of DNA recombination at centromere,DNA biosynthetic process,mitotic DNA replication initiation,synthesis of RNA primer involved in mitotic DNA replication;Ontology_id=GO:0000785,GO:0005658,GO:0035861,GO:0140445,GO:0003682,GO:0003688,GO:0003697,GO:0003887,G
Fv_160_3.g2154	256	ID=Fv_160_3.g2154;Description=hypothetical protein FVER53590_03218 [Fusarium
Fv_160_3.g2155	1359	ID=Fv_160_3.g2155;Description=hypothetical protein FVEG_03221 [Fusarium verticillioides 7600];Gene=pho4;Ontology_term=extracellular region,fungal-type cell wall,cell wall-bounded periplasmic space,thiamine phosphate phosphatase activity,thiamine salvage;Ontology_id=GO:0005576,GO:0009277,GO:0030287,GO:0042131,GO:0036172;Enzyme_code=EC:3.1.3.2,EC:3.1.3.100;Enzy
Fv_160_3.g2156	212	ID=Fv_160_3.g2156;Description=hypothetical protein FOXG_04919 [Fusarium oxysporum f. sp. lycopersici 4287];Gene=CHMP5;Ontology_term=fungal-type vacuole membrane,late endosome to vacuole transport via multivesicular body
Fv_160_3.g2157	957	ID=Fv_160_3.g2157;Description=hypothetical protein FVER53263_03223 [Fusarium verticillioides]

Fv_160_3.g2158	640	ID=Fv_160_3.g2158;Description=hypothetical protein FVEG_03224 [Fusarium verticillioides 7600]
Fv_160_3.g2159	627	ID=Fv_160_3.g2159;Description=hypothetical protein FVER53590_03225 [Fusarium verticillioides];Gene=FNAPI_7656;Ontology_term=nucleus,DNA binding,zinc ion binding,DNA-binding transcription factor activity, RNA polymerase II-specific,regulation of transcription by RNA polymerase
Fv_160_3.g2160	448	ID=Fv_160_3.g2160;Description=hypothetical protein FVER14953_03226 [Fusarium verticillioides];Gene=FNAPI_7655;Ontology_term=PCNA complex,transmembrane transport,UV-damage excision repair,transmembrane transporter activity,positive regulation of DNA-directed DNA polymerase activity,enterobactin binding,lipid binding,positive regulation of endoplasmic reticulum unfolded protein response,protein folding,endoplasmic reticulum lumen,DNA polymerase processivity factor activity,extracellular space,protein binding,metal ion binding,membrane,DNA repair-dependent chromatin remodeling,error-free translesion synthesis,isomerase
Fv_160_3.g2161	285	ID=Fv_160_3.g2161;Description=hypothetical protein FVER53263_03227 [Fusarium verticillioides];Gene=FANTH_1462;Ontology_term=nuclear outer membrane;Ontology_id=GO:0005640
Fv_160_3.g2162	344	ID=Fv_160_3.g2162;Description=alcohol dehydrogenase [Fusarium verticillioides 7600]
Fv_160_3.g2163	1317	ID=Fv_160_3.g2163;Description=CMGC/DYRK protein kinase [Fusarium verticillioides 7600];Gene=FTJAE_5781;Ontology_term=protein serine/threonine kinase activity,ATP
Fv_160_3.g2164	128	ID=Fv_160_3.g2164;Description=hypothetical protein FVER14953_03231 [Fusarium verticillioides];Gene=FNAPI_7652;Ontology_term=lyase activity,metal ion binding,iron-sulfur cluster binding,lysine biosynthetic
Fv_160_3.g2165	207	ID=Fv_160_3.g2165;Description=hypothetical protein FVER53590_03232 [Fusarium verticillioides];Gene=FFUJ_06944;Ontology_term=membrane,glycosyltransferase
Fv_160_3.g2166	977	ID=Fv_160_3.g2166;Description=hypothetical protein FVER14953_03233 [Fusarium verticillioides];Gene=scd1-1;Ontology_term=guanyl-nucleotide exchange factor activity,intracellular signal transduction;Ontology_id=GO:0005085,GO:0035556
Fv_160_3.g2167	118	ID=Fv_160_3.g2167;Description=hypothetical protein FVER14953_03234 [Fusarium verticillioides];Gene=Forpe1208_v008118;Ontology_term=mitochondrion,structural constituent of ribosome,mitochondrial
Fv_160_3.g2168	168	ID=Fv_160_3.g2168;Description=hypothetical protein FVER53590_03235 [Fusarium verticillioides];Gene=FMUND_13815;Ontology_term=membrane,metal ion binding;Ontology_id=GO:0016020,GO:0046872
Fv_160_3.g2169	520	ID=Fv_160_3.g2169;Description=hypothetical protein FVEG_03236 [Fusarium verticillioides 7600]
Fv_160_3.g2170	186	ID=Fv_160_3.g2170;Description=hypothetical protein FVEG_03237 [Fusarium verticillioides 7600]
Fv_160_3.g2171	161	ID=Fv_160_3.g2171;Description=hypothetical protein FVER53263_20202 [Fusarium verticillioides]
Fv_160_3.g2172	387	ID=Fv_160_3.g2172;Description=hypothetical protein FVER53263_03238 [Fusarium verticillioides];Gene=FVER53263_03238;Ontology_term=protein tyrosine phosphatase activity,protein tyrosine/serine/threonine phosphatase

Fv_160_3.g2173	620	ID=Fv_160_3.g2173;Description=hypothetical protein FVER14953_03239 [Fusarium verticillioides];Gene=PDC1;Ontology_term=cytoplasm,branched-chain-2-oxoacid decarboxylase activity,indolepyruvate decarboxylase activity,aromatic amino acid family catabolic process to alcohol via Ehrlich pathway,glycolytic fermentation to ethanol;Ontology_id=GO:0005737,GO:0047433,GO:0047434,GO:0000949,GO:0019655;Enzyme_code=EC:4.1.1.74,EC:4.1.1.72;Enzy
Fv_160_3.g2174	530	ID=Fv_160_3.g2174;Description=hypothetical protein FVER53590_03240 [Fusarium verticillioides];Gene=1374;Ontology_term=aspartic-type endopeptidase
Fv_160_3.g2175	339	ID=Fv_160_3.g2175;Description=hypothetical protein FVER14953_03241 [Fusarium verticillioides];Gene=1373
Fv_160_3.g2176	879	ID=Fv_160_3.g2176;Description=hypothetical protein FVER14953_03242 [Fusarium verticillioides];Gene=FPCIR_8074;Ontology_term=membrane,oligopeptide transmembrane transporter activity,protein
Fv_160_3.g2177	2474	ID=Fv_160_3.g2177;Description=hypothetical protein FVEG_03243 [Fusarium verticillioides 7600];Gene=FPCIR_8075;Ontology_term=peroxisomal membrane,catalytic activity,peroxisome
Fv_160_3.g2178	197	ID=Fv_160_3.g2178;Description=hypothetical protein FANTH_1445 [Fusarium
Fv_160_3.g2179	1014	ID=Fv_160_3.g2179;Description=hypothetical protein FVER53590_03244 [Fusarium verticillioides];Gene=FPANT_5407;Ontology_term=protein phosphatase binding;Ontology_id=GO:0019903
Fv_160_3.g2180	396	ID=Fv_160_3.g2180;Description=subtilisin [Fusarium verticillioides 7600];Gene=FPANT_5408;Ontology_term=cellular anatomical entity,serine-type endopeptidase
Fv_160_3.g2181	788	ID=Fv_160_3.g2181;Description=hypothetical protein FVEG_03246 [Fusarium verticillioides 7600];Gene=FPANT_5409;Ontology_term=DNA binding,protein binding,transcription coactivator activity,spliceosomal complex disassembly,mating type determination,spliceosomal complex,positive regulation of transcription by RNA polymerase II,positive regulation of induction of conjugation with cellular fusion,positive regulation of meiotic cell
Fv_160_3.g2182	878	ID=Fv_160_3.g2182;Description=hypothetical protein FVEG_03247 [Fusarium verticillioides 7600];Gene=JEQ12_018810;Ontology_term=extracellular region,nucleus,cytoskeleton,supramolecular complex,RNA binding,hormone activity,ATP binding,metal ion binding,microtubule plus-end binding,microtubule plus end polymerase,poly(A) RNA polymerase activity,mRNA processing,spindle organization,signal transduction,establishment or maintenance of microtubule cytoskeleton polarity,RNA 3'-end processing,microtubule polymerization;Ontology_id=GO:0005576,GO:0005634,GO:0005856,GO:0099080,GO:0003723,GO:0005179,GO:0005524,GO:00468
Fv_160_3.g2183	498	ID=Fv_160_3.g2183;Description=hypothetical protein FVER53590_03248 [Fusarium
Fv_160_3.g2184	3205	ID=Fv_160_3.g2184;Description=hypothetical protein FVER53590_03249 [Fusarium verticillioides];Gene=FPCIR_8083;Ontology_term=Golgi apparatus,lipid transport,protein retention in Golgi apparatus,late endosome
Fv_160_3.g2185	650	ID=Fv_160_3.g2185;Description=CNT family concentrative nucleoside transporter [Fusarium verticillioides 7600];Gene=FPANT_13779;Ontology_term=plasma membrane,nucleoside transmembrane transporter activity,nucleoside
Fv_160_3.g2186	736	ID=Fv_160_3.g2186;Description=hypothetical protein FVER53590_03251 [Fusarium
Fv_160_3.g2187	695	ID=Fv_160_3.g2187;Description=hypothetical protein FVEG_03253 [Fusarium verticillioides

Fv_160_3.g2188	277	ID=Fv_160_3.g2188;Description=hypothetical protein FVER53590_03255 [Fusarium verticillioides];Gene=FPRO_00021;Ontology_term=metalloendopeptidase activity,zinc ion binding,carbohydrate binding,proteolysis,cell
Fv_160_3.g2189	784	ID=Fv_160_3.g2189;Description=hypothetical protein FVER53590_03256 [Fusarium verticillioides];Gene=HZS61_009011;Ontology_term=S-adenosylmethionine-dependent methyltransferase
Fv_160_3.g2190	456	ID=Fv_160_3.g2190;Description=hypothetical protein FVEG_03257 [Fusarium verticillioides 7600];Gene=Forpe1208_v008138;Ontology_term=nucleus,DNA binding,zinc ion binding,pyridoxal phosphate binding,transaminase activity,DNA-binding transcription factor activity, RNA polymerase II-specific,regulation of transcription by RNA polymerase
Fv_160_3.g2191	603	ID=Fv_160_3.g2191;Description=hypothetical protein FVEG_03258 [Fusarium verticillioides 7600];Gene=FOTG_12296;Ontology_term=nucleus,DNA binding,zinc ion binding,DNA-binding transcription factor activity, RNA
Fv_160_3.g2192	287	ID=Fv_160_3.g2192;Description=hypothetical protein FVER53590_03259 [Fusarium verticillioides]
Fv_160_3.g2193	156	ID=Fv_160_3.g2193;Description=uncharacterized protein FTJAE_808 [Fusarium tjaetaba];Gene=1356;Ontology_term=fungal-type vacuole membrane,ATP binding,ATP hydrolysis activity,ABC-type phytochelatin transporter activity,phytochelatin 2 import into vacuole,cadmium ion import into vacuole,glutathione transmembrane import into vacuole,cellular detoxification of cadmium ion;Ontology_id=GO:0000329,GO:0005524,GO:0016887,GO:0044604,GO:0036246,GO:0036249,GO:0071996,GO:0098849;Enzyme_code=EC:7.2.2,EC:7.4.2.5,EC:3.6.1.15;Enzyme_name=Catalysing the translocation of inorganic cations,bacterial ABC-type protein
Fv_160_3.g2194	397	ID=Fv_160_3.g2194;Description=hypothetical protein FVER53590_03260 [Fusarium verticillioides];Gene=FVER53590_03260;Ontology_term=extracellular space,cytoplasm,neurohypophyseal hormone activity,cellulose binding,carbohydrate metabolic process,signal transduction,response to mechanical stimulus,response to food,response to external biotic stimulus,positive regulation of cold-induced
Fv_160_3.g2195	497	ID=Fv_160_3.g2195;Description=hypothetical protein FVEG_03261 [Fusarium verticillioides 7600];Gene=csbC;Ontology_term=nucleosome,nucleus,pericentric heterochromatin,membrane,DNA binding,transmembrane transporter activity,structural constituent of chromatin,transmembrane
Fv_160_3.g2196	523	ID=Fv_160_3.g2196;Description=hypothetical protein FVER53590_03262 [Fusarium verticillioides];Gene=FMUND_1435;Ontology_term=membrane,monooxygenase activity,iron ion binding,oxidoreductase activity, acting on paired donors, with incorporation or reduction of molecular oxygen,heme binding;Ontology_id=GO:0016020,GO:0004497,GO:0005506,GO:0016705,GO:0020037;Enzyme_code=EC:1.14;Enzyme_name=Actin
Fv_160_3.g2197	342	ID=Fv_160_3.g2197;Description=hypothetical protein FVER14953_03263 [Fusarium verticillioides];Gene=deoB;Ontology_term=uroporphyrinogen-III synthase activity,uroporphyrinogen III biosynthetic process,protoporphyrinogen IX biosynthetic
Fv_160_3.g2198	177	ID=Fv_160_3.g2198;Description=hypothetical protein FVEG_15226 [Fusarium verticillioides 7600];Gene=BFJ71_g14560;Ontology_term=endoplasmic reticulum membrane;Ontology_id=GO:0005789

Fv_160_3.g2199	893	ID=Fv_160_3.g2199;Description=hypothetical protein FVER53263_03264 [Fusarium verticillioides];Gene=FPCIR_8095;Ontology_term=ligase
Fv_160_3.g2200	315	ID=Fv_160_3.g2200;Description=hypothetical protein FVEG_03265 [Fusarium verticillioides 7600];Gene=FPANT_596;Ontology_term=protein kinase activity,ATP
Fv_160_3.g2201	593	ID=Fv_160_3.g2201;Description=hypothetical protein FVEG_15227 [Fusarium verticillioides 7600]
Fv_160_3.g2202	562	ID=Fv_160_3.g2202;Description=hypothetical protein FVER14953_03266 [Fusarium
Fv_160_3.g2203	638	ID=Fv_160_3.g2203;Description=hypothetical protein FVEG_15228 [Fusarium verticillioides 7600];Gene=FVER53590_28310;Ontology_term=nucleus,DNA binding,zinc ion binding,membrane,DNA-binding transcription factor activity, RNA polymerase II-specific,regulation of transcription by RNA polymerase
Fv_160_3.g2204	350	ID=Fv_160_3.g2204;Description=hypothetical protein FVEG_03267 [Fusarium verticillioides 7600];Gene=FOC1_g10010701;Ontology_term=nucleosome,pericentric heterochromatin,mating-type region heterochromatin,chromosome, subtelomeric region,nucleolar peripheral inclusion body,DNA binding,D-amino-acid oxidase activity,transferase activity, transferring alkyl or aryl (other than methyl) groups,pyridoxal phosphate binding,structural constituent of chromatin,protein heterodimerization activity,FAD binding,chromatin-protein adaptor activity,DNA damage response,transsulfuration,heterochromatin boundary formation,D-amino acid metabolic process,mitotic sister chromatid biorientation;Ontology_id=GO:0000786,GO:0005721,GO:0031934,GO:0099115,GO:0140602,GO:0003677,GO:0003884,GO:0016765
Fv_160_3.g2205	364	ID=Fv_160_3.g2205;Description=hypothetical protein FVER14953_03268 [Fusarium verticillioides];Gene=FFUJ_06899;Ontology_term=endoplasmic reticulum membrane,perinuclear region of cytoplasm,D-amino-acid oxidase activity,GTPase activity,protein binding,GTP binding,FAD binding,innate immune response,D-amino acid metabolic process;Ontology_id=GO:0005789,GO:0048471,GO:0003884,GO:0003924,GO:0005515,GO:0005525,GO:0071949,GO:0045087,GO:
Fv_160_3.g2206	534	ID=Fv_160_3.g2206;Description=peptide transporter ptr2 [Fusarium mundagurra];Gene=FPHYL_4195;Ontology_term=nuclear membrane,nuclear pore central transport channel,nuclear localization sequence binding,structural constituent of nuclear pore,transmembrane transporter activity,protein import into nucleus,oligopeptide transport,transmembrane
Fv_160_3.g2207	545	ID=Fv_160_3.g2207;Description=f-box domain-containing protein [Fusarium denticulatum]
Fv_160_3.g2208	680	ID=Fv_160_3.g2208;Description=hypothetical protein FPANT_8892 [Fusarium pseudoanthophilum]
Fv_160_3.g2209	442	ID=Fv_160_3.g2209;Description=hypothetical protein FCOIX_2748 [Fusarium coicis]
Fv_160_3.g2210	745	ID=Fv_160_3.g2210;Description=hypothetical protein FVER14953_03272 [Fusarium verticillioides];Gene=FACUT_3918;Ontology_term=membrane,D-arabinono-1,4-lactone oxidase activity,FAD
Fv_160_3.g2211	344	ID=Fv_160_3.g2211;Description=hypothetical protein FVER53590_03273 [Fusarium verticillioides];Gene=NUM1;Ontology_term=endoplasmic reticulum,cellular bud tip,cell cortex,membrane,D-arabinono-1,4-lactone oxidase activity,phospholipid binding,tubulin binding,FAD binding,mitochondrion inheritance,microtubule cytoskeleton organization,mitochondrial fission,nuclear migration along microtubule,maintenance of protein location in cell cortex;Ontology_id=GO:0005783,GO:0005934,GO:0005938,GO:0016020,GO:0003885,GO:0005543,GO:0015631,GO:0071949,GO:0

Fv_160_3.g2212	887	ID=Fv_160_3.g2212;Description=hypothetical protein FVER14953_03274 [Fusarium verticillioides];Gene=SPC110;Ontology_term=nucleus,spindle pole body;Ontology_id=GO:0005634,GO:0005816
Fv_160_3.g2213	350	ID=Fv_160_3.g2213;Description=hypothetical protein FVER14953_03275 [Fusarium verticillioides];Gene=FVER53590_03275;Ontology_term=nucleus,cytosol,protein serine/threonine kinase activity,ATP binding,protein homodimerization activity,glycerol-3-phosphate dehydrogenase [NAD(P)+] activity,NAD binding,carbohydrate metabolic process,NADH oxidation,intracellular water homeostasis,phosphorylation,glycerol-3-phosphate catabolic process,cellular response to osmotic stress;Ontology_id=GO:0005634,GO:0005829,GO:0004674,GO:0005524,GO:0042803,GO:0047952,GO:0051287,GO:0005975,GO:00
Fv_160_3.g2214	159	ID=Fv_160_3.g2214;Description=hypothetical protein FVEG_03276 [Fusarium verticillioides 7600]
Fv_160_3.g2215	369	ID=Fv_160_3.g2215;Description=hypothetical protein FVER14953_03277 [Fusarium verticillioides];Gene=FPHYL_4182;Ontology_term=cellular anatomical entity,asparaginase activity,asparagine catabolic
Fv_160_3.g2216	480	ID=Fv_160_3.g2216;Description=hypothetical protein FVEG_15230 [Fusarium verticillioides 7600];Gene=FPHYL_4181;Ontology_term=nucleus,DNA-binding transcription factor activity, RNA polymerase II-specific,zinc ion
Fv_160_3.g2217	538	ID=Fv_160_3.g2217;Description=hypothetical protein FVER53590_03278 [Fusarium verticillioides];Gene=Focb16_v011159;Ontology_term=membrane,transmembrane transporter activity,transmembrane
Fv_160_3.g2218	606	ID=Fv_160_3.g2218;Description=hypothetical protein FVER53590_03279 [Fusarium verticillioides];Gene=FPANT_8905;Ontology_term=alpha-glucosidase activity,maltose metabolic process,carbohydrate catabolic
Fv_160_3.g2219	736	ID=Fv_160_3.g2219;Description=heterokaryon incompatibility protein (het-6OR allele) [Fusarium fujikuroi]
Fv_160_3.g2220	572	ID=Fv_160_3.g2220;Description=hypothetical protein FVER53590_03282 [Fusarium verticillioides];Gene=FRV6_02446;Ontology_term=isomerase
Fv_160_3.g2221	300	ID=Fv_160_3.g2221;Description=hypothetical protein FVER14953_03284 [Fusarium verticillioides];Gene=1329
Fv_160_3.g2222	484	ID=Fv_160_3.g2222;Description=hypothetical protein FVER53590_03286 [Fusarium
Fv_160_3.g2223	312	ID=Fv_160_3.g2223;Description=hypothetical protein FVER53590_03287 [Fusarium verticillioides];Gene=FFUJ_06878;Ontology_term=membrane,catalytic activity,biosynthetic
Fv_160_3.g2224	286	ID=Fv_160_3.g2224;Description=hypothetical protein FVER53263_03289 [Fusarium
Fv_160_3.g2225	275	ID=Fv_160_3.g2225;Description=hypothetical protein J7337_006191 [Fusarium
Fv_160_3.g2226	134	ID=Fv_160_3.g2226;Description=hypothetical protein FVEG_03290 [Fusarium verticillioides 7600];Gene=xseA;Ontology_term=RNA
Fv_160_3.g2227	220	ID=Fv_160_3.g2227;Description=hypothetical protein FVEG_15232 [Fusarium verticillioides
Fv_160_3.g2228	337	ID=Fv_160_3.g2228;Description=hypothetical protein FVEG_03291 [Fusarium verticillioides 7600]
Fv_160_3.g2229	87	ID=Fv_160_3.g2229;Description=hypothetical protein FVER53590_28307 [Fusarium verticillioides]

Fv_160_3.g2230	219	ID=Fv_160_3.g2230;Description=hypothetical protein FVER14953_03292 [Fusarium verticillioides];Gene=NUP49;Ontology_term=negative regulation of peptide hormone secretion,positive regulation of lactation,hormone activity,nuclear localization sequence binding,positive regulation of fatty acid biosynthetic process,nuclear pore central transport channel,prolactin receptor binding,extracellular space,nuclear membrane,metal ion binding,protein import into nucleus,positive regulation of gene expression,negative regulation of apoptotic process,blastocyst formation,structural constituent of nuclear pore,negative regulation of luteinizing hormone secretion,negative regulation of nitric oxide mediated signal
Fv_160_3.g2231	146	ID=Fv_160_3.g2231;Description=hypothetical protein FVER14953_20106 [Fusarium verticillioides]
Fv_160_3.g2232	241	ID=Fv_160_3.g2232;Description=hypothetical protein FVER53590_03295 [Fusarium
Fv_160_3.g2233	287	ID=Fv_160_3.g2233;Description=hypothetical protein FVEG_03296 [Fusarium verticillioides
Fv_160_3.g2234	544	ID=Fv_160_3.g2234;Description=hypothetical protein FVER14953_03297 [Fusarium verticillioides];Gene=FOQG_14240;Ontology_term=membrane,monooxygenase activity,iron ion binding,oxidoreductase activity, acting on paired donors, with incorporation or reduction of molecular oxygen,heme binding;Ontology_id=GO:0016020,GO:0004497,GO:0005506,GO:0016705,GO:0020037;Enzyme_code=EC:1.14;Enzyme_name=Actin
Fv_160_3.g2235	483	ID=Fv_160_3.g2235;Description=hypothetical protein FVER53590_03298 [Fusarium verticillioides];Gene=FDENT_778;Ontology_term=nucleus,membrane,DNA-binding transcription factor activity, RNA polymerase II-
Fv_160_3.g2236	677	ID=Fv_160_3.g2236;Description=hypothetical protein FDENT_777 [Fusarium denticulatum];Gene=FPANT_2130;Ontology_term=ATP binding,oxidoreductase activity,ATP hydrolysis
Fv_160_3.g2237	220	ID=Fv_160_3.g2237;Description=hypothetical protein FVEG_15236 [Fusarium verticillioides 7600];Gene=FDENT_776;Ontology_term=negative regulation of endocytosis,zinc ion binding,regulation of sphingolipid biosynthetic process,Golgi apparatus,protein serine/threonine kinase activity,plasma membrane,DNA-binding transcription factor activity, RNA polymerase II-specific,regulation of transcription by RNA polymerase II,nucleus,DNA binding,ATP binding,protein binding,phosphorylation,intracellular signal transduction,protein serine kinase activity;Ontology_id=GO:0045806,GO:0008270,GO:0090153,GO:0005794,GO:0004674,GO:0005886,GO:0000981,GO:0006357,GO:0
Fv_160_3.g2238	499	ID=Fv_160_3.g2238;Description=hypothetical protein FVEG_03301 [Fusarium verticillioides 7600];Gene=FCIRC_2634;Ontology_term=nucleus,DNA binding,zinc ion binding,membrane,DNA-binding transcription factor activity, RNA polymerase II-specific,regulation of transcription by RNA polymerase
Fv_160_3.g2239	510	ID=Fv_160_3.g2239;Description=hypothetical protein FVER14953_03302 [Fusarium verticillioides];Gene=FFC1_02387;Ontology_term=nuclear pore,membrane,transmembrane transporter activity,protein transport,intracellular transport,mRNA transport,transmembrane
Fv_160_3.g2240	402	ID=Fv_160_3.g2240;Description=beta-lactamase [Fusarium verticillioides 7600]
Fv_160_3.g2241	1485	ID=Fv_160_3.g2241;Description=hypothetical protein FVER14953_03304 [Fusarium verticillioides]
Fv_160_3.g2242	144	ID=Fv_160_3.g2242;Description=hypothetical protein FVER53590_03305 [Fusarium verticillioides]

Fv_160_3.g2243	675	ID=Fv_160_3.g2243;Description=hypothetical protein FVEG_03306 [Fusarium verticillioides 7600];Gene=FOVG_05618;Ontology_term=phosphoribosylaminoimidazole carboxylase activity,ATP binding,5-amino-4-imidazole carboxylate lyase activity,metal ion binding,'de novo' IMP biosynthetic
Fv_160_3.g2244	232	ID=Fv_160_3.g2244;Description=hypothetical protein FVEG_03307 [Fusarium verticillioides 7600]
Fv_160_3.g2245	167	ID=Fv_160_3.g2245;Description=hypothetical protein FVEG_15238 [Fusarium verticillioides 7600];Gene=FPRO_07395
Fv_160_3.g2246	227	ID=Fv_160_3.g2246;Description=hypothetical protein FVER14953_03308 [Fusarium verticillioides];Gene=FFUJ_06855;Ontology_term=carboxylic ester hydrolase
Fv_160_3.g2247	127	ID=Fv_160_3.g2247;Description=hypothetical protein FVEG_15239 [Fusarium verticillioides 7600];Gene=BFJ72_g11153;Ontology_term=membrane,monooxygenase activity,iron ion binding,oxidoreductase activity, acting on paired donors, with incorporation or reduction of molecular oxygen,heme binding;Ontology_id=GO:0016020,GO:0004497,GO:0005506,GO:0016705,GO:0020037;Enzyme_code=EC:1.14;Enzyme_name=Actin
Fv_160_3.g2248	909	ID=Fv_160_3.g2248;Description=hypothetical protein FVER53590_03309 [Fusarium verticillioides];Gene=FVER53590_03309;Ontology_term=nucleus,DNA binding,monooxygenase activity,iron ion binding,zinc ion binding,oxidoreductase activity, acting on paired donors, with incorporation or reduction of molecular oxygen,heme binding,DNA-templated
Fv_160_3.g2249	534	ID=Fv_160_3.g2249;Description=hypothetical protein FVER14953_03311 [Fusarium verticillioides];Gene=FMAN_06989;Ontology_term=mitochondrial matrix,homogentisate 1,2-dioxygenase activity,ornithine carbamoyltransferase activity,amino acid binding,metal ion binding,urea cycle,L-phenylalanine catabolic process,tyrosine metabolic process,citrulline biosynthetic process,arginine biosynthetic process via ornithine;Ontology_id=GO:0005759,GO:0004411,GO:0004585,GO:0016597,GO:0046872,GO:0000050,GO:0006559,GO:0006570,GO
Fv_160_3.g2250	432	ID=Fv_160_3.g2250;Description=hypothetical protein FVER53263_03312 [Fusarium verticillioides];Gene=FPHYL_5040;Ontology_term=isomerase activity,3,4-dihydroxybenzoate catabolic
Fv_160_3.g2251	442	ID=Fv_160_3.g2251;Description=hypothetical protein FVER53263_03313 [Fusarium verticillioides];Gene=FTJAE_9495;Ontology_term=mitochondrion,succinyl-CoA:3-oxo-acid CoA-transferase activity,ketone body
Fv_160_3.g2252	434	ID=Fv_160_3.g2252;Description=hypothetical protein FVEG_03314 [Fusarium verticillioides 7600];Gene=FTJAE_9496;Ontology_term=extracellular region,transferase
Fv_160_3.g2253	407	ID=Fv_160_3.g2253;Description=hypothetical protein FVER53263_03315 [Fusarium verticillioides];Gene=BFJ63_vAg10168;Ontology_term=acyltransferase activity, transferring groups other than amino-acyl
Fv_160_3.g2254	416	ID=Fv_160_3.g2254;Description=hypothetical protein FVEG_15241 [Fusarium verticillioides 7600];Gene=FMEXI_7709;Ontology_term=oxidoreductase activity,metal ion

Fv_160_3.g2255	545	ID=Fv_160_3.g2255;Description=hypothetical protein FVER14953_03316 [Fusarium verticillioides];Gene=FMEXI_7708;Ontology_term=membrane,aminoacyl-tRNA editing activity,leucine-tRNA ligase activity,ATP binding,transmembrane transporter activity,leucyl-tRNA aminoacylation,transmembrane transport,aminoacyl-tRNA metabolism involved in translational fidelity;Ontology_id=GO:0016020,GO:0002161,GO:0004823,GO:0005524,GO:0022857,GO:0006429,GO:0055085,GO:0106074;Enzy
Fv_160_3.g2256	1344	ID=Fv_160_3.g2256;Description=hypothetical protein FVEG_03317 [Fusarium verticillioides 7600];Gene=FVEG_03317;Ontology_term=plasma membrane,ATP binding,ATP hydrolysis activity,ABC-type transporter activity,transmembrane transport;Ontology_id=GO:0005886,GO:0005524,GO:0016887,GO:0140359,GO:0055085;Enzyme_code=EC:7.2.2,EC:3.6.1.15;Enzym
Fv_160_3.g2257	605	ID=Fv_160_3.g2257;Description=hypothetical protein FVER53590_03318 [Fusarium verticillioides];Gene=FocTR4_00007989;Ontology_term=plasma membrane,ATP binding,ATP hydrolysis activity,ABC-type transporter activity,transmembrane transport;Ontology_id=GO:0005886,GO:0005524,GO:0016887,GO:0140359,GO:0055085;Enzyme_code=EC:7.2.2,EC:3.6.1.15;Enzym
Fv_160_3.g2258	454	ID=Fv_160_3.g2258;Description=salicylate hydroxylase [Fusarium verticillioides 7600];Gene=BFJ72_g11184;Ontology_term=FAD binding,transcription initiation-coupled chromatin remodeling,NuA4 histone acetyltransferase complex,mitotic actomyosin contractile ring, proximal layer,mitotic actomyosin contractile ring, intermediate layer,mitotic actomyosin contractile ring, distal actin filament layer,Swr1 complex,mating projection tip,structural constituent of cytoskeleton,actin filament,endocytosis,mitotic actomyosin contractile ring contraction,ATP binding,protein binding,perinuclear region of cytoplasm,prospore membrane,oxidoreductase activity,DNA repair-dependent chromatin remodeling,Ino80 complex,actin cortical patch,hydrolase activity;Ontology_id=GO:0071949,GO:0045815,GO:0035267,GO:0120104,GO:0120105,GO:0120106,GO:0000812,GO:0043332,GO:0
Fv_160_3.g2259	541	ID=Fv_160_3.g2259;Description=Zn(II)2Cys6 transcription factor [Fusarium pseudoanthophilum];Gene=FPHYL_5031;Ontology_term=nucleus,DNA binding,zinc ion binding,DNA-binding transcription factor
Fv_160_3.g2260	310	ID=Fv_160_3.g2260;Description=hypothetical protein FVER53590_03321 [Fusarium verticillioides]
Fv_160_3.g2261	1358	ID=Fv_160_3.g2261;Description=hypothetical protein FVER14953_03322 [Fusarium verticillioides]
Fv_160_3.g2262	267	ID=Fv_160_3.g2262;Description=hypothetical protein FVER53590_03323 [Fusarium verticillioides]
Fv_160_3.g2263	386	ID=Fv_160_3.g2263;Description=major facilitator superfamily transporter [Fusarium pseudocircinatum];Gene=FPRO05_07492;Ontology_term=incipient cellular bud site,cellular bud tip,cellular bud neck,membrane,cell periphery,transmembrane transporter activity,transmembrane transport,negative regulation of mitotic
Fv_160_3.g2264	125	ID=Fv_160_3.g2264;Description=hypothetical protein FVER53263_20216 [Fusarium verticillioides];Gene=BKA59DRAFT_487549;Ontology_term=isocitrate lyase activity,methylisocitrate lyase activity,metal ion binding,carboxylic acid metabolic

Fv_160_3.g2265	738	ID=Fv_160_3.g2265;Description=hypothetical protein FVER53263_20217 [Fusarium verticillioides];Gene=aguA;Ontology_term=xylan catabolic process,nucleus,DNA binding,zinc ion binding,extracellular region,DNA-binding transcription factor activity, RNA polymerase II-specific,alpha-glucuronidase activity,regulation of transcription by RNA polymerase
Fv_160_3.g2266	390	ID=Fv_160_3.g2266;Description=alpha-glucuronidase [Fusarium verticillioides 7600];Gene=aguA;Ontology_term=extracellular region,alpha-glucuronidase activity,xylan catabolic
Fv_160_3.g2267	288	ID=Fv_160_3.g2267;Description=alpha-glucuronidase precursor [Fusarium sp. MPI-SDFR-AT-0072];Gene=aguA;Ontology_term=extracellular region,alpha-glucuronidase activity,xylan catabolic
Fv_160_3.g2268	198	ID=Fv_160_3.g2268;Description=hypothetical protein FVER53263_20219 [Fusarium verticillioides]
Fv_160_3.g2269	254	ID=Fv_160_3.g2269;Description=hypothetical protein FVER53263_03325 [Fusarium verticillioides]
Fv_160_3.g2270	309	ID=Fv_160_3.g2270;Description=hypothetical protein FVER53590_29956 [Fusarium verticillioides];Gene=C2S_14591;Ontology_term=tryptophan-tRNA ligase activity,ATP binding,inositol monophosphate 1-phosphatase activity,metal ion binding,inositol monophosphate 3-phosphatase activity,inositol monophosphate 4-phosphatase activity,inositol biosynthetic process,tryptophanyl-tRNA aminoacylation,phosphatidylinositol phosphate biosynthetic process;Ontology_id=GO:0004830,GO:0005524,GO:0008934,GO:0046872,GO:0052832,GO:0052833,GO:0006021,GO:0006436,GO:
Fv_160_3.g2271	414	ID=Fv_160_3.g2271;Description=transcriptional regulatory moc3 [Fusarium globosum];Gene=FSUBG_13531;Ontology_term=zinc ion binding,interleukin-2-mediated signaling pathway,response to antibiotic,response to cycloheximide,ribosome,DNA-binding transcription factor activity, RNA polymerase II-specific,translation,regulation of transcription by RNA polymerase II,regulation of T cell tolerance induction,nucleus,interleukin-2 receptor activity,structural constituent of ribosome,membrane,ribonucleoprotein complex,interleukin-2 binding;Ontology_id=GO:0008270,GO:0038110,GO:0046677,GO:0046898,GO:0005840,GO:0000981,GO:0006412,GO:0006357,GO:0
Fv_160_3.g2272	536	ID=Fv_160_3.g2272;Description=hypothetical protein FVER53590_29955 [Fusarium verticillioides];Gene=FGLOB1_5854;Ontology_term=mitochondrial inner membrane,GTPase activity,GTP binding,transmembrane transporter activity,tRNA methylation,transmembrane transport,mitochondrial tRNA wobble uridine modification;Ontology_id=GO:0005743,GO:0003924,GO:0005525,GO:0022857,GO:0030488,GO:0055085,GO:0070899;Enzyme_cod
Fv_160_3.g2273	249	ID=Fv_160_3.g2273;Description=hypothetical protein FVER53263_03327 [Fusarium verticillioides];Gene=FMEXI_10944;Ontology_term=signal transduction,integrin binding,cytokine activity,fever generation,defense response to bacterium,interleukin-1 receptor binding,lysosome,extracellular space,positive regulation of cell division,positive regulation of type II interferon production,regulation of defense response to virus by host,cytosol,positive regulation of T-helper 1 cell cytokine production,vascular endothelial growth factor production,ligase activity;Ontology_id=GO:0007165,GO:0005178,GO:0005125,GO:0001660,GO:0042742,GO:0005149,GO:0005764,GO:0005615,GO:0

Fv_160_3.g2274	153	ID=Fv_160_3.g2274;Description=hypothetical protein FVEG_03328 [Fusarium verticillioides 7600];Gene=FACUT_5858;Ontology_term=monooxygenase activity,iron ion binding,methyltransferase activity,oxidoreductase activity, acting on paired donors, with incorporation or reduction of molecular oxygen,heme binding,methylation;Ontology_id=GO:0004497,GO:0005506,GO:0008168,GO:0016705,GO:0020037,GO:0032259;Enzyme_code=EC:
Fv_160_3.g2275	1696	ID=Fv_160_3.g2275;Description=hypothetical protein FVER53263_03329 [Fusarium verticillioides];Gene=FVER53590_03329;Ontology_term=membrane,monooxygenase activity,iron ion binding,methyltransferase activity,oxidoreductase activity, acting on paired donors, with incorporation or reduction of molecular oxygen,heme binding,methylation;Ontology_id=GO:0016020,GO:0004497,GO:0005506,GO:0008168,GO:0016705,GO:0020037,GO:0032259;Enzy
Fv_160_3.g2276	494	ID=Fv_160_3.g2276;Description=hypothetical protein FVER53590_03331 [Fusarium verticillioides];Gene=Forpe1208_v008228;Ontology_term=membrane,transmembrane transporter activity,carbohydrate
Fv_160_3.g2277	904	ID=Fv_160_3.g2277;Description=hypothetical protein FVEG_03332 [Fusarium verticillioides 7600];Gene=Forpe1208_v008229;Ontology_term=alpha-L-rhamnosidase activity,carbohydrate metabolic
Fv_160_3.g2278	679	ID=Fv_160_3.g2278;Description=hypothetical protein FVER53263_03333 [Fusarium verticillioides];Gene=Q09048;Ontology_term=S-adenosylmethionine-dependent methyltransferase
Fv_160_3.g2279	571	ID=Fv_160_3.g2279;Description=hypothetical protein FVEG_03334 [Fusarium verticillioides 7600]
Fv_160_3.g2280	294	ID=Fv_160_3.g2280;Description=hypothetical protein FVER53263_03334 [Fusarium verticillioides]
Fv_160_3.g2281	846	ID=Fv_160_3.g2281;Description=hypothetical protein FCOIX_5324 [Fusarium coicis]
Fv_160_3.g2282	431	ID=Fv_160_3.g2282;Description=hypothetical protein FVEG_03338 [Fusarium verticillioides 7600];Gene=FPANT_7928;Ontology_term=nucleus,DNA-binding transcription factor activity, RNA polymerase II-specific,zinc ion
Fv_160_3.g2283	399	ID=Fv_160_3.g2283;Description=hypothetical protein FVER53263_20222 [Fusarium verticillioides]
Fv_160_3.g2284	499	ID=Fv_160_3.g2284;Description=hypothetical protein J7337_006262 [Fusarium musae];Gene=FVEG_03339;Ontology_term=oxidoreductase activity, acting on the aldehyde or oxo group of donors, NAD or NADP as
Fv_160_3.g2285	250	ID=Fv_160_3.g2285;Description=3-oxoacyl-[acyl-carrier protein] reductase [Fusarium verticillioides 7600]
Fv_160_3.g2286	756	ID=Fv_160_3.g2286;Description=hypothetical protein FVEG_03341 [Fusarium verticillioides 7600];Gene=LW93_8039;Ontology_term=magnesium ion binding,3-isopropylmalate dehydrogenase activity,NAD binding,leucine biosynthetic process;Ontology_id=GO:0000287,GO:0003862,GO:0051287,GO:0009098;Enzyme_code=EC:1.1.1.85;Enzyme_name=3-
Fv_160_3.g2287	210	ID=Fv_160_3.g2287;Description=hypothetical protein FVER53263_03342 [Fusarium verticillioides];Gene=BFJ70_g13097;Ontology_term=extracellular space,membrane,hormone activity,growth factor activity,carboxy-lyase activity,ossification,signal
Fv_160_3.g2288	205	ID=Fv_160_3.g2288;Description=hypothetical protein FVEG_03343 [Fusarium verticillioides 7600];Gene=FTJAE_9452;Ontology_term=mitochondrial inner membrane,zinc ion binding,ferrous iron transmembrane transporter activity,oxidoreductase activity,mRNA processing,iron import into the
Fv_160_3.g2289	779	ID=Fv_160_3.g2289;Description=hypothetical protein FVER14953_03344 [Fusarium verticillioides]

Fv_160_3.g2290	374	ID=Fv_160_3.g2290;Description=hypothetical protein FVER53263_03345 [Fusarium verticillioides];Gene=FFUJ_06801;Ontology_term=membrane,urea transmembrane transporter activity,urea transmembrane
Fv_160_3.g2291	311	ID=Fv_160_3.g2291;Description=fungal-specific transcription factor domain-containing protein [Fusarium oxysporum]
Fv_160_3.g2292	132	ID=Fv_160_3.g2292;Description=hypothetical protein FVEG_03346 [Fusarium verticillioides 7600];Gene=MSM1;Ontology_term=extracellular space,mitochondrial matrix,methionine-tRNA ligase activity,hormone activity,ossification,methionyl-tRNA aminoacylation,signal
Fv_160_3.g2293	272	ID=Fv_160_3.g2293;Description=hypothetical protein FVEG_15245 [Fusarium verticillioides 7600]
Fv_160_3.g2294	129	ID=Fv_160_3.g2294;Description=hypothetical protein FVEG_15246 [Fusarium verticillioides
Fv_160_3.g2295	744	ID=Fv_160_3.g2295;Description=hypothetical protein FVER14953_03347 [Fusarium verticillioides];Gene=FVER53263_03347;Ontology_term=metal ion binding,linoleate 11-lipoxygenase activity,lipid oxidation,linoleic acid metabolic
Fv_160_3.g2296	374	ID=Fv_160_3.g2296;Description=hypothetical protein FVER53263_03348 [Fusarium verticillioides];Gene=FPANT_7918;Ontology_term=catalase activity,heme binding,response to oxidative stress,cellular oxidant
Fv_160_3.g2297	224	ID=Fv_160_3.g2297;Description=hypothetical protein BFJ63_vAg10130 [Fusarium oxysporum f. sp. narcissi];Gene=1271;Ontology_term=membrane,protein serine/threonine kinase activity,ATP binding,transmembrane transporter activity,transmembrane
Fv_160_3.g2298	1168	ID=Fv_160_3.g2298;Description=Nacht domain-containing protein [Fusarium mundagurra];Gene=FTJAE_9445;Ontology_term=catalase activity,heme binding,response to oxidative stress,cellular oxidant
Fv_160_3.g2299	816	ID=Fv_160_3.g2299;Description=helicase-like transcription factor [Fusarium napiforme];Gene=BFJ70_g13055;Ontology_term=nucleus,helicase activity,ATP binding,hydrolase activity,ATP-dependent chromatin remodeler activity,chromatin
Fv_160_3.g2300	902	ID=Fv_160_3.g2300;Description=hypothetical protein FNYG_07974 [Fusarium nygamai];Gene=CEP51_004583;Ontology_term=copper ion binding,oxidoreductase
Fv_160_3.g2301	353	ID=Fv_160_3.g2301;Description=hypothetical protein FVEG_03353 [Fusarium verticillioides 7600];Gene=BFJ70_g13054;Ontology_term=oxidoreductase activity, acting on
Fv_160_3.g2302	368	ID=Fv_160_3.g2302;Description=hypothetical protein FVER14953_03354 [Fusarium verticillioides];Gene=ADH7-1;Ontology_term=zinc ion binding,oxidoreductase activity, acting on the CH-OH group of donors, NAD or NADP as
Fv_160_3.g2303	295	ID=Fv_160_3.g2303;Description=chaperone hchA [Fusarium coicis];Gene=BFJ65_g6182;Ontology_term=nucleus,DNA binding,zinc ion binding,protein deglycase activity,DNA-binding transcription factor activity, RNA polymerase II-specific,regulation of transcription by RNA polymerase
Fv_160_3.g2304	683	ID=Fv_160_3.g2304;Description=hypothetical protein FVER14953_03356 [Fusarium verticillioides];Gene=F25303_12209;Ontology_term=nucleus,DNA binding,zinc ion binding,membrane,DNA-binding transcription factor activity, RNA polymerase II-specific,regulation of transcription by RNA polymerase

Fv_160_3.g2305	246	ID=Fv_160_3.g2305;Description=hypothetical protein FVEG_15248 [Fusarium verticillioides 7600];Gene=CD3G;Ontology_term=extracellular region,alpha-beta T cell receptor complex,mating pheromone activity,transmembrane signaling receptor activity,pheromone-dependent signal transduction involved in conjugation with cellular fusion,adaptive immune response,establishment or maintenance of cell polarity,cell surface receptor signaling
Fv_160_3.g2306	654	ID=Fv_160_3.g2306;Description=hypothetical protein FVEG_03357 [Fusarium verticillioides 7600];Gene=FFC1_02297;Ontology_term=mitochondrial inner membrane,mitochondrial large ribosomal subunit,structural
Fv_160_3.g2307	274	ID=Fv_160_3.g2307;Description=hypothetical protein FVER53263_20225 [Fusarium verticillioides]
Fv_160_3.g2308	1050	ID=Fv_160_3.g2308;Description=hypothetical protein FVER53263_03359 [Fusarium verticillioides]
Fv_160_3.g2309	388	ID=Fv_160_3.g2309;Description=hypothetical protein FVEG_03360 [Fusarium verticillioides 7600];Gene=1244
Fv_160_3.g2310	257	ID=Fv_160_3.g2310;Description=protoporphyrinogen oxidase [Fusarium sp. NRRL 52700];Gene=BFJ65_g7607;Ontology_term=membrane,ATP binding,oxidoreductase activity,cellular biosynthetic
Fv_160_3.g2311	529	ID=Fv_160_3.g2311;Description=hypothetical protein FVER53590_03362 [Fusarium verticillioides];Gene=C2S_12271;Ontology_term=alpha-beta T cell receptor complex,polygalacturonase activity,transmembrane signaling receptor activity,adaptive immune response,carbohydrate metabolic process,cell surface receptor signaling pathway,cell wall
Fv_160_3.g2312	1359	ID=Fv_160_3.g2312;Description=hypothetical protein FVER53590_29051 [Fusarium
Fv_160_3.g2313	152	ID=Fv_160_3.g2313;Description=hypothetical protein FVER53590_03366 [Fusarium verticillioides]
Fv_160_3.g2314	360	ID=Fv_160_3.g2314;Description=hypothetical protein FVER53263_03367 [Fusarium verticillioides];Gene=TIM44;Ontology_term=mitochondrial inner membrane,protein-folding chaperone binding,protein import into
Fv_160_3.g2315	288	ID=Fv_160_3.g2315;Description=signaling ykoW [Fusarium tjaetaba];Gene=FVEG_15250;Ontology_term=cellular anatomical entity,glucan 1,4-alpha-glucosidase activity,monooxygenase activity,iron ion binding,oxidoreductase activity, acting on paired donors, with incorporation or reduction of molecular oxygen,heme binding,carbohydrate metabolic process;Ontology_id=GO:0110165,GO:0004339,GO:0004497,GO:0005506,GO:0016705,GO:0020037,GO:0005975;Enzyme_code=EC
Fv_160_3.g2316	898	ID=Fv_160_3.g2316;Description=hypothetical protein FVER53590_03368 [Fusarium
Fv_160_3.g2317	204	ID=Fv_160_3.g2317;Description=hypothetical protein FVEG_15254 [Fusarium verticillioides
Fv_160_3.g2318	228	ID=Fv_160_3.g2318;Description=hypothetical protein FVER14953_03370 [Fusarium verticillioides];Gene=FMUND_8549;Ontology_term=acyltransferase activity, transferring groups other than amino-acyl
Fv_160_3.g2319	656	ID=Fv_160_3.g2319;Description=hypothetical protein FVER14953_03371 [Fusarium verticillioides];Gene=FNAPI_1938;Ontology_term=acyltransferase activity, transferring groups other than amino-acyl
Fv_160_3.g2320	113	ID=Fv_160_3.g2320;Description=hypothetical protein FVEG_03372 [Fusarium verticillioides 7600]
Fv_160_3.g2321	356	ID=Fv_160_3.g2321;Description=hypothetical protein FVER53263_03373 [Fusarium verticillioides]
Fv_160_3.g2322	532	ID=Fv_160_3.g2322;Description=hypothetical protein FVEG_15255 [Fusarium verticillioides 7600];Gene=C2S_5803;Ontology_term=membrane,metal ion transmembrane transporter activity,magnesium ion

Fv_160_3.g2323	991	ID=Fv_160_3.g2323;Description=hypothetical protein FVER53263_03374 [Fusarium verticillioides];Gene=FVER53590_03374;Ontology_term=hydrolase
Fv_160_3.g2324	550	ID=Fv_160_3.g2324;Description=hypothetical protein FVER14953_03375 [Fusarium verticillioides];Gene=FANTH_4123;Ontology_term=nucleus,membrane,DNA binding,zinc ion binding,2,3-dihydro-2,3-dihydroxybenzoate dehydrogenase activity,transmembrane transporter activity,DNA-templated transcription,siderophore biosynthetic process,transmembrane transport,nitrogen compound transport;Ontology_id=GO:0005634,GO:0016020,GO:0003677,GO:0008270,GO:0008667,GO:0022857,GO:0006351,GO:0019290,GO
Fv_160_3.g2325	609	ID=Fv_160_3.g2325;Description=hypothetical protein FVER53590_03376 [Fusarium verticillioides];Gene=1224
Fv_160_3.g2326	307	ID=Fv_160_3.g2326;Description=hypothetical protein FVER53590_03377 [Fusarium verticillioides]
Fv_160_3.g2327	707	ID=Fv_160_3.g2327;Description=hypothetical protein FVER53590_03378 [Fusarium verticillioides];Gene=FTJAE_5496;Ontology_term=serine-type endopeptidase
Fv_160_3.g2328	2036	ID=Fv_160_3.g2328;Description=hypothetical protein FVER53590_03379 [Fusarium verticillioides];Gene=Fsuc_13636;Ontology_term=3-oxoacyl-[acyl-carrier-protein] synthase activity,hydrolase activity,phosphopantetheine binding,fatty acid biosynthetic
Fv_160_3.g2329	489	ID=Fv_160_3.g2329;Description=hypothetical protein FVER53263_03380 [Fusarium verticillioides];Gene=FNYG_08004;Ontology_term=3-oxoacyl-[acyl-carrier-protein] synthase activity,monooxygenase activity,hydrolase activity,FAD binding,fatty acid biosynthetic process;Ontology_id=GO:0004315,GO:0004497,GO:0016787,GO:0071949,GO:0006633;Enzyme_code=EC:2.3.1.41,EC:1,EC:3;Enzym
Fv_160_3.g2330	453	ID=Fv_160_3.g2330;Description=hypothetical protein FVEG_03381 [Fusarium verticillioides 7600];Gene=FVER53590_03381;Ontology_term=O-methyltransferase activity,S-adenosylmethionine-dependent methyltransferase activity,methylation,secondary metabolite biosynthetic
Fv_160_3.g2331	326	ID=Fv_160_3.g2331;Description=hypothetical protein FVER14953_03383 [Fusarium verticillioides]
Fv_160_3.g2332	749	ID=Fv_160_3.g2332;Description=hypothetical protein FVER53590_03382 [Fusarium verticillioides];Gene=bik5;Ontology_term=nucleus,DNA binding,zinc ion binding,DNA-binding transcription factor activity, RNA
Fv_160_3.g2333	482	ID=Fv_160_3.g2333;Description=hypothetical protein FVEG_03384 [Fusarium verticillioides 7600];Gene=F25303_5786;Ontology_term=membrane,transmembrane transporter activity,transmembrane
Fv_160_3.g2334	473	ID=Fv_160_3.g2334;Description=hypothetical protein FVER14953_20455 [Fusarium verticillioides];Gene=FPHYL_3191;Ontology_term=nucleus,DNA-binding transcription factor activity, RNA polymerase II-specific,zinc
Fv_160_3.g2335	179	ID=Fv_160_3.g2335;Description=hypothetical protein FVEG_03386 [Fusarium verticillioides 7600];Gene=FPHYL_3190;Ontology_term=cellular anatomical entity,serine-type endopeptidase activity,N,N-dimethylaniline monooxygenase activity,flavin adenine dinucleotide binding,NADP binding,proteolysis;Ontology_id=GO:0110165,GO:0004252,GO:0004499,GO:0050660,GO:0050661,GO:0006508;Enzyme_code=EC:3.

Fv_160_3.g2336	653	ID=Fv_160_3.g2336;Description=hypothetical protein FVER53590_03387 [Fusarium verticillioides];Gene=FGLOB1_4510;Ontology_term=membrane,serine-type endopeptidase activity,N,N-dimethylaniline monooxygenase activity,flavin adenine dinucleotide binding,NADP binding,proteolysis;Ontology_id=GO:0016020,GO:0004252,GO:0004499,GO:0050660,GO:0050661,GO:0006508;Enzyme_code=EC:3.
Fv_160_3.g2337	845	ID=Fv_160_3.g2337;Description=hypothetical protein FVER53263_03388 [Fusarium verticillioides];Gene=FMAN_06926;Ontology_term=nucleus,membrane,mannosyltransferase activity,DNA binding,zinc ion binding,2,3-dihydro-2,3-dihydroxybenzoate dehydrogenase activity,oxidoreductase activity, acting on single donors with incorporation of molecular oxygen, incorporation of two atoms of oxygen,transmembrane transporter activity,DNA-templated transcription,siderophore biosynthetic process,transmembrane transport,nitrogen compound transport;Ontology_id=GO:0005634,GO:0016020,GO:0000030,GO:0003677,GO:0008270,GO:0008667,GO:0016702,GO:0022857,GO:0006351,GO:0019290,GO:0055085,GO:0071705;Enzyme_code=EC:1.13.11,EC:1.3.1.28,EC:2.4.1;Enzyme_name=Acting on single
Fv_160_3.g2338	514	ID=Fv_160_3.g2338;Description=lignostilbene dioxygenase [Fusarium pseudocircinatum];Gene=FPCIR_10925;Ontology_term=nucleus,DNA binding,zinc ion binding,oxidoreductase activity, acting on single donors with incorporation of molecular oxygen, incorporation of two atoms of oxygen,DNA-templated transcription;Ontology_id=GO:0005634,GO:0003677,GO:0008270,GO:0016702,GO:0006351;Enzyme_code=EC:1.13.11;Enzyme_na
Fv_160_3.g2339	1001	ID=Fv_160_3.g2339;Description=cutinase transcription factor 1 beta [Fusarium tjaetaba];Gene=FVER53590_03391;Ontology_term=nucleus,DNA binding,zinc ion binding,DNA-binding transcription factor activity,
Fv_160_3.g2340	327	ID=Fv_160_3.g2340;Description=hypothetical protein FVER14953_03392 [Fusarium verticillioides]
Fv_160_3.g2341	738	ID=Fv_160_3.g2341;Description=hypothetical protein FVEG_03393 [Fusarium verticillioides 7600];Gene=FRV6_02589;Ontology_term=nucleus,DNA binding,zinc ion binding,membrane,DNA-binding transcription factor activity, RNA polymerase II-specific,regulation of transcription by RNA polymerase
Fv_160_3.g2342	296	ID=Fv_160_3.g2342;Description=hypothetical protein FVER14953_03394 [Fusarium verticillioides];Gene=FPANT_7808;Ontology_term=hydrolase
Fv_160_3.g2343	234	ID=Fv_160_3.g2343;Description=cutinase [Fusarium verticillioides 7600];Gene=BFJ72_g11261;Ontology_term=extracellular region,cutinase activity;Ontology_id=GO:0005576,GO:0050525;Enzyme_code=EC:3.1.1.74;Enzyme_name=cutinase
Fv_160_3.g2344	324	ID=Fv_160_3.g2344;Description=hypothetical protein FVEG_03396 [Fusarium verticillioides 7600];Gene=MET8;Ontology_term=endoplasmic reticulum,acylglycerol lipase
Fv_160_3.g2345	665	ID=Fv_160_3.g2345;Description=hypothetical protein FVEG_03397 [Fusarium verticillioides 7600];Gene=FANTH_4141;Ontology_term=nucleus,DNA binding,zinc ion binding,DNA-binding transcription factor activity, RNA polymerase II-specific,regulation of transcription by RNA polymerase
Fv_160_3.g2346	448	ID=Fv_160_3.g2346;Description=hypothetical protein FVEG_03398 [Fusarium verticillioides 7600];Gene=FANTH_4142;Ontology_term=triglyceride lipase activity,lipid catabolic

Fv_160_3.g2347	463	ID=Fv_160_3.g2347;Description=hypothetical protein FVER14953_03399 [Fusarium verticillioides];Gene=FDENT_11732;Ontology_term=oxidoreductase activity,FAD binding,biosynthetic
Fv_160_3.g2348	347	ID=Fv_160_3.g2348;Description=gentisate 1,2-dioxygenase [Fusarium verticillioides 7600];Gene=FCIRC_3765;Ontology_term=dioxygenase
Fv_160_3.g2349	483	ID=Fv_160_3.g2349;Description=hypothetical protein FVEG_03401 [Fusarium verticillioides 7600];Gene=FNAPI_1970;Ontology_term=membrane,oxidoreductase activity, acting on the aldehyde or oxo group of donors, NAD or NADP as acceptor,transmembrane transporter activity,transmembrane
Fv_160_3.g2350	489	ID=Fv_160_3.g2350;Description=hypothetical protein FVER53263_03402 [Fusarium verticillioides];Gene=FNAPI_1971;Ontology_term=extracellular space,mitochondrial matrix,membrane,growth hormone receptor binding,hormone activity,iron ion binding,oxidoreductase activity, acting on the aldehyde or oxo group of donors, NAD or NADP as acceptor,transmembrane transporter activity,iron-sulfur cluster assembly,transmembrane transport,protein maturation by [4Fe-4S] cluster
Fv_160_3.g2351	490	ID=Fv_160_3.g2351;Description=hypothetical protein FVEG_03403 [Fusarium verticillioides 7600]
Fv_160_3.g2352	503	ID=Fv_160_3.g2352;Description=hypothetical protein FVER53263_03404 [Fusarium verticillioides];Gene=FOQG_16022;Ontology_term=membrane,cyclin-dependent protein serine/threonine kinase regulator activity,transmembrane transporter activity,regulation of protein phosphorylation,mitotic cell cycle phase transition,transmembrane
Fv_160_3.g2353	526	ID=Fv_160_3.g2353;Description=hypothetical protein FVER14953_03405 [Fusarium verticillioides];Gene=FDC1;Ontology_term=cytoplasm,carboxy-lyase activity,metal ion binding,ferulate metabolic process,cinnamic acid catabolic
Fv_160_3.g2354	245	ID=Fv_160_3.g2354;Description=3-octaprenyl-4-hydroxybenzoate carboxy-lyase UbiX [Fusarium verticillioides 7600];Gene=PAD1;Ontology_term=mitochondrion,lyase activity,flavin prenyltransferase
Fv_160_3.g2355	347	ID=Fv_160_3.g2355;Description=hypothetical protein FVER14953_03407 [Fusarium verticillioides]
Fv_160_3.g2356	392	ID=Fv_160_3.g2356;Description=sarcosine oxidase [Fusarium verticillioides 7600];Gene=FTJAE_13324;Ontology_term=oxidoreductase activity,flavin adenine dinucleotide
Fv_160_3.g2357	542	ID=Fv_160_3.g2357;Description=hypothetical protein FVER53590_03409 [Fusarium verticillioides];Gene=FANTH_4153;Ontology_term=membrane,transmembrane transporter activity,transmembrane
Fv_160_3.g2358	417	ID=Fv_160_3.g2358;Description=hypothetical protein FVER53590_03410 [Fusarium verticillioides];Gene=FNAPI_12798;Ontology_term=dioxygenase activity,nucleus,DNA binding,zinc ion binding,glycine betaine biosynthetic process from choline,iron ion binding,choline monooxygenase activity,2 iron, 2 sulfur cluster binding,DNA-binding transcription factor activity, RNA polymerase II-specific,regulation of transcription by RNA polymerase II;Ontology_id=GO:0051213,GO:0005634,GO:0003677,GO:0008270,GO:0019285,GO:0005506,GO:0019133,GO:0051537,GO:000098
Fv_160_3.g2359	131	ID=Fv_160_3.g2359;Description=hypothetical protein FVER53263_03411 [Fusarium verticillioides];Gene=FCIRC_11453;Ontology_term=disulfide oxidoreductase activity,metal ion binding,iron-sulfur cluster

Fv_160_3.g2360	597	ID=Fv_160_3.g2360;Description=hypothetical protein FVER53590_03412 [Fusarium verticillioides];Gene=FFUJ_06714;Ontology_term=nucleus,DNA binding,zinc ion binding,signal transduction,electron transfer activity,lactation,electron transport chain,extracellular region,2 iron, 2 sulfur cluster binding,hormone activity,DNA-binding transcription factor activity, RNA polymerase II-specific,regulation of transcription by RNA polymerase II;Ontology_id=GO:0005634,GO:0003677,GO:0008270,GO:0007165,GO:0009055,GO:0007595,GO:0022900,GO:0005576,GO:005153
Fv_160_3.g2361	425	ID=Fv_160_3.g2361;Description=hypothetical protein FVER53590_03413 [Fusarium verticillioides];Gene=FOQG_17100;Ontology_term=lyase activity,pyridoxal phosphate binding,biosynthetic
Fv_160_3.g2362	425	ID=Fv_160_3.g2362;Description=hypothetical protein FVER53590_03414 [Fusarium verticillioides];Gene=FACUT_13382;Ontology_term=nucleus,zinc ion binding,pyridoxal phosphate binding,transmembrane transport,transmembrane transporter activity,membrane,transferase activity,DNA-binding transcription factor activity, RNA polymerase II-specific,regulation of transcription by RNA polymerase
Fv_160_3.g2363	1015	ID=Fv_160_3.g2363;Description=hypothetical protein FVER14953_03415 [Fusarium verticillioides];Gene=FocTR4_00007866;Ontology_term=secondary metabolite biosynthetic process;Ontology_id=GO:0044550
Fv_160_3.g2364	427	ID=Fv_160_3.g2364;Description=hypothetical protein FVEG_03416 [Fusarium verticillioides 7600];Gene=FVEG_03416;Ontology_term=periplasmic space,lyase
Fv_160_3.g2365	441	ID=Fv_160_3.g2365;Description=hypothetical protein FVER53263_03417 [Fusarium verticillioides];Gene=FPHYL_3160;Ontology_term=oxidoreductase activity, acting on the CH-CH group of donors,flavin adenine
Fv_160_3.g2366	453	ID=Fv_160_3.g2366;Description=hypothetical protein FVEG_15259 [Fusarium verticillioides
Fv_160_3.g2367	631	ID=Fv_160_3.g2367;Description=hypothetical protein FVER53590_03418 [Fusarium
Fv_160_3.g2368	655	ID=Fv_160_3.g2368;Description=hypothetical protein FVEG_15260 [Fusarium verticillioides 7600];Gene=BFJ63_vAg14434;Ontology_term=nucleus,DNA binding,zinc ion binding,DNA-binding transcription factor activity, RNA polymerase II-specific,regulation of transcription by RNA polymerase
Fv_160_3.g2369	628	ID=Fv_160_3.g2369;Description=hypothetical protein FVER53263_03420 [Fusarium verticillioides]
Fv_160_3.g2370	541	ID=Fv_160_3.g2370;Description=hypothetical protein FVEG_03421 [Fusarium verticillioides 7600];Gene=FDENT_6917;Ontology_term=transferase
Fv_160_3.g2371	249	ID=Fv_160_3.g2371;Description=oxidoreductase [Fusarium verticillioides 7600];Gene=FTJAE_13305;Ontology_term=membrane,iron ion binding,oxidoreductase activity,lipid biosynthetic process,cellular lipid metabolic
Fv_160_3.g2372	560	ID=Fv_160_3.g2372;Description=Zinc finger C2H2 type domain containing [Fusarium acutatum];Gene=LW93_7974;Ontology_term=nucleus,DNA binding,zinc ion binding,DNA-binding transcription factor activity, RNA

Fv_160_3.g2373	355	ID=Fv_160_3.g2373;Description=hypothetical protein FVER53590_03423 [Fusarium verticillioides];Gene=MET4;Ontology_term=nucleus,Cbf1-Met4-Met28 complex,RNA polymerase II transcription regulatory region sequence-specific DNA binding,DNA-binding transcription activator activity, RNA polymerase II-specific,transcription coactivator activity,zinc ion binding,oxidoreductase activity,identical protein binding,methionine biosynthetic process,cysteine biosynthetic process,regulation of sulfur metabolic process,positive regulation of transcription by RNA polymerase II,response to arsenic-containing substance,response to cadmium
Fv_160_3.g2374	563	ID=Fv_160_3.g2374;Description=hypothetical protein FVER53263_03424 [Fusarium verticillioides];Gene=FTJAE_13302;Ontology_term=mitochondrial membrane,proton-transporting ATP synthase complex, coupling factor F(o),2-isopropylmalate synthase activity,protein binding,zinc ion binding,lipid binding,proton transmembrane transporter activity,oxidoreductase activity,FAD binding,leucine biosynthetic process,proton motive force-driven ATP synthesis,proton transmembrane
Fv_160_4.g2375	471	ID=Fv_160_4.g2375;Description=hypothetical protein FVEG_11298 [Fusarium verticillioides 7600]
Fv_160_4.g2376	225	ID=Fv_160_4.g2376;Description=hypothetical protein J7337_011833 [Fusarium musae];Gene=BFJ63_vAg13587;Ontology_term=plasma membrane,host cell surface receptor binding,DNA dealkylation involved in
Fv_160_4.g2377	143	ID=Fv_160_4.g2377;Description=hypothetical protein J7337_011832 [Fusarium musae];Gene=FOQG_09918;Ontology_term=histidine phosphotransfer kinase activity,protein histidine kinase binding,phosphorelay signal transduction system;Ontology_id=GO:0009927,GO:0043424,GO:0000160;Enzyme_code=EC:2.7.1;Enzyme_name=Transferring
Fv_160_4.g2378	370	ID=Fv_160_4.g2378;Description=hypothetical protein J7337_011831 [Fusarium musae]
Fv_160_4.g2379	1296	ID=Fv_160_4.g2379;Description=elongator complex protein 1 [Fusarium verticillioides 7600];Gene=FOMG_10283;Ontology_term=nucleus,cytoplasm,elongator holoenzyme complex,tRNA wobble uridine
Fv_160_4.g2380	1026	ID=Fv_160_4.g2380;Description=hypothetical protein FVEG_11303 [Fusarium verticillioides 7600];Gene=AU210_011524;Ontology_term=cellular anatomical entity,cytoskeletal regulatory protein
Fv_160_4.g2381	703	ID=Fv_160_4.g2381;Description=hypothetical protein FVER14953_11304 [Fusarium
Fv_160_4.g2382	629	ID=Fv_160_4.g2382;Description=hypothetical protein FVEG_11305 [Fusarium verticillioides 7600];Gene=BFJ63_vAg13598;Ontology_term=mitochondrial inner membrane,calcium ion binding,transmembrane
Fv_160_4.g2383	339	ID=Fv_160_4.g2383;Description=cyclin UMe3 [Fusarium phyllophilum];Gene=FVER53263_11306;Ontology_term=nucleus,cyclin-dependent protein serine/threonine kinase regulator activity,regulation of transcription by RNA polymerase
Fv_160_4.g2384	431	ID=Fv_160_4.g2384;Description=hypothetical protein FVER14953_11307 [Fusarium verticillioides];Gene=FACUT_2403;Ontology_term=cyclin-dependent protein serine/threonine kinase regulator activity,regulation of
Fv_160_4.g2385	349	ID=Fv_160_4.g2385;Description=hypothetical protein FVEG_11308 [Fusarium verticillioides 7600];Gene=FNAPI_10015;Ontology_term=membrane,catalytic activity,molybdenum ion binding,pyridoxal phosphate
Fv_160_4.g2386	184	ID=Fv_160_4.g2386;Description=hypothetical protein FOXG_13882 [Fusarium oxysporum f. sp. lycopersici 4287]
Fv_160_4.g2387	112	ID=Fv_160_4.g2387;Description=hypothetical protein FVER14953_11310 [Fusarium verticillioides]

Fv_160_4.g2388	280	ID=Fv_160_4.g2388;Description=hypothetical protein FVEG_11311 [Fusarium verticillioides 7600];Gene=FACUT_6409;Ontology_term=mitochondrial inner membrane,S-adenosyl-L-methionine transmembrane transporter
Fv_160_4.g2389	1020	ID=Fv_160_4.g2389;Description=hypothetical protein FVER53590_11312 [Fusarium
Fv_160_4.g2390	589	ID=Fv_160_4.g2390;Description=ATP-dependent RNA helicase HAS1 [Fusarium verticillioides 7600];Gene=FVER53263_11313;Ontology_term=nuclear envelope,nucleolus,preribosome, large subunit precursor,small-subunit processome,RNA binding,RNA helicase activity,ATP binding,ATP hydrolysis activity,identical protein binding,maturation of SSU-rRNA from tricistronic rRNA transcript (SSU-rRNA, 5.8S rRNA, LSU-rRNA),maturation of LSU-rRNA from tricistronic rRNA transcript (SSU-rRNA, 5.8S rRNA, LSU-rRNA),snoRNA release from pre-
Fv_160_4.g2391	563	ID=Fv_160_4.g2391;Description=hypothetical protein FVEG_11314 [Fusarium verticillioides 7600];Gene=AU210_011535;Ontology_term=RNA binding,alcohol dehydrogenase (NAD+) activity,alcohol metabolic process;Ontology_id=GO:0003723,GO:0004022,GO:0006066;Enzyme_code=EC:1.1.1.1,EC:1.1.1.71;Enzyme_name=alcohol
Fv_160_4.g2392	283	ID=Fv_160_4.g2392;Description=hypothetical protein FVEG_11315 [Fusarium verticillioides 7600];Gene=FPANT_13504;Ontology_term=membrane,UDP-glycosyltransferase
Fv_160_4.g2393	98	ID=Fv_160_4.g2393;Description=hypothetical protein FVER14953_20399 [Fusarium verticillioides];Gene=FVER53263_11316;Ontology_term=UDP-glycosyltransferase
Fv_160_4.g2394	105	ID=Fv_160_4.g2394;Description=hypothetical protein FVER14953_11316 [Fusarium verticillioides];Gene=FMAN_12448;Ontology_term=UDP-glycosyltransferase
Fv_160_4.g2395	137	ID=Fv_160_4.g2395;Description=hypothetical protein FVEG_17000 [Fusarium verticillioides 7600];Gene=BFJ68_g8524;Ontology_term=UDP-glycosyltransferase
Fv_160_4.g2396	406	ID=Fv_160_4.g2396;Description=hypothetical protein FVEG_11317 [Fusarium verticillioides 7600];Gene=FMEXI_4738;Ontology_term=aspartic-type endopeptidase
Fv_160_4.g2397	442	ID=Fv_160_4.g2397;Description=hypothetical protein FOXG_13893 [Fusarium oxysporum f. sp. lycopersici 4287];Gene=F25303_6732;Ontology_term=6-phosphofructo-2-kinase activity,fructose-2,6-bisphosphate 2-phosphatase activity,ATP binding,fructose metabolic process,fructose 2,6-bisphosphate metabolic process,glucose metabolic process,carbohydrate phosphorylation;Ontology_id=GO:0003873,GO:0004331,GO:0005524,GO:0006000,GO:0006003,GO:0006006,GO:0046835;Enzyme_code=EC:3.1.3.23,EC:3.1.3.46,EC:2.7.1.105;Enzyme_name=sugar-phosphatase,fructose-2,6-bisphosphate 2-phosphatase,6-
Fv_160_4.g2398	549	ID=Fv_160_4.g2398;Description=hypothetical protein FCOIX_4990 [Fusarium
Fv_160_4.g2399	405	ID=Fv_160_4.g2399;Description=hypothetical protein FCOIX_4989 [Fusarium coicis]
Fv_160_4.g2400	752	ID=Fv_160_4.g2400;Description=hypothetical protein FVEG_11322 [Fusarium verticillioides 7600]
Fv_160_4.g2401	847	ID=Fv_160_4.g2401;Description=hypothetical protein FVEG_11323 [Fusarium verticillioides 7600]
Fv_160_4.g2402	89	ID=Fv_160_4.g2402;Description=hypothetical protein J7337_011808 [Fusarium
Fv_160_4.g2403	1069	ID=Fv_160_4.g2403;Description=hypothetical protein FVEG_11325 [Fusarium verticillioides 7600];Gene=FPANT_13549;Ontology_term=cytoplasm,intracellular membrane-bounded organelle,intracellular protein

Fv_160_4.g2404	973	ID=Fv_160_4.g2404;Description=ATPase [Fusarium acutatum];Gene=FPANT_13548;Ontology_term=membrane,ATP binding,ATP hydrolysis activity,ABC-type transporter activity,transmembrane transport;Ontology_id=GO:0016020,GO:0005524,GO:0016887,GO:0140359,GO:0055085;Enzyme_code=EC:7.2.2,EC:3.6.1.15;Enzym
Fv_160_4.g2405	1359	ID=Fv_160_4.g2405;Description=hypothetical protein FVER14953_11327 [Fusarium verticillioides];Gene=FMAN_12459;Ontology_term=membrane,ATP binding,ATP hydrolysis activity,ABC-type transporter activity,transmembrane transport;Ontology_id=GO:0016020,GO:0005524,GO:0016887,GO:0140359,GO:0055085;Enzyme_code=EC:7.2.2,EC:3.6.1.15;Enzym
Fv_160_4.g2406	421	ID=Fv_160_4.g2406;Description=hypothetical protein FVEG_11328 [Fusarium verticillioides 7600];Gene=FVER53263_11328;Ontology_term=oxidoreductase
Fv_160_4.g2407	457	ID=Fv_160_4.g2407;Description=hypothetical protein FVER14953_11329 [Fusarium verticillioides];Gene=FNAPI_3960;Ontology_term=membrane,alcohol dehydrogenase (NAD+) activity,acyltransferase activity,alcohol metabolic process,siderophore biosynthetic process;Ontology_id=GO:0016020,GO:0004022,GO:0016746,GO:0006066,GO:0019290;Enzyme_code=EC:2.3,EC:1.1.1.1,EC:1.1.1.71;
Fv_160_4.g2408	575	ID=Fv_160_4.g2408;Description=long-chain acyl-CoA synthetase [Fusarium verticillioides 7600];Gene=sidI-0;Ontology_term=ligase activity;Ontology_id=GO:0016874;Enzyme_code=EC:6;Enzyme_name=Ligases
Fv_160_4.g2409	442	ID=Fv_160_4.g2409;Description=hypothetical protein FVER53590_11331 [Fusarium verticillioides];Gene=Adh;Ontology_term=membrane,alcohol dehydrogenase (NAD+) activity,alcohol metabolic
Fv_160_4.g2410	1163	ID=Fv_160_4.g2410;Description=hypothetical protein J7337_011800 [Fusarium musae]
Fv_160_4.g2411	404	ID=Fv_160_4.g2411;Description=hypothetical protein FVER14953_11333 [Fusarium verticillioides];Gene=FVER53263_11333;Ontology_term=Ino80 complex,alcohol dehydrogenase (NAD+) activity,alcohol metabolic process,chromatin remodeling;Ontology_id=GO:0031011,GO:0004022,GO:0006066,GO:0006338;Enzyme_code=EC:1.1.1.1,EC:1.1.1.71;Enzyme_name=
Fv_160_4.g2412	391	ID=Fv_160_4.g2412;Description=hypothetical protein FVEG_11334 [Fusarium verticillioides 7600];Gene=BFJ72_g12363;Ontology_term=peroxisomal membrane,protein import into peroxisome matrix,
Fv_160_4.g2413	1298	ID=Fv_160_4.g2413;Description=hypothetical protein FVER14953_11335 [Fusarium verticillioides];Gene=FVER53263_11335;Ontology_term=chromatin remodeling;Ontology_id=GO:0006338
Fv_160_4.g2414	221	ID=Fv_160_4.g2414;Description=hypothetical protein FVER53263_11336 [Fusarium verticillioides];Gene=FGADI_6453;Ontology_term=GTPase activity,GTP
Fv_160_4.g2415	307	ID=Fv_160_4.g2415;Description=BRX1-like protein [Fusarium tjaetaba];Gene=FVER53263_11337;Ontology_term=nucleolus,preribosome, large subunit precursor,5S rRNA binding,rRNA primary transcript binding,ribosomal large subunit assembly,endonucleolytic cleavage in ITS1 upstream of 5.8S rRNA from tricistronic rRNA transcript (SSU-rRNA, 5.8S rRNA, LSU-rRNA),exonucleolytic trimming to generate mature 5'-end of 5.8S rRNA from tricistronic rRNA
Fv_160_4.g2416	431	ID=Fv_160_4.g2416;Description=hypothetical protein FVEG_11338 [Fusarium verticillioides 7600]

Fv_160_4.g2417	736	ID=Fv_160_4.g2417;Description=hypothetical protein FVER14953_11339 [Fusarium verticillioides];Gene=C2S_10160;Ontology_term=pericentric heterochromatin,nucleolus,mating-type region heterochromatin,rDNA heterochromatin,HDA1 complex,SHREC complex,chromosome, subtelomeric region,heterochromatin island,chromatin binding,alcohol dehydrogenase (NAD+) activity,histone H3K14 deacetylase activity,identical protein binding,negative regulation of transcription by RNA polymerase II,rDNA heterochromatin formation,alcohol metabolic process,negative regulation of transcription by transcription factor localization,silent mating-type cassette heterochromatin formation,positive regulation of transcription by RNA polymerase II,positive regulation of pericentric heterochromatin formation;Ontology_id=GO:0005721,GO:0005730,GO:0031934,GO:0033553,GO:0070823,GO:0070824,GO:0099115,GO:1990342,G
Fv_160_4.g2418	511	ID=Fv_160_4.g2418;Description=histone deacetylase [Fusarium coicis]
Fv_160_4.g2419	722	ID=Fv_160_4.g2419;Description=hypothetical protein J7337_011791 [Fusarium musae]
Fv_160_4.g2420	784	ID=Fv_160_4.g2420;Description=hypothetical protein J7337_011790 [Fusarium musae];Gene=FPANT_2779;Ontology_term=nucleus,metal ion binding,chromatin
Fv_160_4.g2421	621	ID=Fv_160_4.g2421;Description=hypothetical protein FVEG_11343 [Fusarium verticillioides 7600];Gene=FOXYS1_11171;Ontology_term=nucleus,alcohol dehydrogenase (NAD+) activity,alcohol metabolic process;Ontology_id=GO:0005634,GO:0004022,GO:0006066;Enzyme_code=EC:1.1.1.1,EC:1.1.1.71;Enzyme_name=alcohol
Fv_160_4.g2422	523	ID=Fv_160_4.g2422;Description=hypothetical protein FVEG_11344 [Fusarium verticillioides 7600];Gene=BFJ65_g12302;Ontology_term=Golgi apparatus,membrane,phosphoenolpyruvate transmembrane transporter activity,phosphoenolpyruvate transmembrane import into Golgi
Fv_160_4.g2423	2651	ID=Fv_160_4.g2423;Description=hypothetical protein J7337_011787 [Fusarium musae];Gene=FOBC_13690;Ontology_term=nucleus,DNA repair,replication fork
Fv_160_4.g2424	718	ID=Fv_160_4.g2424;Description=hypothetical protein FVEG_17008 [Fusarium verticillioides 7600];Gene=C2S_10171;Ontology_term=nucleus,membrane,DNA-binding transcription factor activity, RNA polymerase II-specific,alcohol dehydrogenase (NAD+) activity,ATP binding,zinc ion binding,ABC-type xenobiotic transporter activity,ATP hydrolysis activity,alcohol metabolic process,regulation of transcription by RNA polymerase II,xenobiotic transport,transmembrane transport;Ontology_id=GO:0005634,GO:0016020,GO:0000981,GO:0004022,GO:0005524,GO:0008270,GO:0008559,GO:0016887,GO:0006066,GO:0006357,GO:0042908,GO:0055085;Enzyme_code=EC:7.6.2.2,EC:7.2.2,EC:3.6.1.15,EC:1.1.1.1,EC:1.1.1.71;Enzyme_nam
Fv_160_4.g2425	512	ID=Fv_160_4.g2425;Description=hypothetical protein FVEG_11348 [Fusarium verticillioides 7600];Gene=FVER53263_11348;Ontology_term=membrane,transmembrane transporter activity,transmembrane
Fv_160_4.g2426	169	ID=Fv_160_4.g2426;Description=cytochrome c oxidase polypeptide 5, mitochondrial [Fusarium verticillioides 7600];Gene=FOTG_07978;Ontology_term=mitochondrial respiratory chain complex IV,cytochrome-c oxidase activity,mitochondrial electron transport, cytochrome c to oxygen,proton transmembrane

Fv_160_4.g2427	602	ID=Fv_160_4.g2427;Description=hypothetical protein FVER14953_11350 [Fusarium verticillioides];Gene=Forpi1262_v009653;Ontology_term=ATP binding,peptidase activity,ATP hydrolysis activity,proteolysis;Ontology_id=GO:0005524,GO:0008233,GO:0016887,GO:0006508;Enzyme_code=EC:3.4,EC:3.6.1.15;Enzyme_na
Fv_160_4.g2428	705	ID=Fv_160_4.g2428;Description=1,4-alpha-glucan-branching enzyme [Fusarium verticillioides 7600];Gene=F25303_6765;Ontology_term=1,4-alpha-glucan branching enzyme activity,hydrolase activity, hydrolyzing O-glycosyl compounds,cation binding,1,4-alpha-glucan branching enzyme activity (using a glucosylated glycogenin as primer for glycogen synthesis),glycogen biosynthetic
Fv_160_4.g2429	514	ID=Fv_160_4.g2429;Description=ATP synthase subunit beta, mitochondrial [Fusarium verticillioides 7600];Gene=F53441_7393;Ontology_term=mitochondrial proton-transporting ATP synthase, catalytic core,ATP binding,ATP hydrolysis activity,ADP binding,proton-transporting ATP synthase activity, rotational mechanism,proton-transporting ATPase activity, rotational mechanism,proton motive force-driven mitochondrial ATP synthesis,proton transmembrane transport;Ontology_id=GO:0005754,GO:0005524,GO:0016887,GO:0043531,GO:0046933,GO:0046961,GO:0042776,GO:1902600;En
Fv_160_4.g2430	509	ID=Fv_160_4.g2430;Description=hypothetical protein FVER14953_11353 [Fusarium verticillioides];Gene=BFJ70_g2151;Ontology_term=nucleus,DNA-binding transcription factor activity, RNA polymerase II-specific,zinc
Fv_160_4.g2431	1011	ID=Fv_160_4.g2431;Description=hypothetical protein FVEG_11354 [Fusarium verticillioides 7600];Gene=FCIRC_11379;Ontology_term=ATP binding,ATP hydrolysis
Fv_160_4.g2432	645	ID=Fv_160_4.g2432;Description=transcription initiation factor TFIIH subunit 1 [Fusarium verticillioides 7600];Gene=FPANT_12614;Ontology_term=transcription factor TFIIH core complex,translation initiation factor activity,nucleotide-excision repair,DNA-templated transcription,translational
Fv_160_4.g2433	1051	ID=Fv_160_4.g2433;Description=oxoglutarate dehydrogenase (succinyl-transferring), E1 component [Fusarium verticillioides 7600];Gene=FGADI_6431;Ontology_term=mitochondrial oxoglutarate dehydrogenase complex,mitochondrial nucleoid,oxoglutarate dehydrogenase (succinyl-transferring) activity,thiamine pyrophosphate binding,tricarboxylic acid cycle,2-oxoglutarate metabolic process;Ontology_id=GO:0009353,GO:0042645,GO:0004591,GO:0030976,GO:0006099,GO:0006103;Enzyme_code=EC:1.2.4.2;Enzy
Fv_160_4.g2434	443	ID=Fv_160_4.g2434;Description=hypothetical protein FVER14953_11357 [Fusarium verticillioides];Gene=CIA1;Ontology_term=CIA complex,iron-sulfur cluster assembly;Ontology_id=GO:0097361,GO:0016226
Fv_160_4.g2435	398	ID=Fv_160_4.g2435;Description=hypothetical protein FVER53263_11358 [Fusarium verticillioides];Gene=FTJAE_8230;Ontology_term=membrane,transmembrane transporter activity,transmembrane
Fv_160_4.g2436	290	ID=Fv_160_4.g2436;Description=hypothetical protein FVEG_11359 [Fusarium verticillioides 7600]
Fv_160_4.g2437	938	ID=Fv_160_4.g2437;Description=hypothetical protein FVEG_11360 [Fusarium verticillioides 7600];Gene=FPCIR_3608;Ontology_term=monooxygenase
Fv_160_4.g2438	639	ID=Fv_160_4.g2438;Description=hypothetical protein FVEG_11361 [Fusarium verticillioides 7600];Gene=BFJ70_g2143;Ontology_term=membrane,monooxygenase activity,FAD binding,biosynthetic

Fv_160_4.g2439	1031	ID=Fv_160_4.g2439;Description=hypothetical protein FVER14953_11362 [Fusarium verticillioides];Gene=FVER53263_11362;Ontology_term=cysteine-type peptidase activity,ubiquitin-like protein peptidase
Fv_160_4.g2440	323	ID=Fv_160_4.g2440;Description=hypothetical protein FVEG_11363 [Fusarium verticillioides 7600]
Fv_160_4.g2441	147	ID=Fv_160_4.g2441;Description=hypothetical protein FVEG_17011 [Fusarium verticillioides 7600]
Fv_160_4.g2442	253	ID=Fv_160_4.g2442;Description=glycerol-3-phosphate dehydrogenase [Fusarium verticillioides 7600];Gene=Forpi1262_v009667;Ontology_term=glycerol-3-phosphate dehydrogenase complex,glycerol-3-phosphate dehydrogenase (quinone) activity,glycerol-3-phosphate metabolic
Fv_160_4.g2443	278	ID=Fv_160_4.g2443;Description=hypothetical protein FVER14953_11364 [Fusarium verticillioides];Gene=FOMG_10361;Ontology_term=glycerol-3-phosphate dehydrogenase complex,membrane,glycerol-3-phosphate dehydrogenase (quinone) activity,glycerol-3-phosphate metabolic
Fv_160_4.g2444	881	ID=Fv_160_4.g2444;Description=hypothetical protein FVER14953_11365 [Fusarium verticillioides];Gene=FTJAE_11186;Ontology_term=glycerol-3-phosphate dehydrogenase complex,membrane,glycerol-3-phosphate dehydrogenase (quinone) activity,glycerol-3-phosphate metabolic
Fv_160_4.g2445	1014	ID=Fv_160_4.g2445;Description=leucine carboxyl methyltransferase 2 [Fusarium verticillioides 7600];Gene=FVER53263_11366;Ontology_term=S-adenosylmethionine-dependent methyltransferase activity,tRNA
Fv_160_4.g2446	458	ID=Fv_160_4.g2446;Description=autophagy protein [Fusarium pseudocircinatum]
Fv_160_4.g2447	697	ID=Fv_160_4.g2447;Description=hypothetical protein FVER14953_11368 [Fusarium verticillioides];Gene=FOMG_10365;Ontology_term=nucleus,protein tyrosine phosphatase activity,protein tyrosine/serine/threonine phosphatase activity,dephosphorylation,nuclear DNA
Fv_160_4.g2448	748	ID=Fv_160_4.g2448;Description=hypothetical protein FVER14953_11369 [Fusarium verticillioides];Gene=FGLOB1_11774;Ontology_term=nucleus,polytene chromosome interband,centrosome,endoplasmic reticulum chaperone complex,perinuclear region of cytoplasm,protein folding chaperone complex,DNA binding,DNA helicase activity,insulin receptor binding,ATP binding,ATP hydrolysis activity,TPR domain binding,unfolded protein binding,ATP-dependent protein folding chaperone,positive regulation of neuroblast proliferation,nucleotide-excision repair,protein folding,centrosome cycle,negative regulation of cell population proliferation,response to heat,cold acclimation,pole plasm mRNA localization,DNA duplex unwinding,proteasome assembly,regulation of circadian sleep/wake cycle, sleep,positive regulation of insulin receptor signaling pathway,RISC complex
Fv_160_4.g2449	1008	ID=Fv_160_4.g2449;Description=hypothetical protein FVER53590_11370 [Fusarium
Fv_160_4.g2450	401	ID=Fv_160_4.g2450;Description=hypothetical protein FVEG_11371 [Fusarium verticillioides 7600]
Fv_160_4.g2451	485	ID=Fv_160_4.g2451;Description=hypothetical protein FVER14953_11372 [Fusarium verticillioides];Gene=FCIRC_9911;Ontology_term=U4/U6 x U5 tri-snRNP complex,mRNA cis splicing, via

Fv_160_4.g2452	1676	ID=Fv_160_4.g2452;Description=hypothetical protein FVER14953_11373 [Fusarium verticillioides];Gene=FCIRC_9910;Ontology_term=DNA-directed RNA polymerase complex,nucleus,DNA binding,DNA-directed 5'-3' RNA polymerase activity,DNA-templated
Fv_160_4.g2453	136	ID=Fv_160_4.g2453;Description=hypothetical protein HG531_000382 [Fusarium graminearum];Gene=FGLOB1_11769;Ontology_term=nucleosome,nucleus,DNA binding,structural constituent of chromatin,protein
Fv_160_4.g2454	103	ID=Fv_160_4.g2454;Description=unnamed protein product, partial [Fusarium graminearum];Gene=FOYG_11191;Ontology_term=nucleosome,nucleus,DNA binding,structural constituent of chromatin,protein
Fv_160_4.g2455	623	ID=Fv_160_4.g2455;Description=hypothetical protein J7337_011755 [Fusarium
Fv_160_4.g2456	1569	ID=Fv_160_4.g2456;Description=hypothetical protein FVER14953_11377 [Fusarium verticillioides];Gene=FOC4_g10005967;Ontology_term=cytoplasm,3-dehydroquinate dehydratase activity,3-dehydroquinate synthase activity,3-phosphoshikimate 1-carboxyvinyltransferase activity,shikimate 3-dehydrogenase (NADP+) activity,shikimate kinase activity,ATP binding,metal ion binding,amino acid biosynthetic process,aromatic amino acid family biosynthetic process,chorismate biosynthetic process,phosphorylation;Ontology_id=GO:0005737,GO:0003855,GO:0003856,GO:0003866,GO:0004764,GO:0004765,GO:0005524,
Fv_160_4.g2457	262	ID=Fv_160_4.g2457;Description=hypothetical protein J7337_011753 [Fusarium musae];Gene=FGADI_6530;Ontology_term=plasma membrane,cell surface,side of membrane,ribonucleoprotein complex,RNA binding,G protein-coupled photoreceptor activity,G protein-coupled receptor signaling pathway,visual perception,phototransduction,detection of visible light;Ontology_id=GO:0005886,GO:0009986,GO:0098552,GO:1990904,GO:0003723,GO:0008020,GO:0007186,GO:0007601,GO:000
Fv_160_4.g2458	253	ID=Fv_160_4.g2458;Description=vacuolar atp synthase subunit d [Fusarium tjaetaba];Gene=FOC1_g10010667;Ontology_term=proton-transporting ATPase activity, rotational mechanism,proton
Fv_160_4.g2459	822	ID=Fv_160_4.g2459;Description=hypothetical protein FVER14953_10476 [Fusarium verticillioides];Gene=FOYG_12330;Ontology_term=Prp19 complex,post-mRNA release spliceosomal complex,mRNA splicing, via
Fv_160_4.g2460	93	ID=Fv_160_4.g2460;Description=hypothetical protein FVEG_10475 [Fusarium verticillioides 7600]
Fv_160_4.g2461	977	ID=Fv_160_4.g2461;Description=hypothetical protein FVER14953_10474 [Fusarium verticillioides];Gene=FTJAE_13227;Ontology_term=fungal-type vacuole membrane,nuclear periphery,PAS complex,G protein-coupled photoreceptor activity,phosphatidylinositol-3,5-bisphosphate 5-phosphatase activity,G protein-coupled receptor signaling pathway,visual perception,phototransduction, UV,detection of visible light,absorption of UV light,phosphatidylinositol-3-phosphate biosynthetic process,phosphatidylinositol
Fv_160_4.g2462	1048	ID=Fv_160_4.g2462;Description=hypothetical protein FVER53590_10471 [Fusarium verticillioides];Gene=FSUBG_13124;Ontology_term=RNA binding,nucleotidyltransferase activity,tRNA 3'-terminal CCA
Fv_160_4.g2463	1598	ID=Fv_160_4.g2463;Description=hypothetical protein FVER14953_10470 [Fusarium verticillioides];Gene=FTJAE_13225;Ontology_term=Golgi apparatus,guanyl-nucleotide exchange factor activity,regulation of ARF
Fv_160_4.g2464	491	ID=Fv_160_4.g2464;Description=mannan polymerase II complex ANP1 subunit [Fusarium

Fv_160_4.g2465	283	ID=Fv_160_4.g2465;Description=hypothetical protein FVEG_10467 [Fusarium verticillioides 7600];Gene=AU210_011614;Ontology_term=nuclear exosome (RNase complex),nucleolus,vacuole,membrane,symbiont-containing vacuole membrane,rRNA processing;Ontology_id=GO:0000176,GO:0005730,GO:0005773,GO:0016020,GO:0020005,GO:0006364
Fv_160_4.g2466	959	ID=Fv_160_4.g2466;Description=hypothetical protein FVEG_10466 [Fusarium verticillioides
Fv_160_4.g2467	284	ID=Fv_160_4.g2467;Description=hypothetical protein FVER14953_10465 [Fusarium verticillioides];Gene=FACUT_2209;Ontology_term=tRNA-intron endonuclease complex,tRNA-intron endonuclease activity,nucleic acid binding,lyase activity,tRNA-type intron splice site recognition and cleavage;Ontology_id=GO:0000214,GO:0000213,GO:0003676,GO:0016829,GO:0000379;Enzyme_code=EC:3.1.31.1,EC:3.1.27,EC:4.6
Fv_160_4.g2468	378	ID=Fv_160_4.g2468;Description=hypothetical protein FVER14953_10464 [Fusarium verticillioides];Gene=FOTG_06729;Ontology_term=DNA-directed RNA polymerase complex,nucleus,cytoplasm,elongator
Fv_160_4.g2469	148	ID=Fv_160_4.g2469;Description=DNA-directed RNA polymerase I, II, and III subunit RPABC3 [Fusarium oxysporum f. sp. lycopersici 4287];Gene=FOTG_06728;Ontology_term=RNA polymerase II, core complex,RNA polymerase III complex,RNA polymerase I complex,RNA polymerase I activity,RNA polymerase II activity,RNA polymerase III activity,RNA-dependent RNA polymerase activity,RNA-templated transcription,transcription initiation at RNA polymerase I promoter,transcription elongation by RNA polymerase I,termination of RNA polymerase I transcription,transcription initiation at RNA polymerase II promoter,transcription elongation by RNA polymerase II,transcription initiation at RNA polymerase III promoter,termination of RNA polymerase III transcription,tRNA transcription by RNA polymerase
Fv_160_4.g2470	332	ID=Fv_160_4.g2470;Description=hypothetical protein FVEG_10462 [Fusarium verticillioides 7600];Gene=FNAPI_13092;Ontology_term=kinase
Fv_160_4.g2471	197	ID=Fv_160_4.g2471;Description=hypothetical protein FVEG_10461 [Fusarium verticillioides 7600]
Fv_160_4.g2472	676	ID=Fv_160_4.g2472;Description=hypothetical protein FVER14953_10460 [Fusarium verticillioides];Gene=FMEXI_14160;Ontology_term=Golgi apparatus,membrane,ATP binding,nucleoside diphosphate phosphatase activity,metal ion
Fv_160_4.g2473	510	ID=Fv_160_4.g2473;Description=putative lactose regulatory [Fusarium denticulatum];Gene=FTJAE_13214;Ontology_term=synaptonemal complex,ligase activity,metal ion binding,ubiquitin protein ligase activity,reciprocal meiotic
Fv_160_4.g2474	252	ID=Fv_160_4.g2474;Description=hypothetical protein FVEG_10459 [Fusarium verticillioides 7600];Gene=FRV6_13634;Ontology_term=nucleus,DNA-binding transcription factor activity, RNA polymerase II-specific,zinc ion
Fv_160_4.g2475	533	ID=Fv_160_4.g2475;Description=hypothetical protein FVER14953_10458 [Fusarium verticillioides];Gene=FVER53263_10458;Ontology_term=NEDD8 activating enzyme activity,protein
Fv_160_4.g2476	564	ID=Fv_160_4.g2476;Description=hypothetical protein FNYG_03229 [Fusarium nygamai];Gene=FPCIR_7439;Ontology_term=membrane,transmembrane transporter activity,transmembrane
Fv_160_4.g2477	757	ID=Fv_160_4.g2477;Description=hypothetical protein FVER14953_10456 [Fusarium verticillioides]

Fv_160_4.g2478	332	ID=Fv_160_4.g2478;Description=hypothetical protein J7337_011732 [Fusarium musae];Gene=FANTH_4266;Ontology_term=U2-type
Fv_160_4.g2479	665	ID=Fv_160_4.g2479;Description=transcription initiation factor TFIIF subunit alpha [Fusarium verticillioides 7600];Gene=FMEXI_887;Ontology_term=nucleus,DNA binding,translation initiation factor activity,transcription initiation at RNA polymerase II promoter,translational initiation,positive regulation of transcription elongation by RNA polymerase
Fv_160_4.g2480	1055	ID=Fv_160_4.g2480;Description=hypothetical protein FVER53590_10453 [Fusarium verticillioides];Gene=FMAN_12539;Ontology_term=poly(A) RNA polymerase
Fv_160_4.g2481	573	ID=Fv_160_4.g2481;Description=hypothetical protein FVEG_16803 [Fusarium verticillioides 7600]
Fv_160_4.g2482	503	ID=Fv_160_4.g2482;Description=ribose-phosphate pyrophosphokinase [Fusarium verticillioides 7600];Gene=FTJAE_13205;Ontology_term=ribose phosphate diphosphokinase complex,magnesium ion binding,ribose phosphate diphosphokinase activity,ATP binding,kinase activity,5-phosphoribose 1-diphosphate biosynthetic process,ribonucleoside monophosphate biosynthetic process,nucleotide biosynthetic process,phosphorylation,fungal-type cell wall organization;Ontology_id=GO:0002189,GO:0000287,GO:0004749,GO:0005524,GO:0016301,GO:0006015,GO:0009156,GO:0009165,
Fv_160_4.g2483	271	ID=Fv_160_4.g2483;Description=oxidoreductase [Fusarium verticillioides 7600];Gene=FTJAE_13204;Ontology_term=oxidoreductase activity,cellular biosynthetic process;Ontology_id=GO:0016491,GO:0044249;Enzyme_code=EC:1;Enzyme_name=Oxidoreductases
Fv_160_4.g2484	456	ID=Fv_160_4.g2484;Description=hypothetical protein FVER53263_10450 [Fusarium
Fv_160_4.g2485	436	ID=Fv_160_4.g2485;Description=e3 ubiquitin ligase RAD18 [Fusarium coicis];Gene=HZS61_016434;Ontology_term=nucleus,single-stranded DNA binding,ligase activity,metal ion binding,ubiquitin protein ligase activity,postreplication repair,protein ubiquitination;Ontology_id=GO:0005634,GO:0003697,GO:0016874,GO:0046872,GO:0061630,GO:0006301,GO:0016567;Enzyme_co
Fv_160_4.g2486	447	ID=Fv_160_4.g2486;Description=hypothetical protein FVER53590_10448 [Fusarium verticillioides];Gene=FPANT_2084;Ontology_term=delta DNA polymerase complex,DNA
Fv_160_4.g2487	479	ID=Fv_160_4.g2487;Description=hypothetical protein FVER53590_10447 [Fusarium verticillioides];Gene=FOXG_11370;Ontology_term=transaminase activity,pyridoxal phosphate binding,carbohydrate metabolic process,biosynthetic
Fv_160_4.g2488	543	ID=Fv_160_4.g2488;Description=hypothetical protein FVEG_10446 [Fusarium verticillioides 7600];Gene=FVER53263_10446;Ontology_term=pyridoxal phosphate binding,carbohydrate metabolic process,biosynthetic
Fv_160_4.g2489	498	ID=Fv_160_4.g2489;Description=hypothetical protein FVER53263_10445 [Fusarium verticillioides];Gene=F25303_5289;Ontology_term=nucleus,pericentric heterochromatin,NuA4 histone acetyltransferase complex,site of double-strand break,histone H3K4 acetyltransferase activity,peptide 2-hydroxyisobutyryltransferase activity,peptide crotonyltransferase activity,peptide butyryltransferase activity,DNA repair,regulation of DNA-templated transcription,positive regulation of heterochromatin formation,DNA repair-dependent chromatin
Fv_160_4.g2490	319	ID=Fv_160_4.g2490;Description=RNA exonuclease 4 [Fusarium verticillioides 7600];Gene=FOC1_g10010632;Ontology_term=nucleic acid binding,3'-5' exonuclease activity,rRNA

Fv_160_4.g2491	156	ID=Fv_160_4.g2491;Description=ribonuclease H2 subunit C [Fusarium verticillioides 7600];Gene=FOPG_11591;Ontology_term=ribonuclease H2 complex,RNA catabolic process;Ontology_id=GO:0032299,GO:0006401
Fv_160_4.g2492	276	ID=Fv_160_4.g2492;Description=hypothetical protein FVER53590_10442 [Fusarium verticillioides]
Fv_160_4.g2493	289	ID=Fv_160_4.g2493;Description=hypothetical protein FVEG_10441 [Fusarium verticillioides 7600];Gene=FANTH_4252;Ontology_term=oxidoreductase activity,cellular biosynthetic
Fv_160_4.g2494	442	ID=Fv_160_4.g2494;Description=hypothetical protein FVER14953_10440 [Fusarium verticillioides];Gene=BFJ65_g12247;Ontology_term=phosphoribosylamine-glycine ligase activity,ATP binding,metal ion binding,'de novo' IMP biosynthetic process,purine nucleobase biosynthetic
Fv_160_4.g2495	586	ID=Fv_160_4.g2495;Description=hypothetical protein FVEG_10439 [Fusarium verticillioides 7600];Gene=BFJ63_vAg10783;Ontology_term=membrane,monooxygenase activity,iron ion binding,oxidoreductase activity, acting on paired donors, with incorporation or reduction of molecular oxygen,heme binding;Ontology_id=GO:0016020,GO:0004497,GO:0005506,GO:0016705,GO:0020037;Enzyme_code=EC:1.14;Enzyme_name=Actin
Fv_160_4.g2496	296	ID=Fv_160_4.g2496;Description=hypothetical protein FVER14953_10438 [Fusarium verticillioides];Gene=BFJ63_vAg10771;Ontology_term=oxidoreductase activity,cellular biosynthetic
Fv_160_4.g2497	358	ID=Fv_160_4.g2497;Description=hypothetical protein FVEG_10437 [Fusarium verticillioides 7600];Gene=xdhA-1;Ontology_term=zinc ion binding,oxidoreductase activity, acting on the CH-OH group of donors, NAD or NADP as acceptor,cellular biosynthetic process;Ontology_id=GO:0008270,GO:0016616,GO:0044249;Enzyme_code=EC:1.1.1;Enzyme_name=Acting on the CH-OH group of
Fv_160_4.g2498	489	ID=Fv_160_4.g2498;Description=hypothetical protein FVER14953_10436 [Fusarium verticillioides];Gene=FOXG_11381;Ontology_term=zinc ion binding,oxidoreductase activity,metallodipeptidase activity,proteolysis;Ontology_id=GO:0008270,GO:0016491,GO:0070573,GO:0006508;Enzyme_code=EC:3.4.13,EC:1;Enzyme_name=
Fv_160_4.g2499	1125	ID=Fv_160_4.g2499;Description=potassium/sodium efflux P-type ATPase, fungal-type [Fusarium verticillioides 7600];Gene=BFJ63_vAg10750;Ontology_term=membrane,ATP binding,ATP hydrolysis activity,ATPase-coupled monoatomic cation transmembrane transporter activity,sodium ion transport,monoatomic cation transmembrane transport;Ontology_id=GO:0016020,GO:0005524,GO:0016887,GO:0019829,GO:0006814,GO:0098655;Enzyme_code=EC:7.2.2,EC:3.
Fv_160_4.g2500	316	ID=Fv_160_4.g2500;Description=hypothetical protein FVEG_10434 [Fusarium verticillioides 7600]
Fv_160_4.g2501	416	ID=Fv_160_4.g2501;Description=acetyl-CoA acyltransferase [Fusarium verticillioides 7600];Gene=FOYG_12380;Ontology_term=acyltransferase activity, transferring groups other than amino-acyl
Fv_160_4.g2502	801	ID=Fv_160_4.g2502;Description=hypothetical protein FVER14953_10432 [Fusarium verticillioides];Gene=FPHYL_9244;Ontology_term=membrane,monoatomic anion transmembrane transporter activity,monoatomic
Fv_160_4.g2503	684	ID=Fv_160_4.g2503;Description=hypothetical protein FVER14953_10429 [Fusarium
Fv_160_4.g2504	239	ID=Fv_160_4.g2504;Description=hypothetical protein FVER14953_10428 [Fusarium verticillioides];Gene=FDENT_2040;Ontology_term=carboxypeptidase
Fv_160_4.g2505	787	ID=Fv_160_4.g2505;Description=hypothetical protein FVER14953_10427 [Fusarium verticillioides]

Fv_160_4.g2506	455	ID=Fv_160_4.g2506;Description=hypothetical protein FVER14953_10426 [Fusarium verticillioides];Gene=FOXG_11391;Ontology_term=endoplasmic reticulum,membrane,Golgi to plasma membrane transport vesicle,phosphatidate cytidylyltransferase activity,phosphatidylserine metabolic process,CDP-diacylglycerol biosynthetic process,phosphatidylinositol metabolic
Fv_160_4.g2507	448	ID=Fv_160_4.g2507;Description=hypothetical protein FVER14953_10425 [Fusarium verticillioides];Gene=FPHYL_9250;Ontology_term=transferase
Fv_160_4.g2508	675	ID=Fv_160_4.g2508;Description=hypothetical protein J7337_011702 [Fusarium musae];Gene=FVER53263_10424;Ontology_term=nucleotide binding,hydrolase activity,nucleotide catabolic
Fv_160_4.g2509	275	ID=Fv_160_4.g2509;Description=hypothetical protein FVER14953_20098 [Fusarium
Fv_160_4.g2510	334	ID=Fv_160_4.g2510;Description=hypothetical protein FVER14953_10423 [Fusarium
Fv_160_4.g2511	523	ID=Fv_160_4.g2511;Description=hypothetical protein FVEG_10422 [Fusarium verticillioides 7600];Gene=FOMG_13891;Ontology_term=membrane,glycosyltransferase
Fv_160_4.g2512	605	ID=Fv_160_4.g2512;Description=UDPglucose 6-dehydrogenase [Fusarium verticillioides 7600];Gene=FANTH_4230;Ontology_term=UDP-glucose 6-dehydrogenase activity,NAD binding,polysaccharide biosynthetic process,UDP-glucuronate biosynthetic
Fv_160_4.g2513	271	ID=Fv_160_4.g2513;Description=hypothetical protein FVER14953_10420 [Fusarium verticillioides]
Fv_160_4.g2514	284	ID=Fv_160_4.g2514;Description=hypothetical protein FVER53590_10419 [Fusarium verticillioides];Gene=BFJ69_g3040;Ontology_term=extracellular region,chitosanase activity,polysaccharide catabolic
Fv_160_4.g2515	341	ID=Fv_160_4.g2515;Description=hypothetical protein FVER14953_10418 [Fusarium verticillioides];Gene=Acp26Aa;Ontology_term=extracellular space,identical protein binding,behavior,mating,positive regulation of ovulation,positive regulation of octopamine signaling
Fv_160_4.g2516	157	ID=Fv_160_4.g2516;Description=hypothetical protein FVEG_16797 [Fusarium verticillioides
Fv_160_4.g2517	223	ID=Fv_160_4.g2517;Description=hypothetical protein FVEG_10417 [Fusarium verticillioides 7600]
Fv_160_4.g2518	83	ID=Fv_160_4.g2518;Description=hypothetical protein FVEG_16796 [Fusarium verticillioides 7600]
Fv_160_4.g2519	351	ID=Fv_160_4.g2519;Description=hypothetical protein FVEG_10416 [Fusarium verticillioides
Fv_160_4.g2520	409	ID=Fv_160_4.g2520;Description=hypothetical protein FVEG_10415 [Fusarium verticillioides 7600];Gene=F25303_12807;Ontology_term=chromosome, centromeric region,nucleus;Ontology_id=GO:0000775,GO:0005634
Fv_160_4.g2521	1141	ID=Fv_160_4.g2521;Description=hypothetical protein FVER53590_10414 [Fusarium verticillioides]
Fv_160_4.g2522	332	ID=Fv_160_4.g2522;Description=alcohol dehydrogenase (NADP+) [Fusarium verticillioides 7600];Gene=FCIRC_4542;Ontology_term=oxidoreductase
Fv_160_4.g2523	257	ID=Fv_160_4.g2523;Description=hypothetical protein FPRO05_08881 [Fusarium proliferatum];Gene=FVER53590_10412;Ontology_term=ferrochelatase activity,precorrin-2 dehydrogenase activity,siroheme biosynthetic

Fv_160_4.g2524	679	ID=Fv_160_4.g2524;Description=hypothetical protein FVEG_10411 [Fusarium verticillioides 7600];Gene=F25303_12803;Ontology_term=nuclear speck,DNA binding,pre-mRNA binding,metal ion binding,mRNA splicing, via spliceosome,DNA repair,cell differentiation,female sex determination,female sex differentiation,epithelium regeneration,regulation
Fv_160_4.g2525	792	ID=Fv_160_4.g2525;Description=hypothetical protein FVEG_10410 [Fusarium verticillioides 7600];Gene=FNAPI_3894;Ontology_term=RNA polymerase II transcription regulator complex,DNA-binding transcription activator activity, RNA polymerase II-specific,DNA binding,sporulation resulting in formation of a cellular spore,positive regulation of
Fv_160_4.g2526	438	ID=Fv_160_4.g2526;Description=hypothetical protein FVER53590_27560 [Fusarium verticillioides]
Fv_160_4.g2527	181	ID=Fv_160_4.g2527;Description=acetyltransferase [Fusarium verticillioides 7600];Gene=AU210_011679;Ontology_term=acyltransferase activity, transferring groups other than amino-acyl
Fv_160_4.g2528	498	ID=Fv_160_4.g2528;Description=hypothetical protein J7337_011683 [Fusarium musae];Gene=FTJAE_4052;Ontology_term=membrane,transmembrane transporter activity,transmembrane
Fv_160_4.g2529	278	ID=Fv_160_4.g2529;Description=hypothetical protein FVER14953_10407 [Fusarium verticillioides];Gene=FTJAE_4053;Ontology_term=S-adenosylmethionine-dependent methyltransferase
Fv_160_4.g2530	450	ID=Fv_160_4.g2530;Description=hypothetical protein FVER53590_27561 [Fusarium verticillioides];Gene=FVER53590_27561;Ontology_term=membrane,transmembrane transporter activity,transmembrane
Fv_160_4.g2531	387	ID=Fv_160_4.g2531;Description=alcohol dehydrogenase [Fusarium verticillioides 7600];Gene=FTJAE_4055;Ontology_term=zinc ion binding,oxidoreductase activity;Ontology_id=GO:0008270,GO:0016491;Enzyme_code=EC:1;Enzyme_name=Oxidoreductases
Fv_160_4.g2532	184	ID=Fv_160_4.g2532;Description=hypothetical protein FVER14953_10404 [Fusarium verticillioides];Gene=FVER53263_10404;Ontology_term=extracellular region,alcohol dehydrogenase (NAD+) activity,alcohol metabolic process;Ontology_id=GO:0005576,GO:0004022,GO:0006066;Enzyme_code=EC:1.1.1.1,EC:1.1.1.71;Enzyme_name=alcohol
Fv_160_4.g2533	280	ID=Fv_160_4.g2533;Description=hypothetical protein J7337_011678 [Fusarium musae];Gene=FVER53263_10403;Ontology_term=cytoplasm,glutathione transferase activity,glutathione dehydrogenase (ascorbate) activity,cellular oxidant detoxification;Ontology_id=GO:0005737,GO:0004364,GO:0045174,GO:0098869;Enzyme_code=EC:2.5.1.18,EC:1.8.5.1;Enzyme_nam
Fv_160_4.g2534	363	ID=Fv_160_4.g2534;Description=hypothetical protein FVER14953_10402 [Fusarium
Fv_160_4.g2535	149	ID=Fv_160_4.g2535;Description=hypothetical protein FVEG_16794 [Fusarium verticillioides
Fv_160_4.g2536	256	ID=Fv_160_4.g2536;Description=transcriptional regulator [Fusarium verticillioides 7600]
Fv_160_4.g2537	391	ID=Fv_160_4.g2537;Description=hypothetical protein FVER14953_10400 [Fusarium verticillioides];Gene=FNAPI_3882;Ontology_term=monooxygenase activity,oxidoreductase activity, acting on paired donors, with incorporation or reduction of molecular
Fv_160_4.g2538	472	ID=Fv_160_4.g2538;Description=hypothetical protein FVER53590_10399 [Fusarium verticillioides];Gene=DTH-2;Ontology_term=nucleus,DNA-binding transcription factor activity, RNA polymerase II-specific,DNA binding,regulation of
Fv_160_4.g2539	203	ID=Fv_160_4.g2539;Description=hypothetical protein FVER53590_10398 [Fusarium verticillioides]

Fv_160_4.g2540	330	ID=Fv_160_4.g2540;Description=hypothetical protein FVER14953_10397 [Fusarium verticillioides];Gene=CEK26_013117;Ontology_term=nucleus,DNA-binding transcription factor activity, RNA polymerase II-specific,zinc ion binding,dioxygenase activity,regulation of transcription by RNA polymerase
Fv_160_4.g2541	984	ID=Fv_160_4.g2541;Description=hypothetical protein DER46DRAFT_627205 [Fusarium sp. MPI-SDFR-AT-0072]
Fv_160_4.g2542	137	ID=Fv_160_4.g2542;Description=hypothetical protein FVEG_10395 [Fusarium verticillioides 7600];Gene=FDENT_14161;Ontology_term=carbon-sulfur lyase activity,metal ion
Fv_160_4.g2543	506	ID=Fv_160_4.g2543;Description=MFS transporter, SHS family, lactate transporter [Fusarium verticillioides 7600];Gene=F25303_2876;Ontology_term=membrane,transmembrane transporter activity,transmembrane
Fv_160_4.g2544	689	ID=Fv_160_4.g2544;Description=hypothetical protein FVEG_16790 [Fusarium verticillioides 7600];Gene=FocTR4_00014191;Ontology_term=nucleus,DNA binding,zinc ion binding,DNA-binding transcription factor activity, RNA polymerase II-specific,regulation of transcription by RNA polymerase
Fv_160_4.g2545	472	ID=Fv_160_4.g2545;Description=seryl-tRNA synthetase [Fusarium verticillioides 7600];Gene=FPANT_12910;Ontology_term=serine-tRNA ligase activity,ATP binding,seryl-tRNA
Fv_160_4.g2546	1978	ID=Fv_160_4.g2546;Description=Ankyrin-1 [Fusarium oxysporum f. sp. cubense race 1];Gene=FSARC_5791;Ontology_term=ATP hydrolysis activity;Ontology_id=GO:0016887;Enzyme_code=EC:3.6.1.15;Enzyme_name=nucleoside-triphosphate phosphatase
Fv_160_4.g2547	564	ID=Fv_160_4.g2547;Description=hypothetical protein FVER14953_10390 [Fusarium verticillioides];Gene=FVER53590_10390;Ontology_term=ATP hydrolysis
Fv_160_4.g2548	807	ID=Fv_160_4.g2548;Description=hypothetical protein FVER53590_10389 [Fusarium verticillioides]
Fv_160_4.g2549	257	ID=Fv_160_4.g2549;Description=hypothetical protein FVER14953_10388 [Fusarium verticillioides]
Fv_160_4.g2550	335	ID=Fv_160_4.g2550;Description=23S rRNA (-N6)-methyltransferase [Fusarium acutatum];Gene=FNAPI_6645;Ontology_term=membrane,S-adenosylmethionine-dependent methyltransferase activity,cellular aromatic compound metabolic process,methylation,cellular nitrogen compound metabolic process,primary metabolic process,heterocycle metabolic process,organic cyclic compound metabolic process;Ontology_id=GO:0016020,GO:0008757,GO:0006725,GO:0032259,GO:0034641,GO:0044238,GO:0046483,GO:1901360;Enzy
Fv_160_4.g2551	481	ID=Fv_160_4.g2551;Description=23S rRNA (-N6)-methyltransferase [Fusarium verticillioides 7600];Gene=FNAPI_6644;Ontology_term=S-adenosylmethionine-dependent methyltransferase activity,cellular aromatic compound metabolic process,methylation,cellular nitrogen compound metabolic process,primary metabolic process,heterocycle metabolic process,organic cyclic compound metabolic
Fv_160_4.g2552	401	ID=Fv_160_4.g2552;Description=hypothetical protein FVER14953_10385 [Fusarium verticillioides];Gene=FNAPI_6643;Ontology_term=phytanoyl-CoA dioxygenase activity,fatty acid alpha-
Fv_160_4.g2553	494	ID=Fv_160_4.g2553;Description=hypothetical protein J7337_011659 [Fusarium musae];Gene=FOTG_06629;Ontology_term=oxidoreductase activity, acting on the aldehyde or oxo group of donors, NAD or NADP as
Fv_160_4.g2554	409	ID=Fv_160_4.g2554;Description=hypothetical protein FVEG_10383 [Fusarium verticillioides 7600]

Fv_160_4.g2555	294	ID=Fv_160_4.g2555;Description=hypothetical protein FVER14953_10382 [Fusarium verticillioides];Gene=FNAPI_6640;Ontology_term=5'-nucleotidase activity,pseudouridine 5'-phosphatase
Fv_160_4.g2556	288	ID=Fv_160_4.g2556;Description=hypothetical protein FVER53590_10381 [Fusarium verticillioides];Gene=en;Ontology_term=nucleus,RNA polymerase II cis-regulatory region sequence-specific DNA binding,DNA-binding transcription repressor activity, RNA polymerase II-specific,negative regulation of transcription by RNA polymerase II,segment polarity determination,neuroblast fate determination,axon guidance,ventral midline development,imaginal disc-derived wing vein specification,gonad development,negative regulation of gene expression,genital disc anterior/posterior pattern formation,spiracle morphogenesis, open tracheal system,negative regulation of neuron apoptotic process,positive regulation of transcription by RNA polymerase
Fv_160_4.g2557	784	ID=Fv_160_4.g2557;Description=hypothetical protein FVER53263_10380 [Fusarium verticillioides];Gene=FFUJ_09638;Ontology_term=nucleus,DNA-binding transcription factor activity, RNA polymerase II-specific,zinc
Fv_160_4.g2558	433	ID=Fv_160_4.g2558;Description=hypothetical protein FVEG_10379 [Fusarium verticillioides 7600]
Fv_160_4.g2559	247	ID=Fv_160_4.g2559;Description=hypothetical protein J7337_011653 [Fusarium musae]
Fv_160_4.g2560	562	ID=Fv_160_4.g2560;Description=aldehyde dehydrogenase (NAD+) [Fusarium verticillioides 7600];Gene=BFJ65_g13015;Ontology_term=oxidoreductase activity, acting on the aldehyde or oxo group of donors, NAD or NADP as
Fv_160_4.g2561	567	ID=Fv_160_4.g2561;Description=hypothetical protein FVEG_10376 [Fusarium verticillioides 7600];Gene=FOTG_06619;Ontology_term=membrane,transmembrane transporter activity,transmembrane
Fv_160_4.g2562	375	ID=Fv_160_4.g2562;Description=hypothetical protein FVER53590_10375 [Fusarium verticillioides];Gene=FNAPI_6633;Ontology_term=pantoate-beta-alanine ligase activity,pantothenate biosynthetic
Fv_160_4.g2563	835	ID=Fv_160_4.g2563;Description=hypothetical protein FVER53590_10374 [Fusarium verticillioides];Gene=FNAPI_6632;Ontology_term=membrane,O-acyltransferase activity,cellular lipid metabolic
Fv_160_4.g2564	228	ID=Fv_160_4.g2564;Description=ribonuclease P protein subunit POP4 [Fusarium verticillioides 7600];Gene=BFJ65_g12387;Ontology_term=ribonuclease MRP complex,nucleus,cytoplasm,ribonuclease P complex,endonuclease activity,ribonuclease P RNA binding,tRNA 5'-leader
Fv_160_4.g2565	1459	ID=Fv_160_4.g2565;Description=hypothetical protein FVEG_10372 [Fusarium verticillioides 7600]
Fv_160_4.g2566	1031	ID=Fv_160_4.g2566;Description=hypothetical protein FVER14953_10371 [Fusarium verticillioides]
Fv_160_4.g2567	565	ID=Fv_160_4.g2567;Description=hypothetical protein FVER14953_10370 [Fusarium verticillioides];Gene=FFUJ_09628;Ontology_term=membrane,transmembrane transport;Ontology_id=GO:0016020,GO:0055085
Fv_160_4.g2568	1055	ID=Fv_160_4.g2568;Description=elongation factor 3 [Fusarium verticillioides 7600];Gene=28952854;Ontology_term=cytoskeleton,RNA binding,translation elongation factor activity,ATP binding,ATP hydrolysis activity,translational
Fv_160_4.g2569	1256	ID=Fv_160_4.g2569;Description=hypothetical protein FVEG_10368 [Fusarium verticillioides 7600]

Fv_160_4.g2570	222	ID=Fv_160_4.g2570;Description=hypothetical protein FVER14953_10367 [Fusarium verticillioides];Gene=FOTG_06608;Ontology_term=RNA-dependent RNA polymerase activity,phosphoprotein phosphatase activity,RNA-templated
Fv_160_4.g2571	691	ID=Fv_160_4.g2571;Description=hypothetical protein J7337_011639 [Fusarium
Fv_160_4.g2572	1014	ID=Fv_160_4.g2572;Description=hypothetical protein FVER53590_10365 [Fusarium verticillioides];Gene=FPANT_7230;Ontology_term=ATP binding,ATP hydrolysis
Fv_160_4.g2573	315	ID=Fv_160_4.g2573;Description=hypothetical protein FVER14953_10364 [Fusarium verticillioides];Gene=FTJAE_1250;Ontology_term=extracellular region,IgE binding;Ontology_id=GO:0005576,GO:0019863
Fv_160_4.g2574	1000	ID=Fv_160_4.g2574;Description=hypothetical protein FVER53590_10363 [Fusarium verticillioides]
Fv_160_4.g2575	458	ID=Fv_160_4.g2575;Description=hypothetical protein FVER53590_10362 [Fusarium verticillioides]
Fv_160_4.g2576	436	ID=Fv_160_4.g2576;Description=hypothetical protein FVER53590_10361 [Fusarium verticillioides];Gene=HZS61_016526;Ontology_term=hydrolase activity,lipid metabolic
Fv_160_4.g2577	1422	ID=Fv_160_4.g2577;Description=hypothetical protein FVEG_10359 [Fusarium verticillioides 7600];Gene=FVER53263_10359;Ontology_term=mitochondrion,membrane,pyruvate dehydrogenase complex,dihydrolipoyllysine-residue acetyltransferase activity,acetyl-CoA biosynthetic process from
Fv_160_4.g2578	457	ID=Fv_160_4.g2578;Description=dihydrolipoamide acetyltransferase component pyruvate dehydrogenase complex [Fusarium verticillioides 7600];Gene=AU210_011732;Ontology_term=mitochondrial pyruvate dehydrogenase complex,membrane,dihydrolipoyllysine-residue acetyltransferase activity,acetyl-CoA biosynthetic process from
Fv_160_4.g2579	824	ID=Fv_160_4.g2579;Description=hypothetical protein FVER53590_10357 [Fusarium verticillioides];Gene=FFUJ_09616;Ontology_term=nucleus,DNA binding,zinc ion binding,DNA-binding transcription factor activity,
Fv_160_4.g2580	521	ID=Fv_160_4.g2580;Description=hypothetical protein FVEG_10356 [Fusarium verticillioides 7600]
Fv_160_4.g2581	1833	ID=Fv_160_4.g2581;Description=hypothetical protein FVEG_10355 [Fusarium verticillioides 7600];Gene=FTJAE_1242;Ontology_term=Golgi apparatus,guanyl-nucleotide exchange factor activity,protein transport,regulation of
Fv_160_4.g2582	369	ID=Fv_160_4.g2582;Description=hypothetical protein FVEG_10354 [Fusarium verticillioides 7600]
Fv_160_4.g2583	265	ID=Fv_160_4.g2583;Description=hypothetical protein FOC4_g10008097 [Fusarium odoratissimum];Gene=Forpi1262_v009813;Ontology_term=RNA polymerase III complex,transcription by RNA polymerase
Fv_160_4.g2584	452	ID=Fv_160_4.g2584;Description=hypothetical protein FVEG_10352 [Fusarium verticillioides 7600];Gene=FPANT_12810;Ontology_term=nuclear pore,nuclear localization sequence binding,structural constituent of nuclear
Fv_160_4.g2585	330	ID=Fv_160_4.g2585;Description=hypothetical protein FVEG_10351 [Fusarium verticillioides
Fv_160_4.g2586	267	ID=Fv_160_4.g2586;Description=hypothetical protein FVER53590_10350 [Fusarium verticillioides]
Fv_160_4.g2587	258	ID=Fv_160_4.g2587;Description=hypothetical protein FVEG_10349 [Fusarium verticillioides 7600]

Fv_160_4.g2588	270	ID=Fv_160_4.g2588;Description=hypothetical protein FVEG_10348 [Fusarium verticillioides 7600];Gene=FCIRC_1534;Ontology_term=membrane,ATP-dependent peptidase activity,metalloendopeptidase activity,ATP binding,proteolysis;Ontology_id=GO:0016020,GO:0004176,GO:0004222,GO:0005524,GO:0006508;Enzyme_code=EC:3.4.24;Enzyme
Fv_160_4.g2589	242	ID=Fv_160_4.g2589;Description=hypothetical protein FVEG_10347 [Fusarium verticillioides 7600];Gene=FCIRC_1533;Ontology_term=nuclear speck,S-adenosylmethionine-dependent methyltransferase activity,mRNA processing,cell differentiation,methylation,female sex
Fv_160_4.g2590	767	ID=Fv_160_4.g2590;Description=hypothetical protein FVER53590_10346 [Fusarium verticillioides];Gene=FNAPI_10358;Ontology_term=membrane,oxidoreductase activity,monoatomic ion
Fv_160_4.g2591	381	ID=Fv_160_4.g2591;Description=hypothetical protein FVER14953_10345 [Fusarium
Fv_160_4.g2592	281	ID=Fv_160_4.g2592;Description=glutathione S-transferase [Fusarium verticillioides 7600];Gene=FACUT_917;Ontology_term=transferase
Fv_160_4.g2593	974	ID=Fv_160_4.g2593;Description=hypothetical protein FVER14953_10343 [Fusarium
Fv_160_4.g2594	484	ID=Fv_160_4.g2594;Description=hypothetical protein FVEG_10342 [Fusarium verticillioides 7600];Gene=FACUT_914;Ontology_term=peroxisome,plasma membrane,urate oxidase activity,fluoride transmembrane transporter activity,purine nucleobase metabolic process,urate catabolic process,fluoride transmembrane transport;Ontology_id=GO:0005777,GO:0005886,GO:0004846,GO:1903425,GO:0006144,GO:0019628,GO:1903424;Enzyme_code=E
Fv_160_4.g2595	228	ID=Fv_160_4.g2595;Description=hypothetical protein FVER14953_20570 [Fusarium verticillioides];Gene=urg2;Ontology_term=glycosyltransferase
Fv_160_4.g2596	984	ID=Fv_160_4.g2596;Description=hypothetical protein FVER53590_10341 [Fusarium verticillioides];Gene=FNAPI_9753;Ontology_term=nucleus,DNA binding,DNA ligase (ATP) activity,ATP binding,DNA replication,DNA recombination,DNA ligation involved in DNA repair,DNA biosynthetic
Fv_160_4.g2597	607	ID=Fv_160_4.g2597;Description=hypothetical protein FVEG_10340 [Fusarium verticillioides 7600];Gene=FGLOB1_547;Ontology_term=membrane,metal ion transmembrane transporter activity,magnesium ion
Fv_160_4.g2598	562	ID=Fv_160_4.g2598;Description=alkaline phosphatase [Fusarium verticillioides 7600];Gene=FNAPI_9755;Ontology_term=membrane,alkaline phosphatase activity,oxidoreductase activity,metal ion
Fv_160_4.g2599	634	ID=Fv_160_4.g2599;Description=hypothetical protein FVER14953_10337 [Fusarium verticillioides];Gene=FVER53263_10337;Ontology_term=protein tyrosine phosphatase activity,protein tyrosine/serine/threonine phosphatase
Fv_160_4.g2600	227	ID=Fv_160_4.g2600;Description=alkaline phosphatase [Fusarium verticillioides 7600]
Fv_160_4.g2601	116	ID=Fv_160_4.g2601;Description=50S ribosomal protein L34e [Fusarium verticillioides 7600];Gene=AU210_011756;Ontology_term=ribosome,ribonucleoprotein complex,structural constituent of
Fv_160_4.g2602	129	ID=Fv_160_4.g2602;Description=hypothetical protein FVEG_10335 [Fusarium verticillioides 7600]
Fv_160_4.g2603	208	ID=Fv_160_4.g2603;Description=hypothetical protein FOXG_11487 [Fusarium oxysporum f. sp. lycopersici 4287]

Fv_160_4.g2604	475	ID=Fv_160_4.g2604;Description=hypothetical protein FVER53590_10333 [Fusarium verticillioides];Gene=FACUT_904;Ontology_term=chitin binding,hydrolase activity, acting on carbon-nitrogen (but not peptide) bonds,carbohydrate metabolic
Fv_160_4.g2605	771	ID=Fv_160_4.g2605;Description=hypothetical protein FVER14953_10332 [Fusarium verticillioides];Gene=CEK26_013180;Ontology_term=plasma membrane,chitin synthase activity,conidium
Fv_160_4.g2606	460	ID=Fv_160_4.g2606;Description=hypothetical protein FVER14953_10331 [Fusarium verticillioides];Gene=FNAPI_9763;Ontology_term=oxidoreductase activity, acting on the CH-OH group of donors, NAD or NADP as acceptor,oxidoreductase activity, acting on the CH-CH group of donors, NAD or NADP as acceptor,NAD binding,polysaccharide biosynthetic
Fv_160_4.g2607	349	ID=Fv_160_4.g2607;Description=hypothetical protein J7337_011603 [Fusarium musae];Gene=FNAPI_9764;Ontology_term=extracellular region,chitinase activity,chitin binding,polysaccharide catabolic process,chitin catabolic
Fv_160_4.g2608	314	ID=Fv_160_4.g2608;Description=hypothetical protein FVER14953_10329 [Fusarium verticillioides]
Fv_160_4.g2609	463	ID=Fv_160_4.g2609;Description=hypothetical protein FVEG_10328 [Fusarium verticillioides 7600];Gene=FNAPI_9766;Ontology_term=checkpoint clamp complex,DNA damage checkpoint signaling,DNA
Fv_160_4.g2610	362	ID=Fv_160_4.g2610;Description=hypothetical protein FVEG_10327 [Fusarium verticillioides 7600]
Fv_160_4.g2611	446	ID=Fv_160_4.g2611;Description=uncharacterized protein FOBCDRAFT_279331 [Fusarium oxysporum Fo47];Gene=FVER53263_10325;Ontology_term=prephenate dehydrogenase (NADP+) activity,prephenate dehydrogenase (NAD+) activity,tyrosine biosynthetic
Fv_160_4.g2612	420	ID=Fv_160_4.g2612;Description=hypothetical protein FVEG_10324 [Fusarium verticillioides 7600];Gene=Adh;Ontology_term=cytosol,protein-containing complex,alcohol dehydrogenase (NAD+) activity,acetaldehyde dehydrogenase (acetylating) activity,protein homodimerization activity,ethanol oxidation,acetaldehyde metabolic process,NADH metabolic process,alcohol catabolic process,behavioral response to ethanol;Ontology_id=GO:0005829,GO:0032991,GO:0004022,GO:0008774,GO:0042803,GO:0006069,GO:0006117,GO:0006734,GO:
Fv_160_4.g2613	130	ID=Fv_160_4.g2613;Description=60S ribosomal protein L32 [Fusarium oxysporum f. sp. lycopersici 4287];Gene=FOC1_g10010503;Ontology_term=ribosome,ribonucleoprotein complex,structural constituent of
Fv_160_4.g2614	142	ID=Fv_160_4.g2614;Description=40S ribosomal protein S16 [Fusarium verticillioides 7600];Gene=CEP54_013527;Ontology_term=cytosol,ribosome,ribonucleoprotein complex,structural constituent of ribosome,alcohol dehydrogenase (NAD+) activity,acetaldehyde dehydrogenase (acetylating) activity,protein homodimerization activity,ethanol oxidation,acetaldehyde metabolic process,translation,NADH metabolic process,alcohol catabolic process,behavioral response to ethanol;Ontology_id=GO:0005829,GO:0005840,GO:1990904,GO:0003735,GO:0004022,GO:0008774,GO:0042803,GO:0006069,GO:
Fv_160_4.g2615	512	ID=Fv_160_4.g2615;Description=hypothetical protein FVER14953_10321 [Fusarium verticillioides]

Fv_160_4.g2616	419	ID=Fv_160_4.g2616;Description=hypothetical protein FVEG_10320 [Fusarium verticillioides 7600];Gene=FOMG_13772;Ontology_term=regulation of DNA-templated transcription;Ontology_id=GO:0006355
Fv_160_4.g2617	561	ID=Fv_160_4.g2617;Description=ATP-dependent RNA helicase DBP2 [Fusarium verticillioides 7600];Gene=DBP2;Ontology_term=cytosol,protein-containing complex,nucleic acid binding,RNA helicase activity,alcohol dehydrogenase (NAD+) activity,ATP binding,acetaldehyde dehydrogenase (acetylating) activity,hydrolase activity,protein homodimerization activity,ethanol oxidation,acetaldehyde metabolic process,NADH metabolic process,alcohol catabolic process,behavioral response to ethanol;Ontology_id=GO:0005829,GO:0032991,GO:0003676,GO:0003724,GO:0004022,GO:0005524,GO:0008774,GO:0016787,GO:
Fv_160_4.g2618	467	ID=Fv_160_4.g2618;Description=hypothetical protein FVER53590_10317 [Fusarium verticillioides];Gene=BNA5;Ontology_term=cytoplasm,pyridoxal phosphate binding,kynureninase activity,3-hydroxykynureninase activity,tryptophan catabolic process,quinolinate biosynthetic process,'de novo' NAD biosynthetic process from tryptophan,anthranilate metabolic process,L-kynurenine catabolic process;Ontology_id=GO:0005737,GO:0030170,GO:0030429,GO:0061981,GO:0006569,GO:0019805,GO:0034354,GO:0043420,GO:
Fv_160_4.g2619	577	ID=Fv_160_4.g2619;Description=NADH dehydrogenase [Fusarium verticillioides 7600];Gene=FPRO_13262;Ontology_term=cytosol,membrane,protein-containing complex,NADH dehydrogenase activity,alcohol dehydrogenase (NAD+) activity,acetaldehyde dehydrogenase (acetylating) activity,protein homodimerization activity,ethanol oxidation,NADH oxidation,acetaldehyde metabolic process,NADH metabolic process,alcohol catabolic process,behavioral response to ethanol;Ontology_id=GO:0005829,GO:0016020,GO:0032991,GO:0003954,GO:0004022,GO:0008774,GO:0042803,GO:0006069,GO:
Fv_160_4.g2620	487	ID=Fv_160_4.g2620;Description=hypothetical protein FVER14953_10315 [Fusarium
Fv_160_4.g2621	503	ID=Fv_160_4.g2621;Description=hypothetical protein LB503_007356 [Fusarium chuoi];Gene=AU210_011777;Ontology_term=phosphomethylpyrimidine kinase activity,thiaminase activity,thiamine biosynthetic process,phosphorylation;Ontology_id=GO:0008972,GO:0050334,GO:0009228,GO:0016310;Enzyme_code=EC:2.7.4.7,EC:3.5.99.2;Enzyme_name=phosphooxymethylpyrimidine kinase,aminopyrimidine aminohydrolase
Fv_160_4.g2622	1388	ID=Fv_160_4.g2622;Description=hypothetical protein FVER14953_10313 [Fusarium verticillioides];Gene=F25303_7445;Ontology_term=TORC2 complex,phosphomethylpyrimidine kinase activity,thiamine biosynthetic process,TOR
Fv_160_4.g2623	302	ID=Fv_160_4.g2623;Description=uricase [Fusarium verticillioides 7600];Gene=AU210_011779;Ontology_term=peroxisome,urate oxidase activity,purine nucleobase metabolic process,urate catabolic
Fv_160_4.g2624	179	ID=Fv_160_4.g2624;Description=50S ribosomal protein L13 [Fusarium verticillioides 7600];Gene=Forpi1262_v009854;Ontology_term=fungal-type vacuole,mitochondrial large ribosomal subunit,structural constituent
Fv_160_4.g2625	248	ID=Fv_160_4.g2625;Description=hypothetical protein FVEG_10310 [Fusarium verticillioides 7600];Gene=FVER53263_10310;Ontology_term=copper ion binding,superoxide metabolic

Fv_160_4.g2626	512	ID=Fv_160_4.g2626;Description=hypothetical protein FVER14953_10309 [Fusarium verticillioides]
Fv_160_4.g2627	646	ID=Fv_160_4.g2627;Description=hypothetical protein FVER14953_10308 [Fusarium verticillioides];Gene=FVER53590_10308;Ontology_term=cellular anatomical entity,glutamate-tRNA ligase activity,ATP binding,zinc ion binding,glutamyl-tRNA
Fv_160_4.g2628	434	ID=Fv_160_4.g2628;Description=DnaJ like subfamily A member 2 [Fusarium verticillioides 7600];Gene=BFJ65_g12189;Ontology_term=ATP binding,Hsp70 protein binding,metal ion binding,unfolded protein binding,protein
Fv_160_4.g2629	260	ID=Fv_160_4.g2629;Description=hypothetical protein FVEG_10306 [Fusarium verticillioides 7600];Gene=FCIRC_1494;Ontology_term=ESCRT II complex,nuclear body,Mei2 nuclear dot complex,carbon catabolite repression of transcription from RNA polymerase II promoter by glucose,karyogamy involved in conjugation with cellular fusion,protein targeting to vacuole,protein transport to vacuole involved in ubiquitin-dependent protein catabolic process via the multivesicular body sorting
Fv_160_4.g2630	281	ID=Fv_160_4.g2630;Description=hypothetical protein FOXG_11518 [Fusarium oxysporum f. sp. lycopersici 4287];Gene=BN851_0124780;Ontology_term=membrane;Ontology_id=GO:0016020
Fv_160_4.g2631	960	ID=Fv_160_4.g2631;Description=hypothetical protein FVER53263_10304 [Fusarium
Fv_160_4.g2632	1078	ID=Fv_160_4.g2632;Description=NAD-specific glutamate dehydrogenase [Fusarium verticillioides 7600];Gene=FVER53263_10303;Ontology_term=glutamate dehydrogenase (NAD+) activity,glutamate catabolic process to 2-oxoglutarate;Ontology_id=GO:0004352,GO:0019551;Enzyme_code=EC:1.4.1.3,EC:1.4.1.2;Enzyme_name=glutamate dehydrogenase
Fv_160_4.g2633	155	ID=Fv_160_4.g2633;Description=hypothetical protein FVEG_10302 [Fusarium verticillioides 7600];Gene=BFJ69_g13871;Ontology_term=mitochondrial outer membrane translocase complex,protein transmembrane transporter activity,deaminase activity,protein import into mitochondrial matrix,protein insertion into mitochondrial outer membrane;Ontology_id=GO:0005742,GO:0008320,GO:0019239,GO:0030150,GO:0045040;Enzyme_code=EC:3.5;Enzyme_name=Act
Fv_160_4.g2634	337	ID=Fv_160_4.g2634;Description=hypothetical protein FVER14953_10301 [Fusarium verticillioides];Gene=FPCIR_41;Ontology_term=deaminase
Fv_160_4.g2635	906	ID=Fv_160_4.g2635;Description=hypothetical protein FVER14953_10300 [Fusarium verticillioides];Gene=FNAPI_12504;Ontology_term=mitochondrial outer membrane,GTPase activity,GTP binding,mitochondrion organization;Ontology_id=GO:0005741,GO:0003924,GO:0005525,GO:0007005;Enzyme_code=EC:3.6.1.15;Enzyme_name=nucleosid
Fv_160_4.g2636	227	ID=Fv_160_4.g2636;Description=hypothetical protein FVEG_10299 [Fusarium verticillioides 7600];Gene=FGLOB1_14639;Ontology_term=ESCRT III complex,protein transport to vacuole involved in ubiquitin-dependent protein catabolic process via the multivesicular body sorting pathway,protein retention in Golgi apparatus,intralumenal vesicle
Fv_160_4.g2637	576	ID=Fv_160_4.g2637;Description=hypothetical protein FVER14953_10298 [Fusarium verticillioides];Gene=BFJ63_vAg11046;Ontology_term=protein serine/threonine phosphatase activity,metal ion
Fv_160_4.g2638	1208	ID=Fv_160_4.g2638;Description=hypothetical protein FVER14953_10297 [Fusarium verticillioides]
Fv_160_4.g2639	1018	ID=Fv_160_4.g2639;Description=hypothetical protein J7337_011569 [Fusarium musae];Gene=FTJAE_1183;Ontology_term=nucleus,DNA-binding transcription factor activity, RNA polymerase II-specific,zinc ion

Fv_160_4.g2640	249	ID=Fv_160_4.g2640;Description=transcription initiation factor TFIID/TFIIF subunit [Fusarium verticillioides 7600];Gene=FNAPI_9566;Ontology_term=transcription factor TFIID complex,transcription factor TFIIF complex,SWI/SNF complex,Ino80 complex,translation initiation factor activity,chromatin remodeling,regulation of transcription by RNA polymerase
Fv_160_4.g2641	242	ID=Fv_160_4.g2641;Description=hypothetical protein FVEG_10294 [Fusarium verticillioides 7600];Gene=BFJ65_g12222;Ontology_term=nucleocytoplasmic transport;Ontology_id=GO:0006913
Fv_160_4.g2642	675	ID=Fv_160_4.g2642;Description=hypothetical protein FVEG_10293 [Fusarium verticillioides 7600]
Fv_160_4.g2643	290	ID=Fv_160_4.g2643;Description=hypothetical protein FVEG_10292 [Fusarium verticillioides 7600];Gene=BFJ65_g12465;Ontology_term=actin filament,cell cortex,ATP binding,hydrolase activity,mitotic cytokinesis,meiosis II cytokinesis,regulation of G protein-coupled receptor signaling pathway,embryo development ending in birth or egg hatching,cortical actin cytoskeleton organization,locomotion;Ontology_id=GO:0005884,GO:0005938,GO:0005524,GO:0016787,GO:0000281,GO:0007111,GO:0008277,
Fv_160_4.g2644	359	ID=Fv_160_4.g2644;Description=hypothetical protein FPSE_00124 [Fusarium pseudograminearum CS3096];Gene=C2S_2759;Ontology_term=membrane,signal transduction;Ontology_id=GO:0016020,GO:0007165
Fv_160_4.g2645	578	ID=Fv_160_4.g2645;Description=dimethylaniline monooxygenase [Fusarium tjaetaba];Gene=FPCIR_30;Ontology_term=striated muscle thin filament,monooxygenase activity,ATP binding,hydrolase activity,mitotic cytokinesis,embryo development ending in birth or egg hatching,cortical actin cytoskeleton organization,cellular biosynthetic process,organonitrogen compound biosynthetic process;Ontology_id=GO:0005865,GO:0004497,GO:0005524,GO:0016787,GO:0000281,GO:0009792,GO:0030866,GO:0044249,GO:1901566;Enzyme_code=EC:1,EC:3;Enzyme_name=Oxidoreductases,Hydrolases
Fv_160_4.g2646	313	ID=Fv_160_4.g2646;Description=hypothetical protein FVER14953_10290 [Fusarium verticillioides];Gene=FPCIR_30;Ontology_term=monooxygenase
Fv_160_4.g2647	493	ID=Fv_160_4.g2647;Description=hypothetical protein J7337_011560 [Fusarium musae];Gene=FVER53263_10288;Ontology_term=kinase activity,signal
Fv_160_4.g2648	650	ID=Fv_160_4.g2648;Description=hypothetical protein FVER53263_10287 [Fusarium verticillioides];Gene=F25303_5555;Ontology_term=extracellular space,membrane,SPOTS complex,serine C-palmitoyltransferase activity,oxygen carrier activity,copper ion binding,oxidoreductase activity,pyridoxal phosphate binding,chloride ion binding,oxygen transport,sphingolipid biosynthetic process;Ontology_id=GO:0005615,GO:0016020,GO:0035339,GO:0004758,GO:0005344,GO:0005507,GO:0016491,GO:0030170,GO:
Fv_160_4.g2649	699	ID=Fv_160_4.g2649;Description=acetoacetate-CoA ligase [Fusarium verticillioides 7600];Gene=BFJ65_g12467;Ontology_term=acetoacetate-CoA ligase activity,lipid metabolic
Fv_160_4.g2650	482	ID=Fv_160_4.g2650;Description=hypothetical protein FVEG_10285 [Fusarium verticillioides 7600];Gene=HCE;Ontology_term=extracellular space,oxygen carrier activity,copper ion binding,oxidoreductase activity,chloride ion binding,oxygen
Fv_160_4.g2651	120	ID=Fv_160_4.g2651;Description=hypothetical protein FVEG_16777 [Fusarium verticillioides

Fv_160_4.g2652	86	ID=Fv_160_4.g2652;Description=hypothetical protein FVEG_16776 [Fusarium verticillioides 7600]
Fv_160_4.g2653	477	ID=Fv_160_4.g2653;Description=hypothetical protein FVER53590_10284 [Fusarium verticillioides];Gene=FNAPI_9578;Ontology_term=serine-type carboxypeptidase
Fv_160_4.g2654	284	ID=Fv_160_4.g2654;Description=N-acetylglucosaminylphosphatidylinositol deacetylase [Fusarium verticillioides 7600];Gene=BFJ63_vAg11016;Ontology_term=N-acetylglucosaminylphosphatidylinositol deacetylase activity;Ontology_id=GO:0000225;Enzyme_code=EC:3.5.1.89;Enzyme_name=N-acetylglucosaminylphosphatidylinositol deacetylase
Fv_160_4.g2655	205	ID=Fv_160_4.g2655;Description=hypothetical protein FVEG_16775 [Fusarium verticillioides
Fv_160_4.g2656	264	ID=Fv_160_4.g2656;Description=hypothetical protein FVEG_10282 [Fusarium verticillioides
Fv_160_4.g2657	945	ID=Fv_160_4.g2657;Description=CPA1 family monovalent cation:H+ antiporter [Fusarium verticillioides 7600];Gene=FVEG_10281;Ontology_term=plasma membrane,membrane raft,sodium:proton antiporter activity,response to osmotic stress,intracellular potassium ion homeostasis,sodium ion export across plasma membrane,regulation of membrane potential,potassium ion export across plasma membrane,proton export across plasma
Fv_160_4.g2658	268	ID=Fv_160_4.g2658;Description=hypothetical protein FVER14953_10280 [Fusarium verticillioides]
Fv_160_4.g2659	305	ID=Fv_160_4.g2659;Description=hypothetical protein FVER14953_10279 [Fusarium verticillioides]
Fv_160_4.g2660	334	ID=Fv_160_4.g2660;Description=hypothetical protein FVEG_10278 [Fusarium verticillioides 7600];Gene=FPANT_8367;Ontology_term=methyltransferase
Fv_160_4.g2661	506	ID=Fv_160_4.g2661;Description=hypothetical protein FVER14953_10277 [Fusarium verticillioides];Gene=FCIRC_5656;Ontology_term=membrane,monooxygenase activity,iron ion binding,methyltransferase activity,oxidoreductase activity, acting on paired donors, with incorporation or reduction of molecular oxygen,heme binding,methylation;Ontology_id=GO:0016020,GO:0004497,GO:0005506,GO:0008168,GO:0016705,GO:0020037,GO:0032259;Enzy
Fv_160_4.g2662	861	ID=Fv_160_4.g2662;Description=hypothetical protein J7337_011545 [Fusarium musae];Gene=FTJAE_1162;Ontology_term=cytoplasm,zinc ion binding;Ontology_id=GO:0005737,GO:0008270
Fv_160_4.g2663	249	ID=Fv_160_4.g2663;Description=hypothetical protein FVEG_10274 [Fusarium verticillioides
Fv_160_4.g2664	266	ID=Fv_160_4.g2664;Description=YPC1-alkaline Ceramidase [Fusarium phyllophilum];Gene=AU210_011820;Ontology_term=membrane,hydrolase activity, acting on carbon-nitrogen (but not peptide) bonds, in linear amides,ceramide metabolic
Fv_160_4.g2665	194	ID=Fv_160_4.g2665;Description=hypothetical protein J7337_011542 [Fusarium musae];Gene=hgl2;Ontology_term=plasma membrane,carbohydrate binding,cell adhesion;Ontology_id=GO:0005886,GO:0030246,GO:0007155
Fv_160_4.g2666	526	ID=Fv_160_4.g2666;Description=galactokinase [Fusarium verticillioides 7600];Gene=FSUBG_940;Ontology_term=cytoplasm,galactokinase activity,ATP binding,galactose metabolic process,sterol biosynthetic process,carbohydrate
Fv_160_4.g2667	542	ID=Fv_160_4.g2667;Description=hypothetical protein FVEG_10270 [Fusarium verticillioides
Fv_160_4.g2668	613	ID=Fv_160_4.g2668;Description=hypothetical protein FVER14953_10269 [Fusarium verticillioides]

Fv_160_4.g2669	108	ID=Fv_160_4.g2669;Description=uncharacterized protein FFB14_08156 [Fusarium fujikuroi];Gene=FDENT_14186;Ontology_term=monooxygenase
Fv_160_4.g2670	670	ID=Fv_160_4.g2670;Description=hypothetical protein H9Q72_006037 [Fusarium xylarioides]
Fv_160_4.g2671	226	ID=Fv_160_4.g2671;Description=hypothetical protein FVEG_10268 [Fusarium verticillioides 7600];Gene=FOC1_g10010446;Ontology_term=acyltransferase activity, transferring groups other than amino-acyl
Fv_160_4.g2672	144	ID=Fv_160_4.g2672;Description=hypothetical protein FVER14953_00020 [Fusarium verticillioides];Gene=dddW;Ontology_term=lyase activity;Ontology_id=GO:0016829;Enzyme_code=EC:4;Enzyme_name=Lyases
Fv_160_4.g2673	684	ID=Fv_160_4.g2673;Description=acetolactate synthase I/II/III large subunit [Fusarium verticillioides 7600];Gene=BFJ65_g12004;Ontology_term=acetolactate synthase complex,magnesium ion binding,acetolactate synthase activity,pyruvate decarboxylase activity,thiamine pyrophosphate binding,flavin adenine dinucleotide binding,isoleucine biosynthetic process,valine biosynthetic process;Ontology_id=GO:0005948,GO:0000287,GO:0003984,GO:0004737,GO:0030976,GO:0050660,GO:0009097,GO:0009099;Enzy
Fv_160_4.g2674	310	ID=Fv_160_4.g2674;Description=hypothetical protein FVER53263_10266 [Fusarium
Fv_160_4.g2675	275	ID=Fv_160_4.g2675;Description=hypothetical protein Forpe1208_v013280 [Fusarium oxysporum f. sp. rapae]
Fv_160_4.g2676	296	ID=Fv_160_4.g2676;Description=hypothetical protein FVER53590_10265 [Fusarium verticillioides];Gene=C2S_2727;Ontology_term=carboxylic ester hydrolase
Fv_160_4.g2677	68	ID=Fv_160_4.g2677;Description=hypothetical protein H9Q71_002509 [Fusarium xylarioides]
Fv_160_4.g2678	404	ID=Fv_160_4.g2678;Description=hypothetical protein J7337_011529 [Fusarium musae];Gene=FNAPI_13888;Ontology_term=O-methyltransferase activity,S-adenosylmethionine-dependent methyltransferase activity,methylation,secondary metabolite biosynthetic
Fv_160_4.g2679	396	ID=Fv_160_4.g2679;Description=hypothetical protein J7337_011528 [Fusarium musae];Gene=FANTH_5572;Ontology_term=S-adenosylmethionine-dependent methyltransferase
Fv_160_4.g2680	104	ID=Fv_160_4.g2680;Description=hypothetical protein FPHYL_13673 [Fusarium
Fv_160_4.g2681	644	ID=Fv_160_4.g2681;Description=hypothetical protein FVER14953_10261 [Fusarium verticillioides]

Fv_160_4.g2682	536	ID=Fv_160_4.g2682;Description=hypothetical protein FVER14953_10260 [Fusarium verticillioides];Gene=ced-4;Ontology_term=endopeptidase activator activity,muscle cell cellular homeostasis,regulation of development, heterochronic,embryo development ending in birth or egg hatching,caspase binding,regulation of cell size,negative regulation of execution phase of apoptosis,caspase complex,magnesium ion binding,ATP binding,activation of cysteine-type endopeptidase activity,cytosol,positive regulation of protein processing,activation of cysteine-type endopeptidase activity involved in apoptotic process,identical protein binding,defense response to Gram-negative bacterium,actin filament depolymerization,cysteine-type endopeptidase activator activity involved in apoptotic process,positive regulation of apoptotic process involved in development,nucleus,BH3 domain binding,positive regulation of synapse pruning,BH1 domain binding,perinuclear region of cytoplasm,regulation of protein stability,embryonic morphogenesis,mitochondrion,membrane,regulation of cell adhesion;Ontology_id=GO:0061133,GO:0046716,GO:0040034,GO:0009792,GO:0089720,GO:0008361,GO:1900118,GO:0008303,GO
Fv_160_4.g2683	197	ID=Fv_160_4.g2683;Description=hypothetical protein FVEG_10258 [Fusarium verticillioides 7600]
Fv_160_4.g2684	130	ID=Fv_160_4.g2684;Description=hypothetical protein FVEG_10257 [Fusarium verticillioides 7600]
Fv_160_4.g2685	204	ID=Fv_160_4.g2685;Description=PUA domain-containing protein [Fusarium verticillioides 7600];Gene=C2S_2718;Ontology_term=cytosol,RNA binding,ribosome
Fv_160_4.g2686	82	ID=Fv_160_4.g2686;Description=hypothetical protein FOXG_11570 [Fusarium oxysporum f. sp. lycopersici 4287]
Fv_160_4.g2687	273	ID=Fv_160_4.g2687;Description=hypothetical protein J7337_011520 [Fusarium
Fv_160_4.g2688	1168	ID=Fv_160_4.g2688;Description=hypothetical protein FVER53263_10253 [Fusarium verticillioides];Gene=FPANT_2317;Ontology_term=collagen trimer,cytoplasm;Ontology_id=GO:0005581,GO:0005737
Fv_160_4.g2689	208	ID=Fv_160_4.g2689;Description=hypothetical protein LZL87_003887 [Fusarium oxysporum];Gene=FDENT_303;Ontology_term=cell division site,old growing cell tip,new growing cell tip,GTPase activity,GTP binding,small GTPase mediated signal transduction,establishment or maintenance of actin cytoskeleton polarity,regulation of fungal-type cell wall (1->3)-alpha-glucan biosynthetic process,negative regulation of glucose mediated signaling pathway;Ontology_id=GO:0032153,GO:0035840,GO:0035841,GO:0003924,GO:0005525,GO:0007264,GO:0030950,GO:0070610,GO:
Fv_160_4.g2690	153	ID=Fv_160_4.g2690;Description=RING finger domain-containing protein [Fusarium phyllophilum];Gene=FOC1_g10010426;Ontology_term=mitochondrion,structural constituent of ribosome,mitochondrial
Fv_160_4.g2691	429	ID=Fv_160_4.g2691;Description=hypothetical protein J7337_011516 [Fusarium musae]
Fv_160_4.g2692	253	ID=Fv_160_4.g2692;Description=hypothetical protein FVEG_10249 [Fusarium verticillioides 7600]
Fv_160_4.g2693	727	ID=Fv_160_4.g2693;Description=hypothetical protein FVER14953_10248 [Fusarium verticillioides];Gene=FVER53263_10248;Ontology_term=transketolase activity,metal ion binding,purine nucleotide metabolic process,carbohydrate derivative metabolic
Fv_160_4.g2694	293	ID=Fv_160_4.g2694;Description=hypothetical protein FVEG_10247 [Fusarium verticillioides 7600];Gene=Forpi1262_v009923;Ontology_term=oxidoreductase activity,cellular biosynthetic
Fv_160_4.g2695	441	ID=Fv_160_4.g2695;Description=hypothetical protein FVER14953_10246 [Fusarium verticillioides]

Fv_160_4.g2696	516	ID=Fv_160_4.g2696;Description=hypothetical protein FVEG_10245 [Fusarium verticillioides 7600];Gene=FPANT_2309;Ontology_term=membrane,transmembrane transporter activity,carbohydrate transport,transmembrane
Fv_160_4.g2697	604	ID=Fv_160_4.g2697;Description=hypothetical protein FVER53590_10244 [Fusarium verticillioides];Gene=BFJ65_g11961;Ontology_term=nucleus,DNA-binding transcription factor activity, RNA polymerase II-specific,glycerone kinase activity,ATP binding,zinc ion binding,triokinase activity,regulation of transcription by RNA polymerase II,phosphorylation,anaerobic glycerol catabolic process;Ontology_id=GO:0005634,GO:0000981,GO:0004371,GO:0005524,GO:0008270,GO:0050354,GO:0006357,GO:0016310,GO:
Fv_160_4.g2698	553	ID=Fv_160_4.g2698;Description=hypothetical protein FVEG_10243 [Fusarium verticillioides 7600];Gene=C2S_2705;Ontology_term=nucleus,DNA-binding transcription factor activity, RNA polymerase II-specific,glycerone kinase activity,ATP binding,zinc ion binding,regulation of transcription by RNA polymerase II,phosphorylation,anaerobic glycerol
Fv_160_4.g2699	279	ID=Fv_160_4.g2699;Description=hypothetical protein FVER53263_10242 [Fusarium verticillioides];Gene=FANTH_5990;Ontology_term=nucleus,membrane,DNA-binding transcription factor activity, RNA polymerase II-specific,glycerone kinase activity,ATP binding,zinc ion binding,regulation of transcription by RNA polymerase II,phosphorylation,anaerobic glycerol catabolic process;Ontology_id=GO:0005634,GO:0016020,GO:0000981,GO:0004371,GO:0005524,GO:0008270,GO:0006357,GO:0016310,GO:
Fv_160_4.g2700	220	ID=Fv_160_4.g2700;Description=hypothetical protein FVEG_10241 [Fusarium verticillioides 7600];Gene=FMUND_7819;Ontology_term=anatomical structure development;Ontology_id=GO:0048856
Fv_160_4.g2701	316	ID=Fv_160_4.g2701;Description=hypothetical protein FVER14953_10240 [Fusarium
Fv_160_4.g2702	81	ID=Fv_160_4.g2702;Description=hypothetical protein LB506_008802 [Fusarium annulatum]
Fv_160_4.g2703	173	ID=Fv_160_4.g2703;Description=hypothetical protein FVER53590_25050 [Fusarium
Fv_160_4.g2704	486	ID=Fv_160_4.g2704;Description=hypothetical protein FVER14953_10238 [Fusarium verticillioides];Gene=FPCIR_7848;Ontology_term=glycosyltransferase
Fv_160_4.g2705	481	ID=Fv_160_4.g2705;Description=hypothetical protein J7337_011503 [Fusarium musae];Gene=FDENT_316;Ontology_term=nucleic
Fv_160_4.g2706	789	ID=Fv_160_4.g2706;Description=hypothetical protein FVER53590_10235 [Fusarium verticillioides];Gene=FDENT_317;Ontology_term=ATP binding,ATP hydrolysis
Fv_160_4.g2707	920	ID=Fv_160_4.g2707;Description=hypothetical protein FVEG_10234 [Fusarium verticillioides
Fv_160_4.g2708	714	ID=Fv_160_4.g2708;Description=potassium transporter TRK-1 [Fusarium tjaetaba];Gene=FPHYL_9581;Ontology_term=plasma membrane,potassium ion transmembrane transporter activity,intracellular potassium ion homeostasis,potassium ion
Fv_160_4.g2709	108	ID=Fv_160_4.g2709;Description=hypothetical protein FVEG_10231 [Fusarium verticillioides 7600];Gene=FGADI_809;Ontology_term=plasma membrane,potassium ion transmembrane transporter activity,intracellular
Fv_160_4.g2710	1094	ID=Fv_160_4.g2710;Description=hypothetical protein FVER53263_10229 [Fusarium verticillioides];Gene=BFJ70_g15000;Ontology_term=membrane,hydrolase activity,transmembrane

Fv_160_4.g2711	507	ID=Fv_160_4.g2711;Description=unnamed protein product [Fusarium fujikuroi];Gene=FVER53263_10227;Ontology_term=protein serine/threonine kinase activity,ATP binding,phosphorylation,mRNA cis splicing, via spliceosome;Ontology_id=GO:0004674,GO:0005524,GO:0016310,GO:0045292;Enzyme_code=EC:2.7.11.1;Enzyme_name=non-
Fv_160_4.g2712	821	ID=Fv_160_4.g2712;Description=CMGC/DYRK/PRP4 protein kinase [Fusarium verticillioides 7600];Gene=FOC1_g10010403;Ontology_term=protein serine/threonine kinase activity,ATP binding,phosphorylation,mRNA cis splicing, via
Fv_160_4.g2713	68	ID=Fv_160_4.g2713;Description=hypothetical protein FVEG_10225 [Fusarium verticillioides 7600]
Fv_160_4.g2714	269	ID=Fv_160_4.g2714;Description=regulatory role in lipid phosphate metabolism [Fusarium
Fv_160_4.g2715	706	ID=Fv_160_4.g2715;Description=hypothetical protein FVEG_10222 [Fusarium verticillioides 7600]
Fv_160_4.g2716	78	ID=Fv_160_4.g2716;Description=hypothetical protein IWW34DRAFT_820985 [Fusarium oxysporum f. sp. albedinis]
Fv_160_4.g2717	189	ID=Fv_160_4.g2717;Description=hypothetical protein FVEG_10221 [Fusarium verticillioides 7600]
Fv_160_4.g2718	253	ID=Fv_160_4.g2718;Description=hypothetical protein FVER53263_10220 [Fusarium verticillioides]
Fv_160_4.g2719	249	ID=Fv_160_4.g2719;Description=hypothetical protein FVER53590_10219 [Fusarium verticillioides]
Fv_160_4.g2720	683	ID=Fv_160_4.g2720;Description=hypothetical protein FNAPI_10783 [Fusarium napiforme]
Fv_160_4.g2721	280	ID=Fv_160_4.g2721;Description=hypothetical protein FVER53263_10216 [Fusarium verticillioides];Gene=deg-1;Ontology_term=ligand-gated sodium channel activity,response to acidic pH,sodium ion transmembrane transport,response to
Fv_160_4.g2722	288	ID=Fv_160_4.g2722;Description=3-oxoacyl-[acyl-carrier protein] reductase [Fusarium verticillioides 7600]
Fv_160_4.g2723	240	ID=Fv_160_4.g2723;Description=dolichol-phosphate mannosyltransferase [Fusarium verticillioides 7600];Gene=FTJAE_6197;Ontology_term=endoplasmic reticulum,dolichyl-phosphate beta-D-mannosyltransferase
Fv_160_4.g2724	155	ID=Fv_160_4.g2724;Description=hypothetical protein FVEG_16756 [Fusarium verticillioides 7600]
Fv_160_4.g2725	236	ID=Fv_160_4.g2725;Description=hypothetical protein FVER53590_10213 [Fusarium verticillioides]
Fv_160_4.g2726	278	ID=Fv_160_4.g2726;Description=hypothetical protein FVER53263_10212 [Fusarium verticillioides];Gene=FVER53263_10212;Ontology_term=kinase activity,protein-ribulosamine 3-kinase
Fv_160_4.g2727	177	ID=Fv_160_4.g2727;Description=hypothetical protein FVER14953_10211 [Fusarium verticillioides];Gene=FOC4_g10007934;Ontology_term=catalytic activity,nucleobase-containing compound metabolic
Fv_160_4.g2728	1281	ID=Fv_160_4.g2728;Description=hypothetical protein LB503_007259 [Fusarium chuoi];Gene=BFJ70_g15939;Ontology_term=endoplasmic reticulum membrane,hydrolase activity, acting on ester bonds,protein
Fv_160_4.g2729	1013	ID=Fv_160_4.g2729;Description=hypothetical protein FVEG_10208 [Fusarium verticillioides 7600];Gene=FVER53590_10208;Ontology_term=membrane,calcium-activated cation channel activity,monoatomic cation
Fv_160_4.g2730	484	ID=Fv_160_4.g2730;Description=hypothetical protein FVER53590_10207 [Fusarium verticillioides]
Fv_160_4.g2731	1132	ID=Fv_160_4.g2731;Description=hypothetical protein FVER14953_10206 [Fusarium
Fv_160_4.g2732	239	ID=Fv_160_4.g2732;Description=hypothetical protein FVEG_10205 [Fusarium verticillioides 7600];Gene=FVER53263_10205;Ontology_term=nucleus,preribosome, large subunit precursor,preribosome, small subunit

Fv_160_4.g2733	389	ID=Fv_160_4.g2733;Description=GPN-loop GTPase [Fusarium verticillioides 7600];Gene=FVER53263_10204;Ontology_term=nucleus,cytoplasm,GTPase activity,GTP binding,ATP hydrolysis activity,protein import into nucleus,mitotic sister chromatid cohesion;Ontology_id=GO:0005634,GO:0005737,GO:0003924,GO:0005525,GO:0016887,GO:0006606,GO:0007064;Enzyme_code=E
Fv_160_4.g2734	72	ID=Fv_160_4.g2734;Description=DNA-directed RNA polymerase and III subunit RPABC4 [Fusarium sp. NRRL 25303];Gene=FIESC28_01716;Ontology_term=DNA-directed RNA polymerase complex,DNA binding,DNA-directed 5'-3' RNA polymerase activity,zinc ion binding,DNA-templated
Fv_160_4.g2735	195	ID=Fv_160_4.g2735;Description=hypothetical protein FVER53590_10202 [Fusarium
Fv_160_4.g2736	311	ID=Fv_160_4.g2736;Description=hypothetical protein FVER53263_10201 [Fusarium verticillioides];Gene=FFUJ_09460;Ontology_term=oxygen carrier activity,oxygen binding,heme binding,metal ion binding,protein
Fv_160_4.g2737	145	ID=Fv_160_4.g2737;Description=hypothetical protein J7337_011473 [Fusarium musae]
Fv_160_4.g2738	991	ID=Fv_160_4.g2738;Description=hypothetical protein FVER14953_10199 [Fusarium verticillioides]
Fv_160_4.g2739	159	ID=Fv_160_4.g2739;Description=ubiquitin-conjugating enzyme E2 D/E [Fusarium verticillioides 7600];Gene=FMEXI_9640;Ontology_term=ligase activity;Ontology_id=GO:0016874;Enzyme_code=EC:6;Enzyme_name=Ligases
Fv_160_4.g2740	701	ID=Fv_160_4.g2740;Description=hypothetical protein FVEG_10197 [Fusarium verticillioides 7600];Gene=FANTH_9820;Ontology_term=exomer complex,Golgi to vacuole transport,Golgi to plasma membrane protein
Fv_160_4.g2741	2012	ID=Fv_160_4.g2741;Description=hypothetical protein FVER14953_10196 [Fusarium verticillioides];Gene=FPCIR_8392;Ontology_term=nucleus,chromatin organization;Ontology_id=GO:0005634,GO:0006325
Fv_160_4.g2742	290	ID=Fv_160_4.g2742;Description=hypothetical protein FVER14953_21678 [Fusarium verticillioides]
Fv_160_4.g2743	485	ID=Fv_160_4.g2743;Description=ATP-dependent rRNA helicase RRP3 [Fusarium verticillioides 7600];Gene=FACUT_11171;Ontology_term=nucleic acid binding,RNA helicase activity,ATP binding,hydrolase activity,rRNA
Fv_160_4.g2744	454	ID=Fv_160_4.g2744;Description=hypothetical protein FVEG_10194 [Fusarium verticillioides 7600];Gene=FTJAE_6175;Ontology_term=rRNA binding,rRNA processing;Ontology_id=GO:0019843,GO:0006364
Fv_160_4.g2745	479	ID=Fv_160_4.g2745;Description=hypothetical protein FVEG_10193 [Fusarium verticillioides 7600];Gene=FPHYL_12658;Ontology_term=protein serine/threonine phosphatase activity,metal ion
Fv_160_4.g2746	712	ID=Fv_160_4.g2746;Description=hypothetical protein FVER53590_10192 [Fusarium verticillioides];Gene=FVEG_10192;Ontology_term=cytoplasm,microtubule,membrane,structural constituent of cytoskeleton,oxidoreductase activity, acting on the CH-OH group of donors, NAD or NADP as acceptor,NAD binding,cytoskeleton organization;Ontology_id=GO:0005737,GO:0005874,GO:0016020,GO:0005200,GO:0016616,GO:0051287,GO:0007010;Enzyme_cod
Fv_160_4.g2747	340	ID=Fv_160_4.g2747;Description=hypothetical protein FVER14953_10191 [Fusarium verticillioides];Gene=FVEG_10191;Ontology_term=oxidoreductase activity, acting on the CH-OH group of donors, NAD or NADP as
Fv_160_4.g2748	299	ID=Fv_160_4.g2748;Description=hypothetical protein J7337_011460 [Fusarium musae]
Fv_160_4.g2749	396	ID=Fv_160_4.g2749;Description=hypothetical protein FVEG_10190 [Fusarium verticillioides

Fv_160_4.g2750	583	ID=Fv_160_4.g2750;Description=hypothetical protein FVER14953_10189 [Fusarium verticillioides];Gene=FDENT_8106;Ontology_term=transferase
Fv_160_4.g2751	146	ID=Fv_160_4.g2751;Description=hypothetical protein FVER14953_10188 [Fusarium verticillioides]
Fv_160_4.g2752	152	ID=Fv_160_4.g2752;Description=hypothetical protein FVER14953_10187 [Fusarium
Fv_160_4.g2753	656	ID=Fv_160_4.g2753;Description=hypothetical protein FVEG_16745 [Fusarium verticillioides 7600]
Fv_160_4.g2754	324	ID=Fv_160_4.g2754;Description=hypothetical protein FVER14953_21679 [Fusarium verticillioides]
Fv_160_4.g2755	526	ID=Fv_160_4.g2755;Description=hypothetical protein FVER53590_30364 [Fusarium verticillioides];Gene=FPANT_6184;Ontology_term=transferase
Fv_160_4.g2756	314	ID=Fv_160_4.g2756;Description=hypothetical protein FVEG_16743 [Fusarium verticillioides 7600];Gene=FDENT_8100;Ontology_term=hydrolase
Fv_160_4.g2757	330	ID=Fv_160_4.g2757;Description=hypothetical protein FVEG_10184 [Fusarium verticillioides 7600];Gene=FTJAE_11026;Ontology_term=S-adenosylmethionine-dependent methyltransferase
Fv_160_4.g2758	432	ID=Fv_160_4.g2758;Description=hypothetical protein FVEG_10183 [Fusarium verticillioides 7600]
Fv_160_4.g2759	286	ID=Fv_160_4.g2759;Description=endo-1,3(4)-beta-glucanase [Fusarium verticillioides 7600];Gene=FNAPI_8435;Ontology_term=hydrolase activity, hydrolyzing O-glycosyl compounds,carbohydrate metabolic
Fv_160_4.g2760	221	ID=Fv_160_4.g2760;Description=hypothetical protein FVEG_16742 [Fusarium verticillioides
Fv_160_4.g2761	448	ID=Fv_160_4.g2761;Description=hypothetical protein FVER53590_10181 [Fusarium verticillioides];Gene=FVEG_10181;Ontology_term=FMN binding,oxidoreductase
Fv_160_4.g2762	185	ID=Fv_160_4.g2762;Description=hypothetical protein FVEG_16741 [Fusarium verticillioides 7600];Gene=FMAN_12823;Ontology_term=membrane,monooxygenase activity,iron ion binding,oxidoreductase activity, acting on paired donors, with incorporation or reduction of molecular oxygen,heme binding;Ontology_id=GO:0016020,GO:0004497,GO:0005506,GO:0016705,GO:0020037;Enzyme_code=EC:1.14;Enzyme_name=Actin
Fv_160_4.g2763	386	ID=Fv_160_4.g2763;Description=hypothetical protein FVEG_10180 [Fusarium verticillioides 7600]
Fv_160_4.g2764	378	ID=Fv_160_4.g2764;Description=hypothetical protein FVEG_16740 [Fusarium verticillioides 7600]
Fv_160_4.g2765	422	ID=Fv_160_4.g2765;Description=hypothetical protein FVER14953_10179 [Fusarium verticillioides];Gene=CEK26_013329;Ontology_term=ATP binding,ATP hydrolysis
Fv_160_4.g2766	1428	ID=Fv_160_4.g2766;Description=hypothetical protein FVER53590_10178 [Fusarium verticillioides]

Fv_160_4.g2767	333	ID=Fv_160_4.g2767;Description=hypothetical protein FVEG_16739 [Fusarium verticillioides 7600];Gene=FMUND_11899;Ontology_term=positive regulation of MAP kinase activity,protein phosphatase binding,oxidoreductase activity, acting on the CH-OH group of donors, NAD or NADP as acceptor,protein serine/threonine phosphatase complex,positive regulation of oocyte development,positive regulation of meiotic cell cycle process involved in oocyte maturation,spermatogenesis,fatty acid metabolic process,masculinization of hermaphroditic germ-line,regulation of germ cell proliferation,sex differentiation,cytoplasm,protein localization to chromatin,positive regulation of gene expression,membrane,male somatic sex determination,Cul2-RING ubiquitin ligase complex,NAD+
Fv_160_4.g2768	340	ID=Fv_160_4.g2768;Description=hypothetical protein FVEG_10177 [Fusarium verticillioides 7600];Gene=FTJAE_11015;Ontology_term=nucleotide binding,oxidoreductase activity, acting on
Fv_160_4.g2769	528	ID=Fv_160_4.g2769;Description=hypothetical protein FVER14953_21680 [Fusarium verticillioides];Gene=FMEXI_7781;Ontology_term=transferase
Fv_160_4.g2770	1093	ID=Fv_160_4.g2770;Description=hypothetical protein FNAPI_7517 [Fusarium napiforme];Gene=FTJAE_11013;Ontology_term=transferase
Fv_160_4.g2771	356	ID=Fv_160_4.g2771;Description=hypothetical protein FVEG_10175 [Fusarium verticillioides 7600];Gene=F52700_11390;Ontology_term=S-adenosylmethionine-dependent methyltransferase
Fv_160_4.g2772	344	ID=Fv_160_4.g2772;Description=hypothetical protein FVER53590_10174 [Fusarium verticillioides];Gene=F53441_7144;Ontology_term=S-adenosylmethionine-dependent methyltransferase activity,carbon-oxygen lyase activity, acting on
Fv_160_4.g2773	150	ID=Fv_160_4.g2773;Description=hypothetical protein FNYG_11949 [Fusarium nygamai]
Fv_160_4.g2774	414	ID=Fv_160_4.g2774;Description=hypothetical protein FVEG_10172 [Fusarium verticillioides 7600]
Fv_160_4.g2775	482	ID=Fv_160_4.g2775;Description=hypothetical protein FVER53263_10171 [Fusarium verticillioides]
Fv_160_4.g2776	677	ID=Fv_160_4.g2776;Description=hypothetical protein J7337_011434 [Fusarium musae]
Fv_160_4.g2777	535	ID=Fv_160_4.g2777;Description=hypothetical protein J7337_011433 [Fusarium musae]
Fv_160_4.g2778	168	ID=Fv_160_4.g2778;Description=hypothetical protein FVEG_10167 [Fusarium verticillioides 7600];Gene=gpd-1;Ontology_term=cytosol,glyceraldehyde-3-phosphate dehydrogenase (NAD+) (phosphorylating) activity,adenyl nucleotide binding,glycolytic process;Ontology_id=GO:0005829,GO:0004365,GO:0030554,GO:0006096;Enzyme_code=EC:1.2.1.59,EC:1.2.1.12;Enzyme_name=gly
Fv_160_4.g2779	220	ID=Fv_160_4.g2779;Description=hypothetical protein FVEG_16736 [Fusarium verticillioides 7600]
Fv_160_4.g2780	298	ID=Fv_160_4.g2780;Description=hypothetical protein FVEG_10166 [Fusarium verticillioides
Fv_160_4.g2781	244	ID=Fv_160_4.g2781;Description=hypothetical protein FVER53263_10165 [Fusarium verticillioides];Gene=FANTH_9783;Ontology_term=signal peptidase complex,serine-type endopeptidase activity,signal peptide processing,protein targeting to

Fv_160_4.g2782	98	ID=Fv_160_4.g2782;Description=hypothetical protein FVEG_10164 [Fusarium verticillioides 7600];Gene=gpd-3;Ontology_term=cytosol,glyceraldehyde-3-phosphate dehydrogenase (NAD+) (phosphorylating) activity,NADP binding,NAD binding,glucose metabolic process,glycolytic process;Ontology_id=GO:0005829,GO:0004365,GO:0050661,GO:0051287,GO:0006006,GO:0006096;Enzyme_code=EC:1.2.1.59,EC:1
Fv_160_4.g2783	331	ID=Fv_160_4.g2783;Description=hypothetical protein FVEG_10163 [Fusarium verticillioides
Fv_160_4.g2784	290	ID=Fv_160_4.g2784;Description=hypothetical protein FVEG_10162 [Fusarium verticillioides 7600];Gene=gpd-4;Ontology_term=cytosol,glyceraldehyde-3-phosphate dehydrogenase (NAD+) (phosphorylating) activity,NADP binding,NAD binding,glucose metabolic process,glycolytic process;Ontology_id=GO:0005829,GO:0004365,GO:0050661,GO:0051287,GO:0006006,GO:0006096;Enzyme_code=EC:1.2.1.59,EC:1
Fv_160_4.g2785	200	ID=Fv_160_4.g2785;Description=hypothetical protein FVEG_10161 [Fusarium verticillioides 7600]
Fv_160_4.g2786	753	ID=Fv_160_4.g2786;Description=hypothetical protein FVEG_10160 [Fusarium verticillioides 7600];Gene=FNAPI_10637;Ontology_term=TBP-class protein binding,transcription preinitiation complex
Fv_160_4.g2787	575	ID=Fv_160_4.g2787;Description=hypothetical protein FVER14953_10159 [Fusarium verticillioides]
Fv_160_4.g2788	805	ID=Fv_160_4.g2788;Description=hypothetical protein J7337_011423 [Fusarium musae];Gene=FNYG_12870;Ontology_term=nucleosome,nucleus,cytoskeleton,DNA binding,microtubule motor activity,ATP binding,microtubule binding,structural constituent of chromatin,protein heterodimerization activity,nucleosome
Fv_160_4.g2789	970	ID=Fv_160_4.g2789;Description=hypothetical protein FVER53590_10155 [Fusarium verticillioides]
Fv_160_4.g2790	257	ID=Fv_160_4.g2790;Description=hypothetical protein FVEG_10154 [Fusarium verticillioides
Fv_160_4.g2791	418	ID=Fv_160_4.g2791;Description=phosphoglycerate kinase [Fusarium verticillioides 7600];Gene=HZS61_016722;Ontology_term=mitochondrion,phosphoglycerate kinase activity,ATP binding,gluconeogenesis,glycolytic process,phosphorylation;Ontology_id=GO:0005739,GO:0004618,GO:0005524,GO:0006094,GO:0006096,GO:0016310;Enzyme_code
Fv_160_4.g2792	87	ID=Fv_160_4.g2792;Description=26 proteasome complex subunit DSS1 [Fusarium oxysporum f. sp. lycopersici 4287];Gene=FANTH_8013;Ontology_term=nucleus,proteasome regulatory particle, lid subcomplex,double-strand break repair via homologous recombination,mRNA export from nucleus,proteasome
Fv_160_4.g2793	190	ID=Fv_160_4.g2793;Description=hypothetical protein FVEG_10152 [Fusarium verticillioides 7600];Gene=FANTH_8012;Ontology_term=phosphoprotein phosphatase
Fv_160_4.g2794	277	ID=Fv_160_4.g2794;Description=hypothetical protein FVER14953_10151 [Fusarium verticillioides];Gene=FRV6_13976;Ontology_term=nucleus,cytoplasm,catalytic activity,unfolded protein binding,response to
Fv_160_4.g2795	117	ID=Fv_160_4.g2795;Description=hypothetical protein FVER53590_30370 [Fusarium verticillioides]
Fv_160_4.g2796	357	ID=Fv_160_4.g2796;Description=hypothetical protein FVEG_16730 [Fusarium verticillioides 7600]
Fv_160_4.g2797	247	ID=Fv_160_4.g2797;Description=hypothetical protein FVER14953_20405 [Fusarium
Fv_160_4.g2798	532	ID=Fv_160_4.g2798;Description=hypothetical protein FVEG_10149 [Fusarium verticillioides 7600];Gene=FVER53263_10149;Ontology_term=oxidoreductase activity,FAD

Fv_160_4.g2799	422	ID=Fv_160_4.g2799;Description=hypothetical protein FVER14953_10148 [Fusarium verticillioides];Gene=F25303_1311;Ontology_term=membrane,metal ion transmembrane transporter activity,metal ion
Fv_160_4.g2800	123	ID=Fv_160_4.g2800;Description=f-box domain-containing protein [Fusarium pseudoanthophilum]
Fv_160_4.g2801	197	ID=Fv_160_4.g2801;Description=hypothetical protein FVER14953_10147 [Fusarium verticillioides]
Fv_160_4.g2802	509	ID=Fv_160_4.g2802;Description=hypothetical protein FVER53590_10146 [Fusarium verticillioides];Gene=FMUND_11865;Ontology_term=endonuclease activity,exonuclease
Fv_160_4.g2803	327	ID=Fv_160_4.g2803;Description=hypothetical protein FVER53590_10145 [Fusarium verticillioides];Gene=FPRO05_08616;Ontology_term=hydrolase
Fv_160_4.g2804	297	ID=Fv_160_4.g2804;Description=hypothetical protein FVEG_10144 [Fusarium verticillioides 7600]
Fv_160_4.g2805	546	ID=Fv_160_4.g2805;Description=hypothetical protein FVEG_10143 [Fusarium verticillioides 7600];Gene=FTJAE_13621;Ontology_term=membrane,amino acid transport,transmembrane
Fv_160_4.g2806	1057	ID=Fv_160_4.g2806;Description=pre-mRNA-splicing factor cwf19 [Fusarium coicis];Gene=P01512;Ontology_term=Prp19 complex,extracellular region,U2-type spliceosomal complex,cell-cell junction,basolateral plasma membrane,apical plasma membrane,lateral plasma membrane,receptor complex,post-mRNA release spliceosomal complex,epidermal growth factor receptor activity,ATP binding,regulation of DNA-templated transcription,epidermal growth factor receptor signaling pathway,positive regulation of cell population proliferation,determination of adult lifespan,phosphorylation,neurogenesis,sleep,male genitalia development,ovulation,positive regulation of proteasomal ubiquitin-dependent protein catabolic process,positive regulation of vulval development,defense response to bacterium,negative regulation of apoptotic process,innate immune response,vulval cell fate specification;Ontology_id=GO:0000974,GO:0005576,GO:0005684,GO:0005911,GO:0016323,GO:0016324,GO:0016328,GO:0043235,
Fv_160_4.g2807	870	ID=Fv_160_4.g2807;Description=hypothetical protein FVER53590_10140 [Fusarium verticillioides];Gene=BFJ70_g7504;Ontology_term=hydrolase
Fv_160_4.g2808	339	ID=Fv_160_4.g2808;Description=hypothetical protein FVEG_10139 [Fusarium verticillioides 7600];Gene=F25303_1302;Ontology_term=membrane,iron ion binding,oxidoreductase activity,lipid biosynthetic process,cellular lipid metabolic
Fv_160_4.g2809	699	ID=Fv_160_4.g2809;Description=hypothetical protein FVER53590_10138 [Fusarium
Fv_160_4.g2810	252	ID=Fv_160_4.g2810;Description=hypothetical protein FDENT_5030 [Fusarium
Fv_160_4.g2811	254	ID=Fv_160_4.g2811;Description=hypothetical protein J7337_011404 [Fusarium musae];Gene=FANTH_7995;Ontology_term=double-
Fv_160_4.g2812	243	ID=Fv_160_4.g2812;Description=hypothetical protein FVEG_10136 [Fusarium verticillioides 7600]
Fv_160_4.g2813	428	ID=Fv_160_4.g2813;Description=L-lactate dehydrogenase (cytochrome) [Fusarium verticillioides 7600];Gene=FPANT_12479;Ontology_term=monooxygenase activity,FMN
Fv_160_4.g2814	728	ID=Fv_160_4.g2814;Description=hypothetical protein FVEG_10134 [Fusarium verticillioides 7600];Gene=FSUBG_8046;Ontology_term=nucleus,DNA binding,zinc ion binding,DNA-binding transcription factor activity, RNA

Fv_160_4.g2815	193	ID=Fv_160_4.g2815;Description=hypothetical protein FVEG_10133, partial [Fusarium verticillioides 7600];Gene=FOXG_11690;Ontology_term=protein kinase activity,ATP
Fv_160_4.g2816	309	ID=Fv_160_4.g2816;Description=hypothetical protein FVEG_16727 [Fusarium verticillioides 7600]
Fv_160_4.g2817	350	ID=Fv_160_4.g2817;Description=hypothetical protein FVEG_10132 [Fusarium verticillioides 7600];Gene=FPRO_13483;Ontology_term=hydrolase
Fv_160_4.g2818	582	ID=Fv_160_4.g2818;Description=AAT family amino acid transporter [Fusarium verticillioides 7600];Gene=FPRO_13482;Ontology_term=membrane,transmembrane transporter activity,amino acid transport,transmembrane
Fv_160_4.g2819	402	ID=Fv_160_4.g2819;Description=hypothetical protein FVEG_10131 [Fusarium verticillioides
Fv_160_4.g2820	1087	ID=Fv_160_4.g2820;Description=hypothetical protein FVER53590_10129 [Fusarium verticillioides];Gene=FVER53263_10129;Ontology_term=amidase activity,indoleacetamide hydrolase
Fv_160_4.g2821	497	ID=Fv_160_4.g2821;Description=hypothetical protein FVER53263_10128 [Fusarium verticillioides];Gene=FTJAE_6494;Ontology_term=membrane,transmembrane transporter activity,transmembrane
Fv_160_4.g2822	446	ID=Fv_160_4.g2822;Description=hypothetical protein FVER53263_10127 [Fusarium verticillioides];Gene=FVER53263_10127;Ontology_term=sulfuric ester hydrolase
Fv_160_4.g2823	553	ID=Fv_160_4.g2823;Description=hypothetical protein FVEG_10126 [Fusarium verticillioides 7600];Gene=FFUJ_09360;Ontology_term=nucleus,DNA binding,zinc ion binding,DNA-binding transcription factor activity, RNA
Fv_160_4.g2824	501	ID=Fv_160_4.g2824;Description=hypothetical protein FVER14953_10125 [Fusarium verticillioides];Gene=FOXB_15255;Ontology_term=plasma membrane,glycerophosphodiester transmembrane transporter activity,glycerol-3-phosphate transmembrane transporter activity,glycerophosphodiester transmembrane transport,glycerol-3-
Fv_160_4.g2825	552	ID=Fv_160_4.g2825;Description=hypothetical protein FVER53263_10124 [Fusarium verticillioides];Gene=FocTR4_00014508;Ontology_term=monooxygenase activity,iron ion binding,oxidoreductase activity, acting on paired donors, with incorporation or reduction of molecular oxygen,heme binding;Ontology_id=GO:0004497,GO:0005506,GO:0016705,GO:0020037;Enzyme_code=EC:1.14;Enzyme_name=Acting on paired
Fv_160_4.g2826	162	ID=Fv_160_4.g2826;Description=hypothetical protein FVEG_10124 [Fusarium verticillioides 7600]
Fv_160_4.g2827	510	ID=Fv_160_4.g2827;Description=hypothetical protein FVER53263_10123 [Fusarium verticillioides];Gene=FTJAE_6496;Ontology_term=membrane,S-adenosylmethionine-dependent methyltransferase
Fv_160_4.g2828	208	ID=Fv_160_4.g2828;Description=hypothetical protein FVEG_10122 [Fusarium verticillioides
Fv_160_4.g2829	428	ID=Fv_160_4.g2829;Description=hypothetical protein FVER14953_10121 [Fusarium verticillioides];Gene=FFC1_09015;Ontology_term=arylesterase
Fv_160_4.g2830	525	ID=Fv_160_4.g2830;Description=hypothetical protein FVER53590_10120 [Fusarium verticillioides];Gene=FFC1_09016;Ontology_term=N,N-dimethylaniline monooxygenase activity,flavin adenine dinucleotide binding,NADP binding;Ontology_id=GO:0004499,GO:0050660,GO:0050661;Enzyme_code=EC:1.14.13.8;Enzyme_name=flavin-

Fv_160_4.g2831	288	ID=Fv_160_4.g2831;Description=hypothetical protein FVER53590_10119 [Fusarium verticillioides];Gene=FVER53590_10119;Ontology_term=metallopeptidase
Fv_160_4.g2832	161	ID=Fv_160_4.g2832;Description=hypothetical protein FVEG_10118 [Fusarium verticillioides 7600]
Fv_160_4.g2833	269	ID=Fv_160_4.g2833;Description=hypothetical protein FVEG_16724 [Fusarium verticillioides 7600]
Fv_160_4.g2834	572	ID=Fv_160_4.g2834;Description=hypothetical protein FVEG_10117 [Fusarium verticillioides 7600]
Fv_160_4.g2835	358	ID=Fv_160_4.g2835;Description=hypothetical protein FVEG_10116 [Fusarium verticillioides 7600];Gene=BFJ69_g4370;Ontology_term=extracellular region,lysozyme activity,metabolic process,killing of cells of another organism,defense response to
Fv_160_4.g2836	569	ID=Fv_160_4.g2836;Description=hypothetical protein FVER53590_10115 [Fusarium verticillioides];Gene=FOXB_15179;Ontology_term=extracellular region,metallocarboxypeptidase activity,metal ion
Fv_160_4.g2837	703	ID=Fv_160_4.g2837;Description=hypothetical protein FVER53263_10114 [Fusarium
Fv_160_4.g2838	73	ID=Fv_160_4.g2838;Description=hypothetical protein FVEG_10113 [Fusarium verticillioides 7600]
Fv_160_4.g2839	255	ID=Fv_160_4.g2839;Description=hypothetical protein FVER14953_10112 [Fusarium verticillioides];Gene=BFJ65_g12840;Ontology_term=extracellular region,membrane;Ontology_id=GO:0005576,GO:0016020
Fv_160_4.g2840	580	ID=Fv_160_4.g2840;Description=hypothetical protein FVER53263_10111 [Fusarium verticillioides];Gene=LOX2;Ontology_term=nucleus,DNA-binding transcription factor activity, RNA polymerase II-specific,DNA binding,oxidoreductase activity,FAD binding,regulation of transcription by RNA polymerase
Fv_160_4.g2841	395	ID=Fv_160_4.g2841;Description=hypothetical protein FVER53263_10110 [Fusarium verticillioides];Gene=FVER53263_10110;Ontology_term=membrane,arylesterase
Fv_160_4.g2842	743	ID=Fv_160_4.g2842;Description=hypothetical protein FVER14953_10109 [Fusarium verticillioides];Gene=opsB-3;Ontology_term=aspartic-type endopeptidase
Fv_160_4.g2843	274	ID=Fv_160_4.g2843;Description=hypothetical protein FVER14953_20413 [Fusarium verticillioides];Gene=FDENT_2427;Ontology_term=lyase activity,amino acid metabolic
Fv_160_4.g2844	353	ID=Fv_160_4.g2844;Description=threonine aldolase [Fusarium verticillioides 7600];Gene=FCIRC_7339;Ontology_term=lyase activity,amino acid metabolic process;Ontology_id=GO:0016829,GO:0006520;Enzyme_code=EC:4;Enzyme_name=Lyases
Fv_160_4.g2845	496	ID=Fv_160_4.g2845;Description=hypothetical protein FVEG_10107 [Fusarium verticillioides 7600];Gene=FOIG_03925;Ontology_term=membrane,DNA-binding transcription factor activity,transmembrane transporter activity,regulation of DNA-templated transcription,transmembrane
Fv_160_4.g2846	501	ID=Fv_160_4.g2846;Description=hypothetical protein FVER53590_10106 [Fusarium verticillioides];Gene=FOTG_10294;Ontology_term=membrane,monooxygenase activity,iron ion binding,oxidoreductase activity, acting on paired donors, with incorporation or reduction of molecular oxygen,heme binding;Ontology_id=GO:0016020,GO:0004497,GO:0005506,GO:0016705,GO:0020037;Enzyme_code=EC:1.14;Enzyme_name=Actin

Fv_160_4.g2847	459	ID=Fv_160_4.g2847;Description=hypothetical protein FVEG_10105 [Fusarium verticillioides 7600];Gene=FVER53590_10105;Ontology_term=membrane,chloride channel activity,chloride transmembrane
Fv_160_4.g2848	355	ID=Fv_160_4.g2848;Description=hypothetical protein FVEG_10104 [Fusarium verticillioides 7600];Gene=FOMG_14175;Ontology_term=oxidoreductase
Fv_160_4.g2849	242	ID=Fv_160_4.g2849;Description=hypothetical protein FVEG_10103 [Fusarium verticillioides 7600];Gene=LOX2;Ontology_term=nucleus,RNA polymerase II cis-regulatory region sequence-specific DNA binding,DNA-binding transcription factor activity, RNA polymerase II-specific,hydrolase activity,regulation of transcription by RNA polymerase
Fv_160_4.g2850	306	ID=Fv_160_4.g2850;Description=hypothetical protein FVER14953_10102 [Fusarium verticillioides];Gene=AU210_012008;Ontology_term=extracellular region,pectate lyase
Fv_160_4.g2851	324	ID=Fv_160_4.g2851;Description=pectate lyase plyB [Fusarium verticillioides 7600];Gene=AU210_012007;Ontology_term=extracellular region,pectate lyase activity,polysaccharide catabolic
Fv_160_4.g2852	258	ID=Fv_160_4.g2852;Description=hypothetical protein FVER53263_10100 [Fusarium verticillioides];Gene=FANTH_2509;Ontology_term=cellular anatomical entity,hydrolase activity, acting on glycosyl
Fv_160_4.g2853	543	ID=Fv_160_4.g2853;Description=hypothetical protein FVER53590_10099 [Fusarium verticillioides];Gene=FMAN_12932;Ontology_term=membrane,mannosyl-oligosaccharide 1,2-alpha-mannosidase activity,calcium ion binding,carbohydrate metabolic process,ubiquitin-dependent ERAD pathway;Ontology_id=GO:0016020,GO:0004571,GO:0005509,GO:0005975,GO:0030433;Enzyme_code=EC:3.2.1.113,EC:3.2.1.24;Enz
Fv_160_4.g2854	419	ID=Fv_160_4.g2854;Description=hypothetical protein FVER14953_10098 [Fusarium verticillioides];Gene=FMUND_586;Ontology_term=hydrolase activity, acting on glycosyl bonds,xylan catabolic
Fv_160_4.g2855	1002	ID=Fv_160_4.g2855;Description=hypothetical protein FVER14953_10096 [Fusarium verticillioides];Gene=FSUBG_12787;Ontology_term=beta-galactosidase activity,carbohydrate metabolic
Fv_160_4.g2856	445	ID=Fv_160_4.g2856;Description=hypothetical protein FVER14953_10095 [Fusarium
Fv_160_4.g2857	390	ID=Fv_160_4.g2857;Description=hypothetical protein FVEG_10094 [Fusarium verticillioides 7600];Gene=FVEG_10094;Ontology_term=collagen trimer,hydrolase activity, acting on ester
Fv_160_4.g2858	250	ID=Fv_160_4.g2858;Description=hypothetical protein FVER53263_10093 [Fusarium verticillioides];Gene=FTJAE_7570;Ontology_term=membrane,GTP binding;Ontology_id=GO:0016020,GO:0005525
Fv_160_4.g2859	361	ID=Fv_160_4.g2859;Description=hypothetical protein FVER53263_10092 [Fusarium verticillioides];Gene=FNYG_12929;Ontology_term=acyltransferase activity, transferring groups other than amino-acyl
Fv_160_4.g2860	114	ID=Fv_160_4.g2860;Description=hypothetical protein FVEG_10091 [Fusarium verticillioides 7600]
Fv_160_4.g2861	285	ID=Fv_160_4.g2861;Description=hypothetical protein FVER53263_10090 [Fusarium verticillioides];Gene=FPCIR_3470;Ontology_term=S-adenosylmethionine-dependent methyltransferase
Fv_160_4.g2862	615	ID=Fv_160_4.g2862;Description=hypothetical protein FVEG_10089 [Fusarium verticillioides 7600];Gene=FDENT_2409;Ontology_term=membrane,transmembrane transporter activity,carbohydrate transport,transmembrane

Fv_160_4.g2863	528	ID=Fv_160_4.g2863;Description=hypothetical protein FVER14953_10088 [Fusarium verticillioides];Gene=AU210_011996;Ontology_term=membrane,transmembrane transporter activity,transmembrane
Fv_160_4.g2864	262	ID=Fv_160_4.g2864;Description=2-deoxy-D-gluconate 3-dehydrogenase [Fusarium verticillioides 7600];Gene=FOVG_15225;Ontology_term=oxidoreductase activity,cellular biosynthetic
Fv_160_4.g2865	383	ID=Fv_160_4.g2865;Description=putative starvation sensing protein rspA [Fusarium fujikuroi];Gene=CEK26_013416;Ontology_term=2-dehydropantoate 2-reductase activity,galactonate dehydratase activity,amino acid catabolic process,pantothenate biosynthetic process,D-galactonate catabolic process;Ontology_id=GO:0008677,GO:0008869,GO:0009063,GO:0015940,GO:0034194;Enzyme_code=EC:1.1.1.169,EC:4.2.1.6;Enzy
Fv_160_4.g2866	323	ID=Fv_160_4.g2866;Description=2-dehydropantoate 2-reductase [Fusarium verticillioides 7600];Gene=BFJ70_g7560;Ontology_term=membrane,2-dehydropantoate 2-reductase activity,galactonate dehydratase activity,amino acid catabolic process,pantothenate biosynthetic process,D-galactonate catabolic process;Ontology_id=GO:0016020,GO:0008677,GO:0008869,GO:0009063,GO:0015940,GO:0034194;Enzyme_code=EC:1.1.1.169,EC:
Fv_160_4.g2867	226	ID=Fv_160_4.g2867;Description=hypothetical protein FVER14953_10084 [Fusarium
Fv_160_4.g2868	690	ID=Fv_160_4.g2868;Description=hypothetical protein FVER14953_10083 [Fusarium verticillioides];Gene=FMUND_565;Ontology_term=hydrolase activity, hydrolyzing O-glycosyl compounds,carbohydrate metabolic
Fv_160_4.g2869	535	ID=Fv_160_4.g2869;Description=hypothetical protein FVER14953_10082 [Fusarium verticillioides];Gene=FVEG_10082;Ontology_term=hydrolase activity, hydrolyzing O-glycosyl compounds,carbohydrate metabolic
Fv_160_4.g2870	317	ID=Fv_160_4.g2870;Description=hypothetical protein FPANT_5043 [Fusarium
Fv_160_4.g2871	531	ID=Fv_160_4.g2871;Description=hypothetical protein FVER53263_10080 [Fusarium verticillioides];Gene=FPANT_5042;Ontology_term=membrane,transmembrane transporter activity,carbohydrate
Fv_160_4.g2872	281	ID=Fv_160_4.g2872;Description=pyrroline-5-carboxylate reductase [Fusarium verticillioides 7600];Gene=FANTH_2495;Ontology_term=pyrroline-5-carboxylate reductase activity,L-proline biosynthetic
Fv_160_4.g2873	222	ID=Fv_160_4.g2873;Description=hypothetical protein FVER14953_10078 [Fusarium verticillioides];Gene=FOVG_15250;Ontology_term=monoatomic cation homeostasis,inorganic ion
Fv_160_4.g2874	465	ID=Fv_160_4.g2874;Description=hypothetical protein FVEG_10077 [Fusarium verticillioides 7600]
Fv_160_4.g2875	637	ID=Fv_160_4.g2875;Description=hypothetical protein FVEG_10076 [Fusarium verticillioides 7600];Gene=FPRO_13516;Ontology_term=monooxygenase
Fv_160_4.g2876	1451	ID=Fv_160_4.g2876;Description=hypothetical protein FVEG_16719 [Fusarium verticillioides 7600];Gene=FPRO05_08551;Ontology_term=ATP hydrolysis activity,nucleoside metabolic
Fv_160_4.g2877	328	ID=Fv_160_4.g2877;Description=hypothetical protein FVER14953_10073 [Fusarium verticillioides]
Fv_160_4.g2878	233	ID=Fv_160_4.g2878;Description=hypothetical protein FVER14953_10072 [Fusarium verticillioides]
Fv_160_4.g2879	183	ID=Fv_160_4.g2879;Description=hypothetical protein FVEG_10071 [Fusarium verticillioides 7600];Gene=FMUND_576;Ontology_term=phospholipase A2 activity,phospholipid metabolic process,arachidonic acid

Fv_160_4.g2880	527	ID=Fv_160_4.g2880;Description=hypothetical protein FVEG_10070 [Fusarium verticillioides 7600];Gene=FVEG_10070;Ontology_term=monooxygenase activity,iron ion binding,oxidoreductase activity, acting on paired donors, with incorporation or reduction of molecular oxygen,heme binding;Ontology_id=GO:0004497,GO:0005506,GO:0016705,GO:0020037;Enzyme_code=EC:1.14;Enzyme_name=Acting on paired
Fv_160_4.g2881	460	ID=Fv_160_4.g2881;Description=hypothetical protein FVEG_10069 [Fusarium verticillioides 7600];Gene=FOTG_10272;Ontology_term=nucleus,DNA-binding transcription factor activity, RNA polymerase II-specific,zinc ion
Fv_160_4.g2882	105	ID=Fv_160_4.g2882;Description=hypothetical protein FVEG_10068 [Fusarium verticillioides 7600];Gene=FOTG_10260;Ontology_term=oxidoreductase activity,FAD
Fv_160_4.g2883	231	ID=Fv_160_4.g2883;Description=hypothetical protein FVER53590_10067 [Fusarium verticillioides]
Fv_160_4.g2884	693	ID=Fv_160_4.g2884;Description=related to ferric reductase Fre2p [Fusarium fujikuroi IMI 58289];Gene=FPRO05_06193;Ontology_term=peptidase activity,monoatomic ion
Fv_160_4.g2885	136	ID=Fv_160_4.g2885;Description=hypothetical protein H9Q69_000426 [Fusarium xylarioides]
Fv_160_4.g2886	424	ID=Fv_160_4.g2886;Description=hypothetical protein FVEG_10062 [Fusarium verticillioides 7600]
Fv_160_4.g2887	292	ID=Fv_160_4.g2887;Description=hypothetical protein FVEG_10061 [Fusarium verticillioides 7600];Gene=FOMG_14123;Ontology_term=extracellular region,catalytic activity,lipid transporter activity,nutrient reservoir
Fv_160_4.g2888	118	ID=Fv_160_4.g2888;Description=hypothetical protein FOXG_11794 [Fusarium oxysporum f. sp. lycopersici 4287]
Fv_160_4.g2889	306	ID=Fv_160_4.g2889;Description=hypothetical protein FVER14953_10059 [Fusarium verticillioides];Gene=FNAPI_2685;Ontology_term=kinase activity,regulation of DNA-templated
Fv_160_4.g2890	418	ID=Fv_160_4.g2890;Description=hypothetical protein FCOIX_1175 [Fusarium coicis];Gene=FACUT_5027;Ontology_term=transferase activity;Ontology_id=GO:0016740;Enzyme_code=EC:2;Enzyme_name=Transferases
Fv_160_4.g2891	152	ID=Fv_160_4.g2891;Description=hypothetical protein FVER14953_10058 [Fusarium verticillioides]
Fv_160_4.g2892	241	ID=Fv_160_4.g2892;Description=hypothetical protein FVER14953_10057 [Fusarium verticillioides]
Fv_160_4.g2893	313	ID=Fv_160_4.g2893;Description=hypothetical protein FVEG_16716 [Fusarium verticillioides 7600];Gene=FCIRC_10763;Ontology_term=metalloendopeptidase activity,zinc ion
Fv_160_4.g2894	87	ID=Fv_160_4.g2894;Description=hypothetical protein FVEG_10056 [Fusarium verticillioides 7600]
Fv_160_4.g2895	258	ID=Fv_160_4.g2895;Description=hypothetical protein FVEG_10054 [Fusarium verticillioides 7600]
Fv_160_4.g2896	344	ID=Fv_160_4.g2896;Description=hypothetical protein FVEG_10053 [Fusarium verticillioides 7600];Gene=CEK26_013528;Ontology_term=nuclear outer membrane,transferase
Fv_160_4.g2897	288	ID=Fv_160_4.g2897;Description=hypothetical protein FVER14953_10052 [Fusarium verticillioides];Gene=FMEXI_13294;Ontology_term=nuclear outer membrane;Ontology_id=GO:0005640
Fv_160_4.g2898	97	ID=Fv_160_4.g2898;Description=hypothetical protein FVEG_10051 [Fusarium verticillioides 7600];Gene=FCIRC_10767
Fv_160_4.g2899	507	ID=Fv_160_4.g2899;Description=hypothetical protein FVER14953_10050 [Fusarium verticillioides];Gene=FOXB_01418;Ontology_term=oxidoreductase activity, acting on the aldehyde or oxo group of donors, NAD or

Fv_160_4.g2900	500	ID=Fv_160_4.g2900;Description=hypothetical protein FVER53263_10049 [Fusarium verticillioides];Gene=FOMG_14132;Ontology_term=oxidoreductase activity, acting on the aldehyde or oxo group of donors, NAD or
Fv_160_4.g2901	249	ID=Fv_160_4.g2901;Description=hypothetical protein FVEG_10048 [Fusarium verticillioides 7600]
Fv_160_4.g2902	534	ID=Fv_160_4.g2902;Description=hypothetical protein FVEG_10047 [Fusarium verticillioides 7600];Gene=FNAPI_2695;Ontology_term=membrane,transmembrane transporter activity,carbohydrate transport,transmembrane
Fv_160_4.g2903	324	ID=Fv_160_4.g2903;Description=hypothetical protein FVEG_10046 [Fusarium verticillioides 7600];Gene=FNAPI_2696;Ontology_term=arylformamidase activity,tryptophan catabolic process to
Fv_160_4.g2904	389	ID=Fv_160_4.g2904;Description=hypothetical protein FVER14953_10044 [Fusarium verticillioides];Gene=FNAPI_2697;Ontology_term=cilium,membrane;Ontology_id=GO:0005929,GO:0016020
Fv_160_4.g2905	1269	ID=Fv_160_4.g2905;Description=hypothetical protein FVER14953_20957 [Fusarium
Fv_160_4.g2906	507	ID=Fv_160_4.g2906;Description=hypothetical protein FVER53590_10043 [Fusarium verticillioides];Gene=FDENT_2367;Ontology_term=mitochondrial inner membrane,transmembrane transporter
Fv_160_4.g2907	313	ID=Fv_160_4.g2907;Description=hypothetical protein FVEG_10042 [Fusarium verticillioides 7600];Gene=FOC4_g10007743;Ontology_term=mitochondrial inner membrane,transmembrane
Fv_160_4.g2908	777	ID=Fv_160_4.g2908;Description=hypothetical protein FVER53590_10041 [Fusarium verticillioides];Gene=FPRO05_06172;Ontology_term=nucleus,DNA binding,zinc ion binding,DNA-binding transcription factor activity, RNA polymerase II-specific,regulation of transcription by RNA polymerase
Fv_160_4.g2909	412	ID=Fv_160_4.g2909;Description=hypothetical protein FVER53263_10040 [Fusarium verticillioides];Gene=FPRO05_06171;Ontology_term=isomerase
Fv_160_4.g2910	441	ID=Fv_160_4.g2910;Description=hypothetical protein J7337_011315 [Fusarium musae];Gene=C2S_12793;Ontology_term=transferase
Fv_160_4.g2911	581	ID=Fv_160_4.g2911;Description=hypothetical protein LB505_009360 [Fusarium chuoi];Gene=C2S_12792;Ontology_term=membrane,transmembrane transporter activity,transmembrane transport,nitrogen
Fv_160_4.g2912	783	ID=Fv_160_4.g2912;Description=hypothetical protein FVER14953_10037 [Fusarium verticillioides];Gene=C2S_12791;Ontology_term=nucleus,DNA binding,zinc ion binding,membrane,cilium,DNA-binding transcription factor activity, RNA polymerase II-specific,regulation of transcription by RNA polymerase
Fv_160_4.g2913	328	ID=Fv_160_4.g2913;Description=hypothetical protein FVER14953_10036 [Fusarium verticillioides];Gene=cda1;Ontology_term=hydrolase activity, acting on carbon-nitrogen (but not peptide) bonds,carbohydrate metabolic process;Ontology_id=GO:0016810,GO:0005975;Enzyme_code=EC:3.5;Enzyme_name=Acting on carbon-nitrogen bonds,
Fv_160_4.g2914	591	ID=Fv_160_4.g2914;Description=hypothetical protein FVEG_10035 [Fusarium verticillioides 7600];Gene=HZS61_016810;Ontology_term=membrane,transmembrane transporter activity,transmembrane
Fv_160_4.g2915	370	ID=Fv_160_4.g2915;Description=NADPH2 dehydrogenase [Fusarium verticillioides 7600];Gene=FMUND_554;Ontology_term=FMN binding,oxidoreductase activity;Ontology_id=GO:0010181,GO:0016491;Enzyme_code=EC:1;Enzyme_name=Oxidoreductases

Fv_160_4.g2916	542	ID=Fv_160_4.g2916;Description=hypothetical protein FVER14953_10033 [Fusarium verticillioides]
Fv_160_4.g2917	542	ID=Fv_160_4.g2917;Description=hypothetical protein FVER14953_10032 [Fusarium verticillioides];Gene=FPCIR_3416;Ontology_term=membrane,mannosyl-oligosaccharide 1,2-alpha-mannosidase activity,calcium ion binding,carbohydrate metabolic process,ubiquitin-dependent ERAD pathway;Ontology_id=GO:0016020,GO:0004571,GO:0005509,GO:0005975,GO:0030433;Enzyme_code=EC:3.2.1.113,EC:3.2.1.24;Enz
Fv_160_4.g2918	139	ID=Fv_160_4.g2918;Description=hypothetical protein FVEG_10031 [Fusarium verticillioides 7600]
Fv_160_4.g2919	141	ID=Fv_160_4.g2919;Description=hypothetical protein FVEG_10030 [Fusarium verticillioides 7600]
Fv_160_4.g2920	322	ID=Fv_160_4.g2920;Description=hypothetical protein FVEG_10029 [Fusarium verticillioides 7600];Gene=FVER53263_10029;Ontology_term=oxidoreductase activity,chlorophyllase activity,chlorophyll catabolic
Fv_160_4.g2921	583	ID=Fv_160_4.g2921;Description=hypothetical protein FVEG_10028 [Fusarium verticillioides 7600];Gene=FOBC_14171;Ontology_term=nucleus,DNA binding,zinc ion binding,membrane,DNA-binding transcription factor activity, RNA polymerase II-specific,regulation of transcription by RNA polymerase
Fv_160_4.g2922	323	ID=Fv_160_4.g2922;Description=hypothetical protein FVER14953_10027 [Fusarium verticillioides]
Fv_160_4.g2923	511	ID=Fv_160_4.g2923;Description=hypothetical protein FVER14953_10026 [Fusarium verticillioides];Gene=CEK26_013515;Ontology_term=extracellular region,membrane,lipid transporter activity,transmembrane transporter activity,nutrient reservoir activity,lipid transport,transmembrane
Fv_160_4.g2924	807	ID=Fv_160_4.g2924;Description=hypothetical protein FVEG_10025 [Fusarium verticillioides 7600];Gene=FDENT_11646;Ontology_term=hydrolase activity, hydrolyzing O-glycosyl compounds,cellulose catabolic
Fv_160_4.g2925	819	ID=Fv_160_4.g2925;Description=hypothetical protein FVER14953_10024 [Fusarium verticillioides];Gene=FOVG_15286;Ontology_term=beta-glucosidase activity,scopolin beta-glucosidase activity,cellulose catabolic
Fv_160_4.g2926	350	ID=Fv_160_4.g2926;Description=hypothetical protein FVER53263_10023 [Fusarium verticillioides];Gene=FOC1_g10003288;Ontology_term=dioxygenase
Fv_160_4.g2927	316	ID=Fv_160_4.g2927;Description=hypothetical protein FVEG_10022 [Fusarium verticillioides 7600];Gene=FMEXI_12194;Ontology_term=serine-type peptidase
Fv_160_4.g2928	436	ID=Fv_160_4.g2928;Description=hypothetical protein FVER14953_10021 [Fusarium verticillioides];Gene=FNYG_11510;Ontology_term=oxidoreductase activity,FAD binding,biosynthetic
Fv_160_4.g2929	756	ID=Fv_160_4.g2929;Description=hypothetical protein FVEG_10020 [Fusarium verticillioides 7600];Gene=unc-93;Ontology_term=zinc ion binding,striated muscle dense body,glycolytic process,regulation of potassium ion transport,glucose metabolic process,glyceraldehyde-3-phosphate dehydrogenase (NAD+) (phosphorylating) activity,plasma membrane,DNA-binding transcription factor activity, RNA polymerase II-specific,regulation of transcription by RNA polymerase II,nucleus,DNA binding,NADP binding,NAD binding,regulation of muscle contraction,potassium channel regulator activity;Ontology_id=GO:0008270,GO:0055120,GO:0006096,GO:0043266,GO:0006006,GO:0004365,GO:0005886,GO:0000981,GO:0006357,GO:0005634,GO:0003677,GO:0050661,GO:0051287,GO:0006937,GO:0015459;Enzyme_code=EC:1.2.1.59,EC:1.2.1.12;Enzy

Fv_160_4.g2930	542	ID=Fv_160_4.g2930;Description=hypothetical protein FVER14953_10019 [Fusarium verticillioides];Gene=FGLOB1_12726;Ontology_term=membrane,transmembrane transporter activity,transmembrane
Fv_160_4.g2931	363	ID=Fv_160_4.g2931;Description=hypothetical protein J7337_011296 [Fusarium musae];Gene=GAPG;Ontology_term=oxidoreductase activity;Ontology_id=GO:0016491;Enzyme_code=EC:1;Enzyme_name=Oxidoreductases
Fv_160_4.g2932	684	ID=Fv_160_4.g2932;Description=hypothetical protein FVEG_10018 [Fusarium verticillioides 7600];Gene=FMAN_13009;Ontology_term=nucleus,glycosome,DNA-binding transcription factor activity, RNA polymerase II-specific,glyceraldehyde-3-phosphate dehydrogenase (NAD+) (phosphorylating) activity,zinc ion binding,NADP binding,NAD binding,glucose metabolic process,glycolytic process,regulation of transcription by RNA polymerase II;Ontology_id=GO:0005634,GO:0020015,GO:0000981,GO:0004365,GO:0008270,GO:0050661,GO:0051287,GO:0006006,GO:000609
Fv_160_4.g2933	403	ID=Fv_160_4.g2933;Description=hypothetical protein FVER53590_10017 [Fusarium verticillioides];Gene=FGLOB1_12723;Ontology_term=iron ion binding,hydrolase activity, acting on carbon-nitrogen (but not peptide) bonds,choline monooxygenase activity,2 iron, 2 sulfur cluster binding,glycine betaine biosynthetic process from choline;Ontology_id=GO:0005506,GO:0016810,GO:0019133,GO:0051537,GO:0019285;Enzyme_code=EC:1.14.15.7,EC:3.5;Enzyme_
Fv_160_4.g2934	603	ID=Fv_160_4.g2934;Description=hypothetical protein FVER14953_10016 [Fusarium verticillioides];Gene=FTJAE_9341;Ontology_term=N,N-dimethylaniline monooxygenase activity,flavin adenine dinucleotide binding,NADP binding;Ontology_id=GO:0004499,GO:0050660,GO:0050661;Enzyme_code=EC:1.14.13.8;Enzyme_name=flavin-
Fv_160_4.g2935	176	ID=Fv_160_4.g2935;Description=hypothetical protein FVEG_10015 [Fusarium verticillioides 7600]
Fv_160_4.g2936	538	ID=Fv_160_4.g2936;Description=hypothetical protein FVER53263_10014 [Fusarium verticillioides];Gene=FNYG_11501;Ontology_term=mitochondrion,cytosol,membrane,dihydrofolate reductase activity,thymidylate synthase activity,transmembrane transporter activity,dTMP biosynthetic process,one-carbon metabolic process,methylation,tetrahydrofolate biosynthetic process,transmembrane transport;Ontology_id=GO:0005739,GO:0005829,GO:0016020,GO:0004146,GO:0004799,GO:0022857,GO:0006231,GO:0006730,GO
Fv_160_4.g2937	840	ID=Fv_160_4.g2937;Description=hypothetical protein FVER53590_10013 [Fusarium verticillioides];Gene=HZS61_016842;Ontology_term=alpha-glucosidase activity,maltose metabolic process,carbohydrate catabolic
Fv_160_4.g2938	538	ID=Fv_160_4.g2938;Description=hypothetical protein FVEG_10012 [Fusarium verticillioides 7600];Gene=FDENT_215;Ontology_term=cytosol,membrane,axon,dendrite,dihydropyrimidinase activity,transmembrane transporter activity,filamin binding,protein homodimerization activity,protein heterodimerization activity,pyrimidine nucleobase catabolic process,motor neuron axon guidance,regulation of cell migration,establishment or maintenance of actin cytoskeleton polarity,establishment or maintenance of microtubule cytoskeleton polarity,negative regulation of microtubule polymerization,netrin-activated signaling pathway,transmembrane transport,chemorepulsion of axon,sensory neuron axon guidance,regulation of dorsal/ventral axon
Fv_160_4.g2939	502	ID=Fv_160_4.g2939;Description=sucrose utilization SUC1 [Fusarium tjaetaba];Gene=FFC1_09146;Ontology_term=nucleus,DNA binding,zinc ion binding,DNA-binding transcription factor activity, RNA polymerase II-specific,regulation of transcription by RNA

Fv_160_4.g2940	889	ID=Fv_160_4.g2940;Description=transcriptional regulatory [Fusarium coicis];Gene=FDENT_218;Ontology_term=nucleus,DNA binding,zinc ion binding,DNA-binding transcription factor activity, RNA polymerase II-specific,regulation of transcription by RNA
Fv_160_4.g2941	383	ID=Fv_160_4.g2941;Description=hypothetical protein FVER14953_10009 [Fusarium verticillioides];Gene=FANTH_2439;Ontology_term=fungal-type cell wall,structural constituent of cell
Fv_160_4.g2942	499	ID=Fv_160_4.g2942;Description=hypothetical protein FVER14953_10007 [Fusarium verticillioides];Gene=FNYG_15477;Ontology_term=membrane,cytosolic large ribosomal subunit,structural constituent of ribosome,transmembrane transporter activity,cytoplasmic translational elongation,transmembrane
Fv_160_4.g2943	263	ID=Fv_160_4.g2943;Description=hypothetical protein FVER14953_20911 [Fusarium verticillioides];Gene=AU210_012086;Ontology_term=nucleus,DNA-binding transcription factor activity, RNA polymerase II-
Fv_160_4.g2944	947	ID=Fv_160_4.g2944;Description=hypothetical protein FVEG_10005 [Fusarium verticillioides 7600];Gene=LW93_366;Ontology_term=nucleus,membrane,DNA-binding transcription factor activity, RNA polymerase II-specific,zinc ion binding,metal ion transmembrane transporter activity,regulation of transcription by RNA polymerase II,magnesium
Fv_160_4.g2945	279	ID=Fv_160_4.g2945;Description=hypothetical protein FVER14953_10004 [Fusarium
Fv_160_4.g2946	91	ID=Fv_160_4.g2946;Description=aldehyde reductase II [Fusarium napiforme]
Fv_160_4.g2947	358	ID=Fv_160_4.g2947;Description=hypothetical protein FVER14953_10003 [Fusarium
Fv_160_4.g2948	472	ID=Fv_160_4.g2948;Description=hypothetical protein FPANT_12056 [Fusarium pseudoanthophilum];Gene=FOTG_10194;Ontology_term=nucleus,DNA-binding transcription factor activity, RNA polymerase II-
Fv_160_4.g2949	863	ID=Fv_160_4.g2949;Description=hypothetical protein FVEG_10001 [Fusarium verticillioides 7600];Gene=HZS61_016852;Ontology_term=membrane,sulfuric ester hydrolase
Fv_160_4.g2950	156	ID=Fv_160_4.g2950;Description=hypothetical protein FVER53263_20464 [Fusarium verticillioides];Gene=FACUT_8360;Ontology_term=kinase
Fv_160_4.g2951	212	ID=Fv_160_4.g2951;Description=hypothetical protein FVER14953_10000 [Fusarium verticillioides]
Fv_160_4.g2952	858	ID=Fv_160_4.g2952;Description=hypothetical protein FVER53590_09999 [Fusarium verticillioides]
Fv_160_4.g2953	93	ID=Fv_160_4.g2953;Description=hypothetical protein FVEG_09998 [Fusarium verticillioides 7600]
Fv_160_4.g2954	160	ID=Fv_160_4.g2954;Description=hypothetical protein FVER53590_09997 [Fusarium verticillioides]
Fv_160_4.g2955	224	ID=Fv_160_4.g2955;Description=hypothetical protein FVER14953_09996 [Fusarium verticillioides];Gene=P27891;Ontology_term=nucleosome,nucleus,DNA binding,structural constituent of chromatin,protein
Fv_160_4.g2956	224	ID=Fv_160_4.g2956;Description=hypothetical protein FVER53590_09995 [Fusarium verticillioides];Gene=FTJAE_9361;Ontology_term=acyltransferase activity, transferring groups other than amino-acyl
Fv_160_4.g2957	247	ID=Fv_160_4.g2957;Description=hypothetical protein FVER53590_09994 [Fusarium verticillioides];Gene=FGADI_10127;Ontology_term=membrane,ATP binding,ATP hydrolysis activity,ABC-type transporter activity,transmembrane transport;Ontology_id=GO:0016020,GO:0005524,GO:0016887,GO:0140359,GO:0055085;Enzyme_code=EC:7.2.2,EC:3.6.1.15;Enzym

Fv_160_4.g2958	115	ID=Fv_160_4.g2958;Description=ABC transporter bea3 [Fusarium musae];Gene=LW93_388;Ontology_term=nucleosome,nucleus,membrane,DNA binding,ATP binding,ATP hydrolysis activity,structural constituent of chromatin,protein heterodimerization activity,ABC-type transporter activity,transmembrane transport;Ontology_id=GO:0000786,GO:0005634,GO:0016020,GO:0003677,GO:0005524,GO:0016887,GO:0030527,GO:0046982,GO
Fv_160_4.g2959	709	ID=Fv_160_4.g2959;Description=hypothetical protein FVER14953_21058 [Fusarium verticillioides];Gene=FPANT_13888;Ontology_term=dimethylallyltranstransferase activity,geranyltranstransferase activity,isoprenoid biosynthetic process,ketone biosynthetic process,mycotoxin biosynthetic process,alcohol biosynthetic process,organic cyclic compound biosynthetic
Fv_160_4.g2960	342	ID=Fv_160_4.g2960;Description=hypothetical protein FVEG_09992 [Fusarium verticillioides 7600];Gene=FVER53590_09992;Ontology_term=membrane,transferase activity,isoprenoid biosynthetic process,ketone biosynthetic process,mycotoxin biosynthetic process,alcohol biosynthetic process,organic cyclic compound biosynthetic
Fv_160_4.g2961	641	ID=Fv_160_4.g2961;Description=hypothetical protein FVEG_09991 [Fusarium verticillioides 7600];Gene=FOBC_14252;Ontology_term=nucleus,DNA-binding transcription factor activity, RNA polymerase II-specific,zinc ion binding,oxidoreductase activity,regulation of transcription by RNA polymerase
Fv_160_4.g2962	490	ID=Fv_160_4.g2962;Description=hypothetical protein FVER53590_09990 [Fusarium verticillioides];Gene=F52700_13626;Ontology_term=oxidoreductase
Fv_160_4.g2963	243	ID=Fv_160_4.g2963;Description=hypothetical protein FVEG_09989 [Fusarium verticillioides 7600]
Fv_160_4.g2964	380	ID=Fv_160_4.g2964;Description=hypothetical protein FVER14953_09988 [Fusarium verticillioides]
Fv_160_4.g2965	278	ID=Fv_160_4.g2965;Description=hypothetical protein FVEG_09987 [Fusarium verticillioides 7600];Gene=FNAPI_651;Ontology_term=monooxygenase activity,FAD binding,biosynthetic
Fv_160_4.g2966	670	ID=Fv_160_4.g2966;Description=hypothetical protein FVER14953_09986 [Fusarium verticillioides];Gene=AU210_012118;Ontology_term=monooxygenase activity,FAD binding,biosynthetic
Fv_160_4.g2967	240	ID=Fv_160_4.g2967;Description=hypothetical protein FVER53263_09985 [Fusarium
Fv_160_4.g2968	348	ID=Fv_160_4.g2968;Description=hypothetical protein FPRO06_11044 [Fusarium
Fv_160_4.g2969	94	ID=Fv_160_4.g2969;Description=hypothetical protein FVEG_16701 [Fusarium verticillioides 7600]
Fv_160_4.g2970	344	ID=Fv_160_4.g2970;Description=hypothetical protein FVER53263_09983 [Fusarium verticillioides];Gene=A0A866WKL3;Ontology_term=zinc ion binding,oxidoreductase
Fv_160_4.g2971	285	ID=Fv_160_4.g2971;Description=hypothetical protein FVER53263_09982 [Fusarium verticillioides];Gene=rutD-1;Ontology_term=hydrolase activity;Ontology_id=GO:0016787;Enzyme_code=EC:3;Enzyme_name=Hydrolases
Fv_160_4.g2972	409	ID=Fv_160_4.g2972;Description=hypothetical protein FVEG_09981 [Fusarium verticillioides 7600];Gene=BFJ68_g14223;Ontology_term=FMN binding,oxidoreductase

Fv_160_4.g2973	370	ID=Fv_160_4.g2973;Description=cytochrome P450 oxidoreductase [Fusarium verticillioides 7600];Gene=FOPG_17121;Ontology_term=nucleus,endoplasmic reticulum membrane,RNA binding,iron ion binding,heme binding,fatty acid alpha-hydroxylase activity,mRNA processing,fatty acid biosynthetic
Fv_160_4.g2974	314	ID=Fv_160_4.g2974;Description=hypothetical protein FVEG_09979 [Fusarium verticillioides 7600]
Fv_160_4.g2975	275	ID=Fv_160_4.g2975;Description=hypothetical protein FVEG_09978 [Fusarium verticillioides 7600];Gene=A0A2L2TEB4;Ontology_term=oxidoreductase activity,cellular biosynthetic
Fv_160_4.g2976	697	ID=Fv_160_4.g2976;Description=hypothetical protein FVEG_09977 [Fusarium verticillioides 7600];Gene=BKA59DRAFT_492634;Ontology_term=membrane,transferase activity,metal ion binding,purine nucleotide metabolic process,carbohydrate derivative metabolic
Fv_160_4.g2977	327	ID=Fv_160_4.g2977;Description=hypothetical protein FVEG_09976 [Fusarium verticillioides 7600];Gene=F53441_6751;Ontology_term=arylformamidase activity,tryptophan catabolic process to
Fv_160_4.g2978	424	ID=Fv_160_4.g2978;Description=hypothetical protein FVEG_09975 [Fusarium verticillioides 7600]
Fv_160_4.g2979	188	ID=Fv_160_4.g2979;Description=hypothetical protein FVER53263_09974 [Fusarium verticillioides]
Fv_160_4.g2980	497	ID=Fv_160_4.g2980;Description=hypothetical protein FVEG_16700 [Fusarium verticillioides 7600];Gene=FSUBG_12335;Ontology_term=membrane,transmembrane transporter activity,transmembrane
Fv_160_4.g2981	803	ID=Fv_160_4.g2981;Description=hypothetical protein FVER53590_30484 [Fusarium verticillioides];Gene=CEP51_003142;Ontology_term=nucleus,DNA binding,zinc ion binding,DNA-binding transcription factor activity, RNA polymerase II-specific,regulation of transcription by RNA polymerase
Fv_160_4.g2982	313	ID=Fv_160_4.g2982;Description=hypothetical protein FVEG_09972 [Fusarium verticillioides 7600];Gene=6959;Ontology_term=S-adenosylmethionine-dependent methyltransferase activity,oxidoreductase activity,methylation;Ontology_id=GO:0008757,GO:0016491,GO:0032259;Enzyme_code=EC:1,EC:2.1.1;Enzyme_name=Oxidoreducta
Fv_160_4.g2983	415	ID=Fv_160_4.g2983;Description=cytochrome P450 oxidoreductase [Fusarium verticillioides 7600];Gene=FNAPI_661;Ontology_term=iron ion binding,hydrolase activity, acting on carbon-nitrogen (but not peptide) bonds,choline monooxygenase activity,heme binding,2 iron, 2 sulfur cluster binding,glycine betaine biosynthetic process from choline;Ontology_id=GO:0005506,GO:0016810,GO:0019133,GO:0020037,GO:0051537,GO:0019285;Enzyme_code=EC:1.14.15.7,EC:
Fv_160_4.g2984	1021	ID=Fv_160_4.g2984;Description=oxidoreductase [Fusarium pseudoanthophilum];Gene=FPANT_11679;Ontology_term=monooxygenase activity,iron ion binding,oxidoreductase activity, acting on paired donors, with incorporation or reduction of molecular oxygen,hydrolase activity, acting on carbon-nitrogen (but not peptide) bonds,heme
Fv_160_4.g2985	700	ID=Fv_160_4.g2985;Description=hypothetical protein FVER53590_09968 [Fusarium verticillioides];Gene=FTJAE_9386;Ontology_term=nucleus,DNA binding,zinc ion binding,DNA-binding transcription factor activity,
Fv_160_4.g2986	351	ID=Fv_160_4.g2986;Description=hypothetical protein FVER53590_09967 [Fusarium verticillioides];Gene=FNYG_11760;Ontology_term=S-adenosylmethionine-dependent methyltransferase

Fv_160_4.g2987	421	ID=Fv_160_4.g2987;Description=hypothetical protein FVER53590_25486 [Fusarium verticillioides];Gene=FDENT_259;Ontology_term=transferase activity, transferring alkyl or aryl (other than methyl) groups,alkaloid metabolic process;Ontology_id=GO:0016765,GO:0009820;Enzyme_code=EC:2.5.1;Enzyme_name=Transferring alkyl or aryl groups,
Fv_160_4.g2988	648	ID=Fv_160_4.g2988;Description=hypothetical protein FVER53590_09965 [Fusarium
Fv_160_5.g2989	335	ID=Fv_160_5.g2989;Description=FLO9-like protein [Fusarium subglutinans];Gene=F25303_12485;Ontology_term=cell
Fv_160_5.g2990	134	ID=Fv_160_5.g2990;Description=hypothetical protein F52700_4276 [Fusarium sp. NRRL 52700]
Fv_160_5.g2991	246	ID=Fv_160_5.g2991;Description=hypothetical protein F52700_4277 [Fusarium sp. NRRL 52700]
Fv_160_5.g2992	452	ID=Fv_160_5.g2992;Description=hypothetical protein FVER14953_06582 [Fusarium
Fv_160_5.g2993	397	ID=Fv_160_5.g2993;Description=integral membrane [Fusarium
Fv_160_5.g2994	495	ID=Fv_160_5.g2994;Description=hypothetical protein FVEG_06584 [Fusarium verticillioides 7600];Gene=Focb16_v014329;Ontology_term=nucleus,membrane,mannosyltransferase activity,RNA polymerase II cis-regulatory region sequence-specific DNA binding,nuclear receptor activity,zinc ion binding,heme binding,FAD binding,negative regulation of transcription by RNA polymerase II,regulation of ecdysteroid metabolic process,hormone-mediated signaling pathway,ecdysis, chitin-based cuticle,intracellular receptor signaling pathway,response to ecdysone,positive regulation of transcription by RNA polymerase II,oogenesis,negative regulation of peptidoglycan recognition protein signaling
Fv_160_5.g2995	514	ID=Fv_160_5.g2995;Description=hypothetical protein FVEG_06586 [Fusarium verticillioides 7600];Gene=FSUBG_13322;Ontology_term=nucleus,DNA-binding transcription factor activity, RNA polymerase II-specific,zinc ion
Fv_160_5.g2996	547	ID=Fv_160_5.g2996;Description=hypothetical protein J7337_009238 [Fusarium musae];Gene=FOC1_g10012206;Ontology_term=membrane,transmembrane transporter activity,transmembrane
Fv_160_5.g2997	592	ID=Fv_160_5.g2997;Description=hypothetical protein FVER53590_06588 [Fusarium verticillioides];Gene=FTJAE_7197;Ontology_term=alpha-glucosidase activity,maltose metabolic process,carbohydrate catabolic
Fv_160_5.g2998	434	ID=Fv_160_5.g2998;Description=hypothetical protein FVEG_06589 [Fusarium verticillioides 7600]
Fv_160_5.g2999	152	ID=Fv_160_5.g2999;Description=hypothetical protein FVEG_06590 [Fusarium verticillioides 7600]
Fv_160_5.g3000	348	ID=Fv_160_5.g3000;Description=hypothetical protein FVEG_06591 [Fusarium verticillioides 7600];Gene=FNAPI_5258;Ontology_term=oxidoreductase
Fv_160_5.g3001	480	ID=Fv_160_5.g3001;Description=hypothetical protein FVEG_06592 [Fusarium verticillioides
Fv_160_5.g3002	167	ID=Fv_160_5.g3002;Description=hypothetical protein LB505_014044 [Fusarium chuoi];Gene=FMAN_09913;Ontology_term=DNA binding,acyltransferase activity, transferring groups other than amino-acyl
Fv_160_5.g3003	800	ID=Fv_160_5.g3003;Description=hypothetical protein FVER53263_06594 [Fusarium verticillioides];Gene=FTJAE_14149;Ontology_term=glutamate dehydrogenase [NAD(P)+] activity,amino acid metabolic
Fv_160_5.g3004	343	ID=Fv_160_5.g3004;Description=hypothetical protein FVER53263_06595 [Fusarium verticillioides];Gene=F66182_2369;Ontology_term=S-adenosylmethionine-dependent methyltransferase

Fv_160_5.g3005	545	ID=Fv_160_5.g3005;Description=hypothetical protein FVER53590_26489 [Fusarium verticillioides];Gene=azaF-0;Ontology_term=ligase activity;Ontology_id=GO:0016874;Enzyme_code=EC:6;Enzyme_name=Ligases
Fv_160_5.g3006	140	ID=Fv_160_5.g3006;Description=hypothetical protein FVEG_15899 [Fusarium verticillioides 7600];Gene=FNAPI_5254;Ontology_term=oxidoreductase
Fv_160_5.g3007	413	ID=Fv_160_5.g3007;Description=CMGC/SRPK protein kinase [Fusarium oxysporum NRRL 32931];Gene=F66182_2512;Ontology_term=protein kinase activity,ATP
Fv_160_5.g3008	1022	ID=Fv_160_5.g3008;Description=hypothetical protein FVEG_06599 [Fusarium verticillioides
Fv_160_5.g3009	138	ID=Fv_160_5.g3009;Description=hypothetical protein FVER53263_20822 [Fusarium verticillioides]
Fv_160_5.g3010	113	ID=Fv_160_5.g3010;Description=hypothetical protein AU210_010146 [Fusarium oxysporum f. sp. radicis-cucumerinum]
Fv_160_5.g3011	368	ID=Fv_160_5.g3011;Description=hypothetical protein FNYG_07130 [Fusarium nygamai];Gene=FPCIR_12915;Ontology_term=nucleic acid binding,zinc ion binding;Ontology_id=GO:0003676,GO:0008270
Fv_160_5.g3012	197	ID=Fv_160_5.g3012;Description=uncharacterized protein FSUBG_12346 [Fusarium subglutinans]
Fv_160_5.g3013	530	ID=Fv_160_5.g3013;Description=hypothetical protein J7337_009251 [Fusarium musae]
Fv_160_5.g3014	421	ID=Fv_160_5.g3014;Description=hypothetical protein FMUND_13621 [Fusarium
Fv_160_5.g3015	260	ID=Fv_160_5.g3015;Description=hypothetical protein FVER14953_20009 [Fusarium verticillioides]
Fv_160_5.g3016	610	ID=Fv_160_5.g3016;Description=hypothetical protein FVER14953_21762 [Fusarium verticillioides]
Fv_160_5.g3017	217	ID=Fv_160_5.g3017;Description=hypothetical protein FPANT_2427 [Fusarium pseudoanthophilum];Gene=Ace;Ontology_term=cytoplasm,plasma membrane,acetylcholinesterase activity,protein homodimerization activity,sulfate binding,acetylcholine catabolic process,chemical synaptic transmission,choline catabolic process;Ontology_id=GO:0005737,GO:0005886,GO:0003990,GO:0042803,GO:0043199,GO:0006581,GO:0007268,GO:0042426;Enzy
Fv_160_5.g3018	231	ID=Fv_160_5.g3018;Description=hypothetical protein H9Q70_000071 [Fusarium xylarioides];Gene=MAL63;Ontology_term=binding,regulation of DNA-templated
Fv_160_5.g3019	195	ID=Fv_160_5.g3019;Description=hypothetical protein H9Q70_000072 [Fusarium xylarioides]
Fv_160_5.g3020	326	ID=Fv_160_5.g3020;Description=hypothetical protein FPANT_2431 [Fusarium pseudoanthophilum];Gene=nAChRbeta1;Ontology_term=neuron projection,postsynaptic membrane,acetylcholine-gated monoatomic cation-selective channel activity,monoatomic ion transport,synaptic transmission, cholinergic,regulation of postsynaptic
Fv_160_5.g3021	224	ID=Fv_160_5.g3021;Description=hypothetical protein H9Q73_000380 [Fusarium xylarioides]
Fv_160_5.g3022	674	ID=Fv_160_5.g3022;Description=hypothetical protein J7337_009252 [Fusarium musae];Gene=FMEXI_13790;Ontology_term=nucleus,ATP binding,hydrolase activity,ATP-dependent chromatin remodeler
Fv_160_5.g3023	245	ID=Fv_160_5.g3023;Description=uncharacterized protein FTJAE_3043 [Fusarium tjaetaba]
Fv_160_5.g3024	406	ID=Fv_160_5.g3024;Description=hypothetical protein FVER14953_06607 [Fusarium verticillioides]
Fv_160_5.g3025	141	ID=Fv_160_5.g3025;Description=---NA---
Fv_160_5.g3026	519	ID=Fv_160_5.g3026;Description=hypothetical protein H9Q71_000172 [Fusarium xylarioides]

Fv_160_5.g3027	84	ID=Fv_160_5.g3027;Description=hypothetical protein FPCIR_12923 [Fusarium pseudocircinatum]
Fv_160_5.g3028	649	ID=Fv_160_5.g3028;Description=hypothetical protein H9Q71_000165 [Fusarium xylarioides]
Fv_160_5.g3029	499	ID=Fv_160_5.g3029;Description=hypothetical protein H9Q71_000167 [Fusarium xylarioides]
Fv_160_5.g3030	402	ID=Fv_160_5.g3030;Description=hypothetical protein H9Q71_000168 [Fusarium
Fv_160_5.g3031	731	ID=Fv_160_5.g3031;Description=hypothetical protein H9Q71_000169 [Fusarium xylarioides]
Fv_160_5.g3032	643	ID=Fv_160_5.g3032;Description=hypothetical protein H9Q71_000170 [Fusarium xylarioides]
Fv_160_5.g3033	473	ID=Fv_160_5.g3033;Description=hypothetical protein FPCIR_12922 [Fusarium pseudocircinatum]
Fv_160_5.g3034	724	ID=Fv_160_5.g3034;Description=hypothetical protein FPHYL_13378 [Fusarium phyllophilum]
Fv_160_5.g3035	548	ID=Fv_160_5.g3035;Description=hypothetical protein FNAPI_9694 [Fusarium napiforme];Gene=ced-4;Ontology_term=embryo development,regulation of biological quality,peptidase activator activity involved in apoptotic process,anion binding,BH domain binding,regulation of developmental process,adenyl ribonucleotide binding,regulation of cysteine-type endopeptidase activity involved in apoptotic process,response to external stimulus,metal ion binding,cytoplasm,intracellular membrane-bounded organelle,negative regulation of apoptotic process,positive regulation of apoptotic process,cellular component organization,protein-containing complex,positive regulation of cysteine-type endopeptidase
Fv_160_5.g3036	376	ID=Fv_160_5.g3036;Description=hypothetical protein H9Q70_000086 [Fusarium xylarioides]
Fv_160_5.g3037	684	ID=Fv_160_5.g3037;Description=hypothetical protein LZL87_002559 [Fusarium oxysporum]
Fv_160_5.g3038	442	ID=Fv_160_5.g3038;Description=hypothetical protein FPANT_2441 [Fusarium pseudoanthophilum];Gene=HMLALPHA1;Ontology_term=nucleus,transcription coactivator activity,DNA binding, bending,regulation of transcription by RNA polymerase II,positive regulation of mating-type specific transcription, DNA-
Fv_160_5.g3039	116	ID=Fv_160_5.g3039;Description=hypothetical protein FNAPI_11529 [Fusarium napiforme];Gene=cal-1;Ontology_term=calcium ion
Fv_160_5.g3040	373	ID=Fv_160_5.g3040;Description=hypothetical protein J7337_009266 [Fusarium musae];Gene=FPANT_13203
Fv_160_5.g3041	370	ID=Fv_160_5.g3041;Description=hypothetical protein FPANT_13202 [Fusarium pseudoanthophilum]
Fv_160_5.g3042	157	ID=Fv_160_5.g3042;Description=hypothetical protein H9Q71_000130 [Fusarium xylarioides]
Fv_160_5.g3043	565	ID=Fv_160_5.g3043;Description=hypothetical protein FVER14953_06612 [Fusarium verticillioides]
Fv_160_5.g3044	81	ID=Fv_160_5.g3044;Description=hypothetical protein H9Q70_000004 [Fusarium xylarioides]
Fv_160_5.g3045	70	ID=Fv_160_5.g3045;Description=hypothetical protein FVER53590_06607 [Fusarium verticillioides]
Fv_160_5.g3046	230	ID=Fv_160_5.g3046;Description=hypothetical protein FNAPI_11525 [Fusarium napiforme]
Fv_160_5.g3047	615	ID=Fv_160_5.g3047;Description=uncharacterized protein FTJAE_12523 [Fusarium tjaetaba]

Fv_160_5.g3048	210	ID=Fv_160_5.g3048;Description=Uncharacterized protein Y057_7546 [Fusarium fujikuroi];Gene=cdk-1;Ontology_term=protein kinase binding,positive regulation of meiotic nuclear division,anterior/posterior axis specification, embryo,mitotic cell cycle process,nematode male tail tip morphogenesis,brahma complex,cyclin-dependent protein serine/threonine kinase activity,cell division,oocyte maturation,organic cyclic compound binding,cytoplasm,positive regulation of proteasomal ubiquitin-dependent protein catabolic process,peptidyl-serine phosphorylation,positive regulation of protein serine/threonine kinase activity,peptidyl-threonine
Fv_160_5.g3049	223	ID=Fv_160_5.g3049;Description=hypothetical protein J7337_009270 [Fusarium musae]
Fv_160_5.g3050	207	ID=Fv_160_5.g3050;Description=hypothetical protein FVER14953_06622 [Fusarium verticillioides];Gene=BFJ65_g11024;Ontology_term=hydrolase
Fv_160_5.g3051	897	ID=Fv_160_5.g3051;Description=hypothetical protein FVER53263_06623 [Fusarium verticillioides]
Fv_160_5.g3052	556	ID=Fv_160_5.g3052;Description=hypothetical protein FVER53263_06625 [Fusarium verticillioides];Gene=FOXB_06914;Ontology_term=extracellular region,peptidase activity,hydrolase activity, acting on carbon-nitrogen (but not peptide) bonds, in linear amidines,proteolysis;Ontology_id=GO:0005576,GO:0008233,GO:0016813,GO:0006508;Enzyme_code=EC:3.5.3,EC:3.4;Enzyme_nam
Fv_160_5.g3053	521	ID=Fv_160_5.g3053;Description=signal recognition particle protein [Fusarium verticillioides 7600];Gene=FCIRC_6404;Ontology_term=endoplasmic reticulum,signal recognition particle, endoplasmic reticulum targeting,GTPase activity,GTP binding,7S RNA binding,ATP hydrolysis activity,SRP-dependent cotranslational protein targeting to membrane;Ontology_id=GO:0005783,GO:0005786,GO:0003924,GO:0005525,GO:0008312,GO:0016887,GO:0006614;Enzyme_code
Fv_160_5.g3054	267	ID=Fv_160_5.g3054;Description=hypothetical protein FVEG_06627 [Fusarium verticillioides 7600]
Fv_160_5.g3055	517	ID=Fv_160_5.g3055;Description=hypothetical protein FVEG_15904 [Fusarium verticillioides 7600];Gene=FPCIR_13619;Ontology_term=hydrolase
Fv_160_5.g3056	360	ID=Fv_160_5.g3056;Description=alcohol dehydrogenase (NADP+) [Fusarium verticillioides 7600];Gene=FVEG_06629;Ontology_term=zinc ion binding,oxidoreductase activity, acting on the CH-OH group of donors, NAD or
Fv_160_5.g3057	226	ID=Fv_160_5.g3057;Description=hypothetical protein FVEG_06630 [Fusarium verticillioides 7600];Gene=Ald1;Ontology_term=collagen trimer,cytosol,Z disc,M band,fructose-bisphosphate aldolase activity,structural constituent of cuticle,glycolytic process,mesoderm development,fructose 1,6-bisphosphate metabolic process,glucose homeostasis;Ontology_id=GO:0005581,GO:0005829,GO:0030018,GO:0031430,GO:0004332,GO:0042302,GO:0006096,GO:0007498,
Fv_160_5.g3058	243	ID=Fv_160_5.g3058;Description=hypothetical protein FVEG_06631 [Fusarium verticillioides 7600]
Fv_160_5.g3059	334	ID=Fv_160_5.g3059;Description=porphobilinogen deaminase [Fusarium verticillioides 7600];Gene=BFJ63_vAg11146;Ontology_term=hydroxymethylbilane synthase activity,protoporphyrinogen IX biosynthetic
Fv_160_5.g3060	168	ID=Fv_160_5.g3060;Description=glycine cleavage system H protein [Fusarium verticillioides 7600];Gene=FFB14_13402;Ontology_term=mitochondrion,glycine cleavage complex,glycine decarboxylation via glycine cleavage

Fv_160_5.g3061	291	ID=Fv_160_5.g3061;Description=rab family, other [Fusarium verticillioides 7600];Gene=FFB14_13401;Ontology_term=GTPase activity,GTP binding;Ontology_id=GO:0003924,GO:0005525;Enzyme_code=EC:3.6.1.15;Enzyme_name=nucleoside-triphosphate
Fv_160_5.g3062	188	ID=Fv_160_5.g3062;Description=hypothetical protein FVEG_06635 [Fusarium verticillioides
Fv_160_5.g3063	78	ID=Fv_160_5.g3063;Description=hypothetical protein FCOIX_402 [Fusarium
Fv_160_5.g3064	150	ID=Fv_160_5.g3064;Description=hypothetical protein FVEG_06636 [Fusarium verticillioides
Fv_160_5.g3065	353	ID=Fv_160_5.g3065;Description=hypothetical protein FVER53590_26494 [Fusarium verticillioides];Gene=LEU4;Ontology_term=mitochondrion,2-isopropylmalate synthase activity,zinc ion binding,leucine biosynthetic
Fv_160_5.g3066	361	ID=Fv_160_5.g3066;Description=hypothetical protein FVER53263_06638 [Fusarium verticillioides];Gene=F52700_2511;Ontology_term=kinase
Fv_160_5.g3067	254	ID=Fv_160_5.g3067;Description=thymidylate kinase [Fusarium tjaetaba];Gene=FVEG_06639;Ontology_term=thymidylate kinase activity,ATP binding,dTDP biosynthetic process,phosphorylation,nucleoside monophosphate phosphorylation;Ontology_id=GO:0004798,GO:0005524,GO:0006233,GO:0016310,GO:0046940;Enzyme_code=EC:2.7.4.4,EC:2.7.4.9 ;Enzyme_name=nucleoside-phosphate kinase,dTMP kinase
Fv_160_5.g3068	283	ID=Fv_160_5.g3068;Description=formyltetrahydrofolate deformylase [Fusarium verticillioides 7600];Gene=FOC1_g10012307;Ontology_term=formyltetrahydrofolate deformylase activity,'de novo' IMP biosynthetic process,amino acid metabolic process,one-carbon metabolic
Fv_160_5.g3069	475	ID=Fv_160_5.g3069;Description=hypothetical protein FVER53590_06641 [Fusarium verticillioides];Gene=FRV6_12027;Ontology_term=nucleus,DNA-binding transcription factor activity, RNA polymerase II-specific,zinc
Fv_160_5.g3070	826	ID=Fv_160_5.g3070;Description=related to integral membrane protein [Fusarium fujikuroi];Gene=FGLOB1_13902;Ontology_term=nucleus,membrane,DNA-binding transcription factor activity, RNA polymerase II-specific,transcription corepressor activity,zinc ion binding,regulation of transcription by RNA polymerase II,negative regulation of
Fv_160_5.g3071	560	ID=Fv_160_5.g3071;Description=hypothetical protein FVEG_06643 [Fusarium verticillioides 7600];Gene=BFJ63_vAg11124;Ontology_term=transcription corepressor activity,negative regulation of DNA-templated
Fv_160_5.g3072	448	ID=Fv_160_5.g3072;Description=AP-1 complex subunit mu-1 [Fusarium oxysporum f. sp. lycopersici 4287];Gene=CEP52_006122;Ontology_term=endosome,cytosol,AP-1 adaptor complex,Golgi to endosome transport,Golgi to vacuole transport,retrograde transport, endosome to Golgi,Golgi to plasma membrane protein transport,endosome to plasma membrane
Fv_160_5.g3073	298	ID=Fv_160_5.g3073;Description=hypothetical protein FVER53263_06645 [Fusarium verticillioides]
Fv_160_5.g3074	463	ID=Fv_160_5.g3074;Description=hypothetical protein FVER53590_06646 [Fusarium verticillioides];Gene=FOPG_10072;Ontology_term=nucleus,endonuclease activity,protein-lysine N-methyltransferase activity,nucleotide-excision repair,response to UV,methylation;Ontology_id=GO:0005634,GO:0004519,GO:0016279,GO:0006289,GO:0009411,GO:0032259;Enzyme_code=EC:3.1,E

Fv_160_5.g3075	611	ID=Fv_160_5.g3075;Description=hypothetical protein FVER53590_06647 [Fusarium verticillioides];Gene=FMAN_09979;Ontology_term=nucleus,mitochondrion,membrane,heteroduplex DNA loop binding,5' deoxyribonuclease (pyrimidine dimer) activity,base-excision repair,nucleotide-excision repair,pyrimidine dimer repair,mismatch repair,mitochondrial DNA repair,UV-damage excision repair;Ontology_id=GO:0005634,GO:0005739,GO:0016020,GO:0000404,GO:1990043,GO:0006284,GO:0006289,GO:0006290,GO:00
Fv_160_5.g3076	82	ID=Fv_160_5.g3076;Description=hypothetical protein FVEG_15911 [Fusarium verticillioides 7600];Gene=FOC4_g10008290;Ontology_term=membrane,endonuclease activity,nucleotide-excision repair,response to
Fv_160_5.g3077	1068	ID=Fv_160_5.g3077;Description=hypothetical protein FVER14953_06648 [Fusarium verticillioides];Gene=FNAPI_7046;Ontology_term=membrane,ATP binding,ATP hydrolysis activity,ATPase-coupled monoatomic cation transmembrane transporter activity,sodium ion transport,monoatomic cation transmembrane transport;Ontology_id=GO:0016020,GO:0005524,GO:0016887,GO:0019829,GO:0006814,GO:0098655;Enzyme_code=EC:7.2.2,EC:3.
Fv_160_5.g3078	72	ID=Fv_160_5.g3078;Description=hypothetical protein FVEG_06649 [Fusarium verticillioides 7600];Gene=FOXB_06938
Fv_160_5.g3079	260	ID=Fv_160_5.g3079;Description=hypothetical protein FVEG_06650 [Fusarium verticillioides 7600];Gene=BFJ65_g11299;Ontology_term=protein phosphatase inhibitor activity,regulation of signal
Fv_160_5.g3080	119	ID=Fv_160_5.g3080;Description=hypothetical protein FVEG_15912 [Fusarium verticillioides 7600]
Fv_160_5.g3081	268	ID=Fv_160_5.g3081;Description=60s ribosomal L2 (mitochondrial) [Fusarium pseudocircinatum];Gene=FCIRC_1017;Ontology_term=ribosome,ribonucleoprotein complex,structural constituent of
Fv_160_5.g3082	1890	ID=Fv_160_5.g3082;Description=uncharacterized protein FSUBG_6826 [Fusarium subglutinans];Gene=FPANT_9395;Ontology_term=membrane,metal ion transmembrane transporter activity,magnesium ion
Fv_160_5.g3083	314	ID=Fv_160_5.g3083;Description=hypothetical protein FVER53263_06655 [Fusarium verticillioides];Gene=FVER53263_06655;Ontology_term=membrane,metal ion transmembrane transporter activity,magnesium ion
Fv_160_5.g3084	551	ID=Fv_160_5.g3084;Description=hypothetical protein FVER53590_26498 [Fusarium verticillioides]
Fv_160_5.g3085	430	ID=Fv_160_5.g3085;Description=hypothetical protein FVER53263_06656 [Fusarium verticillioides];Gene=FMUND_11275;Ontology_term=gene expression,cellular nitrogen compound metabolic process,primary
Fv_160_5.g3086	251	ID=Fv_160_5.g3086;Description=hypothetical protein FVER53590_06657 [Fusarium verticillioides];Gene=FPANT_13327;Ontology_term=mitochondrial large ribosomal subunit,structural constituent of ribosome,ribonuclease III activity,RNA processing,mitochondrial
Fv_160_5.g3087	233	ID=Fv_160_5.g3087;Description=hypothetical protein FVER53263_06658 [Fusarium
Fv_160_5.g3088	1101	ID=Fv_160_5.g3088;Description=hypothetical protein FVEG_06659 [Fusarium verticillioides 7600];Gene=HZS61_017103;Ontology_term=membrane,NAD(P)+ transhydrogenase activity,proton transmembrane
Fv_160_5.g3089	128	ID=Fv_160_5.g3089;Description=hypothetical protein J7337_009317 [Fusarium musae];Gene=LW93_5190;Ontology_term=mitochondrial inner membrane,respirasome,mitochondrial respiratory chain complex I

Fv_160_5.g3090	488	ID=Fv_160_5.g3090;Description=hypothetical protein FVER53590_06661 [Fusarium verticillioides];Gene=FOIG_04815;Ontology_term=mitochondrial membrane,ATP binding,CDP-diacylglycerol-glycerol-3-phosphate 3-phosphatidyltransferase activity,cardiolipin biosynthetic
Fv_160_5.g3091	320	ID=Fv_160_5.g3091;Description=hypothetical protein FVEG_06662 [Fusarium verticillioides 7600];Gene=FOYG_11571;Ontology_term=cytosolic small ribosomal subunit,structural constituent of
Fv_160_5.g3092	437	ID=Fv_160_5.g3092;Description=hypothetical protein FVEG_06663 [Fusarium verticillioides
Fv_160_5.g3093	317	ID=Fv_160_5.g3093;Description=hypothetical protein FVER53263_06664 [Fusarium verticillioides];Gene=FPHYL_5590;Ontology_term=DNA-binding transcription factor activity,regulation of DNA-templated
Fv_160_5.g3094	371	ID=Fv_160_5.g3094;Description=hypothetical protein FVER53263_06665 [Fusarium verticillioides];Gene=C2S_1725;Ontology_term=nuclear lumen,DNA binding,DNA replication,DNA repair,DNA
Fv_160_5.g3095	1270	ID=Fv_160_5.g3095;Description=Rho-GTPase-activating 6 [Fusarium pseudocircinatum];Gene=rga6;Ontology_term=GTPase activator activity,signal transduction;Ontology_id=GO:0005096,GO:0007165
Fv_160_5.g3096	517	ID=Fv_160_5.g3096;Description=hypothetical protein FVEG_06667 [Fusarium verticillioides 7600];Gene=SNF4;Ontology_term=kinase
Fv_160_5.g3097	573	ID=Fv_160_5.g3097;Description=hypothetical protein FVER53263_06668 [Fusarium verticillioides];Gene=FTJAE_7461;Ontology_term=fungal-type vacuole membrane,phagophore assembly site,membrane raft,nucleus-vacuole junction,Myo2p-Vac17p-Vac8p transport complex,identical protein binding,protein-membrane adaptor activity,vacuole inheritance,autophagosome assembly,pexophagy,ribophagy,piecemeal microautophagy of the nucleus,vacuole fusion, non-autophagic,nucleus-vacuole junction assembly,protein localization to membrane raft;Ontology_id=GO:0000329,GO:0000407,GO:0045121,GO:0071561,GO:0071563,GO:0042802,GO:0043495,GO:0000011,GO:0000
Fv_160_5.g3098	168	ID=Fv_160_5.g3098;Description=hypothetical protein FVEG_15918 [Fusarium verticillioides 7600];Gene=FOC1_g10012339;Ontology_term=mitochondrial inner membrane peptidase complex,serine-type endopeptidase activity,signal peptide processing,protein processing involved in protein targeting to
Fv_160_5.g3099	293	ID=Fv_160_5.g3099;Description=translation initiation factor IF-3 [Fusarium verticillioides 7600];Gene=FPANT_4607;Ontology_term=translation initiation factor activity,translational
Fv_160_5.g3100	170	ID=Fv_160_5.g3100;Description=hypothetical protein FVEG_06670 [Fusarium verticillioides 7600];Gene=FSUBG_6809;Ontology_term=nucleus,cytoplasm;Ontology_id=GO:0005634,GO:0005737
Fv_160_5.g3101	608	ID=Fv_160_5.g3101;Description=hypothetical protein FVER14953_06671 [Fusarium verticillioides]
Fv_160_5.g3102	385	ID=Fv_160_5.g3102;Description=hypothetical protein FVEG_06673 [Fusarium verticillioides 7600];Gene=FOIG_04828;Ontology_term=oxidoreductase activity, acting on the CH-OH group of donors, NAD or NADP as
Fv_160_5.g3103	518	ID=Fv_160_5.g3103;Description=hypothetical protein FVER14953_06674 [Fusarium verticillioides];Gene=FOYG_11558;Ontology_term=nucleus,histone H3 acetyltransferase activity,chromatin remodeling,regulation of transcription from RNA polymerase II promoter in response to

Fv_160_5.g3104	344	ID=Fv_160_5.g3104;Description=hypothetical protein FVER14953_06675 [Fusarium verticillioides]
Fv_160_5.g3105	286	ID=Fv_160_5.g3105;Description=hypothetical protein FVER14953_06676 [Fusarium verticillioides]
Fv_160_5.g3106	296	ID=Fv_160_5.g3106;Description=hypothetical protein FVEG_06677 [Fusarium verticillioides 7600];Gene=TIM44;Ontology_term=mitochondrial inner membrane,transferase activity,protein-folding chaperone binding,protein import into mitochondrial
Fv_160_5.g3107	137	ID=Fv_160_5.g3107;Description=hypothetical protein FVER14953_06678 [Fusarium verticillioides]
Fv_160_5.g3108	463	ID=Fv_160_5.g3108;Description=transcription initiation factor TFIID subunit 6 [Fusarium verticillioides 7600];Gene=FPANT_4616;Ontology_term=SAGA complex,transcription factor TFIID complex,cytosol,SLIK (SAGA-like) complex,chromatin binding,translation initiation factor activity,protein serine/threonine kinase activity,ATP binding,RNA polymerase II general transcription initiation factor activity,identical protein binding,protein heterodimerization activity,molecular adaptor activity,RNA polymerase II-specific DNA-binding transcription factor binding,chromatin organization,translational initiation,positive regulation of transcription by RNA polymerase II,RNA polymerase II preinitiation complex assembly,positive regulation of DNA-templated transcription
Fv_160_5.g3109	187	ID=Fv_160_5.g3109;Description=hypothetical protein FVEG_06680 [Fusarium verticillioides 7600];Gene=FMEXI_358;Ontology_term=succinate dehydrogenase complex,succinate dehydrogenase activity,electron transfer activity,metal ion binding,tricarboxylic acid cycle,electron transport
Fv_160_5.g3110	97	ID=Fv_160_5.g3110;Description=hypothetical protein FVER14953_20976 [Fusarium
Fv_160_5.g3111	100	ID=Fv_160_5.g3111;Description=hypothetical protein J7337_009339 [Fusarium
Fv_160_5.g3112	707	ID=Fv_160_5.g3112;Description=hypothetical protein J7337_009340 [Fusarium musae];Gene=FTJAE_13456;Ontology_term=nucleus,DNA binding,zinc ion binding,DNA-binding transcription factor activity, RNA
Fv_160_5.g3113	451	ID=Fv_160_5.g3113;Description=tRNA dimethylallyltransferase [Fusarium verticillioides 7600];Gene=FOIG_04839;Ontology_term=cytoplasm,ATP binding,tRNA dimethylallyltransferase activity,tRNA
Fv_160_5.g3114	476	ID=Fv_160_5.g3114;Description=protein transporter SEC61 subunit alpha [Fusarium oxysporum f. sp. lycopersici 4287];Gene=CEP51_006220;Ontology_term=fungal-type vacuole,Sec61 translocon complex,signal sequence binding,protein-transporting ATPase activity,peptide transmembrane transporter activity,SRP-dependent cotranslational protein targeting to membrane, translocation,ubiquitin-dependent ERAD pathway,retrograde protein transport, ER to cytosol,post-translational protein targeting to membrane, translocation,amide transport,protein transmembrane import into intracellular organelle,misfolded protein transport;Ontology_id=GO:0000324,GO:0005784,GO:0005048,GO:0015450,GO:1904680,GO:0006616,GO:0030433,GO:0030970,GO
Fv_160_5.g3115	1684	ID=Fv_160_5.g3115;Description=hypothetical protein FVER14953_06686 [Fusarium verticillioides];Gene=F25303_9689;Ontology_term=membrane,ATP binding,ATP hydrolysis activity,ABC-type transporter activity,transmembrane transport;Ontology_id=GO:0016020,GO:0005524,GO:0016887,GO:0140359,GO:0055085;Enzyme_code=EC:7.2.2,EC:3.6.1.15;Enzym

Fv_160_5.g3116	391	ID=Fv_160_5.g3116;Description=hypothetical protein FVER14953_06687 [Fusarium verticillioides];Gene=C2S_1704;Ontology_term=mitochondrial inner membrane,mitochondrial large ribosomal subunit,structural
Fv_160_5.g3117	321	ID=Fv_160_5.g3117;Description=hypothetical protein FVEG_06688 [Fusarium verticillioides 7600];Gene=FMUND_11305;Ontology_term=membrane,RNA polymerase II transcription regulator complex,RNA polymerase II transcription regulatory region sequence-specific DNA binding,DNA-binding transcription factor activity, RNA polymerase II-specific,protein homodimerization activity,protein heterodimerization activity,neuroblast fate determination,central nervous system development,peripheral nervous system development,chaeta morphogenesis,vesicle-mediated transport,neuron differentiation,negative regulation of mitotic nuclear division,positive regulation of transcription by RNA polymerase II,regulation of neurogenesis,epithelial cell proliferation involved in Malpighian tubule morphogenesis,Malpighian tubule tip cell
Fv_160_5.g3118	612	ID=Fv_160_5.g3118;Description=hypothetical protein FVER14953_06689 [Fusarium verticillioides];Gene=FOXB_14960;Ontology_term=nucleus,mitochondrial large ribosomal subunit,heterotrimeric G-protein complex,axon,neuronal cell body,non-motile cilium,DNA-binding transcription factor activity, RNA polymerase II-specific,G protein-coupled receptor binding,DNA binding,structural constituent of ribosome,GTPase activity,GTP binding,G-protein beta/gamma-subunit complex binding,metal ion binding,regulation of transcription by RNA polymerase II,adenylate cyclase-modulating G protein-coupled receptor signaling pathway,olfactory learning,positive regulation of gene expression,mitochondrial translation,G protein-coupled receptor signaling pathway involved in defense response to Gram-negative
Fv_160_5.g3119	1557	ID=Fv_160_5.g3119;Description=phospholipid-translocating ATPase [Fusarium verticillioides 7600];Gene=FVER53590_06690;Ontology_term=membrane,RNA polymerase II transcription regulator complex,magnesium ion binding,RNA polymerase II transcription regulatory region sequence-specific DNA binding,DNA-binding transcription factor activity, RNA polymerase II-specific,ATP binding,ATP hydrolysis activity,protein homodimerization activity,protein heterodimerization activity,phosphatidylethanolamine flippase activity,neuroblast fate determination,central nervous system development,peripheral nervous system development,chaeta morphogenesis,phospholipid transport,neuron differentiation,lipid translocation,negative regulation of mitotic nuclear division,positive regulation of transcription by RNA polymerase II,regulation of neurogenesis,epithelial cell proliferation involved in Malpighian tubule morphogenesis,Malpighian tubule tip cell differentiation,regulation of neural precursor cell
Fv_160_5.g3120	237	ID=Fv_160_5.g3120;Description=vesicle-associated membrane protein 7 [Fusarium verticillioides 7600];Gene=FCIRC_8702;Ontology_term=membrane,vesicle-mediated transport;Ontology_id=GO:0016020,GO:0016192
Fv_160_5.g3121	761	ID=Fv_160_5.g3121;Description=hypothetical protein J7337_009349 [Fusarium musae];Gene=FPCIR_13273;Ontology_term=mitochondrial matrix,RNA helicase activity,ATP binding,hydrolase activity, acting on acid

Fv_160_5.g3122	316	ID=Fv_160_5.g3122;Description=hypothetical protein FVEG_06693 [Fusarium verticillioides 7600];Gene=BFJ72_g2691;Ontology_term=mitochondrial inner membrane,mitochondrial large ribosomal subunit,structural constituent of ribosome,glyceraldehyde-3-phosphate dehydrogenase (NAD+) (phosphorylating) activity,protein tyrosine phosphatase activity,protein tyrosine/serine/threonine phosphatase activity,NADP binding,glycolytic process,translation,dephosphorylation;Ontology_id=GO:0005743,GO:0005762,GO:0003735,GO:0004365,GO:0004725,GO:0008138,GO:0050661,GO:0006096,GO:0006412,GO:0016311;Enzyme_code=EC:1.2.1.59,EC:1.2.1.12,EC:3.1.3.48;Enzyme_name=glyceraldehy
Fv_160_5.g3123	547	ID=Fv_160_5.g3123;Description=hypothetical protein FVEG_06694 [Fusarium verticillioides 7600];Gene=FACUT_13762;Ontology_term=protein tyrosine/serine/threonine phosphatase
Fv_160_5.g3124	338	ID=Fv_160_5.g3124;Description=hypothetical protein FVER14953_06695 [Fusarium verticillioides]
Fv_160_5.g3125	1500	ID=Fv_160_5.g3125;Description=hypothetical protein FVEG_06696 [Fusarium verticillioides 7600];Gene=FCIRC_12048;Ontology_term=ATP binding,DNA binding,RNA binding,ribonuclease III activity,metal ion binding,helicase activity,RNA processing,regulation of defense response to virus;Ontology_id=GO:0005524,GO:0003677,GO:0003723,GO:0004525,GO:0046872,GO:0004386,GO:0006396,GO:0050688;Enzyme
Fv_160_5.g3126	685	ID=Fv_160_5.g3126;Description=hypothetical protein FVER14953_06697 [Fusarium verticillioides];Gene=FMUND_12081;Ontology_term=GTPase activity,cysteine-type deubiquitinase activity,GTP binding,ubiquitin-dependent protein catabolic process;Ontology_id=GO:0003924,GO:0004843,GO:0005525,GO:0006511;Enzyme_code=EC:3.6.1.15,EC:3.4.19.12;Enzyme_name=n
Fv_160_5.g3127	248	ID=Fv_160_5.g3127;Description=ubiquitin carboxyl-terminal hydrolase L3 [Fusarium verticillioides 7600];Gene=FFUJ_08934;Ontology_term=cysteine-type deubiquitinase activity,ubiquitin-dependent protein catabolic
Fv_160_5.g3128	748	ID=Fv_160_5.g3128;Description=hypothetical protein FVEG_06699 [Fusarium verticillioides 7600]
Fv_160_5.g3129	317	ID=Fv_160_5.g3129;Description=cysteine sulfinate desulfinase [Fusarium verticillioides 7600];Gene=MRS1;Ontology_term=mitochondrial matrix,ribonucleoprotein complex,crossed form four-way junction DNA binding,Y-form DNA binding,RNA binding,DNA endonuclease activity,flap-structured DNA binding,mitochondrial genome maintenance,Group I intron splicing,mitochondrial RNA processing,DNA metabolic process,mRNA processing;Ontology_id=GO:0005759,GO:1990904,GO:0000402,GO:0000403,GO:0003723,GO:0004520,GO:0070336,GO:0000002,G
Fv_160_5.g3130	296	ID=Fv_160_5.g3130;Description=hypothetical protein J7337_009359 [Fusarium musae];Gene=FCIRC_11768;Ontology_term=oxidoreductase activity,cellular biosynthetic
Fv_160_5.g3131	764	ID=Fv_160_5.g3131;Description=hypothetical protein FVER14953_06702 [Fusarium verticillioides];Gene=FPHYL_13835;Ontology_term=oxidoreductase
Fv_160_5.g3132	451	ID=Fv_160_5.g3132;Description=hypothetical protein FVEG_06703 [Fusarium verticillioides 7600];Gene=nbs;Ontology_term=nucleosome,nucleus,mitochondrial inner membrane,DNA binding,ferrous iron transmembrane transporter activity,structural constituent of chromatin,protein heterodimerization activity,DNA repair,regulation of DNA-templated transcription,mRNA processing,iron import into the

Fv_160_5.g3133	1205	ID=Fv_160_5.g3133;Description=hypothetical protein FPRO05_11877 [Fusarium proliferatum];Gene=FPRO05_11877;Ontology_term=catalytic activity,nucleoside metabolic
Fv_160_5.g3134	491	ID=Fv_160_5.g3134;Description=hypothetical protein J7337_009363 [Fusarium musae];Gene=FVEG_06704;Ontology_term=aminopeptidase activity,metalloexopeptidase activity,metal ion
Fv_160_5.g3135	404	ID=Fv_160_5.g3135;Description=hypothetical protein FVEG_15927 [Fusarium verticillioides 7600]
Fv_160_5.g3136	362	ID=Fv_160_5.g3136;Description=hypothetical protein FVEG_06705 [Fusarium verticillioides 7600]
Fv_160_5.g3137	672	ID=Fv_160_5.g3137;Description=hypothetical protein FVEG_06706 [Fusarium verticillioides
Fv_160_5.g3138	351	ID=Fv_160_5.g3138;Description=hypothetical protein FVEG_06707 [Fusarium verticillioides 7600];Gene=FOXYS1_7024;Ontology_term=S-adenosylmethionine-dependent methyltransferase
Fv_160_5.g3139	510	ID=Fv_160_5.g3139;Description=hypothetical protein FVEG_06708 [Fusarium verticillioides 7600];Gene=FTJAE_13512;Ontology_term=hydrolase activity, acting on carbon-nitrogen (but not peptide)
Fv_160_5.g3140	287	ID=Fv_160_5.g3140;Description=hypothetical protein J7337_009371 [Fusarium musae];Gene=FPANT_6042;Ontology_term=membrane,kinase activity,oxidoreductase activity,hydrolase activity, acting on carbon-nitrogen (but not peptide) bonds,phosphorylation;Ontology_id=GO:0016020,GO:0016301,GO:0016491,GO:0016810,GO:0016310;Enzyme_code=EC:1,EC:2.7,E
Fv_160_5.g3141	343	ID=Fv_160_5.g3141;Description=hypothetical protein FVER14953_06710 [Fusarium verticillioides];Gene=FNAPI_9358;Ontology_term=membrane,beta-1,4-mannosylglycoprotein 4-beta-N-acetylglucosaminyltransferase activity;Ontology_id=GO:0016020,GO:0003830;Enzyme_code=EC:2.4.1.144;Enzyme_name=beta-1,4-
Fv_160_5.g3142	246	ID=Fv_160_5.g3142;Description=hypothetical protein FVER14953_06711 [Fusarium verticillioides];Gene=FNAPI_9359;Ontology_term=membrane,kinase
Fv_160_5.g3143	377	ID=Fv_160_5.g3143;Description=hypothetical protein FVER14953_06712 [Fusarium verticillioides];Gene=FRV6_12106;Ontology_term=membrane,oxidoreductase
Fv_160_5.g3144	429	ID=Fv_160_5.g3144;Description=hypothetical protein FVER53263_12563 [Fusarium verticillioides];Gene=FTJAE_10576;Ontology_term=membrane,monooxygenase activity,iron ion binding,methyltransferase activity,oxidoreductase activity, acting on paired donors, with incorporation or reduction of molecular oxygen,heme binding,methylation;Ontology_id=GO:0016020,GO:0004497,GO:0005506,GO:0008168,GO:0016705,GO:0020037,GO:0032259;Enzy
Fv_160_5.g3145	105	ID=Fv_160_5.g3145;Description=thioredoxin [Fusarium verticillioides 7600];Gene=FCIRC_11753;Ontology_term=protein-disulfide reductase activity;Ontology_id=GO:0015035;Enzyme_code=EC:1.8;Enzyme_name=Acting on a sulfur group of donors
Fv_160_5.g3146	103	ID=Fv_160_5.g3146;Description=hypothetical protein FVER53263_06715 [Fusarium verticillioides]
Fv_160_5.g3147	896	ID=Fv_160_5.g3147;Description=amino-acid permease inda1 [Fusarium coicis];Gene=BFJ70_g2627;Ontology_term=membrane,amino acid transport,transmembrane
Fv_160_5.g3148	378	ID=Fv_160_5.g3148;Description=hypothetical protein FVEG_06717 [Fusarium verticillioides 7600]

Fv_160_5.g3149	770	ID=Fv_160_5.g3149;Description=mitotic spindle assembly checkpoint protein MAD1 [Fusarium verticillioides 7600];Gene=FNYG_13695;Ontology_term=nucleus,mitotic spindle assembly checkpoint
Fv_160_5.g3150	495	ID=Fv_160_5.g3150;Description=hypothetical protein J7337_009382 [Fusarium musae];Gene=FNAPI_9369;Ontology_term=mitochondrial inner membrane,phosphatidate cytidylyltransferase activity,tryptophan-tRNA ligase activity,ATP binding,tryptophanyl-tRNA aminoacylation,CDP-diacylglycerol biosynthetic process,cardiolipin biosynthetic process;Ontology_id=GO:0005743,GO:0004605,GO:0004830,GO:0005524,GO:0006436,GO:0016024,GO:0032049;Enzyme_code=EC
Fv_160_5.g3151	890	ID=Fv_160_5.g3151;Description=hypothetical protein FVER53590_06720 [Fusarium verticillioides]
Fv_160_5.g3152	453	ID=Fv_160_5.g3152;Description=hypothetical protein FVER14953_06721 [Fusarium verticillioides];Gene=FTJAE_2591;Ontology_term=protein kinase activity,ATP
Fv_160_5.g3153	1634	ID=Fv_160_5.g3153;Description=hypothetical protein FVER14953_06722 [Fusarium verticillioides];Gene=FTJAE_2592;Ontology_term=cytosol,RQC complex,ribosomal large subunit binding,metal ion binding,ubiquitin protein ligase activity,protein ubiquitination,rescue of stalled ribosome,ribosome-associated ubiquitin-dependent protein catabolic
Fv_160_5.g3154	1050	ID=Fv_160_5.g3154;Description=hypothetical protein FVEG_06723 [Fusarium verticillioides
Fv_160_5.g3155	97	ID=Fv_160_5.g3155;Description=hypothetical protein FOXB_14357 [Fusarium oxysporum f. sp. conglutinans Fo5176]
Fv_160_5.g3156	521	ID=Fv_160_5.g3156;Description=hypothetical protein FVEG_06725 [Fusarium verticillioides 7600];Gene=IMP3;Ontology_term=small ribosomal subunit,structural constituent of ribosome,rRNA binding;Ontology_id=GO:0015935,GO:0003735,GO:0019843
Fv_160_5.g3157	186	ID=Fv_160_5.g3157;Description=hypothetical protein FVEG_06726 [Fusarium verticillioides 7600]
Fv_160_5.g3158	1037	ID=Fv_160_5.g3158;Description=hypothetical protein FVER53590_06728 [Fusarium verticillioides];Gene=FVEG_06728;Ontology_term=incipient cellular bud site,cellular bud neck contractile ring,myosin II complex,myosin filament,microfilament motor activity,hydrolase activity, hydrolyzing O-glycosyl compounds,ATP binding,myosin II light chain binding,actin filament binding,polysaccharide catabolic process,septum digestion after cytokinesis,primary cell septum biogenesis,mitotic actomyosin contractile ring contraction,mitotic actomyosin contractile ring assembly,protein localization to mitotic actomyosin contractile
Fv_160_5.g3159	81	ID=Fv_160_5.g3159;Description=hypothetical protein FVER14953_20472 [Fusarium verticillioides]
Fv_160_5.g3160	74	ID=Fv_160_5.g3160;Description=hypothetical protein FVER14953_20471 [Fusarium verticillioides]
Fv_160_5.g3161	120	ID=Fv_160_5.g3161;Description=hypothetical protein FVEG_06731 [Fusarium verticillioides 7600]
Fv_160_5.g3162	1307	ID=Fv_160_5.g3162;Description=hypothetical protein FVER14953_20470 [Fusarium verticillioides]
Fv_160_5.g3163	823	ID=Fv_160_5.g3163;Description=hypothetical protein FVEG_06732 [Fusarium verticillioides 7600]
Fv_160_5.g3164	476	ID=Fv_160_5.g3164;Description=hypothetical protein FVER14953_06733 [Fusarium verticillioides];Gene=FMUND_3204;Ontology_term=membrane,oxidoreductase activity, acting on the aldehyde or oxo group of
Fv_160_5.g3165	121	ID=Fv_160_5.g3165;Description=hypothetical protein FVEG_15933 [Fusarium verticillioides 7600];Gene=NAM8;Ontology_term=commitment complex,positive regulation of mRNA splicing, via spliceosome,cytosol,mRNA
Fv_160_5.g3166	183	ID=Fv_160_5.g3166;Description=hypothetical protein FVEG_06734 [Fusarium verticillioides 7600]

Fv_160_5.g3167	411	ID=Fv_160_5.g3167;Description=hypothetical protein FVEG_06735 [Fusarium verticillioides 7600];Gene=FCIRC_6888;Ontology_term=fumarylacetoacetase activity,metal ion binding,L-phenylalanine catabolic process,tyrosine catabolic
Fv_160_5.g3168	718	ID=Fv_160_5.g3168;Description=hypothetical protein FVEG_06736 [Fusarium verticillioides 7600];Gene=FNAPI_9387;Ontology_term=monooxygenase activity,carboxy-lyase activity,FAD binding,biosynthetic
Fv_160_5.g3169	592	ID=Fv_160_5.g3169;Description=hypothetical protein FVER14953_06737 [Fusarium verticillioides];Gene=FVEG_06737;Ontology_term=dipeptidyl-peptidase activity;Ontology_id=GO:0008239;Enzyme_code=EC:3.4.14.1,EC:3.4.14.5,EC:3.4.14.4,EC:3.4.14.2;Enzyme_name=dipeptidyl-
Fv_160_5.g3170	110	ID=Fv_160_5.g3170;Description=hypothetical protein FVEG_06738 [Fusarium verticillioides 7600]
Fv_160_5.g3171	300	ID=Fv_160_5.g3171;Description=hypothetical protein J7337_009402 [Fusarium musae]
Fv_160_5.g3172	290	ID=Fv_160_5.g3172;Description=hypothetical protein FVER53590_06740 [Fusarium verticillioides];Gene=F25303_5643;Ontology_term=ferrous iron binding,zinc ion binding,oxidoreductase activity, acting on single donors with incorporation of molecular oxygen,dioxygenase activity,cellular aromatic compound metabolic process;Ontology_id=GO:0008198,GO:0008270,GO:0016701,GO:0051213,GO:0006725;Enzyme_code=EC:1.13;Enzyme_name=Actin
Fv_160_5.g3173	865	ID=Fv_160_5.g3173;Description=hypothetical protein FVER53263_06741 [Fusarium verticillioides];Gene=FOYG_11472;Ontology_term=nucleus,chromosome,protein-DNA complex,DNA-binding transcription factor activity, RNA polymerase II-specific,protein binding,zinc ion binding,DNA binding, bending,nucleosome binding,MutSalpha complex binding,maintenance of transcriptional fidelity during transcription elongation by RNA polymerase III,DNA repair,chromatin remodeling,regulation of transcription by RNA polymerase II,negative regulation of DNA binding,RNA polymerase II preinitiation complex assembly,RNA polymerase III preinitiation complex
Fv_160_5.g3174	110	ID=Fv_160_5.g3174;Description=cyanovirin-N [Fusarium tjaetaba]
Fv_160_5.g3175	532	ID=Fv_160_5.g3175;Description=hypothetical protein FVER14953_06743 [Fusarium verticillioides];Gene=C2S_1644;Ontology_term=nucleus,chromosome,striated muscle myosin thick filament,membrane,myosin II complex,A band,microfilament motor activity,ATP binding,DNA binding, bending,transmembrane transporter activity,actin filament binding,maintenance of transcriptional fidelity during transcription elongation by RNA polymerase III,DNA repair,chromatin remodeling,transcription by RNA polymerase II,muscle contraction,locomotory behavior,sarcomere organization,transmembrane transport,positive regulation of ovulation,RNA polymerase III preinitiation complex
Fv_160_5.g3176	786	ID=Fv_160_5.g3176;Description=hypothetical protein FVEG_06745 [Fusarium verticillioides 7600]
Fv_160_5.g3177	336	ID=Fv_160_5.g3177;Description=uncharacterized protein FTJAE_2619 [Fusarium tjaetaba];Gene=pal-1;Ontology_term=condensed nuclear chromosome,RNA polymerase II transcription regulatory region sequence-specific DNA binding,DNA-binding transcription factor activity, RNA polymerase II-specific,protein binding,ectodermal cell fate commitment,embryo development ending in birth or egg hatching,embryonic pattern specification,muscle cell fate commitment,positive regulation of apoptotic process,positive regulation of transcription by RNA polymerase II,positive regulation of miRNA

Fv_160_5.g3178	296	ID=Fv_160_5.g3178;Description=hypothetical protein FVER53263_06747 [Fusarium verticillioides];Gene=NPL3;Ontology_term=membrane,RNA binding,transferase
Fv_160_5.g3179	612	ID=Fv_160_5.g3179;Description=hypothetical protein LB504_008825 [Fusarium proliferatum];Gene=AU210_010304;Ontology_term=S-adenosylmethionine-dependent methyltransferase
Fv_160_5.g3180	374	ID=Fv_160_5.g3180;Description=hypothetical protein FVER14953_06749 [Fusarium verticillioides];Gene=FVEG_06749;Ontology_term=proteasome complex,nucleus,ribonucleoprotein complex,DNA-binding transcription factor activity, RNA polymerase II-specific,DNA binding,regulation of transcription by RNA polymerase II,DNA
Fv_160_5.g3181	331	ID=Fv_160_5.g3181;Description=hypothetical protein FVER14953_06750 [Fusarium verticillioides];Gene=SEC63;Ontology_term=Sec62/Sec63 complex,translocon complex,protein binding,protein transmembrane transporter activity,palmitoyl-(protein) hydrolase activity,SRP-dependent cotranslational protein targeting to membrane,post-translational protein targeting to membrane, translocation,macromolecule depalmitoylation;Ontology_id=GO:0031207,GO:0071256,GO:0005515,GO:0008320,GO:0008474,GO:0006614,GO:0031204,GO:0098
Fv_160_5.g3182	420	ID=Fv_160_5.g3182;Description=hypothetical protein FVER53263_06751 [Fusarium verticillioides];Gene=LW93_5291;Ontology_term=apical plasma membrane,ATP binding,ABC-type xenobiotic transporter activity,efflux transmembrane transporter activity,kinase activity,cyclin-dependent protein serine/threonine kinase regulator activity,ATP hydrolysis activity,regulation of transcription by RNA polymerase II,response to metal ion,phosphorylation,xenobiotic transport,innate immune response,defense response to Gram-negative bacterium,transmembrane transport,response to nematicide;Ontology_id=GO:0016324,GO:0005524,GO:0008559,GO:0015562,GO:0016301,GO:0016538,GO:0016887,GO:0006357,GO:0010038,GO:0016310,GO:0042908,GO:0045087,GO:0050829,GO:0055085,GO:0093002;Enzyme_code=EC:2.7,EC:7.6.2.2,EC:7.2.
Fv_160_5.g3183	569	ID=Fv_160_5.g3183;Description=hypothetical protein FVER53263_06752 [Fusarium verticillioides];Gene=FFUJ_08869;Ontology_term=Golgi apparatus,plasma membrane,protein serine/threonine kinase activity,protein binding,ATP binding,protein tyrosine/serine/threonine phosphatase activity,protein serine kinase activity,phosphorylation,dephosphorylation,intracellular signal transduction,negative regulation of endocytosis,regulation of sphingolipid biosynthetic
Fv_160_5.g3184	284	ID=Fv_160_5.g3184;Description=hypothetical protein FVER53263_06753 [Fusarium
Fv_160_5.g3185	215	ID=Fv_160_5.g3185;Description=hypothetical protein FVER53263_06754 [Fusarium
Fv_160_5.g3186	342	ID=Fv_160_5.g3186;Description=hypothetical protein FVEG_06755 [Fusarium verticillioides 7600];Gene=FOIG_04926;Ontology_term=oxidoreductase activity,cellular biosynthetic
Fv_160_5.g3187	349	ID=Fv_160_5.g3187;Description=hypothetical protein FVEG_06756 [Fusarium verticillioides 7600];Gene=TFS1;Ontology_term=fungal-type vacuole lumen,fungal-type vacuole membrane,serine-type endopeptidase inhibitor activity,phospholipid binding,regulation of proteolysis,regulation of Ras protein signal
Fv_160_5.g3188	441	ID=Fv_160_5.g3188;Description=hypothetical protein FVEG_06757 [Fusarium verticillioides 7600]

Fv_160_5.g3189	222	ID=Fv_160_5.g3189;Description=hypothetical protein FVEG_06758 [Fusarium verticillioides 7600];Gene=C2S_1627;Ontology_term=phosphatase
Fv_160_5.g3190	677	ID=Fv_160_5.g3190;Description=hypothetical protein FVEG_06759 [Fusarium verticillioides 7600];Gene=FVEG_06759;Ontology_term=nucleus,DNA binding,zinc ion binding,proteolysis,peptidase activity,DNA-binding transcription factor activity, RNA polymerase II-specific,regulation of transcription by RNA polymerase
Fv_160_5.g3191	321	ID=Fv_160_5.g3191;Description=hypothetical protein FVER14953_06760 [Fusarium verticillioides]
Fv_160_5.g3192	410	ID=Fv_160_5.g3192;Description=L-lactate dehydrogenase (cytochrome) [Fusarium verticillioides 7600];Gene=FPANT_5958;Ontology_term=FMN binding,oxidoreductase
Fv_160_5.g3193	838	ID=Fv_160_5.g3193;Description=hypothetical protein FVER53590_06762 [Fusarium verticillioides]
Fv_160_5.g3194	196	ID=Fv_160_5.g3194;Description=hypothetical protein FVEG_06763 [Fusarium verticillioides 7600]
Fv_160_5.g3195	1862	ID=Fv_160_5.g3195;Description=hypothetical protein FVER53263_06765 [Fusarium verticillioides]
Fv_160_5.g3196	237	ID=Fv_160_5.g3196;Description=hypothetical protein FVER53263_06767 [Fusarium verticillioides];Gene=FNAPI_6500;Ontology_term=protein serine/threonine kinase activity,ATP binding,acyltransferase activity, transferring groups other than amino-acyl
Fv_160_5.g3197	324	ID=Fv_160_5.g3197;Description=hypothetical protein FVEG_06768 [Fusarium verticillioides 7600];Gene=FOC1_g10012447;Ontology_term=membrane,iron ion binding,oxidoreductase activity,lipid biosynthetic process,cellular lipid metabolic
Fv_160_5.g3198	1024	ID=Fv_160_5.g3198;Description=hypothetical protein FVER14953_06769 [Fusarium verticillioides];Gene=FMUND_9390;Ontology_term=membrane,ATP binding,ATP hydrolysis activity,ATPase-coupled monoatomic cation transmembrane transporter activity,sodium ion transport,monoatomic cation transmembrane transport;Ontology_id=GO:0016020,GO:0005524,GO:0016887,GO:0019829,GO:0006814,GO:0098655;Enzyme_code=EC:7.2.2,EC:3.
Fv_160_5.g3199	448	ID=Fv_160_5.g3199;Description=hypothetical protein FVEG_06770 [Fusarium verticillioides 7600];Gene=CLP1;Ontology_term=mRNA cleavage factor complex,ATP binding,polynucleotide 5'-hydroxyl-kinase activity,mRNA 3'-end
Fv_160_5.g3200	436	ID=Fv_160_5.g3200;Description=hypothetical protein FVER14953_06771 [Fusarium verticillioides];Gene=FVER53590_06771;Ontology_term=ARC complex,regulatory ncRNA-mediated gene
Fv_160_5.g3201	413	ID=Fv_160_5.g3201;Description=transcriptional regulator ARG82 [Fusarium coicis];Gene=FSUBG_3045;Ontology_term=kinase activity,phosphorylation,inositol phosphate biosynthetic
Fv_160_5.g3202	168	ID=Fv_160_5.g3202;Description=hypothetical protein FVEG_06773 [Fusarium verticillioides

Fv_160_5.g3203	1196	ID=Fv_160_5.g3203;Description=adenosinetriphosphatase [Fusarium verticillioides 7600];Gene=FPANT_5968;Ontology_term=U2-type catalytic step 2 spliceosome,U2-type post-spliceosomal complex,second spliceosomal transesterification activity,nucleic acid binding,RNA helicase activity,ATP binding,hydrolase activity,generation of catalytic spliceosome for second transesterification step,spliceosomal complex disassembly;Ontology_id=GO:0071007,GO:0071021,GO:0000386,GO:0003676,GO:0003724,GO:0005524,GO:0016787,GO:0000350,
Fv_160_5.g3204	664	ID=Fv_160_5.g3204;Description=hypothetical protein FVEG_06775 [Fusarium verticillioides 7600]
Fv_160_5.g3205	175	ID=Fv_160_5.g3205;Description=hypothetical protein FVEG_15941 [Fusarium verticillioides 7600];Gene=FFUJ_08847;Ontology_term=extracellular region,membrane,antibacterial humoral response,innate immune
Fv_160_5.g3206	455	ID=Fv_160_5.g3206;Description=hypothetical protein FVER14953_06777 [Fusarium verticillioides]
Fv_160_5.g3207	285	ID=Fv_160_5.g3207;Description=hypothetical protein FVEG_06778 [Fusarium verticillioides 7600];Gene=FNAPI_6489;Ontology_term=extracellular region,membrane,chitinase activity,chitin binding,polysaccharide catabolic process,chitin catabolic
Fv_160_5.g3208	1477	ID=Fv_160_5.g3208;Description=hypothetical protein FVER53590_06779 [Fusarium verticillioides];Gene=MDCFG851_LOCUS338073;Ontology_term=extracellular region,chitinase activity,chitin binding,polysaccharide catabolic process,chitin catabolic
Fv_160_5.g3209	589	ID=Fv_160_5.g3209;Description=hypothetical protein FVEG_06780 [Fusarium verticillioides 7600];Gene=FPHYL_12042;Ontology_term=regulation of cell shape,motor neuron axon guidance,actin filament organization,neuronal cell body,cell projection,cytoplasmic side of plasma membrane,cell chemotaxis,GTP binding,cortical cytoskeleton organization,neuron migration,nematode larval development,endonuclease activity,GTPase activity,apoptotic process,cytosol,cytoplasmic vesicle,regulation of synaptic transmission, GABAergic,netrin-activated signaling pathway,left/right axis specification,negative regulation of microtubule polymerization,protein kinase binding,small GTPase mediated signal transduction,positive regulation of engulfment of apoptotic cell,positive regulation of distal tip cell migration,embryonic body morphogenesis,nematode male tail tip morphogenesis,chemorepulsion of axon,regulation of dorsal/ventral axon guidance,gastrulation,axon extension involved in axon guidance,nucleic acid binding,establishment of mitotic spindle orientation,cytoskeleton,regulation of actin cytoskeleton organization,metal ion binding,establishment or maintenance of actin cytoskeleton polarity,axon
Fv_160_5.g3210	1114	ID=Fv_160_5.g3210;Description=hypothetical protein FVER53263_06782 [Fusarium verticillioides];Gene=FNAPI_6602;Ontology_term=nucleus,ATP binding,hydrolase activity,metal ion binding,ATP-dependent chromatin remodeler activity,chromatin
Fv_160_5.g3211	246	ID=Fv_160_5.g3211;Description=GMP synthase (glutamine-hydrolysing) [Fusarium verticillioides 7600];Gene=FNAPI_6601;Ontology_term=glutamine metabolic process;Ontology_id=GO:0006541

Fv_160_5.g3212	393	ID=Fv_160_5.g3212;Description=hypothetical protein FVEG_06784 [Fusarium verticillioides 7600];Gene=FNAPI_6600;Ontology_term=motor neuron axon guidance,brain segmentation,RNA polymerase II cis-regulatory region sequence-specific DNA binding,peripheral nervous system development,DNA-binding transcription activator activity, RNA polymerase II-specific,positive regulation of transcription by RNA polymerase II,polyphosphate catabolic process,regulation of dendrite morphogenesis,nucleus,metal ion binding,cytoplasm,exopolyphosphatase activity,positive regulation of antimicrobial peptide biosynthetic
Fv_160_5.g3213	645	ID=Fv_160_5.g3213;Description=hypothetical protein FVER53263_06785 [Fusarium verticillioides];Gene=FNAPI_6599;Ontology_term=mitochondrial inner membrane,mitochondrial gene
Fv_160_5.g3214	1427	ID=Fv_160_5.g3214;Description=hypothetical protein FVER14953_06786 [Fusarium verticillioides];Gene=FTJAE_8670;Ontology_term=DNA-directed RNA polymerase complex,mitochondrion,DNA binding,DNA-directed 5'-3' RNA polymerase activity,DNA-templated
Fv_160_5.g3215	390	ID=Fv_160_5.g3215;Description=methionyl-tRNA synthetase [Fusarium verticillioides 7600];Gene=FOVG_10380;Ontology_term=tRNA binding,aminoacyl-tRNA ligase
Fv_160_5.g3216	868	ID=Fv_160_5.g3216;Description=hypothetical protein FVEG_06788 [Fusarium verticillioides 7600];Gene=AU210_010338;Ontology_term=nucleus,helicase activity,ATP binding,hydrolase activity,ATP-dependent chromatin remodeler activity,chromatin
Fv_160_5.g3217	149	ID=Fv_160_5.g3217;Description=hypothetical protein FVEG_15942 [Fusarium verticillioides 7600]
Fv_160_5.g3218	1844	ID=Fv_160_5.g3218;Description=hypothetical protein FVER14953_06789 [Fusarium verticillioides]
Fv_160_5.g3219	329	ID=Fv_160_5.g3219;Description=NADPH2:quinone reductase [Fusarium verticillioides 7600];Gene=ZTA1-0;Ontology_term=NADPH:quinone reductase activity,zinc ion
Fv_160_5.g3220	364	ID=Fv_160_5.g3220;Description=UDP-glucose 4-epimerase [Fusarium acutatum];Gene=FGLOB1_12637;Ontology_term=UDP-glucose 4-epimerase activity,galactose metabolic
Fv_160_5.g3221	1029	ID=Fv_160_5.g3221;Description=hypothetical protein FVER53590_06792 [Fusarium
Fv_160_5.g3222	795	ID=Fv_160_5.g3222;Description=ATP-dependent RNA helicase DBP4 [Fusarium verticillioides 7600];Gene=FVER53590_06793;Ontology_term=nucleolus,small-subunit processome,RNA binding,RNA helicase activity,ATP binding,ATP hydrolysis activity,identical protein binding,rRNA processing;Ontology_id=GO:0005730,GO:0032040,GO:0003723,GO:0003724,GO:0005524,GO:0016887,GO:0042802,GO:0006364;E
Fv_160_5.g3223	689	ID=Fv_160_5.g3223;Description=hypothetical protein FVEG_06794 [Fusarium verticillioides 7600];Gene=FNAPI_6589;Ontology_term=nucleolus,small-subunit processome,RNA binding,rRNA
Fv_160_5.g3224	386	ID=Fv_160_5.g3224;Description=hypothetical protein FVEG_06795 [Fusarium verticillioides 7600];Gene=FOC4_g10008448;Ontology_term=S-adenosylmethionine-dependent methyltransferase
Fv_160_5.g3225	219	ID=Fv_160_5.g3225;Description=hypothetical protein FVEG_06796 [Fusarium verticillioides 7600]

Fv_160_5.g3226	479	ID=Fv_160_5.g3226;Description=hypothetical protein FVER14953_06797 [Fusarium verticillioides];Gene=FPANT_12449;Ontology_term=cAMP response element binding protein binding,RNA polymerase II cis-regulatory region sequence-specific DNA binding,DNA-binding transcription activator activity, RNA polymerase II-specific,mucosal immune response,positive regulation of transcription by RNA polymerase II,dendrite morphogenesis,nucleoplasm,wing disc anterior/posterior pattern formation,negative regulation of transcription by RNA polymerase II,labial disc development,cytosol,regulation of cell proliferation involved in compound eye morphogenesis,cuticle pattern formation,protein kinase binding,negative regulation of hippo signaling,spiracle morphogenesis, open tracheal system,protein homodimerization activity,positive regulation of G1/S transition of mitotic cell cycle,segment polarity determination,smoothened signaling pathway,N,N-dimethylaniline monooxygenase activity,NADP binding,heart formation,metal ion binding,positive regulation of epithelial cell differentiation,flavin adenine dinucleotide binding,Hedgehog signaling complex,genital disc anterior/posterior pattern
Fv_160_5.g3227	663	ID=Fv_160_5.g3227;Description=hypothetical protein FVEG_06798 [Fusarium verticillioides 7600];Gene=AU210_010348;Ontology_term=nucleus,DNA-binding transcription factor activity, RNA polymerase II-specific,zinc ion
Fv_160_5.g3228	117	ID=Fv_160_5.g3228;Description=hypothetical protein FVEG_06799 [Fusarium verticillioides 7600]
Fv_160_5.g3229	399	ID=Fv_160_5.g3229;Description=hypothetical protein LB504_008782 [Fusarium proliferatum];Gene=FPHYL_11737;Ontology_term=glycosyltransferase
Fv_160_5.g3230	479	ID=Fv_160_5.g3230;Description=hypothetical protein FVEG_06801 [Fusarium verticillioides
Fv_160_5.g3231	126	ID=Fv_160_5.g3231;Description=hypothetical protein FVEG_06802 [Fusarium verticillioides
Fv_160_5.g3232	434	ID=Fv_160_5.g3232;Description=hypothetical protein FVER53590_06803 [Fusarium verticillioides];Gene=FVEG_06803;Ontology_term=oxidoreductase activity,flavin adenine dinucleotide
Fv_160_5.g3233	140	ID=Fv_160_5.g3233;Description=hypothetical protein FVEG_06804 [Fusarium verticillioides 7600]
Fv_160_5.g3234	307	ID=Fv_160_5.g3234;Description=hypothetical protein FVER14953_06805 [Fusarium verticillioides];Gene=OYE1;Ontology_term=FMN binding,oxidoreductase activity;Ontology_id=GO:0010181,GO:0016491;Enzyme_code=EC:1;Enzyme_name=Oxidoreductases
Fv_160_5.g3235	211	ID=Fv_160_5.g3235;Description=hypothetical protein FVEG_06806 [Fusarium verticillioides
Fv_160_5.g3236	289	ID=Fv_160_5.g3236;Description=hypothetical protein FVEG_06807 [Fusarium verticillioides 7600]
Fv_160_5.g3237	105	ID=Fv_160_5.g3237;Description=hypothetical protein FVEG_06808 [Fusarium verticillioides 7600]
Fv_160_5.g3238	629	ID=Fv_160_5.g3238;Description=hypothetical protein FVEG_06809 [Fusarium verticillioides 7600];Gene=FFUJ_08815;Ontology_term=nucleus,DNA-binding transcription factor activity, RNA polymerase II-specific,zinc ion
Fv_160_5.g3239	177	ID=Fv_160_5.g3239;Description=hypothetical protein FVEG_06810 [Fusarium verticillioides 7600];Gene=Forpi1262_v006684;Ontology_term=endoplasmic reticulum membrane;Ontology_id=GO:0005789
Fv_160_5.g3240	296	ID=Fv_160_5.g3240;Description=hypothetical protein FVEG_06811 [Fusarium verticillioides

Fv_160_5.g3241	297	ID=Fv_160_5.g3241;Description=hypothetical protein FVER14953_06812 [Fusarium verticillioides];Gene=BFJ72_g2608;Ontology_term=nucleus,RNA binding,ATP binding,S-adenosylmethionine-dependent methyltransferase activity,metal ion binding,poly(A) RNA polymerase activity,mRNA processing,RNA 3'-end processing,methylation;Ontology_id=GO:0005634,GO:0003723,GO:0005524,GO:0008757,GO:0046872,GO:1990817,GO:0006397,G
Fv_160_5.g3242	1403	ID=Fv_160_5.g3242;Description=hypothetical protein FVER14953_06813 [Fusarium verticillioides];Gene=BFJ72_g2607;Ontology_term=nuclear pore,structural constituent of nuclear pore,nucleocytoplasmic
Fv_160_5.g3243	334	ID=Fv_160_5.g3243;Description=3-ketoacyl-CoA reductase [Fusarium verticillioides 7600];Gene=FPCIR_11507;Ontology_term=endoplasmic reticulum membrane,ketoreductase activity,very-long-chain 3-oxoacyl-CoA reductase activity,sphingolipid biosynthetic process,fatty acid elongation,very long-chain fatty acid biosynthetic
Fv_160_5.g3244	170	ID=Fv_160_5.g3244;Description=hypothetical protein FVEG_06815 [Fusarium verticillioides 7600];Gene=FOPG_03959;Ontology_term=endoplasmic reticulum membrane;Ontology_id=GO:0005789
Fv_160_5.g3245	382	ID=Fv_160_5.g3245;Description=galactose-1-phosphate uridylyltransferase [Fusarium verticillioides 7600];Gene=FNAPI_6566;Ontology_term=peroxisomal membrane,UDP-glucose:hexose-1-phosphate uridylyltransferase activity,zinc ion binding,peroxisome organization,galactose catabolic process via UDP-
Fv_160_5.g3246	536	ID=Fv_160_5.g3246;Description=transcriptional adapter 2-alpha [Fusarium verticillioides 7600];Gene=FPANT_13426;Ontology_term=nucleus,transcription coactivator activity,zinc ion binding,regulation of transcription by RNA polymerase II,regulation of histone acetylation,positive regulation of DNA-templated
Fv_160_5.g3247	142	ID=Fv_160_5.g3247;Description=hypothetical protein FCOIX_7542 [Fusarium
Fv_160_5.g3248	118	ID=Fv_160_5.g3248;Description=hypothetical protein FVEG_15945 [Fusarium verticillioides
Fv_160_5.g3249	239	ID=Fv_160_5.g3249;Description=hypothetical protein FVER14953_06818 [Fusarium
Fv_160_5.g3250	89	ID=Fv_160_5.g3250;Description=hypothetical protein BFJ72_g2673 [Fusarium proliferatum];Gene=Focb16_v014604
Fv_160_5.g3251	146	ID=Fv_160_5.g3251;Description=hypothetical protein FVEG_06819 [Fusarium verticillioides 7600];Gene=C2S_13919;Ontology_term=thiosulfate sulfurtransferase activity,phosphatase
Fv_160_5.g3252	272	ID=Fv_160_5.g3252;Description=hydrolase-HD superfamily protein [Fusarium verticillioides 7600];Gene=BFJ65_g11597;Ontology_term=5'-deoxynucleotidase activity,metal ion
Fv_160_5.g3253	468	ID=Fv_160_5.g3253;Description=hypothetical protein FVEG_06821 [Fusarium verticillioides 7600]
Fv_160_5.g3254	274	ID=Fv_160_5.g3254;Description=related to chitin binding protein [Fusarium fujikuroi IMI 58289];Gene=FGADI_11235;Ontology_term=hydrolase activity, acting on glycosyl bonds,hydrolase activity, acting on carbon-nitrogen (but not peptide) bonds,xylan catabolic
Fv_160_5.g3255	818	ID=Fv_160_5.g3255;Description=hypothetical protein FVER53263_06823 [Fusarium verticillioides];Gene=FPCIR_11517;Ontology_term=nucleus,DNA binding,zinc ion binding,DNA-binding transcription factor activity, RNA polymerase II-specific,regulation of transcription by RNA polymerase

Fv_160_5.g3256	734	ID=Fv_160_5.g3256;Description=hypothetical protein FVER14953_06824 [Fusarium verticillioides];Gene=FOQG_03937;Ontology_term=GTPase activity,GTP binding,organelle
Fv_160_5.g3257	712	ID=Fv_160_5.g3257;Description=hypothetical protein J7337_009488 [Fusarium musae];Gene=FANTH_6671;Ontology_term=membrane,inorganic phosphate transmembrane transporter activity,phosphate ion
Fv_160_5.g3258	492	ID=Fv_160_5.g3258;Description=homoserine O-acetyltransferase [Fusarium verticillioides 7600];Gene=FOQG_03935;Ontology_term=acyltransferase activity, transferring groups other than amino-acyl groups,biosynthetic
Fv_160_5.g3259	2430	ID=Fv_160_5.g3259;Description=hypothetical protein FVER14953_06827 [Fusarium verticillioides];Gene=FVEG_06827;Ontology_term=THO complex,mRNA processing,mRNA export from
Fv_160_5.g3260	350	ID=Fv_160_5.g3260;Description=hypothetical protein FVEG_06828 [Fusarium verticillioides 7600];Gene=FOC1_g10012505;Ontology_term=membrane,phosphoric diester hydrolase activity,lipid metabolic
Fv_160_5.g3261	324	ID=Fv_160_5.g3261;Description=cell wall integrity and stress response component [Fusarium verticillioides 7600];Gene=FVEG_06829;Ontology_term=nucleus,cytosol,membrane,magnesium ion binding,pyruvate decarboxylase activity,thiamine pyrophosphate binding,branched-chain-2-oxoacid decarboxylase activity,indolepyruvate decarboxylase activity,phenylpyruvate decarboxylase activity,aromatic amino acid family catabolic process to alcohol via Ehrlich pathway,ethanol metabolic process,L-phenylalanine catabolic process,tryptophan catabolic process,branched-chain amino acid catabolic process;Ontology_id=GO:0005634,GO:0005829,GO:0016020,GO:0000287,GO:0004737,GO:0030976,GO:0047433,GO:0047434,GO:0050177,GO:0000949,GO:0006067,GO:0006559,GO:0006569,GO:0009083;Enzyme_code=EC:4.1.1.1,EC:4.1.1.43,EC:4.1.1.74,EC:4.1.
Fv_160_5.g3262	1172	ID=Fv_160_5.g3262;Description=hypothetical protein FVER14953_06830 [Fusarium
Fv_160_5.g3263	892	ID=Fv_160_5.g3263;Description=hypothetical protein FVEG_06831 [Fusarium verticillioides 7600];Gene=FPRO_10592;Ontology_term=membrane,calcium-activated cation channel activity,monoatomic cation transmembrane
Fv_160_5.g3264	467	ID=Fv_160_5.g3264;Description=hypothetical protein FVEG_06832 [Fusarium verticillioides 7600]
Fv_160_5.g3265	626	ID=Fv_160_5.g3265;Description=hypothetical protein FVER14953_06833 [Fusarium verticillioides];Gene=ERG6;Ontology_term=mitochondrion,endoplasmic reticulum,lipid droplet,chitin-based extracellular matrix,sterol 24-C-methyltransferase activity,structural constituent of chitin-based larval cuticle,oxidoreductase activity, acting on CH-OH group of donors,identical protein binding,flavin adenine dinucleotide binding,ergosterol biosynthetic process,larval chitin-based cuticle
Fv_160_5.g3266	362	ID=Fv_160_5.g3266;Description=pyruvate formate lyase activating enzyme [Fusarium verticillioides 7600];Gene=FPRO05_12006;Ontology_term=lyase activity,metal ion binding,iron-sulfur cluster
Fv_160_5.g3267	315	ID=Fv_160_5.g3267;Description=hypothetical protein FVER14953_20488 [Fusarium verticillioides]
Fv_160_5.g3268	255	ID=Fv_160_5.g3268;Description=hypothetical protein FVEG_06836 [Fusarium verticillioides 7600];Gene=FVEG_06836;Ontology_term=nucleus,membrane,DNA binding,endonuclease activity,DNA transposition,endocytosis,DNA

Fv_160_5.g3269	331	ID=Fv_160_5.g3269;Description=hypothetical protein FVEG_06837 [Fusarium verticillioides 7600];Gene=FMEXI_11091;Ontology_term=cytosol,eukaryotic translation initiation factor 2B complex,guanyl-nucleotide exchange factor complex,translation initiation factor activity,phosphoenolpyruvate carboxykinase (ATP) activity,guanyl-nucleotide exchange factor activity,protein binding,ATP binding,cytoplasmic translational initiation,gluconeogenesis,regulation of translational initiation,positive regulation of cellular response to amino acid starvation,negative regulation of TORC1 signaling;Ontology_id=GO:0005829,GO:0005851,GO:0032045,GO:0003743,GO:0004612,GO:0005085,GO:0005515,GO:0005524,GO:
Fv_160_5.g3270	855	ID=Fv_160_5.g3270;Description=hypothetical protein FVER14953_06838 [Fusarium verticillioides];Gene=FOVG_10441;Ontology_term=S-adenosylmethionine-dependent methyltransferase
Fv_160_5.g3271	820	ID=Fv_160_5.g3271;Description=hypothetical protein J7337_009502 [Fusarium musae];Gene=FNAPI_5535;Ontology_term=maltose metabolic process,nucleus,DNA binding,mitochondrial cytochrome c oxidase assembly,translation initiation factor activity,zinc ion binding,maltose alpha-glucosidase activity,positive regulation of mitochondrial translation,translational initiation,mitochondrial inner membrane,DNA-binding transcription factor activity, RNA polymerase II-specific,regulation of transcription by RNA polymerase II;Ontology_id=GO:0000023,GO:0005634,GO:0003677,GO:0033617,GO:0003743,GO:0008270,GO:0032450,GO:0070131,GO:000641
Fv_160_5.g3272	505	ID=Fv_160_5.g3272;Description=hypothetical protein FVEG_06841 [Fusarium verticillioides 7600];Gene=FTJAE_1000;Ontology_term=spindle pole,gamma-tubulin complex,cytoplasm,spindle pole body,microtubule,gamma-tubulin binding,microtubule
Fv_160_5.g3273	788	ID=Fv_160_5.g3273;Description=hypothetical protein FVER14953_06842 [Fusarium verticillioides];Gene=FNAPI_5533;Ontology_term=spindle pole,gamma-tubulin complex,cytoplasm,spindle pole body,microtubule,gamma-tubulin binding,microtubule
Fv_160_5.g3274	516	ID=Fv_160_5.g3274;Description=hypothetical protein FVEG_06843 [Fusarium verticillioides 7600]
Fv_160_5.g3275	1228	ID=Fv_160_5.g3275;Description=hypothetical protein FVER53590_06844 [Fusarium verticillioides]
Fv_160_5.g3276	267	ID=Fv_160_5.g3276;Description=hypothetical protein FVEG_06846 [Fusarium verticillioides 7600];Gene=FPANT_10613;Ontology_term=nucleus,myosin complex,A band,myosin filament,nucleic acid binding,cytoskeletal protein binding,RNA polymerase II CTD heptapeptide repeat phosphatase activity,myosin phosphatase activity,SH3 domain binding,protein kinase binding,RNA polymerase core enzyme binding,metal ion binding,regulation of transcription by RNA polymerase II,skeletal muscle myosin thick filament assembly,locomotion,positive regulation of ovulation,positive regulation of sarcomere
Fv_160_5.g3277	147	ID=Fv_160_5.g3277;Description=40S ribosomal protein S17 [Fusarium oxysporum f. sp. lycopersici 4287];Gene=CEP52_001393;Ontology_term=ribosome,ribonucleoprotein complex,structural constituent of
Fv_160_5.g3278	435	ID=Fv_160_5.g3278;Description=hypothetical protein FVER14953_06847 [Fusarium verticillioides]
Fv_160_5.g3279	302	ID=Fv_160_5.g3279;Description=queuine tRNA-ribosyltransferase [Fusarium verticillioides 7600];Gene=FCIRC_11048;Ontology_term=cytoplasm,tRNA-guanosine(34) queuine transglycosylase activity,metal ion binding,tRNA-guanine

Fv_160_5.g3280	333	ID=Fv_160_5.g3280;Description=hypothetical protein FVER14953_06849 [Fusarium verticillioides];Gene=FDENT_11758;Ontology_term=transferase
Fv_160_5.g3281	1167	ID=Fv_160_5.g3281;Description=hypothetical protein FVER53590_06850 [Fusarium verticillioides];Gene=FPHYL_9360;Ontology_term=regulation of DNA-templated transcription;Ontology_id=GO:0006355
Fv_160_5.g3282	309	ID=Fv_160_5.g3282;Description=hypothetical protein FVER14953_06851 [Fusarium verticillioides];Gene=AU210_010404;Ontology_term=histidinol-phosphatase activity,histidine biosynthetic
Fv_160_5.g3283	1030	ID=Fv_160_5.g3283;Description=hypothetical protein FVER53590_06852 [Fusarium
Fv_160_5.g3284	1059	ID=Fv_160_5.g3284;Description=hypothetical protein FVER53590_06853 [Fusarium verticillioides];Gene=FPCIR_6140;Ontology_term=plasma membrane,osmosensor activity,osmosensory signaling pathway via Sho1
Fv_160_5.g3285	207	ID=Fv_160_5.g3285;Description=hypothetical protein FVEG_06854 [Fusarium verticillioides 7600];Gene=FGADI_9741;Ontology_term=endoplasmic reticulum membrane,cytosol,axon,dendrite,dihydropyrimidinase activity,monooxygenase activity,iron ion binding,oxidoreductase activity, acting on paired donors, with incorporation or reduction of molecular oxygen,flavin-dependent sulfhydryl oxidase activity,heme binding,filamin binding,protein homodimerization activity,protein heterodimerization activity,pyrimidine nucleobase catabolic process,motor neuron axon guidance,regulation of cell migration,establishment or maintenance of actin cytoskeleton polarity,establishment or maintenance of microtubule cytoskeleton polarity,negative regulation of microtubule polymerization,netrin-activated signaling pathway,chemorepulsion of axon,sensory neuron axon guidance,regulation of dorsal/ventral axon guidance;Ontology_id=GO:0005789,GO:0005829,GO:0030424,GO:0030425,GO:0004157,GO:0004497,GO:0005506,GO:0016705,GO
Fv_160_5.g3286	229	ID=Fv_160_5.g3286;Description=proteasome component PRE3 [Fusarium oxysporum f. sp. lycopersici 4287];Gene=BHE90_008904;Ontology_term=nucleus,proteasome core complex, beta-subunit complex,proteasome storage granule,threonine-type endopeptidase activity,hydrolase activity, acting on carbon-nitrogen (but not peptide) bonds,proteasomal ubiquitin-independent protein catabolic process,proteasome-mediated ubiquitin-dependent protein catabolic process;Ontology_id=GO:0005634,GO:0019774,GO:0034515,GO:0004298,GO:0016810,GO:0010499,GO:0043161;Enzyme_code=EC
Fv_160_5.g3287	488	ID=Fv_160_5.g3287;Description=hypothetical protein FVEG_06857 [Fusarium verticillioides
Fv_160_5.g3288	288	ID=Fv_160_5.g3288;Description=hypothetical protein FVER53263_06858 [Fusarium verticillioides];Gene=F52700_1673;Ontology_term=monooxygenase activity,melanin biosynthetic
Fv_160_5.g3289	586	ID=Fv_160_5.g3289;Description=hypothetical protein FVER53590_06859 [Fusarium verticillioides];Gene=FTJAE_984;Ontology_term=tyrosinase activity,melanin biosynthetic

Fv_160_5.g3290	1232	ID=Fv_160_5.g3290;Description=DNA-directed RNA polymerase I subunit RPA2 [Fusarium verticillioides 7600];Gene=unc-93;Ontology_term=transcription initiation at RNA polymerase I promoter,striated muscle dense body,transcription elongation by RNA polymerase I,regulation of potassium ion transport,termination of RNA polymerase I transcription,plasma membrane,DNA-binding transcription factor activity, RNA polymerase II-specific,regulation of transcription by RNA polymerase II,DNA binding,RNA polymerase I complex,ribonucleoside binding,RNA polymerase I activity,regulation of muscle contraction,potassium channel regulator
Fv_160_5.g3291	184	ID=Fv_160_5.g3291;Description=arf/Sar family, other [Fusarium oxysporum f. sp. lycopersici 4287];Gene=CDV31_001391;Ontology_term=membrane,GTPase activity,GTP binding,protein
Fv_160_5.g3292	376	ID=Fv_160_5.g3292;Description=hypothetical protein FVER14953_06863 [Fusarium verticillioides];Gene=Focb16_v014647;Ontology_term=membrane,beta-1,4-mannosylglycoprotein 4-beta-N-acetylglucosaminyltransferase activity;Ontology_id=GO:0016020,GO:0003830;Enzyme_code=EC:2.4.1.144;Enzyme_name=beta-1,4-
Fv_160_5.g3293	385	ID=Fv_160_5.g3293;Description=threonine aldolase [Fusarium verticillioides 7600];Gene=FOPG_03902;Ontology_term=lyase activity,amino acid metabolic process;Ontology_id=GO:0016829,GO:0006520;Enzyme_code=EC:4;Enzyme_name=Lyases
Fv_160_5.g3294	687	ID=Fv_160_5.g3294;Description=hypothetical protein FVER14953_06865 [Fusarium verticillioides];Gene=BFJ70_g2640;Ontology_term=extracellular region,cytoplasmic vesicle,vesicle lumen,yolk granule,alpha,alpha-trehalase activity,lipid transporter activity,nutrient reservoir activity,trehalose metabolic process,lipid transport;Ontology_id=GO:0005576,GO:0031410,GO:0031983,GO:0042718,GO:0004555,GO:0005319,GO:0045735,GO:0005991,GO
Fv_160_5.g3295	152	ID=Fv_160_5.g3295;Description=hypothetical protein FANTH_6709 [Fusarium anthophilum]
Fv_160_5.g3296	910	ID=Fv_160_5.g3296;Description=hypothetical protein FVER14953_06867 [Fusarium verticillioides];Gene=FTJAE_13998;Ontology_term=extracellular region,nucleus,vacuolar membrane,aminopeptidase activity,lipid transporter activity,protein binding,serine-type peptidase activity,dipeptidyl-peptidase activity,sequence-specific DNA binding,nutrient reservoir activity,chromatin remodeling,proteolysis,lipid transport,cellular response to phosphate starvation,positive regulation of phosphate metabolic process,positive regulation of transcription by RNA polymerase II;Ontology_id=GO:0005576,GO:0005634,GO:0005774,GO:0004177,GO:0005319,GO:0005515,GO:0008236,GO:0008239,GO:0043565,GO:0045735,GO:0006338,GO:0006508,GO:0006869,GO:0016036,GO:0045937,GO:0045944;Enzyme_code=EC:3.4.11,EC:3.4.14.1,
Fv_160_5.g3297	1683	ID=Fv_160_5.g3297;Description=hypothetical protein J7337_009528 [Fusarium musae];Gene=chc1;Ontology_term=acetylcholine-gated channel complex,clathrin coat of trans-Golgi network vesicle,clathrin coat of coated pit,neuron projection,postsynaptic membrane,clathrin complex,structural molecule activity,acetylcholine receptor activity,acetylcholine-gated monoatomic cation-selective channel activity,clathrin light chain binding,intracellular protein transport,synaptic transmission, cholinergic,vesicle-mediated transport,excitatory postsynaptic potential,monoatomic cation transmembrane transport;Ontology_id=GO:0005892,GO:0030130,GO:0030132,GO:0043005,GO:0045211,GO:0071439,GO:0005198,GO:0015464,GO
Fv_160_5.g3298	189	ID=Fv_160_5.g3298;Description=hypothetical protein FVEG_06869 [Fusarium verticillioides 7600]

Fv_160_5.g3299	570	ID=Fv_160_5.g3299;Description=hypothetical protein FVEG_06870 [Fusarium verticillioides 7600];Gene=FTJAE_13995;Ontology_term=nucleus,membrane,DNA-binding transcription factor activity, RNA polymerase II-specific,zinc ion binding,transmembrane transporter activity,regulation of transcription by RNA polymerase II,transmembrane
Fv_160_5.g3300	628	ID=Fv_160_5.g3300;Description=hypothetical protein FVEG_06871 [Fusarium verticillioides 7600];Gene=BFJ69_g5175;Ontology_term=nucleus,membrane,DNA-binding transcription factor activity, RNA polymerase II-specific,zinc ion binding,transmembrane transporter activity,regulation of transcription by RNA polymerase II,transmembrane
Fv_160_5.g3301	259	ID=Fv_160_5.g3301;Description=hypothetical protein FVER14953_06872 [Fusarium verticillioides];Gene=FOC1_g10012548;Ontology_term=mitochondrion,membrane,mitochondrial cytochrome c oxidase
Fv_160_5.g3302	334	ID=Fv_160_5.g3302;Description=hypothetical protein FVER14953_06873 [Fusarium verticillioides];Gene=FPANT_487;Ontology_term=oxidoreductase activity, acting on the CH-OH group of donors, NAD or NADP as
Fv_160_5.g3303	449	ID=Fv_160_5.g3303;Description=adenosylhomocysteinase [Fusarium oxysporum f. sp. lycopersici 4287];Gene=AU210_010426;Ontology_term=adenosylhomocysteinase activity,triglyceride metabolic process,phosphatidylcholine biosynthetic process,one-carbon metabolic process,S-adenosylhomocysteine catabolic
Fv_160_5.g3304	364	ID=Fv_160_5.g3304;Description=translation initiation factor 3 subunit H [Fusarium verticillioides 7600];Gene=FMEXI_14024;Ontology_term=eukaryotic translation initiation factor 3 complex,eukaryotic 43S preinitiation complex,eukaryotic 48S preinitiation complex,translation initiation factor activity,metallopeptidase activity,formation of cytoplasmic
Fv_160_5.g3305	219	ID=Fv_160_5.g3305;Description=hypothetical protein FVEG_06876 [Fusarium verticillioides 7600]
Fv_160_5.g3306	315	ID=Fv_160_5.g3306;Description=hypothetical protein FVEG_06877 [Fusarium verticillioides 7600];Gene=AU210_010429;Ontology_term=extracellular region,mitochondrial inner membrane,cytoplasmic vesicle,vesicle lumen,yolk granule,lipid transporter activity,nutrient reservoir activity,lipid transport,transmembrane
Fv_160_5.g3307	394	ID=Fv_160_5.g3307;Description=hypothetical protein FVER53263_06878 [Fusarium verticillioides];Gene=FOC4_g10008536;Ontology_term=myosin phosphatase activity,metal ion
Fv_160_5.g3308	278	ID=Fv_160_5.g3308;Description=succinate dehydrogenase [ubiquinone] iron-sulfur subunit, mitochondrial [Fusarium verticillioides 7600];Gene=FOC4_g10008537;Ontology_term=mitochondrial inner membrane,succinate dehydrogenase (ubiquinone) activity,electron transfer activity,metal ion binding,2 iron, 2 sulfur cluster binding,3 iron, 4 sulfur cluster binding,4 iron, 4 sulfur cluster binding,fumarate reductase (menaquinone),tricarboxylic acid cycle,electron transport chain;Ontology_id=GO:0005743,GO:0008177,GO:0009055,GO:0046872,GO:0051537,GO:0051538,GO:0051539,GO:0102040,GO:00
Fv_160_5.g3309	92	ID=Fv_160_5.g3309;Description=hypothetical protein FVEG_15951 [Fusarium verticillioides 7600]
Fv_160_5.g3310	479	ID=Fv_160_5.g3310;Description=hypothetical protein FVEG_06880 [Fusarium verticillioides
Fv_160_5.g3311	598	ID=Fv_160_5.g3311;Description=alpha-1,3-glucosyltransferase [Fusarium verticillioides 7600];Gene=FTJAE_12457;Ontology_term=endoplasmic reticulum membrane,dolichyl pyrophosphate Man9GlcNAc2 alpha-1,3-glucosyltransferase activity,dolichyl pyrophosphate Glc1Man9GlcNAc2 alpha-1,3-glucosyltransferase activity,oligosaccharide-lipid intermediate biosynthetic

Fv_160_5.g3312	381	ID=Fv_160_5.g3312;Description=hypothetical protein FVER14953_06882 [Fusarium verticillioides];Gene=FTJAE_12458;Ontology_term=nucleus,cytoplasm,membrane,Pho85-Pho80 CDK-cyclin complex,cyclin-dependent protein serine/threonine kinase regulator activity,protein kinase binding,negative regulation of transcription by RNA polymerase II,negative regulation of macroautophagy,intracellular monoatomic cation homeostasis,regulation of protein localization,vacuole fusion, non-autophagic,negative regulation of phosphate metabolic process,negative regulation of calcium-
Fv_160_5.g3313	110	ID=Fv_160_5.g3313;Description=50S ribosomal protein LP2 [Fusarium oxysporum f. sp. lycopersici 4287];Gene=FPANT_476;Ontology_term=cytosolic large ribosomal subunit,structural constituent of ribosome,cytoplasmic
Fv_160_5.g3314	415	ID=Fv_160_5.g3314;Description=hypothetical protein FVER53263_06884 [Fusarium verticillioides]
Fv_160_5.g3315	599	ID=Fv_160_5.g3315;Description=hypothetical protein J7337_009543 [Fusarium musae];Gene=FOXB_13760;Ontology_term=RNA binding,metal ion binding;Ontology_id=GO:0003723,GO:0046872
Fv_160_5.g3316	193	ID=Fv_160_5.g3316;Description=DNA-directed RNA polymerase III subunit RPC8 [Fusarium oxysporum f. sp. lycopersici 4287];Gene=FCIRC_3698;Ontology_term=chromatin,RNA polymerase III complex,RNA polymerase III activity,DNA binding,transcription initiation at RNA polymerase III promoter,termination of RNA polymerase III transcription,tRNA transcription by RNA polymerase
Fv_160_5.g3317	260	ID=Fv_160_5.g3317;Description=hypothetical protein FVEG_06888 [Fusarium verticillioides
Fv_160_5.g3318	223	ID=Fv_160_5.g3318;Description=hypothetical protein FVEG_06889 [Fusarium verticillioides 7600];Gene=FGLOB1_11649;Ontology_term=signal peptidase complex,signal peptide
Fv_160_5.g3319	1164	ID=Fv_160_5.g3319;Description=hypothetical protein FVEG_06890 [Fusarium verticillioides 7600];Gene=FVER53590_06890;Ontology_term=nucleus,CIA complex,DNA repair,iron-sulfur cluster assembly,protein maturation by
Fv_160_5.g3320	335	ID=Fv_160_5.g3320;Description=hypothetical protein FVER14953_06891 [Fusarium verticillioides];Gene=BFJ68_g191;Ontology_term=mitochondrion,protein-containing complex
Fv_160_5.g3321	723	ID=Fv_160_5.g3321;Description=hypothetical protein FVER14953_06892 [Fusarium verticillioides];Gene=FTJAE_12467;Ontology_term=membrane,lipid metabolic process;Ontology_id=GO:0016020,GO:0006629
Fv_160_5.g3322	1389	ID=Fv_160_5.g3322;Description=peroxisomal biogenesis factor 6 [Fusarium verticillioides 7600];Gene=FTJAE_12468;Ontology_term=peroxisomal membrane,cytosol,ATP binding,ATP hydrolysis activity,peroxisome organization;Ontology_id=GO:0005778,GO:0005829,GO:0005524,GO:0016887,GO:0007031;Enzyme_code=EC:3.6.1.15;Enzyme_na
Fv_160_5.g3323	480	ID=Fv_160_5.g3323;Description=hypothetical protein FVER14953_06894 [Fusarium verticillioides]
Fv_160_5.g3324	195	ID=Fv_160_5.g3324;Description=hypothetical protein FVEG_06895 [Fusarium verticillioides
Fv_160_5.g3325	521	ID=Fv_160_5.g3325;Description=cyclopropane-fatty-acyl-phospholipid synthase [Fusarium verticillioides 7600];Gene=AU210_010448;Ontology_term=membrane,S-adenosylmethionine-dependent methyltransferase
Fv_160_5.g3326	283	ID=Fv_160_5.g3326;Description=mesaconyl-C4 CoA hydratase [Fusarium verticillioides 7600]
Fv_160_5.g3327	1064	ID=Fv_160_5.g3327;Description=hypothetical protein FVER53590_06898 [Fusarium verticillioides];Gene=FNYG_04809;Ontology_term=endoplasmic reticulum membrane,mannose-ethanolamine phosphotransferase

Fv_160_5.g3328	877	ID=Fv_160_5.g3328;Description=hypothetical protein FVEG_06899 [Fusarium verticillioides
Fv_160_5.g3329	383	ID=Fv_160_5.g3329;Description=hypothetical protein FVER53590_06900 [Fusarium verticillioides];Gene=FACUT_5638;Ontology_term=hydrolase activity, hydrolyzing O-glycosyl compounds,polysaccharide catabolic
Fv_160_5.g3330	628	ID=Fv_160_5.g3330;Description=hypothetical protein FVEG_06901 [Fusarium verticillioides 7600];Gene=BFJ69_g5242;Ontology_term=membrane,ATP binding,ATP hydrolysis activity,ABC-type transporter activity,transmembrane transport;Ontology_id=GO:0016020,GO:0005524,GO:0016887,GO:0140359,GO:0055085;Enzyme_code=EC:7.2.2,EC:3.6.1.15;Enzym
Fv_160_5.g3331	678	ID=Fv_160_5.g3331;Description=hypothetical protein FVEG_06902 [Fusarium verticillioides 7600];Gene=FVEG_06902;Ontology_term=nucleus,DNA binding,zinc ion binding,DNA-binding transcription factor activity, RNA
Fv_160_5.g3332	641	ID=Fv_160_5.g3332;Description=hypothetical protein FVER53590_06904 [Fusarium verticillioides];Gene=FTJAE_12478;Ontology_term=plasma membrane,protein kinase activity,ATP binding,P-type proton-exporting transporter activity,ATP hydrolysis activity,proton export across plasma membrane;Ontology_id=GO:0005886,GO:0004672,GO:0005524,GO:0008553,GO:0016887,GO:0120029;Enzyme_code=EC:2.7.1,EC:
Fv_160_5.g3333	72	ID=Fv_160_5.g3333;Description=hypothetical protein FOXG_20025 [Fusarium oxysporum f. sp. lycopersici 4287]
Fv_160_5.g3334	1011	ID=Fv_160_5.g3334;Description=hypothetical protein FVER53590_06905 [Fusarium verticillioides];Gene=FDENT_2749;Ontology_term=plasma membrane,protein kinase activity,ATP binding,P-type sodium transporter activity,P-type potassium transmembrane transporter activity,metal ion binding,response to pH,response to salt stress,phosphorylation,monoatomic ion transmembrane transport;Ontology_id=GO:0005886,GO:0004672,GO:0005524,GO:0008554,GO:0008556,GO:0046872,GO:0009268,GO:0009651,GO
Fv_160_5.g3335	489	ID=Fv_160_5.g3335;Description=hypothetical protein FVER53590_06906 [Fusarium verticillioides];Gene=ENA1;Ontology_term=plasma membrane,nucleotide binding,protein kinase activity,P-type sodium transporter activity,P-type potassium transmembrane transporter activity,metal ion binding,response to pH,response to salt stress,monoatomic ion transmembrane transport;Ontology_id=GO:0005886,GO:0000166,GO:0004672,GO:0008554,GO:0008556,GO:0046872,GO:0009268,GO:0009651,GO
Fv_160_5.g3336	385	ID=Fv_160_5.g3336;Description=hypothetical protein FVEG_06907 [Fusarium verticillioides 7600];Gene=FVEG_06907;Ontology_term=endoplasmic reticulum membrane;Ontology_id=GO:0005789
Fv_160_5.g3337	142	ID=Fv_160_5.g3337;Description=hypothetical protein FVEG_06908 [Fusarium verticillioides 7600];Gene=POL3;Ontology_term=nucleus,nucleotide binding,DNA binding,DNA-directed DNA polymerase activity,metal ion binding,4 iron, 4 sulfur cluster binding,DNA replication,DNA biosynthetic process;Ontology_id=GO:0005634,GO:0000166,GO:0003677,GO:0003887,GO:0046872,GO:0051539,GO:0006260,GO:0071897;Enzy
Fv_160_5.g3338	592	ID=Fv_160_5.g3338;Description=hypothetical protein FVEG_06909 [Fusarium verticillioides
Fv_160_5.g3339	809	ID=Fv_160_5.g3339;Description=hypothetical protein FVER53590_06910 [Fusarium verticillioides]

Fv_160_5.g3340	272	ID=Fv_160_5.g3340;Description=hypothetical protein FVER53590_06911 [Fusarium verticillioides];Gene=BFJ65_g11881;Ontology_term=dephospho-CoA kinase activity,ATP binding,coenzyme A biosynthetic process,phosphorylation;Ontology_id=GO:0004140,GO:0005524,GO:0015937,GO:0016310;Enzyme_code=EC:2.7.1.24;Enzyme_nam
Fv_160_5.g3341	455	ID=Fv_160_5.g3341;Description=hypothetical protein FVER53590_06911 [Fusarium verticillioides];Gene=FOXB_13735;Ontology_term=dephospho-CoA kinase activity,ATP binding,coenzyme A biosynthetic
Fv_160_5.g3342	472	ID=Fv_160_5.g3342;Description=hypothetical protein J7337_009570 [Fusarium musae];Gene=6959;Ontology_term=membrane,transmembrane transporter activity,transmembrane
Fv_160_5.g3343	284	ID=Fv_160_5.g3343;Description=TIM23 translocase [Fusarium phyllophilum];Gene=FOQG_03838;Ontology_term=TIM23 mitochondrial import inner membrane translocase complex,acetyl-CoA C-acyltransferase activity,protein transmembrane transporter activity,mitochondrion targeting sequence binding,protein import into mitochondrial
Fv_160_5.g3344	363	ID=Fv_160_5.g3344;Description=hypothetical protein FVEG_06916 [Fusarium verticillioides
Fv_160_5.g3345	416	ID=Fv_160_5.g3345;Description=hypothetical protein FVEG_06917 [Fusarium verticillioides 7600];Gene=NUDT12;Ontology_term=NAD+ diphosphatase activity,NADH pyrophosphatase activity,metal ion
Fv_160_5.g3346	223	ID=Fv_160_5.g3346;Description=hypothetical protein FVEG_06918 [Fusarium verticillioides 7600]
Fv_160_5.g3347	227	ID=Fv_160_5.g3347;Description=hypothetical protein FVEG_06919 [Fusarium verticillioides 7600]
Fv_160_5.g3348	536	ID=Fv_160_5.g3348;Description=hypothetical protein FVEG_06920 [Fusarium verticillioides 7600];Gene=FNAPI_221;Ontology_term=membrane,amino acid transport,transmembrane
Fv_160_5.g3349	1061	ID=Fv_160_5.g3349;Description=hypothetical protein FVER53590_06922 [Fusarium verticillioides]
Fv_160_5.g3350	296	ID=Fv_160_5.g3350;Description=hypothetical protein FVEG_06923 [Fusarium verticillioides 7600]
Fv_160_5.g3351	1543	ID=Fv_160_5.g3351;Description=hypothetical protein FVEG_06924 [Fusarium verticillioides 7600];Gene=BFJ65_g10680;Ontology_term=membrane,ATP binding,ATP hydrolysis activity,ABC-type transporter activity,transmembrane transport;Ontology_id=GO:0016020,GO:0005524,GO:0016887,GO:0140359,GO:0055085;Enzyme_code=EC:7.2.2,EC:3.6.1.15;Enzym
Fv_160_5.g3352	592	ID=Fv_160_5.g3352;Description=hypothetical protein FVEG_06926 [Fusarium verticillioides 7600]
Fv_160_5.g3353	308	ID=Fv_160_5.g3353;Description=hypothetical protein FVEG_06927 [Fusarium verticillioides 7600];Gene=FACUT_5616;Ontology_term=nucleus,DNA-binding transcription factor activity,protein dimerization activity,regulation
Fv_160_5.g3354	418	ID=Fv_160_5.g3354;Description=hypothetical protein FVEG_06928 [Fusarium verticillioides 7600];Gene=FNAPI_11997;Ontology_term=cytoplasm,RNA binding,metal ion binding,ubiquitin protein ligase activity,galactose metabolic process,proteasome-mediated ubiquitin-dependent protein catabolic process,negative regulation of gluconeogenesis,RNA polymerase III

Fv_160_5.g3355	484	ID=Fv_160_5.g3355;Description=hypothetical protein FVEG_06929 [Fusarium verticillioides 7600];Gene=BFJ65_g10980;Ontology_term=nucleus,CCR4-NOT complex,nucleic acid binding,poly(A)-specific ribonuclease activity,mRNA catabolic process;Ontology_id=GO:0005634,GO:0030014,GO:0003676,GO:0004535,GO:0006402;Enzyme_code=EC:3.1.15,EC:3.1.13.4;Enzym
Fv_160_5.g3356	96	ID=Fv_160_5.g3356;Description=hypothetical protein FVEG_06930 [Fusarium verticillioides 7600];Gene=BFJ65_g11155;Ontology_term=mitochondrial inner membrane,respirasome,electron transport
Fv_160_5.g3357	525	ID=Fv_160_5.g3357;Description=hypothetical protein FVEG_06931 [Fusarium verticillioides 7600]
Fv_160_5.g3358	211	ID=Fv_160_5.g3358;Description=hypothetical protein FVEG_06932 [Fusarium verticillioides
Fv_160_5.g3359	563	ID=Fv_160_5.g3359;Description=hypothetical protein FVEG_06933 [Fusarium verticillioides 7600]
Fv_160_5.g3360	574	ID=Fv_160_5.g3360;Description=ubiquitin-like 1-activating enzyme E1 A [Fusarium verticillioides 7600];Gene=FPRO_10691;Ontology_term=Cul2-RING ubiquitin ligase complex,ubiquitin-like modifier activating enzyme activity,cyclin binding,anterior/posterior axis specification, embryo,embryo development ending in birth or egg hatching,anaphase-promoting complex-dependent catabolic process,positive regulation of proteasomal ubiquitin-dependent protein catabolic process,protein modification process,sister chromosome movement towards spindle pole involved in meiotic sister chromatid segregation,positive regulation of metaphase/anaphase transition of meiotic cell
Fv_160_5.g3361	1311	ID=Fv_160_5.g3361;Description=hypothetical protein FVER53590_06935 [Fusarium verticillioides]
Fv_160_5.g3362	342	ID=Fv_160_5.g3362;Description=hypothetical protein FVEG_06936 [Fusarium verticillioides 7600];Gene=FVEG_06936;Ontology_term=rRNA (uridine-N3-)-methyltransferase activity,rRNA base
Fv_160_5.g3363	919	ID=Fv_160_5.g3363;Description=hypothetical protein FVER14953_06937 [Fusarium verticillioides];Gene=FVEG_06937;Ontology_term=nucleus,chromosome,ATP binding,D-alanine-D-alanine ligase activity,metal ion binding,histone H3K36 trimethyltransferase activity,chromatin remodeling,regulation of DNA-templated transcription,methylation;Ontology_id=GO:0005634,GO:0005694,GO:0005524,GO:0008716,GO:0046872,GO:0140955,GO:0006338,
Fv_160_5.g3364	403	ID=Fv_160_5.g3364;Description=hypothetical protein FVER53263_06938 [Fusarium verticillioides];Gene=BFJ65_g11365;Ontology_term=nucleus,chromosome,ATP binding,D-alanine-D-alanine ligase activity,metal ion binding,histone H3K36 methyltransferase activity,chromatin remodeling,regulation of DNA-templated transcription,methylation;Ontology_id=GO:0005634,GO:0005694,GO:0005524,GO:0008716,GO:0046872,GO:0046975,GO:0006338,
Fv_160_5.g3365	253	ID=Fv_160_5.g3365;Description=hypothetical protein FVER14953_06939 [Fusarium verticillioides]
Fv_160_5.g3366	502	ID=Fv_160_5.g3366;Description=4-aminobutyrate aminotransferase [Fusarium verticillioides 7600];Gene=C2S_6859;Ontology_term=pyridoxal phosphate binding,4-aminobutyrate:2-oxoglutarate transaminase activity,gamma-aminobutyric acid metabolic
Fv_160_5.g3367	867	ID=Fv_160_5.g3367;Description=hypothetical protein J7337_009594 [Fusarium musae];Gene=FNYG_04849;Ontology_term=membrane,voltage-gated chloride channel activity,chloride transmembrane

Fv_160_5.g3368	616	ID=Fv_160_5.g3368;Description=hypothetical protein FVEG_06942 [Fusarium verticillioides 7600];Gene=PRE2;Ontology_term=cytoplasm,proteasome core complex, beta-subunit complex,threonine-type endopeptidase activity,proteasomal ubiquitin-independent protein catabolic process,proteasome-mediated ubiquitin-dependent protein catabolic
Fv_160_5.g3369	818	ID=Fv_160_5.g3369;Description=hypothetical protein FVER14953_06943 [Fusarium verticillioides];Gene=FDENT_2718;Ontology_term=membrane,Golgi transport complex,glycosome,phosphoglycerate kinase activity,ATP binding,glycolytic process,intra-Golgi vesicle-mediated transport,protein transport,phosphorylation;Ontology_id=GO:0016020,GO:0017119,GO:0020015,GO:0004618,GO:0005524,GO:0006096,GO:0006891
Fv_160_5.g3370	270	ID=Fv_160_5.g3370;Description=unnamed protein product [Fusarium fujikuroi];Gene=FDENT_2717;Ontology_term=DNA-directed RNA polymerase complex,nucleolus,cytoplasm,proteasome core complex, beta-subunit complex,DNA binding,isomerase activity,DNA-templated transcription,proteasomal ubiquitin-independent protein catabolic process,proteasome-mediated ubiquitin-dependent protein catabolic
Fv_160_5.g3371	439	ID=Fv_160_5.g3371;Description=hypothetical protein FVEG_06945 [Fusarium verticillioides 7600];Gene=FDENT_2716;Ontology_term=DNA-directed RNA polymerase complex,nucleolus,DNA binding,DNA-templated
Fv_160_5.g3372	627	ID=Fv_160_5.g3372;Description=CAMK kinase [Fusarium coicis];Gene=FOQG_03803;Ontology_term=protein kinase activity,ATP binding,phosphorylation;Ontology_id=GO:0004672,GO:0005524,GO:0016310;Enzyme_code=EC:2.7.1;Enzyme_name=Transferring
Fv_160_5.g3373	194	ID=Fv_160_5.g3373;Description=hypothetical protein FVER53263_06948 [Fusarium verticillioides];Gene=FNYG_04855;Ontology_term=nucleus,DNA-binding transcription factor activity, RNA polymerase II-specific,zinc
Fv_160_5.g3374	952	ID=Fv_160_5.g3374;Description=hypothetical protein FVER53590_06949 [Fusarium verticillioides];Gene=FVEG_06949;Ontology_term=phagophore assembly site membrane,protein serine/threonine kinase activity,ATP binding,autophagy,protein transport,phosphorylation;Ontology_id=GO:0034045,GO:0004674,GO:0005524,GO:0006914,GO:0015031,GO:0016310;Enzyme_cod
Fv_160_5.g3375	437	ID=Fv_160_5.g3375;Description=ornithine aminotransferase [Fusarium verticillioides 7600];Gene=FNAPI_8875;Ontology_term=cytosol,ornithine aminotransferase activity,pyridoxal phosphate binding,ornithine metabolic process,arginine catabolic process to proline via ornithine,L-proline biosynthetic
Fv_160_5.g3376	613	ID=Fv_160_5.g3376;Description=GPI mannosyltransferase 3 [Fusarium verticillioides 7600];Gene=FNAPI_8874;Ontology_term=endoplasmic reticulum membrane,glycosyltransferase
Fv_160_5.g3377	956	ID=Fv_160_5.g3377;Description=hypothetical protein FVER53590_06952 [Fusarium verticillioides];Gene=FNAPI_8873;Ontology_term=GTPase activator activity,signal
Fv_160_5.g3378	87	ID=Fv_160_5.g3378;Description=mitochondrial import inner membrane translocase subunit TIM9 [Fusarium oxysporum f. sp. lycopersici 4287];Gene=CDV31_003172;Ontology_term=mitochondrial inner membrane,metal ion binding,protein
Fv_160_5.g3379	1821	ID=Fv_160_5.g3379;Description=hypothetical protein FVEG_15967 [Fusarium verticillioides 7600];Gene=FPANT_7709;Ontology_term=nuclear pore,structural constituent of nuclear pore,nucleocytoplasmic transport,protein

Fv_160_5.g3380	761	ID=Fv_160_5.g3380;Description=anthranilate synthase component 2 [Fusarium verticillioides 7600];Gene=FTJAE_37;Ontology_term=anthranilate synthase activity,indole-3-glycerol-phosphate synthase activity,phosphoribosylanthranilate isomerase activity,tryptophan biosynthetic process,glutamine metabolic process;Ontology_id=GO:0004049,GO:0004425,GO:0004640,GO:0000162,GO:0006541;Enzyme_code=EC:4.1.1.48,EC:4.1.3.27,EC:5.
Fv_160_5.g3381	454	ID=Fv_160_5.g3381;Description=hypothetical protein FVER14953_06957 [Fusarium verticillioides]
Fv_160_5.g3382	346	ID=Fv_160_5.g3382;Description=hypothetical protein FVER53590_06958 [Fusarium verticillioides];Gene=CEK26_010953;Ontology_term=endoplasmic reticulum membrane,plasma membrane,transmembrane receptor protein tyrosine kinase activity,protein homodimerization activity,protein transport,internal genitalia morphogenesis,dendrite self-avoidance;Ontology_id=GO:0005789,GO:0005886,GO:0004714,GO:0042803,GO:0015031,GO:0035260,GO:0070593;Enzyme_code=
Fv_160_5.g3383	175	ID=Fv_160_5.g3383;Description=pre-mRNA-splicing factor CWC21 [Fusarium verticillioides
Fv_160_5.g3384	417	ID=Fv_160_5.g3384;Description=hypothetical protein FVEG_06960 [Fusarium verticillioides 7600];Gene=BFJ63_vAg8144;Ontology_term=magnesium ion binding,ATP binding,cell
Fv_160_5.g3385	300	ID=Fv_160_5.g3385;Description=hypothetical protein FVER53263_06961 [Fusarium verticillioides]
Fv_160_5.g3386	139	ID=Fv_160_5.g3386;Description=hypothetical protein FVEG_15968 [Fusarium verticillioides 7600]
Fv_160_5.g3387	353	ID=Fv_160_5.g3387;Description=uncharacterized protein NECHADRAFT_71077 [Fusarium vanettenii 77-13-4];Gene=FHETE_89;Ontology_term=heterotrimeric G-protein complex,G protein-coupled receptor binding,GTPase activity,GTP binding,G-protein beta/gamma-subunit complex binding,G protein-coupled receptor signaling
Fv_160_5.g3388	234	ID=Fv_160_5.g3388;Description=hypothetical protein FVER53590_06963 [Fusarium verticillioides];Gene=FVEG_06963;Ontology_term=nuclear-transcribed mRNA catabolic process, non-stop
Fv_160_5.g3389	262	ID=Fv_160_5.g3389;Description=hypothetical protein FVEG_06964 [Fusarium verticillioides 7600];Gene=F25303_4536;Ontology_term=spliceosomal complex,membrane,RNA binding,zinc ion binding,mRNA splicing, via
Fv_160_5.g3390	337	ID=Fv_160_5.g3390;Description=hypothetical protein FVER53590_06965 [Fusarium
Fv_160_5.g3391	821	ID=Fv_160_5.g3391;Description=cell division control protein 48 [Fusarium oxysporum f. sp. lycopersici 4287];Gene=FPHYL_9131;Ontology_term=ATP binding,ATP hydrolysis activity,response to stimulus,cell division;Ontology_id=GO:0005524,GO:0016887,GO:0050896,GO:0051301;Enzyme_code=EC:3.6.1.15;Enzyme_name=nucleoside-
Fv_160_5.g3392	160	ID=Fv_160_5.g3392;Description=hypothetical protein FVEG_06967 [Fusarium verticillioides 7600]
Fv_160_5.g3393	1009	ID=Fv_160_5.g3393;Description=hypothetical protein FVER14953_06970 [Fusarium verticillioides];Gene=FTJAE_23;Ontology_term=membrane,ATP binding,ATP hydrolysis activity,ABC-type transporter activity,transmembrane transport;Ontology_id=GO:0016020,GO:0005524,GO:0016887,GO:0140359,GO:0055085;Enzyme_code=EC:7.2.2,EC:3.6.1.15;Enzym

Fv_160_5.g3394	474	ID=Fv_160_5.g3394;Description=acyl-CoA-dependent ceramide synthase [Fusarium verticillioides 7600];Gene=CEK26_010964;Ontology_term=nucleus,cytoplasm,membrane,nuclear localization sequence binding,sphingosine N-acyltransferase activity,nuclear import signal receptor activity,mRNA export from nucleus,protein import into nucleus,plasma membrane organization,regulation of mitotic nuclear division,ceramide biosynthetic process,regulation of protein desumoylation;Ontology_id=GO:0005634,GO:0005737,GO:0016020,GO:0008139,GO:0050291,GO:0061608,GO:0006406,GO:00066
Fv_160_5.g3395	779	ID=Fv_160_5.g3395;Description=hypothetical protein FVER53590_06972 [Fusarium
Fv_160_5.g3396	710	ID=Fv_160_5.g3396;Description=hypothetical protein FVER53263_06973 [Fusarium
Fv_160_5.g3397	820	ID=Fv_160_5.g3397;Description=hypothetical protein FVER14953_06974 [Fusarium verticillioides];Gene=PIF1;Ontology_term=nucleolus,mitochondrion,rDNA heterochromatin,site of double-strand break,chromosome, telomeric repeat region,RNA binding,ATP binding,ATP hydrolysis activity,single-stranded DNA helicase activity,5'-3' DNA/RNA helicase activity,5'-3' DNA helicase activity,G-quadruplex DNA binding,ATP-dependent protein-DNA complex displacement activity,flap-structured DNA binding,DNA/DNA annealing activity,telomere maintenance,DNA recombination,DNA duplex unwinding,mitochondrial DNA repair,G-quadruplex DNA unwinding,DNA strand elongation involved in mitotic DNA replication,removal of RNA primer involved in mitotic DNA replication,mitotic recombination-dependent replication fork processing;Ontology_id=GO:0005730,GO:0005739,GO:0033553,GO:0035861,GO:0140445,GO:0003723,GO:0005524,GO:0016887,G
Fv_160_5.g3398	674	ID=Fv_160_5.g3398;Description=hypothetical protein FVER53590_30361 [Fusarium verticillioides]
Fv_160_5.g3399	795	ID=Fv_160_5.g3399;Description=hypothetical protein FVEG_06976 [Fusarium verticillioides 7600];Gene=FNAPI_10835;Ontology_term=chromosome, telomeric region,nucleus,protein serine/threonine kinase activity,ATP binding,ubiquitin protein ligase binding,protein serine kinase activity,DNA repair,chromatin organization,ubiquitin-dependent protein catabolic
Fv_160_5.g3400	2939	ID=Fv_160_5.g3400;Description=hypothetical protein FVER53590_06977 [Fusarium verticillioides];Gene=FNAPI_10834;Ontology_term=chromosome, telomeric region,nucleus,protein serine/threonine kinase activity,ATP binding,protein serine kinase activity,DNA repair,chromatin organization,phosphorylation;Ontology_id=GO:0000781,GO:0005634,GO:0004674,GO:0005524,GO:0106310,GO:0006281,GO:0006
Fv_160_5.g3401	1662	ID=Fv_160_5.g3401;Description=hypothetical protein FVER53590_07007 [Fusarium verticillioides];Gene=FNAPI_10833;Ontology_term=DNA binding,zinc ion binding,membrane,nuclear pore,DNA-binding transcription factor activity, RNA polymerase II-specific,regulation of transcription by RNA polymerase
Fv_160_5.g3402	475	ID=Fv_160_5.g3402;Description=hypothetical protein FVER53263_07008 [Fusarium verticillioides];Gene=FPANT_223;Ontology_term=ligase activity,metal ion
Fv_160_5.g3403	1139	ID=Fv_160_5.g3403;Description=DNA polymerase gamma, mitochondrial [Fusarium verticillioides 7600];Gene=FPANT_222;Ontology_term=gamma DNA polymerase complex,DNA binding,DNA-directed DNA polymerase activity,mitochondrial DNA replication,DNA biosynthetic

Fv_160_5.g3404	599	ID=Fv_160_5.g3404;Description=hypothetical protein FVEG_07010 [Fusarium verticillioides 7600];Gene=BFJ68_g15762;Ontology_term=cellular response to oxidative stress;Ontology_id=GO:0034599
Fv_160_5.g3405	1150	ID=Fv_160_5.g3405;Description=hypothetical protein FVER53590_07011 [Fusarium verticillioides];Gene=FVER53590_07011;Ontology_term=sterol 3-beta-glucosyltransferase activity,carbohydrate metabolic process,lipid glycosylation;Ontology_id=GO:0016906,GO:0005975,GO:0030259;Enzyme_code=EC:2.4.1.173;Enzyme_name=sterol
Fv_160_5.g3406	345	ID=Fv_160_5.g3406;Description=hypothetical protein FVER53590_07012 [Fusarium verticillioides];Gene=FNAPI_3289;Ontology_term=spliceosomal complex,mRNA splicing, via
Fv_160_5.g3407	1526	ID=Fv_160_5.g3407;Description=hypothetical protein FVER53590_07013 [Fusarium verticillioides];Gene=FACUT_1758;Ontology_term=NuA4 histone acetyltransferase complex,chromatin
Fv_160_5.g3408	643	ID=Fv_160_5.g3408;Description=hypothetical protein FVER14953_07014 [Fusarium verticillioides];Gene=FACUT_1759;Ontology_term=fungal-type vacuole membrane,early endosome,late endosome,trans-Golgi network,sodium:proton antiporter activity,potassium:proton antiporter activity,vacuolar acidification,sodium ion transmembrane transport,potassium ion transmembrane transport,proton transmembrane
Fv_160_5.g3409	213	ID=Fv_160_5.g3409;Description=ADP-ribosylation factor like protein 1 [Fusarium verticillioides 7600];Gene=FLAG1_05295;Ontology_term=Golgi apparatus,nuclear outer membrane-endoplasmic reticulum membrane network,GTPase activity,GTP binding,intracellular protein transport,protein localization to Golgi apparatus,response to endoplasmic reticulum stress,Golgi to plasma membrane protein transport;Ontology_id=GO:0005794,GO:0042175,GO:0003924,GO:0005525,GO:0006886,GO:0034067,GO:0034976,GO:0043001;En
Fv_160_5.g3410	293	ID=Fv_160_5.g3410;Description=exosome complex component RRP45 [Fusarium verticillioides 7600];Gene=FNAPI_3285;Ontology_term=nuclear exosome (RNase complex),cytoplasmic exosome (RNase complex),nucleolus,exonucleolytic trimming to generate mature 3'-end of 5.8S rRNA from tricistronic rRNA transcript (SSU-rRNA, 5.8S rRNA, LSU-rRNA),U1 snRNA 3'-end processing,U4 snRNA 3'-end processing,U5 snRNA 3'-end processing,nuclear polyadenylation-
Fv_160_5.g3411	237	ID=Fv_160_5.g3411;Description=hypothetical protein FVEG_07017 [Fusarium verticillioides 7600];Gene=HZS61_017414;Ontology_term=Prp19 complex,post-mRNA release spliceosomal complex,RNA binding,mRNA splicing,
Fv_160_5.g3412	596	ID=Fv_160_5.g3412;Description=hypothetical protein FVER53590_07018 [Fusarium verticillioides];Gene=HZS61_017415;Ontology_term=xylulokinase activity,ATP binding,D-xylose metabolic process,carbohydrate
Fv_160_5.g3413	597	ID=Fv_160_5.g3413;Description=hypothetical protein FVEG_07019 [Fusarium verticillioides 7600];Gene=MMT2;Ontology_term=membrane,monoatomic cation transmembrane transporter activity,intracellular monoatomic cation homeostasis,monoatomic cation transmembrane transport,inorganic ion
Fv_160_5.g3414	400	ID=Fv_160_5.g3414;Description=hypothetical protein FVEG_07020 [Fusarium verticillioides 7600];Gene=FMUND_13116;Ontology_term=transferase activity,metal ion binding,NAD+
Fv_160_5.g3415	196	ID=Fv_160_5.g3415;Description=hypothetical protein FVEG_16000 [Fusarium verticillioides

Fv_160_5.g3416	2090	ID=Fv_160_5.g3416;Description=hypothetical protein FVER14953_07021 [Fusarium verticillioides];Gene=FANTH_14469;Ontology_term=nucleus,cysteine-type endopeptidase activity,nuclear division,proteolysis,cell division,nuclear chromosome
Fv_160_5.g3417	1054	ID=Fv_160_5.g3417;Description=hypothetical protein FVER53263_07022 [Fusarium verticillioides];Gene=FNAPI_3278;Ontology_term=nucleus,DNA binding,zinc ion binding,DNA-binding transcription factor activity, RNA polymerase II-specific,regulation of transcription by RNA polymerase
Fv_160_5.g3418	658	ID=Fv_160_5.g3418;Description=hypothetical protein FVER14953_07023 [Fusarium verticillioides]
Fv_160_5.g3419	235	ID=Fv_160_5.g3419;Description=rab family, other [Fusarium verticillioides 7600];Gene=FOTG_03934;Ontology_term=GTPase activity,GTP binding;Ontology_id=GO:0003924,GO:0005525;Enzyme_code=EC:3.6.1.15;Enzyme_name=nucleoside-triphosphate
Fv_160_5.g3420	927	ID=Fv_160_5.g3420;Description=hypothetical protein FVEG_07025 [Fusarium verticillioides 7600]
Fv_160_5.g3421	977	ID=Fv_160_5.g3421;Description=hypothetical protein FVER14953_07026 [Fusarium verticillioides];Gene=FOTG_03932;Ontology_term=nucleus,trans-Golgi network,1-phosphatidylinositol 4-kinase activity,endocytosis,phosphorylation,positive regulation of Golgi to plasma membrane protein transport,phosphatidylinositol phosphate biosynthetic process,phosphatidylinositol-mediated signaling,positive regulation of protein secretion,microlipophagy,positive regulation of autophagosome
Fv_160_5.g3422	328	ID=Fv_160_5.g3422;Description=hypothetical protein FVER53590_07027 [Fusarium verticillioides];Gene=FNYG_04904;Ontology_term=protein heterodimerization activity;Ontology_id=GO:0046982
Fv_160_5.g3423	422	ID=Fv_160_5.g3423;Description=hypothetical protein FVER14953_07028 [Fusarium verticillioides];Gene=HZS61_017426;Ontology_term=membrane,hydrolase
Fv_160_5.g3424	209	ID=Fv_160_5.g3424;Description=hypothetical protein FVER53590_29409 [Fusarium verticillioides]
Fv_160_5.g3425	166	ID=Fv_160_5.g3425;Description=hypothetical protein FVER14953_07029 [Fusarium verticillioides];Gene=FGADI_7202;Ontology_term=endoplasmic reticulum membrane;Ontology_id=GO:0005789
Fv_160_5.g3426	116	ID=Fv_160_5.g3426;Description=40S ribosomal protein S20 [Fusarium verticillioides 7600];Gene=FOYG_11193;Ontology_term=cytosolic small ribosomal subunit,RNA binding,structural constituent of ribosome,maturation of SSU-rRNA from tricistronic rRNA transcript (SSU-rRNA, 5.8S rRNA, LSU-
Fv_160_5.g3427	823	ID=Fv_160_5.g3427;Description=hypothetical protein FVER14953_07032 [Fusarium verticillioides]
Fv_160_5.g3428	103	ID=Fv_160_5.g3428;Description=unnamed protein product, partial [Fusarium graminearum];Gene=FOYG_11191;Ontology_term=nucleosome,nucleus,DNA binding,structural constituent of chromatin,protein
Fv_160_5.g3429	162	ID=Fv_160_5.g3429;Description=hypothetical protein J7337_009656 [Fusarium musae];Gene=CEP52_009058;Ontology_term=cytoplasm,endoplasmic reticulum to Golgi vesicle-mediated
Fv_160_5.g3430	110	ID=Fv_160_5.g3430;Description=ribosomal subunit l31 [Fusarium pseudoanthophilum];Gene=CEP52_009057;Ontology_term=mitochondrial large ribosomal subunit,structural constituent of

Fv_160_5.g3431	173	ID=Fv_160_5.g3431;Description=hypothetical protein FVEG_07036 [Fusarium verticillioides 7600];Gene=FMUND_11745;Ontology_term=nucleus,cytoplasm,nuclear cap binding complex,RNA cap binding,zinc ion binding,sequence-specific DNA binding,rRNA processing,mRNA cis splicing, via
Fv_160_5.g3432	890	ID=Fv_160_5.g3432;Description=hypothetical protein FVER14953_07037 [Fusarium verticillioides];Gene=FGLOB1_13935;Ontology_term=nuclear pore,structural constituent of nuclear pore,ribosomal large subunit export from nucleus,ribosomal small subunit export from nucleus,protein transport,mRNA
Fv_160_5.g3433	382	ID=Fv_160_5.g3433;Description=hypothetical protein FVER14953_07038 [Fusarium verticillioides]
Fv_160_5.g3434	223	ID=Fv_160_5.g3434;Description=hypothetical protein FVEG_07039 [Fusarium verticillioides 7600];Gene=FDENT_5819;Ontology_term=endoplasmic reticulum membrane,cholestenol delta-isomerase activity,sterol metabolic
Fv_160_5.g3435	376	ID=Fv_160_5.g3435;Description=hypothetical protein FVEG_07040 [Fusarium verticillioides 7600];Gene=FCIRC_9607;Ontology_term=isomerase
Fv_160_5.g3436	207	ID=Fv_160_5.g3436;Description=hypothetical protein FVER14953_07041 [Fusarium verticillioides];Gene=FVER53590_07041;Ontology_term=gamma-glutamylcyclotransferase
Fv_160_5.g3437	905	ID=Fv_160_5.g3437;Description=STE/STE11 protein kinase [Fusarium verticillioides 7600];Gene=HZS61_017439;Ontology_term=cytoplasm,mevalonate kinase activity,protein kinase activity,ATP binding,signal transduction,sterol biosynthetic process,phosphorylation,isopentenyl diphosphate biosynthetic process, mevalonate
Fv_160_5.g3438	355	ID=Fv_160_5.g3438;Description=hypothetical protein FVER53263_07043 [Fusarium verticillioides];Gene=BFJ68_g1078;Ontology_term=nucleus,membrane,RNA polymerase II cis-regulatory region sequence-specific DNA binding,DNA-binding transcription activator activity, RNA polymerase II-specific,protein homodimerization activity,epithelial cell morphogenesis,amino acid transport,ganglion mother cell fate determination,ventral cord development,tracheal outgrowth, open tracheal system,R3/R4 cell fate commitment,chitin-based embryonic cuticle biosynthetic process,fibroblast growth factor receptor signaling pathway,regulation of tube length, open tracheal system,rhabdomere development,regulation of cell population proliferation,positive regulation of transcription by RNA polymerase II,regulation of neurogenesis,transmembrane transport,membrane organization,regulation of wound
Fv_160_5.g3439	128	ID=Fv_160_5.g3439;Description=hypothetical protein FVER14953_07044 [Fusarium verticillioides];Gene=FOTG_03914;Ontology_term=nucleus,DNA-binding transcription factor activity, RNA polymerase II-specific,zinc ion binding,transaminase activity,pyridoxal phosphate binding,regulation of transcription by RNA polymerase
Fv_160_5.g3440	255	ID=Fv_160_5.g3440;Description=hypothetical protein FVER53263_07044 [Fusarium verticillioides];Gene=FOTG_03914;Ontology_term=transaminase activity,pyridoxal phosphate
Fv_160_5.g3441	236	ID=Fv_160_5.g3441;Description=hypothetical protein FVEG_16003 [Fusarium verticillioides 7600];Gene=FOIG_05198;Ontology_term=nucleus,DNA-binding transcription factor activity, RNA polymerase II-specific,zinc ion binding,transaminase activity,pyridoxal phosphate binding,regulation of transcription by RNA polymerase
Fv_160_5.g3442	168	ID=Fv_160_5.g3442;Description=hypothetical protein FVER14953_07046 [Fusarium verticillioides]

Fv_160_5.g3443	331	ID=Fv_160_5.g3443;Description=hypothetical protein FVEG_07047 [Fusarium verticillioides 7600];Gene=FOTG_03911;Ontology_term=chorismate mutase activity,prephenate dehydratase activity,L-phenylalanine biosynthetic process;Ontology_id=GO:0004106,GO:0004664,GO:0009094;Enzyme_code=EC:4.2.1.51,EC:5.4.99.5;Enzyme_name=prephenate
Fv_160_5.g3444	302	ID=Fv_160_5.g3444;Description=hypothetical protein FOXG_09416 [Fusarium oxysporum f. sp. lycopersici 4287];Gene=Forpi1262_v006479;Ontology_term=transaminase
Fv_160_5.g3445	257	ID=Fv_160_5.g3445;Description=hypothetical protein FVER14953_07049 [Fusarium verticillioides];Gene=FDENT_5828;Ontology_term=mitochondrion,ribosome,DNA strand exchange activity,single-stranded DNA
Fv_160_5.g3446	924	ID=Fv_160_5.g3446;Description=hsp98-like protein [Fusarium verticillioides 7600];Gene=F25303_8479;Ontology_term=cytoplasm,protein aggregate center,nucleolar peripheral inclusion body,ATP binding,ATP hydrolysis activity,misfolded protein binding,protein refolding,protein unfolding,cellular heat acclimation,cellular response to misfolded
Fv_160_5.g3447	1073	ID=Fv_160_5.g3447;Description=hypothetical protein FPANT_5200 [Fusarium pseudoanthophilum];Gene=FCIRC_11477;Ontology_term=endoplasmic reticulum membrane,mannosyltransferase
Fv_160_5.g3448	972	ID=Fv_160_5.g3448;Description=hypothetical protein FVER14953_07053 [Fusarium verticillioides];Gene=FVER53590_07053;Ontology_term=nucleus,DNA binding,cysteine-type endopeptidase
Fv_160_5.g3449	411	ID=Fv_160_5.g3449;Description=hypothetical protein FVEG_07056 [Fusarium verticillioides 7600];Gene=PKNOX2;Ontology_term=nucleus,DNA binding,regulation of DNA-templated
Fv_160_5.g3450	825	ID=Fv_160_5.g3450;Description=hypothetical protein FVER53590_07057 [Fusarium verticillioides];Gene=FOC4_g10008701;Ontology_term=nucleus,DNA binding,zinc ion binding,DNA-binding transcription factor activity, RNA polymerase II-specific,regulation of transcription by RNA polymerase
Fv_160_5.g3451	303	ID=Fv_160_5.g3451;Description=hypothetical protein FVEG_16006 [Fusarium verticillioides 7600];Gene=FMEXI_6262;Ontology_term=membrane,iron ion binding,oxidoreductase activity, acting on paired donors, with incorporation or reduction of molecular oxygen,L-ascorbic acid binding,dioxygenase activity;Ontology_id=GO:0016020,GO:0005506,GO:0016705,GO:0031418,GO:0051213;Enzyme_code=EC:1.14;Enzyme_name=Actin
Fv_160_5.g3452	200	ID=Fv_160_5.g3452;Description=hypothetical protein FVER53590_28989 [Fusarium verticillioides];Gene=FACUT_1801;Ontology_term=aspartic-type endopeptidase
Fv_160_5.g3453	466	ID=Fv_160_5.g3453;Description=hypothetical protein FVEG_07058 [Fusarium verticillioides 7600];Gene=FPANT_61;Ontology_term=negative regulation of transcription by RNA polymerase II,nucleus,aspartic-type endopeptidase activity,protein homodimerization activity,reciprocal meiotic recombination,protein heterodimerization activity,RNA polymerase II cis-regulatory region sequence-specific DNA binding,proteolysis,anterior/posterior pattern specification,DNA-binding transcription repressor activity, RNA polymerase II-specific,regulation of neurogenesis;Ontology_id=GO:0000122,GO:0005634,GO:0004190,GO:0042803,GO:0007131,GO:0046982,GO:0000978,GO:000650

Fv_160_5.g3454	170	ID=Fv_160_5.g3454;Description=V-type H+-transporting ATPase 16kDa proteolipid subunit [Fusarium verticillioides 7600];Gene=FMUND_11766;Ontology_term=vacuolar membrane,proton-transporting V-type ATPase, V0 domain,proton-transporting ATPase activity, rotational mechanism,proton transmembrane
Fv_160_5.g3455	964	ID=Fv_160_5.g3455;Description=hypothetical protein FVEG_07060 [Fusarium verticillioides 7600];Gene=Forpi1262_v006466;Ontology_term=membrane,metal ion binding,intracellular protein transport,vesicle-mediated
Fv_160_5.g3456	431	ID=Fv_160_5.g3456;Description=hypothetical protein FVEG_07061 [Fusarium verticillioides 7600];Gene=FNAPI_9654;Ontology_term=membrane,S-adenosylmethionine-dependent methyltransferase activity,metal ion binding,intracellular protein transport,vesicle-mediated
Fv_160_5.g3457	451	ID=Fv_160_5.g3457;Description=dTDP-glucose 4,6-dehydratase [Fusarium verticillioides 7600];Gene=BFJ68_g1057;Ontology_term=extracellular region,hemoglobin complex,oxygen carrier activity,dTDP-glucose 4,6-dehydratase activity,oxygen binding,heme binding,metal ion binding,nucleotide-sugar metabolic process,oxygen transport;Ontology_id=GO:0005576,GO:0005833,GO:0005344,GO:0008460,GO:0019825,GO:0020037,GO:0046872,GO:0009225,GO
Fv_160_5.g3458	415	ID=Fv_160_5.g3458;Description=hypothetical protein FVEG_07063 [Fusarium verticillioides 7600];Gene=HZS61_017458;Ontology_term=extracellular region,nucleolus,hemoglobin complex,preribosome, large subunit precursor,oxygen carrier activity,oxygen binding,heme binding,rRNA primary transcript binding,metal ion binding,ribosomal large subunit export from nucleus,maturation of LSU-rRNA from tricistronic rRNA transcript (SSU-rRNA, 5.8S rRNA, LSU-rRNA),maturation of 5.8S rRNA from tricistronic rRNA transcript (SSU-rRNA, 5.8S rRNA, LSU-rRNA),oxygen
Fv_160_5.g3459	669	ID=Fv_160_5.g3459;Description=hypothetical protein FVEG_07064 [Fusarium verticillioides 7600];Gene=FANTH_7355;Ontology_term=nuclear pre-replicative complex,nucleolus,Noc2p-Noc3p complex,chromatin binding,ribosomal large subunit assembly,pre-replicative complex assembly involved in nuclear cell cycle DNA replication,DNA
Fv_160_5.g3460	568	ID=Fv_160_5.g3460;Description=hypothetical protein FVER14953_07065 [Fusarium verticillioides]
Fv_160_5.g3461	239	ID=Fv_160_5.g3461;Description=hypothetical protein FVEG_16009 [Fusarium verticillioides 7600];Gene=FANTH_7353;Ontology_term=rRNA processing,tRNA processing;Ontology_id=GO:0006364,GO:0008033
Fv_160_5.g3462	2588	ID=Fv_160_5.g3462;Description=hypothetical protein FVER14953_07066 [Fusarium verticillioides];Gene=FFC1_14265;Ontology_term=extracellular region,hemoglobin complex,cysteine-type deubiquitinase activity,oxygen carrier activity,oxygen binding,heme binding,metal ion binding,oxygen transport;Ontology_id=GO:0005576,GO:0005833,GO:0004843,GO:0005344,GO:0019825,GO:0020037,GO:0046872,GO:0015671;En
Fv_160_5.g3463	509	ID=Fv_160_5.g3463;Description=hypothetical protein FVER14953_07067 [Fusarium verticillioides]
Fv_160_5.g3464	383	ID=Fv_160_5.g3464;Description=hypothetical protein FVER14953_07068 [Fusarium verticillioides];Gene=FACUT_1813;Ontology_term=kinase
Fv_160_5.g3465	822	ID=Fv_160_5.g3465;Description=hypothetical protein FNYG_04947 [Fusarium nygamai];Gene=AU210_010588;Ontology_term=TORC2 complex,kinase
Fv_160_5.g3466	198	ID=Fv_160_5.g3466;Description=hypothetical protein FVEG_07070 [Fusarium verticillioides

Fv_160_5.g3467	350	ID=Fv_160_5.g3467;Description=hypothetical protein FVEG_07071 [Fusarium verticillioides 7600];Gene=Focb16_v014824;Ontology_term=canonical NF-kappaB signal transduction;Ontology_id=GO:0007249
Fv_160_5.g3468	454	ID=Fv_160_5.g3468;Description=hypothetical protein FVEG_07072 [Fusarium verticillioides 7600];Gene=FDENT_4475;Ontology_term=Golgi apparatus,membrane,metal ion transmembrane transporter activity,zinc ion
Fv_160_5.g3469	674	ID=Fv_160_5.g3469;Description=hypothetical protein FVER53590_07073 [Fusarium verticillioides];Gene=FOXB_12759;Ontology_term=intracellular membrane-bounded organelle,pyruvate dehydrogenase (acetyl-transferring) activity,acetyl-CoA biosynthetic process from
Fv_160_5.g3470	409	ID=Fv_160_5.g3470;Description=hypothetical protein LB506_003357, partial [Fusarium annulatum];Gene=FOTG_03875;Ontology_term=mitochondrial pyruvate dehydrogenase complex,mitochondrial nucleoid,pyruvate dehydrogenase (acetyl-transferring) activity,acetyl-CoA biosynthetic process from pyruvate,pseudohyphal
Fv_160_5.g3471	879	ID=Fv_160_5.g3471;Description=hypothetical protein FVER53590_07075 [Fusarium verticillioides];Gene=FVER53590_07075;Ontology_term=DNA binding,DNA ligase (ATP) activity,ATP binding,DNA replication,DNA repair,DNA recombination,DNA biosynthetic
Fv_160_5.g3472	908	ID=Fv_160_5.g3472;Description=hypothetical protein FVER14953_07076 [Fusarium verticillioides];Gene=FANTH_7342;Ontology_term=membrane,voltage-gated chloride channel activity,chloride transmembrane
Fv_160_5.g3473	628	ID=Fv_160_5.g3473;Description=hypothetical protein FVER53590_07077 [Fusarium verticillioides]
Fv_160_5.g3474	478	ID=Fv_160_5.g3474;Description=hypothetical protein FVER14953_07078 [Fusarium verticillioides];Gene=FOPG_02027;Ontology_term=oxidoreductase activity,flavin adenine dinucleotide
Fv_160_5.g3475	202	ID=Fv_160_5.g3475;Description=hypothetical protein FVER14953_20890 [Fusarium verticillioides];Gene=FACUT_1824;Ontology_term=vacuolar proton-transporting V-type ATPase complex
Fv_160_5.g3476	469	ID=Fv_160_5.g3476;Description=hypothetical protein FVER14953_07079 [Fusarium verticillioides];Gene=FNAPI_3421;Ontology_term=membrane,transmembrane transporter activity,transmembrane
Fv_160_5.g3477	241	ID=Fv_160_5.g3477;Description=cell division control protein 42 [Fusarium verticillioides 7600];Gene=HZS61_017478;Ontology_term=cytoplasm,plasma membrane,membrane-bounded organelle,G protein activity,GTP binding,establishment or maintenance of cell polarity,small GTPase mediated signal transduction,regulation of cellular component organization,cell
Fv_160_5.g3478	411	ID=Fv_160_5.g3478;Description=hypothetical protein FVER14953_07082 [Fusarium verticillioides];Gene=FPANT_13723;Ontology_term=hydrolase activity, acting on carbon-nitrogen (but not peptide) bonds, in linear amidines,metal ion binding;Ontology_id=GO:0016813,GO:0046872;Enzyme_code=EC:3.5.3;Enzyme_name=Acting on carbon-
Fv_160_5.g3479	786	ID=Fv_160_5.g3479;Description=hypothetical protein FVEG_07083 [Fusarium verticillioides
Fv_160_5.g3480	525	ID=Fv_160_5.g3480;Description=hypothetical protein FVER14953_07084 [Fusarium verticillioides];Gene=FNYG_04962;Ontology_term=3-phytase
Fv_160_5.g3481	800	ID=Fv_160_5.g3481;Description=hypothetical protein FVER53590_07085 [Fusarium verticillioides]

Fv_160_5.g3482	238	ID=Fv_160_5.g3482;Description=hypothetical protein FVEG_07086 [Fusarium verticillioides 7600]
Fv_160_5.g3483	232	ID=Fv_160_5.g3483;Description=hypothetical protein FVEG_07087 [Fusarium verticillioides 7600];Gene=FOC4_g10008736;Ontology_term=extracellular region,hemoglobin complex,membrane,oxygen carrier activity,oxygen binding,heme binding,metal ion binding,oxygen
Fv_160_5.g3484	342	ID=Fv_160_5.g3484;Description=hypothetical protein FVEG_16012 [Fusarium verticillioides 7600]
Fv_160_5.g3485	398	ID=Fv_160_5.g3485;Description=Ras-like G [Fusarium subglutinans];Gene=FOTG_03858;Ontology_term=fungal-type vacuole membrane,extracellular region,Gtr1-Gtr2 GTPase complex,GTPase activity,GTP binding;Ontology_id=GO:0000329,GO:0005576,GO:1990131,GO:0003924,GO:0005525;Enzyme_code=EC:3.6.1.15;Enzyme_name=n
Fv_160_5.g3486	761	ID=Fv_160_5.g3486;Description=hypothetical protein FVER53590_07090 [Fusarium verticillioides];Gene=FACUT_9481;Ontology_term=anaphase-promoting complex,cell cycle,anaphase-promoting complex-dependent
Fv_160_5.g3487	523	ID=Fv_160_5.g3487;Description=hypothetical protein FVER53590_07091 [Fusarium verticillioides];Gene=FVER53263_07091;Ontology_term=Golgi membrane,COPI vesicle coat,retrograde vesicle-mediated transport,
Fv_160_5.g3488	825	ID=Fv_160_5.g3488;Description=hypothetical protein FVEG_07092 [Fusarium verticillioides 7600]
Fv_160_5.g3489	399	ID=Fv_160_5.g3489;Description=hypothetical protein FOXG_09464 [Fusarium oxysporum f. sp. lycopersici 4287];Gene=Focb16_v014846;Ontology_term=membrane,magnesium ion transmembrane transporter activity,magnesium ion
Fv_160_5.g3490	260	ID=Fv_160_5.g3490;Description=hypothetical protein LZL87_002860 [Fusarium oxysporum];Gene=FOYG_11117;Ontology_term=cytosolic small ribosomal subunit,preribosome, small subunit precursor,RNA binding,structural constituent of ribosome,DNA-(apurinic or apyrimidinic site) endonuclease activity,ribosomal small subunit export
Fv_160_5.g3491	774	ID=Fv_160_5.g3491;Description=hypothetical protein FVEG_07095 [Fusarium verticillioides 7600];Gene=FOC4_g10008744;Ontology_term=phosphatidylinositol-3-phosphate phosphatase
Fv_160_5.g3492	609	ID=Fv_160_5.g3492;Description=phenylalanyl-tRNA synthetase, beta subunit [Fusarium verticillioides 7600];Gene=FCIRC_7115;Ontology_term=cytoplasm,magnesium ion binding,RNA binding,phenylalanine-tRNA ligase activity,ATP binding,phenylalanyl-tRNA
Fv_160_5.g3493	623	ID=Fv_160_5.g3493;Description=hypothetical protein FVER14953_07097 [Fusarium verticillioides];Gene=C2S_10051;Ontology_term=protein phosphatase type 2A complex,chromosome, centromeric region,nucleus,spindle pole body,cellular bud tip,cellular bud neck,mating projection tip,FAR/SIN/STRIPAK complex,mitotic actomyosin contractile ring,protein serine/threonine phosphatase activity,regulation of translation,mitotic spindle assembly checkpoint signaling,establishment or maintenance of cytoskeleton polarity,negative regulation of septation initiation signaling,asymmetric protein localization to old mitotic spindle pole body,meiotic centromeric cohesion protection in anaphase
Fv_160_5.g3494	248	ID=Fv_160_5.g3494;Description=hypothetical protein FVEG_07098 [Fusarium verticillioides 7600];Gene=EFM4;Ontology_term=cytoplasm,protein-lysine N-methyltransferase activity,vesicle-mediated

Fv_160_5.g3495	246	ID=Fv_160_5.g3495;Description=eukaryotic translation initiation factor 6 [Fusarium oxysporum f. sp. lycopersici 4287];Gene=FG05428.1;Ontology_term=nucleolus,cytoplasm,preribosome, large subunit precursor,translation initiation factor activity,protein-lysine N-methyltransferase activity,ribosomal large subunit binding,ribosomal subunit export from nucleus,maturation of LSU-rRNA from tricistronic rRNA transcript (SSU-rRNA, 5.8S rRNA, LSU-rRNA),maturation of 5.8S rRNA from tricistronic rRNA transcript (SSU-rRNA, 5.8S rRNA, LSU-rRNA),translational initiation,vesicle-mediated transport,methylation,cytosolic ribosome assembly,assembly of large subunit precursor of preribosome;Ontology_id=GO:0005730,GO:0005737,GO:0030687,GO:0003743,GO:0016279,GO:0043023,GO:0000054,GO:0000463
Fv_160_5.g3496	1031	ID=Fv_160_5.g3496;Description=hypothetical protein FVER14953_07100 [Fusarium verticillioides]
Fv_160_5.g3497	499	ID=Fv_160_5.g3497;Description=hypothetical protein FVER53263_21051 [Fusarium verticillioides];Gene=FRV6_12479;Ontology_term=chromosome, telomeric region,extracellular region,nucleus,cytosol,membrane,perinuclear region of cytoplasm,monooxygenase activity,GTPase activator activity,iron ion binding,oxidoreductase activity, acting on paired donors, with incorporation or reduction of molecular oxygen,heme binding,small GTPase binding,ribosomal subunit export from nucleus,RNA import into nucleus,rRNA export from nucleus,tRNA export from nucleus,protein import into nucleus,subtelomeric heterochromatin formation;Ontology_id=GO:0000781,GO:0005576,GO:0005634,GO:0005829,GO:0016020,GO:0048471,GO:0004497,GO:0005096,G
Fv_160_5.g3498	423	ID=Fv_160_5.g3498;Description=translation initiation factor 5 [Fusarium verticillioides 7600];Gene=F25303_8754;Ontology_term=extracellular region,eukaryotic 48S preinitiation complex,multi-eIF complex,translation initiation factor activity,GDP-dissociation inhibitor activity,GTPase activator activity,GTP binding,translation initiation factor binding,formation of cytoplasmic translation initiation complex,cytosolic ribosome assembly,negative regulation of translational
Fv_160_5.g3499	815	ID=Fv_160_5.g3499;Description=hypothetical protein FVEG_07103 [Fusarium verticillioides 7600]
Fv_160_5.g3500	1439	ID=Fv_160_5.g3500;Description=hypothetical protein FVER14953_07104 [Fusarium verticillioides];Gene=FOC4_g10008756;Ontology_term=U2-type spliceosomal complex,helicase activity,mRNA cis splicing, via
Fv_160_5.g3501	1505	ID=Fv_160_5.g3501;Description=translation initiation factor 4G [Fusarium verticillioides 7600];Gene=FVEG_07105;Ontology_term=RNA binding,translation initiation factor activity,translational
Fv_160_5.g3502	1455	ID=Fv_160_5.g3502;Description=hypothetical protein FVER53263_07106 [Fusarium verticillioides];Gene=FACUT_1988;Ontology_term=nucleus,mitochondrion,nucleotide binding,DNA binding,DNA-directed DNA polymerase activity,DNA biosynthetic process,mitotic DNA replication
Fv_160_5.g3503	755	ID=Fv_160_5.g3503;Description=hypothetical protein FVEG_07107 [Fusarium verticillioides 7600]
Fv_160_5.g3504	313	ID=Fv_160_5.g3504;Description=hypothetical protein FVEG_07108 [Fusarium verticillioides
Fv_160_5.g3505	905	ID=Fv_160_5.g3505;Description=CMGC/DYRK/YAK protein kinase [Fusarium verticillioides 7600];Gene=FVEG_07109;Ontology_term=membrane,protein kinase activity,ATP
Fv_160_5.g3506	474	ID=Fv_160_5.g3506;Description=hypothetical protein HZS61_017507 [Fusarium oxysporum f. sp. conglutinans];Gene=BFJ65_g11353;Ontology_term=RNA binding;Ontology_id=GO:0003723

Fv_160_5.g3507	382	ID=Fv_160_5.g3507;Description=hypothetical protein FVEG_07112 [Fusarium verticillioides 7600]
Fv_160_5.g3508	396	ID=Fv_160_5.g3508;Description=hypothetical protein FVER14953_07113 [Fusarium verticillioides]
Fv_160_5.g3509	380	ID=Fv_160_5.g3509;Description=hypothetical protein FVER53263_07114 [Fusarium verticillioides];Gene=RNA1;Ontology_term=chromosome, telomeric region,nucleus,cytosol,perinuclear region of cytoplasm,tRNA binding,GTPase activator activity,nucleotidyltransferase activity,small GTPase binding,ribosomal subunit export from nucleus,tRNA wobble uridine modification,RNA import into nucleus,rRNA export from nucleus,tRNA export from nucleus,protein import into nucleus,subtelomeric heterochromatin formation,protein urmylation,tRNA thio-modification;Ontology_id=GO:0000781,GO:0005634,GO:0005829,GO:0048471,GO:0000049,GO:0005096,GO:0016779,GO:0031267,
Fv_160_5.g3510	389	ID=Fv_160_5.g3510;Description=hypothetical protein FVEG_07115 [Fusarium verticillioides 7600];Gene=ncs2;Ontology_term=cytoplasm,tRNA binding,nucleotidyltransferase activity,tRNA wobble uridine modification,protein urmylation,tRNA thio-
Fv_160_5.g3511	310	ID=Fv_160_5.g3511;Description=solute carrier family 25 member 46 [Fusarium mexicanum];Gene=BFJ69_g13430;Ontology_term=mitochondrial inner membrane,sulfate transmembrane transporter activity,oxaloacetate transmembrane transporter activity,isopropylmalate transmembrane transporter activity,malonate(1-) transmembrane transporter activity,isopropylmalate transport,malonic acid transmembrane transport,oxaloacetate(2-)
Fv_160_5.g3512	384	ID=Fv_160_5.g3512;Description=hypothetical protein FVER53590_07117 [Fusarium verticillioides];Gene=FACUT_1978;Ontology_term=nucleus,methyltransferase
Fv_160_5.g3513	414	ID=Fv_160_5.g3513;Description=hypothetical protein FVEG_07118 [Fusarium verticillioides 7600]
Fv_160_5.g3514	141	ID=Fv_160_5.g3514;Description=hypothetical protein FVEG_07119 [Fusarium verticillioides 7600];Gene=FOQG_03639;Ontology_term=oenocyte delamination,identical protein binding,ommatidial rotation,cytosolic small ribosomal subunit,haltere development,epidermal growth factor receptor binding,receptor antagonist activity,eye-antennal disc morphogenesis,imaginal disc-derived wing vein specification,dorsal/ventral axis specification,ribosomal small subunit assembly,visual perception,negative regulation of epidermal growth factor receptor signaling pathway,translation,anterior/posterior axis specification,extracellular space,SRP-dependent cotranslational protein targeting to membrane,structural constituent of ribosome,signal recognition particle,negative regulation of ERK1 and ERK2 cascade,negative regulation of stem cell differentiation,positive regulation of apoptotic process,negative regulation of neurogenesis,genital disc
Fv_160_5.g3515	409	ID=Fv_160_5.g3515;Description=Ribonucleotide-diphosphate reductase (RNR), small subunit [Fusarium musae];Gene=ROX3;Ontology_term=membrane,mediator complex,transcription coregulator activity,oxidoreductase activity,regulation of transcription by RNA polymerase II,deoxyribonucleotide biosynthetic
Fv_160_5.g3516	284	ID=Fv_160_5.g3516;Description=hypothetical protein FVER14953_07121 [Fusarium

Fv_160_5.g3517	829	ID=Fv_160_5.g3517;Description=hypothetical protein FVER14953_07122 [Fusarium verticillioides];Gene=FNAPI_4215;Ontology_term=cytoplasm,RNA binding,manganese ion binding,5'-(N(7)-methylguanosine 5'-triphospho)-[mRNA] hydrolase activity,nuclear-transcribed mRNA catabolic process, nonsense-mediated decay,deadenylation-dependent decapping of nuclear-transcribed
Fv_160_5.g3518	382	ID=Fv_160_5.g3518;Description=hypothetical protein FVEG_07123 [Fusarium verticillioides
Fv_160_5.g3519	260	ID=Fv_160_5.g3519;Description=hypothetical protein FVEG_07124 [Fusarium verticillioides 7600]
Fv_160_5.g3520	456	ID=Fv_160_5.g3520;Description=hypothetical protein FVER14953_07125 [Fusarium verticillioides];Gene=FDENT_6719;Ontology_term=protein serine/threonine kinase activity,ATP
Fv_160_5.g3521	523	ID=Fv_160_5.g3521;Description=hypothetical protein FVEG_07126 [Fusarium verticillioides 7600];Gene=FTJAE_13148;Ontology_term=mitochondrion,protein-arginine omega-N symmetric methyltransferase activity,methylation;Ontology_id=GO:0005739,GO:0035243,GO:0032259;Enzyme_code=EC:2.1.1.320;Enzyme_name=type II protein
Fv_160_5.g3522	540	ID=Fv_160_5.g3522;Description=hypothetical protein FVER14953_07127 [Fusarium verticillioides];Gene=AU210_010646;Ontology_term=membrane,cytosolic large ribosomal subunit,structural constituent of ribosome,protein binding,large ribosomal subunit rRNA binding,ribosomal large subunit assembly,cytoplasmic
Fv_160_5.g3523	579	ID=Fv_160_5.g3523;Description=hypothetical protein FVER14953_07128 [Fusarium verticillioides];Gene=FOTG_03816;Ontology_term=nucleus,peptidyl-prolyl cis-trans isomerase activity,ubiquitin-protein transferase activity,protein folding,protein
Fv_160_5.g3524	617	ID=Fv_160_5.g3524;Description=hypothetical protein FVER14953_20490 [Fusarium verticillioides];Gene=FCIRC_3423;Ontology_term=nucleus,DNA-binding transcription factor activity, RNA polymerase II-specific,zinc
Fv_160_5.g3525	382	ID=Fv_160_5.g3525;Description=hypothetical protein FVEG_16021 [Fusarium verticillioides 7600];Gene=F25303_10464;Ontology_term=hydrolase activity, hydrolyzing O-glycosyl compounds,carbohydrate metabolic
Fv_160_5.g3526	293	ID=Fv_160_5.g3526;Description=xyloglucan endo-transglycosylase [Fusarium coicis];Gene=FVER53590_29283;Ontology_term=cytosolic large ribosomal subunit,structural constituent of ribosome,hydrolase activity, hydrolyzing O-glycosyl compounds,oxidoreductase activity,cytoplasmic translational elongation,carbohydrate metabolic
Fv_160_5.g3527	804	ID=Fv_160_5.g3527;Description=hypothetical protein FVER14953_07130 [Fusarium verticillioides];Gene=BFJ65_g11134;Ontology_term=nucleus,DNA binding,zinc ion binding,DNA-templated
Fv_160_5.g3528	1219	ID=Fv_160_5.g3528;Description=hypothetical protein FVER14953_07132 [Fusarium verticillioides];Gene=FSUBG_11432;Ontology_term=RNA polymerase III complex,alcohol dehydrogenase (NAD+) activity,guanyl-nucleotide exchange factor activity,alcohol metabolic process,transcription by RNA polymerase III,small GTPase mediated signal transduction,cell division;Ontology_id=GO:0005666,GO:0004022,GO:0005085,GO:0006066,GO:0006383,GO:0007264,GO:0051301;Enzyme_code=EC:
Fv_160_5.g3529	277	ID=Fv_160_5.g3529;Description=hypothetical protein FVEG_07133 [Fusarium verticillioides 7600]

Fv_160_5.g3530	544	ID=Fv_160_5.g3530;Description=hypothetical protein FVER53590_07134 [Fusarium verticillioides];Gene=FPCIR_8859;Ontology_term=nucleus,DNA-binding transcription factor activity, RNA polymerase II-specific,zinc
Fv_160_5.g3531	410	ID=Fv_160_5.g3531;Description=SAGA-associated factor 73 [Fusarium verticillioides 7600];Gene=FOYG_11071;Ontology_term=SAGA complex,mitochondrial inner membrane;Ontology_id=GO:0000124,GO:0005743
Fv_160_5.g3532	326	ID=Fv_160_5.g3532;Description=hypothetical protein FVEG_07137 [Fusarium verticillioides 7600];Gene=FPHYL_14084;Ontology_term=SAGA complex,mitochondrial inner membrane;Ontology_id=GO:0000124,GO:0005743
Fv_160_5.g3533	355	ID=Fv_160_5.g3533;Description=hypothetical protein FVER14953_07138 [Fusarium verticillioides];Gene=FNAPI_13527;Ontology_term=nucleus,metal ion binding,ubiquitin protein ligase
Fv_160_5.g3534	369	ID=Fv_160_5.g3534;Description=hypothetical protein FVEG_07139 [Fusarium verticillioides 7600];Gene=FNAPI_13526;Ontology_term=acyltransferase activity,phospholipid metabolic
Fv_160_5.g3535	323	ID=Fv_160_5.g3535;Description=Protein STE50 [Fusarium oxysporum f. sp. albedinis];Gene=F25303_9843;Ontology_term=nucleus,cellular bud neck,Pho85-Pho80 CDK-cyclin complex,cyclin-dependent protein serine/threonine kinase activity,ATP binding,negative regulation of transcription by RNA polymerase II,DNA damage response,positive regulation of macroautophagy,negative regulation of macroautophagy,phosphorylation,regulation of protein stability,regulation of establishment or maintenance of cell polarity,regulation of protein localization,negative regulation of glycogen biosynthetic process,negative regulation of phosphate metabolic process,regulation of nucleocytoplasmic transport,negative regulation of calcium-mediated signaling,regulation of cell division,lipid homeostasis,positive regulation of phospholipid biosynthetic process,regulation of cell cycle phase
Fv_160_5.g3536	635	ID=Fv_160_5.g3536;Description=hypothetical protein FVEG_07141 [Fusarium verticillioides
Fv_160_5.g3537	216	ID=Fv_160_5.g3537;Description=hypothetical protein FVEG_07142 [Fusarium verticillioides 7600]
Fv_160_5.g3538	489	ID=Fv_160_5.g3538;Description=hypothetical protein FVER53590_07143 [Fusarium
Fv_160_5.g3539	635	ID=Fv_160_5.g3539;Description=poly(A) polymerase [Fusarium verticillioides 7600];Gene=FOC4_g10008801;Ontology_term=cytosol,ribosome,mRNA cleavage and polyadenylation specificity factor complex,Mei2 nuclear dot complex,nuclear exosome focus,magnesium ion binding,RNA binding,structural constituent of ribosome,ATP binding,poly(A) RNA polymerase activity,mRNA polyadenylation,translation,sno(s)RNA 3'-end processing,nuclear-transcribed mRNA catabolic process, meiosis-specific
Fv_160_5.g3540	617	ID=Fv_160_5.g3540;Description=hypothetical protein FVEG_07145 [Fusarium verticillioides 7600];Gene=FOC4_g10008802;Ontology_term=nucleus,DNA-binding transcription factor activity,sequence-specific DNA
Fv_160_5.g3541	1263	ID=Fv_160_5.g3541;Description=protein TIF31 [Fusarium verticillioides 7600];Gene=CLU1;Ontology_term=cytoplasm,RNA binding,translation initiation factor activity,translational initiation,mitochondrion organization,organelle
Fv_160_5.g3542	801	ID=Fv_160_5.g3542;Description=hypothetical protein FVEG_07147 [Fusarium verticillioides 7600]
Fv_160_5.g3543	585	ID=Fv_160_5.g3543;Description=ferric-chelate reductase [Fusarium pseudoanthophilum];Gene=FTJAE_12348;Ontology_term=membrane,ferric-chelate reductase activity,iron ion

Fv_160_5.g3544	414	ID=Fv_160_5.g3544;Description=hypothetical protein FVER53590_07149 [Fusarium verticillioides];Gene=AU210_010668;Ontology_term=Golgi membrane,pyrimidine nucleotide-sugar transmembrane transporter
Fv_160_5.g3545	110	ID=Fv_160_5.g3545;Description=peptidylprolyl isomerase [Fusarium oxysporum f. sp. lycopersici 4287];Gene=FMUND_9162;Ontology_term=membrane,isomerase activity,thiol oxidase activity,metal ion
Fv_160_5.g3546	436	ID=Fv_160_5.g3546;Description=hypothetical protein FVEG_07151 [Fusarium verticillioides
Fv_160_5.g3547	138	ID=Fv_160_5.g3547;Description=hypothetical protein J7337_009771 [Fusarium musae];Gene=FPANT_6309;Ontology_term=tRNA-intron endonuclease complex,nucleic acid binding,endonuclease activity,tRNA-type intron splice site recognition and cleavage;Ontology_id=GO:0000214,GO:0003676,GO:0004519,GO:0000379;Enzyme_code=EC:3.1;Enzyme_name=Acting on ester
Fv_160_5.g3548	106	ID=Fv_160_5.g3548;Description=hypothetical protein FVEG_07152 [Fusarium verticillioides 7600];Gene=FVER53590_07152;Ontology_term=zinc ion binding,oxidoreductase
Fv_160_5.g3549	238	ID=Fv_160_5.g3549;Description=hypothetical protein J7337_009773 [Fusarium musae];Gene=FVER53590_07152;Ontology_term=zinc ion binding,oxidoreductase
Fv_160_5.g3550	491	ID=Fv_160_5.g3550;Description=hypothetical protein FVER14953_07153 [Fusarium verticillioides];Gene=hry;Ontology_term=RNA polymerase II, core complex,DNA-binding transcription factor activity, RNA polymerase II-specific,transcription corepressor binding,DNA-directed 5'-3' RNA polymerase activity,RNA-dependent RNA polymerase activity,zinc ion binding,E-box binding,negative regulation of transcription by RNA polymerase II,RNA-templated transcription,transcription initiation at RNA polymerase II promoter,transcription elongation by RNA polymerase II,open tracheal system development,salivary gland morphogenesis,anterior/posterior pattern specification,translesion synthesis,tube morphogenesis,trunk segmentation,regulation of neurogenesis,membrane organization;Ontology_id=GO:0005665,GO:0000981,GO:0001222,GO:0003899,GO:0003968,GO:0008270,GO:0070888,GO:0000122,
Fv_160_5.g3551	867	ID=Fv_160_5.g3551;Description=hypothetical protein FVER53263_07154 [Fusarium verticillioides];Gene=FVER53590_07154;Ontology_term=calcium-dependent cysteine-type endopeptidase
Fv_160_5.g3552	340	ID=Fv_160_5.g3552;Description=hypothetical protein FVEG_07156 [Fusarium verticillioides 7600];Gene=FOC4_g10008815;Ontology_term=mitochondrial inner membrane,peroxisomal membrane,adenine nucleotide transmembrane transporter activity,fatty acid beta-oxidation,peroxisome organization,ATP transport,transmembrane
Fv_160_5.g3553	619	ID=Fv_160_5.g3553;Description=protein VTS1 [Fusarium verticillioides 7600];Gene=FOPG_01937;Ontology_term=P-body,cytosol,mRNA binding,regulation of mRNA stability;Ontology_id=GO:0000932,GO:0005829,GO:0003729,GO:0043488
Fv_160_5.g3554	923	ID=Fv_160_5.g3554;Description=hypothetical protein FVER53590_07159 [Fusarium verticillioides];Gene=FOPG_01936;Ontology_term=transcription by RNA polymerase III;Ontology_id=GO:0006383
Fv_160_6.g3555	102	ID=Fv_160_6.g3555;Description=hypothetical protein FVEG_04849 [Fusarium verticillioides 7600]
Fv_160_6.g3556	209	ID=Fv_160_6.g3556;Description=hypothetical protein FVER53590_25132 [Fusarium verticillioides]
Fv_160_6.g3557	467	ID=Fv_160_6.g3557;Description=hypothetical protein FVEG_04848 [Fusarium verticillioides 7600];Gene=FTJAE_8693;Ontology_term=transaminase activity,pyridoxal phosphate binding,biosynthetic

Fv_160_6.g3558	138	ID=Fv_160_6.g3558;Description=hypothetical protein FVER53263_04847 [Fusarium verticillioides];Gene=418
Fv_160_6.g3559	281	ID=Fv_160_6.g3559;Description=hypothetical protein FVEG_04846 [Fusarium verticillioides 7600];Gene=FVEG_04846;Ontology_term=DNA-binding transcription factor activity,regulation of DNA-templated
Fv_160_6.g3560	505	ID=Fv_160_6.g3560;Description=hypothetical protein FVER53590_25130 [Fusarium verticillioides];Gene=FPANT_11435;Ontology_term=chromosome segregation,CENP-A containing chromatin
Fv_160_6.g3561	394	ID=Fv_160_6.g3561;Description=hypothetical protein J7337_006881 [Fusarium musae];Gene=FVEG_04845;Ontology_term=hydrolase activity, acting on ester
Fv_160_6.g3562	896	ID=Fv_160_6.g3562;Description=ribonucleoside-diphosphate reductase large chain [Fusarium verticillioides 7600];Gene=FOTG_02228;Ontology_term=ribonucleoside-diphosphate reductase activity, thioredoxin disulfide as acceptor,ATP binding,DNA replication,deoxyribonucleotide biosynthetic
Fv_160_6.g3563	314	ID=Fv_160_6.g3563;Description=hypothetical protein FVEG_04843 [Fusarium verticillioides
Fv_160_6.g3564	363	ID=Fv_160_6.g3564;Description=pirin (iron-binding nuclear protein) [Fusarium verticillioides 7600]
Fv_160_6.g3565	556	ID=Fv_160_6.g3565;Description=hypothetical protein FVEG_04841 [Fusarium verticillioides 7600];Gene=3244;Ontology_term=mitochondrial outer membrane,cytosol,flavin adenine dinucleotide binding,aliphatic amine oxidase activity,phenethylamine:oxygen oxidoreductase (deaminating) activity,monoamine oxidase activity,positive regulation of signal transduction,dopamine catabolic process;Ontology_id=GO:0005741,GO:0005829,GO:0050660,GO:0052595,GO:0052596,GO:0097621,GO:0009967,GO:0042420;Enzy
Fv_160_6.g3566	240	ID=Fv_160_6.g3566;Description=hypothetical protein FVEG_04840 [Fusarium verticillioides 7600];Gene=H2AZ1;Ontology_term=nucleosome,nucleus,DNA binding,structural constituent of chromatin,protein
Fv_160_6.g3567	182	ID=Fv_160_6.g3567;Description=hypothetical protein FVEG_04839 [Fusarium verticillioides 7600]
Fv_160_6.g3568	623	ID=Fv_160_6.g3568;Description=hypothetical protein FVEG_04838 [Fusarium verticillioides 7600];Gene=FTJAE_12973;Ontology_term=guanylate cyclase complex, soluble,membrane,synapse,guanylate cyclase activity,copper ion binding,GTP binding,oxidoreductase activity,heme binding,cGMP biosynthetic process,monoatomic ion transport,nitric oxide-cGMP-mediated signaling pathway,response to oxygen levels,cellular response to nitric oxide,trans-synaptic signaling by nitric oxide, modulating synaptic
Fv_160_6.g3569	355	ID=Fv_160_6.g3569;Description=high-affinity iron transporter [Fusarium verticillioides 7600];Gene=FTJAE_12974;Ontology_term=high-affinity iron permease complex,iron ion transmembrane transporter activity,iron ion
Fv_160_6.g3570	474	ID=Fv_160_6.g3570;Description=hypothetical protein FVEG_04836 [Fusarium verticillioides 7600]
Fv_160_6.g3571	662	ID=Fv_160_6.g3571;Description=hypothetical protein FVEG_04835 [Fusarium verticillioides 7600];Gene=FNAPI_7237;Ontology_term=T cell costimulation,positive regulation of inflammatory response,myoblast differentiation,positive regulation of viral entry into host cell,laminin binding,protein serine/threonine phosphatase activity,extracellular space,metal ion binding,plasma cell differentiation,positive regulation of apoptotic process,galectin complex,cytosol,carbohydrate

Fv_160_6.g3572	770	ID=Fv_160_6.g3572;Description=hypothetical protein FVEG_04834 [Fusarium verticillioides 7600]
Fv_160_6.g3573	383	ID=Fv_160_6.g3573;Description=cytochrome P450 oxidoreductase [Fusarium verticillioides 7600];Gene=FPANT_773;Ontology_term=nucleus,endoplasmic reticulum membrane,RNA binding,monooxygenase activity,iron ion binding,heme binding,fatty acid alpha-hydroxylase activity,mRNA processing,fatty acid biosynthetic process;Ontology_id=GO:0005634,GO:0005789,GO:0003723,GO:0004497,GO:0005506,GO:0020037,GO:0080132,GO:0006397,GO:
Fv_160_6.g3574	566	ID=Fv_160_6.g3574;Description=hypothetical protein FVEG_04832 [Fusarium verticillioides 7600];Gene=FFB14_13862;Ontology_term=nucleus,membrane,RNA binding,iron ion binding,oxidoreductase activity,heme binding,U1 snRNP binding,mRNA processing,lipid biosynthetic process,cellular lipid metabolic process;Ontology_id=GO:0005634,GO:0016020,GO:0003723,GO:0005506,GO:0016491,GO:0020037,GO:1990446,GO:0006397,GO:
Fv_160_6.g3575	683	ID=Fv_160_6.g3575;Description=hypothetical protein FVER53263_04831 [Fusarium verticillioides]
Fv_160_6.g3576	330	ID=Fv_160_6.g3576;Description=hypothetical protein FVER53590_04830 [Fusarium verticillioides];Gene=metK;Ontology_term=extracellular space,growth hormone receptor binding,hormone activity,metal ion binding,signal transduction,cell population proliferation,negative regulation of gene expression,positive regulation of peptidyl-threonine phosphorylation,positive regulation of triglyceride biosynthetic process,insulin secretion,response to food,negative regulation of urine volume,negative regulation of apoptotic process,positive regulation of cholesterol biosynthetic process,negative regulation of fatty acid biosynthetic process,positive regulation of fatty acid biosynthetic process,positive regulation of peptidyl-tyrosine phosphorylation,positive regulation of lipid catabolic process,renal sodium ion absorption,positive regulation of phospholipid biosynthetic process,positive regulation of diacylglycerol biosynthetic process,negative regulation of protein acetylation,positive regulation of lactation,response to L-arginine,positive regulation of feeding behavior,negative regulation of
Fv_160_6.g3577	308	ID=Fv_160_6.g3577;Description=hypothetical protein J7337_006864 [Fusarium musae];Gene=HZS61_012223;Ontology_term=membrane,phosphotransferase activity, for other substituted phosphate groups,phospholipid biosynthetic
Fv_160_6.g3578	366	ID=Fv_160_6.g3578;Description=orotidine 5'-phosphate decarboxylase [Fusarium verticillioides 7600];Gene=HZS61_012222;Ontology_term=nucleus,DNA binding,zinc ion binding,'de novo' pyrimidine nucleobase biosynthetic process,orotidine-5'-phosphate decarboxylase activity,cytosol,'de novo' UMP biosynthetic process,DNA-binding transcription factor activity, RNA polymerase II-specific,regulation of transcription by RNA polymerase II;Ontology_id=GO:0005634,GO:0003677,GO:0008270,GO:0006207,GO:0004590,GO:0005829,GO:0044205,GO:0000981,GO:000635
Fv_160_6.g3579	730	ID=Fv_160_6.g3579;Description=hypothetical protein FVER53590_04827 [Fusarium verticillioides];Gene=FTJAE_5221;Ontology_term=nucleus,DNA binding,zinc ion binding,'de novo' pyrimidine nucleobase biosynthetic process,orotidine-5'-phosphate decarboxylase activity,'de novo' UMP biosynthetic process,DNA-binding transcription factor activity, RNA polymerase II-specific,regulation of transcription by RNA polymerase II;Ontology_id=GO:0005634,GO:0003677,GO:0008270,GO:0006207,GO:0004590,GO:0044205,GO:0000981,GO:0006357;Enzyme_co

Fv_160_6.g3580	477	ID=Fv_160_6.g3580;Description=hypothetical protein FVEG_04826 [Fusarium verticillioides 7600];Gene=FMAN_05825;Ontology_term=nucleus,RNA polymerase II cis-regulatory region sequence-specific DNA binding,protein binding,metal ion binding,positive regulation of transcription by RNA polymerase II,positive regulation of thiamine biosynthetic
Fv_160_6.g3581	275	ID=Fv_160_6.g3581;Description=signal recognition particle subunit SRP19 [Fusarium verticillioides 7600];Gene=CEK26_007180;Ontology_term=signal recognition particle, endoplasmic reticulum targeting,7S RNA binding,SRP-
Fv_160_6.g3582	1332	ID=Fv_160_6.g3582;Description=dynactin 1 [Fusarium verticillioides 7600];Gene=FNAPI_7227;Ontology_term=microtubule,dynein
Fv_160_6.g3583	673	ID=Fv_160_6.g3583;Description=hypothetical protein FVER14953_04822 [Fusarium verticillioides];Gene=FNAPI_7226;Ontology_term=nucleus,microtubule,dynein complex,DNA
Fv_160_6.g3584	458	ID=Fv_160_6.g3584;Description=RuvB-like helicase 1 [Fusarium verticillioides 7600];Gene=FMUND_943;Ontology_term=Swr1 complex,Ino80 complex,ASTRA complex,R2TP complex,ATP binding,ATP hydrolysis activity,3'-5' DNA helicase activity,5'-3' DNA helicase activity,box C/D snoRNP assembly,DNA repair,chromatin remodeling,regulation of transcription by RNA polymerase II,DNA duplex unwinding;Ontology_id=GO:0000812,GO:0031011,GO:0070209,GO:0097255,GO:0005524,GO:0016887,GO:0043138,GO:0043139,G
Fv_160_6.g3585	1303	ID=Fv_160_6.g3585;Description=phospholipid-translocating ATPase [Fusarium verticillioides 7600];Gene=FPANT_762;Ontology_term=membrane,magnesium ion binding,ATP binding,ATP hydrolysis activity,phosphatidylethanolamine flippase activity,phospholipid transport,lipid translocation;Ontology_id=GO:0016020,GO:0000287,GO:0005524,GO:0016887,GO:0090555,GO:0015914,GO:0034204;Enzyme_cod
Fv_160_6.g3586	1504	ID=Fv_160_6.g3586;Description=hypothetical protein LB504_006721 [Fusarium proliferatum];Gene=FPANT_761;Ontology_term=membrane,oxidoreductase
Fv_160_6.g3587	1339	ID=Fv_160_6.g3587;Description=hypothetical protein FVER53590_04817 [Fusarium verticillioides];Gene=BFJ70_g8362;Ontology_term=membrane,chloride:monoatomic cation symporter activity,chloride
Fv_160_6.g3588	539	ID=Fv_160_6.g3588;Description=hypothetical protein FVER53263_04816 [Fusarium verticillioides]
Fv_160_6.g3589	371	ID=Fv_160_6.g3589;Description=hypothetical protein FVEG_04815 [Fusarium verticillioides 7600]
Fv_160_6.g3590	104	ID=Fv_160_6.g3590;Description=hypothetical protein FVEG_04814 [Fusarium verticillioides 7600]
Fv_160_6.g3591	924	ID=Fv_160_6.g3591;Description=multiple ankyrin repeats single kh domain-containing protein [Fusarium pseudoanthophilum];Gene=383;Ontology_term=extracellular region,hormone activity,metal ion binding,signal transduction,positive
Fv_160_6.g3592	574	ID=Fv_160_6.g3592;Description=hypothetical protein FVER14953_04812 [Fusarium verticillioides];Gene=FVEG_04812;Ontology_term=peptidase
Fv_160_6.g3593	545	ID=Fv_160_6.g3593;Description=guanosine-diphosphatase [Fusarium verticillioides 7600];Gene=FMAN_05811;Ontology_term=ATP binding,inflammatory response,extracellular space,cysteine-type endopeptidase inhibitor activity,UDP phosphatase activity,GDP phosphatase activity,negative regulation of blood coagulation,membrane,Golgi apparatus,vasodilation,positive regulation of cytosolic calcium ion concentration;Ontology_id=GO:0005524,GO:0006954,GO:0005615,GO:0004869,GO:0045134,GO:0004382,GO:0030195,GO:001602

Fv_160_6.g3594	365	ID=Fv_160_6.g3594;Description=hypothetical protein FVEG_04810 [Fusarium verticillioides 7600];Gene=FOQG_02518;Ontology_term=magnesium chelatase
Fv_160_6.g3595	205	ID=Fv_160_6.g3595;Description=hypothetical protein FZEAL_7235 [Fusarium zealandicum];Gene=BHE90_008866;Ontology_term=vacuolar membrane,late endosome membrane,GTPase activity,GTP binding,oxidoreductase activity, acting on the CH-CH group of donors,flavin adenine dinucleotide binding;Ontology_id=GO:0005774,GO:0031902,GO:0003924,GO:0005525,GO:0016627,GO:0050660;Enzyme_code=EC:1.3,EC:3.6.1.
Fv_160_6.g3596	452	ID=Fv_160_6.g3596;Description=acyl-CoA dehydrogenase [Fusarium verticillioides 7600];Gene=FANTH_5691;Ontology_term=3-oxoacyl-[acyl-carrier-protein] synthase activity,oxidoreductase activity, acting on the CH-CH group of donors,hydrolase activity,phosphopantetheine binding,flavin adenine dinucleotide binding,fatty acid biosynthetic process,polyketide biosynthetic process,lactone biosynthetic process;Ontology_id=GO:0004315,GO:0016627,GO:0016787,GO:0031177,GO:0050660,GO:0006633,GO:0030639,GO:1901336;Enzy
Fv_160_6.g3597	106	ID=Fv_160_6.g3597;Description=hypothetical protein FVEG_04807 [Fusarium verticillioides 7600];Gene=mcsB;Ontology_term=extracellular space,secretory granule,galanin receptor activity,neuropeptide hormone activity,type 1 galanin receptor binding,type 2 galanin receptor binding,type 3 galanin receptor binding,neuropeptide signaling
Fv_160_6.g3598	371	ID=Fv_160_6.g3598;Description=hypothetical protein FVEG_04806 [Fusarium verticillioides 7600];Gene=BFJ68_g10045;Ontology_term=GTPase activity,GTP binding,cytoplasmic translation,positive regulation of cellular response to amino acid
Fv_160_6.g3599	404	ID=Fv_160_6.g3599;Description=hypothetical protein FVER14953_04805 [Fusarium verticillioides];Gene=FMAN_05805;Ontology_term=Prp19 complex,post-mRNA release spliceosomal complex,RNA binding,metal ion
Fv_160_6.g3600	284	ID=Fv_160_6.g3600;Description=urease accessory protein [Fusarium verticillioides 7600];Gene=AU210_004190;Ontology_term=membrane,nickel cation binding,nitrogen compound metabolic
Fv_160_6.g3601	682	ID=Fv_160_6.g3601;Description=hypothetical protein FVER53263_04804 [Fusarium verticillioides]
Fv_160_6.g3602	1600	ID=Fv_160_6.g3602;Description=hypothetical protein FVER14953_04803 [Fusarium verticillioides];Gene=BFJ69_g3590;Ontology_term=eukaryotic translation initiation factor 2alpha kinase activity,ATP binding,DNA damage checkpoint signaling,phosphorylation,regulation of protein metabolic
Fv_160_6.g3603	224	ID=Fv_160_6.g3603;Description=ubiquitin-conjugating enzyme E2 R [Fusarium verticillioides 7600];Gene=C2S_10737;Ontology_term=nucleus,cytoplasm,SCF ubiquitin ligase complex,ubiquitin protein ligase activity,G1/S transition of mitotic cell cycle,G2/M transition of mitotic cell cycle,positive regulation of glucose transmembrane transport,silent mating-type cassette heterochromatin formation,SCF-dependent proteasomal ubiquitin-dependent protein catabolic process;Ontology_id=GO:0005634,GO:0005737,GO:0019005,GO:0061630,GO:0000082,GO:0000086,GO:0010828,GO:0030466,GO:
Fv_160_6.g3604	904	ID=Fv_160_6.g3604;Description=hypothetical protein FVEG_04801 [Fusarium verticillioides 7600]

Fv_160_6.g3605	368	ID=Fv_160_6.g3605;Description=DnaJ like subfamily B member 4 [Fusarium verticillioides 7600];Gene=FOQG_02507;Ontology_term=nucleus,cytosolic small ribosomal subunit,protein aggregate center,unfolded protein binding,misfolded protein binding,translational initiation,protein folding,tRNA import into nucleus,misfolded protein transport,nuclear protein quality control by the ubiquitin-proteasome system,mitochondria-associated ubiquitin-dependent protein
Fv_160_6.g3606	245	ID=Fv_160_6.g3606;Description=phosphoglycolate phosphatase [Fusarium verticillioides 7600];Gene=FNAPI_7204;Ontology_term=phosphatase
Fv_160_6.g3607	453	ID=Fv_160_6.g3607;Description=initiator tRNA phosphoribosyl transferase [Fusarium verticillioides 7600];Gene=FOMG_03915;Ontology_term=tRNA A64-2'-O-ribosylphosphate transferase activity,charged-tRNA amino acid
Fv_160_6.g3608	416	ID=Fv_160_6.g3608;Description=oxidoreductase [Fusarium verticillioides 7600];Gene=FOMG_03916;Ontology_term=nucleotide
Fv_160_6.g3609	330	ID=Fv_160_6.g3609;Description=hypothetical protein FVEG_04796 [Fusarium verticillioides 7600];Gene=FOC1_g10014329;Ontology_term=lyase activity;Ontology_id=GO:0016829;Enzyme_code=EC:4;Enzyme_name=Lyases
Fv_160_6.g3610	571	ID=Fv_160_6.g3610;Description=hypothetical protein J7337_006829 [Fusarium musae];Gene=FNAPI_7200;Ontology_term=membrane,transmembrane transporter activity,transmembrane
Fv_160_6.g3611	280	ID=Fv_160_6.g3611;Description=hypothetical protein FVEG_04794 [Fusarium verticillioides 7600]
Fv_160_6.g3612	281	ID=Fv_160_6.g3612;Description=hypothetical protein FVEG_04793 [Fusarium verticillioides
Fv_160_6.g3613	215	ID=Fv_160_6.g3613;Description=hypothetical protein FVEG_04792 [Fusarium verticillioides 7600];Gene=FTJAE_5187;Ontology_term=nucleolus,preribosome, large subunit precursor,ribosomal large subunit
Fv_160_6.g3614	428	ID=Fv_160_6.g3614;Description=hypothetical protein FVER14953_04791 [Fusarium verticillioides];Gene=FTJAE_5186;Ontology_term=membrane,oxidoreductase
Fv_160_6.g3615	171	ID=Fv_160_6.g3615;Description=glutathione peroxidase [Fusarium verticillioides 7600];Gene=FGADI_7064;Ontology_term=glutathione peroxidase activity,thioredoxin-dependent peroxiredoxin activity,response to oxidative stress,cellular oxidant detoxification;Ontology_id=GO:0004602,GO:0140824,GO:0006979,GO:0098869;Enzyme_code=EC:1.11.1.24,EC:1.11.1.9;Enzyme_na
Fv_160_6.g3616	580	ID=Fv_160_6.g3616;Description=hypothetical protein FVEG_04789 [Fusarium verticillioides 7600];Gene=FOMG_03924;Ontology_term=cytoskeleton,microtubule motor activity,ATP binding,microtubule binding,microtubule-
Fv_160_6.g3617	85	ID=Fv_160_6.g3617;Description=hypothetical protein J7337_006822 [Fusarium musae]
Fv_160_6.g3618	859	ID=Fv_160_6.g3618;Description=hypothetical protein FVER53263_04788 [Fusarium verticillioides];Gene=FocTR4_00002886;Ontology_term=nucleus,DNA-binding transcription factor activity, RNA polymerase II-

Fv_160_6.g3619	254	ID=Fv_160_6.g3619;Description=hypothetical protein FVER14953_04787 [Fusarium verticillioides];Gene=1032;Ontology_term=heterotrimeric G-protein complex,GTPase activity,GTP binding,transferase activity,G-protein beta/gamma-subunit complex binding,G protein-coupled serotonin receptor binding,mu-type opioid receptor binding,metal ion binding,corticotropin-releasing hormone receptor 1 binding,glutamine metabolic process,adenylate cyclase-modulating G protein-coupled receptor signaling pathway,dopamine receptor signaling pathway;Ontology_id=GO:0005834,GO:0003924,GO:0005525,GO:0016740,GO:0031683,GO:0031821,GO:0031852,GO:0046872,GO:
Fv_160_6.g3620	447	ID=Fv_160_6.g3620;Description=hypothetical protein J7337_006819 [Fusarium musae];Gene=FPANT_6608;Ontology_term=cytoplasm,glutathione hydrolase complex,transferase activity,glutathione hydrolase activity,glutamine metabolic process,glutathione catabolic process;Ontology_id=GO:0005737,GO:0061672,GO:0016740,GO:0036374,GO:0006541,GO:0006751;Enzyme_code=EC:2,EC:3.4.19.1
Fv_160_6.g3621	719	ID=Fv_160_6.g3621;Description=hypothetical protein FVER14953_04785 [Fusarium verticillioides]
Fv_160_6.g3622	507	ID=Fv_160_6.g3622;Description=hypothetical protein J7337_006817 [Fusarium musae];Gene=FNAPI_11822;Ontology_term=membrane,heme oxygenase (decyclizing) activity,heme
Fv_160_6.g3623	435	ID=Fv_160_6.g3623;Description=hypothetical protein FVER53590_04783 [Fusarium verticillioides];Gene=FOYG_01527;Ontology_term=membrane,transmembrane transporter activity,transmembrane
Fv_160_6.g3624	282	ID=Fv_160_6.g3624;Description=hypothetical protein FVEG_04782 [Fusarium verticillioides 7600]
Fv_160_6.g3625	297	ID=Fv_160_6.g3625;Description=hypothetical protein J7337_006814 [Fusarium musae];Gene=FMOD;Ontology_term=extracellular space,cytoplasm,actin cytoskeleton,protein binding,ATP binding,hydrolase activity,phagocytosis,collagen fibril organization,response to
Fv_160_6.g3626	538	ID=Fv_160_6.g3626;Description=hypothetical protein FVER53263_04780 [Fusarium verticillioides];Gene=FMAN_05778;Ontology_term=nucleus,spindle pole body,membrane,transmembrane transporter
Fv_160_6.g3627	1467	ID=Fv_160_6.g3627;Description=hypothetical protein FVER53590_04778 [Fusarium verticillioides];Gene=FACUT_1716;Ontology_term=plasma membrane,ATP binding,ATP hydrolysis activity,ABC-type transporter activity,transmembrane transport;Ontology_id=GO:0005886,GO:0005524,GO:0016887,GO:0140359,GO:0055085;Enzyme_code=EC:7.2.2,EC:3.6.1.15;Enzym
Fv_160_6.g3628	918	ID=Fv_160_6.g3628;Description=hypothetical protein FVER53590_04776 [Fusarium verticillioides]
Fv_160_6.g3629	622	ID=Fv_160_6.g3629;Description=hypothetical protein FVER14953_04775 [Fusarium
Fv_160_6.g3630	347	ID=Fv_160_6.g3630;Description=hypothetical protein FVEG_04774 [Fusarium verticillioides 7600];Gene=3182;Ontology_term=nucleic acid binding,endonuclease activity,metal ion
Fv_160_6.g3631	124	ID=Fv_160_6.g3631;Description=hypothetical protein HZ326_0131 [Fusarium oxysporum f. sp. albedinis]
Fv_160_6.g3632	982	ID=Fv_160_6.g3632;Description=hypothetical protein FVER14953_04773 [Fusarium
Fv_160_6.g3633	248	ID=Fv_160_6.g3633;Description=hypothetical protein FVEG_04772 [Fusarium verticillioides 7600]
Fv_160_6.g3634	270	ID=Fv_160_6.g3634;Description=hypothetical protein FVER14953_04771 [Fusarium verticillioides]

Fv_160_6.g3635	558	ID=Fv_160_6.g3635;Description=hypothetical protein FVEG_04770 [Fusarium verticillioides 7600];Gene=FOYG_01539;Ontology_term=zinc ion binding,Golgi apparatus,aminopeptidase activity,acute-phase response,plasma membrane,metallopeptidase activity,negative regulation of bone mineralization,extracellular space,cysteine-type endopeptidase inhibitor activity,proteolysis,extracellular membrane-bounded organelle,vesicle,regulation of inflammatory response,positive regulation of
Fv_160_6.g3636	498	ID=Fv_160_6.g3636;Description=hypothetical protein FVER14953_04769 [Fusarium verticillioides]
Fv_160_6.g3637	170	ID=Fv_160_6.g3637;Description=hypothetical protein FVEG_04768 [Fusarium verticillioides 7600]
Fv_160_6.g3638	260	ID=Fv_160_6.g3638;Description=hypothetical protein FVER14953_04767 [Fusarium verticillioides]
Fv_160_6.g3639	813	ID=Fv_160_6.g3639;Description=hypothetical protein FVER53590_04766 [Fusarium verticillioides]
Fv_160_6.g3640	237	ID=Fv_160_6.g3640;Description=hypothetical protein FVEG_04765 [Fusarium verticillioides 7600]
Fv_160_6.g3641	144	ID=Fv_160_6.g3641;Description=hypothetical protein FVER14953_04764 [Fusarium verticillioides]
Fv_160_6.g3642	493	ID=Fv_160_6.g3642;Description=hypothetical protein FVEG_04763 [Fusarium verticillioides 7600];Gene=340
Fv_160_6.g3643	948	ID=Fv_160_6.g3643;Description=hypothetical protein FVEG_04762 [Fusarium verticillioides 7600];Gene=NUP49;Ontology_term=nuclear membrane,nuclear pore central transport channel,nuclear localization sequence binding,structural constituent of nuclear pore,protein import into
Fv_160_6.g3644	365	ID=Fv_160_6.g3644;Description=formate dehydrogenase [Fusarium verticillioides 7600];Gene=FTJAE_9008;Ontology_term=cytosol,membrane,formate dehydrogenase (NAD+) activity,oxidoreductase activity, acting on the CH-OH group of donors, NAD or NADP as acceptor,NAD binding,formate catabolic process;Ontology_id=GO:0005829,GO:0016020,GO:0008863,GO:0016616,GO:0051287,GO:0042183;Enzyme_code=EC:1.1.1,EC:1.17
Fv_160_6.g3645	610	ID=Fv_160_6.g3645;Description=hypothetical protein FVEG_04760 [Fusarium verticillioides 7600];Gene=FocTR4_00002852;Ontology_term=membrane,transmembrane transporter activity,transmembrane
Fv_160_6.g3646	107	ID=Fv_160_6.g3646;Description=hypothetical protein FVER14953_04759 [Fusarium verticillioides];Gene=3169

Fv_160_6.g3647	520	ID=Fv_160_6.g3647;Description=hypothetical protein FVEG_04758 [Fusarium verticillioides 7600];Gene=FTJAE_9011;Ontology_term=male gonad development,Golgi apparatus,prostate epithelial cord arborization involved in prostate glandular acinus morphogenesis,DNA-binding transcription activator activity, RNA polymerase II-specific,positive regulation of DNA-binding transcription factor activity,regulation of branching involved in prostate gland morphogenesis,prostate epithelial cord elongation,transcription coactivator binding,transcription regulator complex,RNA polymerase II preinitiation complex assembly,positive regulation of nitric-oxide synthase activity,antral ovarian follicle growth,TBP-class protein binding,protein kinase binding,zinc ion binding,epithelial cell proliferation involved in mammary gland duct elongation,negative regulation of DNA-binding transcription factor activity,plasma membrane,nucleus,negative regulation of gene expression,euchromatin,protein localization to chromatin,positive regulation of phospholipase C activity,regulation of inflammatory response,nuclear estrogen receptor binding,14-3-3 protein binding,vagina development,positive regulation of nitric oxide biosynthetic process,estrogen response element binding,negative regulation of miRNA transcription,uterus development,regulation of epithelial cell apoptotic process,mammary gland branching involved in pregnancy,epithelial cell development,steroid binding,negative regulation of canonical NF-kappaB signal transduction,positive regulation of transcription by RNA polymerase II,negative regulation of transcription by RNA polymerase II,cellular response to estradiol stimulus,stem cell differentiation,mammary gland alveolus development,nuclear receptor activity,positive regulation of fibroblast proliferation,TFIIB-class transcription factor binding,intracellular estrogen receptor signaling pathway,identical protein binding,nuclear estrogen receptor activity,regulation of toll-like receptor signaling pathway,chromatin binding,phospholipase C-activating G protein-coupled receptor signaling pathway,beta-catenin binding,cellular response to estrogen stimulus,positive regulation of cytosolic calcium ion concentration,transcription corepressor binding,androgen metabolic
Fv_160_6.g3648	889	ID=Fv_160_6.g3648;Description=hypothetical protein FVEG_04757 [Fusarium verticillioides 7600];Gene=FVEG_04757;Ontology_term=endoplasmic reticulum,membrane,kinase activity,nucleotidyltransferase activity,phosphorylation;Ontology_id=GO:0005783,GO:0016020,GO:0016301,GO:0016779,GO:0016310;Enzyme_code=EC:2.7.7;Enz
Fv_160_6.g3649	578	ID=Fv_160_6.g3649;Description=hypothetical protein J7337_006791 [Fusarium musae];Gene=FACUT_1736;Ontology_term=nuclear origin of replication recognition complex,DNA replication origin binding,DNA
Fv_160_6.g3650	216	ID=Fv_160_6.g3650;Description=HAD superfamily (subfamily IIIA) phosphatase [Fusarium verticillioides 7600];Gene=FMAN_05757;Ontology_term=phosphatidylglycerophosphatase
Fv_160_6.g3651	354	ID=Fv_160_6.g3651;Description=hypothetical protein FVER14953_04755 [Fusarium verticillioides];Gene=2330
Fv_160_6.g3652	177	ID=Fv_160_6.g3652;Description=hypothetical protein FVEG_04754 [Fusarium verticillioides 7600];Gene=F53441_7013;Ontology_term=nuclear pore,TIM22 mitochondrial import inner membrane insertion complex,protein transmembrane transporter activity,phosphatidylglycerophosphatase activity,mitochondrion targeting sequence binding,protein insertion into mitochondrial inner membrane,mRNA transport,protein transmembrane transport;Ontology_id=GO:0005643,GO:0042721,GO:0008320,GO:0008962,GO:0030943,GO:0045039,GO:0051028,GO:0071806;En

Fv_160_6.g3653	378	ID=Fv_160_6.g3653;Description=endo-1,3(4)-beta-glucanase [Fusarium verticillioides 7600];Gene=C2S_13383;Ontology_term=membrane,anthranilate synthase activity,indole-3-glycerol-phosphate synthase activity,hydrolase activity, hydrolyzing O-glycosyl compounds,phosphoribosylanthranilate isomerase activity,tryptophan biosynthetic process,carbohydrate metabolic process,glutamine metabolic process;Ontology_id=GO:0016020,GO:0004049,GO:0004425,GO:0004553,GO:0004640,GO:0000162,GO:0005975,GO:0006541;Enzy
Fv_160_6.g3654	170	ID=Fv_160_6.g3654;Description=hypothetical protein FVEG_04752 [Fusarium verticillioides
Fv_160_6.g3655	670	ID=Fv_160_6.g3655;Description=hypothetical protein FVEG_04751 [Fusarium verticillioides 7600];Gene=rglB;Ontology_term=extracellular region,carbohydrate binding,rhamnogalacturonan endolyase activity,polysaccharide catabolic process,cell wall
Fv_160_6.g3656	585	ID=Fv_160_6.g3656;Description=SNW protein 1 [Fusarium verticillioides 7600];Gene=FGADI_12349;Ontology_term=Prp19 complex,post-mRNA release spliceosomal complex,RNA binding,molecular adaptor activity,mRNA cis splicing, via
Fv_160_6.g3657	210	ID=Fv_160_6.g3657;Description=hypothetical protein FVEG_04749 [Fusarium verticillioides

Fv_160_6.g3658	375	ID=Fv_160_6.g3658;Description=3-isopropylmalate dehydrogenase [Fusarium proliferatum];Gene=FNAPI_6017;Ontology_term=positive regulation of heart rate,peptide hormone secretion,cellular response to human chorionic gonadotropin stimulus,cardiac neural crest cell migration involved in outflow tract morphogenesis,positive regulation of mitotic nuclear division,sympathetic neuron axon guidance,regulation of pH,dorsal/ventral pattern formation,cellular response to follicle-stimulating hormone stimulus,cellular response to luteinizing hormone stimulus,response to hypoxia,in utero embryonic development,phosphoglycerate kinase activity,protein kinase A signaling,cytosol,calcium-mediated signaling,negative regulation of protein metabolic process,neural crest cell fate commitment,glyoxylate cycle,nitric oxide transport,semaphorin-plexin signaling pathway involved in axon guidance,glycolytic process,respiratory gaseous exchange by respiratory system,regulation of systemic arterial blood pressure by endothelin,cytokine activity,meiotic cell cycle process involved in oocyte maturation,body fluid secretion,3-isopropylmalate dehydrogenase activity,regulation of glucose transmembrane transport,endothelin A receptor binding,negative regulation of nitric-oxide synthase biosynthetic process,endothelin B receptor binding,metal ion binding,prostaglandin biosynthetic process,vein smooth muscle contraction,phospholipase D-activating G protein-coupled receptor signaling pathway,artery smooth muscle contraction,positive regulation of cell growth involved in cardiac muscle cell development,positive regulation of cell migration,cartilage development,axonogenesis involved in innervation,adenylate cyclase-inhibiting G protein-coupled receptor signaling pathway,leucine biosynthetic process,middle ear morphogenesis,positive regulation of transcription by RNA polymerase II,protein kinase C deactivation,hormone activity,positive regulation of smooth muscle contraction,positive regulation of smooth muscle cell proliferation,negative regulation of transcription by RNA polymerase II,ATP binding,positive regulation of renal sodium excretion,positive regulation of cell size,positive regulation of chemokine-mediated signaling pathway,branching involved in blood vessel morphogenesis,calcium ion transmembrane transport,canonical Wnt signaling pathway,phosphorylation,cellular response to hydrogen peroxide,regulation of vasoconstriction,positive regulation of urine volume,endothelin receptor signaling pathway involved in heart process,positive regulation of sarcomere organization,positive regulation of hormone secretion,pharyngeal arch artery morphogenesis,embryonic heart tube development,rhythmic excitation,protein kinase C-activating G protein-coupled receptor signaling pathway,canonical NF-kappaB signal transduction,mitochondrion organization,intracellular calcium ion homeostasis,positive regulation of cytosolic calcium ion concentration,renal sodium ion absorption,noradrenergic neuron differentiation,ERK1 and ERK2 cascade,extracellular space,thyroid gland development,response to amphetamine,axon
Fv_160_6.g3659	645	ID=Fv_160_6.g3659;Description=hypothetical protein FVEG_04747 [Fusarium verticillioides 7600];Gene=FTJAE_9022;Ontology_term=membrane,phosphoribosylaminoimidazolesuccinocarboxamide synthase activity,protein binding,ATP binding,'de novo' IMP biosynthetic process,rRNA processing;Ontology_id=GO:0016020,GO:0004639,GO:0005515,GO:0005524,GO:0006189,GO:0006364;Enzyme_code=EC:6.3.2.6;Enzyme_name=phosphoribosylaminoimidazolesuccinocarboxamide synthase

Fv_160_6.g3660	300	ID=Fv_160_6.g3660;Description=hypothetical protein FVEG_04746 [Fusarium verticillioides 7600];Gene=FVEG_04746;Ontology_term=membrane,phosphoribosylaminoimidazolesuccinocarboxamide synthase activity,protein binding,ATP binding,'de novo' IMP biosynthetic process,rRNA processing;Ontology_id=GO:0016020,GO:0004639,GO:0005515,GO:0005524,GO:0006189,GO:0006364;Enzyme_code=EC:6.3.2.6;Enzyme_name=phosphoribosylaminoimidazolesuccinocarboxamide synthase
Fv_160_6.g3661	189	ID=Fv_160_6.g3661;Description=hypothetical protein FVEG_04745 [Fusarium verticillioides 7600];Gene=FCIRC_6228;Ontology_term=mitochondrial matrix,oxidoreductase activity, acting on NAD(P)H, heme protein as acceptor,2 iron, 2 sulfur cluster binding,ubiquinone biosynthetic process,heme A biosynthetic process,iron-sulfur cluster assembly,P450-containing electron transport
Fv_160_6.g3662	489	ID=Fv_160_6.g3662;Description=hypothetical protein FVER14953_04744 [Fusarium verticillioides];Gene=FCIRC_6229;Ontology_term=mitochondrial inner membrane,diarylpropane peroxidase activity,heme binding,metal ion binding,cellular response to oxidative stress,hydrogen peroxide catabolic process,lignin catabolic process,cellular oxidant
Fv_160_6.g3663	275	ID=Fv_160_6.g3663;Description=endoglucanase [Fusarium verticillioides 7600];Gene=FACUT_8518;Ontology_term=extracellular
Fv_160_6.g3664	384	ID=Fv_160_6.g3664;Description=hypothetical protein FVER53590_04742 [Fusarium
Fv_160_6.g3665	707	ID=Fv_160_6.g3665;Description=hypothetical protein FVEG_04741 [Fusarium verticillioides 7600];Gene=FNAPI_6024;Ontology_term=membrane,cysteine-type deubiquitinase
Fv_160_6.g3666	398	ID=Fv_160_6.g3666;Description=hypothetical protein CEK27_007123 [Fusarium
Fv_160_6.g3667	753	ID=Fv_160_6.g3667;Description=hypothetical protein FVER53590_04739 [Fusarium verticillioides];Gene=NHP6B;Ontology_term=nucleus,chromosome,DNA binding, bending,maintenance of transcriptional fidelity during transcription elongation by RNA polymerase III,DNA repair,chromatin remodeling,transcription by RNA polymerase II,RNA
Fv_160_6.g3668	566	ID=Fv_160_6.g3668;Description=serine/threonine-protein phosphatase 2B catalytic subunit [Fusarium oxysporum f. sp. lycopersici 4287];Gene=FVEG_04738;Ontology_term=calcineurin complex,mitotic actomyosin contractile ring, intermediate layer,calmodulin binding,myosin phosphatase activity,calmodulin-dependent protein phosphatase activity,regulation of cell morphogenesis,cellular response to calcium ion,fungal-type cell wall organization or biogenesis,calcineurin-mediated signaling,positive regulation of mitotic division septum assembly,positive regulation of mitotic actomyosin contractile ring contraction,negative regulation of calcium ion import across plasma
Fv_160_6.g3669	290	ID=Fv_160_6.g3669;Description=oxidoreductase [Fusarium verticillioides 7600];Gene=FVEG_04737;Ontology_term=oxidoreductase activity,cellular biosynthetic process;Ontology_id=GO:0016491,GO:0044249;Enzyme_code=EC:1;Enzyme_name=Oxidoreductases
Fv_160_6.g3670	487	ID=Fv_160_6.g3670;Description=hypothetical protein FVEG_04736 [Fusarium verticillioides 7600];Gene=BFJ68_g11143;Ontology_term=iron ion binding,trimethyllysine dioxygenase activity,carnitine biosynthetic
Fv_160_6.g3671	149	ID=Fv_160_6.g3671;Description=hypothetical protein FVEG_15534 [Fusarium verticillioides 7600];Gene=HZS61_012137;Ontology_term=proteasome complex,proteasome assembly;Ontology_id=GO:0000502,GO:0043248

Fv_160_6.g3672	1211	ID=Fv_160_6.g3672;Description=hypothetical protein FVEG_04735 [Fusarium verticillioides 7600];Gene=FMAN_05735;Ontology_term=nucleolus,small-subunit processome,nucleic acid binding,RNA helicase activity,ATP binding,hydrolase activity,maturation of SSU-rRNA from tricistronic rRNA transcript (SSU-rRNA, 5.8S rRNA, LSU-
Fv_160_6.g3673	163	ID=Fv_160_6.g3673;Description=3-oxoacyl-[acyl-carrier protein] reductase [Fusarium verticillioides 7600]
Fv_160_6.g3674	320	ID=Fv_160_6.g3674;Description=hypothetical protein FVEG_04734 [Fusarium verticillioides 7600];Gene=FANTH_6144;Ontology_term=postsynaptic density,calcium-dependent cell-cell adhesion via plasma membrane cell adhesion molecules,adherens junction organization,cell-cell junction assembly,blood vessel morphogenesis,brain development,homophilic cell adhesion via plasma membrane adhesion molecules,neuron projection,regulation of transcription by RNA polymerase II,adherens junction,synapse assembly,transcription coregulator activity,apical part of cell,cytoplasm,regulation of axonogenesis,calcium ion binding,cell-cell adhesion mediated by cadherin,cadherin binding,mediator complex,intercalated disc,catenin complex,desmosome,regulation of synaptic transmission,
Fv_160_6.g3675	252	ID=Fv_160_6.g3675;Description=hypothetical protein FVER53590_04733 [Fusarium verticillioides]
Fv_160_6.g3676	396	ID=Fv_160_6.g3676;Description=hypothetical protein HG530_008959 [Fusarium avenaceum];Gene=FOXG_07808;Ontology_term=argininosuccinate synthase activity,ATP binding,arginine biosynthetic
Fv_160_6.g3677	1753	ID=Fv_160_6.g3677;Description=hypothetical protein FVER53590_04731 [Fusarium verticillioides];Gene=DSC1;Ontology_term=postsynaptic density,calcium-dependent cell-cell adhesion via plasma membrane cell adhesion molecules,adherens junction organization,cell-cell junction assembly,blood vessel morphogenesis,brain development,homophilic cell adhesion via plasma membrane adhesion molecules,neuron projection,adherens junction,synapse assembly,apical part of cell,cytoplasm,regulation of axonogenesis,calcium ion binding,cell-cell adhesion mediated by cadherin,cadherin binding,intercalated disc,catenin complex,desmosome,regulation of synaptic transmission,
Fv_160_6.g3678	84	ID=Fv_160_6.g3678;Description=Uncharacterized protein HZ326_0062 [Fusarium oxysporum f. sp. albedinis];Gene=F66182_4799;Ontology_term=nucleosome,nucleus,membrane,DNA binding,structural constituent of
Fv_160_6.g3679	453	ID=Fv_160_6.g3679;Description=hypothetical protein FVER53263_04729 [Fusarium verticillioides];Gene=FNYG_12717;Ontology_term=eukaryotic translation initiation factor 2B complex,translation initiation factor activity,guanyl-nucleotide exchange factor activity,cytoplasmic translational
Fv_160_6.g3680	452	ID=Fv_160_6.g3680;Description=arginine biosynthesis ArgJ, mitochondrial [Fusarium verticillioides 7600];Gene=308;Ontology_term=nucleosome,nucleus,mitochondrial matrix,DNA binding,acetyl-CoA:L-glutamate N-acetyltransferase activity,glutamate N-acetyltransferase activity,structural constituent of chromatin,protein heterodimerization activity,methione N-acyltransferase activity,arginine biosynthetic process,ornithine biosynthetic process;Ontology_id=GO:0000786,GO:0005634,GO:0005759,GO:0003677,GO:0004042,GO:0004358,GO:0030527,GO:0046982,GO:

Fv_160_6.g3681	227	ID=Fv_160_6.g3681;Description=imidazoleglycerol-phosphate dehydratase [Fusarium verticillioides 7600];Gene=FNAPI_6038;Ontology_term=nucleus,cytoplasm,plasma membrane,desmosome,imidazoleglycerol-phosphate dehydratase activity,calcium ion binding,gamma-catenin binding,histidine biosynthetic process,homophilic cell adhesion via plasma membrane adhesion molecules;Ontology_id=GO:0005634,GO:0005737,GO:0005886,GO:0030057,GO:0004424,GO:0005509,GO:0045295,GO:0000105,G
Fv_160_6.g3682	616	ID=Fv_160_6.g3682;Description=anaphase-promoting complex subunit 6 [Fusarium verticillioides 7600];Gene=C2S_9456;Ontology_term=ATP hydrolysis activity,zinc ion binding,anaphase-promoting complex,positive regulation of mitotic sister chromatid separation,pericentric heterochromatin,heme binding,hydrogen peroxide catabolic process,lignin catabolic process,ATP binding,RNA binding,RNA helicase activity,nuclear-transcribed mRNA catabolic process, nonsense-mediated decay,cytoplasm,diarylpropane peroxidase activity,cellular oxidant detoxification,anaphase-promoting complex-dependent catabolic process,cellular response to oxidative stress;Ontology_id=GO:0016887,GO:0008270,GO:0005680,GO:1901970,GO:0005721,GO:0020037,GO:0042744,GO:0046274,GO:00
Fv_160_6.g3683	169	ID=Fv_160_6.g3683;Description=hypothetical protein FVER53590_28416 [Fusarium
Fv_160_6.g3684	697	ID=Fv_160_6.g3684;Description=hypothetical protein FVER53590_04725 [Fusarium verticillioides];Gene=FMAN_05724;Ontology_term=membrane,aminoacyl-tRNA editing activity,leucine-tRNA ligase activity,ATP binding,diarylpropane peroxidase activity,heme binding,acetoacetate-CoA ligase activity,metal ion binding,leucyl-tRNA aminoacylation,lipid metabolic process,cellular response to oxidative stress,hydrogen peroxide catabolic process,lignin catabolic process,cellular oxidant detoxification,aminoacyl-tRNA metabolism involved in translational fidelity;Ontology_id=GO:0016020,GO:0002161,GO:0004823,GO:0005524,GO:0016690,GO:0020037,GO:0030729,GO:0046872,GO:0
Fv_160_6.g3685	3875	ID=Fv_160_6.g3685;Description=hypothetical protein J7337_006758 [Fusarium musae];Gene=FVEG_04724;Ontology_term=chromosome, telomeric region,nucleus,Smc5-Smc6 complex,site of double-strand break,damaged DNA binding,single-stranded DNA binding,protein binding,ATP binding,ATP hydrolysis activity,SUMO transferase activity,double-strand break repair via homologous recombination,protein sumoylation,regulation of telomere maintenance,chromosome separation,resolution of DNA recombination intermediates,chromatin looping,DNA double-strand break attachment to nuclear
Fv_160_6.g3686	167	ID=Fv_160_6.g3686;Description=hypothetical protein FVEG_04723 [Fusarium verticillioides 7600];Gene=3134;Ontology_term=acetate-CoA ligase activity,ATP binding,AMP binding,acetyl-CoA biosynthetic process from
Fv_160_6.g3687	383	ID=Fv_160_6.g3687;Description=hypothetical protein FVER14953_04722 [Fusarium verticillioides];Gene=2361;Ontology_term=incipient cellular bud site,cellular bud neck contractile ring,myosin II complex,myosin filament,microfilament motor activity,ATP binding,myosin II light chain binding,actin filament binding,septum digestion after cytokinesis,primary cell septum biogenesis,mitotic actomyosin contractile ring contraction,mitotic actomyosin contractile ring assembly,protein localization to mitotic actomyosin contractile

Fv_160_6.g3688	1152	ID=Fv_160_6.g3688;Description=hypothetical protein FOVG_07459 [Fusarium oxysporum f. sp. pisi HDV247];Gene=FFUJ_13281;Ontology_term=extracellular region,mitochondrial inner membrane,respirasome,metalloendopeptidase activity,hydrolase activity, hydrolyzing O-glycosyl compounds,cellulose binding,metal ion binding,proteolysis,cellulose catabolic process;Ontology_id=GO:0005576,GO:0005743,GO:0070469,GO:0004222,GO:0004553,GO:0030248,GO:0046872,GO:0006508,GO:
Fv_160_6.g3689	1155	ID=Fv_160_6.g3689;Description=hypothetical protein FVER53263_04721 [Fusarium verticillioides];Gene=FNYG_12707;Ontology_term=cysteine-type deubiquitinase activity,proteasome-mediated ubiquitin-dependent
Fv_160_6.g3690	318	ID=Fv_160_6.g3690;Description=methylglutaconyl-CoA hydratase [Fusarium verticillioides 7600];Gene=fer4;Ontology_term=mitochondrial outer membrane,endoplasmic reticulum membrane,cytosol,isomerase activity,enzyme binding,heme binding,metal ion
Fv_160_6.g3691	649	ID=Fv_160_6.g3691;Description=hypothetical protein FVER53590_04719 [Fusarium
Fv_160_6.g3692	126	ID=Fv_160_6.g3692;Description=hypothetical protein FVEG_04718 [Fusarium verticillioides 7600]
Fv_160_6.g3693	272	ID=Fv_160_6.g3693;Description=hypothetical protein NW758_009276 [Fusarium
Fv_160_6.g3694	222	ID=Fv_160_6.g3694;Description=hypothetical protein J7337_006749 [Fusarium
Fv_160_6.g3695	316	ID=Fv_160_6.g3695;Description=hypothetical protein FVEG_15530 [Fusarium verticillioides 7600];Gene=FTJAE_4384;Ontology_term=mitochondrion,membrane,glutamyl-tRNA(Gln) amidotransferase complex,ATP binding,transferase activity,glutaminyl-tRNA synthase (glutamine-hydrolyzing) activity,mitochondrial translation,glutaminyl-tRNAGln biosynthesis via
Fv_160_6.g3696	617	ID=Fv_160_6.g3696;Description=hypothetical protein FVER14953_04715 [Fusarium verticillioides];Gene=FTJAE_4383;Ontology_term=mitochondrion,glutamyl-tRNA(Gln) amidotransferase complex,ATP binding,transferase activity,glutaminyl-tRNA synthase (glutamine-hydrolyzing) activity,mitochondrial translation,glutaminyl-tRNAGln biosynthesis via
Fv_160_6.g3697	362	ID=Fv_160_6.g3697;Description=hypothetical protein FVER53590_28414 [Fusarium
Fv_160_6.g3698	264	ID=Fv_160_6.g3698;Description=hypothetical protein FVEG_04713 [Fusarium verticillioides 7600];Gene=3123;Ontology_term=tryptophan-tRNA ligase activity,ATP binding,tryptophanyl-tRNA
Fv_160_6.g3699	1333	ID=Fv_160_6.g3699;Description=hypothetical protein FVER53590_04712 [Fusarium verticillioides];Gene=BFJ68_g15598;Ontology_term=protein targeting to vacuole,vesicle-mediated
Fv_160_6.g3700	1943	ID=Fv_160_6.g3700;Description=hypothetical protein FVER53263_04711 [Fusarium verticillioides];Gene=FNAPI_6057;Ontology_term=anaphase-promoting complex;Ontology_id=GO:0005680
Fv_160_6.g3701	439	ID=Fv_160_6.g3701;Description=eukaryotic translation initiation factor 3 subunit E [Fusarium verticillioides 7600];Gene=INT6;Ontology_term=eukaryotic 43S preinitiation complex,eukaryotic 48S preinitiation complex,eukaryotic translation initiation factor 3 complex, eIF3e,translation initiation factor activity,formation of cytoplasmic translation initiation
Fv_160_6.g3702	172	ID=Fv_160_6.g3702;Description=hypothetical protein FVEG_04709 [Fusarium verticillioides 7600];Gene=FNYG_12694;Ontology_term=mitochondrion,ribosome,ribonucleoprotein complex,structural constituent of

Fv_160_6.g3703	1064	ID=Fv_160_6.g3703;Description=hypothetical protein FVER53263_04708 [Fusarium
Fv_160_6.g3704	396	ID=Fv_160_6.g3704;Description=hypothetical protein FVEG_04707 [Fusarium verticillioides 7600];Gene=AU210_004090;Ontology_term=cAMP response element binding,positive regulation of fat cell differentiation,protein serine/threonine kinase activity,positive regulation of transcription by RNA polymerase II,circadian rhythm,DNA-binding transcription factor activity, RNA polymerase II-specific,positive regulation of lipid biosynthetic process,maturation of SSU-rRNA from tricistronic rRNA transcript (SSU-rRNA, 5.8S rRNA, LSU-rRNA),ATP binding,snoRNA binding,ATF4-CREB1 transcription factor complex,negative regulation of apoptotic process,response to glucagon,protein
Fv_160_6.g3705	967	ID=Fv_160_6.g3705;Description=hypothetical protein J7337_006738 [Fusarium
Fv_160_6.g3706	531	ID=Fv_160_6.g3706;Description=cytochrome P450 monooxygenase [Fusarium bulbicola];Gene=FOXYS1_4733;Ontology_term=nuclear pore,membrane,monooxygenase activity,iron ion binding,oxidoreductase activity, acting on paired donors, with incorporation or reduction of molecular oxygen,heme binding;Ontology_id=GO:0005643,GO:0016020,GO:0004497,GO:0005506,GO:0016705,GO:0020037;Enzyme_code=EC:1.14;Enzyme
Fv_160_6.g3707	636	ID=Fv_160_6.g3707;Description=hypothetical protein FVER53590_04703 [Fusarium verticillioides];Gene=FVEG_04703;Ontology_term=nuclear pore,membrane,monooxygenase activity,iron ion binding,oxidoreductase activity, acting on paired donors, with incorporation or reduction of molecular oxygen,heme binding;Ontology_id=GO:0005643,GO:0016020,GO:0004497,GO:0005506,GO:0016705,GO:0020037;Enzyme_code=EC:1.14;Enzyme
Fv_160_6.g3708	652	ID=Fv_160_6.g3708;Description=hypothetical protein FVER53590_04702 [Fusarium
Fv_160_6.g3709	458	ID=Fv_160_6.g3709;Description=hypothetical protein FVER14953_04701 [Fusarium verticillioides]
Fv_160_6.g3710	302	ID=Fv_160_6.g3710;Description=hypothetical protein FVER53263_04700 [Fusarium verticillioides]
Fv_160_6.g3711	2211	ID=Fv_160_6.g3711;Description=hypothetical protein FVER14953_04699 [Fusarium verticillioides];Gene=FPRO05_06983;Ontology_term=mitochondrion,epsilon DNA polymerase complex,nucleotide binding,DNA binding,DNA-directed DNA polymerase activity,zinc ion binding,4 iron, 4 sulfur cluster binding,DNA replication,DNA repair,DNA biosynthetic
Fv_160_6.g3712	169	ID=Fv_160_6.g3712;Description=hypothetical protein J7337_006731 [Fusarium musae];Gene=radA;Ontology_term=mitochondrial inner membrane,mitochondrial cytochrome c oxidase assembly;Ontology_id=GO:0005743,GO:0033617
Fv_160_6.g3713	306	ID=Fv_160_6.g3713;Description=hypothetical protein FVER53263_04697 [Fusarium verticillioides]
Fv_160_6.g3714	173	ID=Fv_160_6.g3714;Description=hypothetical protein FVEG_04696 [Fusarium verticillioides 7600];Gene=mcsB
Fv_160_6.g3715	182	ID=Fv_160_6.g3715;Description=hypothetical protein FVEG_04695 [Fusarium verticillioides 7600];Gene=FACUT_9619;Ontology_term=membrane,copper ion transmembrane transporter activity,copper ion transmembrane
Fv_160_6.g3716	651	ID=Fv_160_6.g3716;Description=hypothetical protein FVER14953_04694 [Fusarium verticillioides];Gene=FTJAE_4174;Ontology_term=mitochondrial inner membrane,ferric-chelate reductase activity,ferrous iron transmembrane transporter activity,mRNA processing,iron import into the

Fv_160_6.g3717	340	ID=Fv_160_6.g3717;Description=hypothetical protein FVER53263_04693 [Fusarium verticillioides];Gene=FMEXI_12060;Ontology_term=isomerase activity,carbohydrate binding,carbohydrate metabolic
Fv_160_6.g3718	440	ID=Fv_160_6.g3718;Description=hypothetical protein FVEG_04692 [Fusarium verticillioides 7600]
Fv_160_6.g3719	903	ID=Fv_160_6.g3719;Description=phosphatidylinositol class q [Fusarium
Fv_160_6.g3720	363	ID=Fv_160_6.g3720;Description=hypothetical protein FVER14953_04689 [Fusarium verticillioides];Gene=FVER53263_04689;Ontology_term=cytoplasm,tubulin binding,post-chaperonin tubulin folding
Fv_160_6.g3721	148	ID=Fv_160_6.g3721;Description=hypothetical protein FVEG_04688 [Fusarium verticillioides 7600];Gene=MED9;Ontology_term=mediator complex,transcription coregulator activity,regulation of transcription by RNA
Fv_160_6.g3722	525	ID=Fv_160_6.g3722;Description=2,3-bisphosphoglycerate-independent phosphoglycerate mutase [Fusarium verticillioides 7600];Gene=FOXYS1_12274;Ontology_term=mitochondrial inner membrane,mitochondrial large ribosomal subunit,respiratory chain complex IV,structural constituent of ribosome,manganese ion binding,2,3-bisphosphoglycerate-independent phosphoglycerate mutase activity,glucose catabolic process,glycolytic process,mitochondrial translation;Ontology_id=GO:0005743,GO:0005762,GO:0045277,GO:0003735,GO:0030145,GO:0046537,GO:0006007,GO:0006096,G
Fv_160_6.g3723	992	ID=Fv_160_6.g3723;Description=hypothetical protein FVER14953_04686 [Fusarium verticillioides];Gene=F25303_8;Ontology_term=nucleus,cytoplasm,regulation of DNA-templated transcription,circadian
Fv_160_6.g3724	549	ID=Fv_160_6.g3724;Description=hypothetical protein FVER53263_04685 [Fusarium verticillioides];Gene=FNYG_12672;Ontology_term=mitochondrial inner membrane,mitochondrial large ribosomal subunit,prenylcysteine oxidase activity,structural constituent of ribosome,lyase activity,translation,prenylcysteine catabolic process;Ontology_id=GO:0005743,GO:0005762,GO:0001735,GO:0003735,GO:0016829,GO:0006412,GO:0030328;Enzyme_code=EC
Fv_160_6.g3725	808	ID=Fv_160_6.g3725;Description=hypothetical protein FVER53590_04684 [Fusarium verticillioides]
Fv_160_6.g3726	534	ID=Fv_160_6.g3726;Description=Alpha,alpha-trehalose-phosphate synthase [UDP-forming] [Fusarium odoratissimum];Gene=FocTR4_00002764;Ontology_term=alpha,alpha-trehalose-phosphate synthase complex (UDP-forming),alpha,alpha-trehalose-phosphate synthase (UDP-forming) activity,trehalose biosynthetic process,ascospore formation,trehalose metabolism in response to
Fv_160_6.g3727	810	ID=Fv_160_6.g3727;Description=hypothetical protein FVEG_04682 [Fusarium verticillioides 7600];Gene=FTJAE_4185;Ontology_term=nuclear exosome (RNase complex),nucleotide binding,3'-5'-RNA exonuclease activity,nucleic acid binding,exonucleolytic trimming to generate mature 3'-end of 5.8S rRNA from tricistronic rRNA transcript (SSU-
Fv_160_6.g3728	150	ID=Fv_160_6.g3728;Description=hypothetical protein FVEG_04681 [Fusarium verticillioides 7600];Gene=FOC1_g10014450;Ontology_term=proton-transporting ATP synthase complex, coupling factor F(o),lipid binding,proton transmembrane transporter activity,proton motive force-driven ATP synthesis,proton transmembrane
Fv_160_6.g3729	146	ID=Fv_160_6.g3729;Description=ATP synthase subunit 9, mitochondrial [Fusarium oxysporum f. sp. lycopersici 4287];Gene=FPHYL_3391;Ontology_term=proton-transporting ATP synthase complex, coupling factor F(o),lipid binding,proton transmembrane transporter activity,proton motive force-driven ATP synthesis,proton transmembrane

Fv_160_6.g3730	653	ID=Fv_160_6.g3730;Description=hypothetical protein FVEG_04679 [Fusarium verticillioides 7600];Gene=FOIG_03608;Ontology_term=chromosome,mitochondrial large ribosomal subunit,structural constituent of ribosome,histone acetyltransferase activity,chromatin remodeling,regulation of DNA-templated transcription,mitochondrial
Fv_160_6.g3731	683	ID=Fv_160_6.g3731;Description=hypothetical protein FVER14953_04678 [Fusarium verticillioides];Gene=3092
Fv_160_6.g3732	576	ID=Fv_160_6.g3732;Description=26S proteasome regulatory subunit N3 [Fusarium verticillioides 7600];Gene=FNAPI_179;Ontology_term=proteasome complex,enzyme regulator activity,regulation of protein catabolic
Fv_160_6.g3733	686	ID=Fv_160_6.g3733;Description=hypothetical protein FVER14953_04676 [Fusarium verticillioides];Gene=FNAPI_178;Ontology_term=SAGA complex,transcription factor TFIID complex,SLIK (SAGA-like) complex,translation initiation factor activity,protein heterodimerization activity,DNA-templated transcription initiation,translational
Fv_160_6.g3734	259	ID=Fv_160_6.g3734;Description=hypothetical protein FVEG_04675 [Fusarium verticillioides 7600];Gene=FNAPI_177;Ontology_term=nucleoplasm,mitochondrial respiratory chain complex I,hydrolase activity,mitochondrial respiratory chain complex I
Fv_160_6.g3735	185	ID=Fv_160_6.g3735;Description=hypothetical protein FVER53590_04674 [Fusarium
Fv_160_6.g3736	512	ID=Fv_160_6.g3736;Description=hypothetical protein FVER53263_04672 [Fusarium verticillioides];Gene=FocTR4_00002754;Ontology_term=nucleus,DNA repair,chromatin organization,regulation of DNA-templated
Fv_160_6.g3737	87	ID=Fv_160_6.g3737;Description=hypothetical protein LB506_004160 [Fusarium annulatum];Gene=FMEXI_12041;Ontology_term=large ribosomal subunit,structural constituent of
Fv_160_6.g3738	655	ID=Fv_160_6.g3738;Description=ATP-citrate synthase subunit 1 [Fusarium pseudocircinatum];Gene=FTJAE_4195;Ontology_term=cytoplasm,large ribosomal subunit,structural constituent of ribosome,ATP citrate synthase activity,ATP binding,lyase activity,metal ion binding,tricarboxylic acid cycle,translation,lipid metabolic process;Ontology_id=GO:0005737,GO:0015934,GO:0003735,GO:0003878,GO:0005524,GO:0016829,GO:0046872,GO:0006099,GO:
Fv_160_6.g3739	489	ID=Fv_160_6.g3739;Description=ATP-citrate synthase subunit 2 [Fusarium verticillioides 7600];Gene=F52700_13083;Ontology_term=nucleotide binding,ATP citrate synthase activity,lyase activity,tricarboxylic acid
Fv_160_6.g3740	490	ID=Fv_160_6.g3740;Description=hypothetical protein FVEG_04666 [Fusarium verticillioides 7600];Gene=FPRO_05927;Ontology_term=plasma membrane,fungal-type cell wall,side of membrane,transferase
Fv_160_6.g3741	338	ID=Fv_160_6.g3741;Description=hypothetical protein FVEG_04665 [Fusarium verticillioides 7600];Gene=FOPG_06708;Ontology_term=cell cortex,septin cytoskeleton,GTP binding,cell
Fv_160_6.g3742	361	ID=Fv_160_6.g3742;Description=GTP-binding protein beta subunit [Fusarium coicis]
Fv_160_6.g3743	202	ID=Fv_160_6.g3743;Description=hypothetical protein FVER14953_04662 [Fusarium verticillioides];Gene=FGLOB1_11116;Ontology_term=mitochondrial respiratory chain complex I,DNA synthesis involved in DNA
Fv_160_6.g3744	365	ID=Fv_160_6.g3744;Description=hypothetical protein FVEG_04661 [Fusarium verticillioides 7600];Gene=FVER53590_04661;Ontology_term=malate synthase activity,hydrolase activity,glyoxylate cycle,tricarboxylic acid cycle,tRNA-guanine

Fv_160_6.g3745	685	ID=Fv_160_6.g3745;Description=hypothetical protein FVER53263_04660 [Fusarium verticillioides];Gene=FSUBG_13453;Ontology_term=mitochondrial respiratory chain complex I,SUMO activating enzyme complex,ATP binding,SUMO activating enzyme activity,metal ion binding,protein sumoylation,mitochondrial respiratory chain
Fv_160_6.g3746	225	ID=Fv_160_6.g3746;Description=hypothetical protein FOXG_07735 [Fusarium oxysporum f. sp. lycopersici 4287];Gene=FVEG_04659;Ontology_term=oxidoreductase activity,cellular biosynthetic
Fv_160_6.g3747	968	ID=Fv_160_6.g3747;Description=sphingoid base-phosphate phosphatase [Fusarium globosum];Gene=FGADI_9773;Ontology_term=membrane,Elg1 RFC-like complex,DNA binding,ATP binding,ATP hydrolysis activity,DNA strand elongation involved in DNA replication,lipid metabolic process,DNA clamp unloading;Ontology_id=GO:0016020,GO:0031391,GO:0003677,GO:0005524,GO:0016887,GO:0006271,GO:0006629,GO:0090618;En
Fv_160_6.g3748	468	ID=Fv_160_6.g3748;Description=Replication factor C subunit 3 [Fusarium pseudoanthophilum];Gene=FNYG_13321;Ontology_term=membrane,Elg1 RFC-like complex,DNA binding,ATP binding,ATP hydrolysis activity,DNA strand elongation involved in DNA replication,lipid metabolic process,protein homooligomerization,DNA clamp unloading;Ontology_id=GO:0016020,GO:0031391,GO:0003677,GO:0005524,GO:0016887,GO:0006271,GO:0006629,GO:0051260,G
Fv_160_6.g3749	673	ID=Fv_160_6.g3749;Description=hypothetical protein FCOIX_12327 [Fusarium coicis]
Fv_160_6.g3750	268	ID=Fv_160_6.g3750;Description=hypothetical protein FVEG_04654 [Fusarium verticillioides
Fv_160_6.g3751	269	ID=Fv_160_6.g3751;Description=hypothetical protein FVEG_04653 [Fusarium verticillioides 7600];Gene=241;Ontology_term=mitochondrial inner membrane,mitochondrial intermembrane space,microtubule,phosphatidylserine binding,GTPase activity,microtubule binding,phosphatidic acid binding,cardiolipin binding,mitochondrion inheritance,mitochondrial genome maintenance;Ontology_id=GO:0005743,GO:0005758,GO:0005874,GO:0001786,GO:0003924,GO:0008017,GO:0070300,GO:1901612
Fv_160_6.g3752	134	ID=Fv_160_6.g3752;Description=hypothetical protein FVER53590_04652 [Fusarium verticillioides];Gene=FVEG_04652;Ontology_term=DNA binding,DNA repair,kinetochore
Fv_160_6.g3753	311	ID=Fv_160_6.g3753;Description=ADP,ATP carrier protein [Fusarium odoratissimum NRRL 54006];Gene=FGLOB1_11106;Ontology_term=mitochondrial inner membrane,ATP:ADP antiporter activity,mitochondrial ADP
Fv_160_6.g3754	96	ID=Fv_160_6.g3754;Description=40S ribosomal protein S25 [Fusarium verticillioides 7600];Gene=F66182_8051;Ontology_term=ribosome,ribonucleoprotein complex;Ontology_id=GO:0005840,GO:1990904
Fv_160_6.g3755	213	ID=Fv_160_6.g3755;Description=40S ribosomal protein S5 [Fusarium verticillioides 7600];Gene=CEP52_005153;Ontology_term=cytosolic small ribosomal subunit,RNA binding,structural constituent of ribosome,rRNA
Fv_160_6.g3756	277	ID=Fv_160_6.g3756;Description=hypothetical protein FVEG_04648 [Fusarium verticillioides 7600];Gene=FVEG_04648;Ontology_term=glucosamine 6-phosphate N-acetyltransferase activity,UDP-N-acetylglucosamine
Fv_160_6.g3757	235	ID=Fv_160_6.g3757;Description=hypothetical protein FVEG_04647 [Fusarium verticillioides 7600];Gene=3066
Fv_160_6.g3758	2030	ID=Fv_160_6.g3758;Description=hypothetical protein FVER53590_04646 [Fusarium verticillioides];Gene=FNYG_13331;Ontology_term=cytoplasm,guanyl-nucleotide exchange factor activity,small GTPase mediated

Fv_160_6.g3759	154	ID=Fv_160_6.g3759;Description=hypothetical protein J7337_006684 [Fusarium musae]
Fv_160_6.g3760	257	ID=Fv_160_6.g3760;Description=hypothetical protein FVEG_04644 [Fusarium verticillioides 7600];Gene=FNYG_13332;Ontology_term=ATP binding,ATP-dependent protein folding chaperone,protein
Fv_160_6.g3761	635	ID=Fv_160_6.g3761;Description=hypothetical protein FVEG_04643 [Fusarium verticillioides 7600];Gene=FFC1_06420;Ontology_term=ubiquitin-protein transferase activity,ATP binding,zinc ion binding,lyase activity,ATP-dependent protein folding chaperone,protein folding,protein ubiquitination,monocarboxylic acid metabolic process;Ontology_id=GO:0004842,GO:0005524,GO:0008270,GO:0016829,GO:0140662,GO:0006457,GO:0016567,GO:0032787;Enzy
Fv_160_6.g3762	709	ID=Fv_160_6.g3762;Description=hypothetical protein FVER14953_04642 [Fusarium verticillioides];Gene=FOXG_07718;Ontology_term=mitochondrial respiratory chain complex I,mitochondrial matrix,ATP binding,ATP hydrolysis activity,ABC-type transporter activity,mitochondrial electron transport, NADH to ubiquinone,mitochondrial respiratory chain complex I assembly,transmembrane transport;Ontology_id=GO:0005747,GO:0005759,GO:0005524,GO:0016887,GO:0140359,GO:0006120,GO:0032981,GO:0055085;En
Fv_160_6.g3763	86	ID=Fv_160_6.g3763;Description=hypothetical protein FVEG_04641 [Fusarium verticillioides 7600];Gene=F52700_13060;Ontology_term=nucleus,cytoplasm,ferrous iron binding,oxidoreductase activity,iron chaperone activity,tRNA wobble base 5-methoxycarbonylmethyl-2-thiouridinylation,protein histidyl modification to
Fv_160_6.g3764	78	ID=Fv_160_6.g3764;Description=uncharacterized protein FFUJ_13203 [Fusarium fujikuroi IMI
Fv_160_6.g3765	1112	ID=Fv_160_6.g3765;Description=leucyl-tRNA synthetase, cytoplasmic [Fusarium verticillioides 7600];Gene=FVEG_04639;Ontology_term=cytoplasm,aminoacyl-tRNA editing activity,leucine-tRNA ligase activity,ATP binding,leucyl-tRNA aminoacylation,aminoacyl-tRNA metabolism involved in translational fidelity,regulation of TORC1 signaling;Ontology_id=GO:0005737,GO:0002161,GO:0004823,GO:0005524,GO:0006429,GO:0106074,GO:1903432;Enzyme_code=E
Fv_160_6.g3766	142	ID=Fv_160_6.g3766;Description=hypothetical protein FVEG_04638 [Fusarium verticillioides
Fv_160_6.g3767	203	ID=Fv_160_6.g3767;Description=hypothetical protein FVEG_04636 [Fusarium verticillioides 7600];Gene=FVEG_04636;Ontology_term=mitochondrion,ferroxidase activity,ferric iron binding,intracellular iron ion homeostasis,iron-sulfur cluster assembly,iron ion transmembrane
Fv_160_6.g3768	751	ID=Fv_160_6.g3768;Description=hypothetical protein FVER53590_04634 [Fusarium verticillioides];Gene=Forpi1262_v004048;Ontology_term=membrane,transmembrane transporter activity,carbohydrate
Fv_160_6.g3769	296	ID=Fv_160_6.g3769;Description=hypothetical protein FVEG_04632 [Fusarium verticillioides
Fv_160_6.g3770	468	ID=Fv_160_6.g3770;Description=transcription initiation factor TFIIH subunit 2 [Fusarium verticillioides 7600];Gene=BFJ63_vAg4326;Ontology_term=translation initiation factor activity,zinc ion binding,transcription factor TFIIH core complex,nucleotide-excision repair,transcription factor TFIIH holo complex,translational initiation,regulation of transcription by RNA

Fv_160_6.g3771	424	ID=Fv_160_6.g3771;Description=4-hydroxyphenylpyruvate dioxygenase [Fusarium verticillioides 7600];Gene=FACUT_2528;Ontology_term=cytosol,external encapsulating structure,4-hydroxyphenylpyruvate dioxygenase activity,glyceraldehyde-3-phosphate dehydrogenase (NAD+) (phosphorylating) activity,metal ion binding,NADP binding,NAD binding,glucose metabolic process,glycolytic process,L-phenylalanine catabolic process,tyrosine catabolic process;Ontology_id=GO:0005829,GO:0030312,GO:0003868,GO:0004365,GO:0046872,GO:0050661,GO:0051287,GO:0006006,GO:0006096,GO:0006559,GO:0006572;Enzyme_code=EC:1.2.1.59,EC:1.2.1.12,EC:1.13.11.27;Enzyme_name=glyceraldehyde-3-
Fv_160_6.g3772	265	ID=Fv_160_6.g3772;Description=hypothetical protein FVEG_04629 [Fusarium verticillioides 7600]
Fv_160_6.g3773	159	ID=Fv_160_6.g3773;Description=hypothetical protein H9Q70_013738 [Fusarium xylarioides];Gene=FANTH_6242;Ontology_term=ribosome,ribonucleoprotein complex,structural constituent of
Fv_160_6.g3774	405	ID=Fv_160_6.g3774;Description=hypothetical protein FVEG_04627 [Fusarium verticillioides
Fv_160_6.g3775	519	ID=Fv_160_6.g3775;Description=hypothetical protein FVEG_04626 [Fusarium verticillioides 7600];Gene=FDENT_6828;Ontology_term=oxidoreductase
Fv_160_6.g3776	2563	ID=Fv_160_6.g3776;Description=hypothetical protein FVER53590_04624 [Fusarium verticillioides];Gene=FDENT_6829;Ontology_term=membrane,phosphorelay sensor kinase activity,protein serine/threonine kinase activity,ATP binding,phosphorelay signal transduction system,phosphorylation;Ontology_id=GO:0016020,GO:0000155,GO:0004674,GO:0005524,GO:0000160,GO:0016310;Enzyme_code=
Fv_160_6.g3777	466	ID=Fv_160_6.g3777;Description=hypothetical protein FVER53590_04621 [Fusarium verticillioides]
Fv_160_6.g3778	1061	ID=Fv_160_6.g3778;Description=hypothetical protein FVEG_15515 [Fusarium verticillioides 7600]
Fv_160_6.g3779	814	ID=Fv_160_6.g3779;Description=hypothetical protein FVER14953_04618 [Fusarium verticillioides]
Fv_160_6.g3780	360	ID=Fv_160_6.g3780;Description=Allantoicase [Fusarium musae];Gene=FNYG_13354;Ontology_term=allantoicase activity,allantoin catabolic process;Ontology_id=GO:0004037,GO:0000256;Enzyme_code=EC:3.5.3.4;Enzyme_name=allantoicase
Fv_160_6.g3781	250	ID=Fv_160_6.g3781;Description=cytochrome B-561 [Fusarium tjaetaba];Gene=HZS61_012026;Ontology_term=membrane,transmembrane monodehydroascorbate reductase
Fv_160_6.g3782	2072	ID=Fv_160_6.g3782;Description=hypothetical protein FVEG_04615 [Fusarium verticillioides 7600];Gene=FVEG_04615;Ontology_term=phosphorelay sensor kinase activity,phosphorelay signal transduction system,phosphorylation;Ontology_id=GO:0000155,GO:0000160,GO:0016310;Enzyme_code=EC:2.7.3,EC:2.7.13.3;Enzyme_name=Tra
Fv_160_6.g3783	483	ID=Fv_160_6.g3783;Description=hypothetical protein FVER14953_04614 [Fusarium verticillioides];Gene=FVEG_04614;Ontology_term=zinc ion binding,positive regulation of epidermal growth factor receptor signaling pathway,3',5'-cyclic-GMP phosphodiesterase activity,positive regulation of MAPK cascade,visual perception,photoreceptor disc membrane,cGMP binding,photoreceptor outer segment membrane,molecular adaptor activity,positive regulation of G protein-coupled receptor signaling
Fv_160_6.g3784	212	ID=Fv_160_6.g3784;Description=hypothetical protein FVEG_04613 [Fusarium verticillioides 7600];Gene=FOQG_02302;Ontology_term=double-stranded RNA binding;Ontology_id=GO:0003725

Fv_160_6.g3785	538	ID=Fv_160_6.g3785;Description=T-complex protein 1 subunit gamma [Fusarium verticillioides 7600];Gene=FANTH_6254;Ontology_term=chaperonin-containing T-complex,ATP binding,ATP hydrolysis activity,unfolded protein binding,ATP-dependent protein folding chaperone,chaperone mediated protein folding independent of
Fv_160_6.g3786	150	ID=Fv_160_6.g3786;Description=impact like [Fusarium tjaetaba]
Fv_160_6.g3787	586	ID=Fv_160_6.g3787;Description=hypothetical protein FCOIX_9918 [Fusarium coicis]
Fv_160_6.g3788	135	ID=Fv_160_6.g3788;Description=hypothetical protein FVER14953_20658 [Fusarium verticillioides];Gene=F25303_1622;Ontology_term=extracellular region,mitochondrial inner membrane,respirasome,alpha-galactosidase activity,NADH dehydrogenase (ubiquinone) activity,oxidoreductase activity, acting on CH-OH group of donors,metal ion binding,flavin adenine dinucleotide binding,2 iron, 2 sulfur cluster binding,4 iron, 4 sulfur cluster binding,carbohydrate metabolic process,ATP synthesis coupled electron transport;Ontology_id=GO:0005576,GO:0005743,GO:0070469,GO:0004557,GO:0008137,GO:0016614,GO:0046872,GO:0050660,GO
Fv_160_6.g3789	392	ID=Fv_160_6.g3789;Description=hypothetical protein FVEG_04610 [Fusarium verticillioides 7600];Gene=FTJAE_9870;Ontology_term=extracellular region,mitochondrial inner membrane,respirasome,alpha-galactosidase activity,NADH dehydrogenase (ubiquinone) activity,oxidoreductase activity, acting on CH-OH group of donors,metal ion binding,flavin adenine dinucleotide binding,2 iron, 2 sulfur cluster binding,4 iron, 4 sulfur cluster binding,carbohydrate metabolic process,ATP synthesis coupled electron transport;Ontology_id=GO:0005576,GO:0005743,GO:0070469,GO:0004557,GO:0008137,GO:0016614,GO:0046872,GO:0050660,GO
Fv_160_6.g3790	177	ID=Fv_160_6.g3790;Description=hypothetical protein FVEG_04609 [Fusarium verticillioides 7600]
Fv_160_6.g3791	297	ID=Fv_160_6.g3791;Description=hypothetical protein FVEG_04608 [Fusarium verticillioides 7600];Gene=FPRO05_07062;Ontology_term=nucleus,mitochondrion,translation elongation factor activity,GTPase activity,GTP binding,mitochondrial translational
Fv_160_6.g3792	868	ID=Fv_160_6.g3792;Description=hypothetical protein FVEG_04607 [Fusarium verticillioides
Fv_160_6.g3793	538	ID=Fv_160_6.g3793;Description=hypothetical protein FVEG_04606 [Fusarium verticillioides 7600];Gene=MDM1;Ontology_term=cytoplasm,membrane,nucleus-vacuole junction,phosphatidylinositol-3-phosphate
Fv_160_6.g3794	897	ID=Fv_160_6.g3794;Description=hypothetical protein FVER53590_04605 [Fusarium verticillioides]
Fv_160_6.g3795	163	ID=Fv_160_6.g3795;Description=ubiquitin-conjugating enzyme E2-18 kDa [Fusarium oxysporum f. sp. lycopersici 4287];Gene=FSPOR_330;Ontology_term=Doa10p ubiquitin ligase complex,Hrd1p ubiquitin ligase ERAD-L complex,CUE1-UBC7 ubiquitin-conjugating enzyme complex,ATP binding,ubiquitin conjugating enzyme activity,chromatin organization,ubiquitin-dependent ERAD pathway,fungal-type cell wall organization;Ontology_id=GO:0000837,GO:0000839,GO:1990389,GO:0005524,GO:0061631,GO:0006325,GO:0030433,GO:0031505;
Fv_160_6.g3796	159	ID=Fv_160_6.g3796;Description=hypothetical protein FVER14953_04603 [Fusarium verticillioides]
Fv_160_6.g3797	117	ID=Fv_160_6.g3797;Description=hypothetical protein FGLOB1_14049, partial [Fusarium globosum]

Fv_160_6.g3798	388	ID=Fv_160_6.g3798;Description=hypothetical protein FVEG_04601 [Fusarium verticillioides 7600];Gene=3022;Ontology_term=external side of plasma membrane,protein serine/threonine kinase activity,ATP binding,T cell mediated immunity,cell surface receptor signaling pathway,cytotoxic T cell differentiation;Ontology_id=GO:0009897,GO:0004674,GO:0005524,GO:0002456,GO:0007166,GO:0045065;Enzyme_code=EC:2.7.11
Fv_160_6.g3799	203	ID=Fv_160_6.g3799;Description=hypothetical protein FVEG_04600 [Fusarium verticillioides
Fv_160_6.g3800	238	ID=Fv_160_6.g3800;Description=nucleoside-diphosphate kinase [Fusarium verticillioides 7600];Gene=FNYG_01719;Ontology_term=membrane,nucleoside diphosphate kinase activity,ATP binding,GTP biosynthetic process,UTP biosynthetic process,CTP biosynthetic process,phosphorylation;Ontology_id=GO:0016020,GO:0004550,GO:0005524,GO:0006183,GO:0006228,GO:0006241,GO:0016310;E
Fv_160_6.g3801	963	ID=Fv_160_6.g3801;Description=alpha 1,3-glucosidase [Fusarium verticillioides 7600];Gene=FOC1_g10014527;Ontology_term=carbohydrate binding,Glc2Man9GlcNAc2 oligosaccharide glucosidase activity,carbohydrate metabolic process,N-glycan
Fv_160_6.g3802	860	ID=Fv_160_6.g3802;Description=hypothetical protein FVEG_04597 [Fusarium verticillioides 7600];Gene=FPHYL_619;Ontology_term=endoplasmic reticulum membrane,perinuclear endoplasmic reticulum,calcium ion binding,stretch-activated, monoatomic cation-selective, calcium channel activity,intracellular calcium ion homeostasis,cell volume
Fv_160_6.g3803	1628	ID=Fv_160_6.g3803;Description=hypothetical protein FVER53590_04596 [Fusarium verticillioides]
Fv_160_6.g3804	185	ID=Fv_160_6.g3804;Description=hypothetical protein FVEG_04595 [Fusarium verticillioides 7600]
Fv_160_6.g3805	344	ID=Fv_160_6.g3805;Description=chitinase [Fusarium verticillioides 7600];Gene=FPCIR_206;Ontology_term=membrane,chitinase activity,carbohydrate metabolic
Fv_160_6.g3806	296	ID=Fv_160_6.g3806;Description=hypothetical protein FVEG_04593 [Fusarium verticillioides 7600];Gene=F25303_1606;Ontology_term=membrane,carbohydrate metabolic process;Ontology_id=GO:0016020,GO:0005975
Fv_160_6.g3807	358	ID=Fv_160_6.g3807;Description=hypothetical protein FVER53590_04592 [Fusarium verticillioides]
Fv_160_6.g3808	93	ID=Fv_160_6.g3808;Description=hypothetical protein FVER14953_06658 [Fusarium verticillioides];Gene=fliP;Ontology_term=incipient cellular bud site,cellular bud neck,membrane,cell periphery,guanyl-nucleotide exchange factor activity,protein binding,axial cellular bud site selection,bipolar cellular bud site selection,small GTPase mediated
Fv_160_6.g3809	342	ID=Fv_160_6.g3809;Description=Dimethyladenosine transferase [Fusarium proliferatum];Gene=FVER53263_04591;Ontology_term=preribosome, small subunit precursor,RNA binding,18S rRNA (adenine(1779)-N(6)/adenine(1780)-N(6))-dimethyltransferase activity,maturation of SSU-rRNA from tricistronic rRNA transcript (SSU-rRNA, 5.8S rRNA, LSU-rRNA),rRNA
Fv_160_6.g3810	636	ID=Fv_160_6.g3810;Description=hypothetical protein FVER53590_04590 [Fusarium verticillioides]
Fv_160_6.g3811	115	ID=Fv_160_6.g3811;Description=Small nuclear ribonucleoprotein Sm D3 [Fusarium oxysporum f. sp. cubense race 1];Gene=HZS61_012002;Ontology_term=commitment complex,U5 snRNP,U1 snRNP,U2 snRNP,cytosol,U4/U6 x U5 tri-snRNP complex,U2-type prespliceosome,post-mRNA release spliceosomal complex,mRNA binding,spliceosomal snRNP

Fv_160_6.g3812	754	ID=Fv_160_6.g3812;Description=tRNA (adenine57-N1/adenine58-N1)-methyltransferase [Fusarium verticillioides 7600];Gene=FNYG_01729;Ontology_term=tRNA (m1A) methyltransferase complex,tRNA (adenine-N1)-methyltransferase activity,tRNA methylation;Ontology_id=GO:0031515,GO:0016429,GO:0030488;Enzyme_code=EC:2.1.1;Enzyme_name=Transferring
Fv_160_6.g3813	800	ID=Fv_160_6.g3813;Description=cysteinyl-tRNA synthetase [Fusarium verticillioides 7600];Gene=FOYG_01736;Ontology_term=cysteine-tRNA ligase activity,ATP binding,metal ion binding,cysteinyl-tRNA
Fv_160_6.g3814	477	ID=Fv_160_6.g3814;Description=hypothetical protein FVER14953_04586 [Fusarium verticillioides];Gene=FCIRC_10378;Ontology_term=mitochondrial intermembrane space,respirasome,electron transfer activity,heme binding,transmembrane transporter activity,metal ion binding,electron transport chain,transmembrane
Fv_160_6.g3815	408	ID=Fv_160_6.g3815;Description=hypothetical protein FVEG_04585 [Fusarium verticillioides 7600]
Fv_160_6.g3816	196	ID=Fv_160_6.g3816;Description=hypothetical protein FVEG_04584 [Fusarium verticillioides 7600]
Fv_160_6.g3817	693	ID=Fv_160_6.g3817;Description=hypothetical protein FVEG_04583 [Fusarium verticillioides 7600];Gene=FVER53590_04583;Ontology_term=nucleus,DNA binding,zinc ion binding,DNA-binding transcription factor activity, RNA polymerase II-specific,regulation of transcription by RNA polymerase
Fv_160_6.g3818	157	ID=Fv_160_6.g3818;Description=hypothetical protein FVER14953_20663 [Fusarium verticillioides];Gene=FOIG_03707;Ontology_term=membrane,monooxygenase
Fv_160_6.g3819	549	ID=Fv_160_6.g3819;Description=hypothetical protein FVER14953_20661 [Fusarium verticillioides];Gene=FPRO05_07087;Ontology_term=nucleus,mitochondrial respiratory chain complex III,DNA-binding transcription factor activity, RNA polymerase II-specific,monooxygenase activity,iron ion binding,ubiquinol-cytochrome-c reductase activity,zinc ion binding,oxidoreductase activity, acting on paired donors, with incorporation or reduction of molecular oxygen,heme binding,mitochondrial electron transport, ubiquinol to cytochrome c,regulation of transcription by RNA polymerase II,proton transmembrane transport;Ontology_id=GO:0005634,GO:0005750,GO:0000981,GO:0004497,GO:0005506,GO:0008121,GO:0008270,GO:0016705,GO
Fv_160_6.g3820	305	ID=Fv_160_6.g3820;Description=hypothetical protein FVEG_04581 [Fusarium verticillioides 7600]
Fv_160_6.g3821	193	ID=Fv_160_6.g3821;Description=hypothetical protein FVER14953_04580 [Fusarium verticillioides];Gene=FVER53590_04580;Ontology_term=guanylate kinase activity,ATP binding,phosphorylation,GMP metabolic process,GDP metabolic
Fv_160_6.g3822	525	ID=Fv_160_6.g3822;Description=hypothetical protein FVER14953_04579 [Fusarium verticillioides];Gene=FNAPI_5942;Ontology_term=membrane,mitochondrial processing peptidase complex,metalloendopeptidase activity,protein binding,ceramide glucosyltransferase activity,metal ion binding,endopeptidase activator activity,dihydroceramide glucosyltransferase activity,protein processing involved in protein targeting to mitochondrion,sphingolipid metabolic process;Ontology_id=GO:0016020,GO:0017087,GO:0004222,GO:0005515,GO:0008120,GO:0046872,GO:0061133,GO:0102769,GO:
Fv_160_6.g3823	176	ID=Fv_160_6.g3823;Description=hypothetical protein FVER53263_04578 [Fusarium verticillioides];Gene=Forpe1208_v005129;Ontology_term=carbon-sulfur lyase activity,metal ion

Fv_160_6.g3824	604	ID=Fv_160_6.g3824;Description=hypothetical protein FVER14953_04577 [Fusarium verticillioides];Gene=FPANT_12700;Ontology_term=tRNA-intron endonuclease complex,tRNA-intron endonuclease activity,nucleic acid binding,tRNA splicing, via endonucleolytic cleavage and ligation;Ontology_id=GO:0000214,GO:0000213,GO:0003676,GO:0006388;Enzyme_code=EC:3.1.31.1,EC:3.1.27,EC:4.6.1.16;Enzyme_
Fv_160_6.g3825	508	ID=Fv_160_6.g3825;Description=hypothetical protein FVER14953_20662 [Fusarium verticillioides];Gene=FOPG_15099;Ontology_term=nucleus,tRNA-intron endonuclease activity,nucleic acid binding,metal ion binding,dioxygenase activity,tRNA splicing, via endonucleolytic cleavage and ligation;Ontology_id=GO:0005634,GO:0000213,GO:0003676,GO:0046872,GO:0051213,GO:0006388;Enzyme_code=EC:3.1.31.1,EC:1
Fv_160_6.g3826	469	ID=Fv_160_6.g3826;Description=hypothetical protein FVEG_04576 [Fusarium verticillioides 7600];Gene=FMUND_2061;Ontology_term=nucleus,DNA-binding transcription factor activity, RNA polymerase II-specific,DNA binding,zinc ion binding,protein-folding chaperone binding,maltose metabolic process,regulation of transcription by RNA polymerase
Fv_160_6.g3827	796	ID=Fv_160_6.g3827;Description=lanosterol synthase [Fusarium verticillioides 7600];Gene=FPANT_12703;Ontology_term=lipid droplet,intramolecular transferase activity,triterpenoid biosynthetic
Fv_160_6.g3828	411	ID=Fv_160_6.g3828;Description=hypothetical protein FVER14953_04574 [Fusarium verticillioides];Gene=FOPG_15101;Ontology_term=chromosome,DNA binding,DNA topoisomerase type II (double strand cut, ATP-hydrolyzing) activity,ATP binding,metal ion binding,DNA metabolic
Fv_160_6.g3829	187	ID=Fv_160_6.g3829;Description=hypothetical protein FVEG_04573 [Fusarium verticillioides 7600]
Fv_160_6.g3830	804	ID=Fv_160_6.g3830;Description=hypothetical protein FVER53590_04572 [Fusarium verticillioides];Gene=FVER53590_04572;Ontology_term=nucleus,DNA binding,ATP binding,zinc ion binding,hydrolase activity,ligase activity,DNA-templated
Fv_160_6.g3831	1237	ID=Fv_160_6.g3831;Description=hypothetical protein FVEG_04571 [Fusarium verticillioides 7600];Gene=FNAPI_5951;Ontology_term=nucleus,DNA binding,ATP binding,zinc ion binding,hydrolase activity,lyase activity,ligase activity,DNA-templated transcription;Ontology_id=GO:0005634,GO:0003677,GO:0005524,GO:0008270,GO:0016787,GO:0016829,GO:0016874,GO:0006351
Fv_160_6.g3832	552	ID=Fv_160_6.g3832;Description=hypothetical protein FVEG_04570 [Fusarium verticillioides 7600];Gene=MAG1;Ontology_term=large ribosomal subunit,membrane,nucleic acid binding,structural constituent of ribosome,alkylbase DNA N-glycosylase activity,methyltransferase activity,transmembrane transporter activity,base-excision repair, AP site formation,translation,macromolecule methylation,transmembrane transport;Ontology_id=GO:0015934,GO:0016020,GO:0003676,GO:0003735,GO:0003905,GO:0008168,GO:0022857,GO:0006285,GO
Fv_160_6.g3833	262	ID=Fv_160_6.g3833;Description=hypothetical protein FVEG_04569 [Fusarium verticillioides 7600];Gene=FOYG_01757;Ontology_term=carbohydrate metabolic process;Ontology_id=GO:0005975

Fv_160_6.g3834	231	ID=Fv_160_6.g3834;Description=hypothetical protein FVEG_04568 [Fusarium verticillioides 7600];Gene=FOYG_01758;Ontology_term=large ribosomal subunit,membrane,structural constituent of ribosome,alkylbase DNA N-glycosylase activity,S-adenosylmethionine-dependent methyltransferase activity,base-excision repair, AP site formation,translation;Ontology_id=GO:0015934,GO:0016020,GO:0003735,GO:0003905,GO:0008757,GO:0006285,GO:0006412;Enz
Fv_160_6.g3835	89	ID=Fv_160_6.g3835;Description=hypothetical protein J7337_006609 [Fusarium musae];Gene=2990;Ontology_term=S-adenosylmethionine-dependent methyltransferase
Fv_160_6.g3836	316	ID=Fv_160_6.g3836;Description=hypothetical protein FVER14953_04566 [Fusarium verticillioides];Gene=FOTG_02529;Ontology_term=oxidoreductase
Fv_160_6.g3837	211	ID=Fv_160_6.g3837;Description=hypothetical protein FVER14953_04565 [Fusarium verticillioides]
Fv_160_6.g3838	701	ID=Fv_160_6.g3838;Description=hypothetical protein FVER14953_04564 [Fusarium verticillioides];Gene=FPANT_12714;Ontology_term=DNA-binding transcription factor activity, RNA polymerase II-specific,ATP binding,zinc ion binding,ATP hydrolysis activity,regulation of transcription by RNA polymerase
Fv_160_6.g3839	269	ID=Fv_160_6.g3839;Description=hypothetical protein FVEG_04563 [Fusarium verticillioides
Fv_160_6.g3840	218	ID=Fv_160_6.g3840;Description=hypothetical protein FVEG_04562 [Fusarium verticillioides 7600];Gene=FOIG_03729;Ontology_term=cytosolic large ribosomal subunit,RNA binding,acyltransferase activity, transferring groups other than amino-acyl groups,ribosome
Fv_160_6.g3841	559	ID=Fv_160_6.g3841;Description=hypothetical protein FVEG_04561 [Fusarium verticillioides 7600];Gene=FOTG_02535;Ontology_term=1-alkyl-2-acetylglycerophosphocholine esterase activity,lipid catabolic
Fv_160_6.g3842	159	ID=Fv_160_6.g3842;Description=hypothetical protein FVEG_15503 [Fusarium verticillioides 7600]
Fv_160_6.g3843	202	ID=Fv_160_6.g3843;Description=hypothetical protein FVEG_04560 [Fusarium verticillioides 7600];Gene=FTJAE_13239;Ontology_term=nucleus,DNA binding,zinc ion binding,DNA-binding transcription factor activity, RNA polymerase II-specific,regulation of transcription by RNA polymerase
Fv_160_6.g3844	538	ID=Fv_160_6.g3844;Description=hypothetical protein FVER14953_04559 [Fusarium verticillioides];Gene=FVEG_04559;Ontology_term=hydrolase
Fv_160_6.g3845	679	ID=Fv_160_6.g3845;Description=hypothetical protein FNAPI_1817 [Fusarium napiforme];Gene=152;Ontology_term=mitochondrial proton-transporting ATP synthase complex, coupling factor F(o),protein binding,lipid binding,proton transmembrane transporter activity,proton motive force-driven ATP synthesis,proton transmembrane
Fv_160_6.g3846	393	ID=Fv_160_6.g3846;Description=hypothetical protein FVER14953_04558 [Fusarium verticillioides];Gene=BRN;Ontology_term=extracellular region,nucleic acid binding,endonuclease activity,RNA nuclease activity,FMN binding,oxidoreductase activity,defense response to Gram-positive bacterium;Ontology_id=GO:0005576,GO:0003676,GO:0004519,GO:0004540,GO:0010181,GO:0016491,GO:0050830;Enzyme_code=
Fv_160_6.g3847	422	ID=Fv_160_6.g3847;Description=hypothetical protein FVEG_15501 [Fusarium verticillioides 7600];Gene=FTJAE_9819;Ontology_term=Golgi membrane,glycosyltransferase

Fv_160_6.g3848	516	ID=Fv_160_6.g3848;Description=hypothetical protein FVEG_04557 [Fusarium verticillioides 7600];Gene=FPANT_488;Ontology_term=membrane,transmembrane transporter activity,transmembrane
Fv_160_6.g3849	1268	ID=Fv_160_6.g3849;Description=hypothetical protein FVER53590_04556 [Fusarium verticillioides];Gene=KRS1;Ontology_term=cytosol,tRNA binding,nucleotide binding,lysine-tRNA ligase activity,lysyl-tRNA
Fv_160_6.g3850	575	ID=Fv_160_6.g3850;Description=hypothetical protein FVER53590_04555 [Fusarium verticillioides]
Fv_160_6.g3851	1356	ID=Fv_160_6.g3851;Description=hypothetical protein J7337_006595 [Fusarium musae];Gene=FPHYL_571;Ontology_term=SCF
Fv_160_6.g3852	600	ID=Fv_160_6.g3852;Description=hypothetical protein FVER14953_04553 [Fusarium verticillioides]
Fv_160_6.g3853	518	ID=Fv_160_6.g3853;Description=hypothetical protein FVEG_04552 [Fusarium verticillioides 7600]
Fv_160_6.g3854	103	ID=Fv_160_6.g3854;Description=hypothetical protein FOXG_07623 [Fusarium oxysporum f. sp. lycopersici 4287];Gene=apc13;Ontology_term=anaphase-promoting complex,cell cycle,cell
Fv_160_6.g3855	221	ID=Fv_160_6.g3855;Description=hypothetical protein J7337_006591 [Fusarium musae];Gene=FGADI_9863;Ontology_term=endoplasmic reticulum,Golgi apparatus,TRAPP complex,Golgi vesicle
Fv_160_6.g3856	635	ID=Fv_160_6.g3856;Description=hypothetical protein FVER14953_04549 [Fusarium verticillioides];Gene=FVEG_04549;Ontology_term=mitochondrial inner membrane,endoplasmic reticulum,Golgi apparatus,TRAPP complex,ATP binding,ATP hydrolysis activity,Golgi vesicle transport;Ontology_id=GO:0005743,GO:0005783,GO:0005794,GO:0030008,GO:0005524,GO:0016887,GO:0048193;Enzyme_code=E
Fv_160_6.g3857	349	ID=Fv_160_6.g3857;Description=hypothetical protein FVEG_04548 [Fusarium verticillioides 7600];Gene=LSDV138;Ontology_term=microtubule,microtubule motor activity,ATP binding,microtubule binding,microtubule-based
Fv_160_6.g3858	1224	ID=Fv_160_6.g3858;Description=hypothetical protein FVER14953_04547 [Fusarium verticillioides];Gene=FPANT_497;Ontology_term=phosphatase activity,phosphatidylinositol
Fv_160_6.g3859	111	ID=Fv_160_6.g3859;Description=hypothetical protein FCOIX_7994 [Fusarium
Fv_160_6.g3860	526	ID=Fv_160_6.g3860;Description=hypothetical protein FVER14953_20172 [Fusarium verticillioides];Gene=FMUND_2091;Ontology_term=intracellular calcium ion homeostasis;Ontology_id=GO:0006874
Fv_160_6.g3861	658	ID=Fv_160_6.g3861;Description=hypothetical protein FVER14953_04543 [Fusarium verticillioides];Gene=FMUND_2092;Ontology_term=protein phosphatase type 2A complex,kinetochore,nucleus,spindle pole body,cellular bud neck,DNA binding,protein phosphatase regulator activity,DNA repair,septin ring disassembly,sister chromatid biorientation,mitotic spindle orientation checkpoint signaling,cellular bud neck septin ring organization,meiotic sister chromatid cohesion, centromeric,establishment of protein localization to chromosome,positive regulation of autophagosome assembly;Ontology_id=GO:0000159,GO:0000776,GO:0005634,GO:0005816,GO:0005935,GO:0003677,GO:0019888,GO:0006281,GO
Fv_160_6.g3862	603	ID=Fv_160_6.g3862;Description=hypothetical protein J7337_006584 [Fusarium musae];Gene=FPANT_10898;Ontology_term=transcription factor TFIIIC complex,nucleus,DNA binding,transcription initiation at RNA

Fv_160_6.g3863	392	ID=Fv_160_6.g3863;Description=hypothetical protein FDENT_7322 [Fusarium denticulatum];Gene=FCIRC_1660;Ontology_term=transcription factor TFIIIC complex,nucleus,DNA binding,transferase activity,transcription initiation at RNA polymerase III
Fv_160_6.g3864	546	ID=Fv_160_6.g3864;Description=palmitoyltransferase PFA3 [Fusarium verticillioides 7600];Gene=FPANT_10896;Ontology_term=cytoplasm,membrane,RNA binding,5'-3' RNA exonuclease activity,protein-cysteine S-palmitoyltransferase activity,nuclear-transcribed mRNA catabolic process, nonsense-mediated decay,rRNA processing;Ontology_id=GO:0005737,GO:0016020,GO:0003723,GO:0004534,GO:0019706,GO:0000184,GO:0006364;Enzyme_code=
Fv_160_6.g3865	224	ID=Fv_160_6.g3865;Description=hypothetical protein FVER53590_29294 [Fusarium verticillioides];Gene=FPCIR_12391;Ontology_term=hydrolase
Fv_160_6.g3866	1093	ID=Fv_160_6.g3866;Description=hypothetical protein FVER53590_04539 [Fusarium verticillioides];Gene=FVEG_04539;Ontology_term=phosphatidylinositol phospholipase C activity,lipid catabolic process,intracellular
Fv_160_6.g3867	855	ID=Fv_160_6.g3867;Description=hypothetical protein FVER14953_04537 [Fusarium verticillioides];Gene=E9PA17;Ontology_term=kinetochore,nucleus,cytoplasm,membrane,mitotic cell cycle,cell
Fv_160_6.g3868	1050	ID=Fv_160_6.g3868;Description=hypothetical protein FVER53263_04536 [Fusarium verticillioides];Gene=FCIRC_1665;Ontology_term=metallopeptidase activity,metal ion
Fv_160_6.g3869	463	ID=Fv_160_6.g3869;Description=myosin-like coiled-coil protein-domain-containing protein [Fusarium sp. MPI-SDFR-AT-0072];Gene=FANTH_1680;Ontology_term=endoplasmic reticulum membrane,metalloendopeptidase activity,syntaxin binding,metal ion binding,CAAX-box protein
Fv_160_6.g3870	443	ID=Fv_160_6.g3870;Description=hypothetical protein FVEG_04534 [Fusarium verticillioides
Fv_160_6.g3871	1006	ID=Fv_160_6.g3871;Description=mitochondrial presequence protease [Fusarium verticillioides 7600];Gene=FMUND_972;Ontology_term=mitochondrial intermembrane space,mitochondrial matrix,metalloendopeptidase activity,metal ion binding,protein processing,proteolysis involved in protein catabolic
Fv_160_6.g3872	448	ID=Fv_160_6.g3872;Description=ornithine decarboxylase [Fusarium verticillioides 7600];Gene=FOC1_g10014604;Ontology_term=catalytic activity,polyamine biosynthetic
Fv_160_6.g3873	1074	ID=Fv_160_6.g3873;Description=hypothetical protein FVER14953_04531 [Fusarium verticillioides];Gene=FPANT_10887;Ontology_term=nucleus,mitochondrial outer membrane,pore complex,DNA binding,ATP binding,protein transmembrane transporter activity,porin activity,hydrolase activity,metal ion binding,ATP-dependent chromatin remodeler activity,chromatin remodeling,protein import into mitochondrial matrix;Ontology_id=GO:0005634,GO:0005741,GO:0046930,GO:0003677,GO:0005524,GO:0008320,GO:0015288,GO:0016787,GO:0
Fv_160_6.g3874	670	ID=Fv_160_6.g3874;Description=hypothetical protein FVEG_04530 [Fusarium verticillioides 7600]
Fv_160_6.g3875	346	ID=Fv_160_6.g3875;Description=hypothetical protein FVEG_04529 [Fusarium verticillioides
Fv_160_6.g3876	128	ID=Fv_160_6.g3876;Description=hypothetical protein J7337_006571 [Fusarium musae];Gene=BKA59DRAFT_512394;Ontology_term=ribosome,ribonucleoprotein complex,structural constituent of

Fv_160_6.g3877	793	ID=Fv_160_6.g3877;Description=hypothetical protein FVEG_04527 [Fusarium verticillioides 7600];Gene=FMAN_05526;Ontology_term=GTPase activity,GTP
Fv_160_6.g3878	478	ID=Fv_160_6.g3878;Description=acid phosphatase [Fusarium verticillioides
Fv_160_6.g3879	226	ID=Fv_160_6.g3879;Description=hypothetical protein FVEG_04525 [Fusarium verticillioides 7600];Gene=FOYG_01812;Ontology_term=aminoacyl-tRNA ligase activity,N-acetyltransferase
Fv_160_6.g3880	589	ID=Fv_160_6.g3880;Description=hypothetical protein FVER53590_04524 [Fusarium verticillioides];Gene=FFUJ_13081;Ontology_term=methionine-tRNA ligase activity,ATP binding,methionyl-tRNA
Fv_160_6.g3881	169	ID=Fv_160_6.g3881;Description=hypothetical protein FVEG_04523 [Fusarium verticillioides 7600];Gene=FMUND_982;Ontology_term=mitochondrion,membrane,cytochrome-c oxidase activity,nucleocytoplasmic transport,aerobic electron transport chain,proton transmembrane transport;Ontology_id=GO:0005739,GO:0016020,GO:0004129,GO:0006913,GO:0019646,GO:1902600;Enzyme_code=EC:1.9,EC:7.1.
Fv_160_6.g3882	510	ID=Fv_160_6.g3882;Description=hypothetical protein J7337_006565 [Fusarium musae];Gene=FocTR4_00002589;Ontology_term=protein prenyltransferase
Fv_160_6.g3883	508	ID=Fv_160_6.g3883;Description=mevalonate kinase [Fusarium verticillioides 7600];Gene=FOC1_g10014615;Ontology_term=cytoplasm,mevalonate kinase activity,ATP binding,sterol biosynthetic process,phosphorylation,isopentenyl diphosphate biosynthetic process, mevalonate
Fv_160_6.g3884	139	ID=Fv_160_6.g3884;Description=hypothetical protein FVEG_15495 [Fusarium verticillioides
Fv_160_6.g3885	209	ID=Fv_160_6.g3885;Description=hypothetical protein FVER14953_04520 [Fusarium verticillioides];Gene=109
Fv_160_6.g3886	79	ID=Fv_160_6.g3886;Description=ribosomal L38e [Fusarium tjaetaba];Gene=FOYG_01817;Ontology_term=ribosome,ribonucleoprotein complex,structural constituent of
Fv_160_6.g3887	74	ID=Fv_160_6.g3887;Description=hypothetical protein J7337_006561 [Fusarium musae];Gene=2940
Fv_160_6.g3888	424	ID=Fv_160_6.g3888;Description=hypothetical protein FVER14953_04517 [Fusarium verticillioides]
Fv_160_6.g3889	350	ID=Fv_160_6.g3889;Description=2-nitropropane dioxygenase [Fusarium tjaetaba];Gene=FVEG_04516;Ontology_term=nitronate monooxygenase activity,dioxygenase
Fv_160_6.g3890	629	ID=Fv_160_6.g3890;Description=hypothetical protein FVER53590_04515 [Fusarium verticillioides];Gene=FTJAE_13710;Ontology_term=mitochondrial proton-transporting ATP synthase complex,proton-transporting ATP synthase complex, catalytic core F(1),L-threonine ammonia-lyase activity,protein binding,ATP hydrolysis activity,nitronate monooxygenase activity,proton-transporting ATP synthase activity, rotational mechanism,dioxygenase activity,L-serine catabolic process,threonine catabolic process,isoleucine biosynthetic process,proton motive force-driven mitochondrial ATP synthesis;Ontology_id=GO:0005753,GO:0045261,GO:0004794,GO:0005515,GO:0016887,GO:0018580,GO:0046933,GO:0051213,GO:0006565,GO:0006567,GO:0009097,GO:0042776;Enzyme_code=EC:7.1.2.2,EC:1.13.12.16,EC:4.3.1.19,EC:6,EC:3.6.1.15;Enzyme_nam
Fv_160_6.g3891	502	ID=Fv_160_6.g3891;Description=hypothetical protein FVEG_04514 [Fusarium verticillioides 7600];Gene=FTJAE_13711;Ontology_term=delta24-sterol reductase activity,FAD

Fv_160_6.g3892	145	ID=Fv_160_6.g3892;Description=hypothetical protein FVEG_04513 [Fusarium verticillioides 7600];Gene=LSDV104;Ontology_term=mitochondrial proton-transporting ATP synthase, catalytic core,plasma membrane,protein binding,ATP binding,ADP binding,proton-transporting ATP synthase activity, rotational mechanism,proton motive force-driven ATP synthesis,proton transmembrane transport;Ontology_id=GO:0005754,GO:0005886,GO:0005515,GO:0005524,GO:0043531,GO:0046933,GO:0015986,GO:1902600;En
Fv_160_6.g3893	79	ID=Fv_160_6.g3893;Description=hypothetical protein FOXG_07583 [Fusarium oxysporum f. sp. lycopersici 4287];Gene=FIESC28_10055;Ontology_term=membrane;Ontology_id=GO:0016020
Fv_160_6.g3894	855	ID=Fv_160_6.g3894;Description=hypothetical protein FVEG_15492 [Fusarium verticillioides
Fv_160_6.g3895	256	ID=Fv_160_6.g3895;Description=hypothetical protein FVER14953_20129 [Fusarium verticillioides];Gene=FPANT_6252;Ontology_term=membrane,nucleic acid binding,zinc ion
Fv_160_6.g3896	203	ID=Fv_160_6.g3896;Description=hypothetical protein FVEG_04510 [Fusarium verticillioides 7600]
Fv_160_6.g3897	672	ID=Fv_160_6.g3897;Description=hypothetical protein FVEG_04509 [Fusarium verticillioides 7600];Gene=FOMG_04262;Ontology_term=nucleus,DNA binding,zinc ion binding,DNA-binding transcription factor activity, RNA polymerase II-specific,regulation of transcription by RNA polymerase
Fv_160_6.g3898	767	ID=Fv_160_6.g3898;Description=hypothetical protein FVER14953_04507 [Fusarium verticillioides];Gene=FTJAE_10797;Ontology_term=nucleus,DNA binding,zinc ion binding,DNA-binding transcription factor activity, RNA polymerase II-specific,regulation of transcription by RNA polymerase
Fv_160_6.g3899	745	ID=Fv_160_6.g3899;Description=hypothetical protein FVER14953_04506 [Fusarium verticillioides];Gene=FTJAE_10796;Ontology_term=nucleus,NAD+ ADP-ribosyltransferase activity,nucleotidyltransferase activity;Ontology_id=GO:0005634,GO:0003950,GO:0016779;Enzyme_code=EC:2.4.2.30,EC:2.7.7;Enzyme_name=NAD(+) ADP-
Fv_160_6.g3900	506	ID=Fv_160_6.g3900;Description=hypothetical protein FVER14953_04505 [Fusarium verticillioides]
Fv_160_6.g3901	1755	ID=Fv_160_6.g3901;Description=hypothetical protein FVEG_04504 [Fusarium verticillioides 7600];Gene=95;Ontology_term=cytoplasm,ribosomal small subunit biogenesis;Ontology_id=GO:0005737,GO:0042274
Fv_160_6.g3902	601	ID=Fv_160_6.g3902;Description=hypothetical protein FVEG_04503 [Fusarium verticillioides 7600];Gene=FOTG_02606;Ontology_term=nucleus,DNA-binding transcription factor activity,regulation of DNA-templated
Fv_160_6.g3903	289	ID=Fv_160_6.g3903;Description=hypothetical protein FVER53263_04502 [Fusarium verticillioides];Gene=FCIRC_4479;Ontology_term=Golgi apparatus,membrane,Golgi organization,protein
Fv_160_6.g3904	301	ID=Fv_160_6.g3904;Description=hypothetical protein FVEG_04501 [Fusarium verticillioides 7600];Gene=FNAPI_8510;Ontology_term=ribonucleoprotein complex,isocitrate lyase activity,hydrolase activity,metal ion binding,monocarboxylic acid metabolic
Fv_160_6.g3905	297	ID=Fv_160_6.g3905;Description=ribosomal L1 [Fusarium phyllophilum];Gene=FNAPI_8510;Ontology_term=ribonucleoprotein complex,isocitrate lyase activity,nucleotidase activity,metal ion binding,monocarboxylic acid metabolic process;Ontology_id=GO:1990904,GO:0004451,GO:0008252,GO:0046872,GO:0032787;Enzyme_code=EC:4.1.3.1,EC:3.1.3;Enzyme_

Fv_160_6.g3906	244	ID=Fv_160_6.g3906;Description=hypothetical protein FDENT_4310 [Fusarium denticulatum];Gene=2923
Fv_160_6.g3907	691	ID=Fv_160_6.g3907;Description=hypothetical protein FVEG_04499 [Fusarium verticillioides 7600]
Fv_160_6.g3908	104	ID=Fv_160_6.g3908;Description=hypothetical protein FVER53263_04499 [Fusarium verticillioides]
Fv_160_6.g3909	142	ID=Fv_160_6.g3909;Description=hypothetical protein FVEG_04498 [Fusarium verticillioides 7600]
Fv_160_6.g3910	425	ID=Fv_160_6.g3910;Description=hypothetical protein FVER53590_04497 [Fusarium verticillioides]
Fv_160_6.g3911	745	ID=Fv_160_6.g3911;Description=hypothetical protein FVER53263_04496 [Fusarium verticillioides]
Fv_160_6.g3912	660	ID=Fv_160_6.g3912;Description=hypothetical protein J7337_006536 [Fusarium musae]
Fv_160_6.g3913	232	ID=Fv_160_6.g3913;Description=hypothetical protein FVER53263_04494 [Fusarium verticillioides];Gene=87;Ontology_term=plasmid
Fv_160_6.g3914	282	ID=Fv_160_6.g3914;Description=hypothetical protein FVER14953_04493 [Fusarium verticillioides];Gene=efp;Ontology_term=chromosome, telomeric region,endoplasmic reticulum membrane,polysome,nuclear membrane,G-protein alpha-subunit binding,mRNA binding,pheromone-dependent signal transduction involved in conjugation with cellular fusion,silent mating-type cassette heterochromatin formation,subtelomeric heterochromatin
Fv_160_6.g3915	276	ID=Fv_160_6.g3915;Description=hypothetical protein FVER14953_04492 [Fusarium verticillioides]
Fv_160_6.g3916	191	ID=Fv_160_6.g3916;Description=hypothetical protein FVEG_15489 [Fusarium verticillioides 7600]
Fv_160_6.g3917	163	ID=Fv_160_6.g3917;Description=hypothetical protein FVER14953_04491 [Fusarium verticillioides]
Fv_160_6.g3918	177	ID=Fv_160_6.g3918;Description=hypothetical protein J7337_006530 [Fusarium
Fv_160_6.g3919	362	ID=Fv_160_6.g3919;Description=hypothetical protein FVER14953_04490 [Fusarium verticillioides];Gene=80
Fv_160_6.g3920	225	ID=Fv_160_6.g3920;Description=hypothetical protein FVEG_04489 [Fusarium verticillioides 7600]
Fv_160_6.g3921	741	ID=Fv_160_6.g3921;Description=hypothetical protein FVER14953_04488 [Fusarium verticillioides];Gene=HOM2;Ontology_term=aspartate-semialdehyde dehydrogenase activity,protein dimerization activity,NADP binding,NAD binding,methionine biosynthetic process,threonine biosynthetic process,lysine biosynthetic process via diaminopimelate,isoleucine biosynthetic process;Ontology_id=GO:0004073,GO:0046983,GO:0050661,GO:0051287,GO:0009086,GO:0009088,GO:0009089,GO:0009097;Enzy
Fv_160_6.g3922	256	ID=Fv_160_6.g3922;Description=hypothetical protein FVER14953_04487 [Fusarium verticillioides];Gene=FPRO05_07181;Ontology_term=ribosome,ribonucleoprotein complex,structural constituent of
Fv_160_6.g3923	748	ID=Fv_160_6.g3923;Description=hypothetical protein FVER14953_04486 [Fusarium verticillioides];Gene=FACUT_2629;Ontology_term=nucleus,mRNA processing;Ontology_id=GO:0005634,GO:0006397
Fv_160_6.g3924	274	ID=Fv_160_6.g3924;Description=enoyl-CoA hydratase [Fusarium verticillioides 7600];Gene=CEK26_006862;Ontology_term=mitochondrion,ATP binding,glucose binding,fructose metabolic process,glucose metabolic process,phosphorylation,regulation of transcription by glucose,glucose import,fructose import across plasma
Fv_160_6.g3925	626	ID=Fv_160_6.g3925;Description=hypothetical protein FVEG_04484 [Fusarium verticillioides 7600];Gene=76;Ontology_term=orotidine-5'-phosphate decarboxylase activity,'de novo' pyrimidine nucleobase biosynthetic process,'de novo' UMP biosynthetic

Fv_160_6.g3926	414	ID=Fv_160_6.g3926;Description=hypothetical protein J7337_006522 [Fusarium musae]
Fv_160_6.g3927	603	ID=Fv_160_6.g3927;Description=hypothetical protein FVEG_04482 [Fusarium verticillioides 7600];Gene=75;Ontology_term=nucleus,DNA-binding transcription factor activity, RNA polymerase II-specific,zinc ion
Fv_160_6.g3928	581	ID=Fv_160_6.g3928;Description=hypothetical protein FVER53263_20350 [Fusarium verticillioides];Gene=BFJ63_vAg11462;Ontology_term=cytoplasm,membrane,magnesium ion binding,inorganic diphosphate phosphatase activity,phosphate-containing compound metabolic
Fv_160_6.g3929	923	ID=Fv_160_6.g3929;Description=serine/threonine protein kinase [Fusarium verticillioides 7600];Gene=FNAPI_8402;Ontology_term=protein serine/threonine kinase activity,ATP
Fv_160_6.g3930	356	ID=Fv_160_6.g3930;Description=hypothetical protein FVEG_04480 [Fusarium verticillioides 7600];Gene=FOC1_g10014665;Ontology_term=fungal-type vacuole membrane,nucleosome,nucleus,Gtr1-Gtr2 GTPase complex,DNA binding,GTPase activity,GTP binding,structural constituent of chromatin,protein heterodimerization activity,negative regulation of TORC1 signaling,positive regulation of TORC1 signaling;Ontology_id=GO:0000329,GO:0000786,GO:0005634,GO:1990131,GO:0003677,GO:0003924,GO:0005525,GO:0030527,GO:
Fv_160_6.g3931	409	ID=Fv_160_6.g3931;Description=hypothetical protein FVER53590_04479 [Fusarium verticillioides]
Fv_160_6.g3932	123	ID=Fv_160_6.g3932;Description=hypothetical protein FNYG_08708 [Fusarium nygamai];Gene=FNAPI_8399;Ontology_term=mitochondrial outer membrane,membrane insertase activity,establishment of protein
Fv_160_6.g3933	484	ID=Fv_160_6.g3933;Description=hypothetical protein J7337_006515 [Fusarium musae];Gene=FPRO05_07192;Ontology_term=mitochondrial outer membrane,membrane insertase activity,establishment of
Fv_160_6.g3934	529	ID=Fv_160_6.g3934;Description=hypothetical protein FVEG_04476 [Fusarium verticillioides 7600];Gene=gp64;Ontology_term=cytoplasm,phosphoribosyl-AMP cyclohydrolase activity,ATP binding,zinc ion binding,histidine biosynthetic
Fv_160_6.g3935	371	ID=Fv_160_6.g3935;Description=hypothetical protein FVEG_04475 [Fusarium verticillioides 7600];Gene=65
Fv_160_6.g3936	300	ID=Fv_160_6.g3936;Description=hypothetical protein FVEG_04475 [Fusarium verticillioides 7600]
Fv_160_6.g3937	529	ID=Fv_160_6.g3937;Description=hypothetical protein FVEG_04474 [Fusarium verticillioides 7600];Gene=FOPG_04508;Ontology_term=membrane,transmembrane transporter activity,amino acid transport,transmembrane
Fv_160_6.g3938	137	ID=Fv_160_6.g3938;Description=hypothetical protein FVEG_15485 [Fusarium verticillioides 7600]
Fv_160_6.g3939	402	ID=Fv_160_6.g3939;Description=ATP-dependent RNA helicase FAL1 [Fusarium oxysporum f. sp. lycopersici 4287];Gene=HZS61_011891;Ontology_term=nucleolus,RNA binding,RNA helicase activity,translation initiation factor activity,ATP binding,ATP hydrolysis activity,rRNA processing,translational initiation;Ontology_id=GO:0005730,GO:0003723,GO:0003724,GO:0003743,GO:0005524,GO:0016887,GO:0006364,GO:0006413;Enz
Fv_160_6.g3940	501	ID=Fv_160_6.g3940;Description=hypothetical protein J7337_006508 [Fusarium musae];Gene=sap61;Ontology_term=spliceosomal complex,U2 snRNP,RNA binding,zinc ion binding,mRNA splicing, via

Fv_160_6.g3941	319	ID=Fv_160_6.g3941;Description=hypothetical protein FVEG_04471 [Fusarium verticillioides 7600];Gene=RAG1;Ontology_term=nucleosome,membrane,Slx1-Slx4 complex,DNA binding,5'-flap endonuclease activity,transmembrane transporter activity,structural constituent of chromatin,protein heterodimerization activity,DNA repair,DNA recombination,carbohydrate transport,transmembrane transport;Ontology_id=GO:0000786,GO:0016020,GO:0033557,GO:0003677,GO:0017108,GO:0022857,GO:0030527,GO:0046982,GO
Fv_160_6.g3942	1724	ID=Fv_160_6.g3942;Description=hypothetical protein FVER53590_04468 [Fusarium verticillioides];Gene=FPANT_4318;Ontology_term=nucleus,DNA binding,methyltransferase activity,metal ion binding,methylation;Ontology_id=GO:0005634,GO:0003677,GO:0008168,GO:0046872,GO:0032259;Enzyme_code=EC:2.1.1;Enzyme
Fv_160_6.g3943	315	ID=Fv_160_6.g3943;Description=hypothetical protein FVER14953_04467 [Fusarium verticillioides];Gene=FPRO_05718;Ontology_term=DNA binding,GTP cyclohydrolase I activity,helicase activity,ATP binding,7,8-dihydroneopterin 3'-triphosphate biosynthetic process,tetrahydrofolate biosynthetic process,folic acid biosynthetic
Fv_160_6.g3944	320	ID=Fv_160_6.g3944;Description=hypothetical protein FVEG_04466 [Fusarium verticillioides 7600];Gene=BFJ63_vAg11446;Ontology_term=transcription factor TFIID complex,translation initiation factor activity,protein heterodimerization activity,translational initiation,RNA polymerase II preinitiation complex
Fv_160_6.g3945	268	ID=Fv_160_6.g3945;Description=hypothetical protein FVEG_04465 [Fusarium verticillioides 7600];Gene=BALF4 (provisional)
Fv_160_6.g3946	619	ID=Fv_160_6.g3946;Description=hypothetical protein FVER53590_04464 [Fusarium verticillioides];Gene=HEM3;Ontology_term=hydroxymethylbilane synthase activity,protoporphyrinogen IX biosynthetic
Fv_160_6.g3947	206	ID=Fv_160_6.g3947;Description=hypothetical protein FVER53590_04463 [Fusarium verticillioides]
Fv_160_6.g3948	348	ID=Fv_160_6.g3948;Description=hypothetical protein FVER53590_04462 [Fusarium verticillioides]
Fv_160_6.g3949	127	ID=Fv_160_6.g3949;Description=hypothetical protein FVER53263_20352 [Fusarium verticillioides];Gene=54;Ontology_term=membrane,DNA binding;Ontology_id=GO:0016020,GO:0003677
Fv_160_6.g3950	341	ID=Fv_160_6.g3950;Description=hypothetical protein FVER14953_04461 [Fusarium verticillioides];Gene=FCIRC_4700;Ontology_term=nucleotide binding,oxidoreductase activity, acting on
Fv_160_6.g3951	250	ID=Fv_160_6.g3951;Description=murein transglycosylase [Fusarium verticillioides 7600];Gene=FPANT_4308;Ontology_term=cellulase activity,polysaccharide catabolic
Fv_160_6.g3952	422	ID=Fv_160_6.g3952;Description=heterokaryon incompatibility het-6 [Fusarium mundagurra]
Fv_160_6.g3953	586	ID=Fv_160_6.g3953;Description=MFS transporter, SIT family, siderophore-iron:H+ symporter [Fusarium verticillioides 7600];Gene=1999;Ontology_term=membrane,transmembrane transporter activity,transmembrane
Fv_160_6.g3954	444	ID=Fv_160_6.g3954;Description=hypothetical protein FVER14953_04457 [Fusarium verticillioides];Gene=FOC4_g10012404;Ontology_term=chitin binding,hydrolase activity, acting on glycosyl bonds,hydrolase activity, acting on carbon-nitrogen (but not peptide) bonds,xylan catabolic process;Ontology_id=GO:0008061,GO:0016798,GO:0016810,GO:0045493;Enzyme_code=EC:3.2,EC:3.5;Enzyme_name=Glycosylases,

Fv_160_6.g3955	210	ID=Fv_160_6.g3955;Description=hypothetical protein FVEG_04456 [Fusarium verticillioides 7600];Gene=FVER53590_04457;Ontology_term=chitin binding,hydrolase activity, acting on carbon-nitrogen (but not peptide) bonds,carbohydrate metabolic
Fv_160_6.g3956	143	ID=Fv_160_6.g3956;Description=hypothetical protein FVER53263_04455 [Fusarium
Fv_160_6.g3957	841	ID=Fv_160_6.g3957;Description=hypothetical protein FVER14953_04454 [Fusarium verticillioides];Gene=ORF43;Ontology_term=Golgi membrane,DNA-directed RNA polymerase complex,COPI vesicle coat,DNA binding,DNA-directed 5'-3' RNA polymerase activity,structural molecule activity,ribonucleoside binding,ubiquitin binding,DNA-templated transcription,intracellular protein transport,endoplasmic reticulum to Golgi vesicle-mediated transport,retrograde vesicle-mediated transport, Golgi to endoplasmic reticulum,intracellular mRNA localization,late endosome to vacuole transport via multivesicular body sorting
Fv_160_6.g3958	635	ID=Fv_160_6.g3958;Description=AGC/AKT protein kinase [Fusarium verticillioides 7600];Gene=FPHYL_8040;Ontology_term=chromatin,cytoplasm,protein serine/threonine kinase activity,ATP binding,phosphorylation,positive regulation of transcription by RNA polymerase II,positive regulation of induction of conjugation with cellular fusion,negative regulation of TORC2
Fv_160_6.g3959	552	ID=Fv_160_6.g3959;Description=glucose-6-phosphate isomerase [Fusarium verticillioides 7600];Gene=FMAN_05438;Ontology_term=mitochondrion,glucose-6-phosphate isomerase activity,carbohydrate derivative binding,gluconeogenesis,glycolytic process,carbohydrate derivative metabolic
Fv_160_6.g3960	391	ID=Fv_160_6.g3960;Description=mannitol-1-phosphate dehydrogenase [Fusarium oxysporum f. sp. lycopersici 4287];Gene=FMAN_05437;Ontology_term=mannitol-1-phosphate 5-dehydrogenase activity,mannitol metabolic
Fv_160_6.g3961	465	ID=Fv_160_6.g3961;Description=hypothetical protein FVER14953_04450 [Fusarium verticillioides]
Fv_160_6.g3962	106	ID=Fv_160_6.g3962;Description=hypothetical protein FVER14953_20996 [Fusarium verticillioides];Gene=HHT2;Ontology_term=nucleosome,nucleus,DNA binding,isomerase activity,structural constituent of chromatin,protein heterodimerization
Fv_160_6.g3963	148	ID=Fv_160_6.g3963;Description=hypothetical protein FVEG_04449 [Fusarium verticillioides 7600]
Fv_160_6.g3964	109	ID=Fv_160_6.g3964;Description=hypothetical protein FVER14953_04448 [Fusarium verticillioides]
Fv_160_6.g3965	304	ID=Fv_160_6.g3965;Description=uncharacterized protein FTJAE_13389 [Fusarium tjaetaba]
Fv_160_6.g3966	540	ID=Fv_160_6.g3966;Description=sugar transporter stl1 [Fusarium pseudocircinatum];Gene=FOYG_01912;Ontology_term=membrane,transmembrane transporter activity,carbohydrate
Fv_160_6.g3967	369	ID=Fv_160_6.g3967;Description=hypothetical protein FVER53590_04446 [Fusarium verticillioides]
Fv_160_6.g3968	1003	ID=Fv_160_6.g3968;Description=hypothetical protein FVER53590_04444 [Fusarium verticillioides];Gene=FVEG_15480;Ontology_term=serine-type endopeptidase
Fv_160_6.g3969	2059	ID=Fv_160_6.g3969;Description=hypothetical protein FVER53263_04442 [Fusarium verticillioides];Gene=FVER53590_04442;Ontology_term=cytoplasm,zinc ion binding;Ontology_id=GO:0005737,GO:0008270

Fv_160_6.g3970	415	ID=Fv_160_6.g3970;Description=hypothetical protein FVER14953_04440 [Fusarium verticillioides];Gene=ORF31
Fv_160_6.g3971	256	ID=Fv_160_6.g3971;Description=hypothetical protein FVER53590_04439 [Fusarium verticillioides];Gene=ORF30
Fv_160_6.g3972	368	ID=Fv_160_6.g3972;Description=hypothetical protein FPANT_6849 [Fusarium pseudoanthophilum];Gene=FVEG_04438;Ontology_term=S-adenosylmethionine-dependent methyltransferase
Fv_160_6.g3973	200	ID=Fv_160_6.g3973;Description=hypothetical protein FVEG_04437 [Fusarium verticillioides 7600];Gene=3642
Fv_160_6.g3974	119	ID=Fv_160_6.g3974;Description=hypothetical protein FVEG_04436 [Fusarium verticillioides 7600]
Fv_160_6.g3975	384	ID=Fv_160_6.g3975;Description=hypothetical protein FVEG_04435 [Fusarium verticillioides 7600];Gene=FTJAE_13375;Ontology_term=S-adenosylmethionine-dependent methyltransferase
Fv_160_6.g3976	425	ID=Fv_160_6.g3976;Description=hypothetical protein FVER14953_21773 [Fusarium
Fv_160_6.g3977	496	ID=Fv_160_6.g3977;Description=hypothetical protein NM208_g6748 [Fusarium
Fv_160_6.g3978	303	ID=Fv_160_6.g3978;Description=hypothetical protein F52700_6272 [Fusarium sp. NRRL 52700]
Fv_160_6.g3979	682	ID=Fv_160_6.g3979;Description=hypothetical protein FVER14953_04432 [Fusarium verticillioides];Gene=FOYG_01923;Ontology_term=serine-type peptidase
Fv_160_6.g3980	685	ID=Fv_160_6.g3980;Description=hypothetical protein FVER53263_04428 [Fusarium verticillioides]
Fv_160_6.g3981	481	ID=Fv_160_6.g3981;Description=hypothetical protein J7337_006466 [Fusarium musae];Gene=FACUT_7409;Ontology_term=membrane,transmembrane transporter activity,transmembrane
Fv_160_6.g3982	595	ID=Fv_160_6.g3982;Description=hypothetical protein FVEG_04426 [Fusarium verticillioides 7600];Gene=FocTR4_00002481
Fv_160_6.g3983	338	ID=Fv_160_6.g3983;Description=hypothetical protein FVER14953_04425 [Fusarium verticillioides];Gene=ORF20
Fv_160_6.g3984	615	ID=Fv_160_6.g3984;Description=hypothetical protein FVEG_04424 [Fusarium verticillioides 7600];Gene=LW93_8191;Ontology_term=nucleus,DNA binding,zinc ion binding,membrane,DNA-binding transcription factor activity, RNA polymerase II-specific,regulation of transcription by RNA polymerase
Fv_160_6.g3985	300	ID=Fv_160_6.g3985;Description=L-fuculose-phosphate aldolase [Fusarium verticillioides 7600];Gene=FNAPI_9825;Ontology_term=nucleus,DNA binding,zinc ion binding,DNA-binding transcription factor activity, RNA
Fv_160_6.g3986	465	ID=Fv_160_6.g3986;Description=hypothetical protein FVEG_04422 [Fusarium verticillioides 7600];Gene=FTJAE_10697;Ontology_term=membrane,hydrolase activity, hydrolyzing O-glycosyl compounds,carbohydrate metabolic
Fv_160_6.g3987	491	ID=Fv_160_6.g3987;Description=hypothetical protein FVEG_04421 [Fusarium verticillioides 7600];Gene=pckA;Ontology_term=hydrolase activity, hydrolyzing O-glycosyl compounds,toxin activity,carbohydrate metabolic process,modulation of process of another
Fv_160_6.g3988	633	ID=Fv_160_6.g3988;Description=hypothetical protein FVER53590_29044 [Fusarium verticillioides];Gene=FOXG_19495;Ontology_term=nucleus,DNA binding,zinc ion binding,DNA-binding transcription factor activity, RNA polymerase II-specific,regulation of transcription by RNA polymerase
Fv_160_6.g3989	222	ID=Fv_160_6.g3989;Description=hypothetical protein J7337_006458 [Fusarium musae];Gene=FNAPI_13678;Ontology_term=transferase

Fv_160_6.g3990	253	ID=Fv_160_6.g3990;Description=hypothetical protein FVER53590_04418 [Fusarium verticillioides]
Fv_160_6.g3991	519	ID=Fv_160_6.g3991;Description=hypothetical protein FNYG_08769 [Fusarium nygamai];Gene=FMAN_05405;Ontology_term=primary cell septum,extracellular region,plasma membrane,fungal-type cell wall,nuclear periphery,membrane raft,nucleotide binding,DNA binding,DNA-directed DNA polymerase activity,monooxygenase activity,iron ion binding,protein binding,oxidoreductase activity, acting on paired donors, with incorporation or reduction of molecular oxygen,heme binding,glucanosyltransferase activity,DNA replication,fungal-type cell wall organization,heterochromatin formation,DNA biosynthetic process,fungal-type cell wall (1->3)-beta-D-glucan biosynthetic process,regulation of response to endoplasmic reticulum stress;Ontology_id=GO:0000936,GO:0005576,GO:0005886,GO:0009277,GO:0034399,GO:0045121,GO:0000166,GO:0003677,GO:00
Fv_160_6.g3992	720	ID=Fv_160_6.g3992;Description=hypothetical protein FVEG_04416 [Fusarium verticillioides 7600];Gene=FMAN_05404;Ontology_term=nucleus,DNA binding,zinc ion binding,DNA-binding transcription factor activity, RNA polymerase II-specific,regulation of transcription by RNA polymerase
Fv_160_6.g3993	106	ID=Fv_160_6.g3993;Description=hypothetical protein FVER53263_04415 [Fusarium verticillioides];Gene=dlg1;Ontology_term=nucleolus,ribonucleoprotein complex,RNA binding,pseudouridine synthesis,rRNA
Fv_160_6.g3994	429	ID=Fv_160_6.g3994;Description=fumarylacetoacetase [Fusarium verticillioides 7600];Gene=BFJ72_g6709;Ontology_term=fumarylacetoacetase activity,metal ion binding,L-phenylalanine catabolic process,tyrosine catabolic
Fv_160_6.g3995	875	ID=Fv_160_6.g3995;Description=hypothetical protein FVER53263_04412 [Fusarium verticillioides];Gene=hmgA-6;Ontology_term=nucleus,membrane,DNA binding,homogentisate 1,2-dioxygenase activity,zinc ion binding,oxidoreductase activity, acting on the CH-OH group of donors, NAD or NADP as acceptor,NAD binding,DNA-templated transcription,L-phenylalanine catabolic process,tyrosine metabolic process;Ontology_id=GO:0005634,GO:0016020,GO:0003677,GO:0004411,GO:0008270,GO:0016616,GO:0051287,GO:0006351,GO:
Fv_160_6.g3996	339	ID=Fv_160_6.g3996;Description=hypothetical protein LB503_004962, partial [Fusarium chuoi];Gene=FMUND_14050;Ontology_term=protein kinase activity,ATP
Fv_160_6.g3997	532	ID=Fv_160_6.g3997;Description=hypothetical protein FVEG_04410 [Fusarium verticillioides 7600];Gene=FSUBG_11379;Ontology_term=hydrolase activity, acting on carbon-nitrogen (but not peptide)
Fv_160_6.g3998	238	ID=Fv_160_6.g3998;Description=transcriptional regulatory moc3 [Fusarium tjaetaba];Gene=FOC1_g10003998;Ontology_term=nucleus,membrane,DNA-binding transcription factor activity, RNA polymerase II-specific,zinc ion binding,hydrolase activity, acting on carbon-nitrogen (but not peptide) bonds,transmembrane transporter activity,regulation of transcription by RNA polymerase II,transmembrane transport;Ontology_id=GO:0005634,GO:0016020,GO:0000981,GO:0008270,GO:0016810,GO:0022857,GO:0006357,GO:0055085;En

Fv_160_6.g3999	519	ID=Fv_160_6.g3999;Description=hypothetical protein FVER53590_29042 [Fusarium verticillioides];Gene=FOIG_16089;Ontology_term=nucleus,membrane,DNA-binding transcription factor activity, RNA polymerase II-specific,zinc ion binding,hydrolase activity, acting on carbon-nitrogen (but not peptide) bonds,transmembrane transporter activity,regulation of transcription by RNA polymerase II,transmembrane transport;Ontology_id=GO:0005634,GO:0016020,GO:0000981,GO:0008270,GO:0016810,GO:0022857,GO:0006357,GO:0055085;En
Fv_160_6.g4000	509	ID=Fv_160_6.g4000;Description=hypothetical protein J7337_006448 [Fusarium musae];Gene=FPCIR_6050;Ontology_term=membrane,transmembrane transporter activity,transmembrane
Fv_160_6.g4001	419	ID=Fv_160_6.g4001;Description=hypothetical protein FVEG_04406 [Fusarium verticillioides 7600];Gene=FVEG_04406;Ontology_term=transaminase activity,pyridoxal phosphate binding,biosynthetic
Fv_160_6.g4002	454	ID=Fv_160_6.g4002;Description=hypothetical protein FVEG_04405 [Fusarium verticillioides 7600];Gene=FVEG_04405;Ontology_term=membrane,transmembrane transporter activity,transmembrane
Fv_160_6.g4003	632	ID=Fv_160_6.g4003;Description=hypothetical protein FVEG_04404 [Fusarium verticillioides 7600];Gene=BFJ63_vAg10131;Ontology_term=nucleus,DNA binding,zinc ion binding,DNA-binding transcription factor activity, RNA polymerase II-specific,regulation of transcription by RNA polymerase
Fv_160_6.g4004	794	ID=Fv_160_6.g4004;Description=hypothetical protein FVER53590_04403 [Fusarium verticillioides];Gene=FPCIR_6044;Ontology_term=GTPase activity,GTP binding,organelle
Fv_160_6.g4005	469	ID=Fv_160_6.g4005;Description=hypothetical protein FVEG_15473 [Fusarium verticillioides 7600];Gene=gB
Fv_160_6.g4006	574	ID=Fv_160_6.g4006;Description=hypothetical protein FVEG_04402 [Fusarium verticillioides 7600];Gene=FFB14_15512;Ontology_term=serine-type carboxypeptidase
Fv_160_6.g4007	260	ID=Fv_160_6.g4007;Description=hypothetical protein FVER14953_20948 [Fusarium verticillioides];Gene=FPANT_13229;Ontology_term=cysteine-type deubiquitinase activity,ubiquitin-dependent protein catabolic
Fv_160_6.g4008	398	ID=Fv_160_6.g4008;Description=hypothetical protein FVER53590_04400 [Fusarium verticillioides];Gene=FGLOB1_13728;Ontology_term=O-methyltransferase activity,S-adenosylmethionine-dependent methyltransferase activity,methylation,secondary metabolite biosynthetic
Fv_160_6.g4009	365	ID=Fv_160_6.g4009;Description=hypothetical protein FGADI_698 [Fusarium
Fv_160_6.g4010	706	ID=Fv_160_6.g4010;Description=hypothetical protein FVER14953_04398 [Fusarium verticillioides];Gene=cprA-2;Ontology_term=mitochondrial outer membrane,endoplasmic reticulum membrane,plasma membrane,NADPH-hemoprotein reductase activity,FMN binding,flavin adenine dinucleotide binding,NADP binding,ergosterol biosynthetic process;Ontology_id=GO:0005741,GO:0005789,GO:0005886,GO:0003958,GO:0010181,GO:0050660,GO:0050661,GO:0006696;Enzy
Fv_160_6.g4011	273	ID=Fv_160_6.g4011;Description=hypothetical protein FVER14953_04397 [Fusarium verticillioides]
Fv_160_6.g4012	493	ID=Fv_160_6.g4012;Description=hypothetical protein FVER14953_04396 [Fusarium verticillioides];Gene=FNAPI_49;Ontology_term=oxidoreductase activity, acting on the aldehyde or oxo group of donors, NAD or

Fv_160_6.g4013	672	ID=Fv_160_6.g4013;Description=hypothetical protein FVER14953_04394 [Fusarium verticillioides];Gene=FMUND_10534;Ontology_term=copper ion binding,primary amine oxidase activity,quinone binding,amine
Fv_160_6.g4014	529	ID=Fv_160_6.g4014;Description=hypothetical protein FVEG_04393 [Fusarium verticillioides 7600];Gene=FVER53590_04393;Ontology_term=membrane,transmembrane transporter activity,amino acid
Fv_160_6.g4015	709	ID=Fv_160_6.g4015;Description=hypothetical protein FVEG_04392 [Fusarium verticillioides 7600];Gene=FVER53590_04392;Ontology_term=nucleus,DNA binding,zinc ion binding,DNA-binding transcription factor activity, RNA polymerase II-specific,regulation of transcription by RNA polymerase
Fv_160_6.g4016	485	ID=Fv_160_6.g4016;Description=seryl-tRNA synthetase [Fusarium verticillioides 7600];Gene=FVER53590_04391;Ontology_term=serine-tRNA ligase activity,ATP binding,seryl-tRNA
Fv_160_6.g4017	665	ID=Fv_160_6.g4017;Description=hypothetical protein FCOIX_7704 [Fusarium coicis]
Fv_160_6.g4018	421	ID=Fv_160_6.g4018;Description=hypothetical protein FVEG_04390 [Fusarium verticillioides 7600];Gene=F25303_2443;Ontology_term=monooxygenase activity,FAD binding,biosynthetic
Fv_160_6.g4019	465	ID=Fv_160_6.g4019;Description=hypothetical protein FVER14953_04389 [Fusarium verticillioides];Gene=FGLOB1_13738;Ontology_term=oxidoreductase activity,FAD
Fv_160_6.g4020	673	ID=Fv_160_6.g4020;Description=hypothetical protein J7337_006430 [Fusarium musae];Gene=BHE90_009800;Ontology_term=membrane,serine-type peptidase
Fv_160_6.g4021	261	ID=Fv_160_6.g4021;Description=hypothetical protein FVEG_04387 [Fusarium verticillioides
Fv_160_6.g4022	373	ID=Fv_160_6.g4022;Description=hypothetical protein FVEG_15470 [Fusarium verticillioides 7600];Gene=FMUND_10548;Ontology_term=oxidoreductase activity, acting on
Fv_160_6.g4023	782	ID=Fv_160_6.g4023;Description=hypothetical protein FVEG_15469 [Fusarium verticillioides 7600];Gene=FMUND_10547;Ontology_term=membrane,oxidoreductase activity,FAD binding,biosynthetic
Fv_160_6.g4024	500	ID=Fv_160_6.g4024;Description=hypothetical protein FVEG_04385 [Fusarium verticillioides 7600];Gene=FMUND_10546;Ontology_term=membrane,transmembrane transporter activity,transmembrane
Fv_160_6.g4025	486	ID=Fv_160_6.g4025;Description=hypothetical protein FVEG_04384 [Fusarium verticillioides 7600];Gene=FNAPI_39;Ontology_term=membrane,transmembrane transporter activity,transmembrane
Fv_160_6.g4026	747	ID=Fv_160_6.g4026;Description=hypothetical protein FVEG_04383 [Fusarium verticillioides 7600]
Fv_160_6.g4027	827	ID=Fv_160_6.g4027;Description=hypothetical protein FVER53263_04382 [Fusarium verticillioides];Gene=FVER53590_04382;Ontology_term=membrane,serine-type peptidase
Fv_160_6.g4028	710	ID=Fv_160_6.g4028;Description=hypothetical protein FVER53263_04381 [Fusarium
Fv_160_6.g4029	239	ID=Fv_160_6.g4029;Description=hypothetical protein FVEG_15466 [Fusarium verticillioides
Fv_160_6.g4030	572	ID=Fv_160_6.g4030;Description=hypothetical protein FVEG_15465 [Fusarium verticillioides 7600];Gene=FFC1_06733;Ontology_term=nucleus,DNA-binding transcription factor activity, RNA polymerase II-specific,zinc ion
Fv_160_6.g4031	358	ID=Fv_160_6.g4031;Description=hypothetical protein FVER14953_04380 [Fusarium verticillioides]

Fv_160_6.g4032	595	ID=Fv_160_6.g4032;Description=hypothetical protein FVEG_04379 [Fusarium verticillioides 7600];Gene=F25303_14069
Fv_160_6.g4033	353	ID=Fv_160_6.g4033;Description=hypothetical protein FVER53590_29036 [Fusarium verticillioides]
Fv_160_6.g4034	510	ID=Fv_160_6.g4034;Description=allantoate transporter [Fusarium coicis];Gene=FNAPI_12435;Ontology_term=membrane,transmembrane transporter activity,transmembrane
Fv_160_6.g4035	547	ID=Fv_160_6.g4035;Description=hypothetical protein FNYG_13800 [Fusarium nygamai];Gene=FPANT_12965;Ontology_term=membrane,transmembrane transporter activity,transmembrane
Fv_160_6.g4036	369	ID=Fv_160_6.g4036;Description=hypothetical protein FVEG_04374 [Fusarium verticillioides
Fv_160_6.g4037	224	ID=Fv_160_6.g4037;Description=hypothetical protein FVER14953_04373 [Fusarium verticillioides]
Fv_160_6.g4038	457	ID=Fv_160_6.g4038;Description=hypothetical protein FVER53590_04372 [Fusarium verticillioides]
Fv_160_6.g4039	615	ID=Fv_160_6.g4039;Description=hypothetical protein FVER14953_04371 [Fusarium verticillioides]
Fv_160_6.g4040	362	ID=Fv_160_6.g4040;Description=hypothetical protein FVEG_15463 [Fusarium verticillioides 7600]
Fv_160_6.g4041	356	ID=Fv_160_6.g4041;Description=Uncharacterized protein Y057_2620 [Fusarium fujikuroi];Gene=FGLOB1_12337;Ontology_term=nitrilase activity,cyanide hydratase activity,cyanide catabolic
Fv_160_6.g4042	629	ID=Fv_160_6.g4042;Description=hypothetical protein FVER14953_04369 [Fusarium verticillioides];Gene=FACUT_10910;Ontology_term=copper ion binding,primary amine oxidase activity,transferase activity,quinone binding,amine metabolic
Fv_160_6.g4043	772	ID=Fv_160_6.g4043;Description=hypothetical protein FVEG_04368 [Fusarium verticillioides 7600];Gene=FMAN_05327;Ontology_term=copper ion binding,primary amine oxidase activity,quinone binding,amine metabolic
Fv_160_6.g4044	984	ID=Fv_160_6.g4044;Description=hypothetical protein FVER14953_04367 [Fusarium verticillioides]
Fv_160_6.g4045	205	ID=Fv_160_6.g4045;Description=hypothetical protein FVER53590_04366 [Fusarium verticillioides]
Fv_160_6.g4046	82	ID=Fv_160_6.g4046;Description=hypothetical protein FVEG_15461 [Fusarium verticillioides 7600]
Fv_160_6.g4047	133	ID=Fv_160_6.g4047;Description=hypothetical protein FVEG_04365 [Fusarium verticillioides 7600]
Fv_160_6.g4048	439	ID=Fv_160_6.g4048;Description=hypothetical protein FVEG_04364 [Fusarium verticillioides 7600];Gene=FVER53590_04364;Ontology_term=nucleus,DNA-binding transcription factor activity, RNA polymerase II-specific,zinc
Fv_160_6.g4049	303	ID=Fv_160_6.g4049;Description=hypothetical protein FVEG_15459 [Fusarium verticillioides 7600];Gene=FMAN_05319;Ontology_term=nucleus,membrane,DNA-binding transcription factor activity, RNA polymerase II-
Fv_160_6.g4050	188	ID=Fv_160_6.g4050;Description=hypothetical protein FVEG_04362 [Fusarium verticillioides 7600]
Fv_160_6.g4051	298	ID=Fv_160_6.g4051;Description=hypothetical protein FVER53263_04361 [Fusarium verticillioides];Gene=FOQG_16285;Ontology_term=zinc ion binding,oxidoreductase
Fv_160_6.g4052	287	ID=Fv_160_6.g4052;Description=hypothetical protein FVEG_04360 [Fusarium verticillioides 7600];Gene=FRV6_11753;Ontology_term=DNA binding,DNA-binding transcription factor activity,regulation of transcription by RNA
Fv_160_6.g4053	445	ID=Fv_160_6.g4053;Description=hypothetical protein FVER14953_04359 [Fusarium verticillioides];Gene=FSUBG_11909;Ontology_term=glycosyltransferase

Fv_160_6.g4054	593	ID=Fv_160_6.g4054;Description=hypothetical protein FVEG_04358 [Fusarium verticillioides 7600];Gene=FPHYL_7191;Ontology_term=alpha-glucosidase activity,maltose metabolic process,carbohydrate catabolic
Fv_160_6.g4055	190	ID=Fv_160_6.g4055;Description=hypothetical protein FVER14953_04357 [Fusarium verticillioides]
Fv_160_6.g4056	375	ID=Fv_160_6.g4056;Description=hypothetical protein FVER53263_04356 [Fusarium verticillioides];Gene=FDENT_11897;Ontology_term=cytoplasm,tyrosine-tRNA ligase activity,ATP binding,tyrosyl-tRNA
Fv_160_6.g4057	791	ID=Fv_160_6.g4057;Description=hypothetical protein FVER53590_04355 [Fusarium verticillioides];Gene=FMUND_10578;Ontology_term=nucleus,DNA binding,tyrosine-tRNA ligase activity,ATP binding,regulation of DNA-templated transcription,tyrosyl-tRNA
Fv_160_6.g4058	254	ID=Fv_160_6.g4058;Description=hypothetical protein FVER14953_05879 [Fusarium verticillioides];Gene=FVER53590_26451;Ontology_term=nucleus,DNA-binding transcription factor activity, RNA polymerase II-specific,zinc ion binding,hydrolase activity,regulation of transcription by RNA polymerase
Fv_160_6.g4059	667	ID=Fv_160_6.g4059;Description=P-loop containing protein [Fusarium coicis];Gene=FMAN_05305;Ontology_term=proteasome complex,nucleus,DNA-binding transcription factor activity, RNA polymerase II-specific,ATP binding,zinc ion binding,ATP hydrolysis activity,regulation of transcription by RNA polymerase
Fv_160_6.g4060	581	ID=Fv_160_6.g4060;Description=heterokaryon incompatibility protein het-E-1 [Fusarium coicis];Gene=FGLOB1_10929;Ontology_term=nucleus,DNA-binding transcription factor activity, RNA polymerase II-specific,zinc ion
Fv_160_6.g4061	155	ID=Fv_160_6.g4061;Description=hypothetical protein FVER14953_04351 [Fusarium
Fv_160_6.g4062	371	ID=Fv_160_6.g4062;Description=hypothetical protein FVER53263_04351 [Fusarium verticillioides]
Fv_160_6.g4063	555	ID=Fv_160_6.g4063;Description=hypothetical protein FVER53263_04350 [Fusarium verticillioides]
Fv_160_6.g4064	545	ID=Fv_160_6.g4064;Description=hypothetical protein FVER53263_04349 [Fusarium verticillioides];Gene=AU210_003712;Ontology_term=nucleus,DNA binding,zinc ion binding,DNA-binding transcription factor activity, RNA polymerase II-specific,regulation of transcription by RNA polymerase
Fv_160_6.g4065	447	ID=Fv_160_6.g4065;Description=hypothetical protein FVER53263_04348 [Fusarium verticillioides];Gene=FOQG_14382;Ontology_term=membrane,oxidoreductase activity,FAD binding,biosynthetic
Fv_160_6.g4066	536	ID=Fv_160_6.g4066;Description=hypothetical protein FOTG_11780 [Fusarium oxysporum f. sp. vasinfectum 25433];Gene=FOQG_14383;Ontology_term=membrane,monooxygenase activity,iron ion binding,oxidoreductase activity, acting on paired donors, with incorporation or reduction of molecular oxygen,heme binding;Ontology_id=GO:0016020,GO:0004497,GO:0005506,GO:0016705,GO:0020037;Enzyme_code=EC:1.14;Enzyme_name=Actin
Fv_160_6.g4067	486	ID=Fv_160_6.g4067;Description=hypothetical protein FVEG_04346 [Fusarium verticillioides 7600];Gene=FPHYL_7174;Ontology_term=oxidoreductase activity, acting on the aldehyde or oxo group of donors, NAD or NADP as

Fv_160_6.g4068	546	ID=Fv_160_6.g4068;Description=hypothetical protein FVEG_04345 [Fusarium verticillioides 7600];Gene=LSDX1-1;Ontology_term=monooxygenase activity,oxidoreductase activity, acting on single donors with incorporation of molecular oxygen, incorporation of two atoms of oxygen,metal ion binding;Ontology_id=GO:0004497,GO:0016702,GO:0046872;Enzyme_code=EC:1.13.11;Enzyme_name=Acting on single donors with
Fv_160_6.g4069	341	ID=Fv_160_6.g4069;Description=hypothetical protein FVEG_04344 [Fusarium verticillioides 7600];Gene=FPRO05_07341;Ontology_term=hydrolase activity,carboxy-lyase
Fv_160_6.g4070	362	ID=Fv_160_6.g4070;Description=hypothetical protein FVER14953_04343 [Fusarium verticillioides];Gene=FMEXI_89;Ontology_term=zinc ion binding,oxidoreductase activity,hydrolase
Fv_160_6.g4071	273	ID=Fv_160_6.g4071;Description=hypothetical protein FVEG_04342 [Fusarium verticillioides
Fv_160_6.g4072	168	ID=Fv_160_6.g4072;Description=hypothetical protein FVEG_15453 [Fusarium verticillioides 7600];Gene=FPHYL_7180;Ontology_term=nucleus,membrane,DNA binding,zinc ion binding,DNA-templated
Fv_160_6.g4073	712	ID=Fv_160_6.g4073;Description=hypothetical protein FVER53590_04341 [Fusarium verticillioides];Gene=FFC1_06780;Ontology_term=nucleus,DNA binding,zinc ion binding,membrane,DNA-binding transcription factor activity, RNA polymerase II-specific,regulation of transcription by RNA polymerase
Fv_160_6.g4074	503	ID=Fv_160_6.g4074;Description=hypothetical protein FVEG_04340 [Fusarium verticillioides 7600];Gene=FNAPI_6954;Ontology_term=membrane,monooxygenase activity,iron ion binding,methyltransferase activity,oxidoreductase activity, acting on paired donors, with incorporation or reduction of molecular oxygen,heme binding,methylation;Ontology_id=GO:0016020,GO:0004497,GO:0005506,GO:0008168,GO:0016705,GO:0020037,GO:0032259;Enzy
Fv_160_6.g4075	254	ID=Fv_160_6.g4075;Description=cytochrome P450 monooxygenase [Fusarium denticulatum];Gene=FDENT_11912;Ontology_term=monooxygenase activity,iron ion binding,oxidoreductase activity, acting on paired donors, with incorporation or reduction of molecular oxygen,heme binding;Ontology_id=GO:0004497,GO:0005506,GO:0016705,GO:0020037;Enzyme_code=EC:1.14;Enzyme_name=Acting on paired
Fv_160_6.g4076	524	ID=Fv_160_6.g4076;Description=hypothetical protein FVEG_04338 [Fusarium verticillioides 7600];Gene=LW93_8090;Ontology_term=membrane,transmembrane transporter activity,transmembrane
Fv_160_6.g4077	321	ID=Fv_160_6.g4077;Description=hypothetical protein FVEG_04337 [Fusarium verticillioides 7600];Gene=FRV6_06783;Ontology_term=isomerase
Fv_160_6.g4078	663	ID=Fv_160_6.g4078;Description=hypothetical protein FVEG_04336 [Fusarium verticillioides 7600];Gene=FOZG_04606;Ontology_term=nucleus,DNA binding,zinc ion binding,membrane,DNA-binding transcription factor activity, RNA polymerase II-specific,regulation of transcription by RNA polymerase
Fv_160_6.g4079	297	ID=Fv_160_6.g4079;Description=hypothetical protein FVER53590_30448 [Fusarium verticillioides];Gene=A0A2L2TEB4;Ontology_term=oxidoreductase activity,cellular biosynthetic
Fv_160_6.g4080	493	ID=Fv_160_6.g4080;Description=hypothetical protein FVEG_04334 [Fusarium verticillioides 7600];Gene=FOZG_17877;Ontology_term=oxidoreductase activity, acting on the aldehyde or oxo group of donors, NAD or NADP as

Fv_160_6.g4081	586	ID=Fv_160_6.g4081;Description=hypothetical protein FVEG_04333 [Fusarium verticillioides 7600];Gene=F25303_2002;Ontology_term=N,N-dimethylaniline monooxygenase activity,nucleotidyltransferase activity,flavin adenine dinucleotide binding,NADP binding;Ontology_id=GO:0004499,GO:0016779,GO:0050660,GO:0050661;Enzyme_code=EC:1.14.13.8,EC:2.7.7;Enzyme_name=flavi
Fv_160_6.g4082	260	ID=Fv_160_6.g4082;Description=hypothetical protein FVEG_04332 [Fusarium verticillioides 7600];Gene=FOBC_16496;Ontology_term=feruloyl esterase activity,metal ion
Fv_160_6.g4083	245	ID=Fv_160_6.g4083;Description=hypothetical protein FVER14953_04331 [Fusarium verticillioides]
Fv_160_6.g4084	381	ID=Fv_160_6.g4084;Description=hypothetical protein FVER53590_04330 [Fusarium verticillioides];Gene=FGLOB1_14528;Ontology_term=1-alkyl-2-acetylglycerophosphocholine esterase
Fv_160_6.g4085	135	ID=Fv_160_6.g4085;Description=hypothetical protein FNAPI_6949 [Fusarium napiforme]
Fv_160_6.g4086	137	ID=Fv_160_6.g4086;Description=hypothetical protein FNAPI_6948 [Fusarium napiforme]
Fv_160_6.g4087	489	ID=Fv_160_6.g4087;Description=hypothetical protein FVER14953_04329 [Fusarium verticillioides];Gene=FVEG_04329;Ontology_term=membrane,monooxygenase activity,iron ion binding,oxidoreductase activity, acting on paired donors, with incorporation or reduction of molecular oxygen,heme binding;Ontology_id=GO:0016020,GO:0004497,GO:0005506,GO:0016705,GO:0020037;Enzyme_code=EC:1.14;Enzyme_name=Actin
Fv_160_6.g4088	413	ID=Fv_160_6.g4088;Description=hypothetical protein FVEG_04328 [Fusarium verticillioides 7600];Gene=FPHYL_7162;Ontology_term=membrane,oxidoreductase activity,transmembrane transporter activity,transmembrane transport;Ontology_id=GO:0016020,GO:0016491,GO:0022857,GO:0055085;Enzyme_code=EC:1;Enzyme_name=Oxidoreductases
Fv_160_6.g4089	662	ID=Fv_160_6.g4089;Description=hypothetical protein FVER53590_04327 [Fusarium verticillioides]
Fv_160_6.g4090	164	ID=Fv_160_6.g4090;Description=hypothetical protein FVER53590_04326 [Fusarium verticillioides]
Fv_160_6.g4091	539	ID=Fv_160_6.g4091;Description=hypothetical protein FVEG_04325 [Fusarium verticillioides 7600]
Fv_160_6.g4092	497	ID=Fv_160_6.g4092;Description=hypothetical protein FVER14953_04324 [Fusarium verticillioides]
Fv_160_6.g4093	520	ID=Fv_160_6.g4093;Description=hypothetical protein FVER14953_04323 [Fusarium verticillioides];Gene=FGADI_9564;Ontology_term=membrane,transmembrane transporter activity,transmembrane
Fv_160_6.g4094	518	ID=Fv_160_6.g4094;Description=uncharacterized protein FPRN_05378 [Fusarium proliferatum];Gene=FOBC_11183;Ontology_term=nucleus,membrane,DNA binding,zinc ion binding,DNA-templated
Fv_160_6.g4095	678	ID=Fv_160_6.g4095;Description=hypothetical protein FVER53590_04320 [Fusarium verticillioides];Gene=FOQG_13681;Ontology_term=hydrolase
Fv_160_6.g4096	277	ID=Fv_160_6.g4096;Description=hypothetical protein FVER14953_04319 [Fusarium verticillioides];Gene=FDENT_11936;Ontology_term=dioxygenase
Fv_160_6.g4097	307	ID=Fv_160_6.g4097;Description=hypothetical protein FVER14953_04318 [Fusarium verticillioides];Gene=FPCIR_5955;Ontology_term=dioxygenase

Fv_160_6.g4098	495	ID=Fv_160_6.g4098;Description=hypothetical protein FVER53263_04316 [Fusarium verticillioides];Gene=FOQG_16212;Ontology_term=membrane,transmembrane transporter activity,transmembrane
Fv_160_6.g4099	455	ID=Fv_160_6.g4099;Description=hypothetical protein FVER14953_04315 [Fusarium verticillioides];Gene=FRV6_15304;Ontology_term=membrane,transmembrane transporter activity,transmembrane
Fv_160_6.g4100	1417	ID=Fv_160_6.g4100;Description=hypothetical protein FPCIR_5952 [Fusarium pseudocircinatum];Gene=FNAPI_1351;Ontology_term=nucleus,DNA binding,zinc ion binding,iron ion binding,oxidoreductase activity, acting on paired donors, with incorporation or reduction of molecular oxygen,membrane,monooxygenase activity,DNA-binding transcription factor activity, RNA polymerase II-specific,heme binding,regulation of transcription by RNA polymerase II;Ontology_id=GO:0005634,GO:0003677,GO:0008270,GO:0005506,GO:0016705,GO:0016020,GO:0004497,GO:0000981,GO:002003
Fv_160_6.g4101	350	ID=Fv_160_6.g4101;Description=hypothetical protein FVER53263_04313 [Fusarium verticillioides];Gene=FNAPI_1352;Ontology_term=membrane,transmembrane transporter activity,transmembrane
Fv_160_6.g4102	733	ID=Fv_160_6.g4102;Description=hypothetical protein FVER14953_04312 [Fusarium verticillioides]
Fv_160_6.g4103	264	ID=Fv_160_6.g4103;Description=heterokaryon incompatibility 6 OR allele [Fusarium pseudocircinatum]
Fv_160_6.g4104	493	ID=Fv_160_6.g4104;Description=hypothetical protein FVER53590_04310 [Fusarium verticillioides];Gene=FPANT_12203;Ontology_term=acid phosphatase activity,metal ion
Fv_160_6.g4105	710	ID=Fv_160_6.g4105;Description=hypothetical protein FVER53590_04309 [Fusarium verticillioides];Gene=FMAN_16061;Ontology_term=nucleus,DNA binding,zinc ion binding,iron ion binding,oxidoreductase activity, acting on paired donors, with incorporation or reduction of molecular oxygen,membrane,monooxygenase activity,DNA-binding transcription factor activity, RNA polymerase II-specific,heme binding,regulation of transcription by RNA polymerase II;Ontology_id=GO:0005634,GO:0003677,GO:0008270,GO:0005506,GO:0016705,GO:0016020,GO:0004497,GO:0000981,GO:002003
Fv_160_6.g4106	554	ID=Fv_160_6.g4106;Description=hypothetical protein FVEG_04308 [Fusarium verticillioides 7600];Gene=FMUND_1556;Ontology_term=nucleus,DNA binding,zinc ion binding,iron ion binding,oxidoreductase activity, acting on paired donors, with incorporation or reduction of molecular oxygen,membrane,methyltransferase activity,monooxygenase activity,DNA-binding transcription factor activity, RNA polymerase II-specific,heme binding,regulation of transcription by RNA polymerase II,methylation;Ontology_id=GO:0005634,GO:0003677,GO:0008270,GO:0005506,GO:0016705,GO:0016020,GO:0008168,GO:000449
Fv_160_6.g4107	368	ID=Fv_160_6.g4107;Description=hypothetical protein FPANT_12200 [Fusarium pseudoanthophilum]
Fv_160_6.g4108	451	ID=Fv_160_6.g4108;Description=hypothetical protein FVEG_04306 [Fusarium verticillioides 7600];Gene=FPCIR_5945;Ontology_term=acyltransferase
Fv_160_6.g4109	510	ID=Fv_160_6.g4109;Description=hypothetical protein FVER53590_04305 [Fusarium verticillioides];Gene=FOVG_16086;Ontology_term=nucleus,membrane,DNA-binding transcription factor activity, RNA polymerase II-specific,zinc ion binding,transmembrane transporter activity,regulation of transcription by RNA polymerase II,carbohydrate

Fv_160_6.g4110	599	ID=Fv_160_6.g4110;Description=hypothetical protein FVER53590_04304 [Fusarium verticillioides];Gene=FOXG_17553;Ontology_term=hydrolase activity, hydrolyzing O-glycosyl compounds,carbohydrate metabolic
Fv_160_6.g4111	764	ID=Fv_160_6.g4111;Description=hypothetical protein FVEG_04303 [Fusarium verticillioides 7600];Gene=FPRO05_07370;Ontology_term=nucleus,DNA binding,zinc ion binding,DNA-binding transcription factor activity, RNA polymerase II-specific,regulation of transcription by RNA polymerase
Fv_160_6.g4112	451	ID=Fv_160_6.g4112;Description=hypothetical protein FVER53590_04302 [Fusarium verticillioides];Gene=BFJ71_g17088;Ontology_term=nucleus,zinc ion binding,acyltransferase activity,oxidoreductase activity,secondary metabolite biosynthetic process,phosphopantetheine binding,S-adenosylmethionine-dependent methyltransferase activity,DNA-binding transcription factor activity, RNA polymerase II-specific,regulation of transcription by RNA polymerase
Fv_160_6.g4113	599	ID=Fv_160_6.g4113;Description=hypothetical protein FGLOB1_13237 [Fusarium globosum];Gene=AU210_016206
Fv_160_6.g4114	1118	ID=Fv_160_6.g4114;Description=hypothetical protein IL306_010380 [Fusarium sp. DS 682];Gene=FGADI_1054;Ontology_term=vacuolar membrane,endosome membrane,catalytic activity,nucleoside metabolic
Fv_160_6.g4115	371	ID=Fv_160_6.g4115;Description=uncharacterized protein FSUBG_13864 [Fusarium subglutinans];Gene=FRV6_08856;Ontology_term=kinesin complex,microtubule,ADP
Fv_160_6.g4116	561	ID=Fv_160_6.g4116;Description=hypothetical protein AU210_014678 [Fusarium oxysporum f. sp. radicis-cucumerinum]
Fv_160_6.g4117	590	ID=Fv_160_6.g4117;Description=hypothetical protein FOCG_17561 [Fusarium oxysporum f. sp. radicis-lycopersici 26381]
Fv_160_6.g4118	1497	ID=Fv_160_6.g4118;Description=hypothetical protein AU210_016206 [Fusarium oxysporum f. sp. radicis-cucumerinum];Gene=BFJ69_g11651;Ontology_term=kinesin complex,membrane;Ontology_id=GO:0005871,GO:0016020
Fv_160_6.g4119	1310	ID=Fv_160_6.g4119;Description=hypothetical protein AU210_016210 [Fusarium oxysporum f. sp. radicis-cucumerinum];Gene=BKA59DRAFT_536342;Ontology_term=kinesin complex,membrane;Ontology_id=GO:0005871,GO:0016020
Fv_160_6.g4120	394	ID=Fv_160_6.g4120;Description=hypothetical protein FGLOB1_13238 [Fusarium globosum]
Fv_160_6.g4121	323	ID=Fv_160_6.g4121;Description=hypothetical protein FMUND_608 [Fusarium mundagurra]
Fv_160_6.g4122	398	ID=Fv_160_6.g4122;Description=hypothetical protein KAF25_000231 [Fusarium avenaceum]
Fv_160_6.g4123	919	ID=Fv_160_6.g4123;Description=kinesin light chain [Fusarium austroafricanum];Gene=BFJ68_g3248;Ontology_term=catalytic activity,ADP binding,nucleoside metabolic process;Ontology_id=GO:0003824,GO:0043531,GO:0009116
Fv_160_6.g4124	224	ID=Fv_160_6.g4124;Description=hypothetical protein FVER53263_20791 [Fusarium verticillioides]
Fv_160_6.g4125	139	ID=Fv_160_6.g4125;Description=hypothetical protein FGLOB1_5740 [Fusarium globosum]
Fv_160_6.g4126	77	ID=Fv_160_6.g4126;Description=hypothetical protein FOWG_17306 [Fusarium oxysporum f. sp. lycopersici MN25]
Fv_160_6.g4127	771	ID=Fv_160_6.g4127;Description=patatin phospholipase [Fusarium mundagurra];Gene=FOQG_16671;Ontology_term=hydrolase activity,metal ion binding,lipid catabolic process,glycerolipid metabolic
Fv_160_6.g4128	326	ID=Fv_160_6.g4128;Description=FAD-binding domain-containing protein [Fusarium globosum];Gene=FMEXI_10966;Ontology_term=membrane,ferric-chelate reductase activity,iron ion

Fv_160_6.g4129	107	ID=Fv_160_6.g4129;Description=hypothetical protein FGLOB1_5737 [Fusarium globosum]
Fv_160_6.g4130	722	ID=Fv_160_6.g4130;Description=dnaj domain-containing protein [Fusarium globosum]
Fv_160_6.g4131	654	ID=Fv_160_6.g4131;Description=hypothetical protein FGLOB1_5733, partial [Fusarium globosum];Gene=AU210_016658;Ontology_term=DNA binding,calcium-dependent cysteine-type endopeptidase
Fv_160_6.g4132	386	ID=Fv_160_6.g4132;Description=hypothetical protein NW763_014652 [Fusarium
Fv_160_6.g4133	690	ID=Fv_160_6.g4133;Description=hypothetical protein NW753_010615 [Fusarium oxysporum];Gene=FALBO_5853;Ontology_term=mitochondrial inner membrane,asparaginase activity,ATP binding,ATP hydrolysis activity,asparagine metabolic process;Ontology_id=GO:0005743,GO:0004067,GO:0005524,GO:0016887,GO:0006528;Enzyme_code=EC:3.5.1.1,EC:3.6.1.15;Enzym
Fv_160_6.g4134	375	ID=Fv_160_6.g4134;Description=hypothetical protein FOXG_15587 [Fusarium oxysporum f. sp. lycopersici
Fv_160_6.g4135	575	ID=Fv_160_6.g4135;Description=hypothetical protein F66182_10805 [Fusarium sp. NRRL 66182]
Fv_160_6.g4136	167	ID=Fv_160_6.g4136;Description=hypothetical protein FOXG_22075 [Fusarium oxysporum f. sp. lycopersici 4287]
Fv_160_6.g4137	406	ID=Fv_160_6.g4137;Description=hypothetical protein FNYG_02361 [Fusarium nygamai]
Fv_160_6.g4138	314	ID=Fv_160_6.g4138;Description=serine threonine kinase [Fusarium mundagurra];Gene=FPSE_07327;Ontology_term=protein serine/threonine kinase activity,ATP
Fv_160_6.g4139	316	ID=Fv_160_6.g4139;Description=hypothetical protein FGLOB1_4125 [Fusarium globosum]
Fv_160_6.g4140	467	ID=Fv_160_6.g4140;Description=hypothetical protein FMUND_624 [Fusarium mundagurra]
Fv_160_6.g4141	788	ID=Fv_160_6.g4141;Description=hypothetical protein FGLOB1_14806 [Fusarium globosum]
Fv_160_7.g4142	324	ID=Fv_160_7.g4142;Description=hypothetical protein FVER14953_12406 [Fusarium verticillioides]
Fv_160_7.g4143	499	ID=Fv_160_7.g4143;Description=hypothetical protein FVEG_12405 [Fusarium verticillioides 7600];Gene=AU210_013768;Ontology_term=membrane,monooxygenase activity,iron ion binding,oxidoreductase activity, acting on paired donors, with incorporation or reduction of molecular oxygen,heme binding;Ontology_id=GO:0016020,GO:0004497,GO:0005506,GO:0016705,GO:0020037;Enzyme_code=EC:1.14;Enzyme_name=Actin
Fv_160_7.g4144	294	ID=Fv_160_7.g4144;Description=hypothetical protein FVER53263_12404 [Fusarium verticillioides];Gene=FSUBG_10189;Ontology_term=monooxygenase
Fv_160_7.g4145	487	ID=Fv_160_7.g4145;Description=uncharacterized protein FTJAE_4631 [Fusarium tjaetaba]
Fv_160_7.g4146	549	ID=Fv_160_7.g4146;Description=hypothetical protein FVEG_12401 [Fusarium verticillioides 7600]
Fv_160_7.g4147	681	ID=Fv_160_7.g4147;Description=hypothetical protein FVER53590_12400 [Fusarium verticillioides]
Fv_160_7.g4148	380	ID=Fv_160_7.g4148;Description=hypothetical protein FVER53590_12399 [Fusarium verticillioides];Gene=FMAN_06725;Ontology_term=metallopeptidase
Fv_160_7.g4149	118	ID=Fv_160_7.g4149;Description=hypothetical protein FVEG_12398 [Fusarium verticillioides
Fv_160_7.g4150	129	ID=Fv_160_7.g4150;Description=hypothetical protein FVER53263_12397 [Fusarium verticillioides]
Fv_160_7.g4151	182	ID=Fv_160_7.g4151;Description=hypothetical protein FVER53590_12396 [Fusarium verticillioides]

Fv_160_7.g4152	761	ID=Fv_160_7.g4152;Description=DNA excision repair protein ERCC-3 [Fusarium verticillioides 7600];Gene=BFJ72_g713;Ontology_term=DNA binding,DNA helicase activity,ATP binding,hydrolase activity,nucleotide-excision repair,transcription initiation at RNA polymerase II promoter,DNA duplex
Fv_160_7.g4153	1762	ID=Fv_160_7.g4153;Description=hypothetical protein J7337_007739 [Fusarium musae];Gene=FACUT_807;Ontology_term=oxidoreductase activity,hydrolase activity,metal ion binding,lipid catabolic process,cellular biosynthetic process,glycerolipid metabolic
Fv_160_7.g4154	291	ID=Fv_160_7.g4154;Description=hypothetical protein FVEG_17244 [Fusarium verticillioides 7600]
Fv_160_7.g4155	517	ID=Fv_160_7.g4155;Description=hypothetical protein FVER53590_12391 [Fusarium verticillioides];Gene=FNYG_10044;Ontology_term=membrane,monooxygenase activity,iron ion binding,methyltransferase activity,oxidoreductase activity, acting on paired donors, with incorporation or reduction of molecular oxygen,heme binding,methylation;Ontology_id=GO:0016020,GO:0004497,GO:0005506,GO:0008168,GO:0016705,GO:0020037,GO:0032259;Enzy
Fv_160_7.g4156	243	ID=Fv_160_7.g4156;Description=hypothetical protein FVER53590_12390 [Fusarium verticillioides];Gene=F25303_4588;Ontology_term=FMN binding,oxidoreductase
Fv_160_7.g4157	394	ID=Fv_160_7.g4157;Description=hypothetical protein FVER53590_29068 [Fusarium verticillioides];Gene=FVER53590_29068;Ontology_term=membrane,FMN binding,oxidoreductase
Fv_160_7.g4158	321	ID=Fv_160_7.g4158;Description=hypothetical protein FVER14953_12389 [Fusarium verticillioides]
Fv_160_7.g4159	335	ID=Fv_160_7.g4159;Description=hypothetical protein FVEG_12388 [Fusarium verticillioides 7600]
Fv_160_7.g4160	441	ID=Fv_160_7.g4160;Description=hypothetical protein FVER53590_12387 [Fusarium verticillioides]
Fv_160_7.g4161	1168	ID=Fv_160_7.g4161;Description=hypothetical protein FVEG_12386 [Fusarium verticillioides
Fv_160_7.g4162	323	ID=Fv_160_7.g4162;Description=hypothetical protein FVER14953_12385 [Fusarium verticillioides];Gene=HZS61_010686;Ontology_term=catalytic activity,biosynthetic process;Ontology_id=GO:0003824,GO:0009058
Fv_160_7.g4163	271	ID=Fv_160_7.g4163;Description=hypothetical protein FVER53263_12384 [Fusarium verticillioides];Gene=FNAPI_12481;Ontology_term=transferase
Fv_160_7.g4164	546	ID=Fv_160_7.g4164;Description=hypothetical protein FVER53590_12383 [Fusarium
Fv_160_7.g4165	422	ID=Fv_160_7.g4165;Description=hypothetical protein FVER53263_12382 [Fusarium verticillioides];Gene=AU210_013811;Ontology_term=monooxygenase activity,FAD binding,biosynthetic
Fv_160_7.g4166	545	ID=Fv_160_7.g4166;Description=hypothetical protein FVER53590_12381 [Fusarium verticillioides];Gene=FSUBG_11453;Ontology_term=aspartic-type endopeptidase
Fv_160_7.g4167	522	ID=Fv_160_7.g4167;Description=hypothetical protein FVEG_12380 [Fusarium verticillioides 7600];Gene=FOYG_14294;Ontology_term=membrane,monooxygenase activity,iron ion binding,oxidoreductase activity, acting on paired donors, with incorporation or reduction of molecular oxygen,heme binding;Ontology_id=GO:0016020,GO:0004497,GO:0005506,GO:0016705,GO:0020037;Enzyme_code=EC:1.14;Enzyme_name=Actin

Fv_160_7.g4168	875	ID=Fv_160_7.g4168;Description=hypothetical protein FVER53590_12379 [Fusarium verticillioides];Gene=F25303_4577;Ontology_term=membrane,sulfuric ester hydrolase
Fv_160_7.g4169	363	ID=Fv_160_7.g4169;Description=hypothetical protein FVER53590_12378 [Fusarium
Fv_160_7.g4170	394	ID=Fv_160_7.g4170;Description=hypothetical protein FVER53263_12377 [Fusarium verticillioides];Gene=FMAN_06703;Ontology_term=membrane,transmembrane signaling receptor activity,cell surface receptor
Fv_160_7.g4171	605	ID=Fv_160_7.g4171;Description=hypothetical protein FVER53590_29067 [Fusarium verticillioides];Gene=FMUND_15380;Ontology_term=membrane,catalytic activity,nucleoside metabolic
Fv_160_7.g4172	512	ID=Fv_160_7.g4172;Description=hypothetical protein FVER14953_12375 [Fusarium verticillioides];Gene=FPRO_07010;Ontology_term=membrane,hydrolase activity,transmembrane transporter
Fv_160_7.g4173	294	ID=Fv_160_7.g4173;Description=hypothetical protein FVEG_17238 [Fusarium verticillioides 7600];Gene=prlL-3;Ontology_term=membrane,oxidoreductase activity, acting on the aldehyde or oxo group of donors, NAD or NADP as acceptor,hydrolase activity,transmembrane transporter activity,transmembrane transport;Ontology_id=GO:0016020,GO:0016620,GO:0016787,GO:0022857,GO:0055085;Enzyme_code=EC:3,EC:1.2.1;Enzyme_nam
Fv_160_7.g4174	339	ID=Fv_160_7.g4174;Description=hypothetical protein FVEG_12373 [Fusarium verticillioides
Fv_160_7.g4175	598	ID=Fv_160_7.g4175;Description=hypothetical protein FVER14953_12372 [Fusarium verticillioides];Gene=FACUT_828;Ontology_term=oxidoreductase activity, acting on CH-OH group of donors,flavin adenine
Fv_160_7.g4176	104	ID=Fv_160_7.g4176;Description=hypothetical protein J7337_007719 [Fusarium musae]
Fv_160_7.g4177	111	ID=Fv_160_7.g4177;Description=hypothetical protein FVER53263_21097 [Fusarium verticillioides]
Fv_160_7.g4178	125	ID=Fv_160_7.g4178;Description=hypothetical protein FVER14953_12370 [Fusarium verticillioides]
Fv_160_7.g4179	128	ID=Fv_160_7.g4179;Description=hypothetical protein FPCIR_1469 [Fusarium pseudocircinatum]
Fv_160_7.g4180	226	ID=Fv_160_7.g4180;Description=hypothetical protein FVER14953_20054 [Fusarium verticillioides];Gene=FNAPI_12316;Ontology_term=S-adenosylmethionine-dependent methyltransferase
Fv_160_7.g4181	662	ID=Fv_160_7.g4181;Description=hypothetical protein FVER53590_12368 [Fusarium verticillioides];Gene=FVER53263_12368;Ontology_term=extracellular region,carbohydrate binding,rhamnogalacturonan endolyase activity,polysaccharide catabolic process,cell wall
Fv_160_7.g4182	77	ID=Fv_160_7.g4182;Description=hypothetical protein FVEG_12367 [Fusarium verticillioides 7600]
Fv_160_7.g4183	82	ID=Fv_160_7.g4183;Description=uncharacterized protein FTJAE_14213 [Fusarium tjaetaba];Gene=FNAPI_12318
Fv_160_7.g4184	88	ID=Fv_160_7.g4184;Description=hypothetical protein FVEG_17235 [Fusarium verticillioides 7600];Gene=FNAPI_12318
Fv_160_7.g4185	73	ID=Fv_160_7.g4185;Description=integral membrane protein [Fusarium
Fv_160_7.g4186	678	ID=Fv_160_7.g4186;Description=hypothetical protein FDENT_14079 [Fusarium
Fv_160_7.g4187	425	ID=Fv_160_7.g4187;Description=hypothetical protein FVER14953_12365 [Fusarium verticillioides];Gene=FGADI_7541;Ontology_term=L-aspartate:2-oxoglutarate aminotransferase activity,pyridoxal phosphate binding,amino acid metabolic process,biosynthetic

Fv_160_7.g4188	259	ID=Fv_160_7.g4188;Description=hypothetical protein FVER53590_12364 [Fusarium verticillioides];Gene=FMUND_14528;Ontology_term=oxidoreductase activity,cellular biosynthetic
Fv_160_7.g4189	444	ID=Fv_160_7.g4189;Description=hypothetical protein FVER53590_12363 [Fusarium verticillioides]
Fv_160_7.g4190	502	ID=Fv_160_7.g4190;Description=hypothetical protein FVEG_12362 [Fusarium verticillioides 7600];Gene=FOPG_10161;Ontology_term=membrane,transmembrane transporter activity,transmembrane
Fv_160_7.g4191	179	ID=Fv_160_7.g4191;Description=hypothetical protein FVER53263_12361 [Fusarium verticillioides]
Fv_160_7.g4192	560	ID=Fv_160_7.g4192;Description=hypothetical protein FVEG_12360 [Fusarium verticillioides 7600];Gene=FVEG_12360;Ontology_term=plasma membrane,sodium:proton antiporter activity,sodium ion export across plasma membrane,regulation of membrane potential,proton export across plasma
Fv_160_7.g4193	502	ID=Fv_160_7.g4193;Description=hypothetical protein FVER14953_20056 [Fusarium verticillioides];Gene=FFB14_12248;Ontology_term=membrane,transmembrane transporter activity,transmembrane
Fv_160_7.g4194	660	ID=Fv_160_7.g4194;Description=hypothetical protein FVER14953_12358 [Fusarium verticillioides];Gene=FRV6_15636;Ontology_term=copper ion binding,oxidoreductase
Fv_160_7.g4195	429	ID=Fv_160_7.g4195;Description=hypothetical protein FVER53263_12357 [Fusarium verticillioides]
Fv_160_7.g4196	376	ID=Fv_160_7.g4196;Description=hypothetical protein FVER53263_12356 [Fusarium
Fv_160_7.g4197	378	ID=Fv_160_7.g4197;Description=hypothetical protein FVER14953_12355 [Fusarium
Fv_160_7.g4198	134	ID=Fv_160_7.g4198;Description=hypothetical protein FVEG_12354 [Fusarium verticillioides 7600];Gene=FDENT_13360;Ontology_term=extracellular region,metallocarboxypeptidase activity,zinc ion
Fv_160_7.g4199	538	ID=Fv_160_7.g4199;Description=hypothetical protein FVEG_12353 [Fusarium verticillioides 7600];Gene=FMUND_14517;Ontology_term=extracellular region,metallocarboxypeptidase activity,zinc ion
Fv_160_7.g4200	145	ID=Fv_160_7.g4200;Description=hypothetical protein FVEG_12352 [Fusarium verticillioides 7600]
Fv_160_7.g4201	429	ID=Fv_160_7.g4201;Description=hypothetical protein FVER53263_12351 [Fusarium
Fv_160_7.g4202	538	ID=Fv_160_7.g4202;Description=hypothetical protein FVER53263_12350 [Fusarium verticillioides];Gene=FGLOB1_14616;Ontology_term=oxidoreductase activity,FAD
Fv_160_7.g4203	723	ID=Fv_160_7.g4203;Description=hypothetical protein FVER14953_12349 [Fusarium verticillioides];Gene=FVEG_12349;Ontology_term=membrane,monooxygenase activity,iron ion binding,dipeptidyl-peptidase activity,oxidoreductase activity, acting on paired donors, with incorporation or reduction of molecular oxygen,heme binding;Ontology_id=GO:0016020,GO:0004497,GO:0005506,GO:0008239,GO:0016705,GO:0020037;Enzyme_code=EC:3.4.14.1,EC:1.14,EC:3.4.14.5,EC:3.4.14.4,EC:3.4.14.2;Enzyme_name=dipeptidyl-peptidase I,Acting on paired donors, with incorporation or
Fv_160_7.g4204	688	ID=Fv_160_7.g4204;Description=hypothetical protein FVER14953_20058 [Fusarium verticillioides];Gene=FTJAE_6893;Ontology_term=nucleus,DNA binding,zinc ion binding,membrane,DNA-binding transcription factor activity, RNA polymerase II-specific,regulation of transcription by RNA polymerase

Fv_160_7.g4205	456	ID=Fv_160_7.g4205;Description=hypothetical protein FVER14953_12348 [Fusarium verticillioides];Gene=FPANT_9472;Ontology_term=membrane,monooxygenase activity,FAD binding,biosynthetic
Fv_160_7.g4206	365	ID=Fv_160_7.g4206;Description=hypothetical protein FVER14953_12347 [Fusarium verticillioides];Gene=Focb16_v015399;Ontology_term=hydrolase
Fv_160_7.g4207	220	ID=Fv_160_7.g4207;Description=hypothetical protein FVER53263_12346 [Fusarium verticillioides];Gene=FTJAE_6890;Ontology_term=extracellular region,cutinase
Fv_160_7.g4208	733	ID=Fv_160_7.g4208;Description=hypothetical protein FVER14953_12345 [Fusarium verticillioides];Gene=FNAPI_3475;Ontology_term=mitochondrion,arylformamidase activity,tryptophan catabolic process to
Fv_160_7.g4209	534	ID=Fv_160_7.g4209;Description=hypothetical protein FVER14953_12344 [Fusarium verticillioides];Gene=FOXG_13187;Ontology_term=membrane,transmembrane transporter activity,transmembrane
Fv_160_7.g4210	1129	ID=Fv_160_7.g4210;Description=hypothetical protein FCOIX_9035 [Fusarium coicis];Gene=FPANT_10986;Ontology_term=mitochondrion,arylformamidase activity,tryptophan catabolic process to
Fv_160_7.g4211	754	ID=Fv_160_7.g4211;Description=xyloglucanase, partial [Fusarium napiforme];Gene=FNAPI_3475;Ontology_term=mitochondrion,arylformamidase activity,polygalacturonase activity,carbohydrate metabolic process,tryptophan catabolic process to kynurenine,cell wall organization;Ontology_id=GO:0005739,GO:0004061,GO:0004650,GO:0005975,GO:0019441,GO:0071555;Enzyme_code=EC:3.5.1.9,
Fv_160_7.g4212	862	ID=Fv_160_7.g4212;Description=hypothetical protein FVER53590_12337 [Fusarium verticillioides];Gene=FVER53263_12337;Ontology_term=membrane,alpha-L-rhamnosidase activity,carbohydrate metabolic
Fv_160_7.g4213	408	ID=Fv_160_7.g4213;Description=hypothetical protein FVER53263_12336 [Fusarium verticillioides]
Fv_160_7.g4214	671	ID=Fv_160_7.g4214;Description=hypothetical protein FVER53590_12335 [Fusarium verticillioides]
Fv_160_7.g4215	423	ID=Fv_160_7.g4215;Description=hypothetical protein FVER14953_12334 [Fusarium verticillioides];Gene=FNYG_09983;Ontology_term=ADP binding,metal ion binding;Ontology_id=GO:0043531,GO:0046872
Fv_160_7.g4216	468	ID=Fv_160_7.g4216;Description=hypothetical protein FVER53590_12333 [Fusarium verticillioides];Gene=FOZG_15592;Ontology_term=kinesin complex,microtubule,ADP binding,metal ion
Fv_160_7.g4217	385	ID=Fv_160_7.g4217;Description=hypothetical protein FVEG_12332 [Fusarium verticillioides 7600]
Fv_160_7.g4218	324	ID=Fv_160_7.g4218;Description=hypothetical protein FVER53263_12331 [Fusarium verticillioides]
Fv_160_7.g4219	139	ID=Fv_160_7.g4219;Description=hypothetical protein J7337_007681 [Fusarium
Fv_160_7.g4220	680	ID=Fv_160_7.g4220;Description=hypothetical protein FVER14953_12329 [Fusarium verticillioides];Gene=F25303_5522;Ontology_term=membrane,monoatomic anion transmembrane transporter activity,monoatomic
Fv_160_7.g4221	276	ID=Fv_160_7.g4221;Description=hypothetical protein FVEG_12327 [Fusarium verticillioides
Fv_160_7.g4222	465	ID=Fv_160_7.g4222;Description=oxalate decarboxylase [Fusarium verticillioides 7600];Gene=FVER53263_12326;Ontology_term=metal ion binding,oxalate metabolic
Fv_160_7.g4223	129	ID=Fv_160_7.g4223;Description=hypothetical protein J7337_007676 [Fusarium musae]

Fv_160_7.g4224	540	ID=Fv_160_7.g4224;Description=hypothetical protein FVEG_12325 [Fusarium verticillioides 7600];Gene=BFJ68_g5323;Ontology_term=membrane,transmembrane transporter activity,transmembrane
Fv_160_7.g4225	218	ID=Fv_160_7.g4225;Description=hypothetical protein FVEG_12324 [Fusarium verticillioides 7600];Gene=FVER53590_12325;Ontology_term=membrane,transmembrane transporter activity,transmembrane
Fv_160_7.g4226	534	ID=Fv_160_7.g4226;Description=hypothetical protein FVEG_17227 [Fusarium verticillioides 7600];Gene=FTJAE_6870;Ontology_term=membrane,transmembrane transporter activity,transmembrane
Fv_160_7.g4227	296	ID=Fv_160_7.g4227;Description=hypothetical protein FVER14953_12323 [Fusarium verticillioides];Gene=BFJ63_vAg9809;Ontology_term=membrane,monooxygenase activity,iron ion binding,oxidoreductase activity, acting on paired donors, with incorporation or reduction of molecular oxygen,heme binding;Ontology_id=GO:0016020,GO:0004497,GO:0005506,GO:0016705,GO:0020037;Enzyme_code=EC:1.14;Enzyme_name=Actin
Fv_160_7.g4228	93	ID=Fv_160_7.g4228;Description=hypothetical protein FVEG_17224 [Fusarium verticillioides 7600];Gene=FMUND_2906;Ontology_term=membrane,S-adenosylmethionine-dependent methyltransferase
Fv_160_7.g4229	330	ID=Fv_160_7.g4229;Description=hypothetical protein FVEG_12322 [Fusarium verticillioides 7600];Gene=FSUBG_12048;Ontology_term=S-adenosylmethionine-dependent methyltransferase
Fv_160_7.g4230	571	ID=Fv_160_7.g4230;Description=hypothetical protein FVEG_12321 [Fusarium verticillioides 7600];Gene=AU210_013844;Ontology_term=membrane,transmembrane transporter activity,transmembrane
Fv_160_7.g4231	160	ID=Fv_160_7.g4231;Description=hypothetical protein FVEG_17222 [Fusarium verticillioides 7600];Gene=Forpi1262_v018305
Fv_160_7.g4232	151	ID=Fv_160_7.g4232;Description=hypothetical protein FVER53590_30003 [Fusarium verticillioides]
Fv_160_7.g4233	196	ID=Fv_160_7.g4233;Description=hypothetical protein FVEG_17220 [Fusarium verticillioides 7600];Gene=AU210_013846;Ontology_term=membrane,hydrolase
Fv_160_7.g4234	214	ID=Fv_160_7.g4234;Description=hypothetical protein FVER14953_12319 [Fusarium verticillioides];Gene=FMAN_06648;Ontology_term=ether hydrolase
Fv_160_7.g4235	182	ID=Fv_160_7.g4235;Description=hypothetical protein LB505_005877 [Fusarium chuoi];Gene=FMAN_06648;Ontology_term=ether hydrolase activity;Ontology_id=GO:0016803;Enzyme_code=EC:3.3.2;Enzyme_name=Acting on ether bonds
Fv_160_7.g4236	253	ID=Fv_160_7.g4236;Description=hypothetical protein FVEG_12318 [Fusarium verticillioides 7600];Gene=FocTR4_00016015;Ontology_term=nucleus,DNA-binding transcription factor activity, RNA polymerase II-specific,zinc
Fv_160_7.g4237	368	ID=Fv_160_7.g4237;Description=hypothetical protein FVEG_12317 [Fusarium verticillioides 7600];Gene=FVEG_12317;Ontology_term=oxidoreductase activity, acting on
Fv_160_7.g4238	537	ID=Fv_160_7.g4238;Description=hypothetical protein FVER53590_12316 [Fusarium verticillioides];Gene=FPRO_06949;Ontology_term=membrane,transmembrane transporter activity,transmembrane
Fv_160_7.g4239	290	ID=Fv_160_7.g4239;Description=hypothetical protein J7337_007664 [Fusarium musae];Gene=FSUBG_12058;Ontology_term=kinase activity,phosphorylation;Ontology_id=GO:0016301,GO:0016310;Enzyme_code=EC:2.7;Enzyme_name=Transferring phosphorus-
Fv_160_7.g4240	259	ID=Fv_160_7.g4240;Description=hypothetical protein FVEG_12315 [Fusarium verticillioides 7600]

Fv_160_7.g4241	381	ID=Fv_160_7.g4241;Description=hypothetical protein FMEXI_3662 [Fusarium mexicanum];Gene=FPCIR_1418;Ontology_term=hydrolase
Fv_160_7.g4242	231	ID=Fv_160_7.g4242;Description=hypothetical protein FVER14953_21697 [Fusarium verticillioides];Gene=FPCIR_1417;Ontology_term=membrane,kinase
Fv_160_7.g4243	412	ID=Fv_160_7.g4243;Description=hypothetical protein FVER53590_12312 [Fusarium verticillioides];Gene=FNAPI_1454;Ontology_term=kinase
Fv_160_7.g4244	998	ID=Fv_160_7.g4244;Description=hypothetical protein FCOIX_1087 [Fusarium coicis]
Fv_160_7.g4245	521	ID=Fv_160_7.g4245;Description=hypothetical protein FVER53263_20008 [Fusarium
Fv_160_7.g4246	133	ID=Fv_160_7.g4246;Description=hypothetical protein FVEG_17212 [Fusarium verticillioides 7600]
Fv_160_7.g4247	338	ID=Fv_160_7.g4247;Description=hypothetical protein FVEG_12309 [Fusarium verticillioides 7600];Gene=FVER53590_12309;Ontology_term=metal ion binding,dioxygenase activity,small molecule biosynthetic process,organic cyclic compound biosynthetic
Fv_160_7.g4248	389	ID=Fv_160_7.g4248;Description=hypothetical protein FVER53590_12308 [Fusarium verticillioides]
Fv_160_7.g4249	269	ID=Fv_160_7.g4249;Description=hypothetical protein FVER53263_12307 [Fusarium
Fv_160_7.g4250	192	ID=Fv_160_7.g4250;Description=hypothetical protein FVER53263_12306 [Fusarium verticillioides];Gene=FVEG_12306;Ontology_term=NADPH dehydrogenase activity,FMN binding,NADP
Fv_160_7.g4251	311	ID=Fv_160_7.g4251;Description=hypothetical protein FVER53263_12305 [Fusarium verticillioides];Gene=FPOA_02426
Fv_160_7.g4252	717	ID=Fv_160_7.g4252;Description=hypothetical protein FVEG_12304 [Fusarium verticillioides 7600]
Fv_160_7.g4253	647	ID=Fv_160_7.g4253;Description=phospholipase C [Fusarium verticillioides 7600];Gene=FVER53263_12302;Ontology_term=hydrolase activity, acting on ester
Fv_160_7.g4254	617	ID=Fv_160_7.g4254;Description=hypothetical protein FVER14953_12301 [Fusarium verticillioides];Gene=FPANT_12725;Ontology_term=membrane,copper ion binding,S-adenosylmethionine-dependent methyltransferase activity,oxidoreductase
Fv_160_7.g4255	386	ID=Fv_160_7.g4255;Description=hypothetical protein FVER14953_12300 [Fusarium verticillioides];Gene=FOVG_12686;Ontology_term=membrane,copper ion binding,S-adenosylmethionine-dependent methyltransferase activity,oxidoreductase
Fv_160_7.g4256	420	ID=Fv_160_7.g4256;Description=hypothetical protein FVER14953_12299 [Fusarium verticillioides];Gene=FNAPI_1442;Ontology_term=hydrolase activity, hydrolyzing O-glycosyl compounds,polysaccharide catabolic
Fv_160_7.g4257	226	ID=Fv_160_7.g4257;Description=hypothetical protein FVER14953_21417 [Fusarium verticillioides]
Fv_160_7.g4258	1794	ID=Fv_160_7.g4258;Description=unnamed protein product [Fusarium fujikuroi]
Fv_160_7.g4259	131	ID=Fv_160_7.g4259;Description=hypothetical protein FVER14953_12295 [Fusarium verticillioides];Gene=FOYG_14360;Ontology_term=RNA binding,RNA endonuclease activity,lyase activity,ribonuclease T1

Fv_160_7.g4260	289	ID=Fv_160_7.g4260;Description=hypothetical protein FVER53590_12294 [Fusarium verticillioides];Gene=FVER53590_12294;Ontology_term=alpha-galactosidase
Fv_160_7.g4261	616	ID=Fv_160_7.g4261;Description=hypothetical protein FVEG_12293 [Fusarium verticillioides 7600];Gene=FVEG_12293;Ontology_term=oxidoreductase
Fv_160_7.g4262	240	ID=Fv_160_7.g4262;Description=alpha-acetolactate decarboxylase [Fusarium verticillioides 7600];Gene=budA;Ontology_term=acetolactate decarboxylase activity,acetoin biosynthetic
Fv_160_7.g4263	561	ID=Fv_160_7.g4263;Description=hypothetical protein FVEG_12291 [Fusarium verticillioides 7600];Gene=BFJ65_g16541;Ontology_term=oxidoreductase activity,FAD
Fv_160_7.g4264	340	ID=Fv_160_7.g4264;Description=hypothetical protein FVEG_12290 [Fusarium verticillioides
Fv_160_7.g4265	540	ID=Fv_160_7.g4265;Description=hypothetical protein FVEG_12289 [Fusarium verticillioides 7600];Gene=FNAPI_1432;Ontology_term=membrane,alpha-galactosidase activity,carbohydrate binding,carbohydrate metabolic process;Ontology_id=GO:0016020,GO:0004557,GO:0030246,GO:0005975;Enzyme_code=EC:3.2.1.22;Enzyme_name=alpha-
Fv_160_7.g4266	337	ID=Fv_160_7.g4266;Description=hypothetical protein J7337_007632 [Fusarium musae]
Fv_160_7.g4267	511	ID=Fv_160_7.g4267;Description=hypothetical protein FVER53590_12287 [Fusarium verticillioides];Gene=FOC4_g10005701;Ontology_term=acyltransferase
Fv_160_7.g4268	656	ID=Fv_160_7.g4268;Description=hypothetical protein FVER14953_12286 [Fusarium verticillioides];Gene=FPHYL_10171;Ontology_term=membrane,acyltransferase
Fv_160_7.g4269	479	ID=Fv_160_7.g4269;Description=hypothetical protein FVER14953_12285 [Fusarium
Fv_160_7.g4270	85	ID=Fv_160_7.g4270;Description=uncharacterized protein FOIG_10366 [Fusarium odoratissimum NRRL
Fv_160_7.g4271	177	ID=Fv_160_7.g4271;Description=hypothetical protein FVEG_12282 [Fusarium verticillioides 7600];Gene=FFB14_12335;Ontology_term=ribonuclease III activity,RNA
Fv_160_7.g4272	544	ID=Fv_160_7.g4272;Description=hypothetical protein FVER14953_12281 [Fusarium verticillioides]
Fv_160_7.g4273	248	ID=Fv_160_7.g4273;Description=trypsin [Fusarium verticillioides 7600];Gene=FMAN_06604;Ontology_term=serine-type endopeptidase activity,proteolysis;Ontology_id=GO:0004252,GO:0006508;Enzyme_code=EC:3.4.21;Enzyme_name=Acting on
Fv_160_7.g4274	104	ID=Fv_160_7.g4274;Description=hypothetical protein FVEG_17206 [Fusarium verticillioides 7600]
Fv_160_7.g4275	322	ID=Fv_160_7.g4275;Description=pectate lyase [Fusarium verticillioides 7600];Gene=FSUBG_10574;Ontology_term=extracellular region,pectate lyase activity,polysaccharide catabolic
Fv_160_7.g4276	470	ID=Fv_160_7.g4276;Description=hypothetical protein FVEG_12278 [Fusarium verticillioides 7600]
Fv_160_7.g4277	554	ID=Fv_160_7.g4277;Description=hypothetical protein FVER53263_12277 [Fusarium verticillioides];Gene=FPHYL_10182;Ontology_term=nucleus,DNA binding,zinc ion binding,DNA-binding transcription factor activity, RNA polymerase II-specific,regulation of transcription by RNA polymerase
Fv_160_7.g4278	510	ID=Fv_160_7.g4278;Description=hypothetical protein FVEG_12276 [Fusarium verticillioides 7600];Gene=FPANT_801;Ontology_term=membrane,transmembrane transporter activity,transmembrane

Fv_160_7.g4279	481	ID=Fv_160_7.g4279;Description=beta-glucosidase [Fusarium verticillioides 7600];Gene=FFC1_05397;Ontology_term=hydrolase activity, hydrolyzing O-glycosyl compounds,carbohydrate metabolic
Fv_160_7.g4280	260	ID=Fv_160_7.g4280;Description=hypothetical protein FVEG_12274 [Fusarium verticillioides 7600]
Fv_160_7.g4281	273	ID=Fv_160_7.g4281;Description=hypothetical protein FVER14953_12273 [Fusarium verticillioides];Gene=FTJAE_12367;Ontology_term=hydrolase
Fv_160_7.g4282	538	ID=Fv_160_7.g4282;Description=hypothetical protein FVER14953_12272 [Fusarium verticillioides];Gene=Forpe1208_v011557;Ontology_term=mitochondrion,aconitate hydratase activity,metal ion binding,4 iron, 4 sulfur cluster binding,tricarboxylic acid
Fv_160_7.g4283	806	ID=Fv_160_7.g4283;Description=hypothetical protein FVER14953_12271 [Fusarium verticillioides];Gene=BFJ65_g16437;Ontology_term=mitochondrion,aconitate hydratase activity,metal ion binding,4 iron, 4 sulfur cluster binding,tricarboxylic acid
Fv_160_7.g4284	813	ID=Fv_160_7.g4284;Description=hypothetical protein FVER53263_12270 [Fusarium verticillioides];Gene=BFJ65_g16438;Ontology_term=nucleus,DNA binding,zinc ion binding,DNA-binding transcription factor activity, RNA polymerase II-specific,regulation of transcription by RNA polymerase
Fv_160_7.g4285	621	ID=Fv_160_7.g4285;Description=hypothetical protein FPANT_807 [Fusarium pseudoanthophilum];Gene=FPCIR_4559;Ontology_term=mitochondrial inner membrane,hydrolase activity,transmembrane
Fv_160_7.g4286	315	ID=Fv_160_7.g4286;Description=hypothetical protein FVER14953_20795 [Fusarium verticillioides];Gene=FSUBG_10563;Ontology_term=mitochondrial inner membrane,hydrolase activity,transmembrane
Fv_160_7.g4287	218	ID=Fv_160_7.g4287;Description=hypothetical protein FVER14953_12267 [Fusarium verticillioides]
Fv_160_7.g4288	480	ID=Fv_160_7.g4288;Description=hypothetical protein FVER14953_12266 [Fusarium verticillioides];Gene=FMEXI_3707;Ontology_term=membrane,hydrolase activity, hydrolyzing O-glycosyl compounds,transmembrane transporter activity,cellulose catabolic process,transmembrane
Fv_160_7.g4289	838	ID=Fv_160_7.g4289;Description=hypothetical protein FVER53590_25030 [Fusarium verticillioides];Gene=FVER53590_25030;Ontology_term=membrane,beta-glucosidase activity,transmembrane transporter activity,scopolin beta-glucosidase activity,cellulose catabolic process,transmembrane
Fv_160_7.g4290	678	ID=Fv_160_7.g4290;Description=hypothetical protein FVEG_17203 [Fusarium verticillioides 7600];Gene=FRV6_15725;Ontology_term=nucleus,DNA binding,zinc ion binding,DNA-binding transcription factor activity, RNA
Fv_160_7.g4291	512	ID=Fv_160_7.g4291;Description=hypothetical protein FVER14953_12263 [Fusarium verticillioides];Gene=FOYG_14397;Ontology_term=membrane,transmembrane transporter activity,carbohydrate
Fv_160_7.g4292	415	ID=Fv_160_7.g4292;Description=hypothetical protein FVEG_17202 [Fusarium verticillioides
Fv_160_7.g4293	151	ID=Fv_160_7.g4293;Description=hypothetical protein FVER14953_12262 [Fusarium verticillioides];Gene=F25303_7217;Ontology_term=nitrogen compound metabolic process;Ontology_id=GO:0006807

Fv_160_7.g4294	140	ID=Fv_160_7.g4294;Description=hypothetical protein FVER14953_20796 [Fusarium verticillioides];Gene=Focb16_v005092;Ontology_term=nitrogen compound metabolic process;Ontology_id=GO:0006807
Fv_160_7.g4295	493	ID=Fv_160_7.g4295;Description=hypothetical protein FVER14953_12261 [Fusarium verticillioides];Gene=FDENT_7131;Ontology_term=membrane,aspartic-type endopeptidase
Fv_160_7.g4296	254	ID=Fv_160_7.g4296;Description=hypothetical protein FVER14953_12260 [Fusarium verticillioides];Gene=FMUND_15218;Ontology_term=membrane,protein kinase activity,ATP binding,phosphorylation,transmembrane
Fv_160_7.g4297	138	ID=Fv_160_7.g4297;Description=hypothetical protein FVER53590_25027 [Fusarium verticillioides];Gene=FOIG_00062;Ontology_term=hydrolase
Fv_160_7.g4298	500	ID=Fv_160_7.g4298;Description=hypothetical protein FVER14953_20791 [Fusarium verticillioides];Gene=FPRO05_08061;Ontology_term=hydrolase activity, acting on carbon-nitrogen (but not peptide)
Fv_160_7.g4299	540	ID=Fv_160_7.g4299;Description=hypothetical protein FVEG_12258 [Fusarium verticillioides 7600];Gene=BFJ65_g16258;Ontology_term=membrane,transmembrane transporter activity,transmembrane
Fv_160_7.g4300	554	ID=Fv_160_7.g4300;Description=hypothetical protein FVER14953_12257 [Fusarium verticillioides]
Fv_160_7.g4301	799	ID=Fv_160_7.g4301;Description=hypothetical protein FVEG_17197 [Fusarium verticillioides 7600];Gene=FPRO05_08064;Ontology_term=nucleus,DNA-binding transcription factor activity, RNA polymerase II-specific,zinc ion
Fv_160_7.g4302	480	ID=Fv_160_7.g4302;Description=hypothetical protein FVER53590_12256 [Fusarium verticillioides];Gene=BFJ65_g16587;Ontology_term=monooxygenase activity,iron ion binding,oxidoreductase activity, acting on paired donors, with incorporation or reduction of molecular oxygen,heme binding;Ontology_id=GO:0004497,GO:0005506,GO:0016705,GO:0020037;Enzyme_code=EC:1.14;Enzyme_name=Acting on paired
Fv_160_7.g4303	227	ID=Fv_160_7.g4303;Description=hypothetical protein FVER14953_12255 [Fusarium
Fv_160_7.g4304	248	ID=Fv_160_7.g4304;Description=hypothetical protein FVER14953_12254 [Fusarium verticillioides];Gene=BFJ72_g438;Ontology_term=amino-acid racemase activity,nitrogen compound metabolic
Fv_160_7.g4305	639	ID=Fv_160_7.g4305;Description=hypothetical protein FVER14953_12253 [Fusarium verticillioides];Gene=LW93_9547;Ontology_term=hydrolase activity, acting on carbon-nitrogen (but not peptide)
Fv_160_7.g4306	190	ID=Fv_160_7.g4306;Description=hypothetical protein FVER14953_12252 [Fusarium verticillioides]
Fv_160_7.g4307	586	ID=Fv_160_7.g4307;Description=hypothetical protein FVER53263_12251 [Fusarium verticillioides];Gene=FOXG_13286;Ontology_term=membrane,monooxygenase activity,FAD binding,biosynthetic
Fv_160_7.g4308	748	ID=Fv_160_7.g4308;Description=hypothetical protein FVER14953_12250 [Fusarium verticillioides];Gene=noxC;Ontology_term=membrane,ferric-chelate reductase activity,calcium ion binding,iron ion
Fv_160_7.g4309	588	ID=Fv_160_7.g4309;Description=hypothetical protein FVER14953_12249 [Fusarium
Fv_160_7.g4310	394	ID=Fv_160_7.g4310;Description=hypothetical protein FVER14953_12248 [Fusarium verticillioides]

Fv_160_7.g4311	558	ID=Fv_160_7.g4311;Description=hypothetical protein FVEG_12247 [Fusarium verticillioides 7600];Gene=FPCIR_4586;Ontology_term=oxidoreductase activity,cellular biosynthetic
Fv_160_7.g4312	246	ID=Fv_160_7.g4312;Description=hypothetical protein FVER53590_12246 [Fusarium verticillioides];Gene=BFJ68_g5377;Ontology_term=oxidoreductase activity,cellular biosynthetic
Fv_160_7.g4313	262	ID=Fv_160_7.g4313;Description=hypothetical protein FVER14953_12245 [Fusarium verticillioides];Gene=FNAPI_7450;Ontology_term=serine-type endopeptidase
Fv_160_7.g4314	441	ID=Fv_160_7.g4314;Description=hypothetical protein FVER14953_12244 [Fusarium verticillioides];Gene=FCIRC_5535;Ontology_term=serine-type endopeptidase
Fv_160_7.g4315	569	ID=Fv_160_7.g4315;Description=hypothetical protein FVER14953_12243 [Fusarium verticillioides];Gene=FCIRC_5536;Ontology_term=membrane,amino acid transport,transmembrane
Fv_160_7.g4316	277	ID=Fv_160_7.g4316;Description=hypothetical protein FVER53590_12242 [Fusarium verticillioides]
Fv_160_7.g4317	244	ID=Fv_160_7.g4317;Description=hypothetical protein FVER14953_12241 [Fusarium verticillioides]
Fv_160_7.g4318	535	ID=Fv_160_7.g4318;Description=hypothetical protein LB504_005999 [Fusarium proliferatum];Gene=FPANT_456;Ontology_term=membrane,oxidoreductase activity, acting on the CH-OH group of donors, NAD or NADP as acceptor,transmembrane transporter activity,NAD binding,transmembrane
Fv_160_7.g4319	355	ID=Fv_160_7.g4319;Description=hypothetical protein FVER14953_12239 [Fusarium verticillioides];Gene=FPANT_455;Ontology_term=membrane,oxidoreductase activity, acting on the CH-OH group of donors, NAD or NADP as acceptor,transmembrane transporter activity,NAD binding,transmembrane
Fv_160_7.g4320	658	ID=Fv_160_7.g4320;Description=hypothetical protein FVER14953_12238 [Fusarium verticillioides];Gene=FVER53263_12238;Ontology_term=oxidoreductase activity, acting on CH-OH group of donors,flavin adenine
Fv_160_7.g4321	148	ID=Fv_160_7.g4321;Description=hypothetical protein FVEG_17191 [Fusarium verticillioides 7600]
Fv_160_7.g4322	223	ID=Fv_160_7.g4322;Description=hypothetical protein FVER14953_12237 [Fusarium verticillioides]
Fv_160_7.g4323	467	ID=Fv_160_7.g4323;Description=hypothetical protein FVER14953_12236 [Fusarium verticillioides]
Fv_160_7.g4324	456	ID=Fv_160_7.g4324;Description=hypothetical protein FVER14953_12235 [Fusarium verticillioides];Gene=FOXB_14331;Ontology_term=peroxidase activity,metal ion binding,cellular oxidant
Fv_160_7.g4325	235	ID=Fv_160_7.g4325;Description=hypothetical protein FVER14953_20788 [Fusarium verticillioides];Gene=FTJAE_1140;Ontology_term=hydrolase
Fv_160_7.g4326	672	ID=Fv_160_7.g4326;Description=hypothetical protein FVER14953_12234 [Fusarium verticillioides];Gene=FOIG_10295;Ontology_term=membrane,metalloendopeptidase activity,metal ion binding,proteolysis,integrin-mediated signaling
Fv_160_7.g4327	376	ID=Fv_160_7.g4327;Description=hypothetical protein FVER53263_12233 [Fusarium verticillioides];Gene=FNYG_09684;Ontology_term=oxidoreductase

Fv_160_7.g4328	638	ID=Fv_160_7.g4328;Description=hypothetical protein FVEG_12232 [Fusarium verticillioides 7600];Gene=BFJ65_g16301;Ontology_term=nucleus,DNA binding,zinc ion binding,DNA-binding transcription factor activity, RNA polymerase II-specific,regulation of transcription by RNA polymerase
Fv_160_7.g4329	250	ID=Fv_160_7.g4329;Description=hypothetical protein FVER14953_12231 [Fusarium verticillioides]
Fv_160_7.g4330	563	ID=Fv_160_7.g4330;Description=hypothetical protein FVEG_12230 [Fusarium verticillioides 7600];Gene=BFJ65_g16302;Ontology_term=nucleus,DNA binding,zinc ion binding,DNA-binding transcription factor activity, RNA polymerase II-specific,regulation of transcription by RNA polymerase
Fv_160_7.g4331	286	ID=Fv_160_7.g4331;Description=hypothetical protein FVER14953_12229 [Fusarium verticillioides]
Fv_160_7.g4332	220	ID=Fv_160_7.g4332;Description=cysteine dioxygenase [Fusarium verticillioides 7600];Gene=FPRO05_08096;Ontology_term=iron ion binding,cysteine dioxygenase activity;Ontology_id=GO:0005506,GO:0017172;Enzyme_code=EC:1.13.11.20;Enzyme_name=cysteine
Fv_160_7.g4333	668	ID=Fv_160_7.g4333;Description=hypothetical protein FVEG_12227 [Fusarium verticillioides 7600];Gene=FNAPI_7468;Ontology_term=nucleus,DNA binding,zinc ion binding,DNA-binding transcription factor activity, RNA
Fv_160_7.g4334	649	ID=Fv_160_7.g4334;Description=hypothetical protein FVER53590_12226 [Fusarium verticillioides];Gene=FPCIR_4606;Ontology_term=nucleus,DNA binding,zinc ion binding,DNA-binding transcription factor activity,
Fv_160_7.g4335	355	ID=Fv_160_7.g4335;Description=hypothetical protein FVEG_12225 [Fusarium verticillioides 7600]
Fv_160_7.g4336	504	ID=Fv_160_7.g4336;Description=hypothetical protein FVER14953_12224 [Fusarium verticillioides];Gene=FMAN_06537;Ontology_term=membrane,transmembrane transporter activity,transmembrane
Fv_160_7.g4337	683	ID=Fv_160_7.g4337;Description=hypothetical protein FVER14953_12223 [Fusarium verticillioides];Gene=FOVG_12580;Ontology_term=copper ion binding,primary amine oxidase activity,quinone binding,amine
Fv_160_7.g4338	451	ID=Fv_160_7.g4338;Description=hypothetical protein FVER53263_12222 [Fusarium verticillioides]
Fv_160_7.g4339	217	ID=Fv_160_7.g4339;Description=hypothetical protein FVER53590_12222 [Fusarium verticillioides]
Fv_160_7.g4340	617	ID=Fv_160_7.g4340;Description=hypothetical protein FVEG_12221 [Fusarium verticillioides 7600];Gene=FFC1_05461;Ontology_term=dipeptidyl-peptidase activity;Ontology_id=GO:0008239;Enzyme_code=EC:3.4.14.1,EC:3.4.14.5,EC:3.4.14.4,EC:3.4.14.2;Enzyme_name=dipeptidyl-
Fv_160_7.g4341	165	ID=Fv_160_7.g4341;Description=hypothetical protein J7337_007550 [Fusarium musae];Gene=FCIRC_3721;Ontology_term=membrane,transmembrane transporter activity,scytalone dehydratase activity,melanin metabolic process,transmembrane
Fv_160_7.g4342	547	ID=Fv_160_7.g4342;Description=hypothetical protein FVER14953_12219 [Fusarium verticillioides];Gene=BFJ68_g5266;Ontology_term=membrane,transmembrane transporter activity,scytalone dehydratase activity,melanin metabolic process,transmembrane
Fv_160_7.g4343	441	ID=Fv_160_7.g4343;Description=hypothetical protein FVER53590_12218 [Fusarium verticillioides];Gene=FVEG_12218;Ontology_term=transferase activity, transferring alkyl or aryl (other than methyl) groups,alkaloid metabolic process;Ontology_id=GO:0016765,GO:0009820;Enzyme_code=EC:2.5.1;Enzyme_name=Transferring alkyl or aryl groups,

Fv_160_7.g4344	328	ID=Fv_160_7.g4344;Description=hypothetical protein FVER53590_12217 [Fusarium verticillioides];Gene=FVER53590_12217;Ontology_term=hydrolase
Fv_160_7.g4345	531	ID=Fv_160_7.g4345;Description=hypothetical protein FVER53263_12216 [Fusarium verticillioides];Gene=FVER53263_12216;Ontology_term=membrane,monooxygenase activity,iron ion binding,oxidoreductase activity, acting on paired donors, with incorporation or reduction of molecular oxygen,heme binding;Ontology_id=GO:0016020,GO:0004497,GO:0005506,GO:0016705,GO:0020037;Enzyme_code=EC:1.14;Enzyme_name=Actin
Fv_160_7.g4346	294	ID=Fv_160_7.g4346;Description=hypothetical protein FVER53590_12215 [Fusarium
Fv_160_7.g4347	509	ID=Fv_160_7.g4347;Description=hypothetical protein FVER53590_12214 [Fusarium verticillioides]
Fv_160_7.g4348	511	ID=Fv_160_7.g4348;Description=hypothetical protein FVEG_12213 [Fusarium verticillioides 7600];Gene=FOC4_g10005603;Ontology_term=peroxidase activity,UDP-glycosyltransferase activity,metal ion binding,cellular oxidant
Fv_160_7.g4349	255	ID=Fv_160_7.g4349;Description=hypothetical protein FVER14953_12212 [Fusarium verticillioides];Gene=FPANT_12996;Ontology_term=peroxidase activity,glycosyltransferase activity,metal ion binding,cellular biosynthetic process,cellular oxidant detoxification;Ontology_id=GO:0004601,GO:0016757,GO:0046872,GO:0044249,GO:0098869;Enzyme_code=EC:2.4,EC:1.11.1;Enzym
Fv_160_7.g4350	274	ID=Fv_160_7.g4350;Description=hypothetical protein FVER53263_12211 [Fusarium verticillioides];Gene=BFJ72_g637;Ontology_term=peroxidase activity,metal ion binding,cellular biosynthetic process,cellular oxidant detoxification;Ontology_id=GO:0004601,GO:0046872,GO:0044249,GO:0098869;Enzyme_code=EC:1.11.1;Enzyme_name=Acting on
Fv_160_7.g4351	644	ID=Fv_160_7.g4351;Description=hypothetical protein FVER53263_12210 [Fusarium verticillioides]
Fv_160_7.g4352	621	ID=Fv_160_7.g4352;Description=hypothetical protein FVER14953_12209 [Fusarium verticillioides];Gene=FNAPI_5763;Ontology_term=extracellular region,cellulose binding,pectate lyase activity,polysaccharide
Fv_160_7.g4353	389	ID=Fv_160_7.g4353;Description=hypothetical protein FVER14953_21207 [Fusarium
Fv_160_7.g4354	937	ID=Fv_160_7.g4354;Description=carboxylesterase type B [Fusarium denticulatum];Gene=FPCIR_4625;Ontology_term=membrane,hydrolase activity,transmembrane transporter activity,transmembrane
Fv_160_7.g4355	460	ID=Fv_160_7.g4355;Description=hypothetical protein FVER53590_12204 [Fusarium verticillioides]
Fv_160_7.g4356	371	ID=Fv_160_7.g4356;Description=hypothetical protein FVER14953_12203 [Fusarium verticillioides];Gene=CEK26_007846;Ontology_term=nucleus,membrane,transcription coactivator activity,catalytic activity,DNA binding, bending,molybdenum ion binding,pyridoxal phosphate binding,regulation of transcription by RNA polymerase II,positive
Fv_160_7.g4357	477	ID=Fv_160_7.g4357;Description=hypothetical protein FVEG_12202 [Fusarium verticillioides 7600];Gene=FPRO_06818;Ontology_term=oxidoreductase
Fv_160_7.g4358	130	ID=Fv_160_7.g4358;Description=hypothetical protein FVER53263_12201 [Fusarium
Fv_160_7.g4359	242	ID=Fv_160_7.g4359;Description=hypothetical protein J7337_007530 [Fusarium musae];Gene=FNAPI_5753;Ontology_term=membrane,transmembrane transporter activity,transmembrane

Fv_160_7.g4360	551	ID=Fv_160_7.g4360;Description=hypothetical protein FVER53590_12199 [Fusarium verticillioides];Gene=F25303_4448;Ontology_term=cytoplasm,membrane,intracellular organelle,DNA-binding transcription factor activity, RNA polymerase II-specific,transmembrane transporter activity,cytidine kinase activity,CMP biosynthetic process,UMP salvage,CTP salvage,transmembrane transport;Ontology_id=GO:0005737,GO:0016020,GO:0043229,GO:0000981,GO:0022857,GO:0043771,GO:0009224,GO:0044206,GO
Fv_160_7.g4361	288	ID=Fv_160_7.g4361;Description=hypothetical protein FVEG_12198 [Fusarium verticillioides
Fv_160_7.g4362	447	ID=Fv_160_7.g4362;Description=hypothetical protein FVEG_17182 [Fusarium verticillioides 7600];Gene=FVEG_17182;Ontology_term=nucleus,DNA-binding transcription factor activity, RNA polymerase II-specific,zinc ion
Fv_160_7.g4363	286	ID=Fv_160_7.g4363;Description=hypothetical protein FVEG_12197 [Fusarium verticillioides
Fv_160_7.g4364	273	ID=Fv_160_7.g4364;Description=hypothetical protein FVER14953_12196 [Fusarium verticillioides];Gene=FTJAE_1100;Ontology_term=S-adenosylmethionine-dependent methyltransferase
Fv_160_7.g4365	208	ID=Fv_160_7.g4365;Description=hypothetical protein FVER53590_25022 [Fusarium verticillioides]
Fv_160_7.g4366	489	ID=Fv_160_7.g4366;Description=hypothetical protein FVEG_12195 [Fusarium verticillioides 7600];Gene=FVEG_12195;Ontology_term=membrane,transmembrane transporter activity,transmembrane
Fv_160_7.g4367	413	ID=Fv_160_7.g4367;Description=carboxypeptidase A4 [Fusarium verticillioides 7600];Gene=F25303_4442;Ontology_term=extracellular region,metallocarboxypeptidase activity,zinc ion
Fv_160_7.g4368	575	ID=Fv_160_7.g4368;Description=hypothetical protein FVEG_12193 [Fusarium verticillioides 7600];Gene=FTJAE_1096;Ontology_term=nucleus,DNA binding,zinc ion binding,DNA-binding transcription factor activity, RNA
Fv_160_7.g4369	536	ID=Fv_160_7.g4369;Description=hypothetical protein FVEG_12192 [Fusarium verticillioides 7600];Gene=FTJAE_1095;Ontology_term=cytoplasm,membrane,dihydroorotase activity,zinc ion binding,transmembrane transporter activity,'de novo' pyrimidine nucleobase biosynthetic process,'de novo' UMP biosynthetic process,transmembrane
Fv_160_7.g4370	584	ID=Fv_160_7.g4370;Description=hypothetical protein FVEG_12191 [Fusarium verticillioides 7600]
Fv_160_7.g4371	338	ID=Fv_160_7.g4371;Description=hypothetical protein FVER14953_12190 [Fusarium verticillioides]
Fv_160_7.g4372	608	ID=Fv_160_7.g4372;Description=hypothetical protein FVER14953_12189 [Fusarium verticillioides];Gene=FNAPI_5741;Ontology_term=alpha-L-fucosidase activity,fucose metabolic
Fv_160_7.g4373	275	ID=Fv_160_7.g4373;Description=hypothetical protein FNAPI_5740 [Fusarium napiforme]
Fv_160_7.g4374	454	ID=Fv_160_7.g4374;Description=hypothetical protein FVER53263_20634 [Fusarium verticillioides]
Fv_160_7.g4375	340	ID=Fv_160_7.g4375;Description=hypothetical protein FVER53263_12188 [Fusarium verticillioides];Gene=FPANT_630;Ontology_term=endonuclease activity,exonuclease
Fv_160_7.g4376	520	ID=Fv_160_7.g4376;Description=hypothetical protein FVER53263_12187 [Fusarium verticillioides];Gene=F25303_4432;Ontology_term=membrane,monooxygenase activity,iron ion binding,oxidoreductase activity, acting on paired donors, with incorporation or reduction of molecular oxygen,heme binding;Ontology_id=GO:0016020,GO:0004497,GO:0005506,GO:0016705,GO:0020037;Enzyme_code=EC:1.14;Enzyme_name=Actin

Fv_160_7.g4377	151	ID=Fv_160_7.g4377;Description=hypothetical protein FVER53590_25020 [Fusarium verticillioides]
Fv_160_7.g4378	498	ID=Fv_160_7.g4378;Description=hypothetical protein FVER53263_12186 [Fusarium verticillioides];Gene=FOXB_12374;Ontology_term=membrane,monooxygenase activity,iron ion binding,oxidoreductase activity, acting on paired donors, with incorporation or reduction of molecular oxygen,heme binding;Ontology_id=GO:0016020,GO:0004497,GO:0005506,GO:0016705,GO:0020037;Enzyme_code=EC:1.14;Enzyme_name=Actin
Fv_160_7.g4379	437	ID=Fv_160_7.g4379;Description=hypothetical protein FPANT_633 [Fusarium pseudoanthophilum]
Fv_160_7.g4380	629	ID=Fv_160_7.g4380;Description=alkaline phosphatase [Fusarium verticillioides 7600]
Fv_160_7.g4381	749	ID=Fv_160_7.g4381;Description=hypothetical protein FOYG_14512 [Fusarium oxysporum NRRL 32931];Gene=FOXYS1_3495;Ontology_term=O-methyltransferase activity,S-adenosylmethionine-dependent methyltransferase activity,methylation;Ontology_id=GO:0008171,GO:0008757,GO:0032259;Enzyme_code=EC:2.1.1;Enzyme_name=Transferring one-
Fv_160_7.g4382	1119	ID=Fv_160_7.g4382;Description=hypothetical protein J7337_007509 [Fusarium musae];Gene=CSN1S1;Ontology_term=extracellular region,cytoplasm,membrane,dihydroorotate dehydrogenase activity,S-adenosylmethionine-dependent methyltransferase activity,'de novo' pyrimidine nucleobase biosynthetic process,lipid metabolic process,'de novo' UMP biosynthetic process;Ontology_id=GO:0005576,GO:0005737,GO:0016020,GO:0004152,GO:0008757,GO:0006207,GO:0006629,GO:0044205;Enzyme_code=EC:1.3,EC:2.1.1;Enzyme_name=Acting on the CH-CH group of donors,Transferring one-carbon groups
Fv_160_7.g4383	361	ID=Fv_160_7.g4383;Description=hypothetical protein J7337_007508 [Fusarium musae];Gene=FTJAE_1083;Ontology_term=S-adenosylmethionine-dependent methyltransferase
Fv_160_7.g4384	247	ID=Fv_160_7.g4384;Description=hypothetical protein FVEG_12180 [Fusarium verticillioides 7600];Gene=FVEG_12180;Ontology_term=hydrolase
Fv_160_7.g4385	642	ID=Fv_160_7.g4385;Description=hypothetical protein FVEG_12179 [Fusarium verticillioides 7600];Gene=DAL80;Ontology_term=negative regulation of transcription by RNA polymerase II,nucleus,zinc ion binding,RNA polymerase II cis-regulatory region sequence-specific DNA binding,nitrogen catabolite repression of transcription,positive regulation of transcription by RNA polymerase II,DNA-binding transcription factor activity, RNA polymerase II-specific,nitrate
Fv_160_7.g4386	326	ID=Fv_160_7.g4386;Description=hypothetical protein J7337_007505 [Fusarium musae];Gene=FMUND_8349;Ontology_term=methyltransferase
Fv_160_7.g4387	297	ID=Fv_160_7.g4387;Description=hypothetical protein FVER53263_12177 [Fusarium verticillioides];Gene=FPANT_640;Ontology_term=cytosolic large ribosomal subunit,RNA binding,methyltransferase activity,methylation,ribosome
Fv_160_7.g4388	1062	ID=Fv_160_7.g4388;Description=hypothetical protein FVER14953_12176 [Fusarium verticillioides]
Fv_160_7.g4389	585	ID=Fv_160_7.g4389;Description=hypothetical protein FVEG_12175 [Fusarium verticillioides 7600];Gene=FPANT_642;Ontology_term=membrane,transmembrane transporter activity,transmembrane
Fv_160_7.g4390	373	ID=Fv_160_7.g4390;Description=hypothetical protein FVEG_12174 [Fusarium verticillioides 7600];Gene=FOPG_11380;Ontology_term=lyase activity,metal ion

Fv_160_7.g4391	470	ID=Fv_160_7.g4391;Description=hypothetical protein FVER14953_12173 [Fusarium verticillioides];Gene=FPRO05_08151;Ontology_term=cytosol,membrane,tRNA binding,lysine-tRNA ligase activity,ATP binding,lysyl-tRNA
Fv_160_7.g4392	427	ID=Fv_160_7.g4392;Description=hypothetical protein FVER53590_12172 [Fusarium verticillioides];Gene=FPANT_13857;Ontology_term=membrane,monooxygenase activity,iron ion binding,oxidoreductase activity, acting on paired donors, with incorporation or reduction of molecular oxygen,heme binding;Ontology_id=GO:0016020,GO:0004497,GO:0005506,GO:0016705,GO:0020037;Enzyme_code=EC:1.14;Enzyme_name=Actin
Fv_160_7.g4393	276	ID=Fv_160_7.g4393;Description=hypothetical protein FVER53263_11808 [Fusarium verticillioides];Gene=FPRO_06780;Ontology_term=ubiquitin protein ligase activity,zinc ion binding,oxidoreductase activity, acting on paired donors, with incorporation or reduction of molecular oxygen,monooxygenase activity,DNA-binding transcription factor activity, RNA polymerase II-specific,heme binding,regulation of transcription by RNA polymerase II,nucleus,DNA binding,ubiquitin-dependent protein catabolic process via the N-end rule pathway,iron ion binding,membrane,protein ubiquitination;Ontology_id=GO:0061630,GO:0008270,GO:0016705,GO:0004497,GO:0000981,GO:0020037,GO:0006357,GO:0005634,GO:0003677,GO:0071596,GO:0005506,GO:0016020,GO:0016567;Enzyme_code=EC:1.14,EC:2.3.2;Enzyme_name=Acting on paired
Fv_160_7.g4394	329	ID=Fv_160_7.g4394;Description=hypothetical protein FNAPI_10456 [Fusarium napiforme];Gene=FACUT_11207
Fv_160_7.g4395	579	ID=Fv_160_7.g4395;Description=hypothetical protein FVER53590_11809 [Fusarium verticillioides]
Fv_160_7.g4396	83	ID=Fv_160_7.g4396;Description=hypothetical protein FVER14953_11810 [Fusarium verticillioides];Gene=1130
Fv_160_7.g4397	218	ID=Fv_160_7.g4397;Description=hypothetical protein FVEG_11811 [Fusarium verticillioides 7600]
Fv_160_7.g4398	618	ID=Fv_160_7.g4398;Description=hypothetical protein FVER14953_11812 [Fusarium verticillioides];Gene=FCIRC_2214;Ontology_term=membrane,monooxygenase activity,ATP binding,ATP hydrolysis activity;Ontology_id=GO:0016020,GO:0004497,GO:0005524,GO:0016887;Enzyme_code=EC:1,EC:3.6.1.15;Enzyme_name=Oxidoredu
Fv_160_7.g4399	1466	ID=Fv_160_7.g4399;Description=hypothetical protein FVER53263_11813 [Fusarium verticillioides];Gene=FDENT_3838;Ontology_term=ATP hydrolysis activity,nucleus,DNA binding,ATP binding,zinc ion binding,transmembrane transport,ABC-type transporter activity,membrane,DNA-binding transcription factor activity, RNA polymerase II-specific,regulation of transcription by RNA polymerase II;Ontology_id=GO:0016887,GO:0005634,GO:0003677,GO:0005524,GO:0008270,GO:0055085,GO:0140359,GO:0016020,GO:000098
Fv_160_7.g4400	701	ID=Fv_160_7.g4400;Description=hypothetical protein FVER53590_11815 [Fusarium verticillioides];Gene=FVER53590_11815;Ontology_term=nucleus,DNA binding,zinc ion binding,lyase activity,tricarboxylic acid cycle,DNA-binding transcription factor activity, RNA polymerase II-specific,regulation of transcription by RNA polymerase
Fv_160_7.g4401	496	ID=Fv_160_7.g4401;Description=hypothetical protein FVER14953_11816 [Fusarium verticillioides];Gene=FNAPI_10448;Ontology_term=nucleus,DNA binding,zinc ion binding,lyase activity,tricarboxylic acid cycle,DNA-binding transcription factor activity, RNA polymerase II-specific,regulation of transcription by RNA polymerase

Fv_160_7.g4402	332	ID=Fv_160_7.g4402;Description=hypothetical protein LB503_004534 [Fusarium chuoi];Gene=FOXB_08608;Ontology_term=binding,regulation of DNA-templated
Fv_160_7.g4403	296	ID=Fv_160_7.g4403;Description=hypothetical protein FVEG_17105 [Fusarium verticillioides 7600];Gene=FTJAE_1062;Ontology_term=fungal-type vacuole,membrane,aspartic-type endopeptidase activity,transmembrane transporter activity,proteolysis,autophagy,transmembrane transport;Ontology_id=GO:0000324,GO:0016020,GO:0004190,GO:0022857,GO:0006508,GO:0006914,GO:0055085;Enzyme_code=E
Fv_160_7.g4404	239	ID=Fv_160_7.g4404;Description=hypothetical protein FVER53263_11817 [Fusarium verticillioides];Gene=FOPG_11371;Ontology_term=membrane,transmembrane transporter activity,transmembrane
Fv_160_7.g4405	242	ID=Fv_160_7.g4405;Description=hypothetical protein FVER53590_29295 [Fusarium verticillioides];Gene=FOXG_13383;Ontology_term=oxidoreductase activity,cellular biosynthetic
Fv_160_7.g4406	732	ID=Fv_160_7.g4406;Description=hypothetical protein FVEG_11819 [Fusarium verticillioides 7600];Gene=FPRO05_08162;Ontology_term=nucleus,zinc ion binding,oxidoreductase activity,DNA-binding transcription factor activity, RNA polymerase II-specific,regulation of transcription by RNA polymerase
Fv_160_7.g4407	558	ID=Fv_160_7.g4407;Description=hypothetical protein FVEG_11820 [Fusarium verticillioides 7600];Gene=FVER53263_11820;Ontology_term=membrane,N,N-dimethylaniline monooxygenase activity,flavin adenine dinucleotide binding,NADP
Fv_160_7.g4408	270	ID=Fv_160_7.g4408;Description=hypothetical protein J7337_007485 [Fusarium musae];Gene=F25303_8932;Ontology_term=oxidoreductase activity,cellular biosynthetic
Fv_160_7.g4409	665	ID=Fv_160_7.g4409;Description=hypothetical protein FVER14953_11822 [Fusarium verticillioides]
Fv_160_7.g4410	440	ID=Fv_160_7.g4410;Description=hypothetical protein FVER53263_11823 [Fusarium verticillioides]
Fv_160_7.g4411	373	ID=Fv_160_7.g4411;Description=hypothetical protein J7337_007481 [Fusarium musae];Gene=FVER53590_11824;Ontology_term=nucleus,cytosol,ribonucleoprotein complex,hydrolase activity,carboxy-lyase activity,translation,protein ubiquitination;Ontology_id=GO:0005634,GO:0005829,GO:1990904,GO:0016787,GO:0016831,GO:0006412,GO:0016567;Enzyme_co
Fv_160_7.g4412	242	ID=Fv_160_7.g4412;Description=hypothetical protein FVEG_11825 [Fusarium verticillioides 7600];Gene=FTJAE_1051;Ontology_term=serine-type peptidase activity,hydrolase activity, acting on glycosyl bonds,proteolysis,xylan catabolic
Fv_160_7.g4413	545	ID=Fv_160_7.g4413;Description=hypothetical protein FOC4_g10005524 [Fusarium odoratissimum]
Fv_160_7.g4414	207	ID=Fv_160_7.g4414;Description=hypothetical protein FVER53590_11827 [Fusarium verticillioides]
Fv_160_7.g4415	358	ID=Fv_160_7.g4415;Description=hypothetical protein FVER14953_11828 [Fusarium verticillioides]
Fv_160_7.g4416	196	ID=Fv_160_7.g4416;Description=3-octaprenyl-4-hydroxybenzoate carboxy-lyase UbiX [Fusarium verticillioides 7600];Gene=PAD1;Ontology_term=mitochondrion,lyase activity,flavin prenyltransferase

Fv_160_7.g4417	503	ID=Fv_160_7.g4417;Description=hypothetical protein FVEG_11829 [Fusarium verticillioides 7600];Gene=FDC1;Ontology_term=cytoplasm,carboxy-lyase activity,metal ion binding,ferulate metabolic process,cinnamic acid catabolic
Fv_160_7.g4418	571	ID=Fv_160_7.g4418;Description=hypothetical protein FVEG_11830 [Fusarium verticillioides 7600];Gene=F25303_7966;Ontology_term=nucleus,DNA binding,zinc ion binding,DNA-binding transcription factor activity, RNA polymerase II-specific,regulation of transcription by RNA polymerase
Fv_160_7.g4419	299	ID=Fv_160_7.g4419;Description=hypothetical protein FVEG_11831 [Fusarium verticillioides
Fv_160_7.g4420	106	ID=Fv_160_7.g4420;Description=hypothetical protein FVER14953_11832 [Fusarium verticillioides];Gene=TOM40;Ontology_term=mitochondrial outer membrane,pore complex,protein transmembrane transporter
Fv_160_7.g4421	706	ID=Fv_160_7.g4421;Description=hypothetical protein FVER14953_11833 [Fusarium verticillioides]
Fv_160_7.g4422	303	ID=Fv_160_7.g4422;Description=hypothetical protein FVER14953_11834 [Fusarium verticillioides];Gene=UBC1;Ontology_term=glutamine synthetase activity,ATP binding,ubiquitin conjugating enzyme activity,nitrogen compound metabolic
Fv_160_7.g4423	448	ID=Fv_160_7.g4423;Description=hypothetical protein FVEG_11835 [Fusarium verticillioides 7600];Gene=BFJ69_g14410;Ontology_term=glutamine synthetase activity,nitrogen compound metabolic
Fv_160_7.g4424	530	ID=Fv_160_7.g4424;Description=hypothetical protein FVER53590_11836 [Fusarium verticillioides];Gene=FVER53590_11836;Ontology_term=nucleus,cytoplasm,nucleotide binding,glutamine synthetase activity,ubiquitin activating enzyme activity,protein ubiquitination;Ontology_id=GO:0005634,GO:0005737,GO:0000166,GO:0004356,GO:0004839,GO:0016567;Enzyme_code=EC:6.2.1.
Fv_160_7.g4425	77	ID=Fv_160_7.g4425;Description=hypothetical protein FVEG_17114 [Fusarium verticillioides 7600]
Fv_160_7.g4426	191	ID=Fv_160_7.g4426;Description=hypothetical protein FVEG_11837 [Fusarium verticillioides
Fv_160_7.g4427	294	ID=Fv_160_7.g4427;Description=hypothetical protein FVEG_11838 [Fusarium verticillioides 7600];Gene=FANTH_2691;Ontology_term=sulfur dioxygenase activity,glutathione metabolic
Fv_160_7.g4428	322	ID=Fv_160_7.g4428;Description=hypothetical protein FVEG_11839 [Fusarium verticillioides 7600];Gene=FMAN_06439;Ontology_term=plasma membrane,transcription coactivator activity,secondary active sulfate transmembrane transporter activity,protein dimerization activity,carbon catabolite regulation of transcription from RNA polymerase II promoter,positive regulation of DNA-templated transcription,sulfate transmembrane
Fv_160_7.g4429	756	ID=Fv_160_7.g4429;Description=hypothetical protein FVER14953_11840 [Fusarium verticillioides];Gene=FTJAE_10530;Ontology_term=membrane,secondary active sulfate transmembrane transporter activity,monoatomic anion transmembrane transporter activity,monoatomic anion transmembrane transport,sulfate
Fv_160_7.g4430	1027	ID=Fv_160_7.g4430;Description=hypothetical protein FVER14953_11841 [Fusarium verticillioides];Gene=FNAPI_6302;Ontology_term=membrane,oxidoreductase

Fv_160_7.g4431	262	ID=Fv_160_7.g4431;Description=enoyl-CoA hydratase [Fusarium verticillioides 7600];Gene=rpsG;Ontology_term=nucleus,cytoplasm,transaldolase activity,carbohydrate metabolic process,pentose-phosphate shunt, non-oxidative
Fv_160_7.g4432	378	ID=Fv_160_7.g4432;Description=hypothetical protein FVER14953_11843 [Fusarium verticillioides]
Fv_160_7.g4433	639	ID=Fv_160_7.g4433;Description=hypothetical protein FVER53263_20048 [Fusarium verticillioides]
Fv_160_7.g4434	540	ID=Fv_160_7.g4434;Description=hypothetical protein FVER53263_11844 [Fusarium verticillioides];Gene=TY2A-C;Ontology_term=retrotransposon nucleocapsid,cytoplasm,membrane,magnesium ion binding,RNA binding,acetolactate synthase activity,monooxygenase activity,iron ion binding,oxidoreductase activity, acting on paired donors, with incorporation or reduction of molecular oxygen,heme binding,thiamine pyrophosphate binding,flavin adenine dinucleotide binding,isoleucine biosynthetic process,valine biosynthetic process,retrotransposition;Ontology_id=GO:0000943,GO:0005737,GO:0016020,GO:0000287,GO:0003723,GO:0003984,GO:0004497,GO:0005506,GO:0016705,GO:0020037,GO:0030976,GO:0050660,GO:0009097,GO:0009099,GO:0032197;Enzyme_code=EC:2.2.1.6,
Fv_160_7.g4435	395	ID=Fv_160_7.g4435;Description=hypothetical protein FVER53263_20049 [Fusarium
Fv_160_7.g4436	563	ID=Fv_160_7.g4436;Description=hypothetical protein FVEG_11845 [Fusarium verticillioides 7600];Gene=F25303_7984;Ontology_term=nucleus,mitochondrion,DNA-binding transcription factor activity, RNA polymerase II-specific,RNA binding,L-threonine ammonia-lyase activity,zinc ion binding,pyridoxal phosphate binding,regulation of transcription by RNA polymerase II,L-serine catabolic process,threonine catabolic process,isoleucine biosynthetic process;Ontology_id=GO:0005634,GO:0005739,GO:0000981,GO:0003723,GO:0004794,GO:0008270,GO:0030170,GO:0006357,GO:
Fv_160_7.g4437	358	ID=Fv_160_7.g4437;Description=hypothetical protein FVEG_11846 [Fusarium verticillioides 7600];Gene=F25303_7985;Ontology_term=oxidoreductase activity,cellular biosynthetic
Fv_160_7.g4438	382	ID=Fv_160_7.g4438;Description=hypothetical protein FVEG_11847 [Fusarium verticillioides 7600];Gene=FANTH_2680;Ontology_term=membrane,hydrolase
Fv_160_7.g4439	488	ID=Fv_160_7.g4439;Description=hypothetical protein FVEG_11848 [Fusarium verticillioides 7600];Gene=FMAN_06428;Ontology_term=membrane,viral envelope,host cell plasma membrane,virion membrane,tRNA binding,aminoacyl-tRNA editing activity,N,N-dimethylaniline monooxygenase activity,isoleucine-tRNA ligase activity,structural molecule activity,ATP binding,flavin adenine dinucleotide binding,NADP binding,isoleucyl-tRNA aminoacylation,virion attachment to host cell,viral entry into host cell,aminoacyl-tRNA metabolism involved in translational fidelity;Ontology_id=GO:0016020,GO:0019031,GO:0020002,GO:0055036,GO:0000049,GO:0002161,GO:0004499,GO:0004822,GO:0
Fv_160_7.g4440	383	ID=Fv_160_7.g4440;Description=endoglucanase type F [Fusarium denticulatum];Gene=FMAN_06427;Ontology_term=extracellular region,cellulose binding,endo-1,4-beta-xylanase activity,polysaccharide catabolic
Fv_160_7.g4441	319	ID=Fv_160_7.g4441;Description=hypothetical protein FVER53590_25087 [Fusarium verticillioides]
Fv_160_7.g4442	298	ID=Fv_160_7.g4442;Description=hypothetical protein FVEG_17118 [Fusarium verticillioides 7600];Gene=FPANT_1075;Ontology_term=heme binding,dioxygenase

Fv_160_7.g4443	386	ID=Fv_160_7.g4443;Description=hypothetical protein FVER14953_11850 [Fusarium verticillioides];Gene=FDENT_3879;Ontology_term=transferase
Fv_160_7.g4444	1406	ID=Fv_160_7.g4444;Description=hypothetical protein FNAPI_5737 [Fusarium napiforme]
Fv_160_7.g4445	449	ID=Fv_160_7.g4445;Description=hypothetical protein FNAPI_5736 [Fusarium napiforme];Gene=FVER53263_11852;Ontology_term=fungal-type vacuole membrane,mitochondrion,tRNA binding,translation initiation factor activity,GTPase activity,GTP binding,mitochondrial translational initiation;Ontology_id=GO:0000329,GO:0005739,GO:0000049,GO:0003743,GO:0003924,GO:0005525,GO:0070124;Enzyme_code=E
Fv_160_7.g4446	190	ID=Fv_160_7.g4446;Description=hypothetical protein FVEG_17119 [Fusarium verticillioides 7600];Gene=YCL020W;Ontology_term=cytoplasm,RNA binding;Ontology_id=GO:0005737,GO:0003723
Fv_160_7.g4447	186	ID=Fv_160_7.g4447;Description=hypothetical protein FVEG_17120 [Fusarium verticillioides 7600]
Fv_160_7.g4448	566	ID=Fv_160_7.g4448;Description=hypothetical protein FVER53263_11854 [Fusarium verticillioides];Gene=BFJ65_g16544;Ontology_term=copper ion binding,oxidoreductase
Fv_160_7.g4449	168	ID=Fv_160_7.g4449;Description=hypothetical protein FVEG_17121 [Fusarium verticillioides 7600];Gene=FACUT_4818;Ontology_term=acyltransferase activity, transferring groups other than amino-acyl
Fv_160_7.g4450	482	ID=Fv_160_7.g4450;Description=hypothetical protein FVEG_17122 [Fusarium verticillioides 7600];Gene=whiA;Ontology_term=membrane,transmembrane transporter activity,transmembrane
Fv_160_7.g4451	519	ID=Fv_160_7.g4451;Description=hypothetical protein FVER53263_11855 [Fusarium verticillioides];Gene=FNAPI_5731;Ontology_term=membrane,monooxygenase activity,iron ion binding,oxidoreductase activity, acting on paired donors, with incorporation or reduction of molecular oxygen,heme binding;Ontology_id=GO:0016020,GO:0004497,GO:0005506,GO:0016705,GO:0020037;Enzyme_code=EC:1.14;Enzyme_name=Actin
Fv_160_7.g4452	408	ID=Fv_160_7.g4452;Description=hypothetical protein FVEG_11856 [Fusarium verticillioides 7600];Gene=FNAPI_5730;Ontology_term=membrane,monooxygenase activity,iron ion binding,zinc ion binding,oxidoreductase activity, acting on paired donors, with incorporation or reduction of molecular oxygen,heme binding;Ontology_id=GO:0016020,GO:0004497,GO:0005506,GO:0008270,GO:0016705,GO:0020037;Enzyme_code=EC:1.14;Enzyme

Fv_160_7.g4453	518	ID=Fv_160_7.g4453;Description=cytochrome P450 oxidoreductase [Fusarium verticillioides 7600];Gene=FMAN_06416;Ontology_term=growth factor activity,interleukin-2-mediated signaling pathway,oxidoreductase activity, acting on paired donors, with incorporation or reduction of molecular oxygen,interleukin-2 receptor binding,extrinsic apoptotic signaling pathway in absence of ligand,positive regulation of tyrosine phosphorylation of STAT protein,positive regulation of transcription by RNA polymerase II,negative regulation of lymphocyte proliferation,iron ion binding,positive regulation of type II interferon production,positive regulation of interleukin-17 production,positive regulation of plasma cell differentiation,negative regulation of inflammatory response,positive regulation of isotype switching to IgG isotypes,cytokine activity,positive regulation of activated T cell proliferation,monooxygenase activity,heme binding,extracellular space,regulation of T cell homeostatic proliferation,membrane,negative regulation of B cell apoptotic process,positive regulation of B cell proliferation,negative regulation of T-helper 17 cell differentiation,regulation of CD4-positive, alpha-beta T cell
Fv_160_7.g4454	353	ID=Fv_160_7.g4454;Description=hypothetical protein FVER14953_11858 [Fusarium verticillioides]
Fv_160_7.g4455	404	ID=Fv_160_7.g4455;Description=hypothetical protein FVER14953_11859 [Fusarium verticillioides];Gene=FFB14_12532;Ontology_term=endoplasmic reticulum membrane,UDP-N-acetylglucosamine-dolichyl-phosphate N-acetylglucosaminephosphotransferase activity,monooxygenase activity,glycosyltransferase activity,FAD binding,dolichol-linked oligosaccharide biosynthetic process;Ontology_id=GO:0005789,GO:0003975,GO:0004497,GO:0016757,GO:0071949,GO:0006488;Enzyme_code=EC:1,EC:2.4,EC:2
Fv_160_7.g4456	348	ID=Fv_160_7.g4456;Description=hypothetical protein FVER53263_11861 [Fusarium verticillioides];Gene=HZS61_010966;Ontology_term=hydrolase
Fv_160_7.g4457	602	ID=Fv_160_7.g4457;Description=hypothetical protein FVEG_11861 [Fusarium verticillioides 7600];Gene=FRV6_15910;Ontology_term=nucleus,cytoplasm,microtubule,DNA-binding transcription factor activity, RNA polymerase II-specific,GTPase activity,structural constituent of cytoskeleton,GTP binding,zinc ion binding,regulation of transcription by RNA polymerase II,cytoskeleton organization,microtubule-based process;Ontology_id=GO:0005634,GO:0005737,GO:0005874,GO:0000981,GO:0003924,GO:0005200,GO:0005525,GO:0008270,GO:
Fv_160_7.g4458	413	ID=Fv_160_7.g4458;Description=salicylate hydroxylase [Fusarium verticillioides 7600];Gene=FACUT_4826;Ontology_term=monooxygenase activity,FAD binding,biosynthetic
Fv_160_7.g4459	485	ID=Fv_160_7.g4459;Description=hypothetical protein FVER14953_11863 [Fusarium verticillioides];Gene=FACUT_4827;Ontology_term=membrane,transmembrane transporter activity,transmembrane
Fv_160_7.g4460	336	ID=Fv_160_7.g4460;Description=hypothetical protein FVER53263_11864 [Fusarium verticillioides];Gene=FVER53263_11864;Ontology_term=nucleus,DNA binding,zinc ion binding,indole-3-acetonitrile nitrilase activity,DNA-templated
Fv_160_7.g4461	618	ID=Fv_160_7.g4461;Description=pathway-specific regulatory nit-4 [Fusarium pseudoanthophilum];Gene=nirA-1;Ontology_term=nucleus,DNA binding,zinc ion binding,nitrilase activity,DNA-binding transcription factor activity, RNA polymerase II-specific,regulation of transcription by RNA polymerase

Fv_160_7.g4462	495	ID=Fv_160_7.g4462;Description=hypothetical protein FVEG_11866 [Fusarium verticillioides 7600];Gene=FOBC_15990;Ontology_term=nucleus,DNA-binding transcription factor activity, RNA polymerase II-specific,zinc ion
Fv_160_7.g4463	346	ID=Fv_160_7.g4463;Description=hypothetical protein FVER53590_11867 [Fusarium verticillioides];Gene=HZS61_010972;Ontology_term=nucleus,DNA-binding transcription factor activity, RNA polymerase II-specific,hydrolase activity, hydrolyzing O-glycosyl compounds,zinc ion binding,polysaccharide catabolic process,regulation of
Fv_160_7.g4464	335	ID=Fv_160_7.g4464;Description=methyltransferase [Fusarium mundagurra];Gene=FMAN_06405;Ontology_term=S-adenosylmethionine-dependent methyltransferase activity,hydrolase activity, acting on ester bonds,methylation;Ontology_id=GO:0008757,GO:0016788,GO:0032259;Enzyme_code=EC:3.1,EC:2.1.1;Enzyme_name=Acting on
Fv_160_7.g4465	292	ID=Fv_160_7.g4465;Description=hypothetical protein FVER53263_11869 [Fusarium verticillioides];Gene=FVER53590_11869;Ontology_term=S-adenosylmethionine-dependent methyltransferase activity,hydrolase activity, acting on ester
Fv_160_7.g4466	331	ID=Fv_160_7.g4466;Description=heme-binding peroxidase [Fusarium verticillioides 7600];Gene=FPANT_11267;Ontology_term=aspartate-semialdehyde dehydrogenase activity,peroxidase activity,heme binding,metal ion binding,protein dimerization activity,NADP binding,NAD binding,methionine biosynthetic process,threonine biosynthetic process,lysine biosynthetic process via diaminopimelate,isoleucine biosynthetic process,cellular response to oxidative stress,cellular oxidant
Fv_160_7.g4467	534	ID=Fv_160_7.g4467;Description=hypothetical protein FCOIX_2047 [Fusarium coicis];Gene=FDENT_3900;Ontology_term=membrane,metal ion transmembrane transporter activity,magnesium ion
Fv_160_7.g4468	694	ID=Fv_160_7.g4468;Description=hypothetical protein FVEG_11872 [Fusarium verticillioides 7600]
Fv_160_7.g4469	404	ID=Fv_160_7.g4469;Description=hypothetical protein J7337_007425 [Fusarium musae];Gene=FVER53263_11873;Ontology_term=oxidoreductase
Fv_160_7.g4470	468	ID=Fv_160_7.g4470;Description=hypothetical protein FVEG_11874 [Fusarium verticillioides 7600];Gene=FOQG_13350;Ontology_term=oxidoreductase activity,phosphatase
Fv_160_7.g4471	348	ID=Fv_160_7.g4471;Description=hypothetical protein FVEG_11875 [Fusarium verticillioides
Fv_160_7.g4472	462	ID=Fv_160_7.g4472;Description=hypothetical protein FVEG_11876 [Fusarium verticillioides 7600];Gene=F25303_8017;Ontology_term=membrane,anthranilate phosphoribosyltransferase activity,transmembrane transporter activity,tryptophan biosynthetic process,transmembrane
Fv_160_7.g4473	349	ID=Fv_160_7.g4473;Description=hypothetical protein FVEG_11877 [Fusarium verticillioides
Fv_160_7.g4474	738	ID=Fv_160_7.g4474;Description=hypothetical protein FVEG_11878 [Fusarium verticillioides 7600];Gene=F25303_8019;Ontology_term=plasma membrane,arsenite transmembrane transporter activity,arsenite
Fv_160_7.g4475	1323	ID=Fv_160_7.g4475;Description=serine/threonine protein kinase [Fusarium verticillioides 7600];Gene=FACUT_7546;Ontology_term=protein serine/threonine kinase activity,ATP binding,phosphorylation,intracellular signal transduction;Ontology_id=GO:0004674,GO:0005524,GO:0016310,GO:0035556;Enzyme_code=EC:2.7.11.1;Enzyme_name=non-

Fv_160_7.g4476	548	ID=Fv_160_7.g4476;Description=hypothetical protein FVEG_11881 [Fusarium verticillioides 7600];Gene=FOPG_14463;Ontology_term=protein kinase activity,ATP
Fv_160_7.g4477	408	ID=Fv_160_7.g4477;Description=hypothetical protein FVEG_11882 [Fusarium verticillioides 7600];Gene=FACUT_7544;Ontology_term=aspartic-type endopeptidase
Fv_160_7.g4478	458	ID=Fv_160_7.g4478;Description=hypothetical protein FVEG_11883 [Fusarium verticillioides 7600];Gene=FMUND_7239;Ontology_term=nucleus,DNA-binding transcription factor activity, RNA polymerase II-specific,zinc ion
Fv_160_7.g4479	564	ID=Fv_160_7.g4479;Description=hypothetical protein FVER53263_11884 [Fusarium verticillioides]
Fv_160_7.g4480	262	ID=Fv_160_7.g4480;Description=hypothetical protein FVEG_11885 [Fusarium verticillioides 7600]
Fv_160_7.g4481	548	ID=Fv_160_7.g4481;Description=hypothetical protein FVEG_11886 [Fusarium verticillioides 7600];Gene=BFJ69_g7045;Ontology_term=membrane,imidazoleglycerol-phosphate dehydratase activity,transmembrane transporter activity,metal ion binding,histidine biosynthetic process,transmembrane
Fv_160_7.g4482	557	ID=Fv_160_7.g4482;Description=hypothetical protein FVEG_11887 [Fusarium verticillioides 7600];Gene=FOXYS1_221;Ontology_term=nucleus,DNA-binding transcription factor activity, RNA polymerase II-specific,zinc ion
Fv_160_7.g4483	360	ID=Fv_160_7.g4483;Description=hypothetical protein FVEG_11888 [Fusarium verticillioides 7600];Gene=FOYG_00846;Ontology_term=nucleus,DNA-binding transcription factor activity, RNA polymerase II-specific,zinc ion
Fv_160_7.g4484	342	ID=Fv_160_7.g4484;Description=hypothetical protein FVER53263_20594 [Fusarium verticillioides];Gene=ephA-1;Ontology_term=nitrilase activity,nitrogen compound metabolic
Fv_160_7.g4485	308	ID=Fv_160_7.g4485;Description=hypothetical protein FVEG_17125 [Fusarium verticillioides 7600];Gene=FPRO05_08240;Ontology_term=nucleosome,nucleus,nitrilase activity,DNA binding,structural constituent of chromatin,protein heterodimerization activity,nitrogen compound metabolic
Fv_160_7.g4486	499	ID=Fv_160_7.g4486;Description=hypothetical protein FVER53263_11889 [Fusarium verticillioides];Gene=FOIG_16353;Ontology_term=feruloyl esterase activity,metal ion
Fv_160_7.g4487	309	ID=Fv_160_7.g4487;Description=hypothetical protein FVEG_11890 [Fusarium verticillioides 7600]
Fv_160_7.g4488	435	ID=Fv_160_7.g4488;Description=hypothetical protein FVER14953_11891 [Fusarium verticillioides];Gene=FMEXI_8352;Ontology_term=retrotransposon nucleocapsid,cytoplasm,RNA binding,acyltransferase activity,hydrolase activity,retrotransposition;Ontology_id=GO:0000943,GO:0005737,GO:0003723,GO:0016746,GO:0016787,GO:0032197;Enzyme_cod
Fv_160_7.g4489	568	ID=Fv_160_7.g4489;Description=hypothetical protein FVEG_11892 [Fusarium verticillioides 7600];Gene=BFJ63_vAg7397;Ontology_term=nucleus,DNA-binding transcription factor activity, RNA polymerase II-specific,zinc ion
Fv_160_7.g4490	385	ID=Fv_160_7.g4490;Description=hypothetical protein FVEG_11894 [Fusarium verticillioides 7600];Gene=ZRT1;Ontology_term=membrane,zinc ion transmembrane transporter activity,zinc ion transmembrane
Fv_160_7.g4491	345	ID=Fv_160_7.g4491;Description=alcohol dehydrogenase [Fusarium verticillioides 7600];Gene=FMUND_14538;Ontology_term=zinc ion binding,oxidoreductase activity;Ontology_id=GO:0008270,GO:0016491;Enzyme_code=EC:1;Enzyme_name=Oxidoreductases

Fv_160_7.g4492	666	ID=Fv_160_7.g4492;Description=hypothetical protein FVEG_11895 [Fusarium verticillioides 7600]
Fv_160_7.g4493	197	ID=Fv_160_7.g4493;Description=hypothetical protein FVEG_11896 [Fusarium verticillioides 7600];Gene=HEM12;Ontology_term=uroporphyrinogen decarboxylase activity,protoporphyrinogen IX biosynthetic
Fv_160_7.g4494	469	ID=Fv_160_7.g4494;Description=hypothetical protein FVEG_11897 [Fusarium verticillioides 7600];Gene=FDENT_503;Ontology_term=transaminase activity,pyridoxal phosphate
Fv_160_7.g4495	455	ID=Fv_160_7.g4495;Description=hypothetical protein FVER14953_11899 [Fusarium verticillioides];Gene=FDENT_504;Ontology_term=membrane,transmembrane transporter activity,transmembrane
Fv_160_7.g4496	246	ID=Fv_160_7.g4496;Description=hypothetical protein FVEG_11900 [Fusarium verticillioides 7600];Gene=FVEG_11900;Ontology_term=phosphatase
Fv_160_7.g4497	604	ID=Fv_160_7.g4497;Description=hypothetical protein FVEG_11901 [Fusarium verticillioides 7600];Gene=FVER53590_11901;Ontology_term=oxidoreductase activity, acting on CH-OH group of donors,flavin adenine
Fv_160_7.g4498	778	ID=Fv_160_7.g4498;Description=hypothetical protein FVER14953_11902 [Fusarium verticillioides];Gene=FVEG_11902;Ontology_term=carbohydrate metabolic process;Ontology_id=GO:0005975
Fv_160_7.g4499	75	ID=Fv_160_7.g4499;Description=hypothetical protein FVEG_17126 [Fusarium verticillioides 7600];Gene=1033
Fv_160_7.g4500	272	ID=Fv_160_7.g4500;Description=hypothetical protein FVER53263_11903 [Fusarium verticillioides]
Fv_160_7.g4501	311	ID=Fv_160_7.g4501;Description=hypothetical protein FVEG_11904 [Fusarium verticillioides 7600]
Fv_160_7.g4502	537	ID=Fv_160_7.g4502;Description=hypothetical protein FVER53590_11906 [Fusarium
Fv_160_7.g4503	332	ID=Fv_160_7.g4503;Description=hypothetical protein FVEG_11906 [Fusarium verticillioides 7600]
Fv_160_7.g4504	567	ID=Fv_160_7.g4504;Description=hypothetical protein FVER53590_11907 [Fusarium verticillioides];Gene=HHT2;Ontology_term=nucleosome,nucleus,DNA binding,oxidoreductase activity,structural constituent of chromatin,protein heterodimerization activity,FAD
Fv_160_7.g4505	361	ID=Fv_160_7.g4505;Description=hypothetical protein FVEG_11908 [Fusarium verticillioides
Fv_160_7.g4506	95	ID=Fv_160_7.g4506;Description=kinetochore complex Sim4 subunit Fta4 [Fusarium globosum];Gene=Forpe1208_v004359;Ontology_term=nucleosome,nucleus,membrane,DNA binding,structural constituent of
Fv_160_7.g4507	456	ID=Fv_160_7.g4507;Description=hypothetical protein FVER14953_11910 [Fusarium verticillioides];Gene=FPRO05_08261;Ontology_term=membrane,transmembrane transporter activity,transmembrane
Fv_160_7.g4508	205	ID=Fv_160_7.g4508;Description=hypothetical protein FVER14953_11911 [Fusarium verticillioides];Gene=1022
Fv_160_7.g4509	738	ID=Fv_160_7.g4509;Description=hypothetical protein FVER14953_11912 [Fusarium verticillioides];Gene=FVEG_11912;Ontology_term=metalloendopeptidase activity,2,3-bisphosphoglycerate-dependent phosphoglycerate mutase activity,metal ion binding,glycolytic process,proteolysis;Ontology_id=GO:0004222,GO:0046538,GO:0046872,GO:0006096,GO:0006508;Enzyme_code=EC:3.4.24,EC:5.4.
Fv_160_7.g4510	495	ID=Fv_160_7.g4510;Description=hypothetical protein FVEG_11913 [Fusarium verticillioides 7600]

Fv_160_7.g4511	820	ID=Fv_160_7.g4511;Description=hypothetical protein FVEG_11914 [Fusarium verticillioides 7600];Gene=BFJ69_g14641;Ontology_term=helicase activity,zinc ion binding,transferase
Fv_160_7.g4512	345	ID=Fv_160_7.g4512;Description=hypothetical protein FVER14953_11915 [Fusarium verticillioides]
Fv_160_7.g4513	315	ID=Fv_160_7.g4513;Description=hypothetical protein FVEG_11916 [Fusarium verticillioides 7600];Gene=FACUT_7508;Ontology_term=DNA-binding transcription factor activity,regulation of DNA-templated
Fv_160_7.g4514	403	ID=Fv_160_7.g4514;Description=hypothetical protein FVER53263_11917 [Fusarium verticillioides];Gene=leuC
Fv_160_7.g4515	186	ID=Fv_160_7.g4515;Description=hypothetical protein FVER14953_21177 [Fusarium verticillioides];Gene=TPS1;Ontology_term=chromatin,nucleus,cytosol,cAMP-dependent protein kinase complex,alpha,alpha-trehalose-phosphate synthase (UDP-forming) activity,cAMP-dependent protein kinase activity,ATP binding,protein serine kinase activity,invasive growth in response to glucose limitation,trehalose biosynthetic process,Ras protein signal transduction,negative regulation of cytoplasmic mRNA processing body assembly,protein kinase A signaling,phosphorylation,negative regulation of cytoplasmic
Fv_160_7.g4516	594	ID=Fv_160_7.g4516;Description=hypothetical protein FVEG_11918 [Fusarium verticillioides 7600];Gene=FOC1_g10005350;Ontology_term=membrane,transmembrane transporter activity,carbohydrate
Fv_160_7.g4517	365	ID=Fv_160_7.g4517;Description=hypothetical protein FVER14953_11919 [Fusarium verticillioides];Gene=FOYG_00879;Ontology_term=carbohydrate metabolic process;Ontology_id=GO:0005975
Fv_160_7.g4518	309	ID=Fv_160_7.g4518;Description=hypothetical protein FVEG_17130 [Fusarium verticillioides 7600];Gene=FVER53590_25077;Ontology_term=oxidoreductase activity,protein dimerization
Fv_160_7.g4519	427	ID=Fv_160_7.g4519;Description=hypothetical protein FVER53263_11921 [Fusarium verticillioides]
Fv_160_7.g4520	377	ID=Fv_160_7.g4520;Description=hypothetical protein FVEG_11922 [Fusarium verticillioides 7600];Gene=FOXG_03958;Ontology_term=S-adenosylmethionine-dependent methyltransferase
Fv_160_7.g4521	279	ID=Fv_160_7.g4521;Description=hypothetical protein FVER14953_11923 [Fusarium verticillioides];Gene=FTJAE_4031;Ontology_term=hydrolase
Fv_160_7.g4522	487	ID=Fv_160_7.g4522;Description=hypothetical protein FVEG_11924 [Fusarium verticillioides 7600];Gene=GCD2;Ontology_term=organonitrogen compound biosynthetic process;Ontology_id=GO:1901566
Fv_160_7.g4523	300	ID=Fv_160_7.g4523;Description=hypothetical protein FVEG_17131 [Fusarium verticillioides 7600];Gene=FTJAE_4033;Ontology_term=oxidoreductase
Fv_160_7.g4524	444	ID=Fv_160_7.g4524;Description=hypothetical protein FVER53263_11926 [Fusarium verticillioides];Gene=FVER53590_11926;Ontology_term=eukaryotic translation initiation factor 2B complex,guanyl-nucleotide exchange factor complex,translation initiation factor activity,guanyl-nucleotide exchange factor activity,protein binding,oxidoreductase activity,pyridoxal phosphate binding,cytoplasmic translational initiation,regulation of translational initiation,transsulfuration,negative regulation of cellular response to amino acid
Fv_160_7.g4525	321	ID=Fv_160_7.g4525;Description=hypothetical protein FVER53263_11927 [Fusarium verticillioides]

Fv_160_7.g4526	526	ID=Fv_160_7.g4526;Description=hypothetical protein FVEG_11928 [Fusarium verticillioides 7600];Gene=FMUND_15601;Ontology_term=fungal-type vacuole lumen,fungal-type vacuole membrane,serine-type endopeptidase inhibitor activity,iron ion binding,phospholipid binding,oxidoreductase activity, acting on paired donors, with incorporation or reduction of molecular oxygen, reduced flavin or flavoprotein as one donor, and incorporation of one atom of oxygen,heme binding,regulation of proteolysis,regulation of Ras protein signal
Fv_160_7.g4527	566	ID=Fv_160_7.g4527;Description=hypothetical protein FVER14953_21190 [Fusarium verticillioides];Gene=FVER53590_25076;Ontology_term=membrane,transmembrane transporter activity,transmembrane
Fv_160_7.g4528	357	ID=Fv_160_7.g4528;Description=hypothetical protein FVER14953_11931 [Fusarium verticillioides];Gene=FTJAE_11851;Ontology_term=nucleus,nucleotide binding,DNA binding,oxidoreductase activity, acting on NAD(P)H,DNA-templated transcription
Fv_160_7.g4529	4075	ID=Fv_160_7.g4529;Description=hypothetical protein FVEG_11932 [Fusarium verticillioides 7600];Gene=FACUT_7704;Ontology_term=3-oxoacyl-[acyl-carrier-protein] synthase activity,S-adenosylmethionine-dependent methyltransferase activity,oxidoreductase activity,phosphopantetheine binding,fatty acid biosynthetic process,heterocycle biosynthetic process,methylation,amide biosynthetic process,secondary metabolite biosynthetic process,organic cyclic compound biosynthetic
Fv_160_7.g4530	546	ID=Fv_160_7.g4530;Description=beta-fructofuranosidase [Fusarium verticillioides 7600];Gene=FTJAE_11853;Ontology_term=hydrolase activity, hydrolyzing O-glycosyl compounds,carbohydrate metabolic
Fv_160_7.g4531	542	ID=Fv_160_7.g4531;Description=hypothetical protein FVEG_17132 [Fusarium verticillioides 7600];Gene=FOQG_13054;Ontology_term=membrane,transmembrane transporter activity,transmembrane
Fv_160_7.g4532	398	ID=Fv_160_7.g4532;Description=hypothetical protein FVER53263_11934 [Fusarium verticillioides];Gene=FCIRC_12996;Ontology_term=oxidoreductase
Fv_160_7.g4533	453	ID=Fv_160_7.g4533;Description=hypothetical protein FVEG_17133 [Fusarium verticillioides 7600];Gene=FVER53590_25074;Ontology_term=transaminase activity,pyridoxal phosphate binding,biosynthetic
Fv_160_7.g4534	919	ID=Fv_160_7.g4534;Description=hypothetical protein FVER53590_11936 [Fusarium verticillioides]
Fv_160_7.g4535	231	ID=Fv_160_7.g4535;Description=aspartate racemase [Fusarium verticillioides 7600];Gene=FDENT_547;Ontology_term=nucleus,chromosome,double-stranded telomeric DNA binding,protein binding,amino-acid racemase activity,cell cycle,telomere maintenance via telomere
Fv_160_7.g4536	447	ID=Fv_160_7.g4536;Description=hypothetical protein FVER14953_11938 [Fusarium verticillioides]
Fv_160_7.g4537	430	ID=Fv_160_7.g4537;Description=hypothetical protein FVEG_11939 [Fusarium verticillioides 7600];Gene=metK;Ontology_term=nucleus,cytoplasm,transaldolase activity,carbohydrate metabolic process,pentose-phosphate shunt, non-oxidative

Fv_160_7.g4538	572	ID=Fv_160_7.g4538;Description=hypothetical protein FVER53590_11940 [Fusarium verticillioides];Gene=FVER53263_11940;Ontology_term=N,N-dimethylaniline monooxygenase activity,flavin adenine dinucleotide binding,NADP binding;Ontology_id=GO:0004499,GO:0050660,GO:0050661;Enzyme_code=EC:1.14.13.8;Enzyme_name=flavin-
Fv_160_7.g4539	389	ID=Fv_160_7.g4539;Description=hypothetical protein FVER14953_11941 [Fusarium verticillioides];Gene=FTJAE_11862;Ontology_term=oligosaccharyltransferase complex;Ontology_id=GO:0008250
Fv_160_7.g4540	342	ID=Fv_160_7.g4540;Description=carbon-nitrogen hydrolase [Fusarium oxysporum Fo47];Gene=BFJ68_g2125;Ontology_term=nitrilase activity,nitrogen compound metabolic
Fv_160_7.g4541	423	ID=Fv_160_7.g4541;Description=hypothetical protein FVEG_17135 [Fusarium verticillioides 7600];Gene=FTJAE_11864;Ontology_term=transferase activity, transferring alkyl or aryl (other than methyl) groups,alkaloid metabolic process;Ontology_id=GO:0016765,GO:0009820;Enzyme_code=EC:2.5.1;Enzyme_name=Transferring alkyl or aryl groups,
Fv_160_7.g4542	283	ID=Fv_160_7.g4542;Description=hypothetical protein FVEG_17136 [Fusarium verticillioides 7600]
Fv_160_7.g4543	326	ID=Fv_160_7.g4543;Description=hypothetical protein FVER14953_11943 [Fusarium verticillioides]
Fv_160_7.g4544	286	ID=Fv_160_7.g4544;Description=endo-1,3(4)-beta-glucanase [Fusarium verticillioides 7600];Gene=FNAPI_3641;Ontology_term=hydrolase activity, hydrolyzing O-glycosyl compounds,carbohydrate metabolic
Fv_160_7.g4545	332	ID=Fv_160_7.g4545;Description=hypothetical protein FVER53590_11945 [Fusarium
Fv_160_7.g4546	456	ID=Fv_160_7.g4546;Description=hypothetical protein FVER14953_11946 [Fusarium verticillioides];Gene=FDENT_558;Ontology_term=membrane,transmembrane transporter activity,transmembrane
Fv_160_7.g4547	190	ID=Fv_160_7.g4547;Description=hypothetical protein FVEG_11947 [Fusarium verticillioides 7600];Gene=FANTH_5205;Ontology_term=membrane,transmembrane transporter activity,transmembrane
Fv_160_7.g4548	601	ID=Fv_160_7.g4548;Description=L-ascorbate oxidase [Fusarium verticillioides 7600];Gene=FTJAE_11873;Ontology_term=copper ion binding,oxidoreductase activity;Ontology_id=GO:0005507,GO:0016491;Enzyme_code=EC:1;Enzyme_name=Oxidoreductases
Fv_160_7.g4549	305	ID=Fv_160_7.g4549;Description=hypothetical protein FVEG_11949 [Fusarium verticillioides 7600];Gene=ZRT1;Ontology_term=nucleus,DNA binding,zinc ion binding,membrane,zinc ion transmembrane transporter activity,zinc ion transmembrane transport,DNA-binding transcription factor activity, RNA polymerase II-specific,regulation of transcription by
Fv_160_7.g4550	529	ID=Fv_160_7.g4550;Description=hypothetical protein FVEG_17138 [Fusarium verticillioides 7600];Gene=FOIG_14574;Ontology_term=nucleus,DNA binding,zinc ion binding,DNA-binding transcription factor activity, RNA
Fv_160_7.g4551	656	ID=Fv_160_7.g4551;Description=hypothetical protein FVER14953_11950 [Fusarium verticillioides]
Fv_160_7.g4552	573	ID=Fv_160_7.g4552;Description=hypothetical protein FVER53590_11951 [Fusarium verticillioides];Gene=FVER53263_11951;Ontology_term=extracellular space,membrane,growth hormone receptor binding,hormone activity,transmembrane transporter activity,metal ion binding,signal transduction,insulin secretion,positive regulation of superoxide

Fv_160_7.g4553	1436	ID=Fv_160_7.g4553;Description=hypothetical protein FVER53590_11952 [Fusarium verticillioides];Gene=FVER53263_11952;Ontology_term=plasma membrane,water channel activity,glycerol channel activity,hydrolase activity, acting on ester bonds,conjugation with cellular fusion,glycerol metabolic process,water transport,arsenite transport,glycerol transmembrane transport;Ontology_id=GO:0005886,GO:0015250,GO:0015254,GO:0016788,GO:0000747,GO:0006071,GO:0006833,GO:0015700,GO
Fv_160_7.g4554	293	ID=Fv_160_7.g4554;Description=hypothetical protein FVER14953_11953 [Fusarium verticillioides]
Fv_160_7.g4555	496	ID=Fv_160_7.g4555;Description=hypothetical protein FVER53590_11954 [Fusarium verticillioides];Gene=F25303_589;Ontology_term=peroxidase activity,binding,cellular process,response to
Fv_160_7.g4556	503	ID=Fv_160_7.g4556;Description=catalase [Fusarium verticillioides 7600];Gene=FCIRC_7756;Ontology_term=catalase activity,heme binding,metal ion binding,response to oxidative stress,alkaloid metabolic process,hydrogen peroxide catabolic process,cellular oxidant
Fv_160_7.g4557	560	ID=Fv_160_7.g4557;Description=hypothetical protein FVER14953_11956 [Fusarium verticillioides];Gene=FVER53590_11956;Ontology_term=oxidoreductase activity,molybdenum ion
Fv_160_7.g4558	310	ID=Fv_160_7.g4558;Description=hypothetical protein FVEG_11957 [Fusarium verticillioides 7600];Gene=FVER53590_11957;Ontology_term=S-adenosylmethionine-dependent methyltransferase
Fv_160_7.g4559	438	ID=Fv_160_7.g4559;Description=hypothetical protein FVER53263_11958 [Fusarium verticillioides];Gene=FOIG_14582;Ontology_term=membrane,malate transmembrane transporter activity,malate transmembrane
Fv_160_7.g4560	102	ID=Fv_160_7.g4560;Description=hypothetical protein FVER53263_11959 [Fusarium verticillioides]
Fv_160_7.g4561	392	ID=Fv_160_7.g4561;Description=hypothetical protein FVEG_17139 [Fusarium verticillioides
Fv_160_7.g4562	175	ID=Fv_160_7.g4562;Description=hypothetical protein FVEG_11961 [Fusarium verticillioides 7600]
Fv_160_7.g4563	254	ID=Fv_160_7.g4563;Description=hypothetical protein FVER53263_11962 [Fusarium verticillioides];Gene=selD;Ontology_term=cellular anatomical entity,fructose-bisphosphate aldolase activity,zinc ion binding,oxidoreductase activity,glycolytic process,cellular biosynthetic process;Ontology_id=GO:0110165,GO:0004332,GO:0008270,GO:0016491,GO:0006096,GO:0044249;Enzyme_code=EC:1,EC:4.1.2.13
Fv_160_7.g4564	321	ID=Fv_160_7.g4564;Description=hypothetical protein FVEG_11963 [Fusarium verticillioides 7600]
Fv_160_7.g4565	69	ID=Fv_160_7.g4565;Description=uncharacterized protein FFUJ_14658 [Fusarium fujikuroi IMI 58289]
Fv_160_7.g4566	308	ID=Fv_160_7.g4566;Description=hypothetical protein FVEG_11964 [Fusarium verticillioides 7600];Gene=FANTH_5187;Ontology_term=hydrolase activity, hydrolyzing O-glycosyl compounds,carbohydrate metabolic
Fv_160_7.g4567	527	ID=Fv_160_7.g4567;Description=hypothetical protein FVEG_11965 [Fusarium verticillioides 7600];Gene=FOC4_g10003645;Ontology_term=membrane,transmembrane transporter activity,amino acid
Fv_160_7.g4568	1376	ID=Fv_160_7.g4568;Description=hypothetical protein FVEG_11966 [Fusarium verticillioides 7600]
Fv_160_7.g4569	274	ID=Fv_160_7.g4569;Description=hypothetical protein FVER14953_11967 [Fusarium verticillioides];Gene=FCIRC_7745;Ontology_term=oxidoreductase activity,cellular biosynthetic

Fv_160_7.g4570	549	ID=Fv_160_7.g4570;Description=hypothetical protein FVER53590_11968 [Fusarium verticillioides];Gene=FVEG_11968;Ontology_term=nucleus,DNA-binding transcription factor activity, RNA polymerase II-specific,zinc
Fv_160_7.g4571	313	ID=Fv_160_7.g4571;Description=3-hydroxybutyrate dehydrogenase [Fusarium verticillioides 7600];Gene=C2S_7938;Ontology_term=beta-fructofuranosidase activity,oxidoreductase activity,carbohydrate metabolic process,cellular biosynthetic
Fv_160_7.g4572	535	ID=Fv_160_7.g4572;Description=hypothetical protein FVER53590_25070 [Fusarium verticillioides];Gene=FACUT_7739;Ontology_term=membrane,N,N-dimethylaniline monooxygenase activity,flavin adenine dinucleotide binding,NADP
Fv_160_7.g4573	702	ID=Fv_160_7.g4573;Description=primary-amine oxidase [Fusarium tjaetaba];Gene=AMO-1;Ontology_term=copper ion binding,primary amine oxidase activity,quinone binding,amine metabolic
Fv_160_7.g4574	555	ID=Fv_160_7.g4574;Description=hypothetical protein FVEG_11972 [Fusarium verticillioides 7600];Gene=ERG9;Ontology_term=endoplasmic reticulum,membrane,beta-fructofuranosidase activity,transmembrane transporter activity,ergosterol biosynthetic process,isoprenoid biosynthetic process,farnesyl diphosphate metabolic process,transmembrane transport;Ontology_id=GO:0005783,GO:0016020,GO:0004564,GO:0022857,GO:0006696,GO:0008299,GO:0045338,GO:0055085;Enzyme_code=EC:3.2.1.26;Enzyme_name=beta-fructofuranosidase
Fv_160_7.g4575	501	ID=Fv_160_7.g4575;Description=aldehyde dehydrogenase (NAD+) [Fusarium verticillioides 7600];Gene=FVER53263_11973;Ontology_term=oxidoreductase activity, acting on the aldehyde or oxo group of donors, NAD or
Fv_160_7.g4576	361	ID=Fv_160_7.g4576;Description=hypothetical protein FVER14953_11974 [Fusarium verticillioides];Gene=FTJAE_1260;Ontology_term=hydrolase
Fv_160_7.g4577	545	ID=Fv_160_7.g4577;Description=hypothetical protein FVER14953_21184 [Fusarium verticillioides]
Fv_160_7.g4578	98	ID=Fv_160_7.g4578;Description=hypothetical protein FVEG_17141 [Fusarium verticillioides
Fv_160_7.g4579	304	ID=Fv_160_7.g4579;Description=hypothetical protein FVER14953_11976 [Fusarium verticillioides]
Fv_160_7.g4580	335	ID=Fv_160_7.g4580;Description=hypothetical protein FVER14953_11977 [Fusarium
Fv_160_7.g4581	294	ID=Fv_160_7.g4581;Description=hypothetical protein FVER14953_11978 [Fusarium verticillioides]
Fv_160_7.g4582	492	ID=Fv_160_7.g4582;Description=hypothetical protein FVEG_11979 [Fusarium verticillioides 7600];Gene=FVER53590_11979;Ontology_term=membrane,transmembrane transporter activity,transmembrane
Fv_160_7.g4583	346	ID=Fv_160_7.g4583;Description=hypothetical protein FVER14953_11980 [Fusarium verticillioides];Gene=FPCIR_55;Ontology_term=oxidoreductase activity, acting on the CH-OH group of donors, NAD or NADP as
Fv_160_7.g4584	246	ID=Fv_160_7.g4584;Description=hypothetical protein FVER14953_11981 [Fusarium verticillioides];Gene=FDENT_3030;Ontology_term=membrane,mating-type factor pheromone receptor activity,transferase activity,G protein-coupled receptor signaling
Fv_160_7.g4585	698	ID=Fv_160_7.g4585;Description=acyl-CoA oxidase [Fusarium verticillioides 7600];Gene=FCIRC_7811;Ontology_term=peroxisome,acyl-CoA oxidase activity,FAD binding,fatty acid beta-oxidation using acyl-CoA

Fv_160_7.g4586	378	ID=Fv_160_7.g4586;Description=hypothetical protein FVER53590_11983 [Fusarium verticillioides];Gene=FPCIR_52;Ontology_term=membrane,phosphoric diester hydrolase activity,lipid metabolic
Fv_160_7.g4587	443	ID=Fv_160_7.g4587;Description=hypothetical protein FVEG_11985 [Fusarium verticillioides 7600];Gene=FVER53263_11985;Ontology_term=membrane,transmembrane transporter activity,transmembrane
Fv_160_7.g4588	325	ID=Fv_160_7.g4588;Description=hypothetical protein FVEG_11986 [Fusarium verticillioides 7600]
Fv_160_7.g4589	249	ID=Fv_160_7.g4589;Description=hypothetical protein FVEG_11987 [Fusarium verticillioides 7600]
Fv_160_7.g4590	487	ID=Fv_160_7.g4590;Description=hypothetical protein FVEG_11988 [Fusarium verticillioides 7600];Gene=938;Ontology_term=ATP binding,ATP-dependent protein folding chaperone,protein folding;Ontology_id=GO:0005524,GO:0140662,GO:0006457
Fv_160_7.g4591	535	ID=Fv_160_7.g4591;Description=hypothetical protein J7337_007307 [Fusarium musae];Gene=FocTR4_00003424;Ontology_term=membrane,transmembrane transporter activity,transmembrane
Fv_160_7.g4592	181	ID=Fv_160_7.g4592;Description=hypothetical protein FVEG_11990 [Fusarium verticillioides 7600];Gene=FTJAE_1282;Ontology_term=glucosamine 6-phosphate N-acetyltransferase activity,UDP-N-acetylglucosamine
Fv_160_7.g4593	543	ID=Fv_160_7.g4593;Description=hypothetical protein FVER53590_11991 [Fusarium verticillioides];Gene=HYE67_002828;Ontology_term=nucleotide binding,acyltransferase activity, transferring groups other than
Fv_160_7.g4594	1074	ID=Fv_160_7.g4594;Description=hypothetical protein FPCIR_2317 [Fusarium pseudocircinatum]
Fv_160_7.g4595	266	ID=Fv_160_7.g4595;Description=hypothetical protein FVEG_17143 [Fusarium verticillioides
Fv_160_7.g4596	316	ID=Fv_160_7.g4596;Description=hypothetical protein FVEG_11994 [Fusarium verticillioides 7600];Gene=FPCIR_2315;Ontology_term=arylformamidase activity,tryptophan catabolic process to
Fv_160_7.g4597	681	ID=Fv_160_7.g4597;Description=hypothetical protein FVEG_11995 [Fusarium verticillioides 7600];Gene=FPRO05_08353;Ontology_term=ATP hydrolysis activity,zinc ion binding,fungal-type vacuole membrane,serine-type endopeptidase activity,7S RNA binding,endoplasmic reticulum,aminopeptidase activity,dipeptidyl-peptidase activity,plasma membrane,DNA-binding transcription factor activity, RNA polymerase II-specific,signal recognition particle, endoplasmic reticulum targeting,regulation of transcription by RNA polymerase II,GTP binding,nucleus,DNA binding,SRP-dependent cotranslational protein targeting to membrane,GTPase activity,protein processing;Ontology_id=GO:0016887,GO:0008270,GO:0000329,GO:0004252,GO:0008312,GO:0005783,GO:0004177,GO:0008239,GO:0005886,GO:0000981,GO:0005786,GO:0006357,GO:0005525,GO:0005634,GO:0003677,GO:0006614,GO:0003924,GO:0016485;E
Fv_160_7.g4598	258	ID=Fv_160_7.g4598;Description=hypothetical protein FVER53263_11996 [Fusarium verticillioides]
Fv_160_7.g4599	302	ID=Fv_160_7.g4599;Description=hypothetical protein FVEG_11997 [Fusarium verticillioides 7600]
Fv_160_7.g4600	371	ID=Fv_160_7.g4600;Description=hypothetical protein FVER53590_11998 [Fusarium verticillioides]
Fv_160_7.g4601	359	ID=Fv_160_7.g4601;Description=hypothetical protein FVER14953_11999 [Fusarium verticillioides];Gene=FNAPI_2795;Ontology_term=oxidoreductase
Fv_160_7.g4602	561	ID=Fv_160_7.g4602;Description=hypothetical protein FVER53590_12000 [Fusarium verticillioides]

Fv_160_7.g4603	692	ID=Fv_160_7.g4603;Description=hypothetical protein FVEG_12002 [Fusarium verticillioides 7600];Gene=FGADI_6243;Ontology_term=extracellular region,membrane,chitinase activity,chitin binding,polysaccharide catabolic process,chitin catabolic
Fv_160_7.g4604	472	ID=Fv_160_7.g4604;Description=hypothetical protein FVEG_17144 [Fusarium verticillioides 7600]
Fv_160_7.g4605	255	ID=Fv_160_7.g4605;Description=hypothetical protein H9Q70_010283 [Fusarium xylarioides];Gene=FPRO_06551;Ontology_term=pantothenate kinase activity,ATP binding,coenzyme A biosynthetic
Fv_160_7.g4606	543	ID=Fv_160_7.g4606;Description=hypothetical protein FVEG_12004 [Fusarium verticillioides 7600];Gene=FVER53263_12004;Ontology_term=hydrolase activity,lipid catabolic process,glycerolipid metabolic
Fv_160_7.g4607	106	ID=Fv_160_7.g4607;Description=hypothetical protein FVEG_12005 [Fusarium verticillioides 7600]
Fv_160_7.g4608	609	ID=Fv_160_7.g4608;Description=hypothetical protein FVEG_12006 [Fusarium verticillioides 7600];Gene=HZS61_014028;Ontology_term=membrane,transmembrane transporter activity,transmembrane
Fv_160_7.g4609	261	ID=Fv_160_7.g4609;Description=hypothetical protein FVEG_12007 [Fusarium verticillioides 7600];Gene=F52700_8803
Fv_160_7.g4610	347	ID=Fv_160_7.g4610;Description=hypothetical protein FVER53590_12008 [Fusarium verticillioides]
Fv_160_7.g4611	1533	ID=Fv_160_7.g4611;Description=hypothetical protein FVER14953_12009 [Fusarium verticillioides];Gene=3648
Fv_160_7.g4612	832	ID=Fv_160_7.g4612;Description=hypothetical protein FVER53263_12010 [Fusarium verticillioides];Gene=3647;Ontology_term=RNA helicase activity,translation initiation factor activity,protein serine/threonine kinase activity,ATP binding,hydrolase activity,translational initiation,phosphorylation;Ontology_id=GO:0003724,GO:0003743,GO:0004674,GO:0005524,GO:0016787,GO:0006413,GO:0016310;
Fv_160_7.g4613	459	ID=Fv_160_7.g4613;Description=hypothetical protein FVER53590_12011 [Fusarium verticillioides];Gene=BFJ68_g2145;Ontology_term=protein kinase activity,ATP
Fv_160_7.g4614	210	ID=Fv_160_7.g4614;Description=hypothetical protein FVEG_12012 [Fusarium verticillioides 7600]
Fv_160_7.g4615	864	ID=Fv_160_7.g4615;Description=hypothetical protein FVEG_12013 [Fusarium verticillioides 7600];Gene=3643;Ontology_term=zinc ion binding,positive regulation of kinetochore assembly,protein serine/threonine kinase activator activity,positive regulation of meiotic DNA double-strand break formation,chromosome segregation,positive regulation of DNA replication initiation,chromosome, centromeric region,chromatin,DNA replication origin binding,negative regulation of exit from mitosis,centrosome,protein binding,positive regulation of meiosis I,positive regulation of protein phosphorylation,membrane,Dbf4-dependent protein kinase complex,mitotic DNA replication checkpoint

Fv_160_7.g4616	375	ID=Fv_160_7.g4616;Description=hypothetical protein FVER14953_12015 [Fusarium verticillioides];Gene=araB;Ontology_term=ATP hydrolysis activity,muscle cell cellular homeostasis,calcium ion-transporting ATPase complex,protein homodimerization activity,adenylate cyclase-activating adrenergic receptor signaling pathway involved in heart process,regulation of relaxation of muscle,regulation of cardiac muscle cell contraction,negative regulation of ATPase-coupled calcium transmembrane transporter activity,translational initiation,cardiac muscle tissue development,nucleus,ATP binding,acrosome assembly,mitochondrial membrane,Notch signaling pathway,RNA helicase activity,ATPase inhibitor activity,single-stranded RNA binding,negative regulation of heart rate,regulation of the force of heart contraction by cardiac conduction,regulation of cardiac muscle contraction by regulation of the release of sequestered calcium ion,negative regulation of calcium ion import into sarcoplasmic reticulum,sarcoplasmic reticulum
Fv_160_7.g4617	363	ID=Fv_160_7.g4617;Description=hypothetical protein FVER53590_12016 [Fusarium
Fv_160_7.g4618	105	ID=Fv_160_7.g4618;Description=hypothetical protein FVER53263_12018 [Fusarium verticillioides];Gene=FVER53263_12018;Ontology_term=endoplasmic reticulum membrane,iron ion binding,heme binding,transmembrane transporter activity,testosterone 6-beta-hydroxylase activity,aromatase activity,progesterone metabolic process,transmembrane transport,oxidative demethylation,organic hydroxy compound metabolic process;Ontology_id=GO:0005789,GO:0005506,GO:0020037,GO:0022857,GO:0050649,GO:0070330,GO:0042448,GO:0055085,GO:
Fv_160_7.g4619	583	ID=Fv_160_7.g4619;Description=hypothetical protein J7337_007281 [Fusarium musae];Gene=BFJ72_g506;Ontology_term=membrane,transmembrane transporter activity,transmembrane
Fv_160_7.g4620	1061	ID=Fv_160_7.g4620;Description=hypothetical protein FVER53263_12020 [Fusarium verticillioides]
Fv_160_7.g4621	289	ID=Fv_160_7.g4621;Description=hypothetical protein FVEG_12021 [Fusarium verticillioides 7600];Gene=BFJ69_g1050;Ontology_term=hydrolase
Fv_160_7.g4622	202	ID=Fv_160_7.g4622;Description=hypothetical protein FVER14953_20274 [Fusarium verticillioides];Gene=3635
Fv_160_7.g4623	717	ID=Fv_160_7.g4623;Description=hypothetical protein FCOIX_9043 [Fusarium coicis];Gene=905;Ontology_term=membrane,transmembrane transporter activity,transmembrane
Fv_160_7.g4624	522	ID=Fv_160_7.g4624;Description=hypothetical protein FVEG_12022 [Fusarium verticillioides 7600];Gene=sod2;Ontology_term=membrane,superoxide dismutase activity,transmembrane transporter activity,metal ion binding,removal of superoxide radicals,transmembrane
Fv_160_7.g4625	490	ID=Fv_160_7.g4625;Description=hypothetical protein FVER14953_12023 [Fusarium verticillioides];Gene=FPANT_8718;Ontology_term=mitochondrial inner membrane,holocytochrome-c synthase activity,antiporter activity,xenobiotic transmembrane transporter activity,metal ion binding,xenobiotic detoxification by transmembrane export across
Fv_160_7.g4626	397	ID=Fv_160_7.g4626;Description=agmatinase 1 [Fusarium verticillioides 7600];Gene=FOXG_03837;Ontology_term=hydrolase activity, acting on carbon-nitrogen (but not peptide) bonds, in linear amidines,metal ion
Fv_160_7.g4627	351	ID=Fv_160_7.g4627;Description=hypothetical protein FVER53590_25062 [Fusarium verticillioides]

Fv_160_7.g4628	369	ID=Fv_160_7.g4628;Description=hypothetical protein FVER53263_20611 [Fusarium verticillioides];Gene=FVEG_17150;Ontology_term=early endosome,clathrin-coated pit,external side of plasma membrane,recycling endosome,scavenger receptor activity,coreceptor activity,C-C chemokine receptor activity,C-X-C chemokine receptor activity,C-C chemokine binding,C-X-C chemokine binding,angiogenesis,vasculogenesis,immune response,cell adhesion,G protein-coupled receptor signaling pathway,positive regulation of cytosolic calcium ion concentration,negative regulation of cell population proliferation,calcium-mediated signaling,oculomotor nerve development,receptor internalization,cell chemotaxis,chemokine-mediated signaling pathway,positive regulation of ERK1 and ERK2 cascade,negative regulation of intrinsic apoptotic signaling
Fv_160_7.g4629	562	ID=Fv_160_7.g4629;Description=hypothetical protein FNAPI_94 [Fusarium napiforme];Gene=FVER53263_12027;Ontology_term=zinc ion binding,signal transduction,regulation of systemic arterial blood pressure by endothelin,hormone activity,DNA-binding transcription factor activity, RNA polymerase II-specific,regulation of transcription by RNA polymerase II,intracellular calcium ion homeostasis,nucleus,DNA binding,extracellular space,endothelin B receptor binding,vein smooth muscle contraction,regulation of
Fv_160_7.g4630	132	ID=Fv_160_7.g4630;Description=hypothetical protein FVEG_12026 [Fusarium verticillioides 7600];Gene=896
Fv_160_7.g4631	705	ID=Fv_160_7.g4631;Description=hypothetical protein FVER53590_12027 [Fusarium verticillioides];Gene=FOYG_01009;Ontology_term=nucleus,DNA binding,zinc ion binding,cytoplasm,DNA-binding transcription factor activity, RNA polymerase II-specific,regulation of transcription by RNA polymerase
Fv_160_7.g4632	286	ID=Fv_160_7.g4632;Description=monocarboxylate transporter [Fusarium denticulatum];Gene=FPANT_6916;Ontology_term=membrane,transmembrane transporter activity,transmembrane
Fv_160_7.g4633	544	ID=Fv_160_7.g4633;Description=hypothetical protein FVEG_17152 [Fusarium verticillioides 7600];Gene=FPANT_6917
Fv_160_7.g4634	467	ID=Fv_160_7.g4634;Description=hypothetical protein FVEG_17152 [Fusarium verticillioides 7600];Gene=890
Fv_160_7.g4635	128	ID=Fv_160_7.g4635;Description=hypothetical protein FVER14953_20270 [Fusarium
Fv_160_7.g4636	561	ID=Fv_160_7.g4636;Description=hypothetical protein FVEG_12030 [Fusarium verticillioides 7600];Gene=3618;Ontology_term=membrane,SWI/SNF superfamily-type complex,DNA binding,peptidyl-prolyl cis-trans isomerase activity,helicase activity,ATP binding,hydrolase activity,histone binding,ATP-dependent chromatin remodeler activity,chromatin remodeling,regulation of DNA-templated transcription,protein folding,transmembrane transport;Ontology_id=GO:0016020,GO:0070603,GO:0003677,GO:0003755,GO:0004386,GO:0005524,GO:0016787,GO:0042393,GO
Fv_160_7.g4637	284	ID=Fv_160_7.g4637;Description=hypothetical protein FVER14953_12031 [Fusarium verticillioides];Gene=FOXG_03827;Ontology_term=nucleic acid binding,zinc ion binding,mRNA splicing, via
Fv_160_7.g4638	251	ID=Fv_160_7.g4638;Description=esterase/lipase [Fusarium verticillioides 7600];Gene=FNAPI_100;Ontology_term=hydrolase activity;Ontology_id=GO:0016787;Enzyme_code=EC:3;Enzyme_name=Hydrolases
Fv_160_7.g4639	965	ID=Fv_160_7.g4639;Description=hypothetical protein J7337_007267 [Fusarium
Fv_160_7.g4640	1098	ID=Fv_160_7.g4640;Description=hypothetical protein FVEG_17153 [Fusarium verticillioides 7600];Gene=FocTR4_00003369;Ontology_term=nucleus,cytoskeleton,membrane,small GTPase binding,protein import into

Fv_160_7.g4641	177	ID=Fv_160_7.g4641;Description=hypothetical protein FVEG_12036 [Fusarium verticillioides
Fv_160_7.g4642	328	ID=Fv_160_7.g4642;Description=hypothetical protein FVER14953_12037 [Fusarium verticillioides]
Fv_160_7.g4643	98	ID=Fv_160_7.g4643;Description=hypothetical protein FVEG_17155, partial [Fusarium verticillioides 7600]
Fv_160_7.g4644	718	ID=Fv_160_7.g4644;Description=hypothetical protein FVEG_12038 [Fusarium verticillioides 7600]
Fv_160_7.g4645	1032	ID=Fv_160_7.g4645;Description=F-box and WD-40 domain-containing protein CDC4 [Fusarium verticillioides 7600];Gene=FFUJ_14563;Ontology_term=cell division;Ontology_id=GO:0051301
Fv_160_7.g4646	974	ID=Fv_160_7.g4646;Description=hypothetical protein FVER14953_12040 [Fusarium verticillioides]
Fv_160_7.g4647	559	ID=Fv_160_7.g4647;Description=hypothetical protein FVER53590_12041 [Fusarium verticillioides];Gene=pkwA;Ontology_term=kinase
Fv_160_7.g4648	523	ID=Fv_160_7.g4648;Description=hypothetical protein FVEG_12042 [Fusarium verticillioides
Fv_160_7.g4649	405	ID=Fv_160_7.g4649;Description=hypothetical protein FNAPI_111 [Fusarium napiforme];Gene=3605;Ontology_term=membrane,catalase activity,protein kinase activity,heme binding,metal ion binding,response to oxidative stress,phosphorylation,hydrogen peroxide catabolic process,cellular oxidant detoxification;Ontology_id=GO:0016020,GO:0004096,GO:0004672,GO:0020037,GO:0046872,GO:0006979,GO:0016310,GO:004274
Fv_160_7.g4650	897	ID=Fv_160_7.g4650;Description=hypothetical protein FVER14953_12044 [Fusarium verticillioides];Gene=DUG2;Ontology_term=cytoplasm,nuclear periphery,glutathione hydrolase complex,glutathione hydrolase activity,identical protein binding,glutathione catabolic
Fv_160_7.g4651	304	ID=Fv_160_7.g4651;Description=hypothetical protein FVER53590_12045 [Fusarium
Fv_160_7.g4652	296	ID=Fv_160_7.g4652;Description=1-acylglycerone phosphate reductase [Fusarium verticillioides 7600];Gene=BFJ72_g526;Ontology_term=oxidoreductase activity,cellular biosynthetic
Fv_160_7.g4653	552	ID=Fv_160_7.g4653;Description=hypothetical protein FVEG_12047 [Fusarium verticillioides 7600];Gene=FocTR4_00003354;Ontology_term=membrane,metal ion transmembrane transporter activity,metal ion
Fv_160_7.g4654	228	ID=Fv_160_7.g4654;Description=hypothetical protein FVER14953_12048 [Fusarium verticillioides]
Fv_160_7.g4655	923	ID=Fv_160_7.g4655;Description=trehalase [Fusarium circinatum];Gene=FPRO05_08409;Ontology_term=nucleus,membrane,DNA-binding transcription factor activity, RNA polymerase II-specific,zinc ion binding,S-adenosylmethionine-dependent methyltransferase activity,regulation of transcription by RNA polymerase
Fv_160_7.g4656	475	ID=Fv_160_7.g4656;Description=hypothetical protein FVER53590_12051 [Fusarium verticillioides];Gene=SLU7;Ontology_term=nucleus,mitochondrial respiratory chain complex III,mitochondrial intermembrane space,transaminase activity,pyridoxal phosphate
Fv_160_7.g4657	532	ID=Fv_160_7.g4657;Description=hypothetical protein FVER53590_12052 [Fusarium verticillioides];Gene=FMAN_06207;Ontology_term=extracellular region,metallocarboxypeptidase activity,zinc ion
Fv_160_7.g4658	294	ID=Fv_160_7.g4658;Description=hypothetical protein FVER14953_12053 [Fusarium verticillioides];Gene=FANTH_6720;Ontology_term=extracellular region,chitinase activity,polysaccharide catabolic process,chitin

Fv_160_7.g4659	205	ID=Fv_160_7.g4659;Description=hypothetical protein FVEG_12054 [Fusarium verticillioides 7600];Gene=FMEXI_14072;Ontology_term=carbon-sulfur lyase activity,metal ion
Fv_160_7.g4660	226	ID=Fv_160_7.g4660;Description=hypothetical protein FNAPI_123 [Fusarium napiforme];Gene=CPS1
Fv_160_7.g4661	815	ID=Fv_160_7.g4661;Description=hypothetical protein FCOIX_9079 [Fusarium coicis];Gene=mraY;Ontology_term=DNA-binding transcription factor activity,regulation of DNA-templated transcription;Ontology_id=GO:0003700,GO:0006355
Fv_160_7.g4662	441	ID=Fv_160_7.g4662;Description=hypothetical protein J7337_007246 [Fusarium musae];Gene=FNYG_03919;Ontology_term=oxidoreductase activity,flavin adenine dinucleotide
Fv_160_7.g4663	366	ID=Fv_160_7.g4663;Description=hypothetical protein FVER53590_12058 [Fusarium verticillioides];Gene=FVEG_12058;Ontology_term=nucleus,DNA-binding transcription factor activity, RNA polymerase II-specific,zinc
Fv_160_7.g4664	340	ID=Fv_160_7.g4664;Description=hypothetical protein FVER53590_12059 [Fusarium verticillioides]
Fv_160_7.g4665	877	ID=Fv_160_7.g4665;Description=hypothetical protein FVEG_12060 [Fusarium verticillioides 7600];Gene=FNYG_03916;Ontology_term=Golgi apparatus,membrane,transferase activity,carbohydrate metabolic
Fv_160_7.g4666	326	ID=Fv_160_7.g4666;Description=hypothetical protein FVER53263_12061 [Fusarium verticillioides]
Fv_160_7.g4667	325	ID=Fv_160_7.g4667;Description=hypothetical protein FVER14953_12062 [Fusarium verticillioides];Gene=FGLOB1_5190;Ontology_term=arylamine N-acetyltransferase activity,NAD-dependent histone deacetylase activity,metal ion binding,NAD+ binding,heterochromatin formation;Ontology_id=GO:0004060,GO:0017136,GO:0046872,GO:0070403,GO:0031507;Enzyme_code=EC:3.5.1.98,EC:2.3.1.5;Enzy
Fv_160_7.g4668	614	ID=Fv_160_7.g4668;Description=hypothetical protein FVEG_17157 [Fusarium verticillioides 7600];Gene=BFJ69_g1184;Ontology_term=nucleus,DNA binding,zinc ion binding,DNA-binding transcription factor activity, RNA polymerase II-specific,regulation of transcription by RNA polymerase
Fv_160_7.g4669	402	ID=Fv_160_7.g4669;Description=omega-6 fatty acid desaturase (delta-12 desaturase) [Fusarium verticillioides 7600];Gene=HZS61_013972;Ontology_term=membrane,oxidoreductase activity,lipid metabolic
Fv_160_7.g4670	834	ID=Fv_160_7.g4670;Description=hypothetical protein FVEG_12066 [Fusarium verticillioides
Fv_160_7.g4671	328	ID=Fv_160_7.g4671;Description=hypothetical protein FVEG_12067 [Fusarium verticillioides 7600];Gene=FACUT_8905;Ontology_term=cellulase activity,polysaccharide catabolic
Fv_160_7.g4672	226	ID=Fv_160_7.g4672;Description=hypothetical protein FVER14953_12068 [Fusarium verticillioides];Gene=BFJ68_g5950;Ontology_term=membrane,acyltransferase activity, transferring groups other than amino-acyl
Fv_160_7.g4673	567	ID=Fv_160_7.g4673;Description=phosphate:H+ symporter [Fusarium verticillioides 7600];Gene=FACUT_8903;Ontology_term=membrane,inorganic phosphate transmembrane transporter activity,phosphate ion
Fv_160_7.g4674	148	ID=Fv_160_7.g4674;Description=hypothetical protein FVEG_12070 [Fusarium verticillioides 7600];Gene=argD
Fv_160_7.g4675	236	ID=Fv_160_7.g4675;Description=hypothetical protein FVER53590_25058 [Fusarium verticillioides];Gene=FACUT_8901;Ontology_term=mitochondrion,nucleotide binding,succinate-CoA ligase (ADP-forming) activity,tricarboxylic acid

Fv_160_7.g4676	580	ID=Fv_160_7.g4676;Description=CTP synthase [Fusarium verticillioides 7600];Gene=FACUT_13034;Ontology_term=CTP synthase activity,ATP binding,glutamine metabolic process,'de novo' CTP biosynthetic
Fv_160_7.g4677	295	ID=Fv_160_7.g4677;Description=hypothetical protein FVEG_12073 [Fusarium verticillioides 7600];Gene=FANTH_3033;Ontology_term=CTP synthase activity,ATP binding,tricarboxylic acid cycle,glutamine metabolic process,'de novo' CTP biosynthetic
Fv_160_7.g4678	289	ID=Fv_160_7.g4678;Description=hypothetical protein FVEG_17158 [Fusarium verticillioides 7600];Gene=848;Ontology_term=endoplasmic reticulum membrane,endoplasmic reticulum-Golgi intermediate compartment,Golgi apparatus,cytosol,COPII-coated ER to Golgi transport vesicle,CTP synthase activity,serine-tRNA ligase activity,ATP binding,cytoplasmic translation,seryl-tRNA aminoacylation,glutamine metabolic process,intracellular protein transport,endoplasmic reticulum to Golgi vesicle-mediated transport,Golgi organization,'de novo' CTP biosynthetic process;Ontology_id=GO:0005789,GO:0005793,GO:0005794,GO:0005829,GO:0030134,GO:0003883,GO:0004828,GO:0005524,GO:
Fv_160_7.g4679	402	ID=Fv_160_7.g4679;Description=hypothetical protein FVER53590_12074 [Fusarium verticillioides];Gene=FTJAE_9990;Ontology_term=glycosyltransferase
Fv_160_7.g4680	494	ID=Fv_160_7.g4680;Description=hypothetical protein FVEG_12075 [Fusarium verticillioides 7600];Gene=FSUBG_3694;Ontology_term=glycosyltransferase
Fv_160_7.g4681	367	ID=Fv_160_7.g4681;Description=hypothetical protein FVER53590_12076 [Fusarium verticillioides];Gene=FNAPI_145;Ontology_term=membrane,3-beta-hydroxy-delta5-steroid dehydrogenase activity,steroid biosynthetic process;Ontology_id=GO:0016020,GO:0003854,GO:0006694;Enzyme_code=EC:1.1.1.145;Enzyme_name=3beta-
Fv_160_7.g4682	126	ID=Fv_160_7.g4682;Description=hypothetical protein FVEG_12077 [Fusarium verticillioides 7600];Gene=3574
Fv_160_7.g4683	640	ID=Fv_160_7.g4683;Description=hypothetical protein FVER53590_12078 [Fusarium verticillioides];Gene=FGADI_10469;Ontology_term=metallopeptidase activity,defense
Fv_160_7.g4684	157	ID=Fv_160_7.g4684;Description=hypothetical protein FVEG_12079 [Fusarium verticillioides 7600]
Fv_160_7.g4685	402	ID=Fv_160_7.g4685;Description=hypothetical protein FVER14953_12080 [Fusarium verticillioides];Gene=FVEG_12080;Ontology_term=membrane,oxidoreductase
Fv_160_7.g4686	527	ID=Fv_160_7.g4686;Description=hypothetical protein FVEG_12081 [Fusarium verticillioides 7600];Gene=FVEG_12081;Ontology_term=mitochondrial respiratory chain complex IV,transmembrane transporter activity,mitochondrial electron transport, cytochrome c to oxygen,transmembrane
Fv_160_7.g4687	549	ID=Fv_160_7.g4687;Description=hypothetical protein FVER14953_12082 [Fusarium verticillioides];Gene=FOXYS1_15;Ontology_term=plasma membrane,sodium:proton antiporter activity,sodium ion export across plasma membrane,regulation of membrane potential,proton export across plasma
Fv_160_7.g4688	533	ID=Fv_160_7.g4688;Description=hypothetical protein J7337_007218 [Fusarium musae];Gene=FMUND_9769;Ontology_term=phosphatase

Fv_160_7.g4689	241	ID=Fv_160_7.g4689;Description=2-haloacid dehalogenase [Fusarium verticillioides 7600];Gene=FNAPI_3101;Ontology_term=phosphatase
Fv_160_7.g4690	1462	ID=Fv_160_7.g4690;Description=nitrate reductase (NADH) [Fusarium phyllophilum];Gene=FVEG_12090;Ontology_term=extracellular region,molybdenum ion binding,nitrate reductase (NADPH) activity,nitrate
Fv_160_7.g4691	329	ID=Fv_160_7.g4691;Description=Glycerol 2-dehydrogenase (NADP(+)) [Fusarium musae];Gene=Forpi1262_v004588;Ontology_term=oxidoreductase
Fv_160_7.g4692	494	ID=Fv_160_7.g4692;Description=hypothetical protein FVEG_12092 [Fusarium verticillioides 7600];Gene=Focb16_v007192;Ontology_term=transferase
Fv_160_7.g4693	244	ID=Fv_160_7.g4693;Description=hypothetical protein FVER53263_12093 [Fusarium verticillioides];Gene=FPANT_9420;Ontology_term=lyase
Fv_160_7.g4694	281	ID=Fv_160_7.g4694;Description=hypothetical protein FVEG_12094 [Fusarium verticillioides 7600]
Fv_160_7.g4695	798	ID=Fv_160_7.g4695;Description=hypothetical protein FPCIR_1979 [Fusarium pseudocircinatum];Gene=FOXB_07451;Ontology_term=nucleus,DNA binding,regulation of DNA-templated
Fv_160_7.g4696	350	ID=Fv_160_7.g4696;Description=hypothetical protein FVER53590_12097 [Fusarium verticillioides];Gene=FPHYL_7041;Ontology_term=S-adenosylmethionine-dependent methyltransferase
Fv_160_7.g4697	306	ID=Fv_160_7.g4697;Description=hypothetical protein FVEG_17163 [Fusarium verticillioides 7600];Gene=FMUND_9777;Ontology_term=hydrolase
Fv_160_7.g4698	292	ID=Fv_160_7.g4698;Description=hypothetical protein FVER14953_12098 [Fusarium verticillioides];Gene=FDENT_8759;Ontology_term=transferase
Fv_160_7.g4699	447	ID=Fv_160_7.g4699;Description=hypothetical protein FVER53590_12100 [Fusarium verticillioides];Gene=FOQG_02926;Ontology_term=nucleus,membrane,DNA-binding transcription factor activity, RNA polymerase II-specific,SNAP receptor activity,zinc ion binding,regulation of transcription by RNA polymerase II,retrograde vesicle-mediated
Fv_160_7.g4700	92	ID=Fv_160_7.g4700;Description=hypothetical protein FVEG_12100 [Fusarium verticillioides 7600];Gene=FOQG_02924;Ontology_term=nucleus,membrane,DNA-binding transcription factor activity, RNA polymerase II-specific,calmodulin binding,zinc ion binding,calmodulin-dependent protein phosphatase activity,regulation of transcription by RNA polymerase II,calcineurin-mediated signaling;Ontology_id=GO:0005634,GO:0016020,GO:0000981,GO:0005516,GO:0008270,GO:0033192,GO:0006357,GO:0097720;Enz
Fv_160_7.g4701	83	ID=Fv_160_7.g4701;Description=hypothetical protein FVEG_12101 [Fusarium verticillioides 7600];Gene=BFJ63_vAg12433;Ontology_term=nucleus,membrane,DNA binding,orotate phosphoribosyltransferase activity,orotidine-5'-phosphate decarboxylase activity,'de novo' pyrimidine nucleobase biosynthetic process,regulation of DNA-templated transcription,'de novo' UMP biosynthetic process;Ontology_id=GO:0005634,GO:0016020,GO:0003677,GO:0004588,GO:0004590,GO:0006207,GO:0006355,GO:0044205;Enzy

Fv_160_7.g4702	601	ID=Fv_160_7.g4702;Description=hypothetical protein FVER53590_12102 [Fusarium verticillioides];Gene=FTJAE_10013;Ontology_term=nucleus,DNA binding,regulation of DNA-templated
Fv_160_7.g4703	1046	ID=Fv_160_7.g4703;Description=diaminopropionate ammonia-lyase [Fusarium coicis];Gene=F25303_6479;Ontology_term=extracellular region,peptidase activity,succinyl-diaminopimelate desuccinylase activity,lyase activity,proteolysis,lysine biosynthetic process via diaminopimelate;Ontology_id=GO:0005576,GO:0008233,GO:0009014,GO:0016829,GO:0006508,GO:0009089;Enzyme_code=EC:3.4,
Fv_160_7.g4704	449	ID=Fv_160_7.g4704;Description=diaminopropionate ammonia-lyase [Fusarium coicis];Gene=FPANT_9410;Ontology_term=extracellular region,peptidase activity,lyase activity,proteolysis,lysine biosynthetic process via
Fv_160_7.g4705	503	ID=Fv_160_7.g4705;Description=hypothetical protein FVEG_12106 [Fusarium verticillioides 7600];Gene=FVER53263_12106;Ontology_term=membrane,transmembrane transporter activity,transmembrane
Fv_160_7.g4706	221	ID=Fv_160_7.g4706;Description=hypothetical protein FVEG_12107 [Fusarium verticillioides 7600]
Fv_160_7.g4707	530	ID=Fv_160_7.g4707;Description=hypothetical protein FVEG_17167 [Fusarium verticillioides 7600];Gene=826;Ontology_term=nucleus,signal peptidase complex,DNA-binding transcription factor activity, RNA polymerase II-specific,serine-type endopeptidase activity,protein binding,ATP binding,zinc ion binding,ATP-dependent chromatin remodeler activity,chromatin remodeling,regulation of transcription by RNA polymerase II,signal peptide processing,protein targeting to ER;Ontology_id=GO:0005634,GO:0005787,GO:0000981,GO:0004252,GO:0005515,GO:0005524,GO:0008270,GO:0140658,GO:0006
Fv_160_7.g4708	79	ID=Fv_160_7.g4708;Description=hypothetical protein FVEG_12108 [Fusarium verticillioides 7600];Gene=FGRA07_10732;Ontology_term=arylesterase
Fv_160_7.g4709	406	ID=Fv_160_7.g4709;Description=hypothetical protein FVEG_12109 [Fusarium verticillioides 7600]
Fv_160_7.g4710	392	ID=Fv_160_7.g4710;Description=hypothetical protein FVEG_12110 [Fusarium verticillioides 7600];Gene=FOXB_04126;Ontology_term=membrane,arylesterase activity,oxidoreductase activity,cellular biosynthetic
Fv_160_7.g4711	300	ID=Fv_160_7.g4711;Description=hypothetical protein FVER14953_20739 [Fusarium verticillioides];Gene=FOBC_03569;Ontology_term=membrane,arylesterase activity,oxidoreductase activity,cellular biosynthetic
Fv_160_7.g4712	205	ID=Fv_160_7.g4712;Description=hypothetical protein FVEG_12111 [Fusarium verticillioides 7600]
Fv_160_7.g4713	772	ID=Fv_160_7.g4713;Description=hypothetical protein FVER53590_12112 [Fusarium verticillioides]
Fv_160_7.g4714	682	ID=Fv_160_7.g4714;Description=hypothetical protein FVEG_12113 [Fusarium verticillioides 7600];Gene=BFJ63_vAg12444;Ontology_term=membrane,transmembrane transporter activity,transmembrane

Fv_160_7.g4715	214	ID=Fv_160_7.g4715;Description=hypothetical protein FVER14953_12114 [Fusarium verticillioides];Gene=FPANT_6707;Ontology_term=fungal-type vacuole lumen,chromatin,extracellular space,nucleus,mitochondrion,cytosol,plasma membrane,protein-glutamine gamma-glutamyltransferase activity,serine-type endopeptidase activity,calcium ion binding,GTP binding,protein-glutamine glutaminase activity,histone serotonyltransferase activity,histone dopaminyltransferase activity,peptide noradrenalinyltransferase activity,peptide histaminyltransferase activity,pexophagy,chromatin remodeling,proteolysis,protein catabolic process in the vacuole,phospholipase C-activating G protein-coupled receptor signaling pathway,peptide cross-linking,protein deamination,sporulation resulting in formation of a cellular spore,positive regulation of apoptotic process,positive regulation of GTPase activity,positive regulation of small GTPase mediated signal transduction,protein homooligomerization,bone development,cellular response to dopamine,cellular response to serotonin,regulation of apoptotic cell clearance;Ontology_id=GO:0000328,GO:0000785,GO:0005615,GO:0005634,GO:0005739,GO:0005829,GO:0005886,GO:0003810,G
Fv_160_7.g4716	734	ID=Fv_160_7.g4716;Description=hypothetical protein FVER53590_12115 [Fusarium verticillioides];Gene=FPANT_6708;Ontology_term=nucleus,DNA-binding transcription factor activity, RNA polymerase II-specific,zinc
Fv_160_7.g4717	268	ID=Fv_160_7.g4717;Description=globin-like protein [Fusarium tjaetaba];Gene=FOQG_02904;Ontology_term=oxygen binding,heme
Fv_160_7.g4718	883	ID=Fv_160_7.g4718;Description=hypothetical protein FVER14953_12117 [Fusarium verticillioides];Gene=FOQG_02903;Ontology_term=catalytic activity,trehalose biosynthetic
Fv_160_7.g4719	713	ID=Fv_160_7.g4719;Description=hypothetical protein FVEG_12118 [Fusarium verticillioides 7600];Gene=TPS1;Ontology_term=positive regulation of ER to Golgi vesicle-mediated transport,mitochondria-associated endoplasmic reticulum membrane,trehalose biosynthetic process,Golgi membrane,endoplasmic reticulum membrane,GTP binding,nuclear envelope organization,cellular response to heat,alpha,alpha-trehalose-phosphate synthase complex (UDP-forming),GTPase activity,alpha,alpha-trehalose-phosphate synthase (UDP-forming) activity,positive regulation of protein exit from endoplasmic reticulum,COPII vesicle coat,zinc ion binding,DNA-binding transcription factor activity, RNA polymerase II-specific,mitochondrial membrane organization,mitochondrial fission,regulation of COPII vesicle coating,regulation of transcription by RNA polymerase II,nucleus,DNA binding,protein binding,cellular response to desiccation,trehalose-phosphatase activity,mitochondrion,trehalose metabolism in response to stress,endoplasmic reticulum exit
Fv_160_7.g4720	744	ID=Fv_160_7.g4720;Description=hypothetical protein FVEG_12119 [Fusarium verticillioides 7600];Gene=814;Ontology_term=cytoplasm,heterotrimeric G-protein complex,photoreceptor disc membrane,protein binding,signaling receptor complex adaptor activity,adenylate cyclase-activating dopamine receptor signaling pathway,cellular
Fv_160_7.g4721	581	ID=Fv_160_7.g4721;Description=hypothetical protein FVEG_12121 [Fusarium verticillioides
Fv_160_7.g4722	439	ID=Fv_160_7.g4722;Description=hypothetical protein FVER14953_12122 [Fusarium verticillioides];Gene=FGLOB1_503;Ontology_term=membrane,Golgi transport complex,intra-Golgi vesicle-mediated
Fv_160_7.g4723	396	ID=Fv_160_7.g4723;Description=hypothetical protein FVER14953_12123 [Fusarium verticillioides];Gene=BFJ63_vAg12455;Ontology_term=actin filament binding;Ontology_id=GO:0051015

Fv_160_7.g4724	310	ID=Fv_160_7.g4724;Description=hypothetical protein FVEG_12124 [Fusarium verticillioides 7600];Gene=Forpi1262_v004555;Ontology_term=endonuclease activity,exonuclease
Fv_160_7.g4725	323	ID=Fv_160_7.g4725;Description=hypothetical protein FVEG_12125 [Fusarium verticillioides 7600]
Fv_160_7.g4726	142	ID=Fv_160_7.g4726;Description=hypothetical protein FVEG_17170 [Fusarium verticillioides 7600];Gene=F53441_1175;Ontology_term=carbon-sulfur lyase activity,metal ion
Fv_160_7.g4727	309	ID=Fv_160_7.g4727;Description=hypothetical protein FCOIX_6632 [Fusarium coicis];Gene=RPS28A;Ontology_term=nucleosome,nucleus,endoplasmic reticulum,ribosome,membrane,DNA binding,structural constituent of ribosome,single strand break repair,nucleosome disassembly,translation,spermatid nucleus elongation,positive regulation of protein processing,flagellated sperm motility,heterochromatin formation,sperm DNA condensation,negative regulation of DNA-templated
Fv_160_7.g4728	1337	ID=Fv_160_7.g4728;Description=hypothetical protein FVER53590_12127 [Fusarium verticillioides]
Fv_160_7.g4729	3263	ID=Fv_160_7.g4729;Description=hypothetical protein FVEG_17172 [Fusarium verticillioides 7600]
Fv_160_7.g4730	311	ID=Fv_160_7.g4730;Description=hypothetical protein FVER53590_12131 [Fusarium verticillioides]
Fv_160_7.g4731	282	ID=Fv_160_7.g4731;Description=hypothetical protein FVER53590_12132 [Fusarium verticillioides];Gene=FACUT_10083;Ontology_term=oxidoreductase
Fv_160_7.g4732	444	ID=Fv_160_7.g4732;Description=hypothetical protein FVER14953_12133 [Fusarium verticillioides];Gene=FPCIR_5427;Ontology_term=membrane,transmembrane transporter activity,transmembrane
Fv_160_7.g4733	846	ID=Fv_160_7.g4733;Description=hypothetical protein FVEG_12134 [Fusarium verticillioides 7600];Gene=FACUT_10082;Ontology_term=membrane,transmembrane transporter activity,transmembrane
Fv_160_7.g4734	270	ID=Fv_160_7.g4734;Description=SAC3/GANP/Nin1/mts3/eIF-3 p25 family-domain-containing protein [Fusarium oxysporum Fo47];Gene=FACUT_10081;Ontology_term=nucleus,proteasome regulatory particle,RNA binding,ATP binding,metal ion binding,poly(A) RNA polymerase activity,mRNA processing,proteolysis,RNA 3'-end processing;Ontology_id=GO:0005634,GO:0005838,GO:0003723,GO:0005524,GO:0046872,GO:1990817,GO:0006397,GO:0006508,GO:0031123;Enzyme_code=EC:2.7.7.19;Enzyme_name=polynucleotide adenylyltransferase
Fv_160_7.g4735	1220	ID=Fv_160_7.g4735;Description=coatomer protein complex, subunit alpha (xenin) [Fusarium verticillioides 7600];Gene=FNAPI_8258;Ontology_term=Golgi membrane,COPI vesicle coat,structural molecule activity,lipid binding,ubiquitin binding,sterol transfer activity,intracellular protein transport,exocytosis,endoplasmic reticulum to Golgi vesicle-mediated transport,retrograde vesicle-mediated transport, Golgi to endoplasmic reticulum,endocytosis,intracellular mRNA localization,sterol transport,maintenance of cell polarity,piecemeal microautophagy of the nucleus,intermembrane lipid transfer;Ontology_id=GO:0000139,GO:0030126,GO:0005198,GO:0008289,GO:0043130,GO:0120015,GO:0006886,GO:0006887,GO:
Fv_160_7.g4736	1464	ID=Fv_160_7.g4736;Description=hypothetical protein FVER53590_12137 [Fusarium verticillioides];Gene=FNAPI_8259;Ontology_term=endoplasmic reticulum lumen,UDP-glucose:glycoprotein glucosyltransferase

Fv_160_7.g4737	1024	ID=Fv_160_7.g4737;Description=hypothetical protein FVER53590_12138 [Fusarium verticillioides];Gene=FFB14_12850;Ontology_term=zinc ion binding,sequence-specific DNA binding,regulation of DNA-templated
Fv_160_7.g4738	1096	ID=Fv_160_7.g4738;Description=hypothetical protein FVER53590_12139 [Fusarium verticillioides];Gene=FPANT_6729;Ontology_term=mitochondrial inner membrane,mitochondrial large ribosomal subunit,cytoskeleton,nuclear periphery,structural constituent of ribosome,nuclear localization sequence binding,small GTPase binding,nuclear import signal receptor activity,mRNA export from nucleus,protein import into nucleus,regulation of mitotic nuclear division,mitochondrial translation,regulation of protein
Fv_160_7.g4739	532	ID=Fv_160_7.g4739;Description=hypothetical protein FCOIX_6643 [Fusarium coicis];Gene=FFB14_12852;Ontology_term=fungal-type vacuole membrane,ribosome,late endosome membrane,membrane raft,Ragulator complex,ribonucleoprotein complex,structural constituent of ribosome,protein binding,regulation of receptor recycling,translation,vacuolar acidification,lysosome organization,protein transport,microautophagy,regulation of mitotic metaphase/anaphase transition,positive regulation of TOR signaling,endocytic recycling,cellular response to glucose starvation,cholesterol homeostasis,positive regulation of MAPK cascade,cellular response to amino acid stimulus,protein localization to
Fv_160_7.g4740	534	ID=Fv_160_7.g4740;Description=hypothetical protein FVEG_12141 [Fusarium verticillioides 7600]
Fv_160_7.g4741	348	ID=Fv_160_7.g4741;Description=hypothetical protein FVER14953_12142 [Fusarium verticillioides];Gene=FRV6_05965;Ontology_term=hydrolase activity, hydrolyzing O-glycosyl compounds,carbohydrate metabolic
Fv_160_7.g4742	516	ID=Fv_160_7.g4742;Description=serine palmitoyltransferase [Fusarium verticillioides 7600];Gene=FOVG_07917;Ontology_term=transferase activity,pyridoxal phosphate binding,biosynthetic
Fv_160_7.g4743	1941	ID=Fv_160_7.g4743;Description=hypothetical protein FVER53590_12144 [Fusarium verticillioides];Gene=FPCIR_10709;Ontology_term=1,3-beta-D-glucan synthase complex,1,3-beta-D-glucan synthase activity,(1->3)-beta-D-glucan biosynthetic
Fv_160_7.g4744	699	ID=Fv_160_7.g4744;Description=hypothetical protein FVER14953_12146 [Fusarium verticillioides]
Fv_160_7.g4745	197	ID=Fv_160_7.g4745;Description=hypothetical protein FVEG_12147 [Fusarium verticillioides
Fv_160_7.g4746	110	ID=Fv_160_7.g4746;Description=hit family 1 [Fusarium acutatum];Gene=FOXG_03717;Ontology_term=nucleus,mitochondrion,kinase activity,adenosine 5'-monophosphoramidase activity,lagging strand elongation,base-excision repair,nucleotide metabolic process,phosphorylation;Ontology_id=GO:0005634,GO:0005739,GO:0016301,GO:0043530,GO:0006273,GO:0006284,GO:0009117,
Fv_160_7.g4747	586	ID=Fv_160_7.g4747;Description=hypothetical protein FVEG_12149 [Fusarium verticillioides 7600];Gene=Forpi1262_v004533;Ontology_term=membrane,transferase
Fv_160_7.g4748	204	ID=Fv_160_7.g4748;Description=DASH complex subunit Dam1-domain-containing protein [Fusarium oxysporum];Gene=FANTH_3100;Ontology_term=cytoplasm,microtubule,DASH complex,mitotic spindle,attachment of spindle
Fv_160_7.g4749	119	ID=Fv_160_7.g4749;Description=hypothetical protein FVEG_12151 [Fusarium verticillioides 7600];Gene=FOXG_03714;Ontology_term=nucleic acid binding,zinc ion binding;Ontology_id=GO:0003676,GO:0008270

Fv_160_7.g4750	788	ID=Fv_160_7.g4750;Description=hypothetical protein FVER53590_12152 [Fusarium verticillioides];Gene=BFJ63_vAg12478;Ontology_term=cytosol,mitochondrial nucleoid,double-stranded DNA binding,single-stranded DNA binding,aconitate hydratase activity,metal ion binding,4 iron, 4 sulfur cluster binding,mitochondrial genome maintenance,tricarboxylic acid
Fv_160_7.g4751	267	ID=Fv_160_7.g4751;Description=hypothetical protein FVER53590_12153 [Fusarium verticillioides];Gene=FACUT_10064;Ontology_term=membrane,serine-type endopeptidase activity,ATP hydrolysis activity,DNA replication initiation,cell division;Ontology_id=GO:0016020,GO:0004252,GO:0016887,GO:0006270,GO:0051301;Enzyme_code=EC:3.4.21,EC:3.6.1.15;Enzym
Fv_160_7.g4752	593	ID=Fv_160_7.g4752;Description=glycosylphosphatidylinositol transamidase [Fusarium verticillioides 7600];Gene=FGADI_10733;Ontology_term=GPI-anchor transamidase complex;Ontology_id=GO:0042765
Fv_160_7.g4753	381	ID=Fv_160_7.g4753;Description=26S proteasome regulatory subunit N9 [Fusarium verticillioides 7600];Gene=HZS61_013897;Ontology_term=proteasome regulatory particle, lid subcomplex,cytosolic large ribosomal subunit,aminoacyl-tRNA editing activity,structural constituent of ribosome,leucine-tRNA ligase activity,ATP binding,cytoplasmic translation,leucyl-tRNA aminoacylation,aminoacyl-tRNA metabolism involved in translational fidelity;Ontology_id=GO:0008541,GO:0022625,GO:0002161,GO:0003735,GO:0004823,GO:0005524,GO:0002181,GO:0006429,GO:0
Fv_160_7.g4754	647	ID=Fv_160_7.g4754;Description=NADH-ubiquinone oxidoreductase 40 kDa subunit mitochondrial [Fusarium coicis];Gene=EFM5;Ontology_term=cytoplasm,protein-lysine N-methyltransferase
Fv_160_7.g4755	514	ID=Fv_160_7.g4755;Description=hypothetical protein J7337_007149 [Fusarium musae];Gene=FACUT_10060;Ontology_term=cysteine-type deubiquitinase
Fv_160_7.g4756	289	ID=Fv_160_7.g4756;Description=hypothetical protein FVEG_12159 [Fusarium verticillioides 7600];Gene=780;Ontology_term=ribosome,ribonucleoprotein complex,structural constituent of
Fv_160_7.g4757	581	ID=Fv_160_7.g4757;Description=hypothetical protein FVEG_12160 [Fusarium verticillioides
Fv_160_7.g4758	76	ID=Fv_160_7.g4758;Description=hypothetical protein FVEG_12161 [Fusarium verticillioides
Fv_160_7.g4759	805	ID=Fv_160_7.g4759;Description=DNA repair protein rhp54 [Fusarium verticillioides 7600];Gene=28945808;Ontology_term=nucleus,ATP binding,hydrolase activity, acting on acid anhydrides,ATP-dependent chromatin remodeler activity,chromatin
Fv_160_7.g4760	328	ID=Fv_160_7.g4760;Description=hypothetical protein FVER53590_12163 [Fusarium verticillioides];Gene=FocTR4_00003238;Ontology_term=mitochondrial inner membrane,transmembrane
Fv_160_7.g4761	421	ID=Fv_160_7.g4761;Description=hypothetical protein FVER53590_12164 [Fusarium verticillioides]

Fv_160_7.g4762	341	ID=Fv_160_7.g4762;Description=hypothetical protein FVER53590_12165 [Fusarium verticillioides];Gene=FOQG_02849;Ontology_term=extracellular space,membrane,nucleotide binding,hormone activity,zinc ion binding,histone binding,adenosine 5'-monophosphoramidase activity,thyroid hormone generation,signal transduction,nucleotide metabolic process,hormone biosynthetic process;Ontology_id=GO:0005615,GO:0016020,GO:0000166,GO:0005179,GO:0008270,GO:0042393,GO:0043530,GO:0006590,GO:
Fv_160_7.g4763	701	ID=Fv_160_7.g4763;Description=hypothetical protein FVER53590_12166 [Fusarium verticillioides];Gene=HZS61_013886;Ontology_term=extrinsic component of intraperoxisomal membrane,transmembrane transporter activity,peroxisome inheritance,transmembrane transport,nitrogen compound
Fv_160_7.g4764	375	ID=Fv_160_7.g4764;Description=hypothetical protein FVER53590_12167 [Fusarium
Fv_160_7.g4765	368	ID=Fv_160_7.g4765;Description=hypothetical protein FVER14953_12168 [Fusarium verticillioides];Gene=BFJ70_g11283;Ontology_term=cytoplasm,cysteine-type deubiquitinase
Fv_160_7.g4766	917	ID=Fv_160_7.g4766;Description=hypothetical protein FVER14953_12169 [Fusarium verticillioides];Gene=FocTR4_00003237;Ontology_term=RSC-type complex,chromatin binding,chromatin remodeling,transcription
Fv_160_7.g4767	627	ID=Fv_160_7.g4767;Description=hypothetical protein FVER53590_12170 [Fusarium verticillioides];Gene=HZS61_013883;Ontology_term=oxoglutarate dehydrogenase complex,dihydrolipoyllysine-residue succinyltransferase activity,tricarboxylic acid cycle,L-lysine catabolic process to acetyl-CoA via
Fv_160_7.g4768	206	ID=Fv_160_7.g4768;Description=hypothetical protein FVER53590_12171 [Fusarium verticillioides]
Fv_160_8.g4769	77	ID=Fv_160_8.g4769;Description=hypothetical protein FVER53590_28405 [Fusarium
Fv_160_8.g4770	1066	ID=Fv_160_8.g4770;Description=bifunctional P-450:NADPH-P450 reductase [Fusarium verticillioides 7600];Gene=FTJAE_5075;Ontology_term=NADPH-hemoprotein reductase activity,iron ion binding,FMN binding,heme binding,aromatase activity;Ontology_id=GO:0003958,GO:0005506,GO:0010181,GO:0020037,GO:0070330;Enzyme_code=EC:1.6.2.4,EC:1.14.14.1;Enzy
Fv_160_8.g4771	522	ID=Fv_160_8.g4771;Description=hypothetical protein J7337_010719 [Fusarium musae]
Fv_160_8.g4772	89	ID=Fv_160_8.g4772;Description=hypothetical protein FVEG_16062 [Fusarium verticillioides
Fv_160_8.g4773	383	ID=Fv_160_8.g4773;Description=hypothetical protein FVER53590_07272 [Fusarium verticillioides];Gene=FPANT_2827;Ontology_term=ribosome,double-stranded RNA binding,ribonuclease III activity,rRNA processing;Ontology_id=GO:0005840,GO:0003725,GO:0004525,GO:0006364;Enzyme_code=EC:3.1.30,EC:3.1.26.3;Enzyme_name=A
Fv_160_8.g4774	821	ID=Fv_160_8.g4774;Description=phospholipase A2 [Fusarium verticillioides 7600];Gene=FPANT_2826;Ontology_term=membrane,lysophospholipase activity,phosphatidyl phospholipase B activity,phospholipid
Fv_160_8.g4775	398	ID=Fv_160_8.g4775;Description=hypothetical protein FVER53590_07274 [Fusarium verticillioides];Gene=Forpi1262_v004983;Ontology_term=nucleus,ribonucleoprotein complex,RNA binding,RNA

Fv_160_8.g4776	297	ID=Fv_160_8.g4776;Description=myo-inositol-1(or 4)-monophosphatase [Fusarium verticillioides 7600];Gene=FPANT_2824;Ontology_term=nucleus,membrane,RNA polymerase II cis-regulatory region sequence-specific DNA binding,DNA-binding transcription factor activity, RNA polymerase II-specific,inositol monophosphate 1-phosphatase activity,metal ion binding,protein dimerization activity,inositol monophosphate 3-phosphatase activity,inositol monophosphate 4-phosphatase activity,negative regulation of transcription by RNA polymerase II,inositol biosynthetic process,Notch signaling pathway,somatic muscle development,anterior/posterior pattern specification,R7 cell differentiation,phosphatidylinositol phosphate biosynthetic process,R1/R6 cell differentiation,regulation of neurogenesis,regulation of neuroblast
Fv_160_8.g4777	726	ID=Fv_160_8.g4777;Description=3-phytase [Fusarium denticulatum];Gene=FNAPI_10107;Ontology_term=membrane,inositol monophosphate 1-phosphatase activity,3-phytase activity,metal ion binding,phosphatidylinositol phosphate biosynthetic process;Ontology_id=GO:0016020,GO:0008934,GO:0016158,GO:0046872,GO:0046854;Enzyme_code=EC:3.1.3.25,EC:3.1.3.8;Enzym
Fv_160_8.g4778	452	ID=Fv_160_8.g4778;Description=hypothetical protein FVEG_07278 [Fusarium verticillioides 7600]
Fv_160_8.g4779	1131	ID=Fv_160_8.g4779;Description=hypothetical protein FVER14953_07279 [Fusarium verticillioides]
Fv_160_8.g4780	1862	ID=Fv_160_8.g4780;Description=hypothetical protein FVER14953_07280 [Fusarium verticillioides];Gene=FTJAE_5086;Ontology_term=plasma membrane,myosin complex,cytoskeletal motor activity,actin binding,chitin synthase activity,ATP binding,conidium
Fv_160_8.g4781	1780	ID=Fv_160_8.g4781;Description=chitin synthase [Fusarium verticillioides 7600];Gene=FFC1_11719;Ontology_term=plasma membrane,myosin complex,cytoskeletal motor activity,chitin synthase activity,ATP binding,conidium formation;Ontology_id=GO:0005886,GO:0016459,GO:0003774,GO:0004100,GO:0005524,GO:0048315;Enzyme_code=EC:2.4.1.16;E
Fv_160_8.g4782	415	ID=Fv_160_8.g4782;Description=hypothetical protein FVER14953_07282 [Fusarium verticillioides];Gene=FTJAE_5088;Ontology_term=mitochondrial matrix,protein kinase activity,ATP
Fv_160_8.g4783	71	ID=Fv_160_8.g4783;Description=hypothetical protein FVEG_07283 [Fusarium verticillioides 7600];Gene=FSUBG_11775;Ontology_term=mitochondrial matrix,protein kinase activity,ATP
Fv_160_8.g4784	536	ID=Fv_160_8.g4784;Description=hypothetical protein FVER14953_07284 [Fusarium verticillioides];Gene=FSUBG_11774;Ontology_term=membrane,monooxygenase activity,iron ion binding,oxidoreductase activity, acting on paired donors, with incorporation or reduction of molecular oxygen,heme binding;Ontology_id=GO:0016020,GO:0004497,GO:0005506,GO:0016705,GO:0020037;Enzyme_code=EC:1.14;Enzyme_name=Actin
Fv_160_8.g4785	192	ID=Fv_160_8.g4785;Description=hypothetical protein FVEG_16064 [Fusarium verticillioides 7600]
Fv_160_8.g4786	282	ID=Fv_160_8.g4786;Description=hypothetical protein FVEG_07285 [Fusarium verticillioides 7600];Gene=FNAPI_13505;Ontology_term=Golgi membrane,small GTPase binding,vesicle-mediated

Fv_160_8.g4787	856	ID=Fv_160_8.g4787;Description=hypothetical protein FVER53590_07286 [Fusarium verticillioides];Gene=FRV6_05491;Ontology_term=positive regulation of translational termination,ribosome,positive regulation of translational elongation,RNA polymerase I preinitiation complex assembly,RNA binding,RNA polymerase I core promoter sequence-specific DNA binding,structural constituent of ribosome,translation elongation factor activity,metal ion binding,RNA polymerase I core factor complex,cytoplasm,ribosome binding,ribonucleoprotein
Fv_160_8.g4788	662	ID=Fv_160_8.g4788;Description=hypothetical protein FVER53590_07290 [Fusarium verticillioides];Gene=FVER53263_07290;Ontology_term=nucleosome,nucleus,Golgi apparatus,double-stranded DNA binding,protein binding,structural constituent of chromatin,nucleosomal DNA binding,nucleosome assembly,protein transport,chromosome condensation,heterochromatin formation,negative regulation of DNA
Fv_160_8.g4789	416	ID=Fv_160_8.g4789;Description=hypothetical protein FVEG_07291 [Fusarium verticillioides 7600];Gene=FACUT_958;Ontology_term=GTPase activator activity,vesicle-mediated
Fv_160_8.g4790	1310	ID=Fv_160_8.g4790;Description=hypothetical protein FVER53590_07293 [Fusarium verticillioides]
Fv_160_8.g4791	1055	ID=Fv_160_8.g4791;Description=translation initiation factor 5B [Fusarium verticillioides 7600];Gene=FVER53590_07294;Ontology_term=translation initiation factor activity,GTPase activity,GTP binding,translational initiation;Ontology_id=GO:0003743,GO:0003924,GO:0005525,GO:0006413;Enzyme_code=EC:3.6.1.15;Enzyme_name=nucleoside-
Fv_160_8.g4792	783	ID=Fv_160_8.g4792;Description=hsp88-like protein [Fusarium verticillioides 7600];Gene=FNYG_15256;Ontology_term=ATP binding,ATP-dependent protein folding chaperone,protein folding;Ontology_id=GO:0005524,GO:0140662,GO:0006457
Fv_160_8.g4793	768	ID=Fv_160_8.g4793;Description=chitin synthase [Fusarium denticulatum];Gene=FTJAE_5098;Ontology_term=plasma membrane,chitin synthase activity,conidium
Fv_160_8.g4794	1122	ID=Fv_160_8.g4794;Description=hypothetical protein FVEG_07297 [Fusarium verticillioides 7600];Gene=FSUBG_11763;Ontology_term=cytoplasm,RNA binding,nuclear-transcribed mRNA catabolic process, nonsense-
Fv_160_8.g4795	907	ID=Fv_160_8.g4795;Description=nitrate reductase [NADPH] [Fusarium verticillioides 7600];Gene=FTJAE_5100;Ontology_term=heme binding,molybdenum ion binding,molybdopterin cofactor binding,nitrate reductase (NADPH) activity,nitric oxide biosynthetic process,nitrate assimilation;Ontology_id=GO:0020037,GO:0030151,GO:0043546,GO:0050464,GO:0006809,GO:0042128;Enzyme_code=EC:1.7.1.2,E
Fv_160_8.g4796	470	ID=Fv_160_8.g4796;Description=hypothetical protein FVER14953_07299 [Fusarium verticillioides];Gene=FNAPI_4113;Ontology_term=vacuolar membrane,calcium:proton antiporter activity,calcium ion
Fv_160_8.g4797	1097	ID=Fv_160_8.g4797;Description=hypothetical protein J7337_010691 [Fusarium musae];Gene=FNAPI_4114;Ontology_term=nucleic acid binding,helicase activity,ATP binding,hydrolase activity,intracellular
Fv_160_8.g4798	438	ID=Fv_160_8.g4798;Description=DEAD/DEAH box helicase [Fusarium verticillioides 7600];Gene=FACUT_949;Ontology_term=nucleic acid binding,helicase activity,ATP binding,hydrolase activity,intracellular
Fv_160_8.g4799	299	ID=Fv_160_8.g4799;Description=uncharacterized protein FSUBG_13713 [Fusarium subglutinans];Gene=FACUT_948;Ontology_term=Golgi membrane,endoplasmic reticulum to Golgi vesicle-mediated

Fv_160_8.g4800	659	ID=Fv_160_8.g4800;Description=hypothetical protein FVEG_07303 [Fusarium verticillioides 7600];Gene=Adh;Ontology_term=alcohol dehydrogenase (NAD+) activity,alcohol metabolic
Fv_160_8.g4801	528	ID=Fv_160_8.g4801;Description=hypothetical protein FVEG_16068 [Fusarium verticillioides 7600];Gene=pyp2;Ontology_term=cytoplasm,alcohol dehydrogenase (NAD+) activity,protein binding,MAP kinase tyrosine phosphatase activity,alcohol metabolic process,cell cycle,dephosphorylation,cell division,negative regulation of p38MAPK cascade;Ontology_id=GO:0005737,GO:0004022,GO:0005515,GO:0033550,GO:0006066,GO:0007049,GO:0016311,GO:0051301,GO:
Fv_160_8.g4802	603	ID=Fv_160_8.g4802;Description=hypothetical protein FOFC_06149 [Fusarium oxysporum];Gene=FOXB_10101;Ontology_term=mitochondrial inner membrane,RNA polymerase I core promoter sequence-specific DNA binding,RNA polymerase I general transcription initiation factor activity,RNA polymerase I preinitiation complex
Fv_160_8.g4803	325	ID=Fv_160_8.g4803;Description=hypothetical protein FVEG_07305 [Fusarium verticillioides 7600];Gene=FTJAE_9781;Ontology_term=membrane organization,posterior head segmentation,cell morphogenesis,periodic partitioning by pair rule gene,trunk segmentation,protein dimerization activity,DNA-binding transcription repressor activity, RNA polymerase II-specific,open tracheal system development,salivary gland morphogenesis,negative regulation of transcription by RNA polymerase II,transcription corepressor binding,nucleus,response to hypoxia,arginine biosynthetic process,mitochondrion,tube morphogenesis,transferase activity,regulation of neurogenesis,E-box
Fv_160_8.g4804	661	ID=Fv_160_8.g4804;Description=hypothetical protein FVER53590_07306 [Fusarium verticillioides];Gene=FVEG_07306;Ontology_term=mitochondrion,acetyl-CoA:L-glutamate N-acetyltransferase activity,methione N-acyltransferase activity,arginine biosynthetic
Fv_160_8.g4805	450	ID=Fv_160_8.g4805;Description=hypothetical protein FVER14953_07307 [Fusarium
Fv_160_8.g4806	307	ID=Fv_160_8.g4806;Description=hypothetical protein FVER14953_07308 [Fusarium verticillioides];Gene=FVEG_07308;Ontology_term=proton-transporting V-type ATPase, V0 domain,oxidoreductase activity, acting on the CH-OH group of donors, NAD or NADP as acceptor,proton-transporting ATPase activity, rotational mechanism,NADP binding,NAD binding,proton transmembrane transport;Ontology_id=GO:0033179,GO:0016616,GO:0046961,GO:0050661,GO:0051287,GO:1902600;Enzyme_code=EC:1.1.1,EC:7.
Fv_160_8.g4807	74	ID=Fv_160_8.g4807;Description=V-type H+-transporting ATPase subunit H [Fusarium oxysporum f. sp. lycopersici 4287];Gene=A0A2L2T4R6;Ontology_term=proton-transporting V-type ATPase, V0 domain,proton-transporting ATPase activity, rotational mechanism,proton transmembrane
Fv_160_8.g4808	287	ID=Fv_160_8.g4808;Description=hypothetical protein FVEG_16070 [Fusarium verticillioides 7600];Gene=FANTH_5242;Ontology_term=Golgi membrane,endoplasmic reticulum membrane,cortical endoplasmic reticulum,sphingolipid metabolic process,sterol metabolic process,intracellular sterol transport,regulation of plasma membrane
Fv_160_8.g4809	627	ID=Fv_160_8.g4809;Description=hypothetical protein FVER53590_07310 [Fusarium verticillioides]
Fv_160_8.g4810	909	ID=Fv_160_8.g4810;Description=hypothetical protein FVER53263_07311 [Fusarium verticillioides];Gene=BFJ72_g14340;Ontology_term=nucleus,DNA binding,zinc ion binding,DNA-templated

Fv_160_8.g4811	981	ID=Fv_160_8.g4811;Description=GPI ethanolamine phosphate transferase 1 [Fusarium verticillioides 7600];Gene=FACUT_936;Ontology_term=endoplasmic reticulum membrane,DNA binding,mannose-ethanolamine phosphotransferase activity,cell wall
Fv_160_8.g4812	467	ID=Fv_160_8.g4812;Description=hypothetical protein FVER14953_07316 [Fusarium verticillioides];Gene=FOC4_g10006814;Ontology_term=endoplasmic reticulum membrane,monopolin complex,DNA binding,mannose-ethanolamine phosphotransferase activity,cell wall
Fv_160_8.g4813	408	ID=Fv_160_8.g4813;Description=adenosinetriphosphatase [Fusarium verticillioides 7600];Gene=FCIRC_5634;Ontology_term=mitochondrial outer membrane,peroxisomal membrane,ATP binding,ATP hydrolysis activity,membrane protein dislocase activity,protein targeting to mitochondrion,protein hexamerization,extraction of mislocalized protein from mitochondrial outer membrane;Ontology_id=GO:0005741,GO:0005778,GO:0005524,GO:0016887,GO:0140567,GO:0006626,GO:0034214,GO:0140570;E
Fv_160_8.g4814	462	ID=Fv_160_8.g4814;Description=cystathionine beta-lyase [Fusarium verticillioides 7600];Gene=FTJAE_9771;Ontology_term=cystathionine beta-lyase activity,pyridoxal phosphate binding,transsulfuration,'de novo' L-methionine biosynthetic
Fv_160_8.g4815	475	ID=Fv_160_8.g4815;Description=hypothetical protein FVEG_07319 [Fusarium verticillioides
Fv_160_8.g4816	574	ID=Fv_160_8.g4816;Description=hypothetical protein FVEG_07320 [Fusarium verticillioides 7600];Gene=Forpi1262_v005027;Ontology_term=membrane,transmembrane transporter activity,transmembrane
Fv_160_8.g4817	1123	ID=Fv_160_8.g4817;Description=hypothetical protein FVER14953_07321 [Fusarium verticillioides];Gene=bst-1;Ontology_term=endoplasmic reticulum membrane,hydrolase activity, acting on ester bonds,lyase activity,protein transport;Ontology_id=GO:0005789,GO:0016788,GO:0016829,GO:0015031;Enzyme_code=EC:3.1,EC:4;Enzyme_name=Acting on
Fv_160_8.g4818	1035	ID=Fv_160_8.g4818;Description=hypothetical protein J7337_010671 [Fusarium musae];Gene=CEK26_011988;Ontology_term=cytoplasm,mismatch repair complex,alcohol dehydrogenase (NAD+) activity,glutathione transferase activity,glutathione peroxidase activity,ATP binding,ATP hydrolysis activity,DDT-dehydrochlorinase activity,mismatched DNA binding,ATP-dependent DNA damage sensor activity,alcohol metabolic process,mismatch repair,glutathione metabolic process,meiosis I cell cycle process,cellular oxidant detoxification;Ontology_id=GO:0005737,GO:0032300,GO:0004022,GO:0004364,GO:0004602,GO:0005524,GO:0016887,GO:001883
Fv_160_8.g4819	591	ID=Fv_160_8.g4819;Description=hypothetical protein FVEG_07323 [Fusarium verticillioides
Fv_160_8.g4820	450	ID=Fv_160_8.g4820;Description=ferredoxin-NADP+ reductase [Fusarium verticillioides 7600];Gene=FOC4_g10006803;Ontology_term=mitochondrion,NADPH-adrenodoxin reductase
Fv_160_8.g4821	347	ID=Fv_160_8.g4821;Description=hypothetical protein FVER53590_07325 [Fusarium verticillioides];Gene=FACUT_926;Ontology_term=mitochondrial matrix,electron transfer activity,flavin adenine dinucleotide binding,electron transport
Fv_160_8.g4822	672	ID=Fv_160_8.g4822;Description=hypothetical protein FVER14953_07326 [Fusarium verticillioides]

Fv_160_8.g4823	610	ID=Fv_160_8.g4823;Description=carnitine O-acetyltransferase [Fusarium verticillioides 7600];Gene=FOXG_04209;Ontology_term=acyltransferase
Fv_160_8.g4824	539	ID=Fv_160_8.g4824;Description=hypothetical protein FVEG_07328 [Fusarium verticillioides 7600];Gene=HZS61_014325;Ontology_term=actin binding,cytoskeleton organization;Ontology_id=GO:0003779,GO:0007010
Fv_160_8.g4825	478	ID=Fv_160_8.g4825;Description=hypothetical protein FVEG_07329 [Fusarium verticillioides 7600];Gene=FNAPI_4600;Ontology_term=kinase
Fv_160_8.g4826	880	ID=Fv_160_8.g4826;Description=minichromosome maintenance protein 3 [Fusarium verticillioides 7600];Gene=FCIRC_13202;Ontology_term=chromosome, telomeric region,nuclear pre-replicative complex,cytoplasm,replication fork protection complex,MCM complex,CMG complex,chromatin binding,DNA replication origin binding,ATP binding,ATP hydrolysis activity,single-stranded DNA helicase activity,MCM complex binding,double-strand break repair via break-induced replication,pre-replicative complex assembly involved in nuclear cell cycle DNA replication,DNA unwinding involved in DNA replication,DNA strand elongation involved in DNA replication,premeiotic DNA replication,regulation of DNA-templated DNA replication initiation,silent mating-type cassette heterochromatin formation,subtelomeric heterochromatin formation,mitotic DNA replication
Fv_160_8.g4827	632	ID=Fv_160_8.g4827;Description=exocyst complex component exo70 [Fusarium musae];Gene=FPHYL_12184;Ontology_term=exocyst,cellular bud neck,phosphatidylinositol-4,5-bisphosphate
Fv_160_8.g4828	1207	ID=Fv_160_8.g4828;Description=hypothetical protein FVER53590_07333 [Fusarium verticillioides];Gene=FPHYL_12185;Ontology_term=nucleus,RNA binding,ATP binding,poly(A) RNA polymerase activity,mRNA processing,RNA 3'-end
Fv_160_8.g4829	337	ID=Fv_160_8.g4829;Description=hypothetical protein FVER14953_07334 [Fusarium verticillioides]
Fv_160_8.g4830	771	ID=Fv_160_8.g4830;Description=protein transporter SEC23 [Fusarium oxysporum f. sp. lycopersici 4287];Gene=FMUND_11802;Ontology_term=Golgi membrane,endoplasmic reticulum membrane,ribosome,COPII vesicle coat,ribonucleoprotein complex,structural constituent of ribosome,zinc ion binding,translation,intracellular protein transport,COPII-
Fv_160_8.g4831	165	ID=Fv_160_8.g4831;Description=60S ribosomal protein L12 [Fusarium oxysporum f. sp. lycopersici 4287];Gene=BFJ72_g13491;Ontology_term=ribosome,ribonucleoprotein complex,structural constituent of
Fv_160_8.g4832	1497	ID=Fv_160_8.g4832;Description=hypothetical protein FVER53590_07337 [Fusarium verticillioides];Gene=FACUT_10448;Ontology_term=glycosyltransferase
Fv_160_8.g4833	281	ID=Fv_160_8.g4833;Description=myb-like DNA-binding protein FlbD [Fusarium verticillioides
Fv_160_8.g4834	441	ID=Fv_160_8.g4834;Description=hypothetical protein FVEG_07341 [Fusarium verticillioides 7600];Gene=Adh;Ontology_term=U2-type catalytic step 1 spliceosome,U2-type catalytic step 2 spliceosome,post-mRNA release spliceosomal complex,first spliceosomal transesterification activity,second spliceosomal transesterification activity,alcohol dehydrogenase (NAD+) activity,protein binding,generation of catalytic spliceosome for second transesterification step,alcohol metabolic process;Ontology_id=GO:0071006,GO:0071007,GO:0071014,GO:0000384,GO:0000386,GO:0004022,GO:0005515,GO:0000350,GO:

Fv_160_8.g4835	473	ID=Fv_160_8.g4835;Description=hypothetical protein FVEG_16076 [Fusarium verticillioides 7600];Gene=FANTH_11369;Ontology_term=mitochondrial inner membrane,respirasome;Ontology_id=GO:0005743,GO:0070469
Fv_160_8.g4836	104	ID=Fv_160_8.g4836;Description=NADH dehydrogenase (ubiquinone) Fe-S protein 5 [Fusarium verticillioides 7600];Gene=FVER53263_07342;Ontology_term=mitochondrial inner
Fv_160_8.g4837	921	ID=Fv_160_8.g4837;Description=hypothetical protein FVEG_07343 [Fusarium verticillioides 7600];Gene=FTJAE_11697;Ontology_term=endoplasmic reticulum,retrograde vesicle-mediated transport, Golgi to endoplasmic
Fv_160_8.g4838	350	ID=Fv_160_8.g4838;Description=hypothetical protein FVEG_07344 [Fusarium verticillioides
Fv_160_8.g4839	249	ID=Fv_160_8.g4839;Description=Short-chain dehydrogenase/reductase aba4 [Fusarium musae]
Fv_160_8.g4840	190	ID=Fv_160_8.g4840;Description=acetyltransferase [Fusarium verticillioides 7600];Gene=FNYG_01918;Ontology_term=acyltransferase activity, transferring groups other than amino-acyl
Fv_160_8.g4841	376	ID=Fv_160_8.g4841;Description=hypothetical protein J7337_010649 [Fusarium musae]
Fv_160_8.g4842	609	ID=Fv_160_8.g4842;Description=cell division cycle 20-like protein 1, cofactor-APC complex [Fusarium verticillioides 7600];Gene=FOC4_g10006779;Ontology_term=positive regulation of anaphase-promoting complex-dependent catabolic process,anaphase-promoting complex,dihydroorotase activity,'de novo' UMP biosynthetic process,deactivation of mitotic spindle assembly checkpoint,'de novo' pyrimidine nucleobase biosynthetic process,metal ion binding,cytoplasm,positive regulation of protein ubiquitination,ubiquitin ligase activator activity,cyclin binding,anaphase-promoting complex binding,positive regulation of mitotic actomyosin contractile ring disassembly,negative regulation of mitotic spindle pole body
Fv_160_8.g4843	566	ID=Fv_160_8.g4843;Description=hypothetical protein FVER53590_07349 [Fusarium verticillioides];Gene=BFJ69_g11563;Ontology_term=membrane,acyltransferase activity,lipid metabolic
Fv_160_8.g4844	1260	ID=Fv_160_8.g4844;Description=structural maintenance-chromosome 1 [Fusarium verticillioides 7600];Gene=FVER53590_07350;Ontology_term=nucleus,cohesin complex,ATP binding,ATP hydrolysis activity,sister chromatid cohesion,cell
Fv_160_8.g4845	376	ID=Fv_160_8.g4845;Description=hypothetical protein FVER53590_07351 [Fusarium verticillioides];Gene=FVER53590_07351;Ontology_term=translation initiation factor activity,transcription initiation at RNA
Fv_160_8.g4846	474	ID=Fv_160_8.g4846;Description=hypothetical protein FVEG_07352 [Fusarium verticillioides 7600]
Fv_160_8.g4847	276	ID=Fv_160_8.g4847;Description=hypothetical protein FVER14953_07353 [Fusarium verticillioides]
Fv_160_8.g4848	644	ID=Fv_160_8.g4848;Description=hypothetical protein FVER53590_07354 [Fusarium verticillioides];Gene=FVER53590_07354;Ontology_term=endoplasmic reticulum,signal recognition particle, endoplasmic reticulum targeting,7S RNA binding,SRP-dependent cotranslational protein targeting to
Fv_160_8.g4849	1406	ID=Fv_160_8.g4849;Description=hypothetical protein FVER53590_07355 [Fusarium verticillioides];Gene=FOMG_02946;Ontology_term=guanyl-nucleotide exchange factor activity,regulation of ARF protein signal
Fv_160_8.g4850	557	ID=Fv_160_8.g4850;Description=hypothetical protein FVER53590_07357 [Fusarium verticillioides];Gene=BFJ70_g14385;Ontology_term=extracellular region,metallocarboxypeptidase activity,zinc ion

Fv_160_8.g4851	149	ID=Fv_160_8.g4851;Description=glutathione S-transferase [Fusarium verticillioides 7600];Gene=FMAN_11390;Ontology_term=membrane,transferase
Fv_160_8.g4852	584	ID=Fv_160_8.g4852;Description=hypothetical protein FVER53263_07358 [Fusarium verticillioides];Gene=FNAPI_12988;Ontology_term=nucleus,DNA binding,DNA-directed DNA polymerase activity,metal ion binding,DNA repair,DNA biosynthetic
Fv_160_8.g4853	405	ID=Fv_160_8.g4853;Description=hypothetical protein FVER14953_07359 [Fusarium verticillioides];Gene=FOMG_02942;Ontology_term=RNA 7-methylguanosine cap binding,mRNA
Fv_160_8.g4854	1740	ID=Fv_160_8.g4854;Description=glycosyl hydrolase family 47-domain-containing protein [Fusarium oxysporum];Gene=FPANT_8113;Ontology_term=cytosol,endomembrane system,membrane coat,mannosyl-oligosaccharide 1,2-alpha-mannosidase activity,calcium ion binding,oxidoreductase activity,carbohydrate metabolic process,intracellular protein transport,endosomal transport,cytosolic transport,ubiquitin-dependent ERAD pathway;Ontology_id=GO:0005829,GO:0012505,GO:0030117,GO:0004571,GO:0005509,GO:0016491,GO:0005975,GO:0006886,GO:
Fv_160_8.g4855	149	ID=Fv_160_8.g4855;Description=uncharacterized protein NECHADRAFT_72399 [Fusarium vanettenii 77-13-4];Gene=F66182_9080;Ontology_term=calcium ion binding;Ontology_id=GO:0005509
Fv_160_8.g4856	344	ID=Fv_160_8.g4856;Description=syntaxin 16 [Fusarium verticillioides 7600];Gene=FCIRC_3474;Ontology_term=trans-Golgi network,endosome membrane,SNARE complex,SNAP receptor activity,mannosyl-inositol phosphorylceramide metabolic process,Golgi to vacuole transport,endocytosis,vesicle fusion,protein secretion,protein exit from endoplasmic
Fv_160_8.g4857	844	ID=Fv_160_8.g4857;Description=hypothetical protein FVEG_07364 [Fusarium verticillioides 7600]
Fv_160_8.g4858	69	ID=Fv_160_8.g4858;Description=hypothetical protein FVEG_07365 [Fusarium verticillioides 7600];Gene=BFJ68_g13399;Ontology_term=mitochondrial inner membrane;Ontology_id=GO:0005743
Fv_160_8.g4859	238	ID=Fv_160_8.g4859;Description=hypothetical protein J7337_010630 [Fusarium musae];Gene=FOYG_00549;Ontology_term=endosome,clathrin coat of trans-Golgi network vesicle,clathrin coat of coated pit,actin cortical patch,structural molecule activity,intracellular protein transport,exocytosis,clathrin-dependent
Fv_160_8.g4860	410	ID=Fv_160_8.g4860;Description=hypothetical protein FVEG_07367 [Fusarium verticillioides 7600];Gene=FTJAE_11720;Ontology_term=RNA polymerase II C-terminal domain binding,mRNA 3'-end
Fv_160_8.g4861	798	ID=Fv_160_8.g4861;Description=hypothetical protein FVER14953_07368 [Fusarium verticillioides];Gene=FTJAE_11721;Ontology_term=mitochondrion,membrane,ATP binding,ATP hydrolysis activity,ABC-type transporter activity,transmembrane transport;Ontology_id=GO:0005739,GO:0016020,GO:0005524,GO:0016887,GO:0140359,GO:0055085;Enzyme_code=EC:7.2.2,EC:3.
Fv_160_8.g4862	299	ID=Fv_160_8.g4862;Description=hypothetical protein FVEG_07369 [Fusarium verticillioides 7600];Gene=FCIRC_3468;Ontology_term=peptidase

Fv_160_8.g4863	454	ID=Fv_160_8.g4863;Description=hypothetical protein FVER14953_07370 [Fusarium verticillioides];Gene=FPHYL_13291;Ontology_term=nucleus,membrane,DNA binding,alcohol dehydrogenase (NAD+) activity,monoatomic cation transmembrane transporter activity,alcohol metabolic process,regulation of DNA-templated transcription,intracellular monoatomic cation homeostasis,monoatomic cation transmembrane transport,inorganic ion homeostasis;Ontology_id=GO:0005634,GO:0016020,GO:0003677,GO:0004022,GO:0008324,GO:0006066,GO:0006355,GO:0030003,
Fv_160_8.g4864	790	ID=Fv_160_8.g4864;Description=hypothetical protein FVER53263_07372 [Fusarium verticillioides];Gene=FVER53590_07372;Ontology_term=glycosyltransferase
Fv_160_8.g4865	839	ID=Fv_160_8.g4865;Description=E3 ubiquitin-protein ligase synoviolin [Fusarium verticillioides 7600];Gene=FNAPI_2355;Ontology_term=membrane,zinc ion binding,ubiquitin-dependent protein catabolic process,protein
Fv_160_8.g4866	254	ID=Fv_160_8.g4866;Description=hypothetical protein FVEG_07374 [Fusarium verticillioides 7600]
Fv_160_8.g4867	632	ID=Fv_160_8.g4867;Description=zinc finger protein [Fusarium coicis]
Fv_160_8.g4868	406	ID=Fv_160_8.g4868;Description=hypothetical protein FVER14953_07377 [Fusarium
Fv_160_8.g4869	1189	ID=Fv_160_8.g4869;Description=hypothetical protein FVER53590_07379 [Fusarium
Fv_160_8.g4870	1195	ID=Fv_160_8.g4870;Description=hypothetical protein FVER53590_07380 [Fusarium verticillioides];Gene=FocTR4_00003841;Ontology_term=adherens junction,extrinsic component of cytoplasmic side of plasma membrane,non-membrane spanning protein tyrosine kinase activity,ATP binding,phospholipid binding,dorsal closure, elongation of leading edge cells,axon guidance,vesicle-mediated transport,phosphorylation,dorsal closure, amnioserosa morphology change,actin filament bundle
Fv_160_8.g4871	722	ID=Fv_160_8.g4871;Description=hypothetical protein FVER14953_07381 [Fusarium verticillioides];Gene=FACUT_2409;Ontology_term=Golgi membrane,DNA binding,intra-Golgi vesicle-mediated
Fv_160_8.g4872	264	ID=Fv_160_8.g4872;Description=hypothetical protein FVEG_07382 [Fusarium verticillioides 7600]
Fv_160_8.g4873	853	ID=Fv_160_8.g4873;Description=hypothetical protein FVEG_07383 [Fusarium verticillioides 7600]
Fv_160_8.g4874	185	ID=Fv_160_8.g4874;Description=60S ribosomal protein L17 [Fusarium verticillioides 7600];Gene=FCIRC_5679;Ontology_term=large ribosomal subunit,structural constituent of ribosome,translation;Ontology_id=GO:0015934,GO:0003735,GO:0006412
Fv_160_8.g4875	319	ID=Fv_160_8.g4875;Description=hypothetical protein FVEG_07385 [Fusarium verticillioides 7600];Gene=FMEXI_14240;Ontology_term=plasma membrane,receptor complex,nucleotide binding,RNA binding,fibroblast growth factor receptor activity,protein binding,S-adenosylmethionine-dependent methyltransferase activity,rRNA processing,transmembrane receptor protein tyrosine kinase signaling pathway,epithelial cell migration, open tracheal system,secondary branching, open tracheal system,positive regulation of cell population proliferation,glial cell migration,phosphorylation,negative regulation of axon extension,methylation,positive regulation of melanization defense response,terminal cell fate specification, open tracheal system,tracheal pit formation in open tracheal system,genital disc development,negative regulation of crystal cell differentiation,negative regulation of plasmatocyte differentiation;Ontology_id=GO:0005886,GO:0043235,GO:0000166,GO:0003723,GO:0005007,GO:0005515,GO:0008757,GO:000636

Fv_160_8.g4876	517	ID=Fv_160_8.g4876;Description=hypothetical protein FVEG_07386 [Fusarium verticillioides 7600];Gene=FOMG_02915;Ontology_term=membrane,monooxygenase activity,iron ion binding,oxidoreductase activity, acting on paired donors, with incorporation or reduction of molecular oxygen,heme binding;Ontology_id=GO:0016020,GO:0004497,GO:0005506,GO:0016705,GO:0020037;Enzyme_code=EC:1.14;Enzyme_name=Actin
Fv_160_8.g4877	725	ID=Fv_160_8.g4877;Description=hypothetical protein FVER53590_07387 [Fusarium verticillioides];Gene=FNAPI_2366;Ontology_term=cysteine-type endopeptidase
Fv_160_8.g4878	616	ID=Fv_160_8.g4878;Description=hypothetical protein J7337_010610 [Fusarium musae];Gene=FocTR4_00003851;Ontology_term=oxidoreductase
Fv_160_8.g4879	205	ID=Fv_160_8.g4879;Description=hypothetical protein FOXG_04267 [Fusarium oxysporum f. sp. lycopersici 4287];Gene=BFJ69_g8254;Ontology_term=cytoplasm,peptide-methionine (S)-S-oxide reductase activity,oxidoreductase activity, acting on NAD(P)H, nitrogenous group as acceptor,L-methionine-(S)-S-oxide reductase activity,sulfur amino acid metabolic process,determination of adult lifespan,cellular response to oxidative stress;Ontology_id=GO:0005737,GO:0008113,GO:0016657,GO:0036456,GO:0000096,GO:0008340,GO:0034599;Enzyme_code=EC:1
Fv_160_8.g4880	540	ID=Fv_160_8.g4880;Description=phosphatidylserine decarboxylase [Fusarium verticillioides 7600];Gene=PSD1;Ontology_term=mitochondrial inner membrane,phosphatidylserine decarboxylase activity,mitochondrial electron transport, ubiquinol to cytochrome c,phosphatidylethanolamine biosynthetic process,phosphatidylcholine biosynthetic process,positive regulation of mitochondrial fusion,positive regulation of protein processing,protein autoprocessing;Ontology_id=GO:0005743,GO:0004609,GO:0006122,GO:0006646,GO:0006656,GO:0010636,GO:0010954,GO:00165
Fv_160_8.g4881	766	ID=Fv_160_8.g4881;Description=hypothetical protein FVER14953_07391 [Fusarium
Fv_160_8.g4882	323	ID=Fv_160_8.g4882;Description=ubiquitin carboxyl-terminal hydrolase L5 [Fusarium verticillioides 7600];Gene=FSUBG_10166;Ontology_term=proteasome complex,cysteine-type deubiquitinase activity,deNEDDylase activity,cytoplasm protein quality control by the ubiquitin-proteasome
Fv_160_8.g4883	617	ID=Fv_160_8.g4883;Description=MFS transporter, AGZA family, xanthine/uracil permease [Fusarium verticillioides 7600];Gene=FCIRC_5688;Ontology_term=membrane,nucleobase transmembrane transporter activity,purine nucleobase
Fv_160_8.g4884	586	ID=Fv_160_8.g4884;Description=hypothetical protein FVEG_07394 [Fusarium verticillioides 7600]
Fv_160_8.g4885	667	ID=Fv_160_8.g4885;Description=hypothetical protein FVER53263_20394 [Fusarium verticillioides];Gene=Yp1;Ontology_term=extracellular space,orotate phosphoribosyltransferase activity,sex differentiation,lipid catabolic process,'de novo' UMP biosynthetic
Fv_160_8.g4886	459	ID=Fv_160_8.g4886;Description=hypothetical protein FVEG_07395 [Fusarium verticillioides 7600];Gene=BFJ63_vAg1959;Ontology_term=membrane,G protein-coupled receptor activity,cell surface receptor signaling pathway,G protein-coupled receptor signaling pathway,anatomical structure
Fv_160_8.g4887	228	ID=Fv_160_8.g4887;Description=hypothetical protein FVEG_07396 [Fusarium verticillioides 7600]
Fv_160_8.g4888	821	ID=Fv_160_8.g4888;Description=hypothetical protein FVER53263_07397 [Fusarium verticillioides];Gene=sima

Fv_160_8.g4889	443	ID=Fv_160_8.g4889;Description=hypothetical protein FVEG_07398 [Fusarium verticillioides 7600]
Fv_160_8.g4890	273	ID=Fv_160_8.g4890;Description=gluconate 5-dehydrogenase [Fusarium verticillioides 7600];Gene=FPHYL_9790;Ontology_term=oxidoreductase activity,cellular biosynthetic
Fv_160_8.g4891	421	ID=Fv_160_8.g4891;Description=hypothetical protein FVEG_07400 [Fusarium verticillioides 7600];Gene=FACUT_2429;Ontology_term=S-adenosylmethionine-dependent methyltransferase
Fv_160_8.g4892	140	ID=Fv_160_8.g4892;Description=hypothetical protein FVEG_07401 [Fusarium verticillioides 7600];Gene=AU210_005080;Ontology_term=proteasome complex,proteasome assembly;Ontology_id=GO:0000502,GO:0043248
Fv_160_8.g4893	943	ID=Fv_160_8.g4893;Description=hypothetical protein FVER53590_07402 [Fusarium verticillioides];Gene=AU210_005081;Ontology_term=cytoplasm,nuclear periphery,mitotic spindle midzone,small GTPase
Fv_160_8.g4894	559	ID=Fv_160_8.g4894;Description=hypothetical protein FVEG_07406 [Fusarium verticillioides 7600];Gene=FVEG_07406;Ontology_term=nucleus,DNA-binding transcription factor activity, RNA polymerase II-specific,zinc ion
Fv_160_8.g4895	412	ID=Fv_160_8.g4895;Description=protein ssh-4 [Fusarium verticillioides 7600];Gene=FOYG_00505;Ontology_term=vacuolar membrane,endosome membrane;Ontology_id=GO:0005774,GO:0010008
Fv_160_8.g4896	95	ID=Fv_160_8.g4896;Description=hypothetical protein FOXYS1_12050, partial [Fusarium oxysporum];Gene=FOYG_00505;Ontology_term=nucleus,vacuolar membrane,endosome membrane,DNA-binding transcription factor activity, RNA polymerase II-specific,sequence-specific DNA binding,regulation of glutamine family amino acid metabolic process,cellular response to starvation,regulation of mitochondrion organization,positive regulation of transcription by RNA
Fv_160_8.g4897	385	ID=Fv_160_8.g4897;Description=hypothetical protein FVER14953_07408 [Fusarium
Fv_160_8.g4898	617	ID=Fv_160_8.g4898;Description=hypothetical protein FVEG_07409 [Fusarium verticillioides 7600]
Fv_160_8.g4899	298	ID=Fv_160_8.g4899;Description=hypothetical protein FVEG_07410 [Fusarium verticillioides 7600];Gene=FMUND_11998;Ontology_term=endonuclease activity,exonuclease
Fv_160_8.g4900	263	ID=Fv_160_8.g4900;Description=hypothetical protein FVEG_07411 [Fusarium verticillioides 7600];Gene=grh;Ontology_term=nucleus,RNA polymerase II cis-regulatory region sequence-specific DNA binding,DNA-binding transcription activator activity, RNA polymerase II-specific,serine-type peptidase activity,protein homodimerization activity,metal ion binding,epithelial cell morphogenesis,proteolysis,ganglion mother cell fate determination,ventral cord development,tracheal outgrowth, open tracheal system,R3/R4 cell fate commitment,chitin-based embryonic cuticle biosynthetic process,fibroblast growth factor receptor signaling pathway,regulation of tube length, open tracheal system,rhabdomere development,regulation of cell population proliferation,positive regulation of transcription by RNA polymerase II,regulation of neurogenesis,membrane organization,regulation of wound
Fv_160_8.g4901	2075	ID=Fv_160_8.g4901;Description=hypothetical protein FVER53590_07412 [Fusarium verticillioides]

Fv_160_8.g4902	549	ID=Fv_160_8.g4902;Description=hypothetical protein FVEG_07413 [Fusarium verticillioides 7600];Gene=BFJ68_g15576;Ontology_term=cytoplasm,peptide-methionine (S)-S-oxide reductase activity,hydrolase activity,L-methionine-(S)-S-oxide reductase activity,sulfur amino acid metabolic process,determination of adult lifespan,cellular response to oxidative
Fv_160_8.g4903	250	ID=Fv_160_8.g4903;Description=hypothetical protein FVEG_07414 [Fusarium verticillioides 7600];Gene=FNAPI_6668;Ontology_term=DNA binding,zinc ion binding;Ontology_id=GO:0003677,GO:0008270
Fv_160_8.g4904	1365	ID=Fv_160_8.g4904;Description=hypothetical protein FVER14953_07415 [Fusarium verticillioides];Gene=FNAPI_6667;Ontology_term=S-adenosylmethionine-dependent methyltransferase
Fv_160_8.g4905	424	ID=Fv_160_8.g4905;Description=hypothetical protein FVER53590_07416 [Fusarium verticillioides];Gene=FNAPI_6666;Ontology_term=tRNA (m1A) methyltransferase complex,tRNA (adenine-N1)-methyltransferase
Fv_160_8.g4906	887	ID=Fv_160_8.g4906;Description=hypothetical protein FVEG_07417 [Fusarium verticillioides 7600];Gene=PROK;Ontology_term=cellular anatomical entity,serine-type endopeptidase activity,metal ion
Fv_160_8.g4907	739	ID=Fv_160_8.g4907;Description=hypothetical protein FVER14953_07418 [Fusarium verticillioides];Gene=FVEG_07418;Ontology_term=protein serine/threonine kinase activity,ATP
Fv_160_8.g4908	553	ID=Fv_160_8.g4908;Description=hypothetical protein FVER14953_07419 [Fusarium verticillioides]
Fv_160_8.g4909	398	ID=Fv_160_8.g4909;Description=hypothetical protein FVEG_07420 [Fusarium verticillioides 7600]
Fv_160_8.g4910	500	ID=Fv_160_8.g4910;Description=DNA primase large subunit [Fusarium verticillioides 7600];Gene=FACUT_3716;Ontology_term=alpha DNA polymerase:primase complex,DNA binding,metal ion binding,4 iron, 4 sulfur
Fv_160_8.g4911	92	ID=Fv_160_8.g4911;Description=hypothetical protein FOXG_04299 [Fusarium oxysporum f. sp. lycopersici 4287]
Fv_160_8.g4912	76	ID=Fv_160_8.g4912;Description=hypothetical protein FVEG_07424 [Fusarium verticillioides 7600]
Fv_160_8.g4913	134	ID=Fv_160_8.g4913;Description=hypothetical protein FVEG_07426 [Fusarium verticillioides 7600]
Fv_160_8.g4914	495	ID=Fv_160_8.g4914;Description=aldehyde dehydrogenase (NAD+) [Fusarium verticillioides 7600];Gene=FANTH_3540;Ontology_term=acetyl-CoA C-acyltransferase activity,oxidoreductase activity, acting on the aldehyde or oxo group of donors, NAD or NADP as acceptor,dioxygenase activity;Ontology_id=GO:0003988,GO:0016620,GO:0051213;Enzyme_code=EC:2.3.1.16,EC:1.2.1;Enzyme_name=acetyl-CoA C-
Fv_160_8.g4915	441	ID=Fv_160_8.g4915;Description=hypothetical protein FVEG_07428 [Fusarium verticillioides 7600];Gene=ydcJ-1;Ontology_term=dioxygenase activity;Ontology_id=GO:0051213;Enzyme_code=EC:1;Enzyme_name=Oxidoreductases
Fv_160_8.g4916	142	ID=Fv_160_8.g4916;Description=hypothetical protein FVER14953_07429 [Fusarium verticillioides]
Fv_160_8.g4917	241	ID=Fv_160_8.g4917;Description=hypothetical protein FVER14953_07430 [Fusarium verticillioides]
Fv_160_8.g4918	188	ID=Fv_160_8.g4918;Description=hypothetical protein FVEG_07431 [Fusarium verticillioides 7600];Gene=FACUT_3707;Ontology_term=succinate dehydrogenase complex,succinate dehydrogenase activity,electron transfer activity,metal ion binding,tricarboxylic acid cycle,electron transport

Fv_160_8.g4919	370	ID=Fv_160_8.g4919;Description=hypothetical protein FVEG_07432 [Fusarium verticillioides 7600];Gene=FCIRC_8602;Ontology_term=Golgi membrane,nucleus,endosome,cleavage furrow leading edge,ferric iron binding,oxidoreductase activity, acting on single donors with incorporation of molecular oxygen, incorporation of two atoms of oxygen,mitotic cytokinesis,cellular aromatic compound metabolic process;Ontology_id=GO:0000139,GO:0005634,GO:0005768,GO:0090689,GO:0008199,GO:0016702,GO:0000281,GO:0006725;Enzy
Fv_160_8.g4920	446	ID=Fv_160_8.g4920;Description=hypothetical protein FVER14953_07433 [Fusarium verticillioides];Gene=eEF2;Ontology_term=cytosol,intracellular organelle,ribonucleoprotein complex,translation elongation factor activity,GTPase activity,GTP binding,ribosome binding,translational elongation;Ontology_id=GO:0005829,GO:0043229,GO:1990904,GO:0003746,GO:0003924,GO:0005525,GO:0043022,GO:0006414;E
Fv_160_8.g4921	172	ID=Fv_160_8.g4921;Description=hypothetical protein FVER53590_07434 [Fusarium verticillioides];Gene=eEF1alpha2;Ontology_term=eukaryotic translation elongation factor 1 complex,translation elongation factor activity,GTPase activity,GTP binding,translational
Fv_160_8.g4922	368	ID=Fv_160_8.g4922;Description=hypothetical protein FVER53590_07435 [Fusarium verticillioides];Gene=FVEG_07435;Ontology_term=Golgi membrane,endoplasmic reticulum membrane,phagophore assembly site membrane,GTPase activity,GTP binding,autophagosome assembly,intracellular protein transport,endoplasmic reticulum to Golgi vesicle-mediated transport;Ontology_id=GO:0000139,GO:0005789,GO:0034045,GO:0003924,GO:0005525,GO:0000045,GO:0006886,GO:0006888;En
Fv_160_8.g4923	587	ID=Fv_160_8.g4923;Description=hypothetical protein FVER53590_07436 [Fusarium verticillioides];Gene=eEF1alpha1;Ontology_term=cytoplasm,translation elongation factor activity,GTPase activity,GTP binding,translational
Fv_160_8.g4924	202	ID=Fv_160_8.g4924;Description=hypothetical protein FVEG_07438 [Fusarium verticillioides 7600];Gene=FVEG_07438;Ontology_term=Golgi membrane,endoplasmic reticulum membrane,phagophore assembly site membrane,GTPase activity,GTP binding,acyltransferase activity, transferring groups other than amino-acyl groups,autophagosome assembly,intracellular protein transport,endoplasmic reticulum to Golgi vesicle-mediated transport;Ontology_id=GO:0000139,GO:0005789,GO:0034045,GO:0003924,GO:0005525,GO:0016747,GO:0000045,GO:0006886,GO
Fv_160_8.g4925	500	ID=Fv_160_8.g4925;Description=hypothetical protein FVER53263_07439 [Fusarium verticillioides];Gene=FVEG_07439;Ontology_term=membrane,transmembrane transporter activity,transmembrane
Fv_160_8.g4926	638	ID=Fv_160_8.g4926;Description=hypothetical protein FVEG_16101 [Fusarium verticillioides 7600];Gene=F25303_7413;Ontology_term=nucleus,plasma membrane,DNA-binding transcription factor activity, RNA polymerase II-specific,GTPase activity,GTP binding,zinc ion binding,oxidoreductase activity, acting on the CH-OH group of donors, NAD or NADP as acceptor,NAD+ binding,regulation of transcription by RNA polymerase II,fatty acid metabolic process,intracellular protein transport,Golgi to plasma membrane transport,vesicle docking involved in exocytosis,protein secretion,signaling;Ontology_id=GO:0005634,GO:0005886,GO:0000981,GO:0003924,GO:0005525,GO:0008270,GO:0016616,GO:00

Fv_160_8.g4927	287	ID=Fv_160_8.g4927;Description=hypothetical protein FVER14953_20333 [Fusarium verticillioides];Gene=FOVG_08619;Ontology_term=oxidoreductase activity, acting on the CH-OH group of donors, NAD or NADP as acceptor,NAD+ binding,fatty acid metabolic
Fv_160_8.g4928	403	ID=Fv_160_8.g4928;Description=hypothetical protein FVER14953_07441 [Fusarium verticillioides];Gene=FGLOB1_4;Ontology_term=monooxygenase
Fv_160_8.g4929	403	ID=Fv_160_8.g4929;Description=hypothetical protein FVEG_07442 [Fusarium verticillioides 7600];Gene=F25303_7415;Ontology_term=microtubule,membrane,GTPase activity,GTP
Fv_160_8.g4930	764	ID=Fv_160_8.g4930;Description=hypothetical protein FNAPI_1113 [Fusarium napiforme];Gene=vma2;Ontology_term=vacuolar proton-transporting V-type ATPase, V1 domain,protein binding,ATP binding,ATP hydrolysis activity,proton-transporting ATPase activity, rotational mechanism,vacuolar acidification,ATP metabolic process,proton transmembrane transport;Ontology_id=GO:0000221,GO:0005515,GO:0005524,GO:0016887,GO:0046961,GO:0007035,GO:0046034,GO:1902600;En
Fv_160_8.g4931	272	ID=Fv_160_8.g4931;Description=hypothetical protein FVEG_16105 [Fusarium verticillioides 7600];Gene=FNAPI_1112;Ontology_term=membrane,transmembrane transporter activity,transmembrane
Fv_160_8.g4932	247	ID=Fv_160_8.g4932;Description=hypothetical protein FVEG_16105 [Fusarium verticillioides 7600];Gene=FOPG_15176;Ontology_term=membrane,transmembrane transporter activity,transmembrane
Fv_160_8.g4933	318	ID=Fv_160_8.g4933;Description=hypothetical protein FVEG_07446 [Fusarium verticillioides 7600];Gene=BFJ69_g10674;Ontology_term=hydrolase activity,nitrogen compound metabolic
Fv_160_8.g4934	660	ID=Fv_160_8.g4934;Description=hypothetical protein FVEG_07447 [Fusarium verticillioides 7600];Gene=vma3;Ontology_term=vacuolar proton-transporting V-type ATPase, V0 domain,fungal-type vacuole membrane,nucleus,DNA-binding transcription factor activity, RNA polymerase II-specific,zinc ion binding,ATP hydrolysis activity,proton-transporting ATPase activity, rotational mechanism,regulation of transcription by RNA polymerase II,proton transmembrane
Fv_160_8.g4935	656	ID=Fv_160_8.g4935;Description=amidohydrolase ytcJ-like [Fusarium verticillioides 7600];Gene=C2S_4409;Ontology_term=hydrolase activity, acting on carbon-nitrogen (but not peptide) bonds;Ontology_id=GO:0016810;Enzyme_code=EC:3.5;Enzyme_name=Acting
Fv_160_8.g4936	361	ID=Fv_160_8.g4936;Description=hypothetical protein FVEG_07449 [Fusarium verticillioides 7600];Gene=FPANT_4519;Ontology_term=membrane,antiporter activity,proton transmembrane
Fv_160_8.g4937	565	ID=Fv_160_8.g4937;Description=hypothetical protein FVER53590_07450 [Fusarium verticillioides];Gene=HZS61_014435;Ontology_term=membrane,antiporter activity,proton transmembrane
Fv_160_8.g4938	544	ID=Fv_160_8.g4938;Description=cysteine synthase A [Fusarium verticillioides 7600]
Fv_160_8.g4939	566	ID=Fv_160_8.g4939;Description=hypothetical protein FVEG_16107 [Fusarium verticillioides 7600];Gene=FNYG_01818;Ontology_term=membrane,transmembrane transporter activity,transmembrane
Fv_160_8.g4940	289	ID=Fv_160_8.g4940;Description=hypothetical protein FVER53590_07455 [Fusarium verticillioides];Gene=Forpi1262_v005155;Ontology_term=fructose-bisphosphate aldolase activity,zinc ion binding,glycolytic

Fv_160_8.g4941	253	ID=Fv_160_8.g4941;Description=glutathione S-transferase [Fusarium verticillioides 7600];Gene=F25303_7426;Ontology_term=transferase
Fv_160_8.g4942	446	ID=Fv_160_8.g4942;Description=phosphatidylserine decarboxylase [Fusarium verticillioides 7600];Gene=FNAPI_1102;Ontology_term=phosphatidylserine decarboxylase activity,phospholipid biosynthetic
Fv_160_8.g4943	543	ID=Fv_160_8.g4943;Description=hypothetical protein FVEG_07458 [Fusarium verticillioides 7600];Gene=puc1;Ontology_term=cell division,nucleus,cyclin-dependent protein serine/threonine kinase regulator activity,positive regulation of G1/S transition of mitotic cell cycle,cytoplasm,cyclin-dependent protein kinase holoenzyme complex,signaling,spindle pole body,negative regulation of cell cycle switching, mitotic to meiotic cell
Fv_160_8.g4944	379	ID=Fv_160_8.g4944;Description=hypothetical protein FVER14953_20330 [Fusarium verticillioides];Gene=FNAPI_1100;Ontology_term=hydrolase activity,lyase
Fv_160_8.g4945	574	ID=Fv_160_8.g4945;Description=hypothetical protein FVEG_07459 [Fusarium verticillioides 7600]
Fv_160_8.g4946	232	ID=Fv_160_8.g4946;Description=hypothetical protein FVEG_07460 [Fusarium verticillioides 7600]
Fv_160_8.g4947	719	ID=Fv_160_8.g4947;Description=hypothetical protein J7337_010539 [Fusarium musae]
Fv_160_8.g4948	294	ID=Fv_160_8.g4948;Description=hypothetical protein FVER14953_07462 [Fusarium verticillioides]
Fv_160_8.g4949	110	ID=Fv_160_8.g4949;Description=hypothetical protein FVEG_07463 [Fusarium verticillioides 7600]
Fv_160_8.g4950	481	ID=Fv_160_8.g4950;Description=hypothetical protein FVEG_07464 [Fusarium verticillioides
Fv_160_8.g4951	182	ID=Fv_160_8.g4951;Description=hypothetical protein FVEG_07465 [Fusarium verticillioides 7600];Gene=MED11;Ontology_term=mediator complex,transcription coregulator activity,regulation of transcription by RNA
Fv_160_8.g4952	758	ID=Fv_160_8.g4952;Description=hypothetical protein J7337_010534 [Fusarium musae];Gene=FCIRC_6104;Ontology_term=nucleus,translation initiation factor activity,translational
Fv_160_8.g4953	561	ID=Fv_160_8.g4953;Description=hypothetical protein FVER53263_07467 [Fusarium verticillioides];Gene=FocTR4_00003950;Ontology_term=prospore membrane,endosome,cytosol,retromer complex,phosphatidylinositol binding,intracellular protein transport,ascospore-type prospore membrane formation,retrograde
Fv_160_8.g4954	159	ID=Fv_160_8.g4954;Description=hypothetical protein FVER14953_07468 [Fusarium verticillioides];Gene=FPANT_13368;Ontology_term=mitochondrial large ribosomal subunit,structural constituent of
Fv_160_8.g4955	169	ID=Fv_160_8.g4955;Description=hypothetical protein FVER53590_07469 [Fusarium verticillioides];Gene=FNAPI_1089;Ontology_term=FMN binding,oxidoreductase
Fv_160_8.g4956	700	ID=Fv_160_8.g4956;Description=molecular chaperone HtpG [Fusarium verticillioides 7600];Gene=FocTR4_00003953;Ontology_term=ATP binding,ATP hydrolysis activity,unfolded protein binding,ATP-dependent protein folding chaperone,protein

Fv_160_8.g4957	607	ID=Fv_160_8.g4957;Description=sterol O-acyltransferase [Fusarium verticillioides 7600];Gene=FMUND_12059;Ontology_term=endoplasmic reticulum membrane,plasma membrane,cortical endoplasmic reticulum,perinuclear endoplasmic reticulum,transmembrane receptor protein tyrosine kinase activity,O-acyltransferase activity,neurogenesis,internal genitalia morphogenesis,lipid droplet formation;Ontology_id=GO:0005789,GO:0005886,GO:0032541,GO:0097038,GO:0004714,GO:0008374,GO:0022008,GO:0035260,G
Fv_160_8.g4958	331	ID=Fv_160_8.g4958;Description=hypothetical protein FVER14953_07472 [Fusarium verticillioides]
Fv_160_8.g4959	265	ID=Fv_160_8.g4959;Description=hypothetical protein J7337_010527 [Fusarium musae]
Fv_160_8.g4960	1909	ID=Fv_160_8.g4960;Description=DNA helicase INO80 [Fusarium verticillioides 7600];Gene=FPHYL_8260;Ontology_term=Ino80 complex,DNA binding,helicase activity,ATP binding,hydrolase activity,ATP-dependent chromatin remodeler activity,DNA repair,chromatin remodeling,DNA-templated transcription;Ontology_id=GO:0031011,GO:0003677,GO:0004386,GO:0005524,GO:0016787,GO:0140658,GO:0006281,GO:0006338
Fv_160_8.g4961	618	ID=Fv_160_8.g4961;Description=ABC transporter ATP-binding protein ARB1 [Fusarium verticillioides 7600];Gene=BFJ69_g10683;Ontology_term=nucleus,ATP binding,ATP hydrolysis activity,ribosomal small subunit export from nucleus,protein quality control for misfolded or incompletely synthesized
Fv_160_8.g4962	196	ID=Fv_160_8.g4962;Description=hypothetical protein FVER14953_07476 [Fusarium verticillioides];Gene=RyR;Ontology_term=ryanodine-sensitive calcium-release channel activity,heart contraction,muscle contraction,regulation of hemocyte proliferation,response to anesthetic,release of sequestered calcium ion into cytosol,sarcoplasmic
Fv_160_8.g4963	540	ID=Fv_160_8.g4963;Description=hypothetical protein FVEG_07477 [Fusarium verticillioides 7600];Gene=FSUBG_4909;Ontology_term=primary cell septum,cellular bud scar,plasma membrane,COPII-coated ER to Golgi transport vesicle,nuclear periphery,membrane raft,side of membrane,1,3-beta-glucanosyltransferase activity,filamentous growth,fungal-type cell wall organization,heterochromatin formation,regulation of response to endoplasmic reticulum stress;Ontology_id=GO:0000936,GO:0005621,GO:0005886,GO:0030134,GO:0034399,GO:0045121,GO:0098552,GO:0042124,GO:00
Fv_160_8.g4964	689	ID=Fv_160_8.g4964;Description=hypothetical protein FPCIR_10821 [Fusarium pseudocircinatum]
Fv_160_8.g4965	737	ID=Fv_160_8.g4965;Description=hypothetical protein FVEG_07478 [Fusarium verticillioides 7600];Gene=FocTR4_00003964;Ontology_term=serine-type peptidase
Fv_160_8.g4966	369	ID=Fv_160_8.g4966;Description=hypothetical protein FVER14953_21614 [Fusarium verticillioides];Gene=FVER53263_21081;Ontology_term=membrane,phospholipid metabolic
Fv_160_8.g4967	388	ID=Fv_160_8.g4967;Description=hypothetical protein FVER14953_07480 [Fusarium verticillioides]
Fv_160_8.g4968	150	ID=Fv_160_8.g4968;Description=uncharacterized protein FFUJ_13757 [Fusarium fujikuroi IMI 58289];Gene=FNAPI_2850;Ontology_term=membrane,kinase

Fv_160_8.g4969	661	ID=Fv_160_8.g4969;Description=26S protease regulatory subunit 4 [Fusarium proliferatum];Gene=FOC4_g10006633;Ontology_term=chromatin,nucleus,proteasome regulatory particle, base subcomplex,ATP binding,peptidase activity,ATP hydrolysis activity,proteasome-activating activity,protein-containing complex localization,proteasome-mediated ubiquitin-dependent protein catabolic process,peptide catabolic process,nonfunctional rRNA decay,proteasome regulatory particle assembly,positive regulation of proteasomal protein catabolic process;Ontology_id=GO:0000785,GO:0005634,GO:0008540,GO:0005524,GO:0008233,GO:0016887,GO:0036402,GO:0031503,GO:
Fv_160_8.g4970	329	ID=Fv_160_8.g4970;Description=hypothetical protein LB504_010602 [Fusarium proliferatum];Gene=HZS61_014469;Ontology_term=mitochondrion,membrane,nucleotide binding,succinate-CoA ligase (ADP-forming) activity,transmembrane transporter activity,2-(3-amino-3-carboxypropyl)histidine synthase activity,tricarboxylic acid cycle,transmembrane transport;Ontology_id=GO:0005739,GO:0016020,GO:0000166,GO:0004775,GO:0022857,GO:0090560,GO:0006099,GO:0055085;En
Fv_160_8.g4971	468	ID=Fv_160_8.g4971;Description=hypothetical protein FVER53263_07485 [Fusarium verticillioides];Gene=FPCIR_10829;Ontology_term=membrane,transmembrane transporter activity,2-(3-amino-3-carboxypropyl)histidine synthase activity,transmembrane
Fv_160_8.g4972	507	ID=Fv_160_8.g4972;Description=hypothetical protein FVEG_07486 [Fusarium verticillioides 7600];Gene=F25303_3433;Ontology_term=membrane,protein binding,protein serine/threonine kinase activator activity,meiotic cell
Fv_160_8.g4973	484	ID=Fv_160_8.g4973;Description=hypothetical protein FVER14953_07487 [Fusarium
Fv_160_8.g4974	391	ID=Fv_160_8.g4974;Description=hypothetical protein FVEG_07488 [Fusarium verticillioides
Fv_160_8.g4975	245	ID=Fv_160_8.g4975;Description=hypothetical protein FVEG_07489 [Fusarium verticillioides 7600];Gene=da
Fv_160_8.g4976	336	ID=Fv_160_8.g4976;Description=hypothetical protein FVEG_07490 [Fusarium verticillioides 7600];Gene=FNYG_01509;Ontology_term=carbohydrate metabolic process,zinc ion binding,positive regulation of pheromone response MAPK cascade,oxidoreductase activity,guanyl-nucleotide exchange factor activity,Ras protein signal transduction,hydrolase activity, hydrolyzing O-glycosyl compounds,plasma membrane,mating projection actin fusion focus;Ontology_id=GO:0005975,GO:0008270,GO:0062038,GO:0016491,GO:0005085,GO:0007265,GO:0004553,GO:0005886,GO:19
Fv_160_8.g4977	444	ID=Fv_160_8.g4977;Description=hypothetical protein FVER53263_07491 [Fusarium verticillioides];Gene=FPANT_12747;Ontology_term=zinc ion binding,oxidoreductase
Fv_160_8.g4978	1085	ID=Fv_160_8.g4978;Description=hypothetical protein FVER53590_29572 [Fusarium verticillioides]
Fv_160_8.g4979	138	ID=Fv_160_8.g4979;Description=hypothetical protein FVER53590_07492 [Fusarium verticillioides];Gene=A0A2L2TJC4
Fv_160_8.g4980	317	ID=Fv_160_8.g4980;Description=hypothetical protein FVER14953_07493 [Fusarium verticillioides]
Fv_160_8.g4981	766	ID=Fv_160_8.g4981;Description=hypothetical protein FVEG_16128 [Fusarium verticillioides 7600]
Fv_160_8.g4982	548	ID=Fv_160_8.g4982;Description=hypothetical protein FVER53590_07496 [Fusarium verticillioides]

Fv_160_8.g4983	306	ID=Fv_160_8.g4983;Description=hypothetical protein FVEG_07497 [Fusarium verticillioides 7600];Gene=CEK26_011815;Ontology_term=cytoplasm,protein-DNA complex,RNA polymerase II transcription regulator complex,RNA polymerase II cis-regulatory region sequence-specific DNA binding,DNA-binding transcription factor activity, RNA polymerase II-specific,transcription coactivator activity,protein kinase activity,ATP binding,protein heterodimerization activity,DNA-binding transcription factor binding,eye development,brain development,peripheral nervous system development,salivary gland boundary specification,oenocyte development,somatic muscle development,phosphorylation,regulation of cell fate specification,positive regulation of transcription by RNA polymerase II,embryonic organ development,neuron development;Ontology_id=GO:0005737,GO:0032993,GO:0090575,GO:0000978,GO:0000981,GO:0003713,GO:0004672,GO:000552
Fv_160_8.g4984	572	ID=Fv_160_8.g4984;Description=hypothetical protein LZL87_007948 [Fusarium oxysporum];Gene=FCIRC_12413;Ontology_term=mitochondrial inner membrane,tRNA binding,metal ion binding,4 iron, 4 sulfur cluster binding,tRNA uridine(34) acetyltransferase activity,tRNA
Fv_160_8.g4985	449	ID=Fv_160_8.g4985;Description=hypothetical protein FVER53263_21079 [Fusarium verticillioides];Gene=FVER53590_29573;Ontology_term=mitochondrial inner membrane;Ontology_id=GO:0005743
Fv_160_8.g4986	372	ID=Fv_160_8.g4986;Description=hypothetical protein FVER14953_07499 [Fusarium verticillioides]
Fv_160_8.g4987	698	ID=Fv_160_8.g4987;Description=hypothetical protein FVEG_07500 [Fusarium verticillioides
Fv_160_8.g4988	619	ID=Fv_160_8.g4988;Description=hypothetical protein FVER14953_07501 [Fusarium verticillioides];Gene=FACUT_3638;Ontology_term=S-adenosylmethionine-dependent methyltransferase
Fv_160_8.g4989	69	ID=Fv_160_8.g4989;Description=hypothetical protein FVEG_07502 [Fusarium verticillioides 7600];Gene=CEP53_003494;Ontology_term=respiratory electron transport chain;Ontology_id=GO:0022904
Fv_160_8.g4990	448	ID=Fv_160_8.g4990;Description=hypothetical protein FVER53590_07503 [Fusarium verticillioides]
Fv_160_8.g4991	947	ID=Fv_160_8.g4991;Description=hypothetical protein FVER53263_07504 [Fusarium
Fv_160_8.g4992	197	ID=Fv_160_8.g4992;Description=hypothetical protein J7337_010490 [Fusarium musae]
Fv_160_8.g4993	203	ID=Fv_160_8.g4993;Description=hypothetical protein FVEG_07506 [Fusarium verticillioides 7600]
Fv_160_8.g4994	1075	ID=Fv_160_8.g4994;Description=hypothetical protein FVER53263_07507 [Fusarium verticillioides]
Fv_160_8.g4995	403	ID=Fv_160_8.g4995;Description=hypothetical protein FVER53590_07508 [Fusarium verticillioides]
Fv_160_8.g4996	161	ID=Fv_160_8.g4996;Description=hypothetical protein FVER53263_07509 [Fusarium verticillioides]
Fv_160_8.g4997	259	ID=Fv_160_8.g4997;Description=hypothetical protein FVER14953_07510 [Fusarium verticillioides];Gene=Forpi1262_v005215;Ontology_term=superoxide dismutase activity,metal ion binding,removal of superoxide
Fv_160_8.g4998	542	ID=Fv_160_8.g4998;Description=hypothetical protein FVER14953_07511 [Fusarium verticillioides]
Fv_160_8.g4999	266	ID=Fv_160_8.g4999;Description=hypothetical protein FVEG_07512 [Fusarium verticillioides 7600]
Fv_160_8.g5000	817	ID=Fv_160_8.g5000;Description=kinesin family member C1 [Fusarium verticillioides 7600];Gene=FOBC_02877;Ontology_term=microtubule,microtubule motor activity,ATP binding,microtubule binding,microtubule-

Fv_160_8.g5001	322	ID=Fv_160_8.g5001;Description=hypothetical protein FVEG_07514 [Fusarium verticillioides 7600];Gene=FVEG_07514;Ontology_term=membrane,acyltransferase
Fv_160_8.g5002	598	ID=Fv_160_8.g5002;Description=dihydroxy-acid dehydratase, mitochondrial [Fusarium verticillioides 7600];Gene=FPCIR_10859;Ontology_term=plasma membrane,ATP-dependent diacylglycerol kinase activity,dihydroxy-acid dehydratase activity,calcium ion binding,ATP binding,2 iron, 2 sulfur cluster binding,protein kinase C-activating G protein-coupled receptor signaling pathway,isoleucine biosynthetic process,valine biosynthetic process,intracellular signal transduction,diacylglycerol metabolic process,lipid
Fv_160_8.g5003	261	ID=Fv_160_8.g5003;Description=hypothetical protein FVEG_07516 [Fusarium verticillioides 7600];Gene=FVEG_07516;Ontology_term=dihydroxy-acid dehydratase activity,metal ion binding,2 iron, 2 sulfur cluster binding,isoleucine biosynthetic process,valine biosynthetic
Fv_160_8.g5004	451	ID=Fv_160_8.g5004;Description=hypothetical protein FVEG_16132 [Fusarium verticillioides 7600]
Fv_160_8.g5005	260	ID=Fv_160_8.g5005;Description=ATP-dependent Clp protease, protease subunit [Fusarium verticillioides 7600];Gene=FVEG_07517;Ontology_term=ATP-dependent peptidase activity,serine-type endopeptidase activity,proteolysis;Ontology_id=GO:0004176,GO:0004252,GO:0006508;Enzyme_code=EC:3.4.21;Enzyme_name=Acting on peptide
Fv_160_8.g5006	409	ID=Fv_160_8.g5006;Description=hypothetical protein FVEG_07518 [Fusarium verticillioides 7600];Gene=FCIRC_11972;Ontology_term=nucleoplasm,nucleolus,cytosolic small ribosomal subunit,neuronal cell body,RNA polymerase II cis-regulatory region sequence-specific DNA binding,DNA-binding transcription factor activity, RNA polymerase II-specific,structural constituent of ribosome,alpha-galactosidase activity,small ribosomal subunit rRNA binding,DNA-binding transcription factor binding,negative regulation of transcription by RNA polymerase II,cytoplasmic translation,carbohydrate metabolic process,specification of segmental identity, head,anterior/posterior pattern specification,posterior head segmentation,positive regulation of transcription by RNA polymerase II,head morphogenesis,positive regulation of apoptotic process
Fv_160_8.g5007	475	ID=Fv_160_8.g5007;Description=hypothetical protein FVER14953_07519 [Fusarium verticillioides];Gene=HZS61_014501;Ontology_term=methyltransferase activity,metal ion binding,fructose 6-phosphate aldolase activity,fructose-1-phosphatase activity,methylation;Ontology_id=GO:0008168,GO:0046872,GO:0097023,GO:0103026,GO:0032259;Enzyme_code=EC:4.1.2,EC:2.1.1
Fv_160_8.g5008	108	ID=Fv_160_8.g5008;Description=hypothetical protein FVEG_07520 [Fusarium verticillioides 7600]
Fv_160_8.g5009	603	ID=Fv_160_8.g5009;Description=hypothetical protein FVEG_07521 [Fusarium verticillioides 7600];Gene=FPRO_11692;Ontology_term=nucleus,DNA-binding transcription factor activity, RNA polymerase II-specific,zinc ion

Fv_160_8.g5010	325	ID=Fv_160_8.g5010;Description=hypothetical protein FVEG_07522 [Fusarium verticillioides 7600];Gene=CEK26_011789;Ontology_term=cytoplasm,microtubule,plasma membrane,neuromuscular junction,sperm individualization complex,mitotic spindle,presynapse,actin binding,GTPase activity,GTP binding,microtubule binding,lyase activity,conditioned taste aversion,morphogenesis of a polarized epithelium,negative regulation of protein phosphorylation,sperm individualization,border follicle cell migration,epithelial cell migration, open tracheal system,salivary gland morphogenesis,short-term memory,proboscis extension reflex,olfactory learning,positive regulation of autophagy,synaptic vesicle budding from presynaptic endocytic zone membrane,extracellular matrix organization,larval feeding behavior,positive regulation of actin filament polymerization,adherens junction maintenance,regulation of tube architecture, open tracheal system,syncytial blastoderm mitotic cell cycle,regulation of growth,compound eye retinal cell programmed cell death,dorsal appendage formation,regulation of short-term neuronal synaptic plasticity,synaptic vesicle membrane organization,regulation of synapse structure or activity,clathrin-dependent endocytosis involved in vitellogenesis,protein localization to plasma membrane,membrane fission,presynaptic dense core vesicle exocytosis,clathrin-dependent synaptic vesicle endocytosis,bulk synaptic vesicle endocytosis,mitotic actomyosin contractile ring
Fv_160_8.g5011	528	ID=Fv_160_8.g5011;Description=hypothetical protein FVEG_07523 [Fusarium verticillioides 7600];Gene=FACUT_3618;Ontology_term=membrane,transmembrane transporter activity,transmembrane
Fv_160_8.g5012	381	ID=Fv_160_8.g5012;Description=hypothetical protein FVEG_16133 [Fusarium verticillioides 7600];Gene=shi;Ontology_term=cytoplasm,microtubule,plasma membrane,neuromuscular junction,sperm individualization complex,mitotic spindle,presynapse,actin binding,GTPase activity,GTP binding,microtubule binding,conditioned taste aversion,morphogenesis of a polarized epithelium,negative regulation of protein phosphorylation,sperm individualization,border follicle cell migration,epithelial cell migration, open tracheal system,salivary gland morphogenesis,short-term memory,proboscis extension reflex,olfactory learning,positive regulation of autophagy,synaptic vesicle budding from presynaptic endocytic zone membrane,extracellular matrix organization,larval feeding behavior,positive regulation of actin filament polymerization,adherens junction maintenance,regulation of tube architecture, open tracheal system,syncytial blastoderm mitotic cell cycle,regulation of growth,compound eye retinal cell programmed cell death,dorsal appendage formation,regulation of short-term neuronal synaptic plasticity,synaptic vesicle membrane organization,regulation of synapse structure or activity,clathrin-dependent endocytosis involved in vitellogenesis,protein localization to plasma membrane,membrane fission,presynaptic dense core vesicle exocytosis,clathrin-dependent synaptic vesicle endocytosis,bulk synaptic vesicle endocytosis,mitotic actomyosin contractile ring assembly;Ontology_id=GO:0005737,GO:0005874,GO:0005886,GO:0031594,GO:0070864,GO:0072686,GO:0098793,GO:0003779,GO
Fv_160_8.g5013	165	ID=Fv_160_8.g5013;Description=hypothetical protein FVEG_16134 [Fusarium verticillioides 7600]
Fv_160_8.g5014	490	ID=Fv_160_8.g5014;Description=hypothetical protein FVER53590_07524 [Fusarium verticillioides];Gene=C2S_4328;Ontology_term=membrane,transmembrane transporter activity,carbohydrate
Fv_160_8.g5015	535	ID=Fv_160_8.g5015;Description=hypothetical protein FVER53590_07525 [Fusarium verticillioides];Gene=FVEG_07525;Ontology_term=hydrolase activity, hydrolyzing O-glycosyl compounds,carbohydrate metabolic

Fv_160_8.g5016	739	ID=Fv_160_8.g5016;Description=hypothetical protein FVER14953_07526 [Fusarium verticillioides];Gene=FTJAE_13566;Ontology_term=nucleus,DNA binding,zinc ion binding,DNA-binding transcription factor activity, RNA polymerase II-specific,regulation of transcription by RNA polymerase
Fv_160_8.g5017	588	ID=Fv_160_8.g5017;Description=5'-nucleotidase [Fusarium verticillioides 7600];Gene=FVEG_07528;Ontology_term=membrane,nucleotide binding,hydrolase activity,nucleotide catabolic
Fv_160_8.g5018	494	ID=Fv_160_8.g5018;Description=hypothetical protein J7337_010467 [Fusarium musae];Gene=FOBC_02859;Ontology_term=membrane,cytosolic large ribosomal subunit,structural constituent of ribosome,rRNA binding,transmembrane transporter activity,cytoplasmic translation,transmembrane
Fv_160_8.g5019	420	ID=Fv_160_8.g5019;Description=hypothetical protein FVER14953_07530 [Fusarium verticillioides];Gene=F25303_3388;Ontology_term=membrane,acyltransferase
Fv_160_8.g5020	431	ID=Fv_160_8.g5020;Description=hypothetical protein FVER53590_07531 [Fusarium verticillioides];Gene=FANTH_7472;Ontology_term=NAD+ kinase activity,NADP biosynthetic process,phosphorylation,NAD metabolic
Fv_160_8.g5021	648	ID=Fv_160_8.g5021;Description=lysophospholipase [Fusarium verticillioides 7600];Gene=FACUT_3608;Ontology_term=lysophospholipase activity,phosphatidyl phospholipase B activity,phospholipid catabolic
Fv_160_8.g5022	362	ID=Fv_160_8.g5022;Description=oxidoreductase [Fusarium verticillioides 7600];Gene=FTJAE_7120;Ontology_term=nucleotide
Fv_160_8.g5023	253	ID=Fv_160_8.g5023;Description=hypothetical protein FVER14953_07534 [Fusarium verticillioides];Gene=CEK26_011778;Ontology_term=cytosolic large ribosomal subunit,DNA binding,structural constituent of ribosome,transferase activity,cytoplasmic translation,DNA transposition,DNA
Fv_160_8.g5024	193	ID=Fv_160_8.g5024;Description=hypothetical protein FVEG_07535 [Fusarium verticillioides
Fv_160_8.g5025	315	ID=Fv_160_8.g5025;Description=hypothetical protein FVEG_07536 [Fusarium verticillioides 7600];Gene=FNAPI_9203;Ontology_term=lipid droplet,membrane,aminopeptidase activity,metalloexopeptidase activity,lipase activity,metal ion binding,proteolysis,lipid storage;Ontology_id=GO:0005811,GO:0016020,GO:0004177,GO:0008235,GO:0016298,GO:0046872,GO:0006508,GO:0019915;Enzy
Fv_160_8.g5026	1034	ID=Fv_160_8.g5026;Description=hypothetical protein FVER53590_07537 [Fusarium verticillioides];Gene=FNYG_01462;Ontology_term=membrane,aminopeptidase activity,metalloexopeptidase activity,metal ion binding,proteolysis;Ontology_id=GO:0016020,GO:0004177,GO:0008235,GO:0046872,GO:0006508;Enzyme_code=EC:3.4.11;Enzyme
Fv_160_8.g5027	552	ID=Fv_160_8.g5027;Description=branchpoint-bridging [Fusarium acutatum];Gene=FGADI_12973;Ontology_term=spliceosomal complex,membrane,zinc ion binding,transmembrane transporter activity,pre-mRNA branch point binding,mRNA splicing, via spliceosome,transmembrane
Fv_160_8.g5028	536	ID=Fv_160_8.g5028;Description=hypothetical protein FVER53590_07539 [Fusarium verticillioides];Gene=FVEG_07539;Ontology_term=membrane,transmembrane transporter activity,transmembrane

Fv_160_8.g5029	608	ID=Fv_160_8.g5029;Description=hypothetical protein FVER53590_07540 [Fusarium verticillioides];Gene=FPHYL_14099;Ontology_term=nucleus,membrane,RNA binding,zinc ion binding,transmembrane transporter activity,mRNA processing,RNA splicing,transmembrane
Fv_160_8.g5030	707	ID=Fv_160_8.g5030;Description=hypothetical protein FVER53263_07541 [Fusarium verticillioides]
Fv_160_8.g5031	488	ID=Fv_160_8.g5031;Description=hypothetical protein FVEG_16135 [Fusarium verticillioides 7600]
Fv_160_8.g5032	88	ID=Fv_160_8.g5032;Description=hypothetical protein FVER14953_21671 [Fusarium verticillioides]
Fv_160_8.g5033	71	ID=Fv_160_8.g5033;Description=hypothetical protein FVEG_16137 [Fusarium verticillioides 7600]
Fv_160_8.g5034	707	ID=Fv_160_8.g5034;Description=hypothetical protein FVER53590_07543 [Fusarium verticillioides]
Fv_160_8.g5035	582	ID=Fv_160_8.g5035;Description=hypothetical protein FVER14953_07544 [Fusarium verticillioides];Gene=HZS61_014523;Ontology_term=lipid metabolic process;Ontology_id=GO:0006629
Fv_160_8.g5036	669	ID=Fv_160_8.g5036;Description=hypothetical protein FVEG_07545 [Fusarium verticillioides 7600];Gene=HZS61_014524;Ontology_term=nucleus,DNA binding,zinc ion binding,membrane,DNA-binding transcription factor activity, RNA polymerase II-specific,regulation of transcription by RNA polymerase
Fv_160_8.g5037	300	ID=Fv_160_8.g5037;Description=hypothetical protein FVEG_07546 [Fusarium verticillioides
Fv_160_8.g5038	461	ID=Fv_160_8.g5038;Description=hypothetical protein FVER53263_07547 [Fusarium verticillioides];Gene=rad15;Ontology_term=nucleus,membrane;Ontology_id=GO:0005634,GO:0016020
Fv_160_8.g5039	409	ID=Fv_160_8.g5039;Description=hypothetical protein FVER53590_07548 [Fusarium verticillioides];Gene=FVEG_07548;Ontology_term=imaginal disc-derived wing morphogenesis,DNA integration,oocyte microtubule cytoskeleton polarization,neuronal cell body,protein phosphorylation,cAMP-dependent protein kinase complex,sprouting of injured axon,ATP binding,dendrite,AMP-activated protein kinase activity,oxidoreductase activity,protein kinase A signaling,thermosensory behavior,cytosol,protein serine kinase activity,positive regulation of hippo signaling,cAMP-dependent protein kinase activity,olfactory learning,regulation of apoptotic process,negative regulation of smoothened signaling pathway,anterior/posterior pattern specification, imaginal disc,plasma membrane,regulation of bicoid mRNA localization,negative regulation of nucleocytoplasmic transport,DNA transposition,cellular response to ethanol,nucleus,DNA binding,regulation of pole plasm oskar mRNA localization,protein binding,anesthesia-resistant memory,regulation of actin cytoskeleton organization,positive regulation of gene expression,regulation of circadian sleep/wake cycle, sleep,positive regulation of smoothened signaling pathway,modulation of chemical synaptic transmission,positive regulation of proteasomal ubiquitin-dependent protein catabolic process,cAMP-mediated signaling;Ontology_id=GO:0007476,GO:0015074,GO:0008103,GO:0043025,GO:0006468,GO:0005952,GO:0048682,GO:0005524,GO:
Fv_160_8.g5040	1003	ID=Fv_160_8.g5040;Description=hypothetical protein FVER53590_07549 [Fusarium verticillioides];Gene=FVEG_07549;Ontology_term=nucleus,DNA-binding transcription factor activity, RNA polymerase II-specific,zinc
Fv_160_8.g5041	460	ID=Fv_160_8.g5041;Description=hypothetical protein FVER53590_07550 [Fusarium verticillioides]
Fv_160_8.g5042	969	ID=Fv_160_8.g5042;Description=hypothetical protein FVER53590_07551 [Fusarium verticillioides];Gene=FDENT_4957;Ontology_term=phosphatase activity,phosphatidylinositol

Fv_160_8.g5043	219	ID=Fv_160_8.g5043;Description=hypothetical protein FVEG_07552 [Fusarium verticillioides 7600];Gene=Forpe1208_v003886;Ontology_term=intracellular organelle,enzyme activator activity,deadenylation-dependent
Fv_160_8.g5044	261	ID=Fv_160_8.g5044;Description=protein hob3 [Fusarium oxysporum f. sp. lycopersici 4287];Gene=F66182_9185;Ontology_term=medial cortex,cell cortex of cell tip,endocytosis,actin filament organization,actin cortical patch localization,mitotic actomyosin contractile ring
Fv_160_8.g5045	198	ID=Fv_160_8.g5045;Description=PTH1 family peptidyl-tRNA hydrolase [Fusarium verticillioides 7600];Gene=FOXB_10364;Ontology_term=aminoacyl-tRNA hydrolase
Fv_160_8.g5046	466	ID=Fv_160_8.g5046;Description=ammonium transporter MEPa [Fusarium pseudoanthophilum];Gene=F25303_1721;Ontology_term=plasma membrane,ammonium transmembrane transporter
Fv_160_8.g5047	742	ID=Fv_160_8.g5047;Description=hypothetical protein FVER14953_07556 [Fusarium verticillioides];Gene=BFJ70_g9382;Ontology_term=negative regulation of protein import into nucleus,positive regulation of G1/S transition of mitotic cell cycle,protein serine/threonine kinase activity,negative regulation of cell cycle switching, mitotic to meiotic cell cycle,negative regulation of transcription by RNA polymerase II,nucleus,ATP binding,protein binding,negative regulation of induction of conjugation with cellular fusion,cytoplasm,autophagy,phosphorylation,ligase activity,intracellular signal transduction,protein serine kinase
Fv_160_8.g5048	385	ID=Fv_160_8.g5048;Description=hypothetical protein FVER14953_07557 [Fusarium verticillioides];Gene=FPANT_12795;Ontology_term=membrane,intramolecular lyase
Fv_160_8.g5049	109	ID=Fv_160_8.g5049;Description=glutaredoxin 3 [Fusarium verticillioides 7600];Gene=FGADI_2874;Ontology_term=oxidoreductase activity;Ontology_id=GO:0016491;Enzyme_code=EC:1;Enzyme_name=Oxidoreductases
Fv_160_8.g5050	305	ID=Fv_160_8.g5050;Description=hypothetical protein FVEG_07559 [Fusarium verticillioides 7600];Gene=FDENT_4949;Ontology_term=cytoplasm,intracellular membrane-bounded organelle,protein-folding chaperone
Fv_160_8.g5051	238	ID=Fv_160_8.g5051;Description=60s ribosomal l16 [Fusarium subglutinans];Gene=FGADI_2872;Ontology_term=large ribosomal subunit,structural constituent of ribosome,translation;Ontology_id=GO:0015934,GO:0003735,GO:0006412
Fv_160_8.g5052	202	ID=Fv_160_8.g5052;Description=hypothetical protein FVEG_07561 [Fusarium verticillioides 7600]
Fv_160_8.g5053	174	ID=Fv_160_8.g5053;Description=hypothetical protein FVER53590_07562 [Fusarium verticillioides]
Fv_160_8.g5054	272	ID=Fv_160_8.g5054;Description=hypothetical protein FVER53590_07564 [Fusarium verticillioides]
Fv_160_8.g5055	67	ID=Fv_160_8.g5055;Description=---NA---
Fv_160_8.g5056	321	ID=Fv_160_8.g5056;Description=hypothetical protein FVER14953_07567 [Fusarium verticillioides]
Fv_160_8.g5057	437	ID=Fv_160_8.g5057;Description=hypothetical protein FVEG_07568 [Fusarium verticillioides 7600];Gene=FACUT_13150;Ontology_term=clathrin-coated pit,clathrin adaptor complex,intracellular protein

Fv_160_8.g5058	477	ID=Fv_160_8.g5058;Description=hypothetical protein FVER14953_21280 [Fusarium verticillioides];Gene=FTJAE_7086;Ontology_term=ATP hydrolysis activity,imaginal disc-derived leg segmentation,transcription factor TFIIH core complex,positive regulation of mitotic recombination,nucleotide-excision repair,sex comb development,chaeta development,positive regulation of transcription by RNA polymerase II,transcription factor TFIIH holo complex,damaged DNA binding,nucleotide-excision repair factor 3 complex,ATP binding,DNA helicase activity,transcription coregulator activity,metal ion binding,RNA polymerase II general transcription initiation factor activity,mediator complex,iron-sulfur cluster
Fv_160_8.g5059	222	ID=Fv_160_8.g5059;Description=hypothetical protein FVEG_07570 [Fusarium verticillioides 7600];Gene=FPCIR_7170;Ontology_term=oxidoreductase
Fv_160_8.g5060	503	ID=Fv_160_8.g5060;Description=hypothetical protein FVER14953_07571 [Fusarium verticillioides];Gene=FTJAE_7084;Ontology_term=cyclin-dependent protein kinase holoenzyme complex,chromosome, centromeric region,nucleus,cytoplasm,spindle midzone,mitotic spindle,single-stranded DNA endodeoxyribonuclease activity,single-stranded DNA binding,aminopeptidase activity,protein binding,cyclin-dependent protein serine/threonine kinase regulator activity,metal ion binding,embryonic development via the syncytial blastoderm,mitotic chromosome movement towards spindle pole,attachment of spindle microtubules to kinetochore,syncytial blastoderm mitotic cell cycle,regulation of chromatin binding,mitotic cell cycle phase transition,germ-line cyst formation,midbody abscission,nucleotide-excision repair involved in interstrand cross-link repair;Ontology_id=GO:0000307,GO:0000775,GO:0005634,GO:0005737,GO:0051233,GO:0072686,GO:0000014,GO:0003697,GO:00
Fv_160_8.g5061	145	ID=Fv_160_8.g5061;Description=hypothetical protein FVEG_07572 [Fusarium verticillioides 7600]
Fv_160_8.g5062	245	ID=Fv_160_8.g5062;Description=hypothetical protein FVEG_07573 [Fusarium verticillioides 7600];Gene=FPANT_12735;Ontology_term=mitochondrial intermembrane space,cytosol,mRNA cleavage stimulating factor complex,side of membrane,protein tyrosine phosphatase activity,electron transfer activity,heme binding,metal ion binding,signal transduction,dephosphorylation,electron transport chain;Ontology_id=GO:0005758,GO:0005829,GO:0005848,GO:0098552,GO:0004725,GO:0009055,GO:0020037,GO:0046872,GO:00
Fv_160_8.g5063	153	ID=Fv_160_8.g5063;Description=hypothetical protein FDENT_8867 [Fusarium
Fv_160_8.g5064	604	ID=Fv_160_8.g5064;Description=hypothetical protein FVEG_07575 [Fusarium verticillioides 7600]
Fv_160_8.g5065	1450	ID=Fv_160_8.g5065;Description=calcium-independent phospholipase A2 [Fusarium napiforme];Gene=FOQG_09132;Ontology_term=catalytic activity,ADP binding,nucleoside metabolic
Fv_160_8.g5066	503	ID=Fv_160_8.g5066;Description=cholesterol 7-alpha-monooxygenase [Fusarium napiforme];Gene=FOQG_09131;Ontology_term=membrane,monooxygenase activity,iron ion binding,oxidoreductase activity, acting on paired donors, with incorporation or reduction of molecular oxygen,heme binding;Ontology_id=GO:0016020,GO:0004497,GO:0005506,GO:0016705,GO:0020037;Enzyme_code=EC:1.14;Enzyme_name=Actin

Fv_160_8.g5067	403	ID=Fv_160_8.g5067;Description=hypothetical protein FVEG_07579 [Fusarium verticillioides 7600];Gene=FOC4_g10006526;Ontology_term=membrane,monooxygenase activity,iron ion binding,O-acyltransferase activity,oxidoreductase activity, acting on paired donors, with incorporation or reduction of molecular oxygen,heme binding,lipid metabolic
Fv_160_8.g5068	544	ID=Fv_160_8.g5068;Description=hypothetical protein FVER14953_07580 [Fusarium verticillioides];Gene=FOTG_01979;Ontology_term=membrane,monooxygenase activity,iron ion binding,oxidoreductase activity, acting on paired donors, with incorporation or reduction of molecular oxygen,heme binding;Ontology_id=GO:0016020,GO:0004497,GO:0005506,GO:0016705,GO:0020037;Enzyme_code=EC:1.14;Enzyme_name=Actin
Fv_160_8.g5069	463	ID=Fv_160_8.g5069;Description=hypothetical protein FVER53590_07580 [Fusarium verticillioides];Gene=FVER53590_07580;Ontology_term=membrane,monooxygenase activity,iron ion binding,oxidoreductase activity, acting on paired donors, with incorporation or reduction of molecular oxygen,heme binding;Ontology_id=GO:0016020,GO:0004497,GO:0005506,GO:0016705,GO:0020037;Enzyme_code=EC:1.14;Enzyme_name=Actin
Fv_160_8.g5070	376	ID=Fv_160_8.g5070;Description=hypothetical protein FVER14953_07582 [Fusarium verticillioides];Gene=FACUT_13140;Ontology_term=membrane,O-acyltransferase activity,lipid metabolic
Fv_160_8.g5071	251	ID=Fv_160_8.g5071;Description=hypothetical protein FVER14953_07583 [Fusarium
Fv_160_8.g5072	441	ID=Fv_160_8.g5072;Description=hypothetical protein FNYG_01418 [Fusarium nygamai];Gene=FOXB_01807;Ontology_term=monooxygenase activity,iron ion binding,oxidoreductase activity, acting on paired donors, with incorporation or reduction of molecular oxygen,heme binding;Ontology_id=GO:0004497,GO:0005506,GO:0016705,GO:0020037;Enzyme_code=EC:1.14;Enzyme_name=Acting on paired
Fv_160_8.g5073	541	ID=Fv_160_8.g5073;Description=hypothetical protein FVEG_07585 [Fusarium verticillioides 7600];Gene=FOTG_01974;Ontology_term=membrane,monooxygenase activity,iron ion binding,methyltransferase activity,oxidoreductase activity, acting on paired donors, with incorporation or reduction of molecular oxygen,heme binding,methylation;Ontology_id=GO:0016020,GO:0004497,GO:0005506,GO:0008168,GO:0016705,GO:0020037,GO:0032259;Enzy
Fv_160_8.g5074	290	ID=Fv_160_8.g5074;Description=cytochrome P450 [Fusarium oxysporum];Gene=HZS61_014556;Ontology_term=membrane,monooxygenase activity,iron ion binding,methyltransferase activity,oxidoreductase activity, acting on paired donors, with incorporation or reduction of molecular oxygen,heme binding,methylation;Ontology_id=GO:0016020,GO:0004497,GO:0005506,GO:0008168,GO:0016705,GO:0020037,GO:0032259;Enzy
Fv_160_8.g5075	413	ID=Fv_160_8.g5075;Description=hypothetical protein FVEG_07587 [Fusarium verticillioides 7600];Gene=FPANT_13661;Ontology_term=binding,oxidoreductase
Fv_160_8.g5076	360	ID=Fv_160_8.g5076;Description=hypothetical protein FCOIX_9 [Fusarium coicis]

Fv_160_8.g5077	408	ID=Fv_160_8.g5077;Description=hypothetical protein FCOIX_8 [Fusarium coicis];Gene=pho4;Ontology_term=extracellular region,fungal-type cell wall,cell wall-bounded periplasmic space,thiamine phosphate phosphatase activity,thiamine salvage;Ontology_id=GO:0005576,GO:0009277,GO:0030287,GO:0042131,GO:0036172;Enzyme_code=EC:3.1.3.2,EC:3.1.3.100;Enzyme_name=acid phosphatase,thiamine phosphate phosphatase
Fv_160_8.g5078	437	ID=Fv_160_8.g5078;Description=uncharacterized protein Y057_12177 [Fusarium fujikuroi];Gene=pho2;Ontology_term=cytoplasm,membrane,chitin-based extracellular matrix,alkaline phosphatase activity,structural constituent of chitin-based larval cuticle,phosphoglycolate phosphatase activity,cellularization,larval chitin-based cuticle development,cellular
Fv_160_8.g5079	98	ID=Fv_160_8.g5079;Description=hypothetical protein J7337_010412 [Fusarium
Fv_160_8.g5080	387	ID=Fv_160_8.g5080;Description=hypothetical protein FVER53590_07588 [Fusarium verticillioides]
Fv_160_8.g5081	275	ID=Fv_160_8.g5081;Description=hypothetical protein FVER53590_29058 [Fusarium verticillioides]
Fv_160_8.g5082	322	ID=Fv_160_8.g5082;Description=killer toxin alpha beta [Fusarium coicis];Gene=FOQG_09120;Ontology_term=extracellular region,membrane,chitinase activity,chitin binding,polysaccharide catabolic process,chitin catabolic process;Ontology_id=GO:0005576,GO:0016020,GO:0004568,GO:0008061,GO:0000272,GO:0006032;Enzyme_code=EC:3.2.1.14;Enz
Fv_160_8.g5083	501	ID=Fv_160_8.g5083;Description=hypothetical protein FVER53590_07590 [Fusarium verticillioides];Gene=ct;Ontology_term=nucleus,RNA polymerase II transcription regulatory region sequence-specific DNA binding,DNA-binding transcription factor activity, RNA polymerase II-specific,negative regulation of transcription by RNA polymerase II,compound eye morphogenesis,central nervous system development,peripheral nervous system development,sensory perception of sound,female gonad development,imaginal disc-derived wing margin morphogenesis,spiracle morphogenesis, open tracheal system,antennal joint development,oogenesis,formation of a compartment boundary,Malpighian tubule bud morphogenesis,dendrite guidance,dendrite
Fv_160_8.g5084	494	ID=Fv_160_8.g5084;Description=ankyrin 3 [Fusarium coicis];Gene=pap1;Ontology_term=chromatin,cytoplasm,RNA polymerase II transcription regulator complex,RNA polymerase II cis-regulatory region sequence-specific DNA binding,DNA-binding transcription activator activity, RNA polymerase II-specific,protein binding,DNA binding, bending,cellular response to oxidative stress,positive regulation of transcription by RNA polymerase
Fv_160_8.g5085	590	ID=Fv_160_8.g5085;Description=hypothetical protein FCOIX_6201 [Fusarium coicis];Gene=Sgs4;Ontology_term=adhesive extracellular matrix,puparial adhesion;Ontology_id=GO:0062130,GO:0007594
Fv_160_8.g5086	350	ID=Fv_160_8.g5086;Description=hypothetical protein FACUT_8404 [Fusarium acutatum]
Fv_160_8.g5087	281	ID=Fv_160_8.g5087;Description=hypothetical protein FVER14953_07597 [Fusarium verticillioides];Gene=FANTH_14082;Ontology_term=nucleoplasm,nucleolus,rDNA binding,double-stranded RNA binding,monooxygenase activity,ribonuclease III activity,RNA stem-loop binding,nuclear-transcribed mRNA catabolic process,rRNA processing,termination of RNA polymerase II transcription, exosome-dependent,U2 snRNA 3'-end processing,U4 snRNA 3'-end processing,melanin biosynthetic process,meiotic cell cycle,box C/D RNA 5'-end

Fv_160_8.g5088	467	ID=Fv_160_8.g5088;Description=hypothetical protein FVER53263_07598 [Fusarium verticillioides];Gene=FFUJ_13879;Ontology_term=cysteine dioxygenase
Fv_160_8.g5089	428	ID=Fv_160_8.g5089;Description=hypothetical protein FVER14953_07599 [Fusarium verticillioides];Gene=FOMG_02638;Ontology_term=phosphatidylserine decarboxylase activity,phospholipid biosynthetic
Fv_160_8.g5090	709	ID=Fv_160_8.g5090;Description=hypothetical protein FVEG_07600 [Fusarium verticillioides
Fv_160_8.g5091	222	ID=Fv_160_8.g5091;Description=hypothetical protein FPANT_7550 [Fusarium pseudoanthophilum];Gene=BFJ63_vAg2609;Ontology_term=cytoplasm,membrane,polyamine oxidase
Fv_160_8.g5092	585	ID=Fv_160_8.g5092;Description=hypothetical protein FVEG_07601 [Fusarium verticillioides 7600];Gene=FVEG_07601;Ontology_term=peroxisome,membrane,superoxide dismutase activity,copper ion binding,protein homodimerization activity,monoatomic ion transport,determination of adult lifespan,removal of superoxide radicals,regulation of synaptic plasticity,regulation of autophagy of mitochondrion,regulation of terminal button organization;Ontology_id=GO:0005777,GO:0016020,GO:0004784,GO:0005507,GO:0042803,GO:0006811,GO:0008340,GO:0019430,
Fv_160_8.g5093	332	ID=Fv_160_8.g5093;Description=hypothetical protein FVER14953_21159 [Fusarium verticillioides];Gene=FVER53590_25484;Ontology_term=protein serine/threonine kinase inhibitor activity,signal transduction involved in cell cycle switching, mitotic to meiotic cell cycle,protein binding,high-affinity iron permease complex,iron ion transmembrane transporter activity,negative regulation of conjugation with zygote,sporulation resulting in formation of a cellular
Fv_160_8.g5094	267	ID=Fv_160_8.g5094;Description=hypothetical protein FVER14953_07603 [Fusarium
Fv_160_8.g5095	522	ID=Fv_160_8.g5095;Description=hypothetical protein FVER53263_07604 [Fusarium verticillioides];Gene=FVEG_07604;Ontology_term=membrane,transmembrane transporter activity,transmembrane
Fv_160_8.g5096	518	ID=Fv_160_8.g5096;Description=hypothetical protein FVER14953_07605 [Fusarium verticillioides];Gene=FANTH_14091;Ontology_term=mitochondrion,membrane,succinyl-CoA:3-oxo-acid CoA-transferase activity,transmembrane transporter activity,ketone body catabolic process,transmembrane
Fv_160_8.g5097	438	ID=Fv_160_8.g5097;Description=hypothetical protein CEK27_011728 [Fusarium fujikuroi];Gene=AU210_005280;Ontology_term=Tor2-Mei2-Ste11 complex,zinc ion binding,transmembrane transport,transmembrane transporter activity,metallocarboxypeptidase activity,nuclear chromosome,CoA-transferase activity,poly(U) RNA binding,extracellular region,signal transduction involved in cell cycle switching, mitotic to meiotic cell cycle,protein binding,cytoplasm,negative regulation of conjugation with zygote,membrane,proteolysis,Mei2 nuclear dot complex,positive regulation of metaphase/anaphase transition of meiosis II,lncRNA binding;Ontology_id=GO:0034064,GO:0008270,GO:0055085,GO:0022857,GO:0004181,GO:0000228,GO:0008410,GO:0008266,GO:0
Fv_160_8.g5098	283	ID=Fv_160_8.g5098;Description=hypothetical protein FPHYL_5394 [Fusarium phyllophilum]
Fv_160_8.g5099	296	ID=Fv_160_8.g5099;Description=hypothetical protein FVER14953_07608 [Fusarium verticillioides];Gene=Forpi1262_v005303;Ontology_term=mitochondrial inner membrane,mitochondrial

Fv_160_8.g5100	728	ID=Fv_160_8.g5100;Description=hypothetical protein FVEG_07609 [Fusarium verticillioides 7600];Gene=FOTG_01955;Ontology_term=nucleus,DNA-binding transcription factor activity, RNA polymerase II-specific,zinc ion
Fv_160_8.g5101	467	ID=Fv_160_8.g5101;Description=hypothetical protein J7337_010381 [Fusarium musae];Gene=HZS61_014568;Ontology_term=membrane,transmembrane transporter activity,transmembrane
Fv_160_8.g5102	591	ID=Fv_160_8.g5102;Description=hypothetical protein FVER53263_07611 [Fusarium
Fv_160_8.g5103	177	ID=Fv_160_8.g5103;Description=hypothetical protein FVER14953_21164 [Fusarium verticillioides]
Fv_160_8.g5104	377	ID=Fv_160_8.g5104;Description=hypothetical protein FVEG_07612 [Fusarium verticillioides 7600];Gene=mat2-Pc;Ontology_term=nucleus,DNA binding,protein binding,transcription coactivator activity,mating type determination,positive regulation of transcription by RNA polymerase II,positive regulation of induction of conjugation with cellular fusion,positive
Fv_160_8.g5105	348	ID=Fv_160_8.g5105;Description=cyclin H [Fusarium verticillioides 7600];Gene=FTJAE_6077;Ontology_term=cytoplasm,cyclin-dependent protein serine/threonine kinase regulator activity,regulation of transcription by RNA polymerase
Fv_160_8.g5106	404	ID=Fv_160_8.g5106;Description=hypothetical protein J7337_010375 [Fusarium musae];Gene=FTJAE_6076;Ontology_term=cytoplasm,acyltransferase
Fv_160_8.g5107	435	ID=Fv_160_8.g5107;Description=hypothetical protein FVER14953_07615 [Fusarium verticillioides]
Fv_160_8.g5108	2257	ID=Fv_160_8.g5108;Description=hypothetical protein FVER14953_07616 [Fusarium verticillioides];Gene=FVEG_07616;Ontology_term=plasma membrane,helicase activity,ATP binding,ATP hydrolysis activity,ABC-type transporter activity,transmembrane transport;Ontology_id=GO:0005886,GO:0004386,GO:0005524,GO:0016887,GO:0140359,GO:0055085;Enzyme_code=EC:7.2.2,EC:3.
Fv_160_8.g5109	752	ID=Fv_160_8.g5109;Description=NFX1-type zinc finger-containing protein [Fusarium mundagurra];Gene=F52700_6614;Ontology_term=helicase activity,ATP binding,ATP hydrolysis
Fv_160_8.g5110	368	ID=Fv_160_8.g5110;Description=hypothetical protein FVER53263_07617 [Fusarium verticillioides];Gene=FNAPI_6764;Ontology_term=membrane,saccharopine dehydrogenase (NAD+, L-lysine-forming) activity,transmembrane transporter activity,lysine biosynthetic process via aminoadipic acid,transmembrane
Fv_160_8.g5111	179	ID=Fv_160_8.g5111;Description=hypothetical protein FVER53263_07618 [Fusarium verticillioides];Gene=prlL-10;Ontology_term=membrane,transmembrane transporter activity,transmembrane
Fv_160_8.g5112	530	ID=Fv_160_8.g5112;Description=hypothetical protein FVER14953_07619 [Fusarium verticillioides];Gene=FPCIR_11704;Ontology_term=membrane,oxidoreductase activity,heme binding,transmembrane transporter activity,metal ion binding,transmembrane
Fv_160_8.g5113	450	ID=Fv_160_8.g5113;Description=hypothetical protein FVER14953_07620 [Fusarium verticillioides];Gene=FTJAE_13799;Ontology_term=mitochondrial outer membrane,oxidoreductase activity,heme binding,metal ion

Fv_160_8.g5114	549	ID=Fv_160_8.g5114;Description=hypothetical protein J7337_010363 [Fusarium musae];Gene=FOVG_17550;Ontology_term=monooxygenase activity,iron ion binding,oxidoreductase activity, acting on paired donors, with incorporation or reduction of molecular oxygen,heme binding;Ontology_id=GO:0004497,GO:0005506,GO:0016705,GO:0020037;Enzyme_code=EC:1.14;Enzyme_name=Acting on paired
Fv_160_8.g5115	1373	ID=Fv_160_8.g5115;Description=hypothetical protein FVEG_07622 [Fusarium verticillioides 7600];Gene=FOXG_04493;Ontology_term=plasma membrane,ATP binding,ATP hydrolysis activity,ABC-type transporter activity,transmembrane transport;Ontology_id=GO:0005886,GO:0005524,GO:0016887,GO:0140359,GO:0055085;Enzyme_code=EC:7.2.2,EC:3.6.1.15;Enzym
Fv_160_8.g5116	286	ID=Fv_160_8.g5116;Description=hypothetical protein FVER53263_07624 [Fusarium verticillioides]
Fv_160_8.g5117	177	ID=Fv_160_8.g5117;Description=hypothetical protein FPCIR_11559 [Fusarium pseudocircinatum]
Fv_160_8.g5118	233	ID=Fv_160_8.g5118;Description=hypothetical protein FNYG_01377 [Fusarium nygamai]
Fv_160_8.g5119	1837	ID=Fv_160_8.g5119;Description=hypothetical protein H9Q74_013689 [Fusarium xylarioides];Gene=FCULG_00002715
Fv_160_8.g5120	297	ID=Fv_160_8.g5120;Description=hypothetical protein H9Q71_013691 [Fusarium xylarioides];Gene=FHETE_2245;Ontology_term=peptidase
Fv_160_8.g5121	1129	ID=Fv_160_8.g5121;Description=hypothetical protein H9Q71_013689 [Fusarium xylarioides];Gene=FCULG_00002713;Ontology_term=peptidase
Fv_160_8.g5122	158	ID=Fv_160_8.g5122;Description=hypothetical protein FMUND_15424 [Fusarium mundagurra];Gene=hhf1
Fv_160_8.g5123	299	ID=Fv_160_8.g5123;Description=hypothetical protein J7337_010357 [Fusarium musae]
Fv_160_8.g5124	131	ID=Fv_160_8.g5124;Description=hypothetical protein FVER53590_07628 [Fusarium verticillioides];Gene=shakB;Ontology_term=plasma membrane,gap junction,gap junction channel activity,photoreceptor activity,regulation of membrane depolarization,monoatomic ion transport,phototransduction,jump response,intercellular transport,cell communication by electrical coupling,gap junction assembly,transmembrane
Fv_160_8.g5125	172	ID=Fv_160_8.g5125;Description=hypothetical protein FVER14953_07629 [Fusarium verticillioides]
Fv_160_8.g5126	74	ID=Fv_160_8.g5126;Description=---NA---
Fv_160_8.g5127	319	ID=Fv_160_8.g5127;Description=hypothetical protein FVER14953_07631 [Fusarium verticillioides]
Fv_160_8.g5128	273	ID=Fv_160_8.g5128;Description=hypothetical protein FMUND_9422 [Fusarium mundagurra]
Fv_160_8.g5129	652	ID=Fv_160_8.g5129;Description=hypothetical protein J7337_010353 [Fusarium musae];Gene=FPHYL_3968;Ontology_term=ribonucleoprotein complex,helicase activity,ATP binding,hydrolase
Fv_160_8.g5130	455	ID=Fv_160_8.g5130;Description=hypothetical protein FVER53263_07633 [Fusarium verticillioides];Gene=FSUBG_9443;Ontology_term=membrane,transmembrane transporter activity,transmembrane
Fv_160_8.g5131	807	ID=Fv_160_8.g5131;Description=hypothetical protein FVER53590_07634 [Fusarium verticillioides]
Fv_160_8.g5132	270	ID=Fv_160_8.g5132;Description=hypothetical protein FVEG_07635 [Fusarium verticillioides 7600]
Fv_160_8.g5133	245	ID=Fv_160_8.g5133;Description=hypothetical protein FVER53263_07636 [Fusarium verticillioides]

Fv_160_8.g5134	222	ID=Fv_160_8.g5134;Description=hypothetical protein FVER53263_20169 [Fusarium verticillioides]
Fv_160_8.g5135	269	ID=Fv_160_8.g5135;Description=hypothetical protein FVER53263_07637 [Fusarium verticillioides]
Fv_160_8.g5136	174	ID=Fv_160_8.g5136;Description=hypothetical protein FVER14953_20894 [Fusarium verticillioides];Gene=FSUBG_9449
Fv_160_8.g5137	201	ID=Fv_160_8.g5137;Description=hypothetical protein FVER53263_07638 [Fusarium verticillioides]
Fv_160_8.g5138	324	ID=Fv_160_8.g5138;Description=hypothetical protein FVER14953_07639 [Fusarium verticillioides]
Fv_160_8.g5139	177	ID=Fv_160_8.g5139;Description=hypothetical protein FVEG_07640 [Fusarium verticillioides 7600];Gene=FDENT_5194;Ontology_term=nucleic acid binding,zinc ion binding;Ontology_id=GO:0003676,GO:0008270
Fv_160_8.g5140	277	ID=Fv_160_8.g5140;Description=hypothetical protein FVER14953_07641 [Fusarium
Fv_160_8.g5141	67	ID=Fv_160_8.g5141;Description=Uncharacterized protein HZ326_4323 [Fusarium oxysporum f. sp. albedinis];Gene=tug1;Ontology_term=equatorial microtubule organizing center,gamma-tubulin ring complex,nucleus,cytoplasm,spindle,microtubule,gamma-tubulin small complex,interphase microtubule organizing center,mating projection tip,half bridge of mitotic spindle pole body,inner plaque of mitotic spindle pole body,outer plaque of mitotic spindle pole body,old mitotic spindle pole body,structural constituent of cytoskeleton,protein binding,GTP binding,microtubule nucleator activity,meiotic spindle organization,microtubule nucleation,cytoplasmic microtubule organization,mitotic spindle midzone assembly,mitotic cytokinesis, division site
Fv_160_8.g5142	508	ID=Fv_160_8.g5142;Description=hypothetical protein FVER14953_07642 [Fusarium verticillioides];Gene=FTJAE_8293;Ontology_term=membrane,transmembrane transporter activity,transmembrane
Fv_160_8.g5143	309	ID=Fv_160_8.g5143;Description=hypothetical protein FVER53590_07643 [Fusarium verticillioides];Gene=FOC4_g10000643;Ontology_term=hydrolase
Fv_160_8.g5144	350	ID=Fv_160_8.g5144;Description=hypothetical protein FVER53590_07644 [Fusarium verticillioides];Gene=FANTH_6484;Ontology_term=nucleotide binding,oxidoreductase activity, acting on
Fv_160_8.g5145	635	ID=Fv_160_8.g5145;Description=hypothetical protein FVER53590_07645 [Fusarium verticillioides];Gene=FOVG_15063;Ontology_term=nucleus,DNA binding,zinc ion binding,DNA-binding transcription factor activity, RNA polymerase II-specific,regulation of transcription by RNA polymerase
Fv_160_8.g5146	618	ID=Fv_160_8.g5146;Description=hypothetical protein FVER53590_07646 [Fusarium verticillioides];Gene=FVEG_07646;Ontology_term=N,N-dimethylaniline monooxygenase activity,flavin adenine dinucleotide binding,NADP binding;Ontology_id=GO:0004499,GO:0050660,GO:0050661;Enzyme_code=EC:1.14.13.8;Enzyme_name=flavin-
Fv_160_8.g5147	618	ID=Fv_160_8.g5147;Description=hypothetical protein FGLOB1_9288 [Fusarium globosum];Gene=Forpe1208_v003747;Ontology_term=extracellular region,membrane,DNA binding,S-adenosylmethionine-dependent methyltransferase activity,DNA transposition,DNA
Fv_160_8.g5148	766	ID=Fv_160_8.g5148;Description=hypothetical protein FVER53263_07651 [Fusarium
Fv_160_8.g5149	224	ID=Fv_160_8.g5149;Description=hypothetical protein FVER53590_07652 [Fusarium verticillioides];Gene=FTJAE_8286;Ontology_term=transferase

Fv_160_8.g5150	368	ID=Fv_160_8.g5150;Description=hypothetical protein FVER14953_07653 [Fusarium verticillioides];Gene=FTJAE_8285;Ontology_term=aminopeptidase activity,metalloexopeptidase activity,metal ion
Fv_160_8.g5151	350	ID=Fv_160_8.g5151;Description=hypothetical protein FVER14953_20895 [Fusarium verticillioides]
Fv_160_8.g5152	537	ID=Fv_160_8.g5152;Description=hypothetical protein FVEG_07655 [Fusarium verticillioides 7600];Gene=FMUND_15814;Ontology_term=membrane,transmembrane transporter activity,transmembrane
Fv_160_8.g5153	319	ID=Fv_160_8.g5153;Description=hypothetical protein FVER14953_07656 [Fusarium
Fv_160_8.g5154	356	ID=Fv_160_8.g5154;Description=hypothetical protein FVER14953_07657 [Fusarium verticillioides];Gene=FANTH_6473;Ontology_term=monooxygenase
Fv_160_8.g5155	1302	ID=Fv_160_8.g5155;Description=hypothetical protein FVER53590_07658 [Fusarium verticillioides];Gene=FVEG_07658;Ontology_term=catalytic activity,nucleoside metabolic
Fv_160_8.g5156	97	ID=Fv_160_8.g5156;Description=hypothetical protein FVER53590_30458 [Fusarium verticillioides];Gene=FMUND_12327
Fv_160_8.g5157	567	ID=Fv_160_8.g5157;Description=hypothetical protein FVER53590_07659 [Fusarium
Fv_160_8.g5158	130	ID=Fv_160_8.g5158;Description=hypothetical protein FVER53590_07660 [Fusarium verticillioides]
Fv_160_8.g5159	355	ID=Fv_160_8.g5159;Description=hypothetical protein FVER53590_30459 [Fusarium verticillioides];Gene=BFJ72_g10051;Ontology_term=membrane,transmembrane transporter activity,transmembrane
Fv_160_8.g5160	104	ID=Fv_160_8.g5160;Description=hypothetical protein FVER53590_07661 [Fusarium verticillioides];Gene=CEK26_011647;Ontology_term=nucleus,DNA binding,zinc ion binding,DNA integration,DNA-binding transcription factor activity, RNA polymerase II-specific,regulation of transcription by RNA polymerase
Fv_160_8.g5161	460	ID=Fv_160_8.g5161;Description=hypothetical protein FVER53590_30460 [Fusarium verticillioides];Gene=CEK26_011647;Ontology_term=nucleus,DNA binding,zinc ion binding,DNA integration,DNA-binding transcription factor activity, RNA polymerase II-specific,regulation of transcription by RNA polymerase
Fv_160_8.g5162	359	ID=Fv_160_8.g5162;Description=hypothetical protein FVEG_07662 [Fusarium verticillioides 7600];Gene=FTJAE_8275;Ontology_term=chromatin,cytoplasm,nuclear periphery,new mitotic spindle pole body,mitotic spindle midzone,protein binding,zinc ion binding,oxidoreductase activity,cyclin-dependent protein serine/threonine kinase activator activity,mitotic cell cycle phase transition,meiotic nuclear division,positive regulation of mitotic sister chromatid biorientation;Ontology_id=GO:0000785,GO:0005737,GO:0034399,GO:0071958,GO:1990023,GO:0005515,GO:0008270,GO:0016491
Fv_160_8.g5163	384	ID=Fv_160_8.g5163;Description=hypothetical protein FVEG_07663 [Fusarium verticillioides 7600]
Fv_160_8.g5164	299	ID=Fv_160_8.g5164;Description=hypothetical protein FVEG_07664 [Fusarium verticillioides 7600]
Fv_160_8.g5165	354	ID=Fv_160_8.g5165;Description=hypothetical protein FVEG_16161 [Fusarium verticillioides
Fv_160_8.g5166	350	ID=Fv_160_8.g5166;Description=hypothetical protein FVEG_07665 [Fusarium verticillioides 7600]
Fv_160_8.g5167	192	ID=Fv_160_8.g5167;Description=hypothetical protein FVEG_07666 [Fusarium verticillioides 7600];Gene=FPRO_11493;Ontology_term=S-adenosylmethionine-dependent methyltransferase

Fv_160_8.g5168	511	ID=Fv_160_8.g5168;Description=hypothetical protein FVER53263_20698 [Fusarium verticillioides];Gene=FVEG_07667;Ontology_term=endoplasmic reticulum lumen,nuclear membrane,endoplasmic reticulum chaperone complex,nucleotide binding,ATP hydrolysis activity,transmembrane transporter activity,heat shock protein binding,unfolded protein binding,ATP-dependent protein folding chaperone,ubiquitin-dependent ERAD pathway,IRE1-mediated unfolded protein response,protein refolding,transmembrane
Fv_160_8.g5169	631	ID=Fv_160_8.g5169;Description=hypothetical protein FVER14953_07668 [Fusarium verticillioides];Gene=FGADI_8124;Ontology_term=hydrolase activity, acting on carbon-nitrogen (but not peptide) bonds,carbohydrate metabolic process;Ontology_id=GO:0016810,GO:0005975;Enzyme_code=EC:3.5;Enzyme_name=Acting on
Fv_160_8.g5170	297	ID=Fv_160_8.g5170;Description=hypothetical protein FVER14953_07669 [Fusarium verticillioides];Gene=FPANT_8934;Ontology_term=hydrolase activity, acting on carbon-nitrogen (but not peptide) bonds, in linear amidines,metal ion binding;Ontology_id=GO:0016813,GO:0046872;Enzyme_code=EC:3.5.3;Enzyme_name=Acting on carbon-
Fv_160_8.g5171	387	ID=Fv_160_8.g5171;Description=hypothetical protein FVER14953_07670 [Fusarium verticillioides];Gene=CEK26_011636;Ontology_term=hydrolase activity, acting on carbon-nitrogen (but not peptide) bonds, in linear amidines,metal ion binding;Ontology_id=GO:0016813,GO:0046872;Enzyme_code=EC:3.5.3;Enzyme_name=Acting on carbon-
Fv_160_8.g5172	1237	ID=Fv_160_8.g5172;Description=uncharacterized protein FTJAE_8265 [Fusarium tjaetaba];Gene=FVER53590_07671;Ontology_term=nucleus,DNA-binding transcription factor activity, RNA polymerase II-
Fv_160_8.g5173	1368	ID=Fv_160_8.g5173;Description=related to vegetatible incompatibility protein HET-E-1 [Fusarium oxysporum];Gene=F52700_4127;Ontology_term=glycosphingolipid binding,imaginal disc-derived leg segmentation,nucleoside metabolic process,imaginal disc-derived wing margin morphogenesis,Notch binding,receptor ligand activity,apical cortex,larval salivary gland morphogenesis,crystal cell differentiation,cell surface,wing disc anterior/posterior pattern formation,Notch signaling pathway,catalytic activity,calcium ion binding,germ-line stem cell population maintenance,lateral inhibition,second mitotic wave involved in compound eye morphogenesis,axolemma,glial cell proliferation,eye-antennal disc development,cuticle pattern formation,apical plasma membrane,negative regulation of lamellocyte
Fv_160_8.g5174	873	ID=Fv_160_8.g5174;Description=hypothetical protein FVEG_07673 [Fusarium verticillioides 7600];Gene=FTJAE_8263;Ontology_term=membrane,calcium-activated cation channel activity,monoatomic cation transmembrane
Fv_160_8.g5175	188	ID=Fv_160_8.g5175;Description=hypothetical protein FVEG_16162 [Fusarium verticillioides 7600]
Fv_160_8.g5176	169	ID=Fv_160_8.g5176;Description=hypothetical protein FVER53263_20338 [Fusarium verticillioides];Gene=FPCIR_1758;Ontology_term=hydrolase
Fv_160_8.g5177	505	ID=Fv_160_8.g5177;Description=hypothetical protein FVEG_01619 [Fusarium verticillioides 7600];Gene=LW93_4531;Ontology_term=hydrolase activity, hydrolyzing O-glycosyl compounds,polysaccharide catabolic
Fv_160_8.g5178	604	ID=Fv_160_8.g5178;Description=hypothetical protein FVEG_14878 [Fusarium verticillioides 7600];Gene=FNAPI_5324;Ontology_term=nucleus,DNA-binding transcription factor activity, RNA polymerase II-specific,zinc ion

Fv_160_8.g5179	521	ID=Fv_160_8.g5179;Description=hypothetical protein FVER14953_01618 [Fusarium verticillioides];Gene=FocTR4_00009604;Ontology_term=membrane,transmembrane transporter activity,transmembrane
Fv_160_8.g5180	630	ID=Fv_160_8.g5180;Description=hypothetical protein FVER14953_01617 [Fusarium verticillioides];Gene=F25303_10739;Ontology_term=lipid droplet,cytosol,fatty acid synthase complex,membrane,fatty acid synthase activity,[acyl-carrier-protein] S-acetyltransferase activity,[acyl-carrier-protein] S-malonyltransferase activity,(3R)-hydroxypalmitoyl-[acyl-carrier-protein] dehydratase activity,enoyl-[acyl-carrier-protein] reductase (NADH) activity,enoyl-[acyl-carrier-protein] reductase (NADPH, B-specific) activity,oleoyl-[acyl-carrier-protein] hydrolase activity,fatty-acyl-CoA synthase activity,protein binding,(3R)-hydroxymyristoyl-[acyl-carrier-protein] dehydratase activity,myristoyl-[acyl-carrier-protein] hydrolase activity,palmitoyl-[acyl-carrier-protein] hydrolase activity,palmitoyltransferase activity,long-chain fatty acid biosynthetic process;Ontology_id=GO:0005811,GO:0005829,GO:0005835,GO:0016020,GO:0004312,GO:0004313,GO:0004314,GO:0004317,GO:0004318,GO:0004319,GO:0004320,GO:0004321,GO:0005515,GO:0008659,GO:0016295,GO:0016296,GO:0016409,GO:0042759;Enz
Fv_160_8.g5181	143	ID=Fv_160_8.g5181;Description=hypothetical protein FVEG_01616 [Fusarium verticillioides 7600];Gene=FPRO_10075;Ontology_term=cytosolic large ribosomal subunit,structural constituent of ribosome,protein kinase activator activity,ribonucleoprotein complex binding,cytoplasmic translation,translational elongation,hemolysis by symbiont of host
Fv_160_8.g5182	486	ID=Fv_160_8.g5182;Description=hypothetical protein FVEG_01615 [Fusarium verticillioides 7600];Gene=FAA1;Ontology_term=catalytic activity,small molecule metabolic process;Ontology_id=GO:0003824,GO:0044281
Fv_160_8.g5183	382	ID=Fv_160_8.g5183;Description=hypothetical protein FVER14953_01614 [Fusarium verticillioides];Gene=FPRO05_12549;Ontology_term=cytosolic large ribosomal subunit,structural constituent of ribosome,monooxygenase activity,protein binding,large ribosomal subunit rRNA binding,ribosomal large subunit
Fv_160_8.g5184	532	ID=Fv_160_8.g5184;Description=hypothetical protein FVER14953_01613 [Fusarium verticillioides];Gene=FVEG_01613;Ontology_term=membrane,monooxygenase activity,iron ion binding,oxidoreductase activity, acting on paired donors, with incorporation or reduction of molecular oxygen,heme binding;Ontology_id=GO:0016020,GO:0004497,GO:0005506,GO:0016705,GO:0020037;Enzyme_code=EC:1.14;Enzyme_name=Actin
Fv_160_8.g5185	362	ID=Fv_160_8.g5185;Description=hypothetical protein FVEG_01612 [Fusarium verticillioides 7600];Gene=FocTR4_00009609;Ontology_term=membrane,monooxygenase activity,iron ion binding,oxidoreductase activity, acting on paired donors, with incorporation or reduction of molecular oxygen,heme binding;Ontology_id=GO:0016020,GO:0004497,GO:0005506,GO:0016705,GO:0020037;Enzyme_code=EC:1.14;Enzyme_name=Actin
Fv_160_8.g5186	507	ID=Fv_160_8.g5186;Description=hypothetical protein FVER14953_01611 [Fusarium verticillioides];Gene=FVEG_01611;Ontology_term=membrane,monooxygenase activity,iron ion binding,oxidoreductase activity, acting on paired donors, with incorporation or reduction of molecular oxygen,heme binding;Ontology_id=GO:0016020,GO:0004497,GO:0005506,GO:0016705,GO:0020037;Enzyme_code=EC:1.14;Enzyme_name=Actin

Fv_160_8.g5187	449	ID=Fv_160_8.g5187;Description=hypothetical protein FVER14953_01610 [Fusarium verticillioides];Gene=FPANT_7076;Ontology_term=monooxygenase activity,orotate phosphoribosyltransferase activity,orotidine-5'-phosphate decarboxylase activity,iron ion binding,oxidoreductase activity, acting on paired donors, with incorporation or reduction of molecular oxygen,isomerase activity,heme binding,'de novo' pyrimidine nucleobase biosynthetic process,'de novo' UMP biosynthetic process;Ontology_id=GO:0004497,GO:0004588,GO:0004590,GO:0005506,GO:0016705,GO:0016853,GO:0020037,GO:0006207,GO:
Fv_160_8.g5188	351	ID=Fv_160_8.g5188;Description=Pth11-like integral membrane [Fusarium sp. NRRL
Fv_160_8.g5189	916	ID=Fv_160_8.g5189;Description=magnesium-translocating P-type ATPase [Fusarium verticillioides 7600];Gene=FNAPI_5312;Ontology_term=plasma membrane,ATP binding,P-type magnesium transporter activity,ATP hydrolysis activity,magnesium ion transmembrane transport;Ontology_id=GO:0005886,GO:0005524,GO:0015444,GO:0016887,GO:1903830;Enzyme_code=EC:7.2.2.14,EC:3.6.1.15;Enz
Fv_160_8.g5190	223	ID=Fv_160_8.g5190;Description=hypothetical protein FVER53590_01607 [Fusarium verticillioides];Gene=FMUND_10722;Ontology_term=pyridoxamine phosphate oxidase activity,FMN binding,pyridoxine biosynthetic process,pyridoxal phosphate biosynthetic
Fv_160_8.g5191	112	ID=Fv_160_8.g5191;Description=hypothetical protein FVEG_01606 [Fusarium verticillioides 7600];Gene=FACUT_12193;Ontology_term=protein-disulfide reductase (NAD(P))
Fv_160_8.g5192	227	ID=Fv_160_8.g5192;Description=hypothetical protein FVEG_01605 [Fusarium verticillioides 7600]
Fv_160_8.g5193	351	ID=Fv_160_8.g5193;Description=hypothetical protein FVER53590_01604 [Fusarium
Fv_160_8.g5194	455	ID=Fv_160_8.g5194;Description=hypothetical protein FVEG_01603 [Fusarium verticillioides 7600];Gene=BFJ69_g6020;Ontology_term=nucleus,DNA-binding transcription factor activity, RNA polymerase II-specific,zinc ion
Fv_160_8.g5195	761	ID=Fv_160_8.g5195;Description=hypothetical protein FNAPI_5306 [Fusarium napiforme];Gene=FOXYS1_6023;Ontology_term=cell cortex,actin cytoskeleton,membrane,actin monomer binding,transmembrane transporter activity,myosin I heavy chain binding,mitotic cytokinesis,exocytosis,pinocytosis,hyperosmotic response,sorocarp development,positive regulation of actin filament polymerization,secretion of lysosomal enzymes,sequestering of actin monomers,actin nucleation,cell motility,negative regulation of phagocytosis,transmembrane transport;Ontology_id=GO:0005938,GO:0015629,GO:0016020,GO:0003785,GO:0022857,GO:0032037,GO:0000281,GO:0006887,GO
Fv_160_8.g5196	588	ID=Fv_160_8.g5196;Description=hypothetical protein FVEG_01601 [Fusarium verticillioides 7600];Gene=FOQG_10095;Ontology_term=membrane,transmembrane transporter activity,transmembrane
Fv_160_8.g5197	462	ID=Fv_160_8.g5197;Description=hypothetical protein FVEG_01600 [Fusarium verticillioides 7600];Gene=FSUBG_12158;Ontology_term=cell cortex,actin cytoskeleton,membrane,early phagosome,actin monomer binding,myosin I heavy chain binding,mitotic cytokinesis,exocytosis,pinocytosis,sexual reproduction,sorocarp development,positive regulation of actin filament polymerization,secretion of lysosomal enzymes,sequestering of actin monomers,actin nucleation,cell motility,negative regulation of

Fv_160_8.g5198	333	ID=Fv_160_8.g5198;Description=hypothetical protein FVEG_01599 [Fusarium verticillioides 7600];Gene=FPCIR_1776;Ontology_term=kinase
Fv_160_8.g5199	104	ID=Fv_160_8.g5199;Description=hypothetical protein H9Q70_008583 [Fusarium xylarioides]
Fv_160_8.g5200	110	ID=Fv_160_8.g5200;Description=hypothetical protein FVEG_14876 [Fusarium verticillioides 7600]
Fv_160_8.g5201	284	ID=Fv_160_8.g5201;Description=hypothetical protein FVEG_14875 [Fusarium verticillioides 7600];Gene=FNYG_14472;Ontology_term=nucleus,DNA binding,catalytic activity,base-excision repair, AP site
Fv_160_8.g5202	259	ID=Fv_160_8.g5202;Description=hypothetical protein FVER53590_01597 [Fusarium verticillioides];Gene=Forpe1208_v009457;Ontology_term=peroxiredoxin activity,cellular oxidant
Fv_160_8.g5203	270	ID=Fv_160_8.g5203;Description=hypothetical protein FVER53590_01596 [Fusarium verticillioides];Gene=FTJAE_6440;Ontology_term=thiamine metabolic process;Ontology_id=GO:0006772
Fv_160_8.g5204	473	ID=Fv_160_8.g5204;Description=L-lactate dehydrogenase (cytochrome) [Fusarium acutatum];Gene=FocTR4_00009627;Ontology_term=oxidoreductase
Fv_160_8.g5205	257	ID=Fv_160_8.g5205;Description=hypothetical protein LB504_003852 [Fusarium proliferatum];Gene=FFUJ_06602
Fv_160_8.g5206	795	ID=Fv_160_8.g5206;Description=hypothetical protein FVER14953_01594 [Fusarium verticillioides]
Fv_160_8.g5207	280	ID=Fv_160_8.g5207;Description=hypothetical protein FVEG_14873 [Fusarium verticillioides 7600];Gene=FTJAE_10024;Ontology_term=nucleic acid binding,3'-5' exonuclease activity,nucleobase-containing compound
Fv_160_8.g5208	351	ID=Fv_160_8.g5208;Description=alcohol dehydrogenase [Fusarium verticillioides 7600];Gene=C2S_12849;Ontology_term=plasma membrane,zinc ion binding,water channel activity,glycerol channel activity,oxidoreductase activity,conjugation with cellular fusion,glycerol metabolic process,water transport,arsenite transport,glycerol transmembrane transport;Ontology_id=GO:0005886,GO:0008270,GO:0015250,GO:0015254,GO:0016491,GO:0000747,GO:0006071,GO:0006833,GO
Fv_160_8.g5209	358	ID=Fv_160_8.g5209;Description=hypothetical protein H9Q70_008571 [Fusarium xylarioides];Gene=FANTH_14509;Ontology_term=zinc ion binding,oxidoreductase
Fv_160_8.g5210	799	ID=Fv_160_8.g5210;Description=hypothetical protein FVEG_01591 [Fusarium verticillioides 7600];Gene=FOBC_10706;Ontology_term=nucleus,DNA binding,zinc ion binding,DNA-binding transcription factor activity, RNA
Fv_160_8.g5211	440	ID=Fv_160_8.g5211;Description=hypothetical protein FVER53263_01590 [Fusarium verticillioides];Gene=FNAPI_5335;Ontology_term=plasma membrane,eisosome,nucleotide binding,uracil:monoatomic cation
Fv_160_8.g5212	555	ID=Fv_160_8.g5212;Description=hypothetical protein FVER14953_01589 [Fusarium verticillioides];Gene=FACUT_12218;Ontology_term=membrane,transmembrane transporter activity,transmembrane
Fv_160_8.g5213	184	ID=Fv_160_8.g5213;Description=hypothetical protein FVER14953_01588 [Fusarium verticillioides]
Fv_160_8.g5214	590	ID=Fv_160_8.g5214;Description=hypothetical protein FVEG_01587 [Fusarium verticillioides 7600];Gene=FTJAE_10031;Ontology_term=membrane,transmembrane transporter activity,transmembrane
Fv_160_8.g5215	710	ID=Fv_160_8.g5215;Description=heterokaryon incompatibility protein [Fusarium coicis];Gene=FALBO_6840

Fv_160_8.g5216	345	ID=Fv_160_8.g5216;Description=hypothetical protein H9Q71_000627 [Fusarium xylarioides];Gene=GAC1;Ontology_term=mitotic spindle assembly checkpoint signaling,heat shock protein binding,ATP binding,protein phosphatase type 1 complex,response to heat,meiotic cell cycle,regulation of glycogen biosynthetic process,membrane,protein kinase activity,protein phosphatase 1 binding,glycogen binding,protein phosphatase regulator activity;Ontology_id=GO:0007094,GO:0031072,GO:0005524,GO:0000164,GO:0009408,GO:0051321,GO:0005979,GO:0016020,GO:0
Fv_160_8.g5217	365	ID=Fv_160_8.g5217;Description=hypothetical protein FVER53590_29917 [Fusarium
Fv_160_8.g5218	278	ID=Fv_160_8.g5218;Description=hypothetical protein FVER53590_01582 [Fusarium
Fv_160_8.g5219	178	ID=Fv_160_8.g5219;Description=hypothetical protein FVER53590_01581 [Fusarium verticillioides];Gene=SUP35;Ontology_term=cytoplasmic stress granule,translation release factor complex,translation release factor activity,GTPase activity,identical protein binding,nuclear-transcribed mRNA catabolic process, deadenylation-dependent decay,translational
Fv_160_8.g5220	320	ID=Fv_160_8.g5220;Description=hypothetical protein FVEG_01580 [Fusarium verticillioides 7600]
Fv_160_8.g5221	241	ID=Fv_160_8.g5221;Description=hypothetical protein FVER53590_01579 [Fusarium verticillioides];Gene=LW93_13576;Ontology_term=cytosolic small ribosomal subunit,RNA binding,structural constituent of ribosome,phosphatase
Fv_160_8.g5222	462	ID=Fv_160_8.g5222;Description=hypothetical protein FVEG_01578 [Fusarium verticillioides 7600];Gene=FVER53263_01578;Ontology_term=oxidoreductase
Fv_160_8.g5223	488	ID=Fv_160_8.g5223;Description=hypothetical protein FVER53590_01577 [Fusarium verticillioides];Gene=FNYG_06894;Ontology_term=membrane,transmembrane transporter activity,transmembrane
Fv_160_8.g5224	499	ID=Fv_160_8.g5224;Description=succinate-semialdehyde dehydrogenase [Fusarium verticillioides 7600];Gene=FPANT_12964;Ontology_term=fungal-type vacuole lumen,extracellular region,nucleus,multivesicular body,endoplasmic reticulum,plasma membrane,COPII-coated ER to Golgi transport vesicle,DNA-binding transcription factor activity, RNA polymerase II-specific,zinc ion binding,amino acid transmembrane transporter activity,oxidoreductase activity, acting on the aldehyde or oxo group of donors, NAD or NADP as acceptor,regulation of transcription by RNA polymerase II,phenylalanine transport,proline transmembrane transport,polyamine transmembrane
Fv_160_8.g5225	100	ID=Fv_160_8.g5225;Description=hydrophobin 4 [Fusarium verticillioides];Gene=FMUND_13009;Ontology_term=extracellular
Fv_160_8.g5226	276	ID=Fv_160_8.g5226;Description=hypothetical protein FVER53590_01574 [Fusarium verticillioides]
Fv_160_8.g5227	651	ID=Fv_160_8.g5227;Description=hypothetical protein FVER53590_01573 [Fusarium verticillioides];Gene=FNAPI_11964;Ontology_term=extracellular region,membrane;Ontology_id=GO:0005576,GO:0016020
Fv_160_8.g5228	549	ID=Fv_160_8.g5228;Description=hypothetical protein FVER53590_01572 [Fusarium verticillioides];Gene=BFJ72_g13053;Ontology_term=membrane,UDP-glycosyltransferase
Fv_160_8.g5229	467	ID=Fv_160_8.g5229;Description=hypothetical protein FOZG_09412 [Fusarium oxysporum Fo47]

Fv_160_8.g5230	552	ID=Fv_160_8.g5230;Description=quinate permease [Fusarium verticillioides 7600];Gene=HZS61_014864;Ontology_term=membrane,transmembrane transporter activity,transmembrane
Fv_160_8.g5231	169	ID=Fv_160_8.g5231;Description=hypothetical protein BFJ65_g10596 [Fusarium oxysporum f. sp. cepae];Gene=BFJ68_g3016;Ontology_term=oxidoreductase activity,cellular biosynthetic
Fv_160_8.g5232	362	ID=Fv_160_8.g5232;Description=hypothetical protein FVEG_14864 [Fusarium verticillioides 7600];Gene=FOPG_14050;Ontology_term=membrane,ferric-chelate reductase activity,iron ion
Fv_160_8.g5233	385	ID=Fv_160_8.g5233;Description=hypothetical protein FVEG_14863 [Fusarium verticillioides 7600];Gene=FOXYS1_13341;Ontology_term=ferric-chelate reductase activity,oxidoreductase activity, acting on the aldehyde or oxo group of donors, NAD or NADP as acceptor,iron ion
Fv_160_8.g5234	582	ID=Fv_160_8.g5234;Description=hypothetical protein FVEG_01563 [Fusarium verticillioides 7600];Gene=FCULG_00011757;Ontology_term=DNA binding,hydrolase activity,lipid catabolic process,glycerolipid metabolic
Fv_160_8.g5235	3265	ID=Fv_160_8.g5235;Description=hypothetical protein J7337_007937 [Fusarium musae];Gene=FSUBG_9394;Ontology_term=hydrolase activity,lipid catabolic process,glycerolipid metabolic
Fv_160_8.g5236	1592	ID=Fv_160_8.g5236;Description=hypothetical protein FVER53263_01559 [Fusarium verticillioides]
Fv_160_8.g5237	379	ID=Fv_160_8.g5237;Description=hypothetical protein FVEG_01557 [Fusarium verticillioides 7600];Gene=FocTR4_00009549;Ontology_term=aminopeptidase activity,metalloexopeptidase activity,metal ion binding,proteolysis;Ontology_id=GO:0004177,GO:0008235,GO:0046872,GO:0006508;Enzyme_code=EC:3.4.11;Enzyme_name=Actin
Fv_160_8.g5238	1243	ID=Fv_160_8.g5238;Description=hypothetical protein FVEG_14860 [Fusarium verticillioides 7600]
Fv_160_8.g5239	748	ID=Fv_160_8.g5239;Description=hypothetical protein FVEG_01555 [Fusarium verticillioides 7600]
Fv_160_8.g5240	142	ID=Fv_160_8.g5240;Description=hypothetical protein FVER14953_01554 [Fusarium verticillioides]
Fv_160_8.g5241	384	ID=Fv_160_8.g5241;Description=hypothetical protein FVER14953_01553 [Fusarium verticillioides];Gene=FRV6_11745;Ontology_term=dioxygenase
Fv_160_8.g5242	513	ID=Fv_160_8.g5242;Description=hypothetical protein FVER53590_01552 [Fusarium verticillioides];Gene=F52700_290;Ontology_term=cytoplasm,metal ion binding;Ontology_id=GO:0005737,GO:0046872
Fv_160_8.g5243	566	ID=Fv_160_8.g5243;Description=hypothetical protein FVEG_01551 [Fusarium verticillioides 7600]
Fv_160_8.g5244	316	ID=Fv_160_8.g5244;Description=alcohol dehydrogenase [Fusarium verticillioides 7600];Gene=BHE90_003456
Fv_160_8.g5245	180	ID=Fv_160_8.g5245;Description=hypothetical protein FVER53263_01549 [Fusarium verticillioides];Gene=FGADI_11533;Ontology_term=carbonate dehydratase activity,zinc ion
Fv_160_8.g5246	268	ID=Fv_160_8.g5246;Description=hypothetical protein J7337_007927 [Fusarium musae];Gene=FACUT_3193;Ontology_term=hydrolase
Fv_160_8.g5247	1544	ID=Fv_160_8.g5247;Description=hypothetical protein FVER14953_01548 [Fusarium verticillioides]
Fv_160_8.g5248	306	ID=Fv_160_8.g5248;Description=hypothetical protein LB506_002098 [Fusarium annulatum];Gene=HHF1;Ontology_term=nucleosome,nucleus,DNA binding,structural constituent of chromatin,protein

Fv_160_8.g5249	365	ID=Fv_160_8.g5249;Description=hypothetical protein FVEG_01546 [Fusarium verticillioides 7600];Gene=HHT2;Ontology_term=nucleosome,nucleus,DNA binding,structural constituent of chromatin,protein heterodimerization
Fv_160_8.g5250	644	ID=Fv_160_8.g5250;Description=hypothetical protein J7337_007922 [Fusarium musae];Gene=FPRO_10163;Ontology_term=membrane,transmembrane transporter activity,transmembrane
Fv_160_8.g5251	523	ID=Fv_160_8.g5251;Description=3-hydroxyaspartate ammonia-lyase [Fusarium coicis];Gene=FRV6_10356;Ontology_term=lyase activity;Ontology_id=GO:0016829;Enzyme_code=EC:4;Enzyme_name=Lyases
Fv_160_8.g5252	929	ID=Fv_160_8.g5252;Description=hypothetical protein FVEG_01541 [Fusarium verticillioides 7600];Gene=F25303_1763;Ontology_term=cytosol,membrane,protein-containing complex,alcohol dehydrogenase (NAD+) activity,serine-type endopeptidase activity,acetaldehyde dehydrogenase (acetylating) activity,protein homodimerization activity,ethanol oxidation,acetaldehyde metabolic process,proteolysis,NADH metabolic process,alcohol catabolic process,behavioral response to ethanol;Ontology_id=GO:0005829,GO:0016020,GO:0032991,GO:0004022,GO:0004252,GO:0008774,GO:0042803,GO:0006069,GO:
Fv_160_8.g5253	471	ID=Fv_160_8.g5253;Description=hypothetical protein FVEG_14857 [Fusarium verticillioides 7600];Gene=FVEG_14857;Ontology_term=cytoplasm,aspartic-type endopeptidase activity,metal ion
Fv_160_8.g5254	707	ID=Fv_160_8.g5254;Description=hypothetical protein FVEG_01538 [Fusarium verticillioides 7600]
Fv_160_8.g5255	495	ID=Fv_160_8.g5255;Description=tartrate transporter [Fusarium pseudoanthophilum];Gene=FOQG_10183;Ontology_term=nucleus,DNA binding,zinc ion binding,transmembrane transport,transmembrane transporter activity,catalytic activity,membrane,DNA-binding transcription factor activity, RNA polymerase
Fv_160_8.g5256	598	ID=Fv_160_8.g5256;Description=hypothetical protein FVER53590_30311 [Fusarium verticillioides];Gene=FVEG_01536;Ontology_term=nucleus,DNA binding,zinc ion binding,transmembrane transport,transmembrane transporter activity,membrane,DNA-binding transcription factor activity, RNA polymerase II-specific,regulation of transcription by
Fv_160_8.g5257	310	ID=Fv_160_8.g5257;Description=hypothetical protein FVER53263_01535 [Fusarium verticillioides];Gene=FFUJ_06657;Ontology_term=nucleus,DNA-binding transcription factor activity, RNA polymerase II-specific,catalytic activity,zinc ion binding,regulation of transcription by RNA polymerase II,positive regulation of gene
Fv_160_8.g5258	323	ID=Fv_160_8.g5258;Description=hypothetical protein FVER14953_01534 [Fusarium verticillioides];Gene=FNAPI_5048;Ontology_term=oxidoreductase activity, acting on the CH-OH group of donors, NAD or NADP as
Fv_160_8.g5259	1180	ID=Fv_160_8.g5259;Description=hypothetical protein FANTH_2347 [Fusarium anthophilum];Gene=BFJ68_g3118;Ontology_term=nucleus,membrane,DNA binding,zinc ion binding,transmembrane transporter activity,DNA-templated transcription,transmembrane
Fv_160_8.g5260	312	ID=Fv_160_8.g5260;Description=hypothetical protein FVEG_01532 [Fusarium verticillioides 7600]
Fv_160_8.g5261	329	ID=Fv_160_8.g5261;Description=hypothetical protein J7337_007904 [Fusarium musae];Gene=BFJ69_g6031;Ontology_term=cellular anatomical entity,serine-type endopeptidase
Fv_160_8.g5262	304	ID=Fv_160_8.g5262;Description=hypothetical protein FVER14953_01529 [Fusarium verticillioides]

Fv_160_8.g5263	661	ID=Fv_160_8.g5263;Description=hypothetical protein FVEG_01528 [Fusarium verticillioides 7600];Gene=FTJAE_367;Ontology_term=nucleus,membrane,DNA-binding transcription factor activity, RNA polymerase II-
Fv_160_8.g5264	530	ID=Fv_160_8.g5264;Description=murein transglycosylase [Fusarium verticillioides 7600];Gene=FocTR4_00009528;Ontology_term=hydrolase activity, hydrolyzing O-glycosyl compounds,polysaccharide catabolic
Fv_160_8.g5265	540	ID=Fv_160_8.g5265;Description=hypothetical protein FVEG_01526 [Fusarium verticillioides 7600];Gene=FocTR4_00009527;Ontology_term=membrane,alpha,alpha-trehalose-phosphate synthase (UDP-forming) activity,methylenetetrahydrofolate dehydrogenase (NADP+) activity,transmembrane transporter activity,trehalose biosynthetic process,one-carbon metabolic process,transmembrane transport;Ontology_id=GO:0016020,GO:0003825,GO:0004488,GO:0022857,GO:0005992,GO:0006730,GO:0055085;Enzyme_code=E
Fv_160_8.g5266	312	ID=Fv_160_8.g5266;Description=hypothetical protein FVEG_01525 [Fusarium verticillioides 7600];Gene=FVEG_01525;Ontology_term=plasma membrane,side of membrane,GTPase activity,methylenetetrahydrofolate dehydrogenase (NADP+) activity,GTP binding,transmembrane transporter activity,G-protein beta/gamma-subunit complex binding,glucanosyltransferase activity,one-carbon metabolic process,G protein-coupled receptor signaling pathway,transmembrane transport,cellular component organization or biogenesis,fungal-type cell wall organization or biogenesis;Ontology_id=GO:0005886,GO:0098552,GO:0003924,GO:0004488,GO:0005525,GO:0022857,GO:0031683,GO:0042123,G
Fv_160_8.g5267	552	ID=Fv_160_8.g5267;Description=hypothetical protein FVEG_01524 [Fusarium verticillioides 7600];Gene=FMEXI_13292;Ontology_term=membrane,transferase
Fv_160_8.g5268	284	ID=Fv_160_8.g5268;Description=hypothetical protein FVEG_01523 [Fusarium verticillioides 7600];Gene=FVER53263_01523;Ontology_term=GTPase activity,methylenetetrahydrofolate dehydrogenase (NADP+) activity,oxygen carrier activity,GTP binding,heme binding,G-protein beta/gamma-subunit complex binding,metal ion binding,G protein-coupled receptor signaling pathway,oxygen transport;Ontology_id=GO:0003924,GO:0004488,GO:0005344,GO:0005525,GO:0020037,GO:0031683,GO:0046872,GO:0007186,GO
Fv_160_8.g5269	643	ID=Fv_160_8.g5269;Description=hypothetical protein FNAPI_5061 [Fusarium
Fv_160_8.g5270	483	ID=Fv_160_8.g5270;Description=hypothetical protein FVER53590_01520 [Fusarium verticillioides];Gene=FRV6_10299;Ontology_term=membrane,alditol:NADP+ 1-oxidoreductase activity,arabinose catabolic process,cellular response to oxidative stress,D-xylose catabolic
Fv_160_8.g5271	346	ID=Fv_160_8.g5271;Description=hypothetical protein FPRO05_12632 [Fusarium proliferatum];Gene=HAP4;Ontology_term=mitochondrial respirasome,CCAAT-binding factor complex,DNA binding,transcription coactivator activity,RNA polymerase II-specific DNA-binding transcription factor binding,carbon catabolite activation of transcription from RNA polymerase II promoter,regulation of carbohydrate metabolic process,regulation of cellular
Fv_160_8.g5272	514	ID=Fv_160_8.g5272;Description=hypothetical protein BFJ67_g12704 [Fusarium oxysporum f. sp. cepae];Gene=BFJ63_vAg12158;Ontology_term=regulation of DNA-templated transcription;Ontology_id=GO:0006355

Fv_160_8.g5273	533	ID=Fv_160_8.g5273;Description=hypothetical protein FVER53263_01519 [Fusarium verticillioides];Gene=FOVG_10082;Ontology_term=membrane,transmembrane transporter activity,transmembrane
Fv_160_8.g5274	525	ID=Fv_160_8.g5274;Description=hypothetical protein FVER53590_01518 [Fusarium verticillioides];Gene=FACUT_1229;Ontology_term=membrane,monooxygenase activity,iron ion binding,oxidoreductase activity, acting on paired donors, with incorporation or reduction of molecular oxygen,heme binding;Ontology_id=GO:0016020,GO:0004497,GO:0005506,GO:0016705,GO:0020037;Enzyme_code=EC:1.14;Enzyme_name=Actin
Fv_160_8.g5275	1016	ID=Fv_160_8.g5275;Description=hypothetical protein NW762_006379 [Fusarium torreyae];Gene=CEP54_011481
Fv_160_8.g5276	294	ID=Fv_160_8.g5276;Description=hypothetical protein NW762_006378 [Fusarium torreyae];Gene=BFJ68_g16773
Fv_160_8.g5277	81	ID=Fv_160_8.g5277;Description=hypothetical protein IL306_005372 [Fusarium sp. DS 682]
Fv_160_8.g5278	479	ID=Fv_160_8.g5278;Description=hypothetical protein F53441_13151 [Fusarium
Fv_160_8.g5279	524	ID=Fv_160_8.g5279;Description=hypothetical protein F53441_13149 [Fusarium austroafricanum]
Fv_160_8.g5280	247	ID=Fv_160_8.g5280;Description=hypothetical protein FNAPI_5066 [Fusarium napiforme];Gene=AU210_011205;Ontology_term=oxidoreductase activity,cellular biosynthetic
Fv_160_8.g5281	652	ID=Fv_160_8.g5281;Description=hypothetical protein FVER53263_14120 [Fusarium verticillioides];Gene=AU210_011227;Ontology_term=N,N-dimethylaniline monooxygenase activity,flavin adenine dinucleotide binding,NADP binding,cellular biosynthetic process,organonitrogen compound biosynthetic
Fv_160_8.g5282	773	ID=Fv_160_8.g5282;Description=hypothetical protein FVER53590_14121 [Fusarium verticillioides];Gene=FNAPI_9785;Ontology_term=nucleus,DNA binding,peroxisome,fatty acid beta-oxidation,zinc ion binding,oxidoreductase activity, acting on the CH-CH group of donors,flavin adenine dinucleotide binding,DNA-binding transcription factor activity, RNA polymerase II-specific,regulation of transcription by RNA polymerase II;Ontology_id=GO:0005634,GO:0003677,GO:0005777,GO:0006635,GO:0008270,GO:0016627,GO:0050660,GO:0000981,GO:000635
Fv_160_8.g5283	302	ID=Fv_160_8.g5283;Description=hypothetical protein FVER53590_14122 [Fusarium verticillioides];Gene=FNAPI_9786;Ontology_term=monooxygenase activity,hydrolase
Fv_160_8.g5284	382	ID=Fv_160_8.g5284;Description=hypothetical protein FVER53263_14123 [Fusarium verticillioides];Gene=FOPG_14120;Ontology_term=nucleotide binding,oxidoreductase activity, acting on
Fv_160_8.g5285	201	ID=Fv_160_8.g5285;Description=hypothetical protein FVEG_14124 [Fusarium verticillioides 7600];Gene=FOXG_17344;Ontology_term=oxidoreductase activity, acting on the CH-CH group of donors,flavin adenine dinucleotide
Fv_160_8.g5286	487	ID=Fv_160_8.g5286;Description=hypothetical protein FVER53263_14125 [Fusarium verticillioides];Gene=FOIG_11261;Ontology_term=membrane,monooxygenase activity,iron ion binding,oxidoreductase activity, acting on paired donors, with incorporation or reduction of molecular oxygen,heme binding;Ontology_id=GO:0016020,GO:0004497,GO:0005506,GO:0016705,GO:0020037;Enzyme_code=EC:1.14;Enzyme_name=Actin
Fv_160_8.g5287	347	ID=Fv_160_8.g5287;Description=hypothetical protein FVER53590_25676 [Fusarium verticillioides]
Fv_160_8.g5288	798	ID=Fv_160_8.g5288;Description=hypothetical protein FVER53590_14127 [Fusarium verticillioides]

Fv_160_8.g5289	637	ID=Fv_160_8.g5289;Description=hypothetical protein FVER53263_14128 [Fusarium verticillioides];Gene=FTJAE_413;Ontology_term=hydrolase activity,carbohydrate metabolic
Fv_160_8.g5290	434	ID=Fv_160_8.g5290;Description=hypothetical protein FVEG_14129 [Fusarium verticillioides 7600];Gene=FVER53263_14129;Ontology_term=membrane,transmembrane transporter activity,transmembrane
Fv_160_8.g5291	729	ID=Fv_160_8.g5291;Description=hypothetical protein FVEG_14130 [Fusarium verticillioides 7600];Gene=FTJAE_411;Ontology_term=nucleus,DNA binding,zinc ion binding,DNA-templated
Fv_160_8.g5292	489	ID=Fv_160_8.g5292;Description=hypothetical protein FVEG_14131 [Fusarium verticillioides 7600]
Fv_160_8.g5293	459	ID=Fv_160_8.g5293;Description=hypothetical protein FVER53263_20302 [Fusarium verticillioides]
Fv_160_8.g5294	676	ID=Fv_160_8.g5294;Description=hypothetical protein FVEG_14132 [Fusarium verticillioides 7600];Gene=FPANT_10962;Ontology_term=nucleus,DNA binding,zinc ion binding,DNA-binding transcription factor activity, RNA polymerase II-specific,regulation of transcription by RNA polymerase
Fv_160_8.g5295	282	ID=Fv_160_8.g5295;Description=hypothetical protein FVEG_14133 [Fusarium verticillioides 7600];Gene=FANTH_8453;Ontology_term=cytoplasm,membrane,phosphoribosyl-AMP cyclohydrolase activity,ATP binding,zinc ion binding,transferase activity,histidine biosynthetic process;Ontology_id=GO:0005737,GO:0016020,GO:0004635,GO:0005524,GO:0008270,GO:0016740,GO:0000105;Enzyme_code=EC
Fv_160_8.g5296	336	ID=Fv_160_8.g5296;Description=hypothetical protein FVEG_14134 [Fusarium verticillioides 7600]
Fv_160_8.g5297	400	ID=Fv_160_8.g5297;Description=hypothetical protein FVER14953_14135 [Fusarium verticillioides];Gene=FANTH_8451;Ontology_term=catalytic activity,thiamine pyrophosphate
Fv_160_8.g5298	977	ID=Fv_160_8.g5298;Description=hypothetical protein FVER53263_14136 [Fusarium verticillioides];Gene=FCIRC_6429;Ontology_term=carbohydrate binding,maltose alpha-glucosidase activity,maltose metabolic
Fv_160_8.g5299	478	ID=Fv_160_8.g5299;Description=hypothetical protein FVER53590_14137 [Fusarium verticillioides];Gene=FNAPI_4103;Ontology_term=membrane,oxidoreductase activity,transmembrane transporter activity,transmembrane
Fv_160_8.g5300	407	ID=Fv_160_8.g5300;Description=hypothetical protein FVER53263_14138 [Fusarium verticillioides];Gene=BFJ63_vAg12125;Ontology_term=dioxygenase
Fv_160_8.g5301	404	ID=Fv_160_8.g5301;Description=hypothetical protein FVER14953_14139 [Fusarium
Fv_160_8.g5302	607	ID=Fv_160_8.g5302;Description=hypothetical protein FVEG_14140 [Fusarium verticillioides 7600];Gene=BFJ68_g2982;Ontology_term=oxidoreductase activity, acting on CH-OH group of donors,flavin adenine dinucleotide
Fv_160_8.g5303	545	ID=Fv_160_8.g5303;Description=Monocarboxylate transporter 12 [Fusarium oxysporum f. sp. cubense race 1];Gene=FPANT_10954;Ontology_term=membrane,oxidoreductase activity, acting on CH-OH group of donors,transmembrane transporter activity,flavin adenine dinucleotide binding,transmembrane

Fv_160_8.g5304	502	ID=Fv_160_8.g5304;Description=hypothetical protein FVEG_14141 [Fusarium verticillioides 7600];Gene=FPANT_10953;Ontology_term=membrane,monooxygenase activity,iron ion binding,oxidoreductase activity, acting on paired donors, with incorporation or reduction of molecular oxygen,heme binding;Ontology_id=GO:0016020,GO:0004497,GO:0005506,GO:0016705,GO:0020037;Enzyme_code=EC:1.14;Enzyme_name=Actin
Fv_160_8.g5305	470	ID=Fv_160_8.g5305;Description=hypothetical protein FVEG_14142 [Fusarium verticillioides 7600];Gene=FRV6_10315;Ontology_term=membrane,transmembrane transporter activity,transmembrane
Fv_160_8.g5306	419	ID=Fv_160_8.g5306;Description=hypothetical protein FVEG_14143 [Fusarium verticillioides 7600];Gene=FDENT_13632;Ontology_term=nucleus,DNA binding,zinc ion binding,DNA-binding transcription factor activity, RNA polymerase II-specific,regulation of transcription by RNA polymerase
Fv_160_8.g5307	429	ID=Fv_160_8.g5307;Description=hypothetical protein FVEG_14144 [Fusarium verticillioides 7600];Gene=F53441_10733;Ontology_term=O-methyltransferase activity,S-adenosylmethionine-dependent methyltransferase activity,methylation,secondary metabolite biosynthetic
Fv_160_9.g5308	289	ID=Fv_160_9.g5308;Description=hypothetical protein FVER14953_03431 [Fusarium verticillioides]
Fv_160_9.g5309	97	ID=Fv_160_9.g5309;Description=hypothetical protein FVER53590_25511 [Fusarium verticillioides];Gene=FNYG_11298
Fv_160_9.g5310	104	ID=Fv_160_9.g5310;Description=hypothetical protein FVER14953_21569 [Fusarium verticillioides]
Fv_160_9.g5311	390	ID=Fv_160_9.g5311;Description=hypothetical protein FVER53263_03432 [Fusarium verticillioides];Gene=FPANT_1935;Ontology_term=monooxygenase activity,iron ion binding,2 iron, 2 sulfur cluster
Fv_160_9.g5312	154	ID=Fv_160_9.g5312;Description=hypothetical protein FVEG_03433 [Fusarium verticillioides 7600]
Fv_160_9.g5313	326	ID=Fv_160_9.g5313;Description=hypothetical protein FVER53263_03434 [Fusarium verticillioides]
Fv_160_9.g5314	79	ID=Fv_160_9.g5314;Description=hypothetical protein FVEG_15263 [Fusarium verticillioides 7600];Gene=FOIG_13691;Ontology_term=argininosuccinate lyase activity,arginine biosynthetic process via
Fv_160_9.g5315	496	ID=Fv_160_9.g5315;Description=hypothetical protein FVER53263_20620 [Fusarium verticillioides]
Fv_160_9.g5316	749	ID=Fv_160_9.g5316;Description=hypothetical protein FVER53263_03435 [Fusarium verticillioides]
Fv_160_9.g5317	568	ID=Fv_160_9.g5317;Description=hypothetical protein FVER53263_03436 [Fusarium verticillioides];Gene=FMAN_03624;Ontology_term=membrane,transmembrane transporter activity,transmembrane
Fv_160_9.g5318	401	ID=Fv_160_9.g5318;Description=hypothetical protein FVER53263_03437 [Fusarium verticillioides];Gene=2208;Ontology_term=extracellular region,lactose synthase activity,calcium ion binding,glycerate kinase activity,lactose biosynthetic process,organic acid phosphorylation;Ontology_id=GO:0005576,GO:0004461,GO:0005509,GO:0008887,GO:0005989,GO:0031388;Enzyme_code=EC:2.7.
Fv_160_9.g5319	91	ID=Fv_160_9.g5319;Description=hypothetical protein FNYG_05729 [Fusarium nygamai];Gene=2209
Fv_160_9.g5320	767	ID=Fv_160_9.g5320;Description=hypothetical protein FVER53263_03438 [Fusarium verticillioides];Gene=FVEG_03438;Ontology_term=alpha-L-fucosidase activity,carbohydrate metabolic

Fv_160_9.g5321	549	ID=Fv_160_9.g5321;Description=hypothetical protein FVEG_15266 [Fusarium verticillioides 7600];Gene=BFJ63_vAg15821;Ontology_term=membrane,hydrolase activity, hydrolyzing O-glycosyl compounds,transmembrane transporter activity,carbohydrate metabolic process,transmembrane
Fv_160_9.g5322	495	ID=Fv_160_9.g5322;Description=beta-glucosidase [Fusarium verticillioides 7600];Gene=FPANT_1946;Ontology_term=membrane,hydrolase activity, hydrolyzing O-glycosyl compounds,transmembrane transporter activity,carbohydrate metabolic process,transmembrane
Fv_160_9.g5323	835	ID=Fv_160_9.g5323;Description=hypothetical protein FVEG_03442 [Fusarium verticillioides 7600];Gene=F52700_3216;Ontology_term=membrane,hydrolase activity, hydrolyzing O-glycosyl compounds,transmembrane transporter activity,carbohydrate metabolic process,transmembrane
Fv_160_9.g5324	329	ID=Fv_160_9.g5324;Description=hypothetical protein FVEG_03443 [Fusarium verticillioides
Fv_160_9.g5325	298	ID=Fv_160_9.g5325;Description=hypothetical protein FVER14953_03444 [Fusarium verticillioides];Gene=truA;Ontology_term=extracellular region,retinol binding;Ontology_id=GO:0005576,GO:0019841
Fv_160_9.g5326	229	ID=Fv_160_9.g5326;Description=hypothetical protein FVER14953_03446 [Fusarium verticillioides];Gene=FMAN_03633;Ontology_term=membrane,ATP binding,nucleobase-containing compound kinase activity,nucleobase-containing compound metabolic
Fv_160_9.g5327	353	ID=Fv_160_9.g5327;Description=hypothetical protein FVER14953_03447 [Fusarium verticillioides];Gene=FPRO_03805;Ontology_term=membrane,catalytic activity,metal ion binding,iron-sulfur cluster
Fv_160_9.g5328	512	ID=Fv_160_9.g5328;Description=hypothetical protein FVEG_03448 [Fusarium verticillioides 7600];Gene=FPRO05_10181;Ontology_term=membrane,transmembrane transporter activity,transmembrane
Fv_160_9.g5329	635	ID=Fv_160_9.g5329;Description=hypothetical protein FVEG_03449 [Fusarium verticillioides 7600];Gene=FOVG_17032;Ontology_term=nucleus,membrane,DNA-binding transcription factor activity, RNA polymerase II-specific,zinc ion binding,transmembrane transporter activity,regulation of transcription by RNA polymerase II,transmembrane
Fv_160_9.g5330	507	ID=Fv_160_9.g5330;Description=hypothetical protein FVEG_15267 [Fusarium verticillioides 7600];Gene=FTJAE_12543;Ontology_term=oxidoreductase
Fv_160_9.g5331	228	ID=Fv_160_9.g5331;Description=hypothetical protein FVER14953_21566 [Fusarium verticillioides];Gene=FTJAE_12544;Ontology_term=acyltransferase activity, transferring groups other than amino-acyl
Fv_160_9.g5332	275	ID=Fv_160_9.g5332;Description=hypothetical protein FVEG_03450 [Fusarium verticillioides 7600];Gene=FNAPI_7765;Ontology_term=phosphoric diester hydrolase activity,lipid metabolic
Fv_160_9.g5333	203	ID=Fv_160_9.g5333;Description=hypothetical protein J7337_003472 [Fusarium
Fv_160_9.g5334	568	ID=Fv_160_9.g5334;Description=hypothetical protein FVER14953_03453 [Fusarium verticillioides];Gene=FSUBG_12447;Ontology_term=membrane,transmembrane transporter activity,transmembrane
Fv_160_9.g5335	317	ID=Fv_160_9.g5335;Description=hypothetical protein FPCIR_3165 [Fusarium pseudocircinatum];Gene=FEQUK3_LOCUS2211;Ontology_term=cellular anatomical entity,regulation of DNA-templated

Fv_160_9.g5336	154	ID=Fv_160_9.g5336;Description=hypothetical protein FVEG_15270 [Fusarium verticillioides 7600]
Fv_160_9.g5337	500	ID=Fv_160_9.g5337;Description=hypothetical protein FVER53263_03454 [Fusarium verticillioides];Gene=FSUBG_12449;Ontology_term=membrane,transmembrane transporter activity,transmembrane
Fv_160_9.g5338	940	ID=Fv_160_9.g5338;Description=arylsulfatase [Fusarium oxysporum];Gene=FPANT_10623;Ontology_term=nucleus,DNA-binding transcription factor activity, RNA polymerase II-specific,zinc ion binding,sulfuric ester hydrolase activity,regulation of transcription by RNA polymerase
Fv_160_9.g5339	568	ID=Fv_160_9.g5339;Description=hypothetical protein FVEG_03457 [Fusarium verticillioides 7600]
Fv_160_9.g5340	398	ID=Fv_160_9.g5340;Description=polysaccharide synthase [Fusarium pseudoanthophilum];Gene=FPSE_07856;Ontology_term=membrane,transferase
Fv_160_9.g5341	538	ID=Fv_160_9.g5341;Description=hypothetical protein FVER14953_21570 [Fusarium verticillioides];Gene=FVEG_03459;Ontology_term=membrane,transmembrane transporter activity,carbohydrate
Fv_160_9.g5342	852	ID=Fv_160_9.g5342;Description=hypothetical protein FVER53263_03461 [Fusarium verticillioides];Gene=FOMG_17221;Ontology_term=nucleus,DNA-binding transcription factor activity, RNA polymerase II-
Fv_160_9.g5343	543	ID=Fv_160_9.g5343;Description=hypothetical protein FVER14953_03462 [Fusarium verticillioides];Gene=FocTR4_00007822;Ontology_term=membrane,transmembrane transporter activity,transmembrane
Fv_160_9.g5344	366	ID=Fv_160_9.g5344;Description=hypothetical protein FVER53590_03463 [Fusarium verticillioides];Gene=FSUBG_12300;Ontology_term=mannan endo-1,4-beta-mannosidase activity,mannan catabolic
Fv_160_9.g5345	269	ID=Fv_160_9.g5345;Description=hypothetical protein FVEG_03464 [Fusarium verticillioides 7600];Gene=F52700_3235;Ontology_term=hydrolase
Fv_160_9.g5346	395	ID=Fv_160_9.g5346;Description=hypothetical protein FVER53263_03465 [Fusarium verticillioides]
Fv_160_9.g5347	870	ID=Fv_160_9.g5347;Description=pppde peptidase [Fusarium tjaetaba];Gene=FTJAE_7483;Ontology_term=RNA binding,RNA helicase activity,ATP binding,hydrolase activity,inorganic cation transmembrane transporter activity,metal ion transport;Ontology_id=GO:0003723,GO:0003724,GO:0005524,GO:0016787,GO:0022890,GO:0030001;Enzyme_code=EC:3.6.4.13;En
Fv_160_9.g5348	223	ID=Fv_160_9.g5348;Description=hypothetical protein FVER53263_03469 [Fusarium verticillioides];Gene=FVER53590_03468;Ontology_term=nucleic acid binding,RNA helicase activity,ATP binding,hydrolase
Fv_160_9.g5349	291	ID=Fv_160_9.g5349;Description=hypothetical protein FVEG_15272 [Fusarium verticillioides 7600];Gene=BFJ63_vAg13379;Ontology_term=membrane,keratin filament;Ontology_id=GO:0016020,GO:0045095
Fv_160_9.g5350	377	ID=Fv_160_9.g5350;Description=hypothetical protein FVEG_03470 [Fusarium verticillioides 7600];Gene=FGLOB1_11957;Ontology_term=extracellular region,pectate lyase activity,polysaccharide catabolic
Fv_160_9.g5351	437	ID=Fv_160_9.g5351;Description=hypothetical protein FVER53263_20627 [Fusarium verticillioides];Gene=BFJ68_g12763;Ontology_term=hydrolase activity, hydrolyzing O-glycosyl compounds,polysaccharide catabolic
Fv_160_9.g5352	279	ID=Fv_160_9.g5352;Description=hypothetical protein FPCIR_3183 [Fusarium pseudocircinatum]

Fv_160_9.g5353	304	ID=Fv_160_9.g5353;Description=alcohol dehydrogenase [Fusarium tjaetaba];Gene=FSPOR_11869;Ontology_term=oxidoreductase activity;Ontology_id=GO:0016491;Enzyme_code=EC:1;Enzyme_name=Oxidoreductases
Fv_160_9.g5354	313	ID=Fv_160_9.g5354;Description=hypothetical protein FVER53263_03471 [Fusarium verticillioides]
Fv_160_9.g5355	703	ID=Fv_160_9.g5355;Description=hypothetical protein FVER53263_03472 [Fusarium verticillioides];Gene=FNAPI_3323;Ontology_term=nucleus,membrane,DNA-binding transcription factor activity, RNA polymerase II-
Fv_160_9.g5356	236	ID=Fv_160_9.g5356;Description=hypothetical protein J7337_003492 [Fusarium
Fv_160_9.g5357	548	ID=Fv_160_9.g5357;Description=hypothetical protein FVEG_03475 [Fusarium verticillioides 7600];Gene=FNAPI_3322;Ontology_term=membrane,transmembrane transporter activity,transmembrane
Fv_160_9.g5358	522	ID=Fv_160_9.g5358;Description=hypothetical protein FVER53263_03476 [Fusarium verticillioides];Gene=FMAN_03606;Ontology_term=membrane,transmembrane transporter activity,amino acid
Fv_160_9.g5359	1705	ID=Fv_160_9.g5359;Description=hypothetical protein FVER53590_03477 [Fusarium verticillioides];Gene=FSUBG_12312;Ontology_term=cell surface,cell adhesion;Ontology_id=GO:0009986,GO:0007155
Fv_160_9.g5360	821	ID=Fv_160_9.g5360;Description=hypothetical protein FVER53263_03479 [Fusarium verticillioides];Gene=BFJ63_vAg13389;Ontology_term=membrane,transmembrane transporter activity,transmembrane
Fv_160_9.g5361	671	ID=Fv_160_9.g5361;Description=transforming growth factor-beta-induced ig-h3 [Fusarium phyllophilum]
Fv_160_9.g5362	438	ID=Fv_160_9.g5362;Description=hypothetical protein FVER14953_03482 [Fusarium verticillioides];Gene=FMAN_03602;Ontology_term=histidinol dehydrogenase activity,metal ion binding,NAD binding,histidine
Fv_160_9.g5363	193	ID=Fv_160_9.g5363;Description=hypothetical protein FVEG_03483 [Fusarium verticillioides 7600];Gene=FDENT_12019;Ontology_term=oxidoreductase activity, acting on the CH-OH group of donors, NAD or NADP as acceptor,metal ion binding,NAD binding,histidine biosynthetic
Fv_160_9.g5364	1421	ID=Fv_160_9.g5364;Description=hypothetical protein FVEG_15274 [Fusarium verticillioides 7600];Gene=Focb16_v011449;Ontology_term=nucleus,DNA binding,zinc ion binding,transmembrane transport,transmembrane transporter activity,membrane,DNA-binding transcription factor activity, RNA polymerase II-specific,regulation of transcription by
Fv_160_9.g5365	706	ID=Fv_160_9.g5365;Description=hypothetical protein FVER14953_03486 [Fusarium verticillioides];Gene=FTJAE_7471;Ontology_term=membrane,oxidoreductase activity,carboxy-lyase activity,FAD binding,biosynthetic process;Ontology_id=GO:0016020,GO:0016491,GO:0016831,GO:0071949,GO:0009058;Enzyme_code=EC:4.1.1,EC:1;Enzyme_name
Fv_160_9.g5366	255	ID=Fv_160_9.g5366;Description=3-oxoacyl-[acyl-carrier protein] reductase [Fusarium verticillioides 7600]
Fv_160_9.g5367	473	ID=Fv_160_9.g5367;Description=hypothetical protein FVER53263_03487 [Fusarium
Fv_160_9.g5368	519	ID=Fv_160_9.g5368;Description=transcriptional activator UGA3 [Fusarium coicis];Gene=FNAPI_3344;Ontology_term=nucleus,zinc ion binding,pyridoxal phosphate binding,transaminase activity,DNA-binding transcription factor activity, RNA polymerase II-specific,regulation of transcription by RNA polymerase

Fv_160_9.g5369	529	ID=Fv_160_9.g5369;Description=aromatic amino acid aminotransferase I [Fusarium verticillioides 7600];Gene=Forpi1262_v005577;Ontology_term=nucleus,zinc ion binding,pyridoxal phosphate binding,transaminase activity,DNA-binding transcription factor activity, RNA polymerase II-specific,regulation of transcription by RNA polymerase
Fv_160_9.g5370	565	ID=Fv_160_9.g5370;Description=hypothetical protein FVEG_03490 [Fusarium verticillioides
Fv_160_9.g5371	289	ID=Fv_160_9.g5371;Description=2 4-dihydroxyhept-2-ene-1 7-dioic acid aldolase [Fusarium tjaetaba];Gene=FVER53590_03491;Ontology_term=catalytic activity;Ontology_id=GO:0003824
Fv_160_9.g5372	282	ID=Fv_160_9.g5372;Description=hypothetical protein FVEG_03492 [Fusarium verticillioides
Fv_160_9.g5373	713	ID=Fv_160_9.g5373;Description=hypothetical protein FVEG_15276 [Fusarium verticillioides 7600];Gene=FMUND_14307;Ontology_term=catalytic activity,nucleoside metabolic process;Ontology_id=GO:0003824,GO:0009116
Fv_160_9.g5374	308	ID=Fv_160_9.g5374;Description=hypothetical protein FVER53263_03493 [Fusarium verticillioides];Gene=FACUT_1758;Ontology_term=NuA4 histone acetyltransferase complex,catalytic activity,chromatin
Fv_160_9.g5375	577	ID=Fv_160_9.g5375;Description=hypothetical protein FVER53263_03494 [Fusarium verticillioides];Gene=FMAN_05192;Ontology_term=extracellular space,membrane,serine-type endopeptidase activity,metal ion binding,proteolysis;Ontology_id=GO:0005615,GO:0016020,GO:0004252,GO:0046872,GO:0006508;Enzyme_code=EC:3.4.21;Enzyme
Fv_160_9.g5376	433	ID=Fv_160_9.g5376;Description=hypothetical protein FVEG_03495 [Fusarium verticillioides 7600];Gene=FFUJ_03644;Ontology_term=anthranilate synthase activity,aspartic-type endopeptidase activity,indole-3-glycerol-phosphate synthase activity,tryptophan biosynthetic process,proteolysis,glutamine metabolic process;Ontology_id=GO:0004049,GO:0004190,GO:0004425,GO:0000162,GO:0006508,GO:0006541;Enzyme_code=EC:4.1.1.48,EC:3
Fv_160_9.g5377	98	ID=Fv_160_9.g5377;Description=hypothetical protein FVEG_15278 [Fusarium verticillioides 7600];Gene=F52700_6557;Ontology_term=metallopeptidase
Fv_160_9.g5378	102	ID=Fv_160_9.g5378;Description=hypothetical protein FVEG_15281 [Fusarium verticillioides 7600];Gene=CDV36_003917
Fv_160_9.g5379	994	ID=Fv_160_9.g5379;Description=hypothetical protein LCI18_010700 [Fusarium solani-melongenae];Gene=INSB
Fv_160_9.g5380	105	ID=Fv_160_9.g5380;Description=hypothetical protein FVEG_03498 [Fusarium verticillioides 7600]
Fv_160_9.g5381	435	ID=Fv_160_9.g5381;Description=hypothetical protein FVEG_03499 [Fusarium verticillioides 7600];Gene=1274;Ontology_term=signal transduction,integrin binding,cytokine activity,fever generation,defense response to bacterium,interleukin-1 receptor binding,lysosome,extracellular space,positive regulation of cell division,positive regulation of type II interferon production,regulation of defense response to virus by host,cytosol,positive regulation of T-helper 1 cell cytokine production,vascular endothelial growth factor
Fv_160_9.g5382	286	ID=Fv_160_9.g5382;Description=hypothetical protein FVEG_03500 [Fusarium verticillioides 7600];Gene=1273;Ontology_term=exonuclease activity;Ontology_id=GO:0004527;Enzyme_code=EC:3.1;Enzyme_name=Acting on
Fv_160_9.g5383	168	ID=Fv_160_9.g5383;Description=uncharacterized protein FFUJ_03767 [Fusarium fujikuroi IMI 58289];Gene=2173
Fv_160_9.g5384	306	ID=Fv_160_9.g5384;Description=hypothetical protein FVER14953_03501 [Fusarium

Fv_160_9.g5385	229	ID=Fv_160_9.g5385;Description=hypothetical protein FVER14953_20177 [Fusarium verticillioides];Gene=FPANT_12359;Ontology_term=acyltransferase activity, transferring groups other than amino-acyl
Fv_160_9.g5386	315	ID=Fv_160_9.g5386;Description=hypothetical protein FVER14953_03502 [Fusarium verticillioides];Gene=FPCIR_7099;Ontology_term=hydrolase
Fv_160_9.g5387	489	ID=Fv_160_9.g5387;Description=hypothetical protein FVER14953_03503 [Fusarium verticillioides]
Fv_160_9.g5388	1135	ID=Fv_160_9.g5388;Description=NACHT domain-containing protein [Fusarium coicis];Gene=IGF2;Ontology_term=extracellular space,hormone activity,growth factor activity,ossification,signal
Fv_160_9.g5389	234	ID=Fv_160_9.g5389;Description=hypothetical protein FVEG_15285 [Fusarium verticillioides 7600];Gene=HZS61_013263;Ontology_term=regulation of DNA-templated transcription;Ontology_id=GO:0006355
Fv_160_9.g5390	111	ID=Fv_160_9.g5390;Description=hypothetical protein FVEG_03505 [Fusarium verticillioides 7600];Gene=recA
Fv_160_9.g5391	344	ID=Fv_160_9.g5391;Description=hypothetical protein FVER14953_03506 [Fusarium
Fv_160_9.g5392	328	ID=Fv_160_9.g5392;Description=hypothetical protein FVEG_03507 [Fusarium verticillioides
Fv_160_9.g5393	602	ID=Fv_160_9.g5393;Description=hypothetical protein FVER14953_03508 [Fusarium verticillioides]
Fv_160_9.g5394	239	ID=Fv_160_9.g5394;Description=hypothetical protein FVER53590_03509 [Fusarium
Fv_160_9.g5395	266	ID=Fv_160_9.g5395;Description=L-xylulose reductase [Fusarium verticillioides 7600]
Fv_160_9.g5396	507	ID=Fv_160_9.g5396;Description=hypothetical protein FVER53590_03511 [Fusarium verticillioides];Gene=FOC4_g10001642;Ontology_term=nucleus,membrane,RNA polymerase II cis-regulatory region sequence-specific DNA binding,DNA-binding transcription factor activity, RNA polymerase II-specific,catalytic activity,heme binding,transmembrane transporter activity,protein homodimerization activity,metal ion binding,kidney development,liver development,regulation of transcription by RNA polymerase II,tryptophan catabolic process to kynurenine,insulin secretion,endocrine pancreas development,positive regulation of DNA-templated transcription,genitalia
Fv_160_9.g5397	425	ID=Fv_160_9.g5397;Description=hypothetical protein FVER14953_03512 [Fusarium verticillioides];Gene=FOC4_g10001643;Ontology_term=membrane,heme binding,transmembrane transporter activity,metal ion binding,dioxygenase activity,tryptophan catabolic process to kynurenine,transmembrane
Fv_160_9.g5398	487	ID=Fv_160_9.g5398;Description=kynureninase 2 [Fusarium verticillioides 7600];Gene=BNA5;Ontology_term=cytoplasm,pyridoxal phosphate binding,kynureninase activity,3-hydroxykynureninase activity,tryptophan catabolic process,quinolinate biosynthetic process,'de novo' NAD biosynthetic process from tryptophan,anthranilate metabolic process,L-kynurenine catabolic process;Ontology_id=GO:0005737,GO:0030170,GO:0030429,GO:0061981,GO:0006569,GO:0019805,GO:0034354,GO:0043420,GO:0097053;Enzyme_code=EC:3.7.1.3;Enzyme_name=kynureninase
Fv_160_9.g5399	696	ID=Fv_160_9.g5399;Description=hypothetical protein FVER14953_03514 [Fusarium verticillioides];Gene=FVEG_03514;Ontology_term=nucleus,DNA binding,zinc ion binding,membrane,DNA-binding transcription factor activity, RNA polymerase II-specific,regulation of transcription by RNA polymerase
Fv_160_9.g5400	798	ID=Fv_160_9.g5400;Description=hypothetical protein FVEG_03515 [Fusarium verticillioides 7600];Gene=2154;Ontology_term=metal

Fv_160_9.g5401	115	ID=Fv_160_9.g5401;Description=hypothetical protein FVER14953_20175 [Fusarium verticillioides];Gene=2154;Ontology_term=membrane,ion channel regulator activity,potassium ion transport,sodium ion
Fv_160_9.g5402	158	ID=Fv_160_9.g5402;Description=hypothetical protein FVEG_03516 [Fusarium verticillioides 7600]
Fv_160_9.g5403	527	ID=Fv_160_9.g5403;Description=hypothetical protein FVER14953_03517 [Fusarium verticillioides]
Fv_160_9.g5404	564	ID=Fv_160_9.g5404;Description=hypothetical protein FVEG_03518 [Fusarium verticillioides 7600];Gene=FPCIR_12716;Ontology_term=extracellular space,membrane,serine-type endopeptidase activity,plasminogen activation,regulation of cell adhesion mediated by integrin,fibrinolysis;Ontology_id=GO:0005615,GO:0016020,GO:0004252,GO:0031639,GO:0033628,GO:0042730;Enzyme_code=EC:3
Fv_160_9.g5405	224	ID=Fv_160_9.g5405;Description=pectate lyase [Fusarium verticillioides 7600];Gene=FPANT_9819;Ontology_term=extracellular region,pectate lyase activity;Ontology_id=GO:0005576,GO:0030570;Enzyme_code=EC:4.2.2.2;Enzyme_name=pectate lyase
Fv_160_9.g5406	353	ID=Fv_160_9.g5406;Description=hypothetical protein FVEG_03520 [Fusarium verticillioides 7600];Gene=F52700_8346;Ontology_term=cellular anatomical entity;Ontology_id=GO:0110165
Fv_160_9.g5407	404	ID=Fv_160_9.g5407;Description=hypothetical protein FVEG_15287 [Fusarium verticillioides 7600];Gene=FocTR4_00007723;Ontology_term=nucleus,DNA-binding transcription factor activity, RNA polymerase II-specific,zinc
Fv_160_9.g5408	224	ID=Fv_160_9.g5408;Description=hypothetical protein FVEG_03521 [Fusarium verticillioides 7600]
Fv_160_9.g5409	257	ID=Fv_160_9.g5409;Description=hypothetical protein FVEG_03522 [Fusarium verticillioides 7600];Gene=FPRO_03718;Ontology_term=hydrolase activity, acting on ester
Fv_160_9.g5410	133	ID=Fv_160_9.g5410;Description=hypothetical protein FVEG_03523 [Fusarium verticillioides 7600];Gene=FTJAE_621;Ontology_term=extracellular region,cellulase activity,polysaccharide catabolic
Fv_160_9.g5411	125	ID=Fv_160_9.g5411;Description=hypothetical protein FVER14953_03524 [Fusarium
Fv_160_9.g5412	134	ID=Fv_160_9.g5412;Description=killer toxin Kp4 SMK-like core [Fusarium tjaetaba];Gene=FPCIR_12705;Ontology_term=extracellular
Fv_160_9.g5413	799	ID=Fv_160_9.g5413;Description=hypothetical protein J7337_003545 [Fusarium musae];Gene=FVEG_03526;Ontology_term=oxidoreductase activity, acting on CH-OH group of donors,flavin adenine dinucleotide
Fv_160_9.g5414	302	ID=Fv_160_9.g5414;Description=hypothetical protein FVEG_03527 [Fusarium verticillioides 7600];Gene=FVER53590_03527;Ontology_term=nucleus,DNA-binding transcription factor activity, RNA polymerase II-specific,zinc ion binding,oxidoreductase activity, acting on CH-OH group of donors,flavin adenine dinucleotide binding,regulation of transcription
Fv_160_9.g5415	546	ID=Fv_160_9.g5415;Description=hypothetical protein FVEG_03528 [Fusarium verticillioides 7600]
Fv_160_9.g5416	247	ID=Fv_160_9.g5416;Description=hypothetical protein FVER53263_03529 [Fusarium verticillioides]
Fv_160_9.g5417	280	ID=Fv_160_9.g5417;Description=hypothetical protein FVER14953_03530 [Fusarium verticillioides]
Fv_160_9.g5418	449	ID=Fv_160_9.g5418;Description=hypothetical protein FVER53590_03534 [Fusarium verticillioides];Gene=Forpe1208_v005526;Ontology_term=membrane,tyrosinase activity,hydrolase activity, hydrolyzing O-glycosyl compounds,carbohydrate metabolic process,melanin biosynthetic

Fv_160_9.g5419	767	ID=Fv_160_9.g5419;Description=hypothetical protein FVEG_03534 [Fusarium verticillioides 7600];Gene=FVER53263_03534;Ontology_term=tyrosinase activity,melanin biosynthetic
Fv_160_9.g5420	277	ID=Fv_160_9.g5420;Description=hypothetical protein FVER53263_03535 [Fusarium verticillioides]
Fv_160_9.g5421	510	ID=Fv_160_9.g5421;Description=hypothetical protein FVEG_03536 [Fusarium verticillioides 7600]
Fv_160_9.g5422	1360	ID=Fv_160_9.g5422;Description=metal-nicotianamine transporter YSL11 [Fusarium denticulatum];Gene=FPRO_03706;Ontology_term=membrane,oligopeptide transmembrane transporter activity,oligopeptide
Fv_160_9.g5423	513	ID=Fv_160_9.g5423;Description=hypothetical protein FVER53263_03539 [Fusarium verticillioides];Gene=FNYG_11673;Ontology_term=membrane,transmembrane transporter activity,transmembrane
Fv_160_9.g5424	486	ID=Fv_160_9.g5424;Description=hypothetical protein FVEG_03540 [Fusarium verticillioides 7600];Gene=FPCIR_9391;Ontology_term=oxidoreductase
Fv_160_9.g5425	292	ID=Fv_160_9.g5425;Description=hypothetical protein J7337_003563 [Fusarium musae];Gene=C2S_106;Ontology_term=extracellular space,membrane;Ontology_id=GO:0005615,GO:0016020
Fv_160_9.g5426	355	ID=Fv_160_9.g5426;Description=hypothetical protein FVEG_03541 [Fusarium verticillioides 7600]
Fv_160_9.g5427	250	ID=Fv_160_9.g5427;Description=hypothetical protein FVER14953_03542 [Fusarium verticillioides]
Fv_160_9.g5428	139	ID=Fv_160_9.g5428;Description=hypothetical protein FVER14953_03543 [Fusarium verticillioides];Gene=FOC1_g10002091;Ontology_term=extracellular space,aminoacyl-tRNA ligase activity,polyketide metabolic
Fv_160_9.g5429	357	ID=Fv_160_9.g5429;Description=hypothetical protein FVER53590_03544 [Fusarium verticillioides];Gene=FNAPI_8674;Ontology_term=dorsal closure,hemocyte migration,protein kinase binding,torso signaling pathway,negative regulation of hippo signaling,ommatidial rotation,cell leading edge,positive regulation of cell-cell adhesion,positive regulation of sevenless signaling pathway,substrate-dependent cell migration, cell extension,plasma membrane,GTP binding,nucleus,germ-line stem cell population maintenance,G protein activity,cellular response to cAMP,positive regulation of smoothened signaling pathway,GDP binding,imaginal disc-derived wing hair organization,cytosol,Rap protein signal transduction,adherens junction assembly,regulation of embryonic cell shape,negative regulation of synaptic vesicle exocytosis;Ontology_id=GO:0007391,GO:0035099,GO:0019901,GO:0008293,GO:0035331,GO:0016318,GO:0031252,GO:0022409,G
Fv_160_9.g5430	546	ID=Fv_160_9.g5430;Description=hypothetical protein FVER53590_03545 [Fusarium verticillioides];Gene=FTJAE_3494;Ontology_term=oxidoreductase activity,FAD
Fv_160_9.g5431	503	ID=Fv_160_9.g5431;Description=hypothetical protein FVER53590_03546 [Fusarium verticillioides]
Fv_160_9.g5432	693	ID=Fv_160_9.g5432;Description=hypothetical protein FVER53590_03547 [Fusarium verticillioides];Gene=FTJAE_3492;Ontology_term=cytoplasm,membrane,aminoacyl-tRNA ligase activity,ATP binding,tRNA aminoacylation for protein
Fv_160_9.g5433	157	ID=Fv_160_9.g5433;Description=hypothetical protein FVER53590_03548 [Fusarium verticillioides]
Fv_160_9.g5434	384	ID=Fv_160_9.g5434;Description=hypothetical protein FNAPI_8669 [Fusarium napiforme]
Fv_160_9.g5435	391	ID=Fv_160_9.g5435;Description=hypothetical protein FVER53590_03550 [Fusarium

Fv_160_9.g5436	188	ID=Fv_160_9.g5436;Description=hypothetical protein FVER53590_03551 [Fusarium verticillioides]
Fv_160_9.g5437	124	ID=Fv_160_9.g5437;Description=hypothetical protein FPRO05_10077 [Fusarium proliferatum]
Fv_160_9.g5438	680	ID=Fv_160_9.g5438;Description=hypothetical protein FVER53590_03555 [Fusarium verticillioides]
Fv_160_9.g5439	551	ID=Fv_160_9.g5439;Description=hypothetical protein FVER53590_03556 [Fusarium verticillioides]
Fv_160_9.g5440	102	ID=Fv_160_9.g5440;Description=hypothetical protein FVER53590_29546 [Fusarium verticillioides]
Fv_160_9.g5441	503	ID=Fv_160_9.g5441;Description=hypothetical protein FVEG_03557 [Fusarium verticillioides 7600];Gene=FACUT_4282;Ontology_term=cytosol,plasma membrane,mannosyl-oligosaccharide 1,2-alpha-mannosidase activity,MAP kinase kinase kinase activity,calcium ion binding,ATP binding,kinase binding,protein serine kinase activity,instar larval development,carbohydrate metabolic process,epidermal growth factor receptor signaling pathway,spermatogenesis,border follicle cell migration,terminal region determination,gastrulation,epithelial cell migration, open tracheal system,terminal branching, open tracheal system,imaginal disc-derived wing vein specification,dorsal/ventral axis specification, ovarian follicular epithelium,positive regulation of cell population proliferation,torso signaling pathway,fibroblast growth factor receptor signaling pathway,cellular response to starvation,negative regulation of macroautophagy,ubiquitin-dependent ERAD pathway,regulation of cellular pH,lamellocyte differentiation,wing and notum subfield formation,regulation of multicellular organism growth,compound eye photoreceptor development,sevenless signaling pathway,positive regulation of photoreceptor cell differentiation,protein autophosphorylation,dorsal appendage formation,vascular endothelial growth factor receptor signaling pathway,ERK1 and ERK2 cascade,negative regulation of apoptotic signaling pathway;Ontology_id=GO:0005829,GO:0005886,GO:0004571,GO:0004709,GO:0005509,GO:0005524,GO:0019900,GO:0106310,GO:
Fv_160_9.g5442	264	ID=Fv_160_9.g5442;Description=hypothetical protein FVEG_03558 [Fusarium verticillioides 7600];Gene=FTJAE_3482;Ontology_term=nucleolus,small-subunit processome,rRNA
Fv_160_9.g5443	594	ID=Fv_160_9.g5443;Description=hypothetical protein FVEG_03559 [Fusarium verticillioides
Fv_160_9.g5444	269	ID=Fv_160_9.g5444;Description=hypothetical protein FVEG_03560 [Fusarium verticillioides 7600];Gene=FOPG_12617;Ontology_term=mitochondrial inner membrane;Ontology_id=GO:0005743
Fv_160_9.g5445	493	ID=Fv_160_9.g5445;Description=hypothetical protein J7337_003579 [Fusarium musae];Gene=LW93_14269;Ontology_term=oxidoreductase activity,metal ion
Fv_160_9.g5446	289	ID=Fv_160_9.g5446;Description=hypothetical protein FVEG_03562 [Fusarium verticillioides 7600];Gene=2118
Fv_160_9.g5447	401	ID=Fv_160_9.g5447;Description=hypothetical protein FVER53263_03563 [Fusarium
Fv_160_9.g5448	353	ID=Fv_160_9.g5448;Description=D-xylulose reductase A [Fusarium verticillioides 7600];Gene=xdhA-2;Ontology_term=zinc ion binding,oxidoreductase activity, acting on the CH-OH group of donors, NAD or NADP as
Fv_160_9.g5449	170	ID=Fv_160_9.g5449;Description=hypothetical protein FVEG_03565 [Fusarium verticillioides 7600]
Fv_160_9.g5450	269	ID=Fv_160_9.g5450;Description=hypothetical protein FVEG_03566 [Fusarium verticillioides

Fv_160_9.g5451	1087	ID=Fv_160_9.g5451;Description=hypothetical protein J7337_003585 [Fusarium musae];Gene=FNAPI_8654;Ontology_term=membrane,ATP binding,ATP hydrolysis activity,ATPase-coupled monoatomic cation transmembrane transporter activity,sodium ion transport,monoatomic cation transmembrane transport;Ontology_id=GO:0016020,GO:0005524,GO:0016887,GO:0019829,GO:0006814,GO:0098655;Enzyme_code=EC:7.2.2,EC:3.
Fv_160_9.g5452	137	ID=Fv_160_9.g5452;Description=hypothetical protein FVEG_03570 [Fusarium verticillioides 7600]
Fv_160_9.g5453	891	ID=Fv_160_9.g5453;Description=hypothetical protein FVEG_03571 [Fusarium verticillioides 7600]
Fv_160_9.g5454	155	ID=Fv_160_9.g5454;Description=26S proteasome regulatory subunit N7 [Fusarium mexicanum];Gene=HZS61_013312;Ontology_term=proteasome complex,nucleus,cytoplasm,ribosome,ribonucleoprotein complex,mRNA binding,structural constituent of ribosome,RNA
Fv_160_9.g5455	858	ID=Fv_160_9.g5455;Description=hypothetical protein FVER14953_03574 [Fusarium verticillioides];Gene=groS;Ontology_term=nucleoplasm,hemoglobin complex,proteasome core complex, alpha-subunit complex,oxygen carrier activity,beta-glucosidase activity,oxygen binding,heme binding,metal ion binding,scopolin beta-glucosidase activity,oxygen transport,cellulose catabolic process,proteasome-mediated ubiquitin-dependent protein catabolic process;Ontology_id=GO:0005654,GO:0005833,GO:0019773,GO:0005344,GO:0008422,GO:0019825,GO:0020037,GO:0046872,GO:
Fv_160_9.g5456	208	ID=Fv_160_9.g5456;Description=hypothetical protein FVEG_15293 [Fusarium verticillioides
Fv_160_9.g5457	239	ID=Fv_160_9.g5457;Description=hypothetical protein FVER53590_03575 [Fusarium verticillioides];Gene=FDENT_7706;Ontology_term=endoplasmic reticulum membrane,protein-disulfide reductase activity,thiol oxidase activity,FAD binding,protein folding in endoplasmic
Fv_160_9.g5458	237	ID=Fv_160_9.g5458;Description=hypothetical protein FVEG_03576 [Fusarium verticillioides 7600];Gene=FDENT_7706;Ontology_term=axonogenesis involved in innervation,protein-disulfide reductase activity,RNA polymerase II cis-regulatory region sequence-specific DNA binding,male courtship behavior,axon guidance,DNA-binding transcription repressor activity, RNA polymerase II-specific,positive regulation of transcription by RNA polymerase II,regulation of DNA-binding transcription factor activity,endoplasmic reticulum membrane,negative regulation of transcription by RNA polymerase II,negative regulation of axonogenesis,negative regulation of neuroblast proliferation,dendrite guidance,thiol oxidase activity,FAD binding,asymmetric neuroblast division resulting in ganglion mother cell formation,sensory perception of taste,regulation of R7 cell differentiation,R7 cell fate commitment,positive regulation of neuroblast proliferation,peripheral nervous system development,apical cortex,cell dedifferentiation,brain development,plasma membrane,ganglion mother cell fate determination,nucleus,negative regulation of gene expression,synapse assembly,positive regulation of gene expression,G1 to G0 transition,basal cortex,glial cell differentiation,regulation of protein localization to nucleus,protein folding in endoplasmic
Fv_160_9.g5459	600	ID=Fv_160_9.g5459;Description=hypothetical protein FVER14953_03577 [Fusarium verticillioides];Gene=FMAN_03508;Ontology_term=endoplasmic reticulum membrane,protein-disulfide reductase activity,thiol oxidase activity,FAD binding,protein folding in endoplasmic

Fv_160_9.g5460	114	ID=Fv_160_9.g5460;Description=cyclin-dependent kinase regulatory subunit CKS1 [Fusarium oxysporum f. sp. lycopersici 4287];Gene=CEP52_009310;Ontology_term=cyclin-dependent protein kinase holoenzyme complex,zinc ion binding,kinase activity,protein-containing complex binding,cyclin-dependent protein serine/threonine kinase activator activity,cell cycle,phosphorylation,cell division;Ontology_id=GO:0000307,GO:0008270,GO:0016301,GO:0044877,GO:0061575,GO:0007049,GO:0016310,GO:0051301;Enzy
Fv_160_9.g5461	324	ID=Fv_160_9.g5461;Description=hypothetical protein FVER14953_03579 [Fusarium verticillioides];Gene=USB1;Ontology_term=nucleus,lyase activity,poly(U)-specific exoribonuclease activity, producing 3' uridine cyclic phosphate ends,U6 snRNA 3'-end
Fv_160_9.g5462	420	ID=Fv_160_9.g5462;Description=hypothetical protein FVEG_03580 [Fusarium verticillioides 7600]
Fv_160_9.g5463	1427	ID=Fv_160_9.g5463;Description=hypothetical protein FVEG_03581 [Fusarium verticillioides 7600];Gene=FMAN_03504;Ontology_term=extracellular space,nucleus,mitochondrial membrane,proton-transporting ATP synthase complex, coupling factor F(o),nucleic acid binding,growth factor activity,lipid binding,proton transmembrane transporter activity,CXCR4 chemokine receptor binding,proton motive force-driven ATP synthesis,maintenance of gastrointestinal epithelium,negative regulation of gastric acid secretion,chemokine-mediated signaling pathway,proton transmembrane transport;Ontology_id=GO:0005615,GO:0005634,GO:0031966,GO:0045263,GO:0003676,GO:0008083,GO:0008289,GO:0015078,GO
Fv_160_9.g5464	595	ID=Fv_160_9.g5464;Description=hypothetical protein FVER14953_03582 [Fusarium verticillioides];Gene=BFJ65_g17875;Ontology_term=membrane,cobalamin binding;Ontology_id=GO:0016020,GO:0031419
Fv_160_9.g5465	347	ID=Fv_160_9.g5465;Description=hypothetical protein FVEG_03583 [Fusarium verticillioides 7600];Gene=deoC;Ontology_term=extracellular space,sodium:potassium-exchanging ATPase complex,cytokine activity,type I interferon receptor binding,adaptive immune response,T cell activation involved in immune response,natural killer cell activation involved in immune response,potassium ion transport,sodium ion transport,humoral immune response,cytokine-mediated signaling pathway,B cell differentiation,positive regulation of peptidyl-serine phosphorylation of STAT protein,B cell proliferation,response to exogenous dsRNA,defense response to
Fv_160_9.g5466	498	ID=Fv_160_9.g5466;Description=hypothetical protein FVER14953_03584 [Fusarium verticillioides];Gene=FNAPI_8543;Ontology_term=glycosyltransferase
Fv_160_9.g5467	291	ID=Fv_160_9.g5467;Description=cytochrome-b5 reductase [Fusarium verticillioides 7600];Gene=mcr1-0;Ontology_term=mitochondrial outer membrane,cytochrome-b5 reductase activity, acting on
Fv_160_9.g5468	1306	ID=Fv_160_9.g5468;Description=hypothetical protein FVER14953_03586 [Fusarium verticillioides];Gene=FFUJ_03865;Ontology_term=nucleus,aggresome,amphisome,DNA binding,protein kinase C binding,zinc ion binding,hydrolase activity,K63-linked polyubiquitin modification-dependent protein binding,mitophagy,endosome organization,sperm mitochondrion organization,aggrephagy;Ontology_id=GO:0005634,GO:0016235,GO:0044753,GO:0003677,GO:0005080,GO:0008270,GO:0016787,

Fv_160_9.g5469	643	ID=Fv_160_9.g5469;Description=hypothetical protein FVEG_03587 [Fusarium verticillioides 7600];Gene=FFUJ_03866;Ontology_term=nucleus,DNA binding,extracellular space,zinc ion binding,serine-type endopeptidase inhibitor activity,identical protein binding,regulation of blood coagulation,heparin binding,protease binding,DNA-binding transcription factor activity, RNA polymerase II-specific,regulation of transcription by RNA polymerase
Fv_160_9.g5470	286	ID=Fv_160_9.g5470;Description=hypothetical protein FVEG_03588 [Fusarium verticillioides 7600];Gene=FNAPI_11579;Ontology_term=nucleosome,nucleus,cytoplasm,membrane,DNA binding,phosphogluconate dehydrogenase (decarboxylating) activity,NADP binding,pentose-phosphate shunt,nucleus organization,spermatid development,D-gluconate metabolic process,chromosome condensation,mycotoxin biosynthetic process;Ontology_id=GO:0000786,GO:0005634,GO:0005737,GO:0016020,GO:0003677,GO:0004616,GO:0050661,GO:0006098,GO:
Fv_160_9.g5471	369	ID=Fv_160_9.g5471;Description=monooxygenase [Fusarium coicis];Gene=FTJAE_14007;Ontology_term=nucleus,membrane,aggresome,amphisome,DNA binding,monooxygenase activity,protein kinase C binding,zinc ion binding,K63-linked polyubiquitin modification-dependent protein binding,mitophagy,endosome organization,sperm mitochondrion
Fv_160_9.g5472	477	ID=Fv_160_9.g5472;Description=acyltransferase [Fusarium mundagurra];Gene=FFC1_09300;Ontology_term=nucleosome,nucleus,endoplasmic reticulum membrane,DNA binding,metalloendopeptidase activity,acyltransferase activity, transferring groups other than amino-acyl groups,metal ion binding,7-beta-hydroxysteroid dehydrogenase (NADP+) activity,11-beta-hydroxysteroid dehydrogenase (NADP+) activity,cortisol dehydrogenase activity,proteolysis,steroid metabolic process,chromosome condensation,sperm DNA condensation;Ontology_id=GO:0000786,GO:0005634,GO:0005789,GO:0003677,GO:0004222,GO:0016747,GO:0046872,GO:004702
Fv_160_9.g5473	674	ID=Fv_160_9.g5473;Description=hypothetical protein FVEG_03590 [Fusarium verticillioides 7600];Gene=FFC1_09301;Ontology_term=nucleosome,nucleus,endoplasmic reticulum membrane,DNA binding,metalloendopeptidase activity,acyltransferase activity, transferring groups other than amino-acyl groups,metal ion binding,7-beta-hydroxysteroid dehydrogenase (NADP+) activity,11-beta-hydroxysteroid dehydrogenase (NADP+) activity,cortisol dehydrogenase activity,proteolysis,steroid metabolic process,chromosome condensation,sperm DNA condensation;Ontology_id=GO:0000786,GO:0005634,GO:0005789,GO:0003677,GO:0004222,GO:0016747,GO:0046872,GO:0047022,GO:0070524,GO:0102196,GO:0006508,GO:0008202,GO:0030261,GO:0035092;Enzyme_code=EC:3.4.24,EC:1.1.1.146,EC:1.1.1.201
Fv_160_9.g5474	230	ID=Fv_160_9.g5474;Description=hypothetical protein FVEG_03591 [Fusarium verticillioides 7600];Gene=dnaG;Ontology_term=membrane,mycotoxin biosynthetic process;Ontology_id=GO:0016020,GO:0043386
Fv_160_9.g5475	487	ID=Fv_160_9.g5475;Description=hypothetical protein FVEG_03592 [Fusarium verticillioides 7600];Gene=BFJ72_g12216;Ontology_term=membrane,transmembrane transporter activity,transmembrane
Fv_160_9.g5476	267	ID=Fv_160_9.g5476;Description=hypothetical protein FVER53590_03593 [Fusarium verticillioides]
Fv_160_9.g5477	430	ID=Fv_160_9.g5477;Description=hypothetical protein FVEG_03594 [Fusarium verticillioides 7600]
Fv_160_9.g5478	316	ID=Fv_160_9.g5478;Description=hypothetical protein CEK27_005197 [Fusarium fujikuroi];Gene=BFJ70_g2293;Ontology_term=ATP

Fv_160_9.g5479	214	ID=Fv_160_9.g5479;Description=hypothetical protein FVEG_03595 [Fusarium verticillioides 7600];Gene=Forpi1262_v005686;Ontology_term=endoplasmic reticulum membrane,lyase activity,fatty acid biosynthetic
Fv_160_9.g5480	728	ID=Fv_160_9.g5480;Description=heterokaryon incompatibility (het-6OR allele) [Fusarium coicis]
Fv_160_9.g5481	326	ID=Fv_160_9.g5481;Description=hypothetical protein FVER53263_20034 [Fusarium verticillioides];Gene=FMAN_03468;Ontology_term=membrane,S-adenosylmethionine-dependent methyltransferase activity,oxidoreductase activity, acting on the CH-CH group of donors,flavin adenine dinucleotide binding,methylation;Ontology_id=GO:0016020,GO:0008757,GO:0016627,GO:0050660,GO:0032259;Enzyme_code=EC:1.3,EC:2.1.1;E
Fv_160_9.g5482	599	ID=Fv_160_9.g5482;Description=zinc finger transcription factor ace1 [Fusarium napiforme];Gene=BFJ63_vAg10650;Ontology_term=nucleus,DNA-binding transcription factor activity, RNA polymerase II-
Fv_160_9.g5483	617	ID=Fv_160_9.g5483;Description=hypothetical protein FOTG_08937 [Fusarium oxysporum f. sp. vasinfectum 25433]
Fv_160_9.g5484	188	ID=Fv_160_9.g5484;Description=hypothetical protein FVER53263_03601 [Fusarium verticillioides];Gene=FPANT_5948;Ontology_term=isomerase
Fv_160_9.g5485	78	ID=Fv_160_9.g5485;Description=hypothetical protein FVER53263_03602 [Fusarium
Fv_160_9.g5486	564	ID=Fv_160_9.g5486;Description=hypothetical protein FVER53590_03604 [Fusarium verticillioides];Gene=FNAPI_11558;Ontology_term=extracellular region,membrane,exonuclease activity,hormone activity,signal transduction,lactation,cell
Fv_160_9.g5487	133	ID=Fv_160_9.g5487;Description=hypothetical protein J7337_003615 [Fusarium
Fv_160_9.g5488	179	ID=Fv_160_9.g5488;Description=hypothetical protein FNAPI_11560 [Fusarium napiforme];Gene=1181;Ontology_term=PRC1 complex,zinc ion binding,sequence-specific DNA binding,promoter-specific chromatin binding,negative regulation of transcription by RNA polymerase II,anterior/posterior axis specification,heterochromatin formation,syncytial blastoderm mitotic cell
Fv_160_9.g5489	482	ID=Fv_160_9.g5489;Description=proline dehydrogenase [Fusarium verticillioides 7600];Gene=Forpi1262_v005676;Ontology_term=proline dehydrogenase activity,proline catabolic
Fv_160_9.g5490	197	ID=Fv_160_9.g5490;Description=hypothetical protein FVEG_03608 [Fusarium verticillioides 7600];Gene=FOC4_g10001424;Ontology_term=spindle pole,outer kinetochore,nuclear envelope,centrosome,centriole,kinetochore microtubule,cytosol,midbody,mitotic spindle midzone,protein serine/threonine kinase activity,protein binding,ATP binding,protein serine kinase activity,actomyosin contractile ring assembly,protein phosphorylation,mitotic spindle organization,spindle assembly involved in female meiosis II,centrosome cycle,male meiosis cytokinesis,pronuclear fusion,negative regulation of neuroblast proliferation,larval somatic muscle development,protein localization,male germline ring canal formation,astral microtubule nucleation,sperm aster formation,pronuclear migration,embryonic heart tube development,oocyte fate commitment,meiotic spindle midzone assembly,attachment of mitotic spindle microtubules to kinetochore;Ontology_id=GO:0000922,GO:0000940,GO:0005635,GO:0005813,GO:0005814,GO:0005828,GO:0005829,GO:0030496,
Fv_160_9.g5491	1135	ID=Fv_160_9.g5491;Description=hypothetical protein FVEG_03609 [Fusarium verticillioides 7600];Gene=FCIRC_3546;Ontology_term=nucleus,DNA binding,zinc ion binding,DNA-binding transcription factor activity, RNA

Fv_160_9.g5492	308	ID=Fv_160_9.g5492;Description=kinesin family member 18 19 [Fusarium denticulatum];Gene=FOVG_03928;Ontology_term=cytoskeleton,DNA binding,microtubule motor activity,ATP binding,microtubule
Fv_160_9.g5493	1031	ID=Fv_160_9.g5493;Description=kinesin family member 18/19 [Fusarium verticillioides 7600];Gene=FFC1_09321;Ontology_term=cytoskeleton,DNA binding,microtubule motor activity,ATP binding,microtubule binding,metal ion binding,microtubule-based
Fv_160_9.g5494	292	ID=Fv_160_9.g5494;Description=hypothetical protein FVEG_03612 [Fusarium verticillioides 7600];Gene=2074;Ontology_term=plasma membrane,calcium,diacylglycerol-dependent serine/threonine kinase activity,ATP binding,zinc ion binding,protein serine kinase activity,protein phosphorylation,regulation of hemocyte proliferation,intracellular signal transduction,positive regulation of clathrin-dependent endocytosis;Ontology_id=GO:0005886,GO:0004698,GO:0005524,GO:0008270,GO:0106310,GO:0006468,GO:0035206,GO:0035556,
Fv_160_9.g5495	444	ID=Fv_160_9.g5495;Description=hypothetical protein FVEG_03613 [Fusarium verticillioides 7600]
Fv_160_9.g5496	295	ID=Fv_160_9.g5496;Description=hypothetical protein FVER53590_03617 [Fusarium verticillioides]
Fv_160_9.g5497	132	ID=Fv_160_9.g5497;Description=hypothetical protein FVEG_03618 [Fusarium verticillioides
Fv_160_9.g5498	452	ID=Fv_160_9.g5498;Description=hypothetical protein FVER53263_03619 [Fusarium verticillioides]
Fv_160_9.g5499	1124	ID=Fv_160_9.g5499;Description=hypothetical protein FVER53590_03621 [Fusarium verticillioides]
Fv_160_9.g5500	89	ID=Fv_160_9.g5500;Description=hypothetical protein FVEG_15301 [Fusarium verticillioides 7600]
Fv_160_9.g5501	254	ID=Fv_160_9.g5501;Description=hypothetical protein FVER14953_03623 [Fusarium verticillioides]
Fv_160_9.g5502	473	ID=Fv_160_9.g5502;Description=hypothetical protein FVER14953_03624 [Fusarium verticillioides]
Fv_160_9.g5503	284	ID=Fv_160_9.g5503;Description=hypothetical protein FVER14953_03625 [Fusarium verticillioides];Gene=FVEG_03625;Ontology_term=membrane,hydrolase activity, acting on glycosyl bonds,transmembrane
Fv_160_9.g5504	344	ID=Fv_160_9.g5504;Description=hypothetical protein FVEG_15302 [Fusarium verticillioides 7600]
Fv_160_9.g5505	518	ID=Fv_160_9.g5505;Description=hypothetical protein FVER53263_03626 [Fusarium verticillioides];Gene=FOPG_11260;Ontology_term=membrane,transmembrane transporter activity,carbohydrate
Fv_160_9.g5506	159	ID=Fv_160_9.g5506;Description=hypothetical protein FVER14953_03627 [Fusarium verticillioides];Gene=1164;Ontology_term=membrane,transmembrane transporter activity,transmembrane
Fv_160_9.g5507	279	ID=Fv_160_9.g5507;Description=hypothetical protein FVEG_03628 [Fusarium verticillioides 7600];Gene=fabG-9;Ontology_term=oxidoreductase activity,cellular biosynthetic
Fv_160_9.g5508	535	ID=Fv_160_9.g5508;Description=hypothetical protein FVEG_03629 [Fusarium verticillioides 7600];Gene=FANTH_5087;Ontology_term=N,N-dimethylaniline monooxygenase activity,flavin adenine dinucleotide binding,NADP binding;Ontology_id=GO:0004499,GO:0050660,GO:0050661;Enzyme_code=EC:1.14.13.8;Enzyme_name=flavin-containing
Fv_160_9.g5509	324	ID=Fv_160_9.g5509;Description=hypothetical protein J7337_003641 [Fusarium musae];Gene=FOXB_14655;Ontology_term=hydrolase

Fv_160_9.g5510	703	ID=Fv_160_9.g5510;Description=hypothetical protein FVER14953_03631 [Fusarium verticillioides];Gene=FANTH_5085;Ontology_term=nucleus,DNA binding,zinc ion binding,DNA-binding transcription factor activity, RNA polymerase II-specific,regulation of transcription by RNA polymerase
Fv_160_9.g5511	398	ID=Fv_160_9.g5511;Description=hypothetical protein FVEG_03632 [Fusarium verticillioides 7600];Gene=FDENT_9112;Ontology_term=nucleus,DNA binding,zinc ion binding,DNA-templated
Fv_160_9.g5512	499	ID=Fv_160_9.g5512;Description=hypothetical protein FVER14953_03633 [Fusarium verticillioides];Gene=BFJ63_vAg15116;Ontology_term=membrane,transmembrane transporter activity,transmembrane
Fv_160_9.g5513	429	ID=Fv_160_9.g5513;Description=hypothetical protein FVEG_03634 [Fusarium verticillioides 7600];Gene=FPCIR_11148;Ontology_term=nucleus,DNA-binding transcription factor activity, RNA polymerase II-specific,zinc ion
Fv_160_9.g5514	521	ID=Fv_160_9.g5514;Description=hypothetical protein FVER53590_27566 [Fusarium verticillioides];Gene=FPRO_03607;Ontology_term=nucleus,DNA-binding transcription factor activity, RNA polymerase II-specific,zinc
Fv_160_9.g5515	790	ID=Fv_160_9.g5515;Description=ENTH domain-containing protein [Fusarium mundagurra];Gene=FVEG_03635;Ontology_term=membrane,clathrin-coated vesicle,1-phosphatidylinositol binding,clathrin
Fv_160_9.g5516	363	ID=Fv_160_9.g5516;Description=CDK-activating kinase assembly factor MAT1 [Fusarium verticillioides 7600];Gene=FVEG_03636;Ontology_term=transcription factor TFIIH holo complex,kinase activity,metal ion binding,cyclin-dependent protein serine/threonine kinase activator activity,nucleotide-excision repair,phosphorylation;Ontology_id=GO:0005675,GO:0016301,GO:0046872,GO:0061575,GO:0006289,GO:0016310;Enzyme_code=
Fv_160_9.g5517	188	ID=Fv_160_9.g5517;Description=hypothetical protein FVEG_03637 [Fusarium verticillioides 7600]
Fv_160_9.g5518	636	ID=Fv_160_9.g5518;Description=hypothetical protein FVER53263_03638 [Fusarium verticillioides]
Fv_160_9.g5519	263	ID=Fv_160_9.g5519;Description=hypothetical protein FVEG_03639 [Fusarium verticillioides 7600];Gene=Forpi1262_v005710;Ontology_term=glycoprotein metabolic process;Ontology_id=GO:0009100
Fv_160_9.g5520	343	ID=Fv_160_9.g5520;Description=related to endoplasmic reticulum retention receptor [Fusarium fujikuroi IMI 58289];Gene=BFJ69_g11397;Ontology_term=endoplasmic reticulum membrane,ER retention sequence binding,protein retention in
Fv_160_9.g5521	268	ID=Fv_160_9.g5521;Description=adenylate kinase [Fusarium verticillioides 7600];Gene=ADK2;Ontology_term=mitochondrial matrix,adenylate kinase activity,ATP binding,GTP binding,nucleoside triphosphate adenylate kinase activity,ADP biosynthetic process,phosphorylation,AMP metabolic process,GTP metabolic process,ITP metabolic process,nucleoside monophosphate phosphorylation;Ontology_id=GO:0005759,GO:0004017,GO:0005524,GO:0005525,GO:0046899,GO:0006172,GO:0016310,GO:0046033,GO:0046039,GO:0046041,GO:0046940;Enzyme_code=EC:2.7.4.4,EC:2.7.4.3,EC:2.7.4.10;Enzyme_name=nucleoside-phosphate
Fv_160_9.g5522	600	ID=Fv_160_9.g5522;Description=hypothetical protein FVEG_03642 [Fusarium verticillioides 7600];Gene=FMAN_03437;Ontology_term=hydrolase activity, hydrolyzing O-glycosyl compounds,carbohydrate metabolic
Fv_160_9.g5523	552	ID=Fv_160_9.g5523;Description=hypothetical protein FVEG_03643 [Fusarium verticillioides 7600];Gene=FMAN_03436;Ontology_term=mitochondrial inner membrane,ribosome

Fv_160_9.g5524	460	ID=Fv_160_9.g5524;Description=suppressor of mrs2-1 mutation [Fusarium coicis];Gene=FNAPI_11215;Ontology_term=extracellular region,mitochondrial inner membrane,retinol binding,phosphatidylinositol binding,ribosome
Fv_160_9.g5525	847	ID=Fv_160_9.g5525;Description=hypothetical protein FVER14953_03645 [Fusarium verticillioides];Gene=FNAPI_11216;Ontology_term=membrane,serine-type endopeptidase
Fv_160_9.g5526	315	ID=Fv_160_9.g5526;Description=hypothetical protein FVEG_03646 [Fusarium verticillioides 7600];Gene=FVEG_03646;Ontology_term=mitochondrial inner membrane,inorganic phosphate transmembrane transporter
Fv_160_9.g5527	66	ID=Fv_160_9.g5527;Description=hypothetical protein FVEG_03647 [Fusarium verticillioides 7600];Gene=2042
Fv_160_9.g5528	514	ID=Fv_160_9.g5528;Description=hypothetical protein FVER14953_03648 [Fusarium verticillioides];Gene=BFJ72_g13392;Ontology_term=dipeptidase activity,cysteine-type exopeptidase
Fv_160_9.g5529	621	ID=Fv_160_9.g5529;Description=hypothetical protein FVEG_03649 [Fusarium verticillioides 7600];Gene=FVEG_03649;Ontology_term=nucleus,DNA binding,zinc ion binding,proteolysis,peptidase activity,DNA-binding transcription factor activity, RNA polymerase II-specific,regulation of transcription by RNA polymerase
Fv_160_9.g5530	446	ID=Fv_160_9.g5530;Description=hypothetical protein J7337_003665 [Fusarium musae]
Fv_160_9.g5531	555	ID=Fv_160_9.g5531;Description=uncharacterized protein FTJAE_12079 [Fusarium
Fv_160_9.g5532	488	ID=Fv_160_9.g5532;Description=hypothetical protein FVEG_03652 [Fusarium verticillioides
Fv_160_9.g5533	78	ID=Fv_160_9.g5533;Description=hypothetical protein FNAPI_11227 [Fusarium napiforme]
Fv_160_9.g5534	125	ID=Fv_160_9.g5534;Description=hypothetical protein FVEG_03654 [Fusarium verticillioides 7600]
Fv_160_9.g5535	602	ID=Fv_160_9.g5535;Description=hypothetical protein FVER14953_03655 [Fusarium verticillioides];Gene=FOC4_g10005778;Ontology_term=membrane,metal ion transmembrane transporter activity,metal ion
Fv_160_9.g5536	736	ID=Fv_160_9.g5536;Description=hypothetical protein FVER14953_03656 [Fusarium
Fv_160_9.g5537	580	ID=Fv_160_9.g5537;Description=hypothetical protein J7337_003672 [Fusarium musae];Gene=FVEG_03657;Ontology_term=glutathione hydrolase activity,leukotriene C4 gamma-glutamyl transferase activity,glutathione catabolic
Fv_160_9.g5538	228	ID=Fv_160_9.g5538;Description=hypothetical protein CEK27_005104 [Fusarium fujikuroi];Gene=2032;Ontology_term=extracellular space,membrane,transport vesicle,neuron projection,serine-type endopeptidase activity,peptide hormone processing;Ontology_id=GO:0005615,GO:0016020,GO:0030133,GO:0043005,GO:0004252,GO:0016486;Enzyme_code=EC:3.4.21;En
Fv_160_9.g5539	683	ID=Fv_160_9.g5539;Description=hypothetical protein FVEG_03659 [Fusarium verticillioides 7600];Gene=FVEG_03659;Ontology_term=nucleus,DNA binding,zinc ion binding,DNA-binding transcription factor activity, RNA
Fv_160_9.g5540	293	ID=Fv_160_9.g5540;Description=hypothetical protein FVEG_03660 [Fusarium verticillioides 7600];Gene=FVER53590_03660;Ontology_term=ferric iron binding,catechol 1,2-dioxygenase activity,catechol-containing compound
Fv_160_9.g5541	747	ID=Fv_160_9.g5541;Description=hypothetical protein FMUND_5615 [Fusarium mundagurra];Gene=FANTH_5055;Ontology_term=membrane,ferric iron binding,catechol 1,2-dioxygenase activity,catechol-containing compound metabolic

Fv_160_9.g5542	404	ID=Fv_160_9.g5542;Description=hypothetical protein FVER14953_03661 [Fusarium verticillioides];Gene=FCIRC_2798;Ontology_term=monooxygenase activity,FAD binding,biosynthetic
Fv_160_9.g5543	257	ID=Fv_160_9.g5543;Description=hypothetical protein FVEG_03662 [Fusarium verticillioides 7600]
Fv_160_9.g5544	311	ID=Fv_160_9.g5544;Description=hypothetical protein FVEG_03663 [Fusarium verticillioides 7600]
Fv_160_9.g5545	345	ID=Fv_160_9.g5545;Description=hypothetical protein FVEG_03664 [Fusarium verticillioides 7600];Gene=msopa;Ontology_term=extracellular space,defense response;Ontology_id=GO:0005615,GO:0006952
Fv_160_9.g5546	525	ID=Fv_160_9.g5546;Description=hypothetical protein FVEG_15313 [Fusarium verticillioides 7600];Gene=FFC1_09421;Ontology_term=membrane,acyltransferase activity, transferring groups other than amino-acyl
Fv_160_9.g5547	949	ID=Fv_160_9.g5547;Description=uncharacterized protein LW93_14374 [Fusarium fujikuroi];Gene=CEK26_005092;Ontology_term=membrane,amidase activity,phosphoric diester hydrolase activity,acyltransferase activity, transferring groups other than amino-acyl groups,indoleacetamide hydrolase activity,lipid metabolic process;Ontology_id=GO:0016020,GO:0004040,GO:0008081,GO:0016747,GO:0043864,GO:0006629;Enzyme_code=EC:3.1.4,EC:3.5.
Fv_160_9.g5548	446	ID=Fv_160_9.g5548;Description=hypothetical protein FVEG_03668 [Fusarium verticillioides 7600];Gene=FDENT_5608;Ontology_term=amidase activity,phosphoric diester hydrolase activity,acyltransferase activity, transferring groups other than amino-acyl groups,indoleacetamide hydrolase activity,lipid metabolic process;Ontology_id=GO:0004040,GO:0008081,GO:0016747,GO:0043864,GO:0006629;Enzyme_code=EC:3.1.4,EC:3.5.1.4,EC:2.3.1;E
Fv_160_9.g5549	256	ID=Fv_160_9.g5549;Description=hypothetical protein FVER14953_03669 [Fusarium verticillioides];Gene=nullo;Ontology_term=adherens junction,cell-cell junction assembly,cellularization,regulation of cell shape,morphogenesis of embryonic epithelium,cleavage furrow
Fv_160_9.g5550	411	ID=Fv_160_9.g5550;Description=hypothetical protein FVEG_15314 [Fusarium verticillioides 7600];Gene=FSUBG_2078;Ontology_term=nucleus,DNA-binding transcription factor activity, RNA polymerase II-specific,DNA
Fv_160_9.g5551	226	ID=Fv_160_9.g5551;Description=hypothetical protein FCIRC_2051 [Fusarium circinatum];Gene=FSUBG_2078;Ontology_term=extracellular region,nucleus,DNA-binding transcription factor activity, RNA polymerase II-specific,DNA binding,peptidase activity,zinc ion binding,regulation of transcription by RNA polymerase II,proteolysis;Ontology_id=GO:0005576,GO:0005634,GO:0000981,GO:0003677,GO:0008233,GO:0008270,GO:0006357,GO:0006508
Fv_160_9.g5552	431	ID=Fv_160_9.g5552;Description=hypothetical protein FVER14953_03671 [Fusarium verticillioides];Gene=FNYG_01055;Ontology_term=extracellular region,peptidase
Fv_160_9.g5553	551	ID=Fv_160_9.g5553;Description=hypothetical protein FVER14953_03672 [Fusarium verticillioides];Gene=FTJAE_5274;Ontology_term=extracellular region,peptidase activity,acyltransferase activity,proteolysis;Ontology_id=GO:0005576,GO:0008233,GO:0016746,GO:0006508;Enzyme_code=EC:2.3,EC:3.4;Enzyme_name=A
Fv_160_9.g5554	266	ID=Fv_160_9.g5554;Description=hypothetical protein FVER14953_03673 [Fusarium
Fv_160_9.g5555	105	ID=Fv_160_9.g5555;Description=hypothetical protein FVEG_03674 [Fusarium verticillioides 7600]

Fv_160_9.g5556	559	ID=Fv_160_9.g5556;Description=hypothetical protein FVEG_03675 [Fusarium verticillioides 7600];Gene=FNAPI_6523;Ontology_term=plasma membrane,very-low-density lipoprotein particle,chylomicron,lipoprotein lipase activity,heparin binding,phospholipase A1 activity,apolipoprotein binding,metal ion binding,phosphatidylserine 1-acylhydrolase activity,1-acyl-2-lysophosphatidylserine acylhydrolase activity,fatty acid biosynthetic process,response to glucose,triglyceride catabolic process,very-low-density lipoprotein particle
Fv_160_9.g5557	613	ID=Fv_160_9.g5557;Description=hypothetical protein FVER14953_03676 [Fusarium verticillioides];Gene=FFUJ_03962;Ontology_term=phosphatidylinositol 3-kinase complex, class III, type II;Ontology_id=GO:0034272
Fv_160_9.g5558	154	ID=Fv_160_9.g5558;Description=hypothetical protein FVEG_03677 [Fusarium verticillioides 7600];Gene=FPCIR_13916;Ontology_term=hydrolase
Fv_160_9.g5559	827	ID=Fv_160_9.g5559;Description=hypothetical protein FVEG_03678 [Fusarium verticillioides 7600];Gene=Forpi1262_v005751;Ontology_term=nucleus,DNA binding,zinc ion binding,DNA-binding transcription factor activity, RNA polymerase II-specific,regulation of transcription by RNA polymerase
Fv_160_9.g5560	457	ID=Fv_160_9.g5560;Description=hypothetical protein FVEG_03679 [Fusarium verticillioides 7600];Gene=FTJAE_5284;Ontology_term=nucleus,DNA-binding transcription factor activity, RNA polymerase II-specific,zinc ion
Fv_160_9.g5561	239	ID=Fv_160_9.g5561;Description=hypothetical protein FVEG_03680 [Fusarium verticillioides 7600]
Fv_160_9.g5562	146	ID=Fv_160_9.g5562;Description=hypothetical protein FVER53263_03681 [Fusarium verticillioides]
Fv_160_9.g5563	191	ID=Fv_160_9.g5563;Description=hypothetical protein FVEG_03682 [Fusarium verticillioides 7600]
Fv_160_9.g5564	170	ID=Fv_160_9.g5564;Description=hypothetical protein FVER14953_03683 [Fusarium verticillioides]
Fv_160_9.g5565	284	ID=Fv_160_9.g5565;Description=hypothetical protein FVEG_03684 [Fusarium verticillioides 7600];Gene=FPCIR_13957;Ontology_term=metallopeptidase
Fv_160_9.g5566	123	ID=Fv_160_9.g5566;Description=hypothetical protein FVEG_03685 [Fusarium verticillioides 7600];Gene=FNAPI_6533;Ontology_term=extracellular region,fungal-type cell wall,membrane,structural constituent of cell
Fv_160_9.g5567	334	ID=Fv_160_9.g5567;Description=hypothetical protein FVEG_03686 [Fusarium verticillioides 7600]
Fv_160_9.g5568	341	ID=Fv_160_9.g5568;Description=hypothetical protein FVEG_03687 [Fusarium verticillioides
Fv_160_9.g5569	342	ID=Fv_160_9.g5569;Description=hypothetical protein FVEG_15317 [Fusarium verticillioides 7600];Gene=FGADI_9245;Ontology_term=nucleus,DNA-binding transcription factor activity, RNA polymerase II-specific,zinc ion
Fv_160_9.g5570	255	ID=Fv_160_9.g5570;Description=hypothetical protein FVEG_03688 [Fusarium verticillioides 7600];Gene=FVER53590_03688;Ontology_term=nucleus,DNA-binding transcription factor activity, RNA polymerase II-specific,zinc
Fv_160_9.g5571	272	ID=Fv_160_9.g5571;Description=hypothetical protein FVEG_15318 [Fusarium verticillioides 7600]
Fv_160_9.g5572	126	ID=Fv_160_9.g5572;Description=hypothetical protein FVEG_03689 [Fusarium verticillioides 7600];Gene=FGLOB1_11747;Ontology_term=extracellular region,fungal-type cell wall,structural constituent of cell
Fv_160_9.g5573	201	ID=Fv_160_9.g5573;Description=hypothetical protein FVEG_15319 [Fusarium verticillioides
Fv_160_9.g5574	214	ID=Fv_160_9.g5574;Description=hypothetical protein FVEG_03690 [Fusarium verticillioides 7600]

Fv_160_9.g5575	1292	ID=Fv_160_9.g5575;Description=hypothetical protein FVER53263_03691 [Fusarium verticillioides];Gene=F25303_2586;Ontology_term=positive regulation of TOR signaling;Ontology_id=GO:0032008
Fv_160_9.g5576	193	ID=Fv_160_9.g5576;Description=hypothetical protein J7337_003705 [Fusarium musae];Gene=FVER53590_03693;Ontology_term=growth factor activity,negative regulation of release of cytochrome c from mitochondria,positive regulation of cell population proliferation,insulin-like growth factor receptor signaling pathway,GDP metabolic process,positive regulation of glucose import,GMP metabolic process,guanylate kinase activity,hormone activity,positive regulation of glycogen biosynthetic process,insulin-like growth factor receptor binding,exocytic vesicle,ATP binding,extracellular space,regulation of protein phosphorylation,alphav-beta3 integrin-IGF-1-IGF1R complex,positive regulation of phosphatidylinositol 3-kinase/protein kinase B signal
Fv_160_9.g5577	595	ID=Fv_160_9.g5577;Description=hypothetical protein FVER53263_03694 [Fusarium verticillioides];Gene=F25303_2584;Ontology_term=oxidoreductase
Fv_160_9.g5578	2285	ID=Fv_160_9.g5578;Description=hypothetical protein FVER53263_03695 [Fusarium verticillioides];Gene=MtnA;Ontology_term=growth factor activity,negative regulation of release of cytochrome c from mitochondria,positive regulation of cell population proliferation,insulin-like growth factor receptor signaling pathway,positive regulation of glucose import,fatty acid biosynthetic process,hormone activity,positive regulation of glycogen biosynthetic process,insulin-like growth factor receptor binding,exocytic vesicle,3-oxoacyl-[acyl-carrier-protein] synthase activity,extracellular space,metal ion binding,regulation of protein phosphorylation,secondary metabolite biosynthetic process,alphav-beta3 integrin-IGF-1-IGF1R complex,phosphopantetheine binding,positive regulation of phosphatidylinositol 3-kinase/protein kinase B signal
Fv_160_9.g5579	387	ID=Fv_160_9.g5579;Description=hypothetical protein FVER53263_03696 [Fusarium verticillioides];Gene=FANTH_5013;Ontology_term=O-methyltransferase activity,S-adenosylmethionine-dependent methyltransferase activity,methylation,secondary metabolite biosynthetic
Fv_160_9.g5580	527	ID=Fv_160_9.g5580;Description=hypothetical protein FVEG_03697 [Fusarium verticillioides 7600];Gene=FOXG_05818;Ontology_term=monooxygenase activity,oxidoreductase activity, acting on NAD(P)H,FAD
Fv_160_9.g5581	369	ID=Fv_160_9.g5581;Description=hypothetical protein FVEG_03698 [Fusarium verticillioides 7600];Gene=CEK26_005060;Ontology_term=oxidoreductase activity, acting on NAD(P)H,FAD binding,biosynthetic
Fv_160_9.g5582	265	ID=Fv_160_9.g5582;Description=hypothetical protein FVEG_03699 [Fusarium verticillioides
Fv_160_9.g5583	329	ID=Fv_160_9.g5583;Description=hypothetical protein FVER53263_03700 [Fusarium verticillioides];Gene=FTJAE_963;Ontology_term=nucleus,DNA-binding transcription factor activity, RNA polymerase II-specific,DNA binding,zinc ion binding,regulation of transcription by RNA polymerase II,aflatoxin biosynthetic

Fv_160_9.g5584	374	ID=Fv_160_9.g5584;Description=hypothetical protein FVER14953_03702 [Fusarium verticillioides];Gene=1988;Ontology_term=extracellular space,nucleus,cytosol,cytokine activity,interleukin-1 receptor binding,copper ion binding,lyase activity,fever generation,connective tissue replacement involved in inflammatory response wound healing,intracellular sodium ion homeostasis,immune response,negative regulation of cell population proliferation,positive regulation of vascular endothelial growth factor production,cytokine-mediated signaling pathway,positive regulation of interleukin-2 production,positive regulation of immature T cell proliferation in thymus,cellular response to heat,ectopic germ cell programmed cell death,positive regulation of angiogenesis,positive regulation of mitotic nuclear division,positive regulation of transcription by RNA polymerase II,response to copper ion,positive regulation of protein secretion,positive regulation of cell division,cellular response to lipopolysaccharide,extrinsic apoptotic signaling pathway in absence of ligand;Ontology_id=GO:0005615,GO:0005634,GO:0005829,GO:0005125,GO:0005149,GO:0005507,GO:0016829,GO:0001660,GO:00
Fv_160_9.g5585	519	ID=Fv_160_9.g5585;Description=hypothetical protein FVER53263_03702 [Fusarium verticillioides];Gene=FVEG_03702;Ontology_term=lyase
Fv_160_9.g5586	817	ID=Fv_160_9.g5586;Description=hypothetical protein FVER53263_03703 [Fusarium verticillioides]
Fv_160_9.g5587	302	ID=Fv_160_9.g5587;Description=hypothetical protein J7337_003716 [Fusarium musae];Gene=FVER53590_27572;Ontology_term=transmembrane transport,transmembrane transporter activity,positive regulation of octopamine signaling pathway,cytoplasm,membrane,positive regulation of ovulation,extracellular
Fv_160_9.g5588	271	ID=Fv_160_9.g5588;Description=hypothetical protein FVEG_03705 [Fusarium verticillioides 7600];Gene=rplX;Ontology_term=membrane,hydrolase activity, acting on carbon-nitrogen (but not peptide) bonds, in linear amides,ceramide metabolic
Fv_160_9.g5589	557	ID=Fv_160_9.g5589;Description=host-specific ak-toxin akt2 [Fusarium coicis]
Fv_160_9.g5590	520	ID=Fv_160_9.g5590;Description=hypothetical protein FVEG_03707 [Fusarium verticillioides 7600]
Fv_160_9.g5591	152	ID=Fv_160_9.g5591;Description=hypothetical protein FVEG_03708 [Fusarium verticillioides 7600]
Fv_160_9.g5592	500	ID=Fv_160_9.g5592;Description=hypothetical protein FVEG_03709 [Fusarium verticillioides 7600];Gene=FFUJ_03998;Ontology_term=extracellular region,membrane,monooxygenase activity,iron ion binding,oxidoreductase activity, acting on paired donors, with incorporation or reduction of molecular oxygen,heme binding,mating behavior;Ontology_id=GO:0005576,GO:0016020,GO:0004497,GO:0005506,GO:0016705,GO:0020037,GO:0007617;Enzyme_code=E
Fv_160_9.g5593	229	ID=Fv_160_9.g5593;Description=hypothetical protein FVEG_03710 [Fusarium verticillioides 7600]
Fv_160_9.g5594	1183	ID=Fv_160_9.g5594;Description=hypothetical protein FVER14953_03711 [Fusarium verticillioides];Gene=HGR1;Ontology_term=endoplasmic reticulum membrane,hydroxymethylglutaryl-CoA reductase (NADPH) activity,isoprenoid biosynthetic process,coenzyme A metabolic
Fv_160_9.g5595	569	ID=Fv_160_9.g5595;Description=myosin-crossreactive antigen [Fusarium verticillioides 7600];Gene=FPRO_03519;Ontology_term=oleate hydratase activity,FAD binding,fatty acid metabolic

Fv_160_9.g5596	631	ID=Fv_160_9.g5596;Description=hypothetical protein FVER53590_03712 [Fusarium verticillioides];Gene=FTJAE_952;Ontology_term=metal ion binding,oleate hydratase activity,FAD binding,fatty acid metabolic
Fv_160_9.g5597	451	ID=Fv_160_9.g5597;Description=hypothetical protein FVER53590_03715 [Fusarium verticillioides];Gene=FTJAE_951;Ontology_term=ubiquitin-protein transferase activity,zinc ion binding,protein
Fv_160_9.g5598	418	ID=Fv_160_9.g5598;Description=hypothetical protein FVER14953_03716 [Fusarium verticillioides];Gene=FPANT_3559;Ontology_term=membrane,transferase
Fv_160_9.g5599	1047	ID=Fv_160_9.g5599;Description=hypothetical protein FVEG_03717 [Fusarium verticillioides 7600];Gene=FMUND_12569;Ontology_term=membrane,calcium-activated cation channel activity,monoatomic cation
Fv_160_9.g5600	694	ID=Fv_160_9.g5600;Description=hypothetical protein FVER53263_03718 [Fusarium verticillioides];Gene=F25303_2563;Ontology_term=metalloendopeptidase activity,metal ion
Fv_160_9.g5601	340	ID=Fv_160_9.g5601;Description=hypothetical protein FVEG_03719 [Fusarium verticillioides 7600];Gene=F25303_2562;Ontology_term=negative regulation of extrinsic apoptotic signaling pathway via death domain receptors,negative regulation by host of viral transcription,zinc ion binding,protein homodimerization activity,erythrocyte homeostasis,heme oxygenase (decyclizing) activity,regulation of transcription from RNA polymerase II promoter in response to iron,positive regulation of canonical NF-kappaB signal transduction,heme catabolic process,response to nicotine,heme oxidation,heme binding,negative regulation of viral life cycle,wound healing involved in inflammatory response,endoplasmic reticulum membrane,intracellular iron ion homeostasis,positive regulation of smooth muscle cell proliferation,nucleus,response to oxidative stress,perinuclear region of cytoplasm,cellular response to arsenic-containing substance,cellular response to cadmium ion,negative regulation of smooth muscle cell
Fv_160_9.g5602	268	ID=Fv_160_9.g5602;Description=hypothetical protein FVEG_03720 [Fusarium verticillioides 7600];Gene=F25303_2561;Ontology_term=nuclear exosome (RNase complex),cytoplasmic exosome (RNase complex),chromatin,nucleolus,exonuclease activity,exonucleolytic trimming to generate mature 3'-end of 5.8S rRNA from tricistronic rRNA transcript (SSU-rRNA, 5.8S rRNA, LSU-rRNA),rRNA catabolic process,termination of RNA polymerase II transcription, exosome-dependent,U1 snRNA 3'-end processing,U4 snRNA 3'-end processing,U5 snRNA 3'-end processing,nuclear-transcribed mRNA catabolic process, 3'-5' exonucleolytic nonsense-mediated decay,nuclear mRNA surveillance of mRNA 3'-end processing,nuclear polyadenylation-dependent tRNA catabolic process,nuclear polyadenylation-dependent CUT catabolic process,polyadenylation-
Fv_160_9.g5603	503	ID=Fv_160_9.g5603;Description=hypothetical protein FVER14953_03721 [Fusarium verticillioides];Gene=FocTR4_00007525;Ontology_term=protein disulfide isomerase
Fv_160_9.g5604	393	ID=Fv_160_9.g5604;Description=hypothetical protein FVER14953_03722 [Fusarium verticillioides];Gene=BFJ65_g8156;Ontology_term=endosome membrane,protein transport,late endosome to vacuole transport via
Fv_160_9.g5605	316	ID=Fv_160_9.g5605;Description=hypothetical protein FVER14953_03723 [Fusarium verticillioides]
Fv_160_9.g5606	522	ID=Fv_160_9.g5606;Description=mitochondrial import inner membrane translocase subunit TIM50 [Fusarium verticillioides 7600];Gene=FVEG_03724;Ontology_term=TIM23 mitochondrial import inner membrane translocase complex,protein

Fv_160_9.g5607	126	ID=Fv_160_9.g5607;Description=hypothetical protein FVEG_03725 [Fusarium verticillioides 7600]
Fv_160_9.g5608	151	ID=Fv_160_9.g5608;Description=hypothetical protein FVEG_03726 [Fusarium verticillioides 7600]
Fv_160_9.g5609	301	ID=Fv_160_9.g5609;Description=hypothetical protein FVEG_03727, partial [Fusarium verticillioides 7600];Gene=FNAPI_5213;Ontology_term=acyltransferase activity,hydrolase activity, acting on ester
Fv_160_9.g5610	558	ID=Fv_160_9.g5610;Description=hypothetical protein FVEG_03728 [Fusarium verticillioides 7600];Gene=FOXG_19131;Ontology_term=extracellular space,nucleus,DNA-binding transcription factor activity, RNA polymerase II-specific,triglyceride lipase activity,zinc ion binding,all-trans-retinyl-palmitate hydrolase, all-trans-retinol forming activity,regulation of transcription by RNA polymerase II,lipid catabolic process,retinol metabolic process;Ontology_id=GO:0005615,GO:0005634,GO:0000981,GO:0004806,GO:0008270,GO:0047376,GO:0006357,GO:0016042,GO:
Fv_160_9.g5611	259	ID=Fv_160_9.g5611;Description=hypothetical protein FVEG_15325 [Fusarium verticillioides 7600]
Fv_160_9.g5612	196	ID=Fv_160_9.g5612;Description=hypothetical protein FVEG_03730 [Fusarium verticillioides 7600];Gene=HBQ1;Ontology_term=hemoglobin complex,haptoglobin-hemoglobin complex,peroxidase activity,oxygen carrier activity,iron ion binding,oxygen binding,heme binding,haptoglobin binding,organic acid binding,oxygen transport,hydrogen peroxide catabolic process,cellular oxidant detoxification;Ontology_id=GO:0005833,GO:0031838,GO:0004601,GO:0005344,GO:0005506,GO:0019825,GO:0020037,GO:003172
Fv_160_9.g5613	572	ID=Fv_160_9.g5613;Description=hypothetical protein J7337_003740 [Fusarium musae];Gene=FVER53590_03733;Ontology_term=membrane,transmembrane transporter activity,transmembrane
Fv_160_9.g5614	546	ID=Fv_160_9.g5614;Description=hypothetical protein J7337_003741 [Fusarium musae];Gene=FVER53590_03733;Ontology_term=membrane,transmembrane transporter activity,transmembrane
Fv_160_9.g5615	69	ID=Fv_160_9.g5615;Description=hypothetical protein FVER14953_20772 [Fusarium verticillioides];Gene=F25303_2552;Ontology_term=protein serine/threonine kinase activity,ATP
Fv_160_9.g5616	573	ID=Fv_160_9.g5616;Description=hypothetical protein J7337_003742 [Fusarium musae];Gene=F25303_2551;Ontology_term=membrane,transmembrane transporter activity,transmembrane
Fv_160_9.g5617	451	ID=Fv_160_9.g5617;Description=hypothetical protein FVEG_03734 [Fusarium verticillioides 7600];Gene=FVEG_03734;Ontology_term=heterotrimeric G-protein complex,GTPase activity,insulin-like growth factor receptor binding,methyltransferase activity,adenylate cyclase activator activity,guanyl nucleotide binding,G-protein beta/gamma-subunit complex binding,beta-2 adrenergic receptor binding,D1 dopamine receptor binding,mu-type opioid receptor binding,ionotropic glutamate receptor binding,ion binding,corticotropin-releasing hormone receptor 1 binding,adenylate cyclase-activating dopamine receptor signaling pathway,sensory perception of chemical stimulus,sexual reproduction,methylation,positive regulation of GTPase activity,positive regulation of cAMP-mediated signaling,adenylate cyclase-activating adrenergic receptor signaling pathway;Ontology_id=GO:0005834,GO:0003924,GO:0005159,GO:0008168,GO:0010856,GO:0019001,GO:0031683,GO:0031698,GO:

Fv_160_9.g5618	470	ID=Fv_160_9.g5618;Description=hypothetical protein J7337_003744 [Fusarium musae];Gene=FNAPI_276;Ontology_term=extracellular space,nucleoplasm,Golgi apparatus,external side of plasma membrane,signaling receptor binding,identical protein binding,behavior,natural killer cell activation,positive regulation of type II interferon production,positive regulation of interleukin-8 production,positive regulation of tumor necrosis factor production,heterotypic cell-cell adhesion,natural killer cell mediated cytotoxicity,positive regulation of ovulation,positive regulation of octopamine signaling
Fv_160_9.g5619	292	ID=Fv_160_9.g5619;Description=hypothetical protein FVER14953_03736 [Fusarium verticillioides];Gene=rplM;Ontology_term=peptidase
Fv_160_9.g5620	317	ID=Fv_160_9.g5620;Description=hypothetical protein FVEG_03737 [Fusarium verticillioides 7600];Gene=FVEG_03737;Ontology_term=cellular anatomical entity,serine-type endopeptidase
Fv_160_9.g5621	409	ID=Fv_160_9.g5621;Description=hypothetical protein FVER14953_03738 [Fusarium verticillioides];Gene=Acp26Ab;Ontology_term=extracellular space,cytoplasm,mating
Fv_160_9.g5622	951	ID=Fv_160_9.g5622;Description=hypothetical protein FVEG_03739 [Fusarium verticillioides 7600];Gene=FPCIR_5801;Ontology_term=protein kinase activity,ATP
Fv_160_9.g5623	414	ID=Fv_160_9.g5623;Description=hypothetical protein FNAPI_270 [Fusarium napiforme];Gene=FMUND_9222;Ontology_term=protein kinase activity,ATP
Fv_160_9.g5624	510	ID=Fv_160_9.g5624;Description=hypothetical protein FVEG_03740 [Fusarium verticillioides 7600];Gene=BFJ68_g6005;Ontology_term=membrane,monooxygenase activity,iron ion binding,methyltransferase activity,oxidoreductase activity, acting on paired donors, with incorporation or reduction of molecular oxygen,heme binding,methylation;Ontology_id=GO:0016020,GO:0004497,GO:0005506,GO:0008168,GO:0016705,GO:0020037,GO:0032259;Enzy
Fv_160_9.g5625	378	ID=Fv_160_9.g5625;Description=hypothetical protein FVER14953_03741 [Fusarium
Fv_160_9.g5626	440	ID=Fv_160_9.g5626;Description=hypothetical protein FVEG_15327 [Fusarium verticillioides 7600];Gene=1052;Ontology_term=extracellular region,mating behavior;Ontology_id=GO:0005576,GO:0007617
Fv_160_9.g5627	795	ID=Fv_160_9.g5627;Description=CMGC protein kinase [Fusarium oxysporum NRRL 32931];Gene=F25303_5579;Ontology_term=extracellular space,nucleus,DNA binding,protein kinase activity,ATP binding,zinc ion binding,identical protein binding,DNA-templated transcription,phosphorylation,sexual reproduction,sperm competition;Ontology_id=GO:0005615,GO:0005634,GO:0003677,GO:0004672,GO:0005524,GO:0008270,GO:0042802,GO:0006351,
Fv_160_9.g5628	686	ID=Fv_160_9.g5628;Description=hypothetical protein FVEG_15328 [Fusarium verticillioides 7600];Gene=FCIRC_2406;Ontology_term=nucleus,DNA binding,zinc ion binding,DNA-binding transcription factor activity, RNA
Fv_160_9.g5629	699	ID=Fv_160_9.g5629;Description=hypothetical protein FVEG_03744 [Fusarium verticillioides 7600];Gene=FVER53263_03744;Ontology_term=nucleus,DNA binding,zinc ion binding,DNA-binding transcription factor activity, RNA polymerase II-specific,regulation of transcription by RNA polymerase

Fv_160_9.g5630	326	ID=Fv_160_9.g5630;Description=hypothetical protein FVEG_03745 [Fusarium verticillioides 7600];Gene=FVEG_03745;Ontology_term=FMN binding,oxidoreductase
Fv_160_9.g5631	461	ID=Fv_160_9.g5631;Description=hypothetical protein FVEG_03746 [Fusarium verticillioides 7600];Gene=FVEG_03746;Ontology_term=nucleus,DNA binding,zinc ion binding,proline dehydrogenase activity,transaminase activity,proline catabolic process,DNA-binding transcription factor activity, RNA polymerase II-specific,regulation of transcription by RNA polymerase II;Ontology_id=GO:0005634,GO:0003677,GO:0008270,GO:0004657,GO:0008483,GO:0006562,GO:0000981,GO:0006357;Enzyme_co
Fv_160_9.g5632	586	ID=Fv_160_9.g5632;Description=1-pyrroline-5-carboxylate dehydrogenase [Fusarium verticillioides 7600];Gene=FCIRC_2403;Ontology_term=1-pyrroline-5-carboxylate dehydrogenase activity,proline catabolic process to
Fv_160_9.g5633	743	ID=Fv_160_9.g5633;Description=hypothetical protein FVEG_15329 [Fusarium verticillioides 7600];Gene=ecdB-6;Ontology_term=nucleus,DNA binding,zinc ion binding,DNA-binding transcription factor activity, RNA polymerase II-
Fv_160_9.g5634	353	ID=Fv_160_9.g5634;Description=hypothetical protein FVER53263_03748 [Fusarium verticillioides];Gene=FVEG_03748;Ontology_term=membrane,nucleotide binding;Ontology_id=GO:0016020,GO:0000166
Fv_160_9.g5635	525	ID=Fv_160_9.g5635;Description=hypothetical protein FVEG_03749 [Fusarium verticillioides 7600];Gene=C2S_305;Ontology_term=membrane,transmembrane transporter activity,transmembrane
Fv_160_9.g5636	373	ID=Fv_160_9.g5636;Description=hypothetical protein FVER53263_03750 [Fusarium
Fv_160_9.g5637	379	ID=Fv_160_9.g5637;Description=hypothetical protein FNYG_00975 [Fusarium nygamai]
Fv_160_9.g5638	72	ID=Fv_160_9.g5638;Description=hypothetical protein FVER53590_03752 [Fusarium verticillioides];Gene=BFJ68_g5993;Ontology_term=nucleus,DNA-binding transcription factor activity, RNA polymerase II-specific,zinc
Fv_160_9.g5639	721	ID=Fv_160_9.g5639;Description=hypothetical protein FVEG_15330 [Fusarium verticillioides 7600];Gene=FANTH_3582;Ontology_term=nucleus,DNA binding,zinc ion binding,DNA-templated
Fv_160_9.g5640	311	ID=Fv_160_9.g5640;Description=hypothetical protein FVER14953_03753 [Fusarium verticillioides];Gene=FDENT_9220;Ontology_term=ATP binding,kinase
Fv_160_9.g5641	369	ID=Fv_160_9.g5641;Description=hypothetical protein FVEG_03754 [Fusarium verticillioides 7600];Gene=FFC1_09522;Ontology_term=magnesium ion binding,D-malate dehydrogenase (decarboxylating) activity,NAD
Fv_160_9.g5642	659	ID=Fv_160_9.g5642;Description=hypothetical protein FVEG_03755 [Fusarium verticillioides 7600];Gene=FMAN_03314;Ontology_term=ubiquitin-protein transferase activity,zinc ion binding,ligase activity,protein
Fv_160_9.g5643	580	ID=Fv_160_9.g5643;Description=hypothetical protein FVEG_03756 [Fusarium verticillioides 7600];Gene=clpX;Ontology_term=cytosol,plasma membrane,ATP-dependent diacylglycerol kinase activity,calcium ion binding,ATP binding,transmembrane transporter activity,phosphatidic acid biosynthetic process,protein kinase C-activating G protein-coupled receptor signaling pathway,carbohydrate transport,intracellular signal transduction,diacylglycerol metabolic process,lipid phosphorylation,transmembrane

Fv_160_9.g5644	631	ID=Fv_160_9.g5644;Description=hypothetical protein FVEG_03757 [Fusarium verticillioides 7600];Gene=osm1;Ontology_term=FMN binding,oxidoreductase activity;Ontology_id=GO:0010181,GO:0016491;Enzyme_code=EC:1;Enzyme_name=Oxidoreductases
Fv_160_9.g5645	202	ID=Fv_160_9.g5645;Description=hypothetical protein FVEG_03758 [Fusarium verticillioides 7600]
Fv_160_9.g5646	652	ID=Fv_160_9.g5646;Description=MADS-box transcription factor, other eukaryote [Fusarium verticillioides 7600];Gene=BFJ65_g8044;Ontology_term=nucleus,RNA polymerase II transcription regulatory region sequence-specific DNA binding,protein dimerization activity,positive regulation of transcription by RNA polymerase
Fv_160_9.g5647	1225	ID=Fv_160_9.g5647;Description=hypothetical protein FVER14953_03760 [Fusarium verticillioides];Gene=1935;Ontology_term=smooth endoplasmic reticulum,outflow tract morphogenesis,transcription by RNA polymerase II,striated muscle contraction,skin development,ATP binding,translation initiation factor activity,chromatin remodeling,transcription factor TFIID complex,protein homotetramerization,cellular response to calcium ion,skeletal muscle fiber development,sarcolemma,calcium ion binding,ryanodine receptor complex,RNA polymerase II general transcription initiation factor binding,cellular response to caffeine,TBP-class protein binding,translational initiation,voltage-gated calcium channel activity,ossification involved in bone maturation,Z disc,histone acetyltransferase activity,calmodulin binding,ryanodine-sensitive calcium-release channel activity,release of sequestered calcium ion into cytosol by sarcoplasmic reticulum,terminal cisterna;Ontology_id=GO:0005790,GO:0003151,GO:0006366,GO:0006941,GO:0043588,GO:0005524,GO:0003743,GO:0006338,GO:
Fv_160_9.g5648	315	ID=Fv_160_9.g5648;Description=hypothetical protein FVEG_03761 [Fusarium verticillioides
Fv_160_9.g5649	602	ID=Fv_160_9.g5649;Description=hypothetical protein FVEG_03762 [Fusarium verticillioides 7600];Gene=1933;Ontology_term=DNA binding,zinc ion binding,protein kinase CK2 complex,cytoplasm,PcG protein complex,Wnt signaling pathway,protein kinase regulator activity,DNA-binding transcription factor activity, RNA polymerase II-specific,regulation of transcription by RNA polymerase
Fv_160_9.g5650	221	ID=Fv_160_9.g5650;Description=NADH dehydrogenase (ubiquinone) Fe-S protein 7 [Fusarium verticillioides 7600];Gene=FocTR4_00007477;Ontology_term=NADH dehydrogenase (ubiquinone) activity,metal ion binding,quinone binding,4 iron, 4 sulfur cluster binding,electron transport chain;Ontology_id=GO:0008137,GO:0046872,GO:0048038,GO:0051539,GO:0022900;Enzyme_code=EC:7.1.1.2,EC:1.6.5.11,EC:1.6.5.
Fv_160_9.g5651	543	ID=Fv_160_9.g5651;Description=protein RDR1 [Fusarium verticillioides 7600];Gene=F25303_5599;Ontology_term=lipid droplet,endoplasmic reticulum lumen,nucleus,DNA binding,zinc ion binding,lipid catabolic process,carboxylesterase activity,DNA-binding transcription factor activity, RNA polymerase II-specific,regulation of transcription by RNA polymerase II;Ontology_id=GO:0005811,GO:0005788,GO:0005634,GO:0003677,GO:0008270,GO:0016042,GO:0106435,GO:0000981,GO:000635
Fv_160_9.g5652	434	ID=Fv_160_9.g5652;Description=hypothetical protein FVEG_03764 [Fusarium verticillioides 7600];Gene=HZS61_013478;Ontology_term=membrane,transmembrane transporter activity,transmembrane
Fv_160_9.g5653	172	ID=Fv_160_9.g5653;Description=LOW QUALITY PROTEIN: hypothetical protein FVEG_15332 [Fusarium verticillioides 7600]
Fv_160_9.g5654	260	ID=Fv_160_9.g5654;Description=hypothetical protein FVEG_15333 [Fusarium verticillioides

Fv_160_9.g5655	533	ID=Fv_160_9.g5655;Description=hypothetical protein FVEG_15334 [Fusarium verticillioides 7600];Gene=1927;Ontology_term=cytoplasm,actin filament,protein homodimerization activity,protein heterodimerization activity,actin filament binding,actin filament organization,cardiac muscle
Fv_160_9.g5656	409	ID=Fv_160_9.g5656;Description=hypothetical protein FVER14953_03767 [Fusarium verticillioides];Gene=FVEG_03767;Ontology_term=oxidoreductase activity,FAD binding,biosynthetic
Fv_160_9.g5657	107	ID=Fv_160_9.g5657;Description=hypothetical protein FVEG_03768 [Fusarium verticillioides 7600];Gene=FTJAE_897;Ontology_term=monooxygenase
Fv_160_9.g5658	608	ID=Fv_160_9.g5658;Description=hypothetical protein FVEG_03769 [Fusarium verticillioides 7600];Gene=FTJAE_896;Ontology_term=membrane,transmembrane transporter activity,transmembrane
Fv_160_9.g5659	504	ID=Fv_160_9.g5659;Description=hypothetical protein FVEG_03770 [Fusarium verticillioides 7600];Gene=FVER53590_03770;Ontology_term=nucleus,DNA-binding transcription factor activity, RNA polymerase II-specific,monooxygenase activity,iron ion binding,zinc ion binding,oxidoreductase activity, acting on paired donors, with incorporation or reduction of molecular oxygen,heme binding,regulation of transcription by RNA polymerase II;Ontology_id=GO:0005634,GO:0000981,GO:0004497,GO:0005506,GO:0008270,GO:0016705,GO:0020037,GO:0006357;Enzyme_co
Fv_160_9.g5660	206	ID=Fv_160_9.g5660;Description=hypothetical protein FVER14953_03771 [Fusarium verticillioides];Gene=BFJ69_g4826;Ontology_term=lyase activity,metal ion binding,terpenoid biosynthetic
Fv_160_9.g5661	428	ID=Fv_160_9.g5661;Description=hypothetical protein FVER14953_03772 [Fusarium verticillioides];Gene=FNAPI_234;Ontology_term=membrane,monooxygenase activity,FAD binding,biosynthetic
Fv_160_9.g5662	300	ID=Fv_160_9.g5662;Description=hypothetical protein FVER53590_03773 [Fusarium verticillioides];Gene=F66182_4216;Ontology_term=extracellular region,cytoplasm,membrane,3-oxoacyl-[acyl-carrier-protein] synthase activity,S-adenosylmethionine-dependent methyltransferase activity,oxidoreductase activity,phosphopantetheine binding,fatty acid biosynthetic process,secondary metabolite biosynthetic process,transmembrane transport;Ontology_id=GO:0005576,GO:0005737,GO:0016020,GO:0004315,GO:0008757,GO:0016491,GO:0031177,GO:0006633,GO
Fv_160_9.g5663	564	ID=Fv_160_9.g5663;Description=hypothetical protein FVEG_03774 [Fusarium verticillioides 7600];Gene=FNAPI_233;Ontology_term=membrane,transmembrane transport;Ontology_id=GO:0016020,GO:0055085
Fv_160_9.g5664	1466	ID=Fv_160_9.g5664;Description=hypothetical protein FVER14953_03775 [Fusarium verticillioides];Gene=FNAPI_232;Ontology_term=membrane,lysozyme activity,ATP binding,ATP hydrolysis activity,ABC-type transporter activity,transmembrane transport;Ontology_id=GO:0016020,GO:0003796,GO:0005524,GO:0016887,GO:0140359,GO:0055085;Enzyme_code=EC:7.2.2,EC:3.
Fv_160_9.g5665	583	ID=Fv_160_9.g5665;Description=hypothetical protein FVER53263_03776 [Fusarium verticillioides];Gene=FMUND_9184;Ontology_term=nucleus,DNA binding,zinc ion binding,membrane,DNA-binding transcription factor activity, RNA polymerase II-specific,regulation of transcription by RNA polymerase

Fv_160_9.g5666	94	ID=Fv_160_9.g5666;Description=hypothetical protein FVEG_15336 [Fusarium verticillioides 7600];Gene=LysD;Ontology_term=extracellular space,lysozyme activity,chitinase activity,metabolic process,killing of cells of another organism,defense response to Gram-negative
Fv_160_9.g5667	621	ID=Fv_160_9.g5667;Description=hypothetical protein FVEG_03777 [Fusarium verticillioides
Fv_160_9.g5668	289	ID=Fv_160_9.g5668;Description=hypothetical protein FVEG_03778 [Fusarium verticillioides 7600];Gene=FMUND_9182;Ontology_term=acrosomal vesicle,larval serum protein complex,mitochondrion,cytosol,plasma membrane,cAMP-dependent protein kinase complex,sperm flagellum,germinal vesicle,perinuclear region of cytoplasm,AMP-activated protein kinase activity,cAMP-dependent protein kinase activity,ATP binding,protein kinase A regulatory subunit binding,nutrient reservoir activity,protein serine kinase activity,protein kinase A signaling,phosphorylation,cellular response to heat,negative regulation of meiotic cell cycle process involved in oocyte maturation,negative regulation of TORC1 signaling;Ontology_id=GO:0001669,GO:0005616,GO:0005739,GO:0005829,GO:0005886,GO:0005952,GO:0036126,GO:0042585,GO:
Fv_160_9.g5669	791	ID=Fv_160_9.g5669;Description=hypothetical protein FVER53263_03779 [Fusarium verticillioides];Gene=1912
Fv_160_9.g5670	530	ID=Fv_160_9.g5670;Description=hypothetical protein FVER14953_03780 [Fusarium verticillioides];Gene=PRKACB;Ontology_term=larval serum protein complex,nucleus,cytosol,plasma membrane,cAMP-dependent protein kinase complex,SCF ubiquitin ligase complex,magnesium ion binding,AMP-activated protein kinase activity,cAMP-dependent protein kinase activity,ATP binding,nutrient reservoir activity,protein serine kinase activity,protein phosphorylation,protein kinase A signaling,SCF-dependent proteasomal ubiquitin-dependent protein catabolic process,negative regulation of TORC1 signaling;Ontology_id=GO:0005616,GO:0005634,GO:0005829,GO:0005886,GO:0005952,GO:0019005,GO:0000287,GO:0004679,GO:
Fv_160_9.g5671	298	ID=Fv_160_9.g5671;Description=hypothetical protein FVER14953_03781 [Fusarium verticillioides];Gene=FTJAE_883;Ontology_term=hydrolase
Fv_160_9.g5672	805	ID=Fv_160_9.g5672;Description=hypothetical protein FVER53590_03782 [Fusarium verticillioides];Gene=F25303_5620;Ontology_term=mitochondrial inner membrane,mitochondrial transmembrane
Fv_160_9.g5673	270	ID=Fv_160_9.g5673;Description=hypothetical protein J7337_003792 [Fusarium
Fv_160_9.g5674	365	ID=Fv_160_9.g5674;Description=hypothetical protein FOXG_05914 [Fusarium oxysporum f. sp. lycopersici 4287]
Fv_160_9.g5675	553	ID=Fv_160_9.g5675;Description=hypothetical protein FVER14953_03786 [Fusarium verticillioides];Gene=FNYG_00939;Ontology_term=nucleus,cytoplasm,membrane,DNA binding,helicase activity,zinc ion transmembrane transporter activity,ATP binding,ATP hydrolysis activity,ATP-dependent chromatin remodeler activity,DNA repair,chromatin remodeling,DNA-templated transcription termination,female meiotic nuclear division,spermatogenesis,microtubule anchoring at centrosome,zinc ion transmembrane
Fv_160_9.g5676	216	ID=Fv_160_9.g5676;Description=hypothetical protein FVEG_03787 [Fusarium verticillioides 7600]
Fv_160_9.g5677	287	ID=Fv_160_9.g5677;Description=hypothetical protein FVER53263_03788 [Fusarium
Fv_160_9.g5678	861	ID=Fv_160_9.g5678;Description=cutinase transcription factor 1 alpha [Fusarium coicis];Gene=FMUND_9171;Ontology_term=nucleus,DNA binding,zinc ion binding,DNA-binding transcription factor activity, RNA

Fv_160_9.g5679	203	ID=Fv_160_9.g5679;Description=hypothetical protein FVEG_03790 [Fusarium verticillioides 7600];Gene=FANTH_5440;Ontology_term=CIA complex,iron-sulfur cluster assembly,protein maturation by [4Fe-4S] cluster
Fv_160_9.g5680	983	ID=Fv_160_9.g5680;Description=elongation factor 2 [Fusarium verticillioides 7600];Gene=FTJAE_7186;Ontology_term=protein localization to nuclear envelope,meiosis I cytokinesis,gonad morphogenesis,negative regulation of cell population proliferation,nuclear migration,terminal branching, open tracheal system,Prp19 complex,male meiosis cytokinesis,nuclear pore localization,GTP binding,U5 snRNA binding,translation elongation factor activity,post-mRNA release spliceosomal complex,compound eye morphogenesis,GTPase activity,U4/U6 x U5 tri-snRNP complex,negative regulation of immune response,positive regulation of cytokinesis, actomyosin contractile ring assembly,positive regulation of protein localization to nucleus,mitotic nuclear membrane reassembly,chitin-based cuticle development,U5 snRNP,spindle pole,generation of catalytic spliceosome for first transesterification step,structural constituent of cytoskeleton,digestive tract morphogenesis,positive regulation of spindle assembly,chromatin binding,nuclear envelope lumen,translational elongation,signaling receptor binding,spliceosome conformational change to release U4 (or U4atac) and U1 (or U11),spliceosomal tri-snRNP complex assembly,nuclear inner membrane,central nervous system development,spermatocyte division,lamin filament,cytoplasm,heterochromatin formation,positive regulation of actin filament polymerization,spindle envelope;Ontology_id=GO:0090435,GO:0007110,GO:0035262,GO:0008285,GO:0007097,GO:0007430,GO:0000974,GO:0007112,GO
Fv_160_9.g5681	396	ID=Fv_160_9.g5681;Description=acetyl-CoA C-acetyltransferase [Fusarium verticillioides 7600];Gene=1900;Ontology_term=lateral plasma membrane,regulation of calcium ion transmembrane transport,positive regulation of potassium ion import across plasma membrane,intracellular sodium ion homeostasis,sodium:potassium-exchanging ATPase complex,cell adhesion,positive regulation of P-type sodium:potassium-exchanging transporter activity,ATPase activator activity,intracellular potassium ion homeostasis,acetyl-CoA C-acyltransferase activity,sperm flagellum,protein kinase binding,positive regulation of sodium ion export across plasma membrane,proton transmembrane transport,membrane repolarization,organelle membrane,intracellular calcium ion homeostasis,T-tubule,innate immune response,ATP metabolic process,regulation of gene expression,establishment or maintenance of transmembrane electrochemical gradient,intercalated disc,regulation of cardiac muscle contraction by calcium ion signaling,protein stabilization,basolateral plasma membrane,protein-macromolecule adaptor activity,apical plasma membrane,protein localization to plasma membrane,relaxation of cardiac muscle,ATPase
Fv_160_9.g5682	1492	ID=Fv_160_9.g5682;Description=hypothetical protein FVER53590_03793 [Fusarium verticillioides];Gene=F25303_2542;Ontology_term=endoplasmic reticulum membrane,nuclear membrane,transcription corepressor activity,karyogamy involved in conjugation with cellular fusion,negative regulation of DNA-templated transcription,nuclear
Fv_160_9.g5683	481	ID=Fv_160_9.g5683;Description=hypothetical protein FVER53263_03794 [Fusarium verticillioides];Gene=FNAPI_6225;Ontology_term=endoplasmic reticulum membrane,nuclear membrane,karyogamy involved in
Fv_160_9.g5684	504	ID=Fv_160_9.g5684;Description=hypothetical protein FVEG_03795 [Fusarium verticillioides 7600];Gene=FNYG_00929;Ontology_term=protein dimerization activity;Ontology_id=GO:0046983

Fv_160_9.g5685	503	ID=Fv_160_9.g5685;Description=xaa-Pro aminopeptidase [Fusarium verticillioides 7600];Gene=FNAPI_4567;Ontology_term=regulation of multicellular organism growth,regulation of epidermal growth factor-activated receptor activity,axo-dendritic transport,adult locomotory behavior,heparin binding,Golgi apparatus,cell adhesion,visual learning,perikaryon,extracellular region,Golgi-associated vesicle,growth cone,regulation of translation,signaling receptor activator activity,early endosome,intracellular copper ion homeostasis,clathrin-coated pit,apoptotic process,metalloaminopeptidase activity,manganese ion binding,axon midline choice point recognition,ionotropic glutamate receptor signaling pathway,dendrite development,collateral sprouting in absence of injury,endoplasmic reticulum,endocytosis,signaling receptor binding,positive regulation of mitotic cell cycle,mRNA polyadenylation,cell surface,nucleus,DNA binding,mating behavior,serine-type endopeptidase inhibitor activity,central nervous system development,Notch signaling pathway,mitochondrion,membrane raft,extracellular matrix organization,regulation of synapse structure or activity,protein processing,recycling endosome,neuron remodeling,protein stabilization;Ontology_id=GO:0040014,GO:0007176,GO:0008088,GO:0008344,GO:0008201,GO:0005794,GO:0007155,GO:0008542,GO:0043204,GO:0005576,GO:0005798,GO:0030426,GO:0006417,GO:0030546,GO:0005769,GO:0006878,GO:0005905,GO:0006915,
Fv_160_9.g5686	629	ID=Fv_160_9.g5686;Description=hypothetical protein FVEG_03797 [Fusarium verticillioides 7600];Gene=FNYG_00927;Ontology_term=nucleus,nucleic acid binding,zinc ion binding,kinase activity,phosphorylation;Ontology_id=GO:0005634,GO:0003676,GO:0008270,GO:0016301,GO:0016310;Enzyme_code=EC:2.7;Enzy
Fv_160_9.g5687	723	ID=Fv_160_9.g5687;Description=hypothetical protein FVER14953_03798 [Fusarium verticillioides];Gene=Forpe1208_v005767;Ontology_term=cytoplasm,ammonia-lyase activity,L-phenylalanine catabolic
Fv_160_9.g5688	158	ID=Fv_160_9.g5688;Description=autophagy-like protein 12 [Fusarium verticillioides 7600];Gene=FGLOB1_602;Ontology_term=phagophore assembly site membrane,autophagosome assembly,protein
Fv_160_9.g5689	345	ID=Fv_160_9.g5689;Description=hypothetical protein FVER53590_03800 [Fusarium verticillioides];Gene=FVEG_03800;Ontology_term=3-methyl-2-oxobutanoate hydroxymethyltransferase activity,methyltransferase activity,pantothenate biosynthetic process,methylation;Ontology_id=GO:0003864,GO:0008168,GO:0015940,GO:0032259;Enzyme_code=EC:2.1.2.11,EC:2.1.1;Enzyme_
Fv_160_9.g5690	208	ID=Fv_160_9.g5690;Description=hypothetical protein H9Q70_000710 [Fusarium xylarioides];Gene=FOC1_g10011954;Ontology_term=ATP binding,transferase
Fv_160_9.g5691	346	ID=Fv_160_9.g5691;Description=hypothetical protein FVEG_03802 [Fusarium verticillioides 7600];Gene=FTJAE_7175;Ontology_term=mRNA splicing, via spliceosome;Ontology_id=GO:0000398
Fv_160_9.g5692	295	ID=Fv_160_9.g5692;Description=hypothetical protein FVEG_03803 [Fusarium verticillioides 7600];Gene=FTJAE_7174;Ontology_term=transferase activity,protein-malonyllysine demalonylase activity,protein-succinyllysine desuccinylase activity,metal ion binding,NAD+ binding;Ontology_id=GO:0016740,GO:0036054,GO:0036055,GO:0046872,GO:0070403;Enzyme_code=EC:3.5.1,EC:2;Enzyme_name=
Fv_160_9.g5693	211	ID=Fv_160_9.g5693;Description=uncharacterized protein FTJAE_7172 [Fusarium tjaetaba]
Fv_160_9.g5694	238	ID=Fv_160_9.g5694;Description=hypothetical protein FVEG_03804 [Fusarium verticillioides 7600]

Fv_160_9.g5695	189	ID=Fv_160_9.g5695;Description=hypothetical protein FOXG_05936 [Fusarium oxysporum f. sp. lycopersici 4287];Gene=BFJ68_g5972;Ontology_term=acyltransferase activity, transferring groups other than amino-acyl
Fv_160_9.g5696	375	ID=Fv_160_9.g5696;Description=23S rRNA (-2'-O)-methyltransferase [Fusarium verticillioides 7600];Gene=FMUND_12525;Ontology_term=extracellular space,Golgi apparatus,cell projection,perikaryon,hormone activity,hormone receptor binding,tRNA 2'-O-methyltransferase activity,tRNA (cytidine(32)-2'-O)-methyltransferase activity,wobble position ribose methylation,cytoplasmic translation,negative regulation of systemic arterial blood pressure,cGMP biosynthetic process,receptor guanylyl cyclase signaling pathway,neuropeptide signaling pathway,female pregnancy,protein transport,cGMP-mediated
Fv_160_9.g5697	1719	ID=Fv_160_9.g5697;Description=hypothetical protein FVER53263_03807 [Fusarium verticillioides];Gene=FNYG_00915;Ontology_term=Golgi apparatus,protein transport;Ontology_id=GO:0005794,GO:0015031
Fv_160_9.g5698	323	ID=Fv_160_9.g5698;Description=ribose-phosphate pyrophosphokinase 3 [Fusarium oxysporum f. sp. lycopersici 4287];Gene=FHETE_5090;Ontology_term=ribose phosphate diphosphokinase complex,magnesium ion binding,ribose phosphate diphosphokinase activity,ATP binding,kinase activity,5-phosphoribose 1-diphosphate biosynthetic process,ribonucleoside monophosphate biosynthetic process,nucleotide biosynthetic process,phosphorylation,fungal-type cell wall organization;Ontology_id=GO:0002189,GO:0000287,GO:0004749,GO:0005524,GO:0016301,GO:0006015,GO:0009156,GO:0009165,
Fv_160_9.g5699	620	ID=Fv_160_9.g5699;Description=hypothetical protein FVEG_03809 [Fusarium verticillioides
Fv_160_9.g5700	558	ID=Fv_160_9.g5700;Description=hypothetical protein FVEG_03811 [Fusarium verticillioides 7600];Gene=FNAPI_9007;Ontology_term=membrane,transmembrane transport;Ontology_id=GO:0016020,GO:0055085
Fv_160_9.g5701	848	ID=Fv_160_9.g5701;Description=hypothetical protein FVER14953_03812 [Fusarium verticillioides]
Fv_160_9.g5702	140	ID=Fv_160_9.g5702;Description=ubiquitin-conjugating enzyme E2 [Fusarium verticillioides 7600]
Fv_160_9.g5703	402	ID=Fv_160_9.g5703;Description=cell division cycle 20-like protein 1, cofactor-APC complex [Fusarium verticillioides 7600];Gene=FPANT_4726;Ontology_term=nucleus,membrane,RNA polymerase II cis-regulatory region sequence-specific DNA binding,DNA-binding transcription factor activity, RNA polymerase II-specific,metal ion binding,negative regulation of transcription by RNA polymerase II,zygotic determination of anterior/posterior axis, embryo,neuroblast fate determination,axon guidance,muscle organ development,heterochromatin formation,regulation of hemocyte proliferation,trunk segmentation,regulation of development, heterochronic,compound eye development,regulation of neurogenesis,cell division,Malpighian tubule bud morphogenesis,regulation of neural precursor cell
Fv_160_9.g5704	348	ID=Fv_160_9.g5704;Description=hypothetical protein J7337_003822 [Fusarium musae]
Fv_160_9.g5705	356	ID=Fv_160_9.g5705;Description=hypothetical protein FVEG_03816 [Fusarium verticillioides

Fv_160_9.g5706	476	ID=Fv_160_9.g5706;Description=hypothetical protein FVER14953_03817 [Fusarium verticillioides];Gene=FNYG_00906;Ontology_term=oxygen binding,zinc ion binding,oxygen transport,regulation of DNA repair,regulation of DNA-templated transcription,sequence-specific DNA binding,oxygen carrier activity,heme binding,hemoglobin complex,nucleus,carbohydrate metabolic process,DNA-binding transcription factor activity,poly(ADP-ribose) glycohydrolase activity;Ontology_id=GO:0019825,GO:0008270,GO:0015671,GO:0006282,GO:0006355,GO:0043565,GO:0005344,GO:0020037,GO:0
Fv_160_9.g5707	328	ID=Fv_160_9.g5707;Description=pectate lyase [Fusarium verticillioides 7600];Gene=FMUND_12514;Ontology_term=extracellular region,pectate lyase activity,polysaccharide catabolic
Fv_160_9.g5708	362	ID=Fv_160_9.g5708;Description=hypothetical protein FVEG_03819 [Fusarium verticillioides 7600];Gene=FNYG_00904;Ontology_term=nucleus,DNA-binding transcription repressor activity, RNA polymerase II-specific,metalloendopeptidase activity,nuclear receptor activity,zinc ion binding,estrogen response element binding,DNA-binding transcription factor binding,negative regulation of transcription by RNA polymerase II,proteolysis,regulation of mitotic nuclear division,zygotic determination of anterior/posterior axis, embryo,epithelial cell migration, open tracheal system,intracellular receptor signaling pathway,trunk
Fv_160_9.g5709	427	ID=Fv_160_9.g5709;Description=hypothetical protein FVEG_03820 [Fusarium verticillioides 7600];Gene=FocTR4_00007416;Ontology_term=membrane,hydrolase activity, hydrolyzing O-glycosyl compounds,carbohydrate metabolic process,cell wall
Fv_160_9.g5710	546	ID=Fv_160_9.g5710;Description=hypothetical protein FVEG_15341 [Fusarium verticillioides 7600];Gene=968;Ontology_term=extracellular region,retinol binding,identical protein
Fv_160_9.g5711	791	ID=Fv_160_9.g5711;Description=hypothetical protein FVER53263_03821 [Fusarium verticillioides];Gene=FCIRC_2469;Ontology_term=membrane,oligopeptide transmembrane transporter activity,protein
Fv_160_9.g5712	485	ID=Fv_160_9.g5712;Description=Cross-pathway control 1 [Fusarium mundagurra];Gene=FTJAE_7153;Ontology_term=DNA-binding transcription factor activity,regulation of DNA-templated transcription;Ontology_id=GO:0003700,GO:0006355
Fv_160_9.g5713	322	ID=Fv_160_9.g5713;Description=hypothetical protein J7337_003831 [Fusarium musae]
Fv_160_9.g5714	365	ID=Fv_160_9.g5714;Description=hypothetical protein FVER14953_03824 [Fusarium verticillioides];Gene=FPHYL_7372;Ontology_term=aspartate kinase activity,homoserine dehydrogenase activity,NADP binding,methionine biosynthetic process,threonine biosynthetic process,isoleucine biosynthetic process;Ontology_id=GO:0004072,GO:0004412,GO:0050661,GO:0009086,GO:0009088,GO:0009097;Enzyme_code=EC:1.1.1.3,EC:2.
Fv_160_9.g5715	107	ID=Fv_160_9.g5715;Description=hypothetical protein FVEG_15342 [Fusarium verticillioides
Fv_160_9.g5716	1518	ID=Fv_160_9.g5716;Description=hypothetical protein FVER53263_03826 [Fusarium verticillioides];Gene=TRM8;Ontology_term=nucleus,membrane,tRNA binding,tRNA (guanine(46)-N7)-methyltransferase activity,phosphatidylinositol binding,tRNA (guanine-N7)-methylation;Ontology_id=GO:0005634,GO:0016020,GO:0000049,GO:0008176,GO:0035091,GO:0106004;Enzyme_code=EC:2.1.1.33;

Fv_160_9.g5717	308	ID=Fv_160_9.g5717;Description=putative methyltransferase-domain-containing protein [Fusarium oxysporum];Gene=FOXG_05959;Ontology_term=nucleus,membrane,tRNA binding,tRNA (guanine(46)-N7)-methyltransferase activity,phosphatidylinositol binding,tRNA (guanine-N7)-methylation;Ontology_id=GO:0005634,GO:0016020,GO:0000049,GO:0008176,GO:0035091,GO:0106004;Enzyme_code=EC:2.1.1.33;
Fv_160_9.g5718	235	ID=Fv_160_9.g5718;Description=hypothetical protein FVER14953_03828 [Fusarium verticillioides];Gene=FGADI_8333;Ontology_term=peroxisomal membrane,peroxisome
Fv_160_9.g5719	342	ID=Fv_160_9.g5719;Description=fructose-1,6-bisphosphatase [Fusarium verticillioides 7600];Gene=FGADI_8332;Ontology_term=fructose 1,6-bisphosphate 1-phosphatase activity,metal ion binding,carbohydrate metabolic process;Ontology_id=GO:0042132,GO:0046872,GO:0005975;Enzyme_code=EC:3.1.3.23,EC:3.1.3.11;Enzyme_name=sugar-
Fv_160_9.g5720	317	ID=Fv_160_9.g5720;Description=hypothetical protein FVEG_03830 [Fusarium verticillioides 7600]
Fv_160_9.g5721	277	ID=Fv_160_9.g5721;Description=hypothetical protein J7337_003840 [Fusarium musae];Gene=FNAPI_9028;Ontology_term=DNA-binding transcription factor activity,regulation of DNA-templated transcription;Ontology_id=GO:0003700,GO:0006355
Fv_160_9.g5722	205	ID=Fv_160_9.g5722;Description=hypothetical protein J7337_003841 [Fusarium musae]
Fv_160_9.g5723	361	ID=Fv_160_9.g5723;Description=hypothetical protein FVEG_03833 [Fusarium verticillioides 7600];Gene=F52700_10190;Ontology_term=CCR4-NOT complex,mRNA catabolic process;Ontology_id=GO:0030014,GO:0006402
Fv_160_9.g5724	1154	ID=Fv_160_9.g5724;Description=hypothetical protein FVER53263_03834 [Fusarium verticillioides];Gene=1856;Ontology_term=nucleus,RNA polymerase II cis-regulatory region sequence-specific DNA binding,DNA-binding transcription factor activity, RNA polymerase II-specific,transcription corepressor activity,protein serine/threonine kinase activity,ATP binding,protein serine kinase activity,axon guidance,chemosensory jump behavior,synaptic target recognition,phosphorylation,negative regulation of DNA-templated transcription,positive regulation of transcription by RNA polymerase II,detection of chemical stimulus involved in sensory perception of smell,dendrite
Fv_160_9.g5725	1135	ID=Fv_160_9.g5725;Description=dipeptidyl-peptidase III [Fusarium verticillioides 7600];Gene=FTJAE_7142;Ontology_term=nucleic acid binding,mRNA processing;Ontology_id=GO:0003676,GO:0006397
Fv_160_9.g5726	540	ID=Fv_160_9.g5726;Description=hypothetical protein FVER14953_03837 [Fusarium verticillioides];Gene=FTJAE_7141;Ontology_term=nucleus,membrane,RNA polymerase II cis-regulatory region sequence-specific DNA binding,DNA-binding transcription factor activity, RNA polymerase II-specific,monooxygenase activity,iron ion binding,oxidoreductase activity, acting on paired donors, with incorporation or reduction of molecular oxygen,heme binding,regulation of transcription by RNA polymerase II,compartment pattern specification,neuroblast fate determination,imaginal disc-derived wing vein specification,neuron differentiation,wing disc anterior/posterior pattern formation,defense response to fungus;Ontology_id=GO:0005634,GO:0016020,GO:0000978,GO:0000981,GO:0004497,GO:0005506,GO:0016705,GO:0020037,GO:0
Fv_160_9.g5727	465	ID=Fv_160_9.g5727;Description=hypothetical protein FVER14953_03838 [Fusarium verticillioides]

Fv_160_9.g5728	305	ID=Fv_160_9.g5728;Description=hypothetical protein FVER14953_03839 [Fusarium verticillioides];Gene=F25303_2496;Ontology_term=positive regulation of TORC1 signaling,nuclear pore outer ring,COPII-coated vesicle budding,structural molecule activity,positive regulation of ER to Golgi vesicle-mediated transport,endoplasmic reticulum,GATOR2 complex,nuclear pore localization,positive regulation of DNA-templated transcription,positive regulation of protein exit from endoplasmic reticulum,ubiquitin-dependent ERAD pathway,COPII vesicle coat,mRNA
Fv_160_9.g5729	337	ID=Fv_160_9.g5729;Description=hypothetical protein FVEG_03840 [Fusarium verticillioides 7600];Gene=950;Ontology_term=mitochondrial matrix,pyruvate dehydrogenase complex,pyruvate dehydrogenase (acetyl-transferring) activity,glucose metabolic process,acetyl-CoA biosynthetic process from pyruvate,tricarboxylic acid
Fv_160_9.g5730	740	ID=Fv_160_9.g5730;Description=hypothetical protein FVEG_03841 [Fusarium verticillioides
Fv_160_9.g5731	246	ID=Fv_160_9.g5731;Description=hypothetical protein FVEG_03842 [Fusarium verticillioides 7600];Gene=AU210_002497;Ontology_term=mitochondrial inner membrane,ATP binding,ATP hydrolysis
Fv_160_9.g5732	656	ID=Fv_160_9.g5732;Description=hypothetical protein FVER14953_03843 [Fusarium verticillioides];Gene=BFJ70_g15583;Ontology_term=mitochondrial inner membrane,ATP binding,ATP hydrolysis
Fv_160_9.g5733	456	ID=Fv_160_9.g5733;Description=hypothetical protein FVER14953_03844 [Fusarium verticillioides];Gene=FVEG_03844;Ontology_term=hydroxymethylglutaryl-CoA synthase activity,acetyl-CoA metabolic process,farnesyl diphosphate biosynthetic process, mevalonate
Fv_160_9.g5734	541	ID=Fv_160_9.g5734;Description=delta24-sterol reductase [Fusarium verticillioides 7600];Gene=FVEG_03845;Ontology_term=delta24-sterol reductase activity,FAD
Fv_160_9.g5735	477	ID=Fv_160_9.g5735;Description=hypothetical protein FVER14953_03846 [Fusarium verticillioides];Gene=FPHYL_9150;Ontology_term=hydrolase
Fv_160_9.g5736	369	ID=Fv_160_9.g5736;Description=hypothetical protein FVEG_03847 [Fusarium verticillioides 7600];Gene=FPHYL_9151;Ontology_term=peroxisomal membrane,protein import into peroxisome matrix,
Fv_160_9.g5737	727	ID=Fv_160_9.g5737;Description=kinetochore protein NDC80 [Fusarium verticillioides 7600];Gene=FVEG_03848;Ontology_term=nucleus,Ndc80 complex,cell division,attachment of mitotic spindle microtubules to
Fv_160_9.g5738	414	ID=Fv_160_9.g5738;Description=kinetochore NDC80 [Fusarium tjaetaba];Gene=FMEXI_14094;Ontology_term=Ndc80 complex,zinc ion binding,N-acylphosphatidylethanolamine-specific phospholipase D activity,cell division,attachment of mitotic spindle microtubules to
Fv_160_9.g5739	70	ID=Fv_160_9.g5739;Description=hypothetical protein FVER14953_20498 [Fusarium verticillioides]
Fv_160_9.g5740	386	ID=Fv_160_9.g5740;Description=hypothetical protein FVER14953_03850 [Fusarium verticillioides];Gene=FOMG_11872;Ontology_term=nucleus,damaged DNA binding,double-strand break repair,DNA
Fv_160_9.g5741	431	ID=Fv_160_9.g5741;Description=hypothetical protein FVER14953_03851 [Fusarium verticillioides]
Fv_160_9.g5742	450	ID=Fv_160_9.g5742;Description=hypothetical protein FVER14953_03852 [Fusarium verticillioides];Gene=FNAPI_9049;Ontology_term=L-fuconate dehydratase activity,amino acid catabolic process,carbohydrate

Fv_160_9.g5743	293	ID=Fv_160_9.g5743;Description=hypothetical protein FCOIX_6740 [Fusarium coicis];Gene=FANTH_8760;Ontology_term=endoplasmic reticulum membrane,hydrolase
Fv_160_9.g5744	426	ID=Fv_160_9.g5744;Description=hypothetical protein FVER14953_03854 [Fusarium verticillioides];Gene=BFJ65_g8361;Ontology_term=endoplasmic reticulum membrane,alpha-1,6-mannosyltransferase
Fv_160_9.g5745	122	ID=Fv_160_9.g5745;Description=hypothetical protein FVER14953_20496 [Fusarium verticillioides];Gene=FANTH_8759
Fv_160_9.g5746	1273	ID=Fv_160_9.g5746;Description=hypothetical protein FVER14953_03855 [Fusarium verticillioides];Gene=FVEG_03855;Ontology_term=membrane,sterol transport;Ontology_id=GO:0016020,GO:0015918
Fv_160_9.g5747	531	ID=Fv_160_9.g5747;Description=hypothetical protein FVER53590_03856 [Fusarium
Fv_160_9.g5748	230	ID=Fv_160_9.g5748;Description=hypothetical protein FVER14953_03857 [Fusarium
Fv_160_9.g5749	290	ID=Fv_160_9.g5749;Description=hypothetical protein J7337_003868 [Fusarium musae];Gene=FCIRC_6349;Ontology_term=peroxisomal membrane,respiratory chain complex I,NADH dehydrogenase (ubiquinone) activity,FMN binding,metal ion binding,NAD binding,4 iron, 4 sulfur cluster binding,peroxisome fission,electron transport chain;Ontology_id=GO:0005778,GO:0045271,GO:0008137,GO:0010181,GO:0046872,GO:0051287,GO:0051539,GO:0016559,GO:00
Fv_160_9.g5750	486	ID=Fv_160_9.g5750;Description=NADH2 dehydrogenase (ubiquinone) flavo 1 precursor [Fusarium napiforme];Gene=FDENT_33;Ontology_term=mitochondrial inner membrane,peroxisomal membrane,respiratory chain complex I,NADH dehydrogenase (ubiquinone) activity,FMN binding,metal ion binding,NAD binding,4 iron, 4 sulfur cluster binding,peroxisome fission,electron transport chain;Ontology_id=GO:0005743,GO:0005778,GO:0045271,GO:0008137,GO:0010181,GO:0046872,GO:0051287,GO:0051539,GO:00
Fv_160_9.g5751	194	ID=Fv_160_9.g5751;Description=dynactin 6 [Fusarium verticillioides 7600];Gene=BFJ65_g9099;Ontology_term=cytoplasm,dynactin complex,mannose-1-phosphate guanylyltransferase (GTP) activity,acyltransferase activity;Ontology_id=GO:0005737,GO:0005869,GO:0004475,GO:0016746;Enzyme_code=EC:2.3,EC:2.7.7.13;Enzyme_name=Acyltran
Fv_160_9.g5752	278	ID=Fv_160_9.g5752;Description=hypothetical protein FVEG_03861 [Fusarium verticillioides
Fv_160_9.g5753	808	ID=Fv_160_9.g5753;Description=hypothetical protein FVEG_03862 [Fusarium verticillioides 7600];Gene=FNAPI_9534;Ontology_term=membrane,transmembrane transporter activity,transmembrane
Fv_160_9.g5754	495	ID=Fv_160_9.g5754;Description=hypothetical protein FVER14953_03863 [Fusarium verticillioides];Gene=BFJ65_g9096;Ontology_term=nucleus,mitochondrion,alcohol dehydrogenase (NAD+) activity,zinc ion binding,hydroxyacid-oxoacid transhydrogenase activity,negative regulation of growth,cellular response to zinc ion;Ontology_id=GO:0005634,GO:0005739,GO:0004022,GO:0008270,GO:0047988,GO:0045926,GO:0071294;Enzyme_code=EC:1.1.
Fv_160_9.g5755	1546	ID=Fv_160_9.g5755;Description=hypothetical protein FVEG_03864 [Fusarium verticillioides 7600];Gene=HZS61_013576;Ontology_term=exocyst,exocytosis;Ontology_id=GO:0000145,GO:0006887
Fv_160_9.g5756	918	ID=Fv_160_9.g5756;Description=hypothetical protein FVEG_03865 [Fusarium verticillioides 7600]
Fv_160_9.g5757	1866	ID=Fv_160_9.g5757;Description=hypothetical protein FVER53590_03867 [Fusarium verticillioides];Gene=FVER53263_03867;Ontology_term=ligase activity,ubiquitin protein ligase activity,ubiquitin-dependent protein

Fv_160_9.g5758	449	ID=Fv_160_9.g5758;Description=2-oxoisovalerate dehydrogenase E1 component, alpha subunit [Fusarium verticillioides 7600];Gene=FVEG_03868;Ontology_term=3-methyl-2-oxobutanoate dehydrogenase (2-methylpropanoyl-transferring) activity;Ontology_id=GO:0003863;Enzyme_code=EC:1.2.4.4;Enzyme_name=3-methyl-2-oxobutanoate dehydrogenase (2-
Fv_160_9.g5759	307	ID=Fv_160_9.g5759;Description=hypothetical protein FVER14953_03869 [Fusarium verticillioides];Gene=COQ5;Ontology_term=mitochondrial matrix,extrinsic component of mitochondrial inner membrane,2-octaprenyl-6-methoxy-1,4-benzoquinone methylase activity,2-hexaprenyl-6-methoxy-1,4-benzoquinone methyltransferase activity,ubiquinone biosynthetic process,aerobic respiration,methylation;Ontology_id=GO:0005759,GO:0031314,GO:0043333,GO:0043334,GO:0006744,GO:0009060,GO:0032259;E
Fv_160_9.g5760	680	ID=Fv_160_9.g5760;Description=hypothetical protein FVER14953_03870 [Fusarium verticillioides]
Fv_160_9.g5761	897	ID=Fv_160_9.g5761;Description=hypothetical protein FVER14953_03871 [Fusarium verticillioides]
Fv_160_9.g5762	102	ID=Fv_160_9.g5762;Description=hypothetical protein FVEG_03872 [Fusarium verticillioides 7600]
Fv_160_9.g5763	290	ID=Fv_160_9.g5763;Description=NADH-ubiquinone oxidoreductase 30.4 kDa subunit, mitochondrial [Fusarium verticillioides 7600];Gene=Forpe1208_v005845;Ontology_term=NADH dehydrogenase (ubiquinone) activity,electron transport chain;Ontology_id=GO:0008137,GO:0022900;Enzyme_code=EC:7.1.1.2,EC:1.6.5.11,EC:1.6.5.2;Enzyme_name=NADH:ubiquinone
Fv_160_9.g5764	1549	ID=Fv_160_9.g5764;Description=CCR4-NOT transcription complex subunit 4 [Fusarium verticillioides 7600];Gene=916;Ontology_term=CCR4-NOT complex,RNA binding,ubiquitin-protein transferase
Fv_160_9.g5765	740	ID=Fv_160_9.g5765;Description=hypothetical protein FVEG_03876 [Fusarium verticillioides 7600];Gene=FPCIR_8251;Ontology_term=membrane,potassium channel activity,potassium ion transmembrane
Fv_160_9.g5766	361	ID=Fv_160_9.g5766;Description=hypothetical protein J7337_003885 [Fusarium musae]
Fv_160_9.g5767	194	ID=Fv_160_9.g5767;Description=hypothetical protein FVER14953_03878 [Fusarium verticillioides]
Fv_160_9.g5768	446	ID=Fv_160_9.g5768;Description=hypothetical protein FVER14953_03879 [Fusarium verticillioides];Gene=1814;Ontology_term=protein phosphatase type 2A complex,chromatin,nucleus,cytoplasm,peptidyl-prolyl cis-trans isomerase activity,phosphatase activator activity,protein phosphatase regulator activity,G1/S transition of mitotic cell cycle,DNA repair,regulation of transcription by RNA polymerase II,autophagy,mitotic spindle organization;Ontology_id=GO:0000159,GO:0000785,GO:0005634,GO:0005737,GO:0003755,GO:0019211,GO:0019888,GO:0000082,
Fv_160_9.g5769	815	ID=Fv_160_9.g5769;Description=hypothetical protein FVEG_03880 [Fusarium verticillioides
Fv_160_9.g5770	1241	ID=Fv_160_9.g5770;Description=hypothetical protein FVER14953_03881 [Fusarium verticillioides]
Fv_160_9.g5771	804	ID=Fv_160_9.g5771;Description=hypothetical protein FVEG_15353 [Fusarium verticillioides 7600];Gene=FPANT_11745;Ontology_term=hydrolase activity, hydrolyzing O-glycosyl compounds,carbohydrate metabolic

Fv_160_9.g5772	494	ID=Fv_160_9.g5772;Description=hypothetical protein FVEG_03883 [Fusarium verticillioides 7600];Gene=FPANT_11746;Ontology_term=polytene chromosome,activator ecdysone receptor complex,dendrite,RNA polymerase II cis-regulatory region sequence-specific DNA binding,core promoter sequence-specific DNA binding,DNA-binding transcription activator activity, RNA polymerase II-specific,nuclear steroid receptor activity,nuclear receptor activity,signaling receptor binding,zinc ion binding,ecdysone binding,protein heterodimerization activity,ecdysone biosynthetic process,border follicle cell migration,metamorphosis,mushroom body development,neuron remodeling,intracellular receptor signaling pathway,pupariation,regulation of development, heterochronic,response to starvation,steroid hormone mediated signaling pathway,negative regulation of cell differentiation,negative regulation of DNA-templated transcription,positive regulation of transcription by RNA polymerase II,regulation of organ growth,dendrite morphogenesis,cellular response to ecdysone;Ontology_id=GO:0005700,GO:0008232,GO:0030425,GO:0000978,GO:0001046,GO:0001228,GO:0003707,GO:0004879,G
Fv_160_9.g5773	354	ID=Fv_160_9.g5773;Description=hypothetical protein FVER53590_03884 [Fusarium
Fv_160_9.g5774	429	ID=Fv_160_9.g5774;Description=hypothetical protein FVEG_03885 [Fusarium verticillioides 7600];Gene=Hrb87F;Ontology_term=heterochromatin,polytene chromosome puff,cytoplasm,membrane,omega speckle,ribonucleoprotein complex,mRNA 3'-UTR binding,poly(G) binding,sequence-specific DNA binding,regulation of alternative mRNA splicing, via spliceosome,compound eye morphogenesis,female gonad development,response to heat,response to starvation,positive regulation of mRNA splicing, via
Fv_160_9.g5775	281	ID=Fv_160_9.g5775;Description=hypothetical protein J7337_003895 [Fusarium musae];Gene=905
Fv_160_9.g5776	748	ID=Fv_160_9.g5776;Description=hypothetical protein FVEG_03887 [Fusarium verticillioides
Fv_160_9.g5777	109	ID=Fv_160_9.g5777;Description=hypothetical protein FOXG_06021 [Fusarium oxysporum f. sp. lycopersici
Fv_160_9.g5778	418	ID=Fv_160_9.g5778;Description=hypothetical protein FVER53590_03889 [Fusarium verticillioides];Gene=FVEG_03889;Ontology_term=copper ion binding,oxidoreductase
Fv_160_9.g5779	437	ID=Fv_160_9.g5779;Description=hypothetical protein FVER14953_03890 [Fusarium verticillioides];Gene=BFJ68_g7852;Ontology_term=transferase activity,NAD-dependent histone deacetylase activity,metal ion binding,NAD+ binding,chromatin
Fv_160_9.g5780	146	ID=Fv_160_9.g5780;Description=Negative cofactor 2 complex subunit beta [Fusarium agapanthi];Gene=FPHYL_4480;Ontology_term=core promoter sequence-specific DNA binding,transcription coactivator activity,protein heterodimerization activity,negative regulation of transcription by RNA polymerase III,transcription corepressor activity,negative cofactor 2 complex,general transcription initiation factor activity,negative regulation of RNA polymerase II transcription preinitiation complex assembly,chromatin binding,TBP-class protein binding,positive regulation of transcription by RNA
Fv_160_9.g5781	250	ID=Fv_160_9.g5781;Description=hypothetical protein FVEG_03892 [Fusarium verticillioides 7600]
Fv_160_9.g5782	405	ID=Fv_160_9.g5782;Description=hypothetical protein FVEG_03893 [Fusarium verticillioides 7600];Gene=FOYG_04507;Ontology_term=oxidoreductase
Fv_160_9.g5783	106	ID=Fv_160_9.g5783;Description=hypothetical protein FPCIR_2423 [Fusarium pseudocircinatum]

Fv_160_9.g5784	342	ID=Fv_160_9.g5784;Description=homocysteine S-methyltransferase [Fusarium verticillioides 7600];Gene=MHT1;Ontology_term=extracellular space,cytokine activity,type II interferon receptor binding,methyltransferase activity,metal ion binding,microglial cell activation,macrophage activation involved in immune response,positive regulation of autophagy,positive regulation of epithelial cell migration,macrophage differentiation,positive regulation of protein-containing complex assembly,methylation,negative regulation of interleukin-17 production,positive regulation of chemokine production,positive regulation of interleukin-12 production,positive regulation of interleukin-23 production,positive regulation of interleukin-6 production,positive regulation of CD4-positive, CD25-positive, alpha-beta regulatory T cell differentiation involved in immune response,positive regulation of peptidyl-serine phosphorylation of STAT protein,positive regulation of smooth muscle cell apoptotic process,type III interferon-mediated signaling pathway,positive regulation of protein import into nucleus,positive regulation of tyrosine phosphorylation of STAT protein,positive regulation of nitric oxide biosynthetic process,positive regulation of osteoclast differentiation,negative regulation of DNA-templated transcription,astrocyte activation,negative regulation of smooth muscle cell proliferation,positive regulation of inflammatory response,positive regulation of neurogenesis,regulation of insulin secretion,positive regulation of membrane protein ectodomain proteolysis,defense response to virus,positive regulation of killing of cells of another organism,type II interferon-mediated signaling pathway,positive regulation of fructose 1,6-bisphosphate metabolic process,positive regulation of vitamin D biosynthetic process,positive regulation of protein deacetylation,extrinsic apoptotic signaling pathway,positive regulation of amyloid-beta formation,positive regulation of tumor necrosis factor (ligand) superfamily member 11
Fv_160_9.g5785	174	ID=Fv_160_9.g5785;Description=hypothetical protein FVEG_03895 [Fusarium verticillioides 7600]
Fv_160_9.g5786	125	ID=Fv_160_9.g5786;Description=hypothetical protein FVEG_15358 [Fusarium verticillioides 7600];Gene=FMEXI_12297;Ontology_term=hydrolase
Fv_160_9.g5787	164	ID=Fv_160_9.g5787;Description=hypothetical protein FVEG_15359 [Fusarium verticillioides 7600];Gene=FGLOB1_14196;Ontology_term=nucleus,ATP binding,hydrolase activity,ATP-dependent chromatin remodeler activity,chromatin
Fv_160_9.g5788	142	ID=Fv_160_9.g5788;Description=hypothetical protein FVEG_15360 [Fusarium verticillioides
Fv_160_9.g5789	623	ID=Fv_160_9.g5789;Description=retrograde regulation protein 2 [Fusarium verticillioides 7600];Gene=E(spl)mgamma-HLH;Ontology_term=negative regulation of transcription by RNA polymerase II,nucleus,Notch signaling pathway,RNA polymerase II cis-regulatory region sequence-specific DNA binding,anterior/posterior pattern specification,positive regulation of neuroblast proliferation,wing disc dorsal/ventral pattern formation,protein dimerization activity,neuronal stem cell population maintenance,DNA-binding transcription factor binding,DNA-binding transcription factor activity, RNA polymerase II-
Fv_160_9.g5790	317	ID=Fv_160_9.g5790;Description=hypothetical protein FVER14953_03898 [Fusarium verticillioides];Gene=FNYG_00819;Ontology_term=S-adenosylmethionine-dependent methyltransferase activity,hydrolase activity,methylation;Ontology_id=GO:0008757,GO:0016787,GO:0032259;Enzyme_code=EC:3,EC:2.1.1;Enzyme_name=Hydrolases,Tr
Fv_160_9.g5791	221	ID=Fv_160_9.g5791;Description=hypothetical protein FVEG_15361 [Fusarium verticillioides 7600];Gene=FNYG_00818;Ontology_term=S-adenosylmethionine-dependent methyltransferase

Fv_160_9.g5792	211	ID=Fv_160_9.g5792;Description=hypothetical protein FVEG_03900 [Fusarium verticillioides
Fv_160_9.g5793	351	ID=Fv_160_9.g5793;Description=hypothetical protein FVER14953_03901 [Fusarium verticillioides];Gene=E(spl)mbeta-HLH;Ontology_term=negative regulation of transcription by RNA polymerase II,nucleus,transmembrane transport,Notch signaling pathway,transmembrane transporter activity,RNA polymerase II cis-regulatory region sequence-specific DNA binding,membrane,anterior/posterior pattern specification,positive regulation of neuroblast proliferation,protein dimerization
Fv_160_9.g5794	452	ID=Fv_160_9.g5794;Description=hypothetical protein FVEG_03902 [Fusarium verticillioides 7600];Gene=FNYG_00816;Ontology_term=membrane,transmembrane transporter activity,transmembrane
Fv_160_9.g5795	233	ID=Fv_160_9.g5795;Description=hypothetical protein FVEG_03903 [Fusarium verticillioides 7600]
Fv_160_9.g5796	367	ID=Fv_160_9.g5796;Description=hypothetical protein FVEG_03904 [Fusarium verticillioides 7600];Gene=FPANT_11768;Ontology_term=monooxygenase activity,iron ion binding,oxidoreductase activity, acting on paired donors, with incorporation or reduction of molecular oxygen,heme binding;Ontology_id=GO:0004497,GO:0005506,GO:0016705,GO:0020037;Enzyme_code=EC:1.14;Enzyme_name=Acting on paired
Fv_160_9.g5797	413	ID=Fv_160_9.g5797;Description=pectate lyase C [Fusarium verticillioides 7600];Gene=FOTG_03779;Ontology_term=lyase activity;Ontology_id=GO:0016829;Enzyme_code=EC:4;Enzyme_name=Lyases
Fv_160_9.g5798	245	ID=Fv_160_9.g5798;Description=hypothetical protein FVER14953_03906 [Fusarium verticillioides];Gene=FOTG_03778;Ontology_term=hydrolase activity, hydrolyzing O-glycosyl compounds,carbohydrate metabolic
Fv_160_9.g5799	549	ID=Fv_160_9.g5799;Description=hypothetical protein FVEG_03907 [Fusarium verticillioides 7600];Gene=FMEXI_12290;Ontology_term=nucleosome,extracellular region,polytene chromosome,membrane,RCAF complex,transmembrane transporter activity,peptidase inhibitor activity,structural constituent of chromatin,nucleosomal DNA binding,protein heterodimerization activity,nucleosome assembly,transmembrane
Fv_160_9.g5800	449	ID=Fv_160_9.g5800;Description=hypothetical protein FVER14953_03908 [Fusarium verticillioides];Gene=FTJAE_12015;Ontology_term=hydrolase activity, hydrolyzing O-glycosyl compounds,carbohydrate metabolic
Fv_160_9.g5801	221	ID=Fv_160_9.g5801;Description=hypothetical protein FVER53263_03909 [Fusarium verticillioides]
Fv_160_9.g5802	389	ID=Fv_160_9.g5802;Description=hypothetical protein FVER14953_03910 [Fusarium verticillioides];Gene=His2A;Ontology_term=nucleosome,nucleus,polytene chromosome band,DNA binding,structural constituent of chromatin,protein heterodimerization activity,nucleosome assembly,larval somatic muscle
Fv_160_9.g5803	400	ID=Fv_160_9.g5803;Description=hypothetical protein FVEG_03911 [Fusarium verticillioides 7600];Gene=FVEG_03911;Ontology_term=cysteine-type endopeptidase activity,proteolysis,apoptotic
Fv_160_9.g5804	288	ID=Fv_160_9.g5804;Description=hypothetical protein FVEG_03912 [Fusarium verticillioides 7600];Gene=1779;Ontology_term=nucleosome,nucleus,double-stranded DNA binding,protein binding,structural constituent of chromatin,nucleosomal DNA binding,nucleosome assembly,chromosome condensation,heterochromatin formation,negative
Fv_160_9.g5805	396	ID=Fv_160_9.g5805;Description=hypothetical protein FVER14953_03913 [Fusarium verticillioides];Gene=FMUND_1008;Ontology_term=lyase

Fv_160_9.g5806	386	ID=Fv_160_9.g5806;Description=hypothetical protein FVER14953_03914 [Fusarium
Fv_160_9.g5807	1003	ID=Fv_160_9.g5807;Description=hypothetical protein FVEG_03915 [Fusarium verticillioides 7600];Gene=FocTR4_00007313;Ontology_term=nucleus,DNA binding,zinc ion binding,DNA-templated
Fv_160_9.g5808	739	ID=Fv_160_9.g5808;Description=hypothetical protein FVER53590_03915 [Fusarium verticillioides];Gene=Focb16_v011857;Ontology_term=nucleus,DNA binding,zinc ion binding,DNA-binding transcription factor activity, RNA polymerase II-specific,regulation of transcription by RNA polymerase
Fv_160_9.g5809	311	ID=Fv_160_9.g5809;Description=hypothetical protein FVER53263_03917 [Fusarium verticillioides]
Fv_160_9.g5810	519	ID=Fv_160_9.g5810;Description=aromatic-L-amino-acid decarboxylase [Fusarium verticillioides 7600];Gene=LW93_14646;Ontology_term=FMN binding,fumarate reductase (NADH)
Fv_160_9.g5811	281	ID=Fv_160_9.g5811;Description=hypothetical protein FVER53263_03920 [Fusarium verticillioides];Gene=FDENT_702;Ontology_term=membrane,transmembrane transporter activity,transmembrane
Fv_160_9.g5812	486	ID=Fv_160_9.g5812;Description=hypothetical protein FVEG_03921 [Fusarium verticillioides 7600];Gene=FNAPI_6863;Ontology_term=troponin complex,membrane,ceramide glucosyltransferase activity,dihydroceramide glucosyltransferase activity,sphingolipid metabolic
Fv_160_9.g5813	507	ID=Fv_160_9.g5813;Description=hypothetical protein FVEG_03922 [Fusarium verticillioides 7600];Gene=FVEG_03922;Ontology_term=membrane,transmembrane transporter activity,transmembrane
Fv_160_9.g5814	274	ID=Fv_160_9.g5814;Description=hypothetical protein FVER53263_03923 [Fusarium verticillioides]
Fv_160_9.g5815	794	ID=Fv_160_9.g5815;Description=hypothetical protein FVER14953_03924 [Fusarium verticillioides];Gene=FSUBG_13057;Ontology_term=Mre11 complex,double-strand break repair,mitotic G2 DNA damage checkpoint
Fv_160_9.g5816	382	ID=Fv_160_9.g5816;Description=hypothetical protein FVEG_03925 [Fusarium verticillioides 7600];Gene=map
Fv_160_9.g5817	742	ID=Fv_160_9.g5817;Description=hypothetical protein J7337_003935 [Fusarium musae]
Fv_160_9.g5818	524	ID=Fv_160_9.g5818;Description=hypothetical protein FVEG_03927 [Fusarium verticillioides 7600];Gene=UOX;Ontology_term=mitochondrion,peroxisome,cytosol,urate oxidase activity,purine nucleobase metabolic process,protein import into peroxisome matrix,urate catabolic process,protein insertion into mitochondrial outer membrane;Ontology_id=GO:0005739,GO:0005777,GO:0005829,GO:0004846,GO:0006144,GO:0016558,GO:0019628,GO:0045040;E
Fv_160_10.g5819	2448	ID=Fv_160_10.g5819;Description=hypothetical protein FVER53590_14171 [Fusarium verticillioides]
Fv_160_10.g5820	988	ID=Fv_160_10.g5820;Description=hypothetical protein DER44DRAFT_842020 [Fusarium oxysporum];Gene=HZS61_017557;Ontology_term=nucleus,nucleic acid binding,5'-3' RNA exonuclease activity,zinc ion binding,DNA-templated transcription termination,mRNA processing;Ontology_id=GO:0005634,GO:0003676,GO:0004534,GO:0008270,GO:0006353,GO:0006397;Enzyme_code=EC:3.1.13,EC:
Fv_160_10.g5821	1069	ID=Fv_160_10.g5821;Description=hypothetical protein J7337_009783 [Fusarium musae];Gene=FPCIR_7280;Ontology_term=Golgi membrane,intracellular protein transport,vesicle fusion with Golgi apparatus;Ontology_id=GO:0000139,GO:0006886,GO:0048280

Fv_160_10.g5822	519	ID=Fv_160_10.g5822;Description=protein arginine N-methyltransferase 3 [Fusarium verticillioides 7600];Gene=FNYG_13165;Ontology_term=Golgi membrane,extracellular region,lysosome,ribosome,ribonucleoprotein complex,structural constituent of ribosome,protein-arginine N-methyltransferase activity,1,6-alpha-L-fucosidase activity,fucose metabolic process,translation,intracellular protein transport,glycoside catabolic process,methylation,vesicle fusion with Golgi apparatus,response to
Fv_160_10.g5823	698	ID=Fv_160_10.g5823;Description=hypothetical protein FVER53590_14166 [Fusarium verticillioides];Gene=FTJAE_13435;Ontology_term=membrane,oligopeptide transmembrane transporter activity,oligopeptide
Fv_160_10.g5824	2502	ID=Fv_160_10.g5824;Description=hypothetical protein FVER53263_11378 [Fusarium verticillioides];Gene=FSUBG_12738;Ontology_term=tRNA binding,aminoacyl-tRNA editing activity,isoleucine-tRNA ligase activity,ATP binding,isoleucyl-tRNA aminoacylation,aminoacyl-tRNA metabolism involved in translational fidelity;Ontology_id=GO:0000049,GO:0002161,GO:0004822,GO:0005524,GO:0006428,GO:0106074;Enzyme_code=EC:6.1.1.5,EC:3.1
Fv_160_10.g5825	1071	ID=Fv_160_10.g5825;Description=RAV1 [Fusarium napiforme];Gene=FOTG_06746;Ontology_term=cytosol,tRNA binding,aminoacyl-tRNA editing activity,isoleucine-tRNA ligase activity,ATP binding,isoleucyl-tRNA aminoacylation,aminoacyl-tRNA metabolism involved in translational fidelity;Ontology_id=GO:0005829,GO:0000049,GO:0002161,GO:0004822,GO:0005524,GO:0006428,GO:0106074;Enzyme_code=EC:
Fv_160_10.g5826	369	ID=Fv_160_10.g5826;Description=calcium binding protein 39 [Fusarium oxysporum f. sp. lycopersici 4287];Gene=28953951;Ontology_term=nucleus,cytoplasm,cell division site,non-growing cell tip,new mitotic spindle pole body,protein localization to medial cortex,regulation of establishment or maintenance of bipolar cell polarity regulating cell
Fv_160_10.g5827	500	ID=Fv_160_10.g5827;Description=hypothetical protein FVEG_11384 [Fusarium verticillioides 7600];Gene=abpA;Ontology_term=contractile vacuole,proteasome complex,cytosol,actin filament,cell junction,cortical actin cytoskeleton,extracellular matrix,pseudopodium,cell leading edge,phagocytic vesicle,macropinocytic cup,protein serine/threonine kinase activity,structural constituent of cytoskeleton,calcium ion binding,ATP binding,protein-macromolecule adaptor activity,actin filament binding,phagocytosis,hyperosmotic response,cellular response to starvation,phosphorylation,sorocarp development,cell motility,actin filament bundle assembly,actin crosslink formation;Ontology_id=GO:0000331,GO:0000502,GO:0005829,GO:0005884,GO:0030054,GO:0030864,GO:0031012,GO:0031143,G
Fv_160_10.g5828	808	ID=Fv_160_10.g5828;Description=AGC/PDK1 protein kinase [Fusarium verticillioides 7600];Gene=FOTG_06749;Ontology_term=protein serine/threonine kinase activity,ATP
Fv_160_10.g5829	329	ID=Fv_160_10.g5829;Description=hypothetical protein FVER14953_11386 [Fusarium verticillioides]
Fv_160_10.g5830	282	ID=Fv_160_10.g5830;Description=hypothetical protein FVEG_11387 [Fusarium verticillioides 7600]
Fv_160_10.g5831	263	ID=Fv_160_10.g5831;Description=syntaxin 8 [Fusarium verticillioides

Fv_160_10.g5832	758	ID=Fv_160_10.g5832;Description=hypothetical protein FVEG_11389 [Fusarium verticillioides 7600];Gene=FFC1_14809;Ontology_term=nucleosome,phagocytic cup,nucleus,actin filament,cell-cell junction,cortical actin cytoskeleton,pseudopodium,early phagosome,phagolysosome,cell pole,intranuclear rod,leading edge of lamellipodium,macropinocytic cup,DNA binding,structural constituent of cytoskeleton,ATP binding,anaphase-promoting complex binding,hydrolase activity,myosin binding,structural constituent of chromatin,protein heterodimerization activity,ubiquitin-protein transferase activator activity,mitotic cytokinesis,cell morphogenesis,plasma membrane repair,chromatin organization,phagocytosis,chemotaxis,hyperosmotic response,cytoskeleton organization,phototaxis,response to cAMP;Ontology_id=GO:0000786,GO:0001891,GO:0005634,GO:0005884,GO:0005911,GO:0030864,GO:0031143,GO:0032009,GO:00
Fv_160_10.g5833	363	ID=Fv_160_10.g5833;Description=hypothetical protein FVEG_11390 [Fusarium verticillioides 7600];Gene=FVEG_11390;Ontology_term=extracellular region,chitinase activity,chitin binding,polysaccharide catabolic process,chitin catabolic
Fv_160_10.g5834	402	ID=Fv_160_10.g5834;Description=geranylgeranyl transferase type-2 subunit beta [Fusarium verticillioides 7600];Gene=FTJAE_13588;Ontology_term=Rab-protein geranylgeranyltransferase complex,Rab geranylgeranyltransferase activity;Ontology_id=GO:0005968,GO:0004663;Enzyme_code=EC:2.5.1.60;Enzyme_name=protein geranylgeranyltransferase type II
Fv_160_10.g5835	1370	ID=Fv_160_10.g5835;Description=hypothetical protein FVER53590_11392 [Fusarium verticillioides];Gene=FVEG_11392;Ontology_term=nucleus,DNA binding;Ontology_id=GO:0005634,GO:0003677
Fv_160_10.g5836	399	ID=Fv_160_10.g5836;Description=4-hydroxybenzoate hexaprenyltransferase [Fusarium verticillioides 7600];Gene=FSUBG_11896;Ontology_term=mitochondrial inner membrane,4-hydroxybenzoate decaprenyltransferase activity,4-hydroxybenzoate octaprenyltransferase activity,4-hydroxybenzoate nonaprenyltransferase activity,ubiquinone biosynthetic process,terpenoid biosynthetic
Fv_160_10.g5837	145	ID=Fv_160_10.g5837;Description=hypothetical protein FVER53263_11394 [Fusarium verticillioides];Gene=BFJ68_g15039;Ontology_term=small ribosomal subunit,structural constituent of
Fv_160_10.g5838	269	ID=Fv_160_10.g5838;Description=hypothetical protein FVEG_11395 [Fusarium verticillioides 7600]
Fv_160_10.g5839	96	ID=Fv_160_10.g5839;Description=hypothetical protein FVEG_11396 [Fusarium verticillioides 7600]
Fv_160_10.g5840	893	ID=Fv_160_10.g5840;Description=hypothetical protein FVER14953_11397 [Fusarium verticillioides];Gene=FDENT_11317;Ontology_term=cytoplasm,cytoskeleton-dependent intracellular
Fv_160_10.g5841	256	ID=Fv_160_10.g5841;Description=adenylate kinase 1 [Fusarium verticillioides 7600];Gene=FG10737.1;Ontology_term=mitochondrial intermembrane space,cytosol,adenylate kinase activity,ATP binding,ADP biosynthetic process,DNA replication initiation,phosphorylation,AMP metabolic process,ATP metabolic process,nucleoside monophosphate phosphorylation;Ontology_id=GO:0005758,GO:0005829,GO:0004017,GO:0005524,GO:0006172,GO:0006270,GO:0016310,GO:0046
Fv_160_10.g5842	341	ID=Fv_160_10.g5842;Description=26S proteasome regulatory subunit rpn-8 [Fusarium verticillioides 7600];Gene=FNAPI_2210;Ontology_term=proteasome regulatory particle, lid subcomplex,proteasome storage granule,mitotic spindle midzone,metallopeptidase activity,proteasome-mediated ubiquitin-dependent protein catabolic

Fv_160_10.g5843	408	ID=Fv_160_10.g5843;Description=coproporphyrinogen III oxidase [Fusarium verticillioides 7600];Gene=FNYG_13188;Ontology_term=membrane,coproporphyrinogen oxidase activity,protoporphyrinogen IX biosynthetic
Fv_160_10.g5844	120	ID=Fv_160_10.g5844;Description=autophagy-like protein 8 [Fusarium verticillioides 7600];Gene=NECHADRAFT_100430;Ontology_term=autophagosome membrane,Cvt vesicle membrane,autophagy,protein
Fv_160_10.g5845	329	ID=Fv_160_10.g5845;Description=hypothetical protein FVEG_11402 [Fusarium verticillioides 7600];Gene=FOTG_06768;Ontology_term=S-adenosylmethionine-dependent methyltransferase
Fv_160_10.g5846	732	ID=Fv_160_10.g5846;Description=hypothetical protein FVEG_11403 [Fusarium verticillioides 7600];Gene=FocTR4_00013074;Ontology_term=plasma membrane,mating projection tip;Ontology_id=GO:0005886,GO:0043332
Fv_160_10.g5847	717	ID=Fv_160_10.g5847;Description=tryptophan synthase [Fusarium verticillioides 7600];Gene=FOMG_08408;Ontology_term=tryptophan synthase activity,tryptophan biosynthetic
Fv_160_10.g5848	255	ID=Fv_160_10.g5848;Description=hypothetical protein FPANT_3796 [Fusarium pseudoanthophilum];Gene=Focb16_v014944;Ontology_term=ribosome,ribonucleoprotein complex,structural constituent of
Fv_160_10.g5849	354	ID=Fv_160_10.g5849;Description=hypothetical protein FVER14953_11406 [Fusarium verticillioides]
Fv_160_10.g5850	438	ID=Fv_160_10.g5850;Description=aspartate mitochondrial [Fusarium denticulatum];Gene=FPCIR_7251;Ontology_term=L-aspartate:2-oxoglutarate aminotransferase activity,pyridoxal phosphate binding,amino acid metabolic process,biosynthetic process;Ontology_id=GO:0004069,GO:0030170,GO:0006520,GO:0009058;Enzyme_code=EC:2.6.1.1;Enzyme_name=aspartate
Fv_160_10.g5851	576	ID=Fv_160_10.g5851;Description=hypothetical protein FVER14953_11408 [Fusarium verticillioides];Gene=BFJ65_g11167;Ontology_term=membrane,transmembrane transporter activity,transmembrane
Fv_160_10.g5852	500	ID=Fv_160_10.g5852;Description=hypothetical protein FVER14953_11409 [Fusarium verticillioides]
Fv_160_10.g5853	577	ID=Fv_160_10.g5853;Description=hypothetical protein FVEG_11410 [Fusarium verticillioides 7600]
Fv_160_10.g5854	1197	ID=Fv_160_10.g5854;Description=hypothetical protein FVER53590_11411 [Fusarium verticillioides]
Fv_160_10.g5855	524	ID=Fv_160_10.g5855;Description=hypothetical protein FVER14953_11412 [Fusarium verticillioides];Gene=FNYG_13200;Ontology_term=membrane,phosphatidate phosphatase
Fv_160_10.g5856	416	ID=Fv_160_10.g5856;Description=hypothetical protein FVER14953_11413 [Fusarium verticillioides];Gene=FVER53263_11413;Ontology_term=phosphatidate phosphatase
Fv_160_10.g5857	254	ID=Fv_160_10.g5857;Description=DUF636 domain protein [Fusarium tjaetaba];Gene=FPANT_3805;Ontology_term=carbon-sulfur lyase activity,metal ion binding;Ontology_id=GO:0016846,GO:0046872;Enzyme_code=EC:4.4;Enzyme_name=Carbon-sulfur lyases
Fv_160_10.g5858	280	ID=Fv_160_10.g5858;Description=hypothetical protein FVEG_11415 [Fusarium verticillioides 7600]

Fv_160_10.g5859	135	ID=Fv_160_10.g5859;Description=hypothetical protein FVEG_11417 [Fusarium verticillioides 7600];Gene=AU210_010723;Ontology_term=polytene chromosome,ribosome,activator ecdysone receptor complex,mitochondrial oxoglutarate dehydrogenase complex,membrane,dendrite,RNA polymerase II cis-regulatory region sequence-specific DNA binding,core promoter sequence-specific DNA binding,DNA-binding transcription activator activity, RNA polymerase II-specific,nuclear steroid receptor activity,nuclear receptor activity,signaling receptor binding,zinc ion binding,ecdysone binding,protein heterodimerization activity,2-oxoglutarate metabolic process,ecdysone biosynthetic process,border follicle cell migration,metamorphosis,protein transport,mushroom body development,neuron remodeling,intracellular receptor signaling pathway,pupariation,regulation of development, heterochronic,response to starvation,steroid hormone mediated signaling pathway,negative regulation of cell differentiation,negative regulation of DNA-templated transcription,positive regulation of transcription by RNA polymerase II,regulation of organ growth,dendrite morphogenesis,cellular response to
Fv_160_10.g5860	781	ID=Fv_160_10.g5860;Description=protein arginine N-methyltransferase 5 [Fusarium verticillioides 7600];Gene=FVER53590_11418;Ontology_term=protein-arginine N-methyltransferase activity,kinase activity,phosphorylation,methylation;Ontology_id=GO:0016274,GO:0016301,GO:0016310,GO:0032259;Enzyme_code=EC:2.7,EC:2.1
Fv_160_10.g5861	767	ID=Fv_160_10.g5861;Description=pre-mRNA-splicing factor ATP-dependent RNA helicase prp43 [Fusarium verticillioides 7600];Gene=FRV6_12587;Ontology_term=U2-type spliceosomal complex,nucleolus,small-subunit processome,post-mRNA release spliceosomal complex,nucleic acid binding,RNA helicase activity,ATP binding,hydrolase activity,spliceosomal complex disassembly,maturation of SSU-rRNA from tricistronic rRNA transcript (SSU-rRNA, 5.8S rRNA, LSU-rRNA),maturation of LSU-rRNA from tricistronic rRNA transcript (SSU-rRNA, 5.8S rRNA, LSU-rRNA),maturation of 5.8S rRNA from tricistronic rRNA transcript (SSU-rRNA, 5.8S rRNA, LSU-
Fv_160_10.g5862	113	ID=Fv_160_10.g5862;Description=hypothetical protein FVEG_11420 [Fusarium verticillioides 7600];Gene=SEC6;Ontology_term=exocyst,cellular bud tip,cellular bud neck,SNARE binding,Golgi to plasma membrane transport,vesicle docking involved in exocytosis,protein transport,negative regulation of SNARE complex assembly,exocyst
Fv_160_10.g5863	514	ID=Fv_160_10.g5863;Description=hypothetical protein FVER14953_11421 [Fusarium verticillioides];Gene=FNAPI_11467;Ontology_term=phosphatidate phosphatase
Fv_160_10.g5864	909	ID=Fv_160_10.g5864;Description=hypothetical protein FVEG_11422 [Fusarium verticillioides 7600]

Fv_160_10.g5865	348	ID=Fv_160_10.g5865;Description=hypothetical protein FVER14953_11423 [Fusarium verticillioides];Gene=comA;Ontology_term=Golgi membrane,endosome,centrosome,rough endoplasmic reticulum membrane,endoplasmic reticulum chaperone complex,phagocytic vesicle,protein folding chaperone complex,nucleotide binding,actin monomer binding,insulin receptor binding,structural constituent of cell wall,ATP hydrolysis activity,TPR domain binding,unfolded protein binding,disordered domain specific binding,ATP-dependent protein folding chaperone,positive regulation of neuroblast proliferation,protein folding,centrosome cycle,negative regulation of cell population proliferation,response to heat,pole plasm mRNA localization,defense response to bacterium,regulation of circadian sleep/wake cycle, sleep,response to cycloheximide,protein stabilization,response to cAMP,RISC complex assembly,membrane bending;Ontology_id=GO:0000139,GO:0005768,GO:0005813,GO:0030867,GO:0034663,GO:0045335,GO:0101031,GO:0000166,GO:
Fv_160_10.g5866	595	ID=Fv_160_10.g5866;Description=hypothetical protein J7337_009829 [Fusarium musae];Gene=FPANT_11877;Ontology_term=chromosome, centromeric region,nucleus,DNA
Fv_160_10.g5867	384	ID=Fv_160_10.g5867;Description=hypothetical protein J7337_009830 [Fusarium musae];Gene=capA-1;Ontology_term=nucleus,cytoplasm,cell surface,membrane,SNAP receptor activity,cAMP binding,intracellular protein transport,organelle organization,vesicle-mediated transport,membrane
Fv_160_10.g5868	246	ID=Fv_160_10.g5868;Description=hypothetical protein FVEG_11427 [Fusarium verticillioides 7600];Gene=Focb16_v014966;Ontology_term=cytoplasm,spindle microtubule,DASH complex,attachment of spindle microtubules to
Fv_160_10.g5869	216	ID=Fv_160_10.g5869;Description=hypothetical protein FVER14953_21232 [Fusarium verticillioides];Gene=FVEG_17022;Ontology_term=oxidoreductase activity,isomerase
Fv_160_10.g5870	357	ID=Fv_160_10.g5870;Description=hypothetical protein FVER53590_30345 [Fusarium verticillioides]
Fv_160_10.g5871	452	ID=Fv_160_10.g5871;Description=DNA replication regulator SLD2 [Fusarium verticillioides 7600];Gene=FVER53590_11428;Ontology_term=nucleus,DNA replication initiation,cell
Fv_160_10.g5872	1332	ID=Fv_160_10.g5872;Description=hypothetical protein FVER14953_11429 [Fusarium verticillioides];Gene=FVER53590_11429;Ontology_term=nucleus,DNA binding,chromatin binding,S-adenosylmethionine-dependent methyltransferase
Fv_160_10.g5873	1046	ID=Fv_160_10.g5873;Description=hypothetical protein FVEG_11430 [Fusarium verticillioides 7600];Gene=FDENT_11180;Ontology_term=ATP binding,ATP hydrolysis
Fv_160_10.g5874	471	ID=Fv_160_10.g5874;Description=hypothetical protein FVER14953_11431 [Fusarium verticillioides];Gene=F25303_6103;Ontology_term=centrosome,cytoplasmic dynein complex,cytoplasmic microtubule,cell cortex,early phagosome membrane,phagolysosome membrane,ATPase complex,GTPase activator activity,ATP binding,microtubule binding,minus-end-directed microtubule motor activity,ATP hydrolysis activity,identical protein binding,CTPase activity,dynein intermediate chain binding,dynein light intermediate chain binding,nuclear migration,cytoplasmic microtubule organization,minus-end-directed vesicle transport along microtubule,mitotic spindle assembly,phagosome

Fv_160_10.g5875	421	ID=Fv_160_10.g5875;Description=26S protease regulatory subunit 6B [Fusarium verticillioides 7600];Gene=FNAPI_12779;Ontology_term=chromatin,proteasome regulatory particle, base subcomplex,nuclear periphery,ATP binding,peptidase activity,ATP hydrolysis activity,identical protein binding,proteasome-mediated ubiquitin-dependent protein catabolic process,positive regulation of RNA polymerase II transcription preinitiation complex assembly,proteasome regulatory particle
Fv_160_10.g5876	128	ID=Fv_160_10.g5876;Description=hypothetical protein FVEG_11433 [Fusarium verticillioides 7600]
Fv_160_10.g5877	438	ID=Fv_160_10.g5877;Description=hypothetical protein FVER53590_11434 [Fusarium verticillioides]
Fv_160_10.g5878	612	ID=Fv_160_10.g5878;Description=hypothetical protein FVER14953_11435 [Fusarium verticillioides]
Fv_160_10.g5879	211	ID=Fv_160_10.g5879;Description=hypothetical protein FVER14953_11436 [Fusarium verticillioides];Gene=FVER53263_11436;Ontology_term=mitochondrial respiratory chain complex IV,cytochrome-c oxidase activity,structural constituent of cell wall,response to hypoxia,mitochondrial electron transport, cytochrome c to oxygen,fungal-type cell wall organization,proton transmembrane transport;Ontology_id=GO:0005751,GO:0004129,GO:0005199,GO:0001666,GO:0006123,GO:0031505,GO:1902600;Enzyme_code=E
Fv_160_10.g5880	596	ID=Fv_160_10.g5880;Description=hypothetical protein FVER53590_11437 [Fusarium verticillioides]
Fv_160_10.g5881	131	ID=Fv_160_10.g5881;Description=endoribonuclease L-PSP [Fusarium verticillioides 7600];Gene=cryS;Ontology_term=lysosome,early phagosome,esterosome membrane,carboxylic ester hydrolase activity,mitotic cytokinesis,endocytosis,sorocarp development;Ontology_id=GO:0005764,GO:0032009,GO:0033118,GO:0052689,GO:0000281,GO:0006897,GO:0030587;Enzyme_co
Fv_160_10.g5882	322	ID=Fv_160_10.g5882;Description=hypothetical protein FVEG_11440 [Fusarium verticillioides 7600]
Fv_160_10.g5883	121	ID=Fv_160_10.g5883;Description=hypothetical protein FVER14953_11441 [Fusarium verticillioides]
Fv_160_10.g5884	89	ID=Fv_160_10.g5884;Description=hypothetical protein FVEG_17030 [Fusarium verticillioides 7600]
Fv_160_10.g5885	308	ID=Fv_160_10.g5885;Description=hypothetical protein FVER14953_11442 [Fusarium verticillioides]
Fv_160_10.g5886	356	ID=Fv_160_10.g5886;Description=hypothetical protein FVER14953_11443 [Fusarium verticillioides];Gene=FTJAE_10552;Ontology_term=lyase activity,metal ion
Fv_160_10.g5887	227	ID=Fv_160_10.g5887;Description=hypothetical protein FVER14953_11444 [Fusarium verticillioides];Gene=FTJAE_10551;Ontology_term=zinc ion binding,S-(hydroxymethyl)glutathione synthase activity,formaldehyde
Fv_160_10.g5888	197	ID=Fv_160_10.g5888;Description=Laccase-2 [Fusarium odoratissimum];Gene=FOC4_g10004525;Ontology_term=copper ion binding,oxidoreductase activity;Ontology_id=GO:0005507,GO:0016491;Enzyme_code=EC:1;Enzyme_name=Oxidoreductases
Fv_160_10.g5889	498	ID=Fv_160_10.g5889;Description=hypothetical protein FVER53590_11448 [Fusarium verticillioides];Gene=FNYG_05047;Ontology_term=nucleus,DNA-binding transcription factor activity, RNA polymerase II-specific,zinc
Fv_160_10.g5890	222	ID=Fv_160_10.g5890;Description=hypothetical protein FVEG_11449 [Fusarium verticillioides 7600];Gene=FOZG_15031;Ontology_term=kinetochore,mitotic sister chromatid segregation;Ontology_id=GO:0000776,GO:0000070

Fv_160_10.g5891	337	ID=Fv_160_10.g5891;Description=hypothetical protein FVEG_11450 [Fusarium verticillioides 7600];Gene=FVEG_11450;Ontology_term=prospore membrane,nucleus,cell division site,cell tip,phosphatidylcholine transporter activity,phosphatidylinositol transfer activity,phospholipid transport,ascospore-type prospore-specific spindle pole body
Fv_160_10.g5892	419	ID=Fv_160_10.g5892;Description=hypothetical protein FVEG_11451 [Fusarium verticillioides
Fv_160_10.g5893	886	ID=Fv_160_10.g5893;Description=hypothetical protein FVER14953_11452 [Fusarium verticillioides]
Fv_160_10.g5894	396	ID=Fv_160_10.g5894;Description=saccharopepsin [Fusarium musae];Gene=FNAPI_10952;Ontology_term=nucleus,Z disc,aspartic-type endopeptidase activity,unfolded protein binding,proteolysis,response to heat,regulation of autophagy,regulation of translational initiation by eIF2 alpha phosphorylation,protein refolding,chaperone-mediated protein folding,positive regulation of protein lipidation;Ontology_id=GO:0005634,GO:0030018,GO:0004190,GO:0051082,GO:0006508,GO:0009408,GO:0010506,GO:0010998,GO
Fv_160_10.g5895	839	ID=Fv_160_10.g5895;Description=hypothetical protein FVER53590_11454 [Fusarium verticillioides]
Fv_160_10.g5896	110	ID=Fv_160_10.g5896;Description=hypothetical protein FVEG_11455 [Fusarium verticillioides 7600]
Fv_160_10.g5897	380	ID=Fv_160_10.g5897;Description=hypothetical protein FVEG_11456 [Fusarium verticillioides
Fv_160_10.g5898	280	ID=Fv_160_10.g5898;Description=hypothetical protein FVEG_11457 [Fusarium verticillioides
Fv_160_10.g5899	696	ID=Fv_160_10.g5899;Description=hypothetical protein FVER14953_11458 [Fusarium verticillioides];Gene=D2;Ontology_term=cell cortex,esterosome membrane,carboxylic ester hydrolase activity,vesicle-mediated
Fv_160_10.g5900	395	ID=Fv_160_10.g5900;Description=flap endonuclease 1 [Fusarium verticillioides 7600];Gene=FVER53263_11459;Ontology_term=nucleoplasm,nucleolus,mitochondrion,magnesium ion binding,DNA binding,5'-3' exonuclease activity,5'-flap endonuclease activity,base-excision repair,DNA replication, removal of RNA primer;Ontology_id=GO:0005654,GO:0005730,GO:0005739,GO:0000287,GO:0003677,GO:0008409,GO:0017108,GO:0006284,GO:0
Fv_160_10.g5901	548	ID=Fv_160_10.g5901;Description=acyl-CoA dehydrogenase [Fusarium verticillioides 7600];Gene=CEK26_011206;Ontology_term=cytoplasm,dihydroorotate dehydrogenase activity,flavin adenine dinucleotide binding,'de novo' pyrimidine nucleobase biosynthetic process,'de novo' UMP biosynthetic
Fv_160_10.g5902	523	ID=Fv_160_10.g5902;Description=ATP-dependent RNA helicase DHH1 [Fusarium verticillioides 7600];Gene=28954024;Ontology_term=nucleic acid binding,RNA helicase activity,ATP binding,hydrolase
Fv_160_10.g5903	966	ID=Fv_160_10.g5903;Description=hypothetical protein FVER14953_11463 [Fusarium verticillioides];Gene=FRV6_12633;Ontology_term=phosphatidylinositol kinase activity,phosphorylation,phosphatidylinositol metabolic process;Ontology_id=GO:0052742,GO:0016310,GO:0046488;Enzyme_code=EC:2.7.1;Enzyme_name=Transferring
Fv_160_10.g5904	134	ID=Fv_160_10.g5904;Description=hypothetical protein FVEG_11464 [Fusarium verticillioides 7600];Gene=FNAPI_210;Ontology_term=mitochondrion,respiratory chain complex IV;Ontology_id=GO:0005739,GO:0045277

Fv_160_10.g5905	204	ID=Fv_160_10.g5905;Description=hypothetical protein FVEG_11465 [Fusarium verticillioides 7600];Gene=FOTG_06849;Ontology_term=nucleus,endoplasmic reticulum,Golgi apparatus,membrane,RNA polymerase II cis-regulatory region sequence-specific DNA binding,DNA-binding transcription factor activity, RNA polymerase II-specific,transcription corepressor activity,antiporter activity,phosphoenolpyruvate transmembrane transporter activity,endoplasmic reticulum to Golgi vesicle-mediated transport,axon guidance,chemosensory jump behavior,synaptic target recognition,negative regulation of DNA-templated transcription,positive regulation of transcription by RNA polymerase II,detection of chemical stimulus involved in sensory perception of smell,dendrite guidance,phosphoenolpyruvate transmembrane import into Golgi
Fv_160_10.g5906	962	ID=Fv_160_10.g5906;Description=hypothetical protein FVEG_11466 [Fusarium verticillioides 7600];Gene=FTJAE_10533;Ontology_term=mRNA cleavage and polyadenylation specificity factor complex,RNA binding,mRNA
Fv_160_10.g5907	425	ID=Fv_160_10.g5907;Description=cAMP-independent regulatory protein [Fusarium coicis]
Fv_160_10.g5908	532	ID=Fv_160_10.g5908;Description=asparaginyl-tRNA synthetase [Fusarium verticillioides 7600];Gene=FPCIR_7194;Ontology_term=nucleus,mitochondrion,nucleic acid binding,ribokinase activity,asparagine-tRNA ligase activity,ATP binding,metal ion binding,D-ribose catabolic process,carbohydrate phosphorylation,mitochondrial asparaginyl-tRNA aminoacylation;Ontology_id=GO:0005634,GO:0005739,GO:0003676,GO:0004747,GO:0004816,GO:0005524,GO:0046872,GO:00193
Fv_160_10.g5909	341	ID=Fv_160_10.g5909;Description=hypothetical protein FVEG_11469 [Fusarium verticillioides 7600]
Fv_160_10.g5910	1038	ID=Fv_160_10.g5910;Description=hypothetical protein FVER14953_11470 [Fusarium verticillioides];Gene=AU210_010775;Ontology_term=incipient cellular bud site,Golgi membrane,endoplasmic reticulum membrane,plasma membrane,cellular bud neck,cell cortex,COPII vesicle coat,guanyl-nucleotide exchange factor activity,protein binding,zinc ion binding,intracellular protein transport,endoplasmic reticulum to Golgi vesicle-mediated transport,axial cellular bud site selection,bipolar cellular bud site selection,Ras protein signal
Fv_160_10.g5911	221	ID=Fv_160_10.g5911;Description=hypothetical protein FVER14953_11471 [Fusarium verticillioides];Gene=FCULG_00010754;Ontology_term=nucleosome,nucleus,DNA binding,structural constituent of
Fv_160_10.g5912	617	ID=Fv_160_10.g5912;Description=hypothetical protein FVER53590_11472 [Fusarium verticillioides]
Fv_160_10.g5913	71	ID=Fv_160_10.g5913;Description=hypothetical protein FOMA001_g10930 [Fusarium oxysporum f. sp. matthiolae]
Fv_160_10.g5914	552	ID=Fv_160_10.g5914;Description=hypothetical protein FVEG_11474 [Fusarium verticillioides
Fv_160_10.g5915	426	ID=Fv_160_10.g5915;Description=glutaryl-CoA dehydrogenase [Fusarium verticillioides 7600];Gene=BFJ68_g13156;Ontology_term=endoplasmic reticulum membrane,alpha-1,2-mannosyltransferase activity,oxidoreductase activity, acting on the CH-CH group of donors,flavin adenine dinucleotide binding,plasmid maintenance,GPI anchor biosynthetic

Fv_160_10.g5916	147	ID=Fv_160_10.g5916;Description=hypothetical protein FPSE_08852 [Fusarium pseudograminearum CS3096];Gene=FNAPI_199;Ontology_term=cytoplasm,nuclear SCF ubiquitin ligase complex,ATP binding,ubiquitin conjugating enzyme activity,SCF-dependent proteasomal ubiquitin-dependent protein catabolic process,SREBP signaling pathway,positive regulation of mitotic metaphase/anaphase transition;Ontology_id=GO:0005737,GO:0043224,GO:0005524,GO:0061631,GO:0031146,GO:0032933,GO:0045842;Enzyme_code=
Fv_160_10.g5917	576	ID=Fv_160_10.g5917;Description=NADPH oxidase [Fusarium verticillioides 7600];Gene=FGADI_11751;Ontology_term=membrane,ferric-chelate reductase activity,iron ion
Fv_160_10.g5918	525	ID=Fv_160_10.g5918;Description=hypothetical protein FVER14953_11479 [Fusarium
Fv_160_10.g5919	493	ID=Fv_160_10.g5919;Description=hypothetical protein FVEG_11480 [Fusarium verticillioides 7600];Gene=FVEG_11480;Ontology_term=nucleus,membrane,DNA binding,transmembrane transporter activity,metal ion
Fv_160_10.g5920	139	ID=Fv_160_10.g5920;Description=hypothetical protein FVEG_11481 [Fusarium verticillioides 7600]
Fv_160_10.g5921	372	ID=Fv_160_10.g5921;Description=hypothetical protein J7337_009881 [Fusarium musae];Gene=psvA;Ontology_term=nucleus,vesicle membrane,spore wall,ribonucleoprotein complex,RNA binding,cellulose binding,cell differentiation,sporulation resulting in formation of a cellular
Fv_160_10.g5922	688	ID=Fv_160_10.g5922;Description=hypothetical protein FVEG_11483 [Fusarium verticillioides 7600];Gene=AU210_010788;Ontology_term=nucleus,DNA-binding transcription factor activity, RNA polymerase II-specific,zinc ion
Fv_160_10.g5923	1499	ID=Fv_160_10.g5923;Description=uncharacterized protein FMAN_10576 [Fusarium mangiferae]
Fv_160_10.g5924	479	ID=Fv_160_10.g5924;Description=hypothetical protein FVEG_11487 [Fusarium verticillioides
Fv_160_10.g5925	1087	ID=Fv_160_10.g5925;Description=hypothetical protein FVER53590_11488 [Fusarium verticillioides]
Fv_160_10.g5926	433	ID=Fv_160_10.g5926;Description=hypothetical protein FVER14953_11489 [Fusarium verticillioides];Gene=FNAPI_189;Ontology_term=anaphase-promoting complex,membrane,cell cycle,anaphase-promoting complex-
Fv_160_10.g5927	110	ID=Fv_160_10.g5927;Description=anaphase-promoting complex subunit 10 [Fusarium verticillioides 7600];Gene=FPHYL_8573;Ontology_term=anaphase-promoting complex,cell cycle,anaphase-promoting complex-dependent
Fv_160_10.g5928	539	ID=Fv_160_10.g5928;Description=hypothetical protein FVEG_11490 [Fusarium verticillioides 7600];Gene=FPCIR_7590;Ontology_term=membrane,channel activity,transmembrane
Fv_160_10.g5929	69	ID=Fv_160_10.g5929;Description=hypothetical protein FVEG_17036 [Fusarium verticillioides
Fv_160_10.g5930	195	ID=Fv_160_10.g5930;Description=hypothetical protein FVEG_11491 [Fusarium verticillioides 7600]
Fv_160_10.g5931	415	ID=Fv_160_10.g5931;Description=hypothetical protein FVEG_11492 [Fusarium verticillioides 7600];Gene=FTJAE_7791;Ontology_term=aspartic-type endopeptidase
Fv_160_10.g5932	1005	ID=Fv_160_10.g5932;Description=hypothetical protein FVER14953_11493 [Fusarium verticillioides];Gene=FTJAE_7790;Ontology_term=ATP binding,ATP-dependent protein folding chaperone,protein
Fv_160_10.g5933	420	ID=Fv_160_10.g5933;Description=hypothetical protein FVER14953_11494 [Fusarium verticillioides];Gene=FMUND_1657;Ontology_term=protein heterodimerization activity;Ontology_id=GO:0046982

Fv_160_10.g5934	78	ID=Fv_160_10.g5934;Description=hypothetical protein FVEG_11495 [Fusarium verticillioides 7600];Gene=AU210_010800;Ontology_term=protein heterodimerization activity;Ontology_id=GO:0046982
Fv_160_10.g5935	138	ID=Fv_160_10.g5935;Description=prefoldin subunit 4 [Fusarium oxysporum f. sp. lycopersici 4287];Gene=F53441_8786;Ontology_term=prefoldin complex,unfolded protein binding,protein
Fv_160_10.g5936	464	ID=Fv_160_10.g5936;Description=hypothetical protein FVEG_11497 [Fusarium verticillioides 7600];Gene=FPCIR_7582;Ontology_term=nuclear speck,phototaxis,response to cAMP,protein kinase B binding,sequence-specific DNA binding,nucleolus,phagocytic cup,chemotaxis to cAMP,mRNA splicing, via spliceosome,actin crosslink formation,RNA binding,thermotaxis,macropinocytic cup,sorocarp development,cell migration,cortical actin cytoskeleton,transmembrane transport,transmembrane transporter activity,cell leading edge,regulation of pseudopodium assembly,anterior cell cortex,posterior cell cortex,actin filament,hyperosmotic response,actin filament binding,lateral cell cortex,phagocytosis,small GTPase binding,lamellipodium assembly,pseudopodium,protein-containing complex,mitogen-activated protein kinase binding;Ontology_id=GO:0016607,GO:0042331,GO:0051591,GO:0043422,GO:0043565,GO:0005730,GO:0001891,GO:0043327,GO:0
Fv_160_10.g5937	1028	ID=Fv_160_10.g5937;Description=hypothetical protein FVEG_17038 [Fusarium verticillioides 7600]
Fv_160_10.g5938	766	ID=Fv_160_10.g5938;Description=hypothetical protein FVER53590_11500 [Fusarium verticillioides];Gene=FNAPI_1486;Ontology_term=5-methyltetrahydropteroyltriglutamate-homocysteine S-methyltransferase activity,zinc ion binding,L-methionine biosynthetic process from homoserine via O-acetyl-L-homoserine and cystathionine,methylation,adenine biosynthetic
Fv_160_10.g5939	616	ID=Fv_160_10.g5939;Description=hypothetical protein FVER14953_11501 [Fusarium
Fv_160_10.g5940	350	ID=Fv_160_10.g5940;Description=hypothetical protein FVER53590_11502 [Fusarium verticillioides];Gene=FVER53590_11502;Ontology_term=nucleolus,mitochondrial inner membrane,RNA binding,RNA helicase activity,ATP binding,hydrolase activity,rRNA
Fv_160_10.g5941	358	ID=Fv_160_10.g5941;Description=hypothetical protein FVEG_11503 [Fusarium verticillioides
Fv_160_10.g5942	79	ID=Fv_160_10.g5942;Description=uncharacterized protein FTJAE_7780 [Fusarium tjaetaba];Gene=FTJAE_7780;Ontology_term=endoplasmic reticulum membrane,transferase
Fv_160_10.g5943	261	ID=Fv_160_10.g5943;Description=hypothetical protein J7337_009903 [Fusarium
Fv_160_10.g5944	211	ID=Fv_160_10.g5944;Description=hypothetical protein FVEG_11505 [Fusarium verticillioides 7600];Gene=FGADI_10661;Ontology_term=lipase activity,Hsp90 protein
Fv_160_10.g5945	228	ID=Fv_160_10.g5945;Description=hypothetical protein FVEG_11506 [Fusarium verticillioides
Fv_160_10.g5946	154	ID=Fv_160_10.g5946;Description=hypothetical protein FVEG_11508 [Fusarium verticillioides 7600]
Fv_160_10.g5947	175	ID=Fv_160_10.g5947;Description=hypothetical protein FVEG_11509 [Fusarium verticillioides 7600];Gene=FSUBG_664;Ontology_term=tRNA-specific adenosine-34 deaminase activity,tRNA wobble adenosine to inosine editing;Ontology_id=GO:0052717,GO:0002100;Enzyme_code=EC:3.5.4.33,EC:3.5.4.4;Enzyme_name=tRNA(adenine(34))

Fv_160_10.g5948	1607	ID=Fv_160_10.g5948;Description=hypothetical protein FVEG_11510 [Fusarium verticillioides 7600];Gene=BFJ63_vAg10561;Ontology_term=nucleus,transcription coregulator activity,regulation of DNA-templated
Fv_160_10.g5949	211	ID=Fv_160_10.g5949;Description=phosphatidylinositol glycan, class H [Fusarium verticillioides 7600];Gene=FNAPI_1497;Ontology_term=glycosylphosphatidylinositol-N-acetylglucosaminyltransferase (GPI-GnT)
Fv_160_10.g5950	546	ID=Fv_160_10.g5950;Description=hypothetical protein FVEG_11511 [Fusarium verticillioides 7600];Gene=F25303_6379;Ontology_term=RNA binding,pseudouridine synthase activity,pseudouridine synthesis,tRNA
Fv_160_10.g5951	187	ID=Fv_160_10.g5951;Description=hypothetical protein FVEG_11512 [Fusarium verticillioides 7600];Gene=rhoA-0;Ontology_term=GTPase activity,GTP binding,small GTPase mediated signal
Fv_160_10.g5952	150	ID=Fv_160_10.g5952;Description=hypothetical protein FVEG_11513 [Fusarium verticillioides 7600];Gene=FOC4_g10004601;Ontology_term=RNA polymerase III complex,nucleotide binding,transcription initiation at RNA
Fv_160_10.g5953	461	ID=Fv_160_10.g5953;Description=hypothetical protein FVER14953_11514 [Fusarium verticillioides];Gene=FCIRC_10332;Ontology_term=ribose phosphate diphosphokinase complex,magnesium ion binding,ribose phosphate diphosphokinase activity,kinase activity,5-phosphoribose 1-diphosphate biosynthetic process,nucleotide biosynthetic process,phosphorylation,fungal-type cell wall organization;Ontology_id=GO:0002189,GO:0000287,GO:0004749,GO:0016301,GO:0006015,GO:0009165,GO:0016310,GO:0031505;
Fv_160_10.g5954	1180	ID=Fv_160_10.g5954;Description=hypothetical protein FVER53590_11515 [Fusarium verticillioides];Gene=FPRO05_01060;Ontology_term=NAD+ ADP-ribosyltransferase
Fv_160_10.g5955	657	ID=Fv_160_10.g5955;Description=hypothetical protein FVER14953_11516 [Fusarium
Fv_160_10.g5956	246	ID=Fv_160_10.g5956;Description=hypothetical protein FVEG_11517 [Fusarium verticillioides 7600];Gene=FVER53590_11517;Ontology_term=methyltransferase
Fv_160_10.g5957	865	ID=Fv_160_10.g5957;Description=hypothetical protein FVER14953_11518 [Fusarium verticillioides]
Fv_160_10.g5958	136	ID=Fv_160_10.g5958;Description=50S ribosomal protein L26e [Fusarium verticillioides 7600];Gene=F53441_4087;Ontology_term=extracellular region,mitochondrial inner membrane,fungal-type cell wall,large ribosomal subunit,side of membrane,RNA binding,structural constituent of ribosome,structural constituent of cell wall,copper ion binding,translation,fungal-type cell wall
Fv_160_10.g5959	261	ID=Fv_160_10.g5959;Description=cytochrome c oxidase assembly protein subunit 11 [Fusarium verticillioides 7600];Gene=FVER53263_11520;Ontology_term=mitochondrial inner membrane,mitochondrial intermembrane space,mitochondrial ribosome,copper ion binding,aerobic respiration,protein-containing complex
Fv_160_10.g5960	538	ID=Fv_160_10.g5960;Description=hypothetical protein FVER53590_11521 [Fusarium verticillioides];Gene=F25303_6389;Ontology_term=endoplasmic reticulum,signal recognition particle, endoplasmic reticulum targeting,membrane,GTPase activity,GTP binding,7S RNA binding,ATP hydrolysis activity,SRP-dependent cotranslational protein targeting to

Fv_160_10.g5961	531	ID=Fv_160_10.g5961;Description=hypothetical protein FPANT_6070 [Fusarium pseudoanthophilum];Gene=C2S_10260;Ontology_term=larval serum protein complex,membrane,oxidoreductase activity,nutrient reservoir
Fv_160_10.g5962	227	ID=Fv_160_10.g5962;Description=vesicle transport through interaction with t-SNAREs 1 [Fusarium verticillioides 7600];Gene=C2S_10261;Ontology_term=cytoplasm,membrane,intracellular membrane-bounded organelle,intracellular protein transport,vesicle-mediated transport;Ontology_id=GO:0005737,GO:0016020,GO:0043231,GO:0006886,GO:0016192
Fv_160_10.g5963	554	ID=Fv_160_10.g5963;Description=hypothetical protein FVEG_11524 [Fusarium verticillioides 7600];Gene=FVER53263_11524;Ontology_term=cytoplasm,membrane,intracellular membrane-bounded organelle,ribonucleoprotein complex,RNA binding,intracellular protein transport,vesicle-mediated
Fv_160_10.g5964	700	ID=Fv_160_10.g5964;Description=hypothetical protein FVER14953_11525 [Fusarium
Fv_160_10.g5965	811	ID=Fv_160_10.g5965;Description=hypothetical protein FVEG_11526 [Fusarium verticillioides 7600];Gene=FVEG_11526;Ontology_term=nucleus,DNA binding,zinc ion binding,DNA-binding transcription factor activity, RNA polymerase II-specific,lysozyme activity,regulation of transcription by RNA polymerase
Fv_160_10.g5966	209	ID=Fv_160_10.g5966;Description=hypothetical protein FVER53590_11527 [Fusarium verticillioides]
Fv_160_10.g5967	458	ID=Fv_160_10.g5967;Description=hypothetical protein FVER14953_11528 [Fusarium verticillioides];Gene=FVER53590_11528;Ontology_term=cytoplasm,spindle,microtubule,microtubule associated complex,midbody,protein binding,acyltransferase activity, transferring groups other than amino-acyl groups,mitotic cell cycle,centrosome cycle;Ontology_id=GO:0005737,GO:0005819,GO:0005874,GO:0005875,GO:0030496,GO:0005515,GO:0016747,GO:0000278,GO:000
Fv_160_10.g5968	295	ID=Fv_160_10.g5968;Description=hypothetical protein FVEG_11529 [Fusarium verticillioides 7600];Gene=mam;Ontology_term=chromosome, centromeric region,chromatin,polytene chromosome puff,nuclear speck,CSL-Notch-Mastermind transcription factor complex,transcription coactivator activity,heart morphogenesis,chromosome segregation,Notch signaling pathway,neuroblast fate determination,ventral midline development,apposition of dorsal and ventral imaginal disc-derived wing surfaces,mesodermal cell fate determination,asymmetric cell division,follicle cell of egg chamber development,germ-line stem cell population maintenance,CENP-A containing chromatin assembly,somatic stem cell population maintenance,positive regulation of transcription by RNA polymerase II,lateral inhibition,neuron fate
Fv_160_10.g5969	82	ID=Fv_160_10.g5969;Description=copper metallochaperone ATX1 [Fusarium oxysporum Fo47];Gene=FSUBG_684;Ontology_term=extracellular region,plasma membrane,cellular bud neck,side of membrane,identical protein binding,metal ion binding,flocculation,invasive growth in response to glucose limitation,pseudohyphal growth,homotypic cell-cell adhesion,cell adhesion involved in single-species biofilm formation,single-species surface biofilm formation,cell-cell self recognition;Ontology_id=GO:0005576,GO:0005886,GO:0005935,GO:0098552,GO:0042802,GO:0046872,GO:0000128,GO:0001403,
Fv_160_10.g5970	353	ID=Fv_160_10.g5970;Description=hypothetical protein FVER14953_11531 [Fusarium verticillioides];Gene=BFJ63_vAg10550;Ontology_term=alcohol dehydrogenase (NAD+) activity,zinc ion

Fv_160_10.g5971	439	ID=Fv_160_10.g5971;Description=actin 3 [Fusarium mexicanum];Gene=28954090;Ontology_term=Arp2/3 protein complex,actin cortical patch,cell division site,cell cortex of cell tip,ATP binding,actin filament binding,mitotic cytokinesis,actin filament polymerization,Arp2/3 complex-mediated actin nucleation,actin cortical patch
Fv_160_10.g5972	600	ID=Fv_160_10.g5972;Description=hypothetical protein FVEG_11533 [Fusarium verticillioides 7600];Gene=FRV6_12702;Ontology_term=threonine-type endopeptidase
Fv_160_10.g5973	356	ID=Fv_160_10.g5973;Description=transcription initiation factor TFIIH subunit 3 [Fusarium verticillioides 7600];Gene=FCIRC_3228;Ontology_term=transcription factor TFIIH core complex,transcription factor TFIIH holo complex,cell cortex,actin cytoskeleton,translation initiation factor activity,actin monomer binding,RNA polymerase II CTD heptapeptide repeat kinase activity,myosin I heavy chain binding,metal ion binding,mitotic cytokinesis,nucleotide-excision repair,regulation of transcription by RNA polymerase II,translational initiation,exocytosis,pinocytosis,hyperosmotic response,sorocarp development,positive regulation of actin filament polymerization,secretion of lysosomal enzymes,sequestering of actin monomers,actin nucleation,cell motility,negative regulation of phagocytosis;Ontology_id=GO:0000439,GO:0005675,GO:0005938,GO:0015629,GO:0003743,GO:0003785,GO:0008353,GO:0032037
Fv_160_10.g5974	704	ID=Fv_160_10.g5974;Description=hypothetical protein FVER53263_11535 [Fusarium
Fv_160_10.g5975	542	ID=Fv_160_10.g5975;Description=hypothetical protein FVER14953_11536 [Fusarium verticillioides];Gene=FOTG_06939;Ontology_term=exonuclease activity,tRNA (uracil(54)-C5)-methyltransferase activity, S-adenosyl methionine-dependent,tRNA
Fv_160_10.g5976	586	ID=Fv_160_10.g5976;Description=hypothetical protein FVER14953_11538 [Fusarium verticillioides];Gene=FOXB_14056;Ontology_term=glutathione hydrolase activity,leukotriene C4 gamma-glutamyl transferase activity,glutathione catabolic
Fv_160_10.g5977	532	ID=Fv_160_10.g5977;Description=alpha-1,2-mannosyltransferase alg-11 [Fusarium verticillioides 7600];Gene=FPRO_11063;Ontology_term=endoplasmic reticulum membrane,GDP-Man:Man3GlcNAc2-PP-Dol alpha-1,2-mannosyltransferase activity,dolichol-linked oligosaccharide biosynthetic
Fv_160_10.g5978	353	ID=Fv_160_10.g5978;Description=hypothetical protein FVER53590_25946 [Fusarium verticillioides];Gene=FOPG_14571;Ontology_term=membrane,membrane insertase activity,establishment of protein localization to
Fv_160_10.g5979	262	ID=Fv_160_10.g5979;Description=syntaxin 7 [Fusarium verticillioides 7600];Gene=BFJ69_g15231;Ontology_term=membrane,vesicle-mediated transport;Ontology_id=GO:0016020,GO:0016192
Fv_160_10.g5980	1068	ID=Fv_160_10.g5980;Description=unnamed protein product, partial [Fusarium graminearum];Gene=FPHYL_1856;Ontology_term=nuclear pore,nuclear membrane,protein transport,mRNA
Fv_160_10.g5981	199	ID=Fv_160_10.g5981;Description=hypothetical protein FVER53590_11543 [Fusarium verticillioides]
Fv_160_10.g5982	583	ID=Fv_160_10.g5982;Description=hypothetical protein FVEG_11544 [Fusarium verticillioides 7600];Gene=FSUBG_698;Ontology_term=nucleus,DNA-binding transcription factor activity,sequence-specific DNA binding,regulation

Fv_160_10.g5983	855	ID=Fv_160_10.g5983;Description=hypothetical protein FVER14953_11546 [Fusarium verticillioides];Gene=FVER53263_11546;Ontology_term=chromatin,nucleoplasm,nuclear replication fork,DNA damage
Fv_160_10.g5984	393	ID=Fv_160_10.g5984;Description=hypothetical protein FVEG_11547 [Fusarium verticillioides 7600]
Fv_160_10.g5985	981	ID=Fv_160_10.g5985;Description=hypothetical protein FVEG_11548 [Fusarium verticillioides 7600]
Fv_160_10.g5986	102	ID=Fv_160_10.g5986;Description=hypothetical protein FVEG_11549 [Fusarium verticillioides 7600];Gene=SUC3;Ontology_term=beta-fructofuranosidase activity,carbohydrate metabolic
Fv_160_10.g5987	202	ID=Fv_160_10.g5987;Description=hypothetical protein FPSE_09831 [Fusarium pseudograminearum CS3096];Gene=ypt-1;Ontology_term=cytosolic large ribosomal subunit,structural constituent of ribosome,GTPase activity,GTP binding,cytoplasmic translational
Fv_160_10.g5988	289	ID=Fv_160_10.g5988;Description=hypothetical protein FVEG_11551 [Fusarium verticillioides 7600]
Fv_160_10.g5989	169	ID=Fv_160_10.g5989;Description=hypothetical protein FVEG_11552 [Fusarium verticillioides 7600];Gene=lsm1;Ontology_term=P-body,nucleus,Lsm1-7-Pat1 complex,ribonucleoprotein complex,RNA cap binding,chromatin binding,mRNA binding,deadenylation-dependent decapping of nuclear-transcribed mRNA,mRNA
Fv_160_10.g5990	163	ID=Fv_160_10.g5990;Description=hypothetical protein FVEG_11553 [Fusarium verticillioides 7600];Gene=A0A2L2U304;Ontology_term=endopolyphosphatase activity,diphosphoinositol-polyphosphate diphosphatase activity,bis(5'-adenosyl)-hexaphosphatase activity,bis(5'-adenosyl)-pentaphosphatase activity,inositol phosphate phosphatase activity,phosphodiesterase decapping endonuclease activity,polyphosphate catabolic process,inositol phosphate catabolic process,diadenosine pentaphosphate catabolic process,diadenosine hexaphosphate catabolic process,adenosine 5'-(hexahydrogen pentaphosphate) catabolic
Fv_160_10.g5991	349	ID=Fv_160_10.g5991;Description=hypothetical protein FVER14953_11554 [Fusarium verticillioides];Gene=Forpi1262_v007998;Ontology_term=arginyl-tRNA--protein transferase
Fv_160_10.g5992	339	ID=Fv_160_10.g5992;Description=hypothetical protein FVER53590_11555 [Fusarium verticillioides];Gene=C2S_7905;Ontology_term=actin filament binding,nuclear exosome (RNase complex),regulation of myoblast fusion,actin cytoskeleton,actin filament organization,nucleolus,arginyl-tRNA--protein transferase
Fv_160_10.g5993	243	ID=Fv_160_10.g5993;Description=hypothetical protein FVEG_11556 [Fusarium verticillioides
Fv_160_10.g5994	106	ID=Fv_160_10.g5994;Description=cytochrome c [Fusarium oxysporum f. sp. lycopersici 4287];Gene=FFB14_14874;Ontology_term=mitochondrial intermembrane space,respirasome,electron transfer activity,heme binding,metal ion binding,electron transport
Fv_160_10.g5995	754	ID=Fv_160_10.g5995;Description=hypothetical protein FVER53590_11558 [Fusarium verticillioides];Gene=FVER53263_11558;Ontology_term=ATP binding,ATP hydrolysis
Fv_160_10.g5996	201	ID=Fv_160_10.g5996;Description=hypothetical protein FVEG_11559 [Fusarium verticillioides

Fv_160_10.g5997	323	ID=Fv_160_10.g5997;Description=serine/threonine protein kinase [Fusarium verticillioides 7600];Gene=FPRO_11085;Ontology_term=protein serine/threonine kinase activity,protein-ribulosamine 3-kinase activity,phosphorylation;Ontology_id=GO:0004674,GO:0102193,GO:0016310;Enzyme_code=EC:2.7.1.172,EC:2.7.11.1;Enzyme_nam
Fv_160_10.g5998	79	ID=Fv_160_10.g5998;Description=hypothetical protein FVER14953_21220 [Fusarium verticillioides]
Fv_160_10.g5999	447	ID=Fv_160_10.g5999;Description=succinyl-CoA ligase [GDP-forming] subunit beta, mitochondrial [Fusarium verticillioides 7600];Gene=F25303_3047;Ontology_term=mitochondrion,magnesium ion binding,succinate-CoA ligase (ADP-forming) activity,ATP binding,tricarboxylic acid cycle,succinyl-CoA metabolic process;Ontology_id=GO:0005739,GO:0000287,GO:0004775,GO:0005524,GO:0006099,GO:0006104;Enzyme_code=EC:6.2.1.5;Enzy
Fv_160_10.g6000	162	ID=Fv_160_10.g6000;Description=hypothetical protein FVEG_11562 [Fusarium verticillioides 7600];Gene=F25303_3046;Ontology_term=nucleolus,cytosolic small ribosomal subunit,vesicle,small-subunit processome,structural constituent of ribosome,methylated-DNA-[protein]-cysteine S-methyltransferase activity,rRNA binding,DNA repair,translation,methylation,ribosomal small subunit biogenesis;Ontology_id=GO:0005730,GO:0022627,GO:0031982,GO:0032040,GO:0003735,GO:0003908,GO:0019843,GO:0006281,G
Fv_160_10.g6001	245	ID=Fv_160_10.g6001;Description=hypothetical protein J7337_009963 [Fusarium musae];Gene=F25303_3045;Ontology_term=translation release factor activity,GTPase activity,GTP binding,iron-sulfur cluster binding,nuclear-transcribed mRNA catabolic process, deadenylation-dependent decay,translational termination,iron-sulfur cluster assembly;Ontology_id=GO:0003747,GO:0003924,GO:0005525,GO:0051536,GO:0000288,GO:0006415,GO:0016226;Enzyme_code=E
Fv_160_10.g6002	488	ID=Fv_160_10.g6002;Description=hypothetical protein J7337_009964 [Fusarium musae];Gene=rpl8;Ontology_term=membrane,cytosolic large ribosomal subunit,RNA binding,structural constituent of ribosome,transmembrane transporter activity,cytoplasmic translation,transmembrane
Fv_160_10.g6003	1209	ID=Fv_160_10.g6003;Description=hypothetical protein FVEG_11565 [Fusarium verticillioides 7600];Gene=FOC4_g10004657;Ontology_term=guanyl-nucleotide exchange factor activity,small GTPase mediated signal
Fv_160_10.g6004	652	ID=Fv_160_10.g6004;Description=hypothetical protein FVEG_11567 [Fusarium verticillioides
Fv_160_10.g6005	575	ID=Fv_160_10.g6005;Description=Amino-acid permease inda1 [Fusarium musae];Gene=FVER53263_11568;Ontology_term=extracellular space,cytoplasm,membrane,identical protein binding,amino acid transport,behavior,transmembrane transport,positive regulation of ovulation,positive regulation of octopamine signaling
Fv_160_10.g6006	561	ID=Fv_160_10.g6006;Description=hypothetical protein FVEG_11569 [Fusarium verticillioides 7600];Gene=FACUT_6926;Ontology_term=membrane,transmembrane transport;Ontology_id=GO:0016020,GO:0055085
Fv_160_10.g6007	679	ID=Fv_160_10.g6007;Description=hypothetical protein FVER53590_11570 [Fusarium verticillioides];Gene=FPANT_11635;Ontology_term=nucleus,DNA binding,zinc ion binding,DNA-binding transcription factor activity, RNA polymerase II-specific,regulation of transcription by RNA polymerase

Fv_160_10.g6008	327	ID=Fv_160_10.g6008;Description=hypothetical protein FVER14953_11572 [Fusarium verticillioides];Gene=THI4;Ontology_term=extracellular space,nucleus,cytosol,iron ion binding,pentosyltransferase activity,identical protein binding,mating,thiamine biosynthetic process,sperm competition,thiazole biosynthetic process,positive regulation of ovulation;Ontology_id=GO:0005615,GO:0005634,GO:0005829,GO:0005506,GO:0016763,GO:0042802,GO:0007618,GO:0009228,GO
Fv_160_10.g6009	461	ID=Fv_160_10.g6009;Description=alanine-glyoxylate transaminase/(R)-3-amino-2-methylpropionate-pyruvate transaminase
Fv_160_10.g6010	1079	ID=Fv_160_10.g6010;Description=hypothetical protein FVEG_11574 [Fusarium verticillioides 7600];Gene=FANTH_5642;Ontology_term=kinetochore,spindle pole body,nuclear periphery,nuclear export signal receptor activity,U6 snRNA binding,U1 snRNA binding,U2 snRNA binding,U4 snRNA binding,U5 snRNA binding,small GTPase binding,nuclear import signal receptor activity,ribosomal large subunit export from nucleus,mRNA export from nucleus,protein export from nucleus,tRNA processing,protein localization to kinetochore,import into nucleus;Ontology_id=GO:0000776,GO:0005816,GO:0034399,GO:0005049,GO:0017070,GO:0030619,GO:0030620,GO:0030621,GO:
Fv_160_10.g6011	461	ID=Fv_160_10.g6011;Description=hypothetical protein FVEG_11575 [Fusarium verticillioides 7600];Gene=FNAPI_8555;Ontology_term=extracellular region,nucleus,cytosolic small ribosomal subunit,DNA-binding transcription factor activity, RNA polymerase II-specific,RNA binding,zinc ion binding,regulation of transcription by RNA polymerase II,sexual reproduction,positive regulation of octopamine signaling
Fv_160_10.g6012	635	ID=Fv_160_10.g6012;Description=ATP-dependent rRNA helicase SPB4 [Fusarium verticillioides 7600];Gene=BFJ72_g12384;Ontology_term=nucleolus,RNA binding,RNA helicase activity,ATP binding,ATP hydrolysis activity,rRNA processing;Ontology_id=GO:0005730,GO:0003723,GO:0003724,GO:0005524,GO:0016887,GO:0006364;Enzyme_code=EC:3.6.4.13,EC:3.6.1.15;Enzyme_name=RNA helicase,nucleoside-triphosphate phosphatase
Fv_160_10.g6013	400	ID=Fv_160_10.g6013;Description=phosphatidate cytidylyltransferase [Fusarium verticillioides 7600];Gene=FNAPI_8553;Ontology_term=membrane,ATP-dependent diacylglycerol kinase activity,nucleotidyltransferase activity;Ontology_id=GO:0016020,GO:0004143,GO:0016779;Enzyme_code=EC:2.7.7,EC:2.7.1.107;Enzyme_name=Transferring
Fv_160_10.g6014	185	ID=Fv_160_10.g6014;Description=hypothetical protein FVEG_11578 [Fusarium verticillioides
Fv_160_10.g6015	163	ID=Fv_160_10.g6015;Description=hypothetical protein FVEG_11579 [Fusarium verticillioides 7600]
Fv_160_10.g6016	201	ID=Fv_160_10.g6016;Description=hypothetical protein FVEG_11580 [Fusarium verticillioides 7600]
Fv_160_10.g6017	374	ID=Fv_160_10.g6017;Description=hypothetical protein FVER53263_11581 [Fusarium verticillioides];Gene=AU210_010885;Ontology_term=extracellular space,spliceosomal complex,cytoplasm,mating,RNA splicing,positive regulation of ovulation,positive regulation of octopamine signaling
Fv_160_10.g6018	772	ID=Fv_160_10.g6018;Description=vesicle-fusing ATPase [Fusarium verticillioides 7600];Gene=FPANT_11624;Ontology_term=cytoplasm,ATP binding,ATP hydrolysis activity,protein transport,SNARE complex disassembly;Ontology_id=GO:0005737,GO:0005524,GO:0016887,GO:0015031,GO:0035494;Enzyme_code=EC:3.6.1.15;Enzyme_na
Fv_160_10.g6019	1816	ID=Fv_160_10.g6019;Description=hypothetical protein FVER53263_11583 [Fusarium verticillioides];Gene=FCIRC_10997;Ontology_term=guanyl-nucleotide exchange factor activity,small GTPase mediated signal

Fv_160_10.g6020	293	ID=Fv_160_10.g6020;Description=hypothetical protein LB506_003633 [Fusarium annulatum];Gene=RPS0;Ontology_term=cytosolic small ribosomal subunit,structural constituent of ribosome,ribosomal small subunit
Fv_160_10.g6021	318	ID=Fv_160_10.g6021;Description=hypothetical protein FVEG_11585 [Fusarium verticillioides 7600];Gene=FNAPI_11303;Ontology_term=extracellular region,cytoplasm,ribosome,ribonucleoprotein complex,structural constituent of ribosome,translation,sexual reproduction,sperm competition,positive regulation of octopamine signaling
Fv_160_10.g6022	364	ID=Fv_160_10.g6022;Description=V-type proton ATPase subunit D [Fusarium verticillioides 7600];Gene=FCIRC_10994;Ontology_term=vacuolar proton-transporting V-type ATPase, V0 domain,fungal-type vacuole membrane,proton-transporting ATPase activity, rotational mechanism,vacuolar transport,proton transmembrane
Fv_160_10.g6023	592	ID=Fv_160_10.g6023;Description=nucleolar NOP58 [Fusarium pseudoanthophilum];Gene=FOC4_g10004679;Ontology_term=nucleolus,box C/D RNP complex,small-subunit processome,snoRNA
Fv_160_10.g6024	356	ID=Fv_160_10.g6024;Description=hypothetical protein FVEG_11588 [Fusarium verticillioides
Fv_160_10.g6025	569	ID=Fv_160_10.g6025;Description=hypothetical protein FVEG_11589 [Fusarium verticillioides 7600];Gene=FVER53263_11589;Ontology_term=monooxygenase activity,iron ion binding,oxidoreductase activity, acting on paired donors, with incorporation or reduction of molecular oxygen,heme binding;Ontology_id=GO:0004497,GO:0005506,GO:0016705,GO:0020037;Enzyme_code=EC:1.14;Enzyme_name=Acting on paired
Fv_160_10.g6026	698	ID=Fv_160_10.g6026;Description=Iron-sulfur clusters transporter atm1, mitochondrial [Fusarium musae];Gene=FVEG_11590;Ontology_term=membrane,ATP binding,ATP hydrolysis activity,ABC-type transporter activity,transmembrane transport;Ontology_id=GO:0016020,GO:0005524,GO:0016887,GO:0140359,GO:0055085;Enzyme_code=EC:7.2.2,EC:3.6.1.15;Enzym
Fv_160_10.g6027	427	ID=Fv_160_10.g6027;Description=hypothetical protein J7337_009995 [Fusarium musae];Gene=FOXB_06509;Ontology_term=UDP-glycosyltransferase activity;Ontology_id=GO:0008194;Enzyme_code=EC:2.4;Enzyme_name=Glycosyltransferases
Fv_160_10.g6028	164	ID=Fv_160_10.g6028;Description=hypothetical protein FVEG_17058 [Fusarium verticillioides
Fv_160_10.g6029	270	ID=Fv_160_10.g6029;Description=hypothetical protein FVER14953_11592 [Fusarium verticillioides];Gene=Acp26Aa;Ontology_term=positive regulation of octopamine signaling pathway,cellular anatomical
Fv_160_10.g6030	1829	ID=Fv_160_10.g6030;Description=hypothetical protein FVER14953_11593 [Fusarium verticillioides];Gene=FDENT_9415;Ontology_term=ATP binding,hydrolase activity,lyase activity,ligase activity,metal ion binding;Ontology_id=GO:0005524,GO:0016787,GO:0016829,GO:0016874,GO:0046872;Enzyme_code=EC:3,EC:4,EC:6;Enzyme_name
Fv_160_10.g6031	674	ID=Fv_160_10.g6031;Description=hypothetical protein FVER14953_11594 [Fusarium verticillioides];Gene=FPHYL_1222;Ontology_term=nucleus,DNA binding,zinc ion binding,DNA-binding transcription factor activity, RNA polymerase II-specific,regulation of transcription by RNA polymerase
Fv_160_10.g6032	396	ID=Fv_160_10.g6032;Description=hypothetical protein FVEG_11595 [Fusarium verticillioides 7600];Gene=C2S_7865;Ontology_term=nucleus,DNA binding,zinc ion binding,DNA-binding transcription factor activity, RNA

Fv_160_10.g6033	273	ID=Fv_160_10.g6033;Description=hypothetical protein FVEG_11596 [Fusarium verticillioides 7600];Gene=CEK26_011337;Ontology_term=fungal-type vacuole lumen,fungal-type vacuole membrane,sperm flagellum,serine-type endopeptidase inhibitor activity,phospholipid binding,spermatid development,regulation of proteolysis,regulation of Ras protein
Fv_160_10.g6034	504	ID=Fv_160_10.g6034;Description=glycosyltransferase family 31 [Fusarium pseudocircinatum];Gene=CEK26_011338;Ontology_term=membrane,sperm flagellum,glycosyltransferase activity,spermatid
Fv_160_10.g6035	505	ID=Fv_160_10.g6035;Description=hypothetical protein J7337_010003 [Fusarium musae];Gene=FVER53263_11598;Ontology_term=membrane,homoserine kinase activity,ATP binding,transmembrane transporter activity,threonine biosynthetic process,homoserine metabolic process,phosphorylation,transmembrane
Fv_160_10.g6036	349	ID=Fv_160_10.g6036;Description=hypothetical protein FVEG_11599 [Fusarium verticillioides 7600];Gene=FOQG_05413;Ontology_term=oxidoreductase activity, acting on the CH-CH group of donors, NAD or NADP as
Fv_160_10.g6037	316	ID=Fv_160_10.g6037;Description=hypothetical protein J7337_010005 [Fusarium musae];Gene=FVER53590_11600;Ontology_term=nucleosome,DNA binding,threonine synthase activity,pyridoxal phosphate binding,structural constituent of chromatin,nucleosome assembly,threonine biosynthetic process,anatomical structure development;Ontology_id=GO:0000786,GO:0003677,GO:0004795,GO:0030170,GO:0030527,GO:0006334,GO:0009088,GO:004885
Fv_160_10.g6038	514	ID=Fv_160_10.g6038;Description=hypothetical protein FVEG_11601 [Fusarium verticillioides 7600];Gene=HZS61_017774;Ontology_term=membrane,transmembrane transporter activity,carbohydrate
Fv_160_10.g6039	425	ID=Fv_160_10.g6039;Description=hypothetical protein FVEG_11602 [Fusarium verticillioides 7600];Gene=THS1;Ontology_term=cytoplasm,threonine-tRNA ligase activity,ion binding,threonyl-tRNA
Fv_160_10.g6040	244	ID=Fv_160_10.g6040;Description=hypothetical protein J7337_010008 [Fusarium musae]
Fv_160_10.g6041	312	ID=Fv_160_10.g6041;Description=hypothetical protein FVEG_11604 [Fusarium verticillioides 7600]
Fv_160_10.g6042	390	ID=Fv_160_10.g6042;Description=saccharopine dehydrogenase (NAD+, L-lysine forming) [Fusarium verticillioides 7600];Gene=HZS61_017779;Ontology_term=saccharopine dehydrogenase (NAD+, L-lysine-forming) activity,lysine biosynthetic process via aminoadipic acid;Ontology_id=GO:0004754,GO:0019878;Enzyme_code=EC:1.5.1.7;Enzyme_name=saccharopine
Fv_160_10.g6043	157	ID=Fv_160_10.g6043;Description=hypothetical protein FVER14953_11606 [Fusarium verticillioides]
Fv_160_10.g6044	171	ID=Fv_160_10.g6044;Description=hypothetical protein FVER14953_21398 [Fusarium verticillioides]
Fv_160_10.g6045	292	ID=Fv_160_10.g6045;Description=WW domain-containing protein [Fusarium globosum];Gene=TIF2;Ontology_term=RNA helicase activity,translation initiation factor activity,ATP binding,hydrolase activity,translational initiation,positive regulation of gene expression,aggregation involved in sorocarp
Fv_160_10.g6046	295	ID=Fv_160_10.g6046;Description=hypothetical protein FVER14953_11608 [Fusarium
Fv_160_10.g6047	117	ID=Fv_160_10.g6047;Description=hypothetical protein J7337_010015 [Fusarium musae]
Fv_160_10.g6048	502	ID=Fv_160_10.g6048;Description=hypothetical protein J7337_010016 [Fusarium musae];Gene=fray;Ontology_term=nucleus,cytoplasm,binding,larval somatic muscle development,determination of muscle
Fv_160_10.g6049	143	ID=Fv_160_10.g6049;Description=hypothetical protein FVEG_11611 [Fusarium verticillioides 7600]

Fv_160_10.g6050	165	ID=Fv_160_10.g6050;Description=hypothetical protein FVER53263_11612 [Fusarium verticillioides]
Fv_160_10.g6051	242	ID=Fv_160_10.g6051;Description=hypothetical protein FVER53263_11613 [Fusarium verticillioides]
Fv_160_10.g6052	327	ID=Fv_160_10.g6052;Description=ATPase [Fusarium circinatum];Gene=FOC4_g10004711;Ontology_term=membrane,ATP binding,S-adenosylmethionine-dependent methyltransferase activity,ATP hydrolysis activity,ABC-type transporter activity,methylation,transmembrane transport;Ontology_id=GO:0016020,GO:0005524,GO:0008757,GO:0016887,GO:0140359,GO:0032259,GO:0055085;Enzyme_code=E
Fv_160_10.g6053	1323	ID=Fv_160_10.g6053;Description=hypothetical protein FVER14953_11615 [Fusarium verticillioides];Gene=FVER53590_11615;Ontology_term=membrane,ATP binding,ATP hydrolysis activity,ABC-type transporter activity,transmembrane transport;Ontology_id=GO:0016020,GO:0005524,GO:0016887,GO:0140359,GO:0055085;Enzyme_code=EC:7.2.2,EC:3.6.1.15;Enzym
Fv_160_10.g6054	527	ID=Fv_160_10.g6054;Description=allantoinase [Fusarium verticillioides 7600];Gene=FPHYL_6293;Ontology_term=allantoinase activity,metal ion binding,allantoin assimilation
Fv_160_10.g6055	455	ID=Fv_160_10.g6055;Description=hexaprenyl pyrophosphate synthase, mitochondrial [Fusarium verticillioides 7600];Gene=FOVG_11072;Ontology_term=dimethylallyltranstransferase activity,geranyltranstransferase activity,isoprenoid biosynthetic process,ketone biosynthetic process,mycotoxin biosynthetic process,alcohol biosynthetic process,organic cyclic compound biosynthetic
Fv_160_10.g6056	275	ID=Fv_160_10.g6056;Description=hypothetical protein FVER14953_11618 [Fusarium verticillioides];Gene=FOVG_11073;Ontology_term=endoplasmic reticulum membrane,vacuolar proton-transporting V-type ATPase
Fv_160_10.g6057	265	ID=Fv_160_10.g6057;Description=hypothetical protein J7337_010024 [Fusarium musae];Gene=FPANT_3605;Ontology_term=endoplasmic reticulum membrane,nuclear speck,pre-mRNA binding,mRNA splicing, via spliceosome,cell differentiation,female sex determination,female sex differentiation,vacuolar proton-transporting V-type ATPase complex assembly,epithelium regeneration,regulation of stem cell
Fv_160_10.g6058	966	ID=Fv_160_10.g6058;Description=hypothetical protein FVER14953_11619 [Fusarium verticillioides]
Fv_160_10.g6059	336	ID=Fv_160_10.g6059;Description=hypothetical protein FPHYL_6289 [Fusarium
Fv_160_10.g6060	340	ID=Fv_160_10.g6060;Description=hypothetical protein FVEG_17062 [Fusarium verticillioides 7600];Gene=FVEG_17062;Ontology_term=nucleus,membrane,DNA-binding transcription factor activity, RNA polymerase II-
Fv_160_10.g6061	238	ID=Fv_160_10.g6061;Description=hypothetical protein FVER53590_11622 [Fusarium verticillioides];Gene=FGADI_9994;Ontology_term=endoplasmic reticulum membrane,cholestenol delta-isomerase activity,sterol
Fv_160_10.g6062	959	ID=Fv_160_10.g6062;Description=hypothetical protein FVER14953_11624 [Fusarium verticillioides];Gene=FMAN_10711;Ontology_term=membrane,helicase activity,transferase

Fv_160_10.g6063	896	ID=Fv_160_10.g6063;Description=allantoate permease [Fusarium tjaetaba];Gene=F25303_1675;Ontology_term=extracellular region,nucleolus,membrane,box C/D RNP complex,small-subunit processome,hydrolase activity, hydrolyzing O-glycosyl compounds,chitin binding,transmembrane transporter activity,snoRNA binding,polysaccharide catabolic process,chitin catabolic process,ribosome biogenesis,transmembrane transport;Ontology_id=GO:0005576,GO:0005730,GO:0016020,GO:0031428,GO:0032040,GO:0004553,GO:0008061,GO:0022857,GO
Fv_160_10.g6064	399	ID=Fv_160_10.g6064;Description=chitinase [Fusarium verticillioides 7600];Gene=FMUND_11986;Ontology_term=extracellular region,nucleolus,membrane,box C/D RNP complex,small-subunit processome,chitinase activity,chitin binding,transmembrane transporter activity,snoRNA binding,polysaccharide catabolic process,chitin catabolic process,ribosome biogenesis,transmembrane transport;Ontology_id=GO:0005576,GO:0005730,GO:0016020,GO:0031428,GO:0032040,GO:0004568,GO:0008061,GO:0022857,GO :0030515,GO:0000272,GO:0006032,GO:0042254,GO:0055085;Enzyme_code=EC:3.2.1.14;Enzyme_name=chitinase
Fv_160_10.g6065	511	ID=Fv_160_10.g6065;Description=hypothetical protein FVER53590_11633 [Fusarium verticillioides];Gene=FCIRC_9988;Ontology_term=extracellular region,nucleolus,membrane,box C/D RNP complex,small-subunit processome,hydrolase activity, hydrolyzing O-glycosyl compounds,chitin binding,transmembrane transporter activity,snoRNA binding,polysaccharide catabolic process,snoRNA guided rRNA 2'-O-methylation,box C/D RNA 3'-end processing,chitin catabolic process,transmembrane
Fv_160_10.g6066	154	ID=Fv_160_10.g6066;Description=hypothetical protein FNYG_05331 [Fusarium nygamai];Gene=FMUND_11987;Ontology_term=ribosome,ribonucleoprotein complex,structural constituent of
Fv_160_10.g6067	117	ID=Fv_160_10.g6067;Description=unnamed protein product [Fusarium graminearum];Gene=FSUBG_785;Ontology_term=ribosome,ribonucleoprotein complex,structural constituent of
Fv_160_10.g6068	602	ID=Fv_160_10.g6068;Description=hypothetical protein FVEG_11636 [Fusarium verticillioides 7600];Gene=AU210_010935;Ontology_term=chromosome,ATP binding,chromosome
Fv_160_10.g6069	488	ID=Fv_160_10.g6069;Description=hypothetical protein FVER53263_11637 [Fusarium
Fv_160_10.g6070	1484	ID=Fv_160_10.g6070;Description=hypothetical protein FVER14953_11639 [Fusarium verticillioides];Gene=FMUND_11989;Ontology_term=nucleus,chromosome,ATP binding,ATP hydrolysis activity,chromosome organization,cell
Fv_160_10.g6071	305	ID=Fv_160_10.g6071;Description=hypothetical protein FVEG_11640 [Fusarium verticillioides
Fv_160_10.g6072	383	ID=Fv_160_10.g6072;Description=hypothetical protein FVER53263_11641 [Fusarium verticillioides];Gene=C2S_7828;Ontology_term=extracellular region,membrane,egg chorion,oxoglutarate dehydrogenase complex,dihydrolipoyllysine-residue succinyltransferase activity,tricarboxylic acid cycle,L-lysine catabolic process to acetyl-CoA via saccharopine,dorsal appendage formation;Ontology_id=GO:0005576,GO:0016020,GO:0042600,GO:0045252,GO:0004149,GO:0006099,GO:0033512,GO:0046843;En

Fv_160_10.g6073	434	ID=Fv_160_10.g6073;Description=hypothetical protein FVER53590_11642 [Fusarium verticillioides];Gene=FNAPI_398;Ontology_term=oxoglutarate dehydrogenase complex,dihydrolipoyllysine-residue succinyltransferase activity,tricarboxylic acid cycle,L-lysine catabolic process to acetyl-CoA via
Fv_160_10.g6074	317	ID=Fv_160_10.g6074;Description=hypothetical protein FVER53590_11643 [Fusarium verticillioides]
Fv_160_10.g6075	777	ID=Fv_160_10.g6075;Description=homoaconitase, mitochondrial [Fusarium verticillioides 7600];Gene=FNYG_05321;Ontology_term=mitochondrion,homoaconitate hydratase activity,metal ion binding,4 iron, 4 sulfur cluster binding,lysine biosynthetic process via aminoadipic
Fv_160_10.g6076	595	ID=Fv_160_10.g6076;Description=acyl dehydrogenase [Fusarium mundagurra];Gene=mmgC;Ontology_term=oxidoreductase activity, acting on the CH-CH group of donors,flavin adenine dinucleotide
Fv_160_10.g6077	1181	ID=Fv_160_10.g6077;Description=beta-n-acetylhexosaminidase [Fusarium denticulatum];Gene=FVEG_11647;Ontology_term=beta-N-acetylhexosaminidase activity,N-acetyl-beta-D-galactosaminidase activity,carbohydrate metabolic
Fv_160_10.g6078	567	ID=Fv_160_10.g6078;Description=hypothetical protein FVER14953_11648 [Fusarium
Fv_160_10.g6079	545	ID=Fv_160_10.g6079;Description=GNT1 alphan-acetylglucosamine transferase [Fusarium coicis];Gene=FGLOB1_11539;Ontology_term=transferase
Fv_160_10.g6080	1599	ID=Fv_160_10.g6080;Description=ankyrin repeat domain-containing protein [Fusarium pseudocircinatum];Gene=FDENT_9363;Ontology_term=membrane,transferase
Fv_160_10.g6081	406	ID=Fv_160_10.g6081;Description=hypothetical protein FVEG_17067 [Fusarium verticillioides 7600];Gene=FVEG_17067;Ontology_term=binding,oxidoreductase
Fv_160_10.g6082	527	ID=Fv_160_10.g6082;Description=hypothetical protein FVEG_11655 [Fusarium verticillioides 7600];Gene=FNAPI_411;Ontology_term=membrane,monooxygenase activity,iron ion binding,oxidoreductase activity, acting on paired donors, with incorporation or reduction of molecular oxygen,heme binding;Ontology_id=GO:0016020,GO:0004497,GO:0005506,GO:0016705,GO:0020037;Enzyme_code=EC:1.14;Enzyme_name=Actin
Fv_160_10.g6083	1303	ID=Fv_160_10.g6083;Description=major facilitator superfamily transporter [Fusarium subglutinans];Gene=Hrb87F;Ontology_term=nucleosome,heterochromatin,polytene chromosome puff,cytoplasm,membrane,omega speckle,ribonucleoprotein complex,mRNA 3'-UTR binding,helicase activity,ATP binding,hydrolase activity,transmembrane transporter activity,structural constituent of chromatin,poly(G) binding,sequence-specific DNA binding,protein-containing complex binding,metal ion binding,protein heterodimerization activity,ATP-dependent chromatin remodeler activity,regulation of alternative mRNA splicing, via spliceosome,compound eye morphogenesis,chromatin remodeling,female gonad development,response to heat,response to starvation,positive regulation of mRNA splicing, via spliceosome,transmembrane transport;Ontology_id=GO:0000786,GO:0000792,GO:0005703,GO:0005737,GO:0016020,GO:0035062,GO:1990904,GO:0003730,GO
Fv_160_10.g6084	824	ID=Fv_160_10.g6084;Description=hypothetical protein FVEG_17068 [Fusarium verticillioides 7600];Gene=FPANT_4021;Ontology_term=GTPase activity,GTP

Fv_160_10.g6085	572	ID=Fv_160_10.g6085;Description=hypothetical protein FVEG_17068 [Fusarium verticillioides 7600];Gene=FPANT_4021;Ontology_term=GTPase activity,GTP
Fv_160_10.g6086	521	ID=Fv_160_10.g6086;Description=hypothetical protein FVEG_11661 [Fusarium verticillioides 7600]
Fv_160_10.g6087	265	ID=Fv_160_10.g6087;Description=hypothetical protein FVER14953_11662 [Fusarium verticillioides];Gene=Fbp1;Ontology_term=nucleosome,nucleus,DNA binding,structural constituent of chromatin,nucleosome
Fv_160_10.g6088	839	ID=Fv_160_10.g6088;Description=hypothetical protein FVEG_11663 [Fusarium verticillioides 7600];Gene=FVER53263_11663;Ontology_term=nucleus,DNA binding,zinc ion binding,mitochondrion,membrane,DNA-binding transcription factor activity, RNA polymerase II-specific,regulation of transcription by RNA polymerase
Fv_160_10.g6089	538	ID=Fv_160_10.g6089;Description=hypothetical protein FVER14953_11664 [Fusarium verticillioides];Gene=FPRO_11195;Ontology_term=nucleosome,nucleus,membrane,DNA binding,hydrolase activity, hydrolyzing O-glycosyl compounds,zinc ion binding,structural constituent of chromatin,protein heterodimerization activity,carbohydrate metabolic process,DNA-templated transcription;Ontology_id=GO:0000786,GO:0005634,GO:0016020,GO:0003677,GO:0004553,GO:0008270,GO:0030527,GO:0046982
Fv_160_10.g6090	203	ID=Fv_160_10.g6090;Description=hypothetical protein FVEG_11665 [Fusarium verticillioides 7600]
Fv_160_10.g6091	395	ID=Fv_160_10.g6091;Description=hypothetical protein FVER14953_11666 [Fusarium verticillioides]
Fv_160_10.g6092	232	ID=Fv_160_10.g6092;Description=hypothetical protein FVER14953_11667 [Fusarium verticillioides];Gene=LOC115483234;Ontology_term=nucleosome,nucleus,DNA binding,structural constituent of chromatin,protein
Fv_160_10.g6093	660	ID=Fv_160_10.g6093;Description=hypothetical protein FVER53590_26478 [Fusarium verticillioides];Gene=FFUJ_08219;Ontology_term=nucleus,polytene chromosome puff,tryptophan biosynthetic process,metal ion binding,single-stranded RNA binding,regulation of mRNA splicing, via spliceosome,chitin synthase activity,conidium formation,plasma membrane,anthranilate synthase activity,single-stranded DNA binding;Ontology_id=GO:0005634,GO:0005703,GO:0000162,GO:0046872,GO:0003727,GO:0048024,GO:0004100,GO:0048315,GO:0
Fv_160_10.g6094	413	ID=Fv_160_10.g6094;Description=hypothetical protein FVER53263_21137 [Fusarium verticillioides];Gene=C2S_7805;Ontology_term=membrane,oxidoreductase activity, acting on the CH-OH group of donors, NAD or NADP as acceptor,oxidoreductase activity, acting on the CH-CH group of donors, NAD or NADP as acceptor,NAD binding,polysaccharide biosynthetic
Fv_160_10.g6095	1101	ID=Fv_160_10.g6095;Description=prostaglandin-endoperoxide synthase 1 [Fusarium verticillioides 7600];Gene=BFJ68_g15327;Ontology_term=monooxygenase activity,peroxidase activity,iron ion binding,oxidoreductase activity, acting on paired donors, with incorporation or reduction of molecular oxygen,heme binding,dioxygenase activity,response to oxidative stress,cellular oxidant detoxification;Ontology_id=GO:0004497,GO:0004601,GO:0005506,GO:0016705,GO:0020037,GO:0051213,GO:0006979,GO:009886
Fv_160_10.g6096	527	ID=Fv_160_10.g6096;Description=hypothetical protein FVER53263_11671 [Fusarium verticillioides];Gene=BFJ63_vAg13730;Ontology_term=K63-linked deubiquitinase activity,metal-dependent deubiquitinase

Fv_160_10.g6097	227	ID=Fv_160_10.g6097;Description=hypothetical protein FVER53263_11672 [Fusarium verticillioides];Gene=Abl;Ontology_term=tryptophan synthase activity,tryptophan biosynthetic
Fv_160_10.g6098	123	ID=Fv_160_10.g6098;Description=prefoldin beta subunit [Fusarium verticillioides 7600];Gene=FGADI_13336;Ontology_term=prefoldin complex,unfolded protein binding,protein
Fv_160_10.g6099	431	ID=Fv_160_10.g6099;Description=vacuolar protein sorting-associated protein 4 [Fusarium verticillioides 7600];Gene=AU210_010964;Ontology_term=plasma membrane,ESCRT IV complex,ATP binding,ATP hydrolysis activity,protein homodimerization activity,sterol metabolic process,late endosome to vacuole transport via multivesicular body sorting pathway,multivesicular body assembly,protein retention in Golgi apparatus,reticulophagy,late endosome to lysosome transport via multivesicular body sorting pathway,intralumenal vesicle formation,membrane fission,autophagosome maturation;Ontology_id=GO:0005886,GO:1990621,GO:0005524,GO:0016887,GO:0042803,GO:0016125,GO:0032511,GO:0036258,
Fv_160_10.g6100	594	ID=Fv_160_10.g6100;Description=hypothetical protein FVEG_11675 [Fusarium verticillioides 7600];Gene=FTJAE_9067;Ontology_term=RNA binding,S-adenosylmethionine-dependent methyltransferase activity,rRNA processing,methylation;Ontology_id=GO:0003723,GO:0008757,GO:0006364,GO:0032259;Enzyme_code=EC:2.1.1;Enzyme_name=Tr
Fv_160_10.g6101	641	ID=Fv_160_10.g6101;Description=hypothetical protein FVEG_11676 [Fusarium verticillioides 7600];Gene=CXT1-1;Ontology_term=transferase activity;Ontology_id=GO:0016740;Enzyme_code=EC:2;Enzyme_name=Transferases
Fv_160_10.g6102	325	ID=Fv_160_10.g6102;Description=arginase [Fusarium verticillioides 7600];Gene=FTJAE_9065;Ontology_term=arginase activity,metal ion binding,urea cycle,arginine metabolic
Fv_160_10.g6103	204	ID=Fv_160_10.g6103;Description=hypothetical protein FVEG_11678 [Fusarium verticillioides
Fv_160_10.g6104	428	ID=Fv_160_10.g6104;Description=homocitrate synthase, mitochondrial [Fusarium verticillioides 7600];Gene=Forpe1208_v000346;Ontology_term=homocitrate synthase activity,lysine biosynthetic process via aminoadipic
Fv_160_10.g6105	74	ID=Fv_160_10.g6105;Description=hypothetical protein FVEG_11680 [Fusarium verticillioides 7600]
Fv_160_10.g6106	1498	ID=Fv_160_10.g6106;Description=hypothetical protein FVER53263_11681 [Fusarium verticillioides];Gene=FNAPI_3400;Ontology_term=cytoplasm,microtubule organizing center;Ontology_id=GO:0005737,GO:0005815
Fv_160_10.g6107	478	ID=Fv_160_10.g6107;Description=hypothetical protein FVER14953_11682 [Fusarium verticillioides]
Fv_160_10.g6108	170	ID=Fv_160_10.g6108;Description=hypothetical protein FVEG_11683 [Fusarium verticillioides 7600];Gene=tra;Ontology_term=nuclear speck,mRNA processing,cell differentiation,female sex
Fv_160_10.g6109	92	ID=Fv_160_10.g6109;Description=hypothetical protein FVEG_11684 [Fusarium verticillioides 7600]
Fv_160_10.g6110	638	ID=Fv_160_10.g6110;Description=hypothetical protein FVEG_11685 [Fusarium verticillioides 7600];Gene=FVER53263_11685;Ontology_term=nucleus,DNA binding,zinc ion binding,DNA-binding transcription factor activity, RNA polymerase II-specific,regulation of transcription by RNA polymerase
Fv_160_10.g6111	672	ID=Fv_160_10.g6111;Description=hypothetical protein FVER53590_11686 [Fusarium verticillioides];Gene=FACUT_9088;Ontology_term=glycosyltransferase
Fv_160_10.g6112	239	ID=Fv_160_10.g6112;Description=hypothetical protein FVEG_11687 [Fusarium verticillioides

Fv_160_10.g6113	350	ID=Fv_160_10.g6113;Description=hypothetical protein FVER53263_11688 [Fusarium verticillioides];Gene=FVER53590_11688;Ontology_term=inorganic cation transmembrane transporter activity,metal ion
Fv_160_10.g6114	465	ID=Fv_160_10.g6114;Description=hypothetical protein FVER53590_11689 [Fusarium
Fv_160_10.g6115	752	ID=Fv_160_10.g6115;Description=hypothetical protein FNYG_05274 [Fusarium nygamai];Gene=FNAPI_8853;Ontology_term=membrane,DNA binding,DNA transposition,DNA
Fv_160_10.g6116	417	ID=Fv_160_10.g6116;Description=---NA---
Fv_160_10.g6117	477	ID=Fv_160_10.g6117;Description=hypothetical protein CEP54_012910 [Fusarium duplospermum]
Fv_160_10.g6118	367	ID=Fv_160_10.g6118;Description=hypothetical protein J7337_010089 [Fusarium musae];Gene=FGLOB1_12548;Ontology_term=monooxygenase
Fv_160_10.g6119	187	ID=Fv_160_10.g6119;Description=GNAT family n-acetyltransferase [Fusarium coicis];Gene=FVEG_17072;Ontology_term=acyltransferase activity, transferring groups other than amino-acyl
Fv_160_10.g6120	369	ID=Fv_160_10.g6120;Description=hypothetical protein FVEG_11692 [Fusarium verticillioides 7600]
Fv_160_10.g6121	613	ID=Fv_160_10.g6121;Description=hypothetical protein FVER53590_11693 [Fusarium verticillioides];Gene=C2S_11086;Ontology_term=nucleosome,nucleus,membrane,DNA binding,transmembrane transporter activity,structural constituent of chromatin,protein heterodimerization activity,transmembrane
Fv_160_10.g6122	568	ID=Fv_160_10.g6122;Description=hypothetical protein J7337_010094 [Fusarium musae]
Fv_160_10.g6123	778	ID=Fv_160_10.g6123;Description=hypothetical protein FVER14953_11695 [Fusarium verticillioides];Gene=FMUND_13329;Ontology_term=vacuolar membrane,serine-type peptidase activity,dipeptidyl-peptidase activity,proteolysis;Ontology_id=GO:0005774,GO:0008236,GO:0008239,GO:0006508;Enzyme_code=EC:3.4.14.1,EC:3.4.14.5,EC:3.4.14.4,EC:3.4.14.2;Enzyme_name=dipeptidyl-peptidase I,dipeptidyl-peptidase IV,dipeptidyl-peptidase III,dipeptidyl-peptidase II
Fv_160_10.g6124	755	ID=Fv_160_10.g6124;Description=hypothetical protein J7337_010096 [Fusarium musae]
Fv_160_10.g6125	348	ID=Fv_160_10.g6125;Description=hypothetical protein FVER14953_11697 [Fusarium verticillioides];Gene=FOTG_19089;Ontology_term=glucosidase activity,arabinogalactan endo-1,4-beta-galactosidase
Fv_160_10.g6126	243	ID=Fv_160_10.g6126;Description=hypothetical protein FVEG_11698 [Fusarium verticillioides 7600];Gene=FMAN_10777;Ontology_term=nucleic acid binding,3'-5' exonuclease activity,nucleobase-containing compound
Fv_160_10.g6127	528	ID=Fv_160_10.g6127;Description=hypothetical protein FPRO03_03767 [Fusarium proliferatum]
Fv_160_10.g6128	311	ID=Fv_160_10.g6128;Description=hypothetical protein FVER14953_11700 [Fusarium verticillioides];Gene=CEK26_011424;Ontology_term=extracellular region,membrane,carboxylic ester hydrolase activity,xylan
Fv_160_10.g6129	354	ID=Fv_160_10.g6129;Description=hypothetical protein FVER53263_21006 [Fusarium verticillioides];Gene=FVEG_17077;Ontology_term=S-adenosylmethionine-dependent methyltransferase
Fv_160_10.g6130	411	ID=Fv_160_10.g6130;Description=hypothetical protein FVER14953_11703 [Fusarium verticillioides];Gene=FVEG_11703;Ontology_term=membrane,monooxygenase activity,FAD binding,biosynthetic
Fv_160_10.g6131	396	ID=Fv_160_10.g6131;Description=hypothetical protein FVER14953_11704 [Fusarium

Fv_160_10.g6132	1404	ID=Fv_160_10.g6132;Description=hypothetical protein H9Q72_005155 [Fusarium xylarioides];Gene=FMUND_13313;Ontology_term=membrane,transmembrane transporter activity,transmembrane
Fv_160_10.g6133	572	ID=Fv_160_10.g6133;Description=hypothetical protein FVEG_11707 [Fusarium verticillioides 7600];Gene=AU210_010997;Ontology_term=nucleus,DNA binding,zinc ion binding,DNA-binding transcription factor activity, RNA polymerase II-specific,regulation of transcription by RNA polymerase
Fv_160_10.g6134	520	ID=Fv_160_10.g6134;Description=hypothetical protein FVEG_11708 [Fusarium verticillioides 7600];Gene=FPANT_11866;Ontology_term=sulfuric ester hydrolase
Fv_160_10.g6135	1078	ID=Fv_160_10.g6135;Description=hypothetical protein FVER53263_11709 [Fusarium verticillioides];Gene=LOC115626326;Ontology_term=alcohol dehydrogenase (NAD+) activity,alcohol metabolic
Fv_160_10.g6136	666	ID=Fv_160_10.g6136;Description=hypothetical protein FVEG_11710 [Fusarium verticillioides 7600];Gene=TY2A-C;Ontology_term=DNA binding,zinc ion binding,RNA binding,retrotransposition,cytoplasm,retrotransposon nucleocapsid,DNA-binding transcription factor activity, RNA polymerase II-specific,regulation of transcription by RNA polymerase
Fv_160_10.g6137	331	ID=Fv_160_10.g6137;Description=hypothetical protein FVER53263_11711 [Fusarium verticillioides]
Fv_160_10.g6138	884	ID=Fv_160_10.g6138;Description=putative ZDHHC-type palmitoyltransferase 6 [Fusarium bulbicola];Gene=F52700_2852;Ontology_term=kinase
Fv_160_10.g6139	504	ID=Fv_160_10.g6139;Description=hypothetical protein FVEG_11713 [Fusarium verticillioides 7600];Gene=Pp1alpha-96A;Ontology_term=myosin phosphatase activity;Ontology_id=GO:0017018;Enzyme_code=EC:3.1.3.16;Enzyme_name=protein-
Fv_160_10.g6140	232	ID=Fv_160_10.g6140;Description=hypothetical protein FVEG_17079 [Fusarium verticillioides 7600];Gene=FDENT_2271;Ontology_term=carbon-sulfur lyase activity,metal ion
Fv_160_10.g6141	524	ID=Fv_160_10.g6141;Description=N1-acetylpolyamine oxidase [Fusarium verticillioides 7600];Gene=FPRO05_13628;Ontology_term=oxidoreductase
Fv_160_10.g6142	340	ID=Fv_160_10.g6142;Description=hypothetical protein FVEG_11715 [Fusarium verticillioides 7600];Gene=FOC4_g10002412;Ontology_term=membrane,protein dimerization activity;Ontology_id=GO:0016020,GO:0046983
Fv_160_10.g6143	1026	ID=Fv_160_10.g6143;Description=hypothetical protein FAGAP_7889, partial [Fusarium agapanthi];Gene=FSUBG_11921
Fv_160_10.g6144	432	ID=Fv_160_10.g6144;Description=hypothetical protein FVER14953_11717 [Fusarium verticillioides];Gene=FNYG_02330;Ontology_term=hydrolase activity, acting on carbon-nitrogen (but not peptide)
Fv_160_10.g6145	334	ID=Fv_160_10.g6145;Description=hypothetical protein FVEG_11719 [Fusarium verticillioides
Fv_160_10.g6146	270	ID=Fv_160_10.g6146;Description=hypothetical protein FVER53263_11720 [Fusarium verticillioides]
Fv_160_10.g6147	179	ID=Fv_160_10.g6147;Description=hypothetical protein FVER14953_11721 [Fusarium verticillioides];Gene=FMUND_7081;Ontology_term=nucleus,cytoplasm,nucleotide binding,ubiquitin activating enzyme activity,dioxygenase activity,protein ubiquitination;Ontology_id=GO:0005634,GO:0005737,GO:0000166,GO:0004839,GO:0051213,GO:0016567;Enzyme_code=EC:6.2.1.

Fv_160_10.g6148	230	ID=Fv_160_10.g6148;Description=hypothetical protein J7337_010125 [Fusarium musae];Gene=PpY-55A;Ontology_term=nucleus,cytoplasm,myosin phosphatase activity,metal ion binding,protein dephosphorylation;Ontology_id=GO:0005634,GO:0005737,GO:0017018,GO:0046872,GO:0006470;Enzyme_code=EC:3.1.3.16;Enzy
Fv_160_10.g6149	108	ID=Fv_160_10.g6149;Description=hypothetical protein FNAPI_10060 [Fusarium napiforme];Gene=UBC1;Ontology_term=ATP binding,ubiquitin conjugating enzyme activity;Ontology_id=GO:0005524,GO:0061631;Enzyme_code=EC:2.3.2.23;Enzyme_name=E2
Fv_160_10.g6150	80	ID=Fv_160_10.g6150;Description=hypothetical protein FVER53263_21008 [Fusarium verticillioides]
Fv_160_10.g6151	250	ID=Fv_160_10.g6151;Description=hypothetical protein FVER53263_11724 [Fusarium verticillioides]
Fv_160_10.g6152	286	ID=Fv_160_10.g6152;Description=hypothetical protein FVEG_11725 [Fusarium verticillioides 7600]
Fv_160_10.g6153	396	ID=Fv_160_10.g6153;Description=hypothetical protein FVER53590_11726 [Fusarium
Fv_160_10.g6154	706	ID=Fv_160_10.g6154;Description=heterokaryon incompatibility protein [Fusarium napiforme]
Fv_160_10.g6155	320	ID=Fv_160_10.g6155;Description=ribonuclease p mrp [Fusarium napiforme]
Fv_160_10.g6156	1335	ID=Fv_160_10.g6156;Description=hypothetical protein FVEG_11729 [Fusarium verticillioides 7600]
Fv_160_10.g6157	638	ID=Fv_160_10.g6157;Description=hypothetical protein FVER14953_11730 [Fusarium verticillioides]
Fv_160_10.g6158	495	ID=Fv_160_10.g6158;Description=hypothetical protein FVEG_11731 [Fusarium verticillioides 7600];Gene=FPANT_245;Ontology_term=nucleus,cytosol,ribonucleoprotein complex,oxidoreductase activity,FAD binding,translation,protein
Fv_160_10.g6159	101	ID=Fv_160_10.g6159;Description=hypothetical protein FVEG_17084 [Fusarium verticillioides 7600];Gene=RPS31;Ontology_term=membrane,ribosomal subunit,structural constituent of
Fv_160_10.g6160	1091	ID=Fv_160_10.g6160;Description=heterokaryon incompatibility protein [Fusarium sp. NRRL
Fv_160_10.g6161	329	ID=Fv_160_10.g6161;Description=hypothetical protein FVEG_17087 [Fusarium verticillioides
Fv_160_10.g6162	513	ID=Fv_160_10.g6162;Description=hypothetical protein J7337_010135 [Fusarium musae];Gene=FVER53263_11734;Ontology_term=membrane,monooxygenase activity,iron ion binding,methyltransferase activity,oxidoreductase activity, acting on paired donors, with incorporation or reduction of molecular oxygen,heme binding,methylation;Ontology_id=GO:0016020,GO:0004497,GO:0005506,GO:0008168,GO:0016705,GO:0020037,GO:0032259;Enzy
Fv_160_10.g6163	81	ID=Fv_160_10.g6163;Description=hypothetical protein FVER14953_11735 [Fusarium verticillioides];Gene=FVER53263_11735;Ontology_term=spliceosomal complex,monooxygenase activity,mRNA splicing, via
Fv_160_10.g6164	794	ID=Fv_160_10.g6164;Description=hypothetical protein FVER53590_11736 [Fusarium verticillioides]
Fv_160_10.g6165	115	ID=Fv_160_10.g6165;Description=hypothetical protein FVEG_11737 [Fusarium verticillioides 7600]
Fv_160_10.g6166	259	ID=Fv_160_10.g6166;Description=hypothetical protein FVER14953_11738 [Fusarium verticillioides]
Fv_160_10.g6167	375	ID=Fv_160_10.g6167;Description=hypothetical protein FVER14953_13398 [Fusarium verticillioides];Gene=LW93_6082;Ontology_term=membrane,hydrolase
Fv_160_10.g6168	142	ID=Fv_160_10.g6168;Description=hypothetical protein FVER53263_11739 [Fusarium verticillioides]

Fv_160_10.g6169	426	ID=Fv_160_10.g6169;Description=hypothetical protein FVEG_11740 [Fusarium verticillioides 7600];Gene=BFJ72_g10915;Ontology_term=nucleus,DNA-binding transcription factor activity, RNA polymerase II-specific,zinc ion
Fv_160_10.g6170	332	ID=Fv_160_10.g6170;Description=hypothetical protein FVER53590_11741 [Fusarium verticillioides]
Fv_160_10.g6171	335	ID=Fv_160_10.g6171;Description=hypothetical protein FVEG_17089 [Fusarium verticillioides 7600]
Fv_160_10.g6172	369	ID=Fv_160_10.g6172;Description=hypothetical protein FVEG_11742 [Fusarium verticillioides 7600];Gene=lepG-1;Ontology_term=nucleotide binding,oxidoreductase activity, acting on
Fv_160_10.g6173	407	ID=Fv_160_10.g6173;Description=glycolipid 2-alpha-mannosyltransferase [Fusarium coicis];Gene=FCIRC_2028;Ontology_term=membrane,mannosyltransferase
Fv_160_10.g6174	241	ID=Fv_160_10.g6174;Description=hypothetical protein FVER14953_11744 [Fusarium verticillioides];Gene=FVER53263_11744;Ontology_term=mitochondrion,membrane;Ontology_id=GO:0005739,GO:0016020
Fv_160_10.g6175	244	ID=Fv_160_10.g6175;Description=hypothetical protein FVEG_11745 [Fusarium verticillioides 7600]
Fv_160_10.g6176	877	ID=Fv_160_10.g6176;Description=hypothetical protein FVER53590_11746 [Fusarium verticillioides];Gene=FVEG_11746;Ontology_term=membrane,sterol 3-beta-glucosyltransferase activity,carbohydrate metabolic process,lipid
Fv_160_10.g6177	361	ID=Fv_160_10.g6177;Description=hypothetical protein FVER53590_11750 [Fusarium verticillioides];Gene=FANTH_9622;Ontology_term=maleylacetate reductase activity,metal ion
Fv_160_10.g6178	510	ID=Fv_160_10.g6178;Description=hypothetical protein FVEG_11751 [Fusarium verticillioides 7600];Gene=FPHYL_4409;Ontology_term=membrane,transmembrane transporter activity,transmembrane
Fv_160_10.g6179	694	ID=Fv_160_10.g6179;Description=hypothetical protein FVER53590_11753 [Fusarium verticillioides];Gene=FGLOB1_9309;Ontology_term=nucleus,DNA-binding transcription factor activity, RNA polymerase II-
Fv_160_10.g6180	338	ID=Fv_160_10.g6180;Description=hypothetical protein FVER53590_30471 [Fusarium verticillioides];Gene=FCIRC_1464;Ontology_term=cytoplasm,NADPH dehydrogenase activity,FMN binding,5-oxoprolinase (ATP-hydrolyzing) activity,NADP binding,glutathione metabolic process;Ontology_id=GO:0005737,GO:0003959,GO:0010181,GO:0017168,GO:0050661,GO:0006749;Enzyme_code=EC:3.5.2.9,EC:1.
Fv_160_10.g6181	437	ID=Fv_160_10.g6181;Description=hypothetical protein FVER14953_11755 [Fusarium verticillioides];Gene=FVER53263_11755;Ontology_term=NADPH dehydrogenase activity,FMN binding,NADP
Fv_160_10.g6182	633	ID=Fv_160_10.g6182;Description=hypothetical protein FVER14953_11756 [Fusarium verticillioides];Gene=moxY-3;Ontology_term=nuclear chromosome,nucleolus,cytosolic small ribosomal subunit,small-subunit processome,structural constituent of ribosome,N,N-dimethylaniline monooxygenase activity,flavin adenine dinucleotide binding,NADP binding,small ribosomal subunit rRNA binding,cytoplasmic translation,ribosomal small subunit biogenesis;Ontology_id=GO:0000228,GO:0005730,GO:0022627,GO:0032040,GO:0003735,GO:0004499,GO:0050660,GO:0050661,G
Fv_160_10.g6183	296	ID=Fv_160_10.g6183;Description=hypothetical protein FVER14953_11757 [Fusarium verticillioides];Gene=FVER53263_11757;Ontology_term=oxidoreductase activity,cellular biosynthetic

Fv_160_10.g6184	254	ID=Fv_160_10.g6184;Description=acetoacetate decarboxylase [Fusarium verticillioides 7600];Gene=FVER53263_11758;Ontology_term=carboxy-lyase
Fv_160_10.g6185	257	ID=Fv_160_10.g6185;Description=hypothetical protein FVEG_11759 [Fusarium verticillioides 7600];Gene=FPANT_10104;Ontology_term=membrane,oxidoreductase activity,transmembrane transporter activity,amino acid transport,cellular biosynthetic process,transmembrane
Fv_160_10.g6186	584	ID=Fv_160_10.g6186;Description=hypothetical protein FVER14953_20233 [Fusarium verticillioides];Gene=FPANT_10105;Ontology_term=membrane,monooxygenase activity,iron ion binding,oxidoreductase activity, acting on paired donors, with incorporation or reduction of molecular oxygen,heme binding;Ontology_id=GO:0016020,GO:0004497,GO:0005506,GO:0016705,GO:0020037;Enzyme_code=EC:1.14;Enzyme_name=Actin
Fv_160_10.g6187	7779	ID=Fv_160_10.g6187;Description=hypothetical protein FVER53263_11762 [Fusarium verticillioides];Gene=FNAPI_959;Ontology_term=membrane,isomerase activity,ligase activity,phosphopantetheine binding,organic substance biosynthetic
Fv_160_10.g6188	1473	ID=Fv_160_10.g6188;Description=hypothetical protein FVER53263_11763 [Fusarium verticillioides];Gene=F25303_9856;Ontology_term=plasma membrane,copper ion binding,ATP binding,primary amine oxidase activity,ATP hydrolysis activity,quinone binding,ABC-type transporter activity,amine metabolic process,transmembrane transport;Ontology_id=GO:0005886,GO:0005507,GO:0005524,GO:0008131,GO:0016887,GO:0048038,GO:0140359,GO:0009308,GO
Fv_160_10.g6189	791	ID=Fv_160_10.g6189;Description=hypothetical protein FVER53263_11763 [Fusarium verticillioides];Gene=FPRO05_13669;Ontology_term=plasma membrane,copper ion binding,ATP binding,primary amine oxidase activity,ATP hydrolysis activity,quinone binding,ABC-type transporter activity,amine metabolic process,transmembrane transport;Ontology_id=GO:0005886,GO:0005507,GO:0005524,GO:0008131,GO:0016887,GO:0048038,GO:0140359,GO:0009308,GO
Fv_160_10.g6190	265	ID=Fv_160_10.g6190;Description=alcohol dehydrogenase [Fusarium verticillioides 7600]
Fv_160_10.g6191	357	ID=Fv_160_10.g6191;Description=hypothetical protein FVER14953_11766 [Fusarium verticillioides];Gene=FVER53263_11766;Ontology_term=nucleotide binding,oxidoreductase activity, acting on
Fv_160_10.g6192	655	ID=Fv_160_10.g6192;Description=hypothetical protein FVEG_17090 [Fusarium verticillioides 7600];Gene=FTJAE_7592;Ontology_term=nucleotide binding,nucleus,DNA binding,zinc ion binding,oxidoreductase activity, acting on NAD(P)H,DNA-binding transcription factor activity, RNA polymerase II-specific,regulation of transcription by RNA polymerase
Fv_160_10.g6193	562	ID=Fv_160_10.g6193;Description=hypothetical protein FVEG_11767 [Fusarium verticillioides 7600];Gene=FVER53263_11767;Ontology_term=membrane,transmembrane transport;Ontology_id=GO:0016020,GO:0055085
Fv_160_10.g6194	458	ID=Fv_160_10.g6194;Description=hypothetical protein FVER14953_11768 [Fusarium verticillioides];Gene=FVER53263_11768;Ontology_term=triglyceride lipase activity,lipid catabolic

Fv_160_10.g6195	1058	ID=Fv_160_10.g6195;Description=hypothetical protein FVER53590_30468 [Fusarium verticillioides];Gene=FFC1_14419;Ontology_term=DNA repair,mitotic G2 DNA damage checkpoint signaling,FMN binding,regulation of nuclear cell cycle DNA replication,mitotic sister chromatid cohesion,telomere maintenance,heme binding,protein binding,subtelomeric heterochromatin formation,maintenance of DNA repeat elements,iron ion binding,NADPH-hemoprotein reductase activity,aromatase activity,replication fork protection complex,anaphase-promoting complex binding,mitotic intra-S DNA damage checkpoint signaling,nuclear periphery,silent mating-type cassette heterochromatin formation,mitotic DNA replication checkpoint signaling;Ontology_id=GO:0006281,GO:0007095,GO:0010181,GO:0033262,GO:0007064,GO:0000723,GO:0020037,GO:0005515,GO:
Fv_160_10.g6196	532	ID=Fv_160_10.g6196;Description=hypothetical protein FVER14953_11771 [Fusarium verticillioides];Gene=FACUT_10569;Ontology_term=N,N-dimethylaniline monooxygenase activity,flavin adenine dinucleotide binding,NADP binding;Ontology_id=GO:0004499,GO:0050660,GO:0050661;Enzyme_code=EC:1.14.13.8;Enzyme_name=flavin-
Fv_160_10.g6197	640	ID=Fv_160_10.g6197;Description=choline dehydrogenase [Fusarium verticillioides 7600];Gene=FNYG_12808;Ontology_term=oxidoreductase activity, acting on CH-OH group of donors,flavin adenine dinucleotide
Fv_160_10.g6198	598	ID=Fv_160_10.g6198;Description=hypothetical protein FVER53590_11773 [Fusarium verticillioides]
Fv_160_10.g6199	378	ID=Fv_160_10.g6199;Description=hypothetical protein FVER53590_11774 [Fusarium verticillioides];Gene=FOMG_16297;Ontology_term=oxidoreductase activity, acting on the CH-OH group of donors, NAD or NADP as
Fv_160_10.g6200	425	ID=Fv_160_10.g6200;Description=hypothetical protein FVEG_11775 [Fusarium verticillioides 7600]
Fv_160_10.g6201	356	ID=Fv_160_10.g6201;Description=hypothetical protein FVEG_11776 [Fusarium verticillioides 7600];Gene=Focb16_v015329;Ontology_term=membrane,S-adenosylmethionine-dependent methyltransferase
Fv_160_10.g6202	293	ID=Fv_160_10.g6202;Description=hypothetical protein FVER53590_30467 [Fusarium
Fv_160_10.g6203	594	ID=Fv_160_10.g6203;Description=hypothetical protein FVEG_11777 [Fusarium verticillioides 7600];Gene=FVER53263_11777;Ontology_term=ATP binding,ATP-dependent protein folding chaperone,protein
Fv_160_10.g6204	328	ID=Fv_160_10.g6204;Description=hypothetical protein FVEG_11778 [Fusarium verticillioides 7600];Gene=FANTH_9596;Ontology_term=membrane,GTP binding;Ontology_id=GO:0016020,GO:0005525
Fv_160_10.g6205	517	ID=Fv_160_10.g6205;Description=hypothetical protein FVEG_11779 [Fusarium verticillioides 7600];Gene=FANTH_9596;Ontology_term=membrane,GTP binding;Ontology_id=GO:0016020,GO:0005525
Fv_160_10.g6206	242	ID=Fv_160_10.g6206;Description=hypothetical protein FVEG_11780 [Fusarium verticillioides 7600]
Fv_160_10.g6207	1805	ID=Fv_160_10.g6207;Description=hypothetical protein J7337_010182 [Fusarium musae]
Fv_160_10.g6208	168	ID=Fv_160_10.g6208;Description=hypothetical protein FVER53590_30466 [Fusarium
Fv_160_10.g6209	203	ID=Fv_160_10.g6209;Description=hypothetical protein FVER14953_11782 [Fusarium verticillioides]
Fv_160_10.g6210	984	ID=Fv_160_10.g6210;Description=hypothetical protein FVER53263_11783 [Fusarium verticillioides];Gene=FMAN_10864;Ontology_term=nucleus,ATP binding,glycosyltransferase activity,hydrolase activity,ATP-dependent chromatin remodeler activity,chromatin

Fv_160_10.g6211	1452	ID=Fv_160_10.g6211;Description=alpha-L-fucosidase 2 [Fusarium phyllophilum];Gene=FTJAE_7580;Ontology_term=membrane,DNA binding,G protein-coupled photoreceptor activity,ATP hydrolysis activity,G protein-coupled receptor signaling pathway,visual perception,phototransduction, UV,detection of visible light,absorption of UV light;Ontology_id=GO:0016020,GO:0003677,GO:0008020,GO:0016887,GO:0007186,GO:0007601,GO:0007604,GO:0009584,GO:001
Fv_160_10.g6212	660	ID=Fv_160_10.g6212;Description=hypothetical protein FVER53590_11786 [Fusarium verticillioides]
Fv_160_10.g6213	359	ID=Fv_160_10.g6213;Description=endopolygalacturonase 1 [Fusarium verticillioides 7600];Gene=pg5;Ontology_term=polygalacturonase activity,pectin catabolic process,cell wall
Fv_160_10.g6214	428	ID=Fv_160_10.g6214;Description=hypothetical protein F52700_13565 [Fusarium sp. NRRL 52700];Gene=FMAN_10887
Fv_160_10.g6215	572	ID=Fv_160_10.g6215;Description=hypothetical protein FVER14953_11788 [Fusarium verticillioides];Gene=FVER53263_11788;Ontology_term=membrane,transmembrane transporter activity,transmembrane
Fv_160_10.g6216	547	ID=Fv_160_10.g6216;Description=hypothetical protein FVEG_11789 [Fusarium verticillioides 7600];Gene=FMUND_466;Ontology_term=hydrolase activity, hydrolyzing O-glycosyl compounds,carbohydrate metabolic
Fv_160_10.g6217	524	ID=Fv_160_10.g6217;Description=hypothetical protein FVEG_11790 [Fusarium verticillioides 7600];Gene=FTJAE_13655;Ontology_term=cytoplasm,membrane,RNA polymerase II transcription regulator complex,transcription cis-regulatory region binding,DNA-binding transcription factor activity,transmembrane transporter activity,molecular adaptor activity,response to singlet oxygen,carbohydrate transport,response to heat,positive regulation of transcription by RNA polymerase II,transmembrane transport,regulation of endoplasmic reticulum unfolded protein
Fv_160_10.g6218	297	ID=Fv_160_10.g6218;Description=3-hydroxybutyrate dehydrogenase [Fusarium verticillioides 7600];Gene=FTJAE_13654;Ontology_term=oxidoreductase activity,cellular biosynthetic
Fv_160_10.g6219	559	ID=Fv_160_10.g6219;Description=hypothetical protein FVER14953_11792 [Fusarium verticillioides];Gene=FGLOB1_3578;Ontology_term=N,N-dimethylaniline monooxygenase activity,flavin adenine dinucleotide binding,NADP binding;Ontology_id=GO:0004499,GO:0050660,GO:0050661;Enzyme_code=EC:1.14.13.8;Enzyme_name=flavin-
Fv_160_10.g6220	557	ID=Fv_160_10.g6220;Description=hypothetical protein FVEG_11793 [Fusarium verticillioides 7600];Gene=CEK26_011526;Ontology_term=nucleus,membrane,DNA-binding transcription factor activity, RNA polymerase II-specific,glycerone kinase activity,ATP binding,zinc ion binding,transmembrane transporter activity,regulation of transcription by RNA polymerase II,phosphorylation,anaerobic glycerol catabolic process,transmembrane transport;Ontology_id=GO:0005634,GO:0016020,GO:0000981,GO:0004371,GO:0005524,GO:0008270,GO:0022857,GO:0006357,GO
Fv_160_10.g6221	494	ID=Fv_160_10.g6221;Description=hypothetical protein FVER14953_11794 [Fusarium verticillioides];Gene=FRV6_11742;Ontology_term=nucleus,membrane,DNA-binding transcription factor activity, RNA polymerase II-specific,zinc ion binding,transmembrane transporter activity,regulation of transcription by RNA polymerase II,transmembrane
Fv_160_10.g6222	687	ID=Fv_160_10.g6222;Description=hypothetical protein FVEG_11795 [Fusarium verticillioides 7600];Gene=FANTH_9576;Ontology_term=nucleus,DNA binding,zinc ion binding,DNA-binding transcription factor activity, RNA polymerase II-specific,regulation of transcription by RNA polymerase

Fv_160_10.g6223	469	ID=Fv_160_10.g6223;Description=WSC domain protein [Fusarium sp. NRRL 52700];Gene=FACUT_9374;Ontology_term=nucleus,DNA-binding transcription factor activity, RNA polymerase II-specific,zinc ion binding,regulation of transcription by RNA polymerase
Fv_160_10.g6224	570	ID=Fv_160_10.g6224;Description=hypothetical protein FVER53590_30464 [Fusarium verticillioides];Gene=FANTH_9574;Ontology_term=hydrolase activity, acting on carbon-nitrogen (but not peptide) bonds,nucleus,DNA binding,zinc ion binding,DNA-binding transcription factor activity, RNA polymerase II-specific,regulation of transcription by RNA polymerase
Fv_160_10.g6225	444	ID=Fv_160_10.g6225;Description=hypothetical protein FVER14953_11797 [Fusarium verticillioides];Gene=FANTH_9573;Ontology_term=hydrolase activity, acting on carbon-nitrogen (but not peptide)
Fv_160_10.g6226	503	ID=Fv_160_10.g6226;Description=hypothetical protein FVEG_11798 [Fusarium verticillioides 7600];Gene=FGADI_10248;Ontology_term=membrane,oxidoreductase activity,transmembrane transporter activity,cellular biosynthetic process,transmembrane
Fv_160_10.g6227	570	ID=Fv_160_10.g6227;Description=hypothetical protein FVER14953_11799 [Fusarium verticillioides];Gene=FVER53263_11799;Ontology_term=membrane,amino acid transport,transmembrane
Fv_160_10.g6228	219	ID=Fv_160_10.g6228;Description=hypothetical protein FVER53590_30463 [Fusarium verticillioides]
Fv_160_10.g6229	457	ID=Fv_160_10.g6229;Description=acetyl synthetase [Fusarium denticulatum]
Fv_160_10.g6230	496	ID=Fv_160_10.g6230;Description=hypothetical protein FVER14953_11801 [Fusarium verticillioides];Gene=FNAPI_11743;Ontology_term=membrane,transmembrane transporter activity,transmembrane
Fv_160_10.g6231	470	ID=Fv_160_10.g6231;Description=hypothetical protein FVER53590_11802 [Fusarium verticillioides];Gene=FOMG_16275;Ontology_term=membrane,catalytic activity,FAD
Fv_160_10.g6232	388	ID=Fv_160_10.g6232;Description=hypothetical protein FVER53263_11802 [Fusarium verticillioides];Gene=FNAPI_11745;Ontology_term=nucleus,DNA-binding transcription factor activity, RNA polymerase II-specific,zinc ion binding,oxidoreductase activity,FAD binding,regulation of transcription by RNA polymerase
Fv_160_10.g6233	476	ID=Fv_160_10.g6233;Description=hypothetical protein FVEG_11803 [Fusarium verticillioides 7600];Gene=FSUBG_11365;Ontology_term=nucleus,DNA-binding transcription factor activity, RNA polymerase II-specific,zinc ion binding,oxidoreductase activity,regulation of transcription by RNA polymerase
Fv_160_10.g6234	555	ID=Fv_160_10.g6234;Description=hypothetical protein FVER53590_11804 [Fusarium verticillioides];Gene=FMUND_11821;Ontology_term=membrane,transmembrane transport;Ontology_id=GO:0016020,GO:0055085
Fv_160_10.g6235	240	ID=Fv_160_10.g6235;Description=hypothetical protein FVER14953_11805 [Fusarium verticillioides];Gene=CEK26_011514;Ontology_term=cyclin-dependent protein serine/threonine kinase regulator activity,phosphatase activity,regulation of protein phosphorylation,mitotic cell cycle phase
Fv_160_10.g6236	684	ID=Fv_160_10.g6236;Description=hypothetical protein FVEG_11806 [Fusarium verticillioides 7600];Gene=FOQG_15241;Ontology_term=nucleus,DNA binding,zinc ion binding,DNA-binding transcription factor activity, RNA polymerase II-specific,regulation of transcription by RNA polymerase

Fv_160_10.g6237	448	ID=Fv_160_10.g6237;Description=hypothetical protein FVER14953_11807 [Fusarium verticillioides];Gene=FNAPI_11749;Ontology_term=nucleolus,cytoplasm,RNA helicase activity,mRNA binding,ATP binding,oxidoreductase activity,ATP hydrolysis activity,FAD binding,mRNA splicing, via spliceosome,regulation of translation,regulatory ncRNA-mediated gene silencing,regulation of defense response to virus;Ontology_id=GO:0005730,GO:0005737,GO:0003724,GO:0003729,GO:0005524,GO:0016491,GO:0016887,GO:0071949,GO:000
Fv_160_10.g6238	558	ID=Fv_160_10.g6238;Description=hypothetical protein FVER14953_20227 [Fusarium verticillioides]
Fv_160_11.g6239	255	ID=Fv_160_11.g6239;Description=hypothetical protein FVER53590_08751 [Fusarium verticillioides];Gene=FVER53263_08752;Ontology_term=alpha-glucosidase activity,maltose metabolic process,carbohydrate
Fv_160_11.g6240	253	ID=Fv_160_11.g6240;Description=hypothetical protein FVER53590_08750 [Fusarium verticillioides]
Fv_160_11.g6241	533	ID=Fv_160_11.g6241;Description=hypothetical protein FVER14953_08749 [Fusarium verticillioides];Gene=C2S_3309;Ontology_term=monooxygenase activity,iron ion binding,oxidoreductase activity, acting on paired donors, with incorporation or reduction of molecular oxygen,heme binding;Ontology_id=GO:0004497,GO:0005506,GO:0016705,GO:0020037;Enzyme_code=EC:1.14;Enzyme_name=Acting on paired
Fv_160_11.g6242	75	ID=Fv_160_11.g6242;Description=hypothetical protein FVEG_16417 [Fusarium verticillioides 7600];Gene=F25303_3734;Ontology_term=oxidoreductase activity,metal ion
Fv_160_11.g6243	522	ID=Fv_160_11.g6243;Description=hypothetical protein FVER14953_08748 [Fusarium verticillioides]
Fv_160_11.g6244	460	ID=Fv_160_11.g6244;Description=hypothetical protein FVER14953_08747 [Fusarium verticillioides];Gene=FTJAE_6054;Ontology_term=methyltransferase
Fv_160_11.g6245	118	ID=Fv_160_11.g6245;Description=hypothetical protein FVEG_16415 [Fusarium verticillioides 7600];Gene=FTJAE_6054;Ontology_term=methyltransferase
Fv_160_11.g6246	128	ID=Fv_160_11.g6246;Description=hypothetical protein FVEG_08746 [Fusarium verticillioides 7600];Gene=ORF 46
Fv_160_11.g6247	477	ID=Fv_160_11.g6247;Description=hypothetical protein FVER14953_08745 [Fusarium verticillioides];Gene=FOIG_14265;Ontology_term=membrane,monooxygenase activity,iron ion binding,oxidoreductase activity, acting on paired donors, with incorporation or reduction of molecular oxygen,heme binding;Ontology_id=GO:0016020,GO:0004497,GO:0005506,GO:0016705,GO:0020037;Enzyme_code=EC:1.14;Enzyme_name=Actin
Fv_160_11.g6248	538	ID=Fv_160_11.g6248;Description=hypothetical protein FVER53590_08745 [Fusarium verticillioides];Gene=FTJAE_6056;Ontology_term=nucleus,DNA-binding transcription factor activity, RNA polymerase II-specific,monooxygenase activity,iron ion binding,zinc ion binding,oxidoreductase activity, acting on paired donors, with incorporation or reduction of molecular oxygen,heme binding,regulation of transcription by RNA polymerase II;Ontology_id=GO:0005634,GO:0000981,GO:0004497,GO:0005506,GO:0008270,GO:0016705,GO:0020037,GO:0006357;Enzyme_co
Fv_160_11.g6249	511	ID=Fv_160_11.g6249;Description=hypothetical protein FVEG_08744 [Fusarium verticillioides 7600];Gene=FNAPI_12766;Ontology_term=membrane,transmembrane transporter activity,transmembrane

Fv_160_11.g6250	423	ID=Fv_160_11.g6250;Description=salicylate hydroxylase [Fusarium verticillioides 7600];Gene=FPCIR_3779;Ontology_term=oxidoreductase activity,FAD binding,biosynthetic
Fv_160_11.g6251	450	ID=Fv_160_11.g6251;Description=hypothetical protein FVER14953_08742 [Fusarium verticillioides];Gene=FNAPI_12768;Ontology_term=FMN binding,oxidoreductase
Fv_160_11.g6252	1150	ID=Fv_160_11.g6252;Description=hypothetical protein FVER53263_08740 [Fusarium verticillioides];Gene=FACUT_3836;Ontology_term=protein serine/threonine kinase activity,ATP binding,FMN binding,oxidoreductase activity,phosphorylation;Ontology_id=GO:0004674,GO:0005524,GO:0010181,GO:0016491,GO:0016310;Enzyme_code=EC:2.7.11.1,EC:1;Enzyme_name=non-specific serine/threonine protein kinase,Oxidoreductases
Fv_160_11.g6253	69	ID=Fv_160_11.g6253;Description=hypothetical protein FVER14953_21013 [Fusarium verticillioides];Gene=CEP54_002882
Fv_160_11.g6254	743	ID=Fv_160_11.g6254;Description=serine/threonine protein kinase [Fusarium verticillioides 7600];Gene=FOXG_09738;Ontology_term=protein serine/threonine kinase activity,ATP
Fv_160_11.g6255	340	ID=Fv_160_11.g6255;Description=hypothetical protein FVEG_08738 [Fusarium verticillioides 7600];Gene=FVEG_08738;Ontology_term=protein kinase activity,ATP binding,phosphoric diester hydrolase activity,lipid metabolic process,phosphorylation;Ontology_id=GO:0004672,GO:0005524,GO:0008081,GO:0006629,GO:0016310;Enzyme_code=EC:3.1.4,EC:2.7.1;Enzyme_name=Acting on ester bonds,Transferring phosphorus-containing groups
Fv_160_11.g6256	538	ID=Fv_160_11.g6256;Description=hypothetical protein FVER53590_08737 [Fusarium verticillioides];Gene=FVEG_08737;Ontology_term=membrane,transmembrane transporter activity,transmembrane
Fv_160_11.g6257	239	ID=Fv_160_11.g6257;Description=hypothetical protein FVEG_08736 [Fusarium verticillioides 7600];Gene=FCIRC_3127;Ontology_term=membrane,oxidoreductase activity,FAD binding,biosynthetic
Fv_160_11.g6258	510	ID=Fv_160_11.g6258;Description=hypothetical protein FVER53590_08735 [Fusarium verticillioides];Gene=FOTG_14054;Ontology_term=membrane,oxidoreductase activity,transmembrane transporter activity,FAD binding,biosynthetic process,transmembrane
Fv_160_11.g6259	355	ID=Fv_160_11.g6259;Description=hypothetical protein FVER53590_08734 [Fusarium verticillioides];Gene=ORF56;Ontology_term=extracellular region,nucleus,DNA binding,pectate lyase
Fv_160_11.g6260	449	ID=Fv_160_11.g6260;Description=hypothetical protein FVEG_08733 [Fusarium verticillioides 7600];Gene=FTJAE_6065;Ontology_term=mannan endo-1,6-alpha-mannosidase activity,carbohydrate catabolic process;Ontology_id=GO:0008496,GO:0016052;Enzyme_code=EC:3.2.1.101,EC:3.2.1.24;Enzyme_name=mannan endo-1,6-alpha-
Fv_160_11.g6261	107	ID=Fv_160_11.g6261;Description=hypothetical protein FVEG_08732 [Fusarium verticillioides 7600]
Fv_160_11.g6262	605	ID=Fv_160_11.g6262;Description=tetracycline resistance protein [Fusarium denticulatum];Gene=BFJ63_vAg5927;Ontology_term=membrane,transmembrane transporter activity,transmembrane
Fv_160_11.g6263	267	ID=Fv_160_11.g6263;Description=hypothetical protein FVEG_16410 [Fusarium verticillioides

Fv_160_11.g6264	576	ID=Fv_160_11.g6264;Description=hypothetical protein FVEG_16409 [Fusarium verticillioides 7600];Gene=FACUT_3826;Ontology_term=nucleus,DNA-binding transcription factor activity, RNA polymerase II-specific,zinc ion binding,oxidoreductase activity,FAD binding,regulation of transcription by RNA polymerase
Fv_160_11.g6265	271	ID=Fv_160_11.g6265;Description=hypothetical protein FVEG_16408 [Fusarium verticillioides
Fv_160_11.g6266	408	ID=Fv_160_11.g6266;Description=hypothetical protein FVEG_08729 [Fusarium verticillioides 7600];Gene=FPANT_6262;Ontology_term=nucleus,DNA-binding transcription factor activity, RNA polymerase II-specific,zinc ion binding,oxidoreductase activity,FAD binding,regulation of transcription by RNA polymerase
Fv_160_11.g6267	137	ID=Fv_160_11.g6267;Description=hypothetical protein FVEG_16407 [Fusarium verticillioides
Fv_160_11.g6268	94	ID=Fv_160_11.g6268;Description=hypothetical protein FVER14953_21011 [Fusarium verticillioides]
Fv_160_11.g6269	122	ID=Fv_160_11.g6269;Description=hypothetical protein FVEG_08728 [Fusarium verticillioides 7600]
Fv_160_11.g6270	318	ID=Fv_160_11.g6270;Description=hypothetical protein FVEG_08727 [Fusarium verticillioides 7600]
Fv_160_11.g6271	340	ID=Fv_160_11.g6271;Description=hypothetical protein J7337_012610 [Fusarium musae]
Fv_160_11.g6272	306	ID=Fv_160_11.g6272;Description=hypothetical protein FVEG_08725 [Fusarium verticillioides 7600]
Fv_160_11.g6273	363	ID=Fv_160_11.g6273;Description=hypothetical protein FVER14953_08724 [Fusarium verticillioides];Gene=FSUBG_13212;Ontology_term=S-adenosylmethionine-dependent methyltransferase
Fv_160_11.g6274	342	ID=Fv_160_11.g6274;Description=hypothetical protein FVER53263_08723 [Fusarium verticillioides];Gene=FOMG_14401;Ontology_term=hydrolase activity, hydrolyzing O-glycosyl compounds,carbohydrate metabolic
Fv_160_11.g6275	602	ID=Fv_160_11.g6275;Description=hypothetical protein FVEG_08722 [Fusarium verticillioides 7600];Gene=FDENT_679;Ontology_term=membrane,transmembrane transporter activity,transmembrane
Fv_160_11.g6276	534	ID=Fv_160_11.g6276;Description=hypothetical protein FVER14953_08721 [Fusarium verticillioides];Gene=BFJ63_vAg5918;Ontology_term=membrane,transmembrane transporter activity,transmembrane
Fv_160_11.g6277	696	ID=Fv_160_11.g6277;Description=hypothetical protein FVER53590_08719 [Fusarium verticillioides];Gene=FPHYL_4093;Ontology_term=nucleus,DNA binding,zinc ion binding,DNA-binding transcription factor activity, RNA polymerase II-specific,regulation of transcription by RNA polymerase
Fv_160_11.g6278	349	ID=Fv_160_11.g6278;Description=hypothetical protein FVEG_08718 [Fusarium verticillioides 7600];Gene=His1;Ontology_term=nucleosome,nucleus,DNA binding,structural constituent of chromatin,nucleosome
Fv_160_11.g6279	412	ID=Fv_160_11.g6279;Description=hypothetical protein J7337_012620 [Fusarium musae];Gene=FPRO_14242;Ontology_term=nucleosome,nucleus,DNA binding,monooxygenase activity,structural constituent of chromatin,protein heterodimerization activity,FAD binding,biosynthetic
Fv_160_11.g6280	601	ID=Fv_160_11.g6280;Description=hypothetical protein FVEG_08716 [Fusarium verticillioides 7600];Gene=FVEG_08716;Ontology_term=membrane,transmembrane transporter activity,transmembrane
Fv_160_11.g6281	346	ID=Fv_160_11.g6281;Description=hypothetical protein FVEG_08715 [Fusarium verticillioides 7600]

Fv_160_11.g6282	588	ID=Fv_160_11.g6282;Description=hypothetical protein FVEG_08714 [Fusarium verticillioides 7600];Gene=FANTH_565;Ontology_term=dipeptidyl-peptidase activity;Ontology_id=GO:0008239;Enzyme_code=EC:3.4.14.1,EC:3.4.14.5,EC:3.4.14.4,EC:3.4.14.2;Enzyme_name=dipeptidyl-
Fv_160_11.g6283	210	ID=Fv_160_11.g6283;Description=hypothetical protein FVEG_08713 [Fusarium verticillioides 7600]
Fv_160_11.g6284	575	ID=Fv_160_11.g6284;Description=hypothetical protein FVEG_08712 [Fusarium verticillioides 7600];Gene=FTJAE_9120;Ontology_term=membrane,transmembrane transporter activity,transmembrane
Fv_160_11.g6285	1209	ID=Fv_160_11.g6285;Description=hypothetical protein FVER53590_08711 [Fusarium verticillioides]
Fv_160_11.g6286	503	ID=Fv_160_11.g6286;Description=hypothetical protein FVER14953_08710 [Fusarium verticillioides]
Fv_160_11.g6287	349	ID=Fv_160_11.g6287;Description=hypothetical protein FVER14953_08709 [Fusarium verticillioides];Gene=FOQG_11062;Ontology_term=hydrolase
Fv_160_11.g6288	476	ID=Fv_160_11.g6288;Description=hypothetical protein FVEG_08708 [Fusarium verticillioides 7600];Gene=LSDV080;Ontology_term=nucleosome,nucleus,DNA binding,structural constituent of chromatin,protein
Fv_160_11.g6289	609	ID=Fv_160_11.g6289;Description=hypothetical protein FVEG_08707 [Fusarium verticillioides 7600];Gene=FPRO_14252;Ontology_term=oxidoreductase activity, acting on CH-OH group of donors,flavin adenine dinucleotide
Fv_160_11.g6290	461	ID=Fv_160_11.g6290;Description=hypothetical protein FVEG_16404 [Fusarium verticillioides 7600];Gene=80;Ontology_term=nucleosome,nucleus,DNA binding,structural constituent of chromatin,protein heterodimerization
Fv_160_11.g6291	220	ID=Fv_160_11.g6291;Description=hypothetical protein FVER14953_08706 [Fusarium verticillioides];Gene=FNAPI_1750;Ontology_term=mannose-1-phosphate guanylyltransferase (GTP) activity,acetyltransferase
Fv_160_11.g6292	114	ID=Fv_160_11.g6292;Description=hypothetical protein FVEG_08705 [Fusarium verticillioides
Fv_160_11.g6293	233	ID=Fv_160_11.g6293;Description=hypothetical protein FVEG_08704 [Fusarium verticillioides 7600]
Fv_160_11.g6294	516	ID=Fv_160_11.g6294;Description=cellobiose dehydrogenase [Fusarium agapanthi];Gene=FPHYL_4074;Ontology_term=monooxygenase activity,iron ion binding,oxidoreductase activity, acting on paired donors, with incorporation or reduction of molecular oxygen,heme binding;Ontology_id=GO:0004497,GO:0005506,GO:0016705,GO:0020037;Enzyme_code=EC:1.14;Enzyme_name=Acting on paired
Fv_160_11.g6295	793	ID=Fv_160_11.g6295;Description=hypothetical protein FVER14953_08702 [Fusarium verticillioides];Gene=FPANT_387;Ontology_term=oxidoreductase activity, acting on CH-OH group of donors,flavin adenine
Fv_160_11.g6296	302	ID=Fv_160_11.g6296;Description=hypothetical protein FVEG_08701 [Fusarium verticillioides 7600]
Fv_160_11.g6297	593	ID=Fv_160_11.g6297;Description=hypothetical protein FVEG_08700 [Fusarium verticillioides 7600];Gene=FDENT_657;Ontology_term=membrane,transmembrane transporter activity,transmembrane
Fv_160_11.g6298	428	ID=Fv_160_11.g6298;Description=hypothetical protein FVEG_08699 [Fusarium verticillioides 7600];Gene=F25303_3683;Ontology_term=acyltransferase activity,siderophore biosynthetic
Fv_160_11.g6299	290	ID=Fv_160_11.g6299;Description=hypothetical protein FVER14953_08698 [Fusarium verticillioides];Gene=LSDV091

Fv_160_11.g6300	2067	ID=Fv_160_11.g6300;Description=hypothetical protein FVER14953_08697 [Fusarium verticillioides];Gene=FOVG_13174;Ontology_term=isomerase activity,ligase activity,phosphopantetheine binding,organic substance biosynthetic
Fv_160_11.g6301	488	ID=Fv_160_11.g6301;Description=hypothetical protein FVER53590_08696 [Fusarium
Fv_160_11.g6302	610	ID=Fv_160_11.g6302;Description=hypothetical protein FVER14953_08695 [Fusarium
Fv_160_11.g6303	654	ID=Fv_160_11.g6303;Description=hypothetical protein FVEG_08694 [Fusarium verticillioides 7600];Gene=FOC4_g10009769;Ontology_term=oxidoreductase activity,FAD
Fv_160_11.g6304	405	ID=Fv_160_11.g6304;Description=hypothetical protein FVEG_08692 [Fusarium verticillioides
Fv_160_11.g6305	342	ID=Fv_160_11.g6305;Description=hypothetical protein FVER14953_08691 [Fusarium
Fv_160_11.g6306	502	ID=Fv_160_11.g6306;Description=hypothetical protein J7337_012650 [Fusarium musae];Gene=FOQG_11085;Ontology_term=FAD
Fv_160_11.g6307	384	ID=Fv_160_11.g6307;Description=hypothetical protein FVEG_08689 [Fusarium verticillioides
Fv_160_11.g6308	168	ID=Fv_160_11.g6308;Description=hypothetical protein FVEG_08688 [Fusarium verticillioides 7600]
Fv_160_11.g6309	503	ID=Fv_160_11.g6309;Description=mannitol 2-dehydrogenase [Fusarium verticillioides 7600];Gene=FDENT_645;Ontology_term=oxidoreductase
Fv_160_11.g6310	256	ID=Fv_160_11.g6310;Description=hypothetical protein FVER14953_08686 [Fusarium verticillioides]
Fv_160_11.g6311	312	ID=Fv_160_11.g6311;Description=iron-dependent peroxidase [Fusarium verticillioides 7600];Gene=FMEXI_764;Ontology_term=peroxidase activity,heme binding,cellular oxidant
Fv_160_11.g6312	280	ID=Fv_160_11.g6312;Description=hypothetical protein FVER53590_08684 [Fusarium verticillioides];Gene=FOZG_14043;Ontology_term=protein dimerization activity;Ontology_id=GO:0046983
Fv_160_11.g6313	373	ID=Fv_160_11.g6313;Description=hypothetical protein FVER53263_20762 [Fusarium verticillioides];Gene=BFJ63_vAg5829;Ontology_term=carbohydrate metabolic process;Ontology_id=GO:0005975
Fv_160_11.g6314	299	ID=Fv_160_11.g6314;Description=hypothetical protein FVER53590_08683 [Fusarium verticillioides];Gene=FPCIR_7370;Ontology_term=mitochondrial proton-transporting ATP synthase, catalytic core,plasma membrane,protein binding,ATP binding,oxidoreductase activity,ADP binding,proton-transporting ATP synthase activity, rotational mechanism,proton motive force-driven ATP synthesis,proton transmembrane transport;Ontology_id=GO:0005754,GO:0005886,GO:0005515,GO:0005524,GO:0016491,GO:0043531,GO:0046933,GO:0015986,GO
Fv_160_11.g6315	219	ID=Fv_160_11.g6315;Description=hypothetical protein FVER53590_08682 [Fusarium verticillioides]
Fv_160_11.g6316	674	ID=Fv_160_11.g6316;Description=hypothetical protein FVEG_16398 [Fusarium verticillioides 7600]
Fv_160_11.g6317	414	ID=Fv_160_11.g6317;Description=hypothetical protein FVER14953_08679 [Fusarium verticillioides];Gene=FOQG_11097;Ontology_term=cellular anatomical entity,serine-type endopeptidase

Fv_160_11.g6318	526	ID=Fv_160_11.g6318;Description=hypothetical protein FVER53590_08678 [Fusarium verticillioides];Gene=FNAPI_11733;Ontology_term=membrane,monooxygenase activity,iron ion binding,oxidoreductase activity, acting on paired donors, with incorporation or reduction of molecular oxygen,heme binding;Ontology_id=GO:0016020,GO:0004497,GO:0005506,GO:0016705,GO:0020037;Enzyme_code=EC:1.14;Enzyme_name=Actin
Fv_160_11.g6319	554	ID=Fv_160_11.g6319;Description=ARCA [Fusarium coicis];Gene=FOVG_13152;Ontology_term=nucleus,membrane,DNA-binding transcription factor activity, RNA polymerase II-specific,zinc ion binding,regulation of transcription by RNA polymerase
Fv_160_11.g6320	496	ID=Fv_160_11.g6320;Description=hypothetical protein FVER53590_08676 [Fusarium verticillioides];Gene=FOIG_14186;Ontology_term=copper ion binding,nitrite reductase (NO-forming) activity,nitrogen compound
Fv_160_11.g6321	372	ID=Fv_160_11.g6321;Description=hypothetical protein FVER14953_08675 [Fusarium verticillioides];Gene=NUP-0;Ontology_term=catalytic activity,nucleoside metabolic process,transmembrane
Fv_160_11.g6322	98	ID=Fv_160_11.g6322;Description=hypothetical protein FVER14953_21339 [Fusarium verticillioides];Gene=BFJ65_g13498;Ontology_term=membrane,catalytic activity,nucleoside metabolic process,transmembrane
Fv_160_11.g6323	356	ID=Fv_160_11.g6323;Description=hypothetical protein FVER14953_08674 [Fusarium
Fv_160_11.g6324	291	ID=Fv_160_11.g6324;Description=3-oxoacyl-[acyl-carrier protein] reductase [Fusarium verticillioides
Fv_160_11.g6325	933	ID=Fv_160_11.g6325;Description=hypothetical protein FVER14953_08672 [Fusarium verticillioides];Gene=FNAPI_11727;Ontology_term=ATP binding,ATP hydrolysis activity,cell
Fv_160_11.g6326	187	ID=Fv_160_11.g6326;Description=hypothetical protein FVER53590_08671 [Fusarium verticillioides]
Fv_160_11.g6327	852	ID=Fv_160_11.g6327;Description=hypothetical protein FVER53590_08670 [Fusarium verticillioides]
Fv_160_11.g6328	732	ID=Fv_160_11.g6328;Description=OPT family small oligopeptide transporter [Fusarium verticillioides 7600];Gene=FMUND_10437;Ontology_term=membrane,oligopeptide transmembrane transporter activity,protein
Fv_160_11.g6329	693	ID=Fv_160_11.g6329;Description=hypothetical protein FVER53263_08668 [Fusarium verticillioides];Gene=FDENT_624;Ontology_term=hydrolase activity, hydrolyzing O-glycosyl compounds,carbohydrate metabolic
Fv_160_11.g6330	328	ID=Fv_160_11.g6330;Description=hypothetical protein FVEG_08667 [Fusarium verticillioides 7600];Gene=115
Fv_160_11.g6331	598	ID=Fv_160_11.g6331;Description=POT family proton-dependent oligopeptide transporter [Fusarium verticillioides 7600];Gene=FDENT_622;Ontology_term=plasma membrane,tripeptide transmembrane transporter activity,dipeptide transmembrane transporter activity,dipeptide transmembrane transport,tripeptide
Fv_160_11.g6332	558	ID=Fv_160_11.g6332;Description=hypothetical protein FVEG_08665 [Fusarium verticillioides 7600];Gene=117
Fv_160_11.g6333	544	ID=Fv_160_11.g6333;Description=hypothetical protein FVEG_08664 [Fusarium verticillioides 7600];Gene=FPHYL_10736;Ontology_term=membrane,transmembrane transporter activity,transmembrane
Fv_160_11.g6334	376	ID=Fv_160_11.g6334;Description=hypothetical protein J7337_012679 [Fusarium musae]
Fv_160_11.g6335	187	ID=Fv_160_11.g6335;Description=hypothetical protein FVER53590_08662 [Fusarium verticillioides];Gene=120
Fv_160_11.g6336	148	ID=Fv_160_11.g6336;Description=hypothetical protein FVEG_16393 [Fusarium verticillioides 7600]
Fv_160_11.g6337	125	ID=Fv_160_11.g6337;Description=hypothetical protein FCOIX_108 [Fusarium coicis]

Fv_160_11.g6338	150	ID=Fv_160_11.g6338;Description=---NA---
Fv_160_11.g6339	321	ID=Fv_160_11.g6339;Description=hypothetical protein FVEG_08660 [Fusarium verticillioides 7600];Gene=FOIG_14165;Ontology_term=hydrolase
Fv_160_11.g6340	1021	ID=Fv_160_11.g6340;Description=beta-galactosidase [Fusarium verticillioides 7600];Gene=FOIG_14164;Ontology_term=beta-galactosidase activity,carbohydrate metabolic
Fv_160_11.g6341	365	ID=Fv_160_11.g6341;Description=hypothetical protein FVEG_08658 [Fusarium verticillioides 7600]
Fv_160_11.g6342	459	ID=Fv_160_11.g6342;Description=hypothetical protein FVEG_08657 [Fusarium verticillioides 7600]
Fv_160_11.g6343	468	ID=Fv_160_11.g6343;Description=hypothetical protein FVER14953_08656 [Fusarium verticillioides];Gene=FDENT_611;Ontology_term=membrane,transmembrane transporter activity,transmembrane
Fv_160_11.g6344	1002	ID=Fv_160_11.g6344;Description=hypothetical protein FVEG_08655 [Fusarium verticillioides
Fv_160_11.g6345	422	ID=Fv_160_11.g6345;Description=hypothetical protein FVEG_08654 [Fusarium verticillioides 7600]
Fv_160_11.g6346	474	ID=Fv_160_11.g6346;Description=hypothetical protein FVER53590_08653 [Fusarium verticillioides];Gene=FACUT_3517;Ontology_term=membrane,transmembrane transporter activity,transmembrane
Fv_160_11.g6347	552	ID=Fv_160_11.g6347;Description=hypothetical protein FVEG_08652 [Fusarium verticillioides 7600];Gene=FPRO05_00390;Ontology_term=nucleus,DNA binding,zinc ion binding,sulfuric ester hydrolase activity,DNA-binding transcription factor activity, RNA polymerase II-specific,regulation of transcription by RNA polymerase
Fv_160_11.g6348	1001	ID=Fv_160_11.g6348;Description=hypothetical protein FVER53263_08651 [Fusarium verticillioides];Gene=FANTH_5322;Ontology_term=nucleus,DNA binding,zinc ion binding,DNA-binding transcription factor activity, RNA polymerase II-specific,regulation of transcription by RNA polymerase
Fv_160_11.g6349	753	ID=Fv_160_11.g6349;Description=hypothetical protein J7337_012695 [Fusarium musae];Gene=C2S_3408;Ontology_term=beta-glucosidase activity,scopolin beta-glucosidase activity,cellulose catabolic
Fv_160_11.g6350	1028	ID=Fv_160_11.g6350;Description=nicotinamide mononucleotide permease [Fusarium napiforme];Gene=BFJ65_g13334;Ontology_term=membrane,transmembrane transporter activity,transmembrane
Fv_160_11.g6351	210	ID=Fv_160_11.g6351;Description=hypothetical protein FVEG_08647 [Fusarium verticillioides
Fv_160_11.g6352	340	ID=Fv_160_11.g6352;Description=hypothetical protein FVER53263_08646 [Fusarium verticillioides]
Fv_160_11.g6353	172	ID=Fv_160_11.g6353;Description=hypothetical protein FVER14953_08645 [Fusarium verticillioides]
Fv_160_11.g6354	481	ID=Fv_160_11.g6354;Description=hypothetical protein FVEG_08644 [Fusarium verticillioides 7600];Gene=FOTG_12690;Ontology_term=nucleus,DNA-binding transcription factor activity, RNA polymerase II-specific,zinc ion
Fv_160_11.g6355	1019	ID=Fv_160_11.g6355;Description=hypothetical protein FVER53590_08643 [Fusarium
Fv_160_11.g6356	892	ID=Fv_160_11.g6356;Description=hypothetical protein FVER53590_08642 [Fusarium verticillioides];Gene=FPANT_9120;Ontology_term=chitin synthase activity,molecular adaptor activity,apoptotic process,hippo signaling,mRNA cis splicing, via
Fv_160_11.g6357	299	ID=Fv_160_11.g6357;Description=hypothetical protein FVEG_16384 [Fusarium verticillioides 7600]

Fv_160_11.g6358	1297	ID=Fv_160_11.g6358;Description=hypothetical protein FVEG_08641 [Fusarium verticillioides 7600];Gene=FVER53590_08641;Ontology_term=membrane,ATP binding,ATP hydrolysis activity,ABC-type transporter activity,transmembrane transport;Ontology_id=GO:0016020,GO:0005524,GO:0016887,GO:0140359,GO:0055085;Enzyme_code=EC:7.2.2,EC:3.6.1.15;Enzym
Fv_160_11.g6359	624	ID=Fv_160_11.g6359;Description=hypothetical protein FVER53590_08640 [Fusarium verticillioides];Gene=FVER53590_08640;Ontology_term=nucleus,DNA-binding transcription factor activity, RNA polymerase II-
Fv_160_11.g6360	855	ID=Fv_160_11.g6360;Description=hypothetical protein FVEG_08639 [Fusarium verticillioides 7600];Gene=FVEG_08639;Ontology_term=membrane,sulfuric ester hydrolase
Fv_160_11.g6361	153	ID=Fv_160_11.g6361;Description=hypothetical protein FVER53590_08638 [Fusarium verticillioides];Gene=146
Fv_160_11.g6362	495	ID=Fv_160_11.g6362;Description=oxalate decarboxylase [Fusarium coicis];Gene=FPHYL_9062;Ontology_term=metal ion binding,oxalate metabolic process;Ontology_id=GO:0046872,GO:0033609
Fv_160_11.g6363	327	ID=Fv_160_11.g6363;Description=hypothetical protein FPHYL_9061 [Fusarium phyllophilum]
Fv_160_11.g6364	262	ID=Fv_160_11.g6364;Description=hypothetical protein FVEG_08634 [Fusarium verticillioides 7600];Gene=BKA59DRAFT_512915;Ontology_term=catalytic activity;Ontology_id=GO:0003824
Fv_160_11.g6365	181	ID=Fv_160_11.g6365;Description=hypothetical protein FVEG_16382 [Fusarium verticillioides
Fv_160_11.g6366	430	ID=Fv_160_11.g6366;Description=hypothetical protein FVEG_08633 [Fusarium verticillioides 7600]
Fv_160_11.g6367	218	ID=Fv_160_11.g6367;Description=hypothetical protein FVER53590_08632 [Fusarium verticillioides];Gene=FRV6_14889;Ontology_term=transferase
Fv_160_11.g6368	476	ID=Fv_160_11.g6368;Description=hypothetical protein FVER53590_08631 [Fusarium verticillioides]
Fv_160_11.g6369	318	ID=Fv_160_11.g6369;Description=hypothetical protein FVEG_08630 [Fusarium verticillioides 7600];Gene=BFJ68_g4389;Ontology_term=ferric iron binding,catechol 1,2-dioxygenase activity,catechol-containing compound
Fv_160_11.g6370	268	ID=Fv_160_11.g6370;Description=hypothetical protein FVER14953_08629 [Fusarium verticillioides];Gene=28951375;Ontology_term=membrane,structural constituent of eye lens,morphogenesis of an epithelium,lens
Fv_160_11.g6371	503	ID=Fv_160_11.g6371;Description=hypothetical protein FVEG_16381 [Fusarium verticillioides 7600];Gene=157
Fv_160_11.g6372	322	ID=Fv_160_11.g6372;Description=hypothetical protein FVER53263_08628 [Fusarium
Fv_160_11.g6373	394	ID=Fv_160_11.g6373;Description=hypothetical protein FVEG_08627 [Fusarium verticillioides 7600];Gene=FVEG_08627;Ontology_term=nucleus,RNA polymerase II cis-regulatory region sequence-specific DNA binding,nuclear receptor activity,zinc ion binding,oxidoreductase activity,heme binding,negative regulation of transcription by RNA polymerase II,regulation of ecdysteroid metabolic process,hormone-mediated signaling pathway,ecdysis, chitin-based cuticle,intracellular receptor signaling pathway,response to ecdysone,positive regulation of transcription by RNA polymerase II,oogenesis,negative regulation of peptidoglycan recognition protein signaling
Fv_160_11.g6374	305	ID=Fv_160_11.g6374;Description=hypothetical protein FVER14953_08626 [Fusarium verticillioides];Gene=FOTG_15471;Ontology_term=membrane,zinc ion binding,oxidoreductase

Fv_160_11.g6375	359	ID=Fv_160_11.g6375;Description=hypothetical protein FVEG_08625 [Fusarium verticillioides 7600];Gene=FVEG_08625;Ontology_term=membrane,zinc ion binding,oxidoreductase
Fv_160_11.g6376	506	ID=Fv_160_11.g6376;Description=hypothetical protein FVER14953_08624 [Fusarium verticillioides];Gene=BFJ69_g276;Ontology_term=membrane,transmembrane transporter activity,transmembrane
Fv_160_11.g6377	413	ID=Fv_160_11.g6377;Description=hypothetical protein FVEG_16379 [Fusarium verticillioides
Fv_160_11.g6378	226	ID=Fv_160_11.g6378;Description=hypothetical protein FNAPI_6979 [Fusarium napiforme];Gene=FVER53263_08623
Fv_160_11.g6379	318	ID=Fv_160_11.g6379;Description=hypothetical protein FVEG_16378 [Fusarium verticillioides 7600];Gene=161
Fv_160_11.g6380	323	ID=Fv_160_11.g6380;Description=hypothetical protein J7337_012720 [Fusarium
Fv_160_11.g6381	719	ID=Fv_160_11.g6381;Description=hypothetical protein FVER53590_08621 [Fusarium verticillioides];Gene=FDENT_2603;Ontology_term=nucleus,DNA binding,zinc ion binding,DNA-binding transcription factor activity, RNA polymerase II-specific,regulation of transcription by RNA polymerase
Fv_160_11.g6382	239	ID=Fv_160_11.g6382;Description=hypothetical protein FVER53590_08620 [Fusarium verticillioides]
Fv_160_11.g6383	398	ID=Fv_160_11.g6383;Description=hypothetical protein FVER53590_08619 [Fusarium verticillioides];Gene=FOTG_15482;Ontology_term=FMN binding,oxidoreductase
Fv_160_11.g6384	175	ID=Fv_160_11.g6384;Description=hypothetical protein FVER53590_08618 [Fusarium verticillioides];Gene=FDENT_2606;Ontology_term=carbon-sulfur lyase activity,metal ion
Fv_160_11.g6385	365	ID=Fv_160_11.g6385;Description=hypothetical protein FVER53263_08617 [Fusarium verticillioides]
Fv_160_11.g6386	371	ID=Fv_160_11.g6386;Description=hypothetical protein FVEG_08616 [Fusarium verticillioides 7600]
Fv_160_11.g6387	592	ID=Fv_160_11.g6387;Description=hypothetical protein FVER14953_08615 [Fusarium verticillioides];Gene=C2S_3449;Ontology_term=membrane,acyltransferase activity, transferring groups other than amino-acyl
Fv_160_11.g6388	288	ID=Fv_160_11.g6388;Description=hypothetical protein FVER53263_08614 [Fusarium verticillioides];Gene=FOVG_13072;Ontology_term=oxidoreductase
Fv_160_11.g6389	264	ID=Fv_160_11.g6389;Description=hypothetical protein FVEG_16375 [Fusarium verticillioides 7600]
Fv_160_11.g6390	86	ID=Fv_160_11.g6390;Description=hypothetical protein FVEG_08613 [Fusarium verticillioides 7600]
Fv_160_11.g6391	459	ID=Fv_160_11.g6391;Description=hypothetical protein FVER14953_08612 [Fusarium
Fv_160_11.g6392	348	ID=Fv_160_11.g6392;Description=hypothetical protein J7337_012734 [Fusarium musae];Gene=FMUND_459
Fv_160_11.g6393	460	ID=Fv_160_11.g6393;Description=hypothetical protein FVER53263_08610 [Fusarium
Fv_160_11.g6394	157	ID=Fv_160_11.g6394;Description=hypothetical protein FVEG_08609 [Fusarium verticillioides 7600]
Fv_160_11.g6395	833	ID=Fv_160_11.g6395;Description=hypothetical protein FVER53590_08607 [Fusarium verticillioides];Gene=178;Ontology_term=neuron projection,postsynaptic membrane,transmembrane signaling receptor activity,acetylcholine-gated monoatomic cation-selective channel activity,synaptic transmission, cholinergic,monoatomic ion
Fv_160_11.g6396	598	ID=Fv_160_11.g6396;Description=hypothetical protein FVEG_16372 [Fusarium verticillioides 7600]

Fv_160_11.g6397	596	ID=Fv_160_11.g6397;Description=hypothetical protein FVER53590_08606 [Fusarium verticillioides];Gene=FACUT_11291;Ontology_term=membrane,transmembrane transporter activity,transmembrane
Fv_160_11.g6398	574	ID=Fv_160_11.g6398;Description=hypothetical protein FVER53590_08605 [Fusarium verticillioides];Gene=FVEG_08605;Ontology_term=hydrolase activity, hydrolyzing O-glycosyl compounds,carbohydrate metabolic
Fv_160_11.g6399	355	ID=Fv_160_11.g6399;Description=hypothetical protein FVER53263_08604 [Fusarium verticillioides];Gene=FVER53263_08604;Ontology_term=methyltransferase
Fv_160_11.g6400	266	ID=Fv_160_11.g6400;Description=hypothetical protein FVER53590_08603 [Fusarium verticillioides]
Fv_160_11.g6401	373	ID=Fv_160_11.g6401;Description=hypothetical protein FVEG_16371 [Fusarium verticillioides 7600]
Fv_160_11.g6402	645	ID=Fv_160_11.g6402;Description=hypothetical protein FVER53590_29945 [Fusarium verticillioides];Gene=AU210_013228;Ontology_term=nucleus,DNA binding,zinc ion binding,DNA-binding transcription factor activity, RNA polymerase II-specific,regulation of transcription by RNA polymerase
Fv_160_11.g6403	477	ID=Fv_160_11.g6403;Description=hypothetical protein FVEG_08601 [Fusarium verticillioides 7600];Gene=FOVG_13050;Ontology_term=gamma-glutamylcyclotransferase
Fv_160_11.g6404	302	ID=Fv_160_11.g6404;Description=hypothetical protein J7337_012746 [Fusarium musae]
Fv_160_11.g6405	232	ID=Fv_160_11.g6405;Description=hypothetical protein FVEG_08599 [Fusarium verticillioides 7600];Gene=FNYG_05442;Ontology_term=gamma-glutamylcyclotransferase
Fv_160_11.g6406	372	ID=Fv_160_11.g6406;Description=hypothetical protein FVER14953_08598 [Fusarium verticillioides];Gene=186
Fv_160_11.g6407	486	ID=Fv_160_11.g6407;Description=hypothetical protein FVER53590_08597 [Fusarium
Fv_160_11.g6408	246	ID=Fv_160_11.g6408;Description=hypothetical protein FVER53263_08596 [Fusarium verticillioides];Gene=FDENT_2636;Ontology_term=NAD glycohydrolase
Fv_160_11.g6409	79	ID=Fv_160_11.g6409;Description=hypothetical protein FVEG_16369 [Fusarium verticillioides 7600]
Fv_160_11.g6410	512	ID=Fv_160_11.g6410;Description=hypothetical protein FVEG_08595 [Fusarium verticillioides 7600];Gene=FPANT_1528;Ontology_term=membrane,transmembrane transporter activity,transmembrane
Fv_160_11.g6411	683	ID=Fv_160_11.g6411;Description=hypothetical protein FVEG_08594 [Fusarium verticillioides 7600]
Fv_160_11.g6412	272	ID=Fv_160_11.g6412;Description=hypothetical protein FVEG_08593 [Fusarium verticillioides 7600];Gene=192
Fv_160_11.g6413	463	ID=Fv_160_11.g6413;Description=hypothetical protein J7337_012754 [Fusarium musae];Gene=FTJAE_4609;Ontology_term=hydrolase
Fv_160_11.g6414	338	ID=Fv_160_11.g6414;Description=hypothetical protein FVER53590_08591 [Fusarium verticillioides];Gene=FACUT_11274;Ontology_term=hydrolase
Fv_160_11.g6415	146	ID=Fv_160_11.g6415;Description=hypothetical protein FVEG_08590 [Fusarium verticillioides 7600];Gene=FOXG_09905;Ontology_term=amino acid metabolic process;Ontology_id=GO:0006520
Fv_160_11.g6416	230	ID=Fv_160_11.g6416;Description=hypothetical protein FVER53590_08589 [Fusarium verticillioides];Gene=FOMG_15386;Ontology_term=carboxy-lyase activity,thiamine pyrophosphate binding,metal ion

Fv_160_11.g6417	220	ID=Fv_160_11.g6417;Description=pyruvate decarboxylase [Fusarium verticillioides 7600];Gene=FVER53590_25274;Ontology_term=carboxy-lyase activity,thiamine pyrophosphate
Fv_160_11.g6418	1139	ID=Fv_160_11.g6418;Description=hypothetical protein FVEG_08588 [Fusarium verticillioides 7600];Gene=FMEXI_3250;Ontology_term=nucleus,DNA binding,zinc ion binding,sulfuric ester hydrolase activity,DNA-binding transcription factor activity, RNA polymerase II-specific,regulation of transcription by RNA polymerase
Fv_160_11.g6419	528	ID=Fv_160_11.g6419;Description=hypothetical protein FVEG_08587 [Fusarium verticillioides 7600];Gene=FNAPI_8347;Ontology_term=membrane,transmembrane transporter activity,transmembrane
Fv_160_11.g6420	412	ID=Fv_160_11.g6420;Description=hypothetical protein FVER14953_08586 [Fusarium verticillioides]
Fv_160_11.g6421	559	ID=Fv_160_11.g6421;Description=hypothetical protein FVER53590_08585 [Fusarium verticillioides];Gene=FOC4_g10009913;Ontology_term=membrane,transmembrane transporter activity,transmembrane
Fv_160_11.g6422	582	ID=Fv_160_11.g6422;Description=hypothetical protein FVEG_08584 [Fusarium verticillioides 7600];Gene=F25303_11276;Ontology_term=hydrolase activity, hydrolyzing O-glycosyl compounds,carbohydrate metabolic
Fv_160_11.g6423	309	ID=Fv_160_11.g6423;Description=hypothetical protein FVER53590_08583 [Fusarium
Fv_160_11.g6424	374	ID=Fv_160_11.g6424;Description=hypothetical protein FVEG_16365 [Fusarium verticillioides
Fv_160_11.g6425	420	ID=Fv_160_11.g6425;Description=hypothetical protein FVEG_16364 [Fusarium verticillioides
Fv_160_11.g6426	554	ID=Fv_160_11.g6426;Description=hypothetical protein FVEG_08581 [Fusarium verticillioides 7600];Gene=FPRO_14395;Ontology_term=extracellular space,secretory granule,transport vesicle membrane,transmembrane
Fv_160_11.g6427	277	ID=Fv_160_11.g6427;Description=hypothetical protein FVEG_08580 [Fusarium verticillioides 7600]
Fv_160_11.g6428	730	ID=Fv_160_11.g6428;Description=hypothetical protein FVEG_16362 [Fusarium verticillioides 7600];Gene=FOXG_09926;Ontology_term=nucleus,DNA binding,zinc ion binding,DNA-binding transcription factor activity, RNA polymerase II-specific,regulation of transcription by RNA polymerase
Fv_160_11.g6429	361	ID=Fv_160_11.g6429;Description=hypothetical protein FVER14953_08577 [Fusarium
Fv_160_11.g6430	676	ID=Fv_160_11.g6430;Description=hypothetical protein FVER14953_08576 [Fusarium verticillioides];Gene=FTJAE_4594;Ontology_term=zinc ion binding,positive regulation of epidermal growth factor receptor signaling pathway,3',5'-cyclic-GMP phosphodiesterase activity,positive regulation of MAPK cascade,visual perception,photoreceptor disc membrane,photoreceptor outer segment membrane,DNA-binding transcription factor activity, RNA polymerase II-specific,positive regulation of G protein-coupled receptor signaling pathway,regulation of transcription by RNA polymerase II,nucleus,DNA binding,cGMP binding,molecular adaptor
Fv_160_11.g6431	175	ID=Fv_160_11.g6431;Description=hypothetical protein FVEG_16361 [Fusarium verticillioides 7600];Gene=FVEG_16361;Ontology_term=nucleus,DNA-binding transcription factor activity, RNA polymerase II-specific,zinc ion
Fv_160_11.g6432	187	ID=Fv_160_11.g6432;Description=hypothetical protein FVEG_08575 [Fusarium verticillioides 7600]
Fv_160_11.g6433	703	ID=Fv_160_11.g6433;Description=hypothetical protein FDENT_2658 [Fusarium denticulatum];Gene=FOPG_08198;Ontology_term=chromosome,membrane,nucleotide binding,protein kinase

Fv_160_11.g6434	238	ID=Fv_160_11.g6434;Description=hypothetical protein FVER53590_08573 [Fusarium verticillioides]
Fv_160_11.g6435	255	ID=Fv_160_11.g6435;Description=hypothetical protein FVEG_08572 [Fusarium verticillioides 7600];Gene=FPCIR_5529;Ontology_term=hydrolase
Fv_160_11.g6436	408	ID=Fv_160_11.g6436;Description=hypothetical protein FVEG_08571 [Fusarium verticillioides 7600];Gene=FRV6_14975;Ontology_term=nucleus,DNA-binding transcription factor activity, RNA polymerase II-specific,zinc ion
Fv_160_11.g6437	156	ID=Fv_160_11.g6437;Description=hypothetical protein FVER53590_08570 [Fusarium verticillioides];Gene=FTJAE_4587;Ontology_term=zinc ion binding,sequence-specific DNA binding,regulation of DNA-templated
Fv_160_11.g6438	1527	ID=Fv_160_11.g6438;Description=hypothetical protein FVER14953_08568 [Fusarium verticillioides];Gene=FVER53590_08568;Ontology_term=membrane,oligopeptide transmembrane transporter activity,protein
Fv_160_11.g6439	876	ID=Fv_160_11.g6439;Description=hypothetical protein FVER53590_08568 [Fusarium verticillioides];Gene=FPANT_134;Ontology_term=cytosol,cell surface,apical plasma membrane,nuclear membrane,chromaffin granule membrane,perinuclear region of cytoplasm,calcium ion binding,calcium-dependent phospholipid binding,heparin binding,carbohydrate binding,chondroitin sulfate binding,oligopeptide transmembrane transporter activity,identical protein binding,calcium-dependent protein binding,NF-kappaB binding,regulation of transcription by RNA polymerase II,Notch signaling pathway,protein transport,epithelial cell differentiation,negative regulation of interleukin-8 production,oligopeptide transmembrane transport;Ontology_id=GO:0005829,GO:0009986,GO:0016324,GO:0031965,GO:0042584,GO:0048471,GO:0005509,GO:0005544,GO
Fv_160_11.g6440	259	ID=Fv_160_11.g6440;Description=hypothetical protein FVER14953_08566 [Fusarium verticillioides];Gene=217;Ontology_term=acetylcholine-gated channel complex,neuron projection,postsynaptic membrane,acetylcholine receptor activity,acetylcholine-gated monoatomic cation-selective channel activity,synaptic transmission, cholinergic,response to insecticide,excitatory postsynaptic potential,monoatomic cation transmembrane
Fv_160_11.g6441	682	ID=Fv_160_11.g6441;Description=hypothetical protein FVER14953_08565 [Fusarium verticillioides];Gene=FTJAE_4583;Ontology_term=copper ion binding,primary amine oxidase activity,quinone binding,amine
Fv_160_11.g6442	383	ID=Fv_160_11.g6442;Description=hypothetical protein FVEG_08564 [Fusarium verticillioides
Fv_160_11.g6443	194	ID=Fv_160_11.g6443;Description=hypothetical protein FVEG_08563 [Fusarium verticillioides 7600];Gene=220
Fv_160_11.g6444	561	ID=Fv_160_11.g6444;Description=hypothetical protein FVER14953_21103 [Fusarium verticillioides];Gene=FPANT_139;Ontology_term=membrane,DNA helicase activity,ATP binding,hydrolase activity,telomere maintenance,DNA repair,DNA recombination,DNA duplex
Fv_160_11.g6445	319	ID=Fv_160_11.g6445;Description=hypothetical protein FVEG_08561 [Fusarium verticillioides 7600];Gene=FOYG_13282;Ontology_term=S-adenosylmethionine-dependent methyltransferase

Fv_160_11.g6446	533	ID=Fv_160_11.g6446;Description=hypothetical protein FPRO03_13589 [Fusarium proliferatum];Gene=FNYG_12254;Ontology_term=nucleus,DNA binding,zinc ion binding,transmembrane transport,transmembrane transporter activity,membrane,lyase activity,carbohydrate transport,amino acid metabolic process,S-adenosylmethionine-dependent methyltransferase activity,DNA-binding transcription factor activity, RNA polymerase II-specific,regulation of transcription by RNA polymerase
Fv_160_11.g6447	579	ID=Fv_160_11.g6447;Description=hypothetical protein FVEG_16356 [Fusarium verticillioides 7600];Gene=FOMG_15331;Ontology_term=nucleus,DNA binding,zinc ion binding,transmembrane transport,transmembrane transporter activity,membrane,DNA-binding transcription factor activity, RNA polymerase II-specific,regulation of transcription by
Fv_160_11.g6448	207	ID=Fv_160_11.g6448;Description=hypothetical protein FVER14953_08559 [Fusarium verticillioides]
Fv_160_11.g6449	220	ID=Fv_160_11.g6449;Description=hypothetical protein FVER14953_08558 [Fusarium verticillioides]
Fv_160_11.g6450	1049	ID=Fv_160_11.g6450;Description=hypothetical protein FVER14953_08557 [Fusarium verticillioides];Gene=FPANT_145;Ontology_term=beta-galactosidase complex,beta-galactosidase activity,carbohydrate binding,carbohydrate metabolic process,cell wall organization,organic substance catabolic
Fv_160_11.g6451	261	ID=Fv_160_11.g6451;Description=hypothetical protein FVEG_08556 [Fusarium verticillioides
Fv_160_11.g6452	681	ID=Fv_160_11.g6452;Description=hypothetical protein FVER14953_08555 [Fusarium verticillioides];Gene=gaoA;Ontology_term=galactose oxidase
Fv_160_11.g6453	285	ID=Fv_160_11.g6453;Description=hypothetical protein FVER14953_08554 [Fusarium verticillioides]
Fv_160_11.g6454	379	ID=Fv_160_11.g6454;Description=hypothetical protein FVER14953_08553 [Fusarium verticillioides];Gene=FNYG_12262;Ontology_term=extracellular region,mitochondrial respiratory chain complex I,mitochondrial matrix,mitochondrial electron transport, NADH to ubiquinone,antibacterial humoral response,mitochondrial respiratory chain complex I assembly,innate immune response,defense response to Gram-positive
Fv_160_11.g6455	760	ID=Fv_160_11.g6455;Description=hypothetical protein FVER53590_08552 [Fusarium verticillioides]
Fv_160_11.g6456	202	ID=Fv_160_11.g6456;Description=hypothetical protein FDENT_8425 [Fusarium denticulatum]
Fv_160_11.g6457	1368	ID=Fv_160_11.g6457;Description=hypothetical protein FVER14953_08551 [Fusarium verticillioides]
Fv_160_11.g6458	343	ID=Fv_160_11.g6458;Description=hypothetical protein LB505_011828 [Fusarium chuoi];Gene=FNYG_12264;Ontology_term=oxidoreductase activity,glycosyltransferase
Fv_160_11.g6459	281	ID=Fv_160_11.g6459;Description=alcohol dehydrogenase [Fusarium verticillioides 7600];Gene=FNYG_12265;Ontology_term=oxidoreductase
Fv_160_11.g6460	276	ID=Fv_160_11.g6460;Description=hypothetical protein FVER14953_08548 [Fusarium verticillioides]
Fv_160_11.g6461	571	ID=Fv_160_11.g6461;Description=hypothetical protein FVER53590_08547 [Fusarium verticillioides];Gene=BFJ70_g4063;Ontology_term=membrane,transmembrane transporter activity,transmembrane
Fv_160_11.g6462	376	ID=Fv_160_11.g6462;Description=hypothetical protein FVER53590_08546 [Fusarium verticillioides];Gene=FOIG_08665;Ontology_term=nucleus,DNA-binding transcription factor activity, RNA polymerase II-specific,zinc

Fv_160_11.g6463	1085	ID=Fv_160_11.g6463;Description=hypothetical protein FVER53590_08545 [Fusarium verticillioides];Gene=FCIRC_3379;Ontology_term=oxidoreductase activity,phosphopantetheine binding,secondary metabolite biosynthetic process,organic substance biosynthetic
Fv_160_11.g6464	325	ID=Fv_160_11.g6464;Description=hypothetical protein FVEG_08544 [Fusarium verticillioides 7600];Gene=FPANT_157;Ontology_term=nucleus,zinc ion binding,oxidoreductase activity,secondary metabolite biosynthetic process,phosphopantetheine binding,DNA-binding transcription factor activity, RNA polymerase II-specific,regulation of transcription
Fv_160_11.g6465	217	ID=Fv_160_11.g6465;Description=hypothetical protein FVER14953_21101 [Fusarium verticillioides];Gene=FPANT_158;Ontology_term=specification of segmental identity, labial segment,specification of segmental identity, maxillary segment,proboscis development,DNA-binding transcription factor activity, RNA polymerase II-specific,regulation of transcription by RNA polymerase II,nucleus,DNA binding,response to oxidative stress,mitochondrial respiratory chain complex I,oxidoreductase activity,secondary metabolite biosynthetic process,phosphopantetheine binding,mitochondrial respiratory chain complex I
Fv_160_11.g6466	444	ID=Fv_160_11.g6466;Description=hypothetical protein FVEG_08543 [Fusarium verticillioides 7600];Gene=FMUND_389;Ontology_term=oxidoreductase
Fv_160_11.g6467	203	ID=Fv_160_11.g6467;Description=hypothetical protein FVER53590_08542 [Fusarium verticillioides];Gene=FACUT_11651;Ontology_term=oxidoreductase
Fv_160_11.g6468	211	ID=Fv_160_11.g6468;Description=hypothetical protein FVEG_16351 [Fusarium verticillioides 7600]
Fv_160_11.g6469	950	ID=Fv_160_11.g6469;Description=hypothetical protein FVER14953_08541 [Fusarium verticillioides]
Fv_160_11.g6470	508	ID=Fv_160_11.g6470;Description=hypothetical protein FVER53590_08540 [Fusarium verticillioides];Gene=BFJ68_g4578;Ontology_term=monooxygenase activity,iron ion binding,methyltransferase activity,oxidoreductase activity, acting on paired donors, with incorporation or reduction of molecular oxygen,heme binding,methylation;Ontology_id=GO:0004497,GO:0005506,GO:0008168,GO:0016705,GO:0020037,GO:0032259;Enzyme_code=EC:
Fv_160_11.g6471	357	ID=Fv_160_11.g6471;Description=hypothetical protein FVER14953_08539 [Fusarium verticillioides]
Fv_160_11.g6472	297	ID=Fv_160_11.g6472;Description=beta-1,6-n contains WSC domain-containing protein [Fusarium
Fv_160_11.g6473	142	ID=Fv_160_11.g6473;Description=hypothetical protein FVER14953_08537 [Fusarium
Fv_160_11.g6474	474	ID=Fv_160_11.g6474;Description=hypothetical protein FVER14953_08536 [Fusarium verticillioides];Gene=mntP
Fv_160_11.g6475	370	ID=Fv_160_11.g6475;Description=NADPH2 dehydrogenase [Fusarium verticillioides 7600];Gene=FOTG_12382;Ontology_term=mitochondrial respiratory chain complex I,FMN binding,oxidoreductase activity,mitochondrial respiratory chain complex I
Fv_160_11.g6476	514	ID=Fv_160_11.g6476;Description=hypothetical protein FVER14953_08534 [Fusarium
Fv_160_11.g6477	70	ID=Fv_160_11.g6477;Description=---NA---
Fv_160_11.g6478	139	ID=Fv_160_11.g6478;Description=hypothetical protein FVER14953_21100 [Fusarium verticillioides]
Fv_160_11.g6479	331	ID=Fv_160_11.g6479;Description=hypothetical protein FVER53590_08531 [Fusarium verticillioides]

Fv_160_11.g6480	365	ID=Fv_160_11.g6480;Description=hypothetical protein FVER53263_08530 [Fusarium
Fv_160_11.g6481	858	ID=Fv_160_11.g6481;Description=hypothetical protein FVER14953_08529 [Fusarium verticillioides];Gene=FPHYL_5905;Ontology_term=membrane,hydrolase
Fv_160_11.g6482	245	ID=Fv_160_11.g6482;Description=hypothetical protein FPANT_8190 [Fusarium
Fv_160_11.g6483	196	ID=Fv_160_11.g6483;Description=hypothetical protein FVEG_08527 [Fusarium verticillioides 7600];Gene=FNYG_12286;Ontology_term=mitochondrial respiratory chain complex I,DNA binding,acyltransferase activity, transferring groups other than amino-acyl groups,mitochondrial respiratory chain complex I
Fv_160_11.g6484	352	ID=Fv_160_11.g6484;Description=hypothetical protein FVEG_08526 [Fusarium verticillioides 7600];Gene=FNYG_12287;Ontology_term=oxidoreductase activity, acting on the CH-CH group of donors, NAD or NADP as
Fv_160_11.g6485	379	ID=Fv_160_11.g6485;Description=hypothetical protein FVER14953_08525 [Fusarium
Fv_160_11.g6486	428	ID=Fv_160_11.g6486;Description=hypothetical protein FVER53263_08524 [Fusarium
Fv_160_11.g6487	400	ID=Fv_160_11.g6487;Description=hypothetical protein FVER14953_08523 [Fusarium verticillioides];Gene=FPANT_8185;Ontology_term=hydrolase
Fv_160_11.g6488	581	ID=Fv_160_11.g6488;Description=hypothetical protein FVEG_08522 [Fusarium verticillioides 7600];Gene=FocTR4_00015503;Ontology_term=membrane,DNA helicase activity,ATP binding,ATP hydrolysis activity,transmembrane transporter activity,telomere maintenance,DNA repair,DNA recombination,carbohydrate transport,DNA duplex unwinding,transmembrane transport;Ontology_id=GO:0016020,GO:0003678,GO:0005524,GO:0016887,GO:0022857,GO:0000723,GO:0006281,GO:0006310,GO
Fv_160_11.g6489	200	ID=Fv_160_11.g6489;Description=hypothetical protein FVER14953_08521 [Fusarium
Fv_160_11.g6490	509	ID=Fv_160_11.g6490;Description=hypothetical protein FVER14953_08520 [Fusarium verticillioides];Gene=FNYG_12296;Ontology_term=membrane,glycosyltransferase
Fv_160_11.g6491	246	ID=Fv_160_11.g6491;Description=hypothetical protein FVER14953_08519 [Fusarium verticillioides];Gene=LW93_223;Ontology_term=peptidase
Fv_160_11.g6492	551	ID=Fv_160_11.g6492;Description=hypothetical protein FVER14953_08518 [Fusarium verticillioides];Gene=FVEG_08518;Ontology_term=hydrolase
Fv_160_11.g6493	258	ID=Fv_160_11.g6493;Description=enoyl-CoA hydratase [Fusarium verticillioides 7600];Gene=FVEG_08517;Ontology_term=catalytic
Fv_160_11.g6494	992	ID=Fv_160_11.g6494;Description=hypothetical protein FVER14953_08516 [Fusarium verticillioides];Gene=FVEG_08516;Ontology_term=membrane,oxidoreductase
Fv_160_11.g6495	754	ID=Fv_160_11.g6495;Description=hypothetical protein FVEG_08515 [Fusarium verticillioides 7600];Gene=FPANT_13407;Ontology_term=nucleus,DNA binding,zinc ion binding,DNA-binding transcription factor activity, RNA polymerase II-specific,regulation of transcription by RNA polymerase

Fv_160_11.g6496	516	ID=Fv_160_11.g6496;Description=glycerol kinase [Fusarium verticillioides 7600];Gene=FOMG_15275;Ontology_term=glycerol kinase activity,ATP binding,glycerol-3-phosphate metabolic process,phosphorylation,glycerol catabolic process;Ontology_id=GO:0004370,GO:0005524,GO:0006072,GO:0016310,GO:0019563;Enzyme_code=EC:2.7.1.30;Enzyme_name=gl
Fv_160_11.g6497	331	ID=Fv_160_11.g6497;Description=aquaglyceroporin like protein, other eukaryote [Fusarium oxysporum f. sp. conglutinans race 2 54008];Gene=FPANT_13405;Ontology_term=membrane,channel activity,transmembrane
Fv_160_11.g6498	682	ID=Fv_160_11.g6498;Description=hypothetical protein FVER53263_08511 [Fusarium verticillioides];Gene=FMUND_12181;Ontology_term=glycerol-3-phosphate dehydrogenase complex,membrane,glycerol-3-phosphate dehydrogenase (quinone) activity,glycerol-3-phosphate metabolic
Fv_160_11.g6499	364	ID=Fv_160_11.g6499;Description=hypothetical protein FVEG_08510 [Fusarium verticillioides 7600]
Fv_160_11.g6500	452	ID=Fv_160_11.g6500;Description=hypothetical protein FVER14953_08508 [Fusarium verticillioides]
Fv_160_11.g6501	210	ID=Fv_160_11.g6501;Description=hypothetical protein FVEG_08507 [Fusarium verticillioides 7600];Gene=FVEG_08507;Ontology_term=endoplasmic reticulum membrane,nucleoside diphosphate kinase activity,protein C-terminal S-isoprenylcysteine carboxyl O-methyltransferase activity,ATP binding,GTP biosynthetic process,UTP biosynthetic process,CTP biosynthetic process,phosphorylation,methylation;Ontology_id=GO:0005789,GO:0004550,GO:0004671,GO:0005524,GO:0006183,GO:0006228,G
Fv_160_11.g6502	696	ID=Fv_160_11.g6502;Description=hypothetical protein FVEG_08506 [Fusarium verticillioides 7600];Gene=FNAPI_4411;Ontology_term=transcription coactivator activity,positive regulation of DNA-templated
Fv_160_11.g6503	268	ID=Fv_160_11.g6503;Description=hypothetical protein FVER53590_08505 [Fusarium verticillioides]
Fv_160_11.g6504	555	ID=Fv_160_11.g6504;Description=hypothetical protein FVER14953_21108 [Fusarium verticillioides];Gene=F25303_10173;Ontology_term=kinase
Fv_160_11.g6505	218	ID=Fv_160_11.g6505;Description=hypothetical protein FVER14953_21096 [Fusarium verticillioides]
Fv_160_11.g6506	138	ID=Fv_160_11.g6506;Description=hypothetical protein FVER14953_21095 [Fusarium verticillioides];Gene=FVER53263_08504;Ontology_term=oxidoreductase
Fv_160_11.g6507	255	ID=Fv_160_11.g6507;Description=hypothetical protein FVER14953_08504 [Fusarium verticillioides];Gene=FOZG_14283;Ontology_term=oxidoreductase
Fv_160_11.g6508	298	ID=Fv_160_11.g6508;Description=hypothetical protein FVER14953_08503 [Fusarium verticillioides];Gene=FVEG_08503;Ontology_term=membrane,transmembrane transport;Ontology_id=GO:0016020,GO:0055085
Fv_160_11.g6509	457	ID=Fv_160_11.g6509;Description=hypothetical protein FVEG_16342 [Fusarium verticillioides 7600];Gene=FVEG_16342;Ontology_term=DNA binding,oxidoreductase activity,ADP binding,DNA transposition,nucleoside metabolic process,DNA
Fv_160_11.g6510	731	ID=Fv_160_11.g6510;Description=hypothetical protein FVEG_16341 [Fusarium verticillioides 7600];Gene=FPRO_14485;Ontology_term=oxidoreductase activity,ATP hydrolysis activity,ADP binding,nucleoside metabolic process;Ontology_id=GO:0016491,GO:0016887,GO:0043531,GO:0009116;Enzyme_code=EC:1,EC:3.6.1.15;Enzyme_name=Oxidored

Fv_160_11.g6511	370	ID=Fv_160_11.g6511;Description=hypothetical protein FVER14953_08498 [Fusarium
Fv_160_11.g6512	503	ID=Fv_160_11.g6512;Description=hypothetical protein FVEG_16340 [Fusarium verticillioides
Fv_160_11.g6513	566	ID=Fv_160_11.g6513;Description=hypothetical protein FVER53590_08497 [Fusarium
Fv_160_11.g6514	633	ID=Fv_160_11.g6514;Description=hypothetical protein FVER53263_08497 [Fusarium
Fv_160_11.g6515	338	ID=Fv_160_11.g6515;Description=uncharacterized protein FTJAE_3350 [Fusarium tjaetaba]
Fv_160_11.g6516	291	ID=Fv_160_11.g6516;Description=hypothetical protein FANTH_7623 [Fusarium anthophilum]
Fv_160_11.g6517	430	ID=Fv_160_11.g6517;Description=hypothetical protein FVEG_16336 [Fusarium verticillioides 7600];Gene=FPRO_14495;Ontology_term=hydrolase
Fv_160_11.g6518	318	ID=Fv_160_11.g6518;Description=hypothetical protein FVER53590_25261 [Fusarium verticillioides];Gene=FGLOB1_13506;Ontology_term=arylformamidase activity,tryptophan catabolic process to
Fv_160_11.g6519	302	ID=Fv_160_11.g6519;Description=hypothetical protein FVEG_08490 [Fusarium verticillioides 7600];Gene=FGLOB1_13506;Ontology_term=arylformamidase activity,tryptophan catabolic process to
Fv_160_11.g6520	503	ID=Fv_160_11.g6520;Description=hypothetical protein FVER53590_08489 [Fusarium verticillioides];Gene=FGLOB1_13505;Ontology_term=membrane,transmembrane transporter activity,carbohydrate
Fv_160_11.g6521	675	ID=Fv_160_11.g6521;Description=hypothetical protein FVER14953_08488 [Fusarium verticillioides];Gene=FNAPI_4430;Ontology_term=arylformamidase activity,tryptophan catabolic process to
Fv_160_11.g6522	437	ID=Fv_160_11.g6522;Description=Homocitrate synthase [Fusarium napiforme];Gene=FTJAE_3340;Ontology_term=homocitrate synthase activity,lysine biosynthetic process via aminoadipic
Fv_160_11.g6523	919	ID=Fv_160_11.g6523;Description=hypothetical protein FVEG_08486 [Fusarium verticillioides 7600];Gene=FNAPI_4432;Ontology_term=endoplasmic reticulum membrane,iron ion binding,sulfuric ester hydrolase activity,heme binding,aromatase activity,xenobiotic metabolic process,arachidonic acid metabolic process;Ontology_id=GO:0005789,GO:0005506,GO:0008484,GO:0020037,GO:0070330,GO:0006805,GO:0019369;Enzyme_code=EC
Fv_160_11.g6524	314	ID=Fv_160_11.g6524;Description=hypothetical protein FVER53590_08485 [Fusarium verticillioides]
Fv_160_11.g6525	575	ID=Fv_160_11.g6525;Description=hypothetical protein FVER53263_08484 [Fusarium verticillioides];Gene=FOMG_15244;Ontology_term=vacuolar membrane,calcium:proton antiporter activity,calcium ion
Fv_160_11.g6526	398	ID=Fv_160_11.g6526;Description=CMGC/SRPK protein kinase [Fusarium verticillioides 7600];Gene=rplV;Ontology_term=protein serine/threonine kinase activity,ATP
Fv_160_11.g6527	481	ID=Fv_160_11.g6527;Description=hypothetical protein FVEG_08482 [Fusarium verticillioides 7600]
Fv_160_11.g6528	833	ID=Fv_160_11.g6528;Description=aminotriazole resistance protein [Fusarium globosum];Gene=FPANT_11684;Ontology_term=membrane,transmembrane transporter activity,transmembrane
Fv_160_11.g6529	631	ID=Fv_160_11.g6529;Description=hypothetical protein FVER53590_08478 [Fusarium verticillioides]
Fv_160_11.g6530	357	ID=Fv_160_11.g6530;Description=hypothetical protein NW760_001086 [Fusarium oxysporum]

Fv_160_11.g6531	586	ID=Fv_160_11.g6531;Description=hypothetical protein FVER53590_08477 [Fusarium verticillioides];Gene=FGADI_6155;Ontology_term=membrane,transmembrane transporter activity,transmembrane
Fv_160_11.g6532	213	ID=Fv_160_11.g6532;Description=hypothetical protein FVEG_08476 [Fusarium verticillioides 7600]
Fv_160_11.g6533	505	ID=Fv_160_11.g6533;Description=hypothetical protein FVER53263_08475 [Fusarium verticillioides];Gene=DSC1;Ontology_term=postsynaptic density,calcium-dependent cell-cell adhesion via plasma membrane cell adhesion molecules,adherens junction organization,cell-cell junction assembly,blood vessel morphogenesis,brain development,homophilic cell adhesion via plasma membrane adhesion molecules,neuron projection,adherens junction,synapse assembly,apical part of cell,cytoplasm,regulation of axonogenesis,calcium ion binding,cell-cell adhesion mediated by cadherin,cadherin binding,intercalated disc,catenin complex,desmosome,regulation of synaptic transmission,
Fv_160_11.g6534	189	ID=Fv_160_11.g6534;Description=hypothetical protein FVER53263_08474 [Fusarium verticillioides]
Fv_160_11.g6535	512	ID=Fv_160_11.g6535;Description=hypothetical protein FVEG_08473 [Fusarium verticillioides 7600]
Fv_160_11.g6536	281	ID=Fv_160_11.g6536;Description=hypothetical protein FVEG_16332 [Fusarium verticillioides
Fv_160_11.g6537	484	ID=Fv_160_11.g6537;Description=hypothetical protein FVEG_08472 [Fusarium verticillioides 7600];Gene=FVER53590_08472;Ontology_term=nucleus,DNA-binding transcription factor activity, RNA polymerase II-specific,zinc
Fv_160_11.g6538	260	ID=Fv_160_11.g6538;Description=hypothetical protein FVER14953_08471 [Fusarium verticillioides];Gene=FNAPI_4446;Ontology_term=nucleus,DNA-binding transcription factor activity, RNA polymerase II-specific,zinc ion binding,ligase activity,regulation of transcription by RNA polymerase
Fv_160_11.g6539	417	ID=Fv_160_11.g6539;Description=hypothetical protein FVEG_08470 [Fusarium verticillioides 7600];Gene=F25303_3558;Ontology_term=chitinase activity,chitin binding,carbohydrate metabolic
Fv_160_11.g6540	337	ID=Fv_160_11.g6540;Description=hypothetical protein FVER14953_08469 [Fusarium verticillioides];Gene=FOMG_15232;Ontology_term=monooxygenase activity,metal ion binding,dioxygenase activity,small molecule biosynthetic process,organic cyclic compound biosynthetic
Fv_160_11.g6541	754	ID=Fv_160_11.g6541;Description=hypothetical protein FVEG_08468 [Fusarium verticillioides 7600];Gene=FOMG_15231;Ontology_term=nucleus,DNA-binding transcription factor activity, RNA polymerase II-specific,zinc ion
Fv_160_11.g6542	423	ID=Fv_160_11.g6542;Description=hypothetical protein FVER14953_08467 [Fusarium
Fv_160_11.g6543	337	ID=Fv_160_11.g6543;Description=hypothetical protein FVEG_08466 [Fusarium verticillioides
Fv_160_11.g6544	187	ID=Fv_160_11.g6544;Description=hypothetical protein FVER53590_08465 [Fusarium verticillioides]
Fv_160_11.g6545	697	ID=Fv_160_11.g6545;Description=hypothetical protein FVEG_08464 [Fusarium verticillioides 7600];Gene=FOXB_16551;Ontology_term=membrane,oxidoreductase activity,transition metal ion
Fv_160_11.g6546	410	ID=Fv_160_11.g6546;Description=hypothetical protein FVER53590_08463 [Fusarium verticillioides];Gene=FANTH_8099;Ontology_term=hydrolase activity, hydrolyzing O-glycosyl compounds,polysaccharide catabolic
Fv_160_11.g6547	328	ID=Fv_160_11.g6547;Description=oxidoreductase [Fusarium verticillioides 7600];Gene=FNAPI_4453;Ontology_term=hydrolase activity, hydrolyzing O-glycosyl compounds,polysaccharide catabolic

Fv_160_11.g6548	168	ID=Fv_160_11.g6548;Description=hypothetical protein FVEG_16330 [Fusarium verticillioides 7600];Gene=FNAPI_4453;Ontology_term=hydrolase activity, hydrolyzing O-glycosyl compounds,polysaccharide catabolic
Fv_160_11.g6549	551	ID=Fv_160_11.g6549;Description=hypothetical protein FVEG_08461 [Fusarium verticillioides 7600];Gene=ACHE-1;Ontology_term=hydrolase activity;Ontology_id=GO:0016787;Enzyme_code=EC:3;Enzyme_name=Hydrolases
Fv_160_11.g6550	386	ID=Fv_160_11.g6550;Description=hypothetical protein FVEG_08460 [Fusarium verticillioides 7600];Gene=FVER53590_08460;Ontology_term=FMN binding,oxidoreductase
Fv_160_11.g6551	126	ID=Fv_160_11.g6551;Description=hypothetical protein FVEG_08459 [Fusarium verticillioides 7600];Gene=2340
Fv_160_11.g6552	178	ID=Fv_160_11.g6552;Description=hypothetical protein FVEG_16329 [Fusarium verticillioides 7600]
Fv_160_11.g6553	618	ID=Fv_160_11.g6553;Description=hypothetical protein FVER53590_08457 [Fusarium verticillioides];Gene=FPANT_5727;Ontology_term=nucleus,DNA binding,zinc ion binding,DNA-binding transcription factor activity, RNA polymerase II-specific,regulation of transcription by RNA polymerase
Fv_160_11.g6554	375	ID=Fv_160_11.g6554;Description=hypothetical protein FVER53263_08456 [Fusarium verticillioides];Gene=FPHYL_12704;Ontology_term=ligase
Fv_160_11.g6555	250	ID=Fv_160_11.g6555;Description=hypothetical protein FVEG_08455 [Fusarium verticillioides 7600];Gene=326

Fv_160_11.g6556	517	ID=Fv_160_11.g6556;Description=hypothetical protein FVEG_08454 [Fusarium verticillioides 7600];Gene=FPRO05_00192;Ontology_term=positive regulation of heart rate,peptide hormone secretion,cellular response to human chorionic gonadotropin stimulus,cardiac neural crest cell migration involved in outflow tract morphogenesis,positive regulation of mitotic nuclear division,sympathetic neuron axon guidance,regulation of pH,dorsal/ventral pattern formation,cellular response to follicle-stimulating hormone stimulus,cellular response to luteinizing hormone stimulus,response to hypoxia,oxidoreductase activity,in utero embryonic development,protein kinase A signaling,calcium-mediated signaling,negative regulation of protein metabolic process,neural crest cell fate commitment,nitric oxide transport,semaphorin-plexin signaling pathway involved in axon guidance,respiratory gaseous exchange by respiratory system,regulation of systemic arterial blood pressure by endothelin,cytokine activity,meiotic cell cycle process involved in oocyte maturation,body fluid secretion,regulation of glucose transmembrane transport,endothelin A receptor binding,negative regulation of nitric-oxide synthase biosynthetic process,endothelin B receptor binding,prostaglandin biosynthetic process,vein smooth muscle contraction,phospholipase D-activating G protein-coupled receptor signaling pathway,artery smooth muscle contraction,positive regulation of cell growth involved in cardiac muscle cell development,positive regulation of cell migration,cartilage development,axonogenesis involved in innervation,adenylate cyclase-inhibiting G protein-coupled receptor signaling pathway,middle ear morphogenesis,positive regulation of transcription by RNA polymerase II,protein kinase C deactivation,hormone activity,positive regulation of smooth muscle contraction,positive regulation of smooth muscle cell proliferation,negative regulation of transcription by RNA polymerase II,positive regulation of renal sodium excretion,positive regulation of cell size,positive regulation of chemokine-mediated signaling pathway,branching involved in blood vessel morphogenesis,calcium ion transmembrane transport,canonical Wnt signaling pathway,cellular response to hydrogen peroxide,regulation of vasoconstriction,positive regulation of urine volume,endothelin receptor signaling pathway involved in heart process,positive regulation of sarcomere organization,positive regulation of hormone secretion,pharyngeal arch artery morphogenesis,embryonic heart tube development,rhythmic excitation,protein kinase C-activating G protein-coupled receptor signaling pathway,canonical NF-kappaB signal transduction,mitochondrion organization,intracellular calcium ion homeostasis,positive regulation of cytosolic calcium ion concentration,renal sodium ion absorption,noradrenergic neuron differentiation,ERK1 and ERK2 cascade,extracellular space,thyroid gland development,response to
Fv_160_11.g6557	355	ID=Fv_160_11.g6557;Description=hypothetical protein FVER53590_29372 [Fusarium verticillioides];Gene=FOYG_13172;Ontology_term=dioxygenase
Fv_160_11.g6558	378	ID=Fv_160_11.g6558;Description=polygalacturonase [Fusarium verticillioides 7600];Gene=FVER53590_08451;Ontology_term=endoplasmic reticulum lumen,polygalacturonase activity,calcium ion binding,unfolded protein binding,protein folding,pectin catabolic process,cell wall

Fv_160_11.g6559	470	ID=Fv_160_11.g6559;Description=hypothetical protein FVER14953_08450 [Fusarium verticillioides];Gene=FVER53590_08450;Ontology_term=centrosome,centriole,cell cortex,rhabdomere,midbody,myosin V complex,myosin VI complex,myosin VII complex,mitotic spindle pole,calcium ion binding,channel regulator activity,ligase activity,enzyme regulator activity,myosin V binding,myosin VI head/neck binding,protein phosphorylation,centriole replication,sensory perception of sound,sensory perception of smell,metarhodopsin inactivation,adaptation of rhodopsin mediated signaling,actin filament-based movement,salivary gland cell autophagic cell death,rhabdomere development,muscle cell cellular homeostasis,kinetochore organization,cellular response to ethanol,photoreceptor cell axon guidance;Ontology_id=GO:0005813,GO:0005814,GO:0005938,GO:0016028,GO:0030496,GO:0031475,GO:0031476,GO:0031477,GO
Fv_160_11.g6560	244	ID=Fv_160_11.g6560;Description=hypothetical protein FVEG_16328 [Fusarium verticillioides 7600];Gene=FVER53590_08450;Ontology_term=protein modification process;Ontology_id=GO:0036211
Fv_160_11.g6561	271	ID=Fv_160_11.g6561;Description=hypothetical protein FVEG_08449 [Fusarium verticillioides 7600]
Fv_160_11.g6562	101	ID=Fv_160_11.g6562;Description=hypothetical protein FVEG_16326 [Fusarium verticillioides 7600];Gene=BFJ63_vAg16897;Ontology_term=membrane,magnesium ion binding,D-malate dehydrogenase (decarboxylating) activity,NAD binding;Ontology_id=GO:0016020,GO:0000287,GO:0046553,GO:0051287;Enzyme_code=EC:1.1.1.83;Enzyme_name=D-
Fv_160_11.g6563	361	ID=Fv_160_11.g6563;Description=hypothetical protein FVER14953_08447 [Fusarium verticillioides];Gene=BFJ63_vAg16896;Ontology_term=magnesium ion binding,D-malate dehydrogenase (decarboxylating) activity,NAD binding;Ontology_id=GO:0000287,GO:0046553,GO:0051287;Enzyme_code=EC:1.1.1.83;Enzyme_name=D-malate
Fv_160_11.g6564	299	ID=Fv_160_11.g6564;Description=haloacetate dehalogenase [Fusarium verticillioides
Fv_160_11.g6565	607	ID=Fv_160_11.g6565;Description=hypothetical protein FVEG_08445 [Fusarium verticillioides 7600];Gene=F25303_3586;Ontology_term=membrane,inorganic phosphate transmembrane transporter activity,symporter
Fv_160_11.g6566	436	ID=Fv_160_11.g6566;Description=hypothetical protein FVER14953_08444 [Fusarium verticillioides];Gene=FRV6_15109;Ontology_term=oxidoreductase activity, acting on the CH-OH group of donors, NAD or NADP as acceptor,oxidoreductase activity, acting on the CH-CH group of donors, NAD or NADP as acceptor,NAD binding,polysaccharide biosynthetic
Fv_160_11.g6567	751	ID=Fv_160_11.g6567;Description=hypothetical protein FVER53263_08443 [Fusarium verticillioides];Gene=FNYG_12373;Ontology_term=plasma membrane,chitin synthase activity,conidium
Fv_160_11.g6568	510	ID=Fv_160_11.g6568;Description=hypothetical protein FVER14953_08442 [Fusarium
Fv_160_11.g6569	574	ID=Fv_160_11.g6569;Description=hypothetical protein FVER53590_08441 [Fusarium verticillioides];Gene=FOXG_10063;Ontology_term=plasma membrane,myo-inositol transmembrane transporter activity,carbohydrate transport,translocation of molecules into host,myo-inositol import across plasma
Fv_160_11.g6570	152	ID=Fv_160_11.g6570;Description=hypothetical protein J7337_012898 [Fusarium
Fv_160_11.g6571	373	ID=Fv_160_11.g6571;Description=hypothetical protein FVER14953_08439 [Fusarium

Fv_160_11.g6572	489	ID=Fv_160_11.g6572;Description=hypothetical protein FVER53263_08438 [Fusarium verticillioides];Gene=FVEG_08438;Ontology_term=membrane,monooxygenase activity,iron ion binding,oxidoreductase activity, acting on paired donors, with incorporation or reduction of molecular oxygen,heme binding;Ontology_id=GO:0016020,GO:0004497,GO:0005506,GO:0016705,GO:0020037;Enzyme_code=EC:1.14;Enzyme_name=Actin
Fv_160_11.g6573	478	ID=Fv_160_11.g6573;Description=hypothetical protein FVER53590_08437 [Fusarium verticillioides];Gene=FPHYL_11083;Ontology_term=negative regulation of bone mineralization,extracellular space,cysteine-type endopeptidase inhibitor activity,extracellular membrane-bounded organelle,vesicle,regulation of inflammatory response,Golgi apparatus,positive regulation of phagocytosis,acute-phase response,plasma
Fv_160_11.g6574	137	ID=Fv_160_11.g6574;Description=ethanolamine utilization [Fusarium sp. NRRL 52700]
Fv_160_11.g6575	80	ID=Fv_160_11.g6575;Description=hypothetical protein FVEG_16325 [Fusarium verticillioides 7600]
Fv_160_11.g6576	575	ID=Fv_160_11.g6576;Description=hypothetical protein FVEG_08435 [Fusarium verticillioides 7600];Gene=FMUND_5900;Ontology_term=translation initiation factor activity,translational
Fv_160_11.g6577	202	ID=Fv_160_11.g6577;Description=hypothetical protein FVER14953_08434 [Fusarium verticillioides];Gene=CG5363;Ontology_term=protein serine/threonine kinase activity,ATP
Fv_160_11.g6578	199	ID=Fv_160_11.g6578;Description=hypothetical protein FVEG_08433 [Fusarium verticillioides 7600]
Fv_160_11.g6579	273	ID=Fv_160_11.g6579;Description=hypothetical protein FVER14953_08432 [Fusarium verticillioides];Gene=FGF2B;Ontology_term=growth factor activity,branch elongation involved in ureteric bud branching,fibroblast growth factor receptor signaling pathway,integrin binding,heparin binding,positive regulation of cholesterol biosynthetic process,positive regulation of transcription by RNA polymerase II,nucleoplasm,wound healing,cellular response to heat,positive regulation of cell division,cytosol,extracellular matrix,positive regulation of epithelial cell proliferation,positive regulation of endothelial cell migration,lung development,positive regulation of sprouting angiogenesis,S100 protein binding,fibroblast growth factor receptor binding,extracellular space,regulation of endothelial tube morphogenesis,cell cortex,positive regulation of protein phosphorylation,positive regulation of ERK1 and ERK2 cascade,organ induction,cell differentiation,regulation of endothelial cell
Fv_160_11.g6580	434	ID=Fv_160_11.g6580;Description=hypothetical protein FVEG_08431 [Fusarium verticillioides 7600];Gene=FNYG_12385;Ontology_term=cell division,FAD binding,oxidoreductase activity, acting on paired donors, with incorporation or reduction of molecular oxygen,positive regulation of cell cycle G2/M phase transition,iron ion binding,protein tyrosine phosphatase activity,monooxygenase activity,heme binding;Ontology_id=GO:0051301,GO:0071949,GO:0016705,GO:1902751,GO:0005506,GO:0004725,GO:0004497,GO:0020037;Enzy
Fv_160_11.g6581	539	ID=Fv_160_11.g6581;Description=hypothetical protein FVEG_08430 [Fusarium verticillioides 7600];Gene=FVER53590_08431;Ontology_term=fibrinogen complex,membrane,collagen-containing extracellular matrix,monooxygenase activity,signaling receptor binding,iron ion binding,oxidoreductase activity, acting on paired donors, with incorporation or reduction of molecular oxygen,heme binding,FAD binding,cell-matrix adhesion,protein polymerization,platelet aggregation;Ontology_id=GO:0005577,GO:0016020,GO:0062023,GO:0004497,GO:0005102,GO:0005506,GO:0016705,GO:0020037,

Fv_160_11.g6582	512	ID=Fv_160_11.g6582;Description=hypothetical protein FPANT_6384 [Fusarium pseudoanthophilum];Gene=351;Ontology_term=protein serine/threonine kinase activity,oxidoreductase activity,FAD
Fv_160_11.g6583	408	ID=Fv_160_11.g6583;Description=hypothetical protein FVEG_08427 [Fusarium verticillioides 7600];Gene=FPANT_6383;Ontology_term=oxidoreductase activity,FAD binding,cellular biosynthetic
Fv_160_11.g6584	350	ID=Fv_160_11.g6584;Description=hypothetical protein FVEG_16324 [Fusarium verticillioides 7600];Gene=FVER53263_20017;Ontology_term=oxidoreductase activity,cellular biosynthetic
Fv_160_11.g6585	266	ID=Fv_160_11.g6585;Description=hypothetical protein FVEG_16323 [Fusarium verticillioides 7600];Gene=FPHYL_11094;Ontology_term=oxidoreductase activity,cellular biosynthetic
Fv_160_11.g6586	270	ID=Fv_160_11.g6586;Description=hypothetical protein FVER53263_08426 [Fusarium verticillioides]
Fv_160_11.g6587	2434	ID=Fv_160_11.g6587;Description=hypothetical protein FVER14953_08425 [Fusarium verticillioides];Gene=Fsub_11242;Ontology_term=3-oxoacyl-[acyl-carrier-protein] synthase activity,oxidoreductase activity,phosphopantetheine binding,fatty acid biosynthetic process,secondary metabolite biosynthetic process;Ontology_id=GO:0004315,GO:0016491,GO:0031177,GO:0006633,GO:0044550;Enzyme_code=EC:2.3.1.41,EC:1;Enzyme_na
Fv_160_11.g6588	380	ID=Fv_160_11.g6588;Description=hypothetical protein FVEG_16322 [Fusarium verticillioides
Fv_160_11.g6589	641	ID=Fv_160_11.g6589;Description=hypothetical protein FVER14953_08422 [Fusarium verticillioides];Gene=FPANT_11840;Ontology_term=oxidoreductase activity,FAD
Fv_160_11.g6590	321	ID=Fv_160_11.g6590;Description=hypothetical protein FVER14953_08421 [Fusarium verticillioides];Gene=BFJ69_g12116;Ontology_term=hydrolase activity, hydrolyzing O-glycosyl compounds,carbohydrate metabolic
Fv_160_11.g6591	110	ID=Fv_160_11.g6591;Description=hypothetical protein FVER53263_08420 [Fusarium verticillioides];Gene=FFUJ_11038;Ontology_term=transferase
Fv_160_11.g6592	292	ID=Fv_160_11.g6592;Description=hypothetical protein FVER53263_08419 [Fusarium verticillioides];Gene=FPRO_14571;Ontology_term=trans-aconitate 2-methyltransferase
Fv_160_11.g6593	687	ID=Fv_160_11.g6593;Description=hypothetical protein J7337_012922 [Fusarium musae];Gene=BFJ70_g17288;Ontology_term=nucleus,membrane,DNA-binding transcription factor activity, RNA polymerase II-
Fv_160_11.g6594	632	ID=Fv_160_11.g6594;Description=related to C6 finger domain protein [Fusarium proliferatum];Gene=FOMG_15188;Ontology_term=nucleus,DNA-binding transcription factor activity, RNA polymerase II-specific,zinc
Fv_160_11.g6595	263	ID=Fv_160_11.g6595;Description=putative secondary metabolism biosynthetic enzyme [Fusarium musae]
Fv_160_11.g6596	410	ID=Fv_160_11.g6596;Description=hypothetical protein FVER53263_08415 [Fusarium
Fv_160_11.g6597	113	ID=Fv_160_11.g6597;Description=hypothetical protein FVER53263_08414 [Fusarium
Fv_160_11.g6598	456	ID=Fv_160_11.g6598;Description=hypothetical protein FVER14953_08413 [Fusarium verticillioides]
Fv_160_11.g6599	274	ID=Fv_160_11.g6599;Description=TDG/mug DNA glycosylase [Fusarium verticillioides 7600];Gene=BFJ72_g11262;Ontology_term=mismatch base pair DNA N-glycosylase activity,base-excision repair, AP site

Fv_160_11.g6600	1171	ID=Fv_160_11.g6600;Description=hypothetical protein FVEG_08411 [Fusarium verticillioides 7600];Gene=FVER53590_08411;Ontology_term=lysophospholipase activity,phosphatidyl phospholipase B activity,phospholipid
Fv_160_11.g6601	584	ID=Fv_160_11.g6601;Description=hypothetical protein FVER53590_08411 [Fusarium verticillioides];Gene=FOPG_15518;Ontology_term=lysophospholipase activity,phosphatidyl phospholipase B activity,phospholipid
Fv_160_11.g6602	726	ID=Fv_160_11.g6602;Description=hypothetical protein FVER53590_29375 [Fusarium verticillioides];Gene=Chc;Ontology_term=clathrin coat of trans-Golgi network vesicle,clathrin coat of coated pit,clathrin complex,structural molecule activity,clathrin light chain binding,intracellular protein transport,vesicle-mediated
Fv_160_11.g6603	594	ID=Fv_160_11.g6603;Description=hypothetical protein FVER53590_29376 [Fusarium verticillioides]
Fv_160_11.g6604	209	ID=Fv_160_11.g6604;Description=hypothetical protein FVEG_08410 [Fusarium verticillioides 7600];Gene=FMAN_13946;Ontology_term=extracellular region,egg chorion,cysteine-type endopeptidase activity,structural constituent of egg chorion,proteolysis,vitelline membrane formation involved in chorion-containing eggshell formation,egg chorion assembly;Ontology_id=GO:0005576,GO:0042600,GO:0004197,GO:0005213,GO:0006508,GO:0007305,GO:0007306;Enzyme_code=E
Fv_160_11.g6605	357	ID=Fv_160_11.g6605;Description=NADPH2:quinone reductase [Fusarium verticillioides 7600];Gene=FCIRC_13413;Ontology_term=membrane,oxidoreductase activity, acting on
Fv_160_11.g6606	466	ID=Fv_160_11.g6606;Description=hypothetical protein FVEG_08408 [Fusarium verticillioides
Fv_160_11.g6607	425	ID=Fv_160_11.g6607;Description=hypothetical protein FVER53590_08407 [Fusarium verticillioides];Gene=FPCIR_1865;Ontology_term=acyltransferase
Fv_160_11.g6608	1047	ID=Fv_160_11.g6608;Description=DAL5-Allantoate ureidosuccinate permease [Fusarium coicis];Gene=BFJ65_g13620;Ontology_term=membrane,transmembrane transporter activity,transmembrane
Fv_160_11.g6609	406	ID=Fv_160_11.g6609;Description=hypothetical protein FVER53590_08403 [Fusarium verticillioides];Gene=F25303_3631;Ontology_term=aspartic-type endopeptidase
Fv_160_11.g6610	412	ID=Fv_160_11.g6610;Description=hypothetical protein FVEG_08402 [Fusarium verticillioides 7600];Gene=F25303_3630;Ontology_term=aspartic-type endopeptidase
Fv_160_11.g6611	147	ID=Fv_160_11.g6611;Description=hypothetical protein FVER53590_29377 [Fusarium
Fv_160_11.g6612	739	ID=Fv_160_11.g6612;Description=hypothetical protein FVEG_08400 [Fusarium verticillioides 7600];Gene=FVER53590_08400;Ontology_term=membrane,oligopeptide transmembrane transporter activity,protein
Fv_160_11.g6613	768	ID=Fv_160_11.g6613;Description=hypothetical protein FVER14953_08399 [Fusarium verticillioides];Gene=C2S_11370;Ontology_term=membrane,oligopeptide transmembrane transporter activity,protein
Fv_160_11.g6614	438	ID=Fv_160_11.g6614;Description=hypothetical protein FVER14953_08398 [Fusarium verticillioides];Gene=FACUT_2706;Ontology_term=plasma membrane,cell surface,neuromuscular junction,axonal growth cone,side of membrane,oxidoreductase activity,flavin adenine dinucleotide binding,homophilic cell adhesion via plasma membrane adhesion molecules,axonal fasciculation,synaptic target attraction;Ontology_id=GO:0005886,GO:0009986,GO:0031594,GO:0044295,GO:0098552,GO:0016491,GO:0050660,GO:0007156,G

Fv_160_11.g6615	305	ID=Fv_160_11.g6615;Description=hypothetical protein FVEG_08397 [Fusarium verticillioides 7600];Gene=380
Fv_160_11.g6616	449	ID=Fv_160_11.g6616;Description=hypothetical protein FVER14953_08396 [Fusarium verticillioides];Gene=FOVG_18770;Ontology_term=phosphatidylserine decarboxylase activity,phospholipid biosynthetic
Fv_160_11.g6617	197	ID=Fv_160_11.g6617;Description=hypothetical protein FVER14953_08395 [Fusarium verticillioides];Gene=382;Ontology_term=regulation of acrosome reaction,external side of plasma membrane,lactose synthase activity,protein N-linked glycosylation,epithelial cell development,cell adhesion,Golgi trans cisterna,lactose biosynthetic process,N-acetyllactosamine synthase activity,beta-tubulin binding,manganese ion binding,angiogenesis involved in wound healing,identical protein binding,penetration of zona pellucida,Golgi cisterna membrane,positive regulation of epithelial cell proliferation involved in wound healing,development of secondary sexual characteristics,alpha-tubulin binding,galactose metabolic process,protein galactosylation,macrophage migration,beta-N-acetylglucosaminylglycopeptide beta-1,4-galactosyltransferase activity,extracellular space,binding of sperm to zona pellucida,brush border membrane,acute inflammatory response,negative regulation of epithelial cell proliferation,lipid metabolic process,positive regulation of apoptotic process,extracellular matrix organization,filopodium,desmosome,protein-containing complex,basolateral plasma membrane;Ontology_id=GO:0060046,GO:0009897,GO:0004461,GO:0006487,GO:0002064,GO:0007155,GO:0000138,GO:0005989,G
Fv_160_11.g6618	502	ID=Fv_160_11.g6618;Description=hypothetical protein FVEG_08394 [Fusarium verticillioides 7600];Gene=C2S_11375;Ontology_term=membrane,transmembrane transporter activity,transmembrane
Fv_160_11.g6619	303	ID=Fv_160_11.g6619;Description=hypothetical protein FVEG_08393 [Fusarium verticillioides
Fv_160_11.g6620	152	ID=Fv_160_11.g6620;Description=hypothetical protein FVEG_08392 [Fusarium verticillioides 7600];Gene=FPHYL_6657;Ontology_term=hydrolase activity, acting on glycosyl bonds,metabolic
Fv_160_11.g6621	264	ID=Fv_160_11.g6621;Description=hypothetical protein FVEG_08391 [Fusarium verticillioides 7600];Gene=NECHADRAFT_53546;Ontology_term=metalloendopeptidase activity,zinc ion binding,carbohydrate binding,proteolysis,cell
Fv_160_11.g6622	433	ID=Fv_160_11.g6622;Description=hypothetical protein FVER14953_08390 [Fusarium verticillioides]
Fv_160_11.g6623	866	ID=Fv_160_11.g6623;Description=hypothetical protein FVER53263_08389 [Fusarium verticillioides]
Fv_160_11.g6624	482	ID=Fv_160_11.g6624;Description=hypothetical protein FVER53263_08388 [Fusarium verticillioides];Gene=FANTH_8166;Ontology_term=carboxy-lyase activity,pyridoxal phosphate binding,amino acid metabolic
Fv_160_11.g6625	210	ID=Fv_160_11.g6625;Description=peroxiredoxin (alkyl hydroperoxide reductase subunit C) [Fusarium verticillioides 7600];Gene=FOPG_14021;Ontology_term=peroxiredoxin activity,cellular oxidant
Fv_160_11.g6626	527	ID=Fv_160_11.g6626;Description=hypothetical protein FVER53263_20064 [Fusarium verticillioides];Gene=394
Fv_160_11.g6627	550	ID=Fv_160_11.g6627;Description=hypothetical protein FVER53590_08386 [Fusarium verticillioides];Gene=HBB;Ontology_term=hemoglobin complex,histone acetyltransferase activity,oxygen carrier activity,oxygen binding,heme binding,metal ion binding,chromatin remodeling,oxygen transport;Ontology_id=GO:0005833,GO:0004402,GO:0005344,GO:0019825,GO:0020037,GO:0046872,GO:0006338,GO:0015671;En

Fv_160_11.g6628	413	ID=Fv_160_11.g6628;Description=histone acetyltransferase [Fusarium musae];Gene=FCIRC_8526;Ontology_term=membrane,histone acetyltransferase activity,chromatin
Fv_160_11.g6629	271	ID=Fv_160_11.g6629;Description=hypothetical protein J7337_012961 [Fusarium musae]
Fv_160_11.g6630	1255	ID=Fv_160_11.g6630;Description=hypothetical protein J7337_012962 [Fusarium musae];Gene=FRV6_11664;Ontology_term=protein kinase activity,ATP binding;Ontology_id=GO:0004672,GO:0005524;Enzyme_code=EC:2.7.1;Enzyme_name=Transferring phosphorus-
Fv_160_11.g6631	581	ID=Fv_160_11.g6631;Description=uncharacterized protein FRV6_11666 [Fusarium oxysporum];Gene=FRV6_11666;Ontology_term=nucleotide binding,kinase
Fv_160_11.g6632	329	ID=Fv_160_11.g6632;Description=hypothetical protein FVEG_08384 [Fusarium verticillioides 7600];Gene=FVER53590_08384;Ontology_term=membrane,transmembrane transporter activity,transmembrane
Fv_160_11.g6633	599	ID=Fv_160_11.g6633;Description=hypothetical protein FVER53263_08383 [Fusarium verticillioides]
Fv_160_11.g6634	512	ID=Fv_160_11.g6634;Description=hypothetical protein FVEG_08382 [Fusarium verticillioides 7600];Gene=FNAPI_1013;Ontology_term=membrane,transmembrane transporter activity,transmembrane
Fv_160_11.g6635	605	ID=Fv_160_11.g6635;Description=arylsulfatase [Fusarium verticillioides 7600];Gene=FACUT_2725;Ontology_term=sulfuric ester hydrolase activity;Ontology_id=GO:0008484;Enzyme_code=EC:3.1.6;Enzyme_name=Acting on ester bonds
Fv_160_11.g6636	734	ID=Fv_160_11.g6636;Description=hypothetical protein FVEG_08380 [Fusarium verticillioides 7600];Gene=FOVG_18432;Ontology_term=nucleus,DNA binding,zinc ion binding,DNA-binding transcription factor activity, RNA polymerase II-specific,regulation of transcription by RNA polymerase
Fv_160_11.g6637	125	ID=Fv_160_11.g6637;Description=hypothetical protein FVEG_16305 [Fusarium verticillioides 7600];Gene=CDV36_002726;Ontology_term=sulfuric ester hydrolase
Fv_160_11.g6638	272	ID=Fv_160_11.g6638;Description=hypothetical protein FVER14953_08379 [Fusarium verticillioides];Gene=FDENT_11218;Ontology_term=dioxygenase activity,toxin activity,modulation of process of another
Fv_160_11.g6639	358	ID=Fv_160_11.g6639;Description=hypothetical protein FCOIX_12134 [Fusarium coicis];Gene=FVER53590_08379;Ontology_term=toxin activity,modulation of process of another
Fv_160_11.g6640	189	ID=Fv_160_11.g6640;Description=hypothetical protein FVER14953_08378 [Fusarium verticillioides];Gene=FCIRC_4447;Ontology_term=membrane,kinase activity,transmembrane transporter activity,phosphorylation,transmembrane
Fv_160_11.g6641	579	ID=Fv_160_11.g6641;Description=dual specificity catalytic domain-containing protein [Fusarium coicis];Gene=FPRO05_00117;Ontology_term=membrane,protein tyrosine phosphatase activity,protein tyrosine/serine/threonine phosphatase
Fv_160_11.g6642	499	ID=Fv_160_11.g6642;Description=hypothetical protein FVER14953_08375 [Fusarium verticillioides];Gene=F25303_7138;Ontology_term=membrane,transmembrane transporter activity,transmembrane
Fv_160_11.g6643	403	ID=Fv_160_11.g6643;Description=hypothetical protein FVER14953_08374 [Fusarium verticillioides];Gene=FNAPI_1024;Ontology_term=catalytic activity,nucleoside metabolic

Fv_160_11.g6644	260	ID=Fv_160_11.g6644;Description=hypothetical protein FVEG_08373 [Fusarium verticillioides 7600];Gene=FANTH_8188;Ontology_term=glutamic-type endopeptidase
Fv_160_11.g6645	205	ID=Fv_160_11.g6645;Description=hypothetical protein FVER14953_08372 [Fusarium verticillioides]
Fv_160_11.g6646	224	ID=Fv_160_11.g6646;Description=hypothetical protein FVER53590_08371 [Fusarium verticillioides]
Fv_160_11.g6647	512	ID=Fv_160_11.g6647;Description=hypothetical protein FVER14953_06002 [Fusarium verticillioides];Gene=FPHYL_11257;Ontology_term=membrane,transmembrane transporter activity,transmembrane
Fv_160_11.g6648	503	ID=Fv_160_11.g6648;Description=hypothetical protein FVER14953_08370 [Fusarium verticillioides];Gene=FVEG_08370;Ontology_term=membrane,transmembrane transporter activity,transmembrane
Fv_160_11.g6649	604	ID=Fv_160_11.g6649;Description=hypothetical protein FVEG_08369 [Fusarium verticillioides 7600];Gene=BFJ72_g7210;Ontology_term=magnesium ion binding,pyruvate decarboxylase activity,thiamine pyrophosphate
Fv_160_11.g6650	346	ID=Fv_160_11.g6650;Description=hypothetical protein FVER53590_08368 [Fusarium verticillioides];Gene=FOXB_13599;Ontology_term=membrane,nucleotide binding,oxidoreductase activity, acting on
Fv_160_11.g6651	161	ID=Fv_160_11.g6651;Description=hypothetical protein FVEG_16301 [Fusarium verticillioides 7600]
Fv_160_11.g6652	538	ID=Fv_160_11.g6652;Description=hypothetical protein FVER53263_08367 [Fusarium verticillioides];Gene=BFJ70_g4163;Ontology_term=membrane,catalytic activity,FAD
Fv_160_11.g6653	722	ID=Fv_160_11.g6653;Description=hypothetical protein FVER53263_08366 [Fusarium verticillioides];Gene=FOYG_13073;Ontology_term=nucleus,DNA binding,zinc ion binding,DNA-binding transcription factor activity, RNA polymerase II-specific,regulation of transcription by RNA polymerase
Fv_160_11.g6654	831	ID=Fv_160_11.g6654;Description=hypothetical protein FVER14953_08365 [Fusarium verticillioides];Gene=FMAN_13989;Ontology_term=beta-glucosidase activity,scopolin beta-glucosidase activity,cellulose catabolic
Fv_160_11.g6655	525	ID=Fv_160_11.g6655;Description=hypothetical protein FVER14953_08364 [Fusarium verticillioides];Gene=FNAPI_2774;Ontology_term=membrane,transmembrane transporter activity,transmembrane
Fv_160_11.g6656	546	ID=Fv_160_11.g6656;Description=MFS transporter, SP family, general alpha glucoside:H+ symporter [Fusarium verticillioides 7600];Gene=FNAPI_2773;Ontology_term=membrane,transmembrane transporter activity,transmembrane
Fv_160_11.g6657	475	ID=Fv_160_11.g6657;Description=hypothetical protein FVER53263_08362 [Fusarium verticillioides];Gene=F25303_7951;Ontology_term=oxidoreductase
Fv_160_11.g6658	497	ID=Fv_160_11.g6658;Description=hypothetical protein FVEG_08361 [Fusarium verticillioides 7600];Gene=FOMG_15860;Ontology_term=cysteine-type endopeptidase inhibitor activity,carbohydrate metabolic
Fv_160_11.g6659	640	ID=Fv_160_11.g6659;Description=hypothetical protein FVER53263_08360 [Fusarium verticillioides];Gene=FACUT_2747;Ontology_term=nucleus,DNA-binding transcription factor activity, RNA polymerase II-specific,zinc ion binding,carbohydrate metabolic process,regulation of transcription by RNA polymerase

Fv_160_11.g6660	814	ID=Fv_160_11.g6660;Description=hypothetical protein FVER53263_08359 [Fusarium verticillioides];Gene=FACUT_2748;Ontology_term=nucleus,DNA-binding transcription factor activity, RNA polymerase II-specific,zinc ion binding,carbohydrate metabolic process,regulation of transcription by RNA polymerase
Fv_160_11.g6661	628	ID=Fv_160_11.g6661;Description=hypothetical protein FVEG_08358 [Fusarium verticillioides 7600];Gene=FPANT_4869;Ontology_term=zinc ion binding,cyclin-dependent protein serine/threonine kinase regulator activity,snRNA 3'-end processing,imaginal disc-derived leg segmentation,sex comb development,chaeta development,positive regulation of transcription by RNA polymerase II,DNA-binding transcription factor activity, RNA polymerase II-specific,DNA binding,positive regulation of autophagy,protein binding,transcription coregulator activity,mediator
Fv_160_11.g6662	508	ID=Fv_160_11.g6662;Description=hypothetical protein FVER53590_08357 [Fusarium verticillioides];Gene=FPRO05_00093;Ontology_term=Prp19 complex,spliceosomal complex,nucleolus,lysosomal membrane,autophagosome,plasma membrane,dendrite,melanosome,terminal bouton,presynaptic cytosol,postsynaptic cytosol,ATP binding,ATP hydrolysis activity,transmembrane transporter activity,protein-macromolecule adaptor activity,heat shock protein binding,ATP-dependent protein folding chaperone,clathrin-uncoating ATPase activity,mRNA processing,RNA splicing,synaptic vesicle uncoating,protein refolding,negative regulation of DNA-templated transcription,chaperone cofactor-dependent protein refolding,transmembrane transport,late endosomal microautophagy,protein targeting to lysosome involved in chaperone-mediated autophagy,chaperone-mediated autophagy translocation complex disassembly,slow axonal transport;Ontology_id=GO:0000974,GO:0005681,GO:0005730,GO:0005765,GO:0005776,GO:0005886,GO:0030425,GO:0042470,GO
Fv_160_11.g6663	803	ID=Fv_160_11.g6663;Description=hypothetical protein FVER14953_08356 [Fusarium verticillioides];Gene=FGLOB1_767;Ontology_term=cyclin-dependent protein kinase holoenzyme complex,chromosome, centromeric region,nucleus,cytoplasm,spindle midzone,mitotic spindle,protein binding,cyclin-dependent protein serine/threonine kinase regulator activity,sterol 3-beta-glucosyltransferase activity,embryonic development via the syncytial blastoderm,carbohydrate metabolic process,mitotic chromosome movement towards spindle pole,attachment of spindle microtubules to kinetochore,lipid glycosylation,syncytial blastoderm mitotic cell cycle,regulation of chromatin binding,mitotic cell cycle phase transition,germ-line cyst formation,midbody
Fv_160_11.g6664	802	ID=Fv_160_11.g6664;Description=hypothetical protein FVER53263_08355 [Fusarium verticillioides];Gene=FNYG_12463;Ontology_term=carbohydrate metabolic process;Ontology_id=GO:0005975
Fv_160_11.g6665	356	ID=Fv_160_11.g6665;Description=hypothetical protein FVEG_16300 [Fusarium verticillioides 7600]
Fv_160_11.g6666	432	ID=Fv_160_11.g6666;Description=hypothetical protein FVER53590_08354 [Fusarium verticillioides]
Fv_160_11.g6667	440	ID=Fv_160_11.g6667;Description=CMGC protein kinase [Fusarium verticillioides 7600];Gene=FNYG_12473;Ontology_term=chromosome, telomeric region,protein serine/threonine kinase activity,ATP binding,phosphorylation;Ontology_id=GO:0000781,GO:0004674,GO:0005524,GO:0016310;Enzyme_code=EC:2.7.11.1;Enzyme_nam

Fv_160_11.g6668	486	ID=Fv_160_11.g6668;Description=hypothetical protein FVEG_08352 [Fusarium verticillioides 7600];Gene=FOYG_13052;Ontology_term=cytoplasm,insulin receptor complex,axon,insulin receptor activity,insulin-like growth factor receptor activity,structural molecule activity,insulin-like growth factor binding,ATP binding,phosphatidylinositol 3-kinase binding,insulin receptor substrate binding,insulin receptor signaling pathway,negative regulation of apoptotic process,negative regulation of MAPK cascade,positive regulation of MAPK cascade,regulation of JNK cascade,protein autophosphorylation,insulin-like growth factor receptor signaling pathway,positive regulation of phosphatidylinositol 3-kinase/protein kinase B signal transduction,cellular response to glucose
Fv_160_11.g6669	553	ID=Fv_160_11.g6669;Description=hypothetical protein FVEG_08351 [Fusarium verticillioides 7600];Gene=FDENT_8907;Ontology_term=membrane,transmembrane transporter activity,transmembrane
Fv_160_11.g6670	216	ID=Fv_160_11.g6670;Description=hypothetical protein FVER53263_08350 [Fusarium
Fv_160_11.g6671	220	ID=Fv_160_11.g6671;Description=hypothetical protein FVEG_08349 [Fusarium verticillioides
Fv_160_11.g6672	149	ID=Fv_160_11.g6672;Description=hypothetical protein FVEG_08348 [Fusarium verticillioides 7600];Gene=459
Fv_160_11.g6673	185	ID=Fv_160_11.g6673;Description=hypothetical protein FVER14953_08347 [Fusarium verticillioides]
Fv_160_11.g6674	1350	ID=Fv_160_11.g6674;Description=hypothetical protein FVEG_08346 [Fusarium verticillioides 7600];Gene=FMUND_8515;Ontology_term=vesicle membrane,hydrolase activity, hydrolyzing O-glycosyl compounds,structural molecule activity,carbohydrate metabolic process,nucleoside metabolic
Fv_160_11.g6675	511	ID=Fv_160_11.g6675;Description=xylosidase glycosyl hydrolase [Fusarium pseudoanthophilum];Gene=FNYG_12480;Ontology_term=hydrolase activity, hydrolyzing O-glycosyl compounds,carbohydrate
Fv_160_11.g6676	449	ID=Fv_160_11.g6676;Description=hypothetical protein FVEG_08344 [Fusarium verticillioides 7600];Gene=FNYG_12481;Ontology_term=hydrolase activity, hydrolyzing O-glycosyl compounds,carbohydrate binding,xylan
Fv_160_11.g6677	526	ID=Fv_160_11.g6677;Description=hypothetical protein FVEG_08343 [Fusarium verticillioides 7600];Gene=BFJ65_g13611;Ontology_term=membrane,transmembrane transporter activity,transmembrane
Fv_160_11.g6678	389	ID=Fv_160_11.g6678;Description=hypothetical protein FVEG_08342 [Fusarium verticillioides 7600];Gene=FPRO_14682;Ontology_term=hydrolase activity,carbohydrate metabolic
Fv_160_11.g6679	547	ID=Fv_160_11.g6679;Description=hypothetical protein FVER14953_08341 [Fusarium verticillioides]
Fv_160_11.g6680	605	ID=Fv_160_11.g6680;Description=hypothetical protein FVEG_16298 [Fusarium verticillioides 7600];Gene=FOXG_10181;Ontology_term=nucleus,DNA binding,zinc ion binding,DNA-binding transcription factor activity, RNA polymerase II-specific,regulation of transcription by RNA polymerase
Fv_160_11.g6681	123	ID=Fv_160_11.g6681;Description=hypothetical protein FVEG_08340 [Fusarium verticillioides 7600]
Fv_160_11.g6682	528	ID=Fv_160_11.g6682;Description=hypothetical protein FVER14953_08339 [Fusarium verticillioides];Gene=FDENT_8919;Ontology_term=membrane,transmembrane transport;Ontology_id=GO:0016020,GO:0055085
Fv_160_11.g6683	337	ID=Fv_160_11.g6683;Description=hypothetical protein FVEG_08338 [Fusarium verticillioides 7600]

Fv_160_11.g6684	286	ID=Fv_160_11.g6684;Description=hypothetical protein FVEG_08337 [Fusarium verticillioides 7600];Gene=FNYG_12089;Ontology_term=membrane,oxidoreductase activity,cellular biosynthetic
Fv_160_11.g6685	174	ID=Fv_160_11.g6685;Description=hypothetical protein FVER53263_20072 [Fusarium verticillioides]
Fv_160_11.g6686	117	ID=Fv_160_11.g6686;Description=hypothetical protein FVER53263_08336 [Fusarium verticillioides];Gene=FDENT_8917;Ontology_term=oxidoreductase activity,cellular biosynthetic
Fv_160_11.g6687	558	ID=Fv_160_11.g6687;Description=hypothetical protein FVEG_16296 [Fusarium verticillioides 7600];Gene=Dhod;Ontology_term=mitochondrial inner membrane,mitochondrial intermembrane space,dihydroorotate dehydrogenase (quinone) activity,'de novo' pyrimidine nucleobase biosynthetic process,'de novo' UMP biosynthetic
Fv_160_11.g6688	590	ID=Fv_160_11.g6688;Description=hypothetical protein FVEG_08335 [Fusarium verticillioides 7600];Gene=FPRO_14719;Ontology_term=nucleus,DNA-binding transcription factor activity, RNA polymerase II-specific,zinc ion
Fv_160_11.g6689	955	ID=Fv_160_11.g6689;Description=hypothetical protein FVER53590_08334 [Fusarium verticillioides];Gene=BFJ69_g7277;Ontology_term=catalytic activity,nucleoside metabolic
Fv_160_11.g6690	520	ID=Fv_160_11.g6690;Description=hypothetical protein FVEG_08333 [Fusarium verticillioides 7600]
Fv_160_11.g6691	405	ID=Fv_160_11.g6691;Description=hypothetical protein FVER14953_08332 [Fusarium verticillioides];Gene=FPRO05_00071;Ontology_term=FMN binding,oxidoreductase
Fv_160_11.g6692	298	ID=Fv_160_11.g6692;Description=hypothetical protein FVER14953_08331 [Fusarium verticillioides];Gene=nAChRalpha2;Ontology_term=extracellular space,nucleus,cytosol,acetylcholine-gated channel complex,neuron projection,postsynaptic membrane,cytokine activity,interleukin-1 receptor binding,copper ion binding,acetylcholine receptor activity,acetylcholine-gated monoatomic cation-selective channel activity,fever generation,connective tissue replacement involved in inflammatory response wound healing,intracellular sodium ion homeostasis,immune response,synaptic transmission, cholinergic,negative regulation of cell population proliferation,positive regulation of vascular endothelial growth factor production,cytokine-mediated signaling pathway,positive regulation of interleukin-2 production,positive regulation of immature T cell proliferation in thymus,cellular response to heat,ectopic germ cell programmed cell death,positive regulation of angiogenesis,positive regulation of mitotic nuclear division,positive regulation of transcription by RNA polymerase II,response to copper ion,positive regulation of protein secretion,positive regulation of cell division,excitatory postsynaptic potential,cellular response to lipopolysaccharide,extrinsic apoptotic signaling pathway in absence of ligand,monoatomic cation transmembrane
Fv_160_11.g6693	195	ID=Fv_160_11.g6693;Description=hypothetical protein FVER14953_08330 [Fusarium
Fv_160_11.g6694	291	ID=Fv_160_11.g6694;Description=hypothetical protein FVER53590_29388 [Fusarium verticillioides]
Fv_160_11.g6695	563	ID=Fv_160_11.g6695;Description=hypothetical protein FVER53263_20075 [Fusarium verticillioides];Gene=FPCIR_1799;Ontology_term=membrane,transmembrane transport;Ontology_id=GO:0016020,GO:0055085
Fv_160_11.g6696	889	ID=Fv_160_11.g6696;Description=c6 zinc finger domain-containing protein [Fusarium coicis];Gene=FTJAE_3172;Ontology_term=nucleus,DNA-binding transcription factor activity, RNA polymerase II-specific,iron ion binding,zinc ion binding,oxidoreductase activity,2 iron, 2 sulfur cluster binding,regulation of transcription by RNA polymerase

Fv_160_11.g6697	396	ID=Fv_160_11.g6697;Description=hypothetical protein FVER53263_08325 [Fusarium verticillioides];Gene=FFUJ_11128;Ontology_term=iron ion binding,choline monooxygenase activity,2 iron, 2 sulfur cluster binding,glycine betaine biosynthetic process from
Fv_160_11.g6698	622	ID=Fv_160_11.g6698;Description=hypothetical protein FVER53590_08324 [Fusarium verticillioides];Gene=FNAPI_2740;Ontology_term=monooxygenase activity,FAD binding,biosynthetic
Fv_160_11.g6699	283	ID=Fv_160_11.g6699;Description=hypothetical protein FVER53590_08323 [Fusarium
Fv_160_11.g6700	788	ID=Fv_160_11.g6700;Description=hypothetical protein FVER53590_08322 [Fusarium verticillioides];Gene=FVEG_16293;Ontology_term=nucleus,DNA binding,zinc ion binding,transferase activity,DNA-binding transcription factor activity, RNA polymerase II-specific,regulation of transcription by RNA polymerase
Fv_160_11.g6701	598	ID=Fv_160_11.g6701;Description=hypothetical protein FVER14953_08321 [Fusarium verticillioides];Gene=FOPG_15441;Ontology_term=magnesium ion binding,pyruvate decarboxylase activity,thiamine pyrophosphate
Fv_160_11.g6702	182	ID=Fv_160_11.g6702;Description=hypothetical protein FVEG_08320 [Fusarium verticillioides 7600];Gene=FVER53590_08320;Ontology_term=acyltransferase activity, transferring groups other than amino-acyl
Fv_160_11.g6703	260	ID=Fv_160_11.g6703;Description=hypothetical protein FVER53590_08319 [Fusarium verticillioides]
Fv_160_11.g6704	742	ID=Fv_160_11.g6704;Description=hypothetical protein FVER53590_08318 [Fusarium verticillioides];Gene=FACUT_10944;Ontology_term=oxidoreductase
Fv_160_11.g6705	459	ID=Fv_160_11.g6705;Description=hypothetical protein FVER14953_08317 [Fusarium verticillioides];Gene=468;Ontology_term=negative regulation of extrinsic apoptotic signaling pathway in absence of ligand,growth factor activity,positive regulation of organ growth,T cell lineage commitment,cytokine activity,positive regulation of B cell differentiation,positive regulation of cytokine-mediated signaling pathway,regulation of peptidyl-tyrosine phosphorylation,interleukin-7 receptor binding,extracellular space,immune response,bone resorption,homeostasis of number of cells within a tissue,positive regulation of T cell differentiation,positive regulation of B cell proliferation,positive regulation of chemokine
Fv_160_11.g6706	494	ID=Fv_160_11.g6706;Description=hypothetical protein FVER53590_08316 [Fusarium verticillioides];Gene=FANTH_1939;Ontology_term=membrane,transmembrane transporter activity,transmembrane
Fv_160_11.g6707	143	ID=Fv_160_11.g6707;Description=hypothetical protein FVEG_08315 [Fusarium verticillioides 7600]
Fv_160_11.g6708	439	ID=Fv_160_11.g6708;Description=hypothetical protein FVER53590_29390 [Fusarium verticillioides]
Fv_160_11.g6709	111	ID=Fv_160_11.g6709;Description=hypothetical protein FVER14953_20360 [Fusarium verticillioides]
Fv_160_11.g6710	767	ID=Fv_160_11.g6710;Description=hypothetical protein FVER14953_20359 [Fusarium verticillioides];Gene=F52700_1301;Ontology_term=dimethylallyltranstransferase activity,geranyltranstransferase activity,isoprenoid biosynthetic process,ketone biosynthetic process,mycotoxin biosynthetic process,alcohol biosynthetic process,organic cyclic compound biosynthetic

Fv_160_11.g6711	407	ID=Fv_160_11.g6711;Description=negative acting factor [Fusarium tjaetaba];Gene=FFC1_10142;Ontology_term=nucleus,DNA-binding transcription factor activity, RNA polymerase II-specific,zinc ion binding,regulation of transcription by RNA polymerase
Fv_160_11.g6712	195	ID=Fv_160_11.g6712;Description=hypothetical protein FVER14953_20358 [Fusarium verticillioides];Gene=472;Ontology_term=dioxygenase
Fv_160_11.g6713	305	ID=Fv_160_11.g6713;Description=hypothetical protein FVER53590_29395 [Fusarium
Fv_160_11.g6714	164	ID=Fv_160_11.g6714;Description=hypothetical protein FVER53590_29396 [Fusarium verticillioides]
Fv_160_11.g6715	195	ID=Fv_160_11.g6715;Description=hypothetical protein FVER14953_20356 [Fusarium
Fv_160_11.g6716	316	ID=Fv_160_11.g6716;Description=hypothetical protein FVER53590_29398 [Fusarium verticillioides];Gene=FVER53590_29398;Ontology_term=NADPH dehydrogenase activity,FMN binding,NADP
Fv_160_11.g6717	303	ID=Fv_160_11.g6717;Description=hypothetical protein FVER14953_20355 [Fusarium verticillioides]
Fv_160_11.g6718	278	ID=Fv_160_11.g6718;Description=hypothetical protein FVER53590_08312 [Fusarium verticillioides]
Fv_160_11.g6719	170	ID=Fv_160_11.g6719;Description=hypothetical protein FDENT_8941 [Fusarium denticulatum]
Fv_160_11.g6720	667	ID=Fv_160_11.g6720;Description=hypothetical protein FVEG_08310 [Fusarium verticillioides 7600];Gene=BFJ72_g7264;Ontology_term=nucleus,membrane,DNA-binding transcription factor activity, RNA polymerase II-specific,monooxygenase activity,hydrolase activity, hydrolyzing O-glycosyl compounds,zinc ion binding,transmembrane transporter activity,carbohydrate metabolic process,regulation of transcription by RNA polymerase II,transmembrane transport;Ontology_id=GO:0005634,GO:0016020,GO:0000981,GO:0004497,GO:0004553,GO:0008270,GO:0022857,GO:0005975,GO
Fv_160_11.g6721	306	ID=Fv_160_11.g6721;Description=monophenol monooxygenase [Fusarium napiforme];Gene=FVEG_08309;Ontology_term=membrane,monooxygenase activity,transmembrane transporter
Fv_160_11.g6722	216	ID=Fv_160_11.g6722;Description=hypothetical protein FVEG_08307 [Fusarium verticillioides 7600];Gene=F53441_469;Ontology_term=membrane,monoatomic cation transmembrane transporter activity,acyltransferase activity,hydrolase activity,catalytic activity, acting on a protein,intracellular monoatomic cation homeostasis,monoatomic cation transmembrane transport,inorganic ion homeostasis;Ontology_id=GO:0016020,GO:0008324,GO:0016746,GO:0016787,GO:0140096,GO:0030003,GO:0098655,GO:0098771;
Fv_160_11.g6723	413	ID=Fv_160_11.g6723;Description=hypothetical protein FVEG_08306 [Fusarium verticillioides 7600];Gene=FMUND_10996;Ontology_term=membrane,acyltransferase activity, transferring groups other than amino-acyl
Fv_160_11.g6724	287	ID=Fv_160_11.g6724;Description=hypothetical protein FVER14953_08305 [Fusarium verticillioides];Gene=FVEG_08305;Ontology_term=iron ion binding,oxidoreductase activity, acting on paired donors, with incorporation or reduction of molecular oxygen,L-ascorbic acid binding,dioxygenase activity;Ontology_id=GO:0005506,GO:0016705,GO:0031418,GO:0051213;Enzyme_code=EC:1.14;Enzyme_name=Acting on paired
Fv_160_11.g6725	710	ID=Fv_160_11.g6725;Description=hypothetical protein FVEG_08304 [Fusarium verticillioides 7600];Gene=FMUND_10994;Ontology_term=metalloendopeptidase activity,metal ion

Fv_160_11.g6726	260	ID=Fv_160_11.g6726;Description=hypothetical protein FANTH_12220 [Fusarium anthophilum];Gene=FVEG_08303;Ontology_term=iron ion binding,oxidoreductase activity, acting on paired donors, with incorporation or reduction of molecular oxygen,L-ascorbic acid binding,dioxygenase activity;Ontology_id=GO:0005506,GO:0016705,GO:0031418,GO:0051213;Enzyme_code=EC:1.14;Enzyme_name=Acting on paired
Fv_160_11.g6727	213	ID=Fv_160_11.g6727;Description=hypothetical protein FVEG_16292 [Fusarium verticillioides 7600];Gene=F66182_8878;Ontology_term=membrane,mycotoxin biosynthetic process;Ontology_id=GO:0016020,GO:0043386
Fv_160_11.g6728	510	ID=Fv_160_11.g6728;Description=hypothetical protein FVEG_16289 [Fusarium verticillioides 7600];Gene=FPANT_8917;Ontology_term=mitochondrial inner membrane,monooxygenase activity,iron ion binding,methyltransferase activity,oxidoreductase activity, acting on paired donors, with incorporation or reduction of molecular oxygen,heme
Fv_160_11.g6729	427	ID=Fv_160_11.g6729;Description=hypothetical protein FVER53590_08302 [Fusarium verticillioides];Gene=FMEXI_13667;Ontology_term=membrane,monooxygenase activity,transmembrane transporter activity,transmembrane
Fv_160_11.g6730	302	ID=Fv_160_11.g6730;Description=hypothetical protein FVER14953_08301 [Fusarium verticillioides];Gene=FCIRC_11006;Ontology_term=membrane,monooxygenase activity,hydrolase activity, hydrolyzing O-glycosyl compounds,transmembrane transporter activity,carbohydrate metabolic process,cellular biosynthetic process,transmembrane transport;Ontology_id=GO:0016020,GO:0004497,GO:0004553,GO:0022857,GO:0005975,GO:0044249,GO:0055085;Enzyme_code=E
Fv_160_11.g6731	85	ID=Fv_160_11.g6731;Description=hypothetical protein FVEG_08300 [Fusarium verticillioides 7600]
Fv_160_11.g6732	247	ID=Fv_160_11.g6732;Description=hypothetical protein FVEG_08299 [Fusarium verticillioides 7600];Gene=FOXYS1_13010;Ontology_term=oxidoreductase activity,cellular biosynthetic
Fv_160_11.g6733	394	ID=Fv_160_11.g6733;Description=hypothetical protein FVER53263_08298 [Fusarium verticillioides];Gene=BFJ69_g7320;Ontology_term=membrane,monoatomic cation transmembrane transporter activity,intracellular monoatomic cation homeostasis,monoatomic cation transmembrane transport,inorganic ion
Fv_160_11.g6734	365	ID=Fv_160_11.g6734;Description=hypothetical protein FVEG_08297 [Fusarium verticillioides 7600];Gene=C2S_10470;Ontology_term=sarcoplasmic reticulum,molting cycle, chitin-based cuticle,smooth endoplasmic reticulum,endoplasmic reticulum membrane,response to oxidative stress,secretory granule membrane,calcium ion binding,flight behavior,positive regulation of ecdysteroid secretion,nuclear division,sensory perception of smell,release of sequestered calcium ion into cytosol,stabilization of membrane potential,sensory perception of taste,mushroom body development,fatty acid homeostasis,inositol 1,4,5-trisphosphate-sensitive calcium-release channel activity,phosphatidylinositol binding,salivary gland cell autophagic cell death,cellular response to starvation,phospholipase C-activating G protein-coupled receptor signaling pathway,visual perception,plasma membrane,regulation of feeding behavior,cellular response to ethanol,larval feeding behavior,axonal growth cone,negative regulation of lipid storage,inositol 1,4,5 trisphosphate

Fv_160_11.g6735	632	ID=Fv_160_11.g6735;Description=hypothetical protein FVER14953_08296 [Fusarium verticillioides];Gene=AU210_013558;Ontology_term=nucleus,DNA binding,zinc ion binding,transmembrane transport,transmembrane transporter activity,membrane,DNA-binding transcription factor activity, RNA polymerase II-
Fv_160_11.g6736	530	ID=Fv_160_11.g6736;Description=hypothetical protein FVEG_08295 [Fusarium verticillioides 7600];Gene=CEP54_009661;Ontology_term=amidase activity,indoleacetamide hydrolase
Fv_160_11.g6737	719	ID=Fv_160_11.g6737;Description=hypothetical protein FVEG_08294 [Fusarium verticillioides 7600];Gene=AU210_012139;Ontology_term=nucleus,DNA binding,zinc ion binding,DNA-binding transcription factor activity, RNA polymerase II-specific,regulation of transcription by RNA polymerase
Fv_160_11.g6738	445	ID=Fv_160_11.g6738;Description=salicylate hydroxylase [Fusarium verticillioides 7600];Gene=FTJAE_3692;Ontology_term=membrane,monooxygenase activity,FAD binding,biosynthetic
Fv_160_11.g6739	471	ID=Fv_160_11.g6739;Description=hypothetical protein FVEG_08292 [Fusarium verticillioides
Fv_160_11.g6740	399	ID=Fv_160_11.g6740;Description=hypothetical protein FVEG_08291 [Fusarium verticillioides 7600];Gene=MBL1;Ontology_term=hydrolase activity,metal ion
Fv_160_11.g6741	272	ID=Fv_160_11.g6741;Description=dienelactone hydrolase [Fusarium subglutinans];Gene=FVER53263_08290;Ontology_term=hydrolase
Fv_160_11.g6742	807	ID=Fv_160_11.g6742;Description=hypothetical protein FVEG_16285 [Fusarium verticillioides 7600];Gene=FCULG_00012390;Ontology_term=membrane,amidase activity,indoleacetamide hydrolase
Fv_160_11.g6743	303	ID=Fv_160_11.g6743;Description=hypothetical protein FVER14953_08287 [Fusarium verticillioides];Gene=FSUBG_12795;Ontology_term=membrane,oxidoreductase
Fv_160_11.g6744	144	ID=Fv_160_11.g6744;Description=hypothetical protein FVER14953_21673 [Fusarium
Fv_160_11.g6745	705	ID=Fv_160_11.g6745;Description=hypothetical protein FVEG_14159 [Fusarium verticillioides 7600];Gene=FSUBG_14048;Ontology_term=nucleus,DNA binding,zinc ion binding,DNA-binding transcription factor activity, RNA polymerase II-specific,regulation of transcription by RNA polymerase
Fv_160_11.g6746	459	ID=Fv_160_11.g6746;Description=hypothetical protein FVEG_14160 [Fusarium verticillioides 7600];Gene=FVEG_14160;Ontology_term=hydrolase activity, hydrolyzing O-glycosyl compounds,transferase activity,carbohydrate metabolic
Fv_160_11.g6747	521	ID=Fv_160_11.g6747;Description=hypothetical protein FVER53590_14161 [Fusarium verticillioides];Gene=CEP51_004188;Ontology_term=membrane,transmembrane transporter activity,transmembrane
Fv_160_11.g6748	316	ID=Fv_160_11.g6748;Description=hypothetical protein FVER14953_21701 [Fusarium verticillioides]
Fv_160_11.g6749	279	ID=Fv_160_11.g6749;Description=uncharacterized protein FVRRES_00078 [Fusarium venenatum];Gene=BFJ65_g17358;Ontology_term=membrane,transmembrane transporter activity,transmembrane
Fv_160_11.g6750	192	ID=Fv_160_11.g6750;Description=hypothetical protein FVEG_17757 [Fusarium verticillioides 7600];Gene=CDV36_010657;Ontology_term=membrane,transmembrane transporter activity,transmembrane

Fv_160_11.g6751	296	ID=Fv_160_11.g6751;Description=hypothetical protein FVER53263_14164 [Fusarium verticillioides];Gene=CEP52_005581;Ontology_term=mitochondrial inner membrane,binding,regulation of DNA-templated
Fv_160_11.g6752	456	ID=Fv_160_11.g6752;Description=hypothetical protein FVEG_14165 [Fusarium verticillioides 7600];Gene=FOXG_17351;Ontology_term=mitochondrion,acyltransferase
Fv_160_11.g6753	897	ID=Fv_160_11.g6753;Description=hypothetical protein FVEG_17758 [Fusarium verticillioides 7600];Gene=BFJ69_g11997;Ontology_term=nucleus,DNA binding,zinc ion binding,DNA-binding transcription factor activity, RNA polymerase II-specific,regulation of transcription by RNA polymerase
Fv_160_12.g6754	891	ID=Fv_160_12.g6754;Description=hypothetical protein FVER14953_02568 [Fusarium verticillioides];Gene=FVEG_02568;Ontology_term=cyclin-dependent protein kinase holoenzyme complex,nucleus,eukaryotic translation initiation factor 3 complex,RNA binding,translation initiation factor activity,cyclin-dependent protein serine/threonine kinase regulator activity,sterol 3-beta-glucosyltransferase activity,translation initiation factor binding,G2/M transition of mitotic cell cycle,regulation of protein phosphorylation,carbohydrate metabolic process,translational initiation,meiosis II,positive regulation of mitotic spindle pole body separation,lipid glycosylation,cell division,regulation of mitotic spindle
Fv_160_12.g6755	349	ID=Fv_160_12.g6755;Description=hypothetical protein FVEG_02567 [Fusarium verticillioides 7600]
Fv_160_12.g6756	399	ID=Fv_160_12.g6756;Description=hypothetical protein FVEG_02566 [Fusarium verticillioides
Fv_160_12.g6757	122	ID=Fv_160_12.g6757;Description=hypothetical protein FVEG_15097 [Fusarium verticillioides
Fv_160_12.g6758	505	ID=Fv_160_12.g6758;Description=hypothetical protein FVEG_02565 [Fusarium verticillioides 7600];Gene=FNYG_12540;Ontology_term=catalytic activity,nucleobase-containing compound metabolic
Fv_160_12.g6759	702	ID=Fv_160_12.g6759;Description=hypothetical protein J7337_008904 [Fusarium musae];Gene=FDENT_2805;Ontology_term=nucleus,DNA-binding transcription factor activity, RNA polymerase II-specific,zinc ion
Fv_160_12.g6760	1055	ID=Fv_160_12.g6760;Description=hypothetical protein FVER53263_02563 [Fusarium verticillioides]
Fv_160_12.g6761	321	ID=Fv_160_12.g6761;Description=hypothetical protein J7337_008902 [Fusarium musae];Gene=F52700_10911;Ontology_term=nucleic acid binding,methyltransferase activity,nitrogen compound metabolic process,macromolecule methylation,primary metabolic
Fv_160_12.g6762	199	ID=Fv_160_12.g6762;Description=hypothetical protein FVER53263_02561 [Fusarium
Fv_160_12.g6763	156	ID=Fv_160_12.g6763;Description=hypothetical protein FVEG_02560 [Fusarium verticillioides
Fv_160_12.g6764	269	ID=Fv_160_12.g6764;Description=tyrosine phosphatase [Fusarium circinatum];Gene=FMAN_08627;Ontology_term=extracellular region,glucan 1,4-alpha-glucosidase activity,phosphoprotein phosphatase activity,starch binding,polysaccharide catabolic process,dephosphorylation;Ontology_id=GO:0005576,GO:0004339,GO:0004721,GO:2001070,GO:0000272,GO:0016311;Enzyme_code=EC:3.2.1.3,EC:3.1.3;Enzyme_name=glucan 1,4-alpha-glucosidase,Acting on ester bonds
Fv_160_12.g6765	320	ID=Fv_160_12.g6765;Description=hypothetical protein FVEG_02558 [Fusarium verticillioides 7600]
Fv_160_12.g6766	843	ID=Fv_160_12.g6766;Description=osomolarity two-component system, response regulator SSK1 [Fusarium verticillioides 7600];Gene=FVEG_02557;Ontology_term=phosphorelay signal transduction system;Ontology_id=GO:0000160

Fv_160_12.g6767	202	ID=Fv_160_12.g6767;Description=hypothetical protein FVEG_02556 [Fusarium verticillioides 7600]
Fv_160_12.g6768	327	ID=Fv_160_12.g6768;Description=alpha-N-arabinofuranosidase [Fusarium verticillioides 7600];Gene=FNAPI_3860;Ontology_term=hydrolase activity, hydrolyzing O-glycosyl compounds,carbohydrate metabolic
Fv_160_12.g6769	1116	ID=Fv_160_12.g6769;Description=esterase/lipase [Fusarium verticillioides 7600];Gene=FNAPI_3861;Ontology_term=lipid metabolic
Fv_160_12.g6770	251	ID=Fv_160_12.g6770;Description=glucosamine-phosphate n-acetyltransferase [Fusarium sp. NRRL 52700];Gene=FNAPI_3862;Ontology_term=glucosamine 6-phosphate N-acetyltransferase activity,UDP-N-acetylglucosamine biosynthetic process;Ontology_id=GO:0004343,GO:0006048;Enzyme_code=EC:2.3.1.4;Enzyme_name=glucosamine-phosphate N-
Fv_160_12.g6771	260	ID=Fv_160_12.g6771;Description=hypothetical protein FVER53263_02552 [Fusarium verticillioides];Gene=FOVG_08904;Ontology_term=oxidoreductase activity,cellular biosynthetic
Fv_160_12.g6772	380	ID=Fv_160_12.g6772;Description=L-iditol 2-dehydrogenase [Fusarium verticillioides 7600];Gene=FGADI_7420;Ontology_term=zinc ion binding,oxidoreductase activity, acting on the CH-OH group of donors, NAD or NADP as
Fv_160_12.g6773	309	ID=Fv_160_12.g6773;Description=alcohol dehydrogenase [Fusarium verticillioides 7600];Gene=FOVG_08906;Ontology_term=oxidoreductase
Fv_160_12.g6774	322	ID=Fv_160_12.g6774;Description=syntaxin 5 [Fusarium verticillioides 7600];Gene=HZS61_015754;Ontology_term=membrane,vesicle-mediated transport;Ontology_id=GO:0016020,GO:0016192
Fv_160_12.g6775	2674	ID=Fv_160_12.g6775;Description=hypothetical protein FVEG_02548 [Fusarium verticillioides 7600]
Fv_160_12.g6776	399	ID=Fv_160_12.g6776;Description=nitrogen metabolic regulatory protein [Fusarium denticulatum];Gene=2798
Fv_160_12.g6777	504	ID=Fv_160_12.g6777;Description=hypothetical protein FVEG_02544 [Fusarium verticillioides 7600];Gene=FMEXI_14151;Ontology_term=nucleus,membrane,DNA binding,zinc ion binding,antiporter activity,xenobiotic transmembrane transporter activity,DNA-templated transcription,xenobiotic detoxification by transmembrane export across the
Fv_160_12.g6778	673	ID=Fv_160_12.g6778;Description=hypothetical protein FVEG_02543 [Fusarium verticillioides 7600];Gene=FOMG_13153;Ontology_term=guanyl-nucleotide exchange factor activity;Ontology_id=GO:0005085
Fv_160_12.g6779	961	ID=Fv_160_12.g6779;Description=dolichyl-phosphate-mannose-protein mannosyltransferase [Fusarium verticillioides 7600];Gene=FNAPI_3872;Ontology_term=nucleus,endoplasmic reticulum membrane,DNA-binding transcription factor activity, RNA polymerase II-specific,DNA binding,dolichyl-phosphate-mannose-protein mannosyltransferase activity,DNA recombination,regulation of transcription by RNA polymerase II,anterior/posterior pattern specification,DNA integration;Ontology_id=GO:0005634,GO:0005789,GO:0000981,GO:0003677,GO:0004169,GO:0006310,GO:0006357,GO:0009952,G
Fv_160_12.g6780	529	ID=Fv_160_12.g6780;Description=hypothetical protein FVEG_02541 [Fusarium verticillioides 7600]
Fv_160_12.g6781	534	ID=Fv_160_12.g6781;Description=hypothetical protein FVER53263_02540 [Fusarium verticillioides];Gene=RP20_CCG028131;Ontology_term=spliceosomal complex,U2 snRNP,RNA binding,dihydrofolate reductase activity,NADP binding,glycine biosynthetic process,one-carbon metabolic process,mRNA cis splicing, via spliceosome,tetrahydrofolate biosynthetic

Fv_160_12.g6782	176	ID=Fv_160_12.g6782;Description=sorting nexin-3 [Fusarium oxysporum f. sp. pisi HDV247];Gene=FMEXI_14282;Ontology_term=Golgi membrane,phosphatidylinositol-3-phosphate
Fv_160_12.g6783	932	ID=Fv_160_12.g6783;Description=hypothetical protein LB505_007760 [Fusarium chuoi];Gene=mts;Ontology_term=nucleus,mitochondrial inner membrane,DNA-binding transcription factor activity, RNA polymerase II-specific,zinc ion binding,regulation of transcription by RNA polymerase II,transmembrane
Fv_160_12.g6784	133	ID=Fv_160_12.g6784;Description=small nuclear ribonucleoprotein [Fusarium verticillioides 7600];Gene=FVEG_02536;Ontology_term=P-body,U5 snRNP,U6 snRNP,nucleolus,sno(s)RNA-containing ribonucleoprotein complex,U4/U6 x U5 tri-snRNP complex,Lsm1-7-Pat1 complex,poly(U) RNA binding,U2 snRNA binding,deadenylation-dependent decapping of nuclear-transcribed mRNA,mRNA splicing, via spliceosome,maturation of SSU-
Fv_160_12.g6785	591	ID=Fv_160_12.g6785;Description=hypothetical protein FVEG_02535 [Fusarium verticillioides 7600];Gene=PAN3;Ontology_term=PAN complex,RNA binding,ATP binding,metal ion binding,nuclear-transcribed mRNA poly(A) tail
Fv_160_12.g6786	518	ID=Fv_160_12.g6786;Description=hypothetical protein FVEG_02534 [Fusarium verticillioides 7600];Gene=2809;Ontology_term=extracellular space,serine-type endopeptidase
Fv_160_12.g6787	499	ID=Fv_160_12.g6787;Description=hypothetical protein FVER14953_02533 [Fusarium verticillioides];Gene=FCIRC_10862;Ontology_term=mitochondrial matrix,alcohol dehydrogenase (NAD+) activity,zinc ion binding,phosphatidylinositol binding,ubiquitin binding,endocytosis,actin filament organization,vacuolar transport,protein transport;Ontology_id=GO:0005759,GO:0004022,GO:0008270,GO:0035091,GO:0043130,GO:0006897,GO:0007015,GO:0007034,GO
Fv_160_12.g6788	551	ID=Fv_160_12.g6788;Description=hypothetical protein FVEG_02532 [Fusarium verticillioides 7600]
Fv_160_12.g6789	466	ID=Fv_160_12.g6789;Description=hypothetical protein FVEG_02531 [Fusarium verticillioides 7600];Gene=FOVG_08929;Ontology_term=nucleus,DNA-binding transcription factor activity, RNA polymerase II-specific,zinc ion
Fv_160_12.g6790	260	ID=Fv_160_12.g6790;Description=hypothetical protein FVEG_02528 [Fusarium verticillioides 7600];Gene=FOC1_g10008490;Ontology_term=S-adenosylmethionine-dependent methyltransferase
Fv_160_12.g6791	246	ID=Fv_160_12.g6791;Description=hypothetical protein FVER14953_02527 [Fusarium verticillioides];Gene=C2S_13133;Ontology_term=sedoheptulose-bisphosphatase activity,ribose phosphate biosynthetic
Fv_160_12.g6792	119	ID=Fv_160_12.g6792;Description=hypothetical protein FVEG_15081 [Fusarium verticillioides 7600]
Fv_160_12.g6793	99	ID=Fv_160_12.g6793;Description=hypothetical protein FVEG_15080 [Fusarium verticillioides 7600]
Fv_160_12.g6794	563	ID=Fv_160_12.g6794;Description=ubiquitin thiolesterase [Fusarium verticillioides 7600];Gene=FVEG_02525;Ontology_term=cysteine-type deubiquitinase activity,proteasome-mediated ubiquitin-dependent protein
Fv_160_12.g6795	169	ID=Fv_160_12.g6795;Description=uncharacterized protein NECHADRAFT_67698 [Fusarium vanettenii 77-13-4];Gene=HZS61_015732;Ontology_term=Arp2/3 protein complex,actin cortical patch,actin filament binding,molecular adaptor activity,actin filament polymerization,Arp2/3 complex-mediated actin nucleation,actin cortical patch
Fv_160_12.g6796	796	ID=Fv_160_12.g6796;Description=uncharacterized protein FTJAE_763 [Fusarium

Fv_160_12.g6797	415	ID=Fv_160_12.g6797;Description=hypothetical protein FVER53263_02522 [Fusarium verticillioides];Gene=FCIRC_4854;Ontology_term=endoplasmic reticulum membrane,phosphatidylinositol transfer activity,metal ion binding,phospholipid transport,intermembrane lipid
Fv_160_12.g6798	786	ID=Fv_160_12.g6798;Description=histone-lysine N-methyltransferase ASH1L [Fusarium verticillioides 7600];Gene=FTJAE_761;Ontology_term=nucleus,chromosome,protein serine/threonine kinase activity,ATP binding,histone methyltransferase activity,chromatin remodeling,phosphorylation,methylation;Ontology_id=GO:0005634,GO:0005694,GO:0004674,GO:0005524,GO:0042054,GO:00063
Fv_160_12.g6799	497	ID=Fv_160_12.g6799;Description=hypothetical protein FVER14953_02518 [Fusarium verticillioides];Gene=FFUJ_05656;Ontology_term=nucleus,mitochondrion,cytosol,membrane,external encapsulating structure,damaged DNA binding,glyceraldehyde-3-phosphate dehydrogenase (NAD+) (phosphorylating) activity,protein-cysteine S-palmitoyltransferase activity,metal ion binding,NADP binding,NAD binding,glucose metabolic process,glycolytic process,error-free translesion synthesis;Ontology_id=GO:0005634,GO:0005739,GO:0005829,GO:0016020,GO:0030312,GO:0003684,GO:0004365,GO:0019706,GO:0046872,GO:0050661,GO:0051287,GO:0006006,GO:0006096,GO:0070987;Enzyme_code=EC:1.2.1.59,EC:1.2.1.12,EC:2.3.1.225;Enz
Fv_160_12.g6800	683	ID=Fv_160_12.g6800;Description=hypothetical protein J7337_008859 [Fusarium musae];Gene=FPHYL_5866;Ontology_term=nucleus,mitochondrion,damaged DNA binding,transferase activity,metal ion binding,error-free translesion
Fv_160_12.g6801	732	ID=Fv_160_12.g6801;Description=uncharacterized protein FTJAE_758 [Fusarium tjaetaba]
Fv_160_12.g6802	195	ID=Fv_160_12.g6802;Description=hypothetical protein FVEG_15073 [Fusarium verticillioides 7600]
Fv_160_12.g6803	270	ID=Fv_160_12.g6803;Description=hypothetical protein FVEG_02516 [Fusarium verticillioides 7600];Gene=Forpe1208_v008613;Ontology_term=peroxidase activity,metal ion binding,cellular oxidant
Fv_160_12.g6804	548	ID=Fv_160_12.g6804;Description=hypothetical protein FVER53263_02515 [Fusarium verticillioides];Gene=P09805;Ontology_term=chitinase activity,chitin binding,toxin activity,polysaccharide catabolic process,chitin catabolic process,modulation of process of another
Fv_160_12.g6805	176	ID=Fv_160_12.g6805;Description=hypothetical protein FVEG_15072 [Fusarium verticillioides 7600]
Fv_160_12.g6806	1066	ID=Fv_160_12.g6806;Description=hypothetical protein FVEG_02513 [Fusarium verticillioides 7600];Gene=FTJAE_754;Ontology_term=nucleus,small GTPase binding,intracellular protein
Fv_160_12.g6807	150	ID=Fv_160_12.g6807;Description=hypothetical protein FVEG_02512 [Fusarium verticillioides 7600];Gene=FPRO05_13308;Ontology_term=lyase activity,pyridoxal phosphate
Fv_160_12.g6808	295	ID=Fv_160_12.g6808;Description=cystathionine beta-lyase [Fusarium tjaetaba];Gene=FTJAE_753;Ontology_term=lyase activity,pyridoxal phosphate

Fv_160_12.g6809	446	ID=Fv_160_12.g6809;Description=IMP-specific 5'-nucleotidase 1 [Fusarium oxysporum f. sp. lycopersici 4287];Gene=FOXG_03639;Ontology_term=nucleotide binding,magnesium ion binding,IMP 5'-nucleotidase activity,inosine salvage,nucleotide metabolic
Fv_160_12.g6810	474	ID=Fv_160_12.g6810;Description=hypothetical protein FVEG_02509 [Fusarium verticillioides 7600]
Fv_160_12.g6811	232	ID=Fv_160_12.g6811;Description=hypothetical protein FVEG_02508 [Fusarium verticillioides 7600];Gene=FTJAE_750;Ontology_term=membrane,calcium ion binding;Ontology_id=GO:0016020,GO:0005509
Fv_160_12.g6812	455	ID=Fv_160_12.g6812;Description=hypothetical protein FVER14953_02507 [Fusarium verticillioides]
Fv_160_12.g6813	294	ID=Fv_160_12.g6813;Description=endoglucanase c [Fusarium tjaetaba]
Fv_160_12.g6814	562	ID=Fv_160_12.g6814;Description=HAL protein kinase [Fusarium verticillioides 7600];Gene=FFUJ_05668;Ontology_term=extracellular region,protein kinase activity,calcium ion binding,ATP binding,phosphorylation;Ontology_id=GO:0005576,GO:0004672,GO:0005509,GO:0005524,GO:0016310;Enzyme_code=EC:2.7.1;Enz
Fv_160_12.g6815	983	ID=Fv_160_12.g6815;Description=hypothetical protein FVER53263_02503 [Fusarium verticillioides];Gene=FVEG_02503;Ontology_term=endoplasmic reticulum,membrane,DNA binding,lipid binding,lipid
Fv_160_12.g6816	1882	ID=Fv_160_12.g6816;Description=hypothetical protein FVER53263_02502 [Fusarium verticillioides];Gene=BFJ69_g621;Ontology_term=cell cortex,phospholipid binding,maintenance of protein location in cell
Fv_160_12.g6817	315	ID=Fv_160_12.g6817;Description=hypothetical protein FVER53590_02500 [Fusarium verticillioides];Gene=FPRO_09155;Ontology_term=membrane,alpha-1,6-mannosyltransferase activity,carbohydrate derivative
Fv_160_12.g6818	289	ID=Fv_160_12.g6818;Description=hypothetical protein FVER53590_02499 [Fusarium verticillioides];Gene=FTJAE_742;Ontology_term=spliceosomal complex,membrane,nucleotide binding,DNA binding,DNA-directed DNA polymerase activity,metal ion binding,DNA replication,mRNA processing,RNA splicing,DNA biosynthetic process,carbohydrate derivative metabolic process;Ontology_id=GO:0005681,GO:0016020,GO:0000166,GO:0003677,GO:0003887,GO:0046872,GO:0006260,GO:0006397,GO:
Fv_160_12.g6819	347	ID=Fv_160_12.g6819;Description=E2-like enzyme [Fusarium musae];Gene=HZS61_015710;Ontology_term=phagophore assembly site,mitochondrion,cytosol,phagophore,Atg8-family ligase activity,autophagosome assembly,autophagy of mitochondrion,protein targeting to membrane,piecemeal microautophagy of the nucleus;Ontology_id=GO:0000407,GO:0005739,GO:0005829,GO:0061908,GO:0019776,GO:0000045,GO:0000422,GO:0006612,GO:
Fv_160_12.g6820	668	ID=Fv_160_12.g6820;Description=hypothetical protein FVER53263_02497 [Fusarium verticillioides]
Fv_160_12.g6821	1694	ID=Fv_160_12.g6821;Description=hypothetical protein FVER53590_02496 [Fusarium verticillioides];Gene=FCIRC_2243;Ontology_term=nucleus,mitochondrion,zeta DNA polymerase complex,nucleotide binding,DNA binding,DNA-directed DNA polymerase activity,metal ion binding,4 iron, 4 sulfur cluster binding,DNA replication,translesion synthesis;Ontology_id=GO:0005634,GO:0005739,GO:0016035,GO:0000166,GO:0003677,GO:0003887,GO:0046872,GO:0051539,GO
Fv_160_12.g6822	467	ID=Fv_160_12.g6822;Description=mRNA splicing protein [Fusarium musae];Gene=FOXB_10032;Ontology_term=spliceosomal complex,pre-mRNA 3'-splice site binding,mRNA splicing, via spliceosome;Ontology_id=GO:0005681,GO:0030628,GO:0000398

Fv_160_12.g6823	191	ID=Fv_160_12.g6823;Description=40S ribosomal protein S9 [Fusarium oxysporum f. sp. rapae];Gene=HYE68_000915;Ontology_term=small ribosomal subunit,structural constituent of ribosome,rRNA
Fv_160_12.g6824	160	ID=Fv_160_12.g6824;Description=50S ribosomal protein L21e [Fusarium oxysporum f. sp. lycopersici 4287];Gene=FOC1_g10008454;Ontology_term=ribosome,structural constituent of
Fv_160_12.g6825	1054	ID=Fv_160_12.g6825;Description=cytoskeleton assembly control SLA2 [Fusarium sp. NRRL 52700];Gene=FANTH_1121;Ontology_term=incipient cellular bud site,actin binding,clathrin binding,endocytosis,phospholipid binding,actin cortical patch assembly,actin cortical patch,negative regulation of Arp2/3 complex-mediated actin
Fv_160_12.g6826	382	ID=Fv_160_12.g6826;Description=mating-type protein MAT-1 [Fusarium verticillioides 7600];Gene=FVEG_02491;Ontology_term=nucleus,DNA binding, bending,positive regulation of mating-type specific transcription,
Fv_160_12.g6827	433	ID=Fv_160_12.g6827;Description=hypothetical protein FVEG_02490 [Fusarium verticillioides
Fv_160_12.g6828	201	ID=Fv_160_12.g6828;Description=hypothetical protein FVEG_02489 [Fusarium verticillioides 7600];Gene=FVER53263_02489;Ontology_term=nucleus,cytoplasm,magnesium ion binding,DNA binding,3-isopropylmalate dehydrogenase activity,NAD binding,leucine biosynthetic process;Ontology_id=GO:0005634,GO:0005737,GO:0000287,GO:0003677,GO:0003862,GO:0051287,GO:0009098;Enzyme_code=EC
Fv_160_12.g6829	601	ID=Fv_160_12.g6829;Description=hypothetical protein FVER53263_02488 [Fusarium verticillioides];Gene=FPANT_6828;Ontology_term=DNA binding,endonuclease activity,zinc ion binding,DNA
Fv_160_12.g6830	127	ID=Fv_160_12.g6830;Description=cytochrome c oxidase subunit 6a [Fusarium verticillioides 7600];Gene=HZS61_015698;Ontology_term=mitochondrial respiratory chain complex IV,cytochrome-c oxidase activity,symporter activity,enzyme regulator activity,mitochondrial electron transport, cytochrome c to oxygen,carbohydrate transport,mitochondrial respirasome assembly,proton transmembrane transport;Ontology_id=GO:0005751,GO:0004129,GO:0015293,GO:0030234,GO:0006123,GO:0008643,GO:0097250,GO:1902600;En
Fv_160_12.g6831	786	ID=Fv_160_12.g6831;Description=hypothetical protein FVER14953_02486 [Fusarium verticillioides];Gene=FNAPI_2102;Ontology_term=anaphase-promoting complex,spindle,cell cycle,protein ubiquitination,cell
Fv_160_12.g6832	263	ID=Fv_160_12.g6832;Description=hypothetical protein FVER14953_02485 [Fusarium verticillioides]
Fv_160_12.g6833	307	ID=Fv_160_12.g6833;Description=hypothetical protein J7337_008827 [Fusarium musae]
Fv_160_12.g6834	522	ID=Fv_160_12.g6834;Description=hypothetical protein FVEG_15068 [Fusarium verticillioides 7600];Gene=FOYG_10150;Ontology_term=membrane,transmembrane transporter activity,transmembrane
Fv_160_12.g6835	948	ID=Fv_160_12.g6835;Description=hypothetical protein FVEG_02481 [Fusarium verticillioides
Fv_160_12.g6836	487	ID=Fv_160_12.g6836;Description=hypothetical protein FVEG_02480 [Fusarium verticillioides 7600];Gene=FVER53263_02480;Ontology_term=endoplasmic reticulum membrane,cellular response to oxidative
Fv_160_12.g6837	257	ID=Fv_160_12.g6837;Description=hypothetical protein FPHYL_1333 [Fusarium phyllophilum];Gene=F25303_4684;Ontology_term=endoplasmic reticulum membrane,cellular response to oxidative

Fv_160_12.g6838	132	ID=Fv_160_12.g6838;Description=hypothetical protein J7337_008820 [Fusarium musae];Gene=FSUBG_7652;Ontology_term=translation initiation factor activity,translational
Fv_160_12.g6839	511	ID=Fv_160_12.g6839;Description=hypothetical protein FVER14953_02477 [Fusarium verticillioides];Gene=FACUT_3094;Ontology_term=cytoplasm,tRNA (uridine) methyltransferase activity,tRNA nucleoside ribose
Fv_160_12.g6840	169	ID=Fv_160_12.g6840;Description=hypothetical protein FVEG_02476 [Fusarium verticillioides 7600]
Fv_160_12.g6841	345	ID=Fv_160_12.g6841;Description=hypothetical protein FCOIX_11209 [Fusarium coicis]
Fv_160_12.g6842	98	ID=Fv_160_12.g6842;Description=hypothetical protein FVEG_15065 [Fusarium verticillioides 7600];Gene=MELT;Ontology_term=extracellular region,other organism cell membrane,pore complex,protein kinase inhibitor activity,protein binding,lipid binding,porin activity,toxin activity,monoatomic ion transport,killing of cells of another organism,modulation of process of another organism,transmembrane
Fv_160_12.g6843	935	ID=Fv_160_12.g6843;Description=hypothetical protein FVEG_02474 [Fusarium verticillioides
Fv_160_12.g6844	574	ID=Fv_160_12.g6844;Description=sulfate adenylyltransferase [Fusarium verticillioides 7600];Gene=28945716;Ontology_term=cytoplasm,adenylylsulfate kinase activity,sulfate adenylyltransferase (ATP) activity,ATP binding,sulfate assimilation,methionine biosynthetic process,cysteine biosynthetic process,hydrogen sulfide biosynthetic process;Ontology_id=GO:0005737,GO:0004020,GO:0004781,GO:0005524,GO:0000103,GO:0009086,GO:0019344,GO:0070814;Enzy
Fv_160_12.g6845	917	ID=Fv_160_12.g6845;Description=hypothetical protein FVEG_02471 [Fusarium verticillioides 7600];Gene=FANTH_1141;Ontology_term=nucleus,DNA binding;Ontology_id=GO:0005634,GO:0003677
Fv_160_12.g6846	577	ID=Fv_160_12.g6846;Description=hypothetical protein FVEG_02470 [Fusarium verticillioides
Fv_160_12.g6847	622	ID=Fv_160_12.g6847;Description=WD-repeat protein [Fusarium denticulatum]
Fv_160_12.g6848	70	ID=Fv_160_12.g6848;Description=protein transporter Sec61 subunit gamma [Fusarium oxysporum f. sp. lycopersici 4287];Gene=FG08871.1;Ontology_term=endoplasmic reticulum membrane,protein transmembrane transporter activity,protein
Fv_160_12.g6849	365	ID=Fv_160_12.g6849;Description=hypothetical protein J7337_008809 [Fusarium musae];Gene=BFJ68_g10683;Ontology_term=nucleus,ribonucleoprotein complex,nucleic acid binding,mRNA
Fv_160_12.g6850	236	ID=Fv_160_12.g6850;Description=uncharacterized protein FTJAE_6313 [Fusarium tjaetaba]
Fv_160_12.g6851	381	ID=Fv_160_12.g6851;Description=hypothetical protein FDENT_8708 [Fusarium
Fv_160_12.g6852	712	ID=Fv_160_12.g6852;Description=hypothetical protein FVER14953_02464 [Fusarium verticillioides];Gene=FNAPI_2124;Ontology_term=ribonucleoprotein complex,nucleic acid binding,helicase activity,ATP
Fv_160_12.g6853	478	ID=Fv_160_12.g6853;Description=hypothetical protein FVER14953_02463 [Fusarium verticillioides]
Fv_160_12.g6854	521	ID=Fv_160_12.g6854;Description=hypothetical protein FVER53263_02462 [Fusarium verticillioides];Gene=COQ4;Ontology_term=extrinsic component of mitochondrial inner membrane,ubiquinone biosynthetic
Fv_160_12.g6855	736	ID=Fv_160_12.g6855;Description=ubiquinone biosynthesis coq-4 mitochondrial [Fusarium coicis];Gene=COQ4;Ontology_term=extrinsic component of mitochondrial inner membrane,ubiquinone biosynthetic
Fv_160_12.g6856	129	ID=Fv_160_12.g6856;Description=programmed cell death protein 5 [Fusarium verticillioides

Fv_160_12.g6857	462	ID=Fv_160_12.g6857;Description=hypothetical protein FVER14953_02458 [Fusarium verticillioides];Gene=FNYG_11452;Ontology_term=DNA binding,oxidoreductase
Fv_160_12.g6858	185	ID=Fv_160_12.g6858;Description=hypothetical protein FVEG_02457 [Fusarium verticillioides 7600];Gene=FOPG_03518;Ontology_term=nucleus,RNA binding,rRNA
Fv_160_12.g6859	564	ID=Fv_160_12.g6859;Description=asparaginyl-tRNA synthetase [Fusarium verticillioides 7600];Gene=FGADI_6671;Ontology_term=asparagine-tRNA ligase activity,ATP binding,asparaginyl-tRNA
Fv_160_12.g6860	147	ID=Fv_160_12.g6860;Description=hypothetical protein FVEG_02455 [Fusarium verticillioides 7600];Gene=P05472;Ontology_term=DNA-directed RNA polymerase complex,mitochondrial intermembrane space,DNA binding,DNA-directed 5'-3' RNA polymerase activity,ribonucleoside binding,DNA-templated transcription,aerobic respiration;Ontology_id=GO:0000428,GO:0005758,GO:0003677,GO:0003899,GO:0032549,GO:0006351,GO:0009060;Enzyme_code
Fv_160_12.g6861	455	ID=Fv_160_12.g6861;Description=hypothetical protein FVEG_02454 [Fusarium verticillioides
Fv_160_12.g6862	371	ID=Fv_160_12.g6862;Description=hypothetical protein FVEG_02453 [Fusarium verticillioides 7600];Gene=FNAPI_2134;Ontology_term=methyltransferase
Fv_160_12.g6863	241	ID=Fv_160_12.g6863;Description=hypothetical protein FVER14953_02452 [Fusarium
Fv_160_12.g6864	601	ID=Fv_160_12.g6864;Description=hypothetical protein J7337_008793 [Fusarium musae];Gene=RAG1;Ontology_term=nucleus,membrane,DNA-binding transcription factor activity, RNA polymerase II-specific,zinc ion binding,transmembrane transporter activity,regulation of transcription by RNA polymerase II,carbohydrate
Fv_160_12.g6865	312	ID=Fv_160_12.g6865;Description=hypothetical protein FVER14953_02450 [Fusarium verticillioides];Gene=FTJAE_6298;Ontology_term=mitochondrial inner membrane;Ontology_id=GO:0005743
Fv_160_12.g6866	528	ID=Fv_160_12.g6866;Description=hypothetical protein FVER14953_02449 [Fusarium verticillioides];Gene=FMAN_08737;Ontology_term=nucleus,chromosome,cytosol,glucose-6-phosphate isomerase activity,protein heterodimerization activity,carbohydrate derivative binding,gluconeogenesis,glycolytic process,carbohydrate derivative metabolic process;Ontology_id=GO:0005634,GO:0005694,GO:0005829,GO:0004347,GO:0046982,GO:0097367,GO:0006094,GO:0006096,GO:
Fv_160_12.g6867	134	ID=Fv_160_12.g6867;Description=hypothetical protein FVER14953_02448 [Fusarium verticillioides];Gene=FVEG_02448;Ontology_term=membrane,GTPase activity,GTP binding,G-protein beta/gamma-subunit complex binding,adenylate cyclase-modulating G protein-coupled receptor signaling
Fv_160_12.g6868	199	ID=Fv_160_12.g6868;Description=hypothetical protein FPSE_08260 [Fusarium pseudograminearum CS3096];Gene=FCIRC_3254;Ontology_term=GTPase activity,GTP binding,small GTPase mediated signal
Fv_160_12.g6869	324	ID=Fv_160_12.g6869;Description=hypothetical protein FVER14953_02446 [Fusarium verticillioides];Gene=Forpe1208_v008679;Ontology_term=nucleosome,membrane,ribonucleoprotein complex,DNA binding,RNA binding,guanylate cyclase activity,protein kinase activity,ATP binding,GTP binding,structural constituent of chromatin,cGMP biosynthetic process,nucleosome assembly;Ontology_id=GO:0000786,GO:0016020,GO:1990904,GO:0003677,GO:0003723,GO:0004383,GO:0004672,GO:0005524,GO

Fv_160_12.g6870	716	ID=Fv_160_12.g6870;Description=hypothetical protein FVER14953_02445 [Fusarium verticillioides]
Fv_160_12.g6871	309	ID=Fv_160_12.g6871;Description=hypothetical protein FVER53590_02444 [Fusarium
Fv_160_12.g6872	454	ID=Fv_160_12.g6872;Description=hypothetical protein FVER53590_29431 [Fusarium verticillioides];Gene=FGLOB1_9735;Ontology_term=glycosyltransferase
Fv_160_12.g6873	715	ID=Fv_160_12.g6873;Description=hypothetical protein FVER53590_02442 [Fusarium verticillioides];Gene=FOIG_12065;Ontology_term=DNA photolyase activity,DNA
Fv_160_12.g6874	555	ID=Fv_160_12.g6874;Description=2-methylcitrate dehydratase [Fusarium sp. NRRL 25303];Gene=HZS61_015657;Ontology_term=2-methylcitrate dehydratase activity,2 iron, 2 sulfur cluster binding,propionate metabolic process, methylcitrate cycle;Ontology_id=GO:0047547,GO:0051537,GO:0019679;Enzyme_code=EC:4.2.1.79;Enzyme_name=2-methylcitrate dehydratase
Fv_160_12.g6875	264	ID=Fv_160_12.g6875;Description=hypothetical protein FVEG_02440 [Fusarium verticillioides 7600];Gene=rplT
Fv_160_12.g6876	251	ID=Fv_160_12.g6876;Description=hypothetical protein FVER53590_02439 [Fusarium verticillioides]
Fv_160_12.g6877	747	ID=Fv_160_12.g6877;Description=hypothetical protein FVER53590_29432 [Fusarium verticillioides];Gene=FDENT_8681;Ontology_term=acrosomal membrane,cell adhesion,fusion of sperm to egg plasma membrane
Fv_160_12.g6878	1287	ID=Fv_160_12.g6878;Description=hypothetical protein FVER14953_02437 [Fusarium verticillioides]
Fv_160_12.g6879	665	ID=Fv_160_12.g6879;Description=hypothetical protein FVEG_02436 [Fusarium verticillioides 7600];Gene=FVEG_02436;Ontology_term=ATP binding,ATP hydrolysis
Fv_160_12.g6880	110	ID=Fv_160_12.g6880;Description=hypothetical protein FVEG_02435 [Fusarium verticillioides 7600]
Fv_160_12.g6881	422	ID=Fv_160_12.g6881;Description=hypothetical protein FVER53590_02434 [Fusarium verticillioides];Gene=FNAPI_2153;Ontology_term=DNA binding,DNA transposition,DNA
Fv_160_12.g6882	642	ID=Fv_160_12.g6882;Description=hypothetical protein FVEG_02433 [Fusarium verticillioides 7600];Gene=FOIG_12074;Ontology_term=cytoplasm,endonuclease
Fv_160_12.g6883	172	ID=Fv_160_12.g6883;Description=hypothetical protein FVER53590_07003 [Fusarium verticillioides]
Fv_160_12.g6884	322	ID=Fv_160_12.g6884;Description=CMGC/SRPK protein kinase [Fusarium verticillioides 7600];Gene=FFUJ_05744;Ontology_term=protein kinase activity,ATP
Fv_160_12.g6885	862	ID=Fv_160_12.g6885;Description=hypothetical protein FVEG_02431 [Fusarium verticillioides
Fv_160_12.g6886	587	ID=Fv_160_12.g6886;Description=hypothetical protein FVER53590_02430 [Fusarium verticillioides];Gene=BFJ63_vAg4804;Ontology_term=membrane,transmembrane transporter activity,transmembrane
Fv_160_12.g6887	576	ID=Fv_160_12.g6887;Description=hypothetical protein FVER14953_02429 [Fusarium verticillioides];Gene=FDENT_8671;Ontology_term=ligase
Fv_160_12.g6888	516	ID=Fv_160_12.g6888;Description=hypothetical protein FVER14953_02428 [Fusarium verticillioides];Gene=FMAN_08759;Ontology_term=cytoplasm,cytoskeleton,DNA binding,aspartic-type endopeptidase activity,ATP binding,DNA recombination,proteolysis,DNA integration;Ontology_id=GO:0005737,GO:0005856,GO:0003677,GO:0004190,GO:0005524,GO:0006310,GO:0006508,GO:0015074;E

Fv_160_12.g6889	367	ID=Fv_160_12.g6889;Description=hypothetical protein J7337_008769 [Fusarium musae];Gene=Focb16_v013834;Ontology_term=mitochondrion,proton-transporting ATP synthase complex
Fv_160_12.g6890	916	ID=Fv_160_12.g6890;Description=hypothetical protein FVEG_02426 [Fusarium verticillioides 7600];Gene=FANTH_6771;Ontology_term=Golgi membrane,COPI vesicle coat,structural molecule activity,intracellular protein
Fv_160_12.g6891	283	ID=Fv_160_12.g6891;Description=hypothetical protein FVER14953_02424 [Fusarium verticillioides]
Fv_160_12.g6892	195	ID=Fv_160_12.g6892;Description=hypothetical protein FVER53590_02423 [Fusarium verticillioides];Gene=2919
Fv_160_12.g6893	478	ID=Fv_160_12.g6893;Description=hypothetical protein FVER14953_02422 [Fusarium verticillioides];Gene=FGLOB1_10404;Ontology_term=membrane,transmembrane transporter activity,transmembrane
Fv_160_12.g6894	270	ID=Fv_160_12.g6894;Description=hypothetical protein FVER53590_02421 [Fusarium verticillioides];Gene=2921
Fv_160_12.g6895	690	ID=Fv_160_12.g6895;Description=hypothetical protein FVER53263_02420 [Fusarium verticillioides];Gene=FFUJ_05755;Ontology_term=nucleus,DNA binding,zinc ion binding,DNA-binding transcription factor activity, RNA polymerase II-specific,regulation of transcription by RNA polymerase
Fv_160_12.g6896	212	ID=Fv_160_12.g6896;Description=hypothetical protein J7337_008763 [Fusarium musae]
Fv_160_12.g6897	530	ID=Fv_160_12.g6897;Description=hypothetical protein FVEG_02418 [Fusarium verticillioides 7600];Gene=FPHYL_1070;Ontology_term=membrane,transmembrane transporter activity,transmembrane
Fv_160_12.g6898	263	ID=Fv_160_12.g6898;Description=hypothetical protein FVER53263_02417 [Fusarium verticillioides]
Fv_160_12.g6899	126	ID=Fv_160_12.g6899;Description=hypothetical protein FVER14953_02416 [Fusarium verticillioides]
Fv_160_12.g6900	195	ID=Fv_160_12.g6900;Description=hypothetical protein FVEG_02415 [Fusarium verticillioides 7600]
Fv_160_12.g6901	290	ID=Fv_160_12.g6901;Description=hypothetical protein FVER53590_02414 [Fusarium verticillioides]
Fv_160_12.g6902	711	ID=Fv_160_12.g6902;Description=hypothetical protein FVEG_02413 [Fusarium verticillioides 7600]
Fv_160_12.g6903	795	ID=Fv_160_12.g6903;Description=hypothetical protein FVER53590_02412 [Fusarium verticillioides];Gene=FVER53263_02412;Ontology_term=nucleus,DNA binding,zinc ion binding,DNA-binding transcription factor activity, RNA polymerase II-specific,regulation of transcription by RNA polymerase
Fv_160_12.g6904	492	ID=Fv_160_12.g6904;Description=hypothetical protein FVER14953_02411 [Fusarium verticillioides];Gene=ugl-1;Ontology_term=hydrolase activity,carbohydrate metabolic
Fv_160_12.g6905	1406	ID=Fv_160_12.g6905;Description=hypothetical protein FVER14953_02410 [Fusarium verticillioides];Gene=FNAPI_2174;Ontology_term=membrane,ATP binding,ATP hydrolysis activity,ABC-type transporter activity,transmembrane transport;Ontology_id=GO:0016020,GO:0005524,GO:0016887,GO:0140359,GO:0055085;Enzyme_code=EC:7.2.2,EC:3.6.1.15;Enzym
Fv_160_12.g6906	693	ID=Fv_160_12.g6906;Description=hypothetical protein FVEG_02409 [Fusarium verticillioides 7600];Gene=FOIG_12098;Ontology_term=nucleus,DNA binding,zinc ion binding,membrane,DNA-binding transcription factor activity, RNA polymerase II-specific,regulation of transcription by RNA polymerase

Fv_160_12.g6907	232	ID=Fv_160_12.g6907;Description=hypothetical protein FVER53590_25977 [Fusarium verticillioides];Gene=FTJAE_6258;Ontology_term=acyltransferase activity, transferring groups other than amino-acyl
Fv_160_12.g6908	403	ID=Fv_160_12.g6908;Description=hypothetical protein FVER53590_02407 [Fusarium verticillioides];Gene=BFJ68_g15147;Ontology_term=metallopeptidase
Fv_160_12.g6909	215	ID=Fv_160_12.g6909;Description=hypothetical protein FVEG_15057 [Fusarium verticillioides 7600]
Fv_160_12.g6910	495	ID=Fv_160_12.g6910;Description=hypothetical protein FVER14953_02406 [Fusarium verticillioides];Gene=FVER53590_02406;Ontology_term=triglyceride lipase activity,lipid catabolic
Fv_160_12.g6911	663	ID=Fv_160_12.g6911;Description=hypothetical protein DER46DRAFT_635113 [Fusarium sp. MPI-SDFR-AT-0072];Gene=FVER53590_02406;Ontology_term=triglyceride lipase activity,lipid catabolic
Fv_160_12.g6912	263	ID=Fv_160_12.g6912;Description=hypothetical protein FVER14953_02404 [Fusarium verticillioides];Gene=FNYG_11397;Ontology_term=hydrolase
Fv_160_12.g6913	499	ID=Fv_160_12.g6913;Description=hypothetical protein FVEG_02403 [Fusarium verticillioides 7600];Gene=FTJAE_6252;Ontology_term=oxidoreductase activity,FAD
Fv_160_12.g6914	1261	ID=Fv_160_12.g6914;Description=hypothetical protein FVEG_02402 [Fusarium verticillioides 7600];Gene=FNAPI_2182;Ontology_term=membrane,ATP binding,ATP hydrolysis activity,ABC-type transporter activity,transmembrane transport;Ontology_id=GO:0016020,GO:0005524,GO:0016887,GO:0140359,GO:0055085;Enzyme_code=EC:7.2.2,EC:3.6.1.15;Enzym
Fv_160_12.g6915	620	ID=Fv_160_12.g6915;Description=hypothetical protein FVER53263_02401 [Fusarium verticillioides];Gene=FCIRC_3302;Ontology_term=nucleus,DNA binding,zinc ion binding,DNA-binding transcription factor activity, RNA polymerase II-specific,regulation of transcription by RNA polymerase
Fv_160_12.g6916	151	ID=Fv_160_12.g6916;Description=cofilin [Fusarium verticillioides 7600];Gene=BFJ63_vAg840;Ontology_term=actin cytoskeleton,nuclear matrix,actin binding,actin filament
Fv_160_12.g6917	613	ID=Fv_160_12.g6917;Description=hypothetical protein FVEG_02399 [Fusarium verticillioides 7600]
Fv_160_12.g6918	660	ID=Fv_160_12.g6918;Description=hypothetical protein FVER53263_02398 [Fusarium verticillioides];Gene=FNAPI_2186;Ontology_term=nucleus,DNA binding,zinc ion binding,DNA-binding transcription factor activity, RNA polymerase II-specific,regulation of transcription by RNA polymerase
Fv_160_12.g6919	560	ID=Fv_160_12.g6919;Description=hypothetical protein FVER14953_02397 [Fusarium verticillioides];Gene=FFC1_04286;Ontology_term=molybdenum ion binding,pyridoxal phosphate binding,dioxygenase activity,2 iron,
Fv_160_12.g6920	419	ID=Fv_160_12.g6920;Description=hypothetical protein FVEG_15054 [Fusarium verticillioides 7600]
Fv_160_12.g6921	149	ID=Fv_160_12.g6921;Description=hypothetical protein FVEG_15053 [Fusarium verticillioides 7600]
Fv_160_12.g6922	86	ID=Fv_160_12.g6922;Description=hypothetical protein FVEG_02395 [Fusarium verticillioides 7600]

Fv_160_12.g6923	512	ID=Fv_160_12.g6923;Description=hypothetical protein FVEG_02394 [Fusarium verticillioides 7600];Gene=COX3;Ontology_term=mitochondrion,membrane,cytochrome-c oxidase activity,aerobic electron transport chain,proton transmembrane transport;Ontology_id=GO:0005739,GO:0016020,GO:0004129,GO:0019646,GO:1902600;Enzyme_code=EC:1.9,EC:7.1.1.9;Enzyme_n
Fv_160_12.g6924	426	ID=Fv_160_12.g6924;Description=hypothetical protein FVEG_02393 [Fusarium verticillioides 7600];Gene=FVEG_02393;Ontology_term=oxidoreductase activity,FAD binding,biosynthetic
Fv_160_12.g6925	1110	ID=Fv_160_12.g6925;Description=hypothetical protein FVER53263_02392 [Fusarium verticillioides];Gene=FNYG_11386;Ontology_term=protein localization to plasma membrane;Ontology_id=GO:0072659
Fv_160_12.g6926	190	ID=Fv_160_12.g6926;Description=hypothetical protein FVEG_02391 [Fusarium verticillioides
Fv_160_12.g6927	207	ID=Fv_160_12.g6927;Description=ras superfamily [Fusarium subglutinans];Gene=HZS61_015613;Ontology_term=plasma membrane,GTPase activity,GTP binding,signal
Fv_160_12.g6928	354	ID=Fv_160_12.g6928;Description=hypothetical protein FVER53590_02389 [Fusarium verticillioides];Gene=F25303_3925;Ontology_term=oxidoreductase activity,cellular biosynthetic
Fv_160_12.g6929	538	ID=Fv_160_12.g6929;Description=hypothetical protein FVEG_02387 [Fusarium verticillioides 7600];Gene=scw1;Ontology_term=RNA
Fv_160_12.g6930	619	ID=Fv_160_12.g6930;Description=hypothetical protein FVEG_02386 [Fusarium verticillioides 7600];Gene=FFC1_04296;Ontology_term=nucleus,DNA-binding transcription factor activity, RNA polymerase II-specific,zinc ion
Fv_160_12.g6931	434	ID=Fv_160_12.g6931;Description=hypothetical protein FVEG_02385 [Fusarium verticillioides 7600];Gene=2954;Ontology_term=intermediate filament,S-adenosylmethionine-dependent methyltransferase
Fv_160_12.g6932	84	ID=Fv_160_12.g6932;Description=splicing factor 3B subunit 10-domain-containing protein [Fusarium oxysporum];Gene=FOXG_03518;Ontology_term=U2 snRNP,mRNA splicing, via spliceosome;Ontology_id=GO:0005686,GO:0000398
Fv_160_12.g6933	308	ID=Fv_160_12.g6933;Description=hypothetical protein FVEG_02383 [Fusarium verticillioides 7600]
Fv_160_12.g6934	1223	ID=Fv_160_12.g6934;Description=hypothetical protein FVER53263_02382 [Fusarium verticillioides];Gene=FPCIR_115;Ontology_term=mitochondrial inner membrane;Ontology_id=GO:0005743
Fv_160_12.g6935	460	ID=Fv_160_12.g6935;Description=elongation factor 1-alpha [Fusarium verticillioides 7600];Gene=FOTG_01933;Ontology_term=translation elongation factor activity,GTPase activity,GTP binding,translational
Fv_160_12.g6936	450	ID=Fv_160_12.g6936;Description=hypothetical protein FVEG_02380 [Fusarium verticillioides 7600];Gene=FVER53590_02380;Ontology_term=membrane,transmembrane transporter activity,transmembrane
Fv_160_12.g6937	559	ID=Fv_160_12.g6937;Description=hypothetical protein FVEG_02379 [Fusarium verticillioides 7600];Gene=Forpe1208_v008745;Ontology_term=membrane,monooxygenase activity,iron ion binding,oxidoreductase activity, acting on paired donors, with incorporation or reduction of molecular oxygen,heme binding;Ontology_id=GO:0016020,GO:0004497,GO:0005506,GO:0016705,GO:0020037;Enzyme_code=EC:1.14;Enzyme_name=Actin
Fv_160_12.g6938	529	ID=Fv_160_12.g6938;Description=hypothetical protein FVEG_15050 [Fusarium verticillioides 7600];Gene=Forpi1262_v008700;Ontology_term=nucleus,DNA-binding transcription factor activity, RNA polymerase II-specific,zinc

Fv_160_12.g6939	230	ID=Fv_160_12.g6939;Description=hypothetical protein FVEG_02378 [Fusarium verticillioides 7600];Gene=FPRO05_09460;Ontology_term=nucleus,cytoplasm;Ontology_id=GO:0005634,GO:0005737
Fv_160_12.g6940	289	ID=Fv_160_12.g6940;Description=hypothetical protein FVEG_02377 [Fusarium verticillioides 7600];Gene=F25303_4412;Ontology_term=kinetochore,nucleus,centromere complex
Fv_160_12.g6941	579	ID=Fv_160_12.g6941;Description=hypothetical protein FVEG_02376 [Fusarium verticillioides 7600];Gene=HZS61_015599;Ontology_term=nucleus,membrane,DNA-binding transcription factor activity, RNA polymerase II-specific,DNA binding,acetyl-CoA transmembrane transporter activity,regulation of transcription by RNA polymerase II,acetyl-CoA
Fv_160_12.g6942	291	ID=Fv_160_12.g6942;Description=hypothetical protein FVER14953_20445 [Fusarium
Fv_160_12.g6943	463	ID=Fv_160_12.g6943;Description=hypothetical protein FVER53590_02375 [Fusarium verticillioides]
Fv_160_12.g6944	1207	ID=Fv_160_12.g6944;Description=hypothetical protein FVER53590_02373 [Fusarium verticillioides];Gene=FPHYL_39;Ontology_term=ligase activity;Ontology_id=GO:0016874;Enzyme_code=EC:6;Enzyme_name=Ligases
Fv_160_12.g6945	502	ID=Fv_160_12.g6945;Description=hypothetical protein FVEG_02372 [Fusarium verticillioides 7600];Gene=FNYG_11364;Ontology_term=membrane,monooxygenase activity,iron ion binding,oxidoreductase activity, acting on paired donors, with incorporation or reduction of molecular oxygen,heme binding;Ontology_id=GO:0016020,GO:0004497,GO:0005506,GO:0016705,GO:0020037;Enzyme_code=EC:1.14;Enzyme_name=Actin
Fv_160_12.g6946	601	ID=Fv_160_12.g6946;Description=hypothetical protein FVER14953_02371 [Fusarium verticillioides];Gene=Q04948;Ontology_term=nucleus,cytoplasm,intermediate filament,DNA-binding transcription factor activity, RNA polymerase II-specific,structural molecule activity,zinc ion binding,regulation of transcription by RNA polymerase
Fv_160_12.g6947	107	ID=Fv_160_12.g6947;Description=hypothetical protein FVEG_15045 [Fusarium verticillioides 7600]
Fv_160_12.g6948	187	ID=Fv_160_12.g6948;Description=hypothetical protein FVER53263_02370 [Fusarium verticillioides]
Fv_160_12.g6949	359	ID=Fv_160_12.g6949;Description=hypothetical protein FVEG_02369 [Fusarium verticillioides 7600]
Fv_160_12.g6950	1155	ID=Fv_160_12.g6950;Description=hypothetical protein FVER14953_02367 [Fusarium verticillioides]
Fv_160_12.g6951	301	ID=Fv_160_12.g6951;Description=hypothetical protein FVEG_02366 [Fusarium verticillioides
Fv_160_12.g6952	360	ID=Fv_160_12.g6952;Description=receptor expression-enhancing 4 [Fusarium
Fv_160_12.g6953	304	ID=Fv_160_12.g6953;Description=hypothetical protein FVEG_02364 [Fusarium verticillioides 7600];Gene=FNAPI_3696;Ontology_term=mitochondrial chromosome,peroxisomal matrix,glyoxysome,single-stranded DNA binding,malate synthase activity,mitochondrial genome maintenance,recombinational repair,glyoxylate cycle,tricarboxylic acid cycle,interstrand cross-link repair;Ontology_id=GO:0000262,GO:0005782,GO:0009514,GO:0003697,GO:0004474,GO:0000002,GO:0000725,GO:0006097,GO:00
Fv_160_12.g6954	669	ID=Fv_160_12.g6954;Description=hypothetical protein FVER14953_02363 [Fusarium verticillioides];Gene=FNAPI_3695;Ontology_term=biotin-[acetyl-CoA-carboxylase] ligase activity,biotin-[pyruvate-carboxylase] ligase activity,protein modification
Fv_160_12.g6955	232	ID=Fv_160_12.g6955;Description=hypothetical protein FVEG_15041 [Fusarium verticillioides

Fv_160_12.g6956	332	ID=Fv_160_12.g6956;Description=hypothetical protein FVER53590_25951 [Fusarium
Fv_160_12.g6957	476	ID=Fv_160_12.g6957;Description=hypothetical protein FVEG_02361 [Fusarium verticillioides
Fv_160_12.g6958	138	ID=Fv_160_12.g6958;Description=mitochondrial import inner membrane translocase subunit TIM16 [Fusarium verticillioides 7600];Gene=BFJ65_g10481;Ontology_term=PAM complex, Tim23 associated import motor,protein domain specific binding,protein
Fv_160_12.g6959	658	ID=Fv_160_12.g6959;Description=hypothetical protein FVER14953_02359 [Fusarium verticillioides];Gene=rap1gds1-a;Ontology_term=guanyl-nucleotide exchange factor activity;Ontology_id=GO:0005085
Fv_160_12.g6960	212	ID=Fv_160_12.g6960;Description=50S ribosomal protein L13e [Fusarium verticillioides 7600];Gene=BFJ65_g10485;Ontology_term=ribosome,ribonucleoprotein complex,structural constituent of
Fv_160_12.g6961	272	ID=Fv_160_12.g6961;Description=hypothetical protein FVEG_02357 [Fusarium verticillioides
Fv_160_12.g6962	304	ID=Fv_160_12.g6962;Description=hypothetical protein FVEG_15038 [Fusarium verticillioides 7600]
Fv_160_12.g6963	364	ID=Fv_160_12.g6963;Description=phospho-2-dehydro-3-deoxyheptonate aldolase, tyrosine-inhibited [Fusarium verticillioides 7600];Gene=FPRO05_09483;Ontology_term=3-deoxy-7-phosphoheptulonate synthase activity,amino acid biosynthetic process,aromatic amino acid family biosynthetic
Fv_160_12.g6964	154	ID=Fv_160_12.g6964;Description=hypothetical protein FVEG_02353 [Fusarium verticillioides
Fv_160_12.g6965	380	ID=Fv_160_12.g6965;Description=hypothetical protein J7337_008696 [Fusarium musae];Gene=FNAPI_3685;Ontology_term=mitochondrion,ribosome,cytoskeleton,ribonucleoprotein complex,structural constituent of cytoskeleton,ATP binding,hydrolase activity,mitotic cytokinesis,embryo development ending in birth or egg hatching,cortical actin cytoskeleton
Fv_160_12.g6966	1011	ID=Fv_160_12.g6966;Description=aminopeptidase [Fusarium verticillioides 7600];Gene=FNAPI_3684;Ontology_term=aminopeptidase activity,metallopeptidase activity,zinc ion
Fv_160_12.g6967	1021	ID=Fv_160_12.g6967;Description=H+-transporting ATPase [Fusarium verticillioides 7600];Gene=BFJ63_vAg699;Ontology_term=plasma membrane,ATP binding,P-type proton-exporting transporter activity,ATP hydrolysis activity,proton export across plasma membrane;Ontology_id=GO:0005886,GO:0005524,GO:0008553,GO:0016887,GO:0120029;Enzyme_code=EC:7.1.2.1,EC:7.2.2,EC:3.6
Fv_160_12.g6968	121	ID=Fv_160_12.g6968;Description=hypothetical protein J7337_008693 [Fusarium
Fv_160_12.g6969	533	ID=Fv_160_12.g6969;Description=hypothetical protein FVER14953_02348 [Fusarium verticillioides];Gene=Focb16_v013757;Ontology_term=membrane,transmembrane transporter activity,transmembrane
Fv_160_12.g6970	381	ID=Fv_160_12.g6970;Description=hypothetical protein J7337_008691 [Fusarium musae];Gene=Forpe1208_v008776;Ontology_term=actin filament,cell cortex,ATP binding,hydrolase activity,metal ion binding,dioxygenase activity,mitotic cytokinesis,meiosis II cytokinesis,embryo development ending in birth or egg hatching,cortical actin cytoskeleton
Fv_160_12.g6971	572	ID=Fv_160_12.g6971;Description=hypothetical protein FVEG_02346 [Fusarium verticillioides 7600];Gene=BFJ69_g710;Ontology_term=nucleus,DNA-binding transcription factor activity, RNA polymerase II-specific,zinc ion

Fv_160_12.g6972	352	ID=Fv_160_12.g6972;Description=hypothetical protein FVER14953_02345 [Fusarium verticillioides];Gene=P04829;Ontology_term=cytoplasm,cytoskeleton,ATP binding,hydrolase
Fv_160_12.g6973	343	ID=Fv_160_12.g6973;Description=hypothetical protein FVEG_02344 [Fusarium verticillioides 7600];Gene=FOXB_13430;Ontology_term=oxidoreductase
Fv_160_12.g6974	531	ID=Fv_160_12.g6974;Description=hypothetical protein FVEG_02343 [Fusarium verticillioides 7600];Gene=HNM1-8;Ontology_term=membrane,transmembrane transporter activity,amino acid transport,transmembrane
Fv_160_12.g6975	550	ID=Fv_160_12.g6975;Description=hypothetical protein H9Q70_005149 [Fusarium xylarioides]
Fv_160_12.g6976	155	ID=Fv_160_12.g6976;Description=hypothetical protein FVER14953_02342 [Fusarium
Fv_160_12.g6977	249	ID=Fv_160_12.g6977;Description=hypothetical protein FVEG_02341 [Fusarium verticillioides 7600];Gene=FOIG_13032;Ontology_term=transferase activity,glutamine metabolic
Fv_160_12.g6978	569	ID=Fv_160_12.g6978;Description=hypothetical protein FVER53590_02340 [Fusarium verticillioides];Gene=CZF1;Ontology_term=nucleus,DNA-binding transcription factor activity, RNA polymerase II-specific,zinc ion
Fv_160_12.g6979	503	ID=Fv_160_12.g6979;Description=serine hydroxymethyltransferase, mitochondrial [Fusarium oxysporum f. sp. lycopersici 4287];Gene=FOTG_01878;Ontology_term=glycine hydroxymethyltransferase activity,methyltransferase activity,pyridoxal phosphate binding,glycine biosynthetic process from serine,methylation,tetrahydrofolate interconversion;Ontology_id=GO:0004372,GO:0008168,GO:0030170,GO:0019264,GO:0032259,GO:0035999;Enzyme_code=EC:2.1.2
Fv_160_12.g6980	172	ID=Fv_160_12.g6980;Description=hypothetical protein J7337_008681 [Fusarium musae];Gene=FVEG_02338;Ontology_term=mitochondrion,glycine cleavage complex,glycine decarboxylation via glycine cleavage
Fv_160_12.g6981	1048	ID=Fv_160_12.g6981;Description=glycine dehydrogenase [Fusarium verticillioides 7600];Gene=FVEG_02337;Ontology_term=mitochondrion,glycine dehydrogenase (decarboxylating) activity,glycine catabolic
Fv_160_12.g6982	635	ID=Fv_160_12.g6982;Description=hypothetical protein FVER53263_02336 [Fusarium verticillioides];Gene=F25303_6522;Ontology_term=mitochondrial inner membrane,peroxisomal membrane,respirasome,ubiquinol-cytochrome-c reductase activity,metal ion binding,2 iron, 2 sulfur cluster binding,peroxisome organization,electron transport chain,proton transmembrane transport;Ontology_id=GO:0005743,GO:0005778,GO:0070469,GO:0008121,GO:0046872,GO:0051537,GO:0007031,GO:0022900,GO
Fv_160_12.g6983	330	ID=Fv_160_12.g6983;Description=hypothetical protein FVER53590_25952 [Fusarium verticillioides];Gene=FFUJ_05845;Ontology_term=peroxisomal membrane,peroxisome
Fv_160_12.g6984	479	ID=Fv_160_12.g6984;Description=hypothetical protein FVER53263_02335 [Fusarium verticillioides]
Fv_160_12.g6985	421	ID=Fv_160_12.g6985;Description=hypothetical protein FVER53263_02335 [Fusarium verticillioides]
Fv_160_12.g6986	688	ID=Fv_160_12.g6986;Description=hypothetical protein FVER53590_02334 [Fusarium verticillioides]
Fv_160_12.g6987	236	ID=Fv_160_12.g6987;Description=hypothetical protein FVEG_02333 [Fusarium verticillioides 7600];Gene=FANTH_832;Ontology_term=acyltransferase activity, transferring groups other than amino-acyl

Fv_160_12.g6988	470	ID=Fv_160_12.g6988;Description=homogentisate 1,2-dioxygenase [Fusarium verticillioides 7600];Gene=FTJAE_11169;Ontology_term=homogentisate 1,2-dioxygenase activity,metal ion binding,L-phenylalanine catabolic process,tyrosine metabolic
Fv_160_12.g6989	177	ID=Fv_160_12.g6989;Description=hypothetical protein FVEG_02331 [Fusarium verticillioides 7600]
Fv_160_12.g6990	458	ID=Fv_160_12.g6990;Description=hypothetical protein FVER53590_02330 [Fusarium verticillioides]
Fv_160_12.g6991	679	ID=Fv_160_12.g6991;Description=hypothetical protein FOMG_12889 [Fusarium oxysporum f. sp. melonis 26406];Gene=3010;Ontology_term=mitochondrial intermembrane space,respirasome,electron transfer activity,heme binding,metal ion binding,electron transport
Fv_160_12.g6992	355	ID=Fv_160_12.g6992;Description=hypothetical protein FVER53263_02327 [Fusarium verticillioides]
Fv_160_12.g6993	278	ID=Fv_160_12.g6993;Description=hypothetical protein FVEG_02326 [Fusarium verticillioides
Fv_160_12.g6994	86	ID=Fv_160_12.g6994;Description=hypothetical protein FVEG_02325 [Fusarium verticillioides
Fv_160_12.g6995	489	ID=Fv_160_12.g6995;Description=hypothetical protein FVEG_15030 [Fusarium verticillioides 7600];Gene=FVEG_15030;Ontology_term=nucleus,DNA binding,zinc ion binding,membrane,DNA-binding transcription factor activity, RNA polymerase II-specific,regulation of transcription by RNA polymerase
Fv_160_12.g6996	341	ID=Fv_160_12.g6996;Description=hypothetical protein FVER14953_02323 [Fusarium verticillioides]
Fv_160_12.g6997	823	ID=Fv_160_12.g6997;Description=hypothetical protein FVEG_02322 [Fusarium verticillioides 7600];Gene=FTJAE_11159;Ontology_term=nucleus,damaged DNA binding,nucleotide-excision
Fv_160_12.g6998	1625	ID=Fv_160_12.g6998;Description=hypothetical protein FVEG_02321 [Fusarium verticillioides 7600];Gene=FNAPI_3652;Ontology_term=membrane,ATP binding,ATP hydrolysis activity,ABC-type transporter activity,transmembrane transport;Ontology_id=GO:0016020,GO:0005524,GO:0016887,GO:0140359,GO:0055085;Enzyme_code=EC:7.2.2,EC:3.6.1.15;Enzym
Fv_160_12.g6999	251	ID=Fv_160_12.g6999;Description=hypothetical protein FVER53263_02320 [Fusarium verticillioides]
Fv_160_12.g7000	332	ID=Fv_160_12.g7000;Description=mitochondrial 2-oxoglutarate/malate carrier protein [Fusarium oxysporum f. sp. lycopersici 4287];Gene=FVEG_02319;Ontology_term=mitochondrial inner membrane,transmembrane
Fv_160_12.g7001	464	ID=Fv_160_12.g7001;Description=hypothetical protein FVER53590_02318 [Fusarium verticillioides]
Fv_160_12.g7002	508	ID=Fv_160_12.g7002;Description=hypothetical protein FVER53590_02317 [Fusarium verticillioides];Gene=FMUND_10679;Ontology_term=membrane,monooxygenase activity,iron ion binding,oxidoreductase activity, acting on paired donors, with incorporation or reduction of molecular oxygen,heme binding;Ontology_id=GO:0016020,GO:0004497,GO:0005506,GO:0016705,GO:0020037;Enzyme_code=EC:1.14;Enzyme_name=Actin
Fv_160_12.g7003	448	ID=Fv_160_12.g7003;Description=hypothetical protein FVEG_02316 [Fusarium verticillioides 7600];Gene=FVEG_02316;Ontology_term=acyltransferase activity, transferring groups other than amino-acyl groups,biosynthetic
Fv_160_12.g7004	901	ID=Fv_160_12.g7004;Description=transcriptional activator acu-15 [Fusarium denticulatum];Gene=FVEG_02315;Ontology_term=nucleus,DNA binding,zinc ion binding,DNA-binding transcription factor activity,

Fv_160_12.g7005	130	ID=Fv_160_12.g7005;Description=hypothetical protein FVEG_15021 [Fusarium verticillioides
Fv_160_12.g7006	535	ID=Fv_160_12.g7006;Description=hypothetical protein FVEG_02314 [Fusarium verticillioides 7600];Gene=tom70;Ontology_term=nucleus,mitochondrial outer membrane,protein-transporting ATPase activity,mitochondrion targeting sequence binding,protein import into mitochondrial matrix,protein insertion into mitochondrial inner membrane;Ontology_id=GO:0005634,GO:0005741,GO:0015450,GO:0030943,GO:0030150,GO:0045039;Enzyme_code=EC:7.2.2;Enz
Fv_160_12.g7007	296	ID=Fv_160_12.g7007;Description=ATP-binding protein [Fusarium verticillioides 7600];Gene=FNAPI_5353;Ontology_term=ATP binding,iron-sulfur cluster binding,ATP-dependent FeS chaperone activity,iron-sulfur cluster
Fv_160_12.g7008	278	ID=Fv_160_12.g7008;Description=hypothetical protein FVEG_02313 [Fusarium verticillioides 7600];Gene=FOXG_03439;Ontology_term=DNA-binding transcription factor activity,negative regulation of DNA-templated
Fv_160_12.g7009	614	ID=Fv_160_12.g7009;Description=hypothetical protein FVEG_02312 [Fusarium verticillioides 7600]
Fv_160_12.g7010	694	ID=Fv_160_12.g7010;Description=hypothetical protein FVER14953_02311 [Fusarium verticillioides];Gene=nuo-51;Ontology_term=mitochondrial respiratory chain complex I,NADH dehydrogenase (ubiquinone) activity,FMN binding,kinase activity,metal ion binding,NAD binding,4 iron, 4 sulfur cluster binding,phosphorylation,electron transport chain;Ontology_id=GO:0005747,GO:0008137,GO:0010181,GO:0016301,GO:0046872,GO:0051287,GO:0051539,GO:0016310,GO:00
Fv_160_12.g7011	318	ID=Fv_160_12.g7011;Description=cytochrome c heme lyase [Fusarium verticillioides 7600];Gene=BFJ65_g10003;Ontology_term=mitochondrial inner membrane,mitochondrial intermembrane space,holocytochrome-c synthase activity,metal ion
Fv_160_12.g7012	190	ID=Fv_160_12.g7012;Description=hypothetical protein FVEG_02309 [Fusarium verticillioides 7600];Gene=nuo78;Ontology_term=mitochondrial inner membrane,respirasome,NADH dehydrogenase (ubiquinone) activity,metal ion binding,2 iron, 2 sulfur cluster binding,4 iron, 4 sulfur cluster binding,ATP synthesis coupled electron transport;Ontology_id=GO:0005743,GO:0070469,GO:0008137,GO:0046872,GO:0051537,GO:0051539,GO:0042773;Enzyme_code=E
Fv_160_12.g7013	552	ID=Fv_160_12.g7013;Description=hypothetical protein FVEG_02308 [Fusarium verticillioides 7600];Gene=Forpi1262_v008777;Ontology_term=nucleic acid binding;Ontology_id=GO:0003676
Fv_160_12.g7014	802	ID=Fv_160_12.g7014;Description=hypothetical protein FVEG_02307 [Fusarium verticillioides 7600]
Fv_160_12.g7015	1137	ID=Fv_160_12.g7015;Description=PAB-dependent poly(A)-specific ribonuclease subunit PAN2 [Fusarium verticillioides 7600];Gene=PAN2;Ontology_term=PAN complex,nucleic acid binding,poly(A)-specific ribonuclease activity,metal ion binding,nuclear-transcribed mRNA poly(A) tail shortening,mRNA processing;Ontology_id=GO:0031251,GO:0003676,GO:0004535,GO:0046872,GO:0000289,GO:0006397;Enzyme_code=EC:3.1.15,EC:
Fv_160_12.g7016	320	ID=Fv_160_12.g7016;Description=hypothetical protein FVEG_02304 [Fusarium verticillioides 7600];Gene=FGADI_10434;Ontology_term=S-adenosylmethionine-dependent methyltransferase

Fv_160_12.g7017	747	ID=Fv_160_12.g7017;Description=hypothetical protein FVEG_15018 [Fusarium verticillioides 7600];Gene=FOXG_03429;Ontology_term=FAD binding,nitrate reductase (NADPH) activity,nucleus,DNA binding,zinc ion binding,molybdenum ion binding,molybdopterin cofactor binding,DNA-binding transcription factor activity, RNA polymerase II-specific,heme binding,regulation of transcription by RNA polymerase II,nitrate assimilation;Ontology_id=GO:0071949,GO:0050464,GO:0005634,GO:0003677,GO:0008270,GO:0030151,GO:0043546,GO:0000981,
Fv_160_12.g7018	425	ID=Fv_160_12.g7018;Description=hypothetical protein J7337_008640 [Fusarium musae];Gene=AU210_009460;Ontology_term=nucleus,DNA binding,DNA-binding transcription factor activity,copper ion
Fv_160_12.g7019	98	ID=Fv_160_12.g7019;Description=hypothetical protein FVEG_15015 [Fusarium verticillioides 7600]
Fv_160_12.g7020	351	ID=Fv_160_12.g7020;Description=2-dehydropantoate 2-reductase [Fusarium verticillioides 7600];Gene=FDENT_5409;Ontology_term=2-dehydropantoate 2-reductase activity,pantothenate biosynthetic
Fv_160_12.g7021	1529	ID=Fv_160_12.g7021;Description=hypothetical protein FVEG_02296 [Fusarium verticillioides 7600];Gene=FACUT_10190;Ontology_term=phosphorelay sensor kinase activity,ATP binding,photoreceptor activity,phosphorelay signal transduction system,regulation of DNA-templated transcription,detection of visible light,phosphorylation;Ontology_id=GO:0000155,GO:0005524,GO:0009881,GO:0000160,GO:0006355,GO:0009584,GO:0016310;Enz
Fv_160_12.g7022	341	ID=Fv_160_12.g7022;Description=casein kinase II subunit beta-1 [Fusarium verticillioides 7600];Gene=FNAPI_5367;Ontology_term=protein kinase CK2 complex,kinase activity,protein kinase regulator activity,phosphorylation;Ontology_id=GO:0005956,GO:0016301,GO:0019887,GO:0016310;Enzyme_code=EC:2.7;Enzyme_name=Tra
Fv_160_12.g7023	369	ID=Fv_160_12.g7023;Description=hypothetical protein J7337_008636 [Fusarium musae]
Fv_160_12.g7024	287	ID=Fv_160_12.g7024;Description=hypothetical protein FVEG_02293 [Fusarium verticillioides
Fv_160_12.g7025	617	ID=Fv_160_12.g7025;Description=hypothetical protein FVEG_02292 [Fusarium verticillioides 7600]
Fv_160_12.g7026	705	ID=Fv_160_12.g7026;Description=hypothetical protein FVER53590_02291 [Fusarium verticillioides];Gene=FOXG_03419;Ontology_term=translation release factor activity,GTPase activity,GTP binding,nuclear-transcribed mRNA catabolic process, deadenylation-dependent decay,translational
Fv_160_12.g7027	589	ID=Fv_160_12.g7027;Description=phosphoenolpyruvate carboxykinase [ATP] [Fusarium verticillioides 7600];Gene=FMAN_08904;Ontology_term=phosphoenolpyruvate carboxykinase (ATP) activity,ATP binding,kinase activity,gluconeogenesis,phosphorylation;Ontology_id=GO:0004612,GO:0005524,GO:0016301,GO:0006094,GO:0016310;Enzyme_code=EC:4.1.1.49,EC:2.7,EC:4.1.1.32;Enzyme_name=phosphoenolpyruvate carboxykinase (ATP),Transferring phosphorus-containing
Fv_160_12.g7028	607	ID=Fv_160_12.g7028;Description=hypothetical protein FVER14953_02288 [Fusarium verticillioides];Gene=FPANT_1507;Ontology_term=nucleic acid binding,RNA helicase activity,ATP binding,hydrolase
Fv_160_12.g7029	342	ID=Fv_160_12.g7029;Description=hypothetical protein FVER53590_02287 [Fusarium verticillioides];Gene=IPI1;Ontology_term=nucleus,Rix1 complex,rRNA
Fv_160_12.g7030	136	ID=Fv_160_12.g7030;Description=hypothetical protein FVEG_02286 [Fusarium verticillioides 7600]

Fv_160_12.g7031	1223	ID=Fv_160_12.g7031;Description=hypothetical protein FVEG_02285 [Fusarium verticillioides 7600];Gene=FPCIR_9744;Ontology_term=nucleus,endoplasmic reticulum membrane,ATP binding,hydrolase activity,protein folding chaperone,ATP-dependent chromatin remodeler activity,chromatin remodeling,protein folding,protein insertion into ER
Fv_160_12.g7032	115	ID=Fv_160_12.g7032;Description=hypothetical protein FOXG_03413 [Fusarium oxysporum f. sp. lycopersici 4287];Gene=F25303_2793;Ontology_term=nucleus,endoplasmic reticulum membrane,ATP binding,hydrolase activity,protein folding chaperone,ATP-dependent chromatin remodeler activity,chromatin remodeling,protein folding,protein insertion into ER
Fv_160_12.g7033	1156	ID=Fv_160_12.g7033;Description=hypothetical protein FVEG_02283 [Fusarium verticillioides 7600];Gene=BFJ65_g9363;Ontology_term=nucleus,ATP binding,hydrolase activity,ATP-dependent chromatin remodeler activity,chromatin
Fv_160_12.g7034	615	ID=Fv_160_12.g7034;Description=NAD-dependent aldehyde dehydrogenase [Fusarium verticillioides 7600];Gene=FOXB_11011;Ontology_term=oxidoreductase activity, acting on the aldehyde or oxo group of donors, NAD or NADP as acceptor,reciprocal meiotic recombination;Ontology_id=GO:0016620,GO:0007131;Enzyme_code=EC:1.2.1;Enzyme_name=Acting on
Fv_160_12.g7035	1522	ID=Fv_160_12.g7035;Description=hypothetical protein FVER14953_02281 [Fusarium verticillioides];Gene=FVER53590_02281;Ontology_term=endoplasmic reticulum lumen,endoplasmic reticulum membrane,magnesium ion binding,calcium ion binding,ATP binding,lyase activity,ATP hydrolysis activity,carbohydrate binding,unfolded protein binding,phosphatidylethanolamine flippase activity,nucleobase-containing compound metabolic process,protein folding,response to heat,regulation of gene expression,programmed cell death,phospholipid transport,defecation,ubiquitin-dependent ERAD pathway,endoplasmic reticulum unfolded protein response,hemidesmosome assembly,lipid translocation,response to ethanol,multicellular organismal reproductive process;Ontology_id=GO:0005788,GO:0005789,GO:0000287,GO:0005509,GO:0005524,GO:0016829,GO:0016887,GO:0030246,GO:
Fv_160_12.g7036	633	ID=Fv_160_12.g7036;Description=hypothetical protein FVER14953_02280 [Fusarium verticillioides];Gene=3055
Fv_160_12.g7037	229	ID=Fv_160_12.g7037;Description=V-type proton ATPase subunit E [Fusarium verticillioides 7600];Gene=FCIRC_4645;Ontology_term=vacuolar proton-transporting V-type ATPase, V1 domain,fungal-type vacuole membrane,proton-transporting ATPase activity, rotational mechanism,proton transmembrane
Fv_160_12.g7038	519	ID=Fv_160_12.g7038;Description=hypothetical protein FVER14953_02278 [Fusarium verticillioides];Gene=FPANT_1517;Ontology_term=membrane,magnesium ion transmembrane transporter activity,magnesium ion
Fv_160_12.g7039	754	ID=Fv_160_12.g7039;Description=hypothetical protein FVER53263_02277 [Fusarium verticillioides];Gene=P08915;Ontology_term=collagen trimer,egg chorion,annular furrow extracellular matrix,structural constituent of egg chorion,structural constituent of collagen and cuticulin-based cuticle,chorion-containing eggshell formation,post-embryonic body morphogenesis,cuticle development involved in collagen and cuticulin-based cuticle molting
Fv_160_12.g7040	353	ID=Fv_160_12.g7040;Description=hypothetical protein FVEG_02276 [Fusarium verticillioides
Fv_160_12.g7041	875	ID=Fv_160_12.g7041;Description=hypothetical protein FVER14953_02275 [Fusarium
Fv_160_12.g7042	291	ID=Fv_160_12.g7042;Description=pirin (iron-binding nuclear protein) [Fusarium verticillioides

Fv_160_12.g7043	859	ID=Fv_160_12.g7043;Description=hypothetical protein FVER14953_02273 [Fusarium verticillioides];Gene=FVER53590_02273;Ontology_term=extracellular region,nucleus,axon,egg chorion,perikaryon,RNA polymerase II cis-regulatory region sequence-specific DNA binding,DNA-binding transcription factor activity, RNA polymerase II-specific,RNA polymerase II intronic transcription regulatory region sequence-specific DNA binding,3-dehydroquinate dehydratase activity,chitinase activity,structural constituent of egg chorion,chitin binding,zinc ion binding,negative regulation of transcription by RNA polymerase II,polysaccharide catabolic process,chitin catabolic process,spermatid development,chorion-containing eggshell formation,epidermal cell fate specification,quinate metabolic process,stem cell population maintenance,positive regulation of transcription by RNA polymerase II,3,4-dihydroxybenzoate biosynthetic process;Ontology_id=GO:0005576,GO:0005634,GO:0030424,GO:0042600,GO:0043204,GO:0000978,GO:0000981,GO:0001162,GO:
Fv_160_12.g7044	1267	ID=Fv_160_12.g7044;Description=hypothetical protein FVEG_02272 [Fusarium verticillioides 7600];Gene=FOTG_01799;Ontology_term=lipid binding,lipid transport;Ontology_id=GO:0008289,GO:0006869
Fv_160_12.g7045	353	ID=Fv_160_12.g7045;Description=hypothetical protein LB505_011076 [Fusarium chuoi];Gene=ACOT8-1;Ontology_term=shikimate 3-dehydrogenase (NADP+) activity,quinate 3-dehydrogenase (NAD+) activity,acyl-CoA hydrolase activity,acyl-CoA metabolic process,chorismate biosynthetic process,quinate metabolic process,shikimate metabolic process,3,4-dihydroxybenzoate biosynthetic process;Ontology_id=GO:0004764,GO:0030266,GO:0047617,GO:0006637,GO:0009423,GO:0019630,GO:0019632,GO:0046279;Enzyme_code=EC:1.1.1.25,EC:1.1.1.24,EC:3.1.2.20,EC:1.1.1.282;Enzyme_name=shikimate dehydrogenase (NADP(+)),quinate/shikimate
Fv_160_12.g7046	190	ID=Fv_160_12.g7046;Description=hypothetical protein FVEG_02270 [Fusarium verticillioides 7600];Gene=FNAPI_3609;Ontology_term=membrane,egg chorion,aspartic-type endopeptidase activity,structural constituent of egg chorion,carbohydrate:proton symporter activity,proteolysis,chorion-containing eggshell formation,quinate metabolic process,carbohydrate transmembrane transport,proton transmembrane transport;Ontology_id=GO:0016020,GO:0042600,GO:0004190,GO:0005213,GO:0005351,GO:0006508,GO:0007304,GO:0019630,GO
Fv_160_12.g7047	465	ID=Fv_160_12.g7047;Description=hypothetical protein FVEG_02268 [Fusarium verticillioides 7600];Gene=FOXB_11025;Ontology_term=membrane,aspartic-type endopeptidase
Fv_160_12.g7048	820	ID=Fv_160_12.g7048;Description=hypothetical protein FVEG_02267 [Fusarium verticillioides 7600];Gene=Forpi1262_v008811;Ontology_term=nucleic acid binding,metal ion binding;Ontology_id=GO:0003676,GO:0046872
Fv_160_12.g7049	164	ID=Fv_160_12.g7049;Description=molybdopterin synthase catalytic subunit [Fusarium verticillioides 7600];Gene=cnxH;Ontology_term=cytosol,molybdopterin synthase complex,molybdopterin synthase activity,Mo-molybdopterin cofactor biosynthetic
Fv_160_12.g7050	147	ID=Fv_160_12.g7050;Description=hypothetical protein FVEG_02266 [Fusarium verticillioides 7600]
Fv_160_12.g7051	865	ID=Fv_160_12.g7051;Description=hypothetical protein FVEG_02265 [Fusarium verticillioides 7600];Gene=F25303_2812;Ontology_term=membrane,ferric-chelate reductase activity,iron ion
Fv_160_12.g7052	111	ID=Fv_160_12.g7052;Description=uncharacterized protein BKA55DRAFT_230716 [Fusarium redolens]

Fv_160_12.g7053	333	ID=Fv_160_12.g7053;Description=hypothetical protein FVEG_02264 [Fusarium verticillioides 7600];Gene=F25303_2814;Ontology_term=nuclear exosome (RNase complex),nucleolus,exonuclease activity,rRNA
Fv_160_12.g7054	496	ID=Fv_160_12.g7054;Description=hypothetical protein FVEG_02263 [Fusarium verticillioides 7600];Gene=FGADI_10875;Ontology_term=GDP-dissociation inhibitor activity,transferase activity,small GTPase mediated signal
Fv_160_12.g7055	684	ID=Fv_160_12.g7055;Description=hypothetical protein FVEG_02262 [Fusarium verticillioides 7600];Gene=rec8;Ontology_term=nucleus,cohesin complex,sister chromatid
Fv_160_12.g7056	549	ID=Fv_160_12.g7056;Description=hypothetical protein FVEG_02261 [Fusarium verticillioides 7600];Gene=FOBC_10044;Ontology_term=membrane,transmembrane transporter activity,transmembrane
Fv_160_12.g7057	230	ID=Fv_160_12.g7057;Description=hypothetical protein FVER14953_02260 [Fusarium verticillioides];Gene=3076;Ontology_term=cytosolic small ribosomal subunit,mRNA binding,structural constituent of
Fv_160_12.g7058	160	ID=Fv_160_12.g7058;Description=V-type proton ATPase proteolipid subunit 2 [Fusarium verticillioides 7600];Gene=FOPG_00802;Ontology_term=vacuolar proton-transporting V-type ATPase, V0 domain,fungal-type vacuole,proton-transporting ATPase activity, rotational mechanism,proton transmembrane
Fv_160_12.g7059	1236	ID=Fv_160_12.g7059;Description=hypothetical protein FVER53590_02258 [Fusarium verticillioides];Gene=FOPG_00803;Ontology_term=cellular anatomical entity,guanyl-nucleotide exchange factor activity,intracellular
Fv_160_12.g7060	627	ID=Fv_160_12.g7060;Description=hypothetical protein FVER53590_02257 [Fusarium verticillioides];Gene=FOQG_01418;Ontology_term=membrane,hydrolase
Fv_160_12.g7061	346	ID=Fv_160_12.g7061;Description=hypothetical protein J7337_008598 [Fusarium
Fv_160_12.g7062	347	ID=Fv_160_12.g7062;Description=hypothetical protein J7337_008597 [Fusarium musae];Gene=FPANT_1701;Ontology_term=hydrolase
Fv_160_12.g7063	1711	ID=Fv_160_12.g7063;Description=hypothetical protein FVEG_02254 [Fusarium verticillioides 7600];Gene=FPANT_1702;Ontology_term=membrane,guanyl-nucleotide exchange factor activity,intracellular signal
Fv_160_12.g7064	177	ID=Fv_160_12.g7064;Description=putative mitochondrial pyruvate carrier 2 [Fusarium oxysporum f. sp. rapae];Gene=FGADI_10885;Ontology_term=mitochondrial inner membrane,mitochondrial pyruvate transmembrane
Fv_160_12.g7065	94	ID=Fv_160_12.g7065;Description=NADH-ubiquinone oxidoreductase 10.5 kDa subunit [Fusarium verticillioides 7600];Gene=FGADI_10886;Ontology_term=mitochondrial inner membrane,respirasome;Ontology_id=GO:0005743,GO:0070469
Fv_160_12.g7066	1622	ID=Fv_160_12.g7066;Description=hypothetical protein FVER53590_02251 [Fusarium verticillioides]
Fv_160_12.g7067	139	ID=Fv_160_12.g7067;Description=hypothetical protein FVEG_02249 [Fusarium verticillioides 7600];Gene=FGADI_10888;Ontology_term=mitochondrion,oxidoreductase
Fv_160_12.g7068	112	ID=Fv_160_12.g7068;Description=hypothetical protein FVEG_02248 [Fusarium verticillioides 7600];Gene=AU210_009412;Ontology_term=ribosome,structural constituent of
Fv_160_12.g7069	147	ID=Fv_160_12.g7069;Description=hypothetical protein NW761_003780 [Fusarium oxysporum];Gene=FCIRC_11225;Ontology_term=ribosome,ribonucleoprotein complex,RNA binding,structural constituent of

Fv_160_12.g7070	209	ID=Fv_160_12.g7070;Description=nascent polypeptide-associated complex subunit alpha [Fusarium verticillioides 7600];Gene=F25303_2831;Ontology_term=mitochondrial proton-transporting ATP synthase complex, coupling factor F(o),nucleus,nascent polypeptide-associated complex,polysomal ribosome,structural constituent of ribosome,proton transmembrane transporter activity,protein tag activity,ubiquitin protein ligase binding,metal ion binding,translation,protein transport,proton motive force-driven ATP synthesis,protein ubiquitination,modification-dependent protein catabolic process,proton transmembrane
Fv_160_12.g7071	173	ID=Fv_160_12.g7071;Description=ATP synthase subunit D, mitochondrial [Fusarium verticillioides 7600];Gene=FCIRC_11223;Ontology_term=mitochondrial proton-transporting ATP synthase, stator stalk,nucleus,nascent polypeptide-associated complex,proton-transporting ATP synthase activity, rotational mechanism,protein transport,proton motive force-driven ATP synthesis,proton transmembrane transport;Ontology_id=GO:0000274,GO:0005634,GO:0005854,GO:0046933,GO:0015031,GO:0015986,GO:1902600;Enzyme_code=E
Fv_160_12.g7072	336	ID=Fv_160_12.g7072;Description=hypothetical protein FVEG_02244 [Fusarium verticillioides 7600];Gene=FCIRC_11222;Ontology_term=hydrolase
Fv_160_12.g7073	149	ID=Fv_160_12.g7073;Description=ubiquitin-conjugating enzyme E2 35 [Fusarium oxysporum f. sp. lycopersici 4287];Gene=FPANT_1712;Ontology_term=fungal-type vacuole membrane,nucleus,ubiquitin conjugating enzyme complex,ubiquitin-protein transferase activity,ATP binding,postreplication repair,free ubiquitin chain polymerization,protein targeting to vacuolar membrane;Ontology_id=GO:0000329,GO:0005634,GO:0031371,GO:0004842,GO:0005524,GO:0006301,GO:0010994,GO:0044395;E
Fv_160_12.g7074	1451	ID=Fv_160_12.g7074;Description=hypothetical protein FVEG_02242 [Fusarium verticillioides 7600]
Fv_160_12.g7075	287	ID=Fv_160_12.g7075;Description=hypothetical protein FVER53590_02241 [Fusarium verticillioides]
Fv_160_12.g7076	318	ID=Fv_160_12.g7076;Description=hypothetical protein FVEG_02241 [Fusarium verticillioides 7600];Gene=XYNA;Ontology_term=endo-1,4-beta-xylanase activity,xylan catabolic
Fv_160_12.g7077	526	ID=Fv_160_12.g7077;Description=phosphatidylinositol glycan, class C [Fusarium verticillioides 7600];Gene=FPRO05_09592;Ontology_term=membrane,egg chorion,structural constituent of egg chorion,chorion-containing
Fv_160_12.g7078	196	ID=Fv_160_12.g7078;Description=hypothetical protein FVER14953_02239 [Fusarium
Fv_160_12.g7079	331	ID=Fv_160_12.g7079;Description=hypothetical protein FVEG_02238 [Fusarium verticillioides 7600];Gene=FVEG_02238;Ontology_term=hydrolase activity,lyase
Fv_160_12.g7080	172	ID=Fv_160_12.g7080;Description=uncharacterized protein FMAN_08959 [Fusarium mangiferae];Gene=FMAN_08959;Ontology_term=positive regulation of MAP kinase activity,protein phosphatase binding,protein serine/threonine phosphatase complex,positive regulation of oocyte development,positive regulation of meiotic cell cycle process involved in oocyte maturation,spermatogenesis,regulation of DNA-templated transcription,masculinization of hermaphroditic germ-line,regulation of germ cell proliferation,sex differentiation,transcription coregulator activity,cytoplasm,protein localization to chromatin,positive regulation of gene expression,mediator complex,male somatic sex determination,Cul2-RING ubiquitin ligase complex;Ontology_id=GO:0043406,GO:0019903,GO:0008287,GO:0060282,GO:1904146,GO:0007283,GO:0006355,GO:0042006,GO:

Fv_160_12.g7081	110	ID=Fv_160_12.g7081;Description=DNA-directed RNA polymerase III subunit RPC10 [Fusarium verticillioides 7600];Gene=FOTG_01757;Ontology_term=DNA-directed RNA polymerase complex,nucleolus,nucleic acid binding,DNA-directed 5'-3' RNA polymerase activity,zinc ion binding,DNA-templated
Fv_160_12.g7082	208	ID=Fv_160_12.g7082;Description=hypothetical protein FVEG_02236 [Fusarium verticillioides 7600]
Fv_160_12.g7083	269	ID=Fv_160_12.g7083;Description=hypothetical protein FVER53263_02235 [Fusarium verticillioides]
Fv_160_12.g7084	747	ID=Fv_160_12.g7084;Description=hypothetical protein FOXG_03359 [Fusarium oxysporum f. sp. lycopersici 4287];Gene=FNYG_14542;Ontology_term=ATP binding,ATP-dependent protein folding chaperone,protein
Fv_160_12.g7085	606	ID=Fv_160_12.g7085;Description=hypothetical protein FVER14953_02233 [Fusarium verticillioides];Gene=FGLOB1_14614;Ontology_term=heterotrimeric G-protein complex,axon,egg chorion,neuronal cell body,non-motile cilium,G protein-coupled receptor binding,GTPase activity,glycerol kinase activity,structural constituent of egg chorion,ATP binding,GTP binding,G-protein beta/gamma-subunit complex binding,metal ion binding,glycerol-3-phosphate metabolic process,adenylate cyclase-modulating G protein-coupled receptor signaling pathway,chorion-containing eggshell formation,olfactory learning,positive regulation of gene expression,phosphorylation,glycerol catabolic process,G protein-coupled receptor signaling pathway involved in defense response to Gram-negative bacterium;Ontology_id=GO:0005834,GO:0030424,GO:0042600,GO:0043025,GO:0097730,GO:0001664,GO:0003924,GO:0004370,G
Fv_160_12.g7086	148	ID=Fv_160_12.g7086;Description=hypothetical protein FVEG_02232 [Fusarium verticillioides 7600]
Fv_160_12.g7087	246	ID=Fv_160_12.g7087;Description=hypothetical protein FVEG_02231 [Fusarium verticillioides 7600]
Fv_160_12.g7088	731	ID=Fv_160_12.g7088;Description=hypothetical protein FVER14953_02230 [Fusarium verticillioides];Gene=FOTG_01748;Ontology_term=endosome membrane,phosphatidylinositol binding,ubiquitin binding,metal ion binding,vacuolar transport,vesicle-mediated transport,establishment of protein localization,organic substance
Fv_160_12.g7089	1056	ID=Fv_160_12.g7089;Description=hypothetical protein FVEG_02228 [Fusarium verticillioides 7600];Gene=FVEG_02228;Ontology_term=cellular anatomical entity,cysteine-type deubiquitinase
Fv_160_12.g7090	793	ID=Fv_160_12.g7090;Description=long-chain acyl-CoA synthetase [Fusarium verticillioides 7600]
Fv_160_12.g7091	674	ID=Fv_160_12.g7091;Description=hypothetical protein FVEG_02225 [Fusarium verticillioides 7600];Gene=FMEXI_4916;Ontology_term=membrane,egg chorion,structural constituent of egg chorion,chorion-containing eggshell
Fv_160_12.g7092	628	ID=Fv_160_12.g7092;Description=hypothetical protein FVER53590_02224 [Fusarium verticillioides];Gene=MED17;Ontology_term=mediator complex,transcription coregulator activity,regulation of transcription by RNA
Fv_160_12.g7093	1132	ID=Fv_160_12.g7093;Description=hypothetical protein FVEG_15006 [Fusarium verticillioides 7600];Gene=BFJ72_g9420;Ontology_term=nucleus,helicase activity,ATP binding,hydrolase activity,metal ion binding,ATP-dependent chromatin remodeler activity,chromatin
Fv_160_12.g7094	775	ID=Fv_160_12.g7094;Description=ectonucleotide pyrophosphatase/phosphodiesterase family member 1/3 [Fusarium verticillioides 7600];Gene=FMUND_12889;Ontology_term=nucleus,membrane,DNA-binding transcription factor activity, RNA polymerase II-specific,zinc ion binding,regulation of transcription by RNA polymerase

Fv_160_12.g7095	411	ID=Fv_160_12.g7095;Description=hypothetical protein FVEG_02219 [Fusarium verticillioides 7600];Gene=FOPG_00848;Ontology_term=nucleosome,nucleus,mitochondrial matrix,membrane,DNA-binding transcription factor activity, RNA polymerase II-specific,DNA binding,RNA binding,tyrosine-tRNA ligase activity,ATP binding,zinc ion binding,structural constituent of chromatin,protein heterodimerization activity,nucleosome assembly,regulation of transcription by RNA polymerase II,mRNA processing,tyrosyl-tRNA
Fv_160_12.g7096	304	ID=Fv_160_12.g7096;Description=hypothetical protein J7337_008561 [Fusarium musae];Gene=hslV;Ontology_term=nucleosome,nucleus,DNA binding,structural constituent of chromatin,protein
Fv_160_12.g7097	664	ID=Fv_160_12.g7097;Description=hypothetical protein FVER14953_02217 [Fusarium verticillioides];Gene=FVEG_02217;Ontology_term=membrane,mannan endo-1,6-alpha-mannosidase activity,carbohydrate catabolic process;Ontology_id=GO:0016020,GO:0008496,GO:0016052;Enzyme_code=EC:3.2.1.101,EC:3.2.1.24;Enzyme_name=mannan endo-
Fv_160_12.g7098	727	ID=Fv_160_12.g7098;Description=hypothetical protein FVEG_15004 [Fusarium verticillioides 7600];Gene=Forpe1208_v008911;Ontology_term=nucleus,DNA-binding transcription factor activity, RNA polymerase II-specific,zinc
Fv_160_12.g7099	117	ID=Fv_160_12.g7099;Description=vesicle-associated membrane protein 4 [Fusarium oxysporum f. sp. lycopersici 4287];Gene=FOYG_09839;Ontology_term=membrane,vesicle-mediated transport;Ontology_id=GO:0016020,GO:0016192
Fv_160_12.g7100	523	ID=Fv_160_12.g7100;Description=N-acetyl-gamma-glutamyl-phosphate reductase [Fusarium verticillioides 7600];Gene=AU210_009382;Ontology_term=mitochondrial matrix,N-acetyl-gamma-glutamyl-phosphate reductase activity,acetylglutamate kinase activity,regulation of DNA-templated transcription,arginine biosynthetic process,phosphorylation;Ontology_id=GO:0005759,GO:0003942,GO:0003991,GO:0006355,GO:0006526,GO:0016310;Enzyme_code
Fv_160_12.g7101	123	ID=Fv_160_12.g7101;Description=hypothetical protein FVER53263_20952 [Fusarium
Fv_160_12.g7102	303	ID=Fv_160_12.g7102;Description=RNA-binding with serine-rich domain protein [Fusarium tjaetaba];Gene=rnps1-a;Ontology_term=nucleus,RNA binding;Ontology_id=GO:0005634,GO:0003723
Fv_160_12.g7103	91	ID=Fv_160_12.g7103;Description=hypothetical protein FVEG_15002 [Fusarium verticillioides 7600]
Fv_160_12.g7104	404	ID=Fv_160_12.g7104;Description=hypothetical protein FVEG_02210 [Fusarium verticillioides 7600]
Fv_160_12.g7105	481	ID=Fv_160_12.g7105;Description=hypothetical protein FVER53263_02209 [Fusarium verticillioides];Gene=FOTG_01726;Ontology_term=membrane,hydrolase activity, hydrolyzing O-glycosyl compounds,carbohydrate
Fv_160_12.g7106	884	ID=Fv_160_12.g7106;Description=hypothetical protein FVEG_02208 [Fusarium verticillioides 7600];Gene=FTJAE_2490;Ontology_term=membrane,transferase
Fv_160_12.g7107	1110	ID=Fv_160_12.g7107;Description=elongation factor 3 [Fusarium verticillioides 7600];Gene=BFJ63_vAg754;Ontology_term=ATP hydrolysis activity,translation termination factor activity,translational elongation,polysome,ATP binding,chromatin remodeling,RNA binding,regulation of translational termination,translation elongation factor activity,cytoplasm,ribosome binding,ribosomal small subunit biogenesis,poly(A)+ mRNA export from nucleus;Ontology_id=GO:0016887,GO:0008079,GO:0006414,GO:0005844,GO:0005524,GO:0006338,GO:0003723,GO:0006449,GO:

Fv_160_12.g7108	678	ID=Fv_160_12.g7108;Description=palmitoyltransferase ERF2 [Fusarium verticillioides 7600];Gene=BFJ68_g13344;Ontology_term=membrane,protein-cysteine S-palmitoyltransferase
Fv_160_12.g7109	127	ID=Fv_160_12.g7109;Description=hypothetical protein FVEG_02204 [Fusarium verticillioides
Fv_160_12.g7110	478	ID=Fv_160_12.g7110;Description=hypothetical protein FVEG_02203 [Fusarium verticillioides 7600];Gene=HZS61_015435;Ontology_term=L-phosphoserine phosphatase activity,metal ion binding,L-serine biosynthetic
Fv_160_12.g7111	316	ID=Fv_160_12.g7111;Description=hypothetical protein LB503_006846 [Fusarium chuoi];Gene=HZS61_015434;Ontology_term=phosphoadenylyl-sulfate reductase (thioredoxin) activity,methyltransferase activity,glutathione biosynthetic process,tRNA processing,cysteine biosynthetic process,sulfate assimilation, phosphoadenylyl sulfate reduction by phosphoadenylyl-sulfate reductase (thioredoxin),methylation;Ontology_id=GO:0004604,GO:0008168,GO:0006750,GO:0008033,GO:0019344,GO:0019379,GO:0032259;
Fv_160_12.g7112	313	ID=Fv_160_12.g7112;Description=hypothetical protein FVER14953_20899 [Fusarium verticillioides];Gene=FACUT_1999;Ontology_term=methyltransferase activity,tRNA
Fv_160_12.g7113	261	ID=Fv_160_12.g7113;Description=ochre suppressor tyr-tRNA [Fusarium tjaetaba]
Fv_160_12.g7114	618	ID=Fv_160_12.g7114;Description=hypothetical protein DER46DRAFT_624219 [Fusarium sp. MPI-SDFR-AT-0072];Gene=FOXB_11783;Ontology_term=membrane,ferric-chelate reductase activity,transition metal ion
Fv_160_12.g7115	447	ID=Fv_160_12.g7115;Description=hypothetical protein FVEG_02199 [Fusarium verticillioides 7600];Gene=FPANT_10425;Ontology_term=nucleus,membrane,Smc5-Smc6 complex,ferric-chelate reductase activity,transition metal ion transport,DNA repair,DNA
Fv_160_12.g7116	842	ID=Fv_160_12.g7116;Description=hypothetical protein FVEG_15000 [Fusarium verticillioides 7600];Gene=FPANT_10425;Ontology_term=nucleus,membrane,Smc5-Smc6 complex,DNA
Fv_160_12.g7117	274	ID=Fv_160_12.g7117;Description=hypothetical protein QSH57_012572 [Fusarium oxysporum f. sp. vasinfectum];Gene=FPANT_10425;Ontology_term=nucleus,membrane,Smc5-Smc6 complex,hydrolase activity, hydrolyzing O-glycosyl compounds,DNA repair,cellulose catabolic
Fv_160_12.g7118	354	ID=Fv_160_12.g7118;Description=hypothetical protein FVER14953_20897 [Fusarium
Fv_160_12.g7119	916	ID=Fv_160_12.g7119;Description=phosphatidylinositol 3-kinase [Fusarium verticillioides 7600];Gene=FOYG_09818;Ontology_term=ATP binding,1-phosphatidylinositol-3-kinase activity,phosphorylation,phosphatidylinositol-3-phosphate biosynthetic process,phosphatidylinositol-mediated
Fv_160_12.g7120	387	ID=Fv_160_12.g7120;Description=mitochondrial trans-2-enoyl-CoA reductase [Fusarium verticillioides 7600];Gene=Forpe1208_v008933;Ontology_term=mitochondrion,enoyl-[acyl-carrier-protein] reductase activity,fatty acid biosynthetic process,aerobic
Fv_160_12.g7121	234	ID=Fv_160_12.g7121;Description=hypothetical protein FVER14953_02192 [Fusarium verticillioides];Gene=fucI
Fv_160_12.g7122	305	ID=Fv_160_12.g7122;Description=hypothetical protein FVEG_02191 [Fusarium verticillioides 7600]

Fv_160_12.g7123	587	ID=Fv_160_12.g7123;Description=hypothetical protein FVEG_02190 [Fusarium verticillioides 7600];Gene=FNAPI_13846;Ontology_term=membrane,solute:inorganic anion antiporter activity,monoatomic anion
Fv_160_12.g7124	339	ID=Fv_160_12.g7124;Description=hypothetical protein FVEG_02189 [Fusarium verticillioides
Fv_160_12.g7125	712	ID=Fv_160_12.g7125;Description=CTI6 Cyc8-Tup1 interacting, partial [Fusarium coicis];Gene=C2S_3969;Ontology_term=diarylpropane peroxidase activity,heme binding,metal ion binding,cellular response to oxidative stress,hydrogen peroxide catabolic process,lignin catabolic process,cellular oxidant
Fv_160_12.g7126	528	ID=Fv_160_12.g7126;Description=hypothetical protein FVER14953_02187 [Fusarium verticillioides];Gene=3139;Ontology_term=extracellular region,structural molecule activity;Ontology_id=GO:0005576,GO:0005198
Fv_160_12.g7127	1526	ID=Fv_160_12.g7127;Description=hypothetical protein FVER53263_02186 [Fusarium verticillioides]
Fv_160_12.g7128	503	ID=Fv_160_12.g7128;Description=hypothetical protein FVEG_02185 [Fusarium verticillioides 7600]
Fv_160_12.g7129	345	ID=Fv_160_12.g7129;Description=gluconate 5-dehydrogenase [Fusarium verticillioides 7600];Gene=FCIRC_2720;Ontology_term=nucleosome,nucleus,DNA binding,oxidoreductase activity,structural constituent of chromatin,protein heterodimerization activity,cellular biosynthetic
Fv_160_13.g7130	584	ID=Fv_160_13.g7130;Description=hypothetical protein FVER53590_08752 [Fusarium verticillioides];Gene=FPANT_6948;Ontology_term=alpha-glucosidase activity,maltose metabolic process,carbohydrate catabolic
Fv_160_13.g7131	521	ID=Fv_160_13.g7131;Description=hypothetical protein J7337_012585 [Fusarium musae];Gene=FPANT_6949;Ontology_term=membrane,transmembrane transporter activity,transmembrane
Fv_160_13.g7132	509	ID=Fv_160_13.g7132;Description=hypothetical protein FVEG_08754 [Fusarium verticillioides 7600];Gene=BFJ63_vAg5939;Ontology_term=nucleus,DNA binding,zinc ion binding,DNA-binding transcription factor activity, RNA polymerase II-specific,regulation of transcription by RNA polymerase
Fv_160_13.g7133	352	ID=Fv_160_13.g7133;Description=hypothetical protein FVER14953_08755 [Fusarium verticillioides];Gene=BFJ63_vAg5938;Ontology_term=nucleus,membrane,DNA binding,zinc ion binding,transferase activity, transferring alkyl or aryl (other than methyl) groups,DNA-templated transcription;Ontology_id=GO:0005634,GO:0016020,GO:0003677,GO:0008270,GO:0016765,GO:0006351;Enzyme_code=EC:2.5.1;E
Fv_160_13.g7134	444	ID=Fv_160_13.g7134;Description=hypothetical protein FVEG_16419 [Fusarium verticillioides 7600];Gene=FOXG_09719;Ontology_term=membrane,oxidoreductase
Fv_160_13.g7135	431	ID=Fv_160_13.g7135;Description=hypothetical protein FVER53590_08756 [Fusarium
Fv_160_13.g7136	452	ID=Fv_160_13.g7136;Description=hypothetical protein FVEG_08757 [Fusarium verticillioides 7600];Gene=FRV6_14745;Ontology_term=peroxidase activity,metal ion binding,cellular oxidant
Fv_160_13.g7137	455	ID=Fv_160_13.g7137;Description=hypothetical protein FVER53263_20753 [Fusarium verticillioides];Gene=FNAPI_4532;Ontology_term=oxidoreductase activity,heme binding,metal ion
Fv_160_13.g7138	415	ID=Fv_160_13.g7138;Description=hypothetical protein FVER53590_08758 [Fusarium verticillioides]

Fv_160_13.g7139	532	ID=Fv_160_13.g7139;Description=hypothetical protein FVEG_08760 [Fusarium verticillioides 7600];Gene=FNAPI_4534;Ontology_term=vacuolar membrane,amino acid
Fv_160_13.g7140	435	ID=Fv_160_13.g7140;Description=oxidoreductase [Fusarium verticillioides 7600];Gene=FOC1_g10007160;Ontology_term=nucleotide binding,nucleus,DNA binding,zinc ion binding,DNA-binding transcription factor activity, RNA polymerase II-specific,regulation of transcription by RNA polymerase
Fv_160_13.g7141	349	ID=Fv_160_13.g7141;Description=Uncharacterized protein LW93_3711 [Fusarium fujikuroi];Gene=FFC1_10691;Ontology_term=nucleus,DNA binding,zinc ion binding,DNA-binding transcription factor activity, RNA
Fv_160_13.g7142	324	ID=Fv_160_13.g7142;Description=hypothetical protein FVER53263_08761 [Fusarium verticillioides];Gene=FPANT_6959;Ontology_term=membrane,oxidoreductase
Fv_160_13.g7143	351	ID=Fv_160_13.g7143;Description=hypothetical protein FVER14953_08762 [Fusarium verticillioides];Gene=ORF31
Fv_160_13.g7144	187	ID=Fv_160_13.g7144;Description=hypothetical protein FVEG_16424 [Fusarium verticillioides 7600]
Fv_160_13.g7145	427	ID=Fv_160_13.g7145;Description=hypothetical protein J7337_012571 [Fusarium musae];Gene=FACUT_3858;Ontology_term=oxidoreductase activity,FAD binding,biosynthetic
Fv_160_13.g7146	250	ID=Fv_160_13.g7146;Description=hypothetical protein H9Q70_011505 [Fusarium xylarioides];Gene=FRV6_14734;Ontology_term=membrane,hydrolase
Fv_160_13.g7147	292	ID=Fv_160_13.g7147;Description=hypothetical protein FVEG_08765 [Fusarium verticillioides 7600]
Fv_160_13.g7148	82	ID=Fv_160_13.g7148;Description=hypothetical protein FVER53263_20750 [Fusarium verticillioides]
Fv_160_13.g7149	442	ID=Fv_160_13.g7149;Description=hypothetical protein FVER53590_08766 [Fusarium
Fv_160_13.g7150	284	ID=Fv_160_13.g7150;Description=hypothetical protein FVER14953_08767 [Fusarium verticillioides]
Fv_160_13.g7151	638	ID=Fv_160_13.g7151;Description=hypothetical protein FVEG_08768 [Fusarium verticillioides 7600];Gene=FPRO_14186;Ontology_term=nucleus,membrane,DNA-binding transcription factor activity, RNA polymerase II-specific,peroxidase activity,zinc ion binding,regulation of transcription by RNA polymerase II,cellular oxidant detoxification;Ontology_id=GO:0005634,GO:0016020,GO:0000981,GO:0004601,GO:0008270,GO:0006357,GO:0098869;Enzyme_co
Fv_160_13.g7152	282	ID=Fv_160_13.g7152;Description=hypothetical protein FVEG_16427 [Fusarium verticillioides 7600];Gene=FOQG_15097;Ontology_term=nucleus,DNA-binding transcription factor activity, RNA polymerase II-specific,peroxidase activity,zinc ion binding,regulation of transcription by RNA polymerase II,cellular oxidant
Fv_160_13.g7153	520	ID=Fv_160_13.g7153;Description=hypothetical protein FVER14953_08769 [Fusarium verticillioides];Gene=BFJ68_g12023;Ontology_term=nucleus,DNA-binding transcription factor activity, RNA polymerase II-specific,peroxidase activity,zinc ion binding,regulation of transcription by RNA polymerase II,cellular oxidant
Fv_160_13.g7154	88	ID=Fv_160_13.g7154;Description=hypothetical protein DER46DRAFT_393790 [Fusarium sp. MPI-SDFR-AT-
Fv_160_13.g7155	359	ID=Fv_160_13.g7155;Description=hypothetical protein FVER53263_08770 [Fusarium verticillioides];Gene=FDENT_1217;Ontology_term=nucleus,DNA binding,carboxypeptidase activity,zinc ion binding,DNA-binding transcription factor activity, RNA polymerase II-specific,regulation of transcription by RNA polymerase

Fv_160_13.g7156	959	ID=Fv_160_13.g7156;Description=transcriptional activator Mut3p [Fusarium coicis];Gene=FMEXI_1341;Ontology_term=nucleus,DNA binding,zinc ion binding,DNA-binding transcription factor activity, RNA polymerase II-specific,regulation of transcription by RNA
Fv_160_13.g7157	565	ID=Fv_160_13.g7157;Description=hypothetical protein FVER14953_08773 [Fusarium
Fv_160_13.g7158	300	ID=Fv_160_13.g7158;Description=hypothetical protein FVEG_08774 [Fusarium verticillioides 7600];Gene=FOXYS1_13583;Ontology_term=ferric iron binding,catechol 1,2-dioxygenase activity,catechol-containing compound
Fv_160_13.g7159	710	ID=Fv_160_13.g7159;Description=hypothetical protein J7337_012555 [Fusarium musae];Gene=FPRO_14177;Ontology_term=L-serine ammonia-lyase activity,metal ion binding,4 iron, 4 sulfur cluster
Fv_160_13.g7160	688	ID=Fv_160_13.g7160;Description=hypothetical protein FVEG_16430 [Fusarium verticillioides 7600];Gene=274;Ontology_term=L-serine ammonia-lyase activity,metal ion binding,4 iron, 4 sulfur cluster
Fv_160_13.g7161	1732	ID=Fv_160_13.g7161;Description=hypothetical protein FVER53263_08776 [Fusarium verticillioides]
Fv_160_13.g7162	497	ID=Fv_160_13.g7162;Description=hypothetical protein FVEG_08777 [Fusarium verticillioides 7600];Gene=F25303_3763;Ontology_term=membrane,transmembrane transporter activity,intracellular phosphate ion
Fv_160_13.g7163	348	ID=Fv_160_13.g7163;Description=hypothetical protein FVEG_08778 [Fusarium verticillioides 7600];Gene=FNAPI_4554;Ontology_term=nucleotide binding,oxidoreductase activity, acting on
Fv_160_13.g7164	309	ID=Fv_160_13.g7164;Description=hypothetical protein FVEG_08779 [Fusarium verticillioides 7600];Gene=FSUBG_8860;Ontology_term=acyltransferase activity,hydrolase
Fv_160_13.g7165	423	ID=Fv_160_13.g7165;Description=hypothetical protein FVER53590_08780 [Fusarium verticillioides];Gene=FVER53590_08780;Ontology_term=extracellular region,metallocarboxypeptidase activity,zinc ion binding,proteolysis;Ontology_id=GO:0005576,GO:0004181,GO:0008270,GO:0006508;Enzyme_code=EC:3.4.17;Enzyme_name=Actin
Fv_160_13.g7166	298	ID=Fv_160_13.g7166;Description=hypothetical protein FVER53590_29541 [Fusarium verticillioides];Gene=ORF13
Fv_160_13.g7167	331	ID=Fv_160_13.g7167;Description=alcohol dehydrogenase [Fusarium verticillioides 7600]
Fv_160_13.g7168	224	ID=Fv_160_13.g7168;Description=hypothetical protein FVEG_08782 [Fusarium verticillioides 7600];Gene=FocTR4_00015190;Ontology_term=NAD glycohydrolase
Fv_160_13.g7169	242	ID=Fv_160_13.g7169;Description=hypothetical protein FVER14953_08783 [Fusarium verticillioides];Gene=FPRO_14167;Ontology_term=extracellular region,hydrolase
Fv_160_13.g7170	391	ID=Fv_160_13.g7170;Description=hypothetical protein FVEG_08784 [Fusarium verticillioides
Fv_160_13.g7171	606	ID=Fv_160_13.g7171;Description=hypothetical protein FVER14953_08785 [Fusarium verticillioides];Gene=FPRO_14165;Ontology_term=hydrolase
Fv_160_13.g7172	534	ID=Fv_160_13.g7172;Description=hypothetical protein FVER14953_08786 [Fusarium verticillioides];Gene=FPANT_5628;Ontology_term=monooxygenase activity,iron ion binding,oxidoreductase activity, acting on paired donors, with incorporation or reduction of molecular oxygen,heme binding;Ontology_id=GO:0004497,GO:0005506,GO:0016705,GO:0020037;Enzyme_code=EC:1.14;Enzyme_name=Acting on paired

Fv_160_13.g7173	340	ID=Fv_160_13.g7173;Description=hypothetical protein FVER53590_29540 [Fusarium verticillioides];Gene=FVER53590_29540;Ontology_term=nucleus,DNA-binding transcription factor activity, RNA polymerase II-
Fv_160_13.g7174	492	ID=Fv_160_13.g7174;Description=hypothetical protein FVEG_08787 [Fusarium verticillioides 7600];Gene=Lgr4;Ontology_term=nucleus,DNA-binding transcription factor activity, RNA polymerase II-specific,zinc ion
Fv_160_13.g7175	258	ID=Fv_160_13.g7175;Description=hypothetical protein FVEG_16438 [Fusarium verticillioides 7600];Gene=FOVG_13295;Ontology_term=nucleus,DNA-binding transcription factor activity, RNA polymerase II-specific,zinc ion binding,acyltransferase activity, transferring groups other than amino-acyl groups,regulation of transcription by RNA polymerase
Fv_160_13.g7176	200	ID=Fv_160_13.g7176;Description=hypothetical protein FVEG_16439 [Fusarium verticillioides 7600];Gene=FPRO_14160;Ontology_term=acyltransferase activity, transferring groups other than amino-acyl
Fv_160_13.g7177	359	ID=Fv_160_13.g7177;Description=hypothetical protein FVER53590_08789 [Fusarium verticillioides]
Fv_160_13.g7178	273	ID=Fv_160_13.g7178;Description=aquaporin rerated protein, other eukaryote [Fusarium verticillioides 7600];Gene=FOC4_g10009648;Ontology_term=membrane,channel activity,transmembrane
Fv_160_13.g7179	580	ID=Fv_160_13.g7179;Description=hypothetical protein FVEG_08791 [Fusarium verticillioides
Fv_160_13.g7180	287	ID=Fv_160_13.g7180;Description=hypothetical protein FVER14953_08792 [Fusarium verticillioides]
Fv_160_13.g7181	1765	ID=Fv_160_13.g7181;Description=hypothetical protein FVER53263_08793 [Fusarium verticillioides]
Fv_160_13.g7182	328	ID=Fv_160_13.g7182;Description=hypothetical protein FVEG_08794 [Fusarium verticillioides 7600];Gene=FPRO_14154;Ontology_term=membrane,transferase
Fv_160_13.g7183	322	ID=Fv_160_13.g7183;Description=hypothetical protein FVEG_08795 [Fusarium verticillioides 7600]
Fv_160_13.g7184	269	ID=Fv_160_13.g7184;Description=hypothetical protein FVEG_08796 [Fusarium verticillioides 7600];Gene=FNAPI_7840;Ontology_term=nucleotide binding,oxidoreductase activity, acting on
Fv_160_13.g7185	516	ID=Fv_160_13.g7185;Description=hypothetical protein FVEG_08797 [Fusarium verticillioides 7600];Gene=FOQG_13440;Ontology_term=nucleus,cytosolic small ribosomal subunit,DNA-binding transcription factor activity, RNA polymerase II-specific,RNA binding,structural constituent of ribosome,zinc ion binding,regulation of transcription by RNA polymerase
Fv_160_13.g7186	364	ID=Fv_160_13.g7186;Description=hypothetical protein J7337_012529 [Fusarium musae];Gene=FOIG_13482;Ontology_term=cytoplasm,microtubule,membrane,structural constituent of cytoskeleton,GTP binding,glycosyltransferase activity,hydrolase activity,metal ion binding,cytoskeleton organization,microtubule-based process;Ontology_id=GO:0005737,GO:0005874,GO:0016020,GO:0005200,GO:0005525,GO:0016757,GO:0016787,GO:0046872,GO:
Fv_160_13.g7187	380	ID=Fv_160_13.g7187;Description=hypothetical protein FVER53263_08799 [Fusarium verticillioides]
Fv_160_13.g7188	230	ID=Fv_160_13.g7188;Description=hypothetical protein FVEG_08800 [Fusarium verticillioides
Fv_160_13.g7189	391	ID=Fv_160_13.g7189;Description=hypothetical protein FCOIX_1505 [Fusarium coicis]
Fv_160_13.g7190	351	ID=Fv_160_13.g7190;Description=hypothetical protein FVEG_08803 [Fusarium verticillioides 7600]

Fv_160_13.g7191	334	ID=Fv_160_13.g7191;Description=ADP-ribosylglycohydrolase [Fusarium verticillioides 7600];Gene=FVEG_08804;Ontology_term=cytosolic large ribosomal subunit,RNA binding,structural constituent of ribosome,hydrolase activity,cytoplasmic
Fv_160_13.g7192	627	ID=Fv_160_13.g7192;Description=hypothetical protein FVEG_08805 [Fusarium verticillioides 7600];Gene=FPHYL_1954;Ontology_term=nucleus,DNA binding,zinc ion binding,membrane,DNA-binding transcription factor activity, RNA polymerase II-specific,regulation of transcription by RNA polymerase
Fv_160_13.g7193	271	ID=Fv_160_13.g7193;Description=oxidoreductase [Fusarium verticillioides 7600];Gene=FNAPI_7850;Ontology_term=nucleus,membrane,DNA binding,zinc ion binding,oxidoreductase activity,DNA-templated
Fv_160_13.g7194	345	ID=Fv_160_13.g7194;Description=hypothetical protein FVER53590_08807 [Fusarium verticillioides];Gene=FSUBG_11569;Ontology_term=nucleolus,cytosolic small ribosomal subunit,vesicle,small-subunit processome,structural constituent of ribosome,lyase activity,rRNA binding,translation,ribosomal small subunit
Fv_160_13.g7195	246	ID=Fv_160_13.g7195;Description=hypothetical protein FVER53590_08808 [Fusarium verticillioides]
Fv_160_13.g7196	520	ID=Fv_160_13.g7196;Description=hypothetical protein FVER53590_08809 [Fusarium verticillioides];Gene=FPCIR_10509;Ontology_term=membrane,transmembrane transporter activity,transmembrane
Fv_160_13.g7197	423	ID=Fv_160_13.g7197;Description=glucarate dehydratase [Fusarium verticillioides 7600];Gene=gudD;Ontology_term=metal ion
Fv_160_13.g7198	82	ID=Fv_160_13.g7198;Description=hypothetical protein FVEG_16444 [Fusarium verticillioides 7600]
Fv_160_13.g7199	169	ID=Fv_160_13.g7199;Description=hypothetical protein J7337_012516 [Fusarium musae]
Fv_160_13.g7200	431	ID=Fv_160_13.g7200;Description=hypothetical protein FVER53590_08812 [Fusarium verticillioides];Gene=FSUBG_11563;Ontology_term=membrane,malate transmembrane transporter activity,malate transmembrane
Fv_160_13.g7201	356	ID=Fv_160_13.g7201;Description=hypothetical protein FVER14953_08813 [Fusarium verticillioides]
Fv_160_13.g7202	605	ID=Fv_160_13.g7202;Description=hypothetical protein FVEG_08814 [Fusarium verticillioides 7600];Gene=rasD;Ontology_term=cytosol,plasma membrane,G protein activity,protein kinase inhibitor activity,GTP binding,GDP binding,filamin binding,protein kinase B binding,mitogen-activated protein kinase binding,mitotic cytokinesis,chemotaxis,Ras protein signal transduction,phototaxis,thermotaxis,ERK1 and ERK2 cascade,adenylate cyclase-activating G protein-coupled cAMP receptor signaling
Fv_160_13.g7203	617	ID=Fv_160_13.g7203;Description=hypothetical protein FVEG_08815 [Fusarium verticillioides 7600]
Fv_160_13.g7204	364	ID=Fv_160_13.g7204;Description=hypothetical protein FVER14953_08816 [Fusarium
Fv_160_13.g7205	610	ID=Fv_160_13.g7205;Description=hypothetical protein FVEG_08817 [Fusarium verticillioides 7600];Gene=FMUND_10943;Ontology_term=oxidoreductase activity, acting on CH-OH group of donors,flavin adenine dinucleotide
Fv_160_13.g7206	342	ID=Fv_160_13.g7206;Description=hypothetical protein FVER53590_29589 [Fusarium verticillioides];Gene=FNAPI_7863;Ontology_term=S-adenosylmethionine-dependent methyltransferase
Fv_160_13.g7207	453	ID=Fv_160_13.g7207;Description=hypothetical protein FVER53590_08819 [Fusarium verticillioides];Gene=FMEXI_5458;Ontology_term=S-adenosylmethionine-dependent methyltransferase

Fv_160_13.g7208	757	ID=Fv_160_13.g7208;Description=hypothetical protein FVER53590_08820 [Fusarium verticillioides];Gene=FACUT_6309;Ontology_term=cytosolic large ribosomal subunit,DNA binding,structural constituent of
Fv_160_13.g7209	329	ID=Fv_160_13.g7209;Description=hypothetical protein FVER14953_08821 [Fusarium
Fv_160_13.g7210	467	ID=Fv_160_13.g7210;Description=hypothetical protein FVER14953_08822 [Fusarium verticillioides]
Fv_160_13.g7211	233	ID=Fv_160_13.g7211;Description=hypothetical protein FVEG_08823 [Fusarium verticillioides 7600]
Fv_160_13.g7212	842	ID=Fv_160_13.g7212;Description=alpha-glucuronidase [Fusarium verticillioides 7600];Gene=aguA;Ontology_term=extracellular region,alpha-glucuronidase activity,xylan catabolic
Fv_160_13.g7213	462	ID=Fv_160_13.g7213;Description=endoglucanase type B [Fusarium verticillioides 7600];Gene=FOQG_13471;Ontology_term=extracellular region,cellulase activity,cellulose binding,cellulose catabolic
Fv_160_13.g7214	177	ID=Fv_160_13.g7214;Description=hypothetical protein J7337_012500 [Fusarium musae];Gene=FOQG_13472;Ontology_term=oxidoreductase activity, acting on
Fv_160_13.g7215	885	ID=Fv_160_13.g7215;Description=hypothetical protein FVEG_08826 [Fusarium verticillioides 7600];Gene=FMEXI_5451;Ontology_term=oxidoreductase activity, acting on
Fv_160_13.g7216	209	ID=Fv_160_13.g7216;Description=hypothetical protein FVEG_08827 [Fusarium verticillioides 7600];Gene=FPHYL_938;Ontology_term=oxidoreductase
Fv_160_13.g7217	327	ID=Fv_160_13.g7217;Description=hypothetical protein FVEG_16448 [Fusarium verticillioides
Fv_160_13.g7218	233	ID=Fv_160_13.g7218;Description=endo-1,4-beta-xylanase [Fusarium verticillioides 7600];Gene=FANTH_6562;Ontology_term=endo-1,4-beta-xylanase activity,xylan catabolic
Fv_160_13.g7219	448	ID=Fv_160_13.g7219;Description=hypothetical protein FVER53263_08829 [Fusarium verticillioides]
Fv_160_13.g7220	939	ID=Fv_160_13.g7220;Description=hypothetical protein FVER14953_08830 [Fusarium verticillioides]
Fv_160_13.g7221	247	ID=Fv_160_13.g7221;Description=hypothetical protein FVER53590_08831 [Fusarium verticillioides]
Fv_160_13.g7222	499	ID=Fv_160_13.g7222;Description=hypothetical protein FVER14953_08832 [Fusarium verticillioides]
Fv_160_13.g7223	292	ID=Fv_160_13.g7223;Description=hypothetical protein FVER53590_08833 [Fusarium verticillioides];Gene=FRV6_14647;Ontology_term=hydrolase activity, acting on ester
Fv_160_13.g7224	98	ID=Fv_160_13.g7224;Description=hypothetical protein FVEG_16451 [Fusarium verticillioides 7600]
Fv_160_13.g7225	307	ID=Fv_160_13.g7225;Description=hypothetical protein FVER14953_08834 [Fusarium verticillioides]
Fv_160_13.g7226	342	ID=Fv_160_13.g7226;Description=hypothetical protein FVER14953_08835 [Fusarium verticillioides];Gene=FVEG_08835;Ontology_term=nucleotide binding,oxidoreductase activity, acting on
Fv_160_13.g7227	530	ID=Fv_160_13.g7227;Description=hypothetical protein FVER14953_08836 [Fusarium verticillioides];Gene=FVEG_08836;Ontology_term=hydrolase activity, hydrolyzing O-glycosyl compounds,carbohydrate metabolic
Fv_160_13.g7228	75	ID=Fv_160_13.g7228;Description=hypothetical protein FVEG_16452 [Fusarium verticillioides 7600]
Fv_160_13.g7229	540	ID=Fv_160_13.g7229;Description=hypothetical protein FVER53590_08837 [Fusarium verticillioides]

Fv_160_13.g7230	284	ID=Fv_160_13.g7230;Description=hypothetical protein FVER14953_08838 [Fusarium verticillioides];Gene=FPRO_14100;Ontology_term=S-adenosylmethionine-dependent methyltransferase
Fv_160_13.g7231	110	ID=Fv_160_13.g7231;Description=hypothetical protein FVER14953_08839 [Fusarium verticillioides]
Fv_160_13.g7232	142	ID=Fv_160_13.g7232;Description=hypothetical protein FVER53590_08840 [Fusarium verticillioides]
Fv_160_13.g7233	529	ID=Fv_160_13.g7233;Description=hypothetical protein FVER14953_08841 [Fusarium verticillioides];Gene=FOMG_14571;Ontology_term=membrane,transmembrane transporter activity,carbohydrate
Fv_160_13.g7234	837	ID=Fv_160_13.g7234;Description=hypothetical protein FVER14953_08842 [Fusarium verticillioides];Gene=FRV6_14635;Ontology_term=DNA-directed RNA polymerase complex,nucleus,DNA binding,DNA-directed 5'-3' RNA polymerase activity,beta-glucosidase activity,metal ion binding,scopolin beta-glucosidase activity,transcription by RNA polymerase II,cellulose catabolic process;Ontology_id=GO:0000428,GO:0005634,GO:0003677,GO:0003899,GO:0008422,GO:0046872,GO:0102483,GO:0006366,GO:
Fv_160_13.g7235	142	ID=Fv_160_13.g7235;Description=hypothetical protein FVEG_16454 [Fusarium verticillioides 7600];Gene=BHE90_002873;Ontology_term=carbon-sulfur lyase activity,metal ion
Fv_160_13.g7236	763	ID=Fv_160_13.g7236;Description=hypothetical protein FVEG_08843 [Fusarium verticillioides 7600];Gene=FNAPI_7589;Ontology_term=nucleus,DNA binding,zinc ion binding,carbon-sulfur lyase activity,hydrolase activity, hydrolyzing O-glycosyl compounds,cellulose catabolic process,DNA-binding transcription factor activity, RNA polymerase II-specific,regulation of transcription by RNA polymerase II;Ontology_id=GO:0005634,GO:0003677,GO:0008270,GO:0016846,GO:0004553,GO:0030245,GO:0000981,GO:0006357;Enzyme_co
Fv_160_13.g7237	292	ID=Fv_160_13.g7237;Description=5-carboxymethyl-2-hydroxymuconate isomerase [Fusarium verticillioides 7600];Gene=FVEG_08844;Ontology_term=isomerase
Fv_160_13.g7238	327	ID=Fv_160_13.g7238;Description=gentisate 1,2-dioxygenase [Fusarium verticillioides 7600];Gene=FFUJ_10568;Ontology_term=dioxygenase
Fv_160_13.g7239	239	ID=Fv_160_13.g7239;Description=hypothetical protein FVEG_08846 [Fusarium verticillioides 7600];Gene=PK2;Ontology_term=membrane,protein serine/threonine kinase activity,ATP binding,protein serine kinase activity,phosphorylation;Ontology_id=GO:0016020,GO:0004674,GO:0005524,GO:0106310,GO:0016310;Enzyme_code=EC:2.7.11.1;E
Fv_160_13.g7240	1105	ID=Fv_160_13.g7240;Description=hypothetical protein FVER14953_08848 [Fusarium verticillioides]
Fv_160_13.g7241	555	ID=Fv_160_13.g7241;Description=hypothetical protein FVER14953_08850 [Fusarium verticillioides]
Fv_160_13.g7242	816	ID=Fv_160_13.g7242;Description=hypothetical protein FVER53590_08851 [Fusarium verticillioides];Gene=mx3-0;Ontology_term=GTPase activity,GTP binding,organelle
Fv_160_13.g7243	352	ID=Fv_160_13.g7243;Description=hypothetical protein FVER14953_08852 [Fusarium
Fv_160_13.g7244	344	ID=Fv_160_13.g7244;Description=hypothetical protein FVER14953_20805 [Fusarium verticillioides]
Fv_160_13.g7245	621	ID=Fv_160_13.g7245;Description=hypothetical protein FVER14953_08853 [Fusarium verticillioides];Gene=FNYG_12166;Ontology_term=translation initiation factor activity,formation of translation preinitiation

Fv_160_13.g7246	387	ID=Fv_160_13.g7246;Description=hypothetical protein FVEG_08854 [Fusarium verticillioides 7600]
Fv_160_13.g7247	479	ID=Fv_160_13.g7247;Description=hypothetical protein FVEG_08855 [Fusarium verticillioides 7600];Gene=28951156;Ontology_term=negative regulation of signal transduction,intracellular signal
Fv_160_13.g7248	669	ID=Fv_160_13.g7248;Description=hypothetical protein FVER14953_20806 [Fusarium
Fv_160_13.g7249	285	ID=Fv_160_13.g7249;Description=hypothetical protein FVER14953_08856 [Fusarium verticillioides]
Fv_160_13.g7250	649	ID=Fv_160_13.g7250;Description=hypothetical protein J7337_012464 [Fusarium musae];Gene=FVER53590_08858;Ontology_term=periplasmic space,lyase
Fv_160_13.g7251	490	ID=Fv_160_13.g7251;Description=hypothetical protein FVEG_08858 [Fusarium verticillioides 7600];Gene=FVEG_08858;Ontology_term=periplasmic space,lyase
Fv_160_13.g7252	134	ID=Fv_160_13.g7252;Description=hypothetical protein J7337_012462 [Fusarium musae]
Fv_160_13.g7253	572	ID=Fv_160_13.g7253;Description=hypothetical protein FVER14953_08860 [Fusarium verticillioides];Gene=FOXG_09608;Ontology_term=oxidoreductase activity,FAD
Fv_160_13.g7254	422	ID=Fv_160_13.g7254;Description=hypothetical protein FVEG_08861 [Fusarium verticillioides 7600]
Fv_160_13.g7255	702	ID=Fv_160_13.g7255;Description=hypothetical protein FVEG_08862 [Fusarium verticillioides 7600];Gene=FTJAE_10478;Ontology_term=oxidoreductase
Fv_160_13.g7256	928	ID=Fv_160_13.g7256;Description=hypothetical protein FVER14953_20807 [Fusarium verticillioides];Gene=FANTH_12049;Ontology_term=cytosol,phosphomevalonate kinase activity,ATP binding,S-adenosylmethionine-dependent methyltransferase activity,oxidoreductase activity,glycosyltransferase activity,FAD binding,cholesterol biosynthetic process,phosphorylation,isopentenyl diphosphate biosynthetic process, mevalonate pathway;Ontology_id=GO:0005829,GO:0004631,GO:0005524,GO:0008757,GO:0016491,GO:0016757,GO:0071949,GO:0006695,GO:
Fv_160_13.g7257	538	ID=Fv_160_13.g7257;Description=hypothetical protein FVEG_16467 [Fusarium verticillioides 7600];Gene=FFB14_09799;Ontology_term=membrane,ferric-chelate reductase activity,iron ion
Fv_160_13.g7258	174	ID=Fv_160_13.g7258;Description=hypothetical protein FVER14953_08865 [Fusarium verticillioides]
Fv_160_13.g7259	525	ID=Fv_160_13.g7259;Description=hypothetical protein FVEG_08866 [Fusarium verticillioides 7600];Gene=FVEG_08866;Ontology_term=membrane,transmembrane transporter activity,glucan endo-1,3-alpha-glucosidase activity,transmembrane
Fv_160_13.g7260	943	ID=Fv_160_13.g7260;Description=hypothetical protein FVER14953_08867 [Fusarium verticillioides];Gene=FVER53263_08867;Ontology_term=membrane,transmembrane transporter activity,glucan endo-1,3-alpha-glucosidase activity,transmembrane
Fv_160_13.g7261	484	ID=Fv_160_13.g7261;Description=hypothetical protein FNAPI_7564 [Fusarium napiforme]
Fv_160_13.g7262	471	ID=Fv_160_13.g7262;Description=hypothetical protein FVEG_08868 [Fusarium verticillioides 7600];Gene=FVEG_08868;Ontology_term=Golgi apparatus,membrane,transferase activity,transmembrane transporter activity,carbohydrate metabolic process,transmembrane

Fv_160_13.g7263	873	ID=Fv_160_13.g7263;Description=hypothetical protein FVER53590_08869 [Fusarium verticillioides];Gene=FPRO_14066;Ontology_term=Golgi apparatus,membrane,transferase activity,transmembrane transporter activity,carbohydrate metabolic process,transmembrane
Fv_160_13.g7264	534	ID=Fv_160_13.g7264;Description=hypothetical protein FVER14953_08870 [Fusarium verticillioides];Gene=FNYG_12144;Ontology_term=hydrolase
Fv_160_13.g7265	112	ID=Fv_160_13.g7265;Description=hypothetical protein FVEG_16469 [Fusarium verticillioides 7600];Gene=glpV;Ontology_term=cytoplasm,nucleotide binding,glycogen phosphorylase activity,pyridoxal phosphate binding,linear malto-oligosaccharide phosphorylase activity,SHG alpha-glucan phosphorylase activity,glycogen catabolic process,response to cAMP;Ontology_id=GO:0005737,GO:0000166,GO:0008184,GO:0030170,GO:0102250,GO:0102499,GO:0005980,GO:0051591;Enzym
Fv_160_13.g7266	95	ID=Fv_160_13.g7266;Description=hypothetical protein FVEG_08871 [Fusarium verticillioides
Fv_160_13.g7267	214	ID=Fv_160_13.g7267;Description=hypothetical protein FVER14953_08872 [Fusarium
Fv_160_13.g7268	79	ID=Fv_160_13.g7268;Description=hypothetical protein FCOIX_1431 [Fusarium coicis]
Fv_160_13.g7269	197	ID=Fv_160_13.g7269;Description=hypothetical protein FVEG_08873 [Fusarium verticillioides 7600];Gene=FFC1_10829;Ontology_term=protein kinase activity,ATP
Fv_160_13.g7270	367	ID=Fv_160_13.g7270;Description=hypothetical protein FVER53263_11740 [Fusarium verticillioides];Gene=BFJ68_g13256;Ontology_term=nucleus,DNA-binding transcription factor activity, RNA polymerase II-
Fv_160_13.g7271	304	ID=Fv_160_13.g7271;Description=hypothetical protein FVER53263_08874 [Fusarium verticillioides]
Fv_160_13.g7272	311	ID=Fv_160_13.g7272;Description=hypothetical protein FVEG_08875 [Fusarium verticillioides 7600]
Fv_160_13.g7273	395	ID=Fv_160_13.g7273;Description=hypothetical protein FVEG_08876 [Fusarium verticillioides 7600];Gene=FOPG_09551;Ontology_term=nucleus,DNA-binding transcription factor activity, RNA polymerase II-specific,zinc ion
Fv_160_13.g7274	415	ID=Fv_160_13.g7274;Description=chitinase 1 [Fusarium verticillioides 7600];Gene=FTJAE_10444;Ontology_term=chitinase activity,positive regulation of translational termination,chitin binding,RNA binding,polysaccharide catabolic process,translation elongation factor activity,chitin catabolic process,cytoplasm,ribosome binding,extracellular region,positive regulation of translational elongation;Ontology_id=GO:0004568,GO:0045905,GO:0008061,GO:0003723,GO:0000272,GO:0003746,GO:0006032,GO:0005737,GO:0043022,GO:0005576,GO:0045901;Enzyme_code=EC:3.2.1.14;Enzyme_name=chitinase
Fv_160_13.g7275	470	ID=Fv_160_13.g7275;Description=hypothetical protein FVER53263_08878 [Fusarium
Fv_160_13.g7276	429	ID=Fv_160_13.g7276;Description=hypothetical protein FVER53590_08879 [Fusarium verticillioides];Gene=FVEG_08879;Ontology_term=polyketide metabolic process;Ontology_id=GO:0030638
Fv_160_13.g7277	217	ID=Fv_160_13.g7277;Description=hypothetical protein FVEG_16472 [Fusarium verticillioides 7600]
Fv_160_13.g7278	361	ID=Fv_160_13.g7278;Description=hypothetical protein FVEG_08880 [Fusarium verticillioides
Fv_160_13.g7279	347	ID=Fv_160_13.g7279;Description=hypothetical protein FVER14953_08881 [Fusarium verticillioides]
Fv_160_13.g7280	228	ID=Fv_160_13.g7280;Description=hypothetical protein FVER14953_08883 [Fusarium verticillioides]
Fv_160_13.g7281	179	ID=Fv_160_13.g7281;Description=hypothetical protein FVEG_08884 [Fusarium verticillioides

Fv_160_13.g7282	425	ID=Fv_160_13.g7282;Description=hypothetical protein FVER14953_08885 [Fusarium verticillioides]
Fv_160_13.g7283	575	ID=Fv_160_13.g7283;Description=hypothetical protein FVER14953_08886 [Fusarium verticillioides];Gene=FDENT_4916;Ontology_term=membrane,ligase
Fv_160_13.g7284	595	ID=Fv_160_13.g7284;Description=hypothetical protein FVEG_08887 [Fusarium verticillioides 7600];Gene=FTJAE_10433;Ontology_term=membrane,transmembrane transporter activity,transmembrane
Fv_160_13.g7285	443	ID=Fv_160_13.g7285;Description=hypothetical protein FVER53263_08888 [Fusarium verticillioides];Gene=FPRO_14043;Ontology_term=hydrolase activity,polyketide metabolic
Fv_160_13.g7286	521	ID=Fv_160_13.g7286;Description=hypothetical protein FVEG_08889 [Fusarium verticillioides 7600];Gene=FOMG_13584;Ontology_term=membrane,transmembrane transporter activity,transmembrane
Fv_160_13.g7287	866	ID=Fv_160_13.g7287;Description=hypothetical protein FVER53263_08890 [Fusarium verticillioides];Gene=FVER53590_08890;Ontology_term=membrane,beta-glucosidase activity,transmembrane transporter activity,scopolin beta-glucosidase activity,cellulose catabolic process,transmembrane
Fv_160_13.g7288	679	ID=Fv_160_13.g7288;Description=sialidase-1 [Fusarium verticillioides 7600];Gene=gaoB;Ontology_term=galactose oxidase activity;Ontology_id=GO:0045480;Enzyme_code=EC:1.1.3.9;Enzyme_name=galactose oxidase
Fv_160_13.g7289	403	ID=Fv_160_13.g7289;Description=hypothetical protein FVEG_08892 [Fusarium verticillioides 7600];Gene=FOMG_13581;Ontology_term=O-methyltransferase activity,S-adenosylmethionine-dependent methyltransferase activity,methylation,secondary metabolite biosynthetic
Fv_160_13.g7290	331	ID=Fv_160_13.g7290;Description=hypothetical protein FVEG_16474 [Fusarium verticillioides 7600];Gene=FOC4_g10009516;Ontology_term=S-adenosylmethionine-dependent methyltransferase
Fv_160_13.g7291	238	ID=Fv_160_13.g7291;Description=hypothetical protein FVER14953_08895 [Fusarium verticillioides];Gene=MEF-1;Ontology_term=cytoplasm,membrane,translation elongation factor activity,GTPase activity,GTP binding,translational elongation;Ontology_id=GO:0005737,GO:0016020,GO:0003746,GO:0003924,GO:0005525,GO:0006414;Enzyme_code=EC:3.6.1.15;E
Fv_160_13.g7292	118	ID=Fv_160_13.g7292;Description=hypothetical protein FVEG_08896 [Fusarium verticillioides 7600]
Fv_160_13.g7293	547	ID=Fv_160_13.g7293;Description=hypothetical protein FVER53263_08897 [Fusarium verticillioides];Gene=FPRO_14035;Ontology_term=plasma membrane,aminopeptidase activity,acyltransferase activity,host cell surface receptor binding,adhesion of symbiont to host
Fv_160_13.g7294	147	ID=Fv_160_13.g7294;Description=hypothetical protein FCOIX_13506 [Fusarium coicis]
Fv_160_13.g7295	435	ID=Fv_160_13.g7295;Description=uncharacterized protein FFM5_15001 [Fusarium fujikuroi];Gene=FGADI_11478;Ontology_term=extracellular space,serine-type endopeptidase inhibitor activity,hormone
Fv_160_13.g7296	177	ID=Fv_160_13.g7296;Description=hypothetical protein FVEG_17725 [Fusarium verticillioides

Fv_160_13.g7297	432	ID=Fv_160_13.g7297;Description=hypothetical protein FVER53590_14083 [Fusarium verticillioides];Gene=FTJAE_10422;Ontology_term=membrane,monooxygenase activity,iron ion binding,oxidoreductase activity, acting on paired donors, with incorporation or reduction of molecular oxygen,heme binding;Ontology_id=GO:0016020,GO:0004497,GO:0005506,GO:0016705,GO:0020037;Enzyme_code=EC:1.14;Enzyme_name=Actin
Fv_160_13.g7298	564	ID=Fv_160_13.g7298;Description=hypothetical protein J7337_012413 [Fusarium
Fv_160_13.g7299	396	ID=Fv_160_13.g7299;Description=hypothetical protein FVER14953_14085 [Fusarium verticillioides]
Fv_160_13.g7300	494	ID=Fv_160_13.g7300;Description=hypothetical protein FVEG_17729 [Fusarium verticillioides 7600];Gene=FOQG_12132;Ontology_term=nucleus,DNA-binding transcription factor activity, RNA polymerase II-specific,zinc ion
Fv_160_13.g7301	306	ID=Fv_160_13.g7301;Description=hypothetical protein FVEG_14086 [Fusarium verticillioides 7600];Gene=FNYG_12111;Ontology_term=oxidoreductase activity,cellular biosynthetic
Fv_160_13.g7302	223	ID=Fv_160_13.g7302;Description=hypothetical protein FVER53263_14087 [Fusarium verticillioides]
Fv_160_13.g7303	199	ID=Fv_160_13.g7303;Description=hypothetical protein FVER14953_14088 [Fusarium
Fv_160_13.g7304	421	ID=Fv_160_13.g7304;Description=hypothetical protein FVEG_14089 [Fusarium verticillioides 7600];Gene=FVER53263_14089;Ontology_term=membrane,lipid metabolic process;Ontology_id=GO:0016020,GO:0006629
Fv_160_13.g7305	182	ID=Fv_160_13.g7305;Description=hypothetical protein FVER14953_14090 [Fusarium verticillioides]
Fv_160_13.g7306	535	ID=Fv_160_13.g7306;Description=hypothetical protein FVER14953_14091 [Fusarium verticillioides];Gene=FVER53263_14091;Ontology_term=hydrolase
Fv_160_13.g7307	178	ID=Fv_160_13.g7307;Description=hypothetical protein FVER14953_14092 [Fusarium verticillioides];Gene=BFJ65_g13630;Ontology_term=metallopeptidase activity,carbohydrate
Fv_160_13.g7308	414	ID=Fv_160_13.g7308;Description=hypothetical protein FVEG_14093 [Fusarium verticillioides
Fv_160_13.g7309	169	ID=Fv_160_13.g7309;Description=hypothetical protein FVER53590_28985 [Fusarium verticillioides];Gene=FVER53263_21092;Ontology_term=acyltransferase activity, transferring groups other than amino-acyl
Fv_160_13.g7310	252	ID=Fv_160_13.g7310;Description=hypothetical protein FVEG_14094 [Fusarium verticillioides 7600]
Fv_160_13.g7311	642	ID=Fv_160_13.g7311;Description=hypothetical protein FVER53590_14095 [Fusarium verticillioides];Gene=FCIRC_5714;Ontology_term=nucleus,DNA binding,zinc ion binding,DNA-binding transcription factor activity, RNA polymerase II-specific,regulation of transcription by RNA polymerase
Fv_160_13.g7312	487	ID=Fv_160_13.g7312;Description=hypothetical protein J7337_012397 [Fusarium musae];Gene=FPANT_11723;Ontology_term=membrane,3-beta-hydroxy-delta5-steroid dehydrogenase activity,isomerase activity,steroid biosynthetic
Fv_160_13.g7313	478	ID=Fv_160_13.g7313;Description=hypothetical protein J7337_012396 [Fusarium musae];Gene=BFJ65_g13344;Ontology_term=membrane,transmembrane transporter activity,transmembrane
Fv_160_13.g7314	525	ID=Fv_160_13.g7314;Description=hypothetical protein FVEG_14098 [Fusarium verticillioides 7600];Gene=FOBC_15085;Ontology_term=membrane,transmembrane transporter activity,transmembrane

Fv_160_13.g7315	470	ID=Fv_160_13.g7315;Description=hypothetical protein FVER14953_14099 [Fusarium
Fv_160_13.g7316	387	ID=Fv_160_13.g7316;Description=hypothetical protein FVER53263_14100 [Fusarium verticillioides];Gene=FNAPI_7951;Ontology_term=aminopeptidase
Fv_160_13.g7317	384	ID=Fv_160_13.g7317;Description=hypothetical protein FVER14953_14101 [Fusarium
Fv_160_13.g7318	357	ID=Fv_160_13.g7318;Description=hypothetical protein FVER53590_14102 [Fusarium verticillioides];Gene=FTJAE_10400;Ontology_term=3-beta-hydroxy-delta5-steroid dehydrogenase activity,steroid biosynthetic
Fv_160_13.g7319	659	ID=Fv_160_13.g7319;Description=hypothetical protein FVER53590_30308 [Fusarium verticillioides];Gene=BFJ69_g11100;Ontology_term=nucleus,DNA binding,zinc ion binding,DNA-binding transcription factor activity, RNA polymerase II-specific,regulation of transcription by RNA polymerase
Fv_160_13.g7320	486	ID=Fv_160_13.g7320;Description=hypothetical protein FVEG_17733 [Fusarium verticillioides 7600];Gene=FANTH_11993;Ontology_term=nucleus,DNA binding,zinc ion binding,ligase activity,DNA-templated
Fv_160_13.g7321	249	ID=Fv_160_13.g7321;Description=hypothetical protein FVER53263_14107 [Fusarium verticillioides];Gene=F52700_9755;Ontology_term=hydrolase
Fv_160_13.g7322	343	ID=Fv_160_13.g7322;Description=hypothetical protein FVEG_14108 [Fusarium verticillioides 7600];Gene=FVER53263_14108;Ontology_term=lysosome,early phagosome,esterosome membrane,oxidoreductase activity,carboxylic ester hydrolase activity,mitotic cytokinesis,endocytosis,sorocarp development;Ontology_id=GO:0005764,GO:0032009,GO:0033118,GO:0016491,GO:0052689,GO:0000281,GO:0006897,GO:003058
Fv_160_13.g7323	481	ID=Fv_160_13.g7323;Description=hypothetical protein J7337_012386 [Fusarium musae]
Fv_160_13.g7324	494	ID=Fv_160_13.g7324;Description=hypothetical protein FVEG_14110 [Fusarium verticillioides 7600];Gene=FDENT_4877;Ontology_term=kinase
Fv_160_13.g7325	445	ID=Fv_160_13.g7325;Description=hypothetical protein FNAPI_7944 [Fusarium napiforme];Gene=FOBC_15098;Ontology_term=membrane,phosphoric diester hydrolase activity,lipid metabolic
Fv_160_13.g7326	401	ID=Fv_160_13.g7326;Description=hypothetical protein FVEG_14112 [Fusarium verticillioides
Fv_160_13.g7327	358	ID=Fv_160_13.g7327;Description=hypothetical protein FVER53263_14113 [Fusarium verticillioides];Gene=FOIG_14682;Ontology_term=S-adenosylmethionine-dependent methyltransferase
Fv_160_13.g7328	676	ID=Fv_160_13.g7328;Description=hypothetical protein FVEG_14114 [Fusarium verticillioides
Fv_160_13.g7329	668	ID=Fv_160_13.g7329;Description=hypothetical protein FVER53263_14115 [Fusarium verticillioides];Gene=dhcA;Ontology_term=centrosome,cytoplasmic dynein complex,cytoplasmic microtubule,cell cortex,early phagosome membrane,phagolysosome membrane,ATPase complex,ATP binding,microtubule binding,minus-end-directed microtubule motor activity,ATP hydrolysis activity,identical protein binding,CTPase activity,dynein intermediate chain binding,dynein light intermediate chain binding,nuclear migration,cytoplasmic microtubule organization,minus-end-directed vesicle transport along microtubule,mitotic spindle assembly,phagosome

Fv_160_13.g7330	186	ID=Fv_160_13.g7330;Description=hypothetical protein FVER14953_14116 [Fusarium verticillioides];Gene=AU210_012847;Ontology_term=chorismate mutase activity,chorismate metabolic
Fv_160_13.g7331	263	ID=Fv_160_13.g7331;Description=hypothetical protein FVER14953_13588 [Fusarium verticillioides]
Fv_160_13.g7332	126	ID=Fv_160_13.g7332;Description=hypothetical protein FVER53263_13589 [Fusarium verticillioides];Gene=FVER53590_13590;Ontology_term=glutathione transferase
Fv_160_13.g7333	346	ID=Fv_160_13.g7333;Description=glutathione S-transferase [Fusarium verticillioides 7600];Gene=FNAPI_4765;Ontology_term=glutathione transferase
Fv_160_13.g7334	828	ID=Fv_160_13.g7334;Description=hypothetical protein FVEG_13591 [Fusarium verticillioides 7600];Gene=FVEG_13591;Ontology_term=nucleus,DNA binding,zinc ion binding,DNA-binding transcription factor activity, RNA polymerase II-specific,regulation of transcription by RNA polymerase
Fv_160_13.g7335	242	ID=Fv_160_13.g7335;Description=hypothetical protein FVER14953_13592 [Fusarium verticillioides]
Fv_160_13.g7336	2558	ID=Fv_160_13.g7336;Description=hypothetical protein FVEG_13593 [Fusarium verticillioides 7600]
Fv_160_13.g7337	784	ID=Fv_160_13.g7337;Description=hypothetical protein FVEG_13594 [Fusarium verticillioides 7600];Gene=FOIG_14671;Ontology_term=membrane,oligopeptide transmembrane transporter activity,protein
Fv_160_13.g7338	483	ID=Fv_160_13.g7338;Description=hypothetical protein FVEG_13595 [Fusarium verticillioides
Fv_160_13.g7339	430	ID=Fv_160_13.g7339;Description=hypothetical protein FVER14953_13596 [Fusarium verticillioides];Gene=FTJAE_10687;Ontology_term=membrane,transmembrane transport;Ontology_id=GO:0016020,GO:0055085
Fv_160_13.g7340	122	ID=Fv_160_13.g7340;Description=hypothetical protein FVEG_17559 [Fusarium verticillioides
Fv_160_13.g7341	311	ID=Fv_160_13.g7341;Description=gluconate 5-dehydrogenase [Fusarium verticillioides 7600];Gene=FOPG_15072;Ontology_term=oxidoreductase activity,cellular biosynthetic
Fv_160_13.g7342	154	ID=Fv_160_13.g7342;Description=hypothetical protein FVEG_13598 [Fusarium verticillioides 7600]
Fv_160_13.g7343	239	ID=Fv_160_13.g7343;Description=DNA polymerase alpha subunit B [Fusarium verticillioides 7600]
Fv_160_13.g7344	203	ID=Fv_160_13.g7344;Description=hypothetical protein FVEG_17560 [Fusarium verticillioides 7600]
Fv_160_13.g7345	178	ID=Fv_160_13.g7345;Description=hypothetical protein FVER14953_13600 [Fusarium verticillioides]
Fv_160_13.g7346	117	ID=Fv_160_13.g7346;Description=hypothetical protein FVEG_13601 [Fusarium verticillioides 7600]
Fv_160_13.g7347	274	ID=Fv_160_13.g7347;Description=hypothetical protein FVEG_13602 [Fusarium verticillioides 7600];Gene=FVER53263_13602;Ontology_term=nucleic acid binding,3'-5' exonuclease activity,nucleobase-containing compound
Fv_160_13.g7348	559	ID=Fv_160_13.g7348;Description=hypothetical protein FVER53590_13604 [Fusarium verticillioides];Gene=FANTH_8674;Ontology_term=nucleus,DNA-binding transcription factor activity, RNA polymerase II-specific,zinc
Fv_160_13.g7349	413	ID=Fv_160_13.g7349;Description=hypothetical protein FVEG_13605 [Fusarium verticillioides 7600];Gene=FPHYL_7099;Ontology_term=plasma membrane,side of membrane,aspartic-type endopeptidase activity,proteolysis,cell adhesion;Ontology_id=GO:0005886,GO:0098552,GO:0004190,GO:0006508,GO:0007155;Enzyme_code=EC:3.4.23;Enzyme_name=A

Fv_160_13.g7350	465	ID=Fv_160_13.g7350;Description=hypothetical protein FVEG_13606 [Fusarium verticillioides 7600];Gene=FMAN_13328;Ontology_term=cellular anatomical entity,metal ion binding,regulation of DNA-templated
Fv_160_13.g7351	158	ID=Fv_160_13.g7351;Description=hypothetical protein FVEG_17563 [Fusarium verticillioides 7600];Gene=FOC4_g10002753;Ontology_term=oxidoreductase activity,metal ion
Fv_160_13.g7352	231	ID=Fv_160_13.g7352;Description=hypothetical protein FVEG_13607 [Fusarium verticillioides 7600];Gene=FANTH_8671;Ontology_term=peroxidase activity,metal ion binding,cellular oxidant
Fv_160_13.g7353	118	ID=Fv_160_13.g7353;Description=hypothetical protein FVEG_13608 [Fusarium verticillioides
Fv_160_13.g7354	254	ID=Fv_160_13.g7354;Description=hypothetical protein FVEG_13609 [Fusarium verticillioides 7600]
Fv_160_13.g7355	261	ID=Fv_160_13.g7355;Description=hypothetical protein FVEG_13610 [Fusarium verticillioides 7600];Gene=BFJ65_g13443;Ontology_term=nucleus,cytosol,plasma membrane,cell cortex,actin rod,cell leading edge,side of membrane,adenosylhomocysteinase activity,oxidoreductase activity,cAMP binding,one-carbon metabolic process,cell adhesion,establishment or maintenance of cell polarity,S-adenosylmethionine cycle,cellular biosynthetic process;Ontology_id=GO:0005634,GO:0005829,GO:0005886,GO:0005938,GO:0031002,GO:0031252,GO:0098552,GO:0004013,GO:
Fv_160_13.g7356	509	ID=Fv_160_13.g7356;Description=hypothetical protein FVEG_13611 [Fusarium verticillioides 7600];Gene=FNAPI_4787;Ontology_term=membrane,transmembrane transporter activity,transmembrane
Fv_160_13.g7357	386	ID=Fv_160_13.g7357;Description=hypothetical protein FVER14953_13612 [Fusarium
Fv_160_13.g7358	617	ID=Fv_160_13.g7358;Description=hypothetical protein FVER53590_13613 [Fusarium verticillioides];Gene=FACUT_8828;Ontology_term=oxidoreductase activity, acting on CH-OH group of donors,flavin adenine
Fv_160_13.g7359	482	ID=Fv_160_13.g7359;Description=hypothetical protein FVEG_13614 [Fusarium verticillioides 7600];Gene=FCIRC_9316;Ontology_term=serine-type carboxypeptidase
Fv_160_13.g7360	167	ID=Fv_160_13.g7360;Description=hypothetical protein FVEG_13615 [Fusarium verticillioides 7600]
Fv_160_13.g7361	123	ID=Fv_160_13.g7361;Description=hypothetical protein FVEG_13618 [Fusarium verticillioides 7600]
Fv_160_13.g7362	158	ID=Fv_160_13.g7362;Description=hypothetical protein FVEG_13619 [Fusarium verticillioides 7600]
Fv_160_13.g7363	225	ID=Fv_160_13.g7363;Description=hypothetical protein FVEG_13620 [Fusarium verticillioides 7600]
Fv_160_13.g7364	110	ID=Fv_160_13.g7364;Description=hypothetical protein FVEG_13621 [Fusarium verticillioides 7600];Gene=act18;Ontology_term=cytoplasm,actin cytoskeleton,structural constituent of cytoskeleton,ATP binding,hydrolase activity,myosin binding,phagocytosis,cytoskeleton organization,response to
Fv_160_13.g7365	135	ID=Fv_160_13.g7365;Description=hypothetical protein FCOIX_1660 [Fusarium coicis]
Fv_160_13.g7366	340	ID=Fv_160_13.g7366;Description=hypothetical protein FVER14953_13622 [Fusarium verticillioides]
Fv_160_13.g7367	73	ID=Fv_160_13.g7367;Description=Nucleoporin NUP85 [Fusarium oxysporum f. sp. albedinis];Gene=CEP54_003479
Fv_160_13.g7368	383	ID=Fv_160_13.g7368;Description=hypothetical protein FVEG_13623 [Fusarium verticillioides 7600]
Fv_160_13.g7369	256	ID=Fv_160_13.g7369;Description=hypothetical protein FVER53590_13624 [Fusarium verticillioides];Gene=FACUT_8836;Ontology_term=hydrolase

Fv_160_13.g7370	243	ID=Fv_160_13.g7370;Description=hypothetical protein FVER53263_13625 [Fusarium verticillioides]
Fv_160_13.g7371	410	ID=Fv_160_13.g7371;Description=hypothetical protein FVER53590_13626 [Fusarium verticillioides];Gene=FDENT_7670;Ontology_term=nucleus,DNA-binding transcription factor activity, RNA polymerase II-specific,DNA helicase activity,ATP binding,zinc ion binding,ATP hydrolysis activity,telomere maintenance,DNA repair,DNA recombination,regulation of transcription by RNA polymerase II,DNA duplex unwinding;Ontology_id=GO:0005634,GO:0000981,GO:0003678,GO:0005524,GO:0008270,GO:0016887,GO:0000723,GO:0006281,G
Fv_160_13.g7372	1032	ID=Fv_160_13.g7372;Description=5'-3' DNA helicase [Fusarium napiforme];Gene=FACUT_8838;Ontology_term=nucleus,DNA-binding transcription factor activity, RNA polymerase II-specific,DNA helicase activity,ATP binding,zinc ion binding,oxidoreductase activity, acting on the CH-OH group of donors, NAD or NADP as acceptor,ATP hydrolysis activity,telomere maintenance,DNA repair,DNA recombination,regulation of transcription by RNA polymerase II,DNA duplex unwinding;Ontology_id=GO:0005634,GO:0000981,GO:0003678,GO:0005524,GO:0008270,GO:0016616,GO:0016887,GO:0000723,G
Fv_160_13.g7373	243	ID=Fv_160_13.g7373;Description=hypothetical protein J7337_012342 [Fusarium musae];Gene=FFB14_09679;Ontology_term=nucleus,DNA-binding transcription factor activity, RNA polymerase II-specific,zinc ion binding,oxidoreductase activity, acting on the CH-OH group of donors, NAD or NADP as acceptor,regulation of transcription by RNA
Fv_160_13.g7374	632	ID=Fv_160_13.g7374;Description=hypothetical protein FVER14953_13630 [Fusarium verticillioides];Gene=BFJ63_vAg10355;Ontology_term=extracellular space,metalloendopeptidase activity,zinc ion
Fv_160_13.g7375	514	ID=Fv_160_13.g7375;Description=hypothetical protein FVEG_13632 [Fusarium verticillioides 7600];Gene=CEK26_002148;Ontology_term=phagocytic cup,extracellular space,actin filament,cell-cell junction,cortical actin cytoskeleton,pseudopodium,early phagosome,phagolysosome,cell pole,intranuclear rod,leading edge of lamellipodium,macropinocytic cup,metalloendopeptidase activity,structural constituent of cytoskeleton,oxygen carrier activity,ATP binding,zinc ion binding,myosin binding,oxygen binding,heme binding,mitotic cytokinesis,cell morphogenesis,plasma membrane repair,phagocytosis,chemotaxis,hyperosmotic response,cytoskeleton organization,oxygen transport,phototaxis,response to cAMP;Ontology_id=GO:0001891,GO:0005615,GO:0005884,GO:0005911,GO:0030864,GO:0031143,GO:0032009,GO:0032010,GO:00
Fv_160_13.g7376	198	ID=Fv_160_13.g7376;Description=hypothetical protein FVEG_13633 [Fusarium verticillioides 7600];Gene=C2S_9373;Ontology_term=membrane,mycotoxin biosynthetic process;Ontology_id=GO:0016020,GO:0043386
Fv_160_13.g7377	135	ID=Fv_160_13.g7377;Description=hypothetical protein FPCIR_9656 [Fusarium pseudocircinatum]
Fv_160_13.g7378	782	ID=Fv_160_13.g7378;Description=hypothetical protein FVEG_13635 [Fusarium verticillioides 7600];Gene=FVER53263_13635;Ontology_term=cytoplasm,microtubule,membrane,GTPase activity,structural constituent of cytoskeleton,GTP binding,oligopeptide transmembrane transporter activity,metal ion binding,cytoskeleton organization,microtubule-based process,protein transport,oligopeptide transmembrane transport;Ontology_id=GO:0005737,GO:0005874,GO:0016020,GO:0003924,GO:0005200,GO:0005525,GO:0035673,GO:0046872,GO
Fv_160_13.g7379	808	ID=Fv_160_13.g7379;Description=hypothetical protein FVER14953_13636 [Fusarium

Fv_160_13.g7380	430	ID=Fv_160_13.g7380;Description=hypothetical protein FVER53590_13637 [Fusarium verticillioides];Gene=FPANT_3380;Ontology_term=transaminase
Fv_160_13.g7381	230	ID=Fv_160_13.g7381;Description=cutinase 3 [Fusarium verticillioides 7600];Gene=AU210_012802;Ontology_term=extracellular region,cutinase activity;Ontology_id=GO:0005576,GO:0050525;Enzyme_code=EC:3.1.1.74;Enzyme_name=cutinase
Fv_160_13.g7382	270	ID=Fv_160_13.g7382;Description=alanyl-tRNA synthetase [Fusarium verticillioides 7600];Gene=FVER53263_13639;Ontology_term=nucleic acid binding,alanine-tRNA ligase activity,ATP binding,alanyl-tRNA
Fv_160_13.g7383	537	ID=Fv_160_13.g7383;Description=hypothetical protein FVEG_17565 [Fusarium verticillioides 7600];Gene=FDENT_7660;Ontology_term=monooxygenase activity,iron ion binding,methyltransferase activity,oxidoreductase activity, acting on paired donors, with incorporation or reduction of molecular oxygen,heme binding,methylation;Ontology_id=GO:0004497,GO:0005506,GO:0008168,GO:0016705,GO:0020037,GO:0032259;Enzyme_code=EC:
Fv_160_13.g7384	685	ID=Fv_160_13.g7384;Description=hypothetical protein FANTH_8643 [Fusarium anthophilum];Gene=FPCIR_9649;Ontology_term=monooxygenase activity,iron ion binding,methyltransferase activity,oxidoreductase activity, acting on paired donors, with incorporation or reduction of molecular oxygen,heme binding,methylation;Ontology_id=GO:0004497,GO:0005506,GO:0008168,GO:0016705,GO:0020037,GO:0032259;Enzyme_code=EC:
Fv_160_13.g7385	275	ID=Fv_160_13.g7385;Description=hypothetical protein FVER14953_13641 [Fusarium verticillioides];Gene=FTJAE_10643;Ontology_term=myosin complex,myofibril,myosin filament,actin binding,ATP binding,serine-type peptidase activity,hydrolase activity, acting on glycosyl bonds,proteolysis,xylan catabolic process;Ontology_id=GO:0016459,GO:0030016,GO:0032982,GO:0003779,GO:0005524,GO:0008236,GO:0016798,GO:0006508,GO:
Fv_160_13.g7386	164	ID=Fv_160_13.g7386;Description=hypothetical protein FVEG_13642 [Fusarium verticillioides 7600]
Fv_160_13.g7387	240	ID=Fv_160_13.g7387;Description=hypothetical protein FVEG_13643 [Fusarium verticillioides 7600]
Fv_160_13.g7388	183	ID=Fv_160_13.g7388;Description=hypothetical protein FVEG_13644 [Fusarium verticillioides 7600]
Fv_160_13.g7389	232	ID=Fv_160_13.g7389;Description=uncharacterized protein FMAN_13278 [Fusarium
Fv_160_13.g7390	228	ID=Fv_160_13.g7390;Description=hypothetical protein FVEG_13646 [Fusarium verticillioides
Fv_160_13.g7391	884	ID=Fv_160_13.g7391;Description=hypothetical protein FVER14953_13647 [Fusarium verticillioides];Gene=FNYG_10682;Ontology_term=membrane,sulfuric ester hydrolase
Fv_160_13.g7392	299	ID=Fv_160_13.g7392;Description=hypothetical protein FVEG_13648 [Fusarium verticillioides 7600];Gene=FNYG_10683;Ontology_term=cell surface,membrane,oxidoreductase activity, acting on the aldehyde or oxo group of donors, NAD or NADP as acceptor,NAD
Fv_160_13.g7393	341	ID=Fv_160_13.g7393;Description=hypothetical protein FVER53263_13649 [Fusarium verticillioides];Gene=FMUND_9260;Ontology_term=ATP binding,hydrolase activity,ATP-dependent protein folding

Fv_160_13.g7394	604	ID=Fv_160_13.g7394;Description=hypothetical protein FVER14953_13650 [Fusarium verticillioides];Gene=FNYG_10685;Ontology_term=alcohol dehydrogenase (NAD+) activity,ATP binding,ATP-dependent protein folding chaperone,alcohol metabolic process,protein folding;Ontology_id=GO:0004022,GO:0005524,GO:0140662,GO:0006066,GO:0006457;Enzyme_code=EC:1.1.1.1,EC:1.1.1.71;Enzyme
Fv_160_13.g7395	500	ID=Fv_160_13.g7395;Description=hypothetical protein FVEG_13651 [Fusarium verticillioides 7600]
Fv_160_13.g7396	493	ID=Fv_160_13.g7396;Description=hypothetical protein FVER53590_13652 [Fusarium verticillioides];Gene=FOQG_17066;Ontology_term=alcohol dehydrogenase (NAD+) activity,GTP binding,alcohol metabolic process;Ontology_id=GO:0004022,GO:0005525,GO:0006066;Enzyme_code=EC:1.1.1.1,EC:1.1.1.71;Enzyme_name=alcohol
Fv_160_13.g7397	210	ID=Fv_160_13.g7397;Description=potassium channel beta subunit [Fusarium napiforme]
Fv_160_13.g7398	349	ID=Fv_160_13.g7398;Description=potassium channel beta subunit [Fusarium mundagurra]
Fv_160_13.g7399	801	ID=Fv_160_13.g7399;Description=hypothetical protein FVER53590_13654 [Fusarium verticillioides];Gene=FPANT_10821;Ontology_term=nucleus,membrane,DNA-binding transcription factor activity, RNA polymerase II-specific,DNA binding,transmembrane transporter activity,negative regulation of transcription by RNA polymerase II,compound eye photoreceptor cell differentiation,eye-antennal disc morphogenesis,leg disc proximal/distal pattern formation,visual perception,sensory organ boundary specification,eye pigment granule organization,chaeta morphogenesis,response to stimulus,transmembrane
Fv_160_13.g7400	180	ID=Fv_160_13.g7400;Description=hypothetical protein J7337_012315 [Fusarium musae];Gene=FMAN_13290;Ontology_term=oxidoreductase
Fv_160_13.g7401	610	ID=Fv_160_13.g7401;Description=hypothetical protein FVER53263_13656 [Fusarium verticillioides];Gene=FOIG_16166;Ontology_term=oxidoreductase activity, acting on CH-OH group of donors,flavin adenine
Fv_160_13.g7402	852	ID=Fv_160_13.g7402;Description=hypothetical protein FVER14953_13657 [Fusarium verticillioides];Gene=FVER53263_13657;Ontology_term=cytoplasm,beta-glucosidase activity,protein-macromolecule adaptor activity,actin filament binding,glucan endo-1,3-beta-glucanase activity, C-3 substituted reducing group,glucan endo-1,4-beta-glucanase activity, C-3 substituted reducing group,polysaccharide catabolic process,cell wall organization;Ontology_id=GO:0005737,GO:0008422,GO:0030674,GO:0051015,GO:0052861,GO:0052862,GO:0000272,GO:0071555;
Fv_160_13.g7403	288	ID=Fv_160_13.g7403;Description=hypothetical protein FVEG_13658 [Fusarium verticillioides 7600];Gene=FNAPI_7723;Ontology_term=hydrolase
Fv_160_13.g7404	1030	ID=Fv_160_13.g7404;Description=hypothetical protein FVER14953_13659 [Fusarium verticillioides];Gene=FNAPI_7724;Ontology_term=beta-glucosidase activity,carbohydrate binding,maltose alpha-glucosidase activity,scopolin beta-glucosidase activity,maltose metabolic
Fv_160_13.g7405	534	ID=Fv_160_13.g7405;Description=hypothetical protein FVER53590_13660 [Fusarium verticillioides];Gene=P62777;Ontology_term=nucleosome,nucleus,membrane,DNA binding,transmembrane transporter activity,structural constituent of chromatin,protein heterodimerization activity,transmembrane

Fv_160_13.g7406	312	ID=Fv_160_13.g7406;Description=hypothetical protein FVER14953_20602 [Fusarium
Fv_160_13.g7407	630	ID=Fv_160_13.g7407;Description=hypothetical protein J7337_012308 [Fusarium musae];Gene=FPHYL_5668;Ontology_term=membrane,oxidoreductase activity, acting on the CH-CH group of donors, NAD or NADP as acceptor,sterol biosynthetic
Fv_160_13.g7408	478	ID=Fv_160_13.g7408;Description=hypothetical protein FVEG_13662 [Fusarium verticillioides 7600];Gene=FOIG_16145;Ontology_term=membrane,oxidoreductase activity, acting on the CH-CH group of donors, NAD or NADP as acceptor,sterol biosynthetic
Fv_160_13.g7409	374	ID=Fv_160_13.g7409;Description=hypothetical protein FNYG_10671 [Fusarium nygamai];Gene=BFJ72_g9574;Ontology_term=membrane,serine-type endopeptidase activity,delta24-sterol reductase activity,FAD binding,proteolysis,sterol biosynthetic process;Ontology_id=GO:0016020,GO:0004252,GO:0050614,GO:0071949,GO:0006508,GO:0016126;Enzyme_code=EC:1.3.1.72,EC:3
Fv_160_13.g7410	1205	ID=Fv_160_13.g7410;Description=hypothetical protein FPANT_4159 [Fusarium pseudoanthophilum];Gene=FOXB_12728;Ontology_term=membrane,serine-type endopeptidase activity,oxidoreductase activity, acting on the CH-CH group of donors, NAD or NADP as acceptor,FAD binding,proteolysis,sterol biosynthetic process;Ontology_id=GO:0016020,GO:0004252,GO:0016628,GO:0071949,GO:0006508,GO:0016126;Enzyme_code=EC:3.4.21,EC:1.3
Fv_160_13.g7411	249	ID=Fv_160_13.g7411;Description=hypothetical protein J7337_012304 [Fusarium musae]
Fv_160_13.g7412	991	ID=Fv_160_13.g7412;Description=hypothetical protein H9Q72_009287 [Fusarium xylarioides]
Fv_160_13.g7413	299	ID=Fv_160_13.g7413;Description=hypothetical protein FVEG_13666 [Fusarium verticillioides 7600]
Fv_160_13.g7414	246	ID=Fv_160_13.g7414;Description=hypothetical protein FVEG_13667 [Fusarium verticillioides 7600]
Fv_160_13.g7415	171	ID=Fv_160_13.g7415;Description=hypothetical protein FVER53590_13668 [Fusarium verticillioides];Gene=Lsp1beta;Ontology_term=larval serum protein complex,metallopeptidase activity,nutrient reservoir activity,proteolysis;Ontology_id=GO:0005616,GO:0008237,GO:0045735,GO:0006508;Enzyme_code=EC:3.4;Enzyme_name=Acting
Fv_160_13.g7416	123	ID=Fv_160_13.g7416;Description=hypothetical protein FVER53590_25665 [Fusarium verticillioides];Gene=FVEG_17582;Ontology_term=nucleus,DNA-binding transcription factor activity, RNA polymerase II-specific,zinc
Fv_160_13.g7417	576	ID=Fv_160_13.g7417;Description=hypothetical protein FVER53590_25664 [Fusarium verticillioides];Gene=FPANT_4151;Ontology_term=protein kinase activity,ATP
Fv_160_13.g7418	412	ID=Fv_160_13.g7418;Description=NEK kinase [Fusarium pseudoanthophilum];Gene=FGADI_5041
Fv_160_13.g7419	291	ID=Fv_160_13.g7419;Description=calcium calmodulin-dependent kinase [Fusarium pseudoanthophilum];Gene=FPANT_4149;Ontology_term=kinase
Fv_160_13.g7420	145	ID=Fv_160_13.g7420;Description=hypothetical protein FOXG_16569 [Fusarium oxysporum f. sp. lycopersici 4287]
Fv_160_13.g7421	576	ID=Fv_160_13.g7421;Description=hypothetical protein FVEG_13670 [Fusarium verticillioides 7600];Gene=FMUND_9280;Ontology_term=ligase activity;Ontology_id=GO:0016874;Enzyme_code=EC:6;Enzyme_name=Ligases

Fv_160_13.g7422	262	ID=Fv_160_13.g7422;Description=hypothetical protein FVER53263_13671 [Fusarium verticillioides];Gene=cnox2;Ontology_term=nucleus,DNA-binding transcription factor activity, RNA polymerase II-specific,DNA
Fv_160_13.g7423	247	ID=Fv_160_13.g7423;Description=hypothetical protein FVER53263_13672 [Fusarium verticillioides]
Fv_160_13.g7424	547	ID=Fv_160_13.g7424;Description=hypothetical protein FVER53263_13673 [Fusarium verticillioides]
Fv_160_13.g7425	844	ID=Fv_160_13.g7425;Description=hypothetical protein FVEG_13674 [Fusarium verticillioides 7600]
Fv_160_13.g7426	360	ID=Fv_160_13.g7426;Description=hypothetical protein FVEG_13675 [Fusarium verticillioides 7600]
Fv_160_13.g7427	534	ID=Fv_160_13.g7427;Description=hypothetical protein FNYG_10652 [Fusarium nygamai];Gene=AU210_012755;Ontology_term=UDP-glycosyltransferase
Fv_160_13.g7428	444	ID=Fv_160_13.g7428;Description=hypothetical protein FVER53590_13676 [Fusarium verticillioides];Gene=FRV6_14437;Ontology_term=monooxygenase activity,UDP-glycosyltransferase activity,FMN
Fv_160_13.g7429	230	ID=Fv_160_13.g7429;Description=hypothetical protein FVER14953_13677 [Fusarium verticillioides]
Fv_160_13.g7430	286	ID=Fv_160_13.g7430;Description=hypothetical protein FVEG_13678 [Fusarium verticillioides 7600]
Fv_160_13.g7431	210	ID=Fv_160_13.g7431;Description=hypothetical protein FVEG_13679 [Fusarium verticillioides 7600]
Fv_160_13.g7432	313	ID=Fv_160_13.g7432;Description=hypothetical protein J7337_012285 [Fusarium musae];Gene=FVER53590_13680;Ontology_term=oxidoreductase activity,cellular biosynthetic
Fv_160_13.g7433	454	ID=Fv_160_13.g7433;Description=hypothetical protein FVER14953_13681 [Fusarium verticillioides];Gene=BFJ68_g13663;Ontology_term=nucleus,DNA-binding transcription factor activity, RNA polymerase II-
Fv_160_13.g7434	409	ID=Fv_160_13.g7434;Description=hypothetical protein FVEG_13682 [Fusarium verticillioides 7600];Gene=FMUND_9292;Ontology_term=cytoplasm,superoxide dismutase activity,triglyceride lipase activity,copper ion binding,lipid catabolic process,removal of superoxide radicals;Ontology_id=GO:0005737,GO:0004784,GO:0004806,GO:0005507,GO:0016042,GO:0019430;Enzyme_code=EC:1.15.1.1,EC:3
Fv_160_13.g7435	183	ID=Fv_160_13.g7435;Description=hypothetical protein FVEG_13683 [Fusarium verticillioides 7600];Gene=FPCIR_12838;Ontology_term=acyltransferase activity, transferring groups other than amino-acyl
Fv_160_13.g7436	534	ID=Fv_160_13.g7436;Description=hypothetical protein FVER14953_13684 [Fusarium verticillioides];Gene=AU210_012747;Ontology_term=membrane,transmembrane transporter activity,transmembrane
Fv_160_13.g7437	377	ID=Fv_160_13.g7437;Description=acetylornithine deacetylase [Fusarium verticillioides 7600];Gene=FPHYL_5691;Ontology_term=extracellular region,carboxypeptidase activity,lyase
Fv_160_13.g7438	381	ID=Fv_160_13.g7438;Description=hypothetical protein FVER14953_13686 [Fusarium verticillioides];Gene=FCIRC_1410;Ontology_term=S-adenosylmethionine-dependent methyltransferase
Fv_160_13.g7439	338	ID=Fv_160_13.g7439;Description=hypothetical protein FVER14953_13687 [Fusarium verticillioides];Gene=P24635;Ontology_term=cytoplasm,microtubule,structural constituent of cytoskeleton,GTP binding,hydrolase activity,cytoskeleton organization,microtubule-based

Fv_160_13.g7440	234	ID=Fv_160_13.g7440;Description=hypothetical protein FVER53590_13688 [Fusarium verticillioides];Gene=FVER53263_13688;Ontology_term=amino-acid racemase activity,nitrogen compound metabolic
Fv_160_13.g7441	142	ID=Fv_160_13.g7441;Description=hypothetical protein FVER14953_13689 [Fusarium verticillioides]
Fv_160_13.g7442	643	ID=Fv_160_13.g7442;Description=hypothetical protein FVER14953_13690 [Fusarium verticillioides];Gene=AU210_012733;Ontology_term=S-adenosylmethionine-dependent methyltransferase
Fv_160_13.g7443	590	ID=Fv_160_13.g7443;Description=hypothetical protein FVER14953_13691 [Fusarium verticillioides];Gene=FNAPI_6700;Ontology_term=membrane,nucleobase transmembrane transporter activity,purine nucleobase
Fv_160_13.g7444	182	ID=Fv_160_13.g7444;Description=hypothetical protein FVEG_13692 [Fusarium verticillioides 7600]
Fv_160_13.g7445	219	ID=Fv_160_13.g7445;Description=hypothetical protein FVER53263_13693 [Fusarium verticillioides];Gene=FNAPI_6698;Ontology_term=catalytic activity,nucleobase-containing compound metabolic
Fv_160_13.g7446	1214	ID=Fv_160_13.g7446;Description=hypothetical protein FVER53590_13694 [Fusarium verticillioides];Gene=FNYG_10634;Ontology_term=phosphorelay sensor kinase activity,phosphorelay signal transduction system,phosphorylation;Ontology_id=GO:0000155,GO:0000160,GO:0016310;Enzyme_code=EC:2.7.3,EC:2.7.13.3;Enzyme_name=Tra
Fv_160_13.g7447	384	ID=Fv_160_13.g7447;Description=hypothetical protein FVEG_13695 [Fusarium verticillioides
Fv_160_13.g7448	752	ID=Fv_160_13.g7448;Description=hypothetical protein FOXYS1_2322 [Fusarium
Fv_160_13.g7449	1317	ID=Fv_160_13.g7449;Description=hypothetical protein FVER53263_13698 [Fusarium verticillioides];Gene=FCIRC_1423;Ontology_term=protein serine/threonine kinase activity,ATP
Fv_160_13.g7450	573	ID=Fv_160_13.g7450;Description=hypothetical protein FVEG_13699 [Fusarium verticillioides 7600];Gene=BFJ72_g9552;Ontology_term=membrane,protein kinase activity,ATP
Fv_160_13.g7451	604	ID=Fv_160_13.g7451;Description=hypothetical protein J7337_012268 [Fusarium musae];Gene=FACUT_6717;Ontology_term=RNA binding,helicase activity,ATP binding,hydrolase
Fv_160_13.g7452	347	ID=Fv_160_13.g7452;Description=hypothetical protein FVEG_13701 [Fusarium verticillioides 7600];Gene=FDENT_4199
Fv_160_13.g7453	502	ID=Fv_160_13.g7453;Description=hypothetical protein FVEG_13702 [Fusarium verticillioides 7600];Gene=F25303_6620;Ontology_term=membrane,monooxygenase activity,iron ion binding,oxidoreductase activity, acting on paired donors, with incorporation or reduction of molecular oxygen,heme binding;Ontology_id=GO:0016020,GO:0004497,GO:0005506,GO:0016705,GO:0020037;Enzyme_code=EC:1.14;Enzyme_name=Actin
Fv_160_13.g7454	969	ID=Fv_160_13.g7454;Description=hypothetical protein FVER53590_13703 [Fusarium verticillioides];Gene=FPCIR_13309;Ontology_term=serine-type endopeptidase activity,monooxygenase activity,iron ion binding,oxidoreductase activity, acting on paired donors, with incorporation or reduction of molecular oxygen,heme binding,proteolysis;Ontology_id=GO:0004252,GO:0004497,GO:0005506,GO:0016705,GO:0020037,GO:0006508;Enzyme_code=EC:3.
Fv_160_13.g7455	543	ID=Fv_160_13.g7455;Description=hypothetical protein FVER53590_13704 [Fusarium verticillioides];Gene=FACUT_6713;Ontology_term=serine-type endopeptidase
Fv_160_13.g7456	165	ID=Fv_160_13.g7456;Description=hypothetical protein FVER14953_13705 [Fusarium verticillioides]

Fv_160_13.g7457	362	ID=Fv_160_13.g7457;Description=hypothetical protein H9Q71_005508 [Fusarium xylarioides]
Fv_160_13.g7458	219	ID=Fv_160_13.g7458;Description=hypothetical protein FVEG_13706 [Fusarium verticillioides 7600]
Fv_160_13.g7459	359	ID=Fv_160_13.g7459;Description=hypothetical protein FVER14953_13707 [Fusarium verticillioides]
Fv_160_13.g7460	292	ID=Fv_160_13.g7460;Description=hypothetical protein FVEG_13708 [Fusarium verticillioides 7600];Gene=P21896;Ontology_term=nucleosome,nucleus,DNA binding,structural constituent of chromatin,protein
Fv_160_13.g7461	195	ID=Fv_160_13.g7461;Description=heterokaryon incompatibility protein [Fusarium mundagurra]
Fv_160_13.g7462	535	ID=Fv_160_13.g7462;Description=hypothetical protein FVER53590_13709 [Fusarium verticillioides];Gene=FCIRC_1434;Ontology_term=nucleosome,nucleus,membrane,DNA-binding transcription factor activity, RNA polymerase II-specific,DNA binding,zinc ion binding,transmembrane transporter activity,structural constituent of chromatin,protein heterodimerization activity,regulation of transcription by RNA polymerase II,transmembrane
Fv_160_13.g7463	696	ID=Fv_160_13.g7463;Description=hypothetical protein FVER14953_20609 [Fusarium verticillioides];Gene=FNAPI_6676;Ontology_term=nucleus,DNA-binding transcription factor activity, RNA polymerase II-specific,zinc
Fv_160_13.g7464	344	ID=Fv_160_13.g7464;Description=hypothetical protein FVER53263_13711 [Fusarium verticillioides];Gene=FOMG_13384;Ontology_term=extracellular region,pectate lyase activity,polysaccharide catabolic
Fv_160_13.g7465	2498	ID=Fv_160_13.g7465;Description=hypothetical protein FVER14953_20612 [Fusarium verticillioides];Gene=FANTH_443;Ontology_term=3-oxoacyl-[acyl-carrier-protein] synthase activity,S-adenosylmethionine-dependent methyltransferase activity,oxidoreductase activity,phosphopantetheine binding,fatty acid biosynthetic process,secondary metabolite biosynthetic
Fv_160_13.g7466	256	ID=Fv_160_13.g7466;Description=hypothetical protein FVEG_13716 [Fusarium verticillioides 7600];Gene=FVER53590_13716;Ontology_term=S-adenosylmethionine-dependent methyltransferase
Fv_160_13.g7467	532	ID=Fv_160_13.g7467;Description=hypothetical protein FVER53263_13717 [Fusarium verticillioides];Gene=FVER53590_13717;Ontology_term=extracellular region,hemoglobin complex,membrane,oxygen carrier activity,copper ion binding,oxidoreductase activity,oxygen binding,heme binding,oxygen transport;Ontology_id=GO:0005576,GO:0005833,GO:0016020,GO:0005344,GO:0005507,GO:0016491,GO:0019825,GO:0020037,GO
Fv_160_13.g7468	364	ID=Fv_160_13.g7468;Description=hypothetical protein FVEG_13718 [Fusarium verticillioides 7600];Gene=O3G_MSEX005174;Ontology_term=extracellular region,vacuolar membrane,hemoglobin complex,proton-transporting V-type ATPase, V0 domain,oxygen carrier activity,oxygen binding,heme binding,metal ion binding,proton-transporting ATPase activity, rotational mechanism,oxygen transport,proton transmembrane transport;Ontology_id=GO:0005576,GO:0005774,GO:0005833,GO:0033179,GO:0005344,GO:0019825,GO:0020037,GO:0046872,GO
Fv_160_13.g7469	1298	ID=Fv_160_13.g7469;Description=hypothetical protein FVER53590_13719 [Fusarium verticillioides];Gene=FTJAE_4097;Ontology_term=membrane,monooxygenase activity,iron ion binding,lipid biosynthetic process,cellular lipid metabolic

Fv_160_13.g7470	373	ID=Fv_160_13.g7470;Description=Alkylglycerol monooxygenase [Fusarium tjaetaba];Gene=FTJAE_4097;Ontology_term=membrane,monooxygenase activity,iron ion binding,lipid biosynthetic process,cellular
Fv_160_13.g7471	538	ID=Fv_160_13.g7471;Description=hypothetical protein FVER53590_13721 [Fusarium verticillioides]
Fv_160_13.g7472	310	ID=Fv_160_13.g7472;Description=hypothetical protein FNAPI_13935 [Fusarium
Fv_160_13.g7473	408	ID=Fv_160_13.g7473;Description=hypothetical protein LB503_012610 [Fusarium chuoi];Gene=F25303_6600;Ontology_term=ATP hydrolysis activity,ADP binding,lipid catabolic process,glycerolipid metabolic
Fv_160_13.g7474	1193	ID=Fv_160_13.g7474;Description=hypothetical protein J7337_012244 [Fusarium musae]
Fv_160_13.g7475	205	ID=Fv_160_13.g7475;Description=hypothetical protein FVER53590_13725 [Fusarium verticillioides]
Fv_160_13.g7476	275	ID=Fv_160_13.g7476;Description=hypothetical protein FPANT_6553 [Fusarium pseudoanthophilum]
Fv_160_13.g7477	293	ID=Fv_160_13.g7477;Description=hypothetical protein FVER53590_13726 [Fusarium
Fv_160_13.g7478	455	ID=Fv_160_13.g7478;Description=hypothetical protein FVER53590_13727 [Fusarium verticillioides];Gene=FVER53590_13727;Ontology_term=nucleus,membrane,DNA-binding transcription factor activity, RNA polymerase II-specific,DNA binding,zinc ion binding,transmembrane transporter activity,regulation of transcription by RNA
Fv_160_13.g7479	279	ID=Fv_160_13.g7479;Description=hypothetical protein FVER14953_13728 [Fusarium verticillioides]
Fv_160_13.g7480	473	ID=Fv_160_13.g7480;Description=amino acid transporter [Fusarium sp. NRRL
Fv_160_13.g7481	343	ID=Fv_160_13.g7481;Description=hypothetical protein FVEG_13730 [Fusarium verticillioides 7600];Gene=FCIRC_1452;Ontology_term=oxidoreductase
Fv_160_13.g7482	844	ID=Fv_160_13.g7482;Description=hypothetical protein FVER53590_13731 [Fusarium verticillioides]
Fv_160_13.g7483	382	ID=Fv_160_13.g7483;Description=hypothetical protein FVER53263_13732 [Fusarium verticillioides];Gene=FVER53263_13732;Ontology_term=nucleotide binding,oxidoreductase activity, acting on
Fv_160_13.g7484	211	ID=Fv_160_13.g7484;Description=hypothetical protein FVEG_13733 [Fusarium verticillioides 7600]
Fv_160_13.g7485	229	ID=Fv_160_13.g7485;Description=hypothetical protein FVER53590_25118 [Fusarium
Fv_160_13.g7486	186	ID=Fv_160_13.g7486;Description=hypothetical protein FVER14953_20281 [Fusarium
Fv_160_13.g7487	388	ID=Fv_160_13.g7487;Description=hypothetical protein FVER53590_13734 [Fusarium verticillioides];Gene=FVER53263_13734;Ontology_term=nucleotide binding,oxidoreductase activity, acting on
Fv_160_13.g7488	1224	ID=Fv_160_13.g7488;Description=heterokaryon incompatibility protein het-E-1 [Fusarium tjaetaba];Gene=FOIG_14761;Ontology_term=catalytic activity,nucleoside metabolic process;Ontology_id=GO:0003824,GO:0009116
Fv_160_13.g7489	355	ID=Fv_160_13.g7489;Description=hypothetical protein FVEG_13736 [Fusarium verticillioides 7600]
Fv_160_13.g7490	531	ID=Fv_160_13.g7490;Description=hypothetical protein FVER53590_13737 [Fusarium verticillioides];Gene=FFUJ_10316;Ontology_term=nucleus,DNA-binding transcription factor activity, RNA polymerase II-specific,zinc
Fv_160_13.g7491	353	ID=Fv_160_13.g7491;Description=hypothetical protein FVER14953_13738 [Fusarium
Fv_160_13.g7492	551	ID=Fv_160_13.g7492;Description=hypothetical protein FVER53590_13739 [Fusarium verticillioides]
Fv_160_13.g7493	386	ID=Fv_160_13.g7493;Description=hypothetical protein FVEG_13740 [Fusarium verticillioides 7600]

Fv_160_13.g7494	966	ID=Fv_160_13.g7494;Description=RCO3 glucose transporter [Fusarium sp. NRRL 52700];Gene=FOXG_16482;Ontology_term=membrane,transmembrane transporter activity,carbohydrate transport,transmembrane
Fv_160_13.g7495	531	ID=Fv_160_13.g7495;Description=hypothetical protein J7337_012235 [Fusarium musae];Gene=FPHYL_1425;Ontology_term=proton-transporting V-type ATPase, V1 domain,ATP binding,ligase activity,metal ion binding,proton-transporting ATPase activity, rotational mechanism,dioxygenase activity,ATP metabolic process,proton transmembrane transport;Ontology_id=GO:0033180,GO:0005524,GO:0016874,GO:0046872,GO:0046961,GO:0051213,GO:0046034,GO:1902600;En
Fv_160_13.g7496	544	ID=Fv_160_13.g7496;Description=hypothetical protein FVEG_13743 [Fusarium verticillioides 7600]
Fv_160_13.g7497	525	ID=Fv_160_13.g7497;Description=hypothetical protein FVEG_13745 [Fusarium verticillioides 7600];Gene=FVER53263_13745;Ontology_term=membrane,transmembrane transporter activity,transmembrane
Fv_160_13.g7498	253	ID=Fv_160_13.g7498;Description=hypothetical protein FVEG_13746 [Fusarium verticillioides 7600]
Fv_160_13.g7499	380	ID=Fv_160_13.g7499;Description=hypothetical protein FVEG_13747 [Fusarium verticillioides 7600];Gene=AU210_012668;Ontology_term=nucleosome,nucleus,membrane,DNA binding,spermatogenesis,cell
Fv_160_13.g7500	528	ID=Fv_160_13.g7500;Description=hypothetical protein FVEG_13748 [Fusarium verticillioides 7600];Gene=BFJ63_vAg14610;Ontology_term=membrane,transmembrane transporter activity,transmembrane
Fv_160_13.g7501	681	ID=Fv_160_13.g7501;Description=hypothetical protein FVER14953_13749 [Fusarium verticillioides];Gene=BFJ70_g11481;Ontology_term=copper ion binding,primary amine oxidase activity,quinone binding,amine
Fv_160_13.g7502	788	ID=Fv_160_13.g7502;Description=hypothetical protein FVER14953_13750 [Fusarium verticillioides];Gene=FDENT_2965;Ontology_term=membrane,metal ion transmembrane transporter activity,magnesium ion
Fv_160_13.g7503	843	ID=Fv_160_13.g7503;Description=hypothetical protein FVEG_17597 [Fusarium verticillioides 7600];Gene=FPRO_13806;Ontology_term=extracellular region,inorganic cation transmembrane transporter activity,metal ion
Fv_160_13.g7504	627	ID=Fv_160_13.g7504;Description=hypothetical protein J7337_012215 [Fusarium musae]
Fv_160_13.g7505	433	ID=Fv_160_13.g7505;Description=uncharacterized protein FTJAE_4133 [Fusarium tjaetaba]
Fv_160_13.g7506	510	ID=Fv_160_13.g7506;Description=hypothetical protein J7337_012213 [Fusarium musae];Gene=FMEXI_14380;Ontology_term=membrane,monooxygenase activity,iron ion binding,oxidoreductase activity, acting on paired donors, with incorporation or reduction of molecular oxygen,heme binding;Ontology_id=GO:0016020,GO:0004497,GO:0005506,GO:0016705,GO:0020037;Enzyme_code=EC:1.14;Enzyme_name=Actin
Fv_160_13.g7507	231	ID=Fv_160_13.g7507;Description=hypothetical protein FVER14953_13756 [Fusarium
Fv_160_13.g7508	242	ID=Fv_160_13.g7508;Description=hypothetical protein FVER14953_13757 [Fusarium verticillioides];Gene=FANTH_4471;Ontology_term=ribosome,ribonucleoprotein complex,structural constituent of
Fv_160_13.g7509	485	ID=Fv_160_13.g7509;Description=adenylosuccinate lyase [Fusarium verticillioides 7600];Gene=FANTH_4470;Ontology_term=N6-(1,2-dicarboxyethyl)AMP AMP-lyase (fumarate-forming) activity,(S)-2-(5-amino-1-(5-phospho-D-ribosyl)imidazole-4-carboxamido) succinate lyase (fumarate-forming) activity,'de novo' IMP biosynthetic process,'de novo' AMP biosynthetic

Fv_160_13.g7510	302	ID=Fv_160_13.g7510;Description=glycosyl hydrolase family 17 [Fusarium verticillioides 7600];Gene=FNYG_10572;Ontology_term=hydrolase activity, hydrolyzing O-glycosyl compounds,carbohydrate metabolic
Fv_160_13.g7511	969	ID=Fv_160_13.g7511;Description=hypothetical protein FVEG_13760 [Fusarium verticillioides 7600]
Fv_160_13.g7512	473	ID=Fv_160_13.g7512;Description=hypothetical protein FVER53590_13761 [Fusarium verticillioides];Gene=FOMG_13320;Ontology_term=phosphatase
Fv_160_13.g7513	263	ID=Fv_160_13.g7513;Description=hypothetical protein FVER53590_13762 [Fusarium verticillioides]
Fv_160_13.g7514	627	ID=Fv_160_13.g7514;Description=hypothetical protein IWW34DRAFT_911760 [Fusarium oxysporum f. sp. albedinis]
Fv_160_13.g7515	264	ID=Fv_160_13.g7515;Description=uncharacterized protein FMAN_13156 [Fusarium mangiferae]
Fv_160_13.g7516	264	ID=Fv_160_13.g7516;Description=hypothetical protein FVER53590_25113 [Fusarium verticillioides]
Fv_160_13.g7517	1257	ID=Fv_160_13.g7517;Description=chitinase [Fusarium globosum];Gene=CEP51_000292;Ontology_term=extracellular region,chitinase activity,chitin binding,polysaccharide catabolic process,chitin catabolic
Fv_160_13.g7518	427	ID=Fv_160_13.g7518;Description=hypothetical protein FVER53590_13765 [Fusarium verticillioides]
Fv_160_13.g7519	685	ID=Fv_160_13.g7519;Description=hypothetical protein FVER53590_13766 [Fusarium
Fv_160_13.g7520	281	ID=Fv_160_13.g7520;Description=hypothetical protein FVEG_13767 [Fusarium verticillioides 7600]
Fv_160_13.g7521	282	ID=Fv_160_13.g7521;Description=hypothetical protein FVEG_17601 [Fusarium verticillioides 7600]
Fv_160_13.g7522	566	ID=Fv_160_13.g7522;Description=hypothetical protein FVER53590_13769 [Fusarium verticillioides]
Fv_160_13.g7523	472	ID=Fv_160_13.g7523;Description=hypothetical protein FVER14953_20198 [Fusarium verticillioides]
Fv_160_13.g7524	258	ID=Fv_160_13.g7524;Description=hypothetical protein FVER14953_13770 [Fusarium verticillioides]
Fv_160_13.g7525	555	ID=Fv_160_13.g7525;Description=hypothetical protein FVEG_13771 [Fusarium verticillioides 7600];Gene=FPCIR_6191;Ontology_term=membrane,peroxidase activity,heme binding,metal ion binding,response to oxidative stress,cellular oxidant
Fv_160_13.g7526	125	ID=Fv_160_13.g7526;Description=hypothetical protein FVER14953_20199 [Fusarium verticillioides]
Fv_160_13.g7527	300	ID=Fv_160_13.g7527;Description=hypothetical protein FVER14953_13773 [Fusarium verticillioides]
Fv_160_13.g7528	350	ID=Fv_160_13.g7528;Description=hypothetical protein FVER14953_13775 [Fusarium verticillioides];Gene=FCIRC_6525;Ontology_term=membrane,zinc ion binding,oxidoreductase
Fv_160_13.g7529	107	ID=Fv_160_13.g7529;Description=hypothetical protein LZL87_013447 [Fusarium
Fv_160_13.g7530	338	ID=Fv_160_13.g7530;Description=hypothetical protein FVEG_13776 [Fusarium verticillioides 7600];Gene=FVER53263_13776;Ontology_term=monooxygenase
Fv_160_13.g7531	276	ID=Fv_160_13.g7531;Description=hypothetical protein FVEG_13777 [Fusarium verticillioides 7600]
Fv_160_13.g7532	878	ID=Fv_160_13.g7532;Description=hypothetical protein FVEG_13778 [Fusarium verticillioides 7600];Gene=FVER53263_13778;Ontology_term=serine-type endopeptidase

Fv_160_13.g7533	1176	ID=Fv_160_13.g7533;Description=hypothetical protein FVEG_17605 [Fusarium verticillioides 7600];Gene=CEK26_001997;Ontology_term=phosphatidylinositol phospholipase C activity,lipid catabolic process,intracellular signal transduction;Ontology_id=GO:0004435,GO:0016042,GO:0035556;Enzyme_code=EC:3.1.4.11;Enzyme_name=phosphoinositide
Fv_160_13.g7534	321	ID=Fv_160_13.g7534;Description=hypothetical protein FAUST_11741 [Fusarium austroamericanum];Gene=FGLOB1_5513;Ontology_term=dioxygenase
Fv_160_13.g7535	290	ID=Fv_160_13.g7535;Description=hypothetical protein FVER53590_13783 [Fusarium verticillioides];Gene=FAUST_11740;Ontology_term=serine-type peptidase
Fv_160_13.g7536	281	ID=Fv_160_13.g7536;Description=hypothetical protein FVEG_13784 [Fusarium verticillioides 7600];Gene=FPOA_13850;Ontology_term=nucleic acid binding,serine-type peptidase activity,oxidoreductase activity,proteolysis;Ontology_id=GO:0003676,GO:0008236,GO:0016491,GO:0006508;Enzyme_code=EC:3.4,EC:1;Enzyme_name=Acti
Fv_160_13.g7537	586	ID=Fv_160_13.g7537;Description=hypothetical protein FVEG_17606 [Fusarium verticillioides 7600];Gene=FOIG_15451;Ontology_term=nucleus,DNA binding,zinc ion binding,DNA-binding transcription factor activity, RNA polymerase II-specific,regulation of transcription by RNA polymerase
Fv_160_13.g7538	753	ID=Fv_160_13.g7538;Description=hypothetical protein FVER53263_13786 [Fusarium verticillioides];Gene=FVER53263_13786;Ontology_term=oxidoreductase activity,FAD
Fv_160_13.g7539	507	ID=Fv_160_13.g7539;Description=hypothetical protein FVER53590_13787 [Fusarium verticillioides];Gene=FNYG_10550;Ontology_term=membrane,monooxygenase activity,iron ion binding,oxidoreductase activity, acting on paired donors, with incorporation or reduction of molecular oxygen,heme binding,transmembrane transporter activity,transmembrane
Fv_160_13.g7540	457	ID=Fv_160_13.g7540;Description=hypothetical protein FVEG_13788 [Fusarium verticillioides 7600];Gene=FVER53263_13789;Ontology_term=cellular anatomical entity,monooxygenase activity,iron ion binding,oxidoreductase activity, acting on paired donors, with incorporation or reduction of molecular oxygen,heme binding;Ontology_id=GO:0110165,GO:0004497,GO:0005506,GO:0016705,GO:0020037;Enzyme_code=EC:1.14;Enzyme_name=Actin
Fv_160_13.g7541	550	ID=Fv_160_13.g7541;Description=cytochrome P450 oxidoreductase [Fusarium verticillioides 7600];Gene=FPCIR_13549;Ontology_term=monooxygenase activity,iron ion binding,oxidoreductase activity, acting on paired donors, with incorporation or reduction of molecular oxygen,heme binding;Ontology_id=GO:0004497,GO:0005506,GO:0016705,GO:0020037;Enzyme_code=EC:1.14;Enzyme_name=Acting on paired
Fv_160_13.g7542	452	ID=Fv_160_13.g7542;Description=cytochrome-b5 reductase [Fusarium verticillioides 7600];Gene=BFJ69_g13541;Ontology_term=mitochondrial outer membrane,oxidoreductase activity,heme binding,metal ion
Fv_160_13.g7543	528	ID=Fv_160_13.g7543;Description=hypothetical protein FVEG_13792 [Fusarium verticillioides 7600];Gene=HZS61_005524;Ontology_term=nucleus,mitochondrial outer membrane,DNA-binding transcription factor activity, RNA polymerase II-specific,zinc ion binding,oxidoreductase activity,heme binding,regulation of transcription by RNA polymerase

Fv_160_13.g7544	603	ID=Fv_160_13.g7544;Description=hypothetical protein FVER14953_13793 [Fusarium verticillioides];Gene=FTJAE_10146;Ontology_term=oxidoreductase activity, acting on the CH-OH group of donors, NAD or NADP as acceptor,NAD+ binding,fatty acid metabolic
Fv_160_13.g7545	372	ID=Fv_160_13.g7545;Description=hypothetical protein FVEG_17607 [Fusarium verticillioides 7600];Gene=BFJ69_g13540;Ontology_term=membrane,transmembrane transporter activity,transmembrane
Fv_160_13.g7546	473	ID=Fv_160_13.g7546;Description=hypothetical protein FVER14953_13796 [Fusarium verticillioides];Gene=CEK26_001986;Ontology_term=membrane,transmembrane transporter activity,transmembrane
Fv_160_13.g7547	697	ID=Fv_160_13.g7547;Description=hypothetical protein FVEG_13797 [Fusarium verticillioides 7600];Gene=FTJAE_10148;Ontology_term=nucleus,DNA binding,zinc ion binding,membrane,DNA-binding transcription factor activity, RNA polymerase II-specific,regulation of transcription by RNA polymerase
Fv_160_13.g7548	740	ID=Fv_160_13.g7548;Description=hypothetical protein FNYG_10542 [Fusarium nygamai];Gene=FNYG_10542;Ontology_term=copper ion binding,primary amine oxidase activity,quinone binding,amine metabolic
Fv_160_13.g7549	315	ID=Fv_160_13.g7549;Description=NAD(P)-binding protein [Fusarium pseudoanthophilum]
Fv_160_13.g7550	355	ID=Fv_160_13.g7550;Description=alcohol sorbitol dehydrogenase [Fusarium tjaetaba];Gene=F25303_818;Ontology_term=zinc ion binding,oxidoreductase activity;Ontology_id=GO:0008270,GO:0016491;Enzyme_code=EC:1;Enzyme_name=Oxidoreductases
Fv_160_13.g7551	575	ID=Fv_160_13.g7551;Description=hypothetical protein FNYG_10539 [Fusarium nygamai];Gene=FPCIR_13558;Ontology_term=N,N-dimethylaniline monooxygenase activity,flavin adenine dinucleotide binding,NADP
Fv_160_13.g7552	422	ID=Fv_160_13.g7552;Description=hypothetical protein FNYG_10538 [Fusarium nygamai];Gene=P42577;Ontology_term=cytoplasm,membrane,ferroxidase activity,monooxygenase activity,ferric iron binding,FAD binding,iron ion transport,intracellular iron ion homeostasis,biosynthetic process;Ontology_id=GO:0005737,GO:0016020,GO:0004322,GO:0004497,GO:0008199,GO:0071949,GO:0006826,GO:0006879,GO:
Fv_160_13.g7553	317	ID=Fv_160_13.g7553;Description=hypothetical protein FVEG_13803 [Fusarium verticillioides 7600];Gene=FVER53263_13803;Ontology_term=extracellular region,neuropeptide hormone activity,hydrolase activity,glucose metabolic process,neuropeptide signaling
Fv_160_13.g7554	390	ID=Fv_160_13.g7554;Description=hypothetical protein FVER53263_13804 [Fusarium
Fv_160_13.g7555	360	ID=Fv_160_13.g7555;Description=hypothetical protein FVEG_13805 [Fusarium verticillioides 7600]
Fv_160_13.g7556	270	ID=Fv_160_13.g7556;Description=hypothetical protein FVEG_13806 [Fusarium verticillioides 7600];Gene=FTJAE_10159;Ontology_term=aminoacyltransferase activity,catalytic activity, acting on a
Fv_160_13.g7557	298	ID=Fv_160_13.g7557;Description=hypothetical protein FVER53263_13807 [Fusarium verticillioides];Gene=FVER53590_13807;Ontology_term=oxidoreductase activity,cellular biosynthetic
Fv_160_13.g7558	176	ID=Fv_160_13.g7558;Description=hypothetical protein FVEG_13808 [Fusarium verticillioides 7600];Gene=SDH1-1;Ontology_term=scytalone dehydratase activity,melanin metabolic

Fv_160_13.g7559	570	ID=Fv_160_13.g7559;Description=hypothetical protein FVER53263_13809 [Fusarium verticillioides];Gene=FUS6-3;Ontology_term=membrane,transmembrane transporter activity,transmembrane
Fv_160_13.g7560	432	ID=Fv_160_13.g7560;Description=hypothetical protein FVEG_17608 [Fusarium verticillioides
Fv_160_13.g7561	439	ID=Fv_160_13.g7561;Description=hypothetical protein FVER53590_13810 [Fusarium verticillioides]
Fv_160_13.g7562	394	ID=Fv_160_13.g7562;Description=hypothetical protein FVER53590_13811 [Fusarium verticillioides];Gene=FNAPI_4059;Ontology_term=cellular anatomical entity,serine-type endopeptidase
Fv_160_13.g7563	290	ID=Fv_160_13.g7563;Description=hypothetical protein FVER53590_13812 [Fusarium verticillioides];Gene=FNAPI_4058;Ontology_term=S-adenosylmethionine-dependent methyltransferase
Fv_160_13.g7564	277	ID=Fv_160_13.g7564;Description=hypothetical protein FVER53590_13813 [Fusarium verticillioides];Gene=FCULG_00011159;Ontology_term=hydrolase
Fv_160_13.g7565	110	ID=Fv_160_13.g7565;Description=hypothetical protein FVEG_17610 [Fusarium verticillioides 7600]
Fv_160_13.g7566	370	ID=Fv_160_13.g7566;Description=hypothetical protein FVER14953_13815 [Fusarium verticillioides];Gene=462
Fv_160_13.g7567	584	ID=Fv_160_13.g7567;Description=hypothetical protein FVEG_17611 [Fusarium verticillioides 7600];Gene=BFJ72_g13460;Ontology_term=membrane,transmembrane transporter activity,transmembrane
Fv_160_13.g7568	892	ID=Fv_160_13.g7568;Description=hypothetical protein FVER14953_13817 [Fusarium verticillioides];Gene=FVER53590_13817;Ontology_term=exo-1,4-beta-D-glucosaminidase activity,carbohydrate metabolic
Fv_160_13.g7569	171	ID=Fv_160_13.g7569;Description=hypothetical protein FVER14953_13818 [Fusarium verticillioides];Gene=FGADI_10249;Ontology_term=nucleus,DNA binding,zinc ion binding,hydrolase activity, acting on carbon-nitrogen (but not peptide) bonds,DNA-templated transcription;Ontology_id=GO:0005634,GO:0003677,GO:0008270,GO:0016810,GO:0006351;Enzyme_code=EC:3.5;Enzyme_name=A
Fv_160_13.g7570	272	ID=Fv_160_13.g7570;Description=hypothetical protein FVER14953_13819 [Fusarium verticillioides];Gene=FGLOB1_6634;Ontology_term=hydrolase activity, acting on carbon-nitrogen (but not peptide) bonds,carbohydrate metabolic process;Ontology_id=GO:0016810,GO:0005975;Enzyme_code=EC:3.5;Enzyme_name=Acting on
Fv_160_13.g7571	459	ID=Fv_160_13.g7571;Description=hypothetical protein FVER14953_13820 [Fusarium verticillioides]
Fv_160_13.g7572	348	ID=Fv_160_13.g7572;Description=hypothetical protein FVER53590_13821 [Fusarium verticillioides];Gene=FOIG_15386;Ontology_term=cytoplasm,phosphoglycerate kinase activity,ATP binding,hydrolase activity,glycolytic process,phosphorylation;Ontology_id=GO:0005737,GO:0004618,GO:0005524,GO:0016787,GO:0006096,GO:0016310;Enzyme_code
Fv_160_13.g7573	509	ID=Fv_160_13.g7573;Description=hypothetical protein FVER14953_13822 [Fusarium verticillioides];Gene=FNYG_10235;Ontology_term=nucleus,membrane,phosphorelay sensor kinase activity,DNA-binding transcription factor activity, RNA polymerase II-specific,zinc ion binding,transmembrane transporter activity,feruloyl esterase activity,phosphorelay signal transduction system,regulation of transcription by RNA polymerase II,phosphorylation,transmembrane transport;Ontology_id=GO:0005634,GO:0016020,GO:0000155,GO:0000981,GO:0008270,GO:0022857,GO:0030600,GO:0000160,GO

Fv_160_13.g7574	592	ID=Fv_160_13.g7574;Description=hypothetical protein FVEG_17614 [Fusarium verticillioides 7600];Gene=FOIG_15388;Ontology_term=nucleus,membrane,phosphorelay sensor kinase activity,DNA-binding transcription factor activity, RNA polymerase II-specific,zinc ion binding,transmembrane transporter activity,phosphorelay signal transduction system,regulation of transcription by RNA polymerase II,phosphorylation,transmembrane transport;Ontology_id=GO:0005634,GO:0016020,GO:0000155,GO:0000981,GO:0008270,GO:0022857,GO:0000160,GO:0006357,GO
Fv_160_13.g7575	589	ID=Fv_160_13.g7575;Description=hypothetical protein FVER53590_13823 [Fusarium verticillioides];Gene=FVEG_13823;Ontology_term=membrane,DNA binding,site-specific DNA-methyltransferase (cytosine-N4-specific) activity,transmembrane transporter activity,feruloyl esterase activity,metal ion binding,transmembrane transport,N-4 methylation of
Fv_160_13.g7576	503	ID=Fv_160_13.g7576;Description=hypothetical protein FVEG_13824 [Fusarium verticillioides 7600];Gene=FNAPI_4048;Ontology_term=transferase
Fv_160_13.g7577	354	ID=Fv_160_13.g7577;Description=hypothetical protein FVEG_17615 [Fusarium verticillioides 7600];Gene=FOTG_10732;Ontology_term=extracellular region,proteolysis;Ontology_id=GO:0005576,GO:0006508
Fv_160_13.g7578	509	ID=Fv_160_13.g7578;Description=hypothetical protein FVEG_17616 [Fusarium verticillioides 7600];Gene=BFJ71_g3798;Ontology_term=nucleus,DNA-binding transcription factor activity, RNA polymerase II-specific,zinc ion
Fv_160_13.g7579	308	ID=Fv_160_13.g7579;Description=hypothetical protein FVER14953_13825 [Fusarium
Fv_160_13.g7580	340	ID=Fv_160_13.g7580;Description=hypothetical protein FVER14953_13826 [Fusarium verticillioides]
Fv_160_13.g7581	421	ID=Fv_160_13.g7581;Description=hypothetical protein FVER14953_13827 [Fusarium verticillioides];Gene=FVER53590_13827;Ontology_term=nitric oxide dioxygenase activity,oxygen binding,heme
Fv_160_13.g7582	354	ID=Fv_160_13.g7582;Description=formaldehyde dehydrogenase [Fusarium napiforme];Gene=C2S_14085;Ontology_term=membrane,zinc ion binding,oxidoreductase
Fv_160_13.g7583	532	ID=Fv_160_13.g7583;Description=Quinic acid utilization activator [Fusarium oxysporum f. sp. conglutinans];Gene=qutA-0;Ontology_term=nucleus,DNA-binding transcription factor activity, RNA polymerase II-specific,zinc ion binding,regulation of
Fv_160_13.g7584	585	ID=Fv_160_13.g7584;Description=myosin-crossreactive antigen [Fusarium verticillioides 7600];Gene=FRV6_14259;Ontology_term=oleate hydratase activity,FAD binding,fatty acid metabolic
Fv_160_13.g7585	405	ID=Fv_160_13.g7585;Description=hypothetical protein FVEG_13831 [Fusarium verticillioides 7600];Gene=C2S_14081;Ontology_term=membrane,transmembrane signaling receptor activity,cell surface receptor signaling
Fv_160_13.g7586	381	ID=Fv_160_13.g7586;Description=hypothetical protein FVER14953_13832 [Fusarium verticillioides]
Fv_160_13.g7587	312	ID=Fv_160_13.g7587;Description=hypothetical protein FVER53590_13833 [Fusarium verticillioides]
Fv_160_13.g7588	305	ID=Fv_160_13.g7588;Description=hypothetical protein J7337_012149 [Fusarium musae]

Fv_160_13.g7589	550	ID=Fv_160_13.g7589;Description=hypothetical protein FVEG_13835 [Fusarium verticillioides 7600];Gene=FMAN_13081;Ontology_term=membrane,monooxygenase activity,iron ion binding,methyltransferase activity,oxidoreductase activity, acting on paired donors, with incorporation or reduction of molecular oxygen,heme binding,methylation;Ontology_id=GO:0016020,GO:0004497,GO:0005506,GO:0008168,GO:0016705,GO:0020037,GO:0032259;Enzy
Fv_160_13.g7590	483	ID=Fv_160_13.g7590;Description=hypothetical protein FVER14953_13836 [Fusarium verticillioides];Gene=FPANT_13720;Ontology_term=nucleosome,nucleus,DNA binding,oxidoreductase activity, acting on the aldehyde or oxo group of donors, NAD or NADP as acceptor,structural constituent of chromatin,protein heterodimerization
Fv_160_13.g7591	372	ID=Fv_160_13.g7591;Description=long-chain alcohol dehydrogenase [Fusarium denticulatum];Gene=FMUND_11832;Ontology_term=nucleus,DNA binding,zinc ion binding,oxidoreductase activity,DNA-templated transcription;Ontology_id=GO:0005634,GO:0003677,GO:0008270,GO:0016491,GO:0006351;Enzyme_code=EC:1;Enzyme_name=Oxi
Fv_160_13.g7592	790	ID=Fv_160_13.g7592;Description=hypothetical protein FVEG_13838 [Fusarium verticillioides 7600];Gene=FNYG_10220;Ontology_term=nucleus,DNA binding,zinc ion binding,oxidoreductase activity,membrane,DNA-binding transcription factor activity, RNA polymerase II-specific,regulation of transcription by RNA polymerase
Fv_160_14.g7593	365	ID=Fv_160_14.g7593;Description=C-3 sterol dehydrogenase 3-beta-hydroxysteroid dehydrogenase and related dehydrogenase
Fv_160_14.g7594	282	ID=Fv_160_14.g7594;Description=hypothetical protein FPCIR_1084 [Fusarium pseudocircinatum];Gene=FNAPI_10757;Ontology_term=incipient cellular bud site,nucleus,cellular bud tip,F-actin capping protein complex,actin cortical patch,medial cortex,mating projection tip,actin body,chromatin binding,actin filament binding,filamentous growth,actin cortical patch organization,barbed-end actin filament capping,mitotic actomyosin contractile ring contraction,mitotic actomyosin contractile ring
Fv_160_14.g7595	426	ID=Fv_160_14.g7595;Description=hypothetical protein FVEG_01354 [Fusarium verticillioides
Fv_160_14.g7596	653	ID=Fv_160_14.g7596;Description=autophagy-like protein 22 [Fusarium verticillioides 7600];Gene=FGLOB1_11704;Ontology_term=vacuolar membrane,autophagy,amino acid transmembrane export from
Fv_160_14.g7597	165	ID=Fv_160_14.g7597;Description=hypothetical protein FVER53263_01352 [Fusarium verticillioides]
Fv_160_14.g7598	303	ID=Fv_160_14.g7598;Description=hypothetical protein FVER53590_01351 [Fusarium verticillioides];Gene=TkR99D;Ontology_term=plasma membrane,tachykinin receptor activity,tachykinin receptor signaling pathway,neuropeptide signaling pathway,olfactory behavior,negative regulation of synaptic transmission,detection of chemical stimulus involved in sensory perception of smell,positive regulation of sensory perception of
Fv_160_14.g7599	316	ID=Fv_160_14.g7599;Description=hypothetical protein FVEG_01350 [Fusarium verticillioides 7600];Gene=FOIG_00639;Ontology_term=catalytic activity,metal ion binding;Ontology_id=GO:0003824,GO:0046872
Fv_160_14.g7600	108	ID=Fv_160_14.g7600;Description=hypothetical protein FVEG_14818 [Fusarium verticillioides 7600]
Fv_160_14.g7601	303	ID=Fv_160_14.g7601;Description=2,4-dihydroxyhept-2-ene-1,7-dioic acid aldolase [Fusarium oxysporum f. sp. lycopersici 4287];Gene=FSARC_12524;Ontology_term=DNA-directed RNA polymerase complex,nucleus,nucleic acid binding,catalytic

Fv_160_14.g7602	172	ID=Fv_160_14.g7602;Description=DNA-directed RNA polymerase II subunit RPB7 [Fusarium oxysporum f. sp. lycopersici 4287];Gene=FOMG_00826;Ontology_term=P-body,extracellular region,RNA polymerase II, core complex,RPB4-RPB7 complex,single-stranded DNA binding,single-stranded RNA binding,RNA-dependent RNA polymerase activity,translation initiation factor binding,nuclear-transcribed mRNA catabolic process, exonucleolytic,RNA-templated transcription,transcription initiation at RNA polymerase II promoter,transcription elongation by RNA polymerase II,regulation of septum digestion after cytokinesis,antibacterial humoral response,innate immune response,positive regulation of translational initiation,defense response to Gram-positive bacterium,positive regulation of nuclear-transcribed mRNA poly(A) tail
Fv_160_14.g7603	188	ID=Fv_160_14.g7603;Description=hypothetical protein FVEG_01346 [Fusarium verticillioides
Fv_160_14.g7604	136	ID=Fv_160_14.g7604;Description=hypothetical protein FVEG_14815 [Fusarium verticillioides 7600]
Fv_160_14.g7605	385	ID=Fv_160_14.g7605;Description=hypothetical protein FVER53263_01345 [Fusarium verticillioides]
Fv_160_14.g7606	1175	ID=Fv_160_14.g7606;Description=carbamoyl-phosphate synthase arginine-specific large chain [Fusarium verticillioides 7600];Gene=FPCIR_13729;Ontology_term=carbamoyl-phosphate synthase complex,carbamoyl-phosphate synthase (glutamine-hydrolyzing) activity,ATP binding,metal ion binding,pyrimidine nucleotide biosynthetic process,arginine biosynthetic process;Ontology_id=GO:0005951,GO:0004088,GO:0005524,GO:0046872,GO:0006221,GO:0006526;Enzyme_code=EC:6.3.5.5;Enzy
Fv_160_14.g7607	646	ID=Fv_160_14.g7607;Description=nuclear protein localization protein 4 [Fusarium verticillioides 7600];Gene=FPCIR_13728;Ontology_term=endoplasmic reticulum membrane,nuclear membrane,perinuclear region of cytoplasm,ubiquitin-dependent protein catabolic process,protein transport,mRNA
Fv_160_14.g7608	222	ID=Fv_160_14.g7608;Description=hypothetical protein FVEG_01342 [Fusarium verticillioides 7600];Gene=FPCIR_13727;Ontology_term=Sec62/Sec63 complex,post-translational protein targeting to membrane,
Fv_160_14.g7609	147	ID=Fv_160_14.g7609;Description=hypothetical protein FVEG_01341 [Fusarium verticillioides 7600]
Fv_160_14.g7610	701	ID=Fv_160_14.g7610;Description=hypothetical protein FVER53263_01340 [Fusarium verticillioides];Gene=FOXB_11671;Ontology_term=nuclear origin of replication recognition complex,DNA binding,DNA
Fv_160_14.g7611	576	ID=Fv_160_14.g7611;Description=hypothetical protein FVER53590_01339 [Fusarium verticillioides];Gene=FVEG_01339;Ontology_term=mitochondrion,anterograde axonal
Fv_160_14.g7612	2385	ID=Fv_160_14.g7612;Description=pre-mRNA-splicing factor brr2 [Fusarium tjaetaba];Gene=FNAPI_6379;Ontology_term=nucleic acid binding,helicase activity,ATP binding,ATP hydrolysis activity,mRNA processing;Ontology_id=GO:0003676,GO:0004386,GO:0005524,GO:0016887,GO:0006397;Enzyme_code=EC:3.6.1.15;Enzyme_name
Fv_160_14.g7613	516	ID=Fv_160_14.g7613;Description=hypothetical protein FVER14953_01337 [Fusarium verticillioides]
Fv_160_14.g7614	382	ID=Fv_160_14.g7614;Description=hypothetical protein FVER14953_01336 [Fusarium verticillioides];Gene=FPANT_2628;Ontology_term=Elg1 RFC-like complex,DNA binding,chromatin binding,ATP binding,ATP hydrolysis activity,DNA strand elongation involved in DNA replication,mitotic DNA replication checkpoint signaling,UV-damage excision repair,DNA clamp

Fv_160_14.g7615	447	ID=Fv_160_14.g7615;Description=hypothetical protein FVER53590_01335 [Fusarium verticillioides];Gene=Cp18;Ontology_term=extracellular region,cytoplasm,egg chorion,arylsulfatase activity,structural constituent of egg chorion,metal ion
Fv_160_14.g7616	264	ID=Fv_160_14.g7616;Description=hypothetical protein FVEG_01334 [Fusarium verticillioides
Fv_160_14.g7617	168	ID=Fv_160_14.g7617;Description=hypothetical protein FVEG_01333 [Fusarium verticillioides 7600]
Fv_160_14.g7618	531	ID=Fv_160_14.g7618;Description=hypothetical protein FVEG_01332 [Fusarium verticillioides
Fv_160_14.g7619	1157	ID=Fv_160_14.g7619;Description=hypothetical protein FVEG_01331 [Fusarium verticillioides 7600];Gene=FMAN_00721;Ontology_term=GTPase activator activity,metal ion binding,signal
Fv_160_14.g7620	375	ID=Fv_160_14.g7620;Description=Drug resistance protein [Fusarium oxysporum f. sp. albedinis];Gene=FTJAE_12852;Ontology_term=PRC1 complex,DNA binding,3-beta-hydroxy-delta5-steroid dehydrogenase activity,zinc ion binding,promoter-specific chromatin binding,negative regulation of transcription by RNA polymerase II,steroid biosynthetic process,heterochromatin formation;Ontology_id=GO:0035102,GO:0003677,GO:0003854,GO:0008270,GO:1990841,GO:0000122,GO:0006694,GO:0031507;En
Fv_160_14.g7621	451	ID=Fv_160_14.g7621;Description=hypothetical protein FVEG_01329 [Fusarium verticillioides 7600]
Fv_160_14.g7622	309	ID=Fv_160_14.g7622;Description=hypothetical protein FVEG_01328 [Fusarium verticillioides 7600];Gene=FVEG_01328;Ontology_term=nucleus,cytoplasm,cytoskeleton,DNA binding,ATP binding,hydrolase
Fv_160_14.g7623	261	ID=Fv_160_14.g7623;Description=20S proteasome subunit beta 7 [Fusarium verticillioides 7600];Gene=FVEG_01327;Ontology_term=nucleus,proteasome core complex, beta-subunit complex,proteasome storage granule,proteasomal ubiquitin-independent protein catabolic process,proteasome-mediated ubiquitin-dependent protein catabolic
Fv_160_14.g7624	699	ID=Fv_160_14.g7624;Description=glucosamine-fructose-6-phosphate aminotransferase [Fusarium verticillioides 7600];Gene=FOMG_00848;Ontology_term=glutamine-fructose-6-phosphate transaminase (isomerizing) activity,carbohydrate derivative binding,UDP-N-acetylglucosamine biosynthetic process,glutamine metabolic
Fv_160_14.g7625	391	ID=Fv_160_14.g7625;Description=26S protease subunit rpt4 [Fusarium oxysporum f. sp. lycopersici 4287];Gene=FOMG_00849;Ontology_term=nucleus,proteasome regulatory particle, base subcomplex,ATP binding,peptidase activity,ATP hydrolysis activity,protein domain specific binding,ubiquitin protein ligase binding,nucleotide-excision repair,ubiquitin-dependent ERAD pathway,positive regulation of transcription elongation by RNA polymerase II,positive regulation of RNA polymerase II transcription preinitiation complex assembly,proteasome regulatory particle assembly;Ontology_id=GO:0005634,GO:0008540,GO:0005524,GO:0008233,GO:0016887,GO:0019904,GO:0031625,GO:0006289,GO
Fv_160_14.g7626	192	ID=Fv_160_14.g7626;Description=hypothetical protein FVEG_01324 [Fusarium verticillioides 7600];Gene=FANTH_138;Ontology_term=exon-exon junction complex disassembly;Ontology_id=GO:1903259

Fv_160_14.g7627	1014	ID=Fv_160_14.g7627;Description=Ca2+-transporting ATPase [Fusarium pseudoanthophilum];Gene=FPANT_13411;Ontology_term=mitochondrial respiratory chain complex III,P-type calcium transporter activity,ATP binding,ATP hydrolysis activity,mitochondrial electron transport, ubiquinol to cytochrome c,calcium ion transmembrane transport;Ontology_id=GO:0005750,GO:0005388,GO:0005524,GO:0016887,GO:0006122,GO:0070588;Enzyme_code=EC:7.2.2.10,EC
Fv_160_14.g7628	2259	ID=Fv_160_14.g7628;Description=hypothetical protein FVER53590_01321 [Fusarium verticillioides];Gene=FTJAE_13258;Ontology_term=mitochondrial respiratory chain complex III,P-type calcium transporter activity,ATP binding,zinc ion binding,ligase activity,ATP hydrolysis activity,ubiquitin protein ligase activity,mitochondrial electron transport, ubiquinol to cytochrome c,protein ubiquitination,calcium ion transmembrane transport,ubiquitin-dependent protein catabolic process via the N-end rule
Fv_160_14.g7629	521	ID=Fv_160_14.g7629;Description=hypothetical protein FVEG_01320 [Fusarium verticillioides 7600]
Fv_160_14.g7630	333	ID=Fv_160_14.g7630;Description=hypothetical protein FVEG_01319 [Fusarium verticillioides
Fv_160_14.g7631	233	ID=Fv_160_14.g7631;Description=hypothetical protein FVER14953_01318 [Fusarium
Fv_160_14.g7632	458	ID=Fv_160_14.g7632;Description=hypothetical protein FVEG_01317 [Fusarium verticillioides 7600];Gene=F25303_2322;Ontology_term=collagen trimer,mitochondrial inner membrane;Ontology_id=GO:0005581,GO:0005743
Fv_160_14.g7633	641	ID=Fv_160_14.g7633;Description=AGC NDR NDR kinase [Fusarium tjaetaba];Gene=FOXB_11693;Ontology_term=incipient cellular bud site,nucleus,cellular bud tip,cellular bud neck,cell division site,growing cell tip,mating projection tip,cell cortex of cell tip,serine/threonine protein kinase complex,protein serine/threonine kinase activity,ATP binding,identical protein binding,septum digestion after cytokinesis,budding cell apical bud growth,phosphorylation,cortical actin cytoskeleton organization,establishment or maintenance of actin cytoskeleton polarity,regulation of fungal-type cell wall biogenesis,positive regulation of exocytosis,regulation of protein secretion,regulation of fungal-type cell wall organization,RAM/MOR signaling pathway,cellular response to salt stress,maintenance of protein location in cell cortex of cell tip,positive regulation of establishment or maintenance of bipolar cell polarity regulating cell shape;Ontology_id=GO:0000131,GO:0005634,GO:0005934,GO:0005935,GO:0032153,GO:0035838,GO:0043332,GO:0051285,GO:19
Fv_160_14.g7634	240	ID=Fv_160_14.g7634;Description=hypothetical protein FVEG_01315 [Fusarium verticillioides 7600];Gene=BFJ68_g9750;Ontology_term=spliceosomal complex,U2 snRNP,nucleic acid binding,zinc ion binding,mRNA
Fv_160_14.g7635	436	ID=Fv_160_14.g7635;Description=glutamate-1-semialdehyde 2,1-aminomutase [Fusarium verticillioides 7600];Gene=FOVG_01319;Ontology_term=transaminase activity,pyridoxal phosphate
Fv_160_14.g7636	544	ID=Fv_160_14.g7636;Description=hypothetical protein FVEG_01313 [Fusarium verticillioides 7600];Gene=FANTH_148;Ontology_term=nucleolus,preribosome, large subunit precursor,RNA binding,GTPase activity,GTP binding,ribosomal large subunit export from nucleus,maturation of LSU-rRNA from tricistronic rRNA transcript (SSU-rRNA, 5.8S rRNA, LSU-

Fv_160_14.g7637	983	ID=Fv_160_14.g7637;Description=hypothetical protein FVEG_01312 [Fusarium verticillioides 7600];Gene=FANTH_149;Ontology_term=exosome (RNase complex),nucleus,RNA binding,exonuclease activity,RNA nuclease activity,RNA metabolic
Fv_160_14.g7638	633	ID=Fv_160_14.g7638;Description=hypothetical protein FVEG_01311 [Fusarium verticillioides 7600]
Fv_160_14.g7639	308	ID=Fv_160_14.g7639;Description=hypothetical protein J7337_000710 [Fusarium musae];Gene=FOTG_05727;Ontology_term=protein heterodimerization activity;Ontology_id=GO:0046982
Fv_160_14.g7640	265	ID=Fv_160_14.g7640;Description=hypothetical protein FVER53590_01309 [Fusarium
Fv_160_14.g7641	636	ID=Fv_160_14.g7641;Description=hypothetical protein FVER14953_01307 [Fusarium verticillioides]
Fv_160_14.g7642	203	ID=Fv_160_14.g7642;Description=hypothetical protein FVEG_01306 [Fusarium verticillioides 7600]
Fv_160_14.g7643	541	ID=Fv_160_14.g7643;Description=hypothetical protein FVER14953_01305 [Fusarium verticillioides]
Fv_160_14.g7644	422	ID=Fv_160_14.g7644;Description=hypothetical protein FVER14953_01304 [Fusarium verticillioides];Gene=F52700_10070;Ontology_term=lyase activity,pyridoxal phosphate
Fv_160_14.g7645	491	ID=Fv_160_14.g7645;Description=hypothetical protein FVEG_01303 [Fusarium verticillioides 7600];Gene=FVEG_01303;Ontology_term=nucleus,DNA-binding transcription factor activity, RNA polymerase II-specific,zinc ion
Fv_160_14.g7646	318	ID=Fv_160_14.g7646;Description=hypothetical protein FVER14953_01302 [Fusarium verticillioides];Gene=F25303_2337;Ontology_term=voltage-gated sodium channel complex,nucleolus,aminoacyl-tRNA ligase activity,voltage-gated sodium channel activity,rRNA binding,voltage-gated monoatomic cation channel activity,ribosomal large subunit assembly,maturation of LSU-rRNA,sensory perception of smell,neuronal action potential,regulation of monoatomic ion transmembrane transport,sodium ion transmembrane transport,olfactory behavior,membrane depolarization during action potential;Ontology_id=GO:0001518,GO:0005730,GO:0004812,GO:0005248,GO:0019843,GO:0022843,GO:0000027,GO:0000470,GO
Fv_160_14.g7647	638	ID=Fv_160_14.g7647;Description=hypothetical protein FVEG_01301 [Fusarium verticillioides 7600];Gene=FNAPI_12679;Ontology_term=peroxisome,protein transport;Ontology_id=GO:0005777,GO:0015031
Fv_160_14.g7648	492	ID=Fv_160_14.g7648;Description=hypothetical protein FVEG_01300 [Fusarium verticillioides 7600];Gene=Forpi1262_v001279;Ontology_term=protein dimerization activity;Ontology_id=GO:0046983
Fv_160_14.g7649	453	ID=Fv_160_14.g7649;Description=hypothetical protein FVER14953_01299 [Fusarium verticillioides];Gene=FMAN_00753;Ontology_term=nucleus,DNA-binding transcription factor activity, RNA polymerase II-
Fv_160_14.g7650	375	ID=Fv_160_14.g7650;Description=hypothetical protein FVEG_01298 [Fusarium verticillioides 7600];Gene=Forpe1208_v001245;Ontology_term=nucleotide binding,oxidoreductase activity, acting on
Fv_160_14.g7651	96	ID=Fv_160_14.g7651;Description=hypothetical protein FVEG_14803 [Fusarium verticillioides 7600]
Fv_160_14.g7652	311	ID=Fv_160_14.g7652;Description=hypothetical protein FVEG_01297 [Fusarium verticillioides 7600]
Fv_160_14.g7653	282	ID=Fv_160_14.g7653;Description=hypothetical protein FVEG_01296 [Fusarium verticillioides 7600];Gene=FTJAE_13525;Ontology_term=membrane,oxidoreductase

Fv_160_14.g7654	91	ID=Fv_160_14.g7654;Description=hypothetical protein FVEG_01295 [Fusarium verticillioides 7600];Gene=FPHYL_1911;Ontology_term=oxidoreductase
Fv_160_14.g7655	418	ID=Fv_160_14.g7655;Description=hypothetical protein FVER14953_01294 [Fusarium verticillioides];Gene=FTJAE_13523;Ontology_term=endoplasmic reticulum,adenyl-nucleotide exchange factor activity,ATP binding,methyltransferase activity,metal ion binding,ATP-dependent protein folding chaperone,protein folding,methylation;Ontology_id=GO:0005783,GO:0000774,GO:0005524,GO:0008168,GO:0046872,GO:0140662,GO:0006457,GO:0
Fv_160_14.g7656	530	ID=Fv_160_14.g7656;Description=SET and MYND protein [Fusarium verticillioides 7600];Gene=FTJAE_13522;Ontology_term=methyltransferase activity,metal ion
Fv_160_14.g7657	717	ID=Fv_160_14.g7657;Description=hypothetical protein FVEG_01292 [Fusarium verticillioides 7600];Gene=FVER53590_01292;Ontology_term=nucleic acid binding,exonuclease activity,RNA metabolic
Fv_160_14.g7658	1142	ID=Fv_160_14.g7658;Description=hypothetical protein FVER14953_01291 [Fusarium
Fv_160_14.g7659	967	ID=Fv_160_14.g7659;Description=hypothetical protein FVER53263_01290 [Fusarium verticillioides];Gene=FCIRC_1795;Ontology_term=mismatch repair complex,ATP binding,ATP hydrolysis activity,mismatched DNA binding,ATP-dependent DNA damage sensor activity,mismatch repair,meiosis I cell cycle process;Ontology_id=GO:0032300,GO:0005524,GO:0016887,GO:0030983,GO:0140664,GO:0006298,GO:0061982;Enzyme_code=EC
Fv_160_14.g7660	196	ID=Fv_160_14.g7660;Description=6,7-dimethyl-8-ribityllumazine synthase [Fusarium verticillioides 7600];Gene=FGADI_4276;Ontology_term=riboflavin synthase complex,6,7-dimethyl-8-ribityllumazine synthase activity,riboflavin biosynthetic process;Ontology_id=GO:0009349,GO:0000906,GO:0009231;Enzyme_code=EC:2.5.1.78;Enzyme_name=6,7-dimethyl-8-
Fv_160_14.g7661	909	ID=Fv_160_14.g7661;Description=hypothetical protein FVER14953_01288 [Fusarium verticillioides]
Fv_160_14.g7662	588	ID=Fv_160_14.g7662;Description=hypothetical protein FVER14953_01287 [Fusarium verticillioides];Gene=FOC1_g10015516;Ontology_term=membrane,monoatomic cation transmembrane transporter activity,intracellular monoatomic cation homeostasis,monoatomic cation transmembrane transport,inorganic ion
Fv_160_14.g7663	1938	ID=Fv_160_14.g7663;Description=hypothetical protein FVER14953_01286 [Fusarium verticillioides];Gene=FOC1_g10015517;Ontology_term=plasma membrane,1-phosphatidylinositol 4-kinase activity,autophagy of mitochondrion,phosphorylation,cortical actin cytoskeleton organization,phosphatidylinositol phosphate biosynthetic process,phosphatidylinositol-mediated signaling,regulation of fungal-type cell wall organization,autophagosome-lysosome fusion,microlipophagy;Ontology_id=GO:0005886,GO:0004430,GO:0000422,GO:0016310,GO:0030866,GO:0046854,GO:0048015,GO
Fv_160_14.g7664	264	ID=Fv_160_14.g7664;Description=20S proteasome subunit beta 6 [Fusarium oxysporum f. sp. lycopersici 4287];Gene=MDCFG202_LOCUS20932;Ontology_term=proteasome core complex,proteolysis involved in protein catabolic
Fv_160_14.g7665	620	ID=Fv_160_14.g7665;Description=hypothetical protein FVEG_01284 [Fusarium verticillioides
Fv_160_14.g7666	214	ID=Fv_160_14.g7666;Description=hypothetical protein FVER14953_01283 [Fusarium verticillioides]

Fv_160_14.g7667	348	ID=Fv_160_14.g7667;Description=DNA repair protein rhp51 [Fusarium verticillioides 7600];Gene=FOMG_00903;Ontology_term=chromosome, centromeric region,nucleus,site of double-strand break,DNA strand exchange activity,double-stranded DNA binding,single-stranded DNA binding,ATP binding,ATP hydrolysis activity,identical protein binding,ATP-dependent DNA damage sensor activity,Swi5-Sfr1 complex binding,meiotic strand invasion,telomere maintenance,DNA recombinase assembly,mitotic recombination,mating type switching,interstrand cross-link repair,protein homooligomerization,stalled replication fork localization to nuclear periphery,mitotic recombination-dependent replication fork processing,double-strand break repair involved in meiotic recombination;Ontology_id=GO:0000775,GO:0005634,GO:0035861,GO:0000150,GO:0003690,GO:0003697,GO:0005524,GO:00168
Fv_160_14.g7668	1161	ID=Fv_160_14.g7668;Description=hypothetical protein FVEG_01281 [Fusarium verticillioides 7600];Gene=FVER53590_01281;Ontology_term=nuclear pore,nucleocytoplasmic transport,macromolecule localization,macromolecule metabolic process,organic substance transport,nitrogen compound
Fv_160_14.g7669	321	ID=Fv_160_14.g7669;Description=hypothetical protein FOXG_00236 [Fusarium oxysporum f. sp. lycopersici 4287];Gene=FOMG_00905;Ontology_term=endosome,cytosol,retromer, cargo-selective complex,protein transporter activity,intracellular protein transport,retrograde transport, endosome to Golgi,protein retention in Golgi
Fv_160_14.g7670	211	ID=Fv_160_14.g7670;Description=60S ribosomal protein L9B [Fusarium musae];Gene=FOMG_00906;Ontology_term=ribosome,ribonucleoprotein complex,structural constituent of ribosome,rRNA
Fv_160_14.g7671	397	ID=Fv_160_14.g7671;Description=hypothetical protein FVEG_01278 [Fusarium verticillioides 7600];Gene=FocTR4_00001434;Ontology_term=nucleosome,nucleus,membrane,DNA binding,structural constituent of
Fv_160_14.g7672	479	ID=Fv_160_14.g7672;Description=V-type H+-transporting ATPase 54 kDa subunit [Fusarium verticillioides 7600];Gene=FPRO05_12667;Ontology_term=vacuolar proton-transporting V-type ATPase, V1 domain,proton-transporting ATPase activity, rotational mechanism,proton transmembrane
Fv_160_14.g7673	441	ID=Fv_160_14.g7673;Description=hypothetical protein FVER53263_01276 [Fusarium verticillioides];Gene=FPANT_357;Ontology_term=mitochondrion,aminomethyltransferase activity,methyltransferase activity,transaminase activity,glycine catabolic process,methylation;Ontology_id=GO:0005739,GO:0004047,GO:0008168,GO:0008483,GO:0006546,GO:0032259;Enzyme_code=EC:
Fv_160_14.g7674	711	ID=Fv_160_14.g7674;Description=hypothetical protein FVER14953_01275 [Fusarium verticillioides];Gene=FPRO_00804;Ontology_term=vacuolar membrane,endopolyphosphatase
Fv_160_14.g7675	788	ID=Fv_160_14.g7675;Description=hypothetical protein FVER14953_01274 [Fusarium
Fv_160_14.g7676	539	ID=Fv_160_14.g7676;Description=hypothetical protein FVEG_01273 [Fusarium verticillioides 7600];Gene=FFUJ_01336;Ontology_term=nucleus,DNA-binding transcription factor activity, RNA polymerase II-specific,zinc ion
Fv_160_14.g7677	567	ID=Fv_160_14.g7677;Description=hypothetical protein FVER14953_01272 [Fusarium verticillioides]
Fv_160_14.g7678	552	ID=Fv_160_14.g7678;Description=hypothetical protein FocTR4_00001426 [Fusarium oxysporum f. sp. cubense];Gene=FOMG_00916;Ontology_term=carbon-sulfur lyase activity,metal ion

Fv_160_14.g7679	1215	ID=Fv_160_14.g7679;Description=hypothetical protein FVEG_01269 [Fusarium verticillioides 7600]
Fv_160_14.g7680	802	ID=Fv_160_14.g7680;Description=hypothetical protein FVEG_01268 [Fusarium verticillioides 7600]
Fv_160_14.g7681	576	ID=Fv_160_14.g7681;Description=delta-1-pyrroline-5-carboxylate dehydrogenase, mitochondrial [Fusarium verticillioides 7600];Gene=FOPG_03738;Ontology_term=1-pyrroline-5-carboxylate dehydrogenase activity,proline catabolic process to glutamate;Ontology_id=GO:0003842,GO:0010133;Enzyme_code=EC:1.2.1.88;Enzyme_name=L-glutamate gamma-semialdehyde
Fv_160_14.g7682	407	ID=Fv_160_14.g7682;Description=hypothetical protein FVEG_01266 [Fusarium verticillioides 7600];Gene=FMUND_10416;Ontology_term=protein dimerization activity;Ontology_id=GO:0046983
Fv_160_14.g7683	331	ID=Fv_160_14.g7683;Description=hypothetical protein FVEG_01265 [Fusarium verticillioides 7600];Gene=FNYG_13431;Ontology_term=fungal-type vacuole membrane,endoplasmic reticulum membrane,intracellular
Fv_160_14.g7684	884	ID=Fv_160_14.g7684;Description=hypothetical protein FVER14953_01264 [Fusarium verticillioides];Gene=FMAN_00787;Ontology_term=protein kinase activity,ATP binding,organelle
Fv_160_14.g7685	206	ID=Fv_160_14.g7685;Description=hypothetical protein FVER14953_01263 [Fusarium verticillioides];Gene=FSUBG_12971;Ontology_term=cytoplasm,ribosome;Ontology_id=GO:0005737,GO:0005840
Fv_160_14.g7686	260	ID=Fv_160_14.g7686;Description=ribosome biogenesis protein n [Fusarium oxysporum f. sp. lycopersici 4287];Gene=CEP51_003197;Ontology_term=nucleolus,ribosome,preribosome, large subunit precursor,protein-arginine N-methyltransferase activity,maturation of LSU-rRNA from tricistronic rRNA transcript (SSU-rRNA, 5.8S rRNA, LSU-rRNA),maturation of 5.8S rRNA from tricistronic rRNA transcript (SSU-rRNA, 5.8S rRNA, LSU-rRNA),methylation;Ontology_id=GO:0005730,GO:0005840,GO:0030687,GO:0016274,GO:0000463,GO:0000466,GO:0032259;Enzym
Fv_160_14.g7687	345	ID=Fv_160_14.g7687;Description=protein arginine N-methyltransferase 1 [Fusarium verticillioides 7600];Gene=FOMG_00926;Ontology_term=nucleus,protein-arginine omega-N monomethyltransferase activity,protein-arginine omega-N asymmetric methyltransferase activity,identical protein binding,mRNA export from nucleus,methylation,positive regulation of transcription elongation by RNA polymerase II,folic acid biosynthetic process,negative regulation of termination of DNA-templated transcription;Ontology_id=GO:0005634,GO:0035241,GO:0035242,GO:0042802,GO:0006406,GO:0032259,GO:0032968,GO:0046656
Fv_160_14.g7688	537	ID=Fv_160_14.g7688;Description=phosphoacetylglucosamine mutase [Fusarium tjaetaba];Gene=FOMG_00927;Ontology_term=magnesium ion binding,phosphoacetylglucosamine mutase activity,carbohydrate metabolic process,UDP-N-acetylglucosamine biosynthetic

Fv_160_14.g7689	581	ID=Fv_160_14.g7689;Description=hypothetical protein FVER14953_01259 [Fusarium verticillioides];Gene=FNAPI_6931;Ontology_term=regulation of synaptic activity,delayed rectifier potassium channel activity,larval locomotory behavior,resolution of meiotic recombination intermediates,crossover junction DNA endonuclease activity,learning or memory,mating behavior, sex discrimination,regulation of monoatomic ion transmembrane transport,action potential,replication fork processing,mitotic intra-S DNA damage checkpoint signaling,mitotic DNA replication checkpoint signaling,positive regulation of membrane potential,cellular response to dopamine,positive regulation of circadian sleep/wake cycle, sleep,potassium ion transmembrane transport,sensory perception of taste,double-strand break repair via break-induced replication,detection of visible light,voltage-gated potassium channel complex,protein homooligomerization,visual perception,nucleus,DNA binding,DNA catabolic process,protein binding,proboscis extension reflex,metal ion binding,axon extension,Holliday junction resolvase complex,courtship behavior,behavioral response to ether;Ontology_id=GO:0060025,GO:0005251,GO:0008345,GO:0000712,GO:0008821,GO:0007611,GO:0048047,GO:0034765,GO:00
Fv_160_14.g7690	234	ID=Fv_160_14.g7690;Description=riboflavin synthase, alpha subunit [Fusarium verticillioides 7600];Gene=28942578;Ontology_term=riboflavin synthase activity,riboflavin biosynthetic
Fv_160_14.g7691	373	ID=Fv_160_14.g7691;Description=hypothetical protein J7337_000763 [Fusarium musae];Gene=Sh;Ontology_term=positive regulation of membrane potential,cellular response to dopamine,positive regulation of circadian sleep/wake cycle, sleep,potassium ion transmembrane transport,regulation of synaptic activity,sensory perception of taste,detection of visible light,voltage-gated potassium channel complex,delayed rectifier potassium channel activity,protein homooligomerization,larval locomotory behavior,learning or memory,protein binding,proboscis extension reflex,axon extension,mating behavior, sex discrimination,courtship behavior,regulation of monoatomic ion transmembrane transport,action potential,behavioral response to ether;Ontology_id=GO:0045838,GO:1903351,GO:0045938,GO:0071805,GO:0060025,GO:0050909,GO:0009584,GO:0008076,GO:00
Fv_160_14.g7692	227	ID=Fv_160_14.g7692;Description=hypothetical protein FVEG_01256 [Fusarium verticillioides 7600];Gene=28942580;Ontology_term=polysome,cytoplasmic translation,negative regulation of protein phosphorylation,negative regulation of protein-containing complex assembly,positive regulation of cellular response to amino acid starvation,dephosphorylation,protein kinase inhibitor activity,protein tyrosine/serine/threonine phosphatase
Fv_160_14.g7693	683	ID=Fv_160_14.g7693;Description=hypothetical protein FVEG_01255 [Fusarium verticillioides 7600];Gene=Forpi1262_v001234;Ontology_term=nucleolus,RNA binding,rRNA
Fv_160_14.g7694	396	ID=Fv_160_14.g7694;Description=hypothetical protein FVER14953_01254 [Fusarium verticillioides];Gene=FPRO_00826;Ontology_term=positive regulation of membrane potential,cellular response to dopamine,positive regulation of circadian sleep/wake cycle, sleep,potassium ion transmembrane transport,regulation of synaptic activity,sensory perception of taste,detection of visible light,voltage-gated potassium channel complex,delayed rectifier potassium channel activity,protein homooligomerization,larval locomotory behavior,learning or memory,protein binding,proboscis extension reflex,axon extension,mating behavior, sex discrimination,courtship behavior,regulation of monoatomic ion transmembrane transport,action potential,behavioral response to ether,hydrolase

Fv_160_14.g7695	586	ID=Fv_160_14.g7695;Description=hypothetical protein FVEG_01253 [Fusarium verticillioides 7600]
Fv_160_14.g7696	158	ID=Fv_160_14.g7696;Description=DNA-directed RNA polymerase II subunit RPB4 [Fusarium oxysporum f. sp. lycopersici 4287];Gene=FACUT_6606;Ontology_term=P-body,RNA polymerase II, core complex,RPB4-RPB7 complex,nucleotide binding,single-stranded DNA binding,single-stranded RNA binding,DNA-directed 5'-3' RNA polymerase activity,RNA-dependent RNA polymerase activity,translation initiation factor binding,nuclear-transcribed mRNA catabolic process, deadenylation-dependent decay,RNA-templated transcription,transcription initiation at RNA polymerase II promoter,transcription elongation by RNA polymerase II,recruitment of 3'-end processing factors to RNA polymerase II holoenzyme complex,positive regulation of translational initiation;Ontology_id=GO:0000932,GO:0005665,GO:1990328,GO:0000166,GO:0003697,GO:0003727,GO:0003899,GO:0003968,GO
Fv_160_14.g7697	152	ID=Fv_160_14.g7697;Description=hypothetical protein FVER53590_25712 [Fusarium verticillioides];Gene=FFUJ_01313;Ontology_term=endoplasmic reticulum,Golgi apparatus,cytosolic large ribosomal subunit,TRAPP complex,structural constituent of ribosome,cytoplasmic translational elongation,endoplasmic reticulum to Golgi vesicle-mediated
Fv_160_14.g7698	289	ID=Fv_160_14.g7698;Description=26S proteasome regulatory subunit N10 [Fusarium verticillioides 7600];Gene=FACUT_6607;Ontology_term=proteasome regulatory particle, base subcomplex,ubiquitin-dependent protein catabolic
Fv_160_14.g7699	359	ID=Fv_160_14.g7699;Description=hypothetical protein FVEG_01250 [Fusarium verticillioides 7600];Gene=FPANT_332;Ontology_term=chromosome, centromeric region,nucleus,cell cycle,cell
Fv_160_14.g7700	200	ID=Fv_160_14.g7700;Description=Translation machinery-associated protein 22 [Fusarium musae];Gene=FVEG_01249;Ontology_term=cytoplasm,ribosome,ribonucleoprotein complex,translation initiation factor
Fv_160_14.g7701	987	ID=Fv_160_14.g7701;Description=hypothetical protein FVER14953_01248 [Fusarium verticillioides];Gene=FocTR4_00001401;Ontology_term=S-adenosylmethionine-dependent methyltransferase
Fv_160_14.g7702	306	ID=Fv_160_14.g7702;Description=hypothetical protein FVEG_01247 [Fusarium verticillioides 7600];Gene=FOPG_03716;Ontology_term=mitochondrial inner membrane;Ontology_id=GO:0005743
Fv_160_14.g7703	80	ID=Fv_160_14.g7703;Description=NEDD8 [Fusarium oxysporum f. sp. lycopersici 4287];Gene=F66182_4338;Ontology_term=membrane,protein tag activity,protein
Fv_160_14.g7704	382	ID=Fv_160_14.g7704;Description=hypothetical protein J7337_000774 [Fusarium musae];Gene=Forpi1262_v001223;Ontology_term=elongin complex,transcription elongation by RNA polymerase
Fv_160_14.g7705	372	ID=Fv_160_14.g7705;Description=transcription initiation factor TFIIF subunit beta [Fusarium verticillioides 7600];Gene=FTJAE_224;Ontology_term=transcription factor TFIIF complex,DNA binding,translation initiation factor activity,transcription initiation at RNA polymerase II promoter,translational
Fv_160_14.g7706	301	ID=Fv_160_14.g7706;Description=hypothetical protein FVEG_01243 [Fusarium verticillioides 7600];Gene=FocTR4_00001398;Ontology_term=nucleus,DNA binding,transcription initiation at RNA polymerase II
Fv_160_14.g7707	634	ID=Fv_160_14.g7707;Description=hypothetical protein FVEG_01241 [Fusarium verticillioides

Fv_160_14.g7708	708	ID=Fv_160_14.g7708;Description=hypothetical protein FVEG_01240 [Fusarium verticillioides 7600];Gene=FPANT_323;Ontology_term=extracellular region,chitinase activity,chitin binding,polysaccharide catabolic process,chitin catabolic
Fv_160_14.g7709	702	ID=Fv_160_14.g7709;Description=hypothetical protein FVEG_01239 [Fusarium verticillioides
Fv_160_14.g7710	492	ID=Fv_160_14.g7710;Description=6-phosphogluconate dehydrogenase, decarboxylating 1 [Fusarium verticillioides 7600];Gene=FCIRC_1850;Ontology_term=phosphogluconate dehydrogenase (decarboxylating) activity,NADP binding,pentose-phosphate shunt, oxidative branch,D-gluconate catabolic process,glycolytic process via Entner-Doudoroff Pathway;Ontology_id=GO:0004616,GO:0050661,GO:0009051,GO:0046177,GO:0061688;Enzyme_code=EC:1.1.1.44;Enzyme_name=
Fv_160_14.g7711	350	ID=Fv_160_14.g7711;Description=hypothetical protein J7337_000781 [Fusarium musae];Gene=FOXYS1_10051;Ontology_term=membrane,RNA binding;Ontology_id=GO:0016020,GO:0003723
Fv_160_14.g7712	622	ID=Fv_160_14.g7712;Description=hypothetical protein FVEG_01236 [Fusarium verticillioides
Fv_160_14.g7713	696	ID=Fv_160_14.g7713;Description=hypothetical protein FVER53263_01235 [Fusarium verticillioides];Gene=FSUBG_10873;Ontology_term=hydrolase activity,carbohydrate metabolic
Fv_160_14.g7714	828	ID=Fv_160_14.g7714;Description=hypothetical protein FVER53590_01234 [Fusarium verticillioides];Gene=hxB;Ontology_term=Mo-molybdopterin cofactor sulfurase activity,lyase activity,molybdenum ion binding,pyridoxal phosphate binding,molybdenum cofactor sulfurtransferase activity,Mo-molybdopterin cofactor biosynthetic process;Ontology_id=GO:0008265,GO:0016829,GO:0030151,GO:0030170,GO:0102867,GO:0006777;Enzyme_code=EC:2.8.1.9,EC:4;
Fv_160_14.g7715	898	ID=Fv_160_14.g7715;Description=hypothetical protein FNYG_13399 [Fusarium nygamai];Gene=FGLOB1_1092;Ontology_term=nucleus,mRNA processing;Ontology_id=GO:0005634,GO:0006397
Fv_160_14.g7716	356	ID=Fv_160_14.g7716;Description=Pin2-interacting protein X1 [Fusarium verticillioides
Fv_160_14.g7717	71	ID=Fv_160_14.g7717;Description=hypothetical protein FVEG_14797 [Fusarium verticillioides 7600]
Fv_160_14.g7718	511	ID=Fv_160_14.g7718;Description=phenylalanyl-tRNA synthetase alpha chain [Fusarium verticillioides 7600];Gene=FPRO05_12713;Ontology_term=phenylalanine-tRNA ligase complex,tRNA binding,aminoacyl-tRNA editing activity,phenylalanine-tRNA ligase activity,ATP binding,metal ion binding,phenylalanyl-tRNA aminoacylation,aminoacyl-tRNA metabolism involved in translational fidelity;Ontology_id=GO:0009328,GO:0000049,GO:0002161,GO:0004826,GO:0005524,GO:0046872,GO:0006432,GO:0106074;Enzy
Fv_160_14.g7719	374	ID=Fv_160_14.g7719;Description=hypothetical protein FVEG_01230 [Fusarium verticillioides 7600];Gene=FACUT_6629;Ontology_term=extracellular region,egg chorion,Dsc E3 ubiquitin ligase complex,serine-type endopeptidase activity,structural constituent of egg chorion,chorion-containing eggshell formation,dorsal/ventral axis specification,zymogen activation,SREBP signaling pathway,Toll receptor ligand protein activation cascade;Ontology_id=GO:0005576,GO:0042600,GO:0044695,GO:0004252,GO:0005213,GO:0007304,GO:0009950,GO:0031638,GO:

Fv_160_14.g7720	404	ID=Fv_160_14.g7720;Description=hypothetical protein J7337_000790 [Fusarium musae];Gene=FVEG_01229;Ontology_term=nucleolus,90S preribosome,small-subunit processome,RNA endonuclease activity,enzyme activator activity,endonucleolytic cleavage in ITS1 to separate SSU-rRNA from 5.8S rRNA and LSU-rRNA from tricistronic rRNA transcript (SSU-rRNA, 5.8S rRNA, LSU-rRNA),endonucleolytic cleavage to generate mature 5'-end of SSU-rRNA from (SSU-rRNA, 5.8S rRNA, LSU-rRNA),endonucleolytic cleavage in 5'-ETS of tricistronic rRNA transcript (SSU-rRNA, 5.8S rRNA, LSU-rRNA),regulation of rRNA
Fv_160_14.g7721	855	ID=Fv_160_14.g7721;Description=hypothetical protein FVER53263_01228 [Fusarium verticillioides]
Fv_160_14.g7722	232	ID=Fv_160_14.g7722;Description=hypothetical protein FVEG_01227 [Fusarium verticillioides 7600];Gene=TAF3;Ontology_term=transcription factor TFIID complex,translation initiation factor activity,protein heterodimerization
Fv_160_14.g7723	702	ID=Fv_160_14.g7723;Description=hypothetical protein FVER53590_01226 [Fusarium verticillioides];Gene=FVEG_01226;Ontology_term=egg chorion,structural constituent of egg chorion,serine-type peptidase activity,protein heterodimerization activity,proteolysis,chorion-containing eggshell
Fv_160_14.g7724	180	ID=Fv_160_14.g7724;Description=chromatin modification-like protein EAF6 [Fusarium verticillioides 7600];Gene=FocTR4_00001379;Ontology_term=nucleus,NuA4 histone acetyltransferase complex,DNA repair,chromatin
Fv_160_14.g7725	282	ID=Fv_160_14.g7725;Description=hypothetical protein J7337_000796 [Fusarium musae];Gene=F53441_7100;Ontology_term=nucleus,mRNA processing;Ontology_id=GO:0005634,GO:0006397
Fv_160_14.g7726	229	ID=Fv_160_14.g7726;Description=26S proteasome non-ATPase regulatory subunit 9 [Fusarium verticillioides 7600];Gene=FNAPI_6896;Ontology_term=proteasome complex,proteolysis,apoptotic process,proteasome regulatory particle
Fv_160_14.g7727	216	ID=Fv_160_14.g7727;Description=unnamed protein product, partial [Fusarium graminearum];Gene=FOVG_01216;Ontology_term=nucleus,GTPase activity,GTP binding,nucleocytoplasmic transport,protein transport;Ontology_id=GO:0005634,GO:0003924,GO:0005525,GO:0006913,GO:0015031;Enzyme_code=EC:3.6.1.15;Enzyme_name=
Fv_160_14.g7728	584	ID=Fv_160_14.g7728;Description=hypothetical protein FVER14953_01221 [Fusarium verticillioides];Gene=FNYG_13386;Ontology_term=nucleus,glycosome,DNA-binding transcription factor activity, RNA polymerase II-specific,DNA binding,glyceraldehyde-3-phosphate dehydrogenase (NAD+) (phosphorylating) activity,NADP binding,NAD binding,glucose metabolic process,glycolytic process,regulation of transcription by RNA polymerase II;Ontology_id=GO:0005634,GO:0020015,GO:0000981,GO:0003677,GO:0004365,GO:0050661,GO:0051287,GO:0006006,GO:000609
Fv_160_14.g7729	521	ID=Fv_160_14.g7729;Description=hypothetical protein FVER14953_01220 [Fusarium verticillioides];Gene=FPRO05_12722;Ontology_term=NAD+ kinase activity,NADP biosynthetic process,phosphorylation,NAD
Fv_160_14.g7730	250	ID=Fv_160_14.g7730;Description=hypothetical protein FVEG_01219 [Fusarium verticillioides 7600];Gene=BFJ72_g13552;Ontology_term=nuclear exosome (RNase complex),nucleolus,rRNA
Fv_160_14.g7731	427	ID=Fv_160_14.g7731;Description=hypothetical protein FVER14953_01218 [Fusarium verticillioides];Gene=FVEG_01218;Ontology_term=cytoplasm,peptidyl-prolyl cis-trans isomerase activity,phosphatase activator
Fv_160_14.g7732	530	ID=Fv_160_14.g7732;Description=hypothetical protein FVER53590_01217 [Fusarium verticillioides]

Fv_160_14.g7733	325	ID=Fv_160_14.g7733;Description=hypothetical protein FVEG_01216 [Fusarium verticillioides 7600]
Fv_160_14.g7734	293	ID=Fv_160_14.g7734;Description=hypothetical protein FVER53590_25763 [Fusarium verticillioides];Gene=9558
Fv_160_14.g7735	496	ID=Fv_160_14.g7735;Description=hypothetical protein FVEG_14794 [Fusarium verticillioides 7600]
Fv_160_14.g7736	232	ID=Fv_160_14.g7736;Description=hypothetical protein FVEG_01213 [Fusarium verticillioides 7600]
Fv_160_14.g7737	232	ID=Fv_160_14.g7737;Description=hypothetical protein FVER14953_01212 [Fusarium verticillioides];Gene=FNYG_13376;Ontology_term=ribonucleoprotein complex,RNA binding;Ontology_id=GO:1990904,GO:0003723
Fv_160_14.g7738	173	ID=Fv_160_14.g7738;Description=60S ribosomal protein L11 [Fusarium oxysporum f. sp. lycopersici 4287];Gene=FACUT_6648;Ontology_term=ribosome,ribonucleoprotein complex,structural constituent of
Fv_160_14.g7739	552	ID=Fv_160_14.g7739;Description=ATP synthase subunit alpha, mitochondrial [Fusarium verticillioides 7600];Gene=FOBC_01649;Ontology_term=proton-transporting ATP synthase complex, catalytic core F(1),ATP binding,proton-transporting ATP synthase activity, rotational mechanism,proton motive force-driven ATP synthesis,proton transmembrane
Fv_160_14.g7740	350	ID=Fv_160_14.g7740;Description=hypothetical protein FVEG_01208 [Fusarium verticillioides 7600];Gene=FPRO05_12746;Ontology_term=cytoplasm,ATP binding,ATP-dependent NAD(P)H-hydrate dehydratase activity,nicotinamide nucleotide metabolic
Fv_160_14.g7741	386	ID=Fv_160_14.g7741;Description=hypothetical protein FVEG_01207 [Fusarium verticillioides 7600]
Fv_160_14.g7742	103	ID=Fv_160_14.g7742;Description=hypothetical protein FVEG_14793 [Fusarium verticillioides
Fv_160_14.g7743	472	ID=Fv_160_14.g7743;Description=hypothetical protein FVEG_01206 [Fusarium verticillioides 7600];Gene=Focb16_v000865;Ontology_term=metallopeptidase
Fv_160_14.g7744	292	ID=Fv_160_14.g7744;Description=hypothetical protein FVEG_01204 [Fusarium verticillioides 7600]
Fv_160_14.g7745	910	ID=Fv_160_14.g7745;Description=hypothetical protein FVER53590_01203 [Fusarium verticillioides];Gene=FNAPI_6875;Ontology_term=nucleus,DNA binding,zinc ion binding,DNA-binding transcription factor activity, RNA polymerase II-specific,regulation of transcription by RNA polymerase
Fv_160_14.g7746	173	ID=Fv_160_14.g7746;Description=hypothetical protein FVEG_01202 [Fusarium verticillioides 7600]
Fv_160_14.g7747	1500	ID=Fv_160_14.g7747;Description=hypothetical protein FVER53590_01201 [Fusarium verticillioides];Gene=FNYG_13363;Ontology_term=GTPase activator activity,phosphatidylinositol binding,signal
Fv_160_14.g7748	196	ID=Fv_160_14.g7748;Description=hypothetical protein FVER14953_01200 [Fusarium verticillioides]
Fv_160_14.g7749	1146	ID=Fv_160_14.g7749;Description=family sulfate permease [Fusarium coicis];Gene=FOXYS1_13097;Ontology_term=membrane,secondary active sulfate transmembrane transporter activity,monoatomic anion transmembrane transporter activity,monoatomic anion transmembrane transport,sulfate transmembrane
Fv_160_14.g7750	482	ID=Fv_160_14.g7750;Description=hypothetical protein FVER14953_01197 [Fusarium verticillioides]
Fv_160_14.g7751	497	ID=Fv_160_14.g7751;Description=hypothetical protein J7337_000821 [Fusarium
Fv_160_14.g7752	211	ID=Fv_160_14.g7752;Description=hypothetical protein FVEG_01196 [Fusarium verticillioides

Fv_160_14.g7753	642	ID=Fv_160_14.g7753;Description=glycosyl transferase gt-a type structural fold [Fusarium coicis];Gene=FPANT_8509;Ontology_term=membrane,transferase
Fv_160_14.g7754	258	ID=Fv_160_14.g7754;Description=hypothetical protein FVER14953_01193 [Fusarium verticillioides];Gene=FTJAE_278;Ontology_term=transferase
Fv_160_14.g7755	463	ID=Fv_160_14.g7755;Description=peptidylprolyl isomerase fpr4 [Fusarium musae];Gene=C2S_8818;Ontology_term=extracellular region,cilium,membrane,peptidyl-prolyl cis-trans isomerase
Fv_160_14.g7756	832	ID=Fv_160_14.g7756;Description=hypothetical protein FVER53590_01191 [Fusarium verticillioides];Gene=FNAPI_12239;Ontology_term=protein serine/threonine kinase activity,ATP
Fv_160_14.g7757	540	ID=Fv_160_14.g7757;Description=hypothetical protein FVER14953_01190 [Fusarium verticillioides];Gene=FNAPI_12238;Ontology_term=membrane,sphingomyelin phosphodiesterase activity,sphingolipid metabolic
Fv_160_14.g7758	214	ID=Fv_160_14.g7758;Description=hypothetical protein FVEG_01189 [Fusarium verticillioides 7600]
Fv_160_14.g7759	471	ID=Fv_160_14.g7759;Description=hypothetical protein J7337_000829 [Fusarium musae];Gene=FCIRC_8624;Ontology_term=RNA
Fv_160_14.g7760	323	ID=Fv_160_14.g7760;Description=hypothetical protein FVER14953_01187 [Fusarium verticillioides];Gene=FPRO05_12755;Ontology_term=chromosome, centromeric region,nucleus,cilium,membrane,kinetochore
Fv_160_14.g7761	169	ID=Fv_160_14.g7761;Description=deoxyuridine 5'-triphosphate nucleotidohydrolase [Fusarium verticillioides 7600];Gene=FOIG_00826;Ontology_term=magnesium ion binding,dUTP diphosphatase activity,dUMP biosynthetic process,dUTP catabolic
Fv_160_14.g7762	285	ID=Fv_160_14.g7762;Description=hypothetical protein FVER53263_01185 [Fusarium verticillioides];Gene=FACUT_5749;Ontology_term=pyridoxal kinase activity,pyridoxal 5'-phosphate
Fv_160_14.g7763	259	ID=Fv_160_14.g7763;Description=hypothetical protein FVER53263_01184 [Fusarium verticillioides];Gene=FFUJ_01235;Ontology_term=chromosome, telomeric region,chromatin,nucleoplasm,membrane,site of double-strand break,nuclear replication fork,PCNA complex,DNA binding,myosin binding,DNA polymerase processivity factor activity,identical protein binding,meiotic mismatch repair,leading strand elongation,nucleotide-excision repair,silent mating-type cassette heterochromatin formation,subtelomeric heterochromatin formation,establishment of mitotic sister chromatid cohesion,maintenance of DNA trinucleotide repeats,error-prone translesion synthesis,positive regulation of DNA repair,positive regulation of DNA replication,UV-damage excision repair,error-free translesion synthesis,mitotic DNA replication lagging strand
Fv_160_14.g7764	617	ID=Fv_160_14.g7764;Description=hypothetical protein FVEG_14789 [Fusarium verticillioides 7600];Gene=FPRO05_12758;Ontology_term=nucleus,plasma membrane,myosin binding,cell-cell
Fv_160_14.g7765	746	ID=Fv_160_14.g7765;Description=hypothetical protein FVER14953_01181 [Fusarium verticillioides]
Fv_160_14.g7766	6896	ID=Fv_160_14.g7766;Description=hypothetical protein FVEG_01180 [Fusarium verticillioides 7600]

Fv_160_14.g7767	549	ID=Fv_160_14.g7767;Description=hypothetical protein FOXB_07498 [Fusarium oxysporum f. sp. conglutinans Fo5176];Gene=Forpe1208_v001127;Ontology_term=membrane,monooxygenase activity,iron ion binding,oxidoreductase activity, acting on paired donors, with incorporation or reduction of molecular oxygen,heme binding;Ontology_id=GO:0016020,GO:0004497,GO:0005506,GO:0016705,GO:0020037;Enzyme_code=EC:1.14;Enzyme_name=Actin
Fv_160_14.g7768	545	ID=Fv_160_14.g7768;Description=hypothetical protein FVER14953_01177 [Fusarium verticillioides];Gene=FACUT_5743;Ontology_term=ligase
Fv_160_14.g7769	873	ID=Fv_160_14.g7769;Description=hypothetical protein FVER53263_01175 [Fusarium verticillioides];Gene=FGADI_10200;Ontology_term=ubiquitin protein ligase binding,ubiquitin-dependent protein catabolic
Fv_160_14.g7770	434	ID=Fv_160_14.g7770;Description=hypothetical protein FVEG_01174 [Fusarium verticillioides 7600]
Fv_160_14.g7771	167	ID=Fv_160_14.g7771;Description=hypothetical protein FVEG_01172 [Fusarium verticillioides 7600];Gene=FANTH_5891;Ontology_term=membrane,vacuolar proton-transporting V-type ATPase complex
Fv_160_14.g7772	710	ID=Fv_160_14.g7772;Description=hypothetical protein FVEG_01171 [Fusarium verticillioides
Fv_160_14.g7773	2000	ID=Fv_160_14.g7773;Description=hypothetical protein FVER14953_01170 [Fusarium verticillioides];Gene=FACUT_5738;Ontology_term=cytoplasm,membrane,guanyl-nucleotide exchange factor
Fv_160_14.g7774	1137	ID=Fv_160_14.g7774;Description=hypothetical protein FVER53590_01169 [Fusarium verticillioides];Gene=FOXG_00349;Ontology_term=chromosome, telomeric region,nucleus,mitochondrion,DNA binding,telomerase RNA reverse transcriptase activity,metal ion binding,RNA-templated DNA biosynthetic
Fv_160_14.g7775	539	ID=Fv_160_14.g7775;Description=hypothetical protein FVEG_01168 [Fusarium verticillioides 7600];Gene=FTJAE_300;Ontology_term=membrane,O-acyltransferase activity,lipid metabolic
Fv_160_14.g7776	423	ID=Fv_160_14.g7776;Description=hypothetical protein FVEG_01167 [Fusarium verticillioides 7600];Gene=FocTR4_00001310;Ontology_term=nucleus,DNA-binding transcription factor activity, RNA polymerase II-specific,zinc
Fv_160_14.g7777	404	ID=Fv_160_14.g7777;Description=hypothetical protein FVER14953_01166 [Fusarium verticillioides]
Fv_160_14.g7778	296	ID=Fv_160_14.g7778;Description=hypothetical protein FVEG_01165 [Fusarium verticillioides 7600];Gene=FTJAE_303;Ontology_term=mitochondrial inner membrane,transmembrane
Fv_160_14.g7779	376	ID=Fv_160_14.g7779;Description=hypothetical protein FVER14953_01164 [Fusarium verticillioides];Gene=BFJ70_g3492;Ontology_term=hydrolase activity, hydrolyzing N-glycosyl compounds,nucleobase-containing compound metabolic process,organonitrogen compound metabolic
Fv_160_14.g7780	545	ID=Fv_160_14.g7780;Description=hypothetical protein FVEG_01163 [Fusarium verticillioides
Fv_160_14.g7781	1141	ID=Fv_160_14.g7781;Description=hypothetical protein FVER53590_01162 [Fusarium verticillioides];Gene=FPCIR_8580;Ontology_term=nucleus,mitochondrion,damaged DNA binding,nucleotidyltransferase activity,carbohydrate metabolic process,error-prone translesion synthesis,error-free translesion synthesis;Ontology_id=GO:0005634,GO:0005739,GO:0003684,GO:0016779,GO:0005975,GO:0042276,GO:0070987;Enzyme_code=E
Fv_160_14.g7782	222	ID=Fv_160_14.g7782;Description=ER membrane protein SH3 [Fusarium verticillioides

Fv_160_14.g7783	325	ID=Fv_160_14.g7783;Description=thymidylate synthase [Fusarium verticillioides 7600];Gene=FACUT_5727;Ontology_term=thymidylate synthase activity,dTMP biosynthetic process,dTTP biosynthetic
Fv_160_14.g7784	331	ID=Fv_160_14.g7784;Description=amino-acid transporter Arg-13 [Fusarium verticillioides 7600];Gene=FACUT_5726;Ontology_term=mitochondrial inner membrane,L-ornithine transmembrane transporter activity,arginine
Fv_160_14.g7785	185	ID=Fv_160_14.g7785;Description=benzodiazapine receptor [Fusarium verticillioides
Fv_160_14.g7786	603	ID=Fv_160_14.g7786;Description=cell division control protein 6 [Fusarium verticillioides 7600];Gene=FNAPI_7535;Ontology_term=DNA binding,ATP hydrolysis activity,DNA replication initiation,DNA transposition,DNA integration,cell
Fv_160_14.g7787	856	ID=Fv_160_14.g7787;Description=hypothetical protein FVEG_01155 [Fusarium verticillioides 7600]
Fv_160_14.g7788	260	ID=Fv_160_14.g7788;Description=molecular chaperone HscB [Fusarium verticillioides 7600];Gene=FTJAE_314;Ontology_term=ATPase activator activity,protein-folding chaperone binding,[2Fe-2S] cluster
Fv_160_14.g7789	256	ID=Fv_160_14.g7789;Description=hypothetical protein J7337_000859 [Fusarium musae];Gene=FOC1_g10015755;Ontology_term=D-ribulose-phosphate 3-epimerase activity,iron ion binding,heme binding,L-ascorbic acid binding,peptidyl-proline dioxygenase activity,carbohydrate metabolic process,pentose-phosphate shunt;Ontology_id=GO:0004750,GO:0005506,GO:0020037,GO:0031418,GO:0031543,GO:0005975,GO:0006098;Enzyme_code=EC:1.
Fv_160_14.g7790	488	ID=Fv_160_14.g7790;Description=hypothetical protein FVER53590_01152 [Fusarium verticillioides];Gene=Forpi1262_v001135;Ontology_term=D-ribulose-phosphate 3-epimerase activity,oxidoreductase activity,heme binding,metal ion binding,carbohydrate metabolic process,pentose-phosphate shunt;Ontology_id=GO:0004750,GO:0016491,GO:0020037,GO:0046872,GO:0005975,GO:0006098;Enzyme_code=EC:1,EC:5.1.3.1;En
Fv_160_14.g7791	343	ID=Fv_160_14.g7791;Description=hypothetical protein FVEG_01151 [Fusarium verticillioides 7600];Gene=FMAN_00897;Ontology_term=fungal-type vacuole membrane;Ontology_id=GO:0000329
Fv_160_14.g7792	249	ID=Fv_160_14.g7792;Description=hypothetical protein FVEG_01150 [Fusarium verticillioides 7600];Gene=FVER53590_01150;Ontology_term=regulation of DNA-templated transcription;Ontology_id=GO:0006355
Fv_160_14.g7793	1248	ID=Fv_160_14.g7793;Description=pre-mRNA-processing ATP-dependent RNA helicase PRP5 [Fusarium tjaetaba];Gene=FVEG_01149;Ontology_term=nucleic acid binding,RNA helicase activity,ATP binding,hydrolase
Fv_160_14.g7794	120	ID=Fv_160_14.g7794;Description=hypothetical protein FVEG_01148 [Fusarium verticillioides 7600]
Fv_160_14.g7795	632	ID=Fv_160_14.g7795;Description=hypothetical protein FVER14953_01147 [Fusarium verticillioides]
Fv_160_14.g7796	125	ID=Fv_160_14.g7796;Description=hypothetical protein FNYG_09057 [Fusarium nygamai];Gene=FACUT_5715;Ontology_term=cytosolic large ribosomal subunit,structural constituent of ribosome,maturation of LSU-
Fv_160_14.g7797	228	ID=Fv_160_14.g7797;Description=hypothetical protein FVER14953_01145 [Fusarium verticillioides];Gene=FNAPI_7545;Ontology_term=Golgi membrane,Golgi medial cisterna,cis-Golgi network,SNARE complex,SNAP receptor activity,intracellular protein transport,endoplasmic reticulum to Golgi vesicle-mediated transport,vesicle
Fv_160_14.g7798	346	ID=Fv_160_14.g7798;Description=hypothetical protein FVER14953_01144 [Fusarium verticillioides]

Fv_160_14.g7799	488	ID=Fv_160_14.g7799;Description=hypothetical protein FVER14953_01143 [Fusarium verticillioides];Gene=FNAPI_7547;Ontology_term=acyltransferase
Fv_160_14.g7800	231	ID=Fv_160_14.g7800;Description=gluconokinase [Fusarium verticillioides 7600];Gene=FNAPI_7548;Ontology_term=ATP binding,gluconokinase activity,phosphorylation,D-gluconate catabolic
Fv_160_14.g7801	707	ID=Fv_160_14.g7801;Description=dipeptidyl-peptidase III [Fusarium verticillioides 7600];Gene=BFJ69_g9780;Ontology_term=cytoplasm,aminopeptidase activity,metalloexopeptidase activity,dipeptidyl-peptidase activity,metal ion binding,proteolysis;Ontology_id=GO:0005737,GO:0004177,GO:0008235,GO:0008239,GO:0046872,GO:0006508;Enzyme_code=EC:3.
Fv_160_14.g7802	221	ID=Fv_160_14.g7802;Description=hypothetical protein J7337_000872 [Fusarium musae];Gene=C2S_13077;Ontology_term=ribosome,ribonucleoprotein complex,structural constituent of
Fv_160_14.g7803	797	ID=Fv_160_14.g7803;Description=heat shock protein 78, mitochondrial [Fusarium verticillioides 7600];Gene=FTJAE_328;Ontology_term=ATP binding,ATP hydrolysis
Fv_160_14.g7804	183	ID=Fv_160_14.g7804;Description=uncharacterized protein NECHADRAFT_102366 [Fusarium vanettenii 77-13-4];Gene=C2S_13075;Ontology_term=Golgi apparatus,GTPase activity,GTP binding,protein transport,vesicle-mediated transport;Ontology_id=GO:0005794,GO:0003924,GO:0005525,GO:0015031,GO:0016192;Enzyme_code=EC:3.6.1.15;Enzyme_name=
Fv_160_14.g7805	203	ID=Fv_160_14.g7805;Description=hypothetical protein FVEG_01137 [Fusarium verticillioides 7600];Gene=C2S_13074;Ontology_term=endoplasmic reticulum,membrane,COPII-coated ER to Golgi transport vesicle,protein retention in ER lumen,endoplasmic reticulum to Golgi vesicle-mediated transport,vesicle
Fv_160_14.g7806	986	ID=Fv_160_14.g7806;Description=hypothetical protein FVER53263_01136 [Fusarium
Fv_160_14.g7807	300	ID=Fv_160_14.g7807;Description=hypothetical protein FVEG_01135 [Fusarium verticillioides 7600]
Fv_160_14.g7808	376	ID=Fv_160_14.g7808;Description=hypothetical protein FVEG_01134 [Fusarium verticillioides 7600];Gene=FNAPI_7556;Ontology_term=U2-type spliceosomal complex,U2 snRNP,RNA binding,mRNA splicing, via
Fv_160_14.g7809	389	ID=Fv_160_14.g7809;Description=hypothetical protein FVEG_01133 [Fusarium verticillioides 7600];Gene=FNAPI_7557;Ontology_term=nucleus,cytoplasm,ribosome,ribonucleoprotein complex,RNA binding,structural constituent of ribosome,mRNA splicing, via
Fv_160_14.g7810	231	ID=Fv_160_14.g7810;Description=elongation factor 1-beta [Fusarium oxysporum f. sp. lycopersici 4287];Gene=FTJAE_335;Ontology_term=eukaryotic translation elongation factor 1 complex,translation elongation factor
Fv_160_14.g7811	214	ID=Fv_160_14.g7811;Description=DNA replication complex GINS protein PSF1 [Fusarium verticillioides 7600];Gene=FTJAE_336;Ontology_term=GINS complex,replication fork protection complex,CMG complex,double-strand break repair via break-induced replication,DNA unwinding involved in DNA replication,mitotic DNA replication initiation,DNA strand elongation
Fv_160_14.g7812	1082	ID=Fv_160_14.g7812;Description=hypothetical protein FVEG_01130 [Fusarium verticillioides
Fv_160_14.g7813	364	ID=Fv_160_14.g7813;Description=hypothetical protein FVER14953_20351 [Fusarium verticillioides]

Fv_160_14.g7814	943	ID=Fv_160_14.g7814;Description=hypothetical protein FVER14953_01127 [Fusarium verticillioides];Gene=FMEXI_13094;Ontology_term=microtubule,microtubule motor activity,ATP binding,microtubule
Fv_160_14.g7815	611	ID=Fv_160_14.g7815;Description=hypothetical protein FVER53263_01126 [Fusarium
Fv_160_14.g7816	488	ID=Fv_160_14.g7816;Description=hypothetical protein J7337_000886 [Fusarium musae];Gene=FNAPI_12438;Ontology_term=transcription factor TFIID complex,troponin complex,translation initiation factor activity,transcription initiation at RNA polymerase II promoter,translational
Fv_160_14.g7817	328	ID=Fv_160_14.g7817;Description=hypothetical protein FVEG_14782 [Fusarium verticillioides 7600];Gene=FDENT_9613;Ontology_term=transcription factor TFIID complex,transcription initiation at RNA polymerase II
Fv_160_14.g7818	313	ID=Fv_160_14.g7818;Description=hypothetical protein FVEG_01124 [Fusarium verticillioides 7600]
Fv_160_14.g7819	527	ID=Fv_160_14.g7819;Description=eburicol 14-alpha-demethylase [Fusarium verticillioides 7600];Gene=FTJAE_344;Ontology_term=muscle cell cellular homeostasis,oxidoreductase activity, acting on paired donors, with incorporation or reduction of molecular oxygen,mesoderm development,DNA integration,sarcomere organization,methyltransferase activity,muscle organ morphogenesis,troponin complex,monooxygenase activity,tropomyosin binding,mitochondrion organization,heme binding,DNA transposition,intracellular calcium ion homeostasis,nucleus,DNA binding,iron ion binding,calcium ion binding,membrane,muscle thin filament assembly,regulation of cardiac muscle contraction by calcium ion signaling,methylation;Ontology_id=GO:0046716,GO:0016705,GO:0007498,GO:0015074,GO:0045214,GO:0008168,GO:0048644,GO:
Fv_160_14.g7820	405	ID=Fv_160_14.g7820;Description=hypothetical protein FVER53263_01122 [Fusarium
Fv_160_14.g7821	513	ID=Fv_160_14.g7821;Description=nucleolin [Fusarium verticillioides 7600];Gene=FPANT_1228;Ontology_term=nuclear envelope,nucleolus,small-subunit processome,RNA binding,nuclear localization sequence binding,single-stranded telomeric DNA
Fv_160_14.g7822	372	ID=Fv_160_14.g7822;Description=mitochondrial ribosomal L37p [Fusarium napiforme];Gene=FTJAE_13984;Ontology_term=mitochondrion,ribosome,ribonucleoprotein
Fv_160_14.g7823	415	ID=Fv_160_14.g7823;Description=basic proline-rich [Fusarium pseudoanthophilum];Gene=FVEG_01118;Ontology_term=actin
Fv_160_14.g7824	412	ID=Fv_160_14.g7824;Description=hypothetical protein FVEG_01117 [Fusarium verticillioides 7600];Gene=FocTR4_00001258;Ontology_term=extracellular region,membrane,ferroxidase activity,ferric iron binding,iron ion transport,intracellular iron ion
Fv_160_14.g7825	289	ID=Fv_160_14.g7825;Description=hypothetical protein FNYG_09087 [Fusarium nygamai]
Fv_160_14.g7826	306	ID=Fv_160_14.g7826;Description=hypothetical protein FVER53590_01115 [Fusarium verticillioides]
Fv_160_14.g7827	684	ID=Fv_160_14.g7827;Description=hypothetical protein FVER53590_01114 [Fusarium verticillioides];Gene=PGPA;Ontology_term=membrane,ATP binding,ABC-type xenobiotic transporter activity,ATP hydrolysis activity,xenobiotic transport,response to antibiotic,transmembrane transport;Ontology_id=GO:0016020,GO:0005524,GO:0008559,GO:0016887,GO:0042908,GO:0046677,GO:0055085;Enzyme_code=E
Fv_160_14.g7828	343	ID=Fv_160_14.g7828;Description=hypothetical protein FVEG_01113 [Fusarium verticillioides 7600]

Fv_160_14.g7829	540	ID=Fv_160_14.g7829;Description=hypothetical protein FVEG_01112 [Fusarium verticillioides 7600];Gene=FOYG_06948;Ontology_term=extracellular region,carbohydrate binding,rhamnogalacturonan endolyase activity,polysaccharide catabolic process,cell wall
Fv_160_14.g7830	77	ID=Fv_160_14.g7830;Description=hypothetical protein FVEG_14780 [Fusarium verticillioides
Fv_160_14.g7831	342	ID=Fv_160_14.g7831;Description=hypothetical protein FVER14953_01111 [Fusarium verticillioides]
Fv_160_14.g7832	454	ID=Fv_160_14.g7832;Description=hypothetical protein FVEG_01109 [Fusarium verticillioides 7600];Gene=FPCIR_13737;Ontology_term=calcium ion binding,calcium-dependent phospholipid
Fv_160_14.g7833	659	ID=Fv_160_14.g7833;Description=hypothetical protein FVER14953_01108 [Fusarium verticillioides]
Fv_160_14.g7834	364	ID=Fv_160_14.g7834;Description=hypothetical protein FVEG_01107 [Fusarium verticillioides 7600]
Fv_160_14.g7835	629	ID=Fv_160_14.g7835;Description=hypothetical protein FVER53590_01106 [Fusarium verticillioides];Gene=twe;Ontology_term=male meiotic nuclear division,spermatogenesis,protein dephosphorylation,regulation of syncytial blastoderm mitotic cell cycle,positive regulation of G2/MI transition of meiotic cell cycle,cell division,nucleus,protein binding,cytoplasm,protein tyrosine phosphatase activity,meiotic nuclear membrane disassembly,spindle,positive regulation of G2/M transition of mitotic cell cycle;Ontology_id=GO:0007140,GO:0007283,GO:0006470,GO:0007348,GO:0110032,GO:0051301,GO:0005634,GO:0005515,GO:000
Fv_160_14.g7836	594	ID=Fv_160_14.g7836;Description=hypothetical protein FVER53590_01105 [Fusarium verticillioides]
Fv_160_14.g7837	618	ID=Fv_160_14.g7837;Description=hypothetical protein FVER14953_01103 [Fusarium verticillioides]
Fv_160_14.g7838	175	ID=Fv_160_14.g7838;Description=hypothetical protein FVER53263_21119 [Fusarium verticillioides]
Fv_160_14.g7839	485	ID=Fv_160_14.g7839;Description=hypothetical protein FVEG_01101 [Fusarium verticillioides 7600];Gene=FOIG_00915;Ontology_term=nucleus,cytoplasm,RNA polymerase II transcription regulatory region sequence-specific DNA binding,DNA-binding transcription factor activity, RNA polymerase II-specific,zinc ion binding,protein homodimerization activity,protein heterodimerization activity,ventral furrow formation,salivary gland morphogenesis,Malpighian tubule morphogenesis,ectoderm and mesoderm interaction,mesodermal cell fate specification,heart development,regulation of striated muscle tissue development,actomyosin structure organization,positive regulation of transcription by RNA polymerase II,muscle cell development;Ontology_id=GO:0005634,GO:0005737,GO:0000977,GO:0000981,GO:0008270,GO:0042803,GO:0046982,GO:000737
Fv_160_14.g7840	1142	ID=Fv_160_14.g7840;Description=hypothetical protein FVEG_01099 [Fusarium verticillioides 7600]
Fv_160_14.g7841	804	ID=Fv_160_14.g7841;Description=hypothetical protein FVEG_01098 [Fusarium verticillioides 7600]
Fv_160_14.g7842	1134	ID=Fv_160_14.g7842;Description=hypothetical protein FVEG_01097 [Fusarium verticillioides 7600];Gene=FACUT_5317;Ontology_term=nucleus,helicase activity,ATP binding,hydrolase activity,ATP-dependent chromatin remodeler activity,chromatin
Fv_160_14.g7843	284	ID=Fv_160_14.g7843;Description=hypothetical protein FVER53590_01097 [Fusarium verticillioides];Gene=FVER53590_01097;Ontology_term=nucleus,ATP binding,hydrolase activity,ATP-dependent chromatin remodeler activity,chromatin

Fv_160_14.g7844	407	ID=Fv_160_14.g7844;Description=hypothetical protein FVEG_01095 [Fusarium verticillioides 7600];Gene=FPRO05_12835;Ontology_term=oxidoreductase
Fv_160_14.g7845	858	ID=Fv_160_14.g7845;Description=hypothetical protein FVER53263_01092 [Fusarium verticillioides];Gene=FNAPI_882;Ontology_term=nucleus,ATP binding,oxidoreductase activity,DNA repair,cell
Fv_160_14.g7846	595	ID=Fv_160_14.g7846;Description=bifunctional purine biosynthesis protein ADE17 [Fusarium verticillioides 7600];Gene=BFJ68_g4026;Ontology_term=cytosol,IMP cyclohydrolase activity,phosphoribosylaminoimidazolecarboxamide formyltransferase activity,'de novo' IMP biosynthetic process;Ontology_id=GO:0005829,GO:0003937,GO:0004643,GO:0006189;Enzyme_code=EC:3.5.4.10,EC:2.1.2.3;Enzyme_name=IMP
Fv_160_14.g7847	247	ID=Fv_160_14.g7847;Description=hypothetical protein FVEG_01090 [Fusarium verticillioides 7600]
Fv_160_14.g7848	421	ID=Fv_160_14.g7848;Description=hypothetical protein FVEG_01089 [Fusarium verticillioides 7600]
Fv_160_14.g7849	227	ID=Fv_160_14.g7849;Description=hypothetical protein FVER14953_01088 [Fusarium verticillioides]
Fv_160_14.g7850	808	ID=Fv_160_14.g7850;Description=hypothetical protein FVER14953_01087 [Fusarium verticillioides];Gene=FPANT_1957;Ontology_term=DNA binding,DNA-directed DNA polymerase activity,DNA repair,DNA biosynthetic
Fv_160_14.g7851	521	ID=Fv_160_14.g7851;Description=ammonium transporter MEPa [Fusarium pseudoanthophilum];Gene=FPANT_1958;Ontology_term=plasma membrane,ammonium transmembrane transporter
Fv_160_14.g7852	339	ID=Fv_160_14.g7852;Description=hypothetical protein FVEG_01085 [Fusarium verticillioides
Fv_160_14.g7853	1151	ID=Fv_160_14.g7853;Description=hypothetical protein FVER14953_01084 [Fusarium verticillioides]
Fv_160_14.g7854	951	ID=Fv_160_14.g7854;Description=hypothetical protein FVEG_01083 [Fusarium verticillioides
Fv_160_14.g7855	774	ID=Fv_160_14.g7855;Description=hypothetical protein FVEG_14773 [Fusarium verticillioides 7600]
Fv_160_14.g7856	1516	ID=Fv_160_14.g7856;Description=hypothetical protein FVER53590_01079 [Fusarium verticillioides];Gene=FNAPI_11848;Ontology_term=Golgi apparatus,membrane,carboxypeptidase activity,protein transport,intracellular
Fv_160_14.g7857	697	ID=Fv_160_14.g7857;Description=hypothetical protein FVER53590_01078 [Fusarium verticillioides];Gene=RpL40;Ontology_term=nucleus,cytoplasm,ribosome,ribonucleoprotein complex,structural constituent of
Fv_160_14.g7858	603	ID=Fv_160_14.g7858;Description=amino acid transporter AAT family [Fusarium coicis];Gene=FFUJ_01137;Ontology_term=membrane,mannosyl-oligosaccharide 1,2-alpha-mannosidase activity,calcium ion binding,transmembrane transporter activity,carbohydrate metabolic process,amino acid transport,ubiquitin-dependent ERAD pathway,transmembrane
Fv_160_14.g7859	689	ID=Fv_160_14.g7859;Description=hypothetical protein FVER14953_01076 [Fusarium verticillioides];Gene=FPHYL_7470;Ontology_term=membrane,mannosyl-oligosaccharide 1,2-alpha-mannosidase activity,calcium ion binding,transmembrane transporter activity,carbohydrate metabolic process,amino acid transport,ubiquitin-dependent ERAD pathway,transmembrane transport;Ontology_id=GO:0016020,GO:0004571,GO:0005509,GO:0022857,GO:0005975,GO:0006865,GO:0030433,GO:0055085;En

Fv_160_14.g7860	233	ID=Fv_160_14.g7860;Description=hypothetical protein FVEG_14772 [Fusarium verticillioides 7600];Gene=FOYG_06913;Ontology_term=membrane,ubiquitin-like protein transferase
Fv_160_14.g7861	203	ID=Fv_160_14.g7861;Description=U2 small nuclear ribonucleoprotein B'' [Fusarium verticillioides 7600];Gene=FOC1_g10015732;Ontology_term=ribonucleoprotein complex,RNA binding;Ontology_id=GO:1990904,GO:0003723
Fv_160_14.g7862	363	ID=Fv_160_14.g7862;Description=actin like protein 2/3 complex, subunit 1A/1B [Fusarium verticillioides 7600];Gene=FPHYL_7472;Ontology_term=actin filament binding,ubiquitin binding,actin cortical patch organization,positive regulation of Arp2/3 complex-mediated actin nucleation,actin cortical patch,Arp2/3 protein
Fv_160_14.g7863	125	ID=Fv_160_14.g7863;Description=TdcF protein [Fusarium verticillioides 7600];Gene=RpL40;Ontology_term=nucleus,cytoplasm,ribosome,ribonucleoprotein complex,structural constituent of
Fv_160_14.g7864	770	ID=Fv_160_14.g7864;Description=dolichyl-phosphate-mannose-protein mannosyltransferase [Fusarium verticillioides 7600];Gene=C2S_12392;Ontology_term=endoplasmic reticulum membrane,dolichyl-phosphate-mannose-protein mannosyltransferase activity;Ontology_id=GO:0005789,GO:0004169;Enzyme_code=EC:2.4.1.109;Enzyme_name=dolichyl-phosphate-
Fv_160_14.g7865	406	ID=Fv_160_14.g7865;Description=hypothetical protein FVER14953_01071 [Fusarium verticillioides];Gene=FOC1_g10015736;Ontology_term=membrane,glycosyltransferase
Fv_160_14.g7866	305	ID=Fv_160_14.g7866;Description=hypothetical protein FVEG_01069 [Fusarium verticillioides 7600]
Fv_160_14.g7867	759	ID=Fv_160_14.g7867;Description=zinc finger transcription factor ace1 [Fusarium verticillioides 7600];Gene=Q04621;Ontology_term=nucleosome,nucleus,DNA binding,spermatogenesis,cell
Fv_160_14.g7868	538	ID=Fv_160_14.g7868;Description=hypothetical protein FVEG_01066 [Fusarium verticillioides 7600];Gene=FOYG_06904;Ontology_term=asparagine synthase (glutamine-hydrolyzing) activity,asparagine biosynthetic
Fv_160_14.g7869	260	ID=Fv_160_14.g7869;Description=hypothetical protein FCOIX_8592 [Fusarium coicis];Gene=FCIRC_12258;Ontology_term=membrane,asparagine synthase (glutamine-hydrolyzing) activity,asparagine biosynthetic
Fv_160_14.g7870	303	ID=Fv_160_14.g7870;Description=hypothetical protein FVEG_01064 [Fusarium verticillioides 7600]
Fv_160_14.g7871	412	ID=Fv_160_14.g7871;Description=hypothetical protein FVEG_01063 [Fusarium verticillioides
Fv_160_14.g7872	438	ID=Fv_160_14.g7872;Description=hypothetical protein FVER14953_01062 [Fusarium verticillioides];Gene=FOXG_00449;Ontology_term=transferase
Fv_160_14.g7873	159	ID=Fv_160_14.g7873;Description=hypothetical protein FVEG_14770 [Fusarium verticillioides 7600]
Fv_160_14.g7874	411	ID=Fv_160_14.g7874;Description=hypothetical protein J7337_000940 [Fusarium musae];Gene=FVER53590_01061;Ontology_term=anthranilate phosphoribosyltransferase activity,tryptophan biosynthetic
Fv_160_14.g7875	346	ID=Fv_160_14.g7875;Description=transcription initiation factor TFIIB [Fusarium verticillioides 7600];Gene=FPANT_1979;Ontology_term=nucleus,transcription preinitiation complex,RNA polymerase II complex recruiting activity,translation initiation factor activity,RNA polymerase II general transcription initiation factor activity,TBP-class protein binding,transcription open complex formation at RNA polymerase II promoter,transcriptional start site selection at RNA polymerase

Fv_160_14.g7876	437	ID=Fv_160_14.g7876;Description=L-asparaginase [Fusarium verticillioides 7600];Gene=FTJAE_7043;Ontology_term=cellular anatomical entity,asparaginase activity,asparagine catabolic
Fv_160_14.g7877	1442	ID=Fv_160_14.g7877;Description=hypothetical protein FVER14953_01058 [Fusarium verticillioides];Gene=FGLOB1_14507;Ontology_term=membrane,viral occlusion body,magnesium ion binding,structural molecule activity,ATP binding,ATP hydrolysis activity,phosphatidylethanolamine flippase activity,phospholipid transport,lipid translocation;Ontology_id=GO:0016020,GO:0039679,GO:0000287,GO:0005198,GO:0005524,GO:0016887,GO:0090555,GO:0015914
Fv_160_14.g7878	416	ID=Fv_160_14.g7878;Description=hypothetical protein FVEG_01057 [Fusarium verticillioides 7600];Gene=FOIG_00958;Ontology_term=oxidoreductase
Fv_160_14.g7879	586	ID=Fv_160_14.g7879;Description=hypothetical protein FVEG_01056 [Fusarium verticillioides 7600];Gene=Forpi1262_v001048;Ontology_term=chromosome, telomeric region,nucleus,cytoplasm,membrane,transmembrane transporter activity,ubiquitin binding,transmembrane
Fv_160_14.g7880	925	ID=Fv_160_14.g7880;Description=hypothetical protein FVEG_01055 [Fusarium verticillioides 7600];Gene=FNAPI_11018;Ontology_term=chromosome, telomeric region,nucleus,cytoplasm,ubiquitin
Fv_160_14.g7881	402	ID=Fv_160_14.g7881;Description=hypothetical protein FVEG_01054 [Fusarium verticillioides 7600]
Fv_160_14.g7882	643	ID=Fv_160_14.g7882;Description=hypothetical protein FVER53263_01053 [Fusarium verticillioides];Gene=FDENT_6969;Ontology_term=oxygen sensor activity,poly(A) binding,ferrous iron binding,regulation of translational fidelity,nuclear-transcribed mRNA catabolic process, deadenylation-dependent decay,negative regulation of SREBP signaling pathway,nucleus,L-ascorbic acid binding,regulation of translational termination,side of membrane,protein sequestering activity,cellular response to hypoxia,positive regulation of proteasomal ubiquitin-dependent protein catabolic process,transferase activity,peptidyl-proline dioxygenase activity;Ontology_id=GO:0019826,GO:0008143,GO:0008198,GO:0006450,GO:0000288,GO:2000639,GO:0005634,GO:0031418,GO:0
Fv_160_14.g7883	154	ID=Fv_160_14.g7883;Description=CFEM domain-containing protein [Fusarium pseudocircinatum];Gene=FVER53590_01052;Ontology_term=side of membrane,iron ion binding,L-ascorbic acid binding,peptidyl-proline dioxygenase
Fv_160_14.g7884	403	ID=Fv_160_14.g7884;Description=hypothetical protein FVER53590_01051 [Fusarium verticillioides];Gene=FVER53590_01051;Ontology_term=protein methylesterase
Fv_160_14.g7885	480	ID=Fv_160_14.g7885;Description=hypothetical protein FVEG_01050 [Fusarium verticillioides 7600];Gene=FVER53590_01050;Ontology_term=nucleus,DNA-binding transcription factor activity, RNA polymerase II-specific,zinc
Fv_160_14.g7886	136	ID=Fv_160_14.g7886;Description=hypothetical protein FVEG_01049 [Fusarium verticillioides
Fv_160_14.g7887	564	ID=Fv_160_14.g7887;Description=hypothetical protein FVEG_01048 [Fusarium verticillioides 7600];Gene=BFJ68_g10244;Ontology_term=cell division,positive regulation of cell cycle G2/M phase transition,protein tyrosine phosphatase activity;Ontology_id=GO:0051301,GO:1902751,GO:0004725;Enzyme_code=EC:3.1.3.48;Enzyme_name=protein-
Fv_160_14.g7888	553	ID=Fv_160_14.g7888;Description=hypothetical protein FVEG_01046 [Fusarium verticillioides 7600]

Fv_160_14.g7889	2958	ID=Fv_160_14.g7889;Description=papaya ringspot virus poly [Fusarium agapanthi];Gene=FPRO05_11569;Ontology_term=lyase activity;Ontology_id=GO:0016829;Enzyme_code=EC:4;Enzyme_name=Lyases
Fv_160_14.g7890	956	ID=Fv_160_14.g7890;Description=nicotinamide mononucleotide permease [Fusarium mundagurra];Gene=FGADI_5453;Ontology_term=membrane,transmembrane transporter activity,transmembrane
Fv_160_14.g7891	484	ID=Fv_160_14.g7891;Description=hypothetical protein FVEG_01041 [Fusarium verticillioides 7600];Gene=FMEXI_6709;Ontology_term=lyase activity;Ontology_id=GO:0016829;Enzyme_code=EC:4;Enzyme_name=Lyases
Fv_160_14.g7892	180	ID=Fv_160_14.g7892;Description=hypothetical protein FVER14953_01040 [Fusarium
Fv_160_14.g7893	730	ID=Fv_160_14.g7893;Description=SFH1 subunit of RSC complex [Fusarium denticulatum];Gene=C2S_12360;Ontology_term=nuclear chromosome,proton-transporting V-type ATPase, V1 domain,SWI/SNF superfamily-type complex,zinc ion binding,proton-transporting ATPase activity, rotational mechanism,chromatin remodeling,regulation of DNA-templated transcription,proton transmembrane transport;Ontology_id=GO:0000228,GO:0033180,GO:0070603,GO:0008270,GO:0046961,GO:0006338,GO:0006355,GO:1902600;En
Fv_160_14.g7894	514	ID=Fv_160_14.g7894;Description=exoglucanase type C [Fusarium verticillioides 7600];Gene=FTJAE_10299;Ontology_term=extracellular region,nucleus,RNA polymerase II cis-regulatory region sequence-specific DNA binding,DNA-binding transcription factor activity, RNA polymerase II-specific,hydrolase activity, hydrolyzing O-glycosyl compounds,cellulose binding,embryonic development via the syncytial blastoderm,regulation of transcription by RNA polymerase II,axonogenesis,ventral cord development,neuroblast development,cellulose catabolic process,spiracle morphogenesis, open tracheal system,brain segmentation,anterior head segmentation,dendrite
Fv_160_14.g7895	505	ID=Fv_160_14.g7895;Description=hypothetical protein FVER53590_01036 [Fusarium verticillioides];Gene=FOYG_06867;Ontology_term=membrane,transmembrane transporter activity,transmembrane
Fv_160_14.g7896	635	ID=Fv_160_14.g7896;Description=hypothetical protein FVEG_01035 [Fusarium verticillioides 7600];Gene=FTJAE_10301;Ontology_term=nucleus,DNA binding,zinc ion binding,DNA-binding transcription factor activity, RNA polymerase II-specific,regulation of transcription by RNA polymerase
Fv_160_14.g7897	933	ID=Fv_160_14.g7897;Description=hypothetical protein FVER53590_01034 [Fusarium verticillioides];Gene=FMAN_01007;Ontology_term=nucleus,DNA binding,RNA-directed DNA polymerase activity,zinc ion binding,RNA-templated DNA biosynthetic process,DNA-binding transcription factor activity, RNA polymerase II-specific,regulation of transcription by RNA polymerase
Fv_160_14.g7898	683	ID=Fv_160_14.g7898;Description=hypothetical protein FVER53590_01033 [Fusarium verticillioides]
Fv_160_14.g7899	552	ID=Fv_160_14.g7899;Description=hypothetical protein FNYG_09155 [Fusarium nygamai]
Fv_160_14.g7900	271	ID=Fv_160_14.g7900;Description=hypothetical protein FVER14953_01031 [Fusarium verticillioides];Gene=FNAPI_4026;Ontology_term=metal ion binding,superoxide metabolic
Fv_160_14.g7901	87	ID=Fv_160_14.g7901;Description=hypothetical protein FVEG_01030 [Fusarium verticillioides

Fv_160_14.g7902	298	ID=Fv_160_14.g7902;Description=oxidoreductase [Fusarium verticillioides 7600];Gene=Forpe1208_v001005;Ontology_term=oxidoreductase activity,cellular biosynthetic
Fv_160_14.g7903	585	ID=Fv_160_14.g7903;Description=hypothetical protein FVER53590_29025 [Fusarium verticillioides];Gene=FPHYL_8303;Ontology_term=oxidoreductase activity,cellular biosynthetic
Fv_160_14.g7904	211	ID=Fv_160_14.g7904;Description=hypothetical protein FVER14953_01028 [Fusarium
Fv_160_14.g7905	783	ID=Fv_160_14.g7905;Description=hypothetical protein FVER53590_01027 [Fusarium verticillioides];Gene=FMAN_01015;Ontology_term=guanyl-nucleotide exchange factor activity,ubiquitin binding,vesicle-mediated
Fv_160_14.g7906	241	ID=Fv_160_14.g7906;Description=hypothetical protein FVER53263_01026 [Fusarium verticillioides];Gene=FPANT_12317;Ontology_term=ligase
Fv_160_14.g7907	767	ID=Fv_160_14.g7907;Description=hypothetical protein FVER14953_01025 [Fusarium
Fv_160_14.g7908	1154	ID=Fv_160_14.g7908;Description=hypothetical protein FVEG_01024 [Fusarium verticillioides 7600]
Fv_160_14.g7909	992	ID=Fv_160_14.g7909;Description=hypothetical protein FVER53590_01023 [Fusarium verticillioides];Gene=FTJAE_10313;Ontology_term=nucleolus,DNA binding,regulation of DNA-templated
Fv_160_14.g7910	293	ID=Fv_160_14.g7910;Description=hypothetical protein FNYG_09165 [Fusarium nygamai];Gene=FNAPI_4015;Ontology_term=nucleus,proteasome core complex, alpha-subunit complex,proteasome storage granule,mRNA binding,proteasomal ubiquitin-independent protein catabolic process,proteasome-mediated ubiquitin-dependent
Fv_160_14.g7911	258	ID=Fv_160_14.g7911;Description=hypothetical protein FVEG_01021 [Fusarium verticillioides 7600]
Fv_160_14.g7912	418	ID=Fv_160_14.g7912;Description=hypothetical protein FVEG_01020 [Fusarium verticillioides 7600];Gene=C2S_9327;Ontology_term=chromosome, telomeric region,cytosol,nuclear periphery,GTPase activator activity,small GTPase binding,ribosomal subunit export from nucleus,RNA import into nucleus,rRNA export from nucleus,tRNA export from nucleus,protein import into nucleus,subtelomeric heterochromatin formation,positive regulation of protein export from
Fv_160_14.g7913	322	ID=Fv_160_14.g7913;Description=cytochrome c1, heme protein, mitochondrial [Fusarium oxysporum f. sp. lycopersici 4287];Gene=FGLOB1_11866;Ontology_term=mitochondrial respiratory chain complex III,ubiquinol-cytochrome-c reductase activity,heme binding,metal ion binding,mitochondrial electron transport, ubiquinol to cytochrome c,proton transmembrane transport;Ontology_id=GO:0005750,GO:0008121,GO:0020037,GO:0046872,GO:0006122,GO:1902600;Enzyme_code=EC:1.10,EC:7.1
Fv_160_14.g7914	228	ID=Fv_160_14.g7914;Description=hypothetical protein FVEG_01018 [Fusarium verticillioides 7600]
Fv_160_14.g7915	440	ID=Fv_160_14.g7915;Description=hypothetical protein LB506_000471, partial [Fusarium annulatum];Gene=FGADI_5442;Ontology_term=proteasome regulatory particle, base subcomplex,ATP binding,peptidase activity,ATP hydrolysis activity,ubiquitin protein ligase binding,proteasome-mediated ubiquitin-dependent protein catabolic process,positive regulation of protein catabolic process,positive regulation of RNA polymerase II transcription preinitiation complex assembly,proteasome regulatory particle

Fv_160_14.g7916	537	ID=Fv_160_14.g7916;Description=T-complex protein 1 subunit delta [Fusarium verticillioides 7600];Gene=FGADI_5441;Ontology_term=chaperonin-containing T-complex,ATP binding,ATP hydrolysis activity,unfolded protein binding,ATP-dependent protein folding chaperone,chaperone mediated protein folding independent of
Fv_160_14.g7917	125	ID=Fv_160_14.g7917;Description=hypothetical protein FVER14953_01015 [Fusarium verticillioides]
Fv_160_14.g7918	76	ID=Fv_160_14.g7918;Description=cytochrome c oxidase assembly protein subunit 17 [Fusarium verticillioides 7600];Gene=FOYG_06847;Ontology_term=mitochondrial intermembrane space,copper ion binding,copper chaperone
Fv_160_14.g7919	336	ID=Fv_160_14.g7919;Description=hypothetical protein J7337_000987 [Fusarium musae];Gene=FTJAE_10323;Ontology_term=hydrolase
Fv_160_14.g7920	348	ID=Fv_160_14.g7920;Description=hypothetical protein FVER14953_01012 [Fusarium verticillioides]
Fv_160_14.g7921	534	ID=Fv_160_14.g7921;Description=hypothetical protein FVER14953_01011 [Fusarium verticillioides]
Fv_160_14.g7922	1084	ID=Fv_160_14.g7922;Description=hypothetical protein FVER14953_01010 [Fusarium verticillioides]
Fv_160_14.g7923	1669	ID=Fv_160_14.g7923;Description=bloom syndrome protein [Fusarium verticillioides 7600];Gene=FVEG_01009;Ontology_term=nucleic acid binding,ATP binding,hydrolase activity,3'-5' DNA helicase activity,DNA replication,DNA repair,DNA recombination,DNA duplex unwinding;Ontology_id=GO:0003676,GO:0005524,GO:0016787,GO:0043138,GO:0006260,GO:0006281,GO:0006310,GO:0032508;E
Fv_160_14.g7924	1852	ID=Fv_160_14.g7924;Description=hypothetical protein FVEG_01008 [Fusarium verticillioides 7600]
Fv_160_14.g7925	196	ID=Fv_160_14.g7925;Description=hypothetical protein FVEG_01007 [Fusarium verticillioides
Fv_160_14.g7926	1090	ID=Fv_160_14.g7926;Description=fanconi anemia group M protein [Fusarium verticillioides 7600];Gene=FPANT_12337;Ontology_term=nucleus,DNA binding,ATP binding,ATP hydrolysis activity,3'-5' DNA helicase activity,DNA repair,DNA duplex unwinding;Ontology_id=GO:0005634,GO:0003677,GO:0005524,GO:0016887,GO:0043138,GO:0006281,GO:0032508;Enzyme_code=
Fv_160_14.g7927	658	ID=Fv_160_14.g7927;Description=hypothetical protein FVEG_01005 [Fusarium verticillioides 7600];Gene=FOC1_g10015804;Ontology_term=nucleoplasm,mRNA transport;Ontology_id=GO:0005654,GO:0051028
Fv_160_14.g7928	743	ID=Fv_160_14.g7928;Description=hypothetical protein FVER14953_01004 [Fusarium
Fv_160_14.g7929	657	ID=Fv_160_14.g7929;Description=hypothetical protein FVEG_01003 [Fusarium verticillioides 7600];Gene=FTJAE_10333;Ontology_term=protein dimerization activity;Ontology_id=GO:0046983
Fv_160_14.g7930	97	ID=Fv_160_14.g7930;Description=hypothetical protein J7337_000997 [Fusarium musae]

Fv_160_14.g7931	658	ID=Fv_160_14.g7931;Description=hypothetical protein J7337_000998 [Fusarium musae];Gene=FPANT_3508;Ontology_term=kinetochore,nucleoplasm,RENT complex,mitotic spindle pole body,mitotic actomyosin contractile ring, intermediate layer,nucleolar peripheral inclusion body,mitotic spindle midzone,protein serine/threonine phosphatase activity,protein tyrosine phosphatase activity,protein tyrosine/serine/threonine phosphatase activity,autophagy of mitochondrion,DNA damage response,dephosphorylation,positive regulation of septation initiation signaling,positive regulation of exit from mitosis,protein localization to kinetochore,mitotic cytokinesis checkpoint signaling,meiotic spindle disassembly,cellular response to osmotic stress,positive regulation of mitotic sister chromatid biorientation,mitotic actomyosin contractile ring maturation,protein localization to mitotic spindle pole body,positive regulation of mitotic spindle elongation,positive regulation of mitotic actomyosin contractile ring assembly,positive regulation of autophagosome
Fv_160_14.g7932	332	ID=Fv_160_14.g7932;Description=hypothetical protein FVER53590_01000 [Fusarium verticillioides];Gene=FPHYL_8275;Ontology_term=membrane,protein kinase activity,ATP binding,ATP hydrolysis activity,ABC-type transporter activity,transmembrane transport;Ontology_id=GO:0016020,GO:0004672,GO:0005524,GO:0016887,GO:0140359,GO:0055085;Enzyme_code=EC:2.7.1,EC:7.
Fv_160_14.g7933	1527	ID=Fv_160_14.g7933;Description=leptomycin B resistance pmd1 [Fusarium mundagurra];Gene=FGADI_12003;Ontology_term=membrane,protein kinase activity,ATP binding,ATP hydrolysis activity,ABC-type transporter activity,transmembrane transport;Ontology_id=GO:0016020,GO:0004672,GO:0005524,GO:0016887,GO:0140359,GO:0055085;Enzyme_code=EC:2.7.1,EC:7.
Fv_160_14.g7934	194	ID=Fv_160_14.g7934;Description=hypothetical protein FVEG_00997 [Fusarium verticillioides 7600];Gene=FVEG_00997;Ontology_term=acyltransferase activity, transferring groups other than amino-acyl
Fv_160_14.g7935	609	ID=Fv_160_14.g7935;Description=hypothetical protein FVEG_00996 [Fusarium verticillioides 7600];Gene=FOC1_g10015813;Ontology_term=membrane,transmembrane transporter activity,transmembrane
Fv_160_14.g7936	302	ID=Fv_160_14.g7936;Description=hypothetical protein FOXG_00518 [Fusarium oxysporum f. sp. lycopersici 4287];Gene=FOC1_g10015814;Ontology_term=endoplasmic reticulum membrane;Ontology_id=GO:0005789
Fv_160_14.g7937	528	ID=Fv_160_14.g7937;Description=hypothetical protein J7337_001005 [Fusarium musae];Gene=FPHYL_8271;Ontology_term=nucleus,DNA binding,catalytic activity,DNA
Fv_160_14.g7938	136	ID=Fv_160_14.g7938;Description=RNA-binding (RRM superfamily) [Fusarium coicis];Gene=FOYG_06826;Ontology_term=U2
Fv_160_14.g7939	458	ID=Fv_160_14.g7939;Description=hypothetical protein FVER14953_00992 [Fusarium
Fv_160_14.g7940	575	ID=Fv_160_14.g7940;Description=hypothetical protein FVEG_00991 [Fusarium verticillioides 7600];Gene=FTJAE_10343;Ontology_term=protein kinase activity,ATP
Fv_160_14.g7941	685	ID=Fv_160_14.g7941;Description=hypothetical protein FVEG_14760 [Fusarium verticillioides 7600];Gene=FCIRC_4786;Ontology_term=nucleus,membrane,DNA-binding transcription factor activity, RNA polymerase II-

Fv_160_14.g7942	385	ID=Fv_160_14.g7942;Description=hypothetical protein FVER14953_00988 [Fusarium verticillioides];Gene=FTJAE_10345;Ontology_term=alcohol dehydrogenase (NAD+) activity,structural constituent of eye lens,alcohol metabolic process;Ontology_id=GO:0004022,GO:0005212,GO:0006066;Enzyme_code=EC:1.1.1.1,EC:1.1.1.71;Enzyme_name=alcohol
Fv_160_14.g7943	593	ID=Fv_160_14.g7943;Description=hypothetical protein FVER14953_00987 [Fusarium verticillioides];Gene=FPCIR_13509;Ontology_term=membrane,3-phytase
Fv_160_14.g7944	513	ID=Fv_160_14.g7944;Description=hypothetical protein FVEG_00986 [Fusarium verticillioides 7600];Gene=FNAPI_3982;Ontology_term=plasma membrane,ammonium transmembrane transporter activity,ammonium
Fv_160_15.g7945	462	ID=Fv_160_15.g7945;Description=squalene synthase [Fusarium verticillioides 7600];Gene=FNAPI_4610;Ontology_term=endoplasmic reticulum membrane,farnesyl-diphosphate farnesyltransferase activity,squalene synthase activity,ergosterol biosynthetic process;Ontology_id=GO:0005789,GO:0004310,GO:0051996,GO:0006696;Enzyme_code=EC:2.5.1.29,EC:2.5.1.21;Enzyme_name=ge
Fv_160_15.g7946	442	ID=Fv_160_15.g7946;Description=hypothetical protein FVEG_03930 [Fusarium verticillioides 7600];Gene=FACUT_4925;Ontology_term=cellular anatomical entity,serine-type endopeptidase
Fv_160_15.g7947	224	ID=Fv_160_15.g7947;Description=hypothetical protein FVER53590_03931 [Fusarium verticillioides];Gene=862;Ontology_term=membrane organization,O-methyltransferase activity,posterior head segmentation,cell morphogenesis,periodic partitioning by pair rule gene,trunk segmentation,protein dimerization activity,DNA-binding transcription repressor activity, RNA polymerase II-specific,S-adenosylmethionine-dependent methyltransferase activity,open tracheal system development,salivary gland morphogenesis,negative regulation of transcription by RNA polymerase II,transcription corepressor binding,nucleus,response to hypoxia,tube morphogenesis,regulation of neurogenesis,methylation,E-box
Fv_160_15.g7948	622	ID=Fv_160_15.g7948;Description=hypothetical protein FVEG_03932 [Fusarium verticillioides 7600]
Fv_160_15.g7949	422	ID=Fv_160_15.g7949;Description=hypothetical protein FVER53590_03933 [Fusarium verticillioides];Gene=FVEG_03933;Ontology_term=endoplasmic reticulum membrane,glycolipid mannosyltransferase activity,alpha-
Fv_160_15.g7950	260	ID=Fv_160_15.g7950;Description=hypothetical protein FVEG_03934 [Fusarium verticillioides 7600];Gene=FPANT_11966;Ontology_term=nucleus,sequence-specific DNA binding;Ontology_id=GO:0005634,GO:0043565
Fv_160_15.g7951	836	ID=Fv_160_15.g7951;Description=hypothetical protein FVEG_03935 [Fusarium verticillioides 7600]
Fv_160_15.g7952	275	ID=Fv_160_15.g7952;Description=unnamed protein product [Fusarium fujikuroi];Gene=FPANT_11964;Ontology_term=fungal-type vacuole,mitochondrion,endoplasmic reticulum,membrane,apoptotic process,calcium-mediated signaling,endoplasmic reticulum unfolded protein
Fv_160_15.g7953	383	ID=Fv_160_15.g7953;Description=hypothetical protein FOXG_06069 [Fusarium oxysporum f. sp. lycopersici 4287];Gene=FPANT_11963;Ontology_term=cell cortex,septin cytoskeleton,GTP
Fv_160_15.g7954	381	ID=Fv_160_15.g7954;Description=hypothetical protein FVEG_03938 [Fusarium verticillioides 7600];Gene=C2S_11649;Ontology_term=fungal-type vacuole,endosome,phosphatidylinositol-5-phosphate binding,phosphatidylinositol-3-phosphate binding,phosphatidylinositol-4-phosphate binding,phosphatidylinositol-3,5-bisphosphate

Fv_160_15.g7955	323	ID=Fv_160_15.g7955;Description=hypothetical protein FVEG_03939 [Fusarium verticillioides 7600];Gene=FNAPI_4620;Ontology_term=RNA binding,mRNA splicing, via spliceosome;Ontology_id=GO:0003723,GO:0000398
Fv_160_15.g7956	524	ID=Fv_160_15.g7956;Description=hypothetical protein FVEG_15367 [Fusarium verticillioides 7600];Gene=FDENT_721;Ontology_term=cytoplasm,cytoplasmic dynein complex,microtubule,ATP binding,microtubule-based
Fv_160_15.g7957	720	ID=Fv_160_15.g7957;Description=hypothetical protein J7337_003949 [Fusarium
Fv_160_15.g7958	412	ID=Fv_160_15.g7958;Description=hypothetical protein FVEG_03943 [Fusarium verticillioides 7600];Gene=FDENT_723;Ontology_term=phagophore assembly site,autophagosome assembly,protein
Fv_160_15.g7959	416	ID=Fv_160_15.g7959;Description=hypothetical protein FVEG_03944 [Fusarium verticillioides 7600];Gene=His1;Ontology_term=phagophore assembly site,nucleosome,nucleus,double-stranded DNA binding,protein binding,structural constituent of chromatin,nucleosomal DNA binding,autophagosome assembly,nucleosome assembly,protein transport,chromosome condensation,heterochromatin formation,negative regulation of DNA recombination;Ontology_id=GO:0000407,GO:0000786,GO:0005634,GO:0003690,GO:0005515,GO:0030527,GO:0031492,GO:00000
Fv_160_15.g7960	552	ID=Fv_160_15.g7960;Description=hypothetical protein FVEG_03945 [Fusarium verticillioides 7600];Gene=BFJ72_g1728;Ontology_term=extracellular space,basolateral plasma membrane,thyroid-stimulating hormone receptor activity,ferric iron binding,G protein-coupled peptide receptor activity,protein-containing complex binding,intracellular iron ion homeostasis,cell surface receptor signaling pathway,adenylate cyclase-activating G protein-coupled receptor signaling pathway,hormone-mediated signaling pathway,iron ion transmembrane transport,thyroid-stimulating hormone signaling pathway,cellular response to glycoprotein,cellular response to thyrotropin-releasing
Fv_160_15.g7961	507	ID=Fv_160_15.g7961;Description=hypothetical protein FVEG_03946 [Fusarium verticillioides 7600];Gene=FPANT_11955;Ontology_term=membrane,ubiquitin-like modifier activating enzyme
Fv_160_15.g7962	724	ID=Fv_160_15.g7962;Description=hypothetical protein FVER53590_03948 [Fusarium verticillioides]
Fv_160_15.g7963	596	ID=Fv_160_15.g7963;Description=hypothetical protein FVER53263_03949 [Fusarium verticillioides];Gene=847;Ontology_term=cytoplasm,glutathione transferase activity,glutathione peroxidase activity,DDT-dehydrochlorinase activity,glutathione metabolic process,cellular oxidant detoxification;Ontology_id=GO:0005737,GO:0004364,GO:0004602,GO:0018833,GO:0006749,GO:0098869;Enzyme_code=EC:2.5.1.1
Fv_160_15.g7964	276	ID=Fv_160_15.g7964;Description=hypothetical protein J7337_003956 [Fusarium musae];Gene=1747;Ontology_term=calcium ion binding,transition metal ion binding,keratinization,intermediate filament
Fv_160_15.g7965	179	ID=Fv_160_15.g7965;Description=hypothetical protein FVEG_03951 [Fusarium verticillioides
Fv_160_15.g7966	732	ID=Fv_160_15.g7966;Description=hypothetical protein FVER14953_03952 [Fusarium verticillioides];Gene=FMEXI_14121;Ontology_term=protein kinase activity,ATP
Fv_160_15.g7967	741	ID=Fv_160_15.g7967;Description=hypothetical protein FVER14953_03953 [Fusarium verticillioides];Gene=FDENT_732;Ontology_term=chromosome, telomeric region,nucleus,DNA binding,regulation of DNA-templated

Fv_160_15.g7968	1499	ID=Fv_160_15.g7968;Description=hypothetical protein FVER14953_03954 [Fusarium verticillioides];Gene=FVEG_03954;Ontology_term=transcription factor TFIID complex,translation initiation factor
Fv_160_15.g7969	506	ID=Fv_160_15.g7969;Description=hypothetical protein FVER14953_03955 [Fusarium verticillioides];Gene=FVEG_03955;Ontology_term=actin cytoskeleton,SAS acetyltransferase complex,organelle
Fv_160_15.g7970	616	ID=Fv_160_15.g7970;Description=hypothetical protein FVEG_03956 [Fusarium verticillioides 7600];Gene=F25303_6434;Ontology_term=nucleus,actin cytoskeleton,SAS acetyltransferase complex,organelle
Fv_160_15.g7971	227	ID=Fv_160_15.g7971;Description=hypothetical protein FVER53590_03957 [Fusarium verticillioides];Gene=BFJ68_g7897;Ontology_term=vacuolar membrane,endosome membrane,phosphatidylinositol-3-phosphate
Fv_160_15.g7972	204	ID=Fv_160_15.g7972;Description=hypothetical protein FVER14953_03958 [Fusarium verticillioides];Gene=FDENT_7593;Ontology_term=transferase
Fv_160_15.g7973	435	ID=Fv_160_15.g7973;Description=hypothetical protein FVEG_03959 [Fusarium verticillioides 7600];Gene=FOTG_03715;Ontology_term=membrane,phosphotransferase activity, for other substituted phosphate groups,phospholipid biosynthetic
Fv_160_15.g7974	397	ID=Fv_160_15.g7974;Description=hypothetical protein FVEG_03960 [Fusarium verticillioides 7600];Gene=FOTG_03714;Ontology_term=peptidase activity,glutaminyl-peptide cyclotransferase activity,metal ion binding,proteolysis;Ontology_id=GO:0008233,GO:0016603,GO:0046872,GO:0006508;Enzyme_code=EC:3.4,EC:2.3.2.5;Enzyme_nam
Fv_160_15.g7975	318	ID=Fv_160_15.g7975;Description=hypothetical protein FVEG_03961 [Fusarium verticillioides 7600];Gene=FNAPI_4248;Ontology_term=arylamine N-acetyltransferase
Fv_160_15.g7976	282	ID=Fv_160_15.g7976;Description=hypothetical protein FVEG_03962 [Fusarium verticillioides 7600];Gene=HZS61_013665;Ontology_term=oligosaccharyltransferase complex,transferase
Fv_160_15.g7977	355	ID=Fv_160_15.g7977;Description=hypothetical protein FVER53263_03963 [Fusarium verticillioides];Gene=FPANT_5243;Ontology_term=hydrolase
Fv_160_15.g7978	569	ID=Fv_160_15.g7978;Description=glycylpeptide N-tetradecanoyltransferase [Fusarium verticillioides 7600];Gene=FNYG_00757;Ontology_term=cytosol,glycylpeptide N-tetradecanoyltransferase
Fv_160_15.g7979	392	ID=Fv_160_15.g7979;Description=hypothetical protein FVER53263_03965 [Fusarium verticillioides];Gene=FVEG_03965;Ontology_term=membrane,hydrolase
Fv_160_15.g7980	751	ID=Fv_160_15.g7980;Description=hypothetical protein FVEG_03966 [Fusarium verticillioides 7600];Gene=FVEG_03966;Ontology_term=Swr1 complex,ATP-dependent H2AZ histone chaperone activity,chromatin
Fv_160_15.g7981	1442	ID=Fv_160_15.g7981;Description=hypothetical protein FVER14953_03967 [Fusarium verticillioides]
Fv_160_15.g7982	605	ID=Fv_160_15.g7982;Description=hypothetical protein FVEG_03968 [Fusarium verticillioides 7600]
Fv_160_15.g7983	224	ID=Fv_160_15.g7983;Description=hypothetical protein FVER14953_03969 [Fusarium
Fv_160_15.g7984	896	ID=Fv_160_15.g7984;Description=hypothetical protein FVER53590_03970 [Fusarium verticillioides];Gene=FNAPI_4257;Ontology_term=calcium ion binding,zinc ion binding;Ontology_id=GO:0005509,GO:0008270

Fv_160_15.g7985	406	ID=Fv_160_15.g7985;Description=hypothetical protein FVER53590_03971 [Fusarium verticillioides];Gene=1727
Fv_160_15.g7986	235	ID=Fv_160_15.g7986;Description=AraC family transcriptional regulator [Fusarium verticillioides 7600];Gene=ada;Ontology_term=DNA repair,zinc ion binding,DNA-binding transcription factor activity,regulation of DNA-templated transcription,methyltransferase activity,sequence-specific DNA binding,methylation;Ontology_id=GO:0006281,GO:0008270,GO:0003700,GO:0006355,GO:0008168,GO:0043565,GO:0032259;Enzy
Fv_160_15.g7987	521	ID=Fv_160_15.g7987;Description=hypothetical protein FVEG_15375 [Fusarium verticillioides 7600];Gene=Focb16_v011911;Ontology_term=membrane,sphingosine N-acyltransferase activity,ceramide biosynthetic
Fv_160_15.g7988	641	ID=Fv_160_15.g7988;Description=hypothetical protein FVEG_03974 [Fusarium verticillioides 7600];Gene=FDENT_7609;Ontology_term=ligase activity;Ontology_id=GO:0016874;Enzyme_code=EC:6;Enzyme_name=Ligases
Fv_160_15.g7989	389	ID=Fv_160_15.g7989;Description=alpha 1,6-mannosyltransferase [Fusarium verticillioides 7600];Gene=FOYG_04606;Ontology_term=alpha-1,6-mannosyltransferase activity,carbohydrate derivative metabolic
Fv_160_15.g7990	264	ID=Fv_160_15.g7990;Description=hypothetical protein FVEG_03976 [Fusarium verticillioides 7600]
Fv_160_15.g7991	168	ID=Fv_160_15.g7991;Description=hypothetical protein FVEG_03977 [Fusarium verticillioides 7600];Gene=FVEG_03977;Ontology_term=ribonuclease MRP complex,nucleolar ribonuclease P complex,nucleic acid binding,ribonuclease P activity,tRNA 5'-leader
Fv_160_15.g7992	303	ID=Fv_160_15.g7992;Description=hypothetical protein FVEG_03978 [Fusarium verticillioides 7600]
Fv_160_15.g7993	354	ID=Fv_160_15.g7993;Description=hypothetical protein FVER53263_03979 [Fusarium verticillioides];Gene=hemA
Fv_160_15.g7994	82	ID=Fv_160_15.g7994;Description=hypothetical protein J7337_003985 [Fusarium musae];Gene=FOMG_12021;Ontology_term=proton-transporting ATP synthase complex, coupling factor F(o),proton transmembrane transporter activity,proton motive force-driven ATP synthesis,proton transmembrane
Fv_160_15.g7995	260	ID=Fv_160_15.g7995;Description=hypothetical protein FVEG_03981 [Fusarium verticillioides 7600];Gene=FMUND_5603;Ontology_term=cytoplasm,heterotrimeric G-protein complex,photoreceptor disc membrane,protein binding,hydrolase activity,signaling receptor complex adaptor activity,adenylate cyclase-activating dopamine receptor signaling pathway,cellular response to prostaglandin E
Fv_160_15.g7996	481	ID=Fv_160_15.g7996;Description=26S proteasome regulatory subunit N5 [Fusarium verticillioides 7600];Gene=C2S_11692;Ontology_term=COP9 signalosome,proteasome regulatory particle, lid subcomplex,proteasome storage granule,proteasome-mediated ubiquitin-dependent protein catabolic
Fv_160_15.g7997	171	ID=Fv_160_15.g7997;Description=hypothetical protein FVEG_03983 [Fusarium verticillioides 7600];Gene=BFJ70_g9694;Ontology_term=cytoplasm,spindle microtubule,DASH complex,attachment of spindle microtubules to
Fv_160_15.g7998	324	ID=Fv_160_15.g7998;Description=SHO1 osmosensor [Fusarium verticillioides 7600];Gene=FDENT_7618;Ontology_term=plasma
Fv_160_15.g7999	203	ID=Fv_160_15.g7999;Description=hypothetical protein FVEG_03985 [Fusarium verticillioides 7600];Gene=FCIRC_12225;Ontology_term=dynactin complex,cytoskeleton-dependent

Fv_160_15.g8000	526	ID=Fv_160_15.g8000;Description=aspartate/glutamate transporter [Fusarium oxysporum Fo47];Gene=FOYG_04619;Ontology_term=fungal-type vacuole membrane,L-histidine transmembrane transporter activity,L-tyrosine transmembrane transporter activity,L-glutamate transmembrane transporter activity,L-lysine transmembrane transporter activity,L-serine transmembrane transporter activity,L-arginine transmembrane transporter activity,L-histidine transmembrane import into vacuole,L-tyrosine transmembrane import into vacuole,L-glutamate transmembrane import into vacuole,L-serine transmembrane import into vacuole,L-arginine transmembrane import into vacuole,L-glutamate import involved in cellular response to nitrogen starvation,L-lysine import into vacuole involved in cellular response to nitrogen
Fv_160_15.g8001	114	ID=Fv_160_15.g8001;Description=ribosomal protein S16, mitochondrial [Fusarium verticillioides 7600];Gene=FMUND_543;Ontology_term=mitochondrial small ribosomal subunit,structural constituent of
Fv_160_15.g8002	236	ID=Fv_160_15.g8002;Description=hypothetical protein J7337_003993 [Fusarium musae];Gene=FMUND_542;Ontology_term=mitochondrion,nucleotide binding,metal ion binding,NADHX epimerase activity,NADPHX epimerase
Fv_160_15.g8003	5397	ID=Fv_160_15.g8003;Description=hypothetical protein FVEG_03990 [Fusarium verticillioides
Fv_160_15.g8004	1355	ID=Fv_160_15.g8004;Description=hypothetical protein FVER14953_03991 [Fusarium verticillioides];Gene=BFJ72_g1969;Ontology_term=phosphoribosylformylglycinamidine synthase activity,ATP binding,metal ion binding,'de novo' IMP biosynthetic process,glutamine metabolic
Fv_160_15.g8005	545	ID=Fv_160_15.g8005;Description=hypothetical protein FVER14953_20874 [Fusarium verticillioides];Gene=HZS61_012300;Ontology_term=membrane,transmembrane transporter activity,carbohydrate
Fv_160_15.g8006	892	ID=Fv_160_15.g8006;Description=hypothetical protein FVEG_03993 [Fusarium verticillioides 7600]
Fv_160_15.g8007	232	ID=Fv_160_15.g8007;Description=hypothetical protein FVER14953_03994 [Fusarium verticillioides];Gene=FocTR4_00007239;Ontology_term=protein modification process;Ontology_id=GO:0036211
Fv_160_15.g8008	129	ID=Fv_160_15.g8008;Description=hypothetical protein FVEG_03995 [Fusarium verticillioides 7600];Gene=TNP2;Ontology_term=nucleosome,nucleolus,DNA binding,zinc ion binding,acrosome reaction,penetration of zona pellucida,positive regulation of protein processing,sperm DNA
Fv_160_15.g8009	411	ID=Fv_160_15.g8009;Description=lipoyl synthase, mitochondrial [Fusarium verticillioides 7600];Gene=FNYG_00727;Ontology_term=mitochondrion,lipoate synthase activity,metal ion binding,4 iron, 4 sulfur cluster binding,lipoate biosynthetic
Fv_160_15.g8010	768	ID=Fv_160_15.g8010;Description=hypothetical protein FVER53590_03997 [Fusarium verticillioides];Gene=FNAPI_12964;Ontology_term=polygalacturonase
Fv_160_15.g8011	338	ID=Fv_160_15.g8011;Description=hypothetical protein J7337_004004 [Fusarium musae]
Fv_160_15.g8012	947	ID=Fv_160_15.g8012;Description=37S ribosomal Rsm22 [Fusarium pseudocircinatum];Gene=FMUND_532;Ontology_term=mitochondrion,S-adenosylmethionine-dependent methyltransferase activity,translation,methylation;Ontology_id=GO:0005739,GO:0008757,GO:0006412,GO:0032259;Enzyme_code=EC:2.1.1;Enzyme_n

Fv_160_15.g8013	575	ID=Fv_160_15.g8013;Description=hypothetical protein FVER14953_04001 [Fusarium verticillioides];Gene=BFJ72_g1739;Ontology_term=cytoplasm,calcineurin complex,uroporphyrin-III C-methyltransferase activity,calmodulin binding,oxidoreductase activity,myosin phosphatase activity,calmodulin-dependent protein phosphatase activity,metal ion binding,sulfate assimilation,adaptation of signaling pathway by response to pheromone involved in conjugation with cellular fusion,intracellular monoatomic ion homeostasis,siroheme biosynthetic process,regulation of cell morphogenesis,fungal-type cell wall organization,methylation,positive regulation of DNA-templated transcription,calcineurin-mediated signaling;Ontology_id=GO:0005737,GO:0005955,GO:0004851,GO:0005516,GO:0016491,GO:0017018,GO:0033192,GO:0046872,GO:
Fv_160_15.g8014	602	ID=Fv_160_15.g8014;Description=uncharacterized protein FTJAE_6597 [Fusarium tjaetaba];Gene=purE
Fv_160_15.g8015	108	ID=Fv_160_15.g8015;Description=hypothetical protein FVER53263_04002 [Fusarium verticillioides]
Fv_160_15.g8016	429	ID=Fv_160_15.g8016;Description=hypothetical protein FVEG_04003 [Fusarium verticillioides 7600];Gene=F25303_7644;Ontology_term=catalytic activity,pyridoxal phosphate binding,biosynthetic
Fv_160_15.g8017	305	ID=Fv_160_15.g8017;Description=hypothetical protein J7337_004011 [Fusarium musae];Gene=BFJ72_g1948;Ontology_term=chromosome, centromeric region,spindle pole,nucleus,cytoplasm,actin cytoskeleton,phosphoribosylaminoimidazolesuccinocarboxamide synthase activity,ATP binding,myosin phosphatase activity,metal ion binding,'de novo' IMP biosynthetic process,organelle organization;Ontology_id=GO:0000775,GO:0000922,GO:0005634,GO:0005737,GO:0015629,GO:0004639,GO:0005524,GO:0017018,
Fv_160_15.g8018	559	ID=Fv_160_15.g8018;Description=hypothetical protein FVER53590_25870 [Fusarium verticillioides];Gene=FOIG_05959;Ontology_term=nucleus,DNA binding,zinc ion binding,DNA-binding transcription factor activity, RNA polymerase II-specific,regulation of transcription by RNA polymerase
Fv_160_15.g8019	875	ID=Fv_160_15.g8019;Description=hypothetical protein FVEG_04005 [Fusarium verticillioides 7600];Gene=FOC1_g10011316;Ontology_term=nuclear envelope,cytosol,nuclear periphery,NLS-dependent protein nuclear import complex,mitotic spindle midzone,guanyl-nucleotide exchange factor activity,small GTPase binding,protein-containing complex binding,nuclear import signal receptor activity,importin-alpha family protein binding,disordered domain specific binding,NLS-bearing protein import into nucleus,protein targeting to membrane,phosphatidylcholine biosynthetic process,regulation of nucleocytoplasmic transport,nuclear pore complex assembly,regulation of protein
Fv_160_15.g8020	533	ID=Fv_160_15.g8020;Description=hypothetical protein FVEG_04006 [Fusarium verticillioides 7600]
Fv_160_15.g8021	783	ID=Fv_160_15.g8021;Description=hypothetical protein FVER53590_04007 [Fusarium verticillioides];Gene=FOC1_g10011314;Ontology_term=cytoplasm,6-phosphofructokinase activity,ATP binding,metal ion binding,fructose 6-phosphate metabolic process,phosphorylation,glycolytic process through fructose-6-
Fv_160_15.g8022	561	ID=Fv_160_15.g8022;Description=hypothetical protein FVER53590_04008 [Fusarium verticillioides]

Fv_160_15.g8023	354	ID=Fv_160_15.g8023;Description=hypothetical protein FVEG_15386 [Fusarium verticillioides 7600];Gene=FMEXI_10505;Ontology_term=immunological synapse,basolateral plasma membrane,synaptic vesicle membrane,presynaptic membrane,cell projection,P-type calcium transporter activity,calmodulin binding,ATP binding,ATP hydrolysis activity,metal ion binding,negative regulation of cytokine production,regulation of vascular associated smooth muscle contraction,regulation of blood pressure,positive regulation of bone mineralization,regulation of cytosolic calcium ion concentration,negative regulation of cytosolic calcium ion concentration,positive regulation of calcium ion transport,calcium ion transmembrane transport,regulation of cellular response to insulin stimulus;Ontology_id=GO:0001772,GO:0016323,GO:0030672,GO:0042734,GO:0042995,GO:0005388,GO:0005516,GO:0005524,GO:
Fv_160_15.g8024	597	ID=Fv_160_15.g8024;Description=hypothetical protein FVEG_15387 [Fusarium verticillioides
Fv_160_15.g8025	578	ID=Fv_160_15.g8025;Description=hypothetical protein FVEG_04012 [Fusarium verticillioides 7600];Gene=FPANT_101;Ontology_term=membrane,transmembrane transporter activity,transmembrane
Fv_160_15.g8026	681	ID=Fv_160_15.g8026;Description=hypothetical protein FVEG_04013 [Fusarium verticillioides
Fv_160_15.g8027	282	ID=Fv_160_15.g8027;Description=hypothetical protein FVER14953_20692 [Fusarium verticillioides];Gene=F25303_8866;Ontology_term=positive regulation of canonical NF-kappaB signal transduction,B cell activation,positive regulation of NF-kappaB transcription factor activity,post-translational protein modification,positive regulation of angiogenesis,ATP binding,chromatin remodeling,positive regulation of DNA-templated transcription,positive regulation of vascular endothelial growth factor receptor signaling pathway,apoptotic process,phosphorylation,protein serine kinase activity,zinc ion binding,adaptive immune response,chromatin binding,negative regulation of insulin receptor signaling pathway,calcium,diacylglycerol-dependent serine/threonine kinase activity,regulation of transcription by RNA polymerase II,positive regulation of B cell receptor signaling pathway,nucleus,negative regulation of glucose transmembrane transport,nuclear androgen receptor binding,histone binding,cytoplasm,membrane,nuclear receptor coactivator activity,histone H3T6 kinase activity;Ontology_id=GO:0043123,GO:0042113,GO:0051092,GO:0043687,GO:0045766,GO:0005524,GO:0006338,GO:0045893,GO:0
Fv_160_15.g8028	436	ID=Fv_160_15.g8028;Description=hypothetical protein FVEG_04014 [Fusarium verticillioides 7600];Gene=FOMG_12064;Ontology_term=nucleus,DNA-binding transcription factor activity, RNA polymerase II-specific,zinc ion
Fv_160_15.g8029	552	ID=Fv_160_15.g8029;Description=T-complex protein 1 subunit epsilon [Fusarium verticillioides 7600];Gene=HZS61_012323;Ontology_term=chaperonin-containing T-complex,ATP binding,ATP hydrolysis activity,unfolded protein binding,ATP-dependent protein folding chaperone,chaperone mediated protein folding independent of
Fv_160_15.g8030	794	ID=Fv_160_15.g8030;Description=elongation factor 1-alpha [Fusarium verticillioides 7600];Gene=FACUT_11070;Ontology_term=cytoplasm,translation elongation factor activity,GTPase activity,GTP binding,translational elongation,regulation of

Fv_160_15.g8031	454	ID=Fv_160_15.g8031;Description=hypothetical protein FVER14953_04017 [Fusarium verticillioides];Gene=hisG;Ontology_term=positive regulation of canonical NF-kappaB signal transduction,B cell activation,positive regulation of NF-kappaB transcription factor activity,post-translational protein modification,positive regulation of angiogenesis,ATP binding,chromatin remodeling,positive regulation of DNA-templated transcription,positive regulation of vascular endothelial growth factor receptor signaling pathway,apoptotic process,phosphorylation,protein serine kinase activity,zinc ion binding,adaptive immune response,chromatin binding,negative regulation of insulin receptor signaling pathway,calcium,diacylglycerol-dependent serine/threonine kinase activity,regulation of transcription by RNA polymerase II,positive regulation of B cell receptor signaling pathway,nucleus,negative regulation of glucose transmembrane transport,nuclear androgen receptor binding,histone binding,cytoplasm,membrane,nuclear receptor coactivator activity,histone H3T6 kinase activity;Ontology_id=GO:0043123,GO:0042113,GO:0051092,GO:0043687,GO:0045766,GO:0005524,GO:0006338,GO:0045893,GO:0
Fv_160_15.g8032	356	ID=Fv_160_15.g8032;Description=hypothetical protein FVER14953_04018 [Fusarium verticillioides]
Fv_160_15.g8033	663	ID=Fv_160_15.g8033;Description=glucose-regulated protein [Fusarium verticillioides 7600];Gene=FPANT_94;Ontology_term=nuclear membrane,luminal surveillance complex,perinuclear endoplasmic reticulum lumen,cortical endoplasmic reticulum lumen,ATP binding,protein-transporting ATPase activity,ATP hydrolysis activity,unfolded protein binding,ATP-dependent protein folding chaperone,karyogamy involved in conjugation with cellular fusion,protein folding,SRP-dependent cotranslational protein targeting to membrane, translocation,ubiquitin-dependent ERAD pathway,post-translational protein targeting to membrane, translocation,IRE1-mediated unfolded protein response,fungal-type cell wall beta-glucan biosynthetic
Fv_160_15.g8034	443	ID=Fv_160_15.g8034;Description=hypothetical protein FVER14953_04020 [Fusarium verticillioides]
Fv_160_15.g8035	379	ID=Fv_160_15.g8035;Description=hypothetical protein FVEG_04021 [Fusarium verticillioides 7600]
Fv_160_15.g8036	602	ID=Fv_160_15.g8036;Description=hypothetical protein FVER14953_04022 [Fusarium verticillioides];Gene=FMAN_03039;Ontology_term=endonuclease activity,exonuclease
Fv_160_15.g8037	470	ID=Fv_160_15.g8037;Description=nicotinate (nicotinamide) nucleotide adenylyltransferase [Fusarium coicis];Gene=BFJ68_g7256;Ontology_term=nicotinamide-nucleotide adenylyltransferase activity,nicotinate-nucleotide adenylyltransferase activity,ATP binding,NAD biosynthetic
Fv_160_15.g8038	598	ID=Fv_160_15.g8038;Description=hypothetical protein FVEG_04024 [Fusarium verticillioides 7600];Gene=FNAPI_5214;Ontology_term=plasma membrane,potassium ion transmembrane transporter activity,oxidoreductase activity,potassium ion transmembrane
Fv_160_15.g8039	567	ID=Fv_160_15.g8039;Description=hypothetical protein FVER14953_04025 [Fusarium verticillioides];Gene=FNAPI_5215;Ontology_term=plasma membrane,potassium ion transmembrane transporter activity,oxidoreductase activity,potassium ion transmembrane
Fv_160_15.g8040	124	ID=Fv_160_15.g8040;Description=hypothetical protein FVEG_04026 [Fusarium verticillioides 7600]

Fv_160_15.g8041	451	ID=Fv_160_15.g8041;Description=hypothetical protein FVER14953_04027 [Fusarium verticillioides];Gene=FACUT_11060;Ontology_term=protein serine/threonine kinase activity,ATP
Fv_160_15.g8042	237	ID=Fv_160_15.g8042;Description=hypothetical protein FPCIR_8135 [Fusarium pseudocircinatum]
Fv_160_15.g8043	615	ID=Fv_160_15.g8043;Description=hypothetical protein FVER53590_04029 [Fusarium verticillioides]
Fv_160_15.g8044	357	ID=Fv_160_15.g8044;Description=lactoylglutathione lyase [Fusarium verticillioides 7600];Gene=BFJ65_g8031;Ontology_term=lactoylglutathione lyase activity,metal ion
Fv_160_15.g8045	569	ID=Fv_160_15.g8045;Description=hypothetical protein FVER14953_04031 [Fusarium verticillioides];Gene=Forpe1208_v005997;Ontology_term=phosphoglycerate dehydrogenase activity,NAD binding,L-serine
Fv_160_15.g8046	569	ID=Fv_160_15.g8046;Description=hypothetical protein FVEG_04032 [Fusarium verticillioides 7600];Gene=C2S_8729;Ontology_term=extracellular region,membrane,nucleic acid binding,ribonuclease A activity,lyase activity,identical protein binding,metabolic process,magnesium ion homeostasis,defense response to Gram-positive bacterium;Ontology_id=GO:0005576,GO:0016020,GO:0003676,GO:0004522,GO:0016829,GO:0042802,GO:0008152,GO:0010960,G
Fv_160_15.g8047	364	ID=Fv_160_15.g8047;Description=hypothetical protein FVER14953_04034 [Fusarium verticillioides];Gene=thyX;Ontology_term=nucleus,DNA-binding transcription factor activity, RNA polymerase II-specific,sequence-specific DNA binding,regulation of glutamine family amino acid metabolic process,cellular response to starvation,regulation of mitochondrion organization,positive regulation of transcription by RNA polymerase II,positive regulation of oxidative
Fv_160_15.g8048	804	ID=Fv_160_15.g8048;Description=hypothetical protein FVER14953_04035 [Fusarium verticillioides];Gene=FPCIR_8141;Ontology_term=actin binding,small GTPase binding,actin cytoskeleton
Fv_160_15.g8049	228	ID=Fv_160_15.g8049;Description=hypothetical protein FVEG_04037 [Fusarium verticillioides 7600]
Fv_160_15.g8050	255	ID=Fv_160_15.g8050;Description=hypothetical protein FOXG_06182 [Fusarium oxysporum f. sp. lycopersici 4287];Gene=FOMG_12086;Ontology_term=extracellular region,nucleic acid binding,transcription coactivator activity,endonuclease activity,positive regulation of DNA-templated
Fv_160_15.g8051	134	ID=Fv_160_15.g8051;Description=ubiquionol cytochrome C reductase hinge [Fusarium napiforme];Gene=FACUT_11050;Ontology_term=nucleus,mitochondrial respiratory chain complex III,RNA polymerase II cis-regulatory region sequence-specific DNA binding,DNA-binding transcription activator activity, RNA polymerase II-specific,actin binding,protein homodimerization activity,epithelial cell morphogenesis,mitochondrial electron transport, ubiquinol to cytochrome c,ganglion mother cell fate determination,ventral cord development,tracheal outgrowth, open tracheal system,R3/R4 cell fate commitment,chitin-based embryonic cuticle biosynthetic process,fibroblast growth factor receptor signaling pathway,regulation of tube length, open tracheal system,rhabdomere development,regulation of cell population proliferation,positive regulation of transcription by RNA polymerase II,regulation of neurogenesis,membrane organization,regulation of wound
Fv_160_15.g8052	487	ID=Fv_160_15.g8052;Description=hypothetical protein FVER14953_04040 [Fusarium verticillioides];Gene=Forpi1262_v006105;Ontology_term=membrane,transmembrane transporter activity,transmembrane

Fv_160_15.g8053	475	ID=Fv_160_15.g8053;Description=hypothetical protein FVEG_04041 [Fusarium verticillioides 7600];Gene=gcvH;Ontology_term=cytoplasm,RNA binding,arylamine N-acetyltransferase activity,peptide-methionine (S)-S-oxide reductase activity,L-methionine-(S)-S-oxide reductase activity,sulfur amino acid metabolic process,determination of adult lifespan,cellular response to oxidative stress;Ontology_id=GO:0005737,GO:0003723,GO:0004060,GO:0008113,GO:0036456,GO:0000096,GO:0008340,GO:0034599;Enzym
Fv_160_15.g8054	1272	ID=Fv_160_15.g8054;Description=hypothetical protein FVER14953_04042 [Fusarium
Fv_160_15.g8055	862	ID=Fv_160_15.g8055;Description=hypothetical protein FVER14953_04043 [Fusarium verticillioides];Gene=FOQG_04508;Ontology_term=incipient cellular bud site,extracellular space,nucleus,cytoplasm,mating projection tip,serine-type endopeptidase inhibitor activity,ATP binding,histone H2BS14 kinase activity,vacuole inheritance,negative regulation of transcription by RNA polymerase II,pheromone-dependent signal transduction involved in conjugation with cellular fusion,signal transduction involved in filamentous growth,invasive growth in response to glucose limitation,chromatin remodeling,regulation of exit from mitosis,budding cell apical bud growth,bipolar cellular bud site selection,pseudohyphal growth,osmosensory signaling pathway via Sho1 osmosensor,negative regulation of gene expression,phosphorylation,stress granule assembly,sterol import,positive regulation of apoptotic process;Ontology_id=GO:0000131,GO:0005615,GO:0005634,GO:0005737,GO:0043332,GO:0004867,GO:0005524,GO:0044025,GO:
Fv_160_15.g8056	117	ID=Fv_160_15.g8056;Description=hypothetical protein FVEG_15390 [Fusarium verticillioides 7600]
Fv_160_15.g8057	362	ID=Fv_160_15.g8057;Description=hypothetical protein FVEG_04044 [Fusarium verticillioides 7600];Gene=FMUND_488;Ontology_term=oxidoreductase activity,cellular biosynthetic
Fv_160_15.g8058	938	ID=Fv_160_15.g8058;Description=hypothetical protein FVEG_04045 [Fusarium verticillioides 7600];Gene=FPCIR_8151;Ontology_term=DNA binding,regulation of DNA-templated
Fv_160_15.g8059	561	ID=Fv_160_15.g8059;Description=hypothetical protein FVEG_04046 [Fusarium verticillioides 7600]
Fv_160_15.g8060	203	ID=Fv_160_15.g8060;Description=hypothetical protein FVEG_15392 [Fusarium verticillioides 7600]
Fv_160_15.g8061	406	ID=Fv_160_15.g8061;Description=hypothetical protein FVEG_04047 [Fusarium verticillioides 7600]
Fv_160_15.g8062	97	ID=Fv_160_15.g8062;Description=blocked early in transport 1 [Fusarium oxysporum f. sp. lycopersici 4287];Gene=F53441_2141;Ontology_term=Golgi trans cisterna,SNARE complex,SNAP receptor activity,intracellular protein transport,endoplasmic reticulum to Golgi vesicle-mediated transport,intra-Golgi vesicle-mediated transport,vesicle
Fv_160_15.g8063	558	ID=Fv_160_15.g8063;Description=hypothetical protein FVEG_04049 [Fusarium verticillioides 7600];Gene=BFJ72_g1915;Ontology_term=GTPase activator activity,metal ion binding;Ontology_id=GO:0005096,GO:0046872
Fv_160_15.g8064	202	ID=Fv_160_15.g8064;Description=hypothetical protein FVEG_04050 [Fusarium verticillioides 7600];Gene=F25303_8833;Ontology_term=peptide-methionine (R)-S-oxide reductase activity,metal ion binding,response to oxidative stress,protein
Fv_160_15.g8065	225	ID=Fv_160_15.g8065;Description=hypothetical protein FVEG_04051 [Fusarium verticillioides 7600]

Fv_160_15.g8066	386	ID=Fv_160_15.g8066;Description=hypothetical protein FVER53590_04052 [Fusarium verticillioides];Gene=FSUBG_4443;Ontology_term=acyltransferase activity, transferring groups other than amino-acyl
Fv_160_15.g8067	418	ID=Fv_160_15.g8067;Description=3-ketoacyl thiolase peroxisomal [Fusarium pseudoanthophilum];Gene=FPCIR_8160;Ontology_term=acetyl-CoA C-acyltransferase
Fv_160_15.g8068	464	ID=Fv_160_15.g8068;Description=hypothetical protein FVER53590_04054 [Fusarium verticillioides]
Fv_160_15.g8069	149	ID=Fv_160_15.g8069;Description=golgi apparatus membrane protein TVP18 [Fusarium verticillioides 7600];Gene=FMEXI_13050;Ontology_term=Golgi membrane;Ontology_id=GO:0000139
Fv_160_15.g8070	944	ID=Fv_160_15.g8070;Description=hypothetical protein FVER53263_04056 [Fusarium verticillioides]
Fv_160_15.g8071	398	ID=Fv_160_15.g8071;Description=hypothetical protein FVEG_04057 [Fusarium verticillioides 7600];Gene=TfIIS;Ontology_term=nucleus,DNA binding,translation elongation factor activity,zinc ion binding,transcription
Fv_160_15.g8072	1575	ID=Fv_160_15.g8072;Description=hypothetical protein FVER53263_04058 [Fusarium verticillioides];Gene=FNAPI_5246;Ontology_term=nucleus,chromosome,DNA binding,ATP binding,ATP hydrolysis activity,5'-flap endonuclease activity,single-stranded DNA helicase activity,metal ion binding,4 iron, 4 sulfur cluster binding,DNA repair,DNA duplex unwinding,DNA replication, Okazaki fragment processing;Ontology_id=GO:0005634,GO:0005694,GO:0003677,GO:0005524,GO:0016887,GO:0017108,GO:0017116,GO:0046872,G
Fv_160_15.g8073	1040	ID=Fv_160_15.g8073;Description=hypothetical protein FVER53263_04059 [Fusarium verticillioides];Gene=FACUT_309;Ontology_term=cytosol,membrane,intracellular organelle,ribonucleoprotein complex,translation elongation factor activity,GTPase activity,GTP binding,ribosome binding,translational elongation;Ontology_id=GO:0005829,GO:0016020,GO:0043229,GO:1990904,GO:0003746,GO:0003924,GO:0005525,GO:0043022,G
Fv_160_15.g8074	116	ID=Fv_160_15.g8074;Description=hypothetical protein FOXG_06207 [Fusarium oxysporum f. sp. lycopersici 4287];Gene=Forpi1262_v006127;Ontology_term=mitochondrial inner membrane;Ontology_id=GO:0005743
Fv_160_15.g8075	408	ID=Fv_160_15.g8075;Description=branched-chain amino acid aminotransferase [Fusarium verticillioides 7600];Gene=Forpi1262_v006128;Ontology_term=mitochondrial matrix,eukaryotic translation elongation factor 1 complex,translation elongation factor activity,GTPase activity,GTP binding,L-leucine:2-oxoglutarate aminotransferase activity,L-leucine transaminase activity,L-valine transaminase activity,L-isoleucine transaminase activity,translational elongation,isoleucine biosynthetic process,leucine biosynthetic process,valine biosynthetic process;Ontology_id=GO:0005759,GO:0005853,GO:0003746,GO:0003924,GO:0005525,GO:0050048,GO:0052654,GO:0052655,GO:
Fv_160_15.g8076	915	ID=Fv_160_15.g8076;Description=hypothetical protein FVER14953_04062 [Fusarium verticillioides];Gene=FVER53590_04062;Ontology_term=protein kinase activity,ATP

Fv_160_15.g8077	487	ID=Fv_160_15.g8077;Description=hypothetical protein J7337_004069 [Fusarium musae];Gene=F25303_8822;Ontology_term=nucleus,pericentric heterochromatin,mating-type region heterochromatin,rDNA heterochromatin,chromosome, subtelomeric region,transferase activity,histone H3K14 deacetylase activity,metal ion binding,NAD-dependent histone H3K9 deacetylase activity,NAD-dependent histone H4K16 deacetylase activity,NAD+ binding,histone H3K4 deacetylase activity,negative regulation of transcription by RNA polymerase II,heterochromatin formation;Ontology_id=GO:0005634,GO:0005721,GO:0031934,GO:0033553,GO:0099115,GO:0016740,GO:0031078,GO:0046872,G
Fv_160_15.g8078	1338	ID=Fv_160_15.g8078;Description=Ca2+-transporting ATPase [Fusarium verticillioides 7600];Gene=FPCIR_8171;Ontology_term=membrane,P-type calcium transporter activity,ATP binding,ATP hydrolysis activity,calcium ion transmembrane transport;Ontology_id=GO:0016020,GO:0005388,GO:0005524,GO:0016887,GO:0070588;Enzyme_code=EC:7.2.2.10,EC:3.6.1.15;Enz
Fv_160_15.g8079	1185	ID=Fv_160_15.g8079;Description=hypothetical protein FVEG_15395 [Fusarium verticillioides 7600];Gene=FOXB_07167;Ontology_term=hydrolase activity,ligase activity,protein modification
Fv_160_15.g8080	232	ID=Fv_160_15.g8080;Description=hypothetical protein FVER14953_04067 [Fusarium verticillioides];Gene=BFJ69_g4130;Ontology_term=cytoplasm,microtubule,DASH complex,mitotic spindle,mitotic cell
Fv_160_15.g8081	416	ID=Fv_160_15.g8081;Description=hypothetical protein FVEG_04068 [Fusarium verticillioides 7600];Gene=FMAN_02995;Ontology_term=nucleus,ribonuclease H2 complex;Ontology_id=GO:0005634,GO:0032299
Fv_160_15.g8082	770	ID=Fv_160_15.g8082;Description=hypothetical protein FVER53590_04069 [Fusarium verticillioides];Gene=FCIRC_6889;Ontology_term=nucleus,double-stranded DNA binding,single-stranded DNA binding,DNA
Fv_160_15.g8083	369	ID=Fv_160_15.g8083;Description=hypothetical protein FVEG_04070 [Fusarium verticillioides 7600];Gene=FNAPI_4950;Ontology_term=lyase activity;Ontology_id=GO:0016829;Enzyme_code=EC:4;Enzyme_name=Lyases
Fv_160_15.g8084	572	ID=Fv_160_15.g8084;Description=hypothetical protein FVEG_04071 [Fusarium verticillioides 7600];Gene=FVER53263_04071;Ontology_term=monooxygenase
Fv_160_15.g8085	215	ID=Fv_160_15.g8085;Description=hypothetical protein FVEG_04072 [Fusarium verticillioides 7600];Gene=FNAPI_4952;Ontology_term=acyltransferase activity, transferring groups other than amino-acyl
Fv_160_15.g8086	717	ID=Fv_160_15.g8086;Description=hypothetical protein FVEG_04074 [Fusarium verticillioides 7600];Gene=FOPG_00407;Ontology_term=nucleus,DNA binding,zinc ion binding,DNA-templated
Fv_160_15.g8087	577	ID=Fv_160_15.g8087;Description=hypothetical protein FVER14953_04075 [Fusarium verticillioides];Gene=BFJ69_g4195;Ontology_term=membrane,S-adenosylmethionine-dependent methyltransferase
Fv_160_15.g8088	261	ID=Fv_160_15.g8088;Description=hypothetical protein FVER14953_04076 [Fusarium verticillioides]
Fv_160_15.g8089	2000	ID=Fv_160_15.g8089;Description=hypothetical protein FVER53590_04077 [Fusarium verticillioides];Gene=FPCIR_8183;Ontology_term=extracellular region,membrane,serine-type endopeptidase inhibitor activity,blood
Fv_160_15.g8090	1127	ID=Fv_160_15.g8090;Description=hypothetical protein FVEG_04078 [Fusarium verticillioides 7600];Gene=FPCIR_8184;Ontology_term=GTPase activator activity,regulation of mitotic

Fv_160_15.g8091	750	ID=Fv_160_15.g8091;Description=hypothetical protein FVER53263_04079 [Fusarium verticillioides];Gene=FOYG_04727;Ontology_term=cytosolic ribosome,ATP binding,ATP hydrolysis activity,translation initiation factor binding,ribosome binding,regulation of translational elongation,positive regulation of phosphorylation,cellular response to histidine,positive regulation of translational initiation in response to starvation;Ontology_id=GO:0022626,GO:0005524,GO:0016887,GO:0031369,GO:0043022,GO:0006448,GO:0042327,GO:0071232,G
Fv_160_15.g8092	117	ID=Fv_160_15.g8092;Description=hypothetical protein FVEG_04080 [Fusarium verticillioides 7600];Gene=708
Fv_160_15.g8093	446	ID=Fv_160_15.g8093;Description=Tubulin beta chain [Fusarium albosuccineum];Gene=FOYG_04729;Ontology_term=cytoplasm,ribosome,microtubule,structural constituent of ribosome,GTPase activity,structural constituent of cytoskeleton,GTP binding,rRNA binding,metal ion binding,translation,cytoskeleton organization,microtubule-based
Fv_160_15.g8094	263	ID=Fv_160_15.g8094;Description=hypothetical protein FVEG_04082 [Fusarium verticillioides 7600];Gene=FNAPI_4961;Ontology_term=mitochondrial large ribosomal subunit,microtubule,structural constituent of ribosome,GTPase activity,structural constituent of cytoskeleton,GTP binding,rRNA binding,metal ion binding,translation,cytoskeleton organization,microtubule-based process;Ontology_id=GO:0005762,GO:0005874,GO:0003735,GO:0003924,GO:0005200,GO:0005525,GO:0019843,GO:0046872,GO:
Fv_160_15.g8095	357	ID=Fv_160_15.g8095;Description=3'(2'),5'-bisphosphate nucleotidase [Fusarium verticillioides 7600];Gene=FOC1_g10011239;Ontology_term=membrane,inositol-1,4-bisphosphate 1-phosphatase activity,3'(2'),5'-bisphosphate nucleotidase activity,metal ion binding,inositol-1,3,4-trisphosphate 1-phosphatase activity,sulfate assimilation,methionine biosynthetic process,hyperosmotic salinity response,inositol phosphate metabolic process,phosphatidylinositol phosphate biosynthetic
Fv_160_15.g8096	107	ID=Fv_160_15.g8096;Description=hypothetical protein FVEG_04084 [Fusarium verticillioides 7600];Gene=FVER53590_04084;Ontology_term=mitochondrion,mitochondrial proton-transporting ATP synthase complex
Fv_160_15.g8097	1086	ID=Fv_160_15.g8097;Description=hypothetical protein FVER53590_04085 [Fusarium verticillioides]
Fv_160_15.g8098	82	ID=Fv_160_15.g8098;Description=hypothetical protein F25303_8800 [Fusarium sp. NRRL 25303]
Fv_160_15.g8099	325	ID=Fv_160_15.g8099;Description=hypothetical protein FVEG_04086 [Fusarium verticillioides 7600];Gene=F25303_8799;Ontology_term=cytoplasm,ATPase activator activity,protein-folding chaperone binding,protein
Fv_160_15.g8100	159	ID=Fv_160_15.g8100;Description=hypothetical protein FVEG_15398 [Fusarium verticillioides
Fv_160_15.g8101	645	ID=Fv_160_15.g8101;Description=hypothetical protein FVEG_04087 [Fusarium verticillioides 7600]
Fv_160_15.g8102	147	ID=Fv_160_15.g8102;Description=SWF1-like protein [Fusarium denticulatum];Gene=FOXYS1_6424;Ontology_term=membrane,protein-cysteine S-palmitoyltransferase
Fv_160_15.g8103	781	ID=Fv_160_15.g8103;Description=hypothetical protein FVER53590_04089 [Fusarium verticillioides];Gene=FTJAE_4471;Ontology_term=kinase activity,phosphotransferase activity, alcohol group as
Fv_160_15.g8104	587	ID=Fv_160_15.g8104;Description=hypothetical protein FVEG_04090 [Fusarium verticillioides 7600];Gene=1600

Fv_160_15.g8105	500	ID=Fv_160_15.g8105;Description=hypothetical protein FVEG_04091 [Fusarium verticillioides 7600];Gene=2688
Fv_160_15.g8106	281	ID=Fv_160_15.g8106;Description=hypothetical protein FVEG_04092 [Fusarium verticillioides 7600];Gene=FTJAE_4473;Ontology_term=mitochondrial inner membrane,copper ion binding,copper chaperone activity,intracellular copper ion homeostasis,respiratory chain complex IV
Fv_160_15.g8107	443	ID=Fv_160_15.g8107;Description=hypothetical protein FVEG_04093 [Fusarium verticillioides 7600];Gene=FNYG_00629;Ontology_term=Set1C/COMPASS complex,metal ion binding;Ontology_id=GO:0048188,GO:0046872
Fv_160_15.g8108	502	ID=Fv_160_15.g8108;Description=hypothetical protein FVEG_04094 [Fusarium verticillioides
Fv_160_15.g8109	327	ID=Fv_160_15.g8109;Description=hypothetical protein FVER14953_04095 [Fusarium
Fv_160_15.g8110	173	ID=Fv_160_15.g8110;Description=probable NADH dehydrogenase (ubiquinone) 22K chain precursor [Fusarium fujikuroi IMI 58289];Gene=FEQUK3_LOCUS10503;Ontology_term=mitochondrial inner membrane,respirasome,mitochondrial electron transport, NADH to ubiquinone;Ontology_id=GO:0005743,GO:0070469,GO:0006120
Fv_160_15.g8111	336	ID=Fv_160_15.g8111;Description=hypothetical protein FVEG_04097 [Fusarium verticillioides 7600];Gene=FocTR4_00007103;Ontology_term=extracellular region,plasma membrane,transmembrane receptor protein tyrosine kinase activity,protein binding,internal genitalia morphogenesis,dendrite self-
Fv_160_15.g8112	294	ID=Fv_160_15.g8112;Description=nicotinate-nucleotide diphosphorylase (carboxylating) [Fusarium verticillioides 7600];Gene=FCIRC_13963;Ontology_term=nicotinate-nucleotide diphosphorylase (carboxylating) activity,NAD biosynthetic process;Ontology_id=GO:0004514,GO:0009435;Enzyme_code=EC:2.4.2.19;Enzyme_name=nicotinate-nucleotide diphosphorylase
Fv_160_15.g8113	310	ID=Fv_160_15.g8113;Description=hypothetical protein FOXG_06247 [Fusarium oxysporum f. sp. lycopersici 4287];Gene=FGADI_1087;Ontology_term=GTPase activity,GTP
Fv_160_15.g8114	1001	ID=Fv_160_15.g8114;Description=hypothetical protein FVER53263_04100 [Fusarium verticillioides]
Fv_160_15.g8115	294	ID=Fv_160_15.g8115;Description=hypothetical protein FVEG_04101 [Fusarium verticillioides 7600]
Fv_160_15.g8116	616	ID=Fv_160_15.g8116;Description=hypothetical protein FVEG_04102 [Fusarium verticillioides 7600];Gene=RyR;Ontology_term=ryanodine-sensitive calcium-release channel activity,heart contraction,muscle contraction,regulation of hemocyte proliferation,response to anesthetic,release of sequestered calcium ion into cytosol,sarcoplasmic
Fv_160_15.g8117	456	ID=Fv_160_15.g8117;Description=carbamoyl-phosphate synthase arginine-specific small chain, mitochondrial [Fusarium verticillioides 7600];Gene=fliP;Ontology_term=carbamoyl-phosphate synthase complex,carbamoyl-phosphate synthase (glutamine-hydrolyzing) activity,'de novo' pyrimidine nucleobase biosynthetic process,pyrimidine nucleotide biosynthetic process,arginine biosynthetic process,glutamine metabolic process;Ontology_id=GO:0005951,GO:0004088,GO:0006207,GO:0006221,GO:0006526,GO:0006541;Enzyme_code=EC:6.3.5.5;Enzy
Fv_160_15.g8118	258	ID=Fv_160_15.g8118;Description=probable electron transfer flavoprotein beta chain [Fusarium fujikuroi IMI 58289];Gene=Etfb-1;Ontology_term=mitochondrial matrix,electron transfer activity,electron transport
Fv_160_15.g8119	465	ID=Fv_160_15.g8119;Description=hypothetical protein FVER53590_04105 [Fusarium verticillioides];Gene=FCIRC_11800;Ontology_term=argininosuccinate lyase activity,GTPase activator activity,arginine biosynthetic

Fv_160_15.g8120	369	ID=Fv_160_15.g8120;Description=argininosuccinate lyase [Fusarium coicis];Gene=FOC1_g10011216;Ontology_term=endosome,trans-Golgi network,cytoskeleton,actin binding,argininosuccinate lyase activity,GTPase activator activity,endoplasmic reticulum to Golgi vesicle-mediated transport,retrograde vesicle-mediated transport, Golgi to endoplasmic reticulum,actin filament organization,arginine biosynthetic process via ornithine,Golgi to plasma membrane protein
Fv_160_15.g8121	408	ID=Fv_160_15.g8121;Description=hypothetical protein FVEG_04107 [Fusarium verticillioides 7600];Gene=FFUJ_04424;Ontology_term=DNA binding,RNA binding;Ontology_id=GO:0003677,GO:0003723
Fv_160_15.g8122	603	ID=Fv_160_15.g8122;Description=NADPH reductase TAH18 [Fusarium verticillioides 7600];Gene=TAH18;Ontology_term=mitochondrion,NADPH-hemoprotein reductase activity,FMN binding,flavin adenine dinucleotide binding,NADP binding,iron-sulfur cluster
Fv_160_15.g8123	847	ID=Fv_160_15.g8123;Description=hypothetical protein FVER53590_04109 [Fusarium verticillioides];Gene=FVEG_04109;Ontology_term=membrane,hydrolase
Fv_160_15.g8124	704	ID=Fv_160_15.g8124;Description=hypothetical protein FVEG_04110 [Fusarium verticillioides
Fv_160_15.g8125	1123	ID=Fv_160_15.g8125;Description=hypothetical protein FVER53590_04111 [Fusarium verticillioides];Gene=FocTR4_00007089;Ontology_term=proteasome regulatory particle, base subcomplex,ubiquitin-dependent
Fv_160_15.g8126	347	ID=Fv_160_15.g8126;Description=hypothetical protein FVER14953_04113 [Fusarium verticillioides];Gene=675
Fv_160_15.g8127	620	ID=Fv_160_15.g8127;Description=hypothetical protein FVEG_04114 [Fusarium verticillioides 7600];Gene=FTJAE_4493;Ontology_term=SHREC complex,heterochromatin formation;Ontology_id=GO:0070824,GO:0031507
Fv_160_15.g8128	331	ID=Fv_160_15.g8128;Description=hypothetical protein FVER14953_04115 [Fusarium verticillioides];Gene=FNYG_00607;Ontology_term=nucleus,Smc5-Smc6 complex,transferase activity,metal ion binding,DNA repair,DNA
Fv_160_15.g8129	432	ID=Fv_160_15.g8129;Description=hypothetical protein FVER14953_04116 [Fusarium
Fv_160_15.g8130	550	ID=Fv_160_15.g8130;Description=hypothetical protein FVEG_04117 [Fusarium verticillioides 7600];Gene=FNAPI_10239;Ontology_term=vacuolar membrane,endosome membrane,Set1C/COMPASS complex,Lid2 complex,methyltransferase activity,methylation,negative regulation of gene expression,
Fv_160_15.g8131	557	ID=Fv_160_15.g8131;Description=hypothetical protein FVEG_04118 [Fusarium verticillioides 7600];Gene=sre1;Ontology_term=nucleus,DNA-binding transcription factor activity,zinc ion binding,sequence-specific DNA
Fv_160_15.g8132	1192	ID=Fv_160_15.g8132;Description=hypothetical protein FVEG_15409 [Fusarium verticillioides 7600];Gene=HZS61_012420;Ontology_term=nucleus,DNA binding,regulation of DNA-templated
Fv_160_15.g8133	831	ID=Fv_160_15.g8133;Description=hypothetical protein FVEG_04121 [Fusarium verticillioides 7600]
Fv_160_15.g8134	567	ID=Fv_160_15.g8134;Description=hypothetical protein FVEG_04122 [Fusarium verticillioides 7600];Gene=BFJ69_g7484;Ontology_term=membrane,transmembrane transporter activity,transmembrane
Fv_160_15.g8135	292	ID=Fv_160_15.g8135;Description=hypothetical protein FVEG_04123 [Fusarium verticillioides 7600]

Fv_160_15.g8136	171	ID=Fv_160_15.g8136;Description=hypothetical protein FVEG_04124 [Fusarium verticillioides 7600]
Fv_160_15.g8137	383	ID=Fv_160_15.g8137;Description=hypothetical protein J7337_004131 [Fusarium musae];Gene=cbiN
Fv_160_15.g8138	621	ID=Fv_160_15.g8138;Description=methylenetetrahydrofolate reductase (NADPH) [Fusarium verticillioides 7600];Gene=FOTG_03518;Ontology_term=extracellular region,cytoplasm,protein-DNA complex,synapse,RNA polymerase II transcription regulator complex,RNA polymerase II cis-regulatory region sequence-specific DNA binding,DNA-binding transcription factor activity, RNA polymerase II-specific,transcription coactivator activity,methylenetetrahydrofolate reductase (NAD(P)H) activity,protein heterodimerization activity,DNA-binding transcription factor binding,eye development,tachykinin receptor signaling pathway,neuropeptide signaling pathway,chemical synaptic transmission,brain development,peripheral nervous system development,salivary gland boundary specification,oenocyte development,somatic muscle development,methionine biosynthetic process,tetrahydrofolate interconversion,regulation of cell fate specification,positive regulation of transcription by RNA polymerase II,embryonic organ development,neuron
Fv_160_15.g8139	684	ID=Fv_160_15.g8139;Description=hypothetical protein FVER14953_04127 [Fusarium verticillioides];Gene=BFJ69_g7462;Ontology_term=nucleus,RNA binding,translation elongation factor activity,GTPase activity,GTP binding,metal ion binding,mRNA processing,translational elongation;Ontology_id=GO:0005634,GO:0003723,GO:0003746,GO:0003924,GO:0005525,GO:0046872,GO:0006397,GO:0006414;E
Fv_160_15.g8140	844	ID=Fv_160_15.g8140;Description=elongation factor 2 [Fusarium verticillioides 7600];Gene=FOTG_03516;Ontology_term=RNA binding,translation elongation factor activity,GTPase activity,GTP binding,metal ion binding,translational elongation;Ontology_id=GO:0003723,GO:0003746,GO:0003924,GO:0005525,GO:0046872,GO:0006414;Enzyme_code=EC:3.6.1.15;E
Fv_160_15.g8141	3213	ID=Fv_160_15.g8141;Description=hypothetical protein FVER53590_04129 [Fusarium verticillioides];Gene=FPHYL_2428;Ontology_term=membrane,fermentation;Ontology_id=GO:0016020,GO:0006113
Fv_160_15.g8142	587	ID=Fv_160_15.g8142;Description=hypothetical protein FVEG_04130 [Fusarium verticillioides
Fv_160_15.g8143	357	ID=Fv_160_15.g8143;Description=dihydroorotase, homodimeric type [Fusarium verticillioides 7600];Gene=FOIG_05818;Ontology_term=dihydroorotase activity,metal ion binding,'de novo' pyrimidine nucleobase biosynthetic process,'de novo' UMP biosynthetic

Fv_160_15.g8144	724	ID=Fv_160_15.g8144;Description=hypothetical protein FVEG_04132 [Fusarium verticillioides 7600];Gene=F25303_7049;Ontology_term=nucleus,early endosome,plasma membrane,cell surface,endocytic vesicle,apical cortex,DNA-binding transcription factor activity, RNA polymerase II-specific,Notch binding,calcium ion binding,zinc ion binding,glycosphingolipid binding,receptor ligand activity,regulation of transcription by RNA polymerase II,actin filament organization,Notch signaling pathway,border follicle cell migration,neuroblast fate determination,peripheral nervous system development,apposition of dorsal and ventral imaginal disc-derived wing surfaces,mesoderm development,positive regulation of cell population proliferation,glial cell migration,asymmetric cell division,chaeta morphogenesis,second mitotic wave involved in compound eye morphogenesis,follicle cell of egg chamber stalk formation,germ-line stem cell population maintenance,oocyte localization involved in germarium-derived egg chamber formation,imaginal disc-derived leg segmentation,compound eye photoreceptor development,R7 cell differentiation,positive regulation of mitotic cell cycle,lateral inhibition,compound eye retinal cell programmed cell death,R3/R4 cell differentiation,wing disc anterior/posterior pattern formation,wing disc dorsal/ventral pattern formation,neuron fate specification,antennal morphogenesis,stem cell differentiation,regulation of establishment of planar
Fv_160_15.g8145	1032	ID=Fv_160_15.g8145;Description=hypothetical protein FVEG_04133 [Fusarium verticillioides 7600];Gene=FMAN_02933;Ontology_term=exocyst,exocytosis,Golgi to plasma membrane transport,protein
Fv_160_15.g8146	381	ID=Fv_160_15.g8146;Description=hypothetical protein LB504_002876 [Fusarium proliferatum];Gene=FFUJ_04449;Ontology_term=magnesium ion binding,isocitrate dehydrogenase (NAD+) activity,NAD binding,tricarboxylic acid
Fv_160_15.g8147	1050	ID=Fv_160_15.g8147;Description=hypothetical protein FVEG_04135 [Fusarium verticillioides 7600]
Fv_160_15.g8148	674	ID=Fv_160_15.g8148;Description=hypothetical protein FVER14953_04136 [Fusarium verticillioides];Gene=FPHYL_13825;Ontology_term=isomerase
Fv_160_15.g8149	782	ID=Fv_160_15.g8149;Description=hypothetical protein FVER14953_04137 [Fusarium verticillioides];Gene=BFJ68_g7236;Ontology_term=membrane,potassium ion transmembrane transporter activity,potassium ion
Fv_160_15.g8150	468	ID=Fv_160_15.g8150;Description=hypothetical protein FVER14953_04138 [Fusarium verticillioides];Gene=FANTH_9226;Ontology_term=UDP-N-acetylglucosamine transferase complex,UDP-N-acetylglucosamine-dolichyl-phosphate N-acetylglucosaminephosphotransferase activity,potassium ion transmembrane transporter activity,glycosyltransferase activity,dolichol-linked oligosaccharide biosynthetic process,aerobic respiration,potassium ion transmembrane
Fv_160_15.g8151	907	ID=Fv_160_15.g8151;Description=hypothetical protein FVER14953_04139 [Fusarium verticillioides];Gene=FVER53263_04139;Ontology_term=methyltransferase
Fv_160_15.g8152	180	ID=Fv_160_15.g8152;Description=hypothetical protein FVEG_04141 [Fusarium verticillioides 7600];Gene=FANTH_9224;Ontology_term=fungal-type vacuole lumen,sterol binding,intracellular sterol

Fv_160_15.g8153	123	ID=Fv_160_15.g8153;Description=V-type proton ATPase subunit F [Fusarium verticillioides 7600];Gene=FANTH_9223;Ontology_term=vacuolar membrane,proton-transporting V-type ATPase, V1 domain,proton-transporting ATPase activity, rotational mechanism,proton transmembrane
Fv_160_15.g8154	457	ID=Fv_160_15.g8154;Description=hypothetical protein FVER53263_21002 [Fusarium
Fv_160_15.g8155	950	ID=Fv_160_15.g8155;Description=hypothetical protein FPHYL_11025 [Fusarium
Fv_160_15.g8156	777	ID=Fv_160_15.g8156;Description=3-isopropylmalate dehydratase [Fusarium verticillioides 7600];Gene=HZS61_012443;Ontology_term=3-isopropylmalate dehydratase complex,3-isopropylmalate dehydratase activity,metal ion binding,4 iron, 4 sulfur cluster binding,leucine biosynthetic
Fv_160_15.g8157	309	ID=Fv_160_15.g8157;Description=hypothetical protein FVEG_04145 [Fusarium verticillioides 7600];Gene=HZS61_012444;Ontology_term=epithelial tube branching involved in lung morphogenesis,tubulin binding,protein transport,dystroglycan binding,branching involved in salivary gland morphogenesis,protein-containing complex binding,regulation of neurotransmitter receptor localization to postsynaptic specialization membrane,heart morphogenesis,myelination in peripheral nervous system,nerve maturation,postsynaptic membrane,sarcolemma,calcium ion binding,regulation of synaptic plasticity,dystroglycan complex,lamellipodium,regulation of gastrulation,regulation of synapse organization,protein dimerization activity,laminin-1 binding,morphogenesis of an epithelial sheet,S-adenosylmethionine-dependent methyltransferase activity,GABA-ergic synapse,cell-cell junction,structural constituent of muscle,retrograde trans-synaptic signaling by trans-synaptic protein complex,actin binding,filopodium,positive regulation of basement membrane assembly involved in embryonic body morphogenesis,cellular response to organic cyclic compound,negative regulation of cell migration,negative regulation of phosphatidylinositol 3-kinase/protein kinase B signal transduction,viral entry into host cell,microtubule anchoring,negative regulation of MAPK cascade,external side of plasma membrane,alpha-actinin binding,nucleoplasm,regulation of epithelial to mesenchymal transition,basement membrane,focal adhesion,vinculin binding,commissural neuron axon guidance,contractile ring,response to muscle activity,SH2 domain binding,extracellular space,cytoskeleton,photoreceptor ribbon synapse,membrane protein ectodomain proteolysis,cytoplasm,membrane raft,node of Ranvier,virus receptor activity,methylation;Ontology_id=GO:0060441,GO:0015631,GO:0015031,GO:0002162,GO:0060445,GO:0044877,GO:0098696,GO:0
Fv_160_15.g8158	195	ID=Fv_160_15.g8158;Description=Neuronal calcium sensor 1 [Fusarium musae];Gene=FOC1_g10011178;Ontology_term=calcium ion binding,S-adenosylmethionine-dependent methyltransferase activity,kinase activity,phosphorylation;Ontology_id=GO:0005509,GO:0008757,GO:0016301,GO:0016310;Enzyme_code=EC:2.7,EC:2.1.1;Enzyme_n
Fv_160_15.g8159	853	ID=Fv_160_15.g8159;Description=hypothetical protein FVER14953_04147 [Fusarium verticillioides];Gene=FMEXI_3826;Ontology_term=nucleus,endoplasmic reticulum membrane,NADPH-hemoprotein reductase activity,FMN binding,flavin adenine dinucleotide binding,NADP binding,DNA replication initiation,cell cycle,cell division;Ontology_id=GO:0005634,GO:0005789,GO:0003958,GO:0010181,GO:0050660,GO:0050661,GO:0006270,GO:0007049,GO:

Fv_160_15.g8160	321	ID=Fv_160_15.g8160;Description=hypothetical protein FVEG_15414 [Fusarium verticillioides 7600];Gene=FMEXI_3827;Ontology_term=plasma membrane,myelin sheath,S-adenosylmethionine-dependent methyltransferase activity,structural constituent of myelin sheath,inflammatory response,positive regulation of gene expression,astrocyte development,central nervous system myelination,methylation,long-chain fatty acid biosynthetic process,cell maturation,axon development;Ontology_id=GO:0005886,GO:0043209,GO:0008757,GO:0019911,GO:0006954,GO:0010628,GO:0014002,GO:002201
Fv_160_15.g8161	785	ID=Fv_160_15.g8161;Description=hypothetical protein FVER14953_04148 [Fusarium verticillioides];Gene=FMEXI_3828;Ontology_term=nucleus,DNA binding,zinc ion binding,DNA-binding transcription factor activity, RNA polymerase II-specific,regulation of transcription by RNA polymerase
Fv_160_15.g8162	591	ID=Fv_160_15.g8162;Description=hypothetical protein J7337_004154 [Fusarium musae];Gene=FVER53590_04150;Ontology_term=membrane,transmembrane transporter activity,transmembrane
Fv_160_15.g8163	119	ID=Fv_160_15.g8163;Description=hypothetical protein FVER14953_04151 [Fusarium verticillioides];Gene=murF;Ontology_term=cytoplasm,microtubule,plasma membrane,neuromuscular junction,sperm individualization complex,mitotic spindle,presynapse,actin binding,GTPase activity,GTP binding,microtubule binding,conditioned taste aversion,morphogenesis of a polarized epithelium,negative regulation of protein phosphorylation,sperm individualization,border follicle cell migration,epithelial cell migration, open tracheal system,salivary gland morphogenesis,short-term memory,proboscis extension reflex,olfactory learning,positive regulation of autophagy,synaptic vesicle budding from presynaptic endocytic zone membrane,extracellular matrix organization,larval feeding behavior,positive regulation of actin filament polymerization,adherens junction maintenance,regulation of tube architecture, open tracheal system,syncytial blastoderm mitotic cell cycle,regulation of growth,compound eye retinal cell programmed cell death,dorsal appendage formation,regulation of short-term neuronal synaptic plasticity,synaptic vesicle membrane organization,regulation of synapse structure or activity,clathrin-dependent endocytosis involved in vitellogenesis,protein localization to plasma membrane,membrane fission,presynaptic dense core vesicle exocytosis,clathrin-dependent synaptic vesicle endocytosis,bulk synaptic vesicle endocytosis,mitotic actomyosin contractile ring
Fv_160_15.g8164	129	ID=Fv_160_15.g8164;Description=hypothetical protein FVEG_04152 [Fusarium verticillioides 7600];Gene=637
Fv_160_15.g8165	418	ID=Fv_160_15.g8165;Description=hypothetical protein FVEG_04153 [Fusarium verticillioides 7600];Gene=FNAPI_9425;Ontology_term=cytoplasm,COP9 signalosome,metallopeptidase
Fv_160_15.g8166	428	ID=Fv_160_15.g8166;Description=hypothetical protein FVER14953_04154 [Fusarium verticillioides];Gene=FTJAE_4530;Ontology_term=zinc ion binding,oxidoreductase activity, acting on the CH-OH group of donors,
Fv_160_15.g8167	608	ID=Fv_160_15.g8167;Description=hypothetical protein J7337_004157 [Fusarium musae];Gene=FPANT_11304;Ontology_term=prospore membrane,cytosol,retromer complex,phosphatidylinositol binding,intracellular protein transport,ascospore-type prospore membrane formation,retrograde transport, endosome to
Fv_160_15.g8168	285	ID=Fv_160_15.g8168;Description=Mss4p nuclear export [Fusarium musae];Gene=FPANT_11305;Ontology_term=nucleus,protein

Fv_160_15.g8169	260	ID=Fv_160_15.g8169;Description=chorismate mutase [Fusarium verticillioides 7600];Gene=BFJ68_g8424;Ontology_term=nucleus,chorismate mutase activity,tyrosine binding,tryptophan binding,tyrosine biosynthetic process,L-phenylalanine biosynthetic process,chorismate metabolic
Fv_160_15.g8170	1140	ID=Fv_160_15.g8170;Description=mitochondrial division 1 [Fusarium napiforme]
Fv_160_15.g8171	514	ID=Fv_160_15.g8171;Description=hypothetical protein FVER14953_04159 [Fusarium verticillioides]
Fv_160_15.g8172	1180	ID=Fv_160_15.g8172;Description=hypothetical protein FVER53590_04160 [Fusarium
Fv_160_15.g8173	993	ID=Fv_160_15.g8173;Description=hypothetical protein FVER53590_04161 [Fusarium verticillioides]
Fv_160_15.g8174	229	ID=Fv_160_15.g8174;Description=hypothetical protein H9Q71_014481, partial [Fusarium xylarioides]
Fv_160_15.g8175	270	ID=Fv_160_15.g8175;Description=mutator-like element transposase [Fusarium tjaetaba];Gene=FOPG_00506;Ontology_term=methylmalonate-semialdehyde dehydrogenase (acylating, NAD)
Fv_160_15.g8176	4347	ID=Fv_160_15.g8176;Description=hypothetical protein FVER53263_04163 [Fusarium verticillioides];Gene=FNAPI_9435;Ontology_term=cytoplasm,microtubule,dynein complex,ATP binding,minus-end-directed microtubule motor activity,ATP hydrolysis activity,dynein intermediate chain binding,dynein light intermediate chain binding,microtubule-based
Fv_160_15.g8177	546	ID=Fv_160_15.g8177;Description=hypothetical protein FVER53590_04164 [Fusarium verticillioides]
Fv_160_15.g8178	155	ID=Fv_160_15.g8178;Description=hypothetical protein FVEG_04165 [Fusarium verticillioides 7600]
Fv_160_15.g8179	287	ID=Fv_160_15.g8179;Description=hypothetical protein FVER14953_04166 [Fusarium
Fv_160_15.g8180	1514	ID=Fv_160_15.g8180;Description=hypothetical protein FVEG_04167 [Fusarium verticillioides 7600];Gene=FMAN_02898;Ontology_term=membrane,ATP binding,ATP hydrolysis activity,ABC-type transporter activity,transmembrane transport;Ontology_id=GO:0016020,GO:0005524,GO:0016887,GO:0140359,GO:0055085;Enzyme_code=EC:7.2.2,EC:3.6.1.15;Enzym
Fv_160_15.g8181	357	ID=Fv_160_15.g8181;Description=CMGC/MAPK/P38 protein kinase [Fusarium oxysporum f. sp. lycopersici 4287];Gene=HZS61_012465;Ontology_term=nucleus,cytoplasm,MAP kinase activity,ATP binding,protein serine kinase activity,phosphorylation,stress-activated MAPK cascade;Ontology_id=GO:0005634,GO:0005737,GO:0004707,GO:0005524,GO:0106310,GO:0016310,GO:0051403;Enzyme_code=EC
Fv_160_15.g8182	885	ID=Fv_160_15.g8182;Description=starch phosphorylase [Fusarium verticillioides 7600];Gene=FNAPI_12976;Ontology_term=glycogen phosphorylase activity,pyridoxal phosphate binding,linear malto-oligosaccharide phosphorylase activity,SHG alpha-glucan phosphorylase activity,carbohydrate metabolic
Fv_160_15.g8183	355	ID=Fv_160_15.g8183;Description=guanine nucleotide-binding protein alpha-3 subunit [Fusarium verticillioides 7600];Gene=HZS61_012467;Ontology_term=heterotrimeric G-protein complex,G protein-coupled receptor binding,GTPase activity,GTP binding,G-protein beta/gamma-subunit complex binding,G protein-coupled receptor signaling
Fv_160_15.g8184	723	ID=Fv_160_15.g8184;Description=hypothetical protein FVEG_04171 [Fusarium verticillioides 7600]

Fv_160_15.g8185	770	ID=Fv_160_15.g8185;Description=Cullin 1 [Fusarium verticillioides 7600];Gene=FOYG_04829;Ontology_term=nuclear SCF ubiquitin ligase complex,DNA replication origin binding,protein-macromolecule adaptor activity,ubiquitin protein ligase binding,ubiquitin protein ligase activity,G1/S transition of mitotic cell cycle,G2/M transition of mitotic cell cycle,positive regulation of glucose transmembrane transport,silent mating-type cassette heterochromatin formation,SCF-dependent proteasomal ubiquitin-dependent protein catabolic process;Ontology_id=GO:0043224,GO:0003688,GO:0030674,GO:0031625,GO:0061630,GO:0000082,GO:0000086,GO:0010828,GO:
Fv_160_15.g8186	377	ID=Fv_160_15.g8186;Description=hypothetical protein FVEG_04173 [Fusarium verticillioides
Fv_160_15.g8187	117	ID=Fv_160_15.g8187;Description=hypothetical protein FVEG_04174 [Fusarium verticillioides 7600];Gene=FDENT_13416;Ontology_term=glycosyltransferase
[truncated: 1,520,246 more chars]
