# Supplementary material for: Genomic Insights into Fusarium verticillioides Diversity: The Genome of Two Clinical Isolates and Their Demethylase Inhibitor Fungicides Susceptibility
Source: Pathogens. 2024 Dec 3;13(12):1062. doi: 10.3390/pathogens13121062 (PMC11728828; doi:10.3390/pathogens13121062)
Supplement: Supplementary file 1 [file pathogens-13-01062-s001.zip › Table S3.pdf]

ID	Length	Description. GO
contig00001.g1	324	ID=contig00001.g1;Description=hypothetical protein FVEG_03430 [Fusarium verticillioides 7600]
contig00001.g2	1080	ID=contig00001.g2;Description=PFS domain-containing protein [Colletotrichum tofieldiae];Gene=CSPAE12_05395;Ontology_term=nucleobase-containing small molecule metabolic process,catalytic activity,carbohydrate derivative metabolic process;Ontology_id=GO:0055086,GO:0003824,GO:1901135
contig00001.g3	8529	ID=contig00001.g3;Description=vegetative incompatibility protein HET-E-1 [Colletotrichum spaethianum];Gene=CI238_13108
contig00001.g4	4054	ID=contig00001.g4;Description=related to ALCOHOL DEHYDROGENASE I-ADH1 [Fusarium proliferatum ET1];Gene=F52700_8545;Ontology_term=oxidoreductase activity;Ontology_id=GO:0016491;Enzyme_code=EC:1,EC:1;Enzyme_name=Oxidoreductases,Oxidoreductases
contig00001.g5	2129	ID=contig00001.g5;Description=zinc finger C2H2 type domain-containing protein [Fusarium napiforme];Gene=LW93_7974;Ontology_term=nucleus,DNA binding,regulation of DNA-templated transcription,transcription regulator activity;Ontology_id=GO:0005634,GO:0003677,GO:0006355,GO:0140110
contig00001.g6	750	ID=contig00001.g6;Description=oxidoreductase [Fusarium verticillioides 7600];Gene=FPANT_7828;Ontology_term=oxidoreductase activity,lipid metabolic process;Ontology_id=GO:0016491,GO:0006629;Enzyme_code=EC:1,EC:1,EC:1.14.18.9;Enzyme_name=Oxidoreductases,Oxidoreductases,4alpha-methylsterol monooxygenase
contig00001.g7	1679	ID=contig00001.g7;Description=acyl transferase carnitine dehydratase [Fusarium napiforme];Gene=FDENT_6917;Ontology_term=transferase activity;Ontology_id=GO:0016740;Enzyme_code=EC:2,EC:2;Enzyme_name=Transferases,Transferases
contig00001.g8	2142	ID=contig00001.g8;Description=DUF1446 domain-containing protein [Fusarium coicis];Gene=FVEG_15260;Ontology_term=nucleus,DNA binding,regulation of DNA-templated transcription,transcription regulator activity;Ontology_id=GO:0005634,GO:0003677,GO:0006355,GO:0140110
contig00001.g9	1604	ID=contig00001.g9;Description=transcriptional activator Mut3p [Fusarium coicis];Gene=FVEG_15260;Ontology_term=nucleus,DNA binding,regulation of DNA-templated transcription,transcription regulator activity;Ontology_id=GO:0005634,GO:0003677,GO:0006355,GO:0140110

contig00001.g10	1896	ID=contig00001.g10;Description=IQ calmodulin-binding motif [Fusarium tjaetaba];Gene=FVER53590_03418;Ontology_term=catalytic activity;Ontology_id=GO:0003824
contig00001.g11	951	ID=contig00001.g11;Description=hypothetical protein FVEG_15259 [Fusarium verticillioides 7600]
contig00001.g12	1537	ID=contig00001.g12;Description=acyl dehydrogenase [Fusarium tjaetaba];Gene=BFJ63_vAg16446;Ontology_term=oxidoreductase activity;Ontology_id=GO:0016491;Enzyme_code=EC:1.3,EC:1.3,EC:1;Enzyme_name=Acting on the CH-CH group of donors,Acting on the CH-CH group of donors,Oxidoreductases
contig00001.g13	1337	ID=contig00001.g13;Description=hypothetical protein J7337_006336 [Fusarium musae];Gene=FVEG_03416;Ontology_term=lyase activity;Ontology_id=GO:0016829;Enzyme_code=EC:4,EC:4;Enzyme_name=Lyases,Lyases
contig00001.g14	3048	ID=contig00001.g14;Description=putative NRPS-like protein biosynthetic cluster [Fusarium musae];Gene=FTJAE_13313;Ontology_term=secondary metabolite biosynthetic process;Ontology_id=GO:0044550
contig00001.g15	1391	ID=contig00001.g15;Description=putative secondary metabolism biosynthetic enzyme [Fusarium musae];Gene=FACUT_13382;Ontology_term=pyridoxal phosphate binding,biosynthetic process;Ontology_id=GO:0030170,GO:0009058;Enzyme_code=EC:2.3.1.47;Enzyme_name=8-amino-7-oxononanoate synthase
contig00001.g16	1278	ID=contig00001.g16;Description=cystathionine beta-lyase [Fusarium verticillioides 7600];Gene=FVER53263_03413;Ontology_term=nucleus,sulfur compound metabolic process,lyase activity,amino acid metabolic process,transferase activity;Ontology_id=GO:0005634,GO:0006790,GO:0016829,GO:0006520,GO:0016740;Enzyme_code=EC:4,EC:2.5.1,EC:2.5.1.48,EC:4.4.1.8,EC:2,EC:4.4,EC:4,EC:4.4.1.1;Enzyme_name=Lyases,Transferring alkyl or aryl groups, other than methyl groups,cystathionine gamma-synthase,Carbon-sulfur lyases,Transferases,Carbon-sulfur lyases,Lyases,cystathionine gamma-lyase

contig00001.g17	1958	ID=contig00001.g17;Description=Putative dioxygenase subunit alpha yeaW [Fusarium oxysporum f. sp. cubense race 1];Gene=FOMG_16361;Ontology_term=nucleus,DNA binding,generation of precursor metabolites and energy,oxidoreductase activity,cellular modified amino acid metabolic process,regulation of DNA-templated transcription,transcription regulator activity;Ontology_id=GO:0005634,GO:0003677,GO:0006091,GO:0016491,GO:0006575,GO:0006355,GO:0140110;Enzyme_code=EC:1.14.15.7,EC:1.14.15.7,EC:1,EC:1.14.15,EC:1.14;Enzyme_name=choline monooxygenase,choline monooxygenase,Oxidoreductases,Acting on paired donors, with incorporation or reduction of molecular oxygen. The oxygen incorporated need not be derived from O2,Acting on paired donors, with incorporation or reduction of molecular oxygen. The oxygen incorporated need not be derived from O2
contig00001.g18	456	ID=contig00001.g18;Description=monothiol glutaredoxin [Fusarium verticillioides 7600];Gene=FCIRC_11453;Ontology_term=nucleus,catalytic activity, acting on a protein,sulfur compound metabolic process,cytoskeleton organization,oxidoreductase activity,cytosol,protein maturation,chromatin organization,muscle system process,circulatory system process;Ontology_id=GO:0005634,GO:0140096,GO:0006790,GO:0007010,GO:0016491,GO:0005829,GO:0051604,GO:0006325,GO:0003012,GO:0003013;Enzyme_code=EC:1.8,EC:1,EC:1.8;Enzyme_name=Acting on a sulfur group of donors,Oxidoreductases,Acting on a sulfur group of donors
contig00001.g19	1317	ID=contig00001.g19;Description=cytochrome P450 oxidoreductase [Fusarium verticillioides 7600];Gene=FOC1_g10003720;Ontology_term=nucleus,DNA binding,oxidoreductase activity,cellular modified amino acid metabolic process,regulation of DNA-templated transcription,transcription regulator activity;Ontology_id=GO:0005634,GO:0003677,GO:0016491,GO:0006575,GO:0006355,GO:0140110;Enzyme_code=EC:1.14.15.7,EC:1.14.15.7,EC:1,EC:1.14.15,EC:1.14;Enzyme_name=choline monooxygenase,choline monooxygenase,Oxidoreductases,Acting on paired donors, with incorporation or reduction of molecular oxygen. The oxygen incorporated need not be derived from O2,Acting on paired donors, with incorporation or reduction of molecular oxygen. The oxygen incorporated need not be derived from O2
contig00001.g20	2051	ID=contig00001.g20;Description=synaptic vesicle transporter SV2 [Fusarium subglutinans];Gene=FANTH_4153;Ontology_term=transmembrane transport,transporter activity;Ontology_id=GO:0055085,GO:0005215

contig00001.g21	1179	ID=contig00001.g21;Description=sarcosine oxidase [Fusarium verticillioides 7600];Gene=FTJAE_13324;Ontology_term=oxidoreductase activity;Ontology_id=GO:0016491;Enzyme_code=EC:1,EC:1;Enzyme_name=Oxidoreductases,Oxidoreductases
contig00001.g22	1044	ID=contig00001.g22;Description=yukJ [Fusarium pseudocircinatum]
contig00001.g23	738	ID=contig00001.g23;Description=3-octaprenyl-4-hydroxybenzoate carboxy-lyase UbiX [Fusarium verticillioides 7600];Gene=PAD1;Ontology_term=mitochondrion,lyase activity,transferase activity;Ontology_id=GO:0005739,GO:0016829,GO:0016740;Enzyme_code=EC:4,EC:2.5.1.129,EC:2.5.1,EC:2,EC:4,EC:2.5.1.129;Enzyme_name=Lyases,flavin prenyltransferase,Transferring alkyl or aryl groups, other than methyl groups,Transferases,Lyases,flavin prenyltransferase
contig00001.g24	1581	ID=contig00001.g24;Description=Ferulic acid decarboxylase 1 [Fusarium oxysporum f. sp. narcissi];Gene=FDC1;Ontology_term=lyase activity;Ontology_id=GO:0016829;Enzyme_code=EC:4.1.1,EC:4.1.1,EC:4.1,EC:4.1.1.102,EC:4;Enzyme_name=Carbon-carbon lyases,Carbon-carbon lyases,Carbon-carbon lyases,phenacrylate decarboxylase,Lyases
contig00001.g25	1571	ID=contig00001.g25;Description=major facilitator superfamily transporter [Fusarium subglutinans];Gene=FPCIR_8814;Ontology_term=transmembrane transport,transporter activity;Ontology_id=GO:0055085,GO:0005215
contig00001.g26	997	ID=contig00001.g26;Description=hypothetical protein FVEG_03403 [Fusarium verticillioides 7600]
contig00001.g27	1590	ID=contig00001.g27;Description=major facilitator superfamily transporter [Fusarium pseudoanthophilum];Gene=FNYG_08026;Ontology_term=transmembrane transport,oxidoreductase activity,transporter activity;Ontology_id=GO:0055085,GO:0016491,GO:0005215;Enzyme_code=EC:1.2.1,EC:1.2,EC:1,EC:1.2.1;Enzyme_name=Acting on the aldehyde or oxo group of donors,Acting on the aldehyde or oxo group of donors,Oxidoreductases,Acting on the aldehyde or oxo group of donors
contig00001.g28	1683	ID=contig00001.g28;Description=salicylaldehyde dehydrogenase [Fusarium subglutinans];Gene=FNAPI_1970;Ontology_term=oxidoreductase activity;Ontology_id=GO:0016491;Enzyme_code=EC:1.2.1,EC:1.2,EC:1,EC:1.2.1;Enzyme_name=Acting on the aldehyde or oxo group of donors,Acting on the aldehyde or oxo group of donors,Oxidoreductases,Acting on the aldehyde or oxo group of donors

contig00001.g29	1153	ID=contig00001.g29;Description=gentisate 1,2-dioxygenase [Fusarium verticillioides 7600];Gene=FNYG_08024;Ontology_term=oxidoreductase activity;Ontology_id=GO:0016491;Enzyme_code=EC:1,EC:1,EC:1.13.11.4;Enzyme_name=Oxidoreductases,Oxidoreductases,gentisate 1,2-dioxygenase
contig00001.g30	1597	ID=contig00001.g30;Description=salicylate hydroxylase [Fusarium verticillioides 7600];Gene=FSUBG_13398;Ontology_term=oxidoreductase activity;Ontology_id=GO:0016491;Enzyme_code=EC:1,EC:1,EC:1.14.13.1;Enzyme_name=Oxidoreductases,Oxidoreductases,salicylate 1-monooxygenase
contig00001.g31	1347	ID=contig00001.g31;Description=lipase 2 [Fusarium tjaetaba];Gene=FPCIR_10929;Ontology_term=lipid metabolic process,extracellular region,hydrolase activity;Ontology_id=GO:0006629,GO:0005576,GO:0016787;Enzyme_code=EC:3.1.1.3,EC:3.1,EC:3,EC:3.1.1,EC:3.1.1.3;Enzyme_name=triacylglycerol lipase,Acting on ester bonds,Hydrolases,Acting on ester bonds,triacylglycerol lipase
contig00001.g32	2369	ID=contig00001.g32;Description=cutinase transcription factor 1 alpha [Fusarium tjaetaba];Gene=FPOA_13265;Ontology_term=nucleus,DNA binding,regulation of DNA-templated transcription,transcription regulator activity;Ontology_id=GO:0005634,GO:0003677,GO:0006355,GO:0140110
contig00001.g33	978	ID=contig00001.g33;Description=hypothetical protein FVER14953_03396 [Fusarium verticillioides]
contig00001.g34	813	ID=contig00001.g34;Description=putative cutinase precursor [Fusarium fujikuroi];Gene=C2S_12241;Ontology_term=extracellular region,hydrolase activity;Ontology_id=GO:0005576,GO:0016787;Enzyme_code=EC:3.1.1.74,EC:3.1,EC:3,EC:3.1.1.74,EC:3.1.1;Enzyme_name=cutinase,Acting on ester bonds,Hydrolases,cutinase,Acting on ester bonds
contig00001.g35	1069	ID=contig00001.g35;Description=fumarylacetoacetate hydrolase [Fusarium tjaetaba];Gene=FPANT_7808;Ontology_term=hydrolase activity;Ontology_id=GO:0016787;Enzyme_code=EC:3,EC:3;Enzyme_name=Hydrolases,Hydrolases
contig00001.g36	3753	ID=contig00001.g36;Description=trans-anethole oxidase [Fusarium pseudocircinatum]

contig00001.g37	4030	ID=contig00001.g37;Description=cutinase transcription factor 1 beta [Fusarium fujikuroi];Gene=FVER53590_03391;Ontology_term=nucleus,DNA binding,structural molecule activity,regulation of DNA-templated transcription,ribosome,transcription regulator activity;Ontology_id=GO:0005634,GO:0003677,GO:0005198,GO:0006355,GO:0005840,GO:0140110
contig00001.g38	3151	ID=contig00001.g38;Description=Zinc finger C2H2-type [Fusarium oxysporum f. sp. vasinfectum];Gene=FOMG_16330;Ontology_term=nucleus,DNA binding,DNA-templated transcription;Ontology_id=GO:0005634,GO:0003677,GO:0006351
contig00001.g39	2690	ID=contig00001.g39;Description=C2H2 zinc finger [Fusarium coicis];Gene=FOC1_g10003756;Ontology_term=nucleus,DNA binding,oxidoreductase activity,DNA-templated transcription;Ontology_id=GO:0005634,GO:0003677,GO:0016491,GO:0006351;Enzyme_code=EC:1.13.11,EC:1.13.11,EC:1.13,EC:1;Enzyme_name=Acting on single donors with incorporation of molecular oxygen (oxygenases). The oxygen incorporated need not be derived from O2,Acting on single donors with incorporation of molecular oxygen (oxygenases). The oxygen incorporated need not be derived from O2,Acting on single donors with incorporation of molecular oxygen (oxygenases). The oxygen incorporated need not be derived from O2,Oxidoreductases
contig00001.g40	3973	ID=contig00001.g40;Description=C2H2 zinc finger [Fusarium mexicanum];Gene=FACUT_4377;Ontology_term=nucleus,DNA binding,catalytic activity, acting on a protein,oxidoreductase activity,DNA-templated transcription,hydrolase activity;Ontology_id=GO:0005634,GO:0003677,GO:0140096,GO:0016491,GO:0006351,GO:0016787;Enzyme_code=EC:3.4.21,EC:1.14.13.8;Enzyme_name=Acting on peptide bonds (peptidases),flavin-containing monooxygenase
contig00001.g41	1422	ID=contig00001.g41;Description=C6 transcription factor [Fusarium tjaetaba];Gene=FVER53590_29055;Ontology_term=nucleus,regulation of DNA-templated transcription,transcription regulator activity;Ontology_id=GO:0005634,GO:0006355,GO:0140110
contig00001.g42	1883	ID=contig00001.g42;Description=bikaverin cluster-efflux pump [Fusarium fujikuroi];Gene=FFC1_02266;Ontology_term=transmembrane transport,transporter activity;Ontology_id=GO:0055085,GO:0005215

contig00001.g43	2365	ID=contig00001.g43;Description=Bikaverin cluster transcription factor bik5 [Fusarium musae];Gene=bik5;Ontology_term=nucleus,DNA binding,regulation of DNA-templated transcription,transcription regulator activity;Ontology_id=GO:0005634,GO:0003677,GO:0006355,GO:0140110
contig00001.g44	981	ID=contig00001.g44;Description=bikaverin cluster-transcription factor enhancer [Fusarium fujikuroi]
contig00001.g45	1464	ID=contig00001.g45;Description=bikaverin cluster-O-methyltransferase [Fusarium subglutinans];Gene=FANTH_4131;Ontology_term=transferase activity;Ontology_id=GO:0016740;Enzyme_code=EC:2.1.1,EC:2.1,EC:2.1.1.293,EC:2,EC:2.1.1;Enzyme_name=Transferring one-carbon groups,Transferring one-carbon groups,6-hydroxytryprostatin B O-methyltransferase,Transferases,Transferring one-carbon groups
contig00001.g46	1574	ID=contig00001.g46;Description=FAD-dependent monooxygenase bik2 [Fusarium musae];Gene=FVER53590_03380;Ontology_term=oxidoreductase activity;Ontology_id=GO:0016491;Enzyme_code=EC:1,EC:1;Enzyme_name=Oxidoreductases,Oxidoreductases
contig00001.g47	6277	ID=contig00001.g47;Description=Interface between microtubules and kinetochore protein [Fusarium musae];Gene=Fth_03108;Ontology_term=lipid metabolic process,transferase activity,hydrolase activity;Ontology_id=GO:0006629,GO:0016740,GO:0016787;Enzyme_code=EC:2.3.1.41,EC:3;Enzyme_name=beta-ketoacyl-[acyl-carrier-protein] synthase I,Hydrolases
contig00001.g48	2124	ID=contig00001.g48;Description=subtilisin [Fusarium tjaetaba];Gene=FTJAE_5496;Ontology_term=catalytic activity, acting on a protein,hydrolase activity;Ontology_id=GO:0140096,GO:0016787;Enzyme_code=EC:3.4.21,EC:3.4.21,EC:3.4,EC:3;Enzyme_name=Acting on peptide bonds (peptidases),Acting on peptide bonds (peptidases),Acting on peptide bonds (peptidases),Hydrolases
contig00001.g49	1029	ID=contig00001.g49;Description=peptidase cysteine serine trypsin [Fusarium napiforme]
contig00001.g50	2295	ID=contig00001.g50;Description=subtilisin [Fusarium coicis]
contig00001.g51	1826	ID=contig00001.g51;Description=uracil permease [Fusarium subglutinans];Gene=FVEG_03375;Ontology_term=transmembrane transport,transporter activity;Ontology_id=GO:0055085,GO:0005215
contig00001.g52	2976	ID=contig00001.g52;Description=d-amino acid hydantoin hydrolase (hydantoinase) [Fusarium napiforme];Gene=FVER53590_03374;Ontology_term=hydrolase activity;Ontology_id=GO:0016787;Enzyme_code=EC:3,EC:3;Enzyme_name=Hydrolases,Hydrolases

contig00001.g53	1539	ID=contig00001.g53;Description=hypothetical protein FVEG_15255 [Fusarium verticillioides 7600];Gene=FMUND_3843;Ontology_term=transmembrane transport,transporter activity;Ontology_id=GO:0055085,GO:0005215
contig00001.g54	1137	ID=contig00001.g54;Description=Heterokaryon incompatibility protein 6, OR allele [Fusarium oxysporum f. sp. rapae]
contig00001.g55	395	ID=contig00001.g55;Description=hypothetical protein FVEG_03372 [Fusarium verticillioides 7600]
contig00001.g56	1971	ID=contig00001.g56;Description=heterokaryon incompatibility 6 OR allele [Fusarium tjaetaba];Gene=FNAPI_1938;Ontology_term=transferase activity;Ontology_id=GO:0016740;Enzyme_code=EC:2.3.1,EC:2.3,EC:2,EC:2.3.1;Enzyme_name=Acyltransferases,Acyltransferases,Transferases,Acyltransferases
contig00001.g57	687	ID=contig00001.g57;Description=hypothetical protein FVER14953_03370 [Fusarium verticillioides];Gene=FVER53590_03370;Ontology_term=transferase activity;Ontology_id=GO:0016740;Enzyme_code=EC:2.3.1,EC:2.3,EC:2,EC:2.3.1;Enzyme_name=Acyltransferases,Acyltransferases,Transferases,Acyltransferases
contig00001.g58	615	ID=contig00001.g58;Description=nucleosome binding protein [Fusarium coicis];Gene=C2S_12260;Ontology_term=membrane;Ontology_id=GO:0016020
contig00001.g59	2704	ID=contig00001.g59;Description=signaling ykoW [Fusarium coicis];Gene=FNYG_07991;Ontology_term=membrane;Ontology_id=GO:0016020
contig00001.g60	1212	ID=contig00001.g60;Description=signaling ykoW [Fusarium tjaetaba];Gene=FNYG_07990;Ontology_term=oxidoreductase activity;Ontology_id=GO:0016491;Enzyme_code=EC:1.14,EC:1,EC:1.14;Enzyme_name=Acting on paired donors, with incorporation or reduction of molecular oxygen. The oxygen incorporated need not be derived from O2,Oxidoreductases,Acting on paired donors, with incorporation or reduction of molecular oxygen. The oxygen incorporated need not be derived from O2
contig00001.g61	1133	ID=contig00001.g61;Description=hypothetical protein FVER53263_03367 [Fusarium verticillioides];Gene=TIM44;Ontology_term=transmembrane transport,mitochondrion,intracellular protein transport;Ontology_id=GO:0055085,GO:0005739,GO:0006886
contig00001.g62	555	ID=contig00001.g62;Description=related to RF2 protein [Fusarium proliferatum ET1]
contig00001.g63	4177	ID=contig00001.g63;Description=hypothetical protein FVER53590_29051 [Fusarium verticillioides];Gene=FSUBG_13666;Ontology_term=membrane;Ontology_id=GO:0016020

contig00001.g64	1787	ID=contig00001.g64;Description=murein transglycosylase [Fusarium verticillioides 7600];Gene=FPANT_7905;Ontology_term=carbohydrate metabolic process,cell wall organization or biogenesis,hydrolase activity;Ontology_id=GO:0005975,GO:0071554,GO:0016787;Enzyme_code=EC:3.2.1.15,EC:3.2,EC:3,EC:3.2.1.171,EC:3.2.1.15,EC:3.2.1;Enzyme_name=endo-polygalacturonase,Glycosylases,Hydrolases,rhamnogalacturonan hydrolase,endo-polygalacturonase,Glycosylases
contig00001.g65	987	ID=contig00001.g65;Description=protoporphyrinogen oxidase [Fusarium subglutinans]
contig00001.g66	985	ID=contig00001.g66;Description=hypothetical protein FVEG_03360 [Fusarium verticillioides 7600]
contig00001.g67	1005	ID=contig00001.g67;Description=hypothetical protein FVEG_03357 [Fusarium verticillioides 7600];Gene=FNAPI_1927;Ontology_term=membrane;Ontology_id=GO:0016020
contig00001.g68	2390	ID=contig00001.g68;Description=transcriptional regulatory [Fusarium mundagurra];Gene=FVER53590_03356;Ontology_term=nucleus,DNA binding,regulation of DNA-templated transcription,transcription regulator activity;Ontology_id=GO:0005634,GO:0003677,GO:0006355,GO:0140110
contig00001.g69	888	ID=contig00001.g69;Description=chaperone hchA [Fusarium tjaetaba];Gene=FTJAE_12693;Ontology_term=nucleus,DNA binding,catalytic activity, acting on a protein,regulation of DNA-templated transcription,transcription regulator activity,hydrolase activity;Ontology_id=GO:0005634,GO:0003677,GO:0140096,GO:0006355,GO:0140110,GO:0016787;Enzyme_code=EC:3.5.1.124,EC:3.5.1,EC:3.5,EC:3,EC:3.5.1.124;Enzyme_name=protein deglycase,Acting on carbon-nitrogen bonds, other than peptide bonds,Acting on carbon-nitrogen bonds, other than peptide bonds,Hydrolases,protein deglycase
contig00001.g70	1215	ID=contig00001.g70;Description=alcohol dehydrogenase, class C [Fusarium fujikuroi];Gene=FMUND_8538;Ontology_term=oxidoreductase activity;Ontology_id=GO:0016491;Enzyme_code=EC:1.1.1,EC:1.1,EC:1,EC:1.1.1,EC:1.1.1.2;Enzyme_name=Acting on the CH-OH group of donors,Acting on the CH-OH group of donors,Oxidoreductases,Acting on the CH-OH group of donors,alcohol dehydrogenase (NADP(+))
contig00001.g71	1062	ID=contig00001.g71;Description=zinc-binding alcohol dehydrogenase domain protein [Fusarium tjaetaba];Gene=FMUND_8537;Ontology_term=oxidoreductase activity;Ontology_id=GO:0016491;Enzyme_code=EC:1.6,EC:1.6,EC:1;Enzyme_name=Acting on NADH or NADPH,Acting on NADH or NADPH,Oxidoreductases

contig00001.g72	5082	ID=contig00001.g72;Description=Cupredoxin [Fusarium coicis];Gene=FOYG_17133;Ontology_term=oxidoreductase activity;Ontology_id=GO:0016491;Enzyme_code=EC:1,EC:1;Enzyme_name=Oxidoreductases,Oxidoreductases
contig00001.g73	3005	ID=contig00001.g73;Description=helicase-like transcription factor protein [Fusarium fujikuroi];Gene=BFJ70_g13055;Ontology_term=ATP-dependent activity,catalytic activity;Ontology_id=GO:0140657,GO:0003824
contig00001.g74	5448	ID=contig00001.g74;Description=Nacht domain-containing protein [Fusarium phyllophilum]
contig00001.g75	1180	ID=contig00001.g75;Description=catalase [Fusarium verticillioides 7600];Gene=FVER53263_03348;Ontology_term=antioxidant activity,oxidoreductase activity;Ontology_id=GO:0016209,GO:0016491;Enzyme_code=EC:1.11.1.6,EC:1.11,EC:1,EC:1.11.1,EC:1.11.1.6;Enzyme_name=catalase,Acting on a peroxide as acceptor,Oxidoreductases,Acting on a peroxide as acceptor,catalase
contig00001.g76	2292	ID=contig00001.g76;Description=lipoxygenase 1 [Fusarium coicis];Gene=FVER53263_03347;Ontology_term=oxidoreductase activity,lipid metabolic process;Ontology_id=GO:0016491,GO:0006629;Enzyme_code=EC:1.13.11.45,EC:1.13.11,EC:1.13,EC:1.13.11.45,EC:1;Enzyme_name=linoleate 11-lipoxygenase,Acting on single donors with incorporation of molecular oxygen (oxygenases). The oxygen incorporated need not be derived from O2,Acting on single donors with incorporation of molecular oxygen (oxygenases). The oxygen incorporated need not be derived from O2,linoleate 11-lipoxygenase,Oxidoreductases
contig00001.g77	390	ID=contig00001.g77;Description=DUF2470 domain [Fusarium agapanthi];Gene=FDENT_10558;Ontology_term=membrane;Ontology_id=GO:0016020
contig00001.g78	819	ID=contig00001.g78;Description=hypothetical protein FVEG_15245 [Fusarium verticillioides 7600]
contig00001.g79	453	ID=contig00001.g79;Description=hypothetical protein J7337_006269 [Fusarium musae];Gene=MSM1;Ontology_term=extracellular space,mitochondrion,tRNA metabolic process,amino acid metabolic process,receptor ligand activity,catalytic activity, acting on RNA,ligase activity;Ontology_id=GO:0005615,GO:0005739,GO:0006399,GO:0006520,GO:0048018,GO:0140098,GO:0016874;Enzyme_code=EC:6.1.1.10,EC:6.1,EC:6.1.1.10,EC:6.1.1,EC:6;Enzyme_name=methionine--tRNA ligase,Forming carbon-oxygen bonds,methionine--tRNA ligase,Forming carbon-oxygen bonds,Ligases

contig00001.g80	1799	ID=contig00001.g80;Description=probable DUR3-Urea permease [Fusarium fujikuroi];Gene=FPCIR_9778;Ontology_term=transmembrane transport,transporter activity;Ontology_id=GO:0055085,GO:0005215
contig00001.g81	2416	ID=contig00001.g81;Description=hypothetical protein FVER53263_03344 [Fusarium verticillioides]
contig00001.g82	822	ID=contig00001.g82;Description=ADH2-Alcohol dehydrogenase II [Fusarium fujikuroi];Gene=C2S_8451;Ontology_term=mRNA metabolic process,transmembrane transport,oxidoreductase activity,mitochondrion,transporter activity;Ontology_id=GO:0016071,GO:0055085,GO:0016491,GO:0005739,GO:0005215;Enzyme_code=EC:1,EC:1,EC:1.1.1.1;Enzyme_name=Oxidoreductases,Oxidoreductases,alcohol dehydrogenase
contig00001.g83	1135	ID=contig00001.g83;Description=acetoacetate decarboxylase [Fusarium oxysporum f. sp. lycopersici 4287];Gene=FDENT_735;Ontology_term=lyase activity;Ontology_id=GO:0016829;Enzyme_code=EC:4.1.1;Enzyme_name=Carbon-carbon lyases
contig00001.g84	2707	ID=contig00001.g84;Description=probable 3-isopropylmalate dehydrogenase beta [Fusarium proliferatum ET1];Gene=FPRO_07332;Ontology_term=oxidoreductase activity,amino acid metabolic process;Ontology_id=GO:0016491,GO:0006520;Enzyme_code=EC:1.1.1.85,EC:1.1,EC:1.1.1.85,EC:1,EC:1.1;Enzyme_name=3-isopropylmalate dehydrogenase,Acting on the CH-OH group of donors,3-isopropylmalate dehydrogenase,Oxidoreductases,Acting on the CH-OH group of donors
contig00001.g85	858	ID=contig00001.g85;Description=3-oxoacyl-[acyl-carrier protein] reductase [Fusarium verticillioides 7600]
contig00001.g86	1552	ID=contig00001.g86;Description=succinate-semialdehyde dehydrogenase [Fusarium verticillioides 7600];Gene=FVEG_03339;Ontology_term=oxidoreductase activity;Ontology_id=GO:0016491;Enzyme_code=EC:1.2.1,EC:1.2,EC:1,EC:1.2.1;Enzyme_name=Acting on the aldehyde or oxo group of donors,Acting on the aldehyde or oxo group of donors,Oxidoreductases,Acting on the aldehyde or oxo group of donors

contig00001.g87	1200	ID=contig00001.g87;Description=hypothetical protein FVER53590_29954 [Fusarium verticillioides];Ontology_term=ribosome biogenesis,transferase activity,catalytic activity, acting on RNA;Ontology_id=GO:0042254,GO:0016740,GO:0140098;Enzyme_code=EC:2.1,EC:2,EC:2.1.1,EC:2.1.1.312;Enzyme_name=Transferring one-carbon groups,Transferases,Transferring one-carbon groups,25S rRNA (uracil(2843)-N(3))-methyltransferase
contig00001.g88	4435	ID=contig00001.g88;Description=hypothetical protein FVEG_03337 [Fusarium verticillioides 7600];Gene=FNAPI_12835;Ontology_term=oxidoreductase activity;Ontology_id=GO:0016491;Enzyme_code=EC:1,EC:1;Enzyme_name=Oxidoreductases,Oxidoreductases
contig00001.g89	885	ID=contig00001.g89;Description=hypothetical protein FVER53263_03334 [Fusarium verticillioides]
contig00001.g90	1980	ID=contig00001.g90;Description=hypothetical protein FVER14953_03334 [Fusarium verticillioides]
contig00001.g91	2154	ID=contig00001.g91;Description=hypothetical protein FVER14953_03333 [Fusarium verticillioides]
contig00001.g92	1894	ID=contig00001.g92;Description=hypothetical protein J7337_006255 [Fusarium musae]
contig00001.g93	541	ID=contig00001.g93;Description=general substrate transporter [Fusarium oxysporum Fo47];Gene=FOTG_08262;Ontology_term=transmembrane transport,transporter activity;Ontology_id=GO:0055085,GO:0005215
contig00001.g94	7132	ID=contig00001.g94;Description=pisatin demethylase cytochrome P450 [Fusarium tjaetaba];Gene=FTJAE_9464;Ontology_term=oxidoreductase activity,transferase activity;Ontology_id=GO:0016491,GO:0016740;Enzyme_code=EC:1.14,EC:2.1.1,EC:2.1,EC:1,EC:1.14,EC:2,EC:2.1.1;Enzyme_name=Acting on paired donors, with incorporation or reduction of molecular oxygen. The oxygen incorporated need not be derived from O2,Transferring one-carbon groups,Transferring one-carbon groups,Oxidoreductases,Acting on paired donors, with incorporation or reduction of molecular oxygen. The oxygen incorporated need not be derived from O2,Transferases,Transferring one-carbon groups
contig00001.g95	726	ID=contig00001.g95;Description=RNA ligase cyclic nucleotide phosphodiesterase [Fusarium tjaetaba];Gene=1274;Ontology_term=defense response to other organism,inflammatory response,extracellular space,cytosol,receptor ligand activity,immune system process,ligase activity,lysosome;Ontology_id=GO:0098542,GO:0006954,GO:0005615,GO:0005829,GO:0048018,GO:0002376,GO:0016874,GO:0005764;Enzyme_code=EC:6,EC:6;Enzyme_name=Ligases,Ligases

contig00001.g96	1825	ID=contig00001.g96;Description=probable Maltose permease [Fusarium fujikuroi IMI 58289];Gene=FFUJ_06812;Ontology_term=transmembrane transport,mitochondrial gene expression,GTPase activity,mitochondrion,tRNA metabolic process,transporter activity;Ontology_id=GO:0055085,GO:0140053,GO:0003924,GO:0005739,GO:0006399,GO:0005215;Enzyme_code=EC:3.6.1.15,EC:3.6.1,EC:3.6,EC:3,EC:3.6.1.15;Enzyme_name=nucleoside-triphosphate phosphatase,Acting on acid anhydrides,Acting on acid anhydrides,Hydrolases,nucleoside-triphosphate phosphatase
contig00001.g97	979	ID=contig00001.g97;Description=quinic acid utilization QUTG (inositol-1(or 4)-monophosphatase) [Fusarium tjaetaba];Gene=C2S_14591;Ontology_term=lipid metabolic process,hydrolase activity;Ontology_id=GO:0006629,GO:0016787;Enzyme_code=EC:3.1.3.25;Enzyme_name=inositol-phosphate phosphatase
contig00001.g98	765	ID=contig00001.g98;Description=putative arabinosidase [Fusarium oxysporum Fo47]
contig00001.g99	597	ID=contig00001.g99;Description=arabinosidase [Fusarium tjaetaba]
contig00001.g100	516	ID=contig00001.g100;Description=alpha-glucuronidase [Fusarium verticillioides 7600];Gene=aguA;Ontology_term=carbohydrate metabolic process,cell wall organization or biogenesis,extracellular region,hydrolase activity;Ontology_id=GO:0005975,GO:0071554,GO:0005576,GO:0016787;Enzyme_code=EC:3.2.1.139,EC:3.2.1.139,EC:3.2,EC:3,EC:3.2.1;Enzyme_name=alpha-glucuronidase,alpha-glucuronidase,Glycosylases,Hydrolases,Glycosylases
contig00001.g101	4809	ID=contig00001.g101;Description=fungal specific transcription factor factor domain protein [Fusarium tjaetaba];Gene=FPRO_07352;Ontology_term=carbohydrate metabolic process,nucleus,DNA binding,regulation of DNA-templated transcription,cell wall organization or biogenesis,extracellular region,transcription regulator activity,hydrolase activity;Ontology_id=GO:0005975,GO:0005634,GO:0003677,GO:0006355,GO:0071554,GO:0005576,GO:0140110,GO:0016787;Enzyme_code=EC:3.2.1.139,EC:3.2.1.139,EC:3.2,EC:3,EC:3.2.1;Enzyme_name=alpha-glucuronidase,alpha-glucuronidase,Glycosylases,Hydrolases,Glycosylases
contig00001.g102	378	ID=contig00001.g102;Description=isocitrate lyase [Fusarium tjaetaba];Gene=FVER53263_20216;Ontology_term=lyase activity;Ontology_id=GO:0016829;Enzyme_code=EC:4.1.3.1,EC:4.1.3.30,EC:4.1.3.1,EC:4.1,EC:4,EC:4.1.3.30,EC:4.1.3;Enzyme_name=isocitrate lyase,methylisocitrate lyase,isocitrate lyase,Carbon-carbon lyases,Lyases,methylisocitrate lyase,Carbon-carbon lyases

contig00001.g103	1261	ID=contig00001.g103;Description=major facilitator superfamily transporter [Fusarium tjaetaba];Gene=FOMG_06120;Ontology_term=transmembrane transport,transporter activity;Ontology_id=GO:0055085,GO:0005215
contig00001.g104	804	ID=contig00001.g104;Description=hypothetical protein FVER53590_03323 [Fusarium verticillioides]
contig00001.g105	3741	ID=contig00001.g105;Description=tetratricopeptide-like helical [Fusarium tjaetaba]
contig00001.g106	1095	ID=contig00001.g106;Description=related to nitrogen metabolic regulation protein nmr [Fusarium proliferatum ET1]
contig00001.g107	1874	ID=contig00001.g107;Description=Zn(II)Cys6 transcriptional activator [Fusarium mundagurra];Gene=FOQG_14194;Ontology_term=nucleus,DNA binding,regulation of DNA-templated transcription,transcription regulator activity;Ontology_id=GO:0005634,GO:0003677,GO:0006355,GO:0140110
contig00001.g108	1420	ID=contig00001.g108;Description=salicylate hydroxylase [Fusarium verticillioides 7600];Gene=FVER53590_03319;Ontology_term=oxidoreductase activity;Ontology_id=GO:0016491;Enzyme_code=EC:1;Enzyme_name=Oxidoreductases
contig00001.g109	1937	ID=contig00001.g109;Description=drug facilitator PEP5 [Fusarium tjaetaba];Gene=FVER53590_03318;Ontology_term=transmembrane transport,ATP-dependent activity,plasma membrane,transporter activity,hydrolase activity;Ontology_id=GO:0055085,GO:0140657,GO:0005886,GO:0005215,GO:0016787;Enzyme_code=EC:7.2.2,EC:3.6.1.15;Enzyme_name=Catalysing the translocation of inorganic cations,nucleoside-triphosphate phosphatase
contig00001.g110	4841	ID=contig00001.g110;Description=related to multidrug resistance protein [Fusarium fujikuroi];Gene=FVEG_03317;Ontology_term=transmembrane transport,ATP-dependent activity,transporter activity,plasma membrane,hydrolase activity;Ontology_id=GO:0055085,GO:0140657,GO:0005215,GO:0005886,GO:0016787;Enzyme_code=EC:7.2.2,EC:3.6.1.15,EC:3.6.1,EC:3.6,EC:3,EC:7.2.2,EC:7,EC:3.6.1.15;Enzyme_name=Catalysing the translocation of inorganic cations,nucleoside-triphosphate phosphatase,Acting on acid anhydrides,Acting on acid anhydrides,Hydrolases,Catalysing the translocation of inorganic cations,Translocases,nucleoside-triphosphate phosphatase
contig00001.g111	1888	ID=contig00001.g111;Description=HOL1 substrate-H+ antiporter [Fusarium coicis];Gene=FPANT_2151;Ontology_term=transmembrane transport,transporter activity;Ontology_id=GO:0055085,GO:0005215

contig00001.g112	1251	ID=contig00001.g112;Description=alcohol dehydrogenase [Fusarium tjaetaba];Gene=FVEG_15241;Ontology_term=oxidoreductase activity;Ontology_id=GO:0016491;Enzyme_code=EC:1,EC:1;Enzyme_name=Oxidoreductases,Oxidoreductases
contig00001.g113	1384	ID=contig00001.g113;Description=acetyl-CoA acyltransferase [Fusarium verticillioides 7600];Gene=FNAPI_1585;Ontology_term=transferase activity;Ontology_id=GO:0016740;Enzyme_code=EC:2.3.1;Enzyme_name=Acyltransferases
contig00001.g114	1566	ID=contig00001.g114;Description=alpha-methylacyl-coa racemase [Fusarium tjaetaba];Gene=FTJAE_9496;Ontology_term=catalytic activity;Ontology_id=GO:0003824;Enzyme_code=EC:2.8.3.13;Enzyme_name=succinate--hydroxymethylglutarate CoA-transferase
contig00001.g115	1835	ID=contig00001.g115;Description=putative succinyl-:3-ketoacid-coenzyme A mitochondrial precursor [Fusarium denticulatum];Gene=FPCIR_9757;Ontology_term=generation of precursor metabolites and energy,mitochondrion,lipid metabolic process,transferase activity;Ontology_id=GO:0006091,GO:0005739,GO:0006629,GO:0016740;Enzyme_code=EC:2.8.3.5,EC:2.8.3.5,EC:2,EC:2.8.3,EC:2.8;Enzyme_name=3-oxoacid CoA-transferase,3-oxoacid CoA-transferase,Transferases,Transferring sulfur-containing groups,Transferring sulfur-containing groups
contig00001.g116	1705	ID=contig00001.g116;Description=3-carboxy-cis,cis-muconate cycloisomerase [Fusarium verticillioides 7600];Gene=FPHYL_5040;Ontology_term=isomerase activity;Ontology_id=GO:0016853;Enzyme_code=EC:5,EC:5;Enzyme_name=Isomerases,Isomerases
contig00001.g117	1701	ID=contig00001.g117;Description=homogentisate 1,2-dioxygenase [Fusarium verticillioides 7600];Gene=FPCIR_9759;Ontology_term=oxidoreductase activity,amino acid metabolic process;Ontology_id=GO:0016491,GO:0006520;Enzyme_code=EC:1.13.11.5,EC:1.13.11,EC:1.13,EC:1,EC:1.13.11.5;Enzyme_name=homogentisate 1,2-dioxygenase,Acting on single donors with incorporation of molecular oxygen (oxygenases). The oxygen incorporated need not be derived from O2,Acting on single donors with incorporation of molecular oxygen (oxygenases). The oxygen incorporated need not be derived from O2,Oxidoreductases,homogentisate 1,2-dioxygenase

contig00001.g118	3063	ID=contig00001.g118;Description=oxidoreductase [Fusarium globosum];Gene=FVER53590_03309;Ontology_term=oxidoreductase activity;Ontology_id=GO:0016491;Enzyme_code=EC:1.14,EC:1,EC:1.14;Enzyme_name=Acting on paired donors, with incorporation or reduction of molecular oxygen. The oxygen incorporated need not be derived from O2,Oxidoreductases,Acting on paired donors, with incorporation or reduction of molecular oxygen. The oxygen incorporated need not be derived from O2
contig00001.g119	506	ID=contig00001.g119;Description=oxidoreductase [Fusarium tjaetaba];Gene=FPHYL_5042;Ontology_term=nucleus,DNA binding,oxidoreductase activity,DNA-templated transcription;Ontology_id=GO:0005634,GO:0003677,GO:0016491,GO:0006351;Enzyme_code=EC:1.14,EC:1,EC:1.14;Enzyme_name=Acting on paired donors, with incorporation or reduction of molecular oxygen. The oxygen incorporated need not be derived from O2,Oxidoreductases,Acting on paired donors, with incorporation or reduction of molecular oxygen. The oxygen incorporated need not be derived from O2
contig00001.g120	786	ID=contig00001.g120;Description=probable acetylxylan esterase precursor [Fusarium fujikuroi];Gene=FFUJ_06855;Ontology_term=hydrolase activity;Ontology_id=GO:0016787;Enzyme_code=EC:3.1.1,EC:3.1,EC:3,EC:3.1.1.72,EC:3.1.1;Enzyme_name=Acting on ester bonds,Acting on ester bonds,Hydrolases,acetylxylan esterase,Acting on ester bonds
contig00001.g121	1466	ID=contig00001.g121;Description=Het-eN [Fusarium subglutinans]
contig00001.g122	699	ID=contig00001.g122;Description=secreted in xylem 2-1 [Fusarium proliferatum]
contig00001.g123	1881	ID=contig00001.g123;Description=phosphoribosylaminoimidazole carboxylase [Fusarium phyllophilum];Gene=FOVG_05618;Ontology_term=nucleobase-containing small molecule metabolic process,lyase activity,carbohydrate derivative metabolic process;Ontology_id=GO:0055086,GO:0016829,GO:1901135;Enzyme_code=EC:4.1.1.21,EC:4.1.1,EC:4.1,EC:4,EC:4.1.1.21;Enzyme_name=phosphoribosylaminoimidazole carboxylase,Carbon-carbon lyases,Carbon-carbon lyases,Lyases,phosphoribosylaminoimidazole carboxylase
contig00001.g124	806	ID=contig00001.g124;Description=hypothetical protein FVER14953_20108 [Fusarium verticillioides]
contig00001.g125	570	ID=contig00001.g125;Description=hypothetical protein FVER53590_03305 [Fusarium verticillioides]

contig00001.g126	4569	ID=contig00001.g126;Description=hypothetical protein FVER53590_03304 [Fusarium verticillioides]
contig00001.g127	980	ID=contig00001.g127;Description=beta-lactamase [Fusarium verticillioides 7600]
contig00001.g128	1844	ID=contig00001.g128;Description=HXT3-low-affinity hexose facilitator [Fusarium tjaetaba];Gene=FNYG_05982;Ontology_term=transmembrane transport,transporter activity;Ontology_id=GO:0055085,GO:0005215
contig00001.g129	1467	ID=contig00001.g129;Description=hypothetical protein FVER53263_03301 [Fusarium verticillioides];Gene=FPHYL_5019;Ontology_term=nucleus,DNA binding,regulation of DNA-templated transcription,transcription regulator activity;Ontology_id=GO:0005634,GO:0003677,GO:0006355,GO:0140110
contig00001.g130	855	ID=contig00001.g130;Description=uncharacterized protein FTJAE_10928 [Fusarium tjaetaba];Gene=FANTH_1383;Ontology_term=nucleus,DNA binding,DNA-templated transcription;Ontology_id=GO:0005634,GO:0003677,GO:0006351
contig00001.g131	1244	ID=contig00001.g131;Description=beta-lactamase [Fusarium verticillioides 7600];Gene=FPHYL_5017;Ontology_term=ATP-dependent activity,hydrolase activity;Ontology_id=GO:0140657,GO:0016787;Enzyme_code=EC:3.6.1.15;Enzyme_name=nucleoside-triphosphate phosphatase
contig00001.g132	990	ID=contig00001.g132;Description=hypothetical protein FVER53590_13312 [Fusarium verticillioides];Gene=FMUND_5108;Ontology_term=oxidoreductase activity;Ontology_id=GO:0016491;Enzyme_code=EC:1,EC:1;Enzyme_name=Oxidoreductases,Oxidoreductases
contig00001.g133	1452	ID=contig00001.g133;Description=C6 zinc finger domain [Fusarium acutatum];Gene=FACUT_3890;Ontology_term=nucleus,regulation of DNA-templated transcription,transcription regulator activity;Ontology_id=GO:0005634,GO:0006355,GO:0140110
contig00001.g134	1905	ID=contig00001.g134;Description=TRI13-cytochrome P450 [Fusarium tjaetaba];Gene=FDENT_779;Ontology_term=oxidoreductase activity;Ontology_id=GO:0016491;Enzyme_code=EC:1.14,EC:1,EC:1.14;Enzyme_name=Acting on paired donors, with incorporation or reduction of molecular oxygen. The oxygen incorporated need not be derived from O2,Oxidoreductases,Acting on paired donors, with incorporation or reduction of molecular oxygen. The oxygen incorporated need not be derived from O2

contig00001.g135	686	ID=contig00001.g135;Description=integral membrane protein [Fusarium tjaetaba];Gene=FVER53590_03296;Ontology_term=side of membrane;Ontology_id=GO:0098552
contig00001.g136	822	ID=contig00001.g136;Description=Zinc finger protein [Fusarium oxysporum f. sp. albedinis];Gene=BFJ63_vAg16756;Ontology_term=membrane;Ontology_id=GO:0016020
contig00001.g137	441	ID=contig00001.g137;Description=hypothetical protein FVER14953_20106 [Fusarium verticillioides]
contig00001.g138	1981	ID=contig00001.g138;Description=amidase signature domain protein [Fusarium subglutinans]
contig00001.g139	1433	ID=contig00001.g139;Description=heterokaryon incompatibility protein [Fusarium tjaetaba];Gene=F25303_1843;Ontology_term=toxin activity;Ontology_id=GO:0090729
contig00001.g140	718	ID=contig00001.g140;Description=hypothetical protein FVEG_03292 [Fusarium verticillioides 7600];Gene=FNAPI_1610;Ontology_term=membrane;Ontology_id=GO:0016020
contig00001.g141	240	ID=contig00001.g141;Description=hypothetical protein FVER53590_28307 [Fusarium verticillioides]
contig00001.g142	1014	ID=contig00001.g142;Description=tol [Fusarium tjaetaba]
contig00001.g143	1895	ID=contig00001.g143;Description=hypothetical protein FVEG_15232 [Fusarium verticillioides 7600]
contig00001.g144	483	ID=contig00001.g144;Description=phenazine biosynthesis [Fusarium sp. NRRL 25303];Gene=FTJAE_771;Ontology_term=membrane;Ontology_id=GO:0016020
contig00001.g145	932	ID=contig00001.g145;Description=hypothetical protein FVER53590_03288 [Fusarium verticillioides];Gene=FTJAE_771;Ontology_term=membrane;Ontology_id=GO:0016020
contig00001.g146	939	ID=contig00001.g146;Description=phenazine biosynthesis protein phzC [Fusarium fujikuroi];Gene=FFUJ_06878;Ontology_term=catalytic activity;Ontology_id=GO:0003824
contig00001.g147	1100	ID=contig00001.g147;Description=hypothetical protein FVER53590_03286 [Fusarium verticillioides];Gene=FVEG_03286;Ontology_term=metal ion binding;Ontology_id=GO:0046872
contig00001.g148	903	ID=contig00001.g148;Description=hypothetical protein FVER53590_03284 [Fusarium verticillioides];Gene=1329

contig00001.g149	2321	ID=contig00001.g149;Description=disulfide-isomerase precursor [Colletotrichum scovillei];Ontology_term=catalytic activity, acting on a protein,protein-containing complex assembly,protein catabolic process,carbohydrate derivative metabolic process,cellular modified amino acid metabolic process,amino acid metabolic process,endoplasmic reticulum,extracellular region,protein folding,plasma membrane,oxidoreductase activity,signaling,programmed cell death,isomerase activity;Ontology_id=GO:0140096,GO:0065003,GO:0030163,GO:1901135,GO:0006575,GO:0006520,GO:0005783,GO:0005576,GO:0006457,GO:0005886,GO:0016491,GO:0023052,GO:0012501,GO:0016853;Enzyme_code=EC:5.3.4.1,EC:1.14.11,EC:1,EC:1.8,EC:1.14,EC:5.3,EC:5.3.4,EC:5,EC:1.14.11.2;Enzyme_name=protein disulfide-isomerase,Acting on paired donors, with incorporation or reduction of molecular oxygen. The oxygen incorporated need not be derived from O2,Oxidoreductases,Acting on a sulfur group of donors,Acting on paired donors, with incorporation or reduction of molecular oxygen. The oxygen incorporated need not be derived from O2,Intramolecular oxidoreductases,Intramolecular oxidoreductases,Isomerases,procollagen-proline 4-dioxygenase
contig00001.g150	4831	ID=contig00001.g150;Description=canalicular multispecific organic anion transporter 1 [Fusarium mundagurra];Gene=FVER53590_03281;Ontology_term=transmembrane transport,ATP-dependent activity,transporter activity,plasma membrane,hydrolase activity;Ontology_id=GO:0055085,GO:0140657,GO:0005215,GO:0005886,GO:0016787;Enzyme_code=EC:7.2.2,EC:3.6.1.15,EC:3.6.1,EC:3.6,EC:3,EC:7.2.2,EC:7,EC:3.6.1.15;Enzyme_name=Catalysing the translocation of inorganic cations,nucleoside-triphosphate phosphatase,Acting on acid anhydrides,Acting on acid anhydrides,Hydrolases,Catalysing the translocation of inorganic cations,Translocases,nucleoside-triphosphate phosphatase
contig00001.g151	1986	ID=contig00001.g151;Description=heterokaryon incompatibility 6 OR allele [Fusarium pseudoanthophilum]
contig00001.g152	1821	ID=contig00001.g152;Description=alpha-glucosidase [Fusarium verticillioides 7600];Gene=FPANT_8905;Ontology_term=carbohydrate metabolic process,hydrolase activity;Ontology_id=GO:0005975,GO:0016787;Enzyme_code=EC:3.2.1.20,EC:3.2,EC:3.2.1.20,EC:3.2.1.10,EC:3,EC:3.2.1;Enzyme_name=alpha-glucosidase,Glycosylases,alpha-glucosidase,oligo-1,6-glucosidase,Hydrolases,Glycosylases
contig00001.g153	1721	ID=contig00001.g153;Description=general substrate transporter [Fusarium redolens];Gene=AU210_007390;Ontology_term=transmembrane transport,transporter activity;Ontology_id=GO:0055085,GO:0005215

contig00001.g154	1443	ID=contig00001.g154;Description=transcription activator amyR [Fusarium tjaetaba];Gene=BFJ70_g1161;Ontology_term=nucleus,regulation of DNA-templated transcription,transcription regulator activity;Ontology_id=GO:0005634,GO:0006355,GO:0140110
contig00001.g155	1110	ID=contig00001.g155;Description=putative ASP3-1-L-asparaginase II [Fusarium fujikuroi];Gene=FANTH_1406;Ontology_term=amino acid metabolic process,hydrolase activity;Ontology_id=GO:0006520,GO:0016787;Enzyme_code=EC:3.5.1.1,EC:3.5.1.1,EC:3.5.1,EC:3.5,EC:3;Enzyme_name=asparaginase,asparaginase,Acting on carbon-nitrogen bonds, other than peptide bonds,Acting on carbon-nitrogen bonds, other than peptide bonds,Hydrolases
contig00001.g156	570	ID=contig00001.g156;Description=actin monomer binding protein [Fusarium coicis];Gene=NUD1;Ontology_term=nuclear envelope,cytoskeleton organization,microtubule organizing center,mitotic nuclear division,chromosome segregation,molecular adaptor activity,meiotic nuclear division;Ontology_id=GO:0005635,GO:0007010,GO:0005815,GO:0140014,GO:0007059,GO:0060090,GO:0140013
contig00001.g157	1101	ID=contig00001.g157;Description=serine/threonine protein kinase [Fusarium verticillioides 7600];Gene=FPHYL_4184;Ontology_term=catalytic activity, acting on a protein,transferase activity;Ontology_id=GO:0140096,GO:0016740;Enzyme_code=EC:2.7.11.1,EC:2.7.1,EC:2.7.11.1,EC:2.7,EC:2;Enzyme_name=non-specific serine/threonine protein kinase,Transferring phosphorus-containing groups,non-specific serine/threonine protein kinase,Transferring phosphorus-containing groups,Transferases
contig00001.g158	2756	ID=contig00001.g158;Description=hypothetical protein FVER53590_03274 [Fusarium verticillioides]
contig00001.g159	1035	ID=contig00001.g159;Description=heterokaryon incompatibility (het-6OR allele) [Fusarium tjaetaba];Gene=FMUND_1446;Ontology_term=oxidoreductase activity;Ontology_id=GO:0016491;Enzyme_code=EC:1.1.3.37,EC:1.1,EC:1,EC:1.1.3.37,EC:1.1.3;Enzyme_name=D-arabinono-1,4-lactone oxidase,Acting on the CH-OH group of donors,Oxidoreductases,D-arabinono-1,4-lactone oxidase,Acting on the CH-OH group of donors

contig00001.g160	2486	ID=contig00001.g160;Description=heterokaryon incompatibility protein (het-6OR allele) [Fusarium denticulatum];Gene=FSUBG_6333;Ontology_term=oxidoreductase activity;Ontology_id=GO:0016491;Enzyme_code=EC:1.1.3.37,EC:1.1,EC:1,EC:1.1.3.37,EC:1.1.3;Enzyme_name=D-arabinono-1,4-lactone oxidase,Acting on the CH-OH group of donors,Oxidoreductases,D-arabinono-1,4-lactone oxidase,Acting on the CH-OH group of donors
contig00001.g161	454	ID=contig00001.g161;Description=cyanate hydratase [Fusarium mundagurra];Gene=tug1;Ontology_term=nucleus,microtubule organizing center,cytoskeleton organization,protein-containing complex assembly,structural molecule activity,mitotic nuclear division,reproductive process,chromosome segregation,cytokinesis;Ontology_id=GO:0005634,GO:0005815,GO:0007010,GO:0065003,GO:0005198,GO:0140014,GO:0022414,GO:0007059,GO:0000910
contig00001.g162	6336	ID=contig00001.g162;Description=f-box domain-containing protein [Fusarium denticulatum]
contig00001.g163	1196	ID=contig00001.g163;Description=esterase lipase [Fusarium sp. NRRL 52700];Gene=BFJ72_g9736;Ontology_term=hydrolase activity;Ontology_id=GO:0016787;Enzyme_code=EC:3,EC:3;Enzyme_name=Hydrolases,Hydrolases
contig00001.g164	1245	ID=contig00001.g164;Description=FAD dependent oxidoreductase [Fusarium redolens];Gene=FOC1_g10010701;Ontology_term=oxidoreductase activity,amino acid metabolic process;Ontology_id=GO:0016491,GO:0006520;Enzyme_code=EC:1.4.3.3,EC:1.4.3.21,EC:1.4,EC:1.4.3,EC:1,EC:1.4.3.1,EC:1.4.3.3,EC:1.4.3.21;Enzyme_name=D-amino-acid oxidase,primary-amine oxidase,Acting on the CH-NH2 group of donors,Acting on the CH-NH2 group of donors,Oxidoreductases,D-aspartate oxidase,D-amino-acid oxidase,primary-amine oxidase
contig00001.g165	1975	ID=contig00001.g165;Description=related to DAL81-transcriptional activator for allantoin and GABA catabolic genes [Fusarium mangiferae];Gene=FOC4_g10013257;Ontology_term=nucleus,DNA binding,regulation of DNA-templated transcription,transcription regulator activity;Ontology_id=GO:0005634,GO:0003677,GO:0006355,GO:0140110
contig00001.g166	1910	ID=contig00001.g166;Description=regulator of G signaling superfamily [Fusarium napiforme];Gene=FPANT_598;Ontology_term=nucleus,DNA binding,regulation of DNA-templated transcription,transcription regulator activity;Ontology_id=GO:0005634,GO:0003677,GO:0006355,GO:0140110

contig00001.g167	1788	ID=contig00001.g167;Description=related to beta transducin-like protein [Fusarium fujikuroi]
contig00001.g168	2744	ID=contig00001.g168;Description=ubiquitin ligase [Fusarium coicis];Gene=FPCIR_8095;Ontology_term=ligase activity;Ontology_id=GO:0016874;Enzyme_code=EC:6,EC:6;Enzyme_name=Ligases,Ligases
contig00001.g169	642	ID=contig00001.g169;Description=transmembrane protein 6/97 [Fusarium oxysporum];Gene=FVER53263_20205;Ontology_term=endoplasmic reticulum;Ontology_id=GO:0005783
contig00001.g170	1076	ID=contig00001.g170;Description=Ff.00g067290.m01.CDS01 [Fusarium sp. VM40];Gene=BKA59DRAFT_550138;Ontology_term=isomerase activity;Ontology_id=GO:0016853;Enzyme_code=EC:5,EC:5;Enzyme_name=Isomerases,Isomerases
contig00001.g171	1833	ID=contig00001.g171;Description=cytochrome P450 [Fusarium sp. MPI-SDFR-AT-0072];Gene=FVER53590_03262;Ontology_term=oxidoreductase activity;Ontology_id=GO:0016491;Enzyme_code=EC:1.14,EC:1,EC:1.14;Enzyme_name=Acting on paired donors, with incorporation or reduction of molecular oxygen. The oxygen incorporated need not be derived from O2,Oxidoreductases,Acting on paired donors, with incorporation or reduction of molecular oxygen. The oxygen incorporated need not be derived from O2
contig00001.g172	1689	ID=contig00001.g172;Description=related to ascus development protein 3 [Fusarium proliferatum ET1];Gene=FPRO_07453;Ontology_term=transmembrane transport,transporter activity;Ontology_id=GO:0055085,GO:0005215
contig00001.g173	1194	ID=contig00001.g173;Description=cellulose-binding family II [Fusarium pseudoanthophilum];Gene=FVER53590_03260;Ontology_term=carbohydrate metabolic process,extracellular space,receptor ligand activity;Ontology_id=GO:0005975,GO:0005615,GO:0048018
contig00001.g174	961	ID=contig00001.g174;Description=hypothetical protein J7337_006159 [Fusarium musae]
contig00001.g175	2005	ID=contig00001.g175;Description=hypothetical protein FVEG_03258 [Fusarium verticillioides 7600];Gene=FMEXI_10591;Ontology_term=nucleus,DNA binding,regulation of DNA-templated transcription,transferase activity,transcription regulator activity;Ontology_id=GO:0005634,GO:0003677,GO:0006355,GO:0016740,GO:0140110;Enzyme_code=EC:2.6.1,EC:2.6.1,EC:2,EC:2.6;Enzyme_name=Transferring nitrogenous groups,Transferring nitrogenous groups,Transferases,Transferring nitrogenous groups

contig00001.g176	1371	ID=contig00001.g176;Description=ornithine aminotransferase [Fusarium tjaetaba];Gene=FMUND_1428;Ontology_term=transferase activity;Ontology_id=GO:0016740;Enzyme_code=EC:2.6.1,EC:2.6.1,EC:2,EC:2.6;Enzyme_name=Transferring nitrogenous groups,Transferring nitrogenous groups,Transferases,Transferring nitrogenous groups
contig00001.g177	2943	ID=contig00001.g177;Description=HET domain-containing protein [Fusarium keratoplasticum]
contig00001.g178	834	ID=contig00001.g178;Description=Peptidase M12A astacin [Fusarium acutatum];Gene=FACUT_4681;Ontology_term=catalytic activity, acting on a protein,cell adhesion,hydrolase activity;Ontology_id=GO:0140096,GO:0007155,GO:0016787;Enzyme_code=EC:3.4.24,EC:3.4.24,EC:3.4,EC:3;Enzyme_name=Acting on peptide bonds (peptidases),Acting on peptide bonds (peptidases),Acting on peptide bonds (peptidases),Hydrolases
contig00001.g179	2088	ID=contig00001.g179;Description=hypothetical protein FVEG_03253 [Fusarium verticillioides 7600];Gene=FMUND_1427;Ontology_term=membrane;Ontology_id=GO:0016020
contig00001.g180	2267	ID=contig00001.g180;Description=AHMP1-like protein [Fusarium tjaetaba];Gene=FPANT_13778;Ontology_term=membrane;Ontology_id=GO:0016020
contig00001.g181	2005	ID=contig00001.g181;Description=CNT family concentrative nucleoside transporter [Fusarium proliferatum];Gene=FVEG_03250;Ontology_term=transmembrane transport,transporter activity,plasma membrane;Ontology_id=GO:0055085,GO:0005215,GO:0005886
contig00001.g182	9665	ID=contig00001.g182;Description=VPS13-like vacuolar sorting protein [Fusarium denticulatum];Gene=FPCIR_8083;Ontology_term=membrane organization,vesicle-mediated transport,endosome,mitochondrion,anatomical structure development,cell differentiation,reproductive process,Golgi apparatus,intracellular protein transport,mitochondrion organization;Ontology_id=GO:0061024,GO:0016192,GO:0005768,GO:0005739,GO:0048856,GO:0030154,GO:0022414,GO:0005794,GO:0006886,GO:0007005
contig00001.g183	1600	ID=contig00001.g183;Description=MNN4-regulates the mannosylphosphorylation [Fusarium subglutinans];Gene=FVER53263_03248;Ontology_term=membrane;Ontology_id=GO:0016020

contig00001.g184	2689	ID=contig00001.g184;Description=microtubule associated protein [Fusarium fujikuroi];Gene=FNYG_06047;Ontology_term=catalytic activity, acting on a protein,cytoskeleton organization,protein-containing complex assembly,establishment or maintenance of cell polarity,structural molecule activity,anatomical structure development,nuclear chromosome,chromosome segregation,intracellular protein transport,microtubule-based movement,microtubule organizing center,mitotic nuclear division,transferase activity,cytoskeletal protein binding;Ontology_id=GO:0140096,GO:0007010,GO:0065003,GO:0007163,GO:0005198,GO:0048856,GO:0000228,GO:0007059,GO:0006886,GO:0007018,GO:0005815,GO:0140014,GO:0016740,GO:0008092;Enzyme_code=EC:2,EC:2;Enzyme_name=Transferases,Transferases
contig00001.g185	2367	ID=contig00001.g185;Description=tuftelin-interacting 11 [Fusarium napiforme];Gene=FNAPI_1850;Ontology_term=nucleus,mRNA metabolic process,RNA binding,protein-containing complex assembly,mitochondrion;Ontology_id=GO:0005634,GO:0016071,GO:0003723,GO:0065003,GO:0005739
contig00001.g186	1283	ID=contig00001.g186;Description=subtilisin [Fusarium verticillioides 7600];Gene=FPCIR_8078;Ontology_term=catalytic activity, acting on a protein,hydrolase activity;Ontology_id=GO:0140096,GO:0016787;Enzyme_code=EC:3.4.21;Enzyme_name=Acting on peptide bonds (peptidases)
contig00001.g187	3143	ID=contig00001.g187;Description=Sit4p-associated protein [Fusarium subglutinans];Gene=FPANT_5407;Ontology_term=molecular function regulator activity,signaling,mitochondrion,mitotic nuclear division,nuclear chromosome,tRNA metabolic process,chromosome segregation,nucleoplasm;Ontology_id=GO:0098772,GO:0023052,GO:0005739,GO:0140014,GO:0000228,GO:0006399,GO:0007059,GO:0005654
contig00001.g188	498	ID=contig00001.g188;Description=extragenic suppressor of kinetochore 1 [Fusarium subglutinans];Ontology_term=molecular function regulator activity,signaling,mitochondrion,mitotic nuclear division,nuclear chromosome,tRNA metabolic process,chromosome segregation,nucleoplasm;Ontology_id=GO:0098772,GO:0023052,GO:0005739,GO:0140014,GO:0000228,GO:0006399,GO:0007059,GO:0005654
contig00001.g189	7471	ID=contig00001.g189;Description=NRPS protein [Fusarium musae];Gene=FGLOB1_2434;Ontology_term=catalytic activity;Ontology_id=GO:0003824

contig00001.g190	2791	ID=contig00001.g190;Description=sexual differentiation process isp4 [Fusarium coicis];Gene=FOVG_05690;Ontology_term=transmembrane transport,transporter activity;Ontology_id=GO:0055085,GO:0005215
contig00001.g191	1069	ID=contig00001.g191;Description=retinol dehydrogenase 11 [Fusarium coicis]
contig00001.g192	1759	ID=contig00001.g192;Description=hypothetical protein FVEG_03240 [Fusarium verticillioides 7600]
contig00001.g193	2053	ID=contig00001.g193;Description=zinc finger protein [Fusarium tjaetaba]
contig00001.g194	1673	ID=contig00001.g194;Description=Tyrosine phosphatase YVH1 [Fusarium tjaetaba];Gene=YVH1;Ontology_term=catalytic activity, acting on a protein,hydrolase activity;Ontology_id=GO:0140096,GO:0016787;Enzyme_code=EC:3.1.3.48;Enzyme_name=protein-tyrosine-phosphatase
contig00001.g195	486	ID=contig00001.g195;Description=hypothetical protein FVEG_15218 [Fusarium verticillioides 7600]
contig00001.g196	727	ID=contig00001.g196;Description=AAA-ATPase vps4-associated 1 domain-containing protein [Fusarium coicis];Gene=rpa1;Ontology_term=DNA binding,carbohydrate metabolic process,generation of precursor metabolites and energy,sulfur compound metabolic process,regulation of DNA-templated transcription,nucleolus,catalytic activity, acting on RNA,transferase activity,vitamin metabolic process;Ontology_id=GO:0003677,GO:0005975,GO:0006091,GO:0006790,GO:0006355,GO:0005730,GO:0140098,GO:0016740,GO:0006766;Enzyme_code=EC:2.7.7.6,EC:2.7,EC:2,EC:2.7.7,EC:2.7.7.6;Enzyme_name=DNA-directed RNA polymerase,Transferring phosphorus-containing groups,Transferases,Transferring phosphorus-containing groups,DNA-directed RNA polymerase
contig00001.g197	1657	ID=contig00001.g197;Description=related to structure-specific recognition proteins [Fusarium mangiferae]
contig00001.g198	614	ID=contig00001.g198;Description=litaf zinc finger [Fusarium tjaetaba];Gene=FMUND_13815;Ontology_term=membrane,metal ion binding;Ontology_id=GO:0016020,GO:0046872
contig00001.g199	357	ID=contig00001.g199;Description=37s ribosomal mrp10 [Fusarium tjaetaba];Gene=Forpe1208_v008118;Ontology_term=mitochondrial gene expression,mitochondrion,structural molecule activity,ribosome;Ontology_id=GO:0140053,GO:0005739,GO:0005198,GO:0005840

contig00001.g200	3126	ID=contig00001.g200;Description=Scd1-like protein [Fusarium sp. NRRL 25303];Gene=pac1;Ontology_term=cytoskeleton organization,DNA-templated transcription,establishment or maintenance of cell polarity,anatomical structure development,lyase activity,ribosome biogenesis,reproductive process,cytokinesis,amino acid metabolic process,nucleolus,plasma membrane,nucleoplasm,DNA binding,mRNA metabolic process,cytoskeleton,RNA binding,vesicle-mediated transport,molecular function regulator activity,snRNA metabolic process,signaling,mitotic nuclear division,catalytic activity, acting on RNA,hydrolase activity;Ontology_id=GO:0007010,GO:0006351,GO:0007163,GO:0048856,GO:0016829,GO:0042254,GO:0022414,GO:0000910,GO:0006520,GO:0005730,GO:0005886,GO:0005654,GO:0003677,GO:0016071,GO:0005856,GO:0003723,GO:0016192,GO:0098772,GO:0016073,GO:0023052,GO:0140014,GO:0140098,GO:0016787;Enzyme_code=EC:4.1.1,EC:3.1.30,EC:3.1.26.3,EC:4.1.1,EC:3.1.30,EC:4.1,EC:3.1,EC:3,EC:3.1.26.3,EC:3.1.26,EC:4;Enzyme_name=Carbon-carbon lyases,Acting on ester bonds,ribonuclease III,Carbon-carbon lyases,Acting on ester bonds,Carbon-carbon lyases,Acting on ester bonds,Hydrolases,ribonuclease III,Acting on ester bonds,Lyases
contig00001.g201	624	ID=contig00001.g201;Description=Dolichyl-phosphate-mannose mannosyltransferase 2 [Fusarium pseudocircinatum];Gene=FPCIR_8061;Ontology_term=transferase activity;Ontology_id=GO:0016740;Enzyme_code=EC:2.4,EC:2.4,EC:2;Enzyme_name=Glycosyltransferases,Glycosyltransferases,Transferases
contig00001.g202	4080	ID=contig00001.g202;Description=serine/threonine protein kinase [Fusarium oxysporum f. sp. lycopersici 4287];Gene=FOMG_06230;Ontology_term=catalytic activity, acting on a protein,transferase activity;Ontology_id=GO:0140096,GO:0016740;Enzyme_code=EC:2.7.11.1,EC:2.7.1,EC:2.7.11.1,EC:2.7,EC:2;Enzyme_name=non-specific serine/threonine protein kinase,Transferring phosphorus-containing groups,non-specific serine/threonine protein kinase,Transferring phosphorus-containing groups,Transferases
contig00001.g203	1150	ID=contig00001.g203;Description=alcohol dehydrogenase [Fusarium verticillioides 7600]
contig00001.g204	858	ID=contig00001.g204;Description=Transmembrane protein 53-A [Fusarium odoratissimum];Gene=FOXG_04914;Ontology_term=nuclear envelope;Ontology_id=GO:0005635

contig00001.g205	1440	ID=contig00001.g205;Description=related to monocarboxylate transporter 2 [Fusarium fujikuroi];Gene=FNAPI_7655;Ontology_term=DNA repair,transmembrane transport,DNA replication,lipid binding,endoplasmic reticulum,protein folding,chromatin organization,transporter activity,nucleus,extracellular space,molecular function regulator activity,signaling,isomerase activity;Ontology_id=GO:0006281,GO:0055085,GO:0006260,GO:0008289,GO:0005783,GO:0006457,GO:0006325,GO:0005215,GO:0005634,GO:0005615,GO:0098772,GO:0023052,GO:0016853;Enzyme_code=EC:5,EC:5;Enzyme_name=Isomerases,Isomerases
contig00001.g206	2029	ID=contig00001.g206;Description=hypothetical protein FVER14953_03225 [Fusarium verticillioides];Gene=FNAPI_7656;Ontology_term=nucleus,DNA binding,regulation of DNA-templated transcription,transcription regulator activity;Ontology_id=GO:0005634,GO:0003677,GO:0006355,GO:0140110
contig00001.g207	1812	ID=contig00001.g207;Description=kinesin light chain 2 [Fusarium sp. NRRL 25303]
contig00001.g208	2894	ID=contig00001.g208;Description=putative f-box WD-repeat [Fusarium tjaetaba]
contig00001.g209	639	ID=contig00001.g209;Description=Snf7 family [Fusarium redolens];Gene=CHMP5;Ontology_term=vesicle-mediated transport,intracellular protein transport,vacuole;Ontology_id=GO:0016192,GO:0006886,GO:0005773
contig00001.g210	4250	ID=contig00001.g210;Description=hypothetical protein FVEG_03221 [Fusarium verticillioides 7600]
contig00001.g211	1874	ID=contig00001.g211;Description=hypothetical protein FVER14953_03218 [Fusarium verticillioides];Gene=MDCFG851_LOCUS455429;Ontology_term=catalytic activity;Ontology_id=GO:0003824
contig00001.g212	1842	ID=contig00001.g212;Description=hypothetical protein FVEG_03217 [Fusarium verticillioides 7600]
contig00001.g213	2080	ID=contig00001.g213;Description=hypothetical protein FGLOB1_12346 [Fusarium globosum];Gene=FTJAE_5771;Ontology_term=membrane;Ontology_id=GO:0016020
contig00001.g214	981	ID=contig00001.g214;Description=metallo beta lactamase [Fusarium tjaetaba]
contig00001.g215	462	ID=contig00001.g215;Description=hypothetical protein J7337_006111 [Fusarium musae]
contig00001.g216	798	ID=contig00001.g216;Description=putative WD repeat-containing protein [Fusarium oxysporum f. sp. albedinis]
contig00001.g217	1782	ID=contig00001.g217;Description=conidial development fluffy [Fusarium tjaetaba];Gene=FNAPI_12319;Ontology_term=nucleus,regulation of DNA-templated transcription,transcription regulator activity;Ontology_id=GO:0005634,GO:0006355,GO:0140110

contig00001.g218	270	ID=contig00001.g218;Description=uncharacterized protein FRV6_02369 [Fusarium oxysporum]
contig00001.g219	1414	ID=contig00001.g219;Description=hypothetical protein FVEG_03206 [Fusarium verticillioides 7600];Gene=FVEG_03206;Ontology_term=membrane;Ontology_id=GO:0016020
contig00001.g220	2986	ID=contig00001.g220;Description=tpa inducible [Fusarium napiforme];Gene=FVER53590_03205;Ontology_term=mRNA metabolic process;Ontology_id=GO:0016071
contig00001.g221	4493	ID=contig00001.g221;Description=heterokaryon incompatibility protein het-E-1 [Fusarium tjaetaba]
contig00001.g222	476	ID=contig00001.g222;Description=hypothetical protein FVEG_03203 [Fusarium verticillioides 7600];Gene=FNAPI_663;Ontology_term=nucleus,catalytic activity, acting on DNA,ATP-dependent activity,chromatin organization,hydrolase activity;Ontology_id=GO:0005634,GO:0140097,GO:0140657,GO:0006325,GO:0016787;Enzyme_code=EC:3;Enzyme_name=Hydrolases
contig00001.g223	358	ID=contig00001.g223;Description=hypothetical protein FVER53590_03202 [Fusarium verticillioides]
contig00001.g224	1452	ID=contig00001.g224;Description=kinesin light chain [Fusarium tjaetaba];Gene=FVEG_15208;Ontology_term=ATP-dependent activity,hydrolase activity;Ontology_id=GO:0140657,GO:0016787;Enzyme_code=EC:3.6.1.15,EC:3.6.1,EC:3.6,EC:3,EC:3.6.1.15;Enzyme_name=nucleoside-triphosphate phosphatase,Acting on acid anhydrides,Acting on acid anhydrides,Hydrolases,nucleoside-triphosphate phosphatase
contig00001.g225	1194	ID=contig00001.g225;Description=nucleoside phosphorylase [Fusarium tjaetaba];Gene=CEK26_008093;Ontology_term=DNA repair,catalytic activity, acting on a protein,nucleobase-containing small molecule metabolic process,DNA replication,carbohydrate derivative metabolic process,nuclear chromosome,receptor ligand activity,nucleolus,chromatin organization,mitotic cell cycle,defense response to other organism,extracellular space,telomere organization,transferase activity,meiotic nuclear division;Ontology_id=GO:0006281,GO:0140096,GO:0055086,GO:0006260,GO:1901135,GO:0000228,GO:0048018,GO:0005730,GO:0006325,GO:0000278,GO:0098542,GO:0005615,GO:0032200,GO:0016740,GO:0140013;Enzyme_code=EC:2.7.11.1,EC:2.7.1,EC:2.7.11.1,EC:2.7,EC:2;Enzyme_name=non-specific serine/threonine protein kinase,Transferring phosphorus-containing groups,non-specific serine/threonine protein kinase,Transferring phosphorus-containing groups,Transferases

contig00001.g226	1781	ID=contig00001.g226;Description=general substrate transporter [Fusarium oxysporum Fo47];Gene=FOC1_g10010765;Ontology_term=transmembrane transport,transporter activity;Ontology_id=GO:0055085,GO:0005215
contig00001.g227	1557	ID=contig00001.g227;Description=bifunctional polynucleotide phosphatase kinase [Fusarium pseudoanthophilum];Gene=FDENT_9079;Ontology_term=DNA repair,nucleus,DNA binding,transferase activity,hydrolase activity;Ontology_id=GO:0006281,GO:0005634,GO:0003677,GO:0016740,GO:0016787;Enzyme_code=EC:2.7,EC:2.7.1,EC:3.1,EC:2.7,EC:2,EC:3,EC:3.1.3.32,EC:2.7.1.78,EC:3.1.3;Enzyme_name=Transferring phosphorus-containing groups,Transferring phosphorus-containing groups,Acting on ester bonds,Transferring phosphorus-containing groups,Transferases,Hydrolases,polynucleotide 3'-phosphatase,polynucleotide 5'-hydroxyl-kinase,Acting on ester bonds
contig00001.g228	887	ID=contig00001.g228;Description=mediator complex subunit Med18 [Fusarium tjaetaba];Gene=MED18;Ontology_term=nucleus,regulation of DNA-templated transcription,transcription regulator activity,molecular adaptor activity;Ontology_id=GO:0005634,GO:0006355,GO:0140110,GO:0060090
contig00001.g229	814	ID=contig00001.g229;Description=DUF1264 domain protein [Fusarium fujikuroi];Gene=IFNT5;Ontology_term=DNA repair,signaling,nuclear chromosome,mitotic cell cycle;Ontology_id=GO:0006281,GO:0023052,GO:0000228,GO:0000278
contig00001.g230	1389	ID=contig00001.g230;Description=hypothetical protein FVEG_03196 [Fusarium verticillioides]
contig00001.g231	5560	ID=contig00001.g231;Description=hypothetical protein FVEG_03195 [Fusarium verticillioides 7600]
contig00001.g232	2012	ID=contig00001.g232;Description=cytochrome P450 monooxygenase [Fusarium coicis];Gene=FPANT_5204;Ontology_term=oxidoreductase activity;Ontology_id=GO:0016491;Enzyme_code=EC:1,EC:1;Enzyme_name=Oxidoreductases,Oxidoreductases
contig00001.g233	2171	ID=contig00001.g233;Description=myo-inositol transport ITR1 [Fusarium coicis];Gene=FVEG_03193;Ontology_term=DNA repair,transmembrane transport,molecular function regulator activity,signaling,nuclear chromosome,telomere organization,transporter activity,mitotic cell cycle,molecular adaptor activity;Ontology_id=GO:0006281,GO:0055085,GO:0098772,GO:0023052,GO:0000228,GO:0032200,GO:0005215,GO:0000278,GO:0060090

contig00001.g234	2102	ID=contig00001.g234;Description=hypothetical protein FVER53590_03191 [Fusarium verticillioides];Gene=FPANT_12868;Ontology_term=membrane;Ontology_id=GO:0016020
contig00001.g235	987	ID=contig00001.g235;Description=maltose permease [Fusarium beomiforme];Gene=FVEG_03190
contig00001.g236	792	ID=contig00001.g236;Description=bacterial alpha-L-rhamnosidase domain protein [Fusarium tjaetaba];Gene=F25303_9327;Ontology_term=carbohydrate metabolic process,hydrolase activity;Ontology_id=GO:0005975,GO:0016787;Enzyme_code=EC:3.2.1.40,EC:3.2,EC:3.2.1.40,EC:3,EC:3.2.1;Enzyme_name=alpha-L-rhamnosidase,Glycosylases,alpha-L-rhamnosidase,Hydrolases,Glycosylases
contig00001.g237	1967	ID=contig00001.g237;Description=major facilitator superfamily transporter [Fusarium tjaetaba];Gene=FPHYL_10698;Ontology_term=carbohydrate metabolic process,transmembrane transport,transporter activity,hydrolase activity;Ontology_id=GO:0005975,GO:0055085,GO:0005215,GO:0016787;Enzyme_code=EC:3,EC:3;Enzyme_name=Hydrolases,Hydrolases
contig00001.g238	1285	ID=contig00001.g238;Description=alcohol dehydrogenase [Fusarium verticillioides 7600]
contig00001.g239	860	ID=contig00001.g239;Description=alcohol dehydrogenase [Fusarium acutatum];Ontology_term=transmembrane transport,oxidoreductase activity;Ontology_id=GO:0055085,GO:0016491;Enzyme_code=EC:1.1,EC:1,EC:1.1.1.90,EC:1.1.1;Enzyme_name=Acting on the CH-OH group of donors,Oxidoreductases,aryl-alcohol dehydrogenase,Acting on the CH-OH group of donors
contig00001.g240	1322	ID=contig00001.g240;Description=NADH oxidase [Fusarium tjaetaba];Gene=FNAPI_1200;Ontology_term=oxidoreductase activity;Ontology_id=GO:0016491;Enzyme_code=EC:1,EC:1;Enzyme_name=Oxidoreductases,Oxidoreductases
contig00001.g241	1588	ID=contig00001.g241;Description=benzoate 4-monooxygenase [Fusarium verticillioides 7600];Gene=FVER53590_03183;Ontology_term=oxidoreductase activity;Ontology_id=GO:0016491;Enzyme_code=EC:1.14,EC:1,EC:1.14,EC:1.14.13.12;Enzyme_name=Acting on paired donors, with incorporation or reduction of molecular oxygen. The oxygen incorporated need not be derived from O2,Oxidoreductases,Acting on paired donors, with incorporation or reduction of molecular oxygen. The oxygen incorporated need not be derived from O2,Acting on paired donors, with incorporation or reduction of molecular oxygen. The oxygen incorporated need not be derived from O2
contig00001.g242	576	ID=contig00001.g242;Description=hypothetical protein FCOIX_3702 [Fusarium coicis];Gene=FPANT_3928;Ontology_term=membrane;Ontology_id=GO:0016020

contig00001.g243	1068	ID=contig00001.g243;Description=hypothetical protein FVER53263_03182 [Fusarium verticillioides];Gene=Forpe1208_v008070;Ontology_term=membrane;Ontology_id=GO:0016020
contig00001.g244	317	ID=contig00001.g244;Description=hypothetical protein FVEG_15204 [Fusarium verticillioides 7600]
contig00001.g245	2004	ID=contig00001.g245;Description=Metacaspase-1 [Fusarium oxysporum f. sp. raphani];Gene=FVEG_03181;Ontology_term=catalytic activity, acting on a protein,hydrolase activity;Ontology_id=GO:0140096,GO:0016787;Enzyme_code=EC:3.4.22,EC:3.4.22,EC:3.4,EC:3;Enzyme_name=Acting on peptide bonds (peptidases),Acting on peptide bonds (peptidases),Acting on peptide bonds (peptidases),Hydrolases
contig00001.g246	3176	ID=contig00001.g246;Description=30S ribosomal S17P protein [Fusarium tjaetaba];Gene=FVER53263_20642;Ontology_term=lyase activity;Ontology_id=GO:0016829;Enzyme_code=EC:4.2.1.1,EC:4.2.1,EC:4.2.1.1,EC:4.2,EC:4;Enzyme_name=carbonic anhydrase,Carbon-oxygen lyases,carbonic anhydrase,Carbon-oxygen lyases,Lyases
contig00001.g247	786	ID=contig00001.g247;Description=carbonic anhydrase [Fusarium oxysporum];Gene=FOXYS1_7357;Enzyme_code=EC:4.2.1.1;Enzyme_name=carbonic anhydrase
contig00001.g248	1147	ID=contig00001.g248;Description=hypothetical protein FVEG_03180 [Fusarium verticillioides 7600];Gene=FVEG_03180;Ontology_term=membrane;Ontology_id=GO:0016020
contig00001.g249	3226	ID=contig00001.g249;Description=RNA-binding protein [Fusarium coicis];Gene=F25303_9316;Ontology_term=membrane;Ontology_id=GO:0016020
contig00001.g250	858	ID=contig00001.g250;Description=hypothetical protein FVER14953_20680 [Fusarium verticillioides]
contig00001.g251	294	ID=contig00001.g251;Description=structural toxin [Fusarium tjaetaba]
contig00001.g252	2322	ID=contig00001.g252;Description=hypothetical protein FVEG_03175 [Fusarium verticillioides];Gene=FVER53590_03175;Ontology_term=nucleus,catalytic activity, acting on DNA,ATP-dependent activity,regulation of DNA-templated transcription,chromatin organization,transcription regulator activity,hydrolase activity;Ontology_id=GO:0005634,GO:0140097,GO:0140657,GO:0006355,GO:0006325,GO:0140110,GO:0016787;Enzyme_code=EC:3,EC:3;Enzyme_name=Hydrolases,Hydrolases
contig00001.g253	1401	ID=contig00001.g253;Description=TOL [Fusarium tjaetaba]
contig00001.g254	3346	ID=contig00001.g254;Description=early growth response 1-B [Fusarium tjaetaba]

contig00001.g255	3499	ID=contig00001.g255;Description=helicase-like transcription factor [Fusarium tjaetaba];Gene=Forpi1262_v011533;Ontology_term=nucleus,catalytic activity, acting on DNA,ATP-dependent activity,chromatin organization,hydrolase activity;Ontology_id=GO:0005634,GO:0140097,GO:0140657,GO:0006325,GO:0016787;Enzyme_code=EC:3,EC:3;Enzyme_name=Hydrolases,Hydrolases
contig00001.g256	3589	ID=contig00001.g256;Description=WSC domain protein [Fusarium tjaetaba]
contig00001.g257	1122	ID=contig00001.g257;Description=hypothetical protein FVER53590_03170 [Fusarium verticillioides];Enzyme_code=EC:2.5.1.18;Enzyme_name=glutathione transferase
contig00001.g258	600	ID=contig00001.g258;Description=WSC domain protein [Fusarium tjaetaba]
contig00001.g259	693	ID=contig00001.g259;Description=glutathione S-transferase [Fusarium tjaetaba];Gene=FTJAE_5723;Ontology_term=nucleus,DNA binding,regulation of DNA-templated transcription,transferase activity,transcription regulator activity;Ontology_id=GO:0005634,GO:0003677,GO:0006355,GO:0016740,GO:0140110;Enzyme_code=EC:2,EC:2.5.1.18,EC:2;Enzyme_name=Transferases,glutathione transferase,Transferases
contig00001.g260	2036	ID=contig00001.g260;Description=transcriptional activator CMR1 [Fusarium fujikuroi];Gene=FOXYS1_7368;Ontology_term=nucleus,DNA binding,regulation of DNA-templated transcription,transcription regulator activity;Ontology_id=GO:0005634,GO:0003677,GO:0006355,GO:0140110
contig00001.g261	900	ID=contig00001.g261;Description=2'-hydroxyisoflavone reductase [Fusarium pseudoanthophilum];Gene=FPCIR_11617;Ontology_term=oxidoreductase activity;Ontology_id=GO:0016491;Enzyme_code=EC:1,EC:1;Enzyme_name=Oxidoreductases,Oxidoreductases
contig00001.g262	1434	ID=contig00001.g262;Description=oxidoreductase [Fusarium tjaetaba];Gene=FOTG_14571;Ontology_term=oxidoreductase activity;Ontology_id=GO:0016491;Enzyme_code=EC:1.6,EC:1.6,EC:1;Enzyme_name=Acting on NADH or NADPH,Acting on NADH or NADPH,Oxidoreductases
contig00001.g263	265	ID=contig00001.g263;Description=hypothetical protein FVEG_15199 [Fusarium verticillioides 7600]

contig00001.g264	960	ID=contig00001.g264;Description=hydrolase or acyltransferase (alpha beta hydrolase superfamily) [Fusarium tjaetaba];Gene=FTJAE_5719;Ontology_term=extracellular space,inflammatory response,oxidoreductase activity,transferase activity,immune system process,hydrolase activity;Ontology_id=GO:0005615,GO:0006954,GO:0016491,GO:0016740,GO:0002376,GO:0016787;Enzyme_code=EC:2.3,EC:1,EC:3,EC:2.3,EC:1,EC:2,EC:3;Enzyme_name=Acyltransferases,Oxidoreductases,Hydrolases,Acyltransferases,Oxidoreductases,Transferases,Hydrolases
contig00001.g265	1401	ID=contig00001.g265;Description=6-hydroxy-D-nicotine oxidase [Fusarium coicis];Gene=FNYG_06135;Ontology_term=oxidoreductase activity;Ontology_id=GO:0016491;Enzyme_code=EC:1,EC:1;Enzyme_name=Oxidoreductases,Oxidoreductases
contig00001.g266	1644	ID=contig00001.g266;Description=aerobactin siderophore biosynthesis protein iucB [Fusarium fujikuroi];Gene=FFB14_01983;Ontology_term=cytoplasmic translation,structural molecule activity,cytosol,transferase activity,ribosome;Ontology_id=GO:0002181,GO:0005198,GO:0005829,GO:0016740,GO:0005840;Enzyme_code=EC:2.3,EC:2.3,EC:2;Enzyme_name=Acyltransferases,Acyltransferases,Transferases
contig00001.g267	1480	ID=contig00001.g267;Description=COQ2-para-hydroxybenzoate-polyprenyltransferase [Fusarium tjaetaba];Gene=FNYG_06137;Ontology_term=transmembrane transport,mitochondrion,transferase activity,transporter activity;Ontology_id=GO:0055085,GO:0005739,GO:0016740,GO:0005215;Enzyme_code=EC:2,EC:2;Enzyme_name=Transferases,Transferases
contig00001.g268	1228	ID=contig00001.g268;Description=diphosphomevalonate decarboxylase [Fusarium proliferatum];Gene=FMAN_07150;Ontology_term=nucleobase-containing small molecule metabolic process,sulfur compound metabolic process,lipid metabolic process,lyase activity,cytosol,carbohydrate derivative metabolic process;Ontology_id=GO:0055086,GO:0006790,GO:0006629,GO:0016829,GO:0005829,GO:1901135;Enzyme_code=EC:4.1.1.33;Enzyme_name=diphosphomevalonate decarboxylase
contig00001.g269	1400	ID=contig00001.g269;Description=UPF0420 protein [Fusarium tjaetaba]

contig00001.g270	843	ID=contig00001.g270;Description=cytochrome c heme-lyase [Fusarium verticillioides 7600];Gene=FOTG_14562;Ontology_term=catalytic activity, acting on a protein,protein-containing complex assembly,mitochondrion,lyase activity,mitochondrion organization;Ontology_id=GO:0140096,GO:0065003,GO:0005739,GO:0016829,GO:0007005;Enzyme_code=EC:4.4.1.17,EC:4.4.1.17,EC:4.4,EC:4;Enzyme_name=holocytochrome-c synthase,holocytochrome-c synthase,Carbon-sulfur lyases,Lyases
contig00001.g271	1898	ID=contig00001.g271;Description=Clavaminate synthase [Fusarium pseudoanthophilum];Gene=FVEG_03160;Ontology_term=oxidoreductase activity;Ontology_id=GO:0016491;Enzyme_code=EC:1,EC:1;Enzyme_name=Oxidoreductases,Oxidoreductases
contig00001.g272	1395	ID=contig00001.g272;Description=FAD NAD(P)-binding protein [Fusarium tjaetaba];Gene=FPANT_11107;Ontology_term=oxidoreductase activity;Ontology_id=GO:0016491;Enzyme_code=EC:1,EC:1;Enzyme_name=Oxidoreductases,Oxidoreductases
contig00001.g273	1685	ID=contig00001.g273;Description=o-acetylhomoserine (thiol)-lyase [Fusarium tjaetaba];Gene=FMUND_7290;Ontology_term=sulfur compound metabolic process,lyase activity,amino acid metabolic process,transferase activity;Ontology_id=GO:0006790,GO:0016829,GO:0006520,GO:0016740;Enzyme_code=EC:2.5.1,EC:4;Enzyme_name=Transferring alkyl or aryl groups, other than methyl groups,Lyases
contig00001.g274	1221	ID=contig00001.g274;Description=GA4 desaturase [Fusarium tjaetaba];Gene=FDENT_11427;Ontology_term=oxidoreductase activity;Ontology_id=GO:0016491;Enzyme_code=EC:1,EC:1;Enzyme_name=Oxidoreductases,Oxidoreductases
contig00001.g275	1136	ID=contig00001.g275;Description=ankyrin repeat domain-containing protein [Fusarium coicis]
contig00001.g276	1140	ID=contig00001.g276;Description=hypothetical protein FVEG_03155 [Fusarium verticillioides 7600];Gene=FVER53263_03154;Ontology_term=ATP binding,metal ion binding;Ontology_id=GO:0005524,GO:0046872
contig00001.g277	2083	ID=contig00001.g277;Description=glutathione synthetase ATP-binding protein [Fusarium coicis];Gene=FNYG_06148;Ontology_term=ATP binding,metal ion binding;Ontology_id=GO:0005524,GO:0046872

contig00001.g278	1476	ID=contig00001.g278;Description=o-acetylhomoserine (thiol)-lyase [Fusarium tjaetaba];Gene=FVEG_03153;Ontology_term=sulfur compound metabolic process,lyase activity,amino acid metabolic process,transferase activity;Ontology_id=GO:0006790,GO:0016829,GO:0006520,GO:0016740;Enzyme_code=EC:2.5.1,EC:4,EC:2.5.1,EC:2.5.1.49,EC:2.5.1.47,EC:2,EC:4;Enzyme_name=Transferring alkyl or aryl groups, other than methyl groups,Lyases,Transferring alkyl or aryl groups, other than methyl groups,O-acetylhomoserine aminocarboxypropyltransferase,cysteine synthase,Transferases,Lyases
contig00001.g279	1017	ID=contig00001.g279;Description=unnamed protein product [Fusarium fujikuroi];Gene=LW93_12358;Ontology_term=amino acid metabolic process,transferase activity;Ontology_id=GO:0006520,GO:0016740;Enzyme_code=EC:2.5.1.43,EC:2.1.1,EC:2.1,EC:2.5.1,EC:2,EC:2.5.1.43,EC:2.1.1;Enzyme_name=nicotianamine synthase,Transferring one-carbon groups,Transferring one-carbon groups,Transferring alkyl or aryl groups, other than methyl groups,Transferases,nicotianamine synthase,Transferring one-carbon groups
contig00001.g280	1717	ID=contig00001.g280;Description=major facilitator superfamily transporter [Fusarium tjaetaba];Gene=FVEG_03151;Ontology_term=transmembrane transport,transporter activity;Ontology_id=GO:0055085,GO:0005215
contig00001.g281	1247	ID=contig00001.g281;Description=homoserine O-acetyltransferase [Fusarium verticillioides 7600];Gene=FVER53590_03150;Ontology_term=sulfur compound metabolic process,amino acid metabolic process,transferase activity;Ontology_id=GO:0006790,GO:0006520,GO:0016740;Enzyme_code=EC:2.3.1,EC:2.3,EC:2.3.1.31,EC:2,EC:2.3.1;Enzyme_name=Acyltransferases,Acyltransferases,homoserine O-acetyltransferase,Transferases,Acyltransferases
contig00001.g282	4191	ID=contig00001.g282;Description=ABC transporter [Fusarium tjaetaba];Gene=FVER53263_03149;Ontology_term=transmembrane transport,ATP-dependent activity,transporter activity,plasma membrane,hydrolase activity;Ontology_id=GO:0055085,GO:0140657,GO:0005215,GO:0005886,GO:0016787;Enzyme_code=EC:7.2.2,EC:3.6.1.15,EC:3.6.1,EC:3.6,EC:7.6.2.2,EC:3,EC:7.2.2,EC:7,EC:3.6.1.15;Enzyme_name=Catalysing the translocation of inorganic cations,nucleoside-triphosphate phosphatase,Acting on acid anhydrides,Acting on acid anhydrides,ABC-type xenobiotic transporter,Hydrolases,Catalysing the translocation of inorganic cations,Translocases,nucleoside-triphosphate phosphatase
contig00001.g283	610	ID=contig00001.g283;Description=unnamed protein product [Fusarium fujikuroi];Gene=1455

contig00001.g284	1421	ID=contig00001.g284;Description=protein BTN1 [Fusarium verticillioides 7600];Gene=FOXYS1_7332;Ontology_term=transmembrane transport,vacuole;Ontology_id=GO:0055085,GO:0005773
contig00001.g285	1311	ID=contig00001.g285;Description=galactosyl transferase [Fusarium agapanthi];Gene=FGLOB1_2885;Ontology_term=transferase activity;Ontology_id=GO:0016740;Enzyme_code=EC:2.4,EC:2.4,EC:2;Enzyme_name=Glycosyltransferases,Glycosyltransferases,Transferases
contig00001.g286	1632	ID=contig00001.g286;Description=polyamine transporter 4 [Fusarium tjaetaba];Gene=FDENT_11415;Ontology_term=transmembrane transport,transporter activity;Ontology_id=GO:0055085,GO:0005215
contig00001.g287	1281	ID=contig00001.g287;Description=inorganic pyrophosphatase [Fusarium flagelliforme];Gene=FNYG_06158;Ontology_term=hydrolase activity;Ontology_id=GO:0016787;Enzyme_code=EC:3.6.1.1,EC:3.6.1.1,EC:3.6.1,EC:3.6,EC:3;Enzyme_name=inorganic diphosphatase,inorganic diphosphatase,Acting on acid anhydrides,Acting on acid anhydrides,Hydrolases
contig00001.g288	468	ID=contig00001.g288;Description=hypothetical protein FVEG_03143 [Fusarium verticillioides 7600]
contig00001.g289	417	ID=contig00001.g289;Description=hypothetical protein FVER14953_03142 [Fusarium verticillioides]
contig00001.g290	422	ID=contig00001.g290;Description=hypothetical protein FVER14953_03141 [Fusarium verticillioides]
contig00001.g291	1837	ID=contig00001.g291;Description=endo-1,4-beta-xylanase [Fusarium verticillioides 7600];Gene=FTJAE_5693;Ontology_term=carbohydrate metabolic process,cell wall organization or biogenesis,extracellular region,hydrolase activity;Ontology_id=GO:0005975,GO:0071554,GO:0005576,GO:0016787;Enzyme_code=EC:3.2.1.8,EC:3.2,EC:3.2.1.8,EC:3,EC:3.2.1;Enzyme_name=endo-1,4-beta-xylanase,Glycosylases,endo-1,4-beta-xylanase,Hydrolases,Glycosylases
contig00001.g292	1257	ID=contig00001.g292;Description=hypothetical protein FVER53590_03139 [Fusarium verticillioides]

contig00001.g293	1036	ID=contig00001.g293;Description=related to FAD1-flavin adenine dinucleotide (FAD) synthetase [Fusarium fujikuroi];Gene=BFJ72_g1508;Ontology_term=nucleobase-containing small molecule metabolic process,carbohydrate derivative metabolic process,transferase activity;Ontology_id=GO:0055086,GO:1901135,GO:0016740;Enzyme_code=EC:2.7,EC:2,EC:2.7.7.2,EC:2.7.7;Enzyme_name=Transferring phosphorus-containing groups,Transferases,FAD synthase,Transferring phosphorus-containing groups
contig00001.g294	918	ID=contig00001.g294;Description=hypothetical protein FVER53590_29316 [Fusarium verticillioides]
contig00001.g295	228	ID=contig00001.g295;Description=hypothetical protein FVER53263_03137 [Fusarium verticillioides]
contig00001.g296	324	ID=contig00001.g296;Description=hypothetical protein FVEG_03136 [Fusarium verticillioides 7600]
contig00001.g297	631	ID=contig00001.g297;Description=hypothetical protein FVER53590_03135 [Fusarium verticillioides]
contig00001.g298	1296	ID=contig00001.g298;Description=hypothetical protein FVEG_15194 [Fusarium verticillioides 7600]
contig00001.g299	1596	ID=contig00001.g299;Description=PiT family inorganic phosphate transporter [Fusarium verticillioides 7600];Gene=FMUND_13428;Ontology_term=transmembrane transport,transporter activity;Ontology_id=GO:0055085,GO:0005215
contig00001.g300	393	ID=contig00001.g300;Description=FMP21 [Fusarium tjaetaba];Ontology_term=protein-containing complex assembly,mitochondrion,mitochondrion organization;Ontology_id=GO:0065003,GO:0005739,GO:0007005
contig00001.g301	3465	ID=contig00001.g301;Description=transcriptional regulatory RXT3 [Fusarium tjaetaba]
contig00001.g302	978	ID=contig00001.g302;Description=hypothetical protein FVER53263_03129 [Fusarium verticillioides];Gene=FDENT_10313;Ontology_term=catalytic activity;Ontology_id=GO:0003824
contig00001.g303	280	ID=contig00001.g303;Description=hypothetical protein FVEG_03128 [Fusarium verticillioides 7600]
contig00001.g304	1161	ID=contig00001.g304;Description=hypothetical protein FVER53590_03127 [Fusarium verticillioides];Gene=Forpi1262_v011576;Ontology_term=membrane;Ontology_id=GO:0016020
contig00001.g305	906	ID=contig00001.g305;Description=hypothetical protein FVEG_03126 [Fusarium verticillioides 7600]

contig00001.g306	469	ID=contig00001.g306;Description=glyoxalase bleomycin resistance dioxygenase [Fusarium subglutinans];Gene=FPHYL_12072;Ontology_term=oxidoreductase activity;Ontology_id=GO:0016491;Enzyme_code=EC:1,EC:1;Enzyme_name=Oxidoreductases,Oxidoreductases
contig00001.g307	1915	ID=contig00001.g307;Description=laccase precursor [Fusarium tjaetaba];Gene=FMEXI_5361;Ontology_term=oxidoreductase activity;Ontology_id=GO:0016491;Enzyme_code=EC:1,EC:1;Enzyme_name=Oxidoreductases,Oxidoreductases
contig00001.g308	1796	ID=contig00001.g308;Description=AAT family amino acid transporter [Fusarium oxysporum NRRL 32931];Gene=FOZG_10746;Ontology_term=transmembrane transport,transporter activity;Ontology_id=GO:0055085,GO:0005215
contig00001.g309	946	ID=contig00001.g309;Description=oxidoreductase containing protein [Fusarium tjaetaba];Gene=FocTR4_00008207;Ontology_term=oxidoreductase activity;Ontology_id=GO:0016491;Enzyme_code=EC:1.14,EC:1,EC:1.14;Enzyme_name=Acting on paired donors, with incorporation or reduction of molecular oxygen. The oxygen incorporated need not be derived from O2,Oxidoreductases,Acting on paired donors, with incorporation or reduction of molecular oxygen. The oxygen incorporated need not be derived from O2
contig00001.g310	2631	ID=contig00001.g310;Description=Cyclin-dependent kinase catalytic subunit [Fusarium musae];Gene=FTJAE_5675;Ontology_term=RNA binding,mRNA metabolic process,protein-containing complex assembly,ATP-dependent activity,catalytic activity, acting on RNA,hydrolase activity;Ontology_id=GO:0003723,GO:0016071,GO:0065003,GO:0140657,GO:0140098,GO:0016787;Enzyme_code=EC:3,EC:3.6.1,EC:3.6.4.13,EC:3.6,EC:3,EC:3.6.1.15;Enzyme_name=Hydrolases,Acting on acid anhydrides,RNA helicase,Acting on acid anhydrides,Hydrolases,nucleoside-triphosphate phosphatase
contig00001.g311	1353	ID=contig00001.g311;Description=SCJ1 protein [Fusarium fujikuroi];Gene=PPH3;Ontology_term=nucleus,catalytic activity, acting on a protein,protein catabolic process,signaling,cytosol,endoplasmic reticulum,protein folding,hydrolase activity;Ontology_id=GO:0005634,GO:0140096,GO:0030163,GO:0023052,GO:0005829,GO:0005783,GO:0006457,GO:0016787;Enzyme_code=EC:3.1.3.16,EC:3.1.3.16,EC:3.1,EC:3,EC:3.1.3;Enzyme_name=protein-serine/threonine phosphatase,protein-serine/threonine phosphatase,Acting on ester bonds,Hydrolases,Acting on ester bonds
contig00001.g312	3313	ID=contig00001.g312;Description=hypothetical protein J7337_006023 [Fusarium musae]

contig00001.g313	1225	ID=contig00001.g313;Description=COPII-coated vesicle (Erv41) [Fusarium tjaetaba];Gene=FVEG_03119;Ontology_term=vesicle-mediated transport,cytoplasmic vesicle,endoplasmic reticulum,Golgi apparatus,intracellular protein transport;Ontology_id=GO:0016192,GO:0031410,GO:0005783,GO:0005794,GO:0006886
contig00001.g314	1544	ID=contig00001.g314;Description=mannan endo-1 6-alpha-mannosidase [Fusarium tjaetaba];Gene=FVEG_03118;Ontology_term=carbohydrate metabolic process,hydrolase activity;Ontology_id=GO:0005975,GO:0016787;Enzyme_code=EC:3.2.1.101,EC:3.2.1.24;Enzyme_name=mannan endo-1,6-alpha-mannosidase,alpha-mannosidase
contig00001.g315	420	ID=contig00001.g315;Description=hypothetical protein FVER14953_03117 [Fusarium verticillioides]
contig00001.g316	1449	ID=contig00001.g316;Description=actin-like protein [Fusarium odoratissimum NRRL 54006];Ontology_term=cytoskeleton,cytoskeleton organization,establishment or maintenance of cell polarity,signaling,mitotic nuclear division,structural molecule activity,chromosome segregation,microtubule-based movement,meiotic nuclear division;Ontology_id=GO:0005856,GO:0007010,GO:0007163,GO:0023052,GO:0140014,GO:0005198,GO:0007059,GO:0007018,GO:0140013
contig00001.g317	2307	ID=contig00001.g317;Description=MBP1-transcription factor subunit of the MBF factor [Fusarium tjaetaba];Gene=FPCIR_11902;Ontology_term=DNA binding,anatomical structure development,cell differentiation,general transcription initiation factor activity,nuclear chromosome,regulation of DNA-templated transcription,reproductive process,transcription regulator activity,mitotic cell cycle;Ontology_id=GO:0003677,GO:0048856,GO:0030154,GO:0140223,GO:0000228,GO:0006355,GO:0022414,GO:0140110,GO:0000278
contig00001.g318	915	ID=contig00001.g318;Description=hypothetical protein FVEG_03114 [Fusarium verticillioides 7600];Gene=FPCIR_11903;Ontology_term=membrane;Ontology_id=GO:0016020
contig00001.g319	1659	ID=contig00001.g319;Description=hypothetical protein F25303_9048 [Fusarium sp. NRRL 25303]

contig00001.g320	4856	ID=contig00001.g320;Description=STE/STE11/CDC15 protein kinase [Fusarium verticillioides 7600];Gene=FVEG_15183;Ontology_term=catalytic activity, acting on a protein,microtubule organizing center,cytoskeleton organization,signaling,mitotic nuclear division,cytokinesis,cytosol,reproductive process,transferase activity,programmed cell death;Ontology_id=GO:0140096,GO:0005815,GO:0007010,GO:0023052,GO:0140014,GO:0000910,GO:0005829,GO:0022414,GO:0016740,GO:0012501;Enzyme_code=EC:2.7.1,EC:2.7.1,EC:2.7.11.1,EC:2.7,EC:2;Enzyme_name=Transferring phosphorus-containing groups,Transferring phosphorus-containing groups,non-specific serine/threonine protein kinase,Transferring phosphorus-containing groups,Transferases
contig00001.g321	1723	ID=contig00001.g321;Description=hypothetical protein FVEG_03110 [Fusarium verticillioides 7600]
contig00001.g322	2028	ID=contig00001.g322;Description=tetrahydrofolylpolyglutamate synthase [Fusarium tjaetaba];Gene=FVER53590_03109;Ontology_term=mitochondrion,amino acid metabolic process,cellular modified amino acid metabolic process,ligase activity;Ontology_id=GO:0005739,GO:0006520,GO:0006575,GO:0016874;Enzyme_code=EC:6.3.2.17,EC:6.3.2.17,EC:6.3,EC:6.3.2,EC:6,EC:6.3.2.12;Enzyme_name=tetrahydrofolate synthase,tetrahydrofolate synthase,Forming carbon-nitrogen bonds,Forming carbon-nitrogen bonds,Ligases,dihydrofolate synthase
contig00001.g323	799	ID=contig00001.g323;Description=DUF218 domain-containing protein [Fusarium coicis];Ontology_term=catalytic activity;Ontology_id=GO:0003824
contig00001.g324	1060	ID=contig00001.g324;Description=hypothetical protein FVEG_03108 [Fusarium verticillioides 7600]
contig00001.g325	1350	ID=contig00001.g325;Description=er membrane protein [Fusarium pseudoanthophilum];Gene=FMAN_07208;Ontology_term=membrane organization,cytoskeleton organization,protein-containing complex assembly,lipid metabolic process,endoplasmic reticulum;Ontology_id=GO:0061024,GO:0007010,GO:0065003,GO:0006629,GO:0005783
contig00001.g326	1603	ID=contig00001.g326;Description=neutral amino acid permease [Fusarium fujikuroi];Gene=FGADI_10059;Ontology_term=cytoskeleton,cytoskeleton organization,protein-containing complex assembly,cytokinesis,cytosol,lipid binding,cytoskeletal protein binding,mitotic cell cycle;Ontology_id=GO:0005856,GO:0007010,GO:0065003,GO:0000910,GO:0005829,GO:0008289,GO:0008092,GO:0000278

contig00001.g327	1243	ID=contig00001.g327;Description=putative fatty acid desaturase (mld) [Fusarium fujikuroi];Gene=FPCIR_13816;Ontology_term=nucleus,DNA binding,oxidoreductase activity,lipid metabolic process,DNA recombination,reproductive process;Ontology_id=GO:0005634,GO:0003677,GO:0016491,GO:0006629,GO:0006310,GO:0022414;Enzyme_code=EC:1.14.19.17;Enzyme_name=sphingolipid 4-desaturase
contig00001.g328	1192	ID=contig00001.g328;Description=PapD-like protein [Fusarium redolens];Gene=FMEXI_5340
contig00001.g329	750	ID=contig00001.g329;Description=hypothetical protein FVEG_03103 [Fusarium verticillioides 7600]
contig00001.g330	1106	ID=contig00001.g330;Description=UBX domain-containing protein 1 [Fusarium oxysporum f. sp. rapae]
contig00001.g331	560	ID=contig00001.g331;Description=putative RPP1A-60S large subunit acidic ribosomal protein a1 [Fusarium fujikuroi];Gene=BFJ68_g1771;Ontology_term=ATP-dependent activity,structural molecule activity,ribosome,hydrolase activity;Ontology_id=GO:0140657,GO:0005198,GO:0005840,GO:0016787;Enzyme_code=EC:3;Enzyme_name=Hydrolases
contig00001.g332	1569	ID=contig00001.g332;Description=UPF0662 domain-containing protein [Fusarium denticulatum];Ontology_term=nucleus;Ontology_id=GO:0005634
contig00001.g333	594	ID=contig00001.g333;Description=dimeric alpha-beta barrel [Fusarium bulbicola];Gene=top1
contig00001.g334	901	ID=contig00001.g334;Description=hypothetical protein FVEG_03098 [Fusarium verticillioides 7600];Gene=FNAPI_5104;Ontology_term=membrane;Ontology_id=GO:0016020
contig00001.g335	366	ID=contig00001.g335;Description=actin polymerization [Fusarium tjaetaba]
contig00001.g336	1758	ID=contig00001.g336;Description=putative fatty acid transporter FAT2 [Fusarium sp. NRRL 25303];Gene=FNAPI_5106;Ontology_term=peroxisome,RNA binding,nucleobase-containing small molecule metabolic process,sulfur compound metabolic process,carbohydrate derivative metabolic process,ligase activity;Ontology_id=GO:0005777,GO:0003723,GO:0055086,GO:0006790,GO:1901135,GO:0016874;Enzyme_code=EC:6,EC:6.2.1.8,EC:6.2,EC:6,EC:6.2.1;Enzyme_name=Ligases,oxalate--CoA ligase,Forming carbon-sulfur bonds,Ligases,Forming carbon-sulfur bonds

contig00001.g337	2457	ID=contig00001.g337;Description=BZZ1-Myo3 5p-Bee1p-Vrp1p actin assembly complex component [Fusarium tjaetaba];Gene=FOIG_09259;Ontology_term=molecular function regulator activity,vesicle-mediated transport,cytoskeleton,cytoskeleton organization,protein-containing complex assembly,lipid binding,plasma membrane;Ontology_id=GO:0098772,GO:0016192,GO:0005856,GO:0007010,GO:0065003,GO:0008289,GO:0005886
contig00001.g338	1053	ID=contig00001.g338;Description=fungal specific transcription factor factor [Fusarium subglutinans]
contig00001.g339	501	ID=contig00001.g339;Description=methyltransferase [Fusarium verticillioides 7600];Gene=FVEG_03092;Ontology_term=transferase activity;Ontology_id=GO:0016740;Enzyme_code=EC:2.1.1,EC:2.1,EC:2,EC:2.1.1;Enzyme_name=Transferring one-carbon groups,Transferring one-carbon groups,Transferases,Transferring one-carbon groups
contig00001.g340	1731	ID=contig00001.g340;Description=major facilitator superfamily domain-containing protein [Fusarium redolens];Gene=FDENT_3272;Ontology_term=transmembrane transport,transporter activity,plasma membrane;Ontology_id=GO:0055085,GO:0005215,GO:0005886
contig00001.g341	2562	ID=contig00001.g341;Description=hypothetical protein FVER14953_03090 [Fusarium verticillioides]
contig00001.g342	297	ID=contig00001.g342;Description=hypothetical protein FVEG_03089 [Fusarium verticillioides 7600]
contig00001.g343	1684	ID=contig00001.g343;Description=putative tripeptidyl-peptidase SED2 [Fusarium oxysporum f. sp. albedinis]
contig00001.g344	621	ID=contig00001.g344;Description=ribosomal protein YmL20 precursor, mitochondrial [Fusarium fujikuroi];Gene=1509;Ontology_term=mitochondrial gene expression,nucleobase-containing small molecule metabolic process,mitochondrion,structural molecule activity,carbohydrate derivative metabolic process,cell wall organization or biogenesis,ribosome,transferase activity;Ontology_id=GO:0140053,GO:0055086,GO:0005739,GO:0005198,GO:1901135,GO:0071554,GO:0005840,GO:0016740;Enzyme_code=EC:2.7.6.1,EC:2.7.6.1,EC:2.7.6,EC:2.7,EC:2;Enzyme_name=ribose-phosphate diphosphokinase,ribose-phosphate diphosphokinase,Transferring phosphorus-containing groups,Transferring phosphorus-containing groups,Transferases

contig00001.g345	1191	ID=contig00001.g345;Description=hypothetical protein FVER14953_03086 [Fusarium verticillioides]
contig00001.g346	3714	ID=contig00001.g346;Description=BUD2-GTPase-activating for Bud1p Rsr1p [Fusarium tjaetaba];Gene=FNAPI_5115;Ontology_term=molecular function regulator activity,cytoskeleton organization,cell wall,establishment or maintenance of cell polarity,signaling,mitotic nuclear division,cytokinesis,chromosome segregation,plasma membrane;Ontology_id=GO:0098772,GO:0007010,GO:0005618,GO:0007163,GO:0023052,GO:0140014,GO:0000910,GO:0007059,GO:0005886
contig00001.g347	1182	ID=contig00001.g347;Description=peptidyl-prolyl cis-trans isomerase D [Fusarium verticillioides 7600];Gene=FFB14_02068;Ontology_term=catalytic activity, acting on a protein,anatomical structure development,reproductive process,protein folding,plastid,isomerase activity,thylakoid;Ontology_id=GO:0140096,GO:0048856,GO:0022414,GO:0006457,GO:0009536,GO:0016853,GO:0009579;Enzyme_code=EC:5.2.1.8,EC:5.2.1.8,EC:5.2,EC:5;Enzyme_name=peptidylprolyl isomerase,peptidylprolyl isomerase,Cis-trans-isomerases,Isomerases
contig00001.g348	795	ID=contig00001.g348;Description=D-tyrosyl-tRNA(Tyr) deacylase [Fusarium pseudocircinatum];Gene=DTD1;Ontology_term=RNA binding,lipid metabolic process,tRNA metabolic process,endoplasmic reticulum,transferase activity,catalytic activity, acting on RNA,hydrolase activity;Ontology_id=GO:0003723,GO:0006629,GO:0006399,GO:0005783,GO:0016740,GO:0140098,GO:0016787;Enzyme_code=EC:3.1.1.96,EC:2.7.8;Enzyme_name=D-aminoacyl-tRNA deacylase,Transferring phosphorus-containing groups
contig00001.g349	2049	ID=contig00001.g349;Description=NOT3-general negative regulator of transcription subunit 3 [Fusarium tjaetaba];Gene=FMUND_14151;Ontology_term=nucleus,mRNA metabolic process,regulation of DNA-templated transcription;Ontology_id=GO:0005634,GO:0016071,GO:0006355
contig00001.g350	757	ID=contig00001.g350;Description=egg shell [Fusarium tjaetaba]

contig00001.g351	2187	ID=contig00001.g351;Description=hypothetical protein FCOIX_9665 [Fusarium coicis];Gene=vma3;Ontology_term=transmembrane transport,ATP-dependent activity,vacuole,transporter activity,hydrolase activity;Ontology_id=GO:0055085,GO:0140657,GO:0005773,GO:0005215,GO:0016787;Enzyme_code=EC:7.2.2,EC:3.6.1.15,EC:3.6.1,EC:3.6,EC:3,EC:7.2.2,EC:7,EC:3.6.1.15;Enzyme_name=Catalysing the translocation of inorganic cations,nucleoside-triphosphate phosphatase,Acting on acid anhydrides,Acting on acid anhydrides,Hydrolases,Catalysing the translocation of inorganic cations,Translocases,nucleoside-triphosphate phosphatase
contig00001.g352	1868	ID=contig00001.g352;Description=USO1-intracellular transport [Fusarium tjaetaba]
contig00001.g353	1654	ID=contig00001.g353;Description=isocitrate dehydrogenase (NADP), mitochondrial [Fusarium proliferatum];Gene=FACUT_4728;Ontology_term=peroxisome,generation of precursor metabolites and energy,nucleobase-containing small molecule metabolic process,oxidoreductase activity,mitochondrion,lipid metabolic process,amino acid metabolic process,cytosol;Ontology_id=GO:0005777,GO:0006091,GO:0055086,GO:0016491,GO:0005739,GO:0006629,GO:0006520,GO:0005829;Enzyme_code=EC:1.1.1.42,EC:1.1,EC:1.1.1.42,EC:1,EC:1.1.1;Enzyme_name=isocitrate dehydrogenase (NADP(+)),Acting on the CH-OH group of donors,isocitrate dehydrogenase (NADP(+)),Oxidoreductases,Acting on the CH-OH group of donors
contig00001.g354	1077	ID=contig00001.g354;Description=IEC3 subunit of the Ino80 complex, chromatin re-modelling-domain-containing protein [Fusarium oxysporum];Gene=FNAPI_12548;Ontology_term=nuclear chromosome,chromatin organization;Ontology_id=GO:0000228,GO:0006325
contig00001.g355	2326	ID=contig00001.g355;Description=SLY1 [Fusarium tjaetaba];Gene=FDENT_3286;Ontology_term=membrane organization,vesicle-mediated transport,protein-containing complex assembly,signaling,cytoplasmic vesicle,cytosol,Golgi apparatus,endoplasmic reticulum,intracellular protein transport;Ontology_id=GO:0061024,GO:0016192,GO:0065003,GO:0023052,GO:0031410,GO:0005829,GO:0005794,GO:0005783,GO:0006886
contig00001.g356	4404	ID=contig00001.g356;Description=hypothetical protein FVER14953_03075 [Fusarium verticillioides];Gene=FPANT_116;Ontology_term=metal ion binding;Ontology_id=GO:0046872

contig00001.g357	913	ID=contig00001.g357;Description=translin [Fusarium subglutinans];Gene=FOC1_g10010899;Ontology_term=nucleus,DNA binding,RNA binding,tRNA metabolic process;Ontology_id=GO:0005634,GO:0003677,GO:0003723,GO:0006399
contig00001.g358	1533	ID=contig00001.g358;Description=tripeptidyl-peptidase I [Fusarium coicis]
contig00001.g359	2068	ID=contig00001.g359;Description=related to tripeptidyl-peptidase I [Fusarium proliferatum ET1];Gene=FGADI_10028;Ontology_term=extracellular space,catalytic activity, acting on a protein,hydrolase activity;Ontology_id=GO:0005615,GO:0140096,GO:0016787;Enzyme_code=EC:3.4.21,EC:3.4.21,EC:3.4,EC:3,EC:3.4.14.9;Enzyme_name=Acting on peptide bonds (peptidases),Acting on peptide bonds (peptidases),Acting on peptide bonds (peptidases),Hydrolases,tripeptidyl-peptidase I
contig00001.g360	1097	ID=contig00001.g360;Description=nitrogen metabolic regulation nmr [Fusarium tjaetaba]
contig00001.g361	231	ID=contig00001.g361;Description=hypothetical protein FVEG_15177 [Fusarium verticillioides 7600]
contig00001.g362	261	ID=contig00001.g362;Description=hypothetical protein FVER14953_21273 [Fusarium verticillioides]
contig00001.g363	1615	ID=contig00001.g363;Description=hypothetical protein FVEG_03070 [Fusarium verticillioides 7600];Gene=FNAPI_10525;Ontology_term=membrane;Ontology_id=GO:0016020
contig00001.g364	1599	ID=contig00001.g364;Description=xylan 1,4-beta-xylosidase [Fusarium verticillioides 7600];Gene=FVER53590_29970;Ontology_term=carbohydrate metabolic process,hydrolase activity;Ontology_id=GO:0005975,GO:0016787;Enzyme_code=EC:3.2.1,EC:3.2,EC:3,EC:3.2.1,EC:3.2.1.37;Enzyme_name=Glycosylases,Glycosylases,Hydrolases,Glycosylases,xylan 1,4-beta-xylosidase
contig00001.g365	1632	ID=contig00001.g365;Description=heterokaryon incompatibility protein het-E-1 [Fusarium pseudoanthophilum]
contig00001.g366	837	ID=contig00001.g366;Description=methyltransferase type 11 [Fusarium coicis];Gene=FPHYL_1752;Ontology_term=transferase activity;Ontology_id=GO:0016740;Enzyme_code=EC:2.1.1,EC:2.1,EC:2,EC:2.1.1;Enzyme_name=Transferring one-carbon groups,Transferring one-carbon groups,Transferases,Transferring one-carbon groups

contig00001.g367	4056	ID=contig00001.g367;Description=transcriptional activator ARO80 [Fusarium beomiforme];Gene=FACUT_4716;Ontology_term=nucleus,DNA binding,oxidoreductase activity,regulation of DNA-templated transcription,transcription regulator activity;Ontology_id=GO:0005634,GO:0003677,GO:0016491,GO:0006355,GO:0140110;Enzyme_code=EC:1,EC:1;Enzyme_name=Oxidoreductases,Oxidoreductases
contig00001.g368	1777	ID=contig00001.g368;Description=sugar transporter STL1 [Fusarium subglutinans];Gene=FSUBG_4014;Ontology_term=transmembrane transport,transporter activity;Ontology_id=GO:0055085,GO:0005215
contig00001.g369	1049	ID=contig00001.g369;Description=gluconolactonase [Fusarium verticillioides 7600];Gene=FRV6_02213;Ontology_term=hydrolase activity;Ontology_id=GO:0016787;Enzyme_code=EC:3,EC:3.1.1.17,EC:3;Enzyme_name=Hydrolases,gluconolactonase,Hydrolases
contig00001.g370	2067	ID=contig00001.g370;Description=hypothetical protein J7337_005965 [Fusarium musae]
contig00001.g371	660	ID=contig00001.g371;Description=infection structure specific [Fusarium tjaetaba];Gene=FDENT_3301;Ontology_term=membrane;Ontology_id=GO:0016020
contig00001.g372	2390	ID=contig00001.g372;Description=hypothetical protein FVER14953_03060 [Fusarium verticillioides]
contig00001.g373	2828	ID=contig00001.g373;Description=chitin synthase 3 [Fusarium verticillioides 7600];Gene=FACUT_4708;Ontology_term=carbohydrate metabolic process,anatomical structure development,cell differentiation,cytokinesis,cytoplasmic vesicle,carbohydrate derivative metabolic process,reproductive process,cell wall organization or biogenesis,transferase activity,mitotic cell cycle,plasma membrane;Ontology_id=GO:0005975,GO:0048856,GO:0030154,GO:0000910,GO:0031410,GO:1901135,GO:0022414,GO:0071554,GO:0016740,GO:0000278,GO:0005886;Enzyme_code=EC:2.4.1.16,EC:2.4,EC:2.4.1.16,EC:2,EC:2.4.1;Enzyme_name=chitin synthase,Glycosyltransferases,chitin synthase,Transferases,Glycosyltransferases
contig00001.g374	1724	ID=contig00001.g374;Description=TKL kinase [Fusarium tjaetaba];Gene=BFJ65_g6750;Ontology_term=catalytic activity, acting on a protein,transferase activity;Ontology_id=GO:0140096,GO:0016740;Enzyme_code=EC:2.7.1;Enzyme_name=Transferring phosphorus-containing groups
contig00001.g375	1259	ID=contig00001.g375;Description=hypothetical protein FVER53590_03056 [Fusarium verticillioides];Gene=FACUT_3252;Ontology_term=membrane;Ontology_id=GO:0016020

contig00001.g376	1149	ID=contig00001.g376;Description=hypothetical protein FVEG_03055 [Fusarium verticillioides 7600]
contig00001.g377	1719	ID=contig00001.g377;Description=hypothetical protein FVEG_03054 [Fusarium verticillioides 7600]
contig00001.g378	431	ID=contig00001.g378;Description=hypothetical protein FVEG_03053 [Fusarium verticillioides 7600]
contig00001.g379	879	ID=contig00001.g379;Description=panthothenate kinase uridine kinase-related [Fusarium tjaetaba];Gene=FVEG_03052;Ontology_term=nucleobase-containing small molecule metabolic process,carbohydrate derivative metabolic process,transferase activity;Ontology_id=GO:0055086,GO:1901135,GO:0016740;Enzyme_code=EC:2.7,EC:2.7,EC:2;Enzyme_name=Transferring phosphorus-containing groups,Transferring phosphorus-containing groups,Transferases
contig00001.g380	1177	ID=contig00001.g380;Description=fmHP [Fusarium tjaetaba]
contig00001.g381	1514	ID=contig00001.g381;Description=methionyl aminopeptidase [Fusarium mexicanum];Gene=Forpe1208_v007945;Ontology_term=catalytic activity, acting on a protein,hydrolase activity;Ontology_id=GO:0140096,GO:0016787;Enzyme_code=EC:3.4.11.18;Enzyme_name=methionyl aminopeptidase
contig00001.g382	555	ID=contig00001.g382;Description=preli msf1 [Fusarium tjaetaba];Gene=Forpe1208_v007944;Ontology_term=membrane organization,mitochondrion,lipid metabolic process,anatomical structure development,mitochondrion organization,transporter activity;Ontology_id=GO:0061024,GO:0005739,GO:0006629,GO:0048856,GO:0007005,GO:0005215
contig00001.g383	1098	ID=contig00001.g383;Description=dynactin 5 [Fusarium pseudocircinatum];Gene=FPCIR_1181;Ontology_term=cytoskeleton;Ontology_id=GO:0005856
contig00001.g384	640	ID=contig00001.g384;Description=dynactin 5 [Fusarium tjaetaba];Gene=FCIRC_12856;Ontology_term=cytoskeleton,transferase activity;Ontology_id=GO:0005856,GO:0016740;Enzyme_code=EC:2.7.7.13;Enzyme_name=mannose-1-phosphate guanylyltransferase
contig00001.g385	803	ID=contig00001.g385;Description=hypothetical protein J7337_005948 [Fusarium musae]

contig00001.g386	1596	ID=contig00001.g386;Description=major facilitator superfamily transporter [Fusarium tjaetaba];Gene=F25303_220;Ontology_term=transmembrane transport,vesicle-mediated transport,vacuole,transporter activity,plasma membrane;Ontology_id=GO:0055085,GO:0016192,GO:0005773,GO:0005215,GO:0005886
contig00001.g387	1840	ID=contig00001.g387;Description=general amidase [Fusarium tjaetaba];Gene=FMAN_07270;Ontology_term=hydrolase activity;Ontology_id=GO:0016787;Enzyme_code=EC:3.5.1.4,EC:3.5.1,EC:3.5.1.4,EC:3.5,EC:3;Enzyme_name=amidase,Acting on carbon-nitrogen bonds, other than peptide bonds,amidase,Acting on carbon-nitrogen bonds, other than peptide bonds,Hydrolases
contig00001.g388	1530	ID=contig00001.g388;Description=Subtilisin-like proteinase Spm1 [Fusarium musae];Gene=FNAPI_8059;Ontology_term=catalytic activity, acting on a protein,transmembrane transport,protein-containing complex assembly,cytokinesis,regulation of DNA-templated transcription,mitotic cell cycle,nucleus,autophagy,signaling,mitochondrion,cytosol,cell wall organization or biogenesis,transferase activity;Ontology_id=GO:0140096,GO:0055085,GO:0065003,GO:0000910,GO:0006355,GO:0000278,GO:0005634,GO:0006914,GO:0023052,GO:0005739,GO:0005829,GO:0071554,GO:0016740;Enzyme_code=EC:2.7.11.1,EC:2.7.11.24,EC:2.7.1,EC:2.7.11.1,EC:2.7.11.24,EC:2.7,EC:2;Enzyme_name=non-specific serine/threonine protein kinase,mitogen-activated protein kinase,Transferring phosphorus-containing groups,non-specific serine/threonine protein kinase,mitogen-activated protein kinase,Transferring phosphorus-containing groups,Transferases
contig00001.g389	593	ID=contig00001.g389;Description=low molecular weight phosphotyrosine phosphatase [Fusarium globosum];Gene=FOPG_03037;Ontology_term=catalytic activity, acting on a protein,hydrolase activity;Ontology_id=GO:0140096,GO:0016787;Enzyme_code=EC:3.1.3.48,EC:3.1.3.2;Enzyme_name=protein-tyrosine-phosphatase,acid phosphatase
contig00001.g390	450	ID=contig00001.g390;Description=glyoxalase-like domain protein [Fusarium tjaetaba]
contig00001.g391	699	ID=contig00001.g391;Description=UPS1 mitochondrial intermembrane space [Fusarium tjaetaba];Gene=FocTR4_00008306;Ontology_term=membrane organization,mitochondrion,lipid metabolic process,transporter activity,mitochondrion organization;Ontology_id=GO:0061024,GO:0005739,GO:0006629,GO:0005215,GO:0007005;Enzyme_code=EC:4.1.3.38;Enzyme_name=aminodeoxychorismate lyase

contig00001.g392	1456	ID=contig00001.g392;Description=lipase serine esterase [Fusarium tjaetaba];Gene=FVER53263_03039;Ontology_term=lipid metabolic process;Ontology_id=GO:0006629
contig00001.g393	1959	ID=contig00001.g393;Description=REX3-RNA exonuclease member of the family of 3'-5' exonuclease [Fusarium tjaetaba];Gene=FNYG_06264;Ontology_term=nucleus,snRNA metabolic process,regulatory ncRNA-mediated gene silencing,ribosome biogenesis,catalytic activity, acting on RNA,hydrolase activity;Ontology_id=GO:0005634,GO:0016073,GO:0031047,GO:0042254,GO:0140098,GO:0016787;Enzyme_code=EC:3.1,EC:3.1,EC:3.1.13,EC:3,EC:3.1.15;Enzyme_name=Acting on ester bonds,Acting on ester bonds,Acting on ester bonds,Hydrolases,Acting on ester bonds
contig00001.g394	893	ID=contig00001.g394;Description=prohibitin PHB1 [Fusarium subglutinans];Gene=FGADI_13038;Ontology_term=peroxisome,membrane organization,mitochondrion,anatomical structure development,protein folding,mitochondrion organization;Ontology_id=GO:0005777,GO:0061024,GO:0005739,GO:0048856,GO:0006457,GO:0007005
contig00001.g395	725	ID=contig00001.g395;Description=ubiquinol-cytochrome c reductase subunit 8 [Fusarium proliferatum];Gene=FOMG_06460;Ontology_term=generation of precursor metabolites and energy,mitochondrion;Ontology_id=GO:0006091,GO:0005739
contig00001.g396	779	ID=contig00001.g396;Description=H ACA ribonucleoprotein complex subunit 2 [Fusarium phyllophilum];Gene=FOC1_g10010941;Ontology_term=ribosome biogenesis;Ontology_id=GO:0042254
contig00001.g397	512	ID=contig00001.g397;Description=related to UPF0591 membrane protein C15E1.02c [Fusarium proliferatum ET1];Gene=FOC1_g10010942;Ontology_term=membrane;Ontology_id=GO:0016020
contig00001.g398	1536	ID=contig00001.g398;Description=NLI interacting factor-like phosphatase-domain-containing protein [Fusarium redolens];Gene=FPANT_9188;Ontology_term=lipid droplet,membrane organization,catalytic activity, acting on a protein,lipid metabolic process,signaling,mitochondrion,cell differentiation,anatomical structure development,reproductive process,hydrolase activity;Ontology_id=GO:0005811,GO:0061024,GO:0140096,GO:0006629,GO:0023052,GO:0005739,GO:0030154,GO:0048856,GO:0022414,GO:0016787;Enzyme_code=EC:3.1.3,EC:3.1.3.16,EC:3.1,EC:3,EC:3.1.3;Enzyme_name=Acting on ester bonds,protein-serine/threonine phosphatase,Acting on ester bonds,Hydrolases,Acting on ester bonds

contig00001.g399	1642	ID=contig00001.g399;Description=hypothetical protein J7337_005934 [Fusarium musae]
contig00001.g400	1468	ID=contig00001.g400;Description=translation initiation factor eIF-2B subunit beta [Fusarium verticillioides 7600];Gene=FPANT_9187;Ontology_term=translation regulator activity,RNA binding,molecular function regulator activity,cytoplasmic translation,mitochondrion,cytosol;Ontology_id=GO:0045182,GO:0003723,GO:0098772,GO:0002181,GO:0005739,GO:0005829
contig00001.g401	2217	ID=contig00001.g401;Description=hypothetical protein FVEG_03030 [Fusarium verticillioides 7600]
contig00001.g402	729	ID=contig00001.g402;Description=acetyltransferase [Fusarium verticillioides 7600];Gene=FCIRC_12874;Ontology_term=cytosol,protein maturation,transferase activity,ribosome,chromatin organization;Ontology_id=GO:0005829,GO:0051604,GO:0016740,GO:0005840,GO:0006325;Enzyme_code=EC:2.3.1.255,EC:2.3,EC:2,EC:2.3.1.255,EC:2.3.1;Enzyme_name=N-terminal amino-acid N(alpha)-acetyltransferase NatA,Acyltransferases,Transferases,N-terminal amino-acid N(alpha)-acetyltransferase NatA,Acyltransferases
contig00001.g403	1621	ID=contig00001.g403;Description=3-oxoacyl-[acyl-carrier-protein] reductase [Fusarium fujikuroi];Gene=FCIRC_12875;Ontology_term=oxidoreductase activity,DNA recombination,meiotic nuclear division;Ontology_id=GO:0016491,GO:0006310,GO:0140013;Enzyme_code=EC:1;Enzyme_name=Oxidoreductases
contig00001.g404	717	ID=contig00001.g404;Description=NADH dehydrogenase [Fusarium sp. NRRL 25303];Ontology_term=mitochondrion;Ontology_id=GO:0005739
contig00001.g405	755	ID=contig00001.g405;Description=peroxiredoxin Q BCP [Fusarium denticulatum];Gene=FPCIR_1203;Ontology_term=nucleus,antioxidant activity,oxidoreductase activity;Ontology_id=GO:0005634,GO:0016209,GO:0016491;Enzyme_code=EC:1,EC:1.11,EC:1,EC:1.11.1.24,EC:1.11.1,EC:1.11.1.15;Enzyme_name=Oxidoreductases,Acting on a peroxide as acceptor,Oxidoreductases,thioredoxin-dependent peroxiredoxin,Acting on a peroxide as acceptor,Acting on a peroxide as acceptor
contig00001.g406	1297	ID=contig00001.g406;Description=folate transporter carrier (mitochondrial) [Fusarium coicis];Gene=FOC1_g10010953;Ontology_term=transmembrane transport,mitochondrion,transporter activity;Ontology_id=GO:0055085,GO:0005739,GO:0005215

contig00001.g407	605	ID=contig00001.g407;Description=ubiquinol-cytochrome-c reductase [Fusarium coicis];Gene=BFJ68_g1710;Ontology_term=generation of precursor metabolites and energy,nucleobase-containing small molecule metabolic process,protein-containing complex assembly,mitochondrion,carbohydrate derivative metabolic process,mitochondrion organization;Ontology_id=GO:0006091,GO:0055086,GO:0065003,GO:0005739,GO:1901135,GO:0007005
contig00001.g408	2969	ID=contig00001.g408;Description=ubiquitin thiolesterase [Fusarium verticillioides 7600];Gene=FNYG_06279;Ontology_term=catalytic activity, acting on a protein,vesicle-mediated transport,RNA binding,protein catabolic process,autophagy,cytosol,organelle,hydrolase activity;Ontology_id=GO:0140096,GO:0016192,GO:0003723,GO:0030163,GO:0006914,GO:0005829,GO:0043226,GO:0016787;Enzyme_code=EC:3.4.19.12,EC:3.4,EC:3,EC:3.4.19.12;Enzyme_name=ubiquitinyl hydrolase 1,Acting on peptide bonds (peptidases),Hydrolases,ubiquitinyl hydrolase 1
contig00001.g409	2132	ID=contig00001.g409;Description=ubiquitin thiolesterase [Fusarium tjaetaba];Gene=FVER53590_03022;Ontology_term=nucleus,DNA repair;Ontology_id=GO:0005634,GO:0006281
contig00001.g410	1869	ID=contig00001.g410;Description=MYHalpha3 protein (DNA repair enzyme) [Fusarium fujikuroi];Gene=FDENT_8554;Ontology_term=DNA binding,DNA repair,nucleus,catalytic activity, acting on DNA,signaling,hydrolase activity;Ontology_id=GO:0003677,GO:0006281,GO:0005634,GO:0140097,GO:0023052,GO:0016787;Enzyme_code=EC:3.2.2.31,EC:3.2,EC:3.2.2.31,EC:3,EC:3.2.2.23,EC:3.2.2;Enzyme_name=adenine glycosylase,Glycosylases,adenine glycosylase,Hydrolases,DNA-formamidopyrimidine glycosylase,Glycosylases
contig00001.g411	1977	ID=contig00001.g411;Description=tol [Fusarium tjaetaba]
contig00001.g412	1230	ID=contig00001.g412;Description=C4-dicarboxylate transport mae1 [Fusarium tjaetaba];Gene=FTJAE_1381;Ontology_term=transmembrane transport,transporter activity;Ontology_id=GO:0055085,GO:0005215
contig00001.g413	855	ID=contig00001.g413;Description=beta-lactamase [Fusarium verticillioides 7600]
contig00001.g414	1162	ID=contig00001.g414;Description=zinc knuckle [Colletotrichum incanum];Gene=PCL_05335;Ontology_term=nucleic acid binding,zinc ion binding;Ontology_id=GO:0003676,GO:0008270

contig00001.g415	1706	ID=contig00001.g415;Description=acyl-CoA dehydrogenase [Fusarium verticillioides 7600];Gene=FOTG_03208;Ontology_term=oxidoreductase activity;Ontology_id=GO:0016491;Enzyme_code=EC:1.3.8,EC:1.3,EC:1,EC:1.3.8;Enzyme_name=Acting on the CH-CH group of donors,Acting on the CH-CH group of donors,Oxidoreductases,Acting on the CH-CH group of donors
contig00001.g416	1670	ID=contig00001.g416;Description=hypothetical protein FVEG_03013 [Fusarium verticillioides 7600];Gene=FDENT_8560;Ontology_term=oxidoreductase activity;Ontology_id=GO:0016491;Enzyme_code=EC:1;Enzyme_name=Oxidoreductases
contig00001.g417	6748	ID=contig00001.g417;Description=autophagy- protein 2 [Fusarium musae];Gene=FTJAE_1385;Ontology_term=autophagy,cytosol,endoplasmic reticulum,lipid binding,intracellular protein transport,mitochondrion organization;Ontology_id=GO:0006914,GO:0005829,GO:0005783,GO:0008289,GO:0006886,GO:0007005
contig00001.g418	1018	ID=contig00001.g418;Description=galactinol synthase [Fusarium tjaetaba];Gene=FOC1_g10010964;Ontology_term=transferase activity;Ontology_id=GO:0016740;Enzyme_code=EC:2.4;Enzyme_name=Glycosyltransferases
contig00001.g419	666	ID=contig00001.g419;Description=hypothetical protein LZL87_003334 [Fusarium oxysporum]
contig00001.g420	1186	ID=contig00001.g420;Description=putative SONA [Fusarium fujikuroi];Gene=1582;Ontology_term=nuclear envelope,nucleocytoplasmic transport,RNA binding,ribosome biogenesis,cytosol,intracellular protein transport;Ontology_id=GO:0005635,GO:0006913,GO:0003723,GO:0042254,GO:0005829,GO:0006886
contig00001.g421	1089	ID=contig00001.g421;Description=capsule polysaccharide biosynthesis [Fusarium tjaetaba];Gene=FPRO05_07805;Ontology_term=membrane;Ontology_id=GO:0016020
contig00001.g422	1042	ID=contig00001.g422;Description=dihydrodipicolinate synthase [Fusarium tjaetaba];Gene=FVER53263_03007;Ontology_term=lyase activity;Ontology_id=GO:0016829;Enzyme_code=EC:4,EC:4,EC:4.1.2.53;Enzyme_name=Lyases,Lyases,2-keto-3-deoxy-L-rhamnonate aldolase

contig00001.g423	1173	ID=contig00001.g423;Description=homoserine kinase [Trichoderma gamsii];Gene=TASIC1_0007043100;Ontology_term=sulfur compound metabolic process,lipid metabolic process,amino acid metabolic process,transferase activity;Ontology_id=GO:0006790,GO:0006629,GO:0006520,GO:0016740;Enzyme_code=EC:2.7.1.39,EC:2.7.1,EC:2.7.1.39,EC:2.7,EC:2;Enzyme_name=homoserine kinase,Transferring phosphorus-containing groups,homoserine kinase,Transferring phosphorus-containing groups,Transferases
contig00001.g424	476	ID=contig00001.g424;Description=rot1 PRECURSOR [Fusarium subglutinans];Gene=FOYG_07924;Ontology_term=extracellular region;Ontology_id=GO:0005576
contig00001.g425	1052	ID=contig00001.g425;Description=hypothetical protein FVER14953_03004 [Fusarium verticillioides]
contig00001.g426	1323	ID=contig00001.g426;Description=hypothetical protein FVER14953_03003 [Fusarium verticillioides]
contig00001.g427	1668	ID=contig00001.g427;Description=DUF6 domain protein [Fusarium fujikuroi];Gene=FPHYL_12746;Ontology_term=nucleus,DNA binding,RNA binding,protein-containing complex assembly,cytoplasmic translation,structural molecule activity,regulation of DNA-templated transcription,cytosol,ribosome biogenesis,ribosome;Ontology_id=GO:0005634,GO:0003677,GO:0003723,GO:0065003,GO:0002181,GO:0005198,GO:0006355,GO:0005829,GO:0042254,GO:0005840
contig00001.g428	1117	ID=contig00001.g428;Description=potassium channel beta subunit [Fusarium subglutinans];Gene=FOC1_g10010974;Ontology_term=oxidoreductase activity;Ontology_id=GO:0016491;Enzyme_code=EC:1,EC:1;Enzyme_name=Oxidoreductases,Oxidoreductases
contig00001.g429	2429	ID=contig00001.g429;Description=complex I intermediate-associated CIA84 precursor [Fusarium tjaetaba];Ontology_term=RNA binding,mitochondrion,hydrolase activity;Ontology_id=GO:0003723,GO:0005739,GO:0016787;Enzyme_code=EC:3.1,EC:3;Enzyme_name=Acting on ester bonds,Hydrolases
contig00001.g430	689	ID=contig00001.g430;Description=ribosomal S18 [Fusarium tjaetaba];Gene=FNYG_06301;Ontology_term=mitochondrial gene expression,mitochondrion,structural molecule activity,cytosol,ribosome;Ontology_id=GO:0140053,GO:0005739,GO:0005198,GO:0005829,GO:0005840

contig00001.g431	607	ID=contig00001.g431;Description=U3 small nucleolar ribonucleoprotein IMP3 [Fusarium oxysporum f. sp. lycopersici 4287];Gene=FVEG_02998;Ontology_term=RNA binding,structural molecule activity,ribosome biogenesis,nucleolus,ribosome;Ontology_id=GO:0003723,GO:0005198,GO:0042254,GO:0005730,GO:0005840
contig00001.g432	956	ID=contig00001.g432;Description=phospholipid methyltransferase [Fusarium tjaetaba];Gene=FNAPI_9557;Ontology_term=transferase activity;Ontology_id=GO:0016740;Enzyme_code=EC:2.1.1,EC:2.1,EC:2,EC:2.1.1;Enzyme_name=Transferring one-carbon groups,Transferring one-carbon groups,Transferases,Transferring one-carbon groups
contig00001.g433	3093	ID=contig00001.g433;Description=DNA repair RAD26 [Fusarium tjaetaba];Gene=FVER53263_02996;Ontology_term=nucleus,catalytic activity, acting on DNA,ATP-dependent activity,chromatin organization,hydrolase activity;Ontology_id=GO:0005634,GO:0140097,GO:0140657,GO:0006325,GO:0016787;Enzyme_code=EC:3,EC:3;Enzyme_name=Hydrolases,Hydrolases
contig00001.g434	802	ID=contig00001.g434;Description=glycosylphosphatidylinositol anchor biosynthesis protein 11 [Fusarium proliferatum];Gene=FOC1_g10010980;Ontology_term=oxidoreductase activity,lipid metabolic process,carbohydrate derivative metabolic process,endoplasmic reticulum,transferase activity;Ontology_id=GO:0016491,GO:0006629,GO:1901135,GO:0005783,GO:0016740;Enzyme_code=EC:1,EC:2.7,EC:2,EC:2.7.8;Enzyme_name=Oxidoreductases,Transferring phosphorus-containing groups,Transferases,Transferring phosphorus-containing groups
contig00001.g435	314	ID=contig00001.g435;Description=Ff.00g069630.m01.CDS01 [Fusarium sp. VM40];Gene=FOXYS1_9317;Ontology_term=membrane;Ontology_id=GO:0016020
contig00001.g436	2228	ID=contig00001.g436;Description=ubiquitin-like modifier-activating enzyme atg-7 [Fusarium verticillioides 7600];Gene=FOQG_06782;Ontology_term=catalytic activity, acting on a protein,ATP-dependent activity,autophagy,mitochondrion,cytosol,transferase activity,intracellular protein transport,mitochondrion organization,ligase activity,hydrolase activity;Ontology_id=GO:0140096,GO:0140657,GO:0006914,GO:0005739,GO:0005829,GO:0016740,GO:0006886,GO:0007005,GO:0016874,GO:0016787;Enzyme_code=EC:6.2,EC:3.5,EC:3.5.4.12,EC:2,EC:6.2,EC:3.5,EC:3,EC:6;Enzyme_name=Forming carbon-sulfur bonds,Acting on carbon-nitrogen bonds, other than peptide bonds,dCMP deaminase,Transferases,Forming carbon-sulfur bonds,Acting on carbon-nitrogen bonds, other than peptide bonds,Hydrolases,Ligases

contig00001.g437	1309	ID=contig00001.g437;Description=dCMP deaminase [Fusarium verticillioides 7600];Gene=FVEG_15163;Ontology_term=catalytic activity, acting on a protein,ATP-dependent activity,autophagy,mitochondrion,cytosol,transferase activity,intracellular protein transport,mitochondrion organization,ligase activity,hydrolase activity;Ontology_id=GO:0140096,GO:0140657,GO:0006914,GO:0005739,GO:0005829,GO:0016740,GO:0006886,GO:0007005,GO:0016874,GO:0016787;Enzyme_code=EC:6.2,EC:3.5,EC:3.5.4.12,EC:2,EC:6.2,EC:3.5,EC:3,EC:6;Enzyme_name=Forming carbon-sulfur bonds,Acting on carbon-nitrogen bonds, other than peptide bonds,dCMP deaminase,Transferases,Forming carbon-sulfur bonds,Acting on carbon-nitrogen bonds, other than peptide bonds,Hydrolases,Ligases
contig00001.g438	3496	ID=contig00001.g438;Description=WEE/WEE-UNCLASSIFIED protein kinase [Fusarium verticillioides 7600];Gene=FTJAE_1406;Ontology_term=catalytic activity, acting on a protein,transferase activity;Ontology_id=GO:0140096,GO:0016740;Enzyme_code=EC:2.7.1,EC:2.7.1,EC:2.7.11.1,EC:2.7,EC:2;Enzyme_name=Transferring phosphorus-containing groups,Transferring phosphorus-containing groups,non-specific serine/threonine protein kinase,Transferring phosphorus-containing groups,Transferases
contig00001.g439	1435	ID=contig00001.g439;Description=fad dependent oxidoreductase [Fusarium tjaetaba];Gene=FNYG_06311;Ontology_term=oxidoreductase activity;Ontology_id=GO:0016491;Enzyme_code=EC:1,EC:1;Enzyme_name=Oxidoreductases,Oxidoreductases
contig00001.g440	1919	ID=contig00001.g440;Description=4-coumarate ligase [Fusarium tjaetaba];Gene=FTJAE_1408;Ontology_term=ligase activity;Ontology_id=GO:0016874;Enzyme_code=EC:6,EC:6.2.1.12,EC:6;Enzyme_name=Ligases,4-coumarate--CoA ligase,Ligases

contig00001.g441	1267	ID=contig00001.g441;Description=putative NADH-ubiquinone oxidoreductase, mitochondrial precursor [Fusarium fujikuroi];Gene=FDENT_1758;Ontology_term=generation of precursor metabolites and energy,nucleobase-containing small molecule metabolic process,oxidoreductase activity,mitochondrion,carbohydrate derivative metabolic process,transporter activity;Ontology_id=GO:0006091,GO:0055086,GO:0016491,GO:0005739,GO:1901135,GO:0005215;Enzyme_code=EC:1,EC:7.1.1.2,EC:1.6,EC:1,EC:1.6.5.11,EC:1.6.5,EC:1.6.99.3,EC:7,EC:1.6.5.3,EC:1.6.5.2;Enzyme_name=Oxidoreductases,NADH:ubiquinone reductase (H(+)-translocating),Acting on NADH or NADPH,Oxidoreductases,Acting on NADH or NADPH,Acting on NADH or NADPH,Deleted entry,Translocases,Acting on NADH or NADPH,NAD(P)H dehydrogenase (quinone)
contig00001.g442	1719	ID=contig00001.g442;Description=nitrile-specifier 5 [Fusarium tjaetaba]
contig00001.g443	1517	ID=contig00001.g443;Description=hypothetical protein FVEG_02984 [Fusarium verticillioides 7600];Gene=FVER53263_02984;Ontology_term=vesicle-mediated transport,endosome;Ontology_id=GO:0016192,GO:0005768
contig00001.g444	2403	ID=contig00001.g444;Description=vacuolar fusion CCZ1 like [Fusarium denticulatum];Gene=FNAPI_9731;Ontology_term=vesicle-mediated transport,endosome;Ontology_id=GO:0016192,GO:0005768
contig00001.g445	870	ID=contig00001.g445;Description=rasp f 7 allergen [Fusarium tjaetaba]
contig00001.g446	1000	ID=contig00001.g446;Description=N2 N2-dimethylguanosine tRNA methyltransferase [Fusarium tjaetaba];Gene=EFM6;Ontology_term=catalytic activity, acting on a protein,transferase activity;Ontology_id=GO:0140096,GO:0016740;Enzyme_code=EC:2.1.1,EC:2.1,EC:2,EC:2.1.1;Enzyme_name=Transferring one-carbon groups,Transferring one-carbon groups,Transferases,Transferring one-carbon groups
contig00001.g447	2715	ID=contig00001.g447;Description=Axial budding pattern 2 [Fusarium tjaetaba];Gene=FPANT_13533;Ontology_term=cytoskeleton,establishment or maintenance of cell polarity,cytokinesis,vacuole,mitotic cell cycle,plasma membrane;Ontology_id=GO:0005856,GO:0007163,GO:0000910,GO:0005773,GO:0000278,GO:0005886

contig00001.g448	2087	ID=contig00001.g448;Description=Protein phosphatase 2C 2 [Fusarium oxysporum];Gene=FOC1_g10010996;Ontology_term=nucleus,catalytic activity, acting on a protein,signaling,hydrolase activity;Ontology_id=GO:0005634,GO:0140096,GO:0023052,GO:0016787;Enzyme_code=EC:3.1.3.16,EC:3.1.3.16,EC:3.1,EC:3,EC:3.1.3;Enzyme_name=protein-serine/threonine phosphatase,protein-serine/threonine phosphatase,Acting on ester bonds,Hydrolases,Acting on ester bonds
contig00001.g449	2538	ID=contig00001.g449;Description=hypothetical protein FVER14953_02978 [Fusarium verticillioides]
contig00001.g450	1556	ID=contig00001.g450;Description=meiotically up-regulated 65 [Fusarium pseudoanthophilum];Gene=PYR3;Ontology_term=nucleobase-containing small molecule metabolic process,anatomical structure development,cell differentiation,reproductive process,carbohydrate derivative metabolic process,cell wall organization or biogenesis,hydrolase activity;Ontology_id=GO:0055086,GO:0048856,GO:0030154,GO:0022414,GO:1901135,GO:0071554,GO:0016787;Enzyme_code=EC:3.5.2.3,EC:3.5.2.3,EC:3.5.2,EC:3.5,EC:3;Enzyme_name=dihydroorotase,dihydroorotase,Acting on carbon-nitrogen bonds, other than peptide bonds,Acting on carbon-nitrogen bonds, other than peptide bonds,Hydrolases
contig00001.g451	525	ID=contig00001.g451;Description=calcofluor white hypersensitive [Fusarium sp. NRRL 25303]
contig00001.g452	2793	ID=contig00001.g452;Description=U4/U6 x U5 tri-snRNP complex subunit Prp1 [Fusarium oxysporum];Gene=FVEG_02975;Ontology_term=nucleus,mRNA metabolic process,protein-containing complex assembly;Ontology_id=GO:0005634,GO:0016071,GO:0065003
contig00001.g453	2571	ID=contig00001.g453;Description=vacuolar VAC7 [Fusarium pseudoanthophilum];Gene=FPRO05_07837;Ontology_term=lipid metabolic process,vacuole;Ontology_id=GO:0006629,GO:0005773
contig00001.g454	1923	ID=contig00001.g454;Description=hypothetical protein FVEG_02973 [Fusarium verticillioides 7600]
contig00001.g455	583	ID=contig00001.g455;Description=60S ribosomal protein L30 [Fusarium verticillioides 7600];Gene=rpl-30;Ontology_term=RNA binding,mRNA metabolic process,structural molecule activity,cytosol,ribosome biogenesis,ribosome;Ontology_id=GO:0003723,GO:0016071,GO:0005198,GO:0005829,GO:0042254,GO:0005840

contig00001.g456	881	ID=contig00001.g456;Description=60S ribosomal protein L10-A [Fusarium oxysporum f. sp. lycopersici 4287];Gene=FOMG_06524;Ontology_term=structural molecule activity,ribosome;Ontology_id=GO:0005198,GO:0005840
contig00001.g457	1704	ID=contig00001.g457;Description=hypothetical protein FVER14953_02970 [Fusarium verticillioides]
contig00001.g458	2802	ID=contig00001.g458;Description=hypothetical protein FVER53263_02969 [Fusarium verticillioides]
contig00001.g459	1587	ID=contig00001.g459;Description=spermidine synthase [Fusarium odoratissimum NRRL 54006];Gene=FVEG_15160;Ontology_term=transferase activity;Ontology_id=GO:0016740;Enzyme_code=EC:2.1.1;Enzyme_name=Transferring one-carbon groups
contig00001.g460	1011	ID=contig00001.g460;Description=nitrilase [Fusarium verticillioides 7600];Gene=NIT3;Ontology_term=detoxification,hydrolase activity;Ontology_id=GO:0098754,GO:0016787;Enzyme_code=EC:3.5.1,EC:3.5.1,EC:3.5.1.3,EC:3.5,EC:3;Enzyme_name=Acting on carbon-nitrogen bonds, other than peptide bonds,Acting on carbon-nitrogen bonds, other than peptide bonds,omega-amidase,Acting on carbon-nitrogen bonds, other than peptide bonds,Hydrolases
contig00001.g461	613	ID=contig00001.g461;Description=hypothetical protein FVER53263_02965 [Fusarium verticillioides]
contig00001.g462	1118	ID=contig00001.g462;Description=WD40-repeat-containing domain protein [Fusarium redolens];Gene=FDENT_1778;Ontology_term=transmembrane transport,cytoskeleton organization,establishment or maintenance of cell polarity,Golgi apparatus,transporter activity,plasma membrane,nucleus,molecular function regulator activity,endosome,ATP-dependent activity,signaling,cytosol,cell wall organization or biogenesis,hydrolase activity;Ontology_id=GO:0055085,GO:0007010,GO:0007163,GO:0005794,GO:0005215,GO:0005886,GO:0005634,GO:0098772,GO:0005768,GO:0140657,GO:0023052,GO:0005829,GO:0071554,GO:0016787;Enzyme_code=EC:7.2.2,EC:3.6.1.15,EC:3.6.1,EC:3.6,EC:3,EC:7.2.2,EC:7,EC:3.6.1.15;Enzyme_name=Catalysing the translocation of inorganic cations,nucleoside-triphosphate phosphatase,Acting on acid anhydrides,Acting on acid anhydrides,Hydrolases,Catalysing the translocation of inorganic cations,Translocases,nucleoside-triphosphate phosphatase
contig00001.g463	349	ID=contig00001.g463;Description=conidiation 6 (con-6) [Fusarium tjaetaba];Gene=rpsO
contig00001.g464	609	ID=contig00001.g464;Description=integral membrane [Fusarium agapanthi];Gene=FOPG_02957;Ontology_term=membrane;Ontology_id=GO:0016020
contig00001.g465	770	ID=contig00001.g465;Description=unnamed protein product [Fusarium fujikuroi]

contig00001.g466	1022	ID=contig00001.g466;Description=nucleophile aminohydrolase [Fusarium flagelliforme];Gene=FCIRC_12710;Ontology_term=nucleus,protein catabolic process,mitochondrion,mitotic nuclear division,chromosome segregation,cytosol;Ontology_id=GO:0005634,GO:0030163,GO:0005739,GO:0140014,GO:0007059,GO:0005829;Enzyme_code=EC:3.4.25.1;Enzyme_name=proteasome endopeptidase complex
contig00001.g467	1185	ID=contig00001.g467;Description=proteasome subunit alpha type 6 [Fusarium mundagurra];Gene=FMUND_3997;Ontology_term=proteasome complex;Ontology_id=GO:0000502
contig00001.g468	966	ID=contig00001.g468;Description=phytanoyl dioxygenase family [Fusarium tjaetaba];Gene=FNAPI_3311;Ontology_term=oxidoreductase activity;Ontology_id=GO:0016491;Enzyme_code=EC:1,EC:1;Enzyme_name=Oxidoreductases,Oxidoreductases
contig00001.g469	332	ID=contig00001.g469;Description=pre-mrna splicing factor [Fusarium tjaetaba];Gene=FOQG_06818;Ontology_term=membrane;Ontology_id=GO:0016020
contig00001.g470	1359	ID=contig00001.g470;Description=IFRD domain-containing protein [Fusarium denticulatum];Ontology_term=nucleus;Ontology_id=GO:0005634
contig00001.g471	2240	ID=contig00001.g471;Description=sarcolemmal membrane-associated [Fusarium tjaetaba]
contig00001.g472	2122	ID=contig00001.g472;Description=hypothetical protein FVER14953_02954 [Fusarium verticillioides];Gene=FVEG_02954;Ontology_term=cellular anatomical entity;Ontology_id=GO:0110165
contig00001.g473	942	ID=contig00001.g473;Description=RRP7-like protein [Fusarium pseudocircinatum];Ontology_term=protein-containing complex assembly,ribosome biogenesis,nucleolus;Ontology_id=GO:0065003,GO:0042254,GO:0005730
contig00001.g474	741	ID=contig00001.g474;Description=RRP7-like protein [Fusarium tjaetaba];Gene=FTJAE_1440;Ontology_term=nucleic acid binding,zinc ion binding;Ontology_id=GO:0003676,GO:0008270
contig00001.g475	1087	ID=contig00001.g475;Description=RRP7-like protein [Fusarium tjaetaba];Gene=FVER53263_02952;Ontology_term=nucleic acid binding,zinc ion binding;Ontology_id=GO:0003676,GO:0008270

contig00001.g476	1688	ID=contig00001.g476;Description=glutamate--ammonia ligase [Fusarium musae];Gene=FANTH_6991;Ontology_term=nucleus,amino acid metabolic process,ligase activity;Ontology_id=GO:0005634,GO:0006520,GO:0016874;Enzyme_code=EC:6.3.1.2,EC:6.3,EC:6.3.1,EC:6.3.1.2,EC:6;Enzyme_name=glutamine synthetase,Forming carbon-nitrogen bonds,Forming carbon-nitrogen bonds,glutamine synthetase,Ligases
contig00001.g477	1241	ID=contig00001.g477;Description=transmembrane protein [Fusarium coicis];Gene=FOPG_02945;Ontology_term=membrane;Ontology_id=GO:0016020
contig00001.g478	2349	ID=contig00001.g478;Description=transcription factor [Fusarium tjaetaba];Gene=FPHYL_3095;Ontology_term=nucleus,DNA binding,regulation of DNA-templated transcription,transcription regulator activity;Ontology_id=GO:0005634,GO:0003677,GO:0006355,GO:0140110
contig00001.g479	3037	ID=contig00001.g479;Description=formate-tetrahydrofolate ligase [Fusarium verticillioides 7600];Gene=FACUT_10637;Ontology_term=DNA binding,nucleus,nucleobase-containing small molecule metabolic process,oxidoreductase activity,mitochondrion,cellular modified amino acid metabolic process,vitamin metabolic process,ligase activity,hydrolase activity;Ontology_id=GO:0003677,GO:0005634,GO:0055086,GO:0016491,GO:0005739,GO:0006575,GO:0006766,GO:0016874,GO:0016787;Enzyme_code=EC:6.3.4.3,EC:3.5.4.9,EC:1.5.1.5,EC:3.5.4,EC:1.5,EC:1,EC:6.3.4.3,EC:6.3,EC:3.5,EC:3,EC:3.5.4.9,EC:6,EC:1.5.1,EC:1.5.1.5;Enzyme_name=formate--tetrahydrofolate ligase,methenyltetrahydrofolate cyclohydrolase,methylenetetrahydrofolate dehydrogenase (NADP(+)),Acting on carbon-nitrogen bonds, other than peptide bonds,Acting on the CH-NH group of donors,Oxidoreductases,formate--tetrahydrofolate ligase,Forming carbon-nitrogen bonds,Acting on carbon-nitrogen bonds, other than peptide bonds,Hydrolases,methenyltetrahydrofolate cyclohydrolase,Ligases,Acting on the CH-NH group of donors,methylenetetrahydrofolate dehydrogenase (NADP(+))
contig00001.g480	1190	ID=contig00001.g480;Description=WD40-repeat-containing domain protein [Fusarium oxysporum Fo47];Gene=tif-34;Ontology_term=nucleus,translation regulator activity,RNA binding,protein-containing complex assembly,cytoplasmic translation;Ontology_id=GO:0005634,GO:0045182,GO:0003723,GO:0065003,GO:0002181

contig00001.g481	3575	ID=contig00001.g481;Description=probable ATPase component of chromatin remodeling complex (ISW1) [Fusarium fujikuroi];Gene=FFUJ_07229;Ontology_term=catalytic activity, acting on DNA,protein-containing complex assembly,anatomical structure development,chromosome segregation,regulation of DNA-templated transcription,nuclear chromosome,chromatin organization,nucleolus,DNA binding,ATP-dependent activity,cell differentiation,telomere organization,hydrolase activity;Ontology_id=GO:0140097,GO:0065003,GO:0048856,GO:0007059,GO:0006355,GO:0000228,GO:0006325,GO:0005730,GO:0003677,GO:0140657,GO:0030154,GO:0032200,GO:0016787;Enzyme_code=EC:3,EC:3.6.1,EC:3.6,EC:3,EC:3.6.1.15;Enzyme_name=Hydrolases,Acting on acid anhydrides,Acting on acid anhydrides,Hydrolases,nucleoside-triphosphate phosphatase
contig00001.g482	732	ID=contig00001.g482;Description=hypothetical protein FOXG_05188 [Fusarium oxysporum f. sp. lycopersici 4287];Gene=SLC9A1;Ontology_term=generation of precursor metabolites and energy,transmembrane transport,nucleobase-containing small molecule metabolic process,protein-containing complex assembly,anatomical structure development,regulation of DNA-templated transcription,carbohydrate derivative metabolic process,lipid binding,muscle system process,circulatory system process,transporter activity,plasma membrane,nucleoplasm,carbohydrate metabolic process,signaling,cell differentiation,transferase activity;Ontology_id=GO:0006091,GO:0055085,GO:0055086,GO:0065003,GO:0048856,GO:0006355,GO:1901135,GO:0008289,GO:0003012,GO:0003013,GO:0005215,GO:0005886,GO:0005654,GO:0005975,GO:0023052,GO:0030154,GO:0016740;Enzyme_code=EC:2.7.1.40,EC:2.7.1,EC:2.7,EC:2,EC:2.7.1.40;Enzyme_name=pyruvate kinase,Transferring phosphorus-containing groups,Transferring phosphorus-containing groups,Transferases,pyruvate kinase
contig00001.g483	727	ID=contig00001.g483;Description=probable RRM-type RNA binding protein [Fusarium proliferatum ET1];Gene=FDENT_1799;Ontology_term=RNA binding,mRNA metabolic process,nuclear chromosome,nucleolus,nucleoplasm;Ontology_id=GO:0003723,GO:0016071,GO:0000228,GO:0005730,GO:0005654

contig00001.g484	2827	ID=contig00001.g484;Description=E3 ubiquitin-protein ligase hulA [Fusarium verticillioides 7600];Gene=FocTR4_00008406;Ontology_term=catalytic activity, acting on a protein,cytoskeleton organization,protein catabolic process,regulation of DNA-templated transcription,ribosome biogenesis,tRNA metabolic process,Golgi apparatus,lipid binding,ribosome,chromatin organization,mitochondrion organization,nucleus,nucleocytoplasmic transport,endosome,vesicle-mediated transport,autophagy,lipid metabolic process,cytosol,transferase activity,ligase activity;Ontology_id=GO:0140096,GO:0007010,GO:0030163,GO:0006355,GO:0042254,GO:0006399,GO:0005794,GO:0008289,GO:0005840,GO:0006325,GO:0007005,GO:0005634,GO:0006913,GO:0005768,GO:0016192,GO:0006914,GO:0006629,GO:0005829,GO:0016740,GO:0016874;Enzyme_code=EC:6,EC:2.3.2;Enzyme_name=Ligases,Acyltransferases
contig00001.g485	2956	ID=contig00001.g485;Description=hypothetical protein IL306_001366 [Fusarium sp. DS 682];Gene=FOYG_07988;Ontology_term=RNA binding;Ontology_id=GO:0003723
contig00001.g486	2092	ID=contig00001.g486;Description=GTPase-activating protein [Fusarium oxysporum];Ontology_term=membrane organization,vesicle-mediated transport,molecular function regulator activity,mitochondrion,Golgi apparatus,intracellular protein transport;Ontology_id=GO:0061024,GO:0016192,GO:0098772,GO:0005739,GO:0005794,GO:0006886
contig00001.g487	1683	ID=contig00001.g487;Description=beta-1 2-xylosyltransferase 1 [Fusarium tjaetaba];Gene=FPANT_11416;Ontology_term=transferase activity;Ontology_id=GO:0016740;Enzyme_code=EC:2,EC:2;Enzyme_name=Transferases,Transferases
contig00001.g488	794	ID=contig00001.g488;Description=NADH-ubiquinone oxidoreductase kDa subunit mitochondrial [Fusarium subglutinans];Gene=FDENT_1804;Ontology_term=generation of precursor metabolites and energy,oxidoreductase activity,transporter activity;Ontology_id=GO:0006091,GO:0016491,GO:0005215;Enzyme_code=EC:7.1.1.2,EC:1.6.5.11,EC:1.6.5.2;Enzyme_name=NADH:ubiquinone reductase (H(+)-translocating),Acting on NADH or NADPH,NAD(P)H dehydrogenase (quinone)
contig00001.g489	1801	ID=contig00001.g489;Description=adenosine deaminase [Fusarium verticillioides 7600];Gene=FPHYL_3108;Ontology_term=hydrolase activity;Ontology_id=GO:0016787;Enzyme_code=EC:3.5,EC:3.5,EC:3,EC:3.5.4.4;Enzyme_name=Acting on carbon-nitrogen bonds, other than peptide bonds,Acting on carbon-nitrogen bonds, other than peptide bonds,Hydrolases,adenosine deaminase

contig00001.g490	489	ID=contig00001.g490;Description=hypothetical protein FVEG_15153 [Fusarium verticillioides 7600]
contig00001.g491	2018	ID=contig00001.g491;Description=acyl dehydrogenase [Fusarium tjaetaba];Gene=FDENT_10447;Ontology_term=oxidoreductase activity;Ontology_id=GO:0016491;Enzyme_code=EC:1.3,EC:1.3,EC:1;Enzyme_name=Acting on the CH-CH group of donors,Acting on the CH-CH group of donors,Oxidoreductases
contig00001.g492	1023	ID=contig00001.g492;Description=isoflavone reductase family [Fusarium tjaetaba]
contig00001.g493	4171	ID=contig00001.g493;Description=5-oxoprolinase (ATP-hydrolysing) [Fusarium verticillioides 7600];Gene=FDENT_10445;Ontology_term=sulfur compound metabolic process,detoxification,cellular modified amino acid metabolic process,cytosol,hydrolase activity;Ontology_id=GO:0006790,GO:0098754,GO:0006575,GO:0005829,GO:0016787;Enzyme_code=EC:3,EC:3.5.2,EC:3,EC:3.5,EC:3.5.2.9;Enzyme_name=Hydrolases,Acting on carbon-nitrogen bonds, other than peptide bonds,Hydrolases,Acting on carbon-nitrogen bonds, other than peptide bonds,5-oxoprolinase (ATP-hydrolyzing)
contig00001.g494	275	ID=contig00001.g494;Description=related to ubiquitin [Fusarium proliferatum ET1];Gene=FVEG_02932;Ontology_term=nucleus,catalytic activity, acting on a protein,cytoplasmic translation,structural molecule activity,cytosol,ribosome,transferase activity;Ontology_id=GO:0005634,GO:0140096,GO:0002181,GO:0005198,GO:0005829,GO:0005840,GO:0016740;Enzyme_code=EC:2.7.11.1,EC:2.7.1,EC:2.7.11.1,EC:2.7,EC:2;Enzyme_name=non-specific serine/threonine protein kinase,Transferring phosphorus-containing groups,non-specific serine/threonine protein kinase,Transferring phosphorus-containing groups,Transferases
contig00001.g495	1243	ID=contig00001.g495;Description=endonuclease III [Fusarium tjaetaba];Gene=NTH1;Ontology_term=DNA repair,nucleus,DNA binding,catalytic activity, acting on DNA,DNA replication,mitochondrion,lyase activity,mitochondrion organization,hydrolase activity;Ontology_id=GO:0006281,GO:0005634,GO:0003677,GO:0140097,GO:0006260,GO:0005739,GO:0016829,GO:0007005,GO:0016787;Enzyme_code=EC:4.2.99.18,EC:3.1,EC:3.2.2,EC:3.2,EC:4.2.99.18,EC:3.1,EC:4.2,EC:3,EC:4,EC:3.2.2.23,EC:3.2.2;Enzyme_name=DNA-(apurinic or apyrimidinic site) lyase,Acting on ester bonds,Glycosylases,Glycosylases,DNA-(apurinic or apyrimidinic site) lyase,Acting on ester bonds,Carbon-oxygen lyases,Hydrolases,Lyases,DNA-formamidopyrimidine glycosylase,Glycosylases

contig00001.g496	1445	ID=contig00001.g496;Description=S-(hydroxymethyl)glutathione dehydrogenase [Fusarium proliferatum];Gene=FCIRC_4622;Ontology_term=generation of precursor metabolites and energy,oxidoreductase activity,detoxification;Ontology_id=GO:0006091,GO:0016491,GO:0098754;Enzyme_code=EC:1.1.1.284,EC:1.1.1.1,EC:1.1.1.71;Enzyme_name=S-(hydroxymethyl)glutathione dehydrogenase,alcohol dehydrogenase,alcohol dehydrogenase [NAD(P)(+)]
contig00001.g497	3325	ID=contig00001.g497;Description=nuclear pore complex subunit nup85 [Fusarium tjaetaba];Gene=FPANT_5913;Ontology_term=nuclear envelope,nucleocytoplasmic transport,structural molecule activity;Ontology_id=GO:0005635,GO:0006913,GO:0005198
contig00001.g498	2570	ID=contig00001.g498;Description=aconitate hydratase, mitochondrial [Fusarium verticillioides 7600];Gene=F25303_3144;Ontology_term=generation of precursor metabolites and energy,mitochondrion,lyase activity;Ontology_id=GO:0006091,GO:0005739,GO:0016829;Enzyme_code=EC:4.2.1.3;Enzyme_name=aconitate hydratase
contig00001.g499	1572	ID=contig00001.g499;Description=hypothetical protein FVEG_02927 [Fusarium verticillioides 7600]
contig00001.g500	3302	ID=contig00001.g500;Description=SCY1 protein kinase [Fusarium verticillioides 7600];Gene=FMAN_07429;Ontology_term=catalytic activity, acting on a protein,transferase activity;Ontology_id=GO:0140096,GO:0016740;Enzyme_code=EC:2.7.1;Enzyme_name=Transferring phosphorus-containing groups
contig00001.g501	831	ID=contig00001.g501;Description=L-2,4-diaminobutyrate decarboxylase [Fusarium denticulatum];Gene=FDENT_10437;Ontology_term=lyase activity;Ontology_id=GO:0016829;Enzyme_code=EC:4.1.1;Enzyme_name=Carbon-carbon lyases
contig00001.g502	1616	ID=contig00001.g502;Description=l diaminobutyrate decarboxylase [Fusarium tjaetaba];Gene=FVER53590_02923;Ontology_term=lyase activity;Ontology_id=GO:0016829;Enzyme_code=EC:4.1.1,EC:4.1.1,EC:4.1,EC:4;Enzyme_name=Carbon-carbon lyases,Carbon-carbon lyases,Carbon-carbon lyases,Lyases
contig00001.g503	306	ID=contig00001.g503;Description=probable cytochrome b5 [Fusarium proliferatum ET1];Gene=Forpe1208_v007823;Ontology_term=metal ion binding,heme binding;Ontology_id=GO:0046872,GO:0020037

contig00001.g504	1222	ID=contig00001.g504;Description=mitochondrial metalloendopeptidase OMA1 [Fusarium tjaetaba];Gene=FPANT_5905;Ontology_term=catalytic activity, acting on a protein,protein catabolic process,signaling,mitochondrion,mitochondrion organization,hydrolase activity;Ontology_id=GO:0140096,GO:0030163,GO:0023052,GO:0005739,GO:0007005,GO:0016787;Enzyme_code=EC:3.4.24,EC:3.4.24,EC:3.4,EC:3;Enzyme_name=Acting on peptide bonds (peptidases),Acting on peptide bonds (peptidases),Acting on peptide bonds (peptidases),Hydrolases
contig00001.g505	672	ID=contig00001.g505;Description=DUF985 domain protein [Fusarium tjaetaba];Ontology_term=nucleus;Ontology_id=GO:0005634
contig00001.g506	1311	ID=contig00001.g506;Description=methyltransferase [Fusarium tjaetaba];Gene=FDENT_10433;Ontology_term=transferase activity;Ontology_id=GO:0016740;Enzyme_code=EC:2.1.1,EC:2.1,EC:2,EC:2.1.1;Enzyme_name=Transferring one-carbon groups,Transferring one-carbon groups,Transferases,Transferring one-carbon groups
contig00001.g507	5359	ID=contig00001.g507;Description=kinesin family member 1/13/14 [Fusarium verticillioides 7600];Gene=FNAPI_4666;Ontology_term=cytoskeleton,ATP-dependent activity,cytoskeletal protein binding,microtubule-based movement,cytoskeletal motor activity;Ontology_id=GO:0005856,GO:0140657,GO:0008092,GO:0007018,GO:0003774
contig00001.g508	646	ID=contig00001.g508;Description=---NA---
contig00001.g509	2024	ID=contig00001.g509;Description=leukotriene A-4 hydrolase [Fusarium verticillioides 7600];Gene=FMUND_6669;Ontology_term=nucleus,catalytic activity, acting on a protein,endosome,protein catabolic process,lipid metabolic process,intracellular protein transport,vacuole,hydrolase activity;Ontology_id=GO:0005634,GO:0140096,GO:0005768,GO:0030163,GO:0006629,GO:0006886,GO:0005773,GO:0016787;Enzyme_code=EC:3.3.2.10,EC:3.4,EC:3.3.2,EC:3.4.11,EC:3.3.2.6,EC:3.3.2.10,EC:3.4,EC:3.3,EC:3;Enzyme_name=soluble epoxide hydrolase,Acting on peptide bonds (peptidases),Acting on ether bonds,Acting on peptide bonds (peptidases),leukotriene-A4 hydrolase,soluble epoxide hydrolase,Acting on peptide bonds (peptidases),Acting on ether bonds,Hydrolases
contig00001.g510	2155	ID=contig00001.g510;Description=hypothetical protein FVEG_02915 [Fusarium verticillioides 7600]
contig00001.g511	3273	ID=contig00001.g511;Description=PX domain protein [Fusarium tjaetaba];Gene=FTJAE_6571;Ontology_term=lipid binding;Ontology_id=GO:0008289

contig00001.g512	555	ID=contig00001.g512;Description=transcriptional Coactivator p15 family [Fusarium pseudocircinatum];Gene=FVER53590_02913;Ontology_term=catalytic activity, acting on a protein,protein-containing complex assembly,anatomical structure development,regulation of DNA-templated transcription,cell adhesion,cell motility,nucleus,DNA binding,signaling,transferase activity,transcription regulator activity,molecular adaptor activity,hydrolase activity;Ontology_id=GO:0140096,GO:0065003,GO:0048856,GO:0006355,GO:0007155,GO:0048870,GO:0005634,GO:0003677,GO:0023052,GO:0016740,GO:0140110,GO:0060090,GO:0016787;Enzyme_code=EC:3.1,EC:2.7.11.13,EC:2.7.1,EC:3.1,EC:2.7.11.13,EC:2.7.11.1,EC:2.7,EC:2,EC:3;Enzyme_name=Acting on ester bonds,protein kinase C,Transferring phosphorus-containing groups,Acting on ester bonds,protein kinase C,non-specific serine/threonine protein kinase,Transferring phosphorus-containing groups,Transferases,Hydrolases
contig00001.g513	1798	ID=contig00001.g513;Description=MFS siderochrome iron transporter 1 [Fusarium oxysporum f. sp. rapae];Gene=HZS61_003808;Ontology_term=transmembrane transport,transporter activity;Ontology_id=GO:0055085,GO:0005215
contig00001.g514	1130	ID=contig00001.g514;Description=chaperone DNAJ [Fusarium coicis];Gene=FVEG_02910;Ontology_term=membrane;Ontology_id=GO:0016020
contig00001.g515	973	ID=contig00001.g515;Description=plc-like phosphodiesterase TIM beta alpha-barrel domain protein [Fusarium tjaetaba];Gene=FDENT_10425;Ontology_term=lipid metabolic process,hydrolase activity;Ontology_id=GO:0006629,GO:0016787;Enzyme_code=EC:3.1.4,EC:3.1.4,EC:3.1,EC:3;Enzyme_name=Acting on ester bonds,Acting on ester bonds,Acting on ester bonds,Hydrolases
contig00001.g516	1580	ID=contig00001.g516;Description=cellobiose dehydrogenase [Fusarium tjaetaba];Gene=FACUT_3462;Ontology_term=membrane;Ontology_id=GO:0016020
contig00001.g517	744	ID=contig00001.g517;Description=ureidoglycolate hydrolase [Fusarium verticillioides 7600];Gene=FNAPI_4675;Ontology_term=nucleobase-containing small molecule metabolic process,lyase activity,hydrolase activity;Ontology_id=GO:0055086,GO:0016829,GO:0016787;Enzyme_code=EC:3.5.1.116,EC:4.3.2.3,EC:3.5.1.116,EC:3.5.1,EC:4.3,EC:4.3.2.3,EC:3.5,EC:3,EC:4,EC:4.3.2;Enzyme_name=ureidoglycolate amidohydrolase,ureidoglycolate lyase,ureidoglycolate amidohydrolase,Acting on carbon-nitrogen bonds, other than peptide bonds,Carbon-nitrogen lyases,ureidoglycolate lyase,Acting on carbon-nitrogen bonds, other than peptide bonds,Hydrolases,Lyases,Carbon-nitrogen lyases

contig00001.g518	1073	ID=contig00001.g518;Description=ureidoglycolate hydrolase [Fusarium tjaetaba];Gene=PPP1CA;Ontology_term=carbohydrate metabolic process,generation of precursor metabolites and energy,catalytic activity, acting on a protein,molecular function regulator activity,signaling,lipid binding,nucleolus,nucleoplasm,hydrolase activity;Ontology_id=GO:0005975,GO:0006091,GO:0140096,GO:0098772,GO:0023052,GO:0008289,GO:0005730,GO:0005654,GO:0016787;Enzyme_code=EC:3.1.3.16,EC:3.1.3.16,EC:3.1,EC:3,EC:3.1.3;Enzyme_name=protein-serine/threonine phosphatase,protein-serine/threonine phosphatase,Acting on ester bonds,Hydrolases,Acting on ester bonds
contig00001.g519	733	ID=contig00001.g519;Description=probable RPL42B-ribosomal protein L36a.e [Fusarium fujikuroi];Gene=FANTH_7034;Ontology_term=structural molecule activity,ribosome;Ontology_id=GO:0005198,GO:0005840
contig00001.g520	1047	ID=contig00001.g520;Description=coatomer epsilon subunit [Fusarium tjaetaba];Gene=FPANT_2381;Ontology_term=vesicle-mediated transport,structural molecule activity,cytoplasmic vesicle,Golgi apparatus,intracellular protein transport;Ontology_id=GO:0016192,GO:0005198,GO:0031410,GO:0005794,GO:0006886
contig00001.g521	507	ID=contig00001.g521;Description=multiprotein-bridging factor 1 [Fusarium verticillioides 7600];Gene=FOMG_06608;Ontology_term=DNA binding;Ontology_id=GO:0003677
contig00001.g522	527	ID=contig00001.g522;Description=Mss4-like protein [Fusarium redolens];Gene=FOC1_g10012664
contig00001.g523	1339	ID=contig00001.g523;Description=gpi anchored protein [Fusarium tjaetaba]
contig00001.g524	2073	ID=contig00001.g524;Description=PET56-rRNA (guanosine-2'-O-)-methyltransferase [Fusarium tjaetaba];Gene=FDENT_10415;Ontology_term=RNA binding,mitochondrial gene expression,mitochondrion,ribosome biogenesis,transferase activity,catalytic activity, acting on RNA;Ontology_id=GO:0003723,GO:0140053,GO:0005739,GO:0042254,GO:0016740,GO:0140098;Enzyme_code=EC:2.1.1,EC:2.1.1.185,EC:2.1,EC:2,EC:2.1.1;Enzyme_name=Transferring one-carbon groups,23S rRNA (guanosine(2251)-2'-O)-methyltransferase,Transferring one-carbon groups,Transferases,Transferring one-carbon groups

contig00001.g525	5335	ID=contig00001.g525;Description=CHD1-transcriptional regulator [Fusarium tjaetaba];Gene=FDENT_10414;Ontology_term=catalytic activity, acting on a protein,catalytic activity, acting on DNA,ATP-dependent activity,nuclear chromosome,regulation of DNA-templated transcription,transferase activity,chromatin organization,hydrolase activity,nucleoplasm;Ontology_id=GO:0140096,GO:0140097,GO:0140657,GO:0000228,GO:0006355,GO:0016740,GO:0006325,GO:0016787,GO:0005654;Enzyme_code=EC:3,EC:2.3,EC:3.6.1,EC:2,EC:3.6,EC:3,EC:3.6.1.15,EC:2.3.2;Enzyme_name=Hydrolases,Acyltransferases,Acting on acid anhydrides,Transferases,Acting on acid anhydrides,Hydrolases,nucleoside-triphosphate phosphatase,Acyltransferases
contig00001.g526	1178	ID=contig00001.g526;Description=CHD1-transcriptional regulator [Fusarium napiforme];Gene=FNYG_05861;Ontology_term=nucleus,catalytic activity, acting on DNA,ATP-dependent activity,chromatin organization,hydrolase activity;Ontology_id=GO:0005634,GO:0140097,GO:0140657,GO:0006325,GO:0016787;Enzyme_code=EC:3,EC:3;Enzyme_name=Hydrolases,Hydrolases
contig00001.g527	1569	ID=contig00001.g527;Description=signalosome complex subunit 3 COP9 [Fusarium tjaetaba];Ontology_term=nucleus,protein catabolic process,anatomical structure development,reproductive process;Ontology_id=GO:0005634,GO:0030163,GO:0048856,GO:0022414
contig00001.g528	877	ID=contig00001.g528;Description=SUR7 [Fusarium tjaetaba];Gene=FNAPI_4686;Ontology_term=nucleobase-containing small molecule metabolic process,lipid metabolic process,transferase activity,plasma membrane,hydrolase activity;Ontology_id=GO:0055086,GO:0006629,GO:0016740,GO:0005886,GO:0016787;Enzyme_code=EC:3.1.1.32,EC:3.1.1.23,EC:3.1.1.5,EC:2.7.7,EC:3.1.1.4,EC:3.1,EC:3.1.1.32,EC:2.7,EC:2,EC:3.1.1.23,EC:3,EC:3.1.1.5,EC:2.7.7,EC:3.1.1,EC:3.1.1.4,EC:3.1.1.3;Enzyme_name=phospholipase A1,acylglycerol lipase,lysophospholipase,Transferring phosphorus-containing groups,phospholipase A2,Acting on ester bonds,phospholipase A1,Transferring phosphorus-containing groups,Transferases,acylglycerol lipase,Hydrolases,lysophospholipase,Transferring phosphorus-containing groups,Acting on ester bonds,phospholipase A2,triacylglycerol lipase

contig00001.g529	1335	ID=contig00001.g529;Description=BSC1 Transcript encoded by this ORF shows a high level of stop codon bypass [Fusarium acutatum];Gene=FPANT_2371;Ontology_term=lipid metabolic process,hydrolase activity;Ontology_id=GO:0006629,GO:0016787;Enzyme_code=EC:3.1.4,EC:3.1.4,EC:3.1,EC:3;Enzyme_name=Acting on ester bonds,Acting on ester bonds,Acting on ester bonds,Hydrolases
contig00001.g530	2604	ID=contig00001.g530;Description=hypothetical protein FVER14953_02893 [Fusarium verticillioides];Gene=FPANT_2370;Ontology_term=membrane;Ontology_id=GO:0016020
contig00001.g531	2324	ID=contig00001.g531;Description=calcium spray [Fusarium tjaetaba];Gene=FPHYL_3150;Ontology_term=transmembrane transport,vesicle-mediated transport,mitochondrion,lipid metabolic process,endoplasmic reticulum,cell wall organization or biogenesis,protein folding,vacuole,transporter activity,plasma membrane;Ontology_id=GO:0055085,GO:0016192,GO:0005739,GO:0006629,GO:0005783,GO:0071554,GO:0006457,GO:0005773,GO:0005215,GO:0005886
contig00001.g532	1848	ID=contig00001.g532;Description=WetA-like protein [Fusarium tjaetaba];Ontology_term=carbohydrate metabolic process,anatomical structure development,cell differentiation,reproductive process,regulation of DNA-templated transcription,cell wall organization or biogenesis;Ontology_id=GO:0005975,GO:0048856,GO:0030154,GO:0022414,GO:0006355,GO:0071554
contig00001.g533	1557	ID=contig00001.g533;Description=DUF124 domain protein [Fusarium tjaetaba];Gene=FVER53590_02889;Ontology_term=mitochondrion;Ontology_id=GO:0005739
contig00001.g534	1875	ID=contig00001.g534;Description=hypothetical protein FVEG_02888 [Fusarium verticillioides 7600]
contig00001.g535	1716	ID=contig00001.g535;Description=PR domain protein [Fusarium tjaetaba];Gene=FMEXI_10857;Ontology_term=regulation of DNA-templated transcription,transcription regulator activity;Ontology_id=GO:0006355,GO:0140110
contig00001.g536	2538	ID=contig00001.g536;Description=Sex-determining transformer 1 [Fusarium tjaetaba]
contig00001.g537	597	ID=contig00001.g537;Description=DAP1 Damage response [Fusarium pseudoanthophilum]
contig00001.g538	1616	ID=contig00001.g538;Description=hypothetical protein J7337_005790 [Fusarium musae];Gene=FOBC_08450;Ontology_term=membrane;Ontology_id=GO:0016020

contig00001.g539	1664	ID=contig00001.g539;Description=DNA repair and recombination protein RAD52 [Fusarium verticillioides 7600];Gene=ATG22;Ontology_term=DNA repair,catalytic activity, acting on DNA,transmembrane transport,protein-containing complex assembly,DNA replication,DNA recombination,nuclear chromosome,vacuole,mitotic cell cycle,nucleoplasm,DNA binding,autophagy,cell differentiation,telomere organization,meiotic nuclear division;Ontology_id=GO:0006281,GO:0140097,GO:0055085,GO:0065003,GO:0006260,GO:0006310,GO:0000228,GO:0005773,GO:0000278,GO:0005654,GO:0003677,GO:0006914,GO:0030154,GO:0032200,GO:0140013
contig00001.g540	1003	ID=contig00001.g540;Description=Tethering factor for nuclear proteasome sts1 [Fusarium musae];Gene=FPCIR_11749;Ontology_term=cytoskeleton organization,protein-containing complex assembly,protein catabolic process,nuclear chromosome,chromosome segregation,lipid binding,chromatin organization,nuclear envelope,mitotic nuclear division,molecular adaptor activity;Ontology_id=GO:0007010,GO:0065003,GO:0030163,GO:0000228,GO:0007059,GO:0008289,GO:0006325,GO:0005635,GO:0140014,GO:0060090
contig00001.g541	1269	ID=contig00001.g541;Description=splicing factor [Fusarium tjaetaba];Gene=FPCIR_11748;Ontology_term=RNA binding,ribosome biogenesis,nucleolus;Ontology_id=GO:0003723,GO:0042254,GO:0005730
contig00001.g542	799	ID=contig00001.g542;Description=uncharacterized protein FOIG_07276 [Fusarium odoratissimum NRRL 54006];Gene=FOIG_07276;Ontology_term=oxidoreductase activity;Ontology_id=GO:0016491;Enzyme_code=EC:1;Enzyme_name=Oxidoreductases
contig00001.g543	1980	ID=contig00001.g543;Description=hypothetical protein FVEG_02879 [Fusarium verticillioides 7600]
contig00001.g544	1896	ID=contig00001.g544;Description=AIR2 [Fusarium tjaetaba];Gene=FNAPI_4842;Ontology_term=nucleic acid binding,zinc ion binding;Ontology_id=GO:0003676,GO:0008270
contig00001.g545	766	ID=contig00001.g545;Description=ORMDL family-domain-containing protein [Fusarium oxysporum];Gene=FOMG_06638;Ontology_term=endoplasmic reticulum;Ontology_id=GO:0005783

contig00001.g546	1455	ID=contig00001.g546;Description=related to cyclin-dependent kinase inhibitor 1c [Fusarium fujikuroi];Gene=MXR2;Ontology_term=oxidoreductase activity,transferase activity;Ontology_id=GO:0016491,GO:0016740;Enzyme_code=EC:2.7,EC:1.8.4.12,EC:1,EC:2.7,EC:1.8,EC:1.8.4,EC:2,EC:1.8.4.12;Enzyme_name=Transferring phosphorus-containing groups,peptide-methionine (R)-S-oxide reductase,Oxidoreductases,Transferring phosphorus-containing groups,Acting on a sulfur group of donors,Acting on a sulfur group of donors,Transferases,peptide-methionine (R)-S-oxide reductase
contig00001.g547	870	ID=contig00001.g547;Description=S-adenosyl-L-methionine-dependent methyltransferase [Fusarium sp. MPI-SDFR-AT-0072];Gene=FVER53263_02875;Ontology_term=transferase activity;Ontology_id=GO:0016740;Enzyme_code=EC:2.1.1,EC:2.1,EC:2,EC:2.1.1;Enzyme_name=Transferring one-carbon groups,Transferring one-carbon groups,Transferases,Transferring one-carbon groups
contig00001.g548	710	ID=contig00001.g548;Description=palI [Fusarium subglutinans];Gene=FTJAE_11761;Ontology_term=plasma membrane;Ontology_id=GO:0005886
contig00001.g549	837	ID=contig00001.g549;Description=hypothetical protein FVEG_02873 [Fusarium verticillioides 7600]
contig00001.g550	2075	ID=contig00001.g550;Description=mediator subunit med1 [Fusarium tjaetaba];Gene=FNYG_09423;Ontology_term=regulation of DNA-templated transcription,transcription regulator activity,molecular adaptor activity,nucleoplasm;Ontology_id=GO:0006355,GO:0140110,GO:0060090,GO:0005654
contig00001.g551	1277	ID=contig00001.g551;Description=methionyl-tRNA formyltransferase [Fusarium verticillioides 7600];Gene=FMUND_12307;Ontology_term=mitochondrial gene expression,mitochondrion,tRNA metabolic process,catalytic activity, acting on RNA,transferase activity;Ontology_id=GO:0140053,GO:0005739,GO:0006399,GO:0140098,GO:0016740;Enzyme_code=EC:2.1.2.9,EC:2.1,EC:2,EC:2.1.2,EC:2.1.2.9;Enzyme_name=methionyl-tRNA formyltransferase,Transferring one-carbon groups,Transferases,Transferring one-carbon groups,methionyl-tRNA formyltransferase

contig00001.g552	847	ID=contig00001.g552;Description=carboxypeptidase D [Fusarium verticillioides 7600];Gene=FPCIR_11737;Ontology_term=catalytic activity, acting on a protein,transferase activity,hydrolase activity;Ontology_id=GO:0140096,GO:0016740,GO:0016787;Enzyme_code=EC:3.4,EC:2.1.1,EC:2.1,EC:3.4,EC:2,EC:3,EC:2.1.1.321,EC:2.1.1;Enzyme_name=Acting on peptide bonds (peptidases),Transferring one-carbon groups,Transferring one-carbon groups,Acting on peptide bonds (peptidases),Transferases,Hydrolases,type III protein arginine methyltransferase,Transferring one-carbon groups
contig00001.g553	1968	ID=contig00001.g553;Description=Cell death protease [Fusarium musae];Gene=FNAPI_4851;Ontology_term=catalytic activity, acting on a protein,protein catabolic process,anatomical structure development,cell differentiation,Golgi apparatus,protein maturation,programmed cell death,vacuole,hydrolase activity;Ontology_id=GO:0140096,GO:0030163,GO:0048856,GO:0030154,GO:0005794,GO:0051604,GO:0012501,GO:0005773,GO:0016787;Enzyme_code=EC:3.4.16,EC:3.4,EC:3.4.16,EC:3,EC:3.4.16.6;Enzyme_name=Acting on peptide bonds (peptidases),Acting on peptide bonds (peptidases),Acting on peptide bonds (peptidases),Hydrolases,carboxypeptidase D
contig00001.g554	1051	ID=contig00001.g554;Description=Fe-S cluster assembly DRE2 [Fusarium subglutinans];Gene=BFJ63_vAg12851;Ontology_term=generation of precursor metabolites and energy,sulfur compound metabolic process,oxidoreductase activity,mitochondrion,cytosol,programmed cell death;Ontology_id=GO:0006091,GO:0006790,GO:0016491,GO:0005739,GO:0005829,GO:0012501;Enzyme_code=EC:1;Enzyme_name=Oxidoreductases
contig00001.g555	1703	ID=contig00001.g555;Description=hexamer-binding HEXBP [Fusarium tjaetaba];Gene=FPANT_1391;Ontology_term=nucleic acid binding,zinc ion binding;Ontology_id=GO:0003676,GO:0008270
contig00001.g556	1860	ID=contig00001.g556;Description=transcription factor atf1+ [Fusarium coicis];Gene=FOZG_11040;Ontology_term=DNA recombination,regulation of DNA-templated transcription,nuclear chromosome,chromatin organization,mitotic cell cycle,DNA binding,RNA binding,signaling,cytosol,cell wall organization or biogenesis,transcription regulator activity,meiotic nuclear division;Ontology_id=GO:0006310,GO:0006355,GO:0000228,GO:0006325,GO:0000278,GO:0003677,GO:0003723,GO:0023052,GO:0005829,GO:0071554,GO:0140110,GO:0140013

contig00001.g557	1676	ID=contig00001.g557;Description=kinetochore mis13 [Fusarium tjaetaba];Gene=FTJAE_11752;Ontology_term=cytoskeleton,mitotic nuclear division,chromosome segregation,nuclear chromosome,meiotic nuclear division;Ontology_id=GO:0005856,GO:0140014,GO:0007059,GO:0000228,GO:0140013
contig00001.g558	709	ID=contig00001.g558;Description=tryptophanyl-tRNA synthetase [Fusarium denticulatum];Gene=FACUT_3419;Ontology_term=generation of precursor metabolites and energy,nucleobase-containing small molecule metabolic process,protein-containing complex assembly,anatomical structure development,reproductive process,regulation of DNA-templated transcription,tRNA metabolic process,amino acid metabolic process,carbohydrate derivative metabolic process,mitochondrion organization,mitochondrion,cell differentiation,catalytic activity, acting on RNA,ligase activity;Ontology_id=GO:0006091,GO:0055086,GO:0065003,GO:0048856,GO:0022414,GO:0006355,GO:0006399,GO:0006520,GO:1901135,GO:0007005,GO:0005739,GO:0030154,GO:0140098,GO:0016874;Enzyme_code=EC:6.1.1.2,EC:6.1.1.11,EC:6.1.1.2,EC:6.1,EC:6.1.1.11,EC:6,EC:6.1.1;Enzyme_name=tryptophan--tRNA ligase,serine--tRNA ligase,tryptophan--tRNA ligase,Forming carbon-oxygen bonds,serine--tRNA ligase,Ligases,Forming carbon-oxygen bonds
contig00001.g559	1529	ID=contig00001.g559;Description=tryptophanyl-tRNA synthetase [Fusarium verticillioides 7600];Gene=FVER53263_02863;Ontology_term=tRNA metabolic process,amino acid metabolic process,catalytic activity, acting on RNA,ligase activity;Ontology_id=GO:0006399,GO:0006520,GO:0140098,GO:0016874;Enzyme_code=EC:6.1.1.2;Enzyme_name=tryptophan--tRNA ligase
contig00001.g560	803	ID=contig00001.g560;Description=endoplasmic reticulum vesicle protein 25 [Fusarium proliferatum];Gene=F25303_7679;Ontology_term=vesicle-mediated transport,cytoplasmic vesicle;Ontology_id=GO:0016192,GO:0031410
contig00001.g561	882	ID=contig00001.g561;Description=U1 small nuclear ribonucleoprotein C [Fusarium tjaetaba];Gene=MAC_03840;Ontology_term=nucleus,mRNA metabolic process,RNA binding,protein-containing complex assembly;Ontology_id=GO:0005634,GO:0016071,GO:0003723,GO:0065003
contig00001.g562	3054	ID=contig00001.g562;Description=hypothetical protein FVER53263_02860 [Fusarium verticillioides]
contig00001.g563	859	ID=contig00001.g563;Description=beta endoglucanase [Fusarium coicis]
contig00001.g564	528	ID=contig00001.g564;Description=hypothetical protein FVEG_02858 [Fusarium verticillioides 7600]

contig00001.g565	624	ID=contig00001.g565;Description=ypgQ [Fusarium tjaetaba];Gene=FMAN_07496;Ontology_term=hydrolase activity;Ontology_id=GO:0016787;Enzyme_code=EC:3,EC:3;Enzyme_name=Hydrolases,Hydrolases
contig00001.g566	969	ID=contig00001.g566;Description=SRP40-like suppressor protein [Fusarium heterosporum]
contig00001.g567	1091	ID=contig00001.g567;Description=amidohydrolase family [Fusarium tjaetaba];Gene=FVEG_02855;Ontology_term=carbohydrate metabolic process,hydrolase activity;Ontology_id=GO:0005975,GO:0016787;Enzyme_code=EC:3,EC:3.1.1.65,EC:3;Enzyme_name=Hydrolases,L-rhamnono-1,4-lactonase,Hydrolases
contig00001.g568	2130	ID=contig00001.g568;Description=kinetoplast-associated KAP [Fusarium tjaetaba];Gene=FTJAE_11741;Ontology_term=cytoskeleton organization,protein-containing complex assembly,establishment or maintenance of cell polarity,anatomical structure development,chromosome segregation,nuclear chromosome,microtubule-based movement,intracellular protein transport,microtubule organizing center,mitotic nuclear division,cytoskeletal protein binding;Ontology_id=GO:0007010,GO:0065003,GO:0007163,GO:0048856,GO:0007059,GO:0000228,GO:0007018,GO:0006886,GO:0005815,GO:0140014,GO:0008092
contig00001.g569	1740	ID=contig00001.g569;Description=cell pattern formation-associated stuA [Fusarium tjaetaba];Gene=FTJAE_11740;Ontology_term=nucleus,DNA binding,anatomical structure development,cell differentiation,reproductive process,regulation of DNA-templated transcription,transcription regulator activity;Ontology_id=GO:0005634,GO:0003677,GO:0048856,GO:0030154,GO:0022414,GO:0006355,GO:0140110
contig00001.g570	854	ID=contig00001.g570;Description=DNA repair REV1 [Fusarium tjaetaba]
contig00001.g571	2208	ID=contig00001.g571;Description=propionyl-CoA synthetase [Fusarium verticillioides 7600];Enzyme_code=EC:6.2.1.17;Enzyme_name=propionate--CoA ligase
contig00001.g572	509	ID=contig00001.g572;Description=putative clock-controlled protein 6 (CCG-6) [Fusarium fujikuroi];Gene=B4FUQ3;Ontology_term=structural molecule activity,cell wall organization or biogenesis;Ontology_id=GO:0005198,GO:0071554
contig00001.g573	1061	ID=contig00001.g573;Description=putative NADPH quinone oxidoreductase PIG3 [Fusarium fujikuroi];Gene=FVEG_02847;Ontology_term=oxidoreductase activity;Ontology_id=GO:0016491;Enzyme_code=EC:1;Enzyme_name=Oxidoreductases

contig00001.g574	1112	ID=contig00001.g574;Description=dienelactone hydrolase [Fusarium tjaetaba];Gene=FVEG_02846;Ontology_term=hydrolase activity;Ontology_id=GO:0016787;Enzyme_code=EC:3,EC:3;Enzyme_name=Hydrolases,Hydrolases
contig00001.g575	1979	ID=contig00001.g575;Description=replication factor A1 [Fusarium verticillioides 7600];Gene=FDENT_8991;Ontology_term=nucleus,DNA binding,DNA repair,DNA replication,DNA recombination;Ontology_id=GO:0005634,GO:0003677,GO:0006281,GO:0006260,GO:0006310
contig00001.g576	1455	ID=contig00001.g576;Description=glutamate-5-semialdehyde dehydrogenase [Fusarium verticillioides 7600];Gene=FVEG_02844;Ontology_term=nucleus,oxidoreductase activity,mitochondrion,amino acid metabolic process;Ontology_id=GO:0005634,GO:0016491,GO:0005739,GO:0006520;Enzyme_code=EC:1.2.1.41,EC:1.2,EC:1,EC:1.2.1.41,EC:1.2.1;Enzyme_name=glutamate-5-semialdehyde dehydrogenase,Acting on the aldehyde or oxo group of donors,Oxidoreductases,glutamate-5-semialdehyde dehydrogenase,Acting on the aldehyde or oxo group of donors
contig00001.g577	1781	ID=contig00001.g577;Description=quinidine resistance 3 [Fusarium pseudocircinatum];Gene=FPCIR_6956;Ontology_term=transmembrane transport,anatomical structure development,cell differentiation,reproductive process,cell wall organization or biogenesis,transporter activity,plasma membrane;Ontology_id=GO:0055085,GO:0048856,GO:0030154,GO:0022414,GO:0071554,GO:0005215,GO:0005886
contig00001.g578	1273	ID=contig00001.g578;Description=threonine dehydratase [Fusarium verticillioides 7600];Ontology_term=lyase activity,amino acid metabolic process,isomerase activity;Ontology_id=GO:0016829,GO:0006520,GO:0016853;Enzyme_code=EC:5.1.1.18,EC:5.1.1,EC:4.3,EC:4.3.1.17,EC:4.3.1.18,EC:5.1,EC:4.3.1.19,EC:4,EC:5,EC:5.1.1.10,EC:4.3.1;Enzyme_name=serine racemase,Racemases and epimerases,Carbon-nitrogen lyases,L-serine ammonia-lyase,D-serine ammonia-lyase,Racemases and epimerases,threonine ammonia-lyase,Lyases,Isomerases,amino-acid racemase,Carbon-nitrogen lyases
contig00001.g579	1346	ID=contig00001.g579;Description=Bifunctional acetohydroxyacid reductoisomerase [Loxospora ochrophaea];Gene=VSDG_04266;Ontology_term=oxidoreductase activity,mitochondrion,amino acid metabolic process;Ontology_id=GO:0016491,GO:0005739,GO:0006520;Enzyme_code=EC:1.1.1.86;Enzyme_name=ketol-acid reductoisomerase (NADP(+))

contig00001.g580	777	ID=contig00001.g580;Description=chitin synthase [Fusarium subglutinans];Gene=FMEXI_10901;Ontology_term=catalytic activity, acting on a protein,anatomical structure development,cell differentiation,carbohydrate derivative metabolic process,transferase activity,plasma membrane;Ontology_id=GO:0140096,GO:0048856,GO:0030154,GO:1901135,GO:0016740,GO:0005886;Enzyme_code=EC:2.4.1.16,EC:2.3,EC:2.4,EC:2.4.1.16,EC:2,EC:2.3.2.34,EC:2.3.2,EC:2.4.1;Enzyme_name=chitin synthase,Acyltransferases,Glycosyltransferases,chitin synthase,Transferases,E2 NEDD8-conjugating enzyme,Acyltransferases,Glycosyltransferases
contig00001.g581	2822	ID=contig00001.g581;Description=chitin synthase 1 [Fusarium verticillioides 7600];Gene=FGADI_12027;Ontology_term=anatomical structure development,cell differentiation,carbohydrate derivative metabolic process,cell wall organization or biogenesis,transferase activity,plasma membrane;Ontology_id=GO:0048856,GO:0030154,GO:1901135,GO:0071554,GO:0016740,GO:0005886;Enzyme_code=EC:2.4.1.16;Enzyme_name=chitin synthase
contig00001.g582	1354	ID=contig00001.g582;Description=OPY2 protein [Fusarium fujikuroi];Gene=C2S_1318;Ontology_term=nucleus,RNA binding,mRNA metabolic process;Ontology_id=GO:0005634,GO:0003723,GO:0016071
contig00001.g583	773	ID=contig00001.g583;Description=protein Ras-2 [Fusarium verticillioides 7600];Gene=ras-2;Ontology_term=GTPase activity,signaling,plasma membrane;Ontology_id=GO:0003924,GO:0023052,GO:0005886;Enzyme_code=EC:3.6.1.15;Enzyme_name=nucleoside-triphosphate phosphatase
contig00001.g584	804	ID=contig00001.g584;Description=haloacid dehalogenase type II [Fusarium tjaetaba];Gene=FPRO_07908;Ontology_term=hydrolase activity;Ontology_id=GO:0016787;Enzyme_code=EC:3.8.1,EC:3.1.3,EC:3.1,EC:3.8.1,EC:3,EC:3.8.1.2,EC:3.8,EC:3.1.3;Enzyme_name=Acting on halide bonds,Acting on ester bonds,Acting on ester bonds,Acting on halide bonds,Hydrolases,(S)-2-haloacid dehalogenase,Acting on halide bonds,Acting on ester bonds
contig00001.g585	4173	ID=contig00001.g585;Description=Anucleate primary sterigmata A [Fusarium tjaetaba];Gene=FVER53590_02834;Ontology_term=lipid binding;Ontology_id=GO:0008289
contig00001.g586	912	ID=contig00001.g586;Description=OTU domain-containing protein [Fusarium coicis];Ontology_term=protein catabolic process;Ontology_id=GO:0030163;Enzyme_code=EC:3.4.19.12;Enzyme_name=ubiquitinyl hydrolase 1

contig00001.g587	925	ID=contig00001.g587;Description=Thiocyanate methyltransferase 1 [Fusarium culmorum];Gene=HYE67_010890;Ontology_term=transferase activity;Ontology_id=GO:0016740;Enzyme_code=EC:2.1.1,EC:2.1,EC:2.1.1.165,EC:2,EC:2.1.1;Enzyme_name=Transferring one-carbon groups,Transferring one-carbon groups,methyl halide transferase,Transferases,Transferring one-carbon groups
contig00001.g588	1740	ID=contig00001.g588;Description=hypothetical protein FVEG_02830 [Fusarium verticillioides 7600]
contig00001.g589	201	ID=contig00001.g589;Description=thiol methyltransferase [Fusarium subglutinans];Gene=FNAPI_2586;Ontology_term=transferase activity;Ontology_id=GO:0016740;Enzyme_code=EC:2.1.1,EC:2.1,EC:2,EC:2.1.1;Enzyme_name=Transferring one-carbon groups,Transferring one-carbon groups,Transferases,Transferring one-carbon groups
contig00001.g590	980	ID=contig00001.g590;Description=mitochondrial outer membrane (Sam35) [Fusarium tjaetaba];Ontology_term=membrane organization,mitochondrion,intracellular protein transport,mitochondrion organization;Ontology_id=GO:0061024,GO:0005739,GO:0006886,GO:0007005
contig00001.g591	2761	ID=contig00001.g591;Description=RANBPM-like protein [Fusarium tjaetaba];Gene=FNAPI_2584;Ontology_term=nucleus,carbohydrate metabolic process,endosome,protein catabolic process,vacuole;Ontology_id=GO:0005634,GO:0005975,GO:0005768,GO:0030163,GO:0005773
contig00001.g592	881	ID=contig00001.g592;Description=INO80 complex, subunit Ies4 [Fusarium oxysporum];Gene=FOXB_10087;Ontology_term=DNA recombination,nuclear chromosome,regulation of DNA-templated transcription,telomere organization,chromatin organization;Ontology_id=GO:0006310,GO:0000228,GO:0006355,GO:0032200,GO:0006325
contig00001.g593	1961	ID=contig00001.g593;Description=hypothetical protein FVEG_02824 [Fusarium verticillioides 7600];Gene=FVEG_02824;Ontology_term=membrane;Ontology_id=GO:0016020
contig00001.g594	1274	ID=contig00001.g594;Description=hypothetical protein FNYG_09467 [Fusarium nygamai];Gene=FNYG_09467;Ontology_term=membrane;Ontology_id=GO:0016020

contig00001.g595	943	ID=contig00001.g595;Description=cell division control protein 14 [Fusarium fujikuroi];Gene=FPANT_12104;Ontology_term=carbohydrate metabolic process,nucleus,microtubule organizing center,cytoskeleton organization,signaling,mitotic nuclear division,cytokinesis,transferase activity;Ontology_id=GO:0005975,GO:0005634,GO:0005815,GO:0007010,GO:0023052,GO:0140014,GO:0000910,GO:0016740;Enzyme_code=EC:2.7.1.15,EC:2.7.1,EC:2.7.1.15,EC:2.7,EC:2;Enzyme_name=ribokinase,Transferring phosphorus-containing groups,ribokinase,Transferring phosphorus-containing groups,Transferases
contig00001.g596	2929	ID=contig00001.g596;Description=major facilitator superfamily transporter [Fusarium subglutinans];Gene=FACUT_3380;Ontology_term=transmembrane transport,transporter activity,hydrolase activity;Ontology_id=GO:0055085,GO:0005215,GO:0016787;Enzyme_code=EC:3.8.1,EC:3.1.3,EC:3.1,EC:3.8.1,EC:3,EC:3.8,EC:3.1.3;Enzyme_name=Acting on halide bonds,Acting on ester bonds,Acting on ester bonds,Acting on halide bonds,Hydrolases,Acting on halide bonds,Acting on ester bonds
contig00001.g597	1257	ID=contig00001.g597;Description=geranylgeranyl pyrophosphate synthase [Fusarium verticillioides 7600];Gene=FPHYL_10647;Ontology_term=lipid metabolic process,transferase activity;Ontology_id=GO:0006629,GO:0016740;Enzyme_code=EC:2.5.1.29,EC:2.5.1.10,EC:2.5.1,EC:2.5.1.29,EC:2,EC:2.5.1.10,EC:2.5.1.1;Enzyme_name=geranylgeranyl diphosphate synthase,(2E,6E)-farnesyl diphosphate synthase,Transferring alkyl or aryl groups, other than methyl groups,geranylgeranyl diphosphate synthase,Transferases,(2E,6E)-farnesyl diphosphate synthase,dimethylallyltranstransferase
contig00001.g598	1535	ID=contig00001.g598;Description=amino acid transporter [Fusarium tjaetaba];Gene=FPANT_8143;Ontology_term=peroxisome,peroxisome organization;Ontology_id=GO:0005777,GO:0007031

contig00001.g599	4107	ID=contig00001.g599;Description=CMGC/DYRK/DYRK2 protein kinase [Fusarium verticillioides 7600];Gene=FPHYL_10649;Ontology_term=catalytic activity, acting on a protein,cytoskeleton organization,establishment or maintenance of cell polarity,anatomical structure development,cytokinesis,reproductive process,lipid binding,mitotic cell cycle,plasma membrane,mitochondrion organization,cytoskeleton,signaling,mitochondrion,cell differentiation,transferase activity,protein localization to plasma membrane;Ontology_id=GO:0140096,GO:0007010,GO:0007163,GO:0048856,GO:0000910,GO:0022414,GO:0008289,GO:0000278,GO:0005886,GO:0007005,GO:0005856,GO:0023052,GO:0005739,GO:0030154,GO:0016740,GO:0072659;Enzyme_code=EC:2.7.1,EC:2.7.1,EC:2.7.12.1,EC:2.7.11.1,EC:2.7,EC:2;Enzyme_name=Transferring phosphorus-containing groups,Transferring phosphorus-containing groups,dual-specificity kinase,non-specific serine/threonine protein kinase,Transferring phosphorus-containing groups,Transferases
contig00001.g600	3045	ID=contig00001.g600;Description=related to Polyamine transport protein [Fusarium fujikuroi IMI 58289];Gene=F52700_152;Ontology_term=membrane;Ontology_id=GO:0016020;Enzyme_code=EC:3.5.1.3;Enzyme_name=omega-amidase
contig00001.g601	3097	ID=contig00001.g601;Description=DEF1-coordinates repair RNA pol II proteolysis in response to DNA damage [Fusarium coicis];Gene=FMUND_9061;Ontology_term=membrane;Ontology_id=GO:0016020
contig00001.g602	852	ID=contig00001.g602;Description=ESCRT-III subunit protein snf7 [Fusarium musae];Gene=FMUND_9060;Ontology_term=membrane organization,transmembrane transport,catalytic activity, acting on DNA,protein catabolic process,regulation of DNA-templated transcription,chromosome,intracellular protein transport,chromatin organization,plasma membrane,nucleus,DNA binding,vesicle-mediated transport,endosome,ATP-dependent activity,histone binding,signaling,cell wall organization or biogenesis,protein maturation,hydrolase activity;Ontology_id=GO:0061024,GO:0055085,GO:0140097,GO:0030163,GO:0006355,GO:0005694,GO:0006886,GO:0006325,GO:0005886,GO:0005634,GO:0003677,GO:0016192,GO:0005768,GO:0140657,GO:0042393,GO:0023052,GO:0071554,GO:0051604,GO:0016787;Enzyme_code=EC:3,EC:3;Enzyme_name=Hydrolases,Hydrolases
contig00001.g603	1426	ID=contig00001.g603;Description=mannan polymerase II complex ANP1 subunit [Fusarium verticillioides 7600]

contig00001.g604	1086	ID=contig00001.g604;Description=DNA-3-methyladenine glycosylase II [Fusarium verticillioides 7600];Gene=FTJAE_2075;Ontology_term=DNA repair,nucleus,DNA binding,catalytic activity, acting on DNA,hydrolase activity;Ontology_id=GO:0006281,GO:0005634,GO:0003677,GO:0140097,GO:0016787;Enzyme_code=EC:3.2,EC:3.2.2.21,EC:3.2.2.20,EC:3,EC:3.2.2;Enzyme_name=Glycosylases,DNA-3-methyladenine glycosylase II,DNA-3-methyladenine glycosylase I,Hydrolases,Glycosylases
contig00001.g605	1307	ID=contig00001.g605;Description=sporulation-specific SPS2 [Fusarium tjaetaba];Gene=FVER53590_02811;Ontology_term=membrane;Ontology_id=GO:0016020
contig00001.g606	1632	ID=contig00001.g606;Description=DNA-damage repair [Fusarium sp. NRRL 52700];Gene=FVEG_02810;Ontology_term=mRNA metabolic process,RNA binding;Ontology_id=GO:0016071,GO:0003723
contig00001.g607	1607	ID=contig00001.g607;Description=ATP-dependent RNA helicase DBP5 [Fusarium verticillioides 7600];Gene=BFJ69_g4072;Ontology_term=nuclear envelope,nucleocytoplasmic transport,ATP-dependent activity,cytoplasmic translation,intracellular protein transport,nucleolus,catalytic activity, acting on RNA,hydrolase activity;Ontology_id=GO:0005635,GO:0006913,GO:0140657,GO:0002181,GO:0006886,GO:0005730,GO:0140098,GO:0016787;Enzyme_code=EC:3.6.4.13,EC:3.6.1,EC:3.6.4.13,EC:3.6,EC:3,EC:3.6.1.15;Enzyme_name=RNA helicase,Acting on acid anhydrides,RNA helicase,Acting on acid anhydrides,Hydrolases,nucleoside-triphosphate phosphatase
contig00001.g608	12291	ID=contig00001.g608;Description=hypothetical protein FVER53590_02808 [Fusarium verticillioides]
contig00001.g609	1851	ID=contig00001.g609;Description=hypothetical protein FVER53590_02806 [Fusarium verticillioides]
contig00001.g610	716	ID=contig00001.g610;Description=50S ribosomal protein L22e [Fusarium verticillioides 7600];Gene=UOX;Ontology_term=oxidoreductase activity,organelle;Ontology_id=GO:0016491,GO:0043226;Enzyme_code=EC:1;Enzyme_name=Oxidoreductases
contig00001.g611	1084	ID=contig00001.g611;Description=carrier protein YMC1, mitochondrial [Fusarium fujikuroi];Gene=FPANT_8130;Ontology_term=transmembrane transport,mitochondrion;Ontology_id=GO:0055085,GO:0005739
contig00001.g612	5079	ID=contig00001.g612;Description=hypothetical protein FVEG_02803 [Fusarium verticillioides 7600]

contig00001.g613	414	ID=contig00001.g613;Description=pgaD domain containing protein [Fusarium agapanthi];Gene=FOVG_06196;Ontology_term=membrane;Ontology_id=GO:0016020
contig00001.g614	348	ID=contig00001.g614;Description=RNA polymerase II transcription factor B subunit 5 [Fusarium oxysporum f. sp. lycopersici 4287];Gene=FMEXI_14084;Ontology_term=DNA repair,DNA-templated transcription,nucleoplasm;Ontology_id=GO:0006281,GO:0006351,GO:0005654
contig00001.g615	922	ID=contig00001.g615;Description=delta3 5-delta2 4-dienoyl isomerase precursor (ech1) [Fusarium tjaetaba];Gene=FVEG_02800;Ontology_term=lipid metabolic process,isomerase activity;Ontology_id=GO:0006629,GO:0016853;Enzyme_code=EC:5,EC:5;Enzyme_name=Isomerases,Isomerases
contig00001.g616	1448	ID=contig00001.g616;Description=1 3-beta-glucanosyltransferase gel1 [Fusarium coicis];Gene=FGADI_9487;Ontology_term=carbohydrate metabolic process,cell wall,anatomical structure development,cell differentiation,reproductive process,cell wall organization or biogenesis,endoplasmic reticulum,transferase activity,extracellular region,plasma membrane;Ontology_id=GO:0005975,GO:0005618,GO:0048856,GO:0030154,GO:0022414,GO:0071554,GO:0005783,GO:0016740,GO:0005576,GO:0005886;Enzyme_code=EC:2,EC:2;Enzyme_name=Transferases,Transferases
contig00001.g617	3129	ID=contig00001.g617;Description=related to OSBP-related protein 7 [Fusarium fujikuroi];Gene=FVER53263_02798;Ontology_term=membrane organization,vesicle-mediated transport,autophagy,establishment or maintenance of cell polarity,reproductive process,cytosol,lipid binding,endoplasmic reticulum,plasma membrane,transporter activity;Ontology_id=GO:0061024,GO:0016192,GO:0006914,GO:0007163,GO:0022414,GO:0005829,GO:0008289,GO:0005783,GO:0005886,GO:0005215
contig00001.g618	2305	ID=contig00001.g618;Description=GTS1-transcription factor of the Gcs1p Glo3p Sps18p family [Fusarium tjaetaba];Gene=FVER53263_02797;Ontology_term=molecular function regulator activity;Ontology_id=GO:0098772
contig00001.g619	947	ID=contig00001.g619;Description=ornithine decarboxylase antizyme [Fusarium tjaetaba];Gene=FOTG_02958;Ontology_term=molecular function regulator activity;Ontology_id=GO:0098772

contig00001.g620	1697	ID=contig00001.g620;Description=imidazole glycerol phosphate synthase hisHF [Fusarium verticillioides 7600];Gene=FDENT_5968;Ontology_term=lyase activity,amino acid metabolic process,transferase activity,hydrolase activity;Ontology_id=GO:0016829,GO:0006520,GO:0016740,GO:0016787;Enzyme_code=EC:3.5.1.2,EC:2.4.2,EC:4.1.3;Enzyme_name=glutaminase,Glycosyltransferases,Carbon-carbon lyases
contig00001.g621	1248	ID=contig00001.g621;Description=transcription factor TFIIIC, tau55-related [Fusarium agapanthi]
contig00001.g622	1509	ID=contig00001.g622;Description=VPS62-vacuolar sorting [Fusarium tjaetaba];Ontology_term=endoplasmic reticulum,intracellular protein transport,vacuole;Ontology_id=GO:0005783,GO:0006886,GO:0005773
contig00001.g623	1349	ID=contig00001.g623;Description=guanine nucleotide-binding protein alpha-2 subunit [Fusarium verticillioides 7600];Gene=FGADI_12544;Ontology_term=GTPase activity,signaling,anatomical structure development,cell differentiation,reproductive process,plasma membrane;Ontology_id=GO:0003924,GO:0023052,GO:0048856,GO:0030154,GO:0022414,GO:0005886;Enzyme_code=EC:3.6.1.15,EC:3.6.1,EC:3.6,EC:3,EC:3.6.1.15;Enzyme_name=nucleoside-triphosphate phosphatase,Acting on acid anhydrides,Acting on acid anhydrides,Hydrolases,nucleoside-triphosphate phosphatase
contig00001.g624	1050	ID=contig00001.g624;Description=MYG1 [Fusarium tjaetaba];Ontology_term=nucleus,mitochondrion,hydrolase activity;Ontology_id=GO:0005634,GO:0005739,GO:0016787;Enzyme_code=EC:3;Enzyme_name=Hydrolases
contig00001.g625	628	ID=contig00001.g625;Description=cAMP-regulated phosphoprotein family protein [Fusarium fujikuroi IMI 58289]
contig00001.g626	2428	ID=contig00001.g626;Description=glutamate-cysteine ligase [Fusarium verticillioides 7600];Gene=FVEG_02789;Ontology_term=sulfur compound metabolic process,cellular modified amino acid metabolic process,ligase activity;Ontology_id=GO:0006790,GO:0006575,GO:0016874;Enzyme_code=EC:6.3.2.2;Enzyme_name=glutamate--cysteine ligase
contig00001.g627	1367	ID=contig00001.g627;Description=uncharacterized protein FOIG_07178 [Fusarium odoratissimum NRRL 54006]
contig00001.g628	249	ID=contig00001.g628;Description=Transposable element Tc3 transposase [Fusarium oxysporum f. sp. albedinis]

contig00001.g629	6281	ID=contig00001.g629;Description=nucleoporin nup61 [Fusarium coicis];Gene=FDENT_11831;Ontology_term=nuclear envelope,cytoskeleton organization,protein-containing complex assembly,microtubule organizing center;Ontology_id=GO:0005635,GO:0007010,GO:0065003,GO:0005815
contig00001.g630	2766	ID=contig00001.g630;Description=ZZ type zinc finger domain protein [Fusarium tjaetaba];Gene=FNAPI_9501;Ontology_term=endosome,autophagy,intracellular protein transport,vacuole;Ontology_id=GO:0005768,GO:0006914,GO:0006886,GO:0005773
contig00001.g631	2221	ID=contig00001.g631;Description=hypothetical protein FVEG_02782 [Fusarium verticillioides 7600];Gene=FDENT_11833;Ontology_term=nucleus,mRNA metabolic process,RNA binding;Ontology_id=GO:0005634,GO:0016071,GO:0003723
contig00001.g632	546	ID=contig00001.g632;Description=hypothetical protein FVEG_02781 [Fusarium verticillioides 7600]
contig00001.g633	2605	ID=contig00001.g633;Description=transketolase [Fusarium verticillioides 7600];Gene=FVEG_02780;Ontology_term=nucleobase-containing small molecule metabolic process,carbohydrate derivative metabolic process,transferase activity;Ontology_id=GO:0055086,GO:1901135,GO:0016740;Enzyme_code=EC:2.2.1.1;Enzyme_name=transketolase
contig00001.g634	1194	ID=contig00001.g634;Description=25S rRNA (uridine(2843)-N(3))-methyltransferase [Fusarium coicis];Gene=FMUND_13547;Ontology_term=ribosome biogenesis,transferase activity,catalytic activity, acting on RNA;Ontology_id=GO:0042254,GO:0016740,GO:0140098;Enzyme_code=EC:2.1.1,EC:2.1,EC:2,EC:2.1.1,EC:2.1.1.312;Enzyme_name=Transferring one-carbon groups,Transferring one-carbon groups,Transferases,Transferring one-carbon groups,25S rRNA (uracil(2843)-N(3))-methyltransferase
contig00001.g635	2958	ID=contig00001.g635;Description=phosphatidylinositide phosphatase SAC2 [Fusarium tjaetaba];Gene=FOTG_02936;Ontology_term=hydrolase activity;Ontology_id=GO:0016787;Enzyme_code=EC:3.1.3,EC:3.1,EC:3,EC:3.1.3;Enzyme_name=Acting on ester bonds,Acting on ester bonds,Hydrolases,Acting on ester bonds

		ID=contig00001.g636;Description=ribonucleoprotein-associated protein [Fusarium proliferatum];Gene=FNAPI_12537;Ontology_term=mRNA metabolic process,RNA binding,oxidoreductase activity,DNA-templated transcription,lipid metabolic process,ribosome biogenesis,transferase activity,nucleolus,ribosome;Ontology_id=GO:0016071,GO:0003723,GO:0016491,GO:0006351,GO:0006629,GO:0042254,GO:0016740,GO:0005730,GO:0005840;Enzyme_code=EC:2.8.1.7,EC:1.3.1,EC:2.8.1,EC:1.3,EC:2.8.1.7,EC:1,EC:2,EC:2.8,EC:1.3.1;Enzyme_name=cysteine desulfurase,Acting on the CH-CH group of donors,Transferring sulfur-containing groups,Acting on the CH-CH group of donors,cysteine desulfurase,Oxidoreductases,Transferases,Transferring sulfur-containing groups,Acting on the CH-CH group of donors
contig00001.g636	548	
		ID=contig00001.g637;Description=related to H+-transporting ATPase lipid-binding protein [Fusarium fujikuroi];Gene=FSPOR_2834;Ontology_term=transmembrane transport,vesicle-mediated transport,ATP-dependent activity,intracellular protein transport,vacuole,transporter activity,hydrolase activity;Ontology_id=GO:0055085,GO:0016192,GO:0140657,GO:0006886,GO:0005773,GO:0005215,GO:0016787;Enzyme_code=EC:7.2.2,EC:3.6.1,EC:7.1.2.1,EC:3.6,EC:3,EC:7.2.2,EC:7,EC:3.6.1.15;Enzyme_name=Catalysing the translocation of inorganic cations,Acting on acid anhydrides,P-type H(+)-exporting transporter,Acting on acid anhydrides,Hydrolases,Catalysing the translocation of inorganic cations,Translocases,nucleoside-triphosphate phosphatase
contig00001.g637	710	
		ID=contig00001.g638;Description=delta24(24(1))-sterol reductase [Fusarium verticillioides 7600];Gene=FACUT_3968;Ontology_term=RNA binding,oxidoreductase activity,DNA-templated transcription,lipid metabolic process,ribosome biogenesis,endoplasmic reticulum,nucleolus,transferase activity;Ontology_id=GO:0003723,GO:0016491,GO:0006351,GO:0006629,GO:0042254,GO:0005783,GO:0005730,GO:0016740;Enzyme_code=EC:1.3.1.72,EC:2.8.1.7,EC:1.3.1.71;Enzyme_name=Delta(24)-sterol reductase,cysteine desulfurase,Delta(24(24(1)))-sterol reductase
contig00001.g638	1847	
contig00001.g639	599	ID=contig00001.g639;Description=mismatched base pair and cruciform dna recognition [Fusarium tjaetaba]

contig00001.g640	1772	ID=contig00001.g640;Description=cysteine desulfurase [Fusarium verticillioides 7600];Gene=FMUND_15869;Ontology_term=nucleus,mitochondrial gene expression,sulfur compound metabolic process,mitochondrion,lyase activity,tRNA metabolic process,cytosol,transferase activity;Ontology_id=GO:0005634,GO:0140053,GO:0006790,GO:0005739,GO:0016829,GO:0006399,GO:0005829,GO:0016740;Enzyme_code=EC:2.8.1.7,EC:2.8.1,EC:2.8.1.7,EC:2,EC:4.4,EC:4,EC:2.8,EC:4.4.1.1;Enzyme_name=cysteine desulfurase,Transferring sulfur-containing groups,cysteine desulfurase,Transferases,Carbon-sulfur lyases,Lyases,Transferring sulfur-containing groups,cystathionine gamma-lyase
contig00001.g641	1504	ID=contig00001.g641;Description=BAR domain-containing protein [Fusarium oxysporum];Gene=FOQG_06520;Ontology_term=membrane organization,molecular function regulator activity,vesicle-mediated transport,cytoskeleton,protein-containing complex assembly,cytoskeleton organization,establishment or maintenance of cell polarity,anatomical structure development,cytosol,cell wall organization or biogenesis,Golgi apparatus,cytoskeletal protein binding;Ontology_id=GO:0061024,GO:0098772,GO:0016192,GO:0005856,GO:0065003,GO:0007010,GO:0007163,GO:0048856,GO:0005829,GO:0071554,GO:0005794,GO:0008092
contig00001.g642	1906	ID=contig00001.g642;Description=hypothetical protein FVER53590_02771 [Fusarium verticillioides]
contig00001.g643	3849	ID=contig00001.g643;Description=hypothetical protein FVEG_02770 [Fusarium verticillioides 7600];Gene=FPANT_2183;Ontology_term=protein heterodimerization activity;Ontology_id=GO:0046982
contig00001.g644	1132	ID=contig00001.g644;Description=major facilitator superfamily domain-containing protein [Fusarium redolens];Gene=FVEG_02768;Ontology_term=transmembrane transport,transporter activity;Ontology_id=GO:0055085,GO:0005215
contig00001.g645	2589	ID=contig00001.g645;Description=fluG [Fusarium tjaetaba];Gene=FNYG_09521;Ontology_term=anatomical structure development,cell differentiation,reproductive process,amino acid metabolic process,programmed cell death,ligase activity,hydrolase activity;Ontology_id=GO:0048856,GO:0030154,GO:0022414,GO:0006520,GO:0012501,GO:0016874,GO:0016787;Enzyme_code=EC:3,EC:6.3.1.2,EC:6.3,EC:3,EC:6.3.1,EC:6.3.1.2,EC:6;Enzyme_name=Hydrolases,glutamine synthetase,Forming carbon-nitrogen bonds,Hydrolases,Forming carbon-nitrogen bonds,glutamine synthetase,Ligases

contig00001.g646	3504	ID=contig00001.g646;Description=osomolarity two-component system, sensor histidine kinase SLN1 [Fusarium verticillioides 7600];Gene=FVER53263_02765;Ontology_term=molecular transducer activity,catalytic activity, acting on a protein,signaling,transferase activity,plasma membrane,small molecule sensor activity;Ontology_id=GO:0060089,GO:0140096,GO:0023052,GO:0016740,GO:0005886,GO:0140299;Enzyme_code=EC:2.7.3,EC:2.7.13.3,EC:2.7.1,EC:2.7.3,EC:2.7,EC:2,EC:2.7.13.3;Enzyme_name=Transferring phosphorus-containing groups,histidine kinase,Transferring phosphorus-containing groups,Transferring phosphorus-containing groups,Transferring phosphorus-containing groups,Transferring phosphorus-containing groups,Transferases,histidine kinase
contig00001.g647	1597	ID=contig00001.g647;Description=cell division CDC50 [Fusarium tjaetaba];Gene=FVER53263_02764;Ontology_term=membrane organization,vesicle-mediated transport,endosome,ATP-dependent activity,signaling,endoplasmic reticulum,Golgi apparatus,intracellular protein transport,transporter activity,plasma membrane,hydrolase activity;Ontology_id=GO:0061024,GO:0016192,GO:0005768,GO:0140657,GO:0023052,GO:0005783,GO:0005794,GO:0006886,GO:0005215,GO:0005886,GO:0016787;Enzyme_code=EC:3.6.1,EC:3.6,EC:7.6.2.1,EC:3,EC:7,EC:7.2.2,EC:3.6.1.15;Enzyme_name=Acting on acid anhydrides,Acting on acid anhydrides,P-type phospholipid transporter,Hydrolases,Translocases,Catalysing the translocation of inorganic cations,nucleoside-triphosphate phosphatase
contig00001.g648	3315	ID=contig00001.g648;Description=FACT complex subunit SPT16 [Fusarium subglutinans];Gene=FACUT_3958;Ontology_term=DNA repair,protein-containing complex assembly,histone binding,DNA replication,regulation of DNA-templated transcription,nuclear chromosome,molecular carrier activity,chromatin organization,nucleoplasm;Ontology_id=GO:0006281,GO:0065003,GO:0042393,GO:0006260,GO:0006355,GO:0000228,GO:0140104,GO:0006325,GO:0005654;Enzyme_code=EC:3.4.11.9;Enzyme_name=Xaa-Pro aminopeptidase
contig00001.g649	682	ID=contig00001.g649;Description=AhpD-like protein [Fusarium oxysporum];Gene=FMEXI_4719;Ontology_term=antioxidant activity,oxidoreductase activity;Ontology_id=GO:0016209,GO:0016491;Enzyme_code=EC:1.11.1,EC:1.11,EC:1,EC:1.11.1;Enzyme_name=Acting on a peroxide as acceptor,Acting on a peroxide as acceptor,Oxidoreductases,Acting on a peroxide as acceptor

contig00001.g650	762	ID=contig00001.g650;Description=hypothetical protein FVEG_02761 [Fusarium verticillioides 7600];Gene=FOMG_06769;Ontology_term=membrane;Ontology_id=GO:0016020
contig00001.g651	789	ID=contig00001.g651;Description=BUD32 protein kinase [Fusarium verticillioides 7600];Gene=FTJAE_10741;Ontology_term=catalytic activity, acting on a protein,establishment or maintenance of cell polarity,anatomical structure development,DNA recombination,regulation of DNA-templated transcription,cytokinesis,tRNA metabolic process,chromosome,mitotic cell cycle,nucleus,ATP-dependent activity,cell differentiation,cytosol,telomere organization,transferase activity,hydrolase activity;Ontology_id=GO:0140096,GO:0007163,GO:0048856,GO:0006310,GO:0006355,GO:0000910,GO:0006399,GO:0005694,GO:0000278,GO:0005634,GO:0140657,GO:0030154,GO:0005829,GO:0032200,GO:0016740,GO:0016787;Enzyme_code=EC:2.7.11.1,EC:2.7.1,EC:3.6.1,EC:2.7.11.1,EC:2.7,EC:2,EC:3.6,EC:3,EC:3.6.1.15;Enzyme_name=non-specific serine/threonine protein kinase,Transferring phosphorus-containing groups,Acting on acid anhydrides,non-specific serine/threonine protein kinase,Transferring phosphorus-containing groups,Transferases,Acting on acid anhydrides,Hydrolases,nucleoside-triphosphate phosphatase
contig00001.g652	1000	ID=contig00001.g652;Description=gamma-glutamylcysteine synthetase light chain [Fusarium sp. NRRL 25303];Gene=FPANT_9582;Ontology_term=sulfur compound metabolic process,cellular modified amino acid metabolic process;Ontology_id=GO:0006790,GO:0006575
contig00001.g653	939	ID=contig00001.g653;Description=AP-1 complex subunit sigma-1 [Fusarium proliferatum];Gene=FNAPI_13801;Ontology_term=vesicle-mediated transport,cytoplasmic vesicle,Golgi apparatus,intracellular protein transport,molecular adaptor activity;Ontology_id=GO:0016192,GO:0031410,GO:0005794,GO:0006886,GO:0060090
contig00001.g654	999	ID=contig00001.g654;Description=MAF1 Protein required for sorting of Mod5p [Fusarium fujikuroi];Gene=FVEG_02757;Ontology_term=vesicle-mediated transport,regulation of DNA-templated transcription,cytoplasmic vesicle,Golgi apparatus,chromatin organization,nucleolus,molecular adaptor activity;Ontology_id=GO:0016192,GO:0006355,GO:0031410,GO:0005794,GO:0006325,GO:0005730,GO:0060090
contig00001.g655	342	ID=contig00001.g655;Description=peptidase yqhT [Fusarium tjaetaba]

contig00001.g656	2130	ID=contig00001.g656;Description=related to U3 snoRNP protein [Fusarium fujikuroi IMI 58289];Gene=FVEG_02756;Ontology_term=ribosome biogenesis,cell adhesion,extracellular region,nucleolus,plasma membrane;Ontology_id=GO:0042254,GO:0007155,GO:0005576,GO:0005730,GO:0005886
contig00001.g657	1739	ID=contig00001.g657;Description=HAT1-interacting factor 1 [Fusarium verticillioides 7600];Ontology_term=protein-containing complex assembly,histone binding,regulation of DNA-templated transcription,nuclear chromosome,telomere organization,chromatin organization,nucleoplasm;Ontology_id=GO:0065003,GO:0042393,GO:0006355,GO:0000228,GO:0032200,GO:0006325,GO:0005654
contig00001.g658	402	ID=contig00001.g658;Description=hypothetical protein FCOIX_5503 [Fusarium coicis]
contig00001.g659	2280	ID=contig00001.g659;Description=3-dehydroquinate dehydratase I [Fusarium verticillioides 7600];Gene=FACUT_12094;Ontology_term=lyase activity;Ontology_id=GO:0016829;Enzyme_code=EC:4.2.1.10,EC:4.2.1,EC:4.2.1.10,EC:4.2,EC:4;Enzyme_name=3-dehydroquinate dehydratase,Carbon-oxygen lyases,3-dehydroquinate dehydratase,Carbon-oxygen lyases,Lyases
contig00001.g660	2988	ID=contig00001.g660;Description=nitrate assimilation regulatory nirA [Fusarium tjaetaba];Gene=FMEXI_4708;Ontology_term=nucleus,DNA binding,regulation of DNA-templated transcription,transcription regulator activity;Ontology_id=GO:0005634,GO:0003677,GO:0006355,GO:0140110;Enzyme_code=EC:4.2.1.10;Enzyme_name=3-dehydroquinate dehydratase
contig00001.g661	1981	ID=contig00001.g661;Description=vacuolar basic amino acid transporter 1 [Fusarium tjaetaba];Gene=FTJAE_8165;Ontology_term=transmembrane transport,transporter activity;Ontology_id=GO:0055085,GO:0005215
contig00001.g662	2862	ID=contig00001.g662;Description=L-fucose permease [Fusarium tjaetaba];Gene=FVEG_02749;Ontology_term=membrane;Ontology_id=GO:0016020
contig00001.g663	1856	ID=contig00001.g663;Description=ferric-chelate reductase [Fusarium tjaetaba];Gene=FVER53590_02748;Ontology_term=oxidoreductase activity;Ontology_id=GO:0016491;Enzyme_code=EC:1.16,EC:1.16,EC:1;Enzyme_name=Oxidizing metal ions,Oxidizing metal ions,Oxidoreductases
contig00001.g664	985	ID=contig00001.g664;Description=3-oxoacyl-reductase [Fusarium denticulatum];Gene=FVEG_02747;Ontology_term=oxidoreductase activity;Ontology_id=GO:0016491;Enzyme_code=EC:1;Enzyme_name=Oxidoreductases

contig00001.g665	988	ID=contig00001.g665;Description=oxidoreductase [Fusarium verticillioides 7600];Gene=FOC1_g10012823;Ontology_term=carbohydrate metabolic process,oxidoreductase activity;Ontology_id=GO:0005975,GO:0016491;Enzyme_code=EC:1,EC:1.1,EC:1.1.1.138,EC:1,EC:1.1.1.289,EC:1.1.1;Enzyme_name=Oxidoreductases,Acting on the CH-OH group of donors,mannitol 2-dehydrogenase (NADP(+)),Oxidoreductases,sorbose reductase,Acting on the CH-OH group of donors
contig00001.g666	1815	ID=contig00001.g666;Description=hypothetical protein FVER53590_02745 [Fusarium verticillioides]
contig00001.g667	1572	ID=contig00001.g667;Description=hypothetical protein FOVG_06264 [Fusarium oxysporum f. sp. pisi HDV247];Gene=FOPG_04902;Ontology_term=membrane;Ontology_id=GO:0016020
contig00001.g668	1289	ID=contig00001.g668;Description=related to phospholipid-translocating ATPase [Fusarium proliferatum ET1];Gene=FPRO_08000;Ontology_term=membrane;Ontology_id=GO:0016020
contig00001.g669	1659	ID=contig00001.g669;Description=hypothetical protein FVER53590_02742 [Fusarium verticillioides]
contig00001.g670	1525	ID=contig00001.g670;Description=triacylglycerol lipase [Fusarium tjaetaba];Gene=dapF;Ontology_term=endoplasmic reticulum,hydrolase activity;Ontology_id=GO:0005783,GO:0016787;Enzyme_code=EC:3.1;Enzyme_name=Acting on ester bonds
contig00001.g671	1388	ID=contig00001.g671;Description=sulfate transporter [Fusarium tjaetaba];Gene=FTJAE_8175;Ontology_term=transporter activity;Ontology_id=GO:0005215
contig00001.g672	3897	ID=contig00001.g672;Description=forkhead box J2 [Fusarium tjaetaba];Gene=FTJAE_8176;Ontology_term=nucleus,DNA binding,regulation of DNA-templated transcription,transcription regulator activity;Ontology_id=GO:0005634,GO:0003677,GO:0006355,GO:0140110
contig00001.g673	2941	ID=contig00001.g673;Description=budding BOI2 [Fusarium tjaetaba];Ontology_term=reproductive process,lipid binding,plasma membrane;Ontology_id=GO:0022414,GO:0008289,GO:0005886
contig00001.g674	3069	ID=contig00001.g674;Description=hypothetical protein FVER53590_02735 [Fusarium verticillioides];Gene=FNAPI_6148;Ontology_term=zinc ion binding;Ontology_id=GO:0008270
contig00001.g675	1683	ID=contig00001.g675;Description=hypothetical protein FVEG_02734 [Fusarium verticillioides 7600]

contig00001.g676	1269	ID=contig00001.g676;Description=hypothetical protein FVEG_02733 [Fusarium verticillioides 7600]
contig00001.g677	738	ID=contig00001.g677;Description=l-isoaspartate(d-aspartate) O-methyltransferase [Fusarium tjaetaba];Gene=FTJAE_9670;Ontology_term=transferase activity;Ontology_id=GO:0016740;Enzyme_code=EC:2.1.1,EC:2.1,EC:2,EC:2.1.1;Enzyme_name=Transferring one-carbon groups,Transferring one-carbon groups,Transferases,Transferring one-carbon groups
contig00001.g678	974	ID=contig00001.g678;Description=microsomal epoxide hydrolase [Fusarium verticillioides 7600];Gene=FVER53263_02732;Ontology_term=hydrolase activity;Ontology_id=GO:0016787;Enzyme_code=EC:3.1.3;Enzyme_name=Acting on ester bonds
contig00001.g679	1131	ID=contig00001.g679;Description=probable 60S ribosomal protein L5 [Fusarium fujikuroi];Gene=FOMG_06802;Ontology_term=carbohydrate metabolic process,RNA binding,protein-containing complex assembly,cytoplasmic translation,structural molecule activity,cytosol,ribosome biogenesis,ribosome,hydrolase activity;Ontology_id=GO:0005975,GO:0003723,GO:0065003,GO:0002181,GO:0005198,GO:0005829,GO:0042254,GO:0005840,GO:0016787;Enzyme_code=EC:3.2.1.26,EC:3.2,EC:3,EC:3.2.1.26,EC:3.2.1;Enzyme_name=beta-fructofuranosidase,Glycosylases,Hydrolases,beta-fructofuranosidase,Glycosylases
contig00001.g680	3645	ID=contig00001.g680;Description=GIP3 [Fusarium subglutinans];Ontology_term=cytoskeleton,molecular function regulator activity,cytoskeleton organization,mitochondrion,structural molecule activity,chromosome segregation,cell wall organization or biogenesis,endoplasmic reticulum;Ontology_id=GO:0005856,GO:0098772,GO:0007010,GO:0005739,GO:0005198,GO:0007059,GO:0071554,GO:0005783
contig00001.g681	2112	ID=contig00001.g681;Description=hypothetical protein J7337_005645 [Fusarium musae];Gene=FPCIR_13251;Ontology_term=membrane;Ontology_id=GO:0016020

contig00001.g682	3735	ID=contig00001.g682;Description=phosphatidylserine decarboxylase [Fusarium tjaetaba];Gene=PSD2;Ontology_term=endosome,lipid metabolic process,lyase activity,cellular modified amino acid metabolic process,Golgi apparatus,lipid binding,protein maturation,vacuole;Ontology_id=GO:0005768,GO:0006629,GO:0016829,GO:0006575,GO:0005794,GO:0008289,GO:0051604,GO:0005773;Enzyme_code=EC:4.1.1.65,EC:4.1.1,EC:4.1,EC:4,EC:4.1.1.65;Enzyme_name=phosphatidylserine decarboxylase,Carbon-carbon lyases,Carbon-carbon lyases,Lyases,phosphatidylserine decarboxylase
contig00001.g683	3262	ID=contig00001.g683;Description=related to C2H2 zinc finger protein [Fusarium fujikuroi IMI 58289];Gene=FOIG_07111;Ontology_term=nucleus,DNA binding,DNA-templated transcription;Ontology_id=GO:0005634,GO:0003677,GO:0006351
contig00001.g684	1486	ID=contig00001.g684;Description=choline-phosphate cytidylyltransferase [Daldinia caldariorum];Gene=THARTR1_00898;Ontology_term=nuclear envelope,lipid metabolic process,Golgi apparatus,transferase activity;Ontology_id=GO:0005635,GO:0006629,GO:0005794,GO:0016740;Enzyme_code=EC:2.7.7.15,EC:2.7,EC:2,EC:2.7.7.15,EC:2.7.7;Enzyme_name=choline-phosphate cytidylyltransferase,Transferring phosphorus-containing groups,Transferases,choline-phosphate cytidylyltransferase,Transferring phosphorus-containing groups
contig00001.g685	427	ID=contig00001.g685;Description=Putative pectate lyase F [Fusarium oxysporum f. sp. cubense race 1];Gene=BFJ71_g15010;Ontology_term=carbohydrate metabolic process,lyase activity,extracellular region;Ontology_id=GO:0005975,GO:0016829,GO:0005576;Enzyme_code=EC:4.2.2.2;Enzyme_name=pectate lyase
contig00001.g686	3679	ID=contig00001.g686;Description=hypothetical protein J7337_005640 [Fusarium musae];Gene=FVEG_02723;Ontology_term=nucleus,regulation of DNA-templated transcription,transcription regulator activity;Ontology_id=GO:0005634,GO:0006355,GO:0140110
contig00001.g687	972	ID=contig00001.g687;Description=hypothetical protein FVER14953_02721 [Fusarium verticillioides];Gene=FVER53263_02721;Ontology_term=DNA-templated transcription;Ontology_id=GO:0006351
contig00001.g688	1443	ID=contig00001.g688;Description=DNA excision repair protein ERCC-8 [Fusarium verticillioides 7600];Gene=FTJAE_9660;Ontology_term=DNA repair,nucleus;Ontology_id=GO:0006281,GO:0005634

contig00001.g689	884	ID=contig00001.g689;Description=autophagy protein 5 [Fusarium verticillioides 7600];Gene=FPCIR_14174;Ontology_term=autophagy,mitochondrion organization;Ontology_id=GO:0006914,GO:0007005
contig00001.g690	587	ID=contig00001.g690;Description=integral membrane protein [Fusarium tjaetaba];Gene=F25303_7467;Ontology_term=membrane;Ontology_id=GO:0016020
contig00001.g691	1096	ID=contig00001.g691;Description=period circadian [Fusarium tjaetaba]
contig00001.g692	1200	ID=contig00001.g692;Description=hypothetical protein FVER53590_02716 [Fusarium verticillioides];Gene=1855
contig00001.g693	551	ID=contig00001.g693;Description=hypothetical protein FVEG_02715 [Fusarium verticillioides 7600];Gene=FOZG_09528;Ontology_term=nucleic acid binding,zinc ion binding;Ontology_id=GO:0003676,GO:0008270
contig00001.g694	1009	ID=contig00001.g694;Description=hypothetical protein FCOIX_11869 [Fusarium coicis]
contig00001.g695	921	ID=contig00001.g695;Description=hypothetical protein F52700_13529 [Fusarium sp. NRRL 52700];Gene=CEK26_008549;Ontology_term=nucleic acid binding,zinc ion binding;Ontology_id=GO:0003676,GO:0008270
contig00001.g696	462	ID=contig00001.g696;Description=mfs monocarboxylate transporter [Fusarium pseudoanthophilum];Gene=FTJAE_9652;Ontology_term=nucleic acid binding,zinc ion binding;Ontology_id=GO:0003676,GO:0008270
contig00001.g697	942	ID=contig00001.g697;Description=Pwp2p [Fusarium acutatum];Gene=FACUT_1349;Ontology_term=ribosome biogenesis,nucleolus;Ontology_id=GO:0042254,GO:0005730
contig00001.g698	1587	ID=contig00001.g698;Description=Pwp2p [Fusarium fujikuroi];Gene=FNYG_07035;Ontology_term=RNA binding,ribosome biogenesis,regulation of DNA-templated transcription,chromosome,nucleolus;Ontology_id=GO:0003723,GO:0042254,GO:0006355,GO:0005694,GO:0005730
contig00001.g699	2888	ID=contig00001.g699;Description=Ingression fic1 [Fusarium tjaetaba];Gene=FMUND_12751;Ontology_term=ribosome biogenesis,nucleolus;Ontology_id=GO:0042254,GO:0005730

contig00001.g700	372	ID=contig00001.g700;Description=GAF protein [Fusarium verticillioides 7600];Gene=FVEG_02712;Ontology_term=nucleus,oxidoreductase activity,cytosol;Ontology_id=GO:0005634,GO:0016491,GO:0005829;Enzyme_code=EC:1.8.4.14,EC:1,EC:1.8,EC:1.8.4,EC:1.8.4.14;Enzyme_name=L-methionine (R)-S-oxide reductase,Oxidoreductases,Acting on a sulfur group of donors,Acting on a sulfur group of donors,L-methionine (R)-S-oxide reductase
contig00001.g701	1089	ID=contig00001.g701;Description=hypothetical protein FVEG_02711 [Fusarium verticillioides 7600];Gene=FPCIR_13360;Ontology_term=mitochondrion;Ontology_id=GO:0005739
contig00001.g702	549	ID=contig00001.g702;Description=probable peroxisomal protein POX18 [Fusarium fujikuroi IMI 58289]
contig00001.g703	1328	ID=contig00001.g703;Description=FMn-dependent 2-nitropropane dioxygenase [Fusarium tjaetaba];Gene=CEK26_008557;Ontology_term=nucleus,oxidoreductase activity,regulation of DNA-templated transcription,transcription regulator activity;Ontology_id=GO:0005634,GO:0016491,GO:0006355,GO:0140110;Enzyme_code=EC:1.13.12.16,EC:1.13,EC:1.13.12.16,EC:1,EC:1.13.12;Enzyme_name=nitronate monooxygenase,Acting on single donors with incorporation of molecular oxygen (oxygenases). The oxygen incorporated need not be derived from O2,nitronate monooxygenase,Oxidoreductases,Acting on single donors with incorporation of molecular oxygen (oxygenases). The oxygen incorporated need not be derived from O2
contig00001.g704	1216	ID=contig00001.g704;Description=potassium channel beta subunit [Fusarium subglutinans]
contig00001.g705	1482	ID=contig00001.g705;Description=CK1/CK1/CK1-G protein kinase [Fusarium proliferatum];Gene=FOXB_02295;Ontology_term=nucleus,catalytic activity, acting on a protein,vesicle-mediated transport,lipid metabolic process,mitochondrion,signaling,anatomical structure development,endoplasmic reticulum,transferase activity,vacuole,plasma membrane;Ontology_id=GO:0005634,GO:0140096,GO:0016192,GO:0006629,GO:0005739,GO:0023052,GO:0048856,GO:0005783,GO:0016740,GO:0005773,GO:0005886;Enzyme_code=EC:2.7.11.1,EC:2.7.1,EC:2.7.11.1,EC:2.7,EC:2;Enzyme_name=non-specific serine/threonine protein kinase,Transferring phosphorus-containing groups,non-specific serine/threonine protein kinase,Transferring phosphorus-containing groups,Transferases
contig00001.g706	793	ID=contig00001.g706;Description=p24 involved in membrane trafficking [Fusarium acutatum];Gene=F52700_13539;Ontology_term=vesicle-mediated transport,mitochondrion,cytoplasmic vesicle,endoplasmic reticulum;Ontology_id=GO:0016192,GO:0005739,GO:0031410,GO:0005783

contig00001.g707	1881	ID=contig00001.g707;Description=SWR1-complex protein 4 [Fusarium verticillioides 7600];Gene=FVEG_02705;Ontology_term=DNA repair,DNA binding,nuclear chromosome,chromatin organization,transcription regulator activity,molecular adaptor activity,nucleoplasm;Ontology_id=GO:0006281,GO:0003677,GO:0000228,GO:0006325,GO:0140110,GO:0060090,GO:0005654
contig00001.g708	2053	ID=contig00001.g708;Description=acetyltransferase (nodulation nodL) [Fusarium pseudocircinatum];Gene=FNYG_07025;Ontology_term=nucleus,regulation of DNA-templated transcription,transferase activity,transcription regulator activity;Ontology_id=GO:0005634,GO:0006355,GO:0016740,GO:0140110;Enzyme_code=EC:2.7.7.13,EC:2.3.1,EC:2.3,EC:2.7,EC:2,EC:2.7.7,EC:2.7.7.13,EC:2.3.1;Enzyme_name=mannose-1-phosphate guanylyltransferase,Acyltransferases,Acyltransferases,Transferring phosphorus-containing groups,Transferases,Transferring phosphorus-containing groups,mannose-1-phosphate guanylyltransferase,Acyltransferases
contig00001.g709	2115	ID=contig00001.g709;Description=hypothetical protein J7337_005618 [Fusarium musae];Gene=FMUND_12944;Ontology_term=nucleus,DNA binding,regulation of DNA-templated transcription,transcription regulator activity;Ontology_id=GO:0005634,GO:0003677,GO:0006355,GO:0140110
contig00001.g710	2762	ID=contig00001.g710;Description=hypothetical protein FNAPI_9961 [Fusarium napiforme]
contig00001.g711	627	ID=contig00001.g711;Description=cwf18 pre-mrna splicing factor [Fusarium tjaetaba];Gene=SPT15;Ontology_term=DNA binding,nucleus,mRNA metabolic process,DNA-templated transcription;Ontology_id=GO:0003677,GO:0005634,GO:0016071,GO:0006351
contig00001.g712	933	ID=contig00001.g712;Description=hypothetical protein FVEG_02700 [Fusarium verticillioides 7600];Gene=1873
contig00001.g713	2660	ID=contig00001.g713;Description=R3H domain-containing protein [Fusarium coicis];Gene=FVER53263_02699;Ontology_term=anatomical structure development,regulation of DNA-templated transcription,receptor ligand activity,lipid binding,vacuole,cell motility,nucleus,wound healing,extracellular space,lipid metabolic process,cell differentiation,cytosol;Ontology_id=GO:0048856,GO:0006355,GO:0048018,GO:0008289,GO:0005773,GO:0048870,GO:0005634,GO:0042060,GO:0005615,GO:0006629,GO:0030154,GO:0005829

contig00001.g714	1190	ID=contig00001.g714;Description=eukaryotic translation initiation factor 3 subunit F [Fusarium proliferatum];Gene=FNAPI_9957;Ontology_term=translation regulator activity,catalytic activity, acting on a protein,protein-containing complex assembly,cytoplasmic translation,hydrolase activity;Ontology_id=GO:0045182,GO:0140096,GO:0065003,GO:0002181,GO:0016787;Enzyme_code=EC:3.4;Enzyme_name=Acting on peptide bonds (peptidases)
contig00001.g715	2264	ID=contig00001.g715;Description=u3 small nucleolar RNA-associated 25 [Fusarium subglutinans];Gene=FNAPI_9956;Ontology_term=RNA binding,ribosome biogenesis,nucleolus;Ontology_id=GO:0003723,GO:0042254,GO:0005730
contig00001.g716	744	ID=contig00001.g716;Description=mitochondrial ribosomal 8 [Fusarium pseudoanthophilum];Gene=FPCIR_13714;Ontology_term=mitochondrial gene expression,mitochondrion,structural molecule activity,ribosome,mitochondrion organization;Ontology_id=GO:0140053,GO:0005739,GO:0005198,GO:0005840,GO:0007005
contig00001.g717	776	ID=contig00001.g717;Description=MICOS complex subunit Mic12 [Fusarium oxysporum f. sp. vasinfectum];Gene=FPANT_1667;Ontology_term=membrane organization,mitochondrion,mitochondrion organization;Ontology_id=GO:0061024,GO:0005739,GO:0007005
contig00001.g718	1222	ID=contig00001.g718;Description=delta-aminolevulinic acid dehydratase [Fusarium subglutinans];Gene=FMUND_15403;Ontology_term=nucleus,lyase activity,cytosol;Ontology_id=GO:0005634,GO:0016829,GO:0005829;Enzyme_code=EC:4.2.1.24,EC:4.2.1,EC:4.2,EC:4,EC:4.2.1.24;Enzyme_name=porphobilinogen synthase,Carbon-oxygen lyases,Carbon-oxygen lyases,Lyases,porphobilinogen synthase
contig00001.g719	1611	ID=contig00001.g719;Description=acyltransferase [Fusarium tjaetaba];Gene=FNAPI_9951;Ontology_term=transferase activity;Ontology_id=GO:0016740;Enzyme_code=EC:2.3.1,EC:2.3,EC:2,EC:2.3.1;Enzyme_name=Acyltransferases,Acyltransferases,Transferases,Acyltransferases
contig00001.g720	1893	ID=contig00001.g720;Description=amidohydrolase ytcJ-like [Fusarium verticillioides 7600];Gene=FNYG_07011;Ontology_term=hydrolase activity;Ontology_id=GO:0016787;Enzyme_code=EC:3.5,EC:3.5,EC:3;Enzyme_name=Acting on carbon-nitrogen bonds, other than peptide bonds,Acting on carbon-nitrogen bonds, other than peptide bonds,Hydrolases

contig00001.g721	895	ID=contig00001.g721;Description=Ff.00g072530.m01.CDS01 [Fusarium sp. VM40];Gene=FVER53590_02690;Ontology_term=catalytic activity, acting on a protein,hydrolase activity;Ontology_id=GO:0140096,GO:0016787;Enzyme_code=EC:3.1.3,EC:3.1,EC:3,EC:3.1.3;Enzyme_name=Acting on ester bonds,Acting on ester bonds,Hydrolases,Acting on ester bonds
contig00001.g722	394	ID=contig00001.g722;Description=hypothetical protein IWW34DRAFT_723782 [Fusarium oxysporum f. sp. albedinis]
contig00001.g723	1964	ID=contig00001.g723;Description=hypothetical protein FVEG_15118 [Fusarium verticillioides 7600]
contig00001.g724	1018	ID=contig00001.g724;Description=n-acetyltransferase NAT2 [Fusarium tjaetaba];Gene=FNAPI_9946;Ontology_term=mitochondrion,protein maturation;Ontology_id=GO:0005739,GO:0051604
contig00001.g725	474	ID=contig00001.g725;Description=hypothetical protein FVER14953_02686 [Fusarium verticillioides]
contig00001.g726	534	ID=contig00001.g726;Description=non-classical export 2 [Fusarium tjaetaba];Gene=F25303_6840;Ontology_term=membrane organization,cytoskeleton organization,mitochondrion,endoplasmic reticulum,cell wall organization or biogenesis,plasma membrane,protein localization to plasma membrane;Ontology_id=GO:0061024,GO:0007010,GO:0005739,GO:0005783,GO:0071554,GO:0005886,GO:0072659
contig00001.g727	405	ID=contig00001.g727;Description=Mim2 [Fusarium oxysporum f. sp. vasinfectum];Gene=FOC1_g10012901;Ontology_term=membrane organization,protein-containing complex assembly,mitochondrion,intracellular protein transport,mitochondrion organization;Ontology_id=GO:0061024,GO:0065003,GO:0005739,GO:0006886,GO:0007005
contig00001.g728	1604	ID=contig00001.g728;Description=translation initiation factor 2 subunit 2 [Fusarium oxysporum f. sp. lycopersici 4287];Gene=FOTG_02815;Ontology_term=translation regulator activity,RNA binding,protein-containing complex assembly,cytoplasmic translation;Ontology_id=GO:0045182,GO:0003723,GO:0065003,GO:0002181

contig00001.g729	4287	ID=contig00001.g729;Description=nuclear pore protein [Fusarium denticulatum];Gene=FNYG_07002;Ontology_term=nuclear envelope,nucleocytoplasmic transport,protein-containing complex assembly,structural molecule activity,regulation of DNA-templated transcription,chromatin organization,intracellular protein transport;Ontology_id=GO:0005635,GO:0006913,GO:0065003,GO:0005198,GO:0006355,GO:0006325,GO:0006886
contig00001.g730	1926	ID=contig00001.g730;Description=ATPase family [Fusarium tjaetaba];Gene=FDENT_11503;Ontology_term=ATP-dependent activity,hydrolase activity;Ontology_id=GO:0140657,GO:0016787;Enzyme_code=EC:3.6.1.15,EC:3.6.1,EC:3.6,EC:3,EC:3.6.4.7,EC:3.6.1.15;Enzyme_name=nucleoside-triphosphate phosphatase,Acting on acid anhydrides,Acting on acid anhydrides,Hydrolases,peroxisome-assembly ATPase,nucleoside-triphosphate phosphatase
contig00001.g731	636	ID=contig00001.g731;Description=hypothetical protein J7337_005596 [Fusarium musae];Gene=FocTR4_00008725;Ontology_term=vesicle-mediated transport;Ontology_id=GO:0016192
contig00001.g732	1302	ID=contig00001.g732;Description=[acyl-carrier-protein] S-malonyltransferase [Fusarium verticillioides 7600];Gene=FVEG_02679;Ontology_term=transferase activity;Ontology_id=GO:0016740;Enzyme_code=EC:2,EC:2,EC:2.3.1.39;Enzyme_name=Transferases,Transferases,[acyl-carrier-protein] S-malonyltransferase
contig00001.g733	1731	ID=contig00001.g733;Description=APM1-AP-1 complex subunit mu1 subunit [Fusarium subglutinans];Gene=FSUBG_13553;Ontology_term=vesicle-mediated transport,intracellular protein transport;Ontology_id=GO:0016192,GO:0006886
contig00001.g734	352	ID=contig00001.g734;Description=exosome complex [Fusarium denticulatum];Gene=FDENT_11499;Ontology_term=membrane;Ontology_id=GO:0016020
contig00001.g735	1053	ID=contig00001.g735;Description=exosome complex component RRP4 [Fusarium tjaetaba];Gene=FVER53263_02676;Ontology_term=mRNA metabolic process,RNA binding,snRNA metabolic process,regulatory ncRNA-mediated gene silencing,nuclear chromosome,ribosome biogenesis,tRNA metabolic process;Ontology_id=GO:0016071,GO:0003723,GO:0016073,GO:0031047,GO:0000228,GO:0042254,GO:0006399

contig00001.g736	1669	ID=contig00001.g736;Description=aldehyde dehydrogenase [Fusarium tjaetaba];Gene=FPANT_9571;Ontology_term=lipid droplet,endosome,oxidoreductase activity,mitochondrion,lipid metabolic process,amino acid metabolic process,endoplasmic reticulum;Ontology_id=GO:0005811,GO:0005768,GO:0016491,GO:0005739,GO:0006629,GO:0006520,GO:0005783;Enzyme_code=EC:1.2.1,EC:1.2,EC:1,EC:1.2.99.6,EC:1.2.1.64,EC:1.2.1.82,EC:1.2.1.5,EC:1.2.1.3,EC:1.2.1;Enzyme_name=Acting on the aldehyde or oxo group of donors,Acting on the aldehyde or oxo group of donors,Oxidoreductases,carboxylate reductase,4-hydroxybenzaldehyde dehydrogenase (NAD(+)),beta-apo-4'-carotenal oxygenase,aldehyde dehydrogenase [NAD(P)(+)],aldehyde dehydrogenase (NAD(+)),Acting on the aldehyde or oxo group of donors
contig00001.g737	2326	ID=contig00001.g737;Description=mitochondrial ribosomal subunit protein-domain-containing protein [Fusarium oxysporum Fo47];Gene=FFUJ_07505;Ontology_term=mitochondrial gene expression,mitochondrion,structural molecule activity,ribosome;Ontology_id=GO:0140053,GO:0005739,GO:0005198,GO:0005840
contig00001.g738	718	ID=contig00001.g738;Description=beta-1,4-N-acetylglucosaminyltransferase [Fusarium verticillioides 7600];Gene=ALG13;Ontology_term=nucleus,lipid metabolic process,protein glycosylation,cytosol,endoplasmic reticulum,transferase activity;Ontology_id=GO:0005634,GO:0006629,GO:0006486,GO:0005829,GO:0005783,GO:0016740;Enzyme_code=EC:2.4.1.141,EC:2.4.1.141,EC:2.4,EC:2,EC:2.4.1;Enzyme_name=N-acetylglucosaminyldiphosphodolichol N-acetylglucosaminyltransferase,N-acetylglucosaminyldiphosphodolichol N-acetylglucosaminyltransferase,Glycosyltransferases,Transferases,Glycosyltransferases
contig00001.g739	1893	ID=contig00001.g739;Description=stress p66 [Fusarium tjaetaba];Gene=FVEG_02671;Ontology_term=nucleus,cytoskeleton,cytoskeleton organization,protein-containing complex assembly,cytokinesis,cytoskeletal protein binding;Ontology_id=GO:0005634,GO:0005856,GO:0007010,GO:0065003,GO:0000910,GO:0008092
contig00001.g740	563	ID=contig00001.g740;Description=CFEM domain-containing protein [Fusarium denticulatum]
contig00001.g741	1890	ID=contig00001.g741;Description=ADA HAT complex component 1 [Fusarium verticillioides 7600]
contig00001.g742	633	ID=contig00001.g742;Description=hypothetical protein FVER14953_21283 [Fusarium verticillioides]

contig00001.g743	466	ID=contig00001.g743;Description=vacuolar transporter chaperone 1 [Fusarium oxysporum f. sp. lycopersici 4287];Gene=FNAPI_9643;Ontology_term=nuclear envelope,RNA binding,autophagy,endoplasmic reticulum,vacuole;Ontology_id=GO:0005635,GO:0003723,GO:0006914,GO:0005783,GO:0005773
contig00001.g744	671	ID=contig00001.g744;Description=Tctex-1 [Fusarium redolens]
contig00001.g745	921	ID=contig00001.g745;Description=f-box domain protein [Fusarium tjaetaba]
contig00001.g746	2442	ID=contig00001.g746;Description=glycosyltransferase family 31 [Fusarium tjaetaba];Gene=FACUT_11815;Ontology_term=transferase activity;Ontology_id=GO:0016740;Enzyme_code=EC:2,EC:2;Enzyme_name=Transferases,Transferases
contig00001.g747	1239	ID=contig00001.g747;Description=arabinose 5-phosphate isomerase [Fusarium tjaetaba];Gene=FNYG_06984;Ontology_term=carbohydrate derivative metabolic process,isomerase activity;Ontology_id=GO:1901135,GO:0016853;Enzyme_code=EC:5,EC:5;Enzyme_name=Isomerases,Isomerases
contig00001.g748	1610	ID=contig00001.g748;Description=hypothetical protein FVEG_02663 [Fusarium verticillioides 7600];Gene=FTJAE_8560;Ontology_term=Golgi apparatus,vacuole,plasma membrane;Ontology_id=GO:0005794,GO:0005773,GO:0005886
contig00001.g749	1840	ID=contig00001.g749;Description=uncharacterized protein FRV6_01793 [Fusarium oxysporum];Gene=FOPG_03151;Ontology_term=nucleus,DNA binding,regulation of DNA-templated transcription,transcription regulator activity;Ontology_id=GO:0005634,GO:0003677,GO:0006355,GO:0140110
contig00001.g750	564	ID=contig00001.g750;Description=hypothetical protein FocTR4_00008746 [Fusarium oxysporum f. sp. cubense]
contig00001.g751	767	ID=contig00001.g751;Description=puromycin n-acetyltransferase [Fusarium beomiforme];Gene=FSPOR_1417;Ontology_term=transferase activity;Ontology_id=GO:0016740;Enzyme_code=EC:2.3.1,EC:2.3,EC:2,EC:2.3.1;Enzyme_name=Acyltransferases,Acyltransferases,Transferases,Acyltransferases
contig00001.g752	3799	ID=contig00001.g752;Description=hypothetical protein FVEG_02660 [Fusarium verticillioides 7600]
contig00001.g753	1093	ID=contig00001.g753;Description=hypothetical protein FVER53590_02659 [Fusarium verticillioides]

contig00001.g754	2461	ID=contig00001.g754;Description=major facilitator superfamily transporter [Fusarium tjaetaba];Gene=FVER53590_02658;Ontology_term=membrane;Ontology_id=GO:0016020
contig00001.g755	2195	ID=contig00001.g755;Description=paxillin 1 [Fusarium tjaetaba];Gene=FVEG_02657;Ontology_term=molecular function regulator activity;Ontology_id=GO:0098772
contig00001.g756	1679	ID=contig00001.g756;Description=myo-inositol-1-phosphate synthase [Fusarium verticillioides 7600];Gene=FVEG_02656;Ontology_term=carbohydrate metabolic process,lipid metabolic process,cell wall,isomerase activity;Ontology_id=GO:0005975,GO:0006629,GO:0005618,GO:0016853;Enzyme_code=EC:5.5.1.4,EC:5,EC:5.5.1.4,EC:5.5.1;Enzyme_name=inositol-3-phosphate synthase,Isomerases,inositol-3-phosphate synthase,Intramolecular lyases
contig00001.g757	3907	ID=contig00001.g757;Description=mediator of replication checkpoint 1 [Fusarium tjaetaba];Gene=FVER53263_02655;Ontology_term=DNA repair,DNA replication,regulation of DNA-templated transcription,nuclear chromosome,chromosome segregation,chromatin organization,DNA binding,signaling,mitotic nuclear division,telomere organization,molecular adaptor activity;Ontology_id=GO:0006281,GO:0006260,GO:0006355,GO:0000228,GO:0007059,GO:0006325,GO:0003677,GO:0023052,GO:0140014,GO:0032200,GO:0060090
contig00001.g758	1903	ID=contig00001.g758;Description=aarF domain-containing kinase [Fusarium verticillioides 7600];Gene=FOVG_06382;Ontology_term=mitochondrion,transferase activity,mitochondrion organization;Ontology_id=GO:0005739,GO:0016740,GO:0007005;Enzyme_code=EC:2.7,EC:2.7,EC:2;Enzyme_name=Transferring phosphorus-containing groups,Transferring phosphorus-containing groups,Transferases
contig00001.g759	725	ID=contig00001.g759;Description=hypothetical protein FVER53263_02653 [Fusarium verticillioides]
contig00001.g760	1510	ID=contig00001.g760;Description=peroxisomal biogenesis factor 19 [Fusarium tjaetaba];Gene=FNYG_06971;Ontology_term=peroxisome,peroxisome organization,cytosol,endoplasmic reticulum,intracellular protein transport;Ontology_id=GO:0005777,GO:0007031,GO:0005829,GO:0005783,GO:0006886

contig00001.g761	633	ID=contig00001.g761;Description=SUMO-conjugating enzyme ubc9 [Fusarium subglutinans];Gene=FCIRC_13257;Ontology_term=catalytic activity, acting on a protein,cytoskeleton organization,ATP-dependent activity,mitotic nuclear division,chromosome segregation,nuclear chromosome,transferase activity,ligase activity;Ontology_id=GO:0140096,GO:0007010,GO:0140657,GO:0140014,GO:0007059,GO:0000228,GO:0016740,GO:0016874;Enzyme_code=EC:2,EC:2.3,EC:2,EC:6.2,EC:6,EC:2.3.2;Enzyme_name=Transferases,Acyltransferases,Transferases,Forming carbon-sulfur bonds,Ligases,Acyltransferases
contig00001.g762	2380	ID=contig00001.g762;Description=asparagine synthetase [Fusarium verticillioides 7600];Gene=FOTG_02778;Ontology_term=amino acid metabolic process,ligase activity;Ontology_id=GO:0006520,GO:0016874;Enzyme_code=EC:6.3.5.4,EC:6.3.5.4,EC:6.3,EC:6,EC:6.3.5;Enzyme_name=asparagine synthase (glutamine-hydrolyzing),asparagine synthase (glutamine-hydrolyzing),Forming carbon-nitrogen bonds,Ligases,Forming carbon-nitrogen bonds
contig00001.g763	1571	ID=contig00001.g763;Description=outer mitochondrial membrane porin [Fusarium tjaetaba];Gene=FNAPI_6830;Ontology_term=transmembrane transport,mitochondrion,transporter activity;Ontology_id=GO:0055085,GO:0005739,GO:0005215
contig00001.g764	908	ID=contig00001.g764;Description=hypothetical protein FVEG_15108 [Fusarium verticillioides 7600];Gene=FOXG_05492;Ontology_term=membrane;Ontology_id=GO:0016020
contig00001.g765	1836	ID=contig00001.g765;Description=mannosyl-oligosaccharide alpha-1 2-mannosidase 1B [Fusarium tjaetaba];Gene=FPRO05_13529;Ontology_term=carbohydrate metabolic process,hydrolase activity;Ontology_id=GO:0005975,GO:0016787;Enzyme_code=EC:3.2.1.113,EC:3.2.1.24,EC:3.2,EC:3.2.1.113,EC:3,EC:3.2.1.24,EC:3.2.1;Enzyme_name=mannosyl-oligosaccharide 1,2-alpha-mannosidase,alpha-mannosidase,Glycosylases,mannosyl-oligosaccharide 1,2-alpha-mannosidase,Hydrolases,alpha-mannosidase,Glycosylases

contig00001.g766	3303	ID=contig00001.g766;Description=regulator-nonsense transcripts 1 [Fusarium verticillioides 7600];Gene=FPANT_5704;Ontology_term=DNA binding,mRNA metabolic process,RNA binding,ATP-dependent activity,DNA recombination,regulation of DNA-templated transcription,catalytic activity, acting on RNA,chromatin organization,hydrolase activity;Ontology_id=GO:0003677,GO:0016071,GO:0003723,GO:0140657,GO:0006310,GO:0006355,GO:0140098,GO:0006325,GO:0016787;Enzyme_code=EC:3.6.4.13,EC:3.6.1,EC:3.6.4.13,EC:3.6,EC:3,EC:3.6.1.15;Enzyme_name=RNA helicase,Acting on acid anhydrides,RNA helicase,Acting on acid anhydrides,Hydrolases,nucleoside-triphosphate phosphatase
contig00001.g767	955	ID=contig00001.g767;Description=putative IMP4 protein [Fusarium fujikuroi];Gene=FNAPI_6833;Ontology_term=DNA binding,RNA binding,amino acid metabolic process,ribosome biogenesis,nucleolus,isomerase activity;Ontology_id=GO:0003677,GO:0003723,GO:0006520,GO:0042254,GO:0005730,GO:0016853;Enzyme_code=EC:5.3.1.24;Enzyme_name=phosphoribosylanthranilate isomerase
contig00001.g768	1088	ID=contig00001.g768;Description=t-SNARE [Fusarium redolens];Gene=FOC1_g10012942;Ontology_term=vesicle-mediated transport;Ontology_id=GO:0016192
contig00001.g769	2051	ID=contig00001.g769;Description=mitochondrial import receptor subunit tom-70 [Fusarium verticillioides 7600];Gene=FPHYL_13380;Ontology_term=membrane organization,transmembrane transport,ATP-dependent activity,mitochondrion,intracellular protein transport,transporter activity,mitochondrion organization;Ontology_id=GO:0061024,GO:0055085,GO:0140657,GO:0005739,GO:0006886,GO:0005215,GO:0007005;Enzyme_code=EC:7.2.2,EC:7.2.2,EC:7;Enzyme_name=Catalysing the translocation of inorganic cations,Catalysing the translocation of inorganic cations,Translocases
contig00001.g770	876	ID=contig00001.g770;Description=aminodeoxychorismate lyase [Fusarium tjaetaba];Gene=FMUND_5822;Ontology_term=mitochondrion,lyase activity,amino acid metabolic process,cellular modified amino acid metabolic process,transferase activity,vitamin metabolic process;Ontology_id=GO:0005739,GO:0016829,GO:0006520,GO:0006575,GO:0016740,GO:0006766;Enzyme_code=EC:4,EC:2.6.1,EC:4.1,EC:4.1.3.38,EC:2,EC:2.6,EC:2.6.1.42,EC:4,EC:4.1.3;Enzyme_name=Lyases,Transferring nitrogenous groups,Carbon-carbon lyases,aminodeoxychorismate lyase,Transferases,Transferring nitrogenous groups,branched-chain-amino-acid transaminase,Lyases,Carbon-carbon lyases
contig00001.g771	2194	ID=contig00001.g771;Description=f-box TPR repeat pof3 [Fusarium coicis]

contig00001.g772	1473	ID=contig00001.g772;Description=GTPase [Fusarium proliferatum];Gene=FGADI_4397;Ontology_term=GTP binding;Ontology_id=GO:0005525
contig00001.g773	1942	ID=contig00001.g773;Description=related to SET domain protein [Fusarium proliferatum ET1];Gene=FGLOB1_9467;Ontology_term=catalytic activity, acting on a protein,transferase activity;Ontology_id=GO:0140096,GO:0016740;Enzyme_code=EC:2.1.1,EC:2.1,EC:2,EC:2.1.1;Enzyme_name=Transferring one-carbon groups,Transferring one-carbon groups,Transferases,Transferring one-carbon groups
contig00001.g774	2728	ID=contig00001.g774;Description=Transcriptional activator protein acu-15 [Fusarium musae];Gene=FVEG_02639;Ontology_term=nucleus,DNA binding,carbohydrate metabolic process,nucleobase-containing small molecule metabolic process,regulation of DNA-templated transcription,transcription regulator activity;Ontology_id=GO:0005634,GO:0003677,GO:0005975,GO:0055086,GO:0006355,GO:0140110
contig00001.g775	1412	ID=contig00001.g775;Description=glycerophosphoryl diester phosphodiesterase [Fusarium verticillioides 7600];Gene=FOXYS1_3159;Ontology_term=lipid metabolic process,hydrolase activity;Ontology_id=GO:0006629,GO:0016787;Enzyme_code=EC:3.1.4;Enzyme_name=Acting on ester bonds
contig00001.g776	2029	ID=contig00001.g776;Description=Pol II transcription elongation factor [Fusarium tjaetaba];Gene=FNAPI_6842;Ontology_term=DNA repair,protein-containing complex assembly,regulation of DNA-templated transcription,chromosome,chromatin organization,nucleoplasm,DNA binding,translation regulator activity,RNA binding,mRNA metabolic process,regulatory ncRNA-mediated gene silencing,transcription regulator activity;Ontology_id=GO:0006281,GO:0065003,GO:0006355,GO:0005694,GO:0006325,GO:0005654,GO:0003677,GO:0045182,GO:0003723,GO:0016071,GO:0031047,GO:0140110
contig00001.g777	764	ID=contig00001.g777;Description=ubiquinone biosynthesis protein CAT5 [Fusarium fujikuroi];Gene=clpX;Ontology_term=extracellular space,catalytic activity, acting on a protein,oxidoreductase activity,mitochondrion,lipid metabolic process,signaling,cytosol,transferase activity,plasma membrane,hydrolase activity;Ontology_id=GO:0005615,GO:0140096,GO:0016491,GO:0005739,GO:0006629,GO:0023052,GO:0005829,GO:0016740,GO:0005886,GO:0016787;Enzyme_code=EC:3.4.21,EC:1.14.99.60,EC:1.14.13,EC:2.7.1.107;Enzyme_name=Acting on peptide bonds (peptidases),3-demethoxyubiquinol 3-hydroxylase,Acting on paired donors, with incorporation or reduction of molecular oxygen. The oxygen incorporated need not be derived from O2,diacylglycerol kinase (ATP)

contig00001.g778	5478	ID=contig00001.g778;Description=phospholipase D [Fusarium tjaetaba];Gene=FVER53263_02633;Ontology_term=nucleus,vesicle-mediated transport,endosome,lipid metabolic process,signaling,anatomical structure development,cell differentiation,reproductive process,lipid binding,hydrolase activity;Ontology_id=GO:0005634,GO:0016192,GO:0005768,GO:0006629,GO:0023052,GO:0048856,GO:0030154,GO:0022414,GO:0008289,GO:0016787;Enzyme_code=EC:3.1.4.4,EC:3.1.4,EC:3.1,EC:3,EC:3.1.4.4;Enzyme_name=phospholipase D,Acting on ester bonds,Acting on ester bonds,Hydrolases,phospholipase D
contig00001.g779	1370	ID=contig00001.g779;Description=hypothetical protein FOZG_09633 [Fusarium oxysporum Fo47];Gene=FOXG_05508;Ontology_term=membrane;Ontology_id=GO:0016020
contig00001.g780	2321	ID=contig00001.g780;Description=hypothetical protein FVER14953_02631 [Fusarium verticillioides];Gene=BFJ72_g11461;Ontology_term=membrane;Ontology_id=GO:0016020
contig00001.g781	2025	ID=contig00001.g781;Description=major facilitator superfamily domain-containing protein [Fusarium oxysporum];Gene=FTJAE_12216;Ontology_term=transmembrane transport,transporter activity;Ontology_id=GO:0055085,GO:0005215
contig00001.g782	1434	ID=contig00001.g782;Description=hypothetical protein FVER14953_02627 [Fusarium verticillioides];Gene=FMUND_5834;Ontology_term=cell cycle;Ontology_id=GO:0007049
contig00001.g783	2702	ID=contig00001.g783;Description=spindle pole body component alp6 [Fusarium tjaetaba];Gene=FTJAE_12214;Ontology_term=microtubule organizing center,cytoskeleton organization,protein-containing complex assembly,establishment or maintenance of cell polarity,structural molecule activity,anatomical structure development,mitotic nuclear division,reproductive process,chromosome segregation,cytoskeletal protein binding;Ontology_id=GO:0005815,GO:0007010,GO:0065003,GO:0007163,GO:0005198,GO:0048856,GO:0140014,GO:0022414,GO:0007059,GO:0008092
contig00001.g784	1504	ID=contig00001.g784;Description=transcriptional coactivator HFI1/ADA1 [Fusarium verticillioides 7600];Gene=FVEG_02623;Ontology_term=regulation of DNA-templated transcription,chromosome,chromatin organization,transcription regulator activity,molecular adaptor activity,nucleoplasm;Ontology_id=GO:0006355,GO:0005694,GO:0006325,GO:0140110,GO:0060090,GO:0005654

contig00001.g785	1245	ID=contig00001.g785;Description=cAMP-dependent protein kinase regulatory subunit [Fusarium verticillioides 7600];Gene=FTJAE_5564;Ontology_term=nucleocytoplasmic transport,molecular function regulator activity,signaling,nuclear chromosome,reproductive process,intracellular protein transport,transferase activity,plasma membrane;Ontology_id=GO:0006913,GO:0098772,GO:0023052,GO:0000228,GO:0022414,GO:0006886,GO:0016740,GO:0005886;Enzyme_code=EC:2.7,EC:2.7,EC:2;Enzyme_name=Transferring phosphorus-containing groups,Transferring phosphorus-containing groups,Transferases
contig00001.g786	660	ID=contig00001.g786;Description=BCAS2 family [Fusarium tjaetaba];Gene=FVER53263_02621;Ontology_term=nucleus,mRNA metabolic process;Ontology_id=GO:0005634,GO:0016071
contig00001.g787	1381	ID=contig00001.g787;Description=F1F0-ATP synthase regulatory factor Stf2 [Fusarium sp. NRRL 25303]
contig00001.g788	4402	ID=contig00001.g788;Description=transcription elongation factor SPT6 [Fusarium verticillioides 7600];Gene=FMUND_5840;Ontology_term=nucleus,DNA binding,nucleocytoplasmic transport,translation regulator activity,mRNA metabolic process,protein-containing complex assembly,histone binding,regulation of DNA-templated transcription,chromosome,chromatin organization,transcription regulator activity;Ontology_id=GO:0005634,GO:0003677,GO:0006913,GO:0045182,GO:0016071,GO:0065003,GO:0042393,GO:0006355,GO:0005694,GO:0006325,GO:0140110
contig00001.g789	1707	ID=contig00001.g789;Description=STE/STE7/MEK1 protein kinase [Fusarium verticillioides 7600];Gene=FCIRC_5359;Ontology_term=catalytic activity, acting on a protein,signaling,anatomical structure development,cell differentiation,reproductive process,cell wall organization or biogenesis,transferase activity,programmed cell death,mitotic cell cycle;Ontology_id=GO:0140096,GO:0023052,GO:0048856,GO:0030154,GO:0022414,GO:0071554,GO:0016740,GO:0012501,GO:0000278;Enzyme_code=EC:2.7.1,EC:2.7.1,EC:2.7.12.1,EC:2.7.11.1,EC:2.7.12.2,EC:2.7,EC:2;Enzyme_name=Transferring phosphorus-containing groups,Transferring phosphorus-containing groups,dual-specificity kinase,non-specific serine/threonine protein kinase,mitogen-activated protein kinase kinase,Transferring phosphorus-containing groups,Transferases
contig00001.g790	940	ID=contig00001.g790;Description=HAD superfamily hydrolase [Fusarium tjaetaba];Gene=FMUND_5842;Ontology_term=hydrolase activity;Ontology_id=GO:0016787;Enzyme_code=EC:3.1.3.74;Enzyme_name=pyridoxal phosphatase

contig00001.g791	509	ID=contig00001.g791;Description=cell division cycle 123 [Fusarium tjaetaba];Gene=FTJAE_5570;Ontology_term=cell division;Ontology_id=GO:0051301
contig00001.g792	3300	ID=contig00001.g792;Description=Lactobacillus histidine kinase [Fusarium acutatum];Gene=FMUND_5843;Ontology_term=transferase activity;Ontology_id=GO:0016740;Enzyme_code=EC:2.7,EC:2.7,EC:2;Enzyme_name=Transferring phosphorus-containing groups,Transferring phosphorus-containing groups,Transferases
contig00001.g793	2388	ID=contig00001.g793;Description=DNA polymerase epsilon subunit 2 [Fusarium verticillioides 7600];Gene=FMAN_07737;Ontology_term=DNA binding,DNA repair,DNA replication,regulation of DNA-templated transcription,nuclear chromosome,chromatin organization,mitotic cell cycle,nucleoplasm;Ontology_id=GO:0003677,GO:0006281,GO:0006260,GO:0006355,GO:0000228,GO:0006325,GO:0000278,GO:0005654;Enzyme_code=EC:2.7.7.7;Enzyme_name=DNA-directed DNA polymerase
contig00001.g794	4451	ID=contig00001.g794;Description=putative serine/threonine protein kinase (SNF1) [Fusarium fujikuroi];Gene=TY2A-C;Ontology_term=cytoskeleton organization,cytoplasmic translation,DNA replication,anatomical structure development,cell adhesion,chromatin organization,intracellular protein transport,vacuole,mitotic cell cycle,nucleoplasm,nucleocytoplasmic transport,RNA binding,molecular function regulator activity,endosome,autophagy,cytosol,cell wall organization or biogenesis,catalytic activity, acting on a protein,establishment or maintenance of cell polarity,regulation of DNA-templated transcription,reproductive process,nuclear envelope,carbohydrate metabolic process,microtubule organizing center,signaling,mitochondrion,transferase activity;Ontology_id=GO:0007010,GO:0002181,GO:0006260,GO:0048856,GO:0007155,GO:0006325,GO:0006886,GO:0005773,GO:0000278,GO:0005654,GO:0006913,GO:0003723,GO:0098772,GO:0005768,GO:0006914,GO:0005829,GO:0071554,GO:0140096,GO:0007163,GO:0006355,GO:0022414,GO:0005635,GO:0005975,GO:0005815,GO:0023052,GO:0005739,GO:0016740;Enzyme_code=EC:2.7.11.11,EC:2.7.1,EC:2.7.11.1,EC:2.7,EC:2,EC:2.7.11.11,EC:2.7.11.22;Enzyme_name=cAMP-dependent protein kinase,Transferring phosphorus-containing groups,non-specific serine/threonine protein kinase,Transferring phosphorus-containing groups,Transferases,cAMP-dependent protein kinase,cyclin-dependent kinase

contig00001.g795	1920	ID=contig00001.g795;Description=isocitrate lyase [Fusarium tjaetaba];Gene=FVER53263_02611;Ontology_term=carbohydrate metabolic process,peroxisome,lipid metabolic process,lyase activity;Ontology_id=GO:0005975,GO:0005777,GO:0006629,GO:0016829;Enzyme_code=EC:4.1.3.1,EC:4.1.3.30,EC:4.1.3.1,EC:4.1,EC:4,EC:4.1.3.30,EC:4.1.3;Enzyme_name=isocitrate lyase,methylisocitrate lyase,isocitrate lyase,Carbon-carbon lyases,Lyases,methylisocitrate lyase,Carbon-carbon lyases
contig00001.g796	2382	ID=contig00001.g796;Description=neutral trehalase [Fusarium odoratissimum NRRL 54006];Gene=FNYG_11771;Ontology_term=carbohydrate metabolic process,cell differentiation,anatomical structure development,reproductive process,hydrolase activity;Ontology_id=GO:0005975,GO:0030154,GO:0048856,GO:0022414,GO:0016787;Enzyme_code=EC:3.2.1.28;Enzyme_name=alpha,alpha-trehalase
contig00001.g797	962	ID=contig00001.g797;Description=yippee family protein [Fusarium coicis];Gene=FANTH_12743;Ontology_term=metal ion binding;Ontology_id=GO:0046872
contig00001.g798	1484	ID=contig00001.g798;Description=cytosolic nonspecific dipeptidase [Fusarium proliferatum];Gene=FOYG_08370;Ontology_term=catalytic activity, acting on a protein,sulfur compound metabolic process,mitochondrion,cellular modified amino acid metabolic process,extracellular matrix,extracellular region,hydrolase activity;Ontology_id=GO:0140096,GO:0006790,GO:0005739,GO:0006575,GO:0031012,GO:0005576,GO:0016787;Enzyme_code=EC:3.4.13,EC:3.4.13,EC:3.4,EC:3,EC:3.4.19;Enzyme_name=Acting on peptide bonds (peptidases),Acting on peptide bonds (peptidases),Acting on peptide bonds (peptidases),Hydrolases,Acting on peptide bonds (peptidases)
contig00001.g799	1959	ID=contig00001.g799;Description=MPE1 Role in mRNA 3' end formation [Fusarium coicis];Gene=FDENT_7495;Ontology_term=catalytic activity, acting on a protein,RNA binding,mRNA metabolic process,cytosol,transferase activity,nucleoplasm;Ontology_id=GO:0140096,GO:0003723,GO:0016071,GO:0005829,GO:0016740,GO:0005654;Enzyme_code=EC:2.3.2,EC:2.3,EC:2,EC:2.3.2;Enzyme_name=Acyltransferases,Acyltransferases,Transferases,Acyltransferases

contig00001.g800	1074	ID=contig00001.g800;Description=ATPase get3 [Fusarium odoratissimum NRRL 54006];Gene=FocTR4_00008802;Ontology_term=membrane organization,vesicle-mediated transport,molecular function regulator activity,protein folding chaperone,ATP-dependent activity,signaling,cytosol,reproductive process,endoplasmic reticulum,intracellular protein transport,hydrolase activity;Ontology_id=GO:0061024,GO:0016192,GO:0098772,GO:0044183,GO:0140657,GO:0023052,GO:0005829,GO:0022414,GO:0005783,GO:0006886,GO:0016787;Enzyme_code=EC:3.6.1.15,EC:3.6.3.16,EC:3.6.1,EC:3.6,EC:3,EC:3.6.1.15;Enzyme_name=nucleoside-triphosphate phosphatase,Acting on acid anhydrides,Acting on acid anhydrides,Acting on acid anhydrides,Hydrolases,nucleoside-triphosphate phosphatase
contig00001.g801	435	ID=contig00001.g801;Description=hydroxyisourate hydrolase [Fusarium verticillioides 7600];Gene=F25303_3202;Ontology_term=nucleobase-containing small molecule metabolic process,hydrolase activity;Ontology_id=GO:0055086,GO:0016787;Enzyme_code=EC:3.5.2.17,EC:3.5.2,EC:3.5,EC:3,EC:3.5.2.17;Enzyme_name=hydroxyisourate hydrolase,Acting on carbon-nitrogen bonds, other than peptide bonds,Acting on carbon-nitrogen bonds, other than peptide bonds,Hydrolases,hydroxyisourate hydrolase
contig00001.g802	569	ID=contig00001.g802;Description=hypothetical protein FVER14953_02604 [Fusarium verticillioides]
contig00001.g803	1935	ID=contig00001.g803;Description=RING finger [Fusarium napiforme];Gene=BFJ68_g10584;Ontology_term=nucleus,catalytic activity, acting on a protein,DNA-templated transcription,transferase activity;Ontology_id=GO:0005634,GO:0140096,GO:0006351,GO:0016740;Enzyme_code=EC:2.3,EC:2,EC:1.3.1.71,EC:2.3.2;Enzyme_name=Acyltransferases,Transferases,Delta(24(24(1)))-sterol reductase,Acyltransferases
contig00001.g804	3264	ID=contig00001.g804;Description=5 AMP-activated kinase subunit beta-2 [Fusarium tjaetaba];Gene=FTJAE_5583;Ontology_term=nucleus,DNA binding,signaling,reproductive process,transferase activity,plasma membrane;Ontology_id=GO:0005634,GO:0003677,GO:0023052,GO:0022414,GO:0016740,GO:0005886;Enzyme_code=EC:2.7,EC:2.7,EC:2;Enzyme_name=Transferring phosphorus-containing groups,Transferring phosphorus-containing groups,Transferases
contig00001.g805	839	ID=contig00001.g805;Description=hypothetical protein FCOIX_6618 [Fusarium coicis]

contig00001.g806	1086	ID=contig00001.g806;Description=endo alpha-1 4 polygalactosaminidase precursor [Fusarium tjaetaba];Gene=FVER53263_02600;Ontology_term=hydrolase activity;Ontology_id=GO:0016787;Enzyme_code=EC:3.2.1.22,EC:3.2,EC:3,EC:3.2.1.22,EC:3.2.1;Enzyme_name=alpha-galactosidase,Glycosylases,Hydrolases,alpha-galactosidase,Glycosylases
contig00001.g807	2905	ID=contig00001.g807;Description=hypothetical protein FOXG_05541 [Fusarium oxysporum f. sp. lycopersici 4287];Gene=FOTG_02727;Ontology_term=nucleus,DNA binding,regulation of DNA-templated transcription,transcription regulator activity;Ontology_id=GO:0005634,GO:0003677,GO:0006355,GO:0140110
contig00001.g808	1488	ID=contig00001.g808;Description=starvation sensing rspA [Fusarium mundagurra];Gene=FANTH_12732;Ontology_term=lyase activity;Ontology_id=GO:0016829;Enzyme_code=EC:4.2.1.90,EC:4.2.1,EC:4.2,EC:4,EC:4.2.1.90;Enzyme_name=L-rhamnonate dehydratase,Carbon-oxygen lyases,Carbon-oxygen lyases,Lyases,L-rhamnonate dehydratase
contig00001.g809	819	ID=contig00001.g809;Description=glucose 1-dehydrogenase [Fusarium odoratissimum NRRL 54006];Ontology_term=carbohydrate metabolic process,oxidoreductase activity;Ontology_id=GO:0005975,GO:0016491;Enzyme_code=EC:1.6,EC:1,EC:1.1.1.378,EC:1.1.1.377,EC:1.1.1.173;Enzyme_name=Acting on NADH or NADPH,Oxidoreductases,L-rhamnose 1-dehydrogenase [NAD(P)(+)],L-rhamnose 1-dehydrogenase (NADP(+)),L-rhamnose 1-dehydrogenase
contig00001.g810	1414	ID=contig00001.g810;Description=CDP-alcohol phosphatidyltransferase class-I family [Fusarium tjaetaba];Gene=FCIRC_5339;Ontology_term=transferase activity;Ontology_id=GO:0016740;Enzyme_code=EC:2,EC:2;Enzyme_name=Transferases,Transferases
contig00001.g811	1121	ID=contig00001.g811;Description=hypothetical protein FVEG_02595 [Fusarium verticillioides 7600]
contig00001.g812	484	ID=contig00001.g812;Description=hypothetical protein FVEG_02594 [Fusarium verticillioides 7600];Gene=FCIRC_5337;Ontology_term=mitochondrion;Ontology_id=GO:0005739

contig00001.g813	1137	ID=contig00001.g813;Description=39S ribosomal protein L22, mitochondrial [Fusarium oxysporum];Gene=FOMG_06968;Ontology_term=nucleus,catalytic activity, acting on a protein,mitochondrial gene expression,ATP-dependent activity,mitochondrion,structural molecule activity,ribosome,ligase activity;Ontology_id=GO:0005634,GO:0140096,GO:0140053,GO:0140657,GO:0005739,GO:0005198,GO:0005840,GO:0016874;Enzyme_code=EC:6.2.1.45,EC:6.2.1.45,EC:6.2,EC:6;Enzyme_name=E1 ubiquitin-activating enzyme,E1 ubiquitin-activating enzyme,Forming carbon-sulfur bonds,Ligases
contig00001.g814	1092	ID=contig00001.g814;Description=hypothetical protein FVEG_02591 [Fusarium verticillioides 7600];Gene=FTJAE_5593;Ontology_term=side of membrane;Ontology_id=GO:0098552
contig00001.g815	3246	ID=contig00001.g815;Description=PRP24-pre-mRNA splicing factor [Fusarium tjaetaba];Gene=FNAPI_12977;Ontology_term=nucleus,RNA binding,mRNA metabolic process,protein-containing complex assembly;Ontology_id=GO:0005634,GO:0003723,GO:0016071,GO:0065003
contig00001.g816	4774	ID=contig00001.g816;Description=DNA RNA helicase (DEAD H box family II) [Fusarium pseudoanthophilum];Gene=FNYG_11793;Ontology_term=RNA binding,ATP-dependent activity,cytoplasmic translation,mitochondrion,catalytic activity, acting on RNA,hydrolase activity;Ontology_id=GO:0003723,GO:0140657,GO:0002181,GO:0005739,GO:0140098,GO:0016787;Enzyme_code=EC:3,EC:3.6.1,EC:3.6.4.13,EC:3.6,EC:3,EC:3.6.1.15;Enzyme_name=Hydrolases,Acting on acid anhydrides,RNA helicase,Acting on acid anhydrides,Hydrolases,nucleoside-triphosphate phosphatase
contig00001.g817	8180	ID=contig00001.g817;Description=translational activator GCN1 [Fusarium tjaetaba];Gene=FPHYL_8184;Ontology_term=cytoskeleton,structural molecule activity,cytosol,ribosome;Ontology_id=GO:0005856,GO:0005198,GO:0005829,GO:0005840
contig00001.g818	790	ID=contig00001.g818;Description=60S ribosomal protein L19A [Fusarium musae];Gene=FOXB_09168;Ontology_term=cytoskeleton,RNA binding,structural molecule activity,cytosol,ribosome;Ontology_id=GO:0005856,GO:0003723,GO:0005198,GO:0005829,GO:0005840
contig00001.g819	1819	ID=contig00001.g819;Description=serine hydroxymethyltransferase, mitochondrial [Fusarium verticillioides 7600];Gene=FSUBG_8674;Ontology_term=amino acid metabolic process,cellular modified amino acid metabolic process,transferase activity;Ontology_id=GO:0006520,GO:0006575,GO:0016740;Enzyme_code=EC:2.1.2.1,EC:2.1.1;Enzyme_name=glycine hydroxymethyltransferase,Transferring one-carbon groups

contig00001.g820	2814	ID=contig00001.g820;Description=alpha-ketoglutarate-dependent dioxygenase alkB like 3 [Fusarium tjaetaba];Gene=FPCIR_9171;Ontology_term=DNA repair,oxidoreductase activity,hydrolase activity;Ontology_id=GO:0006281,GO:0016491,GO:0016787;Enzyme_code=EC:1,EC:3,EC:1,EC:3;Enzyme_name=Oxidoreductases,Hydrolases,Oxidoreductases,Hydrolases
contig00001.g821	975	ID=contig00001.g821;Description=transcription initiation factor TFIID subunit 8 [Fusarium bulbicola];Gene=FCIRC_1103;Ontology_term=regulation of DNA-templated transcription,transcription regulator activity,nucleoplasm;Ontology_id=GO:0006355,GO:0140110,GO:0005654
contig00001.g822	1465	ID=contig00001.g822;Description=guanine nucleotide-binding protein subunit beta-like protein [Colletotrichum tofieldiae];Gene=CEK26_008678;Ontology_term=translation regulator activity;Ontology_id=GO:0045182
contig00001.g823	1164	ID=contig00001.g823;Description=OST3-oligosaccharyltransferase gamma subunit [Fusarium subglutinans];Gene=FANTH_13568;Ontology_term=protein glycosylation,endoplasmic reticulum,transferase activity;Ontology_id=GO:0006486,GO:0005783,GO:0016740;Enzyme_code=EC:2,EC:2.4,EC:2,EC:2.4.99.18,EC:2.4.1;Enzyme_name=Transferases,Glycosyltransferases,Transferases,dolichyl-diphosphooligosaccharide--protein glycotransferase,Glycosyltransferases
contig00001.g824	1772	ID=contig00001.g824;Description=ribosomal RNA large subunit methyltransferase J [Fusarium tjaetaba];Gene=FTJAE_5602;Ontology_term=transferase activity;Ontology_id=GO:0016740;Enzyme_code=EC:2.1.1;Enzyme_name=Transferring one-carbon groups
contig00001.g825	1026	ID=contig00001.g825;Description=ribosomal RNA large subunit methyltransferase J [Fusarium verticillioides 7600];Gene=FVEG_02579;Ontology_term=mitochondrion,ribosome biogenesis,transferase activity,catalytic activity, acting on RNA;Ontology_id=GO:0005739,GO:0042254,GO:0016740,GO:0140098;Enzyme_code=EC:2.1.1,EC:2.1,EC:2.1.1.166,EC:2,EC:2.1.1,EC:2.1.1.168;Enzyme_name=Transferring one-carbon groups,Transferring one-carbon groups,23S rRNA (uridine(2552)-2'-O)-methyltransferase,Transferases,Transferring one-carbon groups,21S rRNA (uridine(2791)-2'-O)-methyltransferase

contig00001.g826	854	ID=contig00001.g826;Description=solute carrier family 25 member 42 [Fusarium oxysporum Fo47];Gene=FOTG_02704;Ontology_term=RNA binding,transmembrane transport,cytoplasmic translation,mitochondrion,structural molecule activity,cytosol,ribosome;Ontology_id=GO:0003723,GO:0055085,GO:0002181,GO:0005739,GO:0005198,GO:0005829,GO:0005840
contig00001.g827	1279	ID=contig00001.g827;Description=mitochondrial carrier [Fusarium tjaetaba];Gene=RPL40A;Ontology_term=nucleus,transmembrane transport,mitochondrion,cytosol,transporter activity;Ontology_id=GO:0005634,GO:0055085,GO:0005739,GO:0005829,GO:0005215
contig00001.g828	1202	ID=contig00001.g828;Description=mRNA splicing factor [Fusarium fujikuroi];Gene=FPANT_10091;Ontology_term=RNA binding;Ontology_id=GO:0003723
contig00001.g829	2289	ID=contig00001.g829;Description=acyl-CoA cholesterol acyltransferase [Fusarium fujikuroi];Gene=RPL40A;Ontology_term=protein-containing complex assembly,cytoplasmic translation,protein catabolic process,structural molecule activity,ribosome biogenesis,endoplasmic reticulum,ribosome,nucleus,nucleocytoplasmic transport,lipid metabolic process,cytosol,protein tag activity,transferase activity;Ontology_id=GO:0065003,GO:0002181,GO:0030163,GO:0005198,GO:0042254,GO:0005783,GO:0005840,GO:0005634,GO:0006913,GO:0006629,GO:0005829,GO:0031386,GO:0016740;Enzyme_code=EC:2.3.1,EC:2.3,EC:2,EC:2.3.1.26,EC:2.3.1;Enzyme_name=Acyltransferases,Acyltransferases,Transferases,sterol O-acyltransferase,Acyltransferases
contig00001.g830	2158	ID=contig00001.g830;Description=calponin homology domain-containing protein [Fusarium redolens];Gene=BFJ63_vAg15445;Ontology_term=cytoskeleton,vesicle-mediated transport,cytoskeleton organization,protein-containing complex assembly,cytokinesis,cytoskeletal protein binding,plasma membrane,molecular adaptor activity;Ontology_id=GO:0005856,GO:0016192,GO:0007010,GO:0065003,GO:0000910,GO:0008092,GO:0005886,GO:0060090
contig00001.g831	861	ID=contig00001.g831;Description=ring finger [Fusarium coicis];Gene=RPS31;Ontology_term=structural molecule activity,ribosome;Ontology_id=GO:0005198,GO:0005840

contig00001.g832	8793	ID=contig00001.g832;Description=DNA-directed RNA polymerase III subunit RPC1 [Fusarium subglutinans];Gene=FSUBG_14002;Ontology_term=DNA binding,catalytic activity, acting on a protein,DNA-templated transcription,tRNA metabolic process,catalytic activity, acting on RNA,transferase activity,hydrolase activity,nucleoplasm;Ontology_id=GO:0003677,GO:0140096,GO:0006351,GO:0006399,GO:0140098,GO:0016740,GO:0016787,GO:0005654;Enzyme_code=EC:3.4,EC:2.7.7.6,EC:3.4,EC:2.7,EC:2,EC:3,EC:2.7.7,EC:2.7.7.6;Enzyme_name=Acting on peptide bonds (peptidases),DNA-directed RNA polymerase,Acting on peptide bonds (peptidases),Transferring phosphorus-containing groups,Transferases,Hydrolases,Transferring phosphorus-containing groups,DNA-directed RNA polymerase
contig00001.g833	1288	ID=contig00001.g833;Description=putative peptidase [Fusarium oxysporum f. sp. rapae];Gene=FDENT_7461;Ontology_term=nucleus,DNA binding,catalytic activity, acting on a protein,hydrolase activity;Ontology_id=GO:0005634,GO:0003677,GO:0140096,GO:0016787;Enzyme_code=EC:3.4,EC:3.4,EC:3;Enzyme_name=Acting on peptide bonds (peptidases),Acting on peptide bonds (peptidases),Hydrolases
contig00002.g834	1007	ID=contig00002.g834;Description=hypothetical protein FOXG_00599 [Fusarium oxysporum f. sp. lycopersici 4287];Gene=FOXG_00599
contig00002.g835	1446	ID=contig00002.g835;Description=cystathionine beta-lyase [Fusarium verticillioides 7600];Gene=FPCIR_3724;Ontology_term=nucleus,sulfur compound metabolic process,lyase activity,amino acid metabolic process,transferase activity;Ontology_id=GO:0005634,GO:0006790,GO:0016829,GO:0006520,GO:0016740;Enzyme_code=EC:4,EC:2.5.1,EC:2.5.1.48,EC:4.4.1.8,EC:2,EC:4.4,EC:4,EC:4.4.1.1;Enzyme_name=Lyases,Transferring alkyl or aryl groups, other than methyl groups,cystathionine gamma-synthase,Carbon-sulfur lyases,Transferases,Carbon-sulfur lyases,Lyases,cystathionine gamma-lyase
contig00002.g836	700	ID=contig00002.g836;Description=hypothetical protein FVEG_00912 [Fusarium verticillioides 7600]
contig00002.g837	999	ID=contig00002.g837;Description=nodulation nodB [Fusarium tjaetaba];Gene=FTJAE_5394;Ontology_term=carbohydrate metabolic process,hydrolase activity;Ontology_id=GO:0005975,GO:0016787;Enzyme_code=EC:3.5,EC:3.5,EC:3;Enzyme_name=Acting on carbon-nitrogen bonds, other than peptide bonds,Acting on carbon-nitrogen bonds, other than peptide bonds,Hydrolases

contig00002.g838	1060	ID=contig00002.g838;Description=terpene synthase [Fusarium tjaetaba];Gene=FVEG_00910;Ontology_term=lyase activity;Ontology_id=GO:0016829;Enzyme_code=EC:4,EC:4;Enzyme_name=Lyases,Lyases
contig00002.g839	903	ID=contig00002.g839;Description=hypothetical protein FVEG_00909 [Fusarium verticillioides 7600]
contig00002.g840	5151	ID=contig00002.g840;Description=adenosinetriphosphatase [Fusarium proliferatum];Gene=FOC1_g10015904;Ontology_term=nucleus,RNA binding,mRNA metabolic process,protein-containing complex assembly,ATP-dependent activity,catalytic activity, acting on RNA,hydrolase activity;Ontology_id=GO:0005634,GO:0003723,GO:0016071,GO:0065003,GO:0140657,GO:0140098,GO:0016787;Enzyme_code=EC:3,EC:3.6.1,EC:3.6.4.13,EC:3.6,EC:3,EC:3.6.1.15;Enzyme_name=Hydrolases,Acting on acid anhydrides,RNA helicase,Acting on acid anhydrides,Hydrolases,nucleoside-triphosphate phosphatase
contig00002.g841	1159	ID=contig00002.g841;Description=hypothetical protein FVEG_00906 [Fusarium verticillioides 7600];Gene=FFUJ_00964;Ontology_term=nucleus;Ontology_id=GO:0005634
contig00002.g842	1709	ID=contig00002.g842;Description=Ff.00g093220.m01.CDS01 [Fusarium sp. VM40]
contig00002.g843	1142	ID=contig00002.g843;Description=programmed cell death (calcium-binding) [Fusarium subglutinans];Gene=BFJ72_g7709;Ontology_term=calcium ion binding;Ontology_id=GO:0005509
contig00002.g844	928	ID=contig00002.g844;Description=cation transporter ChaC [Fusarium oxysporum f. sp. lycopersici 4287];Gene=FOYG_06727;Ontology_term=nucleus,catalytic activity, acting on a protein,sulfur compound metabolic process,detoxification,lyase activity,cellular modified amino acid metabolic process,transferase activity;Ontology_id=GO:0005634,GO:0140096,GO:0006790,GO:0098754,GO:0016829,GO:0006575,GO:0016740;Enzyme_code=EC:4.3.2.7,EC:4.3.2.9,EC:2.3,EC:4.3,EC:2.3.2.5,EC:2,EC:4,EC:4.3.2.7,EC:4.3.2.9,EC:2.3.2,EC:4.3.2;Enzyme_name=glutathione-specific gamma-glutamylcyclotransferase,gamma-glutamylcyclotransferase,Acyltransferases,Carbon-nitrogen lyases,glutaminyl-peptide cyclotransferase,Transferases,Lyases,glutathione-specific gamma-glutamylcyclotransferase,gamma-glutamylcyclotransferase,Acyltransferases,Carbon-nitrogen lyases
contig00002.g845	814	ID=contig00002.g845;Description=RNA recognition motif domain [Fusarium oxysporum f. sp. vasinfectum];Gene=FOPG_01915;Ontology_term=RNA binding;Ontology_id=GO:0003723
contig00002.g846	1898	ID=contig00002.g846;Description=hypothetical protein FVEG_00901 [Fusarium verticillioides 7600]

contig00002.g847	897	ID=contig00002.g847;Description=citrate lyase subunit beta-like protein [Fusarium verticillioides 7600];Gene=FPCIR_3713;Ontology_term=lyase activity;Ontology_id=GO:0016829;Enzyme_code=EC:4,EC:4;Enzyme_name=Lyases,Lyases
contig00002.g848	791	ID=contig00002.g848;Description=Golgi apparatus membrane protein tvp23 [Fusarium musae];Gene=AK830_g8472;Ontology_term=vesicle-mediated transport,Golgi apparatus;Ontology_id=GO:0016192,GO:0005794
contig00002.g849	1247	ID=contig00002.g849;Description=hypothetical protein FVEG_00898 [Fusarium verticillioides 7600];Gene=FVEG_00898;Ontology_term=membrane;Ontology_id=GO:0016020
contig00002.g850	3556	ID=contig00002.g850;Description=DUF726 domain protein [Fusarium tjaetaba];Gene=FVER53263_00897;Ontology_term=hydrolase activity;Ontology_id=GO:0016787;Enzyme_code=EC:3;Enzyme_name=Hydrolases
contig00002.g851	963	ID=contig00002.g851;Description=rRNA-processing UTP23 [Fusarium tjaetaba];Gene=FVER53263_00896;Ontology_term=RNA binding,mitochondrion,ribosome biogenesis,nucleolus;Ontology_id=GO:0003723,GO:0005739,GO:0042254,GO:0005730
contig00002.g852	1203	ID=contig00002.g852;Description=phosphatase 2c 80 [Fusarium subglutinans];Gene=FDENT_12960;Ontology_term=catalytic activity, acting on a protein,ribosome biogenesis,nucleolus,hydrolase activity;Ontology_id=GO:0140096,GO:0042254,GO:0005730,GO:0016787;Enzyme_code=EC:3.1.3,EC:3.1.3.16,EC:3.1,EC:3,EC:3.1.3;Enzyme_name=Acting on ester bonds,protein-serine/threonine phosphatase,Acting on ester bonds,Hydrolases,Acting on ester bonds
contig00002.g853	4872	ID=contig00002.g853;Description=related to CAF120 CCR4 Associated Factor 120 kDa [Fusarium proliferatum];Ontology_term=catalytic activity, acting on a protein,signaling,regulation of DNA-templated transcription,transferase activity,programmed cell death,mitotic cell cycle;Ontology_id=GO:0140096,GO:0023052,GO:0006355,GO:0016740,GO:0012501,GO:0000278;Enzyme_code=EC:2.7.1,EC:2.7.11.1,EC:2.7,EC:2;Enzyme_name=Transferring phosphorus-containing groups,non-specific serine/threonine protein kinase,Transferring phosphorus-containing groups,Transferases

contig00002.g854	3647	ID=contig00002.g854;Description=serine/threonine protein kinase [Fusarium verticillioides 7600];Gene=FPANT_215;Ontology_term=DNA repair,catalytic activity, acting on a protein,nucleobase-containing small molecule metabolic process,DNA replication,reproductive process,intracellular protein transport,mitotic cell cycle,nucleocytoplasmic transport,DNA binding,nucleus,autophagy,signaling,cytosol,transferase activity,programmed cell death;Ontology_id=GO:0006281,GO:0140096,GO:0055086,GO:0006260,GO:0022414,GO:0006886,GO:0000278,GO:0006913,GO:0003677,GO:0005634,GO:0006914,GO:0023052,GO:0005829,GO:0016740,GO:0012501;Enzyme_code=EC:2.7.11.1,EC:2.7.1,EC:2.7.12.1,EC:2.7.11.1,EC:2.7,EC:2;Enzyme_name=non-specific serine/threonine protein kinase,Transferring phosphorus-containing groups,dual-specificity kinase,non-specific serine/threonine protein kinase,Transferring phosphorus-containing groups,Transferases
contig00002.g855	2810	ID=contig00002.g855;Description=hypothetical protein FVER53590_00892 [Fusarium verticillioides]
contig00002.g856	2534	ID=contig00002.g856;Description=oligosaccharyl transferase stt3 subunit [Fusarium musae];Gene=FMUND_10217;Ontology_term=protein glycosylation,endoplasmic reticulum,transferase activity;Ontology_id=GO:0006486,GO:0005783,GO:0016740;Enzyme_code=EC:2.4.99.18,EC:2.4.1,EC:2.4,EC:2,EC:2.4.99.18,EC:2.4.1;Enzyme_name=dolichyl-diphosphooligosaccharide--protein glycotransferase,Glycosyltransferases,Glycosyltransferases,Transferases,dolichyl-diphosphooligosaccharide--protein glycotransferase,Glycosyltransferases
contig00002.g857	820	ID=contig00002.g857;Description=F1F0-ATP synthase subunit E [Fusarium tjaetaba];Gene=FPANT_212;Ontology_term=transmembrane transport,nucleobase-containing small molecule metabolic process,mitochondrion,carbohydrate derivative metabolic process,transferase activity,transporter activity;Ontology_id=GO:0055085,GO:0055086,GO:0005739,GO:1901135,GO:0016740,GO:0005215;Enzyme_code=EC:2.4.99.18,EC:2.4.1;Enzyme_name=dolichyl-diphosphooligosaccharide--protein glycotransferase,Glycosyltransferases
contig00002.g858	2137	ID=contig00002.g858;Description=dynein intermediate chain, cytosolic [Fusarium verticillioides 7600]
contig00002.g859	1093	ID=contig00002.g859;Description=pyridoxine 4-dehydrogenase [Fusarium tjaetaba]
contig00002.g860	954	ID=contig00002.g860;Description=transcription factor [Fusarium napiforme];Gene=FVEG_00887;Ontology_term=metal ion binding;Ontology_id=GO:0046872

contig00002.g861	1868	ID=contig00002.g861;Description=tubulin-specific chaperone E [Fusarium tjaetaba];Ontology_term=protein-containing complex assembly,cytoskeletal protein binding,protein folding;Ontology_id=GO:0065003,GO:0008092,GO:0006457
contig00002.g862	1326	ID=contig00002.g862;Description=hypothetical protein FVEG_00885 [Fusarium verticillioides 7600];Gene=FVER53263_00885;Ontology_term=proteasome binding;Ontology_id=GO:0070628
contig00002.g863	1416	ID=contig00002.g863;Description=1-phosphatidylinositol phosphodiesterase [Fusarium proliferatum];Gene=FVER53263_00884;Ontology_term=lipid metabolic process,hydrolase activity;Ontology_id=GO:0006629,GO:0016787;Enzyme_code=EC:3.1.4,EC:3.1.4,EC:4.6.1.13,EC:3.1,EC:3;Enzyme_name=Acting on ester bonds,Acting on ester bonds,phosphatidylinositol diacylglycerol-lyase,Acting on ester bonds,Hydrolases
contig00002.g864	621	ID=contig00002.g864;Description=hypothetical protein FVER14953_00883 [Fusarium verticillioides]
contig00002.g865	1391	ID=contig00002.g865;Description=methionine adenosyltransferase sam2 [Fusarium oxysporum];Gene=FOYG_06703;Ontology_term=sulfur compound metabolic process,amino acid metabolic process,transferase activity;Ontology_id=GO:0006790,GO:0006520,GO:0016740;Enzyme_code=EC:2.5.1.6;Enzyme_name=methionine adenosyltransferase
contig00002.g866	1060	ID=contig00002.g866;Description=GPN-loop GTPase 3 [Fusarium oxysporum f. sp. cepae];Gene=FCIRC_11267;Ontology_term=nucleocytoplasmic transport,intracellular protein transport,hydrolase activity;Ontology_id=GO:0006913,GO:0006886,GO:0016787;Enzyme_code=EC:3;Enzyme_name=Hydrolases
contig00002.g867	540	ID=contig00002.g867;Description=hypothetical protein LB506_000601 [Fusarium annulatum];Gene=FVER53590_00879;Ontology_term=membrane;Ontology_id=GO:0016020
contig00002.g868	2756	ID=contig00002.g868;Description=hypothetical protein FVER53263_00879 [Fusarium verticillioides]
contig00002.g869	1151	ID=contig00002.g869;Description=methylenetetrahydrofolate dehydrogenase (NAD+) [Fusarium verticillioides 7600];Gene=FOVG_00836;Ontology_term=oxidoreductase activity;Ontology_id=GO:0016491;Enzyme_code=EC:1.5.1.5;Enzyme_name=methylenetetrahydrofolate dehydrogenase (NADP(+))

contig00002.g870	1677	ID=contig00002.g870;Description=MFS transporter, NNP family, nitrate/nitrite transporter [Fusarium verticillioides 7600];Gene=FVEG_00877;Ontology_term=transmembrane transport,nitrogen cycle metabolic process,transporter activity,plasma membrane;Ontology_id=GO:0055085,GO:0071941,GO:0005215,GO:0005886
contig00002.g871	847	ID=contig00002.g871;Description=Ser-Thr-rich glycosyl-phosphatidyl-inositol-anchored membrane family-domain-containing protein [Fusarium redolens]
contig00002.g872	1532	ID=contig00002.g872;Description=WD domain protein [Fusarium falciforme];Ontology_term=reproductive process,nuclear chromosome,regulation of DNA-templated transcription,telomere organization,chromatin organization,nucleoplasm;Ontology_id=GO:0022414,GO:0000228,GO:0006355,GO:0032200,GO:0006325,GO:0005654
contig00002.g873	2228	ID=contig00002.g873;Description=Hsp70 chaperone protein [Fusarium pseudocircinatum];Gene=FVER53590_00874;Ontology_term=nucleotide binding;Ontology_id=GO:0000166
contig00002.g874	1526	ID=contig00002.g874;Description=hypothetical protein FVER14953_00873 [Fusarium verticillioides]
contig00002.g875	1351	ID=contig00002.g875;Description=Repetitive proline-rich cell wall protein 1 [Fusarium oxysporum f. sp. cubense race 1]
contig00002.g876	1041	ID=contig00002.g876;Description=basic proline-rich [Fusarium tjaetaba]
contig00002.g877	642	ID=contig00002.g877;Description=cytidine deaminase [Fusarium verticillioides 7600];Gene=FACUT_11405;Ontology_term=nucleus,catalytic activity, acting on a protein,nucleobase-containing small molecule metabolic process,signaling,carbohydrate derivative metabolic process,cytosol,transferase activity,hydrolase activity;Ontology_id=GO:0005634,GO:0140096,GO:0055086,GO:0023052,GO:1901135,GO:0005829,GO:0016740,GO:0016787;Enzyme_code=EC:2.7.11.25,EC:2.7.11.1,EC:3.5.4.14,EC:3.5.4.5,EC:2.7.11.25,EC:2.7.1,EC:2.7.11.1,EC:3.5.4.14,EC:3.5.4,EC:2.7,EC:2,EC:3.5,EC:3,EC:3.5.4.5;Enzyme_name=mitogen-activated protein kinase kinase kinase,non-specific serine/threonine protein kinase,Acting on carbon-nitrogen bonds, other than peptide bonds,cytidine deaminase,mitogen-activated protein kinase kinase kinase,Transferring phosphorus-containing groups,non-specific serine/threonine protein kinase,Acting on carbon-nitrogen bonds, other than peptide bonds,Acting on carbon-nitrogen bonds, other than peptide bonds,Transferring phosphorus-containing groups,Transferases,Acting on carbon-nitrogen bonds, other than peptide bonds,Hydrolases,cytidine deaminase

contig00002.g878	4290	ID=contig00002.g878;Description=STE STE11 SSK kinase [Fusarium tjaetaba];Gene=FPCIR_4899;Ontology_term=catalytic activity, acting on a protein,nucleobase-containing small molecule metabolic process,cytoskeleton organization,signaling,carbohydrate derivative metabolic process,cytosol,cytoskeletal protein binding,transferase activity,programmed cell death,mitotic cell cycle,hydrolase activity;Ontology_id=GO:0140096,GO:0055086,GO:0007010,GO:0023052,GO:1901135,GO:0005829,GO:0008092,GO:0016740,GO:0012501,GO:0000278,GO:0016787;Enzyme_code=EC:2.7.11.25,EC:2.7.11.1,EC:3.5.4.5,EC:2.7.11.25,EC:2.7.1,EC:2.7.11.1,EC:3.5.4,EC:2.7,EC:2,EC:3,EC:3.5,EC:3.5.4.5;Enzyme_name=mitogen-activated protein kinase kinase kinase,non-specific serine/threonine protein kinase,cytidine deaminase,mitogen-activated protein kinase kinase kinase,Transferring phosphorus-containing groups,non-specific serine/threonine protein kinase,Acting on carbon-nitrogen bonds, other than peptide bonds,Transferring phosphorus-containing groups,Transferases,Hydrolases,Acting on carbon-nitrogen bonds, other than peptide bonds,cytidine deaminase
contig00002.g879	1095	ID=contig00002.g879;Description=2,4-dienoyl-CoA reductase (NADPH2) [Fusarium verticillioides 7600];Gene=FVEG_00866;Ontology_term=peroxisome,oxidoreductase activity,lipid metabolic process,cell differentiation,anatomical structure development,reproductive process;Ontology_id=GO:0005777,GO:0016491,GO:0006629,GO:0030154,GO:0048856,GO:0022414;Enzyme_code=EC:1.3.1.34,EC:1.3,EC:1,EC:1.3.1.34,EC:1.3.1;Enzyme_name=2,4-dienoyl-CoA reductase [(2E)-enoyl-CoA-producing],Acting on the CH-CH group of donors,Oxidoreductases,2,4-dienoyl-CoA reductase [(2E)-enoyl-CoA-producing],Acting on the CH-CH group of donors
contig00002.g880	1465	ID=contig00002.g880;Description=acid phosphatase [Fusarium verticillioides 7600];Gene=FVEG_00865;Ontology_term=membrane;Ontology_id=GO:0016020
contig00002.g881	1987	ID=contig00002.g881;Description=transporter HOL1 [Fusarium tjaetaba];Gene=FDENT_6087;Ontology_term=transmembrane transport,transporter activity;Ontology_id=GO:0055085,GO:0005215
contig00002.g882	2700	ID=contig00002.g882;Description=acetate regulatory DNA binding protein [Fusarium tjaetaba];Gene=FVER53263_00863;Ontology_term=nucleus,DNA binding,regulation of DNA-templated transcription,transcription regulator activity;Ontology_id=GO:0005634,GO:0003677,GO:0006355,GO:0140110
contig00002.g883	3158	ID=contig00002.g883;Description=hypothetical protein FVEG_00860 [Fusarium verticillioides 7600]

contig00002.g884	1625	ID=contig00002.g884;Description=chromosome segregation smc [Fusarium sp. NRRL 25303];Gene=FANTH_11702;Ontology_term=membrane;Ontology_id=GO:0016020
contig00002.g885	2359	ID=contig00002.g885;Description=PTA1-pre-tRNA processing PF I subunit [Fusarium tjaetaba];Gene=FNAPI_2344;Ontology_term=mRNA metabolic process,DNA-templated transcription,tRNA metabolic process,nucleoplasm;Ontology_id=GO:0016071,GO:0006351,GO:0006399,GO:0005654
contig00002.g886	343	ID=contig00002.g886;Description=Cytochrome oxidase assembly protein 3, mitochondrial [Fusarium oxysporum f. sp. cubense race 1];Gene=FOXB_13111;Ontology_term=protein-containing complex assembly,mitochondrion,mitochondrion organization;Ontology_id=GO:0065003,GO:0005739,GO:0007005
contig00002.g887	2056	ID=contig00002.g887;Description=hypothetical protein FVER14953_00857 [Fusarium verticillioides];Gene=FVER53590_00857;Ontology_term=regulation of DNA-templated transcription,nuclear chromosome,chromatin organization,nucleoplasm;Ontology_id=GO:0006355,GO:0000228,GO:0006325,GO:0005654
contig00002.g888	1865	ID=contig00002.g888;Description=nucleolar protein 12 [Fusarium verticillioides 7600];Gene=FPCIR_4910;Ontology_term=RNA binding,ribosome biogenesis,nucleolus;Ontology_id=GO:0003723,GO:0042254,GO:0005730
contig00002.g889	1712	ID=contig00002.g889;Description=Tubulin/FtsZ, GTPase domain-containing protein [Fusarium redolens];Gene=FHETE_11386;Ontology_term=cytoskeleton,cytoskeleton organization,cell differentiation,anatomical structure development,structural molecule activity,reproductive process,hydrolase activity;Ontology_id=GO:0005856,GO:0007010,GO:0030154,GO:0048856,GO:0005198,GO:0022414,GO:0016787;Enzyme_code=EC:3,EC:3;Enzyme_name=Hydrolases,Hydrolases
contig00002.g890	1628	ID=contig00002.g890;Description=amino acid/polyamine transporter I [Fusarium oxysporum];Gene=FDENT_6096
contig00002.g891	1559	ID=contig00002.g891;Description=gamma-glutamylputrescine oxidoreductase [Fusarium coicis];Gene=FNYG_09334;Ontology_term=oxidoreductase activity;Ontology_id=GO:0016491;Enzyme_code=EC:1,EC:1;Enzyme_name=Oxidoreductases,Oxidoreductases

contig00002.g892	1515	ID=contig00002.g892;Description=aldehyde dehydrogenase (NAD+) [Fusarium odoratissimum NRRL 54006];Gene=F25303_1487;Ontology_term=oxidoreductase activity,amino acid metabolic process;Ontology_id=GO:0016491,GO:0006520;Enzyme_code=EC:1.2.1,EC:1.2,EC:1,EC:1.2.1.5,EC:1.2.1.3,EC:1.2.1;Enzyme_name=Acting on the aldehyde or oxo group of donors,Acting on the aldehyde or oxo group of donors,Oxidoreductases,aldehyde dehydrogenase [NAD(P)(+)],aldehyde dehydrogenase (NAD(+)),Acting on the aldehyde or oxo group of donors
contig00002.g893	1170	ID=contig00002.g893;Description=hypothetical protein FVEG_00852 [Fusarium verticillioides 7600];Gene=FVER53263_00852;Ontology_term=membrane;Ontology_id=GO:0016020
contig00002.g894	797	ID=contig00002.g894;Description=40S ribosomal protein S15 [Fusarium oxysporum f. sp. lycopersici 4287];Gene=FOVG_00802;Ontology_term=nucleocytoplasmic transport,RNA binding,protein-containing complex assembly,cytoplasmic translation,structural molecule activity,ribosome biogenesis,cytosol,ribosome,intracellular protein transport;Ontology_id=GO:0006913,GO:0003723,GO:0065003,GO:0002181,GO:0005198,GO:0042254,GO:0005829,GO:0005840,GO:0006886
contig00002.g895	745	ID=contig00002.g895;Description=nadh-ubiquinone oxidoreductase kda subunit [Fusarium mundagurra];Gene=FDENT_6101;Ontology_term=transferase activity;Ontology_id=GO:0016740;Enzyme_code=EC:2.1.1;Enzyme_name=Transferring one-carbon groups
contig00002.g896	702	ID=contig00002.g896;Description=nadh-ubiquinone oxidoreductase kda subunit [Fusarium tjaetaba];Gene=FOVG_00800;Ontology_term=mitochondrion;Ontology_id=GO:0005739
contig00002.g897	318	ID=contig00002.g897;Description=hypothetical protein FVEG_00849 [Fusarium verticillioides 7600]
contig00002.g898	1269	ID=contig00002.g898;Description=NUDIX hydrolase domain protein [Fusarium tjaetaba];Gene=FNAPI_2333;Ontology_term=hydrolase activity;Ontology_id=GO:0016787;Enzyme_code=EC:3,EC:3;Enzyme_name=Hydrolases,Hydrolases
contig00002.g899	552	ID=contig00002.g899;Description=cupin [Fusarium tjaetaba]
contig00002.g900	2782	ID=contig00002.g900;Description=hypothetical protein FVEG_00845 [Fusarium verticillioides 7600];Gene=FMEXI_3061;Ontology_term=membrane;Ontology_id=GO:0016020
contig00002.g901	1698	ID=contig00002.g901;Description=oxidoreductase [Fusarium verticillioides 7600];Gene=FVEG_00844;Ontology_term=nucleotide binding;Ontology_id=GO:0000166

contig00002.g902	691	ID=contig00002.g902;Description=REX2 3`-5` exonuclease [Fusarium beomiforme];Gene=FVEG_00843;Ontology_term=catalytic activity, acting on RNA,hydrolase activity;Ontology_id=GO:0140098,GO:0016787;Enzyme_code=EC:3.1.13,EC:3.1.15;Enzyme_name=Acting on ester bonds,Acting on ester bonds
contig00002.g903	1960	ID=contig00002.g903;Description=phosphoglucomutase [Fusarium verticillioides 7600];Gene=F25303_1498;Ontology_term=carbohydrate metabolic process,generation of precursor metabolites and energy,nucleobase-containing small molecule metabolic process,carbohydrate derivative metabolic process,cytosol,isomerase activity;Ontology_id=GO:0005975,GO:0006091,GO:0055086,GO:1901135,GO:0005829,GO:0016853;Enzyme_code=EC:5.4.2.2,EC:5.4.2.2,EC:5.4,EC:5,EC:5.4.2;Enzyme_name=phosphoglucomutase (alpha-D-glucose-1,6-bisphosphate-dependent),phosphoglucomutase (alpha-D-glucose-1,6-bisphosphate-dependent),Intramolecular transferases,Isomerases,Intramolecular transferases
contig00002.g904	1765	ID=contig00002.g904;Description=hypothetical protein QSH57_001202 [Fusarium oxysporum f. sp. vasinfectum];Gene=Adh;Ontology_term=oxidoreductase activity;Ontology_id=GO:0016491;Enzyme_code=EC:1.1.1.71,EC:1.1,EC:1,EC:1.1.1,EC:1.1.1.71;Enzyme_name=alcohol dehydrogenase [NAD(P)(+)],Acting on the CH-OH group of donors,Oxidoreductases,Acting on the CH-OH group of donors,alcohol dehydrogenase [NAD(P)(+)]
contig00002.g905	464	ID=contig00002.g905;Description=Non-histone chromosomal protein 6 [Fusarium poae];Gene=FEQUK3_LOCUS3014;Ontology_term=nucleus,DNA binding,DNA repair,chromosome;Ontology_id=GO:0005634,GO:0003677,GO:0006281,GO:0005694
contig00002.g906	798	ID=contig00002.g906;Description=hypothetical protein FVEG_00839 [Fusarium verticillioides 7600];Gene=FPANT_2716;Ontology_term=nucleus,protein-containing complex assembly,chromosome;Ontology_id=GO:0005634,GO:0065003,GO:0005694
contig00002.g907	4311	ID=contig00002.g907;Description=oligomeric, coiled-coil, peripheral membrane protein [Fusarium musae];Gene=FPANT_2717;Ontology_term=carbohydrate metabolic process,generation of precursor metabolites and energy,autophagy,structural molecule activity,intracellular protein transport,vacuole,mitochondrion organization,molecular adaptor activity;Ontology_id=GO:0005975,GO:0006091,GO:0006914,GO:0005198,GO:0006886,GO:0005773,GO:0007005,GO:0060090
contig00002.g908	1176	ID=contig00002.g908;Description=hypothetical protein FVER53263_00837 [Fusarium verticillioides]

contig00002.g909	984	ID=contig00002.g909;Description=hypothetical protein FVEG_00836 [Fusarium verticillioides 7600]
contig00002.g910	1866	ID=contig00002.g910;Description=ubiquitin carboxyl-terminal hydrolase 16 [Fusarium tjaetaba];Gene=FNYG_09356;Ontology_term=catalytic activity, acting on a protein,hydrolase activity;Ontology_id=GO:0140096,GO:0016787;Enzyme_code=EC:3.4.19.12,EC:3.4,EC:3,EC:3.4.19.12;Enzyme_name=ubiquitinyl hydrolase 1,Acting on peptide bonds (peptidases),Hydrolases,ubiquitinyl hydrolase 1
contig00002.g911	1410	ID=contig00002.g911;Description=glycosyltransferase family 31 [Fusarium pseudocircinatum];Gene=FNAPI_2320;Ontology_term=transferase activity;Ontology_id=GO:0016740;Enzyme_code=EC:2,EC:2;Enzyme_name=Transferases,Transferases
contig00002.g912	1942	ID=contig00002.g912;Description=transcriptional regulatory [Fusarium tjaetaba];Gene=FTJAE_420;Ontology_term=nucleus,regulation of DNA-templated transcription,transcription regulator activity;Ontology_id=GO:0005634,GO:0006355,GO:0140110
contig00002.g913	867	ID=contig00002.g913;Description=hypothetical protein FVER14953_00833 [Fusarium verticillioides];Gene=FMAN_01208;Ontology_term=nucleus,regulation of DNA-templated transcription,transcription regulator activity;Ontology_id=GO:0005634,GO:0006355,GO:0140110
contig00002.g914	630	ID=contig00002.g914;Description=n-acetyltransferase p20 [Fusarium tjaetaba];Gene=FVEG_00832;Ontology_term=transferase activity;Ontology_id=GO:0016740;Enzyme_code=EC:2.3.1,EC:2.3,EC:2,EC:2.3.1;Enzyme_name=Acyltransferases,Acyltransferases,Transferases,Acyltransferases
contig00002.g915	1508	ID=contig00002.g915;Description=NADH-ubiquinone oxidoreductase 49 kDa subunit, mitochondrial [Fusarium verticillioides 7600];Gene=FANTH_9695;Ontology_term=oxidoreductase activity,mitochondrion,transferase activity;Ontology_id=GO:0016491,GO:0005739,GO:0016740;Enzyme_code=EC:1.6,EC:2.3.1,EC:2.3,EC:1.6,EC:1,EC:2,EC:1.6.99.3,EC:1.6.5.3,EC:2.3.1;Enzyme_name=Acting on NADH or NADPH,Acyltransferases,Acyltransferases,Acting on NADH or NADPH,Oxidoreductases,Transferases,Deleted entry,Acting on NADH or NADPH,Acyltransferases

contig00002.g916	1512	ID=contig00002.g916;Description=ubiquinone biosynthesis monooxygenase Coq6 [Fusarium verticillioides 7600];Gene=COQ6;Ontology_term=oxidoreductase activity,mitochondrion;Ontology_id=GO:0016491,GO:0005739;Enzyme_code=EC:1.14.14,EC:1.14.13,EC:1,EC:1.14.14,EC:1.14,EC:1.14.13;Enzyme_name=Acting on paired donors, with incorporation or reduction of molecular oxygen. The oxygen incorporated need not be derived from O2,Acting on paired donors, with incorporation or reduction of molecular oxygen. The oxygen incorporated need not be derived from O2,Oxidoreductases,Acting on paired donors, with incorporation or reduction of molecular oxygen. The oxygen incorporated need not be derived from O2,Acting on paired donors, with incorporation or reduction of molecular oxygen. The oxygen incorporated need not be derived from O2,Acting on paired donors, with incorporation or reduction of molecular oxygen. The oxygen incorporated need not be derived from O2
contig00002.g917	2129	ID=contig00002.g917;Description=Inner nuclear membrane protein SRC1 [Fusarium oxysporum f. sp. rapae];Gene=FOPG_01827;Ontology_term=nuclear envelope,DNA binding,membrane organization,mitotic nuclear division,chromosome segregation,meiotic nuclear division;Ontology_id=GO:0005635,GO:0003677,GO:0061024,GO:0140014,GO:0007059,GO:0140013
contig00002.g918	930	ID=contig00002.g918;Description=atp3 gamma subunit of the F1 sector of mitochondrial F1F0 ATP synthase [Fusarium musae];Gene=FPANT_2728;Ontology_term=transmembrane transport,nucleobase-containing small molecule metabolic process,protein-containing complex assembly,ATP-dependent activity,mitochondrion,carbohydrate derivative metabolic process,ligase activity,transporter activity;Ontology_id=GO:0055085,GO:0055086,GO:0065003,GO:0140657,GO:0005739,GO:1901135,GO:0016874,GO:0005215;Enzyme_code=EC:7.1.2.2,EC:6,EC:7.2.2;Enzyme_name=H(+)-transporting two-sector ATPase,Ligases,Catalysing the translocation of inorganic cations
contig00002.g919	1769	ID=contig00002.g919;Description=NOC4 nucleolar protein [Fusarium tjaetaba];Gene=FNAPI_2312;Ontology_term=ribosome biogenesis,cytosol,nucleolus;Ontology_id=GO:0042254,GO:0005829,GO:0005730
contig00002.g920	2607	ID=contig00002.g920;Description=metal ion transporter [Fusarium pseudocircinatum];Gene=FPCIR_4944;Ontology_term=transmembrane transport,vacuole,transporter activity;Ontology_id=GO:0055085,GO:0005773,GO:0005215

contig00002.g921	1000	ID=contig00002.g921;Description=related to cleavage stimulation factor 64K chain [Fusarium proliferatum ET1];Gene=FMAN_01216;Ontology_term=mRNA metabolic process,RNA binding,nucleoplasm;Ontology_id=GO:0016071,GO:0003723,GO:0005654
contig00002.g922	1448	ID=contig00002.g922;Description=related to ERV46-component of copii vesicles [Fusarium mangiferae];Gene=FGLOB1_8081;Ontology_term=vesicle-mediated transport,cytoplasmic vesicle,endoplasmic reticulum,Golgi apparatus,intracellular protein transport;Ontology_id=GO:0016192,GO:0031410,GO:0005783,GO:0005794,GO:0006886
contig00002.g923	2322	ID=contig00002.g923;Description=DSF2 Suppressor of mptFive pufFive mutation [Fusarium tjaetaba]
contig00002.g924	911	ID=contig00002.g924;Description=proteasome component PUP1 [Fusarium proliferatum];Gene=FCIRC_9635;Ontology_term=nucleus,catalytic activity, acting on a protein,protein catabolic process,hydrolase activity;Ontology_id=GO:0005634,GO:0140096,GO:0030163,GO:0016787;Enzyme_code=EC:3.4.25;Enzyme_name=Acting on peptide bonds (peptidases)
contig00002.g925	4658	ID=contig00002.g925;Description=nucleoporin [Fusarium pseudocircinatum];Gene=FPCIR_4949;Ontology_term=nuclear envelope,nucleocytoplasmic transport,ribosome biogenesis,intracellular protein transport,transcription regulator activity,molecular adaptor activity;Ontology_id=GO:0005635,GO:0006913,GO:0042254,GO:0006886,GO:0140110,GO:0060090
contig00002.g926	1200	ID=contig00002.g926;Description=DNA-directed RNA polymerase I and III subunit RPAC1 [Fusarium verticillioides 7600];Gene=FOPG_01815;Ontology_term=DNA binding,DNA-templated transcription,tRNA metabolic process,catalytic activity, acting on RNA,transferase activity,nucleolus,nucleoplasm;Ontology_id=GO:0003677,GO:0006351,GO:0006399,GO:0140098,GO:0016740,GO:0005730,GO:0005654;Enzyme_code=EC:2.7.7.6,EC:2.7,EC:2,EC:2.7.7,EC:2.7.7.6;Enzyme_name=DNA-directed RNA polymerase,Transferring phosphorus-containing groups,Transferases,Transferring phosphorus-containing groups,DNA-directed RNA polymerase

contig00002.g927	1539	ID=contig00002.g927;Description=ubiquitin-activating enzyme E1 C [Fusarium verticillioides 7600];Gene=FPHYL_10105;Ontology_term=catalytic activity, acting on a protein,ATP-dependent activity,ligase activity;Ontology_id=GO:0140096,GO:0140657,GO:0016874;Enzyme_code=EC:6.2.1.64,EC:6.2.1.45,EC:6.2.1.64,EC:6.2,EC:6;Enzyme_name=E1 NEDD8-activating enzyme,E1 ubiquitin-activating enzyme,E1 NEDD8-activating enzyme,Forming carbon-sulfur bonds,Ligases
contig00002.g928	3071	ID=contig00002.g928;Description=TEL2 involved in controlling telomere length and position effect [Fusarium tjaetaba];Ontology_term=DNA binding,signaling,nuclear chromosome,telomere organization,chromatin organization,mitotic cell cycle;Ontology_id=GO:0003677,GO:0023052,GO:0000228,GO:0032200,GO:0006325,GO:0000278
contig00002.g929	2517	ID=contig00002.g929;Description=STE/STE20/YSK protein kinase [Fusarium proliferatum];Gene=C2S_11825;Ontology_term=catalytic activity, acting on a protein,oxidoreductase activity,transferase activity;Ontology_id=GO:0140096,GO:0016491,GO:0016740;Enzyme_code=EC:2.7.1,EC:1.1.1.1,EC:1.1.1.71,EC:2.7.1,EC:1.1,EC:1,EC:2.7,EC:2,EC:1.1.1,EC:1.1.1.1,EC:1.1.1.71;Enzyme_name=Transferring phosphorus-containing groups,alcohol dehydrogenase,alcohol dehydrogenase [NAD(P)(+)],Transferring phosphorus-containing groups,Acting on the CH-OH group of donors,Oxidoreductases,Transferring phosphorus-containing groups,Transferases,Acting on the CH-OH group of donors,alcohol dehydrogenase,alcohol dehydrogenase [NAD(P)(+)]
contig00002.g930	2550	ID=contig00002.g930;Description=DNA cross-link repair protein pso2 snm1 [Fusarium tjaetaba];Gene=FNYG_09378;Ontology_term=DNA repair,DNA binding,catalytic activity, acting on DNA,nuclear chromosome,telomere organization,hydrolase activity;Ontology_id=GO:0006281,GO:0003677,GO:0140097,GO:0000228,GO:0032200,GO:0016787;Enzyme_code=EC:3.1,EC:3.1.11,EC:3,EC:3.1.15;Enzyme_name=Acting on ester bonds,Acting on ester bonds,Hydrolases,Acting on ester bonds
contig00002.g931	485	ID=contig00002.g931;Description=U6 snRNA-associated Sm-like protein LSm2 [Fusarium musae];Gene=FNAPI_2300;Ontology_term=RNA binding,mRNA metabolic process,ribosome biogenesis,nucleolus;Ontology_id=GO:0003723,GO:0016071,GO:0042254,GO:0005730

contig00002.g932	2631	ID=contig00002.g932;Description=cullulin 3 [Fusarium fujikuroi];Gene=FACUT_8560;Ontology_term=nucleus,catalytic activity, acting on a protein,oxidoreductase activity,protein catabolic process,transferase activity;Ontology_id=GO:0005634,GO:0140096,GO:0016491,GO:0030163,GO:0016740;Enzyme_code=EC:1.1.1.1,EC:1.1.1.71,EC:1.1,EC:2.3,EC:1,EC:2,EC:1.1.1,EC:2.3.2,EC:1.1.1.1,EC:1.1.1.71;Enzyme_name=alcohol dehydrogenase,alcohol dehydrogenase [NAD(P)(+)],Acting on the CH-OH group of donors,Acyltransferases,Oxidoreductases,Transferases,Acting on the CH-OH group of donors,Acyltransferases,alcohol dehydrogenase,alcohol dehydrogenase [NAD(P)(+)]
contig00002.g933	532	ID=contig00002.g933;Description=small nuclear ribonucleoprotein D1 [Fusarium proliferatum];Gene=FNAPI_2298;Ontology_term=nucleus,RNA binding,mRNA metabolic process,protein-containing complex assembly;Ontology_id=GO:0005634,GO:0003723,GO:0016071,GO:0065003
contig00002.g934	765	ID=contig00002.g934;Description=methyltransferase [Fusarium tjaetaba];Gene=FVEG_00811;Ontology_term=nucleus,catalytic activity, acting on a protein,cytoplasmic translation,transferase activity;Ontology_id=GO:0005634,GO:0140096,GO:0002181,GO:0016740;Enzyme_code=EC:2.1.1,EC:2.1,EC:2.1.1.297,EC:2,EC:2.1.1;Enzyme_name=Transferring one-carbon groups,Transferring one-carbon groups,peptide chain release factor N(5)-glutamine methyltransferase,Transferases,Transferring one-carbon groups
contig00002.g935	7848	ID=contig00002.g935;Description=IQ domain-containing protein containing GTPase activating protein [Fusarium proliferatum];Gene=FOXG_00703;Ontology_term=nucleus,molecular function regulator activity,signaling,anatomical structure development,reproductive process,cytosol;Ontology_id=GO:0005634,GO:0098772,GO:0023052,GO:0048856,GO:0022414,GO:0005829
contig00002.g936	749	ID=contig00002.g936;Description=peroxiredoxin (alkyl hydroperoxide reductase subunit C) [Fusarium oxysporum f. sp. lycopersici 4287];Gene=FOYG_06617;Ontology_term=antioxidant activity,oxidoreductase activity;Ontology_id=GO:0016209,GO:0016491;Enzyme_code=EC:1.11.1.24,EC:1.11,EC:1,EC:1.11.1.24,EC:1.11.1,EC:1.11.1.15;Enzyme_name=thioredoxin-dependent peroxiredoxin,Acting on a peroxide as acceptor,Oxidoreductases,thioredoxin-dependent peroxiredoxin,Acting on a peroxide as acceptor,Acting on a peroxide as acceptor

contig00002.g937	909	ID=contig00002.g937;Description=related to CCAAT-binding factor HAPB protein [Fusarium fujikuroi];Gene=FOVG_00746;Ontology_term=DNA binding,nucleus,generation of precursor metabolites and energy,regulation of DNA-templated transcription,transcription regulator activity;Ontology_id=GO:0003677,GO:0005634,GO:0006091,GO:0006355,GO:0140110
contig00002.g938	2462	ID=contig00002.g938;Description=hypothetical protein FVEG_00806 [Fusarium verticillioides 7600];Gene=FNYG_09389;Ontology_term=nucleus,transmembrane transport,chromatin organization,transporter activity;Ontology_id=GO:0005634,GO:0055085,GO:0006325,GO:0005215
contig00002.g939	1015	ID=contig00002.g939;Description=histone chaperone ASF1 [Fusarium verticillioides 7600];Gene=FSUBG_10156;Ontology_term=protein-containing complex assembly,histone binding,nuclear chromosome,regulation of DNA-templated transcription,cytosol,telomere organization,chromatin organization;Ontology_id=GO:0065003,GO:0042393,GO:0000228,GO:0006355,GO:0005829,GO:0032200,GO:0006325
contig00002.g940	3128	ID=contig00002.g940;Description=mRNA 3'-end-processing protein rna14 [Fusarium oxysporum];Gene=RNA14;Ontology_term=RNA binding,mRNA metabolic process,mitochondrion,cytosol,nucleoplasm;Ontology_id=GO:0003723,GO:0016071,GO:0005739,GO:0005829,GO:0005654
contig00002.g941	3020	ID=contig00002.g941;Description=argonaute like [Fusarium tjaetaba];Gene=FPCIR_4967;Ontology_term=RNA binding,regulatory ncRNA-mediated gene silencing,regulation of DNA-templated transcription,nuclear chromosome,chromatin organization,catalytic activity, acting on RNA,hydrolase activity;Ontology_id=GO:0003723,GO:0031047,GO:0006355,GO:0000228,GO:0006325,GO:0140098,GO:0016787;Enzyme_code=EC:3.1.30,EC:3.1,EC:3,EC:3.1.26;Enzyme_name=Acting on ester bonds,Acting on ester bonds,Hydrolases,Acting on ester bonds
contig00002.g942	1020	ID=contig00002.g942;Description=Collagen alpha-5(VI) chain [Fusarium subglutinans]
contig00002.g943	1820	ID=contig00002.g943;Description=saccharopine dehydrogenase (NADP+, L-glutamate-forming) [Fusarium proliferatum];Gene=Forpi1262_v000795;Ontology_term=generation of precursor metabolites and energy,oxidoreductase activity,amino acid metabolic process;Ontology_id=GO:0006091,GO:0016491,GO:0006520;Enzyme_code=EC:1,EC:2.5.1.16,EC:1,EC:1.5,EC:1.5.1.10,EC:1.5.1;Enzyme_name=Oxidoreductases,spermidine synthase,Oxidoreductases,Acting on the CH-NH group of donors,saccharopine dehydrogenase (NADP(+), L-glutamate-forming),Acting on the CH-NH group of donors

contig00002.g944	3379	ID=contig00002.g944;Description=major facilitator superfamily domain-containing protein [Fusarium oxysporum];Gene=FOIG_01250;Ontology_term=transmembrane transport,transporter activity;Ontology_id=GO:0055085,GO:0005215
contig00002.g945	3087	ID=contig00002.g945;Description=related to SRO77-polarized exocytosis by regulating SNARE function [Fusarium fujikuroi];Gene=FVER53263_00798;Ontology_term=molecular function regulator activity,vesicle-mediated transport,signaling,establishment or maintenance of cell polarity,cytosol,plasma membrane;Ontology_id=GO:0098772,GO:0016192,GO:0023052,GO:0007163,GO:0005829,GO:0005886
contig00002.g946	950	ID=contig00002.g946;Description=zinc finger ZIC 4 [Fusarium tjaetaba]
contig00002.g947	431	ID=contig00002.g947;Description=hypothetical protein LB503_000652 [Fusarium chuoi]
contig00002.g948	279	ID=contig00002.g948;Description=hypothetical protein FCOIX_4630 [Fusarium coicis]
contig00002.g949	378	ID=contig00002.g949;Description=hypothetical protein FPCIR_7071 [Fusarium pseudocircinatum]
contig00002.g950	2336	ID=contig00002.g950;Description=hypothetical protein FVEG_00794 [Fusarium verticillioides 7600];Gene=FPCIR_7072;Ontology_term=nucleus,DNA binding,regulation of DNA-templated transcription,transcription regulator activity;Ontology_id=GO:0005634,GO:0003677,GO:0006355,GO:0140110
contig00002.g951	2408	ID=contig00002.g951;Description=Polynucleotide 5'-hydroxyl-kinase GRC3 [Fusarium oxysporum];Gene=BFJ68_g4873;Ontology_term=tRNA metabolic process,ribosome biogenesis,regulation of DNA-templated transcription,nuclear chromosome,chromatin organization,nucleolus,mRNA metabolic process,ATP-dependent activity,cytosol,transferase activity,hydrolase activity;Ontology_id=GO:0006399,GO:0042254,GO:0006355,GO:0000228,GO:0006325,GO:0005730,GO:0016071,GO:0140657,GO:0005829,GO:0016740,GO:0016787;Enzyme_code=EC:2.7.1,EC:2.7.1,EC:3.6.1,EC:2.7,EC:2,EC:3.6,EC:3,EC:3.6.1.15;Enzyme_name=Transferring phosphorus-containing groups,Transferring phosphorus-containing groups,Acting on acid anhydrides,Transferring phosphorus-containing groups,Transferases,Acting on acid anhydrides,Hydrolases,nucleoside-triphosphate phosphatase
contig00002.g952	2030	ID=contig00002.g952;Description=SRP40-suppressor of mutant AC40 of RNA polymerase I and III [Fusarium tjaetaba];Gene=FNYG_08453;Ontology_term=DNA-templated transcription;Ontology_id=GO:0006351

contig00002.g953	1421	ID=contig00002.g953;Description=aureobasidin-resistance [Fusarium tjaetaba];Gene=FGADI_5100;Ontology_term=lipid metabolic process,Golgi apparatus,endoplasmic reticulum,transferase activity;Ontology_id=GO:0006629,GO:0005794,GO:0005783,GO:0016740;Enzyme_code=EC:2.4,EC:2,EC:2.7.1.227,EC:2.4.1;Enzyme_name=Glycosyltransferases,Transferases,inositol phosphorylceramide synthase,Glycosyltransferases
contig00002.g954	1306	ID=contig00002.g954;Description=CAMK/CAMK1 protein kinase [Fusarium verticillioides 7600];Gene=FOVG_00726;Ontology_term=catalytic activity, acting on a protein,signaling,regulation of DNA-templated transcription,transferase activity,mitotic cell cycle;Ontology_id=GO:0140096,GO:0023052,GO:0006355,GO:0016740,GO:0000278;Enzyme_code=EC:2.7.11.1,EC:2.7.1,EC:2.7.11.1,EC:2.7,EC:2,EC:2.7.11.17;Enzyme_name=non-specific serine/threonine protein kinase,Transferring phosphorus-containing groups,non-specific serine/threonine protein kinase,Transferring phosphorus-containing groups,Transferases,calcium/calmodulin-dependent protein kinase
contig00002.g955	2635	ID=contig00002.g955;Description=RNA-binding protein [Fusarium tjaetaba];Gene=FVEG_00788;Ontology_term=nucleus,RNA binding,mRNA metabolic process,snRNA metabolic process,DNA-templated transcription,regulatory ncRNA-mediated gene silencing,tRNA metabolic process;Ontology_id=GO:0005634,GO:0003723,GO:0016071,GO:0016073,GO:0006351,GO:0031047,GO:0006399
contig00002.g956	1763	ID=contig00002.g956;Description=ferric cupric reductase transmembrane component 2 [Fusarium napiforme];Gene=FOYG_06591;Ontology_term=oxidoreductase activity;Ontology_id=GO:0016491;Enzyme_code=EC:1.16,EC:1.16,EC:1;Enzyme_name=Oxidizing metal ions,Oxidizing metal ions,Oxidoreductases
contig00002.g957	1138	ID=contig00002.g957;Description=eukaryotic integral membrane protein-domain-containing protein [Fusarium redolens];Gene=FVER53263_00786;Ontology_term=vesicle-mediated transport;Ontology_id=GO:0016192
contig00002.g958	2363	ID=contig00002.g958;Description=WD40-repeat-containing domain protein [Fusarium oxysporum Fo47];Gene=FPANT_11921;Ontology_term=nucleus,regulation of DNA-templated transcription,transcription regulator activity,molecular adaptor activity;Ontology_id=GO:0005634,GO:0006355,GO:0140110,GO:0060090
contig00002.g959	479	ID=contig00002.g959;Description=hypothetical protein FVEG_00783 [Fusarium verticillioides 7600]

contig00002.g960	2385	ID=contig00002.g960;Description=Disintegrin and metallo ase domain protein [Fusarium sp. NRRL 52700];Gene=F52700_12551;Ontology_term=catalytic activity, acting on a protein,signaling,hydrolase activity;Ontology_id=GO:0140096,GO:0023052,GO:0016787;Enzyme_code=EC:3.4.24,EC:3.4.24,EC:3.4,EC:3;Enzyme_name=Acting on peptide bonds (peptidases),Acting on peptide bonds (peptidases),Acting on peptide bonds (peptidases),Hydrolases
contig00002.g961	2521	ID=contig00002.g961;Description=acetyl-coenzyme A synthetase [Fusarium proliferatum];Gene=FOC4_g10014746;Ontology_term=generation of precursor metabolites and energy,nucleobase-containing small molecule metabolic process,sulfur compound metabolic process,mitochondrion,cytosol,carbohydrate derivative metabolic process,chromatin organization,extracellular region,nucleolus,ligase activity;Ontology_id=GO:0006091,GO:0055086,GO:0006790,GO:0005739,GO:0005829,GO:1901135,GO:0006325,GO:0005576,GO:0005730,GO:0016874;Enzyme_code=EC:6.2.1.1,EC:6.3,EC:6.2,EC:6.3.1,EC:6.2.1.1,EC:6,EC:6.2.1;Enzyme_name=acetate--CoA ligase,Forming carbon-nitrogen bonds,Forming carbon-sulfur bonds,Forming carbon-nitrogen bonds,acetate--CoA ligase,Ligases,Forming carbon-sulfur bonds
contig00002.g962	1177	ID=contig00002.g962;Description=replication factor C subunit 5 [Fusarium subglutinans];Gene=FNYG_08443;Ontology_term=nucleus,DNA binding,DNA repair,catalytic activity, acting on DNA,ATP-dependent activity,DNA replication,chromosome,hydrolase activity;Ontology_id=GO:0005634,GO:0003677,GO:0006281,GO:0140097,GO:0140657,GO:0006260,GO:0005694,GO:0016787;Enzyme_code=EC:3.6.1.15;Enzyme_name=nucleoside-triphosphate phosphatase
contig00002.g963	861	ID=contig00002.g963;Description=ribosomal L16 precursor mitochondrial [Fusarium tjaetaba];Gene=FPANT_7450;Ontology_term=catalytic activity, acting on a protein,mitochondrial gene expression,RNA binding,mitochondrion,structural molecule activity,ribosome,catalytic activity, acting on RNA,transferase activity;Ontology_id=GO:0140096,GO:0140053,GO:0003723,GO:0005739,GO:0005198,GO:0005840,GO:0140098,GO:0016740;Enzyme_code=EC:2.3,EC:2,EC:2.3.2,EC:2.3.2.12;Enzyme_name=Acyltransferases,Transferases,Acyltransferases,peptidyltransferase

contig00002.g964	3352	ID=contig00002.g964;Description=replication factor C subunit 1 [Fusarium verticillioides 7600];Gene=FDENT_8341;Ontology_term=nucleus,DNA binding,DNA repair,catalytic activity, acting on DNA,ATP-dependent activity,DNA replication,chromosome,hydrolase activity;Ontology_id=GO:0005634,GO:0003677,GO:0006281,GO:0140097,GO:0140657,GO:0006260,GO:0005694,GO:0016787;Enzyme_code=EC:3.6.1.15;Enzyme_name=nucleoside-triphosphate phosphatase
contig00002.g965	3611	ID=contig00002.g965;Description=hypothetical protein FVER53590_00776 [Fusarium verticillioides]
contig00002.g966	7622	ID=contig00002.g966;Description=DNA-binding SNT1 [Fusarium denticulatum];Gene=FNAPI_13904;Ontology_term=DNA binding,nucleus,cytoskeleton,chromosome;Ontology_id=GO:0003677,GO:0005634,GO:0005856,GO:0005694
contig00002.g967	1198	ID=contig00002.g967;Description=Deoxyhypusine synthase [Fusarium flagelliforme];Gene=FVEG_00771;Ontology_term=transferase activity;Ontology_id=GO:0016740;Enzyme_code=EC:2.5.1.46;Enzyme_name=deoxyhypusine synthase
contig00002.g968	901	ID=contig00002.g968;Description=hypothetical protein FVER53590_25647 [Fusarium verticillioides]
contig00002.g969	955	ID=contig00002.g969;Description=d-xylose reductase II III [Fusarium tjaetaba]
contig00002.g970	1276	ID=contig00002.g970;Description=oxidoreductase [Fusarium odoratissimum NRRL 54006];Gene=FOVG_00703;Ontology_term=nucleotide binding;Ontology_id=GO:0000166
contig00002.g971	843	ID=contig00002.g971;Description=tRNA (uracil-5-)-methyltransferase TRM9 [Fusarium pseudoanthophilum];Gene=FNYG_08432;Ontology_term=nucleus,RNA binding,oxidoreductase activity,tRNA metabolic process,transferase activity,catalytic activity, acting on RNA;Ontology_id=GO:0005634,GO:0003723,GO:0016491,GO:0006399,GO:0016740,GO:0140098;Enzyme_code=EC:2.1.1,EC:2.1,EC:1.14.11,EC:1,EC:2,EC:1.14,EC:2.1.1.229,EC:2.1.1;Enzyme_name=Transferring one-carbon groups,Transferring one-carbon groups,Acting on paired donors, with incorporation or reduction of molecular oxygen. The oxygen incorporated need not be derived from O2,Oxidoreductases,Transferases,Acting on paired donors, with incorporation or reduction of molecular oxygen. The oxygen incorporated need not be derived from O2,tRNA (carboxymethyluridine(34)-5-O)-methyltransferase,Transferring one-carbon groups
contig00002.g972	3150	ID=contig00002.g972;Description=DRPLA-like protein [Fusarium denticulatum]

contig00002.g973	1070	ID=contig00002.g973;Description=tubulin specific chaperone cofactor B [Fusarium sp. NRRL 52700];Gene=FPANT_7440;Ontology_term=protein-containing complex assembly,cytoskeletal protein binding,protein folding;Ontology_id=GO:0065003,GO:0008092,GO:0006457
contig00002.g974	1662	ID=contig00002.g974;Description=e3 ubiquitin ligase SDIR1 [Fusarium coicis];Gene=FPCIR_12319;Ontology_term=membrane;Ontology_id=GO:0016020
contig00002.g975	1693	ID=contig00002.g975;Description=uncharacterized protein FSUBG_6463 [Fusarium subglutinans];Gene=FANTH_3907;Ontology_term=membrane;Ontology_id=GO:0016020
contig00002.g976	1176	ID=contig00002.g976;Description=glycoside hydrolase [Fusarium tjaetaba];Gene=FNAPI_5840;Ontology_term=hydrolase activity;Ontology_id=GO:0016787;Enzyme_code=EC:3,EC:3;Enzyme_name=Hydrolases,Hydrolases
contig00002.g977	1551	ID=contig00002.g977;Description=UPC2-like protein [Fusarium napiforme];Gene=FNYG_03728;Ontology_term=nucleus,regulation of DNA-templated transcription,transcription regulator activity;Ontology_id=GO:0005634,GO:0006355,GO:0140110
contig00002.g978	930	ID=contig00002.g978;Description=hypothetical protein FVEG_00759 [Fusarium verticillioides 7600]
contig00002.g979	2100	ID=contig00002.g979;Description=bacterial low temperature requirement A protein-domain-containing protein [Fusarium sp. MPI-SDFR-AT-0072];Gene=FVER53590_00758;Ontology_term=membrane;Ontology_id=GO:0016020
contig00002.g980	391	ID=contig00002.g980;Description=F-type H+-transporting ATPase subunit epsilon [Fusarium odoratissimum NRRL 54006];Gene=FOXG_00758;Ontology_term=nucleobase-containing small molecule metabolic process,mitochondrion,carbohydrate derivative metabolic process,ligase activity,transporter activity;Ontology_id=GO:0055086,GO:0005739,GO:1901135,GO:0016874,GO:0005215;Enzyme_code=EC:7.1.2.2,EC:6;Enzyme_name=H(+)-transporting two-sector ATPase,Ligases
contig00002.g981	2614	ID=contig00002.g981;Description=MAK10 glucose-repressible [Fusarium tjaetaba];Gene=FVEG_00756;Ontology_term=autophagy,protein maturation,transferase activity;Ontology_id=GO:0006914,GO:0051604,GO:0016740;Enzyme_code=EC:2,EC:2.3,EC:2,EC:2.3.1.255,EC:2.3.1;Enzyme_name=Transferases,Acyltransferases,Transferases,N-terminal amino-acid N(alpha)-acetyltransferase NatA,Acyltransferases
contig00002.g982	1149	ID=contig00002.g982;Description=peroxisomal membrane PMP47B [Fusarium mundagurra];Gene=FVEG_00755;Ontology_term=mitochondrion;Ontology_id=GO:0005739

contig00002.g983	4584	ID=contig00002.g983;Description=hypothetical protein FVER14953_00754 [Fusarium verticillioides];Gene=FPCIR_8507;Ontology_term=DNA binding;Ontology_id=GO:0003677
contig00002.g984	1388	ID=contig00002.g984;Description=26S proteasome regulatory subunit N6 [Fusarium oxysporum f. sp. lycopersici 4287];Gene=FGADI_5131;Ontology_term=proteasome complex;Ontology_id=GO:0000502
contig00002.g985	792	ID=contig00002.g985;Description=molecular chaperone GrpE [Fusarium verticillioides 7600];Gene=Forpi1262_v000750;Ontology_term=transmembrane transport,molecular function regulator activity,sulfur compound metabolic process,mitochondrion,intracellular protein transport,protein folding,mitochondrion organization;Ontology_id=GO:0055085,GO:0098772,GO:0006790,GO:0005739,GO:0006886,GO:0006457,GO:0007005
contig00002.g986	917	ID=contig00002.g986;Description=rho family, other [Fusarium verticillioides 7600];Gene=rhoC;Ontology_term=cytoskeleton organization,GTPase activity,signaling,anatomical structure development,cytokinesis,cell wall organization or biogenesis,mitotic cell cycle;Ontology_id=GO:0007010,GO:0003924,GO:0023052,GO:0048856,GO:0000910,GO:0071554,GO:0000278;Enzyme_code=EC:3.6.1.15,EC:3.6.1,EC:3.6,EC:3,EC:3.6.1.15;Enzyme_name=nucleoside-triphosphate phosphatase,Acting on acid anhydrides,Acting on acid anhydrides,Hydrolases,nucleoside-triphosphate phosphatase
contig00002.g987	666	ID=contig00002.g987;Description=transcription elongation factor B, polypeptide 1 [Fusarium verticillioides 7600];Gene=FLONG3_1486;Ontology_term=DNA repair,translation regulator activity,protein catabolic process,regulation of DNA-templated transcription,nucleoplasm;Ontology_id=GO:0006281,GO:0045182,GO:0030163,GO:0006355,GO:0005654
contig00002.g988	1731	ID=contig00002.g988;Description=lariat-debranching enzyme [Fusarium coicis];Gene=FVER53590_00749;Ontology_term=nucleus,mRNA metabolic process,catalytic activity, acting on RNA,hydrolase activity;Ontology_id=GO:0005634,GO:0016071,GO:0140098,GO:0016787;Enzyme_code=EC:3.1,EC:3.1.30,EC:3.1,EC:3,EC:3.1.26;Enzyme_name=Acting on ester bonds,Acting on ester bonds,Acting on ester bonds,Hydrolases,Acting on ester bonds

contig00002.g989	964	ID=contig00002.g989;Description=Mob1/phocein [Fusarium redolens];Gene=FOXB_08366;Ontology_term=nucleus,molecular function regulator activity,cytoskeleton organization,nucleobase-containing small molecule metabolic process,microtubule organizing center,signaling,mitotic nuclear division,cytokinesis,carbohydrate derivative metabolic process;Ontology_id=GO:0005634,GO:0098772,GO:0007010,GO:0055086,GO:0005815,GO:0023052,GO:0140014,GO:0000910,GO:1901135
contig00002.g990	1045	ID=contig00002.g990;Description=ATP synthase F1 delta subunit [Fusarium tjaetaba];Gene=FOXG_00768;Ontology_term=generation of precursor metabolites and energy,transmembrane transport,nucleobase-containing small molecule metabolic process,mitochondrion,structural molecule activity,carbohydrate derivative metabolic process;Ontology_id=GO:0006091,GO:0055085,GO:0055086,GO:0005739,GO:0005198,GO:1901135
contig00002.g991	3506	ID=contig00002.g991;Description=glucosidase I [Fusarium coicis];Gene=FPRO05_03505;Ontology_term=carbohydrate metabolic process,hydrolase activity;Ontology_id=GO:0005975,GO:0016787;Enzyme_code=EC:3.2.1.106,EC:3.2.1.106,EC:3.2,EC:3,EC:3.2.1;Enzyme_name=mannosyl-oligosaccharide glucosidase,mannosyl-oligosaccharide glucosidase,Glycosylases,Hydrolases,Glycosylases
contig00002.g992	1195	ID=contig00002.g992;Description=cis-prenyltransferase [Fusarium irregulare];Gene=AK830_g11263;Ontology_term=lipid metabolic process,transferase activity;Ontology_id=GO:0006629,GO:0016740;Enzyme_code=EC:2.5.1;Enzyme_name=Transferring alkyl or aryl groups, other than methyl groups
contig00002.g993	2187	ID=contig00002.g993;Description=transcriptional regulatory PHO23 [Fusarium coicis];Gene=FMUND_6027;Ontology_term=nucleus,chromosome,chromatin organization;Ontology_id=GO:0005634,GO:0005694,GO:0006325;Enzyme_code=EC:2.5.1.87;Enzyme_name=ditrans,polycis-polyprenyl diphosphate synthase [(2E,6E)-farnesyl
contig00002.g994	1874	ID=contig00002.g994;Description=related to Leucine Rich Repeat domain protein [Fusarium fujikuroi];Ontology_term=SCF ubiquitin ligase complex,intracellular anatomical structure;Ontology_id=GO:0019005,GO:0005622
contig00002.g995	2793	ID=contig00002.g995;Description=V-type proton ATPase subunit A [Fusarium proliferatum];Gene=FNYG_03709;Ontology_term=transmembrane transport,ATP-dependent activity,vacuole,transporter activity;Ontology_id=GO:0055085,GO:0140657,GO:0005773,GO:0005215;Enzyme_code=EC:7.2.2;Enzyme_name=Catalysing the translocation of inorganic cations

contig00002.g996	2945	ID=contig00002.g996;Description=endoribonuclease ysh1 [Fusarium musae];Gene=FOPG_01725;Ontology_term=nucleus,generation of precursor metabolites and energy,mRNA metabolic process,nucleobase-containing small molecule metabolic process,oxidoreductase activity,cytosol;Ontology_id=GO:0005634,GO:0006091,GO:0016071,GO:0055086,GO:0016491,GO:0005829;Enzyme_code=EC:1.2.1.10,EC:1.1.1.1,EC:1.1.1.71;Enzyme_name=acetaldehyde dehydrogenase (acetylating),alcohol dehydrogenase,alcohol dehydrogenase [NAD(P)(+)]
contig00002.g997	1980	ID=contig00002.g997;Description=tpa inducible [Fusarium mundagurra];Gene=FPANT_9287;Ontology_term=DNA-templated transcription,nucleoplasm;Ontology_id=GO:0006351,GO:0005654
contig00002.g998	1277	ID=contig00002.g998;Description=probable nitrilase [Fusarium fujikuroi];Gene=FTJAE_12895;Ontology_term=hydrolase activity;Ontology_id=GO:0016787;Enzyme_code=EC:3.5.1;Enzyme_name=Acting on carbon-nitrogen bonds, other than peptide bonds
contig00002.g999	738	ID=contig00002.g999;Description=actin like protein 2/3 complex, subunit 5 [Fusarium verticillioides 7600];Gene=BFJ68_g14345;Ontology_term=cytoskeleton;Ontology_id=GO:0005856
contig00002.g1000	1126	ID=contig00002.g1000;Description=methyltransferase [Fusarium coicis];Gene=FVER53590_00736;Ontology_term=transferase activity;Ontology_id=GO:0016740;Enzyme_code=EC:2.1.1,EC:2.1,EC:2,EC:2.1.1;Enzyme_name=Transferring one-carbon groups,Transferring one-carbon groups,Transferases,Transferring one-carbon groups
contig00002.g1001	3116	ID=contig00002.g1001;Description=ap-2 complex subunit alpha-2 [Fusarium tjaetaba];Gene=FPANT_1837;Ontology_term=cargo receptor activity,cytoplasmic vesicle,intracellular protein transport,plasma membrane,molecular adaptor activity;Ontology_id=GO:0038024,GO:0031410,GO:0006886,GO:0005886,GO:0060090
contig00002.g1002	942	ID=contig00002.g1002;Description=YggS family pyridoxal phosphate enzyme [Fusarium proliferatum];Gene=FVEG_00734;Ontology_term=amino acid metabolic process,protein maturation;Ontology_id=GO:0006520,GO:0051604
contig00002.g1003	1125	ID=contig00002.g1003;Description=hypothetical protein J7337_001257 [Fusarium musae]
contig00002.g1004	1685	ID=contig00002.g1004;Description=nif-specific regulatory [Fusarium subglutinans];Gene=FANTH_9881;Ontology_term=nucleoplasm;Ontology_id=GO:0005654

contig00002.g1005	849	ID=contig00002.g1005;Description=7alpha-cephem-methoxylase P8 chain [Fusarium tjaetaba];Gene=FVER53263_00731;Ontology_term=oxidoreductase activity;Ontology_id=GO:0016491;Enzyme_code=EC:1,EC:1;Enzyme_name=Oxidoreductases,Oxidoreductases
contig00002.g1006	2008	ID=contig00002.g1006;Description=ars binding 2 [Fusarium tjaetaba];Ontology_term=DNA binding;Ontology_id=GO:0003677
contig00002.g1007	2149	ID=contig00002.g1007;Description=sorbitol transporter [Fusarium pseudocircinatum];Gene=BFJ63_vAg8866;Ontology_term=transmembrane transport,transporter activity;Ontology_id=GO:0055085,GO:0005215
contig00002.g1008	2757	ID=contig00002.g1008;Description=mechanosensitive ion channel family [Fusarium coicis];Gene=TRX2;Ontology_term=transmembrane transport,extracellular region,external encapsulating structure;Ontology_id=GO:0055085,GO:0005576,GO:0030312
contig00002.g1009	1126	ID=contig00002.g1009;Description=dienelactone hydrolase [Colletotrichum orchidophilum];Gene=FVER53590_00726;Ontology_term=hydrolase activity;Ontology_id=GO:0016787;Enzyme_code=EC:3;Enzyme_name=Hydrolases
contig00002.g1010	2780	ID=contig00002.g1010;Description=protein SEY1 [Fusarium verticillioides 7600];Gene=SEY1;Ontology_term=membrane organization,transmembrane transport,vesicle-mediated transport,GTPase activity,endoplasmic reticulum;Ontology_id=GO:0061024,GO:0055085,GO:0016192,GO:0003924,GO:0005783;Enzyme_code=EC:3.6.1.15,EC:3.6.1,EC:3.6,EC:3,EC:3.6.1.15;Enzyme_name=nucleoside-triphosphate phosphatase,Acting on acid anhydrides,Acting on acid anhydrides,Hydrolases,nucleoside-triphosphate phosphatase
contig00002.g1011	1020	ID=contig00002.g1011;Description=ribonuclease H2 subunit A [Fusarium verticillioides 7600];Gene=FNYG_03693;Ontology_term=nucleus,DNA repair,RNA binding,DNA replication,catalytic activity, acting on RNA,mitotic cell cycle,hydrolase activity;Ontology_id=GO:0005634,GO:0006281,GO:0003723,GO:0006260,GO:0140098,GO:0000278,GO:0016787;Enzyme_code=EC:3.1.30,EC:3.1.26.4,EC:3.1.30,EC:3.1,EC:3,EC:3.1.26,EC:3.1.26.4;Enzyme_name=Acting on ester bonds,ribonuclease H,Acting on ester bonds,Acting on ester bonds,Hydrolases,Acting on ester bonds,ribonuclease H
contig00002.g1012	2137	ID=contig00002.g1012;Description=centromere associated protein [Fusarium tjaetaba];Gene=FPCIR_5867;Ontology_term=nucleus,DNA binding,protein-containing complex assembly,structural molecule activity,chromosome,chromatin organization;Ontology_id=GO:0005634,GO:0003677,GO:0065003,GO:0005198,GO:0005694,GO:0006325

contig00002.g1013	2861	ID=contig00002.g1013;Description=mitochondrial escape 2 [Fusarium tjaetaba];Gene=FVEG_00722;Ontology_term=RNA binding,mRNA metabolic process,mitochondrion,mitochondrion organization;Ontology_id=GO:0003723,GO:0016071,GO:0005739,GO:0007005
contig00002.g1014	663	ID=contig00002.g1014;Description=early meiotic induction 1 [Fusarium tjaetaba]
contig00002.g1015	2082	ID=contig00002.g1015;Description=heat shock protein 70 family [Fusarium redolens];Gene=FNAPI_8411;Ontology_term=transmembrane transport,cytoplasmic translation,protein catabolic process,extracellular region,intracellular protein transport,vacuole,plasma membrane,mitochondrion organization,immune system process,defense response to other organism,nucleus,nucleocytoplasmic transport,RNA binding,vesicle-mediated transport,protein folding chaperone,ATP-dependent activity,cell wall,mitochondrion,cytosol,extracellular matrix,hydrolase activity;Ontology_id=GO:0055085,GO:0002181,GO:0030163,GO:0005576,GO:0006886,GO:0005773,GO:0005886,GO:0007005,GO:0002376,GO:0098542,GO:0005634,GO:0006913,GO:0003723,GO:0016192,GO:0044183,GO:0140657,GO:0005618,GO:0005739,GO:0005829,GO:0031012,GO:0016787;Enzyme_code=EC:3.6.1,EC:3.6,EC:3,EC:3.6.1.15;Enzyme_name=Acting on acid anhydrides,Acting on acid anhydrides,Hydrolases,nucleoside-triphosphate phosphatase
contig00002.g1016	816	ID=contig00002.g1016;Description=hypothetical protein FVEG_00719 [Fusarium verticillioides 7600]
contig00002.g1017	2233	ID=contig00002.g1017;Description=multidrug transporter [Fusarium tjaetaba];Gene=F25303_14035;Ontology_term=DNA binding,transmembrane transport,transporter activity;Ontology_id=GO:0003677,GO:0055085,GO:0005215
contig00002.g1018	2690	ID=contig00002.g1018;Description=carnitine O-acetyltransferase [Fusarium odoratissimum NRRL 54006];Gene=FOTG_06294;Ontology_term=mitochondrion,lipid metabolic process,cellular modified amino acid metabolic process,transferase activity;Ontology_id=GO:0005739,GO:0006629,GO:0006575,GO:0016740;Enzyme_code=EC:2.3,EC:2.3,EC:2.3.1.7,EC:2,EC:2.3.1;Enzyme_name=Acyltransferases,Acyltransferases,carnitine O-acetyltransferase,Transferases,Acyltransferases
contig00002.g1019	4395	ID=contig00002.g1019;Description=related to Drosophila pumilio protein and Mpt5p protein [Fusarium fujikuroi];Gene=FVER53590_00716;Ontology_term=mRNA metabolic process,RNA binding,ribosome biogenesis,regulation of DNA-templated transcription,chromatin organization,nucleolus;Ontology_id=GO:0016071,GO:0003723,GO:0042254,GO:0006355,GO:0006325,GO:0005730

contig00002.g1020	3637	ID=contig00002.g1020;Description=DNA-directed RNA polymerase III subunit RPC2 [Fusarium oxysporum f. sp. lycopersici 4287];Gene=BFJ68_g14322;Ontology_term=nucleus,DNA binding,DNA-templated transcription,tRNA metabolic process,chromosome,catalytic activity, acting on RNA,transferase activity;Ontology_id=GO:0005634,GO:0003677,GO:0006351,GO:0006399,GO:0005694,GO:0140098,GO:0016740;Enzyme_code=EC:2.7.7.6;Enzyme_name=DNA-directed RNA polymerase
contig00002.g1021	1320	ID=contig00002.g1021;Description=DNA repair protein RAD57 [Fusarium verticillioides 7600];Gene=FNYG_03683;Ontology_term=DNA repair,catalytic activity, acting on DNA,protein-containing complex assembly,DNA recombination,chromosome,nucleus,DNA binding,ATP-dependent activity,telomere organization,hydrolase activity,meiotic nuclear division,small molecule sensor activity;Ontology_id=GO:0006281,GO:0140097,GO:0065003,GO:0006310,GO:0005694,GO:0005634,GO:0003677,GO:0140657,GO:0032200,GO:0016787,GO:0140013,GO:0140299;Enzyme_code=EC:3.6.1,EC:3.6,EC:3,EC:3.6.1.15;Enzyme_name=Acting on acid anhydrides,Acting on acid anhydrides,Hydrolases,nucleoside-triphosphate phosphatase
contig00002.g1022	960	ID=contig00002.g1022;Description=60S ribosomal protein L15 [Fusarium oxysporum];Gene=FOXG_00802;Ontology_term=RNA binding,cytoplasmic translation,autophagy,structural molecule activity,cytosol,ribosome,nucleolus;Ontology_id=GO:0003723,GO:0002181,GO:0006914,GO:0005198,GO:0005829,GO:0005840,GO:0005730
contig00002.g1023	655	ID=contig00002.g1023;Description=60s ribosomal l15 [Fusarium coicis];Gene=FVER53590_00712;Ontology_term=structural molecule activity,ribosome;Ontology_id=GO:0005198,GO:0005840
contig00002.g1024	2803	ID=contig00002.g1024;Description=PSP1-like suppressor protein [Fusarium denticulatum];Ontology_term=RNA binding,mitochondrion;Ontology_id=GO:0003723,GO:0005739
contig00002.g1025	3289	ID=contig00002.g1025;Description=WD repeat WDR6 [Fusarium tjaetaba]
contig00002.g1026	1020	ID=contig00002.g1026;Description=integral membrane protein [Fusarium phyllophilum];Gene=C2S_5869;Ontology_term=plasma membrane;Ontology_id=GO:0005886
contig00002.g1027	1271	ID=contig00002.g1027;Description=hypothetical protein FVER53590_00705 [Fusarium verticillioides];Gene=ORF45;Ontology_term=extracellular space,reproductive process;Ontology_id=GO:0005615,GO:0022414

contig00002.g1028	1698	ID=contig00002.g1028;Description=protein phosphatase [Fusarium verticillioides 7600];Gene=FVEG_00704;Ontology_term=nucleus,catalytic activity, acting on a protein,signaling,vacuole,plasma membrane,hydrolase activity;Ontology_id=GO:0005634,GO:0140096,GO:0023052,GO:0005773,GO:0005886,GO:0016787;Enzyme_code=EC:3.1.3.16,EC:3.1.3.16,EC:3.1,EC:3,EC:3.1.3;Enzyme_name=protein-serine/threonine phosphatase,protein-serine/threonine phosphatase,Acting on ester bonds,Hydrolases,Acting on ester bonds
contig00002.g1029	1047	ID=contig00002.g1029;Description=hypothetical protein FVER14953_00703 [Fusarium verticillioides]
contig00002.g1030	870	ID=contig00002.g1030;Description=ribosome-recycling factor [Fusarium denticulatum];Gene=FOXB_12065;Ontology_term=translation regulator activity,RNA binding,mitochondrial gene expression,mitochondrion;Ontology_id=GO:0045182,GO:0003723,GO:0140053,GO:0005739
contig00002.g1031	1701	ID=contig00002.g1031;Description=UDP-glucuronosyltransferase 2C1 microsomal [Fusarium tjaetaba];Gene=FVEG_00701;Ontology_term=transferase activity;Ontology_id=GO:0016740;Enzyme_code=EC:2.4,EC:2.4,EC:2;Enzyme_name=Glycosyltransferases,Glycosyltransferases,Transferases
contig00002.g1032	2871	ID=contig00002.g1032;Description=hypothetical protein FOXG_00905 [Fusarium oxysporum f. sp. lycopersici 4287];Gene=FOZG_01659;Ontology_term=molecular function regulator activity,cytoskeleton,cytoskeleton organization,establishment or maintenance of cell polarity,signaling,anatomical structure development,structural molecule activity,reproductive process,cytokinesis,cytoskeletal protein binding,mitotic cell cycle,molecular adaptor activity;Ontology_id=GO:0098772,GO:0005856,GO:0007010,GO:0007163,GO:0023052,GO:0048856,GO:0005198,GO:0022414,GO:0000910,GO:0008092,GO:0000278,GO:0060090
contig00002.g1033	1567	ID=contig00002.g1033;Description=hypothetical protein FVEG_00699 [Fusarium verticillioides 7600]

contig00002.g1034	822	ID=contig00002.g1034;Description=RNA polymerase II subunit A domain phosphatase SSU72 [Fusarium proliferatum];Gene=FVER53263_00698;Ontology_term=catalytic activity, acting on a protein,DNA replication,chromosome segregation,regulation of DNA-templated transcription,chromosome,chromatin organization,nucleoplasm,mRNA metabolic process,signaling,mitotic nuclear division,telomere organization,hydrolase activity;Ontology_id=GO:0140096,GO:0006260,GO:0007059,GO:0006355,GO:0005694,GO:0006325,GO:0005654,GO:0016071,GO:0023052,GO:0140014,GO:0032200,GO:0016787;Enzyme_code=EC:3.1.3.16,EC:3.1.3.48,EC:3.1.3.16,EC:3.1,EC:3.1.3.48,EC:3,EC:3.1.3;Enzyme_name=protein-serine/threonine phosphatase,protein-tyrosine-phosphatase,protein-serine/threonine phosphatase,Acting on ester bonds,protein-tyrosine-phosphatase,Hydrolases,Acting on ester bonds
contig00002.g1035	1296	ID=contig00002.g1035;Description=NAP1-binding 2 [Fusarium tjaetaba];Ontology_term=nucleus,molecular function regulator activity,signaling,cell wall organization or biogenesis,molecular adaptor activity;Ontology_id=GO:0005634,GO:0098772,GO:0023052,GO:0071554,GO:0060090
contig00002.g1036	1169	ID=contig00002.g1036;Description=NADH-cytochrome b5 reductase 2 [Fusarium verticillioides 7600];Gene=FVER53263_00696;Ontology_term=oxidoreductase activity,mitochondrion;Ontology_id=GO:0016491,GO:0005739;Enzyme_code=EC:1.6.2.2;Enzyme_name=cytochrome-b5 reductase
contig00002.g1037	2678	ID=contig00002.g1037;Description=glutamate carboxypeptidase II [Fusarium verticillioides 7600];Gene=FACUT_11483;Ontology_term=catalytic activity, acting on a protein,hydrolase activity;Ontology_id=GO:0140096,GO:0016787;Enzyme_code=EC:3.4,EC:3.4.17.21,EC:3.4,EC:3;Enzyme_name=Acting on peptide bonds (peptidases),glutamate carboxypeptidase II,Acting on peptide bonds (peptidases),Hydrolases
contig00002.g1038	2148	ID=contig00002.g1038;Description=tetracycline resistance TCR1 [Fusarium phyllophilum];Gene=FOC1_g10016205;Ontology_term=transmembrane transport,transporter activity;Ontology_id=GO:0055085,GO:0005215
contig00002.g1039	1350	ID=contig00002.g1039;Description=putative alcohol acetyltransferase FCK4 [Fusarium oxysporum f. sp. albedinis]
contig00002.g1040	1503	ID=contig00002.g1040;Description=hypothetical protein FVER53263_20986 [Fusarium verticillioides]
contig00002.g1041	2048	ID=contig00002.g1041;Description=hypothetical protein NW758_000679 [Fusarium oxysporum]

contig00002.g1042	2600	ID=contig00002.g1042;Description=hypothetical protein FVEG_00690 [Fusarium verticillioides 7600];Gene=FNYG_03660;Ontology_term=membrane;Ontology_id=GO:0016020
contig00002.g1043	334	ID=contig00002.g1043;Description=hypothetical protein CEK27_001276 [Fusarium fujikuroi];Gene=61
contig00002.g1044	1229	ID=contig00002.g1044;Description=nucleoside-diphosphate-sugar epimerase [Fusarium napiforme];Gene=62;Ontology_term=oxidoreductase activity;Ontology_id=GO:0016491;Enzyme_code=EC:1.1.1.1,EC:1.1.1.71;Enzyme_name=alcohol dehydrogenase,alcohol dehydrogenase [NAD(P)(+)]
contig00002.g1045	2471	ID=contig00002.g1045;Description=heat shock protein SSB1 [Fusarium proliferatum];Gene=FOMG_01658;Ontology_term=protein folding chaperone,ATP-dependent activity,cytosol;Ontology_id=GO:0044183,GO:0140657,GO:0005829
contig00002.g1046	601	ID=contig00002.g1046;Description=uncharacterized protein FTJAE_9750 [Fusarium tjaetaba];Gene=F25303_2399;Ontology_term=catalytic activity;Ontology_id=GO:0003824
contig00002.g1047	1118	ID=contig00002.g1047;Description=hypothetical protein FVEG_00686 [Fusarium verticillioides 7600];Gene=FACUT_9515;Ontology_term=membrane;Ontology_id=GO:0016020
contig00002.g1048	774	ID=contig00002.g1048;Description=hypothetical protein FVER14953_00685 [Fusarium verticillioides]
contig00002.g1049	276	ID=contig00002.g1049;Description=uncharacterized protein FTJAE_9753 [Fusarium tjaetaba]
contig00002.g1050	5359	ID=contig00002.g1050;Description=DNA-directed RNA polymerase II subunit RPB1 [Fusarium verticillioides 7600];Gene=FACUT_12914;Ontology_term=nucleus,DNA binding,DNA-templated transcription,catalytic activity, acting on RNA,transferase activity;Ontology_id=GO:0005634,GO:0003677,GO:0006351,GO:0140098,GO:0016740;Enzyme_code=EC:2.7.7.6;Enzyme_name=DNA-directed RNA polymerase
contig00002.g1051	1437	ID=contig00002.g1051;Description=COMPASS component SWD1 [Fusarium tjaetaba];Gene=FGLOB1_12092;Ontology_term=cytoskeleton,regulation of DNA-templated transcription,nuclear chromosome,telomere organization,chromatin organization,nucleoplasm;Ontology_id=GO:0005856,GO:0006355,GO:0000228,GO:0032200,GO:0006325,GO:0005654

contig00002.g1052	1213	ID=contig00002.g1052;Description=tRNA(His) guanylyltransferase [Fusarium subglutinans];Gene=FOVG_00549;Ontology_term=nucleus,tRNA metabolic process,catalytic activity, acting on RNA,transferase activity;Ontology_id=GO:0005634,GO:0006399,GO:0140098,GO:0016740;Enzyme_code=EC:2.7.7,EC:2.7,EC:2,EC:2.7.7,EC:2.7.7.79;Enzyme_name=Transferring phosphorus-containing groups,Transferring phosphorus-containing groups,Transferases,Transferring phosphorus-containing groups,tRNA(His) guanylyltransferase
contig00002.g1053	660	ID=contig00002.g1053;Description=dna-directed rna polymerase ii subunit rpb1 [Fusarium acutatum];Gene=FACUT_12917;Ontology_term=DNA-directed RNA polymerase complex;Ontology_id=GO:0000428
contig00002.g1054	1158	ID=contig00002.g1054;Description=transcription elongation factor S-II [Fusarium tjaetaba];Gene=FPANT_8331;Ontology_term=DNA binding,nucleus,translation regulator activity,mRNA metabolic process,protein-containing complex assembly,tRNA metabolic process,regulation of DNA-templated transcription,transcription regulator activity,molecular adaptor activity;Ontology_id=GO:0003677,GO:0005634,GO:0045182,GO:0016071,GO:0065003,GO:0006399,GO:0006355,GO:0140110,GO:0060090
contig00002.g1055	2490	ID=contig00002.g1055;Description=DUF726 domain-containing protein [Fusarium denticulatum];Gene=FVEG_00678;Ontology_term=vesicle-mediated transport,hydrolase activity;Ontology_id=GO:0016192,GO:0016787;Enzyme_code=EC:3.1,EC:3;Enzyme_name=Acting on ester bonds,Hydrolases
contig00002.g1056	1338	ID=contig00002.g1056;Description=cysteine synthase A [Fusarium verticillioides 7600];Gene=FMEXI_510;Ontology_term=sulfur compound metabolic process,mitochondrion,amino acid metabolic process;Ontology_id=GO:0006790,GO:0005739,GO:0006520;Enzyme_code=EC:2.5.1.47;Enzyme_name=cysteine synthase
contig00002.g1057	1617	ID=contig00002.g1057;Description=hypothetical protein HG530_004456 [Fusarium avenaceum];Gene=FVEG_00676;Ontology_term=catalytic activity, acting on a protein,nucleobase-containing small molecule metabolic process,protein-containing complex assembly,mitochondrion,carbohydrate derivative metabolic process,transferase activity;Ontology_id=GO:0140096,GO:0055086,GO:0065003,GO:0005739,GO:1901135,GO:0016740;Enzyme_code=EC:2.1.1.320,EC:2.1,EC:2.1.1.320,EC:2,EC:2.1.1;Enzyme_name=type II protein arginine methyltransferase,Transferring one-carbon groups,type II protein arginine methyltransferase,Transferases,Transferring one-carbon groups

contig00002.g1058	3088	ID=contig00002.g1058;Description=alanyl-tRNA synthetase [Fusarium verticillioides 7600];Gene=ALA1;Ontology_term=RNA binding,mitochondrial gene expression,mitochondrion,tRNA metabolic process,amino acid metabolic process,catalytic activity, acting on RNA,ligase activity;Ontology_id=GO:0003723,GO:0140053,GO:0005739,GO:0006399,GO:0006520,GO:0140098,GO:0016874;Enzyme_code=EC:6.1.1.7;Enzyme_name=alanine--tRNA ligase
contig00002.g1059	751	ID=contig00002.g1059;Description=hypothetical protein F52700_13453 [Fusarium sp. NRRL 52700];Gene=F52700_13453;Ontology_term=membrane;Ontology_id=GO:0016020
contig00002.g1060	611	ID=contig00002.g1060;Description=ubiquitin ligase sel1 [Fusarium beomiforme];Gene=FACUT_12924;Ontology_term=ligase activity;Ontology_id=GO:0016874;Enzyme_code=EC:6;Enzyme_name=Ligases
contig00002.g1061	2933	ID=contig00002.g1061;Description=hypothetical protein FVEG_00672 [Fusarium verticillioides 7600];Gene=FOQG_03171;Ontology_term=membrane;Ontology_id=GO:0016020
contig00002.g1062	1163	ID=contig00002.g1062;Description=uracil-DNA glycosylase [Fusarium verticillioides 7600];Gene=UNG1;Ontology_term=nucleus,DNA repair,catalytic activity, acting on DNA,mitochondrion,hydrolase activity;Ontology_id=GO:0005634,GO:0006281,GO:0140097,GO:0005739,GO:0016787;Enzyme_code=EC:3.2.2.27,EC:3.2,EC:3,EC:3.2.2.27,EC:3.2.2;Enzyme_name=uracil-DNA glycosylase,Glycosylases,Hydrolases,uracil-DNA glycosylase,Glycosylases
contig00002.g1063	3420	ID=contig00002.g1063;Description=morphogenesis-related MSB1 [Fusarium tjaetaba]
contig00002.g1064	3696	ID=contig00002.g1064;Description=DNA excision repair protein ERCC-5 [Fusarium verticillioides 7600];Gene=FNAPI_12091;Ontology_term=DNA repair,DNA binding,nucleus,catalytic activity, acting on DNA,DNA-templated transcription,hydrolase activity;Ontology_id=GO:0006281,GO:0003677,GO:0005634,GO:0140097,GO:0006351,GO:0016787;Enzyme_code=EC:3.1,EC:3.1,EC:3;Enzyme_name=Acting on ester bonds,Acting on ester bonds,Hydrolases
contig00002.g1065	388	ID=contig00002.g1065;Description=LSM domain protein [Fusarium subglutinans];Gene=C2S_5827;Ontology_term=transferase activity;Ontology_id=GO:0016740;Enzyme_code=EC:2,EC:2;Enzyme_name=Transferases,Transferases

contig00002.g1066	1288	ID=contig00002.g1066;Description=Eisosome component PIL1-domain-containing protein [Fusarium oxysporum];Gene=PIL1;Ontology_term=cytoskeleton,vesicle-mediated transport,mitochondrion,lipid binding,plasma membrane;Ontology_id=GO:0005856,GO:0016192,GO:0005739,GO:0008289,GO:0005886
contig00002.g1067	3481	ID=contig00002.g1067;Description=polycomb group MEDEA [Fusarium tjaetaba];Gene=FVER53263_00666;Ontology_term=catalytic activity, acting on a protein,chromatin organization,transferase activity;Ontology_id=GO:0140096,GO:0006325,GO:0016740;Enzyme_code=EC:2.1.1.356,EC:2.1,EC:2.1.1.43,EC:2,EC:2.1.1,EC:2.1.1.356;Enzyme_name=[histone H3]-lysine(27) N-trimethyltransferase,Transferring one-carbon groups,Transferring one-carbon groups,Transferases,Transferring one-carbon groups,[histone H3]-lysine(27) N-trimethyltransferase
contig00002.g1068	830	ID=contig00002.g1068;Description=endoplasmic reticulum transmembrane [Fusarium subglutinans];Gene=FOPG_00249;Ontology_term=vesicle-mediated transport,endoplasmic reticulum,intracellular protein transport;Ontology_id=GO:0016192,GO:0005783,GO:0006886
contig00002.g1069	1437	ID=contig00002.g1069;Description=DUF1479 domain protein [Fusarium tjaetaba]
contig00002.g1070	926	ID=contig00002.g1070;Description=hypothetical protein FCOIX_10255 [Fusarium coicis]
contig00002.g1071	1495	ID=contig00002.g1071;Description=6-hydroxy-D-nicotine oxidase [Fusarium tjaetaba];Gene=FPCIR_7539;Ontology_term=oxidoreductase activity;Ontology_id=GO:0016491;Enzyme_code=EC:1,EC:1;Enzyme_name=Oxidoreductases,Oxidoreductases
contig00002.g1072	415	ID=contig00002.g1072;Description=zinc-binding domain protein [Fusarium subglutinans]
contig00002.g1073	1229	ID=contig00002.g1073;Description=flavonol synthase [Fusarium tjaetaba];Gene=FPANT_8076;Ontology_term=oxidoreductase activity;Ontology_id=GO:0016491;Enzyme_code=EC:1;Enzyme_name=Oxidoreductases
contig00002.g1074	2986	ID=contig00002.g1074;Description=small nucleolar ribonucleoprotein [Fusarium tjaetaba];Gene=FPANT_8075;Ontology_term=RNA binding,ribosome biogenesis,regulation of DNA-templated transcription,chromosome,nucleolus;Ontology_id=GO:0003723,GO:0042254,GO:0006355,GO:0005694,GO:0005730

contig00002.g1075	399	ID=contig00002.g1075;Description=DNA-directed RNA polymerase I, II, and III subunit RPABC5 [Fusarium odoratissimum NRRL 54006];Gene=FOYG_06449;Ontology_term=DNA-templated transcription,tRNA metabolic process,nucleolus,nucleoplasm;Ontology_id=GO:0006351,GO:0006399,GO:0005730,GO:0005654
contig00002.g1076	2441	ID=contig00002.g1076;Description=hypothetical protein FVEG_14715 [Fusarium verticillioides 7600]
contig00002.g1077	327	ID=contig00002.g1077;Description=hypothetical protein FVER14953_00654 [Fusarium verticillioides]
contig00002.g1078	919	ID=contig00002.g1078;Description=hypothetical protein FVER53590_00654 [Fusarium verticillioides];Gene=FVER53263_00653;Ontology_term=transferase activity;Ontology_id=GO:0016740;Enzyme_code=EC:2.1.1,EC:2.1,EC:2,EC:2.1.1;Enzyme_name=Transferring one-carbon groups,Transferring one-carbon groups,Transferases,Transferring one-carbon groups
contig00002.g1079	1154	ID=contig00002.g1079;Description=rapid response to glucose 1 [Fusarium denticulatum];Gene=FMEXI_5670;Ontology_term=catalytic activity, acting on a protein,cytoplasmic translation,transferase activity;Ontology_id=GO:0140096,GO:0002181,GO:0016740;Enzyme_code=EC:2.1.1,EC:2.1,EC:2,EC:2.1.1;Enzyme_name=Transferring one-carbon groups,Transferring one-carbon groups,Transferases,Transferring one-carbon groups
contig00002.g1080	783	ID=contig00002.g1080;Description=hypothetical protein FVEG_00652 [Fusarium verticillioides 7600]
contig00002.g1081	2121	ID=contig00002.g1081;Description=hypothetical protein FVEG_00651 [Fusarium verticillioides 7600];Gene=FNAPI_8611;Ontology_term=protein ubiquitination;Ontology_id=GO:0016567
contig00002.g1082	673	ID=contig00002.g1082;Description=dynein light chain LC8-type [Fusarium oxysporum f. sp. lycopersici 4287];Gene=FTJAE_13117;Ontology_term=cytoskeleton;Ontology_id=GO:0005856
contig00002.g1083	2835	ID=contig00002.g1083;Description=multiple RNA-binding domain-containing protein 1 [Fusarium graminearum PH-1];Gene=FEQUK3_LOCUS2872;Ontology_term=RNA binding,protein-containing complex assembly,ribosome biogenesis,nucleolus;Ontology_id=GO:0003723,GO:0065003,GO:0042254,GO:0005730

contig00002.g1084	2705	ID=contig00002.g1084;Description=FoabaA [Fusarium oxysporum f. sp. lycopersici];Gene=FPCIR_7527;Ontology_term=nucleus,RNA binding,regulation of DNA-templated transcription,transcription regulator activity;Ontology_id=GO:0005634,GO:0003723,GO:0006355,GO:0140110
contig00002.g1085	1560	ID=contig00002.g1085;Description=hypothetical protein FVEG_00645 [Fusarium verticillioides 7600]
contig00002.g1086	969	ID=contig00002.g1086;Description=peptidyl-prolyl cis-trans isomerase H [Fusarium austroafricanum];Gene=FDENT_11091;Ontology_term=catalytic activity, acting on a protein,protein folding,isomerase activity;Ontology_id=GO:0140096,GO:0006457,GO:0016853;Enzyme_code=EC:5.2.1.8,EC:5.2.1.8,EC:5.2,EC:5;Enzyme_name=peptidylprolyl isomerase,peptidylprolyl isomerase,Cis-trans-isomerases,Isomerases
contig00002.g1087	1436	ID=contig00002.g1087;Description=RING-8 [Fusarium tjaetaba];Gene=FVER53263_00643;Ontology_term=membrane;Ontology_id=GO:0016020
contig00002.g1088	933	ID=contig00002.g1088;Description=hypothetical protein FVEG_00642 [Fusarium verticillioides 7600]
contig00002.g1089	6273	ID=contig00002.g1089;Description=related to VPS8-vacuolar sorting protein, 134 kD [Fusarium fujikuroi];Gene=FPANT_3077;Ontology_term=endosome,vesicle-mediated transport,intracellular protein transport;Ontology_id=GO:0005768,GO:0016192,GO:0006886
contig00002.g1090	964	ID=contig00002.g1090;Description=hypothetical protein FNAPI_8620 [Fusarium napiforme];Gene=FMUND_3843;Ontology_term=transmembrane transport,transporter activity;Ontology_id=GO:0055085,GO:0005215
contig00002.g1091	2011	ID=contig00002.g1091;Description=hypothetical protein FVEG_00638 [Fusarium verticillioides 7600]
contig00002.g1092	1811	ID=contig00002.g1092;Description=hypothetical protein LB504_000555 [Fusarium proliferatum]
contig00002.g1093	964	ID=contig00002.g1093;Description=copii coat assembly sec16 [Fusarium pseudocircinatum]
contig00002.g1094	627	ID=contig00002.g1094;Description=hypothetical protein FVEG_00634 [Fusarium verticillioides 7600]

contig00002.g1095	2079	ID=contig00002.g1095;Description=ATP-dependent RNA helicase ROK1 [Fusarium verticillioides 7600];Gene=FVER53263_00633;Ontology_term=RNA binding,ATP-dependent activity,ribosome biogenesis,catalytic activity, acting on RNA,nucleolus,hydrolase activity;Ontology_id=GO:0003723,GO:0140657,GO:0042254,GO:0140098,GO:0005730,GO:0016787;Enzyme_code=EC:3.6.4.13,EC:3.6.1,EC:3.6.4.13,EC:3.6,EC:3,EC:3.6.1.15;Enzyme_name=RNA helicase,Acting on acid anhydrides,RNA helicase,Acting on acid anhydrides,Hydrolases,nucleoside-triphosphate phosphatase
contig00002.g1096	1540	ID=contig00002.g1096;Description=TOS1 [Fusarium tjaetaba];Ontology_term=carbohydrate metabolic process,cell wall,cell wall organization or biogenesis,transferase activity,vacuole;Ontology_id=GO:0005975,GO:0005618,GO:0071554,GO:0016740,GO:0005773;Enzyme_code=EC:2.4.1.34,EC:2.4,EC:2,EC:2.4.1;Enzyme_name=1,3-beta-glucan synthase,Glycosyltransferases,Transferases,Glycosyltransferases
contig00002.g1097	1941	ID=contig00002.g1097;Description=lysyl-tRNA synthetase, class II [Fusarium verticillioides 7600];Gene=FPANT_3085;Ontology_term=mitochondrial gene expression,transmembrane transport,RNA binding,mitochondrion,tRNA metabolic process,amino acid metabolic process,catalytic activity, acting on RNA,ligase activity;Ontology_id=GO:0140053,GO:0055085,GO:0003723,GO:0005739,GO:0006399,GO:0006520,GO:0140098,GO:0016874;Enzyme_code=EC:6.1.1.6,EC:6.1,EC:6.1.1.6,EC:6.1.1,EC:6;Enzyme_name=lysine--tRNA ligase,Forming carbon-oxygen bonds,lysine--tRNA ligase,Forming carbon-oxygen bonds,Ligases
contig00002.g1098	1489	ID=contig00002.g1098;Description=actin-like protein 4 [Fusarium verticillioides 7600]
contig00002.g1099	960	ID=contig00002.g1099;Description=TDG/mug DNA glycosylase [Fusarium verticillioides 7600];Gene=FPANT_3087;Ontology_term=DNA repair,catalytic activity, acting on DNA,hydrolase activity;Ontology_id=GO:0006281,GO:0140097,GO:0016787;Enzyme_code=EC:3.2.2,EC:3.2,EC:3,EC:3.2.2.29,EC:3.2.2.27,EC:3.2.2;Enzyme_name=Glycosylases,Glycosylases,Hydrolases,thymine-DNA glycosylase,uracil-DNA glycosylase,Glycosylases

contig00002.g1100	1560	ID=contig00002.g1100;Description=UDP-N-acetylglucosamine pyrophosphorylase [Fusarium verticillioides 7600];Gene=FNAPI_8630;Ontology_term=nucleus,nucleobase-containing small molecule metabolic process,cytosol,carbohydrate derivative metabolic process,transferase activity;Ontology_id=GO:0005634,GO:0055086,GO:0005829,GO:1901135,GO:0016740;Enzyme_code=EC:2.7.7,EC:2.7.7.83,EC:2.7.7.23,EC:2.7,EC:2,EC:2.7.7;Enzyme_name=Transferring phosphorus-containing groups,UDP-N-acetylgalactosamine diphosphorylase,UDP-N-acetylglucosamine diphosphorylase,Transferring phosphorus-containing groups,Transferases,Transferring phosphorus-containing groups
contig00002.g1101	1201	ID=contig00002.g1101;Description=thioredoxin reductase [Fusarium verticillioides 7600];Gene=FNAPI_8631;Ontology_term=catalytic activity, acting on a protein,mRNA metabolic process,antioxidant activity,oxidoreductase activity,mitochondrion,anatomical structure development,cell differentiation,reproductive process,cytosol,nucleoplasm;Ontology_id=GO:0140096,GO:0016071,GO:0016209,GO:0016491,GO:0005739,GO:0048856,GO:0030154,GO:0022414,GO:0005829,GO:0005654;Enzyme_code=EC:1.8.1.9,EC:1.8.1.8,EC:1.8.1,EC:1.6,EC:1,EC:1.8,EC:1.6.5.5,EC:1.8.1.9,EC:1.8.1.8;Enzyme_name=thioredoxin-disulfide reductase,protein-disulfide reductase,Acting on a sulfur group of donors,Acting on NADH or NADPH,Oxidoreductases,Acting on a sulfur group of donors,NADPH:quinone reductase,thioredoxin-disulfide reductase,protein-disulfide reductase
contig00002.g1102	2599	ID=contig00002.g1102;Description=Vacuolar protein sorting-associated protein 53 [Fusarium oxysporum];Gene=FOVG_00606;Ontology_term=endosome,vesicle-mediated transport,cytosol,Golgi apparatus;Ontology_id=GO:0005768,GO:0016192,GO:0005829,GO:0005794
contig00002.g1103	4317	ID=contig00002.g1103;Description=hypothetical protein FVEG_14704 [Fusarium verticillioides 7600];Gene=FVEG_14704;Ontology_term=endoplasmic reticulum;Ontology_id=GO:0005783

contig00002.g1104	2399	ID=contig00002.g1104;Description=SMP2 [Fusarium tjaetaba];Ontology_term=lipid droplet,nuclear envelope,membrane organization,DNA binding,generation of precursor metabolites and energy,lipid metabolic process,regulation of DNA-templated transcription,cytosol,vacuole,transcription regulator activity,hydrolase activity,molecular adaptor activity;Ontology_id=GO:0005811,GO:0005635,GO:0061024,GO:0003677,GO:0006091,GO:0006629,GO:0006355,GO:0005829,GO:0005773,GO:0140110,GO:0016787,GO:0060090;Enzyme_code=EC:3.1,EC:3,EC:3.1.3.4,EC:3.1.3;Enzyme_name=Acting on ester bonds,Hydrolases,phosphatidate phosphatase,Acting on ester bonds
contig00002.g1105	2618	ID=contig00002.g1105;Description=histidine biosynthesis trifunctional protein [Fusarium verticillioides 7600];Gene=FPCIR_7506;Ontology_term=oxidoreductase activity,amino acid metabolic process,cytosol,hydrolase activity;Ontology_id=GO:0016491,GO:0006520,GO:0005829,GO:0016787;Enzyme_code=EC:1.1.1.23,EC:3.6.1.31,EC:3.5.4.19,EC:1.1,EC:3.6.1,EC:3.5.4,EC:1,EC:1.1.1.23,EC:3.6,EC:3.6.1.31,EC:3.5,EC:3,EC:1.1.1,EC:3.5.4.19;Enzyme_name=histidinol dehydrogenase,phosphoribosyl-ATP diphosphatase,phosphoribosyl-AMP cyclohydrolase,Acting on the CH-OH group of donors,Acting on acid anhydrides,Acting on carbon-nitrogen bonds, other than peptide bonds,Oxidoreductases,histidinol dehydrogenase,Acting on acid anhydrides,phosphoribosyl-ATP diphosphatase,Acting on carbon-nitrogen bonds, other than peptide bonds,Hydrolases,Acting on the CH-OH group of donors,phosphoribosyl-AMP cyclohydrolase
contig00002.g1106	762	ID=contig00002.g1106;Description=related to TFIID and SAGA subunit [Fusarium fujikuroi];Gene=FOTG_06329;Ontology_term=DNA binding,translation regulator activity,protein-containing complex assembly,structural molecule activity,nuclear chromosome,regulation of DNA-templated transcription,chromatin organization,transcription regulator activity,nucleoplasm,molecular adaptor activity;Ontology_id=GO:0003677,GO:0045182,GO:0065003,GO:0005198,GO:0000228,GO:0006355,GO:0006325,GO:0140110,GO:0005654,GO:0060090
contig00002.g1107	1650	ID=contig00002.g1107;Description=mitochondrial-processing peptidase subunit beta [Fusarium subglutinans];Gene=FCIRC_8944;Ontology_term=catalytic activity, acting on a protein,mitochondrion,protein maturation,hydrolase activity;Ontology_id=GO:0140096,GO:0005739,GO:0051604,GO:0016787;Enzyme_code=EC:3.4.24;Enzyme_name=Acting on peptide bonds (peptidases)

contig00002.g1108	2942	ID=contig00002.g1108;Description=Sip1-like protein [Fusarium tjaetaba];Gene=FPANT_3096;Ontology_term=carbohydrate metabolic process,hydrolase activity;Ontology_id=GO:0005975,GO:0016787;Enzyme_code=EC:3.2.1.22,EC:3.2,EC:3,EC:3.2.1.22,EC:3.2.1;Enzyme_name=alpha-galactosidase,Glycosylases,Hydrolases,alpha-galactosidase,Glycosylases
contig00002.g1109	1845	ID=contig00002.g1109;Description=inosine 5-monophosphate dehydrogenase [Drepanopeziza brunnea f. sp. 'multigermtubi' MB_m1];Gene=OAory_01070310;Ontology_term=RNA binding,nucleobase-containing small molecule metabolic process,oxidoreductase activity,carbohydrate derivative metabolic process,chromosome,plasma membrane;Ontology_id=GO:0003723,GO:0055086,GO:0016491,GO:1901135,GO:0005694,GO:0005886;Enzyme_code=EC:1.1.1.205,EC:1.1,EC:1.1.1.205,EC:1,EC:1.1.1;Enzyme_name=IMP dehydrogenase,Acting on the CH-OH group of donors,IMP dehydrogenase,Oxidoreductases,Acting on the CH-OH group of donors
contig00002.g1110	711	ID=contig00002.g1110;Description=plac8 family [Fusarium tjaetaba]
contig00002.g1111	910	ID=contig00002.g1111;Description=hypothetical protein FVEG_00616 [Fusarium verticillioides 7600];Gene=FVER53590_00616;Ontology_term=nucleus,catalytic activity, acting on a protein,chromatin organization,transferase activity;Ontology_id=GO:0005634,GO:0140096,GO:0006325,GO:0016740;Enzyme_code=EC:2.3.1.48,EC:2.3,EC:2,EC:2.3.1.48,EC:2.3.1.257,EC:2.3.1;Enzyme_name=histone acetyltransferase,Acyltransferases,Transferases,histone acetyltransferase,N-terminal L-serine N(alpha)-acetyltransferase NatD,Acyltransferases
contig00002.g1112	511	ID=contig00002.g1112;Description=LYR motif-containing protein [Fusarium subglutinans]
contig00002.g1113	351	ID=contig00002.g1113;Description=ribosomal l36 [Fusarium tjaetaba];Gene=FVER53590_00615;Ontology_term=structural molecule activity,ribosome;Ontology_id=GO:0005198,GO:0005840
contig00002.g1114	2466	ID=contig00002.g1114;Description=hypothetical protein FVER53590_00614 [Fusarium verticillioides]
contig00002.g1115	1911	ID=contig00002.g1115;Description=hypothetical protein FMUND_3819 [Fusarium mundagurra]
contig00002.g1116	960	ID=contig00002.g1116;Description=UDP-glucuronosyltransferase 2C1 [Fusarium oxysporum f. sp. rapae];Gene=Forpe1208_v000588

contig00002.g1117	1731	ID=contig00002.g1117;Description=Aldehyde/histidinol dehydrogenase [Fusarium oxysporum Fo47];Gene=FCIRC_8952;Ontology_term=oxidoreductase activity,mitochondrion,amino acid metabolic process;Ontology_id=GO:0016491,GO:0005739,GO:0006520;Enzyme_code=EC:1.2.1.16,EC:1.2.1.24,EC:1.2.1.79,EC:1.2.1.16,EC:1.2,EC:1.2.1.24,EC:1.2.1.79,EC:1,EC:1.2.1.20,EC:1.2.1;Enzyme_name=succinate-semialdehyde dehydrogenase [NAD(P)(+)],succinate-semialdehyde dehydrogenase (NAD(+)),succinate-semialdehyde dehydrogenase (NADP(+)),succinate-semialdehyde dehydrogenase [NAD(P)(+)],Acting on the aldehyde or oxo group of donors,succinate-semialdehyde dehydrogenase (NAD(+)),succinate-semialdehyde dehydrogenase (NADP(+)),Oxidoreductases,glutarate-semialdehyde dehydrogenase,Acting on the aldehyde or oxo group of donors
contig00002.g1118	963	ID=contig00002.g1118;Description=succinate-semialdehyde dehydrogenase [Fusarium tjaetaba];Gene=FACUT_2827;Ontology_term=membrane;Ontology_id=GO:0016020
contig00002.g1119	4589	ID=contig00002.g1119;Description=Hybrid signal transduction histidine kinase K [Fusarium tjaetaba];Gene=FVER53263_00610;Ontology_term=catalytic activity, acting on a protein,signaling,transferase activity,small molecule sensor activity;Ontology_id=GO:0140096,GO:0023052,GO:0016740,GO:0140299;Enzyme_code=EC:2.7.3,EC:2.7.13.3,EC:2.7.1,EC:2.7.3,EC:2.7,EC:2,EC:2.7.13.3;Enzyme_name=Transferring phosphorus-containing groups,histidine kinase,Transferring phosphorus-containing groups,Transferring phosphorus-containing groups,Transferring phosphorus-containing groups,Transferases,histidine kinase
contig00002.g1120	1443	ID=contig00002.g1120;Description=transcription factor jumonji [Fusarium tjaetaba];Gene=FPCIR_7492;Ontology_term=transferase activity;Ontology_id=GO:0016740;Enzyme_code=EC:2.1.1,EC:2.1,EC:2,EC:2.1.1;Enzyme_name=Transferring one-carbon groups,Transferring one-carbon groups,Transferases,Transferring one-carbon groups
contig00002.g1121	1306	ID=contig00002.g1121;Description=Pma1 stabilization in the Golgi protein 1 [Fusarium oxysporum f. sp. vasinfectum];Gene=FNAPI_2385;Ontology_term=membrane;Ontology_id=GO:0016020

contig00002.g1122	555	ID=contig00002.g1122;Description=peptidyl-prolyl cis-trans isomerase-like 3 [Fusarium verticillioides 7600];Gene=FDENT_4351;Ontology_term=catalytic activity, acting on a protein,protein folding,isomerase activity;Ontology_id=GO:0140096,GO:0006457,GO:0016853;Enzyme_code=EC:5.2.1.8,EC:5.2.1.8,EC:5.2,EC:5;Enzyme_name=peptidylprolyl isomerase,peptidylprolyl isomerase,Cis-trans-isomerases,Isomerases
contig00002.g1123	909	ID=contig00002.g1123;Description=hypothetical protein FVER53590_00605 [Fusarium verticillioides]
contig00002.g1124	1653	ID=contig00002.g1124;Description=Peptidyl-prolyl isomerase cwc27 [Fusarium musae];Gene=FVER53590_00604;Ontology_term=nucleus,catalytic activity, acting on a protein,protein folding,isomerase activity;Ontology_id=GO:0005634,GO:0140096,GO:0006457,GO:0016853;Enzyme_code=EC:5.2.1.8,EC:5.2.1.8,EC:5.2,EC:5;Enzyme_name=peptidylprolyl isomerase,peptidylprolyl isomerase,Cis-trans-isomerases,Isomerases
contig00002.g1125	2298	ID=contig00002.g1125;Description=heat shock factor [Fusarium tjaetaba];Gene=FNAPI_2389;Ontology_term=DNA binding,mitochondrion,signaling,nuclear chromosome,regulation of DNA-templated transcription,transcription regulator activity;Ontology_id=GO:0003677,GO:0005739,GO:0023052,GO:0000228,GO:0006355,GO:0140110
contig00002.g1126	1233	ID=contig00002.g1126;Description=G1 S-specific cyclin [Fusarium tjaetaba]
contig00002.g1127	2442	ID=contig00002.g1127;Description=j domain protein [Fusarium tjaetaba];Gene=FNYG_03575;Ontology_term=membrane organization,mitochondrion,mitochondrion organization;Ontology_id=GO:0061024,GO:0005739,GO:0007005
contig00002.g1128	1867	ID=contig00002.g1128;Description=CDF family cation efflux system [Fusarium subglutinans];Gene=FMEXI_6033;Ontology_term=transmembrane transport,mitochondrion,detoxification,endoplasmic reticulum,vacuole,transporter activity;Ontology_id=GO:0055085,GO:0005739,GO:0098754,GO:0005783,GO:0005773,GO:0005215
contig00002.g1129	1903	ID=contig00002.g1129;Description=hypothetical protein FVEG_00596 [Fusarium verticillioides 7600]
contig00002.g1130	1033	ID=contig00002.g1130;Description=hypothetical protein FVEG_00595 [Fusarium verticillioides 7600];Ontology_term=nucleus;Ontology_id=GO:0005634

contig00002.g1131	2329	ID=contig00002.g1131;Description=syntaxin 1B/2/3 [Fusarium oxysporum f. sp. vasinfectum 25433];Gene=FOIG_01471;Ontology_term=vesicle-mediated transport;Ontology_id=GO:0016192
contig00002.g1132	714	ID=contig00002.g1132;Description=hypothetical protein FVEG_00593 [Fusarium verticillioides 7600]
contig00002.g1133	1328	ID=contig00002.g1133;Description=chitinase [Fusarium tjaetaba];Gene=FSUBG_3287;Ontology_term=carbohydrate metabolic process,hydrolase activity;Ontology_id=GO:0005975,GO:0016787;Enzyme_code=EC:3.2.1.14,EC:3.2,EC:3,EC:3.2.1.14,EC:3.2.1;Enzyme_name=chitinase,Glycosylases,Hydrolases,chitinase,Glycosylases
contig00002.g1134	1286	ID=contig00002.g1134;Description=methyltransferase [Fusarium sp. NRRL 25303];Gene=FVER53590_00591;Ontology_term=nucleus,RNA binding,mRNA metabolic process,transferase activity;Ontology_id=GO:0005634,GO:0003723,GO:0016071,GO:0016740;Enzyme_code=EC:2.1.1,EC:2.1,EC:2,EC:2.1.1;Enzyme_name=Transferring one-carbon groups,Transferring one-carbon groups,Transferases,Transferring one-carbon groups
contig00002.g1135	759	ID=contig00002.g1135;Description=RNA-binding component of cleavage and polyadenylation factor [Fusarium musae];Gene=FNAPI_2399;Ontology_term=RNA binding,mRNA metabolic process,cytosol,catalytic activity, acting on RNA,hydrolase activity,nucleoplasm;Ontology_id=GO:0003723,GO:0016071,GO:0005829,GO:0140098,GO:0016787,GO:0005654;Enzyme_code=EC:3.1,EC:3;Enzyme_name=Acting on ester bonds,Hydrolases
contig00002.g1136	1588	ID=contig00002.g1136;Description=DUF1776-domain protein [Fusarium tjaetaba];Gene=148;Ontology_term=generation of precursor metabolites and energy,nucleobase-containing small molecule metabolic process,oxidoreductase activity,mitochondrion,cytosol;Ontology_id=GO:0006091,GO:0055086,GO:0016491,GO:0005739,GO:0005829;Enzyme_code=EC:1.2.1.10,EC:1.1.1.1,EC:1.1.1.71,EC:1.2,EC:1.1,EC:1,EC:1.2.1.10,EC:1.1.1,EC:1.1.1.1,EC:1.1.1.71,EC:1.2.1;Enzyme_name=acetaldehyde dehydrogenase (acetylating),alcohol dehydrogenase,alcohol dehydrogenase [NAD(P)(+)],Acting on the aldehyde or oxo group of donors,Acting on the CH-OH group of donors,Oxidoreductases,acetaldehyde dehydrogenase (acetylating),Acting on the CH-OH group of donors,alcohol dehydrogenase,alcohol dehydrogenase [NAD(P)(+)],Acting on the aldehyde or oxo group of donors
contig00002.g1137	1748	ID=contig00002.g1137;Description=amidase signature domain-containing protein [Fusarium oxysporum]

contig00002.g1138	1164	ID=contig00002.g1138;Description=hypothetical protein FVER14953_00588 [Fusarium verticillioides]
contig00002.g1139	522	ID=contig00002.g1139;Description=hypothetical protein FVER14953_20835 [Fusarium verticillioides]
contig00002.g1140	1796	ID=contig00002.g1140;Description=Nonsense-mediated mRNA decay 1 [Fusarium denticulatum];Gene=FPCIR_3280;Ontology_term=catalytic activity,ATP-dependent activity;Ontology_id=GO:0003824,GO:0140657
contig00002.g1141	1591	ID=contig00002.g1141;Description=aldehyde dehydrogenase [Fusarium tjaetaba];Gene=FocTR4_00000668;Ontology_term=oxidoreductase activity;Ontology_id=GO:0016491;Enzyme_code=EC:1.2.1;Enzyme_name=Acting on the aldehyde or oxo group of donors
contig00002.g1142	581	ID=contig00002.g1142;Description=hypothetical protein LZL87_005537 [Fusarium oxysporum]
contig00002.g1143	1218	ID=contig00002.g1143;Description=hypothetical protein FVEG_00584 [Fusarium verticillioides 7600]
contig00002.g1144	2515	ID=contig00002.g1144;Description=RGP1-like protein [Fusarium coicis];Ontology_term=molecular function regulator activity,vesicle-mediated transport,signaling,Golgi apparatus;Ontology_id=GO:0098772,GO:0016192,GO:0023052,GO:0005794
contig00002.g1145	3060	ID=contig00002.g1145;Description=transcriptional repressor rco-1 [Fusarium tjaetaba]
contig00002.g1146	3255	ID=contig00002.g1146;Description=tpa inducible [Fusarium tjaetaba]
contig00002.g1147	1560	ID=contig00002.g1147;Description=phosphatidylinositol N-acetylglucosaminyltransferase gpi3 subunit [Fusarium verticillioides 7600];Gene=BFJ69_g10575;Ontology_term=lipid metabolic process,carbohydrate derivative metabolic process,cytosol,endoplasmic reticulum,transferase activity;Ontology_id=GO:0006629,GO:1901135,GO:0005829,GO:0005783,GO:0016740;Enzyme_code=EC:2.4.1.198,EC:2.4,EC:2,EC:2.4.1,EC:2.4.1.198;Enzyme_name=phosphatidylinositol N-acetylglucosaminyltransferase,Glycosyltransferases,Transferases,Glycosyltransferases,phosphatidylinositol N-acetylglucosaminyltransferase

contig00002.g1148	2703	ID=contig00002.g1148;Description=ATP-dependent RNA helicase dbp6 [Fusarium tjaetaba];Gene=FVEG_00579;Ontology_term=RNA binding,ATP-dependent activity,ribosome biogenesis,catalytic activity, acting on RNA,nucleolus,hydrolase activity;Ontology_id=GO:0003723,GO:0140657,GO:0042254,GO:0140098,GO:0005730,GO:0016787;Enzyme_code=EC:3,EC:3.6.1,EC:3.6.4.13,EC:3.6,EC:3,EC:3.6.1.15;Enzyme_name=Hydrolases,Acting on acid anhydrides,RNA helicase,Acting on acid anhydrides,Hydrolases,nucleoside-triphosphate phosphatase
contig00002.g1149	1783	ID=contig00002.g1149;Description=hypothetical protein FVER14953_00577 [Fusarium verticillioides]
contig00002.g1150	663	ID=contig00002.g1150;Description=integral membrane protein [Fusarium coicis];Gene=FNAPI_12842;Ontology_term=membrane;Ontology_id=GO:0016020
contig00002.g1151	2451	ID=contig00002.g1151;Description=C6 zinc finger domain protein [Fusarium sp. NRRL 25303];Gene=FMUND_4865;Ontology_term=nucleus,DNA binding,regulation of DNA-templated transcription,transcription regulator activity;Ontology_id=GO:0005634,GO:0003677,GO:0006355,GO:0140110
contig00002.g1152	2377	ID=contig00002.g1152;Description=C6 transcription factor [Fusarium tjaetaba];Gene=FNYG_03552;Ontology_term=nucleus,DNA binding,regulation of DNA-templated transcription,transcription regulator activity;Ontology_id=GO:0005634,GO:0003677,GO:0006355,GO:0140110
contig00002.g1153	1594	ID=contig00002.g1153;Description=recQ DNA helicase [Fusarium pseudocircinatum];Gene=FACUT_2790;Ontology_term=nucleus,ATP-dependent activity,DNA recombination,hydrolase activity;Ontology_id=GO:0005634,GO:0140657,GO:0006310,GO:0016787;Enzyme_code=EC:3.6.1.15;Enzyme_name=nucleoside-triphosphate phosphatase
contig00002.g1154	2997	ID=contig00002.g1154;Description=adenylate cyclase [Fusarium fujikuroi];Ontology_term=microtubule organizing center,signaling,establishment or maintenance of cell polarity,anatomical structure development,reproductive process,plasma membrane;Ontology_id=GO:0005815,GO:0023052,GO:0007163,GO:0048856,GO:0022414,GO:0005886
contig00002.g1155	816	ID=contig00002.g1155;Description=hypothetical protein FCOIX_2470 [Fusarium coicis];Gene=FTJAE_2969;Ontology_term=BLOC-1 complex,localization;Ontology_id=GO:0031083,GO:0051179

contig00002.g1156	1044	ID=contig00002.g1156;Description=enoyl reductase [Fusarium verticillioides 7600];Gene=FVEG_00568;Ontology_term=oxidoreductase activity,mitochondrion,lipid metabolic process,endoplasmic reticulum;Ontology_id=GO:0016491,GO:0005739,GO:0006629,GO:0005783;Enzyme_code=EC:1.3,EC:1.3,EC:1,EC:1.3.1.93,EC:1.3.1;Enzyme_name=Acting on the CH-CH group of donors,Acting on the CH-CH group of donors,Oxidoreductases,very-long-chain enoyl-CoA reductase,Acting on the CH-CH group of donors
contig00002.g1157	2412	ID=contig00002.g1157;Description=hypothetical protein FPCIR_13587 [Fusarium pseudocircinatum];Gene=rplI
contig00002.g1158	684	ID=contig00002.g1158;Description=40S ribosomal protein S8 [Fusarium oxysporum f. sp. lycopersici 4287];Gene=FGADI_1461;Ontology_term=cytoplasmic translation,structural molecule activity,ribosome biogenesis,cytosol,ribosome;Ontology_id=GO:0002181,GO:0005198,GO:0042254,GO:0005829,GO:0005840
contig00002.g1159	3171	ID=contig00002.g1159;Description=rab-GTPase-TBC domain-containing protein [Fusarium flagelliforme];Ontology_term=membrane organization,vesicle-mediated transport,molecular function regulator activity,signaling,cytoplasmic vesicle,intracellular protein transport,plasma membrane;Ontology_id=GO:0061024,GO:0016192,GO:0098772,GO:0023052,GO:0031410,GO:0006886,GO:0005886
contig00002.g1160	12137	ID=contig00002.g1160;Description=E3 ubiquitin-protein ligase HUWE1 [Fusarium verticillioides 7600];Gene=FPCIR_13590;Ontology_term=catalytic activity, acting on a protein,transferase activity;Ontology_id=GO:0140096,GO:0016740;Enzyme_code=EC:2.3.2;Enzyme_name=Acyltransferases
contig00002.g1161	1213	ID=contig00002.g1161;Description=Ribosomal protein S6e [Metarhizium album ARSEF 1941];Gene=MAM_06206;Ontology_term=cytoplasmic translation,structural molecule activity,ribosome biogenesis,cytosol,ribosome;Ontology_id=GO:0002181,GO:0005198,GO:0042254,GO:0005829,GO:0005840
contig00002.g1162	441	ID=contig00002.g1162;Description=chromosome transmission fidelity protein 8 [Fusarium verticillioides 7600];Gene=FPCIR_13592;Ontology_term=nucleus,chromosome;Ontology_id=GO:0005634,GO:0005694

contig00002.g1163	1442	ID=contig00002.g1163;Description=diphthamide biosynthesis 1 [Fusarium tjaetaba];Gene=FOXYS1_3972;Ontology_term=cytoplasmic translation,transferase activity;Ontology_id=GO:0002181,GO:0016740;Enzyme_code=EC:2.5.1.108,EC:2.5.1,EC:2,EC:2.5.1.108;Enzyme_name=2-(3-amino-3-carboxypropyl)histidine synthase,Transferring alkyl or aryl groups, other than methyl groups,Transferases,2-(3-amino-3-carboxypropyl)histidine synthase
contig00002.g1164	1872	ID=contig00002.g1164;Description=P-loop containing nucleoside triphosphate hydrolase protein [Fusarium redolens];Gene=FNAPI_2059;Ontology_term=transmembrane transport,protein-containing complex assembly,nucleobase-containing small molecule metabolic process,ATP-dependent activity,autophagy,carbohydrate derivative metabolic process,vacuole,transporter activity,plasma membrane,hydrolase activity;Ontology_id=GO:0055085,GO:0065003,GO:0055086,GO:0140657,GO:0006914,GO:1901135,GO:0005773,GO:0005215,GO:0005886,GO:0016787;Enzyme_code=EC:7.2.2,EC:3.6.1,EC:7.1.2.1,EC:3.6,EC:3,EC:7.2.2,EC:7,EC:3.6.1.15;Enzyme_name=Catalysing the translocation of inorganic cations,Acting on acid anhydrides,P-type H(+)-exporting transporter,Acting on acid anhydrides,Hydrolases,Catalysing the translocation of inorganic cations,Translocases,nucleoside-triphosphate phosphatase
contig00002.g1165	3398	ID=contig00002.g1165;Description=hydroxymethylglutaryl-CoA lyase [Fusarium verticillioides 7600];Gene=FVEG_00558;Ontology_term=cytoskeleton organization,establishment or maintenance of cell polarity,structural molecule activity,lyase activity,anatomical structure development,chromosome segregation,microtubule-based movement,mitochondrion organization,nucleus,microtubule organizing center,mitotic nuclear division,meiotic nuclear division,hydrolase activity;Ontology_id=GO:0007010,GO:0007163,GO:0005198,GO:0016829,GO:0048856,GO:0007059,GO:0007018,GO:0007005,GO:0005634,GO:0005815,GO:0140014,GO:0140013,GO:0016787;Enzyme_code=EC:4.1.3.4,EC:3,EC:4.1,EC:4.1.3.4,EC:3,EC:4,EC:4.1.3;Enzyme_name=hydroxymethylglutaryl-CoA lyase,Hydrolases,Carbon-carbon lyases,hydroxymethylglutaryl-CoA lyase,Hydrolases,Lyases,Carbon-carbon lyases
contig00002.g1166	760	ID=contig00002.g1166;Description=hypothetical protein FVEG_00556 [Fusarium verticillioides 7600];Gene=FVER53263_00556;Ontology_term=membrane;Ontology_id=GO:0016020
contig00002.g1167	1661	ID=contig00002.g1167;Description=RNQ1-prion epigenetic modifier of function [Fusarium subglutinans]

contig00002.g1168	1299	ID=contig00002.g1168;Description=spore coat SP96 precursor [Fusarium tjaetaba]
contig00002.g1169	2911	ID=contig00002.g1169;Description=PBP1-Pab1p interacting [Fusarium tjaetaba];Gene=FDENT_13191;Ontology_term=nucleus,mRNA metabolic process,RNA binding,protein-containing complex assembly,mitochondrion;Ontology_id=GO:0005634,GO:0016071,GO:0003723,GO:0065003,GO:0005739
contig00002.g1170	1547	ID=contig00002.g1170;Description=ubiquinol-cytochrome c reductase core subunit 2 [Fusarium verticillioides 7600];Gene=FLONG3_2142;Ontology_term=generation of precursor metabolites and energy,catalytic activity, acting on a protein,nucleobase-containing small molecule metabolic process,mitochondrion,structural molecule activity,carbohydrate derivative metabolic process,protein maturation,plasma membrane,hydrolase activity;Ontology_id=GO:0006091,GO:0140096,GO:0055086,GO:0005739,GO:0005198,GO:1901135,GO:0051604,GO:0005886,GO:0016787;Enzyme_code=EC:3.4.24,EC:3.4,EC:3;Enzyme_name=Acting on peptide bonds (peptidases),Acting on peptide bonds (peptidases),Hydrolases
contig00002.g1171	2087	ID=contig00002.g1171;Description=DNA-directed RNA polymerase III subunit RPC3 [Fusarium proliferatum];Gene=FDENT_13193;Ontology_term=nucleus,DNA binding,DNA-templated transcription;Ontology_id=GO:0005634,GO:0003677,GO:0006351
contig00002.g1172	1365	ID=contig00002.g1172;Description=YHM1 (mitochondrial carrier) [Fusarium tjaetaba];Gene=FGADI_7264;Ontology_term=transmembrane transport,mitochondrion,transporter activity,mitochondrion organization;Ontology_id=GO:0055085,GO:0005739,GO:0005215,GO:0007005
contig00002.g1173	7163	ID=contig00002.g1173;Description=transport USO1 [Fusarium mundagurra]
contig00002.g1174	336	ID=contig00002.g1174;Description=dash complex subunit dad3 [Fusarium subglutinans];Gene=FGADI_7266;Ontology_term=cytoskeleton,mitotic nuclear division,chromosome segregation,nuclear chromosome,meiotic nuclear division;Ontology_id=GO:0005856,GO:0140014,GO:0007059,GO:0000228,GO:0140013
contig00002.g1175	1569	ID=contig00002.g1175;Description=30S ribosomal protein S5 [Fusarium verticillioides 7600];Gene=186;Ontology_term=RNA binding,mitochondrial gene expression,mitochondrion,structural molecule activity,ribosome;Ontology_id=GO:0003723,GO:0140053,GO:0005739,GO:0005198,GO:0005840
contig00002.g1176	234	ID=contig00002.g1176;Description=uncharacterized protein BKA55DRAFT_658570 [Fusarium redolens];Gene=FNAPI_2047;Ontology_term=membrane;Ontology_id=GO:0016020

contig00002.g1177	1682	ID=contig00002.g1177;Description=aimless (aleA) [Fusarium napiforme];Gene=FNAPI_2046;Ontology_term=molecular function regulator activity,signaling;Ontology_id=GO:0098772,GO:0023052
contig00002.g1178	3125	ID=contig00002.g1178;Description=ras guanine nucleotide exchange factor domain-containing protein [Fusarium oxysporum Fo47];Gene=FTJAE_2946;Ontology_term=molecular function regulator activity,signaling;Ontology_id=GO:0098772,GO:0023052
contig00002.g1179	1299	ID=contig00002.g1179;Description=hypothetical protein FVER53590_00544 [Fusarium verticillioides]
contig00002.g1180	755	ID=contig00002.g1180;Description=zinc finger (HIT type) family [Fusarium tjaetaba]
contig00002.g1181	967	ID=contig00002.g1181;Description=Fe/S biogenesis protein NfuA [Fusarium verticillioides 7600];Gene=FVER53263_00542;Ontology_term=mitochondrion,protein maturation;Ontology_id=GO:0005739,GO:0051604
contig00002.g1182	1183	ID=contig00002.g1182;Description=L-iditol 2-dehydrogenase [Fusarium verticillioides 7600];Gene=FPHYL_9937;Ontology_term=carbohydrate metabolic process,oxidoreductase activity,carbohydrate derivative metabolic process;Ontology_id=GO:0005975,GO:0016491,GO:1901135;Enzyme_code=EC:1.1.1,EC:1.1,EC:1.1.1.14,EC:1,EC:1.1.1.12,EC:1.1.1;Enzyme_name=Acting on the CH-OH group of donors,Acting on the CH-OH group of donors,L-iditol 2-dehydrogenase,Oxidoreductases,L-arabinitol 4-dehydrogenase,Acting on the CH-OH group of donors
contig00002.g1183	710	ID=contig00002.g1183;Description=F-type H+-transporting ATPase subunit G [Fusarium verticillioides 7600];Gene=FocTR4_00000622;Ontology_term=membrane organization,transmembrane transport,nucleobase-containing small molecule metabolic process,mitochondrion,carbohydrate derivative metabolic process,mitochondrion organization,transporter activity;Ontology_id=GO:0061024,GO:0055085,GO:0055086,GO:0005739,GO:1901135,GO:0007005,GO:0005215

contig00002.g1184	1147	ID=contig00002.g1184;Description=methyl transferase [Fusarium sp. NRRL 52700];Gene=F52700_7658;Ontology_term=defense response to other organism,nucleus,sulfur compound metabolic process,anatomical structure development,cell differentiation,reproductive process,transferase activity;Ontology_id=GO:0098542,GO:0005634,GO:0006790,GO:0048856,GO:0030154,GO:0022414,GO:0016740;Enzyme_code=EC:2.1.1,EC:2.1,EC:2,EC:2.1.1;Enzyme_name=Transferring one-carbon groups,Transferring one-carbon groups,Transferases,Transferring one-carbon groups
contig00002.g1185	2020	ID=contig00002.g1185;Description=transcription factor IIA, alpha/beta subunit [Fusarium oxysporum];Gene=FOZG_01738;Ontology_term=translation regulator activity,DNA-templated transcription,nucleoplasm;Ontology_id=GO:0045182,GO:0006351,GO:0005654
contig00002.g1186	1827	ID=contig00002.g1186;Description=class I alpha-mannosidase [Fusarium tjaetaba];Gene=FTJAE_7690;Ontology_term=carbohydrate metabolic process,hydrolase activity;Ontology_id=GO:0005975,GO:0016787;Enzyme_code=EC:3.2.1.113,EC:3.2.1.24,EC:3.2,EC:3.2.1.113,EC:3,EC:3.2.1.24,EC:3.2.1;Enzyme_name=mannosyl-oligosaccharide 1,2-alpha-mannosidase,alpha-mannosidase,Glycosylases,mannosyl-oligosaccharide 1,2-alpha-mannosidase,Hydrolases,alpha-mannosidase,Glycosylases
contig00002.g1187	1861	ID=contig00002.g1187;Description=cell division cycle 20, cofactor-APC complex [Fusarium verticillioides 7600];Gene=FVEG_00536;Ontology_term=nucleus,molecular function regulator activity,protein catabolic process,signaling,mitotic nuclear division,chromosome segregation,meiotic nuclear division;Ontology_id=GO:0005634,GO:0098772,GO:0030163,GO:0023052,GO:0140014,GO:0007059,GO:0140013
contig00002.g1188	2375	ID=contig00002.g1188;Description=ral2 [Fusarium coicis]
contig00002.g1189	2549	ID=contig00002.g1189;Description=zinc finger SFP1 [Fusarium tjaetaba];Ontology_term=nucleus,DNA binding,regulation of DNA-templated transcription,transcription regulator activity;Ontology_id=GO:0005634,GO:0003677,GO:0006355,GO:0140110
contig00002.g1190	751	ID=contig00002.g1190;Description=mitochondrial hypoxia responsive domain protein [Fusarium tjaetaba];Gene=Forpi1262_v000541;Ontology_term=protein-containing complex assembly,mitochondrion,mitochondrion organization;Ontology_id=GO:0065003,GO:0005739,GO:0007005
contig00002.g1191	1518	ID=contig00002.g1191;Description=KEL2-involved in cell fusion morphogenesis [Fusarium napiforme]

contig00002.g1192	2419	ID=contig00002.g1192;Description=tRNA-dihydrouridine synthase 3 [Fusarium verticillioides 7600];Gene=FVER53590_00530;Ontology_term=mRNA metabolic process,oxidoreductase activity,tRNA metabolic process,catalytic activity, acting on RNA;Ontology_id=GO:0016071,GO:0016491,GO:0006399,GO:0140098;Enzyme_code=EC:1.3.1.89;Enzyme_name=tRNA-dihydrouridine(47) synthase [NAD(P)(+)]
contig00002.g1193	1414	ID=contig00002.g1193;Description=peroxisomal membrane protein pex13 [Fusarium fujikuroi];Gene=FOXB_11099;Ontology_term=peroxisome,transmembrane transport,peroxisome organization,intracellular protein transport,transporter activity,molecular adaptor activity;Ontology_id=GO:0005777,GO:0055085,GO:0007031,GO:0006886,GO:0005215,GO:0060090
contig00002.g1194	1273	ID=contig00002.g1194;Description=abc1 containing protein [Fusarium tjaetaba]
contig00002.g1195	2304	ID=contig00002.g1195;Description=chromatin assembly factor 1 subunit b [Fusarium sporotrichioides];Gene=FVER53590_00527;Ontology_term=protein-containing complex assembly,histone binding,DNA replication,nuclear chromosome,cytosol,chromatin organization,mitotic cell cycle;Ontology_id=GO:0065003,GO:0042393,GO:0006260,GO:0000228,GO:0005829,GO:0006325,GO:0000278
contig00002.g1196	1761	ID=contig00002.g1196;Description=general substrate transporter [Ilyonectria robusta];Gene=BKA56DRAFT_617622;Ontology_term=transmembrane transport,transporter activity;Ontology_id=GO:0055085,GO:0005215
contig00002.g1197	1725	ID=contig00002.g1197;Description=arylsulfatase [Fusarium verticillioides 7600];Gene=FVEG_00525;Ontology_term=sulfur compound metabolic process,lipid metabolic process,hydrolase activity;Ontology_id=GO:0006790,GO:0006629,GO:0016787;Enzyme_code=EC:3.1.6,EC:3.1.6,EC:3.1,EC:3,EC:3.1.6.1;Enzyme_name=Acting on ester bonds,Acting on ester bonds,Acting on ester bonds,Hydrolases,arylsulfatase (type I)

contig00002.g1198	4859	ID=contig00002.g1198;Description=ATP-dependent bile acid permease [Fusarium pseudoanthophilum];Gene=FVEG_00524;Ontology_term=transmembrane transport,ATP-dependent activity,transporter activity,hydrolase activity;Ontology_id=GO:0055085,GO:0140657,GO:0005215,GO:0016787;Enzyme_code=EC:7.2.2,EC:3.6.1.15,EC:3.6.1,EC:3.6,EC:3,EC:7.2.2,EC:7,EC:3.6.1.15;Enzyme_name=Catalysing the translocation of inorganic cations,nucleoside-triphosphate phosphatase,Acting on acid anhydrides,Acting on acid anhydrides,Hydrolases,Catalysing the translocation of inorganic cations,Translocases,nucleoside-triphosphate phosphatase
contig00002.g1199	2215	ID=contig00002.g1199;Description=hypothetical protein FVEG_00523 [Fusarium verticillioides 7600];Gene=FVER53263_00523;Ontology_term=nucleus,regulation of DNA-templated transcription,transcription regulator activity;Ontology_id=GO:0005634,GO:0006355,GO:0140110
contig00002.g1200	2523	ID=contig00002.g1200;Description=TOB3 (member of AAA-ATPase family) [Fusarium napiforme];Gene=FVER53263_00522;Ontology_term=ATP-dependent activity,hydrolase activity;Ontology_id=GO:0140657,GO:0016787;Enzyme_code=EC:3.6.1.15,EC:3.6.1,EC:3.6,EC:3,EC:3.6.1.15;Enzyme_name=nucleoside-triphosphate phosphatase,Acting on acid anhydrides,Acting on acid anhydrides,Hydrolases,nucleoside-triphosphate phosphatase
contig00002.g1201	3114	ID=contig00002.g1201;Description=Beclin 1 (coiled-coil myosin-like BCL2-interacting protein) [Fusarium fujikuroi];Gene=FOVG_00428;Ontology_term=autophagy;Ontology_id=GO:0006914
contig00002.g1202	2825	ID=contig00002.g1202;Description=hypothetical protein FVEG_00519 [Fusarium verticillioides 7600];Gene=FNAPI_9169;Ontology_term=nucleus,protein-containing complex assembly,histone binding,chromosome,chromatin organization;Ontology_id=GO:0005634,GO:0065003,GO:0042393,GO:0005694,GO:0006325
contig00002.g1203	1291	ID=contig00002.g1203;Description=CMGC CK2 kinase [Fusarium tjaetaba];Gene=FOZG_01758;Ontology_term=nuclear envelope,catalytic activity, acting on a protein,signaling,cell differentiation,anatomical structure development,cytosol,cytoplasmic vesicle,regulation of DNA-templated transcription,lipid binding,transferase activity,plasma membrane;Ontology_id=GO:0005635,GO:0140096,GO:0023052,GO:0030154,GO:0048856,GO:0005829,GO:0031410,GO:0006355,GO:0008289,GO:0016740,GO:0005886;Enzyme_code=EC:2.7.11.1;Enzyme_name=non-specific serine/threonine protein kinase
contig00002.g1204	1432	ID=contig00002.g1204;Description=hypothetical protein FVER53590_00517 [Fusarium verticillioides]

contig00002.g1205	1622	ID=contig00002.g1205;Description=autophagy protein 14 [Fusarium musae];Gene=FVER53590_00516;Ontology_term=protein-containing complex,cellular anatomical entity;Ontology_id=GO:0032991,GO:0110165
contig00002.g1206	974	ID=contig00002.g1206;Description=autophagy-related 6 [Fusarium denticulatum]
contig00002.g1207	1055	ID=contig00002.g1207;Description=40S ribosomal protein S11 [Cordyceps militaris CM01];Gene=FZEAL_8912;Ontology_term=structural molecule activity,ribosome;Ontology_id=GO:0005198,GO:0005840
contig00002.g1208	4559	ID=contig00002.g1208;Description=kinetochore spc7 [Fusarium tjaetaba]
contig00002.g1209	4161	ID=contig00002.g1209;Description=hypothetical protein FVER53590_00511 [Fusarium verticillioides];Gene=FNAPI_10748;Ontology_term=nuclear envelope;Ontology_id=GO:0005635
contig00002.g1210	1737	ID=contig00002.g1210;Description=udp-galactose transporter like [Fusarium tjaetaba]
contig00002.g1211	858	ID=contig00002.g1211;Description=DUF636 domain protein [Fusarium tjaetaba];Gene=FVEG_00508;Ontology_term=lyase activity;Ontology_id=GO:0016829;Enzyme_code=EC:4.4,EC:4.4,EC:4;Enzyme_name=Carbon-sulfur lyases,Carbon-sulfur lyases,Lyases
contig00002.g1212	1634	ID=contig00002.g1212;Description=folylpolyglutamate synthase [Fusarium verticillioides 7600];Gene=FVEG_00506;Ontology_term=mitochondrion,amino acid metabolic process,cellular modified amino acid metabolic process,endoplasmic reticulum,ligase activity;Ontology_id=GO:0005739,GO:0006520,GO:0006575,GO:0005783,GO:0016874;Enzyme_code=EC:6.3.2.17,EC:6.3.2.17,EC:6.3,EC:6.3.2,EC:6;Enzyme_name=tetrahydrofolate synthase,tetrahydrofolate synthase,Forming carbon-nitrogen bonds,Forming carbon-nitrogen bonds,Ligases
contig00002.g1213	1479	ID=contig00002.g1213;Description=folylpolyglutamate synthetase [Fusarium tjaetaba];Gene=FTJAE_7717;Ontology_term=mitochondrion,cellular modified amino acid metabolic process,ligase activity;Ontology_id=GO:0005739,GO:0006575,GO:0016874;Enzyme_code=EC:6.3.2.17,EC:6.3.2.17,EC:6.3,EC:6.3.2,EC:6;Enzyme_name=tetrahydrofolate synthase,tetrahydrofolate synthase,Forming carbon-nitrogen bonds,Forming carbon-nitrogen bonds,Ligases
contig00002.g1214	2043	ID=contig00002.g1214;Description=mysoin heavy chain [Fusarium tjaetaba];Gene=FTJAE_7718;Ontology_term=mitochondrion;Ontology_id=GO:0005739

contig00002.g1215	902	ID=contig00002.g1215;Description=GTP-binding protein ypt5 [Fusarium proliferatum];Gene=FGLOB1_10714;Ontology_term=membrane organization,endosome,vesicle-mediated transport,GTPase activity;Ontology_id=GO:0061024,GO:0005768,GO:0016192,GO:0003924;Enzyme_code=EC:3.6.1.15;Enzyme_name=nucleoside-triphosphate phosphatase
contig00002.g1216	2255	ID=contig00002.g1216;Description=SAC1 [Fusarium tjaetaba];Gene=FVEG_00503;Ontology_term=lipid metabolic process,mitochondrion,Golgi apparatus,endoplasmic reticulum,hydrolase activity;Ontology_id=GO:0006629,GO:0005739,GO:0005794,GO:0005783,GO:0016787;Enzyme_code=EC:3.1.3.64,EC:3.1.3.95,EC:3.1,EC:3,EC:3.1.3.64,EC:3.1.3.95,EC:3.1.3;Enzyme_name=phosphatidylinositol-3-phosphatase,phosphatidylinositol-3,5-bisphosphate 3-phosphatase,Acting on ester bonds,Hydrolases,phosphatidylinositol-3-phosphatase,phosphatidylinositol-3,5-bisphosphate 3-phosphatase,Acting on ester bonds
contig00002.g1217	3651	ID=contig00002.g1217;Description=hypothetical protein FVEG_00502 [Fusarium verticillioides 7600];Gene=FVEG_00502;Ontology_term=membrane;Ontology_id=GO:0016020
contig00002.g1218	2947	ID=contig00002.g1218;Description=isoleucyl-tRNA synthetase [Fusarium verticillioides 7600];Gene=FVER53263_00501;Ontology_term=RNA binding,tRNA metabolic process,amino acid metabolic process,catalytic activity, acting on RNA,ligase activity,hydrolase activity;Ontology_id=GO:0003723,GO:0006399,GO:0006520,GO:0140098,GO:0016874,GO:0016787;Enzyme_code=EC:6.1.1.5,EC:3.1.1;Enzyme_name=isoleucine--tRNA ligase,Acting on ester bonds
contig00002.g1219	889	ID=contig00002.g1219;Description=hypothetical protein FVEG_00500 [Fusarium verticillioides 7600]
contig00002.g1220	1344	ID=contig00002.g1220;Description=hypothetical protein FVER14953_00498 [Fusarium verticillioides];Gene=FVER53263_00498;Ontology_term=membrane organization,mitochondrion,mitochondrion organization;Ontology_id=GO:0061024,GO:0005739,GO:0007005
contig00002.g1221	1380	ID=contig00002.g1221;Description=phosphatidylinositol glycan, class U [Fusarium verticillioides 7600];Gene=FTJAE_7725;Ontology_term=lipid metabolic process,carbohydrate derivative metabolic process,endoplasmic reticulum;Ontology_id=GO:0006629,GO:1901135,GO:0005783

contig00002.g1222	1974	ID=contig00002.g1222;Description=mRNA-binding ribosome synthesis protein nop7 [Fusarium musae];Gene=NOP7;Ontology_term=RNA binding,DNA replication,ribosome biogenesis,nucleolus,nucleoplasm;Ontology_id=GO:0003723,GO:0006260,GO:0042254,GO:0005730,GO:0005654
contig00002.g1223	1967	ID=contig00002.g1223;Description=hypothetical protein FVER53590_00495 [Fusarium verticillioides]
contig00002.g1224	3591	ID=contig00002.g1224;Description=Kinesin-like protein bimC [Fusarium oxysporum f. sp. cepae];Gene=BFJ65_g1558;Ontology_term=cytoskeleton organization,nuclear chromosome,chromosome segregation,microtubule-based movement,cytoskeletal motor activity,microtubule organizing center,ATP-dependent activity,mitotic nuclear division,cytoskeletal protein binding,hydrolase activity;Ontology_id=GO:0007010,GO:0000228,GO:0007059,GO:0007018,GO:0003774,GO:0005815,GO:0140657,GO:0140014,GO:0008092,GO:0016787;Enzyme_code=EC:3.6.1,EC:3.6,EC:3,EC:5.6.1.3,EC:3.6.1.15;Enzyme_name=Acting on acid anhydrides,Acting on acid anhydrides,Hydrolases,plus-end-directed kinesin ATPase,nucleoside-triphosphate phosphatase
contig00002.g1225	1492	ID=contig00002.g1225;Description=hypothetical protein FVER14953_00493 [Fusarium verticillioides];Gene=FTJAE_7729;Ontology_term=molecular function regulator activity;Ontology_id=GO:0098772
contig00002.g1226	1188	ID=contig00002.g1226;Description=SPRY domain-containing protein [Fusarium oxysporum f. sp. rapae]
contig00002.g1227	2492	ID=contig00002.g1227;Description=vacuolar calcium ion transporter [Fusarium tjaetaba];Gene=FVER53590_00491;Ontology_term=transmembrane transport,vacuole,transporter activity;Ontology_id=GO:0055085,GO:0005773,GO:0005215
contig00002.g1228	3416	ID=contig00002.g1228;Description=CAMK/CAMKL/KIN4 protein kinase [Fusarium oxysporum f. sp. lycopersici 4287];Gene=BFJ68_g14686;Ontology_term=nucleus,catalytic activity, acting on a protein,microtubule organizing center,signaling,mitotic nuclear division,chromosome segregation,transferase activity;Ontology_id=GO:0005634,GO:0140096,GO:0005815,GO:0023052,GO:0140014,GO:0007059,GO:0016740;Enzyme_code=EC:2.7.11.1,EC:2.7.1,EC:2.7.11.1,EC:2.7,EC:2;Enzyme_name=non-specific serine/threonine protein kinase,Transferring phosphorus-containing groups,non-specific serine/threonine protein kinase,Transferring phosphorus-containing groups,Transferases

contig00002.g1229	1570	ID=contig00002.g1229;Description=GPI-anchored wall transfer 1 [Fusarium tjaetaba];Gene=FVER53263_20852;Ontology_term=endoplasmic reticulum,transferase activity;Ontology_id=GO:0005783,GO:0016740;Enzyme_code=EC:2.3.1;Enzyme_name=Acyltransferases
contig00002.g1230	1242	ID=contig00002.g1230;Description=hypothetical protein FVER53263_20853 [Fusarium verticillioides]
contig00002.g1231	1802	ID=contig00002.g1231;Description=polysaccharide synthase Cps1 [Fusarium fujikuroi];Gene=FNAPI_4479;Ontology_term=membrane,carbohydrate binding;Ontology_id=GO:0016020,GO:0030246
contig00002.g1232	1413	ID=contig00002.g1232;Description=hypothetical protein FVER14953_00485 [Fusarium verticillioides]
contig00002.g1233	909	ID=contig00002.g1233;Description=enoyl-CoA hydratase [Fusarium verticillioides 7600]
contig00002.g1234	2018	ID=contig00002.g1234;Description=hypothetical protein FVEG_00483 [Fusarium verticillioides 7600]
contig00002.g1235	1952	ID=contig00002.g1235;Description=D-lactate dehydrogenase (cytochrome) [Fusarium verticillioides 7600];Gene=Forpe1208_v000467;Ontology_term=oxidoreductase activity,mitochondrion;Ontology_id=GO:0016491,GO:0005739;Enzyme_code=EC:1.1,EC:1.1.2.4,EC:1,EC:1.1.2;Enzyme_name=Acting on the CH-OH group of donors,D-lactate dehydrogenase (cytochrome),Oxidoreductases,Acting on the CH-OH group of donors
contig00002.g1236	433	ID=contig00002.g1236;Description=hypothetical protein FVER14953_21349 [Fusarium verticillioides]
contig00002.g1237	1589	ID=contig00002.g1237;Description=UPF0183 domain protein [Fusarium tjaetaba]
contig00002.g1238	1975	ID=contig00002.g1238;Description=amidase [Fusarium tjaetaba];Gene=FVER53590_00480;Ontology_term=catalytic activity;Ontology_id=GO:0003824
contig00002.g1239	1098	ID=contig00002.g1239;Description=beta-flanking [Fusarium pseudocircinatum]
contig00002.g1240	1646	ID=contig00002.g1240;Description=cutinase palindrome-binding protein [Fusarium verticillioides 7600];Gene=FVER53263_00478;Ontology_term=DNA binding,regulation of DNA-templated transcription;Ontology_id=GO:0003677,GO:0006355

contig00002.g1241	3987	ID=contig00002.g1241;Description=DNA repair protein RAD50 [Fusarium verticillioides 7600];Gene=FVER53263_00477;Ontology_term=DNA repair,catalytic activity, acting on DNA,nucleobase-containing small molecule metabolic process,DNA replication,DNA recombination,nuclear chromosome,mitotic cell cycle,DNA binding,ATP-dependent activity,signaling,mitochondrion,telomere organization,transferase activity,hydrolase activity,meiotic nuclear division;Ontology_id=GO:0006281,GO:0140097,GO:0055086,GO:0006260,GO:0006310,GO:0000228,GO:0000278,GO:0003677,GO:0140657,GO:0023052,GO:0005739,GO:0032200,GO:0016740,GO:0016787,GO:0140013;Enzyme_code=EC:3.6.1.15,EC:2.7.4.4,EC:2.7.4.3,EC:3.6.1,EC:2.7,EC:2,EC:3.6,EC:2.7.4,EC:3,EC:3.6.1.15;Enzyme_name=nucleoside-triphosphate phosphatase,nucleoside-phosphate kinase,adenylate kinase,Acting on acid anhydrides,Transferring phosphorus-containing groups,Transferases,Acting on acid anhydrides,Transferring phosphorus-containing groups,Hydrolases,nucleoside-triphosphate phosphatase
contig00002.g1242	1821	ID=contig00002.g1242;Description=DNA repair protein RAD50 [Fusarium verticillioides 7600];Gene=FVER53263_00476;Ontology_term=transmembrane transport,transporter activity;Ontology_id=GO:0055085,GO:0005215
contig00002.g1243	1576	ID=contig00002.g1243;Description=thiol-specific monooxygenase [Fusarium tjaetaba];Gene=FNAPI_4467;Ontology_term=oxidoreductase activity;Ontology_id=GO:0016491;Enzyme_code=EC:1.14.13.8;Enzyme_name=flavin-containing monooxygenase
contig00002.g1244	2241	ID=contig00002.g1244;Description=STB5-SIN3 binding protein [Fusarium tjaetaba];Gene=FNAPI_4466;Ontology_term=nucleus,DNA binding,regulation of DNA-templated transcription,transcription regulator activity;Ontology_id=GO:0005634,GO:0003677,GO:0006355,GO:0140110
contig00002.g1245	1429	ID=contig00002.g1245;Description=hypothetical protein FNAPI_4465 [Fusarium napiforme];Gene=FMAN_01556;Ontology_term=plasma membrane;Ontology_id=GO:0005886

contig00002.g1246	1395	ID=contig00002.g1246;Description=RMD5-like protein [Fusarium denticulatum];Gene=FPANT_6771;Ontology_term=nucleus,peroxisome,carbohydrate metabolic process,catalytic activity, acting on a protein,protein catabolic process,cytosol,transferase activity;Ontology_id=GO:0005634,GO:0005777,GO:0005975,GO:0140096,GO:0030163,GO:0005829,GO:0016740;Enzyme_code=EC:2.3.2,EC:2.3,EC:2,EC:2.3.2;Enzyme_name=Acyltransferases,Acyltransferases,Transferases,Acyltransferases
contig00002.g1247	2748	ID=contig00002.g1247;Description=SIN3 binding STB2 [Fusarium tjaetaba];Ontology_term=nuclear chromosome,nucleoplasm;Ontology_id=GO:0000228,GO:0005654
contig00002.g1248	343	ID=contig00002.g1248;Description=protein transporter tim10 [Fusarium oxysporum];Gene=FOVG_00369;Ontology_term=membrane organization,cytoskeleton,cytoskeleton organization,mitochondrion,lipid binding,intracellular protein transport,transporter activity,mitochondrion organization;Ontology_id=GO:0061024,GO:0005856,GO:0007010,GO:0005739,GO:0008289,GO:0006886,GO:0005215,GO:0007005
contig00002.g1249	1487	ID=contig00002.g1249;Description=aldehyde dehydrogenase [Fusarium subglutinans];Gene=F52700_7723;Ontology_term=oxidoreductase activity;Ontology_id=GO:0016491;Enzyme_code=EC:1.2.1,EC:1.2,EC:1,EC:1.2.1;Enzyme_name=Acting on the aldehyde or oxo group of donors,Acting on the aldehyde or oxo group of donors,Oxidoreductases,Acting on the aldehyde or oxo group of donors
contig00002.g1250	2399	ID=contig00002.g1250;Description=zinc finger transcription factor [Fusarium tjaetaba];Gene=FNYG_13497;Ontology_term=nucleus,regulation of DNA-templated transcription,transcription regulator activity;Ontology_id=GO:0005634,GO:0006355,GO:0140110
contig00002.g1251	1719	ID=contig00002.g1251;Description=hypothetical protein FVEG_00464 [Fusarium verticillioides 7600]

contig00002.g1252	3114	ID=contig00002.g1252;Description=mannosyl-oligosaccharide alpha-1 2-mannosidase [Fusarium tjaetaba];Gene=FNYG_13495;Ontology_term=carbohydrate metabolic process,protein catabolic process,carbohydrate derivative metabolic process,endoplasmic reticulum,Golgi apparatus,hydrolase activity;Ontology_id=GO:0005975,GO:0030163,GO:1901135,GO:0005783,GO:0005794,GO:0016787;Enzyme_code=EC:3.2.1.113,EC:3.2.1.24,EC:3.2,EC:3.2.1.113,EC:3,EC:3.2.1.24,EC:3.2.1;Enzyme_name=mannosyl-oligosaccharide 1,2-alpha-mannosidase,alpha-mannosidase,Glycosylases,mannosyl-oligosaccharide 1,2-alpha-mannosidase,Hydrolases,alpha-mannosidase,Glycosylases
contig00002.g1253	740	ID=contig00002.g1253;Description=adenine phosphoribosyltransferase [Fusarium verticillioides 7600];Gene=FNYG_13494;Ontology_term=nucleus,nucleobase-containing small molecule metabolic process,carbohydrate derivative metabolic process,extracellular matrix,transferase activity,extracellular region;Ontology_id=GO:0005634,GO:0055086,GO:1901135,GO:0031012,GO:0016740,GO:0005576;Enzyme_code=EC:2.4.2.7,EC:2.4.2,EC:2.4,EC:2,EC:2.4.2.7;Enzyme_name=adenine phosphoribosyltransferase,Glycosyltransferases,Glycosyltransferases,Transferases,adenine phosphoribosyltransferase
contig00002.g1254	3917	ID=contig00002.g1254;Description=hypothetical protein FVEG_00461 [Fusarium verticillioides 7600];Gene=FNAPI_1344;Ontology_term=peroxisome,transmembrane transport,peroxisome organization,intracellular protein transport;Ontology_id=GO:0005777,GO:0055085,GO:0007031,GO:0006886
contig00002.g1255	1781	ID=contig00002.g1255;Description=c6 transcription factor [Fusarium napiforme];Gene=FFUJ_00512;Ontology_term=nucleus,DNA binding,regulation of DNA-templated transcription,transcription regulator activity;Ontology_id=GO:0005634,GO:0003677,GO:0006355,GO:0140110
contig00002.g1256	1803	ID=contig00002.g1256;Description=hypothetical protein FVEG_00458 [Fusarium verticillioides 7600];Gene=FVEG_00458;Ontology_term=membrane;Ontology_id=GO:0016020
contig00002.g1257	1859	ID=contig00002.g1257;Description=RING finger membrane [Fusarium sp. NRRL 52700];Gene=FVER53263_00457;Ontology_term=membrane;Ontology_id=GO:0016020
contig00002.g1258	3001	ID=contig00002.g1258;Description=Het-c heterokaryon incompatibility protein [Fusarium tjaetaba]

contig00002.g1259	1557	ID=contig00002.g1259;Description=nonhistone chromosomal [Fusarium acutatum];Gene=FGADI_6035;Ontology_term=DNA binding,regulation of DNA-templated transcription,cytosol,chromosome,nucleolus;Ontology_id=GO:0003677,GO:0006355,GO:0005829,GO:0005694,GO:0005730
contig00002.g1260	832	ID=contig00002.g1260;Description=nucleosomal binding protein [Fusarium fujikuroi];Gene=FSUBG_5522;Ontology_term=membrane;Ontology_id=GO:0016020
contig00002.g1261	634	ID=contig00002.g1261;Description=protein SYM1 [Fusarium odoratissimum NRRL 54006];Gene=FOZG_01835;Ontology_term=mitochondrion;Ontology_id=GO:0005739
contig00002.g1262	1062	ID=contig00002.g1262;Description=DUF1713 domain protein [Fusarium tjaetaba]
contig00002.g1263	3367	ID=contig00002.g1263;Description=killer toxin resistant protein [Fusarium musae];Gene=NAT10;Ontology_term=RNA binding,ribosome biogenesis,tRNA metabolic process,transferase activity,nucleolus;Ontology_id=GO:0003723,GO:0042254,GO:0006399,GO:0016740,GO:0005730;Enzyme_code=EC:2.3.1,EC:2.3,EC:2,EC:2.3.1;Enzyme_name=Acyltransferases,Acyltransferases,Transferases,Acyltransferases
contig00002.g1264	711	ID=contig00002.g1264;Description=hypothetical protein FVEG_00449 [Fusarium verticillioides 7600]
contig00002.g1265	977	ID=contig00002.g1265;Description=hypothetical protein FVER53590_00448 [Fusarium verticillioides];Gene=FOBC_00856;Ontology_term=transferase activity;Ontology_id=GO:0016740;Enzyme_code=EC:2.1.1;Enzyme_name=Transferring one-carbon groups
contig00002.g1266	1032	ID=contig00002.g1266;Description=WSS1 Protein involved in sister chromatid separation and segregation [Fusarium fujikuroi];Gene=BFJ69_g14223;Ontology_term=metal ion binding;Ontology_id=GO:0046872
contig00002.g1267	4267	ID=contig00002.g1267;Description=putative histidine kinase HHK6p [[Gibberella] fujikuroi var. moniliformis];Gene=FVER53263_00446;Ontology_term=catalytic activity, acting on a protein,signaling,transferase activity,small molecule sensor activity;Ontology_id=GO:0140096,GO:0023052,GO:0016740,GO:0140299;Enzyme_code=EC:2.7.3,EC:2.7.13.3,EC:2.7.1,EC:2.7.3,EC:2.7,EC:2,EC:2.7.13.3;Enzyme_name=Transferring phosphorus-containing groups,histidine kinase,Transferring phosphorus-containing groups,Transferring phosphorus-containing groups,Transferring phosphorus-containing groups,Transferases,histidine kinase

contig00002.g1268	3858	ID=contig00002.g1268;Description=hypothetical protein FVEG_00445 [Fusarium verticillioides 7600];Gene=FVER53263_00445;Ontology_term=lipid binding;Ontology_id=GO:0008289
contig00002.g1269	1796	ID=contig00002.g1269;Description=NADPH oxidase heavy chain subunit [Fusarium tjaetaba];Gene=FGADI_6044;Ontology_term=oxidoreductase activity;Ontology_id=GO:0016491;Enzyme_code=EC:1.16;Enzyme_name=Oxidizing metal ions
contig00002.g1270	2845	ID=contig00002.g1270;Description=urease [Fusarium verticillioides 7600];Gene=FCIRC_1358;Ontology_term=nitrogen cycle metabolic process,hydrolase activity;Ontology_id=GO:0071941,GO:0016787;Enzyme_code=EC:3.5.1.5,EC:3.5.1,EC:3.5,EC:3,EC:3.5.1.5;Enzyme_name=urease,Acting on carbon-nitrogen bonds, other than peptide bonds,Acting on carbon-nitrogen bonds, other than peptide bonds,Hydrolases,urease
contig00002.g1271	2032	ID=contig00002.g1271;Description=histone acetyltransferases subunit 3-domain-containing protein [Fusarium redolens];Gene=FNYG_13475;Ontology_term=nuclear chromosome,regulation of DNA-templated transcription,chromatin organization,transcription regulator activity,molecular adaptor activity,nucleoplasm;Ontology_id=GO:0000228,GO:0006355,GO:0006325,GO:0140110,GO:0060090,GO:0005654
contig00002.g1272	2678	ID=contig00002.g1272;Description=s-adenosylmethionine:diacylglycerol 3-amino-3-carboxypropyl transferase [Fusarium tjaetaba];Gene=AU210_001598;Ontology_term=transferase activity;Ontology_id=GO:0016740;Enzyme_code=EC:2.1.1,EC:2.1,EC:2,EC:2.1.1;Enzyme_name=Transferring one-carbon groups,Transferring one-carbon groups,Transferases,Transferring one-carbon groups
contig00002.g1273	679	ID=contig00002.g1273;Description=related to succinate dehydrogenase precursor [Fusarium fujikuroi];Gene=BFJ68_g6850;Ontology_term=mitochondrion;Ontology_id=GO:0005739
contig00002.g1274	1959	ID=contig00002.g1274;Description=tyrosyl-tRNA synthetase [Fusarium verticillioides 7600];Gene=FNYG_13472;Ontology_term=mitochondrial gene expression,RNA binding,mitochondrion,tRNA metabolic process,amino acid metabolic process,cytosol,catalytic activity, acting on RNA,ligase activity;Ontology_id=GO:0140053,GO:0003723,GO:0005739,GO:0006399,GO:0006520,GO:0005829,GO:0140098,GO:0016874;Enzyme_code=EC:6.1.1.1,EC:6.1.1.1,EC:6.1,EC:6.1.1,EC:6;Enzyme_name=tyrosine--tRNA ligase,tyrosine--tRNA ligase,Forming carbon-oxygen bonds,Forming carbon-oxygen bonds,Ligases

contig00002.g1275	1337	ID=contig00002.g1275;Description=nitrogen permease regulator [Fusarium fujikuroi];Gene=28943350;Ontology_term=molecular function regulator activity,signaling;Ontology_id=GO:0098772,GO:0023052
contig00002.g1276	1397	ID=contig00002.g1276;Description=Deubiquitination-protection dph1 [Fusarium tjaetaba];Gene=FVEG_00437;Ontology_term=nucleus,cytoskeleton organization,protein catabolic process,molecular adaptor activity;Ontology_id=GO:0005634,GO:0007010,GO:0030163,GO:0060090
contig00002.g1277	1163	ID=contig00002.g1277;Description=NADPH2:quinone reductase [Fusarium proliferatum];Gene=FANTH_2075;Ontology_term=oxidoreductase activity;Ontology_id=GO:0016491;Enzyme_code=EC:1,EC:1,EC:1.6.5.5;Enzyme_name=Oxidoreductases, Oxidoreductases,NADPH:quinone reductase
contig00002.g1278	3203	ID=contig00002.g1278;Description=nuclear transport factor [Fusarium globosum];Gene=FOXYS1_5447;Ontology_term=nucleus,intracellular protein transport;Ontology_id=GO:0005634,GO:0006886
contig00002.g1279	1497	ID=contig00002.g1279;Description=threonyl-tRNA synthetase [Fusarium verticillioides 7600];Gene=FVER53263_00434;Ontology_term=mitochondrial gene expression,mitochondrion,tRNA metabolic process,amino acid metabolic process,catalytic activity, acting on RNA,ligase activity;Ontology_id=GO:0140053,GO:0005739,GO:0006399,GO:0006520,GO:0140098,GO:0016874;Enzyme_code=EC:6.1.1.3,EC:6.1,EC:6.1.1.3,EC:6.1.1,EC:6;Enzyme_name=threonine--tRNA ligase,Forming carbon-oxygen bonds,threonine--tRNA ligase,Forming carbon-oxygen bonds,Ligases
contig00002.g1280	690	ID=contig00002.g1280;Description=MLX-interacting [Fusarium tjaetaba];Gene=FOMG_01868;Ontology_term=DNA binding,nucleus,lipid metabolic process,regulation of DNA-templated transcription;Ontology_id=GO:0003677,GO:0005634,GO:0006629,GO:0006355
contig00002.g1281	864	ID=contig00002.g1281;Description=septum formation maf [Fusarium fujikuroi];Gene=FPHYL_4293;Ontology_term=hydrolase activity;Ontology_id=GO:0016787;Enzyme_code=EC:3.6.1.9,EC:3.6.1,EC:3.6,EC:3,EC:3.6.1.9;Enzyme_name=nucleotide diphosphatase,Acting on acid anhydrides,Acting on acid anhydrides,Hydrolases,nucleotide diphosphatase

contig00002.g1282	2316	ID=contig00002.g1282;Description=polybromo-1 [Fusarium coicis];Gene=FCIRC_1346;Ontology_term=nucleus,DNA-templated transcription,chromosome,chromatin organization;Ontology_id=GO:0005634,GO:0006351,GO:0005694,GO:0006325
contig00002.g1283	1472	ID=contig00002.g1283;Description=putative Quinone oxidoreductase-like protein 2 like protein [Fusarium oxysporum f. sp. albedinis];Gene=FOIG_13175;Ontology_term=membrane;Ontology_id=GO:0016020
contig00002.g1284	1162	ID=contig00002.g1284;Description=hypothetical protein FVEG_00429 [Fusarium verticillioides 7600];Gene=FNAPI_7402;Ontology_term=membrane;Ontology_id=GO:0016020
contig00002.g1285	2588	ID=contig00002.g1285;Description=Transcriptional regulator WAR1 [Fusarium oxysporum f. sp. rapae];Gene=FOBC_00836;Ontology_term=nucleus,regulation of DNA-templated transcription,transcription regulator activity;Ontology_id=GO:0005634,GO:0006355,GO:0140110
contig00002.g1286	1979	ID=contig00002.g1286;Description=hypothetical protein FVER14953_00425 [Fusarium verticillioides];Gene=Forpe1208_v000416;Ontology_term=membrane;Ontology_id=GO:0016020
contig00002.g1287	2171	ID=contig00002.g1287;Description=arginyl-tRNA synthetase [Fusarium phyllophilum];Gene=FocTR4_00000503;Ontology_term=tRNA metabolic process,amino acid metabolic process,catalytic activity, acting on RNA,ligase activity;Ontology_id=GO:0006399,GO:0006520,GO:0140098,GO:0016874;Enzyme_code=EC:6.1.1.19,EC:6.1,EC:6.1.1,EC:6,EC:6.1.1.19;Enzyme_name=arginine--tRNA ligase,Forming carbon-oxygen bonds,Forming carbon-oxygen bonds,Ligases,arginine--tRNA ligase
contig00002.g1288	315	ID=contig00002.g1288;Description=arginyl-tRNA synthetase [Fusarium sp. NRRL 25303];Gene=Forpi1262_v000443;Ontology_term=tRNA metabolic process,amino acid metabolic process,catalytic activity, acting on RNA,ligase activity;Ontology_id=GO:0006399,GO:0006520,GO:0140098,GO:0016874;Enzyme_code=EC:6.1.1.19,EC:6.1,EC:6.1.1,EC:6,EC:6.1.1.19;Enzyme_name=arginine--tRNA ligase,Forming carbon-oxygen bonds,Forming carbon-oxygen bonds,Ligases,arginine--tRNA ligase

contig00002.g1289	1404	ID=contig00002.g1289;Description=arginyl-tRNA synthetase [Fusarium agapanthi];Gene=Forpi1262_v000443;Ontology_term=tRNA metabolic process,amino acid metabolic process,catalytic activity, acting on RNA,ligase activity;Ontology_id=GO:0006399,GO:0006520,GO:0140098,GO:0016874;Enzyme_code=EC:6.1.1.19,EC:6.1,EC:6.1.1,EC:6,EC:6.1.1.19;Enzyme_name=arginine--tRNA ligase,Forming carbon-oxygen bonds,Forming carbon-oxygen bonds,Ligases,arginine--tRNA ligase
contig00002.g1290	2026	ID=contig00002.g1290;Description=stress-induced-phosphoprotein 1 [Fusarium verticillioides 7600];Gene=FVEG_00423;Ontology_term=RNA binding,molecular function regulator activity,protein folding;Ontology_id=GO:0003723,GO:0098772,GO:0006457
contig00002.g1291	1368	ID=contig00002.g1291;Description=nitric oxide dioxygenase [Fusarium verticillioides 7600];Gene=FTJAE_6373;Ontology_term=oxidoreductase activity;Ontology_id=GO:0016491;Enzyme_code=EC:1.14.12.17,EC:1.14.12,EC:1.14.12.17,EC:1,EC:1.14;Enzyme_name=nitric oxide dioxygenase,Acting on paired donors, with incorporation or reduction of molecular oxygen. The oxygen incorporated need not be derived from O2,nitric oxide dioxygenase,Oxidoreductases,Acting on paired donors, with incorporation or reduction of molecular oxygen. The oxygen incorporated need not be derived from O2
contig00002.g1292	559	ID=contig00002.g1292;Description=hypothetical protein FVEG_00422 [Fusarium verticillioides 7600];Gene=FVER53263_00422;Ontology_term=metal ion binding;Ontology_id=GO:0046872
contig00002.g1293	1714	ID=contig00002.g1293;Description=hypothetical protein FVER53590_00420 [Fusarium verticillioides]
contig00002.g1294	876	ID=contig00002.g1294;Description=glutathione S-transferase [Fusarium tjaetaba];Gene=FPCIR_1555;Ontology_term=transferase activity;Ontology_id=GO:0016740;Enzyme_code=EC:2,EC:2;Enzyme_name=Transferases,Transferases
contig00002.g1295	841	ID=contig00002.g1295;Description=hypothetical protein FVEG_00417 [Fusarium verticillioides 7600]

contig00002.g1296	1479	ID=contig00002.g1296;Description=monooxygenase [Fusarium tjaetaba];Gene=FVER53263_00416;Ontology_term=oxidoreductase activity;Ontology_id=GO:0016491;Enzyme_code=EC:1.14.13.8,EC:1.14.13.8,EC:1,EC:1.14,EC:1.14.13;Enzyme_name=flavin-containing monooxygenase,flavin-containing monooxygenase,Oxidoreductases,Acting on paired donors, with incorporation or reduction of molecular oxygen. The oxygen incorporated need not be derived from O2,Acting on paired donors, with incorporation or reduction of molecular oxygen. The oxygen incorporated need not be derived from O2
contig00002.g1297	1763	ID=contig00002.g1297;Description=pisatin demethylase (cytochrome P450) [Fusarium subglutinans];Gene=FSUBG_5488;Ontology_term=oxidoreductase activity,transferase activity;Ontology_id=GO:0016491,GO:0016740;Enzyme_code=EC:1.14,EC:2.1.1,EC:2.1,EC:1,EC:1.14,EC:2,EC:2.1.1;Enzyme_name=Acting on paired donors, with incorporation or reduction of molecular oxygen. The oxygen incorporated need not be derived from O2,Transferring one-carbon groups,Transferring one-carbon groups,Oxidoreductases,Acting on paired donors, with incorporation or reduction of molecular oxygen. The oxygen incorporated need not be derived from O2,Transferases,Transferring one-carbon groups
contig00002.g1298	1455	ID=contig00002.g1298;Description=hypothetical protein FVER53263_00414 [Fusarium verticillioides];Ontology_term=nucleus,mRNA metabolic process;Ontology_id=GO:0005634,GO:0016071
contig00002.g1299	473	ID=contig00002.g1299;Description=small nuclear ribonucleoprotein G [Fusarium tjaetaba];Gene=FCIRC_1588;Ontology_term=nucleus,mRNA metabolic process;Ontology_id=GO:0005634,GO:0016071
contig00002.g1300	531	ID=contig00002.g1300;Description=arsenate reductase (Arc2) [Fusarium fujikuroi];Gene=P01512;Ontology_term=defense response to other organism,extracellular region,immune system process;Ontology_id=GO:0098542,GO:0005576,GO:0002376;Enzyme_code=EC:2.1.1.137;Enzyme_name=arsenite methyltransferase
contig00002.g1301	2623	ID=contig00002.g1301;Description=beta-glucosidase B [Fusarium tjaetaba];Gene=FVER53263_00412;Ontology_term=carbohydrate metabolic process,hydrolase activity;Ontology_id=GO:0005975,GO:0016787;Enzyme_code=EC:3.2.1.21,EC:3.2,EC:3.2.1.21,EC:3,EC:3.2.1;Enzyme_name=beta-glucosidase,Glycosylases,beta-glucosidase,Hydrolases,Glycosylases

contig00002.g1302	1450	ID=contig00002.g1302;Description=zinc-regulated transporter 2 [Fusarium tjaetaba];Gene=FTJAE_6384;Ontology_term=transmembrane transport,transporter activity;Ontology_id=GO:0055085,GO:0005215
contig00002.g1303	1008	ID=contig00002.g1303;Description=hypothetical protein FVEG_00410 [Fusarium verticillioides 7600];Gene=FTJAE_6385;Ontology_term=inorganic ion homeostasis,monoatomic cation homeostasis;Ontology_id=GO:0098771,GO:0055080
contig00002.g1304	705	ID=contig00002.g1304;Description=hypothetical protein FVEG_14657 [Fusarium verticillioides 7600];Gene=FVER53590_25012;Ontology_term=membrane;Ontology_id=GO:0016020
contig00002.g1305	901	ID=contig00002.g1305;Description=nad dependent epimerase dehydratase family [Fusarium sp. NRRL 52700];Gene=rpmJ;Ontology_term=defense response to other organism,extracellular region,immune system process;Ontology_id=GO:0098542,GO:0005576,GO:0002376
contig00002.g1306	429	ID=contig00002.g1306;Description=mei2-like protein [Fusarium phyllophilum]
contig00002.g1307	751	ID=contig00002.g1307;Description=Ctr copper transporter [Fusarium tjaetaba];Gene=FDENT_6344;Ontology_term=transmembrane transport,transporter activity;Ontology_id=GO:0055085,GO:0005215
contig00002.g1308	1836	ID=contig00002.g1308;Description=C6 transcription factor [Fusarium tjaetaba];Gene=F52700_7779;Ontology_term=nucleus,regulation of DNA-templated transcription,transcription regulator activity;Ontology_id=GO:0005634,GO:0006355,GO:0140110
contig00002.g1309	1019	ID=contig00002.g1309;Description=membrane-associating domain protein [Fusarium tjaetaba];Gene=FOBC_00814;Ontology_term=cell motility,wound healing,extracellular space,lipid metabolic process,anatomical structure development,cell differentiation,regulation of DNA-templated transcription,cytosol,receptor ligand activity,extracellular matrix,nucleoplasm;Ontology_id=GO:0048870,GO:0042060,GO:0005615,GO:0006629,GO:0048856,GO:0030154,GO:0006355,GO:0005829,GO:0048018,GO:0031012,GO:0005654
contig00002.g1310	1785	ID=contig00002.g1310;Description=cytoskeletal adaptor [Fusarium fujikuroi];Ontology_term=vesicle-mediated transport,endosome,cytoskeleton,cytoskeleton organization,lipid binding;Ontology_id=GO:0016192,GO:0005768,GO:0005856,GO:0007010,GO:0008289
contig00002.g1311	420	ID=contig00002.g1311;Description=hypothetical protein FPCIR_1572 [Fusarium pseudocircinatum]

contig00002.g1312	1071	ID=contig00002.g1312;Description=Peptidyl-prolyl cis-trans isomerase [Fusarium oxysporum f. sp. raphani];Gene=Forpi1262_v000421;Ontology_term=catalytic activity, acting on a protein,protein folding,isomerase activity;Ontology_id=GO:0140096,GO:0006457,GO:0016853;Enzyme_code=EC:5.2.1.8;Enzyme_name=peptidylprolyl isomerase
contig00002.g1313	1208	ID=contig00002.g1313;Description=DNA-directed RNA polymerase III subunit RPC6 [Fusarium verticillioides 7600];Gene=FocTR4_00000478;Ontology_term=DNA-templated transcription,mitochondrion,tRNA metabolic process,catalytic activity, acting on RNA,transferase activity,nucleoplasm;Ontology_id=GO:0006351,GO:0005739,GO:0006399,GO:0140098,GO:0016740,GO:0005654;Enzyme_code=EC:2.7,EC:2,EC:2.7.7,EC:2.7.7.6;Enzyme_name=Transferring phosphorus-containing groups,Transferases,Transferring phosphorus-containing groups,DNA-directed RNA polymerase
contig00002.g1314	477	ID=contig00002.g1314;Description=hypothetical protein FVER53590_00401 [Fusarium verticillioides]
contig00002.g1315	2124	ID=contig00002.g1315;Description=histone deacetylase 1/2 [Fusarium verticillioides 7600];Gene=FDENT_6351;Ontology_term=catalytic activity, acting on a protein,DNA replication,DNA recombination,regulation of DNA-templated transcription,nuclear chromosome,chromatin organization,mitotic cell cycle,nucleoplasm,autophagy,transcription regulator activity,meiotic nuclear division,hydrolase activity,molecular adaptor activity;Ontology_id=GO:0140096,GO:0006260,GO:0006310,GO:0006355,GO:0000228,GO:0006325,GO:0000278,GO:0005654,GO:0006914,GO:0140110,GO:0140013,GO:0016787,GO:0060090;Enzyme_code=EC:3.5.1.98,EC:3.5.1,EC:3.5.1.98,EC:3,EC:3.5;Enzyme_name=histone deacetylase,Acting on carbon-nitrogen bonds, other than peptide bonds,histone deacetylase,Hydrolases,Acting on carbon-nitrogen bonds, other than peptide bonds

		ID=contig00002.g1316;Description=JAB1/Mov34/MPN/PAD-1 ubiquitin protease-domain-containing protein [Fusarium redolens];Gene=FOC1_g10016425;Ontology_term=nucleus,catalytic activity, acting on a protein,protein-containing complex assembly,protein catabolic process,mitochondrion,peroxisome organization,cytosol,mitochondrion organization,hydrolase activity;Ontology_id=GO:0005634,GO:0140096,GO:0065003,GO:0030163,GO:0005739,GO:0007031,GO:0005829,GO:0007005,GO:0016787;Enzyme_code=EC:3.4;Enzyme_name=Acting on peptide bonds (peptidases)
contig00002.g1316	1148	
contig00002.g1317	1203	ID=contig00002.g1317;Description=acetylxylan esterase precursor [Fusarium tjaetaba]
contig00002.g1318	830	ID=contig00002.g1318;Description=cutinase-domain-containing protein [Fusarium redolens];Gene=FNAPI_7369;Ontology_term=hydrolase activity;Ontology_id=GO:0016787;Enzyme_code=EC:3.1.1,EC:3.1,EC:3,EC:3.1.1;Enzyme_name=Acting on ester bonds,Acting on ester bonds,Hydrolases,Acting on ester bonds
contig00002.g1319	868	ID=contig00002.g1319;Description=acetylxylan esterase precursor [Fusarium tjaetaba];Gene=axe-2-3;Ontology_term=hydrolase activity;Ontology_id=GO:0016787;Enzyme_code=EC:3.1.1,EC:3.1,EC:3,EC:3.1.1;Enzyme_name=Acting on ester bonds,Acting on ester bonds,Hydrolases,Acting on ester bonds
contig00002.g1320	1412	ID=contig00002.g1320;Description=hypothetical protein FVEG_00395 [Fusarium verticillioides 7600];Gene=2330
contig00002.g1321	2244	ID=contig00002.g1321;Description=CAMK/CAMK1/CAMK1-RCK protein kinase [Fusarium verticillioides 7600];Gene=FOC4_g10014312;Ontology_term=catalytic activity, acting on a protein,signaling,transferase activity,nucleolus,mitotic cell cycle,meiotic nuclear division;Ontology_id=GO:0140096,GO:0023052,GO:0016740,GO:0005730,GO:0000278,GO:0140013;Enzyme_code=EC:2.7.11.1,EC:2.7.1,EC:2.7.11.1,EC:2.7,EC:2,EC:2.7.11.17;Enzyme_name=non-specific serine/threonine protein kinase,Transferring phosphorus-containing groups,non-specific serine/threonine protein kinase,Transferring phosphorus-containing groups,Transferases,calcium/calmodulin-dependent protein kinase
contig00002.g1322	1682	ID=contig00002.g1322;Description=allantoate permease [Fusarium tjaetaba];Gene=FNYG_06787;Ontology_term=transmembrane transport,transporter activity;Ontology_id=GO:0055085,GO:0005215
contig00002.g1323	876	ID=contig00002.g1323;Description=DUF1275 domain-containing protein [Fusarium coicis];Gene=Forpi1262_v000410;Ontology_term=membrane;Ontology_id=GO:0016020

contig00002.g1324	2013	ID=contig00002.g1324;Description=MUC1 extracellular alpha-1 4-glucan glucosidase [Fusarium tjaetaba]
contig00002.g1325	1096	ID=contig00002.g1325;Description=hypothetical protein F52700_7796 [Fusarium sp. NRRL 52700];Gene=FNYG_06784;Ontology_term=membrane;Ontology_id=GO:0016020
contig00002.g1326	1125	ID=contig00002.g1326;Description=novobiocin biosynthesis protein novR [Fusarium fujikuroi]
contig00002.g1327	2041	ID=contig00002.g1327;Description=Dipeptidyl peptidase family member 6 [Fusarium tjaetaba];Gene=FPANT_1916;Ontology_term=catalytic activity, acting on a protein,hydrolase activity;Ontology_id=GO:0140096,GO:0016787;Enzyme_code=EC:3.4.11,EC:3.4.11,EC:3.4,EC:3;Enzyme_name=Acting on peptide bonds (peptidases),Acting on peptide bonds (peptidases),Acting on peptide bonds (peptidases),Hydrolases
contig00002.g1328	1203	ID=contig00002.g1328;Description=hypothetical protein FVEG_00388 [Fusarium verticillioides 7600]
contig00002.g1329	1875	ID=contig00002.g1329;Description=STE protein kinase [Fusarium verticillioides 7600];Gene=FNAPI_7358;Ontology_term=catalytic activity, acting on a protein,transferase activity;Ontology_id=GO:0140096,GO:0016740;Enzyme_code=EC:2.7.1,EC:2.7.1,EC:2.7,EC:2;Enzyme_name=Transferring phosphorus-containing groups,Transferring phosphorus-containing groups,Transferring phosphorus-containing groups,Transferases
contig00002.g1330	715	ID=contig00002.g1330;Description=expression library immunization antigen 1 [Fusarium globosum];Gene=FGADI_3069;Ontology_term=membrane;Ontology_id=GO:0016020
contig00002.g1331	1479	ID=contig00002.g1331;Description=mid region of cactin-domain-containing protein [Fusarium sp. MPI-SDFR-AT-0072];Ontology_term=mRNA metabolic process,nuclear chromosome;Ontology_id=GO:0016071,GO:0000228
contig00002.g1332	2067	ID=contig00002.g1332;Description=forkhead box O4 [Fusarium subglutinans];Gene=FCIRC_1622;Ontology_term=nucleus,DNA binding,regulation of DNA-templated transcription,transcription regulator activity;Ontology_id=GO:0005634,GO:0003677,GO:0006355,GO:0140110
contig00002.g1333	1506	ID=contig00002.g1333;Description=hypothetical protein FVEG_00383 [Fusarium verticillioides 7600];Gene=FNYG_06776;Ontology_term=nucleus,DNA repair;Ontology_id=GO:0005634,GO:0006281

contig00002.g1334	1876	ID=contig00002.g1334;Description=deoxyribodipyrimidine photo-lyase [Fusarium verticillioides 7600];Gene=FDENT_13050;Ontology_term=nucleus,DNA repair,catalytic activity, acting on DNA,RNA binding,mitochondrion,lyase activity;Ontology_id=GO:0005634,GO:0006281,GO:0140097,GO:0003723,GO:0005739,GO:0016829;Enzyme_code=EC:4,EC:4.1.99.3,EC:4.1,EC:4;Enzyme_name=Lyases,deoxyribodipyrimidine photo-lyase,Carbon-carbon lyases,Lyases
contig00002.g1335	338	ID=contig00002.g1335;Description=40s ribosomal protein s21 [Fusarium flagelliforme];Gene=FEQUK3_LOCUS6983;Ontology_term=structural molecule activity,ribosome biogenesis,ribosome;Ontology_id=GO:0005198,GO:0042254,GO:0005840
contig00002.g1336	2670	ID=contig00002.g1336;Description=structure-specific endonuclease subunit SLX4 [Fusarium tjaetaba];Gene=SLX4;Ontology_term=DNA repair,DNA binding,catalytic activity, acting on DNA,DNA replication,DNA recombination,nuclear chromosome,hydrolase activity;Ontology_id=GO:0006281,GO:0003677,GO:0140097,GO:0006260,GO:0006310,GO:0000228,GO:0016787;Enzyme_code=EC:3.1.30,EC:3.1.21,EC:3.1.30,EC:3.1.21,EC:3.1,EC:3;Enzyme_name=Acting on ester bonds,Acting on ester bonds,Acting on ester bonds,Acting on ester bonds,Acting on ester bonds,Hydrolases
contig00002.g1337	2949	ID=contig00002.g1337;Description=hypothetical protein FVEG_00379 [Fusarium verticillioides 7600];Gene=FVER53263_00379;Ontology_term=nucleus,DNA binding,regulation of DNA-templated transcription,transcription regulator activity;Ontology_id=GO:0005634,GO:0003677,GO:0006355,GO:0140110
contig00002.g1338	1053	ID=contig00002.g1338;Description=hypothetical protein FVEG_00378 [Fusarium verticillioides 7600];Gene=FPCIR_1600;Ontology_term=ATP binding,membrane;Ontology_id=GO:0005524,GO:0016020
contig00002.g1339	1098	ID=contig00002.g1339;Description=NUDIX family hydrolase [Fusarium tjaetaba];Gene=FNAPI_7348;Ontology_term=hydrolase activity;Ontology_id=GO:0016787;Enzyme_code=EC:3.6.1,EC:3.6.1,EC:3.6,EC:3;Enzyme_name=Acting on acid anhydrides,Acting on acid anhydrides,Acting on acid anhydrides,Hydrolases
contig00002.g1340	1037	ID=contig00002.g1340;Description=60S ribosomal protein L23 [Colletotrichum truncatum];Gene=D7B24_003697;Ontology_term=oxidoreductase activity,structural molecule activity,ribosome;Ontology_id=GO:0016491,GO:0005198,GO:0005840;Enzyme_code=EC:1;Enzyme_name=Oxidoreductases
contig00002.g1341	4454	ID=contig00002.g1341;Description=hypothetical protein FVEG_00375 [Fusarium verticillioides 7600]

contig00002.g1342	1562	ID=contig00002.g1342;Description=maintenance of telomere capping 1 [Fusarium napiforme];Ontology_term=Golgi apparatus;Ontology_id=GO:0005794
contig00002.g1343	2054	ID=contig00002.g1343;Description=NAD-dependent malic enzyme, mitochondrial [Fusarium oxysporum];Gene=FOQG_03471;Ontology_term=generation of precursor metabolites and energy,oxidoreductase activity,mitochondrion,amino acid metabolic process;Ontology_id=GO:0006091,GO:0016491,GO:0005739,GO:0006520;Enzyme_code=EC:1.1.1.38,EC:1.1.1.39,EC:1.1,EC:1,EC:1.1.1,EC:1.1.1.38,EC:1.1.1.39;Enzyme_name=malate dehydrogenase (oxaloacetate-decarboxylating),malate dehydrogenase (decarboxylating),Acting on the CH-OH group of donors,Oxidoreductases,Acting on the CH-OH group of donors,malate dehydrogenase (oxaloacetate-decarboxylating),malate dehydrogenase (decarboxylating)
contig00002.g1344	1836	ID=contig00002.g1344;Description=related to quinate transport protein [Fusarium fujikuroi IMI 58289];Gene=FFB14_04324;Ontology_term=transmembrane transport,transporter activity;Ontology_id=GO:0055085,GO:0005215
contig00002.g1345	1152	ID=contig00002.g1345;Description=hypothetical protein FVEG_00371 [Fusarium verticillioides 7600];Gene=FVER53590_00371;Ontology_term=ATP binding;Ontology_id=GO:0005524
contig00002.g1346	1302	ID=contig00002.g1346;Description=probable endopeptidase K [Fusarium fujikuroi];Gene=FTJAE_1606;Ontology_term=catalytic activity, acting on a protein,protein catabolic process,carbohydrate derivative metabolic process,protein maturation,extracellular region,immune system process,hydrolase activity;Ontology_id=GO:0140096,GO:0030163,GO:1901135,GO:0051604,GO:0005576,GO:0002376,GO:0016787;Enzyme_code=EC:3.4.21,EC:3.4.21,EC:3.4,EC:3,EC:3.4.21.63;Enzyme_name=Acting on peptide bonds (peptidases),Acting on peptide bonds (peptidases),Acting on peptide bonds (peptidases),Hydrolases,oryzin
contig00002.g1347	2616	ID=contig00002.g1347;Description=beta-mannosidase [Fusarium verticillioides 7600];Gene=FVER53263_00368;Ontology_term=carbohydrate metabolic process,GTPase activity;Ontology_id=GO:0005975,GO:0003924;Enzyme_code=EC:3.6.1.15,EC:3.2.1,EC:3.2,EC:3.6.1,EC:3.6,EC:3,EC:3.2.1.25,EC:3.6.1.15,EC:3.2.1;Enzyme_name=nucleoside-triphosphate phosphatase,Glycosylases,Glycosylases,Acting on acid anhydrides,Acting on acid anhydrides,Hydrolases,beta-mannosidase,nucleoside-triphosphate phosphatase,Glycosylases

contig00002.g1348	1235	ID=contig00002.g1348;Description=P-loop containing nucleoside triphosphate hydrolase protein [Fusarium oxysporum];Gene=FOMG_01942;Ontology_term=endosome,vesicle-mediated transport,GTPase activity,signaling,intracellular protein transport;Ontology_id=GO:0005768,GO:0016192,GO:0003924,GO:0023052,GO:0006886;Enzyme_code=EC:3.6.1.15,EC:3.6.1,EC:3.6,EC:3,EC:3.6.1.15;Enzyme_name=nucleoside-triphosphate phosphatase,Acting on acid anhydrides,Acting on acid anhydrides,Hydrolases,nucleoside-triphosphate phosphatase
contig00002.g1349	1358	ID=contig00002.g1349;Description=homocitrate synthase [Fusarium verticillioides 7600];Gene=FPHYL_372;Ontology_term=transferase activity;Ontology_id=GO:0016740;Enzyme_code=EC:2.3.3;Enzyme_name=Acyltransferases
contig00002.g1350	904	ID=contig00002.g1350;Description=3-hydroxyacyl dehydrogenase [Fusarium napiforme];Gene=BFJ69_g11584;Ontology_term=oxidoreductase activity;Ontology_id=GO:0016491;Enzyme_code=EC:1,EC:1;Enzyme_name=Oxidoreductases,Oxidoreductases
contig00002.g1351	1139	ID=contig00002.g1351;Description=glycerate dehydrogenase [Fusarium tjaetaba];Gene=FNAPI_7336;Ontology_term=oxidoreductase activity;Ontology_id=GO:0016491;Enzyme_code=EC:1.1.1;Enzyme_name=Acting on the CH-OH group of donors
contig00002.g1352	1026	ID=contig00002.g1352;Description=aquaporin [Fusarium subglutinans];Gene=FSUBG_4156;Ontology_term=transmembrane transport,cell differentiation,anatomical structure development,reproductive process,cytosol,endoplasmic reticulum,transporter activity,plasma membrane;Ontology_id=GO:0055085,GO:0030154,GO:0048856,GO:0022414,GO:0005829,GO:0005783,GO:0005215,GO:0005886
contig00002.g1353	576	ID=contig00002.g1353;Description=hypothetical protein FVEG_14645 [Fusarium verticillioides 7600];Gene=FMAN_01667;Ontology_term=membrane;Ontology_id=GO:0016020
contig00002.g1354	2403	ID=contig00002.g1354;Description=nitrogen assimilation transcription factor nit-4 [Fusarium tjaetaba];Gene=FDENT_4990;Ontology_term=nucleus,DNA binding,regulation of DNA-templated transcription,transcription regulator activity;Ontology_id=GO:0005634,GO:0003677,GO:0006355,GO:0140110

contig00002.g1355	891	ID=contig00002.g1355;Description=TIM barrel metal-dependent hydrolase [Fusarium tjaetaba];Gene=FTJAE_1615;Ontology_term=hydrolase activity;Ontology_id=GO:0016787;Enzyme_code=EC:3,EC:3;Enzyme_name=Hydrolases,Hydrolases
contig00002.g1356	668	ID=contig00002.g1356;Description=peptide-methionine (S)-S-oxide reductase [Fusarium verticillioides 7600];Gene=FOVG_00247;Ontology_term=nucleus,sulfur compound metabolic process,oxidoreductase activity,amino acid metabolic process;Ontology_id=GO:0005634,GO:0006790,GO:0016491,GO:0006520;Enzyme_code=EC:1.8.4.11,EC:1,EC:1.8,EC:1.8.4,EC:1.8.4.13,EC:1.8.4.11;Enzyme_name=peptide-methionine (S)-S-oxide reductase,Oxidoreductases,Acting on a sulfur group of donors,Acting on a sulfur group of donors,L-methionine (S)-S-oxide reductase,peptide-methionine (S)-S-oxide reductase
contig00002.g1357	1086	ID=contig00002.g1357;Description=bax Inhibitor family [Fusarium tjaetaba];Gene=FVEG_00359;Ontology_term=membrane;Ontology_id=GO:0016020
contig00002.g1358	2979	ID=contig00002.g1358;Description=f-box LRR-repeat 7 [Fusarium subglutinans]
contig00002.g1359	420	ID=contig00002.g1359;Description=hypothetical protein FVER14953_00356 [Fusarium verticillioides]
contig00002.g1360	1324	ID=contig00002.g1360;Description=hypothetical protein FVEG_14644 [Fusarium verticillioides 7600]
contig00002.g1361	3672	ID=contig00002.g1361;Description=hypothetical protein FVER53590_25709 [Fusarium verticillioides]
contig00002.g1362	1812	ID=contig00002.g1362;Description=threonine ammonia-lyase, biosynthetic [Fusarium verticillioides 7600];Gene=FNAPI_7325;Ontology_term=mitochondrion,lyase activity,amino acid metabolic process;Ontology_id=GO:0005739,GO:0016829,GO:0006520;Enzyme_code=EC:4.3.1.19,EC:4.3,EC:4.3.1.19,EC:4,EC:4.3.1;Enzyme_name=threonine ammonia-lyase,Carbon-nitrogen lyases,threonine ammonia-lyase,Lyases,Carbon-nitrogen lyases
contig00002.g1363	486	ID=contig00002.g1363;Description=hypothetical protein FVEG_00353 [Fusarium verticillioides 7600]
contig00002.g1364	1906	ID=contig00002.g1364;Description=glycosyl hydrolase [Fusarium pseudoanthophilum];Gene=FMUND_4221;Ontology_term=carbohydrate metabolic process,hydrolase activity;Ontology_id=GO:0005975,GO:0016787;Enzyme_code=EC:3,EC:3;Enzyme_name=Hydrolases,Hydrolases

contig00002.g1365	3222	ID=contig00002.g1365;Description=rho coiled-coil associated kinase alpha [Fusarium tjaetaba];Gene=FNAPI_7322;Ontology_term=microtubule organizing center,cytoskeleton organization,transferase activity,cytoskeletal protein binding,mitotic cell cycle;Ontology_id=GO:0005815,GO:0007010,GO:0016740,GO:0008092,GO:0000278;Enzyme_code=EC:2.7,EC:2.7,EC:2;Enzyme_name=Transferring phosphorus-containing groups,Transferring phosphorus-containing groups,Transferases
contig00002.g1366	2337	ID=contig00002.g1366;Description=nucleolar complex 2 [Fusarium tjaetaba];Gene=FTJAE_1626;Ontology_term=RNA binding,mitochondrion,ribosome biogenesis,nucleolus,nucleoplasm;Ontology_id=GO:0003723,GO:0005739,GO:0042254,GO:0005730,GO:0005654
contig00002.g1367	829	ID=contig00002.g1367;Description=VPS28-like protein [Fusarium tjaetaba];Gene=FMEXI_578;Ontology_term=endosome,vesicle-mediated transport,protein catabolic process,intracellular protein transport;Ontology_id=GO:0005768,GO:0016192,GO:0030163,GO:0006886
contig00002.g1368	1269	ID=contig00002.g1368;Description=phosphatidylinositol 4-phosphate 5-kinase [Fusarium sp. NRRL 52700];Gene=MDM12;Ontology_term=membrane organization,protein-containing complex assembly,mitochondrion,lipid binding,endoplasmic reticulum,intracellular protein transport,transferase activity,mitochondrion organization;Ontology_id=GO:0061024,GO:0065003,GO:0005739,GO:0008289,GO:0005783,GO:0006886,GO:0016740,GO:0007005;Enzyme_code=EC:2.7,EC:2.7,EC:2;Enzyme_name=Transferring phosphorus-containing groups,Transferring phosphorus-containing groups,Transferases
contig00002.g1369	7658	ID=contig00002.g1369;Description=1-phosphatidylinositol-3-phosphate 5-kinase [Fusarium verticillioides 7600];Gene=FTJAE_1629;Ontology_term=vesicle-mediated transport,endosome,signaling,lipid metabolic process,mitochondrion,reproductive process,lipid binding,transferase activity,vacuole;Ontology_id=GO:0016192,GO:0005768,GO:0023052,GO:0006629,GO:0005739,GO:0022414,GO:0008289,GO:0016740,GO:0005773;Enzyme_code=EC:2.7.1.150,EC:2.7.1,EC:2.7.1.150,EC:2.7,EC:2;Enzyme_name=1-phosphatidylinositol-3-phosphate 5-kinase,Transferring phosphorus-containing groups,1-phosphatidylinositol-3-phosphate 5-kinase,Transferring phosphorus-containing groups,Transferases
contig00002.g1370	2229	ID=contig00002.g1370;Description=hypothetical protein FVEG_00346 [Fusarium verticillioides 7600]
contig00002.g1371	687	ID=contig00002.g1371;Description=bola protein [Fusarium redolens]

contig00002.g1372	1907	ID=contig00002.g1372;Description=malonyl synthetase [Fusarium tjaetaba]
contig00002.g1373	869	ID=contig00002.g1373;Description=translation initiation factor 4E [Fusarium verticillioides 7600];Gene=F25303_6203;Ontology_term=translation regulator activity,RNA binding;Ontology_id=GO:0045182,GO:0003723
contig00002.g1374	595	ID=contig00002.g1374;Description=hypothetical protein NW758_000967 [Fusarium oxysporum];Gene=FSUBG_4178;Ontology_term=membrane;Ontology_id=GO:0016020
contig00002.g1375	1240	ID=contig00002.g1375;Description=hypothetical protein FVER53590_00342 [Fusarium verticillioides];Gene=FPANT_9237;Ontology_term=membrane;Ontology_id=GO:0016020
contig00002.g1376	5194	ID=contig00002.g1376;Description=hypothetical protein FVER14953_00341 [Fusarium verticillioides];Ontology_term=mitochondrion,plasma membrane;Ontology_id=GO:0005739,GO:0005886
contig00002.g1377	2142	ID=contig00002.g1377;Description=FR, involved in hyphal branching [Fusarium fujikuroi];Gene=392;Ontology_term=DNA repair,vesicle-mediated transport,lipid metabolic process,carbohydrate derivative metabolic process,endoplasmic reticulum,hydrolase activity;Ontology_id=GO:0006281,GO:0016192,GO:0006629,GO:1901135,GO:0005783,GO:0016787;Enzyme_code=EC:3,EC:3;Enzyme_name=Hydrolases,Hydrolases
contig00002.g1378	3120	ID=contig00002.g1378;Description=hypothetical protein FVEG_00338 [Fusarium verticillioides 7600]
contig00002.g1379	1517	ID=contig00002.g1379;Description=cell cycle checkpoint protein [Fusarium verticillioides 7600];Gene=FVEG_00337;Ontology_term=DNA repair,nucleus,signaling;Ontology_id=GO:0006281,GO:0005634,GO:0023052
contig00002.g1380	1307	ID=contig00002.g1380;Description=histone acetyltransferase [Fusarium musae];Gene=FDENT_4619;Ontology_term=nucleus,catalytic activity, acting on a protein,histone binding,cytosol,regulation of DNA-templated transcription,chromosome,transferase activity,chromatin organization,transcription regulator activity,molecular adaptor activity;Ontology_id=GO:0005634,GO:0140096,GO:0042393,GO:0005829,GO:0006355,GO:0005694,GO:0016740,GO:0006325,GO:0140110,GO:0060090;Enzyme_code=EC:2.3.1.48;Enzyme_name=histone acetyltransferase
contig00002.g1381	999	ID=contig00002.g1381;Description=hypothetical protein FVEG_00335 [Fusarium verticillioides 7600]

contig00002.g1382	1348	ID=contig00002.g1382;Description=biotin synthetase [Fusarium proliferatum];Gene=BFJ65_g4;Ontology_term=sulfur compound metabolic process,mitochondrion,transferase activity,vitamin metabolic process;Ontology_id=GO:0006790,GO:0005739,GO:0016740,GO:0006766;Enzyme_code=EC:2.8.1.6,EC:2.8.1,EC:2.8.1.6,EC:2,EC:2.8;Enzyme_name=biotin synthase,Transferring sulfur-containing groups,biotin synthase,Transferases,Transferring sulfur-containing groups
contig00002.g1383	1826	ID=contig00002.g1383;Description=hypothetical protein FNYG_06726 [Fusarium nygamai]
contig00002.g1384	919	ID=contig00002.g1384;Description=mannose-P-dolichol utilization defect 1 [Fusarium verticillioides 7600];Gene=FSUBG_4189;Ontology_term=membrane;Ontology_id=GO:0016020
contig00002.g1385	662	ID=contig00002.g1385;Description=ORF21 [Fusarium tjaetaba]
contig00002.g1386	1323	ID=contig00002.g1386;Description=ORF20 [Fusarium tjaetaba]
contig00002.g1387	4803	ID=contig00002.g1387;Description=RecName: Full=ABC transporter FUM19;AltNameFull=Fumonisin biosynthesis cluster protein 19 [Fusarium verticillioides 7600];Gene=FOXYS1_39;Ontology_term=transmembrane transport,ATP-dependent activity,transporter activity,plasma membrane,hydrolase activity;Ontology_id=GO:0055085,GO:0140657,GO:0005215,GO:0005886,GO:0016787;Enzyme_code=EC:7.2.2,EC:3.6.1.15,EC:3.6.1,EC:3.6,EC:3,EC:7.2.2,EC:7,EC:3.6.1.15;Enzyme_name=Catalysing the translocation of inorganic cations,nucleoside-triphosphate phosphatase,Acting on acid anhydrides,Acting on acid anhydrides,Hydrolases,Catalysing the translocation of inorganic cations,Translocases,nucleoside-triphosphate phosphatase
contig00002.g1388	1479	ID=contig00002.g1388;Description=RecName: Full=Sphingosine N-acyltransferase-like protein FUM18;AltNameFull=Fumonisin biosynthesis cluster protein 18 [Fusarium verticillioides 7600];Gene=FMUND_4248;Ontology_term=lipid metabolic process,endoplasmic reticulum,transferase activity;Ontology_id=GO:0006629,GO:0005783,GO:0016740;Enzyme_code=EC:2.3.1.24,EC:2.3,EC:2,EC:2.3.1.24,EC:2.3.1;Enzyme_name=sphingosine N-acyltransferase,Acyltransferases,Transferases,sphingosine N-acyltransferase,Acyltransferases

contig00002.g1389	933	ID=contig00002.g1389;Description=RecName: Full=Sphingosine N-acyltransferase-like protein FUM17;AltNameFull=Fumonisin biosynthesis cluster protein 17 [Fusarium verticillioides 7600];Gene=FMUND_4249;Ontology_term=membrane organization,nucleus,lipid metabolic process,endoplasmic reticulum,transferase activity;Ontology_id=GO:0061024,GO:0005634,GO:0006629,GO:0005783,GO:0016740;Enzyme_code=EC:2.3.1.24,EC:2.3,EC:2,EC:2.3.1.24,EC:2.3.1;Enzyme_name=sphingosine N-acyltransferase,Acyltransferases,Transferases,sphingosine N-acyltransferase,Acyltransferases
contig00002.g1390	2223	ID=contig00002.g1390;Description=RecName: Full=Acyl-CoA synthetase FUM16;AltNameFull=Long-chain-fatty-acid--CoA ligase FUM16 [Fusarium verticillioides 7600];Gene=FUM16;Ontology_term=lipid droplet,transmembrane transport,nucleobase-containing small molecule metabolic process,sulfur compound metabolic process,ATP-dependent activity,mitochondrion,lipid metabolic process,carbohydrate derivative metabolic process,endoplasmic reticulum,ligase activity,plasma membrane;Ontology_id=GO:0005811,GO:0055085,GO:0055086,GO:0006790,GO:0140657,GO:0005739,GO:0006629,GO:1901135,GO:0005783,GO:0016874,GO:0005886;Enzyme_code=EC:6,EC:6.2,EC:6.2.1.3,EC:6,EC:6.2.1,EC:6.2.1.2;Enzyme_name=Ligases,Forming carbon-sulfur bonds,long-chain-fatty-acid--CoA ligase,Ligases,Forming carbon-sulfur bonds,medium-chain acyl-CoA ligase
contig00002.g1391	1856	ID=contig00002.g1391;Description=RecName: Full=Cytochrome P450 monooxygenase FUM15;AltNameFull=Fumonisin biosynthesis cluster protein 15 [Fusarium verticillioides 7600];Gene=FVEG_14636;Ontology_term=oxidoreductase activity;Ontology_id=GO:0016491;Enzyme_code=EC:1.14,EC:1,EC:1.14;Enzyme_name=Acting on paired donors, with incorporation or reduction of molecular oxygen. The oxygen incorporated need not be derived from O2,Oxidoreductases,Acting on paired donors, with incorporation or reduction of molecular oxygen. The oxygen incorporated need not be derived from O2
contig00002.g1392	1950	ID=contig00002.g1392;Description=RecName: Full=Nonribosomal peptide synthetase 8;AltNameFull=Fumonisin biosynthesis cluster protein 14 [Fusarium verticillioides 7600];Gene=FVER53263_00325;Ontology_term=ligase activity;Ontology_id=GO:0016874;Enzyme_code=EC:6,EC:6;Enzyme_name=Ligases,Ligases

contig00002.g1393	1110	ID=contig00002.g1393;Description=RecName: Full=NAD-dependent epimerase/dehydratase FUM13;AltNameFull=Fumonisin biosynthesis cluster protein 13 [Fusarium verticillioides 7600];Gene=FUM13;Ontology_term=oxidoreductase activity;Ontology_id=GO:0016491;Enzyme_code=EC:1,EC:1;Enzyme_name=Oxidoreductases,Oxidoreductases
contig00002.g1394	1225	ID=contig00002.g1394;Description=Fum2p [[Gibberella] fujikuroi var. moniliformis];Gene=FVER53263_00323;Ontology_term=oxidoreductase activity;Ontology_id=GO:0016491;Enzyme_code=EC:1.14,EC:1,EC:1.14;Enzyme_name=Acting on paired donors, with incorporation or reduction of molecular oxygen. The oxygen incorporated need not be derived from O2,Oxidoreductases,Acting on paired donors, with incorporation or reduction of molecular oxygen. The oxygen incorporated need not be derived from O2
contig00002.g1395	1482	ID=contig00002.g1395;Description=RecName: Full=Acyl-CoA synthetase FUM10;AltNameFull=Fumonisin biosynthesis cluster protein 10 [Fusarium verticillioides 7600];Gene=FUM10;Ontology_term=ligase activity;Ontology_id=GO:0016874;Enzyme_code=EC:6,EC:6.2.1.8,EC:6;Enzyme_name=Ligases,oxalate--CoA ligase,Ligases
contig00002.g1396	903	ID=contig00002.g1396;Description=RecName: Full=Dioxygenase FUM3;AltNameFull=Fumonisin biosynthesis cluster protein 3 [Fusarium verticillioides 7600];Gene=FMUND_4256;Ontology_term=oxidoreductase activity;Ontology_id=GO:0016491;Enzyme_code=EC:1,EC:1;Enzyme_name=Oxidoreductases,Oxidoreductases
contig00002.g1397	2955	ID=contig00002.g1397;Description=FUM8p [[Gibberella] fujikuroi var. moniliformis];Gene=FMUND_4257;Ontology_term=transferase activity;Ontology_id=GO:0016740;Enzyme_code=EC:2.6.1,EC:2.6.1,EC:2.3.1.50,EC:2,EC:2.6;Enzyme_name=Transferring nitrogenous groups,Transferring nitrogenous groups,serine C-palmitoyltransferase,Transferases,Transferring nitrogenous groups
contig00002.g1398	1275	ID=contig00002.g1398;Description=RecName: Full=Dehydrogenase FUM7;AltNameFull=Fumonisin biosynthesis cluster protein 7 [Fusarium verticillioides 7600];Gene=FVER53590_00319;Ontology_term=oxidoreductase activity;Ontology_id=GO:0016491;Enzyme_code=EC:1,EC:1;Enzyme_name=Oxidoreductases,Oxidoreductases

contig00002.g1399	3593	ID=contig00002.g1399;Description=Fum6p [[Gibberella] fujikuroi var. moniliformis];Gene=FVER53263_00317;Ontology_term=oxidoreductase activity;Ontology_id=GO:0016491;Enzyme_code=EC:1.6.2.4,EC:1.14.14.1;Enzyme_name=NADPH--hemoprotein reductase,unspecific monooxygenase
contig00002.g1400	8163	ID=contig00002.g1400;Description=acetyltransferase [Fusarium verticillioides 7600];Gene=FVER53590_00316;Ontology_term=oxidoreductase activity,lipid metabolic process,transferase activity;Ontology_id=GO:0016491,GO:0006629,GO:0016740;Enzyme_code=EC:2.3.1.41,EC:1,EC:2.1.1,EC:2.3.1.41,EC:2.1,EC:2.3,EC:1,EC:2,EC:2.1.1,EC:2.3.1;Enzyme_name=beta-ketoacyl-[acyl-carrier-protein] synthase I,Oxidoreductases,Transferring one-carbon groups,beta-ketoacyl-[acyl-carrier-protein] synthase I,Transferring one-carbon groups,Acyltransferases,Oxidoreductases,Transferases,Transferring one-carbon groups,Acyltransferases
contig00002.g1401	2277	ID=contig00002.g1401;Description=hypothetical protein FVEG_14633 [Fusarium verticillioides 7600];Gene=FVER53263_00314;Ontology_term=nucleus,oxidoreductase activity,regulation of DNA-templated transcription,transcription regulator activity;Ontology_id=GO:0005634,GO:0016491,GO:0006355,GO:0140110;Enzyme_code=EC:1,EC:1;Enzyme_name=Oxidoreductases,Oxidoreductases
contig00002.g1402	1148	ID=contig00002.g1402;Description=Zbd1p [[Gibberella] fujikuroi var. moniliformis];Gene=FPANT_3839;Ontology_term=nucleus,oxidoreductase activity,regulation of DNA-templated transcription,transcription regulator activity;Ontology_id=GO:0005634,GO:0016491,GO:0006355,GO:0140110;Enzyme_code=EC:1;Enzyme_name=Oxidoreductases
contig00002.g1403	975	ID=contig00002.g1403;Description=Znf1p [[Gibberella] fujikuroi var. moniliformis];Gene=FNAPI_4730;Ontology_term=catalytic activity, acting on a protein,transferase activity;Ontology_id=GO:0140096,GO:0016740;Enzyme_code=EC:2.3.2,EC:2.3,EC:2,EC:2.3.2;Enzyme_name=Acyltransferases,Acyltransferases,Transferases,Acyltransferases

contig00002.g1404	1408	ID=contig00002.g1404;Description=protein PNG1 [Fusarium odoratissimum NRRL 54006];Gene=FOC1_g10016503;Ontology_term=nucleus,protein catabolic process,carbohydrate derivative metabolic process,cytosol,hydrolase activity;Ontology_id=GO:0005634,GO:0030163,GO:1901135,GO:0005829,GO:0016787;Enzyme_code=EC:3.5.1,EC:3.5.1.52,EC:3.5,EC:3;Enzyme_name=Acting on carbon-nitrogen bonds, other than peptide bonds,peptide-N(4)-(N-acetyl-beta-glucosaminyl)asparagine amidase,Acting on carbon-nitrogen bonds, other than peptide bonds,Hydrolases
contig00002.g1405	2688	ID=contig00002.g1405;Description=UTP4-u3 snoRNP [Fusarium tjaetaba];Gene=FVER53263_00311;Ontology_term=RNA binding,ribosome biogenesis,regulation of DNA-templated transcription,nucleolus;Ontology_id=GO:0003723,GO:0042254,GO:0006355,GO:0005730
contig00002.g1406	1492	ID=contig00002.g1406;Description=nicotinate phosphoribosyltransferase [Fusarium verticillioides 7600];Gene=FPHYL_314;Ontology_term=nucleus,nucleobase-containing small molecule metabolic process,chromosome,telomere organization,transferase activity,chromatin organization,ligase activity;Ontology_id=GO:0005634,GO:0055086,GO:0005694,GO:0032200,GO:0016740,GO:0006325,GO:0016874;Enzyme_code=EC:2.4,EC:6.3.4.21;Enzyme_name=Glycosyltransferases,nicotinate phosphoribosyltransferase
contig00002.g1407	1237	ID=contig00002.g1407;Description=nicotinate phosphoribosyltransferase [Fusarium tjaetaba];Gene=FVER53590_00309;Ontology_term=nucleus,regulation of DNA-templated transcription,transferase activity,transcription regulator activity;Ontology_id=GO:0005634,GO:0006355,GO:0016740,GO:0140110;Enzyme_code=EC:2.4;Enzyme_name=Glycosyltransferases
contig00002.g1408	1245	ID=contig00002.g1408;Description=protein BTN1 [Fusarium fujikuroi];Gene=FOTG_04607;Ontology_term=vacuole;Ontology_id=GO:0005773
contig00002.g1409	1793	ID=contig00002.g1409;Description=hypothetical protein FVEG_14631 [Fusarium verticillioides 7600];Gene=FVER53263_00305;Ontology_term=membrane;Ontology_id=GO:0016020
contig00002.g1410	2088	ID=contig00002.g1410;Description=hypothetical protein FVEG_00306 [Fusarium verticillioides 7600]

contig00002.g1411	1410	ID=contig00002.g1411;Description=WD-repeat CRB3 [Fusarium agapanthi];Gene=FVER53263_00303;Ontology_term=protein-containing complex assembly,DNA replication,regulation of DNA-templated transcription,nuclear chromosome,ribosome biogenesis,chromatin organization,nucleoplasm;Ontology_id=GO:0065003,GO:0006260,GO:0006355,GO:0000228,GO:0042254,GO:0006325,GO:0005654
contig00002.g1412	550	ID=contig00002.g1412;Description=LYR motif-containing protein 4 [Fusarium oxysporum f. sp. cubense race 1];Gene=FIESC28_00453
contig00002.g1413	2711	ID=contig00002.g1413;Description=hypothetical protein FVER14953_00301 [Fusarium verticillioides]
contig00002.g1414	1069	ID=contig00002.g1414;Description=hypothetical protein FVEG_00300 [Fusarium verticillioides 7600];Gene=FVER53263_00300;Ontology_term=membrane;Ontology_id=GO:0016020
contig00002.g1415	2959	ID=contig00002.g1415;Description=er membrane complex subunit [Fusarium denticulatum];Gene=FTJAE_1661;Ontology_term=endoplasmic reticulum,protein folding;Ontology_id=GO:0005783,GO:0006457
contig00002.g1416	310	ID=contig00002.g1416;Description=hypothetical protein FVEG_00298 [Fusarium verticillioides 7600];Gene=Q01821;Ontology_term=signaling,nervous system process,plasma membrane;Ontology_id=GO:0023052,GO:0050877,GO:0005886
contig00002.g1417	1873	ID=contig00002.g1417;Description=5^ nucleotidase [Fusarium acutatum];Gene=BFJ72_g2928;Ontology_term=nucleobase-containing small molecule metabolic process,cytosol,vacuole,hydrolase activity;Ontology_id=GO:0055086,GO:0005829,GO:0005773,GO:0016787;Enzyme_code=EC:3,EC:3;Enzyme_name=Hydrolases,Hydrolases
contig00002.g1418	1230	ID=contig00002.g1418;Description=solute carrier family 35 member F2 [Fusarium tjaetaba];Gene=FMEXI_541;Ontology_term=transmembrane transport,transporter activity;Ontology_id=GO:0055085,GO:0005215
contig00002.g1419	1154	ID=contig00002.g1419;Description=hypothetical protein FVER14953_00295 [Fusarium verticillioides]
contig00002.g1420	1247	ID=contig00002.g1420;Description=hypothetical protein FVER14953_00294 [Fusarium verticillioides]
contig00002.g1421	1224	ID=contig00002.g1421;Description=hypothetical protein FVEG_00293 [Fusarium verticillioides 7600]

contig00002.g1422	1184	ID=contig00002.g1422;Description=hypothetical protein FVER53590_00292 [Fusarium verticillioides]
contig00002.g1423	281	ID=contig00002.g1423;Description=hypothetical protein FVER14953_20308 [Fusarium verticillioides]
contig00002.g1424	3174	ID=contig00002.g1424;Description=sialidase-1 [Fusarium verticillioides 7600];Gene=gaoC;Ontology_term=oxidoreductase activity;Ontology_id=GO:0016491;Enzyme_code=EC:1.1.3.9,EC:1.1,EC:1,EC:1.1.3.9,EC:1.1.3;Enzyme_name=galactose oxidase,Acting on the CH-OH group of donors,Oxidoreductases,galactose oxidase,Acting on the CH-OH group of donors
contig00002.g1425	4701	ID=contig00002.g1425;Description=hypothetical protein FVEG_14627 [Fusarium verticillioides 7600]
contig00002.g1426	957	ID=contig00002.g1426;Description=indc11 [Fusarium pseudocircinatum]
contig00002.g1427	3984	ID=contig00002.g1427;Description=polynucleotide adenylyltransferase [Fusarium tjaetaba];Gene=FPANT_3867;Ontology_term=mRNA metabolic process,RNA binding,snRNA metabolic process,regulatory ncRNA-mediated gene silencing,transferase activity;Ontology_id=GO:0016071,GO:0003723,GO:0016073,GO:0031047,GO:0016740;Enzyme_code=EC:2.7.7.19,EC:2.7,EC:2,EC:2.7.7,EC:2.7.7.19;Enzyme_name=polynucleotide adenylyltransferase,Transferring phosphorus-containing groups,Transferases,Transferring phosphorus-containing groups,polynucleotide adenylyltransferase
contig00002.g1428	1158	ID=contig00002.g1428;Description=hypothetical protein FVER14953_00287 [Fusarium verticillioides]
contig00002.g1429	879	ID=contig00002.g1429;Description=tsa antioxidant enzyme [Fusarium tjaetaba]
contig00002.g1430	1718	ID=contig00002.g1430;Description=hypothetical protein FNYG_06674 [Fusarium nygamai]
contig00002.g1431	2226	ID=contig00002.g1431;Description=hypothetical protein FVER14953_00286 [Fusarium verticillioides]
contig00002.g1432	2166	ID=contig00002.g1432;Description=hypothetical protein FVER14953_00285 [Fusarium verticillioides]
contig00002.g1433	576	ID=contig00002.g1433;Description=hypothetical protein FVER53263_00284 [Fusarium verticillioides]
contig00002.g1434	2214	ID=contig00002.g1434;Description=hypothetical protein FVER14953_00283 [Fusarium verticillioides]

contig00002.g1435	945	ID=contig00002.g1435;Description=catechol O-methyltransferase [Fusarium odoratissimum NRRL 54006];Gene=FPRO05_03924;Ontology_term=transferase activity;Ontology_id=GO:0016740;Enzyme_code=EC:2.1.1,EC:2.1,EC:2.1.1.6,EC:2,EC:2.1.1;Enzyme_name=Transferring one-carbon groups,Transferring one-carbon groups,catechol O-methyltransferase,Transferases,Transferring one-carbon groups
contig00002.g1436	1562	ID=contig00002.g1436;Description=hypothetical protein FVEG_00281 [Fusarium verticillioides 7600]
contig00002.g1437	2356	ID=contig00002.g1437;Description=zinc finger double-stranded RNA binding protein [Fusarium tjaetaba]
contig00002.g1438	4746	ID=contig00002.g1438;Description=metalloregulatory (zinc-responsiveness transcriptional activator) [Fusarium agapanthi]
contig00002.g1439	570	ID=contig00002.g1439;Description=nudix domain-containing protein [Fusarium denticulatum];Gene=438
contig00002.g1440	831	ID=contig00002.g1440;Description=ankyrin repeat-containing protein [Fusarium subglutinans];Gene=LW93_4624;Ontology_term=nucleobase-containing small molecule metabolic process,carbohydrate derivative metabolic process;Ontology_id=GO:0055086,GO:1901135
contig00002.g1441	7323	ID=contig00002.g1441;Description=Counting factor 60 [Fusarium mundagurra]
contig00002.g1442	4007	ID=contig00002.g1442;Description=heterokaryon incompatibility het-6 [Fusarium tjaetaba]
contig00002.g1443	962	ID=contig00002.g1443;Description=ketoreductase [Fusarium mexicanum]
contig00002.g1444	621	ID=contig00002.g1444;Description=hypothetical protein FVER14953_00266 [Fusarium verticillioides]
contig00002.g1445	378	ID=contig00002.g1445;Description=hypothetical protein FVER14953_00265 [Fusarium verticillioides]
contig00002.g1446	840	ID=contig00002.g1446;Description=hypothetical protein J7337_001687 [Fusarium musae]
contig00002.g1447	558	ID=contig00002.g1447;Description=hypothetical protein FVEG_00263 [Fusarium verticillioides 7600]
contig00002.g1448	2174	ID=contig00002.g1448;Description=choline dehydrogenase [Fusarium tjaetaba];Gene=FMUND_7302;Ontology_term=oxidoreductase activity;Ontology_id=GO:0016491;Enzyme_code=EC:1.1;Enzyme_name=Acting on the CH-OH group of donors
contig00002.g1449	648	ID=contig00002.g1449;Description=hypothetical protein FVER14953_00261 [Fusarium verticillioides]

contig00002.g1450	1319	ID=contig00002.g1450;Description=sterol regulatory element-binding ECM22 [Fusarium tjaetaba];Gene=FOXB_06463;Ontology_term=nucleus,regulation of DNA-templated transcription,transcription regulator activity;Ontology_id=GO:0005634,GO:0006355,GO:0140110
contig00002.g1451	3244	ID=contig00002.g1451;Description=RTA1 [Fusarium tjaetaba];Gene=FVER53263_00259;Ontology_term=transferase activity;Ontology_id=GO:0016740;Enzyme_code=EC:2.4.1.16,EC:2.4,EC:2.4.1.16,EC:2,EC:2.4.1;Enzyme_name=chitin synthase,Glycosyltransferases,chitin synthase,Transferases,Glycosyltransferases
contig00002.g1452	1929	ID=contig00002.g1452;Description=cytochrome P450 [Fusarium oxysporum];Gene=FRV6_03090;Ontology_term=oxidoreductase activity;Ontology_id=GO:0016491;Enzyme_code=EC:1,EC:1;Enzyme_name=Oxidoreductases,Oxidoreductases
contig00002.g1453	714	ID=contig00002.g1453;Description=appr-1-p processing enzyme family [Fusarium phyllophilum]
contig00002.g1454	2406	ID=contig00002.g1454;Description=GTPase-activating of the rho rac family (LRG1) [Fusarium pseudoanthophilum];Gene=FPANT_3905;Ontology_term=molecular function regulator activity;Ontology_id=GO:0098772
contig00002.g1455	909	ID=contig00002.g1455;Description=related to alcohol dehydrogenase homolog Bli-4 [Fusarium mangiferae]
contig00002.g1456	2535	ID=contig00002.g1456;Description=hypothetical protein FVER53590_00253 [Fusarium verticillioides];Gene=FSUBG_4255;Ontology_term=membrane;Ontology_id=GO:0016020
contig00002.g1457	2966	ID=contig00002.g1457;Description=related to beta transducin-like protein [Fusarium proliferatum ET1];Gene=FVER53590_00252;Ontology_term=transferase activity;Ontology_id=GO:0016740;Enzyme_code=EC:2.3.1,EC:2.3,EC:2,EC:5.4.3.2,EC:2.3.1;Enzyme_name=Acyltransferases,Acyltransferases,Transferases,lysine 2,3-aminomutase,Acyltransferases

contig00002.g1458	1423	ID=contig00002.g1458;Description=alpha-glucosidase (maltase) [Fusarium subglutinans];Gene=FANTH_676;Ontology_term=carbohydrate metabolic process,nucleus,DNA binding,lyase activity,regulation of DNA-templated transcription,transcription regulator activity,hydrolase activity;Ontology_id=GO:0005975,GO:0005634,GO:0003677,GO:0016829,GO:0006355,GO:0140110,GO:0016787;Enzyme_code=EC:3.2.1.20,EC:4,EC:3.2,EC:3.2.1.20,EC:3.2.1.40,EC:3,EC:4,EC:3.2.1;Enzyme_name=alpha-glucosidase,Lyases,Glycosylases,alpha-glucosidase,alpha-L-rhamnosidase,Hydrolases,Lyases,Glycosylases
contig00002.g1459	1897	ID=contig00002.g1459;Description=trehalose-6-phosphate hydrolase [Fusarium verticillioides 7600];Gene=FANTH_676;Ontology_term=carbohydrate metabolic process,nucleus,regulation of DNA-templated transcription,transcription regulator activity,hydrolase activity;Ontology_id=GO:0005975,GO:0005634,GO:0006355,GO:0140110,GO:0016787;Enzyme_code=EC:3.2.1.20,EC:3.2,EC:3.2.1.20,EC:3.2.1.10,EC:3,EC:3.2.1,EC:3.2.1.26,EC:3.2.1.48;Enzyme_name=alpha-glucosidase,Glycosylases,alpha-glucosidase,oligo-1,6-glucosidase,Hydrolases,Glycosylases,beta-fructofuranosidase,sucrose alpha-glucosidase
contig00002.g1460	1823	ID=contig00002.g1460;Description=hexose transporter [Fusarium subglutinans];Gene=FVER53263_00248;Ontology_term=transmembrane transport,transporter activity;Ontology_id=GO:0055085,GO:0005215
contig00002.g1461	465	ID=contig00002.g1461;Description=two-component response regulator [Fusarium tjaetaba];Gene=FVER53263_00247;Ontology_term=signaling;Ontology_id=GO:0023052
contig00002.g1462	976	ID=contig00002.g1462;Description=hypothetical protein FVER14953_00246 [Fusarium verticillioides]
contig00002.g1463	1768	ID=contig00002.g1463;Description=ankyrin repeat [Fusarium tjaetaba]
contig00002.g1464	1044	ID=contig00002.g1464;Description=hypothetical protein FVER14953_00244 [Fusarium verticillioides]
contig00002.g1465	4275	ID=contig00002.g1465;Description=heterokaryon incompatibility 6 OR allele [Fusarium tjaetaba]
contig00002.g1466	2216	ID=contig00002.g1466;Description=C6 transcription factor [Fusarium tjaetaba];Gene=FDENT_4064;Ontology_term=nucleus,DNA binding,regulation of DNA-templated transcription,transcription regulator activity;Ontology_id=GO:0005634,GO:0003677,GO:0006355,GO:0140110
contig00002.g1467	1682	ID=contig00002.g1467;Description=hypothetical protein FVER14953_00239 [Fusarium verticillioides];Gene=FPHYL_790;Ontology_term=membrane;Ontology_id=GO:0016020

contig00002.g1468	3328	ID=contig00002.g1468;Description=nonribosomal peptide synthetase [Fusarium denticulatum];Gene=FVEG_00238;Ontology_term=isomerase activity;Ontology_id=GO:0016853;Enzyme_code=EC:5,EC:5;Enzyme_name=Isomerases,Isomerases
contig00002.g1469	957	ID=contig00002.g1469;Description=SDR family [Fusarium tjaetaba]
contig00002.g1470	2144	ID=contig00002.g1470;Description=sodium-dependent serotonin transporter [Fusarium coicis];Gene=FVER53263_00236;Ontology_term=membrane;Ontology_id=GO:0016020
contig00002.g1471	1389	ID=contig00002.g1471;Description=related to microsomal dipeptidase precursor [Fusarium fujikuroi IMI 58289];Gene=LW93_4665;Ontology_term=molecular transducer activity,catalytic activity, acting on a protein,protein catabolic process,signaling,anatomical structure development,cell differentiation,regulation of DNA-templated transcription,reproductive process,transferase activity,programmed cell death,plasma membrane,hydrolase activity;Ontology_id=GO:0060089,GO:0140096,GO:0030163,GO:0023052,GO:0048856,GO:0030154,GO:0006355,GO:0022414,GO:0016740,GO:0012501,GO:0005886,GO:0016787;Enzyme_code=EC:2.7.10.1,EC:3.4.13,EC:2.7.10.1,EC:2.7.1,EC:3.4.13,EC:3.4,EC:3.4.13.19,EC:2.7,EC:2,EC:3;Enzyme_name=receptor protein-tyrosine kinase,Acting on peptide bonds (peptidases),receptor protein-tyrosine kinase,Transferring phosphorus-containing groups,Acting on peptide bonds (peptidases),Acting on peptide bonds (peptidases),membrane dipeptidase,Transferring phosphorus-containing groups,Transferases,Hydrolases
contig00002.g1472	1331	ID=contig00002.g1472;Description=hypothetical protein FVER14953_00233 [Fusarium verticillioides]
contig00002.g1473	1660	ID=contig00002.g1473;Description=DAL5-Allantoate ureidosuccinate permease [Fusarium pseudoanthophilum];Gene=FPANT_3926;Ontology_term=transmembrane transport,transporter activity;Ontology_id=GO:0055085,GO:0005215
contig00002.g1474	1065	ID=contig00002.g1474;Description=putative secondary metabolism biosynthetic enzyme [Fusarium musae];Gene=FDENT_4055;Ontology_term=oxidoreductase activity,lipid metabolic process;Ontology_id=GO:0016491,GO:0006629;Enzyme_code=EC:1.1.1.145,EC:1.1.1.170,EC:1.1,EC:1,EC:1.1.1.145,EC:1.1.1;Enzyme_name=3beta-hydroxy-Delta(5)-steroid dehydrogenase,3beta-hydroxysteroid-4alpha-carboxylate 3-dehydrogenase,Acting on the CH-OH group of donors,Oxidoreductases,3beta-hydroxy-Delta(5)-steroid dehydrogenase,Acting on the CH-OH group of donors

contig00002.g1475	3311	ID=contig00002.g1475;Description=C-3 sterol dehydrogenase [Fusarium globosum];Gene=FGLOB1_2568;Ontology_term=nucleus,nucleobase-containing small molecule metabolic process,oxidoreductase activity,lipid metabolic process,hydrolase activity;Ontology_id=GO:0005634,GO:0055086,GO:0016491,GO:0006629,GO:0016787;Enzyme_code=EC:1.1.1.145,EC:1.1,EC:3.1,EC:3.1.3.106,EC:1,EC:3,EC:1.1.1.145,EC:1.1.1,EC:3.1.3.5,EC:3.1.3;Enzyme_name=3beta-hydroxy-Delta(5)-steroid dehydrogenase,Acting on the CH-OH group of donors,Acting on ester bonds,2-lysophosphatidate phosphatase,Oxidoreductases,Hydrolases,3beta-hydroxy-Delta(5)-steroid dehydrogenase,Acting on the CH-OH group of donors,5'-nucleotidase,Acting on ester bonds
contig00002.g1476	1738	ID=contig00002.g1476;Description=multidrug resistant protein [Fusarium subglutinans];Gene=FNAPI_2476;Ontology_term=transmembrane transport,transporter activity,plasma membrane;Ontology_id=GO:0055085,GO:0005215,GO:0005886
contig00002.g1477	2105	ID=contig00002.g1477;Description=related to cutinase transcription factor 1 beta [Fusarium fujikuroi];Gene=FVEG_00228;Ontology_term=nucleus,DNA binding,regulation of DNA-templated transcription,transcription regulator activity;Ontology_id=GO:0005634,GO:0003677,GO:0006355,GO:0140110
contig00002.g1478	340	ID=contig00002.g1478;Description=conidiation-specific 10 [Fusarium pseudoanthophilum]
contig00002.g1479	314	ID=contig00002.g1479;Description=hypothetical protein FDENT_4050 [Fusarium denticulatum]
contig00002.g1480	1176	ID=contig00002.g1480;Description=prestalk A differentiation A [Fusarium tjaetaba]
contig00002.g1481	1710	ID=contig00002.g1481;Description=hypothetical protein FVEG_00224 [Fusarium verticillioides 7600]
contig00002.g1482	1424	ID=contig00002.g1482;Description=related to monocarboxylate transporter 4 [Fusarium mangiferae];Gene=FACUT_4143;Ontology_term=transmembrane transport,transporter activity;Ontology_id=GO:0055085,GO:0005215
contig00002.g1483	1341	ID=contig00002.g1483;Description=transcription factor [Fusarium pseudoanthophilum];Gene=FPRO_01879;Ontology_term=nucleus,regulation of DNA-templated transcription,transcription regulator activity;Ontology_id=GO:0005634,GO:0006355,GO:0140110
contig00002.g1484	1731	ID=contig00002.g1484;Description=amino acid/polyamine transporter I [Fusarium oxysporum Fo47];Gene=BFJ65_g1723;Ontology_term=transmembrane transport,transporter activity;Ontology_id=GO:0055085,GO:0005215
contig00002.g1485	1301	ID=contig00002.g1485;Description=hypothetical protein FVER14953_00220 [Fusarium verticillioides];Gene=FPANT_12113;Ontology_term=membrane;Ontology_id=GO:0016020

contig00002.g1486	2336	ID=contig00002.g1486;Description=hypothetical protein FVER14953_00218 [Fusarium verticillioides]
contig00002.g1487	1899	ID=contig00002.g1487;Description=choline dehydrogenase [Fusarium tjaetaba];Gene=FNAPI_2488;Ontology_term=oxidoreductase activity;Ontology_id=GO:0016491;Enzyme_code=EC:1.1,EC:1.1,EC:1;Enzyme_name=Acting on the CH-OH group of donors,Acting on the CH-OH group of donors,Oxidoreductases
contig00002.g1488	1366	ID=contig00002.g1488;Description=integral membrane protein PTH11 [Fusarium pseudoanthophilum];Gene=FVEG_00216;Ontology_term=membrane;Ontology_id=GO:0016020
contig00002.g1489	2135	ID=contig00002.g1489;Description=transcriptional regulatory [Fusarium coicis];Gene=FPANT_12108;Ontology_term=nucleus,DNA binding,regulation of DNA-templated transcription,transcription regulator activity;Ontology_id=GO:0005634,GO:0003677,GO:0006355,GO:0140110
contig00002.g1490	1449	ID=contig00002.g1490;Description=monocarboxylate transporter 2 [Fusarium tjaetaba];Gene=FVEG_00214;Ontology_term=transmembrane transport,transporter activity;Ontology_id=GO:0055085,GO:0005215
contig00002.g1491	1620	ID=contig00002.g1491;Description=acetylhydrolase [Fusarium tjaetaba];Gene=FTJAE_1747;Ontology_term=lipid metabolic process,hydrolase activity;Ontology_id=GO:0006629,GO:0016787;Enzyme_code=EC:3.1.1.47,EC:3.1.1.47,EC:3.1,EC:3,EC:3.1.1;Enzyme_name=1-alkyl-2-acetylglycerophosphocholine esterase,1-alkyl-2-acetylglycerophosphocholine esterase,Acting on ester bonds,Hydrolases,Acting on ester bonds
contig00002.g1492	5359	ID=contig00002.g1492;Description=subtilisin-like serine protease [Fusarium tjaetaba];Gene=FPANT_13206;Ontology_term=catalytic activity, acting on a protein,protein catabolic process,autophagy,anatomical structure development,cell differentiation,reproductive process,endoplasmic reticulum,vacuole,hydrolase activity;Ontology_id=GO:0140096,GO:0030163,GO:0006914,GO:0048856,GO:0030154,GO:0022414,GO:0005783,GO:0005773,GO:0016787;Enzyme_code=EC:3.4.21,EC:3.4.21.48,EC:3.4.21,EC:3.4,EC:3;Enzyme_name=Acting on peptide bonds (peptidases),cerevisin,Acting on peptide bonds (peptidases),Acting on peptide bonds (peptidases),Hydrolases

contig00002.g1493	8024	ID=contig00002.g1493;Description=Beige like 1 [Fusarium tjaetaba];Ontology_term=autophagy,mitochondrion,cytosol,cell wall organization or biogenesis,lipid binding,intracellular protein transport,vacuole;Ontology_id=GO:0006914,GO:0005739,GO:0005829,GO:0071554,GO:0008289,GO:0006886,GO:0005773
contig00002.g1494	286	ID=contig00002.g1494;Description=NADH dehydrogenase [Fusarium verticillioides 7600];Gene=FGADI_4143;Ontology_term=mitochondrion;Ontology_id=GO:0005739
contig00002.g1495	1523	ID=contig00002.g1495;Description=oligosaccharyl transferase complex subunit OST4 [Fusarium verticillioides 7600];Gene=FNAPI_2496;Ontology_term=nuclear envelope,protein glycosylation,endoplasmic reticulum,transferase activity;Ontology_id=GO:0005635,GO:0006486,GO:0005783,GO:0016740;Enzyme_code=EC:2,EC:2;Enzyme_name=Transferases,Transferases
contig00002.g1496	783	ID=contig00002.g1496;Description=pre-mRNA-splicing factor ISY1 [Fusarium verticillioides 7600];Gene=FVEG_00206;Ontology_term=nucleus,mRNA metabolic process,protein-containing complex assembly;Ontology_id=GO:0005634,GO:0016071,GO:0065003
contig00002.g1497	2646	ID=contig00002.g1497;Description=hypothetical protein FVEG_00205 [Fusarium verticillioides 7600]
contig00002.g1498	1494	ID=contig00002.g1498;Description=homoserine O-acetyltransferase [Fusarium verticillioides 7600];Gene=FANTH_14502;Ontology_term=sulfur compound metabolic process,amino acid metabolic process,transferase activity;Ontology_id=GO:0006790,GO:0006520,GO:0016740;Enzyme_code=EC:2.3.1.30,EC:2.3.1.30,EC:2.3,EC:2.3.1.31,EC:2,EC:2.3.1;Enzyme_name=serine O-acetyltransferase,serine O-acetyltransferase,Acyltransferases,homoserine O-acetyltransferase,Transferases,Acyltransferases
contig00002.g1499	1114	ID=contig00002.g1499;Description=ankyrin repeat [Fusarium acutatum]
contig00002.g1500	857	ID=contig00002.g1500;Description=Nudix hydrolase 14 chloroplastic [Fusarium tjaetaba];Gene=FMUND_13639;Ontology_term=hydrolase activity;Ontology_id=GO:0016787;Enzyme_code=EC:3,EC:3;Enzyme_name=Hydrolases,Hydrolases

contig00002.g1501	705	ID=contig00002.g1501;Description=ADP-ribose pyrophosphatase [Fusarium verticillioides 7600];Gene=FVER53263_00201;Ontology_term=nucleus,nucleobase-containing small molecule metabolic process,mitochondrion,carbohydrate derivative metabolic process,hydrolase activity;Ontology_id=GO:0005634,GO:0055086,GO:0005739,GO:1901135,GO:0016787;Enzyme_code=EC:3,EC:3.6.1,EC:3.6.1.13,EC:3.6,EC:3;Enzyme_name=Hydrolases,Acting on acid anhydrides,ADP-ribose diphosphatase,Acting on acid anhydrides,Hydrolases
contig00002.g1502	957	ID=contig00002.g1502;Description=xylanase 1 [Fusarium subglutinans];Gene=FNYG_06599;Ontology_term=carbohydrate metabolic process,transmembrane transport,cell wall organization or biogenesis,transporter activity,hydrolase activity;Ontology_id=GO:0005975,GO:0055085,GO:0071554,GO:0005215,GO:0016787;Enzyme_code=EC:3.2.1,EC:3.2,EC:3,EC:3.2.1;Enzyme_name=Glycosylases,Glycosylases,Hydrolases,Glycosylases
contig00002.g1503	2685	ID=contig00002.g1503;Description=hypothetical protein CEK25_001712 [Fusarium fujikuroi];Gene=AU210_001830;Ontology_term=transmembrane transport,transporter activity;Ontology_id=GO:0055085,GO:0005215
contig00002.g1504	1708	ID=contig00002.g1504;Description=hypothetical protein FNYG_06597 [Fusarium nygamai];Gene=FNYG_06597;Ontology_term=transmembrane transport,transporter activity;Ontology_id=GO:0055085,GO:0005215
contig00002.g1505	1568	ID=contig00002.g1505;Description=COP9 signalosome complex subunit 2 [Fusarium verticillioides 7600]
contig00002.g1506	2201	ID=contig00002.g1506;Description=ubiquitin thioesterase otubain [Fusarium coicis];Gene=FPANT_8791;Ontology_term=catalytic activity, acting on a protein,hydrolase activity;Ontology_id=GO:0140096,GO:0016787;Enzyme_code=EC:3.4.19.12,EC:3.4,EC:3,EC:3.4.19.12;Enzyme_name=ubiquitinyl hydrolase 1,Acting on peptide bonds (peptidases),Hydrolases,ubiquitinyl hydrolase 1
contig00002.g1507	1213	ID=contig00002.g1507;Description=trans-aconitate 2-methyltransferase [Fusarium tjaetaba];Gene=FDENT_4020;Ontology_term=transferase activity;Ontology_id=GO:0016740;Enzyme_code=EC:2.1.1,EC:2.1,EC:2,EC:2.1.1;Enzyme_name=Transferring one-carbon groups,Transferring one-carbon groups,Transferases,Transferring one-carbon groups

contig00002.g1508	1539	ID=contig00002.g1508;Description=glutamine synthetase [Fusarium verticillioides 7600];Gene=FVER53590_00192;Ontology_term=amino acid metabolic process,ligase activity;Ontology_id=GO:0006520,GO:0016874;Enzyme_code=EC:6.3.1.2,EC:6.3,EC:6.3.1,EC:6.3.1.2,EC:6;Enzyme_name=glutamine synthetase,Forming carbon-nitrogen bonds,Forming carbon-nitrogen bonds,glutamine synthetase,Ligases
contig00002.g1509	961	ID=contig00002.g1509;Description=glucose 1-dehydrogenase [Fusarium proliferatum];Gene=FMUND_13648;Ontology_term=oxidoreductase activity;Ontology_id=GO:0016491;Enzyme_code=EC:1;Enzyme_name=Oxidoreductases
contig00002.g1510	1844	ID=contig00002.g1510;Description=isocitrate lyase [Fusarium verticillioides 7600];Gene=FVEG_00190;Ontology_term=lyase activity;Ontology_id=GO:0016829;Enzyme_code=EC:4.1.3.1,EC:4.1.3.30;Enzyme_name=isocitrate lyase,methylisocitrate lyase
contig00002.g1511	1519	ID=contig00002.g1511;Description=2-methylcitrate synthase, mitochondrial [Fusarium proliferatum];Gene=Forpe1208_v000211;Ontology_term=generation of precursor metabolites and energy,mitochondrion,transferase activity;Ontology_id=GO:0006091,GO:0005739,GO:0016740;Enzyme_code=EC:2.3.3.5,EC:2.3.3.16;Enzyme_name=2-methylcitrate synthase,citrate synthase (unknown stereospecificity)
contig00002.g1512	573	ID=contig00002.g1512;Description=murein transglycosylase [Fusarium verticillioides 7600];Gene=FNYG_06589;Ontology_term=transferase activity;Ontology_id=GO:0016740;Enzyme_code=EC:2.3.1,EC:2.3,EC:2,EC:2.3.1;Enzyme_name=Acyltransferases,Acyltransferases,Transferases,Acyltransferases
contig00002.g1513	1219	ID=contig00002.g1513;Description=related to putative glycosidases [Fusarium fujikuroi];Gene=FPRO_01911;Ontology_term=carbohydrate metabolic process,hydrolase activity;Ontology_id=GO:0005975,GO:0016787;Enzyme_code=EC:3.2.1,EC:3.2,EC:3,EC:3.2.1;Enzyme_name=Glycosylases,Glycosylases,Hydrolases,Glycosylases
contig00002.g1514	834	ID=contig00002.g1514;Description=glutathione S-transferase [Fusarium verticillioides 7600];Gene=FPANT_7948;Ontology_term=transferase activity;Ontology_id=GO:0016740;Enzyme_code=EC:2,EC:2.5.1.18,EC:2;Enzyme_name=Transferases,glutathione transferase,Transferases
contig00002.g1515	5972	ID=contig00002.g1515;Description=NFX1-type zinc finger-containing protein [Fusarium tjaetaba];Gene=FNYG_06586;Ontology_term=nucleus,catalytic activity,ATP-dependent activity;Ontology_id=GO:0005634,GO:0003824,GO:0140657
contig00002.g1516	540	ID=contig00002.g1516;Description=GTP-binding protein RHO3 [Fusarium tjaetaba]

contig00002.g1517	804	ID=contig00002.g1517;Description=GTP-binding protein RHO3 [Fusarium verticillioides 7600];Gene=FVER53590_00183;Ontology_term=vesicle-mediated transport,cytoskeleton organization,GTPase activity,signaling,establishment or maintenance of cell polarity,anatomical structure development,cytosol,plasma membrane;Ontology_id=GO:0016192,GO:0007010,GO:0003924,GO:0023052,GO:0007163,GO:0048856,GO:0005829,GO:0005886;Enzyme_code=EC:3.6.1,EC:3.6,EC:3,EC:3.6.1.15;Enzyme_name=Acting on acid anhydrides,Acting on acid anhydrides,Hydrolases,nucleoside-triphosphate phosphatase
contig00002.g1518	1140	ID=contig00002.g1518;Description=pre-mRNA-splicing factor CWC25 [Fusarium coicis];Gene=FPCIR_4205;Ontology_term=nucleus,mRNA metabolic process;Ontology_id=GO:0005634,GO:0016071
contig00002.g1519	469	ID=contig00002.g1519;Description=40S ribosomal protein mrp2, mitochondrial [Fusarium solani];Gene=FOYG_05939;Ontology_term=mitochondrial gene expression,mitochondrion,structural molecule activity,ribosome;Ontology_id=GO:0140053,GO:0005739,GO:0005198,GO:0005840
contig00002.g1520	2841	ID=contig00002.g1520;Description=beta-glucosidase [Fusarium verticillioides 7600];Gene=FPCIR_4207;Ontology_term=carbohydrate metabolic process,hydrolase activity;Ontology_id=GO:0005975,GO:0016787;Enzyme_code=EC:3.2.1.21,EC:3.2,EC:3.2.1.21,EC:3,EC:3.2.1;Enzyme_name=beta-glucosidase,Glycosylases,beta-glucosidase,Hydrolases,Glycosylases
contig00002.g1521	1119	ID=contig00002.g1521;Description=glycosyl hydrolase family 88 [Fusarium tjaetaba];Gene=FOYG_05937;Ontology_term=carbohydrate metabolic process,hydrolase activity;Ontology_id=GO:0005975,GO:0016787;Enzyme_code=EC:3,EC:3,EC:3.2.1.172;Enzyme_name=Hydrolases,Hydrolases,unsaturated rhamnogalacturonyl hydrolase
contig00002.g1522	1699	ID=contig00002.g1522;Description=major facilitator superfamily domain-containing protein [Fusarium oxysporum];Gene=FocTR4_00000248;Ontology_term=transmembrane transport,transporter activity;Ontology_id=GO:0055085,GO:0005215
contig00002.g1523	783	ID=contig00002.g1523;Description=hypothetical protein FVEG_00177 [Fusarium verticillioides 7600]
contig00002.g1524	333	ID=contig00002.g1524;Description=hypothetical protein FVEG_00176 [Fusarium verticillioides 7600]
contig00002.g1525	273	ID=contig00002.g1525;Description=L-2-aminoadipate reductase large subunit [Fusarium oxysporum f. sp. albedinis]

contig00002.g1526	491	ID=contig00002.g1526;Description=hypothetical protein FVEG_00175 [Fusarium verticillioides 7600]
contig00002.g1527	378	ID=contig00002.g1527;Description=hypothetical protein FVER14953_20503 [Fusarium verticillioides]
contig00002.g1528	618	ID=contig00002.g1528;Description=hypothetical protein FVER53263_00172 [Fusarium verticillioides];Gene=FPCIR_4216
contig00002.g1529	630	ID=contig00002.g1529;Description=hypothetical protein FVEG_00171 [Fusarium verticillioides 7600]
contig00002.g1530	1545	ID=contig00002.g1530;Description=succinate-semialdehyde dehydrogenase (NADP+) [Fusarium verticillioides 7600];Gene=FOTG_13012;Ontology_term=oxidoreductase activity;Ontology_id=GO:0016491;Enzyme_code=EC:1.2.1,EC:1.2.1.16,EC:1.2,EC:1.2.1.79,EC:1,EC:1.2.1.20,EC:1.2.1;Enzyme_name=Acting on the aldehyde or oxo group of donors,succinate-semialdehyde dehydrogenase [NAD(P)(+)],Acting on the aldehyde or oxo group of donors,succinate-semialdehyde dehydrogenase (NADP(+)),Oxidoreductases,glutarate-semialdehyde dehydrogenase,Acting on the aldehyde or oxo group of donors
contig00002.g1531	639	ID=contig00002.g1531;Description=hypothetical protein FVER14953_20504 [Fusarium verticillioides]
contig00002.g1532	1436	ID=contig00002.g1532;Description=monoamine oxidase [Fusarium verticillioides 7600];Gene=FVER53263_00169;Ontology_term=oxidoreductase activity;Ontology_id=GO:0016491;Enzyme_code=EC:1;Enzyme_name=Oxidoreductases
contig00002.g1533	2684	ID=contig00002.g1533;Description=C6 transcription factor [Fusarium subglutinans];Gene=BFJ71_g11131;Ontology_term=nucleus,DNA binding,regulation of DNA-templated transcription,transcription regulator activity;Ontology_id=GO:0005634,GO:0003677,GO:0006355,GO:0140110
contig00002.g1534	1012	ID=contig00002.g1534;Description=s-adenosyl-L-methionine-dependent methyltransferase [Fusarium tjaetaba];Gene=FPANT_6020;Ontology_term=transferase activity;Ontology_id=GO:0016740;Enzyme_code=EC:2.1.1,EC:2.1,EC:2,EC:2.1.1;Enzyme_name=Transferring one-carbon groups,Transferring one-carbon groups,Transferases,Transferring one-carbon groups
contig00002.g1535	570	ID=contig00002.g1535;Description=hypothetical protein FAGAP_6047 [Fusarium agapanthi];Gene=FCIRC_2962;Ontology_term=membrane;Ontology_id=GO:0016020
contig00002.g1536	3458	ID=contig00002.g1536;Description=hypothetical protein FVER53590_00165 [Fusarium verticillioides]

contig00002.g1537	1807	ID=contig00002.g1537;Description=tetracycline resistance (probable transport) [Fusarium tjaetaba];Gene=FNYG_06565;Ontology_term=transmembrane transport,transporter activity;Ontology_id=GO:0055085,GO:0005215
contig00002.g1538	978	ID=contig00002.g1538;Description=flavoprotein-like protein [Fusarium sp. MPI-SDFR-AT-0072];Gene=FVER53263_00159;Ontology_term=oxidoreductase activity;Ontology_id=GO:0016491;Enzyme_code=EC:1,EC:1;Enzyme_name=Oxidoreductases,Oxidoreductases
contig00002.g1539	1389	ID=contig00002.g1539;Description=heterokaryon incompatibility 6 OR allele [Fusarium tjaetaba]
contig00002.g1540	2302	ID=contig00002.g1540;Description=conidial development fluffy [Fusarium tjaetaba];Gene=FMUND_4090;Ontology_term=nucleus,regulation of DNA-templated transcription,transcription regulator activity;Ontology_id=GO:0005634,GO:0006355,GO:0140110
contig00002.g1541	1109	ID=contig00002.g1541;Description=endo-polygalacturonase 6 [Fusarium tjaetaba];Gene=FOYG_05915;Ontology_term=oxidoreductase activity;Ontology_id=GO:0016491;Enzyme_code=EC:1.3.1,EC:1.3,EC:1,EC:1.3.1;Enzyme_name=Acting on the CH-CH group of donors,Acting on the CH-CH group of donors,Oxidoreductases,Acting on the CH-CH group of donors
contig00002.g1542	1286	ID=contig00002.g1542;Description=hypothetical protein FVEG_14594 [Fusarium verticillioides 7600];Gene=FVEG_14594;Ontology_term=hydrolase activity;Ontology_id=GO:0016787;Enzyme_code=EC:3.2;Enzyme_name=Glycosylases
contig00002.g1543	1585	ID=contig00002.g1543;Description=related to neutral amino acid permease [Fusarium fujikuroi];Gene=FFUJ_14413;Ontology_term=membrane;Ontology_id=GO:0016020
contig00002.g1544	894	ID=contig00002.g1544;Description=related to dihydrodipicolinate synthase [Fusarium proliferatum ET1];Gene=FPRO_01947;Ontology_term=lyase activity;Ontology_id=GO:0016829;Enzyme_code=EC:4,EC:4;Enzyme_name=Lyases,Lyases
contig00002.g1545	2229	ID=contig00002.g1545;Description=ARG81-like transcription factor [Fusarium napiforme];Gene=FANTH_787;Ontology_term=nucleus,regulation of DNA-templated transcription,transcription regulator activity;Ontology_id=GO:0005634,GO:0006355,GO:0140110
contig00002.g1546	1225	ID=contig00002.g1546;Description=related to nucleoside-diphosphate-sugar epimerase [Fusarium fujikuroi]

contig00002.g1547	1551	ID=contig00002.g1547;Description=aldehyde dehydrogenase (NAD+) [Fusarium tjaetaba];Gene=FANTH_785;Ontology_term=oxidoreductase activity,amino acid metabolic process;Ontology_id=GO:0016491,GO:0006520;Enzyme_code=EC:1.2.1,EC:1.2,EC:1,EC:1.2.1.5,EC:1.2.1.3,EC:1.2.1;Enzyme_name=Acting on the aldehyde or oxo group of donors,Acting on the aldehyde or oxo group of donors,Oxidoreductases,aldehyde dehydrogenase [NAD(P)(+)],aldehyde dehydrogenase (NAD(+)),Acting on the aldehyde or oxo group of donors
contig00002.g1548	3108	ID=contig00002.g1548;Description=ankyrin protein [Fusarium tjaetaba]
contig00002.g1549	2566	ID=contig00002.g1549;Description=beta-1 2-xylosyltransferase 1 [Fusarium tjaetaba];Gene=FVER53263_00149;Ontology_term=transferase activity;Ontology_id=GO:0016740;Enzyme_code=EC:2,EC:2;Enzyme_name=Transferases,Transferases
contig00002.g1550	1558	ID=contig00002.g1550;Description=hypothetical protein FVER53590_00147 [Fusarium verticillioides];Gene=FVER53263_00147;Ontology_term=nucleus,regulation of DNA-templated transcription,transcription regulator activity;Ontology_id=GO:0005634,GO:0006355,GO:0140110
contig00002.g1551	777	ID=contig00002.g1551;Description=Alpha/Beta hydrolase protein [Fusarium redolens]
contig00002.g1552	1497	ID=contig00002.g1552;Description=neutral amino acid permease [Fusarium pseudoanthophilum];Gene=FPANT_2888;Ontology_term=membrane;Ontology_id=GO:0016020
contig00002.g1553	1803	ID=contig00002.g1553;Description=hypothetical protein FVER53590_00144 [Fusarium verticillioides]
contig00002.g1554	1821	ID=contig00002.g1554;Description=hypothetical protein FPANT_11311 [Fusarium pseudoanthophilum]
contig00002.g1555	447	ID=contig00002.g1555;Description=hypothetical protein FVEG_00142 [Fusarium verticillioides 7600]
contig00002.g1556	675	ID=contig00002.g1556;Description=Arylesterase [Fusarium oxysporum f. sp. albedinis]
contig00002.g1557	3772	ID=contig00002.g1557;Description=related to ankyrin [Fusarium oxysporum]
contig00002.g1558	2292	ID=contig00002.g1558;Description=hypothetical protein FVER53263_00138 [Fusarium verticillioides]
contig00002.g1559	1564	ID=contig00002.g1559;Description=nicotinamide mononucleotide permease [Fusarium tjaetaba];Gene=FPCIR_8748;Ontology_term=transmembrane transport,transporter activity;Ontology_id=GO:0055085,GO:0005215

contig00002.g1560	832	ID=contig00002.g1560;Description=SGNH hydrolase-type esterase domain-containing protein [Fusarium oxysporum Fo47];Gene=BFJ63_vAg14941;Ontology_term=hydrolase activity;Ontology_id=GO:0016787;Enzyme_code=EC:3;Enzyme_name=Hydrolases
contig00002.g1561	720	ID=contig00002.g1561;Description=hypothetical protein FVEG_00135 [Fusarium verticillioides 7600]
contig00002.g1562	1633	ID=contig00002.g1562;Description=hypothetical protein FVEG_00134 [Fusarium verticillioides 7600]
contig00002.g1563	2223	ID=contig00002.g1563;Description=hypothetical protein FVER53590_00133 [Fusarium verticillioides];Gene=FNYG_10202;Ontology_term=protein folding chaperone,ATP-dependent activity;Ontology_id=GO:0044183,GO:0140657
contig00002.g1564	630	ID=contig00002.g1564;Description=hypothetical protein FVEG_00132 [Fusarium verticillioides 7600]
contig00002.g1565	441	ID=contig00002.g1565;Description=hypothetical protein FVER53263_20152 [Fusarium verticillioides]
contig00002.g1566	2804	ID=contig00002.g1566;Description=heterokaryon incompatibility protein het-E-1 [Fusarium sp. NRRL 52700];Gene=FSARC_14038
contig00002.g1567	3147	ID=contig00002.g1567;Description=flavin-containing protein [Fusarium napiforme];Gene=FPANT_2974;Ontology_term=oxidoreductase activity;Ontology_id=GO:0016491;Enzyme_code=EC:1,EC:1;Enzyme_name=Oxidoreductases,Oxidoreductases
contig00002.g1568	4165	ID=contig00002.g1568;Description=ankyrin 3 [Fusarium fujikuroi]
contig00002.g1569	741	ID=contig00002.g1569;Description=heterokaryon incompatibility (HET) domain protein [Fusarium tjaetaba]
contig00002.g1570	3053	ID=contig00002.g1570;Description=nicotinamide mononucleotide permease [Fusarium phyllophilum];Gene=FPCIR_6758;Ontology_term=catalytic activity, acting on a protein,transmembrane transport,cytoskeleton,ATP-dependent activity,structural molecule activity,cytosol,transferase activity,transporter activity,cytoskeletal motor activity;Ontology_id=GO:0140096,GO:0055085,GO:0005856,GO:0140657,GO:0005198,GO:0005829,GO:0016740,GO:0005215,GO:0003774;Enzyme_code=EC:2.7.11.1,EC:5.6.1.8,EC:2.7.1,EC:2.7.11.1,EC:2.7,EC:2,EC:5.6.1.8;Enzyme_name=non-specific serine/threonine protein kinase,myosin ATPase,Transferring phosphorus-containing groups,non-specific serine/threonine protein kinase,Transferring phosphorus-containing groups,Transferases,myosin ATPase

contig00002.g1571	5212	ID=contig00002.g1571;Description=ankyrin repeat-containing protein [Fusarium napiforme];Gene=FPRO05_04073;Ontology_term=endosome,vacuole;Ontology_id=GO:0005768,GO:0005773
contig00002.g1572	1279	ID=contig00002.g1572;Description=hypothetical protein FVEG_00121 [Fusarium verticillioides 7600];Gene=FMUND_10823;Ontology_term=catalytic activity, acting on a protein,hydrolase activity;Ontology_id=GO:0140096,GO:0016787;Enzyme_code=EC:3.4;Enzyme_name=Acting on peptide bonds (peptidases)
contig00002.g1573	801	ID=contig00002.g1573;Description=hypothetical protein FVER53590_27551 [Fusarium verticillioides]
contig00002.g1574	441	ID=contig00002.g1574;Description=beta-glucosidase [Fusarium napiforme];Gene=FNAPI_12019;Ontology_term=carbohydrate metabolic process,hydrolase activity;Ontology_id=GO:0005975,GO:0016787;Enzyme_code=EC:3.2,EC:3.2,EC:3;Enzyme_name=Glycosylases,Glycosylases,Hydrolases
contig00002.g1575	1941	ID=contig00002.g1575;Description=hypothetical protein FVEG_00119 [Fusarium verticillioides 7600];Gene=FVEG_00119;Ontology_term=nucleus,regulation of DNA-templated transcription,transcription regulator activity;Ontology_id=GO:0005634,GO:0006355,GO:0140110
contig00002.g1576	2646	ID=contig00002.g1576;Description=beta-glucosidase [Fusarium verticillioides 7600];Gene=FVEG_00118;Ontology_term=carbohydrate metabolic process,hydrolase activity;Ontology_id=GO:0005975,GO:0016787;Enzyme_code=EC:3.2.1.21,EC:3.2,EC:3.2.1.21,EC:3,EC:3.2.1;Enzyme_name=beta-glucosidase,Glycosylases,beta-glucosidase,Hydrolases,Glycosylases
contig00002.g1577	1599	ID=contig00002.g1577;Description=sugar transporter [Fusarium pseudoanthophilum];Gene=FVEG_00117;Ontology_term=transmembrane transport,transporter activity;Ontology_id=GO:0055085,GO:0005215
contig00002.g1578	373	ID=contig00002.g1578;Description=cardiolipin synthase [Fusarium denticulatum];Gene=FCIRC_3028;Ontology_term=transmembrane transport,transporter activity,plasma membrane;Ontology_id=GO:0055085,GO:0005215,GO:0005886
contig00002.g1579	3033	ID=contig00002.g1579;Description=hypothetical protein QSH57_001898 [Fusarium oxysporum f. sp. vasinfectum]

contig00002.g1580	876	ID=contig00002.g1580;Description=maleylacetate reductase [Fusarium tjaetaba];Gene=FTJAE_6990;Ontology_term=oxidoreductase activity;Ontology_id=GO:0016491;Enzyme_code=EC:1.3.1.32,EC:1.3,EC:1,EC:1.3.1.32,EC:1.3.1;Enzyme_name=maleylacetate reductase,Acting on the CH-CH group of donors,Oxidoreductases,maleylacetate reductase,Acting on the CH-CH group of donors
contig00002.g1581	927	ID=contig00002.g1581;Description=related to haloacetate dehalogenase H-1 [Fusarium fujikuroi IMI 58289];Gene=CEK26_001799;Ontology_term=catalytic activity;Ontology_id=GO:0003824
contig00002.g1582	1410	ID=contig00002.g1582;Description=hypothetical protein J7337_001834 [Fusarium musae];Gene=FMUND_10831;Ontology_term=membrane;Ontology_id=GO:0016020
contig00002.g1583	654	ID=contig00002.g1583;Description=tpr domain-containing protein [Fusarium denticulatum]
contig00002.g1584	903	ID=contig00002.g1584;Description=TPR domain-containing protein [Fusarium phyllophilum]
contig00002.g1585	816	ID=contig00002.g1585;Description=TPR domain-containing protein [Fusarium pseudoanthophilum]
contig00002.g1586	1282	ID=contig00002.g1586;Description=hypothetical protein FVEG_00110 [Fusarium verticillioides 7600]
contig00002.g1587	4272	ID=contig00002.g1587;Description=fucose-specific lectin [Fusarium tjaetaba];Gene=FPANT_13589;Ontology_term=membrane,carbohydrate binding;Ontology_id=GO:0016020,GO:0030246
contig00002.g1588	1326	ID=contig00002.g1588;Description=hypothetical protein FVER14953_00107 [Fusarium verticillioides];Gene=FVEG_00107;Ontology_term=membrane;Ontology_id=GO:0016020
contig00002.g1589	1843	ID=contig00002.g1589;Description=beta transducin [Fusarium napiforme]
contig00002.g1590	2957	ID=contig00002.g1590;Description=zinc finger C2H2-type integrase DNA-binding protein [Fusarium napiforme];Gene=F25303_3169;Ontology_term=DNA binding,nucleobase-containing small molecule metabolic process,catalytic activity,carbohydrate derivative metabolic process;Ontology_id=GO:0003677,GO:0055086,GO:0003824,GO:1901135
contig00002.g1591	3050	ID=contig00002.g1591;Description=DNA repair recombination RAD5C [Fusarium denticulatum];Gene=F25303_3168;Ontology_term=small molecule binding;Ontology_id=GO:0036094

contig00002.g1592	792	ID=contig00002.g1592;Description=deoxyribose-phosphate aldolase [Fusarium verticillioides 7600];Gene=BFJ65_g883;Ontology_term=nucleobase-containing small molecule metabolic process,lyase activity,carbohydrate derivative metabolic process;Ontology_id=GO:0055086,GO:0016829,GO:1901135;Enzyme_code=EC:4.1.2.4,EC:4.1.2,EC:4.1,EC:4.1.2.4,EC:4;Enzyme_name=deoxyribose-phosphate aldolase,Carbon-carbon lyases,Carbon-carbon lyases,deoxyribose-phosphate aldolase,Lyases
contig00002.g1593	738	ID=contig00002.g1593;Description=arylsulfatase [Fusarium sp. NRRL 52700];Gene=F52700_286;Ontology_term=hydrolase activity;Ontology_id=GO:0016787;Enzyme_code=EC:3.1.6.1,EC:3.1.6,EC:3.1,EC:3,EC:3.1.6.1;Enzyme_name=arylsulfatase (type I),Acting on ester bonds,Acting on ester bonds,Hydrolases,arylsulfatase (type I)
contig00002.g1594	1288	ID=contig00002.g1594;Description=putative sugar transporter [Fusarium venenatum];Gene=FVER53590_00101;Ontology_term=transmembrane transport,transporter activity;Ontology_id=GO:0055085,GO:0005215
contig00002.g1595	1107	ID=contig00002.g1595;Description=NADPH2 dehydrogenase chain OYE2 [Fusarium mexicanum];Gene=FMUND_10840;Ontology_term=oxidoreductase activity,detoxification;Ontology_id=GO:0016491,GO:0098754;Enzyme_code=EC:1,EC:1,EC:1.6.99.1;Enzyme_name=Oxidoreductases,Oxidoreductases,NADPH dehydrogenase
contig00002.g1596	2907	ID=contig00002.g1596;Description=nucleoside diphosphate sugar epimerase [Fusarium tjaetaba]
contig00002.g1597	1489	ID=contig00002.g1597;Description=oviduct-specific glycoprotein [Fusarium heterosporum];Gene=FHETE_6047;Ontology_term=catalytic activity, acting on a protein,hydrolase activity;Ontology_id=GO:0140096,GO:0016787;Enzyme_code=EC:3.4,EC:3.4,EC:3;Enzyme_name=Acting on peptide bonds (peptidases),Acting on peptide bonds (peptidases),Hydrolases
contig00002.g1598	1423	ID=contig00002.g1598;Description=amino-acid oxidase [Fusarium subglutinans];Gene=FPANT_10602;Ontology_term=oxidoreductase activity;Ontology_id=GO:0016491;Enzyme_code=EC:1,EC:1;Enzyme_name=Oxidoreductases,Oxidoreductases
contig00002.g1599	1566	ID=contig00002.g1599;Description=hexose transporter [Fusarium pseudocircinatum];Gene=FVEG_00095;Ontology_term=transmembrane transport,transporter activity;Ontology_id=GO:0055085,GO:0005215
contig00002.g1600	10932	ID=contig00002.g1600;Description=pre-mRNA-splicing regulator WTAP [Fusarium tjaetaba]

contig00003.g1601	4578	ID=contig00003.g1601;Description=cutinase transcription factor 1 beta [Fusarium tjaetaba];Gene=FNYG_10220;Ontology_term=nucleus,DNA binding,oxidoreductase activity,regulation of DNA-templated transcription,transcription regulator activity;Ontology_id=GO:0005634,GO:0003677,GO:0016491,GO:0006355,GO:0140110;Enzyme_code=EC:1,EC:1;Enzyme_name=Oxidoreductases,Oxidoreductases
contig00003.g1602	1621	ID=contig00003.g1602;Description=aldehyde dehydrogenase [Fusarium pseudocircinatum];Gene=FPANT_13720;Ontology_term=oxidoreductase activity;Ontology_id=GO:0016491;Enzyme_code=EC:1.2.1,EC:1.2,EC:1,EC:1.2.1;Enzyme_name=Acting on the aldehyde or oxo group of donors,Acting on the aldehyde or oxo group of donors,Oxidoreductases,Acting on the aldehyde or oxo group of donors
contig00003.g1603	1977	ID=contig00003.g1603;Description=pisatin demethylase cytochrome P450 [Fusarium tjaetaba];Gene=FVEG_13835;Ontology_term=oxidoreductase activity,transferase activity;Ontology_id=GO:0016491,GO:0016740;Enzyme_code=EC:1.14,EC:2.1.1,EC:2.1,EC:1,EC:1.14,EC:1.14.13.176,EC:2,EC:2.1.1;Enzyme_name=Acting on paired donors, with incorporation or reduction of molecular oxygen. The oxygen incorporated need not be derived from O2,Transferring one-carbon groups,Transferring one-carbon groups,Oxidoreductases,Acting on paired donors, with incorporation or reduction of molecular oxygen. The oxygen incorporated need not be derived from O2,Acting on paired donors, with incorporation or reduction of molecular oxygen. The oxygen incorporated need not be derived from O2,Transferases,Transferring one-carbon groups
contig00003.g1604	918	ID=contig00003.g1604;Description=hypothetical protein FVER53590_13834 [Fusarium verticillioides]
contig00003.g1605	939	ID=contig00003.g1605;Description=hypothetical protein FVER53590_13833 [Fusarium verticillioides]
contig00003.g1606	1313	ID=contig00003.g1606;Description=hypothetical protein FVER14953_13832 [Fusarium verticillioides]
contig00003.g1607	1349	ID=contig00003.g1607;Description=G coupled receptor like [Fusarium tjaetaba];Gene=FNAPI_4040;Ontology_term=molecular transducer activity,signaling;Ontology_id=GO:0060089,GO:0023052
contig00003.g1608	1758	ID=contig00003.g1608;Description=myosin-crossreactive antigen [Fusarium verticillioides 7600];Gene=FNYG_10211;Ontology_term=lipid metabolic process,lyase activity;Ontology_id=GO:0006629,GO:0016829;Enzyme_code=EC:4.2.1.53,EC:4.2.1,EC:4.2,EC:4.2.1.53,EC:4;Enzyme_name=oleate hydratase,Carbon-oxygen lyases,Carbon-oxygen lyases,oleate hydratase,Lyases

contig00003.g1609	2282	ID=contig00003.g1609;Description=hypothetical protein FVER53263_20444 [Fusarium verticillioides];Gene=BFJ63_vAg10986;Ontology_term=nucleus,regulation of DNA-templated transcription,transcription regulator activity;Ontology_id=GO:0005634,GO:0006355,GO:0140110
contig00003.g1610	1106	ID=contig00003.g1610;Description=formaldehyde dehydrogenase [Fusarium tjaetaba];Gene=FOVG_15827;Ontology_term=metal ion binding;Ontology_id=GO:0046872
contig00003.g1611	1266	ID=contig00003.g1611;Description=globin-like protein [Fusarium oxysporum Fo47];Gene=FVER53590_13827;Ontology_term=oxidoreductase activity;Ontology_id=GO:0016491;Enzyme_code=EC:1.14.12.17,EC:1.14.12,EC:1.14.12.17,EC:1,EC:1.14;Enzyme_name=nitric oxide dioxygenase,Acting on paired donors, with incorporation or reduction of molecular oxygen. The oxygen incorporated need not be derived from O2,nitric oxide dioxygenase,Oxidoreductases,Acting on paired donors, with incorporation or reduction of molecular oxygen. The oxygen incorporated need not be derived from O2
contig00003.g1612	1023	ID=contig00003.g1612;Description=hypothetical protein J7337_012156 [Fusarium musae]
contig00003.g1613	1173	ID=contig00003.g1613;Description=sphingoid long-chain base transporter RSB1 [Fusarium tjaetaba];Gene=FocTR4_00014748;Ontology_term=membrane;Ontology_id=GO:0016020
contig00003.g1614	1548	ID=contig00003.g1614;Description=hypothetical protein J7337_012158 [Fusarium musae];Gene=FVEG_17616;Ontology_term=molecular transducer activity,nucleus,catalytic activity, acting on a protein,transmembrane transport,regulation of DNA-templated transcription,transferase activity,transcription regulator activity,transporter activity,plasma membrane;Ontology_id=GO:0060089,GO:0005634,GO:0140096,GO:0055085,GO:0006355,GO:0016740,GO:0140110,GO:0005215,GO:0005886;Enzyme_code=EC:2.7.11.23,EC:2.7.11.1,EC:2.7.11.22,EC:2.7.1,EC:2.7.11.23,EC:2.7.11.1,EC:2.7,EC:2,EC:2.7.11.22;Enzyme_name=[RNA-polymerase]-subunit kinase,non-specific serine/threonine protein kinase,cyclin-dependent kinase,Transferring phosphorus-containing groups,[RNA-polymerase]-subunit kinase,non-specific serine/threonine protein kinase,Transferring phosphorus-containing groups,Transferases,cyclin-dependent kinase
contig00003.g1615	1065	ID=contig00003.g1615;Description=hypothetical protein FVEG_17615 [Fusarium verticillioides 7600]
contig00003.g1616	1512	ID=contig00003.g1616;Description=Formyl-coenzyme A transferase [Fusarium tjaetaba];Gene=FVEG_13824;Ontology_term=transferase activity;Ontology_id=GO:0016740;Enzyme_code=EC:2,EC:2;Enzyme_name=Transferases,Transferases

contig00003.g1617	6193	ID=contig00003.g1617;Description=tannase [Fusarium verticillioides 7600];Gene=FVEG_13823;Ontology_term=nucleus,regulation of DNA-templated transcription,transcription regulator activity,hydrolase activity;Ontology_id=GO:0005634,GO:0006355,GO:0140110,GO:0016787;Enzyme_code=EC:3.1.1.73,EC:3.1,EC:3,EC:3.1.1.73,EC:3.1.1;Enzyme_name=feruloyl esterase,Acting on ester bonds,Hydrolases,feruloyl esterase,Acting on ester bonds
contig00003.g1618	1163	ID=contig00003.g1618;Description=alpha beta hydrolase fold-3 [Fusarium tjaetaba];Gene=FVEG_13821;Ontology_term=hydrolase activity;Ontology_id=GO:0016787;Enzyme_code=EC:3;Enzyme_name=Hydrolases
contig00003.g1619	1507	ID=contig00003.g1619;Description=DUF1479 domain protein [Fusarium subglutinans]
contig00003.g1620	921	ID=contig00003.g1620;Description=deacetylase [Fusarium napiforme];Gene=FVER53263_13819;Ontology_term=carbohydrate metabolic process,hydrolase activity;Ontology_id=GO:0005975,GO:0016787;Enzyme_code=EC:3.5,EC:3.5.1.104,EC:3.5,EC:3;Enzyme_name=Acting on carbon-nitrogen bonds, other than peptide bonds,peptidoglycan-N-acetylglucosamine deacetylase,Acting on carbon-nitrogen bonds, other than peptide bonds,Hydrolases
contig00003.g1621	532	ID=contig00003.g1621;Description=hypothetical protein FVEG_13818 [Fusarium verticillioides 7600]
contig00003.g1622	2679	ID=contig00003.g1622;Description=glycoside hydrolase family 2 [Fusarium coicis];Gene=FVER53590_13817;Ontology_term=carbohydrate metabolic process,hydrolase activity;Ontology_id=GO:0005975,GO:0016787;Enzyme_code=EC:3.2.1.165,EC:3.2,EC:3.2.1.165,EC:3,EC:3.2.1;Enzyme_name=exo-1,4-beta-D-glucosaminidase,Glycosylases,exo-1,4-beta-D-glucosaminidase,Hydrolases,Glycosylases
contig00003.g1623	1755	ID=contig00003.g1623;Description=trichothecene efflux pump [Fusarium tjaetaba];Gene=CEK26_003656;Ontology_term=transmembrane transport,transporter activity;Ontology_id=GO:0055085,GO:0005215
contig00003.g1624	1107	ID=contig00003.g1624;Description=aryl-alcohol dehydrogenase AAD14 [Fusarium verticillioides 7600]
contig00003.g1625	976	ID=contig00003.g1625;Description=duf1446 domain protein [Fusarium tjaetaba]
contig00003.g1626	875	ID=contig00003.g1626;Description=endo alpha polygalactosaminidase precursor [Fusarium tjaetaba];Gene=FMEXI_2332;Ontology_term=hydrolase activity;Ontology_id=GO:0016787;Enzyme_code=EC:3,EC:3;Enzyme_name=Hydrolases,Hydrolases

contig00003.g1627	938	ID=contig00003.g1627;Description=s-adenosyl-L-methionine-dependent methyltransferase [Fusarium tjaetaba];Gene=FVEG_13812;Ontology_term=transferase activity;Ontology_id=GO:0016740;Enzyme_code=EC:2.1.1,EC:2.1,EC:2,EC:2.1.1;Enzyme_name=Transferring one-carbon groups,Transferring one-carbon groups,Transferases,Transferring one-carbon groups
contig00003.g1628	1234	ID=contig00003.g1628;Description=alkaline protease (oryzin) [Fusarium tjaetaba];Gene=FVER53263_13811;Ontology_term=catalytic activity, acting on a protein,hydrolase activity;Ontology_id=GO:0140096,GO:0016787;Enzyme_code=EC:3.4.21,EC:3.4.21,EC:3.4,EC:3;Enzyme_name=Acting on peptide bonds (peptidases),Acting on peptide bonds (peptidases),Acting on peptide bonds (peptidases),Hydrolases
contig00003.g1629	1368	ID=contig00003.g1629;Description=hypothetical protein FVER53590_13810 [Fusarium verticillioides]
contig00003.g1630	940	ID=contig00003.g1630;Description=integral membrane protein PTH11 [Fusarium fujikuroi];Gene=FMAN_13108;Ontology_term=membrane;Ontology_id=GO:0016020
contig00003.g1631	1942	ID=contig00003.g1631;Description=Hybrid PKS-NRPS synthetase apdA [Fusarium oxysporum f. sp. albedinis];Gene=F25303_839;Ontology_term=transmembrane transport,transporter activity;Ontology_id=GO:0055085,GO:0005215
contig00003.g1632	611	ID=contig00003.g1632;Description=scytalone dehydratase [Fusarium tjaetaba];Gene=SDH1-1;Ontology_term=lyase activity;Ontology_id=GO:0016829;Enzyme_code=EC:4.2.1.94,EC:4.2.1.94,EC:4.2.1,EC:4.2,EC:4;Enzyme_name=scytalone dehydratase,scytalone dehydratase,Carbon-oxygen lyases,Carbon-oxygen lyases,Lyases
contig00003.g1633	897	ID=contig00003.g1633;Description=peroxisomal short-chain alcohol dehydrogenase [Fusarium acutatum];Gene=BFJ72_g13759;Ontology_term=oxidoreductase activity;Ontology_id=GO:0016491;Enzyme_code=EC:1,EC:1;Enzyme_name=Oxidoreductases,Oxidoreductases
contig00003.g1634	813	ID=contig00003.g1634;Description=chitinase [Fusarium tjaetaba];Gene=FTJAE_10159;Ontology_term=catalytic activity, acting on a protein,transferase activity;Ontology_id=GO:0140096,GO:0016740;Enzyme_code=EC:2.3.2,EC:2.3,EC:2,EC:2.3.2;Enzyme_name=Acyltransferases,Acyltransferases,Transferases,Acyltransferases
contig00003.g1635	1110	ID=contig00003.g1635;Description=hypothetical protein FVER53263_13805 [Fusarium verticillioides]

contig00003.g1636	1249	ID=contig00003.g1636;Description=hypothetical protein FVER53263_13804 [Fusarium verticillioides];Gene=CEK26_001979;Ontology_term=membrane;Ontology_id=GO:0016020
contig00003.g1637	1014	ID=contig00003.g1637;Description=alpha beta-hydrolase [Fusarium pseudoanthophilum];Gene=LW93_10125;Ontology_term=carbohydrate metabolic process,receptor ligand activity,extracellular region,hydrolase activity;Ontology_id=GO:0005975,GO:0048018,GO:0005576,GO:0016787;Enzyme_code=EC:3,EC:3;Enzyme_name=Hydrolases,Hydrolases
contig00003.g1638	2277	ID=contig00003.g1638;Description=sterigmatocystin biosynthesis monooxygenase stcW [Fusarium pseudocircinatum];Gene=FPCIR_13558;Ontology_term=oxidoreductase activity;Ontology_id=GO:0016491;Enzyme_code=EC:1,EC:1;Enzyme_name=Oxidoreductases,Oxidoreductases
contig00003.g1639	1327	ID=contig00003.g1639;Description=alcohol sorbitol dehydrogenase [Fusarium globosum];Gene=P10489;Ontology_term=cytoskeleton,oxidoreductase activity,hydrolase activity;Ontology_id=GO:0005856,GO:0016491,GO:0016787;Enzyme_code=EC:1,EC:3,EC:1.1.1.4,EC:1.1,EC:1.1.1.303,EC:1,EC:3,EC:1.1.1;Enzyme_name=Oxidoreductases,Hydrolases,(R,R)-butanediol dehydrogenase,Acting on the CH-OH group of donors,diacetyl reductase [(R)-acetoin forming],Oxidoreductases,Hydrolases,Acting on the CH-OH group of donors
contig00003.g1640	1183	ID=contig00003.g1640;Description=NAD(P)-binding protein [Fusarium tjaetaba];Ontology_term=lipid droplet,oxidoreductase activity,mitochondrion,protein maturation;Ontology_id=GO:0005811,GO:0016491,GO:0005739,GO:0051604;Enzyme_code=EC:1,EC:1.1.1.300;Enzyme_name=Oxidoreductases,NADP-retinol dehydrogenase
contig00003.g1641	2271	ID=contig00003.g1641;Description=copper amine oxidase 1 [Fusarium tjaetaba];Gene=FMUND_3852;Ontology_term=oxidoreductase activity;Ontology_id=GO:0016491;Enzyme_code=EC:1.4.3.21,EC:1.4,EC:1.4.3,EC:1,EC:1.4.3.21;Enzyme_name=primary-amine oxidase,Acting on the CH-NH2 group of donors,Acting on the CH-NH2 group of donors,Oxidoreductases,primary-amine oxidase
contig00003.g1642	2576	ID=contig00003.g1642;Description=putative transcriptional regulatory protein [Fusarium oxysporum f. sp. conglutinans];Gene=FVER53263_13797;Ontology_term=nucleus,DNA binding,DNA-templated transcription;Ontology_id=GO:0005634,GO:0003677,GO:0006351
contig00003.g1643	1560	ID=contig00003.g1643;Description=related to permease of the major facilitator superfamily [Fusarium proliferatum ET1];Gene=FGLOB1_6656;Ontology_term=transmembrane transport,transporter activity;Ontology_id=GO:0055085,GO:0005215

contig00003.g1644	1215	ID=contig00003.g1644;Description=hypothetical protein FVEG_17607 [Fusarium verticillioides 7600];Gene=FOYG_14020;Ontology_term=membrane;Ontology_id=GO:0016020
contig00003.g1645	1812	ID=contig00003.g1645;Description=alcohol dehydrogenase [Fusarium verticillioides 7600];Gene=FACUT_12802;Ontology_term=oxidoreductase activity,lipid metabolic process;Ontology_id=GO:0016491,GO:0006629;Enzyme_code=EC:1.1.1,EC:1.1,EC:1,EC:1.1.1;Enzyme_name=Acting on the CH-OH group of donors,Acting on the CH-OH group of donors,Oxidoreductases,Acting on the CH-OH group of donors
contig00003.g1646	1326	ID=contig00003.g1646;Description=cytochrome-b5 reductase [Fusarium denticulatum];Gene=FDENT_2934;Ontology_term=nucleus,oxidoreductase activity,mitochondrion,regulation of DNA-templated transcription,transcription regulator activity;Ontology_id=GO:0005634,GO:0016491,GO:0005739,GO:0006355,GO:0140110;Enzyme_code=EC:1,EC:1;Enzyme_name=Oxidoreductases,Oxidoreductases
contig00003.g1647	1406	ID=contig00003.g1647;Description=cytochrome-b5 reductase [Fusarium verticillioides 7600];Gene=FACUT_12804;Ontology_term=oxidoreductase activity,mitochondrion;Ontology_id=GO:0016491,GO:0005739;Enzyme_code=EC:1;Enzyme_name=Oxidoreductases
contig00003.g1648	1759	ID=contig00003.g1648;Description=cytochrome P450 oxidoreductase [Fusarium verticillioides 7600];Gene=FVER53263_13789;Ontology_term=transmembrane transport,oxidoreductase activity,transporter activity;Ontology_id=GO:0055085,GO:0016491,GO:0005215;Enzyme_code=EC:1.14,EC:1,EC:1.14;Enzyme_name=Acting on paired donors, with incorporation or reduction of molecular oxygen. The oxygen incorporated need not be derived from O2,Oxidoreductases,Acting on paired donors, with incorporation or reduction of molecular oxygen. The oxygen incorporated need not be derived from O2
contig00003.g1649	2840	ID=contig00003.g1649;Description=cupin domain protein [Fusarium tjaetaba];Gene=FIE12Z_1723;Ontology_term=oxidoreductase activity;Ontology_id=GO:0016491;Enzyme_code=EC:1;Enzyme_name=Oxidoreductases
contig00003.g1650	3766	ID=contig00003.g1650;Description=related to 1-phosphatidylinositol-4,5-bisphosphate phosphodiesterase [Fusarium proliferatum ET1];Gene=CEK26_001997;Ontology_term=lipid metabolic process,signaling,hydrolase activity;Ontology_id=GO:0006629,GO:0023052,GO:0016787;Enzyme_code=EC:3.1.4.11,EC:3.1.4,EC:3.1,EC:3,EC:3.1.4.11;Enzyme_name=phosphoinositide phospholipase C,Acting on ester bonds,Acting on ester bonds,Hydrolases,phosphoinositide phospholipase C

contig00003.g1651	2825	ID=contig00003.g1651;Description=thermostable alkaline protease precursor [Fusarium phyllophilum];Gene=FVER53263_13778;Ontology_term=catalytic activity, acting on a protein,hydrolase activity;Ontology_id=GO:0140096,GO:0016787;Enzyme_code=EC:3.4.21,EC:3.4.21,EC:3.4,EC:3;Enzyme_name=Acting on peptide bonds (peptidases),Acting on peptide bonds (peptidases),Acting on peptide bonds (peptidases),Hydrolases
contig00003.g1652	831	ID=contig00003.g1652;Description=hypothetical protein FVER53590_13777 [Fusarium verticillioides]
contig00003.g1653	1121	ID=contig00003.g1653;Description=Tyrosinase [Fusarium tjaetaba];Gene=FPANT_3372;Ontology_term=oxidoreductase activity;Ontology_id=GO:0016491;Enzyme_code=EC:1,EC:1,EC:1.14.18.1;Enzyme_name=Oxidoreductases,Oxidoreductases,tyrosinase
contig00003.g1654	1204	ID=contig00003.g1654;Description=related to formaldehyde dehydrogenase [Fusarium proliferatum ET1];Gene=FCIRC_6525;Ontology_term=oxidoreductase activity;Ontology_id=GO:0016491;Enzyme_code=EC:1,EC:1;Enzyme_name=Oxidoreductases,Oxidoreductases
contig00003.g1655	903	ID=contig00003.g1655;Description=short chain dehydrogenase reductase family [Fusarium denticulatum]
contig00003.g1656	2418	ID=contig00003.g1656;Description=hypothetical protein FVER53590_25111 [Fusarium verticillioides];Gene=FMEXI_2298;Ontology_term=antioxidant activity,oxidoreductase activity;Ontology_id=GO:0016209,GO:0016491;Enzyme_code=EC:1.11.1,EC:1.11,EC:1,EC:1.11.1;Enzyme_name=Acting on a peroxide as acceptor,Acting on a peroxide as acceptor,Oxidoreductases,Acting on a peroxide as acceptor
contig00003.g1657	1545	ID=contig00003.g1657;Description=ankyrin [Fusarium tjaetaba];Gene=FNAPI_4088;Ontology_term=antioxidant activity,oxidoreductase activity;Ontology_id=GO:0016209,GO:0016491;Enzyme_code=EC:1.11.1,EC:1.11,EC:1,EC:1.11.1;Enzyme_name=Acting on a peroxide as acceptor,Acting on a peroxide as acceptor,Oxidoreductases,Acting on a peroxide as acceptor
contig00003.g1658	777	ID=contig00003.g1658;Description=hypothetical protein FVER53590_13770 [Fusarium verticillioides]
contig00003.g1659	1419	ID=contig00003.g1659;Description=ankyrin repeat-containing domain protein [Fusarium oxysporum f. sp. albedinis]
contig00003.g1660	1701	ID=contig00003.g1660;Description=ankyrin repeat-containing domain protein [Fusarium oxysporum]

contig00003.g1661	846	ID=contig00003.g1661;Description=hypothetical protein FVEG_13767 [Fusarium verticillioides 7600]
contig00003.g1662	2108	ID=contig00003.g1662;Description=hypothetical protein FNYG_10564 [Fusarium nygamai];Gene=FVER53590_13766;Ontology_term=membrane;Ontology_id=GO:0016020
contig00003.g1663	1323	ID=contig00003.g1663;Description=hypothetical protein FVER53590_13765 [Fusarium verticillioides]
contig00003.g1664	3553	ID=contig00003.g1664;Description=chitinase [Fusarium globosum];Gene=FIE12Z_12577;Ontology_term=carbohydrate metabolic process,carbohydrate derivative metabolic process,extracellular region,hydrolase activity;Ontology_id=GO:0005975,GO:1901135,GO:0005576,GO:0016787;Enzyme_code=EC:3.2.1.14,EC:3.2,EC:3,EC:3.2.1.14,EC:3.2.1;Enzyme_name=chitinase,Glycosylases,Hydrolases,chitinase,Glycosylases
contig00003.g1665	765	ID=contig00003.g1665;Description=hypothetical protein FVER53590_25113 [Fusarium verticillioides]
contig00003.g1666	795	ID=contig00003.g1666;Description=uncharacterized protein FMAN_13156 [Fusarium mangiferae]
contig00003.g1667	1982	ID=contig00003.g1667;Description=activator of stress 1 [Fusarium subglutinans]
contig00003.g1668	842	ID=contig00003.g1668;Description=hypothetical protein FVER53590_13762 [Fusarium verticillioides]
contig00003.g1669	1368	ID=contig00003.g1669;Description=hypothetical protein FVER53590_13761 [Fusarium verticillioides];Gene=FSUBG_7482;Ontology_term=hydrolase activity;Ontology_id=GO:0016787;Enzyme_code=EC:3.1.3,EC:3.1,EC:3,EC:3.1.3;Enzyme_name=Acting on ester bonds,Acting on ester bonds,Hydrolases,Acting on ester bonds
contig00003.g1670	3146	ID=contig00003.g1670;Description=hypothetical protein FVEG_13760 [Fusarium verticillioides 7600]
contig00003.g1671	967	ID=contig00003.g1671;Description=glycosyl hydrolase family 17 [Fusarium verticillioides 7600];Gene=FNYG_10572;Ontology_term=carbohydrate metabolic process,hydrolase activity;Ontology_id=GO:0005975,GO:0016787;Enzyme_code=EC:3.2.1;Enzyme_name=Glycosylases

contig00003.g1672	1901	ID=contig00003.g1672;Description=adenylosuccinate lyase [Fusarium tjaetaba];Gene=FVEG_13758;Ontology_term=nucleobase-containing small molecule metabolic process,lyase activity,carbohydrate derivative metabolic process;Ontology_id=GO:0055086,GO:0016829,GO:1901135;Enzyme_code=EC:4.3.2.2,EC:4.3,EC:4.3.2.2,EC:4,EC:4.3.2;Enzyme_name=adenylosuccinate lyase,Carbon-nitrogen lyases,adenylosuccinate lyase,Lyases,Carbon-nitrogen lyases
contig00003.g1673	774	ID=contig00003.g1673;Description=Rhamnogalacturonan acetylesterase rhgT [Fusarium acutatum];Gene=FNYG_10574;Ontology_term=hydrolase activity;Ontology_id=GO:0016787;Enzyme_code=EC:3,EC:3;Enzyme_name=Hydrolases,Hydrolases
contig00003.g1674	3055	ID=contig00003.g1674;Description=ent-kaurene oxidase [Fusarium pseudocircinatum];Gene=FMEXI_14380;Ontology_term=oxidoreductase activity;Ontology_id=GO:0016491;Enzyme_code=EC:1.14;Enzyme_name=Acting on paired donors, with incorporation or reduction of molecular oxygen. The oxygen incorporated need not be derived from O2
contig00003.g1675	1608	ID=contig00003.g1675;Description=hypothetical protein FVEG_13754 [Fusarium verticillioides 7600]
contig00003.g1676	1887	ID=contig00003.g1676;Description=het-6-heterokaryon incompatibility [Fusarium coicis]
contig00003.g1677	654	ID=contig00003.g1677;Description=ankyrin unc44 [Fusarium subglutinans];Gene=FOXB_17760;Ontology_term=transport;Ontology_id=GO:0006810
contig00003.g1678	525	ID=contig00003.g1678;Description=ankyrin unc44 [Fusarium subglutinans]
contig00003.g1679	309	ID=contig00003.g1679;Description=related to ankyrin [Fusarium proliferatum ET1];Gene=FPRO_13806;Ontology_term=transmembrane transport,transporter activity;Ontology_id=GO:0055085,GO:0005215
contig00003.g1680	3383	ID=contig00003.g1680;Description=related to small s protein [Fusarium proliferatum];Gene=FDENT_2965;Ontology_term=transmembrane transport,transporter activity;Ontology_id=GO:0055085,GO:0005215
contig00003.g1681	2254	ID=contig00003.g1681;Description=primary-amine oxidase [Fusarium verticillioides 7600];Gene=FVER53590_13749;Ontology_term=oxidoreductase activity;Ontology_id=GO:0016491;Enzyme_code=EC:1.4.3.21,EC:1.4,EC:1.4.3,EC:1,EC:1.4.3.21;Enzyme_name=primary-amine oxidase,Acting on the CH-NH2 group of donors,Acting on the CH-NH2 group of donors,Oxidoreductases,primary-amine oxidase

contig00003.g1682	1645	ID=contig00003.g1682;Description=choline permease [Fusarium tjaetaba];Gene=BFJ63_vAg14610;Ontology_term=transmembrane transport,transporter activity;Ontology_id=GO:0055085,GO:0005215
contig00003.g1683	1417	ID=contig00003.g1683;Description=integral membrane protein [Fusarium fujikuroi];Gene=FVEG_13747;Ontology_term=chromosome;Ontology_id=GO:0005694
contig00003.g1684	808	ID=contig00003.g1684;Description=hypothetical protein FVEG_13746 [Fusarium verticillioides 7600]
contig00003.g1685	4357	ID=contig00003.g1685;Description=hypothetical protein FVER14953_20284 [Fusarium verticillioides]
contig00003.g1686	1688	ID=contig00003.g1686;Description=4-hydroxybenzoate benzoate ligase [Fusarium tjaetaba];Gene=FNAPI_1680;Ontology_term=ligase activity;Ontology_id=GO:0016874;Enzyme_code=EC:6,EC:6;Enzyme_name=Ligases,Ligases
contig00003.g1687	1122	ID=contig00003.g1687;Description=Putative oxidoreductase bli-4, mitochondrial [Fusarium odoratissimum]
contig00003.g1688	1608	ID=contig00003.g1688;Description=RCO3 glucose transporter [Fusarium tjaetaba];Gene=FNYG_10594;Ontology_term=transmembrane transport,transporter activity;Ontology_id=GO:0055085,GO:0005215
contig00003.g1689	1161	ID=contig00003.g1689;Description=heterokaryon incompatibility protein [Fusarium globosum]
contig00003.g1690	1656	ID=contig00003.g1690;Description=hypothetical protein FVER14953_13739 [Fusarium verticillioides]
contig00003.g1691	1165	ID=contig00003.g1691;Description=hypothetical protein FVER53263_13738 [Fusarium verticillioides];Gene=FVER53590_13738;Ontology_term=membrane;Ontology_id=GO:0016020
contig00003.g1692	1737	ID=contig00003.g1692;Description=hypothetical protein FVER53590_13737 [Fusarium verticillioides];Gene=FFUJ_10316;Ontology_term=nucleus,regulation of DNA-templated transcription,transcription regulator activity;Ontology_id=GO:0005634,GO:0006355,GO:0140110
contig00003.g1693	1068	ID=contig00003.g1693;Description=hypothetical protein FVEG_13736 [Fusarium verticillioides 7600]
contig00003.g1694	3651	ID=contig00003.g1694;Description=heterokaryon incompatibility protein het-E-1 [Fusarium coicis];Gene=FPRO05_00824;Ontology_term=nucleobase-containing small molecule metabolic process,catalytic activity,carbohydrate derivative metabolic process;Ontology_id=GO:0055086,GO:0003824,GO:1901135

contig00003.g1695	1273	ID=contig00003.g1695;Description=ToxD-like protein [Fusarium mundagurra];Gene=FMUND_3898;Ontology_term=oxidoreductase activity;Ontology_id=GO:0016491;Enzyme_code=EC:1.6;Enzyme_name=Acting on NADH or NADPH
contig00003.g1696	1214	ID=contig00003.g1696;Description=arginase [Fusarium denticulatum];Gene=FDENT_13665
contig00003.g1697	2062	ID=contig00003.g1697;Description=het-6OR heterokaryon incompatibility (het-6OR allele) [Fusarium pseudocircinatum]
contig00003.g1698	1149	ID=contig00003.g1698;Description=ToxD-like protein [Fusarium tjaetaba];Gene=FVER53263_13732;Ontology_term=oxidoreductase activity;Ontology_id=GO:0016491;Enzyme_code=EC:1.6,EC:1.6,EC:1;Enzyme_name=Acting on NADH or NADPH,Acting on NADH or NADPH,Oxidoreductases
contig00003.g1699	2535	ID=contig00003.g1699;Description=hypothetical protein FVER14953_13731 [Fusarium verticillioides]
contig00003.g1700	1089	ID=contig00003.g1700;Description=glycerol dehydrogenase [Fusarium coicis];Gene=FVER53590_13730;Ontology_term=oxidoreductase activity;Ontology_id=GO:0016491;Enzyme_code=EC:1,EC:1,EC:1.1.1.2;Enzyme_name=Oxidoreductases, Oxidoreductases,alcohol dehydrogenase (NADP(+))
contig00003.g1701	1627	ID=contig00003.g1701;Description=N amino acid transport system protein [Fusarium oxysporum f. sp. conglutinans];Gene=F66182_5223;Ontology_term=membrane;Ontology_id=GO:0016020
contig00003.g1702	890	ID=contig00003.g1702;Description=hypothetical protein FVER53263_13728 [Fusarium verticillioides]
contig00003.g1703	1635	ID=contig00003.g1703;Description=Zn(2)-C6 fungal-type DNA-binding domain protein [Fusarium subglutinans];Gene=FVER53590_13727;Ontology_term=nucleus,DNA binding,regulation of DNA-templated transcription,transcription regulator activity;Ontology_id=GO:0005634,GO:0003677,GO:0006355,GO:0140110
contig00003.g1704	933	ID=contig00003.g1704;Description=RTA1-like protein [Fusarium napiforme];Gene=FNAPI_1673;Ontology_term=membrane;Ontology_id=GO:0016020
contig00003.g1705	2880	ID=contig00003.g1705;Description=hypothetical protein FCOIX_11811 [Fusarium coicis]
contig00003.g1706	3637	ID=contig00003.g1706;Description=related to small s protein [Fusarium proliferatum ET1]
contig00003.g1707	665	ID=contig00003.g1707;Description=calcium-independent phospholipase A2 [Fusarium phyllophilum];Gene=FPHYL_1411;Ontology_term=lipid metabolic process;Ontology_id=GO:0006629

contig00003.g1708	384	ID=contig00003.g1708;Description=Nephrocystin-3 [Fusarium oxysporum f. sp. conglutinans];Gene=Pnpla8-3;Ontology_term=lipid metabolic process,hydrolase activity;Ontology_id=GO:0006629,GO:0016787;Enzyme_code=EC:3,EC:3;Enzyme_name=Hydrolases,Hydrolases
contig00003.g1709	978	ID=contig00003.g1709;Description=uncharacterized protein FTJAE_4099 [Fusarium tjaetaba];Gene=F25303_6601;Ontology_term=membrane;Ontology_id=GO:0016020
contig00003.g1710	1665	ID=contig00003.g1710;Description=hypothetical protein FVER53263_13721 [Fusarium verticillioides]
contig00003.g1711	1122	ID=contig00003.g1711;Description=Alkylglycerol monooxygenase [Fusarium tjaetaba];Gene=FTJAE_4097;Ontology_term=oxidoreductase activity,lipid metabolic process;Ontology_id=GO:0016491,GO:0006629;Enzyme_code=EC:1,EC:1;Enzyme_name=Oxidoreductases,Oxidoreductases
contig00003.g1712	3897	ID=contig00003.g1712;Description=Alkylglycerol monooxygenase [Fusarium tjaetaba];Gene=FTJAE_4097;Ontology_term=oxidoreductase activity,lipid metabolic process;Ontology_id=GO:0016491,GO:0006629;Enzyme_code=EC:1,EC:1;Enzyme_name=Oxidoreductases,Oxidoreductases
contig00003.g1713	1232	ID=contig00003.g1713;Description=integral membrane protein [Fusarium pseudocircinatum];Gene=FVER53590_13718;Ontology_term=membrane;Ontology_id=GO:0016020
contig00003.g1714	1643	ID=contig00003.g1714;Description=hypothetical protein FVER14953_13717 [Fusarium verticillioides];Gene=FPANT_6560;Ontology_term=membrane;Ontology_id=GO:0016020
contig00003.g1715	774	ID=contig00003.g1715;Description=Methyltransferase [Fusarium tjaetaba];Gene=FVER53590_13716;Ontology_term=transferase activity;Ontology_id=GO:0016740;Enzyme_code=EC:2.1.1,EC:2.1,EC:2,EC:2.1.1;Enzyme_name=Transferring one-carbon groups,Transferring one-carbon groups,Transferases,Transferring one-carbon groups

contig00003.g1716	7893	ID=contig00003.g1716;Description=polyketide synthase [Fusarium tjaetaba];Gene=FPANT_6562;Ontology_term=oxidoreductase activity,lipid metabolic process,transferase activity;Ontology_id=GO:0016491,GO:0006629,GO:0016740;Enzyme_code=EC:2.3.1.41,EC:1,EC:2.1.1,EC:2.3.1.41,EC:2.1,EC:2.3,EC:1,EC:2,EC:2.1.1,EC:2.3.1;Enzyme_name=beta-ketoacyl-[acyl-carrier-protein] synthase I,Oxidoreductases,Transferring one-carbon groups,beta-ketoacyl-[acyl-carrier-protein] synthase I,Transferring one-carbon groups,Acyltransferases,Oxidoreductases,Transferases,Transferring one-carbon groups,Acyltransferases
contig00003.g1717	774	ID=contig00003.g1717;Description=probable pectin lyase precursor [Fusarium fujikuroi];Gene=FOMG_13384;Ontology_term=carbohydrate metabolic process,lyase activity,extracellular region;Ontology_id=GO:0005975,GO:0016829,GO:0005576;Enzyme_code=EC:4.2.2.2,EC:4.2.2.2,EC:4.2,EC:4.2.2.10,EC:4,EC:4.2.2;Enzyme_name=pectate lyase,pectate lyase,Carbon-oxygen lyases,pectin lyase,Lyases,Carbon-oxygen lyases
contig00003.g1718	405	ID=contig00003.g1718;Description=probable pectin lyase precursor [Fusarium fujikuroi IMI 58289];Gene=FFUJ_10348;Ontology_term=carbohydrate metabolic process,lyase activity,extracellular region;Ontology_id=GO:0005975,GO:0016829,GO:0005576;Enzyme_code=EC:4.2.2.2,EC:4.2.2.2,EC:4.2,EC:4.2.2.10,EC:4,EC:4.2.2;Enzyme_name=pectate lyase,pectate lyase,Carbon-oxygen lyases,pectin lyase,Lyases,Carbon-oxygen lyases
contig00003.g1719	2138	ID=contig00003.g1719;Description=transcriptional regulatory [Fusarium tjaetaba];Gene=FNAPI_6676;Ontology_term=nucleus,regulation of DNA-templated transcription,transcription regulator activity;Ontology_id=GO:0005634,GO:0006355,GO:0140110
contig00003.g1720	1940	ID=contig00003.g1720;Description=hypothetical protein FOXG_16518 [Fusarium oxysporum f. sp. lycopersici 4287];Gene=FOTG_13707;Ontology_term=transmembrane transport,transporter activity;Ontology_id=GO:0055085,GO:0005215
contig00003.g1721	926	ID=contig00003.g1721;Description=hypothetical protein FVEG_13708 [Fusarium verticillioides 7600]
contig00003.g1722	1257	ID=contig00003.g1722;Description=isoprenoid synthase domain-containing protein [Fusarium oxysporum Fo47]
contig00003.g1723	660	ID=contig00003.g1723;Description=hypothetical protein FVEG_13706 [Fusarium verticillioides 7600]

contig00003.g1724	1907	ID=contig00003.g1724;Description=hypothetical protein H9Q71_005508 [Fusarium xylarioides]
contig00003.g1725	1632	ID=contig00003.g1725;Description=hypothetical protein FVER53590_13704 [Fusarium verticillioides]
contig00003.g1726	3174	ID=contig00003.g1726;Description=serine protease [Fusarium mundagurra];Gene=FVEG_13703;Ontology_term=nucleus,catalytic activity, acting on a protein,protein catabolic process,lipid metabolic process,programmed cell death,hydrolase activity;Ontology_id=GO:0005634,GO:0140096,GO:0030163,GO:0006629,GO:0012501,GO:0016787;Enzyme_code=EC:3.4.21,EC:3.4.21,EC:3.4,EC:3;Enzyme_name=Acting on peptide bonds (peptidases),Acting on peptide bonds (peptidases),Acting on peptide bonds (peptidases),Hydrolases
contig00003.g1727	1601	ID=contig00003.g1727;Description=cytochrome P450 monooxygenase 4F5 [Fusarium subglutinans];Gene=FVEG_13702;Ontology_term=oxidoreductase activity;Ontology_id=GO:0016491;Enzyme_code=EC:1.14;Enzyme_name=Acting on paired donors, with incorporation or reduction of molecular oxygen. The oxygen incorporated need not be derived from O2
contig00003.g1728	1297	ID=contig00003.g1728;Description=serine threonine kinase Sgk2 [Fusarium tjaetaba];Gene=FDENT_4199
contig00003.g1729	3825	ID=contig00003.g1729;Description=RNA helicase [Fusarium coicis];Gene=FVER53263_13700;Ontology_term=RNA binding,mitochondrial gene expression,mRNA metabolic process,ATP-dependent activity,mitochondrion,catalytic activity, acting on RNA,nucleolus,hydrolase activity;Ontology_id=GO:0003723,GO:0140053,GO:0016071,GO:0140657,GO:0005739,GO:0140098,GO:0005730,GO:0016787;Enzyme_code=EC:3,EC:3.6.1,EC:3.6.4.13,EC:3.6,EC:3,EC:3.6.1.15;Enzyme_name=Hydrolases,Acting on acid anhydrides,RNA helicase,Acting on acid anhydrides,Hydrolases,nucleoside-triphosphate phosphatase
contig00003.g1730	4146	ID=contig00003.g1730;Description=serine/threonine protein kinase [Fusarium verticillioides 7600];Gene=FMUND_9308;Ontology_term=catalytic activity, acting on a protein,transferase activity;Ontology_id=GO:0140096,GO:0016740;Enzyme_code=EC:2.7.11.1,EC:2.7.1,EC:2.7.11.1,EC:2.7,EC:2;Enzyme_name=non-specific serine/threonine protein kinase,Transferring phosphorus-containing groups,non-specific serine/threonine protein kinase,Transferring phosphorus-containing groups,Transferases

contig00003.g1731	2104	ID=contig00003.g1731;Description=hypothetical protein FCOIX_11780 [Fusarium coicis];Gene=FNAPI_6695;Ontology_term=side of membrane;Ontology_id=GO:0098552
contig00003.g1732	1480	ID=contig00003.g1732;Description=hypothetical protein FVEG_13695 [Fusarium verticillioides 7600];Gene=FVEG_13695;Ontology_term=side of membrane;Ontology_id=GO:0098552
contig00003.g1733	3645	ID=contig00003.g1733;Description=sensory transduction histidine kinase [Fusarium tjaetaba];Gene=FNAPI_6697;Ontology_term=catalytic activity, acting on a protein,signaling,transferase activity,small molecule sensor activity;Ontology_id=GO:0140096,GO:0023052,GO:0016740,GO:0140299;Enzyme_code=EC:2.7.3,EC:2.7.13.3,EC:2.7.1,EC:2.7.3,EC:2.7,EC:2,EC:2.7.13.3;Enzyme_name=Transferring phosphorus-containing groups,histidine kinase,Transferring phosphorus-containing groups,Transferring phosphorus-containing groups,Transferring phosphorus-containing groups,Transferases,histidine kinase
contig00003.g1734	660	ID=contig00003.g1734;Description=cytidine and deoxycytidylate deaminase zinc-binding region [Fusarium tjaetaba];Gene=FNAPI_6698;Ontology_term=catalytic activity;Ontology_id=GO:0003824
contig00003.g1735	599	ID=contig00003.g1735;Description=hypothetical protein FVER53590_13692 [Fusarium verticillioides]
contig00003.g1736	2203	ID=contig00003.g1736;Description=MFS transporter, AGZA family, xanthine/uracil permease [Fusarium verticillioides 7600];Gene=FNAPI_6700;Ontology_term=transmembrane transport,transporter activity;Ontology_id=GO:0055085,GO:0005215
contig00003.g1737	2066	ID=contig00003.g1737;Description=hypothetical protein FVER53590_13690 [Fusarium verticillioides];Gene=FVER53590_13690;Ontology_term=transferase activity;Ontology_id=GO:0016740;Enzyme_code=EC:2.1.1,EC:2.1,EC:2,EC:2.1.1;Enzyme_name=Transferring one-carbon groups,Transferring one-carbon groups,Transferases,Transferring one-carbon groups
contig00003.g1738	429	ID=contig00003.g1738;Description=hypothetical protein FVER53590_13689 [Fusarium verticillioides]
contig00003.g1739	705	ID=contig00003.g1739;Description=aspartate glutamate racemase [Fusarium coicis];Gene=FVER53263_13688;Ontology_term=isomerase activity;Ontology_id=GO:0016853;Enzyme_code=EC:5.1.1.10,EC:5.1.1,EC:5.1,EC:5.1.1.10,EC:5;Enzyme_name=amino-acid racemase,Racemases and epimerases,Racemases and epimerases,amino-acid racemase,Isomerases

contig00003.g1740	1112	ID=contig00003.g1740;Description=NmrA domain-containing protein [Fusarium keratoplasticum]
contig00003.g1741	1247	ID=contig00003.g1741;Description=methyltransferase [Fusarium tjaetaba];Gene=FTJAE_13425;Ontology_term=transferase activity;Ontology_id=GO:0016740;Enzyme_code=EC:2.1.1,EC:2.1,EC:2,EC:2.1.1;Enzyme_name=Transferring one-carbon groups,Transferring one-carbon groups,Transferases,Transferring one-carbon groups
contig00003.g1742	1182	ID=contig00003.g1742;Description=acetylornithine deacetylase [Fusarium verticillioides 7600];Gene=FPHYL_5691;Ontology_term=catalytic activity, acting on a protein,extracellular region,hydrolase activity;Ontology_id=GO:0140096,GO:0005576,GO:0016787;Enzyme_code=EC:3.4,EC:3.4,EC:3,EC:3.5.1.16;Enzyme_name=Acting on peptide bonds (peptidases),Acting on peptide bonds (peptidases),Hydrolases,acetylornithine deacetylase
contig00003.g1743	1944	ID=contig00003.g1743;Description=pantothenate transporter [Penicillium verhagenii];Gene=FANTH_11645;Ontology_term=transmembrane transport,transporter activity;Ontology_id=GO:0055085,GO:0005215
contig00003.g1744	552	ID=contig00003.g1744;Description=acyl-CoA N-acyltransferase [Fusarium sp. MPI-SDFR-AT-0072];Gene=FPCIR_12838;Ontology_term=transferase activity;Ontology_id=GO:0016740;Enzyme_code=EC:2.3.1,EC:2.3,EC:2,EC:2.3.1;Enzyme_name=Acyltransferases,Acyltransferases,Transferases,Acyltransferases
contig00003.g1745	1290	ID=contig00003.g1745;Description=lipase 1 [Fusarium tjaetaba];Gene=FMUND_9292;Ontology_term=lipid metabolic process,hydrolase activity;Ontology_id=GO:0006629,GO:0016787;Enzyme_code=EC:3.1.1.3,EC:3.1,EC:3,EC:3.1.1,EC:3.1.1.3;Enzyme_name=triacylglycerol lipase,Acting on ester bonds,Hydrolases,Acting on ester bonds,triacylglycerol lipase
contig00003.g1746	1365	ID=contig00003.g1746;Description=hypothetical protein FVER53263_13681 [Fusarium verticillioides];Gene=FVER53590_13681;Ontology_term=nucleus,regulation of DNA-templated transcription,transcription regulator activity;Ontology_id=GO:0005634,GO:0006355,GO:0140110
contig00003.g1747	1044	ID=contig00003.g1747;Description=short-chain alcohol dehydrogenase/reductase [Fusarium pseudocircinatum];Gene=FVER53590_13680;Ontology_term=oxidoreductase activity;Ontology_id=GO:0016491;Enzyme_code=EC:1,EC:1;Enzyme_name=Oxidoreductases,Oxidoreductases
contig00003.g1748	491	ID=contig00003.g1748;Description=ARM repeat protein [Fusarium coicis]

contig00003.g1749	963	ID=contig00003.g1749;Description=kelch 8 [Fusarium denticulatum]
contig00003.g1750	693	ID=contig00003.g1750;Description=hypothetical protein FVER14953_13677 [Fusarium verticillioides]
contig00003.g1751	1335	ID=contig00003.g1751;Description=lactate 2-monooxygenase [Fusarium pseudoanthophilum];Gene=FRV6_14437;Ontology_term=oxidoreductase activity,transferase activity;Ontology_id=GO:0016491,GO:0016740;Enzyme_code=EC:2.4,EC:1,EC:2.4,EC:1,EC:2,EC:1.13.12.4;Enzyme_name=Glycosyltransferases,Oxidoreductases,Glycosyltransferases,Oxidoreductases,Transferases,lactate 2-monooxygenase
contig00003.g1752	1648	ID=contig00003.g1752;Description=UDP-glycosyltransferase 84B2 [Fusarium napiforme];Gene=FNAPI_7745;Ontology_term=transferase activity;Ontology_id=GO:0016740;Enzyme_code=EC:2.4,EC:2.4,EC:2;Enzyme_name=Glycosyltransferases,Glycosyltransferases,Transferases
contig00003.g1753	1185	ID=contig00003.g1753;Description=n-acyl homoserine lactonase [Fusarium tjaetaba]
contig00003.g1754	348	ID=contig00003.g1754;Description=hypothetical protein FNYG_10654 [Fusarium nygamai];Gene=FOXB_12008
contig00003.g1755	2349	ID=contig00003.g1755;Description=hypothetical protein FVER53590_13674 [Fusarium verticillioides]
contig00003.g1756	2747	ID=contig00003.g1756;Description=hypothetical protein FVER53590_13673 [Fusarium verticillioides]
contig00003.g1757	836	ID=contig00003.g1757;Description=hypothetical protein FVER53263_13671 [Fusarium verticillioides]
contig00003.g1758	2176	ID=contig00003.g1758;Description=phenylacetyl ligase [Fusarium mundagurra];Gene=FMUND_9280;Ontology_term=ligase activity;Ontology_id=GO:0016874;Enzyme_code=EC:6,EC:6;Enzyme_name=Ligases,Ligases
contig00003.g1759	438	ID=contig00003.g1759;Description=small secreted protein [Fusarium subglutinans]
contig00003.g1760	991	ID=contig00003.g1760;Description=calcium/calmodulin-dependent protein kinase [Colletotrichum abscissum];Gene=CFIO01_07355;Ontology_term=transferase activity;Ontology_id=GO:0016740;Enzyme_code=EC:2.7,EC:2.7.11.1,EC:2.1.1.43,EC:2.7,EC:2;Enzyme_name=Transferring phosphorus-containing groups,non-specific serine/threonine protein kinase,Transferring one-carbon groups,Transferring phosphorus-containing groups,Transferases

contig00003.g1761	1778	ID=contig00003.g1761;Description=calcium/calmodulin-dependent protein kinase [Colletotrichum abscissum];Gene=FPANT_4150;Ontology_term=catalytic activity, acting on a protein,autophagy,transferase activity;Ontology_id=GO:0140096,GO:0006914,GO:0016740;Enzyme_code=EC:2.7.11.1;Enzyme_name=non-specific serine/threonine protein kinase
contig00003.g1762	1831	ID=contig00003.g1762;Description=het-s domain-containing protein [Fusarium pseudoanthophilum];Gene=FPANT_4151;Ontology_term=catalytic activity, acting on a protein,transferase activity;Ontology_id=GO:0140096,GO:0016740;Enzyme_code=EC:2.7.1,EC:2.7.1,EC:2.7,EC:2;Enzyme_name=Transferring phosphorus-containing groups,Transferring phosphorus-containing groups,Transferring phosphorus-containing groups,Transferases
contig00003.g1763	622	ID=contig00003.g1763;Description=metalloprotease 1 [Fusarium pseudoanthophilum];Gene=FPANT_4154;Ontology_term=catalytic activity, acting on a protein,hydrolase activity;Ontology_id=GO:0140096,GO:0016787;Enzyme_code=EC:3.4,EC:3.4,EC:3;Enzyme_name=Acting on peptide bonds (peptidases),Acting on peptide bonds (peptidases),Hydrolases
contig00003.g1764	786	ID=contig00003.g1764;Description=hypothetical protein FCOIX_8119 [Fusarium coicis]
contig00003.g1765	900	ID=contig00003.g1765;Description=hypothetical protein FVEG_13666 [Fusarium verticillioides 7600]
contig00003.g1766	3512	ID=contig00003.g1766;Description=hypothetical protein NW756_008635 [Fusarium oxysporum]
contig00003.g1767	750	ID=contig00003.g1767;Description=hypothetical protein J7337_012304 [Fusarium musae]
contig00003.g1768	4208	ID=contig00003.g1768;Description=glutamyl endopeptidase [Fusarium tjaetaba];Gene=FTJAE_11235;Ontology_term=catalytic activity, acting on a protein,hydrolase activity;Ontology_id=GO:0140096,GO:0016787;Enzyme_code=EC:3.4.21,EC:3.4.21,EC:3.4,EC:3;Enzyme_name=Acting on peptide bonds (peptidases),Acting on peptide bonds (peptidases),Acting on peptide bonds (peptidases),Hydrolases

contig00003.g1769	1354	ID=contig00003.g1769;Description=7-dehydrocholesterol reductase [Fusarium subglutinans];Gene=FNYG_10672;Ontology_term=oxidoreductase activity,lipid metabolic process;Ontology_id=GO:0016491,GO:0006629;Enzyme_code=EC:1.3.1,EC:1.3,EC:1,EC:1.3.1.21,EC:1.3.1;Enzyme_name=Acting on the CH-CH group of donors,Acting on the CH-CH group of donors,Oxidoreductases,7-dehydrocholesterol reductase,Acting on the CH-CH group of donors
contig00003.g1770	3662	ID=contig00003.g1770;Description=hypothetical protein J7337_012308 [Fusarium musae];Gene=FPHYL_5668;Ontology_term=oxidoreductase activity,lipid metabolic process;Ontology_id=GO:0016491,GO:0006629;Enzyme_code=EC:1.3.1,EC:1.3,EC:1,EC:1.3.1;Enzyme_name=Acting on the CH-CH group of donors,Acting on the CH-CH group of donors,Oxidoreductases,Acting on the CH-CH group of donors
contig00003.g1771	1872	ID=contig00003.g1771;Description=aminotriazole resistance [Fusarium tjaetaba];Gene=FPCIR_12859;Ontology_term=transmembrane transport,transporter activity;Ontology_id=GO:0055085,GO:0005215
contig00003.g1772	3312	ID=contig00003.g1772;Description=alpha-glucosidase [Fusarium verticillioides 7600];Gene=FVEG_13659;Ontology_term=carbohydrate metabolic process,hydrolase activity;Ontology_id=GO:0005975,GO:0016787;Enzyme_code=EC:3.2.1.20,EC:3.2.1.21,EC:3.2,EC:3.2.1.20,EC:3.2.1.21,EC:3,EC:3.2.1;Enzyme_name=alpha-glucosidase,beta-glucosidase,Glycosylases,alpha-glucosidase,beta-glucosidase,Hydrolases,Glycosylases
contig00003.g1773	846	ID=contig00003.g1773;Description=alpha beta hydrolase [Fusarium tjaetaba];Gene=FMEXI_2648;Ontology_term=hydrolase activity;Ontology_id=GO:0016787;Enzyme_code=EC:3,EC:3;Enzyme_name=Hydrolases,Hydrolases
contig00003.g1774	2791	ID=contig00003.g1774;Description=glycoside hydrolase family 81 [Fusarium pseudoanthophilum];Gene=FVER53263_13657;Ontology_term=carbohydrate metabolic process,cell wall organization or biogenesis,cytoskeletal protein binding,hydrolase activity,molecular adaptor activity;Ontology_id=GO:0005975,GO:0071554,GO:0008092,GO:0016787,GO:0060090;Enzyme_code=EC:3.2.1.21,EC:3.2.1.6,EC:3.2,EC:3.2.1.21,EC:3,EC:3.2.1.6,EC:3.2.1;Enzyme_name=beta-glucosidase,endo-1,3(4)-beta-glucanase,Glycosylases,beta-glucosidase,Hydrolases,endo-1,3(4)-beta-glucanase,Glycosylases

contig00003.g1775	2162	ID=contig00003.g1775;Description=choline dehydrogenase [Fusarium coicis];Gene=BFJ68_g12862;Ontology_term=oxidoreductase activity;Ontology_id=GO:0016491;Enzyme_code=EC:1.1;Enzyme_name=Acting on the CH-OH group of donors
contig00003.g1776	606	ID=contig00003.g1776;Description=hypothetical protein J7337_012315 [Fusarium musae];Gene=FTJAE_11226;Ontology_term=oxidoreductase activity;Ontology_id=GO:0016491;Enzyme_code=EC:1,EC:1;Enzyme_name=Oxidoreductases,Oxidoreductases
contig00003.g1777	2857	ID=contig00003.g1777;Description=monocarboxylate transporter 8 [Fusarium tjaetaba];Gene=FPANT_10821;Ontology_term=transmembrane transport,transporter activity;Ontology_id=GO:0055085,GO:0005215
contig00003.g1778	3250	ID=contig00003.g1778;Description=NADP-dependent oxidoreductase domain-containing protein [Fusarium redolens]
contig00003.g1779	1233	ID=contig00003.g1779;Description=hypothetical protein BKA60DRAFT_466091 [Fusarium oxysporum];Gene=FOBC_15164;Ontology_term=GTP binding;Ontology_id=GO:0005525
contig00003.g1780	1655	ID=contig00003.g1780;Description=hypothetical protein FVEG_13651 [Fusarium verticillioides 7600]
contig00003.g1781	4095	ID=contig00003.g1781;Description=hsp70 protein [Fusarium fujikuroi];Gene=FFUJ_10414;Ontology_term=protein folding chaperone,oxidoreductase activity,ATP-dependent activity;Ontology_id=GO:0044183,GO:0016491,GO:0140657;Enzyme_code=EC:1.1.1.1,EC:1.1.1.71,EC:1.1,EC:1,EC:1.1.1,EC:1.1.1.1,EC:1.1.1.71;Enzyme_name=alcohol dehydrogenase,alcohol dehydrogenase [NAD(P)(+)],Acting on the CH-OH group of donors,Oxidoreductases,Acting on the CH-OH group of donors,alcohol dehydrogenase,alcohol dehydrogenase [NAD(P)(+)]
contig00003.g1782	1001	ID=contig00003.g1782;Description=related to 2`-hydroxyisoflavone reductase [Fusarium fujikuroi];Gene=FVEG_13648;Ontology_term=oxidoreductase activity;Ontology_id=GO:0016491;Enzyme_code=EC:1;Enzyme_name=Oxidoreductases
contig00003.g1783	2749	ID=contig00003.g1783;Description=hypothetical protein FVEG_13647 [Fusarium verticillioides 7600];Gene=FNYG_10682;Ontology_term=hydrolase activity;Ontology_id=GO:0016787;Enzyme_code=EC:3.1.6,EC:3.1.6,EC:3.1,EC:3;Enzyme_name=Acting on ester bonds,Acting on ester bonds,Acting on ester bonds,Hydrolases

contig00003.g1784	735	ID=contig00003.g1784;Description=mitochondrial hypoxia responsive domain protein [Fusarium tjaetaba];Gene=FVER53263_13646;Ontology_term=protein-containing complex assembly,mitochondrion,mitochondrion organization;Ontology_id=GO:0065003,GO:0005739,GO:0007005
contig00003.g1785	576	ID=contig00003.g1785;Description=Aldehyde reductase 1 [Fusarium oxysporum f. sp. albedinis];Gene=FOMG_13464;Ontology_term=membrane;Ontology_id=GO:0016020
contig00003.g1786	597	ID=contig00003.g1786;Description=hypothetical protein FVEG_13644 [Fusarium verticillioides 7600]
contig00003.g1787	910	ID=contig00003.g1787;Description=hypothetical protein FVEG_13643 [Fusarium verticillioides 7600]
contig00003.g1788	547	ID=contig00003.g1788;Description=small secreted protein [Fusarium tjaetaba]
contig00003.g1789	828	ID=contig00003.g1789;Description=endo-1 4-beta-xylanase B [Fusarium subglutinans];Gene=FSUBG_7573;Ontology_term=carbohydrate metabolic process,cell wall organization or biogenesis,hydrolase activity;Ontology_id=GO:0005975,GO:0071554,GO:0016787;Enzyme_code=EC:3.2,EC:3.2,EC:3;Enzyme_name=Glycosylases,Glycosylases,Hydrolases
contig00003.g1790	2802	ID=contig00003.g1790;Description=hypothetical protein FVER14953_13640 [Fusarium verticillioides];Gene=FOQG_17055;Ontology_term=oxidoreductase activity;Ontology_id=GO:0016491;Enzyme_code=EC:1.14,EC:1,EC:1.14;Enzyme_name=Acting on paired donors, with incorporation or reduction of molecular oxygen. The oxygen incorporated need not be derived from O2,Oxidoreductases,Acting on paired donors, with incorporation or reduction of molecular oxygen. The oxygen incorporated need not be derived from O2
contig00003.g1791	1725	ID=contig00003.g1791;Description=pisatin demethylase cytochrome P450 [Fusarium mundagurra];Gene=FDENT_7660;Ontology_term=oxidoreductase activity,transferase activity;Ontology_id=GO:0016491,GO:0016740;Enzyme_code=EC:1.14,EC:2.1.1;Enzyme_name=Acting on paired donors, with incorporation or reduction of molecular oxygen. The oxygen incorporated need not be derived from O2,Transferring one-carbon groups
contig00003.g1792	813	ID=contig00003.g1792;Description=alanyl-tRNA synthetase [Fusarium verticillioides 7600];Gene=FCIRC_9290;Ontology_term=tRNA metabolic process,amino acid metabolic process,catalytic activity, acting on RNA,ligase activity;Ontology_id=GO:0006399,GO:0006520,GO:0140098,GO:0016874;Enzyme_code=EC:6.1.1.7,EC:6.1,EC:6.1.1.7,EC:6.1.1,EC:6;Enzyme_name=alanine--tRNA ligase,Forming carbon-oxygen bonds,alanine--tRNA ligase,Forming carbon-oxygen bonds,Ligases

contig00003.g1793	754	ID=contig00003.g1793;Description=cutinase 3 [Fusarium proliferatum];Gene=FVER53263_13638;Ontology_term=carbohydrate metabolic process,lipid metabolic process,extracellular region,hydrolase activity;Ontology_id=GO:0005975,GO:0006629,GO:0005576,GO:0016787;Enzyme_code=EC:3.1.1.74,EC:3.1,EC:3,EC:3.1.1.74,EC:3.1.1;Enzyme_name=cutinase,Acting on ester bonds,Hydrolases,cutinase,Acting on ester bonds
contig00003.g1794	1293	ID=contig00003.g1794;Description=aromatic aminotransferase [Fusarium tjaetaba];Gene=FPANT_3380;Ontology_term=transferase activity;Ontology_id=GO:0016740;Enzyme_code=EC:2.6.1,EC:2.6.1,EC:2,EC:2.6;Enzyme_name=Transferring nitrogenous groups,Transferring nitrogenous groups,Transferases,Transferring nitrogenous groups
contig00003.g1795	2538	ID=contig00003.g1795;Description=hypothetical protein FVER14953_13636 [Fusarium verticillioides];Gene=FVEG_13636;Ontology_term=membrane;Ontology_id=GO:0016020
contig00003.g1796	2549	ID=contig00003.g1796;Description=peptide transporter [Fusarium tjaetaba];Gene=FVER53263_13635;Ontology_term=transmembrane transport,transporter activity;Ontology_id=GO:0055085,GO:0005215
contig00003.g1797	624	ID=contig00003.g1797;Description=hypothetical protein FPCIR_9656 [Fusarium pseudocircinatum]
contig00003.g1798	1002	ID=contig00003.g1798;Description=hypothetical protein FVER53590_13633 [Fusarium verticillioides];Gene=FFUJ_10432
contig00003.g1799	2676	ID=contig00003.g1799;Description=putative extracellular elastinolytic metallo ase precursor [Fusarium globosum];Gene=FGLOB1_3396;Ontology_term=extracellular space,catalytic activity, acting on a protein,hydrolase activity;Ontology_id=GO:0005615,GO:0140096,GO:0016787;Enzyme_code=EC:3.4.24,EC:3.4.24,EC:3.4,EC:3;Enzyme_name=Acting on peptide bonds (peptidases),Acting on peptide bonds (peptidases),Acting on peptide bonds (peptidases),Hydrolases
contig00003.g1800	2052	ID=contig00003.g1800;Description=putative extracellular elastinolytic metallo ase precursor [Fusarium denticulatum];Gene=CEK26_002148;Ontology_term=extracellular space,catalytic activity, acting on a protein,molecular carrier activity,hydrolase activity;Ontology_id=GO:0005615,GO:0140096,GO:0140104,GO:0016787;Enzyme_code=EC:3.4.24,EC:3.4.24,EC:3.4,EC:3;Enzyme_name=Acting on peptide bonds (peptidases),Acting on peptide bonds (peptidases),Acting on peptide bonds (peptidases),Hydrolases

contig00003.g1801	2378	ID=contig00003.g1801;Description=short chain dehydrogenase family [Fusarium coicis];Gene=FFUJ_10436;Ontology_term=nucleus,regulation of DNA-templated transcription,transcription regulator activity;Ontology_id=GO:0005634,GO:0006355,GO:0140110
contig00003.g1802	1488	ID=contig00003.g1802;Description=5'-3' DNA helicase [Fusarium subglutinans];Gene=FSUBG_7587;Ontology_term=DNA repair,nucleus,catalytic activity, acting on DNA,ATP-dependent activity,oxidoreductase activity,DNA recombination,regulation of DNA-templated transcription,telomere organization,transcription regulator activity,hydrolase activity;Ontology_id=GO:0006281,GO:0005634,GO:0140097,GO:0140657,GO:0016491,GO:0006310,GO:0006355,GO:0032200,GO:0140110,GO:0016787;Enzyme_code=EC:1.1.1,EC:3.6.1.15,EC:1.1,EC:3.6.1,EC:3.6.4.12,EC:1,EC:3.6,EC:3,EC:1.1.1,EC:3.6.1.15;Enzyme_name=Acting on the CH-OH group of donors,nucleoside-triphosphate phosphatase,Acting on the CH-OH group of donors,Acting on acid anhydrides,DNA helicase,Oxidoreductases,Acting on acid anhydrides,Hydrolases,Acting on the CH-OH group of donors,nucleoside-triphosphate phosphatase
contig00003.g1803	1215	ID=contig00003.g1803;Description=atp-dependent dna helicase pif1 [Fusarium coicis];Gene=FDENT_7670;Ontology_term=DNA repair,catalytic activity, acting on DNA,ATP-dependent activity,DNA recombination,telomere organization,hydrolase activity;Ontology_id=GO:0006281,GO:0140097,GO:0140657,GO:0006310,GO:0032200,GO:0016787;Enzyme_code=EC:3.6.1.15,EC:3.6.1,EC:3.6.4.12,EC:3.6,EC:3,EC:3.6.1.15;Enzyme_name=nucleoside-triphosphate phosphatase,Acting on acid anhydrides,DNA helicase,Acting on acid anhydrides,Hydrolases,nucleoside-triphosphate phosphatase
contig00003.g1804	732	ID=contig00003.g1804;Description=hypothetical protein FVER14953_13625 [Fusarium verticillioides]
contig00003.g1805	824	ID=contig00003.g1805;Description=pyrethroid hydrolase [Fusarium tjaetaba];Gene=FACUT_8836;Ontology_term=hydrolase activity;Ontology_id=GO:0016787;Enzyme_code=EC:3,EC:3;Enzyme_name=Hydrolases,Hydrolases
contig00003.g1806	1152	ID=contig00003.g1806;Description=hypothetical protein FVEG_13623 [Fusarium verticillioides 7600]
contig00003.g1807	1023	ID=contig00003.g1807;Description=ankyrin [Fusarium tjaetaba]
contig00003.g1808	1313	ID=contig00003.g1808;Description=hypothetical protein FCOIX_1660 [Fusarium coicis]

contig00003.g1809	678	ID=contig00003.g1809;Description=hypothetical protein FVEG_13620 [Fusarium verticillioides 7600]
contig00003.g1810	1158	ID=contig00003.g1810;Description=hypothetical protein FVEG_13619 [Fusarium verticillioides 7600]
contig00003.g1811	372	ID=contig00003.g1811;Description=hypothetical protein FVEG_13618 [Fusarium verticillioides 7600]
contig00003.g1812	555	ID=contig00003.g1812;Description=hypothetical protein FVEG_13615 [Fusarium verticillioides 7600]
contig00003.g1813	1715	ID=contig00003.g1813;Description=PRC1-carboxypeptidase y serine-type protease [Fusarium pseudoanthophilum];Gene=FVEG_13614;Ontology_term=catalytic activity, acting on a protein,hydrolase activity;Ontology_id=GO:0140096,GO:0016787;Enzyme_code=EC:3.4.16,EC:3.4,EC:3.4.16,EC:3;Enzyme_name=Acting on peptide bonds (peptidases),Acting on peptide bonds (peptidases),Acting on peptide bonds (peptidases),Hydrolases
contig00003.g1814	1971	ID=contig00003.g1814;Description=alcohol oxidase [Fusarium pseudocircinatum];Gene=FACUT_8828;Ontology_term=oxidoreductase activity;Ontology_id=GO:0016491;Enzyme_code=EC:1.1,EC:1.1,EC:1;Enzyme_name=Acting on the CH-OH group of donors,Acting on the CH-OH group of donors,Oxidoreductases
contig00003.g1815	1393	ID=contig00003.g1815;Description=integral membrane protein PTH11 [Fusarium subglutinans];Gene=FMEXI_2691;Ontology_term=membrane;Ontology_id=GO:0016020
contig00003.g1816	1633	ID=contig00003.g1816;Description=related to TPN1 Pyridoxine transporter [Fusarium proliferatum ET1];Gene=FMEXI_2692;Ontology_term=transmembrane transport,vacuole,transporter activity,plasma membrane;Ontology_id=GO:0055085,GO:0005773,GO:0005215,GO:0005886
contig00003.g1817	834	ID=contig00003.g1817;Description=3-oxoacyl-[acyl-carrier-protein] reductase FabG [Fusarium odoratissimum];Gene=BFJ65_g13443;Ontology_term=oxidoreductase activity;Ontology_id=GO:0016491;Enzyme_code=EC:1,EC:1,EC:1.1.1.100;Enzyme_name=Oxidoreductases,Oxidoreductases,3-oxoacyl-[acyl-carrier-protein] reductase
contig00003.g1818	765	ID=contig00003.g1818;Description=short-chain dehydrogenase reductase SDR [Fusarium mundagurra]
contig00003.g1819	499	ID=contig00003.g1819;Description=hypothetical protein FMUND_14490 [Fusarium mundagurra];Gene=FNAPI_4784;Ontology_term=membrane;Ontology_id=GO:0016020

contig00003.g1820	841	ID=contig00003.g1820;Description=chloroperoxidase [Fusarium acutatum];Gene=FOC4_g10002752;Ontology_term=antioxidant activity,oxidoreductase activity;Ontology_id=GO:0016209,GO:0016491;Enzyme_code=EC:1.11.1,EC:1.11,EC:1,EC:1.11.1;Enzyme_name=Acting on a peroxide as acceptor,Acting on a peroxide as acceptor,Oxidoreductases,Acting on a peroxide as acceptor
contig00003.g1821	518	ID=contig00003.g1821;Description=Alcohol dehydrogenase 4 [Fusarium oxysporum f. sp. cubense race 1];Gene=FOTG_14829;Ontology_term=oxidoreductase activity;Ontology_id=GO:0016491;Enzyme_code=EC:1;Enzyme_name=Oxidoreductases
contig00003.g1822	874	ID=contig00003.g1822;Description=nitrogen assimilation transcription factor nirA [Fusarium tjaetaba];Gene=FTJAE_10676;Ontology_term=oxidoreductase activity;Ontology_id=GO:0016491;Enzyme_code=EC:1,EC:1;Enzyme_name=Oxidoreductases,Oxidoreductases
contig00003.g1823	1242	ID=contig00003.g1823;Description=putative endothiapepsin precursor [Fusarium fujikuroi];Gene=FMAN_13329;Ontology_term=catalytic activity, acting on a protein,cell adhesion,plasma membrane,hydrolase activity;Ontology_id=GO:0140096,GO:0007155,GO:0005886,GO:0016787;Enzyme_code=EC:3.4.23,EC:3.4.23,EC:3.4,EC:3;Enzyme_name=Acting on peptide bonds (peptidases),Acting on peptide bonds (peptidases),Acting on peptide bonds (peptidases),Hydrolases
contig00003.g1824	1837	ID=contig00003.g1824;Description=hypothetical protein FVER53590_13604 [Fusarium verticillioides];Gene=FVEG_17561;Ontology_term=nucleus,regulation of DNA-templated transcription,transcription regulator activity;Ontology_id=GO:0005634,GO:0006355,GO:0140110
contig00003.g1825	878	ID=contig00003.g1825;Description=3' 5' exonuclease [Fusarium pseudoanthophilum];Gene=FVER53263_13602;Ontology_term=hydrolase activity;Ontology_id=GO:0016787;Enzyme_code=EC:3.1,EC:3.1,EC:3;Enzyme_name=Acting on ester bonds,Acting on ester bonds,Hydrolases
contig00003.g1826	354	ID=contig00003.g1826;Description=hypothetical protein FVEG_13601 [Fusarium verticillioides 7600]
contig00003.g1827	537	ID=contig00003.g1827;Description=hypothetical protein FVER14953_13600 [Fusarium verticillioides]
contig00003.g1828	720	ID=contig00003.g1828;Description=DNA polymerase alpha subunit B [Fusarium proliferatum]
contig00003.g1829	561	ID=contig00003.g1829;Description=hypothetical protein FVEG_13598 [Fusarium verticillioides 7600]

contig00003.g1830	1134	ID=contig00003.g1830;Description=gluconate 5-dehydrogenase [Fusarium denticulatum];Gene=FSPOR_3628;Ontology_term=oxidoreductase activity;Ontology_id=GO:0016491;Enzyme_code=EC:1,EC:1;Enzyme_name=Oxidoreductases,Oxidoreductases
contig00003.g1831	369	ID=contig00003.g1831;Description=Sorbose reductase SOU1 [Fusarium tjaetaba];Gene=FMUND_10474;Ontology_term=membrane;Ontology_id=GO:0016020
contig00003.g1832	1449	ID=contig00003.g1832;Description=auxin efflux carrier superfamily transporter [Fusarium globosum];Gene=FPRO05_00688;Ontology_term=transmembrane transport;Ontology_id=GO:0055085
contig00003.g1833	1551	ID=contig00003.g1833;Description=hypothetical protein J7337_012371 [Fusarium musae];Gene=FDENT_3739;Ontology_term=membrane;Ontology_id=GO:0016020
contig00003.g1834	2716	ID=contig00003.g1834;Description=peptide transporter [Fusarium fujikuroi];Gene=FMAN_13339;Ontology_term=transmembrane transport,transporter activity;Ontology_id=GO:0055085,GO:0005215
contig00003.g1835	7837	ID=contig00003.g1835;Description=ankyrin 1 [Fusarium subglutinans]
contig00003.g1836	729	ID=contig00003.g1836;Description=transcription factor [Fusarium tjaetaba]
contig00003.g1837	2525	ID=contig00003.g1837;Description=binuclear zinc cluster transcription factor [Fusarium coicis];Gene=FVER53590_13591;Ontology_term=nucleus,DNA binding,regulation of DNA-templated transcription,transcription regulator activity;Ontology_id=GO:0005634,GO:0003677,GO:0006355,GO:0140110
contig00003.g1838	1087	ID=contig00003.g1838;Description=glutathione S-transferase [Fusarium verticillioides 7600];Gene=FPANT_13070;Ontology_term=transferase activity;Ontology_id=GO:0016740;Enzyme_code=EC:2.5.1.18,EC:2.5.1,EC:2.5.1.18,EC:2,EC:1.8.5.7;Enzyme_name=glutathione transferase,Transferring alkyl or aryl groups, other than methyl groups,glutathione transferase,Transferases,glutathionyl-hydroquinone reductase
contig00003.g1839	381	ID=contig00003.g1839;Description=early nodulin 75 precursor [Fusarium pseudocircinatum];Gene=FVER53590_13590;Ontology_term=transferase activity;Ontology_id=GO:0016740;Enzyme_code=EC:2.5.1.18;Enzyme_name=glutathione transferase
contig00003.g1840	792	ID=contig00003.g1840;Description=catalase [Fusarium tjaetaba]
contig00003.g1841	561	ID=contig00003.g1841;Description=chorismate mutase type II [Fusarium tjaetaba];Gene=FTJAE_10389;Ontology_term=isomerase activity;Ontology_id=GO:0016853;Enzyme_code=EC:5.4.99.5,EC:5.4,EC:5,EC:5.4.99.5;Enzyme_name=chorismate mutase,Intramolecular transferases,Isomerases,chorismate mutase

contig00003.g1842	2007	ID=contig00003.g1842;Description=hypothetical protein FVER53263_14115 [Fusarium verticillioides]
contig00003.g1843	2031	ID=contig00003.g1843;Description=calcium-related spray [Fusarium tjaetaba];Gene=FNYG_15466;Ontology_term=transmembrane transport,vesicle-mediated transport,mitochondrion,lipid metabolic process,endoplasmic reticulum,cell wall organization or biogenesis,protein folding,vacuole,transporter activity,plasma membrane;Ontology_id=GO:0055085,GO:0016192,GO:0005739,GO:0006629,GO:0005783,GO:0071554,GO:0006457,GO:0005773,GO:0005215,GO:0005886
contig00003.g1844	1669	ID=contig00003.g1844;Description=phosphoethanolamine n-methyltransferase 3 [Fusarium subglutinans];Gene=FSUBG_13110;Ontology_term=transferase activity;Ontology_id=GO:0016740;Enzyme_code=EC:2,EC:2;Enzyme_name=Transferases,Transferases
contig00003.g1845	1206	ID=contig00003.g1845;Description=CVNH domain protein [Fusarium tjaetaba];Gene=FNAPI_7943;Ontology_term=mitochondrion;Ontology_id=GO:0005739
contig00003.g1846	1473	ID=contig00003.g1846;Description=hypothetical protein J7337_012384 [Fusarium musae];Gene=FPANT_12180;Ontology_term=lipid metabolic process,hydrolase activity;Ontology_id=GO:0006629,GO:0016787;Enzyme_code=EC:3.1.4,EC:3.1.4,EC:3.1,EC:3;Enzyme_name=Acting on ester bonds,Acting on ester bonds,Acting on ester bonds,Hydrolases
contig00003.g1847	1485	ID=contig00003.g1847;Description=serine threonine kinase [Fusarium denticulatum];Gene=FDENT_4877;Ontology_term=transferase activity;Ontology_id=GO:0016740;Enzyme_code=EC:2.7,EC:2.7,EC:2;Enzyme_name=Transferring phosphorus-containing groups,Transferring phosphorus-containing groups,Transferases
contig00003.g1848	1625	ID=contig00003.g1848;Description=hypothetical protein FVEG_17734 [Fusarium verticillioides 7600]
contig00003.g1849	1032	ID=contig00003.g1849;Description=related to Protein indc11 [Fusarium mangiferae];Gene=FVER53263_14108;Ontology_term=oxidoreductase activity;Ontology_id=GO:0016491;Enzyme_code=EC:1,EC:1;Enzyme_name=Oxidoreductases,Oxidoreductases
contig00003.g1850	504	ID=contig00003.g1850;Description=Alpha/beta hydrolase fold-1 [Fusarium oxysporum]

contig00003.g1851	1561	ID=contig00003.g1851;Description=related to ROD1-O-dinitrobenzene,calcium and zinc resistance protein [Fusarium fujikuroi IMI 58289];Gene=FGADI_2553;Ontology_term=nucleus,DNA binding,vesicle-mediated transport,DNA-templated transcription,signaling,cytosol,reproductive process,plasma membrane;Ontology_id=GO:0005634,GO:0003677,GO:0016192,GO:0006351,GO:0023052,GO:0005829,GO:0022414,GO:0005886
contig00003.g1852	2330	ID=contig00003.g1852;Description=putative transcriptional regulatory protein [Fusarium oxysporum f. sp. cubense];Gene=BFJ69_g11100;Ontology_term=nucleus,DNA binding,regulation of DNA-templated transcription,transcription regulator activity;Ontology_id=GO:0005634,GO:0003677,GO:0006355,GO:0140110
contig00003.g1853	1429	ID=contig00003.g1853;Description=hypothetical protein FVER14953_14102 [Fusarium verticillioides];Gene=FTJAE_10400
contig00003.g1854	1436	ID=contig00003.g1854;Description=related to integral membrane protein [Fusarium mangiferae];Gene=FNYG_15362
contig00003.g1855	1164	ID=contig00003.g1855;Description=D-aminopeptidase [Fusarium verticillioides 7600];Gene=FNAPI_7951;Ontology_term=catalytic activity, acting on a protein,hydrolase activity;Ontology_id=GO:0140096,GO:0016787;Enzyme_code=EC:3.4.11,EC:3.4.11,EC:3.4.11.19,EC:3.4,EC:3;Enzyme_name=Acting on peptide bonds (peptidases),Acting on peptide bonds (peptidases),D-stereospecific aminopeptidase,Acting on peptide bonds (peptidases),Hydrolases
contig00003.g1856	1472	ID=contig00003.g1856;Description=LSB3-possible role in the regulation of actin cytoskeletal organization [Fusarium fujikuroi];Gene=msp1;Ontology_term=plasma membrane;Ontology_id=GO:0005886
contig00003.g1857	2118	ID=contig00003.g1857;Description=related to transporter protein [Fusarium mangiferae];Gene=FOMG_13552
contig00003.g1858	1686	ID=contig00003.g1858;Description=tartrate transporter [Fusarium napiforme];Gene=FPHYL_2061;Ontology_term=transmembrane transport,transporter activity;Ontology_id=GO:0055085,GO:0005215
contig00003.g1859	1563	ID=contig00003.g1859;Description=cholesterol dehydrogenase [Fusarium coicis];Gene=FPANT_11723;Ontology_term=oxidoreductase activity,lipid metabolic process;Ontology_id=GO:0016491,GO:0006629;Enzyme_code=EC:1.1.1.145,EC:1.1,EC:1,EC:1.1.1.145,EC:1.1.1;Enzyme_name=3beta-hydroxy-Delta(5)-steroid dehydrogenase,Acting on the CH-OH group of donors,Oxidoreductases,3beta-hydroxy-Delta(5)-steroid dehydrogenase,Acting on the CH-OH group of donors

contig00003.g1860	2155	ID=contig00003.g1860;Description=related to pathway-specific regulatory protein nit-4 [Fusarium fujikuroi];Gene=FVEG_14095;Ontology_term=nucleus,DNA binding,regulation of DNA-templated transcription,transcription regulator activity;Ontology_id=GO:0005634,GO:0003677,GO:0006355,GO:0140110
contig00003.g1861	815	ID=contig00003.g1861;Description=---NA---
contig00003.g1862	510	ID=contig00003.g1862;Description=GNAT family acetyltransferase [Fusarium tjaetaba];Gene=FVER53263_21092;Ontology_term=transferase activity;Ontology_id=GO:0016740;Enzyme_code=EC:2.3.1,EC:2.3,EC:2,EC:2.3.1;Enzyme_name=Acyltransferases,Acyltransferases,Transferases,Acyltransferases
contig00003.g1863	1648	ID=contig00003.g1863;Description=serine threonine phosphatase 6 regulatory ankyrin repeat subunit A [Fusarium pseudocircinatum];Gene=FVEG_14093;Ontology_term=endosome,vacuole;Ontology_id=GO:0005768,GO:0005773
contig00003.g1864	537	ID=contig00003.g1864;Description=ricin b lectin [Fusarium tjaetaba];Gene=BFJ65_g13630;Ontology_term=catalytic activity, acting on a protein,hydrolase activity;Ontology_id=GO:0140096,GO:0016787;Enzyme_code=EC:3.4,EC:3.4,EC:3;Enzyme_name=Acting on peptide bonds (peptidases),Acting on peptide bonds (peptidases),Hydrolases
contig00003.g1865	1608	ID=contig00003.g1865;Description=triacylglycerol lipase V precursor [Fusarium coicis];Gene=FVER53263_14091;Ontology_term=hydrolase activity;Ontology_id=GO:0016787;Enzyme_code=EC:3,EC:3;Enzyme_name=Hydrolases,Hydrolases
contig00003.g1866	549	ID=contig00003.g1866;Description=competence damage-inducible [Fusarium tjaetaba]
contig00003.g1867	1368	ID=contig00003.g1867;Description=acylamide-delta3(E)-desaturase [Fusarium napiforme];Gene=FGLOB1_12490;Ontology_term=lipid metabolic process;Ontology_id=GO:0006629
contig00003.g1868	600	ID=contig00003.g1868;Description=hypothetical protein FVER14953_14088 [Fusarium verticillioides];Gene=FVEG_14088;Ontology_term=membrane;Ontology_id=GO:0016020
contig00003.g1869	672	ID=contig00003.g1869;Description=hypothetical protein FVER53263_14087 [Fusarium verticillioides]

contig00003.g1870	969	ID=contig00003.g1870;Description=related to ketoreductases [Fusarium fujikuroi];Gene=FOC1_g10002932;Ontology_term=oxidoreductase activity;Ontology_id=GO:0016491;Enzyme_code=EC:1,EC:1;Enzyme_name=Oxidoreductases,Oxidoreductases
contig00003.g1871	947	ID=contig00003.g1871;Description=hypothetical protein FVER53263_20237 [Fusarium verticillioides];Gene=FVEG_17729;Ontology_term=nucleus,regulation of DNA-templated transcription,transcription regulator activity;Ontology_id=GO:0005634,GO:0006355,GO:0140110
contig00003.g1872	1191	ID=contig00003.g1872;Description=hypothetical protein FVER14953_14085 [Fusarium verticillioides]
contig00003.g1873	1695	ID=contig00003.g1873;Description=hypothetical protein J7337_012413 [Fusarium musae];Gene=FVEG_14084;Ontology_term=membrane;Ontology_id=GO:0016020
contig00003.g1874	1777	ID=contig00003.g1874;Description=Isotrichodermin C-15 hydroxylase [Fusarium coicis];Gene=FVEG_14083;Ontology_term=oxidoreductase activity;Ontology_id=GO:0016491;Enzyme_code=EC:1.14,EC:1,EC:1.14;Enzyme_name=Acting on paired donors, with incorporation or reduction of molecular oxygen. The oxygen incorporated need not be derived from O2,Oxidoreductases,Acting on paired donors, with incorporation or reduction of molecular oxygen. The oxygen incorporated need not be derived from O2
contig00003.g1875	534	ID=contig00003.g1875;Description=Isotrichodermin C-15 hydroxylase [Fusarium tjaetaba];Gene=FTJAE_10423;Ontology_term=membrane;Ontology_id=GO:0016020
contig00003.g1876	1308	ID=contig00003.g1876;Description=uncharacterized protein LW93_6009 [Fusarium fujikuroi];Gene=FFC1_15892;Ontology_term=extracellular space,molecular function regulator activity;Ontology_id=GO:0005615,GO:0098772
contig00003.g1877	1735	ID=contig00003.g1877;Description=Alpha/Beta hydrolase protein [Fusarium sp. MPI-SDFR-AT-0072];Gene=FVER53263_08897;Ontology_term=catalytic activity, acting on a protein,hydrolase activity;Ontology_id=GO:0140096,GO:0016787;Enzyme_code=EC:3.4.11,EC:3.4.11,EC:3.4,EC:3;Enzyme_name=Acting on peptide bonds (peptidases),Acting on peptide bonds (peptidases),Acting on peptide bonds (peptidases),Hydrolases
contig00003.g1878	409	ID=contig00003.g1878;Description=hypothetical protein FVEG_08896 [Fusarium verticillioides 7600]

contig00003.g1879	772	ID=contig00003.g1879;Description=hypothetical protein FVER14953_08895 [Fusarium verticillioides];Gene=MEF-1;Ontology_term=translation regulator activity,GTPase activity;Ontology_id=GO:0045182,GO:0003924;Enzyme_code=EC:3.6.1.15,EC:3.6.1,EC:3.6,EC:3,EC:3.6.1.15;Enzyme_name=nucleoside-triphosphate phosphatase,Acting on acid anhydrides,Acting on acid anhydrides,Hydrolases,nucleoside-triphosphate phosphatase
contig00003.g1880	1483	ID=contig00003.g1880;Description=related to methyltransferase [Fusarium fujikuroi IMI 58289];Gene=FLAG1_08838;Ontology_term=transferase activity;Ontology_id=GO:0016740;Enzyme_code=EC:2.1.1,EC:2.1,EC:2,EC:2.1.1;Enzyme_name=Transferring one-carbon groups,Transferring one-carbon groups,Transferases,Transferring one-carbon groups
contig00003.g1881	1297	ID=contig00003.g1881;Description=O-methyltransferase hmp5 [Fusarium oxysporum f. sp. raphani];Gene=FOMG_13581;Ontology_term=transferase activity;Ontology_id=GO:0016740;Enzyme_code=EC:2.1.1,EC:2.1,EC:2,EC:2.1.1;Enzyme_name=Transferring one-carbon groups,Transferring one-carbon groups,Transferases,Transferring one-carbon groups
contig00003.g1882	2040	ID=contig00003.g1882;Description=sialidase-1 [Fusarium proliferatum];Gene=gaoB;Ontology_term=oxidoreductase activity;Ontology_id=GO:0016491;Enzyme_code=EC:1.1.3.9,EC:1.1,EC:1,EC:1.1.3.9,EC:1.1.3;Enzyme_name=galactose oxidase,Acting on the CH-OH group of donors,Oxidoreductases,galactose oxidase,Acting on the CH-OH group of donors
contig00003.g1883	5297	ID=contig00003.g1883;Description=beta-glucosidase [Fusarium verticillioides 7600];Gene=FVER53590_08890;Ontology_term=carbohydrate metabolic process,transmembrane transport,transporter activity,hydrolase activity;Ontology_id=GO:0005975,GO:0055085,GO:0005215,GO:0016787;Enzyme_code=EC:3.2.1.21;Enzyme_name=beta-glucosidase
contig00003.g1884	1438	ID=contig00003.g1884;Description=dienelactone hydrolase [Fusarium heterosporum];Gene=FEQUK3_LOCUS8118;Ontology_term=hydrolase activity;Ontology_id=GO:0016787;Enzyme_code=EC:3,EC:3;Enzyme_name=Hydrolases,Hydrolases
contig00003.g1885	2528	ID=contig00003.g1885;Description=major facilitator superfamily transporter [Fusarium tjaetaba];Gene=FTJAE_10433

contig00003.g1886	1779	ID=contig00003.g1886;Description=4-coumarate ligase [Fusarium tjaetaba];Gene=FDENT_4916;Ontology_term=ligase activity;Ontology_id=GO:0016874;Enzyme_code=EC:6,EC:6.2.1.12,EC:6;Enzyme_name=Ligases,4-coumarate--CoA ligase,Ligases
contig00003.g1887	1329	ID=contig00003.g1887;Description=hypothetical protein FVER53590_08885 [Fusarium verticillioides]
contig00003.g1888	697	ID=contig00003.g1888;Description=hypothetical protein FVEG_08884 [Fusarium verticillioides 7600];Gene=FDENT_4918;Ontology_term=side of membrane;Ontology_id=GO:0098552
contig00003.g1889	687	ID=contig00003.g1889;Description=SLG1 [Fusarium napiforme]
contig00003.g1890	1044	ID=contig00003.g1890;Description=hypothetical protein FVER14953_08881 [Fusarium verticillioides]
contig00003.g1891	1291	ID=contig00003.g1891;Description=integral membrane protein PTH11 [Fusarium coicis];Gene=FNYG_12133;Ontology_term=membrane;Ontology_id=GO:0016020
contig00003.g1892	654	ID=contig00003.g1892;Description=ankyrin repeat domain protein [Fusarium subglutinans]
contig00003.g1893	1458	ID=contig00003.g1893;Description=carboxymethylenebutenolidase [Fusarium pseudocircinatum];Gene=FPHYL_2025;Ontology_term=polyketide metabolic process;Ontology_id=GO:0030638;Enzyme_code=EC:3.1.1.45;Enzyme_name=carboxymethylenebutenolidase
contig00003.g1894	1580	ID=contig00003.g1894;Description=neutral amino acid permease [Fusarium coicis];Gene=FVER53263_08878;Ontology_term=membrane;Ontology_id=GO:0016020
contig00003.g1895	1399	ID=contig00003.g1895;Description=probable endochitinase [Fusarium fujikuroi];Gene=FPCIR_347;Ontology_term=carbohydrate metabolic process,translation regulator activity,RNA binding,carbohydrate derivative metabolic process,extracellular region,hydrolase activity;Ontology_id=GO:0005975,GO:0045182,GO:0003723,GO:1901135,GO:0005576,GO:0016787;Enzyme_code=EC:3.2.1.14;Enzyme_name=chitinase
contig00003.g1896	936	ID=contig00003.g1896;Description=nitrogen metabolite repression nmrA [Fusarium denticulatum]
contig00003.g1897	1047	ID=contig00003.g1897;Description=peroxisomal short-chain alcohol dehydrogenase [Fusarium coicis]

contig00003.g1898	1104	ID=contig00003.g1898;Description=fungal zn(2)-Cys(6) binuclear cluster domain-containing protein [Fusarium pseudoanthophilum];Gene=FVEG_16470;Ontology_term=nucleus,regulation of DNA-templated transcription,transcription regulator activity;Ontology_id=GO:0005634,GO:0006355,GO:0140110
contig00003.g1899	594	ID=contig00003.g1899;Description=dis1-suppressing kinase dsk1 [Fusarium tjaetaba];Gene=FDENT_4929;Ontology_term=catalytic activity, acting on a protein,transferase activity;Ontology_id=GO:0140096,GO:0016740;Enzyme_code=EC:2.7.1,EC:2.7.1,EC:2.7,EC:2;Enzyme_name=Transferring phosphorus-containing groups,Transferring phosphorus-containing groups,Transferring phosphorus-containing groups,Transferases
contig00003.g1900	645	ID=contig00003.g1900;Description=hypothetical protein FVER14953_08872 [Fusarium verticillioides];Gene=FPANT_4439;Ontology_term=membrane;Ontology_id=GO:0016020
contig00003.g1901	342	ID=contig00003.g1901;Description=hypothetical protein FACUT_6253 [Fusarium acutatum];Gene=F25303_4896;Ontology_term=side of membrane;Ontology_id=GO:0098552
contig00003.g1902	1658	ID=contig00003.g1902;Description=cholinesterase precursor [Fusarium tjaetaba];Gene=FNYG_12144;Ontology_term=hydrolase activity;Ontology_id=GO:0016787;Enzyme_code=EC:3,EC:3,EC:3.1.1.8;Enzyme_name=Hydrolases,Hydrolases,cholinesterase
contig00003.g1903	4479	ID=contig00003.g1903;Description=o-acetyltransferase CAS1 [Fusarium mundagurra];Gene=FVEG_08869;Ontology_term=carbohydrate metabolic process,Golgi apparatus,transferase activity;Ontology_id=GO:0005975,GO:0005794,GO:0016740;Enzyme_code=EC:2,EC:2;Enzyme_name=Transferases,Transferases
contig00003.g1904	1017	ID=contig00003.g1904;Description=hypothetical protein FNAPI_7564 [Fusarium napiforme]
contig00003.g1905	3487	ID=contig00003.g1905;Description=glucan endo-1 3-alpha-glucosidase agn1 [Fusarium pseudoanthophilum];Gene=FVER53263_08867;Ontology_term=transmembrane transport,transporter activity,hydrolase activity;Ontology_id=GO:0055085,GO:0005215,GO:0016787;Enzyme_code=EC:3.2.1.59;Enzyme_name=glucan endo-1,3-alpha-glucosidase

contig00003.g1906	1682	ID=contig00003.g1906;Description=TPO4-Proposed vacuolar polyamine transporter [Fusarium tjaetaba];Gene=FVER53263_08867;Ontology_term=transmembrane transport,transporter activity,hydrolase activity;Ontology_id=GO:0055085,GO:0005215,GO:0016787;Enzyme_code=EC:3.2.1.59,EC:3.2,EC:3,EC:3.2.1.59,EC:3.2.1;Enzyme_name=glucan endo-1,3-alpha-glucosidase,Glycosylases,Hydrolases,glucan endo-1,3-alpha-glucosidase,Glycosylases
contig00003.g1907	525	ID=contig00003.g1907;Description=electron transfer DM13 domain protein [Fusarium tjaetaba]
contig00003.g1908	1663	ID=contig00003.g1908;Description=ferric-chelate reductase [Fusarium bulbicola];Gene=FNAPI_7568;Ontology_term=oxidoreductase activity;Ontology_id=GO:0016491;Enzyme_code=EC:1.16;Enzyme_name=Oxidizing metal ions
contig00003.g1909	2787	ID=contig00003.g1909;Description=phosphoribosyl transferase domain-containing protein [Fusarium pseudocircinatum];Gene=FNYG_12154;Ontology_term=nucleobase-containing small molecule metabolic process,sulfur compound metabolic process,oxidoreductase activity,lipid metabolic process,carbohydrate derivative metabolic process,cytosol,transferase activity;Ontology_id=GO:0055086,GO:0006790,GO:0016491,GO:0006629,GO:1901135,GO:0005829,GO:0016740;Enzyme_code=EC:2.7.4.2,EC:1,EC:2.1.1,EC:2.1,EC:2.7.4.2,EC:1,EC:2.7,EC:2,EC:2.7.4,EC:2.1.1;Enzyme_name=phosphomevalonate kinase,Oxidoreductases,Transferring one-carbon groups,Transferring one-carbon groups,phosphomevalonate kinase,Oxidoreductases,Transferring phosphorus-containing groups,Transferases,Transferring phosphorus-containing groups,Transferring one-carbon groups
contig00003.g1910	2317	ID=contig00003.g1910;Description=amino-acid oxidase [Fusarium tjaetaba];Gene=FTJAE_10478;Ontology_term=oxidoreductase activity;Ontology_id=GO:0016491;Enzyme_code=EC:1,EC:1;Enzyme_name=Oxidoreductases,Oxidoreductases
contig00003.g1911	1321	ID=contig00003.g1911;Description=hypothetical protein FVER53263_08861 [Fusarium verticillioides]
contig00003.g1912	1816	ID=contig00003.g1912;Description=6-hydroxy-D-nicotine oxidase [Fusarium tjaetaba];Gene=FVER53590_08860;Ontology_term=oxidoreductase activity;Ontology_id=GO:0016491;Enzyme_code=EC:1,EC:1;Enzyme_name=Oxidoreductases,Oxidoreductases

contig00003.g1913	405	ID=contig00003.g1913;Description=glyoxalase family protein [Fusarium fujikuroi]
contig00003.g1914	1539	ID=contig00003.g1914;Description=GPI anchored [Fusarium coicis];Gene=FPANT_6361;Ontology_term=lyase activity;Ontology_id=GO:0016829;Enzyme_code=EC:4,EC:4;Enzyme_name=Lyases,Lyases
contig00003.g1915	1950	ID=contig00003.g1915;Description=heterokaryon incompatibility protein [Fusarium coicis];Gene=FVER53590_08858;Ontology_term=lyase activity;Ontology_id=GO:0016829;Enzyme_code=EC:4,EC:4;Enzyme_name=Lyases,Lyases
contig00003.g1916	911	ID=contig00003.g1916;Description=phosphoglycerate mutase [Fusarium pseudocircinatum]
contig00003.g1917	2010	ID=contig00003.g1917;Description=hypothetical protein FVER14953_20806 [Fusarium verticillioides];Gene=Forpi1262_v013854;Ontology_term=membrane;Ontology_id=GO:0016020
contig00003.g1918	1573	ID=contig00003.g1918;Description=regulator of G signaling [Fusarium coicis];Gene=FNYG_12164;Ontology_term=signaling;Ontology_id=GO:0023052
contig00003.g1919	1212	ID=contig00003.g1919;Description=BNR Asp-box repeat domain-containing protein [Fusarium mundagurra]
contig00003.g1920	861	ID=contig00003.g1920;Description=ADP-ribosylation factor [Fusarium tjaetaba];Gene=FTJAE_10488;Ontology_term=GTPase activity;Ontology_id=GO:0003924;Enzyme_code=EC:3.6.1.15,EC:3.6.1,EC:3.6,EC:3,EC:3.6.1.15;Enzyme_name=nucleoside-triphosphate phosphatase,Acting on acid anhydrides,Acting on acid anhydrides,Hydrolases,nucleoside-triphosphate phosphatase
contig00003.g1921	1866	ID=contig00003.g1921;Description=translation machinery-associated 64 [Fusarium coicis];Gene=FNYG_12166;Ontology_term=translation regulator activity,RNA binding,protein-containing complex assembly,cytoplasmic translation,ribosome biogenesis;Ontology_id=GO:0045182,GO:0003723,GO:0065003,GO:0002181,GO:0042254
contig00003.g1922	1308	ID=contig00003.g1922;Description=Lactose permease [Fusarium oxysporum f. sp. albedinis]
contig00003.g1923	1159	ID=contig00003.g1923;Description=hypothetical protein FVER14953_08852 [Fusarium verticillioides];Gene=FNAPI_7582;Ontology_term=membrane;Ontology_id=GO:0016020
contig00003.g1924	2594	ID=contig00003.g1924;Description=interferon-regulated resistance GTP-binding protein [Fusarium pseudoanthophilum];Gene=FPANT_6351;Ontology_term=GTPase activity;Ontology_id=GO:0003924;Enzyme_code=EC:3.6.1.15,EC:3.6.1,EC:3.6,EC:3,EC:3.6.1.15;Enzyme_name=nucleoside-triphosphate phosphatase,Acting on acid anhydrides,Acting on acid anhydrides,Hydrolases,nucleoside-triphosphate phosphatase
contig00003.g1925	1668	ID=contig00003.g1925;Description=hypothetical protein FVER14953_08850 [Fusarium verticillioides]

contig00003.g1926	3871	ID=contig00003.g1926;Description=hypothetical protein FVER53263_08848 [Fusarium verticillioides]
contig00003.g1927	768	ID=contig00003.g1927;Description=hypothetical protein FVEG_08846 [Fusarium verticillioides 7600]
contig00003.g1928	1084	ID=contig00003.g1928;Description=gentisate 1,2-dioxygenase [Fusarium verticillioides 7600];Gene=FVER53263_08845;Ontology_term=oxidoreductase activity;Ontology_id=GO:0016491;Enzyme_code=EC:1,EC:1,EC:1.13.11.4;Enzyme_name=Oxidoreductases,Oxidoreductases,gentisate 1,2-dioxygenase
contig00003.g1929	981	ID=contig00003.g1929;Description=5-carboxymethyl-2-hydroxymuconate isomerase [Fusarium verticillioides 7600];Gene=FVER53263_08844;Ontology_term=isomerase activity;Ontology_id=GO:0016853;Enzyme_code=EC:5,EC:5;Enzyme_name=Isomerases,Isomerases
contig00003.g1930	2444	ID=contig00003.g1930;Description=C6 transcription factor [Fusarium tjaetaba];Gene=FNAPI_7589;Ontology_term=nucleus,DNA binding,regulation of DNA-templated transcription,transcription regulator activity;Ontology_id=GO:0005634,GO:0003677,GO:0006355,GO:0140110
contig00003.g1931	930	ID=contig00003.g1931;Description=putative beta-xylosidase [Fusarium oxysporum f. sp. albedinis];Gene=FocTR4_00015117;Ontology_term=lyase activity;Ontology_id=GO:0016829;Enzyme_code=EC:4.4,EC:4.4,EC:4;Enzyme_name=Carbon-sulfur lyases,Carbon-sulfur lyases,Lyases
contig00003.g1932	2613	ID=contig00003.g1932;Description=Beta-glucosidase B [Fusarium oxysporum f. sp. conglutinans];Gene=FocTR4_00015118;Ontology_term=carbohydrate metabolic process,hydrolase activity;Ontology_id=GO:0005975,GO:0016787;Enzyme_code=EC:3.2.1.21,EC:3.2,EC:3.2.1.21,EC:3,EC:3.2.1;Enzyme_name=beta-glucosidase,Glycosylases,beta-glucosidase,Hydrolases,Glycosylases
contig00003.g1933	1794	ID=contig00003.g1933;Description=related to hexose transporter protein [Fusarium fujikuroi];Gene=FVER53263_08841;Ontology_term=transmembrane transport,transporter activity;Ontology_id=GO:0055085,GO:0005215
contig00003.g1934	424	ID=contig00003.g1934;Description=hypothetical protein J7337_012484 [Fusarium musae]
contig00003.g1935	333	ID=contig00003.g1935;Description=hypothetical protein FVER14953_08839 [Fusarium verticillioides]

contig00003.g1936	903	ID=contig00003.g1936;Description=methyltransferase domain-containing protein [Fusarium coicis];Gene=FFUJ_10577;Ontology_term=transferase activity;Ontology_id=GO:0016740;Enzyme_code=EC:2.1.1,EC:2.1,EC:2,EC:2.1.1;Enzyme_name=Transferring one-carbon groups,Transferring one-carbon groups,Transferases,Transferring one-carbon groups
contig00003.g1937	1593	ID=contig00003.g1937;Description=Ammonium transporter 1 [Fusarium coicis]
contig00003.g1938	1593	ID=contig00003.g1938;Description=xylosidase glycosyl hydrolase [Fusarium denticulatum];Gene=FVEG_08836;Ontology_term=carbohydrate metabolic process,hydrolase activity;Ontology_id=GO:0005975,GO:0016787;Enzyme_code=EC:3.2.1,EC:3.2,EC:3,EC:3.2.1;Enzyme_name=Glycosylases,Glycosylases,Hydrolases,Glycosylases
contig00003.g1939	1029	ID=contig00003.g1939;Description=alcohol dehydrogenase [Fusarium subglutinans];Gene=FVER53263_08835;Ontology_term=oxidoreductase activity;Ontology_id=GO:0016491;Enzyme_code=EC:1.6,EC:1.6,EC:1;Enzyme_name=Acting on NADH or NADPH,Acting on NADH or NADPH,Oxidoreductases
contig00003.g1940	924	ID=contig00003.g1940;Description=nucleoside-diphosphate-sugar epimerase [Fusarium tjaetaba]
contig00003.g1941	907	ID=contig00003.g1941;Description=hypothetical protein NW753_013174 [Fusarium oxysporum];Gene=FNYG_12186;Ontology_term=hydrolase activity;Ontology_id=GO:0016787;Enzyme_code=EC:3.1;Enzyme_name=Acting on ester bonds
contig00003.g1942	1550	ID=contig00003.g1942;Description=hypothetical protein FVER53590_08832 [Fusarium verticillioides]
contig00003.g1943	744	ID=contig00003.g1943;Description=hypothetical protein FVER53590_08831 [Fusarium verticillioides]
contig00003.g1944	2820	ID=contig00003.g1944;Description=heterokaryon incompatibility (het-6OR allele) [Fusarium tjaetaba]
contig00003.g1945	1347	ID=contig00003.g1945;Description=hypothetical protein FVER53263_08829 [Fusarium verticillioides]

contig00003.g1946	754	ID=contig00003.g1946;Description=endo-1,4-beta-xylanase [Fusarium verticillioides 7600];Gene=FPCIR_401;Ontology_term=carbohydrate metabolic process,cell wall organization or biogenesis,hydrolase activity;Ontology_id=GO:0005975,GO:0071554,GO:0016787;Enzyme_code=EC:3.2.1.8,EC:3.2,EC:3.2.1.8,EC:3,EC:3.2.1;Enzyme_name=endo-1,4-beta-xylanase,Glycosylases,endo-1,4-beta-xylanase,Hydrolases,Glycosylases
contig00003.g1947	669	ID=contig00003.g1947;Description=integral membrane protein [Fusarium denticulatum];Gene=FNAPI_7874;Ontology_term=membrane;Ontology_id=GO:0016020
contig00003.g1948	630	ID=contig00003.g1948;Description=nadph-dependent fmn reductase [Fusarium tjaetaba];Gene=F52700_9658;Ontology_term=oxidoreductase activity;Ontology_id=GO:0016491;Enzyme_code=EC:1,EC:1;Enzyme_name=Oxidoreductases,Oxidoreductases
contig00003.g1949	2809	ID=contig00003.g1949;Description=hypothetical protein FVEG_08826 [Fusarium verticillioides 7600];Gene=FDENT_1273;Ontology_term=oxidoreductase activity;Ontology_id=GO:0016491;Enzyme_code=EC:1.6,EC:1.6,EC:1;Enzyme_name=Acting on NADH or NADPH,Acting on NADH or NADPH,Oxidoreductases
contig00003.g1950	423	ID=contig00003.g1950;Description=chaperonin 10-like protein [Fusarium oxysporum];Gene=FNYG_05698;Ontology_term=oxidoreductase activity;Ontology_id=GO:0016491;Enzyme_code=EC:1.6,EC:1.6,EC:1;Enzyme_name=Acting on NADH or NADPH,Acting on NADH or NADPH,Oxidoreductases
contig00003.g1951	1591	ID=contig00003.g1951;Description=endoglucanase type B [Fusarium verticillioides 7600];Gene=FMUND_202;Ontology_term=carbohydrate metabolic process,extracellular region,hydrolase activity;Ontology_id=GO:0005975,GO:0005576,GO:0016787;Enzyme_code=EC:3.2.1;Enzyme_name=Glycosylases
contig00003.g1952	2529	ID=contig00003.g1952;Description=alpha-glucuronidase [Fusarium verticillioides 7600];Gene=aguA;Ontology_term=carbohydrate metabolic process,cell wall organization or biogenesis,extracellular region,hydrolase activity;Ontology_id=GO:0005975,GO:0071554,GO:0005576,GO:0016787;Enzyme_code=EC:3.2.1.139,EC:3.2.1.139,EC:3.2,EC:3,EC:3.2.1;Enzyme_name=alpha-glucuronidase,alpha-glucuronidase,Glycosylases,Hydrolases,Glycosylases
contig00003.g1953	764	ID=contig00003.g1953;Description=hypothetical protein IL306_014725 [Fusarium sp. DS 682]
contig00003.g1954	1404	ID=contig00003.g1954;Description=hypothetical protein FVER14953_08822 [Fusarium verticillioides]

contig00003.g1955	990	ID=contig00003.g1955;Description=murein transglycosylase [Fusarium verticillioides 7600];Gene=FVER53263_08821;Ontology_term=extracellular region;Ontology_id=GO:0005576
contig00003.g1956	2274	ID=contig00003.g1956;Description=sensor hoxX [Fusarium tjaetaba];Gene=FACUT_6309;Ontology_term=DNA binding,catalytic activity;Ontology_id=GO:0003677,GO:0003824
contig00003.g1957	1475	ID=contig00003.g1957;Description=hypothetical protein FNYG_05691 [Fusarium nygamai];Gene=FMEXI_5458;Ontology_term=transferase activity;Ontology_id=GO:0016740;Enzyme_code=EC:2.1.1,EC:2.1,EC:2,EC:2.1.1;Enzyme_name=Transferring one-carbon groups,Transferring one-carbon groups,Transferases,Transferring one-carbon groups
contig00003.g1958	1430	ID=contig00003.g1958;Description=methyltransferase like [Fusarium coicis];Gene=FNAPI_7863;Ontology_term=transferase activity;Ontology_id=GO:0016740;Enzyme_code=EC:2.1.1;Enzyme_name=Transferring one-carbon groups
contig00003.g1959	1939	ID=contig00003.g1959;Description=alcohol oxidase [Fusarium coicis];Gene=FVER53590_08817;Ontology_term=oxidoreductase activity;Ontology_id=GO:0016491;Enzyme_code=EC:1.1,EC:1.1,EC:1;Enzyme_name=Acting on the CH-OH group of donors,Acting on the CH-OH group of donors,Oxidoreductases
contig00003.g1960	1258	ID=contig00003.g1960;Description=unnamed protein product [Fusarium fujikuroi];Gene=rasS;Ontology_term=cell motility,vesicle-mediated transport,molecular function regulator activity,cytoskeleton organization,GTPase activity,signaling,anatomical structure development,plasma membrane;Ontology_id=GO:0048870,GO:0016192,GO:0098772,GO:0007010,GO:0003924,GO:0023052,GO:0048856,GO:0005886;Enzyme_code=EC:3.6.1.15,EC:3.6.1,EC:3.6,EC:3,EC:3.6.1.15;Enzyme_name=nucleoside-triphosphate phosphatase,Acting on acid anhydrides,Acting on acid anhydrides,Hydrolases,nucleoside-triphosphate phosphatase
contig00003.g1961	1408	ID=contig00003.g1961;Description=heterokaryon incompatibility (het-6OR allele) [Fusarium tjaetaba]

contig00003.g1962	2073	ID=contig00003.g1962;Description=hypothetical protein FVEG_08814 [Fusarium verticillioides 7600];Gene=rasD;Ontology_term=molecular function regulator activity,GTPase activity,signaling,cytokinesis,cytosol,cytoskeletal protein binding,plasma membrane,mitotic cell cycle;Ontology_id=GO:0098772,GO:0003924,GO:0023052,GO:0000910,GO:0005829,GO:0008092,GO:0005886,GO:0000278;Enzyme_code=EC:3.6.1.15,EC:3.6.1,EC:3.6,EC:3,EC:3.6.1.15;Enzyme_name=nucleoside-triphosphate phosphatase,Acting on acid anhydrides,Acting on acid anhydrides,Hydrolases,nucleoside-triphosphate phosphatase
contig00003.g1963	1121	ID=contig00003.g1963;Description=kinase-like domain-containing protein [Fusarium redolens]
contig00003.g1964	1332	ID=contig00003.g1964;Description=voltage-dependent anion channel-domain-containing protein [Fusarium oxysporum];Gene=FMEXI_59;Ontology_term=transmembrane transport,transporter activity;Ontology_id=GO:0055085,GO:0005215
contig00003.g1965	510	ID=contig00003.g1965;Description=hypothetical protein J7337_012516 [Fusarium musae]
contig00003.g1966	1272	ID=contig00003.g1966;Description=putative glucarate dehydratase [Fusarium oxysporum];Gene=gudD;Ontology_term=metal ion binding;Ontology_id=GO:0046872;Enzyme_code=EC:4.2.1.40;Enzyme_name=glucarate dehydratase
contig00003.g1967	1732	ID=contig00003.g1967;Description=nicotinamide mononucleotide permease [Fusarium tjaetaba];Gene=FPCIR_10509;Ontology_term=transmembrane transport,transporter activity;Ontology_id=GO:0055085,GO:0005215
contig00003.g1968	927	ID=contig00003.g1968;Description=hypothetical protein J7337_012519 [Fusarium musae]
contig00003.g1969	1038	ID=contig00003.g1969;Description=dihydrodipicolinate synthase [Fusarium subglutinans];Gene=FVER53590_08807;Ontology_term=lyase activity;Ontology_id=GO:0016829;Enzyme_code=EC:4,EC:4.1.3.16,EC:4;Enzyme_name=Lyases,4-hydroxy-2-oxoglutarate aldolase,Lyases
contig00003.g1970	924	ID=contig00003.g1970;Description=oxidoreductase [Fusarium verticillioides 7600];Gene=FVER53590_08806;Ontology_term=nucleus,oxidoreductase activity;Ontology_id=GO:0005634,GO:0016491;Enzyme_code=EC:1,EC:1.1,EC:1,EC:1.1.1,EC:1.1.1.276,EC:1.1.1.184,EC:1.1.1.381;Enzyme_name=Oxidoreductases,Acting on the CH-OH group of donors,Oxidoreductases,Acting on the CH-OH group of donors,serine 3-dehydrogenase (NADP(+)),carbonyl reductase (NADPH),3-hydroxy acid dehydrogenase

contig00003.g1971	2198	ID=contig00003.g1971;Description=hypothetical protein FVER53263_08805 [Fusarium verticillioides];Gene=FMUND_10930;Ontology_term=nucleus,DNA binding,DNA-templated transcription;Ontology_id=GO:0005634,GO:0003677,GO:0006351
contig00003.g1972	1005	ID=contig00003.g1972;Description=ADP-ribosylglycohydrolase [Fusarium verticillioides 7600];Gene=FVEG_08804;Ontology_term=hydrolase activity;Ontology_id=GO:0016787;Enzyme_code=EC:3,EC:3;Enzyme_name=Hydrolases,Hydrolases
contig00003.g1973	1056	ID=contig00003.g1973;Description=nad dependent epimerase dehydratase [Fusarium pseudoanthophilum]
contig00003.g1974	2053	ID=contig00003.g1974;Description=hypothetical protein FVEG_08802 [Fusarium verticillioides 7600]
contig00003.g1975	905	ID=contig00003.g1975;Description=GPR1 protein [Fusarium fujikuroi];Gene=FOYG_13590
contig00003.g1976	1710	ID=contig00003.g1976;Description=hypothetical protein FVER53263_08799 [Fusarium verticillioides]
contig00003.g1977	1095	ID=contig00003.g1977;Description=glycosyltransferase family 34 [Fusarium napiforme];Gene=FOIG_13482;Ontology_term=cytoskeleton,cytoskeleton organization,structural molecule activity,transferase activity,hydrolase activity;Ontology_id=GO:0005856,GO:0007010,GO:0005198,GO:0016740,GO:0016787;Enzyme_code=EC:2.4,EC:3,EC:2.4,EC:2,EC:3;Enzyme_name=Glycosyltransferases,Hydrolases,Glycosyltransferases,Transferases,Hydrolases
contig00003.g1978	1670	ID=contig00003.g1978;Description=hypothetical protein FVEG_08797 [Fusarium verticillioides 7600];Gene=FRV6_14688;Ontology_term=nucleus,regulation of DNA-templated transcription,transcription regulator activity;Ontology_id=GO:0005634,GO:0006355,GO:0140110
contig00003.g1979	1143	ID=contig00003.g1979;Description=related to toxD protein [Fusarium proliferatum ET1];Gene=FPRO05_00538;Ontology_term=oxidoreductase activity;Ontology_id=GO:0016491;Enzyme_code=EC:1.6,EC:1.6,EC:1;Enzyme_name=Acting on NADH or NADPH,Acting on NADH or NADPH,Oxidoreductases
contig00003.g1980	969	ID=contig00003.g1980;Description=hypothetical protein FVEG_08795 [Fusarium verticillioides 7600]
contig00003.g1981	987	ID=contig00003.g1981;Description=glycosyltransferase family 28 domain-containing [Fusarium acutatum];Gene=FPRO_14154;Ontology_term=transferase activity;Ontology_id=GO:0016740;Enzyme_code=EC:2,EC:2;Enzyme_name=Transferases,Transferases

contig00003.g1982	5586	ID=contig00003.g1982;Description=ankyrin repeat domain protein [Fusarium subglutinans]
contig00003.g1983	913	ID=contig00003.g1983;Description=hypothetical protein FVEG_08792 [Fusarium verticillioides 7600]
contig00003.g1984	1792	ID=contig00003.g1984;Description=hypothetical protein J7337_012536 [Fusarium musae];Gene=FDENT_1239;Ontology_term=membrane;Ontology_id=GO:0016020
contig00003.g1985	931	ID=contig00003.g1985;Description=aquaporin rerated protein, other eukaryote [Fusarium verticillioides 7600];Gene=FMEXI_37;Ontology_term=transmembrane transport,transporter activity;Ontology_id=GO:0055085,GO:0005215
contig00003.g1986	1919	ID=contig00003.g1986;Description=hypothetical protein FVEG_08789 [Fusarium verticillioides 7600]
contig00003.g1987	1376	ID=contig00003.g1987;Description=hypothetical protein FCIRC_9086 [Fusarium circinatum];Gene=FOIG_13493;Ontology_term=transferase activity;Ontology_id=GO:0016740;Enzyme_code=EC:2.3.1,EC:2.3,EC:2,EC:2.3.1;Enzyme_name=Acyltransferases,Acyltransferases,Transferases,Acyltransferases
contig00003.g1988	1660	ID=contig00003.g1988;Description=Arginine metabolism regulation protein II [Fusarium odoratissimum];Gene=F53441_8795;Ontology_term=nucleus,regulation of DNA-templated transcription,transcription regulator activity;Ontology_id=GO:0005634,GO:0006355,GO:0140110
contig00003.g1989	1130	ID=contig00003.g1989;Description=hypothetical protein FVEG_16437 [Fusarium verticillioides 7600];Gene=FVER53590_29540;Ontology_term=nucleus,regulation of DNA-templated transcription,transcription regulator activity;Ontology_id=GO:0005634,GO:0006355,GO:0140110
contig00003.g1990	1721	ID=contig00003.g1990;Description=cytochrome P450 oxidoreductase [Fusarium verticillioides 7600];Gene=FOVG_13292;Ontology_term=oxidoreductase activity;Ontology_id=GO:0016491;Enzyme_code=EC:1.14,EC:1.14.19.41,EC:1,EC:1.14;Enzyme_name=Acting on paired donors, with incorporation or reduction of molecular oxygen. The oxygen incorporated need not be derived from O2,sterol 22-desaturase,Oxidoreductases,Acting on paired donors, with incorporation or reduction of molecular oxygen. The oxygen incorporated need not be derived from O2
contig00003.g1991	1874	ID=contig00003.g1991;Description=triacylglycerol lipase V precursor [Fusarium tjaetaba];Gene=FCIRC_9082;Ontology_term=hydrolase activity;Ontology_id=GO:0016787;Enzyme_code=EC:3,EC:3;Enzyme_name=Hydrolases,Hydrolases

contig00003.g1992	1052	ID=contig00003.g1992;Description=integral membrane protein pth11 [Fusarium fujikuroi];Gene=FPRO_14166
contig00003.g1993	729	ID=contig00003.g1993;Description=glycoside hydrolase family 61 [Fusarium tjaetaba];Gene=FVER53590_08783;Ontology_term=extracellular region,hydrolase activity;Ontology_id=GO:0005576,GO:0016787;Enzyme_code=EC:3,EC:3;Enzyme_name=Hydrolases,Hydrolases
contig00003.g1994	728	ID=contig00003.g1994;Description=filamentous hemagglutinin / adhesin [Fusarium bulbicola];Gene=FDENT_1228
contig00003.g1995	996	ID=contig00003.g1995;Description=alcohol dehydrogenase [Fusarium verticillioides 7600]
contig00003.g1996	948	ID=contig00003.g1996;Description=hypothetical protein FVER53590_29541 [Fusarium verticillioides]
contig00003.g1997	1427	ID=contig00003.g1997;Description=carboxypeptidase [Fusarium tjaetaba];Gene=FVER53590_08780;Ontology_term=catalytic activity, acting on a protein,extracellular region,hydrolase activity;Ontology_id=GO:0140096,GO:0005576,GO:0016787;Enzyme_code=EC:3.4.17,EC:3.4,EC:3.4.17,EC:3;Enzyme_name=Acting on peptide bonds (peptidases),Acting on peptide bonds (peptidases),Acting on peptide bonds (peptidases),Hydrolases
contig00003.g1998	930	ID=contig00003.g1998;Description=hydrolase or acyltransferase of alpha beta superfamily [Fusarium tjaetaba];Gene=FNAPI_4555;Ontology_term=transferase activity,hydrolase activity;Ontology_id=GO:0016740,GO:0016787;Enzyme_code=EC:2.3,EC:3,EC:2.3,EC:2,EC:3;Enzyme_name=Acyltransferases,Hydrolases,Acyltransferases,Transferases,Hydrolases
contig00003.g1999	1149	ID=contig00003.g1999;Description=chaperonin 10-like protein [Fusarium flagelliforme];Gene=FVER53263_08778;Ontology_term=oxidoreductase activity;Ontology_id=GO:0016491;Enzyme_code=EC:1.6,EC:1.6,EC:1;Enzyme_name=Acting on NADH or NADPH,Acting on NADH or NADPH,Oxidoreductases
contig00003.g2000	1684	ID=contig00003.g2000;Description=MFS phospholipid transporter Git1 [Metarhizium acridum CQMa 102];Gene=MAC_09160;Ontology_term=transmembrane transport,transporter activity;Ontology_id=GO:0055085,GO:0005215
contig00003.g2001	5377	ID=contig00003.g2001;Description=hypothetical protein FVEG_16432 [Fusarium verticillioides 7600]

contig00003.g2002	4489	ID=contig00003.g2002;Description=L-serine dehydratase [Fusarium napiforme];Gene=FNYG_05643;Ontology_term=carbohydrate metabolic process,lyase activity;Ontology_id=GO:0005975,GO:0016829;Enzyme_code=EC:4.3.1.17,EC:4.3.1.17,EC:4.3,EC:4,EC:4.3.1;Enzyme_name=L-serine ammonia-lyase,L-serine ammonia-lyase,Carbon-nitrogen lyases,Lyases,Carbon-nitrogen lyases
contig00003.g2003	1113	ID=contig00003.g2003;Description=Catechol 1,2-dioxygenase [Fusarium oxysporum f. sp. raphani];Gene=FOXB_06341;Ontology_term=oxidoreductase activity;Ontology_id=GO:0016491;Enzyme_code=EC:1.13.11.1,EC:1.13.11,EC:1.13.11.1,EC:1.13,EC:1;Enzyme_name=catechol 1,2-dioxygenase,Acting on single donors with incorporation of molecular oxygen (oxygenases). The oxygen incorporated need not be derived from O2,catechol 1,2-dioxygenase,Acting on single donors with incorporation of molecular oxygen (oxygenases). The oxygen incorporated need not be derived from O2,Oxidoreductases
contig00003.g2004	1854	ID=contig00003.g2004;Description=CAIB BAIF family enzyme [Fusarium tjaetaba];Gene=FNYG_05641;Ontology_term=catalytic activity;Ontology_id=GO:0003824
contig00003.g2005	3543	ID=contig00003.g2005;Description=transcriptional activator Mut3p [Fusarium tjaetaba];Gene=FMEXI_1341;Ontology_term=nucleus,DNA binding,regulation of DNA-templated transcription,transcription regulator activity;Ontology_id=GO:0005634,GO:0003677,GO:0006355,GO:0140110
contig00003.g2006	1080	ID=contig00003.g2006;Description=transcriptional activator Mut3p [Fusarium sp. NRRL 52700];Gene=FVER53590_08770;Ontology_term=nucleus,DNA binding,regulation of DNA-templated transcription,transcription regulator activity,hydrolase activity;Ontology_id=GO:0005634,GO:0003677,GO:0006355,GO:0140110,GO:0016787;Enzyme_code=EC:3,EC:3;Enzyme_name=Hydrolases,Hydrolases
contig00003.g2007	2516	ID=contig00003.g2007;Description=Aromatic peroxygenase [Fusarium oxysporum f. sp. cubense race 1];Gene=FVER53263_08769;Ontology_term=nucleus,antioxidant activity,oxidoreductase activity,regulation of DNA-templated transcription,transcription regulator activity;Ontology_id=GO:0005634,GO:0016209,GO:0016491,GO:0006355,GO:0140110;Enzyme_code=EC:1.11.1,EC:1.11,EC:1,EC:1.11.1,EC:1.11.2.1;Enzyme_name=Acting on a peroxide as acceptor,Acting on a peroxide as acceptor,Oxidoreductases,Acting on a peroxide as acceptor,unspecific peroxygenase

contig00003.g2008	2135	ID=contig00003.g2008;Description=cercosporin resistance [Fusarium tjaetaba];Gene=FVER53263_08768;Ontology_term=nucleus,antioxidant activity,oxidoreductase activity,regulation of DNA-templated transcription,transcription regulator activity;Ontology_id=GO:0005634,GO:0016209,GO:0016491,GO:0006355,GO:0140110;Enzyme_code=EC:1.11.1,EC:1.11,EC:1,EC:1.11.1;Enzyme_name=Acting on a peroxide as acceptor,Acting on a peroxide as acceptor,Oxidoreductases,Acting on a peroxide as acceptor
contig00003.g2009	1015	ID=contig00003.g2009;Description=related to peroxisomal short-chain alcohol dehydrogenase [Fusarium mangiferae]
contig00003.g2010	1329	ID=contig00003.g2010;Description=nitrosoguanidine resistance SNG1 [Fusarium sp. NRRL 25303];Gene=FVER53263_08766;Ontology_term=membrane;Ontology_id=GO:0016020
contig00003.g2011	310	ID=contig00003.g2011;Description=Transcription elongation factor SPT5 [Fusarium oxysporum f. sp. albedinis]
contig00003.g2012	1265	ID=contig00003.g2012;Description=f-box domain protein [Fusarium tjaetaba]
contig00003.g2013	807	ID=contig00003.g2013;Description=Alpha/beta hydrolase fold-1 [Fusarium oxysporum];Gene=Forpi1262_v013324;Ontology_term=hydrolase activity;Ontology_id=GO:0016787;Enzyme_code=EC:3,EC:3;Enzyme_name=Hydrolases,Hydrolases
contig00003.g2014	1385	ID=contig00003.g2014;Description=salicylate hydroxylase [Fusarium verticillioides 7600];Gene=FPCIR_3757;Ontology_term=oxidoreductase activity;Ontology_id=GO:0016491;Enzyme_code=EC:1,EC:1,EC:1.14.13.1;Enzyme_name=Oxidoreductases,Oxidoreductases,salicylate 1-monooxygenase
contig00003.g2015	2444	ID=contig00003.g2015;Description=related to nucleoside-diphosphate-sugar epimerase [Fusarium fujikuroi]
contig00003.g2016	1113	ID=contig00003.g2016;Description=FAD dependent oxidoreductase [Fusarium redolens];Gene=foxred1;Ontology_term=oxidoreductase activity;Ontology_id=GO:0016491;Enzyme_code=EC:1,EC:1;Enzyme_name=Oxidoreductases,Oxidoreductases
contig00003.g2017	3590	ID=contig00003.g2017;Description=related to transcriptional activator Mut3p [Fusarium proliferatum ET1];Gene=FPRO_14195;Ontology_term=regulation of DNA-templated transcription;Ontology_id=GO:0006355

contig00003.g2018	1599	ID=contig00003.g2018;Description=autophagy (Atg22) [Fusarium tjaetaba];Gene=FNAPI_4534;Ontology_term=autophagy,vacuole;Ontology_id=GO:0006914,GO:0005773
contig00003.g2019	1476	ID=contig00003.g2019;Description=hypothetical protein J7337_012579 [Fusarium musae]
contig00003.g2020	1600	ID=contig00003.g2020;Description=L-lactate dehydrogenase (cytochrome) [Fusarium verticillioides 7600];Gene=FVER53263_20753;Ontology_term=oxidoreductase activity;Ontology_id=GO:0016491;Enzyme_code=EC:1;Enzyme_name=Oxidoreductases
contig00003.g2021	1517	ID=contig00003.g2021;Description=oxidase [Fusarium tjaetaba];Gene=FVER53263_08757;Ontology_term=antioxidant activity,oxidoreductase activity;Ontology_id=GO:0016209,GO:0016491;Enzyme_code=EC:1.11.1,EC:1.11,EC:1,EC:1.11.1;Enzyme_name=Acting on a peroxide as acceptor,Acting on a peroxide as acceptor,Oxidoreductases,Acting on a peroxide as acceptor
contig00003.g2022	1555	ID=contig00003.g2022;Description=related to integral membrane protein [Fusarium fujikuroi];Gene=FTJAE_2096;Ontology_term=side of membrane;Ontology_id=GO:0098552
contig00003.g2023	1335	ID=contig00003.g2023;Description=non-ribosomal peptide synthetase [Fusarium tjaetaba];Gene=FVEG_16419;Ontology_term=oxidoreductase activity;Ontology_id=GO:0016491;Enzyme_code=EC:1,EC:1;Enzyme_name=Oxidoreductases,Oxidoreductases
contig00003.g2024	3241	ID=contig00003.g2024;Description=Fumagillin beta-trans-bergamotene synthase [Fusarium oxysporum f. sp. conglutinans];Gene=FOTG_14033;Ontology_term=nucleus,DNA binding,DNA-templated transcription,transferase activity;Ontology_id=GO:0005634,GO:0003677,GO:0006351,GO:0016740;Enzyme_code=EC:2.5.1,EC:2.5.1,EC:2;Enzyme_name=Transferring alkyl or aryl groups, other than methyl groups,Transferring alkyl or aryl groups, other than methyl groups,Transferases
contig00003.g2025	1772	ID=contig00003.g2025;Description=hexose transporter [Fusarium pseudocircinatum];Gene=FOTG_14035;Ontology_term=transmembrane transport,transporter activity;Ontology_id=GO:0055085,GO:0005215
contig00003.g2026	2106	ID=contig00003.g2026;Description=trehalose-6-phosphate hydrolase [Fusarium acutatum];Gene=TRX2;Ontology_term=carbohydrate metabolic process,hydrolase activity;Ontology_id=GO:0005975,GO:0016787;Enzyme_code=EC:3.2.1.20,EC:3.2,EC:3.2.1.20,EC:3.2.1.10,EC:3,EC:3.2.1.26,EC:3.2.1.48,EC:3.2.1;Enzyme_name=alpha-glucosidase,Glycosylases,alpha-glucosidase,oligo-1,6-glucosidase,Hydrolases,beta-fructofuranosidase,sucrose alpha-glucosidase,Glycosylases

contig00003.g2027	814	ID=contig00003.g2027;Description=hypothetical protein FVER53590_08751 [Fusarium verticillioides];Gene=FVER53263_08752;Ontology_term=carbohydrate metabolic process,hydrolase activity;Ontology_id=GO:0005975,GO:0016787;Enzyme_code=EC:3.2.1.20,EC:3.2,EC:3.2.1.20,EC:3,EC:3.2.1;Enzyme_name=alpha-glucosidase,Glycosylases,alpha-glucosidase,Hydrolases,Glycosylases
contig00003.g2028	961	ID=contig00003.g2028;Description=hypothetical protein FVER53263_08750 [Fusarium verticillioides]
contig00003.g2029	1671	ID=contig00003.g2029;Description=related to O-methylsterigmatocystin oxidoreductase [Fusarium proliferatum ET1];Gene=FVEG_08749;Ontology_term=oxidoreductase activity;Ontology_id=GO:0016491;Enzyme_code=EC:1.14,EC:1,EC:1.14,EC:1.14.14.54;Enzyme_name=Acting on paired donors, with incorporation or reduction of molecular oxygen. The oxygen incorporated need not be derived from O2,Oxidoreductases,Acting on paired donors, with incorporation or reduction of molecular oxygen. The oxygen incorporated need not be derived from O2,phenylacetate 2-hydroxylase
contig00003.g2030	1726	ID=contig00003.g2030;Description=hypothetical protein FOXYS1_14865 [Fusarium oxysporum]
contig00003.g2031	1436	ID=contig00003.g2031;Description=unc-45 like b [Fusarium tjaetaba];Gene=FNAPI_13944;Ontology_term=transferase activity;Ontology_id=GO:0016740;Enzyme_code=EC:2.1.1,EC:2.1,EC:2,EC:2.1.1;Enzyme_name=Transferring one-carbon groups,Transferring one-carbon groups,Transferases,Transferring one-carbon groups
contig00003.g2032	672	ID=contig00003.g2032;Description=unc-45 like b [Fusarium tjaetaba];Gene=FTJAE_6054;Ontology_term=transferase activity;Ontology_id=GO:0016740;Enzyme_code=EC:2.1.1,EC:2.1,EC:2,EC:2.1.1;Enzyme_name=Transferring one-carbon groups,Transferring one-carbon groups,Transferases,Transferring one-carbon groups
contig00003.g2033	387	ID=contig00003.g2033;Description=hypothetical protein FVEG_08746 [Fusarium verticillioides 7600];Gene=ORF 46

contig00003.g2034	3788	ID=contig00003.g2034;Description=cytochrome P450 monooxygenase 4ac1 [Fusarium tjaetaba];Gene=FTJAE_6056;Ontology_term=nucleus,oxidoreductase activity,regulation of DNA-templated transcription,transcription regulator activity;Ontology_id=GO:0005634,GO:0016491,GO:0006355,GO:0140110;Enzyme_code=EC:1.14,EC:1,EC:1.14;Enzyme_name=Acting on paired donors, with incorporation or reduction of molecular oxygen. The oxygen incorporated need not be derived from O2,Oxidoreductases,Acting on paired donors, with incorporation or reduction of molecular oxygen. The oxygen incorporated need not be derived from O2
contig00003.g2035	1823	ID=contig00003.g2035;Description=monosaccharide transporter [Fusarium tjaetaba];Gene=FPCIR_3778;Ontology_term=transmembrane transport,transporter activity;Ontology_id=GO:0055085,GO:0005215
contig00003.g2036	1481	ID=contig00003.g2036;Description=salicylate hydroxylase [Fusarium tjaetaba];Gene=FPANT_6939;Ontology_term=oxidoreductase activity;Ontology_id=GO:0016491;Enzyme_code=EC:1,EC:1,EC:1.14.13.1;Enzyme_name=Oxidoreductases,Oxidoreductases,salicylate 1-monooxygenase
contig00003.g2037	1459	ID=contig00003.g2037;Description=oxidoreductase [Fusarium verticillioides 7600];Gene=FNAPI_12768;Ontology_term=catalytic activity, acting on a protein,oxidoreductase activity,transferase activity;Ontology_id=GO:0140096,GO:0016491,GO:0016740;Enzyme_code=EC:2.7.1,EC:1,EC:2.7.1,EC:1,EC:2.7,EC:2;Enzyme_name=Transferring phosphorus-containing groups,Oxidoreductases,Transferring phosphorus-containing groups,Oxidoreductases,Transferring phosphorus-containing groups,Transferases
contig00003.g2038	3924	ID=contig00003.g2038;Description=serine/threonine protein kinase [Fusarium verticillioides 7600];Gene=FMUND_6603;Ontology_term=catalytic activity, acting on a protein,transferase activity;Ontology_id=GO:0140096,GO:0016740;Enzyme_code=EC:2.7.11.1,EC:2.7.1,EC:2.7.11.1,EC:2.7,EC:2;Enzyme_name=non-specific serine/threonine protein kinase,Transferring phosphorus-containing groups,non-specific serine/threonine protein kinase,Transferring phosphorus-containing groups,Transferases

contig00003.g2039	3878	ID=contig00003.g2039;Description=serine/threonine protein kinase [Fusarium odoratissimum NRRL 54006];Gene=FOXG_09738;Ontology_term=catalytic activity, acting on a protein,transferase activity;Ontology_id=GO:0140096,GO:0016740;Enzyme_code=EC:2.7.11.1,EC:2.7.1,EC:2.7.11.1,EC:2.7,EC:2;Enzyme_name=non-specific serine/threonine protein kinase,Transferring phosphorus-containing groups,non-specific serine/threonine protein kinase,Transferring phosphorus-containing groups,Transferases
contig00003.g2040	1775	ID=contig00003.g2040;Description=putative quinate permease [Fusarium oxysporum];Gene=FVEG_08737;Ontology_term=transmembrane transport,transporter activity;Ontology_id=GO:0055085,GO:0005215
contig00003.g2041	1149	ID=contig00003.g2041;Description=related to hydroxylase [Fusarium proliferatum ET1];Gene=FVER53263_08736;Ontology_term=oxidoreductase activity;Ontology_id=GO:0016491;Enzyme_code=EC:1,EC:1;Enzyme_name=Oxidoreductases,Oxidoreductases
contig00003.g2042	1224	ID=contig00003.g2042;Description=hypothetical protein FVER14953_08735 [Fusarium verticillioides];Gene=FVER53263_08735;Ontology_term=transmembrane transport,oxidoreductase activity,transporter activity;Ontology_id=GO:0055085,GO:0016491,GO:0005215;Enzyme_code=EC:1,EC:1;Enzyme_name=Oxidoreductases,Oxidoreductases
contig00003.g2043	1110	ID=contig00003.g2043;Description=pectate lyase [Fusarium verticillioides 7600];Gene=FVER53263_08734;Ontology_term=carbohydrate metabolic process,lyase activity,extracellular region;Ontology_id=GO:0005975,GO:0016829,GO:0005576;Enzyme_code=EC:4.2.2.2,EC:4.2.2.2,EC:4.2,EC:4,EC:4.2.2;Enzyme_name=pectate lyase,pectate lyase,Carbon-oxygen lyases,Lyases,Carbon-oxygen lyases
contig00003.g2044	1456	ID=contig00003.g2044;Description=glycoside hydrolase family 76 [Fusarium tjaetaba];Gene=FTJAE_6065;Ontology_term=carbohydrate metabolic process,hydrolase activity;Ontology_id=GO:0005975,GO:0016787;Enzyme_code=EC:3.2.1.101,EC:3.2.1.24,EC:3.2,EC:3.2.1.101,EC:3,EC:3.2.1.24,EC:3.2.1;Enzyme_name=mannan endo-1,6-alpha-mannosidase,alpha-mannosidase,Glycosylases,mannan endo-1,6-alpha-mannosidase,Hydrolases,alpha-mannosidase,Glycosylases
contig00003.g2045	324	ID=contig00003.g2045;Description=hypothetical protein FVEG_08732 [Fusarium verticillioides 7600]

contig00003.g2046	1717	ID=contig00003.g2046;Description=major facilitator superfamily transporter [Fusarium tjaetaba];Gene=FTJAE_6067;Ontology_term=transmembrane transport,transporter activity;Ontology_id=GO:0055085,GO:0005215
contig00003.g2047	853	ID=contig00003.g2047;Description=related to isoamyl alcohol oxidase [Fusarium fujikuroi IMI 58289];Gene=60;Ontology_term=oxidoreductase activity,extracellular region,external encapsulating structure;Ontology_id=GO:0016491,GO:0005576,GO:0030312;Enzyme_code=EC:1,EC:1;Enzyme_name=Oxidoreductases,Oxidoreductases
contig00003.g2048	1845	ID=contig00003.g2048;Description=isoamyl alcohol oxidase [Fusarium coicis];Gene=FVEG_16409;Ontology_term=oxidoreductase activity;Ontology_id=GO:0016491;Enzyme_code=EC:1,EC:1;Enzyme_name=Oxidoreductases,Oxidoreductases
contig00003.g2049	871	ID=contig00003.g2049;Description=7-aminocholesterol resistance [Fusarium tjaetaba];Gene=FVEG_16408;Ontology_term=membrane;Ontology_id=GO:0016020
contig00003.g2050	1265	ID=contig00003.g2050;Description=C6 finger domain protein [Fusarium tjaetaba];Gene=FVER53590_08729;Ontology_term=nucleus,regulation of DNA-templated transcription,transcription regulator activity;Ontology_id=GO:0005634,GO:0006355,GO:0140110
contig00003.g2051	414	ID=contig00003.g2051;Description=atp-dependent dna helicase [Fusarium napiforme];Gene=FNAPI_1772;Ontology_term=catalytic activity,ATP-dependent activity;Ontology_id=GO:0003824,GO:0140657
contig00003.g2052	1679	ID=contig00003.g2052;Description=hypothetical protein FVER53590_08727 [Fusarium verticillioides]
contig00003.g2053	1023	ID=contig00003.g2053;Description=WW domain protein [Fusarium tjaetaba]
contig00003.g2054	921	ID=contig00003.g2054;Description=nad dependent epimerase dehydratase [Fusarium tjaetaba]
contig00003.g2055	1194	ID=contig00003.g2055;Description=trans-aconitate 2-methyltransferase [Fusarium tjaetaba];Gene=FVER53263_08724;Ontology_term=transferase activity;Ontology_id=GO:0016740;Enzyme_code=EC:2.1.1,EC:2.1,EC:2,EC:2.1.1;Enzyme_name=Transferring one-carbon groups,Transferring one-carbon groups,Transferases,Transferring one-carbon groups

contig00003.g2056	1231	ID=contig00003.g2056;Description=mixed-linked glucanase precursor MLG1 [Fusarium tjaetaba];Gene=FTJAE_9110;Ontology_term=carbohydrate metabolic process,hydrolase activity;Ontology_id=GO:0005975,GO:0016787;Enzyme_code=EC:3.2.1;Enzyme_name=Glycosylases
contig00003.g2057	1806	ID=contig00003.g2057;Description=major facilitator superfamily transporter [Fusarium tjaetaba];Gene=FTJAE_9111;Ontology_term=transmembrane transport,transporter activity;Ontology_id=GO:0055085,GO:0005215
contig00003.g2058	2169	ID=contig00003.g2058;Description=major facilitator superfamily transporter [Fusarium tjaetaba];Gene=FOXB_06375;Ontology_term=transmembrane transport,transporter activity;Ontology_id=GO:0055085,GO:0005215
contig00003.g2059	2286	ID=contig00003.g2059;Description=STB5-transcription factor [Fusarium tjaetaba];Gene=FPHYL_4093;Ontology_term=nucleus,DNA binding,regulation of DNA-templated transcription,transcription regulator activity;Ontology_id=GO:0005634,GO:0003677,GO:0006355,GO:0140110
contig00003.g2060	1152	ID=contig00003.g2060;Description=unnamed protein product [Fusarium graminearum]
contig00003.g2061	1290	ID=contig00003.g2061;Description=salicylate 1-monooxygenase [Fusarium tjaetaba];Gene=FPANT_6273;Ontology_term=oxidoreductase activity;Ontology_id=GO:0016491;Enzyme_code=EC:1,EC:1,EC:1.14.13.1;Enzyme_name=Oxidoreductases,Oxidoreductases,salicylate 1-monooxygenase
contig00003.g2062	2056	ID=contig00003.g2062;Description=transporter HOL1 [Fusarium napiforme];Gene=FVEG_08716;Ontology_term=DNA binding,transmembrane transport,catalytic activity, acting on DNA,transferase activity,transporter activity;Ontology_id=GO:0003677,GO:0055085,GO:0140097,GO:0016740,GO:0005215;Enzyme_code=EC:2.1.1.113,EC:2.1,EC:2,EC:2.1.1.113,EC:2.1.1;Enzyme_name=site-specific DNA-methyltransferase (cytosine-N(4)-specific),Transferring one-carbon groups,Transferases,site-specific DNA-methyltransferase (cytosine-N(4)-specific),Transferring one-carbon groups
contig00003.g2063	1194	ID=contig00003.g2063;Description=hypothetical protein FVEG_08715 [Fusarium verticillioides 7600]

contig00003.g2064	1815	ID=contig00003.g2064;Description=cocaine esterase [Fusarium tjaetaba];Gene=cocE-6;Ontology_term=catalytic activity, acting on a protein,hydrolase activity;Ontology_id=GO:0140096,GO:0016787;Enzyme_code=EC:3.4.14.1,EC:3.4.14.5,EC:3.4.14.4,EC:3.4.14.2,EC:3.4.14.1,EC:3.4,EC:3,EC:3.4.14.5,EC:3.4.14.4,EC:3.4.14.2;Enzyme_name=dipeptidyl-peptidase I,dipeptidyl-peptidase IV,dipeptidyl-peptidase III,dipeptidyl-peptidase II,dipeptidyl-peptidase I,Acting on peptide bonds (peptidases),Hydrolases,dipeptidyl-peptidase IV,dipeptidyl-peptidase III,dipeptidyl-peptidase II
contig00003.g2065	679	ID=contig00003.g2065;Description=related to ThiJ/PfpI family protein [Fusarium mangiferae];Gene=gE
contig00003.g2066	1728	ID=contig00003.g2066;Description=siderophore iron transporter mirB [Fusarium tjaetaba];Gene=FTJAE_9120;Ontology_term=transmembrane transport,transporter activity;Ontology_id=GO:0055085,GO:0005215
contig00003.g2067	3852	ID=contig00003.g2067;Description=putative NRPS-like protein biosynthetic cluster [Fusarium musae]
contig00003.g2068	1561	ID=contig00003.g2068;Description=spore wall maturation DIT1 [Fusarium pseudoanthophilum]
contig00003.g2069	1050	ID=contig00003.g2069;Description=lipase esterase [Fusarium napiforme];Gene=FOQG_11062;Ontology_term=hydrolase activity;Ontology_id=GO:0016787;Enzyme_code=EC:3,EC:3;Enzyme_name=Hydrolases,Hydrolases
contig00003.g2070	1957	ID=contig00003.g2070;Description=hypothetical protein FVEG_08708 [Fusarium verticillioides 7600]
contig00003.g2071	2190	ID=contig00003.g2071;Description=alcohol oxidase [Fusarium tjaetaba];Gene=FDENT_665;Ontology_term=oxidoreductase activity;Ontology_id=GO:0016491;Enzyme_code=EC:1.1;Enzyme_name=Acting on the CH-OH group of donors
contig00003.g2072	2827	ID=contig00003.g2072;Description=hypothetical protein FVEG_16404 [Fusarium verticillioides 7600]
contig00003.g2073	532	ID=contig00003.g2073;Description=RNA-binding domain protein [Fusarium tjaetaba];Gene=FVEG_08705;Ontology_term=RNA binding;Ontology_id=GO:0003723
contig00003.g2074	803	ID=contig00003.g2074;Description=hypothetical protein H9Q70_005656 [Fusarium xylarioides]

contig00003.g2075	1716	ID=contig00003.g2075;Description=cytochrome P450 [Fusarium flagelliforme];Gene=BKA59DRAFT_504235;Ontology_term=sulfur compound metabolic process,oxidoreductase activity,detoxification;Ontology_id=GO:0006790,GO:0016491,GO:0098754;Enzyme_code=EC:1.14,EC:1,EC:1.14,EC:1.14.13,EC:1.14.14.54;Enzyme_name=Acting on paired donors, with incorporation or reduction of molecular oxygen. The oxygen incorporated need not be derived from O2,Oxidoreductases,Acting on paired donors, with incorporation or reduction of molecular oxygen. The oxygen incorporated need not be derived from O2,Acting on paired donors, with incorporation or reduction of molecular oxygen. The oxygen incorporated need not be derived from O2,phenylacetate 2-hydroxylase
contig00003.g2076	2382	ID=contig00003.g2076;Description=cellobiose dehydrogenase [Fusarium tjaetaba];Gene=FPANT_387;Ontology_term=oxidoreductase activity;Ontology_id=GO:0016491;Enzyme_code=EC:1.1,EC:1.1,EC:1.1.99.18,EC:1;Enzyme_name=Acting on the CH-OH group of donors,Acting on the CH-OH group of donors,cellobiose dehydrogenase (acceptor),Oxidoreductases
contig00003.g2077	961	ID=contig00003.g2077;Description=ferri-bacillibactin esterase [Fusarium subglutinans]
contig00003.g2078	1886	ID=contig00003.g2078;Description=major facilitator [Fusarium tjaetaba];Gene=FDENT_657;Ontology_term=transmembrane transport,transporter activity;Ontology_id=GO:0055085,GO:0005215
contig00003.g2079	1287	ID=contig00003.g2079;Description=aerobactin siderophore biosynthesis iucB [Fusarium napiforme];Gene=sidF;Ontology_term=transferase activity;Ontology_id=GO:0016740;Enzyme_code=EC:2.3,EC:2.3,EC:2;Enzyme_name=Acyltransferases,Acyltransferases,Transferases
contig00003.g2080	873	ID=contig00003.g2080;Description=enoyl-CoA hydratase [Fusarium proliferatum];Gene=LSDV091
contig00003.g2081	6260	ID=contig00003.g2081;Description=Non-ribosomal peptide synthetase [Fusarium musae];Gene=FVER53590_08697;Ontology_term=lipid metabolic process,ligase activity,isomerase activity;Ontology_id=GO:0006629,GO:0016874,GO:0016853;Enzyme_code=EC:5,EC:6,EC:5,EC:6;Enzyme_name=Isomerases,Ligases,Isomerases,Ligases
contig00003.g2082	1446	ID=contig00003.g2082;Description=nitrosoguanidine resistance SNG1 [Fusarium denticulatum];Gene=FDENT_653;Ontology_term=membrane;Ontology_id=GO:0016020
contig00003.g2083	1553	ID=contig00003.g2083;Description=hypothetical protein FVEG_16402 [Fusarium verticillioides 7600];Gene=92;Ontology_term=GTP binding;Ontology_id=GO:0005525

contig00003.g2084	330	ID=contig00003.g2084;Description=hypothetical protein FVER53590_28342 [Fusarium verticillioides];Gene=FVER53590_28342;Ontology_term=oxidoreductase activity;Ontology_id=GO:0016491;Enzyme_code=EC:1,EC:1;Enzyme_name=Oxidoreductases,Oxidoreductases
contig00003.g2085	480	ID=contig00003.g2085;Description=isoamyl alcohol oxidase [Fusarium coicis];Gene=FOZG_14032;Ontology_term=oxidoreductase activity;Ontology_id=GO:0016491;Enzyme_code=EC:1,EC:1;Enzyme_name=Oxidoreductases,Oxidoreductases
contig00003.g2086	2283	ID=contig00003.g2086;Description=hypothetical protein FVER53590_28344 [Fusarium verticillioides]
contig00003.g2087	1421	ID=contig00003.g2087;Description=integral membrane protein PTH11 [Fusarium tjaetaba];Gene=FNYG_05552;Ontology_term=membrane;Ontology_id=GO:0016020
contig00003.g2088	1072	ID=contig00003.g2088;Description=piccolo [Fusarium tjaetaba]
contig00003.g2089	1662	ID=contig00003.g2089;Description=FAD-dependent oxygenase [Fusarium tjaetaba];Gene=FTJAE_9142;Ontology_term=FAD binding;Ontology_id=GO:0071949
contig00003.g2090	1421	ID=contig00003.g2090;Description=integral membrane protein PTH11 [Fusarium tjaetaba];Gene=FTJAE_9143;Ontology_term=membrane;Ontology_id=GO:0016020
contig00003.g2091	591	ID=contig00003.g2091;Description=hypothetical protein FNAPI_1733 [Fusarium napiforme]
contig00003.g2092	1512	ID=contig00003.g2092;Description=mannitol 2-dehydrogenase [Fusarium verticillioides 7600];Gene=FPANT_401;Ontology_term=carbohydrate metabolic process,oxidoreductase activity,extracellular region;Ontology_id=GO:0005975,GO:0016491,GO:0005576;Enzyme_code=EC:1,EC:1.1,EC:1,EC:1.1.1.67,EC:1.1.1,EC:1.1.1.255;Enzyme_name=Oxidoreductases,Acting on the CH-OH group of donors,Oxidoreductases,mannitol 2-dehydrogenase,Acting on the CH-OH group of donors,mannitol dehydrogenase
contig00003.g2093	826	ID=contig00003.g2093;Description=PR-1 [Fusarium pseudoanthophilum]
contig00003.g2094	939	ID=contig00003.g2094;Description=iron-dependent peroxidase [Fusarium verticillioides 7600];Gene=FOZG_14042;Ontology_term=antioxidant activity,oxidoreductase activity;Ontology_id=GO:0016209,GO:0016491;Enzyme_code=EC:1.11.1,EC:1.11,EC:1,EC:1.11.1;Enzyme_name=Acting on a peroxide as acceptor,Acting on a peroxide as acceptor,Oxidoreductases,Acting on a peroxide as acceptor
contig00003.g2095	724	ID=contig00003.g2095;Description=hypothetical protein FVEG_08684 [Fusarium verticillioides 7600];Gene=FNYG_05544;Ontology_term=protein dimerization activity;Ontology_id=GO:0046983

contig00003.g2096	1286	ID=contig00003.g2096;Description=transaldolase B [Fusarium tjaetaba];Gene=FVER53263_20762;Ontology_term=carbohydrate metabolic process;Ontology_id=GO:0005975
contig00003.g2097	900	ID=contig00003.g2097;Description=gluconate 5-dehydrogenase [Fusarium verticillioides 7600];Gene=FPCIR_7370;Ontology_term=oxidoreductase activity;Ontology_id=GO:0016491;Enzyme_code=EC:1,EC:1;Enzyme_name=Oxidoreductases,Oxidoreductases
contig00003.g2098	767	ID=contig00003.g2098;Description=hypothetical protein FVER14953_08682 [Fusarium verticillioides]
contig00003.g2099	2184	ID=contig00003.g2099;Description=tenascin X precursor [Fusarium tjaetaba]
contig00003.g2100	1348	ID=contig00003.g2100;Description=endopeptidase K [Fusarium fujikuroi];Gene=FNAPI_11734;Ontology_term=catalytic activity, acting on a protein,protein catabolic process,carbohydrate derivative metabolic process,protein maturation,extracellular region,immune system process,hydrolase activity;Ontology_id=GO:0140096,GO:0030163,GO:1901135,GO:0051604,GO:0005576,GO:0002376,GO:0016787;Enzyme_code=EC:3.4.21,EC:3.4.21,EC:3.4,EC:3,EC:3.4.21.63;Enzyme_name=Acting on peptide bonds (peptidases),Acting on peptide bonds (peptidases),Acting on peptide bonds (peptidases),Hydrolases,oryzin
contig00003.g2101	1678	ID=contig00003.g2101;Description=benzoate 4-monooxygenase cytochrome P450 [Fusarium pseudoanthophilum];Gene=FPANT_410;Ontology_term=oxidoreductase activity;Ontology_id=GO:0016491;Enzyme_code=EC:1.14,EC:1,EC:1.14,EC:1.14.13.12;Enzyme_name=Acting on paired donors, with incorporation or reduction of molecular oxygen. The oxygen incorporated need not be derived from O2,Oxidoreductases,Acting on paired donors, with incorporation or reduction of molecular oxygen. The oxygen incorporated need not be derived from O2,Acting on paired donors, with incorporation or reduction of molecular oxygen. The oxygen incorporated need not be derived from O2
contig00003.g2102	2479	ID=contig00003.g2102;Description=ARCA [Fusarium pseudocircinatum];Gene=FOVG_13152
contig00003.g2103	1538	ID=contig00003.g2103;Description=copper-containing nitrite reductase [Fusarium redolens];Gene=FOBC_14806;Ontology_term=oxidoreductase activity;Ontology_id=GO:0016491;Enzyme_code=EC:1.7.2.1,EC:1.7.2.1,EC:1.7.2,EC:1,EC:1.7;Enzyme_name=nitrite reductase (NO-forming),nitrite reductase (NO-forming),Acting on other nitrogenous compounds as donors,Oxidoreductases,Acting on other nitrogenous compounds as donors

contig00003.g2104	1212	ID=contig00003.g2104;Description=purine nucleoside permease [Fusarium coicis];Gene=FMAN_13637;Ontology_term=transmembrane transport,nucleobase-containing small molecule metabolic process,catalytic activity,carbohydrate derivative metabolic process;Ontology_id=GO:0055085,GO:0055086,GO:0003824,GO:1901135
contig00003.g2105	297	ID=contig00003.g2105;Description=hypothetical protein FVER14953_21339 [Fusarium verticillioides];Gene=FTJAE_8055;Ontology_term=membrane;Ontology_id=GO:0016020
contig00003.g2106	1067	ID=contig00003.g2106;Description=AttH-like domain superfamily [Fusarium oxysporum f. sp. vasinfectum]
contig00003.g2107	973	ID=contig00003.g2107;Description=gluconate 5-dehydrogenase [Fusarium fujikuroi];Gene=C2S_3383;Ontology_term=membrane;Ontology_id=GO:0016020
contig00003.g2108	2802	ID=contig00003.g2108;Description=TOB3 (member of AAA-ATPase family) [Fusarium tjaetaba];Gene=FNYG_05530;Ontology_term=ATP-dependent activity,hydrolase activity;Ontology_id=GO:0140657,GO:0016787;Enzyme_code=EC:3.6.1.15,EC:3.6.1,EC:3.6,EC:3,EC:3.6.1.15;Enzyme_name=nucleoside-triphosphate phosphatase,Acting on acid anhydrides,Acting on acid anhydrides,Hydrolases,nucleoside-triphosphate phosphatase
contig00003.g2109	2707	ID=contig00003.g2109;Description=hypothetical protein FVER53263_08670 [Fusarium verticillioides]
contig00003.g2110	2563	ID=contig00003.g2110;Description=OPT family small oligopeptide transporter [Fusarium tjaetaba];Gene=FNAPI_11723;Ontology_term=transmembrane transport;Ontology_id=GO:0055085
contig00003.g2111	2863	ID=contig00003.g2111;Description=beta-galactosidase [Fusarium tjaetaba];Gene=FTJAE_8064;Ontology_term=carbohydrate metabolic process,hydrolase activity;Ontology_id=GO:0005975,GO:0016787;Enzyme_code=EC:3.2.1,EC:3.2,EC:3,EC:3.2.1;Enzyme_name=Glycosylases,Glycosylases,Hydrolases,Glycosylases
contig00003.g2112	1087	ID=contig00003.g2112;Description=hypothetical protein FVEG_08667 [Fusarium verticillioides 7600]
contig00003.g2113	1856	ID=contig00003.g2113;Description=POT family proton-dependent oligopeptide transporter [Fusarium verticillioides 7600];Gene=FDENT_622;Ontology_term=transmembrane transport,plasma membrane,transporter activity;Ontology_id=GO:0055085,GO:0005886,GO:0005215
contig00003.g2114	1677	ID=contig00003.g2114;Description=general amidase [Fusarium subglutinans];Enzyme_code=EC:3.5.1.4;Enzyme_name=amidase

contig00003.g2115	1898	ID=contig00003.g2115;Description=transporter HOL1 [Fusarium subglutinans];Gene=FVER53590_08664;Ontology_term=transmembrane transport,transporter activity;Ontology_id=GO:0055085,GO:0005215
contig00003.g2116	1244	ID=contig00003.g2116;Description=hypothetical protein FVEG_08663 [Fusarium verticillioides 7600]
contig00003.g2117	564	ID=contig00003.g2117;Description=hypothetical protein FVEG_08662 [Fusarium verticillioides 7600]
contig00003.g2118	551	ID=contig00003.g2118;Description=hypothetical protein FVEG_16393 [Fusarium verticillioides 7600]
contig00003.g2119	1731	ID=contig00003.g2119;Description=hypothetical protein LB505_003607 [Fusarium chuoi]
contig00003.g2120	966	ID=contig00003.g2120;Description=lipase esterase [Fusarium subglutinans];Gene=FTJAE_8074;Ontology_term=hydrolase activity;Ontology_id=GO:0016787;Enzyme_code=EC:3,EC:3;Enzyme_name=Hydrolases,Hydrolases
contig00003.g2121	3425	ID=contig00003.g2121;Description=beta-galactosidase [Fusarium verticillioides 7600];Gene=FVER53263_08659;Ontology_term=carbohydrate metabolic process,vacuole,hydrolase activity;Ontology_id=GO:0005975,GO:0005773,GO:0016787;Enzyme_code=EC:3.2.1.23,EC:3.2,EC:3,EC:3.2.1.23,EC:3.2.1;Enzyme_name=beta-galactosidase,Glycosylases,Hydrolases,beta-galactosidase,Glycosylases
contig00003.g2122	1098	ID=contig00003.g2122;Description=phosphorylcholine phosphatase [Fusarium tjaetaba]
contig00003.g2123	1466	ID=contig00003.g2123;Description=dhh family [Fusarium pseudocircinatum]
contig00003.g2124	1565	ID=contig00003.g2124;Description=multidrug resistance [Fusarium tjaetaba];Gene=FVER53263_08656;Ontology_term=transmembrane transport,vacuole,transporter activity;Ontology_id=GO:0055085,GO:0005773,GO:0005215
contig00003.g2125	3074	ID=contig00003.g2125;Description=hypothetical protein FVER53263_08655 [Fusarium verticillioides];Gene=FPANT_9132;Ontology_term=membrane;Ontology_id=GO:0016020
contig00003.g2126	1270	ID=contig00003.g2126;Description=hypothetical protein FVEG_08654 [Fusarium verticillioides 7600]
contig00003.g2127	1901	ID=contig00003.g2127;Description=quinate transport [Fusarium tjaetaba];Gene=FACUT_3517;Ontology_term=transmembrane transport,transporter activity;Ontology_id=GO:0055085,GO:0005215

contig00003.g2128	1659	ID=contig00003.g2128;Description=extracellular sulfatase sulf-2 [Fusarium coicis];Gene=FocTR4_00015336;Ontology_term=nucleus,DNA binding,regulation of DNA-templated transcription,transcription regulator activity,hydrolase activity;Ontology_id=GO:0005634,GO:0003677,GO:0006355,GO:0140110,GO:0016787;Enzyme_code=EC:3.1.6,EC:3.1.6,EC:3.1,EC:3;Enzyme_name=Acting on ester bonds,Acting on ester bonds,Acting on ester bonds,Hydrolases
contig00003.g2129	3006	ID=contig00003.g2129;Description=nitrate assimilation regulatory nirA [Fusarium tjaetaba];Gene=FTJAE_8083;Ontology_term=nucleus,DNA binding,regulation of DNA-templated transcription,transcription regulator activity;Ontology_id=GO:0005634,GO:0003677,GO:0006355,GO:0140110
contig00003.g2130	2518	ID=contig00003.g2130;Description=Periplasmic beta-glucosidase [Fusarium pseudocircinatum];Gene=FVER53590_08650;Ontology_term=nucleus,DNA binding,carbohydrate metabolic process,DNA-templated transcription,hydrolase activity;Ontology_id=GO:0005634,GO:0003677,GO:0005975,GO:0006351,GO:0016787;Enzyme_code=EC:3.2.1.21,EC:3.2,EC:3.2.1.21,EC:3,EC:3.2.1;Enzyme_name=beta-glucosidase,Glycosylases,beta-glucosidase,Hydrolases,Glycosylases
contig00003.g2131	4205	ID=contig00003.g2131;Description=nicotinamide mononucleotide permease [Fusarium tjaetaba];Gene=FVEG_08648;Ontology_term=nucleus,transmembrane transport,regulation of DNA-templated transcription,transcription regulator activity,transporter activity;Ontology_id=GO:0005634,GO:0055085,GO:0006355,GO:0140110,GO:0005215
contig00003.g2132	633	ID=contig00003.g2132;Description=peroxisomal membrane protein [Fusarium denticulatum];Gene=FOC4_g10009824;Ontology_term=membrane;Ontology_id=GO:0016020
contig00003.g2133	1314	ID=contig00003.g2133;Description=hypothetical protein J7337_012700 [Fusarium musae]
contig00003.g2134	519	ID=contig00003.g2134;Description=hypothetical protein FVER14953_08645 [Fusarium verticillioides]
contig00003.g2135	1093	ID=contig00003.g2135;Description=hypothetical protein FVER53263_08644 [Fusarium verticillioides];Gene=FNAPI_6998;Ontology_term=nucleus,regulation of DNA-templated transcription,transcription regulator activity;Ontology_id=GO:0005634,GO:0006355,GO:0140110
contig00003.g2136	3254	ID=contig00003.g2136;Description=hypothetical protein J7337_012703 [Fusarium musae];Gene=FPANT_9121;Ontology_term=GTP binding;Ontology_id=GO:0005525

contig00003.g2137	2733	ID=contig00003.g2137;Description=atrophin-1 interacting 2 WW domain protein [Fusarium tjaetaba];Gene=FANTH_5315;Ontology_term=signaling,transferase activity,programmed cell death,molecular adaptor activity;Ontology_id=GO:0023052,GO:0016740,GO:0012501,GO:0060090;Enzyme_code=EC:2.4.1.16,EC:2.4,EC:2.4.1.16,EC:2,EC:2.4.1;Enzyme_name=chitin synthase,Glycosyltransferases,chitin synthase,Transferases,Glycosyltransferases
contig00003.g2138	543	ID=contig00003.g2138;Description=Ff.00g064130.m01.CDS01 [Fusarium sp. VM40]
contig00003.g2139	4000	ID=contig00003.g2139;Description=multidrug resistance 1 [Fusarium coicis];Gene=FVER53590_08641;Ontology_term=transmembrane transport,ATP-dependent activity,transporter activity,hydrolase activity;Ontology_id=GO:0055085,GO:0140657,GO:0005215,GO:0016787;Enzyme_code=EC:7.2.2,EC:3.6.1.15,EC:3.6.1,EC:3.6,EC:3,EC:7.2.2,EC:7,EC:3.6.1.15;Enzyme_name=Catalysing the translocation of inorganic cations,nucleoside-triphosphate phosphatase,Acting on acid anhydrides,Acting on acid anhydrides,Hydrolases,Catalysing the translocation of inorganic cations,Translocases,nucleoside-triphosphate phosphatase
contig00003.g2140	1932	ID=contig00003.g2140;Description=transcription factor [Fusarium subglutinans];Gene=FANTH_5312;Ontology_term=nucleus,regulation of DNA-templated transcription,transcription regulator activity;Ontology_id=GO:0005634,GO:0006355,GO:0140110
contig00003.g2141	2568	ID=contig00003.g2141;Description=Lipoteichoic acid synthase-like yvgJ [Fusarium pseudoanthophilum];Gene=FPANT_1150;Ontology_term=hydrolase activity;Ontology_id=GO:0016787;Enzyme_code=EC:3.1.6,EC:3.1.6,EC:3.1,EC:3;Enzyme_name=Acting on ester bonds,Acting on ester bonds,Acting on ester bonds,Hydrolases
contig00003.g2142	462	ID=contig00003.g2142;Description=victoriocin [Fusarium pseudoanthophilum]
contig00003.g2143	1589	ID=contig00003.g2143;Description=oxalate decarboxylase [Fusarium verticillioides 7600];Gene=FVER53263_08636;Ontology_term=metal ion binding,oxalate metabolic process;Ontology_id=GO:0046872,GO:0033609
contig00003.g2144	1524	ID=contig00003.g2144;Description=hypothetical protein FOVG_13101 [Fusarium oxysporum f. sp. pisi HDV247]
contig00003.g2145	846	ID=contig00003.g2145;Description=carboxyphosphonoenolpyruvate phosphonomutase [Fusarium acutatum];Gene=FVEG_08634;Ontology_term=catalytic activity;Ontology_id=GO:0003824
contig00003.g2146	551	ID=contig00003.g2146;Description=transmembrane alpha-helix domain-containing protein [Fusarium coicis];Gene=FPRO_14330;Ontology_term=membrane;Ontology_id=GO:0016020

contig00003.g2147	1230	ID=contig00003.g2147;Description=hypothetical protein FVEG_08633 [Fusarium verticillioides 7600]
contig00003.g2148	766	ID=contig00003.g2148;Description=glutathione S-transferase [Fusarium pseudocircinatum];Gene=FNAPI_6986;Ontology_term=transferase activity;Ontology_id=GO:0016740;Enzyme_code=EC:2,EC:2;Enzyme_name=Transferases,Transferases
contig00003.g2149	1431	ID=contig00003.g2149;Description=hypothetical protein FVER53263_08631 [Fusarium verticillioides]
contig00003.g2150	1009	ID=contig00003.g2150;Description=hydroxyquinol-1 2-dioxygenase [Fusarium subglutinans];Gene=F52700_2731;Ontology_term=oxidoreductase activity;Ontology_id=GO:0016491;Enzyme_code=EC:1.13.11.1,EC:1.13.11,EC:1.13.11.1,EC:1.13,EC:1;Enzyme_name=catechol 1,2-dioxygenase,Acting on single donors with incorporation of molecular oxygen (oxygenases). The oxygen incorporated need not be derived from O2,catechol 1,2-dioxygenase,Acting on single donors with incorporation of molecular oxygen (oxygenases). The oxygen incorporated need not be derived from O2,Oxidoreductases
contig00003.g2151	884	ID=contig00003.g2151;Description=putative membrane protein [Fusarium oxysporum f. sp. conglutinans];Gene=28951375;Ontology_term=membrane;Ontology_id=GO:0016020
contig00003.g2152	1574	ID=contig00003.g2152;Description=related to heterokaryon incompatibility protein (het-6OR allele) [Fusarium fujikuroi];Gene=157
contig00003.g2153	1085	ID=contig00003.g2153;Description=hypothetical protein FVER14953_08628 [Fusarium verticillioides];Gene=156
contig00003.g2154	1412	ID=contig00003.g2154;Description=Clavaminate synthase [Fusarium sp. NRRL 25303];Gene=FVEG_08627;Ontology_term=molecular transducer activity,anatomical structure development,regulation of DNA-templated transcription,reproductive process,immune system process,defense response to other organism,DNA binding,nucleus,oxidoreductase activity,lipid metabolic process,signaling,cell differentiation,transcription regulator activity;Ontology_id=GO:0060089,GO:0048856,GO:0006355,GO:0022414,GO:0002376,GO:0098542,GO:0003677,GO:0005634,GO:0016491,GO:0006629,GO:0023052,GO:0030154,GO:0140110;Enzyme_code=EC:1,EC:1;Enzyme_name=Oxidoreductases,Oxidoreductases
contig00003.g2155	3002	ID=contig00003.g2155;Description=alcohol dehydrogenase I-ADH1 [Fusarium tjaetaba];Gene=FVER53263_08625;Ontology_term=oxidoreductase activity;Ontology_id=GO:0016491;Enzyme_code=EC:1;Enzyme_name=Oxidoreductases

contig00003.g2156	1521	ID=contig00003.g2156;Description=amino acid/polyamine transporter I [Fusarium oxysporum];Gene=FVER53263_08624;Ontology_term=transmembrane transport,transporter activity;Ontology_id=GO:0055085,GO:0005215
contig00003.g2157	1312	ID=contig00003.g2157;Description=hypothetical protein FVER53590_29943 [Fusarium verticillioides];Gene=FVER53263_08623;Ontology_term=membrane;Ontology_id=GO:0016020
contig00003.g2158	1018	ID=contig00003.g2158;Description=hypothetical protein FVER53590_08623 [Fusarium verticillioides];Gene=FVER53263_08623;Ontology_term=membrane;Ontology_id=GO:0016020
contig00003.g2159	1021	ID=contig00003.g2159;Description=PET8 member of the mitochondrial carrier (MCF) family [Fusarium tjaetaba];Gene=FVER53263_08622;Ontology_term=mitochondrion;Ontology_id=GO:0005739
contig00003.g2160	2160	ID=contig00003.g2160;Description=transcription activator acu-15 [Fusarium tjaetaba];Gene=FDENT_2603;Ontology_term=nucleus,DNA binding,regulation of DNA-templated transcription,transcription regulator activity;Ontology_id=GO:0005634,GO:0003677,GO:0006355,GO:0140110
contig00003.g2161	769	ID=contig00003.g2161;Description=plasma membrane heat shock protein [Fusarium oxysporum];Ontology_term=nucleus,catalytic activity, acting on a protein,detoxification,cell wall,lyase activity,extracellular matrix,extracellular region,hydrolase activity;Ontology_id=GO:0005634,GO:0140096,GO:0098754,GO:0005618,GO:0016829,GO:0031012,GO:0005576,GO:0016787;Enzyme_code=EC:4.2.1,EC:4.2.1.130,EC:3.4,EC:4.2,EC:3,EC:4;Enzyme_name=Carbon-oxygen lyases,D-lactate dehydratase,Acting on peptide bonds (peptidases),Carbon-oxygen lyases,Hydrolases,Lyases
contig00003.g2162	1197	ID=contig00003.g2162;Description=NADPH2 dehydrogenase chain OYE2 [Fusarium coicis];Gene=FVER53590_08619;Ontology_term=oxidoreductase activity;Ontology_id=GO:0016491;Enzyme_code=EC:1,EC:1,EC:1.6.99.1;Enzyme_name=Oxidoreductases,Oxidoreductases,NADPH dehydrogenase
contig00003.g2163	528	ID=contig00003.g2163;Description=glutathione-dependent formaldehyde-activating GFA [Fusarium tjaetaba];Gene=FPANT_1169;Ontology_term=lyase activity;Ontology_id=GO:0016829;Enzyme_code=EC:4.4,EC:4.4,EC:4;Enzyme_name=Carbon-sulfur lyases,Carbon-sulfur lyases,Lyases
contig00003.g2164	1192	ID=contig00003.g2164;Description=hypothetical protein J7337_012725 [Fusarium musae]
contig00003.g2165	1399	ID=contig00003.g2165;Description=Ff.00g002060.m01.CDS01 [Fusarium sp. VM40]

contig00003.g2166	1779	ID=contig00003.g2166;Description=hard surface induced 3 (chip3) [Fusarium tjaetaba];Gene=FVER53263_08615;Ontology_term=transferase activity;Ontology_id=GO:0016740;Enzyme_code=EC:2.3.1,EC:2.3,EC:2,EC:2.3.1;Enzyme_name=Acyltransferases,Acyltransferases,Transferases,Acyltransferases
contig00003.g2167	1066	ID=contig00003.g2167;Description=alcohol dehydrogenase (NADP+) [Fusarium verticillioides 7600];Gene=FPRO_14352;Ontology_term=nucleus,carbohydrate metabolic process,oxidoreductase activity;Ontology_id=GO:0005634,GO:0005975,GO:0016491;Enzyme_code=EC:1,EC:1.1.1.21,EC:1.1,EC:1,EC:1.1.1,EC:1.1.1.2;Enzyme_name=Oxidoreductases,aldose reductase,Acting on the CH-OH group of donors,Oxidoreductases,Acting on the CH-OH group of donors,alcohol dehydrogenase (NADP(+))
contig00003.g2168	1735	ID=contig00003.g2168;Description=hypothetical protein H9Q69_009787 [Fusarium xylarioides]
contig00003.g2169	1352	ID=contig00003.g2169;Description=hypothetical protein H9Q69_009788 [Fusarium xylarioides]
contig00003.g2170	447	ID=contig00003.g2170;Description=hypothetical protein FMUND_456 [Fusarium mundagurra]
contig00003.g2171	321	ID=contig00003.g2171;Description=hypothetical protein FPHYL_7332 [Fusarium phyllophilum]
contig00003.g2172	1432	ID=contig00003.g2172;Description=putative neutral amino acid permease [Fusarium fujikuroi];Gene=BFJ68_g4400;Ontology_term=membrane;Ontology_id=GO:0016020
contig00003.g2173	887	ID=contig00003.g2173;Description=DUF895 domain-containing protein [Fusarium pseudocircinatum];Gene=FMUND_459;Ontology_term=membrane;Ontology_id=GO:0016020
contig00003.g2174	1429	ID=contig00003.g2174;Description=putative DUF895 domain membrane protein [Fusarium fujikuroi];Gene=SalF6R;Ontology_term=membrane;Ontology_id=GO:0016020
contig00003.g2175	2502	ID=contig00003.g2175;Description=hypothetical protein FVER14953_08607 [Fusarium verticillioides]
contig00003.g2176	2132	ID=contig00003.g2176;Description=hypothetical protein FVER14953_20342 [Fusarium verticillioides]
contig00003.g2177	1894	ID=contig00003.g2177;Description=photosystem II D2 [Fusarium tjaetaba];Gene=FPCIR_5493;Ontology_term=transmembrane transport,transporter activity;Ontology_id=GO:0055085,GO:0005215

contig00003.g2178	1725	ID=contig00003.g2178;Description=xylosidase arabinosidase [Fusarium acutatum];Gene=FVEG_08605;Ontology_term=carbohydrate metabolic process,hydrolase activity;Ontology_id=GO:0005975,GO:0016787;Enzyme_code=EC:3.2.1,EC:3.2,EC:3,EC:3.2.1;Enzyme_name=Glycosylases,Glycosylases,Hydrolases,Glycosylases
contig00003.g2179	1125	ID=contig00003.g2179;Description=histone-lysine n-methyltransferase ASHR2 [Fusarium coicis];Gene=FVER53263_08604;Ontology_term=transferase activity;Ontology_id=GO:0016740;Enzyme_code=EC:2.1.1,EC:2.1,EC:2,EC:2.1.1;Enzyme_name=Transferring one-carbon groups,Transferring one-carbon groups,Transferases,Transferring one-carbon groups
contig00003.g2180	801	ID=contig00003.g2180;Description=NAD(P)H dehydrogenase (quinone) [Fusarium verticillioides 7600]
contig00003.g2181	1122	ID=contig00003.g2181;Description=ADH3-alcohol dehydrogenase III [Fusarium tjaetaba]
contig00003.g2182	1958	ID=contig00003.g2182;Description=transcription activator acu-15 [Fusarium tjaetaba];Gene=FVEG_16370;Ontology_term=nucleus,DNA binding,regulation of DNA-templated transcription,transcription regulator activity;Ontology_id=GO:0005634,GO:0003677,GO:0006355,GO:0140110
contig00003.g2183	1517	ID=contig00003.g2183;Description=hypothetical protein FVEG_08601 [Fusarium verticillioides 7600];Gene=FTJAE_4618;Ontology_term=lyase activity;Ontology_id=GO:0016829;Enzyme_code=EC:4.3.2.9,EC:4.3,EC:4,EC:4.3.2.9,EC:4.3.2;Enzyme_name=gamma-glutamylcyclotransferase,Carbon-nitrogen lyases,Lyases,gamma-glutamylcyclotransferase,Carbon-nitrogen lyases
contig00003.g2184	1009	ID=contig00003.g2184;Description=swim zinc finger domain-containing protein [Fusarium coicis]
contig00003.g2185	754	ID=contig00003.g2185;Description=AIG2 family [Fusarium tjaetaba];Gene=FACUT_11281;Ontology_term=lyase activity;Ontology_id=GO:0016829;Enzyme_code=EC:4.3.2.9,EC:4.3,EC:4,EC:4.3.2.9,EC:4.3.2;Enzyme_name=gamma-glutamylcyclotransferase,Carbon-nitrogen lyases,Lyases,gamma-glutamylcyclotransferase,Carbon-nitrogen lyases
contig00003.g2186	1282	ID=contig00003.g2186;Description=swim zinc finger domain-containing protein [Fusarium coicis];Gene=186
contig00003.g2187	1567	ID=contig00003.g2187;Description=hypothetical protein FVER53590_08597 [Fusarium verticillioides];Gene=FNAPI_8336;Ontology_term=membrane;Ontology_id=GO:0016020
contig00003.g2188	795	ID=contig00003.g2188;Description=hypothetical protein FVER53263_08596 [Fusarium verticillioides];Gene=FPCIR_5506

contig00003.g2189	1714	ID=contig00003.g2189;Description=hexose transporter protein [Fusarium fujikuroi];Gene=FVER53590_08595;Ontology_term=transmembrane transport,transporter activity;Ontology_id=GO:0055085,GO:0005215
contig00003.g2190	1952	ID=contig00003.g2190;Description=heterokaryon incompatibility protein [Fusarium tjaetaba]
contig00003.g2191	819	ID=contig00003.g2191;Description=related to ketoreductases [Fusarium fujikuroi IMI 58289];Gene=192
contig00003.g2192	1480	ID=contig00003.g2192;Description=related to acyl-CoA thioester hydrolase, mitochondrial precursor [Fusarium proliferatum ET1];Gene=FPRO_14381;Ontology_term=hydrolase activity;Ontology_id=GO:0016787;Enzyme_code=EC:3,EC:3;Enzyme_name=Hydrolases,Hydrolases
contig00003.g2193	1069	ID=contig00003.g2193;Description=acyl- thioester hydrolase mitochondrial precursor [Fusarium acutatum];Gene=F52700_2687;Ontology_term=hydrolase activity;Ontology_id=GO:0016787;Enzyme_code=EC:3;Enzyme_name=Hydrolases
contig00003.g2194	441	ID=contig00003.g2194;Description=COG3602 family [Fusarium tjaetaba];Gene=FNYG_05431;Ontology_term=amino acid metabolic process;Ontology_id=GO:0006520
contig00003.g2195	1210	ID=contig00003.g2195;Description=Pyruvate decarboxylase 1 [Fusarium oxysporum];Gene=FOYG_13329;Ontology_term=nucleus,carbohydrate metabolic process,generation of precursor metabolites and energy,nucleobase-containing small molecule metabolic process,cell wall,lyase activity,amino acid metabolic process,carbohydrate derivative metabolic process,cytosol,extracellular matrix,extracellular region,plasma membrane;Ontology_id=GO:0005634,GO:0005975,GO:0006091,GO:0055086,GO:0005618,GO:0016829,GO:0006520,GO:1901135,GO:0005829,GO:0031012,GO:0005576,GO:0005886;Enzyme_code=EC:4,EC:4.1.1,EC:4.1.1.1,EC:4.1,EC:4,EC:4.1.1.72;Enzyme_name=Lyases,Carbon-carbon lyases,pyruvate decarboxylase,Carbon-carbon lyases,Lyases,branched-chain-2-oxoacid decarboxylase

contig00003.g2196	630	ID=contig00003.g2196;Description=pyruvate decarboxylase [Fusarium verticillioides 7600];Gene=FVER53590_25274;Ontology_term=nucleus,carbohydrate metabolic process,generation of precursor metabolites and energy,nucleobase-containing small molecule metabolic process,cell wall,lyase activity,amino acid metabolic process,carbohydrate derivative metabolic process,cytosol,extracellular matrix,extracellular region,plasma membrane;Ontology_id=GO:0005634,GO:0005975,GO:0006091,GO:0055086,GO:0005618,GO:0016829,GO:0006520,GO:1901135,GO:0005829,GO:0031012,GO:0005576,GO:0005886;Enzyme_code=EC:4.1.1,EC:4.1.1,EC:4.1.1.1,EC:4.1,EC:4,EC:4.1.1.72;Enzyme_name=Carbon-carbon lyases,Carbon-carbon lyases,pyruvate decarboxylase,Carbon-carbon lyases,Lyases,branched-chain-2-oxoacid decarboxylase
contig00003.g2197	4043	ID=contig00003.g2197;Description=n-acetylglucosamine-6-sulfatase [Fusarium pseudocircinatum];Gene=FVER53263_20866;Ontology_term=nucleus,DNA binding,regulation of DNA-templated transcription,transcription regulator activity,hydrolase activity;Ontology_id=GO:0005634,GO:0003677,GO:0006355,GO:0140110,GO:0016787;Enzyme_code=EC:3.1.6,EC:3.1.6,EC:3.1,EC:3;Enzyme_name=Acting on ester bonds,Acting on ester bonds,Acting on ester bonds,Hydrolases
contig00003.g2198	1749	ID=contig00003.g2198;Description=MFS maltose permease [Fusarium pseudocircinatum];Gene=FNAPI_8347;Ontology_term=transmembrane transport,transporter activity;Ontology_id=GO:0055085,GO:0005215
contig00003.g2199	1288	ID=contig00003.g2199;Description=hypothetical protein FPANT_1536 [Fusarium pseudoanthophilum]
contig00003.g2200	1733	ID=contig00003.g2200;Description=MFS transporter, SP family, general alpha glucoside:H+ symporter [Fusarium verticillioides 7600];Gene=FOC4_g10009913;Ontology_term=transmembrane transport,transporter activity;Ontology_id=GO:0055085,GO:0005215
contig00003.g2201	3223	ID=contig00003.g2201;Description=acid beta-fructofuranosidase precursor [Fusarium tjaetaba];Gene=FPHYL_7296;Ontology_term=carbohydrate metabolic process,hydrolase activity;Ontology_id=GO:0005975,GO:0016787;Enzyme_code=EC:3.2.1,EC:3.2,EC:3,EC:3.2.1;Enzyme_name=Glycosylases,Glycosylases,Hydrolases,Glycosylases
contig00003.g2202	2800	ID=contig00003.g2202;Description=related to integral membrane protein [Fusarium proliferatum ET1];Gene=FPRO_14391;Ontology_term=membrane;Ontology_id=GO:0016020

contig00003.g2203	2023	ID=contig00003.g2203;Description=major facilitator superfamily transporter [Fusarium subglutinans];Gene=FUS6-4;Ontology_term=transmembrane transport,transporter activity;Ontology_id=GO:0055085,GO:0005215
contig00003.g2204	834	ID=contig00003.g2204;Description=putative 2-succinyl-6-hydroxy-2,4-cyclohexadiene-1-carboxylate synthase [Fusarium oxysporum f. sp. cubense]
contig00003.g2205	2352	ID=contig00003.g2205;Description=transcription factor [Fusarium tjaetaba];Gene=FNAPI_8357;Ontology_term=nucleus,DNA binding,regulation of DNA-templated transcription,transcription regulator activity;Ontology_id=GO:0005634,GO:0003677,GO:0006355,GO:0140110
contig00003.g2206	1086	ID=contig00003.g2206;Description=oxidoreductase yulF [Fusarium tjaetaba];Gene=FVER53263_08577;Ontology_term=nucleotide binding;Ontology_id=GO:0000166
contig00003.g2207	2227	ID=contig00003.g2207;Description=transcriptional regulatory [Fusarium napiforme];Gene=FPANT_13170;Ontology_term=nucleus,DNA binding,regulation of DNA-templated transcription,transcription regulator activity;Ontology_id=GO:0005634,GO:0003677,GO:0006355,GO:0140110
contig00003.g2208	564	ID=contig00003.g2208;Description=hypothetical protein FVEG_08575 [Fusarium verticillioides 7600]
contig00003.g2209	3862	ID=contig00003.g2209;Description=hypothetical protein FocTR4_00015447, partial [Fusarium oxysporum f. sp. cubense];Gene=FOTG_12433;Ontology_term=membrane;Ontology_id=GO:0016020
contig00003.g2210	717	ID=contig00003.g2210;Description=hypothetical protein FVER53590_08573 [Fusarium verticillioides]
contig00003.g2211	768	ID=contig00003.g2211;Description=alpha beta-hydrolase [Fusarium coicis];Gene=FPCIR_5529;Ontology_term=hydrolase activity;Ontology_id=GO:0016787;Enzyme_code=EC:3,EC:3;Enzyme_name=Hydrolases,Hydrolases
contig00003.g2212	1322	ID=contig00003.g2212;Description=hypothetical protein FVEG_08571 [Fusarium verticillioides 7600];Gene=FPRO_14408;Ontology_term=nucleus,regulation of DNA-templated transcription,transcription regulator activity;Ontology_id=GO:0005634,GO:0006355,GO:0140110
contig00003.g2213	573	ID=contig00003.g2213;Description=GATA zinc finger domain protein [Fusarium tjaetaba];Gene=FPHYL_7280;Ontology_term=DNA binding,regulation of DNA-templated transcription;Ontology_id=GO:0003677,GO:0006355

contig00003.g2214	4748	ID=contig00003.g2214;Description=nacht and tpr domain protein [Fusarium tjaetaba]
contig00003.g2215	2973	ID=contig00003.g2215;Description=OPT family small oligopeptide transporter [Fusarium verticillioides 7600];Gene=FPANT_134;Ontology_term=transmembrane transport,transporter activity;Ontology_id=GO:0055085,GO:0005215
contig00003.g2216	780	ID=contig00003.g2216;Description=hypothetical protein FVER14953_08566 [Fusarium verticillioides]
contig00003.g2217	2199	ID=contig00003.g2217;Description=primary-amine oxidase [Fusarium proliferatum];Gene=FCIRC_3398;Ontology_term=oxidoreductase activity;Ontology_id=GO:0016491;Enzyme_code=EC:1.4.3.21,EC:1.4,EC:1.4.3,EC:1,EC:1.4.3.21;Enzyme_name=primary-amine oxidase,Acting on the CH-NH2 group of donors,Acting on the CH-NH2 group of donors,Oxidoreductases,primary-amine oxidase
contig00003.g2218	1357	ID=contig00003.g2218;Description=integral membrane protein [Fusarium pseudoanthophilum];Gene=FPANT_137;Ontology_term=membrane;Ontology_id=GO:0016020
contig00003.g2219	683	ID=contig00003.g2219;Description=hypothetical protein FVER53590_08563 [Fusarium verticillioides];Gene=220
contig00003.g2220	1798	ID=contig00003.g2220;Description=hypothetical protein FVER14953_21103 [Fusarium verticillioides];Gene=Focb16_v002413;Ontology_term=membrane;Ontology_id=GO:0016020
contig00003.g2221	1345	ID=contig00003.g2221;Description=methyltransferase [Fusarium mundagurra];Gene=FNYG_12253;Ontology_term=transferase activity;Ontology_id=GO:0016740;Enzyme_code=EC:2.1.1,EC:2.1,EC:2,EC:2.1.1;Enzyme_name=Transferring one-carbon groups,Transferring one-carbon groups,Transferases,Transferring one-carbon groups
contig00003.g2222	1743	ID=contig00003.g2222;Description=sugar transporter (hexose transporter) [Fusarium tjaetaba];Gene=FANTH_10575;Ontology_term=nucleus,DNA binding,transmembrane transport,regulation of DNA-templated transcription,transferase activity,transcription regulator activity,transporter activity;Ontology_id=GO:0005634,GO:0003677,GO:0055085,GO:0006355,GO:0016740,GO:0140110,GO:0005215;Enzyme_code=EC:2.1.1,EC:2.1,EC:2,EC:2.1.1;Enzyme_name=Transferring one-carbon groups,Transferring one-carbon groups,Transferases,Transferring one-carbon groups

contig00003.g2223	2122	ID=contig00003.g2223;Description=C6 transcription factor [Fusarium fujikuroi];Gene=CEK26_002557;Ontology_term=nucleus,DNA binding,protein-containing complex assembly,mitochondrion,lyase activity,amino acid metabolic process,regulation of DNA-templated transcription,transcription regulator activity,mitochondrion organization;Ontology_id=GO:0005634,GO:0003677,GO:0065003,GO:0005739,GO:0016829,GO:0006520,GO:0006355,GO:0140110,GO:0007005;Enzyme_code=EC:4,EC:4;Enzyme_name=Lyases,Lyases
contig00003.g2224	824	ID=contig00003.g2224;Description=hypothetical protein H9Q69_000639 [Fusarium xylarioides]
contig00003.g2225	872	ID=contig00003.g2225;Description=hypothetical protein FPRO03_13591 [Fusarium proliferatum]
contig00003.g2226	3248	ID=contig00003.g2226;Description=beta-galactosidase [Fusarium verticillioides 7600];Gene=FPANT_145;Ontology_term=carbohydrate metabolic process,cell wall organization or biogenesis,hydrolase activity;Ontology_id=GO:0005975,GO:0071554,GO:0016787;Enzyme_code=EC:3.2.1.23,EC:3.2,EC:3,EC:3.2.1.23,EC:3.2.1;Enzyme_name=beta-galactosidase,Glycosylases,Hydrolases,beta-galactosidase,Glycosylases
contig00003.g2227	786	ID=contig00003.g2227;Description=P-loop containing nucleoside triphosphate hydrolase protein [Fusarium oxysporum];Gene=BFJ72_g4054;Ontology_term=membrane;Ontology_id=GO:0016020
contig00003.g2228	2046	ID=contig00003.g2228;Description=galactose oxidase [Fusarium verticillioides 7600];Gene=gaoA;Ontology_term=oxidoreductase activity;Ontology_id=GO:0016491;Enzyme_code=EC:1.1.3.9,EC:1.1,EC:1,EC:1.1.3.9,EC:1.1.3;Enzyme_name=galactose oxidase,Acting on the CH-OH group of donors,Oxidoreductases,galactose oxidase,Acting on the CH-OH group of donors
contig00003.g2229	858	ID=contig00003.g2229;Description=chitinase [Fusarium pseudoanthophilum]
contig00003.g2230	1286	ID=contig00003.g2230;Description=integral membrane protein [Fusarium subglutinans];Gene=FSUBG_8497;Ontology_term=membrane;Ontology_id=GO:0016020
contig00003.g2231	2344	ID=contig00003.g2231;Description=hypothetical protein FVER53590_08552 [Fusarium verticillioides]
contig00003.g2232	609	ID=contig00003.g2232;Description=hypothetical protein FDENT_8425 [Fusarium denticulatum]
contig00003.g2233	4107	ID=contig00003.g2233;Description=integral membrane protein [Fusarium pseudoanthophilum]

contig00003.g2234	1032	ID=contig00003.g2234;Description=galactinol synthase [Fusarium fujikuroi];Gene=FVER53590_08550;Ontology_term=oxidoreductase activity,transferase activity;Ontology_id=GO:0016491,GO:0016740;Enzyme_code=EC:2.4,EC:1,EC:2.4,EC:1,EC:2;Enzyme_name=Glycosyltransferases,Oxidoreductases,Glycosyltransferases,Oxidoreductases,Transferases
contig00003.g2235	891	ID=contig00003.g2235;Description=related to 2,5-diketo-D-gluconic acid reductase [Fusarium proliferatum ET1];Gene=FNYG_12265;Ontology_term=oxidoreductase activity;Ontology_id=GO:0016491;Enzyme_code=EC:1,EC:1;Enzyme_name=Oxidoreductases,Oxidoreductases
contig00003.g2236	831	ID=contig00003.g2236;Description=histidine phosphatase superfamily [Fusarium oxysporum]
contig00003.g2237	1716	ID=contig00003.g2237;Description=multidrug resistance [Fusarium pseudocircinatum];Gene=FVER53263_08547;Ontology_term=transmembrane transport,transporter activity;Ontology_id=GO:0055085,GO:0005215
contig00003.g2238	1220	ID=contig00003.g2238;Description=fungal transcriptional regulatory [Fusarium agapanthi];Gene=FOIG_08665;Ontology_term=nucleus,regulation of DNA-templated transcription,transcription regulator activity;Ontology_id=GO:0005634,GO:0006355,GO:0140110
contig00003.g2239	3348	ID=contig00003.g2239;Description=putative NRPS-like protein biosynthetic cluster [Fusarium musae];Gene=FACUT_11649;Ontology_term=secondary metabolite biosynthetic process,organic substance biosynthetic process,phosphopantetheine binding;Ontology_id=GO:0044550,GO:1901576,GO:0031177
contig00003.g2240	480	ID=contig00003.g2240;Description=hypothetical protein FVER14953_08544 [Fusarium verticillioides];Gene=FVEG_08544;Ontology_term=nucleus,regulation of DNA-templated transcription,transcription regulator activity;Ontology_id=GO:0005634,GO:0006355,GO:0140110
contig00003.g2241	1020	ID=contig00003.g2241;Description=thioredoxin reductase [Fusarium pseudoanthophilum];Gene=240;Ontology_term=nucleus,DNA binding,protein-containing complex assembly,oxidoreductase activity,mitochondrion,anatomical structure development,regulation of DNA-templated transcription,transcription regulator activity,mitochondrion organization;Ontology_id=GO:0005634,GO:0003677,GO:0065003,GO:0016491,GO:0005739,GO:0048856,GO:0006355,GO:0140110,GO:0007005;Enzyme_code=EC:1,EC:1;Enzyme_name=Oxidoreductases,Oxidoreductases

contig00003.g2242	1335	ID=contig00003.g2242;Description=sulfide:quinone oxidoreductase mitochondrial precursor [Fusarium tjaetaba];Gene=FTJAE_4561;Ontology_term=oxidoreductase activity;Ontology_id=GO:0016491;Enzyme_code=EC:1,EC:1.8.5.8,EC:1;Enzyme_name=Oxidoreductases, eukaryotic sulfide quinone oxidoreductase,Oxidoreductases
contig00003.g2243	2393	ID=contig00003.g2243;Description=hypothetical protein FVER53590_08542 [Fusarium verticillioides]
contig00003.g2244	2958	ID=contig00003.g2244;Description=hypothetical protein FVER53590_08541 [Fusarium verticillioides]
contig00003.g2245	1689	ID=contig00003.g2245;Description=cytochrome P450 oxidoreductase [Fusarium verticillioides 7600];Gene=FANTH_10555;Ontology_term=oxidoreductase activity,transferase activity;Ontology_id=GO:0016491,GO:0016740;Enzyme_code=EC:1.14,EC:2.1.1,EC:2.1,EC:1,EC:1.14,EC:2,EC:2.1.1;Enzyme_name=Acting on paired donors, with incorporation or reduction of molecular oxygen. The oxygen incorporated need not be derived from O2,Transferring one-carbon groups,Transferring one-carbon groups,Oxidoreductases,Acting on paired donors, with incorporation or reduction of molecular oxygen. The oxygen incorporated need not be derived from O2,Transferases,Transferring one-carbon groups
contig00003.g2246	1137	ID=contig00003.g2246;Description=putative secondary metabolism biosynthetic enzyme [Fusarium musae]
contig00003.g2247	1068	ID=contig00003.g2247;Description=beta-1,6-n contains WSC domain-containing protein [Fusarium denticulatum];Gene=FDENT_8440;Ontology_term=membrane;Ontology_id=GO:0016020
contig00003.g2248	588	ID=contig00003.g2248;Description=hypothetical protein FVER53590_08537 [Fusarium verticillioides];Gene=FVER53263_08538;Ontology_term=membrane;Ontology_id=GO:0016020
contig00003.g2249	1475	ID=contig00003.g2249;Description=hypothetical protein FVER53590_08536 [Fusarium verticillioides]
contig00003.g2250	1113	ID=contig00003.g2250;Description=NADPH2 dehydrogenase [Fusarium verticillioides 7600];Gene=LW93_204;Ontology_term=oxidoreductase activity,detoxification;Ontology_id=GO:0016491,GO:0098754;Enzyme_code=EC:1,EC:1,EC:1.6.99.1;Enzyme_name=Oxidoreductases,Oxidoreductases,NADPH dehydrogenase
contig00003.g2251	1545	ID=contig00003.g2251;Description=hypothetical protein FVER53590_08534 [Fusarium verticillioides];Gene=FPANT_167;Ontology_term=metal ion binding;Ontology_id=GO:0046872

contig00003.g2252	234	ID=contig00003.g2252;Description=hypothetical protein FVER53590_25266 [Fusarium verticillioides]
contig00003.g2253	714	ID=contig00003.g2253;Description=hypothetical protein FVER14953_21100 [Fusarium verticillioides]
contig00003.g2254	3341	ID=contig00003.g2254;Description=hypothetical protein FVER53590_08531 [Fusarium verticillioides];Gene=FVER53590_08530;Ontology_term=membrane;Ontology_id=GO:0016020
contig00003.g2255	2766	ID=contig00003.g2255;Description=glycoside hydrolase family 43 [Fusarium tjaetaba];Gene=FNAPI_861;Ontology_term=hydrolase activity;Ontology_id=GO:0016787;Enzyme_code=EC:3,EC:3;Enzyme_name=Hydrolases,Hydrolases
contig00003.g2256	2060	ID=contig00003.g2256;Description=hypothetical protein FOVG_12941 [Fusarium oxysporum f. sp. pisi HDV247];Gene=FPHYL_5904;Ontology_term=membrane;Ontology_id=GO:0016020
contig00003.g2257	591	ID=contig00003.g2257;Description=transcriptional regulator [Fusarium tjaetaba];Gene=FVER53263_08527;Ontology_term=transferase activity;Ontology_id=GO:0016740;Enzyme_code=EC:2.3.1,EC:2.3,EC:2,EC:2.3.1;Enzyme_name=Acyltransferases,Acyltransferases,Transferases,Acyltransferases
contig00003.g2258	1059	ID=contig00003.g2258;Description=related to endo-polygalacturonase 6 [Fusarium fujikuroi];Gene=FVEG_08526;Ontology_term=oxidoreductase activity,cytosol;Ontology_id=GO:0016491,GO:0005829;Enzyme_code=EC:1.3.1,EC:1.3,EC:1,EC:1.3.1;Enzyme_name=Acting on the CH-CH group of donors,Acting on the CH-CH group of donors,Oxidoreductases,Acting on the CH-CH group of donors
contig00003.g2259	1310	ID=contig00003.g2259;Description=integral membrane protein [Fusarium coicis];Gene=FPANT_8187;Ontology_term=membrane;Ontology_id=GO:0016020
contig00003.g2260	1391	ID=contig00003.g2260;Description=hypothetical protein FVER53590_08524 [Fusarium verticillioides];Gene=FPANT_8186;Ontology_term=membrane;Ontology_id=GO:0016020
contig00003.g2261	1203	ID=contig00003.g2261;Description=bis(5' nucleosyl)-tetraphosphatase symmetrical [Fusarium tjaetaba];Gene=FPANT_8185;Ontology_term=vacuole,hydrolase activity;Ontology_id=GO:0005773,GO:0016787;Enzyme_code=EC:3,EC:3.1,EC:3.6.1.41,EC:3,EC:3.1.3;Enzyme_name=Hydrolases,Acting on ester bonds,bis(5'-nucleosyl)-tetraphosphatase (symmetrical),Hydrolases,Acting on ester bonds
contig00003.g2262	1798	ID=contig00003.g2262;Description=sugar transporter [Fusarium tjaetaba];Gene=FPHYL_12734;Ontology_term=transmembrane transport,transporter activity;Ontology_id=GO:0055085,GO:0005215

contig00003.g2263	1839	ID=contig00003.g2263;Description=hypothetical protein FGLOB1_8134 [Fusarium globosum]
contig00003.g2264	1639	ID=contig00003.g2264;Description=hypothetical protein FVER14953_08520 [Fusarium verticillioides];Gene=FNAPI_4399;Ontology_term=transferase activity;Ontology_id=GO:0016740;Enzyme_code=EC:2.4;Enzyme_name=Glycosyltransferases
contig00003.g2265	986	ID=contig00003.g2265;Description=hypothetical protein FPANT_8180 [Fusarium pseudoanthophilum];Gene=LW93_223
contig00003.g2266	1858	ID=contig00003.g2266;Description=lipase 4 [Fusarium coicis];Gene=FVEG_08518;Ontology_term=hydrolase activity;Ontology_id=GO:0016787;Enzyme_code=EC:3,EC:3;Enzyme_name=Hydrolases,Hydrolases
contig00003.g2267	1197	ID=contig00003.g2267;Description=enoyl hydratase [Fusarium tjaetaba];Gene=FVEG_08517;Ontology_term=catalytic activity;Ontology_id=GO:0003824;Enzyme_code=EC:4.2.1.17;Enzyme_name=enoyl-CoA hydratase
contig00003.g2268	3229	ID=contig00003.g2268;Description=non-ribosomal peptide synthetase [Fusarium tjaetaba];Gene=FVER53263_08516;Ontology_term=oxidoreductase activity;Ontology_id=GO:0016491;Enzyme_code=EC:1,EC:1;Enzyme_name=Oxidoreductases,Oxidoreductases
contig00003.g2269	2366	ID=contig00003.g2269;Description=Zn(II)2Cys6 transcriptional activator [Fusarium napiforme];Gene=FVER53263_08515;Ontology_term=nucleus,DNA binding,regulation of DNA-templated transcription,transcription regulator activity;Ontology_id=GO:0005634,GO:0003677,GO:0006355,GO:0140110
contig00003.g2270	1663	ID=contig00003.g2270;Description=glycerol kinase [Fusarium odoratissimum NRRL 54006];Gene=FOMG_15275;Ontology_term=carbohydrate metabolic process,carbohydrate derivative metabolic process,transferase activity;Ontology_id=GO:0005975,GO:1901135,GO:0016740;Enzyme_code=EC:2.7.1.30;Enzyme_name=glycerol kinase
contig00003.g2271	1100	ID=contig00003.g2271;Description=glycerol channel [Fusarium oxysporum];Gene=FOZG_14274;Ontology_term=transmembrane transport,reproductive process,vacuole,transporter activity,plasma membrane;Ontology_id=GO:0055085,GO:0022414,GO:0005773,GO:0005215,GO:0005886

contig00003.g2272	2097	ID=contig00003.g2272;Description=glycerol-3-phosphate dehydrogenase [Fusarium verticillioides 7600];Gene=FMUND_12181;Ontology_term=carbohydrate metabolic process,nucleobase-containing small molecule metabolic process,oxidoreductase activity,mitochondrion,carbohydrate derivative metabolic process;Ontology_id=GO:0005975,GO:0055086,GO:0016491,GO:0005739,GO:1901135;Enzyme_code=EC:1.1.5.3,EC:1.1,EC:1.1.5.3,EC:1,EC:1.1.5;Enzyme_name=glycerol-3-phosphate dehydrogenase,Acting on the CH-OH group of donors,glycerol-3-phosphate dehydrogenase,Oxidoreductases,Acting on the CH-OH group of donors
contig00003.g2273	1147	ID=contig00003.g2273;Description=hypothetical protein FVEG_08510 [Fusarium verticillioides 7600]
contig00003.g2274	1513	ID=contig00003.g2274;Description=hypothetical protein FVEG_08508 [Fusarium verticillioides 7600]
contig00003.g2275	633	ID=contig00003.g2275;Description=prenyl cysteine carboxyl methyltransferase [Fusarium tjaetaba];Gene=FVEG_08507;Ontology_term=catalytic activity, acting on a protein,endoplasmic reticulum,transferase activity;Ontology_id=GO:0140096,GO:0005783,GO:0016740;Enzyme_code=EC:2.1.1.100,EC:2.1,EC:2.1.1.100,EC:2,EC:2.1.1;Enzyme_name=protein-S-isoprenylcysteine O-methyltransferase,Transferring one-carbon groups,protein-S-isoprenylcysteine O-methyltransferase,Transferases,Transferring one-carbon groups
contig00003.g2276	2091	ID=contig00003.g2276;Description=integral membrane protein [Fusarium napiforme];Gene=FNAPI_4411;Ontology_term=regulation of DNA-templated transcription,transcription regulator activity,molecular adaptor activity;Ontology_id=GO:0006355,GO:0140110,GO:0060090
contig00003.g2277	864	ID=contig00003.g2277;Description=vacuolar ATP synthase subunit C [Fusarium napiforme]
contig00003.g2278	1811	ID=contig00003.g2278;Description=kinase [Fusarium pseudoanthophilum];Gene=FMEXI_10140;Ontology_term=transferase activity;Ontology_id=GO:0016740;Enzyme_code=EC:2.7;Enzyme_name=Transferring phosphorus-containing groups
contig00003.g2279	2008	ID=contig00003.g2279;Description=fungal-specific transcription factor domain-containing protein [Fusarium sp. MPI-SDFR-AT-0072];Gene=FOVG_12923;Ontology_term=nucleus,regulation of DNA-templated transcription,transcription regulator activity;Ontology_id=GO:0005634,GO:0006355,GO:0140110

contig00003.g2280	1299	ID=contig00003.g2280;Description=FAD dependent oxidoreductase-domain-containing protein [Fusarium oxysporum Fo47];Gene=HZS61_002136;Ontology_term=oxidoreductase activity;Ontology_id=GO:0016491;Enzyme_code=EC:1,EC:1;Enzyme_name=Oxidoreductases,Oxidoreductases
contig00003.g2281	952	ID=contig00003.g2281;Description=probable general amino acid permease [Fusarium proliferatum ET1];Gene=FOC4_g10010018;Ontology_term=transmembrane transport;Ontology_id=GO:0055085
contig00003.g2282	3748	ID=contig00003.g2282;Description=gamma-glutamylputrescine oxidoreductase [Fusarium tjaetaba];Gene=BFJ68_g4467;Ontology_term=nucleobase-containing small molecule metabolic process,oxidoreductase activity,carbohydrate derivative metabolic process;Ontology_id=GO:0055086,GO:0016491,GO:1901135;Enzyme_code=EC:1,EC:1;Enzyme_name=Oxidoreductases,Oxidoreductases
contig00003.g2283	1260	ID=contig00003.g2283;Description=hypothetical protein FVER53263_08498 [Fusarium verticillioides];Gene=FPANT_13643;Ontology_term=membrane;Ontology_id=GO:0016020
contig00003.g2284	1512	ID=contig00003.g2284;Description=hypothetical protein FVER14953_08497 [Fusarium verticillioides]
contig00003.g2285	4463	ID=contig00003.g2285;Description=hypothetical protein FVER53590_08497 [Fusarium verticillioides];Gene=FVER53590_08497;Ontology_term=membrane;Ontology_id=GO:0016020
contig00003.g2286	445	ID=contig00003.g2286;Description=uncharacterized protein FTJAE_3349 [Fusarium tjaetaba]
contig00003.g2287	2044	ID=contig00003.g2287;Description=het domain protein [Fusarium tjaetaba]
contig00003.g2288	909	ID=contig00003.g2288;Description=hypothetical protein FVEG_08494 [Fusarium verticillioides 7600]
contig00003.g2289	957	ID=contig00003.g2289;Description=hypothetical protein FVER53590_08493 [Fusarium verticillioides]
contig00003.g2290	1020	ID=contig00003.g2290;Description=hypothetical protein FANTH_7623 [Fusarium anthophilum]
contig00003.g2291	4718	ID=contig00003.g2291;Description=DUF1446 domain protein [Fusarium tjaetaba];Gene=FNAPI_4430;Ontology_term=amino acid metabolic process,hydrolase activity;Ontology_id=GO:0006520,GO:0016787;Enzyme_code=EC:3.5.1.9,EC:3.5.1,EC:3.5,EC:3,EC:3.5.1.9;Enzyme_name=arylformamidase,Acting on carbon-nitrogen bonds, other than peptide bonds,Acting on carbon-nitrogen bonds, other than peptide bonds,Hydrolases,arylformamidase

contig00003.g2292	1908	ID=contig00003.g2292;Description=sugar transporter [Fusarium tjaetaba];Gene=FCIRC_13763;Ontology_term=transmembrane transport,transporter activity;Ontology_id=GO:0055085,GO:0005215
contig00003.g2293	2184	ID=contig00003.g2293;Description=hypothetical protein FPHYL_7950 [Fusarium phyllophilum];Gene=294
contig00003.g2294	1977	ID=contig00003.g2294;Description=Saccharopine dehydrogenase [Fusarium musae];Gene=FDENT_8485;Ontology_term=DNA repair,nucleus,mitochondrion,amino acid metabolic process,chromatin organization,transferase activity;Ontology_id=GO:0006281,GO:0005634,GO:0005739,GO:0006520,GO:0006325,GO:0016740;Enzyme_code=EC:2.3.3.14,EC:2.3.3,EC:2.3,EC:2,EC:2.3.3.14;Enzyme_name=homocitrate synthase,Acyltransferases,Acyltransferases,Transferases,homocitrate synthase
contig00003.g2295	2910	ID=contig00003.g2295;Description=Uncharacterized protein LW93_259 [Fusarium fujikuroi];Gene=FPHYL_7948;Ontology_term=hydrolase activity;Ontology_id=GO:0016787;Enzyme_code=EC:3.1.6,EC:3.1.6,EC:3.1,EC:3;Enzyme_name=Acting on ester bonds,Acting on ester bonds,Acting on ester bonds,Hydrolases
contig00003.g2296	945	ID=contig00003.g2296;Description=related to NmrA-like family protein [Fusarium mangiferae]
contig00003.g2297	1994	ID=contig00003.g2297;Description=Sodium/calcium exchanger membrane region [Fusarium oxysporum f. sp. vasinfectum];Gene=FOXB_08244;Ontology_term=transmembrane transport,vacuole,transporter activity;Ontology_id=GO:0055085,GO:0005773,GO:0005215
contig00003.g2298	1519	ID=contig00003.g2298;Description=CMGC/SRPK protein kinase [Fusarium verticillioides 7600];Gene=FPANT_11686;Ontology_term=catalytic activity, acting on a protein,transferase activity;Ontology_id=GO:0140096,GO:0016740;Enzyme_code=EC:2.7.11.1,EC:2.7.1,EC:2.7.11.1,EC:2.7,EC:2;Enzyme_name=non-specific serine/threonine protein kinase,Transferring phosphorus-containing groups,non-specific serine/threonine protein kinase,Transferring phosphorus-containing groups,Transferases
contig00003.g2299	1642	ID=contig00003.g2299;Description=hypothetical protein LB503_012426 [Fusarium chuoi]
contig00003.g2300	3753	ID=contig00003.g2300;Description=aminotriazole resistance protein [Fusarium tjaetaba];Gene=FPANT_11684;Ontology_term=transmembrane transport,transporter activity;Ontology_id=GO:0055085,GO:0005215
contig00003.g2301	1896	ID=contig00003.g2301;Description=hypothetical protein FVER53590_08478 [Fusarium verticillioides]
contig00003.g2302	1146	ID=contig00003.g2302;Description=hypothetical protein NW760_001086 [Fusarium oxysporum]

contig00003.g2303	2211	ID=contig00003.g2303;Description=related to high affinity methionine permease [Fusarium proliferatum ET1];Gene=FNAPI_4440
contig00003.g2304	2313	ID=contig00003.g2304;Description=hypothetical protein FVEG_08475 [Fusarium verticillioides 7600]
contig00003.g2305	570	ID=contig00003.g2305;Description=hypothetical protein FVER53590_08474 [Fusarium verticillioides]
contig00003.g2306	1575	ID=contig00003.g2306;Description=hypothetical protein FVER53590_08473 [Fusarium verticillioides]
contig00003.g2307	890	ID=contig00003.g2307;Description=hypothetical protein FVER53590_29370 [Fusarium verticillioides];Gene=FNYG_12342;Ontology_term=membrane;Ontology_id=GO:0016020
contig00003.g2308	1328	ID=contig00003.g2308;Description=biotin ligase [Fusarium agapanthi];Gene=FVER53590_08472;Ontology_term=nucleus,regulation of DNA-templated transcription,transcription regulator activity;Ontology_id=GO:0005634,GO:0006355,GO:0140110
contig00003.g2309	840	ID=contig00003.g2309;Description=biotin ligase [Fusarium tjaetaba];Gene=FNAPI_4446;Ontology_term=ligase activity;Ontology_id=GO:0016874;Enzyme_code=EC:6,EC:6;Enzyme_name=Ligases,Ligases
contig00003.g2310	1401	ID=contig00003.g2310;Description=glycosyl hydrolase family 18 [Fusarium tjaetaba];Gene=F25303_3558;Ontology_term=carbohydrate metabolic process,hydrolase activity;Ontology_id=GO:0005975,GO:0016787;Enzyme_code=EC:3,EC:3,EC:3.2.1.14;Enzyme_name=Hydrolases,Hydrolases,chitinase
contig00003.g2311	1014	ID=contig00003.g2311;Description=gibberellin 20-oxidase [Fusarium fujikuroi];Gene=FOMG_15232;Ontology_term=oxidoreductase activity;Ontology_id=GO:0016491;Enzyme_code=EC:1,EC:1;Enzyme_name=Oxidoreductases,Oxidoreductases
contig00003.g2312	2061	ID=contig00003.g2312;Description=Arginine metabolism regulation II [Fusarium coicis];Gene=FMUND_13401;Ontology_term=nucleus,regulation of DNA-templated transcription,transcription regulator activity;Ontology_id=GO:0005634,GO:0006355,GO:0140110
contig00003.g2313	1324	ID=contig00003.g2313;Description=hypothetical protein FVER53590_08467 [Fusarium verticillioides];Gene=FGADI_10112;Ontology_term=membrane;Ontology_id=GO:0016020
contig00003.g2314	1215	ID=contig00003.g2314;Description=hypothetical protein FDENT_12569 [Fusarium denticulatum];Gene=FDENT_12569;Ontology_term=membrane;Ontology_id=GO:0016020
contig00003.g2315	564	ID=contig00003.g2315;Description=poly(A) polymerase pla1 [Fusarium napiforme]

contig00003.g2316	2680	ID=contig00003.g2316;Description=ferric reductase transmembrane component 4 [Fusarium pseudoanthophilum];Gene=FOXB_16551;Ontology_term=oxidoreductase activity;Ontology_id=GO:0016491;Enzyme_code=EC:1,EC:1;Enzyme_name=Oxidoreductases,Oxidoreductases
contig00003.g2317	1385	ID=contig00003.g2317;Description=SPR1-exo-1 3-beta-glucanase precursor [Fusarium beomiforme];Gene=FG01596.1;Ontology_term=carbohydrate metabolic process,hydrolase activity;Ontology_id=GO:0005975,GO:0016787;Enzyme_code=EC:3.2.1,EC:3.2,EC:3.2.1.75,EC:3,EC:3.2.1;Enzyme_name=Glycosylases,Glycosylases,glucan endo-1,6-beta-glucosidase,Hydrolases,Glycosylases
contig00003.g2318	987	ID=contig00003.g2318;Description=oxidoreductase [Fusarium verticillioides 7600];Gene=FNAPI_4453;Ontology_term=carbohydrate metabolic process,hydrolase activity;Ontology_id=GO:0005975,GO:0016787;Enzyme_code=EC:3.2.1,EC:3.2,EC:3,EC:3.2.1;Enzyme_name=Glycosylases,Glycosylases,Hydrolases,Glycosylases
contig00003.g2319	465	ID=contig00003.g2319;Description=oxidoreductase [Fusarium coicis];Gene=FNAPI_4453;Ontology_term=carbohydrate metabolic process,hydrolase activity;Ontology_id=GO:0005975,GO:0016787;Enzyme_code=EC:3.2.1,EC:3.2,EC:3,EC:3.2.1;Enzyme_name=Glycosylases,Glycosylases,Hydrolases,Glycosylases
contig00003.g2320	1656	ID=contig00003.g2320;Description=triacylglycerol lipase II precursor [Fusarium tjaetaba];Gene=FNAPI_4456;Ontology_term=hydrolase activity;Ontology_id=GO:0016787;Enzyme_code=EC:3,EC:3;Enzyme_name=Hydrolases,Hydrolases
contig00003.g2321	1216	ID=contig00003.g2321;Description=probable NADPH2 dehydrogenase chain OYE2 [Fusarium fujikuroi];Gene=MDCFG593_LOCUS567456;Ontology_term=antioxidant activity,oxidoreductase activity,cytosol,molecular carrier activity;Ontology_id=GO:0016209,GO:0016491,GO:0005829,GO:0140104;Enzyme_code=EC:1.11.1,EC:1.11,EC:1,EC:1.11.1,EC:1.6.99.1;Enzyme_name=Acting on a peroxide as acceptor,Acting on a peroxide as acceptor,Oxidoreductases,Acting on a peroxide as acceptor,NADPH dehydrogenase
contig00003.g2322	381	ID=contig00003.g2322;Description=hypothetical protein FVER53590_08459 [Fusarium verticillioides]
contig00003.g2323	574	ID=contig00003.g2323;Description=hypothetical protein FVER53590_29371 [Fusarium verticillioides]

contig00003.g2324	1961	ID=contig00003.g2324;Description=transcription factor [Fusarium sp. NRRL 25303];Gene=FPANT_5727;Ontology_term=nucleus,DNA binding,regulation of DNA-templated transcription,transcription regulator activity;Ontology_id=GO:0005634,GO:0003677,GO:0006355,GO:0140110
contig00003.g2325	1225	ID=contig00003.g2325;Description=HECT-type ubiquitin ligase-interacting creD [Fusarium tjaetaba];Gene=FTJAE_3309;Ontology_term=ligase activity;Ontology_id=GO:0016874;Enzyme_code=EC:6;Enzyme_name=Ligases
contig00003.g2326	803	ID=contig00003.g2326;Description=hypothetical protein FVEG_08455 [Fusarium verticillioides 7600]
contig00003.g2327	1758	ID=contig00003.g2327;Description=lipase 1 [Fusarium coicis];Gene=FNYG_12364;Ontology_term=oxidoreductase activity,hydrolase activity;Ontology_id=GO:0016491,GO:0016787;Enzyme_code=EC:1,EC:3,EC:1,EC:3;Enzyme_name=Oxidoreductases,Hydrolases,Oxidoreductases,Hydrolases
contig00003.g2328	4942	ID=contig00003.g2328;Description=serine threonine phosphatase 6 regulatory ankyrin repeat subunit B [Fusarium mundagurra]
contig00003.g2329	1185	ID=contig00003.g2329;Description=polygalacturonase [Fusarium verticillioides 7600];Gene=F52700_11505;Ontology_term=carbohydrate metabolic process,cell wall organization or biogenesis,hydrolase activity;Ontology_id=GO:0005975,GO:0071554,GO:0016787;Enzyme_code=EC:3.2.1.15,EC:3.2,EC:3,EC:3.2.1.67,EC:3.2.1.15,EC:3.2.1;Enzyme_name=endo-polygalacturonase,Glycosylases,Hydrolases,galacturonan 1,4-alpha-galacturonidase,endo-polygalacturonase,Glycosylases
contig00003.g2330	1413	ID=contig00003.g2330;Description=related to tubulin-tyrosine ligase [Fusarium fujikuroi];Gene=FVER53590_08450;Ontology_term=ligase activity;Ontology_id=GO:0016874;Enzyme_code=EC:6,EC:6;Enzyme_name=Ligases,Ligases
contig00003.g2331	735	ID=contig00003.g2331;Description=hypothetical protein FVEG_16328 [Fusarium verticillioides 7600];Gene=FVER53590_08450;Ontology_term=protein modification process;Ontology_id=GO:0036211
contig00003.g2332	860	ID=contig00003.g2332;Description=hypothetical protein FVEG_08449 [Fusarium verticillioides 7600]

contig00003.g2333	1086	ID=contig00003.g2333;Description=D-malate dehydrogenase [decarboxylating] [Fusarium oxysporum f. sp. cepae];Gene=BFJ68_g10175;Ontology_term=oxidoreductase activity;Ontology_id=GO:0016491;Enzyme_code=EC:1.1.1.83,EC:1.1,EC:1,EC:1.1.1,EC:1.1.1.83;Enzyme_name=D-malate dehydrogenase (decarboxylating),Acting on the CH-OH group of donors,Oxidoreductases,Acting on the CH-OH group of donors,D-malate dehydrogenase (decarboxylating)
contig00003.g2334	900	ID=contig00003.g2334;Description=haloacetate dehalogenase [Fusarium verticillioides 7600];Gene=FACUT_7095;Ontology_term=catalytic activity;Ontology_id=GO:0003824
contig00003.g2335	1934	ID=contig00003.g2335;Description=family inorganic phosphate transporter [Fusarium tjaetaba];Gene=F25303_3586;Ontology_term=transmembrane transport,transporter activity;Ontology_id=GO:0055085,GO:0005215
contig00003.g2336	1517	ID=contig00003.g2336;Description=related to UDP-N-acetyl-D-mannosamine 6-dehydrogenase [Fusarium mangiferae];Gene=FOYG_13161;Ontology_term=carbohydrate metabolic process,oxidoreductase activity;Ontology_id=GO:0005975,GO:0016491;Enzyme_code=EC:1.1.1,EC:1.3.1,EC:1.1,EC:1.3,EC:1,EC:1.1.1,EC:1.3.1;Enzyme_name=Acting on the CH-OH group of donors,Acting on the CH-CH group of donors,Acting on the CH-OH group of donors,Acting on the CH-CH group of donors,Oxidoreductases,Acting on the CH-OH group of donors,Acting on the CH-CH group of donors
contig00003.g2337	2546	ID=contig00003.g2337;Description=chitin synthase [Fusarium pseudoanthophilum];Gene=FPANT_12823;Ontology_term=anatomical structure development,cell differentiation,transferase activity,plasma membrane;Ontology_id=GO:0048856,GO:0030154,GO:0016740,GO:0005886;Enzyme_code=EC:2.4.1.16,EC:2.4,EC:2.4.1.16,EC:2,EC:2.4.1;Enzyme_name=chitin synthase,Glycosyltransferases,chitin synthase,Transferases,Glycosyltransferases
contig00003.g2338	1582	ID=contig00003.g2338;Description=hypothetical protein FVER53590_08442 [Fusarium verticillioides];Gene=FNYG_12374;Ontology_term=membrane;Ontology_id=GO:0016020
contig00003.g2339	1771	ID=contig00003.g2339;Description=MFS transporter, SP family, major inositol transporter [Fusarium verticillioides 7600];Gene=FTJAE_3295;Ontology_term=transmembrane transport,transporter activity,plasma membrane;Ontology_id=GO:0055085,GO:0005215,GO:0005886
contig00003.g2340	459	ID=contig00003.g2340;Description=hypothetical protein FVEG_08440 [Fusarium verticillioides 7600];Gene=FTJAE_3294;Ontology_term=membrane;Ontology_id=GO:0016020

contig00003.g2341	1362	ID=contig00003.g2341;Description=integral membrane protein [Fusarium pseudoanthophilum];Gene=FVER53263_08439;Ontology_term=membrane;Ontology_id=GO:0016020
contig00003.g2342	1595	ID=contig00003.g2342;Description=isotrichodermin C-15 hydroxylase (cytochrome P-450 monooxygenase CYP65A1) [Fusarium fujikuroi];Gene=FVER53263_08438;Ontology_term=oxidoreductase activity;Ontology_id=GO:0016491;Enzyme_code=EC:1.14,EC:1,EC:1.14;Enzyme_name=Acting on paired donors, with incorporation or reduction of molecular oxygen. The oxygen incorporated need not be derived from O2,Oxidoreductases,Acting on paired donors, with incorporation or reduction of molecular oxygen. The oxygen incorporated need not be derived from O2
contig00003.g2343	1377	ID=contig00003.g2343;Description=hypothetical protein FVER53590_08437 [Fusarium verticillioides];Gene=FPCIR_1898;Ontology_term=membrane;Ontology_id=GO:0016020
contig00003.g2344	1261	ID=contig00003.g2344;Description=ethanolamine utilization [Fusarium tjaetaba];Gene=Phb1;Ontology_term=mitochondrion,mitochondrion organization;Ontology_id=GO:0005739,GO:0007005
contig00003.g2345	1776	ID=contig00003.g2345;Description=translation initiation factor IF-2 [Fusarium pseudoanthophilum];Gene=FPCIR_1895;Ontology_term=translation regulator activity;Ontology_id=GO:0045182
contig00003.g2346	658	ID=contig00003.g2346;Description=related to Cupin domain protein [Fusarium fujikuroi];Gene=CG5363;Ontology_term=catalytic activity, acting on a protein,transferase activity;Ontology_id=GO:0140096,GO:0016740;Enzyme_code=EC:2.7.11.1,EC:2.7.1,EC:2.7.11.1,EC:2.7,EC:2;Enzyme_name=non-specific serine/threonine protein kinase,Transferring phosphorus-containing groups,non-specific serine/threonine protein kinase,Transferring phosphorus-containing groups,Transferases
contig00003.g2347	702	ID=contig00003.g2347;Description=cupin [Fusarium subglutinans]
contig00003.g2348	1032	ID=contig00003.g2348;Description=putative secondary metabolism biosynthetic enzyme [Fusarium musae]

contig00003.g2349	1331	ID=contig00003.g2349;Description=fad binding domain-containing protein [Fusarium coicis];Gene=FANTH_8155;Ontology_term=oxidoreductase activity;Ontology_id=GO:0016491;Enzyme_code=EC:1.14,EC:1,EC:1.14;Enzyme_name=Acting on paired donors, with incorporation or reduction of molecular oxygen. The oxygen incorporated need not be derived from O2,Oxidoreductases,Acting on paired donors, with incorporation or reduction of molecular oxygen. The oxygen incorporated need not be derived from O2
contig00003.g2350	1893	ID=contig00003.g2350;Description=cytochrome p450 [Fusarium coicis];Gene=F52700_11524;Ontology_term=oxidoreductase activity;Ontology_id=GO:0016491;Enzyme_code=EC:1.14,EC:1,EC:1.14;Enzyme_name=Acting on paired donors, with incorporation or reduction of molecular oxygen. The oxygen incorporated need not be derived from O2,Oxidoreductases,Acting on paired donors, with incorporation or reduction of molecular oxygen. The oxygen incorporated need not be derived from O2
contig00003.g2351	2366	ID=contig00003.g2351;Description=hypothetical protein FVER14953_08429 [Fusarium verticillioides];Gene=FVER53263_08429;Ontology_term=oxidoreductase activity;Ontology_id=GO:0016491;Enzyme_code=EC:1,EC:1;Enzyme_name=Oxidoreductases,Oxidoreductases
contig00003.g2352	1393	ID=contig00003.g2352;Description=fad binding domain protein [Fusarium tjaetaba];Gene=FPANT_6383;Ontology_term=oxidoreductase activity;Ontology_id=GO:0016491;Enzyme_code=EC:1;Enzyme_name=Oxidoreductases
contig00003.g2353	1098	ID=contig00003.g2353;Description=retinol dehydrogenase 12 [Fusarium denticulatum];Gene=FVER53263_20017;Ontology_term=oxidoreductase activity;Ontology_id=GO:0016491;Enzyme_code=EC:1,EC:1;Enzyme_name=Oxidoreductases,Oxidoreductases
contig00003.g2354	1173	ID=contig00003.g2354;Description=1-acyldihydroxyacetone-phosphate reductase [Fusarium sp. NRRL 52700];Gene=F52700_11526;Ontology_term=oxidoreductase activity;Ontology_id=GO:0016491;Enzyme_code=EC:1,EC:1,EC:1.1.1.101;Enzyme_name=Oxidoreductases,Oxidoreductases,acylglycerone-phosphate reductase
contig00003.g2355	986	ID=contig00003.g2355;Description=putative secondary metabolism biosynthetic enzyme [Fusarium musae]

contig00003.g2356	7723	ID=contig00003.g2356;Description=Type I Iterative PKS [Fusarium musae];Gene=FVER53263_08425;Ontology_term=oxidoreductase activity,lipid metabolic process,transferase activity;Ontology_id=GO:0016491,GO:0006629,GO:0016740;Enzyme_code=EC:2.3.1.41,EC:1,EC:2.3.1.41,EC:2.3,EC:1,EC:2,EC:2.3.1;Enzyme_name=beta-ketoacyl-[acyl-carrier-protein] synthase I,Oxidoreductases,beta-ketoacyl-[acyl-carrier-protein] synthase I,Acyltransferases,Oxidoreductases,Transferases,Acyltransferases
contig00003.g2357	1401	ID=contig00003.g2357;Description=integral membrane protein PTH11 [Fusarium coicis];Gene=BFJ63_vAg17347;Ontology_term=membrane;Ontology_id=GO:0016020
contig00003.g2358	2080	ID=contig00003.g2358;Description=isoamyl alcohol oxidase [Fusarium coicis];Gene=FNYG_12395;Ontology_term=oxidoreductase activity;Ontology_id=GO:0016491;Enzyme_code=EC:1,EC:1;Enzyme_name=Oxidoreductases,Oxidoreductases
contig00003.g2359	966	ID=contig00003.g2359;Description=endo-arabinase [Fusarium phyllophilum];Gene=FOIG_08544;Ontology_term=carbohydrate metabolic process,hydrolase activity;Ontology_id=GO:0005975,GO:0016787;Enzyme_code=EC:3.2.1,EC:3.2,EC:3,EC:3.2.1;Enzyme_name=Glycosylases,Glycosylases,Hydrolases,Glycosylases
contig00003.g2360	663	ID=contig00003.g2360;Description=related to theta class glutathione S-transferase [Fusarium mangiferae];Gene=FNAPI_1061;Ontology_term=transferase activity;Ontology_id=GO:0016740;Enzyme_code=EC:2,EC:2.5.1.18,EC:2;Enzyme_name=Transferases,glutathione transferase,Transferases
contig00003.g2361	879	ID=contig00003.g2361;Description=trans-aconitate 2-methyltransferase [Fusarium verticillioides 7600];Gene=FVEG_08419;Ontology_term=transferase activity;Ontology_id=GO:0016740;Enzyme_code=EC:2.1.1.144,EC:2.1,EC:2.1.1.144,EC:2,EC:2.1.1;Enzyme_name=trans-aconitate 2-methyltransferase,Transferring one-carbon groups,trans-aconitate 2-methyltransferase,Transferases,Transferring one-carbon groups
contig00003.g2362	4917	ID=contig00003.g2362;Description=C6 transcription factor [Fusarium denticulatum];Gene=F52700_11535;Ontology_term=nucleus,regulation of DNA-templated transcription,transcription regulator activity;Ontology_id=GO:0005634,GO:0006355,GO:0140110
contig00003.g2363	792	ID=contig00003.g2363;Description=putative secondary metabolism biosynthetic enzyme [Fusarium musae]

contig00003.g2364	1233	ID=contig00003.g2364;Description=hypothetical protein FVER53590_08415 [Fusarium verticillioides];Gene=FVER53263_08415;Ontology_term=membrane;Ontology_id=GO:0016020
contig00003.g2365	342	ID=contig00003.g2365;Description=hypothetical protein FVER53263_08414 [Fusarium verticillioides]
contig00003.g2366	1426	ID=contig00003.g2366;Description=phospholipase A2 [Fusarium tjaetaba]
contig00003.g2367	825	ID=contig00003.g2367;Description=TDG/mug DNA glycosylase [Fusarium verticillioides 7600];Gene=FNAPI_1055;Ontology_term=DNA repair,catalytic activity, acting on DNA,hydrolase activity;Ontology_id=GO:0006281,GO:0140097,GO:0016787;Enzyme_code=EC:3.2.2,EC:3.2,EC:3,EC:3.2.2.29,EC:3.2.2.27,EC:3.2.2;Enzyme_name=Glycosylases,Glycosylases,Hydrolases,thymine-DNA glycosylase,uracil-DNA glycosylase,Glycosylases
contig00003.g2368	3003	ID=contig00003.g2368;Description=multiple ankyrin repeats single kh domain protein [Fusarium tjaetaba];Gene=FVER53590_08411;Ontology_term=lipid metabolic process,hydrolase activity;Ontology_id=GO:0006629,GO:0016787;Enzyme_code=EC:3.1.1.5,EC:3.1,EC:3,EC:3.1.1.5,EC:3.1.1;Enzyme_name=lysophospholipase,Acting on ester bonds,Hydrolases,lysophospholipase,Acting on ester bonds
contig00003.g2369	2100	ID=contig00003.g2369;Description=phospholipase A2 [Fusarium odoratissimum NRRL 54006];Gene=BFJ68_g10188;Ontology_term=lipid metabolic process,hydrolase activity;Ontology_id=GO:0006629,GO:0016787;Enzyme_code=EC:3.1.1.5,EC:3.1,EC:3,EC:3.1.1.5,EC:3.1.1,EC:3.1.1.4;Enzyme_name=lysophospholipase,Acting on ester bonds,Hydrolases,lysophospholipase,Acting on ester bonds,phospholipase A2
contig00003.g2370	2181	ID=contig00003.g2370;Description=hypothetical protein FVEG_16316 [Fusarium verticillioides 7600];Gene=Chc;Ontology_term=vesicle-mediated transport,structural molecule activity,cytoplasmic vesicle,Golgi apparatus,intracellular protein transport,plasma membrane;Ontology_id=GO:0016192,GO:0005198,GO:0031410,GO:0005794,GO:0006886,GO:0005886

contig00003.g2371	4196	ID=contig00003.g2371;Description=heterokaryon incompatibility protein het-E-1 [Fusarium phyllophilum];Gene=FPRO05_00149;Ontology_term=nucleus,catalytic activity, acting on a protein,regulation of DNA-templated transcription,transcription regulator activity,hydrolase activity;Ontology_id=GO:0005634,GO:0140096,GO:0006355,GO:0140110,GO:0016787;Enzyme_code=EC:3.4.22,EC:3.4.22,EC:3.4,EC:3;Enzyme_name=Acting on peptide bonds (peptidases),Acting on peptide bonds (peptidases),Acting on peptide bonds (peptidases),Hydrolases
contig00003.g2372	612	ID=contig00003.g2372;Description=CHAT domain-containing protein [Fusarium sp. MPI-SDFR-AT-0072];Gene=FPCIR_1871;Ontology_term=nucleus,catalytic activity, acting on a protein,regulation of DNA-templated transcription,transcription regulator activity,hydrolase activity;Ontology_id=GO:0005634,GO:0140096,GO:0006355,GO:0140110,GO:0016787;Enzyme_code=EC:3.4.22,EC:3.4.22,EC:3.4,EC:3;Enzyme_name=Acting on peptide bonds (peptidases),Acting on peptide bonds (peptidases),Acting on peptide bonds (peptidases),Hydrolases
contig00003.g2373	1125	ID=contig00003.g2373;Description=NADPH2:quinone reductase [Fusarium verticillioides 7600];Gene=FTJAE_3266;Ontology_term=oxidoreductase activity;Ontology_id=GO:0016491;Enzyme_code=EC:1.6,EC:1.6,EC:1,EC:1.6.5.5;Enzyme_name=Acting on NADH or NADPH,Acting on NADH or NADPH,Oxidoreductases,NADPH:quinone reductase
contig00003.g2374	1461	ID=contig00003.g2374;Description=U3 small nucleolar RNA-associated protein 17 [Fusarium oxysporum f. sp. albedinis];Gene=F52700_11549;Ontology_term=membrane;Ontology_id=GO:0016020
contig00003.g2375	1332	ID=contig00003.g2375;Description=peptidase acyl-coenzyme a:6-aminopenicillanic acid acyl-transferase [Fusarium tjaetaba];Gene=FPCIR_1865;Ontology_term=transferase activity;Ontology_id=GO:0016740;Enzyme_code=EC:2.3,EC:2.3,EC:2;Enzyme_name=Acyltransferases,Acyltransferases,Transferases
contig00003.g2376	4350	ID=contig00003.g2376;Description=DAL5-Allantoate ureidosuccinate permease [Fusarium mexicanum];Gene=372;Ontology_term=nucleus,transmembrane transport,regulation of DNA-templated transcription,transcription regulator activity,transporter activity;Ontology_id=GO:0005634,GO:0055085,GO:0006355,GO:0140110,GO:0005215

contig00003.g2377	1321	ID=contig00003.g2377;Description=hypothetical protein FVEG_08403 [Fusarium verticillioides 7600];Gene=FPCIR_1863;Ontology_term=catalytic activity, acting on a protein,hydrolase activity;Ontology_id=GO:0140096,GO:0016787;Enzyme_code=EC:3.4.23,EC:3.4.23,EC:3.4,EC:3;Enzyme_name=Acting on peptide bonds (peptidases),Acting on peptide bonds (peptidases),Acting on peptide bonds (peptidases),Hydrolases
contig00003.g2378	1294	ID=contig00003.g2378;Description=hypothetical protein FVEG_08402 [Fusarium verticillioides 7600];Gene=FNAPI_1045;Ontology_term=catalytic activity, acting on a protein,protein catabolic process,extracellular region,hydrolase activity;Ontology_id=GO:0140096,GO:0030163,GO:0005576,GO:0016787;Enzyme_code=EC:3.4.23,EC:3.4.23.18,EC:3.4.23,EC:3.4,EC:3;Enzyme_name=Acting on peptide bonds (peptidases),aspergillopepsin I,Acting on peptide bonds (peptidases),Acting on peptide bonds (peptidases),Hydrolases
contig00003.g2379	444	ID=contig00003.g2379;Description=pyruvate decarboxylase [Fusarium tjaetaba];Gene=FNAPI_1044;Ontology_term=membrane;Ontology_id=GO:0016020
contig00003.g2380	2503	ID=contig00003.g2380;Description=hypothetical protein FVEG_08400 [Fusarium verticillioides 7600];Gene=FVER53590_08400;Ontology_term=transmembrane transport,transporter activity;Ontology_id=GO:0055085,GO:0005215
contig00003.g2381	2473	ID=contig00003.g2381;Description=OPT family small oligopeptide transporter [Fusarium verticillioides 7600];Gene=FVER53263_08399;Ontology_term=transmembrane transport,transporter activity;Ontology_id=GO:0055085,GO:0005215
contig00003.g2382	1474	ID=contig00003.g2382;Description=fad dependent oxidoreductase [Fusarium coicis];Gene=FPANT_1271;Ontology_term=oxidoreductase activity;Ontology_id=GO:0016491;Enzyme_code=EC:1;Enzyme_name=Oxidoreductases
contig00003.g2383	968	ID=contig00003.g2383;Description=hypothetical protein FVEG_08397 [Fusarium verticillioides 7600]
contig00003.g2384	1350	ID=contig00003.g2384;Description=related to phosphatidylserine decarboxylase 2 [Fusarium proliferatum ET1];Gene=FPHYL_6654;Ontology_term=lipid metabolic process,lyase activity;Ontology_id=GO:0006629,GO:0016829;Enzyme_code=EC:4.1.1.65,EC:4.1.1,EC:4.1,EC:4,EC:4.1.1.65;Enzyme_name=phosphatidylserine decarboxylase,Carbon-carbon lyases,Carbon-carbon lyases,Lyases,phosphatidylserine decarboxylase
contig00003.g2385	594	ID=contig00003.g2385;Description=hypothetical protein FVER14953_08395 [Fusarium verticillioides];Enzyme_code=EC:3.1.1.47;Enzyme_name=1-alkyl-2-acetylglycerophosphocholine esterase

contig00003.g2386	1605	ID=contig00003.g2386;Description=tartrate transporter [Fusarium coicis];Gene=FVER53590_08394;Ontology_term=transmembrane transport,transporter activity;Ontology_id=GO:0055085,GO:0005215
contig00003.g2387	509	ID=contig00003.g2387;Description=(trans)glycosidase [Fusarium napiforme]
contig00003.g2388	3354	ID=contig00003.g2388;Description=hypothetical protein FVER14953_08390 [Fusarium verticillioides]
contig00003.g2389	2601	ID=contig00003.g2389;Description=Tol [Fusarium coicis]
contig00003.g2390	1449	ID=contig00003.g2390;Description=l- -diaminobutyrate decarboxylase [Fusarium acutatum];Gene=FPANT_8474;Ontology_term=lyase activity,amino acid metabolic process;Ontology_id=GO:0016829,GO:0006520;Enzyme_code=EC:4.1.1,EC:4.1.1,EC:4.1,EC:4;Enzyme_name=Carbon-carbon lyases,Carbon-carbon lyases,Carbon-carbon lyases,Lyases
contig00003.g2391	701	ID=contig00003.g2391;Description=peroxiredoxin (alkyl hydroperoxide reductase subunit C) [Fusarium verticillioides 7600];Gene=FOPG_14021;Ontology_term=antioxidant activity,oxidoreductase activity;Ontology_id=GO:0016209,GO:0016491;Enzyme_code=EC:1.11.1;Enzyme_name=Acting on a peroxide as acceptor
contig00003.g2392	1584	ID=contig00003.g2392;Description=f-box domain protein [Fusarium tjaetaba]
contig00003.g2393	1653	ID=contig00003.g2393;Description=hypothetical protein LB504_005053 [Fusarium proliferatum]
contig00003.g2394	989	ID=contig00003.g2394;Description=histone acetyltransferase [Fusarium musae];Gene=FCIRC_8526;Ontology_term=catalytic activity, acting on a protein,transmembrane transport,DNA recombination,nuclear chromosome,regulation of DNA-templated transcription,chromatin organization,nucleoplasm,histone binding,cytosol,transferase activity,transcription regulator activity,meiotic nuclear division,molecular adaptor activity;Ontology_id=GO:0140096,GO:0055085,GO:0006310,GO:0000228,GO:0006355,GO:0006325,GO:0005654,GO:0042393,GO:0005829,GO:0016740,GO:0140110,GO:0140013,GO:0060090;Enzyme_code=EC:2.3.1.48,EC:2.3,EC:2,EC:2.3.1.48,EC:2.3.1;Enzyme_name=histone acetyltransferase,Acyltransferases,Transferases,histone acetyltransferase,Acyltransferases
contig00003.g2395	1019	ID=contig00003.g2395;Description=hypothetical protein FVEG_16308 [Fusarium verticillioides 7600]

contig00003.g2396	4495	ID=contig00003.g2396;Description=het-domain-containing protein [Fusarium mundagurra];Gene=FRV6_11664;Ontology_term=catalytic activity, acting on a protein,transferase activity;Ontology_id=GO:0140096,GO:0016740;Enzyme_code=EC:2.7.1,EC:2.7.1,EC:2.7,EC:2;Enzyme_name=Transferring phosphorus-containing groups,Transferring phosphorus-containing groups,Transferring phosphorus-containing groups,Transferases
contig00003.g2397	2245	ID=contig00003.g2397;Description=Cyclin-dependent kinase-like 4 [Fusarium oxysporum f. sp. rapae];Gene=FRV6_11666;Ontology_term=transferase activity;Ontology_id=GO:0016740;Enzyme_code=EC:2.7,EC:2.7,EC:2;Enzyme_name=Transferring phosphorus-containing groups,Transferring phosphorus-containing groups,Transferases
contig00003.g2398	1081	ID=contig00003.g2398;Description=formate transport [Fusarium coicis];Gene=FVER53590_08384;Ontology_term=transmembrane transport,transporter activity;Ontology_id=GO:0055085,GO:0005215
contig00003.g2399	1899	ID=contig00003.g2399;Description=het domain protein [Fusarium tjaetaba]
contig00003.g2400	1745	ID=contig00003.g2400;Description=hexose transporter 2 [Fusarium pseudocircinatum];Gene=FPCIR_1827;Ontology_term=transmembrane transport,transporter activity;Ontology_id=GO:0055085,GO:0005215
contig00003.g2401	1867	ID=contig00003.g2401;Description=arylsulfatase [Fusarium verticillioides 7600];Gene=FVEG_08381;Ontology_term=hydrolase activity;Ontology_id=GO:0016787;Enzyme_code=EC:3.1.6,EC:3.1.6,EC:3.1,EC:3,EC:3.1.6.1;Enzyme_name=Acting on ester bonds,Acting on ester bonds,Acting on ester bonds,Hydrolases,arylsulfatase (type I)
contig00003.g2402	2643	ID=contig00003.g2402;Description=regulator of drug sensitivity 1 [Fusarium napiforme];Gene=FACUT_2726;Ontology_term=nucleus,DNA binding,regulation of DNA-templated transcription,transcription regulator activity;Ontology_id=GO:0005634,GO:0003677,GO:0006355,GO:0140110
contig00003.g2403	2179	ID=contig00003.g2403;Description=dioxygenase [Fusarium coicis];Gene=FPCIR_1830;Ontology_term=oxidoreductase activity;Ontology_id=GO:0016491;Enzyme_code=EC:1,EC:1;Enzyme_name=Oxidoreductases,Oxidoreductases
contig00003.g2404	948	ID=contig00003.g2404;Description=putative ankyrin repeat protein [Fusarium oxysporum f. sp. raphani];Gene=FVER53590_08379;Ontology_term=toxin activity;Ontology_id=GO:0090729

contig00003.g2405	534	ID=contig00003.g2405;Description=shikimate kinase [Fusarium odoratissimum NRRL 54006];Gene=FPCIR_1832;Ontology_term=transmembrane transport,transferase activity,transporter activity;Ontology_id=GO:0055085,GO:0016740,GO:0005215;Enzyme_code=EC:2.7,EC:2.7,EC:2;Enzyme_name=Transferring phosphorus-containing groups,Transferring phosphorus-containing groups,Transferases
contig00003.g2406	2259	ID=contig00003.g2406;Description=dual specificity catalytic domain-containing protein [Fusarium napiforme];Gene=FVER53590_08376;Ontology_term=catalytic activity, acting on a protein,hydrolase activity;Ontology_id=GO:0140096,GO:0016787;Enzyme_code=EC:3.1.3.48,EC:3.1,EC:3.1.3.48,EC:3,EC:3.1.3;Enzyme_name=protein-tyrosine-phosphatase,Acting on ester bonds,protein-tyrosine-phosphatase,Hydrolases,Acting on ester bonds
contig00003.g2407	1657	ID=contig00003.g2407;Description=related to allantoate permease [Fusarium fujikuroi];Gene=F52700_1246;Ontology_term=transmembrane transport,transporter activity;Ontology_id=GO:0055085,GO:0005215
contig00003.g2408	1258	ID=contig00003.g2408;Description=phosphorylase superfamily [Fusarium pseudoanthophilum];Gene=FDENT_11229;Ontology_term=nucleobase-containing small molecule metabolic process,anatomical structure development,structural molecule activity,carbohydrate derivative metabolic process,regulation of DNA-templated transcription,receptor ligand activity,nucleoplasm,cell motility,wound healing,extracellular space,catalytic activity,lipid metabolic process,cell differentiation,cytosol,extracellular matrix;Ontology_id=GO:0055086,GO:0048856,GO:0005198,GO:1901135,GO:0006355,GO:0048018,GO:0005654,GO:0048870,GO:0042060,GO:0005615,GO:0003824,GO:0006629,GO:0030154,GO:0005829,GO:0031012
contig00003.g2409	783	ID=contig00003.g2409;Description=peptidase a4 family [Fusarium subglutinans];Gene=FANTH_8188;Ontology_term=catalytic activity, acting on a protein,hydrolase activity;Ontology_id=GO:0140096,GO:0016787;Enzyme_code=EC:3.4,EC:3.4,EC:3;Enzyme_name=Acting on peptide bonds (peptidases),Acting on peptide bonds (peptidases),Hydrolases
contig00003.g2410	666	ID=contig00003.g2410;Description=hypothetical protein FVER14953_08372 [Fusarium verticillioides]

contig00003.g2411	942	ID=contig00003.g2411;Description=related to phosphoglycerate mutase [Fusarium proliferatum ET1];Enzyme_code=EC:3.1.3.37;Enzyme_name=sedoheptulose-bisphosphatase
contig00003.g2412	1539	ID=contig00003.g2412;Description=tartrate transporter [Fusarium phyllophilum];Gene=FPHYL_11257;Ontology_term=transmembrane transport,transporter activity;Ontology_id=GO:0055085,GO:0005215
contig00003.g2413	1732	ID=contig00003.g2413;Description=tartrate transporter [Fusarium tjaetaba];Gene=FVEG_08370;Ontology_term=transmembrane transport,transporter activity;Ontology_id=GO:0055085,GO:0005215
contig00003.g2414	1908	ID=contig00003.g2414;Description=related to benzoylformate decarboxylase [Fusarium oxysporum];Gene=FOC1_g10004427;Ontology_term=lyase activity;Ontology_id=GO:0016829;Enzyme_code=EC:4.1.1.1,EC:4.1.1,EC:4.1.1.1,EC:4.1,EC:4;Enzyme_name=pyruvate decarboxylase,Carbon-carbon lyases,pyruvate decarboxylase,Carbon-carbon lyases,Lyases
contig00003.g2415	1041	ID=contig00003.g2415;Description=related to oxidoreductase [Fusarium oxysporum];Gene=FOXB_13599;Ontology_term=oxidoreductase activity;Ontology_id=GO:0016491;Enzyme_code=EC:1.6,EC:1.6,EC:1;Enzyme_name=Acting on NADH or NADPH,Acting on NADH or NADPH,Oxidoreductases
contig00003.g2416	486	ID=contig00003.g2416;Description=hypothetical protein FVER53263_20069 [Fusarium verticillioides]
contig00003.g2417	2023	ID=contig00003.g2417;Description=d-lactate dehydrogenase (cytochrome) [Fusarium tjaetaba];Gene=FVER53590_08367;Ontology_term=oxidoreductase activity,mitochondrion;Ontology_id=GO:0016491,GO:0005739;Enzyme_code=EC:1.1,EC:1.1.2.4,EC:1,EC:1.1.2;Enzyme_name=Acting on the CH-OH group of donors,D-lactate dehydrogenase (cytochrome),Oxidoreductases,Acting on the CH-OH group of donors
contig00003.g2418	2399	ID=contig00003.g2418;Description=transcription activator acu-15 [Fusarium tjaetaba];Gene=FVER53590_08366;Ontology_term=nucleus,DNA binding,regulation of DNA-templated transcription,transcription regulator activity;Ontology_id=GO:0005634,GO:0003677,GO:0006355,GO:0140110

contig00003.g2419	2496	ID=contig00003.g2419;Description=glycoside hydrolase family 3 [Fusarium tjaetaba];Gene=FVER53590_08365;Ontology_term=carbohydrate metabolic process,hydrolase activity;Ontology_id=GO:0005975,GO:0016787;Enzyme_code=EC:3.2.1.21,EC:3.2,EC:3.2.1.21,EC:3,EC:3.2.1;Enzyme_name=beta-glucosidase,Glycosylases,beta-glucosidase,Hydrolases,Glycosylases
contig00003.g2420	1686	ID=contig00003.g2420;Description=hexose transporter [Fusarium tjaetaba];Gene=FNAPI_2774;Ontology_term=transmembrane transport,transporter activity;Ontology_id=GO:0055085,GO:0005215
contig00003.g2421	1928	ID=contig00003.g2421;Description=MFS transporter, SP family, general alpha glucoside:H+ symporter [Fusarium proliferatum];Gene=Cyt-c-d;Ontology_term=cellular anatomical entity;Ontology_id=GO:0110165
contig00003.g2422	1428	ID=contig00003.g2422;Description=monoamine oxidase A [Fusarium tjaetaba];Gene=FGLOB1_759;Ontology_term=oxidoreductase activity;Ontology_id=GO:0016491;Enzyme_code=EC:1,EC:1,EC:1.4.3.4;Enzyme_name=Oxidoreductases,Oxidoreductases,monoamine oxidase
contig00003.g2423	1315	ID=contig00003.g2423;Description=related to Transaldolase B [Fusarium fujikuroi];Gene=FFUJ_11119;Ontology_term=carbohydrate metabolic process,molecular function regulator activity;Ontology_id=GO:0005975,GO:0098772;Enzyme_code=EC:2.2.1.2;Enzyme_name=transaldolase
contig00003.g2424	1923	ID=contig00003.g2424;Description=fungal Zn binuclear cluster domain protein [Fusarium tjaetaba];Gene=FACUT_2747;Ontology_term=nucleus,carbohydrate metabolic process,DNA binding,amino acid metabolic process,regulation of DNA-templated transcription,transcription regulator activity,molecular adaptor activity;Ontology_id=GO:0005634,GO:0005975,GO:0003677,GO:0006520,GO:0006355,GO:0140110,GO:0060090
contig00003.g2425	2445	ID=contig00003.g2425;Description=hypothetical protein FVER14953_08359 [Fusarium verticillioides];Gene=FVER53263_08359;Ontology_term=nucleus,carbohydrate metabolic process,regulation of DNA-templated transcription,transcription regulator activity;Ontology_id=GO:0005634,GO:0005975,GO:0006355,GO:0140110

contig00003.g2426	2036	ID=contig00003.g2426;Description=positive regulator of purine utilization [Fusarium tjaetaba];Gene=FPANT_4869;Ontology_term=nucleus,DNA binding,regulation of DNA-templated transcription,transcription regulator activity;Ontology_id=GO:0005634,GO:0003677,GO:0006355,GO:0140110
contig00003.g2427	1605	ID=contig00003.g2427;Description=related to hexose transporter protein [Fusarium fujikuroi IMI 58289];Gene=FVER53263_08357;Ontology_term=transmembrane transport,protein catabolic process,regulation of DNA-templated transcription,nucleolus,intracellular protein transport,microtubule-based movement,transporter activity,plasma membrane,lysosome,vesicle-mediated transport,mRNA metabolic process,protein folding chaperone,ATP-dependent activity,autophagy,cytosol,cytoplasmic vesicle,hydrolase activity,molecular adaptor activity;Ontology_id=GO:0055085,GO:0030163,GO:0006355,GO:0005730,GO:0006886,GO:0007018,GO:0005215,GO:0005886,GO:0005764,GO:0016192,GO:0016071,GO:0044183,GO:0140657,GO:0006914,GO:0005829,GO:0031410,GO:0016787,GO:0060090;Enzyme_code=EC:3.6.1.15,EC:3.6.1,EC:3.6,EC:3,EC:3.6.1.15;Enzyme_name=nucleoside-triphosphate phosphatase,Acting on acid anhydrides,Acting on acid anhydrides,Hydrolases,nucleoside-triphosphate phosphatase
contig00003.g2428	2927	ID=contig00003.g2428;Description=related to sterol glucosyltransferase [Fusarium proliferatum ET1];Gene=LW93_14711;Ontology_term=membrane organization,anatomical structure development,chromosome segregation,cytokinesis,reproductive process,chromosome,microtubule-based movement,nucleus,carbohydrate metabolic process,molecular function regulator activity,cytoskeleton,lipid metabolic process,mitotic nuclear division,cell differentiation,transferase activity;Ontology_id=GO:0061024,GO:0048856,GO:0007059,GO:0000910,GO:0022414,GO:0005694,GO:0007018,GO:0005634,GO:0005975,GO:0098772,GO:0005856,GO:0006629,GO:0140014,GO:0030154,GO:0016740;Enzyme_code=EC:2.4.1.173,EC:2.4.1.173,EC:2.4,EC:2,EC:2.4.1;Enzyme_name=sterol 3beta-glucosyltransferase,sterol 3beta-glucosyltransferase,Glycosyltransferases,Transferases,Glycosyltransferases
contig00003.g2429	641	ID=contig00003.g2429;Description=alpha-L-rhamnosidase C [Fusarium tjaetaba];Gene=FVER53590_08355;Ontology_term=carbohydrate metabolic process;Ontology_id=GO:0005975
contig00003.g2430	1460	ID=contig00003.g2430;Description=urg3 [Fusarium pseudoanthophilum];Ontology_term=catalytic activity;Ontology_id=GO:0003824

contig00003.g2431	1390	ID=contig00003.g2431;Description=CMGC kinase [Fusarium napiforme];Gene=DL769_001740;Ontology_term=catalytic activity, acting on a protein,chromosome,transferase activity;Ontology_id=GO:0140096,GO:0005694,GO:0016740;Enzyme_code=EC:2.7.11.1,EC:2.7.1,EC:2.7.11.1,EC:2.7,EC:2;Enzyme_name=non-specific serine/threonine protein kinase,Transferring phosphorus-containing groups,non-specific serine/threonine protein kinase,Transferring phosphorus-containing groups,Transferases
contig00003.g2432	897	ID=contig00003.g2432;Description=related to ERG6 S-adenosyl-methionine delta-24-sterol-c-methyltransferase [Fusarium fujikuroi];Gene=C2S_10409;Ontology_term=transferase activity;Ontology_id=GO:0016740;Enzyme_code=EC:2.1.1,EC:2.1,EC:2,EC:2.1.1;Enzyme_name=Transferring one-carbon groups,Transferring one-carbon groups,Transferases,Transferring one-carbon groups
contig00003.g2433	2147	ID=contig00003.g2433;Description=hypothetical protein CEK25_002749 [Fusarium fujikuroi];Gene=Sod1;Ontology_term=peroxisome,antioxidant activity,oxidoreductase activity,autophagy,cell junction organization,signaling,mitochondrion organization;Ontology_id=GO:0005777,GO:0016209,GO:0016491,GO:0006914,GO:0034330,GO:0023052,GO:0007005;Enzyme_code=EC:1.15.1.1,EC:1,EC:1.15.1.1,EC:1.15;Enzyme_name=superoxide dismutase,Oxidoreductases,superoxide dismutase,Acting on superoxide as acceptor
contig00003.g2434	1778	ID=contig00003.g2434;Description=hexose transporter [Fusarium tjaetaba];Gene=FTJAE_3195;Ontology_term=transmembrane transport,transporter activity;Ontology_id=GO:0055085,GO:0005215
contig00003.g2435	651	ID=contig00003.g2435;Description=hypothetical protein FVER53263_08350 [Fusarium verticillioides];Gene=FNAPI_2763;Ontology_term=membrane;Ontology_id=GO:0016020
contig00003.g2436	714	ID=contig00003.g2436;Description=hypothetical protein FVEG_08349 [Fusarium verticillioides 7600]
contig00003.g2437	894	ID=contig00003.g2437;Description=hypothetical protein FHETE_11159 [Fusarium heterosporum];Gene=F66182_5494;Ontology_term=membrane;Ontology_id=GO:0016020
contig00003.g2438	923	ID=contig00003.g2438;Description=hypothetical protein FVER14953_08347 [Fusarium verticillioides]

contig00003.g2439	3935	ID=contig00003.g2439;Description=ankyrin protein [Fusarium tjaetaba];Gene=FVER53263_08346;Ontology_term=nucleobase-containing small molecule metabolic process,catalytic activity,structural molecule activity,carbohydrate derivative metabolic process,organelle;Ontology_id=GO:0055086,GO:0003824,GO:0005198,GO:1901135,GO:0043226
contig00003.g2440	1536	ID=contig00003.g2440;Description=murein transglycosylase [Fusarium verticillioides 7600];Gene=FPANT_4881;Ontology_term=carbohydrate metabolic process,nucleobase-containing small molecule metabolic process,carbohydrate derivative metabolic process,hydrolase activity;Ontology_id=GO:0005975,GO:0055086,GO:1901135,GO:0016787;Enzyme_code=EC:3.2.1,EC:3.2,EC:3,EC:3.2.1;Enzyme_name=Glycosylases,Glycosylases,Hydrolases,Glycosylases
contig00003.g2441	1350	ID=contig00003.g2441;Description=endo-1 4-beta-xylanase [Fusarium tjaetaba];Gene=FNYG_12481;Ontology_term=carbohydrate metabolic process,cell wall organization or biogenesis,hydrolase activity;Ontology_id=GO:0005975,GO:0071554,GO:0016787;Enzyme_code=EC:3.2.1,EC:3.2,EC:3,EC:3.2.1;Enzyme_name=Glycosylases,Glycosylases,Hydrolases,Glycosylases
contig00003.g2442	1841	ID=contig00003.g2442;Description=tartrate transporter [Fusarium subglutinans];Gene=FSUBG_7025
contig00003.g2443	1217	ID=contig00003.g2443;Description=glycosyl hydrolase [Fusarium denticulatum];Gene=FOXB_08550;Ontology_term=nucleus,DNA binding,carbohydrate metabolic process,regulation of DNA-templated transcription,transcription regulator activity,hydrolase activity;Ontology_id=GO:0005634,GO:0003677,GO:0005975,GO:0006355,GO:0140110,GO:0016787;Enzyme_code=EC:3,EC:3,EC:3.2.1.172;Enzyme_name=Hydrolases,Hydrolases,unsaturated rhamnogalacturonyl hydrolase
contig00003.g2444	1902	ID=contig00003.g2444;Description=hypothetical protein FVER53590_08341 [Fusarium verticillioides]
contig00003.g2445	2031	ID=contig00003.g2445;Description=hypothetical protein FVEG_16298 [Fusarium verticillioides 7600];Gene=FVEG_16298;Ontology_term=nucleus,DNA binding,regulation of DNA-templated transcription,transcription regulator activity;Ontology_id=GO:0005634,GO:0003677,GO:0006355,GO:0140110
contig00003.g2446	422	ID=contig00003.g2446;Description=4Fe-4S iron-sulfur binding protein [Fusarium tjaetaba]

contig00003.g2447	1909	ID=contig00003.g2447;Description=amino acid permease [Fusarium tjaetaba];Gene=FVER53263_08339;Ontology_term=transmembrane transport;Ontology_id=GO:0055085
contig00003.g2448	1066	ID=contig00003.g2448;Description=Metapyrocatechase 2 [Fusarium acutatum]
contig00003.g2449	1169	ID=contig00003.g2449;Description=1-acyldihydroxyacetone-phosphate reductase [Fusarium sp. NRRL 52700];Gene=FNYG_12089;Ontology_term=oxidoreductase activity;Ontology_id=GO:0016491;Enzyme_code=EC:1,EC:1,EC:1.1.1.101;Enzyme_name=Oxidoreductases,Oxidoreductases,acylglycerone-phosphate reductase
contig00003.g2450	654	ID=contig00003.g2450;Description=hypothetical protein FVEG_16297 [Fusarium verticillioides 7600]
contig00003.g2451	1690	ID=contig00003.g2451;Description=1-acyldihydroxyacetone-phosphate reductase [Fusarium fujikuroi];Gene=FDENT_8917;Ontology_term=inflammatory response,extracellular space,oxidoreductase activity,anatomical structure development,cell differentiation,cytosol,cell adhesion,receptor ligand activity,immune system process,lysosome;Ontology_id=GO:0006954,GO:0005615,GO:0016491,GO:0048856,GO:0030154,GO:0005829,GO:0007155,GO:0048018,GO:0002376,GO:0005764;Enzyme_code=EC:1,EC:1;Enzyme_name=Oxidoreductases,Oxidoreductases
contig00003.g2452	1723	ID=contig00003.g2452;Description=peptidase m61 domain-containing protein [Colletotrichum truncatum]
contig00003.g2453	1745	ID=contig00003.g2453;Description=hypothetical protein FVEG_08335 [Fusarium verticillioides 7600];Gene=FPRO_14719;Ontology_term=nucleus,regulation of DNA-templated transcription,transcription regulator activity;Ontology_id=GO:0005634,GO:0006355,GO:0140110
contig00003.g2454	2867	ID=contig00003.g2454;Description=hypothetical protein FVER53590_08334 [Fusarium verticillioides];Gene=BFJ70_g3948;Ontology_term=nucleobase-containing small molecule metabolic process,catalytic activity,carbohydrate derivative metabolic process;Ontology_id=GO:0055086,GO:0003824,GO:1901135
contig00003.g2455	1300	ID=contig00003.g2455;Description=hypothetical protein FVEG_08333 [Fusarium verticillioides 7600]
contig00003.g2456	1455	ID=contig00003.g2456;Description=NADH oxidase [Fusarium bulbicola];Gene=FVER53590_08332;Ontology_term=catalytic activity;Ontology_id=GO:0003824
contig00003.g2457	952	ID=contig00003.g2457;Description=gEgh 16 [Fusarium tjaetaba]

contig00003.g2458	588	ID=contig00003.g2458;Description=hypothetical protein FVEG_08330 [Fusarium verticillioides 7600]
contig00003.g2459	993	ID=contig00003.g2459;Description=related to novobiocin biosynthesis protein novR [Fusarium fujikuroi]
contig00003.g2460	1985	ID=contig00003.g2460;Description=general amino acid permease [Fusarium tjaetaba];Gene=FTJAE_3173;Ontology_term=transmembrane transport;Ontology_id=GO:0055085
contig00003.g2461	3741	ID=contig00003.g2461;Description=c6 zinc finger domain protein [Fusarium tjaetaba];Gene=FTJAE_3172;Ontology_term=nucleus,oxidoreductase activity,regulation of DNA-templated transcription,transcription regulator activity;Ontology_id=GO:0005634,GO:0016491,GO:0006355,GO:0140110;Enzyme_code=EC:1;Enzyme_name=Oxidoreductases
contig00003.g2462	1409	ID=contig00003.g2462;Description=choline monooxygenase [Fusarium napiforme];Gene=FNAPI_2741;Ontology_term=oxidoreductase activity,cellular modified amino acid metabolic process;Ontology_id=GO:0016491,GO:0006575;Enzyme_code=EC:1.14.15.7,EC:1.14.15.7,EC:1,EC:1.14.15,EC:1.14;Enzyme_name=choline monooxygenase,choline monooxygenase,Oxidoreductases,Acting on paired donors, with incorporation or reduction of molecular oxygen. The oxygen incorporated need not be derived from O2,Acting on paired donors, with incorporation or reduction of molecular oxygen. The oxygen incorporated need not be derived from O2
contig00003.g2463	2065	ID=contig00003.g2463;Description=3-(3-hydroxy-phenyl)propionate 3-hydroxycinnamic acid hydroxylase [Fusarium coicis];Gene=FNAPI_2740;Ontology_term=oxidoreductase activity;Ontology_id=GO:0016491;Enzyme_code=EC:1;Enzyme_name=Oxidoreductases
contig00003.g2464	1082	ID=contig00003.g2464;Description=bifunctional 4-hydroxyphenylacetate degradation enzyme [Fusarium mexicanum];Gene=FPRO05_00080;Ontology_term=catalytic activity;Ontology_id=GO:0003824
contig00003.g2465	3351	ID=contig00003.g2465;Description=fungal specific transcription factor factor [Fusarium mexicanum];Gene=FVEG_16293;Ontology_term=nucleus,DNA binding,regulation of DNA-templated transcription,transcription regulator activity;Ontology_id=GO:0005634,GO:0003677,GO:0006355,GO:0140110

contig00003.g2466	1698	ID=contig00003.g2466;Description=pyruvate decarboxylase [Fusarium oxysporum];Gene=BFJ68_g13566;Ontology_term=generation of precursor metabolites and energy,lyase activity;Ontology_id=GO:0006091,GO:0016829;Enzyme_code=EC:4.1.1.1,EC:4.1.1,EC:4.1.1.1,EC:4.1,EC:4;Enzyme_name=pyruvate decarboxylase,Carbon-carbon lyases,pyruvate decarboxylase,Carbon-carbon lyases,Lyases
contig00003.g2467	549	ID=contig00003.g2467;Description=GNAT family n-acetyltransferase [Fusarium tjaetaba];Gene=FVER53590_08320;Ontology_term=transferase activity;Ontology_id=GO:0016740;Enzyme_code=EC:2.3.1,EC:2.3,EC:2,EC:2.3.1;Enzyme_name=Acyltransferases,Acyltransferases,Transferases,Acyltransferases
contig00003.g2468	837	ID=contig00003.g2468;Description=hypothetical protein FVER14953_08319 [Fusarium verticillioides]
contig00003.g2469	2545	ID=contig00003.g2469;Description=related to L-amino-acid oxidase [Fusarium fujikuroi];Gene=FNYG_12077;Ontology_term=oxidoreductase activity;Ontology_id=GO:0016491;Enzyme_code=EC:1,EC:1;Enzyme_name=Oxidoreductases,Oxidoreductases
contig00003.g2470	1380	ID=contig00003.g2470;Description=hypothetical protein FVER14953_08317 [Fusarium verticillioides]
contig00003.g2471	1678	ID=contig00003.g2471;Description=NCS1 family nucleobase:cation symporter-1 [Fusarium verticillioides 7600];Gene=FTJAE_3163;Ontology_term=transmembrane transport,transporter activity;Ontology_id=GO:0055085,GO:0005215
contig00003.g2472	553	ID=contig00003.g2472;Description=hypothetical protein FVEG_08315 [Fusarium verticillioides 7600]
contig00003.g2473	1422	ID=contig00003.g2473;Description=geranylgeranyl pyrophosphate synthetase [Fusarium proliferatum];Gene=FPRO_14704;Ontology_term=lipid metabolic process,transferase activity;Ontology_id=GO:0006629,GO:0016740;Enzyme_code=EC:2.5.1.10,EC:2.5.1,EC:2,EC:2.5.1.10,EC:2.5.1.1;Enzyme_name=(2E,6E)-farnesyl diphosphate synthase,Transferring alkyl or aryl groups, other than methyl groups,Transferases,(2E,6E)-farnesyl diphosphate synthase,dimethylallyltranstransferase
contig00003.g2474	645	ID=contig00003.g2474;Description=negative acting factor [Fusarium tjaetaba];Gene=FTJAE_3161;Ontology_term=nucleus,regulation of DNA-templated transcription,transcription regulator activity;Ontology_id=GO:0005634,GO:0006355,GO:0140110

contig00003.g2475	588	ID=contig00003.g2475;Description=glyoxalase/Bleomycin resistance protein/Dioxygenase [Fusarium proliferatum];Gene=FNAPI_11522;Ontology_term=oxidoreductase activity;Ontology_id=GO:0016491;Enzyme_code=EC:1,EC:1;Enzyme_name=Oxidoreductases,Oxidoreductases
contig00003.g2476	918	ID=contig00003.g2476;Description=bifunctional 4-hydroxyphenylacetate degradation enzyme [Fusarium tjaetaba];Gene=FPANT_12889;Ontology_term=catalytic activity;Ontology_id=GO:0003824
contig00003.g2477	495	ID=contig00003.g2477;Description=40S ribosomal S3 protein0 [Fusarium tjaetaba]
contig00003.g2478	588	ID=contig00003.g2478;Description=short-chain dehydrogenase reductase family [Fusarium phyllophilum]
contig00003.g2479	1024	ID=contig00003.g2479;Description=NADH-dependent flavin oxidoreductase [Fusarium oxysporum];Gene=FOC4_g10010248;Ontology_term=oxidoreductase activity;Ontology_id=GO:0016491;Enzyme_code=EC:1.6.99.1;Enzyme_name=NADPH dehydrogenase
contig00003.g2480	912	ID=contig00003.g2480;Description=NADP(+)-dependent dehydrogenase [Fusarium pseudoanthophilum]
contig00003.g2481	837	ID=contig00003.g2481;Description=hypothetical protein FVER53590_08312 [Fusarium verticillioides]
contig00003.g2482	562	ID=contig00003.g2482;Description=hypothetical protein FDENT_8941 [Fusarium denticulatum]
contig00003.g2483	2097	ID=contig00003.g2483;Description=C6 finger domain-containing protein [Fusarium globosum];Gene=BFJ72_g7264;Ontology_term=nucleus,regulation of DNA-templated transcription,transcription regulator activity;Ontology_id=GO:0005634,GO:0006355,GO:0140110
contig00003.g2484	1163	ID=contig00003.g2484;Description=related to monophenol monooxygenase [Fusarium fujikuroi];Gene=FVEG_08309;Ontology_term=oxidoreductase activity;Ontology_id=GO:0016491;Enzyme_code=EC:1,EC:1;Enzyme_name=Oxidoreductases,Oxidoreductases

contig00003.g2485	1107	ID=contig00003.g2485;Description=Cation efflux [Fusarium pseudoanthophilum];Gene=FGLOB1_5679;Ontology_term=catalytic activity, acting on a protein,transmembrane transport,mitochondrion,transferase activity,transporter activity,hydrolase activity;Ontology_id=GO:0140096,GO:0055085,GO:0005739,GO:0016740,GO:0005215,GO:0016787;Enzyme_code=EC:2.3,EC:3,EC:2.3,EC:2,EC:3;Enzyme_name=Acyltransferases,Hydrolases,Acyltransferases,Transferases,Hydrolases
contig00003.g2486	982	ID=contig00003.g2486;Description=2OG-Fe(II) oxygenase family Oxidoreductase [Fusarium pseudoanthophilum];Gene=FVER53263_08305;Ontology_term=oxidoreductase activity;Ontology_id=GO:0016491;Enzyme_code=EC:1.14;Enzyme_name=Acting on paired donors, with incorporation or reduction of molecular oxygen. The oxygen incorporated need not be derived from O2
contig00003.g2487	2316	ID=contig00003.g2487;Description=peptidase family M3 [Fusarium globosum];Gene=FMUND_10994;Ontology_term=catalytic activity, acting on a protein,hydrolase activity;Ontology_id=GO:0140096,GO:0016787;Enzyme_code=EC:3.4.24,EC:3.4.24,EC:3.4,EC:3;Enzyme_name=Acting on peptide bonds (peptidases),Acting on peptide bonds (peptidases),Acting on peptide bonds (peptidases),Hydrolases
contig00003.g2488	830	ID=contig00003.g2488;Description=prolyl 4-hydroxylase alpha subunit [Fusarium pseudoanthophilum];Gene=FALBO_16522;Ontology_term=oxidoreductase activity;Ontology_id=GO:0016491;Enzyme_code=EC:1.14;Enzyme_name=Acting on paired donors, with incorporation or reduction of molecular oxygen. The oxygen incorporated need not be derived from O2
contig00003.g2489	813	ID=contig00003.g2489;Description=hypothetical protein FPANT_8916 [Fusarium pseudoanthophilum];Gene=FPANT_8916;Ontology_term=membrane,mycotoxin biosynthetic process;Ontology_id=GO:0016020,GO:0043386

contig00003.g2490	1771	ID=contig00003.g2490;Description=cytochrome P450 [Fusarium oxysporum f. sp. albedinis];Gene=FOC4_g10010239;Ontology_term=oxidoreductase activity,mitochondrion,transferase activity;Ontology_id=GO:0016491,GO:0005739,GO:0016740;Enzyme_code=EC:1.14,EC:2.1.1,EC:2.1,EC:1,EC:1.14,EC:2,EC:2.1.1;Enzyme_name=Acting on paired donors, with incorporation or reduction of molecular oxygen. The oxygen incorporated need not be derived from O2,Transferring one-carbon groups,Transferring one-carbon groups,Oxidoreductases,Acting on paired donors, with incorporation or reduction of molecular oxygen. The oxygen incorporated need not be derived from O2,Transferases,Transferring one-carbon groups
contig00003.g2491	2682	ID=contig00003.g2491;Description=NAD-dependent epimerase dehydratase [Fusarium pseudocircinatum]
contig00003.g2492	909	ID=contig00003.g2492;Description=glucan endo-1 3-beta-glucosidase A1 [Fusarium subglutinans];Gene=FPCIR_11283;Ontology_term=carbohydrate metabolic process,hydrolase activity;Ontology_id=GO:0005975,GO:0016787;Enzyme_code=EC:3.2.1,EC:3.2,EC:3,EC:3.2.1;Enzyme_name=Glycosylases,Glycosylases,Hydrolases,Glycosylases
contig00003.g2493	832	ID=contig00003.g2493;Description=DAL5-Allantoate ureidosuccinate permease [Fusarium pseudocircinatum];Gene=FPCIR_11285;Ontology_term=oxidoreductase activity;Ontology_id=GO:0016491;Enzyme_code=EC:1,EC:1;Enzyme_name=Oxidoreductases,Oxidoreductases
contig00003.g2494	1686	ID=contig00003.g2494;Description=cation efflux family-domain-containing protein [Fusarium oxysporum Fo47];Gene=FVER53590_08298;Ontology_term=transmembrane transport,transporter activity;Ontology_id=GO:0055085,GO:0005215
contig00003.g2495	1441	ID=contig00003.g2495;Description=cation efflux family [Fusarium sp. NRRL 52700];Gene=BFJ71_g8094;Ontology_term=transmembrane transport;Ontology_id=GO:0055085
contig00003.g2496	2336	ID=contig00003.g2496;Description=major facilitator superfamily domain-containing protein [Fusarium oxysporum Fo47];Gene=FVER53590_08296;Ontology_term=transmembrane transport,transporter activity;Ontology_id=GO:0055085,GO:0005215
contig00003.g2497	1867	ID=contig00003.g2497;Description=amidase [Fusarium subglutinans];Gene=FSUBG_12803;Ontology_term=hydrolase activity;Ontology_id=GO:0016787;Enzyme_code=EC:3.5.1.4,EC:3.5.1,EC:3.5.1.4,EC:3.5,EC:3;Enzyme_name=amidase,Acting on carbon-nitrogen bonds, other than peptide bonds,amidase,Acting on carbon-nitrogen bonds, other than peptide bonds,Hydrolases

contig00003.g2498	1933	ID=contig00003.g2498;Description=Zn(II)2Cys6 transcription factor [Fusarium subglutinans];Gene=AU210_012139;Ontology_term=nucleus,DNA binding,regulation of DNA-templated transcription,transcription regulator activity;Ontology_id=GO:0005634,GO:0003677,GO:0006355,GO:0140110
contig00003.g2499	1571	ID=contig00003.g2499;Description=salicylate hydroxylase [Fusarium subglutinans];Gene=FVEG_08293;Ontology_term=oxidoreductase activity;Ontology_id=GO:0016491;Enzyme_code=EC:1,EC:1,EC:1.14.13.1;Enzyme_name=Oxidoreductases,Oxidoreductases,salicylate 1-monooxygenase
contig00003.g2500	1598	ID=contig00003.g2500;Description=amino acid transporter [Fusarium subglutinans];Gene=FVEG_08292;Ontology_term=membrane;Ontology_id=GO:0016020
contig00003.g2501	1200	ID=contig00003.g2501;Description=metallo-beta-lactamase superfamily [Fusarium subglutinans];Gene=BHQ10_009174;Ontology_term=hydrolase activity;Ontology_id=GO:0016787;Enzyme_code=EC:3,EC:3;Enzyme_name=Hydrolases,Hydrolases
contig00003.g2502	1038	ID=contig00003.g2502;Description=dienelactone hydrolase [Fusarium subglutinans];Gene=FVER53263_08290;Ontology_term=hydrolase activity;Ontology_id=GO:0016787;Enzyme_code=EC:3,EC:3;Enzyme_name=Hydrolases,Hydrolases
contig00003.g2503	3477	ID=contig00003.g2503;Description=amidase [Fusarium subglutinans];Gene=FSUBG_12797;Ontology_term=hydrolase activity;Ontology_id=GO:0016787;Enzyme_code=EC:3.5.1.4;Enzyme_name=amidase
contig00003.g2504	912	ID=contig00003.g2504;Description=RecName: Full=NmrA-like family domain-containing oxidoreductase FVEG_08287;AltNameFull=Fusarium detoxification of benzoxazolinone cluster 1 protein FVEG_08287;Short=FDB1 cluster protein FVEG_08287 [Fusarium verticillioides 7600];Gene=FSUBG_12795;Ontology_term=oxidoreductase activity;Ontology_id=GO:0016491;Enzyme_code=EC:1,EC:1;Enzyme_name=Oxidoreductases,Oxidoreductases
contig00003.g2505	2181	ID=contig00003.g2505;Description=c6 zinc finger domain containing [Fusarium acutatum];Gene=FSUBG_14048;Ontology_term=nucleus,DNA binding,regulation of DNA-templated transcription,transcription regulator activity;Ontology_id=GO:0005634,GO:0003677,GO:0006355,GO:0140110

contig00003.g2506	2093	ID=contig00003.g2506;Description=glycoside hydrolase superfamily [Ilyonectria robusta];Gene=FVEG_14160;Ontology_term=carbohydrate metabolic process,hydrolase activity;Ontology_id=GO:0005975,GO:0016787;Enzyme_code=EC:3.2.1,EC:3.2,EC:3,EC:3.2.1;Enzyme_name=Glycosylases,Glycosylases,Hydrolases,Glycosylases
contig00003.g2507	1809	ID=contig00003.g2507;Description=general substrate transporter [Fusarium subglutinans];Gene=FVER53263_14161;Ontology_term=transmembrane transport,transporter activity;Ontology_id=GO:0055085,GO:0005215
contig00003.g2508	255	ID=contig00003.g2508;Description=ribonuclease H-like protein [Fusarium bulbicola];Gene=BFJ68_g16836;Ontology_term=DNA repair,catalytic activity, acting on DNA,ATP-dependent activity,DNA recombination,telomere organization,hydrolase activity;Ontology_id=GO:0006281,GO:0140097,GO:0140657,GO:0006310,GO:0032200,GO:0016787;Enzyme_code=EC:3,EC:3;Enzyme_name=Hydrolases,Hydrolases
contig00003.g2509	987	ID=contig00003.g2509;Description=Zinc finger BED domain-containing protein DAYSLEEPER [Fusarium oxysporum f. sp. raphani];Gene=HZS61_008656;Ontology_term=protein dimerization activity;Ontology_id=GO:0046983
contig00003.g2510	1011	ID=contig00003.g2510;Description=methylglutaconyl hydratase [Fusarium subglutinans];Enzyme_code=EC:4.2.1.18;Enzyme_name=methylglutaconyl-CoA hydratase
contig00003.g2511	935	ID=contig00003.g2511;Description=major facilitator superfamily domain-containing protein [Microdochium trichocladiopsis];Gene=FNYG_09563;Ontology_term=transmembrane transport,transporter activity;Ontology_id=GO:0055085,GO:0005215
contig00003.g2512	579	ID=contig00003.g2512;Description=major facilitator superfamily domain-containing protein [Microdochium trichocladiopsis];Gene=FNYG_09563;Ontology_term=transmembrane transport,transporter activity;Ontology_id=GO:0055085,GO:0005215
contig00003.g2513	891	ID=contig00003.g2513;Description=mitochondrial carrier [Fusarium subglutinans];Gene=FPSE_08119;Ontology_term=transmembrane transport,mitochondrion;Ontology_id=GO:0055085,GO:0005739
contig00003.g2514	1711	ID=contig00003.g2514;Description=transferase family III [Fusarium subglutinans];Gene=FNYG_09565;Ontology_term=mitochondrion,transferase activity;Ontology_id=GO:0005739,GO:0016740;Enzyme_code=EC:2.3,EC:2.3,EC:2,EC:2.8.3.13;Enzyme_name=Acyltransferases,Acyltransferases,Transferases,succinate--hydroxymethylglutarate CoA-transferase

contig00003.g2515	2782	ID=contig00003.g2515;Description=cutinase transcription factor 1 beta [Fusarium subglutinans];Gene=FNYG_09566;Ontology_term=nucleus,DNA binding,regulation of DNA-templated transcription,transcription regulator activity;Ontology_id=GO:0005634,GO:0003677,GO:0006355,GO:0140110
contig00004.g2516	3708	ID=contig00004.g2516;Description=NLRC3 [Fusarium tjaetaba]
contig00004.g2517	4483	ID=contig00004.g2517;Description=hypothetical protein FVER53590_08251 [Fusarium verticillioides];Gene=FOPG_02272;Ontology_term=endosome,vesicle-mediated transport,cytoskeleton,anatomical structure development,cytoskeletal protein binding,plasma membrane;Ontology_id=GO:0005768,GO:0016192,GO:0005856,GO:0048856,GO:0008092,GO:0005886
contig00004.g2518	2319	ID=contig00004.g2518;Description=forkhead sep1 [Fusarium tjaetaba];Gene=FNYG_06454;Ontology_term=DNA binding,cell differentiation,anatomical structure development,regulation of DNA-templated transcription,nuclear chromosome,transcription regulator activity;Ontology_id=GO:0003677,GO:0030154,GO:0048856,GO:0006355,GO:0000228,GO:0140110
contig00004.g2519	872	ID=contig00004.g2519;Description=Autophagy-related protein 101 [Fusarium oxysporum f. sp. raphani];Gene=FOVG_02904;Ontology_term=nucleus,autophagy;Ontology_id=GO:0005634,GO:0006914
contig00004.g2520	592	ID=contig00004.g2520;Description=PEX3-peroxisomal assembly peroxin [Fusarium tjaetaba];Gene=FVER53590_08254;Ontology_term=peroxisome,peroxisome organization;Ontology_id=GO:0005777,GO:0007031
contig00004.g2521	1017	ID=contig00004.g2521;Description=peroxisomal biogenesis factor 3 [Fusarium tjaetaba];Gene=FTJAE_8780;Ontology_term=peroxisome,peroxisome organization,endoplasmic reticulum,intracellular protein transport,molecular adaptor activity;Ontology_id=GO:0005777,GO:0007031,GO:0005783,GO:0006886,GO:0060090
contig00004.g2522	556	ID=contig00004.g2522;Description=multiple myeloma 2 [Fusarium agapanthi];Gene=FOVG_02902;Ontology_term=vesicle-mediated transport,endoplasmic reticulum,Golgi apparatus;Ontology_id=GO:0016192,GO:0005783,GO:0005794

contig00004.g2523	2137	ID=contig00004.g2523;Description=CAMKK/CAMKK-META protein kinase [Fusarium verticillioides 7600];Gene=FPANT_12921;Ontology_term=catalytic activity, acting on a protein,transferase activity;Ontology_id=GO:0140096,GO:0016740;Enzyme_code=EC:2.7.1,EC:2.7.1,EC:2.7.11.1,EC:2.7,EC:2,EC:2.7.11.17;Enzyme_name=Transferring phosphorus-containing groups,Transferring phosphorus-containing groups,non-specific serine/threonine protein kinase,Transferring phosphorus-containing groups,Transferases,calcium/calmodulin-dependent protein kinase
contig00004.g2524	1572	ID=contig00004.g2524;Description=HAL protein kinase [Fusarium verticillioides 7600];Gene=FPANT_12922;Ontology_term=nucleus,catalytic activity, acting on a protein,sulfur compound metabolic process,mitochondrion,signaling,protein maturation,transferase activity,mitotic cell cycle;Ontology_id=GO:0005634,GO:0140096,GO:0006790,GO:0005739,GO:0023052,GO:0051604,GO:0016740,GO:0000278;Enzyme_code=EC:2.7.1,EC:2.7.1,EC:2.7.11.1,EC:2.7,EC:2;Enzyme_name=Transferring phosphorus-containing groups,Transferring phosphorus-containing groups,non-specific serine/threonine protein kinase,Transferring phosphorus-containing groups,Transferases
contig00004.g2525	1356	ID=contig00004.g2525;Description=FAD dependent oxidoreductase [Fusarium oxysporum];Gene=FOC1_g10013888;Ontology_term=oxidoreductase activity;Ontology_id=GO:0016491;Enzyme_code=EC:1,EC:1;Enzyme_name=Oxidoreductases,Oxidoreductases
contig00004.g2526	1363	ID=contig00004.g2526;Description=hypothetical protein FVEG_08259 [Fusarium verticillioides 7600]
contig00004.g2527	596	ID=contig00004.g2527;Description=DNA-directed RNA polymerase I and III subunit RPAC2 [Fusarium proliferatum];Gene=BFJ72_g9201;Ontology_term=extracellular space,DNA-templated transcription,structural molecule activity,tRNA metabolic process,cell adhesion,extracellular matrix,catalytic activity, acting on RNA,transferase activity,nucleolus,nucleoplasm;Ontology_id=GO:0005615,GO:0006351,GO:0005198,GO:0006399,GO:0007155,GO:0031012,GO:0140098,GO:0016740,GO:0005730,GO:0005654;Enzyme_code=EC:2.7.7.6,EC:2.7,EC:2,EC:2.7.7,EC:2.7.7.6;Enzyme_name=DNA-directed RNA polymerase,Transferring phosphorus-containing groups,Transferases,Transferring phosphorus-containing groups,DNA-directed RNA polymerase

contig00004.g2528	678	ID=contig00004.g2528;Description=NADH-ubiquinone oxidoreductase 14.8 kDa subunit [Fusarium oxysporum f. sp. cubense race 1];Gene=FGLOB1_712;Ontology_term=mitochondrion;Ontology_id=GO:0005739
contig00004.g2529	502	ID=contig00004.g2529;Description=G10 [Fusarium tjaetaba];Gene=cwf14;Ontology_term=nucleus,mRNA metabolic process,signaling,establishment or maintenance of cell polarity,tRNA metabolic process,amino acid metabolic process,cytokinesis,catalytic activity, acting on RNA,ligase activity,mitotic cell cycle,hydrolase activity;Ontology_id=GO:0005634,GO:0016071,GO:0023052,GO:0007163,GO:0006399,GO:0006520,GO:0000910,GO:0140098,GO:0016874,GO:0000278,GO:0016787;Enzyme_code=EC:6.1.1.4,EC:3.1.1,EC:3.1,EC:6.1,EC:6.1.1.4,EC:3,EC:6.1.1,EC:6,EC:3.1.1;Enzyme_name=leucine--tRNA ligase,Acting on ester bonds,Acting on ester bonds,Forming carbon-oxygen bonds,leucine--tRNA ligase,Hydrolases,Forming carbon-oxygen bonds,Ligases,Acting on ester bonds
contig00004.g2530	833	ID=contig00004.g2530;Description=Zinc finger protein 511 [Fusarium oxysporum f. sp. rapae]
contig00004.g2531	3167	ID=contig00004.g2531;Description=SMG7 [Fusarium tjaetaba];Gene=FTJAE_8790;Ontology_term=nucleus,mRNA metabolic process;Ontology_id=GO:0005634,GO:0016071
contig00004.g2532	1355	ID=contig00004.g2532;Description=adenosine kinase [Fusarium tjaetaba];Gene=FTJAE_8791;Ontology_term=nucleus,nucleobase-containing small molecule metabolic process,carbohydrate derivative metabolic process,cytosol,transferase activity;Ontology_id=GO:0005634,GO:0055086,GO:1901135,GO:0005829,GO:0016740;Enzyme_code=EC:2.7.1.20,EC:2.7.1,EC:2.7,EC:2,EC:2.7.1.20;Enzyme_name=adenosine kinase,Transferring phosphorus-containing groups,Transferring phosphorus-containing groups,Transferases,adenosine kinase
contig00004.g2533	758	ID=contig00004.g2533;Description=60S ribosomal protein L2 [Fusarium odoratissimum NRRL 54006];Gene=RPL2-0;Ontology_term=RNA binding,cytoplasmic translation,structural molecule activity,cytosol,ribosome;Ontology_id=GO:0003723,GO:0002181,GO:0005198,GO:0005829,GO:0005840
contig00004.g2534	2138	ID=contig00004.g2534;Description=hypothetical protein H9Q70_003086 [Fusarium xylarioides];Gene=FVER53590_08268;Ontology_term=nucleus;Ontology_id=GO:0005634
contig00004.g2535	485	ID=contig00004.g2535;Description=duf1770 domain protein [Fusarium sp. NRRL 52700];Gene=FOXYS1_364;Ontology_term=autophagy,mitochondrion organization,molecular adaptor activity;Ontology_id=GO:0006914,GO:0007005,GO:0060090

contig00004.g2536	1676	ID=contig00004.g2536;Description=hypothetical protein FVER53263_08270 [Fusarium verticillioides];Gene=FVEG_08270;Ontology_term=membrane;Ontology_id=GO:0016020
contig00004.g2537	1861	ID=contig00004.g2537;Description=eukaryotic peptide chain release factor subunit 1 [Fusarium tjaetaba];Gene=FPANT_1628;Ontology_term=defense response to other organism,extracellular space,inflammatory response,translation regulator activity,anatomical structure development,receptor ligand activity,immune system process;Ontology_id=GO:0098542,GO:0005615,GO:0006954,GO:0045182,GO:0048856,GO:0048018,GO:0002376
contig00004.g2538	4098	ID=contig00004.g2538;Description=PH domain protein [Fusarium tjaetaba];Gene=FPCIR_9879;Ontology_term=mitochondrion,regulation of DNA-templated transcription,cytosol,endoplasmic reticulum,transferase activity,transcription regulator activity,plasma membrane,transporter activity,molecular adaptor activity;Ontology_id=GO:0005739,GO:0006355,GO:0005829,GO:0005783,GO:0016740,GO:0140110,GO:0005886,GO:0005215,GO:0060090;Enzyme_code=EC:2.7,EC:2.7,EC:2;Enzyme_name=Transferring phosphorus-containing groups,Transferring phosphorus-containing groups,Transferases
contig00004.g2539	2039	ID=contig00004.g2539;Description=hypothetical protein FVER14953_08273 [Fusarium verticillioides]
contig00004.g2540	5017	ID=contig00004.g2540;Description=putative death-receptor fusion protein-domain-containing protein [Fusarium oxysporum];Ontology_term=tRNA metabolic process;Ontology_id=GO:0006399
contig00004.g2541	795	ID=contig00004.g2541;Description=probable ribosomal protein L10a.e, cytosolic [Fusarium fujikuroi];Gene=BKA59DRAFT_510082;Ontology_term=RNA binding,cytoplasmic translation,structural molecule activity,ribosome biogenesis,cytosol,ribosome;Ontology_id=GO:0003723,GO:0002181,GO:0005198,GO:0042254,GO:0005829,GO:0005840
contig00004.g2542	1810	ID=contig00004.g2542;Description=GPI mannosyltransferase 4 [Fusarium verticillioides 7600];Gene=FNAPI_12382;Ontology_term=lipid metabolic process,carbohydrate derivative metabolic process,endoplasmic reticulum,transferase activity;Ontology_id=GO:0006629,GO:1901135,GO:0005783,GO:0016740;Enzyme_code=EC:2.4,EC:2.4,EC:2,EC:2.4.1;Enzyme_name=Glycosyltransferases,Glycosyltransferases,Transferases,Glycosyltransferases

contig00004.g2543	680	ID=contig00004.g2543;Description=S-phase kinase-associated protein 1 [Fusarium oxysporum f. sp. lycopersici 4287];Gene=FOZG_05349;Ontology_term=cytoskeleton,cytoskeleton organization,protein catabolic process,structural molecule activity,transferase activity,hydrolase activity;Ontology_id=GO:0005856,GO:0007010,GO:0030163,GO:0005198,GO:0016740,GO:0016787;Enzyme_code=EC:2.7,EC:3;Enzyme_name=Transferring phosphorus-containing groups,Hydrolases
contig00004.g2544	786	ID=contig00004.g2544;Description=ADP-ribosylation factor-like protein 1 [Geosmithia morbida];Gene=FOC4_g10011459;Ontology_term=vesicle-mediated transport,autophagy,GTPase activity,cytosol,Golgi apparatus,intracellular protein transport,protein localization to plasma membrane;Ontology_id=GO:0016192,GO:0006914,GO:0003924,GO:0005829,GO:0005794,GO:0006886,GO:0072659;Enzyme_code=EC:3.6.1.15,EC:3.6.1,EC:3.6,EC:3,EC:3.6.1.15;Enzyme_name=nucleoside-triphosphate phosphatase,Acting on acid anhydrides,Acting on acid anhydrides,Hydrolases,nucleoside-triphosphate phosphatase
contig00004.g2545	909	ID=contig00004.g2545;Description=2-dehydropantoate 2-reductase [Fusarium verticillioides 7600];Gene=FVER53263_08280;Ontology_term=nucleus,oxidoreductase activity,cellular modified amino acid metabolic process,vitamin metabolic process;Ontology_id=GO:0005634,GO:0016491,GO:0006575,GO:0006766;Enzyme_code=EC:1.1.1.169,EC:1.1,EC:1,EC:1.1.1.169,EC:1.1.1;Enzyme_name=2-dehydropantoate 2-reductase,Acting on the CH-OH group of donors,Oxidoreductases,2-dehydropantoate 2-reductase,Acting on the CH-OH group of donors
contig00004.g2546	2418	ID=contig00004.g2546;Description=Long-chain-alcohol oxidase [Fusarium oxysporum f. sp. vasinfectum];Gene=FVEG_08281;Ontology_term=oxidoreductase activity;Ontology_id=GO:0016491;Enzyme_code=EC:1.1.3.20,EC:1.1,EC:1,EC:1.1.3.20,EC:1.1.3;Enzyme_name=long-chain-alcohol oxidase,Acting on the CH-OH group of donors,Oxidoreductases,long-chain-alcohol oxidase,Acting on the CH-OH group of donors
contig00004.g2547	1059	ID=contig00004.g2547;Description=hypothetical protein FVEG_08282 [Fusarium verticillioides 7600]
contig00004.g2548	1662	ID=contig00004.g2548;Description=major facilitator superfamily transporter [Fusarium tjaetaba];Gene=FPCIR_9868;Ontology_term=transmembrane transport,transporter activity;Ontology_id=GO:0055085,GO:0005215

contig00004.g2549	2300	ID=contig00004.g2549;Description=acetamidase regulatory protein [Fusarium fujikuroi];Gene=FVER53590_08284;Ontology_term=nucleus,DNA binding,lipid metabolic process,regulation of DNA-templated transcription,transcription regulator activity;Ontology_id=GO:0005634,GO:0003677,GO:0006629,GO:0006355,GO:0140110
contig00004.g2550	2809	ID=contig00004.g2550;Description=high affinity 3',5'-cyclic-nucleotide phosphodiesterase [Fusarium denticulatum];Gene=FNYG_06421;Ontology_term=signaling,hydrolase activity;Ontology_id=GO:0023052,GO:0016787;Enzyme_code=EC:3.1.4.17,EC:3.1.4,EC:3.1.4.17,EC:3.1,EC:3;Enzyme_name=3',5'-cyclic-nucleotide phosphodiesterase,Acting on ester bonds,3',5'-cyclic-nucleotide phosphodiesterase,Acting on ester bonds,Hydrolases
contig00004.g2551	5699	ID=contig00004.g2551;Description=ubiquitin carboxyl-terminal hydrolase [Fusarium napiforme];Gene=3044;Ontology_term=nucleus,catalytic activity, acting on a protein,generation of precursor metabolites and energy,mitochondrion,receptor ligand activity,extracellular region,hydrolase activity;Ontology_id=GO:0005634,GO:0140096,GO:0006091,GO:0005739,GO:0048018,GO:0005576,GO:0016787;Enzyme_code=EC:3.4.19.12,EC:3.4,EC:3,EC:3.4.19.12;Enzyme_name=ubiquitinyl hydrolase 1,Acting on peptide bonds (peptidases),Hydrolases,ubiquitinyl hydrolase 1
contig00004.g2552	3200	ID=contig00004.g2552;Description=insulysin [Fusarium verticillioides 7600];Gene=FDENT_13514;Ontology_term=catalytic activity, acting on a protein,mitochondrion,reproductive process,protein maturation,hydrolase activity;Ontology_id=GO:0140096,GO:0005739,GO:0022414,GO:0051604,GO:0016787;Enzyme_code=EC:3.4.19.12,EC:3.4.24,EC:3.4,EC:3,EC:3.4.24.56,EC:3.4.19.12;Enzyme_name=ubiquitinyl hydrolase 1,Acting on peptide bonds (peptidases),Acting on peptide bonds (peptidases),Hydrolases,insulysin,ubiquitinyl hydrolase 1
contig00004.g2553	855	ID=contig00004.g2553;Description=zinc finger domain protein [Fusarium tjaetaba];Gene=FVER53590_14031;Ontology_term=nucleic acid binding,zinc ion binding;Ontology_id=GO:0003676,GO:0008270
contig00004.g2554	606	ID=contig00004.g2554;Description=NADH dehydrogenase (ubiquinone) Fe-S protein 6 [Fusarium verticillioides 7600];Ontology_term=generation of precursor metabolites and energy,nucleobase-containing small molecule metabolic process,mitochondrion,carbohydrate derivative metabolic process;Ontology_id=GO:0006091,GO:0055086,GO:0005739,GO:1901135
contig00004.g2555	602	ID=contig00004.g2555;Description=family UPF0047 [Hirsutella rhossiliensis];Gene=FOQG_00715

contig00004.g2556	845	ID=contig00004.g2556;Description=nucleotide hydrolase-domain-containing protein [Fusarium flagelliforme];Gene=F25303_9998;Ontology_term=nucleus,RNA binding,mRNA metabolic process;Ontology_id=GO:0005634,GO:0003723,GO:0016071
contig00004.g2557	1827	ID=contig00004.g2557;Description=hypothetical protein FVEG_14034 [Fusarium verticillioides 7600]
contig00004.g2558	387	ID=contig00004.g2558;Description=cytochrome-c oxidase assembly [Fusarium subglutinans];Gene=FNAPI_4158;Ontology_term=protein-containing complex assembly,mitochondrion,mitochondrion organization;Ontology_id=GO:0065003,GO:0005739,GO:0007005
contig00004.g2559	1003	ID=contig00004.g2559;Description=yfcA [Fusarium tjaetaba];Ontology_term=mitochondrial gene expression,mitochondrion;Ontology_id=GO:0140053,GO:0005739
contig00004.g2560	6244	ID=contig00004.g2560;Description=omega-conotoxin-sensitive calcium channel alpha-1 subunit rbB-I [Fusarium tjaetaba];Ontology_term=nucleus,vesicle-mediated transport,cytoplasmic vesicle,intracellular protein transport;Ontology_id=GO:0005634,GO:0016192,GO:0031410,GO:0006886
contig00004.g2561	1543	ID=contig00004.g2561;Description=hypothetical protein Focb16_v008927 [Fusarium oxysporum f. sp. cubense];Gene=FRV6_07636;Ontology_term=RNA binding;Ontology_id=GO:0003723
contig00004.g2562	1708	ID=contig00004.g2562;Description=WD-repeat 8 [Fusarium tjaetaba];Gene=3054;Ontology_term=RNA binding,lyase activity;Ontology_id=GO:0003723,GO:0016829;Enzyme_code=EC:4;Enzyme_name=Lyases
contig00004.g2563	2229	ID=contig00004.g2563;Description=molybdenum cofactor biosynthesis 1 A [Fusarium tjaetaba];Gene=FDENT_13503;Ontology_term=lyase activity;Ontology_id=GO:0016829;Enzyme_code=EC:4.1.99.22,EC:4.6.1.17,EC:4.1.99.22,EC:4.1,EC:4,EC:4.6,EC:4.6.1.17;Enzyme_name=GTP 3',8-cyclase,cyclic pyranopterin monophosphate synthase,GTP 3',8-cyclase,Carbon-carbon lyases,Lyases,Phosphorus-oxygen lyases,cyclic pyranopterin monophosphate synthase
contig00004.g2564	1315	ID=contig00004.g2564;Description=VPS27-vacuolar sorting-associated [Fusarium pseudoanthophilum];Gene=FPANT_13082;Ontology_term=mitochondrion;Ontology_id=GO:0005739

contig00004.g2565	1472	ID=contig00004.g2565;Description=fatty acid elongation [Fusarium pseudocircinatum];Gene=HZS61_009834;Ontology_term=lipid metabolic process,endoplasmic reticulum,transferase activity;Ontology_id=GO:0006629,GO:0005783,GO:0016740;Enzyme_code=EC:2.3.1.199,EC:2.3.1.85,EC:2.3,EC:2,EC:2.3.1.199,EC:2.3.1;Enzyme_name=very-long-chain 3-oxoacyl-CoA synthase,fatty-acid synthase system,Acyltransferases,Transferases,very-long-chain 3-oxoacyl-CoA synthase,Acyltransferases
contig00004.g2566	1680	ID=contig00004.g2566;Description=PRC1-carboxypeptidase y serine-type protease [Fusarium subglutinans];Gene=FPHYL_8627;Ontology_term=catalytic activity, acting on a protein,protein catabolic process,endoplasmic reticulum,extracellular region,vacuole,hydrolase activity;Ontology_id=GO:0140096,GO:0030163,GO:0005783,GO:0005576,GO:0005773,GO:0016787;Enzyme_code=EC:3.4.16,EC:3.4,EC:3.4.16,EC:3,EC:3.4.16.5;Enzyme_name=Acting on peptide bonds (peptidases),Acting on peptide bonds (peptidases),Acting on peptide bonds (peptidases),Hydrolases,carboxypeptidase C
contig00004.g2567	927	ID=contig00004.g2567;Description=SDO1-like protein [Fusarium subglutinans];Gene=FNAPI_13629;Ontology_term=nucleus,RNA binding,molecular function regulator activity,protein-containing complex assembly,ribosome biogenesis;Ontology_id=GO:0005634,GO:0003723,GO:0098772,GO:0065003,GO:0042254
contig00004.g2568	677	ID=contig00004.g2568;Description=ribosomal 40S subunit protein S18B [Aspergillus fumigatus];Gene=LLEC1_07830;Ontology_term=RNA binding,structural molecule activity,ribosome;Ontology_id=GO:0003723,GO:0005198,GO:0005840
contig00004.g2569	1019	ID=contig00004.g2569;Description=hypothetical protein FVER14953_14048 [Fusarium verticillioides]
contig00004.g2570	480	ID=contig00004.g2570;Description=hypothetical protein FCOIX_12223 [Fusarium coicis];Gene=2166;Ontology_term=nucleus,endosome,extracellular region,plasma membrane;Ontology_id=GO:0005634,GO:0005768,GO:0005576,GO:0005886
contig00004.g2571	1932	ID=contig00004.g2571;Description=sucrose transporter SUT1D [Fusarium tjaetaba];Gene=FVEG_14050;Ontology_term=membrane;Ontology_id=GO:0016020
contig00004.g2572	1335	ID=contig00004.g2572;Description=membrane associated eicosanoid glutathione metabolism-like domain-containing protein [Fusarium pseudocircinatum];Gene=BFJ72_g11939;Ontology_term=membrane;Ontology_id=GO:0016020

contig00004.g2573	1521	ID=contig00004.g2573;Description=endo-1,3(4)-beta-glucanase [Fusarium verticillioides 7600];Gene=FVER53590_14052;Ontology_term=carbohydrate metabolic process,hydrolase activity;Ontology_id=GO:0005975,GO:0016787;Enzyme_code=EC:3.2.1,EC:3.2,EC:3,EC:3.2.1;Enzyme_name=Glycosylases,Glycosylases,Hydrolases,Glycosylases
contig00004.g2574	1485	ID=contig00004.g2574;Description=mannan polymerase II complex MNN10 subunit [Fusarium verticillioides 7600];Gene=MNN10;Ontology_term=protein glycosylation,cytokinesis,cell wall organization or biogenesis,endoplasmic reticulum,Golgi apparatus,transferase activity,mitotic cell cycle;Ontology_id=GO:0006486,GO:0000910,GO:0071554,GO:0005783,GO:0005794,GO:0016740,GO:0000278;Enzyme_code=EC:2.4.1,EC:2.4,EC:2,EC:2.4.1;Enzyme_name=Glycosyltransferases,Glycosyltransferases,Transferases,Glycosyltransferases
contig00004.g2575	4760	ID=contig00004.g2575;Description=tRNA N6-adenosine threonylcarbamoyltransferase mitochondrial [Fusarium tjaetaba];Gene=FNYG_14056;Ontology_term=mitochondrion,tRNA metabolic process,transferase activity;Ontology_id=GO:0005739,GO:0006399,GO:0016740;Enzyme_code=EC:2.3.1,EC:2.3,EC:2,EC:2.3.1;Enzyme_name=Acyltransferases,Acyltransferases,Transferases,Acyltransferases
contig00004.g2576	908	ID=contig00004.g2576;Description=proteasome component PRE2 [Fusarium verticillioides 7600];Gene=FPCIR_9192;Ontology_term=nucleus,catalytic activity, acting on a protein,protein-containing complex assembly,protein catabolic process,hydrolase activity;Ontology_id=GO:0005634,GO:0140096,GO:0065003,GO:0030163,GO:0016787;Enzyme_code=EC:3.4.25;Enzyme_name=Acting on peptide bonds (peptidases)
contig00004.g2577	4625	ID=contig00004.g2577;Description=hypothetical protein FOTG_00721 [Fusarium oxysporum f. sp. vasinfectum 25433];Gene=FOZG_05309;Ontology_term=membrane organization,lipid metabolic process,mitochondrion,cytoplasmic vesicle,Golgi apparatus,lipid binding,endoplasmic reticulum,plasma membrane;Ontology_id=GO:0061024,GO:0006629,GO:0005739,GO:0031410,GO:0005794,GO:0008289,GO:0005783,GO:0005886
contig00004.g2578	1988	ID=contig00004.g2578;Description=p-loop containing protein [Fusarium tjaetaba];Gene=FDENT_5295;Ontology_term=DNA binding,catalytic activity, acting on DNA,ATP-dependent activity,hydrolase activity;Ontology_id=GO:0003677,GO:0140097,GO:0140657,GO:0016787;Enzyme_code=EC:3,EC:3;Enzyme_name=Hydrolases,Hydrolases

contig00004.g2579	1919	ID=contig00004.g2579;Description=PIB1-phosphatidylinositol(3)-phosphate binding protein [Fusarium tjaetaba];Gene=FVEG_14060;Ontology_term=catalytic activity, acting on a protein,endosome,protein catabolic process,lipid binding,transferase activity,vacuole;Ontology_id=GO:0140096,GO:0005768,GO:0030163,GO:0008289,GO:0016740,GO:0005773;Enzyme_code=EC:2.3,EC:2,EC:2.3.2;Enzyme_name=Acyltransferases,Transferases,Acyltransferases
contig00004.g2580	2898	ID=contig00004.g2580;Description=transcription factor Pig1p [Fusarium coicis];Gene=FSPOR_8515;Ontology_term=regulation of DNA-templated transcription;Ontology_id=GO:0006355
contig00004.g2581	1260	ID=contig00004.g2581;Description=2-hydroxy-6-oxo-6-phenylhexa-2 4-dienoate hydrolase [Fusarium subglutinans];Gene=F25303_6984;Ontology_term=hydrolase activity;Ontology_id=GO:0016787;Enzyme_code=EC:3,EC:3;Enzyme_name=Hydrolases,Hydrolases
contig00004.g2582	4574	ID=contig00004.g2582;Description=leptomycin B resistance pmd1 [Fusarium tjaetaba];Gene=FPANT_11779;Ontology_term=transmembrane transport,ATP-dependent activity,transporter activity,hydrolase activity;Ontology_id=GO:0055085,GO:0140657,GO:0005215,GO:0016787;Enzyme_code=EC:7.2.2,EC:3.6.1.15,EC:3.6.1,EC:3.6,EC:3,EC:7.2.2,EC:7,EC:3.6.1.15;Enzyme_name=Catalysing the translocation of inorganic cations,nucleoside-triphosphate phosphatase,Acting on acid anhydrides,Acting on acid anhydrides,Hydrolases,Catalysing the translocation of inorganic cations,Translocases,nucleoside-triphosphate phosphatase
contig00004.g2583	1778	ID=contig00004.g2583;Description=histidyl-tRNA synthetase [Fusarium verticillioides 7600];Gene=FNYG_14046;Ontology_term=tRNA metabolic process,amino acid metabolic process,catalytic activity, acting on RNA,ligase activity;Ontology_id=GO:0006399,GO:0006520,GO:0140098,GO:0016874;Enzyme_code=EC:6.1.1.21;Enzyme_name=histidine--tRNA ligase
contig00004.g2584	1246	ID=contig00004.g2584;Description=hypothetical protein FVER14953_05105 [Fusarium verticillioides]

contig00004.g2585	1398	ID=contig00004.g2585;Description=CAMK/CAMK1/CAMK1-CMK protein kinase [Fusarium verticillioides 7600];Gene=FOXG_01946;Ontology_term=catalytic activity, acting on a protein,signaling,cytosol,transferase activity,mitotic cell cycle;Ontology_id=GO:0140096,GO:0023052,GO:0005829,GO:0016740,GO:0000278;Enzyme_code=EC:2.7.11.1,EC:2.7.1,EC:2.7.11.1,EC:2.7,EC:2,EC:2.7.11.17;Enzyme_name=non-specific serine/threonine protein kinase,Transferring phosphorus-containing groups,non-specific serine/threonine protein kinase,Transferring phosphorus-containing groups,Transferases,calcium/calmodulin-dependent protein kinase
contig00004.g2586	610	ID=contig00004.g2586;Description=ubiquitin-conjugating enzyme E2 W [Fusarium verticillioides 7600]
contig00004.g2587	2190	ID=contig00004.g2587;Description=lysine-specific histone demethylase 1 [Fusarium odoratissimum NRRL 54006];Gene=FVEG_05109;Ontology_term=membrane organization,vesicle-mediated transport,endosome,cytoskeleton,protein-containing complex assembly,cytoskeleton organization,autophagy,cytosol,lipid binding,transferase activity,intracellular protein transport,vacuole;Ontology_id=GO:0061024,GO:0016192,GO:0005768,GO:0005856,GO:0065003,GO:0007010,GO:0006914,GO:0005829,GO:0008289,GO:0016740,GO:0006886,GO:0005773;Enzyme_code=EC:2.1.1,EC:2.1,EC:2,EC:2.1.1;Enzyme_name=Transferring one-carbon groups,Transferring one-carbon groups,Transferases,Transferring one-carbon groups
contig00004.g2588	3343	ID=contig00004.g2588;Description=lysine-specific histone demethylase 1 [Fusarium verticillioides 7600];Gene=FVEG_15594;Ontology_term=catalytic activity, acting on a protein,DNA replication,anatomical structure development,regulation of DNA-templated transcription,reproductive process,nuclear chromosome,chromatin organization,DNA binding,vesicle-mediated transport,oxidoreductase activity,cell differentiation,transferase activity;Ontology_id=GO:0140096,GO:0006260,GO:0048856,GO:0006355,GO:0022414,GO:0000228,GO:0006325,GO:0003677,GO:0016192,GO:0016491,GO:0030154,GO:0016740;Enzyme_code=EC:1,EC:2.1.1,EC:2.1,EC:1,EC:1.5.3.16,EC:2,EC:1.5.3.14,EC:2.1.1;Enzyme_name=Oxidoreductases,Transferring one-carbon groups,Transferring one-carbon groups,Oxidoreductases,spermine oxidase,Transferases,polyamine oxidase (propane-1,3-diamine-forming),Transferring one-carbon groups

contig00004.g2589	1316	ID=contig00004.g2589;Description=ATPase, F1 complex, gamma subunit domain-containing protein [Fusarium redolens];Gene=FOVG_02827;Ontology_term=transmembrane transport,nucleobase-containing small molecule metabolic process,ATP-dependent activity,mitochondrion,carbohydrate derivative metabolic process,ligase activity,transporter activity;Ontology_id=GO:0055085,GO:0055086,GO:0140657,GO:0005739,GO:1901135,GO:0016874,GO:0005215;Enzyme_code=EC:7.1.2.2,EC:6,EC:7.2.2;Enzyme_name=H(+)-transporting two-sector ATPase,Ligases,Catalysing the translocation of inorganic cations
contig00004.g2590	3136	ID=contig00004.g2590;Description=DNA topoisomerase I [Fusarium verticillioides 7600];Gene=FDENT_5308;Ontology_term=catalytic activity, acting on DNA,DNA replication,DNA recombination,chromosome segregation,regulation of DNA-templated transcription,nuclear chromosome,nucleolus,chromatin organization,DNA binding,mitotic nuclear division,cytosol,isomerase activity;Ontology_id=GO:0140097,GO:0006260,GO:0006310,GO:0007059,GO:0006355,GO:0000228,GO:0005730,GO:0006325,GO:0003677,GO:0140014,GO:0005829,GO:0016853;Enzyme_code=EC:5.6.2.1,EC:5.6.2.1,EC:5,EC:5.99.1.2;Enzyme_name=DNA topoisomerase,DNA topoisomerase,Isomerases,Other isomerases
contig00004.g2591	1188	ID=contig00004.g2591;Description=licheninase [Fusarium verticillioides 7600];Gene=Forpe1208_v002810;Ontology_term=carbohydrate metabolic process,cell wall,carbohydrate derivative metabolic process,cell wall organization or biogenesis,transferase activity,extracellular region,plasma membrane,hydrolase activity;Ontology_id=GO:0005975,GO:0005618,GO:1901135,GO:0071554,GO:0016740,GO:0005576,GO:0005886,GO:0016787;Enzyme_code=EC:2.4,EC:3.2.1,EC:3.2,EC:2.4,EC:3.2.1.73,EC:2,EC:3,EC:3.2.1;Enzyme_name=Glycosyltransferases,Glycosylases,Glycosylases,Glycosyltransferases,licheninase,Transferases,Hydrolases,Glycosylases
contig00004.g2592	1837	ID=contig00004.g2592;Description=zinc finger protein MSN2/4 [Fusarium verticillioides 7600];Ontology_term=nucleus,DNA binding,cytosol;Ontology_id=GO:0005634,GO:0003677,GO:0005829
contig00004.g2593	2284	ID=contig00004.g2593;Description=meiotically up-regulated 71 [Fusarium tjaetaba];Gene=FVER53590_05116;Ontology_term=cytoplasmic translation,ligase activity;Ontology_id=GO:0002181,GO:0016874;Enzyme_code=EC:6.3.1.14,EC:6.3,EC:6.3.1,EC:6,EC:6.3.1.14;Enzyme_name=diphthine--ammonia ligase,Forming carbon-nitrogen bonds,Forming carbon-nitrogen bonds,Ligases,diphthine--ammonia ligase

contig00004.g2594	1111	ID=contig00004.g2594;Description=aldo keto reductase [Fusarium tjaetaba];Ontology_term=nucleus;Ontology_id=GO:0005634;Enzyme_code=EC:1.1.1.65;Enzyme_name=pyridoxine 4-dehydrogenase
contig00004.g2595	792	ID=contig00004.g2595;Description=DNA-binding SMUBP-2 [Fusarium tjaetaba];Gene=FVER53263_05118;Ontology_term=DNA binding,vacuole;Ontology_id=GO:0003677,GO:0005773
contig00004.g2596	2148	ID=contig00004.g2596;Description=HCS1-DNA helicase A [Fusarium tjaetaba];Gene=FPCIR_12260;Ontology_term=catalytic activity, acting on DNA,ATP-dependent activity,DNA replication,nuclear chromosome,mitotic cell cycle,hydrolase activity;Ontology_id=GO:0140097,GO:0140657,GO:0006260,GO:0000228,GO:0000278,GO:0016787;Enzyme_code=EC:3.6.1.15,EC:3.6.1,EC:3.6.4.12,EC:3.6,EC:3,EC:5.6.2.3,EC:3.6.1.15;Enzyme_name=nucleoside-triphosphate phosphatase,Acting on acid anhydrides,DNA helicase,Acting on acid anhydrides,Hydrolases,DNA 5'-3' helicase,nucleoside-triphosphate phosphatase
contig00004.g2597	2070	ID=contig00004.g2597;Description=hypothetical protein FVEG_05120 [Fusarium verticillioides 7600]
contig00004.g2598	2367	ID=contig00004.g2598;Description=mitochondrial intermediate peptidase [Fusarium coicis];Gene=FDENT_11598;Ontology_term=catalytic activity, acting on a protein,mitochondrion,protein maturation,intracellular protein transport,mitochondrion organization,hydrolase activity;Ontology_id=GO:0140096,GO:0005739,GO:0051604,GO:0006886,GO:0007005,GO:0016787;Enzyme_code=EC:3.4.24,EC:3.4.24,EC:3.4,EC:3,EC:3.4.24.59;Enzyme_name=Acting on peptide bonds (peptidases),Acting on peptide bonds (peptidases),Acting on peptide bonds (peptidases),Hydrolases,mitochondrial intermediate peptidase
contig00004.g2599	857	ID=contig00004.g2599;Description=related to mitochondrial protein [Fusarium fujikuroi IMI 58289];Gene=FNYG_15697;Ontology_term=membrane organization,mitochondrion,mitochondrion organization;Ontology_id=GO:0061024,GO:0005739,GO:0007005

contig00004.g2600	1317	ID=contig00004.g2600;Description=tRNA-dihydrouridine synthase 2 [Fusarium tjaetaba];Gene=FNYG_15696;Ontology_term=nucleus,mRNA metabolic process,oxidoreductase activity,tRNA metabolic process,catalytic activity, acting on RNA;Ontology_id=GO:0005634,GO:0016071,GO:0016491,GO:0006399,GO:0140098;Enzyme_code=EC:1.3.1.89,EC:1.3,EC:1.3.1.91,EC:1,EC:1.3.1.89,EC:1.3.1;Enzyme_name=tRNA-dihydrouridine(47) synthase [NAD(P)(+)],Acting on the CH-CH group of donors,tRNA-dihydrouridine(20) synthase [NAD(P)(+)],Oxidoreductases,tRNA-dihydrouridine(47) synthase [NAD(P)(+)],Acting on the CH-CH group of donors
contig00004.g2601	3559	ID=contig00004.g2601;Description=hypothetical protein FVER14953_05124 [Fusarium verticillioides]
contig00004.g2602	1932	ID=contig00004.g2602;Description=putative oxysterol-binding [Fusarium longipes];Gene=FCULG_00008508;Ontology_term=vesicle-mediated transport,autophagy,lipid metabolic process,establishment or maintenance of cell polarity,cytosol,lipid binding,endoplasmic reticulum;Ontology_id=GO:0016192,GO:0006914,GO:0006629,GO:0007163,GO:0005829,GO:0008289,GO:0005783
contig00004.g2603	751	ID=contig00004.g2603;Description=splicing factor U2AF 23 kDa subunit [Fusarium verticillioides 7600];Gene=F25303_7006;Ontology_term=nucleus,RNA binding,mRNA metabolic process;Ontology_id=GO:0005634,GO:0003723,GO:0016071
contig00004.g2604	2148	ID=contig00004.g2604;Description=ELMO/CED-12 family-domain-containing protein [Fusarium oxysporum Fo47];Gene=FOXG_01967;Ontology_term=programmed cell death;Ontology_id=GO:0012501
contig00004.g2605	1691	ID=contig00004.g2605;Description=COPII vesicles Yip3 [Fusarium pseudocircinatum];Gene=AU210_006427;Ontology_term=vesicle-mediated transport,cytoplasmic vesicle,Golgi apparatus,endoplasmic reticulum;Ontology_id=GO:0016192,GO:0031410,GO:0005794,GO:0005783
contig00004.g2606	568	ID=contig00004.g2606;Description=COPII vesicles Yip3 [Fusarium tjaetaba];Gene=FTJAE_8110;Ontology_term=membrane;Ontology_id=GO:0016020;Enzyme_code=EC:2.3.2.23;Enzyme_name=E2 ubiquitin-conjugating enzyme

contig00004.g2607	1146	ID=contig00004.g2607;Description=mannan polymerase complexes MNN9 subunit [Fusarium tjaetaba];Gene=FMAN_04573;Ontology_term=protein glycosylation,cell wall organization or biogenesis,endoplasmic reticulum,Golgi apparatus,transferase activity;Ontology_id=GO:0006486,GO:0071554,GO:0005783,GO:0005794,GO:0016740;Enzyme_code=EC:2.4,EC:2.4,EC:2,EC:2.4.1.232,EC:2.4.1;Enzyme_name=Glycosyltransferases,Glycosyltransferases,Transferases,initiation-specific alpha-1,6-mannosyltransferase,Glycosyltransferases
contig00004.g2608	1539	ID=contig00004.g2608;Description=RNA and export factor-binding 2 [Fusarium tjaetaba];Gene=FHETE_7767;Ontology_term=nucleocytoplasmic transport,nucleus,RNA binding,regulation of DNA-templated transcription,intracellular protein transport;Ontology_id=GO:0006913,GO:0005634,GO:0003723,GO:0006355,GO:0006886
contig00004.g2609	648	ID=contig00004.g2609;Description=hypothetical protein FVER53263_05133 [Fusarium verticillioides]
contig00004.g2610	1043	ID=contig00004.g2610;Description=hypothetical protein FVEG_05134 [Fusarium verticillioides 7600];Gene=FNYG_15685;Ontology_term=oxidoreductase activity;Ontology_id=GO:0016491;Enzyme_code=EC:1;Enzyme_name=Oxidoreductases
contig00004.g2611	1218	ID=contig00004.g2611;Description=v-type c subunit family [Fusarium tjaetaba]
contig00004.g2612	3483	ID=contig00004.g2612;Description=valyl-tRNA synthetase [Fusarium verticillioides 7600];Gene=FNYG_15683;Ontology_term=cytoplasmic translation,mitochondrion,tRNA metabolic process,amino acid metabolic process,catalytic activity, acting on RNA,ligase activity,hydrolase activity;Ontology_id=GO:0002181,GO:0005739,GO:0006399,GO:0006520,GO:0140098,GO:0016874,GO:0016787;Enzyme_code=EC:6.1.1.9,EC:3.1.1,EC:3.1,EC:6.1,EC:3,EC:6.1.1,EC:6,EC:6.1.1.9,EC:3.1.1;Enzyme_name=valine--tRNA ligase,Acting on ester bonds,Acting on ester bonds,Forming carbon-oxygen bonds,Hydrolases,Forming carbon-oxygen bonds,Ligases,valine--tRNA ligase,Acting on ester bonds
contig00004.g2613	2172	ID=contig00004.g2613;Description=pre-mRNA-splicing ATP-dependent RNA helicase PRP28 [Fusarium verticillioides 7600];Gene=BFJ70_g9122;Ontology_term=mRNA metabolic process,RNA binding,protein-containing complex assembly,ATP-dependent activity,catalytic activity, acting on RNA,nucleolus,hydrolase activity;Ontology_id=GO:0016071,GO:0003723,GO:0065003,GO:0140657,GO:0140098,GO:0005730,GO:0016787;Enzyme_code=EC:3.6.4.13,EC:3.6.1,EC:3.6.4.13,EC:3.6,EC:3,EC:3.6.1.15;Enzyme_name=RNA helicase,Acting on acid anhydrides,RNA helicase,Acting on acid anhydrides,Hydrolases,nucleoside-triphosphate phosphatase

contig00004.g2614	1588	ID=contig00004.g2614;Description=diphthamide biosynthesis protein 4 [Fusarium verticillioides 7600];Gene=FMUND_11559;Ontology_term=mitochondrial gene expression,mitochondrion,structural molecule activity,ribosome;Ontology_id=GO:0140053,GO:0005739,GO:0005198,GO:0005840
contig00004.g2615	1172	ID=contig00004.g2615;Description=tyrosine 3-monooxygenase tryptophan 5-monooxygenase [Fusarium tjaetaba];Gene=F25303_7019;Ontology_term=oxidoreductase activity;Ontology_id=GO:0016491;Enzyme_code=EC:1;Enzyme_name=Oxidoreductases
contig00004.g2616	550	ID=contig00004.g2616;Description=mitochondrial ATPase inhibitor, IATP-domain-containing protein [Fusarium flagelliforme];Gene=FOYG_02924;Ontology_term=molecular function regulator activity,mitochondrion;Ontology_id=GO:0098772,GO:0005739
contig00004.g2617	2204	ID=contig00004.g2617;Description=nucleoporin nsp1 [Fusarium tjaetaba];Gene=FVER53590_05141;Ontology_term=nuclear envelope,nucleocytoplasmic transport,structural molecule activity,ribosome biogenesis,lipid binding,intracellular protein transport,mitotic cell cycle;Ontology_id=GO:0005635,GO:0006913,GO:0005198,GO:0042254,GO:0008289,GO:0006886,GO:0000278
contig00004.g2618	1632	ID=contig00004.g2618;Description=hypothetical protein LB503_011229 [Fusarium chuoi]
contig00004.g2619	1647	ID=contig00004.g2619;Description=eukaryotic translation initiation factor 2 subunit gamma [Fusarium proliferatum];Gene=FACUT_6947;Ontology_term=translation regulator activity,RNA binding,protein-containing complex assembly,cytoplasmic translation,GTPase activity,cytosol,ribosome;Ontology_id=GO:0045182,GO:0003723,GO:0065003,GO:0002181,GO:0003924,GO:0005829,GO:0005840;Enzyme_code=EC:3.6.1.15,EC:3.6.1,EC:3.6,EC:3,EC:3.6.1.15;Enzyme_name=nucleoside-triphosphate phosphatase,Acting on acid anhydrides,Acting on acid anhydrides,Hydrolases,nucleoside-triphosphate phosphatase
contig00004.g2620	1636	ID=contig00004.g2620;Description=hypothetical protein FVEG_05144 [Fusarium verticillioides 7600];Gene=FPCIR_3668;Ontology_term=lyase activity,cytosol,amino acid metabolic process;Ontology_id=GO:0016829,GO:0005829,GO:0006520;Enzyme_code=EC:4.2.1.33;Enzyme_name=3-isopropylmalate dehydratase

contig00004.g2621	1429	ID=contig00004.g2621;Description=ATP-dependent RNA helicase eIF4A [Truncatella angustata];Gene=CSAL01_01106;Ontology_term=translation regulator activity,RNA binding,ATP-dependent activity,cytoplasmic translation,cytosol,nucleolus,ribosome,catalytic activity, acting on RNA,plasma membrane,hydrolase activity;Ontology_id=GO:0045182,GO:0003723,GO:0140657,GO:0002181,GO:0005829,GO:0005730,GO:0005840,GO:0140098,GO:0005886,GO:0016787;Enzyme_code=EC:3.6.4.13,EC:3.6.1,EC:3.6.4.13,EC:3.6,EC:3,EC:3.6.1.15;Enzyme_name=RNA helicase,Acting on acid anhydrides,RNA helicase,Acting on acid anhydrides,Hydrolases,nucleoside-triphosphate phosphatase
contig00004.g2622	1562	ID=contig00004.g2622;Description=phosphatidylserine-specific receptor [Fusarium tjaetaba]
contig00004.g2623	2280	ID=contig00004.g2623;Description=nuclear WD PRL1 [Fusarium tjaetaba];Gene=FPANT_7297;Ontology_term=RNA binding,regulation of DNA-templated transcription;Ontology_id=GO:0003723,GO:0006355
contig00004.g2624	1220	ID=contig00004.g2624;Description=TVP38-like integral membrane protein [Fusarium denticulatum];Gene=FPANT_7296;Ontology_term=vesicle-mediated transport,cytoskeleton organization,mitotic nuclear division,chromosome segregation,Golgi apparatus;Ontology_id=GO:0016192,GO:0007010,GO:0140014,GO:0007059,GO:0005794
contig00004.g2625	1826	ID=contig00004.g2625;Description=hypothetical protein FVEG_05149 [Fusarium verticillioides 7600]
contig00004.g2626	1446	ID=contig00004.g2626;Description=proline iminopeptidase [Fusarium coicis];Gene=FNYG_12067;Ontology_term=catalytic activity, acting on a protein,hydrolase activity;Ontology_id=GO:0140096,GO:0016787;Enzyme_code=EC:3.4.11,EC:3.4.11,EC:3.4,EC:3;Enzyme_name=Acting on peptide bonds (peptidases),Acting on peptide bonds (peptidases),Acting on peptide bonds (peptidases),Hydrolases
contig00004.g2627	5805	ID=contig00004.g2627;Description=DNA polymerase theta subunit [Fusarium coicis];Gene=FACUT_9473;Ontology_term=ATP-dependent activity,hydrolase activity;Ontology_id=GO:0140657,GO:0016787;Enzyme_code=EC:3,EC:3,EC:2.7.7.7;Enzyme_name=Hydrolases,Hydrolases,DNA-directed DNA polymerase
contig00004.g2628	5219	ID=contig00004.g2628;Description=PHD finger [Fusarium phyllophilum];Gene=FNYG_12063;Ontology_term=nucleus;Ontology_id=GO:0005634

contig00004.g2629	1322	ID=contig00004.g2629;Description=NAK protein kinase [Fusarium verticillioides 7600];Gene=FNAPI_12602;Ontology_term=catalytic activity, acting on a protein,signaling,protein maturation,transferase activity,vacuole;Ontology_id=GO:0140096,GO:0023052,GO:0051604,GO:0016740,GO:0005773;Enzyme_code=EC:2.7.11.1,EC:2.7.1,EC:2.7.11.1,EC:2.7,EC:2;Enzyme_name=non-specific serine/threonine protein kinase,Transferring phosphorus-containing groups,non-specific serine/threonine protein kinase,Transferring phosphorus-containing groups,Transferases
contig00004.g2630	4128	ID=contig00004.g2630;Description=alpha-mannosidase [Fusarium verticillioides 7600];Gene=FOTG_00782;Ontology_term=carbohydrate metabolic process,carbohydrate derivative metabolic process,vacuole,hydrolase activity;Ontology_id=GO:0005975,GO:1901135,GO:0005773,GO:0016787;Enzyme_code=EC:3.2.1.24,EC:3.2,EC:3,EC:3.2.1.24,EC:3.2.1;Enzyme_name=alpha-mannosidase,Glycosylases,Hydrolases,alpha-mannosidase,Glycosylases
contig00004.g2631	861	ID=contig00004.g2631;Description=hypothetical protein FVER14953_05156 [Fusarium verticillioides]
contig00004.g2632	3365	ID=contig00004.g2632;Description=MUTS-like protein [Fusarium denticulatum];Gene=FTJAE_4690;Ontology_term=DNA binding,DNA repair,catalytic activity, acting on DNA,ATP-dependent activity,small molecule sensor activity;Ontology_id=GO:0003677,GO:0006281,GO:0140097,GO:0140657,GO:0140299
contig00004.g2633	1046	ID=contig00004.g2633;Description=60S acidic ribosomal protein P0 [Fusarium verticillioides 7600];Gene=FTJAE_4691;Ontology_term=RNA binding,protein-containing complex assembly,cytoplasmic translation,structural molecule activity,cytosol,ribosome biogenesis,ribosome;Ontology_id=GO:0003723,GO:0065003,GO:0002181,GO:0005198,GO:0005829,GO:0042254,GO:0005840
contig00004.g2634	1743	ID=contig00004.g2634;Description=tRNA (guanine-N(7)-)-methyltransferase subunit trm82 [Fusarium tjaetaba];Gene=FVER53590_05160;Ontology_term=nucleus,cytosol,tRNA metabolic process,transferase activity;Ontology_id=GO:0005634,GO:0005829,GO:0006399,GO:0016740;Enzyme_code=EC:2.1.1,EC:2.1,EC:2,EC:2.1.1;Enzyme_name=Transferring one-carbon groups,Transferring one-carbon groups,Transferases,Transferring one-carbon groups

contig00004.g2635	1406	ID=contig00004.g2635;Description=DnaJ like subfamily A member 2 [Fusarium verticillioides 7600];Gene=FOYG_02904;Ontology_term=protein catabolic process,cytosol,regulatory ncRNA-mediated gene silencing,organelle,chromatin organization,protein folding;Ontology_id=GO:0030163,GO:0005829,GO:0031047,GO:0043226,GO:0006325,GO:0006457
contig00004.g2636	1491	ID=contig00004.g2636;Description=hypothetical protein FVEG_05162 [Fusarium verticillioides 7600];Gene=FMAN_04603;Ontology_term=regulation of DNA-templated transcription,transcription regulator activity;Ontology_id=GO:0006355,GO:0140110
contig00004.g2637	2488	ID=contig00004.g2637;Description=Tip elongation 1 [Fusarium tjaetaba];Gene=FCIRC_13502;Ontology_term=cytoskeleton organization,protein-containing complex assembly,establishment or maintenance of cell polarity,anatomical structure development,reproductive process,nuclear chromosome,chromosome segregation,microtubule-based movement,microtubule organizing center,mitotic nuclear division,cytoskeletal protein binding;Ontology_id=GO:0007010,GO:0065003,GO:0007163,GO:0048856,GO:0022414,GO:0000228,GO:0007059,GO:0007018,GO:0005815,GO:0140014,GO:0008092
contig00004.g2638	2349	ID=contig00004.g2638;Description=glycogen [starch] synthase [Fusarium verticillioides 7600];Gene=FOYG_02901;Ontology_term=nucleus,carbohydrate metabolic process,generation of precursor metabolites and energy,mitochondrion,transferase activity;Ontology_id=GO:0005634,GO:0005975,GO:0006091,GO:0005739,GO:0016740;Enzyme_code=EC:2.4.1.11,EC:2.4,EC:2,EC:2.4.1.11,EC:2.4.1;Enzyme_name=glycogen(starch) synthase,Glycosyltransferases,Transferases,glycogen(starch) synthase,Glycosyltransferases
contig00004.g2639	1487	ID=contig00004.g2639;Description=ribosome biogenesis protein ytm1 [Fusarium musae];Gene=YTM1;Ontology_term=ribosome biogenesis,nucleolus,nucleoplasm;Ontology_id=GO:0042254,GO:0005730,GO:0005654
contig00004.g2640	1748	ID=contig00004.g2640;Description=werner syndrome helicase [Fusarium pseudocircinatum];Gene=FPCIR_3647;Ontology_term=ATP-dependent activity,hydrolase activity;Ontology_id=GO:0140657,GO:0016787;Enzyme_code=EC:3.1,EC:3.1.11.1,EC:3.1,EC:3;Enzyme_name=Acting on ester bonds,exodeoxyribonuclease I,Acting on ester bonds,Hydrolases
contig00004.g2641	524	ID=contig00004.g2641;Description=U6 snRNA-associated Sm-like protein LSm5 [Fusarium verticillioides 7600];Gene=LSM5

contig00004.g2642	564	ID=contig00004.g2642;Description=DNA-directed RNA polymerase [Ilyonectria robusta];Gene=F53441_1587;Ontology_term=DNA binding,DNA-templated transcription,catalytic activity, acting on RNA,transferase activity,nucleoplasm;Ontology_id=GO:0003677,GO:0006351,GO:0140098,GO:0016740,GO:0005654;Enzyme_code=EC:2.7.7.6,EC:2.7,EC:2,EC:2.7.7,EC:2.7.7.6;Enzyme_name=DNA-directed RNA polymerase,Transferring phosphorus-containing groups,Transferases,Transferring phosphorus-containing groups,DNA-directed RNA polymerase
contig00004.g2643	717	ID=contig00004.g2643;Description=mitochondrial N(5)-glutamine methyltransferase mtq1 [Fusarium tjaetaba];Gene=FNAPI_6126;Ontology_term=DNA binding,catalytic activity, acting on a protein,mitochondrial gene expression,catalytic activity, acting on DNA,mitochondrion,transferase activity;Ontology_id=GO:0003677,GO:0140096,GO:0140053,GO:0140097,GO:0005739,GO:0016740;Enzyme_code=EC:2.1.1.72,EC:2.1,EC:2,EC:2.1.1.72,EC:2.1.1;Enzyme_name=site-specific DNA-methyltransferase (adenine-specific),Transferring one-carbon groups,Transferases,site-specific DNA-methyltransferase (adenine-specific),Transferring one-carbon groups
contig00004.g2644	2727	ID=contig00004.g2644;Description=AFG3 family protein [Fusarium verticillioides 7600];Gene=BFJ70_g9175;Ontology_term=catalytic activity, acting on a protein,transmembrane transport,protein-containing complex assembly,ATP-dependent activity,cytoplasmic translation,protein catabolic process,mitochondrion,protein maturation,intracellular protein transport,vacuole,mitochondrion organization,hydrolase activity;Ontology_id=GO:0140096,GO:0055085,GO:0065003,GO:0140657,GO:0002181,GO:0030163,GO:0005739,GO:0051604,GO:0006886,GO:0005773,GO:0007005,GO:0016787;Enzyme_code=EC:3.4.24,EC:3.6.1.15,EC:3.4.24,EC:3.6.1,EC:3.4,EC:3.6,EC:3,EC:3.6.1.15;Enzyme_name=Acting on peptide bonds (peptidases),nucleoside-triphosphate phosphatase,Acting on peptide bonds (peptidases),Acting on acid anhydrides,Acting on peptide bonds (peptidases),Acting on acid anhydrides,Hydrolases,nucleoside-triphosphate phosphatase
contig00004.g2645	2135	ID=contig00004.g2645;Description=chloride-bicarbonate anion exchanger AE2 [Fusarium pseudoanthophilum];Gene=FPHYL_11901;Ontology_term=transporter activity;Ontology_id=GO:0005215

contig00004.g2646	3554	ID=contig00004.g2646;Description=replication fork protection complex subunit Tof1/Swi1 [Fusarium oxysporum f. sp. lycopersici MN25];Gene=FVEG_05171;Ontology_term=DNA repair,DNA replication,DNA recombination,reproductive process,chromosome segregation,nuclear chromosome,DNA binding,signaling,cell differentiation,mitotic nuclear division,cytosol;Ontology_id=GO:0006281,GO:0006260,GO:0006310,GO:0022414,GO:0007059,GO:0000228,GO:0003677,GO:0023052,GO:0030154,GO:0140014,GO:0005829
contig00004.g2647	1016	ID=contig00004.g2647;Description=3-hydroxybutyryl-CoA dehydrogenase [Fusarium verticillioides 7600];Gene=FVER53590_05172;Ontology_term=oxidoreductase activity,lipid metabolic process;Ontology_id=GO:0016491,GO:0006629;Enzyme_code=EC:1.1.1;Enzyme_name=Acting on the CH-OH group of donors
contig00004.g2648	1620	ID=contig00004.g2648;Description=homeobox-leucine zipper ROC3 [Fusarium tjaetaba];Gene=FTJAE_4706;Ontology_term=nucleus,DNA binding;Ontology_id=GO:0005634,GO:0003677
contig00004.g2649	1035	ID=contig00004.g2649;Description=mediator of rna polymerase ii transcription subunit 19 [Fusarium tjaetaba];Gene=MED19;Ontology_term=nucleus,regulation of DNA-templated transcription,transcription regulator activity,molecular adaptor activity;Ontology_id=GO:0005634,GO:0006355,GO:0140110,GO:0060090
contig00004.g2650	2965	ID=contig00004.g2650;Description=transcriptional activator Mut3p [Fusarium tjaetaba];Gene=BFJ72_g8089;Ontology_term=nucleus,DNA binding,regulation of DNA-templated transcription,transcription regulator activity;Ontology_id=GO:0005634,GO:0003677,GO:0006355,GO:0140110
contig00004.g2651	2216	ID=contig00004.g2651;Description=hypothetical protein LB504_010067 [Fusarium proliferatum];Gene=FPCIR_3636;Ontology_term=membrane;Ontology_id=GO:0016020
contig00004.g2652	2422	ID=contig00004.g2652;Description=hypothetical protein FVER14953_05179 [Fusarium verticillioides]
contig00004.g2653	2328	ID=contig00004.g2653;Description=hypothetical protein FVEG_05180 [Fusarium verticillioides 7600]
contig00004.g2654	1786	ID=contig00004.g2654;Description=hypothetical protein FVEG_05181 [Fusarium verticillioides 7600];Gene=2250

contig00004.g2655	2123	ID=contig00004.g2655;Description=ATP-dependent RNA helicase DED1 [Fusarium verticillioides 7600];Gene=FOVG_02748;Ontology_term=nucleus,translation regulator activity,RNA binding,mRNA metabolic process,protein-containing complex assembly,ATP-dependent activity,cytoplasmic translation,regulatory ncRNA-mediated gene silencing,catalytic activity, acting on RNA,hydrolase activity;Ontology_id=GO:0005634,GO:0045182,GO:0003723,GO:0016071,GO:0065003,GO:0140657,GO:0002181,GO:0031047,GO:0140098,GO:0016787;Enzyme_code=EC:3.6.4.13;Enzyme_name=RNA helicase
contig00004.g2656	691	ID=contig00004.g2656;Description=proteasome subunit beta type-2 [Fusarium globosum];Gene=FGLOB1_11659;Ontology_term=nucleus,molecular function regulator activity,protein catabolic process,mitotic nuclear division,chromosome segregation,cytosol,endoplasmic reticulum;Ontology_id=GO:0005634,GO:0098772,GO:0030163,GO:0140014,GO:0007059,GO:0005829,GO:0005783;Enzyme_code=EC:3.4.25.1;Enzyme_name=proteasome endopeptidase complex
contig00004.g2657	1749	ID=contig00004.g2657;Description=component of the u3 snoRNP complex [Fusarium tjaetaba];Ontology_term=RNA binding,ribosome biogenesis,nucleolus;Ontology_id=GO:0003723,GO:0042254,GO:0005730
contig00004.g2658	3217	ID=contig00004.g2658;Description=protease ULP2 [Fusarium coicis];Gene=FVEG_15605;Ontology_term=nucleus,catalytic activity, acting on a protein,signaling,mitotic nuclear division,chromosome segregation,hydrolase activity;Ontology_id=GO:0005634,GO:0140096,GO:0023052,GO:0140014,GO:0007059,GO:0016787;Enzyme_code=EC:3.4,EC:3.4,EC:3,EC:3.4.22.68;Enzyme_name=Acting on peptide bonds (peptidases),Acting on peptide bonds (peptidases),Hydrolases,Ulp1 peptidase
contig00004.g2659	784	ID=contig00004.g2659;Description=signal peptidase subunit [Fusarium tjaetaba];Gene=FMAN_04626;Ontology_term=catalytic activity, acting on a protein,protein maturation,endoplasmic reticulum,intracellular protein transport,hydrolase activity;Ontology_id=GO:0140096,GO:0051604,GO:0005783,GO:0006886,GO:0016787;Enzyme_code=EC:3.4,EC:3;Enzyme_name=Acting on peptide bonds (peptidases),Hydrolases

contig00004.g2660	4461	ID=contig00004.g2660;Description=alkaline phosphatase D [Fusarium verticillioides 7600];Gene=FTJAE_4718;Ontology_term=nucleus,mRNA metabolic process,RNA binding,protein-containing complex assembly,regulation of DNA-templated transcription,ribosome biogenesis,catalytic activity, acting on RNA,plasma membrane,mitotic cell cycle,hydrolase activity;Ontology_id=GO:0005634,GO:0016071,GO:0003723,GO:0065003,GO:0006355,GO:0042254,GO:0140098,GO:0005886,GO:0000278,GO:0016787;Enzyme_code=EC:3.1.13,EC:3.1.15,EC:3.1,EC:3.1.13,EC:3,EC:3.1.15;Enzyme_name=Acting on ester bonds,Acting on ester bonds,Acting on ester bonds,Acting on ester bonds,Hydrolases,Acting on ester bonds
contig00004.g2661	1619	ID=contig00004.g2661;Description=putative HAT2-subunit of the major histone acetyltransferase [Fusarium fujikuroi];Gene=FOTG_00817;Ontology_term=protein-containing complex assembly,mitotic nuclear division,chromosome segregation,nuclear chromosome,regulation of DNA-templated transcription,chromatin organization,transferase activity,nucleoplasm;Ontology_id=GO:0065003,GO:0140014,GO:0007059,GO:0000228,GO:0006355,GO:0006325,GO:0016740,GO:0005654;Enzyme_code=EC:2,EC:2;Enzyme_name=Transferases,Transferases
contig00004.g2662	2097	ID=contig00004.g2662;Description=hypothetical protein FVEG_05190 [Fusarium verticillioides 7600]
contig00004.g2663	2277	ID=contig00004.g2663;Description=putative RNA-binding protein C17H9.04c [Fusarium oxysporum];Gene=BFJ68_g11683;Ontology_term=nucleus,RNA binding,catalytic activity, acting on RNA,hydrolase activity;Ontology_id=GO:0005634,GO:0003723,GO:0140098,GO:0016787;Enzyme_code=EC:3.1.13,EC:3.1.15,EC:3.1,EC:3.1.13,EC:3,EC:3.1.15;Enzyme_name=Acting on ester bonds,Acting on ester bonds,Acting on ester bonds,Acting on ester bonds,Hydrolases,Acting on ester bonds
contig00004.g2664	393	ID=contig00004.g2664;Description=Proteolipid membrane potential modulator [Fusarium oxysporum f. sp. vasinfectum];Gene=FVER53590_05193;Ontology_term=membrane;Ontology_id=GO:0016020
contig00004.g2665	1515	ID=contig00004.g2665;Description=hypothetical protein FVEG_05194 [Fusarium verticillioides 7600]

contig00004.g2666	3094	ID=contig00004.g2666;Description=CMGC/CDK/CRK7 protein kinase [Fusarium verticillioides 7600];Gene=FVER53590_05195;Ontology_term=DNA binding,catalytic activity, acting on a protein,mRNA metabolic process,regulation of DNA-templated transcription,chromosome,transferase activity,nucleolus,nucleoplasm;Ontology_id=GO:0003677,GO:0140096,GO:0016071,GO:0006355,GO:0005694,GO:0016740,GO:0005730,GO:0005654;Enzyme_code=EC:2.7.1,EC:2.7.1,EC:2.7.11.23,EC:2.7.11.1,EC:2.7,EC:2,EC:2.7.11.22;Enzyme_name=Transferring phosphorus-containing groups,Transferring phosphorus-containing groups,[RNA-polymerase]-subunit kinase,non-specific serine/threonine protein kinase,Transferring phosphorus-containing groups,Transferases,cyclin-dependent kinase
contig00004.g2667	1578	ID=contig00004.g2667;Description=embryogenesis abundant [Fusarium subglutinans];Gene=FVEG_05196;Ontology_term=lipid metabolic process,transferase activity,hydrolase activity;Ontology_id=GO:0006629,GO:0016740,GO:0016787;Enzyme_code=EC:2.3,EC:3.1,EC:2,EC:3.1.1.23,EC:3,EC:3.1.1;Enzyme_name=Acyltransferases,Acting on ester bonds,Transferases,acylglycerol lipase,Hydrolases,Acting on ester bonds
contig00004.g2668	644	ID=contig00004.g2668;Description=60S ribosomal protein subunit assembly export LOC1 [Fusarium subglutinans];Gene=FMEXI_3592;Ontology_term=RNA binding,ribosome biogenesis,ribosome,nucleolus;Ontology_id=GO:0003723,GO:0042254,GO:0005840,GO:0005730
contig00004.g2669	1133	ID=contig00004.g2669;Description=CDC33-translation initiation factor eIF4E [Fusarium fujikuroi];Gene=C2S_10628;Ontology_term=translation regulator activity,RNA binding;Ontology_id=GO:0045182,GO:0003723
contig00004.g2670	1617	ID=contig00004.g2670;Description=ubiquitin thiolesterase [Fusarium verticillioides 7600];Gene=FTJAE_4728;Ontology_term=nucleus,catalytic activity, acting on a protein,mRNA metabolic process,protein-containing complex assembly,hydrolase activity;Ontology_id=GO:0005634,GO:0140096,GO:0016071,GO:0065003,GO:0016787;Enzyme_code=EC:3.4.19.12,EC:3.4,EC:3,EC:3.4.19.12;Enzyme_name=ubiquitinyl hydrolase 1,Acting on peptide bonds (peptidases),Hydrolases,ubiquitinyl hydrolase 1
contig00004.g2671	2825	ID=contig00004.g2671;Description=UTP13-u3 snoRNP [Fusarium tjaetaba];Gene=FTJAE_4729;Ontology_term=ribosome biogenesis,nucleolus;Ontology_id=GO:0042254,GO:0005730
contig00004.g2672	1742	ID=contig00004.g2672;Description=hypothetical protein FVEG_05201 [Fusarium verticillioides 7600]

contig00004.g2673	1199	ID=contig00004.g2673;Description=dTDP-4-dehydrorhamnose reductase [Fusarium phyllophilum];Gene=mat2b;Ontology_term=catalytic activity, acting on DNA,sulfur compound metabolic process,transferase activity,ligase activity;Ontology_id=GO:0140097,GO:0006790,GO:0016740,GO:0016874;Enzyme_code=EC:2,EC:6.5,EC:2.7.7.1,EC:2,EC:2.5.1.6,EC:6.5,EC:1.1.1.133,EC:6,EC:2.7.7.18;Enzyme_name=Transferases,Forming phosphoric ester bonds,nicotinamide-nucleotide adenylyltransferase,Transferases,methionine adenosyltransferase,Forming phosphoric ester bonds,dTDP-4-dehydrorhamnose reductase,Ligases,nicotinate-nucleotide adenylyltransferase
contig00004.g2674	2852	ID=contig00004.g2674;Description=hypothetical protein J7337_002550 [Fusarium musae];Gene=FVER53590_05203;Ontology_term=catalytic activity, acting on DNA,ligase activity;Ontology_id=GO:0140097,GO:0016874;Enzyme_code=EC:6.5;Enzyme_name=Forming phosphoric ester bonds
contig00004.g2675	1115	ID=contig00004.g2675;Description=farnesyl pyrophosphate synthase [Fusarium proliferatum];Gene=FACUT_9519;Ontology_term=lipid metabolic process,transferase activity;Ontology_id=GO:0006629,GO:0016740;Enzyme_code=EC:2.5.1.10;Enzyme_name=(2E,6E)-farnesyl diphosphate synthase
contig00004.g2676	2159	ID=contig00004.g2676;Description=anaphase promoting complex subunit 8 [Fusarium oxysporum Fo47];Gene=FVER53263_05207;Ontology_term=nucleus,catalytic activity, acting on a protein,protein catabolic process,mitotic nuclear division,chromosome segregation,transferase activity;Ontology_id=GO:0005634,GO:0140096,GO:0030163,GO:0140014,GO:0007059,GO:0016740;Enzyme_code=EC:2.3.2;Enzyme_name=Acyltransferases
contig00004.g2677	870	ID=contig00004.g2677;Description=cell wall SED1 [Fusarium denticulatum]
contig00004.g2678	501	ID=contig00004.g2678;Description=DNase1 protein [Fusarium proliferatum]
contig00004.g2679	3471	ID=contig00004.g2679;Description=VPS54-subunit of VP51-54 complex [Fusarium tjaetaba];Gene=FPCIR_10163;Ontology_term=vesicle-mediated transport,mitochondrion,anatomical structure development,cell differentiation,reproductive process,cytoplasmic vesicle,cytosol,Golgi apparatus,cell wall organization or biogenesis,intracellular protein transport;Ontology_id=GO:0016192,GO:0005739,GO:0048856,GO:0030154,GO:0022414,GO:0031410,GO:0005829,GO:0005794,GO:0071554,GO:0006886

contig00004.g2680	2324	ID=contig00004.g2680;Description=minichromosome maintenance protein 5 (cell division control protein 46) [Fusarium proliferatum];Gene=FOC1_g10013713;Ontology_term=DNA repair,protein-containing complex assembly,DNA replication,DNA recombination,regulation of DNA-templated transcription,nuclear chromosome,chromatin organization,mitotic cell cycle,nucleoplasm,DNA binding,ATP-dependent activity,telomere organization,hydrolase activity;Ontology_id=GO:0006281,GO:0065003,GO:0006260,GO:0006310,GO:0006355,GO:0000228,GO:0006325,GO:0000278,GO:0005654,GO:0003677,GO:0140657,GO:0032200,GO:0016787;Enzyme_code=EC:3.6.1.15,EC:3.6.1,EC:3.6.4.12,EC:3.6,EC:3,EC:3.6.1.15;Enzyme_name=nucleoside-triphosphate phosphatase,Acting on acid anhydrides,DNA helicase,Acting on acid anhydrides,Hydrolases,nucleoside-triphosphate phosphatase
contig00004.g2681	1894	ID=contig00004.g2681;Description=NOL1 NOP2 Sun domain protein [Fusarium tjaetaba];Gene=FPANT_2060;Ontology_term=RNA binding,catalytic activity, acting on RNA,transferase activity;Ontology_id=GO:0003723,GO:0140098,GO:0016740;Enzyme_code=EC:2.1.1;Enzyme_name=Transferring one-carbon groups
contig00004.g2682	1660	ID=contig00004.g2682;Description=WSC domain-containing protein [Fusarium coicis]
contig00004.g2683	1278	ID=contig00004.g2683;Description=sexual development regulator velC [Fusarium tjaetaba];Gene=FPANT_2062;Ontology_term=nucleus,anatomical structure development;Ontology_id=GO:0005634,GO:0048856
contig00004.g2684	1129	ID=contig00004.g2684;Description=hypothetical protein FVER14953_05215 [Fusarium verticillioides];Gene=FSUBG_12565;Ontology_term=membrane;Ontology_id=GO:0016020
contig00004.g2685	4572	ID=contig00004.g2685;Description=ATP-binding cassette, subfamily B (MDR/TAP), member 1 [Fusarium verticillioides 7600];Gene=FPCIR_11825;Ontology_term=transmembrane transport,ATP-dependent activity,transporter activity,hydrolase activity;Ontology_id=GO:0055085,GO:0140657,GO:0005215,GO:0016787;Enzyme_code=EC:7.2.2,EC:3.6.1.15,EC:3.6.1,EC:3.6.3.44,EC:3.6,EC:3,EC:7.2.2,EC:7,EC:3.6.1.15;Enzyme_name=Catalysing the translocation of inorganic cations,nucleoside-triphosphate phosphatase,Acting on acid anhydrides,Acting on acid anhydrides,Acting on acid anhydrides,Hydrolases,Catalysing the translocation of inorganic cations,Translocases,nucleoside-triphosphate phosphatase
contig00004.g2686	900	ID=contig00004.g2686;Description=transmembrane 66 [Fusarium tjaetaba];Gene=FNAPI_11800;Ontology_term=endoplasmic reticulum;Ontology_id=GO:0005783

contig00004.g2687	2584	ID=contig00004.g2687;Description=E3 ubiquitin-protein ligase BRE1 [Fusarium verticillioides 7600];Gene=FOMG_05010;Ontology_term=nucleus,catalytic activity, acting on a protein,transferase activity,chromatin organization;Ontology_id=GO:0005634,GO:0140096,GO:0016740,GO:0006325;Enzyme_code=EC:2.3.2;Enzyme_name=Acyltransferases
contig00004.g2688	1734	ID=contig00004.g2688;Description=transcription factor TFIIIB component B [Fusarium tjaetaba];Ontology_term=nucleus,DNA binding,protein-containing complex assembly,DNA-templated transcription;Ontology_id=GO:0005634,GO:0003677,GO:0065003,GO:0006351
contig00004.g2689	1552	ID=contig00004.g2689;Description=UV excision repair protein Rad23 [Fusarium verticillioides 7600];Gene=FTJAE_12568;Ontology_term=DNA binding,DNA repair,protein catabolic process,cytosol,regulation of DNA-templated transcription,carbohydrate derivative metabolic process,nucleoplasm,hydrolase activity,molecular adaptor activity;Ontology_id=GO:0003677,GO:0006281,GO:0030163,GO:0005829,GO:0006355,GO:1901135,GO:0005654,GO:0016787,GO:0060090;Enzyme_code=EC:3.5.1.52;Enzyme_name=peptide-N(4)-(N-acetyl-beta-glucosaminyl)asparagine amidase
contig00004.g2690	1416	ID=contig00004.g2690;Description=hypothetical protein FVER14953_05221 [Fusarium verticillioides];Gene=FMUND_8703;Ontology_term=membrane;Ontology_id=GO:0016020
contig00004.g2691	1981	ID=contig00004.g2691;Description=cryptochrome [Fusarium verticillioides 7600]
contig00004.g2692	582	ID=contig00004.g2692;Description=tetratricopeptide repeat [Fusarium tjaetaba]
contig00004.g2693	876	ID=contig00004.g2693;Description=u3 snorna associated [Fusarium tjaetaba];Gene=FPANT_3729;Ontology_term=RNA binding,ribosome biogenesis;Ontology_id=GO:0003723,GO:0042254
contig00004.g2694	3527	ID=contig00004.g2694;Description=related to von Willebrand and RING finger domain protein [Fusarium fujikuroi IMI 58289];Gene=alp1;Ontology_term=extracellular space,catalytic activity, acting on a protein,protein catabolic process,carbohydrate derivative metabolic process,hydrolase activity;Ontology_id=GO:0005615,GO:0140096,GO:0030163,GO:1901135,GO:0016787;Enzyme_code=EC:3.4.21,EC:3.4.21,EC:3.4,EC:3;Enzyme_name=Acting on peptide bonds (peptidases),Acting on peptide bonds (peptidases),Acting on peptide bonds (peptidases),Hydrolases

contig00004.g2695	1885	ID=contig00004.g2695;Description=T-complex protein 1 subunit theta [Fusarium proliferatum];Gene=FPANT_11358;Ontology_term=cytoskeleton,protein folding chaperone,ATP-dependent activity,cytosol,hydrolase activity;Ontology_id=GO:0005856,GO:0044183,GO:0140657,GO:0005829,GO:0016787;Enzyme_code=EC:3.6.1.15,EC:3.6.1,EC:3.6,EC:3,EC:3.6.1.15;Enzyme_name=nucleoside-triphosphate phosphatase,Acting on acid anhydrides,Acting on acid anhydrides,Hydrolases,nucleoside-triphosphate phosphatase
contig00004.g2696	979	ID=contig00004.g2696;Description=uncharacterized protein FRV6_07483 [Fusarium oxysporum];Gene=FOQG_00878;Ontology_term=nucleus,DNA binding;Ontology_id=GO:0005634,GO:0003677
contig00004.g2697	1690	ID=contig00004.g2697;Description=Sad1-interacting factor 2 [Fusarium tjaetaba];Gene=FDENT_9891;Ontology_term=mitochondrial gene expression,mitochondrion,vacuole;Ontology_id=GO:0140053,GO:0005739,GO:0005773
contig00004.g2698	1504	ID=contig00004.g2698;Description=Saccharopine dehydrogenase-domain-containing protein [Fusarium redolens];Gene=FVER53263_05229;Ontology_term=oxidoreductase activity;Ontology_id=GO:0016491;Enzyme_code=EC:1,EC:1;Enzyme_name=Oxidoreductases,Oxidoreductases
contig00004.g2699	667	ID=contig00004.g2699;Description=RPL34B [Fusarium tjaetaba];Gene=FANTH_8025;Ontology_term=structural molecule activity,ribosome;Ontology_id=GO:0005198,GO:0005840
contig00004.g2700	2086	ID=contig00004.g2700;Description=ribosomal L37ae [Fusarium mexicanum];Gene=FANTH_8024;Ontology_term=catalytic activity, acting on a protein,regulation of DNA-templated transcription,transferase activity;Ontology_id=GO:0140096,GO:0006355,GO:0016740;Enzyme_code=EC:2.3,EC:2,EC:2.3.2;Enzyme_name=Acyltransferases,Transferases,Acyltransferases

		ID=contig00004.g2701;Description=MRPL17-mitochondrial ribosomal large subunit [Fusarium tjaetaba];Gene=P16126;Ontology_term=peroxisome,mitochondrial gene expression,nucleobase-containing small molecule metabolic process,oxidoreductase activity,mitochondrion,lyase activity,structural molecule activity,carbohydrate derivative metabolic process,cellular modified amino acid metabolic process,ribosome,transferase activity,hydrolase activity;Ontology_id=GO:0005777,GO:0140053,GO:0055086,GO:0016491,GO:0005739,GO:0016829,GO:0005198,GO:1901135,GO:0006575,GO:0005840,GO:0016740,GO:0016787;Enzyme_code=EC:2.1.1.45,EC:4.2.1.19,EC:1.5.1.3,EC:1.7.3.3,EC:3.1.3,EC:4.2.1,EC:2.1.1.45,EC:1.5.1,EC:1.5.1.3,EC:1.7.3.3,EC:3.1.3,EC:2.1,EC:1.7.3,EC:3.1,EC:1.5,EC:1,EC:4.2,EC:2,EC:1.7,EC:3,EC:4,EC:4.2.1.19,EC:2.1.1;Enzyme_name=thymidylate synthase,imidazoleglycerol-phosphate dehydratase,dihydrofolate reductase,factor independent urate hydroxylase,Acting on ester bonds,Carbon-oxygen lyases,thymidylate synthase,Acting on the CH-NH group of donors,dihydrofolate reductase,factor independent urate hydroxylase,Acting on ester bonds,Transferring one-carbon groups,Acting on other nitrogenous compounds as donors,Acting on ester bonds,Acting on the CH-NH group of donors,Oxidoreductases,Carbon-oxygen lyases,Transferases,Acting on other nitrogenous compounds as donors,Hydrolases,Lyases,imidazoleglycerol-phosphate dehydratase,Transferring one-carbon groups
contig00004.g2701	1173	ID=contig00004.g2702;Description=structural maintenance-chromosome 3 (chondroitin sulfate proteoglycan 6) [Fusarium verticillioides 7600];Gene=CEK26_006268;Ontology_term=nucleus,ATP-dependent activity,chromosome,hydrolase activity;Ontology_id=GO:0005634,GO:0140657,GO:0005694,GO:0016787;Enzyme_code=EC:3.6.1.15;Enzyme_name=nucleoside-triphosphate phosphatase
contig00004.g2702	3767	ID=contig00004.g2703;Description=TATA-binding protein-associated factor mot1 [Fusarium musae];Gene=FDENT_9897;Ontology_term=DNA binding,catalytic activity, acting on DNA,protein-containing complex assembly,ATP-dependent activity,mitochondrion,nuclear chromosome,ribosome biogenesis,regulation of DNA-templated transcription,chromatin organization,hydrolase activity;Ontology_id=GO:0003677,GO:0140097,GO:0065003,GO:0140657,GO:0005739,GO:0000228,GO:0042254,GO:0006355,GO:0006325,GO:0016787;Enzyme_code=EC:3.6.1.15,EC:3.6.1,EC:3.6,EC:3,EC:3.6.1.15;Enzyme_name=nucleoside-triphosphate phosphatase,Acting on acid anhydrides,Acting on acid anhydrides,Hydrolases,nucleoside-triphosphate phosphatase
contig00004.g2703	5856	

contig00004.g2704	1638	ID=contig00004.g2704;Description=cell division cycle protein 37 [Fusarium oxysporum f. sp. lycopersici 4287];Gene=FPANT_11349;Ontology_term=nucleus,cytoskeleton organization,signaling,protein folding;Ontology_id=GO:0005634,GO:0007010,GO:0023052,GO:0006457
contig00004.g2705	1089	ID=contig00004.g2705;Description=plc-like phosphodiesterase [Fusarium tjaetaba];Gene=FTJAE_12584;Ontology_term=lipid metabolic process,hydrolase activity;Ontology_id=GO:0006629,GO:0016787;Enzyme_code=EC:3.1.4,EC:3.1.4,EC:3.1,EC:3;Enzyme_name=Acting on ester bonds,Acting on ester bonds,Acting on ester bonds,Hydrolases
contig00004.g2706	1693	ID=contig00004.g2706;Description=succinate-semialdehyde dehydrogenase [Fusarium tjaetaba];Gene=FACUT_3493;Ontology_term=oxidoreductase activity,mitochondrion,amino acid metabolic process;Ontology_id=GO:0016491,GO:0005739,GO:0006520;Enzyme_code=EC:1.2.1.16,EC:1.2.1.24,EC:1.2.1.79,EC:1.2.1.16,EC:1.2,EC:1.2.1.24,EC:1.2.1.79,EC:1,EC:1.2.1.20,EC:1.2.1;Enzyme_name=succinate-semialdehyde dehydrogenase [NAD(P)(+)],succinate-semialdehyde dehydrogenase (NAD(+)),succinate-semialdehyde dehydrogenase (NADP(+)),succinate-semialdehyde dehydrogenase [NAD(P)(+)],Acting on the aldehyde or oxo group of donors,succinate-semialdehyde dehydrogenase (NAD(+)),succinate-semialdehyde dehydrogenase (NADP(+)),Oxidoreductases,glutarate-semialdehyde dehydrogenase,Acting on the aldehyde or oxo group of donors
contig00004.g2707	1433	ID=contig00004.g2707;Description=4-aminobutyrate aminotransferase [Fusarium verticillioides 7600];Gene=FNAPI_11778;Ontology_term=amino acid metabolic process,transferase activity;Ontology_id=GO:0006520,GO:0016740;Enzyme_code=EC:2.6.1.19;Enzyme_name=4-aminobutyrate--2-oxoglutarate transaminase
contig00004.g2708	2770	ID=contig00004.g2708;Description=C6 transcription factor (PrnA) [Fusarium bulbicola];Gene=FCIRC_4337;Ontology_term=nucleus,DNA binding,regulation of DNA-templated transcription,transcription regulator activity;Ontology_id=GO:0005634,GO:0003677,GO:0006355,GO:0140110
contig00004.g2709	1405	ID=contig00004.g2709;Description=DUF1682 domain protein [Fusarium tjaetaba];Gene=Forpe1208_v002932;Ontology_term=endoplasmic reticulum;Ontology_id=GO:0005783

contig00004.g2710	1035	ID=contig00004.g2710;Description=epoxide hydrolase [Fusarium coicis];Gene=FOXB_10420;Ontology_term=catalytic activity;Ontology_id=GO:0003824;Enzyme_code=EC:3.3.2.10,EC:3.1.3.76;Enzyme_name=soluble epoxide hydrolase,lipid-phosphate phosphatase
contig00004.g2711	3186	ID=contig00004.g2711;Description=peroxin 26 [Fusarium tjaetaba];Gene=FMUND_8723;Ontology_term=nucleus,mRNA metabolic process;Ontology_id=GO:0005634,GO:0016071
contig00004.g2712	1694	ID=contig00004.g2712;Description=HASPIN protein kinase [Fusarium verticillioides 7600];Gene=FMUND_8724;Ontology_term=catalytic activity, acting on a protein,chromosome,chromatin organization,transferase activity;Ontology_id=GO:0140096,GO:0005694,GO:0006325,GO:0016740;Enzyme_code=EC:2.7.11.1,EC:2.7.1,EC:2.7.11.1,EC:2.7,EC:2;Enzyme_name=non-specific serine/threonine protein kinase,Transferring phosphorus-containing groups,non-specific serine/threonine protein kinase,Transferring phosphorus-containing groups,Transferases
contig00004.g2713	4202	ID=contig00004.g2713;Description=phospholipid-translocating ATPase [Fusarium verticillioides 7600];Gene=FMUND_8725;Ontology_term=membrane organization,vesicle-mediated transport,protein-containing complex assembly,ATP-dependent activity,ribosome biogenesis,reproductive process,endoplasmic reticulum,Golgi apparatus,intracellular protein transport,transporter activity,plasma membrane,hydrolase activity;Ontology_id=GO:0061024,GO:0016192,GO:0065003,GO:0140657,GO:0042254,GO:0022414,GO:0005783,GO:0005794,GO:0006886,GO:0005215,GO:0005886,GO:0016787;Enzyme_code=EC:7.6.2.1,EC:3.6.1.15,EC:3.6.1,EC:3.6.3.1,EC:7.6.2.1,EC:3.6,EC:3,EC:7,EC:7.2.2,EC:3.6.1.15;Enzyme_name=P-type phospholipid transporter,nucleoside-triphosphate phosphatase,Acting on acid anhydrides,Acting on acid anhydrides,P-type phospholipid transporter,Acting on acid anhydrides,Hydrolases,Translocases,Catalysing the translocation of inorganic cations,nucleoside-triphosphate phosphatase
contig00004.g2714	1057	ID=contig00004.g2714;Description=life-span regulatory factor [Fusarium denticulatum]
contig00004.g2715	2758	ID=contig00004.g2715;Description=hypothetical protein FDENT_9910 [Fusarium denticulatum];Gene=FPANT_13080;Ontology_term=membrane;Ontology_id=GO:0016020
contig00004.g2716	1503	ID=contig00004.g2716;Description=hypothetical protein FVEG_05252 [Fusarium verticillioides 7600]
contig00004.g2717	795	ID=contig00004.g2717;Description=pre-mRNA-splicing factor SYF2 [Fusarium verticillioides 7600];Gene=FSUBG_12124;Ontology_term=nucleus,mRNA metabolic process;Ontology_id=GO:0005634,GO:0016071

contig00004.g2718	923	ID=contig00004.g2718;Description=oxidoreductase [Fusarium acutatum]
contig00004.g2719	854	ID=contig00004.g2719;Description=protein AF-9 [Fusarium verticillioides 7600];Gene=FOPG_02647;Ontology_term=DNA repair,regulation of DNA-templated transcription,nuclear chromosome,telomere organization,chromatin organization,nucleoplasm;Ontology_id=GO:0006281,GO:0006355,GO:0000228,GO:0032200,GO:0006325,GO:0005654
contig00004.g2720	1996	ID=contig00004.g2720;Description=mitochondrial distribution and morphology protein 34 [Fusarium verticillioides 7600];Gene=AU210_006549;Ontology_term=mitochondrion,lipid binding,endoplasmic reticulum,mitochondrion organization;Ontology_id=GO:0005739,GO:0008289,GO:0005783,GO:0007005
contig00004.g2721	1602	ID=contig00004.g2721;Description=inositol-pentakisphosphate 2-kinase [Fusarium napiforme];Gene=FACUT_12062;Ontology_term=transferase activity;Ontology_id=GO:0016740;Enzyme_code=EC:2.7.1.158;Enzyme_name=inositol-pentakisphosphate 2-kinase
contig00004.g2722	1436	ID=contig00004.g2722;Description=nucleoside transporter [Fusarium denticulatum];Gene=FNAPI_8228;Ontology_term=transmembrane transport,transporter activity;Ontology_id=GO:0055085,GO:0005215
contig00004.g2723	2466	ID=contig00004.g2723;Description=tol [Fusarium tjaetaba]
contig00004.g2724	459	ID=contig00004.g2724;Description=metallo-hydrolase oxidoreductase [Fusarium tjaetaba];Gene=FDENT_9918;Ontology_term=hydrolase activity;Ontology_id=GO:0016787;Enzyme_code=EC:3,EC:3;Enzyme_name=Hydrolases,Hydrolases
contig00004.g2725	877	ID=contig00004.g2725;Description=putative 5` Pro-FAR isomerase [Fusarium fujikuroi];Gene=2329;Ontology_term=nucleus,DNA binding,structural molecule activity,amino acid metabolic process,chromosome,isomerase activity;Ontology_id=GO:0005634,GO:0003677,GO:0005198,GO:0006520,GO:0005694,GO:0016853;Enzyme_code=EC:5.3.1.16,EC:5.3.1.16,EC:5.3,EC:5,EC:5.3.1;Enzyme_name=1-(5-phosphoribosyl)-5-,1-(5-phosphoribosyl)-5-,Intramolecular oxidoreductases,Isomerases,Intramolecular oxidoreductases

contig00004.g2726	4374	ID=contig00004.g2726;Description=budding [Fusarium napiforme];Gene=FPCIR_12338;Ontology_term=cytoskeleton,cytoskeleton organization,cell wall,establishment or maintenance of cell polarity,cytokinesis,lipid binding,cell adhesion,mitotic cell cycle;Ontology_id=GO:0005856,GO:0007010,GO:0005618,GO:0007163,GO:0000910,GO:0008289,GO:0007155,GO:0000278
contig00004.g2727	2224	ID=contig00004.g2727;Description=probable coronin [Fusarium fujikuroi IMI 58289];Gene=FPCIR_12338;Ontology_term=cytoskeleton,cytoskeleton organization,cytoplasmic vesicle,cytoskeletal protein binding,molecular adaptor activity;Ontology_id=GO:0005856,GO:0007010,GO:0031410,GO:0008092,GO:0060090
contig00004.g2728	2283	ID=contig00004.g2728;Description=transcription factor STE12 [Fusarium verticillioides 7600];Gene=FACUT_12055;Ontology_term=nucleus,regulation of DNA-templated transcription,transcription regulator activity;Ontology_id=GO:0005634,GO:0006355,GO:0140110
contig00004.g2729	952	ID=contig00004.g2729;Description=putative WD repeat-containing protein [Fusarium oxysporum f. sp. albedinis];Ontology_term=ribosome biogenesis,nucleolus;Ontology_id=GO:0042254,GO:0005730
contig00004.g2730	1017	ID=contig00004.g2730;Description=swr1-complex 5 [Fusarium tjaetaba];Gene=FPANT_7938;Ontology_term=nuclear chromosome,cytosol,chromatin organization,nucleoplasm;Ontology_id=GO:0000228,GO:0005829,GO:0006325,GO:0005654
contig00004.g2731	1707	ID=contig00004.g2731;Description=RRB1-like protein [Fusarium tjaetaba];Gene=FVEG_05270;Ontology_term=nucleus,DNA repair,DNA recombination,chromosome,chromatin organization;Ontology_id=GO:0005634,GO:0006281,GO:0006310,GO:0005694,GO:0006325
contig00004.g2732	4400	ID=contig00004.g2732;Description=component of SWI SNF global transcription activator complex [Fusarium pseudocircinatum];Gene=FACUT_12051;Ontology_term=nucleus,catalytic activity, acting on DNA,ATP-dependent activity,histone binding,regulation of DNA-templated transcription,chromosome,chromatin organization,hydrolase activity;Ontology_id=GO:0005634,GO:0140097,GO:0140657,GO:0042393,GO:0006355,GO:0005694,GO:0006325,GO:0016787;Enzyme_code=EC:3;Enzyme_name=Hydrolases
contig00004.g2733	1425	ID=contig00004.g2733;Description=uncharacterized protein FOIG_05697 [Fusarium odoratissimum NRRL 54006]

contig00004.g2734	2452	ID=contig00004.g2734;Description=gibberellin 20-oxidase [Fusarium tjaetaba];Gene=FNYG_08410;Ontology_term=oxidoreductase activity,lipid metabolic process,lyase activity,endoplasmic reticulum;Ontology_id=GO:0016491,GO:0006629,GO:0016829,GO:0005783;Enzyme_code=EC:1,EC:4,EC:1,EC:4;Enzyme_name=Oxidoreductases,Lyases,Oxidoreductases,Lyases
contig00004.g2735	1137	ID=contig00004.g2735;Description=2-amino-3-carboxymuconate-6-semialdehyde decarboxylase [Fusarium tjaetaba];Gene=FNAPI_8215;Ontology_term=lyase activity,hydrolase activity;Ontology_id=GO:0016829,GO:0016787;Enzyme_code=EC:4.1.1,EC:3,EC:4.1.1,EC:4.1,EC:3,EC:4;Enzyme_name=Carbon-carbon lyases,Hydrolases,Carbon-carbon lyases,Carbon-carbon lyases,Hydrolases,Lyases
contig00004.g2736	861	ID=contig00004.g2736;Description=pre-mRNA-splicing factor cwc23 [Fusarium tjaetaba]
contig00004.g2737	764	ID=contig00004.g2737;Description=uncharacterized protein FOBCDRAFT_258253 [Fusarium oxysporum Fo47]
contig00004.g2738	1188	ID=contig00004.g2738;Description=transferase CAF17 mitochondrial [Fusarium coicis];Gene=FMAN_04712;Ontology_term=sulfur compound metabolic process,mitochondrion,transferase activity;Ontology_id=GO:0006790,GO:0005739,GO:0016740;Enzyme_code=EC:2,EC:2;Enzyme_name=Transferases,Transferases
contig00004.g2739	756	ID=contig00004.g2739;Description=hypothetical protein FVEG_05278 [Fusarium verticillioides 7600]
contig00004.g2740	1620	ID=contig00004.g2740;Description=ATP-dependent RNA helicase DBP8 [Fusarium verticillioides 7600];Gene=FNAPI_13689;Ontology_term=RNA binding,ATP-dependent activity,ribosome biogenesis,catalytic activity, acting on RNA,nucleolus,hydrolase activity;Ontology_id=GO:0003723,GO:0140657,GO:0042254,GO:0140098,GO:0005730,GO:0016787;Enzyme_code=EC:3.6.4.13,EC:3.6.1,EC:3.6.4.13,EC:3.6,EC:3,EC:3.6.1.15;Enzyme_name=RNA helicase,Acting on acid anhydrides,RNA helicase,Acting on acid anhydrides,Hydrolases,nucleoside-triphosphate phosphatase

contig00004.g2741	1908	ID=contig00004.g2741;Description=STE STE7 MKK kinase [Fusarium tjaetaba];Gene=FOQG_00935;Ontology_term=catalytic activity, acting on a protein,autophagy,signaling,cytosol,cell wall organization or biogenesis,transferase activity,programmed cell death,mitotic cell cycle;Ontology_id=GO:0140096,GO:0006914,GO:0023052,GO:0005829,GO:0071554,GO:0016740,GO:0012501,GO:0000278;Enzyme_code=EC:2.7.1,EC:2.7.1,EC:2.7.12.1,EC:2.7.11.1,EC:2.7.12.2,EC:2.7,EC:2;Enzyme_name=Transferring phosphorus-containing groups,Transferring phosphorus-containing groups,dual-specificity kinase,non-specific serine/threonine protein kinase,mitogen-activated protein kinase kinase,Transferring phosphorus-containing groups,Transferases
contig00004.g2742	2343	ID=contig00004.g2742;Description=multisubstrate pseudouridine synthase 7 [Fusarium tjaetaba];Gene=FVEG_05281;Ontology_term=nucleus,RNA binding,mRNA metabolic process,snRNA metabolic process,tRNA metabolic process,ribosome biogenesis,isomerase activity;Ontology_id=GO:0005634,GO:0003723,GO:0016071,GO:0016073,GO:0006399,GO:0042254,GO:0016853;Enzyme_code=EC:5.4,EC:5.4.99.27,EC:5.4,EC:5;Enzyme_name=Intramolecular transferases,tRNA pseudouridine(13) synthase,Intramolecular transferases,Isomerases
contig00004.g2743	1509	ID=contig00004.g2743;Description=PUA-like domain-containing protein [Fusarium oxysporum]
contig00004.g2744	461	ID=contig00004.g2744;Description=peptidyl-prolyl cis-trans isomerase NIMA-interacting 4 [Fusarium proliferatum];Gene=FNYG_08400;Ontology_term=DNA binding,catalytic activity, acting on a protein,ribosome biogenesis,isomerase activity;Ontology_id=GO:0003677,GO:0140096,GO:0042254,GO:0016853;Enzyme_code=EC:5.2.1.8;Enzyme_name=peptidylprolyl isomerase
contig00004.g2745	526	ID=contig00004.g2745;Description=40S ribosomal protein S12 [Fusarium musae];Gene=CDV36_008481;Ontology_term=cytoplasmic translation,structural molecule activity,cytosol,ribosome;Ontology_id=GO:0002181,GO:0005198,GO:0005829,GO:0005840
contig00004.g2746	677	ID=contig00004.g2746;Description=40S ribosomal protein S22 [Fusarium oxysporum f. sp. lycopersici 4287];Gene=FOC1_g10013636;Ontology_term=cytoplasmic translation,structural molecule activity,cytosol,ribosome;Ontology_id=GO:0002181,GO:0005198,GO:0005829,GO:0005840

contig00004.g2747	2175	ID=contig00004.g2747;Description=related to capsular associated protein [Fusarium fujikuroi IMI 58289];Gene=2352;Ontology_term=cytoplasmic translation,structural molecule activity,cytosol,ribosome,transferase activity;Ontology_id=GO:0002181,GO:0005198,GO:0005829,GO:0005840,GO:0016740;Enzyme_code=EC:2;Enzyme_name=Transferases
contig00004.g2748	2940	ID=contig00004.g2748;Description=DNA-directed RNA polymerase II regulator [Fusarium circinatum];Gene=FCIRC_13566;Ontology_term=DNA repair,DNA recombination,ribosome biogenesis,regulation of DNA-templated transcription,chromosome,chromatin organization,mitotic cell cycle,nucleoplasm,mRNA metabolic process,general transcription initiation factor activity,regulatory ncRNA-mediated gene silencing,transcription regulator activity;Ontology_id=GO:0006281,GO:0006310,GO:0042254,GO:0006355,GO:0005694,GO:0006325,GO:0000278,GO:0005654,GO:0016071,GO:0140223,GO:0031047,GO:0140110
contig00004.g2749	945	ID=contig00004.g2749;Description=enoyl hydratase [Fusarium tjaetaba];Gene=FPANT_4836;Ontology_term=catalytic activity;Ontology_id=GO:0003824;Enzyme_code=EC:4.2.1.17;Enzyme_name=enoyl-CoA hydratase
contig00004.g2750	2605	ID=contig00004.g2750;Description=related to vesicular transport-associated repeat protein Tb-291 [Fusarium mangiferae];Gene=FMAN_04725;Ontology_term=signaling;Ontology_id=GO:0023052
contig00004.g2751	1172	ID=contig00004.g2751;Description=cysteine synthase [Fusarium proliferatum];Gene=FSUBG_12675;Ontology_term=sulfur compound metabolic process,amino acid metabolic process;Ontology_id=GO:0006790,GO:0006520
contig00004.g2752	1283	ID=contig00004.g2752;Description=hypothetical protein J7337_002469 [Fusarium musae]
contig00004.g2753	748	ID=contig00004.g2753;Description=ribosomal L19 mitochondrial [Fusarium tjaetaba];Gene=FVEG_05293;Ontology_term=mitochondrial gene expression,mitochondrion,structural molecule activity,ribosome;Ontology_id=GO:0140053,GO:0005739,GO:0005198,GO:0005840
contig00004.g2754	2134	ID=contig00004.g2754;Description=alpha-actinin [Fusarium coicis];Gene=FOTG_00926;Ontology_term=cytoskeletal protein binding;Ontology_id=GO:0008092

contig00004.g2755	1782	ID=contig00004.g2755;Description=d-lactate dehydrogenase (cytochrome) [Fusarium tjaetaba];Gene=FVEG_05295;Ontology_term=generation of precursor metabolites and energy,oxidoreductase activity,mitochondrion,cytoskeletal protein binding;Ontology_id=GO:0006091,GO:0016491,GO:0005739,GO:0008092;Enzyme_code=EC:1.1,EC:1.1.2.4,EC:1.1.99.39,EC:1,EC:1.1.99.40,EC:1.1.2;Enzyme_name=Acting on the CH-OH group of donors,D-lactate dehydrogenase (cytochrome),D-2-hydroxyglutarate dehydrogenase,Oxidoreductases,(R)-2-hydroxyglutarate--pyruvate transhydrogenase,Acting on the CH-OH group of donors
contig00004.g2756	1602	ID=contig00004.g2756;Description=d-lactate dehydrogenase (cytochrome) [Fusarium napiforme];Gene=FDENT_12634;Ontology_term=nucleus,catalytic activity,regulation of DNA-templated transcription,transcription regulator activity;Ontology_id=GO:0005634,GO:0003824,GO:0006355,GO:0140110
contig00004.g2757	1030	ID=contig00004.g2757;Description=N-terminal nucleophile aminohydrolase [Xylona heveae TC161];Gene=DL766_005950;Ontology_term=nucleus,protein catabolic process,mitochondrion,mitotic nuclear division,chromosome segregation,cytosol,hydrolase activity;Ontology_id=GO:0005634,GO:0030163,GO:0005739,GO:0140014,GO:0007059,GO:0005829,GO:0016787;Enzyme_code=EC:3,EC:3.4.25.1,EC:3;Enzyme_name=Hydrolases,proteasome endopeptidase complex,Hydrolases
contig00004.g2758	500	ID=contig00004.g2758;Description=related to HOT13-Zinc-binding mitochondrial intermembrane space protein [Fusarium fujikuroi];Gene=BFJ68_g5752;Ontology_term=zinc ion binding;Ontology_id=GO:0008270
contig00004.g2759	2478	ID=contig00004.g2759;Description=DNA excision repair protein ERCC-3 [Fusarium verticillioides 7600];Gene=FOVG_02627;Ontology_term=DNA repair,catalytic activity, acting on DNA,DNA recombination,regulation of DNA-templated transcription,intracellular protein transport,nucleoplasm,nucleocytoplasmic transport,DNA binding,ATP-dependent activity,cytosol,transcription regulator activity,hydrolase activity;Ontology_id=GO:0006281,GO:0140097,GO:0006310,GO:0006355,GO:0006886,GO:0005654,GO:0006913,GO:0003677,GO:0140657,GO:0005829,GO:0140110,GO:0016787;Enzyme_code=EC:3,EC:3.6.1,EC:3.6.4.12,EC:3.6,EC:3,EC:3.6.1.15;Enzyme_name=Hydrolases,Acting on acid anhydrides,DNA helicase,Acting on acid anhydrides,Hydrolases,nucleoside-triphosphate phosphatase

contig00004.g2760	3008	ID=contig00004.g2760;Description=potassium transporter TRK-1 [Fusarium tjaetaba];Gene=FTJAE_8618;Ontology_term=transmembrane transport,transporter activity,plasma membrane;Ontology_id=GO:0055085,GO:0005215,GO:0005886
contig00004.g2761	2272	ID=contig00004.g2761;Description=hypothetical protein FVER53590_05300 [Fusarium verticillioides];Gene=FOYG_02759;Ontology_term=vesicle-mediated transport,molecular function regulator activity;Ontology_id=GO:0016192,GO:0098772
contig00004.g2762	1934	ID=contig00004.g2762;Description=AMP-binding protein [Fusarium tjaetaba];Enzyme_code=EC:3.2.1.4;Enzyme_name=cellulase
contig00004.g2763	953	ID=contig00004.g2763;Description=members of the aldo keto reductase family [Fusarium tjaetaba];Gene=FOTG_00935;Ontology_term=oxidoreductase activity;Ontology_id=GO:0016491;Enzyme_code=EC:1,EC:1;Enzyme_name=Oxidoreductases,Oxidoreductases
contig00004.g2764	792	ID=contig00004.g2764;Description=Mss4 [Fusarium pseudoanthophilum]
contig00004.g2765	1529	ID=contig00004.g2765;Description=beta-glucosidase [Fusarium verticillioides 7600];Gene=FNAPI_13557;Ontology_term=carbohydrate metabolic process,hydrolase activity;Ontology_id=GO:0005975,GO:0016787;Enzyme_code=EC:3.2.1;Enzyme_name=Glycosylases
contig00004.g2766	864	ID=contig00004.g2766;Description=hypothetical protein FVEG_05305 [Fusarium verticillioides 7600];Gene=FDENT_12624;Ontology_term=transporter activity;Ontology_id=GO:0005215;Enzyme_code=EC:3.4.25.1;Enzyme_name=proteasome endopeptidase complex
contig00004.g2767	2065	ID=contig00004.g2767;Description=hypothetical protein J7337_002456 [Fusarium musae];Gene=FNAPI_8578;Ontology_term=endoplasmic reticulum;Ontology_id=GO:0005783
contig00004.g2768	1534	ID=contig00004.g2768;Description=hypothetical protein FVEG_05307 [Fusarium verticillioides 7600];Gene=FVEG_05307;Ontology_term=membrane;Ontology_id=GO:0016020
contig00004.g2769	3397	ID=contig00004.g2769;Description=sulfite exporter family [Fusarium tjaetaba];Gene=FVEG_05309;Ontology_term=membrane;Ontology_id=GO:0016020
contig00004.g2770	1797	ID=contig00004.g2770;Description=pheromone a factor receptor [Fusarium verticillioides 7600];Gene=FTJAE_8628;Ontology_term=molecular transducer activity,signaling;Ontology_id=GO:0060089,GO:0023052

contig00004.g2771	524	ID=contig00004.g2771;Description=single-stranded DNA-binding protein [Fusarium tjaetaba];Gene=FDENT_2829;Ontology_term=DNA binding,DNA replication,mitochondrion,mitochondrion organization;Ontology_id=GO:0003677,GO:0006260,GO:0005739,GO:0007005
contig00004.g2772	1704	ID=contig00004.g2772;Description=glutathione synthetase [Fusarium verticillioides 7600];Gene=FMAN_04747;Ontology_term=sulfur compound metabolic process,cellular modified amino acid metabolic process,ligase activity;Ontology_id=GO:0006790,GO:0006575,GO:0016874;Enzyme_code=EC:6.3.2.3,EC:6.3.2.3,EC:6.3,EC:6.3.2,EC:6;Enzyme_name=glutathione synthase,glutathione synthase,Forming carbon-nitrogen bonds,Forming carbon-nitrogen bonds,Ligases
contig00004.g2773	5818	ID=contig00004.g2773;Description=DNA repair rhp54 [Fusarium coicis];Gene=FVER53590_05313;Ontology_term=nucleus,DNA binding,catalytic activity, acting on DNA,ATP-dependent activity,chromatin organization,hydrolase activity;Ontology_id=GO:0005634,GO:0003677,GO:0140097,GO:0140657,GO:0006325,GO:0016787;Enzyme_code=EC:3.6.1.15,EC:3.6.1,EC:3.6,EC:3,EC:3.6.1.15;Enzyme_name=nucleoside-triphosphate phosphatase,Acting on acid anhydrides,Acting on acid anhydrides,Hydrolases,nucleoside-triphosphate phosphatase
contig00004.g2774	1930	ID=contig00004.g2774;Description=5-aminolevulinate synthase, mitochondrial [Fusarium musae];Gene=FNYG_08367;Ontology_term=mitochondrion,transferase activity;Ontology_id=GO:0005739,GO:0016740;Enzyme_code=EC:2.3.1.37,EC:2.3,EC:2,EC:2.3.1.37,EC:2.3.1;Enzyme_name=5-aminolevulinate synthase,Acyltransferases,Transferases,5-aminolevulinate synthase,Acyltransferases
contig00004.g2775	2106	ID=contig00004.g2775;Description=uncharacterized protein FOIG_05649 [Fusarium odoratissimum NRRL 54006];Gene=BFJ69_g14377;Ontology_term=nucleus,regulation of DNA-templated transcription,transcription regulator activity;Ontology_id=GO:0005634,GO:0006355,GO:0140110

contig00004.g2776	1757	ID=contig00004.g2776;Description=thiamine-phosphate diphosphorylase [Fusarium tjaetaba];Gene=FVER53263_05316;Ontology_term=sulfur compound metabolic process,cytosol,transferase activity,vitamin metabolic process;Ontology_id=GO:0006790,GO:0005829,GO:0016740,GO:0006766;Enzyme_code=EC:2.7.1.50,EC:2.5.1.3,EC:2.7.1,EC:2.5.1,EC:2.7.1.50,EC:2.7,EC:2,EC:2.5.1.3;Enzyme_name=hydroxyethylthiazole kinase,thiamine phosphate synthase,Transferring phosphorus-containing groups,Transferring alkyl or aryl groups, other than methyl groups,hydroxyethylthiazole kinase,Transferring phosphorus-containing groups,Transferases,thiamine phosphate synthase
contig00004.g2777	1371	ID=contig00004.g2777;Description=NADH dehydrogenase [Fusarium verticillioides 7600];Gene=FPCIR_7329;Ontology_term=nucleobase-containing small molecule metabolic process,oxidoreductase activity,ribosome biogenesis,nucleolus;Ontology_id=GO:0055086,GO:0016491,GO:0042254,GO:0005730;Enzyme_code=EC:1.6,EC:1.6,EC:1,EC:1.6.99.3;Enzyme_name=Acting on NADH or NADPH,Acting on NADH or NADPH,Oxidoreductases,Deleted entry
contig00004.g2778	5524	ID=contig00004.g2778;Description=snoRNA-binding rRNA-processing protein utp10, variant 2 [Fusarium oxysporum];Gene=FVEG_05318;Ontology_term=ribosome biogenesis,nucleolus;Ontology_id=GO:0042254,GO:0005730
contig00004.g2779	966	ID=contig00004.g2779;Description=IAH1 Isoamyl acetate hydrolytic enzyme [Fusarium subglutinans]
contig00004.g2780	1909	ID=contig00004.g2780;Description=ubiquitin carboxyl-terminal hydrolase 19 [Fusarium tjaetaba];Gene=FDENT_2838;Ontology_term=hydrolase activity;Ontology_id=GO:0016787;Enzyme_code=EC:3,EC:3;Enzyme_name=Hydrolases,Hydrolases
contig00004.g2781	779	ID=contig00004.g2781;Description=Isochorismatase domain protein [Fusarium tjaetaba];Gene=FDENT_2839;Ontology_term=hydrolase activity;Ontology_id=GO:0016787;Enzyme_code=EC:3;Enzyme_name=Hydrolases
contig00004.g2782	14772	ID=contig00004.g2782;Description=midasin (AAA ATPase) [Fusarium tjaetaba];Gene=FOXYS1_11160;Ontology_term=protein-containing complex assembly,ATP-dependent activity,mitochondrion,ribosome biogenesis,nucleolus,hydrolase activity,nucleoplasm;Ontology_id=GO:0065003,GO:0140657,GO:0005739,GO:0042254,GO:0005730,GO:0016787,GO:0005654;Enzyme_code=EC:3.6.1.15,EC:3.6.1,EC:3.6,EC:3,EC:3.6.1.15;Enzyme_name=nucleoside-triphosphate phosphatase,Acting on acid anhydrides,Acting on acid anhydrides,Hydrolases,nucleoside-triphosphate phosphatase

contig00004.g2783	627	ID=contig00004.g2783;Description=54S ribosomal L12 mitochondrial [Fusarium tjaetaba];Gene=FFUJ_03205;Ontology_term=carbohydrate metabolic process,mitochondrial gene expression,mitochondrion,structural molecule activity,cell wall organization or biogenesis,extracellular region,ribosome,hydrolase activity;Ontology_id=GO:0005975,GO:0140053,GO:0005739,GO:0005198,GO:0071554,GO:0005576,GO:0005840,GO:0016787;Enzyme_code=EC:3.2.1.15,EC:3.2,EC:3,EC:3.2.1.15,EC:3.2.1;Enzyme_name=endo-polygalacturonase,Glycosylases,Hydrolases,endo-polygalacturonase,Glycosylases
contig00004.g2784	896	ID=contig00004.g2784;Description=CTD kinase subunit gamma [Fusarium tjaetaba];Gene=FPANT_4935;Ontology_term=regulation of DNA-templated transcription,transferase activity,nucleoplasm;Ontology_id=GO:0006355,GO:0016740,GO:0005654;Enzyme_code=EC:2.7;Enzyme_name=Transferring phosphorus-containing groups
contig00004.g2785	1149	ID=contig00004.g2785;Description=pyrroline-5-carboxylate reductase [Fusarium verticillioides 7600];Gene=P5CR-1;Ontology_term=oxidoreductase activity,amino acid metabolic process;Ontology_id=GO:0016491,GO:0006520;Enzyme_code=EC:1.5.1.2,EC:1.5,EC:1,EC:1.5.1.2,EC:1.5.1;Enzyme_name=pyrroline-5-carboxylate reductase,Acting on the CH-NH group of donors,Oxidoreductases,pyrroline-5-carboxylate reductase,Acting on the CH-NH group of donors
contig00004.g2786	1542	ID=contig00004.g2786;Description=histone acetyltransferase type B catalytic subunit [Fusarium verticillioides 7600];Gene=FNAPI_4169;Ontology_term=DNA repair,catalytic activity, acting on a protein,histone binding,nuclear chromosome,regulation of DNA-templated transcription,telomere organization,transferase activity,chromatin organization,nucleoplasm;Ontology_id=GO:0006281,GO:0140096,GO:0042393,GO:0000228,GO:0006355,GO:0032200,GO:0016740,GO:0006325,GO:0005654;Enzyme_code=EC:2.3.1.48,EC:2.3,EC:2,EC:2.3.1.48,EC:2.3.1;Enzyme_name=histone acetyltransferase,Acyltransferases,Transferases,histone acetyltransferase,Acyltransferases
contig00004.g2787	2155	ID=contig00004.g2787;Description=LMBR1-like membrane protein-domain-containing protein [Fusarium redolens];Gene=FVEG_05329;Ontology_term=cobalamin binding,membrane;Ontology_id=GO:0031419,GO:0016020
contig00004.g2788	1812	ID=contig00004.g2788;Description=hypothetical protein FVER53590_05330 [Fusarium verticillioides];Gene=FPCIR_7319;Ontology_term=membrane;Ontology_id=GO:0016020

contig00004.g2789	1875	ID=contig00004.g2789;Description=AGC PKA kinase [Fusarium subglutinans];Gene=FPCIR_7318;Ontology_term=catalytic activity, acting on a protein,generation of precursor metabolites and energy,regulation of DNA-templated transcription,reproductive process,nuclear chromosome,intracellular protein transport,vacuole,mitochondrion organization,carbohydrate metabolic process,nucleocytoplasmic transport,autophagy,signaling,cytosol,transferase activity;Ontology_id=GO:0140096,GO:0006091,GO:0006355,GO:0022414,GO:0000228,GO:0006886,GO:0005773,GO:0007005,GO:0005975,GO:0006913,GO:0006914,GO:0023052,GO:0005829,GO:0016740;Enzyme_code=EC:2.7.11.11,EC:2.7.1,EC:2.7.11.1,EC:2.7,EC:2,EC:2.7.11.11;Enzyme_name=cAMP-dependent protein kinase,Transferring phosphorus-containing groups,non-specific serine/threonine protein kinase,Transferring phosphorus-containing groups,Transferases,cAMP-dependent protein kinase
contig00004.g2790	1162	ID=contig00004.g2790;Description=vesicular integral-membrane protein VIP36 [Fusarium fujikuroi];Gene=FPHYL_13924;Ontology_term=membrane;Ontology_id=GO:0016020
contig00004.g2791	315	ID=contig00004.g2791;Description=mitochondrial import inner membrane translocase subunit TIM13 [Fusarium proliferatum];Gene=FCIRC_1078;Ontology_term=membrane organization,mitochondrion,intracellular protein transport,transporter activity,mitochondrion organization;Ontology_id=GO:0061024,GO:0005739,GO:0006886,GO:0005215,GO:0007005
contig00004.g2792	1203	ID=contig00004.g2792;Description=coiled-coil domain protein [Fusarium tjaetaba];Gene=FTJAE_2729;Ontology_term=mRNA metabolic process,RNA binding;Ontology_id=GO:0016071,GO:0003723
contig00004.g2793	1178	ID=contig00004.g2793;Description=hypothetical protein FVEG_05336 [Fusarium verticillioides 7600]
contig00004.g2794	4010	ID=contig00004.g2794;Description=pre-mRNA-splicing factor RSE1 [Fusarium tjaetaba];Gene=FPANT_13922;Ontology_term=nucleus,mRNA metabolic process,RNA binding,protein-containing complex assembly;Ontology_id=GO:0005634,GO:0016071,GO:0003723,GO:0065003

contig00004.g2795	1620	ID=contig00004.g2795;Description=related to MSS51 protein [Fusarium fujikuroi IMI 58289];Gene=P23631;Ontology_term=translation regulator activity,vesicle-mediated transport,mitochondrial gene expression,signaling,lipid metabolic process,mitochondrion,toxin activity,extracellular region,hydrolase activity;Ontology_id=GO:0045182,GO:0016192,GO:0140053,GO:0023052,GO:0006629,GO:0005739,GO:0090729,GO:0005576,GO:0016787;Enzyme_code=EC:3.1.3.25,EC:3.1,EC:3.1.3.25,EC:3,EC:3.1.3;Enzyme_name=inositol-phosphate phosphatase,Acting on ester bonds,inositol-phosphate phosphatase,Hydrolases,Acting on ester bonds
contig00004.g2796	1813	ID=contig00004.g2796;Description=hypothetical protein FVER14953_05340 [Fusarium verticillioides];Gene=FDENT_2855;Ontology_term=membrane;Ontology_id=GO:0016020
contig00004.g2797	1706	ID=contig00004.g2797;Description=centromere binding B [Fusarium tjaetaba];Gene=FNAPI_13492;Ontology_term=DNA binding;Ontology_id=GO:0003677
contig00004.g2798	1937	ID=contig00004.g2798;Description=GTP binding protein-like protein [Alternaria alternata];Gene=CC77DRAFT_984267;Ontology_term=cytoplasmic translation,GTPase activity,organelle;Ontology_id=GO:0002181,GO:0003924,GO:0043226;Enzyme_code=EC:3.6.1.15,EC:3.6.1,EC:3.6,EC:3,EC:3.6.1.15;Enzyme_name=nucleoside-triphosphate phosphatase,Acting on acid anhydrides,Acting on acid anhydrides,Hydrolases,nucleoside-triphosphate phosphatase
contig00004.g2799	4681	ID=contig00004.g2799;Description=nasopharyngeal carcinoma susceptibility LZ16 [Fusarium coicis]
contig00004.g2800	861	ID=contig00004.g2800;Description=alkylated DNA repair protein alkB like 6 [Fusarium odoratissimum NRRL 54006];Gene=FOC4_g10010525;Ontology_term=nucleus,oxidoreductase activity;Ontology_id=GO:0005634,GO:0016491;Enzyme_code=EC:1,EC:1;Enzyme_name=Oxidoreductases,Oxidoreductases
contig00004.g2801	1034	ID=contig00004.g2801;Description=DFG10 [Fusarium tjaetaba];Gene=FTJAE_2738;Ontology_term=oxidoreductase activity,lipid metabolic process,protein glycosylation,endoplasmic reticulum;Ontology_id=GO:0016491,GO:0006629,GO:0006486,GO:0005783;Enzyme_code=EC:1.3.1.94,EC:1.3.99.5,EC:1.3.1.94,EC:1.3,EC:1,EC:1.3.99.5,EC:1.3.1.22,EC:1.3.1;Enzyme_name=polyprenol reductase,3-oxo-5alpha-steroid 4-dehydrogenase (acceptor),polyprenol reductase,Acting on the CH-CH group of donors,Oxidoreductases,3-oxo-5alpha-steroid 4-dehydrogenase (acceptor),3-oxo-5alpha-steroid 4-dehydrogenase (NADP(+)),Acting on the CH-CH group of donors

contig00004.g2802	2043	ID=contig00004.g2802;Description=beta-1,3 exoglucanase [Fusarium fujikuroi]
contig00004.g2803	3355	ID=contig00004.g2803;Description=translation initiation factor 3 subunit A [Fusarium verticillioides 7600];Gene=TIF32;Ontology_term=translation regulator activity,RNA binding,protein-containing complex assembly,cytoplasmic translation,organelle;Ontology_id=GO:0045182,GO:0003723,GO:0065003,GO:0002181,GO:0043226
contig00004.g2804	337	ID=contig00004.g2804;Description=guanine nucleotide-binding subunit gamma [Fusarium albosuccineum];Gene=FALBO_13196;Ontology_term=signaling;Ontology_id=GO:0023052
contig00004.g2805	469	ID=contig00004.g2805;Description=selenoprotein W-like protein [Fusarium verticillioides 7600]
contig00004.g2806	1225	ID=contig00004.g2806;Description=serine/threonine-protein phosphatase PP1 [Fusarium odoratissimum NRRL 54006];Gene=FOIG_05619;Ontology_term=catalytic activity, acting on a protein,cytoskeleton organization,microtubule organizing center,nucleobase-containing small molecule metabolic process,establishment or maintenance of cell polarity,mitotic nuclear division,chromosome segregation,carbohydrate derivative metabolic process,nucleolus,hydrolase activity;Ontology_id=GO:0140096,GO:0007010,GO:0005815,GO:0055086,GO:0007163,GO:0140014,GO:0007059,GO:1901135,GO:0005730,GO:0016787;Enzyme_code=EC:3.1.3.16,EC:3.1.3.16,EC:3.1,EC:3,EC:3.1.3;Enzyme_name=protein-serine/threonine phosphatase,protein-serine/threonine phosphatase,Acting on ester bonds,Hydrolases,Acting on ester bonds
contig00004.g2807	3578	ID=contig00004.g2807;Description=3 HAT complex component NTO1 [Fusarium tjaetaba];Gene=FANTH_5950;Ontology_term=catalytic activity, acting on a protein,histone binding,chromosome,transferase activity,chromatin organization,nucleoplasm;Ontology_id=GO:0140096,GO:0042393,GO:0005694,GO:0016740,GO:0006325,GO:0005654;Enzyme_code=EC:2.3,EC:2,EC:2.3.2;Enzyme_name=Acyltransferases,Transferases,Acyltransferases
contig00004.g2808	2603	ID=contig00004.g2808;Description=Leucine Rich repeat domain protein [Fusarium tjaetaba]
contig00004.g2809	894	ID=contig00004.g2809;Description=hypothetical protein FVER53590_05354 [Fusarium verticillioides];Gene=FNYG_08332;Ontology_term=membrane;Ontology_id=GO:0016020

contig00004.g2810	1170	ID=contig00004.g2810;Description=pyruvate dehydrogenase complex X precursor dihydrolipoamide acetyltransferase comp [Fusarium tjaetaba];Gene=FNYG_08331;Ontology_term=nucleobase-containing small molecule metabolic process,sulfur compound metabolic process,mitochondrion,structural molecule activity,carbohydrate derivative metabolic process,transferase activity;Ontology_id=GO:0055086,GO:0006790,GO:0005739,GO:0005198,GO:1901135,GO:0016740;Enzyme_code=EC:2.3,EC:2.3,EC:2,EC:2.3.1.12;Enzyme_name=Acyltransferases,Acyltransferases,Transferases,dihydrolipoyllysine-residue acetyltransferase
contig00004.g2811	2240	ID=contig00004.g2811;Description=transcriptional regulatory [Fusarium napiforme];Gene=F25303_11392;Ontology_term=nucleus,DNA binding,regulation of DNA-templated transcription,transcription regulator activity;Ontology_id=GO:0005634,GO:0003677,GO:0006355,GO:0140110
contig00004.g2812	2795	ID=contig00004.g2812;Description=Sexual differentiation process protein isp4 [Fusarium oxysporum];Gene=BFJ70_g8154;Ontology_term=transmembrane transport,Golgi apparatus,transporter activity,plasma membrane;Ontology_id=GO:0055085,GO:0005794,GO:0005215,GO:0005886
contig00004.g2813	2629	ID=contig00004.g2813;Description=Mitochondrial beta-keto-acyl synthase [Fusarium oxysporum];Gene=FVER53590_05360;Ontology_term=mitochondrion,lipid metabolic process,transferase activity;Ontology_id=GO:0005739,GO:0006629,GO:0016740;Enzyme_code=EC:2.3.1.41,EC:2.3.1.41,EC:2.3.1.85,EC:2.3,EC:2,EC:2.3.1.179,EC:2.3.1;Enzyme_name=beta-ketoacyl-[acyl-carrier-protein] synthase I,beta-ketoacyl-[acyl-carrier-protein] synthase I,fatty-acid synthase system,Acyltransferases,Transferases,beta-ketoacyl-[acyl-carrier-protein] synthase II,Acyltransferases
contig00004.g2814	1922	ID=contig00004.g2814;Description=glutathione S-transferase [Fusarium fujikuroi];Gene=FPANT_1815;Ontology_term=oxidoreductase activity,cellular modified amino acid metabolic process,amino acid metabolic process,transferase activity;Ontology_id=GO:0016491,GO:0006575,GO:0006520,GO:0016740;Enzyme_code=EC:2,EC:1.5.1.3,EC:1.5,EC:1,EC:2,EC:1.5.1,EC:1.5.1.3;Enzyme_name=Transferases,dihydrofolate reductase,Acting on the CH-NH group of donors,Oxidoreductases,Transferases,Acting on the CH-NH group of donors,dihydrofolate reductase

contig00004.g2815	1085	ID=contig00004.g2815;Description=oxidoreductase [Fusarium verticillioides 7600];Gene=FPHYL_3070;Ontology_term=oxidoreductase activity;Ontology_id=GO:0016491;Enzyme_code=EC:1,EC:1;Enzyme_name=Oxidoreductases,Oxidoreductases
contig00004.g2816	4899	ID=contig00004.g2816;Description=26S proteasome subunit YTA7 [Fusarium tjaetaba];Gene=FNYG_08323;Ontology_term=ATP-dependent activity,histone binding,nuclear chromosome,regulation of DNA-templated transcription,cytosol,endoplasmic reticulum,chromatin organization,hydrolase activity;Ontology_id=GO:0140657,GO:0042393,GO:0000228,GO:0006355,GO:0005829,GO:0005783,GO:0006325,GO:0016787;Enzyme_code=EC:3.6.1.15,EC:3.6.1,EC:3.6,EC:3,EC:3.6.1.15;Enzyme_name=nucleoside-triphosphate phosphatase,Acting on acid anhydrides,Acting on acid anhydrides,Hydrolases,nucleoside-triphosphate phosphatase
contig00004.g2817	340	ID=contig00004.g2817;Description=hypothetical protein FBEOM_10134 [Fusarium beomiforme]
contig00004.g2818	1549	ID=contig00004.g2818;Description=hypothetical protein FocTR4_00005607 [Fusarium oxysporum f. sp. cubense]
contig00004.g2819	2424	ID=contig00004.g2819;Description=BNI4-like protein [Fusarium coicis];Ontology_term=cytoskeleton,cytokinesis,carbohydrate derivative metabolic process,mitotic cell cycle;Ontology_id=GO:0005856,GO:0000910,GO:1901135,GO:0000278
contig00004.g2820	1575	ID=contig00004.g2820;Description=tRNA-dihydrouridine synthase 1 [Fusarium verticillioides 7600];Gene=FVEG_05370;Ontology_term=nucleus,mRNA metabolic process,oxidoreductase activity,tRNA metabolic process,catalytic activity, acting on RNA;Ontology_id=GO:0005634,GO:0016071,GO:0016491,GO:0006399,GO:0140098;Enzyme_code=EC:1.3.1.89,EC:1.3,EC:1,EC:1.3.1.88,EC:1.3.1.89,EC:1.3.1;Enzyme_name=tRNA-dihydrouridine(47) synthase [NAD(P)(+)],Acting on the CH-CH group of donors,Oxidoreductases,tRNA-dihydrouridine(16/17) synthase [NAD(P)(+)],tRNA-dihydrouridine(47) synthase [NAD(P)(+)],Acting on the CH-CH group of donors
contig00004.g2821	445	ID=contig00004.g2821;Description=pre-mrna splicing factor rna helicase [Fusarium subglutinans];Gene=FCIRC_12127;Ontology_term=ATP-dependent activity,hydrolase activity;Ontology_id=GO:0140657,GO:0016787;Enzyme_code=EC:3;Enzyme_name=Hydrolases
contig00004.g2822	271	ID=contig00004.g2822;Description=hypothetical protein FVEG_15626 [Fusarium verticillioides 7600];Gene=FVEG_15626;Ontology_term=membrane;Ontology_id=GO:0016020

contig00004.g2823	2607	ID=contig00004.g2823;Description=pre-mRNA-splicing factor cwc22 [Fusarium oxysporum];Gene=FNYG_08315;Ontology_term=nucleus,mRNA metabolic process,RNA binding;Ontology_id=GO:0005634,GO:0016071,GO:0003723
contig00004.g2824	346	ID=contig00004.g2824;Description=dolichol phosphate-mannose biosynthesis regulatory [Fusarium flagelliforme];Gene=FANTH_9842;Ontology_term=molecular function regulator activity,lipid metabolic process,endoplasmic reticulum,transferase activity;Ontology_id=GO:0098772,GO:0006629,GO:0005783,GO:0016740;Enzyme_code=EC:2.4;Enzyme_name=Glycosyltransferases
contig00004.g2825	3482	ID=contig00004.g2825;Description=hypothetical protein FVEG_05375 [Fusarium verticillioides 7600];Gene=FNAPI_13953;Ontology_term=autophagy;Ontology_id=GO:0006914
contig00004.g2826	511	ID=contig00004.g2826;Description=fungal specific transcription factor [Fusarium tjaetaba]
contig00004.g2827	2697	ID=contig00004.g2827;Description=Inhibitor of growth 1 [Fusarium subglutinans];Gene=FDENT_7337;Ontology_term=chromosome;Ontology_id=GO:0005694
contig00004.g2828	601	ID=contig00004.g2828;Description=polymer-forming cytoskeletal domain protein [Fusarium tjaetaba]
contig00004.g2829	1131	ID=contig00004.g2829;Description=hypothetical protein FVER53590_05379 [Fusarium verticillioides];Gene=FMEXI_3152;Ontology_term=membrane;Ontology_id=GO:0016020
contig00004.g2830	1420	ID=contig00004.g2830;Description=hypothetical protein FAVG1_07394 [Fusarium avenaceum]
contig00004.g2831	521	ID=contig00004.g2831;Description=hypothetical protein FVER53590_05381 [Fusarium verticillioides]
contig00004.g2832	2213	ID=contig00004.g2832;Description=alpha beta hydrolase fold family [Fusarium denticulatum];Gene=FVER53590_05382;Ontology_term=hydrolase activity;Ontology_id=GO:0016787;Enzyme_code=EC:3,EC:3;Enzyme_name=Hydrolases,Hydrolases
contig00004.g2833	645	ID=contig00004.g2833;Description=50S ribosomal L24e protein [Fusarium tjaetaba];Gene=FOYG_02672;Ontology_term=molecular function regulator activity,RNA binding,protein-containing complex assembly,structural molecule activity,cytosol,ribosome biogenesis,ribosome,nucleolus;Ontology_id=GO:0098772,GO:0003723,GO:0065003,GO:0005198,GO:0005829,GO:0042254,GO:0005840,GO:0005730

contig00004.g2834	421	ID=contig00004.g2834;Description=mitochondrial import inner membrane translocase subunit TIM14 [Fusarium oxysporum f. sp. lycopersici 4287];Gene=FLONG3_3755;Ontology_term=membrane;Ontology_id=GO:0016020
contig00004.g2835	1635	ID=contig00004.g2835;Description=hypothetical protein J7337_002386 [Fusarium musae];Gene=FPANT_9664;Ontology_term=nucleic acid binding;Ontology_id=GO:0003676
contig00004.g2836	2441	ID=contig00004.g2836;Description=ATP-dependent DNA helicase 2 subunit 2 [Fusarium tjaetaba];Gene=FTJAE_9265;Ontology_term=DNA repair,DNA recombination,nuclear chromosome,reproductive process,regulation of DNA-templated transcription,chromatin organization,DNA binding,RNA binding,ATP-dependent activity,cell differentiation,telomere organization,hydrolase activity;Ontology_id=GO:0006281,GO:0006310,GO:0000228,GO:0022414,GO:0006355,GO:0006325,GO:0003677,GO:0003723,GO:0140657,GO:0030154,GO:0032200,GO:0016787;Enzyme_code=EC:3.6.1.15,EC:3.6.1,EC:3.6,EC:3,EC:3.6.1.15;Enzyme_name=nucleoside-triphosphate phosphatase,Acting on acid anhydrides,Acting on acid anhydrides,Hydrolases,nucleoside-triphosphate phosphatase
contig00004.g2837	736	ID=contig00004.g2837;Description=mitochondrial outer membrane protein [Fusarium napiforme];Gene=CEP53_012584;Ontology_term=membrane;Ontology_id=GO:0016020
contig00004.g2838	363	ID=contig00004.g2838;Description=uncharacterized protein FTJAE_9263 [Fusarium tjaetaba]
contig00004.g2839	1074	ID=contig00004.g2839;Description=HAD-like domain-containing protein [Fusarium redolens];Gene=FPCIR_10617;Ontology_term=nucleus,carbohydrate metabolic process,catalytic activity, acting on a protein,detoxification,cytosol,hydrolase activity;Ontology_id=GO:0005634,GO:0005975,GO:0140096,GO:0098754,GO:0005829,GO:0016787;Enzyme_code=EC:3.1.3,EC:3.1.3.18,EC:3.1,EC:3,EC:3.1.3.41,EC:3.1.3,EC:3.1.3.1;Enzyme_name=Acting on ester bonds,phosphoglycolate phosphatase,Acting on ester bonds,Hydrolases,4-nitrophenylphosphatase,Acting on ester bonds,alkaline phosphatase
contig00004.g2840	1220	ID=contig00004.g2840;Description=DUF1295 domain-containing protein [Fusarium pseudoanthophilum];Gene=FMUND_3018;Ontology_term=oxidoreductase activity,lipid metabolic process;Ontology_id=GO:0016491,GO:0006629;Enzyme_code=EC:1;Enzyme_name=Oxidoreductases

contig00004.g2841	2013	ID=contig00004.g2841;Description=DNA topoisomerase III [Fusarium proliferatum];Gene=FOXG_02220;Ontology_term=DNA repair,catalytic activity, acting on DNA,DNA replication,DNA recombination,chromosome segregation,chromosome,nucleus,DNA binding,mitotic nuclear division,telomere organization,meiotic nuclear division,isomerase activity;Ontology_id=GO:0006281,GO:0140097,GO:0006260,GO:0006310,GO:0007059,GO:0005694,GO:0005634,GO:0003677,GO:0140014,GO:0032200,GO:0140013,GO:0016853;Enzyme_code=EC:5.6.2.1,EC:5.6.2.1,EC:5,EC:5.99.1.2;Enzyme_name=DNA topoisomerase,DNA topoisomerase,Isomerases,Other isomerases
contig00004.g2842	1056	ID=contig00004.g2842;Description=DNA-binding proteins Bright/BRCAA1/RBP1 and proteins containing BRIGHT domain [Fusarium oxysporum];Ontology_term=nucleus,DNA binding,regulation of DNA-templated transcription;Ontology_id=GO:0005634,GO:0003677,GO:0006355
contig00004.g2843	2024	ID=contig00004.g2843;Description=pH-response transcription factor pacC/RIM101 [Fusarium verticillioides 7600]
contig00004.g2844	423	ID=contig00004.g2844;Description=hypothetical protein FVEG_05394 [Fusarium verticillioides 7600]
contig00004.g2845	582	ID=contig00004.g2845;Description=hypothetical protein FGADI_3162 [Fusarium gaditjirri];Gene=FOVG_02529;Ontology_term=membrane;Ontology_id=GO:0016020
contig00004.g2846	2435	ID=contig00004.g2846;Description=RNA-binding pumilio [Fusarium coicis];Gene=FDENT_7356;Ontology_term=RNA binding,ribosome biogenesis,nucleolus;Ontology_id=GO:0003723,GO:0042254,GO:0005730
contig00004.g2847	1247	ID=contig00004.g2847;Description=moz represents a chromatin-associated acetyltransferase [Fusarium tjaetaba];Gene=FTJAE_9254;Ontology_term=mitochondrion,transferase activity;Ontology_id=GO:0005739,GO:0016740;Enzyme_code=EC:2,EC:2;Enzyme_name=Transferases,Transferases
contig00004.g2848	850	ID=contig00004.g2848;Description=Kinetochore-associated protein NNF1 [Fusarium oxysporum f. sp. rapae];Gene=HZS61_010127;Ontology_term=mitotic nuclear division,chromosome segregation,nuclear chromosome,meiotic nuclear division;Ontology_id=GO:0140014,GO:0007059,GO:0000228,GO:0140013;Enzyme_code=EC:1.14.18.1;Enzyme_name=tyrosinase

contig00004.g2849	1311	ID=contig00004.g2849;Description=spectrin beta chain [Fusarium denticulatum];Ontology_term=mitochondrion,cytosol;Ontology_id=GO:0005739,GO:0005829
contig00004.g2850	1701	ID=contig00004.g2850;Description=hypothetical protein FVEG_05401 [Fusarium verticillioides 7600];Gene=FPCIR_10606;Ontology_term=RNA binding,organelle;Ontology_id=GO:0003723,GO:0043226
contig00004.g2851	846	ID=contig00004.g2851;Description=mRNA turnover protein 4 [Fusarium verticillioides 7600];Gene=FOPG_02802;Ontology_term=mRNA metabolic process,RNA binding,protein-containing complex assembly,ribosome biogenesis,nucleolus,nucleoplasm;Ontology_id=GO:0016071,GO:0003723,GO:0065003,GO:0042254,GO:0005730,GO:0005654
contig00004.g2852	1513	ID=contig00004.g2852;Description=U1 small nuclear ribonucleoprotein 70kDa [Fusarium verticillioides 7600];Gene=FANTH_12558;Ontology_term=nucleus,mRNA metabolic process,RNA binding,protein-containing complex assembly;Ontology_id=GO:0005634,GO:0016071,GO:0003723,GO:0065003
contig00004.g2853	522	ID=contig00004.g2853;Description=thioredoxin-like protein [Fusarium redolens];Gene=FOPG_02804;Ontology_term=cytoskeleton,mitochondrial gene expression,cytoskeleton organization,protein-containing complex assembly,GTPase activity,mitochondrion,structural molecule activity,ribosome biogenesis,cell adhesion,extracellular region,nucleolus,ribosome;Ontology_id=GO:0005856,GO:0140053,GO:0007010,GO:0065003,GO:0003924,GO:0005739,GO:0005198,GO:0042254,GO:0007155,GO:0005576,GO:0005730,GO:0005840;Enzyme_code=EC:3.6.1.15,EC:3.6.1,EC:3.6,EC:3,EC:3.6.1.15;Enzyme_name=nucleoside-triphosphate phosphatase,Acting on acid anhydrides,Acting on acid anhydrides,Hydrolases,nucleoside-triphosphate phosphatase
contig00004.g2854	1044	ID=contig00004.g2854;Description=triosephosphate isomerase [Fusarium proliferatum];Gene=FVER53263_05405;Ontology_term=molecular transducer activity,carbohydrate metabolic process,generation of precursor metabolites and energy,transmembrane transport,nucleobase-containing small molecule metabolic process,carbohydrate derivative metabolic process,plasma membrane,transporter activity,isomerase activity;Ontology_id=GO:0060089,GO:0005975,GO:0006091,GO:0055085,GO:0055086,GO:1901135,GO:0005886,GO:0005215,GO:0016853;Enzyme_code=EC:5.3.1.1;Enzyme_name=triose-phosphate isomerase

contig00004.g2855	2501	ID=contig00004.g2855;Description=hypothetical protein FMUND_3033 [Fusarium mundagurra]
contig00004.g2856	1496	ID=contig00004.g2856;Description=Sterol regulatory element-binding protein ECM22 [Fusarium odoratissimum];Gene=FocTR4_00005651;Ontology_term=nucleus,regulation of DNA-templated transcription,transcription regulator activity;Ontology_id=GO:0005634,GO:0006355,GO:0140110
contig00004.g2857	1177	ID=contig00004.g2857;Description=hypothetical protein QSH57_007445 [Fusarium oxysporum f. sp. vasinfectum]
contig00004.g2858	2534	ID=contig00004.g2858;Description=hypothetical protein FVER14953_05410 [Fusarium verticillioides];Gene=FOVG_02514;Ontology_term=nucleus,regulation of DNA-templated transcription,transcription regulator activity;Ontology_id=GO:0005634,GO:0006355,GO:0140110
contig00004.g2859	449	ID=contig00004.g2859;Description=uncharacterized protein FTJAE_9242 [Fusarium tjaetaba]
contig00004.g2860	561	ID=contig00004.g2860;Description=hypothetical protein FVEG_15634 [Fusarium verticillioides 7600];Gene=F52700_3852;Ontology_term=membrane;Ontology_id=GO:0016020
contig00004.g2861	342	ID=contig00004.g2861;Description=probable UPF0327 protein NCU06495 [Fusarium proliferatum ET1];Gene=FOMG_04805;Ontology_term=membrane organization,mitochondrion,mitochondrion organization;Ontology_id=GO:0061024,GO:0005739,GO:0007005
contig00004.g2862	4188	ID=contig00004.g2862;Description=nucleolar pre-ribosomal-associated protein [Fusarium tjaetaba];Ontology_term=ribosome biogenesis,nucleolus;Ontology_id=GO:0042254,GO:0005730
contig00004.g2863	1089	ID=contig00004.g2863;Description=3-ketosphinganine reductase [Fusarium tjaetaba];Gene=FVER53263_05415;Ontology_term=lipid droplet,oxidoreductase activity,lipid metabolic process,mitochondrion,carbohydrate derivative metabolic process,endoplasmic reticulum,transferase activity;Ontology_id=GO:0005811,GO:0016491,GO:0006629,GO:0005739,GO:1901135,GO:0005783,GO:0016740;Enzyme_code=EC:1.1.1.102,EC:2.1,EC:1.1,EC:1.6,EC:1,EC:1.1.1.56,EC:2,EC:1.1.1.102,EC:1.1.1,EC:2.1.1,EC:2.1.1.103;Enzyme_name=3-dehydrosphinganine reductase,Transferring one-carbon groups,Acting on the CH-OH group of donors,Acting on NADH or NADPH,Oxidoreductases,ribitol 2-dehydrogenase,Transferases,3-dehydrosphinganine reductase,Acting on the CH-OH group of donors,Transferring one-carbon groups,phosphoethanolamine N-methyltransferase

contig00004.g2864	3758	ID=contig00004.g2864;Description=hypothetical protein FVEG_15635 [Fusarium verticillioides 7600];Gene=FVER53263_05416;Ontology_term=nucleus,regulatory ncRNA-mediated gene silencing,chromatin organization;Ontology_id=GO:0005634,GO:0031047,GO:0006325
contig00004.g2865	1958	ID=contig00004.g2865;Description=1-phosphatidylinositol-4 5-bisphosphate phosphodiesterase 1 [Fusarium pseudocircinatum];Gene=FVEG_05419;Ontology_term=lipid metabolic process,signaling,hydrolase activity;Ontology_id=GO:0006629,GO:0023052,GO:0016787;Enzyme_code=EC:3.1.4.11,EC:3.1.4,EC:3.1,EC:3,EC:3.1.4.11;Enzyme_name=phosphoinositide phospholipase C,Acting on ester bonds,Acting on ester bonds,Hydrolases,phosphoinositide phospholipase C
contig00004.g2866	1007	ID=contig00004.g2866;Description=---NA---
contig00004.g2867	2058	ID=contig00004.g2867;Description=peroxisomal membrane PEX17 [Fusarium coicis]
contig00004.g2868	849	ID=contig00004.g2868;Description=protein-S-isoprenylcysteine O-methyltransferase [Fusarium verticillioides 7600];Gene=FNAPI_7423;Ontology_term=nuclear envelope,catalytic activity, acting on a protein,reproductive process,endoplasmic reticulum,protein maturation,transferase activity;Ontology_id=GO:0005635,GO:0140096,GO:0022414,GO:0005783,GO:0051604,GO:0016740;Enzyme_code=EC:2.1.1.100,EC:2.1,EC:2.1.1.100,EC:2,EC:2.1.1;Enzyme_name=protein-S-isoprenylcysteine O-methyltransferase,Transferring one-carbon groups,protein-S-isoprenylcysteine O-methyltransferase,Transferases,Transferring one-carbon groups
contig00004.g2869	1713	ID=contig00004.g2869;Description=MBOAT, membrane-bound O-acyltransferase family-domain-containing protein [Fusarium redolens];Gene=FVER53263_05426;Ontology_term=endoplasmic reticulum,transferase activity;Ontology_id=GO:0005783,GO:0016740;Enzyme_code=EC:2.3.1,EC:2.3.1.76,EC:2.3,EC:2.3.1.20,EC:2.3.1.75,EC:2,EC:2.3.1;Enzyme_name=Acyltransferases,retinol O-fatty-acyltransferase,Acyltransferases,diacylglycerol O-acyltransferase,long-chain-alcohol O-fatty-acyltransferase,Transferases,Acyltransferases
contig00004.g2870	2205	ID=contig00004.g2870;Description=polyadenylation factor subunit 2 [Fusarium verticillioides 7600];Gene=FPANT_982;Ontology_term=RNA binding,mRNA metabolic process,nuclear chromosome,extracellular region,catalytic activity, acting on RNA,hydrolase activity,nucleoplasm;Ontology_id=GO:0003723,GO:0016071,GO:0000228,GO:0005576,GO:0140098,GO:0016787,GO:0005654;Enzyme_code=EC:3.1,EC:3.1,EC:3;Enzyme_name=Acting on ester bonds,Acting on ester bonds,Hydrolases

contig00004.g2871	1041	ID=contig00004.g2871;Description=endonuclease G, mitochondrial [Fusarium verticillioides 7600];Gene=FVEG_05428;Ontology_term=nucleus,catalytic activity, acting on DNA,mitochondrion,DNA recombination,catalytic activity, acting on RNA,programmed cell death,hydrolase activity;Ontology_id=GO:0005634,GO:0140097,GO:0005739,GO:0006310,GO:0140098,GO:0012501,GO:0016787;Enzyme_code=EC:3.1,EC:3.1,EC:3;Enzyme_name=Acting on ester bonds,Acting on ester bonds,Hydrolases
contig00004.g2872	1727	ID=contig00004.g2872;Description=vacuolar zinc efflux [Fusarium pseudoanthophilum];Gene=FMAN_04855;Ontology_term=transmembrane transport,transporter activity;Ontology_id=GO:0055085,GO:0005215
contig00004.g2873	1562	ID=contig00004.g2873;Description=transcription factor IIIA [Fusarium sp. NRRL 25303]
contig00004.g2874	626	ID=contig00004.g2874;Description=subunit common to RNA polymerases I, II, and III [Purpureocillium takamizusanense];Gene=E4U42_006922;Ontology_term=DNA binding,DNA-templated transcription,tRNA metabolic process,nucleolus,catalytic activity, acting on RNA,transferase activity,nucleoplasm;Ontology_id=GO:0003677,GO:0006351,GO:0006399,GO:0005730,GO:0140098,GO:0016740,GO:0005654;Enzyme_code=EC:2.7.7.6,EC:2.7,EC:2,EC:2.7.7,EC:2.7.7.6;Enzyme_name=DNA-directed RNA polymerase,Transferring phosphorus-containing groups,Transferases,Transferring phosphorus-containing groups,DNA-directed RNA polymerase
contig00004.g2875	965	ID=contig00004.g2875;Description=Alpha/Beta hydrolase protein [Fusarium redolens];Gene=FocTR4_00005676;Ontology_term=detoxification,cytosol,hydrolase activity;Ontology_id=GO:0098754,GO:0005829,GO:0016787;Enzyme_code=EC:3.1.2.12,EC:3.1.1,EC:3.1,EC:3.1.2.12,EC:3,EC:3.1.1,EC:3.1.2;Enzyme_name=S-formylglutathione hydrolase,Acting on ester bonds,Acting on ester bonds,S-formylglutathione hydrolase,Hydrolases,Acting on ester bonds,Acting on ester bonds
contig00004.g2876	2648	ID=contig00004.g2876;Description=docking domain of Afi1 for Arf3 in vesicle trafficking-domain-containing protein [Fusarium redolens];Ontology_term=nucleus,establishment or maintenance of cell polarity,cytokinesis,mitotic cell cycle,plasma membrane;Ontology_id=GO:0005634,GO:0007163,GO:0000910,GO:0000278,GO:0005886
contig00004.g2877	996	ID=contig00004.g2877;Description=myosin regulatory light chain 1 [Fusarium tjaetaba];Gene=FPCIR_13506;Ontology_term=calcium ion binding;Ontology_id=GO:0005509
contig00004.g2878	2607	ID=contig00004.g2878;Description=DnaJ like subfamily C member 7 [Fusarium verticillioides 7600];Gene=FVER53263_05437;Ontology_term=protein folding;Ontology_id=GO:0006457

contig00004.g2879	605	ID=contig00004.g2879;Description=negative transcription regulator [Fusarium tjaetaba];Gene=MED10;Ontology_term=nucleus,regulation of DNA-templated transcription,transcription regulator activity,molecular adaptor activity;Ontology_id=GO:0005634,GO:0006355,GO:0140110,GO:0060090
contig00004.g2880	880	ID=contig00004.g2880;Description=hypothetical protein FVEG_05439 [Fusarium verticillioides 7600]
contig00004.g2881	2406	ID=contig00004.g2881;Description=hypothetical protein FVER53590_05440 [Fusarium verticillioides]
contig00004.g2882	1488	ID=contig00004.g2882;Description=hypothetical protein J7337_002340 [Fusarium musae]
contig00004.g2883	351	ID=contig00004.g2883;Description=3-isopropylmalate dehydrogenase [Fusarium graminearum PH-1];Gene=FAUST_11536;Ontology_term=oxidoreductase activity;Ontology_id=GO:0016491;Enzyme_code=EC:1;Enzyme_name=Oxidoreductases
contig00004.g2884	2151	ID=contig00004.g2884;Description=hypothetical protein FVER14953_05443 [Fusarium verticillioides];Gene=FNAPI_1652;Ontology_term=metal ion binding;Ontology_id=GO:0046872
contig00004.g2885	1926	ID=contig00004.g2885;Description=hypothetical protein FVER14953_05444 [Fusarium verticillioides]
contig00004.g2886	729	ID=contig00004.g2886;Description=hypothetical protein FPANT_967 [Fusarium pseudoanthophilum]
contig00004.g2887	1539	ID=contig00004.g2887;Description=dephospho kinase [Fusarium phyllophilum];Gene=FDENT_6859;Ontology_term=oxidoreductase activity,transferase activity;Ontology_id=GO:0016491,GO:0016740;Enzyme_code=EC:1,EC:2.7,EC:1,EC:2.7,EC:2;Enzyme_name=Oxidoreductases,Transferring phosphorus-containing groups,Oxidoreductases,Transferring phosphorus-containing groups,Transferases
contig00004.g2888	2348	ID=contig00004.g2888;Description=actin [Fusarium pseudoanthophilum];Gene=FNYG_08245;Ontology_term=protein-containing complex assembly,nuclear chromosome,chromatin organization;Ontology_id=GO:0065003,GO:0000228,GO:0006325
contig00004.g2889	1827	ID=contig00004.g2889;Description=major facilitator superfamily transporter [Fusarium tjaetaba];Gene=FVER53263_05448;Ontology_term=transmembrane transport,transporter activity;Ontology_id=GO:0055085,GO:0005215
contig00004.g2890	219	ID=contig00004.g2890;Description=hypothetical protein FVEG_15638 [Fusarium verticillioides 7600]

contig00004.g2891	1656	ID=contig00004.g2891;Description=related to RAY38 protein [Fusarium fujikuroi];Gene=FPCIR_5635;Ontology_term=DNA binding,RNA binding,signaling,telomere organization,programmed cell death;Ontology_id=GO:0003677,GO:0003723,GO:0023052,GO:0032200,GO:0012501
contig00004.g2892	1456	ID=contig00004.g2892;Description=hypothetical protein FVEG_05450 [Fusarium verticillioides 7600]
contig00004.g2893	2232	ID=contig00004.g2893;Description=hypothetical protein J7337_002329 [Fusarium musae]
contig00004.g2894	1526	ID=contig00004.g2894;Description=MEAB-like protein [Fusarium heterosporum];Gene=BKA59DRAFT_510527;Ontology_term=regulation of DNA-templated transcription,transcription regulator activity;Ontology_id=GO:0006355,GO:0140110
contig00004.g2895	458	ID=contig00004.g2895;Description=related to xyloglucan endo-transglycosylase-like protein [Fusarium proliferatum ET1];Gene=FPRO05_06726;Ontology_term=carbohydrate metabolic process,hydrolase activity;Ontology_id=GO:0005975,GO:0016787;Enzyme_code=EC:3.2.1;Enzyme_name=Glycosylases
contig00004.g2896	840	ID=contig00004.g2896;Description=WW domain-containing protein [Fusarium pseudoanthophilum];Gene=3394
contig00004.g2897	2349	ID=contig00004.g2897;Description=hypothetical protein FVER14953_05455 [Fusarium verticillioides];Gene=BFJ69_g6143;Ontology_term=regulation of DNA-templated transcription,nuclear chromosome,ribosome biogenesis,chromatin organization;Ontology_id=GO:0006355,GO:0000228,GO:0042254,GO:0006325
contig00004.g2898	3753	ID=contig00004.g2898;Description=cargo-transport ypp1 [Fusarium pseudocircinatum]
contig00004.g2899	3119	ID=contig00004.g2899;Description=pre-tRNA nuclear export protein [Fusarium musae];Gene=FPHYL_11181;Ontology_term=nuclear envelope,nucleocytoplasmic transport,RNA binding,mitochondrion,tRNA metabolic process,intracellular protein transport;Ontology_id=GO:0005635,GO:0006913,GO:0003723,GO:0005739,GO:0006399,GO:0006886
contig00004.g2900	1066	ID=contig00004.g2900;Description=gpi anchored serine-rich protein [Fusarium tjaetaba];Gene=FPCIR_5625;Ontology_term=membrane;Ontology_id=GO:0016020
contig00004.g2901	1655	ID=contig00004.g2901;Description=neutral amino acid permease [Fusarium napiforme];Gene=FPCIR_5624;Ontology_term=membrane;Ontology_id=GO:0016020

contig00004.g2902	954	ID=contig00004.g2902;Description=RTA1 like family [Fusarium tjaetaba];Gene=FMEXI_12957;Ontology_term=membrane organization,transmembrane transport,ATP-dependent activity,endoplasmic reticulum,vacuole,transporter activity,plasma membrane,hydrolase activity;Ontology_id=GO:0061024,GO:0055085,GO:0140657,GO:0005783,GO:0005773,GO:0005215,GO:0005886,GO:0016787;Enzyme_code=EC:3.6.1,EC:3.6,EC:7.6.2.1,EC:3,EC:7,EC:7.2.2,EC:3.6.1.15;Enzyme_name=Acting on acid anhydrides,Acting on acid anhydrides,P-type phospholipid transporter,Hydrolases,Translocases,Catalysing the translocation of inorganic cations,nucleoside-triphosphate phosphatase
contig00004.g2903	1533	ID=contig00004.g2903;Description=aspartyl aminopeptidase [Fusarium verticillioides 7600];Gene=FVEG_05462;Ontology_term=catalytic activity, acting on a protein,protein folding,extracellular region,vacuole,hydrolase activity;Ontology_id=GO:0140096,GO:0006457,GO:0005576,GO:0005773,GO:0016787;Enzyme_code=EC:3.4.11,EC:3.4.11,EC:3.4,EC:3,EC:3.4.11.21;Enzyme_name=Acting on peptide bonds (peptidases),Acting on peptide bonds (peptidases),Acting on peptide bonds (peptidases),Hydrolases,aspartyl aminopeptidase
contig00004.g2904	756	ID=contig00004.g2904;Description=whi5 like domain protein [Fusarium subglutinans];Gene=FMAN_04888;Ontology_term=nucleus,mitochondrion;Ontology_id=GO:0005634,GO:0005739
contig00004.g2905	4614	ID=contig00004.g2905;Description=gall11 coactivator [Fusarium tjaetaba]
contig00004.g2906	1847	ID=contig00004.g2906;Description=hypothetical protein FVEG_05465 [Fusarium verticillioides 7600];Gene=FPANT_10313;Ontology_term=membrane organization,molecular function regulator activity,vesicle-mediated transport,protein-containing complex assembly,endoplasmic reticulum,Golgi apparatus;Ontology_id=GO:0061024,GO:0098772,GO:0016192,GO:0065003,GO:0005783,GO:0005794
contig00004.g2907	1677	ID=contig00004.g2907;Description=hypothetical protein FVEG_05466 [Fusarium verticillioides 7600];Gene=FPCIR_5618;Ontology_term=regulation of DNA-templated transcription,transcription regulator activity;Ontology_id=GO:0006355,GO:0140110
contig00004.g2908	1010	ID=contig00004.g2908;Description=senescence marker protein 30 [Fusarium fujikuroi];Gene=2512
contig00004.g2909	2184	ID=contig00004.g2909;Description=hypothetical protein FVEG_05468 [Fusarium verticillioides 7600]

contig00004.g2910	814	ID=contig00004.g2910;Description=ATP synthase subunit delta mitochondrial [Fusarium tjaetaba];Gene=FTJAE_6760;Ontology_term=generation of precursor metabolites and energy,transmembrane transport,nucleobase-containing small molecule metabolic process,mitochondrion,carbohydrate derivative metabolic process;Ontology_id=GO:0006091,GO:0055085,GO:0055086,GO:0005739,GO:1901135
contig00004.g2911	1666	ID=contig00004.g2911;Description=alpha-latrocrustotoxin-Lt1a [Fusarium tjaetaba]
contig00004.g2912	648	ID=contig00004.g2912;Description=alpha-latrocrustotoxin-Lt1a [Fusarium tjaetaba]
contig00004.g2913	2142	ID=contig00004.g2913;Description=GTP-binding protein SAR1 [Fusarium coicis];Ontology_term=membrane organization,vesicle-mediated transport,protein-containing complex assembly,GTPase activity,signaling,cytoplasmic vesicle,endoplasmic reticulum,intracellular protein transport,mitochondrion organization;Ontology_id=GO:0061024,GO:0016192,GO:0065003,GO:0003924,GO:0023052,GO:0031410,GO:0005783,GO:0006886,GO:0007005;Enzyme_code=EC:3.6.1,EC:3.6,EC:3,EC:3.6.1.15;Enzyme_name=Acting on acid anhydrides,Acting on acid anhydrides,Hydrolases,nucleoside-triphosphate phosphatase
contig00004.g2914	850	ID=contig00004.g2914;Description=ADP-ribosylation factor family-domain-containing protein [Fusarium sp. MPI-SDFR-AT-0072];Gene=FPHYL_11072;Ontology_term=membrane organization,protein-containing complex assembly,Golgi apparatus,endoplasmic reticulum,intracellular protein transport,mitochondrion organization,vesicle-mediated transport,GTPase activity,signaling,lipid metabolic process,mitochondrion,cytoplasmic vesicle;Ontology_id=GO:0061024,GO:0065003,GO:0005794,GO:0005783,GO:0006886,GO:0007005,GO:0016192,GO:0003924,GO:0023052,GO:0006629,GO:0005739,GO:0031410;Enzyme_code=EC:3.6.1.15,EC:3.1.1.3,EC:3.6.1,EC:3.1,EC:3.6,EC:3,EC:3.6.1.15,EC:3.1.1,EC:3.1.1.3;Enzyme_name=nucleoside-triphosphate phosphatase,triacylglycerol lipase,Acting on acid anhydrides,Acting on ester bonds,Acting on acid anhydrides,Hydrolases,nucleoside-triphosphate phosphatase,Acting on ester bonds,triacylglycerol lipase
contig00004.g2915	2363	ID=contig00004.g2915;Description=probable lipase 5 [Fusarium fujikuroi];Gene=FPHYL_11071;Ontology_term=lipid droplet,lipid metabolic process,detoxification,hydrolase activity;Ontology_id=GO:0005811,GO:0006629,GO:0098754,GO:0016787;Enzyme_code=EC:3.1.1.3,EC:3.1,EC:3,EC:3.1.1,EC:3.1.1.3;Enzyme_name=triacylglycerol lipase,Acting on ester bonds,Hydrolases,Acting on ester bonds,triacylglycerol lipase

contig00004.g2916	2259	ID=contig00004.g2916;Description=translocation protein SEC63 [Fusarium fujikuroi];Gene=FGLOB1_8778;Ontology_term=nuclear envelope,transmembrane transport,mitochondrion,endoplasmic reticulum,intracellular protein transport,transporter activity;Ontology_id=GO:0005635,GO:0055085,GO:0005739,GO:0005783,GO:0006886,GO:0005215
contig00004.g2917	1958	ID=contig00004.g2917;Description=LCHN [Fusarium tjaetaba];Ontology_term=lipid droplet,endoplasmic reticulum;Ontology_id=GO:0005811,GO:0005783
contig00004.g2918	961	ID=contig00004.g2918;Description=1-acylglycerol-3-phosphate O-acyltransferase [Fusarium oxysporum];Gene=FPANT_9917;Ontology_term=lipid droplet,lipid metabolic process,endoplasmic reticulum,transferase activity;Ontology_id=GO:0005811,GO:0006629,GO:0005783,GO:0016740;Enzyme_code=EC:2.3.1.51,EC:2.3.1.51,EC:2.3,EC:2,EC:2.3.1;Enzyme_name=1-acylglycerol-3-phosphate O-acyltransferase,1-acylglycerol-3-phosphate O-acyltransferase,Acyltransferases,Transferases,Acyltransferases
contig00004.g2919	5305	ID=contig00004.g2919;Description=hypothetical protein FVER14953_05479 [Fusarium verticillioides];Gene=FNYG_08214;Ontology_term=cytoskeletal protein binding;Ontology_id=GO:0008092
contig00004.g2920	816	ID=contig00004.g2920;Description=ADP-ribosylation factor-like 2 [Fusarium mundagurra];Gene=HYE68_005046;Ontology_term=cytoskeleton,protein-containing complex assembly,GTPase activity;Ontology_id=GO:0005856,GO:0065003,GO:0003924;Enzyme_code=EC:3.6.1.15,EC:3.6.1,EC:3.6,EC:3,EC:3.6.1.15;Enzyme_name=nucleoside-triphosphate phosphatase,Acting on acid anhydrides,Acting on acid anhydrides,Hydrolases,nucleoside-triphosphate phosphatase
contig00004.g2921	1773	ID=contig00004.g2921;Description=acyl n-acyltransferase [Fusarium tjaetaba];Gene=FTJAE_6770;Ontology_term=transferase activity;Ontology_id=GO:0016740;Enzyme_code=EC:2.3,EC:2.3,EC:2;Enzyme_name=Acyltransferases,Acyltransferases,Transferases
contig00004.g2922	1860	ID=contig00004.g2922;Description=hypothetical protein FVEG_05482 [Fusarium verticillioides 7600]

contig00004.g2923	2373	ID=contig00004.g2923;Description=CMGC/CLK protein kinase [Fusarium proliferatum];Gene=BFJ72_g5904;Ontology_term=nucleus,catalytic activity, acting on a protein,mRNA metabolic process,signaling,regulation of DNA-templated transcription,transferase activity,mitotic cell cycle;Ontology_id=GO:0005634,GO:0140096,GO:0016071,GO:0023052,GO:0006355,GO:0016740,GO:0000278;Enzyme_code=EC:2.7.1,EC:2.7.1,EC:2.7.12.1,EC:2.7.11.1,EC:2.7,EC:2;Enzyme_name=Transferring phosphorus-containing groups,Transferring phosphorus-containing groups,dual-specificity kinase,non-specific serine/threonine protein kinase,Transferring phosphorus-containing groups,Transferases
contig00004.g2924	1112	ID=contig00004.g2924;Description=tricarboxylate carrier [Fusarium bulbicola];Gene=FNAPI_6091;Ontology_term=transmembrane transport,mitochondrion,transporter activity;Ontology_id=GO:0055085,GO:0005739,GO:0005215
contig00004.g2925	2520	ID=contig00004.g2925;Description=ribonuclease Z [Fusarium verticillioides 7600];Gene=FVER53590_05485;Ontology_term=nucleus,mRNA metabolic process,mitochondrial gene expression,mitochondrion,regulatory ncRNA-mediated gene silencing,tRNA metabolic process,catalytic activity, acting on RNA,hydrolase activity;Ontology_id=GO:0005634,GO:0016071,GO:0140053,GO:0005739,GO:0031047,GO:0006399,GO:0140098,GO:0016787;Enzyme_code=EC:3.1.30,EC:3.1.26.11,EC:3.1.30,EC:3.1,EC:3.1.26.11,EC:3,EC:3.1.26;Enzyme_name=Acting on ester bonds,ribonuclease Z,Acting on ester bonds,Acting on ester bonds,ribonuclease Z,Hydrolases,Acting on ester bonds
contig00004.g2926	864	ID=contig00004.g2926;Description=hypothetical protein FVEG_05486 [Fusarium verticillioides 7600];Gene=FPCIR_11352;Ontology_term=oxidoreductase activity;Ontology_id=GO:0016491;Enzyme_code=EC:1,EC:1;Enzyme_name=Oxidoreductases,Oxidoreductases
contig00004.g2927	1794	ID=contig00004.g2927;Description=3 5 cyclic-nucleotide phosphodiesterase [Fusarium tjaetaba];Gene=nuoD;Ontology_term=nucleobase-containing small molecule metabolic process,signaling,carbohydrate derivative metabolic process,mitotic cell cycle,hydrolase activity;Ontology_id=GO:0055086,GO:0023052,GO:1901135,GO:0000278,GO:0016787;Enzyme_code=EC:3.1.4.17,EC:3.1.4.53,EC:3.1.4,EC:3.1.4.17,EC:3.1,EC:3.1.4.35,EC:3,EC:3.1.4.53;Enzyme_name=3',5'-cyclic-nucleotide phosphodiesterase,3',5'-cyclic-AMP phosphodiesterase,Acting on ester bonds,3',5'-cyclic-nucleotide phosphodiesterase,Acting on ester bonds,3',5'-cyclic-GMP phosphodiesterase,Hydrolases,3',5'-cyclic-AMP phosphodiesterase

contig00004.g2928	2985	ID=contig00004.g2928;Description=hypothetical protein FVER14953_05488 [Fusarium verticillioides]
contig00004.g2929	5809	ID=contig00004.g2929;Description=putative NRPS-like protein biosynthetic cluster [Fusarium musae];Gene=FNAPI_6096;Ontology_term=ligase activity;Ontology_id=GO:0016874;Enzyme_code=EC:6;Enzyme_name=Ligases
contig00004.g2930	3447	ID=contig00004.g2930;Description=actin cytoskeleton organization biogenesis [Fusarium napiforme];Gene=FPANT_9906;Ontology_term=cytoskeleton,cytoskeleton organization,lipid metabolic process,hydrolase activity;Ontology_id=GO:0005856,GO:0007010,GO:0006629,GO:0016787;Enzyme_code=EC:3.1.3.4,EC:3.1,EC:3,EC:3.1.3.4,EC:3.1.3;Enzyme_name=phosphatidate phosphatase,Acting on ester bonds,Hydrolases,phosphatidate phosphatase,Acting on ester bonds
contig00004.g2931	2125	ID=contig00004.g2931;Description=2-isopropylmalate synthase [Fusarium verticillioides 7600];Gene=FVEG_05491;Ontology_term=amino acid metabolic process,transferase activity;Ontology_id=GO:0006520,GO:0016740;Enzyme_code=EC:2.3.3.13;Enzyme_name=2-isopropylmalate synthase
contig00004.g2932	688	ID=contig00004.g2932;Description=hypothetical protein FVEG_05492 [Fusarium verticillioides 7600]
contig00004.g2933	2434	ID=contig00004.g2933;Description=nucleolar DEAD-box protein required for synthesis of 60S ribosomal subunit [Fusarium musae];Gene=FVER53590_05493;Ontology_term=RNA binding,protein-containing complex assembly,ATP-dependent activity,ribosome biogenesis,catalytic activity, acting on RNA,nucleolus,hydrolase activity;Ontology_id=GO:0003723,GO:0065003,GO:0140657,GO:0042254,GO:0140098,GO:0005730,GO:0016787;Enzyme_code=EC:3.6.4.13,EC:3.6.1,EC:3.6.4.13,EC:3.6,EC:3,EC:3.6.1.15;Enzyme_name=RNA helicase,Acting on acid anhydrides,RNA helicase,Acting on acid anhydrides,Hydrolases,nucleoside-triphosphate phosphatase
contig00004.g2934	1097	ID=contig00004.g2934;Description=RP/EB family microtubule-associated protein [Fusarium proliferatum];Gene=FDENT_6906;Ontology_term=nucleus,molecular function regulator activity,microtubule organizing center,mitotic nuclear division,chromosome segregation,cytoskeletal protein binding,microtubule-based movement,molecular adaptor activity,meiotic nuclear division;Ontology_id=GO:0005634,GO:0098772,GO:0005815,GO:0140014,GO:0007059,GO:0008092,GO:0007018,GO:0060090,GO:0140013

contig00004.g2935	1086	ID=contig00004.g2935;Description=vacuolar morphogenesis 7 like [Fusarium subglutinans];Gene=FCIRC_13726;Ontology_term=membrane organization,endosome,vesicle-mediated transport,reproductive process,lipid binding,intracellular protein transport,vacuole,molecular adaptor activity;Ontology_id=GO:0061024,GO:0005768,GO:0016192,GO:0022414,GO:0008289,GO:0006886,GO:0005773,GO:0060090
contig00004.g2936	2499	ID=contig00004.g2936;Description=3-hydroxyacyl-CoA dehydrogenase-like protein LAM1 [Fusarium oxysporum f. sp. albedinis];Gene=FACUT_5901;Ontology_term=membrane;Ontology_id=GO:0016020
contig00004.g2937	541	ID=contig00004.g2937;Description=thioredoxin 1 [Fusarium verticillioides 7600]
contig00004.g2938	1017	ID=contig00004.g2938;Description=zinc-binding alcohol dehydrogenase domain protein [Fusarium tjaetaba];Gene=FPANT_9808;Ontology_term=oxidoreductase activity;Ontology_id=GO:0016491;Enzyme_code=EC:1.6,EC:1.6,EC:1;Enzyme_name=Acting on NADH or NADPH,Acting on NADH or NADPH,Oxidoreductases
contig00004.g2939	537	ID=contig00004.g2939;Description=cytochrome P450 monooxygenase [Fusarium tjaetaba];Gene=FPANT_9807;Ontology_term=oxidoreductase activity;Ontology_id=GO:0016491;Enzyme_code=EC:1.14,EC:1,EC:1.14;Enzyme_name=Acting on paired donors, with incorporation or reduction of molecular oxygen. The oxygen incorporated need not be derived from O2,Oxidoreductases,Acting on paired donors, with incorporation or reduction of molecular oxygen. The oxygen incorporated need not be derived from O2
contig00004.g2940	882	ID=contig00004.g2940;Description=cytochrome P450 monooxygenase [Fusarium coicis];Gene=FPANT_9807;Ontology_term=oxidoreductase activity;Ontology_id=GO:0016491;Enzyme_code=EC:1.14,EC:1,EC:1.14;Enzyme_name=Acting on paired donors, with incorporation or reduction of molecular oxygen. The oxygen incorporated need not be derived from O2,Oxidoreductases,Acting on paired donors, with incorporation or reduction of molecular oxygen. The oxygen incorporated need not be derived from O2
contig00004.g2941	313	ID=contig00004.g2941;Description=major facilitator superfamily domain-containing protein [Fusarium sp. MPI-SDFR-AT-0072];Gene=FOC4_g10010689;Ontology_term=transmembrane transport,transporter activity;Ontology_id=GO:0055085,GO:0005215

contig00004.g2942	804	ID=contig00004.g2942;Description=cycloeucalenol cycloisomerase [Fusarium tjaetaba];Gene=FTJAE_6791;Ontology_term=isomerase activity;Ontology_id=GO:0016853;Enzyme_code=EC:5.5.1.9,EC:5,EC:5.5.1.9,EC:5.5.1;Enzyme_name=cycloeucalenol cycloisomerase,Isomerases,cycloeucalenol cycloisomerase,Intramolecular lyases
contig00004.g2943	932	ID=contig00004.g2943;Description=hypothetical protein FVEG_15654 [Fusarium verticillioides 7600];Gene=FNAPI_13570;Ontology_term=nucleus,regulation of DNA-templated transcription,transcription regulator activity;Ontology_id=GO:0005634,GO:0006355,GO:0140110
contig00004.g2944	855	ID=contig00004.g2944;Description=multidrug resistant protein [Fusarium pseudoanthophilum];Gene=FNYG_08190;Ontology_term=transmembrane transport,transporter activity;Ontology_id=GO:0055085,GO:0005215
contig00004.g2945	916	ID=contig00004.g2945;Description=proline iminopeptidase [Fusarium coicis];Gene=FNAPI_13568;Ontology_term=catalytic activity;Ontology_id=GO:0003824;Enzyme_code=EC:3.4.11.5;Enzyme_name=prolyl aminopeptidase
contig00004.g2946	1365	ID=contig00004.g2946;Description=hypothetical protein FVEG_15656 [Fusarium verticillioides 7600]
contig00004.g2947	1014	ID=contig00004.g2947;Description=hypothetical protein FVEG_15658 [Fusarium verticillioides 7600]
contig00004.g2948	1434	ID=contig00004.g2948;Description=hypothetical protein FVEG_05502 [Fusarium verticillioides 7600]
contig00004.g2949	927	ID=contig00004.g2949;Description=WD repeat-containing protein [Fusarium subglutinans]
contig00004.g2950	1132	ID=contig00004.g2950;Description=alcohol dehydrogenase groES-like domain-containing protein [Trichoderma breve];Gene=THAR02_04686;Ontology_term=oxidoreductase activity;Ontology_id=GO:0016491;Enzyme_code=EC:1.1.1,EC:1.1,EC:1,EC:1.1.1,EC:1.1.1.2;Enzyme_name=Acting on the CH-OH group of donors,Acting on the CH-OH group of donors,Oxidoreductases,Acting on the CH-OH group of donors,alcohol dehydrogenase (NADP(+))
contig00004.g2951	501	ID=contig00004.g2951;Description=NADP-dependent alcohol dehydrogenase [Fusarium tjaetaba];Gene=FVEG_05505;Ontology_term=nucleus;Ontology_id=GO:0005634
contig00004.g2952	2226	ID=contig00004.g2952;Description=f-box WD-repeat [Fusarium tjaetaba]

contig00004.g2953	2439	ID=contig00004.g2953;Description=beta-1 3 exoglucanase precursor [Fusarium pseudoanthophilum];Gene=FVER53590_05507;Ontology_term=hydrolase activity;Ontology_id=GO:0016787;Enzyme_code=EC:3.2.1.15,EC:3.2,EC:3,EC:3.2.1.58,EC:3.2.1.15,EC:3.2.1;Enzyme_name=endo-polygalacturonase,Glycosylases,Hydrolases,glucan 1,3-beta-glucosidase,endo-polygalacturonase,Glycosylases
contig00004.g2954	1032	ID=contig00004.g2954;Description=SCS3 inositol phospholipid synthesis [Fusarium subglutinans];Gene=CAF20;Ontology_term=translation regulator activity,lipid metabolic process,endoplasmic reticulum,hydrolase activity;Ontology_id=GO:0045182,GO:0006629,GO:0005783,GO:0016787;Enzyme_code=EC:3.6.1,EC:3.6.1,EC:3.6,EC:2.7.7.53,EC:3;Enzyme_name=Acting on acid anhydrides,Acting on acid anhydrides,Acting on acid anhydrides,ATP adenylyltransferase,Hydrolases
contig00004.g2955	1353	ID=contig00004.g2955;Description=related to amidohydrolase AmhX [Fusarium mangiferae];Gene=FACUT_5883;Ontology_term=catalytic activity, acting on a protein,hydrolase activity;Ontology_id=GO:0140096,GO:0016787;Enzyme_code=EC:3.4.13,EC:3.4.13,EC:3.4,EC:3;Enzyme_name=Acting on peptide bonds (peptidases),Acting on peptide bonds (peptidases),Acting on peptide bonds (peptidases),Hydrolases
contig00004.g2956	534	ID=contig00004.g2956;Description=hypothetical protein FVEG_15661 [Fusarium verticillioides 7600]
contig00004.g2957	1183	ID=contig00004.g2957;Description=zinc-type alcohol dehydrogenase [Fusarium coicis];Gene=FNYG_15762;Ontology_term=oxidoreductase activity;Ontology_id=GO:0016491;Enzyme_code=EC:1,EC:1;Enzyme_name=Oxidoreductases,Oxidoreductases
contig00004.g2958	1719	ID=contig00004.g2958;Description=beta-tubulin [Fusarium fujikuroi IMI 58289];Gene=FOXB_00532;Ontology_term=cytoskeleton,cytoskeleton organization,GTPase activity,structural molecule activity;Ontology_id=GO:0005856,GO:0007010,GO:0003924,GO:0005198;Enzyme_code=EC:3.6.1.15,EC:3.6.1,EC:3.6,EC:3,EC:3.6.1.15;Enzyme_name=nucleoside-triphosphate phosphatase,Acting on acid anhydrides,Acting on acid anhydrides,Hydrolases,nucleoside-triphosphate phosphatase
contig00004.g2959	2282	ID=contig00004.g2959;Description=alkaline phosphatase [Fusarium verticillioides 7600];Enzyme_code=EC:3.1.3.1;Enzyme_name=alkaline phosphatase
contig00004.g2960	1026	ID=contig00004.g2960;Description=Altered inheritance of mitochondria [Fusarium agapanthi];Gene=spoVS

contig00004.g2961	2326	ID=contig00004.g2961;Description=TOB3 (member of AAA-ATPase family) [Fusarium tjaetaba];Gene=FVER53263_05515;Ontology_term=ATP-dependent activity,hydrolase activity;Ontology_id=GO:0140657,GO:0016787;Enzyme_code=EC:3.6.1.15,EC:3.6.1,EC:3.6,EC:3,EC:3.6.1.15;Enzyme_name=nucleoside-triphosphate phosphatase,Acting on acid anhydrides,Acting on acid anhydrides,Hydrolases,nucleoside-triphosphate phosphatase
contig00004.g2962	1632	ID=contig00004.g2962;Description=acetolactate synthase [Fusarium tjaetaba];Gene=FTJAE_8187;Ontology_term=lyase activity;Ontology_id=GO:0016829;Enzyme_code=EC:4.1.1.1,EC:4.1.1,EC:4.1.1.1,EC:4.1,EC:4;Enzyme_name=pyruvate decarboxylase,Carbon-carbon lyases,pyruvate decarboxylase,Carbon-carbon lyases,Lyases
contig00004.g2963	772	ID=contig00004.g2963;Description=hypothetical protein FVER14953_05516 [Fusarium verticillioides];Gene=FVEG_05516;Ontology_term=transferase activity;Ontology_id=GO:0016740;Enzyme_code=EC:2.3.1,EC:2.3,EC:2,EC:2.3.1;Enzyme_name=Acyltransferases,Acyltransferases,Transferases,Acyltransferases
contig00004.g2964	1797	ID=contig00004.g2964;Description=ATP adenylyltransferase [Fusarium verticillioides 7600];Gene=FVEG_05517;Ontology_term=nucleus,nucleobase-containing small molecule metabolic process,carbohydrate derivative metabolic process,transferase activity,hydrolase activity;Ontology_id=GO:0005634,GO:0055086,GO:1901135,GO:0016740,GO:0016787;Enzyme_code=EC:2.7.7.53,EC:3.6.1,EC:2.7.7.5,EC:2.7,EC:3.6,EC:2,EC:2.7.7.53,EC:3,EC:2.7.7;Enzyme_name=ATP adenylyltransferase,Acting on acid anhydrides,sulfate adenylyltransferase (ADP),Transferring phosphorus-containing groups,Acting on acid anhydrides,Transferases,ATP adenylyltransferase,Hydrolases,Transferring phosphorus-containing groups
contig00004.g2965	662	ID=contig00004.g2965;Description=hypothetical protein FVEG_15665 [Fusarium verticillioides 7600];Gene=FACUT_13100
contig00004.g2966	1426	ID=contig00004.g2966;Description=hypothetical protein FVER14953_05518 [Fusarium verticillioides]
contig00004.g2967	1009	ID=contig00004.g2967;Description=mannose 6-phosphate receptor-like protein [Fusarium tjaetaba];Gene=FVEG_05519;Ontology_term=endosome,vesicle-mediated transport;Ontology_id=GO:0005768,GO:0016192

contig00004.g2968	1575	ID=contig00004.g2968;Description=Delta(14)-sterol reductase [Fusarium proliferatum];Gene=FSUBG_478;Ontology_term=oxidoreductase activity,lipid metabolic process;Ontology_id=GO:0016491,GO:0006629;Enzyme_code=EC:1.3.1,EC:1.3,EC:1,EC:1.3.1.70,EC:1.3.1;Enzyme_name=Acting on the CH-CH group of donors,Acting on the CH-CH group of donors,Oxidoreductases,Delta(14)-sterol reductase,Acting on the CH-CH group of donors
contig00004.g2969	2760	ID=contig00004.g2969;Description=beta-glucosidase [Fusarium verticillioides 7600];Gene=FPCIR_2855;Ontology_term=carbohydrate metabolic process,hydrolase activity;Ontology_id=GO:0005975,GO:0016787;Enzyme_code=EC:3.2.1.21,EC:3.2,EC:3.2.1.21,EC:3,EC:3.2.1;Enzyme_name=beta-glucosidase,Glycosylases,beta-glucosidase,Hydrolases,Glycosylases
contig00004.g2970	1467	ID=contig00004.g2970;Description=hypothetical protein FVER53263_21055 [Fusarium verticillioides]
contig00004.g2971	406	ID=contig00004.g2971;Description=hypothetical protein FVEG_05523 [Fusarium verticillioides 7600]
contig00004.g2972	276	ID=contig00004.g2972;Description=hypothetical protein FVEG_05524 [Fusarium verticillioides 7600];Gene=FGLOB1_10188;Ontology_term=membrane;Ontology_id=GO:0016020
contig00004.g2973	1574	ID=contig00004.g2973;Description=UPF0052 domain-containing protein [Fusarium napiforme];Gene=FVER53263_05525;Ontology_term=transferase activity;Ontology_id=GO:0016740;Enzyme_code=EC:2.7.1,EC:2.7.8.28,EC:2.7.1,EC:2.7,EC:2,EC:2.7.8.28;Enzyme_name=Transferring phosphorus-containing groups,2-phospho-L-lactate transferase,Transferring phosphorus-containing groups,Transferring phosphorus-containing groups,Transferases,2-phospho-L-lactate transferase
contig00004.g2974	1113	ID=contig00004.g2974;Description=alcohol dehydrogenase [Fusarium verticillioides 7600];Gene=FPHYL_12527;Ontology_term=generation of precursor metabolites and energy,oxidoreductase activity;Ontology_id=GO:0006091,GO:0016491;Enzyme_code=EC:1,EC:1.1,EC:1,EC:1.1.1,EC:1.1.1.1,EC:1.1.1.71;Enzyme_name=Oxidoreductases,Acting on the CH-OH group of donors,Oxidoreductases,Acting on the CH-OH group of donors,alcohol dehydrogenase,alcohol dehydrogenase [NAD(P)(+)]
contig00004.g2975	750	ID=contig00004.g2975;Description=gpi anchored serine-threonine rich protein [Fusarium subglutinans];Gene=BFJ68_g4142;Ontology_term=membrane;Ontology_id=GO:0016020
contig00004.g2976	1112	ID=contig00004.g2976;Description=isoflavone reductase like IRL [Fusarium tjaetaba]

contig00004.g2977	1744	ID=contig00004.g2977;Description=catalase [Fusarium oxysporum f. sp. lycopersici 4287];Gene=HZS61_010247;Ontology_term=antioxidant activity,oxidoreductase activity;Ontology_id=GO:0016209,GO:0016491;Enzyme_code=EC:1.11.1.6;Enzyme_name=catalase
contig00004.g2978	918	ID=contig00004.g2978;Description=Alpha/Beta hydrolase protein [Fusarium oxysporum Fo47];Gene=3471;Ontology_term=antioxidant activity,oxidoreductase activity;Ontology_id=GO:0016209,GO:0016491;Enzyme_code=EC:1.11.1.6,EC:1.11,EC:1,EC:1.11.1,EC:1.11.1.6;Enzyme_name=catalase,Acting on a peroxide as acceptor,Oxidoreductases,Acting on a peroxide as acceptor,catalase
contig00004.g2979	1242	ID=contig00004.g2979;Description=hypothetical protein FCOIX_12238 [Fusarium coicis];Gene=FDENT_8785;Ontology_term=membrane;Ontology_id=GO:0016020
contig00004.g2980	1668	ID=contig00004.g2980;Description=cercosporin resistance protein [Fusarium denticulatum];Gene=FNYG_04532;Ontology_term=nucleus,regulation of DNA-templated transcription,transcription regulator activity;Ontology_id=GO:0005634,GO:0006355,GO:0140110
contig00004.g2981	1393	ID=contig00004.g2981;Description=transforming growth factor-beta-induced ig-h3 [Fusarium sp. NRRL 25303];Gene=2581
contig00004.g2982	1085	ID=contig00004.g2982;Description=transforming growth factor-beta-induced ig-h3 [Fusarium pseudoanthophilum]
contig00004.g2983	837	ID=contig00004.g2983;Description=hypothetical protein FVEG_05535 [Fusarium verticillioides 7600];Gene=FTJAE_6033;Ontology_term=membrane;Ontology_id=GO:0016020
contig00004.g2984	2502	ID=contig00004.g2984;Description=hypothetical protein FVER53590_05536 [Fusarium verticillioides]
contig00004.g2985	7674	ID=contig00004.g2985;Description=Type I Iterative PKS [Fusarium musae];Gene=FPANT_8593;Ontology_term=oxidoreductase activity,lipid metabolic process,transferase activity;Ontology_id=GO:0016491,GO:0006629,GO:0016740;Enzyme_code=EC:2.3.1.41,EC:1,EC:2.1.1,EC:2.3.1.41,EC:2.1,EC:2.3,EC:1,EC:2,EC:2.1.1,EC:2.3.1;Enzyme_name=beta-ketoacyl-[acyl-carrier-protein] synthase I,Oxidoreductases,Transferring one-carbon groups,beta-ketoacyl-[acyl-carrier-protein] synthase I,Transferring one-carbon groups,Acyltransferases,Oxidoreductases,Transferases,Transferring one-carbon groups,Acyltransferases

contig00004.g2986	1919	ID=contig00004.g2986;Description=cytochrome P450 monooxygenase [Fusarium tjaetaba];Gene=FVEG_05538;Ontology_term=oxidoreductase activity;Ontology_id=GO:0016491;Enzyme_code=EC:1.14,EC:1,EC:1.14;Enzyme_name=Acting on paired donors, with incorporation or reduction of molecular oxygen. The oxygen incorporated need not be derived from O2,Oxidoreductases,Acting on paired donors, with incorporation or reduction of molecular oxygen. The oxygen incorporated need not be derived from O2
contig00004.g2987	720	ID=contig00004.g2987;Description=SAM-dependent methyltransferase [Fusarium pseudocircinatum];Gene=FVEG_05539;Ontology_term=transferase activity;Ontology_id=GO:0016740;Enzyme_code=EC:2.1.1,EC:2.1,EC:2,EC:2.1.1;Enzyme_name=Transferring one-carbon groups,Transferring one-carbon groups,Transferases,Transferring one-carbon groups
contig00004.g2988	1758	ID=contig00004.g2988;Description=carboxylesterase family [Fusarium tjaetaba];Gene=FDENT_8794;Ontology_term=hydrolase activity;Ontology_id=GO:0016787;Enzyme_code=EC:3,EC:3;Enzyme_name=Hydrolases,Hydrolases
contig00004.g2989	1126	ID=contig00004.g2989;Description=acyl thioesterase II [Fusarium tjaetaba];Gene=FTJAE_6027;Ontology_term=peroxisome,nucleobase-containing small molecule metabolic process,sulfur compound metabolic process,lipid metabolic process,mitochondrion,carbohydrate derivative metabolic process,hydrolase activity;Ontology_id=GO:0005777,GO:0055086,GO:0006790,GO:0006629,GO:0005739,GO:1901135,GO:0016787;Enzyme_code=EC:3.1.2.10,EC:3.1.2.20,EC:3.1.2.3,EC:3.1.2.27,EC:3.1,EC:3,EC:3.1.2.10,EC:3.1.2.20,EC:3.1.2.3,EC:3.1.2,EC:3.1.2.2;Enzyme_name=formyl-CoA hydrolase,acyl-CoA hydrolase,succinyl-CoA hydrolase,choloyl-CoA hydrolase,Acting on ester bonds,Hydrolases,formyl-CoA hydrolase,acyl-CoA hydrolase,succinyl-CoA hydrolase,Acting on ester bonds,palmitoyl-CoA hydrolase
contig00004.g2990	1898	ID=contig00004.g2990;Description=cytochrome P450 monooxygenase [Fusarium tjaetaba];Gene=FMUND_5191;Ontology_term=oxidoreductase activity;Ontology_id=GO:0016491;Enzyme_code=EC:1.14,EC:1,EC:1.14;Enzyme_name=Acting on paired donors, with incorporation or reduction of molecular oxygen. The oxygen incorporated need not be derived from O2,Oxidoreductases,Acting on paired donors, with incorporation or reduction of molecular oxygen. The oxygen incorporated need not be derived from O2

contig00004.g2991	1379	ID=contig00004.g2991;Description=Ff.00g064590.m01.CDS01 [Fusarium sp. VM40];Gene=FMUND_5190;Ontology_term=microtubule organizing center,oxidoreductase activity,lipid metabolic process,transferase activity;Ontology_id=GO:0005815,GO:0016491,GO:0006629,GO:0016740;Enzyme_code=EC:1.14,EC:2.3.1,EC:2.3,EC:1,EC:1.14,EC:2,EC:2.3.1;Enzyme_name=Acting on paired donors, with incorporation or reduction of molecular oxygen. The oxygen incorporated need not be derived from O2,Acyltransferases,Acyltransferases,Oxidoreductases,Acting on paired donors, with incorporation or reduction of molecular oxygen. The oxygen incorporated need not be derived from O2,Transferases,Acyltransferases
contig00004.g2992	1848	ID=contig00004.g2992;Description=cytochrome P450 monooxygenase alkane hydroxylase [Fusarium tjaetaba];Gene=FPCIR_2833;Ontology_term=oxidoreductase activity;Ontology_id=GO:0016491;Enzyme_code=EC:1.14,EC:1,EC:1.14;Enzyme_name=Acting on paired donors, with incorporation or reduction of molecular oxygen. The oxygen incorporated need not be derived from O2,Oxidoreductases,Acting on paired donors, with incorporation or reduction of molecular oxygen. The oxygen incorporated need not be derived from O2
contig00004.g2993	1707	ID=contig00004.g2993;Description=dynactin arp1 p62 subunit RO-2 [Fusarium tjaetaba];Gene=FPCIR_2831;Ontology_term=microtubule organizing center,oxidoreductase activity;Ontology_id=GO:0005815,GO:0016491;Enzyme_code=EC:1.14,EC:1,EC:1.14;Enzyme_name=Acting on paired donors, with incorporation or reduction of molecular oxygen. The oxygen incorporated need not be derived from O2,Oxidoreductases,Acting on paired donors, with incorporation or reduction of molecular oxygen. The oxygen incorporated need not be derived from O2
contig00004.g2994	953	ID=contig00004.g2994;Description=ribosomal protein S7e [Fusarium avenaceum];Gene=FNAPI_795;Ontology_term=cytoplasmic translation,structural molecule activity,cytosol,ribosome biogenesis,ribosome;Ontology_id=GO:0002181,GO:0005198,GO:0005829,GO:0042254,GO:0005840

contig00004.g2995	1928	ID=contig00004.g2995;Description=tRNA pseudouridine synthase 1, variant 2 [Fusarium oxysporum];Gene=FVER53263_05547;Ontology_term=nucleus,RNA binding,mRNA metabolic process,snRNA metabolic process,structural molecule activity,tRNA metabolic process,ribosome,isomerase activity;Ontology_id=GO:0005634,GO:0003723,GO:0016071,GO:0016073,GO:0005198,GO:0006399,GO:0005840,GO:0016853;Enzyme_code=EC:5.4;Enzyme_name=Intramolecular transferases
contig00004.g2996	357	ID=contig00004.g2996;Description=hypothetical protein LB504_007883 [Fusarium proliferatum]
contig00004.g2997	2356	ID=contig00004.g2997;Description=F-box and WD-40 domain-containing protein MET30 [Fusarium verticillioides 7600];Ontology_term=nucleus,DNA replication,protein catabolic process,regulation of DNA-templated transcription,mitotic cell cycle,molecular adaptor activity;Ontology_id=GO:0005634,GO:0006260,GO:0030163,GO:0006355,GO:0000278,GO:0060090
contig00004.g2998	759	ID=contig00004.g2998;Description=PPIC-type PPIASE domain protein [Fusarium tjaetaba]
contig00004.g2999	3835	ID=contig00004.g2999;Description=hypothetical protein FVEG_05553 [Fusarium verticillioides 7600];Gene=FNYG_04521;Ontology_term=membrane;Ontology_id=GO:0016020
contig00004.g3000	5050	ID=contig00004.g3000;Description=ERAD-associated e3 ubiquitin ligase doa10 [Fusarium coicis];Gene=FNYG_04520;Ontology_term=nuclear envelope,catalytic activity, acting on a protein,protein catabolic process,endoplasmic reticulum,transferase activity;Ontology_id=GO:0005635,GO:0140096,GO:0030163,GO:0005783,GO:0016740;Enzyme_code=EC:2.3,EC:2,EC:2.3.2.27,EC:2.3.2;Enzyme_name=Acyltransferases,Transferases,RING-type E3 ubiquitin transferase,Acyltransferases
contig00004.g3001	1805	ID=contig00004.g3001;Description=nuclear distribution protein nudE like 1 [Fusarium verticillioides 7600];Gene=FNYG_04519;Ontology_term=cytoskeleton,cytoskeletal protein binding;Ontology_id=GO:0005856,GO:0008092

contig00004.g3002	7069	ID=contig00004.g3002;Description=Acetyl-CoA carboxylase [Fusarium musae];Gene=FOC1_g10009440;Ontology_term=membrane organization,nucleocytoplasmic transport,nucleobase-containing small molecule metabolic process,sulfur compound metabolic process,lipid metabolic process,mitochondrion,carbohydrate derivative metabolic process,cytosol,endoplasmic reticulum,intracellular protein transport,ligase activity;Ontology_id=GO:0061024,GO:0006913,GO:0055086,GO:0006790,GO:0006629,GO:0005739,GO:1901135,GO:0005829,GO:0005783,GO:0006886,GO:0016874;Enzyme_code=EC:6.4.1.2,EC:6.3.4.14,EC:2.1.3.15,EC:6.3,EC:6.4,EC:6,EC:6.4.1.2,EC:6.3.4.14;Enzyme_name=acetyl-CoA carboxylase,biotin carboxylase,acetyl-CoA carboxytransferase,Forming carbon-nitrogen bonds,Forming carbon-carbon bonds,Ligases,acetyl-CoA carboxylase,biotin carboxylase
contig00004.g3003	1593	ID=contig00004.g3003;Description=tetracycline resistance protein (probable transport) [Fusarium tjaetaba];Gene=FPCIR_2822;Ontology_term=transmembrane transport,transporter activity;Ontology_id=GO:0055085,GO:0005215
contig00004.g3004	394	ID=contig00004.g3004;Description=hypothetical protein J7337_002220 [Fusarium musae]
contig00004.g3005	925	ID=contig00004.g3005;Description=coiled-coil domain-containing protein [Fusarium phyllophilum]
contig00004.g3006	3645	ID=contig00004.g3006;Description=hypothetical protein FVEG_05560 [Fusarium verticillioides 7600]
contig00004.g3007	2753	ID=contig00004.g3007;Description=minor extracellular protease vpr [Fusarium coicis];Gene=FNAPI_782;Ontology_term=catalytic activity, acting on a protein,hydrolase activity;Ontology_id=GO:0140096,GO:0016787;Enzyme_code=EC:3.4.21,EC:3.4.21,EC:3.4,EC:3;Enzyme_name=Acting on peptide bonds (peptidases),Acting on peptide bonds (peptidases),Acting on peptide bonds (peptidases),Hydrolases
contig00004.g3008	1238	ID=contig00004.g3008;Description=arsenical-resistance protein [Fusarium verticillioides 7600];Gene=FOPG_04145;Ontology_term=transmembrane transport,transporter activity,plasma membrane;Ontology_id=GO:0055085,GO:0005215,GO:0005886
contig00004.g3009	771	ID=contig00004.g3009;Description=hypothetical protein FVEG_15671 [Fusarium verticillioides 7600]
contig00004.g3010	426	ID=contig00004.g3010;Description=altered inheritance of mitochondria 1 [Fusarium subglutinans];Gene=FVEG_05563;Ontology_term=mitochondrion,protein maturation;Ontology_id=GO:0005739,GO:0051604

contig00004.g3011	2055	ID=contig00004.g3011;Description=arginyl-tRNA synthetase [Fusarium oxysporum];Gene=FDENT_10104;Ontology_term=mitochondrial gene expression,cytoplasmic translation,mitochondrion,tRNA metabolic process,amino acid metabolic process,catalytic activity, acting on RNA,ligase activity;Ontology_id=GO:0140053,GO:0002181,GO:0005739,GO:0006399,GO:0006520,GO:0140098,GO:0016874;Enzyme_code=EC:6.1.1.19,EC:6.1,EC:6.1.1,EC:6,EC:6.1.1.19;Enzyme_name=arginine--tRNA ligase,Forming carbon-oxygen bonds,Forming carbon-oxygen bonds,Ligases,arginine--tRNA ligase
contig00004.g3012	1533	ID=contig00004.g3012;Description=UPF0187 domain protein [Fusarium tjaetaba];Gene=FDENT_10105;Ontology_term=transmembrane transport,transporter activity;Ontology_id=GO:0055085,GO:0005215
contig00004.g3013	1084	ID=contig00004.g3013;Description=hypothetical protein J7337_002211 [Fusarium musae];Gene=FocTR4_00005820;Ontology_term=membrane;Ontology_id=GO:0016020
contig00004.g3014	573	ID=contig00004.g3014;Description=hypothetical protein FVEG_05568 [Fusarium verticillioides 7600];Gene=FPCIR_2811;Ontology_term=regulation of DNA-templated transcription,transcription regulator activity,molecular adaptor activity,nucleoplasm;Ontology_id=GO:0006355,GO:0140110,GO:0060090,GO:0005654
contig00004.g3015	985	ID=contig00004.g3015;Description=TWF1-twinfilin an actin monomer sequestering [Fusarium coicis];Gene=FPCIR_2810;Ontology_term=cell adhesion mediator activity,cell wall,plasma membrane;Ontology_id=GO:0098631,GO:0005618,GO:0005886
contig00004.g3016	1100	ID=contig00004.g3016;Description=TWF1-twinfilin, an actin monomer sequestering protein [Fusarium fujikuroi];Gene=FocTR4_00005823;Ontology_term=cytoskeleton,cytoskeleton organization,protein-containing complex assembly,cytoskeletal protein binding,transferase activity;Ontology_id=GO:0005856,GO:0007010,GO:0065003,GO:0008092,GO:0016740;Enzyme_code=EC:2.7;Enzyme_name=Transferring phosphorus-containing groups
contig00004.g3017	1558	ID=contig00004.g3017;Description=succinate fumarate mitochondrial transporter [Fusarium subglutinans];Gene=FNAPI_772;Ontology_term=transmembrane transport,mitochondrion;Ontology_id=GO:0055085,GO:0005739

contig00004.g3018	3939	ID=contig00004.g3018;Description=bile acid transporter [Fusarium coicis];Gene=FNAPI_771;Ontology_term=transmembrane transport,ATP-dependent activity,transporter activity,hydrolase activity;Ontology_id=GO:0055085,GO:0140657,GO:0005215,GO:0016787;Enzyme_code=EC:7.2.2,EC:3.6.1.15,EC:3.6.1,EC:3.6,EC:3,EC:7.2.2,EC:7,EC:3.6.1.15;Enzyme_name=Catalysing the translocation of inorganic cations,nucleoside-triphosphate phosphatase,Acting on acid anhydrides,Acting on acid anhydrides,Hydrolases,Catalysing the translocation of inorganic cations,Translocases,nucleoside-triphosphate phosphatase
contig00004.g3019	756	ID=contig00004.g3019;Description=gpi anchored protein [Fusarium globosum];Gene=F25303_14082;Ontology_term=membrane;Ontology_id=GO:0016020
contig00004.g3020	1904	ID=contig00004.g3020;Description=ferric reductase (metalloreductase) [Fusarium tjaetaba];Gene=FGLOB1_2983;Ontology_term=oxidoreductase activity;Ontology_id=GO:0016491;Enzyme_code=EC:1.16;Enzyme_name=Oxidizing metal ions
contig00004.g3021	657	ID=contig00004.g3021;Description=kinase [Fusarium phyllophilum];Gene=Forpi1262_v003444;Ontology_term=transferase activity;Ontology_id=GO:0016740;Enzyme_code=EC:2.7,EC:2.7,EC:2;Enzyme_name=Transferring phosphorus-containing groups,Transferring phosphorus-containing groups,Transferases
contig00004.g3022	2553	ID=contig00004.g3022;Description=OPT family small oligopeptide transporter [Fusarium verticillioides 7600];Gene=FVER53590_05578;Ontology_term=transmembrane transport,transporter activity;Ontology_id=GO:0055085,GO:0005215
contig00004.g3023	1035	ID=contig00004.g3023;Description=uncharacterized protein LW93_1628 [Fusarium fujikuroi]
contig00004.g3024	1611	ID=contig00004.g3024;Description=methionyl aminopeptidase [Fusarium verticillioides 7600];Gene=FVER53590_05580;Ontology_term=catalytic activity, acting on a protein,tRNA metabolic process,amino acid metabolic process,catalytic activity, acting on RNA,ligase activity,hydrolase activity;Ontology_id=GO:0140096,GO:0006399,GO:0006520,GO:0140098,GO:0016874,GO:0016787;Enzyme_code=EC:3.4.11.18,EC:6.1.1.4,EC:3.1.1,EC:3.4.11.18,EC:3.4.11,EC:3.1,EC:3.4,EC:6.1,EC:6.1.1.4,EC:3,EC:6.1.1,EC:6,EC:3.1.1;Enzyme_name=methionyl aminopeptidase,leucine--tRNA ligase,Acting on ester bonds,methionyl aminopeptidase,Acting on peptide bonds (peptidases),Acting on ester bonds,Acting on peptide bonds (peptidases),Forming carbon-oxygen bonds,leucine--tRNA ligase,Hydrolases,Forming carbon-oxygen bonds,Ligases,Acting on ester bonds

contig00004.g3025	1020	ID=contig00004.g3025;Description=PHO85 cyclin-7 [Fusarium tjaetaba];Gene=FPCIR_2800;Ontology_term=protein kinase binding;Ontology_id=GO:0019901;Enzyme_code=EC:3.4.13.9;Enzyme_name=Xaa-Pro dipeptidase
contig00004.g3026	2090	ID=contig00004.g3026;Description=related to BENZOYLFORMATE DECARBOXYLASE [Fusarium fujikuroi];Gene=FOXYS1_7634;Ontology_term=ATP-dependent activity,DNA replication,lyase activity,hydrolase activity;Ontology_id=GO:0140657,GO:0006260,GO:0016829,GO:0016787;Enzyme_code=EC:4.1.1.1,EC:3.6.1.15,EC:4.1.1,EC:4.1.1.1,EC:4.1,EC:3.6.1,EC:3.6,EC:3,EC:4,EC:3.6.1.15;Enzyme_name=pyruvate decarboxylase,nucleoside-triphosphate phosphatase,Carbon-carbon lyases,pyruvate decarboxylase,Carbon-carbon lyases,Acting on acid anhydrides,Acting on acid anhydrides,Hydrolases,Lyases,nucleoside-triphosphate phosphatase
contig00004.g3027	1075	ID=contig00004.g3027;Description=NADH-cytochrome b5 reductase 1 [Fusarium verticillioides 7600];Gene=FVEG_05583;Ontology_term=nucleus,generation of precursor metabolites and energy,oxidoreductase activity,mitochondrion,tRNA metabolic process,endoplasmic reticulum,plasma membrane;Ontology_id=GO:0005634,GO:0006091,GO:0016491,GO:0005739,GO:0006399,GO:0005783,GO:0005886;Enzyme_code=EC:1.6.2.2,EC:1.6.2.2,EC:1.6.2,EC:1.6,EC:1;Enzyme_name=cytochrome-b5 reductase,cytochrome-b5 reductase,Acting on NADH or NADPH,Acting on NADH or NADPH,Oxidoreductases
contig00004.g3028	1691	ID=contig00004.g3028;Description=cytochrome p450 [Fusarium coicis];Gene=FPCIR_2797;Ontology_term=oxidoreductase activity;Ontology_id=GO:0016491;Enzyme_code=EC:1.14,EC:1,EC:1.14;Enzyme_name=Acting on paired donors, with incorporation or reduction of molecular oxygen. The oxygen incorporated need not be derived from O2,Oxidoreductases,Acting on paired donors, with incorporation or reduction of molecular oxygen. The oxygen incorporated need not be derived from O2
contig00004.g3029	523	ID=contig00004.g3029;Description=DUF636 domain-containing protein [Fusarium coicis];Gene=LOCC1_G003767;Ontology_term=lyase activity;Ontology_id=GO:0016829;Enzyme_code=EC:4.4;Enzyme_name=Carbon-sulfur lyases
contig00004.g3030	905	ID=contig00004.g3030;Description=2-(S)-hydroxypropyl dehydrogenase [Fusarium mundagurra]

contig00004.g3031	2189	ID=contig00004.g3031;Description=fungal specific transcription factor [Fusarium coicis];Gene=FMUND_5149;Ontology_term=nucleus,DNA binding,regulation of DNA-templated transcription,transcription regulator activity;Ontology_id=GO:0005634,GO:0003677,GO:0006355,GO:0140110
contig00004.g3032	1508	ID=contig00004.g3032;Description=related to 6-hydroxy-D-nicotine oxidase [Fusarium fujikuroi];Gene=FFUJ_03449;Ontology_term=oxidoreductase activity;Ontology_id=GO:0016491;Enzyme_code=EC:1;Enzyme_name=Oxidoreductases
contig00004.g3033	1008	ID=contig00004.g3033;Description=fic doc family [Fusarium tjaetaba];Gene=FVEG_05590;Ontology_term=ATP binding;Ontology_id=GO:0005524
contig00004.g3034	2530	ID=contig00004.g3034;Description=catalase-1 [Fusarium verticillioides 7600];Gene=BFJ69_g1538;Ontology_term=antioxidant activity,oxidoreductase activity,cell wall,anatomical structure development,cell differentiation,cytosol;Ontology_id=GO:0016209,GO:0016491,GO:0005618,GO:0048856,GO:0030154,GO:0005829;Enzyme_code=EC:1.11.1.6,EC:1.11,EC:1,EC:1.11.1,EC:1.11.1.6;Enzyme_name=catalase,Acting on a peroxide as acceptor,Oxidoreductases,Acting on a peroxide as acceptor,catalase
contig00004.g3035	2692	ID=contig00004.g3035;Description=XFP N-terminal domain-containing protein [Fusarium oxysporum];Gene=FVER53590_05592;Ontology_term=carbohydrate metabolic process,lyase activity;Ontology_id=GO:0005975,GO:0016829;Enzyme_code=EC:4.1.2,EC:4.1.2,EC:4.1,EC:4,EC:4.1.2.22,EC:4.1.2.9;Enzyme_name=Carbon-carbon lyases,Carbon-carbon lyases,Carbon-carbon lyases,Lyases,fructose-6-phosphate phosphoketolase,phosphoketolase
contig00004.g3036	404	ID=contig00004.g3036;Description=hypothetical protein FVER53263_05594 [Fusarium verticillioides]
contig00004.g3037	1350	ID=contig00004.g3037;Description=NDP-n-acetyl-D-galactosaminuronic acid dehydrogenase [Fusarium pseudoanthophilum];Gene=FACUT_2094;Ontology_term=carbohydrate metabolic process,oxidoreductase activity;Ontology_id=GO:0005975,GO:0016491;Enzyme_code=EC:1.1.1,EC:1.3.1,EC:1.1,EC:1.3,EC:1,EC:1.1.1,EC:1.3.1;Enzyme_name=Acting on the CH-OH group of donors,Acting on the CH-CH group of donors,Acting on the CH-OH group of donors,Acting on the CH-CH group of donors,Oxidoreductases,Acting on the CH-OH group of donors,Acting on the CH-CH group of donors

contig00004.g3038	1692	ID=contig00004.g3038;Description=related to chitin binding protein [Fusarium proliferatum ET1];Gene=FGLOB1_2970;Ontology_term=carbohydrate metabolic process,hydrolase activity;Ontology_id=GO:0005975,GO:0016787;Enzyme_code=EC:3.5,EC:3.5,EC:3;Enzyme_name=Acting on carbon-nitrogen bonds, other than peptide bonds,Acting on carbon-nitrogen bonds, other than peptide bonds,Hydrolases
contig00004.g3039	2123	ID=contig00004.g3039;Description=chitin synthase 6 [Fusarium tjaetaba];Gene=FVEG_05597;Ontology_term=nucleus,transmembrane transport,anatomical structure development,cell differentiation,regulation of DNA-templated transcription,transferase activity,transcription regulator activity,transporter activity,plasma membrane;Ontology_id=GO:0005634,GO:0055085,GO:0048856,GO:0030154,GO:0006355,GO:0016740,GO:0140110,GO:0005215,GO:0005886;Enzyme_code=EC:2.4.1.16,EC:2.4,EC:2.4.1.16,EC:2,EC:2.4.1;Enzyme_name=chitin synthase,Glycosyltransferases,chitin synthase,Transferases,Glycosyltransferases
contig00004.g3040	2010	ID=contig00004.g3040;Description=hypothetical protein FVER53590_29030 [Fusarium verticillioides];Gene=FMEXI_12329;Ontology_term=nucleus,regulation of DNA-templated transcription,transcription regulator activity;Ontology_id=GO:0005634,GO:0006355,GO:0140110
contig00004.g3041	1715	ID=contig00004.g3041;Description=GABA permease [Fusarium tjaetaba];Gene=FNAPI_751;Ontology_term=transmembrane transport,transporter activity;Ontology_id=GO:0055085,GO:0005215
contig00004.g3042	2099	ID=contig00004.g3042;Description=hypothetical protein FVEG_05599 [Fusarium verticillioides 7600];Gene=FANTH_12406;Ontology_term=membrane;Ontology_id=GO:0016020
contig00004.g3043	2246	ID=contig00004.g3043;Description=hypothetical protein FVER14953_05600 [Fusarium verticillioides]
contig00004.g3044	1396	ID=contig00004.g3044;Description=hypothetical protein FVER14953_20928 [Fusarium verticillioides]
contig00004.g3045	1623	ID=contig00004.g3045;Description=aminopeptidase Y precursor vacuolar [Fusarium subglutinans];Gene=FSUBG_411;Ontology_term=catalytic activity, acting on a protein,hydrolase activity;Ontology_id=GO:0140096,GO:0016787;Enzyme_code=EC:3.4.11;Enzyme_name=Acting on peptide bonds (peptidases)

contig00004.g3046	1603	ID=contig00004.g3046;Description=cystathionine beta-synthase [Fusarium proliferatum];Gene=LW93_1609;Ontology_term=catalytic activity, acting on a protein,RNA binding,sulfur compound metabolic process,mitochondrion,lyase activity,amino acid metabolic process,mitotic cell cycle,hydrolase activity;Ontology_id=GO:0140096,GO:0003723,GO:0006790,GO:0005739,GO:0016829,GO:0006520,GO:0000278,GO:0016787;Enzyme_code=EC:3.4.23,EC:4.2.1.22,EC:4.2.1,EC:3.4.23,EC:4.2.1.22,EC:3.4,EC:4.2,EC:3,EC:4;Enzyme_name=Acting on peptide bonds (peptidases),cystathionine beta-synthase,Carbon-oxygen lyases,Acting on peptide bonds (peptidases),cystathionine beta-synthase,Acting on peptide bonds (peptidases),Carbon-oxygen lyases,Hydrolases,Lyases
contig00004.g3047	855	ID=contig00004.g3047;Description=signal recognition particle receptor beta chain [Fusarium tjaetaba];Gene=FACUT_2083;Ontology_term=GTPase activity,endoplasmic reticulum,intracellular protein transport;Ontology_id=GO:0003924,GO:0005783,GO:0006886;Enzyme_code=EC:3.6.1,EC:3.6,EC:3,EC:3.6.1.15;Enzyme_name=Acting on acid anhydrides,Acting on acid anhydrides,Hydrolases,nucleoside-triphosphate phosphatase
contig00004.g3048	2273	ID=contig00004.g3048;Description=related to AKR1-ankyrin repeat-containing protein [Fusarium fujikuroi];Gene=FVEG_05604;Ontology_term=catalytic activity, acting on a protein,endosome,vesicle-mediated transport,signaling,reproductive process,Golgi apparatus,transferase activity,intracellular protein transport,vacuole,protein localization to plasma membrane;Ontology_id=GO:0140096,GO:0005768,GO:0016192,GO:0023052,GO:0022414,GO:0005794,GO:0016740,GO:0006886,GO:0005773,GO:0072659;Enzyme_code=EC:2.3.1.225,EC:2.3,EC:2,EC:2.3.1.225,EC:2.3.1;Enzyme_name=protein S-acyltransferase,Acyltransferases,Transferases,protein S-acyltransferase,Acyltransferases
contig00004.g3049	1347	ID=contig00004.g3049;Description=integral membrane protein PTH11 [Fusarium tjaetaba];Gene=FVER53590_05605;Ontology_term=membrane;Ontology_id=GO:0016020
contig00004.g3050	748	ID=contig00004.g3050;Description=flavin-binding monooxygenase [Fusarium napiforme];Gene=FVEG_05606;Ontology_term=oxidoreductase activity;Ontology_id=GO:0016491;Enzyme_code=EC:1;Enzyme_name=Oxidoreductases

contig00004.g3051	1654	ID=contig00004.g3051;Description=ACB 4-hydroxyacetophenone monooxygenase [Fusarium pseudoanthophilum];Gene=FPCIR_2778;Ontology_term=oxidoreductase activity;Ontology_id=GO:0016491;Enzyme_code=EC:1.14.13.8,EC:1.14.13.8,EC:1,EC:1.14,EC:1.14.13;Enzyme_name=flavin-containing monooxygenase,flavin-containing monooxygenase,Oxidoreductases,Acting on paired donors, with incorporation or reduction of molecular oxygen. The oxygen incorporated need not be derived from O2,Acting on paired donors, with incorporation or reduction of molecular oxygen. The oxygen incorporated need not be derived from O2
contig00004.g3052	1478	ID=contig00004.g3052;Description=neutral amino acid permease [Fusarium pseudoanthophilum];Gene=FVER53590_05608;Ontology_term=membrane;Ontology_id=GO:0016020
contig00004.g3053	1349	ID=contig00004.g3053;Description=L-pipecolate oxidase [Fusarium tjaetaba];Gene=FNAPI_11006;Ontology_term=oxidoreductase activity;Ontology_id=GO:0016491;Enzyme_code=EC:1,EC:1.5.3.7,EC:1,EC:1.5.3.1;Enzyme_name=Oxidoreductases,L-pipecolate oxidase,Oxidoreductases,sarcosine oxidasee (formaldehyde-forming)
contig00004.g3054	2262	ID=contig00004.g3054;Description=hypothetical protein BFJ72_g4456 [Fusarium proliferatum];Gene=priA;Ontology_term=nucleus,regulation of DNA-templated transcription,transcription regulator activity;Ontology_id=GO:0005634,GO:0006355,GO:0140110
contig00004.g3055	1826	ID=contig00004.g3055;Description=hypothetical protein FMEXI_12313 [Fusarium mexicanum]
contig00004.g3056	1964	ID=contig00004.g3056;Description=hypothetical protein FVER53590_05613 [Fusarium verticillioides];Gene=FVER53263_05613;Ontology_term=membrane;Ontology_id=GO:0016020
contig00004.g3057	768	ID=contig00004.g3057;Description=peptidyl-prolyl cis/trans isomerase [Pochonia chlamydosporia 170];Gene=MAJ_06654;Ontology_term=catalytic activity, acting on a protein,transferase activity,isomerase activity;Ontology_id=GO:0140096,GO:0016740,GO:0016853;Enzyme_code=EC:5.2.1.8,EC:2.7;Enzyme_name=peptidylprolyl isomerase,Transferring phosphorus-containing groups

contig00004.g3058	1034	ID=contig00004.g3058;Description=DNA-directed RNA polymerase II subunit RPB9 [Fusarium odoratissimum NRRL 54006];Gene=FVEG_05615;Ontology_term=DNA-templated transcription,catalytic activity, acting on RNA,transferase activity;Ontology_id=GO:0006351,GO:0140098,GO:0016740;Enzyme_code=EC:2.7.7.6,EC:2.7,EC:2,EC:2.7.7,EC:2.7.7.6;Enzyme_name=DNA-directed RNA polymerase,Transferring phosphorus-containing groups,Transferases,Transferring phosphorus-containing groups,DNA-directed RNA polymerase
contig00004.g3059	451	ID=contig00004.g3059;Description=mitochondrial 54S ribosomal protein YmL38/YmL34 [Fusarium flagelliforme];Gene=CEP53_014571;Ontology_term=mitochondrial gene expression,RNA binding,mitochondrion,structural molecule activity,ribosome;Ontology_id=GO:0140053,GO:0003723,GO:0005739,GO:0005198,GO:0005840
contig00004.g3060	2064	ID=contig00004.g3060;Description=histone-lysine N-methyltransferase SET9 [Fusarium verticillioides 7600];Gene=FocTR4_00005864;Ontology_term=catalytic activity, acting on a protein,signaling,regulation of DNA-templated transcription,nuclear chromosome,transferase activity,chromatin organization,mitotic cell cycle;Ontology_id=GO:0140096,GO:0023052,GO:0006355,GO:0000228,GO:0016740,GO:0006325,GO:0000278;Enzyme_code=EC:2.1.1.372,EC:2.1.1.372,EC:2.1,EC:2.1.1.43,EC:2,EC:2.1.1;Enzyme_name=[histone H4]-lysine(20) N-trimethyltransferase,[histone H4]-lysine(20) N-trimethyltransferase,Transferring one-carbon groups,Transferring one-carbon groups,Transferases,Transferring one-carbon groups
contig00004.g3061	1455	ID=contig00004.g3061;Description=4-hydroxyacetophenone monooxygenase [Fusarium oxysporum f. sp. albedinis];Gene=Q05000;Ontology_term=nucleus,DNA binding,catalytic activity, acting on a protein,cytoskeleton,catalytic activity, acting on DNA,ATP-dependent activity,oxidoreductase activity,transferase activity,chromatin organization,cytoskeletal protein binding,hydrolase activity;Ontology_id=GO:0005634,GO:0003677,GO:0140096,GO:0005856,GO:0140097,GO:0140657,GO:0016491,GO:0016740,GO:0006325,GO:0008092,GO:0016787;Enzyme_code=EC:3.6.1,EC:1.6.99.1,EC:2.1.1,EC:2.1,EC:3.6.1,EC:1.3.1.42,EC:1.6,EC:1,EC:2,EC:3.6,EC:3,EC:1.6.99.1,EC:2.1.1;Enzyme_name=Acting on acid anhydrides,NADPH dehydrogenase,Transferring one-carbon groups,Transferring one-carbon groups,Acting on acid anhydrides,12-oxophytodienoate reductase,Acting on NADH or NADPH,Oxidoreductases,Transferases,Acting on acid anhydrides,Hydrolases,NADPH dehydrogenase,Transferring one-carbon groups

contig00004.g3062	1643	ID=contig00004.g3062;Description=aminopeptidase Y [Fusarium verticillioides 7600];Gene=FPCIR_2767;Ontology_term=catalytic activity, acting on a protein,protein catabolic process,vacuole,hydrolase activity;Ontology_id=GO:0140096,GO:0030163,GO:0005773,GO:0016787;Enzyme_code=EC:3.4.11,EC:3.4.11,EC:3.4,EC:3.4.11.15,EC:3;Enzyme_name=Acting on peptide bonds (peptidases),Acting on peptide bonds (peptidases),Acting on peptide bonds (peptidases),aminopeptidase Y,Hydrolases
contig00004.g3063	917	ID=contig00004.g3063;Description=hypothetical protein FVER53263_05620 [Fusarium verticillioides]
contig00004.g3064	1554	ID=contig00004.g3064;Description=hypothetical protein FVER53590_05621 [Fusarium verticillioides];Gene=FVER53590_05621;Ontology_term=metal ion binding;Ontology_id=GO:0046872
contig00004.g3065	1838	ID=contig00004.g3065;Description=anthranilate synthase component I [Fusarium verticillioides 7600];Gene=FNYG_04453;Ontology_term=biosynthetic process;Ontology_id=GO:0009058
contig00004.g3066	970	ID=contig00004.g3066;Description=hypothetical protein FVER14953_05623 [Fusarium verticillioides]
contig00004.g3067	1051	ID=contig00004.g3067;Description=hypothetical protein FVEG_05624 [Fusarium verticillioides 7600]
contig00004.g3068	882	ID=contig00004.g3068;Description=hypothetical protein FVEG_05625 [Fusarium verticillioides 7600];Gene=FVER53263_05625;Ontology_term=metal ion binding;Ontology_id=GO:0046872
contig00004.g3069	897	ID=contig00004.g3069;Description=hypothetical protein FVER14953_05626 [Fusarium verticillioides]
contig00004.g3070	834	ID=contig00004.g3070;Description=Glyoxalase/Bleomycin resistance protein/Dihydroxybiphenyl dioxygenase [Fusarium oxysporum f. sp. vasinfectum]
contig00004.g3071	561	ID=contig00004.g3071;Description=helix-turn-helix domain protein [Fusarium tjaetaba]
contig00004.g3072	601	ID=contig00004.g3072;Description=related to thioesterase superfamily member 2 [Fusarium fujikuroi IMI 58289];Gene=FVER53590_05629;Ontology_term=hydrolase activity;Ontology_id=GO:0016787;Enzyme_code=EC:3.1.2.10,EC:3.1.2.20,EC:3.1.2.3,EC:3.1,EC:3,EC:3.1.2.10,EC:3.1.2.20,EC:3.1.2.3,EC:3.1.2;Enzyme_name=formyl-CoA hydrolase,acyl-CoA hydrolase,succinyl-CoA hydrolase,Acting on ester bonds,Hydrolases,formyl-CoA hydrolase,acyl-CoA hydrolase,succinyl-CoA hydrolase,Acting on ester bonds

contig00004.g3073	1071	ID=contig00004.g3073;Description=alcohol dehydrogenase [Fusarium verticillioides 7600];Gene=FPCIR_2756;Ontology_term=oxidoreductase activity;Ontology_id=GO:0016491;Enzyme_code=EC:1,EC:1;Enzyme_name=Oxidoreductases,Oxidoreductases
contig00004.g3074	2096	ID=contig00004.g3074;Description=positive regulator of purine utilization [Fusarium coicis];Gene=FVER53263_20502;Ontology_term=nucleus,DNA binding,regulation of DNA-templated transcription,transcription regulator activity;Ontology_id=GO:0005634,GO:0003677,GO:0006355,GO:0140110
contig00004.g3075	1978	ID=contig00004.g3075;Description=dihydroxy-acid dehydratase [Fusarium verticillioides 7600];Gene=FOZG_04735;Ontology_term=lyase activity;Ontology_id=GO:0016829;Enzyme_code=EC:4.2.1,EC:4.2.1,EC:4.2,EC:4,EC:4.2.1.9;Enzyme_name=Carbon-oxygen lyases,Carbon-oxygen lyases,Carbon-oxygen lyases,Lyases,dihydroxy-acid dehydratase
contig00004.g3076	801	ID=contig00004.g3076;Description=short-chain alcohol dehydrogenase [Fusarium tjaetaba];Gene=FNYG_04442;Ontology_term=oxidoreductase activity;Ontology_id=GO:0016491;Enzyme_code=EC:1,EC:1;Enzyme_name=Oxidoreductases,Oxidoreductases
contig00004.g3077	469	ID=contig00004.g3077;Description=stress responsive a b barrel [Fusarium sp. NRRL 25303];Gene=FVER53590_05632;Ontology_term=oxidoreductase activity;Ontology_id=GO:0016491;Enzyme_code=EC:1;Enzyme_name=Oxidoreductases
contig00004.g3078	2715	ID=contig00004.g3078;Description=probable high-affinity nickel transport protein nic1 [Fusarium fujikuroi];Gene=FPHYL_680;Ontology_term=transmembrane transport,transporter activity,plasma membrane;Ontology_id=GO:0055085,GO:0005215,GO:0005886
contig00004.g3079	2958	ID=contig00004.g3079;Description=calpain [Fusarium tjaetaba];Gene=FVER53263_05637;Ontology_term=DNA binding,catalytic activity, acting on a protein,hydrolase activity;Ontology_id=GO:0003677,GO:0140096,GO:0016787;Enzyme_code=EC:3.4.22,EC:3.4.22,EC:3.4,EC:3;Enzyme_name=Acting on peptide bonds (peptidases),Acting on peptide bonds (peptidases),Acting on peptide bonds (peptidases),Hydrolases
contig00004.g3080	1597	ID=contig00004.g3080;Description=autophagy protein 17 [Fusarium musae];Gene=FVER53590_05638;Ontology_term=molecular function regulator activity,autophagy,structural molecule activity,mitochondrion organization,molecular adaptor activity;Ontology_id=GO:0098772,GO:0006914,GO:0005198,GO:0007005,GO:0060090

contig00004.g3081	823	ID=contig00004.g3081;Description=hypothetical protein FVEG_05640 [Fusarium verticillioides 7600]
contig00004.g3082	987	ID=contig00004.g3082;Description=methyltransferase [Fusarium napiforme];Gene=FPHYL_684;Ontology_term=transferase activity;Ontology_id=GO:0016740;Enzyme_code=EC:2.1.1,EC:2.1,EC:2,EC:2.1.1;Enzyme_name=Transferring one-carbon groups,Transferring one-carbon groups,Transferases,Transferring one-carbon groups
contig00004.g3083	1242	ID=contig00004.g3083;Description=hypothetical protein FVER53263_05642 [Fusarium verticillioides]
contig00004.g3084	3855	ID=contig00004.g3084;Description=putative NRPS-like protein biosynthetic cluster [Fusarium musae];Gene=C2S_614;Ontology_term=oxidoreductase activity,isomerase activity;Ontology_id=GO:0016491,GO:0016853;Enzyme_code=EC:1.14.14,EC:5,EC:1,EC:1.14.14,EC:1.14,EC:5;Enzyme_name=Acting on paired donors, with incorporation or reduction of molecular oxygen. The oxygen incorporated need not be derived from O2,Isomerases,Oxidoreductases,Acting on paired donors, with incorporation or reduction of molecular oxygen. The oxygen incorporated need not be derived from O2,Acting on paired donors, with incorporation or reduction of molecular oxygen. The oxygen incorporated need not be derived from O2,Isomerases
contig00004.g3085	1548	ID=contig00004.g3085;Description=AAT family amino acid transporter [Fusarium proliferatum];Gene=FNAPI_10974;Ontology_term=transmembrane transport,mitochondrion,endoplasmic reticulum,transporter activity,plasma membrane;Ontology_id=GO:0055085,GO:0005739,GO:0005783,GO:0005215,GO:0005886
contig00004.g3086	1661	ID=contig00004.g3086;Description=protein PNS1 [Fusarium verticillioides 7600];Gene=FNYG_04431;Ontology_term=transmembrane transport,oxidoreductase activity,transporter activity,plasma membrane;Ontology_id=GO:0055085,GO:0016491,GO:0005215,GO:0005886;Enzyme_code=EC:1.14.14,EC:1,EC:1.14.14,EC:1.14;Enzyme_name=Acting on paired donors, with incorporation or reduction of molecular oxygen. The oxygen incorporated need not be derived from O2,Oxidoreductases,Acting on paired donors, with incorporation or reduction of molecular oxygen. The oxygen incorporated need not be derived from O2,Acting on paired donors, with incorporation or reduction of molecular oxygen. The oxygen incorporated need not be derived from O2

contig00004.g3087	4229	ID=contig00004.g3087;Description=RNA-dependent RNA polymerase 2 [Fusarium napiforme];Gene=FVER53263_05646;Ontology_term=RNA binding,regulatory ncRNA-mediated gene silencing,catalytic activity, acting on RNA,transferase activity;Ontology_id=GO:0003723,GO:0031047,GO:0140098,GO:0016740;Enzyme_code=EC:2.7.7.48,EC:2.7,EC:2,EC:2.7.7.48,EC:2.7.7;Enzyme_name=RNA-directed RNA polymerase,Transferring phosphorus-containing groups,Transferases,RNA-directed RNA polymerase,Transferring phosphorus-containing groups
contig00004.g3088	2637	ID=contig00004.g3088;Description=regulatory protein [Fusarium denticulatum];Gene=FDENT_2481;Ontology_term=nucleus,DNA binding,regulation of DNA-templated transcription,transcription regulator activity;Ontology_id=GO:0005634,GO:0003677,GO:0006355,GO:0140110
contig00004.g3089	1691	ID=contig00004.g3089;Description=RIO kinase 1 [Fusarium subglutinans];Gene=Forpe1208_v003304;Ontology_term=catalytic activity, acting on a protein,mitotic nuclear division,ribosome biogenesis,nuclear chromosome,transferase activity,nucleolus,hydrolase activity;Ontology_id=GO:0140096,GO:0140014,GO:0042254,GO:0000228,GO:0016740,GO:0005730,GO:0016787;Enzyme_code=EC:2.7.11.1,EC:3,EC:2.7.1,EC:2.7.11.1,EC:2.7,EC:2,EC:3;Enzyme_name=non-specific serine/threonine protein kinase,Hydrolases,Transferring phosphorus-containing groups,non-specific serine/threonine protein kinase,Transferring phosphorus-containing groups,Transferases,Hydrolases
contig00004.g3090	1776	ID=contig00004.g3090;Description=aspartic ase precursor [Fusarium acutatum];Gene=FPHYL_692;Ontology_term=catalytic activity, acting on a protein,hydrolase activity;Ontology_id=GO:0140096,GO:0016787;Enzyme_code=EC:3.4.23,EC:3.4.23,EC:3.4,EC:3;Enzyme_name=Acting on peptide bonds (peptidases),Acting on peptide bonds (peptidases),Acting on peptide bonds (peptidases),Hydrolases
contig00004.g3091	1002	ID=contig00004.g3091;Description=hypothetical protein FVEG_05651 [Fusarium verticillioides 7600];Gene=FNAPI_6216;Ontology_term=oxidoreductase activity;Ontology_id=GO:0016491;Enzyme_code=EC:1,EC:1;Enzyme_name=Oxidoreductases,Oxidoreductases
contig00004.g3092	1044	ID=contig00004.g3092;Description=hypothetical protein FVEG_05652 [Fusarium verticillioides 7600]

contig00004.g3093	998	ID=contig00004.g3093;Description=thioredoxin reductase [Fusarium tjaetaba];Gene=FNAPI_6214;Ontology_term=oxidoreductase activity;Ontology_id=GO:0016491;Enzyme_code=EC:1,EC:1;Enzyme_name=Oxidoreductases,Oxidoreductases
contig00004.g3094	1224	ID=contig00004.g3094;Description=hypothetical protein J7337_002129 [Fusarium musae]
contig00004.g3095	1604	ID=contig00004.g3095;Description=xaa-Pro dipeptidase [Fusarium subglutinans];Gene=FOZG_04713;Ontology_term=catalytic activity, acting on a protein,nucleobase-containing small molecule metabolic process,carbohydrate derivative metabolic process,transferase activity,vitamin metabolic process,hydrolase activity;Ontology_id=GO:0140096,GO:0055086,GO:1901135,GO:0016740,GO:0006766,GO:0016787;Enzyme_code=EC:3.4.11,EC:2.7.1.26,EC:3.4.11,EC:2.7.1,EC:2.7.1.26,EC:3.4.13,EC:3.4,EC:2.7,EC:2,EC:3,EC:3.4.13.9;Enzyme_name=Acting on peptide bonds (peptidases),riboflavin kinase,Acting on peptide bonds (peptidases),Transferring phosphorus-containing groups,riboflavin kinase,Acting on peptide bonds (peptidases),Acting on peptide bonds (peptidases),Transferring phosphorus-containing groups,Transferases,Hydrolases,Xaa-Pro dipeptidase
contig00004.g3096	2236	ID=contig00004.g3096;Description=riboflavin kinase [Fusarium napiforme];Gene=FMUND_9857;Ontology_term=catalytic activity, acting on a protein,nucleobase-containing small molecule metabolic process,carbohydrate derivative metabolic process,transferase activity,vitamin metabolic process,hydrolase activity;Ontology_id=GO:0140096,GO:0055086,GO:1901135,GO:0016740,GO:0006766,GO:0016787;Enzyme_code=EC:3.4.11,EC:2.7.1.26,EC:3.4.11,EC:2.7.1,EC:2.7.1.26,EC:3.4,EC:2.7,EC:2,EC:3;Enzyme_name=Acting on peptide bonds (peptidases),riboflavin kinase,Acting on peptide bonds (peptidases),Transferring phosphorus-containing groups,riboflavin kinase,Acting on peptide bonds (peptidases),Transferring phosphorus-containing groups,Transferases,Hydrolases
contig00004.g3097	1977	ID=contig00004.g3097;Description=hypothetical protein FVER14953_05657 [Fusarium verticillioides]
contig00004.g3098	2152	ID=contig00004.g3098;Description=related to serine-type carboxypeptidase Z precursor [Fusarium fujikuroi IMI 58289];Gene=FMEXI_11701;Ontology_term=catalytic activity, acting on a protein,hydrolase activity;Ontology_id=GO:0140096,GO:0016787;Enzyme_code=EC:3.4.16,EC:3.4,EC:3.4.16,EC:3;Enzyme_name=Acting on peptide bonds (peptidases),Acting on peptide bonds (peptidases),Acting on peptide bonds (peptidases),Hydrolases

contig00004.g3099	2418	ID=contig00004.g3099;Description=putative phosphatase regulatory subunit-domain-containing protein [Fusarium oxysporum];Gene=folD
contig00004.g3100	1822	ID=contig00004.g3100;Description=GABA permease [Fusarium tjaetaba];Gene=FVER53590_05661;Ontology_term=transmembrane transport,transporter activity;Ontology_id=GO:0055085,GO:0005215
contig00004.g3101	441	ID=contig00004.g3101;Description=adf-like domain protein [Fusarium tjaetaba];Gene=FCIRC_6789;Ontology_term=cytoskeletal protein binding;Ontology_id=GO:0008092
contig00004.g3102	1673	ID=contig00004.g3102;Description=pantothenate transporter liz1 [Fusarium tjaetaba];Gene=FNAPI_12748;Ontology_term=transmembrane transport,transporter activity;Ontology_id=GO:0055085,GO:0005215
contig00004.g3103	210	ID=contig00004.g3103;Description=hypothetical protein FVER53263_20018 [Fusarium verticillioides]
contig00004.g3104	2257	ID=contig00004.g3104;Description=hypothetical protein FVER14953_05664 [Fusarium verticillioides]
contig00004.g3105	552	ID=contig00004.g3105;Description=hypothetical protein J7337_002117 [Fusarium musae]
contig00004.g3106	2376	ID=contig00004.g3106;Description=hypothetical protein FVER53263_05666 [Fusarium verticillioides];Gene=FPANT_8780;Ontology_term=membrane;Ontology_id=GO:0016020
contig00004.g3107	1278	ID=contig00004.g3107;Description=UDP-glucose 4-epimerase [Fusarium verticillioides 7600];Gene=FNAPI_12744;Ontology_term=carbohydrate metabolic process,isomerase activity;Ontology_id=GO:0005975,GO:0016853;Enzyme_code=EC:5.1.3.2,EC:5.1,EC:5,EC:5.1.3,EC:5.1.3.2 ;Enzyme_name=UDP-glucose 4-epimerase,Racemases and epimerases,Isomerases,Racemases and epimerases,UDP-glucose 4-epimerase
contig00004.g3108	4646	ID=contig00004.g3108;Description=hypothetical protein FVER14953_05669 [Fusarium verticillioides];Gene=FCIRC_6779;Ontology_term=membrane;Ontology_id=GO:0016020
contig00004.g3109	1505	ID=contig00004.g3109;Description=alcohol o-acetyltransferase 1 [Fusarium tjaetaba];Gene=FTJAE_8318;Ontology_term=transferase activity;Ontology_id=GO:0016740;Enzyme_code=EC:2;Enzyme_name=Transferases

contig00004.g3110	1518	ID=contig00004.g3110;Description=lysine decarboxylase [Fusarium tjaetaba];Gene=FMUND_2135;Ontology_term=oxidoreductase activity,hydrolase activity;Ontology_id=GO:0016491,GO:0016787;Enzyme_code=EC:1.14,EC:3,EC:1,EC:1.14,EC:3;Enzyme_name=Acting on paired donors, with incorporation or reduction of molecular oxygen. The oxygen incorporated need not be derived from O2,Hydrolases,Oxidoreductases,Acting on paired donors, with incorporation or reduction of molecular oxygen. The oxygen incorporated need not be derived from O2,Hydrolases
contig00004.g3111	1269	ID=contig00004.g3111;Description=related to pisatin demethylase cytochrome P450 [Fusarium fujikuroi];Gene=FFUJ_03535;Ontology_term=oxidoreductase activity,transferase activity;Ontology_id=GO:0016491,GO:0016740;Enzyme_code=EC:1.14,EC:2.1.1,EC:2.1,EC:1,EC:1.14,EC:2,EC:2.1.1;Enzyme_name=Acting on paired donors, with incorporation or reduction of molecular oxygen. The oxygen incorporated need not be derived from O2,Transferring one-carbon groups,Transferring one-carbon groups,Oxidoreductases,Acting on paired donors, with incorporation or reduction of molecular oxygen. The oxygen incorporated need not be derived from O2,Transferases,Transferring one-carbon groups
contig00004.g3112	1236	ID=contig00004.g3112;Description=putative glycosyl transferase fck3 [Fusarium musae];Gene=FNAPI_2001;Ontology_term=transferase activity;Ontology_id=GO:0016740;Enzyme_code=EC:2.4,EC:2.4,EC:2;Enzyme_name=Glycosyltransferases,Glycosyltransferases,Transferases
contig00004.g3113	1800	ID=contig00004.g3113;Description=ankyrin repeat-containing protein [Fusarium globosum]
contig00004.g3114	428	ID=contig00004.g3114;Description=hypothetical protein FVER53590_25533 [Fusarium verticillioides]
contig00004.g3115	1028	ID=contig00004.g3115;Description=hypothetical protein J7337_002105 [Fusarium musae]
contig00004.g3116	312	ID=contig00004.g3116;Description=hypothetical protein FVER53590_28830 [Fusarium verticillioides]
contig00004.g3117	2202	ID=contig00004.g3117;Description=phosphorylase superfamily [Fusarium phyllophilum];Gene=3588
contig00004.g3118	648	ID=contig00004.g3118;Description=hypothetical protein FVER53590_28831 [Fusarium verticillioides]
contig00004.g3119	1568	ID=contig00004.g3119;Description=murein transglycosylase [Fusarium verticillioides 7600];Gene=FTJAE_8309;Ontology_term=carbohydrate metabolic process,transmembrane transport,transporter activity,hydrolase activity;Ontology_id=GO:0005975,GO:0055085,GO:0005215,GO:0016787;Enzyme_code=EC:3.2.1;Enzyme_name=Glycosylases

contig00004.g3120	1747	ID=contig00004.g3120;Description=hexose transporter [Fusarium napiforme];Gene=BFJ72_g4513;Ontology_term=transmembrane transport,oxidoreductase activity,amino acid metabolic process,transporter activity;Ontology_id=GO:0055085,GO:0016491,GO:0006520,GO:0005215;Enzyme_code=EC:1.1.1.85;Enzyme_name=3-isopropylmalate dehydrogenase
contig00004.g3121	766	ID=contig00004.g3121;Description=hypothetical protein FVEG_05679 [Fusarium verticillioides 7600];Gene=FVER53263_05679;Ontology_term=anatomical structure development;Ontology_id=GO:0048856
contig00004.g3122	3907	ID=contig00004.g3122;Description=hypothetical protein FVEG_05681 [Fusarium verticillioides 7600];Gene=FVEG_05681;Ontology_term=nucleus,catalytic activity, acting on a protein,regulation of DNA-templated transcription,extracellular region,transcription regulator activity,hydrolase activity;Ontology_id=GO:0005634,GO:0140096,GO:0006355,GO:0005576,GO:0140110,GO:0016787;Enzyme_code=EC:3.4,EC:3.4,EC:3;Enzyme_name=Acting on peptide bonds (peptidases),Acting on peptide bonds (peptidases),Hydrolases
contig00004.g3123	1852	ID=contig00004.g3123;Description=vitamin H transporter [Fusarium tjaetaba];Gene=FNYG_04397;Ontology_term=transmembrane transport,transporter activity;Ontology_id=GO:0055085,GO:0005215
contig00004.g3124	921	ID=contig00004.g3124;Description=alcohol dehydrogenase [Fusarium verticillioides 7600];Ontology_term=oxidoreductase activity,detoxification;Ontology_id=GO:0016491,GO:0098754;Enzyme_code=EC:1.2,EC:1,EC:1.2.3,EC:1.2.3.1;Enzyme_name=Acting on the aldehyde or oxo group of donors,Oxidoreductases,Acting on the aldehyde or oxo group of donors,aldehyde oxidase
contig00004.g3125	1902	ID=contig00004.g3125;Description=alcohol dehydrogenase [Fusarium tjaetaba];Gene=FPANT_8762;Ontology_term=transmembrane transport,transporter activity,hydrolase activity;Ontology_id=GO:0055085,GO:0005215,GO:0016787;Enzyme_code=EC:3.2,EC:3.2,EC:3;Enzyme_name=Glycosylases,Glycosylases,Hydrolases
contig00004.g3126	2301	ID=contig00004.g3126;Description=beta-lactamase/transpeptidase-like protein [Fusarium oxysporum Fo47]
contig00004.g3127	1215	ID=contig00004.g3127;Description=hypothetical protein J7337_002091 [Fusarium musae]

contig00004.g3128	965	ID=contig00004.g3128;Description=cytokinin dehydrogenase 3 [Fusarium sp. NRRL 52700];Gene=FOIG_05268;Ontology_term=oxidoreductase activity;Ontology_id=GO:0016491;Enzyme_code=EC:1,EC:1;Enzyme_name=Oxidoreductases,Oxidoreductases
contig00004.g3129	321	ID=contig00004.g3129;Description=hypothetical protein FVER53590_28833 [Fusarium verticillioides]
contig00004.g3130	744	ID=contig00004.g3130;Description=metallopeptidase catalytic domain-containing protein [Fusarium coicis];Gene=FMUND_2159;Ontology_term=catalytic activity, acting on a protein,extracellular matrix,hydrolase activity;Ontology_id=GO:0140096,GO:0031012,GO:0016787;Enzyme_code=EC:3.4.24,EC:3.4.24,EC:3.4,EC:3;Enzyme_name=Acting on peptide bonds (peptidases),Acting on peptide bonds (peptidases),Acting on peptide bonds (peptidases),Hydrolases
contig00004.g3131	267	ID=contig00004.g3131;Description=hypothetical protein FVER53263_20027 [Fusarium verticillioides]
contig00004.g3132	2186	ID=contig00004.g3132;Description=alpha-N-arabinofuranosidase [Fusarium verticillioides 7600];Gene=abfA-1;Ontology_term=carbohydrate metabolic process,transmembrane transport,transporter activity,hydrolase activity;Ontology_id=GO:0005975,GO:0055085,GO:0005215,GO:0016787;Enzyme_code=EC:3.2.1.55;Enzyme_name=non-reducing end alpha-L-arabinofuranosidase
contig00004.g3133	1603	ID=contig00004.g3133;Description=sugar transporter [Fusarium fujikuroi];Gene=LW93_1520;Ontology_term=transmembrane transport,transporter activity;Ontology_id=GO:0055085,GO:0005215
contig00004.g3134	2459	ID=contig00004.g3134;Description=alpha beta-hydrolase [Fusarium tjaetaba];Gene=FVER53263_20028;Ontology_term=hydrolase activity;Ontology_id=GO:0016787;Enzyme_code=EC:3,EC:3;Enzyme_name=Hydrolases,Hydrolases
contig00004.g3135	782	ID=contig00004.g3135;Description=carbohydrate-binding module family 1 [Fusarium sp. NRRL 25303];Gene=FGADI_4374;Ontology_term=membrane;Ontology_id=GO:0016020

contig00004.g3136	1424	ID=contig00004.g3136;Description=kinase-like domain-containing protein [Fusarium redolens];Gene=BFJ68_g10375;Ontology_term=catalytic activity, acting on a protein,transferase activity;Ontology_id=GO:0140096,GO:0016740;Enzyme_code=EC:2.7.1,EC:2.7.1,EC:2.7.11.1,EC:2.7,EC:2;Enzyme_name=Transferring phosphorus-containing groups,Transferring phosphorus-containing groups,non-specific serine/threonine protein kinase,Transferring phosphorus-containing groups,Transferases
contig00004.g3137	1514	ID=contig00004.g3137;Description=hypothetical protein FVEG_05694 [Fusarium verticillioides 7600];Gene=3608;Ontology_term=peroxisome,antioxidant activity,oxidoreductase activity,mitochondrion;Ontology_id=GO:0005777,GO:0016209,GO:0016491,GO:0005739;Enzyme_code=EC:1.11.1.6;Enzyme_name=catalase
contig00004.g3138	1698	ID=contig00004.g3138;Description=nicotinamide mononucleotide permease [Fusarium subglutinans];Gene=FNAPI_11612;Ontology_term=transmembrane transport,transporter activity;Ontology_id=GO:0055085,GO:0005215
contig00004.g3139	1473	ID=contig00004.g3139;Description=n-sulfoglucosamine sulfohydrolase precursor [Fusarium tjaetaba];Gene=FPANT_6299;Ontology_term=hydrolase activity;Ontology_id=GO:0016787;Enzyme_code=EC:3.1.6,EC:3.1.6,EC:3.1,EC:3,EC:3.10.1.1;Enzyme_name=Acting on ester bonds,Acting on ester bonds,Acting on ester bonds,Hydrolases,N-sulfoglucosamine sulfohydrolase
contig00004.g3140	1770	ID=contig00004.g3140;Description=murein transglycosylase [Colletotrichum scovillei];Gene=CFIO01_10450;Ontology_term=carbohydrate metabolic process,cell wall organization or biogenesis,hydrolase activity;Ontology_id=GO:0005975,GO:0071554,GO:0016787;Enzyme_code=EC:3.2.1.15,EC:3.2,EC:3,EC:3.2.1.171,EC:3.2.1.15,EC:3.2.1;Enzyme_name=endo-polygalacturonase,Glycosylases,Hydrolases,rhamnogalacturonan hydrolase,endo-polygalacturonase,Glycosylases
contig00004.g3141	1654	ID=contig00004.g3141;Description=tartrate transporter [Fusarium denticulatum];Gene=FPCIR_2181;Ontology_term=transmembrane transport,transporter activity;Ontology_id=GO:0055085,GO:0005215

contig00004.g3142	2296	ID=contig00004.g3142;Description=amidohydrolase ytcJ-like [Fusarium verticillioides 7600];Gene=FFC1_08003;Ontology_term=hydrolase activity;Ontology_id=GO:0016787;Enzyme_code=EC:3.5,EC:3.5,EC:3;Enzyme_name=Acting on carbon-nitrogen bonds, other than peptide bonds,Acting on carbon-nitrogen bonds, other than peptide bonds,Hydrolases
contig00004.g3143	1272	ID=contig00004.g3143;Description=pectin lyase precursor [Fusarium tjaetaba];Gene=FPANT_6295;Ontology_term=carbohydrate metabolic process,lyase activity,extracellular region;Ontology_id=GO:0005975,GO:0016829,GO:0005576;Enzyme_code=EC:4.2.2.2,EC:4.2.2.2,EC:4.2,EC:4.2.2.10,EC:4,EC:4.2.2;Enzyme_name=pectate lyase,pectate lyase,Carbon-oxygen lyases,pectin lyase,Lyases,Carbon-oxygen lyases
contig00004.g3144	837	ID=contig00004.g3144;Description=hypothetical protein FVEG_05701 [Fusarium verticillioides 7600]
contig00004.g3145	540	ID=contig00004.g3145;Description=hypothetical protein FVEG_05702 [Fusarium verticillioides 7600];Gene=FPANT_6293;Ontology_term=membrane;Ontology_id=GO:0016020
contig00004.g3146	3276	ID=contig00004.g3146;Description=hypothetical protein FVEG_05703 [Fusarium verticillioides 7600]
contig00004.g3147	2901	ID=contig00004.g3147;Description=aminopeptidase 2 [Fusarium tjaetaba];Gene=FPCIR_2190;Ontology_term=catalytic activity, acting on a protein,hydrolase activity;Ontology_id=GO:0140096,GO:0016787;Enzyme_code=EC:3.4.11,EC:3.4.11,EC:3.4,EC:3.4.11.2,EC:3;Enzyme_name=Acting on peptide bonds (peptidases),Acting on peptide bonds (peptidases),Acting on peptide bonds (peptidases),membrane alanyl aminopeptidase,Hydrolases
contig00004.g3148	1169	ID=contig00004.g3148;Description=aminopeptidase 2 [Fusarium coicis];Gene=FPCIR_2190;Ontology_term=catalytic activity, acting on a protein,hydrolase activity;Ontology_id=GO:0140096,GO:0016787;Enzyme_code=EC:3.4.11;Enzyme_name=Acting on peptide bonds (peptidases)
contig00004.g3149	1575	ID=contig00004.g3149;Description=hypothetical protein FVER53263_20029 [Fusarium verticillioides];Gene=FVER53263_20029;Ontology_term=membrane;Ontology_id=GO:0016020
contig00004.g3150	919	ID=contig00004.g3150;Description=uncharacterized protein FTJAE_10822 [Fusarium tjaetaba];Gene=msp1;Ontology_term=plasma membrane;Ontology_id=GO:0005886

contig00004.g3151	1221	ID=contig00004.g3151;Description=hypothetical protein FVER14953_05709 [Fusarium verticillioides]
contig00004.g3152	1605	ID=contig00004.g3152;Description=tartrate transporter [Fusarium subglutinans];Gene=FOXB_03752;Ontology_term=transmembrane transport,transporter activity;Ontology_id=GO:0055085,GO:0005215
contig00004.g3153	4516	ID=contig00004.g3153;Description=D-lactate dehydrogenase (cytochrome) [Fusarium verticillioides 7600];Gene=FVER53263_05713;Ontology_term=nucleus,DNA binding,catalytic activity,DNA-templated transcription;Ontology_id=GO:0005634,GO:0003677,GO:0003824,GO:0006351
contig00004.g3154	396	ID=contig00004.g3154;Description=hypothetical protein FVEG_05714 [Fusarium verticillioides 7600]
contig00004.g3155	246	ID=contig00004.g3155;Description=hypothetical protein FVER53263_05715 [Fusarium verticillioides]
contig00004.g3156	1325	ID=contig00004.g3156;Description=six-hairpin glycosidase [Fusarium coicis];Gene=FPANT_14170;Ontology_term=metabolic process;Ontology_id=GO:0008152
contig00004.g3157	1062	ID=contig00004.g3157;Description=hypothetical protein J7337_002061 [Fusarium musae];Gene=FVER53590_05717;Ontology_term=cell surface;Ontology_id=GO:0009986
contig00004.g3158	1603	ID=contig00004.g3158;Description=major facilitator superfamily domain-containing protein [Fusarium oxysporum Fo47];Gene=FPANT_6285;Ontology_term=transmembrane transport,transporter activity;Ontology_id=GO:0055085,GO:0005215
contig00004.g3159	1526	ID=contig00004.g3159;Description=n-carbamoyl-amino acid hydrolase [Fusarium tjaetaba];Gene=FTJAE_10830;Ontology_term=hydrolase activity;Ontology_id=GO:0016787;Enzyme_code=EC:3.5.3;Enzyme_name=Acting on carbon-nitrogen bonds, other than peptide bonds
contig00004.g3160	2286	ID=contig00004.g3160;Description=transcriptional activator DAL81 [Fusarium pseudoanthophilum];Gene=FNAPI_8246;Ontology_term=nucleus,DNA binding,regulation of DNA-templated transcription,transcription regulator activity,hydrolase activity;Ontology_id=GO:0005634,GO:0003677,GO:0006355,GO:0140110,GO:0016787;Enzyme_code=EC:3.5.3,EC:3.5.3,EC:3.5,EC:3;Enzyme_name=Acting on carbon-nitrogen bonds, other than peptide bonds,Acting on carbon-nitrogen bonds, other than peptide bonds,Acting on carbon-nitrogen bonds, other than peptide bonds,Hydrolases
contig00004.g3161	945	ID=contig00004.g3161;Description=hypothetical protein FVER14953_05722 [Fusarium verticillioides]

contig00004.g3162	5008	ID=contig00004.g3162;Description=related to coenzyme a synthetase [Fusarium fujikuroi];Gene=FVER53263_05723;Ontology_term=membrane,organic substance biosynthetic process,phosphopantetheine binding,cellular biosynthetic process;Ontology_id=GO:0016020,GO:1901576,GO:0031177,GO:0044249
contig00004.g3163	1560	ID=contig00004.g3163;Description=coenzyme a synthetase [Fusarium subglutinans];Gene=FACUT_5209;Ontology_term=transferase activity;Ontology_id=GO:0016740;Enzyme_code=EC:2;Enzyme_name=Transferases
contig00004.g3164	1119	ID=contig00004.g3164;Description=alkaline ceramidase ydc1 [Fusarium oxysporum];Gene=FVEG_05725;Ontology_term=lipid metabolic process,endoplasmic reticulum,transferase activity,vacuole,hydrolase activity;Ontology_id=GO:0006629,GO:0005783,GO:0016740,GO:0005773,GO:0016787;Enzyme_code=EC:3,EC:3.5.1,EC:2.3,EC:3.2,EC:3.5.1.23,EC:2,EC:3,EC:3.5,EC:3.2.1,EC:2.3.1.24,EC:2.3.1;Enzyme_name=Hydrolases,Acting on carbon-nitrogen bonds, other than peptide bonds,Acyltransferases,Glycosylases,ceramidase,Transferases,Hydrolases,Acting on carbon-nitrogen bonds, other than peptide bonds,Glycosylases,sphingosine N-acyltransferase,Acyltransferases
contig00004.g3165	1864	ID=contig00004.g3165;Description=arachidonate 5-lipoxygenase [Fusarium tjaetaba];Gene=FVEG_05726;Ontology_term=oxidoreductase activity,lipid metabolic process;Ontology_id=GO:0016491,GO:0006629;Enzyme_code=EC:1.13.11.45,EC:1.13.11,EC:1.13,EC:1.13.11.45,EC:1;Enzyme_name=linoleate 11-lipoxygenase,Acting on single donors with incorporation of molecular oxygen (oxygenases). The oxygen incorporated need not be derived from O2,Acting on single donors with incorporation of molecular oxygen (oxygenases). The oxygen incorporated need not be derived from O2,linoleate 11-lipoxygenase,Oxidoreductases
contig00004.g3166	1234	ID=contig00004.g3166;Description=salicylate hydroxylase [Fusarium pseudoanthophilum];Gene=FDENT_10766;Ontology_term=oxidoreductase activity;Ontology_id=GO:0016491;Enzyme_code=EC:1;Enzyme_name=Oxidoreductases
contig00004.g3167	1322	ID=contig00004.g3167;Description=salicylate hydroxylase [Fusarium tjaetaba];Gene=FOIG_15583;Ontology_term=oxidoreductase activity;Ontology_id=GO:0016491;Enzyme_code=EC:1,EC:1;Enzyme_name=Oxidoreductases,Oxidoreductases

contig00004.g3168	860	ID=contig00004.g3168;Description=salicylate hydroxylase [Fusarium coicis];Gene=3641;Ontology_term=RNA binding,ATP-dependent activity,catalytic activity, acting on RNA;Ontology_id=GO:0003723,GO:0140657,GO:0140098;Enzyme_code=EC:3.6.4.13;Enzyme_name=RNA helicase
contig00004.g3169	2781	ID=contig00004.g3169;Description=hypothetical protein FVER53263_05729 [Fusarium verticillioides]
contig00004.g3170	3853	ID=contig00004.g3170;Description=gamma hydroxybutyrate dehydrogenase [Fusarium coicis];Gene=FPHYL_3644;Ontology_term=oxidoreductase activity;Ontology_id=GO:0016491;Enzyme_code=EC:1.1.1,EC:1.1,EC:1,EC:1.1.1;Enzyme_name=Acting on the CH-OH group of donors,Acting on the CH-OH group of donors,Oxidoreductases,Acting on the CH-OH group of donors
contig00004.g3171	954	ID=contig00004.g3171;Description=kDa in traX-finO intergenic region [Fusarium acutatum]
contig00004.g3172	1681	ID=contig00004.g3172;Description=monooxygenase [Fusarium tjaetaba];Gene=FANTH_10809;Ontology_term=oxidoreductase activity;Ontology_id=GO:0016491;Enzyme_code=EC:1.14.13.8,EC:1.14.13.8,EC:1,EC:1.14,EC:1.14.13;Enzyme_name=flavin-containing monooxygenase,flavin-containing monooxygenase,Oxidoreductases,Acting on paired donors, with incorporation or reduction of molecular oxygen. The oxygen incorporated need not be derived from O2,Acting on paired donors, with incorporation or reduction of molecular oxygen. The oxygen incorporated need not be derived from O2
contig00004.g3173	1248	ID=contig00004.g3173;Description=hypothetical protein FVER14953_05734 [Fusarium verticillioides]
contig00004.g3174	1830	ID=contig00004.g3174;Description=fungal specific transcription factor [Fusarium denticulatum];Gene=FDENT_10756;Ontology_term=nucleus,DNA binding,oxidoreductase activity,regulation of DNA-templated transcription,transcription regulator activity;Ontology_id=GO:0005634,GO:0003677,GO:0016491,GO:0006355,GO:0140110;Enzyme_code=EC:1,EC:1;Enzyme_name=Oxidoreductases,Oxidoreductases
contig00004.g3175	1277	ID=contig00004.g3175;Description=hypothetical protein FVER53590_05737 [Fusarium verticillioides];Gene=FVER53590_05737;Ontology_term=oxidoreductase activity;Ontology_id=GO:0016491;Enzyme_code=EC:1,EC:1;Enzyme_name=Oxidoreductases,Oxidoreductases
contig00004.g3176	854	ID=contig00004.g3176;Description=hypothetical protein FVEG_05738 [Fusarium verticillioides 7600]

contig00004.g3177	518	ID=contig00004.g3177;Description=Protein transport protein BET1 [Fusarium oxysporum f. sp. albedinis]
contig00004.g3178	693	ID=contig00004.g3178;Description=hypothetical protein FVER53263_05740 [Fusarium verticillioides]
contig00004.g3179	1171	ID=contig00004.g3179;Description=transcriptional regulatory moc3 [Fusarium subglutinans];Gene=FSUBG_12961;Ontology_term=nucleus,oxidoreductase activity,detoxification,regulation of DNA-templated transcription,transcription regulator activity;Ontology_id=GO:0005634,GO:0016491,GO:0098754,GO:0006355,GO:0140110;Enzyme_code=EC:1,EC:1,EC:1.6.99.1;Enzyme_name=Oxidoreductases,Oxidoreductases,NADPH dehydrogenase
contig00004.g3180	1173	ID=contig00004.g3180;Description=dityrosine transporter [Fusarium tjaetaba];Gene=FVEG_15715;Ontology_term=transmembrane transport,transporter activity;Ontology_id=GO:0055085,GO:0005215
contig00004.g3181	1164	ID=contig00004.g3181;Description=transcriptional regulatory moc3 [Fusarium pseudoanthophilum];Gene=FMUND_12404;Ontology_term=nucleus,DNA binding,oxidoreductase activity,regulation of DNA-templated transcription,transcription regulator activity;Ontology_id=GO:0005634,GO:0003677,GO:0016491,GO:0006355,GO:0140110;Enzyme_code=EC:1.13.11,EC:1.13.11,EC:1.13,EC:1;Enzyme_name=Acting on single donors with incorporation of molecular oxygen (oxygenases). The oxygen incorporated need not be derived from O2,Acting on single donors with incorporation of molecular oxygen (oxygenases). The oxygen incorporated need not be derived from O2,Acting on single donors with incorporation of molecular oxygen (oxygenases). The oxygen incorporated need not be derived from O2,Oxidoreductases
contig00004.g3182	2268	ID=contig00004.g3182;Description=hypothetical protein FVER53590_28841 [Fusarium verticillioides];Gene=FOYG_02219;Ontology_term=nucleus,DNA binding,regulation of DNA-templated transcription,transcription regulator activity;Ontology_id=GO:0005634,GO:0003677,GO:0006355,GO:0140110

contig00004.g3183	1569	ID=contig00004.g3183;Description=lignostilbene alpha beta-dioxygenase I [Fusarium coicis];Gene=FocTR4_00006026;Ontology_term=oxidoreductase activity;Ontology_id=GO:0016491;Enzyme_code=EC:1.13.11,EC:1.13.11,EC:1.13,EC:1;Enzyme_name=Acting on single donors with incorporation of molecular oxygen (oxygenases). The oxygen incorporated need not be derived from O2,Acting on single donors with incorporation of molecular oxygen (oxygenases). The oxygen incorporated need not be derived from O2,Acting on single donors with incorporation of molecular oxygen (oxygenases). The oxygen incorporated need not be derived from O2,Oxidoreductases
contig00004.g3184	349	ID=contig00004.g3184;Description=hypothetical protein FVEG_05745 [Fusarium verticillioides 7600];Gene=DAL82;Ontology_term=nucleus,DNA binding,regulation of DNA-templated transcription;Ontology_id=GO:0005634,GO:0003677,GO:0006355
contig00004.g3185	5394	ID=contig00004.g3185;Description=NAM7-nonsense-mediated mRNA decay [Fusarium tjaetaba];Gene=FVER53263_05746;Ontology_term=RNA binding,ATP-dependent activity,regulatory ncRNA-mediated gene silencing,catalytic activity, acting on RNA,transferase activity,hydrolase activity;Ontology_id=GO:0003723,GO:0140657,GO:0031047,GO:0140098,GO:0016740,GO:0016787;Enzyme_code=EC:2.7.7.48,EC:3.6.1.15,EC:3.6.1,EC:2.7,EC:3.6,EC:2,EC:3,EC:2.7.7.48,EC:2.7.7,EC:3.6.1.15;Enzyme_name=RNA-directed RNA polymerase,nucleoside-triphosphate phosphatase,Acting on acid anhydrides,Transferring phosphorus-containing groups,Acting on acid anhydrides,Transferases,Hydrolases,RNA-directed RNA polymerase,Transferring phosphorus-containing groups,nucleoside-triphosphate phosphatase
contig00004.g3186	651	ID=contig00004.g3186;Description=P-loop containing protein [Fusarium tjaetaba]
contig00004.g3187	770	ID=contig00004.g3187;Description=hypothetical protein FVER53590_28842 [Fusarium verticillioides];Gene=FNYG_04340;Ontology_term=membrane;Ontology_id=GO:0016020
contig00004.g3188	523	ID=contig00004.g3188;Description=hypothetical protein FVER53590_05748 [Fusarium verticillioides]
contig00004.g3189	1161	ID=contig00004.g3189;Description=archaeal flagellin n-terminal [Fusarium tjaetaba];Gene=FOYG_02208;Ontology_term=membrane;Ontology_id=GO:0016020
contig00004.g3190	1046	ID=contig00004.g3190;Description=archaeal flagellin N-terminal-like domain-containing protein [Fusarium proliferatum];Gene=F25303_10537;Ontology_term=nucleobase-containing small molecule metabolic process,lyase activity,carbohydrate derivative metabolic process;Ontology_id=GO:0055086,GO:0016829,GO:1901135;Enzyme_code=EC:4.1.1.23;Enzyme_name=orotidine-5'-phosphate decarboxylase

contig00004.g3191	678	ID=contig00004.g3191;Description=hypothetical protein FVER53590_05751 [Fusarium verticillioides]
contig00004.g3192	1301	ID=contig00004.g3192;Description=tetracycline resistance [Fusarium coicis];Gene=FMUND_13626;Ontology_term=oxidoreductase activity;Ontology_id=GO:0016491;Enzyme_code=EC:1,EC:1;Enzyme_name=Oxidoreductases,Oxidoreductases
contig00004.g3193	1227	ID=contig00004.g3193;Description=gpi anchored protein [Fusarium tjaetaba];Gene=FTJAE_7291;Ontology_term=oxidoreductase activity;Ontology_id=GO:0016491;Enzyme_code=EC:1.13.11,EC:1.13.11,EC:1.13,EC:1;Enzyme_name=Acting on single donors with incorporation of molecular oxygen (oxygenases). The oxygen incorporated need not be derived from O2,Acting on single donors with incorporation of molecular oxygen (oxygenases). The oxygen incorporated need not be derived from O2,Acting on single donors with incorporation of molecular oxygen (oxygenases). The oxygen incorporated need not be derived from O2,Oxidoreductases
contig00004.g3194	1412	ID=contig00004.g3194;Description=hypothetical protein H9Q69_006647 [Fusarium xylarioides];Gene=2787
contig00004.g3195	2595	ID=contig00004.g3195;Description=beta-mannosidase [Fusarium tjaetaba];Gene=FNAPI_8763;Ontology_term=carbohydrate metabolic process,extracellular region,hydrolase activity;Ontology_id=GO:0005975,GO:0005576,GO:0016787;Enzyme_code=EC:3.2.1.25,EC:3.2,EC:3,EC:3.2.1.25,EC:3.2.1;Enzyme_name=beta-mannosidase,Glycosylases,Hydrolases,beta-mannosidase,Glycosylases
contig00004.g3196	1544	ID=contig00004.g3196;Description=sugar transporter [Fusarium tjaetaba];Gene=FVER53263_05756;Ontology_term=transmembrane transport,transporter activity;Ontology_id=GO:0055085,GO:0005215
contig00004.g3197	2293	ID=contig00004.g3197;Description=related to pathway-specific regulatory protein nit-4 [Fusarium fujikuroi];Gene=FFC1_08101;Ontology_term=nucleus,DNA binding,regulation of DNA-templated transcription,hydrolase activity;Ontology_id=GO:0005634,GO:0003677,GO:0006355,GO:0016787;Enzyme_code=EC:3.5.5.1;Enzyme_name=nitrilase

contig00004.g3198	1144	ID=contig00004.g3198;Description=cyanide hydratase [Fusarium sp. NRRL 25303];Gene=FVER53590_05759;Ontology_term=nucleus,DNA binding,DNA-templated transcription,hydrolase activity;Ontology_id=GO:0005634,GO:0003677,GO:0006351,GO:0016787;Enzyme_code=EC:3.5.5.1,EC:4.2.1.66,EC:3.5.5,EC:3.5,EC:3,EC:3.5.5.1;Enzyme_name=nitrilase,cyanide hydratase,Acting on carbon-nitrogen bonds, other than peptide bonds,Acting on carbon-nitrogen bonds, other than peptide bonds,Hydrolases,nitrilase
contig00004.g3199	1820	ID=contig00004.g3199;Description=hypothetical protein FCOIX_4172 [Fusarium coicis]
contig00004.g3200	1416	ID=contig00004.g3200;Description=hypothetical protein H9Q69_006643 [Fusarium xylarioides]
contig00004.g3201	1451	ID=contig00004.g3201;Description=aspartic-type signal peptidase [Fusarium tjaetaba];Gene=FPANT_8484;Ontology_term=catalytic activity, acting on a protein,hydrolase activity;Ontology_id=GO:0140096,GO:0016787;Enzyme_code=EC:3.4.23;Enzyme_name=Acting on peptide bonds (peptidases)
contig00004.g3202	1950	ID=contig00004.g3202;Description=indole-3-acetamide hydrolase [Fusarium tjaetaba];Gene=FPANT_8483;Ontology_term=hydrolase activity;Ontology_id=GO:0016787;Enzyme_code=EC:3,EC:3;Enzyme_name=Hydrolases,Hydrolases
contig00004.g3203	1875	ID=contig00004.g3203;Description=tryptophan-2-monooxygenase [Fusarium tjaetaba];Gene=FPANT_8482;Ontology_term=oxidoreductase activity;Ontology_id=GO:0016491;Enzyme_code=EC:1,EC:1;Enzyme_name=Oxidoreductases,Oxidoreductases
contig00004.g3204	1279	ID=contig00004.g3204;Description=integral membrane protein PTH11 [Fusarium pseudoanthophilum];Gene=CEK26_006748;Ontology_term=membrane;Ontology_id=GO:0016020
contig00004.g3205	1911	ID=contig00004.g3205;Description=cell wall anchored [Fusarium coicis];Gene=FVER53263_05767;Ontology_term=membrane;Ontology_id=GO:0016020
contig00004.g3206	1245	ID=contig00004.g3206;Description=hypothetical protein FVEG_15721 [Fusarium verticillioides 7600];Gene=FFC1_08106;Ontology_term=nucleus,regulation of DNA-templated transcription,transcription regulator activity;Ontology_id=GO:0005634,GO:0006355,GO:0140110

contig00004.g3207	2509	ID=contig00004.g3207;Description=hyaluronan synthase [Fusarium pseudocircinatum];Gene=FVEG_05769;Ontology_term=membrane;Ontology_id=GO:0016020
contig00004.g3208	1400	ID=contig00004.g3208;Description=related to hard surface induced protein 3 [Fusarium proliferatum ET1];Gene=LW93_1424;Ontology_term=transferase activity;Ontology_id=GO:0016740;Enzyme_code=EC:2.3.1,EC:2.3,EC:2,EC:2.3.1;Enzyme_name=Acyltransferases,Acyltransferases,Transferases,Acyltransferases
contig00004.g3209	822	ID=contig00004.g3209;Description=polysaccharide deacetylase [Fusarium tjaetaba];Gene=FNYG_06533;Ontology_term=carbohydrate metabolic process,extracellular region,vacuole,hydrolase activity;Ontology_id=GO:0005975,GO:0005576,GO:0005773,GO:0016787;Enzyme_code=EC:3.5,EC:3.5,EC:3;Enzyme_name=Acting on carbon-nitrogen bonds, other than peptide bonds,Acting on carbon-nitrogen bonds, other than peptide bonds,Hydrolases
contig00004.g3210	1546	ID=contig00004.g3210;Description=cytochrome b2 mitochondrial [Fusarium pseudoanthophilum];Gene=FNYG_06532;Ontology_term=oxidoreductase activity;Ontology_id=GO:0016491;Enzyme_code=EC:1,EC:1.1.2.3,EC:1;Enzyme_name=Oxidoreductases,L-lactate dehydrogenase (cytochrome),Oxidoreductases
contig00004.g3211	771	ID=contig00004.g3211;Description=hypothetical protein FVER53590_05774 [Fusarium verticillioides]
contig00004.g3212	1679	ID=contig00004.g3212;Description=glucokinase [Fusarium tjaetaba];Gene=FNYG_06530;Ontology_term=carbohydrate metabolic process,generation of precursor metabolites and energy,nucleobase-containing small molecule metabolic process,carbohydrate derivative metabolic process,transferase activity;Ontology_id=GO:0005975,GO:0006091,GO:0055086,GO:1901135,GO:0016740;Enzyme_code=EC:2.7.1.1;Enzyme_name=hexokinase
contig00004.g3213	1868	ID=contig00004.g3213;Description=c6 finger [Fusarium coicis];Gene=FNAPI_8754;Ontology_term=nucleus,regulation of DNA-templated transcription,transcription regulator activity;Ontology_id=GO:0005634,GO:0006355,GO:0140110

contig00004.g3214	1042	ID=contig00004.g3214;Description=glucosamine-6-phosphate isomerase [Fusarium proliferatum];Gene=FANTH_5131;Ontology_term=carbohydrate metabolic process,cytoskeleton,cytoskeleton organization,structural molecule activity,carbohydrate derivative metabolic process,hydrolase activity,isomerase activity;Ontology_id=GO:0005975,GO:0005856,GO:0007010,GO:0005198,GO:1901135,GO:0016787,GO:0016853;Enzyme_code=EC:3.5.99.6,EC:3.1.3.23,EC:3.1.3.11,EC:5,EC:3.1,EC:3.5.99.6,EC:3.1.3.23,EC:3.5,EC:5.3,EC:3,EC:3.1.3.11,EC:5,EC:5.3.1,EC:3.1.3;Enzyme_name=glucosamine-6-phosphate deaminase,sugar-phosphatase,fructose-bisphosphatase,Isomerases,Acting on ester bonds,glucosamine-6-phosphate deaminase,sugar-phosphatase,Acting on carbon-nitrogen bonds, other than peptide bonds,Intramolecular oxidoreductases,Hydrolases,fructose-bisphosphatase,Isomerases,Intramolecular oxidoreductases,Acting on ester bonds
contig00004.g3215	1824	ID=contig00004.g3215;Description=hypothetical protein FVEG_05778 [Fusarium verticillioides 7600]
contig00004.g3216	1708	ID=contig00004.g3216;Description=hexose transporter [Fusarium tjaetaba];Gene=AU210_007070;Ontology_term=transmembrane transport,transporter activity;Ontology_id=GO:0055085,GO:0005215
contig00004.g3217	3119	ID=contig00004.g3217;Description=beta-galactosidase [Fusarium verticillioides 7600];Gene=FVER53590_05780;Ontology_term=carbohydrate metabolic process,cell wall organization or biogenesis,hydrolase activity;Ontology_id=GO:0005975,GO:0071554,GO:0016787;Enzyme_code=EC:3.2.1.23,EC:3.2,EC:3,EC:3.2.1.23,EC:3.2.1;Enzyme_name=beta-galactosidase,Glycosylases,Hydrolases,beta-galactosidase,Glycosylases
contig00004.g3218	687	ID=contig00004.g3218;Description=2,3-bisphosphoglycerate-dependent phosphoglycerate mutase [Fusarium verticillioides 7600];Gene=FVEG_05781;Ontology_term=carbohydrate metabolic process,generation of precursor metabolites and energy,nucleobase-containing small molecule metabolic process,mitochondrion,cell wall,carbohydrate derivative metabolic process,cytosol,extracellular matrix,extracellular region,isomerase activity;Ontology_id=GO:0005975,GO:0006091,GO:0055086,GO:0005739,GO:0005618,GO:1901135,GO:0005829,GO:0031012,GO:0005576,GO:0016853;Enzyme_code=EC:5.4.2.11,EC:5.4,EC:5,EC:5.4.2.11,EC:5.4.2;Enzyme_name=phosphoglycerate mutase (2,3-diphosphoglycerate-dependent),Intramolecular transferases,Isomerases,phosphoglycerate mutase (2,3-diphosphoglycerate-dependent),Intramolecular transferases

contig00004.g3219	1007	ID=contig00004.g3219;Description=ATP adenylyltransferase II [Fusarium pseudoanthophilum];Gene=FVER53590_05782;Ontology_term=nucleobase-containing small molecule metabolic process,transferase activity;Ontology_id=GO:0055086,GO:0016740;Enzyme_code=EC:2.7.7.53;Enzyme_name=ATP adenylyltransferase
contig00004.g3220	1935	ID=contig00004.g3220;Description=POT family proton-dependent oligopeptide transporter [Fusarium proliferatum];Gene=FOC4_g10001699;Ontology_term=transmembrane transport,vacuole,transporter activity,plasma membrane;Ontology_id=GO:0055085,GO:0005773,GO:0005215,GO:0005886
contig00004.g3221	2191	ID=contig00004.g3221;Description=Rossmann-like alpha beta alpha sandwich fold [Fusarium tjaetaba];Gene=FVEG_15725;Ontology_term=lipid metabolic process,transferase activity;Ontology_id=GO:0006629,GO:0016740;Enzyme_code=EC:2.7.8;Enzyme_name=Transferring phosphorus-containing groups
contig00004.g3222	1546	ID=contig00004.g3222;Description=GDP-mannose 4,6-dehydratase [Fusarium fujikuroi];Gene=FNYG_06520;Ontology_term=carbohydrate metabolic process,transmembrane transport,nucleobase-containing small molecule metabolic process,lyase activity,carbohydrate derivative metabolic process,plasma membrane,transporter activity;Ontology_id=GO:0005975,GO:0055085,GO:0055086,GO:0016829,GO:1901135,GO:0005886,GO:0005215;Enzyme_code=EC:4.2.1.47;Enzyme_name=GDP-mannose 4,6-dehydratase
contig00004.g3223	2183	ID=contig00004.g3223;Description=carnosine synthase 1 [Fusarium sp. NRRL 25303];Gene=FocTR4_00006080;Ontology_term=ATP binding,metal ion binding;Ontology_id=GO:0005524,GO:0046872
contig00004.g3224	1517	ID=contig00004.g3224;Description=Transcription factor radR [Fusarium oxysporum f. sp. conglutinans];Gene=FPHYL_3616;Ontology_term=regulation of DNA-templated transcription,transcription regulator activity;Ontology_id=GO:0006355,GO:0140110
contig00004.g3225	1690	ID=contig00004.g3225;Description=GMP synthase [Fusarium tjaetaba];Gene=FVEG_15726;Ontology_term=membrane;Ontology_id=GO:0016020;Enzyme_code=EC:3.2.1.25;Enzyme_name=beta-mannosidase

contig00004.g3226	1629	ID=contig00004.g3226;Description=salicylate hydroxylase [Fusarium verticillioides 7600];Gene=FVER53263_05789;Ontology_term=carbohydrate metabolic process,nucleus,DNA binding,transmembrane transport,oxidoreductase activity,structural molecule activity,chromosome,transporter activity,isomerase activity;Ontology_id=GO:0005975,GO:0005634,GO:0003677,GO:0055085,GO:0016491,GO:0005198,GO:0005694,GO:0005215,GO:0016853;Enzyme_code=EC:1,EC:5.1.3.3,EC:5.1.3.2,EC:1,EC:5.1,EC:5,EC:5.1.3.3,EC:1.14.13.1,EC:5.1.3,EC:5.1.3.2;Enzyme_name=Oxidoreductases,aldose 1-epimerase,UDP-glucose 4-epimerase,Oxidoreductases,Racemases and epimerases,Isomerases,aldose 1-epimerase,salicylate 1-monooxygenase,Racemases and epimerases,UDP-glucose 4-epimerase
contig00004.g3227	414	ID=contig00004.g3227;Description=related to HNM1-Choline permease [Fusarium fujikuroi];Gene=CEK26_006778;Ontology_term=nucleus,DNA binding,carbohydrate metabolic process,transmembrane transport,structural molecule activity,chromosome,transferase activity,transporter activity;Ontology_id=GO:0005634,GO:0003677,GO:0005975,GO:0055085,GO:0005198,GO:0005694,GO:0016740,GO:0005215;Enzyme_code=EC:2.7.7.12,EC:2.7.7.12,EC:2.7,EC:2,EC:2.7.7;Enzyme_name=UDP-glucose--hexose-1-phosphate uridylyltransferase,UDP-glucose--hexose-1-phosphate uridylyltransferase,Transferring phosphorus-containing groups,Transferases,Transferring phosphorus-containing groups
contig00004.g3228	1733	ID=contig00004.g3228;Description=CYB2-lactate dehydrogenase cytochrome b2 [Fusarium subglutinans];Gene=FVER53590_05790;Ontology_term=oxidoreductase activity;Ontology_id=GO:0016491;Enzyme_code=EC:1,EC:1.1.2.3,EC:1;Enzyme_name=Oxidoreductases,L-lactate dehydrogenase (cytochrome),Oxidoreductases
contig00004.g3229	1590	ID=contig00004.g3229;Description=6-hydroxy-D-nicotine oxidase [Fusarium tjaetaba];Gene=FVEG_05791;Ontology_term=oxidoreductase activity;Ontology_id=GO:0016491;Enzyme_code=EC:1,EC:1;Enzyme_name=Oxidoreductases,Oxidoreductases
contig00004.g3230	1604	ID=contig00004.g3230;Description=3-octaprenyl-4-hydroxybenzoate carboxy-lyase [Fusarium subglutinans];Gene=FDC1;Ontology_term=lyase activity;Ontology_id=GO:0016829;Enzyme_code=EC:4.1.1,EC:4.1.1,EC:4.1,EC:4.1.1.102,EC:4;Enzyme_name=Carbon-carbon lyases,Carbon-carbon lyases,Carbon-carbon lyases,phenacrylate decarboxylase,Lyases

contig00004.g3231	813	ID=contig00004.g3231;Description=3-octaprenyl-4-hydroxybenzoate carboxy-lyase UbiX [Fusarium verticillioides 7600];Gene=PAD1;Ontology_term=RNA binding,mitochondrion,lyase activity,transferase activity;Ontology_id=GO:0003723,GO:0005739,GO:0016829,GO:0016740;Enzyme_code=EC:4,EC:2.5.1.129,EC:4.1.1,EC:2.5.1,EC:4.1,EC:2,EC:4,EC:2.5.1.129;Enzyme_name=Lyases,flavin prenyltransferase,Carbon-carbon lyases,Transferring alkyl or aryl groups, other than methyl groups,Carbon-carbon lyases,Transferases,Lyases,flavin prenyltransferase
contig00004.g3232	2401	ID=contig00004.g3232;Description=hypothetical protein FVEG_05793 [Fusarium verticillioides 7600];Gene=FNYG_06510;Ontology_term=nucleus,regulation of DNA-templated transcription,transcription regulator activity;Ontology_id=GO:0005634,GO:0006355,GO:0140110
contig00004.g3233	1623	ID=contig00004.g3233;Description=NAD-dependent aldehyde dehydrogenase [Fusarium verticillioides 7600];Gene=FMUND_7457;Ontology_term=oxidoreductase activity;Ontology_id=GO:0016491;Enzyme_code=EC:1.2.1,EC:1.2,EC:1,EC:1.2.1;Enzyme_name=Acting on the aldehyde or oxo group of donors,Acting on the aldehyde or oxo group of donors,Oxidoreductases,Acting on the aldehyde or oxo group of donors
contig00004.g3234	1837	ID=contig00004.g3234;Description=benzoylformate decarboxylase [Fusarium subglutinans];Gene=FNAPI_8732;Ontology_term=lyase activity;Ontology_id=GO:0016829;Enzyme_code=EC:4.1.1.1;Enzyme_name=pyruvate decarboxylase
contig00004.g3235	1735	ID=contig00004.g3235;Description=dimethylaniline monooxygenase [Fusarium tjaetaba];Gene=FMUND_7455;Ontology_term=oxidoreductase activity;Ontology_id=GO:0016491;Enzyme_code=EC:1;Enzyme_name=Oxidoreductases
contig00004.g3236	881	ID=contig00004.g3236;Description=D-arabinitol 2-dehydrogenase [Fusarium tjaetaba]
contig00004.g3237	1420	ID=contig00004.g3237;Description=carboxymethylenebutenolidase [Fusarium verticillioides 7600];Gene=FPCIR_13139;Ontology_term=polyketide metabolic process;Ontology_id=GO:0030638;Enzyme_code=EC:3.1.1.45;Enzyme_name=carboxymethylenebutenolidase
contig00004.g3238	1746	ID=contig00004.g3238;Description=probable MFS transporter [Fusarium proliferatum ET1];Gene=BFJ72_g4497;Ontology_term=transmembrane transport,transporter activity;Ontology_id=GO:0055085,GO:0005215

contig00004.g3239	2647	ID=contig00004.g3239;Description=phosphate transport MIR1 [Fusarium subglutinans];Gene=FANTH_9445;Ontology_term=transmembrane transport,mitochondrion,transporter activity;Ontology_id=GO:0055085,GO:0005739,GO:0005215
contig00004.g3240	1839	ID=contig00004.g3240;Description=major facilitator superfamily transporter [Fusarium tjaetaba];Gene=3618;Ontology_term=nucleus,DNA binding,catalytic activity, acting on a protein,transmembrane transport,DNA-templated transcription,protein folding,transporter activity,isomerase activity;Ontology_id=GO:0005634,GO:0003677,GO:0140096,GO:0055085,GO:0006351,GO:0006457,GO:0005215,GO:0016853;Enzyme_code=EC:5.2.1.8;Enzyme_name=peptidylprolyl isomerase
contig00004.g3241	478	ID=contig00004.g3241;Description=hypothetical protein J7337_001939 [Fusarium musae]
contig00005.g3242	1229	ID=contig00005.g3242;Description=beta-n-acetylglucosaminidase [Fusarium subglutinans];Gene=FVER53263_07996;Ontology_term=transferase activity;Ontology_id=GO:0016740;Enzyme_code=EC:2.3.1,EC:2.3,EC:2,EC:2.3.1;Enzyme_name=Acyltransferases,Acyltransferases,Transferases,Acyltransferases
contig00005.g3243	4899	ID=contig00005.g3243;Description=tuberin [Fusarium coicis];Gene=FNYG_03462;Ontology_term=nucleus,transmembrane transport,molecular function regulator activity,signaling,cytosol,mitotic cell cycle;Ontology_id=GO:0005634,GO:0055085,GO:0098772,GO:0023052,GO:0005829,GO:0000278
contig00005.g3244	3242	ID=contig00005.g3244;Description=importin-alpha export receptor [Fusarium tjaetaba];Gene=FNYG_03463;Ontology_term=nuclear envelope,nucleocytoplasmic transport,molecular carrier activity,intracellular protein transport;Ontology_id=GO:0005635,GO:0006913,GO:0140104,GO:0006886
contig00005.g3245	1985	ID=contig00005.g3245;Description=hypothetical protein J7337_002954 [Fusarium musae]
contig00005.g3246	3353	ID=contig00005.g3246;Description=WD repeat-containing protein 26 [Fusarium oxysporum f. sp. cubense];Gene=wdr26;Ontology_term=membrane;Ontology_id=GO:0016020
contig00005.g3247	2363	ID=contig00005.g3247;Description=vacuolar protein sorting-associated protein 1 [Fusarium verticillioides 7600];Gene=F25303_5116;Ontology_term=GTPase activity;Ontology_id=GO:0003924;Enzyme_code=EC:3.6.1.15;Enzyme_name=nucleoside-triphosphate phosphatase

contig00005.g3248	1260	ID=contig00005.g3248;Description=alpha-methylacyl-CoA racemase [Fusarium verticillioides 7600];Gene=FNAPI_11646;Ontology_term=catalytic activity;Ontology_id=GO:0003824;Enzyme_code=EC:5.1.99.4;Enzyme_name=alpha-methylacyl-CoA racemase
contig00005.g3249	1512	ID=contig00005.g3249;Description=putative glutamate dehydrogenase (NADP+) [Fusarium fujikuroi];Gene=FVEG_07988;Ontology_term=nucleus,oxidoreductase activity,mitochondrion,amino acid metabolic process,cytosol,extracellular region;Ontology_id=GO:0005634,GO:0016491,GO:0005739,GO:0006520,GO:0005829,GO:0005576;Enzyme_code=EC:1.4.1.3,EC:1.4.1.4,EC:1.4,EC:1,EC:1.4.1.3,EC:1.4.1,EC:1.4.1.4;Enzyme_name=glutamate dehydrogenase [NAD(P)(+)],glutamate dehydrogenase (NADP(+)),Acting on the CH-NH2 group of donors,Oxidoreductases,glutamate dehydrogenase [NAD(P)(+)],Acting on the CH-NH2 group of donors,glutamate dehydrogenase (NADP(+))
contig00005.g3250	3471	ID=contig00005.g3250;Description=DNA polymerase delta subunit 1 [Fusarium verticillioides 7600];Gene=FVER53263_07987;Ontology_term=DNA repair,DNA binding,catalytic activity, acting on DNA,DNA replication,mitochondrion,nuclear chromosome,transferase activity,mitotic cell cycle,hydrolase activity;Ontology_id=GO:0006281,GO:0003677,GO:0140097,GO:0006260,GO:0005739,GO:0000228,GO:0016740,GO:0000278,GO:0016787;Enzyme_code=EC:2.7.7.7,EC:3.1,EC:3.1.11,EC:2.7,EC:2,EC:3,EC:3.1.15,EC:2.7.7.7,EC:2.7.7;Enzyme_name=DNA-directed DNA polymerase,Acting on ester bonds,Acting on ester bonds,Transferring phosphorus-containing groups,Transferases,Hydrolases,Acting on ester bonds,DNA-directed DNA polymerase,Transferring phosphorus-containing groups
contig00005.g3251	1831	ID=contig00005.g3251;Description=related to membrane protein [Fusarium mangiferae];Gene=FPANT_12219;Ontology_term=catalytic activity, acting on a protein,hydrolase activity;Ontology_id=GO:0140096,GO:0016787;Enzyme_code=EC:3.4.21,EC:3.4.21,EC:3.4,EC:3,EC:2.3.1.225;Enzyme_name=Acting on peptide bonds (peptidases),Acting on peptide bonds (peptidases),Acting on peptide bonds (peptidases),Hydrolases,protein S-acyltransferase
contig00005.g3252	588	ID=contig00005.g3252;Description=rhomboid family membrane [Fusarium tjaetaba];Gene=FNYG_03471;Ontology_term=membrane;Ontology_id=GO:0016020
contig00005.g3253	2410	ID=contig00005.g3253;Description=c6 zinc finger [Fusarium tjaetaba];Gene=FVER53263_07984;Ontology_term=nucleus,regulation of DNA-templated transcription,transcription regulator activity;Ontology_id=GO:0005634,GO:0006355,GO:0140110

contig00005.g3254	2511	ID=contig00005.g3254;Description=TUL1 RING-domain E3 ubiquitin ligase [Fusarium fujikuroi];Gene=2769;Ontology_term=catalytic activity, acting on a protein,protein catabolic process,regulation of DNA-templated transcription,Golgi apparatus,endoplasmic reticulum,vacuole,carbohydrate metabolic process,nucleus,signaling,protein maturation,transferase activity,ligase activity;Ontology_id=GO:0140096,GO:0030163,GO:0006355,GO:0005794,GO:0005783,GO:0005773,GO:0005975,GO:0005634,GO:0023052,GO:0051604,GO:0016740,GO:0016874;Enzyme_code=EC:2.7.1.29,EC:6,EC:2.7.1,EC:2.7.1.29,EC:2.3,EC:2.7,EC:2,EC:6,EC:2.3.2;Enzyme_name=glycerone kinase,Ligases,Transferring phosphorus-containing groups,glycerone kinase,Acyltransferases,Transferring phosphorus-containing groups,Transferases,Ligases,Acyltransferases
contig00005.g3255	436	ID=contig00005.g3255;Description=heat shock factor binding 1 [Fusarium tjaetaba];Gene=FNYG_03474
contig00005.g3256	1175	ID=contig00005.g3256;Description=Protein disulfide-isomerase erp38 [Fusarium torreyae];Gene=FSARC_5243;Ontology_term=catalytic activity, acting on a protein,oxidoreductase activity,endoplasmic reticulum,protein folding,isomerase activity;Ontology_id=GO:0140096,GO:0016491,GO:0005783,GO:0006457,GO:0016853;Enzyme_code=EC:5.3.4.1,EC:5.3.4.1,EC:1,EC:1.8,EC:5.3,EC:5.3.4,EC:5;Enzyme_name=protein disulfide-isomerase,protein disulfide-isomerase,Oxidoreductases,Acting on a sulfur group of donors,Intramolecular oxidoreductases,Intramolecular oxidoreductases,Isomerases
contig00005.g3257	575	ID=contig00005.g3257;Description=40S ribosomal protein S29 [Fusarium odoratissimum NRRL 54006];Gene=FOC4_g10011139;Ontology_term=RNA binding,cytoplasmic translation,structural molecule activity,cytosol,ribosome;Ontology_id=GO:0003723,GO:0002181,GO:0005198,GO:0005829,GO:0005840
contig00005.g3258	1413	ID=contig00005.g3258;Description=elongation factor Tu, mitochondrial [Fusarium verticillioides 7600];Gene=FocTR4_00004965;Ontology_term=translation regulator activity,mitochondrial gene expression,RNA binding,GTPase activity,mitochondrion;Ontology_id=GO:0045182,GO:0140053,GO:0003723,GO:0003924,GO:0005739;Enzyme_code=EC:3.6.1.15,EC:3.6.1,EC:3.6,EC:3,EC:3.6.1.15;Enzyme_name=nucleoside-triphosphate phosphatase,Acting on acid anhydrides,Acting on acid anhydrides,Hydrolases,nucleoside-triphosphate phosphatase
contig00005.g3259	471	ID=contig00005.g3259;Description=trafficking PGA2-domain-containing protein [Fusarium flagelliforme];Gene=FPCIR_1711;Ontology_term=membrane;Ontology_id=GO:0016020

contig00005.g3260	3021	ID=contig00005.g3260;Description=hypothetical protein FVER53263_07977 [Fusarium verticillioides];Gene=FVER53263_07977;Ontology_term=structural molecule activity,ribosome;Ontology_id=GO:0005198,GO:0005840
contig00005.g3261	1273	ID=contig00005.g3261;Description=50S ribosomal protein L4e [Fusarium odoratissimum NRRL 54006];Gene=FOXG_01615;Ontology_term=structural molecule activity,ribosome;Ontology_id=GO:0005198,GO:0005840
contig00005.g3262	2075	ID=contig00005.g3262;Description=aromatic amino acid aminotransferase I [Fusarium tjaetaba];Gene=FVER53263_07975;Ontology_term=transferase activity;Ontology_id=GO:0016740;Enzyme_code=EC:2.6.1;Enzyme_name=Transferring nitrogenous groups
contig00005.g3263	1428	ID=contig00005.g3263;Description=flavin-containing protein [Fusarium tjaetaba];Gene=FPANT_9475;Ontology_term=oxidoreductase activity,cytosol,endoplasmic reticulum,protein folding;Ontology_id=GO:0016491,GO:0005829,GO:0005783,GO:0006457;Enzyme_code=EC:1.14.13.8,EC:1.14.13.8,EC:1,EC:1.14,EC:1.14.13;Enzyme_name=flavin-containing monooxygenase,flavin-containing monooxygenase,Oxidoreductases,Acting on paired donors, with incorporation or reduction of molecular oxygen. The oxygen incorporated need not be derived from O2,Acting on paired donors, with incorporation or reduction of molecular oxygen. The oxygen incorporated need not be derived from O2
contig00005.g3264	4578	ID=contig00005.g3264;Description=hypothetical protein FVER14953_07973 [Fusarium verticillioides];Gene=FVER53263_07973;Ontology_term=DNA binding,chromosome,telomere organization;Ontology_id=GO:0003677,GO:0005694,GO:0032200
contig00005.g3265	2072	ID=contig00005.g3265;Description=malate dehydrogenase (oxaloacetate-decarboxylating)(NADP+) [Fusarium verticillioides 7600];Gene=FNYG_03484;Ontology_term=oxidoreductase activity;Ontology_id=GO:0016491;Enzyme_code=EC:1.1.1.38,EC:1.1.1.39;Enzyme_name=malate dehydrogenase (oxaloacetate-decarboxylating),malate dehydrogenase (decarboxylating)
contig00005.g3266	3207	ID=contig00005.g3266;Description=cutinase transcription factor 1 beta [Fusarium proliferatum];Gene=FMUND_13955;Ontology_term=nucleus,DNA binding,regulation of DNA-templated transcription,transcription regulator activity;Ontology_id=GO:0005634,GO:0003677,GO:0006355,GO:0140110

contig00005.g3267	2886	ID=contig00005.g3267;Description=gas2 containing protein [Fusarium tjaetaba];Gene=FNAPI_4941;Ontology_term=cytoskeleton,cytoskeletal protein binding;Ontology_id=GO:0005856,GO:0008092
contig00005.g3268	2075	ID=contig00005.g3268;Description=GTP-binding protein MMR1 [Fusarium tjaetaba];Gene=FTJAE_8154;Ontology_term=nucleocytoplasmic transport,RNA binding,protein-containing complex assembly,GTPase activity,cell differentiation,anatomical structure development,reproductive process,cytosol,ribosome biogenesis,ribosome,intracellular protein transport;Ontology_id=GO:0006913,GO:0003723,GO:0065003,GO:0003924,GO:0030154,GO:0048856,GO:0022414,GO:0005829,GO:0042254,GO:0005840,GO:0006886;Enzyme_code=EC:3.6.1.15,EC:3.6.1,EC:3.6,EC:3,EC:3.6.1.15;Enzyme_name=nucleoside-triphosphate phosphatase,Acting on acid anhydrides,Acting on acid anhydrides,Hydrolases,nucleoside-triphosphate phosphatase
contig00005.g3269	1281	ID=contig00005.g3269;Description=low-temperature viability protein [Fusarium subglutinans];Gene=FVEG_07967;Ontology_term=nucleocytoplasmic transport,nucleus,transmembrane transport,endosome,ribosome biogenesis,intracellular protein transport,vacuole;Ontology_id=GO:0006913,GO:0005634,GO:0055085,GO:0005768,GO:0042254,GO:0006886,GO:0005773
contig00005.g3270	4224	ID=contig00005.g3270;Description=SAC3/GANP/Nin1/mts3/eIF-3 p25 family-domain-containing protein [Fusarium sp. MPI-SDFR-AT-0072];Ontology_term=nuclear envelope,DNA repair,nucleocytoplasmic transport,mRNA metabolic process,ribosome biogenesis,intracellular protein transport,mitotic cell cycle;Ontology_id=GO:0005635,GO:0006281,GO:0006913,GO:0016071,GO:0042254,GO:0006886,GO:0000278
contig00005.g3271	1423	ID=contig00005.g3271;Description=exo-alpha-sialidase neuraminidase [Fusarium tjaetaba];Gene=FDENT_5839;Ontology_term=membrane;Ontology_id=GO:0016020
contig00005.g3272	1017	ID=contig00005.g3272;Description=endo-arabinase [Fusarium denticulatum];Gene=FDENT_5838;Ontology_term=carbohydrate metabolic process,hydrolase activity;Ontology_id=GO:0005975,GO:0016787;Enzyme_code=EC:3.2.1,EC:3.2,EC:3,EC:3.2.1;Enzyme_name=Glycosylases,Glycosylases,Hydrolases,Glycosylases

contig00005.g3273	1718	ID=contig00005.g3273;Description=pseudouridine synthase [Fusarium flagelliforme];Gene=F52700_6942;Ontology_term=vesicle-mediated transport,mRNA metabolic process,RNA binding,snRNA metabolic process,establishment or maintenance of cell polarity,anatomical structure development,cell differentiation,lyase activity,regulatory ncRNA-mediated gene silencing,ribosome biogenesis,nucleolus,isomerase activity;Ontology_id=GO:0016192,GO:0016071,GO:0003723,GO:0016073,GO:0007163,GO:0048856,GO:0030154,GO:0016829,GO:0031047,GO:0042254,GO:0005730,GO:0016853;Enzyme_code=EC:5.4,EC:4.2.1,EC:4.2.1.70,EC:4.2,EC:5.4,EC:4,EC:5;Enzyme_name=Intramolecular transferases,Carbon-oxygen lyases,pseudouridylate synthase,Carbon-oxygen lyases,Intramolecular transferases,Lyases,Isomerases
contig00005.g3274	1518	ID=contig00005.g3274;Description=hypothetical protein FVER14953_07962 [Fusarium verticillioides];Gene=FVER53263_07962;Ontology_term=membrane;Ontology_id=GO:0016020
contig00005.g3275	3250	ID=contig00005.g3275;Description=related to amino-terminal amidase [Fusarium fujikuroi];Gene=FPCIR_5180;Ontology_term=catalytic activity, acting on a protein,protein catabolic process,mitochondrion,hydrolase activity;Ontology_id=GO:0140096,GO:0030163,GO:0005739,GO:0016787;Enzyme_code=EC:3.5.1.121,EC:3.5.1.122,EC:3.5.1,EC:3.5,EC:3,EC:3.5.1.121,EC:3.5.1.122;Enzyme_name=protein N-terminal asparagine amidohydrolase,protein N-terminal glutamine amidohydrolase,Acting on carbon-nitrogen bonds, other than peptide bonds,Acting on carbon-nitrogen bonds, other than peptide bonds,Hydrolases,protein N-terminal asparagine amidohydrolase,protein N-terminal glutamine amidohydrolase
contig00005.g3276	1790	ID=contig00005.g3276;Description=exo-alpha-sialidase neuraminidase [Fusarium pseudocircinatum];Gene=FPANT_11207;Ontology_term=membrane;Ontology_id=GO:0016020
contig00005.g3277	1179	ID=contig00005.g3277;Description=Transcription factor tau subunit sfc1 [Fusarium oxysporum f. sp. albedinis];Gene=AU210_006050;Ontology_term=endoplasmic reticulum;Ontology_id=GO:0005783
contig00005.g3278	379	ID=contig00005.g3278;Description=imidazoleglycerol-phosphate dehydratase [Fusarium tjaetaba];Gene=FOTG_00330;Ontology_term=membrane;Ontology_id=GO:0016020

contig00005.g3279	1167	ID=contig00005.g3279;Description=replication factor C subunit 4 [Fusarium proliferatum];Gene=FVEG_07957;Ontology_term=DNA repair,DNA binding,catalytic activity, acting on DNA,ATP-dependent activity,DNA replication,chromosome segregation,nuclear chromosome,cytosol,mitotic cell cycle,hydrolase activity;Ontology_id=GO:0006281,GO:0003677,GO:0140097,GO:0140657,GO:0006260,GO:0007059,GO:0000228,GO:0005829,GO:0000278,GO:0016787;Enzyme_code=EC:3.6.1.15,EC:3.6.1,EC:3.6,EC:3,EC:3.6.1.15;Enzyme_name=nucleoside-triphosphate phosphatase,Acting on acid anhydrides,Acting on acid anhydrides,Hydrolases,nucleoside-triphosphate phosphatase
contig00005.g3280	3880	ID=contig00005.g3280;Description=hypothetical protein FCOIX_679 [Fusarium coicis];Gene=FOXYS1_537;Ontology_term=DNA binding,ribosome biogenesis,regulation of DNA-templated transcription,catalytic activity, acting on RNA,nucleolus,transcription regulator activity,hydrolase activity;Ontology_id=GO:0003677,GO:0042254,GO:0006355,GO:0140098,GO:0005730,GO:0140110,GO:0016787;Enzyme_code=EC:3.1;Enzyme_name=Acting on ester bonds
contig00005.g3281	1389	ID=contig00005.g3281;Description=probable nitrogen metabolic regulation protein nmr [Fusarium proliferatum ET1];Ontology_term=nucleus,regulation of DNA-templated transcription;Ontology_id=GO:0005634,GO:0006355
contig00005.g3282	1744	ID=contig00005.g3282;Description=hypothetical protein J7337_002992 [Fusarium musae]
contig00005.g3283	2141	ID=contig00005.g3283;Description=POT family proton-dependent oligopeptide transporter [Fusarium verticillioides 7600];Gene=FTJAE_8140;Ontology_term=transmembrane transport,transporter activity;Ontology_id=GO:0055085,GO:0005215
contig00005.g3284	760	ID=contig00005.g3284;Description=putative C-8 sterol isomerase erg-1 [Fusarium fujikuroi];Gene=F25303_8205;Ontology_term=carbohydrate metabolic process,generation of precursor metabolites and energy,extracellular space,transmembrane transport,lipid metabolic process,anatomical structure development,regulation of DNA-templated transcription,receptor ligand activity,endoplasmic reticulum,programmed cell death,isomerase activity;Ontology_id=GO:0005975,GO:0006091,GO:0005615,GO:0055085,GO:0006629,GO:0048856,GO:0006355,GO:0048018,GO:0005783,GO:0012501,GO:0016853;Enzyme_code=EC:5,EC:5.3,EC:5,EC:5.3.3;Enzyme_name=Isomerases,Intramolecular oxidoreductases,Isomerases,Intramolecular oxidoreductases

contig00005.g3285	2020	ID=contig00005.g3285;Description=Dictyostelium kinase [Fusarium tjaetaba];Gene=FVER53590_07948;Ontology_term=cytosol,nuclear chromosome,chromatin organization,transferase activity;Ontology_id=GO:0005829,GO:0000228,GO:0006325,GO:0016740;Enzyme_code=EC:2.7,EC:3.6.4.13,EC:2.7,EC:2;Enzyme_name=Transferring phosphorus-containing groups,RNA helicase,Transferring phosphorus-containing groups,Transferases
contig00005.g3286	817	ID=contig00005.g3286;Description=nuclear pore complex component [Fusarium tjaetaba];Gene=FNYG_03508;Ontology_term=membrane;Ontology_id=GO:0016020
contig00005.g3287	2731	ID=contig00005.g3287;Description=Alcohol dehydrogenase 1 [Fusarium oxysporum f. sp. cubense];Gene=FOYG_03380;Enzyme_code=EC:1.1.1.1;Enzyme_name=alcohol dehydrogenase
contig00005.g3288	1052	ID=contig00005.g3288;Description=DUF410 domain protein [Fusarium tjaetaba];Gene=FVEG_07945;Ontology_term=membrane organization;Ontology_id=GO:0061024
contig00005.g3289	568	ID=contig00005.g3289;Description=UPF0220 domain [Fusarium acutatum]
contig00005.g3290	708	ID=contig00005.g3290;Description=VPS68-like protein [Fusarium subglutinans];Gene=FVEG_07944;Ontology_term=endosome,vesicle-mediated transport,vacuole;Ontology_id=GO:0005768,GO:0016192,GO:0005773
contig00005.g3291	1248	ID=contig00005.g3291;Description=CSI2 [Fusarium pseudocircinatum];Gene=FACUT_9927;Ontology_term=endoplasmic reticulum,vacuole;Ontology_id=GO:0005783,GO:0005773
contig00005.g3292	942	ID=contig00005.g3292;Description=TFIID and SAGA subunit TAF61 [Fusarium tjaetaba];Gene=FTJAE_8131;Ontology_term=molecular function regulator activity;Ontology_id=GO:0098772
contig00005.g3293	681	ID=contig00005.g3293;Description=CSL4-core component of the 3'-5' exosome [Fusarium mundagurra];Gene=FPCIR_10560;Ontology_term=mRNA metabolic process,RNA binding,regulatory ncRNA-mediated gene silencing,nuclear chromosome,ribosome biogenesis,tRNA metabolic process,nucleolus,catalytic activity, acting on RNA,hydrolase activity;Ontology_id=GO:0016071,GO:0003723,GO:0031047,GO:0000228,GO:0042254,GO:0006399,GO:0005730,GO:0140098,GO:0016787;Enzyme_code=EC:3.1,EC:3.1.13,EC:3,EC:3.1.15;Enzyme_name=Acting on ester bonds,Acting on ester bonds,Hydrolases,Acting on ester bonds

contig00005.g3294	4528	ID=contig00005.g3294;Description=ATPase [Fusarium verticillioides 7600];Gene=F25303_8215;Ontology_term=transmembrane transport,ATP-dependent activity,transporter activity,plasma membrane,hydrolase activity;Ontology_id=GO:0055085,GO:0140657,GO:0005215,GO:0005886,GO:0016787;Enzyme_code=EC:7.2.2,EC:3.6.1.15,EC:3.6.1,EC:3.6,EC:7.6.2.2,EC:3,EC:7.2.2,EC:7,EC:3.6.1.15;Enzyme_name=Catalysing the translocation of inorganic cations,nucleoside-triphosphate phosphatase,Acting on acid anhydrides,Acting on acid anhydrides,ABC-type xenobiotic transporter,Hydrolases,Catalysing the translocation of inorganic cations,Translocases,nucleoside-triphosphate phosphatase
contig00005.g3295	1532	ID=contig00005.g3295;Description=tRNA pseudouridine55 synthase [Fusarium verticillioides 7600];Gene=FOQG_00319;Ontology_term=RNA binding,tRNA metabolic process,isomerase activity;Ontology_id=GO:0003723,GO:0006399,GO:0016853;Enzyme_code=EC:5.4;Enzyme_name=Intramolecular transferases
contig00005.g3296	2514	ID=contig00005.g3296;Description=hypothetical protein FCOIX_696 [Fusarium coicis]
contig00005.g3297	2581	ID=contig00005.g3297;Description=cap binding 80 (Cbp80) [Fusarium tjaetaba];Gene=FVER53590_07937;Ontology_term=nucleocytoplasmic transport,RNA binding;Ontology_id=GO:0006913,GO:0003723
contig00005.g3298	1477	ID=contig00005.g3298;Description=Protein kinase gsk3 [Fusarium keratoplasticum];Gene=BHE90_007117;Ontology_term=catalytic activity, acting on a protein,protein catabolic process,signaling,mitotic nuclear division,cell differentiation,anatomical structure development,reproductive process,chromosome segregation,transferase activity;Ontology_id=GO:0140096,GO:0030163,GO:0023052,GO:0140014,GO:0030154,GO:0048856,GO:0022414,GO:0007059,GO:0016740;Enzyme_code=EC:2.7.12.1,EC:2.7.11.1,EC:2.7.1,EC:2.7.11.26,EC:2.7.12.1,EC:2.7.11.1,EC:2.7,EC:2;Enzyme_name=dual-specificity kinase,non-specific serine/threonine protein kinase,Transferring phosphorus-containing groups,[tau protein] kinase,dual-specificity kinase,non-specific serine/threonine protein kinase,Transferring phosphorus-containing groups,Transferases

contig00005.g3299	2033	ID=contig00005.g3299;Description=6-phosphofructo-2-kinase-domain-containing protein [Fusarium redolens];Gene=BFJ72_g4288;Ontology_term=carbohydrate metabolic process,generation of precursor metabolites and energy,nucleobase-containing small molecule metabolic process,carbohydrate derivative metabolic process,transferase activity,hydrolase activity;Ontology_id=GO:0005975,GO:0006091,GO:0055086,GO:1901135,GO:0016740,GO:0016787;Enzyme_code=EC:2.7.1.105,EC:2.7.1,EC:3.1,EC:2.7,EC:2,EC:3.1.3.23,EC:3.1.3.46,EC:3,EC:2.7.1.105,EC:3.1.3;Enzyme_name=6-phosphofructo-2-kinase,Transferring phosphorus-containing groups,Acting on ester bonds,Transferring phosphorus-containing groups,Transferases,sugar-phosphatase,fructose-2,6-bisphosphate 2-phosphatase,Hydrolases,6-phosphofructo-2-kinase,Acting on ester bonds
contig00005.g3300	1177	ID=contig00005.g3300;Description=mitochondrial import receptor subunit tom-40 [Purpureocillium lilacinum];Gene=PCL_00554;Ontology_term=membrane organization,transmembrane transport,ATP-dependent activity,mitochondrion,intracellular protein transport,transporter activity,mitochondrion organization;Ontology_id=GO:0061024,GO:0055085,GO:0140657,GO:0005739,GO:0006886,GO:0005215,GO:0007005;Enzyme_code=EC:7,EC:7.2.2;Enzyme_name=Translocases,Catalysing the translocation of inorganic cations
contig00005.g3301	2845	ID=contig00005.g3301;Description=hypothetical protein FVEG_07933 [Fusarium verticillioides 7600]
contig00005.g3302	1241	ID=contig00005.g3302;Description=ribosomal L5 precursor mitochondrial [Fusarium tjaetaba];Gene=FVER53263_07932;Ontology_term=RNA binding,mitochondrial gene expression,protein-containing complex assembly,mitochondrion,structural molecule activity,cytosol,ribosome biogenesis,ribosome;Ontology_id=GO:0003723,GO:0140053,GO:0065003,GO:0005739,GO:0005198,GO:0005829,GO:0042254,GO:0005840
contig00005.g3303	642	ID=contig00005.g3303;Description=hsp70-like protein [Fusarium verticillioides 7600];Gene=FVEG_07931;Ontology_term=molecular function regulator activity,protein catabolic process,cytoplasmic translation,cytosol,ribosome;Ontology_id=GO:0098772,GO:0030163,GO:0002181,GO:0005829,GO:0005840
contig00005.g3304	1632	ID=contig00005.g3304;Description=actin [Penicillium citrinum]

contig00005.g3305	644	ID=contig00005.g3305;Description=polypeptide chain release factor [Fusarium tjaetaba];Gene=FVER53263_20514;Ontology_term=translation regulator activity,RNA binding,mitochondrial gene expression,mitochondrion,catalytic activity, acting on RNA,ribosome,hydrolase activity;Ontology_id=GO:0045182,GO:0003723,GO:0140053,GO:0005739,GO:0140098,GO:0005840,GO:0016787;Enzyme_code=EC:3.1,EC:3,EC:3.1.1.29,EC:3.1.1;Enzyme_name=Acting on ester bonds,Hydrolases,aminoacyl-tRNA hydrolase,Acting on ester bonds
contig00005.g3306	507	ID=contig00005.g3306;Description=hypothetical protein FHETE_9546 [Fusarium heterosporum];Gene=AK830_g11893;Ontology_term=endoplasmic reticulum;Ontology_id=GO:0005783
contig00005.g3307	2637	ID=contig00005.g3307;Description=elongator complex protein 2 [Fusarium oxysporum f. sp. lycopersici 4287];Gene=FOZG_05738;Ontology_term=nucleus,tRNA metabolic process,regulation of DNA-templated transcription,cytoskeletal protein binding;Ontology_id=GO:0005634,GO:0006399,GO:0006355,GO:0008092
contig00005.g3308	756	ID=contig00005.g3308;Description=ubiquitin-like modifier 1 [Fusarium mundagurra];Gene=URM1;Ontology_term=nucleus,cytosol,tRNA metabolic process,protein tag activity;Ontology_id=GO:0005634,GO:0005829,GO:0006399,GO:0031386
contig00005.g3309	1587	ID=contig00005.g3309;Description=finger AZF1 [Fusarium tjaetaba];Ontology_term=nucleus,DNA binding,cytosol,regulation of DNA-templated transcription,cell wall organization or biogenesis,transcription regulator activity;Ontology_id=GO:0005634,GO:0003677,GO:0005829,GO:0006355,GO:0071554,GO:0140110
contig00005.g3310	1900	ID=contig00005.g3310;Description=FAD carrier FLX1 [Fusarium tjaetaba];Gene=FLX1;Ontology_term=transmembrane transport,mitochondrion,transporter activity;Ontology_id=GO:0055085,GO:0005739,GO:0005215
contig00005.g3311	1131	ID=contig00005.g3311;Description=spliceosome-associated 49 [Fusarium tjaetaba];Gene=1812;Ontology_term=nucleus,mRNA metabolic process,RNA binding,oxidoreductase activity;Ontology_id=GO:0005634,GO:0016071,GO:0003723,GO:0016491;Enzyme_code=EC:1.1.1.1,EC:1.1.1.71,EC:1.1,EC:1,EC:1.1.1,EC:1.1.1.1,EC:1.1.1.71;Enzyme_name=alcohol dehydrogenase,alcohol dehydrogenase [NAD(P)(+)],Acting on the CH-OH group of donors,Oxidoreductases,Acting on the CH-OH group of donors,alcohol dehydrogenase,alcohol dehydrogenase [NAD(P)(+)]

contig00005.g3312	3412	ID=contig00005.g3312;Description=CYC8-general repressor of transcription [Fusarium tjaetaba];Gene=FVEG_07920;Ontology_term=nucleus,chromosome segregation;Ontology_id=GO:0005634,GO:0007059
contig00005.g3313	3875	ID=contig00005.g3313;Description=SH3-domain protein [Fusarium tjaetaba];Gene=HZS61_009483;Ontology_term=nucleus,chromosome segregation;Ontology_id=GO:0005634,GO:0007059
contig00005.g3314	2049	ID=contig00005.g3314;Description=STE/STE20/YSK protein kinase [Fusarium verticillioides 7600];Gene=FCIRC_10851;Ontology_term=catalytic activity, acting on a protein,microtubule organizing center,cytoskeleton organization,signaling,mitotic nuclear division,cytokinesis,transferase activity,programmed cell death;Ontology_id=GO:0140096,GO:0005815,GO:0007010,GO:0023052,GO:0140014,GO:0000910,GO:0016740,GO:0012501;Enzyme_code=EC:2.7.11.1,EC:2.7.1,EC:2.7.11.1,EC:2.7,EC:2,EC:1.1.99.40;Enzyme_name=non-specific serine/threonine protein kinase,Transferring phosphorus-containing groups,non-specific serine/threonine protein kinase,Transferring phosphorus-containing groups,Transferases,(R)-2-hydroxyglutarate--pyruvate transhydrogenase
contig00005.g3315	1566	ID=contig00005.g3315;Description=protein RAI1 [Fusarium proliferatum];Gene=VDAG_08436;Ontology_term=nucleus,RNA binding,mRNA metabolic process,cytosol,ribosome biogenesis,catalytic activity, acting on RNA,hydrolase activity;Ontology_id=GO:0005634,GO:0003723,GO:0016071,GO:0005829,GO:0042254,GO:0140098,GO:0016787;Enzyme_code=EC:3.6.1,EC:3.1;Enzyme_name=Acting on acid anhydrides,Acting on ester bonds
contig00005.g3316	4943	ID=contig00005.g3316;Description=hypothetical protein FVER53590_07915 [Fusarium verticillioides]
contig00005.g3317	969	ID=contig00005.g3317;Description=cytochrome c oxidase subunit 6, mitochondrial [Fusarium odoratissimum NRRL 54006];Gene=FOIG_02806;Ontology_term=generation of precursor metabolites and energy,mitochondrion;Ontology_id=GO:0006091,GO:0005739
contig00005.g3318	1128	ID=contig00005.g3318;Description=fatty acid elongase 3 [Fusarium oxysporum f. sp. lycopersici 4287];Gene=F25303_3543;Ontology_term=vesicle-mediated transport,lipid metabolic process,endoplasmic reticulum,transferase activity;Ontology_id=GO:0016192,GO:0006629,GO:0005783,GO:0016740;Enzyme_code=EC:2.3.1.199,EC:2.3.1.85,EC:2.3,EC:2,EC:2.3.1.199,EC:2.3.1;Enzyme_name=very-long-chain 3-oxoacyl-CoA synthase,fatty-acid synthase system,Acyltransferases,Transferases,very-long-chain 3-oxoacyl-CoA synthase,Acyltransferases

contig00005.g3319	2009	ID=contig00005.g3319;Description=heterokaryon incompatibility (het-6OR allele) [Fusarium tjaetaba]
contig00005.g3320	1921	ID=contig00005.g3320;Description=f-box protein domain-containing protein [Fusarium denticulatum]
contig00005.g3321	810	ID=contig00005.g3321;Description=hypothetical protein FVER14953_07909 [Fusarium verticillioides]
contig00005.g3322	1158	ID=contig00005.g3322;Description=hypothetical protein FVER53263_07908 [Fusarium verticillioides]
contig00005.g3323	2351	ID=contig00005.g3323;Description=heat shock 70 kDa 12B [Fusarium tjaetaba];Gene=FPANT_5643;Ontology_term=protein folding chaperone,ATP-dependent activity;Ontology_id=GO:0044183,GO:0140657
contig00005.g3324	2439	ID=contig00005.g3324;Description=prolyl tripeptidyl peptidase [Fusarium tjaetaba];Gene=FVER53590_07906;Ontology_term=catalytic activity, acting on a protein,vacuole,hydrolase activity;Ontology_id=GO:0140096,GO:0005773,GO:0016787;Enzyme_code=EC:3.4.14.1,EC:3.4.14.5,EC:3.4.14.4,EC:3.4.14.2,EC:3.4.14.1,EC:3.4,EC:3,EC:3.4.14.5,EC:3.4.14.4,EC:3.4.14.2;Enzyme_name=dipeptidyl-peptidase I,dipeptidyl-peptidase IV,dipeptidyl-peptidase III,dipeptidyl-peptidase II,dipeptidyl-peptidase I,Acting on peptide bonds (peptidases),Hydrolases,dipeptidyl-peptidase IV,dipeptidyl-peptidase III,dipeptidyl-peptidase II
contig00005.g3325	1341	ID=contig00005.g3325;Description=prolyl tripeptidyl peptidase [Fusarium circinatum];Gene=FCIRC_6910;Ontology_term=catalytic activity, acting on a protein,vacuole,hydrolase activity;Ontology_id=GO:0140096,GO:0005773,GO:0016787;Enzyme_code=EC:3.4;Enzyme_name=Acting on peptide bonds (peptidases)
contig00005.g3326	1674	ID=contig00005.g3326;Description=related to dityrosine transporter [Fusarium mangiferae];Gene=FNYG_12594;Ontology_term=transmembrane transport,transporter activity;Ontology_id=GO:0055085,GO:0005215
contig00005.g3327	2275	ID=contig00005.g3327;Description=succinate dehydrogenase (ubiquinone) flavo precursor mitochondrial [Fusarium subglutinans];Gene=F52700_6887;Ontology_term=generation of precursor metabolites and energy,oxidoreductase activity,mitochondrion,lipid binding;Ontology_id=GO:0006091,GO:0016491,GO:0005739,GO:0008289;Enzyme_code=EC:1.3.5.1;Enzyme_name=succinate dehydrogenase

contig00005.g3328	3173	ID=contig00005.g3328;Description=succinate dehydrogenase (ubiquinone) flavoprotein subunit [Fusarium verticillioides 7600];Gene=FACUT_150;Ontology_term=lipid binding;Ontology_id=GO:0008289
contig00005.g3329	1136	ID=contig00005.g3329;Description=septum formation initiator domain protein [Fusarium tjaetaba];Gene=FDENT_3816;Ontology_term=membrane;Ontology_id=GO:0016020
contig00005.g3330	475	ID=contig00005.g3330;Description=rRNA-processing protein cgr1 [Fusarium musae];Gene=FOPG_04849;Ontology_term=ribosome biogenesis,nucleolus;Ontology_id=GO:0042254,GO:0005730
contig00005.g3331	2820	ID=contig00005.g3331;Description=cell wall proline rich protein [Fusarium pseudoanthophilum]
contig00005.g3332	725	ID=contig00005.g3332;Description=hypothetical protein FBULB1_6191 [Fusarium bulbicola];Gene=FNAPI_11973;Ontology_term=protein-containing complex assembly,protein catabolic process,anatomical structure development,regulation of DNA-templated transcription,carbohydrate derivative metabolic process,receptor ligand activity,cell adhesion,intracellular protein transport,vitamin metabolic process,immune system process,defense response to other organism,cell motility,nucleocytoplasmic transport,inflammatory response,extracellular space,autophagy,lipid metabolic process,cell differentiation,programmed cell death;Ontology_id=GO:0065003,GO:0030163,GO:0048856,GO:0006355,GO:1901135,GO:0048018,GO:0007155,GO:0006886,GO:0006766,GO:0002376,GO:0098542,GO:0048870,GO:0006913,GO:0006954,GO:0005615,GO:0006914,GO:0006629,GO:0030154,GO:0012501
contig00005.g3333	881	ID=contig00005.g3333;Description=hypothetical protein FVER53590_29623 [Fusarium verticillioides]
contig00005.g3334	4745	ID=contig00005.g3334;Description=ATPase [Fusarium napiforme];Gene=FNAPI_11976;Ontology_term=transmembrane transport,ATP-dependent activity,mitochondrion,cytosol,transporter activity,plasma membrane,hydrolase activity;Ontology_id=GO:0055085,GO:0140657,GO:0005739,GO:0005829,GO:0005215,GO:0005886,GO:0016787;Enzyme_code=EC:7.2.2,EC:3.6.1.15,EC:3.6.1,EC:3.6,EC:7.6.2.2,EC:3,EC:7.2.2,EC:7,EC:3.6.1.15;Enzyme_name=Catalysing the translocation of inorganic cations,nucleoside-triphosphate phosphatase,Acting on acid anhydrides,Acting on acid anhydrides,ABC-type xenobiotic transporter,Hydrolases,Catalysing the translocation of inorganic cations,Translocases,nucleoside-triphosphate phosphatase

contig00005.g3335	4337	ID=contig00005.g3335;Description=putative ubiquitin-specific processing protease 21 [Fusarium fujikuroi];Gene=FPRO05_05360;Ontology_term=nucleus,peroxisome,DNA repair,catalytic activity, acting on a protein,cytosol,endoplasmic reticulum,hydrolase activity;Ontology_id=GO:0005634,GO:0005777,GO:0006281,GO:0140096,GO:0005829,GO:0005783,GO:0016787;Enzyme_code=EC:3.4.19.12,EC:3.4,EC:3,EC:3.4.19.12;Enzyme_name=ubiquitinyl hydrolase 1,Acting on peptide bonds (peptidases),Hydrolases,ubiquitinyl hydrolase 1
contig00005.g3336	1512	ID=contig00005.g3336;Description=serine/threonine protein kinase [Fusarium proliferatum];Gene=1779;Ontology_term=catalytic activity, acting on a protein,oxidoreductase activity,mitochondrion,transferase activity;Ontology_id=GO:0140096,GO:0016491,GO:0005739,GO:0016740;Enzyme_code=EC:2.7.11.1,EC:1.1.1.1,EC:1.1.1.71,EC:2.7.1,EC:1.1,EC:2.7.11.2,EC:2.7.11.1,EC:1,EC:2.7,EC:2,EC:1.1.1,EC:1.1.1.1,EC:1.1.1.71;Enzyme_name=non-specific serine/threonine protein kinase,alcohol dehydrogenase,alcohol dehydrogenase [NAD(P)(+)],Transferring phosphorus-containing groups,Acting on the CH-OH group of donors,[pyruvate dehydrogenase (acetyl-transferring)] kinase,non-specific serine/threonine protein kinase,Oxidoreductases,Transferring phosphorus-containing groups,Transferases,Acting on the CH-OH group of donors,alcohol dehydrogenase,alcohol dehydrogenase [NAD(P)(+)]
contig00005.g3337	1364	ID=contig00005.g3337;Description=Septin spn4 [Fusarium irregulare];Gene=FG07380.1;Ontology_term=cytoskeleton,cytoskeleton organization,protein-containing complex assembly,GTPase activity,cell wall,establishment or maintenance of cell polarity,structural molecule activity,cytokinesis,lipid binding,mitotic cell cycle,molecular adaptor activity;Ontology_id=GO:0005856,GO:0007010,GO:0065003,GO:0003924,GO:0005618,GO:0007163,GO:0005198,GO:0000910,GO:0008289,GO:0000278,GO:0060090;Enzyme_code=EC:3.6.1.15,EC:3.6.1,EC:3.6,EC:3,EC:3.6.1.15;Enzyme_name=nucleoside-triphosphate phosphatase,Acting on acid anhydrides,Acting on acid anhydrides,Hydrolases,nucleoside-triphosphate phosphatase
contig00005.g3338	2808	ID=contig00005.g3338;Description=periodic tryptophan protein 2 [Fusarium verticillioides 7600];Gene=FCIRC_6924;Ontology_term=RNA binding,protein-containing complex assembly,establishment or maintenance of cell polarity,ribosome biogenesis,nucleolus;Ontology_id=GO:0003723,GO:0065003,GO:0007163,GO:0042254,GO:0005730

contig00005.g3339	1493	ID=contig00005.g3339;Description=hypothetical protein FVER14953_07892 [Fusarium verticillioides];Gene=FDENT_7553;Ontology_term=oxidoreductase activity;Ontology_id=GO:0016491;Enzyme_code=EC:1;Enzyme_name=Oxidoreductases
contig00005.g3340	1049	ID=contig00005.g3340;Description=nucleosome assembly protein [Fusarium tjaetaba];Gene=FMUND_8149;Ontology_term=nucleus,protein-containing complex assembly,chromatin organization;Ontology_id=GO:0005634,GO:0065003,GO:0006325
contig00005.g3341	834	ID=contig00005.g3341;Description=copper homeostasis protein [Fusarium verticillioides 7600]
contig00005.g3342	2171	ID=contig00005.g3342;Description=Alkaline phosphatase H [Fusarium odoratissimum];Gene=FOXB_12152;Ontology_term=oxidoreductase activity,hydrolase activity;Ontology_id=GO:0016491,GO:0016787;Enzyme_code=EC:1.1.1.1,EC:3.1.3.1,EC:1.1.1.71,EC:1.1,EC:3.1,EC:1,EC:3,EC:1.1.1,EC:1.1.1.1,EC:3.1.3.1,EC:1.1.1.71,EC:3.1.3;Enzyme_name=alcohol dehydrogenase,alkaline phosphatase,alcohol dehydrogenase [NAD(P)(+)],Acting on the CH-OH group of donors,Acting on ester bonds,Oxidoreductases,Hydrolases,Acting on the CH-OH group of donors,alcohol dehydrogenase,alkaline phosphatase,alcohol dehydrogenase [NAD(P)(+)],Acting on ester bonds
contig00005.g3343	1426	ID=contig00005.g3343;Description=queuine tRNA-ribosyltransferase [Fusarium verticillioides 7600];Gene=FVER53263_07888;Ontology_term=nucleobase-containing small molecule metabolic process,carbohydrate derivative metabolic process,tRNA metabolic process,catalytic activity, acting on RNA,transferase activity;Ontology_id=GO:0055086,GO:1901135,GO:0006399,GO:0140098,GO:0016740;Enzyme_code=EC:2.4.2.64,EC:2.4.2,EC:2.4,EC:2,EC:2.4.2.29,EC:2.4.2.64;Enzyme_name=tRNA-guanosine(34) queuine transglycosylase,Glycosyltransferases,Glycosyltransferases,Transferases,tRNA-guanosine(34) preQ1 transglycosylase,tRNA-guanosine(34) queuine transglycosylase
contig00005.g3344	447	ID=contig00005.g3344;Description=hypothetical protein FVEG_16205 [Fusarium verticillioides 7600];Gene=FPANT_2639;Ontology_term=nucleobase-containing small molecule metabolic process,carbohydrate derivative metabolic process,tRNA metabolic process,catalytic activity, acting on RNA,transferase activity;Ontology_id=GO:0055086,GO:1901135,GO:0006399,GO:0140098,GO:0016740;Enzyme_code=EC:2.4.2.64,EC:2.4.2,EC:2.4,EC:2,EC:2.4.2.29,EC:2.4.2.64;Enzyme_name=tRNA-guanosine(34) queuine transglycosylase,Glycosyltransferases,Glycosyltransferases,Transferases,tRNA-guanosine(34) preQ1 transglycosylase,tRNA-guanosine(34) queuine transglycosylase

contig00005.g3345	2340	ID=contig00005.g3345;Description=LCCL domain protein [Fusarium tjaetaba];Gene=FPCIR_13240;Ontology_term=vacuole;Ontology_id=GO:0005773
contig00005.g3346	1831	ID=contig00005.g3346;Description=hydrolase [Fusarium tjaetaba];Gene=FNYG_12570;Ontology_term=hydrolase activity;Ontology_id=GO:0016787;Enzyme_code=EC:3,EC:3;Enzyme_name=Hydrolases,Hydrolases
contig00005.g3347	2710	ID=contig00005.g3347;Description=gamma-tubulin complex [Fusarium tjaetaba];Gene=FVER53263_07885;Ontology_term=microtubule organizing center,cytoskeleton organization,protein-containing complex assembly,cytoskeletal protein binding;Ontology_id=GO:0005815,GO:0007010,GO:0065003,GO:0008092
contig00005.g3348	1346	ID=contig00005.g3348;Description=probable alpha-1,2-mannosyltransferase [Fusarium fujikuroi];Gene=UOX;Ontology_term=peroxisome,nucleobase-containing small molecule metabolic process,oxidoreductase activity,transferase activity;Ontology_id=GO:0005777,GO:0055086,GO:0016491,GO:0016740;Enzyme_code=EC:1.7.3.3,EC:2.4.1;Enzyme_name=factor independent urate hydroxylase,Glycosyltransferases
contig00005.g3349	1221	ID=contig00005.g3349;Description=beta-transducin [Fusarium tjaetaba];Ontology_term=mRNA metabolic process;Ontology_id=GO:0016071
contig00005.g3350	2897	ID=contig00005.g3350;Description=nitrate assimilation regulatory nirA [Fusarium tjaetaba];Gene=FPANT_5284;Ontology_term=nucleus,DNA binding,regulation of DNA-templated transcription,transcription regulator activity;Ontology_id=GO:0005634,GO:0003677,GO:0006355,GO:0140110
contig00005.g3351	821	ID=contig00005.g3351;Description=hypothetical protein FVER53590_07881 [Fusarium verticillioides];Gene=FPANT_5285;Ontology_term=membrane;Ontology_id=GO:0016020
contig00005.g3352	747	ID=contig00005.g3352;Description=hypothetical protein FVER53590_07880 [Fusarium verticillioides]
contig00005.g3353	3757	ID=contig00005.g3353;Description=calcium-independent phospholipase [Fusarium tjaetaba];Gene=FVEG_07879;Ontology_term=lipid metabolic process,hydrolase activity;Ontology_id=GO:0006629,GO:0016787;Enzyme_code=EC:3,EC:3;Enzyme_name=Hydrolases,Hydrolases
contig00005.g3354	1760	ID=contig00005.g3354;Description=hypothetical protein FVEG_07877 [Fusarium verticillioides 7600]
contig00005.g3355	650	ID=contig00005.g3355;Description=hypothetical protein FVER53590_26518 [Fusarium verticillioides]

contig00005.g3356	7621	ID=contig00005.g3356;Description=Atypical/ABC1/ABC1-A protein kinase [Fusarium odoratissimum NRRL 54006];Gene=FOYG_03517;Ontology_term=catalytic activity, acting on a protein,ATP-dependent activity,mitochondrion,lipid binding,transferase activity,hydrolase activity;Ontology_id=GO:0140096,GO:0140657,GO:0005739,GO:0008289,GO:0016740,GO:0016787;Enzyme_code=EC:2.7,EC:2.7.1,EC:3.6.1,EC:2.7,EC:3.6,EC:2,EC:3,EC:3.6.1.15;Enzyme_name=Transferring phosphorus-containing groups,Transferring phosphorus-containing groups,Acting on acid anhydrides,Transferring phosphorus-containing groups,Acting on acid anhydrides,Transferases,Hydrolases,nucleoside-triphosphate phosphatase
contig00005.g3357	1744	ID=contig00005.g3357;Description=protein phosphatase 5 [Fusarium oxysporum f. sp. lycopersici 4287];Gene=FPCIR_414;Ontology_term=nucleus,catalytic activity, acting on a protein,signaling,hydrolase activity;Ontology_id=GO:0005634,GO:0140096,GO:0023052,GO:0016787;Enzyme_code=EC:3.1.3.16,EC:3.1.3.16,EC:3.1,EC:3,EC:3.1.3;Enzyme_name=protein-serine/threonine phosphatase,protein-serine/threonine phosphatase,Acting on ester bonds,Hydrolases,Acting on ester bonds
contig00005.g3358	613	ID=contig00005.g3358;Description=SPT4-transcription elongation [Fusarium tjaetaba];Gene=BFJ65_g4449;Ontology_term=nucleus,translation regulator activity,catalytic activity, acting on a protein,regulation of DNA-templated transcription,chromosome,hydrolase activity;Ontology_id=GO:0005634,GO:0045182,GO:0140096,GO:0006355,GO:0005694,GO:0016787;Enzyme_code=EC:3.1.3.16;Enzyme_name=protein-serine/threonine phosphatase
contig00005.g3359	1160	ID=contig00005.g3359;Description=KIN17 [Fusarium tjaetaba]
contig00005.g3360	858	ID=contig00005.g3360;Description=YJU2-Essential nuclear spliceosomal component [Fusarium tjaetaba];Gene=Forpi1262_v002566;Ontology_term=nucleus,mRNA metabolic process,protein-containing complex assembly;Ontology_id=GO:0005634,GO:0016071,GO:0065003
contig00005.g3361	210	ID=contig00005.g3361;Description=hypothetical protein F52700_6847 [Fusarium sp. NRRL 52700]
contig00005.g3362	1173	ID=contig00005.g3362;Description=apoptosis-inducing factor 2 [Fusarium tjaetaba];Gene=FVER53263_07867;Ontology_term=oxidoreductase activity;Ontology_id=GO:0016491;Enzyme_code=EC:1,EC:1;Enzyme_name=Oxidoreductases,Oxidoreductases

contig00005.g3363	1046	ID=contig00005.g3363;Description=enoyl hydratase isomerase [Fusarium coicis];Gene=FPANT_10804;Ontology_term=isomerase activity;Ontology_id=GO:0016853;Enzyme_code=EC:5;Enzyme_name=Isomerases
contig00005.g3364	3308	ID=contig00005.g3364;Description=mediator of RNA polymerase II transcription subunit 5 [Fusarium tjaetaba];Gene=MED5;Ontology_term=mitochondrion,regulation of DNA-templated transcription,transcription regulator activity,molecular adaptor activity,nucleoplasm;Ontology_id=GO:0005739,GO:0006355,GO:0140110,GO:0060090,GO:0005654
contig00005.g3365	5770	ID=contig00005.g3365;Description=hypothetical protein FVEG_07864 [Fusarium verticillioides 7600];Gene=F52700_6843;Ontology_term=DNA binding;Ontology_id=GO:0003677
contig00005.g3366	477	ID=contig00005.g3366;Description=hypothetical protein FVER14953_07863 [Fusarium verticillioides]
contig00005.g3367	1356	ID=contig00005.g3367;Description=hypothetical protein FMUND_8178 [Fusarium mundagurra]
contig00005.g3368	1056	ID=contig00005.g3368;Description=glutamic acid decarboxylase [Fusarium tjaetaba];Gene=FNAPI_7624;Ontology_term=lyase activity;Ontology_id=GO:0016829;Enzyme_code=EC:4.1,EC:4.1,EC:4;Enzyme_name=Carbon-carbon lyases,Carbon-carbon lyases,Lyases
contig00005.g3369	1195	ID=contig00005.g3369;Description=GNT1 alphan-acetylglucosamine transferase [Fusarium tjaetaba];Gene=FMAN_04092;Ontology_term=transferase activity;Ontology_id=GO:0016740;Enzyme_code=EC:2,EC:2;Enzyme_name=Transferases,Transferases
contig00005.g3370	995	ID=contig00005.g3370;Description=kinase [Fusarium subglutinans];Gene=FVEG_07858;Ontology_term=ribosome biogenesis,nucleolus,transferase activity;Ontology_id=GO:0042254,GO:0005730,GO:0016740;Enzyme_code=EC:2.7,EC:2.7,EC:2;Enzyme_name=Transferring phosphorus-containing groups,Transferring phosphorus-containing groups,Transferases
contig00005.g3371	1109	ID=contig00005.g3371;Description=hypothetical protein FVER53590_07857 [Fusarium verticillioides];Ontology_term=catalytic activity, acting on a protein,signaling,plasma membrane,hydrolase activity;Ontology_id=GO:0140096,GO:0023052,GO:0005886,GO:0016787;Enzyme_code=EC:3.1,EC:3,EC:3.1.3;Enzyme_name=Acting on ester bonds,Hydrolases,Acting on ester bonds

contig00005.g3372	1600	ID=contig00005.g3372;Description=elongation factor 1-gamma [Fusarium proliferatum];Gene=FOYG_03539;Ontology_term=translation regulator activity;Ontology_id=GO:0045182
contig00005.g3373	1009	ID=contig00005.g3373;Description=37S ribosomal protein S9, mitochondrial [Fusarium musae];Gene=FVEG_07855;Ontology_term=RNA binding,mitochondrial gene expression,mitochondrion,structural molecule activity,ribosome biogenesis,ribosome;Ontology_id=GO:0003723,GO:0140053,GO:0005739,GO:0005198,GO:0042254,GO:0005840
contig00005.g3374	1237	ID=contig00005.g3374;Description=hypothetical protein LZL87_000254 [Fusarium oxysporum]
contig00005.g3375	1134	ID=contig00005.g3375;Description=probable CNB1-calcineurin B, regulatory subunit [Fusarium fujikuroi];Gene=FOXB_07253;Ontology_term=molecular function regulator activity;Ontology_id=GO:0098772
contig00005.g3376	1997	ID=contig00005.g3376;Description=enhancer-polycomb-like protein 1 [Fusarium verticillioides 7600];Gene=FPCIR_435;Ontology_term=DNA repair,autophagy,nuclear chromosome,regulation of DNA-templated transcription,chromatin organization,nucleoplasm;Ontology_id=GO:0006281,GO:0006914,GO:0000228,GO:0006355,GO:0006325,GO:0005654
contig00005.g3377	1041	ID=contig00005.g3377;Description=glycoprotein endopeptidase KAE1 [Fusarium verticillioides 7600];Gene=BFJ68_g6264;Ontology_term=DNA binding,nucleus,DNA recombination,regulation of DNA-templated transcription,tRNA metabolic process,telomere organization,chromosome,transferase activity;Ontology_id=GO:0003677,GO:0005634,GO:0006310,GO:0006355,GO:0006399,GO:0032200,GO:0005694,GO:0016740;Enzyme_code=EC:2.3.1.234,EC:2.3,EC:2,EC:2.3.1.234,EC:2.3.1;Enzyme_name=N(6)-L-threonylcarbamoyladenine synthase,Acyltransferases,Transferases,N(6)-L-threonylcarbamoyladenine synthase,Acyltransferases
contig00005.g3378	1675	ID=contig00005.g3378;Description=SIP2-dominant suppressor of some ts mutations in RPO21 and PRP4 [Fusarium tjaetaba];Gene=FOPG_06519;Ontology_term=nuclear envelope,molecular function regulator activity,protein-containing complex assembly,signaling,cell adhesion,plasma membrane;Ontology_id=GO:0005635,GO:0098772,GO:0065003,GO:0023052,GO:0007155,GO:0005886

contig00005.g3379	705	ID=contig00005.g3379;Description=hypothetical protein J7337_003100 [Fusarium musae];Gene=FNYG_14970;Ontology_term=membrane;Ontology_id=GO:0016020
contig00005.g3380	1898	ID=contig00005.g3380;Description=CMGC/CDK protein kinase [Fusarium verticillioides 7600];Gene=FocTR4_00004821;Ontology_term=catalytic activity, acting on a protein,oxidoreductase activity,nuclear chromosome,regulation of DNA-templated transcription,cell adhesion,transferase activity,chromatin organization,nucleoplasm;Ontology_id=GO:0140096,GO:0016491,GO:0000228,GO:0006355,GO:0007155,GO:0016740,GO:0006325,GO:0005654;Enzyme_code=EC:2.7,EC:1.1.1.71,EC:2.7.1,EC:1.1,EC:2.7.11.23,EC:2.7.11.1,EC:1,EC:2.7,EC:2,EC:1.1.1,EC:2.7.11.22,EC:1.1.1.71;Enzyme_name=Transferring phosphorus-containing groups,alcohol dehydrogenase [NAD(P)(+)],Transferring phosphorus-containing groups,Acting on the CH-OH group of donors,[RNA-polymerase]-subunit kinase,non-specific serine/threonine protein kinase,Oxidoreductases,Transferring phosphorus-containing groups,Transferases,Acting on the CH-OH group of donors,cyclin-dependent kinase,alcohol dehydrogenase [NAD(P)(+)]
contig00005.g3381	1658	ID=contig00005.g3381;Description=L-asparaginase [Fusarium verticillioides 7600];Gene=FMUND_8192;Ontology_term=amino acid metabolic process,hydrolase activity;Ontology_id=GO:0006520,GO:0016787;Enzyme_code=EC:3.5.1.1,EC:3.5.1.1,EC:3.1.1.47,EC:3.5.1,EC:3.5,EC:3,EC:3.1.1.5;Enzyme_name=asparaginase,asparaginase,1-alkyl-2-acetylglycerophosphocholine esterase,Acting on carbon-nitrogen bonds, other than peptide bonds,Acting on carbon-nitrogen bonds, other than peptide bonds,Hydrolases,lysophospholipase
contig00005.g3382	1404	ID=contig00005.g3382;Description=voltage-dependent anion channel [Fusarium flagelliforme];Gene=F53441_2407;Ontology_term=transmembrane transport,endoplasmic reticulum,transporter activity,plasma membrane;Ontology_id=GO:0055085,GO:0005783,GO:0005215,GO:0005886
contig00005.g3383	905	ID=contig00005.g3383;Description=Increased recombination centers 22-2 [Fusarium tjaetaba];Gene=FVEG_07845;Ontology_term=endoplasmic reticulum;Ontology_id=GO:0005783

contig00005.g3384	4455	ID=contig00005.g3384;Description=rad8 and Rdh54p [Fusarium tjaetaba];Gene=FPANT_3031;Ontology_term=DNA repair,catalytic activity, acting on a protein,catalytic activity, acting on DNA,ATP-dependent activity,mitochondrion,DNA recombination,nuclear chromosome,transferase activity,chromatin organization,hydrolase activity;Ontology_id=GO:0006281,GO:0140096,GO:0140097,GO:0140657,GO:0005739,GO:0006310,GO:0000228,GO:0016740,GO:0006325,GO:0016787;Enzyme_code=EC:3,EC:2.3,EC:3.6.1,EC:2,EC:3.6,EC:3,EC:2.3.2.27,EC:3.6.1.15,EC:2.3.2;Enzyme_name=Hydrolases,Acyltransferases,Acting on acid anhydrides,Transferases,Acting on acid anhydrides,Hydrolases,RING-type E3 ubiquitin transferase,nucleoside-triphosphate phosphatase,Acyltransferases
contig00005.g3385	738	ID=contig00005.g3385;Description=IGR [Fusarium tjaetaba];Gene=FPANT_3030;Ontology_term=mitochondrion,ribosome;Ontology_id=GO:0005739,GO:0005840
contig00005.g3386	584	ID=contig00005.g3386;Description=SRP40-suppressor of mutant AC40 of RNA polymerase I and III [Fusarium tjaetaba];Gene=1716;Ontology_term=regulation of DNA-templated transcription,transcription regulator activity;Ontology_id=GO:0006355,GO:0140110
contig00005.g3387	1166	ID=contig00005.g3387;Description=small acidic family domain protein [Fusarium beomiforme]
contig00005.g3388	343	ID=contig00005.g3388;Description=related to secretory pathway protein YSY6 [Fusarium fujikuroi IMI 58289];Gene=FGADI_1387;Ontology_term=endoplasmic reticulum;Ontology_id=GO:0005783
contig00005.g3389	1906	ID=contig00005.g3389;Description=Ca2+ channel [Fusarium tjaetaba];Gene=FDENT_1606;Ontology_term=transmembrane transport,signaling,endoplasmic reticulum,transporter activity,plasma membrane;Ontology_id=GO:0055085,GO:0023052,GO:0005783,GO:0005215,GO:0005886
contig00005.g3390	775	ID=contig00005.g3390;Description=protein yop-1 [Fusarium austroafricanum];Gene=F66182_579
contig00005.g3391	2843	ID=contig00005.g3391;Description=cephalosporin C regulator 1 [Fusarium pseudoanthophilum];Gene=FPANT_7560;Ontology_term=DNA binding,regulation of DNA-templated transcription,nuclear chromosome,transcription regulator activity;Ontology_id=GO:0003677,GO:0006355,GO:0000228,GO:0140110

contig00005.g3392	1907	ID=contig00005.g3392;Description=aspartate kinase [Fusarium oxysporum f. sp. radicis-lycopersici 26381];Gene=FOTG_00174;Ontology_term=amino acid metabolic process,transferase activity;Ontology_id=GO:0006520,GO:0016740;Enzyme_code=EC:2.7.2.4;Enzyme_name=aspartate kinase
contig00005.g3393	2828	ID=contig00005.g3393;Description=Sls2 [Fusarium coicis];Gene=FVEG_07835;Ontology_term=amino acid metabolic process,transferase activity;Ontology_id=GO:0006520,GO:0016740;Enzyme_code=EC:2.7.2.4;Enzyme_name=aspartate kinase
contig00005.g3394	1292	ID=contig00005.g3394;Description=CMGC/CDK/CDK7 protein kinase [Fusarium oxysporum f. sp. lycopersici 4287];Gene=FVEG_07834;Ontology_term=catalytic activity, acting on a protein,regulation of DNA-templated transcription,transferase activity,nucleoplasm;Ontology_id=GO:0140096,GO:0006355,GO:0016740,GO:0005654;Enzyme_code=EC:2.7.11.23,EC:2.7.11.1;Enzyme_name=[RNA-polymerase]-subunit kinase,non-specific serine/threonine protein kinase
contig00005.g3395	1177	ID=contig00005.g3395;Description=hypothetical protein FPCIR_454 [Fusarium pseudocircinatum]
contig00005.g3396	960	ID=contig00005.g3396;Description=sfk1 [Fusarium tjaetaba];Gene=FVER53263_07832;Ontology_term=cytoskeleton organization,signaling,plasma membrane,lysosome;Ontology_id=GO:0007010,GO:0023052,GO:0005886,GO:0005764
contig00005.g3397	2312	ID=contig00005.g3397;Description=hypothetical protein FVER14953_07831 [Fusarium verticillioides]
contig00005.g3398	1124	ID=contig00005.g3398;Description=Ff.00g127650.m01.CDS01 [Fusarium sp. VM40];Gene=FSARC_195
contig00005.g3399	1395	ID=contig00005.g3399;Description=actin filament-binding protein [Fusarium coicis];Gene=F25303_11067;Ontology_term=cytoskeleton,cytoskeleton organization,tRNA metabolic process,cytoskeletal protein binding,catalytic activity, acting on RNA,transferase activity,molecular adaptor activity;Ontology_id=GO:0005856,GO:0007010,GO:0006399,GO:0008092,GO:0140098,GO:0016740,GO:0060090;Enzyme_code=EC:2.1.1,EC:2.1,EC:2,EC:2.1.1,EC:2.1.1.268;Enzyme_name=Transferring one-carbon groups,Transferring one-carbon groups,Transferases,Transferring one-carbon groups,tRNA(Thr)(cytosine(32)-N(3))-methyltransferase

contig00005.g3400	1307	ID=contig00005.g3400;Description=kinetoplast-associated KAP [Fusarium tjaetaba];Gene=FPHYL_11957;Ontology_term=endoplasmic reticulum,protein folding;Ontology_id=GO:0005783,GO:0006457
contig00005.g3401	4160	ID=contig00005.g3401;Description=kinetoplast-associated KAP [Fusarium pseudocircinatum]
contig00005.g3402	642	ID=contig00005.g3402;Description=cyclase dehydrase family [Fusarium tjaetaba];Gene=FPANT_7571;Ontology_term=generation of precursor metabolites and energy,mitochondrion;Ontology_id=GO:0006091,GO:0005739
contig00005.g3403	3586	ID=contig00005.g3403;Description=AP-3 complex gamma subunit [Fusarium denticulatum];Gene=FPANT_7572;Ontology_term=vesicle-mediated transport,Golgi apparatus,intracellular protein transport;Ontology_id=GO:0016192,GO:0005794,GO:0006886
contig00005.g3404	2693	ID=contig00005.g3404;Description=Rho-GTPase-activating 8 [Fusarium tjaetaba];Gene=FOMG_05708;Ontology_term=molecular function regulator activity,cytoskeleton organization,signaling,establishment or maintenance of cell polarity;Ontology_id=GO:0098772,GO:0007010,GO:0023052,GO:0007163
contig00005.g3405	1521	ID=contig00005.g3405;Description=hypothetical protein FNYG_14804 [Fusarium nygamai]
contig00005.g3406	819	ID=contig00005.g3406;Description=hypothetical protein FVER14953_07821 [Fusarium verticillioides]
contig00005.g3407	1363	ID=contig00005.g3407;Description=tyrosyl-tRNA synthetase [Fusarium verticillioides 7600];Gene=Focb16_v009381;Ontology_term=nucleus,RNA binding,cytoplasmic translation,tRNA metabolic process,amino acid metabolic process,catalytic activity, acting on RNA,ligase activity;Ontology_id=GO:0005634,GO:0003723,GO:0002181,GO:0006399,GO:0006520,GO:0140098,GO:0016874;Enzyme_code=EC:6.1.1.1,EC:6.1.1.1,EC:6.1,EC:6.1.1,EC:6;Enzyme_name=tyrosine--tRNA ligase,tyrosine--tRNA ligase,Forming carbon-oxygen bonds,Forming carbon-oxygen bonds,Ligases
contig00005.g3408	1628	ID=contig00005.g3408;Description=membrane dipeptidase [Fusarium verticillioides 7600];Gene=FTJAE_4959;Ontology_term=catalytic activity, acting on a protein,hydrolase activity;Ontology_id=GO:0140096,GO:0016787;Enzyme_code=EC:3.4.13,EC:3.4.13,EC:3.4,EC:3.4.13.19,EC:3;Enzyme_name=Acting on peptide bonds (peptidases),Acting on peptide bonds (peptidases),Acting on peptide bonds (peptidases),membrane dipeptidase,Hydrolases
contig00005.g3409	472	ID=contig00005.g3409;Description=30S ribosomal S13 protein [Fusarium subglutinans];Gene=BFJ68_g6200;Ontology_term=RNA binding,mitochondrion,structural molecule activity,ribosome;Ontology_id=GO:0003723,GO:0005739,GO:0005198,GO:0005840

contig00005.g3410	985	ID=contig00005.g3410;Description=peptidyl-prolyl cis-trans isomerase B [Fusarium subglutinans];Gene=FNAPI_3781;Ontology_term=catalytic activity, acting on a protein,endoplasmic reticulum,protein folding,vacuole,isomerase activity;Ontology_id=GO:0140096,GO:0005783,GO:0006457,GO:0005773,GO:0016853;Enzyme_code=EC:5.2.1.8,EC:5.2.1.8,EC:5.2,EC:5;Enzyme_name=peptidylprolyl isomerase,peptidylprolyl isomerase,Cis-trans-isomerases,Isomerases
contig00005.g3411	462	ID=contig00005.g3411;Description=protein trm-112 [Fusarium oxysporum f. sp. lycopersici 4287];Gene=FOMG_05715;Ontology_term=catalytic activity, acting on a protein,oxidoreductase activity,ribosome biogenesis,tRNA metabolic process,transferase activity,protein folding,extracellular region,catalytic activity, acting on RNA,hydrolase activity,isomerase activity;Ontology_id=GO:0140096,GO:0016491,GO:0042254,GO:0006399,GO:0016740,GO:0006457,GO:0005576,GO:0140098,GO:0016787,GO:0016853;Enzyme_code=EC:5.2.1.8,EC:3.4,EC:2.1.1,EC:1.1.1.1,EC:1.1.1.71,EC:1.1.1,EC:1.1.1.1,EC:2.1,EC:1.1,EC:5.2.1.8,EC:3.4,EC:5.2,EC:1,EC:2,EC:3,EC:5,EC:2.1.1,EC:1.1.1.71;Enzyme_name=peptidylprolyl isomerase,Acting on peptide bonds (peptidases),Transferring one-carbon groups,alcohol dehydrogenase,alcohol dehydrogenase [NAD(P)(+)],Acting on the CH-OH group of donors,alcohol dehydrogenase,Transferring one-carbon groups,Acting on the CH-OH group of donors,peptidylprolyl isomerase,Acting on peptide bonds (peptidases),Cis-trans-isomerases,Oxidoreductases,Transferases,Hydrolases,Isomerases,Transferring one-carbon groups,alcohol dehydrogenase [NAD(P)(+)]
contig00005.g3412	993	ID=contig00005.g3412;Description=ER BIG1 [Fusarium tjaetaba];Gene=FNYG_10279;Ontology_term=carbohydrate metabolic process,endoplasmic reticulum,cell wall organization or biogenesis;Ontology_id=GO:0005975,GO:0005783,GO:0071554
contig00005.g3413	706	ID=contig00005.g3413;Description=nucleolar 12 [Fusarium napiforme];Gene=FNAPI_3784;Ontology_term=oxidoreductase activity,ribosome biogenesis,nucleolus,hydrolase activity;Ontology_id=GO:0016491,GO:0042254,GO:0005730,GO:0016787;Enzyme_code=EC:1.1.1.1,EC:1.1.1.71,EC:1.1,EC:3.1,EC:1,EC:3,EC:1.1.1,EC:1.1.1.1,EC:1.1.1.71;Enzyme_name=alcohol dehydrogenase,alcohol dehydrogenase [NAD(P)(+)],Acting on the CH-OH group of donors,Acting on ester bonds,Oxidoreductases,Hydrolases,Acting on the CH-OH group of donors,alcohol dehydrogenase,alcohol dehydrogenase [NAD(P)(+)]

contig00005.g3414	701	ID=contig00005.g3414;Description=TPM2-tropomyosin isoform 2 [Fusarium tjaetaba];Gene=FVEG_07813;Ontology_term=membrane organization,cytoskeleton,cytoskeleton organization,reproductive process,cytokinesis,cytoskeletal protein binding,mitotic cell cycle;Ontology_id=GO:0061024,GO:0005856,GO:0007010,GO:0022414,GO:0000910,GO:0008092,GO:0000278
contig00005.g3415	1363	ID=contig00005.g3415;Description=hypothetical protein FVER14953_07812 [Fusarium verticillioides]
contig00005.g3416	3901	ID=contig00005.g3416;Description=histone methyltransferase set1 [Fusarium musae];Gene=FNYG_10275;Ontology_term=catalytic activity, acting on a protein,RNA binding,cell differentiation,anatomical structure development,reproductive process,regulation of DNA-templated transcription,nuclear chromosome,telomere organization,chromatin organization,transferase activity,nucleoplasm;Ontology_id=GO:0140096,GO:0003723,GO:0030154,GO:0048856,GO:0022414,GO:0006355,GO:0000228,GO:0032200,GO:0006325,GO:0016740,GO:0005654;Enzyme_code=EC:2.1.1.354,EC:2.1,EC:2.1.1.43,EC:2,EC:2.1.1.354,EC:2.1.1;Enzyme_name=[histone H3]-lysine(4) N-trimethyltransferase,Transferring one-carbon groups,Transferring one-carbon groups,Transferases,[histone H3]-lysine(4) N-trimethyltransferase,Transferring one-carbon groups
contig00005.g3417	684	ID=contig00005.g3417;Description=apoptosis inhibitor ilp [Fusarium tjaetaba]
contig00005.g3418	677	ID=contig00005.g3418;Description=glutamyl-trna synthetase [Fusarium tjaetaba];Gene=FSUBG_5663;Ontology_term=catalytic activity, acting on RNA,ligase activity;Ontology_id=GO:0140098,GO:0016874;Enzyme_code=EC:6.1.1,EC:6.1,EC:6.1.1,EC:6;Enzyme_name=Forming carbon-oxygen bonds,Forming carbon-oxygen bonds,Forming carbon-oxygen bonds,Ligases
contig00005.g3419	1242	ID=contig00005.g3419;Description=hypothetical protein FVER14953_07808 [Fusarium verticillioides]
contig00005.g3420	921	ID=contig00005.g3420;Description=14-3-3 domain-containing protein [Fusarium oxysporum Fo47]

contig00005.g3421	3260	ID=contig00005.g3421;Description=SREBP cleavage activating [Fusarium tjaetaba];Gene=FTJAE_4972;Ontology_term=signaling,lipid metabolic process,regulation of DNA-templated transcription,cytoplasmic vesicle,Golgi apparatus,endoplasmic reticulum,lipid binding,protein maturation,small molecule sensor activity;Ontology_id=GO:0023052,GO:0006629,GO:0006355,GO:0031410,GO:0005794,GO:0005783,GO:0008289,GO:0051604,GO:0140299
contig00005.g3422	2141	ID=contig00005.g3422;Description=urease accessory protein [Fusarium pseudoanthophilum]
contig00005.g3423	610	ID=contig00005.g3423;Description=putative cyanate lyase [Fusarium fujikuroi];Gene=cyn1;Ontology_term=DNA binding,lyase activity;Ontology_id=GO:0003677,GO:0016829;Enzyme_code=EC:4.3,EC:4.2.1.104;Enzyme_name=Carbon-nitrogen lyases,cyanase
contig00005.g3424	1035	ID=contig00005.g3424;Description=hypothetical protein FVEG_07803 [Fusarium verticillioides 7600];Gene=FVEG_07803;Ontology_term=lipid metabolic process,hydrolase activity;Ontology_id=GO:0006629,GO:0016787;Enzyme_code=EC:3.1.4,EC:3.1.4,EC:3.1,EC:3;Enzyme_name=Acting on ester bonds,Acting on ester bonds,Acting on ester bonds,Hydrolases
contig00005.g3425	1360	ID=contig00005.g3425;Description=related to rRNA processing protein EBP2 [Fusarium proliferatum ET1];Gene=FNYG_10265;Ontology_term=RNA binding,microtubule organizing center,ribosome biogenesis,nucleolus;Ontology_id=GO:0003723,GO:0005815,GO:0042254,GO:0005730
contig00005.g3426	1959	ID=contig00005.g3426;Description=MYND-type zinc finger protein samB [Fusarium oxysporum f. sp. cepae];Gene=FNAPI_11457;Ontology_term=establishment or maintenance of cell polarity,anatomical structure development,cell differentiation,reproductive process;Ontology_id=GO:0007163,GO:0048856,GO:0030154,GO:0022414
contig00005.g3427	1038	ID=contig00005.g3427;Description=hypothetical protein FVEG_07800 [Fusarium verticillioides 7600];Gene=FNAPI_11456;Ontology_term=membrane;Ontology_id=GO:0016020
contig00005.g3428	1511	ID=contig00005.g3428;Description=mitochondrial NADH dehydrogenase subunit 2 [Fusarium bulbicola];Gene=F25303_4500;Ontology_term=transmembrane transport,endoplasmic reticulum,Golgi apparatus,vacuole,transporter activity;Ontology_id=GO:0055085,GO:0005783,GO:0005794,GO:0005773,GO:0005215

contig00005.g3429	2858	ID=contig00005.g3429;Description=dynamin GTPase [Fusarium verticillioides 7600];Gene=BFJ63_vAg10377;Ontology_term=membrane organization,peroxisome,protein-containing complex assembly,oxidoreductase activity,GTPase activity,peroxisome organization,mitochondrion,lipid binding,cytoskeletal protein binding,mitochondrion organization;Ontology_id=GO:0061024,GO:0005777,GO:0065003,GO:0016491,GO:0003924,GO:0007031,GO:0005739,GO:0008289,GO:0008092,GO:0007005;Enzyme_code=EC:1,EC:3.6.1.15,EC:3.6.1,EC:1,EC:3.6,EC:3,EC:3.6.1.15;Enzyme_name=Oxidoreductases,nucleoside-triphosphate phosphatase,Acting on acid anhydrides,Oxidoreductases,Acting on acid anhydrides,Hydrolases,nucleoside-triphosphate phosphatase
contig00005.g3430	2712	ID=contig00005.g3430;Description=probable SPB1-required for ribosome synthesis, putative methylase [Fusarium proliferatum ET1];Gene=FVER53263_07797;Ontology_term=ribosome biogenesis,transferase activity,catalytic activity, acting on RNA,nucleolus;Ontology_id=GO:0042254,GO:0016740,GO:0140098,GO:0005730;Enzyme_code=EC:2.1.1,EC:2.1,EC:2,EC:2.1.1;Enzyme_name=Transferring one-carbon groups,Transferring one-carbon groups,Transferases,Transferring one-carbon groups
contig00005.g3431	1199	ID=contig00005.g3431;Description=phospholipase [Fusarium mundagurra]
contig00005.g3432	5799	ID=contig00005.g3432;Description=hypothetical protein FVER53263_07795 [Fusarium verticillioides];Gene=FNYG_10258;Ontology_term=DNA binding,nucleus,DNA-templated transcription,tRNA metabolic process;Ontology_id=GO:0003677,GO:0005634,GO:0006351,GO:0006399
contig00005.g3433	369	ID=contig00005.g3433;Description=ubiquitin-conjugating enzyme E2 J1 [Fusarium napiforme];Gene=FVER53263_20657;Ontology_term=membrane;Ontology_id=GO:0016020
contig00005.g3434	1072	ID=contig00005.g3434;Description=ubiquitin-conjugating enzyme E2 J1 [Fusarium verticillioides 7600];Gene=FFUJ_02457;Ontology_term=membrane;Ontology_id=GO:0016020;Enzyme_code=EC:2.3.2.23;Enzyme_name=E2 ubiquitin-conjugating enzyme

contig00005.g3435	4423	ID=contig00005.g3435;Description=ATPase [Fusarium verticillioides 7600];Gene=FANTH_13922;Ontology_term=transmembrane transport,ATP-dependent activity,vacuole,transporter activity,plasma membrane,hydrolase activity;Ontology_id=GO:0055085,GO:0140657,GO:0005773,GO:0005215,GO:0005886,GO:0016787;Enzyme_code=EC:7.2.2,EC:3.6.1.15,EC:3.6.1,EC:7.2.2.10,EC:3.6,EC:3,EC:7.2.2,EC:7,EC:3.6.1.15;Enzyme_name=Catalysing the translocation of inorganic cations,nucleoside-triphosphate phosphatase,Acting on acid anhydrides,P-type Ca(2+) transporter,Acting on acid anhydrides,Hydrolases,Catalysing the translocation of inorganic cations,Translocases,nucleoside-triphosphate phosphatase
contig00005.g3436	1542	ID=contig00005.g3436;Description=putative nucleosome assembly protein I [Fusarium fujikuroi];Gene=FDENT_13803;Ontology_term=catalytic activity, acting on DNA,cytoskeleton organization,protein-containing complex assembly,reproductive process,regulation of DNA-templated transcription,nuclear chromosome,ribosome biogenesis,chromatin organization,intracellular protein transport,DNA binding,nucleocytoplasmic transport,cytoskeleton,molecular function regulator activity,histone binding,transferase activity,hydrolase activity;Ontology_id=GO:0140097,GO:0007010,GO:0065003,GO:0022414,GO:0006355,GO:0000228,GO:0042254,GO:0006325,GO:0006886,GO:0003677,GO:0006913,GO:0005856,GO:0098772,GO:0042393,GO:0016740,GO:0016787;Enzyme_code=EC:3.1,EC:2.7.7.49,EC:3.1,EC:2.7,EC:2,EC:3,EC:2.7.7.49,EC:2.7.7;Enzyme_name=Acting on ester bonds,RNA-directed DNA polymerase,Acting on ester bonds,Transferring phosphorus-containing groups,Transferases,Hydrolases,RNA-directed DNA polymerase,Transferring phosphorus-containing groups
contig00005.g3437	1731	ID=contig00005.g3437;Description=D-3-phosphoglycerate dehydrogenase 2 [Fusarium odoratissimum NRRL 54006];Gene=FOBC_05829;Ontology_term=oxidoreductase activity,amino acid metabolic process;Ontology_id=GO:0016491,GO:0006520;Enzyme_code=EC:1.1.1.95,EC:1.1,EC:1,EC:1.1.1.399,EC:1.1.1,EC:1.1.99.2,EC:1.1.1.95;Enzyme_name=phosphoglycerate dehydrogenase,Acting on the CH-OH group of donors,Oxidoreductases,2-oxoglutarate reductase,Acting on the CH-OH group of donors,L-2-hydroxyglutarate dehydrogenase,phosphoglycerate dehydrogenase
contig00005.g3438	5106	ID=contig00005.g3438;Description=myosin MYO2 [Fusarium acutatum];Gene=F25303_4510;Ontology_term=cytoskeleton,cytoskeletal protein binding,cytoskeletal motor activity;Ontology_id=GO:0005856,GO:0008092,GO:0003774

contig00005.g3439	703	ID=contig00005.g3439;Description=dTMP kinase [Fusarium verticillioides 7600];Gene=F25303_4511;Ontology_term=nucleus,nucleobase-containing small molecule metabolic process,ATP-dependent activity,ribosome biogenesis,transferase activity,hydrolase activity;Ontology_id=GO:0005634,GO:0055086,GO:0140657,GO:0042254,GO:0016740,GO:0016787;Enzyme_code=EC:2.7.4.4,EC:2.7.4.3,EC:3.6.1.15,EC:2.7.4.4,EC:2.7.4.3,EC:3.6.1,EC:2.7,EC:2,EC:3.6,EC:2.7.4,EC:3,EC:3.6.1.15;Enzyme_name=nucleoside-phosphate kinase,adenylate kinase,nucleoside-triphosphate phosphatase,nucleoside-phosphate kinase,adenylate kinase,Acting on acid anhydrides,Transferring phosphorus-containing groups,Transferases,Acting on acid anhydrides,Transferring phosphorus-containing groups,Hydrolases,nucleoside-triphosphate phosphatase
contig00005.g3440	833	ID=contig00005.g3440;Description=Ham1 family protein [Fusarium odoratissimum NRRL 54006];Gene=FGADI_1440;Ontology_term=transmembrane transport,nucleobase-containing small molecule metabolic process,anatomical structure development,reproductive process,endoplasmic reticulum,muscle system process,circulatory system process,nucleus,molecular function regulator activity,signaling,cell differentiation,transferase activity,hydrolase activity;Ontology_id=GO:0055085,GO:0055086,GO:0048856,GO:0022414,GO:0005783,GO:0003012,GO:0003013,GO:0005634,GO:0098772,GO:0023052,GO:0030154,GO:0016740,GO:0016787;Enzyme_code=EC:2,EC:3.6.1.9,EC:3.6.1.12,EC:3.6.1.56,EC:3.6.1.23,EC:2.7.4.3,EC:3.6.1.66,EC:3.6.1,EC:2,EC:3.6,EC:3,EC:3.6.1.9;Enzyme_name=Transferases,nucleotide diphosphatase,dCTP diphosphatase,2-hydroxy-dATP diphosphatase,dUTP diphosphatase,adenylate kinase,XTP/dITP diphosphatase,Acting on acid anhydrides,Transferases,Acting on acid anhydrides,Hydrolases,nucleotide diphosphatase
contig00005.g3441	1782	ID=contig00005.g3441;Description=zinc finger with UFM1-specific peptidase domain protein [Fusarium tjaetaba]
contig00005.g3442	711	ID=contig00005.g3442;Description=GTP-binding protein rhb1 [Fusarium subglutinans];Gene=FGLOB1_14738;Ontology_term=transmembrane transport,molecular function regulator activity,vesicle-mediated transport,GTPase activity,signaling,cytosol,mitotic cell cycle,plasma membrane;Ontology_id=GO:0055085,GO:0098772,GO:0016192,GO:0003924,GO:0023052,GO:0005829,GO:0000278,GO:0005886;Enzyme_code=EC:3.6.1.15,EC:3.6.1,EC:3.6,EC:3,EC:3.6.1.15;Enzyme_name=nucleoside-triphosphate phosphatase,Acting on acid anhydrides,Acting on acid anhydrides,Hydrolases,nucleoside-triphosphate phosphatase

contig00005.g3443	2416	ID=contig00005.g3443;Description=wings apart [Fusarium subglutinans]
contig00005.g3444	959	ID=contig00005.g3444;Description=uracil phosphoribosyltransferase [Fusarium odoratissimum NRRL 54006];Gene=FOXG_01418;Ontology_term=nucleobase-containing small molecule metabolic process,carbohydrate derivative metabolic process,cytosol,transferase activity;Ontology_id=GO:0055086,GO:1901135,GO:0005829,GO:0016740;Enzyme_code=EC:2.4,EC:2.4.2,EC:2.4,EC:2.7,EC:2,EC:2.4.2.9;Enzyme_name=Glycosyltransferases,Glycosyltransferases,Glycosyltransferases,Transferring phosphorus-containing groups,Transferases,uracil phosphoribosyltransferase
contig00005.g3445	570	ID=contig00005.g3445;Description=ELF1 [Fusarium tjaetaba];Gene=FNAPI_6259;Ontology_term=nucleus,extracellular region,hydrolase activity;Ontology_id=GO:0005634,GO:0005576,GO:0016787;Enzyme_code=EC:3.1.1.1;Enzyme_name=carboxylesterase
contig00005.g3446	2534	ID=contig00005.g3446;Description=related to bifunctional GTP cyclohydrolase II/3, 4-dihydroxy-2butanone-4-phosphate synthase [Fusarium proliferatum ET1];Gene=FNAPI_6258
contig00005.g3447	495	ID=contig00005.g3447;Description=heterogeneous nuclear ribonucleoprotein g [Fusarium subglutinans];Gene=F53441_3395;Ontology_term=ribonucleoprotein complex;Ontology_id=GO:1990904
contig00005.g3448	1018	ID=contig00005.g3448;Description=40S ribosomal protein S1 [Colletotrichum orbiculare MAFF 240422];Gene=RPS1;Ontology_term=cytoplasmic translation,cell wall,structural molecule activity,ribosome biogenesis,cytosol,ribosome,extracellular region;Ontology_id=GO:0002181,GO:0005618,GO:0005198,GO:0042254,GO:0005829,GO:0005840,GO:0005576
contig00005.g3449	4794	ID=contig00005.g3449;Description=non-ribosomal peptide synthetase [Fusarium tjaetaba];Gene=FVEG_16182;Ontology_term=membrane;Ontology_id=GO:0016020
contig00005.g3450	1036	ID=contig00005.g3450;Description=enoyl-CoA hydratase [Fusarium verticillioides 7600];Gene=F25303_11078;Ontology_term=catalytic activity;Ontology_id=GO:0003824;Enzyme_code=EC:4.2.1.17;Enzyme_name=enoyl-CoA hydratase
contig00005.g3451	1056	ID=contig00005.g3451;Description=hypothetical protein FVEG_07773 [Fusarium verticillioides 7600]
contig00005.g3452	1588	ID=contig00005.g3452;Description=hypothetical protein FVEG_07772 [Fusarium verticillioides 7600];Gene=1649

contig00005.g3453	2337	ID=contig00005.g3453;Description=regulatory alcR [Fusarium tjaetaba];Gene=F25303_11081;Ontology_term=nucleus,regulation of DNA-templated transcription,transcription regulator activity;Ontology_id=GO:0005634,GO:0006355,GO:0140110
contig00005.g3454	1182	ID=contig00005.g3454;Description=hypothetical protein FVER14953_20196 [Fusarium verticillioides]
contig00005.g3455	1802	ID=contig00005.g3455;Description=hypothetical protein FMUND_4440, partial [Fusarium mundagurra]
contig00005.g3456	980	ID=contig00005.g3456;Description=hypothetical protein FVEG_12814 [Fusarium verticillioides 7600]
contig00005.g3457	1203	ID=contig00005.g3457;Description=salicylate hydroxylase [Fusarium tjaetaba];Gene=FFUJ_02433;Ontology_term=oxidoreductase activity;Ontology_id=GO:0016491;Enzyme_code=EC:1,EC:1;Enzyme_name=Oxidoreductases,Oxidoreductases
contig00005.g3458	1611	ID=contig00005.g3458;Description=diphthamide biosynthesis protein 2 [Fusarium verticillioides 7600];Gene=FVEG_12812;Ontology_term=cytoplasmic translation,transferase activity;Ontology_id=GO:0002181,GO:0016740;Enzyme_code=EC:2.5.1.108,EC:2.5.1,EC:2,EC:2.5.1.108;Enzyme_name=2-(3-amino-3-carboxypropyl)histidine synthase,Transferring alkyl or aryl groups, other than methyl groups,Transferases,2-(3-amino-3-carboxypropyl)histidine synthase
contig00005.g3459	660	ID=contig00005.g3459;Description=hypothetical protein FPCIR_13634 [Fusarium pseudocircinatum];Gene=FTJAE_11784;Ontology_term=membrane;Ontology_id=GO:0016020
contig00005.g3460	2577	ID=contig00005.g3460;Description=hypothetical protein FVEG_12810 [Fusarium verticillioides 7600]
contig00005.g3461	533	ID=contig00005.g3461;Description=hypothetical protein FVEG_12809 [Fusarium verticillioides 7600];Gene=FNAPI_10713;Ontology_term=membrane;Ontology_id=GO:0016020
contig00005.g3462	740	ID=contig00005.g3462;Description=integral membrane protein PTH11 [Fusarium coicis];Gene=FVER53263_20368;Ontology_term=membrane;Ontology_id=GO:0016020
contig00005.g3463	996	ID=contig00005.g3463;Description=hypothetical protein FVEG_12806 [Fusarium verticillioides 7600];Gene=BFJ72_g13839;Ontology_term=membrane;Ontology_id=GO:0016020

contig00005.g3464	1263	ID=contig00005.g3464;Description=glycerophosphoryl diester phosphodiesterase [Fusarium verticillioides 7600];Gene=FOC1_g10008094;Ontology_term=lipid metabolic process,hydrolase activity;Ontology_id=GO:0006629,GO:0016787;Enzyme_code=EC:3.1.4,EC:3.1.4,EC:3.1,EC:3.1.4.46,EC:3;Enzyme_name=Acting on ester bonds,Acting on ester bonds,Acting on ester bonds,glycerophosphodiester phosphodiesterase,Hydrolases
contig00005.g3465	1262	ID=contig00005.g3465;Description=extracellular chitinase [Fusarium coicis];Gene=FVER53590_12804;Ontology_term=carbohydrate metabolic process,hydrolase activity;Ontology_id=GO:0005975,GO:0016787;Enzyme_code=EC:3.2.1.14;Enzyme_name=chitinase
contig00005.g3466	3882	ID=contig00005.g3466;Description=putative histidine kinase M232p [[Gibberella] fujikuroi var. moniliformis];Gene=FVER53590_12803;Ontology_term=catalytic activity, acting on a protein,signaling,transferase activity,small molecule sensor activity;Ontology_id=GO:0140096,GO:0023052,GO:0016740,GO:0140299;Enzyme_code=EC:2.7.3,EC:2.7.13.3,EC:2.7.1,EC:2.7.3,EC:2.7,EC:2,EC:2.7.13.3;Enzyme_name=Transferring phosphorus-containing groups,histidine kinase,Transferring phosphorus-containing groups,Transferring phosphorus-containing groups,Transferring phosphorus-containing groups,Transferases,histidine kinase
contig00005.g3467	1341	ID=contig00005.g3467;Description=WD repeat-containing protein jip5 [Fusarium musae];Ontology_term=ribosome biogenesis,nucleolus;Ontology_id=GO:0042254,GO:0005730
contig00005.g3468	777	ID=contig00005.g3468;Description=maleylacetate reductase [Fusarium tjaetaba];Gene=FVEG_12801;Ontology_term=oxidoreductase activity;Ontology_id=GO:0016491;Enzyme_code=EC:1,EC:1;Enzyme_name=Oxidoreductases,Oxidoreductases
contig00005.g3469	1822	ID=contig00005.g3469;Description=NCS2 family nucleobase:cation symporter-2 [Fusarium proliferatum];Gene=FGLOB1_8602;Ontology_term=transmembrane transport,endosome,vacuole,transporter activity,plasma membrane;Ontology_id=GO:0055085,GO:0005768,GO:0005773,GO:0005215,GO:0005886
contig00005.g3470	2959	ID=contig00005.g3470;Description=hypothetical protein FVER53263_12799 [Fusarium verticillioides]
contig00005.g3471	2483	ID=contig00005.g3471;Description=high-affinity methionine permease [Fusarium napiforme];Gene=FNAPI_10723;Ontology_term=transmembrane transport,transporter activity;Ontology_id=GO:0055085,GO:0005215

contig00005.g3472	2642	ID=contig00005.g3472;Description=mRNA-capping enzyme subunit beta [Fusarium tjaetaba];Gene=FNAPI_10724;Ontology_term=nucleus,mRNA metabolic process,ATP-dependent activity,regulation of DNA-templated transcription,hydrolase activity;Ontology_id=GO:0005634,GO:0016071,GO:0140657,GO:0006355,GO:0016787;Enzyme_code=EC:3.1.3.33,EC:3.6.1,EC:3.1,EC:3.6,EC:3,EC:3.1.3.33,EC:3.6.1.15,EC:3.1.3;Enzyme_name=polynucleotide 5'-phosphatase,Acting on acid anhydrides,Acting on ester bonds,Acting on acid anhydrides,Hydrolases,polynucleotide 5'-phosphatase,nucleoside-triphosphate phosphatase,Acting on ester bonds
contig00005.g3473	3575	ID=contig00005.g3473;Description=26S proteasome regulatory subunit N2 [Fusarium verticillioides 7600];Gene=FMUND_4458;Ontology_term=nucleus,molecular function regulator activity,protein-containing complex assembly,protein catabolic process,mitotic nuclear division,chromosome segregation,cytosol,molecular adaptor activity;Ontology_id=GO:0005634,GO:0098772,GO:0065003,GO:0030163,GO:0140014,GO:0007059,GO:0005829,GO:0060090
contig00005.g3474	1821	ID=contig00005.g3474;Description=sulfite oxidase [Fusarium verticillioides 7600];Gene=FPANT_5174;Ontology_term=oxidoreductase activity;Ontology_id=GO:0016491;Enzyme_code=EC:1.7.1.2,EC:1.7.1.3,EC:1.7.99.4,EC:1.7.1.2,EC:1.7.1.1,EC:1.7.1,EC:1.7.1.3,EC:1.8.3.1,EC:1.7.99.4,EC:1,EC:1.7;Enzyme_name=nitrate reductase [NAD(P)H],nitrate reductase (NADPH),Acting on other nitrogenous compounds as donors,nitrate reductase [NAD(P)H],nitrate reductase (NADH),Acting on other nitrogenous compounds as donors,nitrate reductase (NADPH),sulfite oxidase,Acting on other nitrogenous compounds as donors,Oxidoreductases,Acting on other nitrogenous compounds as donors
contig00005.g3475	330	ID=contig00005.g3475;Description=---NA---
contig00005.g3476	534	ID=contig00005.g3476;Description=ankyrin protein 3 [Fusarium denticulatum];Gene=FDENT_3592;Ontology_term=nucleobase-containing small molecule metabolic process,catalytic activity,carbohydrate derivative metabolic process;Ontology_id=GO:0055086,GO:0003824,GO:1901135
contig00005.g3477	987	ID=contig00005.g3477;Description=hypothetical protein FVER14953_12793 [Fusarium verticillioides];Gene=FPANT_5173;Ontology_term=membrane;Ontology_id=GO:0016020
contig00005.g3478	1557	ID=contig00005.g3478;Description=alpha beta hydrolase fold [Fusarium tjaetaba];Gene=FDENT_3594;Ontology_term=hydrolase activity;Ontology_id=GO:0016787;Enzyme_code=EC:3,EC:3;Enzyme_name=Hydrolases,Hydrolases

contig00005.g3479	1858	ID=contig00005.g3479;Description=drug proton antiporter YHK8 [Fusarium coicis];Gene=FVER53263_12791;Ontology_term=transmembrane transport,transporter activity;Ontology_id=GO:0055085,GO:0005215
contig00005.g3480	1115	ID=contig00005.g3480;Description=hypothetical protein FVER14953_12790 [Fusarium verticillioides]
contig00005.g3481	879	ID=contig00005.g3481;Description=hypothetical protein FVEG_17345 [Fusarium verticillioides 7600]
contig00005.g3482	1530	ID=contig00005.g3482;Description=alpha beta hydrolase fold family [Fusarium pseudoanthophilum];Gene=FPHYL_9482;Ontology_term=hydrolase activity;Ontology_id=GO:0016787;Enzyme_code=EC:3,EC:3;Enzyme_name=Hydrolases,Hydrolases
contig00005.g3483	523	ID=contig00005.g3483;Description=cupin domain-containing protein [Fusarium mundagurra]
contig00005.g3484	1560	ID=contig00005.g3484;Description=Protein EXECUTER 2 [Fusarium oxysporum f. sp. raphani];Gene=FOQG_00083;Ontology_term=protein ubiquitination;Ontology_id=GO:0016567
contig00005.g3485	596	ID=contig00005.g3485;Description=DUF757 domain protein [Fusarium tjaetaba]
contig00005.g3486	1698	ID=contig00005.g3486;Description=uncharacterized protein FSUBG_11968 [Fusarium subglutinans];Gene=FSUBG_11968;Ontology_term=membrane;Ontology_id=GO:0016020
contig00005.g3487	1185	ID=contig00005.g3487;Description=phosphopantetheine adenylyltransferase [Fusarium subglutinans];Gene=CAB4;Ontology_term=nucleus,nucleobase-containing small molecule metabolic process,carbohydrate derivative metabolic process,transferase activity;Ontology_id=GO:0005634,GO:0055086,GO:1901135,GO:0016740;Enzyme_code=EC:2.7.7,EC:2.7.1,EC:2.7.7.3,EC:2.7,EC:2,EC:2.7.7,EC:2.7.1.24;Enzyme_name=Transferring phosphorus-containing groups,Transferring phosphorus-containing groups,pantetheine-phosphate adenylyltransferase,Transferring phosphorus-containing groups,Transferases,Transferring phosphorus-containing groups,dephospho-CoA kinase

contig00005.g3488	4215	ID=contig00005.g3488;Description=STE6 [Fusarium subglutinans];Gene=FPANT_2283;Ontology_term=transmembrane transport,ATP-dependent activity,signaling,reproductive process,Golgi apparatus,vacuole,transporter activity,plasma membrane,hydrolase activity;Ontology_id=GO:0055085,GO:0140657,GO:0023052,GO:0022414,GO:0005794,GO:0005773,GO:0005215,GO:0005886,GO:0016787;Enzyme_code=EC:7.2.2,EC:3.6.1.15,EC:3.6.1,EC:3.6.3.44,EC:3.6,EC:3,EC:7.2.2,EC:7,EC:7.4.2.5,EC:3.6.1.15,EC:7.4.2.6;Enzyme_name=Catalysing the translocation of inorganic cations,nucleoside-triphosphate phosphatase,Acting on acid anhydrides,Acting on acid anhydrides,Acting on acid anhydrides,Hydrolases,Catalysing the translocation of inorganic cations,Translocases,bacterial ABC-type protein transporter,nucleoside-triphosphate phosphatase,ABC-type oligopeptide transporter
contig00005.g3489	1415	ID=contig00005.g3489;Description=STE6 [Fusarium sp. NRRL 52700];Gene=F25303_1046;Ontology_term=transmembrane transport,ATP-dependent activity,regulation of DNA-templated transcription,transcription regulator activity,transporter activity,hydrolase activity;Ontology_id=GO:0055085,GO:0140657,GO:0006355,GO:0140110,GO:0005215,GO:0016787;Enzyme_code=EC:7.2.2,EC:3.6.1.15;Enzyme_name=Catalysing the translocation of inorganic cations,nucleoside-triphosphate phosphatase
contig00005.g3490	3769	ID=contig00005.g3490;Description=calcium P-type ATPase [Fusarium tjaetaba];Gene=FDENT_3609;Ontology_term=transmembrane transport,ATP-dependent activity,transporter activity,hydrolase activity;Ontology_id=GO:0055085,GO:0140657,GO:0005215,GO:0016787;Enzyme_code=EC:7.2.2.10,EC:3.6.1.15,EC:3.6.1,EC:7.2.2.10,EC:3.6,EC:3,EC:3.6.3.8,EC:7,EC:7.2.2,EC:3.6.1.15;Enzyme_name=P-type Ca(2+) transporter,nucleoside-triphosphate phosphatase,Acting on acid anhydrides,P-type Ca(2+) transporter,Acting on acid anhydrides,Hydrolases,Acting on acid anhydrides,Translocases,Catalysing the translocation of inorganic cations,nucleoside-triphosphate phosphatase
contig00005.g3491	1350	ID=contig00005.g3491;Description=purine nucleoside permease [Fusarium tjaetaba];Gene=2509;Ontology_term=transmembrane transport,nucleobase-containing small molecule metabolic process,catalytic activity,carbohydrate derivative metabolic process,transporter activity;Ontology_id=GO:0055085,GO:0055086,GO:0003824,GO:1901135,GO:0005215

contig00005.g3492	2374	ID=contig00005.g3492;Description=IKS protein kinase [Fusarium verticillioides 7600];Gene=FVEG_12779;Ontology_term=catalytic activity, acting on a protein,transferase activity;Ontology_id=GO:0140096,GO:0016740;Enzyme_code=EC:2.7.1;Enzyme_name=Transferring phosphorus-containing groups
contig00005.g3493	987	ID=contig00005.g3493;Description=transcription factor ACEII [Fusarium tjaetaba];Gene=FVEG_12778;Ontology_term=nucleus,regulation of DNA-templated transcription,transcription regulator activity;Ontology_id=GO:0005634,GO:0006355,GO:0140110
contig00005.g3494	1876	ID=contig00005.g3494;Description=major facilitator superfamily domain-containing protein [Fusarium redolens];Gene=FVER53263_12777;Ontology_term=transmembrane transport,transporter activity;Ontology_id=GO:0055085,GO:0005215
contig00005.g3495	1672	ID=contig00005.g3495;Description=pyruvate kinase [Fusarium verticillioides 7600];Gene=FOMG_05819;Ontology_term=carbohydrate metabolic process,generation of precursor metabolites and energy,nucleobase-containing small molecule metabolic process,mitochondrion,cell wall,carbohydrate derivative metabolic process,cytosol,transferase activity,plasma membrane;Ontology_id=GO:0005975,GO:0006091,GO:0055086,GO:0005739,GO:0005618,GO:1901135,GO:0005829,GO:0016740,GO:0005886;Enzyme_code=EC:2.7.1.40,EC:2.7.1,EC:2.7,EC:2,EC:2.7.1.40;Enzyme_name=pyruvate kinase,Transferring phosphorus-containing groups,Transferring phosphorus-containing groups,Transferases,pyruvate kinase
contig00005.g3496	463	ID=contig00005.g3496;Description=DUF1748-domain-containing protein [Fusarium bulbicola]
contig00005.g3497	1908	ID=contig00005.g3497;Description=Autophagy protein 28 [Fusarium musae]
contig00005.g3498	2059	ID=contig00005.g3498;Description=HAN11-like protein [Fusarium denticulatum];Ontology_term=nucleus,DNA replication,mitotic cell cycle;Ontology_id=GO:0005634,GO:0006260,GO:0000278

contig00005.g3499	3270	ID=contig00005.g3499;Description=transcription elongation factor spt5 [Fusarium musae];Gene=FPCIR_1318;Ontology_term=DNA repair,anatomical structure development,ribosome biogenesis,receptor ligand activity,nucleoplasm,wound healing,inflammatory response,RNA binding,mRNA metabolic process,mitotic nuclear division,cytosol,transcription regulator activity,regulation of DNA-templated transcription,reproductive process,nuclear chromosome,immune system process,DNA binding,translation regulator activity,extracellular space,mitochondrion,cell differentiation,general transcription initiation factor activity,programmed cell death;Ontology_id=GO:0006281,GO:0048856,GO:0042254,GO:0048018,GO:0005654,GO:0042060,GO:0006954,GO:0003723,GO:0016071,GO:0140014,GO:0005829,GO:0140110,GO:0006355,GO:0022414,GO:0000228,GO:0002376,GO:0003677,GO:0045182,GO:0005615,GO:0005739,GO:0030154,GO:0140223,GO:0012501
contig00005.g3500	736	ID=contig00005.g3500;Description=hypothetical protein FVER53590_12771 [Fusarium verticillioides];Gene=FPANT_2295;Ontology_term=membrane;Ontology_id=GO:0016020
contig00005.g3501	2613	ID=contig00005.g3501;Description=hypothetical protein FVER53590_12770 [Fusarium verticillioides]
contig00005.g3502	1465	ID=contig00005.g3502;Description=Amino acid/polyamine transporter I [Fusarium oxysporum f. sp. vasinfectum];Gene=FPRO05_05186;Ontology_term=transmembrane transport,vacuole,transporter activity,plasma membrane;Ontology_id=GO:0055085,GO:0005773,GO:0005215,GO:0005886
contig00005.g3503	2398	ID=contig00005.g3503;Description=alcohol oxidase [Fusarium verticillioides 7600];Gene=FOMG_05829;Ontology_term=oxidoreductase activity;Ontology_id=GO:0016491;Enzyme_code=EC:1.1,EC:1.1.3.13,EC:1.1,EC:1;Enzyme_name=Acting on the CH-OH group of donors,alcohol oxidase,Acting on the CH-OH group of donors,Oxidoreductases
contig00005.g3504	1178	ID=contig00005.g3504;Description=hypothetical protein FVER53590_12767 [Fusarium verticillioides];Gene=FVEG_12767;Ontology_term=membrane;Ontology_id=GO:0016020
contig00005.g3505	987	ID=contig00005.g3505;Description=integral membrane protein [Fusarium napiforme];Gene=FVER53590_28286;Ontology_term=extracellular space,vesicle-mediated transport,transferase activity,immune system process,plasma membrane;Ontology_id=GO:0005615,GO:0016192,GO:0016740,GO:0002376,GO:0005886;Enzyme_code=EC:2,EC:2;Enzyme_name=Transferases,Transferases

contig00005.g3506	1266	ID=contig00005.g3506;Description=pectinesterase [Fusarium verticillioides 7600];Gene=FVER53263_12766;Ontology_term=carbohydrate metabolic process,cell wall organization or biogenesis,hydrolase activity;Ontology_id=GO:0005975,GO:0071554,GO:0016787;Enzyme_code=EC:3.1.1.11,EC:3.1,EC:3.1.1.11,EC:3,EC:3.1.1;Enzyme_name=pectinesterase,Acting on ester bonds,pectinesterase,Hydrolases,Acting on ester bonds
contig00005.g3507	818	ID=contig00005.g3507;Description=extracellular serine-rich [Fusarium tjaetaba]
contig00005.g3508	1341	ID=contig00005.g3508;Description=hypothetical protein FVER53263_12764 [Fusarium verticillioides];Gene=1593
contig00005.g3509	813	ID=contig00005.g3509;Description=hypothetical protein FNYG_00085 [Fusarium nygamai];Gene=2491
contig00005.g3510	891	ID=contig00005.g3510;Description=aminoglycoside phosphotransferase [Fusarium tjaetaba];Gene=FTJAE_7492;Ontology_term=transferase activity;Ontology_id=GO:0016740;Enzyme_code=EC:2,EC:2;Enzyme_name=Transferases,Transferases
contig00005.g3511	1468	ID=contig00005.g3511;Description=hypothetical protein FVER53263_12762 [Fusarium verticillioides];Gene=FPHYL_12060;Ontology_term=membrane;Ontology_id=GO:0016020
contig00005.g3512	736	ID=contig00005.g3512;Description=peroxidase [Fusarium verticillioides 7600];Gene=FOMG_05839;Ontology_term=antioxidant activity,oxidoreductase activity;Ontology_id=GO:0016209,GO:0016491;Enzyme_code=EC:1.11.1,EC:1.11,EC:1,EC:1.11.1;Enzyme_name=Acting on a peroxide as acceptor,Acting on a peroxide as acceptor,Oxidoreductases,Acting on a peroxide as acceptor
contig00005.g3513	1611	ID=contig00005.g3513;Description=related to beta-lactamase class C and other penicillin binding proteins [Fusarium mangiferae];Gene=dap-1;Ontology_term=catalytic activity, acting on a protein,hydrolase activity;Ontology_id=GO:0140096,GO:0016787;Enzyme_code=EC:3.4.11,EC:3.4.11,EC:3.4,EC:3;Enzyme_name=Acting on peptide bonds (peptidases),Acting on peptide bonds (peptidases),Acting on peptide bonds (peptidases),Hydrolases
contig00005.g3514	3177	ID=contig00005.g3514;Description=ral2 [Fusarium tjaetaba];Ontology_term=signaling,cell differentiation,anatomical structure development,reproductive process,cell adhesion;Ontology_id=GO:0023052,GO:0030154,GO:0048856,GO:0022414,GO:0007155

contig00005.g3515	1993	ID=contig00005.g3515;Description=FAT1-very long-chain fatty acyl synthetase [Fusarium subglutinans];Gene=FVEG_12758;Ontology_term=lipid droplet,peroxisome,ATP-dependent activity,protein catabolic process,lipid metabolic process,endoplasmic reticulum,vacuole,ligase activity,transporter activity,plasma membrane;Ontology_id=GO:0005811,GO:0005777,GO:0140657,GO:0030163,GO:0006629,GO:0005783,GO:0005773,GO:0016874,GO:0005215,GO:0005886;Enzyme_code=EC:6.2.1,EC:6.2,EC:6,EC:6.2.1;Enzyme_name=Forming carbon-sulfur bonds,Forming carbon-sulfur bonds,Ligases,Forming carbon-sulfur bonds
contig00005.g3516	1641	ID=contig00005.g3516;Description=GPI transamidase component PIG-S [Fusarium tjaetaba];Gene=FVEG_12757;Ontology_term=catalytic activity, acting on a protein,lipid metabolic process,carbohydrate derivative metabolic process,endoplasmic reticulum,hydrolase activity;Ontology_id=GO:0140096,GO:0006629,GO:1901135,GO:0005783,GO:0016787;Enzyme_code=EC:3.4.22,EC:3.4,EC:3;Enzyme_name=Acting on peptide bonds (peptidases),Acting on peptide bonds (peptidases),Hydrolases
contig00005.g3517	1294	ID=contig00005.g3517;Description=Alpha/Beta hydrolase protein [Fusarium oxysporum]
contig00005.g3518	1665	ID=contig00005.g3518;Description=sugar transport STP1 [Fusarium napiforme];Gene=FVER53263_12755;Ontology_term=transmembrane transport,transporter activity;Ontology_id=GO:0055085,GO:0005215
contig00005.g3519	1836	ID=contig00005.g3519;Description=related to alpha-L-arabinofuranosidase 1 [Fusarium fujikuroi];Gene=hslO;Ontology_term=carbohydrate metabolic process,hydrolase activity;Ontology_id=GO:0005975,GO:0016787;Enzyme_code=EC:3.2.1.55,EC:3.2,EC:3,EC:3.2.1.55,EC:3.2.1;Enzyme_name=non-reducing end alpha-L-arabinofuranosidase,Glycosylases,Hydrolases,non-reducing end alpha-L-arabinofuranosidase,Glycosylases
contig00005.g3520	1605	ID=contig00005.g3520;Description=BCS1-like protein [Fusarium denticulatum];Gene=FDENT_1161;Ontology_term=ATP-dependent activity,mitochondrion,hydrolase activity;Ontology_id=GO:0140657,GO:0005739,GO:0016787;Enzyme_code=EC:3.6.1.15,EC:3.6.1,EC:3.6,EC:3,EC:3.6.1.15;Enzyme_name=nucleoside-triphosphate phosphatase,Acting on acid anhydrides,Acting on acid anhydrides,Hydrolases,nucleoside-triphosphate phosphatase
contig00005.g3521	1808	ID=contig00005.g3521;Description=hypothetical protein H9Q72_006357 [Fusarium xylarioides]

contig00005.g3522	1365	ID=contig00005.g3522;Description=hypothetical protein FVER53590_12751 [Fusarium verticillioides];Gene=FPCIR_1295;Ontology_term=DNA binding;Ontology_id=GO:0003677
contig00005.g3523	1019	ID=contig00005.g3523;Description=Myb family transcription factor [Fusarium tjaetaba]
contig00005.g3524	1512	ID=contig00005.g3524;Description=protoheme IX farnesyltransferase, mitochondrial [Fusarium verticillioides 7600];Gene=FVER53590_12748;Ontology_term=generation of precursor metabolites and energy,mitochondrion,transferase activity;Ontology_id=GO:0006091,GO:0005739,GO:0016740;Enzyme_code=EC:2.5.1.29,EC:2.5.1.141,EC:2.5.1,EC:2.5.1.29,EC:2.5.1.141,EC:2;Enzyme_name=geranylgeranyl diphosphate synthase,heme o synthase,Transferring alkyl or aryl groups, other than methyl groups,geranylgeranyl diphosphate synthase,heme o synthase,Transferases
contig00005.g3525	1261	ID=contig00005.g3525;Description=transaldolase [Fusarium tjaetaba];Gene=FTJAE_7507;Ontology_term=carbohydrate metabolic process,generation of precursor metabolites and energy,nucleobase-containing small molecule metabolic process,carbohydrate derivative metabolic process,transferase activity;Ontology_id=GO:0005975,GO:0006091,GO:0055086,GO:1901135,GO:0016740;Enzyme_code=EC:2.2.1.2,EC:2.2.1.2,EC:2,EC:2.2.1;Enzyme_name=transaldolase,transaldolase,Transferases,Transferring aldehyde or ketonic groups
contig00005.g3526	6166	ID=contig00005.g3526;Description=transaldolase [Fusarium tjaetaba]
contig00005.g3527	3837	ID=contig00005.g3527;Description=DNA mismatch repair protein MSH6 [Fusarium verticillioides 7600];Gene=FTJAE_7509;Ontology_term=DNA repair,DNA binding,nucleus,catalytic activity, acting on DNA,ATP-dependent activity,DNA replication,reproductive process,hydrolase activity,small molecule sensor activity;Ontology_id=GO:0006281,GO:0003677,GO:0005634,GO:0140097,GO:0140657,GO:0006260,GO:0022414,GO:0016787,GO:0140299;Enzyme_code=EC:3.6.1,EC:3.6,EC:3,EC:3.6.1.15;Enzyme_name=Acting on acid anhydrides,Acting on acid anhydrides,Hydrolases,nucleoside-triphosphate phosphatase
contig00005.g3528	1589	ID=contig00005.g3528;Description=galacturan 1,4-alpha-galacturonidase [Fusarium odoratissimum NRRL 54006];Gene=FMUND_730;Ontology_term=carbohydrate metabolic process,extracellular region,hydrolase activity;Ontology_id=GO:0005975,GO:0005576,GO:0016787;Enzyme_code=EC:3,EC:3.2,EC:3,EC:3.2.1.67,EC:3.2.1.15,EC:3.2.1;Enzyme_name=Hydrolases,Glycosylases,Hydrolases,galacturonan 1,4-alpha-galacturonidase,endo-polygalacturonase,Glycosylases

contig00005.g3529	2594	ID=contig00005.g3529;Description=multidrug resistance [Fusarium coicis];Gene=FVER53263_12743;Ontology_term=transmembrane transport,transporter activity;Ontology_id=GO:0055085,GO:0005215
contig00005.g3530	1385	ID=contig00005.g3530;Description=multidrug resistance [Fusarium tjaetaba];Gene=FACUT_7813;Ontology_term=transmembrane transport,nucleobase-containing small molecule metabolic process,oxidoreductase activity,transporter activity;Ontology_id=GO:0055085,GO:0055086,GO:0016491,GO:0005215;Enzyme_code=EC:1.6,EC:1.6,EC:1;Enzyme_name=Acting on NADH or NADPH,Acting on NADH or NADPH,Oxidoreductases
contig00005.g3531	1716	ID=contig00005.g3531;Description=pyridine nucleotide-disulfide oxidoreductase-domain-containing protein [Fusarium oxysporum f. sp. albedinis];Gene=BFJ63_vAg17648;Ontology_term=nucleobase-containing small molecule metabolic process,oxidoreductase activity;Ontology_id=GO:0055086,GO:0016491;Enzyme_code=EC:1.6,EC:1.6,EC:1,EC:1.6.99.3;Enzyme_name=Acting on NADH or NADPH,Acting on NADH or NADPH,Oxidoreductases,Deleted entry
contig00005.g3532	2940	ID=contig00005.g3532;Description=hypothetical protein FVER14953_12739 [Fusarium verticillioides]
contig00005.g3533	3544	ID=contig00005.g3533;Description=3-hydroxyisobutyrate dehydrogenase [Fusarium napiforme];Gene=FVER53590_12738;Ontology_term=carbohydrate metabolic process,oxidoreductase activity,transferase activity;Ontology_id=GO:0005975,GO:0016491,GO:0016740;Enzyme_code=EC:1,EC:2.7;Enzyme_name=Oxidoreductases,Transferring phosphorus-containing groups
contig00005.g3534	2051	ID=contig00005.g3534;Description=transcriptional activator of protease prtT [Fusarium tjaetaba];Gene=FMEXI_6397;Ontology_term=nucleus,DNA binding,catalytic activity, acting on a protein,transmembrane transport,regulation of DNA-templated transcription,transcription regulator activity,transporter activity,hydrolase activity;Ontology_id=GO:0005634,GO:0003677,GO:0140096,GO:0055085,GO:0006355,GO:0140110,GO:0005215,GO:0016787;Enzyme_code=EC:3.4;Enzyme_name=Acting on peptide bonds (peptidases)
contig00005.g3535	1535	ID=contig00005.g3535;Description=major facilitator superfamily transporter [Fusarium tjaetaba];Gene=FPANT_941;Ontology_term=transmembrane transport,transporter activity;Ontology_id=GO:0055085,GO:0005215

contig00005.g3536	982	ID=contig00005.g3536;Description=YRO2 protein [Fusarium fujikuroi];Gene=FVEG_12735;Ontology_term=molecular transducer activity,transmembrane transport,signaling,transporter activity;Ontology_id=GO:0060089,GO:0055085,GO:0023052,GO:0005215
contig00005.g3537	2396	ID=contig00005.g3537;Description=NTE family protein [Fusarium verticillioides 7600];Gene=FVEG_12734;Ontology_term=lipid metabolic process,hydrolase activity;Ontology_id=GO:0006629,GO:0016787;Enzyme_code=EC:3.1.1.3;Enzyme_name=triacylglycerol lipase
contig00005.g3538	1244	ID=contig00005.g3538;Description=thioredoxin reductase [Fusarium tjaetaba];Gene=FVER53263_12733;Ontology_term=oxidoreductase activity;Ontology_id=GO:0016491;Enzyme_code=EC:1,EC:1;Enzyme_name=Oxidoreductases,Oxidoreductases
contig00005.g3539	1204	ID=contig00005.g3539;Description=transcription co-repressor GAL80 [Fusarium tjaetaba];Gene=FVER53263_12732;Ontology_term=nucleotide binding;Ontology_id=GO:0000166
contig00005.g3540	6751	ID=contig00005.g3540;Description=AAA ATPase domain [Fusarium oxysporum f. sp. vasinfectum];Gene=FNYG_00121;Ontology_term=transmembrane transport,ATP-dependent activity,transporter activity,hydrolase activity;Ontology_id=GO:0055085,GO:0140657,GO:0005215,GO:0016787;Enzyme_code=EC:7.2.2,EC:3.6.1.15,EC:3.6.1,EC:3.6,EC:3,EC:7.2.2,EC:7,EC:3.6.1.15;Enzyme_name=Catalysing the translocation of inorganic cations,nucleoside-triphosphate phosphatase,Acting on acid anhydrides,Acting on acid anhydrides,Hydrolases,Catalysing the translocation of inorganic cations,Translocases,nucleoside-triphosphate phosphatase
contig00005.g3541	660	ID=contig00005.g3541;Description=DUF2625 family protein [Mesorhizobium sp.]
contig00005.g3542	572	ID=contig00005.g3542;Description=---NA---
contig00005.g3543	1089	ID=contig00005.g3543;Description=peptidase s8 s53 subtilisin kexin sedolisin [Fusarium coicis]
contig00005.g3544	1975	ID=contig00005.g3544;Description=related to multidrug resistant protein [Fusarium proliferatum ET1];Gene=FVER53590_12727;Ontology_term=transmembrane transport,transporter activity;Ontology_id=GO:0055085,GO:0005215
contig00005.g3545	962	ID=contig00005.g3545;Description=nucleoside-diphosphate-sugar epimerase [Fusarium mundagurra]

contig00005.g3546	1348	ID=contig00005.g3546;Description=hydroxylase [Fusarium pseudocircinatum];Gene=FPCIR_1270;Ontology_term=oxidoreductase activity;Ontology_id=GO:0016491;Enzyme_code=EC:1;Enzyme_name=Oxidoreductases
contig00005.g3547	1635	ID=contig00005.g3547;Description=hypothetical protein FVEG_12724 [Fusarium verticillioides 7600]
contig00005.g3548	2625	ID=contig00005.g3548;Description=s-layer domain protein [Fusarium tjaetaba]
contig00005.g3549	1211	ID=contig00005.g3549;Description=glycoside hydrolase [Fusarium napiforme];Gene=FVER53263_12722;Ontology_term=carbohydrate metabolic process,transmembrane transport,cell wall organization or biogenesis,transporter activity,hydrolase activity;Ontology_id=GO:0005975,GO:0055085,GO:0071554,GO:0005215,GO:0016787;Enzyme_code=EC:3.2.1.25,EC:3.2.1.78,EC:3.2,EC:3,EC:3.2.1.25,EC:3.2.1.78,EC:3.2.1;Enzyme_name=beta-mannosidase,mannan endo-1,4-beta-mannosidase,Glycosylases,Hydrolases,beta-mannosidase,mannan endo-1,4-beta-mannosidase,Glycosylases
contig00005.g3550	1785	ID=contig00005.g3550;Description=lactose permease [Fusarium tjaetaba];Gene=FMAN_03899;Ontology_term=transmembrane transport,transporter activity;Ontology_id=GO:0055085,GO:0005215
contig00005.g3551	2017	ID=contig00005.g3551;Description=lactose regulatory [Fusarium tjaetaba];Gene=FNYG_00131;Ontology_term=nucleus,DNA binding,regulation of DNA-templated transcription,transcription regulator activity;Ontology_id=GO:0005634,GO:0003677,GO:0006355,GO:0140110
contig00005.g3552	1302	ID=contig00005.g3552;Description=hypothetical protein FVER53263_12719 [Fusarium verticillioides]
contig00005.g3553	1637	ID=contig00005.g3553;Description=large neutral amino acid transporter [Fusarium tjaetaba];Gene=FTJAE_7539;Ontology_term=transmembrane transport,transporter activity;Ontology_id=GO:0055085,GO:0005215
contig00005.g3554	1617	ID=contig00005.g3554;Description=transporter SEO1 [Fusarium tjaetaba];Gene=FVER53590_12717;Ontology_term=transmembrane transport,transporter activity;Ontology_id=GO:0055085,GO:0005215
contig00005.g3555	528	ID=contig00005.g3555;Description=hypothetical protein FVEG_12716 [Fusarium verticillioides 7600]
contig00005.g3556	726	ID=contig00005.g3556;Description=hypothetical protein FPANT_5880 [Fusarium pseudoanthophilum];Gene=hrcA

contig00005.g3557	1557	ID=contig00005.g3557;Description=CAR2-ornithine aminotransferase [Fusarium tjaetaba];Gene=FNYG_00142;Ontology_term=transferase activity;Ontology_id=GO:0016740;Enzyme_code=EC:2.6.1,EC:2.6.1,EC:2,EC:2.6;Enzyme_name=Transferring nitrogenous groups,Transferring nitrogenous groups,Transferases,Transferring nitrogenous groups
contig00005.g3558	3263	ID=contig00005.g3558;Description=multidrug resistance [Fusarium tjaetaba];Gene=FVEG_12712;Ontology_term=transmembrane transport,transporter activity;Ontology_id=GO:0055085,GO:0005215
contig00005.g3559	1672	ID=contig00005.g3559;Description=hypothetical protein FVER53263_12711 [Fusarium verticillioides];Gene=FVER53263_12711;Ontology_term=transmembrane transport,oxidoreductase activity,transporter activity;Ontology_id=GO:0055085,GO:0016491,GO:0005215;Enzyme_code=EC:1.1,EC:1.1,EC:1;Enzyme_name=Acting on the CH-OH group of donors,Acting on the CH-OH group of donors,Oxidoreductases
contig00005.g3560	1900	ID=contig00005.g3560;Description=oxidoreductase like [Fusarium subglutinans];Gene=FANTH_1505
contig00005.g3561	3037	ID=contig00005.g3561;Description=hypothetical protein FOQG_12181 [Fusarium oxysporum f. sp. raphani 54005]
contig00005.g3562	1442	ID=contig00005.g3562;Description=zinc transport [Fusarium tjaetaba];Gene=FNAPI_5489;Ontology_term=transmembrane transport,transporter activity;Ontology_id=GO:0055085,GO:0005215
contig00005.g3563	1121	ID=contig00005.g3563;Description=serine threonine kinase fnkB [Fusarium coicis];Gene=FPHYL_7773;Ontology_term=transferase activity;Ontology_id=GO:0016740;Enzyme_code=EC:2.7;Enzyme_name=Transferring phosphorus-containing groups
contig00005.g3564	1294	ID=contig00005.g3564;Description=Alkylated DNA repair alkB like 8 [Fusarium napiforme];Gene=FVER53263_20434;Ontology_term=regulation of DNA-templated transcription;Ontology_id=GO:0006355
contig00005.g3565	675	ID=contig00005.g3565;Description=Alkylated DNA repair alkB like 8 [Fusarium tjaetaba];Gene=LW93_7830;Ontology_term=demethylation;Ontology_id=GO:0070988;Enzyme_code=EC:2.1.1.229;Enzyme_name=tRNA (carboxymethyluridine(34)-5-O)-methyltransferase

contig00005.g3566	2689	ID=contig00005.g3566;Description=killer toxin subunits alpha beta [Fusarium tjaetaba];Gene=FNYG_00153;Ontology_term=carbohydrate metabolic process,carbohydrate derivative metabolic process,extracellular region,hydrolase activity;Ontology_id=GO:0005975,GO:1901135,GO:0005576,GO:0016787;Enzyme_code=EC:3.2.1.14,EC:3.2,EC:3,EC:3.2.1.14,EC:3.2.1;Enzyme_name=chitinase,Glycosylases,Hydrolases,chitinase,Glycosylases
contig00005.g3567	1076	ID=contig00005.g3567;Description=short-chain dehydrogenase TIC 32 chloroplastic [Fusarium phyllophilum]
contig00005.g3568	391	ID=contig00005.g3568;Description=seed maturation pm25 [Fusarium tjaetaba]
contig00005.g3569	411	ID=contig00005.g3569;Description=hypothetical protein FVEG_17331 [Fusarium verticillioides 7600];Gene=FMEXI_6364;Ontology_term=membrane;Ontology_id=GO:0016020
contig00005.g3570	4154	ID=contig00005.g3570;Description=related to ankyrin [Fusarium fujikuroi];Gene=FFUJ_02313;Ontology_term=transmembrane transport,nucleobase-containing small molecule metabolic process,catalytic activity,carbohydrate derivative metabolic process,transporter activity,plasma membrane;Ontology_id=GO:0055085,GO:0055086,GO:0003824,GO:1901135,GO:0005215,GO:0005886
contig00005.g3571	2075	ID=contig00005.g3571;Description=related to sugar transport protein STP1 [Fusarium fujikuroi];Gene=B0A49_04268;Ontology_term=transmembrane transport,transporter activity;Ontology_id=GO:0055085,GO:0005215
contig00005.g3572	825	ID=contig00005.g3572;Description=hypothetical protein FVER14953_12697 [Fusarium verticillioides]
contig00005.g3573	1603	ID=contig00005.g3573;Description=transcriptional regulatory [Fusarium tjaetaba];Gene=FMUND_686;Ontology_term=nucleus,DNA binding,regulation of DNA-templated transcription,transcription regulator activity;Ontology_id=GO:0005634,GO:0003677,GO:0006355,GO:0140110
contig00005.g3574	2131	ID=contig00005.g3574;Description=hypothetical protein FNYG_00161 [Fusarium nygamai]
contig00005.g3575	676	ID=contig00005.g3575;Description=rab family, other [Fusarium verticillioides 7600];Gene=FTJAE_12623;Ontology_term=GTPase activity;Ontology_id=GO:0003924;Enzyme_code=EC:3.6.1.15;Enzyme_name=nucleoside-triphosphate phosphatase
contig00005.g3576	3550	ID=contig00005.g3576;Description=hypothetical protein FVER53263_12692 [Fusarium verticillioides];Gene=BFJ65_g4746;Ontology_term=membrane;Ontology_id=GO:0016020

contig00005.g3577	1529	ID=contig00005.g3577;Description=3-dehydroquinate synthase [Fusarium verticillioides 7600];Gene=FVER53590_12691;Ontology_term=lyase activity;Ontology_id=GO:0016829;Enzyme_code=EC:4,EC:4.2.3.4,EC:4;Enzyme_name=Lyases,3-dehydroquinate synthase,Lyases
contig00005.g3578	1521	ID=contig00005.g3578;Description=hypothetical protein FVER14953_12690 [Fusarium verticillioides];Gene=FNYG_00164;Ontology_term=ATP binding,metal ion binding,membrane;Ontology_id=GO:0005524,GO:0046872,GO:0016020
contig00005.g3579	1739	ID=contig00005.g3579;Description=hypothetical protein FVER53263_12688 [Fusarium verticillioides];Gene=Forpe1208_v002143;Ontology_term=nucleus,regulation of DNA-templated transcription,transcription regulator activity;Ontology_id=GO:0005634,GO:0006355,GO:0140110
contig00005.g3580	3437	ID=contig00005.g3580;Description=MFS transporter, SP family, sugar:H+ symporter [Fusarium verticillioides 7600];Gene=FNAPI_5510;Ontology_term=transmembrane transport,transporter activity;Ontology_id=GO:0055085,GO:0005215
contig00005.g3581	750	ID=contig00005.g3581;Description=transcriptional regulator [Fusarium verticillioides 7600]
contig00005.g3582	540	ID=contig00005.g3582;Description=gnat family protein acetyltransferase [Fusarium tjaetaba];Gene=FNAPI_5513;Ontology_term=transferase activity;Ontology_id=GO:0016740;Enzyme_code=EC:2.3.1,EC:2.3,EC:2,EC:2.3.1;Enzyme_name=Acyltransferases,Acyltransferases,Transferases,Acyltransferases
contig00005.g3583	2428	ID=contig00005.g3583;Description=c6 finger [Fusarium denticulatum];Gene=FIE12Z_10216;Ontology_term=nucleus,regulation of DNA-templated transcription,transferase activity,transcription regulator activity;Ontology_id=GO:0005634,GO:0006355,GO:0016740,GO:0140110;Enzyme_code=EC:2.3.1,EC:2.3,EC:2,EC:2.3.1;Enzyme_name=Acyltransferases,Acyltransferases,Transferases,Acyltransferases
contig00005.g3584	1189	ID=contig00005.g3584;Description=taurine dioxygenase [Fusarium subglutinans];Gene=FANTH_9277;Ontology_term=oxidoreductase activity;Ontology_id=GO:0016491;Enzyme_code=EC:1,EC:1;Enzyme_name=Oxidoreductases,Oxidoreductases
contig00005.g3585	430	ID=contig00005.g3585;Description=hypothetical protein FVER14953_12682 [Fusarium verticillioides]

contig00005.g3586	1803	ID=contig00005.g3586;Description=dextranase [Fusarium tjaetaba];Gene=FPANT_534;Ontology_term=hydrolase activity;Ontology_id=GO:0016787;Enzyme_code=EC:3.2.1,EC:3.2,EC:3,EC:3.2.1;Enzyme_name=Glycosylases,Glycosylases,Hydrolases,Glycosylases
contig00005.g3587	4788	ID=contig00005.g3587;Description=xanthine dehydrogenase [Fusarium sp. NRRL 25303];Gene=FVER53590_12680;Ontology_term=oxidoreductase activity;Ontology_id=GO:0016491;Enzyme_code=EC:1,EC:1,EC:1.17.1.4,EC:1.17.3.2;Enzyme_name=Oxidoreductases,Oxidoreductases,xanthine dehydrogenase,xanthine oxidase
contig00005.g3588	1383	ID=contig00005.g3588;Description=Secondary metabolism regulator LAE1 [Fusarium oxysporum f. sp. cubense];Gene=BFJ65_g641
contig00005.g3589	3968	ID=contig00005.g3589;Description=hypothetical protein FVER53590_12678 [Fusarium verticillioides]
contig00005.g3590	1929	ID=contig00005.g3590;Description=general amidase [Fusarium tjaetaba];Enzyme_code=EC:3.5.1.4;Enzyme_name=amidase
contig00005.g3591	642	ID=contig00005.g3591;Description=UPF0311 protein [Fusarium oxysporum f. sp. rapae]
contig00005.g3592	1779	ID=contig00005.g3592;Description=major facilitator superfamily transporter [Fusarium tjaetaba];Gene=FPANT_541;Ontology_term=transmembrane transport,transporter activity;Ontology_id=GO:0055085,GO:0005215
contig00005.g3593	1610	ID=contig00005.g3593;Description=transcriptional regulatory protein pro-1 [Fusarium beomiforme];Gene=FOTG_19076;Ontology_term=nucleus,regulation of DNA-templated transcription,transcription regulator activity;Ontology_id=GO:0005634,GO:0006355,GO:0140110
contig00005.g3594	1120	ID=contig00005.g3594;Description=5'/3'-nucleotidase SurE [Fusarium proliferatum];Gene=FNAPI_6243;Ontology_term=extracellular region,hydrolase activity;Ontology_id=GO:0005576,GO:0016787;Enzyme_code=EC:3.1.3,EC:3.1,EC:3,EC:3.1.3.5,EC:3.1.3;Enzyme_name=Acting on ester bonds,Acting on ester bonds,Hydrolases,5'-nucleotidase,Acting on ester bonds
contig00005.g3595	4308	ID=contig00005.g3595;Description=hypothetical protein FANTH_9295 [Fusarium anthophilum];Gene=FMAN_03835;Ontology_term=membrane;Ontology_id=GO:0016020
contig00005.g3596	1648	ID=contig00005.g3596;Description=nadh dehydrogenase [Fusarium pseudoanthophilum];Gene=LW93_7787;Ontology_term=nucleus,DNA binding,regulation of DNA-templated transcription,transcription regulator activity;Ontology_id=GO:0005634,GO:0003677,GO:0006355,GO:0140110

contig00005.g3597	1387	ID=contig00005.g3597;Description=flavo nadh-dependent oxidoreductase [Fusarium napiforme];Gene=BFJ71_g12097;Ontology_term=oxidoreductase activity;Ontology_id=GO:0016491;Enzyme_code=EC:1,EC:1;Enzyme_name=Oxidoreductases,Oxidoreductases
contig00005.g3598	972	ID=contig00005.g3598;Description=alpha-L-arabinofuranosidase axhA [Fusarium proliferatum];Gene=guaA;Ontology_term=carbohydrate metabolic process,cell wall organization or biogenesis,extracellular region,hydrolase activity;Ontology_id=GO:0005975,GO:0071554,GO:0005576,GO:0016787;Enzyme_code=EC:3.1.3.8,EC:3.2.1.55,EC:3.2,EC:3.1,EC:3.1.3.8,EC:3,EC:3.2.1.55,EC:3.2.1,EC:3.1.3;Enzyme_name=3-phytase,non-reducing end alpha-L-arabinofuranosidase,Glycosylases,Acting on ester bonds,3-phytase,Hydrolases,non-reducing end alpha-L-arabinofuranosidase,Glycosylases,Acting on ester bonds
contig00005.g3599	1060	ID=contig00005.g3599;Description=Alpha/Beta hydrolase protein [Fusarium oxysporum Fo47];Gene=AU210_005722
contig00005.g3600	4436	ID=contig00005.g3600;Description=sterigmatocystin biosynthesis monooxygenase stcW [Fusarium globosum];Gene=moxY-11;Ontology_term=oxidoreductase activity;Ontology_id=GO:0016491;Enzyme_code=EC:1.14.13.8,EC:1.14.13.8,EC:1,EC:1.14,EC:1.14.13;Enzyme_name=flavin-containing monooxygenase,flavin-containing monooxygenase,Oxidoreductases,Acting on paired donors, with incorporation or reduction of molecular oxygen. The oxygen incorporated need not be derived from O2,Acting on paired donors, with incorporation or reduction of molecular oxygen. The oxygen incorporated need not be derived from O2
contig00005.g3601	900	ID=contig00005.g3601;Description=hypothetical protein FVER14953_12661 [Fusarium verticillioides];Gene=C2S_8928;Ontology_term=nucleus,DNA binding,regulation of DNA-templated transcription,transcription regulator activity;Ontology_id=GO:0005634,GO:0003677,GO:0006355,GO:0140110
contig00005.g3602	3014	ID=contig00005.g3602;Description=histidine kinase [Fusarium tjaetaba];Gene=FNAPI_8962;Ontology_term=catalytic activity, acting on a protein,signaling,transferase activity,small molecule sensor activity;Ontology_id=GO:0140096,GO:0023052,GO:0016740,GO:0140299;Enzyme_code=EC:2.7.3,EC:2.7.13.3,EC:2.7.1,EC:2.7.3,EC:2.7,EC:2,EC:2.7.13.3;Enzyme_name=Transferring phosphorus-containing groups,histidine kinase,Transferring phosphorus-containing groups,Transferring phosphorus-containing groups,Transferring phosphorus-containing groups,Transferases,histidine kinase

contig00005.g3603	2320	ID=contig00005.g3603;Description=laccase precursor [Fusarium pseudoanthophilum];Gene=FVEG_12658;Ontology_term=oxidoreductase activity;Ontology_id=GO:0016491;Enzyme_code=EC:1;Enzyme_name=Oxidoreductases
contig00005.g3604	4126	ID=contig00005.g3604;Description=glycosyl hydrolases family 31-domain-containing protein [Fusarium oxysporum];Gene=FPANT_552;Ontology_term=hydrolase activity;Ontology_id=GO:0016787;Enzyme_code=EC:3.2,EC:3.2,EC:3;Enzyme_name=Glycosylases,Glycosylases,Hydrolases
contig00005.g3605	4197	ID=contig00005.g3605;Description=Metacaspase-1B [Arthrobotrys entomopaga];Gene=PAC_14638;Ontology_term=catalytic activity, acting on a protein,hydrolase activity;Ontology_id=GO:0140096,GO:0016787;Enzyme_code=EC:3.4.22,EC:3.4.22,EC:3.4,EC:3;Enzyme_name=Acting on peptide bonds (peptidases),Acting on peptide bonds (peptidases),Acting on peptide bonds (peptidases),Hydrolases
contig00005.g3606	3770	ID=contig00005.g3606;Description=CHAT domain-containing protein [Diaporthaceae sp. PMI_573]
contig00005.g3607	1737	ID=contig00005.g3607;Description=vegetative incompatibility het-e-1 [Fusarium tjaetaba];Gene=FOXB_11885;Ontology_term=carbohydrate metabolic process,hydrolase activity;Ontology_id=GO:0005975,GO:0016787;Enzyme_code=EC:3.2.1,EC:3.2,EC:3,EC:3.2.1;Enzyme_name=Glycosylases,Glycosylases,Hydrolases,Glycosylases
contig00005.g3608	2195	ID=contig00005.g3608;Description=related to tol protein [Fusarium fujikuroi]
contig00005.g3609	441	ID=contig00005.g3609;Description=hypothetical protein FVEG_17318 [Fusarium verticillioides 7600]
contig00005.g3610	1186	ID=contig00005.g3610;Description=permease of the major facilitator superfamily [Fusarium tjaetaba];Gene=FNYG_00197;Ontology_term=transmembrane transport,transporter activity;Ontology_id=GO:0055085,GO:0005215
contig00005.g3611	1688	ID=contig00005.g3611;Description=hypothetical protein FVER14953_12651 [Fusarium verticillioides]
contig00005.g3612	2350	ID=contig00005.g3612;Description=hard-surface inducible [Fusarium coicis]
contig00005.g3613	1572	ID=contig00005.g3613;Description=hypothetical protein FVER14953_12647 [Fusarium verticillioides];Gene=FVEG_12647;Ontology_term=mitochondrion,transferase activity;Ontology_id=GO:0005739,GO:0016740;Enzyme_code=EC:2.1.1,EC:2.1,EC:2,EC:2.1.1,EC:2.1.1.114;Enzyme_name=Transferring one-carbon groups,Transferring one-carbon groups,Transferases,Transferring one-carbon groups,polyprenyldihydroxybenzoate methyltransferase

contig00005.g3614	1065	ID=contig00005.g3614;Description=toxD [Fusarium tjaetaba];Gene=FVEG_12646;Ontology_term=oxidoreductase activity;Ontology_id=GO:0016491;Enzyme_code=EC:1.6,EC:1.6,EC:1;Enzyme_name=Acting on NADH or NADPH,Acting on NADH or NADPH,Oxidoreductases
contig00005.g3615	1494	ID=contig00005.g3615;Description=mRNA 3 end-processing YTH1 [Fusarium phyllophilum];Gene=FVER53263_12645;Ontology_term=transferase activity;Ontology_id=GO:0016740;Enzyme_code=EC:2.1.1;Enzyme_name=Transferring one-carbon groups
contig00005.g3616	1351	ID=contig00005.g3616;Description=agglutinin isolectin 1 precursor [Fusarium coicis];Gene=FPRO05_05063;Ontology_term=membrane,chitin binding;Ontology_id=GO:0016020,GO:0008061
contig00005.g3617	1275	ID=contig00005.g3617;Description=glycoside hydrolase [Fusarium coicis];Gene=FVER53263_12643;Ontology_term=carbohydrate metabolic process,hydrolase activity;Ontology_id=GO:0005975,GO:0016787;Enzyme_code=EC:3.2.1.14,EC:3.2,EC:3,EC:3.2.1.14,EC:3.2.1;Enzyme_name=chitinase,Glycosylases,Hydrolases,chitinase,Glycosylases
contig00005.g3618	906	ID=contig00005.g3618;Description=hypothetical protein FVER14953_12642 [Fusarium verticillioides]
contig00005.g3619	1986	ID=contig00005.g3619;Description=phthalate dioxygenase reductase [Fusarium tjaetaba];Gene=FMAN_03812;Ontology_term=oxidoreductase activity;Ontology_id=GO:0016491;Enzyme_code=EC:1,EC:1;Enzyme_name=Oxidoreductases,Oxidoreductases
contig00005.g3620	1312	ID=contig00005.g3620;Description=related to neutral amino acid permease [Fusarium fujikuroi];Gene=sepF;Ontology_term=transmembrane transport,regulation of DNA-templated transcription,endoplasmic reticulum,chromosome,protein folding,microtubule-based movement,transporter activity,DNA binding,nucleus,microtubule organizing center,ATP-dependent activity,autophagy,cell wall organization or biogenesis,transcription regulator activity,hydrolase activity;Ontology_id=GO:0055085,GO:0006355,GO:0005783,GO:0005694,GO:0006457,GO:0007018,GO:0005215,GO:0003677,GO:0005634,GO:0005815,GO:0140657,GO:0006914,GO:0071554,GO:0140110,GO:0016787;Enzyme_code=EC:7.2.2.10,EC:3.6.1.15,EC:3.6.1,EC:7.2.2.10,EC:3.6,EC:3,EC:7,EC:7.2.2,EC:3.6.1.15;Enzyme_name=P-type Ca(2+) transporter,nucleoside-triphosphate phosphatase,Acting on acid anhydrides,P-type Ca(2+) transporter,Acting on acid anhydrides,Hydrolases,Translocases,Catalysing the translocation of inorganic cations,nucleoside-triphosphate phosphatase

contig00005.g3621	1250	ID=contig00005.g3621;Description=alcohol dehydrogenase [Fusarium verticillioides 7600];Gene=FVEG_12638
contig00005.g3622	1026	ID=contig00005.g3622;Description=n-acyl homoserine lactonase [Fusarium coicis]
contig00005.g3623	1038	ID=contig00005.g3623;Description=transcriptional regulatory [Fusarium subglutinans];Gene=FDB2;Ontology_term=nucleus,DNA binding,regulation of DNA-templated transcription,transferase activity,transcription regulator activity;Ontology_id=GO:0005634,GO:0003677,GO:0006355,GO:0016740,GO:0140110;Enzyme_code=EC:2.3.1.5,EC:2.3,EC:2.3.1.5,EC:2,EC:2.3.1;Enzyme_name=arylamine N-acetyltransferase,Acyltransferases,arylamine N-acetyltransferase,Transferases,Acyltransferases
contig00005.g3624	1662	ID=contig00005.g3624;Description=C6 transcription factor [[Gibberella] fujikuroi var. moniliformis];Gene=FDB3;Ontology_term=nucleus,DNA binding,regulation of DNA-templated transcription,transferase activity,transcription regulator activity;Ontology_id=GO:0005634,GO:0003677,GO:0006355,GO:0016740,GO:0140110;Enzyme_code=EC:2.3.1,EC:2.3,EC:2,EC:2.3.1;Enzyme_name=Acyltransferases,Acyltransferases,Transferases,Acyltransferases
contig00005.g3625	1641	ID=contig00005.g3625;Description=triacylglycerol lipase V precursor [Fusarium tjaetaba];Gene=FVER53263_12634;Ontology_term=nucleus,DNA binding,regulation of DNA-templated transcription,transcription regulator activity,hydrolase activity;Ontology_id=GO:0005634,GO:0003677,GO:0006355,GO:0140110,GO:0016787;Enzyme_code=EC:3.1.1.1,EC:3.1,EC:3,EC:3.1.1.1,EC:3.1.1;Enzyme_name=carboxylesterase,Acting on ester bonds,Hydrolases,carboxylesterase,Acting on ester bonds
contig00005.g3626	1861	ID=contig00005.g3626;Description=malonyl synthetase [Fusarium tjaetaba];Gene=FVEG_12633;Ontology_term=ligase activity;Ontology_id=GO:0016874;Enzyme_code=EC:6,EC:6;Enzyme_name=Ligases,Ligases
contig00005.g3627	1849	ID=contig00005.g3627;Description=general amidase [Fusarium fujikuroi];Gene=LW93_7753;Ontology_term=lipid metabolic process;Ontology_id=GO:0006629;Enzyme_code=EC:3.5.1.4;Enzyme_name=amidase
contig00005.g3628	1667	ID=contig00005.g3628;Description=salicylate hydroxylase [Fusarium verticillioides 7600];Gene=nahG-2;Ontology_term=oxidoreductase activity;Ontology_id=GO:0016491;Enzyme_code=EC:1;Enzyme_name=Oxidoreductases

contig00005.g3629	1710	ID=contig00005.g3629;Description=transferase family III [Fusarium tjaetaba];Gene=FVER53590_12629;Ontology_term=transferase activity;Ontology_id=GO:0016740;Enzyme_code=EC:2.3,EC:2.3,EC:2;Enzyme_name=Acyltransferases,Acyltransferases,Transferases
contig00005.g3630	1037	ID=contig00005.g3630;Description=3-hydroxyacyl-CoA dehydrogenase [Fusarium verticillioides 7600];Gene=FVER53590_12628;Ontology_term=transmembrane transport,oxidoreductase activity,lipid metabolic process,transporter activity;Ontology_id=GO:0055085,GO:0016491,GO:0006629,GO:0005215;Enzyme_code=EC:1.1.1,EC:1.1,EC:1,EC:1.1.1.35,EC:1.1.1;Enzyme_name=Acting on the CH-OH group of donors,Acting on the CH-OH group of donors,Oxidoreductases,3-hydroxyacyl-CoA dehydrogenase,Acting on the CH-OH group of donors
contig00005.g3631	2455	ID=contig00005.g3631;Description=transporter MCH4 [Fusarium tjaetaba];Gene=FMUND_8747;Ontology_term=transmembrane transport,transporter activity;Ontology_id=GO:0055085,GO:0005215
contig00005.g3632	975	ID=contig00005.g3632;Description=dienelactone hydrolase [Fusarium oxysporum];Gene=FOXG_15309
contig00005.g3633	683	ID=contig00005.g3633;Description=hypothetical protein FVER53263_12624 [Fusarium verticillioides]
contig00005.g3634	2413	ID=contig00005.g3634;Description=uncharacterized protein FTJAE_3698 [Fusarium tjaetaba]
contig00005.g3635	477	ID=contig00005.g3635;Description=hypothetical protein FVER14953_12622 [Fusarium verticillioides]
contig00005.g3636	1668	ID=contig00005.g3636;Description=multidrug resistance protein fnx1 [Fusarium coicis];Gene=FNAPI_7148;Ontology_term=transmembrane transport,transporter activity;Ontology_id=GO:0055085,GO:0005215
contig00005.g3637	1091	ID=contig00005.g3637;Description=2og-fe oxygenase family [Fusarium subglutinans];Gene=FDENT_3629;Ontology_term=transmembrane transport,oxidoreductase activity,transporter activity;Ontology_id=GO:0055085,GO:0016491,GO:0005215;Enzyme_code=EC:1,EC:1;Enzyme_name=Oxidoreductases,Oxidoreductases
contig00005.g3638	1023	ID=contig00005.g3638;Description=1-aminocyclopropane-1-carboxylate deaminase [Fusarium sp. NRRL 52700];Enzyme_code=EC:3.5.99.7;Enzyme_name=1-aminocyclopropane-1-carboxylate deaminase

contig00005.g3639	1092	ID=contig00005.g3639;Description=gibberellin 20-oxidase [Fusarium tjaetaba];Gene=FGADI_10368;Ontology_term=oxidoreductase activity;Ontology_id=GO:0016491;Enzyme_code=EC:1,EC:1;Enzyme_name=Oxidoreductases,Oxidoreductases
contig00005.g3640	1053	ID=contig00005.g3640;Description=fatty acid hydroxylase superfamily [Fusarium tjaetaba];Gene=FVER53590_12618;Ontology_term=oxidoreductase activity,lipid metabolic process;Ontology_id=GO:0016491,GO:0006629;Enzyme_code=EC:1,EC:1;Enzyme_name=Oxidoreductases,Oxidoreductases
contig00005.g3641	3988	ID=contig00005.g3641;Description=ankyrin repeat domain-containing protein [Fusarium mundagurra]
contig00005.g3642	2011	ID=contig00005.g3642;Description=amino-acid oxidase [Fusarium pseudoanthophilum];Gene=FPANT_2890;Ontology_term=oxidoreductase activity;Ontology_id=GO:0016491;Enzyme_code=EC:1,EC:1;Enzyme_name=Oxidoreductases,Oxidoreductases
contig00005.g3643	2203	ID=contig00005.g3643;Description=amino-acid oxidase [Fusarium tjaetaba];Gene=FPHYL_10625;Ontology_term=oxidoreductase activity;Ontology_id=GO:0016491;Enzyme_code=EC:1,EC:1;Enzyme_name=Oxidoreductases,Oxidoreductases
contig00005.g3644	1731	ID=contig00005.g3644;Description=sugar transport STL1 [Fusarium tjaetaba];Gene=FNYG_00230;Ontology_term=transmembrane transport,transporter activity;Ontology_id=GO:0055085,GO:0005215
contig00005.g3645	723	ID=contig00005.g3645;Description=ThuA-like domain-containing protein [Fusarium sp. MPI-SDFR-AT-0072]
contig00005.g3646	1886	ID=contig00005.g3646;Description=glucose-methanol-choline (gmc) oxidoreductase [Fusarium tjaetaba];Gene=FVER53263_12612;Ontology_term=oxidoreductase activity;Ontology_id=GO:0016491;Enzyme_code=EC:1.1,EC:1.1,EC:1;Enzyme_name=Acting on the CH-OH group of donors,Acting on the CH-OH group of donors,Oxidoreductases
contig00005.g3647	1725	ID=contig00005.g3647;Description=catalase [Fusarium verticillioides 7600];Gene=FVER53263_12611;Ontology_term=peroxisome,antioxidant activity,oxidoreductase activity,cell wall,mitochondrion;Ontology_id=GO:0005777,GO:0016209,GO:0016491,GO:0005618,GO:0005739;Enzyme_code=EC:1.11.1.6,EC:1.11,EC:1,EC:1.11.1,EC:1.11.1.6;Enzyme_name=catalase,Acting on a peroxide as acceptor,Oxidoreductases,Acting on a peroxide as acceptor,catalase

contig00005.g3648	2046	ID=contig00005.g3648;Description=related to tol protein [Fusarium fujikuroi];Gene=FSUBG_6926;Ontology_term=antioxidant activity,oxidoreductase activity;Ontology_id=GO:0016209,GO:0016491;Enzyme_code=EC:1.11.1.6,EC:1.11,EC:1,EC:1.11.1,EC:1.11.1.6;Enzyme_name=catalase,Acting on a peroxide as acceptor,Oxidoreductases,Acting on a peroxide as acceptor,catalase
contig00005.g3649	361	ID=contig00005.g3649;Description=hypothetical protein FVEG_17310 [Fusarium verticillioides 7600]
contig00005.g3650	12277	ID=contig00005.g3650;Description=polyketide synthase [Fusarium tjaetaba];Gene=FVER53263_12610;Ontology_term=oxidoreductase activity,lipid metabolic process,transferase activity;Ontology_id=GO:0016491,GO:0006629,GO:0016740;Enzyme_code=EC:2.3.1.41,EC:1,EC:2.1.1,EC:2.3.1.41,EC:2.1,EC:2.3,EC:1,EC:2,EC:2.1.1,EC:2.3.1;Enzyme_name=beta-ketoacyl-[acyl-carrier-protein] synthase I,Oxidoreductases,Transferring one-carbon groups,beta-ketoacyl-[acyl-carrier-protein] synthase I,Transferring one-carbon groups,Acyltransferases,Oxidoreductases,Transferases,Transferring one-carbon groups,Acyltransferases
contig00005.g3651	1122	ID=contig00005.g3651;Description=hypothetical protein FVEG_12609 [Fusarium verticillioides 7600]
contig00005.g3652	1068	ID=contig00005.g3652;Description=polyketide synthase enoylreductase [Fusarium tjaetaba];Gene=FOTG_15140;Ontology_term=oxidoreductase activity;Ontology_id=GO:0016491;Enzyme_code=EC:1.6,EC:1.6,EC:1;Enzyme_name=Acting on NADH or NADPH,Acting on NADH or NADPH,Oxidoreductases
contig00005.g3653	1362	ID=contig00005.g3653;Description=Zn2Cys6 transcription factor [Fusarium tjaetaba];Gene=FTJAE_3716;Ontology_term=nucleus,regulation of DNA-templated transcription,transcription regulator activity;Ontology_id=GO:0005634,GO:0006355,GO:0140110
contig00005.g3654	2566	ID=contig00005.g3654;Description=Trichosetin synthetase PKS-NRPS1 [Fusarium musae];Gene=FVER53590_12606;Ontology_term=nucleus,DNA binding,regulation of DNA-templated transcription,transcription regulator activity;Ontology_id=GO:0005634,GO:0003677,GO:0006355,GO:0140110
contig00005.g3655	1692	ID=contig00005.g3655;Description=major facilitator superfamily domain-containing protein [Fusarium oxysporum];Gene=FocTR4_00004547;Ontology_term=transmembrane transport,transporter activity;Ontology_id=GO:0055085,GO:0005215

contig00005.g3656	972	ID=contig00005.g3656;Description=putative secondary metabolism biosynthetic enzyme [Fusarium musae];Gene=FMUND_14160;Ontology_term=oxidoreductase activity;Ontology_id=GO:0016491;Enzyme_code=EC:1,EC:1,EC:1.1.1.1;Enzyme_name=Oxidoreductases, Oxidoreductases,alcohol dehydrogenase
contig00005.g3657	1053	ID=contig00005.g3657;Description=hypothetical protein FVEG_17307 [Fusarium verticillioides 7600];Gene=BFJ63_vAg5591;Ontology_term=regulation of DNA-templated transcription,transcription regulator activity;Ontology_id=GO:0006355,GO:0140110
contig00005.g3658	732	ID=contig00005.g3658;Description=acetoacetate decarboxylase [Fusarium verticillioides 7600];Gene=FVEG_12603;Ontology_term=lyase activity;Ontology_id=GO:0016829;Enzyme_code=EC:4.1.1,EC:4.1.1,EC:4.1,EC:4;Enzyme_name=Carbon-carbon lyases,Carbon-carbon lyases,Carbon-carbon lyases,Lyases
contig00005.g3659	719	ID=contig00005.g3659;Description=hypothetical protein F25303_8081 [Fusarium sp. NRRL 25303];Gene=GLAA;Ontology_term=carbohydrate metabolic process,catalytic activity, acting on a protein,anatomical structure development,endoplasmic reticulum,protein maturation,extracellular region,hydrolase activity;Ontology_id=GO:0005975,GO:0140096,GO:0048856,GO:0005783,GO:0051604,GO:0005576,GO:0016787;Enzyme_code=EC:3.4.21,EC:3.2.1.3;Enzyme_name=Acting on peptide bonds (peptidases),glucan 1,4-alpha-glucosidase
contig00005.g3660	821	ID=contig00005.g3660;Description=hypothetical protein FVER53590_26469 [Fusarium verticillioides];Gene=FFUJ_02226
contig00005.g3661	1390	ID=contig00005.g3661;Description=hypothetical protein FVEG_12601 [Fusarium verticillioides 7600]
contig00005.g3662	1540	ID=contig00005.g3662;Description=hypothetical protein FVER53263_12600 [Fusarium verticillioides]
contig00005.g3663	572	ID=contig00005.g3663;Description=hypothetical protein FPANT_3613 [Fusarium pseudoanthophilum];Gene=FTJAE_3727;Ontology_term=membrane;Ontology_id=GO:0016020

contig00005.g3664	3471	ID=contig00005.g3664;Description=putative bifunctional P-450:NADPH-P450 reductase [Fusarium tjaetaba];Gene=FTJAE_3728;Ontology_term=oxidoreductase activity;Ontology_id=GO:0016491;Enzyme_code=EC:1.6.2.4,EC:1.14.14.1,EC:1.6.2.4,EC:1.6.2,EC:1.14.14.1,EC:1.6,EC:1,EC:1.14,EC:1.14.14;Enzyme_name=NADPH--hemoprotein reductase,unspecific monooxygenase,NADPH--hemoprotein reductase,Acting on NADH or NADPH,unspecific monooxygenase,Acting on NADH or NADPH,Oxidoreductases,Acting on paired donors, with incorporation or reduction of molecular oxygen. The oxygen incorporated need not be derived from O2,Acting on paired donors, with incorporation or reduction of molecular oxygen. The oxygen incorporated need not be derived from O2
contig00005.g3665	3960	ID=contig00005.g3665;Description=Sterol uptake control protein 2 [Fusarium oxysporum f. sp. rapae];Gene=FOQG_10859;Ontology_term=nucleus,regulation of DNA-templated transcription,transcription regulator activity;Ontology_id=GO:0005634,GO:0006355,GO:0140110
contig00005.g3666	3798	ID=contig00005.g3666;Description=triacylglycerol lipase II [Fusarium denticulatum];Gene=FMUND_4975;Ontology_term=hydrolase activity;Ontology_id=GO:0016787;Enzyme_code=EC:3,EC:3;Enzyme_name=Hydrolases,Hydrolases
contig00005.g3667	510	ID=contig00005.g3667;Description=histone acetyltransferase HPA2 [Fusarium coicis];Gene=BFJ72_g12087;Ontology_term=nucleus,catalytic activity, acting on a protein,protein-containing complex assembly,detoxification,amino acid metabolic process,transferase activity,chromatin organization;Ontology_id=GO:0005634,GO:0140096,GO:0065003,GO:0098754,GO:0006520,GO:0016740,GO:0006325;Enzyme_code=EC:2.3.1,EC:2.3,EC:2,EC:2.3.1.48,EC:2.3.1.36,EC:2.3.1;Enzyme_name=Acyltransferases,Acyltransferases,Transferases,histone acetyltransferase,D-amino-acid N-acetyltransferase,Acyltransferases
contig00005.g3668	269	ID=contig00005.g3668;Description=hypothetical protein FPANT_3618 [Fusarium pseudoanthophilum]
contig00005.g3669	1128	ID=contig00005.g3669;Description=Transcription factor gsfR2 [Fusarium oxysporum f. sp. rapae];Gene=FPANT_3619;Ontology_term=nucleus,regulation of DNA-templated transcription,transcription regulator activity;Ontology_id=GO:0005634,GO:0006355,GO:0140110

contig00005.g3670	1032	ID=contig00005.g3670;Description=related to 2`-hydroxyisoflavone reductase [Fusarium fujikuroi IMI 58289];Gene=FVER53590_12592;Ontology_term=carbohydrate metabolic process,oxidoreductase activity,hydrolase activity;Ontology_id=GO:0005975,GO:0016491,GO:0016787;Enzyme_code=EC:1,EC:3.2.1.1,EC:3.2,EC:1,EC:3,EC:3.2.1.1,EC:3.2.1;Enzyme_name=Oxidoreductases,alpha-amylase,Glycosylases,Oxidoreductases,Hydrolases,alpha-amylase,Glycosylases
contig00005.g3671	636	ID=contig00005.g3671;Description=chrR cupin-like domain protein [Fusarium tjaetaba];Gene=1427;Ontology_term=lipid binding,extracellular region;Ontology_id=GO:0008289,GO:0005576
contig00005.g3672	1411	ID=contig00005.g3672;Description=putative AC9 transposase [Fusarium oxysporum f. sp. rapae];Gene=FEQUK3_LOCUS15;Ontology_term=membrane,protein dimerization activity;Ontology_id=GO:0016020,GO:0046983
contig00005.g3673	1006	ID=contig00005.g3673;Description=galactinol synthase [Fusarium tjaetaba];Gene=FVER53590_26471;Ontology_term=transferase activity;Ontology_id=GO:0016740;Enzyme_code=EC:2.4;Enzyme_name=Glycosyltransferases
contig00005.g3674	1092	ID=contig00005.g3674;Description=hypothetical protein FVEG_12589 [Fusarium verticillioides 7600];Gene=FVER53263_12589;Ontology_term=catalytic activity, acting on a protein,transferase activity;Ontology_id=GO:0140096,GO:0016740;Enzyme_code=EC:2.7.1,EC:2.7.1,EC:2.7,EC:2;Enzyme_name=Transferring phosphorus-containing groups,Transferring phosphorus-containing groups,Transferring phosphorus-containing groups,Transferases
contig00005.g3675	2008	ID=contig00005.g3675;Description=non-ribosomal peptide synthetase [Fusarium pseudocircinatum];Gene=FVER53263_12588
contig00005.g3676	1778	ID=contig00005.g3676;Description=Zinc-type alcohol dehydrogenase-like protein [Fusarium oxysporum f. sp. albedinis];Gene=FOXG_15269;Ontology_term=nucleus,regulation of DNA-templated transcription,transcription regulator activity;Ontology_id=GO:0005634,GO:0006355,GO:0140110
contig00005.g3677	1959	ID=contig00005.g3677;Description=transporter mfs1 [Fusarium oxysporum];Gene=FocTR4_00004514;Ontology_term=nucleus,DNA binding,transmembrane transport,regulation of DNA-templated transcription,transcription regulator activity,transporter activity;Ontology_id=GO:0005634,GO:0003677,GO:0055085,GO:0006355,GO:0140110,GO:0005215
contig00005.g3678	456	ID=contig00005.g3678;Description=bet V I allergen [Fusarium tjaetaba]

contig00005.g3679	4004	ID=contig00005.g3679;Description=ankyrin [Fusarium subglutinans]
contig00005.g3680	1555	ID=contig00005.g3680;Description=zinc finger domain-containing protein [Fusarium phyllophilum]
contig00005.g3681	1263	ID=contig00005.g3681;Description=hypothetical protein FVER53590_12583 [Fusarium verticillioides]
contig00005.g3682	3070	ID=contig00005.g3682;Description=hypothetical protein FVEG_17302 [Fusarium verticillioides 7600];Gene=FANTH_6463;Ontology_term=membrane;Ontology_id=GO:0016020
contig00005.g3683	810	ID=contig00005.g3683;Description=hypothetical protein FVER14953_12581 [Fusarium verticillioides]
contig00005.g3684	1695	ID=contig00005.g3684;Description=hypothetical protein FVER53263_12580 [Fusarium verticillioides];Gene=FVER53590_12580;Ontology_term=catalytic activity, acting on a protein,transferase activity;Ontology_id=GO:0140096,GO:0016740;Enzyme_code=EC:2.7.1,EC:2.7.1,EC:2.7,EC:2;Enzyme_name=Transferring phosphorus-containing groups,Transferring phosphorus-containing groups,Transferring phosphorus-containing groups,Transferases
contig00005.g3685	4277	ID=contig00005.g3685;Description=serine threonine kinase [Fusarium tjaetaba];Gene=FVER53263_12579;Ontology_term=catalytic activity, acting on a protein,transferase activity;Ontology_id=GO:0140096,GO:0016740;Enzyme_code=EC:2.7.1,EC:2.7.1,EC:2.7,EC:2;Enzyme_name=Transferring phosphorus-containing groups,Transferring phosphorus-containing groups,Transferring phosphorus-containing groups,Transferases
contig00005.g3686	4172	ID=contig00005.g3686;Description=alkaline ceramidase [Fusarium verticillioides 7600];Gene=FVER53263_12578;Ontology_term=lipid metabolic process,hydrolase activity;Ontology_id=GO:0006629,GO:0016787;Enzyme_code=EC:3.5.1.23,EC:3.5.1,EC:3.5.1.23,EC:3.5,EC:3;Enzyme_name=ceramidase,Acting on carbon-nitrogen bonds, other than peptide bonds,ceramidase,Acting on carbon-nitrogen bonds, other than peptide bonds,Hydrolases
contig00005.g3687	1358	ID=contig00005.g3687;Description=monocarboxylate transporter 2 [Fusarium fujikuroi];Gene=FPRO_03938;Ontology_term=transmembrane transport,transporter activity;Ontology_id=GO:0055085,GO:0005215
contig00005.g3688	549	ID=contig00005.g3688;Description=hypothetical protein FVEG_12575 [Fusarium verticillioides 7600]

contig00005.g3689	3717	ID=contig00005.g3689;Description=related to 5-carboxyvanillate decarboxylase [Fusarium fujikuroi];Gene=FPRO_03935;Ontology_term=extracellular space,catalytic activity, acting on a protein,oxidoreductase activity,protein catabolic process,lyase activity,carbohydrate derivative metabolic process,hydrolase activity;Ontology_id=GO:0005615,GO:0140096,GO:0016491,GO:0030163,GO:0016829,GO:1901135,GO:0016787;Enzyme_code=EC:4.1.1,EC:3.4.21,EC:1.2.1,EC:4.1.1,EC:1.2,EC:3.4.21,EC:4.1,EC:3.4,EC:1,EC:3,EC:4,EC:1.2.1;Enzyme_name=Carbon-carbon lyases,Acting on peptide bonds (peptidases),Acting on the aldehyde or oxo group of donors,Carbon-carbon lyases,Acting on the aldehyde or oxo group of donors,Acting on peptide bonds (peptidases),Carbon-carbon lyases,Acting on peptide bonds (peptidases),Oxidoreductases,Hydrolases,Lyases,Acting on the aldehyde or oxo group of donors
contig00005.g3690	453	ID=contig00005.g3690;Description=probable L-PSP endoribonuclease family protein [Fusarium proliferatum ET1];Enzyme_code=EC:3.5.99.5;Enzyme_name=2-aminomuconate deaminase
contig00005.g3691	1066	ID=contig00005.g3691;Description=nuclease S1 precursor [Fusarium tjaetaba];Gene=FACUT_4020;Ontology_term=hydrolase activity;Ontology_id=GO:0016787;Enzyme_code=EC:3.1;Enzyme_name=Acting on ester bonds
contig00005.g3692	585	ID=contig00005.g3692;Description=hypothetical protein FVER53590_12571 [Fusarium verticillioides]
contig00005.g3693	1550	ID=contig00005.g3693;Description=hypothetical protein FPCIR_1050 [Fusarium pseudocircinatum]
contig00005.g3694	446	ID=contig00005.g3694;Description=Endoribonuclease L-PSP/chorismate mutase-like protein [Fusarium sp. MPI-SDFR-AT-0072]
contig00005.g3695	1254	ID=contig00005.g3695;Description=transaminase type I [Fusarium pseudoanthophilum];Gene=FPCIR_1048;Ontology_term=catalytic activity;Ontology_id=GO:0003824
contig00005.g3696	480	ID=contig00005.g3696;Description=arrestin [Fusarium pseudoanthophilum];Gene=FDENT_6223;Ontology_term=catalytic activity;Ontology_id=GO:0003824
contig00005.g3697	1116	ID=contig00005.g3697;Description=HTH-type transcriptional regulator yisV [Fusarium tjaetaba];Gene=BFJ68_g15313;Ontology_term=pyridoxal phosphate binding,biosynthetic process;Ontology_id=GO:0030170,GO:0009058

contig00005.g3698	1664	ID=contig00005.g3698;Description=pisatin demethylase [Fusarium tjaetaba];Gene=FGLOB1_8977;Ontology_term=oxidoreductase activity,transferase activity;Ontology_id=GO:0016491,GO:0016740;Enzyme_code=EC:1.14,EC:2.1.1,EC:2.1,EC:1,EC:1.14,EC:2,EC:2.1.1;Enzyme_name=Acting on paired donors, with incorporation or reduction of molecular oxygen. The oxygen incorporated need not be derived from O2,Transferring one-carbon groups,Transferring one-carbon groups,Oxidoreductases,Acting on paired donors, with incorporation or reduction of molecular oxygen. The oxygen incorporated need not be derived from O2,Transferases,Transferring one-carbon groups
contig00005.g3699	1525	ID=contig00005.g3699;Description=transcriptional activator Mut3p [Fusarium acutatum];Gene=FOXG_21857;Ontology_term=nucleus,DNA binding,regulation of DNA-templated transcription,transcription regulator activity;Ontology_id=GO:0005634,GO:0003677,GO:0006355,GO:0140110
contig00005.g3700	1042	ID=contig00005.g3700;Description=isoflavone reductase like P3 [Fusarium coicis]
contig00005.g3701	473	ID=contig00005.g3701;Description=hypothetical protein FVEG_12560 [Fusarium verticillioides 7600]
contig00005.g3702	684	ID=contig00005.g3702;Description=Xaa-Pro aminopeptidase [Fusarium sp. NRRL 25303];Gene=F25303_8161;Ontology_term=catalytic activity, acting on a protein,hydrolase activity;Ontology_id=GO:0140096,GO:0016787;Enzyme_code=EC:3.4.11,EC:3.4.11,EC:3.4,EC:3;Enzyme_name=Acting on peptide bonds (peptidases),Acting on peptide bonds (peptidases),Acting on peptide bonds (peptidases),Hydrolases
contig00005.g3703	381	ID=contig00005.g3703;Description=alcohol dehydrogenase [Fusarium phyllophilum]
contig00005.g3704	3502	ID=contig00005.g3704;Description=hypothetical protein FVER14953_12558 [Fusarium verticillioides]
contig00005.g3705	1756	ID=contig00005.g3705;Description=related to C6 zink-finger protein PRO1A [Fusarium fujikuroi];Gene=FVEG_12557;Ontology_term=nucleus,regulation of DNA-templated transcription,transcription regulator activity;Ontology_id=GO:0005634,GO:0006355,GO:0140110
contig00005.g3706	2061	ID=contig00005.g3706;Description=glu asp-tRNA amidotransferase subunit A [Fusarium pseudoanthophilum];Gene=FPANT_3654;Ontology_term=transferase activity;Ontology_id=GO:0016740;Enzyme_code=EC:2,EC:2;Enzyme_name=Transferases,Transferases
contig00005.g3707	993	ID=contig00005.g3707;Description=hypothetical protein FVEG_12555 [Fusarium verticillioides 7600]

contig00005.g3708	1638	ID=contig00005.g3708;Description=heterokaryon incompatibility protein-domain-containing protein [Fusarium oxysporum]
contig00005.g3709	3674	ID=contig00005.g3709;Description=hypothetical protein FVER53590_29596 [Fusarium verticillioides];Gene=FVEG_17293;Ontology_term=hydrolase activity;Ontology_id=GO:0016787;Enzyme_code=EC:3;Enzyme_name=Hydrolases
contig00005.g3710	446	ID=contig00005.g3710;Description=DUF718 domain protein [Fusarium tjaetaba];Gene=FPANT_3658;Ontology_term=isomerase activity;Ontology_id=GO:0016853;Enzyme_code=EC:5.1.3,EC:5.1,EC:5,EC:5.1.3;Enzyme_name=Racemases and epimerases,Racemases and epimerases,Isomerases,Racemases and epimerases
contig00005.g3711	1572	ID=contig00005.g3711;Description=related to glucose/galactose transporter [Fusarium fujikuroi IMI 58289];Gene=FFUJ_02138;Ontology_term=transmembrane transport,transporter activity;Ontology_id=GO:0055085,GO:0005215
contig00005.g3712	2540	ID=contig00005.g3712;Description=transcription activator [Fusarium tjaetaba];Gene=FPANT_3660;Ontology_term=nucleus,DNA binding,regulation of DNA-templated transcription,transcription regulator activity;Ontology_id=GO:0005634,GO:0003677,GO:0006355,GO:0140110
contig00005.g3713	1527	ID=contig00005.g3713;Description=mandelate racemase [Fusarium verticillioides 7600];Gene=FVER53263_12549;Ontology_term=carbohydrate metabolic process,lyase activity;Ontology_id=GO:0005975,GO:0016829;Enzyme_code=EC:4.2.1.68,EC:4.2.1,EC:4.2,EC:4,EC:4.2.1.68,EC:4.2.1.146;Enzyme_name=L-fuconate dehydratase,Carbon-oxygen lyases,Carbon-oxygen lyases,Lyases,L-fuconate dehydratase,L-galactonate dehydratase

contig00005.g3714	402	ID=contig00005.g3714;Description=Cofilin-2 [Fusarium oxysporum];Gene=FVEG_12548;Ontology_term=cytoskeleton organization,cell junction organization,anatomical structure development,cytokinesis,cell adhesion,lipid binding,intracellular protein transport,extracellular region,mitotic cell cycle,plastid,nucleocytoplasmic transport,cytosol,transmembrane transport,protein-containing complex assembly,establishment or maintenance of cell polarity,reproductive process,immune system process,plasma membrane,defense response to other organism,cell motility,nucleus,cytoskeleton,signaling,mitochondrion,cell differentiation,cytoskeletal protein binding,programmed cell death;Ontology_id=GO:0007010,GO:0034330,GO:0048856,GO:0000910,GO:0007155,GO:0008289,GO:0006886,GO:0005576,GO:0000278,GO:0009536,GO:0006913,GO:0005829,GO:0055085,GO:0065003,GO:0007163,GO:0022414,GO:0002376,GO:0005886,GO:0098542,GO:0048870,GO:0005634,GO:0005856,GO:0023052,GO:0005739,GO:0030154,GO:0008092,GO:0012501
contig00005.g3715	1158	ID=contig00005.g3715;Description=L-fucose dehydrogenase [Fusarium acutatum];Gene=FOYG_03924;Ontology_term=oxidoreductase activity;Ontology_id=GO:0016491;Enzyme_code=EC:1,EC:1.1,EC:1.1.1.117,EC:1.1.1.116,EC:1,EC:1.1.1;Enzyme_name=Oxidoreductases,Acting on the CH-OH group of donors,D-arabinose 1-dehydrogenase [NAD(P)(+)],D-arabinose 1-dehydrogenase (NAD(+)),Oxidoreductases,Acting on the CH-OH group of donors
contig00005.g3716	1617	ID=contig00005.g3716;Description=carboxypeptidase 2 [Fusarium tjaetaba];Gene=FNYG_00285;Ontology_term=catalytic activity, acting on a protein,extracellular region,hydrolase activity;Ontology_id=GO:0140096,GO:0005576,GO:0016787;Enzyme_code=EC:3.4.17,EC:3.4,EC:3.4.17,EC:3;Enzyme_name=Acting on peptide bonds (peptidases),Acting on peptide bonds (peptidases),Acting on peptide bonds (peptidases),Hydrolases
contig00005.g3717	886	ID=contig00005.g3717;Description=RTM1-like protein [Fusarium denticulatum];Gene=FVER53263_12545;Ontology_term=membrane;Ontology_id=GO:0016020
contig00005.g3718	1911	ID=contig00005.g3718;Description=saponin hydrolase precursor [Fusarium pseudoanthophilum];Gene=FVER53263_12544;Ontology_term=catalytic activity, acting on a protein,hydrolase activity;Ontology_id=GO:0140096,GO:0016787;Enzyme_code=EC:3.4,EC:3.4,EC:3;Enzyme_name=Acting on peptide bonds (peptidases),Acting on peptide bonds (peptidases),Hydrolases

contig00005.g3719	1726	ID=contig00005.g3719;Description=hypothetical protein FCOIX_1875 [Fusarium coicis];Gene=FVER53263_12544;Ontology_term=catalytic activity, acting on a protein,transmembrane transport,transporter activity,hydrolase activity;Ontology_id=GO:0140096,GO:0055085,GO:0005215,GO:0016787;Enzyme_code=EC:3.4;Enzyme_name=Acting on peptide bonds (peptidases)
contig00005.g3720	516	ID=contig00005.g3720;Description=hypothetical protein FPANT_3668 [Fusarium pseudoanthophilum];Gene=FANTH_1866;Ontology_term=membrane;Ontology_id=GO:0016020
contig00005.g3721	798	ID=contig00005.g3721;Description=chloroperoxidase [Fusarium tjaetaba];Gene=FDENT_6252;Ontology_term=antioxidant activity,oxidoreductase activity;Ontology_id=GO:0016209,GO:0016491;Enzyme_code=EC:1.11.1,EC:1.11,EC:1,EC:1.11.1;Enzyme_name=Acting on a peroxide as acceptor,Acting on a peroxide as acceptor,Oxidoreductases,Acting on a peroxide as acceptor
contig00005.g3722	4592	ID=contig00005.g3722;Description=linoleate diol synthase [Fusarium tjaetaba];Gene=FPANT_3670;Ontology_term=antioxidant activity,oxidoreductase activity;Ontology_id=GO:0016209,GO:0016491;Enzyme_code=EC:1.11.1,EC:1.11,EC:1,EC:1.11.1;Enzyme_name=Acting on a peroxide as acceptor,Acting on a peroxide as acceptor,Oxidoreductases,Acting on a peroxide as acceptor
contig00005.g3723	1374	ID=contig00005.g3723;Description=secretory lipase [Fusarium tjaetaba];Gene=FVER53590_12539;Ontology_term=lipid metabolic process,hydrolase activity;Ontology_id=GO:0006629,GO:0016787;Enzyme_code=EC:3.1.1.3,EC:3.1,EC:3,EC:3.1.1,EC:3.1.1.3;Enzyme_name=triacylglycerol lipase,Acting on ester bonds,Hydrolases,Acting on ester bonds,triacylglycerol lipase
contig00005.g3724	1361	ID=contig00005.g3724;Description=related to IBR finger domain protein [Fusarium proliferatum ET1];Gene=FDENT_6255;Ontology_term=catalytic activity, acting on a protein,transferase activity;Ontology_id=GO:0140096,GO:0016740;Enzyme_code=EC:2.3.2,EC:2.3,EC:2,EC:2.3.2;Enzyme_name=Acyltransferases,Acyltransferases,Transferases,Acyltransferases
contig00005.g3725	792	ID=contig00005.g3725;Description=hypothetical protein BFJ69_g6386 [Fusarium oxysporum]
contig00005.g3726	636	ID=contig00005.g3726;Description=cytochrome P450 [Fusarium acutatum];Gene=FVEG_12537;Ontology_term=oxidoreductase activity;Ontology_id=GO:0016491;Enzyme_code=EC:1,EC:1;Enzyme_name=Oxidoreductases,Oxidoreductases

contig00005.g3727	1301	ID=contig00005.g3727;Description=sterol uptake control 2 [Fusarium pseudoanthophilum];Gene=FACUT_3990;Ontology_term=nucleus,DNA binding,regulation of DNA-templated transcription,transcription regulator activity;Ontology_id=GO:0005634,GO:0003677,GO:0006355,GO:0140110
contig00005.g3728	750	ID=contig00005.g3728;Description=Caffeoyl O-methyltransferase 1 [Fusarium tjaetaba];Gene=FVER53263_12535;Ontology_term=transferase activity;Ontology_id=GO:0016740;Enzyme_code=EC:2.1.1,EC:2.1,EC:2,EC:2.1.1;Enzyme_name=Transferring one-carbon groups,Transferring one-carbon groups,Transferases,Transferring one-carbon groups
contig00005.g3729	2414	ID=contig00005.g3729;Description=Fusaric acid cluster transcription factor fub12 [Fusarium musae];Gene=FVER53263_12534;Ontology_term=defense response to other organism,nucleus,DNA binding,extracellular space,reproductive process,regulation of DNA-templated transcription,receptor ligand activity,transcription regulator activity;Ontology_id=GO:0098542,GO:0005634,GO:0003677,GO:0005615,GO:0022414,GO:0006355,GO:0048018,GO:0140110
contig00005.g3730	1879	ID=contig00005.g3730;Description=Efflux pump fub11 [Fusarium musae];Gene=FVER53263_12533;Ontology_term=transmembrane transport,transporter activity;Ontology_id=GO:0055085,GO:0005215
contig00005.g3731	1266	ID=contig00005.g3731;Description=FUB10 [Fusarium sp.];Gene=FGADI_3330;Ontology_term=nucleus,regulation of DNA-templated transcription,transcription regulator activity;Ontology_id=GO:0005634,GO:0006355,GO:0140110
contig00005.g3732	1447	ID=contig00005.g3732;Description=FMN-dependent dehydrogenase [Fusarium oxysporum];Gene=FOC1_g10002868;Enzyme_code=EC:1.1.3.15;Enzyme_name=(S)-2-hydroxy-acid oxidase
contig00005.g3733	3369	ID=contig00005.g3733;Description=FUB8 [Fusarium sp.];Gene=FOQG_10800;Ontology_term=oxidoreductase activity,transferase activity;Ontology_id=GO:0016491,GO:0016740;Enzyme_code=EC:1,EC:2,EC:1,EC:2;Enzyme_name=Oxidoreductases,Transferases,Oxidoreductases,Transferases
contig00005.g3734	1502	ID=contig00005.g3734;Description=FUB7 [Fusarium sp.];Gene=FVER53590_12529;Ontology_term=sulfur compound metabolic process,lyase activity,amino acid metabolic process,transferase activity;Ontology_id=GO:0006790,GO:0016829,GO:0006520,GO:0016740;Enzyme_code=EC:2.5.1,EC:4;Enzyme_name=Transferring alkyl or aryl groups, other than methyl groups,Lyases

contig00005.g3735	1121	ID=contig00005.g3735;Description=Dehydrogenase fub6 [Fusarium musae];Gene=FTJAE_2482;Ontology_term=oxidoreductase activity;Ontology_id=GO:0016491;Enzyme_code=EC:1.3.1,EC:1.3,EC:1,EC:1.3.1;Enzyme_name=Acting on the CH-CH group of donors,Acting on the CH-CH group of donors,Oxidoreductases,Acting on the CH-CH group of donors
contig00005.g3736	974	ID=contig00005.g3736;Description=class II aldolase/adducin domain protein [Fusarium oxysporum Fo47]
contig00005.g3737	1011	ID=contig00005.g3737;Description=Metallo-beta-lactamase L1 type 3 [Fusarium oxysporum f. sp. rapae]
contig00005.g3738	1953	ID=contig00005.g3738;Description=acyl dehydrogenase n-terminal [Fusarium coicis];Gene=FMUND_10743;Ontology_term=carbohydrate metabolic process,catalytic activity, acting on a protein,oxidoreductase activity,transferase activity,hydrolase activity;Ontology_id=GO:0005975,GO:0140096,GO:0016491,GO:0016740,GO:0016787;Enzyme_code=EC:2.7.1,EC:1.3,EC:3.2.1,EC:2.7.1,EC:3.2,EC:1.3,EC:1,EC:2.7,EC:2,EC:3,EC:3.2.1;Enzyme_name=Transferring phosphorus-containing groups,Acting on the CH-CH group of donors,Glycosylases,Transferring phosphorus-containing groups,Glycosylases,Acting on the CH-CH group of donors,Oxidoreductases,Transferring phosphorus-containing groups,Transferases,Hydrolases,Glycosylases
contig00005.g3739	1676	ID=contig00005.g3739;Description=triacylglycerol lipase V precursor [Fusarium pseudoanthophilum];Gene=FVER53263_12524;Ontology_term=hydrolase activity;Ontology_id=GO:0016787;Enzyme_code=EC:3,EC:3;Enzyme_name=Hydrolases,Hydrolases
contig00005.g3740	7423	ID=contig00005.g3740;Description=Reducing polyketide synthase fub1 [Fusarium musae];Gene=FUB1;Ontology_term=oxidoreductase activity,lipid metabolic process,transferase activity;Ontology_id=GO:0016491,GO:0006629,GO:0016740;Enzyme_code=EC:2.3.1.41,EC:1,EC:2.1.1,EC:2.3.1.41,EC:2.1,EC:2.3,EC:1,EC:2,EC:2.1.1,EC:2.3.1;Enzyme_name=beta-ketoacyl-[acyl-carrier-protein] synthase I,Oxidoreductases,Transferring one-carbon groups,beta-ketoacyl-[acyl-carrier-protein] synthase I,Transferring one-carbon groups,Acyltransferases,Oxidoreductases,Transferases,Transferring one-carbon groups,Acyltransferases

contig00005.g3741	2740	ID=contig00005.g3741;Description=aspartate kinase [Fusarium verticillioides 7600];Gene=A0A4D6RJG9;Ontology_term=amino acid metabolic process,transferase activity;Ontology_id=GO:0006520,GO:0016740;Enzyme_code=EC:2.7.2.4,EC:2.7.2,EC:2.7.2.4,EC:2.7,EC:2;Enzyme_name=aspartate kinase,Transferring phosphorus-containing groups,aspartate kinase,Transferring phosphorus-containing groups,Transferases
contig00005.g3742	854	ID=contig00005.g3742;Description=serine hydrolase FSH [Fusarium redolens];Gene=FUB4;Ontology_term=hydrolase activity;Ontology_id=GO:0016787;Enzyme_code=EC:3,EC:3;Enzyme_name=Hydrolases,Hydrolases
contig00005.g3743	1578	ID=contig00005.g3743;Description=homoserine O-acetyltransferase [Fusarium verticillioides 7600];Gene=A0A4D6RKE1;Ontology_term=transferase activity;Ontology_id=GO:0016740;Enzyme_code=EC:2.3.1.31;Enzyme_name=homoserine O-acetyltransferase
contig00005.g3744	3252	ID=contig00005.g3744;Description=Vegetative incompatibility protein HET-E-1 [Fusarium austroafricanum];Gene=FVER53590_12518;Ontology_term=membrane;Ontology_id=GO:0016020
contig00005.g3745	6931	ID=contig00005.g3745;Description=serine/threonine protein kinase [Fusarium verticillioides 7600];Gene=DL764_010377;Ontology_term=catalytic activity, acting on a protein,signaling,transferase activity;Ontology_id=GO:0140096,GO:0023052,GO:0016740;Enzyme_code=EC:2.7.11.1,EC:2.7.1,EC:2.7.11.1,EC:2.7,EC:2;Enzyme_name=non-specific serine/threonine protein kinase,Transferring phosphorus-containing groups,non-specific serine/threonine protein kinase,Transferring phosphorus-containing groups,Transferases
contig00006.g3746	1594	ID=contig00006.g3746;Description=related to Protein esc1 [Fusarium proliferatum ET1];Gene=FNYG_08975;Ontology_term=protein dimerization activity;Ontology_id=GO:0046983
contig00006.g3747	1976	ID=contig00006.g3747;Description=peroxisomal targeting signal receptor [Fusarium subglutinans];Gene=FNAPI_12679;Ontology_term=peroxisome;Ontology_id=GO:0005777
contig00006.g3748	1014	ID=contig00006.g3748;Description=valyl-tRNA synthetase [Fusarium verticillioides 7600];Gene=FVER53263_01302;Ontology_term=RNA binding,protein-containing complex assembly,ribosome biogenesis,catalytic activity, acting on RNA,nucleolus,ligase activity;Ontology_id=GO:0003723,GO:0065003,GO:0042254,GO:0140098,GO:0005730,GO:0016874;Enzyme_code=EC:6.1.1,EC:6.1,EC:6.1.1,EC:6;Enzyme_name=Forming carbon-oxygen bonds,Forming carbon-oxygen bonds,Forming carbon-oxygen bonds,Ligases

contig00006.g3749	1633	ID=contig00006.g3749;Description=Sterol uptake control protein 2 [Fusarium oxysporum f. sp. rapae];Gene=FOXB_13034;Ontology_term=nucleus,regulation of DNA-templated transcription,transcription regulator activity;Ontology_id=GO:0005634,GO:0006355,GO:0140110
contig00006.g3750	1269	ID=contig00006.g3750;Description=cystathionine gamma-lyase [Fusarium verticillioides 7600];Gene=FVEG_01304;Ontology_term=nucleus,sulfur compound metabolic process,lyase activity,amino acid metabolic process,transferase activity;Ontology_id=GO:0005634,GO:0006790,GO:0016829,GO:0006520,GO:0016740;Enzyme_code=EC:4,EC:2.5.1,EC:2.5.1.48,EC:2,EC:4.4,EC:4,EC:4.4.1.1;Enzyme_name=Lyases,Transferring alkyl or aryl groups, other than methyl groups,cystathionine gamma-synthase,Transferases,Carbon-sulfur lyases,Lyases,cystathionine gamma-lyase
contig00006.g3751	1681	ID=contig00006.g3751;Description=hypothetical protein FVER14953_01305 [Fusarium verticillioides]
contig00006.g3752	730	ID=contig00006.g3752;Description=PCI domain protein [Fusarium tjaetaba]
contig00006.g3753	1861	ID=contig00006.g3753;Description=hypothetical protein FVER14953_01307 [Fusarium verticillioides]
contig00006.g3754	798	ID=contig00006.g3754;Description=hypothetical protein FVEG_01309 [Fusarium verticillioides 7600];Gene=FMUND_11524;Ontology_term=membrane;Ontology_id=GO:0016020
contig00006.g3755	1147	ID=contig00006.g3755;Description=DNA polymerase epsilon subunit D [Fusarium pseudoanthophilum];Gene=nAChRbeta2;Ontology_term=molecular transducer activity,transmembrane transport,DNA replication,signaling,nuclear chromosome,regulation of DNA-templated transcription,chromatin organization,nervous system process,transporter activity,mitotic cell cycle,plasma membrane;Ontology_id=GO:0060089,GO:0055085,GO:0006260,GO:0023052,GO:0000228,GO:0006355,GO:0006325,GO:0050877,GO:0005215,GO:0000278,GO:0005886;Enzyme_code=EC:2.7.7.7;Enzyme_name=DNA-directed DNA polymerase
contig00006.g3756	2356	ID=contig00006.g3756;Description=nuclear localization protein NPL6 [Fusarium fujikuroi]

contig00006.g3757	3004	ID=contig00006.g3757;Description=mitotic control dis3+ [Fusarium tjaetaba];Gene=FVER53263_01312;Ontology_term=DNA-templated transcription,tRNA metabolic process,nuclear chromosome,ribosome biogenesis,nucleolus,intracellular protein transport,nucleoplasm,nucleocytoplasmic transport,mRNA metabolic process,RNA binding,mitochondrion,regulatory ncRNA-mediated gene silencing,catalytic activity, acting on RNA,hydrolase activity;Ontology_id=GO:0006351,GO:0006399,GO:0000228,GO:0042254,GO:0005730,GO:0006886,GO:0005654,GO:0006913,GO:0016071,GO:0003723,GO:0005739,GO:0031047,GO:0140098,GO:0016787;Enzyme_code=EC:3.1,EC:3.1,EC:3.1.13,EC:3,EC:3.1.15;Enzyme_name=Acting on ester bonds,Acting on ester bonds,Acting on ester bonds,Hydrolases,Acting on ester bonds
contig00006.g3758	1703	ID=contig00006.g3758;Description=nuclear GTPase [Fusarium subglutinans];Gene=FANTH_148;Ontology_term=nucleocytoplasmic transport,RNA binding,GTPase activity,ribosome biogenesis,nucleolus;Ontology_id=GO:0006913,GO:0003723,GO:0003924,GO:0042254,GO:0005730;Enzyme_code=EC:3.6.1.15;Enzyme_name=nucleoside-triphosphate phosphatase
contig00006.g3759	1415	ID=contig00006.g3759;Description=glutamate-1-semialdehyde 2,1-aminomutase [Fusarium verticillioides 7600];Gene=FVER53263_01314;Ontology_term=transferase activity,isomerase activity;Ontology_id=GO:0016740,GO:0016853;Enzyme_code=EC:2.6.1,EC:2.6.1,EC:5.4,EC:2,EC:2.6,EC:5.4.3,EC:5,EC:5.4.3.8;Enzyme_name=Transferring nitrogenous groups,Transferring nitrogenous groups,Intramolecular transferases,Transferases,Transferring nitrogenous groups,Intramolecular transferases,Isomerases,glutamate-1-semialdehyde 2,1-aminomutase
contig00006.g3760	723	ID=contig00006.g3760;Description=splicing factor 3A subunit 2 [Fusarium tjaetaba];Gene=BFJ68_g9750;Ontology_term=nucleus,RNA binding,mRNA metabolic process,protein-containing complex assembly;Ontology_id=GO:0005634,GO:0003723,GO:0016071,GO:0065003

contig00006.g3761	2109	ID=contig00006.g3761;Description=AGC/NDR/NDR protein kinase [Fusarium odoratissimum NRRL 54006];Gene=FOYG_07173;Ontology_term=membrane organization,catalytic activity, acting on a protein,cytoskeleton organization,establishment or maintenance of cell polarity,anatomical structure development,reproductive process,regulation of DNA-templated transcription,plasma membrane,nucleus,signaling,cell wall organization or biogenesis,transferase activity;Ontology_id=GO:0061024,GO:0140096,GO:0007010,GO:0007163,GO:0048856,GO:0022414,GO:0006355,GO:0005886,GO:0005634,GO:0023052,GO:0071554,GO:0016740;Enzyme_code=EC:2.7.11.1,EC:2.7.1,EC:2.7.11.1,EC:2.7,EC:2;Enzyme_name=non-specific serine/threonine protein kinase,Transferring phosphorus-containing groups,non-specific serine/threonine protein kinase,Transferring phosphorus-containing groups,Transferases
contig00006.g3762	1377	ID=contig00006.g3762;Description=mitochondrial import inner membrane translocase subunit tim-54 [Fusarium tjaetaba];Gene=FVEG_01317;Ontology_term=membrane organization,transmembrane transport,mitochondrion,intracellular protein transport,mitochondrion organization;Ontology_id=GO:0061024,GO:0055085,GO:0005739,GO:0006886,GO:0007005
contig00006.g3763	754	ID=contig00006.g3763;Description=nefa-interacting nuclear nip30 [Fusarium tjaetaba];Gene=FPCIR_11935;Ontology_term=nucleus;Ontology_id=GO:0005634
contig00006.g3764	1002	ID=contig00006.g3764;Description=cyclin-like protein CLG1 [Fusarium fujikuroi];Gene=CEK26_000666;Ontology_term=molecular function regulator activity,autophagy;Ontology_id=GO:0098772,GO:0006914
contig00006.g3765	1643	ID=contig00006.g3765;Description=nuclear localization NPL6 [Fusarium coicis];Ontology_term=DNA binding,nuclear chromosome,regulation of DNA-templated transcription,chromatin organization;Ontology_id=GO:0003677,GO:0000228,GO:0006355,GO:0006325
contig00006.g3766	6579	ID=contig00006.g3766;Description=E3 ubiquitin-protein ligase UBR1 [Fusarium verticillioides 7600];Gene=FTJAE_13258;Ontology_term=nucleus,catalytic activity, acting on a protein,protein catabolic process,signaling,regulation of DNA-templated transcription,transferase activity,ligase activity;Ontology_id=GO:0005634,GO:0140096,GO:0030163,GO:0023052,GO:0006355,GO:0016740,GO:0016874;Enzyme_code=EC:6,EC:2.3.2,EC:2.3,EC:2,EC:6,EC:2.3.2.27,EC:2.3.2;Enzyme_name=Ligases,Acyltransferases,Acyltransferases,Transferases,Ligases,RING-type E3 ubiquitin transferase,Acyltransferases

contig00006.g3767	4493	ID=contig00006.g3767;Description=Ca2+-transporting ATPase [Fusarium pseudoanthophilum];Gene=FVER53263_01323;Ontology_term=generation of precursor metabolites and energy,transmembrane transport,ATP-dependent activity,mitochondrion,transporter activity,hydrolase activity;Ontology_id=GO:0006091,GO:0055085,GO:0140657,GO:0005739,GO:0005215,GO:0016787;Enzyme_code=EC:7.2.2.10,EC:3.6.1.15,EC:3.6.1,EC:7.2.2.10,EC:3.6,EC:3,EC:3.6.3.8,EC:7,EC:7.2.2,EC:3.6.1.15;Enzyme_name=P-type Ca(2+) transporter,nucleoside-triphosphate phosphatase,Acting on acid anhydrides,P-type Ca(2+) transporter,Acting on acid anhydrides,Hydrolases,Acting on acid anhydrides,Translocases,Catalysing the translocation of inorganic cations,nucleoside-triphosphate phosphatase
contig00006.g3768	579	ID=contig00006.g3768;Description=RNA binding protein Pym [Fusarium fujikuroi];Gene=FVER53263_01324;Ontology_term=exon-exon junction complex disassembly;Ontology_id=GO:1903259
contig00006.g3769	1359	ID=contig00006.g3769;Description=26S protease subunit rpt4 protein [Rutstroemia sp. NJR-2017a BBW];Gene=TPAR_00032;Ontology_term=DNA repair,catalytic activity, acting on a protein,protein-containing complex assembly,protein catabolic process,chromosome segregation,regulation of DNA-templated transcription,chromatin organization,nucleus,ATP-dependent activity,mitotic nuclear division,cytosol,hydrolase activity;Ontology_id=GO:0006281,GO:0140096,GO:0065003,GO:0030163,GO:0007059,GO:0006355,GO:0006325,GO:0005634,GO:0140657,GO:0140014,GO:0005829,GO:0016787;Enzyme_code=EC:3.4,EC:3.6.1.15,EC:3.6.1,EC:3.4,EC:3.6,EC:3,EC:5.6.1.5,EC:3.6.1.15;Enzyme_name=Acting on peptide bonds (peptidases),nucleoside-triphosphate phosphatase,Acting on acid anhydrides,Acting on peptide bonds (peptidases),Acting on acid anhydrides,Hydrolases,proteasome ATPase,nucleoside-triphosphate phosphatase
contig00006.g3770	2665	ID=contig00006.g3770;Description=glucosamine-fructose-6-phosphate aminotransferase [Fusarium proliferatum];Gene=FVER53263_01326;Ontology_term=nucleobase-containing small molecule metabolic process,carbohydrate derivative metabolic process,amino acid metabolic process,transferase activity;Ontology_id=GO:0055086,GO:1901135,GO:0006520,GO:0016740;Enzyme_code=EC:2.6.1.16;Enzyme_name=glutamine--fructose-6-phosphate transaminase (isomerizing)

contig00006.g3771	897	ID=contig00006.g3771;Description=nucleophile aminohydrolase [Fusarium redolens];Gene=FVEG_01327;Ontology_term=nucleus,protein-containing complex assembly,protein catabolic process,mitotic nuclear division,chromosome segregation,cytosol,endoplasmic reticulum;Ontology_id=GO:0005634,GO:0065003,GO:0030163,GO:0140014,GO:0007059,GO:0005829,GO:0005783;Enzyme_code=EC:3.4.25.1;Enzyme_name=proteasome endopeptidase complex
contig00006.g3772	986	ID=contig00006.g3772;Description=hmg box [Fusarium mexicanum];Gene=FCIRC_11941;Ontology_term=nucleus,DNA binding;Ontology_id=GO:0005634,GO:0003677
contig00006.g3773	1356	ID=contig00006.g3773;Description=hypothetical protein FVEG_01329 [Fusarium verticillioides 7600]
contig00006.g3774	1189	ID=contig00006.g3774;Description=related to C-3 sterol dehydrogenase (C-4 decarboxylase) [Fusarium fujikuroi];Gene=FFB14_03332;Ontology_term=DNA binding,nucleus,oxidoreductase activity,lipid metabolic process,regulation of DNA-templated transcription,endoplasmic reticulum,chromatin organization;Ontology_id=GO:0003677,GO:0005634,GO:0016491,GO:0006629,GO:0006355,GO:0005783,GO:0006325;Enzyme_code=EC:1.1.1.145,EC:1.1.1.170,EC:1.1,EC:1,EC:1.1.1.145,EC:1.1.1;Enzyme_name=3beta-hydroxy-Delta(5)-steroid dehydrogenase,3beta-hydroxysteroid-4alpha-carboxylate 3-dehydrogenase,Acting on the CH-OH group of donors,Oxidoreductases,3beta-hydroxy-Delta(5)-steroid dehydrogenase,Acting on the CH-OH group of donors
contig00006.g3775	3644	ID=contig00006.g3775;Description=GTPase-activating protein [Fusarium denticulatum];Gene=FDENT_12785;Ontology_term=carbohydrate metabolic process,molecular function regulator activity,cytoskeleton organization,signaling,mitochondrion,anatomical structure development,reproductive process,cytokinesis,cell wall organization or biogenesis,mitotic cell cycle;Ontology_id=GO:0005975,GO:0098772,GO:0007010,GO:0023052,GO:0005739,GO:0048856,GO:0022414,GO:0000910,GO:0071554,GO:0000278
contig00006.g3776	1654	ID=contig00006.g3776;Description=mitochondrial integral membrane protein [Fusarium sp. NRRL 52700];Gene=FDENT_7111;Ontology_term=vacuole,hydrolase activity;Ontology_id=GO:0005773,GO:0016787;Enzyme_code=EC:3;Enzyme_name=Hydrolases

contig00006.g3777	507	ID=contig00006.g3777;Description=hypothetical protein FVEG_01333 [Fusarium verticillioides 7600]
contig00006.g3778	1041	ID=contig00006.g3778;Description=hypothetical protein FPANT_14148 [Fusarium pseudoanthophilum]
contig00006.g3779	1787	ID=contig00006.g3779;Description=ubiquitin-conjugating enzyme [Fusarium fujikuroi];Enzyme_code=EC:2.3.2.23;Enzyme_name=E2 ubiquitin-conjugating enzyme
contig00006.g3780	1306	ID=contig00006.g3780;Description=replication factor C subunit 2 [Fusarium verticillioides 7600];Gene=FVER53263_01336;Ontology_term=DNA repair,DNA binding,catalytic activity, acting on DNA,ATP-dependent activity,DNA replication,signaling,chromosome segregation,nuclear chromosome,mitotic cell cycle,hydrolase activity;Ontology_id=GO:0006281,GO:0003677,GO:0140097,GO:0140657,GO:0006260,GO:0023052,GO:0007059,GO:0000228,GO:0000278,GO:0016787;Enzyme_code=EC:3.6.1.15,EC:3.6.1,EC:3.6,EC:3,EC:3.6.1.15;Enzyme_name=nucleoside-triphosphate phosphatase,Acting on acid anhydrides,Acting on acid anhydrides,Hydrolases,nucleoside-triphosphate phosphatase
contig00006.g3781	1551	ID=contig00006.g3781;Description=hypothetical protein FVER14953_01337 [Fusarium verticillioides]
contig00006.g3782	6684	ID=contig00006.g3782;Description=ATP dependent RNA helicase [Fusarium pseudocircinatum];Gene=FNAPI_6379;Ontology_term=nucleus,mRNA metabolic process,protein-containing complex assembly,ATP-dependent activity,catalytic activity, acting on RNA,hydrolase activity;Ontology_id=GO:0005634,GO:0016071,GO:0065003,GO:0140657,GO:0140098,GO:0016787;Enzyme_code=EC:3.6.1.15,EC:3.6.1,EC:3.6.4.13,EC:3.6,EC:3,EC:3.6.1.15;Enzyme_name=nucleoside-triphosphate phosphatase,Acting on acid anhydrides,RNA helicase,Acting on acid anhydrides,Hydrolases,nucleoside-triphosphate phosphatase
contig00006.g3783	420	ID=contig00006.g3783;Description=pre-mRNA-splicing factor brr2 [Fusarium tjaetaba];Gene=FMUND_1769;Ontology_term=mRNA metabolic process,ATP-dependent activity,mitochondrion,microtubule-based movement,hydrolase activity;Ontology_id=GO:0016071,GO:0140657,GO:0005739,GO:0007018,GO:0016787;Enzyme_code=EC:3.6.1.15;Enzyme_name=nucleoside-triphosphate phosphatase
contig00006.g3784	2084	ID=contig00006.g3784;Description=PWWP domain protein [Fusarium tjaetaba];Gene=FVEG_01339;Ontology_term=mitochondrion,microtubule-based movement;Ontology_id=GO:0005739,GO:0007018

contig00006.g3785	2407	ID=contig00006.g3785;Description=origin recognition complex subunit 3 [Fusarium coicis];Gene=FPCIR_10739;Ontology_term=DNA binding,DNA replication,nuclear chromosome;Ontology_id=GO:0003677,GO:0006260,GO:0000228
contig00006.g3786	444	ID=contig00006.g3786;Description=beta-xylosidase [Fusarium denticulatum]
contig00006.g3787	782	ID=contig00006.g3787;Description=endoplasmic reticulum translocation complex chain SEC66 [Fusarium tjaetaba];Gene=FPCIR_13727;Ontology_term=transmembrane transport,endoplasmic reticulum,intracellular protein transport;Ontology_id=GO:0055085,GO:0005783,GO:0006886
contig00006.g3788	2100	ID=contig00006.g3788;Description=nuclear protein localization protein 4 [Fusarium verticillioides 7600];Gene=FPCIR_13728;Ontology_term=nuclear envelope,cytoskeleton organization,protein catabolic process,mitochondrion,cytosol,endoplasmic reticulum,intracellular protein transport,mitotic cell cycle;Ontology_id=GO:0005635,GO:0007010,GO:0030163,GO:0005739,GO:0005829,GO:0005783,GO:0006886,GO:0000278
contig00006.g3789	3821	ID=contig00006.g3789;Description=carbamoyl-phosphate synthase arginine-specific large chain [Fusarium odoratissimum NRRL 54006];Gene=FNYG_11720;Ontology_term=nucleobase-containing small molecule metabolic process,nitrogen cycle metabolic process,amino acid metabolic process,ligase activity;Ontology_id=GO:0055086,GO:0071941,GO:0006520,GO:0016874;Enzyme_code=EC:6,EC:6.3.5.5,EC:6.3,EC:6,EC:6.3.5,EC:6.3.4.16;Enzyme_name=Ligases,carbamoyl-phosphate synthase (glutamine-hydrolyzing),Forming carbon-nitrogen bonds,Ligases,Forming carbon-nitrogen bonds,carbamoyl-phosphate synthase (ammonia)
contig00006.g3790	1077	ID=contig00006.g3790;Description=hypothetical protein FVER53590_01345 [Fusarium verticillioides]
contig00006.g3791	411	ID=contig00006.g3791;Description=hypothetical protein FVEG_14815 [Fusarium verticillioides 7600]
contig00006.g3792	728	ID=contig00006.g3792;Description=DUF962 domain protein [Fusarium subglutinans];Gene=FOZG_00824;Ontology_term=membrane;Ontology_id=GO:0016020
contig00006.g3793	611	ID=contig00006.g3793;Description=DNA-directed RNA polymerase II subunit [Lachnellula hyalina];Gene=S40288_03516;Ontology_term=DNA binding,mRNA metabolic process,RNA binding,DNA-templated transcription,nucleoplasm;Ontology_id=GO:0003677,GO:0016071,GO:0003723,GO:0006351,GO:0005654

contig00006.g3794	2954	ID=contig00006.g3794;Description=zinc-binding protein [Fusarium fujikuroi];Gene=FOIG_00639;Ontology_term=metal ion binding;Ontology_id=GO:0046872
contig00006.g3795	1021	ID=contig00006.g3795;Description=acetylxylan esterase [Fusarium fujikuroi];Gene=TkR99D;Ontology_term=molecular transducer activity,signaling,nervous system process,plasma membrane;Ontology_id=GO:0060089,GO:0023052,GO:0050877,GO:0005886
contig00006.g3796	498	ID=contig00006.g3796;Description=hypothetical protein FVER53263_01352 [Fusarium verticillioides]
contig00006.g3797	2044	ID=contig00006.g3797;Description=autophagy-like protein 22 [Fusarium verticillioides 7600];Gene=FTJAE_7370;Ontology_term=transmembrane transport,autophagy,vacuole,transporter activity;Ontology_id=GO:0055085,GO:0006914,GO:0005773,GO:0005215
contig00006.g3798	1451	ID=contig00006.g3798;Description=F-actin capping protein [Fusarium oxysporum];Gene=F53441_3925;Ontology_term=cytoskeleton,cytoskeleton organization,protein-containing complex assembly,cytoskeletal protein binding;Ontology_id=GO:0005856,GO:0007010,GO:0065003,GO:0008092
contig00006.g3799	985	ID=contig00006.g3799;Description=F-actin-capping protein subunit beta [Fusarium verticillioides 7600];Gene=FNYG_11731;Ontology_term=nucleus,cytoskeleton,cytoskeleton organization,protein-containing complex assembly,cytokinesis,cytoskeletal protein binding,mitotic cell cycle;Ontology_id=GO:0005634,GO:0005856,GO:0007010,GO:0065003,GO:0000910,GO:0008092,GO:0000278
contig00006.g3800	1287	ID=contig00006.g3800;Description=Epimerase domain-containing protein [Fusarium falciforme]
contig00006.g3801	631	ID=contig00006.g3801;Description=LCCL domain-containing protein [Fusarium denticulatum]
contig00006.g3802	614	ID=contig00006.g3802;Description=cystathionine beta-synthase [Fusarium coicis]
contig00006.g3803	1296	ID=contig00006.g3803;Description=phosphate transport mitochondrial [Fusarium subglutinans];Gene=FPANT_9990;Ontology_term=transmembrane transport,mitochondrion,transporter activity;Ontology_id=GO:0055085,GO:0005739,GO:0005215

contig00006.g3804	2348	ID=contig00006.g3804;Description=ubiquitin thiolesterase [Fusarium verticillioides 7600];Gene=FPHYL_5704;Ontology_term=catalytic activity, acting on a protein,vesicle-mediated transport,protein catabolic process,regulation of DNA-templated transcription,hydrolase activity;Ontology_id=GO:0140096,GO:0016192,GO:0030163,GO:0006355,GO:0016787;Enzyme_code=EC:3.4.19.12,EC:3.4,EC:3,EC:3.4.19.12;Enzyme_name=ubiquitinyl hydrolase 1,Acting on peptide bonds (peptidases),Hydrolases,ubiquitinyl hydrolase 1
contig00006.g3805	469	ID=contig00006.g3805;Description=YOS1 subunit of the Yip1p-Yif1p Complex required for transport between the ER and the Gol [Fusarium tjaetaba];Gene=FPHYL_5705
contig00006.g3806	1765	ID=contig00006.g3806;Description=alpha-1,6-mannosyltransferase [Fusarium verticillioides 7600];Gene=FDENT_7444;Ontology_term=endoplasmic reticulum,transferase activity;Ontology_id=GO:0005783,GO:0016740;Enzyme_code=EC:2.4.1.260;Enzyme_name=dolichyl-P-Man:Man7GlcNAc2-PP-dolichol alpha-1,6-mannosyltransferase
contig00006.g3807	7206	ID=contig00006.g3807;Description=adenylate cyclase [Fusarium verticillioides 7600];Gene=FFUJ_01428;Ontology_term=nucleobase-containing small molecule metabolic process,signaling,mitochondrion,lyase activity,carbohydrate derivative metabolic process,regulation of DNA-templated transcription,cytosol,reproductive process,endoplasmic reticulum,plasma membrane,cyclase activity;Ontology_id=GO:0055086,GO:0023052,GO:0005739,GO:0016829,GO:1901135,GO:0006355,GO:0005829,GO:0022414,GO:0005783,GO:0005886,GO:0009975;Enzyme_code=EC:4.6.1.1,EC:4,EC:4.6,EC:4.6.1.1;Enzyme_name=adenylate cyclase,Lyases,Phosphorus-oxygen lyases,adenylate cyclase
contig00006.g3808	1941	ID=contig00006.g3808;Description=trehalose-6-phosphate hydrolase [Fusarium verticillioides 7600];Gene=B0Z3Y5;Ontology_term=carbohydrate metabolic process,hydrolase activity;Ontology_id=GO:0005975,GO:0016787;Enzyme_code=EC:3.2.1.20,EC:3.2,EC:3.2.1.20,EC:3.2.1.10,EC:3,EC:3.2.1.26,EC:3.2.1.48,EC:3.2.1;Enzyme_name=alpha-glucosidase,Glycosylases,alpha-glucosidase,oligo-1,6-glucosidase,Hydrolases,beta-fructofuranosidase,sucrose alpha-glucosidase,Glycosylases
contig00006.g3809	843	ID=contig00006.g3809;Description=20S proteasome subunit beta3 [Fusarium tjaetaba];Gene=FNAPI_10767;Ontology_term=nucleus,protein catabolic process;Ontology_id=GO:0005634,GO:0030163
contig00006.g3810	1589	ID=contig00006.g3810;Description=UBX domain-containing protein [Fusarium pseudoanthophilum]

contig00006.g3811	681	ID=contig00006.g3811;Description=60S ribosomal protein L36, variant 2 [Pyricularia oryzae];Gene=SPBR_07791;Ontology_term=structural molecule activity,ribosome;Ontology_id=GO:0005198,GO:0005840
contig00006.g3812	573	ID=contig00006.g3812;Description=hypothetical protein FVEG_01368 [Fusarium verticillioides 7600]
contig00006.g3813	1218	ID=contig00006.g3813;Description=triacylglycerol lipase [Fusarium tjaetaba];Gene=FVER53263_01369;Ontology_term=lipid metabolic process;Ontology_id=GO:0006629;Enzyme_code=EC:3.1.1.73;Enzyme_name=feruloyl esterase
contig00006.g3814	1596	ID=contig00006.g3814;Description=probable 14-3-3-like protein [Fusarium fujikuroi]
contig00006.g3815	492	ID=contig00006.g3815;Description=hypothetical protein FVEG_01371 [Fusarium verticillioides 7600]
contig00006.g3816	4123	ID=contig00006.g3816;Description=phosphatidylinositol phosphate phosphatase [Fusarium pseudocircinatum];Gene=FVER53590_01372;Ontology_term=cytoskeleton,lipid metabolic process,signaling,hydrolase activity;Ontology_id=GO:0005856,GO:0006629,GO:0023052,GO:0016787;Enzyme_code=EC:3.1.3.36,EC:3.1,EC:3.1.3.36,EC:3.1.3.56,EC:3,EC:3.1.3.64,EC:3.1.3.86,EC:3.1.3;Enzyme_name=phosphoinositide 5-phosphatase,Acting on ester bonds,phosphoinositide 5-phosphatase,inositol-polyphosphate 5-phosphatase,Hydrolases,phosphatidylinositol-3-phosphatase,phosphatidylinositol-3,4,5-trisphosphate 5-phosphatase,Acting on ester bonds
contig00006.g3817	2339	ID=contig00006.g3817;Description=PRP39-pre-mRNA splicing factor [Fusarium tjaetaba];Gene=FVEG_14824;Ontology_term=RNA processing;Ontology_id=GO:0006396
contig00006.g3818	1077	ID=contig00006.g3818;Description=cytochrome c peroxidase, mitochondrial [Fusarium proliferatum];Gene=Forpe1208_v001322;Ontology_term=antioxidant activity,oxidoreductase activity,mitochondrion;Ontology_id=GO:0016209,GO:0016491,GO:0005739;Enzyme_code=EC:1.11.1,EC:1.11,EC:1,EC:1.11.1.5,EC:1.11.1;Enzyme_name=Acting on a peroxide as acceptor,Acting on a peroxide as acceptor,Oxidoreductases,cytochrome-c peroxidase,Acting on a peroxide as acceptor

contig00006.g3819	1351	ID=contig00006.g3819;Description=oxidoreductase [Fusarium verticillioides 7600];Gene=FACUT_4518;Ontology_term=oxidoreductase activity;Ontology_id=GO:0016491;Enzyme_code=EC:1.6.99.1,EC:1.6,EC:1,EC:1.6.99.1;Enzyme_name=NADPH dehydrogenase,Acting on NADH or NADPH,Oxidoreductases,NADPH dehydrogenase
contig00006.g3820	1623	ID=contig00006.g3820;Description=protein disulfide-isomerase [Fusarium verticillioides 7600];Gene=FPANT_10557;Ontology_term=catalytic activity, acting on a protein,protein-containing complex assembly,protein catabolic process,carbohydrate derivative metabolic process,cellular modified amino acid metabolic process,amino acid metabolic process,endoplasmic reticulum,extracellular region,protein folding,plasma membrane,oxidoreductase activity,signaling,programmed cell death,isomerase activity;Ontology_id=GO:0140096,GO:0065003,GO:0030163,GO:1901135,GO:0006575,GO:0006520,GO:0005783,GO:0005576,GO:0006457,GO:0005886,GO:0016491,GO:0023052,GO:0012501,GO:0016853;Enzyme_code=EC:5.3.4.1,EC:5.3.4.1,EC:1.14.11,EC:1,EC:1.8,EC:1.14,EC:5.3,EC:5.3.4,EC:5,EC:1.14.11.2;Enzyme_name=protein disulfide-isomerase,protein disulfide-isomerase,Acting on paired donors, with incorporation or reduction of molecular oxygen. The oxygen incorporated need not be derived from O2,Oxidoreductases,Acting on a sulfur group of donors,Acting on paired donors, with incorporation or reduction of molecular oxygen. The oxygen incorporated need not be derived from O2,Intramolecular oxidoreductases,Intramolecular oxidoreductases,Isomerases,procollagen-proline 4-dioxygenase
contig00006.g3821	469	ID=contig00006.g3821;Description=lipo leucine-zipper domain protein [Fusarium subglutinans];Gene=FPANT_10558;Ontology_term=membrane;Ontology_id=GO:0016020
contig00006.g3822	845	ID=contig00006.g3822;Description=threonyl-trna synthetase [Fusarium tjaetaba];Gene=FOMG_00791;Ontology_term=nucleus,catalytic activity, acting on RNA,chromatin organization,ligase activity;Ontology_id=GO:0005634,GO:0140098,GO:0006325,GO:0016874;Enzyme_code=EC:6.1.1,EC:6.1,EC:6.1.1,EC:6;Enzyme_name=Forming carbon-oxygen bonds,Forming carbon-oxygen bonds,Forming carbon-oxygen bonds,Ligases
contig00006.g3823	2603	ID=contig00006.g3823;Description=Threonyl-tRNA synthetase, cytoplasmic [Fusarium odoratissimum];Gene=HZS61_000553;Ontology_term=tRNA metabolic process,amino acid metabolic process,catalytic activity, acting on RNA,ligase activity;Ontology_id=GO:0006399,GO:0006520,GO:0140098,GO:0016874;Enzyme_code=EC:6.1.1.3;Enzyme_name=threonine--tRNA ligase

contig00006.g3824	2459	ID=contig00006.g3824;Description=vegetatible incompatibility het-e-1 [Fusarium tjaetaba]
contig00006.g3825	1852	ID=contig00006.g3825;Description=beta-glucosidase btge [Fusarium tjaetaba];Gene=P15518;Ontology_term=cytoskeleton,cytoskeleton organization,cell wall,structural molecule activity;Ontology_id=GO:0005856,GO:0007010,GO:0005618,GO:0005198
contig00006.g3826	3145	ID=contig00006.g3826;Description=mitochondrial cyt-4 [Fusarium tjaetaba];Gene=FNYG_06412;Ontology_term=RNA binding,catalytic activity, acting on RNA,hydrolase activity;Ontology_id=GO:0003723,GO:0140098,GO:0016787;Enzyme_code=EC:3.1;Enzyme_name=Acting on ester bonds
contig00006.g3827	1814	ID=contig00006.g3827;Description=related to negative regulator of glucose-repressible genes [Fusarium proliferatum ET1];Ontology_term=carbohydrate metabolic process,generation of precursor metabolites and energy,molecular function regulator activity,protein catabolic process,signaling,regulation of DNA-templated transcription,chromatin organization;Ontology_id=GO:0005975,GO:0006091,GO:0098772,GO:0030163,GO:0023052,GO:0006355,GO:0006325
contig00006.g3828	1589	ID=contig00006.g3828;Description=tRNA wybutosine-synthesizing protein 2 [Fusarium verticillioides 7600];Gene=FOC1_g10015412;Ontology_term=nucleus,mitochondrion,tRNA metabolic process,catalytic activity, acting on RNA,transferase activity;Ontology_id=GO:0005634,GO:0005739,GO:0006399,GO:0140098,GO:0016740;Enzyme_code=EC:2.5.1.114,EC:2.1.1.228;Enzyme_name=tRNA(Phe) (4-demethylwyosine(37)-C(7)) aminocarboxypropyltransferase,tRNA (guanine(37)-N(1))-methyltransferase
contig00006.g3829	1090	ID=contig00006.g3829;Description=DNA repair family [Fusarium agapanthi];Gene=FNAPI_11513;Ontology_term=DNA repair;Ontology_id=GO:0006281
contig00006.g3830	1077	ID=contig00006.g3830;Description=NUS1 nuclear undecaprenyl pyrophosphate synthase [Fusarium subglutinans];Gene=FGADI_10599;Ontology_term=nuclear envelope,lipid droplet,lipid metabolic process,protein glycosylation,endoplasmic reticulum,transferase activity;Ontology_id=GO:0005635,GO:0005811,GO:0006629,GO:0006486,GO:0005783,GO:0016740;Enzyme_code=EC:2.5.1.87,EC:2.5.1,EC:2,EC:2.5.1.87;Enzyme_name=ditrans,polycis-polyprenyl diphosphate synthase [(2E,6E)-farnesyl,Transferring alkyl or aryl groups, other than methyl groups,Transferases,ditrans,polycis-polyprenyl diphosphate synthase [(2E,6E)-farnesyl

contig00006.g3831	2609	ID=contig00006.g3831;Description=exocyst complex component sec10 [Fusarium tjaetaba];Gene=FOC1_g10015409;Ontology_term=vesicle-mediated transport,intracellular protein transport;Ontology_id=GO:0016192,GO:0006886
contig00006.g3832	1378	ID=contig00006.g3832;Description=tRNA-dihydrouridine synthase 4 [Fusarium verticillioides 7600];Gene=BFJ72_g6136;Ontology_term=mRNA metabolic process,oxidoreductase activity,tRNA metabolic process,catalytic activity, acting on RNA;Ontology_id=GO:0016071,GO:0016491,GO:0006399,GO:0140098;Enzyme_code=EC:1.3.1.90,EC:1.3.1.89,EC:1.3,EC:1.3.1.90,EC:1,EC:1.3.1.89,EC:1.3.1;Enzyme_name=tRNA-dihydrouridine(20a/20b) synthase [NAD(P)(+)],tRNA-dihydrouridine(47) synthase [NAD(P)(+)],Acting on the CH-CH group of donors,tRNA-dihydrouridine(20a/20b) synthase [NAD(P)(+)],Oxidoreductases,tRNA-dihydrouridine(47) synthase [NAD(P)(+)],Acting on the CH-CH group of donors
contig00006.g3833	2175	ID=contig00006.g3833;Description=e3 ubiquitin ligase ARI9 [Fusarium pseudoanthophilum];Gene=FPANT_9719;Ontology_term=catalytic activity, acting on a protein,transferase activity;Ontology_id=GO:0140096,GO:0016740;Enzyme_code=EC:2.3.2,EC:2.3,EC:2,EC:2.3.2;Enzyme_name=Acyltransferases,Acyltransferases,Transferases,Acyltransferases
contig00006.g3834	2367	ID=contig00006.g3834;Description=hypothetical protein FGLOB1_12011 [Fusarium globosum];Gene=FACUT_9882;Ontology_term=protein-containing complex assembly,mitochondrion,mitochondrion organization;Ontology_id=GO:0065003,GO:0005739,GO:0007005
contig00006.g3835	291	ID=contig00006.g3835;Description=ribosomal protein S28e-domain-containing protein [Fusarium oxysporum Fo47];Gene=cnxG;Ontology_term=nucleobase-containing small molecule metabolic process,structural molecule activity,carbohydrate derivative metabolic process,cytosol,ribosome,transferase activity;Ontology_id=GO:0055086,GO:0005198,GO:1901135,GO:0005829,GO:0005840,GO:0016740;Enzyme_code=EC:2.8.1.12,EC:2.8.1,EC:2,EC:2.8.1.12,EC:2.8;Enzyme_name=molybdopterin synthase,Transferring sulfur-containing groups,Transferases,molybdopterin synthase,Transferring sulfur-containing groups
contig00006.g3836	395	ID=contig00006.g3836;Description=40S ribosomal protein S28 [Friedmanniomyces endolithicus];Gene=cnxG;Ontology_term=structural molecule activity,cytosol,ribosome,transferase activity;Ontology_id=GO:0005198,GO:0005829,GO:0005840,GO:0016740;Enzyme_code=EC:2.8.1.12;Enzyme_name=molybdopterin synthase

contig00006.g3837	1387	ID=contig00006.g3837;Description=dynactin subunit 2 [Fusarium tjaetaba];Gene=FNYG_06400;Ontology_term=cytoskeleton;Ontology_id=GO:0005856
contig00006.g3838	1716	ID=contig00006.g3838;Description=RTC4-like domain-containing protein [Fusarium coicis];Gene=FNYG_06399;Ontology_term=nucleus;Ontology_id=GO:0005634
contig00006.g3839	3165	ID=contig00006.g3839;Description=Ca2+ transporting ATPase, sarcoplasmic/endoplasmic reticulum [Fusarium verticillioides 7600];Gene=FNAPI_11503;Ontology_term=transmembrane transport,ATP-dependent activity,transporter activity,hydrolase activity;Ontology_id=GO:0055085,GO:0140657,GO:0005215,GO:0016787;Enzyme_code=EC:7.2.2.10,EC:3.6.1.15;Enzyme_name=P-type Ca(2+) transporter,nucleoside-triphosphate phosphatase
contig00006.g3840	3145	ID=contig00006.g3840;Description=hypothetical protein FVER53590_01398 [Fusarium verticillioides];Gene=FNAPI_11502;Ontology_term=molecular function regulator activity;Ontology_id=GO:0098772
contig00006.g3841	620	ID=contig00006.g3841;Description=shwachman-bodian-diamond syndrome [Fusarium subglutinans];Ontology_term=nucleus,RNA binding,ribosome biogenesis;Ontology_id=GO:0005634,GO:0003723,GO:0042254
contig00006.g3842	611	ID=contig00006.g3842;Description=PRO41 [Fusarium tjaetaba];Gene=FCIRC_7899;Ontology_term=membrane;Ontology_id=GO:0016020
contig00006.g3843	630	ID=contig00006.g3843;Description=optic atrophy 3 protein-domain-containing protein [Fusarium oxysporum Fo47]
contig00006.g3844	3117	ID=contig00006.g3844;Description=PLK protein kinase [Fusarium verticillioides 7600];Gene=FVER53263_01402;Ontology_term=catalytic activity, acting on a protein,cytoskeleton organization,protein catabolic process,DNA recombination,chromosome segregation,regulation of DNA-templated transcription,cytokinesis,intracellular protein transport,nucleocytoplasmic transport,nucleus,DNA binding,molecular function regulator activity,microtubule organizing center,signaling,mitotic nuclear division,transferase activity,meiotic nuclear division;Ontology_id=GO:0140096,GO:0007010,GO:0030163,GO:0006310,GO:0007059,GO:0006355,GO:0000910,GO:0006886,GO:0006913,GO:0005634,GO:0003677,GO:0098772,GO:0005815,GO:0023052,GO:0140014,GO:0016740,GO:0140013;Enzyme_code=EC:2.7.1,EC:2.7.1,EC:2.7.11.1,EC:2.7,EC:2,EC:2.7.11.21;Enzyme_name=Transferring phosphorus-containing groups,Transferring phosphorus-containing groups,non-specific serine/threonine protein kinase,Transferring phosphorus-containing groups,Transferases,polo kinase

contig00006.g3845	3720	ID=contig00006.g3845;Description=putative chitin synthase CHS-4 [Fusarium fujikuroi];Gene=FVEG_01403;Ontology_term=carbohydrate metabolic process,anatomical structure development,cell differentiation,reproductive process,carbohydrate derivative metabolic process,cytoplasmic vesicle,cell wall organization or biogenesis,endoplasmic reticulum,transferase activity,plasma membrane;Ontology_id=GO:0005975,GO:0048856,GO:0030154,GO:0022414,GO:1901135,GO:0031410,GO:0071554,GO:0005783,GO:0016740,GO:0005886;Enzyme_code=EC:2.4.1.16,EC:2.4,EC:2.4.1.16,EC:2,EC:2.4.1;Enzyme_name=chitin synthase,Glycosyltransferases,chitin synthase,Transferases,Glycosyltransferases
contig00006.g3846	2255	ID=contig00006.g3846;Description=protoplast regeneration and killer toxin resistance [Fusarium tjaetaba];Ontology_term=carbohydrate metabolic process,molecular function regulator activity,cell differentiation,anatomical structure development,reproductive process,carbohydrate derivative metabolic process,cell wall organization or biogenesis;Ontology_id=GO:0005975,GO:0098772,GO:0030154,GO:0048856,GO:0022414,GO:1901135,GO:0071554
contig00006.g3847	753	ID=contig00006.g3847;Description=DNA-binding protein [Fusarium tjaetaba];Gene=FMEXI_13516;Ontology_term=DNA binding;Ontology_id=GO:0003677
contig00006.g3848	3118	ID=contig00006.g3848;Description=helicase of the Snf2 Rad54 family [Fusarium coicis];Gene=FMUND_3149;Ontology_term=DNA repair,DNA binding,catalytic activity, acting on DNA,ATP-dependent activity,signaling,mitochondrion,nuclear chromosome,regulation of DNA-templated transcription,telomere organization,chromatin organization,hydrolase activity;Ontology_id=GO:0006281,GO:0003677,GO:0140097,GO:0140657,GO:0023052,GO:0005739,GO:0000228,GO:0006355,GO:0032200,GO:0006325,GO:0016787;Enzyme_code=EC:3,EC:3.6.1,EC:3.6.4.12,EC:3.6,EC:3,EC:3.6.1.15;Enzyme_name=Hydrolases,Acting on acid anhydrides,DNA helicase,Acting on acid anhydrides,Hydrolases,nucleoside-triphosphate phosphatase
contig00006.g3849	478	ID=contig00006.g3849;Description=vacuolar ATPase assembly integral membrane protein vma21 [Fusarium musae];Gene=FOXG_00109;Ontology_term=protein-containing complex assembly,endoplasmic reticulum;Ontology_id=GO:0065003,GO:0005783
contig00006.g3850	1333	ID=contig00006.g3850;Description=hypothetical protein FVEG_01408 [Fusarium verticillioides 7600];Ontology_term=cellular bud,mating projection tip;Ontology_id=GO:0005933,GO:0043332

contig00006.g3851	724	ID=contig00006.g3851;Description=RPL33B [Fusarium tjaetaba];Gene=FGADI_10577;Ontology_term=structural molecule activity,ribosome;Ontology_id=GO:0005198,GO:0005840
contig00006.g3852	1795	ID=contig00006.g3852;Description=RNA polymerase II mediator complex protein [Fusarium sp. NRRL 25303];Gene=FACUT_11802;Ontology_term=generation of precursor metabolites and energy,nucleobase-containing small molecule metabolic process,sulfur compound metabolic process,anatomical structure development,carbohydrate derivative metabolic process,regulation of DNA-templated transcription,cellular modified amino acid metabolic process,immune system process,plasma membrane,carbohydrate metabolic process,nucleus,oxidoreductase activity,lipid metabolic process,cell differentiation,cytosol,transcription regulator activity,molecular adaptor activity;Ontology_id=GO:0006091,GO:0055086,GO:0006790,GO:0048856,GO:1901135,GO:0006355,GO:0006575,GO:0002376,GO:0005886,GO:0005975,GO:0005634,GO:0016491,GO:0006629,GO:0030154,GO:0005829,GO:0140110,GO:0060090;Enzyme_code=EC:1.1.1.49,EC:1.1,EC:1,EC:1.1.1,EC:1.1.1.49,EC:1.1.1.363;Enzyme_name=glucose-6-phosphate dehydrogenase (NADP(+)),Acting on the CH-OH group of donors,Oxidoreductases,Acting on the CH-OH group of donors,glucose-6-phosphate dehydrogenase (NADP(+)),glucose-6-phosphate dehydrogenase [NAD(P)(+)]
contig00006.g3853	3166	ID=contig00006.g3853;Description=mediator of RNA polymerase II transcription subunit 14 [Fusarium tjaetaba];Gene=FTJAE_2151;Ontology_term=carbohydrate metabolic process,generation of precursor metabolites and energy,nucleobase-containing small molecule metabolic process,oxidoreductase activity,carbohydrate derivative metabolic process,regulation of DNA-templated transcription,transcription regulator activity,molecular adaptor activity,nucleoplasm;Ontology_id=GO:0005975,GO:0006091,GO:0055086,GO:0016491,GO:1901135,GO:0006355,GO:0140110,GO:0060090,GO:0005654;Enzyme_code=EC:1.1.1.49,EC:1.1,EC:1,EC:1.1.1,EC:1.1.1.49;Enzyme_name=glucose-6-phosphate dehydrogenase (NADP(+)),Acting on the CH-OH group of donors,Oxidoreductases,Acting on the CH-OH group of donors,glucose-6-phosphate dehydrogenase (NADP(+))

contig00006.g3854	1909	ID=contig00006.g3854;Description=protoporphyrinogen oxidase [Fusarium proliferatum];Gene=FPRO05_04803;Ontology_term=oxidoreductase activity,mitochondrion;Ontology_id=GO:0016491,GO:0005739;Enzyme_code=EC:1.3.3.4,EC:1.3,EC:1,EC:1.3.3.4,EC:1.3.3,EC:1.3.3.15;Enzyme_name=protoporphyrinogen oxidase,Acting on the CH-CH group of donors,Oxidoreductases,protoporphyrinogen oxidase,Acting on the CH-CH group of donors,coproporphyrinogen III oxidase (coproporphyrin-forming)
contig00006.g3855	1515	ID=contig00006.g3855;Description=nuclear poly(A)-binding protein [Fusarium fujikuroi];Gene=FDENT_3042;Ontology_term=nucleus,RNA binding,mRNA metabolic process,cytoskeleton,hydrolase activity;Ontology_id=GO:0005634,GO:0003723,GO:0016071,GO:0005856,GO:0016787;Enzyme_code=EC:3,EC:3;Enzyme_name=Hydrolases,Hydrolases
contig00006.g3856	1894	ID=contig00006.g3856;Description=beta-n-acetylhexosaminidase [Fusarium tjaetaba];Gene=FPANT_13933;Ontology_term=carbohydrate metabolic process,hydrolase activity;Ontology_id=GO:0005975,GO:0016787;Enzyme_code=EC:3.2.1.52,EC:3.2,EC:3.2.1.52,EC:3,EC:3.2.1;Enzyme_name=beta-N-acetylhexosaminidase,Glycosylases,beta-N-acetylhexosaminidase,Hydrolases,Glycosylases
contig00006.g3857	1716	ID=contig00006.g3857;Description=n-alkane-inducible cytochrome P450 [Fusarium fujikuroi];Gene=FVEG_01415;Ontology_term=oxidoreductase activity;Ontology_id=GO:0016491;Enzyme_code=EC:1.14,EC:1,EC:1.14;Enzyme_name=Acting on paired donors, with incorporation or reduction of molecular oxygen. The oxygen incorporated need not be derived from O2,Oxidoreductases,Acting on paired donors, with incorporation or reduction of molecular oxygen. The oxygen incorporated need not be derived from O2
contig00006.g3858	1838	ID=contig00006.g3858;Description=aromatic amino acid aminotransferase I [Fusarium verticillioides 7600];Gene=FNYG_06378;Ontology_term=amino acid metabolic process,transferase activity;Ontology_id=GO:0006520,GO:0016740;Enzyme_code=EC:2.6.1,EC:2.6.1,EC:2.6.1.57,EC:2.6.1.5,EC:2,EC:2.6,EC:2.6.1.27,EC:2.6.1.39;Enzyme_name=Transferring nitrogenous groups,Transferring nitrogenous groups,aromatic-amino-acid transaminase,tyrosine transaminase,Transferases,Transferring nitrogenous groups,tryptophan transaminase,2-aminoadipate transaminase

contig00006.g3859	3366	ID=contig00006.g3859;Description=WD40 repeat protein [Fusarium oxysporum];Gene=FVEG_01417;Ontology_term=molecular function regulator activity,vesicle-mediated transport,signaling,Golgi apparatus,intracellular protein transport;Ontology_id=GO:0098772,GO:0016192,GO:0023052,GO:0005794,GO:0006886
contig00006.g3860	993	ID=contig00006.g3860;Description=casein kinase II subunit beta-2 [Colletotrichum spaethianum];Gene=BFJ68_g7553;Ontology_term=molecular function regulator activity,vesicle-mediated transport,signaling,regulation of DNA-templated transcription,cytosol,Golgi apparatus,intracellular protein transport,nucleolus,transferase activity;Ontology_id=GO:0098772,GO:0016192,GO:0023052,GO:0006355,GO:0005829,GO:0005794,GO:0006886,GO:0005730,GO:0016740;Enzyme_code=EC:2.7,EC:2.7,EC:2;Enzyme_name=Transferring phosphorus-containing groups,Transferring phosphorus-containing groups,Transferases
contig00006.g3861	1452	ID=contig00006.g3861;Description=diacylglycerol pyrophosphate phosphatase [Fusarium tjaetaba];Gene=FACUT_13528;Ontology_term=lipid metabolic process;Ontology_id=GO:0006629
contig00006.g3862	1227	ID=contig00006.g3862;Description=50S ribosomal protein L2 [Fusarium verticillioides 7600];Gene=FCIRC_2664;Ontology_term=RNA binding,mitochondrial gene expression,mitochondrion,structural molecule activity,transferase activity,ribosome;Ontology_id=GO:0003723,GO:0140053,GO:0005739,GO:0005198,GO:0016740,GO:0005840;Enzyme_code=EC:2,EC:2;Enzyme_name=Transferases,Transferases
contig00006.g3863	1609	ID=contig00006.g3863;Description=cyclin B [Fusarium verticillioides 7600];Gene=FPHYL_10549;Ontology_term=DNA repair,cytoskeleton organization,DNA recombination,anatomical structure development,chromosome segregation,nuclear chromosome,nucleolus,nucleoplasm,molecular function regulator activity,microtubule organizing center,signaling,cell differentiation,mitotic nuclear division,meiotic nuclear division;Ontology_id=GO:0006281,GO:0007010,GO:0006310,GO:0048856,GO:0007059,GO:0000228,GO:0005730,GO:0005654,GO:0098772,GO:0005815,GO:0023052,GO:0030154,GO:0140014,GO:0140013
contig00006.g3864	1242	ID=contig00006.g3864;Description=WD40-repeat-containing domain protein [Fusarium sp. MPI-SDFR-AT-0072];Gene=FSARC_11997;Ontology_term=nucleocytoplasmic transport,nucleus,intracellular protein transport;Ontology_id=GO:0006913,GO:0005634,GO:0006886

contig00006.g3865	2837	ID=contig00006.g3865;Description=fungal-specific transcription factor domain-containing protein [Fusarium redolens];Gene=FGADI_10564;Ontology_term=nucleus,DNA binding,regulation of DNA-templated transcription,transcription regulator activity;Ontology_id=GO:0005634,GO:0003677,GO:0006355,GO:0140110
contig00006.g3866	1945	ID=contig00006.g3866;Description=glutathione S-transferase omega-1 [Fusarium tjaetaba];Gene=FACUT_11859;Ontology_term=transferase activity;Ontology_id=GO:0016740;Enzyme_code=EC:2,EC:2;Enzyme_name=Transferases,Transferases
contig00006.g3867	2351	ID=contig00006.g3867;Description=high-affinity phosphate permease phosphate-repressible [Fusarium tjaetaba];Gene=FPANT_8494;Ontology_term=transmembrane transport,transporter activity;Ontology_id=GO:0055085,GO:0005215
contig00006.g3868	3387	ID=contig00006.g3868;Description=Acetokinase family-domain-containing protein [Fusarium oxysporum Fo47];Gene=FDENT_7689;Ontology_term=nucleobase-containing small molecule metabolic process,sulfur compound metabolic process,carbohydrate derivative metabolic process,transferase activity;Ontology_id=GO:0055086,GO:0006790,GO:1901135,GO:0016740;Enzyme_code=EC:2.7.2.1,EC:2.7.2,EC:2.7,EC:2,EC:2.7.2.1;Enzyme_name=acetate kinase,Transferring phosphorus-containing groups,Transferring phosphorus-containing groups,Transferases,acetate kinase
contig00006.g3869	894	ID=contig00006.g3869;Description=Transcriptional regulator NRG1 [Fusarium odoratissimum]
contig00006.g3870	1896	ID=contig00006.g3870;Description=U4 u6 snRNP-associated protein [Fusarium tjaetaba];Gene=FDENT_7691;Ontology_term=nucleus,mRNA metabolic process,RNA binding,protein-containing complex assembly,mitochondrion;Ontology_id=GO:0005634,GO:0016071,GO:0003723,GO:0065003,GO:0005739
contig00006.g3871	2025	ID=contig00006.g3871;Description=pre-mRNA-splicing factor clf-1 [Fusarium verticillioides 7600];Gene=FOC4_g10003046;Ontology_term=DNA binding,nucleus,mRNA metabolic process,protein-containing complex assembly,DNA replication,chromosome;Ontology_id=GO:0003677,GO:0005634,GO:0016071,GO:0065003,GO:0006260,GO:0005694
contig00006.g3872	2127	ID=contig00006.g3872;Description=hypothetical protein FVEG_01431 [Fusarium verticillioides 7600]

contig00006.g3873	1661	ID=contig00006.g3873;Description=major facilitator superfamily transporter [Fusarium tjaetaba];Gene=FACUT_11867;Ontology_term=transmembrane transport,transporter activity;Ontology_id=GO:0055085,GO:0005215
contig00006.g3874	2389	ID=contig00006.g3874;Description=la family [Fusarium tjaetaba];Gene=FPRO_00639;Ontology_term=RNA binding,cytoplasmic translation,organelle;Ontology_id=GO:0003723,GO:0002181,GO:0043226
contig00006.g3875	1136	ID=contig00006.g3875;Description=transport SFT2 [Fusarium tjaetaba];Gene=FPCIR_8680;Ontology_term=vesicle-mediated transport,Golgi apparatus,intracellular protein transport;Ontology_id=GO:0016192,GO:0005794,GO:0006886
contig00006.g3876	1566	ID=contig00006.g3876;Description=related to GATA transcription factor [Fusarium mangiferae];Gene=Forpi1262_v001411;Ontology_term=protein dimerization activity;Ontology_id=GO:0046983
contig00006.g3877	2235	ID=contig00006.g3877;Description=related to RMD11 protein required for sporulation and meiotic nuclear division [Fusarium fujikuroi IMI 58289];Gene=FVEG_01437;Ontology_term=autophagy,signaling,reproductive process,vacuole;Ontology_id=GO:0006914,GO:0023052,GO:0022414,GO:0005773
contig00006.g3878	1366	ID=contig00006.g3878;Description=ethanolamine kinase [Fusarium coicis];Gene=FACUT_13242;Ontology_term=transferase activity;Ontology_id=GO:0016740;Enzyme_code=EC:2.7,EC:2.7,EC:2,EC:2.7.1.82;Enzyme_name=Transferring phosphorus-containing groups,Transferring phosphorus-containing groups,Transferases,ethanolamine kinase
contig00006.g3879	3640	ID=contig00006.g3879;Description=hypothetical protein FPRO06_01369 [Fusarium proliferatum];Gene=ref(2)P;Ontology_term=nucleus,DNA binding,autophagy,vacuole,mitochondrion organization;Ontology_id=GO:0005634,GO:0003677,GO:0006914,GO:0005773,GO:0007005
contig00006.g3880	2082	ID=contig00006.g3880;Description=inorganic phosphate permease [Fusarium pseudoanthophilum];Gene=BFJ68_g7556;Ontology_term=transmembrane transport,transporter activity;Ontology_id=GO:0055085,GO:0005215

contig00006.g3881	5799	ID=contig00006.g3881;Description=AGC protein kinase [Fusarium verticillioides 7600];Gene=FPCIR_8880;Ontology_term=nucleus,catalytic activity, acting on a protein,autophagy,protein catabolic process,signaling,mitotic nuclear division,chromosome segregation,reproductive process,regulation of DNA-templated transcription,transferase activity;Ontology_id=GO:0005634,GO:0140096,GO:0006914,GO:0030163,GO:0023052,GO:0140014,GO:0007059,GO:0022414,GO:0006355,GO:0016740;Enzyme_code=EC:2.7.11.1,EC:2.7.1,EC:2.7.11.1,EC:2.7,EC:2;Enzyme_name=non-specific serine/threonine protein kinase,Transferring phosphorus-containing groups,non-specific serine/threonine protein kinase,Transferring phosphorus-containing groups,Transferases
contig00006.g3882	1376	ID=contig00006.g3882;Description=AP-1-like transcription factor [Fusarium pseudocircinatum];Gene=FOXYS1_13184;Ontology_term=regulation of DNA-templated transcription,transcription regulator activity;Ontology_id=GO:0006355,GO:0140110
contig00006.g3883	1875	ID=contig00006.g3883;Description=eukaryotic translation initiation factor 3 subunit D [Fusarium tjaetaba];Gene=FOC1_g10003150;Ontology_term=translation regulator activity,RNA binding,protein-containing complex assembly,cytoplasmic translation,cytosol;Ontology_id=GO:0045182,GO:0003723,GO:0065003,GO:0002181,GO:0005829
contig00006.g3884	377	ID=contig00006.g3884;Description=hypothetical protein J7337_000581 [Fusarium musae]
contig00006.g3885	2601	ID=contig00006.g3885;Description=cortactin [Fusarium tjaetaba];Gene=FVER53263_01446;Ontology_term=cytoskeleton,cytoskeleton organization,protein-containing complex assembly,cytoskeletal protein binding;Ontology_id=GO:0005856,GO:0007010,GO:0065003,GO:0008092
contig00006.g3886	808	ID=contig00006.g3886;Description=thioredoxin-like protein [Fusarium redolens];Gene=FNAPI_3971;Ontology_term=nucleus,catalytic activity, acting on a protein,sulfur compound metabolic process,cytoskeleton organization,oxidoreductase activity,cytosol,protein maturation,chromatin organization;Ontology_id=GO:0005634,GO:0140096,GO:0006790,GO:0007010,GO:0016491,GO:0005829,GO:0051604,GO:0006325;Enzyme_code=EC:1.8,EC:1,EC:1.8;Enzyme_name=Acting on a sulfur group of donors,Oxidoreductases,Acting on a sulfur group of donors
contig00006.g3887	2736	ID=contig00006.g3887;Description=Pyrrolocin cluster transcription factor fsdR [Fusarium oxysporum f. sp. rapae];Gene=FNAPI_3970;Ontology_term=nucleus,DNA binding,regulation of DNA-templated transcription,transcription regulator activity;Ontology_id=GO:0005634,GO:0003677,GO:0006355,GO:0140110

contig00006.g3888	4229	ID=contig00006.g3888;Description=hypothetical protein FVER14953_01450 [Fusarium verticillioides]
contig00006.g3889	2658	ID=contig00006.g3889;Description=dynamin GTPase [Fusarium verticillioides 7600];Gene=FocTR4_00001632;Ontology_term=membrane organization,peroxisome,protein-containing complex assembly,GTPase activity,autophagy,peroxisome organization,mitochondrion,cytosol,cytoskeletal protein binding,microtubule-based movement,mitochondrion organization,cytoskeletal motor activity;Ontology_id=GO:0061024,GO:0005777,GO:0065003,GO:0003924,GO:0006914,GO:0007031,GO:0005739,GO:0005829,GO:0008092,GO:0007018,GO:0007005,GO:0003774;Enzyme_code=EC:3.6.1.15,EC:3.6.1,EC:3.6,EC:3,EC:3.6.5.5,EC:3.6.1.15;Enzyme_name=nucleoside-triphosphate phosphatase,Acting on acid anhydrides,Acting on acid anhydrides,Hydrolases,dynamin GTPase,nucleoside-triphosphate phosphatase
contig00006.g3890	2444	ID=contig00006.g3890;Description=hydrogen peroxide-inducible protein hic-5 [Fusarium fujikuroi];Gene=FNYG_06345;Ontology_term=receptor ligand activity,extracellular region;Ontology_id=GO:0048018,GO:0005576
contig00006.g3891	410	ID=contig00006.g3891;Description=ubiquitin-related domain-containing protein [Fusarium redolens];Ontology_term=microtubule organizing center,nuclear chromosome,regulation of DNA-templated transcription,telomere organization,protein tag activity,chromatin organization;Ontology_id=GO:0005815,GO:0000228,GO:0006355,GO:0032200,GO:0031386,GO:0006325
contig00006.g3892	4157	ID=contig00006.g3892;Description=hypothetical protein J7337_000573 [Fusarium musae];Gene=F25303_12387;Ontology_term=nuclear envelope,DNA repair,nucleocytoplasmic transport,endosome,protein catabolic process,structural molecule activity,ribosome biogenesis,regulation of DNA-templated transcription,telomere organization,chromatin organization,intracellular protein transport;Ontology_id=GO:0005635,GO:0006281,GO:0006913,GO:0005768,GO:0030163,GO:0005198,GO:0042254,GO:0006355,GO:0032200,GO:0006325,GO:0006886
contig00006.g3893	1141	ID=contig00006.g3893;Description=hypothetical protein FVER14953_01457 [Fusarium verticillioides]
contig00006.g3894	1804	ID=contig00006.g3894;Description=f-box protein [Fusarium coicis]
contig00006.g3895	2733	ID=contig00006.g3895;Description=hypothetical protein FVEG_01459 [Fusarium verticillioides 7600]

contig00006.g3896	1394	ID=contig00006.g3896;Description=v-type h+-transporting atpase subunit c [Colletotrichum scovillei];Gene=FVEG_01460;Ontology_term=molecular function regulator activity,transmembrane transport,ATP-dependent activity,lipid binding,toxin activity,extracellular region,vacuole,transporter activity,hydrolase activity;Ontology_id=GO:0098772,GO:0055085,GO:0140657,GO:0008289,GO:0090729,GO:0005576,GO:0005773,GO:0005215,GO:0016787;Enzyme_code=EC:7.2.2,EC:3.6.1,EC:7.1.2.1,EC:3.6,EC:3,EC:7.2.2,EC:7,EC:3.6.1.15;Enzyme_name=Catalysing the translocation of inorganic cations,Acting on acid anhydrides,P-type H(+)-exporting transporter,Acting on acid anhydrides,Hydrolases,Catalysing the translocation of inorganic cations,Translocases,nucleoside-triphosphate phosphatase
contig00006.g3897	813	ID=contig00006.g3897;Description=probable MET14-ATP adenosine-5`-phosphosulfate 3`-phosphotransferase [Fusarium fujikuroi];Gene=FPHYL_9408;Ontology_term=sulfur compound metabolic process,amino acid metabolic process,transferase activity;Ontology_id=GO:0006790,GO:0006520,GO:0016740;Enzyme_code=EC:2.7.1.25,EC:2.7.1,EC:2.7,EC:2,EC:2.7.1.25;Enzyme_name=adenylyl-sulfate kinase,Transferring phosphorus-containing groups,Transferring phosphorus-containing groups,Transferases,adenylyl-sulfate kinase
contig00006.g3898	929	ID=contig00006.g3898;Description=thioesterase family [Fusarium tjaetaba]
contig00006.g3899	607	ID=contig00006.g3899;Description=hypothetical protein FOXG_00053 [Fusarium oxysporum f. sp. lycopersici 4287]
contig00006.g3900	3169	ID=contig00006.g3900;Description=related to MAK21-protein required for 60S ribosomal subunit biogenesis [Fusarium proliferatum ET1];Gene=FNYG_06334;Ontology_term=nucleus,RNA binding,protein-containing complex assembly,ribosome biogenesis;Ontology_id=GO:0005634,GO:0003723,GO:0065003,GO:0042254
contig00006.g3901	3208	ID=contig00006.g3901;Description=prolyl-tRNA synthetase [Fusarium verticillioides 7600];Gene=FNAPI_6139;Ontology_term=tRNA metabolic process,amino acid metabolic process,catalytic activity, acting on RNA,ligase activity;Ontology_id=GO:0006399,GO:0006520,GO:0140098,GO:0016874;Enzyme_code=EC:6.1.1.15;Enzyme_name=proline--tRNA ligase
contig00006.g3902	1607	ID=contig00006.g3902;Description=hypothetical protein FVER53263_01467 [Fusarium verticillioides]

contig00006.g3903	2322	ID=contig00006.g3903;Description=origin recognition complex subunit 1 [Fusarium verticillioides 7600];Gene=FVER53263_01468;Ontology_term=DNA binding,protein-containing complex assembly,ATP-dependent activity,DNA replication,signaling,nuclear chromosome,regulation of DNA-templated transcription,chromatin organization,mitotic cell cycle,hydrolase activity,nucleoplasm;Ontology_id=GO:0003677,GO:0065003,GO:0140657,GO:0006260,GO:0023052,GO:0000228,GO:0006355,GO:0006325,GO:0000278,GO:0016787,GO:0005654;Enzyme_code=EC:3.6.1.15,EC:3.6.1,EC:3.6,EC:3,EC:3.6.1.15;Enzyme_name=nucleoside-triphosphate phosphatase,Acting on acid anhydrides,Acting on acid anhydrides,Hydrolases,nucleoside-triphosphate phosphatase
contig00006.g3904	1489	ID=contig00006.g3904;Description=pre-mRNA-splicing factor prp46 [Fusarium verticillioides 7600];Gene=FVER53263_01469;Ontology_term=nucleus,mRNA metabolic process;Ontology_id=GO:0005634,GO:0016071
contig00006.g3905	1168	ID=contig00006.g3905;Description=26S proteasome non-ATPase regulatory subunit 10 [Fusarium verticillioides 7600];Gene=FVEG_01470;Ontology_term=nucleus,protein-containing complex assembly,cytosol;Ontology_id=GO:0005634,GO:0065003,GO:0005829
contig00006.g3906	2732	ID=contig00006.g3906;Description=TATA element modulatory factor [Fusarium pseudocircinatum];Gene=FVER53590_01471;Ontology_term=Golgi apparatus;Ontology_id=GO:0005794
contig00006.g3907	9999	ID=contig00006.g3907;Description=hypothetical protein J7337_000556 [Fusarium musae]
contig00006.g3908	594	ID=contig00006.g3908;Description=hypothetical protein FVEG_01475 [Fusarium verticillioides 7600]
contig00006.g3909	2229	ID=contig00006.g3909;Description=calcineurin responsive zinc-finger [Fusarium subglutinans];Ontology_term=nucleus,anatomical structure development,cell differentiation,regulation of DNA-templated transcription;Ontology_id=GO:0005634,GO:0048856,GO:0030154,GO:0006355
contig00006.g3910	1652	ID=contig00006.g3910;Description=alternative oxidase, mitochondrial [Fusarium verticillioides 7600];Gene=FNYG_14311;Ontology_term=oxidoreductase activity,mitochondrion;Ontology_id=GO:0016491,GO:0005739;Enzyme_code=EC:1.10.3,EC:1.10,EC:1,EC:1.10.3;Enzyme_name=Acting on diphenols and related substances as donors,Acting on diphenols and related substances as donors,Oxidoreductases,Acting on diphenols and related substances as donors

contig00006.g3911	1109	ID=contig00006.g3911;Description=translation initiation factor 3 subunit J [Fusarium coicis];Gene=HCR1;Ontology_term=translation regulator activity,protein-containing complex assembly,cytoplasmic translation;Ontology_id=GO:0045182,GO:0065003,GO:0002181
contig00006.g3912	1687	ID=contig00006.g3912;Description=MDM31-mitochondrial Distribution and morphology [Fusarium tjaetaba];Gene=FVER53263_01479;Ontology_term=nucleus,lipid metabolic process,mitochondrion,mitochondrion organization;Ontology_id=GO:0005634,GO:0006629,GO:0005739,GO:0007005
contig00006.g3913	891	ID=contig00006.g3913;Description=pre-rRNA-processing protein PNO1 [Fusarium verticillioides 7600];Gene=FPCIR_13758;Ontology_term=RNA binding,protein-containing complex assembly,ribosome biogenesis,nucleolus;Ontology_id=GO:0003723,GO:0065003,GO:0042254,GO:0005730
contig00006.g3914	1492	ID=contig00006.g3914;Description=enolase [Exophiala aquamarina CBS 119918];Gene=A1O9_01233;Ontology_term=generation of precursor metabolites and energy,catalytic activity, acting on a protein,nucleobase-containing small molecule metabolic process,lyase activity,carbohydrate derivative metabolic process,extracellular region,vacuole,plasma membrane,carbohydrate metabolic process,nucleus,wound healing,cell wall,mitochondrion,cytosol,cell wall organization or biogenesis,extracellular matrix,transferase activity;Ontology_id=GO:0006091,GO:0140096,GO:0055086,GO:0016829,GO:1901135,GO:0005576,GO:0005773,GO:0005886,GO:0005975,GO:0005634,GO:0042060,GO:0005618,GO:0005739,GO:0005829,GO:0071554,GO:0031012,GO:0016740;Enzyme_code=EC:4,EC:4.2.1,EC:2.3,EC:4.2.1.11,EC:4.2,EC:2,EC:4,EC:2.3.2,EC:2.3.2.13;Enzyme_name=Lyases,Carbon-oxygen lyases,Acyltransferases,phosphopyruvate hydratase,Carbon-oxygen lyases,Transferases,Lyases,Acyltransferases,protein-glutamine gamma-glutamyltransferase

contig00006.g3915	3825	ID=contig00006.g3915;Description=BUB protein kinase [Fusarium verticillioides 7600];Gene=FACUT_13181;Ontology_term=catalytic activity, acting on a protein,protein-containing complex assembly,chromosome segregation,nuclear chromosome,chromatin organization,microtubule organizing center,autophagy,signaling,mitotic nuclear division,transferase activity,meiotic nuclear division;Ontology_id=GO:0140096,GO:0065003,GO:0007059,GO:0000228,GO:0006325,GO:0005815,GO:0006914,GO:0023052,GO:0140014,GO:0016740,GO:0140013;Enzyme_code=EC:2.7.1,EC:2.7.1,EC:2.7.11.1,EC:2.7,EC:2;Enzyme_name=Transferring phosphorus-containing groups,Transferring phosphorus-containing groups,non-specific serine/threonine protein kinase,Transferring phosphorus-containing groups,Transferases
contig00006.g3916	1479	ID=contig00006.g3916;Description=ap endonuclease 1 [Fusarium albosuccineum];Gene=FPRO05_04739;Ontology_term=nucleus,DNA binding,DNA repair,catalytic activity, acting on DNA,mitochondrion,transferase activity,hydrolase activity;Ontology_id=GO:0005634,GO:0003677,GO:0006281,GO:0140097,GO:0005739,GO:0016740,GO:0016787;Enzyme_code=EC:3.1,EC:2.3.1.20,EC:3.1.4,EC:2.3,EC:3.1,EC:2.3.1.20,EC:4.2.99.18,EC:3.1.11,EC:2,EC:3,EC:3.1.15,EC:3.1.11.2,EC:2.3.1;Enzyme_name=Acting on ester bonds,diacylglycerol O-acyltransferase,Acting on ester bonds,Acyltransferases,Acting on ester bonds,diacylglycerol O-acyltransferase,DNA-(apurinic or apyrimidinic site) lyase,Acting on ester bonds,Transferases,Hydrolases,Acting on ester bonds,exodeoxyribonuclease III,Acyltransferases
contig00006.g3917	1743	ID=contig00006.g3917;Description=Diacylglycerol O-acyltransferase 2A [Fusarium tjaetaba];Gene=FACUT_13183;Ontology_term=lipid droplet,carbohydrate metabolic process,lipid metabolic process,endoplasmic reticulum,transferase activity;Ontology_id=GO:0005811,GO:0005975,GO:0006629,GO:0005783,GO:0016740;Enzyme_code=EC:2.3.1.20,EC:2.3,EC:2.3.1.20,EC:2,EC:2.3.1.22,EC:2.3.1;Enzyme_name=diacylglycerol O-acyltransferase,Acyltransferases,diacylglycerol O-acyltransferase,Transferases,2-acylglycerol O-acyltransferase,Acyltransferases
contig00006.g3918	1839	ID=contig00006.g3918;Description=transcriptional regulator CRZ1 [Fusarium tjaetaba]

contig00006.g3919	1346	ID=contig00006.g3919;Description=murein transglycosylase [Fusarium verticillioides 7600];Ontology_term=carbohydrate metabolic process,cell wall,mitochondrion,cell wall organization or biogenesis,endoplasmic reticulum,extracellular region,mitochondrion organization,hydrolase activity;Ontology_id=GO:0005975,GO:0005618,GO:0005739,GO:0071554,GO:0005783,GO:0005576,GO:0007005,GO:0016787;Enzyme_code=EC:3.2,EC:3.2.1.21,EC:3,EC:3.2.1;Enzyme_name=Glycosylases,beta-glucosidase,Hydrolases,Glycosylases
contig00006.g3920	1852	ID=contig00006.g3920;Description=related to MFS amino acid transporter [Fusarium mangiferae];Gene=FMAN_00566;Ontology_term=transmembrane transport,transporter activity;Ontology_id=GO:0055085,GO:0005215
contig00006.g3921	1455	ID=contig00006.g3921;Description=endo alpha-1,4 polygalactosaminidase precursor [Fusarium flagelliforme];Gene=FVER53263_01489;Ontology_term=catalytic activity, acting on a protein,regulation of DNA-templated transcription,chromatin organization,hydrolase activity;Ontology_id=GO:0140096,GO:0006355,GO:0006325,GO:0016787;Enzyme_code=EC:3.5.1.98,EC:3.2.1.22,EC:3.5.1,EC:3.2,EC:3.5.1.98,EC:3.5,EC:3,EC:3.2.1.22,EC:3.2.1;Enzyme_name=histone deacetylase,alpha-galactosidase,Acting on carbon-nitrogen bonds, other than peptide bonds,Glycosylases,histone deacetylase,Acting on carbon-nitrogen bonds, other than peptide bonds,Hydrolases,alpha-galactosidase,Glycosylases
contig00006.g3922	1500	ID=contig00006.g3922;Description=histone deacetylase HOS2 [Fusarium odoratissimum NRRL 54006];Gene=FocTR4_00001674;Ontology_term=catalytic activity, acting on a protein,signaling,regulation of DNA-templated transcription,nuclear chromosome,chromatin organization,meiotic nuclear division,hydrolase activity,nucleoplasm;Ontology_id=GO:0140096,GO:0023052,GO:0006355,GO:0000228,GO:0006325,GO:0140013,GO:0016787,GO:0005654;Enzyme_code=EC:3.5.1.98,EC:3.5.1,EC:3.5.1.98,EC:3,EC:3.5;Enzyme_name=histone deacetylase,Acting on carbon-nitrogen bonds, other than peptide bonds,histone deacetylase,Hydrolases,Acting on carbon-nitrogen bonds, other than peptide bonds
contig00006.g3923	1317	ID=contig00006.g3923;Description=component of histone acetyltransferase complex [Fusarium tjaetaba];Gene=FGLOB1_11899;Ontology_term=nucleus,chromosome,chromatin organization,transferase activity;Ontology_id=GO:0005634,GO:0005694,GO:0006325,GO:0016740;Enzyme_code=EC:2,EC:2;Enzyme_name=Transferases,Transferases

contig00006.g3924	1140	ID=contig00006.g3924;Description=MNI1 s-adenosylmethionine-dependent methyltransferase of the seven beta-strand family [Fusarium tjaetaba];Gene=FVER53590_01491;Ontology_term=nucleus,catalytic activity, acting on a protein,protein-containing complex assembly,ribosome biogenesis,transferase activity;Ontology_id=GO:0005634,GO:0140096,GO:0065003,GO:0042254,GO:0016740;Enzyme_code=EC:2.1.1,EC:2.1,EC:2,EC:2.1.1.85,EC:2.1.1;Enzyme_name=Transferring one-carbon groups,Transferring one-carbon groups,Transferases,protein-histidine N-methyltransferase,Transferring one-carbon groups
contig00006.g3925	494	ID=contig00006.g3925;Description=acetate non-utilizing protein 9, mitochondrial [Fusarium verticillioides 7600];Gene=FOTG_05937;Ontology_term=carbohydrate metabolic process,protein-containing complex assembly,mitochondrion,mitochondrion organization;Ontology_id=GO:0005975,GO:0065003,GO:0005739,GO:0007005
contig00006.g3926	2340	ID=contig00006.g3926;Description=ATP-dependent RNA helicase prh1 [Fusarium tjaetaba];Gene=FNAPI_10646;Ontology_term=RNA binding,ATP-dependent activity,ribosome biogenesis,catalytic activity, acting on RNA,nucleolus,hydrolase activity;Ontology_id=GO:0003723,GO:0140657,GO:0042254,GO:0140098,GO:0005730,GO:0016787;Enzyme_code=EC:3.6.4.13,EC:3.6.1,EC:3.6.4.13,EC:3.6,EC:3,EC:3.6.1.15;Enzyme_name=RNA helicase,Acting on acid anhydrides,RNA helicase,Acting on acid anhydrides,Hydrolases,nucleoside-triphosphate phosphatase
contig00006.g3927	1251	ID=contig00006.g3927;Description=synaptosomal-associated 23kDa [Fusarium tjaetaba];Ontology_term=membrane organization,nucleus,vesicle-mediated transport,anatomical structure development,cell differentiation,reproductive process,cytosol,lipid binding,intracellular protein transport,plasma membrane,molecular adaptor activity;Ontology_id=GO:0061024,GO:0005634,GO:0016192,GO:0048856,GO:0030154,GO:0022414,GO:0005829,GO:0008289,GO:0006886,GO:0005886,GO:0060090
contig00006.g3928	2634	ID=contig00006.g3928;Description=GTP-binding protein 2 [Fusarium tjaetaba];Gene=FNAPI_10648;Ontology_term=GTPase activity;Ontology_id=GO:0003924;Enzyme_code=EC:3.6.1.15,EC:3.6.1,EC:3.6,EC:3,EC:3.6.1.15;Enzyme_name=nucleoside-triphosphate phosphatase,Acting on acid anhydrides,Acting on acid anhydrides,Hydrolases,nucleoside-triphosphate phosphatase
contig00006.g3929	445	ID=contig00006.g3929;Description=ER membrane protein complex subunit 6-like [Fusarium oxysporum f. sp. vasinfectum];Gene=FPANT_10346;Ontology_term=endoplasmic reticulum;Ontology_id=GO:0005783

contig00006.g3930	1350	ID=contig00006.g3930;Description=uroporphyrinogen decarboxylase [Fusarium verticillioides 7600];Gene=FNYG_13079;Ontology_term=nucleus,lyase activity,cytosol;Ontology_id=GO:0005634,GO:0016829,GO:0005829;Enzyme_code=EC:4.1.1.37,EC:4.1.1,EC:4.1.1.37,EC:4.1,EC:4;Enzyme_name=uroporphyrinogen decarboxylase,Carbon-carbon lyases,uroporphyrinogen decarboxylase,Carbon-carbon lyases,Lyases
contig00006.g3931	489	ID=contig00006.g3931;Description=hypothetical protein FVEG_01497 [Fusarium verticillioides 7600];Ontology_term=cytoplasm;Ontology_id=GO:0005737
contig00006.g3932	1747	ID=contig00006.g3932;Description=hypothetical protein FGADI_8794 [Fusarium gaditjirri];Gene=FGADI_8794
contig00006.g3933	1189	ID=contig00006.g3933;Description=putative ATP phosphoribosyltransferase [Fusarium fujikuroi];Gene=BFJ63_vAg13675;Ontology_term=transferase activity;Ontology_id=GO:0016740;Enzyme_code=EC:2.4.2.17;Enzyme_name=ATP phosphoribosyltransferase
contig00006.g3934	387	ID=contig00006.g3934;Description=atp phosphoribosyltransferase [Fusarium phyllophilum];Gene=FVER53263_01501;Ontology_term=transferase activity;Ontology_id=GO:0016740;Enzyme_code=EC:2.4,EC:2.4,EC:2;Enzyme_name=Glycosyltransferases,Glycosyltransferases,Transferases
contig00006.g3935	258	ID=contig00006.g3935;Description=calcium channel subunit cch1 [Fusarium pseudocircinatum];Ontology_term=transmembrane transport,signaling,transporter activity,plasma membrane;Ontology_id=GO:0055085,GO:0023052,GO:0005215,GO:0005886
contig00006.g3936	6577	ID=contig00006.g3936;Description=voltage gated Ca2+ channel [Fusarium tjaetaba];Gene=FVER53263_01502;Ontology_term=transmembrane transport,signaling,transporter activity,plasma membrane;Ontology_id=GO:0055085,GO:0023052,GO:0005215,GO:0005886
contig00006.g3937	2384	ID=contig00006.g3937;Description=nasopharyngeal carcinoma susceptibility LZ16 [Fusarium phyllophilum];Gene=FGLOB1_11913;Ontology_term=nucleus,regulation of DNA-templated transcription,chromosome;Ontology_id=GO:0005634,GO:0006355,GO:0005694
contig00006.g3938	3076	ID=contig00006.g3938;Description=mating type 1-2 protein [Fusarium fujikuroi];Gene=FVER53590_01504;Ontology_term=nucleus,DNA binding;Ontology_id=GO:0005634,GO:0003677

contig00006.g3939	3070	ID=contig00006.g3939;Description=DNA mismatch repair protein MSH5 [Fusarium verticillioides 7600];Gene=HZS61_000432;Ontology_term=DNA repair,DNA binding,catalytic activity, acting on DNA,ATP-dependent activity,DNA recombination,chromosome segregation,nuclear chromosome,hydrolase activity,meiotic nuclear division,small molecule sensor activity;Ontology_id=GO:0006281,GO:0003677,GO:0140097,GO:0140657,GO:0006310,GO:0007059,GO:0000228,GO:0016787,GO:0140013,GO:0140299;Enzyme_code=EC:3.6.1,EC:3.6,EC:3,EC:3.6.1.15;Enzyme_name=Acting on acid anhydrides,Acting on acid anhydrides,Hydrolases,nucleoside-triphosphate phosphatase
contig00006.g3940	766	ID=contig00006.g3940;Description=hypothetical protein LB505_013088 [Fusarium chuoi]
contig00006.g3941	930	ID=contig00006.g3941;Description=hypothetical protein FVEG_14850 [Fusarium verticillioides 7600]
contig00006.g3942	1787	ID=contig00006.g3942;Description=UAA transporter family-domain-containing protein [Fusarium redolens];Gene=FOC4_g10003134;Ontology_term=cytoskeleton organization,cytokinesis,regulation of DNA-templated transcription,chromosome segregation,vacuole,nucleus,cytoskeleton,molecular function regulator activity,signaling,mitotic nuclear division,cytosol,meiotic nuclear division;Ontology_id=GO:0007010,GO:0000910,GO:0006355,GO:0007059,GO:0005773,GO:0005634,GO:0005856,GO:0098772,GO:0023052,GO:0140014,GO:0005829,GO:0140013
contig00006.g3943	1453	ID=contig00006.g3943;Description=phosphatase pp2a regulatory subunit b [Fusarium albosuccineum];Gene=FALBO_11700;Ontology_term=carbohydrate metabolic process,transmembrane transport,molecular function regulator activity,carbohydrate derivative metabolic process,cell wall organization or biogenesis,Golgi apparatus,endoplasmic reticulum,transporter activity;Ontology_id=GO:0005975,GO:0055085,GO:0098772,GO:1901135,GO:0071554,GO:0005794,GO:0005783,GO:0005215
contig00006.g3944	1038	ID=contig00006.g3944;Description=related to DUF1680 domain protein [Fusarium oxysporum];Gene=FOTG_05958

contig00006.g3945	3220	ID=contig00006.g3945;Description=AMP deaminase [Fusarium subglutinans];Gene=FVER53263_01511;Ontology_term=nucleobase-containing small molecule metabolic process,carbohydrate derivative metabolic process,hydrolase activity;Ontology_id=GO:0055086,GO:1901135,GO:0016787;Enzyme_code=EC:3.5.4.17,EC:3.5.4.6,EC:3.5.4,EC:3.5,EC:3,EC:3.5.4.17,EC:3.5.4.6;Enzyme_name=adenosine-phosphate deaminase,AMP deaminase,Acting on carbon-nitrogen bonds, other than peptide bonds,Acting on carbon-nitrogen bonds, other than peptide bonds,Hydrolases,adenosine-phosphate deaminase,AMP deaminase
contig00006.g3946	3573	ID=contig00006.g3946;Description=cytoskeleton assembly control protein [Fusarium pseudoanthophilum];Gene=FVER53263_01512;Ontology_term=endosome,vesicle-mediated transport,cytoskeletal protein binding,plasma membrane,molecular adaptor activity;Ontology_id=GO:0005768,GO:0016192,GO:0008092,GO:0005886,GO:0060090
contig00006.g3947	1049	ID=contig00006.g3947;Description=DUF887 domain protein [Fusarium tjaetaba];Gene=F52700_6502;Ontology_term=endoplasmic reticulum;Ontology_id=GO:0005783
contig00006.g3948	1254	ID=contig00006.g3948;Description=Dor1-like family [Fusarium tjaetaba];Gene=FNAPI_13779;Ontology_term=vesicle-mediated transport,Golgi apparatus,intracellular protein transport;Ontology_id=GO:0016192,GO:0005794,GO:0006886
contig00006.g3949	1089	ID=contig00006.g3949;Description=solute carrier family 25 member 38 [Fusarium tjaetaba];Gene=FVER53263_20731;Ontology_term=transmembrane transport,vesicle-mediated transport,protein folding chaperone,ATP-dependent activity,mitochondrion,endoplasmic reticulum,Golgi apparatus,intracellular protein transport,transporter activity,hydrolase activity;Ontology_id=GO:0055085,GO:0016192,GO:0044183,GO:0140657,GO:0005739,GO:0005783,GO:0005794,GO:0006886,GO:0005215,GO:0016787;Enzyme_code=EC:3.6.1.15,EC:3.6.1,EC:3.6,EC:3,EC:3.6.1.15;Enzyme_name=nucleoside-triphosphate phosphatase,Acting on acid anhydrides,Acting on acid anhydrides,Hydrolases,nucleoside-triphosphate phosphatase
contig00006.g3950	1674	ID=contig00006.g3950;Description=Reticulocyte-binding protein 2 like protein a [Fusarium oxysporum f. sp. cubense race 1];Gene=FPCIR_13930;Ontology_term=regulation of DNA-templated transcription,transcription regulator activity;Ontology_id=GO:0006355,GO:0140110
contig00006.g3951	568	ID=contig00006.g3951;Description=prefoldin subunit 1 [Fusarium verticillioides 7600];Gene=FPHYL_14290

contig00006.g3952	3059	ID=contig00006.g3952;Description=transcription factor nirA [Fusarium tjaetaba];Gene=HZS61_000421;Ontology_term=nucleus,DNA binding,regulation of DNA-templated transcription,transcription regulator activity;Ontology_id=GO:0005634,GO:0003677,GO:0006355,GO:0140110
contig00006.g3953	1039	ID=contig00006.g3953;Description=dehydrogenase [Fusarium subglutinans]
contig00006.g3954	336	ID=contig00006.g3954;Description=4-coumarate:coenzyme A ligase [Fusarium beomiforme];Gene=FPCIR_4845;Ontology_term=ligase activity;Ontology_id=GO:0016874;Enzyme_code=EC:6,EC:6;Enzyme_name=Ligases,Ligases
contig00006.g3955	330	ID=contig00006.g3955;Description=small nuclear ribonucleoprotein [Fusarium subglutinans];Gene=Forpe1208_v001465;Ontology_term=nucleus,mRNA metabolic process,RNA binding,protein-containing complex assembly;Ontology_id=GO:0005634,GO:0016071,GO:0003723,GO:0065003
contig00006.g3956	1794	ID=contig00006.g3956;Description=amino acid transporter [Fusarium mundagurra];Gene=FRV6_04360;Ontology_term=transmembrane transport,detoxification,vacuole,transporter activity;Ontology_id=GO:0055085,GO:0098754,GO:0005773,GO:0005215
contig00006.g3957	1043	ID=contig00006.g3957;Description=oxidoreductase [Fusarium verticillioides 7600]
contig00006.g3958	621	ID=contig00006.g3958;Description=hypothetical protein FVEG_09460 [Fusarium verticillioides 7600]
contig00006.g3959	1399	ID=contig00006.g3959;Description=hypothetical protein FVEG_16596 [Fusarium verticillioides 7600]
contig00006.g3960	1035	ID=contig00006.g3960;Description=DUF218 domain protein [Fusarium subglutinans]
contig00006.g3961	2205	ID=contig00006.g3961;Description=nucleolar GTP-binding 2 [Fusarium tjaetaba];Gene=AU210_000536;Ontology_term=nucleocytoplasmic transport,GTPase activity,ribosome biogenesis,intracellular protein transport,nucleolus,nucleoplasm;Ontology_id=GO:0006913,GO:0003924,GO:0042254,GO:0006886,GO:0005730,GO:0005654;Enzyme_code=EC:3.6.1,EC:3.6,EC:3,EC:3.6.1.15;Enzyme_name=Acting on acid anhydrides,Acting on acid anhydrides,Hydrolases,nucleoside-triphosphate phosphatase
contig00006.g3962	909	ID=contig00006.g3962;Description=hypothetical protein FVEG_09463 [Fusarium verticillioides 7600]

		ID=contig00006.g3963;Description=ABC transporter protein [Fusarium fujikuroi];Gene=FVER53590_09464;Ontology_term=transmembrane transport,ATP-dependent activity,endoplasmic reticulum,vacuole,transporter activity,plasma membrane,hydrolase activity;Ontology_id=GO:0055085,GO:0140657,GO:0005783,GO:0005773,GO:0005215,GO:0005886,GO:0016787;Enzyme_code=EC:7.2.2,EC:3.6.1.15,EC:3.6.1,EC:3.6,EC:3,EC:7.2.2,EC:7,EC:3.6.1.15;Enzyme_name=Catalysing the translocation of inorganic cations,nucleoside-triphosphate phosphatase,Acting on acid anhydrides,Acting on acid anhydrides,Hydrolases,Catalysing the translocation of inorganic cations,Translocases,nucleoside-triphosphate phosphatase
contig00006.g3963	3402	
		ID=contig00006.g3964;Description=aspartate aminotransferase [Fusarium subglutinans];Gene=FANTH_10267;Ontology_term=transferase activity;Ontology_id=GO:0016740;Enzyme_code=EC:2.6.1,EC:2.6.1,EC:2,EC:2.6;Enzyme_name=Transferring nitrogenous groups,Transferring nitrogenous groups,Transferases,Transferring nitrogenous groups
contig00006.g3964	1275	
contig00006.g3965	1536	ID=contig00006.g3965;Description=hypothetical protein FPCIR_4834 [Fusarium pseudocircinatum];Gene=FNYG_07630;Ontology_term=catalytic activity, acting on a protein,protein catabolic process,cell wall,endoplasmic reticulum,cell wall organization or biogenesis,extracellular region,plasma membrane,hydrolase activity;Ontology_id=GO:0140096,GO:0030163,GO:0005618,GO:0005783,GO:0071554,GO:0005576,GO:0005886,GO:0016787;Enzyme_code=EC:3.4.23,EC:3.4.23,EC:3.4,EC:3;Enzyme_name=Acting on peptide bonds (peptidases),Acting on peptide bonds (peptidases),Acting on peptide bonds (peptidases),Hydrolases
		ID=contig00006.g3966;Description=Elongation factor G, mitochondrial [Fusarium irregulare];Gene=MEF1;Ontology_term=catalytic activity, acting on a protein,translation regulator activity,RNA binding,mitochondrial gene expression,GTPase activity,mitochondrion,cytosol;Ontology_id=GO:0140096,GO:0045182,GO:0003723,GO:0140053,GO:0003924,GO:0005739,GO:0005829;Enzyme_code=EC:3.4.23,EC:3.6.1.15,EC:3.6.1,EC:3.4.23,EC:3.4,EC:3.6,EC:3,EC:3.6.1.15;Enzyme_name=Acting on peptide bonds (peptidases),nucleoside-triphosphate phosphatase,Acting on acid anhydrides,Acting on peptide bonds (peptidases),Acting on peptide bonds (peptidases),Acting on acid anhydrides,Hydrolases,nucleoside-triphosphate phosphatase
contig00006.g3966	2642	

contig00006.g3967	1127	ID=contig00006.g3967;Description=homoisocitrate dehydrogenase [Fusarium tjaetaba];Gene=FACUT_2959;Ontology_term=oxidoreductase activity,amino acid metabolic process;Ontology_id=GO:0016491,GO:0006520;Enzyme_code=EC:1.1.1.87;Enzyme_name=homoisocitrate dehydrogenase
contig00006.g3968	2420	ID=contig00006.g3968;Description=hypothetical protein FVER53590_09469 [Fusarium verticillioides]
contig00006.g3969	501	ID=contig00006.g3969;Description=30S ribosomal protein S8 [Fusarium proliferatum];Gene=FNAPI_10306;Ontology_term=mitochondrial gene expression,mitochondrion,structural molecule activity,cytosol,ribosome;Ontology_id=GO:0140053,GO:0005739,GO:0005198,GO:0005829,GO:0005840
contig00006.g3970	1815	ID=contig00006.g3970;Description=Kynurenine 3-monooxygenase [Fusarium musae];Gene=BNA4;Ontology_term=nucleobase-containing small molecule metabolic process,oxidoreductase activity,mitochondrion,amino acid metabolic process,plasma membrane;Ontology_id=GO:0055086,GO:0016491,GO:0005739,GO:0006520,GO:0005886;Enzyme_code=EC:1.14.13.9,EC:1.6.3,EC:1.6.3.1,EC:1.14.13.9,EC:1.6,EC:1,EC:1.14,EC:1.14.13;Enzyme_name=kynurenine 3-monooxygenase,Acting on NADH or NADPH,NAD(P)H oxidase (H2O2-forming),kynurenine 3-monooxygenase,Acting on NADH or NADPH,Oxidoreductases,Acting on paired donors, with incorporation or reduction of molecular oxygen. The oxygen incorporated need not be derived from O2,Acting on paired donors, with incorporation or reduction of molecular oxygen. The oxygen incorporated need not be derived from O2
contig00006.g3971	1379	ID=contig00006.g3971;Description=methionyl aminopeptidase [Fusarium verticillioides 7600];Gene=FOXYS1_14274;Ontology_term=catalytic activity, acting on a protein,RNA binding,cytosol,protein maturation,ribosome,hydrolase activity;Ontology_id=GO:0140096,GO:0003723,GO:0005829,GO:0051604,GO:0005840,GO:0016787;Enzyme_code=EC:3.4.11.18,EC:3.4.11.18,EC:3.4.11,EC:3.4,EC:3;Enzyme_name=methionyl aminopeptidase,methionyl aminopeptidase,Acting on peptide bonds (peptidases),Acting on peptide bonds (peptidases),Hydrolases
contig00006.g3972	636	ID=contig00006.g3972;Description=cornichon protein [Colletotrichum tofieldiae];Gene=BFJ63_vAg512;Ontology_term=vesicle-mediated transport,cytoplasmic vesicle,endoplasmic reticulum,intracellular protein transport;Ontology_id=GO:0016192,GO:0031410,GO:0005783,GO:0006886

contig00006.g3973	1560	ID=contig00006.g3973;Description=CDC40-cell division control [Fusarium tjaetaba];Gene=FTJAE_8471;Ontology_term=nucleus,mRNA metabolic process,protein-containing complex assembly,catalytic activity, acting on RNA;Ontology_id=GO:0005634,GO:0016071,GO:0065003,GO:0140098
contig00006.g3974	1281	ID=contig00006.g3974;Description=hypothetical protein FCOIX_3265 [Fusarium coicis]
contig00006.g3975	1211	ID=contig00006.g3975;Description=set 5 [Fusarium tjaetaba];Gene=FTJAE_8473;Ontology_term=transferase activity;Ontology_id=GO:0016740;Enzyme_code=EC:2.1.1,EC:2.1,EC:2,EC:2.1.1;Enzyme_name=Transferring one-carbon groups,Transferring one-carbon groups,Transferases,Transferring one-carbon groups
contig00006.g3976	754	ID=contig00006.g3976;Description=hypothetical protein FVEG_09477 [Fusarium verticillioides 7600]
contig00006.g3977	994	ID=contig00006.g3977;Description=hypothetical protein FVER14953_09478 [Fusarium verticillioides];Gene=FACUT_2968;Ontology_term=membrane;Ontology_id=GO:0016020
contig00006.g3978	830	ID=contig00006.g3978;Description=minor allergen Alt a 7 [Fusarium verticillioides 7600];Gene=FPANT_2518;Ontology_term=oxidoreductase activity;Ontology_id=GO:0016491;Enzyme_code=EC:1.6.5.2;Enzyme_name=NAD(P)H dehydrogenase (quinone)
contig00006.g3979	1439	ID=contig00006.g3979;Description=growth arrest DNA-damage-inducible s-interacting 1 domain-containing protein [Fusarium mundagurra];Gene=FVEG_09480;Ontology_term=membrane;Ontology_id=GO:0016020
contig00006.g3980	1399	ID=contig00006.g3980;Description=related to hydroxyproline-rich glycoprotein precursor [Fusarium proliferatum ET1]
contig00006.g3981	2328	ID=contig00006.g3981;Description=DNA polymerase kappa subunit [Fusarium verticillioides 7600];Gene=FACUT_14023;Ontology_term=DNA binding,DNA repair,catalytic activity, acting on DNA,mitochondrion,nuclear chromosome,transferase activity;Ontology_id=GO:0003677,GO:0006281,GO:0140097,GO:0005739,GO:0000228,GO:0016740;Enzyme_code=EC:2.7.7.7,EC:2.7,EC:2,EC:2.7.7.7,EC:2.7.7;Enzyme_name=DNA-directed DNA polymerase,Transferring phosphorus-containing groups,Transferases,DNA-directed DNA polymerase,Transferring phosphorus-containing groups
contig00006.g3982	2342	ID=contig00006.g3982;Description=TRK1-potassium transporter I [Fusarium subglutinans];Gene=FVER53590_09483;Ontology_term=transmembrane transport,transporter activity,plasma membrane;Ontology_id=GO:0055085,GO:0005215,GO:0005886

contig00006.g3983	822	ID=contig00006.g3983;Description=Peptidyl-prolyl cis-trans isomerase fpr2 [Fusarium musae];Gene=FocTR4_00001738;Ontology_term=nucleus,catalytic activity, acting on a protein,endoplasmic reticulum,protein folding,vacuole,isomerase activity;Ontology_id=GO:0005634,GO:0140096,GO:0005783,GO:0006457,GO:0005773,GO:0016853;Enzyme_code=EC:5,EC:5.2.1.8,EC:5.2,EC:5;Enzyme_name=Isomerases,peptidylprolyl isomerase,Cis-trans-isomerases,Isomerases
contig00006.g3984	339	ID=contig00006.g3984;Description=hypothetical protein FAGAP_5233 [Fusarium agapanthi];Gene=FBP1;Ontology_term=membrane organization,generation of precursor metabolites and energy,nucleobase-containing small molecule metabolic process,regulation of DNA-templated transcription,chromosome,nucleoplasm,nuclear envelope,carbohydrate metabolic process,microtubule organizing center,oxidoreductase activity,signaling,cytosol,transcription regulator activity,hydrolase activity,molecular adaptor activity;Ontology_id=GO:0061024,GO:0006091,GO:0055086,GO:0006355,GO:0005694,GO:0005654,GO:0005635,GO:0005975,GO:0005815,GO:0016491,GO:0023052,GO:0005829,GO:0140110,GO:0016787,GO:0060090;Enzyme_code=EC:3.1.3.23,EC:1.2.1.10,EC:3.1.3.11,EC:1.1.1.1,EC:1.1.1.71,EC:3.1.3.23,EC:1.1.1,EC:1.1.1.1,EC:1.2.1,EC:3.1.3,EC:1.2,EC:1.1,EC:3.1,EC:1,EC:1.2.1.10,EC:3,EC:3.1.3.11,EC:1.1.1.71;Enzyme_name=sugar-phosphatase,acetaldehyde dehydrogenase (acetylating),fructose-bisphosphatase,alcohol dehydrogenase,alcohol dehydrogenase [NAD(P)(+)],sugar-phosphatase,Acting on the CH-OH group of donors,alcohol dehydrogenase,Acting on the aldehyde or oxo group of donors,Acting on ester bonds,Acting on the aldehyde or oxo group of donors,Acting on the CH-OH group of donors,Acting on ester bonds,Oxidoreductases,acetaldehyde dehydrogenase (acetylating),Hydrolases,fructose-bisphosphatase,alcohol dehydrogenase [NAD(P)(+)]
contig00006.g3985	466	ID=contig00006.g3985;Description=RPEL repeat [Fusarium beomiforme]
contig00006.g3986	3803	ID=contig00006.g3986;Description=myosin-1 [Fusarium tjaetaba];Gene=FDENT_10678;Ontology_term=cytoskeleton,cytoskeletal protein binding,cytoskeletal motor activity,hydrolase activity;Ontology_id=GO:0005856,GO:0008092,GO:0003774,GO:0016787;Enzyme_code=EC:3;Enzyme_name=Hydrolases

contig00006.g3987	1290	ID=contig00006.g3987;Description=palmitoyltransferase PFA4 [Fusarium verticillioides 7600];Gene=PFA4;Ontology_term=catalytic activity, acting on a protein,endoplasmic reticulum,transferase activity;Ontology_id=GO:0140096,GO:0005783,GO:0016740;Enzyme_code=EC:2.3.1.225,EC:2.3,EC:2,EC:2.3.1.225,EC:2.3.1;Enzyme_name=protein S-acyltransferase,Acyltransferases,Transferases,protein S-acyltransferase,Acyltransferases
contig00006.g3988	2817	ID=contig00006.g3988;Description=capsular associated protein [Fusarium tjaetaba];Gene=FMAN_00498;Ontology_term=membrane;Ontology_id=GO:0016020
contig00006.g3989	2025	ID=contig00006.g3989;Description=uncharacterized protein FOIG_00405 [Fusarium odoratissimum NRRL 54006]
contig00006.g3990	2370	ID=contig00006.g3990;Description=USO1-intracellular transport [Fusarium acutatum]
contig00006.g3991	830	ID=contig00006.g3991;Description=er membrane complex subunit [Fusarium tjaetaba];Gene=FVEG_09491;Ontology_term=membrane;Ontology_id=GO:0016020
contig00006.g3992	1061	ID=contig00006.g3992;Description=rna-binding fus tls [Fusarium agapanthi]
contig00006.g3993	1385	ID=contig00006.g3993;Description=low temperature requirement A [Fusarium coicis];Gene=FDENT_8890;Ontology_term=membrane;Ontology_id=GO:0016020
contig00006.g3994	1002	ID=contig00006.g3994;Description=hypothetical protein FVER53590_09494 [Fusarium verticillioides]
contig00006.g3995	1691	ID=contig00006.g3995;Description=O-acetylhomoserine (thiol)-lyase [Fusarium verticillioides 7600];Gene=FANTH_10236;Ontology_term=sulfur compound metabolic process,lyase activity,amino acid metabolic process,transferase activity,plasma membrane;Ontology_id=GO:0006790,GO:0016829,GO:0006520,GO:0016740,GO:0005886;Enzyme_code=EC:2.5.1,EC:4,EC:2.5.1,EC:2.5.1.49,EC:2.5.1.47,EC:2,EC:4;Enzyme_name=Transferring alkyl or aryl groups, other than methyl groups,Lyases,Transferring alkyl or aryl groups, other than methyl groups,O-acetylhomoserine aminocarboxypropyltransferase,cysteine synthase,Transferases,Lyases
contig00006.g3996	530	ID=contig00006.g3996;Description=MARVEL-like domain protein [Fusarium tjaetaba];Gene=FTJAE_8494;Ontology_term=membrane;Ontology_id=GO:0016020

contig00006.g3997	6073	ID=contig00006.g3997;Description=long-chain acyl synthetase [Fusarium tjaetaba];Gene=FVER53590_09498;Ontology_term=peroxisome,ATP-dependent activity,lipid metabolic process,mitochondrion,signaling,ligase activity;Ontology_id=GO:0005777,GO:0140657,GO:0006629,GO:0005739,GO:0023052,GO:0016874;Enzyme_code=EC:6.2,EC:6.2.1.3,EC:6,EC:6.2.1.2,EC:6.2.1;Enzyme_name=Forming carbon-sulfur bonds,long-chain-fatty-acid--CoA ligase,Ligases,medium-chain acyl-CoA ligase,Forming carbon-sulfur bonds
contig00006.g3998	1072	ID=contig00006.g3998;Description=mitochondrial DNA replication protein YHM2 [Fusarium verticillioides 7600];Gene=FOXG_11292;Ontology_term=DNA binding,transmembrane transport,mitochondrion,transporter activity,mitochondrion organization;Ontology_id=GO:0003677,GO:0055085,GO:0005739,GO:0005215,GO:0007005
contig00006.g3999	1738	ID=contig00006.g3999;Description=citrate synthase [Fusarium sp. NRRL 52700];Gene=BFJ63_vAg455;Ontology_term=carbohydrate metabolic process,peroxisome,generation of precursor metabolites and energy,nucleobase-containing small molecule metabolic process,sulfur compound metabolic process,GTPase activity,mitochondrion,carbohydrate derivative metabolic process,amino acid metabolic process,transferase activity;Ontology_id=GO:0005975,GO:0005777,GO:0006091,GO:0055086,GO:0006790,GO:0003924,GO:0005739,GO:1901135,GO:0006520,GO:0016740;Enzyme_code=EC:2.3.3.16,EC:3.6.1.15,EC:2.3.3.1,EC:2.3.3,EC:2.3,EC:3.6.1,EC:2,EC:3.6,EC:3,EC:2.3.3.16,EC:3.6.1.15;Enzyme_name=citrate synthase (unknown stereospecificity),nucleoside-triphosphate phosphatase,citrate (Si)-synthase,Acyltransferases,Acyltransferases,Acting on acid anhydrides,Transferases,Acting on acid anhydrides,Hydrolases,citrate synthase (unknown stereospecificity),nucleoside-triphosphate phosphatase
contig00006.g4000	2505	ID=contig00006.g4000;Description=related to RNA binding protein NRD1 protein [Fusarium proliferatum ET1];Gene=F25303_440;Ontology_term=nucleus,mRNA metabolic process,RNA binding,snRNA metabolic process,DNA-templated transcription,regulatory ncRNA-mediated gene silencing,tRNA metabolic process;Ontology_id=GO:0005634,GO:0016071,GO:0003723,GO:0016073,GO:0006351,GO:0031047,GO:0006399

contig00006.g4001	3443	ID=contig00006.g4001;Description=plasma membrane ATPase [Fusarium oxysporum f. sp. lycopersici 4287];Gene=FOQG_05066;Ontology_term=transmembrane transport,protein-containing complex assembly,ATP-dependent activity,mitochondrion,cytosol,transporter activity,plasma membrane,hydrolase activity;Ontology_id=GO:0055085,GO:0065003,GO:0140657,GO:0005739,GO:0005829,GO:0005215,GO:0005886,GO:0016787;Enzyme_code=EC:7.1.2.1,EC:7.2.2,EC:3.6.1.15,EC:3.6.1,EC:7.1.2.1,EC:3.6,EC:3,EC:3.6.3.6,EC:7.2.2,EC:7,EC:3.6.1.15;Enzyme_name=P-type H(+)-exporting transporter,Catalysing the translocation of inorganic cations,nucleoside-triphosphate phosphatase,Acting on acid anhydrides,P-type H(+)-exporting transporter,Acting on acid anhydrides,Hydrolases,Acting on acid anhydrides,Catalysing the translocation of inorganic cations,Translocases,nucleoside-triphosphate phosphatase
contig00006.g4002	1677	ID=contig00006.g4002;Description=myocyte-specific enhancer factor 2d [Fusarium fujikuroi]
contig00006.g4003	291	ID=contig00006.g4003;Description=Monooxygenase aurF [Fusarium oxysporum f. sp. albedinis]
contig00006.g4004	498	ID=contig00006.g4004;Description=hypothetical protein FVER53263_09505 [Fusarium verticillioides]
contig00006.g4005	2040	ID=contig00006.g4005;Description=bZIP transcription factor [Fusarium mexicanum]
contig00006.g4006	3537	ID=contig00006.g4006;Description=related to nucleoporin-interacting protein NIC96 [Fusarium fujikuroi];Gene=FPHYL_9619;Ontology_term=nuclear envelope,nucleocytoplasmic transport,protein-containing complex assembly,structural molecule activity,ribosome biogenesis,intracellular protein transport,mitotic cell cycle;Ontology_id=GO:0005635,GO:0006913,GO:0065003,GO:0005198,GO:0042254,GO:0006886,GO:0000278
contig00006.g4007	2019	ID=contig00006.g4007;Description=D-ribulokinase [Fusarium napiforme];Gene=FGLOB1_11318;Ontology_term=carbohydrate metabolic process,transferase activity;Ontology_id=GO:0005975,GO:0016740;Enzyme_code=EC:2.7.1.47,EC:2.7.1,EC:2.7,EC:2,EC:2.7.1.47;Enzyme_name=D-ribulokinase,Transferring phosphorus-containing groups,Transferring phosphorus-containing groups,Transferases,D-ribulokinase
contig00006.g4008	2820	ID=contig00006.g4008;Description=hypothetical protein J7337_000452 [Fusarium musae]

contig00006.g4009	2417	ID=contig00006.g4009;Description=VTC2 polyphosphate synthetase [Fusarium pseudocircinatum];Gene=FNYG_07586;Ontology_term=membrane organization,autophagy,Golgi apparatus,endoplasmic reticulum,vacuole,plasma membrane;Ontology_id=GO:0061024,GO:0006914,GO:0005794,GO:0005783,GO:0005773,GO:0005886
contig00006.g4010	6445	ID=contig00006.g4010;Description=glutamate synthase [NADPH] [Fusarium verticillioides 7600];Gene=FNYG_07585;Ontology_term=oxidoreductase activity,mitochondrion,amino acid metabolic process;Ontology_id=GO:0016491,GO:0005739,GO:0006520;Enzyme_code=EC:1.4.1.14,EC:1.4.1.14,EC:1.4,EC:1.4.1.13,EC:1,EC:1.4.1;Enzyme_name=glutamate synthase (NADH),glutamate synthase (NADH),Acting on the CH-NH2 group of donors,glutamate synthase (NADPH),Oxidoreductases,Acting on the CH-NH2 group of donors
contig00006.g4011	1145	ID=contig00006.g4011;Description=hypothetical protein FVEG_09513 [Fusarium verticillioides 7600];Gene=FNAPI_7677;Ontology_term=nucleus,protein catabolic process;Ontology_id=GO:0005634,GO:0030163
contig00006.g4012	1858	ID=contig00006.g4012;Description=blocked early in transport 1 [Fusarium globosum];Gene=FVEG_09514;Ontology_term=nucleus,Golgi apparatus;Ontology_id=GO:0005634,GO:0005794
contig00006.g4013	624	ID=contig00006.g4013;Description=hypothetical protein FVEG_09516 [Fusarium verticillioides 7600]
contig00006.g4014	1107	ID=contig00006.g4014;Description=phosphate system cyclin PHO80 [Fusarium verticillioides 7600];Gene=FCIRC_826;Ontology_term=membrane organization,nucleus,molecular function regulator activity,autophagy,signaling,regulation of DNA-templated transcription;Ontology_id=GO:0061024,GO:0005634,GO:0098772,GO:0006914,GO:0023052,GO:0006355
contig00006.g4015	3141	ID=contig00006.g4015;Description=armadillo-type protein [Fusarium sp. MPI-SDFR-AT-0072];Gene=FocTR4_00001775;Ontology_term=nucleus,carbohydrate metabolic process,protein catabolic process,cell differentiation,anatomical structure development,reproductive process,regulation of DNA-templated transcription;Ontology_id=GO:0005634,GO:0005975,GO:0030163,GO:0030154,GO:0048856,GO:0022414,GO:0006355
contig00006.g4016	983	ID=contig00006.g4016;Description=putative plasma membrane protein YRO2 [Fusarium fujikuroi];Gene=FOYG_16254;Ontology_term=membrane;Ontology_id=GO:0016020

contig00006.g4017	1690	ID=contig00006.g4017;Description=velvet protein [Fusarium musae];Gene=FVER53590_09521;Ontology_term=nucleus,anatomical structure development;Ontology_id=GO:0005634,GO:0048856
contig00006.g4018	1434	ID=contig00006.g4018;Description=hypothetical protein FVEG_09522 [Fusarium verticillioides 7600]
contig00006.g4019	4266	ID=contig00006.g4019;Description=SPT23-suppressor of TY retrotransposon [Fusarium fujikuroi];Gene=FNYG_07575;Ontology_term=cytoskeleton organization,anatomical structure development,regulation of DNA-templated transcription,reproductive process,cytokinesis,endoplasmic reticulum,chromosome,chromatin organization,DNA binding,nucleus,mRNA metabolic process,lipid metabolic process,mitotic nuclear division,transcription regulator activity;Ontology_id=GO:0007010,GO:0048856,GO:0006355,GO:0022414,GO:0000910,GO:0005783,GO:0005694,GO:0006325,GO:0003677,GO:0005634,GO:0016071,GO:0006629,GO:0140014,GO:0140110
contig00006.g4020	1664	ID=contig00006.g4020;Description=serine protease [Fusarium tjaetaba];Gene=FTJAE_10104;Ontology_term=catalytic activity, acting on a protein,hydrolase activity;Ontology_id=GO:0140096,GO:0016787;Enzyme_code=EC:3.4,EC:3.4,EC:3;Enzyme_name=Acting on peptide bonds (peptidases),Acting on peptide bonds (peptidases),Hydrolases
contig00006.g4021	1110	ID=contig00006.g4021;Description=RNase H domain protein [Fusarium tjaetaba];Gene=FTJAE_10103;Ontology_term=DNA replication,nuclear chromosome,catalytic activity, acting on RNA,mitotic cell cycle,hydrolase activity;Ontology_id=GO:0006260,GO:0000228,GO:0140098,GO:0000278,GO:0016787;Enzyme_code=EC:3.1.30,EC:3.1.26.4,EC:3.1.30,EC:3.1,EC:3,EC:3.1.26,EC:3.1.26.4;Enzyme_name=Acting on ester bonds,ribonuclease H,Acting on ester bonds,Acting on ester bonds,Hydrolases,Acting on ester bonds,ribonuclease H
contig00006.g4022	1380	ID=contig00006.g4022;Description=tRNA-specific adenosine deaminase subunit tad3 [Fusarium pseudoanthophilum];Gene=FNAPI_7688;Ontology_term=catalytic activity;Ontology_id=GO:0003824

contig00006.g4023	2205	ID=contig00006.g4023;Description=6-phosphofructo-2-kinase [Fusarium tjaetaba];Gene=FOXYS1_15574;Ontology_term=carbohydrate metabolic process,generation of precursor metabolites and energy,nucleobase-containing small molecule metabolic process,carbohydrate derivative metabolic process,transferase activity;Ontology_id=GO:0005975,GO:0006091,GO:0055086,GO:1901135,GO:0016740;Enzyme_code=EC:2.7.1.105,EC:2.7.1,EC:2.7,EC:2,EC:2.7.1.105;Enzyme_name=6-phosphofructo-2-kinase,Transferring phosphorus-containing groups,Transferring phosphorus-containing groups,Transferases,6-phosphofructo-2-kinase
contig00006.g4024	1338	ID=contig00006.g4024;Description=SOF1 [Fusarium tjaetaba];Gene=FVEG_09528;Ontology_term=RNA binding,ribosome biogenesis,nucleolus;Ontology_id=GO:0003723,GO:0042254,GO:0005730
contig00006.g4025	1142	ID=contig00006.g4025;Description=DNA-directed RNA polymerase II subunit RPB3 [Fusarium verticillioides 7600];Gene=FVEG_09529;Ontology_term=DNA binding,DNA-templated transcription,chromosome,catalytic activity, acting on RNA,transferase activity,nucleoplasm;Ontology_id=GO:0003677,GO:0006351,GO:0005694,GO:0140098,GO:0016740,GO:0005654;Enzyme_code=EC:2.7.7.6;Enzyme_name=DNA-directed RNA polymerase
contig00006.g4026	825	ID=contig00006.g4026;Description=Mitochondrial dicarboxylate transporter [Fusarium oxysporum f. sp. cubense race 1];Gene=FVER53590_09530;Ontology_term=DNA binding,transmembrane transport,mitochondrion,cell differentiation,anatomical structure development,cilium,reproductive process,transporter activity;Ontology_id=GO:0003677,GO:0055085,GO:0005739,GO:0030154,GO:0048856,GO:0005929,GO:0022414,GO:0005215
contig00006.g4027	2301	ID=contig00006.g4027;Description=phenylalanyl-tRNA synthetase [Fusarium proliferatum];Gene=FPHYL_6409;Ontology_term=mitochondrial gene expression,RNA binding,mitochondrion,tRNA metabolic process,amino acid metabolic process,catalytic activity, acting on RNA,ligase activity;Ontology_id=GO:0140053,GO:0003723,GO:0005739,GO:0006399,GO:0006520,GO:0140098,GO:0016874;Enzyme_code=EC:6.1.1.20,EC:6.1.1.20,EC:6.1,EC:6.1.1,EC:6;Enzyme_name=phenylalanine--tRNA ligase,phenylalanine--tRNA ligase,Forming carbon-oxygen bonds,Forming carbon-oxygen bonds,Ligases
contig00006.g4028	762	ID=contig00006.g4028;Description=inhibitor of calcineurin [Fusarium tjaetaba];Gene=FOYG_16268;Ontology_term=signaling;Ontology_id=GO:0023052

contig00006.g4029	1581	ID=contig00006.g4029;Description=latex clearing [Fusarium pseudoanthophilum];Gene=F25303_471;Ontology_term=oxidoreductase activity;Ontology_id=GO:0016491;Enzyme_code=EC:1,EC:1;Enzyme_name=Oxidoreductases,Oxidoreductases
contig00006.g4030	1169	ID=contig00006.g4030;Description=Required for respiratory growth mitochondrial [Fusarium sp. NRRL 25303];Gene=FPANT_4360;Ontology_term=mitochondrion;Ontology_id=GO:0005739
contig00006.g4031	1113	ID=contig00006.g4031;Description=short-chain dehydrogenase reductase [Fusarium napiforme];Ontology_term=oxidoreductase activity,mitochondrion;Ontology_id=GO:0016491,GO:0005739;Enzyme_code=EC:1;Enzyme_name=Oxidoreductases
contig00006.g4032	1806	ID=contig00006.g4032;Description=serine/threonine protein kinase [Fusarium verticillioides 7600];Gene=FMUND_6768;Ontology_term=catalytic activity, acting on a protein,transferase activity;Ontology_id=GO:0140096,GO:0016740;Enzyme_code=EC:2.7.11.1,EC:2.7.1,EC:2.7.11.1,EC:2.7,EC:2;Enzyme_name=non-specific serine/threonine protein kinase,Transferring phosphorus-containing groups,non-specific serine/threonine protein kinase,Transferring phosphorus-containing groups,Transferases
contig00006.g4033	509	ID=contig00006.g4033;Description=hypothetical protein FVER14953_09537 [Fusarium verticillioides];Gene=FMUND_6769;Ontology_term=membrane;Ontology_id=GO:0016020
contig00006.g4034	2996	ID=contig00006.g4034;Description=Telomeric DNA-binding factor trf1 [Fusarium tjaetaba];Gene=FVER53263_09538;Ontology_term=DNA binding,nuclear chromosome,regulation of DNA-templated transcription,telomere organization,chromatin organization,transcription regulator activity,molecular adaptor activity;Ontology_id=GO:0003677,GO:0000228,GO:0006355,GO:0032200,GO:0006325,GO:0140110,GO:0060090
contig00006.g4035	796	ID=contig00006.g4035;Description=uncharacterized protein FPRO_00459 [Fusarium proliferatum ET1]
contig00006.g4036	666	ID=contig00006.g4036;Description=hypothetical protein FVER14953_09539 [Fusarium verticillioides]
contig00006.g4037	1332	ID=contig00006.g4037;Description=hypothetical protein FPANT_4367 [Fusarium pseudoanthophilum];Gene=Acp26Aa;Ontology_term=signaling,reproductive process,extracellular region;Ontology_id=GO:0023052,GO:0022414,GO:0005576

contig00006.g4038	831	ID=contig00006.g4038;Description=pyridoxamine phosphate oxidase family [Fusarium tjaetaba];Gene=FACUT_646;Ontology_term=membrane;Ontology_id=GO:0016020
contig00006.g4039	2156	ID=contig00006.g4039;Description=hypothetical protein FVEG_09542 [Fusarium verticillioides 7600];Gene=FNAPI_7970;Ontology_term=RNA binding,mRNA metabolic process;Ontology_id=GO:0003723,GO:0016071
contig00006.g4040	1368	ID=contig00006.g4040;Description=phosphatase [Fusarium subglutinans];Gene=FOYG_16283;Ontology_term=DNA repair,cytoskeleton organization,anatomical structure development,chromosome segregation,nucleolus,mitotic nuclear division,cytosol,cell wall organization or biogenesis,catalytic activity, acting on a protein,nucleobase-containing small molecule metabolic process,sulfur compound metabolic process,establishment or maintenance of cell polarity,carbohydrate derivative metabolic process,nuclear chromosome,tRNA metabolic process,reproductive process,extracellular space,signaling,lipid metabolic process,mitochondrion,transferase activity,external encapsulating structure,hydrolase activity;Ontology_id=GO:0006281,GO:0007010,GO:0048856,GO:0007059,GO:0005730,GO:0140014,GO:0005829,GO:0071554,GO:0140096,GO:0055086,GO:0006790,GO:0007163,GO:1901135,GO:0000228,GO:0006399,GO:0022414,GO:0005615,GO:0023052,GO:0006629,GO:0005739,GO:0016740,GO:0030312,GO:0016787;Enzyme_code=EC:3.1.3.16,EC:2.1.1,EC:2.1,EC:3.1.3.16,EC:3.1,EC:2,EC:3,EC:2.1.1,EC:3.1.3;Enzyme_name=protein-serine/threonine phosphatase,Transferring one-carbon groups,Transferring one-carbon groups,protein-serine/threonine phosphatase,Acting on ester bonds,Transferases,Hydrolases,Transferring one-carbon groups,Acting on ester bonds
contig00006.g4041	1137	ID=contig00006.g4041;Description=tRNA wybutosine-synthesizing protein 2 [Fusarium verticillioides 7600];Gene=FPCIR_3080;Ontology_term=carbohydrate derivative metabolic process,tRNA metabolic process,catalytic activity, acting on RNA,transferase activity;Ontology_id=GO:1901135,GO:0006399,GO:0140098,GO:0016740;Enzyme_code=EC:2.5.1,EC:2.1.1,EC:2.1,EC:2.5.1,EC:2.5.1.114,EC:2,EC:2.1.1;Enzyme_name=Transferring alkyl or aryl groups, other than methyl groups,Transferring one-carbon groups,Transferring one-carbon groups,Transferring alkyl or aryl groups, other than methyl groups,tRNA(Phe) (4-demethylwyosine(37)-C(7)) aminocarboxypropyltransferase,Transferases,Transferring one-carbon groups
contig00006.g4042	664	ID=contig00006.g4042;Description=secretory protein [Fusarium pseudocircinatum];Gene=FVEG_09545;Ontology_term=vacuole;Ontology_id=GO:0005773

contig00006.g4043	1186	ID=contig00006.g4043;Description=origin recognition complex subunit 6 [Fusarium tjaetaba];Gene=FVER53263_09546;Ontology_term=DNA binding,DNA replication,nuclear chromosome;Ontology_id=GO:0003677,GO:0006260,GO:0000228
contig00006.g4044	1371	ID=contig00006.g4044;Description=hypothetical protein FVEG_09547 [Fusarium verticillioides 7600];Gene=Acp26Aa;Ontology_term=extracellular space,signaling,reproductive process;Ontology_id=GO:0005615,GO:0023052,GO:0022414
contig00006.g4045	1015	ID=contig00006.g4045;Description=3-oxoacyl-[acyl-carrier protein] reductase [Fusarium verticillioides 7600];Gene=FFB14_11873;Ontology_term=oxidoreductase activity;Ontology_id=GO:0016491;Enzyme_code=EC:1;Enzyme_name=Oxidoreductases
contig00006.g4046	1150	ID=contig00006.g4046;Description=hypothetical protein FVER53263_09549 [Fusarium verticillioides];Gene=FocTR4_00001811;Ontology_term=oxidoreductase activity;Ontology_id=GO:0016491;Enzyme_code=EC:1,EC:1;Enzyme_name=Oxidoreductases,Oxidoreductases
contig00006.g4047	1510	ID=contig00006.g4047;Description=zinc finger protein [Fusarium verticillioides 7600];Gene=FOQG_05010;Ontology_term=zinc ion binding;Ontology_id=GO:0008270
contig00006.g4048	564	ID=contig00006.g4048;Description=DUF866 domain protein [Fusarium tjaetaba];Gene=FOZG_00516;Ontology_term=ncRNA processing,endoribonuclease complex,ribonucleoprotein complex;Ontology_id=GO:0034470,GO:1902555,GO:1990904
contig00006.g4049	701	ID=contig00006.g4049;Description=DUF866 domain [Fusarium albosuccineum]
contig00006.g4050	2193	ID=contig00006.g4050;Description=pentatricopeptide repeat domain protein [Fusarium tjaetaba]
contig00006.g4051	3705	ID=contig00006.g4051;Description=glycerophosphocholine (ho) phosphodiesterase [Fusarium tjaetaba];Gene=FNYG_07542;Ontology_term=lipid metabolic process,hydrolase activity;Ontology_id=GO:0006629,GO:0016787;Enzyme_code=EC:3.1.4,EC:3.1.4,EC:3.1,EC:3.1.4.46,EC:3,EC:3.1.4.2;Enzyme_name=Acting on ester bonds,Acting on ester bonds,Acting on ester bonds,glycerophosphodiester phosphodiesterase,Hydrolases,glycerophosphocholine phosphodiesterase
contig00006.g4052	894	ID=contig00006.g4052;Description=zinc finger protein [Fusarium tjaetaba]
contig00006.g4053	2498	ID=contig00006.g4053;Description=ATP-dependent metalloprotease [Fusarium verticillioides 7600];Gene=FPANT_4382;Ontology_term=catalytic activity, acting on a protein,ATP-dependent activity,hydrolase activity;Ontology_id=GO:0140096,GO:0140657,GO:0016787;Enzyme_code=EC:3.4.24,EC:3.6.1.15;Enzyme_name=Acting on peptide bonds (peptidases),nucleoside-triphosphate phosphatase

contig00006.g4054	1188	ID=contig00006.g4054;Description=putative AAA protease IAP-1 (mitochondrial intermembrane space) [Fusarium fujikuroi];Gene=C2S_4617;Ontology_term=nucleus,catalytic activity, acting on a protein,extracellular space,ATP-dependent activity,oxidoreductase activity,lipid metabolic process,reproductive process,extracellular matrix,hydrolase activity;Ontology_id=GO:0005634,GO:0140096,GO:0005615,GO:0140657,GO:0016491,GO:0006629,GO:0022414,GO:0031012,GO:0016787;Enzyme_code=EC:3.4.24,EC:3.6.1.15,EC:3.4.24,EC:1.1.1,EC:3.6.1.15,EC:1.2.1,EC:1.2,EC:1.1,EC:3.6.1,EC:3.4,EC:1,EC:3.6,EC:3,EC:1.1.1.265,EC:1.2.1.5,EC:1.1.1.283,EC:1.1.1.184,EC:1.2.1.4,EC:1.2.1.3,EC:1.1.1.71;Enzyme_name=Acting on peptide bonds (peptidases),nucleoside-triphosphate phosphatase,Acting on peptide bonds (peptidases),Acting on the CH-OH group of donors,nucleoside-triphosphate phosphatase,Acting on the aldehyde or oxo group of donors,Acting on the aldehyde or oxo group of donors,Acting on the CH-OH group of donors,Acting on acid anhydrides,Acting on peptide bonds (peptidases),Oxidoreductases,Acting on acid anhydrides,Hydrolases,3-methylbutanal reductase,aldehyde dehydrogenase [NAD(P)(+)],methylglyoxal reductase (NADPH),carbonyl reductase (NADPH),aldehyde dehydrogenase (NADP(+)),aldehyde dehydrogenase (NAD(+)),alcohol dehydrogenase [NAD(P)(+)]
contig00006.g4055	1350	ID=contig00006.g4055;Description=hypothetical protein FVER53590_09558 [Fusarium verticillioides];Gene=FVER53263_09558;Ontology_term=membrane;Ontology_id=GO:0016020
contig00006.g4056	3449	ID=contig00006.g4056;Description=uncharacterized protein FMAN_00427 [Fusarium mangiferae];Gene=Adhr;Ontology_term=oxidoreductase activity;Ontology_id=GO:0016491;Enzyme_code=EC:1,EC:1;Enzyme_name=Oxidoreductases,Oxidoreductases
contig00006.g4057	1362	ID=contig00006.g4057;Description=tRNA-splicing endonuclease [Fusarium tjaetaba];Gene=FTJAE_9929;Ontology_term=nucleus,mitochondrion,tRNA metabolic process,hydrolase activity;Ontology_id=GO:0005634,GO:0005739,GO:0006399,GO:0016787;Enzyme_code=EC:3.1,EC:3.1,EC:3;Enzyme_name=Acting on ester bonds,Acting on ester bonds,Hydrolases
contig00006.g4058	1365	ID=contig00006.g4058;Description=arrestin domain-containing protein [Fusarium denticulatum];Ontology_term=molecular function regulator activity,cytokinesis,mitotic cell cycle;Ontology_id=GO:0098772,GO:0000910,GO:0000278
contig00006.g4059	1964	ID=contig00006.g4059;Description=hypothetical protein FVER53263_09562 [Fusarium verticillioides]

contig00006.g4060	4538	ID=contig00006.g4060;Description=hypothetical protein FVEG_09563 [Fusarium verticillioides 7600]
contig00006.g4061	986	ID=contig00006.g4061;Description=putative 40S ribosomal protein S5 [Fusarium fujikuroi];Gene=28952640;Ontology_term=RNA binding,structural molecule activity,cytosol,ribosome;Ontology_id=GO:0003723,GO:0005198,GO:0005829,GO:0005840
contig00006.g4062	1070	ID=contig00006.g4062;Description=thiosulfate sulfurtransferase [Fusarium tjaetaba];Gene=FNYG_07530;Ontology_term=mitochondrion,tRNA metabolic process,transferase activity;Ontology_id=GO:0005739,GO:0006399,GO:0016740;Enzyme_code=EC:2.8.1.1,EC:2.8.1,EC:2,EC:2.8,EC:2.8.1.2,EC:2.8.1.1;Enzyme_name=thiosulfate sulfurtransferase,Transferring sulfur-containing groups,Transferases,Transferring sulfur-containing groups,3-mercaptopyruvate sulfurtransferase,thiosulfate sulfurtransferase
contig00006.g4063	1746	ID=contig00006.g4063;Description=hypothetical protein FVEG_09567 [Fusarium verticillioides 7600]
contig00006.g4064	1751	ID=contig00006.g4064;Description=hypothetical protein FVEG_09568 [Fusarium verticillioides 7600]
contig00006.g4065	1836	ID=contig00006.g4065;Description=CAMK/CAMKL/CHK1 protein kinase [Fusarium verticillioides 7600];Gene=FOXB_01220;Ontology_term=catalytic activity, acting on a protein,DNA replication,signaling,nuclear chromosome,regulation of DNA-templated transcription,transferase activity,mitotic cell cycle,nucleoplasm,meiotic nuclear division;Ontology_id=GO:0140096,GO:0006260,GO:0023052,GO:0000228,GO:0006355,GO:0016740,GO:0000278,GO:0005654,GO:0140013;Enzyme_code=EC:2.7.11.1,EC:2.7.1,EC:2.7.11.1,EC:2.7,EC:2;Enzyme_name=non-specific serine/threonine protein kinase,Transferring phosphorus-containing groups,non-specific serine/threonine protein kinase,Transferring phosphorus-containing groups,Transferases
contig00006.g4066	648	ID=contig00006.g4066;Description=ubiquitin-conjugating enzyme E2-C [Fusarium tjaetaba];Gene=FTJAE_9921;Ontology_term=catalytic activity, acting on a protein,protein catabolic process,signaling,mitotic nuclear division,chromosome segregation,transferase activity;Ontology_id=GO:0140096,GO:0030163,GO:0023052,GO:0140014,GO:0007059,GO:0016740;Enzyme_code=EC:2.3.2.23;Enzyme_name=E2 ubiquitin-conjugating enzyme
contig00006.g4067	542	ID=contig00006.g4067;Description=50S ribosomal protein L31e [Fusarium oxysporum f. sp. lycopersici 4287];Gene=FCIRC_10531
contig00006.g4068	1191	ID=contig00006.g4068;Description=RAX1 [Fusarium tjaetaba];Gene=FCIRC_10530;Ontology_term=membrane;Ontology_id=GO:0016020

contig00006.g4069	879	ID=contig00006.g4069;Description=ACOB [Fusarium tjaetaba];Ontology_term=nucleus;Ontology_id=GO:0005634
contig00006.g4070	3649	ID=contig00006.g4070;Description=Cu2+-exporting ATPase [Fusarium verticillioides 7600];Gene=FVER53590_09574;Ontology_term=transmembrane transport,ATP-dependent activity,cytoplasmic vesicle,transporter activity,hydrolase activity;Ontology_id=GO:0055085,GO:0140657,GO:0031410,GO:0005215,GO:0016787;Enzyme_code=EC:7.2.2,EC:3.6.1.15;Enzyme_name=Catalysing the translocation of inorganic cations,nucleoside-triphosphate phosphatase
contig00006.g4071	1226	ID=contig00006.g4071;Description=glucose-6-phosphate phosphate translocator [Fusarium tjaetaba];Gene=FVER53590_09575;Ontology_term=membrane;Ontology_id=GO:0016020
contig00006.g4072	2004	ID=contig00006.g4072;Description=sporulation RMD8 [Fusarium sp. NRRL 52700];Gene=BFJ65_g85;Ontology_term=mitochondrial gene expression,mitochondrion,endoplasmic reticulum;Ontology_id=GO:0140053,GO:0005739,GO:0005783
contig00006.g4073	5346	ID=contig00006.g4073;Description=IQ domain-containing protein containing GTPase activating protein [Fusarium verticillioides 7600];Gene=FTJAE_9914;Ontology_term=nuclear envelope,molecular function regulator activity,cytoskeleton organization,microtubule organizing center,protein-containing complex assembly,cytokinesis,cytoskeletal protein binding,mitotic cell cycle;Ontology_id=GO:0005635,GO:0098772,GO:0007010,GO:0005815,GO:0065003,GO:0000910,GO:0008092,GO:0000278
contig00006.g4074	1581	ID=contig00006.g4074;Description=choline-sulfatase [Fusarium verticillioides 7600];Gene=FVER53263_09578;Ontology_term=hydrolase activity;Ontology_id=GO:0016787;Enzyme_code=EC:3.1.6,EC:3.1.6,EC:3.1,EC:3.1.6.6,EC:3;Enzyme_name=Acting on ester bonds,Acting on ester bonds,Acting on ester bonds,choline-sulfatase,Hydrolases
contig00006.g4075	840	ID=contig00006.g4075;Description=mediator of RNA polymerase II transcription subunit 8 [Fusarium subglutinans];Gene=MED8;Ontology_term=DNA binding,structural molecule activity,regulation of DNA-templated transcription,transcription regulator activity,molecular adaptor activity,nucleoplasm;Ontology_id=GO:0003677,GO:0005198,GO:0006355,GO:0140110,GO:0060090,GO:0005654
contig00006.g4076	1047	ID=contig00006.g4076;Description=hypothetical protein FVEG_09580 [Fusarium verticillioides 7600]

contig00006.g4077	688	ID=contig00006.g4077;Description=glutathione-dependent formaldehyde-activating [Fusarium pseudocircinatum];Gene=FocTR4_00001851;Ontology_term=lyase activity;Ontology_id=GO:0016829;Enzyme_code=EC:4.4,EC:4.4,EC:4;Enzyme_name=Carbon-sulfur lyases,Carbon-sulfur lyases,Lyases
contig00006.g4078	1401	ID=contig00006.g4078;Description=hypothetical protein FVEG_09582 [Fusarium verticillioides 7600]
contig00006.g4079	894	ID=contig00006.g4079;Description=ribonuclease P/MRP protein subunit RPP1 [Fusarium proliferatum];Gene=C2S_4591;Ontology_term=RNA binding,mRNA metabolic process,tRNA metabolic process,cytosol,ribosome biogenesis,nucleolus;Ontology_id=GO:0003723,GO:0016071,GO:0006399,GO:0005829,GO:0042254,GO:0005730;Enzyme_code=EC:3.1.26.5;Enzyme_name=ribonuclease P
contig00006.g4080	386	ID=contig00006.g4080;Description=ribonuclease p complex subunit [Fusarium beomiforme]
contig00006.g4081	3485	ID=contig00006.g4081;Description=hypothetical protein FVER53263_09585 [Fusarium verticillioides]
contig00006.g4082	333	ID=contig00006.g4082;Description=hypothetical protein FVEG_09586 [Fusarium verticillioides 7600]
contig00006.g4083	1416	ID=contig00006.g4083;Description=phospho-2-dehydro-3-deoxyheptonate aldolase [Fusarium subglutinans];Gene=BFJ68_g1069;Ontology_term=amino acid metabolic process,transferase activity;Ontology_id=GO:0006520,GO:0016740;Enzyme_code=EC:2.5.1.54,EC:2.5.1,EC:2,EC:2.5.1.54;Enzyme_name=3-deoxy-7-phosphoheptulonate synthase,Transferring alkyl or aryl groups, other than methyl groups,Transferases,3-deoxy-7-phosphoheptulonate synthase
contig00006.g4084	1046	ID=contig00006.g4084;Description=glucose-6-phosphate 1-epimerase [Fusarium tjaetaba];Gene=FACUT_10208;Ontology_term=carbohydrate metabolic process,isomerase activity;Ontology_id=GO:0005975,GO:0016853;Enzyme_code=EC:5.1.3.15;Enzyme_name=glucose-6-phosphate 1-epimerase
contig00006.g4085	2052	ID=contig00006.g4085;Description=sugar transporter [Fusarium tjaetaba];Gene=Focb16_v000414;Ontology_term=transmembrane transport,transporter activity;Ontology_id=GO:0055085,GO:0005215
contig00006.g4086	5370	ID=contig00006.g4086;Description=Telomere length regulator rif1 [Fusarium coicis];Gene=FVEG_09590;Ontology_term=DNA binding,DNA replication,nuclear chromosome,telomere organization,mitotic cell cycle;Ontology_id=GO:0003677,GO:0006260,GO:0000228,GO:0032200,GO:0000278

contig00006.g4087	1587	ID=contig00006.g4087;Description=guanine nucleotide exchange factor synembryn [Fusarium tjaetaba];Gene=FPHYL_2418;Ontology_term=molecular function regulator activity;Ontology_id=GO:0098772
contig00006.g4088	757	ID=contig00006.g4088;Description=Testis-specific chromodomain protein [Fusarium tjaetaba];Gene=FDENT_107;Ontology_term=chromatin organization;Ontology_id=GO:0006325
contig00006.g4089	535	ID=contig00006.g4089;Description=Noranthrone monooxygenase [Fusarium pseudocircinatum];Gene=FMUND_6823;Ontology_term=oxidoreductase activity;Ontology_id=GO:0016491;Enzyme_code=EC:1;Enzyme_name=Oxidoreductases
contig00006.g4090	684	ID=contig00006.g4090;Description=acetyltransferase [Fusarium tjaetaba];Gene=FVER53590_09594;Ontology_term=transferase activity;Ontology_id=GO:0016740;Enzyme_code=EC:2.3.1,EC:2.3,EC:2,EC:2.3.1;Enzyme_name=Acyltransferases,Acyltransferases,Transferases,Acyltransferases
contig00006.g4091	2121	ID=contig00006.g4091;Description=transcription factor [Fusarium coicis];Gene=FNAPI_10187;Ontology_term=nucleus,DNA binding,regulation of DNA-templated transcription,transcription regulator activity;Ontology_id=GO:0005634,GO:0003677,GO:0006355,GO:0140110
contig00006.g4092	642	ID=contig00006.g4092;Description=60S ribosomal protein L20 [Fusarium verticillioides 7600];Gene=F25303_5500;Ontology_term=RNA binding,cytoplasmic translation,structural molecule activity,cytosol,ribosome;Ontology_id=GO:0003723,GO:0002181,GO:0005198,GO:0005829,GO:0005840
contig00006.g4093	1805	ID=contig00006.g4093;Description=protein CSN12 [Fusarium verticillioides 7600];Gene=FPCIR_3030;Ontology_term=DNA binding,nucleocytoplasmic transport,nucleus,mRNA metabolic process,RNA binding,DNA-templated transcription,signaling,reproductive process,intracellular protein transport;Ontology_id=GO:0003677,GO:0006913,GO:0005634,GO:0016071,GO:0003723,GO:0006351,GO:0023052,GO:0022414,GO:0006886
contig00006.g4094	1017	ID=contig00006.g4094;Description=BTB/POZ domain [Fusarium oxysporum f. sp. vasinfectum]

contig00006.g4095	1245	ID=contig00006.g4095;Description=adenylate cyclase [Fusarium subglutinans];Gene=FVEG_09601;Ontology_term=nucleus,molecular function regulator activity,protein catabolic process,signaling,mitotic nuclear division,chromosome segregation;Ontology_id=GO:0005634,GO:0098772,GO:0030163,GO:0023052,GO:0140014,GO:0007059
contig00006.g4096	1316	ID=contig00006.g4096;Description=NAD(P)H-dependent D-xylose reductase xyl1 [Fusarium verticillioides 7600];Gene=FNYG_07496;Ontology_term=carbohydrate metabolic process,oxidoreductase activity;Ontology_id=GO:0005975,GO:0016491;Enzyme_code=EC:1.1.1.21;Enzyme_name=aldose reductase
contig00006.g4097	3093	ID=contig00006.g4097;Description=hypothetical protein FVEG_09603 [Fusarium verticillioides 7600];Gene=FVER53263_09603;Ontology_term=catalytic activity, acting on a protein,transferase activity;Ontology_id=GO:0140096,GO:0016740;Enzyme_code=EC:2.7.1,EC:2.7.1,EC:2.7,EC:2;Enzyme_name=Transferring phosphorus-containing groups,Transferring phosphorus-containing groups,Transferring phosphorus-containing groups,Transferases
contig00006.g4098	1092	ID=contig00006.g4098;Description=hypothetical protein FVEG_09604 [Fusarium verticillioides 7600];Gene=FVER53263_09604;Ontology_term=transmembrane transport,ATP-dependent activity,transporter activity,hydrolase activity;Ontology_id=GO:0055085,GO:0140657,GO:0005215,GO:0016787;Enzyme_code=EC:7.2.2,EC:3.6.1.15,EC:3.6.1,EC:3.6,EC:3,EC:7.2.2,EC:7,EC:3.6.1.15;Enzyme_name=Catalysing the translocation of inorganic cations,nucleoside-triphosphate phosphatase,Acting on acid anhydrides,Acting on acid anhydrides,Hydrolases,Catalysing the translocation of inorganic cations,Translocases,nucleoside-triphosphate phosphatase
contig00006.g4099	2582	ID=contig00006.g4099;Description=peroxisomal ABC transporter [Fusarium napiforme];Gene=C2S_4570;Ontology_term=peroxisome,extracellular space,transmembrane transport,ATP-dependent activity,nutrient reservoir activity,transporter activity,hydrolase activity;Ontology_id=GO:0005777,GO:0005615,GO:0055085,GO:0140657,GO:0045735,GO:0005215,GO:0016787;Enzyme_code=EC:7.2.2,EC:3.6.1.15,EC:3.6.1,EC:3.6,EC:3,EC:7.2.2,EC:7,EC:3.6.1.15;Enzyme_name=Catalysing the translocation of inorganic cations,nucleoside-triphosphate phosphatase,Acting on acid anhydrides,Acting on acid anhydrides,Hydrolases,Catalysing the translocation of inorganic cations,Translocases,nucleoside-triphosphate phosphatase

contig00006.g4100	1593	ID=contig00006.g4100;Description=plasma membrane phosphatase required for sodium stress response [Fusarium fujikuroi];Gene=FVEG_09606;Ontology_term=catalytic activity, acting on a protein,signaling,cytosol,regulation of DNA-templated transcription,plasma membrane,hydrolase activity;Ontology_id=GO:0140096,GO:0023052,GO:0005829,GO:0006355,GO:0005886,GO:0016787;Enzyme_code=EC:3.1.3,EC:3.1.3.16,EC:3.1,EC:3,EC:3.1.3;Enzyme_name=Acting on ester bonds,protein-serine/threonine phosphatase,Acting on ester bonds,Hydrolases,Acting on ester bonds
contig00006.g4101	580	ID=contig00006.g4101;Description=MAG: Arf guanine nucleotide exchange factor sbh1 [Chaenotheca gracillima];Gene=CP532_6472;Ontology_term=endoplasmic reticulum,intracellular protein transport,hydrolase activity;Ontology_id=GO:0005783,GO:0006886,GO:0016787;Enzyme_code=EC:3.1.3;Enzyme_name=Acting on ester bonds
contig00006.g4102	1188	ID=contig00006.g4102;Description=hypothetical protein FVER53590_09608 [Fusarium verticillioides]
contig00006.g4103	4066	ID=contig00006.g4103;Description=sterol 3-beta-glucosyltransferase [Fusarium tjaetaba];Gene=FPCIR_3019;Ontology_term=carbohydrate metabolic process,lipid metabolic process,transferase activity;Ontology_id=GO:0005975,GO:0006629,GO:0016740;Enzyme_code=EC:2.4.1.173,EC:2.4.1.173,EC:2.4,EC:2,EC:2.4.1;Enzyme_name=sterol 3beta-glucosyltransferase,sterol 3beta-glucosyltransferase,Glycosyltransferases,Transferases,Glycosyltransferases
contig00006.g4104	1205	ID=contig00006.g4104;Description=fmn-dependent dehydrogenase [Fusarium tjaetaba];Gene=FOVG_01814;Ontology_term=oxidoreductase activity;Ontology_id=GO:0016491;Enzyme_code=EC:1,EC:1;Enzyme_name=Oxidoreductases,Oxidoreductases
contig00006.g4105	1326	ID=contig00006.g4105;Description=hypothetical protein FVER14953_09612 [Fusarium verticillioides]
contig00006.g4106	906	ID=contig00006.g4106;Description=arginase [Fusarium tjaetaba];Gene=FVER53263_09613;Ontology_term=metal ion binding;Ontology_id=GO:0046872;Enzyme_code=EC:3.5.3.1;Enzyme_name=arginase
contig00006.g4107	2356	ID=contig00006.g4107;Description=choline transporter [Fusarium tjaetaba];Gene=F52700_8217;Ontology_term=transmembrane transport,plasma membrane,transporter activity;Ontology_id=GO:0055085,GO:0005886,GO:0005215

contig00006.g4108	765	ID=contig00006.g4108;Description=GTP-binding protein rab2 [Fusarium subglutinans];Gene=FDENT_127;Ontology_term=GTPase activity;Ontology_id=GO:0003924;Enzyme_code=EC:3.6.1.15,EC:3.6.1,EC:3.6,EC:3,EC:3.6.1.15;Enzyme_name=nucleoside-triphosphate phosphatase,Acting on acid anhydrides,Acting on acid anhydrides,Hydrolases,nucleoside-triphosphate phosphatase
contig00006.g4109	2428	ID=contig00006.g4109;Description=Dolichyl-phosphate-mannose--protein mannosyltransferase 2 [Fusarium oxysporum f. sp. cepae];Gene=PMT2;Ontology_term=catalytic activity, acting on a protein,protein catabolic process,signaling,protein glycosylation,endoplasmic reticulum,cell wall organization or biogenesis,transferase activity,intracellular protein transport,plasma membrane;Ontology_id=GO:0140096,GO:0030163,GO:0023052,GO:0006486,GO:0005783,GO:0071554,GO:0016740,GO:0006886,GO:0005886;Enzyme_code=EC:2.4.1.109,EC:2.4.1.109,EC:2.4,EC:2,EC:2.4.1;Enzyme_name=dolichyl-phosphate-mannose--protein mannosyltransferase,dolichyl-phosphate-mannose--protein mannosyltransferase,Glycosyltransferases,Transferases,Glycosyltransferases
contig00006.g4110	1925	ID=contig00006.g4110;Description=related to Zn-finger protein [Fusarium proliferatum ET1];Gene=FVEG_09617;Ontology_term=nucleus,catalytic activity, acting on a protein,protein catabolic process,transferase activity;Ontology_id=GO:0005634,GO:0140096,GO:0030163,GO:0016740;Enzyme_code=EC:2.3,EC:2,EC:2.3.2;Enzyme_name=Acyltransferases,Transferases,Acyltransferases
contig00006.g4111	339	ID=contig00006.g4111;Description=mitotic-spindle organizing gamma-tubulin ring associated-domain-containing protein [Fusarium flagelliforme];Gene=FEQUK3_LOCUS4159;Ontology_term=microtubule organizing center;Ontology_id=GO:0005815
contig00006.g4112	825	ID=contig00006.g4112;Description=kinetochore protein SPC25 [Fusarium proliferatum];Gene=FACUT_5392;Ontology_term=cytoskeleton organization,protein-containing complex assembly,mitotic nuclear division,structural molecule activity,chromosome segregation,nuclear chromosome,meiotic nuclear division;Ontology_id=GO:0007010,GO:0065003,GO:0140014,GO:0005198,GO:0007059,GO:0000228,GO:0140013
contig00006.g4113	2982	ID=contig00006.g4113;Description=rna polymerase i-specific transcription-initiation factor rrn6 [Fusarium tjaetaba];Gene=FPCIR_3009;Ontology_term=translation regulator activity;Ontology_id=GO:0045182

contig00006.g4114	2073	ID=contig00006.g4114;Description=60S ribosomal protein L3 [Fusarium tjaetaba];Gene=FPCIR_3008;Ontology_term=translation regulator activity,catalytic activity, acting on a protein,mitochondrial gene expression,mitochondrion,structural molecule activity,ribosome,catalytic activity, acting on RNA,transferase activity;Ontology_id=GO:0045182,GO:0140096,GO:0140053,GO:0005739,GO:0005198,GO:0005840,GO:0140098,GO:0016740;Enzyme_code=EC:2.3,EC:2,EC:2.3.2,EC:2.3.2.12;Enzyme_name=Acyltransferases,Transferases,Acyltransferases,peptidyltransferase
contig00006.g4115	707	ID=contig00006.g4115;Description=FMP35 [Fusarium tjaetaba];Gene=FPHYL_12723;Ontology_term=protein-containing complex assembly,mitochondrion,mitochondrion organization;Ontology_id=GO:0065003,GO:0005739,GO:0007005
contig00006.g4116	780	ID=contig00006.g4116;Description=hypothetical protein J7337_000339 [Fusarium musae];Gene=FPCIR_3006;Ontology_term=membrane;Ontology_id=GO:0016020
contig00006.g4117	3753	ID=contig00006.g4117;Description=ankyrin repeat protein nuc-2 [Fusarium verticillioides 7600];Gene=FVER53263_09624;Ontology_term=lipid metabolic process,hydrolase activity;Ontology_id=GO:0006629,GO:0016787;Enzyme_code=EC:3.1.4;Enzyme_name=Acting on ester bonds
contig00006.g4118	1427	ID=contig00006.g4118;Description=CUE domain-containing protein [Fusarium pseudoanthophilum];Gene=F52700_8228;Ontology_term=protein catabolic process,signaling,molecular adaptor activity;Ontology_id=GO:0030163,GO:0023052,GO:0060090
contig00006.g4119	2072	ID=contig00006.g4119;Description=ATP-dependent RNA helicase DHX35 [Fusarium tjaetaba];Gene=FVER53263_09626;Ontology_term=nucleus,mRNA metabolic process,RNA binding,ATP-dependent activity,catalytic activity, acting on RNA,hydrolase activity;Ontology_id=GO:0005634,GO:0016071,GO:0003723,GO:0140657,GO:0140098,GO:0016787;Enzyme_code=EC:3,EC:3.6.1,EC:3.6.4.13,EC:3.6,EC:3,EC:3.6.1.15;Enzyme_name=Hydrolases,Acting on acid anhydrides,RNA helicase,Acting on acid anhydrides,Hydrolases,nucleoside-triphosphate phosphatase
contig00006.g4120	2308	ID=contig00006.g4120;Description=hypothetical protein FPHYL_12728 [Fusarium phyllophilum];Gene=FVEG_09627;Ontology_term=membrane;Ontology_id=GO:0016020
contig00006.g4121	1374	ID=contig00006.g4121;Description=serine-rich protein [Fusarium phyllophilum];Gene=FOXG_11162;Ontology_term=cell adhesion,plasma membrane;Ontology_id=GO:0007155,GO:0005886

contig00006.g4122	2131	ID=contig00006.g4122;Description=hypothetical protein FVER53263_09629 [Fusarium verticillioides]
contig00006.g4123	534	ID=contig00006.g4123;Description=DASH complex subunit DAD2 [Fusarium napiforme];Gene=FOMG_00419;Ontology_term=cytoskeleton organization,protein-containing complex assembly,microtubule organizing center,mitotic nuclear division,chromosome segregation,nuclear chromosome,cytoskeletal protein binding,meiotic nuclear division;Ontology_id=GO:0007010,GO:0065003,GO:0005815,GO:0140014,GO:0007059,GO:0000228,GO:0008092,GO:0140013
contig00006.g4124	2156	ID=contig00006.g4124;Description=Shugoshin [Fusarium tjaetaba];Gene=FPCIR_2998;Ontology_term=nucleus,chromosome segregation,chromosome,meiotic nuclear division;Ontology_id=GO:0005634,GO:0007059,GO:0005694,GO:0140013
contig00006.g4125	722	ID=contig00006.g4125;Description=VPS29-like protein [Fusarium subglutinans];Gene=F25303_5467;Ontology_term=endosome,vesicle-mediated transport,cytosol,intracellular protein transport,molecular adaptor activity,hydrolase activity;Ontology_id=GO:0005768,GO:0016192,GO:0005829,GO:0006886,GO:0060090,GO:0016787;Enzyme_code=EC:3;Enzyme_name=Hydrolases
contig00006.g4126	3796	ID=contig00006.g4126;Description=ribosomal RNA-processing 12 [Fusarium tjaetaba];Gene=FNYG_07464;Ontology_term=nucleus;Ontology_id=GO:0005634
contig00006.g4127	2697	ID=contig00006.g4127;Description=hypothetical protein FVER53590_09634 [Fusarium verticillioides]
contig00006.g4128	4391	ID=contig00006.g4128;Description=heterokaryon incompatibility het-6 [Fusarium pseudoanthophilum];Gene=FMEXI_3958;Ontology_term=regulation of DNA-templated transcription,transcription regulator activity;Ontology_id=GO:0006355,GO:0140110
contig00006.g4129	1806	ID=contig00006.g4129;Description=hypothetical protein FNAPI_12863 [Fusarium napiforme];Gene=FPRO05_04529;Ontology_term=membrane;Ontology_id=GO:0016020

contig00006.g4130	4708	ID=contig00006.g4130;Description=regulator of deoxyribodipyrimidine photo-lyase PHR1 [Fusarium tjaetaba];Gene=FVEG_09639;Ontology_term=catalytic activity, acting on a protein,oxidoreductase activity,lyase activity,chromatin organization;Ontology_id=GO:0140096,GO:0016491,GO:0016829,GO:0006325;Enzyme_code=EC:4,EC:1.14.11.66,EC:1.14.11,EC:1,EC:1.14,EC:4,EC:1.14.11.66;Enzyme_name=Lyases,[histone H3]-trimethyl-L-lysine(9) demethylase,Acting on paired donors, with incorporation or reduction of molecular oxygen. The oxygen incorporated need not be derived from O2,Oxidoreductases,Acting on paired donors, with incorporation or reduction of molecular oxygen. The oxygen incorporated need not be derived from O2,Lyases,[histone H3]-trimethyl-L-lysine(9) demethylase
contig00006.g4131	965	ID=contig00006.g4131;Description=serine/threonine protein kinase [Fusarium verticillioides 7600];Gene=FDENT_149;Ontology_term=catalytic activity, acting on a protein,autophagy,transferase activity;Ontology_id=GO:0140096,GO:0006914,GO:0016740;Enzyme_code=EC:2.6.1,EC:2.7.11.1;Enzyme_name=Transferring nitrogenous groups,non-specific serine/threonine protein kinase
contig00006.g4132	1485	ID=contig00006.g4132;Description=aminotransferase [Fusarium verticillioides 7600];Gene=FPHYL_10050;Ontology_term=catalytic activity, acting on a protein,mitochondrion,amino acid metabolic process,transferase activity;Ontology_id=GO:0140096,GO:0005739,GO:0006520,GO:0016740;Enzyme_code=EC:2.6.1,EC:2.7.1,EC:2.6.1,EC:2.7.1,EC:2.7,EC:2,EC:2.6.1.7,EC:2.6,EC:2.6.1.39;Enzyme_name=Transferring nitrogenous groups,Transferring phosphorus-containing groups,Transferring nitrogenous groups,Transferring phosphorus-containing groups,Transferring phosphorus-containing groups,Transferases,kynurenine--oxoglutarate transaminase,Transferring nitrogenous groups,2-aminoadipate transaminase
contig00006.g4133	4215	ID=contig00006.g4133;Description=xanthine dehydrogenase [Fusarium tjaetaba];Gene=FPANT_3708;Ontology_term=peroxisome,nucleobase-containing small molecule metabolic process,oxidoreductase activity,cytosol;Ontology_id=GO:0005777,GO:0055086,GO:0016491,GO:0005829;Enzyme_code=EC:1.17.1.4,EC:1.2,EC:1.17.1,EC:1.17,EC:1,EC:1.17.1.4,EC:1.17.3.2;Enzyme_name=xanthine dehydrogenase,Acting on the aldehyde or oxo group of donors,Acting on CH or CH2 groups,Acting on CH or CH2 groups,Oxidoreductases,xanthine dehydrogenase,xanthine oxidase

contig00006.g4134	6261	ID=contig00006.g4134;Description=transcription factor [Fusarium tjaetaba];Gene=FPANT_3707;Ontology_term=nucleus,chromosome segregation,meiotic nuclear division;Ontology_id=GO:0005634,GO:0007059,GO:0140013
contig00006.g4135	2538	ID=contig00006.g4135;Description=ZFR1 regulator of fumonisin biosynthesis [Fusarium tjaetaba];Gene=FPANT_3706;Ontology_term=nucleus,DNA binding,regulation of DNA-templated transcription,transcription regulator activity;Ontology_id=GO:0005634,GO:0003677,GO:0006355,GO:0140110
contig00006.g4136	1062	ID=contig00006.g4136;Description=putative AAH1-adenosine deaminase [Fusarium fujikuroi];Gene=AAH1;Ontology_term=nucleus,nucleobase-containing small molecule metabolic process,cell adhesion,hydrolase activity;Ontology_id=GO:0005634,GO:0055086,GO:0007155,GO:0016787;Enzyme_code=EC:3.5.4.2,EC:3.5.4.4,EC:3.5.4,EC:3.5,EC:3,EC:3.5.4.2,EC:3.5.4.4;Enzyme_name=adenine deaminase,adenosine deaminase,Acting on carbon-nitrogen bonds, other than peptide bonds,Acting on carbon-nitrogen bonds, other than peptide bonds,Hydrolases,adenine deaminase,adenosine deaminase
contig00006.g4137	3544	ID=contig00006.g4137;Description=ubiquitin conjugation factor E4 B [Fusarium verticillioides 7600];Gene=F52700_12019;Ontology_term=nucleus,catalytic activity, acting on a protein,protein catabolic process,transferase activity;Ontology_id=GO:0005634,GO:0140096,GO:0030163,GO:0016740;Enzyme_code=EC:2.3.2,EC:2.3,EC:2,EC:2.3.2.27,EC:2.3.2;Enzyme_name=Acyltransferases,Acyltransferases,Transferases,RING-type E3 ubiquitin transferase,Acyltransferases
contig00006.g4138	1590	ID=contig00006.g4138;Description=ubiquitin elongating factor core-domain-containing protein [Fusarium oxysporum Fo47];Gene=FPANT_3703;Ontology_term=zinc ion binding;Ontology_id=GO:0008270
contig00006.g4139	2865	ID=contig00006.g4139;Description=calpain [Fusarium tjaetaba];Gene=BFJ68_g1144;Ontology_term=DNA binding,catalytic activity, acting on a protein,hydrolase activity;Ontology_id=GO:0003677,GO:0140096,GO:0016787;Enzyme_code=EC:3.4.22,EC:3.4.22,EC:3.4,EC:3;Enzyme_name=Acting on peptide bonds (peptidases),Acting on peptide bonds (peptidases),Acting on peptide bonds (peptidases),Hydrolases

contig00006.g4140	1160	ID=contig00006.g4140;Description=cutinase-domain-containing protein [Fusarium redolens];Gene=FMEXI_3946;Ontology_term=extracellular region,hydrolase activity;Ontology_id=GO:0005576,GO:0016787;Enzyme_code=EC:3.1.1.74,EC:3.1,EC:3,EC:3.1.1.74,EC:3.1.1;Enzyme_name=cutinase,Acting on ester bonds,Hydrolases,cutinase,Acting on ester bonds
contig00006.g4141	2270	ID=contig00006.g4141;Description=related to hormone-sensitive lipase [Fusarium proliferatum ET1];Gene=FNAPI_166;Ontology_term=hydrolase activity;Ontology_id=GO:0016787;Enzyme_code=EC:3;Enzyme_name=Hydrolases
contig00006.g4142	2005	ID=contig00006.g4142;Description=glutamate decarboxylase [Fusarium oxysporum f. sp. lycopersici 4287];Gene=FCIRC_9767;Ontology_term=lyase activity,amino acid metabolic process;Ontology_id=GO:0016829,GO:0006520;Enzyme_code=EC:4.1.1.15,EC:4.1.1,EC:4.1.1.15,EC:4.1,EC:4;Enzyme_name=glutamate decarboxylase,Carbon-carbon lyases,glutamate decarboxylase,Carbon-carbon lyases,Lyases
contig00006.g4143	1396	ID=contig00006.g4143;Description=acetylornithine aminotransferase mitochondrial [Fusarium tjaetaba];Gene=FCIRC_9768;Ontology_term=protein-containing complex assembly,mitochondrion,amino acid metabolic process,transferase activity,nervous system process,nucleoplasm;Ontology_id=GO:0065003,GO:0005739,GO:0006520,GO:0016740,GO:0050877,GO:0005654;Enzyme_code=EC:2.6.1.11,EC:2.6.1,EC:2.6.1.11,EC:2.6.1.13,EC:2,EC:2.6;Enzyme_name=acetylornithine transaminase,Transferring nitrogenous groups,acetylornithine transaminase,ornithine aminotransferase,Transferases,Transferring nitrogenous groups
contig00006.g4144	1596	ID=contig00006.g4144;Description=cholesterol 7-alpha-monooxygenase [Fusarium pseudocircinatum];Gene=FCIRC_9769;Ontology_term=oxidoreductase activity;Ontology_id=GO:0016491;Enzyme_code=EC:1.14,EC:1,EC:1.14;Enzyme_name=Acting on paired donors, with incorporation or reduction of molecular oxygen. The oxygen incorporated need not be derived from O2,Oxidoreductases,Acting on paired donors, with incorporation or reduction of molecular oxygen. The oxygen incorporated need not be derived from O2
contig00006.g4145	1078	ID=contig00006.g4145;Description=GPR1 [Fusarium tjaetaba];Gene=FMEXI_3941;Ontology_term=membrane;Ontology_id=GO:0016020
contig00006.g4146	750	ID=contig00006.g4146;Description=hypothetical protein FVEG_09659 [Fusarium verticillioides 7600];Gene=FVER53263_09659;Ontology_term=membrane;Ontology_id=GO:0016020
contig00006.g4147	1258	ID=contig00006.g4147;Description=hypothetical protein FVER14953_21134 [Fusarium verticillioides];Gene=C2S_4519;Ontology_term=membrane;Ontology_id=GO:0016020

contig00006.g4148	2334	ID=contig00006.g4148;Description=hypothetical protein FVEG_09660 [Fusarium verticillioides 7600]
contig00006.g4149	1152	ID=contig00006.g4149;Description=zinc finger protein odd-paired-like (opl) [Fusarium denticulatum]
contig00006.g4150	1823	ID=contig00006.g4150;Description=translation initiation factor eIF-2B subunit gamma [Fusarium verticillioides 7600];Gene=FTJAE_8198;Ontology_term=translation regulator activity;Ontology_id=GO:0045182
contig00006.g4151	1330	ID=contig00006.g4151;Description=hypothetical protein FVEG_09663 [Fusarium verticillioides 7600];Gene=28952551;Ontology_term=membrane;Ontology_id=GO:0016020
contig00006.g4152	3056	ID=contig00006.g4152;Description=positive cofactor 2 [Fusarium tjaetaba];Gene=FNAPI_155;Ontology_term=regulation of DNA-templated transcription,chromosome,transcription regulator activity,molecular adaptor activity;Ontology_id=GO:0006355,GO:0005694,GO:0140110,GO:0060090
contig00006.g4153	1129	ID=contig00006.g4153;Description=autophagy Atg27 [Fusarium tjaetaba];Gene=FDENT_13108;Ontology_term=cytoplasmic vesicle,Golgi apparatus;Ontology_id=GO:0031410,GO:0005794
contig00006.g4154	3919	ID=contig00006.g4154;Description=RNA-directed RNA polymerase [Fusarium tjaetaba];Gene=FDENT_13109;Ontology_term=RNA binding,regulatory ncRNA-mediated gene silencing,catalytic activity, acting on RNA,transferase activity;Ontology_id=GO:0003723,GO:0031047,GO:0140098,GO:0016740;Enzyme_code=EC:2.7.7.48,EC:2.7,EC:2,EC:2.7.7.48,EC:2.7.7;Enzyme_name=RNA-directed RNA polymerase,Transferring phosphorus-containing groups,Transferases,RNA-directed RNA polymerase,Transferring phosphorus-containing groups
contig00006.g4155	1605	ID=contig00006.g4155;Description=3-oxoadipate enol-lactonase I [Fusarium subglutinans];Enzyme_code=EC:2.3.1.9;Enzyme_name=acetyl-CoA C-acetyltransferase
contig00006.g4156	1828	ID=contig00006.g4156;Description=multidrug transporter (major facilitator family) [Fusarium subglutinans];Gene=FSUBG_7993;Ontology_term=transmembrane transport,transporter activity;Ontology_id=GO:0055085,GO:0005215
contig00006.g4157	755	ID=contig00006.g4157;Description=hypothetical protein FPANT_4003 [Fusarium pseudoanthophilum]
contig00006.g4158	716	ID=contig00006.g4158;Description=hypothetical protein FVER53590_09672 [Fusarium verticillioides]

contig00006.g4159	420	ID=contig00006.g4159;Description=hypothetical protein FVEG_09672 [Fusarium verticillioides 7600];Gene=Q24615;Ontology_term=nucleus,DNA binding,regulation of DNA-templated transcription,transcription regulator activity;Ontology_id=GO:0005634,GO:0003677,GO:0006355,GO:0140110
contig00006.g4160	2081	ID=contig00006.g4160;Description=kinase [Fusarium tjaetaba];Gene=FMUND_12209;Ontology_term=catalytic activity, acting on a protein,lyase activity,extracellular region,transferase activity,hydrolase activity;Ontology_id=GO:0140096,GO:0016829,GO:0005576,GO:0016740,GO:0016787;Enzyme_code=EC:2.7.11.1,EC:3.4,EC:4;Enzyme_name=non-specific serine/threonine protein kinase,Acting on peptide bonds (peptidases),Lyases
contig00006.g4161	3533	ID=contig00006.g4161;Description=hypothetical protein FVEG_16635 [Fusarium verticillioides 7600];Gene=FPRO_00318
contig00006.g4162	1131	ID=contig00006.g4162;Description=hypothetical protein FVER53590_09674 [Fusarium verticillioides];Gene=FTJAE_13415;Ontology_term=nucleus,regulation of DNA-templated transcription,transcription regulator activity;Ontology_id=GO:0005634,GO:0006355,GO:0140110
contig00006.g4163	1399	ID=contig00006.g4163;Description=hypothetical protein FVEG_09675 [Fusarium verticillioides 7600]
contig00006.g4164	1132	ID=contig00006.g4164;Description=oxidoreductase [Fusarium verticillioides 7600]
contig00006.g4165	2234	ID=contig00006.g4165;Description=ankyrin repeat domain protein [Fusarium tjaetaba]
contig00006.g4166	1941	ID=contig00006.g4166;Description=membrane ycf1 [Fusarium tjaetaba]
contig00006.g4167	292	ID=contig00006.g4167;Description=hypothetical protein FVEG_16637 [Fusarium verticillioides 7600]
contig00006.g4168	6264	ID=contig00006.g4168;Description=serine threonine phosphatase 6 regulatory ankyrin repeat subunit C [Fusarium tjaetaba];Gene=FVER53590_09679;Ontology_term=catalytic activity, acting on a protein,hydrolase activity;Ontology_id=GO:0140096,GO:0016787;Enzyme_code=EC:3.4.23,EC:3.4.23,EC:3.4,EC:3;Enzyme_name=Acting on peptide bonds (peptidases),Acting on peptide bonds (peptidases),Acting on peptide bonds (peptidases),Hydrolases
contig00006.g4169	1295	ID=contig00006.g4169;Description=putative mitochondrial phosphate transport protein MIR1 [Fusarium fujikuroi];Gene=BFJ63_vAg634;Ontology_term=transmembrane transport,mitochondrion,transporter activity;Ontology_id=GO:0055085,GO:0005739,GO:0005215

contig00006.g4170	2337	ID=contig00006.g4170;Description=related to SDA1 protein, required for normal organization of the actin cytoskeleton [Fusarium fujikuroi];Gene=FGLOB1_5997;Ontology_term=nucleocytoplasmic transport,protein folding chaperone,cytoskeleton organization,ATP-dependent activity,ribosome biogenesis,intracellular protein transport,nucleolus,mitotic cell cycle,hydrolase activity;Ontology_id=GO:0006913,GO:0044183,GO:0007010,GO:0140657,GO:0042254,GO:0006886,GO:0005730,GO:0000278,GO:0016787;Enzyme_code=EC:3.6.1.15,EC:3.6.1,EC:3.6,EC:3,EC:3.6.1.15;Enzyme_name=nucleoside-triphosphate phosphatase,Acting on acid anhydrides,Acting on acid anhydrides,Hydrolases,nucleoside-triphosphate phosphatase
contig00006.g4171	333	ID=contig00006.g4171;Description=hypothetical protein FVEG_09682 [Fusarium verticillioides 7600]
contig00006.g4172	650	ID=contig00006.g4172;Description=hypothetical protein FVEG_09683 [Fusarium verticillioides 7600]
contig00006.g4173	1546	ID=contig00006.g4173;Description=MFS transporter, FHS family, L-fucose permease [Fusarium verticillioides 7600];Gene=F25303_9898;Ontology_term=transmembrane transport,transporter activity;Ontology_id=GO:0055085,GO:0005215
contig00006.g4174	1830	ID=contig00006.g4174;Description=PiT family inorganic phosphate transporter [Fusarium verticillioides 7600];Gene=FPRO05_04491;Ontology_term=transmembrane transport,transporter activity;Ontology_id=GO:0055085,GO:0005215
contig00006.g4175	3644	ID=contig00006.g4175;Description=hypothetical protein H9Q72_012152 [Fusarium xylarioides]
contig00006.g4176	2029	ID=contig00006.g4176;Description=flavin-containing protein [Fusarium tjaetaba];Gene=FVER53590_09687;Ontology_term=nucleus,oxidoreductase activity,tRNA metabolic process;Ontology_id=GO:0005634,GO:0016491,GO:0006399;Enzyme_code=EC:1.14.13.8,EC:1.14.13.8,EC:1,EC:1.14,EC:1.14.13;Enzyme_name=flavin-containing monooxygenase,flavin-containing monooxygenase,Oxidoreductases,Acting on paired donors, with incorporation or reduction of molecular oxygen. The oxygen incorporated need not be derived from O2,Acting on paired donors, with incorporation or reduction of molecular oxygen. The oxygen incorporated need not be derived from O2

contig00006.g4177	1774	ID=contig00006.g4177;Description=tRNA (adenine-N(1)-)-methyltransferase non-catalytic subunit [Fusarium verticillioides 7600];Gene=FVER53263_09688;Ontology_term=nucleus,translation regulator activity,oxidoreductase activity,tRNA metabolic process,transferase activity;Ontology_id=GO:0005634,GO:0045182,GO:0016491,GO:0006399,GO:0016740;Enzyme_code=EC:1.14.13.8,EC:2.1.1;Enzyme_name=flavin-containing monooxygenase,Transferring one-carbon groups
contig00006.g4178	1180	ID=contig00006.g4178;Description=hypothetical protein FVER14953_09689 [Fusarium verticillioides]
contig00006.g4179	1198	ID=contig00006.g4179;Description=dead helicase [Fusarium napiforme];Gene=FNAPI_664;Ontology_term=catalytic activity,ATP-dependent activity;Ontology_id=GO:0003824,GO:0140657
contig00006.g4180	988	ID=contig00006.g4180;Description=speckle-type POZ [Fusarium phyllophilum]
contig00006.g4181	763	ID=contig00006.g4181;Description=actin-related protein 2/3 complex subunit 3 [Ilyonectria robusta];Gene=BKA56DRAFT_67866;Ontology_term=cytoskeleton,vesicle-mediated transport,cytoskeleton organization,protein-containing complex assembly,mitochondrion,establishment or maintenance of cell polarity,anatomical structure development,transferase activity,cytoskeletal protein binding;Ontology_id=GO:0005856,GO:0016192,GO:0007010,GO:0065003,GO:0005739,GO:0007163,GO:0048856,GO:0016740,GO:0008092;Enzyme_code=EC:2.1.1;Enzyme_name=Transferring one-carbon groups
contig00006.g4182	1368	ID=contig00006.g4182;Description=hypothetical protein FVEG_09693 [Fusarium verticillioides 7600]
contig00006.g4183	1755	ID=contig00006.g4183;Description=putative triacylglycerol lipase V precursor [Fusarium fujikuroi];Gene=FVER53590_09694;Ontology_term=hydrolase activity;Ontology_id=GO:0016787;Enzyme_code=EC:3,EC:3;Enzyme_name=Hydrolases,Hydrolases
contig00006.g4184	3382	ID=contig00006.g4184;Description=related to (AAP1)-alanine/arginine aminopeptidase [Fusarium proliferatum ET1];Gene=FPANT_6817;Ontology_term=catalytic activity, acting on a protein,hydrolase activity;Ontology_id=GO:0140096,GO:0016787;Enzyme_code=EC:3.4.11;Enzyme_name=Acting on peptide bonds (peptidases)
contig00006.g4185	3009	ID=contig00006.g4185;Description=t-complex 11 1 [Fusarium tjaetaba]

contig00006.g4186	2890	ID=contig00006.g4186;Description=phosphotyrosine-specific ptp2-like protein [Fusarium oxysporum];Gene=FPANT_11798;Ontology_term=nucleus,catalytic activity, acting on a protein,signaling,reproductive process,regulation of DNA-templated transcription,mitotic cell cycle,hydrolase activity;Ontology_id=GO:0005634,GO:0140096,GO:0023052,GO:0022414,GO:0006355,GO:0000278,GO:0016787;Enzyme_code=EC:3.1.3.48,EC:3.1,EC:3.1.3.48,EC:3,EC:3.1.3;Enzyme_name=protein-tyrosine-phosphatase,Acting on ester bonds,protein-tyrosine-phosphatase,Hydrolases,Acting on ester bonds
contig00006.g4187	414	ID=contig00006.g4187;Description=hypothetical protein FVER14953_09699 [Fusarium verticillioides]
contig00006.g4188	1224	ID=contig00006.g4188;Description=26S protease regulatory subunit 8 [Fusarium proliferatum];Gene=FVEG_09700;Ontology_term=catalytic activity, acting on a protein,ATP-dependent activity,hydrolase activity;Ontology_id=GO:0140096,GO:0140657,GO:0016787;Enzyme_code=EC:3.4,EC:3.6.1.15,EC:3.6.1,EC:3.4,EC:3.6,EC:3,EC:3.6.1.15;Enzyme_name=Acting on peptide bonds (peptidases),nucleoside-triphosphate phosphatase,Acting on acid anhydrides,Acting on peptide bonds (peptidases),Acting on acid anhydrides,Hydrolases,nucleoside-triphosphate phosphatase
contig00006.g4189	3459	ID=contig00006.g4189;Description=Ca2+:H+ antiporter [Fusarium verticillioides 7600];Gene=FOXB_15453;Ontology_term=transmembrane transport,endoplasmic reticulum,vacuole,transporter activity;Ontology_id=GO:0055085,GO:0005783,GO:0005773,GO:0005215
contig00006.g4190	1128	ID=contig00006.g4190;Description=pectin lyase precursor [Fusarium subglutinans];Gene=FPANT_10720;Ontology_term=carbohydrate metabolic process,lyase activity,extracellular region;Ontology_id=GO:0005975,GO:0016829,GO:0005576;Enzyme_code=EC:4.2.2.2,EC:4.2.2.2,EC:4.2,EC:4.2.2.10,EC:4,EC:4.2.2;Enzyme_name=pectate lyase,pectate lyase,Carbon-oxygen lyases,pectin lyase,Lyases,Carbon-oxygen lyases
contig00006.g4191	1389	ID=contig00006.g4191;Description=hypothetical protein FVER14953_09703 [Fusarium verticillioides];Gene=FNYG_08896;Ontology_term=membrane;Ontology_id=GO:0016020
contig00006.g4192	1698	ID=contig00006.g4192;Description=hypothetical protein FNYG_08894 [Fusarium nygamai]
contig00006.g4193	1369	ID=contig00006.g4193;Description=hypothetical protein FVEG_09705 [Fusarium verticillioides 7600]

contig00006.g4194	246	ID=contig00006.g4194;Description=hypothetical protein FDENT_5928 [Fusarium denticulatum]
contig00006.g4195	1336	ID=contig00006.g4195;Description=hypothetical protein FVER53590_25953 [Fusarium verticillioides]
contig00006.g4196	1683	ID=contig00006.g4196;Description=hypothetical protein FVER53590_09706 [Fusarium verticillioides]
contig00006.g4197	4451	ID=contig00006.g4197;Description=cytidylyltransferase family [Fusarium subglutinans];Gene=FVER53590_09708;Ontology_term=nucleobase-containing small molecule metabolic process,transferase activity;Ontology_id=GO:0055086,GO:0016740;Enzyme_code=EC:2.7.7.1,EC:2.7.7.1,EC:2.7,EC:2,EC:2.7.7;Enzyme_name=nicotinamide-nucleotide adenylyltransferase,nicotinamide-nucleotide adenylyltransferase,Transferring phosphorus-containing groups,Transferases,Transferring phosphorus-containing groups
contig00006.g4198	3273	ID=contig00006.g4198;Description=vacuolar sorting PEP5 [Fusarium tjaetaba];Gene=FVER53263_09710;Ontology_term=lysosome organization,membrane organization,catalytic activity, acting on a protein,vesicle-mediated transport,endosome,protein-containing complex assembly,protein catabolic process,transferase activity,chromatin organization,intracellular protein transport,vacuole,molecular adaptor activity;Ontology_id=GO:0007040,GO:0061024,GO:0140096,GO:0016192,GO:0005768,GO:0065003,GO:0030163,GO:0016740,GO:0006325,GO:0006886,GO:0005773,GO:0060090;Enzyme_code=EC:2,EC:2.3,EC:2,EC:2.3.2;Enzyme_name=Transferases,Acyltransferases,Transferases,Acyltransferases
contig00006.g4199	1143	ID=contig00006.g4199;Description=hypothetical protein FAGAP_1566 [Fusarium agapanthi]
contig00006.g4200	2668	ID=contig00006.g4200;Description=DNA mismatch repair protein MLH3 [Fusarium verticillioides 7600];Gene=FPHYL_7509;Ontology_term=DNA repair,catalytic activity, acting on DNA,ATP-dependent activity,hydrolase activity,small molecule sensor activity;Ontology_id=GO:0006281,GO:0140097,GO:0140657,GO:0016787,GO:0140299;Enzyme_code=EC:3.6.1.15;Enzyme_name=nucleoside-triphosphate phosphatase
contig00006.g4201	1016	ID=contig00006.g4201;Description=mannosylphosphorylation MNN4 [Fusarium tjaetaba];Gene=FVER53263_09713;Ontology_term=molecular function regulator activity,protein glycosylation;Ontology_id=GO:0098772,GO:0006486
contig00006.g4202	519	ID=contig00006.g4202;Description=hypothetical protein FVEG_16647 [Fusarium verticillioides 7600]

contig00006.g4203	1684	ID=contig00006.g4203;Description=nicotinamide mononucleotide permease [Fusarium tjaetaba];Gene=FOVG_01924;Ontology_term=transmembrane transport,transporter activity;Ontology_id=GO:0055085,GO:0005215
contig00006.g4204	1069	ID=contig00006.g4204;Description=3-oxo-5-alpha-steroid 4-dehydrogenase [Fusarium tjaetaba];Gene=FNAPI_2894;Ontology_term=catalytic activity, acting on a protein,oxidoreductase activity,lipid metabolic process,hydrolase activity;Ontology_id=GO:0140096,GO:0016491,GO:0006629,GO:0016787;Enzyme_code=EC:1.3.99.5,EC:3.4.19.12;Enzyme_name=3-oxo-5alpha-steroid 4-dehydrogenase (acceptor),ubiquitinyl hydrolase 1
contig00006.g4205	2514	ID=contig00006.g4205;Description=hypothetical protein FVEG_09717 [Fusarium verticillioides 7600];Gene=FVEG_09717;Ontology_term=nucleus,catalytic activity, acting on a protein,oxidoreductase activity,lipid metabolic process,endoplasmic reticulum,plasma membrane,hydrolase activity;Ontology_id=GO:0005634,GO:0140096,GO:0016491,GO:0006629,GO:0005783,GO:0005886,GO:0016787;Enzyme_code=EC:1.3,EC:3.4.19.12,EC:1.3,EC:3.4,EC:1,EC:3,EC:3.4.19.12;Enzyme_name=Acting on the CH-CH group of donors,ubiquitinyl hydrolase 1,Acting on the CH-CH group of donors,Acting on peptide bonds (peptidases),Oxidoreductases,Hydrolases,ubiquitinyl hydrolase 1
contig00006.g4206	1120	ID=contig00006.g4206;Description=Putative J domain-containing protein C17A3.05c [Fusarium oxysporum f. sp. cubense race 1];Gene=FOYG_16470;Ontology_term=catalytic activity, acting on a protein,molecular function regulator activity,protein catabolic process,endoplasmic reticulum,protein folding,hydrolase activity;Ontology_id=GO:0140096,GO:0098772,GO:0030163,GO:0005783,GO:0006457,GO:0016787;Enzyme_code=EC:3.4.19.12,EC:3.4,EC:3,EC:3.4.19.12;Enzyme_name=ubiquitinyl hydrolase 1,Acting on peptide bonds (peptidases),Hydrolases,ubiquitinyl hydrolase 1
contig00006.g4207	1097	ID=contig00006.g4207;Description=endoglucanase 3 precursor [Fusarium subglutinans];Gene=FNAPI_2891;Ontology_term=carbohydrate metabolic process,hydrolase activity;Ontology_id=GO:0005975,GO:0016787;Enzyme_code=EC:3.2.1;Enzyme_name=Glycosylases
contig00006.g4208	425	ID=contig00006.g4208;Description=ribosomal protein S19 [Fusarium graminearum PH-1];Gene=FANTH_2572;Ontology_term=RNA binding,structural molecule activity,ribosome;Ontology_id=GO:0003723,GO:0005198,GO:0005840

contig00006.g4209	612	ID=contig00006.g4209;Description=transcription initiation factor TFIID subunit 10 [Fusarium denticulatum];Gene=FPANT_3519;Ontology_term=translation regulator activity,protein-containing complex assembly,structural molecule activity,nuclear chromosome,regulation of DNA-templated transcription,chromatin organization,transcription regulator activity,nucleoplasm,molecular adaptor activity;Ontology_id=GO:0045182,GO:0065003,GO:0005198,GO:0000228,GO:0006355,GO:0006325,GO:0140110,GO:0005654,GO:0060090
contig00006.g4210	811	ID=contig00006.g4210;Description=ER lumen protein retaining receptor [Fusarium verticillioides 7600];Gene=FANTH_2570;Ontology_term=endoplasmic reticulum;Ontology_id=GO:0005783
contig00006.g4211	798	ID=contig00006.g4211;Description=hypothetical protein FVER14953_09723 [Fusarium verticillioides]
contig00006.g4212	859	ID=contig00006.g4212;Description=histone H2A.Z [Fusarium oxysporum f. sp. lycopersici 4287];Gene=CEP52_007330;Ontology_term=DNA binding,mRNA metabolic process,protein-containing complex assembly,structural molecule activity,nuclear chromosome,regulation of DNA-templated transcription,chromatin organization,nucleoplasm;Ontology_id=GO:0003677,GO:0016071,GO:0065003,GO:0005198,GO:0000228,GO:0006355,GO:0006325,GO:0005654
contig00007.g4213	2329	ID=contig00007.g4213;Description=ribosome biogenesis protein ERB1 [Fusarium verticillioides 7600];Gene=ERB1;Ontology_term=RNA binding,ribosome biogenesis,nucleolus,nucleoplasm;Ontology_id=GO:0003723,GO:0042254,GO:0005730,GO:0005654
contig00007.g4214	1774	ID=contig00007.g4214;Description=brefeldin A resistance [Fusarium tjaetaba];Gene=FOXB_04373;Ontology_term=ATP-dependent activity,hydrolase activity;Ontology_id=GO:0140657,GO:0016787;Enzyme_code=EC:3.6.1.15;Enzyme_name=nucleoside-triphosphate phosphatase

contig00007.g4215	1562	ID=contig00007.g4215;Description=P-loop containing nucleoside triphosphate hydrolase protein [Fusarium redolens];Gene=B0I35DRAFT_452885;Ontology_term=nucleus,catalytic activity, acting on a protein,protein-containing complex assembly,ATP-dependent activity,protein catabolic process,mitotic nuclear division,chromosome segregation,regulation of DNA-templated transcription,cytosol,hydrolase activity;Ontology_id=GO:0005634,GO:0140096,GO:0065003,GO:0140657,GO:0030163,GO:0140014,GO:0007059,GO:0006355,GO:0005829,GO:0016787;Enzyme_code=EC:3.4,EC:3.6.1.15,EC:3.6.1,EC:3.4,EC:3.6,EC:3,EC:5.6.1.5,EC:3.6.1.15;Enzyme_name=Acting on peptide bonds (peptidases),nucleoside-triphosphate phosphatase,Acting on acid anhydrides,Acting on peptide bonds (peptidases),Acting on acid anhydrides,Hydrolases,proteasome ATPase,nucleoside-triphosphate phosphatase
contig00007.g4216	1002	ID=contig00007.g4216;Description=ribosomal 11 precursor mitochondrial [Fusarium acutatum];Ontology_term=mitochondrial gene expression,mitochondrion,structural molecule activity,ribosome;Ontology_id=GO:0140053,GO:0005739,GO:0005198,GO:0005840
contig00007.g4217	722	ID=contig00007.g4217;Description=UPF0132 domain protein [Fusarium subglutinans];Gene=FGADI_12769;Ontology_term=membrane;Ontology_id=GO:0016020
contig00007.g4218	2542	ID=contig00007.g4218;Description=tRNA ligase [Fusarium oxysporum f. sp. cepae];Gene=FVEG_08903;Ontology_term=nuclear envelope,tRNA metabolic process,transferase activity,catalytic activity, acting on RNA,ligase activity,hydrolase activity;Ontology_id=GO:0005635,GO:0006399,GO:0016740,GO:0140098,GO:0016874,GO:0016787;Enzyme_code=EC:2.7.1,EC:3.1.4,EC:6.5.1.3,EC:2.7.1,EC:3.1.4,EC:6.5.1.3,EC:3.1,EC:3.1.4.37,EC:2.7,EC:2,EC:3,EC:6.5,EC:6;Enzyme_name=Transferring phosphorus-containing groups,Acting on ester bonds,RNA ligase (ATP),Transferring phosphorus-containing groups,Acting on ester bonds,RNA ligase (ATP),Acting on ester bonds,2',3'-cyclic-nucleotide 3'-phosphodiesterase,Transferring phosphorus-containing groups,Transferases,Hydrolases,Forming phosphoric ester bonds,Ligases
contig00007.g4219	657	ID=contig00007.g4219;Description=ankyrin repeat-containing protein [Fusarium tjaetaba]
contig00007.g4220	1341	ID=contig00007.g4220;Description=ribonuclease P MRP subunit POP1 [Fusarium subglutinans];Gene=FSUBG_10024;Enzyme_code=EC:3.1.26.5;Enzyme_name=ribonuclease P

contig00007.g4221	1493	ID=contig00007.g4221;Description=S-adenosyl-L-methionine-dependent methyltransferase [Fusarium oxysporum Fo47];Gene=FVER53590_08906;Ontology_term=RNA binding,tRNA metabolic process,transferase activity,catalytic activity, acting on RNA;Ontology_id=GO:0003723,GO:0006399,GO:0016740,GO:0140098;Enzyme_code=EC:2.1.1,EC:2.1,EC:2,EC:2.1.1,EC:2.1.1.214;Enzyme_name=Transferring one-carbon groups,Transferring one-carbon groups,Transferases,Transferring one-carbon groups,tRNA (guanine(10)-N(2))-methyltransferase
contig00007.g4222	1301	ID=contig00007.g4222;Description=VID24-like protein [Fusarium tjaetaba];Ontology_term=carbohydrate metabolic process,protein catabolic process,cytoplasmic vesicle,cytosol,intracellular protein transport;Ontology_id=GO:0005975,GO:0030163,GO:0031410,GO:0005829,GO:0006886
contig00007.g4223	765	ID=contig00007.g4223;Description=Mago nashi protein [Plectosphaerella cucumerina];Gene=F66182_5626
contig00007.g4224	3074	ID=contig00007.g4224;Description=Coatomer subunit beta [Fusarium circinatum];Gene=FOXG_10261;Ontology_term=vesicle-mediated transport,structural molecule activity,cytoplasmic vesicle,Golgi apparatus,intracellular protein transport;Ontology_id=GO:0016192,GO:0005198,GO:0031410,GO:0005794,GO:0006886
contig00007.g4225	998	ID=contig00007.g4225;Description=hypothetical protein FVEG_08910 [Fusarium verticillioides 7600]
contig00007.g4226	1148	ID=contig00007.g4226;Description=Histone-lysine N-methyltransferase, H3 lysine-9 specific dim-5 [Fusarium musae];Gene=FNYG_08587;Ontology_term=nucleus,catalytic activity, acting on a protein,chromosome,transferase activity,chromatin organization;Ontology_id=GO:0005634,GO:0140096,GO:0005694,GO:0016740,GO:0006325;Enzyme_code=EC:2.1.1;Enzyme_name=Transferring one-carbon groups
contig00007.g4227	1272	ID=contig00007.g4227;Description=histone-lysine n-methyltransferase H3 lysine-9 specific dim-5 [Fusarium coicis];Gene=FMUND_14748;Ontology_term=transferase activity;Ontology_id=GO:0016740;Enzyme_code=EC:2.1.1,EC:2.1,EC:2,EC:2.1.1;Enzyme_name=Transferring one-carbon groups,Transferring one-carbon groups,Transferases,Transferring one-carbon groups

		ID=contig00007.g4228;Description=poly A polymerase head domain protein [Fusarium tjaetaba];Gene=FVER53263_08912;Ontology_term=nucleus,RNA binding,mitochondrion,regulatory ncRNA-mediated gene silencing,tRNA metabolic process,catalytic activity, acting on RNA,transferase activity;Ontology_id=GO:0005634,GO:0003723,GO:0005739,GO:0031047,GO:0006399,GO:0140098,GO:0016740;Enzyme_code=EC:2.7.7,EC:2.7.7.72,EC:2.7,EC:2,EC:2.7.7,EC:2.7.7.19;Enzyme_name=Transferring phosphorus-containing groups,CCA tRNA nucleotidyltransferase,Transferring phosphorus-containing groups,Transferases,Transferring phosphorus-containing groups,polynucleotide adenylyltransferase
contig00007.g4228	1593	
		ID=contig00007.g4229;Description=hypothetical protein FVEG_08913 [Fusarium verticillioides 7600]
contig00007.g4229	375	
contig00007.g4230	1035	ID=contig00007.g4230;Description=peroxin 11c [Fusarium tjaetaba]
		ID=contig00007.g4231;Description=hypothetical protein FVEG_08915 [Fusarium verticillioides 7600]
contig00007.g4231	1862	
contig00007.g4232	1090	ID=contig00007.g4232;Description=duf1620 domain protein [Fusarium subglutinans];Gene=FSUBG_213;Ontology_term=membrane;Ontology_id=GO:0016020
contig00007.g4233	873	ID=contig00007.g4233;Description=BTB POZ [Fusarium denticulatum]
contig00007.g4234	1173	ID=contig00007.g4234;Description=hypothetical protein FNAPI_7709 [Fusarium napiforme]
contig00007.g4235	1678	ID=contig00007.g4235;Description=sphingosine kinase [Fusarium verticillioides 7600];Gene=FVER53590_08919;Ontology_term=transferase activity;Ontology_id=GO:0016740;Enzyme_code=EC:2.7;Enzyme_name=Transferring phosphorus-containing groups
contig00007.g4236	2575	ID=contig00007.g4236;Description=acetyltransferase [Fusarium verticillioides 7600];Gene=FOPG_14083;Ontology_term=molecular function regulator activity,mitochondrion,protein maturation,transferase activity;Ontology_id=GO:0098772,GO:0005739,GO:0051604,GO:0016740;Enzyme_code=EC:2,EC:2;Enzyme_name=Transferases,Transferases
contig00007.g4237	2352	ID=contig00007.g4237;Description=ankyrin 3 [Fusarium tjaetaba]
contig00007.g4238	1327	ID=contig00007.g4238;Description=KES1-like protein [Fusarium tjaetaba];Gene=FDENT_358;Ontology_term=lipid binding;Ontology_id=GO:0008289

contig00007.g4239	1258	ID=contig00007.g4239;Description=fructose-bisphosphate aldolase, class II [Fusarium odoratissimum NRRL 54006];Gene=FOC4_g10009448;Ontology_term=carbohydrate metabolic process,generation of precursor metabolites and energy,nucleobase-containing small molecule metabolic process,mitochondrion,lyase activity,cytosol,carbohydrate derivative metabolic process;Ontology_id=GO:0005975,GO:0006091,GO:0055086,GO:0005739,GO:0016829,GO:0005829,GO:1901135;Enzyme_code=EC:4.1.2.13;Enzyme_name=fructose-bisphosphate aldolase
contig00007.g4240	1455	ID=contig00007.g4240;Description=mitochondrial chaperone BCS1 [Fusarium verticillioides 7600];Gene=FPCIR_10639;Ontology_term=membrane organization,transmembrane transport,protein-containing complex assembly,ATP-dependent activity,mitochondrion,mitochondrion organization,transporter activity,hydrolase activity;Ontology_id=GO:0061024,GO:0055085,GO:0065003,GO:0140657,GO:0005739,GO:0007005,GO:0005215,GO:0016787;Enzyme_code=EC:3.6.1.15,EC:3.6.1,EC:3.6,EC:3,EC:3.6.1.15;Enzyme_name=nucleoside-triphosphate phosphatase,Acting on acid anhydrides,Acting on acid anhydrides,Hydrolases,nucleoside-triphosphate phosphatase
contig00007.g4241	1002	ID=contig00007.g4241;Description=mitochondrial chaperone BCS1 [Fusarium tjaetaba]
contig00007.g4242	1787	ID=contig00007.g4242;Description=general amino acid permease agp3 [Fusarium sp. NRRL 52700];Gene=FocTR4_00008865;Ontology_term=transmembrane transport;Ontology_id=GO:0055085
contig00007.g4243	489	ID=contig00007.g4243;Description=hypothetical protein FVER14953_08928 [Fusarium verticillioides]
contig00007.g4244	4053	ID=contig00007.g4244;Description=VPS9 domain protein [Fusarium subglutinans];Gene=FSUBG_10046;Ontology_term=lipid binding;Ontology_id=GO:0008289
contig00007.g4245	1767	ID=contig00007.g4245;Description=hypothetical protein FVER14953_08930 [Fusarium verticillioides]

contig00007.g4246	1512	ID=contig00007.g4246;Description=GTP-binding protein [Fusarium subglutinans];Gene=FOXB_12939;Ontology_term=nucleobase-containing small molecule metabolic process,ATP-dependent activity,mitochondrion,carbohydrate derivative metabolic process,transferase activity,hydrolase activity;Ontology_id=GO:0055086,GO:0140657,GO:0005739,GO:1901135,GO:0016740,GO:0016787;Enzyme_code=EC:3.6.1.15,EC:2.4.2.10,EC:2.4.2,EC:3.6.1,EC:2.4,EC:3.6,EC:2,EC:3,EC:3.6.1.15,EC:2.4.2.10;Enzyme_name=nucleoside-triphosphate phosphatase,orotate phosphoribosyltransferase,Glycosyltransferases,Acting on acid anhydrides,Glycosyltransferases,Acting on acid anhydrides,Transferases,Hydrolases,nucleoside-triphosphate phosphatase,orotate phosphoribosyltransferase
contig00007.g4247	817	ID=contig00007.g4247;Description=UPF0326 domain protein [Fusarium tjaetaba];Gene=FVER53263_08933;Ontology_term=catalytic activity, acting on a protein,hydrolase activity;Ontology_id=GO:0140096,GO:0016787;Enzyme_code=EC:3.4,EC:3.4,EC:3;Enzyme_name=Acting on peptide bonds (peptidases),Acting on peptide bonds (peptidases),Hydrolases
contig00007.g4248	714	ID=contig00007.g4248;Description=37S ribosomal S11 [Fusarium tjaetaba];Gene=FVER53263_08934;Ontology_term=RNA binding,mitochondrial gene expression,protein-containing complex assembly,mitochondrion,structural molecule activity,ribosome biogenesis,ribosome;Ontology_id=GO:0003723,GO:0140053,GO:0065003,GO:0005739,GO:0005198,GO:0042254,GO:0005840
contig00007.g4249	1713	ID=contig00007.g4249;Description=protein translocase subunit [Fusarium oxysporum];Gene=FVEG_08935;Ontology_term=transmembrane transport,mitochondrion,intracellular protein transport,mitochondrion organization,molecular adaptor activity;Ontology_id=GO:0055085,GO:0005739,GO:0006886,GO:0007005,GO:0060090
contig00007.g4250	797	ID=contig00007.g4250;Description=hypothetical protein FVEG_08936 [Fusarium verticillioides 7600]
contig00007.g4251	568	ID=contig00007.g4251;Description=cytochrome b5-like heme/steroid binding domain-containing protein [Fusarium redolens];Gene=FVER53263_08937;Ontology_term=endosome,molecular function regulator activity,lipid metabolic process;Ontology_id=GO:0005768,GO:0098772,GO:0006629

contig00007.g4252	2768	ID=contig00007.g4252;Description=related to glutamate carboxypeptidase II [Fusarium proliferatum ET1];Gene=FPHYL_9927;Ontology_term=catalytic activity, acting on a protein,vesicle-mediated transport,protein catabolic process,intracellular protein transport,vacuole,hydrolase activity;Ontology_id=GO:0140096,GO:0016192,GO:0030163,GO:0006886,GO:0005773,GO:0016787;Enzyme_code=EC:3.4,EC:3.4.17.21,EC:3.4,EC:3;Enzyme_name=Acting on peptide bonds (peptidases),glutamate carboxypeptidase II,Acting on peptide bonds (peptidases),Hydrolases
contig00007.g4253	2868	ID=contig00007.g4253;Description=vacuolar sorting VPS35 [Fusarium napiforme];Gene=FNYG_08562;Ontology_term=vesicle-mediated transport,cytosol;Ontology_id=GO:0016192,GO:0005829
contig00007.g4254	867	ID=contig00007.g4254;Description=Autophagy-related 3 [Fusarium tjaetaba];Gene=groEL;Ontology_term=protein folding chaperone,ATP-dependent activity,isomerase activity;Ontology_id=GO:0044183,GO:0140657,GO:0016853;Enzyme_code=EC:5,EC:5;Enzyme_name=Isomerases,Isomerases
contig00007.g4255	741	ID=contig00007.g4255;Description=uncharacterized protein FTJAE_13406 [Fusarium tjaetaba];Gene=FVER53590_26474;Ontology_term=transferase activity;Ontology_id=GO:0016740;Enzyme_code=EC:2.3.1,EC:2.3,EC:2,EC:2.3.1;Enzyme_name=Acyltransferases,Acyltransferases,Transferases,Acyltransferases
contig00007.g4256	1124	ID=contig00007.g4256;Description=alcohol dehydrogenase [Fusarium verticillioides 7600];Gene=FPANT_575;Ontology_term=oxidoreductase activity,ATP-dependent activity,catalytic activity, acting on RNA,hydrolase activity;Ontology_id=GO:0016491,GO:0140657,GO:0140098,GO:0016787;Enzyme_code=EC:3.6.4.13,EC:1,EC:1.1,EC:3.6.4.13,EC:1,EC:3;Enzyme_name=RNA helicase,Oxidoreductases,Acting on the CH-OH group of donors,RNA helicase,Oxidoreductases,Hydrolases
contig00007.g4257	3729	ID=contig00007.g4257;Description=inositol oxygenase [Fusarium proliferatum];Gene=Forpe1208_v007438;Ontology_term=oxidoreductase activity;Ontology_id=GO:0016491;Enzyme_code=EC:1.13.99.1,EC:1.13,EC:1,EC:1.13.99.1;Enzyme_name=inositol oxygenase,Acting on single donors with incorporation of molecular oxygen (oxygenases). The oxygen incorporated need not be derived from O2,Oxidoreductases,inositol oxygenase

contig00007.g4258	1086	ID=contig00007.g4258;Description=transcription factor atf21 [Fusarium tjaetaba];Gene=FVER53263_08944;Ontology_term=DNA binding,reproductive process,regulation of DNA-templated transcription,transcription regulator activity;Ontology_id=GO:0003677,GO:0022414,GO:0006355,GO:0140110
contig00007.g4259	4133	ID=contig00007.g4259;Description=related to vacuolar membrane protein HMT1 [Fusarium fujikuroi];Gene=FVER53263_08945;Ontology_term=transmembrane transport,ATP-dependent activity,transporter activity,hydrolase activity;Ontology_id=GO:0055085,GO:0140657,GO:0005215,GO:0016787;Enzyme_code=EC:7.2.2,EC:3.6.1.15,EC:3.6.1,EC:3.6,EC:3,EC:7.2.2,EC:7,EC:3.6.1.15;Enzyme_name=Catalysing the translocation of inorganic cations,nucleoside-triphosphate phosphatase,Acting on acid anhydrides,Acting on acid anhydrides,Hydrolases,Catalysing the translocation of inorganic cations,Translocases,nucleoside-triphosphate phosphatase
contig00007.g4260	1543	ID=contig00007.g4260;Description=monocarboxylate transporter 2 [Fusarium coicis];Gene=FVEG_08946;Ontology_term=molecular transducer activity,transmembrane transport,vesicle-mediated transport,signaling,lipid metabolic process,cytosol,extracellular region,transferase activity,transporter activity,plasma membrane;Ontology_id=GO:0060089,GO:0055085,GO:0016192,GO:0023052,GO:0006629,GO:0005829,GO:0005576,GO:0016740,GO:0005215,GO:0005886;Enzyme_code=EC:2.7.1.137,EC:2.7.1,EC:2.7,EC:2,EC:2.7.1.137;Enzyme_name=phosphatidylinositol 3-kinase,Transferring phosphorus-containing groups,Transferring phosphorus-containing groups,Transferases,phosphatidylinositol 3-kinase
contig00007.g4261	591	ID=contig00007.g4261;Description=hypothetical protein FVEG_08947 [Fusarium verticillioides 7600]
contig00007.g4262	3586	ID=contig00007.g4262;Description=zinc finger protein ADR1 [Fusarium verticillioides 7600];Gene=FNYG_08551;Ontology_term=nucleus,DNA binding,DNA-templated transcription;Ontology_id=GO:0005634,GO:0003677,GO:0006351
contig00007.g4263	609	ID=contig00007.g4263;Description=the plant PR-1 class pathogen related [Fusarium tjaetaba];Gene=FTJAE_3141;Ontology_term=extracellular region;Ontology_id=GO:0005576
contig00007.g4264	815	ID=contig00007.g4264;Description=small nuclear ribonucleoprotein E [Fusarium tjaetaba];Gene=FTJAE_3140;Ontology_term=nucleus,mRNA metabolic process;Ontology_id=GO:0005634,GO:0016071

contig00007.g4265	2867	ID=contig00007.g4265;Description=regulatory alcR [Fusarium tjaetaba];Gene=FVER53263_08953;Ontology_term=nucleus,regulation of DNA-templated transcription,transcription regulator activity;Ontology_id=GO:0005634,GO:0006355,GO:0140110
contig00007.g4266	439	ID=contig00007.g4266;Description=hypothetical protein FVEG_08954 [Fusarium verticillioides 7600]
contig00007.g4267	443	ID=contig00007.g4267;Description=hypothetical protein FNAPI_7773 [Fusarium napiforme]
contig00007.g4268	466	ID=contig00007.g4268;Description=hypothetical protein FACUT_3588 [Fusarium acutatum]
contig00007.g4269	3061	ID=contig00007.g4269;Description=arrestin-related trafficking adapter [Fusarium denticulatum];Ontology_term=endosome,vesicle-mediated transport,cytosol,molecular adaptor activity;Ontology_id=GO:0005768,GO:0016192,GO:0005829,GO:0060090
contig00007.g4270	1322	ID=contig00007.g4270;Description=sterol regulatory element binding protein [Fusarium pseudoanthophilum];Gene=FVEG_08958;Ontology_term=nucleus,GTPase activity,regulation of DNA-templated transcription,transcription regulator activity;Ontology_id=GO:0005634,GO:0003924,GO:0006355,GO:0140110;Enzyme_code=EC:3.6.1.15,EC:3.6.1,EC:3.6,EC:3,EC:3.6.1.15;Enzyme_name=nucleoside-triphosphate phosphatase,Acting on acid anhydrides,Acting on acid anhydrides,Hydrolases,nucleoside-triphosphate phosphatase
contig00007.g4271	2101	ID=contig00007.g4271;Description=related to GTP-binding protein 2 [Fusarium oxysporum];Gene=FNAPI_7777;Ontology_term=GTPase activity;Ontology_id=GO:0003924;Enzyme_code=EC:3.6.1.15,EC:3.6.1,EC:3.6,EC:3,EC:3.6.1.15;Enzyme_name=nucleoside-triphosphate phosphatase,Acting on acid anhydrides,Acting on acid anhydrides,Hydrolases,nucleoside-triphosphate phosphatase
contig00007.g4272	599	ID=contig00007.g4272;Description=U4/U6-U5 snRNP complex subunit lsm3 [Fusarium musae];Gene=LSM3;Ontology_term=mRNA metabolic process,RNA binding,protein-containing complex assembly,ribosome biogenesis,chromosome,nucleolus;Ontology_id=GO:0016071,GO:0003723,GO:0065003,GO:0042254,GO:0005694,GO:0005730
contig00007.g4273	2130	ID=contig00007.g4273;Description=hypothetical protein FVER14953_08961 [Fusarium verticillioides];Gene=FPANT_5617;Ontology_term=mitochondrion;Ontology_id=GO:0005739
contig00007.g4274	2075	ID=contig00007.g4274;Description=hypothetical protein J7337_005430 [Fusarium musae]
contig00007.g4275	2761	ID=contig00007.g4275;Description=hypothetical protein FVEG_08963 [Fusarium verticillioides 7600]

contig00007.g4276	3837	ID=contig00007.g4276;Description=ELG1-like protein [Fusarium mundagurra];Gene=FVER53263_08964;Ontology_term=DNA repair,catalytic activity, acting on DNA,DNA replication,DNA recombination,nuclear chromosome,chromosome segregation,DNA binding,ATP-dependent activity,mitochondrion,mitotic nuclear division,telomere organization,hydrolase activity;Ontology_id=GO:0006281,GO:0140097,GO:0006260,GO:0006310,GO:0000228,GO:0007059,GO:0003677,GO:0140657,GO:0005739,GO:0140014,GO:0032200,GO:0016787;Enzyme_code=EC:3.6.1.15,EC:3.6.1,EC:3.6,EC:3,EC:3.6.1.15;Enzyme_name=nucleoside-triphosphate phosphatase,Acting on acid anhydrides,Acting on acid anhydrides,Hydrolases,nucleoside-triphosphate phosphatase
contig00007.g4277	672	ID=contig00007.g4277;Description=hypothetical protein FPCIR_4053 [Fusarium pseudocircinatum]
contig00007.g4278	2104	ID=contig00007.g4278;Description=meiotically up-regulated 80 [Fusarium tjaetaba];Gene=FVER53263_08967;Ontology_term=protein kinase binding;Ontology_id=GO:0019901
contig00007.g4279	1058	ID=contig00007.g4279;Description=CHS7 protein [Fusarium fujikuroi];Gene=BFJ63_vAg15785;Ontology_term=carbohydrate metabolic process,vesicle-mediated transport,carbohydrate derivative metabolic process,cell wall organization or biogenesis,cell adhesion,endoplasmic reticulum,protein folding;Ontology_id=GO:0005975,GO:0016192,GO:1901135,GO:0071554,GO:0007155,GO:0005783,GO:0006457
contig00007.g4280	3189	ID=contig00007.g4280;Description=hypothetical protein FVER53590_08969 [Fusarium verticillioides]
contig00007.g4281	2548	ID=contig00007.g4281;Description=S-M checkpoint control rad4 [Fusarium tjaetaba]
contig00007.g4282	1955	ID=contig00007.g4282;Description=Defective in cullin neddylation 1 [Fusarium tjaetaba]
contig00007.g4283	456	ID=contig00007.g4283;Description=putative ribosomal protein MRP17, mitochondrial [Fusarium fujikuroi];Gene=FNAPI_7789;Ontology_term=RNA binding,structural molecule activity,ribosome;Ontology_id=GO:0003723,GO:0005198,GO:0005840
contig00007.g4284	1135	ID=contig00007.g4284;Description=ribosomal MRP17 mitochondrial [Fusarium tjaetaba];Gene=FPCIR_4060;Ontology_term=biosynthetic process;Ontology_id=GO:0009058

contig00007.g4285	5244	ID=contig00007.g4285;Description=DNA repair protein RAD5 [Fusarium verticillioides 7600];Gene=FVEG_16491;Ontology_term=DNA repair,DNA binding,catalytic activity, acting on a protein,catalytic activity, acting on DNA,protein-containing complex assembly,ATP-dependent activity,nuclear chromosome,transferase activity,chromatin organization,hydrolase activity;Ontology_id=GO:0006281,GO:0003677,GO:0140096,GO:0140097,GO:0065003,GO:0140657,GO:0000228,GO:0016740,GO:0006325,GO:0016787;Enzyme_code=EC:3.6.1,EC:3.6.1,EC:2.3,EC:2,EC:3.6,EC:3,EC:3.6.1.15,EC:2.3.2;Enzyme_name=Acting on acid anhydrides,Acting on acid anhydrides,Acyltransferases,Transferases,Acting on acid anhydrides,Hydrolases,nucleoside-triphosphate phosphatase,Acyltransferases
contig00007.g4286	813	ID=contig00007.g4286;Description=alpha-galactosidase a precursor [Fusarium tjaetaba]
contig00007.g4287	1188	ID=contig00007.g4287;Description=hypothetical protein FVEG_08978 [Fusarium verticillioides 7600]
contig00007.g4288	1592	ID=contig00007.g4288;Description=murein transglycosylase [Fusarium proliferatum];Gene=FVEG_08979;Ontology_term=anatomical structure development,carbohydrate derivative metabolic process,receptor ligand activity,immune system process,defense response to other organism,carbohydrate metabolic process,extracellular space,cytoskeleton,cell wall,cell differentiation,cell wall organization or biogenesis,transferase activity,hydrolase activity;Ontology_id=GO:0048856,GO:1901135,GO:0048018,GO:0002376,GO:0098542,GO:0005975,GO:0005615,GO:0005856,GO:0005618,GO:0030154,GO:0071554,GO:0016740,GO:0016787;Enzyme_code=EC:2.4,EC:3.2.1;Enzyme_name=Glycosyltransferases,Glycosylases
contig00007.g4289	1753	ID=contig00007.g4289;Description=hypothetical protein FOWG_13486 [Fusarium oxysporum f. sp. lycopersici MN25]
contig00007.g4290	1119	ID=contig00007.g4290;Description=hypothetical protein FVEG_08981 [Fusarium verticillioides 7600]
contig00007.g4291	2003	ID=contig00007.g4291;Description=probable dihydroxy-acid dehydratase [Fusarium fujikuroi];Gene=FNAPI_7798;Ontology_term=lyase activity,amino acid metabolic process;Ontology_id=GO:0016829,GO:0006520;Enzyme_code=EC:4.2.1.9;Enzyme_name=dihydroxy-acid dehydratase

contig00007.g4292	2523	ID=contig00007.g4292;Description=sorting nexin mvp-1 [Fusarium oxysporum f. sp. lycopersici 4287];Gene=FMUND_10513;Ontology_term=membrane organization,anatomical structure development,DNA recombination,regulation of DNA-templated transcription,receptor ligand activity,lipid binding,cell adhesion,intracellular protein transport,immune system process,nucleus,inflammatory response,extracellular space,endosome,vesicle-mediated transport,autophagy,cell differentiation,cytosol,programmed cell death;Ontology_id=GO:0061024,GO:0048856,GO:0006310,GO:0006355,GO:0048018,GO:0008289,GO:0007155,GO:0006886,GO:0002376,GO:0005634,GO:0006954,GO:0005615,GO:0005768,GO:0016192,GO:0006914,GO:0030154,GO:0005829,GO:0012501
contig00007.g4293	967	ID=contig00007.g4293;Description=50S ribosomal protein L15 [Fusarium verticillioides 7600];Gene=FMUND_10514;Ontology_term=mitochondrial gene expression,mitochondrion,structural molecule activity,ribosome;Ontology_id=GO:0140053,GO:0005739,GO:0005198,GO:0005840
contig00007.g4294	867	ID=contig00007.g4294;Description=Ser Thr phosphatase superfamily protein [Fusarium denticulatum];Gene=BFJ65_g6405;Ontology_term=hydrolase activity;Ontology_id=GO:0016787;Enzyme_code=EC:3,EC:3;Enzyme_name=Hydrolases,Hydrolases
contig00007.g4295	1739	ID=contig00007.g4295;Description=mannosyltransferase [Fusarium verticillioides 7600];Gene=FCIRC_4768;Ontology_term=protein glycosylation,cell wall organization or biogenesis,Golgi apparatus,endoplasmic reticulum,transferase activity;Ontology_id=GO:0006486,GO:0071554,GO:0005794,GO:0005783,GO:0016740;Enzyme_code=EC:2.4.1,EC:2.4,EC:2,EC:2.4.1;Enzyme_name=Glycosyltransferases,Glycosyltransferases,Transferases,Glycosyltransferases
contig00007.g4296	498	ID=contig00007.g4296;Description=hypothetical protein FPCIR_4072 [Fusarium pseudocircinatum]
contig00007.g4297	989	ID=contig00007.g4297;Description=Cytochrome c oxidase subunit 4, mitochondrial [Fusarium culmorum];Gene=FCULG_00011600;Ontology_term=generation of precursor metabolites and energy,transmembrane transport,nucleobase-containing small molecule metabolic process,mitochondrion,carbohydrate derivative metabolic process;Ontology_id=GO:0006091,GO:0055085,GO:0055086,GO:0005739,GO:1901135

contig00007.g4298	1095	ID=contig00007.g4298;Description=nitronate monooxygenase [Fusarium coicis];Gene=FVER53590_08990;Ontology_term=oxidoreductase activity;Ontology_id=GO:0016491;Enzyme_code=EC:1.13.12.16,EC:1.13,EC:1.13.12.16,EC:1,EC:1.13.12;Enzyme_name=nitronate monooxygenase,Acting on single donors with incorporation of molecular oxygen (oxygenases). The oxygen incorporated need not be derived from O2,nitronate monooxygenase,Oxidoreductases,Acting on single donors with incorporation of molecular oxygen (oxygenases). The oxygen incorporated need not be derived from O2
contig00007.g4299	954	ID=contig00007.g4299;Description=acyl thioesterase ii [Fusarium tjaetaba]
contig00007.g4300	1147	ID=contig00007.g4300;Description=thioesterase thiol ester dehydrase-isomerase [Fusarium pseudoanthophilum];Gene=FPANT_5590;Ontology_term=nucleobase-containing small molecule metabolic process,sulfur compound metabolic process,carbohydrate derivative metabolic process,hydrolase activity,isomerase activity;Ontology_id=GO:0055086,GO:0006790,GO:1901135,GO:0016787,GO:0016853;Enzyme_code=EC:5,EC:3.1.2.10,EC:3.1.2.20,EC:3.1.2.3,EC:3.1,EC:3,EC:5,EC:3.1.2.10,EC:3.1.2.20,EC:3.1.2.3,EC:3.1.2;Enzyme_name=Isomerases,formyl-CoA hydrolase,acyl-CoA hydrolase,succinyl-CoA hydrolase,Acting on ester bonds,Hydrolases,Isomerases,formyl-CoA hydrolase,acyl-CoA hydrolase,succinyl-CoA hydrolase,Acting on ester bonds
contig00007.g4301	2226	ID=contig00007.g4301;Description=hypothetical protein FVER53590_08993 [Fusarium verticillioides];Gene=FMAN_07846;Ontology_term=lipid binding;Ontology_id=GO:0008289
contig00007.g4302	1221	ID=contig00007.g4302;Description=hypothetical protein FVER14953_21241 [Fusarium verticillioides]
contig00007.g4303	577	ID=contig00007.g4303;Description=allantoinase 1 [Fusarium denticulatum];Gene=F52700_9846;Ontology_term=nucleobase-containing small molecule metabolic process;Ontology_id=GO:0055086
contig00007.g4304	1642	ID=contig00007.g4304;Description=pre-mRNA-processing factor 19 [Fusarium verticillioides 7600];Gene=FMEXI_6132;Ontology_term=DNA repair,nucleus,catalytic activity, acting on a protein,mRNA metabolic process,protein-containing complex assembly,mitochondrion,cytosol,transferase activity;Ontology_id=GO:0006281,GO:0005634,GO:0140096,GO:0016071,GO:0065003,GO:0005739,GO:0005829,GO:0016740;Enzyme_code=EC:2.3.2,EC:2.3,EC:2,EC:2.3.2.27,EC:2.3.2;Enzyme_name=Acyltransferases,Acyltransferases,Transferases,RING-type E3 ubiquitin transferase,Acyltransferases

contig00007.g4305	1102	ID=contig00007.g4305;Description=cell cycle control [Fusarium tjaetaba];Gene=FPCIR_4081;Ontology_term=DNA repair,DNA binding,protein catabolic process,nuclear chromosome;Ontology_id=GO:0006281,GO:0003677,GO:0030163,GO:0000228
contig00007.g4306	1806	ID=contig00007.g4306;Description=hypothetical protein FVEG_08997 [Fusarium verticillioides 7600]
contig00007.g4307	1038	ID=contig00007.g4307;Description=related to 2,5-diketo-D-gluconic acid reductase [Fusarium fujikuroi];Gene=FVER53263_08998;Ontology_term=oxidoreductase activity;Ontology_id=GO:0016491;Enzyme_code=EC:1,EC:1;Enzyme_name=Oxidoreductases,Oxidoreductases
contig00007.g4308	1499	ID=contig00007.g4308;Description=translation factor pelota [Fusarium verticillioides 7600];Gene=FVER53263_08999;Ontology_term=mRNA metabolic process;Ontology_id=GO:0016071
contig00007.g4309	3200	ID=contig00007.g4309;Description=WD repeat-containing protein [Fusarium tjaetaba]
contig00007.g4310	402	ID=contig00007.g4310;Description=thioredoxin 1 [Fusarium verticillioides 7600]
contig00007.g4311	1514	ID=contig00007.g4311;Description=ribosome assembly rrb1 [Fusarium tjaetaba];Gene=FVEG_09002;Ontology_term=ribosome biogenesis,nucleolus;Ontology_id=GO:0042254,GO:0005730
contig00007.g4312	1367	ID=contig00007.g4312;Description=GDP-mannose transporter [Fusarium oxysporum f. sp. lycopersici 4287];Gene=FSUBG_6727;Ontology_term=transmembrane transport,mitochondrion,protein glycosylation,cytoplasmic vesicle,endoplasmic reticulum,Golgi apparatus,transporter activity;Ontology_id=GO:0055085,GO:0005739,GO:0006486,GO:0031410,GO:0005783,GO:0005794,GO:0005215
contig00007.g4313	1497	ID=contig00007.g4313;Description=pyruvyl transferase 1 [Fusarium denticulatum];Gene=FDENT_2095;Ontology_term=carbohydrate metabolic process,cell wall organization or biogenesis,Golgi apparatus,transferase activity;Ontology_id=GO:0005975,GO:0071554,GO:0005794,GO:0016740;Enzyme_code=EC:2,EC:2.3,EC:2,EC:2.3.1;Enzyme_name=Transferases,Acyltransferases,Transferases,Acyltransferases
contig00007.g4314	1117	ID=contig00007.g4314;Description=GDP-fucose transporter [Fusarium tjaetaba];Gene=FMUND_796;Ontology_term=membrane;Ontology_id=GO:0016020

contig00007.g4315	1317	ID=contig00007.g4315;Description=hypothetical protein FVEG_09006 [Fusarium verticillioides 7600];Gene=FNAPI_4598;Ontology_term=membrane;Ontology_id=GO:0016020
contig00007.g4316	2235	ID=contig00007.g4316;Description=Bikaverin cluster transcription factor bik5 [Fusarium oxysporum f. sp. cubense];Gene=FOIG_10729;Ontology_term=nucleus,DNA binding,regulation of DNA-templated transcription,transcription regulator activity;Ontology_id=GO:0005634,GO:0003677,GO:0006355,GO:0140110
contig00007.g4317	1146	ID=contig00007.g4317;Description=apoptosis-inducing factor 2 [Fusarium tjaetaba];Gene=FVER53263_09008;Ontology_term=oxidoreductase activity;Ontology_id=GO:0016491;Enzyme_code=EC:1,EC:1;Enzyme_name=Oxidoreductases,Oxidoreductases
contig00007.g4318	1539	ID=contig00007.g4318;Description=secretory lipase [Fusarium tjaetaba];Gene=FTJAE_3085;Ontology_term=lipid metabolic process,hydrolase activity;Ontology_id=GO:0006629,GO:0016787;Enzyme_code=EC:3.1.1.3,EC:3.1,EC:3,EC:3.1.1,EC:3.1.1.3;Enzyme_name=triacylglycerol lipase,Acting on ester bonds,Hydrolases,Acting on ester bonds,triacylglycerol lipase
contig00007.g4319	801	ID=contig00007.g4319;Description=spherulin 1B precursor [Fusarium denticulatum];Gene=FDENT_9153;Ontology_term=extracellular region;Ontology_id=GO:0005576
contig00007.g4320	813	ID=contig00007.g4320;Description=hypothetical protein FVEG_09011 [Fusarium verticillioides 7600]
contig00007.g4321	1263	ID=contig00007.g4321;Description=Alpha-ketoglutarate-dependent xanthine dioxygenase xan-1 [Fusarium musae];Gene=FPANT_1569;Ontology_term=nucleobase-containing small molecule metabolic process,oxidoreductase activity;Ontology_id=GO:0055086,GO:0016491;Enzyme_code=EC:1,EC:1;Enzyme_name=Oxidoreductases,Oxidoreductases
contig00007.g4322	1304	ID=contig00007.g4322;Description=taurine catabolism dioxygenase [Fusarium tjaetaba];Gene=FMUND_787;Ontology_term=oxidoreductase activity;Ontology_id=GO:0016491;Enzyme_code=EC:1,EC:1;Enzyme_name=Oxidoreductases,Oxidoreductases

contig00007.g4323	2391	ID=contig00007.g4323;Description=glycoside hydrolase family 5 [Fusarium tjaetaba];Gene=FNYG_11278;Ontology_term=carbohydrate metabolic process,lipid metabolic process,carbohydrate derivative metabolic process,hydrolase activity;Ontology_id=GO:0005975,GO:0006629,GO:1901135,GO:0016787;Enzyme_code=EC:3.2.1,EC:3.2,EC:3,EC:3.2.1;Enzyme_name=Glycosylases,Glycosylases,Hydrolases,Glycosylases
contig00007.g4324	2415	ID=contig00007.g4324;Description=hypothetical protein FVER14953_09015 [Fusarium verticillioides];Gene=BFJ72_g2063;Ontology_term=membrane;Ontology_id=GO:0016020
contig00007.g4325	1560	ID=contig00007.g4325;Description=tetracycline resistance protein [Fusarium bulbicola];Gene=FOPG_15351;Ontology_term=transmembrane transport,transporter activity;Ontology_id=GO:0055085,GO:0005215
contig00007.g4326	1071	ID=contig00007.g4326;Description=oxidoreductase [Fusarium verticillioides 7600];Gene=FNAPI_4586;Ontology_term=nucleotide binding;Ontology_id=GO:0000166;Enzyme_code=EC:1.1.1.369,EC:1.1.1.18;Enzyme_name=D-chiro-inositol 1-dehydrogenase,inositol 2-dehydrogenase
contig00007.g4327	1718	ID=contig00007.g4327;Description=FAD dependent oxidoreductase [Fusarium mexicanum];Gene=FMEXI_6108;Ontology_term=oxidoreductase activity;Ontology_id=GO:0016491;Enzyme_code=EC:1.14.13.8,EC:1.14.13.8,EC:1,EC:1.14,EC:1.14.13;Enzyme_name=flavin-containing monooxygenase,flavin-containing monooxygenase,Oxidoreductases,Acting on paired donors, with incorporation or reduction of molecular oxygen. The oxygen incorporated need not be derived from O2,Acting on paired donors, with incorporation or reduction of molecular oxygen. The oxygen incorporated need not be derived from O2
contig00007.g4328	1940	ID=contig00007.g4328;Description=FAD dependent oxidoreductase [Fusarium tjaetaba];Gene=FTJAE_3074;Ontology_term=oxidoreductase activity;Ontology_id=GO:0016491;Enzyme_code=EC:1.14.13.8;Enzyme_name=flavin-containing monooxygenase
contig00007.g4329	1664	ID=contig00007.g4329;Description=DUF895 domain protein [Fusarium tjaetaba];Gene=FCIRC_4733;Ontology_term=transmembrane transport,transporter activity;Ontology_id=GO:0055085,GO:0005215
contig00007.g4330	1798	ID=contig00007.g4330;Description=Bac-luciferase domain-containing protein [Fusarium keratoplasticum];Gene=CEP51_009355
contig00007.g4331	1584	ID=contig00007.g4331;Description=related to allantoate transport protein [Fusarium fujikuroi IMI 58289];Gene=FVER53590_09022;Ontology_term=transmembrane transport,transporter activity;Ontology_id=GO:0055085,GO:0005215

contig00007.g4332	435	ID=contig00007.g4332;Description=dgpfaetke family [Fusarium globosum]
contig00007.g4333	894	ID=contig00007.g4333;Description=Crumbs like 3 [Fusarium coicis];Gene=FNYG_11267;Ontology_term=membrane;Ontology_id=GO:0016020
contig00007.g4334	1737	ID=contig00007.g4334;Description=hypothetical protein FVEG_09025 [Fusarium verticillioides 7600];Gene=FNAPI_4578;Ontology_term=oxidoreductase activity;Ontology_id=GO:0016491;Enzyme_code=EC:1.3,EC:1.3,EC:1;Enzyme_name=Acting on the CH-CH group of donors,Acting on the CH-CH group of donors,Oxidoreductases
contig00007.g4335	1293	ID=contig00007.g4335;Description=alpha-1 6-mannosyltransferase HOC1 [Fusarium subglutinans];Gene=FNYG_11265;Ontology_term=carbohydrate derivative metabolic process,transferase activity;Ontology_id=GO:1901135,GO:0016740;Enzyme_code=EC:2.4.1,EC:2.4,EC:2,EC:2.4.1;Enzyme_name=Glycosyltransferases,Glycosyltransferases,Transferases,Glycosyltransferases
contig00007.g4336	1506	ID=contig00007.g4336;Description=hard surface induced 3 (sterol glycosyl transferase) [Fusarium tjaetaba];Gene=FMEXI_6100;Ontology_term=transferase activity;Ontology_id=GO:0016740;Enzyme_code=EC:2.3.1,EC:2.3,EC:2,EC:2.3.1;Enzyme_name=Acyltransferases,Acyltransferases,Transferases,Acyltransferases
contig00007.g4337	1392	ID=contig00007.g4337;Description=hypothetical protein FVEG_09028 [Fusarium verticillioides 7600]
contig00007.g4338	1071	ID=contig00007.g4338;Description=1-aminocyclopropane-1-carboxylate deaminase [Fusarium pseudocircinatum];Enzyme_code=EC:3.5.99.7;Enzyme_name=1-aminocyclopropane-1-carboxylate deaminase
contig00007.g4339	3410	ID=contig00007.g4339;Description=potassium sodium efflux P-type ATPase fungal-type [Fusarium tjaetaba];Gene=ENA2;Ontology_term=transmembrane transport,ATP-dependent activity,transporter activity,hydrolase activity;Ontology_id=GO:0055085,GO:0140657,GO:0005215,GO:0016787;Enzyme_code=EC:7.2.2,EC:3.6.1.15,EC:3.6.1,EC:3.6,EC:3,EC:3.6.3.7,EC:7.2.2,EC:7,EC:3.6.1.15;Enzyme_name=Catalysing the translocation of inorganic cations,nucleoside-triphosphate phosphatase,Acting on acid anhydrides,Acting on acid anhydrides,Hydrolases,Acting on acid anhydrides,Catalysing the translocation of inorganic cations,Translocases,nucleoside-triphosphate phosphatase
contig00007.g4340	831	ID=contig00007.g4340;Description=kinase-like domain protein [Fusarium tjaetaba];Gene=FNAPI_8910;Ontology_term=transferase activity;Ontology_id=GO:0016740;Enzyme_code=EC:2.7,EC:2.7,EC:2;Enzyme_name=Transferring phosphorus-containing groups,Transferring phosphorus-containing groups,Transferases
contig00007.g4341	1375	ID=contig00007.g4341;Description=acriflavine sensitivity control ACR-2 [Fusarium tjaetaba]

contig00007.g4342	1734	ID=contig00007.g4342;Description=major facilitator superfamily transporter [Fusarium denticulatum];Gene=FVER53590_09032;Ontology_term=transmembrane transport,mitochondrion,vacuole,transporter activity,plasma membrane;Ontology_id=GO:0055085,GO:0005739,GO:0005773,GO:0005215,GO:0005886
contig00007.g4343	397	ID=contig00007.g4343;Description=small secreted protein [Fusarium tjaetaba]
contig00007.g4344	593	ID=contig00007.g4344;Description=NTF2-like domain superfamily [Fusarium oxysporum f. sp. vasinfectum]
contig00007.g4345	1758	ID=contig00007.g4345;Description=glycoside hydrolase family 43 [Fusarium tjaetaba];Gene=FTJAE_13832;Ontology_term=carbohydrate metabolic process,cell wall organization or biogenesis,hydrolase activity;Ontology_id=GO:0005975,GO:0071554,GO:0016787;Enzyme_code=EC:3.2.1,EC:3.2,EC:3,EC:3.2.1;Enzyme_name=Glycosylases,Glycosylases,Hydrolases,Glycosylases
contig00007.g4346	891	ID=contig00007.g4346;Description=ribonuclease [Fusarium tjaetaba];Gene=FDENT_12315;Ontology_term=RNA binding,lyase activity,catalytic activity, acting on RNA,hydrolase activity;Ontology_id=GO:0003723,GO:0016829,GO:0140098,GO:0016787;Enzyme_code=EC:3.1,EC:4.6.1.19,EC:3.1,EC:3,EC:4,EC:3.1.27.1,EC:4.6,EC:4.6.1.19;Enzyme_name=Acting on ester bonds,ribonuclease T2,Acting on ester bonds,Hydrolases,Lyases,Acting on ester bonds,Phosphorus-oxygen lyases,ribonuclease T2
contig00007.g4347	542	ID=contig00007.g4347;Description=small secreted protein [Fusarium mundagurra]
contig00007.g4348	1217	ID=contig00007.g4348;Description=hypothetical protein BFJ72_g2351 [Fusarium proliferatum];Gene=FFB14_02750;Ontology_term=membrane;Ontology_id=GO:0016020
contig00007.g4349	1756	ID=contig00007.g4349;Description=Cytochrome P450 monooxygenase trt6 [Fusarium proliferatum];Gene=C2S_13185;Ontology_term=DNA binding,catalytic activity, acting on DNA,oxidoreductase activity,DNA replication,transferase activity;Ontology_id=GO:0003677,GO:0140097,GO:0016491,GO:0006260,GO:0016740;Enzyme_code=EC:1.14,EC:2.7.7.7,EC:1,EC:1.14,EC:2.7,EC:2,EC:2.7.7.7,EC:2.7.7;Enzyme_name=Acting on paired donors, with incorporation or reduction of molecular oxygen. The oxygen incorporated need not be derived from O2,DNA-directed DNA polymerase,Oxidoreductases,Acting on paired donors, with incorporation or reduction of molecular oxygen. The oxygen incorporated need not be derived from O2,Transferring phosphorus-containing groups,Transferases,DNA-directed DNA polymerase,Transferring phosphorus-containing groups

		ID=contig00007.g4350;Description=ent-kaurene oxidase [Fusarium pseudoanthophilum];Gene=F25303_10491;Ontology_term=oxidoreductase activity;Ontology_id=GO:0016491;Enzyme_code=EC:1.14,EC:1,EC:1.14;Enzyme_name=Acting on paired donors, with incorporation or reduction of molecular oxygen. The oxygen incorporated need not be derived from O2,Oxidoreductases,Acting on paired donors, with incorporation or reduction of molecular oxygen. The oxygen incorporated need not be derived from O2
contig00007.g4350	1659	
		ID=contig00007.g4351;Description=hypothetical protein FVEG_09040 [Fusarium verticillioides 7600];Gene=FVEG_09040;Ontology_term=nucleus,regulation of DNA-templated transcription,transcription regulator activity;Ontology_id=GO:0005634,GO:0006355,GO:0140110
contig00007.g4351	2324	
		ID=contig00007.g4352;Description=hypothetical protein FVER14953_09041 [Fusarium verticillioides]
contig00007.g4352	698	
contig00007.g4353	1312	ID=contig00007.g4353;Description=major facilitator superfamily transporter [Fusarium tjaetaba];Gene=FOC4_g10009318;Ontology_term=transmembrane transport,transporter activity;Ontology_id=GO:0055085,GO:0005215
contig00007.g4354	723	ID=contig00007.g4354;Description=Actin-binding FH2 [Fusarium agapanthi]
		ID=contig00007.g4355;Description=metallophosphoesterase domain protein [Fusarium tjaetaba];Gene=FNAPI_367;Ontology_term=hydrolase activity;Ontology_id=GO:0016787;Enzyme_code=EC:3,EC:3;Enzyme_name=Hydrolases,Hydrolases
contig00007.g4355	978	
contig00007.g4356	763	ID=contig00007.g4356;Description=hypothetical protein FACUT_5496 [Fusarium acutatum]
contig00007.g4357	988	ID=contig00007.g4357;Description=isoflavone reductase family [Fusarium tjaetaba]
contig00007.g4358	1647	ID=contig00007.g4358;Description=GABA permease [Fusarium tjaetaba];Gene=FNAPI_364;Ontology_term=transmembrane transport,transporter activity;Ontology_id=GO:0055085,GO:0005215
contig00007.g4359	3265	ID=contig00007.g4359;Description=related to diaminopropionate ammonia-lyase [Fusarium fujikuroi];Gene=FVEG_16508;Ontology_term=catalytic activity, acting on a protein,lyase activity,extracellular region,hydrolase activity;Ontology_id=GO:0140096,GO:0016829,GO:0005576,GO:0016787;Enzyme_code=EC:3.4,EC:4,EC:3.4,EC:3,EC:4;Enzyme_name=Acting on peptide bonds (peptidases),Lyases,Acting on peptide bonds (peptidases),Hydrolases,Lyases
		ID=contig00007.g4360;Description=hypothetical protein FVEG_09047 [Fusarium verticillioides 7600]
contig00007.g4360	1554	

contig00007.g4361	2151	ID=contig00007.g4361;Description=glycoside hydrolase family 2 [Fusarium coicis];Gene=FNYG_11243;Ontology_term=carbohydrate metabolic process,hydrolase activity;Ontology_id=GO:0005975,GO:0016787;Enzyme_code=EC:3.2.1,EC:3.2,EC:3,EC:3.2.1;Enzyme_name=Glycosylases,Glycosylases,Hydrolases,Glycosylases
contig00007.g4362	1888	ID=contig00007.g4362;Description=sporulation-specific Sps2p [Fusarium tjaetaba];Gene=FACUT_5490;Ontology_term=membrane;Ontology_id=GO:0016020
contig00007.g4363	764	ID=contig00007.g4363;Description=GNAT family acetyltransferase [Fusarium pseudoanthophilum];Gene=FVEG_09051;Ontology_term=transferase activity;Ontology_id=GO:0016740;Enzyme_code=EC:2.3.1,EC:2.3,EC:2,EC:2.3.1;Enzyme_name=Acyltransferases,Acyltransferases,Transferases,Acyltransferases
contig00007.g4364	2526	ID=contig00007.g4364;Description=transcriptional regulatory [Fusarium tjaetaba];Gene=FPANT_11327;Ontology_term=nucleus,DNA binding,regulation of DNA-templated transcription,transcription regulator activity;Ontology_id=GO:0005634,GO:0003677,GO:0006355,GO:0140110
contig00007.g4365	2122	ID=contig00007.g4365;Description=hypothetical protein FCOIX_4215 [Fusarium coicis]
contig00007.g4366	831	ID=contig00007.g4366;Description=hypothetical protein FPHYL_6713 [Fusarium phyllophilum]
contig00007.g4367	1363	ID=contig00007.g4367;Description=mitochondrial intermembrane space import and assembly 40 [Fusarium tjaetaba]
contig00007.g4368	3697	ID=contig00007.g4368;Description=nik-1 (Os-1p) [Fusarium tjaetaba];Gene=FNAPI_353;Ontology_term=catalytic activity, acting on a protein,signaling,transferase activity,small molecule sensor activity;Ontology_id=GO:0140096,GO:0023052,GO:0016740,GO:0140299;Enzyme_code=EC:2.7.3,EC:2.7.13.3;Enzyme_name=Transferring phosphorus-containing groups,histidine kinase
contig00007.g4369	720	ID=contig00007.g4369;Description=isoprenylcysteine carboxyl methyltransferase [Fusarium tjaetaba];Gene=FNAPI_352;Ontology_term=catalytic activity, acting on a protein,endoplasmic reticulum,transferase activity;Ontology_id=GO:0140096,GO:0005783,GO:0016740;Enzyme_code=EC:2.1.1.100,EC:2.1,EC:2.1.1.100,EC:2,EC:2.1.1;Enzyme_name=protein-S-isoprenylcysteine O-methyltransferase,Transferring one-carbon groups,protein-S-isoprenylcysteine O-methyltransferase,Transferases,Transferring one-carbon groups

contig00007.g4370	1200	ID=contig00007.g4370;Description=6-aminohexanoate-dimer hydrolase [Fusarium tjaetaba];Gene=FPHYL_6718;Ontology_term=hydrolase activity;Ontology_id=GO:0016787;Enzyme_code=EC:3,EC:3;Enzyme_name=Hydrolases,Hydrolases
contig00007.g4371	1147	ID=contig00007.g4371;Description=isopenicillin-N N-acyltransferase [Fusarium verticillioides 7600];Gene=FOYG_08590;Ontology_term=transferase activity;Ontology_id=GO:0016740;Enzyme_code=EC:2.3;Enzyme_name=Acyltransferases
contig00007.g4372	783	ID=contig00007.g4372;Description=isopenicillin-N N-acyltransferase [Fusarium proliferatum];Gene=F25303_8656;Ontology_term=membrane organization,vesicle-mediated transport,endosome,Golgi apparatus,endoplasmic reticulum,transferase activity,intracellular protein transport,molecular adaptor activity;Ontology_id=GO:0061024,GO:0016192,GO:0005768,GO:0005794,GO:0005783,GO:0016740,GO:0006886,GO:0060090;Enzyme_code=EC:2.3,EC:2.3,EC:2;Enzyme_name=Acyltransferases,Acyltransferases,Transferases
contig00007.g4373	895	ID=contig00007.g4373;Description=thiamine pyrophosphokinase [Fusarium oxysporum];Gene=FPANT_11336;Ontology_term=sulfur compound metabolic process,transferase activity,vitamin metabolic process;Ontology_id=GO:0006790,GO:0016740,GO:0006766;Enzyme_code=EC:2.7.6.2;Enzyme_name=thiamine diphosphokinase
contig00007.g4374	1144	ID=contig00007.g4374;Description=mitochondrial intermembrane space import and assembly 40 [Fusarium subglutinans];Gene=FVER53263_09061;Ontology_term=catalytic activity, acting on a protein,transmembrane transport,oxidoreductase activity,mitochondrion,intracellular protein transport,protein folding,mitochondrion organization,isomerase activity;Ontology_id=GO:0140096,GO:0055085,GO:0016491,GO:0005739,GO:0006886,GO:0006457,GO:0007005,GO:0016853;Enzyme_code=EC:1.8,EC:5.3.4.1,EC:1,EC:1.8,EC:1.8.3,EC:5.3,EC:5.3.4,EC:5,EC:1.8.3.2;Enzyme_name=Acting on a sulfur group of donors,protein disulfide-isomerase,Oxidoreductases,Acting on a sulfur group of donors,Acting on a sulfur group of donors,Intramolecular oxidoreductases,Intramolecular oxidoreductases,Isomerases,thiol oxidase

contig00007.g4375	2313	ID=contig00007.g4375;Description=D-serine dehydratase [Fusarium tjaetaba];Gene=FVEG_09063;Ontology_term=lyase activity,amino acid metabolic process;Ontology_id=GO:0016829,GO:0006520;Enzyme_code=EC:4,EC:4.3,EC:4.3.1.18,EC:4,EC:4.3.1;Enzyme_name=Lyases,Carbon-nitrogen lyases,D-serine ammonia-lyase,Lyases,Carbon-nitrogen lyases
contig00007.g4376	1584	ID=contig00007.g4376;Description=dityrosine transporter [Fusarium tjaetaba];Gene=FPANT_11339;Ontology_term=transmembrane transport,oxidoreductase activity,transporter activity;Ontology_id=GO:0055085,GO:0016491,GO:0005215;Enzyme_code=EC:1.14,EC:1,EC:1.14;Enzyme_name=Acting on paired donors, with incorporation or reduction of molecular oxygen. The oxygen incorporated need not be derived from O2,Oxidoreductases,Acting on paired donors, with incorporation or reduction of molecular oxygen. The oxygen incorporated need not be derived from O2
contig00007.g4377	1552	ID=contig00007.g4377;Description=cytochrome P450 monooxygenase [Fusarium tjaetaba];Gene=FNAPI_345;Ontology_term=transmembrane transport,oxidoreductase activity,transporter activity;Ontology_id=GO:0055085,GO:0016491,GO:0005215;Enzyme_code=EC:1.14;Enzyme_name=Acting on paired donors, with incorporation or reduction of molecular oxygen. The oxygen incorporated need not be derived from O2
contig00007.g4378	849	ID=contig00007.g4378;Description=pisatin demethylase [Fusarium tjaetaba];Gene=FVER53590_09065;Ontology_term=transferase activity;Ontology_id=GO:0016740;Enzyme_code=EC:2.1.1,EC:2.1,EC:2,EC:2.1.1;Enzyme_name=Transferring one-carbon groups,Transferring one-carbon groups,Transferases,Transferring one-carbon groups
contig00007.g4379	969	ID=contig00007.g4379;Description=related to lipase/esterase [Fusarium proliferatum ET1];Gene=FNAPI_343;Ontology_term=hydrolase activity;Ontology_id=GO:0016787;Enzyme_code=EC:3,EC:3;Enzyme_name=Hydrolases,Hydrolases

contig00007.g4380	4063	ID=contig00007.g4380;Description=ATPase [Fusarium verticillioides 7600];Gene=FPHYL_6727;Ontology_term=transmembrane transport,protein-containing complex assembly,ATP-dependent activity,mitochondrion,endoplasmic reticulum,lipid binding,Golgi apparatus,transporter activity,plasma membrane,hydrolase activity;Ontology_id=GO:0055085,GO:0065003,GO:0140657,GO:0005739,GO:0005783,GO:0008289,GO:0005794,GO:0005215,GO:0005886,GO:0016787;Enzyme_code=EC:7.2.2.10,EC:3.6.1.15,EC:3.6.1,EC:7.2.2.10,EC:3.6,EC:3,EC:3.6.3.8,EC:7,EC:7.2.2,EC:3.6.1.15;Enzyme_name=P-type Ca(2+) transporter,nucleoside-triphosphate phosphatase,Acting on acid anhydrides,P-type Ca(2+) transporter,Acting on acid anhydrides,Hydrolases,Acting on acid anhydrides,Translocases,Catalysing the translocation of inorganic cations,nucleoside-triphosphate phosphatase
contig00007.g4381	1352	ID=contig00007.g4381;Description=malate dehydrogenase, mitochondrial [Fusarium verticillioides 7600];Gene=FPANT_11344;Ontology_term=generation of precursor metabolites and energy,oxidoreductase activity;Ontology_id=GO:0006091,GO:0016491;Enzyme_code=EC:1.1.1.37;Enzyme_name=malate dehydrogenase
contig00007.g4382	1575	ID=contig00007.g4382;Description=putative zinc finger motif, C2HC5-type-domain-containing protein [Fusarium oxysporum Fo47];Gene=FPANT_11345;Ontology_term=nucleus,mitochondrion,regulation of DNA-templated transcription,transcription regulator activity,molecular adaptor activity;Ontology_id=GO:0005634,GO:0005739,GO:0006355,GO:0140110,GO:0060090
contig00007.g4383	1761	ID=contig00007.g4383;Description=hypothetical protein FACUT_5467 [Fusarium acutatum];Gene=FDENT_3571;Ontology_term=nucleus;Ontology_id=GO:0005634
contig00007.g4384	873	ID=contig00007.g4384;Description=hypothetical protein FVEG_09071 [Fusarium verticillioides 7600]
contig00007.g4385	1907	ID=contig00007.g4385;Description=cysteine synthase A [Fusarium verticillioides 7600]
contig00007.g4386	1788	ID=contig00007.g4386;Description=tripeptidyl-peptidase sed2 [Fusarium coicis];Gene=FMUND_6306;Ontology_term=extracellular space,catalytic activity, acting on a protein,hydrolase activity;Ontology_id=GO:0005615,GO:0140096,GO:0016787;Enzyme_code=EC:3.4.21,EC:3.4.21,EC:3.4,EC:3,EC:3.4.14.9;Enzyme_name=Acting on peptide bonds (peptidases),Acting on peptide bonds (peptidases),Acting on peptide bonds (peptidases),Hydrolases,tripeptidyl-peptidase I

contig00007.g4387	2966	ID=contig00007.g4387;Description=NAD-dependent histone deacetylase HST3 [Fusarium tjaetaba];Gene=FVEG_09074;Ontology_term=nucleus,DNA repair,catalytic activity, acting on a protein,lipid metabolic process,DNA recombination,regulation of DNA-templated transcription,telomere organization,chromatin organization,transferase activity,hydrolase activity;Ontology_id=GO:0005634,GO:0006281,GO:0140096,GO:0006629,GO:0006310,GO:0006355,GO:0032200,GO:0006325,GO:0016740,GO:0016787;Enzyme_code=EC:2,EC:3.5.1,EC:4.4.1.36,EC:3.5.1.98,EC:2,EC:3.5,EC:3;Enzyme_name=Transferases,Acting on carbon-nitrogen bonds, other than peptide bonds,hercynylcysteine S-oxide lyase,histone deacetylase,Transferases,Acting on carbon-nitrogen bonds, other than peptide bonds,Hydrolases
contig00007.g4388	1462	ID=contig00007.g4388;Description=isopenicillin N epimerase [Fusarium tjaetaba];Gene=BFJ72_g2382;Ontology_term=lyase activity,transferase activity;Ontology_id=GO:0016829,GO:0016740;Enzyme_code=EC:2,EC:4;Enzyme_name=Transferases,Lyases
contig00007.g4389	381	ID=contig00007.g4389;Description=hypothetical protein FVER14953_09076 [Fusarium verticillioides]
contig00007.g4390	1013	ID=contig00007.g4390;Description=thiamine biosynthetic enzyme [Fusarium oxysporum f. sp. lycopersici 4287];Gene=FOYG_08608;Ontology_term=nucleus,sulfur compound metabolic process,mitochondrion,cytosol,transferase activity,vitamin metabolic process,mitochondrion organization;Ontology_id=GO:0005634,GO:0006790,GO:0005739,GO:0005829,GO:0016740,GO:0006766,GO:0007005;Enzyme_code=EC:2.4.2,EC:2.4.2,EC:2.4,EC:2;Enzyme_name=Glycosyltransferases,Glycosyltransferases,Glycosyltransferases,Transferases
contig00007.g4391	786	ID=contig00007.g4391;Description=hypothetical protein FVER53263_09078 [Fusarium verticillioides]
contig00007.g4392	2497	ID=contig00007.g4392;Description=transcriptional regulator Medusa [Fusarium mexicanum]

contig00007.g4393	3002	ID=contig00007.g4393;Description=minichromosome maintenance protein 6 [Fusarium verticillioides 7600];Gene=BFJ63_vAg385;Ontology_term=DNA repair,catalytic activity, acting on DNA,protein-containing complex assembly,DNA replication,DNA recombination,reproductive process,nuclear chromosome,mitotic cell cycle,nucleoplasm,DNA binding,RNA binding,ATP-dependent activity,catalytic activity, acting on RNA,hydrolase activity;Ontology_id=GO:0006281,GO:0140097,GO:0065003,GO:0006260,GO:0006310,GO:0022414,GO:0000228,GO:0000278,GO:0005654,GO:0003677,GO:0003723,GO:0140657,GO:0140098,GO:0016787;Enzyme_code=EC:5.6.2.4,EC:3.6.1.15;Enzyme_name=DNA 3'-5' helicase,nucleoside-triphosphate phosphatase
contig00007.g4394	3833	ID=contig00007.g4394;Description=hypothetical protein FVER14953_09086 [Fusarium verticillioides]
contig00007.g4395	665	ID=contig00007.g4395;Description=mitochondrial F1-F0 ATP synthase subunit F of fungi-domain-containing protein [Fusarium redolens];Gene=FIE12Z_5568;Ontology_term=nucleobase-containing small molecule metabolic process,mitochondrion,carbohydrate derivative metabolic process;Ontology_id=GO:0055086,GO:0005739,GO:1901135
contig00007.g4396	724	ID=contig00007.g4396;Description=NADH dehydrogenase (ubiquinone) Fe-S protein 4 [Fusarium verticillioides 7600];Gene=FTJAE_12423;Ontology_term=generation of precursor metabolites and energy,mitochondrion;Ontology_id=GO:0006091,GO:0005739
contig00007.g4397	1182	ID=contig00007.g4397;Description=LisH motif-containing protein [Fusarium fujikuroi];Gene=AU210_008270;Ontology_term=carbohydrate metabolic process,protein catabolic process;Ontology_id=GO:0005975,GO:0030163
contig00007.g4398	1835	ID=contig00007.g4398;Description=uridine kinase [Fusarium verticillioides 7600];Gene=FOYG_08619;Ontology_term=nucleobase-containing small molecule metabolic process,carbohydrate derivative metabolic process,transferase activity;Ontology_id=GO:0055086,GO:1901135,GO:0016740;Enzyme_code=EC:2.7;Enzyme_name=Transferring phosphorus-containing groups
contig00007.g4399	2110	ID=contig00007.g4399;Description=NADH dehydrogenase [Fusarium verticillioides 7600];Gene=BFJ68_g10893;Ontology_term=peroxisome,nucleobase-containing small molecule metabolic process,oxidoreductase activity,mitochondrion;Ontology_id=GO:0005777,GO:0055086,GO:0016491,GO:0005739;Enzyme_code=EC:1.6,EC:1.6,EC:1,EC:1.6.99.3;Enzyme_name=Acting on NADH or NADPH,Acting on NADH or NADPH,Oxidoreductases,Deleted entry

contig00007.g4400	1370	ID=contig00007.g4400;Description=myosin heavy chain [Fusarium tjaetaba];Gene=rrp16;Ontology_term=protein-containing complex assembly,ribosome biogenesis,nucleolus,nucleoplasm;Ontology_id=GO:0065003,GO:0042254,GO:0005730,GO:0005654
contig00007.g4401	930	ID=contig00007.g4401;Description=Ras-related protein Rab-6B [Fusarium keratoplasticum];Gene=CI238_02368;Ontology_term=GTPase activity;Ontology_id=GO:0003924;Enzyme_code=EC:3.6.1.15;Enzyme_name=nucleoside-triphosphate phosphatase
contig00007.g4402	902	ID=contig00007.g4402;Description=ribose 5-phosphate isomerase A [Fusarium verticillioides 7600];Gene=FVER53263_09094;Ontology_term=nucleus,carbohydrate metabolic process,generation of precursor metabolites and energy,nucleobase-containing small molecule metabolic process,carbohydrate derivative metabolic process,vitamin metabolic process,isomerase activity;Ontology_id=GO:0005634,GO:0005975,GO:0006091,GO:0055086,GO:1901135,GO:0006766,GO:0016853;Enzyme_code=EC:5.3.1.6,EC:5.3,EC:5,EC:5.3.1,EC:5.3.1.6;Enzyme_name=ribose-5-phosphate isomerase,Intramolecular oxidoreductases,Isomerases,Intramolecular oxidoreductases,ribose-5-phosphate isomerase
contig00007.g4403	865	ID=contig00007.g4403;Description=Inositolphosphorylceramide synthase subunit Kei1-domain-containing protein [Fusarium oxysporum Fo47];Gene=FNYG_11199;Ontology_term=molecular function regulator activity,lipid metabolic process;Ontology_id=GO:0098772,GO:0006629
contig00007.g4404	5065	ID=contig00007.g4404;Description=sulfite reductase (ferredoxin) [Fusarium verticillioides 7600];Gene=FOPG_09000;Ontology_term=sulfur compound metabolic process,oxidoreductase activity;Ontology_id=GO:0006790,GO:0016491;Enzyme_code=EC:1.8.1.2;Enzyme_name=assimilatory sulfite reductase (NADPH)

contig00007.g4405	3606	ID=contig00007.g4405;Description=Chitin synthase, class 2 [Fusarium musae];Gene=FPANT_6470;Ontology_term=carbohydrate metabolic process,cytoskeleton,cytoskeleton organization,anatomical structure development,cell differentiation,cytokinesis,carbohydrate derivative metabolic process,cell wall organization or biogenesis,transferase activity,mitotic cell cycle,plasma membrane;Ontology_id=GO:0005975,GO:0005856,GO:0007010,GO:0048856,GO:0030154,GO:0000910,GO:1901135,GO:0071554,GO:0016740,GO:0000278,GO:0005886;Enzyme_code=EC:2.4.1.16,EC:2.4,EC:2.4.1.16,EC:2,EC:2.4.1;Enzyme_name=chitin synthase,Glycosyltransferases,chitin synthase,Transferases,Glycosyltransferases
contig00007.g4406	1948	ID=contig00007.g4406;Description=myo-inositol transport ITR1 [Fusarium tjaetaba];Gene=FPANT_6471;Ontology_term=transmembrane transport,transporter activity;Ontology_id=GO:0055085,GO:0005215
contig00007.g4407	2909	ID=contig00007.g4407;Description=hypothetical protein FVER14953_09099 [Fusarium verticillioides];Gene=FMAN_07956;Ontology_term=hydrolase activity;Ontology_id=GO:0016787;Enzyme_code=EC:3.5,EC:3.5,EC:3;Enzyme_name=Acting on carbon-nitrogen bonds, other than peptide bonds,Acting on carbon-nitrogen bonds, other than peptide bonds,Hydrolases
contig00007.g4408	918	ID=contig00007.g4408;Description=hypothetical protein FVER14953_09100 [Fusarium verticillioides];Gene=FANTH_7387;Ontology_term=oxidoreductase activity;Ontology_id=GO:0016491;Enzyme_code=EC:1.13,EC:1.13,EC:1;Enzyme_name=Acting on single donors with incorporation of molecular oxygen (oxygenases). The oxygen incorporated need not be derived from O2,Acting on single donors with incorporation of molecular oxygen (oxygenases). The oxygen incorporated need not be derived from O2,Oxidoreductases
contig00007.g4409	904	ID=contig00007.g4409;Description=glutamine amidotransferase [Fusarium tjaetaba];Gene=FTJAE_12436;Ontology_term=amino acid metabolic process,transferase activity;Ontology_id=GO:0006520,GO:0016740;Enzyme_code=EC:2,EC:2;Enzyme_name=Transferases,Transferases
contig00007.g4410	1455	ID=contig00007.g4410;Description=CMGC/SRPK protein kinase [Fusarium verticillioides 7600];Gene=FGLOB1_4642;Ontology_term=catalytic activity, acting on a protein,transferase activity;Ontology_id=GO:0140096,GO:0016740;Enzyme_code=EC:2.7.1,EC:2.7.1,EC:2.7.11.1,EC:2.7,EC:2;Enzyme_name=Transferring phosphorus-containing groups,Transferring phosphorus-containing groups,non-specific serine/threonine protein kinase,Transferring phosphorus-containing groups,Transferases

contig00007.g4411	1419	ID=contig00007.g4411;Description=protein LDB19 [Fusarium fujikuroi];Ontology_term=transmembrane transport,vesicle-mediated transport,cytosol,cytoplasmic vesicle,Golgi apparatus,plasma membrane,molecular adaptor activity;Ontology_id=GO:0055085,GO:0016192,GO:0005829,GO:0031410,GO:0005794,GO:0005886,GO:0060090
contig00007.g4412	1293	ID=contig00007.g4412;Description=hypothetical protein FVER53590_09105 [Fusarium verticillioides];Gene=FVER53590_09105;Ontology_term=membrane;Ontology_id=GO:0016020
contig00007.g4413	1203	ID=contig00007.g4413;Description=distribution and morphology protein 10 [Fusarium oxysporum f. sp. albedinis];Gene=FNYG_11189;Ontology_term=membrane;Ontology_id=GO:0016020
contig00007.g4414	1384	ID=contig00007.g4414;Description=mitochondrial distribution and morphology 10 [Fusarium tjaetaba];Gene=MDM10;Ontology_term=membrane organization,protein-containing complex assembly,mitochondrion,endoplasmic reticulum,intracellular protein transport,mitochondrion organization;Ontology_id=GO:0061024,GO:0065003,GO:0005739,GO:0005783,GO:0006886,GO:0007005
contig00007.g4415	1035	ID=contig00007.g4415;Description=membrane zinc transporter [Fusarium tjaetaba];Gene=FVEG_09108;Ontology_term=endosome,vesicle-mediated transport,transmembrane transport,GTPase activity,signaling,cytosol,Golgi apparatus,endoplasmic reticulum,transporter activity,plasma membrane,lysosome;Ontology_id=GO:0005768,GO:0016192,GO:0055085,GO:0003924,GO:0023052,GO:0005829,GO:0005794,GO:0005783,GO:0005215,GO:0005886,GO:0005764;Enzyme_code=EC:3.6.1.15,EC:3.6.1,EC:3.6,EC:3,EC:3.6.1.15;Enzyme_name=nucleoside-triphosphate phosphatase,Acting on acid anhydrides,Acting on acid anhydrides,Hydrolases,nucleoside-triphosphate phosphatase
contig00007.g4416	791	ID=contig00007.g4416;Description=40S ribosomal protein S19 [Paramyrothecium foliicola];Gene=BFJ69_g11872;Ontology_term=structural molecule activity,ribosome;Ontology_id=GO:0005198,GO:0005840
contig00007.g4417	1158	ID=contig00007.g4417;Description=hypothetical protein FVER14953_09110 [Fusarium verticillioides]

contig00007.g4418	2197	ID=contig00007.g4418;Description=px domain protein [Fusarium tjaetaba];Ontology_term=generation of precursor metabolites and energy,oxidoreductase activity,lipid binding;Ontology_id=GO:0006091,GO:0016491,GO:0008289;Enzyme_code=EC:1;Enzyme_name=Oxidoreductases
contig00007.g4419	2763	ID=contig00007.g4419;Description=hypothetical protein FVER53263_09112 [Fusarium verticillioides];Gene=FVER53590_09112;Ontology_term=lipid binding;Ontology_id=GO:0008289
contig00007.g4420	1069	ID=contig00007.g4420;Description=COP9 signalosome complex subunit 5 [Fusarium verticillioides 7600];Gene=FDENT_3533;Ontology_term=nucleus,catalytic activity, acting on a protein,hydrolase activity;Ontology_id=GO:0005634,GO:0140096,GO:0016787;Enzyme_code=EC:3.4;Enzyme_name=Acting on peptide bonds (peptidases)
contig00007.g4421	3171	ID=contig00007.g4421;Description=hypothetical protein FVER53590_09114 [Fusarium verticillioides];Gene=FNAPI_301;Ontology_term=defense response to other organism,extracellular space,receptor ligand activity;Ontology_id=GO:0098542,GO:0005615,GO:0048018
contig00007.g4422	1546	ID=contig00007.g4422;Description=hypothetical protein FVER53263_09115 [Fusarium verticillioides];Gene=FNAPI_300;Ontology_term=membrane;Ontology_id=GO:0016020
contig00007.g4423	854	ID=contig00007.g4423;Description=cel1 precursor [Fusarium tjaetaba];Gene=FNAPI_299;Ontology_term=extracellular region;Ontology_id=GO:0005576
contig00007.g4424	1203	ID=contig00007.g4424;Description=hypothetical protein FVEG_09117 [Fusarium verticillioides 7600]
contig00007.g4425	1362	ID=contig00007.g4425;Description=hypothetical protein FVEG_09119 [Fusarium verticillioides 7600]
contig00007.g4426	1926	ID=contig00007.g4426;Description=maltose permease [Fusarium coicis];Gene=FVER53590_09120;Ontology_term=transmembrane transport,transporter activity;Ontology_id=GO:0055085,GO:0005215
contig00007.g4427	1353	ID=contig00007.g4427;Description=QDE-2-interacting protein [Fusarium tjaetaba];Gene=FDENT_3526;Ontology_term=hydrolase activity;Ontology_id=GO:0016787;Enzyme_code=EC:3.1,EC:3.1,EC:3;Enzyme_name=Acting on ester bonds,Acting on ester bonds,Hydrolases

contig00007.g4428	327	ID=contig00007.g4428;Description=hypothetical protein FVEG_09122 [Fusarium verticillioides 7600];Gene=FPANT_6493;Ontology_term=membrane;Ontology_id=GO:0016020
contig00007.g4429	9129	ID=contig00007.g4429;Description=FMP27 [Fusarium tjaetaba];Gene=FPANT_6494;Ontology_term=mitochondrion;Ontology_id=GO:0005739
contig00007.g4430	793	ID=contig00007.g4430;Description=Histone-lysine N-methyltransferase, H3 lysine-79 specific [Fusarium oxysporum f. sp. albedinis]
contig00007.g4431	2031	ID=contig00007.g4431;Description=Eukaryotic translation initiation factor 4B [Fusarium oxysporum];Gene=sce3;Ontology_term=translation regulator activity,RNA binding,protein-containing complex assembly,cytoplasmic translation,organelle;Ontology_id=GO:0045182,GO:0003723,GO:0065003,GO:0002181,GO:0043226
contig00007.g4432	621	ID=contig00007.g4432;Description=NUDIX domain protein [Fusarium tjaetaba]
contig00007.g4433	4487	ID=contig00007.g4433;Description=hypothetical protein FVER53590_09127 [Fusarium verticillioides];Gene=FTJAE_14128;Ontology_term=catalytic activity;Ontology_id=GO:0003824
contig00007.g4434	1539	ID=contig00007.g4434;Description=eukaryotic translation initiation factor 3 subunit L [Fusarium verticillioides 7600];Gene=FOVG_06729;Ontology_term=translation regulator activity,RNA binding,protein-containing complex assembly,cytoplasmic translation;Ontology_id=GO:0045182,GO:0003723,GO:0065003,GO:0002181
contig00007.g4435	3232	ID=contig00007.g4435;Description=elongation factor 2 [Fusarium verticillioides 7600];Gene=FNYG_11166;Ontology_term=translation regulator activity,GTPase activity,ribosome biogenesis;Ontology_id=GO:0045182,GO:0003924,GO:0042254;Enzyme_code=EC:3.6.1.15;Enzyme_name=nucleoside-triphosphate phosphatase
contig00007.g4436	1485	ID=contig00007.g4436;Description=integral membrane protein PTH11 [Fusarium coicis];Gene=FCIRC_12302;Ontology_term=membrane;Ontology_id=GO:0016020

contig00007.g4437	1730	ID=contig00007.g4437;Description=histone-lysine n-methyltransferase H3 lysine-79 specific [Fusarium tjaetaba];Gene=FVER53590_09134;Ontology_term=DNA repair,nucleus,catalytic activity, acting on a protein,histone binding,signaling,DNA recombination,regulation of DNA-templated transcription,telomere organization,chromatin organization,transferase activity,mitotic cell cycle,meiotic nuclear division;Ontology_id=GO:0006281,GO:0005634,GO:0140096,GO:0042393,GO:0023052,GO:0006310,GO:0006355,GO:0032200,GO:0006325,GO:0016740,GO:0000278,GO:0140013;Enzyme_code=EC:2.1.1.360,EC:2.1,EC:2.1.1.43,EC:2,EC:2.1.1.360,EC:2.1.1;Enzyme_name=[histone H3]-lysine(79) N-trimethyltransferase,Transferring one-carbon groups,Transferring one-carbon groups,Transferases,[histone H3]-lysine(79) N-trimethyltransferase,Transferring one-carbon groups
contig00007.g4438	1535	ID=contig00007.g4438;Description=histone-lysine N-methyltransferase, H3 lysine-79 specific [Fusarium verticillioides 7600];Gene=BFJ69_g11882;Ontology_term=DNA repair,catalytic activity, acting on a protein,DNA recombination,regulation of DNA-templated transcription,chromosome,chromatin organization,mitotic cell cycle,nucleus,histone binding,signaling,telomere organization,transferase activity,meiotic nuclear division;Ontology_id=GO:0006281,GO:0140096,GO:0006310,GO:0006355,GO:0005694,GO:0006325,GO:0000278,GO:0005634,GO:0042393,GO:0023052,GO:0032200,GO:0016740,GO:0140013;Enzyme_code=EC:2.1.1.360,EC:2.1,EC:2.1.1.43,EC:2,EC:2.1.1.360,EC:2.1.1;Enzyme_name=[histone H3]-lysine(79) N-trimethyltransferase,Transferring one-carbon groups,Transferring one-carbon groups,Transferases,[histone H3]-lysine(79) N-trimethyltransferase,Transferring one-carbon groups
contig00007.g4439	747	ID=contig00007.g4439;Description=autophagy protein 16, interacts with Atg12p-Atg5p [Fusarium musae];Gene=FPCIR_6462;Ontology_term=autophagy,vacuole;Ontology_id=GO:0006914,GO:0005773
contig00007.g4440	2819	ID=contig00007.g4440;Description=vacuolar sorting VACUOLELESS1 [Fusarium tjaetaba];Gene=FTJAE_14134;Ontology_term=membrane organization,vesicle-mediated transport,endosome,protein-containing complex assembly,intracellular protein transport,cytoskeletal protein binding,lysosome;Ontology_id=GO:0061024,GO:0016192,GO:0005768,GO:0065003,GO:0006886,GO:0008092,GO:0005764

contig00007.g4441	1919	ID=contig00007.g4441;Description=dma1 component of spindle assembly checkpoint [Fusarium tjaetaba];Ontology_term=catalytic activity, acting on a protein,cytoskeleton organization,protein-containing complex assembly,establishment or maintenance of cell polarity,chromosome segregation,cytokinesis,nucleus,microtubule organizing center,signaling,mitotic nuclear division,transferase activity;Ontology_id=GO:0140096,GO:0007010,GO:0065003,GO:0007163,GO:0007059,GO:0000910,GO:0005634,GO:0005815,GO:0023052,GO:0140014,GO:0016740;Enzyme_code=EC:2.3,EC:2,EC:2.3.2;Enzyme_name=Acyltransferases,Transferases,Acyltransferases
contig00007.g4442	1747	ID=contig00007.g4442;Description=mitochondrial-processing peptidase subunit alpha [Fusarium verticillioides 7600];Gene=FANTH_10402;Ontology_term=catalytic activity, acting on a protein,hydrolase activity;Ontology_id=GO:0140096,GO:0016787;Enzyme_code=EC:3.4.24;Enzyme_name=Acting on peptide bonds (peptidases)
contig00007.g4443	2757	ID=contig00007.g4443;Description=hypothetical protein FVEG_16525 [Fusarium verticillioides 7600]
contig00007.g4444	5514	ID=contig00007.g4444;Description=ribosomal RNA processing RRP5 [Fusarium subglutinans];Gene=FSUBG_222;Ontology_term=RNA binding,protein-containing complex assembly,ribosome biogenesis,nucleolus;Ontology_id=GO:0003723,GO:0065003,GO:0042254,GO:0005730
contig00007.g4445	473	ID=contig00007.g4445;Description=secreted NIS1 [Fusarium subglutinans]
contig00007.g4446	1860	ID=contig00007.g4446;Description=increased rDNA silencing IRS4 [Fusarium tjaetaba];Ontology_term=autophagy,mitochondrion,signaling,regulation of DNA-templated transcription,cell wall organization or biogenesis,lipid binding,chromatin organization;Ontology_id=GO:0006914,GO:0005739,GO:0023052,GO:0006355,GO:0071554,GO:0008289,GO:0006325
contig00007.g4447	1261	ID=contig00007.g4447;Description=hypothetical protein FVEG_09144 [Fusarium verticillioides 7600];Gene=BFJ72_g2283;Ontology_term=membrane;Ontology_id=GO:0016020

contig00007.g4448	2151	ID=contig00007.g4448;Description=glutaminyl-tRNA synthetase [Fusarium mundagurra];Gene=BFJ69_g11862;Ontology_term=RNA binding,cytoplasmic translation,mitochondrion,cytosol,tRNA metabolic process,amino acid metabolic process,catalytic activity, acting on RNA,ligase activity;Ontology_id=GO:0003723,GO:0002181,GO:0005739,GO:0005829,GO:0006399,GO:0006520,GO:0140098,GO:0016874;Enzyme_code=EC:6.1.1.18,EC:6.1,EC:6.1.1,EC:6,EC:6.1.1.18;Enzyme_name=glutamine--tRNA ligase,Forming carbon-oxygen bonds,Forming carbon-oxygen bonds,Ligases,glutamine--tRNA ligase
contig00007.g4449	2904	ID=contig00007.g4449;Description=nitrate assimilation regulatory nirA [Fusarium denticulatum];Gene=FDENT_402;Ontology_term=nucleus,DNA binding,regulation of DNA-templated transcription,transcription regulator activity;Ontology_id=GO:0005634,GO:0003677,GO:0006355,GO:0140110
contig00007.g4450	2417	ID=contig00007.g4450;Description=microtubule associated (Ase1) [Fusarium coicis];Gene=BFJ68_g15444;Ontology_term=cytoskeleton,cytoskeleton organization,cytoskeletal protein binding;Ontology_id=GO:0005856,GO:0007010,GO:0008092
contig00007.g4451	1030	ID=contig00007.g4451;Description=PMU1-high copy suppressor of ts tps2 mutant phenotype [Fusarium tjaetaba]
contig00007.g4452	909	ID=contig00007.g4452;Description=hypothetical protein FVEG_16527 [Fusarium verticillioides 7600]
contig00007.g4453	1586	ID=contig00007.g4453;Description=manganese peroxidase 2 [Fusarium coicis];Gene=FVER53263_09149;Ontology_term=antioxidant activity,oxidoreductase activity;Ontology_id=GO:0016209,GO:0016491;Enzyme_code=EC:1.11.1,EC:1.11,EC:1,EC:1.11.1.13,EC:1.11.1;Enzyme_name=Acting on a peroxide as acceptor,Acting on a peroxide as acceptor,Oxidoreductases,manganese peroxidase,Acting on a peroxide as acceptor
contig00007.g4454	1002	ID=contig00007.g4454;Description=multidrug transporter [Fusarium musae];Gene=sge1;Ontology_term=DNA binding,transmembrane transport,signaling,nuclear chromosome,regulation of DNA-templated transcription,cell adhesion,transcription regulator activity;Ontology_id=GO:0003677,GO:0055085,GO:0023052,GO:0000228,GO:0006355,GO:0007155,GO:0140110

contig00007.g4455	2842	ID=contig00007.g4455;Description=Tyrosyl-DNA phosphodiesterase 1 [Fusarium oxysporum f. sp. raphani];Gene=FVEG_16529;Ontology_term=DNA repair,nucleus,hydrolase activity;Ontology_id=GO:0006281,GO:0005634,GO:0016787;Enzyme_code=EC:3.1.4,EC:3.1.4,EC:3.1,EC:3;Enzyme_name=Acting on ester bonds,Acting on ester bonds,Acting on ester bonds,Hydrolases
contig00007.g4456	1024	ID=contig00007.g4456;Description=CDP-diacylglycerol-glycerol-3-phosphate 3-phosphatidyltransferase [Fusarium verticillioides 7600];Gene=FPHYL_10292;Ontology_term=membrane organization,mitochondrion,lipid metabolic process,transferase activity,intracellular protein transport,mitochondrion organization;Ontology_id=GO:0061024,GO:0005739,GO:0006629,GO:0016740,GO:0006886,GO:0007005;Enzyme_code=EC:2.7.8,EC:2.7.8.41,EC:2.7,EC:2,EC:2.7.8;Enzyme_name=Transferring phosphorus-containing groups,cardiolipin synthase (CMP-forming),Transferring phosphorus-containing groups,Transferases,Transferring phosphorus-containing groups
contig00007.g4457	4250	ID=contig00007.g4457;Description=tRNA uridine 5-carboxymethylaminomethyl modification enzyme GidA [Fusarium verticillioides 7600];Gene=FPANT_5372;Ontology_term=membrane organization,vesicle-mediated transport,endosome,protein-containing complex assembly,tRNA metabolic process,cytosol,Golgi apparatus,intracellular protein transport;Ontology_id=GO:0061024,GO:0016192,GO:0005768,GO:0065003,GO:0006399,GO:0005829,GO:0005794,GO:0006886
contig00007.g4458	1322	ID=contig00007.g4458;Description=maintenance-mitochondrial morphology 1 [Fusarium tjaetaba];Gene=28951977;Ontology_term=membrane organization,protein-containing complex assembly,mitochondrion,endoplasmic reticulum,lipid binding,intracellular protein transport,transporter activity,mitochondrion organization;Ontology_id=GO:0061024,GO:0065003,GO:0005739,GO:0005783,GO:0008289,GO:0006886,GO:0005215,GO:0007005
contig00007.g4459	1160	ID=contig00007.g4459;Description=CAAX prenyl protease 2 [Fusarium tjaetaba];Gene=FNAPI_9445;Ontology_term=catalytic activity, acting on a protein,endoplasmic reticulum,protein maturation,hydrolase activity;Ontology_id=GO:0140096,GO:0005783,GO:0051604,GO:0016787;Enzyme_code=EC:3.4.24;Enzyme_name=Acting on peptide bonds (peptidases)
contig00007.g4460	2663	ID=contig00007.g4460;Description=hypothetical protein FVER53263_09158 [Fusarium verticillioides];Gene=FNAPI_9446;Ontology_term=membrane;Ontology_id=GO:0016020

contig00007.g4461	1907	ID=contig00007.g4461;Description=mboat family [Fusarium tjaetaba];Gene=FNAPI_9447;Ontology_term=transferase activity;Ontology_id=GO:0016740;Enzyme_code=EC:2.3;Enzyme_name=Acyltransferases
contig00007.g4462	1334	ID=contig00007.g4462;Description=GDP-mannose transporter [Fusarium proliferatum];Gene=FVER53263_09160;Ontology_term=transmembrane transport,mitochondrion,protein glycosylation,cytoplasmic vesicle,endoplasmic reticulum,Golgi apparatus,transporter activity;Ontology_id=GO:0055085,GO:0005739,GO:0006486,GO:0031410,GO:0005783,GO:0005794,GO:0005215
contig00007.g4463	1120	ID=contig00007.g4463;Description=related to ketoreductases [Fusarium mangiferae];Gene=rplY
contig00007.g4464	632	ID=contig00007.g4464;Description=1,2-dihydroxy-3-keto-5-methylthiopentene dioxygenase [Fusarium oxysporum f. sp. rapae];Gene=ADI1-0;Ontology_term=nucleus,sulfur compound metabolic process,oxidoreductase activity,amino acid metabolic process;Ontology_id=GO:0005634,GO:0006790,GO:0016491,GO:0006520;Enzyme_code=EC:1.13.11.53,EC:1.13.11.54;Enzyme_name=acireductone dioxygenase (Ni(2+)-requiring),acireductone dioxygenase (Fe(2+)-requiring)
contig00007.g4465	813	ID=contig00007.g4465;Description=F-LANa [Fusarium pseudoanthophilum];Gene=FPHYL_11924;Ontology_term=protein catabolic process,endoplasmic reticulum,intracellular protein transport;Ontology_id=GO:0030163,GO:0005783,GO:0006886
contig00007.g4466	408	ID=contig00007.g4466;Description=putative histone 3 [Fusarium fujikuroi];Gene=FCIRC_3512;Ontology_term=protein-containing complex assembly,structural molecule activity,nuclear chromosome,chromosome segregation,chromatin organization,DNA binding,RNA binding,signaling,mitotic nuclear division,meiotic nuclear division;Ontology_id=GO:0065003,GO:0005198,GO:0000228,GO:0007059,GO:0006325,GO:0003677,GO:0003723,GO:0023052,GO:0140014,GO:0140013
contig00007.g4467	2054	ID=contig00007.g4467;Description=xaa-Pro aminopeptidase [Fusarium verticillioides 7600];Gene=F52700_5700;Ontology_term=catalytic activity, acting on a protein,hydrolase activity;Ontology_id=GO:0140096,GO:0016787;Enzyme_code=EC:3.4.11;Enzyme_name=Acting on peptide bonds (peptidases)
contig00007.g4468	1173	ID=contig00007.g4468;Description=proteophosphoglycan 5 [Fusarium tjaetaba]

contig00007.g4469	1237	ID=contig00007.g4469;Description=RING-box protein 1 [Fusarium verticillioides 7600];Gene=FOPG_08907;Ontology_term=protein catabolic process,endoplasmic reticulum;Ontology_id=GO:0030163,GO:0005783
contig00007.g4470	1922	ID=contig00007.g4470;Description=divalent cation transporter (aluminum resistance ALR1) [Fusarium subglutinans];Gene=FCIRC_3508;Ontology_term=transmembrane transport,mitochondrion,transporter activity;Ontology_id=GO:0055085,GO:0005739,GO:0005215
contig00007.g4471	345	ID=contig00007.g4471;Description=mitochondrial import inner membrane translocase subunit TIM8 [Fusarium tjaetaba];Gene=FOXYS1_14263;Ontology_term=membrane organization,transmembrane transport,mitochondrion,intracellular protein transport,mitochondrion organization,transporter activity;Ontology_id=GO:0061024,GO:0055085,GO:0005739,GO:0006886,GO:0007005,GO:0005215
contig00007.g4472	991	ID=contig00007.g4472;Description=60S ribosomal protein L25-like protein [Ilyonectria robusta];Gene=FALBO_807;Ontology_term=structural molecule activity,ribosome;Ontology_id=GO:0005198,GO:0005840
contig00007.g4473	876	ID=contig00007.g4473;Description=putative methyltransferase-domain-containing protein [Fusarium oxysporum];Gene=FOMG_07297;Ontology_term=ribosome biogenesis,nucleolus,catalytic activity, acting on RNA,transferase activity;Ontology_id=GO:0042254,GO:0005730,GO:0140098,GO:0016740;Enzyme_code=EC:2.1.1,EC:2.1,EC:2.1.1.286,EC:2,EC:2.1.1;Enzyme_name=Transferring one-carbon groups,Transferring one-carbon groups,25S rRNA (adenine(2142)-N(1))-methyltransferase,Transferases,Transferring one-carbon groups
contig00007.g4474	1295	ID=contig00007.g4474;Description=enolase-phosphatase e1 [Fusarium tjaetaba];Gene=FGADI_4881;Ontology_term=sulfur compound metabolic process,amino acid metabolic process,hydrolase activity;Ontology_id=GO:0006790,GO:0006520,GO:0016787;Enzyme_code=EC:3.1.3.77;Enzyme_name=acireductone synthase

contig00007.g4475	1914	ID=contig00007.g4475;Description=cAMP-dependent protein kinase catalytic subunit [Fusarium oxysporum];Gene=FOQG_06056;Ontology_term=nucleus,catalytic activity, acting on a protein,autophagy,signaling,transferase activity;Ontology_id=GO:0005634,GO:0140096,GO:0006914,GO:0023052,GO:0016740;Enzyme_code=EC:2.7.11.1,EC:2.7.1,EC:2.7.11.1,EC:2.7,EC:2;Enzyme_name=non-specific serine/threonine protein kinase,Transferring phosphorus-containing groups,non-specific serine/threonine protein kinase,Transferring phosphorus-containing groups,Transferases
contig00007.g4476	2256	ID=contig00007.g4476;Description=vacuolar amino acid transporter 1 [Fusarium tjaetaba];Gene=FVER53590_09175;Ontology_term=transmembrane transport,vacuole,transporter activity;Ontology_id=GO:0055085,GO:0005773,GO:0005215
contig00007.g4477	880	ID=contig00007.g4477;Description=ribonuclease MRP protein subunit rmp1 [Fusarium bulbicola];Gene=FPANT_5393;Ontology_term=mRNA metabolic process,RNA binding,ribosome biogenesis,nucleolus;Ontology_id=GO:0016071,GO:0003723,GO:0042254,GO:0005730
contig00007.g4478	2026	ID=contig00007.g4478;Description=general transcription repressor [Metarhizium acridum]
contig00007.g4479	1090	ID=contig00007.g4479;Description=C-5 sterol desaturase [Fusarium verticillioides 7600];Gene=FVER53590_09178;Ontology_term=oxidoreductase activity,lipid metabolic process,endoplasmic reticulum;Ontology_id=GO:0016491,GO:0006629,GO:0005783;Enzyme_code=EC:1.14.19,EC:1.14.19.20,EC:1,EC:1.14,EC:1.14.19;Enzyme_name=Acting on paired donors, with incorporation or reduction of molecular oxygen. The oxygen incorporated need not be derived from O2,Delta(7)-sterol 5(6)-desaturase,Oxidoreductases,Acting on paired donors, with incorporation or reduction of molecular oxygen. The oxygen incorporated need not be derived from O2,Acting on paired donors, with incorporation or reduction of molecular oxygen. The oxygen incorporated need not be derived from O2
contig00007.g4480	393	ID=contig00007.g4480;Description=probable protein precursor [Fusarium fujikuroi IMI 58289];Gene=FNAPI_13547;Ontology_term=membrane;Ontology_id=GO:0016020
contig00007.g4481	830	ID=contig00007.g4481;Description=ribosomal protein L28e [Fusarium verticillioides 7600];Gene=28952001

contig00007.g4482	1484	ID=contig00007.g4482;Description=malate dehydrogenase, NAD-dependent [Fusarium verticillioides 7600];Gene=FVER53263_09181;Ontology_term=generation of precursor metabolites and energy,oxidoreductase activity;Ontology_id=GO:0006091,GO:0016491;Enzyme_code=EC:1.1.1.37,EC:1.1,EC:1.1.1.37,EC:1,EC:1.1.1;Enzyme_name=malate dehydrogenase,Acting on the CH-OH group of donors,malate dehydrogenase,Oxidoreductases,Acting on the CH-OH group of donors
contig00007.g4483	828	ID=contig00007.g4483;Description=related to TRS33-TRAPP subunit of 33 kDa involved in targeting and fusion of ER to golgi transp [Fusarium proliferatum ET1];Gene=FMEXI_13393;Ontology_term=endosome,vesicle-mediated transport,autophagy,cytosol,Golgi apparatus,intracellular protein transport;Ontology_id=GO:0005768,GO:0016192,GO:0006914,GO:0005829,GO:0005794,GO:0006886
contig00007.g4484	2394	ID=contig00007.g4484;Description=bifunctional purine biosynthetic ade1 [Fusarium tjaetaba];Gene=FVER53263_09182;Ontology_term=nucleobase-containing small molecule metabolic process,carbohydrate derivative metabolic process,ligase activity;Ontology_id=GO:0055086,GO:1901135,GO:0016874;Enzyme_code=EC:6.3.4.13,EC:6.3.3.1,EC:6.3,EC:6.3.4.13,EC:6,EC:6.3.3.1,EC:6.3.3;Enzyme_name=phosphoribosylamine--glycine ligase,phosphoribosylformylglycinamidine cyclo-ligase,Forming carbon-nitrogen bonds,phosphoribosylamine--glycine ligase,Ligases,phosphoribosylformylglycinamidine cyclo-ligase,Forming carbon-nitrogen bonds
contig00007.g4485	1649	ID=contig00007.g4485;Description=E3 SUMO-protein ligase pli1 [Fusarium odoratissimum];Gene=FMUND_6412;Ontology_term=DNA repair,DNA binding,catalytic activity, acting on a protein,cytoskeleton,DNA recombination,nuclear chromosome,chromosome segregation,telomere organization,transferase activity,ligase activity,meiotic nuclear division;Ontology_id=GO:0006281,GO:0003677,GO:0140096,GO:0005856,GO:0006310,GO:0000228,GO:0007059,GO:0032200,GO:0016740,GO:0016874,GO:0140013;Enzyme_code=EC:6,EC:2.3,EC:2,EC:6,EC:2.3.2;Enzyme_name=Ligases,Acyltransferases,Transferases,Ligases,Acyltransferases
contig00007.g4486	482	ID=contig00007.g4486;Description=family UPF0642 [Fusarium pseudocircinatum];Gene=FOXYS1_2015;Ontology_term=endoplasmic reticulum,transferase activity;Ontology_id=GO:0005783,GO:0016740;Enzyme_code=EC:2,EC:2;Enzyme_name=Transferases,Transferases

ID=contig00007.g4487;Description=ethanolaminephosphotransferase [Fusarium verticillioides 7600];Gene=FOXYS1_2015;Ontology_term=lipid metabolic process,carbohydrate derivative metabolic process,endoplasmic reticulum,transferase activity,plasma membrane;Ontology_id=GO:0006629,GO:1901135,GO:0005783,GO:0016740,GO:0005886;Enzyme_code=EC:2.7.8,EC:2.7,EC:2,EC:2.7.8;Enzyme_name=Transferring phosphorus-containing groups,Transferring phosphorus-containing groups,Transferases,Transferring phosphorus-containing groups

contig00007.g4487 2907

ID=contig00007.g4488;Description=condensin complex subunit 3 [Fusarium proliferatum];Gene=FPRO_08484;Ontology_term=mitotic nuclear division,chromosome segregation,nuclear chromosome,chromatin organization,meiotic nuclear division,hydrolase activity;Ontology_id=GO:0140014,GO:0007059,GO:0000228,GO:0006325,GO:0140013,GO:0016787;Enzyme_code=EC:3.6.1.1,EC:3.6.1.1,EC:3.6.1,EC:3.6,EC:3;Enzyme_name=inorganic diphosphatase,inorganic diphosphatase,Acting on acid anhydrides,Acting on acid anhydrides,Hydrolases

contig00007.g4488 3124

ID=contig00007.g4489;Description=nucleolar NOP4 (NOP77) [Fusarium tjaetaba];Gene=FPCIR_6516;Ontology_term=RNA binding,ribosome biogenesis,nucleolus;Ontology_id=GO:0003723,GO:0042254,GO:0005730

contig00007.g4489 2217

ID=contig00007.g4490;Description=hypothetical protein FVER14953_09188 [Fusarium verticillioides]

contig00007.g4490 972

ID=contig00007.g4491;Description=NIPA 2 [Fusarium acutatum];Gene=FACUT_877;Ontology_term=transmembrane transport,transporter activity;Ontology_id=GO:0055085,GO:0005215

contig00007.g4491 2221

ID=contig00007.g4492;Description=hypothetical protein FVEG_09191 [Fusarium verticillioides 7600]

contig00007.g4492 2430

ID=contig00007.g4493;Description=Allergen Fus c 3 [Fusarium tjaetaba];Gene=FVEG_09192;Ontology_term=protein dimerization activity;Ontology_id=GO:0046983

contig00007.g4493 1476

contig00007.g4494	1922	ID=contig00007.g4494;Description=FACT complex subunit POB3 [Fusarium subglutinans];Gene=FSUBG_169;Ontology_term=DNA repair,DNA binding,protein-containing complex assembly,histone binding,DNA replication,regulation of DNA-templated transcription,nuclear chromosome,chromatin organization,nucleoplasm;Ontology_id=GO:0006281,GO:0003677,GO:0065003,GO:0042393,GO:0006260,GO:0006355,GO:0000228,GO:0006325,GO:0005654
contig00007.g4495	1884	ID=contig00007.g4495;Description=putative lipase atg15 [Fusarium musae];Gene=FNYG_11554;Ontology_term=membrane organization,autophagy,lipid metabolic process,cellular modified amino acid metabolic process,protein maturation,endoplasmic reticulum,vacuole,hydrolase activity;Ontology_id=GO:0061024,GO:0006914,GO:0006629,GO:0006575,GO:0051604,GO:0005783,GO:0005773,GO:0016787;Enzyme_code=EC:3.1,EC:3,EC:3.1.1.5,EC:3.1.1,EC:3.1.1.3;Enzyme_name=Acting on ester bonds,Hydrolases,lysophospholipase,Acting on ester bonds,triacylglycerol lipase
contig00007.g4496	2607	ID=contig00007.g4496;Description=vacuolar sorting-associated protein [Fusarium tjaetaba];Gene=FVEG_09195;Ontology_term=nucleus,catalytic activity, acting on a protein,catalytic activity, acting on DNA,ATP-dependent activity,regulation of DNA-templated transcription,molecular carrier activity,chromatin organization;Ontology_id=GO:0005634,GO:0140096,GO:0140097,GO:0140657,GO:0006355,GO:0140104,GO:0006325
contig00007.g4497	1206	ID=contig00007.g4497;Description=hypothetical protein FVER14953_09196 [Fusarium verticillioides]
contig00007.g4498	1189	ID=contig00007.g4498;Description=C4-hydroxylase [Fusarium verticillioides 7600];Gene=FVER53590_09197;Ontology_term=oxidoreductase activity,lipid metabolic process,carbohydrate derivative metabolic process,endoplasmic reticulum;Ontology_id=GO:0016491,GO:0006629,GO:1901135,GO:0005783;Enzyme_code=EC:1.14.19.17,EC:1,EC:1.14,EC:1.14.19.17,EC:1.14.19,EC:1.14.18.5;Enzyme_name=sphingolipid 4-desaturase,Oxidoreductases,Acting on paired donors, with incorporation or reduction of molecular oxygen. The oxygen incorporated need not be derived from O2,sphingolipid 4-desaturase,Acting on paired donors, with incorporation or reduction of molecular oxygen. The oxygen incorporated need not be derived from O2,sphingolipid C4-monooxygenase
contig00007.g4499	829	ID=contig00007.g4499;Description=translationally-controlled tumor protein [Fusarium verticillioides 7600]
contig00007.g4500	1309	ID=contig00007.g4500;Description=gmp synthase [Fusarium tjaetaba]

contig00007.g4501	1088	ID=contig00007.g4501;Description=MTW1 Determining metaphase spindle length [Fusarium tjaetaba];Gene=FTJAE_12984;Ontology_term=nucleus,chromosome,mitotic cell cycle;Ontology_id=GO:0005634,GO:0005694,GO:0000278
contig00007.g4502	2553	ID=contig00007.g4502;Description=glutathione s-transferase [Fusarium tjaetaba];Gene=FDENT_10569;Ontology_term=transferase activity;Ontology_id=GO:0016740;Enzyme_code=EC:2,EC:2;Enzyme_name=Transferases,Transferases
contig00007.g4503	916	ID=contig00007.g4503;Description=glutathione s-transferase [Fusarium coicis];Gene=FNAPI_573;Ontology_term=antioxidant activity,sulfur compound metabolic process,oxidoreductase activity,cellular modified amino acid metabolic process,endoplasmic reticulum,transferase activity;Ontology_id=GO:0016209,GO:0006790,GO:0016491,GO:0006575,GO:0005783,GO:0016740;Enzyme_code=EC:2.5.1.18,EC:1.11.1.9;Enzyme_name=glutathione transferase,glutathione peroxidase
contig00007.g4504	3293	ID=contig00007.g4504;Description=ITC1-subunit of Isw2 chromatin remodelling complex [Fusarium coicis];Gene=FNYG_11563;Ontology_term=DNA repair,signaling,nuclear chromosome,mitotic cell cycle;Ontology_id=GO:0006281,GO:0023052,GO:0000228,GO:0000278
contig00007.g4505	1625	ID=contig00007.g4505;Description=glucosyltransferase [Fusarium fujikuroi];Gene=LW93_13004;Ontology_term=cell junction organization,structural molecule activity,anatomical structure development,carbohydrate derivative metabolic process,endoplasmic reticulum,cell adhesion,plasma membrane,cell motility,cytoskeleton,lipid metabolic process,cell differentiation,transferase activity,cytoskeletal protein binding;Ontology_id=GO:0034330,GO:0005198,GO:0048856,GO:1901135,GO:0005783,GO:0007155,GO:0005886,GO:0048870,GO:0005856,GO:0006629,GO:0030154,GO:0016740,GO:0008092;Enzyme_code=EC:2.4.1.265;Enzyme_name=dolichyl-P-Glc:Glc1Man9GlcNAc2-PP-dolichol alpha-1,3-glucosyltransferase
contig00007.g4506	1400	ID=contig00007.g4506;Description=EMP47 golgi membrane [Fusarium pseudocircinatum];Gene=FVER53263_09206;Ontology_term=membrane;Ontology_id=GO:0016020

contig00007.g4507	1832	ID=contig00007.g4507;Description=prolidase [Fusarium mundagurra];Gene=FVER53263_09207;Ontology_term=catalytic activity, acting on a protein,hydrolase activity;Ontology_id=GO:0140096,GO:0016787;Enzyme_code=EC:3.4.11;Enzyme_name=Acting on peptide bonds (peptidases)
contig00007.g4508	3092	ID=contig00007.g4508;Description=fungal-specific transcription factor domain-containing protein [Fusarium oxysporum];Gene=FVER53263_09208;Ontology_term=nucleus,DNA binding,regulation of DNA-templated transcription,transcription regulator activity;Ontology_id=GO:0005634,GO:0003677,GO:0006355,GO:0140110
contig00007.g4509	1634	ID=contig00007.g4509;Description=COP9 signalosome complex subunit 1 (G pathway suppressor 1) [Fusarium tjaetaba];Ontology_term=nucleus,anatomical structure development,reproductive process,regulation of DNA-templated transcription,chromatin organization;Ontology_id=GO:0005634,GO:0048856,GO:0022414,GO:0006355,GO:0006325
contig00007.g4510	1091	ID=contig00007.g4510;Description=pre-mrna-splicing factor 18 [Fusarium sporotrichioides];Gene=FG02533.1;Ontology_term=nucleus,mRNA metabolic process,protein-containing complex assembly;Ontology_id=GO:0005634,GO:0016071,GO:0065003
contig00007.g4511	392	ID=contig00007.g4511;Description=NADH:ubiquinone oxidoreductase subunit [Fusarium subglutinans];Gene=BFJ63_vAg298;Ontology_term=membrane;Ontology_id=GO:0016020
contig00007.g4512	7110	ID=contig00007.g4512;Description=pre-mRNA-splicing factor spp42 [Fusarium verticillioides 7600];Gene=FMUND_894;Ontology_term=nucleus,catalytic activity, acting on a protein,mRNA metabolic process,RNA binding,protein-containing complex assembly,catalytic activity, acting on RNA,hydrolase activity;Ontology_id=GO:0005634,GO:0140096,GO:0016071,GO:0003723,GO:0065003,GO:0140098,GO:0016787;Enzyme_code=EC:3.4,EC:3.4,EC:3,EC:3.4.19.12;Enzyme_name=Acting on peptide bonds (peptidases),Acting on peptide bonds (peptidases),Hydrolases,ubiquitinyl hydrolase 1
contig00007.g4513	940	ID=contig00007.g4513;Description=myb DNA-binding domain-containing protein [Fusarium oxysporum]

contig00007.g4514	1308	ID=contig00007.g4514;Description=2-dehydropantoate 2-reductase [Fusarium verticillioides 7600];Gene=FNAPI_562;Ontology_term=oxidoreductase activity,cellular modified amino acid metabolic process,amino acid metabolic process,vitamin metabolic process;Ontology_id=GO:0016491,GO:0006575,GO:0006520,GO:0006766;Enzyme_code=EC:1,EC:1.1,EC:1,EC:1.1.1.169,EC:1.1.1,EC:1.14.14.17;Enzyme_name=Oxidoreductases,Acting on the CH-OH group of donors,Oxidoreductases,2-dehydropantoate 2-reductase,Acting on the CH-OH group of donors,squalene monooxygenase
contig00007.g4515	3489	ID=contig00007.g4515;Description=DNA excision repair protein ERCC-6 [Fusarium verticillioides 7600];Gene=FNYG_11574;Ontology_term=nucleus,DNA repair,catalytic activity, acting on DNA,ATP-dependent activity,chromatin organization,hydrolase activity;Ontology_id=GO:0005634,GO:0006281,GO:0140097,GO:0140657,GO:0006325,GO:0016787;Enzyme_code=EC:3,EC:3.6.1,EC:3.6,EC:3,EC:3.6.1.15;Enzyme_name=Hydrolases,Acting on acid anhydrides,Acting on acid anhydrides,Hydrolases,nucleoside-triphosphate phosphatase
contig00007.g4516	685	ID=contig00007.g4516;Description=40S ribosomal protein S14 [Fusarium verticillioides 7600];Gene=FOVG_06827;Ontology_term=structural molecule activity,ribosome;Ontology_id=GO:0005198,GO:0005840
contig00007.g4517	1442	ID=contig00007.g4517;Description=60S ribosomal protein L7 [Fusarium poae];Gene=FNYG_11576;Ontology_term=structural molecule activity,cytosol,ribosome biogenesis,ribosome;Ontology_id=GO:0005198,GO:0005829,GO:0042254,GO:0005840
contig00007.g4518	682	ID=contig00007.g4518;Description=iron transport protein [Fusarium fujikuroi];Gene=FGLOB1_862;Ontology_term=RNA binding,sulfur compound metabolic process,mitochondrion,protein maturation,extracellular region,catalytic activity, acting on RNA,vitamin metabolic process,hydrolase activity;Ontology_id=GO:0003723,GO:0006790,GO:0005739,GO:0051604,GO:0005576,GO:0140098,GO:0006766,GO:0016787;Enzyme_code=EC:3.1,EC:3.1,EC:3;Enzyme_name=Acting on ester bonds,Acting on ester bonds,Hydrolases
contig00007.g4519	2698	ID=contig00007.g4519;Description=eukaryotic translation initiation factor 3 subunit C [Fusarium odoratissimum NRRL 54006];Gene=NIP1;Ontology_term=translation regulator activity,RNA binding,protein-containing complex assembly,cytoplasmic translation,organelle;Ontology_id=GO:0045182,GO:0003723,GO:0065003,GO:0002181,GO:0043226

contig00007.g4520	1864	ID=contig00007.g4520;Description=30S ribosomal protein S10, mitochondrial [Fusarium verticillioides 7600];Gene=FPHYL_3260;Ontology_term=structural molecule activity,ribosome;Ontology_id=GO:0005198,GO:0005840
contig00007.g4521	1260	ID=contig00007.g4521;Description=tryptophanyl-tRNA synthetase [Fusarium subglutinans];Gene=F25303_4994;Ontology_term=mitochondrial gene expression,mitochondrion,tRNA metabolic process,amino acid metabolic process,catalytic activity, acting on RNA,ligase activity;Ontology_id=GO:0140053,GO:0005739,GO:0006399,GO:0006520,GO:0140098,GO:0016874;Enzyme_code=EC:6.1.1.2,EC:6.1.1.2,EC:6.1,EC:6.1.1,EC:6;Enzyme_name=tryptophan--tRNA ligase,tryptophan--tRNA ligase,Forming carbon-oxygen bonds,Forming carbon-oxygen bonds,Ligases
contig00007.g4522	993	ID=contig00007.g4522;Description=CCAAT-binding transcription factor subunit aab-1 [Fusarium acutatum];Gene=BFJ68_g1222;Ontology_term=DNA binding,nucleus,generation of precursor metabolites and energy,regulation of DNA-templated transcription,chromosome,transcription regulator activity;Ontology_id=GO:0003677,GO:0005634,GO:0006091,GO:0006355,GO:0005694,GO:0140110
contig00007.g4523	4614	ID=contig00007.g4523;Description=member of the karyopherin-beta family nuclear import [Fusarium tjaetaba];Gene=FPANT_9099;Ontology_term=nuclear envelope,nucleocytoplasmic transport,molecular carrier activity,intracellular protein transport,transporter activity;Ontology_id=GO:0005635,GO:0006913,GO:0140104,GO:0006886,GO:0005215
contig00007.g4524	2746	ID=contig00007.g4524;Description=Sec15-like exocyst complex protein [Fusarium denticulatum];Gene=FVER53263_09228;Ontology_term=vesicle-mediated transport,intracellular protein transport;Ontology_id=GO:0016192,GO:0006886
contig00007.g4525	2370	ID=contig00007.g4525;Description=meiosis mei2 [Fusarium tjaetaba];Gene=FNYG_11586;Ontology_term=RNA binding,DNA replication,signaling,nuclear chromosome,chromosome segregation,mitotic cell cycle,meiotic nuclear division;Ontology_id=GO:0003723,GO:0006260,GO:0023052,GO:0000228,GO:0007059,GO:0000278,GO:0140013
contig00007.g4526	2553	ID=contig00007.g4526;Description=SLP1 [Fusarium tjaetaba];Gene=FACUT_5293;Ontology_term=endoplasmic reticulum,protein folding;Ontology_id=GO:0005783,GO:0006457

contig00007.g4527	1328	ID=contig00007.g4527;Description=hypothetical protein FVER53590_09231 [Fusarium verticillioides];Gene=Forpi1262_v014941;Ontology_term=membrane;Ontology_id=GO:0016020
contig00007.g4528	664	ID=contig00007.g4528;Description=hypothetical protein FCOIX_2434 [Fusarium coicis]
contig00007.g4529	873	ID=contig00007.g4529;Description=Valacyclovir hydrolase [Fusarium coicis];Gene=FPANT_1308;Ontology_term=hydrolase activity;Ontology_id=GO:0016787;Enzyme_code=EC:3,EC:3;Enzyme_name=Hydrolases,Hydrolases
contig00007.g4530	405	ID=contig00007.g4530;Description=hypothetical protein FVER14953_09234 [Fusarium verticillioides];Gene=FVER53590_09234;Ontology_term=membrane;Ontology_id=GO:0016020
contig00007.g4531	2699	ID=contig00007.g4531;Description=DNA replication licensing factor mcm2 [Fusarium oxysporum f. sp. cepae];Gene=28952055;Ontology_term=DNA repair,catalytic activity, acting on DNA,protein-containing complex assembly,DNA replication,DNA recombination,reproductive process,nuclear chromosome,mitotic cell cycle,nucleoplasm,DNA binding,ATP-dependent activity,hydrolase activity;Ontology_id=GO:0006281,GO:0140097,GO:0065003,GO:0006260,GO:0006310,GO:0022414,GO:0000228,GO:0000278,GO:0005654,GO:0003677,GO:0140657,GO:0016787;Enzyme_code=EC:3,EC:3.6.4.12,EC:3;Enzyme_name=Hydrolases,DNA helicase,Hydrolases
contig00007.g4532	1756	ID=contig00007.g4532;Description=putative zinc finger in N-recognin-domain-containing protein [Fusarium oxysporum];Gene=BFJ68_g1501;Ontology_term=catalytic activity, acting on a protein,transferase activity;Ontology_id=GO:0140096,GO:0016740;Enzyme_code=EC:2.3.2;Enzyme_name=Acyltransferases
contig00007.g4533	1135	ID=contig00007.g4533;Description=hypothetical protein BFJ71_g2549 [Fusarium oxysporum]
contig00007.g4534	2652	ID=contig00007.g4534;Description=hypothetical protein FVEG_09238 [Fusarium verticillioides 7600];Gene=FNYG_11595;Ontology_term=regulation of DNA-templated transcription,transcription regulator activity;Ontology_id=GO:0006355,GO:0140110
contig00007.g4535	1980	ID=contig00007.g4535;Description=calcium-independent phospholipase A2 [Fusarium tjaetaba];Gene=FNYG_11596;Ontology_term=lipid metabolic process;Ontology_id=GO:0006629;Enzyme_code=EC:2.1.2.1;Enzyme_name=glycine hydroxymethyltransferase

contig00007.g4536	2065	ID=contig00007.g4536;Description=6-hydroxy-D-nicotine oxidase [Fusarium tjaetaba];Gene=FVER53263_20730;Ontology_term=nucleus,regulation of DNA-templated transcription,transcription regulator activity;Ontology_id=GO:0005634,GO:0006355,GO:0140110
contig00007.g4537	1459	ID=contig00007.g4537;Description=6-hydroxy-D-nicotine oxidase [Fusarium tjaetaba];Gene=FVER53590_09241;Ontology_term=oxidoreductase activity;Ontology_id=GO:0016491;Enzyme_code=EC:1,EC:1;Enzyme_name=Oxidoreductases,Oxidoreductases
contig00007.g4538	969	ID=contig00007.g4538;Description=glucose-fructose oxidoreductase [Fusarium verticillioides 7600];Gene=FVER53590_09242;Ontology_term=nucleus,generation of precursor metabolites and energy,nucleobase-containing small molecule metabolic process,oxidoreductase activity;Ontology_id=GO:0005634,GO:0006091,GO:0055086,GO:0016491;Enzyme_code=EC:1.1.99.28,EC:1;Enzyme_name=glucose-fructose oxidoreductase,Oxidoreductases
contig00007.g4539	761	ID=contig00007.g4539;Description=acyl-CoA thioester hydrolase [Fusarium verticillioides 7600];Gene=FOMG_07381;Ontology_term=lipid metabolic process,hydrolase activity;Ontology_id=GO:0006629,GO:0016787;Enzyme_code=EC:3.1.2;Enzyme_name=Acting on ester bonds
contig00007.g4540	1545	ID=contig00007.g4540;Description=pseudouridylate synthase [Fusarium verticillioides 7600];Gene=FDENT_2890;Ontology_term=nucleus,RNA binding,mitochondrion,tRNA metabolic process,ribosome biogenesis,vitamin metabolic process,isomerase activity,hydrolase activity;Ontology_id=GO:0005634,GO:0003723,GO:0005739,GO:0006399,GO:0042254,GO:0006766,GO:0016853,GO:0016787;Enzyme_code=EC:5.4,EC:5.4.99.28,EC:3.5.4,EC:5.4,EC:3.5,EC:3,EC:5;Enzyme_name=Intramolecular transferases,tRNA pseudouridine(32) synthase,Acting on carbon-nitrogen bonds, other than peptide bonds,Intramolecular transferases,Acting on carbon-nitrogen bonds, other than peptide bonds,Hydrolases,Isomerases
contig00007.g4541	1890	ID=contig00007.g4541;Description=hypothetical protein FVEG_16543 [Fusarium verticillioides 7600];Gene=FFUJ_07958;Ontology_term=regulation of DNA-templated transcription,transcription regulator activity;Ontology_id=GO:0006355,GO:0140110
contig00007.g4542	2546	ID=contig00007.g4542;Description=beta-glucosidase [Fusarium verticillioides 7600];Gene=FPANT_1295;Ontology_term=carbohydrate metabolic process,hydrolase activity;Ontology_id=GO:0005975,GO:0016787;Enzyme_code=EC:3.2.1.21;Enzyme_name=beta-glucosidase

contig00007.g4543	1117	ID=contig00007.g4543;Description=hypothetical protein FVEG_09249 [Fusarium verticillioides 7600];Gene=FPANT_1294;Ontology_term=membrane;Ontology_id=GO:0016020
contig00007.g4544	808	ID=contig00007.g4544;Description=kinetochore fta4 [Fusarium tjaetaba];Gene=FPANT_1293;Ontology_term=chromosome;Ontology_id=GO:0005694
contig00007.g4545	1783	ID=contig00007.g4545;Description=beta-carotene 15 15'-dioxygenase [Fusarium tjaetaba];Gene=FPANT_1292;Ontology_term=oxidoreductase activity;Ontology_id=GO:0016491;Enzyme_code=EC:1.13.11;Enzyme_name=Acting on single donors with incorporation of molecular oxygen (oxygenases). The oxygen incorporated need not be derived from O2
contig00007.g4546	650	ID=contig00007.g4546;Description=nitrogen fixation protein [Fusarium denticulatum];Gene=FOBC_09165;Ontology_term=sulfur compound metabolic process,mitochondrion,tRNA metabolic process,protein maturation,transferase activity;Ontology_id=GO:0006790,GO:0005739,GO:0006399,GO:0051604,GO:0016740;Enzyme_code=EC:2,EC:2.8;Enzyme_name=Transferases,Transferring sulfur-containing groups
contig00007.g4547	2040	ID=contig00007.g4547;Description=hypothetical protein FVEG_09253 [Fusarium verticillioides 7600]
contig00007.g4548	450	ID=contig00007.g4548;Description=hypothetical protein FVER53590_09254 [Fusarium verticillioides];Gene=FOYG_08803;Ontology_term=membrane;Ontology_id=GO:0016020
contig00007.g4549	990	ID=contig00007.g4549;Description=hypothetical protein FVER14953_09255 [Fusarium verticillioides]
contig00007.g4550	4001	ID=contig00007.g4550;Description=hypothetical protein FVER14953_09256 [Fusarium verticillioides]
contig00007.g4551	2556	ID=contig00007.g4551;Description=ubiquitin thiolesterase [Fusarium verticillioides 7600];Gene=FANTH_8430;Ontology_term=carbohydrate metabolic process,catalytic activity, acting on a protein,protein catabolic process,hydrolase activity;Ontology_id=GO:0005975,GO:0140096,GO:0030163,GO:0016787;Enzyme_code=EC:3.4.19.12,EC:3.4,EC:3,EC:3.4.19.12;Enzyme_name=ubiquitinyl hydrolase 1,Acting on peptide bonds (peptidases),Hydrolases,ubiquitinyl hydrolase 1

contig00007.g4552	966	ID=contig00007.g4552;Description=DNA-binding protein [Fusarium redolens];Ontology_term=cytoskeleton organization,microtubule organizing center,signaling,mitotic nuclear division,chromosome segregation,cytokinesis,nuclear chromosome,meiotic nuclear division;Ontology_id=GO:0007010,GO:0005815,GO:0023052,GO:0140014,GO:0007059,GO:0000910,GO:0000228,GO:0140013
contig00007.g4553	1162	ID=contig00007.g4553;Description=hypothetical protein FVEG_16546 [Fusarium verticillioides 7600];Gene=F52700_5787;Ontology_term=membrane;Ontology_id=GO:0016020
contig00007.g4554	1850	ID=contig00007.g4554;Description=ATP-dependent RNA helicase DBP3 [Fusarium verticillioides 7600];Gene=FMUND_853;Ontology_term=protein-containing complex assembly,ATP-dependent activity,ribosome biogenesis,catalytic activity, acting on RNA,nucleolus,hydrolase activity;Ontology_id=GO:0065003,GO:0140657,GO:0042254,GO:0140098,GO:0005730,GO:0016787;Enzyme_code=EC:3,EC:3.6.1,EC:3.6.4.13,EC:3.6,EC:3,EC:3.6.1.15;Enzyme_name=Hydrolases,Acting on acid anhydrides,RNA helicase,Acting on acid anhydrides,Hydrolases,nucleoside-triphosphate phosphatase
contig00007.g4555	1683	ID=contig00007.g4555;Description=oligosaccharide transporter [Fusarium fujikuroi];Gene=FOBC_09173;Ontology_term=membrane organization,lipid metabolic process,protein glycosylation,endoplasmic reticulum;Ontology_id=GO:0061024,GO:0006629,GO:0006486,GO:0005783
contig00007.g4556	1790	ID=contig00007.g4556;Description=RCO3-glucose transporter [Fusarium tjaetaba];Gene=FACUT_6491;Ontology_term=transmembrane transport,transporter activity;Ontology_id=GO:0055085,GO:0005215
contig00007.g4557	1248	ID=contig00007.g4557;Description=hypothetical protein FVER14953_09262 [Fusarium verticillioides]
contig00007.g4558	1374	ID=contig00007.g4558;Description=uncharacterized protein FTJAE_3411 [Fusarium tjaetaba]
contig00007.g4559	447	ID=contig00007.g4559;Description=permease [Fusarium flagelliforme];Gene=FIE12Z_8029
contig00007.g4560	7584	ID=contig00007.g4560;Description=modin [Fusarium tjaetaba]

contig00007.g4561	2917	ID=contig00007.g4561;Description=micromolar calcium activated neutral protease 1 (capn1) [Fusarium circinatum];Gene=FCIRC_5318;Ontology_term=DNA binding,catalytic activity, acting on a protein,hydrolase activity;Ontology_id=GO:0003677,GO:0140096,GO:0016787;Enzyme_code=EC:3.4.22,EC:3.4.22,EC:3.4,EC:3;Enzyme_name=Acting on peptide bonds (peptidases),Acting on peptide bonds (peptidases),Acting on peptide bonds (peptidases),Hydrolases
contig00007.g4562	4771	ID=contig00007.g4562;Description=hypothetical protein FVEG_09269 [Fusarium verticillioides 7600];Gene=FFUJ_07978;Ontology_term=membrane;Ontology_id=GO:0016020
contig00007.g4563	1143	ID=contig00007.g4563;Description=hypothetical protein FVER14953_09271 [Fusarium verticillioides]
contig00007.g4564	738	ID=contig00007.g4564;Description=hypothetical protein FVER53590_09272 [Fusarium verticillioides]
contig00007.g4565	2797	ID=contig00007.g4565;Description=Som1 protein [Fusarium coicis];Ontology_term=nucleus,DNA binding,regulation of DNA-templated transcription,cell adhesion,transcription regulator activity;Ontology_id=GO:0005634,GO:0003677,GO:0006355,GO:0007155,GO:0140110
contig00007.g4566	745	ID=contig00007.g4566;Description=probable BET3 [Fusarium fujikuroi];Gene=28952089;Ontology_term=vesicle-mediated transport,endoplasmic reticulum,Golgi apparatus;Ontology_id=GO:0016192,GO:0005783,GO:0005794
contig00007.g4567	1214	ID=contig00007.g4567;Description=putative U5 snRNP-specific protein [Fusarium fujikuroi];Gene=sod1;Ontology_term=nucleus,mRNA metabolic process,antioxidant activity,oxidoreductase activity,mitochondrion,cytosol;Ontology_id=GO:0005634,GO:0016071,GO:0016209,GO:0016491,GO:0005739,GO:0005829;Enzyme_code=EC:1.15.1.1,EC:1,EC:1.15.1.1,EC:1.15;Enzyme_name=superoxide dismutase,Oxidoreductases,superoxide dismutase,Acting on superoxide as acceptor
contig00007.g4568	1356	ID=contig00007.g4568;Description=hypothetical protein FVEG_09276 [Fusarium verticillioides 7600];Gene=Forpe1208_v007110;Ontology_term=membrane;Ontology_id=GO:0016020

contig00007.g4569	768	ID=contig00007.g4569;Description=cellular nucleic acid-binding protein [Fusarium subglutinans];Gene=FOYG_08826;Ontology_term=DNA binding,translation regulator activity,RNA binding,cytoplasmic translation,ribosome;Ontology_id=GO:0003677,GO:0045182,GO:0003723,GO:0002181,GO:0005840
contig00007.g4570	1464	ID=contig00007.g4570;Description=indoleamine 2,3-dioxygenase [Fusarium verticillioides 7600];Gene=FPCIR_11913;Ontology_term=nucleobase-containing small molecule metabolic process,oxidoreductase activity,amino acid metabolic process;Ontology_id=GO:0055086,GO:0016491,GO:0006520;Enzyme_code=EC:1,EC:1.13.11,EC:1.13.11.52,EC:1.13,EC:1;Enzyme_name=Oxidoreductases,Acting on single donors with incorporation of molecular oxygen (oxygenases). The oxygen incorporated need not be derived from O2,indoleamine 2,3-dioxygenase,Acting on single donors with incorporation of molecular oxygen (oxygenases). The oxygen incorporated need not be derived from O2,Oxidoreductases
contig00007.g4571	1763	ID=contig00007.g4571;Description=component of actin cortical patches LAS17 [Fusarium beomiforme];Gene=FOYG_08828;Ontology_term=endosome,protein catabolic process;Ontology_id=GO:0005768,GO:0030163
contig00007.g4572	1194	ID=contig00007.g4572;Description=pheromone alpha factor receptor [Fusarium verticillioides 7600];Gene=FVEG_09280;Ontology_term=molecular transducer activity,protein-containing complex assembly,signaling,reproductive process,plasma membrane;Ontology_id=GO:0060089,GO:0065003,GO:0023052,GO:0022414,GO:0005886
contig00007.g4573	2198	ID=contig00007.g4573;Description=hypothetical protein FVEG_09281 [Fusarium verticillioides 7600]
contig00007.g4574	847	ID=contig00007.g4574;Description=meiotic recombination DMC1 [Fusarium beomiforme]
contig00007.g4575	904	ID=contig00007.g4575;Description=endo-1,3(4)-beta-glucanase [Fusarium verticillioides 7600];Gene=FOQG_05938;Ontology_term=carbohydrate metabolic process,hydrolase activity;Ontology_id=GO:0005975,GO:0016787;Enzyme_code=EC:3.2.1,EC:3.2,EC:3,EC:3.2.1;Enzyme_name=Glycosylases,Glycosylases,Hydrolases,Glycosylases
contig00007.g4576	1206	ID=contig00007.g4576;Description=endoglucanase type K [Fusarium verticillioides 7600];Gene=FDENT_12168;Ontology_term=carbohydrate metabolic process,extracellular region,hydrolase activity;Ontology_id=GO:0005975,GO:0005576,GO:0016787;Enzyme_code=EC:3.2.1.4,EC:3.2,EC:3,EC:3.2.1,EC:3.2.1.4;Enzyme_name=cellulase,Glycosylases,Hydrolases,Glycosylases,cellulase

contig00007.g4577	3907	ID=contig00007.g4577;Description=DNA-directed RNA polymerase II subunit RPB2 [Fusarium verticillioides 7600];Gene=A0A4D6RH16;Ontology_term=nuclear chromosome,regulation of DNA-templated transcription,chromatin organization,nucleoplasm,DNA binding,RNA binding,mitochondrion,regulatory ncRNA-mediated gene silencing,catalytic activity, acting on RNA,transferase activity;Ontology_id=GO:0000228,GO:0006355,GO:0006325,GO:0005654,GO:0003677,GO:0003723,GO:0005739,GO:0031047,GO:0140098,GO:0016740;Enzyme_code=EC:2.7.7.48,EC:2.7.7.6,EC:2.7,EC:2,EC:2.7.7.48,EC:2.7.7,EC:2.7.7.6;Enzyme_name=RNA-directed RNA polymerase,DNA-directed RNA polymerase,Transferring phosphorus-containing groups,Transferases,RNA-directed RNA polymerase,Transferring phosphorus-containing groups,DNA-directed RNA polymerase
contig00007.g4578	327	ID=contig00007.g4578;Description=cytochrome c oxidase assembly factor [Fusarium tjaetaba];Gene=FPANT_11129;Ontology_term=mitochondrion;Ontology_id=GO:0005739
contig00007.g4579	649	ID=contig00007.g4579;Description=V-type ATPase, G subunit [Fusarium verticillioides 7600];Gene=FOVG_06902
contig00007.g4580	1856	ID=contig00007.g4580;Description=nuclear associated [Fusarium tjaetaba];Gene=FTJAE_655;Ontology_term=nucleus;Ontology_id=GO:0005634
contig00007.g4581	6872	ID=contig00007.g4581;Description=translation factor GUF1 mitochondrial [Fusarium pseudoanthophilum];Gene=FVER53263_09291;Ontology_term=oxidoreductase activity,GTPase activity,mitochondrion;Ontology_id=GO:0016491,GO:0003924,GO:0005739;Enzyme_code=EC:1.1,EC:3.6.1.15,EC:1.1,EC:3.6.1,EC:1,EC:3.6,EC:3,EC:3.6.1.15;Enzyme_name=Acting on the CH-OH group of donors,nucleoside-triphosphate phosphatase,Acting on the CH-OH group of donors,Acting on acid anhydrides,Oxidoreductases,Acting on acid anhydrides,Hydrolases,nucleoside-triphosphate phosphatase
contig00007.g4582	871	ID=contig00007.g4582;Description=phosphoglycerate mutase [Fusarium phyllophilum]
contig00007.g4583	1035	ID=contig00007.g4583;Description=related to acetylxylan esterase precursor [Fusarium fujikuroi IMI 58289];Gene=FVEG_09293;Ontology_term=extracellular region,hydrolase activity;Ontology_id=GO:0005576,GO:0016787;Enzyme_code=EC:3.1.1.74,EC:3.1,EC:3,EC:3.1.1.72,EC:3.1.1.74,EC:3.1.1;Enzyme_name=cutinase,Acting on ester bonds,Hydrolases,acetylxylan esterase,cutinase,Acting on ester bonds

contig00007.g4584	3811	ID=contig00007.g4584;Description=Psi-producing oxygenase C [Fusarium coicis];Gene=FPANT_13416;Ontology_term=antioxidant activity,oxidoreductase activity,isomerase activity;Ontology_id=GO:0016209,GO:0016491,GO:0016853;Enzyme_code=EC:1.14,EC:5,EC:1.11.1,EC:1.13.11.60;Enzyme_name=Acting on paired donors, with incorporation or reduction of molecular oxygen. The oxygen incorporated need not be derived from O2,Isomerases,Acting on a peroxide as acceptor,linoleate 8R-lipoxygenase
contig00007.g4585	1860	ID=contig00007.g4585;Description=short chain dehydrogenase reductase family [Fusarium tjaetaba]
contig00007.g4586	1118	ID=contig00007.g4586;Description=Transcription activator AKTR-2 [Fusarium oxysporum f. sp. cubense];Gene=AKTR-2-0;Ontology_term=nucleus,regulation of DNA-templated transcription,transcription regulator activity;Ontology_id=GO:0005634,GO:0006355,GO:0140110
contig00007.g4587	1229	ID=contig00007.g4587;Description=hypothetical protein FVER53590_09297 [Fusarium verticillioides]
contig00007.g4588	669	ID=contig00007.g4588;Description=hypothetical protein FVER53590_29415 [Fusarium verticillioides];Gene=2366
contig00007.g4589	2190	ID=contig00007.g4589;Description=transcriptional regulator [Fusarium pseudocircinatum];Gene=FVER53590_09299;Ontology_term=nucleus,DNA binding,oxidoreductase activity,regulation of DNA-templated transcription,transcription regulator activity;Ontology_id=GO:0005634,GO:0003677,GO:0016491,GO:0006355,GO:0140110;Enzyme_code=EC:1,EC:1;Enzyme_name=Oxidoreductases,Oxidoreductases
contig00007.g4590	1447	ID=contig00007.g4590;Description=NADH oxidase [Fusarium tjaetaba];Gene=FTJAE_665;Ontology_term=nucleus,DNA binding,oxidoreductase activity,regulation of DNA-templated transcription,transcription regulator activity;Ontology_id=GO:0005634,GO:0003677,GO:0016491,GO:0006355,GO:0140110;Enzyme_code=EC:1;Enzyme_name=Oxidoreductases
contig00007.g4591	749	ID=contig00007.g4591;Description=hypothetical protein FGLOB1_14435 [Fusarium globosum]
contig00007.g4592	3117	ID=contig00007.g4592;Description=hypothetical protein FVER53263_09302 [Fusarium verticillioides]

contig00007.g4593	2057	ID=contig00007.g4593;Description=c6 zinc finger [Fusarium tjaetaba];Gene=FNYG_14200;Ontology_term=nucleus,DNA binding,regulation of DNA-templated transcription,transcription regulator activity;Ontology_id=GO:0005634,GO:0003677,GO:0006355,GO:0140110
contig00007.g4594	2284	ID=contig00007.g4594;Description=hypothetical protein FCIRC_6150 [Fusarium circinatum]
contig00007.g4595	1771	ID=contig00007.g4595;Description=putidaredoxin reductase [Fusarium tjaetaba];Gene=FNYG_14198;Ontology_term=nucleus,oxidoreductase activity,mitochondrion,programmed cell death;Ontology_id=GO:0005634,GO:0016491,GO:0005739,GO:0012501;Enzyme_code=EC:1,EC:1.8.1,EC:1.6,EC:1,EC:1.8;Enzyme_name=Oxidoreductases,Acting on a sulfur group of donors,Acting on NADH or NADPH,Oxidoreductases,Acting on a sulfur group of donors
contig00007.g4596	1504	ID=contig00007.g4596;Description=S-(hydroxymethyl)glutathione dehydrogenase/alcohol dehydrogenase [Fusarium verticillioides 7600];Gene=FVER53263_09306;Ontology_term=oxidoreductase activity;Ontology_id=GO:0016491;Enzyme_code=EC:1;Enzyme_name=Oxidoreductases
contig00007.g4597	930	ID=contig00007.g4597;Description=hypothetical protein FVER53263_09307 [Fusarium verticillioides]
contig00007.g4598	1210	ID=contig00007.g4598;Description=hypothetical protein FVEG_09308 [Fusarium verticillioides 7600];Gene=FTJAE_671;Ontology_term=ATP binding;Ontology_id=GO:0005524
contig00007.g4599	977	ID=contig00007.g4599;Description=hydrolase of the alpha beta superfamily [Fusarium tjaetaba];Gene=FTJAE_672;Ontology_term=hydrolase activity;Ontology_id=GO:0016787;Enzyme_code=EC:3;Enzyme_name=Hydrolases
contig00007.g4600	1618	ID=contig00007.g4600;Description=monocarboxylate transporter 4 [Fusarium tjaetaba];Gene=FVER53590_09310;Ontology_term=transmembrane transport,transporter activity;Ontology_id=GO:0055085,GO:0005215
contig00007.g4601	252	ID=contig00007.g4601;Description=hypothetical protein FVEG_16555 [Fusarium verticillioides 7600];Gene=FTJAE_674;Ontology_term=membrane;Ontology_id=GO:0016020
contig00007.g4602	731	ID=contig00007.g4602;Description=hypothetical protein NW765_000204 [Fusarium oxysporum];Gene=F25303_6413;Ontology_term=nucleus,DNA replication,mitotic nuclear division,chromosome segregation;Ontology_id=GO:0005634,GO:0006260,GO:0140014,GO:0007059
contig00007.g4603	1467	ID=contig00007.g4603;Description=f-box domain-containing protein [Fusarium coicis]

contig00007.g4604	1509	ID=contig00007.g4604;Description=alkanesulfonate monooxygenase [Fusarium verticillioides 7600];Gene=FTJAE_677;Ontology_term=oxidoreductase activity;Ontology_id=GO:0016491;Enzyme_code=EC:1.14,EC:1,EC:1.14,EC:1.14.14.5;Enzyme_name=Acting on paired donors, with incorporation or reduction of molecular oxygen. The oxygen incorporated need not be derived from O2,Oxidoreductases,Acting on paired donors, with incorporation or reduction of molecular oxygen. The oxygen incorporated need not be derived from O2,alkanesulfonate monooxygenase
contig00007.g4605	2501	ID=contig00007.g4605;Description=hypothetical protein FVEG_09315 [Fusarium verticillioides 7600];Gene=FPCIR_11425;Ontology_term=nucleus,DNA binding,regulation of DNA-templated transcription,transcription regulator activity;Ontology_id=GO:0005634,GO:0003677,GO:0006355,GO:0140110
contig00007.g4606	1717	ID=contig00007.g4606;Description=major facilitator superfamily domain protein [Fusarium tjaetaba];Gene=FVER53263_09316;Ontology_term=transmembrane transport,transporter activity;Ontology_id=GO:0055085,GO:0005215
contig00007.g4607	1432	ID=contig00007.g4607;Description=acyl-CoA dehydrogenase [Fusarium oxysporum f. sp. lycopersici 4287];Gene=pelB;Ontology_term=carbohydrate metabolic process,oxidoreductase activity,lyase activity,cell wall organization or biogenesis,extracellular region;Ontology_id=GO:0005975,GO:0016491,GO:0016829,GO:0071554,GO:0005576;Enzyme_code=EC:1.3,EC:4.2.2.2,EC:4.2.2.10,EC:1.3,EC:4.2.2.2,EC:1,EC:4.2,EC:4.2.2.10,EC:4,EC:4.2.2;Enzyme_name=Acting on the CH-CH group of donors,pectate lyase,pectin lyase,Acting on the CH-CH group of donors,pectate lyase,Oxidoreductases,Carbon-oxygen lyases,pectin lyase,Lyases,Carbon-oxygen lyases
contig00007.g4608	2033	ID=contig00007.g4608;Description=feebly like [Fusarium napiforme];Gene=FOQG_05905;Ontology_term=antioxidant activity,oxidoreductase activity,lipid metabolic process;Ontology_id=GO:0016209,GO:0016491,GO:0006629;Enzyme_code=EC:1.11.1,EC:1.11,EC:1,EC:1.11.1;Enzyme_name=Acting on a peroxide as acceptor,Acting on a peroxide as acceptor,Oxidoreductases,Acting on a peroxide as acceptor
contig00007.g4609	4918	ID=contig00007.g4609;Description=Nephrocystin-3 [Fusarium globosum]
contig00007.g4610	2791	ID=contig00007.g4610;Description=hypothetical protein FVEG_09320 [Fusarium verticillioides 7600]
contig00007.g4611	597	ID=contig00007.g4611;Description=hypothetical protein FVEG_09321 [Fusarium verticillioides 7600]

contig00007.g4612	4708	ID=contig00007.g4612;Description=ATPase [Fusarium verticillioides 7600];Gene=FPCIR_11429;Ontology_term=membrane organization,DNA repair,transmembrane transport,ATP-dependent activity,mitochondrion,DNA recombination,lipid binding,vacuole,transporter activity,plasma membrane,hydrolase activity;Ontology_id=GO:0061024,GO:0006281,GO:0055085,GO:0140657,GO:0005739,GO:0006310,GO:0008289,GO:0005773,GO:0005215,GO:0005886,GO:0016787;Enzyme_code=EC:7.2.2,EC:3.6.1.15,EC:3.6.1,EC:3.6,EC:7.6.2.1,EC:7.6.2.2,EC:3,EC:7.2.2,EC:7,EC:3.6.1.15;Enzyme_name=Catalysing the translocation of inorganic cations,nucleoside-triphosphate phosphatase,Acting on acid anhydrides,Acting on acid anhydrides,P-type phospholipid transporter,ABC-type xenobiotic transporter,Hydrolases,Catalysing the translocation of inorganic cations,Translocases,nucleoside-triphosphate phosphatase
contig00007.g4613	783	ID=contig00007.g4613;Description=CHK kinase-like [Fusarium oxysporum f. sp. vasinfectum]
contig00007.g4614	1075	ID=contig00007.g4614;Description=integral membrane protein [Fusarium pseudoanthophilum];Gene=HZS61_002762;Ontology_term=membrane;Ontology_id=GO:0016020
contig00007.g4615	893	ID=contig00007.g4615;Description=DUF1275 domain protein [Fusarium tjaetaba];Gene=FANTH_9013;Ontology_term=membrane;Ontology_id=GO:0016020
contig00007.g4616	810	ID=contig00007.g4616;Description=related to putative methyltransferase [Fusarium fujikuroi];Gene=FVER53263_09328;Ontology_term=carbohydrate metabolic process,catalytic activity, acting on a protein,cytoplasmic translation,cytosol,cell wall organization or biogenesis,transferase activity,extracellular region,hydrolase activity;Ontology_id=GO:0005975,GO:0140096,GO:0002181,GO:0005829,GO:0071554,GO:0016740,GO:0005576,GO:0016787;Enzyme_code=EC:2.1.1,EC:3.2.1.15,EC:2.1,EC:3.2,EC:2,EC:3,EC:2.1.1.244,EC:2.1.1,EC:3.2.1.15,EC:3.2.1;Enzyme_name=Transferring one-carbon groups,endo-polygalacturonase,Transferring one-carbon groups,Glycosylases,Transferases,Hydrolases,protein N-terminal methyltransferase,Transferring one-carbon groups,endo-polygalacturonase,Glycosylases
contig00007.g4617	768	ID=contig00007.g4617;Description=putative methyltransferase family protein [Fusarium proliferatum ET1];Gene=FPRO_08620;Ontology_term=catalytic activity, acting on a protein,cytoplasmic translation,cytosol,transferase activity;Ontology_id=GO:0140096,GO:0002181,GO:0005829,GO:0016740;Enzyme_code=EC:2.1.1,EC:2.1,EC:2,EC:2.1.1.244,EC:2.1.1;Enzyme_name=Transferring one-carbon groups,Transferring one-carbon groups,Transferases,protein N-terminal methyltransferase,Transferring one-carbon groups

contig00007.g4618	1545	ID=contig00007.g4618;Description=cytochrome P450 monooxygenase [Fusarium pseudocircinatum];Gene=FOVG_06947;Ontology_term=oxidoreductase activity,transferase activity;Ontology_id=GO:0016491,GO:0016740;Enzyme_code=EC:1.14,EC:2.1.1,EC:2.1,EC:1,EC:1.14,EC:2,EC:2.1.1;Enzyme_name=Acting on paired donors, with incorporation or reduction of molecular oxygen. The oxygen incorporated need not be derived from O2,Transferring one-carbon groups,Transferring one-carbon groups,Oxidoreductases,Acting on paired donors, with incorporation or reduction of molecular oxygen. The oxygen incorporated need not be derived from O2,Transferases,Transferring one-carbon groups
contig00007.g4619	2487	ID=contig00007.g4619;Description=unc-45 like b [Fusarium pseudoanthophilum];Gene=FVER53590_09331;Ontology_term=oxidoreductase activity,transferase activity;Ontology_id=GO:0016491,GO:0016740;Enzyme_code=EC:1.14,EC:2.1.1,EC:2.1,EC:1,EC:1.14,EC:2,EC:2.1.1;Enzyme_name=Acting on paired donors, with incorporation or reduction of molecular oxygen. The oxygen incorporated need not be derived from O2,Transferring one-carbon groups,Transferring one-carbon groups,Oxidoreductases,Acting on paired donors, with incorporation or reduction of molecular oxygen. The oxygen incorporated need not be derived from O2,Transferases,Transferring one-carbon groups
contig00007.g4620	1239	ID=contig00007.g4620;Description=spore coat SP96 precursor [Fusarium tjaetaba]
contig00007.g4621	1876	ID=contig00007.g4621;Description=hypothetical protein J7337_005082 [Fusarium musae]
contig00007.g4622	2372	ID=contig00007.g4622;Description=hypothetical protein H9Q72_010252 [Fusarium xylarioides]
contig00007.g4623	1353	ID=contig00007.g4623;Description=hypothetical protein FVEG_16564 [Fusarium verticillioides 7600]
contig00007.g4624	872	ID=contig00007.g4624;Description=hypothetical protein FVER14953_09339 [Fusarium verticillioides];Gene=FVER53263_09339;Ontology_term=membrane;Ontology_id=GO:0016020
contig00007.g4625	887	ID=contig00007.g4625;Description=krueppel-like factor 11 [Fusarium napiforme]
contig00007.g4626	1428	ID=contig00007.g4626;Description=hypothetical protein FVEG_09342 [Fusarium verticillioides 7600]
contig00007.g4627	1617	ID=contig00007.g4627;Description=hypothetical protein FVER53263_09343 [Fusarium verticillioides];Gene=BFJ70_g16487;Ontology_term=membrane;Ontology_id=GO:0016020

contig00007.g4628	1908	ID=contig00007.g4628;Description=phosphate-repressible phosphate permease [Fusarium proliferatum];Gene=FCIRC_12149;Ontology_term=membrane organization,transmembrane transport,vesicle-mediated transport,endosome,microtubule organizing center,GTPase activity,signaling,endoplasmic reticulum,intracellular protein transport,transporter activity,plasma membrane,protein localization to plasma membrane;Ontology_id=GO:0061024,GO:0055085,GO:0016192,GO:0005768,GO:0005815,GO:0003924,GO:0023052,GO:0005783,GO:0006886,GO:0005215,GO:0005886,GO:0072659;Enzyme_code=EC:3.6.1.15,EC:3.6.1,EC:3.6,EC:3,EC:3.6.1.15;Enzyme_name=nucleoside-triphosphate phosphatase,Acting on acid anhydrides,Acting on acid anhydrides,Hydrolases,nucleoside-triphosphate phosphatase
contig00007.g4629	1176	ID=contig00007.g4629;Description=K P-type ATPase (mediates high-affinity potassium or sodium uptake) [Fusarium tjaetaba]
contig00007.g4630	3748	ID=contig00007.g4630;Description=K, P-type ATPase (mediates high-affinity potassium or sodium uptake) [Fusarium fujikuroi];Gene=FPRO_08640;Ontology_term=ATP-dependent activity,hydrolase activity;Ontology_id=GO:0140657,GO:0016787;Enzyme_code=EC:3.6.1.15,EC:3.6.1,EC:3.6,EC:3.6.3.10,EC:3,EC:3.6.3.9,EC:3.6.1.15;Enzyme_name=nucleoside-triphosphate phosphatase,Acting on acid anhydrides,Acting on acid anhydrides,Acting on acid anhydrides,Hydrolases,Acting on acid anhydrides,nucleoside-triphosphate phosphatase
contig00007.g4631	945	ID=contig00007.g4631;Description=related to SER33 3-phosphoglycerate dehydrogenase [Fusarium fujikuroi];Gene=FPRO_08641;Ontology_term=NAD binding;Ontology_id=GO:0051287
contig00007.g4632	666	ID=contig00007.g4632;Description=hypothetical protein FVER53590_09348 [Fusarium verticillioides]
contig00007.g4633	1197	ID=contig00007.g4633;Description=related to aminopeptidase [Fusarium fujikuroi];Gene=FFB14_03068;Ontology_term=catalytic activity, acting on a protein,lyase activity,hydrolase activity;Ontology_id=GO:0140096,GO:0016829,GO:0016787;Enzyme_code=EC:3.4.11,EC:4.2.1.10,EC:3.4.11,EC:3.4.11.19,EC:4.2.1,EC:3.4,EC:4.2.1.10,EC:4.2,EC:3,EC:4;Enzyme_name=Acting on peptide bonds (peptidases),3-dehydroquinate dehydratase,Acting on peptide bonds (peptidases),D-stereospecific aminopeptidase,Carbon-oxygen lyases,Acting on peptide bonds (peptidases),3-dehydroquinate dehydratase,Carbon-oxygen lyases,Hydrolases,Lyases

contig00007.g4634	1263	ID=contig00007.g4634;Description=hypothetical protein FVEG_09350 [Fusarium verticillioides 7600];Gene=FVER53263_09350;Ontology_term=extracellular region;Ontology_id=GO:0005576
contig00007.g4635	1008	ID=contig00007.g4635;Description=hypothetical protein FVER14953_09351 [Fusarium verticillioides];Gene=FVER53590_09351;Ontology_term=membrane;Ontology_id=GO:0016020
contig00007.g4636	399	ID=contig00007.g4636;Description=galactoside o-acetyltransferase [Fusarium tjaetaba];Gene=FVER53590_09352;Ontology_term=transferase activity;Ontology_id=GO:0016740;Enzyme_code=EC:2.7.7.13,EC:2.3.1,EC:2.3,EC:2.7,EC:2,EC:2.7.7,EC:2.7.7.13,EC:2.3.1;Enzyme_name=mannose-1-phosphate guanylyltransferase,Acyltransferases,Acyltransferases,Transferring phosphorus-containing groups,Transferases,Transferring phosphorus-containing groups,mannose-1-phosphate guanylyltransferase,Acyltransferases
contig00007.g4637	735	ID=contig00007.g4637;Description=hypothetical protein FVEG_09354 [Fusarium verticillioides 7600]
contig00007.g4638	1335	ID=contig00007.g4638;Description=hypothetical protein FVER14953_09355 [Fusarium verticillioides]
contig00007.g4639	366	ID=contig00007.g4639;Description=thioredoxin 1 [Fusarium proliferatum]
contig00007.g4640	1424	ID=contig00007.g4640;Description=integral membrane protein PTH11 [Fusarium globosum];Gene=FOMG_07484;Ontology_term=membrane;Ontology_id=GO:0016020
contig00007.g4641	1305	ID=contig00007.g4641;Description=hypothetical protein FVEG_09358 [Fusarium verticillioides 7600];Gene=FVEG_09358;Ontology_term=nucleus,regulation of DNA-templated transcription,transcription regulator activity;Ontology_id=GO:0005634,GO:0006355,GO:0140110
contig00007.g4642	1669	ID=contig00007.g4642;Description=related to allantoate permease [Fusarium fujikuroi];Gene=BFJ72_g7780;Ontology_term=transmembrane transport,transporter activity;Ontology_id=GO:0055085,GO:0005215
contig00007.g4643	2356	ID=contig00007.g4643;Description=cutinase transcription factor 1 beta [Fusarium coicis];Gene=FANTH_9078;Ontology_term=nucleus,DNA binding,regulation of DNA-templated transcription,transcription regulator activity;Ontology_id=GO:0005634,GO:0003677,GO:0006355,GO:0140110

contig00007.g4644	998	ID=contig00007.g4644;Description=carbohydrate esterase family 1 [Fusarium agapanthi];Gene=FMUND_3090;Ontology_term=carbohydrate metabolic process,cell wall organization or biogenesis,extracellular region,hydrolase activity;Ontology_id=GO:0005975,GO:0071554,GO:0005576,GO:0016787;Enzyme_code=EC:3.1.1.73,EC:3.1,EC:3,EC:3.1.1.73,EC:3.1.1;Enzyme_name=feruloyl esterase,Acting on ester bonds,Hydrolases,feruloyl esterase,Acting on ester bonds
contig00007.g4645	1098	ID=contig00007.g4645;Description=alpha beta hydrolase family domain-containing protein [Fusarium coicis];Gene=FGLOB1_1523;Ontology_term=hydrolase activity;Ontology_id=GO:0016787;Enzyme_code=EC:3,EC:3;Enzyme_name=Hydrolases,Hydrolases
contig00007.g4646	258	ID=contig00007.g4646;Description=hypothetical protein FVER53263_20244 [Fusarium verticillioides]
contig00007.g4647	3304	ID=contig00007.g4647;Description=SNF2-like, N-terminal domain superfamily [Fusarium oxysporum f. sp. vasinfectum];Gene=FOIG_09028;Ontology_term=nucleus,catalytic activity, acting on DNA,ATP-dependent activity,chromatin organization,hydrolase activity;Ontology_id=GO:0005634,GO:0140097,GO:0140657,GO:0006325,GO:0016787;Enzyme_code=EC:3,EC:3;Enzyme_name=Hydrolases,Hydrolases
contig00007.g4648	1059	ID=contig00007.g4648;Description=transcription regulatory SNF2 [Fusarium pseudocircinatum];Gene=FVER53590_09362;Ontology_term=nucleus,catalytic activity, acting on DNA,ATP-dependent activity,regulation of DNA-templated transcription,chromatin organization,transcription regulator activity,hydrolase activity;Ontology_id=GO:0005634,GO:0140097,GO:0140657,GO:0006355,GO:0006325,GO:0140110,GO:0016787;Enzyme_code=EC:3,EC:3;Enzyme_name=Hydrolases,Hydrolases
contig00007.g4649	733	ID=contig00007.g4649;Description=DEAD-like helicase [Fusarium coicis]
contig00007.g4650	1620	ID=contig00007.g4650;Description=hypothetical protein FVER14953_09366 [Fusarium verticillioides]
contig00007.g4651	309	ID=contig00007.g4651;Description=hypothetical protein FVEG_16568 [Fusarium verticillioides 7600]
contig00007.g4652	1519	ID=contig00007.g4652;Description=related to tetracycline resistance proteins [Fusarium fujikuroi];Gene=FHETE_869;Ontology_term=transmembrane transport,transporter activity;Ontology_id=GO:0055085,GO:0005215

contig00007.g4653	1087	ID=contig00007.g4653;Description=alcohol dehydrogenase [Fusarium tjaetaba];Gene=AU210_008528;Ontology_term=oxidoreductase activity;Ontology_id=GO:0016491;Enzyme_code=EC:1.1.1,EC:1.1,EC:1,EC:1.1.1;Enzyme_name=Acting on the CH-OH group of donors,Acting on the CH-OH group of donors,Oxidoreductases,Acting on the CH-OH group of donors
contig00007.g4654	1338	ID=contig00007.g4654;Description=laminaripentaose-producing beta-1,3-glucanase [Fusarium napiforme]
contig00007.g4655	2322	ID=contig00007.g4655;Description=primary-amine oxidase [Fusarium verticillioides 7600];Gene=FNYG_14145;Ontology_term=oxidoreductase activity,detoxification,nitrogen cycle metabolic process;Ontology_id=GO:0016491,GO:0098754,GO:0071941;Enzyme_code=EC:1.4.3.21,EC:1.4,EC:1.4.3,EC:1,EC:1.4.3.21;Enzyme_name=primary-amine oxidase,Acting on the CH-NH2 group of donors,Acting on the CH-NH2 group of donors,Oxidoreductases,primary-amine oxidase
contig00007.g4656	1584	ID=contig00007.g4656;Description=hypothetical protein FVEG_09372 [Fusarium verticillioides 7600];Gene=FVER53263_09372;Ontology_term=regulation of DNA-templated transcription,transcription regulator activity;Ontology_id=GO:0006355,GO:0140110
contig00007.g4657	1335	ID=contig00007.g4657;Description=hypothetical protein FVEG_16569 [Fusarium verticillioides 7600]
contig00007.g4658	3912	ID=contig00007.g4658;Description=Ca2+-transporting ATPase [Fusarium verticillioides 7600];Gene=FVER53590_09373;Ontology_term=transmembrane transport,ATP-dependent activity,transporter activity,hydrolase activity;Ontology_id=GO:0055085,GO:0140657,GO:0005215,GO:0016787;Enzyme_code=EC:7.2.2.10,EC:3.6.1.15,EC:3.6.1,EC:7.2.2.10,EC:3.6,EC:3,EC:3.6.3.8,EC:7,EC:7.2.2,EC:3.6.1.15;Enzyme_name=P-type Ca(2+) transporter,nucleoside-triphosphate phosphatase,Acting on acid anhydrides,P-type Ca(2+) transporter,Acting on acid anhydrides,Hydrolases,Acting on acid anhydrides,Translocases,Catalysing the translocation of inorganic cations,nucleoside-triphosphate phosphatase
contig00007.g4659	3210	ID=contig00007.g4659;Description=hypothetical protein FVER53263_09374 [Fusarium verticillioides]
contig00007.g4660	252	ID=contig00007.g4660;Description=hypothetical protein FVER14953_21590 [Fusarium verticillioides]

contig00007.g4661	1152	ID=contig00007.g4661;Description=trypsin-like serine protease [Fusarium tjaetaba];Gene=FVER53590_09375;Ontology_term=catalytic activity, acting on a protein,hydrolase activity;Ontology_id=GO:0140096,GO:0016787;Enzyme_code=EC:3.4.21,EC:3.4.21,EC:3.4,EC:3;Enzyme_name=Acting on peptide bonds (peptidases),Acting on peptide bonds (peptidases),Acting on peptide bonds (peptidases),Hydrolases
contig00007.g4662	1651	ID=contig00007.g4662;Description=major facilitator superfamily transporter [Fusarium subglutinans];Gene=FACUT_8703;Ontology_term=transmembrane transport,transporter activity;Ontology_id=GO:0055085,GO:0005215
contig00007.g4663	1191	ID=contig00007.g4663;Description=hypothetical protein FVER14953_09378 [Fusarium verticillioides]
contig00007.g4664	1965	ID=contig00007.g4664;Description=heterokaryon incompatibility 6 OR allele [Fusarium tjaetaba]
contig00007.g4665	1821	ID=contig00007.g4665;Description=hypothetical protein FVER53590_09381 [Fusarium verticillioides]
contig00007.g4666	1541	ID=contig00007.g4666;Description=major facilitator superfamily domain-containing protein [Fusarium oxysporum];Gene=FVEG_09382;Ontology_term=transmembrane transport,transporter activity;Ontology_id=GO:0055085,GO:0005215
contig00007.g4667	678	ID=contig00007.g4667;Description=phosphoglycerate mutase family [Fusarium agapanthi]
contig00007.g4668	883	ID=contig00007.g4668;Description=carboxymuconolactone decarboxylase [Fusarium tjaetaba]
contig00007.g4669	498	ID=contig00007.g4669;Description=cupin domain protein [Fusarium tjaetaba]
contig00007.g4670	1189	ID=contig00007.g4670;Description=adenosine deaminase [Fusarium verticillioides 7600];Gene=FVER53590_09385;Ontology_term=nucleobase-containing small molecule metabolic process,hydrolase activity;Ontology_id=GO:0055086,GO:0016787;Enzyme_code=EC:3.5.4.4,EC:3.5.4,EC:3.5,EC:3,EC:3.5.4.4;Enzyme_name=adenosine deaminase,Acting on carbon-nitrogen bonds, other than peptide bonds,Acting on carbon-nitrogen bonds, other than peptide bonds,Hydrolases,adenosine deaminase
contig00007.g4671	219	ID=contig00007.g4671;Description=adenosine deaminase [Fusarium tjaetaba];Gene=FTJAE_12749;Ontology_term=nucleobase-containing small molecule metabolic process,hydrolase activity;Ontology_id=GO:0055086,GO:0016787;Enzyme_code=EC:3.5.4;Enzyme_name=Acting on carbon-nitrogen bonds, other than peptide bonds

contig00007.g4672	779	ID=contig00007.g4672;Description=short-chain dehydrogenases [Fusarium pseudocircinatum]
contig00007.g4673	765	ID=contig00007.g4673;Description=pectate lyase E [Fusarium verticillioides 7600];Gene=FTJAE_12753;Ontology_term=carbohydrate metabolic process,lyase activity,extracellular region;Ontology_id=GO:0005975,GO:0016829,GO:0005576;Enzyme_code=EC:4.2.2.2,EC:4.2.2.2,EC:4.2,EC:4,EC:4.2.2;Enzyme_name=pectate lyase,pectate lyase,Carbon-oxygen lyases,Lyases,Carbon-oxygen lyases
contig00007.g4674	2200	ID=contig00007.g4674;Description=probable flavoprotein involved in K+ transport [Fusarium fujikuroi];Gene=FVER53263_09388;Ontology_term=catalytic activity, acting on a protein,nucleobase-containing small molecule metabolic process,carbohydrate derivative metabolic process,transferase activity;Ontology_id=GO:0140096,GO:0055086,GO:1901135,GO:0016740;Enzyme_code=EC:2.7.1,EC:2.7.1,EC:2.7,EC:2;Enzyme_name=Transferring phosphorus-containing groups,Transferring phosphorus-containing groups,Transferring phosphorus-containing groups,Transferases
contig00007.g4675	5151	ID=contig00007.g4675;Description=AGC/PKA protein kinase [Fusarium verticillioides 7600];Gene=F66182_7748;Ontology_term=catalytic activity, acting on a protein,nucleobase-containing small molecule metabolic process,carbohydrate derivative metabolic process,transferase activity;Ontology_id=GO:0140096,GO:0055086,GO:1901135,GO:0016740;Enzyme_code=EC:2.7.11.1,EC:2.7.1,EC:2.7.11.1,EC:2.7,EC:2;Enzyme_name=non-specific serine/threonine protein kinase,Transferring phosphorus-containing groups,non-specific serine/threonine protein kinase,Transferring phosphorus-containing groups,Transferases
contig00007.g4676	1981	ID=contig00007.g4676;Description=hypothetical protein FVEG_09391 [Fusarium verticillioides 7600];Gene=FPHYL_4047;Ontology_term=oxidoreductase activity;Ontology_id=GO:0016491;Enzyme_code=EC:1.14,EC:1,EC:1.14;Enzyme_name=Acting on paired donors, with incorporation or reduction of molecular oxygen. The oxygen incorporated need not be derived from O2,Oxidoreductases,Acting on paired donors, with incorporation or reduction of molecular oxygen. The oxygen incorporated need not be derived from O2

contig00007.g4677	1761	ID=contig00007.g4677;Description=related to pisatin demethylase cytochrome P450 [Fusarium fujikuroi];Gene=FFC1_03681;Ontology_term=oxidoreductase activity,transferase activity;Ontology_id=GO:0016491,GO:0016740;Enzyme_code=EC:1.14,EC:2.1.1,EC:2.1,EC:1,EC:1.14,EC:2,EC:2.1.1;Enzyme_name=Acting on paired donors, with incorporation or reduction of molecular oxygen. The oxygen incorporated need not be derived from O2,Transferring one-carbon groups,Transferring one-carbon groups,Oxidoreductases,Acting on paired donors, with incorporation or reduction of molecular oxygen. The oxygen incorporated need not be derived from O2,Transferases,Transferring one-carbon groups
contig00007.g4678	1544	ID=contig00007.g4678;Description=lysine decarboxylase [Fusarium tjaetaba];Gene=FVEG_09393;Ontology_term=hydrolase activity;Ontology_id=GO:0016787;Enzyme_code=EC:3,EC:3;Enzyme_name=Hydrolases,Hydrolases
contig00007.g4679	1037	ID=contig00007.g4679;Description=multifunctional beta-oxidation [Fusarium tjaetaba]
contig00007.g4680	1368	ID=contig00007.g4680;Description=toxD [Fusarium tjaetaba];Gene=FVER53590_09395;Ontology_term=oxidoreductase activity;Ontology_id=GO:0016491;Enzyme_code=EC:1.6,EC:1.6,EC:1;Enzyme_name=Acting on NADH or NADPH,Acting on NADH or NADPH,Oxidoreductases
contig00007.g4681	713	ID=contig00007.g4681;Description=lactoylglutathione lyase [Fusarium verticillioides 7600];Gene=FVER53590_09396;Ontology_term=lyase activity;Ontology_id=GO:0016829;Enzyme_code=EC:4.4.1.5,EC:4.4.1.5,EC:4.4,EC:4;Enzyme_name=lactoylglutathione lyase,lactoylglutathione lyase,Carbon-sulfur lyases,Lyases
contig00007.g4682	495	ID=contig00007.g4682;Description=Glyoxalase I [Fusarium globosum];Gene=FGLOB1_6018
contig00007.g4683	6709	ID=contig00007.g4683;Description=heterokaryon incompatibility het-6 [Fusarium tjaetaba];Gene=FVER53263_09397;Ontology_term=oxidoreductase activity;Ontology_id=GO:0016491;Enzyme_code=EC:1.14,EC:1,EC:1.14;Enzyme_name=Acting on paired donors, with incorporation or reduction of molecular oxygen. The oxygen incorporated need not be derived from O2,Oxidoreductases,Acting on paired donors, with incorporation or reduction of molecular oxygen. The oxygen incorporated need not be derived from O2
contig00007.g4684	1032	ID=contig00007.g4684;Description=GA4 desaturase [Fusarium tjaetaba];Gene=FSUBG_9995;Ontology_term=oxidoreductase activity;Ontology_id=GO:0016491;Enzyme_code=EC:1,EC:1;Enzyme_name=Oxidoreductases,Oxidoreductases

contig00007.g4685	1746	ID=contig00007.g4685;Description=ankyrin-2 isoform 2 [Fusarium coicis];Gene=FPCIR_2690;Ontology_term=transferase activity;Ontology_id=GO:0016740;Enzyme_code=EC:2,EC:2;Enzyme_name=Transferases,Transferases
contig00007.g4686	2420	ID=contig00007.g4686;Description=sugar transporter [Fusarium mundagurra];Gene=FNYG_12031;Ontology_term=transmembrane transport,transporter activity;Ontology_id=GO:0055085,GO:0005215
contig00007.g4687	1029	ID=contig00007.g4687;Description=zinc-binding oxidoreductase [Fusarium mexicanum];Gene=FVER53590_09401;Ontology_term=oxidoreductase activity;Ontology_id=GO:0016491;Enzyme_code=EC:1,EC:1;Enzyme_name=Oxidoreductases,Oxidoreductases
contig00007.g4688	1447	ID=contig00007.g4688;Description=related to class V chitinase [Fusarium proliferatum ET1];Gene=rntB;Ontology_term=carbohydrate metabolic process,RNA binding,lyase activity,catalytic activity, acting on RNA,hydrolase activity;Ontology_id=GO:0005975,GO:0003723,GO:0016829,GO:0140098,GO:0016787;Enzyme_code=EC:3.1,EC:3.2.1.14,EC:4.6.1.19,EC:3.2,EC:3.1,EC:3,EC:4,EC:3.2.1.14,EC:4.6,EC:4.6.1.19,EC:3.2.1;Enzyme_name=Acting on ester bonds,chitinase,ribonuclease T2,Glycosylases,Acting on ester bonds,Hydrolases,Lyases,chitinase,Phosphorus-oxygen lyases,ribonuclease T2,Glycosylases
contig00007.g4689	3947	ID=contig00007.g4689;Description=acid phosphatase [Fusarium verticillioides 7600];Gene=FVER53263_09404;Ontology_term=hydrolase activity;Ontology_id=GO:0016787;Enzyme_code=EC:3.1.3.2,EC:3.1,EC:3,EC:3.1.3.2,EC:3.1.3;Enzyme_name=acid phosphatase,Acting on ester bonds,Hydrolases,acid phosphatase,Acting on ester bonds
contig00007.g4690	1045	ID=contig00007.g4690;Description=related to cellulose-binding GDSL lipase/acylhydrolase [Fusarium fujikuroi IMI 58289];Gene=F25303_7624;Ontology_term=hydrolase activity;Ontology_id=GO:0016787;Enzyme_code=EC:3.1,EC:3.1,EC:3;Enzyme_name=Acting on ester bonds,Acting on ester bonds,Hydrolases
contig00007.g4691	574	ID=contig00007.g4691;Description=hypothetical protein FPRO05_05651 [Fusarium proliferatum];Gene=HZS61_002697;Ontology_term=membrane;Ontology_id=GO:0016020
contig00007.g4692	1349	ID=contig00007.g4692;Description=integral membrane protein [Fusarium napiforme];Gene=FVER53263_09407;Ontology_term=membrane;Ontology_id=GO:0016020

contig00007.g4693	1336	ID=contig00007.g4693;Description=integral membrane protein [Fusarium pseudoanthophilum];Gene=FVEG_09408;Ontology_term=membrane;Ontology_id=GO:0016020
contig00007.g4694	1164	ID=contig00007.g4694;Description=hypothetical protein FVER53263_09409 [Fusarium verticillioides]
contig00007.g4695	741	ID=contig00007.g4695;Description=hypothetical protein FVEG_09410 [Fusarium verticillioides 7600]
contig00007.g4696	1580	ID=contig00007.g4696;Description=aldehyde dehydrogenase [Fusarium pseudoanthophilum];Gene=Forpi1262_v015105;Ontology_term=oxidoreductase activity;Ontology_id=GO:0016491;Enzyme_code=EC:1.2.1;Enzyme_name=Acting on the aldehyde or oxo group of donors
contig00007.g4697	4139	ID=contig00007.g4697;Description=major facilitator superfamily transporter [Fusarium mundagurra];Gene=FACUT_6855;Ontology_term=oxidoreductase activity,lipid metabolic process;Ontology_id=GO:0016491,GO:0006629;Enzyme_code=EC:1.3.1,EC:1.3,EC:1,EC:1.3.1;Enzyme_name=Acting on the CH-CH group of donors,Acting on the CH-CH group of donors,Oxidoreductases,Acting on the CH-CH group of donors
contig00007.g4698	1985	ID=contig00007.g4698;Description=heterokaryon incompatibility protein-domain-containing protein [Fusarium oxysporum]
contig00007.g4699	1620	ID=contig00007.g4699;Description=erg24, C-14 sterol reductase [Fusarium oxysporum];Gene=FocTR4_00009391;Ontology_term=oxidoreductase activity,lipid metabolic process,endoplasmic reticulum;Ontology_id=GO:0016491,GO:0006629,GO:0005783;Enzyme_code=EC:1.3.1,EC:1.3,EC:1,EC:1.3.1.70,EC:1.3.1;Enzyme_name=Acting on the CH-CH group of donors,Acting on the CH-CH group of donors,Oxidoreductases,Delta(14)-sterol reductase,Acting on the CH-CH group of donors
contig00007.g4700	1617	ID=contig00007.g4700;Description=homogentisate 1,2-dioxygenase [Fusarium proliferatum];Gene=FPANT_10435;Ontology_term=oxidoreductase activity,amino acid metabolic process;Ontology_id=GO:0016491,GO:0006520;Enzyme_code=EC:1.13.11.5,EC:1.13.11,EC:1.13,EC:1,EC:1.13.11.5;Enzyme_name=homogentisate 1,2-dioxygenase,Acting on single donors with incorporation of molecular oxygen (oxygenases). The oxygen incorporated need not be derived from O2,Acting on single donors with incorporation of molecular oxygen (oxygenases). The oxygen incorporated need not be derived from O2,Oxidoreductases,homogentisate 1,2-dioxygenase

contig00007.g4701	1354	ID=contig00007.g4701;Description=hypothetical protein FVER14953_21583 [Fusarium verticillioides]
contig00007.g4702	1389	ID=contig00007.g4702;Description=hypothetical protein FVER14953_09415 [Fusarium verticillioides];Gene=SDH2;Ontology_term=generation of precursor metabolites and energy,nucleobase-containing small molecule metabolic process,oxidoreductase activity,mitochondrion,carbohydrate derivative metabolic process,vacuole;Ontology_id=GO:0006091,GO:0055086,GO:0016491,GO:0005739,GO:1901135,GO:0005773;Enzyme_code=EC:1.3.5.1,EC:1.3,EC:1,EC:1.3.5.1;Enzyme_name=succinate dehydrogenase,Acting on the CH-CH group of donors,Oxidoreductases,succinate dehydrogenase
contig00007.g4703	3048	ID=contig00007.g4703;Description=hypothetical protein FVEG_09416 [Fusarium verticillioides 7600]
contig00007.g4704	372	ID=contig00007.g4704;Description=hypothetical protein FVEG_09417 [Fusarium verticillioides 7600]
contig00007.g4705	1652	ID=contig00007.g4705;Description=quinate permease [Fusarium subglutinans];Gene=FVER53263_09418;Ontology_term=transmembrane transport,transporter activity;Ontology_id=GO:0055085,GO:0005215
contig00007.g4706	2097	ID=contig00007.g4706;Description=heterokaryon incompatibility (het-6OR allele) [Fusarium tjaetaba];Gene=FVER53590_09419;Ontology_term=oxidoreductase activity;Ontology_id=GO:0016491;Enzyme_code=EC:1.14,EC:1,EC:1.14;Enzyme_name=Acting on paired donors, with incorporation or reduction of molecular oxygen. The oxygen incorporated need not be derived from O2,Oxidoreductases,Acting on paired donors, with incorporation or reduction of molecular oxygen. The oxygen incorporated need not be derived from O2
contig00007.g4707	1518	ID=contig00007.g4707;Description=cytochrome P450 monooxygenase oxidoreductase [Fusarium tjaetaba];Gene=FPANT_10843;Ontology_term=oxidoreductase activity;Ontology_id=GO:0016491;Enzyme_code=EC:1.14,EC:1,EC:1.14;Enzyme_name=Acting on paired donors, with incorporation or reduction of molecular oxygen. The oxygen incorporated need not be derived from O2,Oxidoreductases,Acting on paired donors, with incorporation or reduction of molecular oxygen. The oxygen incorporated need not be derived from O2

contig00007.g4708	1993	ID=contig00007.g4708;Description=C2H2 transcription factor [Fusarium tjaetaba];Gene=FPANT_10842;Ontology_term=nucleus,DNA binding,regulation of DNA-templated transcription,transcription regulator activity;Ontology_id=GO:0005634,GO:0003677,GO:0006355,GO:0140110
contig00007.g4709	1572	ID=contig00007.g4709;Description=HNM1-choline permease [Fusarium napiforme];Gene=BFJ72_g7919;Ontology_term=transmembrane transport,transporter activity;Ontology_id=GO:0055085,GO:0005215
contig00007.g4710	978	ID=contig00007.g4710;Description=phytanoyl-dioxygenase family [Fusarium tjaetaba];Gene=FTJAE_12931;Ontology_term=oxidoreductase activity;Ontology_id=GO:0016491;Enzyme_code=EC:1,EC:1;Enzyme_name=Oxidoreductases,Oxidoreductases
contig00007.g4711	1070	ID=contig00007.g4711;Description=C6 transcription factor [Fusarium agapanthi]
contig00007.g4712	2026	ID=contig00007.g4712;Description=transcriptional activator of proteases prtT [Fusarium napiforme];Gene=FVER53590_26001;Ontology_term=nucleus,catalytic activity, acting on a protein,regulation of DNA-templated transcription,transcription regulator activity,hydrolase activity;Ontology_id=GO:0005634,GO:0140096,GO:0006355,GO:0140110,GO:0016787;Enzyme_code=EC:3.4;Enzyme_name=Acting on peptide bonds (peptidases)
contig00007.g4713	1915	ID=contig00007.g4713;Description=uracil permease [Fusarium tjaetaba];Gene=FGLOB1_6217;Ontology_term=transmembrane transport,transporter activity;Ontology_id=GO:0055085,GO:0005215
contig00007.g4714	5549	ID=contig00007.g4714;Description=TKL protein kinase [Fusarium verticillioides 7600];Gene=CEK26_009195;Ontology_term=catalytic activity, acting on a protein,transferase activity;Ontology_id=GO:0140096,GO:0016740;Enzyme_code=EC:2.7.1,EC:2.7.1,EC:2.7,EC:2;Enzyme_name=Transferring phosphorus-containing groups,Transferring phosphorus-containing groups,Transferring phosphorus-containing groups,Transferases
contig00007.g4715	818	ID=contig00007.g4715;Description=hypothetical protein FNAPI_6164 [Fusarium napiforme];Gene=FAUST_10801;Ontology_term=membrane;Ontology_id=GO:0016020

contig00007.g4716	2292	ID=contig00007.g4716;Description=hypothetical protein FVER14953_09427 [Fusarium verticillioides];Gene=FVER53263_09427;Ontology_term=catalytic activity, acting on a protein,transferase activity,hydrolase activity;Ontology_id=GO:0140096,GO:0016740,GO:0016787;Enzyme_code=EC:2.7.1,EC:3.4.22,EC:2.7.1,EC:3.4.22,EC:3.4,EC:2.7,EC:2,EC:3;Enzyme_name=Transferring phosphorus-containing groups,Acting on peptide bonds (peptidases),Transferring phosphorus-containing groups,Acting on peptide bonds (peptidases),Acting on peptide bonds (peptidases),Transferring phosphorus-containing groups,Transferases,Hydrolases
contig00007.g4717	428	ID=contig00007.g4717;Description=hypothetical protein FVER53263_20322 [Fusarium verticillioides]
contig00007.g4718	2899	ID=contig00007.g4718;Description=transcription factor Cys6 [Fusarium tjaetaba];Gene=FTJAE_12925;Ontology_term=metal ion binding;Ontology_id=GO:0046872
contig00007.g4719	1826	ID=contig00007.g4719;Description=hypothetical protein FVEG_09430 [Fusarium verticillioides 7600]
contig00007.g4720	2079	ID=contig00007.g4720;Description=hypothetical protein FVER53590_09431 [Fusarium verticillioides];Gene=FVER53590_09431;Ontology_term=membrane;Ontology_id=GO:0016020
contig00007.g4721	966	ID=contig00007.g4721;Description=mitochondrial chaperone BCS1 [Fusarium tjaetaba]
contig00007.g4722	944	ID=contig00007.g4722;Description=hypothetical protein FVEG_16586 [Fusarium verticillioides 7600];Gene=FOXB_03864
contig00007.g4723	900	ID=contig00007.g4723;Description=metallo-beta-lactamase superfamily [Fusarium tjaetaba]
contig00007.g4724	906	ID=contig00007.g4724;Description=2'-hydroxyisoflavone reductase [Fusarium tjaetaba];Gene=FPANT_12830;Ontology_term=oxidoreductase activity;Ontology_id=GO:0016491;Enzyme_code=EC:1.2.1,EC:1.2,EC:1,EC:1.2.1;Enzyme_name=Acting on the aldehyde or oxo group of donors,Acting on the aldehyde or oxo group of donors,Oxidoreductases,Acting on the aldehyde or oxo group of donors
contig00007.g4725	525	ID=contig00007.g4725;Description=hypothetical protein FVEG_09435 [Fusarium verticillioides 7600]
contig00007.g4726	1888	ID=contig00007.g4726;Description=MFS transporter, SP family, general alpha glucoside:H+ symporter [Fusarium verticillioides 7600];Gene=FOMG_07584;Ontology_term=transmembrane transport,transporter activity;Ontology_id=GO:0055085,GO:0005215
contig00007.g4727	1626	ID=contig00007.g4727;Description=neutral amino acid permease [Fusarium pseudocircinatum];Gene=FPHYL_2800;Ontology_term=membrane;Ontology_id=GO:0016020

contig00007.g4728	336	ID=contig00007.g4728;Description=hypothetical protein FVER14953_09438 [Fusarium verticillioides]
contig00007.g4729	2528	ID=contig00007.g4729;Description=Pesticidal crystal cry3Ba [Fusarium tjaetaba];Gene=FVER53263_09439;Ontology_term=toxin activity;Ontology_id=GO:0090729
contig00007.g4730	1101	ID=contig00007.g4730;Description=trichothecene biosynthesis [Fusarium tjaetaba]
contig00007.g4731	950	ID=contig00007.g4731;Description=related to ketoreductases [Fusarium proliferatum ET1];Gene=FPCIR_8968;Ontology_term=nucleus,DNA binding,generation of precursor metabolites and energy,oxidoreductase activity,mitochondrion,lipid metabolic process,regulation of DNA-templated transcription,endoplasmic reticulum,transferase activity,transcription regulator activity;Ontology_id=GO:0005634,GO:0003677,GO:0006091,GO:0016491,GO:0005739,GO:0006629,GO:0006355,GO:0005783,GO:0016740,GO:0140110;Enzyme_code=EC:1,EC:2.3.1.85,EC:1.1,EC:2.3,EC:1,EC:2,EC:1.1.1,EC:1.1.1.100,EC:1.3.1.34,EC:2.3.1;Enzyme_name=Oxidoreductases,fatty-acid synthase system,Acting on the CH-OH group of donors,Acyltransferases,Oxidoreductases,Transferases,Acting on the CH-OH group of donors,3-oxoacyl-[acyl-carrier-protein] reductase,2,4-dienoyl-CoA reductase [(2E)-enoyl-CoA-producing],Acyltransferases
contig00007.g4732	2621	ID=contig00007.g4732;Description=glucan endo-1,3-beta-glucosidase [Fusarium verticillioides 7600];Gene=FVER53590_09443;Ontology_term=hydrolase activity;Ontology_id=GO:0016787;Enzyme_code=EC:3.2.1.15;Enzyme_name=endo-polygalacturonase
contig00007.g4733	869	ID=contig00007.g4733;Description=putative uracil permease [Fusarium bulbicola]
contig00007.g4734	1919	ID=contig00007.g4734;Description=uncharacterized protein FTJAE_12908 [Fusarium tjaetaba]
contig00007.g4735	3802	ID=contig00007.g4735;Description=extracellular GDSL-like lipase acylhydrolase [Fusarium pseudoanthophilum];Gene=FPANT_11158;Ontology_term=transmembrane transport,transporter activity,hydrolase activity;Ontology_id=GO:0055085,GO:0005215,GO:0016787;Enzyme_code=EC:3,EC:3;Enzyme_name=Hydrolases,Hydrolases
contig00007.g4736	2203	ID=contig00007.g4736;Description=transcription factor SEF1 [Fusarium tjaetaba];Gene=SEF1-1;Ontology_term=nucleus,transmembrane transport,regulation of DNA-templated transcription,transcription regulator activity,transporter activity;Ontology_id=GO:0005634,GO:0055085,GO:0006355,GO:0140110,GO:0005215

contig00007.g4737	1001	ID=contig00007.g4737;Description=related to RSB1-integral membrane transporter or flippase that may transport LCBs from the cytoplasmic side toward the extracytoplasmic side of the membrane [Fusarium fujikuroi];Gene=FVER53263_20327;Ontology_term=membrane;Ontology_id=GO:0016020
contig00007.g4738	1294	ID=contig00007.g4738;Description=multidrug resistance [Fusarium beomiforme];Gene=FVER53263_09449;Ontology_term=transmembrane transport,transporter activity;Ontology_id=GO:0055085,GO:0005215
contig00007.g4739	1056	ID=contig00007.g4739;Description=Altered inheritance of mitochondria 6 [Fusarium acutatum];Gene=FOIG_09156;Ontology_term=lipid metabolic process,hydrolase activity;Ontology_id=GO:0006629,GO:0016787;Enzyme_code=EC:3.1.4,EC:3.1.4,EC:3.1,EC:3;Enzyme_name=Acting on ester bonds,Acting on ester bonds,Acting on ester bonds,Hydrolases
contig00007.g4740	1191	ID=contig00007.g4740;Description=methyltransferase [Fusarium bulbicola];Gene=FDENT_9719;Ontology_term=transferase activity;Ontology_id=GO:0016740;Enzyme_code=EC:2.1.1,EC:2.1,EC:2,EC:2.1.1;Enzyme_name=Transferring one-carbon groups,Transferring one-carbon groups,Transferases,Transferring one-carbon groups
contig00007.g4741	1698	ID=contig00007.g4741;Description=related to beta-galactosidase [Fusarium oxysporum];Gene=FVEG_16591;Ontology_term=carbohydrate metabolic process,hydrolase activity;Ontology_id=GO:0005975,GO:0016787;Enzyme_code=EC:3.2.1.23,EC:3.2,EC:3,EC:3.2.1.23,EC:3.2.1;Enzyme_name=beta-galactosidase,Glycosylases,Hydrolases,beta-galactosidase,Glycosylases
contig00007.g4742	1227	ID=contig00007.g4742;Description=hypothetical protein J7337_004962 [Fusarium musae]
contig00007.g4743	1770	ID=contig00007.g4743;Description=lactose permease [Fusarium pseudocircinatum];Gene=FMUND_10954;Ontology_term=transmembrane transport,transporter activity;Ontology_id=GO:0055085,GO:0005215
contig00008.g4744	2444	ID=contig00008.g4744;Description=related to tyrosinase precursor [Fusarium oxysporum];Gene=FVER53263_12513;Ontology_term=oxidoreductase activity;Ontology_id=GO:0016491;Enzyme_code=EC:1.14.18.1,EC:1,EC:1.14,EC:1.14.18.1,EC:1.14.18;Enzyme_name=tyrosinase,Oxidoreductases,Acting on paired donors, with incorporation or reduction of molecular oxygen. The oxygen incorporated need not be derived from O2,tyrosinase,Acting on paired donors, with incorporation or reduction of molecular oxygen. The oxygen incorporated need not be derived from O2

contig00008.g4745	1593	ID=contig00008.g4745;Description=l-galactonate dehydratase [Fusarium subglutinans];Gene=FMEXI_12669;Ontology_term=lyase activity;Ontology_id=GO:0016829;Enzyme_code=EC:4.2.1,EC:4.2.1,EC:4.2,EC:4,EC:4.2.1.68,EC:4.2.1.146;Enzyme_name=Carbon-oxygen lyases,Carbon-oxygen lyases,Carbon-oxygen lyases,Lyases,L-fuconate dehydratase,L-galactonate dehydratase
contig00008.g4746	1731	ID=contig00008.g4746;Description=putative C6 finger domain protein [Fusarium sp. MPI-SDFR-AT-0072];Gene=FOTG_11409;Ontology_term=nucleus,DNA binding,regulation of DNA-templated transcription,transcription regulator activity;Ontology_id=GO:0005634,GO:0003677,GO:0006355,GO:0140110
contig00008.g4747	1269	ID=contig00008.g4747;Description=dihydroflavonol-4-reductase [Fusarium verticillioides 7600];Gene=FNAPI_12228;Ontology_term=oxidoreductase activity,lipid metabolic process;Ontology_id=GO:0016491,GO:0006629;Enzyme_code=EC:1.1.1.145,EC:1.1,EC:1,EC:1.1.1.145,EC:1.1.1;Enzyme_name=3beta-hydroxy-Delta(5)-steroid dehydrogenase,Acting on the CH-OH group of donors,Oxidoreductases,3beta-hydroxy-Delta(5)-steroid dehydrogenase,Acting on the CH-OH group of donors
contig00008.g4748	1919	ID=contig00008.g4748;Description=sugar transporter STL1 [Fusarium tjaetaba];Gene=FNAPI_12227;Ontology_term=transmembrane transport,transporter activity;Ontology_id=GO:0055085,GO:0005215
contig00008.g4749	1848	ID=contig00008.g4749;Description=polyamine transporter [Fusarium tjaetaba];Gene=FVER53590_25492;Ontology_term=transmembrane transport,transporter activity;Ontology_id=GO:0055085,GO:0005215
contig00008.g4750	1861	ID=contig00008.g4750;Description=Vanillin dehydrogenase [Fusarium tjaetaba];Gene=TCE0_017f03468;Ontology_term=oxidoreductase activity;Ontology_id=GO:0016491;Enzyme_code=EC:1.2.1,EC:1.2,EC:1,EC:1.2.1;Enzyme_name=Acting on the aldehyde or oxo group of donors,Acting on the aldehyde or oxo group of donors,Oxidoreductases,Acting on the aldehyde or oxo group of donors
contig00008.g4751	1883	ID=contig00008.g4751;Description=acetolactate synthase [Fusarium tjaetaba];Gene=FACUT_10428;Ontology_term=lyase activity;Ontology_id=GO:0016829;Enzyme_code=EC:4.1.1.1,EC:4.1.1,EC:4.1.1.1,EC:4.1,EC:4;Enzyme_name=pyruvate decarboxylase,Carbon-carbon lyases,pyruvate decarboxylase,Carbon-carbon lyases,Lyases

contig00008.g4752	1039	ID=contig00008.g4752;Description=ferric-chelate reductase [Fusarium tjaetaba];Gene=FVER53590_12507;Ontology_term=oxidoreductase activity;Ontology_id=GO:0016491;Enzyme_code=EC:1.16;Enzyme_name=Oxidizing metal ions
contig00008.g4753	1929	ID=contig00008.g4753;Description=siderophore iron transporter [Fusarium tjaetaba];Gene=FVER53590_12506;Ontology_term=transmembrane transport,transporter activity,plasma membrane;Ontology_id=GO:0055085,GO:0005215,GO:0005886
contig00008.g4754	4240	ID=contig00008.g4754;Description=multidrug resistance-associated 1 [Fusarium coicis];Gene=FNYG_10165;Ontology_term=transmembrane transport,ATP-dependent activity,transporter activity,hydrolase activity;Ontology_id=GO:0055085,GO:0140657,GO:0005215,GO:0016787;Enzyme_code=EC:7.2.2,EC:3.6.1.15,EC:3.6.1,EC:3.6,EC:3,EC:7.2.2,EC:7,EC:3.6.1.15;Enzyme_name=Catalysing the translocation of inorganic cations,nucleoside-triphosphate phosphatase,Acting on acid anhydrides,Acting on acid anhydrides,Hydrolases,Catalysing the translocation of inorganic cations,Translocases,nucleoside-triphosphate phosphatase
contig00008.g4755	1299	ID=contig00008.g4755;Description=lysine n-acyltransferase [Fusarium pseudoanthophilum];Gene=AU210_013668;Ontology_term=transferase activity;Ontology_id=GO:0016740;Enzyme_code=EC:2.3,EC:2.3,EC:2;Enzyme_name=Acyltransferases,Acyltransferases,Transferases
contig00008.g4756	14124	ID=contig00008.g4756;Description=NRPS protein [Fusarium musae];Gene=FNAPI_10415;Ontology_term=ligase activity,isomerase activity;Ontology_id=GO:0016874,GO:0016853;Enzyme_code=EC:5,EC:6,EC:5,EC:6;Enzyme_name=Isomerases,Ligases,Isomerases,Ligases
contig00008.g4757	1233	ID=contig00008.g4757;Description=glycoside hydrolase superfamily [Fusarium flagelliforme];Gene=FMEXI_9760;Ontology_term=carbohydrate metabolic process,hydrolase activity;Ontology_id=GO:0005975,GO:0016787;Enzyme_code=EC:3.2.1.8,EC:3.2,EC:3.2.1.8,EC:3,EC:3.2.1;Enzyme_name=endo-1,4-beta-xylanase,Glycosylases,endo-1,4-beta-xylanase,Hydrolases,Glycosylases
contig00008.g4758	1690	ID=contig00008.g4758;Description=related to cytochrome P450 monooxygenase [Fusarium fujikuroi];Gene=FVER53263_12501;Ontology_term=oxidoreductase activity;Ontology_id=GO:0016491;Enzyme_code=EC:1;Enzyme_name=Oxidoreductases

contig00008.g4759	1098	ID=contig00008.g4759;Description=hypothetical protein FVEG_12500 [Fusarium verticillioides 7600];Gene=FVER53263_12500;Ontology_term=membrane;Ontology_id=GO:0016020
contig00008.g4760	1415	ID=contig00008.g4760;Description=S-adenosyl-L-methionine-dependent methyltransferase [Fusarium oxysporum];Gene=FMUND_11954;Ontology_term=transferase activity;Ontology_id=GO:0016740;Enzyme_code=EC:2.1.1,EC:2.1,EC:2,EC:2.1.1;Enzyme_name=Transferring one-carbon groups,Transferring one-carbon groups,Transferases,Transferring one-carbon groups
contig00008.g4761	1521	ID=contig00008.g4761;Description=non-ribosomal peptide synthetase [Fusarium tjaetaba];Gene=FDENT_10533;Ontology_term=oxidoreductase activity;Ontology_id=GO:0016491;Enzyme_code=EC:1,EC:1;Enzyme_name=Oxidoreductases,Oxidoreductases
contig00008.g4762	624	ID=contig00008.g4762;Description=coagulation factor 5 8 type domain protein [Fusarium tjaetaba];Gene=FMAN_06835;Ontology_term=carbohydrate metabolic process;Ontology_id=GO:0005975
contig00008.g4763	792	ID=contig00008.g4763;Description=coagulation factor 5 8 type domain protein [Fusarium tjaetaba];Gene=FTJAE_2201;Ontology_term=carbohydrate metabolic process;Ontology_id=GO:0005975
contig00008.g4764	2031	ID=contig00008.g4764;Description=hypothetical protein FVER53263_12497 [Fusarium verticillioides]
contig00008.g4765	1850	ID=contig00008.g4765;Description=hypothetical protein FVER53590_12496 [Fusarium verticillioides]
contig00008.g4766	1759	ID=contig00008.g4766;Description=probable choline transporter Hnm1 [Fusarium fujikuroi];Gene=F52700_4413;Ontology_term=transmembrane transport,transporter activity;Ontology_id=GO:0055085,GO:0005215
contig00008.g4767	3146	ID=contig00008.g4767;Description=serine/threonine protein kinase [Fusarium verticillioides 7600];Gene=FTJAE_2197;Ontology_term=catalytic activity, acting on a protein,transferase activity;Ontology_id=GO:0140096,GO:0016740;Enzyme_code=EC:2.7.11.1,EC:2.7.1,EC:2.7.11.1,EC:2.7,EC:2;Enzyme_name=non-specific serine/threonine protein kinase,Transferring phosphorus-containing groups,non-specific serine/threonine protein kinase,Transferring phosphorus-containing groups,Transferases

contig00008.g4768	411	ID=contig00008.g4768;Description=serine threonine kinase [Fusarium tjaetaba];Gene=FTJAE_2196;Ontology_term=transferase activity;Ontology_id=GO:0016740;Enzyme_code=EC:2.7;Enzyme_name=Transferring phosphorus-containing groups
contig00008.g4769	603	ID=contig00008.g4769;Description=hypothetical protein FVEG_12493 [Fusarium verticillioides 7600];Gene=FNAPI_10427;Ontology_term=membrane;Ontology_id=GO:0016020
contig00008.g4770	2422	ID=contig00008.g4770;Description=het-s domain-containing protein [Fusarium coicis];Gene=FPHYL_11051;Ontology_term=catalytic activity, acting on a protein,transferase activity;Ontology_id=GO:0140096,GO:0016740;Enzyme_code=EC:2.7.1,EC:2.7.1,EC:2.7,EC:2;Enzyme_name=Transferring phosphorus-containing groups,Transferring phosphorus-containing groups,Transferring phosphorus-containing groups,Transferases
contig00008.g4771	453	ID=contig00008.g4771;Description=hypothetical protein FVEG_12491 [Fusarium verticillioides 7600]
contig00008.g4772	2890	ID=contig00008.g4772;Description=phage tail fiber repeat 2 [Fusarium sporotrichioides]
contig00008.g4773	1530	ID=contig00008.g4773;Description=lignostilbene dioxygenase family [Fusarium tjaetaba];Gene=FGLOB1_5689;Ontology_term=oxidoreductase activity;Ontology_id=GO:0016491;Enzyme_code=EC:1.13.11,EC:1.13.11,EC:1.13,EC:1;Enzyme_name=Acting on single donors with incorporation of molecular oxygen (oxygenases). The oxygen incorporated need not be derived from O2,Acting on single donors with incorporation of molecular oxygen (oxygenases). The oxygen incorporated need not be derived from O2,Acting on single donors with incorporation of molecular oxygen (oxygenases). The oxygen incorporated need not be derived from O2,Oxidoreductases
contig00008.g4774	2203	ID=contig00008.g4774;Description=hypothetical protein FVEG_12488 [Fusarium verticillioides 7600];Gene=FVER53590_12488;Ontology_term=transmembrane transport,transporter activity;Ontology_id=GO:0055085,GO:0005215
contig00008.g4775	1259	ID=contig00008.g4775;Description=Peptidase M35, deuterolysin [Fusarium oxysporum f. sp. vasinfectum];Gene=FocTR4_00015841;Ontology_term=catalytic activity, acting on a protein,extracellular region,hydrolase activity;Ontology_id=GO:0140096,GO:0005576,GO:0016787;Enzyme_code=EC:3.4.24,EC:3.4.24,EC:3.4,EC:3.4.24.39,EC:3;Enzyme_name=Acting on peptide bonds (peptidases),Acting on peptide bonds (peptidases),Acting on peptide bonds (peptidases),deuterolysin,Hydrolases

contig00008.g4776	877	ID=contig00008.g4776;Description=murein transglycosylase [Fusarium verticillioides 7600];Gene=BFJ72_g50;Ontology_term=carbohydrate metabolic process,hydrolase activity;Ontology_id=GO:0005975,GO:0016787;Enzyme_code=EC:3.2.1.4,EC:3.2,EC:3,EC:3.2.1.151,EC:3.2.1,EC:3.2.1.4;Enzyme_name=cellulase,Glycosylases,Hydrolases,xyloglucan-specific endo-beta-1,4-glucanase,Glycosylases,cellulase
contig00008.g4777	884	ID=contig00008.g4777;Description=feruloyl esterase C [Fusarium tjaetaba];Gene=FMAN_06824;Ontology_term=carbohydrate metabolic process,cell wall organization or biogenesis,extracellular region,hydrolase activity;Ontology_id=GO:0005975,GO:0071554,GO:0005576,GO:0016787;Enzyme_code=EC:3.1.1.73,EC:3.1,EC:3,EC:3.1.1.73,EC:3.1.1;Enzyme_name=feruloyl esterase,Acting on ester bonds,Hydrolases,feruloyl esterase,Acting on ester bonds
contig00008.g4778	2457	ID=contig00008.g4778;Description=hypothetical protein FVER53590_12484 [Fusarium verticillioides]
contig00008.g4779	1240	ID=contig00008.g4779;Description=hypothetical protein FVEG_12483 [Fusarium verticillioides 7600]
contig00008.g4780	1474	ID=contig00008.g4780;Description=Soluble quino glucose sorbosone dehydrogenase [Fusarium agapanthi]
contig00008.g4781	699	ID=contig00008.g4781;Description=hypothetical protein FVEG_12481 [Fusarium verticillioides 7600];Gene=FVER53590_12481;Ontology_term=DNA binding,reproductive process,regulation of DNA-templated transcription,transcription regulator activity;Ontology_id=GO:0003677,GO:0022414,GO:0006355,GO:0140110
contig00008.g4782	840	ID=contig00008.g4782;Description=hypothetical protein FVEG_12480 [Fusarium verticillioides 7600];Gene=FVER53590_12480;Ontology_term=protein dimerization activity;Ontology_id=GO:0046983
contig00008.g4783	3282	ID=contig00008.g4783;Description=cytochrome P450 monooxygenase [Fusarium pseudoanthophilum];Gene=FACUT_8;Ontology_term=oxidoreductase activity;Ontology_id=GO:0016491;Enzyme_code=EC:1.14,EC:1,EC:1.14;Enzyme_name=Acting on paired donors, with incorporation or reduction of molecular oxygen. The oxygen incorporated need not be derived from O2,Oxidoreductases,Acting on paired donors, with incorporation or reduction of molecular oxygen. The oxygen incorporated need not be derived from O2

contig00008.g4784	2153	ID=contig00008.g4784;Description=putative transcriptional regulatory protein [Fusarium oxysporum f. sp. cubense];Gene=BFJ68_g13013;Ontology_term=nucleus,DNA binding,regulation of DNA-templated transcription,transcription regulator activity;Ontology_id=GO:0005634,GO:0003677,GO:0006355,GO:0140110
contig00008.g4785	938	ID=contig00008.g4785;Description=hypothetical protein FVEG_12476 [Fusarium verticillioides 7600];Gene=FPHYL_12931;Ontology_term=nucleus,DNA binding,regulation of DNA-templated transcription,transcription regulator activity;Ontology_id=GO:0005634,GO:0003677,GO:0006355,GO:0140110
contig00008.g4786	4470	ID=contig00008.g4786;Description=Cu2+-exporting ATPase [Fusarium verticillioides 7600];Gene=FGLOB1_5674;Ontology_term=nucleus,transmembrane transport,ATP-dependent activity,regulation of DNA-templated transcription,transcription regulator activity,transporter activity,hydrolase activity;Ontology_id=GO:0005634,GO:0055085,GO:0140657,GO:0006355,GO:0140110,GO:0005215,GO:0016787;Enzyme_code=EC:7.2.2,EC:3.6.1.15,EC:3.6.1,EC:3.6,EC:3,EC:7.2.2,EC:7,EC:3.6.1.15,EC:3.6.3.4;Enzyme_name=Catalysing the translocation of inorganic cations,nucleoside-triphosphate phosphatase,Acting on acid anhydrides,Acting on acid anhydrides,Hydrolases,Catalysing the translocation of inorganic cations,Translocases,nucleoside-triphosphate phosphatase,Acting on acid anhydrides
contig00008.g4787	576	ID=contig00008.g4787;Description=hypothetical protein FVEG_12473 [Fusarium verticillioides 7600]
contig00008.g4788	474	ID=contig00008.g4788;Description=hypothetical protein FVEG_17279 [Fusarium verticillioides 7600]
contig00008.g4789	1487	ID=contig00008.g4789;Description=ankyrin repeat domain-containing protein [Fusarium phyllophilum]
contig00008.g4790	1933	ID=contig00008.g4790;Description=related to D-mandelate dehydrogenase [Fusarium proliferatum ET1];Gene=FMUND_10751;Ontology_term=transferase activity;Ontology_id=GO:0016740;Enzyme_code=EC:2,EC:2;Enzyme_name=Transferases,Transferases
contig00008.g4791	585	ID=contig00008.g4791;Description=cupin 2 conserved barrel domain [Fusarium acutatum]
contig00008.g4792	2205	ID=contig00008.g4792;Description=hypothetical protein FVER53590_12469 [Fusarium verticillioides];Gene=FACUT_1;Ontology_term=RNA binding;Ontology_id=GO:0003723
contig00008.g4793	1574	ID=contig00008.g4793;Description=related to quinate transport protein [Fusarium proliferatum ET1];Gene=FVER53263_12468;Ontology_term=transmembrane transport,transporter activity;Ontology_id=GO:0055085,GO:0005215

contig00008.g4794	1278	ID=contig00008.g4794;Description=KES1 [Fusarium subglutinans];Gene=FMUND_10756;Ontology_term=lipid binding;Ontology_id=GO:0008289
contig00008.g4795	396	ID=contig00008.g4795;Description=hypothetical protein FVEG_12466 [Fusarium verticillioides 7600]
contig00008.g4796	965	ID=contig00008.g4796;Description=related to chloroperoxidase [Fusarium fujikuroi];Gene=FPHYL_13549;Ontology_term=antioxidant activity,oxidoreductase activity;Ontology_id=GO:0016209,GO:0016491;Enzyme_code=EC:1.11.1,EC:1.11,EC:1,EC:1.11.1;Enzyme_name=Acting on a peroxide as acceptor,Acting on a peroxide as acceptor,Oxidoreductases,Acting on a peroxide as acceptor
contig00008.g4797	2284	ID=contig00008.g4797;Description=glutamyl endopeptidase [Fusarium sp. NRRL 25303];Gene=FVER53590_25500;Ontology_term=catalytic activity, acting on a protein,hydrolase activity;Ontology_id=GO:0140096,GO:0016787;Enzyme_code=EC:3.4,EC:3.4,EC:3;Enzyme_name=Acting on peptide bonds (peptidases),Acting on peptide bonds (peptidases),Hydrolases
contig00008.g4798	7344	ID=contig00008.g4798;Description=hypothetical protein FVER14953_12463 [Fusarium verticillioides]
contig00008.g4799	2154	ID=contig00008.g4799;Description=hypothetical protein FVER53590_25501 [Fusarium verticillioides]
contig00008.g4800	3093	ID=contig00008.g4800;Description=hypothetical protein FVER53590_12462 [Fusarium verticillioides]
contig00008.g4801	4098	ID=contig00008.g4801;Description=hypothetical protein FVEG_17276 [Fusarium verticillioides 7600]
contig00008.g4802	752	ID=contig00008.g4802;Description=Cerato-platanin-domain-containing protein [Fusarium redolens];Gene=FOBC_15592;Ontology_term=extracellular region;Ontology_id=GO:0005576
contig00008.g4803	564	ID=contig00008.g4803;Description=hypothetical protein FVEG_12459 [Fusarium verticillioides 7600]
contig00008.g4804	2274	ID=contig00008.g4804;Description=hypothetical protein FVEG_12458 [Fusarium verticillioides 7600]
contig00008.g4805	1347	ID=contig00008.g4805;Description=beta-lactamase [Fusarium subglutinans]

contig00008.g4806	1878	ID=contig00008.g4806;Description=steroid monooxygenase [Fusarium subglutinans];Gene=FNYG_10121;Ontology_term=oxidoreductase activity;Ontology_id=GO:0016491;Enzyme_code=EC:1.14.13.8,EC:1.14.13.8,EC:1,EC:1.14,EC:1.14.13;Enzyme_name=flavin-containing monooxygenase,flavin-containing monooxygenase,Oxidoreductases,Acting on paired donors, with incorporation or reduction of molecular oxygen. The oxygen incorporated need not be derived from O2,Acting on paired donors, with incorporation or reduction of molecular oxygen. The oxygen incorporated need not be derived from O2
contig00008.g4807	966	ID=contig00008.g4807;Description=alcohol dehydrogenase like domain protein [Fusarium subglutinans];Gene=FLAG1_10413;Ontology_term=oxidoreductase activity;Ontology_id=GO:0016491;Enzyme_code=EC:1,EC:1,EC:1.1.1.1;Enzyme_name=Oxidoreductases,Oxidoreductases,alcohol dehydrogenase
contig00008.g4808	924	ID=contig00008.g4808;Description=2-hydroxymuconic semialdehyde hydrolase [Fusarium tjaetaba];Gene=FDENT_10502;Ontology_term=hydrolase activity;Ontology_id=GO:0016787;Enzyme_code=EC:3,EC:3;Enzyme_name=Hydrolases,Hydrolases
contig00008.g4809	1278	ID=contig00008.g4809;Description=ferric cupric reductase transmembrane component 7 [Fusarium coicis];Gene=FMUND_1810;Ontology_term=oxidoreductase activity;Ontology_id=GO:0016491;Enzyme_code=EC:1.16,EC:1.16,EC:1;Enzyme_name=Oxidizing metal ions,Oxidizing metal ions,Oxidoreductases
contig00008.g4810	508	ID=contig00008.g4810;Description=hypothetical protein FPCIR_7647 [Fusarium pseudocircinatum];Gene=Forpi1262_v010259;Ontology_term=transmembrane transport,transporter activity;Ontology_id=GO:0055085,GO:0005215
contig00008.g4811	1317	ID=contig00008.g4811;Description=kinase-like domain protein [Fusarium tjaetaba];Gene=FTJAE_9077;Ontology_term=transmembrane transport,transferase activity,transporter activity;Ontology_id=GO:0055085,GO:0016740,GO:0005215;Enzyme_code=EC:2.7,EC:2.7,EC:2;Enzyme_name=Transferring phosphorus-containing groups,Transferring phosphorus-containing groups,Transferases
contig00008.g4812	990	ID=contig00008.g4812;Description=tim barrel metal-dependent [Fusarium tjaetaba];Gene=FTJAE_9078;Ontology_term=hydrolase activity;Ontology_id=GO:0016787;Enzyme_code=EC:3,EC:3;Enzyme_name=Hydrolases,Hydrolases

contig00008.g4813	2918	ID=contig00008.g4813;Description=C6 zinc finger [Fusarium pseudoanthophilum];Gene=FVEG_12449;Ontology_term=nucleus,regulation of DNA-templated transcription,transcription regulator activity;Ontology_id=GO:0005634,GO:0006355,GO:0140110
contig00008.g4814	432	ID=contig00008.g4814;Description=amidase signature enzyme [Fusarium tjaetaba]
contig00008.g4815	1035	ID=contig00008.g4815;Description=NAD(P)-binding protein [Fusarium mundagurra]
contig00008.g4816	405	ID=contig00008.g4816;Description=hypothetical protein FVER14953_12446 [Fusarium verticillioides]
contig00008.g4817	1109	ID=contig00008.g4817;Description=periplasmic alpha-carbonic [Fusarium denticulatum];Gene=FVER53263_12445;Ontology_term=lyase activity;Ontology_id=GO:0016829;Enzyme_code=EC:4.2.1.1;Enzyme_name=carbonic anhydrase
contig00008.g4818	1144	ID=contig00008.g4818;Description=hypothetical protein FVER14953_12444 [Fusarium verticillioides]
contig00008.g4819	441	ID=contig00008.g4819;Description=hypothetical protein FVEG_17268 [Fusarium verticillioides 7600]
contig00008.g4820	783	ID=contig00008.g4820;Description=kinase [Fusarium phyllophilum];Gene=FPANT_5001;Ontology_term=transferase activity;Ontology_id=GO:0016740;Enzyme_code=EC:2.7,EC:2.7,EC:2;Enzyme_name=Transferring phosphorus-containing groups,Transferring phosphorus-containing groups,Transferases
contig00008.g4821	2814	ID=contig00008.g4821;Description=fungistatic metabolite [Fusarium tjaetaba]
contig00008.g4822	1794	ID=contig00008.g4822;Description=related to beta-1,3-glucan binding protein [Fusarium fujikuroi];Gene=BFJ68_g14754;Ontology_term=carbohydrate metabolic process,hydrolase activity;Ontology_id=GO:0005975,GO:0016787;Enzyme_code=EC:3.2.1,EC:3.2,EC:3,EC:3.2.1;Enzyme_name=Glycosylases,Glycosylases,Hydrolases,Glycosylases
contig00008.g4823	3428	ID=contig00008.g4823;Description=glycosyl transferase family group 2-domain-containing protein [Fusarium oxysporum];Gene=BFJ70_g70;Ontology_term=membrane;Ontology_id=GO:0016020
contig00008.g4824	1381	ID=contig00008.g4824;Description=6-hydroxynicotinate 3-monooxygenase [Fusarium denticulatum];Gene=FVER53263_12439;Ontology_term=oxidoreductase activity;Ontology_id=GO:0016491;Enzyme_code=EC:1,EC:1;Enzyme_name=Oxidoreductases,Oxidoreductases

contig00008.g4825	471	ID=contig00008.g4825;Description=6-hydroxynicotinate 3-monooxygenase [Fusarium mundagurra];Gene=FPHYL_240;Ontology_term=oxidoreductase activity;Ontology_id=GO:0016491;Enzyme_code=EC:1,EC:1;Enzyme_name=Oxidoreductases,Oxidoreductases
contig00008.g4826	1655	ID=contig00008.g4826;Description=hypothetical protein J7337_007772 [Fusarium musae]
contig00008.g4827	797	ID=contig00008.g4827;Description=pectate lyase [Fusarium tjaetaba];Gene=FOPG_10096;Ontology_term=lyase activity,extracellular region;Ontology_id=GO:0016829,GO:0005576;Enzyme_code=EC:4.2.2.2,EC:4.2.2.2,EC:4.2,EC:4,EC:4.2.2;Enzyme_name=pectate lyase,pectate lyase,Carbon-oxygen lyases,Lyases,Carbon-oxygen lyases
contig00008.g4828	1627	ID=contig00008.g4828;Description=glutathione S-transferase P 10 [Fusarium tjaetaba];Gene=FTJAE_9097;Ontology_term=transferase activity;Ontology_id=GO:0016740;Enzyme_code=EC:2,EC:5.3.99.2,EC:2.5.1.18,EC:2;Enzyme_name=Transferases,prostaglandin-D synthase,glutathione transferase,Transferases
contig00008.g4829	1274	ID=contig00008.g4829;Description=alpha beta-hydrolase [Fusarium tjaetaba];Gene=FTJAE_9098;Ontology_term=hydrolase activity;Ontology_id=GO:0016787;Enzyme_code=EC:3,EC:3;Enzyme_name=Hydrolases,Hydrolases
contig00008.g4830	6776	ID=contig00008.g4830;Description=hypothetical protein FVER53590_12429 [Fusarium verticillioides];Gene=FVER53590_12429;Ontology_term=nucleus,regulation of DNA-templated transcription,transcription regulator activity;Ontology_id=GO:0005634,GO:0006355,GO:0140110
contig00008.g4831	1704	ID=contig00008.g4831;Description=permease of the major facilitator superfamily [Fusarium bulbicola];Gene=HZS61_010653
contig00008.g4832	3384	ID=contig00008.g4832;Description=related to GABA transport protein [Fusarium proliferatum ET1];Gene=FPRO_07088;Ontology_term=transmembrane transport,transporter activity;Ontology_id=GO:0055085,GO:0005215
contig00008.g4833	3101	ID=contig00008.g4833;Description=serine/threonine protein kinase [Fusarium proliferatum];Gene=FMUND_1787;Ontology_term=catalytic activity, acting on a protein,transferase activity;Ontology_id=GO:0140096,GO:0016740;Enzyme_code=EC:2.7.11.1,EC:2.7.1,EC:2.7.11.1,EC:2.7,EC:2;Enzyme_name=non-specific serine/threonine protein kinase,Transferring phosphorus-containing groups,non-specific serine/threonine protein kinase,Transferring phosphorus-containing groups,Transferases

contig00008.g4834	2514	ID=contig00008.g4834;Description=related to general amino acid permease [Fusarium proliferatum ET1];Gene=FVEG_12424;Ontology_term=transmembrane transport;Ontology_id=GO:0055085
contig00008.g4835	1477	ID=contig00008.g4835;Description=related to beta-mannanase [Fusarium proliferatum ET1];Gene=FNYG_10110;Ontology_term=carbohydrate metabolic process,cell wall organization or biogenesis,extracellular region,hydrolase activity;Ontology_id=GO:0005975,GO:0071554,GO:0005576,GO:0016787;Enzyme_code=EC:3.2.1.25,EC:3.2.1.78,EC:3.2,EC:3,EC:3.2.1.25,EC:3.2.1.78,EC:3.2.1;Enzyme_name=beta-mannosidase,mannan endo-1,4-beta-mannosidase,Glycosylases,Hydrolases,beta-mannosidase,mannan endo-1,4-beta-mannosidase,Glycosylases
contig00008.g4836	898	ID=contig00008.g4836;Description=hypothetical protein FVEG_12422 [Fusarium verticillioides 7600]
contig00008.g4837	1723	ID=contig00008.g4837;Description=major facilitator superfamily domain-containing protein [Fusarium redolens];Gene=FNYG_10112;Ontology_term=transmembrane transport,transporter activity;Ontology_id=GO:0055085,GO:0005215
contig00008.g4838	2077	ID=contig00008.g4838;Description=hypothetical protein FVER14953_12420 [Fusarium verticillioides];Gene=FNYG_10113;Ontology_term=nucleus,DNA binding,regulation of DNA-templated transcription,transcription regulator activity;Ontology_id=GO:0005634,GO:0003677,GO:0006355,GO:0140110
contig00008.g4839	375	ID=contig00008.g4839;Description=hypothetical protein FVEG_12419 [Fusarium verticillioides 7600]
contig00008.g4840	2042	ID=contig00008.g4840;Description=C6 zinc finger domain-containing protein [Fusarium mundagurra];Gene=FVER53590_12418;Ontology_term=nucleus,regulation of DNA-templated transcription,transcription regulator activity;Ontology_id=GO:0005634,GO:0006355,GO:0140110
contig00008.g4841	1607	ID=contig00008.g4841;Description=hypothetical protein FVER53263_12417 [Fusarium verticillioides]
contig00008.g4842	1419	ID=contig00008.g4842;Description=hypothetical protein FPANT_5012 [Fusarium pseudoanthophilum]
contig00008.g4843	767	ID=contig00008.g4843;Description=glycoside hydrolase family 18 [Fusarium pseudoanthophilum];Gene=F53441_6329;Ontology_term=hydrolase activity;Ontology_id=GO:0016787;Enzyme_code=EC:3;Enzyme_name=Hydrolases

contig00008.g4844	3986	ID=contig00008.g4844;Description=aaa family atpase [Fusarium napiforme];Gene=FPHYL_2626;Ontology_term=ATP-dependent activity,hydrolase activity;Ontology_id=GO:0140657,GO:0016787;Enzyme_code=EC:3.6.1.15,EC:3.6.1,EC:3.6,EC:3,EC:3.6.1.15;Enzyme_name=nucleoside-triphosphate phosphatase,Acting on acid anhydrides,Acting on acid anhydrides,Hydrolases,nucleoside-triphosphate phosphatase
contig00008.g4845	2372	ID=contig00008.g4845;Description=hypothetical protein FVEG_12413 [Fusarium verticillioides 7600];Gene=FPANT_12082;Ontology_term=membrane;Ontology_id=GO:0016020
contig00008.g4846	877	ID=contig00008.g4846;Description=hypothetical protein FVER14953_12412 [Fusarium verticillioides]
contig00008.g4847	1162	ID=contig00008.g4847;Description=C6 zinc finger [Fusarium tjaetaba];Gene=FMAN_16040;Ontology_term=nucleus,regulation of DNA-templated transcription,transcription regulator activity;Ontology_id=GO:0005634,GO:0006355,GO:0140110
contig00008.g4848	410	ID=contig00008.g4848;Description=Crystal ET79 [Fusarium napiforme]
contig00008.g4849	2107	ID=contig00008.g4849;Description=nad dependent epimerase [Fusarium globosum]
contig00008.g4850	975	ID=contig00008.g4850;Description=hypothetical protein BFJ72_g356 [Fusarium proliferatum]
contig00008.g4851	1698	ID=contig00008.g4851;Description=cytochrome P450 monooxygenase [Fusarium subglutinans];Gene=FVEG_12405;Ontology_term=oxidoreductase activity;Ontology_id=GO:0016491;Enzyme_code=EC:1.14,EC:1,EC:1.14;Enzyme_name=Acting on paired donors, with incorporation or reduction of molecular oxygen. The oxygen incorporated need not be derived from O2,Oxidoreductases,Acting on paired donors, with incorporation or reduction of molecular oxygen. The oxygen incorporated need not be derived from O2
contig00008.g4852	1087	ID=contig00008.g4852;Description=Tyrosinase [Fusarium pseudoanthophilum];Gene=FVER53590_12404;Ontology_term=oxidoreductase activity;Ontology_id=GO:0016491;Enzyme_code=EC:1,EC:1,EC:1.14.18.1;Enzyme_name=Oxidoreductases,Oxidoreductases,tyrosinase
contig00008.g4853	1646	ID=contig00008.g4853;Description=hypothetical protein FVER53590_12403 [Fusarium verticillioides]
contig00008.g4854	1092	ID=contig00008.g4854;Description=heterokaryon incompatibility protein [Fusarium tjaetaba]

contig00008.g4855	2188	ID=contig00008.g4855;Description=FC receptor [Fusarium sp. NRRL 52700];Ontology_term=cell adhesion,extracellular region,vacuole,plasma membrane;Ontology_id=GO:0007155,GO:0005576,GO:0005773,GO:0005886
contig00008.g4856	1705	ID=contig00008.g4856;Description=hypothetical protein FVER53590_12399 [Fusarium verticillioides];Gene=FSUBG_13894;Ontology_term=catalytic activity, acting on a protein,hydrolase activity;Ontology_id=GO:0140096,GO:0016787;Enzyme_code=EC:3.4,EC:3.4,EC:3;Enzyme_name=Acting on peptide bonds (peptidases),Acting on peptide bonds (peptidases),Hydrolases
contig00008.g4857	420	ID=contig00008.g4857;Description=mannose-binding lectin [Zea mays];Gene=Q45NP5;Ontology_term=response to other organism,membrane,carbohydrate binding;Ontology_id=GO:0051707,GO:0016020,GO:0030246
contig00008.g4858	617	ID=contig00008.g4858;Description=aconitase A isopropylmalate dehydratase small subunit swivel [Fusarium subglutinans]
contig00008.g4859	772	ID=contig00008.g4859;Description=hypothetical protein FVER53263_12396 [Fusarium verticillioides]
contig00008.g4860	2290	ID=contig00008.g4860;Description=transcription factor TFIIH complex ERCC-3 subunit [Fusarium musae];Gene=BFJ72_g713;Ontology_term=DNA repair,catalytic activity, acting on DNA,DNA recombination,regulation of DNA-templated transcription,intracellular protein transport,nucleoplasm,nucleocytoplasmic transport,DNA binding,ATP-dependent activity,cytosol,transcription regulator activity,hydrolase activity;Ontology_id=GO:0006281,GO:0140097,GO:0006310,GO:0006355,GO:0006886,GO:0005654,GO:0006913,GO:0003677,GO:0140657,GO:0005829,GO:0140110,GO:0016787;Enzyme_code=EC:3,EC:3.6.1,EC:3.6.4.12,EC:3.6,EC:3,EC:3.6.1.15;Enzyme_name=Hydrolases,Acting on acid anhydrides,DNA helicase,Acting on acid anhydrides,Hydrolases,nucleoside-triphosphate phosphatase
contig00008.g4861	6489	ID=contig00008.g4861;Description=hypothetical protein FVER53263_12393 [Fusarium verticillioides];Gene=FACUT_807;Ontology_term=oxidoreductase activity,lipid metabolic process,hydrolase activity;Ontology_id=GO:0016491,GO:0006629,GO:0016787;Enzyme_code=EC:1,EC:3,EC:1,EC:3;Enzyme_name=Oxidoreductases,Hydrolases,Oxidoreductases,Hydrolases
contig00008.g4862	1353	ID=contig00008.g4862;Description=hypothetical protein FVEG_17244 [Fusarium verticillioides 7600]

contig00008.g4863	1881	ID=contig00008.g4863;Description=cytochrome P450, family 51 (sterol 14-demethylase) [Fusarium verticillioides 7600];Gene=FVER53590_12391;Ontology_term=oxidoreductase activity,transferase activity;Ontology_id=GO:0016491,GO:0016740;Enzyme_code=EC:1.14,EC:2.1.1,EC:2.1,EC:1.14.13.70,EC:1,EC:1.14,EC:2,EC:2.1.1;Enzyme_name=Acting on paired donors, with incorporation or reduction of molecular oxygen. The oxygen incorporated need not be derived from O2,Transferring one-carbon groups,Transferring one-carbon groups,Acting on paired donors, with incorporation or reduction of molecular oxygen. The oxygen incorporated need not be derived from O2,Oxidoreductases,Acting on paired donors, with incorporation or reduction of molecular oxygen. The oxygen incorporated need not be derived from O2,Transferases,Transferring one-carbon groups
contig00008.g4864	732	ID=contig00008.g4864;Description=hypothetical protein FVER53590_12390 [Fusarium verticillioides];Gene=F25303_4588;Ontology_term=oxidoreductase activity;Ontology_id=GO:0016491;Enzyme_code=EC:1,EC:1;Enzyme_name=Oxidoreductases,Oxidoreductases
contig00008.g4865	1185	ID=contig00008.g4865;Description=hypothetical protein FVER53590_29068 [Fusarium verticillioides];Gene=FVER53590_29068;Ontology_term=oxidoreductase activity;Ontology_id=GO:0016491;Enzyme_code=EC:1,EC:1;Enzyme_name=Oxidoreductases,Oxidoreductases
contig00008.g4866	2659	ID=contig00008.g4866;Description=dehydrogenase reductase sdr family member 12 [Fusarium tjaetaba]
contig00008.g4867	5678	ID=contig00008.g4867;Description=Sex-determining fem-1 [Fusarium tjaetaba];Gene=FCIRC_12057;Ontology_term=membrane;Ontology_id=GO:0016020
contig00008.g4868	1065	ID=contig00008.g4868;Description=phenazine biosynthesis protein [Fusarium verticillioides 7600];Gene=HZS61_010686;Ontology_term=catalytic activity;Ontology_id=GO:0003824
contig00008.g4869	816	ID=contig00008.g4869;Description=acetyltransferase esterase [Fusarium tjaetaba];Gene=FNAPI_12481;Ontology_term=transferase activity;Ontology_id=GO:0016740;Enzyme_code=EC:2,EC:2;Enzyme_name=Transferases,Transferases
contig00008.g4870	1641	ID=contig00008.g4870;Description=hypothetical protein FVER53590_12383 [Fusarium verticillioides];Gene=FVER53590_12383;Ontology_term=membrane;Ontology_id=GO:0016020
contig00008.g4871	1414	ID=contig00008.g4871;Description=zeaxanthin epoxidase chloroplastic [Fusarium pseudoanthophilum];Gene=FOYG_14295

contig00008.g4872	1946	ID=contig00008.g4872;Description=eukaryotic aspartyl protease [Fusarium tjaetaba];Gene=FVER53590_12381;Ontology_term=catalytic activity, acting on a protein,hydrolase activity;Ontology_id=GO:0140096,GO:0016787;Enzyme_code=EC:3.4.23,EC:3.4.23,EC:3.4,EC:3;Enzyme_name=Acting on peptide bonds (peptidases),Acting on peptide bonds (peptidases),Acting on peptide bonds (peptidases),Hydrolases
contig00008.g4873	1872	ID=contig00008.g4873;Description=cytochrome P450 [Fusarium oxysporum Fo47];Gene=FNYG_10033;Ontology_term=oxidoreductase activity;Ontology_id=GO:0016491;Enzyme_code=EC:1.14,EC:1.14.13.175,EC:1,EC:1.14,EC:1.14.14.54;Enzyme_name=Acting on paired donors, with incorporation or reduction of molecular oxygen. The oxygen incorporated need not be derived from O2,Acting on paired donors, with incorporation or reduction of molecular oxygen. The oxygen incorporated need not be derived from O2,Oxidoreductases,Acting on paired donors, with incorporation or reduction of molecular oxygen. The oxygen incorporated need not be derived from O2,phenylacetate 2-hydroxylase
contig00008.g4874	2628	ID=contig00008.g4874;Description=alkaline-phosphatase-like protein [Fusarium oxysporum];Gene=FMUND_5536;Ontology_term=hydrolase activity;Ontology_id=GO:0016787;Enzyme_code=EC:3.1.6,EC:3.1.6,EC:3.1,EC:3;Enzyme_name=Acting on ester bonds,Acting on ester bonds,Acting on ester bonds,Hydrolases
contig00008.g4875	1190	ID=contig00008.g4875;Description=WW domain-containing protein [Fusarium pseudocircinatum];Gene=FVER53263_12378;Ontology_term=membrane;Ontology_id=GO:0016020
contig00008.g4876	1444	ID=contig00008.g4876;Description=G coupled receptor 1 [Fusarium coicis];Gene=FNAPI_4278;Ontology_term=molecular transducer activity,signaling,anatomical structure development;Ontology_id=GO:0060089,GO:0023052,GO:0048856
contig00008.g4877	1959	ID=contig00008.g4877;Description=G coupled receptor 1 [Fusarium mundagurra];Gene=FMUND_15380;Ontology_term=nucleobase-containing small molecule metabolic process,catalytic activity,carbohydrate derivative metabolic process;Ontology_id=GO:0055086,GO:0003824,GO:1901135
contig00008.g4878	1584	ID=contig00008.g4878;Description=probable permease of the major facilitator superfamily [Fusarium fujikuroi];Gene=FNYG_10029;Ontology_term=transmembrane transport,transporter activity;Ontology_id=GO:0055085,GO:0005215

contig00008.g4879	885	ID=contig00008.g4879;Description=amidohydrolase 2 [Fusarium tjaetaba];Gene=FTJAE_13743;Ontology_term=transmembrane transport,transporter activity,hydrolase activity;Ontology_id=GO:0055085,GO:0005215,GO:0016787;Enzyme_code=EC:3,EC:3;Enzyme_name=Hydrolases,Hydrolases
contig00008.g4880	3157	ID=contig00008.g4880;Description=hypothetical protein F53441_10513 [Fusarium austroafricanum];Gene=F53441_10513;Ontology_term=oxidoreductase activity,hydrolase activity;Ontology_id=GO:0016491,GO:0016787;Enzyme_code=EC:3,EC:1.2.1,EC:1.2,EC:1,EC:3,EC:1.2.1;Enzyme_name=Hydrolases,Acting on the aldehyde or oxo group of donors,Acting on the aldehyde or oxo group of donors,Oxidoreductases,Hydrolases,Acting on the aldehyde or oxo group of donors
contig00008.g4881	1955	ID=contig00008.g4881;Description=choline dehydrogenase mitochondrial [Fusarium phyllophilum];Gene=FVER53590_12372;Ontology_term=oxidoreductase activity;Ontology_id=GO:0016491;Enzyme_code=EC:1.1;Enzyme_name=Acting on the CH-OH group of donors
contig00008.g4882	399	ID=contig00008.g4882;Description=hypothetical protein J7337_007719 [Fusarium musae]
contig00008.g4883	2313	ID=contig00008.g4883;Description=hypothetical protein FPCIR_1469 [Fusarium pseudocircinatum]
contig00008.g4884	393	ID=contig00008.g4884;Description=hypothetical protein FNAPI_12315 [Fusarium napiforme]
contig00008.g4885	681	ID=contig00008.g4885;Description=related to O-methyltransferase [Fusarium proliferatum ET1];Gene=FPCIR_1466;Ontology_term=transferase activity;Ontology_id=GO:0016740;Enzyme_code=EC:2.1.1,EC:2.1,EC:2,EC:2.1.1;Enzyme_name=Transferring one-carbon groups,Transferring one-carbon groups,Transferases,Transferring one-carbon groups
contig00008.g4886	1992	ID=contig00008.g4886;Description=rhamnogalacturonate lyase C [Fusarium verticillioides 7600];Gene=FNAPI_12317;Ontology_term=carbohydrate metabolic process,lyase activity,cell wall organization or biogenesis,extracellular region;Ontology_id=GO:0005975,GO:0016829,GO:0071554,GO:0005576;Enzyme_code=EC:4.2.2.23,EC:4.2.2.23,EC:4.2,EC:4,EC:4.2.2;Enzyme_name=rhamnogalacturonan endolyase,rhamnogalacturonan endolyase,Carbon-oxygen lyases,Lyases,Carbon-oxygen lyases
contig00008.g4887	1290	ID=contig00008.g4887;Description=Protein indc11 [Fusarium oxysporum f. sp. albedinis]
contig00008.g4888	2037	ID=contig00008.g4888;Description=integral membrane protein [Fusarium mundagurra];Gene=FPRO05_13480;Ontology_term=membrane;Ontology_id=GO:0016020

contig00008.g4889	1376	ID=contig00008.g4889;Description=aspartate aminotransferase [Fusarium proliferatum];Gene=FNAPI_3497;Ontology_term=amino acid metabolic process,transferase activity;Ontology_id=GO:0006520,GO:0016740;Enzyme_code=EC:2.6.1.1,EC:2.6.1,EC:2,EC:2.6,EC:2.6.1.1;Enzyme_name=aspartate transaminase,Transferring nitrogenous groups,Transferases,Transferring nitrogenous groups,aspartate transaminase
contig00008.g4890	879	ID=contig00008.g4890;Description=Versicolorin reductase [Fusarium coicis];Gene=FNYG_10013;Ontology_term=oxidoreductase activity;Ontology_id=GO:0016491;Enzyme_code=EC:1,EC:1;Enzyme_name=Oxidoreductases,Oxidoreductases
contig00008.g4891	1427	ID=contig00008.g4891;Description=hypothetical protein FOYG_14273 [Fusarium oxysporum NRRL 32931]
contig00008.g4892	1760	ID=contig00008.g4892;Description=nicotinamide mononucleotide permease [Fusarium denticulatum];Gene=FOYG_14272;Ontology_term=transmembrane transport,transporter activity;Ontology_id=GO:0055085,GO:0005215
contig00008.g4893	808	ID=contig00008.g4893;Description=hypothetical protein FVER53263_12361 [Fusarium verticillioides]
contig00008.g4894	1971	ID=contig00008.g4894;Description=Sodium/hydrogen exchanger family-domain-containing protein [Fusarium redolens];Gene=Forpe1208_v011657;Ontology_term=transmembrane transport,transporter activity,plasma membrane;Ontology_id=GO:0055085,GO:0005215,GO:0005886
contig00008.g4895	1706	ID=contig00008.g4895;Description=major facilitator superfamily transporter [Fusarium napiforme];Gene=FFB14_12248;Ontology_term=transmembrane transport,transporter activity;Ontology_id=GO:0055085,GO:0005215
contig00008.g4896	2173	ID=contig00008.g4896;Description=laccase-like multicopper oxidase [Fusarium napiforme];Gene=FPANT_9461;Ontology_term=transmembrane transport,oxidoreductase activity,transporter activity;Ontology_id=GO:0055085,GO:0016491,GO:0005215;Enzyme_code=EC:1,EC:1;Enzyme_name=Oxidoreductases,Oxidoreductases
contig00008.g4897	1290	ID=contig00008.g4897;Description=altered inheritance of mitochondria mitochondrial [Fusarium denticulatum]
contig00008.g4898	1225	ID=contig00008.g4898;Description=dimeric dihydrodiol dehydrogenase [Fusarium acutatum];Gene=FVER53263_12356;Ontology_term=nucleotide binding;Ontology_id=GO:0000166

contig00008.g4899	1331	ID=contig00008.g4899;Description=related to oxidoreductase [Fusarium fujikuroi];Gene=FPANT_9464;Ontology_term=nucleotide binding;Ontology_id=GO:0000166
contig00008.g4900	2881	ID=contig00008.g4900;Description=carboxypeptidase [Fusarium globosum];Gene=BFJ72_g669;Ontology_term=catalytic activity, acting on a protein,extracellular region,hydrolase activity;Ontology_id=GO:0140096,GO:0005576,GO:0016787;Enzyme_code=EC:3.4.17,EC:3.4,EC:3.4.17,EC:3;Enzyme_name=Acting on peptide bonds (peptidases),Acting on peptide bonds (peptidases),Acting on peptide bonds (peptidases),Hydrolases
contig00008.g4901	438	ID=contig00008.g4901;Description=Lactose permease [Fusarium oxysporum f. sp. albedinis]
contig00008.g4902	1335	ID=contig00008.g4902;Description=hypothetical protein FVER53590_12351 [Fusarium verticillioides];Gene=FNYG_10000;Ontology_term=membrane;Ontology_id=GO:0016020
contig00008.g4903	1751	ID=contig00008.g4903;Description=vanillyl-alcohol oxidase [Fusarium tjaetaba];Gene=FNAPI_3482;Ontology_term=oxidoreductase activity;Ontology_id=GO:0016491;Enzyme_code=EC:1,EC:1;Enzyme_name=Oxidoreductases,Oxidoreductases
contig00008.g4904	3260	ID=contig00008.g4904;Description=cytochrome p450 [Fusarium tjaetaba];Gene=FVEG_12349;Ontology_term=catalytic activity, acting on a protein,oxidoreductase activity,hydrolase activity;Ontology_id=GO:0140096,GO:0016491,GO:0016787;Enzyme_code=EC:3.4.14.1,EC:1.14,EC:3.4.14.5,EC:3.4.14.4,EC:3.4.14.2,EC:3.4.14.1,EC:3.4,EC:1,EC:1.14,EC:3,EC:3.4.14.5,EC:3.4.14.4,EC:3.4.14.2;Enzyme_name=dipeptidyl-peptidase I,Acting on paired donors, with incorporation or reduction of molecular oxygen. The oxygen incorporated need not be derived from O2,dipeptidyl-peptidase IV,dipeptidyl-peptidase III,dipeptidyl-peptidase II,dipeptidyl-peptidase I,Acting on peptide bonds (peptidases),Oxidoreductases,Acting on paired donors, with incorporation or reduction of molecular oxygen. The oxygen incorporated need not be derived from O2,Hydrolases,dipeptidyl-peptidase IV,dipeptidyl-peptidase III,dipeptidyl-peptidase II
contig00008.g4905	2330	ID=contig00008.g4905;Description=hypothetical protein FVER53263_21094 [Fusarium verticillioides];Gene=FTJAE_6893;Ontology_term=nucleus,DNA binding,regulation of DNA-templated transcription,transcription regulator activity;Ontology_id=GO:0005634,GO:0003677,GO:0006355,GO:0140110

contig00008.g4906	1380	ID=contig00008.g4906;Description=FAD dependent oxidoreductase [Fusarium tjaetaba];Gene=FPANT_9472;Ontology_term=oxidoreductase activity;Ontology_id=GO:0016491;Enzyme_code=EC:1,EC:1;Enzyme_name=Oxidoreductases,Oxidoreductases
contig00008.g4907	1154	ID=contig00008.g4907;Description=beta-lactamase domain-containing protein [Fusarium mundagurra]
contig00008.g4908	806	ID=contig00008.g4908;Description=cutinase [Fusarium verticillioides 7600];Gene=FPCIR_1447;Ontology_term=carbohydrate metabolic process,lipid metabolic process,extracellular region,hydrolase activity;Ontology_id=GO:0005975,GO:0006629,GO:0005576,GO:0016787;Enzyme_code=EC:3.1.1.74,EC:3.1,EC:3,EC:3.1.1.74,EC:3.1.1;Enzyme_name=cutinase,Acting on ester bonds,Hydrolases,cutinase,Acting on ester bonds
contig00008.g4909	2305	ID=contig00008.g4909;Description=Xyloglucanase [Fusarium musae];Gene=FNAPI_3475;Ontology_term=mitochondrion,amino acid metabolic process,hydrolase activity;Ontology_id=GO:0005739,GO:0006520,GO:0016787;Enzyme_code=EC:3.5.1.9,EC:3.5.1,EC:3.2.1.155,EC:3.5,EC:3,EC:3.5.1.9;Enzyme_name=arylformamidase,Acting on carbon-nitrogen bonds, other than peptide bonds,xyloglucan-specific endo-processive beta-1,4-glucanase,Acting on carbon-nitrogen bonds, other than peptide bonds,Hydrolases,arylformamidase
contig00008.g4910	1749	ID=contig00008.g4910;Description=related to monosaccharide transporter [Fusarium fujikuroi];Gene=FVER53590_12344;Ontology_term=transmembrane transport,transporter activity;Ontology_id=GO:0055085,GO:0005215
contig00008.g4911	1072	ID=contig00008.g4911;Description=cyclase [Fusarium pseudoanthophilum];Gene=FPANT_10987;Ontology_term=amino acid metabolic process,hydrolase activity;Ontology_id=GO:0006520,GO:0016787;Enzyme_code=EC:3.5.1.9,EC:3.5.1,EC:3.5,EC:3,EC:3.5.1.9;Enzyme_name=arylformamidase,Acting on carbon-nitrogen bonds, other than peptide bonds,Acting on carbon-nitrogen bonds, other than peptide bonds,Hydrolases,arylformamidase
contig00008.g4912	1716	ID=contig00008.g4912;Description=zinc finger domain protein [Fusarium subglutinans]
contig00008.g4913	1632	ID=contig00008.g4913;Description=hypothetical protein J7337_007691 [Fusarium musae];Gene=F52700_4585

contig00008.g4914	3717	ID=contig00008.g4914;Description=xyloglucanase [Fusarium napiforme];Gene=FPANT_10986;Ontology_term=mitochondrion,amino acid metabolic process,hydrolase activity;Ontology_id=GO:0005739,GO:0006520,GO:0016787;Enzyme_code=EC:3.5.1.9,EC:3.5.1,EC:3.5,EC:3,EC:3.5.1.9;Enzyme_name=arylformamidase,Acting on carbon-nitrogen bonds, other than peptide bonds,Acting on carbon-nitrogen bonds, other than peptide bonds,Hydrolases,arylformamidase
contig00008.g4915	1644	ID=contig00008.g4915;Description=exopolygalacturonase B [Fusarium pseudoanthophilum];Gene=FNAPI_3475;Ontology_term=carbohydrate metabolic process,mitochondrion,amino acid metabolic process,cell wall organization or biogenesis,hydrolase activity;Ontology_id=GO:0005975,GO:0005739,GO:0006520,GO:0071554,GO:0016787;Enzyme_code=EC:3.5.1.9,EC:3.2.1.15,EC:3.5.1,EC:3.2,EC:3.5,EC:3,EC:3.5.1.9,EC:3.2.1.15,EC:3.2.1;Enzyme_name=arylformamidase,endo-polygalacturonase,Acting on carbon-nitrogen bonds, other than peptide bonds,Glycosylases,Acting on carbon-nitrogen bonds, other than peptide bonds,Hydrolases,arylformamidase,endo-polygalacturonase,Glycosylases
contig00008.g4916	1611	ID=contig00008.g4916;Description=putative exopolygalacturonase B [Fusarium oxysporum f. sp. matthiolae];Gene=FVER53263_12339;Ontology_term=carbohydrate metabolic process,cell wall organization or biogenesis,hydrolase activity;Ontology_id=GO:0005975,GO:0071554,GO:0016787;Enzyme_code=EC:3.2.1.15;Enzyme_name=endo-polygalacturonase
contig00008.g4917	2589	ID=contig00008.g4917;Description=hypothetical protein FVER53590_12337 [Fusarium verticillioides];Gene=FANTH_10115;Ontology_term=carbohydrate metabolic process,hydrolase activity;Ontology_id=GO:0005975,GO:0016787;Enzyme_code=EC:3.2.1.40,EC:3.2,EC:3.2.1.40,EC:3,EC:3.2.1;Enzyme_name=alpha-L-rhamnosidase,Glycosylases,alpha-L-rhamnosidase,Hydrolases,Glycosylases
contig00008.g4918	1281	ID=contig00008.g4918;Description=hypothetical protein FVER53263_12336 [Fusarium verticillioides]
contig00008.g4919	1944	ID=contig00008.g4919;Description=hypothetical protein FVER53590_12335 [Fusarium verticillioides]
contig00008.g4920	4770	ID=contig00008.g4920;Description=hypothetical protein FVEG_12333 [Fusarium verticillioides 7600];Gene=C2S_12687;Ontology_term=ADP binding;Ontology_id=GO:0043531

contig00008.g4921	1158	ID=contig00008.g4921;Description=hypothetical protein FVEG_12332 [Fusarium verticillioides 7600]
contig00008.g4922	975	ID=contig00008.g4922;Description=hypothetical protein FVER53263_12331 [Fusarium verticillioides]
contig00008.g4923	471	ID=contig00008.g4923;Description=probable SnodProt1 precursor [Fusarium fujikuroi];Gene=FCIRC_12765;Ontology_term=extracellular region;Ontology_id=GO:0005576
contig00008.g4924	2095	ID=contig00008.g4924;Description=sulfate transporter family-domain-containing protein [Fusarium oxysporum Fo47];Gene=FPANT_10976;Ontology_term=transmembrane transport,transporter activity;Ontology_id=GO:0055085,GO:0005215
contig00008.g4925	877	ID=contig00008.g4925;Description=DUF1275 domain-containing protein [Fusarium globosum];Gene=FNAPI_1476;Ontology_term=membrane;Ontology_id=GO:0016020
contig00008.g4926	1540	ID=contig00008.g4926;Description=oxalate decarboxylase [Fusarium verticillioides 7600];Gene=oxdC-1;Ontology_term=metal ion binding,oxalate metabolic process;Ontology_id=GO:0046872,GO:0033609
contig00008.g4927	3750	ID=contig00008.g4927;Description=major facilitator superfamily transporter [Fusarium coicis];Gene=Forpe1208_v011613;Ontology_term=transmembrane transport,transporter activity;Ontology_id=GO:0055085,GO:0005215
contig00008.g4928	1751	ID=contig00008.g4928;Description=amino acid transporter [Fusarium subglutinans];Gene=BFJ65_g16365;Ontology_term=transmembrane transport,transporter activity;Ontology_id=GO:0055085,GO:0005215
contig00008.g4929	939	ID=contig00008.g4929;Description=hypothetical protein FVER53590_12323 [Fusarium verticillioides];Gene=FVER53263_12323;Ontology_term=membrane;Ontology_id=GO:0016020
contig00008.g4930	536	ID=contig00008.g4930;Description=---NA---
contig00008.g4931	1497	ID=contig00008.g4931;Description=S-adenosyl-L-methionine-dependent methyltransferase [Fusarium oxysporum Fo47];Gene=FOBC_15739;Ontology_term=transferase activity;Ontology_id=GO:0016740;Enzyme_code=EC:2.1.1,EC:2.1,EC:2,EC:2.1.1;Enzyme_name=Transferring one-carbon groups,Transferring one-carbon groups,Transferases,Transferring one-carbon groups
contig00008.g4932	1814	ID=contig00008.g4932;Description=related to alpha-glucoside transport protein [Fusarium fujikuroi];Gene=AU210_013844;Ontology_term=transmembrane transport,transporter activity;Ontology_id=GO:0055085,GO:0005215

contig00008.g4933	729	ID=contig00008.g4933;Description=GNAT family acetyltransferase [Fusarium agapanthi];Gene=Forpi1262_v018305;Ontology_term=transferase activity;Ontology_id=GO:0016740;Enzyme_code=EC:2;Enzyme_name=Transferases
contig00008.g4934	739	ID=contig00008.g4934;Description=hypothetical protein DER46DRAFT_665461 [Fusarium sp. MPI-SDFR-AT-0072]
contig00008.g4935	700	ID=contig00008.g4935;Description=Alpha/Beta hydrolase protein [Fusarium redolens];Gene=FNAPI_1463;Ontology_term=hydrolase activity;Ontology_id=GO:0016787;Enzyme_code=EC:3;Enzyme_name=Hydrolases
contig00008.g4936	1326	ID=contig00008.g4936;Description=microsomal epoxide hydrolase [Fusarium napiforme];Gene=FMAN_06648;Ontology_term=hydrolase activity;Ontology_id=GO:0016787;Enzyme_code=EC:3.3.2,EC:3.3.2,EC:3.3,EC:3;Enzyme_name=Acting on ether bonds,Acting on ether bonds,Acting on ether bonds,Hydrolases
contig00008.g4937	762	ID=contig00008.g4937;Description=putative C6 finger domain protein [Fusarium sp. NRRL 25303];Gene=FVEG_12318;Ontology_term=nucleus,regulation of DNA-templated transcription,transcription regulator activity;Ontology_id=GO:0005634,GO:0006355,GO:0140110
contig00008.g4938	1107	ID=contig00008.g4938;Description=zinc-binding alcohol dehydrogenase domain protein [Fusarium tjaetaba];Gene=FVEG_12317;Ontology_term=oxidoreductase activity;Ontology_id=GO:0016491;Enzyme_code=EC:1.6,EC:1.6,EC:1;Enzyme_name=Acting on NADH or NADPH,Acting on NADH or NADPH,Oxidoreductases
contig00008.g4939	1675	ID=contig00008.g4939;Description=MFS transporter, SP family, general alpha glucoside:H+ symporter [Fusarium verticillioides 7600];Gene=FNYG_09962;Ontology_term=transmembrane transport,transporter activity;Ontology_id=GO:0055085,GO:0005215
contig00008.g4940	873	ID=contig00008.g4940;Description=kinase-like domain-containing protein [Fusarium redolens];Gene=F25303_14056;Ontology_term=transferase activity;Ontology_id=GO:0016740;Enzyme_code=EC:2.7,EC:2.7,EC:2;Enzyme_name=Transferring phosphorus-containing groups,Transferring phosphorus-containing groups,Transferases
contig00008.g4941	875	ID=contig00008.g4941;Description=hypothetical protein FVER14953_12315 [Fusarium verticillioides];Ontology_term=membrane;Ontology_id=GO:0016020
contig00008.g4942	462	ID=contig00008.g4942;Description=hypothetical protein FVER14953_12313 [Fusarium verticillioides]

contig00008.g4943	2411	ID=contig00008.g4943;Description=kinase-like domain-containing protein [Fusarium pseudoanthophilum];Gene=FNAPI_1454;Ontology_term=transferase activity;Ontology_id=GO:0016740;Enzyme_code=EC:2.7,EC:2.7,EC:2;Enzyme_name=Transferring phosphorus-containing groups,Transferring phosphorus-containing groups,Transferases
contig00008.g4944	3502	ID=contig00008.g4944;Description=hypothetical protein FVEG_17216 [Fusarium verticillioides 7600]
contig00008.g4945	2454	ID=contig00008.g4945;Description=hypothetical protein FVER53263_20008 [Fusarium verticillioides];Gene=FVER53263_20008;Ontology_term=membrane;Ontology_id=GO:0016020
contig00008.g4946	1017	ID=contig00008.g4946;Description=gibberellin 2-beta-dioxygenase 8 [Fusarium tjaetaba];Gene=FVER53590_12309;Ontology_term=oxidoreductase activity;Ontology_id=GO:0016491;Enzyme_code=EC:1,EC:1;Enzyme_name=Oxidoreductases,Oxidoreductases
contig00008.g4947	1287	ID=contig00008.g4947;Description=hypothetical protein FVEG_12308 [Fusarium verticillioides 7600]
contig00008.g4948	810	ID=contig00008.g4948;Description=molybdopterin cofactor [Fusarium tjaetaba];Gene=FTJAE_12402;Ontology_term=membrane;Ontology_id=GO:0016020
contig00008.g4949	1338	ID=contig00008.g4949;Description=transcriptional regulatory moc3 [Fusarium pseudoanthophilum];Gene=FPANT_8298;Ontology_term=nucleus,regulation of DNA-templated transcription,transcription regulator activity;Ontology_id=GO:0005634,GO:0006355,GO:0140110
contig00008.g4950	1309	ID=contig00008.g4950;Description=NADPH dehydrogenase [Fusarium subglutinans];Gene=FMUND_2884;Ontology_term=oxidoreductase activity;Ontology_id=GO:0016491;Enzyme_code=EC:1.6.99.1,EC:1.6,EC:1,EC:1.6.99.1;Enzyme_name=NADPH dehydrogenase,Acting on NADH or NADPH,Oxidoreductases,NADPH dehydrogenase
contig00008.g4951	933	ID=contig00008.g4951;Description=hypothetical protein FVER14953_12305 [Fusarium verticillioides]
contig00008.g4952	2310	ID=contig00008.g4952;Description=hypothetical protein FVEG_12304 [Fusarium verticillioides 7600]
contig00008.g4953	1922	ID=contig00008.g4953;Description=related to non-hemolytic phospholipase C precursor [Fusarium fujikuroi];Gene=FPANT_12724;Ontology_term=hydrolase activity;Ontology_id=GO:0016787;Enzyme_code=EC:3.1;Enzyme_name=Acting on ester bonds

contig00008.g4954	1957	ID=contig00008.g4954;Description=L-ascorbate oxidase [Fusarium tjaetaba];Gene=FPANT_12725;Ontology_term=oxidoreductase activity,transferase activity;Ontology_id=GO:0016491,GO:0016740;Enzyme_code=EC:1,EC:2.1.1;Enzyme_name=Oxidoreductases,Transferring one-carbon groups
contig00008.g4955	1512	ID=contig00008.g4955;Description=multicopper oxidase-domain-containing protein [Fusarium oxysporum Fo47];Gene=BFJ65_g16503;Ontology_term=oxidoreductase activity,transferase activity;Ontology_id=GO:0016491,GO:0016740;Enzyme_code=EC:1,EC:2.1.1;Enzyme_name=Oxidoreductases,Transferring one-carbon groups
contig00008.g4956	1368	ID=contig00008.g4956;Description=beta-16-galactanase [Fusarium acutatum];Gene=FNAPI_1442;Ontology_term=carbohydrate metabolic process,hydrolase activity;Ontology_id=GO:0005975,GO:0016787;Enzyme_code=EC:3.2.1,EC:3.2,EC:3,EC:3.2.1;Enzyme_name=Glycosylases,Glycosylases,Hydrolases,Glycosylases
contig00008.g4957	681	ID=contig00008.g4957;Description=thioesterase superfamily [Fusarium phyllophilum]
contig00008.g4958	5705	ID=contig00008.g4958;Description=heterokaryon incompatibility protein het-E-1 [Fusarium tjaetaba]
contig00008.g4959	504	ID=contig00008.g4959;Description=Guanyl-specific ribonuclease F1 [Fusarium proliferatum ET1];Gene=FOYG_14360;Ontology_term=RNA binding,lyase activity,catalytic activity, acting on RNA,hydrolase activity;Ontology_id=GO:0003723,GO:0016829,GO:0140098,GO:0016787;Enzyme_code=EC:3.1,EC:4.6.1.24,EC:3.1,EC:4.6.1.24,EC:3,EC:4;Enzyme_name=Acting on ester bonds,ribonuclease T1,Acting on ester bonds,ribonuclease T1,Hydrolases,Lyases
contig00008.g4960	919	ID=contig00008.g4960;Description=endo alpha polygalactosaminidase precursor [Fusarium subglutinans];Gene=FVER53590_12294;Ontology_term=hydrolase activity;Ontology_id=GO:0016787;Enzyme_code=EC:3.2.1.22,EC:3.2,EC:3,EC:3.2.1.22,EC:3.2.1;Enzyme_name=alpha-galactosidase,Glycosylases,Hydrolases,alpha-galactosidase,Glycosylases
contig00008.g4961	2045	ID=contig00008.g4961;Description=amino acid oxidase [Fusarium tjaetaba];Gene=FVER53590_12293;Ontology_term=oxidoreductase activity;Ontology_id=GO:0016491;Enzyme_code=EC:1,EC:1;Enzyme_name=Oxidoreductases,Oxidoreductases

contig00008.g4962	723	ID=contig00008.g4962;Description=related to alpha-acetolactate decarboxylase [Fusarium fujikuroi];Gene=FVER53590_12292;Ontology_term=lyase activity;Ontology_id=GO:0016829;Enzyme_code=EC:4.1.1.5,EC:4.1.1,EC:4.1,EC:4.1.1.5,EC:4;Enzyme_name=acetolactate decarboxylase,Carbon-carbon lyases,Carbon-carbon lyases,acetolactate decarboxylase,Lyases
contig00008.g4963	1774	ID=contig00008.g4963;Description=FAD-linked oxidoreductase yvdP [Fusarium coicis];Gene=FVER53263_12291;Ontology_term=oxidoreductase activity;Ontology_id=GO:0016491;Enzyme_code=EC:1,EC:1;Enzyme_name=Oxidoreductases,Oxidoreductases
contig00008.g4964	1125	ID=contig00008.g4964;Description=hypothetical protein FVEG_12290 [Fusarium verticillioides 7600];Gene=FVEG_12290;Ontology_term=membrane;Ontology_id=GO:0016020
contig00008.g4965	1819	ID=contig00008.g4965;Description=alpha-galactosidase D [Fusarium napiforme];Gene=FNAPI_1432;Ontology_term=carbohydrate metabolic process,lipid metabolic process,carbohydrate derivative metabolic process,cell wall organization or biogenesis,hydrolase activity;Ontology_id=GO:0005975,GO:0006629,GO:1901135,GO:0071554,GO:0016787;Enzyme_code=EC:3.2.1.22,EC:3.2,EC:3,EC:3.2.1.22,EC:3.2.1;Enzyme_name=alpha-galactosidase,Glycosylases,Hydrolases,alpha-galactosidase,Glycosylases
contig00008.g4966	1065	ID=contig00008.g4966;Description=7 8-dihydropterin-6-methyl-4-(beta-D-ribofuranosyl) aminobenzene-5 phosphate synthase [Fusarium tjaetaba];Enzyme_code=EC:2.5.1.105;Enzyme_name=7,8-dihydropterin-6-yl-methyl-4-(beta-D-ribofuranosyl)aminobenzene
contig00008.g4967	1870	ID=contig00008.g4967;Description=hypothetical protein FOMG_09662 [Fusarium oxysporum f. sp. melonis 26406];Gene=FOMG_09662;Ontology_term=transferase activity;Ontology_id=GO:0016740;Enzyme_code=EC:2.3.1,EC:2.3,EC:2,EC:2.3.1;Enzyme_name=Acyltransferases,Acyltransferases,Transferases,Acyltransferases
contig00008.g4968	2021	ID=contig00008.g4968;Description=Het-c heterokaryon incompatibility protein [Fusarium denticulatum];Gene=FPANT_789;Ontology_term=transferase activity;Ontology_id=GO:0016740;Enzyme_code=EC:2.3,EC:2.3,EC:2;Enzyme_name=Acyltransferases,Acyltransferases,Transferases
contig00008.g4969	1593	ID=contig00008.g4969;Description=related to oxidoreductase, FAD-binding [Fusarium fujikuroi];Gene=FFC1_05384;Ontology_term=FAD binding;Ontology_id=GO:0071949
contig00008.g4970	258	ID=contig00008.g4970;Description=antifungal defensin [Fusarium globosum]

contig00008.g4971	586	ID=contig00008.g4971;Description=ribonuclease 3 [Fusarium tjaetaba];Gene=FVER53590_12282;Ontology_term=catalytic activity, acting on RNA,hydrolase activity;Ontology_id=GO:0140098,GO:0016787;Enzyme_code=EC:3.1.30,EC:3.1.26.3,EC:3.1.30,EC:3.1,EC:3,EC:3.1.26.3,EC:3.1.26;Enzyme_name=Acting on ester bonds,ribonuclease III,Acting on ester bonds,Acting on ester bonds,Hydrolases,ribonuclease III,Acting on ester bonds
contig00008.g4972	1635	ID=contig00008.g4972;Description=hypothetical protein FVER14953_12281 [Fusarium verticillioides]
contig00008.g4973	849	ID=contig00008.g4973;Description=trypsin [Fusarium verticillioides 7600];Gene=F25303_7237;Ontology_term=catalytic activity, acting on a protein,hydrolase activity;Ontology_id=GO:0140096,GO:0016787;Enzyme_code=EC:3.4.21,EC:3.4.21,EC:3.4.21.4,EC:3.4,EC:3;Enzyme_name=Acting on peptide bonds (peptidases),Acting on peptide bonds (peptidases),trypsin,Acting on peptide bonds (peptidases),Hydrolases
contig00008.g4974	472	ID=contig00008.g4974;Description=succinyl-:3-ketoacid-coenzyme A mitochondrial precursor [Fusarium coicis]
contig00008.g4975	1177	ID=contig00008.g4975;Description=pectate lyase [Fusarium verticillioides 7600];Gene=FVER53263_12279;Ontology_term=carbohydrate metabolic process,lyase activity,extracellular region;Ontology_id=GO:0005975,GO:0016829,GO:0005576;Enzyme_code=EC:4.2.2.2;Enzyme_name=pectate lyase
contig00008.g4976	1828	ID=contig00008.g4976;Description=gpi anchored protein [Fusarium subglutinans]
contig00008.g4977	1784	ID=contig00008.g4977;Description=-L-isoaspartate O-methyltransferase [Fusarium oxysporum f. sp. albedinis];Gene=FPANT_800;Ontology_term=nucleus,DNA binding,regulation of DNA-templated transcription,transcription regulator activity;Ontology_id=GO:0005634,GO:0003677,GO:0006355,GO:0140110
contig00008.g4978	1585	ID=contig00008.g4978;Description=hexose transporter [Fusarium denticulatum];Gene=FDENT_7145;Ontology_term=transmembrane transport,transporter activity;Ontology_id=GO:0055085,GO:0005215
contig00008.g4979	1555	ID=contig00008.g4979;Description=beta-glucosidase [Fusarium verticillioides 7600];Gene=FCIRC_1335;Ontology_term=carbohydrate metabolic process,hydrolase activity;Ontology_id=GO:0005975,GO:0016787;Enzyme_code=EC:3.2.1,EC:3.2,EC:3.2.1.21,EC:3,EC:3.2.1;Enzyme_name=Glycosylases,Glycosylases,beta-glucosidase,Hydrolases,Glycosylases

contig00008.g4980	783	ID=contig00008.g4980;Description=short chain dehydrogenase reductase family oxidoreductase [Fusarium tjaetaba]
contig00008.g4981	822	ID=contig00008.g4981;Description=AB hydrolase superfamily yisY [Fusarium tjaetaba];Gene=FTJAE_12367;Ontology_term=hydrolase activity;Ontology_id=GO:0016787;Enzyme_code=EC:3,EC:3;Enzyme_name=Hydrolases,Hydrolases
contig00008.g4982	4978	ID=contig00008.g4982;Description=aconitate hydratase 1 [Fusarium verticillioides 7600];Gene=FVER53590_12271;Ontology_term=generation of precursor metabolites and energy,mitochondrion,lyase activity;Ontology_id=GO:0006091,GO:0005739,GO:0016829;Enzyme_code=EC:4.2.1.3,EC:4.2.1,EC:4.2.1.3,EC:4.2,EC:4;Enzyme_name=aconitate hydratase,Carbon-oxygen lyases,aconitate hydratase,Carbon-oxygen lyases,Lyases
contig00008.g4983	2420	ID=contig00008.g4983;Description=pyrimidine pathway regulatory 1 [Fusarium pseudoanthophilum];Gene=BFJ68_g5365;Ontology_term=nucleus,DNA binding,regulation of DNA-templated transcription,transcription regulator activity;Ontology_id=GO:0005634,GO:0003677,GO:0006355,GO:0140110
contig00008.g4984	3827	ID=contig00008.g4984;Description=amidohydrolase 2 [Fusarium coicis];Gene=FPCIR_4559;Ontology_term=transmembrane transport,mitochondrion,hydrolase activity;Ontology_id=GO:0055085,GO:0005739,GO:0016787;Enzyme_code=EC:3;Enzyme_name=Hydrolases
contig00008.g4985	756	ID=contig00008.g4985;Description=ribonuclease e inhibitor rraa dimethylmenaquinone methyltransferase [Fusarium tjaetaba];Ontology_term=lyase activity;Ontology_id=GO:0016829;Enzyme_code=EC:4.1.1,EC:4.1,EC:4.1.3.17,EC:4.1.1.112,EC:4,EC:1.1.1.38,EC:1.1.1.40,EC:4.1.3;Enzyme_name=Carbon-carbon lyases,Carbon-carbon lyases,4-hydroxy-4-methyl-2-oxoglutarate aldolase,oxaloacetate decarboxylase,Lyases,malate dehydrogenase (oxaloacetate-decarboxylating),malate dehydrogenase (oxaloacetate-decarboxylating) (NADP(+)),Carbon-carbon lyases
contig00008.g4986	4821	ID=contig00008.g4986;Description=beta-glucosidase [Fusarium tjaetaba];Gene=FVER53590_25030;Ontology_term=carbohydrate metabolic process,transmembrane transport,transporter activity,hydrolase activity;Ontology_id=GO:0005975,GO:0055085,GO:0005215,GO:0016787;Enzyme_code=EC:3.2.1.21,EC:3.2,EC:3.2.1.21,EC:3,EC:3.2.1;Enzyme_name=beta-glucosidase,Glycosylases,beta-glucosidase,Hydrolases,Glycosylases

contig00008.g4987	1568	ID=contig00008.g4987;Description=transcriptional regulatory [Fusarium coicis];Gene=FPRO_06892;Ontology_term=nucleus,DNA binding,regulation of DNA-templated transcription,transcription regulator activity;Ontology_id=GO:0005634,GO:0003677,GO:0006355,GO:0140110
contig00008.g4988	1739	ID=contig00008.g4988;Description=general substrate transporter [Fusarium solani];Gene=FHETE_67;Ontology_term=transmembrane transport,transporter activity;Ontology_id=GO:0055085,GO:0005215
contig00008.g4989	952	ID=contig00008.g4989;Description=omega-amidase NIT2 [Fusarium circinatum];Gene=FNYG_09909;Ontology_term=nitrogen compound metabolic process;Ontology_id=GO:0006807
contig00008.g4990	1482	ID=contig00008.g4990;Description=related to YPS1-aspergillopepsin [Fusarium fujikuroi];Gene=FDENT_7131;Ontology_term=catalytic activity, acting on a protein,protein catabolic process,cell wall,endoplasmic reticulum,cell wall organization or biogenesis,extracellular region,plasma membrane,hydrolase activity;Ontology_id=GO:0140096,GO:0030163,GO:0005618,GO:0005783,GO:0071554,GO:0005576,GO:0005886,GO:0016787;Enzyme_code=EC:3.4.23,EC:3.4.23,EC:3.4,EC:3;Enzyme_name=Acting on peptide bonds (peptidases),Acting on peptide bonds (peptidases),Acting on peptide bonds (peptidases),Hydrolases
contig00008.g4991	765	ID=contig00008.g4991;Description=kinase-like domain protein [Fusarium tjaetaba];Gene=FNAPI_1402;Ontology_term=transferase activity;Ontology_id=GO:0016740;Enzyme_code=EC:2.7,EC:2.7,EC:2;Enzyme_name=Transferring phosphorus-containing groups,Transferring phosphorus-containing groups,Transferases
contig00008.g4992	522	ID=contig00008.g4992;Description=acyl-protein thioesterase [Metarhizium acridum CQMa 102];Gene=FMUND_15217;Ontology_term=hydrolase activity;Ontology_id=GO:0016787;Enzyme_code=EC:3,EC:3;Enzyme_name=Hydrolases,Hydrolases
contig00008.g4993	1668	ID=contig00008.g4993;Description=5-methylthioadenosine s-adenosylhomocysteine deaminase [Fusarium tjaetaba];Gene=FPANT_13280;Ontology_term=hydrolase activity;Ontology_id=GO:0016787;Enzyme_code=EC:3.5,EC:3.5,EC:3;Enzyme_name=Acting on carbon-nitrogen bonds, other than peptide bonds,Acting on carbon-nitrogen bonds, other than peptide bonds,Hydrolases
contig00008.g4994	1623	ID=contig00008.g4994;Description=major Facilitator Superfamily [Fusarium tjaetaba];Gene=FVER53263_12258;Ontology_term=transmembrane transport,transporter activity;Ontology_id=GO:0055085,GO:0005215

contig00008.g4995	1665	ID=contig00008.g4995;Description=hypothetical protein FVER53590_12257 [Fusarium verticillioides];Enzyme_code=EC:3.5.1.4;Enzyme_name=amidase
contig00008.g4996	1263	ID=contig00008.g4996;Description=Zn(2)-C6 fungal-type DNA-binding domain containing protein [Fusarium agapanthi];Gene=F52700_4632;Ontology_term=nucleus,regulation of DNA-templated transcription,transcription regulator activity;Ontology_id=GO:0005634,GO:0006355,GO:0140110
contig00008.g4997	1490	ID=contig00008.g4997;Description=cytochrome P450 [Fusarium oxysporum];Gene=FACUT_3041;Ontology_term=oxidoreductase activity;Ontology_id=GO:0016491;Enzyme_code=EC:1.14,EC:1,EC:1.14;Enzyme_name=Acting on paired donors, with incorporation or reduction of molecular oxygen. The oxygen incorporated need not be derived from O2,Oxidoreductases,Acting on paired donors, with incorporation or reduction of molecular oxygen. The oxygen incorporated need not be derived from O2
contig00008.g4998	734	ID=contig00008.g4998;Description=FAR-17a/AIG1-like protein [Fusarium oxysporum f. sp. albedinis];Gene=FACUT_3040;Ontology_term=membrane,endomembrane system;Ontology_id=GO:0016020,GO:0012505
contig00008.g4999	747	ID=contig00008.g4999;Description=asp Glu Hydantoin racemase domain protein [Fusarium tjaetaba];Gene=BFJ72_g438;Ontology_term=isomerase activity;Ontology_id=GO:0016853;Enzyme_code=EC:5.1.1.10,EC:5.1.1,EC:5.1,EC:5.1.1.10,EC:5;Enzyme_name=amino-acid racemase,Racemases and epimerases,Racemases and epimerases,amino-acid racemase,Isomerases
contig00008.g5000	2752	ID=contig00008.g5000;Description=probable cytosine deaminase and related metal-dependent hydrolases [Fusarium fujikuroi];Gene=FNAPI_7446;Ontology_term=hydrolase activity;Ontology_id=GO:0016787;Enzyme_code=EC:3.5,EC:3.5,EC:3;Enzyme_name=Acting on carbon-nitrogen bonds, other than peptide bonds,Acting on carbon-nitrogen bonds, other than peptide bonds,Hydrolases
contig00008.g5001	543	ID=contig00008.g5001;Description=related to Cupin domain protein [Fusarium fujikuroi]
contig00008.g5002	1968	ID=contig00008.g5002;Description=phenol 2-monooxygenase [Fusarium subglutinans];Gene=FPANT_464;Ontology_term=oxidoreductase activity;Ontology_id=GO:0016491;Enzyme_code=EC:1,EC:1,EC:1.14.13.7;Enzyme_name=Oxidoreductases,Oxidoreductases,phenol 2-monooxygenase (NADPH)

contig00008.g5003	2247	ID=contig00008.g5003;Description=Superoxide-generating NADPH oxidase heavy chain subunit C [Fusarium acutatum];Gene=noxC;Ontology_term=oxidoreductase activity;Ontology_id=GO:0016491;Enzyme_code=EC:1.16,EC:1.6.3.1,EC:1.16,EC:1;Enzyme_name=Oxidizing metal ions,NAD(P)H oxidase (H2O2-forming),Oxidizing metal ions,Oxidoreductases
contig00008.g5004	1917	ID=contig00008.g5004;Description=hypothetical protein FVER53263_12249 [Fusarium verticillioides];Gene=FVER53263_12249;Ontology_term=membrane;Ontology_id=GO:0016020
contig00008.g5005	1185	ID=contig00008.g5005;Description=hypothetical protein FVER53590_12248 [Fusarium verticillioides]
contig00008.g5006	1677	ID=contig00008.g5006;Description=hypothetical protein FVEG_12247 [Fusarium verticillioides 7600];Gene=FPCIR_4586;Ontology_term=oxidoreductase activity;Ontology_id=GO:0016491;Enzyme_code=EC:1,EC:1;Enzyme_name=Oxidoreductases,Oxidoreductases
contig00008.g5007	793	ID=contig00008.g5007;Description=3-oxoacyl-[acyl-carrier protein] reductase [Fusarium oxysporum f. sp. radicis-lycopersici 26381];Gene=BFJ68_g5377;Ontology_term=oxidoreductase activity;Ontology_id=GO:0016491;Enzyme_code=EC:1,EC:1,EC:1.1.1.100;Enzyme_name=Oxidoreductases,Oxidoreductases,3-oxoacyl-[acyl-carrier-protein] reductase
contig00008.g5008	833	ID=contig00008.g5008;Description=subtilisin-like protease 2 [Fusarium napiforme];Gene=FNAPI_7450;Ontology_term=catalytic activity, acting on a protein,hydrolase activity;Ontology_id=GO:0140096,GO:0016787;Enzyme_code=EC:3.4.21;Enzyme_name=Acting on peptide bonds (peptidases)
contig00008.g5009	1583	ID=contig00008.g5009;Description=hypothetical protein J7337_007582 [Fusarium musae];Gene=FCIRC_5535;Ontology_term=catalytic activity, acting on a protein,hydrolase activity;Ontology_id=GO:0140096,GO:0016787;Enzyme_code=EC:3.4.21,EC:3.4.21,EC:3.4,EC:3;Enzyme_name=Acting on peptide bonds (peptidases),Acting on peptide bonds (peptidases),Acting on peptide bonds (peptidases),Hydrolases
contig00008.g5010	1710	ID=contig00008.g5010;Description=proline-specific permease [Fusarium tjaetaba];Gene=FPHYL_12679;Ontology_term=transmembrane transport;Ontology_id=GO:0055085
contig00008.g5011	834	ID=contig00008.g5011;Description=hypothetical protein FVEG_12242 [Fusarium verticillioides 7600]

contig00008.g5012	735	ID=contig00008.g5012;Description=related to acetylxylan esterase [Fusarium proliferatum ET1]
contig00008.g5013	1705	ID=contig00008.g5013;Description=hexose transporter [Fusarium agapanthi];Gene=FVER53263_12240;Ontology_term=transmembrane transport,oxidoreductase activity,transporter activity;Ontology_id=GO:0055085,GO:0016491,GO:0005215;Enzyme_code=EC:1.1.1,EC:1.1,EC:1,EC:1.1.1;Enzyme_name=Acting on the CH-OH group of donors,Acting on the CH-OH group of donors,Oxidoreductases,Acting on the CH-OH group of donors
contig00008.g5014	1125	ID=contig00008.g5014;Description=alcohol dehydrogenase [Fusarium tjaetaba];Gene=FVEG_12239;Ontology_term=oxidoreductase activity;Ontology_id=GO:0016491;Enzyme_code=EC:1.1.1,EC:1.1,EC:1,EC:1.1.1;Enzyme_name=Acting on the CH-OH group of donors,Acting on the CH-OH group of donors,Oxidoreductases,Acting on the CH-OH group of donors
contig00008.g5015	2027	ID=contig00008.g5015;Description=related to choline dehydrogenase [Fusarium fujikuroi];Gene=FVER53263_12238;Ontology_term=oxidoreductase activity;Ontology_id=GO:0016491;Enzyme_code=EC:1.1,EC:1.1,EC:1,EC:1.1.99.1;Enzyme_name=Acting on the CH-OH group of donors,Acting on the CH-OH group of donors,Oxidoreductases,choline dehydrogenase
contig00008.g5016	1429	ID=contig00008.g5016;Description=hypothetical protein FVER14953_12237 [Fusarium verticillioides]
contig00008.g5017	1454	ID=contig00008.g5017;Description=lipase B [Fusarium tjaetaba]
contig00008.g5018	1493	ID=contig00008.g5018;Description=related to oxidase [Fusarium mangiferae];Gene=FVER53263_12235;Ontology_term=antioxidant activity,oxidoreductase activity;Ontology_id=GO:0016209,GO:0016491;Enzyme_code=EC:1.11.1,EC:1.11,EC:1,EC:1.11.1,EC:1.11.2.1;Enzyme_name=Acting on a peroxide as acceptor,Acting on a peroxide as acceptor,Oxidoreductases,Acting on a peroxide as acceptor,unspecific peroxygenase
contig00008.g5019	708	ID=contig00008.g5019;Description=Isochorismatase hydrolase [Fusarium coicis];Gene=FPCIR_4599;Ontology_term=hydrolase activity;Ontology_id=GO:0016787;Enzyme_code=EC:3,EC:3;Enzyme_name=Hydrolases,Hydrolases

contig00008.g5020	2069	ID=contig00008.g5020;Description=ADAM protease [Fusarium denticulatum];Gene=FPANT_449;Ontology_term=catalytic activity, acting on a protein,hydrolase activity;Ontology_id=GO:0140096,GO:0016787;Enzyme_code=EC:3.4.24;Enzyme_name=Acting on peptide bonds (peptidases)
contig00008.g5021	1131	ID=contig00008.g5021;Description=alcohol dehydrogenase [Fusarium coicis];Gene=FNYG_09684;Ontology_term=oxidoreductase activity;Ontology_id=GO:0016491;Enzyme_code=EC:1,EC:1;Enzyme_name=Oxidoreductases,Oxidoreductases
contig00008.g5022	3572	ID=contig00008.g5022;Description=C6 transcription factor asaR [Fusarium oxysporum f. sp. rapae];Gene=FPHYL_12669;Ontology_term=nucleus,DNA binding,regulation of DNA-templated transcription,transcription regulator activity;Ontology_id=GO:0005634,GO:0003677,GO:0006355,GO:0140110
contig00008.g5023	1050	ID=contig00008.g5023;Description=Fusaridione A cluster transcription factor fsdR [Fusarium oxysporum f. sp. conglutinans];Gene=FPANT_445;Ontology_term=nucleus,DNA binding,regulation of DNA-templated transcription,transcription regulator activity;Ontology_id=GO:0005634,GO:0003677,GO:0006355,GO:0140110
contig00008.g5024	909	ID=contig00008.g5024;Description=hypothetical protein FVEG_12229 [Fusarium verticillioides 7600]
contig00008.g5025	765	ID=contig00008.g5025;Description=cysteine dioxygenase [Fusarium verticillioides 7600];Gene=HZS61_010845;Ontology_term=oxidoreductase activity;Ontology_id=GO:0016491;Enzyme_code=EC:1,EC:1.13.11.20,EC:1;Enzyme_name=Oxidoreductases,cysteine dioxygenase,Oxidoreductases
contig00008.g5026	2007	ID=contig00008.g5026;Description=C6 transcription factor [Fusarium tjaetaba];Gene=FNAPI_7468;Ontology_term=nucleus,DNA binding,regulation of DNA-templated transcription,transcription regulator activity;Ontology_id=GO:0005634,GO:0003677,GO:0006355,GO:0140110
contig00008.g5027	1950	ID=contig00008.g5027;Description=heterokaryon incompatibility 6 OR allele [Fusarium tjaetaba];Gene=FPCIR_4606;Ontology_term=nucleus,DNA binding,regulation of DNA-templated transcription,transcription regulator activity;Ontology_id=GO:0005634,GO:0003677,GO:0006355,GO:0140110
contig00008.g5028	1227	ID=contig00008.g5028;Description=related to aldehyde reductase II [Fusarium fujikuroi]

contig00008.g5029	1679	ID=contig00008.g5029;Description=related to putative tartrate transporter [Fusarium fujikuroi];Gene=FNYG_09675;Ontology_term=transmembrane transport,transporter activity;Ontology_id=GO:0055085,GO:0005215
contig00008.g5030	2158	ID=contig00008.g5030;Description=primary-amine oxidase [Fusarium verticillioides 7600];Gene=FNAPI_5777;Ontology_term=oxidoreductase activity;Ontology_id=GO:0016491;Enzyme_code=EC:1.4.3.21;Enzyme_name=primary-amine oxidase
contig00008.g5031	2479	ID=contig00008.g5031;Description=heterokaryon incompatibility 6 OR allele [Fusarium mexicanum]
contig00008.g5032	1998	ID=contig00008.g5032;Description=related to cocaine esterase [Fusarium fujikuroi];Gene=FNYG_09673;Ontology_term=catalytic activity, acting on a protein,hydrolase activity;Ontology_id=GO:0140096,GO:0016787;Enzyme_code=EC:3.4.14.1,EC:3.4.14.5,EC:3.4.14.4,EC:3.4.14.2,EC:3.4.14.1,EC:3.4,EC:3,EC:3.4.14.5,EC:3.4.14.4,EC:3.4.14.2;Enzyme_name=dipeptidyl-peptidase I,dipeptidyl-peptidase IV,dipeptidyl-peptidase III,dipeptidyl-peptidase II,dipeptidyl-peptidase I,Acting on peptide bonds (peptidases),Hydrolases,dipeptidyl-peptidase IV,dipeptidyl-peptidase III,dipeptidyl-peptidase II
contig00008.g5033	3010	ID=contig00008.g5033;Description=related to potential drug facilitator PEP5 [Fusarium mangiferae];Gene=FPHYL_2656;Ontology_term=transmembrane transport,lyase activity,transporter activity;Ontology_id=GO:0055085,GO:0016829,GO:0005215;Enzyme_code=EC:4.2.1.94,EC:4.2.1,EC:4.2,EC:4;Enzyme_name=scytalone dehydratase,Carbon-oxygen lyases,Carbon-oxygen lyases,Lyases
contig00008.g5034	1412	ID=contig00008.g5034;Description=aromatic prenyltransferase [Fusarium tjaetaba];Gene=FVEG_12218;Ontology_term=transferase activity;Ontology_id=GO:0016740;Enzyme_code=EC:2.5.1;Enzyme_name=Transferring alkyl or aryl groups, other than methyl groups
contig00008.g5035	1036	ID=contig00008.g5035;Description=epoxide hydrolase 2 [Fusarium tjaetaba];Gene=FPCIR_4614;Ontology_term=hydrolase activity;Ontology_id=GO:0016787;Enzyme_code=EC:3,EC:3;Enzyme_name=Hydrolases,Hydrolases

contig00008.g5036	1761	ID=contig00008.g5036;Description=cytochrome P450 monooxygenase [Fusarium tjaetaba];Gene=FVER53263_12216;Ontology_term=oxidoreductase activity;Ontology_id=GO:0016491;Enzyme_code=EC:1.14,EC:1,EC:1.14;Enzyme_name=Acting on paired donors, with incorporation or reduction of molecular oxygen. The oxygen incorporated need not be derived from O2,Oxidoreductases,Acting on paired donors, with incorporation or reduction of molecular oxygen. The oxygen incorporated need not be derived from O2
contig00008.g5037	769	ID=contig00008.g5037;Description=Digeranylgeranylglyceryl phosphate synthase [Fusarium tjaetaba];Gene=FOYG_14470;Ontology_term=membrane;Ontology_id=GO:0016020
contig00008.g5038	1666	ID=contig00008.g5038;Description=aminoadipate-semialdehyde dehydrogenase [Fusarium phyllophilum]
contig00008.g5039	2990	ID=contig00008.g5039;Description=2-hydroxyacylsphingosine 1-beta-galactosyltransferase [Fusarium coicis];Gene=FDENT_9032;Ontology_term=antioxidant activity,oxidoreductase activity,transferase activity;Ontology_id=GO:0016209,GO:0016491,GO:0016740;Enzyme_code=EC:2.4,EC:1.11.1,EC:1.11,EC:2.4,EC:1,EC:2,EC:1.11.1;Enzyme_name=Glycosyltransferases,Acting on a peroxide as acceptor,Acting on a peroxide as acceptor,Glycosyltransferases,Oxidoreductases,Transferases,Acting on a peroxide as acceptor
contig00008.g5040	825	ID=contig00008.g5040;Description=short chain alcohol dehydrogenase [Fusarium tjaetaba];Gene=BFJ72_g637;Ontology_term=antioxidant activity,oxidoreductase activity;Ontology_id=GO:0016209,GO:0016491;Enzyme_code=EC:1.11.1,EC:1.11,EC:1,EC:1.11.1;Enzyme_name=Acting on a peroxide as acceptor,Acting on a peroxide as acceptor,Oxidoreductases,Acting on a peroxide as acceptor
contig00008.g5041	1702	ID=contig00008.g5041;Description=vesicular transport protein [Fusarium denticulatum]
contig00008.g5042	1737	ID=contig00008.g5042;Description=bromodomain protein [Fusarium tjaetaba];Gene=FMUND_8380;Ontology_term=carbohydrate metabolic process,lyase activity,extracellular region;Ontology_id=GO:0005975,GO:0016829,GO:0005576;Enzyme_code=EC:4.2.2.2,EC:4.2.2.2,EC:4.2,EC:4.2.2.10,EC:4,EC:4.2.2;Enzyme_name=pectate lyase,pectate lyase,Carbon-oxygen lyases,pectin lyase,Lyases,Carbon-oxygen lyases

contig00008.g5043	6132	ID=contig00008.g5043;Description=integral membrane protein PTH11 [Fusarium napiforme];Gene=FVEG_17184;Ontology_term=hydrolase activity;Ontology_id=GO:0016787;Enzyme_code=EC:3,EC:3;Enzyme_name=Hydrolases,Hydrolases
contig00008.g5044	1383	ID=contig00008.g5044;Description=hypothetical protein FVER53590_12204 [Fusarium verticillioides]
contig00008.g5045	1205	ID=contig00008.g5045;Description=related to MOSC domain protein [Fusarium fujikuroi];Gene=CEK26_007846;Ontology_term=nucleus,DNA binding,catalytic activity,regulation of DNA-templated transcription,reproductive process,transcription regulator activity,molecular adaptor activity;Ontology_id=GO:0005634,GO:0003677,GO:0003824,GO:0006355,GO:0022414,GO:0140110,GO:0060090
contig00008.g5046	1434	ID=contig00008.g5046;Description=hypothetical protein FVEG_12202 [Fusarium verticillioides 7600];Gene=FVER53263_12202;Ontology_term=oxidoreductase activity;Ontology_id=GO:0016491;Enzyme_code=EC:1,EC:1;Enzyme_name=Oxidoreductases,Oxidoreductases
contig00008.g5047	351	ID=contig00008.g5047;Description=sorbose reductase sou1 [Fusarium sp. NRRL 25303];Gene=FFC1_05484;Ontology_term=nucleus,DNA binding;Ontology_id=GO:0005634,GO:0003677
contig00008.g5048	525	ID=contig00008.g5048;Description=PiggyBac transposable element-derived protein 2 [Fusarium oxysporum f. sp. albedinis]
contig00008.g5049	1736	ID=contig00008.g5049;Description=Putative MFS-type transporter PB1E7.08c [Fusarium oxysporum f. sp. cubense race 1];Gene=AU210_013987;Ontology_term=transmembrane transport,transporter activity;Ontology_id=GO:0055085,GO:0005215
contig00008.g5050	922	ID=contig00008.g5050;Description=Rtm1p [Fusarium tjaetaba];Gene=FTJAE_1103;Ontology_term=membrane;Ontology_id=GO:0016020
contig00008.g5051	1117	ID=contig00008.g5051;Description=UPC2-like regulatory protein [Fusarium tjaetaba];Gene=FVER53263_20635;Ontology_term=nucleus,regulation of DNA-templated transcription,transcription regulator activity;Ontology_id=GO:0005634,GO:0006355,GO:0140110
contig00008.g5052	975	ID=contig00008.g5052;Description=RTM1 [Fusarium phyllophilum];Gene=FACUT_2979;Ontology_term=membrane;Ontology_id=GO:0016020

contig00008.g5053	1013	ID=contig00008.g5053;Description=methyltransferase [Fusarium verticillioides 7600];Gene=FVER53590_12196;Ontology_term=transferase activity;Ontology_id=GO:0016740;Enzyme_code=EC:2.1.1,EC:2.1,EC:2,EC:2.1.1,EC:2.1.1.137;Enzyme_name=Transferring one-carbon groups,Transferring one-carbon groups,Transferases,Transferring one-carbon groups,arsenite methyltransferase
contig00008.g5054	731	ID=contig00008.g5054;Description=hypothetical protein FVER53590_25022 [Fusarium verticillioides]
contig00008.g5055	1925	ID=contig00008.g5055;Description=Ion channel regulatory UNC-93 [Fusarium coicis];Gene=FNYG_09643
contig00008.g5056	1454	ID=contig00008.g5056;Description=carboxypeptidase A4 [Fusarium verticillioides 7600];Gene=FNYG_09642;Ontology_term=catalytic activity, acting on a protein,extracellular region,hydrolase activity;Ontology_id=GO:0140096,GO:0005576,GO:0016787;Enzyme_code=EC:3.4.17,EC:3.4,EC:3.4.17,EC:3;Enzyme_name=Acting on peptide bonds (peptidases),Acting on peptide bonds (peptidases),Acting on peptide bonds (peptidases),Hydrolases
contig00008.g5057	1985	ID=contig00008.g5057;Description=Zn(II)2Cys6 transcriptional activator [Fusarium fujikuroi];Gene=FPRO_06806;Ontology_term=DNA binding,nucleus,lipid metabolic process,regulation of DNA-templated transcription,chromatin organization,transcription regulator activity;Ontology_id=GO:0003677,GO:0005634,GO:0006629,GO:0006355,GO:0006325,GO:0140110
contig00008.g5058	1714	ID=contig00008.g5058;Description=probable general amino acid permease [Fusarium fujikuroi];Gene=FTJAE_1095;Ontology_term=transmembrane transport,nucleobase-containing small molecule metabolic process,carbohydrate derivative metabolic process,transporter activity,hydrolase activity;Ontology_id=GO:0055085,GO:0055086,GO:1901135,GO:0005215,GO:0016787;Enzyme_code=EC:3.5.2.3,EC:3.5.2.3,EC:3.5.2,EC:3.5,EC:3;Enzyme_name=dihydroorotase,dihydroorotase,Acting on carbon-nitrogen bonds, other than peptide bonds,Acting on carbon-nitrogen bonds, other than peptide bonds,Hydrolases
contig00008.g5059	1919	ID=contig00008.g5059;Description=hypothetical protein FVER53263_12191 [Fusarium verticillioides];Gene=1152
contig00008.g5060	993	ID=contig00008.g5060;Description=hypothetical protein FVER14953_12190 [Fusarium verticillioides]

contig00008.g5061	1824	ID=contig00008.g5061;Description=alpha-L-fucosidase [Fusarium verticillioides 7600];Gene=FPANT_627;Ontology_term=carbohydrate metabolic process,hydrolase activity;Ontology_id=GO:0005975,GO:0016787;Enzyme_code=EC:3.2.1.51,EC:3.2,EC:3.2.1.51,EC:3,EC:3.2.1;Enzyme_name=alpha-L-fucosidase,Glycosylases,alpha-L-fucosidase,Hydrolases,Glycosylases
contig00008.g5062	989	ID=contig00008.g5062;Description=subtilisin [Fusarium mexicanum]
contig00008.g5063	1453	ID=contig00008.g5063;Description=hypothetical protein FVER53263_20634 [Fusarium verticillioides]
contig00008.g5064	1023	ID=contig00008.g5064;Description=endonuclease exonuclease phosphatase family [Fusarium tjaetaba];Gene=FPCIR_4645;Ontology_term=hydrolase activity;Ontology_id=GO:0016787;Enzyme_code=EC:3.1,EC:3.1,EC:3;Enzyme_name=Acting on ester bonds,Acting on ester bonds,Hydrolases
contig00008.g5065	1563	ID=contig00008.g5065;Description=cytochrome P450 [Fusarium oxysporum f. sp. albedinis];Gene=F25303_4432;Ontology_term=oxidoreductase activity;Ontology_id=GO:0016491;Enzyme_code=EC:1.14,EC:1,EC:1.14;Enzyme_name=Acting on paired donors, with incorporation or reduction of molecular oxygen. The oxygen incorporated need not be derived from O2,Oxidoreductases,Acting on paired donors, with incorporation or reduction of molecular oxygen. The oxygen incorporated need not be derived from O2
contig00008.g5066	456	ID=contig00008.g5066;Description=hypothetical protein FVER53590_25020 [Fusarium verticillioides]
contig00008.g5067	1497	ID=contig00008.g5067;Description=hypothetical protein FVER53263_12186 [Fusarium verticillioides]
contig00008.g5068	1314	ID=contig00008.g5068;Description=hypothetical protein FPANT_633 [Fusarium pseudoanthophilum]
contig00008.g5069	2663	ID=contig00008.g5069;Description=alkaline phosphatase [Fusarium verticillioides 7600];Enzyme_code=EC:3.1.3.1;Enzyme_name=alkaline phosphatase
contig00008.g5070	1801	ID=contig00008.g5070;Description=putative LIM domain-containing serine/threonine-protein [Fusarium oxysporum f. sp. albedinis]
contig00008.g5071	2974	ID=contig00008.g5071;Description=hypothetical protein FVER53263_12182 [Fusarium verticillioides];Gene=FVER53590_12182;Ontology_term=lipid metabolic process;Ontology_id=GO:0006629

contig00008.g5072	1527	ID=contig00008.g5072;Description=hypothetical protein FOXB_12369 [Fusarium oxysporum f. sp. conglutinans Fo5176];Gene=FOBC_15915;Ontology_term=lipid metabolic process,transferase activity;Ontology_id=GO:0006629,GO:0016740;Enzyme_code=EC:2.1.1,EC:2.1,EC:2,EC:2.1.1;Enzyme_name=Transferring one-carbon groups,Transferring one-carbon groups,Transferases,Transferring one-carbon groups
contig00008.g5073	796	ID=contig00008.g5073;Description=acetylesterase [Fusarium tjaetaba];Gene=FVEG_12180;Ontology_term=hydrolase activity;Ontology_id=GO:0016787;Enzyme_code=EC:3,EC:3;Enzyme_name=Hydrolases,Hydrolases
contig00008.g5074	2119	ID=contig00008.g5074;Description=cutinase transcription factor 1 beta [Fusarium tjaetaba];Gene=FCIRC_2197;Ontology_term=nucleus,DNA binding,DNA-templated transcription;Ontology_id=GO:0005634,GO:0003677,GO:0006351
contig00008.g5075	1394	ID=contig00008.g5075;Description=hypothetical protein FDENT_13282 [Fusarium denticulatum];Gene=FTJAE_1080;Ontology_term=transferase activity;Ontology_id=GO:0016740;Enzyme_code=EC:2.1.1,EC:2.1,EC:2,EC:2.1.1;Enzyme_name=Transferring one-carbon groups,Transferring one-carbon groups,Transferases,Transferring one-carbon groups
contig00008.g5076	894	ID=contig00008.g5076;Description=SET domain-containing protein [Fusarium pseudoanthophilum];Gene=FPANT_640;Ontology_term=transferase activity;Ontology_id=GO:0016740;Enzyme_code=EC:2.1.1,EC:2.1,EC:2,EC:2.1.1;Enzyme_name=Transferring one-carbon groups,Transferring one-carbon groups,Transferases,Transferring one-carbon groups
contig00008.g5077	3238	ID=contig00008.g5077;Description=hypothetical protein FVER53263_12176 [Fusarium verticillioides]
contig00008.g5078	1856	ID=contig00008.g5078;Description=major facilitator [Fusarium tjaetaba];Gene=FPCIR_4658;Ontology_term=transmembrane transport,transporter activity;Ontology_id=GO:0055085,GO:0005215
contig00008.g5079	969	ID=contig00008.g5079;Description=epi-isozizaene synthase [Fusarium tjaetaba];Gene=FMUND_8346;Ontology_term=lyase activity;Ontology_id=GO:0016829;Enzyme_code=EC:4,EC:4;Enzyme_name=Lyases,Lyases

		ID=contig00008.g5080;Description=neutral amino acid permease [Fusarium fujikuroi];Gene=mtr-17;Ontology_term=RNA binding,cytosol,tRNA metabolic process,amino acid metabolic process,catalytic activity, acting on RNA,ligase activity;Ontology_id=GO:0003723,GO:0005829,GO:0006399,GO:0006520,GO:0140098,GO:0016874;Enzyme_code=EC:6.1.1.6,EC:6.1,EC:6.1.1.6,EC:6.1.1,EC:6;Enzyme_name=lysine--tRNA ligase,Forming carbon-oxygen bonds,lysine--tRNA ligase,Forming carbon-oxygen bonds,Ligases
contig00008.g5080	1614	
contig00008.g5081	887	ID=contig00008.g5081;Description=hydroxyneurosporene synthase [Fusarium tjaetaba]
		ID=contig00008.g5082;Description=cytochrome P450 monooxygenase [Fusarium tjaetaba];Gene=FVER53590_12172;Ontology_term=oxidoreductase activity;Ontology_id=GO:0016491;Enzyme_code=EC:1.14,EC:1,EC:1.14;Enzyme_name=Acting on paired donors, with incorporation or reduction of molecular oxygen. The oxygen incorporated need not be derived from O2,Oxidoreductases,Acting on paired donors, with incorporation or reduction of molecular oxygen. The oxygen incorporated need not be derived from O2
contig00008.g5082	1458	
contig00008.g5083	2578	ID=contig00008.g5083;Description=hypothetical protein FVER53590_11808 [Fusarium verticillioides];Gene=FVEG_11808;Ontology_term=nucleus,DNA binding,regulation of DNA-templated transcription,transcription regulator activity;Ontology_id=GO:0005634,GO:0003677,GO:0006355,GO:0140110
		ID=contig00008.g5084;Description=hypothetical protein FVER53590_11809 [Fusarium verticillioides]
contig00008.g5084	1740	
contig00008.g5085	279	ID=contig00008.g5085;Description=rapid ALkalinization factor [Fusarium tjaetaba]
contig00008.g5086	657	ID=contig00008.g5086;Description=FAD-dependent pyridine nucleotide-disulfide oxidoreductase [Fusarium tjaetaba]
contig00008.g5087	2028	ID=contig00008.g5087;Description=sterigmatocystin biosynthesis monooxygenase stcW [Fusarium subglutinans];Gene=FCIRC_2214;Ontology_term=oxidoreductase activity;Ontology_id=GO:0016491;Enzyme_code=EC:1,EC:1;Enzyme_name=Oxidoreductases,Oxidoreductases

contig00008.g5088	4625	ID=contig00008.g5088;Description=ATPase [Fusarium verticillioides 7600];Gene=FPCIR_4669;Ontology_term=membrane organization,DNA repair,transmembrane transport,DNA-templated transcription,DNA recombination,lipid binding,vacuole,transporter activity,plasma membrane,nucleus,DNA binding,ATP-dependent activity,mitochondrion,hydrolase activity;Ontology_id=GO:0061024,GO:0006281,GO:0055085,GO:0006351,GO:0006310,GO:0008289,GO:0005773,GO:0005215,GO:0005886,GO:0005634,GO:0003677,GO:0140657,GO:0005739,GO:0016787;Enzyme_code=EC:7.2.2,EC:3.6.1.15,EC:3.6.1,EC:3.6,EC:7.6.2.1,EC:7.6.2.2,EC:3,EC:7.2.2,EC:7,EC:3.6.1.15;Enzyme_name=Catalysing the translocation of inorganic cations,nucleoside-triphosphate phosphatase,Acting on acid anhydrides,Acting on acid anhydrides,P-type phospholipid transporter,ABC-type xenobiotic transporter,Hydrolases,Catalysing the translocation of inorganic cations,Translocases,nucleoside-triphosphate phosphatase
contig00008.g5089	2517	ID=contig00008.g5089;Description=L-Aspartase-like protein [Fusarium oxysporum];Gene=Forpe1208_v011426;Ontology_term=nucleus,DNA binding,generation of precursor metabolites and energy,lyase activity,regulation of DNA-templated transcription,transcription regulator activity;Ontology_id=GO:0005634,GO:0003677,GO:0006091,GO:0016829,GO:0006355,GO:0140110;Enzyme_code=EC:4,EC:4;Enzyme_name=Lyases,Lyases
contig00008.g5090	1546	ID=contig00008.g5090;Description=aspartate ammonia-lyase [Fusarium tjaetaba];Gene=FNAPI_10448;Ontology_term=nucleus,DNA binding,generation of precursor metabolites and energy,lyase activity,regulation of DNA-templated transcription,transcription regulator activity;Ontology_id=GO:0005634,GO:0003677,GO:0006091,GO:0016829,GO:0006355,GO:0140110;Enzyme_code=EC:4;Enzyme_name=Lyases
contig00008.g5091	1686	ID=contig00008.g5091;Description=c6 transcription [Fusarium phyllophilum];Gene=FPHYL_8903;Ontology_term=nucleus,DNA binding,generation of precursor metabolites and energy,lyase activity,regulation of DNA-templated transcription,transcription regulator activity;Ontology_id=GO:0005634,GO:0003677,GO:0006091,GO:0016829,GO:0006355,GO:0140110;Enzyme_code=EC:4,EC:4;Enzyme_name=Lyases,Lyases
contig00008.g5092	1655	ID=contig00008.g5092;Description=related to nicotinamide mononucleotide permease [Fusarium mangiferae];Gene=FOXYS1_660;Ontology_term=transmembrane transport,transporter activity;Ontology_id=GO:0055085,GO:0005215

contig00008.g5093	869	ID=contig00008.g5093;Description=ARG81-transcription factor involved in arginine metabolism [Fusarium acutatum];Gene=FVER53263_20886;Ontology_term=nucleus,oxidoreductase activity,regulation of DNA-templated transcription,transcription regulator activity;Ontology_id=GO:0005634,GO:0016491,GO:0006355,GO:0140110;Enzyme_code=EC:1;Enzyme_name=Oxidoreductases
contig00008.g5094	2245	ID=contig00008.g5094;Description=ARG81-like transcription factor [Fusarium pseudocircinatum];Gene=FPRO05_08162;Ontology_term=nucleus,oxidoreductase activity,regulation of DNA-templated transcription,transcription regulator activity;Ontology_id=GO:0005634,GO:0016491,GO:0006355,GO:0140110;Enzyme_code=EC:1;Enzyme_name=Oxidoreductases
contig00008.g5095	1677	ID=contig00008.g5095;Description=monooxygenase [Fusarium napiforme];Gene=FVER53263_11820;Ontology_term=oxidoreductase activity;Ontology_id=GO:0016491;Enzyme_code=EC:1.14.13.8,EC:1.14.13.8,EC:1,EC:1.14,EC:1.14.13;Enzyme_name=flavin-containing monooxygenase,flavin-containing monooxygenase,Oxidoreductases,Acting on paired donors, with incorporation or reduction of molecular oxygen. The oxygen incorporated need not be derived from O2,Acting on paired donors, with incorporation or reduction of molecular oxygen. The oxygen incorporated need not be derived from O2
contig00008.g5096	813	ID=contig00008.g5096;Description=dehydrogenase [Fusarium agapanthi];Gene=BFJ69_g11287;Ontology_term=oxidoreductase activity;Ontology_id=GO:0016491;Enzyme_code=EC:1,EC:1;Enzyme_name=Oxidoreductases,Oxidoreductases
contig00008.g5097	1998	ID=contig00008.g5097;Description=heterokaryon incompatibility 6 OR allele [Fusarium pseudocircinatum]
contig00008.g5098	1217	ID=contig00008.g5098;Description=hypothetical protein FVER14953_11823 [Fusarium verticillioides]
contig00008.g5099	780	ID=contig00008.g5099;Description=hypothetical protein H9Q70_003347 [Fusarium xylarioides]

contig00008.g5100	1177	ID=contig00008.g5100;Description=2-amino-3-carboxymuconate-6-semialdehyde decarboxylase [Fusarium tjaetaba];Gene=FVER53590_11824;Ontology_term=lyase activity,hydrolase activity;Ontology_id=GO:0016829,GO:0016787;Enzyme_code=EC:4.1.1,EC:3,EC:4.1.1,EC:4.1,EC:3,EC:4,EC:4.1.1.52;Enzyme_name=Carbon-carbon lyases,Hydrolases,Carbon-carbon lyases,Carbon-carbon lyases,Hydrolases,Lyases,6-methylsalicylate decarboxylase
contig00008.g5101	785	ID=contig00008.g5101;Description=endo-1 4-beta-xylanase B [Fusarium tjaetaba];Gene=FVER53590_11825;Ontology_term=carbohydrate metabolic process,catalytic activity, acting on a protein,cell wall organization or biogenesis,hydrolase activity;Ontology_id=GO:0005975,GO:0140096,GO:0071554,GO:0016787;Enzyme_code=EC:3.2,EC:3.4;Enzyme_name=Glycosylases,Acting on peptide bonds (peptidases)
contig00008.g5102	855	ID=contig00008.g5102;Description=hypothetical protein FANTH_13828 [Fusarium anthophilum]
contig00008.g5103	666	ID=contig00008.g5103;Description=hypothetical protein FVER53590_11826 [Fusarium verticillioides]
contig00008.g5104	624	ID=contig00008.g5104;Description=hypothetical protein FVER53263_11827 [Fusarium verticillioides];Gene=ERG20;Ontology_term=lipid metabolic process,endoplasmic reticulum,transferase activity,vacuole,isomerase activity;Ontology_id=GO:0006629,GO:0005783,GO:0016740,GO:0005773,GO:0016853;Enzyme_code=EC:5.3.3.2,EC:2.5.1.10,EC:5.3.3.2,EC:2.5.1,EC:2,EC:5.3,EC:5,EC:2.5.1.10,EC:2.5.1.1,EC:5.3.3;Enzyme_name=isopentenyl-diphosphate Delta-isomerase,(2E,6E)-farnesyl diphosphate synthase,isopentenyl-diphosphate Delta-isomerase,Transferring alkyl or aryl groups, other than methyl groups,Transferases,Intramolecular oxidoreductases,Isomerases,(2E,6E)-farnesyl diphosphate synthase,dimethylallyltranstransferase,Intramolecular oxidoreductases
contig00008.g5105	1077	ID=contig00008.g5105;Description=hypothetical protein FVER14953_11828 [Fusarium verticillioides]
contig00008.g5106	1614	ID=contig00008.g5106;Description=putative 3-polyprenyl-4-hydroxybenzoate decarboxylase and related decarboxylase [Fusarium fujikuroi];Gene=FDC1;Ontology_term=lyase activity;Ontology_id=GO:0016829;Enzyme_code=EC:4.1.1,EC:4.1.1,EC:4.1,EC:4.1.1.102,EC:4;Enzyme_name=Carbon-carbon lyases,Carbon-carbon lyases,Carbon-carbon lyases,phenacrylate decarboxylase,Lyases

contig00008.g5107	1924	ID=contig00008.g5107;Description=Protein RDR1 [Fusarium oxysporum f. sp. cubense race 1];Gene=FGLOB1_13393;Ontology_term=nucleus,DNA binding,regulation of DNA-templated transcription,transcription regulator activity;Ontology_id=GO:0005634,GO:0003677,GO:0006355,GO:0140110
contig00008.g5108	963	ID=contig00008.g5108;Description=hypothetical protein FVEG_11831 [Fusarium verticillioides 7600];Gene=FVER53263_11831;Ontology_term=membrane;Ontology_id=GO:0016020
contig00008.g5109	2914	ID=contig00008.g5109;Description=het-domain protein [Fusarium tjaetaba]
contig00008.g5110	750	ID=contig00008.g5110;Description=dehydrogenase reductase SDR family member 7 precursor [Fusarium subglutinans]
contig00008.g5111	1551	ID=contig00008.g5111;Description=fluG-like protein [Fusarium coicis];Gene=FVEG_11835;Ontology_term=ligase activity;Ontology_id=GO:0016874;Enzyme_code=EC:6.3.1.2,EC:6.3,EC:6.3.1,EC:6.3.1.2,EC:6;Enzyme_name=glutamine synthetase,Forming carbon-nitrogen bonds,Forming carbon-nitrogen bonds,glutamine synthetase,Ligases
contig00008.g5112	1804	ID=contig00008.g5112;Description=hypothetical protein FVER53590_11836 [Fusarium verticillioides];Gene=FVER53590_11836;Ontology_term=ligase activity;Ontology_id=GO:0016874;Enzyme_code=EC:6.3.1.2,EC:6.3,EC:6.3.1,EC:6.3.1.2,EC:6;Enzyme_name=glutamine synthetase,Forming carbon-nitrogen bonds,Forming carbon-nitrogen bonds,glutamine synthetase,Ligases
contig00008.g5113	683	ID=contig00008.g5113;Description=hypothetical protein FVEG_11837 [Fusarium verticillioides 7600];Gene=FRV6_15879;Ontology_term=membrane;Ontology_id=GO:0016020
contig00008.g5114	940	ID=contig00008.g5114;Description=metallo-beta-lactamase domain-containing protein [Fusarium coicis];Gene=FVER53590_11838;Ontology_term=sulfur compound metabolic process,oxidoreductase activity,cellular modified amino acid metabolic process;Ontology_id=GO:0006790,GO:0016491,GO:0006575;Enzyme_code=EC:1.13.11.18,EC:1.13.11,EC:1.13,EC:1,EC:1.13.11.18;Enzyme_name=persulfide dioxygenase,Acting on single donors with incorporation of molecular oxygen (oxygenases). The oxygen incorporated need not be derived from O2,Acting on single donors with incorporation of molecular oxygen (oxygenases). The oxygen incorporated need not be derived from O2,Oxidoreductases,persulfide dioxygenase

contig00008.g5115	969	ID=contig00008.g5115;Description=hypothetical protein FVER14953_11839 [Fusarium verticillioides];Gene=FMAN_06439;Ontology_term=plasma membrane;Ontology_id=GO:0005886
contig00008.g5116	2371	ID=contig00008.g5116;Description=sulfate permease II [Fusarium coicis];Gene=FVER53263_11840;Ontology_term=transmembrane transport,transporter activity;Ontology_id=GO:0055085,GO:0005215
contig00008.g5117	3190	ID=contig00008.g5117;Description=non-ribosomal peptide synthetase [Fusarium fujikuroi];Gene=FVER53263_11841;Ontology_term=oxidoreductase activity;Ontology_id=GO:0016491;Enzyme_code=EC:1,EC:1;Enzyme_name=Oxidoreductases,Oxidoreductases
contig00008.g5118	1103	ID=contig00008.g5118;Description=ClpP/crotonase-like domain-containing protein [Fusarium solani];Enzyme_code=EC:4.2.1.17;Enzyme_name=enoyl-CoA hydratase
contig00008.g5119	1186	ID=contig00008.g5119;Description=3-carboxymuconate cyclase [Fusarium tjaetaba]
contig00008.g5120	1971	ID=contig00008.g5120;Description=beta transducin [Fusarium tjaetaba]
contig00008.g5121	1732	ID=contig00008.g5121;Description=isotrichodermin C-15 hydroxylase (cytochrome P-450 monooxygenase CYP65A1) [Fusarium coicis];Gene=FPANT_1083;Ontology_term=oxidoreductase activity;Ontology_id=GO:0016491;Enzyme_code=EC:1.14,EC:1,EC:1.14;Enzyme_name=Acting on paired donors, with incorporation or reduction of molecular oxygen. The oxygen incorporated need not be derived from O2,Oxidoreductases,Acting on paired donors, with incorporation or reduction of molecular oxygen. The oxygen incorporated need not be derived from O2
contig00008.g5122	1178	ID=contig00008.g5122;Description=hypothetical protein FVER53263_20049 [Fusarium verticillioides];Gene=FTJAE_3948;Ontology_term=membrane;Ontology_id=GO:0016020
contig00008.g5123	1851	ID=contig00008.g5123;Description=sterol uptake control 2 [Fusarium tjaetaba];Gene=FVEG_11845;Ontology_term=nucleus,regulation of DNA-templated transcription,transcription regulator activity;Ontology_id=GO:0005634,GO:0006355,GO:0140110
contig00008.g5124	1077	ID=contig00008.g5124;Description=retinal short-chain dehydrogenase reductase [Fusarium tjaetaba];Gene=FVEG_11846;Ontology_term=oxidoreductase activity;Ontology_id=GO:0016491;Enzyme_code=EC:1,EC:1;Enzyme_name=Oxidoreductases,Oxidoreductases

contig00008.g5125	1149	ID=contig00008.g5125;Description=lipase esterase [Fusarium tjaetaba];Gene=FVER53590_11847;Ontology_term=hydrolase activity;Ontology_id=GO:0016787;Enzyme_code=EC:3,EC:3;Enzyme_name=Hydrolases,Hydrolases
contig00008.g5126	1467	ID=contig00008.g5126;Description=related to monooxygenase [Fusarium fujikuroi];Gene=C2S_5429;Ontology_term=RNA binding,oxidoreductase activity,structural molecule activity,tRNA metabolic process,amino acid metabolic process,catalytic activity, acting on RNA,ligase activity,hydrolase activity;Ontology_id=GO:0003723,GO:0016491,GO:0005198,GO:0006399,GO:0006520,GO:0140098,GO:0016874,GO:0016787;Enzyme_code=EC:1.14.13.8,EC:6.1.1.5,EC:3.1.1,EC:3.1,EC:6.1,EC:1.14.13.8,EC:1,EC:1.14,EC:6.1.1.5,EC:1.14.13,EC:3,EC:6.1.1,EC:6,EC:3.1.1;Enzyme_name=flavin-containing monooxygenase,isoleucine--tRNA ligase,Acting on ester bonds,Acting on ester bonds,Forming carbon-oxygen bonds,flavin-containing monooxygenase,Oxidoreductases,Acting on paired donors, with incorporation or reduction of molecular oxygen. The oxygen incorporated need not be derived from O2,isoleucine--tRNA ligase,Acting on paired donors, with incorporation or reduction of molecular oxygen. The oxygen incorporated need not be derived from O2,Hydrolases,Forming carbon-oxygen bonds,Ligases,Acting on ester bonds
contig00008.g5127	1317	ID=contig00008.g5127;Description=endoglucanase type F [Fusarium proliferatum];Gene=C2S_5428;Ontology_term=carbohydrate metabolic process,cell wall organization or biogenesis,extracellular region,hydrolase activity;Ontology_id=GO:0005975,GO:0071554,GO:0005576,GO:0016787;Enzyme_code=EC:3.2.1.8,EC:3.2,EC:3.2.1.8,EC:3,EC:3.2.1;Enzyme_name=endo-1,4-beta-xylanase,Glycosylases,endo-1,4-beta-xylanase,Hydrolases,Glycosylases
contig00008.g5128	960	ID=contig00008.g5128;Description=isoprenylcysteine alpha-carbonyl methylesterase ICMEL2 [Fusarium pseudoanthophilum]
contig00008.g5129	1605	ID=contig00008.g5129;Description=cupin like protein [Fusarium pseudocircinatum];Gene=FPANT_1075;Ontology_term=oxidoreductase activity;Ontology_id=GO:0016491;Enzyme_code=EC:1,EC:1;Enzyme_name=Oxidoreductases,Oxidoreductases
contig00008.g5130	1215	ID=contig00008.g5130;Description=CRAL-TRIO domain-containing protein [Fusarium sp. MPI-SDFR-AT-0072]
contig00008.g5131	4046	ID=contig00008.g5131;Description=hypothetical protein BFJ72_g573 [Fusarium proliferatum]

contig00008.g5132	1401	ID=contig00008.g5132;Description=hypothetical protein FVEG_11852 [Fusarium verticillioides 7600];Gene=FVER53263_11852;Ontology_term=translation regulator activity,mitochondrial gene expression,RNA binding,GTPase activity,mitochondrion,vacuole;Ontology_id=GO:0045182,GO:0140053,GO:0003723,GO:0003924,GO:0005739,GO:0005773;Enzyme_code=EC:3.6.1.15,EC:3.6.1,EC:3.6,EC:3,EC:3.6.1.15;Enzyme_name=nucleoside-triphosphate phosphatase,Acting on acid anhydrides,Acting on acid anhydrides,Hydrolases,nucleoside-triphosphate phosphatase
contig00008.g5133	622	ID=contig00008.g5133;Description=start-like domain protein [Fusarium tjaetaba]
contig00008.g5134	657	ID=contig00008.g5134;Description=cytochrome P450 monooxygenase [Fusarium coicis]
contig00008.g5135	1803	ID=contig00008.g5135;Description=laccase precursor [Fusarium subglutinans];Gene=FANTH_2671;Ontology_term=oxidoreductase activity;Ontology_id=GO:0016491;Enzyme_code=EC:1,EC:1;Enzyme_name=Oxidoreductases,Oxidoreductases
contig00008.g5136	558	ID=contig00008.g5136;Description=acetyltransferase [Fusarium tjaetaba];Gene=FVEG_17121;Ontology_term=transferase activity;Ontology_id=GO:0016740;Enzyme_code=EC:2.3.1,EC:2.3,EC:2,EC:2.3.1,EC:2.3.1.57;Enzyme_name=Acyltransferases,Acyltransferases,Transferases,Acyltransferases,diamine N-acetyltransferase
contig00008.g5137	1094	ID=contig00008.g5137;Description=tetracycline resistance (probable transport) [Fusarium coicis];Gene=FMAN_06418;Ontology_term=transmembrane transport,transporter activity;Ontology_id=GO:0055085,GO:0005215
contig00008.g5138	1560	ID=contig00008.g5138;Description=benzoate 4-monooxygenase cytochrome P450 [Fusarium tjaetaba];Gene=FNAPI_5731;Ontology_term=oxidoreductase activity;Ontology_id=GO:0016491;Enzyme_code=EC:1.14,EC:1,EC:1.14;Enzyme_name=Acting on paired donors, with incorporation or reduction of molecular oxygen. The oxygen incorporated need not be derived from O2,Oxidoreductases,Acting on paired donors, with incorporation or reduction of molecular oxygen. The oxygen incorporated need not be derived from O2

contig00008.g5139	2920	ID=contig00008.g5139;Description=cytochrome P450 oxidoreductase [Fusarium verticillioides 7600];Gene=FVER53590_11857;Ontology_term=oxidoreductase activity;Ontology_id=GO:0016491;Enzyme_code=EC:1.14,EC:1,EC:1.14,EC:1.14.13.12;Enzyme_name=Acting on paired donors, with incorporation or reduction of molecular oxygen. The oxygen incorporated need not be derived from O2,Oxidoreductases,Acting on paired donors, with incorporation or reduction of molecular oxygen. The oxygen incorporated need not be derived from O2,Acting on paired donors, with incorporation or reduction of molecular oxygen. The oxygen incorporated need not be derived from O2
contig00008.g5140	1062	ID=contig00008.g5140;Description=plant dihydroflavonol-4-reductase [Fusarium tjaetaba]
contig00008.g5141	1267	ID=contig00008.g5141;Description=monooxygenase [Fusarium denticulatum];Gene=FPCIR_4722;Ontology_term=oxidoreductase activity;Ontology_id=GO:0016491;Enzyme_code=EC:1;Enzyme_name=Oxidoreductases
contig00008.g5142	1047	ID=contig00008.g5142;Description=transcriptional activator UGA3 [Fusarium coicis];Gene=FOYG_14591;Ontology_term=hydrolase activity;Ontology_id=GO:0016787;Enzyme_code=EC:3,EC:3;Enzyme_name=Hydrolases,Hydrolases
contig00008.g5143	1860	ID=contig00008.g5143;Description=hypothetical protein FVEG_11861 [Fusarium verticillioides 7600];Gene=FFUJ_14391;Ontology_term=nucleus,cytoskeleton,cytoskeleton organization,GTPase activity,structural molecule activity,regulation of DNA-templated transcription,transcription regulator activity;Ontology_id=GO:0005634,GO:0005856,GO:0007010,GO:0003924,GO:0005198,GO:0006355,GO:0140110;Enzyme_code=EC:3.6.1.15,EC:3.6.1,EC:3.6,EC:3,EC:3.6.1.15;Enzyme_name=nucleoside-triphosphate phosphatase,Acting on acid anhydrides,Acting on acid anhydrides,Hydrolases,nucleoside-triphosphate phosphatase
contig00008.g5144	1242	ID=contig00008.g5144;Description=salicylate hydroxylase [Fusarium verticillioides 7600];Gene=FACUT_4826;Ontology_term=oxidoreductase activity;Ontology_id=GO:0016491;Enzyme_code=EC:1,EC:1,EC:1.14.13.1;Enzyme_name=Oxidoreductases,Oxidoreductases,salicylate 1-monooxygenase
contig00008.g5145	1458	ID=contig00008.g5145;Description=allantoate transport [Fusarium tjaetaba];Gene=FVER53263_11863;Ontology_term=transmembrane transport,transporter activity;Ontology_id=GO:0055085,GO:0005215

contig00008.g5146	1160	ID=contig00008.g5146;Description=aliphatic nitrilase [Fusarium subglutinans];Gene=FCIRC_648;Ontology_term=hydrolase activity;Ontology_id=GO:0016787;Enzyme_code=EC:3.5.5.1,EC:3.5.5,EC:3.5,EC:3,EC:3.5.5.1;Enzyme_name=nitrilase,Acting on carbon-nitrogen bonds, other than peptide bonds,Acting on carbon-nitrogen bonds, other than peptide bonds,Hydrolases,nitrilase
contig00008.g5147	2061	ID=contig00008.g5147;Description=pathway-specific regulatory nit-4 [Fusarium tjaetaba];Gene=FNAPI_5722;Ontology_term=nucleus,DNA binding,DNA-templated transcription,hydrolase activity;Ontology_id=GO:0005634,GO:0003677,GO:0006351,GO:0016787;Enzyme_code=EC:3.5.5.1,EC:3.5.5,EC:3.5,EC:3,EC:3.5.5.1;Enzyme_name=nitrilase,Acting on carbon-nitrogen bonds, other than peptide bonds,Acting on carbon-nitrogen bonds, other than peptide bonds,Hydrolases,nitrilase
contig00008.g5148	1690	ID=contig00008.g5148;Description=thiamine biosynthesis regulatory [Fusarium tjaetaba];Gene=FVEG_11866;Ontology_term=nucleus,regulation of DNA-templated transcription,transcription regulator activity;Ontology_id=GO:0005634,GO:0006355,GO:0140110
contig00008.g5149	1215	ID=contig00008.g5149;Description=endoglucanase EG-II [Fusarium napiforme];Gene=FVEG_11867;Ontology_term=carbohydrate metabolic process,hydrolase activity;Ontology_id=GO:0005975,GO:0016787;Enzyme_code=EC:3.2.1,EC:3.2,EC:3,EC:3.2.1,EC:3.2.1.4;Enzyme_name=Glycosylases,Glycosylases,Hydrolases,Glycosylases,cellulase
contig00008.g5150	1114	ID=contig00008.g5150;Description=methyltransferase [Fusarium subglutinans];Gene=FVEG_11868;Ontology_term=transferase activity;Ontology_id=GO:0016740;Enzyme_code=EC:2.1.1,EC:2.1,EC:2,EC:2.1.1;Enzyme_name=Transferring one-carbon groups,Transferring one-carbon groups,Transferases,Transferring one-carbon groups
contig00008.g5151	978	ID=contig00008.g5151;Description=GDSL-like lipase acylhydrolase [Fusarium tjaetaba];Gene=FVER53590_11869;Ontology_term=hydrolase activity;Ontology_id=GO:0016787;Enzyme_code=EC:3.1,EC:3.1,EC:3;Enzyme_name=Acting on ester bonds,Acting on ester bonds,Hydrolases

contig00008.g5152	1050	ID=contig00008.g5152;Description=putative heme-binding peroxidase [Fusarium fujikuroi];Gene=HOM2;Ontology_term=sulfur compound metabolic process,antioxidant activity,oxidoreductase activity,amino acid metabolic process;Ontology_id=GO:0006790,GO:0016209,GO:0016491,GO:0006520;Enzyme_code=EC:1.2.1.11,EC:1.11.1,EC:1.2,EC:1.11,EC:1.2.1.11,EC:1,EC:1.11.1.5,EC:1.11.1,EC:1.2.1;Enzyme_name=aspartate-semialdehyde dehydrogenase,Acting on a peroxide as acceptor,Acting on the aldehyde or oxo group of donors,Acting on a peroxide as acceptor,aspartate-semialdehyde dehydrogenase,Oxidoreductases,cytochrome-c peroxidase,Acting on a peroxide as acceptor,Acting on the aldehyde or oxo group of donors
contig00008.g5153	1958	ID=contig00008.g5153;Description=hypothetical protein FVEG_11871 [Fusarium verticillioides 7600];Gene=FMUND_7229;Ontology_term=transmembrane transport,transporter activity;Ontology_id=GO:0055085,GO:0005215
contig00008.g5154	2133	ID=contig00008.g5154;Description=tol [Fusarium tjaetaba]
contig00008.g5155	1310	ID=contig00008.g5155;Description=acid phosphatase [Fusarium tjaetaba];Gene=FVER53263_11873;Ontology_term=oxidoreductase activity;Ontology_id=GO:0016491;Enzyme_code=EC:1,EC:1;Enzyme_name=Oxidoreductases,Oxidoreductases
contig00008.g5156	1407	ID=contig00008.g5156;Description=acid phosphatase [Fusarium tjaetaba];Gene=FPCIR_4735;Ontology_term=oxidoreductase activity,hydrolase activity;Ontology_id=GO:0016491,GO:0016787;Enzyme_code=EC:1,EC:3.1.3,EC:3.1,EC:1,EC:3,EC:3.1.3;Enzyme_name=Oxidoreductases,Acting on ester bonds,Acting on ester bonds,Oxidoreductases,Hydrolases,Acting on ester bonds
contig00008.g5157	1155	ID=contig00008.g5157;Description=hypothetical protein CEK27_007762 [Fusarium fujikuroi];Gene=FOQG_13351;Ontology_term=carbohydrate metabolic process,transmembrane transport,mitochondrion,carbohydrate derivative metabolic process;Ontology_id=GO:0005975,GO:0055085,GO:0005739,GO:1901135
contig00008.g5158	1491	ID=contig00008.g5158;Description=DUF985 domain-containing protein [Fusarium bulbicola];Gene=FDENT_3905;Ontology_term=transmembrane transport,transporter activity;Ontology_id=GO:0055085,GO:0005215
contig00008.g5159	1518	ID=contig00008.g5159;Description=hypothetical protein FVEG_11877 [Fusarium verticillioides 7600];Gene=FPHYL_2217;Ontology_term=membrane;Ontology_id=GO:0016020

contig00008.g5160	2322	ID=contig00008.g5160;Description=arsenite efflux transporter like [Fusarium subglutinans];Gene=FVEG_11878;Ontology_term=plasma membrane,transporter activity;Ontology_id=GO:0005886,GO:0005215
contig00008.g5161	4100	ID=contig00008.g5161;Description=serine/threonine protein kinase [Fusarium verticillioides 7600];Gene=FPANT_7261;Ontology_term=catalytic activity, acting on a protein,signaling,transferase activity;Ontology_id=GO:0140096,GO:0023052,GO:0016740;Enzyme_code=EC:2.7.11.1,EC:2.7.1,EC:2.7.11.1,EC:2.7,EC:2;Enzyme_name=non-specific serine/threonine protein kinase,Transferring phosphorus-containing groups,non-specific serine/threonine protein kinase,Transferring phosphorus-containing groups,Transferases
contig00008.g5162	1590	ID=contig00008.g5162;Description=hypothetical protein FVER14953_11881 [Fusarium verticillioides];Gene=FNYG_03761;Ontology_term=catalytic activity, acting on a protein,transferase activity;Ontology_id=GO:0140096,GO:0016740;Enzyme_code=EC:2.7.1,EC:2.7.1,EC:2.7,EC:2;Enzyme_name=Transferring phosphorus-containing groups,Transferring phosphorus-containing groups,Transferring phosphorus-containing groups,Transferases
contig00008.g5163	1227	ID=contig00008.g5163;Description=probable aspartic proteinase precursor [Fusarium fujikuroi];Gene=FNYG_03760;Ontology_term=catalytic activity, acting on a protein,hydrolase activity;Ontology_id=GO:0140096,GO:0016787;Enzyme_code=EC:3.4.23,EC:3.4.23,EC:3.4,EC:3;Enzyme_name=Acting on peptide bonds (peptidases),Acting on peptide bonds (peptidases),Acting on peptide bonds (peptidases),Hydrolases
contig00008.g5164	1377	ID=contig00008.g5164;Description=hypothetical protein FVEG_11883 [Fusarium verticillioides 7600];Gene=FMUND_7239;Ontology_term=nucleus,regulation of DNA-templated transcription,transcription regulator activity;Ontology_id=GO:0005634,GO:0006355,GO:0140110
contig00008.g5165	789	ID=contig00008.g5165;Description=hypothetical protein FVEG_11885 [Fusarium verticillioides 7600]
contig00008.g5166	1703	ID=contig00008.g5166;Description=quinate permease [Fusarium tjaetaba];Gene=FVER53263_11886;Ontology_term=transmembrane transport,transporter activity;Ontology_id=GO:0055085,GO:0005215

contig00008.g5167	1754	ID=contig00008.g5167;Description=Adhesion and hyphal regulator 1 [Fusarium oxysporum f. sp. rapae];Gene=FOXYS1_221;Ontology_term=nucleus,regulation of DNA-templated transcription,transcription regulator activity;Ontology_id=GO:0005634,GO:0006355,GO:0140110
contig00008.g5168	984	ID=contig00008.g5168;Description=transcriptional regulatory [Fusarium napiforme];Gene=FOYG_00846;Ontology_term=nucleus,regulation of DNA-templated transcription,transcription regulator activity;Ontology_id=GO:0005634,GO:0006355,GO:0140110
contig00008.g5169	2758	ID=contig00008.g5169;Description=Alpha/Beta hydrolase protein [Fusarium oxysporum];Gene=FMEXI_8355;Ontology_term=hydrolase activity;Ontology_id=GO:0016787;Enzyme_code=EC:3.5.5.1,EC:3.5.5,EC:3.5,EC:3,EC:3.5.5.1;Enzyme_name=nitrilase,Acting on carbon-nitrogen bonds, other than peptide bonds,Acting on carbon-nitrogen bonds, other than peptide bonds,Hydrolases,nitrilase
contig00008.g5170	1602	ID=contig00008.g5170;Description=feruloyl esterase B precursor [Fusarium mundagurra];Gene=FVEG_11889;Ontology_term=hydrolase activity;Ontology_id=GO:0016787;Enzyme_code=EC:3.1.1.73,EC:3.1,EC:3,EC:3.1.1.73,EC:3.1.1;Enzyme_name=feruloyl esterase,Acting on ester bonds,Hydrolases,feruloyl esterase,Acting on ester bonds
contig00008.g5171	1035	ID=contig00008.g5171;Description=peroxisomal short-chain alcohol dehydrogenase [Fusarium subglutinans]
contig00008.g5172	1308	ID=contig00008.g5172;Description=hydrolase or acyltransferase (alpha beta hydrolase superfamily) [Fusarium tjaetaba];Gene=FDENT_497;Ontology_term=transferase activity,hydrolase activity;Ontology_id=GO:0016740,GO:0016787;Enzyme_code=EC:2.3,EC:3,EC:2.3,EC:2,EC:3;Enzyme_name=Acyltransferases,Hydrolases,Acyltransferases,Transferases,Hydrolases
contig00008.g5173	2013	ID=contig00008.g5173;Description=transcriptional regulatory moc3 [Fusarium napiforme];Gene=FOIG_16356;Ontology_term=nucleus,regulation of DNA-templated transcription,transcription regulator activity;Ontology_id=GO:0005634,GO:0006355,GO:0140110
contig00008.g5174	1158	ID=contig00008.g5174;Description=zinc-regulated transporter 1 [Fusarium tjaetaba];Gene=ZRT1;Ontology_term=transmembrane transport,endoplasmic reticulum,transporter activity,plasma membrane;Ontology_id=GO:0055085,GO:0005783,GO:0005215,GO:0005886

contig00008.g5175	1148	ID=contig00008.g5175;Description=alcohol dehydrogenase [Fusarium verticillioides 7600];Gene=FTJAE_4000;Ontology_term=oxidoreductase activity;Ontology_id=GO:0016491;Enzyme_code=EC:1,EC:1;Enzyme_name=Oxidoreductases,Oxidoreductases
contig00008.g5176	1362	ID=contig00008.g5176;Description=het domain-containing protein [Fusarium coicis]
contig00008.g5177	646	ID=contig00008.g5177;Description=hypothetical protein FVEG_11896 [Fusarium verticillioides 7600]
contig00008.g5178	1521	ID=contig00008.g5178;Description=alanine-glyoxylate aminotransferase AGT2 [Fusarium tjaetaba];Gene=FVER53263_11897;Ontology_term=transferase activity;Ontology_id=GO:0016740;Enzyme_code=EC:2.6.1,EC:2.6.1,EC:2,EC:2.6;Enzyme_name=Transferring nitrogenous groups,Transferring nitrogenous groups,Transferases,Transferring nitrogenous groups
contig00008.g5179	1641	ID=contig00008.g5179;Description=tetracycline resistance [Fusarium tjaetaba];Gene=FDENT_504;Ontology_term=DNA repair,transmembrane transport,catalytic activity, acting on DNA,DNA recombination,chromosome,chromatin organization,transporter activity,nuclear envelope,DNA binding,RNA binding,ATP-dependent activity,telomere organization,hydrolase activity;Ontology_id=GO:0006281,GO:0055085,GO:0140097,GO:0006310,GO:0005694,GO:0006325,GO:0005215,GO:0005635,GO:0003677,GO:0003723,GO:0140657,GO:0032200,GO:0016787;Enzyme_code=EC:3.6.1.15,EC:3.6.1,EC:3.6,EC:3,EC:3.6.1.15;Enzyme_name=nucleoside-triphosphate phosphatase,Acting on acid anhydrides,Acting on acid anhydrides,Hydrolases,nucleoside-triphosphate phosphatase
contig00008.g5180	860	ID=contig00008.g5180;Description=HAD superfamily hydrolase [Fusarium tjaetaba];Gene=FVEG_11900;Ontology_term=hydrolase activity;Ontology_id=GO:0016787;Enzyme_code=EC:3.1.3,EC:3.1,EC:3,EC:3.1.3;Enzyme_name=Acting on ester bonds,Acting on ester bonds,Hydrolases,Acting on ester bonds
contig00008.g5181	2192	ID=contig00008.g5181;Description=glucose-methanol-choline oxidoreductase:gmc oxidoreductase [Fusarium tjaetaba];Gene=FFB14_12576;Ontology_term=oxidoreductase activity;Ontology_id=GO:0016491;Enzyme_code=EC:1.1,EC:1.1,EC:1;Enzyme_name=Acting on the CH-OH group of donors,Acting on the CH-OH group of donors,Oxidoreductases
contig00008.g5182	2515	ID=contig00008.g5182;Description=hypothetical protein FVER14953_11902 [Fusarium verticillioides];Gene=FVEG_11902;Ontology_term=carbohydrate metabolic process;Ontology_id=GO:0005975
contig00008.g5183	918	ID=contig00008.g5183;Description=hypothetical protein J7337_007393 [Fusarium musae]

contig00008.g5184	988	ID=contig00008.g5184;Description=hypothetical protein FVEG_11904 [Fusarium verticillioides 7600]
contig00008.g5185	1614	ID=contig00008.g5185;Description=hypothetical protein FVER53590_11906 [Fusarium verticillioides];Gene=FTJAE_4012;Ontology_term=membrane;Ontology_id=GO:0016020
contig00008.g5186	999	ID=contig00008.g5186;Description=dTDP-glucose 4 6-dehydratase [Fusarium tjaetaba]
contig00008.g5187	1751	ID=contig00008.g5187;Description=probable isoamyl alcohol oxidase [Fusarium fujikuroi];Gene=FFUJ_14724;Ontology_term=oxidoreductase activity;Ontology_id=GO:0016491;Enzyme_code=EC:1;Enzyme_name=Oxidoreductases
contig00008.g5188	1465	ID=contig00008.g5188;Description=integral membrane protein PTH11 [Fusarium fujikuroi];Gene=LW93_9756;Ontology_term=membrane;Ontology_id=GO:0016020
contig00008.g5189	288	ID=contig00008.g5189;Description=kinetochore complex Sim4 subunit Fta4 [Fusarium globosum];Gene=HTA2;Ontology_term=nucleus,DNA binding,structural molecule activity,chromosome;Ontology_id=GO:0005634,GO:0003677,GO:0005198,GO:0005694
contig00008.g5190	1528	ID=contig00008.g5190;Description=monocarboxylate transporter 2 [Fusarium pseudoanthophilum];Gene=FPCIR_12537;Ontology_term=transmembrane transport,transporter activity;Ontology_id=GO:0055085,GO:0005215
contig00008.g5191	722	ID=contig00008.g5191;Description=hypothetical protein FVER14953_11911 [Fusarium verticillioides]
contig00008.g5192	2273	ID=contig00008.g5192;Description=endothelin-converting enzyme 1 [Fusarium coicis];Gene=FNAPI_6446;Ontology_term=catalytic activity, acting on a protein,hydrolase activity;Ontology_id=GO:0140096,GO:0016787;Enzyme_code=EC:3.4.24;Enzyme_name=Acting on peptide bonds (peptidases)
contig00008.g5193	1488	ID=contig00008.g5193;Description=uncharacterized protein FTJAE_4020 [Fusarium tjaetaba]
contig00008.g5194	2523	ID=contig00008.g5194;Description=ATP-dependent RNA helicase DEAH11 [Fusarium oxysporum f. sp. cubense];Gene=BFJ69_g14641;Ontology_term=ATP-dependent activity,transferase activity;Ontology_id=GO:0140657,GO:0016740;Enzyme_code=EC:2,EC:2;Enzyme_name=Transferases,Transferases
contig00008.g5195	399	ID=contig00008.g5195;Description=2-oxoglutarate-dependent ethylene succinate-forming enzyme [Fusarium mundagurra];Gene=FMUND_11701;Ontology_term=oxidoreductase activity;Ontology_id=GO:0016491;Enzyme_code=EC:1,EC:1;Enzyme_name=Oxidoreductases,Oxidoreductases
contig00008.g5196	1038	ID=contig00008.g5196;Description=integral membrane protein PTH11 [Fusarium denticulatum]

contig00008.g5197	1160	ID=contig00008.g5197;Description=hypothetical protein FPHYL_12374 [Fusarium phyllophilum];Gene=FNYG_04078;Ontology_term=regulation of DNA-templated transcription,transcription regulator activity;Ontology_id=GO:0006355,GO:0140110
contig00008.g5198	1265	ID=contig00008.g5198;Description=hypothetical protein FVER53263_11917 [Fusarium verticillioides]
contig00008.g5199	1416	ID=contig00008.g5199;Description=hypothetical protein FPANT_10410 [Fusarium pseudoanthophilum]
contig00008.g5200	1783	ID=contig00008.g5200;Description=transporter [Fusarium tjaetaba];Gene=FNYG_04075;Ontology_term=transmembrane transport,transporter activity;Ontology_id=GO:0055085,GO:0005215
contig00008.g5201	1297	ID=contig00008.g5201;Description=transaldolase [Fusarium verticillioides 7600];Gene=FNYG_04074;Ontology_term=carbohydrate metabolic process;Ontology_id=GO:0005975
contig00008.g5202	982	ID=contig00008.g5202;Description=isoflavone reductase like P3 [Fusarium tjaetaba];Gene=FVER53590_25077;Ontology_term=protein dimerization activity;Ontology_id=GO:0046983
contig00008.g5203	1284	ID=contig00008.g5203;Description=hypothetical protein FVER53263_11921 [Fusarium verticillioides]
contig00008.g5204	1582	ID=contig00008.g5204;Description=related to TAM domain methyltransferase [Fusarium fujikuroi];Gene=FVEG_11922;Ontology_term=nucleus,catalytic activity, acting on a protein,tRNA metabolic process,amino acid metabolic process,transferase activity,chromatin organization,catalytic activity, acting on RNA,ligase activity;Ontology_id=GO:0005634,GO:0140096,GO:0006399,GO:0006520,GO:0016740,GO:0006325,GO:0140098,GO:0016874;Enzyme_code=EC:6.1.1.3,EC:2.3.1.48,EC:2.1.1,EC:2.1,EC:2.3,EC:6.1,EC:6.1.1.3,EC:2,EC:2.3.1.48,EC:6.1.1,EC:6,EC:2.1.1,EC:2.3.1;Enzyme_name=threonine--tRNA ligase,histone acetyltransferase,Transferring one-carbon groups,Transferring one-carbon groups,Acyltransferases,Forming carbon-oxygen bonds,threonine--tRNA ligase,Transferases,histone acetyltransferase,Forming carbon-oxygen bonds,Ligases,Transferring one-carbon groups,Acyltransferases
contig00008.g5205	840	ID=contig00008.g5205;Description=related to Zn-dependent hydrolases of the beta-lactamase fold, putative [Fusarium proliferatum ET1];Gene=FTJAE_4031;Ontology_term=hydrolase activity;Ontology_id=GO:0016787;Enzyme_code=EC:3,EC:3;Enzyme_name=Hydrolases,Hydrolases

contig00008.g5206	1620	ID=contig00008.g5206;Description=multidrug transporter [Fusarium coicis]
contig00008.g5207	903	ID=contig00008.g5207;Description=asparagine oxygenase [Fusarium tjaetaba];Gene=FNYG_04067;Ontology_term=oxidoreductase activity;Ontology_id=GO:0016491;Enzyme_code=EC:1,EC:1;Enzyme_name=Oxidoreductases,Oxidoreductases
contig00008.g5208	1482	ID=contig00008.g5208;Description=translation machinery-associated 64 [Fusarium coicis];Gene=FVER53590_11926;Ontology_term=oxidoreductase activity;Ontology_id=GO:0016491;Enzyme_code=EC:1,EC:1,EC:2.5.1.48;Enzyme_name=Oxidoreductases,Oxidoreductases,cystathionine gamma-synthase
contig00008.g5209	1095	ID=contig00008.g5209;Description=hypothetical protein FVER53263_11927 [Fusarium verticillioides];Gene=FNAPI_6462;Ontology_term=sulfur compound metabolic process,amino acid metabolic process;Ontology_id=GO:0006790,GO:0006520
contig00008.g5210	1581	ID=contig00008.g5210;Description=cytochrome P450 monooxygenase monooxygenase [Fusarium subglutinans];Gene=FPCIR_8708;Ontology_term=oxidoreductase activity;Ontology_id=GO:0016491;Enzyme_code=EC:1.14.14,EC:1,EC:1.14.14,EC:1.14;Enzyme_name=Acting on paired donors, with incorporation or reduction of molecular oxygen. The oxygen incorporated need not be derived from O2,Oxidoreductases,Acting on paired donors, with incorporation or reduction of molecular oxygen. The oxygen incorporated need not be derived from O2,Acting on paired donors, with incorporation or reduction of molecular oxygen. The oxygen incorporated need not be derived from O2
contig00008.g5211	1841	ID=contig00008.g5211;Description=major facilitator superfamily transporter [Fusarium denticulatum];Gene=FVER53590_25076;Ontology_term=transmembrane transport,transporter activity;Ontology_id=GO:0055085,GO:0005215
contig00008.g5212	1074	ID=contig00008.g5212;Description=ToxD-like protein [Fusarium globosum];Gene=F52700_8887;Ontology_term=oxidoreductase activity;Ontology_id=GO:0016491;Enzyme_code=EC:1.6,EC:1.6,EC:1;Enzyme_name=Acting on NADH or NADPH,Acting on NADH or NADPH,Oxidoreductases

		ID=contig00008.g5213;Description=putative Hybrid PKS-NRPS biosynthetic cluster [Fusarium musae];Gene=FACUT_7704;Ontology_term=oxidoreductase activity,lipid metabolic process,transferase activity;Ontology_id=GO:0016491,GO:0006629,GO:0016740;Enzyme_code=EC:2.3.1.41,EC:1,EC:2.1.1,EC:2.3.1.41,EC:2.1,EC:2.3,EC:1,EC:2,EC:2.1.1,EC:2.3.1;Enzyme_name=beta-ketoacyl-[acyl-carrier-protein] synthase I,Oxidoreductases,Transferring one-carbon groups,beta-ketoacyl-[acyl-carrier-protein] synthase I,Transferring one-carbon groups,Acyltransferases,Oxidoreductases,Transferases,Transferring one-carbon groups,Acyltransferases
contig00008.g5213	12714	
contig00008.g5214	1689	ID=contig00008.g5214;Description=beta-fructofuranosidase [Fusarium verticillioides 7600];Gene=FVEG_11933;Ontology_term=carbohydrate metabolic process,mitochondrion,extracellular region,vacuole,hydrolase activity;Ontology_id=GO:0005975,GO:0005739,GO:0005576,GO:0005773,GO:0016787;Enzyme_code=EC:3.2.1,EC:3.2,EC:3.2.1.20,EC:3,EC:3.2.1.7,EC:3.2.1,EC:3.2.1.26,EC:3.2.1.48;Enzyme_name=Glycosylases,Glycosylases,alpha-glucosidase,Hydrolases,inulinase,Glycosylases,beta-fructofuranosidase,sucrose alpha-glucosidase
contig00008.g5215	1719	ID=contig00008.g5215;Description=multidrug resistance fnx1 [Fusarium coicis];Gene=FVER53263_20601;Ontology_term=transmembrane transport,transporter activity;Ontology_id=GO:0055085,GO:0005215
contig00008.g5216	1313	ID=contig00008.g5216;Description=thioredoxin reductase [Fusarium subglutinans];Gene=FOIG_14550;Ontology_term=oxidoreductase activity;Ontology_id=GO:0016491;Enzyme_code=EC:1,EC:1;Enzyme_name=Oxidoreductases,Oxidoreductases
		ID=contig00008.g5217;Description=putative secondary metabolism biosynthetic enzyme [Fusarium musae];Gene=LW93_9788;Ontology_term=sulfur compound metabolic process,lyase activity,transferase activity;Ontology_id=GO:0006790,GO:0016829,GO:0016740;Enzyme_code=EC:2.6.1,EC:2.6.1,EC:2,EC:4.4,EC:2.6,EC:4;Enzyme_name=Transferring nitrogenous groups,Transferring nitrogenous groups,Transferases,Carbon-sulfur lyases,Transferring nitrogenous groups,Lyases
contig00008.g5217	1410	
contig00008.g5218	2760	ID=contig00008.g5218;Description=putative secondary metabolism biosynthetic enzyme [Fusarium musae]

contig00008.g5219	696	ID=contig00008.g5219;Description=aspartate racemase [Fusarium verticillioides 7600];Gene=FDENT_547;Ontology_term=nucleus,DNA binding,telomere organization,chromosome,isomerase activity;Ontology_id=GO:0005634,GO:0003677,GO:0032200,GO:0005694,GO:0016853;Enzyme_code=EC:5.1.1.10,EC:5.1.1,EC:5.1,EC:5.1.1.10,EC:5;Enzyme_name=amino-acid racemase,Racemases and epimerases,Racemases and epimerases,amino-acid racemase,Isomerases
contig00008.g5220	1567	ID=contig00008.g5220;Description=aspartate racemase [Fusarium coicis];Gene=FGLOB1_4628;Ontology_term=isomerase activity;Ontology_id=GO:0016853;Enzyme_code=EC:5.1.1.10,EC:5.1.1,EC:5.1,EC:5.1.1.10,EC:5;Enzyme_name=amino-acid racemase,Racemases and epimerases,Racemases and epimerases,amino-acid racemase,Isomerases
contig00008.g5221	1530	ID=contig00008.g5221;Description=hypothetical protein FVER53590_11939 [Fusarium verticillioides]
contig00008.g5222	1858	ID=contig00008.g5222;Description=sterigmatocystin biosynthesis monooxygenase stcW [Fusarium pseudoanthophilum];Gene=FPANT_9682;Ontology_term=oxidoreductase activity;Ontology_id=GO:0016491;Enzyme_code=EC:1.14.13.8;Enzyme_name=flavin-containing monooxygenase
contig00008.g5223	1318	ID=contig00008.g5223;Description=integral membrane protein PTH11 [Fusarium tjaetaba];Gene=FTJAE_11862;Ontology_term=membrane;Ontology_id=GO:0016020
contig00008.g5224	1085	ID=contig00008.g5224;Description=aliphatic nitrilase [Fusarium tjaetaba];Gene=BFJ69_g12233;Ontology_term=hydrolase activity;Ontology_id=GO:0016787;Enzyme_code=EC:3.5.5.1,EC:3.5.5,EC:3.5,EC:3,EC:3.5.5.1;Enzyme_name=nitrilase,Acting on carbon-nitrogen bonds, other than peptide bonds,Acting on carbon-nitrogen bonds, other than peptide bonds,Hydrolases,nitrilase
contig00008.g5225	1167	ID=contig00008.g5225;Description=tryptophan dimethylallyltransferase 1 [Fusarium tjaetaba];Gene=FTJAE_11864;Ontology_term=transferase activity;Ontology_id=GO:0016740;Enzyme_code=EC:2.5.1,EC:2.5.1,EC:2.5.1.122,EC:2;Enzyme_name=Transferring alkyl or aryl groups, other than methyl groups,Transferring alkyl or aryl groups, other than methyl groups,4-O-dimethylallyl-L-tyrosine synthase,Transferases
contig00008.g5226	1090	ID=contig00008.g5226;Description=Aflatoxin B1 aldehyde reductase member 3 [Fusarium pseudoanthophilum]

contig00008.g5227	916	ID=contig00008.g5227;Description=endo-1,3(4)-beta-glucanase [Fusarium verticillioides 7600];Gene=FVEG_11944;Ontology_term=carbohydrate metabolic process,hydrolase activity;Ontology_id=GO:0005975,GO:0016787;Enzyme_code=EC:3.2.1,EC:3.2,EC:3,EC:3.2.1;Enzyme_name=Glycosylases,Glycosylases,Hydrolases,Glycosylases
contig00008.g5228	1124	ID=contig00008.g5228;Description=sphingoid long-chain base transporter RSB1 [Fusarium tjaetaba];Gene=FVER53590_11945;Ontology_term=membrane;Ontology_id=GO:0016020
contig00008.g5229	2252	ID=contig00008.g5229;Description=MFS transporter asaE [Fusarium oxysporum f. sp. conglutinans];Gene=FNYG_04046;Ontology_term=transmembrane transport,transporter activity;Ontology_id=GO:0055085,GO:0005215
contig00008.g5230	1907	ID=contig00008.g5230;Description=L-ascorbate oxidase [Fusarium verticillioides 7600];Gene=FVER53590_11948;Ontology_term=oxidoreductase activity;Ontology_id=GO:0016491;Enzyme_code=EC:1,EC:1,EC:1.10.3.3;Enzyme_name=Oxidoreductases,Oxidoreductases,L-ascorbate oxidase
contig00008.g5231	918	ID=contig00008.g5231;Description=related to nucleoside-diphosphate-sugar epimerases [Fusarium fujikuroi]
contig00008.g5232	1686	ID=contig00008.g5232;Description=transcription factor SEF1 [Fusarium tjaetaba];Gene=FTJAE_11874;Ontology_term=nucleus,DNA binding,regulation of DNA-templated transcription,transcription regulator activity;Ontology_id=GO:0005634,GO:0003677,GO:0006355,GO:0140110
contig00008.g5233	2109	ID=contig00008.g5233;Description=hypothetical protein FVER53263_11950 [Fusarium verticillioides]
contig00008.g5234	1722	ID=contig00008.g5234;Description=maltose permease MAL61 [Fusarium coicis];Gene=FVER53263_11951;Ontology_term=transmembrane transport,transporter activity;Ontology_id=GO:0055085,GO:0005215
contig00008.g5235	5247	ID=contig00008.g5235;Description=lipolytic G-D-S-L family [Fusarium tjaetaba];Gene=FVER53263_11952;Ontology_term=carbohydrate metabolic process,transmembrane transport,transporter activity,plasma membrane,hydrolase activity;Ontology_id=GO:0005975,GO:0055085,GO:0005215,GO:0005886,GO:0016787;Enzyme_code=EC:3.1,EC:3.1,EC:3;Enzyme_name=Acting on ester bonds,Acting on ester bonds,Hydrolases
contig00008.g5236	942	ID=contig00008.g5236;Description=hypothetical protein FVER14953_11953 [Fusarium verticillioides]

contig00008.g5237	1491	ID=contig00008.g5237;Description=catalase [Fusarium sp. NRRL 25303];Gene=F25303_589;Ontology_term=antioxidant activity,oxidoreductase activity;Ontology_id=GO:0016209,GO:0016491;Enzyme_code=EC:1.11.1,EC:1.11,EC:1,EC:1.11.1;Enzyme_name=Acting on a peroxide as acceptor,Acting on a peroxide as acceptor,Oxidoreductases,Acting on a peroxide as acceptor
contig00008.g5238	2353	ID=contig00008.g5238;Description=probable catalase isozyme P [Fusarium fujikuroi];Gene=FVEG_11955;Ontology_term=antioxidant activity,oxidoreductase activity;Ontology_id=GO:0016209,GO:0016491;Enzyme_code=EC:1.11.1.6;Enzyme_name=catalase
contig00008.g5239	1727	ID=contig00008.g5239;Description=sulfite oxidase [Fusarium verticillioides 7600];Gene=BFJ68_g2223;Ontology_term=oxidoreductase activity;Ontology_id=GO:0016491;Enzyme_code=EC:1;Enzyme_name=Oxidoreductases
contig00008.g5240	933	ID=contig00008.g5240;Description=trans-aconitate 3-methyltransferase [Fusarium tjaetaba];Gene=FVER53590_11957;Ontology_term=transferase activity;Ontology_id=GO:0016740;Enzyme_code=EC:2.1.1,EC:2.1,EC:2,EC:2.1.1.145,EC:2.1.1;Enzyme_name=Transferring one-carbon groups,Transferring one-carbon groups,Transferases,trans-aconitate 3-methyltransferase,Transferring one-carbon groups
contig00008.g5241	1317	ID=contig00008.g5241;Description=related to C4-dicarboxylate transport protein mae1 [Fusarium fujikuroi IMI 58289];Gene=FOIG_14582;Ontology_term=transmembrane transport,transporter activity;Ontology_id=GO:0055085,GO:0005215
contig00008.g5242	425	ID=contig00008.g5242;Description=hypothetical protein FVER14953_11959 [Fusarium verticillioides]
contig00008.g5243	873	ID=contig00008.g5243;Description=cyclic amp receptor 2 [Fusarium subglutinans];Gene=FNAPI_3621;Ontology_term=membrane;Ontology_id=GO:0016020
contig00008.g5244	581	ID=contig00008.g5244;Description=DUF614 domain protein [Fusarium tjaetaba]
contig00008.g5245	817	ID=contig00008.g5245;Description=3-oxoacyl-reductase [Fusarium tjaetaba];Gene=FVER53263_11962;Ontology_term=oxidoreductase activity;Ontology_id=GO:0016491;Enzyme_code=EC:1,EC:1;Enzyme_name=Oxidoreductases,Oxidoreductases
contig00008.g5246	1066	ID=contig00008.g5246;Description=NAD(P)-binding domain protein [Fusarium sp. NRRL 52700]

contig00008.g5247	975	ID=contig00008.g5247;Description=glucan endo-1 3-beta-glucosidase A1 [Fusarium subglutinans];Gene=FOYG_00932;Ontology_term=carbohydrate metabolic process,hydrolase activity;Ontology_id=GO:0005975,GO:0016787;Enzyme_code=EC:3.2.1,EC:3.2,EC:3,EC:3.2.1;Enzyme_name=Glycosylases,Glycosylases,Hydrolases,Glycosylases
contig00008.g5248	1584	ID=contig00008.g5248;Description=cholin permease [Fusarium pseudoanthophilum];Gene=FOC4_g10003645;Ontology_term=transmembrane transport,transporter activity;Ontology_id=GO:0055085,GO:0005215
contig00008.g5249	4178	ID=contig00008.g5249;Description=hypothetical protein FVEG_11966 [Fusarium verticillioides 7600]
contig00008.g5250	972	ID=contig00008.g5250;Description=short-chain alcohol dehydrogenase [Fusarium tjaetaba];Gene=FNYG_04024
contig00008.g5251	1981	ID=contig00008.g5251;Description=transcription factor [Fusarium napiforme];Gene=FVEG_11968;Ontology_term=nucleus,regulation of DNA-templated transcription,transcription regulator activity;Ontology_id=GO:0005634,GO:0006355,GO:0140110
contig00008.g5252	989	ID=contig00008.g5252;Description=15-hydroxyprostaglandin dehydrogenase [Fusarium fujikuroi];Gene=C2S_7938;Ontology_term=carbohydrate metabolic process,oxidoreductase activity,hydrolase activity;Ontology_id=GO:0005975,GO:0016491,GO:0016787;Enzyme_code=EC:1,EC:3.2.1.26;Enzyme_name=Oxidoreductases,beta-fructofuranosidase
contig00008.g5253	1741	ID=contig00008.g5253;Description=monooxygenase [Fusarium tjaetaba];Gene=BFJ72_g409;Ontology_term=oxidoreductase activity;Ontology_id=GO:0016491;Enzyme_code=EC:1.14.13.8,EC:1.14.13.8,EC:1,EC:1.14,EC:1.14.13;Enzyme_name=flavin-containing monooxygenase,flavin-containing monooxygenase,Oxidoreductases,Acting on paired donors, with incorporation or reduction of molecular oxygen. The oxygen incorporated need not be derived from O2,Acting on paired donors, with incorporation or reduction of molecular oxygen. The oxygen incorporated need not be derived from O2
contig00008.g5254	2161	ID=contig00008.g5254;Description=putative peroxisomal amine oxidase (copper-containing) [Fusarium fujikuroi];Gene=FNAPI_10825;Ontology_term=oxidoreductase activity;Ontology_id=GO:0016491;Enzyme_code=EC:1.4.3.21,EC:1.4,EC:1.4.3,EC:1,EC:1.4.3.21;Enzyme_name=primary-amine oxidase,Acting on the CH-NH2 group of donors,Acting on the CH-NH2 group of donors,Oxidoreductases,primary-amine oxidase

contig00008.g5255	1668	ID=contig00008.g5255;Description=choline transporter [Fusarium tjaetaba];Gene=FNAPI_10824;Ontology_term=transmembrane transport,transporter activity;Ontology_id=GO:0055085,GO:0005215
contig00008.g5256	1610	ID=contig00008.g5256;Description=aldehyde dehydrogenase (NAD+) [Fusarium verticillioides 7600];Gene=FMEXI_8264;Ontology_term=oxidoreductase activity;Ontology_id=GO:0016491;Enzyme_code=EC:1.2.1;Enzyme_name=Acting on the aldehyde or oxo group of donors
contig00008.g5257	1136	ID=contig00008.g5257;Description=alpha beta hydrolase fold family [Fusarium tjaetaba];Gene=FTJAE_1260;Ontology_term=hydrolase activity;Ontology_id=GO:0016787;Enzyme_code=EC:3,EC:3;Enzyme_name=Hydrolases,Hydrolases
contig00008.g5258	1683	ID=contig00008.g5258;Description=altered inheritance of mitochondria 6 [Fusarium tjaetaba]
contig00008.g5259	297	ID=contig00008.g5259;Description=30S ribosomal S17P protein [Fusarium subglutinans]
contig00008.g5260	965	ID=contig00008.g5260;Description=isoflavone reductase like [Fusarium tjaetaba]
contig00008.g5261	1095	ID=contig00008.g5261;Description=short chain dehydrogenase [Fusarium pseudoanthophilum];Gene=FPANT_12626;Ontology_term=membrane;Ontology_id=GO:0016020;Enzyme_code=EC:1.1.1.330,EC:1.1.1.62;Enzyme_name=very-long-chain 3-oxoacyl-CoA reductase,17beta-estradiol 17-dehydrogenase
contig00008.g5262	947	ID=contig00008.g5262;Description=gnat family protein [Fusarium tjaetaba]
contig00008.g5263	1683	ID=contig00008.g5263;Description=putative transporter [Fusarium oxysporum f. sp. conglutinans];Gene=BFJ69_g1209;Ontology_term=transmembrane transport,transporter activity;Ontology_id=GO:0055085,GO:0005215
contig00008.g5264	1041	ID=contig00008.g5264;Description=alcohol dehydrogenase [Fusarium verticillioides 7600];Gene=FVEG_11980;Ontology_term=oxidoreductase activity;Ontology_id=GO:0016491;Enzyme_code=EC:1.1.1,EC:1.1,EC:1,EC:1.1.1;Enzyme_name=Acting on the CH-OH group of donors,Acting on the CH-OH group of donors,Oxidoreductases,Acting on the CH-OH group of donors
contig00008.g5265	814	ID=contig00008.g5265;Description=acyl oxidase [Fusarium coicis];Gene=FDENT_3030;Ontology_term=peroxisome,oxidoreductase activity,lipid metabolic process,transferase activity;Ontology_id=GO:0005777,GO:0016491,GO:0006629,GO:0016740;Enzyme_code=EC:2,EC:1.3.3.6;Enzyme_name=Transferases,acyl-CoA oxidase

contig00008.g5266	2097	ID=contig00008.g5266;Description=acyl-CoA oxidase [Fusarium verticillioides 7600];Gene=FPCIR_53;Ontology_term=peroxisome,oxidoreductase activity,lipid metabolic process;Ontology_id=GO:0005777,GO:0016491,GO:0006629;Enzyme_code=EC:1.3.3.6,EC:1.3,EC:1,EC:1.3.3.6,EC:1.3.3;Enzyme_name=acyl-CoA oxidase,Acting on the CH-CH group of donors,Oxidoreductases,acyl-CoA oxidase,Acting on the CH-CH group of donors
contig00008.g5267	1190	ID=contig00008.g5267;Description=hypothetical protein FVEG_11983 [Fusarium verticillioides 7600];Gene=CEK26_007625;Ontology_term=nucleus,lipid metabolic process,regulation of DNA-templated transcription,transcription regulator activity,hydrolase activity;Ontology_id=GO:0005634,GO:0006629,GO:0006355,GO:0140110,GO:0016787;Enzyme_code=EC:3.1.4;Enzyme_name=Acting on ester bonds
contig00008.g5268	1479	ID=contig00008.g5268;Description=transporter MCH4 [Fusarium pseudoanthophilum];Gene=FMEXI_8247;Ontology_term=transmembrane transport,transporter activity;Ontology_id=GO:0055085,GO:0005215
contig00008.g5269	978	ID=contig00008.g5269;Description=light induced alcohol dehydrogenase Bli-4 [Fusarium subglutinans]
contig00008.g5270	804	ID=contig00008.g5270;Description=hypothetical protein FVEG_11987 [Fusarium verticillioides 7600]
contig00008.g5271	1464	ID=contig00008.g5271;Description=hypothetical protein FVEG_11988 [Fusarium verticillioides 7600]
contig00008.g5272	1841	ID=contig00008.g5272;Description=maltose permease [Fusarium tjaetaba];Gene=FNAPI_2784;Ontology_term=transmembrane transport,transporter activity;Ontology_id=GO:0055085,GO:0005215
contig00008.g5273	600	ID=contig00008.g5273;Description=glucosamine-phosphate n-acetyltransferase [Fusarium tjaetaba];Gene=F52700_8822;Ontology_term=nucleus,nucleobase-containing small molecule metabolic process,carbohydrate derivative metabolic process,transferase activity;Ontology_id=GO:0005634,GO:0055086,GO:1901135,GO:0016740;Enzyme_code=EC:2.3.1.4,EC:2.3,EC:2,EC:2.3.1.4,EC:2.3.1;Enzyme_name=glucosamine-phosphate N-acetyltransferase,Acyltransferases,Transferases,glucosamine-phosphate N-acetyltransferase,Acyltransferases
contig00008.g5274	1686	ID=contig00008.g5274;Description=oxidoreductase [Fusarium verticillioides 7600];Gene=FTJAE_1283;Ontology_term=nucleotide binding;Ontology_id=GO:0000166
contig00008.g5275	442	ID=contig00008.g5275;Description=hypothetical protein FVEG_11992 [Fusarium verticillioides 7600]

contig00008.g5276	1332	ID=contig00008.g5276;Description=hypothetical protein FVER53590_11993 [Fusarium verticillioides]
contig00008.g5277	1266	ID=contig00008.g5277;Description=hypothetical protein FVER53590_11993 [Fusarium verticillioides]
contig00008.g5278	801	ID=contig00008.g5278;Description=hypothetical protein FVEG_17143 [Fusarium verticillioides 7600];Gene=FPCIR_2316;Ontology_term=membrane;Ontology_id=GO:0016020
contig00008.g5279	1049	ID=contig00008.g5279;Description=putative cyclase-domain-containing protein [Fusarium redolens];Gene=FVER53263_11994;Ontology_term=amino acid metabolic process,hydrolase activity;Ontology_id=GO:0006520,GO:0016787;Enzyme_code=EC:3.5.1.9,EC:3.5.1,EC:3.5,EC:3,EC:3.5.1.9;Enzyme_name=arylformamidase,Acting on carbon-nitrogen bonds, other than peptide bonds,Acting on carbon-nitrogen bonds, other than peptide bonds,Hydrolases,arylformamidase
contig00008.g5280	1860	ID=contig00008.g5280;Description=probable cutinase transcription factor 1 beta [Fusarium fujikuroi];Gene=FFUJ_14615;Ontology_term=nucleus,DNA binding,catalytic activity, acting on a protein,regulation of DNA-templated transcription,protein maturation,vacuole,transcription regulator activity,plasma membrane,hydrolase activity;Ontology_id=GO:0005634,GO:0003677,GO:0140096,GO:0006355,GO:0051604,GO:0005773,GO:0140110,GO:0005886,GO:0016787;Enzyme_code=EC:3.4.11,EC:3.4.21,EC:3.4.14.1,EC:3.4.14.5,EC:3.4.14.4,EC:3.4.14.2;Enzyme_name=Acting on peptide bonds (peptidases),Acting on peptide bonds (peptidases),dipeptidyl-peptidase I,dipeptidyl-peptidase IV,dipeptidyl-peptidase III,dipeptidyl-peptidase II
contig00008.g5281	938	ID=contig00008.g5281;Description=cis-1 2-dihydro-1 2-dihydroxynaphthalene dehydrogenase [Fusarium coicis]
contig00008.g5282	959	ID=contig00008.g5282;Description=hypothetical protein FVEG_11997 [Fusarium verticillioides 7600]
contig00008.g5283	1215	ID=contig00008.g5283;Description=hypothetical protein FBULB1_184 [Fusarium bulbicola]
contig00008.g5284	1180	ID=contig00008.g5284;Description=alcohol dehydrogenase 5 [Fusarium subglutinans];Gene=FNAPI_2795;Ontology_term=oxidoreductase activity;Ontology_id=GO:0016491;Enzyme_code=EC:1,EC:1,EC:1.1.1.1;Enzyme_name=Oxidoreductases,Oxidoreductases,alcohol dehydrogenase
contig00008.g5285	1698	ID=contig00008.g5285;Description=ankyrin repeats (3 copies) domain protein [Fusarium subglutinans]

contig00008.g5286	1221	ID=contig00008.g5286;Description=hypothetical protein FVEG_12001 [Fusarium verticillioides 7600]
contig00008.g5287	4179	ID=contig00008.g5287;Description=glycoside hydrolase family 18 [Fusarium subglutinans];Gene=F52700_8808;Ontology_term=carbohydrate metabolic process,carbohydrate derivative metabolic process,extracellular region,hydrolase activity;Ontology_id=GO:0005975,GO:1901135,GO:0005576,GO:0016787;Enzyme_code=EC:3.2.1.14,EC:3.2,EC:3,EC:3.2.1.14,EC:3.2.1;Enzyme_name=chitinase,Glycosylases,Hydrolases,chitinase,Glycosylases
contig00008.g5288	818	ID=contig00008.g5288;Description=pantothenate kinase [Fusarium tjaetaba];Gene=FTJAE_1296;Ontology_term=nucleobase-containing small molecule metabolic process,carbohydrate derivative metabolic process,transferase activity;Ontology_id=GO:0055086,GO:1901135,GO:0016740;Enzyme_code=EC:2.7.1.33,EC:2.7.1,EC:2.7,EC:2,EC:2.7.1.33;Enzyme_name=pantothenate kinase,Transferring phosphorus-containing groups,Transferring phosphorus-containing groups,Transferases,pantothenate kinase
contig00008.g5289	1787	ID=contig00008.g5289;Description=calcium-independent phospholipase A2-gamma [Fusarium tjaetaba];Gene=FPCIR_2305;Ontology_term=lipid metabolic process,hydrolase activity;Ontology_id=GO:0006629,GO:0016787;Enzyme_code=EC:3,EC:3;Enzyme_name=Hydrolases,Hydrolases
contig00008.g5290	270	ID=contig00008.g5290;Description=hypothetical protein FVEG_12005 [Fusarium verticillioides 7600]

contig00008.g5291	1923	ID=contig00008.g5291;Description=maltose permease [Fusarium tjaetaba];Gene=FPRO05_08361;Ontology_term=membrane organization,transmembrane transport,cytoskeleton organization,establishment or maintenance of cell polarity,anatomical structure development,cellular modified amino acid metabolic process,amino acid metabolic process,vitamin metabolic process,transporter activity,immune system process,plasma membrane,cell motility,nucleus,molecular function regulator activity,RNA binding,vesicle-mediated transport,cytoskeleton,oxidoreductase activity,GTPase activity,signaling,mitochondrion,cell differentiation,cytosol,cytoplasmic vesicle;Ontology_id=GO:0061024,GO:0055085,GO:0007010,GO:0007163,GO:0048856,GO:0006575,GO:0006520,GO:0006766,GO:0005215,GO:0002376,GO:0005886,GO:0048870,GO:0005634,GO:0098772,GO:0003723,GO:0016192,GO:0005856,GO:0016491,GO:0003924,GO:0023052,GO:0005739,GO:0030154,GO:0005829,GO:0031410;Enzyme_code=EC:1.5.1.3,EC:3.6.1.15,EC:3.6.1,EC:1.5,EC:1,EC:3.6,EC:3,EC:1.5.1,EC:1.5.1.3,EC:3.6.1.15;Enzyme_name=dihydrofolate reductase,nucleoside-triphosphate phosphatase,Acting on acid anhydrides,Acting on the CH-NH group of donors,Oxidoreductases,Acting on acid anhydrides,Hydrolases,Acting on the CH-NH group of donors,dihydrofolate reductase,nucleoside-triphosphate phosphatase
contig00008.g5292	859	ID=contig00008.g5292;Description=hypothetical protein FCOIX_6606 [Fusarium coicis]
contig00008.g5293	1102	ID=contig00008.g5293;Description=hypothetical protein FVER53590_12008 [Fusarium verticillioides]
contig00008.g5294	4650	ID=contig00008.g5294;Description=hypothetical protein FVER14953_12009 [Fusarium verticillioides]
contig00008.g5295	2499	ID=contig00008.g5295;Description=kinase [Fusarium tjaetaba];Gene=FVER53263_12010;Ontology_term=catalytic activity, acting on a protein,transferase activity;Ontology_id=GO:0140096,GO:0016740;Enzyme_code=EC:2.7.11.1,EC:2.7.1,EC:2.7.11.1,EC:2.7,EC:2;Enzyme_name=non-specific serine/threonine protein kinase,Transferring phosphorus-containing groups,non-specific serine/threonine protein kinase,Transferring phosphorus-containing groups,Transferases
contig00008.g5296	1543	ID=contig00008.g5296;Description=CMGC/SRPK protein kinase [Fusarium verticillioides 7600];Gene=BFJ68_g2145;Ontology_term=catalytic activity, acting on a protein,transferase activity;Ontology_id=GO:0140096,GO:0016740;Enzyme_code=EC:2.7.1,EC:2.7.1,EC:2.7,EC:2;Enzyme_name=Transferring phosphorus-containing groups,Transferring phosphorus-containing groups,Transferring phosphorus-containing groups,Transferases

contig00008.g5297	633	ID=contig00008.g5297;Description=hypothetical protein FVEG_12012 [Fusarium verticillioides 7600]
contig00008.g5298	3090	ID=contig00008.g5298;Description=hypothetical protein FVER53263_12014 [Fusarium verticillioides];Gene=FVER53263_12014;Ontology_term=membrane;Ontology_id=GO:0016020
contig00008.g5299	1184	ID=contig00008.g5299;Description=fusarubin cluster-esterase [Fusarium tjaetaba]
contig00008.g5300	1141	ID=contig00008.g5300;Description=PTH11-like integral membrane protein [Fusarium tjaetaba];Gene=FPRO05_08372;Ontology_term=membrane;Ontology_id=GO:0016020
contig00008.g5301	312	ID=contig00008.g5301;Description=hypothetical protein FVER53590_25066 [Fusarium verticillioides]
contig00008.g5302	1480	ID=contig00008.g5302;Description=putative MFS-type transporter [Fusarium oxysporum f. sp. conglutinans];Gene=HZS61_014017;Ontology_term=transmembrane transport,oxidoreductase activity,lipid metabolic process,endoplasmic reticulum,transporter activity;Ontology_id=GO:0055085,GO:0016491,GO:0006629,GO:0005783,GO:0005215;Enzyme_code=EC:1.14.14.1,EC:1.14.14.1,EC:1,EC:1.14.14,EC:1.14;Enzyme_name=unspecific monooxygenase,unspecific monooxygenase,Oxidoreductases,Acting on paired donors, with incorporation or reduction of molecular oxygen. The oxygen incorporated need not be derived from O2,Acting on paired donors, with incorporation or reduction of molecular oxygen. The oxygen incorporated need not be derived from O2
contig00008.g5303	1752	ID=contig00008.g5303;Description=MFS transporter, SIT family, siderophore-iron:H+ symporter [Fusarium odoratissimum NRRL 54006];Gene=FVEG_12019;Ontology_term=nucleus,transmembrane transport,endosome,vacuole,transporter activity,plasma membrane;Ontology_id=GO:0005634,GO:0055085,GO:0005768,GO:0005773,GO:0005215,GO:0005886
contig00008.g5304	3323	ID=contig00008.g5304;Description=putative NRPS-like protein biosynthetic cluster [Fusarium musae]
contig00008.g5305	870	ID=contig00008.g5305;Description=Alpha/Beta hydrolase protein [Fusarium oxysporum];Gene=BFJ69_g1050;Ontology_term=hydrolase activity;Ontology_id=GO:0016787;Enzyme_code=EC:3,EC:3,EC:3.1.1.5;Enzyme_name=Hydrolases,Hydrolases,lysophospholipase
contig00008.g5306	609	ID=contig00008.g5306;Description=hypothetical protein FVER14953_20274 [Fusarium verticillioides]

contig00008.g5307	2218	ID=contig00008.g5307;Description=hypothetical protein J7337_007278 [Fusarium musae]
contig00008.g5308	411	ID=contig00008.g5308;Description=hypothetical protein FNAPI_88 [Fusarium napiforme]
contig00008.g5309	1670	ID=contig00008.g5309;Description=cholin permease [Fusarium globosum];Gene=FOIG_15177;Ontology_term=transmembrane transport,transporter activity;Ontology_id=GO:0055085,GO:0005215
contig00008.g5310	1473	ID=contig00008.g5310;Description=related to ethionine resistance protein [Fusarium fujikuroi];Gene=FPRO05_08381;Ontology_term=transmembrane transport,sulfur compound metabolic process,detoxification,transporter activity;Ontology_id=GO:0055085,GO:0006790,GO:0098754,GO:0005215
contig00008.g5311	1447	ID=contig00008.g5311;Description=putative agmatinase 1 [Fusarium keratoplasticum];Gene=FSARC_953;Ontology_term=hydrolase activity;Ontology_id=GO:0016787;Enzyme_code=EC:3.5.3;Enzyme_name=Acting on carbon-nitrogen bonds, other than peptide bonds
contig00008.g5312	1056	ID=contig00008.g5312;Description=C6 finger domain protein [Fusarium subglutinans]
contig00008.g5313	1110	ID=contig00008.g5313;Description=hypothetical protein FVEG_17150 [Fusarium verticillioides 7600];Gene=FVEG_17150;Ontology_term=membrane;Ontology_id=GO:0016020
contig00008.g5314	3146	ID=contig00008.g5314;Description=hypothetical protein FVER14953_12025 [Fusarium verticillioides]
contig00008.g5315	2419	ID=contig00008.g5315;Description=fungal transcriptional regulatory [Fusarium tjaetaba];Gene=FTJAE_12643;Ontology_term=nucleus,DNA binding,regulation of DNA-templated transcription,transcription regulator activity;Ontology_id=GO:0005634,GO:0003677,GO:0006355,GO:0140110
contig00008.g5316	855	ID=contig00008.g5316;Description=monocarboxylate transporter [Fusarium tjaetaba];Gene=FPANT_6916;Ontology_term=transmembrane transport,mitochondrion,endoplasmic reticulum,vacuole,transporter activity;Ontology_id=GO:0055085,GO:0005739,GO:0005783,GO:0005773,GO:0005215
contig00008.g5317	1998	ID=contig00008.g5317;Description=vegetative incompatibility protein het-E-1 [Fusarium bulbicola];Gene=890
contig00008.g5318	1549	ID=contig00008.g5318;Description=heterokaryon incompatibility protein het-E-1 [Fusarium mundagurra];Gene=890

contig00008.g5319	1821	ID=contig00008.g5319;Description=probable carnitine transport protein [Fusarium fujikuroi];Gene=3618;Ontology_term=catalytic activity, acting on a protein,transmembrane transport,protein folding,isomerase activity;Ontology_id=GO:0140096,GO:0055085,GO:0006457,GO:0016853;Enzyme_code=EC:5.2.1.8,EC:5.2.1.8,EC:5.2,EC:5;Enzyme_name=peptidylprolyl isomerase,peptidylprolyl isomerase,Cis-trans-isomerases,Isomerases
contig00008.g5320	855	ID=contig00008.g5320;Description=formin binding protein [Fusarium tjaetaba];Gene=FPRO05_08393;Ontology_term=mRNA metabolic process;Ontology_id=GO:0016071;Enzyme_code=EC:1.6.99.1;Enzyme_name=NADPH dehydrogenase
contig00008.g5321	865	ID=contig00008.g5321;Description=esterase/lipase [Fusarium verticillioides 7600];Gene=FNAPI_100;Ontology_term=hydrolase activity;Ontology_id=GO:0016787;Enzyme_code=EC:3,EC:3;Enzyme_name=Hydrolases,Hydrolases
contig00008.g5322	2943	ID=contig00008.g5322;Description=TAPT1 like [Fusarium mundagurra];Gene=FMUND_8912;Ontology_term=mitochondrion,endoplasmic reticulum,protein folding;Ontology_id=GO:0005739,GO:0005783,GO:0006457
contig00008.g5323	3590	ID=contig00008.g5323;Description=Importin-beta, N-terminal domain [Fusarium oxysporum f. sp. vasinfectum];Gene=FMUND_8911;Ontology_term=nucleus,nucleocytoplasmic transport,cytoskeleton,intracellular protein transport;Ontology_id=GO:0005634,GO:0006913,GO:0005856,GO:0006886
contig00008.g5324	534	ID=contig00008.g5324;Description=peptidoglycan-binding protein [Fusarium tjaetaba];Gene=FPCIR_2269;Ontology_term=membrane;Ontology_id=GO:0016020
contig00008.g5325	987	ID=contig00008.g5325;Description=dihydroxyacid dehydratase [Fusarium pseudocircinatum]
contig00008.g5326	279	ID=contig00008.g5326;Description=thioesterase family [Fusarium pseudoanthophilum]
contig00008.g5327	2203	ID=contig00008.g5327;Description=putative modin [Fusarium denticulatum]
contig00008.g5328	2235	ID=contig00008.g5328;Description=transcription factor RGM1 [Fusarium tjaetaba];Ontology_term=DNA binding,inflammatory response,nuclear chromosome,regulation of DNA-templated transcription,cytosol,cell adhesion,transcription regulator activity;Ontology_id=GO:0003677,GO:0006954,GO:0000228,GO:0006355,GO:0005829,GO:0007155,GO:0140110

contig00008.g5329	2397	ID=contig00008.g5329;Description=hypothetical protein H9Q72_000344 [Fusarium xylarioides];Gene=FVEG_12118;Ontology_term=nucleus,DNA binding,regulation of DNA-templated transcription,transcription regulator activity;Ontology_id=GO:0005634,GO:0003677,GO:0006355,GO:0140110
contig00008.g5330	2898	ID=contig00008.g5330;Description=trehalose 6-phosphate synthase [Fusarium tjaetaba];Gene=FOQG_02903;Ontology_term=carbohydrate metabolic process,catalytic activity;Ontology_id=GO:0005975,GO:0003824;Enzyme_code=EC:2.4.1.15,EC:3.1.3.12;Enzyme_name=alpha,alpha-trehalose-phosphate synthase (UDP-forming),trehalose-phosphatase
contig00008.g5331	961	ID=contig00008.g5331;Description=globin-like protein [Fusarium tjaetaba];Gene=Forpe1208_v004568;Ontology_term=oxygen binding,heme binding;Ontology_id=GO:0019825,GO:0020037
contig00008.g5332	2212	ID=contig00008.g5332;Description=xylanolytic transcriptional activator xlnR [Fusarium subglutinans];Gene=FSUBG_3662;Ontology_term=nucleus,regulation of DNA-templated transcription,transcription regulator activity;Ontology_id=GO:0005634,GO:0006355,GO:0140110
contig00008.g5333	745	ID=contig00008.g5333;Description=hypothetical protein FVEG_12114 [Fusarium verticillioides 7600];Gene=FFUJ_13712;Ontology_term=membrane;Ontology_id=GO:0016020
contig00008.g5334	2595	ID=contig00008.g5334;Description=myo-inositol transport ITR1 [Fusarium tjaetaba];Gene=FPANT_6706
contig00008.g5335	2360	ID=contig00008.g5335;Description=e3 ubiquitin ligase HACE1 [Fusarium tjaetaba]
contig00008.g5336	1316	ID=contig00008.g5336;Description=hypothetical protein FVEG_17169 [Fusarium verticillioides 7600]
contig00008.g5337	618	ID=contig00008.g5337;Description=hypothetical protein FVEG_12111 [Fusarium verticillioides 7600]
contig00008.g5338	903	ID=contig00008.g5338;Description=gluconate 5-dehydrogenase [Fusarium verticillioides 7600];Gene=FPCIR_5407;Ontology_term=oxidoreductase activity;Ontology_id=GO:0016491;Enzyme_code=EC:1,EC:1;Enzyme_name=Oxidoreductases,Oxidoreductases
contig00008.g5339	1274	ID=contig00008.g5339;Description=serum paraoxonase lactonase 3 [Fusarium tjaetaba];Gene=FNYG_03869;Ontology_term=oxidoreductase activity,hydrolase activity;Ontology_id=GO:0016491,GO:0016787;Enzyme_code=EC:1,EC:3.1.1.2;Enzyme_name=Oxidoreductases,arylesterase

contig00008.g5340	1287	ID=contig00008.g5340;Description=hypothetical protein FVEG_12109 [Fusarium verticillioides 7600]
contig00008.g5341	342	ID=contig00008.g5341;Description=ZNF706/At2g23090 superfamily [Fusarium oxysporum f. sp. vasinfectum]
contig00008.g5342	837	ID=contig00008.g5342;Description=transcriptional regulatory [Fusarium tjaetaba];Gene=FVEG_17167;Ontology_term=nucleus,regulation of DNA-templated transcription,transcription regulator activity;Ontology_id=GO:0005634,GO:0006355,GO:0140110
contig00008.g5343	666	ID=contig00008.g5343;Description=carbohydrate-binding domain-containing protein [Fusarium pseudocircinatum]
contig00008.g5344	1659	ID=contig00008.g5344;Description=DUF895 domain-containing protein [Fusarium napiforme];Gene=FOTG_10989;Ontology_term=transmembrane transport,transporter activity;Ontology_id=GO:0055085,GO:0005215
contig00009.g5345	3316	ID=contig00009.g5345;Description=glutamine amidotransferase [Fusarium verticillioides 7600];Gene=FNAPI_11820;Ontology_term=amino acid metabolic process,transferase activity;Ontology_id=GO:0006520,GO:0016740;Enzyme_code=EC:2,EC:2;Enzyme_name=Transferases,Transferases
contig00009.g5346	2264	ID=contig00009.g5346;Description=lethal(2) denticleless [Fusarium phyllophilum];Ontology_term=nucleus,DNA replication,protein catabolic process,reproductive process,molecular adaptor activity;Ontology_id=GO:0005634,GO:0006260,GO:0030163,GO:0022414,GO:0060090
contig00009.g5347	1524	ID=contig00009.g5347;Description=hypothetical protein J7337_006817 [Fusarium musae];Gene=FNAPI_11822;Ontology_term=oxidoreductase activity;Ontology_id=GO:0016491;Enzyme_code=EC:1.14.14.18,EC:1.14.14.18,EC:1,EC:1.14,EC:1.14.14;Enzyme_name=heme oxygenase (biliverdin-producing),heme oxygenase (biliverdin-producing),Oxidoreductases,Acting on paired donors, with incorporation or reduction of molecular oxygen. The oxygen incorporated need not be derived from O2,Acting on paired donors, with incorporation or reduction of molecular oxygen. The oxygen incorporated need not be derived from O2
contig00009.g5348	1456	ID=contig00009.g5348;Description=hypothetical protein FVEG_04783 [Fusarium verticillioides 7600];Gene=FOYG_01527;Ontology_term=transmembrane transport,mitochondrion,vacuole,transporter activity;Ontology_id=GO:0055085,GO:0005739,GO:0005773,GO:0005215

contig00009.g5349	904	ID=contig00009.g5349;Description=hypothetical protein FVEG_04782 [Fusarium verticillioides 7600]
contig00009.g5350	941	ID=contig00009.g5350;Description=hypothetical protein J7337_006814 [Fusarium musae]
contig00009.g5351	1726	ID=contig00009.g5351;Description=major facilitator superfamily transporter [Fusarium tjaetaba];Gene=F25303_100;Ontology_term=transmembrane transport,transporter activity;Ontology_id=GO:0055085,GO:0005215
contig00009.g5352	5076	ID=contig00009.g5352;Description=multidrug resistance-associated [Fusarium coicis];Gene=F25303_99;Ontology_term=transmembrane transport,ATP-dependent activity,transporter activity,plasma membrane,hydrolase activity;Ontology_id=GO:0055085,GO:0140657,GO:0005215,GO:0005886,GO:0016787;Enzyme_code=EC:7.2.2,EC:3.6.1.15,EC:3.6.1,EC:3.6,EC:3,EC:7.2.2,EC:7,EC:3.6.1.15;Enzyme_name=Catalysing the translocation of inorganic cations,nucleoside-triphosphate phosphatase,Acting on acid anhydrides,Acting on acid anhydrides,Hydrolases,Catalysing the translocation of inorganic cations,Translocases,nucleoside-triphosphate phosphatase
contig00009.g5353	2757	ID=contig00009.g5353;Description=hypothetical protein FVEG_04776 [Fusarium verticillioides 7600]
contig00009.g5354	1969	ID=contig00009.g5354;Description=chromatin assembly complex subunit p90 [Fusarium tjaetaba];Gene=F52700_3455;Ontology_term=nucleus;Ontology_id=GO:0005634
contig00009.g5355	1654	ID=contig00009.g5355;Description=hypothetical protein LB506_004256 [Fusarium annulatum]
contig00009.g5356	3148	ID=contig00009.g5356;Description=exs family [Fusarium pseudocircinatum];Gene=FNAPI_11831;Ontology_term=membrane;Ontology_id=GO:0016020
contig00009.g5357	800	ID=contig00009.g5357;Description=hypothetical protein FVEG_04772 [Fusarium verticillioides 7600]
contig00009.g5358	813	ID=contig00009.g5358;Description=hypothetical protein FVER14953_04771 [Fusarium verticillioides]
contig00009.g5359	1656	ID=contig00009.g5359;Description=aminopeptidase yscI precursor vacuolar [Fusarium tjaetaba];Gene=FTJAE_11201;Ontology_term=catalytic activity, acting on a protein,protein catabolic process,extracellular region,vacuole,hydrolase activity;Ontology_id=GO:0140096,GO:0030163,GO:0005576,GO:0005773,GO:0016787;Enzyme_code=EC:3.4.11,EC:3.4.11,EC:3.4,EC:3,EC:3.4.11.22;Enzyme_name=Acting on peptide bonds (peptidases),Acting on peptide bonds (peptidases),Acting on peptide bonds (peptidases),Hydrolases,aminopeptidase I

contig00009.g5360	1497	ID=contig00009.g5360;Description=hypothetical protein FVER14953_04769 [Fusarium verticillioides]
contig00009.g5361	513	ID=contig00009.g5361;Description=hypothetical protein FVEG_04768 [Fusarium verticillioides 7600]
contig00009.g5362	783	ID=contig00009.g5362;Description=hypothetical protein FVER14953_04767 [Fusarium verticillioides]
contig00009.g5363	3118	ID=contig00009.g5363;Description=hypothetical protein FVER53590_04766 [Fusarium verticillioides]
contig00009.g5364	1267	ID=contig00009.g5364;Description=hypothetical protein DER46DRAFT_675179 [Fusarium sp. MPI-SDFR-AT-0072]
contig00009.g5365	714	ID=contig00009.g5365;Description=hypothetical protein FVEG_04765 [Fusarium verticillioides 7600]
contig00009.g5366	1595	ID=contig00009.g5366;Description=hypothetical protein FVEG_04763 [Fusarium verticillioides 7600]
contig00009.g5367	3060	ID=contig00009.g5367;Description=hypothetical protein FCOIX_12577 [Fusarium coicis]
contig00009.g5368	1595	ID=contig00009.g5368;Description=formate dehydrogenase [Fusarium verticillioides 7600];Gene=BFJ68_g11188;Ontology_term=oxidoreductase activity,cytosol;Ontology_id=GO:0016491,GO:0005829;Enzyme_code=EC:1.1.1,EC:1.17.1.9,EC:1.2.1;Enzyme_name=Acting on the CH-OH group of donors,formate dehydrogenase,Acting on the aldehyde or oxo group of donors
contig00009.g5369	1883	ID=contig00009.g5369;Description=large neutral amino acid transporter [Fusarium subglutinans];Gene=FGLOB1_2808;Ontology_term=transmembrane transport,transporter activity;Ontology_id=GO:0055085,GO:0005215
contig00009.g5370	450	ID=contig00009.g5370;Description=hypothetical protein FOXG_07839 [Fusarium oxysporum f. sp. lycopersici 4287]
contig00009.g5371	1804	ID=contig00009.g5371;Description=hypothetical protein FVEG_04758 [Fusarium verticillioides 7600];Gene=FVEG_04758;Ontology_term=membrane;Ontology_id=GO:0016020

contig00009.g5372	2717	ID=contig00009.g5372;Description=phosphatidate cytidylyltransferase [Fusarium tjaetaba];Gene=FVEG_04757;Ontology_term=lipid metabolic process,protein glycosylation,endoplasmic reticulum,transferase activity;Ontology_id=GO:0006629,GO:0006486,GO:0005783,GO:0016740;Enzyme_code=EC:2.7.7,EC:2.7.1,EC:2.7,EC:2,EC:2.7.7,EC:2.7.1.108,EC:1.3.1.88;Enzyme_name=Transferring phosphorus-containing groups,Transferring phosphorus-containing groups,Transferring phosphorus-containing groups,Transferases,Transferring phosphorus-containing groups,dolichol kinase,tRNA-dihydrouridine(16/17) synthase [NAD(P)(+)]
contig00009.g5373	1787	ID=contig00009.g5373;Description=origin recognition complex subunit 2 [Fusarium verticillioides 7600];Gene=FVER53263_04756;Ontology_term=DNA binding,protein-containing complex assembly,DNA replication,nuclear chromosome,regulation of DNA-templated transcription,telomere organization,chromatin organization,mitotic cell cycle,nucleoplasm;Ontology_id=GO:0003677,GO:0065003,GO:0006260,GO:0000228,GO:0006355,GO:0032200,GO:0006325,GO:0000278,GO:0005654
contig00009.g5374	1170	ID=contig00009.g5374;Description=hypothetical protein FVER14953_04755 [Fusarium verticillioides]
contig00009.g5375	650	ID=contig00009.g5375;Description=Tim17/Tim22/Tim23/Pmp24 family-domain-containing protein [Fusarium redolens];Gene=FOYG_01559;Ontology_term=membrane organization,transmembrane transport,mitochondrion,intracellular protein transport,transporter activity,mitochondrion organization;Ontology_id=GO:0061024,GO:0055085,GO:0005739,GO:0006886,GO:0005215,GO:0007005
contig00009.g5376	1359	ID=contig00009.g5376;Description=putative glycosidase C21B10.07 [Fusarium oxysporum f. sp. albedinis];Gene=FOVG_07494;Ontology_term=carbohydrate metabolic process,hydrolase activity;Ontology_id=GO:0005975,GO:0016787;Enzyme_code=EC:3.2.1,EC:3.2,EC:3,EC:3.2.1;Enzyme_name=Glycosylases,Glycosylases,Hydrolases,Glycosylases
contig00009.g5377	513	ID=contig00009.g5377;Description=integral membrane protein [Fusarium tjaetaba];Gene=Forpe1208_v004963;Ontology_term=membrane;Ontology_id=GO:0016020

contig00009.g5378	2209	ID=contig00009.g5378;Description=rhamnogalacturonate lyase precursor [Fusarium subglutinans];Gene=FNAPI_2730;Ontology_term=carbohydrate metabolic process,lyase activity,cell wall organization or biogenesis,extracellular region;Ontology_id=GO:0005975,GO:0016829,GO:0071554,GO:0005576;Enzyme_code=EC:4.2.2.23,EC:4.2.2.23,EC:4.2,EC:4,EC:4.2.2;Enzyme_name=rhamnogalacturonan endolyase,rhamnogalacturonan endolyase,Carbon-oxygen lyases,Lyases,Carbon-oxygen lyases
contig00009.g5379	1816	ID=contig00009.g5379;Description=SNW protein 1 [Fusarium oxysporum f. sp. lycopersici 4287];Gene=FOXG_07828;Ontology_term=nucleus,mRNA metabolic process,protein-containing complex assembly,regulation of DNA-templated transcription,transcription regulator activity,molecular adaptor activity;Ontology_id=GO:0005634,GO:0016071,GO:0065003,GO:0006355,GO:0140110,GO:0060090
contig00009.g5380	747	ID=contig00009.g5380;Description=Putative membrane protein C17A5.08 [Fusarium odoratissimum];Gene=FNAPI_6016;Ontology_term=vesicle-mediated transport,cytoplasmic vesicle,endoplasmic reticulum,intracellular protein transport;Ontology_id=GO:0016192,GO:0031410,GO:0005783,GO:0006886

contig00009.g5381	1491	ID=contig00009.g5381;Description=3-isopropylmalate dehydrogenase [Fusarium oxysporum f. sp. matthiolae];Gene=FPHYL_3458;Ontology_term=cytoskeleton organization,anatomical structure development,amino acid metabolic process,receptor ligand activity,muscle system process,circulatory system process,renal system process,oxidoreductase activity,mitotic nuclear division,cytosol,generation of precursor metabolites and energy,transmembrane transport,nucleobase-containing small molecule metabolic process,carbohydrate derivative metabolic process,reproductive process,regulation of DNA-templated transcription,mitochondrion organization,cell motility,carbohydrate metabolic process,extracellular space,lipid metabolic process,cell differentiation,transferase activity,endocrine process;Ontology_id=GO:0007010,GO:0048856,GO:0006520,GO:0048018,GO:0003012,GO:0003013,GO:0003014,GO:0016491,GO:0140014,GO:0005829,GO:0006091,GO:0055085,GO:0055086,GO:1901135,GO:0022414,GO:0006355,GO:0007005,GO:0048870,GO:0005975,GO:0005615,GO:0006629,GO:0030154,GO:0016740,GO:0050886;Enzyme_code=EC:1.1.1.85,EC:2.7.2.3,EC:2.7.2,EC:1.1,EC:1.1.1.85,EC:2.7.2.3,EC:1,EC:2.7,EC:2,EC:1.1.1;Enzyme_name=3-isopropylmalate dehydrogenase,phosphoglycerate kinase,Transferring phosphorus-containing groups,Acting on the CH-OH group of donors,3-isopropylmalate dehydrogenase,phosphoglycerate kinase,Oxidoreductases,Transferring phosphorus-containing groups,Transferases,Acting on the CH-OH group of donors
contig00009.g5382	2041	ID=contig00009.g5382;Description=alkaline phosphatase family [Fusarium subglutinans];Gene=FVEG_04747;Ontology_term=nucleobase-containing small molecule metabolic process,carbohydrate derivative metabolic process,ribosome biogenesis,ligase activity;Ontology_id=GO:0055086,GO:1901135,GO:0042254,GO:0016874;Enzyme_code=EC:6.3.2.6,EC:6.3,EC:6.3.2.6,EC:6.3.2,EC:6,EC:3.1.3.1;Enzyme_name=phosphoribosylaminoimidazolesuccinocarboxamide synthase,Forming carbon-nitrogen bonds,phosphoribosylaminoimidazolesuccinocarboxamide synthase,Forming carbon-nitrogen bonds,Ligases,alkaline phosphatase
contig00009.g5383	903	ID=contig00009.g5383;Description=RRP15 Essential protein involved in pre-rRNA processing [Fusarium fujikuroi];Gene=FVEG_04746;Ontology_term=ribosome biogenesis,nucleolus;Ontology_id=GO:0042254,GO:0005730

contig00009.g5384	622	ID=contig00009.g5384;Description=2Fe-2S ferredoxin-type domain-containing protein [Fusarium redolens];Gene=FVER53263_04745;Ontology_term=generation of precursor metabolites and energy,sulfur compound metabolic process,oxidoreductase activity,mitochondrion;Ontology_id=GO:0006091,GO:0006790,GO:0016491,GO:0005739;Enzyme_code=EC:1.6.2,EC:1.6.2,EC:1.6,EC:1;Enzyme_name=Acting on NADH or NADPH,Acting on NADH or NADPH,Acting on NADH or NADPH,Oxidoreductases
contig00009.g5385	1572	ID=contig00009.g5385;Description=mitochondrial fusion and transport ugo1 [Fusarium tjaetaba];Gene=FVER53263_04744;Ontology_term=mitochondrion;Ontology_id=GO:0005739
contig00009.g5386	831	ID=contig00009.g5386;Description=endoglucanase [Fusarium verticillioides 7600];Gene=FMUND_5756;Ontology_term=extracellular region;Ontology_id=GO:0005576;Enzyme_code=EC:1.14.99.54;Enzyme_name=lytic cellulose monooxygenase (C1-hydroxylating)
contig00009.g5387	1155	ID=contig00009.g5387;Description=hypothetical protein FVER53590_04742 [Fusarium verticillioides];Gene=F25303_65;Ontology_term=membrane;Ontology_id=GO:0016020
contig00009.g5388	2124	ID=contig00009.g5388;Description=ubiquitin carboxyl-terminal hydrolase 1 [Fusarium tjaetaba];Gene=FPCIR_5307;Ontology_term=catalytic activity, acting on a protein,endoplasmic reticulum,hydrolase activity;Ontology_id=GO:0140096,GO:0005783,GO:0016787;Enzyme_code=EC:3.4.19.12,EC:3.4,EC:3,EC:3.4.19.12;Enzyme_name=ubiquitinyl hydrolase 1,Acting on peptide bonds (peptidases),Hydrolases,ubiquitinyl hydrolase 1
contig00009.g5389	1446	ID=contig00009.g5389;Description=triose phosphate 3-phosphoglycerate phosphate translocator [Fusarium pseudocircinatum];Gene=FOXG_07819;Ontology_term=membrane;Ontology_id=GO:0016020
contig00009.g5390	2508	ID=contig00009.g5390;Description=complex proteins associated with Set1p component shg1-domain-containing protein [Fusarium oxysporum Fo47]
contig00009.g5391	2245	ID=contig00009.g5391;Description=serine/threonine-protein phosphatase 2B catalytic subunit [Fusarium verticillioides 7600];Gene=FVEG_04738;Ontology_term=catalytic activity, acting on a protein,cytoskeleton,transmembrane transport,cytoskeleton organization,signaling,anatomical structure development,cytokinesis,cell wall organization or biogenesis,mitotic cell cycle,hydrolase activity;Ontology_id=GO:0140096,GO:0005856,GO:0055085,GO:0007010,GO:0023052,GO:0048856,GO:0000910,GO:0071554,GO:0000278,GO:0016787;Enzyme_code=EC:3.1.3.16;Enzyme_name=protein-serine/threonine phosphatase

		ID=contig00009.g5392;Description=Sorbose reductase sou1 [Fusarium irregulare];Gene=FNAPI_6028;Ontology_term=carbohydrate metabolic process,oxidoreductase activity;Ontology_id=GO:0005975,GO:0016491;Enzyme_code=EC:1.1,EC:1.1.1.138,EC:1,EC:1.1.1.289,EC:1.1.1;Enzyme_name=Acting on the CH-OH group of donors,mannitol 2-dehydrogenase (NADP(+)),Oxidoreductases,sorbose reductase,Acting on the CH-OH group of donors
contig00009.g5392	1034	ID=contig00009.g5393;Description=trimethyllysine hydroxylase [Fusarium tjaetaba];Gene=FTJAE_9033;Ontology_term=oxidoreductase activity,cellular modified amino acid metabolic process;Ontology_id=GO:0016491,GO:0006575;Enzyme_code=EC:1.14.11.8,EC:1.14.11,EC:1,EC:1.14,EC:1.14.11.8;Enzyme_name=trimethyllysine dioxygenase,Acting on paired donors, with incorporation or reduction of molecular oxygen. The oxygen incorporated need not be derived from O2,Oxidoreductases,Acting on paired donors, with incorporation or reduction of molecular oxygen. The oxygen incorporated need not be derived from O2,trimethyllysine dioxygenase
contig00009.g5393	1755	ID=contig00009.g5394;Description=ECM16 DEAH-box RNA helicase [Fusarium tjaetaba];Gene=FVEG_04735;Ontology_term=RNA binding,ATP-dependent activity,mitochondrion,ribosome biogenesis,catalytic activity, acting on RNA,nucleolus,hydrolase activity;Ontology_id=GO:0003723,GO:0140657,GO:0005739,GO:0042254,GO:0140098,GO:0005730,GO:0016787;Enzyme_code=EC:3.6.4.13,EC:3.6.1,EC:3.6.4.13,EC:3.6,EC:3,EC:3.6.1.15;Enzyme_name=RNA helicase,Acting on acid anhydrides,RNA helicase,Acting on acid anhydrides,Hydrolases,nucleoside-triphosphate phosphatase
contig00009.g5394	3636	ID=contig00009.g5395;Description=RNA polymerase II transcriptional regulation mediator MED6 [Fusarium coicis];Gene=FPANT_7033;Ontology_term=protein-containing complex assembly,regulation of DNA-templated transcription,transcription regulator activity,molecular adaptor activity,nucleoplasm;Ontology_id=GO:0065003,GO:0006355,GO:0140110,GO:0060090,GO:0005654
contig00009.g5395	1015	ID=contig00009.g5396;Description=multiple myeloma tumor-associated 2 like [Fusarium tjaetaba]
contig00009.g5396	759	

contig00009.g5397	1608	ID=contig00009.g5397;Description=probable arginosuccinate synthetase [Fusarium fujikuroi];Gene=C2S_13360;Ontology_term=amino acid metabolic process,ligase activity;Ontology_id=GO:0006520,GO:0016874;Enzyme_code=EC:6.3.4.5,EC:6.3.4.5,EC:6.3,EC:6;Enzyme_name=argininosuccinate synthase,argininosuccinate synthase,Forming carbon-nitrogen bonds,Ligases
contig00009.g5398	5353	ID=contig00009.g5398;Description=related to NUO-14 NADH2 dehydrogenase (ubiquinone) 14K chain [Fusarium fujikuroi]
contig00009.g5399	375	ID=contig00009.g5399;Description=NADH2 dehydrogenase (ubiquinone) 14K chain [Fusarium subglutinans];Gene=F66182_4799;Ontology_term=cellular anatomical entity;Ontology_id=GO:0110165
contig00009.g5400	1408	ID=contig00009.g5400;Description=translation initiation factor eIF-2B subunit delta [Fusarium verticillioides 7600];Gene=FNYG_12717;Ontology_term=translation regulator activity,RNA binding,molecular function regulator activity,cytoplasmic translation,cytosol;Ontology_id=GO:0045182,GO:0003723,GO:0098772,GO:0002181,GO:0005829
contig00009.g5401	1414	ID=contig00009.g5401;Description=arginine biosynthesis ArgJ, mitochondrial [Fusarium verticillioides 7600];Gene=FVEG_04728;Ontology_term=mitochondrion,amino acid metabolic process,transferase activity;Ontology_id=GO:0005739,GO:0006520,GO:0016740;Enzyme_code=EC:2.3.1.1,EC:2.3.1.35,EC:2.3.1.1,EC:2.3,EC:2,EC:2.3.1.35,EC:2.3.1;Enzyme_name=amino-acid N-acetyltransferase,glutamate N-acetyltransferase,amino-acid N-acetyltransferase,Acyltransferases,Transferases,glutamate N-acetyltransferase,Acyltransferases
contig00009.g5402	742	ID=contig00009.g5402;Description=imidazoleglycerol-phosphate dehydratase [Fusarium verticillioides 7600];Gene=FNAPI_6038;Ontology_term=lyase activity,amino acid metabolic process;Ontology_id=GO:0016829,GO:0006520;Enzyme_code=EC:4.2.1.19,EC:4.2.1,EC:4.2,EC:4,EC:4.2.1.19;Enzyme_name=imidazoleglycerol-phosphate dehydratase,Carbon-oxygen lyases,Carbon-oxygen lyases,Lyases,imidazoleglycerol-phosphate dehydratase
contig00009.g5403	1912	ID=contig00009.g5403;Description=anaphase-promoting complex subunit 6 [Fusarium verticillioides 7600]
contig00009.g5404	510	ID=contig00009.g5404;Description=hypothetical protein FMUND_5775 [Fusarium mundagurra];Gene=FVER53263_20357;Ontology_term=membrane;Ontology_id=GO:0016020

contig00009.g5405	2297	ID=contig00009.g5405;Description=acetoacetate-CoA ligase [Fusarium verticillioides 7600];Gene=FVEG_04725;Ontology_term=lipid metabolic process,ligase activity;Ontology_id=GO:0006629,GO:0016874;Enzyme_code=EC:6.2.1.16,EC:6.2,EC:6,EC:6.2.1,EC:6.2.1.16;Enzyme_name=acetoacetate--CoA ligase,Forming carbon-sulfur bonds,Ligases,Forming carbon-sulfur bonds,acetoacetate--CoA ligase
contig00009.g5406	11771	ID=contig00009.g5406;Description=transformation/transcription domain-associated protein [Fusarium verticillioides 7600];Gene=FOZG_03924;Ontology_term=chromatin organization;Ontology_id=GO:0006325
contig00009.g5407	657	ID=contig00009.g5407;Description=disease resistance aig2 [Fusarium phyllophilum]
contig00009.g5408	1158	ID=contig00009.g5408;Description=related to spore coat protein SP96 precursor [Fusarium fujikuroi]
contig00009.g5409	3913	ID=contig00009.g5409;Description=hypothetical protein CEK26_007086 [Fusarium fujikuroi];Gene=FFUJ_13281;Ontology_term=carbohydrate metabolic process,extracellular region,hydrolase activity;Ontology_id=GO:0005975,GO:0005576,GO:0016787;Enzyme_code=EC:3.2.1,EC:3.2,EC:3,EC:3.2.1;Enzyme_name=Glycosylases,Glycosylases,Hydrolases,Glycosylases
contig00009.g5410	3983	ID=contig00009.g5410;Description=ubiquitin carboxyl-terminal hydrolase 2 [Fusarium pseudoanthophilum];Gene=FNYG_12707;Ontology_term=catalytic activity, acting on a protein,protein catabolic process,hydrolase activity;Ontology_id=GO:0140096,GO:0030163,GO:0016787;Enzyme_code=EC:3.4.19.12,EC:3.4,EC:3,EC:3.4.19.12;Enzyme_name=ubiquitinyl hydrolase 1,Acting on peptide bonds (peptidases),Hydrolases,ubiquitinyl hydrolase 1
contig00009.g5411	1010	ID=contig00009.g5411;Description=methylglutaconyl-CoA hydratase [Fusarium verticillioides 7600];Enzyme_code=EC:4.2.1.18;Enzyme_name=methylglutaconyl-CoA hydratase
contig00009.g5412	2104	ID=contig00009.g5412;Description=hypothetical protein FVER53590_04719 [Fusarium verticillioides];Gene=FPCIR_5283;Ontology_term=membrane;Ontology_id=GO:0016020
contig00009.g5413	438	ID=contig00009.g5413;Description=lea domain protein [Fusarium subglutinans]
contig00009.g5414	874	ID=contig00009.g5414;Description=GPR1/FUN34/yaaH family-domain-containing protein [Fusarium oxysporum Fo47];Gene=FOQG_02408;Ontology_term=membrane;Ontology_id=GO:0016020
contig00009.g5415	669	ID=contig00009.g5415;Description=j domain protein [Fusarium tjaetaba];Gene=FDENT_4450;Ontology_term=mitochondrion;Ontology_id=GO:0005739

contig00009.g5416	951	ID=contig00009.g5416;Description=aspartyl-tRNA(Asn) glutamyl-tRNA (Gln) amidotransferase subunit B [Fusarium pseudocircinatum];Gene=FDENT_4451;Ontology_term=mitochondrial gene expression,mitochondrion,tRNA metabolic process,amino acid metabolic process,transferase activity,catalytic activity, acting on RNA,ligase activity;Ontology_id=GO:0140053,GO:0005739,GO:0006399,GO:0006520,GO:0016740,GO:0140098,GO:0016874;Enzyme_code=EC:6.3.5.7,EC:2,EC:6.3.5.7,EC:2,EC:6.3,EC:6,EC:6.3.5;Enzyme_name=glutaminyl-tRNA synthase (glutamine-hydrolyzing),Transferases,glutaminyl-tRNA synthase (glutamine-hydrolyzing),Transferases,Forming carbon-nitrogen bonds,Ligases,Forming carbon-nitrogen bonds
contig00009.g5417	1854	ID=contig00009.g5417;Description=aspartyl-tRNA(Asn)/glutamyl-tRNA (Gln) amidotransferase subunit B [Fusarium verticillioides 7600];Gene=FTJAE_4383;Ontology_term=mitochondrial gene expression,mitochondrion,tRNA metabolic process,amino acid metabolic process,transferase activity,catalytic activity, acting on RNA,ligase activity;Ontology_id=GO:0140053,GO:0005739,GO:0006399,GO:0006520,GO:0016740,GO:0140098,GO:0016874;Enzyme_code=EC:6.3.5.7,EC:2,EC:6.3.5.6,EC:6.3.5.7,EC:2,EC:6.3,EC:6,EC:6.3.5;Enzyme_name=glutaminyl-tRNA synthase (glutamine-hydrolyzing),Transferases,asparaginyl-tRNA synthase (glutamine-hydrolyzing),glutaminyl-tRNA synthase (glutamine-hydrolyzing),Transferases,Forming carbon-nitrogen bonds,Ligases,Forming carbon-nitrogen bonds
contig00009.g5418	1089	ID=contig00009.g5418;Description=hypothetical protein FVER53590_28414 [Fusarium verticillioides];Gene=FVEG_04714;Ontology_term=membrane;Ontology_id=GO:0016020
contig00009.g5419	896	ID=contig00009.g5419;Description=hypothetical protein FVEG_04713 [Fusarium verticillioides 7600]
contig00009.g5420	4184	ID=contig00009.g5420;Description=vacuolar assembly protein [Fusarium napiforme];Gene=BFJ68_g15598;Ontology_term=membrane organization,vesicle-mediated transport,endosome,protein-containing complex assembly,autophagy,protein maturation,intracellular protein transport,vacuole;Ontology_id=GO:0061024,GO:0016192,GO:0005768,GO:0065003,GO:0006914,GO:0051604,GO:0006886,GO:0005773

contig00009.g5421	5927	ID=contig00009.g5421;Description=anaphase-promoting complex subunit 1 [Fusarium verticillioides 7600];Gene=FVER53263_04711;Ontology_term=protein catabolic process,mitotic nuclear division,chromosome segregation,nuclear chromosome;Ontology_id=GO:0030163,GO:0140014,GO:0007059,GO:0000228
contig00009.g5422	1379	ID=contig00009.g5422;Description=eukaryotic translation initiation factor 3 subunit E [Fusarium subglutinans];Gene=INT6;Ontology_term=nucleus,translation regulator activity,RNA binding,protein-containing complex assembly,cytoplasmic translation,protein catabolic process,cytosol;Ontology_id=GO:0005634,GO:0045182,GO:0003723,GO:0065003,GO:0002181,GO:0030163,GO:0005829
contig00009.g5423	568	ID=contig00009.g5423;Description=mitochondrial large ribosomal subunit l49 [Fusarium tjaetaba];Gene=FPCIR_2259;Ontology_term=mitochondrial gene expression,mitochondrion,structural molecule activity,ribosome;Ontology_id=GO:0140053,GO:0005739,GO:0005198,GO:0005840
contig00009.g5424	3195	ID=contig00009.g5424;Description=SWI1-like protein [Fusarium tjaetaba];Gene=FDENT_8819;Ontology_term=DNA binding;Ontology_id=GO:0003677
contig00009.g5425	1248	ID=contig00009.g5425;Description=related to UTP6-U3 snoRNP protein [Fusarium fujikuroi];Gene=FNYG_12692;Ontology_term=DNA binding,RNA binding,lipid metabolic process,cell differentiation,ribosome biogenesis,regulation of DNA-templated transcription,nucleolus,transcription regulator activity,programmed cell death;Ontology_id=GO:0003677,GO:0003723,GO:0006629,GO:0030154,GO:0042254,GO:0006355,GO:0005730,GO:0140110,GO:0012501
contig00009.g5426	3313	ID=contig00009.g5426;Description=transporter [Fusarium tjaetaba];Gene=FTJAE_4374;Ontology_term=membrane;Ontology_id=GO:0016020
contig00009.g5427	1756	ID=contig00009.g5427;Description=cytochrome P450 monooxygenase [Fusarium bulbicola];Gene=FVEG_04704;Ontology_term=nuclear envelope,oxidoreductase activity;Ontology_id=GO:0005635,GO:0016491;Enzyme_code=EC:1.14;Enzyme_name=Acting on paired donors, with incorporation or reduction of molecular oxygen. The oxygen incorporated need not be derived from O2
contig00009.g5428	2089	ID=contig00009.g5428;Description=nucleoporin NUP42 [Fusarium pseudoanthophilum];Gene=FPANT_7065;Ontology_term=nuclear envelope,oxidoreductase activity;Ontology_id=GO:0005635,GO:0016491;Enzyme_code=EC:1.14;Enzyme_name=Acting on paired donors, with incorporation or reduction of molecular oxygen. The oxygen incorporated need not be derived from O2

contig00009.g5429	2017	ID=contig00009.g5429;Description=activating signal cointegrator 1 complex subunit 2 [Fusarium tjaetaba];Gene=FVEG_04702;Ontology_term=nucleus,regulation of DNA-templated transcription;Ontology_id=GO:0005634,GO:0006355
contig00009.g5430	1436	ID=contig00009.g5430;Description=DNA polymerase epsilon subunit 1 [Fusarium verticillioides 7600]
contig00009.g5431	1005	ID=contig00009.g5431;Description=DNA polymerase epsilon subunit 1 [Fusarium verticillioides 7600]
contig00009.g5432	6868	ID=contig00009.g5432;Description=DNA polymerase epsilon catalytic subunit A [Fusarium verticillioides 7600];Gene=FNAPI_6066;Ontology_term=DNA repair,catalytic activity, acting on DNA,protein-containing complex assembly,DNA replication,DNA recombination,nuclear chromosome,regulation of DNA-templated transcription,reproductive process,chromosome segregation,chromatin organization,DNA binding,RNA binding,signaling,mitochondrion,mitotic nuclear division,regulatory ncRNA-mediated gene silencing,transferase activity,hydrolase activity;Ontology_id=GO:0006281,GO:0140097,GO:0065003,GO:0006260,GO:0006310,GO:0000228,GO:0006355,GO:0022414,GO:0007059,GO:0006325,GO:0003677,GO:0003723,GO:0023052,GO:0005739,GO:0140014,GO:0031047,GO:0016740,GO:0016787;Enzyme_code=EC:2.7.7.7,EC:3.1.11.1,EC:3.1,EC:3.1.11,EC:2.7,EC:2,EC:3,EC:3.1.15,EC:2.7.7.7,EC:2.7.7;Enzyme_name=DNA-directed DNA polymerase,exodeoxyribonuclease I,Acting on ester bonds,Acting on ester bonds,Transferring phosphorus-containing groups,Transferases,Hydrolases,Acting on ester bonds,DNA-directed DNA polymerase,Transferring phosphorus-containing groups
contig00009.g5433	564	ID=contig00009.g5433;Description=Cox20 FAM36A [Fusarium mexicanum];Gene=FMEXI_12727;Ontology_term=protein-containing complex assembly,mitochondrion,mitochondrion organization;Ontology_id=GO:0065003,GO:0005739,GO:0007005
contig00009.g5434	921	ID=contig00009.g5434;Description=hypothetical protein FVER53263_04697 [Fusarium verticillioides]
contig00009.g5435	678	ID=contig00009.g5435;Description=VTS1 [Fusarium coicis]
contig00009.g5436	600	ID=contig00009.g5436;Description=copper transport [Fusarium coicis];Gene=FACUT_9619;Ontology_term=transmembrane transport,endoplasmic reticulum,transporter activity,plasma membrane;Ontology_id=GO:0055085,GO:0005783,GO:0005215,GO:0005886

contig00009.g5437	1956	ID=contig00009.g5437;Description=ferric reductase FRE2 precursor [Fusarium coicis];Gene=FNYG_12681;Ontology_term=oxidoreductase activity;Ontology_id=GO:0016491;Enzyme_code=EC:1.16,EC:1.16,EC:1;Enzyme_name=Oxidizing metal ions,Oxidizing metal ions,Oxidoreductases
contig00009.g5438	1023	ID=contig00009.g5438;Description=aldose 1-epimerase [Fusarium verticillioides 7600];Gene=FTJAE_4175;Ontology_term=carbohydrate metabolic process,isomerase activity;Ontology_id=GO:0005975,GO:0016853;Enzyme_code=EC:5,EC:5,EC:5.1.3.3;Enzyme_name=Isomerases,Isomerases,aldose 1-epimerase
contig00009.g5439	1360	ID=contig00009.g5439;Description=transcription factor [Fusarium tjaetaba]
contig00009.g5440	3736	ID=contig00009.g5440;Description=phosphatidylinositol glycan, class Q [Fusarium verticillioides 7600];Gene=FANTH_6187;Ontology_term=cytoskeletal protein binding;Ontology_id=GO:0008092
contig00009.g5441	1257	ID=contig00009.g5441;Description=tubulin folding cofactor C [Fusarium tjaetaba];Gene=FVER53263_04689;Ontology_term=molecular function regulator activity,protein-containing complex assembly,cytoskeletal protein binding,protein folding;Ontology_id=GO:0098772,GO:0065003,GO:0008092,GO:0006457
contig00009.g5442	447	ID=contig00009.g5442;Description=mediator subunit [Fusarium denticulatum];Gene=MED9;Ontology_term=nucleus,regulation of DNA-templated transcription,transcription regulator activity,molecular adaptor activity;Ontology_id=GO:0005634,GO:0006355,GO:0140110,GO:0060090
contig00009.g5443	1927	ID=contig00009.g5443;Description=2,3-bisphosphoglycerate-independent phosphoglycerate mutase [Fusarium verticillioides 7600];Gene=COX6B1;Ontology_term=carbohydrate metabolic process,generation of precursor metabolites and energy,mitochondrial gene expression,nucleobase-containing small molecule metabolic process,mitochondrion,structural molecule activity,carbohydrate derivative metabolic process,ribosome,isomerase activity;Ontology_id=GO:0005975,GO:0006091,GO:0140053,GO:0055086,GO:0005739,GO:0005198,GO:1901135,GO:0005840,GO:0016853;Enzyme_code=EC:5.4.2.12,EC:5.4,EC:5,EC:5.4.2.12,EC:5.4.2;Enzyme_name=phosphoglycerate mutase (2,3-diphosphoglycerate-independent),Intramolecular transferases,Isomerases,phosphoglycerate mutase (2,3-diphosphoglycerate-independent),Intramolecular transferases
contig00009.g5444	3037	ID=contig00009.g5444;Description=period clock FRQ [Fusarium tjaetaba];Gene=FPANT_8881;Ontology_term=nucleus,regulation of DNA-templated transcription;Ontology_id=GO:0005634,GO:0006355

contig00009.g5445	1857	ID=contig00009.g5445;Description=prenylcysteine oxidase/farnesylcysteine lyase [Fusarium verticillioides 7600];Gene=FPCIR_2239;Ontology_term=oxidoreductase activity,lyase activity,cellular modified amino acid metabolic process;Ontology_id=GO:0016491,GO:0016829,GO:0006575;Enzyme_code=EC:4,EC:1.8.3.5,EC:1,EC:1.8,EC:1.8.3,EC:4,EC:1.8.3.5,EC:1.8.3.6;Enzyme_name=Lyases,prenylcysteine oxidase,Oxidoreductases,Acting on a sulfur group of donors,Acting on a sulfur group of donors,Lyases,prenylcysteine oxidase,farnesylcysteine lyase
contig00009.g5446	2606	ID=contig00009.g5446;Description=vacuolar import and degradation 27 [Fusarium tjaetaba]
contig00009.g5447	1658	ID=contig00009.g5447;Description=alpha,alpha-trehalose-phosphate synthase [Fusarium verticillioides 7600];Gene=FocTR4_00002764;Ontology_term=carbohydrate metabolic process,cell differentiation,anatomical structure development,reproductive process,cytosol,transferase activity,hydrolase activity;Ontology_id=GO:0005975,GO:0030154,GO:0048856,GO:0022414,GO:0005829,GO:0016740,GO:0016787;Enzyme_code=EC:2.4.1.15,EC:2.4.1.15,EC:3.1,EC:2.4,EC:2,EC:3,EC:2.4.1.347,EC:2.4.1,EC:3.1.3;Enzyme_name=alpha,alpha-trehalose-phosphate synthase (UDP-forming),alpha,alpha-trehalose-phosphate synthase (UDP-forming),Acting on ester bonds,Glycosyltransferases,Transferases,Hydrolases,alpha,alpha-trehalose-phosphate synthase (ADP-forming),Glycosyltransferases,Acting on ester bonds
contig00009.g5448	2620	ID=contig00009.g5448;Description=Exosome complex exonuclease rrp6 [Fusarium tjaetaba];Gene=FTJAE_4185;Ontology_term=mRNA metabolic process,snRNA metabolic process,regulatory ncRNA-mediated gene silencing,tRNA metabolic process,nuclear chromosome,ribosome biogenesis,nucleolus,catalytic activity, acting on RNA,nucleoplasm,hydrolase activity;Ontology_id=GO:0016071,GO:0016073,GO:0031047,GO:0006399,GO:0000228,GO:0042254,GO:0005730,GO:0140098,GO:0005654,GO:0016787;Enzyme_code=EC:3.1.13,EC:3.1.15,EC:3.1,EC:3.1.13,EC:3,EC:3.1.15;Enzyme_name=Acting on ester bonds,Acting on ester bonds,Acting on ester bonds,Acting on ester bonds,Hydrolases,Acting on ester bonds
contig00009.g5449	704	ID=contig00009.g5449;Description=Adenosine kinase [Fusarium oxysporum f. sp. albedinis]

contig00009.g5450	626	ID=contig00009.g5450;Description=F-type H+-transporting ATPase subunit C [Fusarium verticillioides 7600];Gene=FocTR4_00002761;Ontology_term=transmembrane transport,nucleobase-containing small molecule metabolic process,ATP-dependent activity,mitochondrion,carbohydrate derivative metabolic process,lipid binding,transporter activity,ligase activity,hydrolase activity;Ontology_id=GO:0055085,GO:0055086,GO:0140657,GO:0005739,GO:1901135,GO:0008289,GO:0005215,GO:0016874,GO:0016787;Enzyme_code=EC:7.1.2.2,EC:3.6.1,EC:3.6,EC:3,EC:6,EC:7,EC:7.2.2,EC:3.6.1.15;Enzyme_name=H(+)-transporting two-sector ATPase,Acting on acid anhydrides,Acting on acid anhydrides,Hydrolases,Ligases,Translocases,Catalysing the translocation of inorganic cations,nucleoside-triphosphate phosphatase
contig00009.g5451	2015	ID=contig00009.g5451;Description=histone acetyltransferase [Fusarium pseudocircinatum];Gene=FNYG_10907;Ontology_term=catalytic activity, acting on a protein,regulation of DNA-templated transcription,nuclear chromosome,telomere organization,chromatin organization,transferase activity,nucleoplasm;Ontology_id=GO:0140096,GO:0006355,GO:0000228,GO:0032200,GO:0006325,GO:0016740,GO:0005654;Enzyme_code=EC:2.3.1.48,EC:2.3,EC:2,EC:2.3.1.48,EC:2.3.1;Enzyme_name=histone acetyltransferase,Acyltransferases,Transferases,histone acetyltransferase,Acyltransferases
contig00009.g5452	2052	ID=contig00009.g5452;Description=hypothetical protein FVER14953_04678 [Fusarium verticillioides]
contig00009.g5453	1850	ID=contig00009.g5453;Description=26S proteasome regulatory subunit N3 [Fusarium verticillioides 7600];Gene=FNYG_10909;Ontology_term=nucleus,molecular function regulator activity,protein catabolic process,mitotic nuclear division,chromosome segregation;Ontology_id=GO:0005634,GO:0098772,GO:0030163,GO:0140014,GO:0007059
contig00009.g5454	2112	ID=contig00009.g5454;Description=Transcription initiation factor TFIID subunit 12 [Fusarium oxysporum];Gene=lysS;Ontology_term=DNA binding,translation regulator activity,protein-containing complex assembly,structural molecule activity,nuclear chromosome,regulation of DNA-templated transcription,chromatin organization,transcription regulator activity,nucleoplasm,molecular adaptor activity;Ontology_id=GO:0003677,GO:0045182,GO:0065003,GO:0005198,GO:0000228,GO:0006355,GO:0006325,GO:0140110,GO:0005654,GO:0060090

contig00009.g5455	932	ID=contig00009.g5455;Description=peptidyl-tRNA hydrolase [Fusarium tjaetaba];Gene=FNAPI_177;Ontology_term=hydrolase activity;Ontology_id=GO:0016787;Enzyme_code=EC:3,EC:3,EC:3.1.1.29;Enzyme_name=Hydrolases,Hydrolases,aminoacyl-tRNA hydrolase
contig00009.g5456	613	ID=contig00009.g5456;Description=Cnl2/NKP2 family protein-domain-containing protein [Fusarium redolens];Gene=FPANT_8870;Ontology_term=chromosome;Ontology_id=GO:0005694
contig00009.g5457	2084	ID=contig00009.g5457;Description=Chromo domain protein Alp13 [Fusarium fujikuroi];Gene=FGLOB1_13626;Ontology_term=DNA repair,protein-containing complex assembly,DNA replication,establishment or maintenance of cell polarity,anatomical structure development,regulation of DNA-templated transcription,nuclear chromosome,chromatin organization,mitochondrion organization,nucleoplasm,translation regulator activity,mitochondrion;Ontology_id=GO:0006281,GO:0065003,GO:0006260,GO:0007163,GO:0048856,GO:0006355,GO:0000228,GO:0006325,GO:0007005,GO:0005654,GO:0045182,GO:0005739
contig00009.g5458	3091	ID=contig00009.g5458;Description=ATP-citrate synthase subunit 1 [Fusarium tjaetaba];Gene=FTJAE_4195;Ontology_term=generation of precursor metabolites and energy,nucleobase-containing small molecule metabolic process,sulfur compound metabolic process,lipid metabolic process,lyase activity,structural molecule activity,carbohydrate derivative metabolic process,cytosol,transferase activity,ribosome;Ontology_id=GO:0006091,GO:0055086,GO:0006790,GO:0006629,GO:0016829,GO:0005198,GO:1901135,GO:0005829,GO:0016740,GO:0005840;Enzyme_code=EC:4,EC:2.3.3.8,EC:2.3.3,EC:2.3,EC:2,EC:4,EC:2.3.3.8;Enzyme_name=Lyases,ATP citrate synthase,Acyltransferases,Acyltransferases,Transferases,Lyases,ATP citrate synthase
contig00009.g5459	1841	ID=contig00009.g5459;Description=ATP-citrate synthase subunit 2 [Fusarium odoratissimum NRRL 54006];Gene=F52700_13083;Ontology_term=generation of precursor metabolites and energy,nucleobase-containing small molecule metabolic process,sulfur compound metabolic process,lipid metabolic process,lyase activity,carbohydrate derivative metabolic process,transferase activity;Ontology_id=GO:0006091,GO:0055086,GO:0006790,GO:0006629,GO:0016829,GO:1901135,GO:0016740;Enzyme_code=EC:4,EC:2.3.3.8,EC:2.3.3,EC:2.3,EC:2,EC:4,EC:2.3.3.8;Enzyme_name=Lyases,ATP citrate synthase,Acyltransferases,Acyltransferases,Transferases,Lyases,ATP citrate synthase

contig00009.g5460	1600	ID=contig00009.g5460;Description=beta (1-3) glucanosyltransferase [Fusarium tjaetaba];Gene=FMAN_05668;Ontology_term=carbohydrate metabolic process,cell wall,anatomical structure development,cell differentiation,reproductive process,cell wall organization or biogenesis,transferase activity,vacuole,plasma membrane;Ontology_id=GO:0005975,GO:0005618,GO:0048856,GO:0030154,GO:0022414,GO:0071554,GO:0016740,GO:0005773,GO:0005886;Enzyme_code=EC:2,EC:2;Enzyme_name=Transferases,Transferases
contig00009.g5461	1203	ID=contig00009.g5461;Description=Septin-type guanine nucleotide-binding (G) domain-containing protein [Fusarium flagelliforme];Gene=FOPG_06708;Ontology_term=membrane organization,cytoskeleton organization,protein-containing complex assembly,establishment or maintenance of cell polarity,structural molecule activity,anatomical structure development,cytokinesis,reproductive process,lipid binding,cytoskeleton,GTPase activity,cell differentiation,mitotic nuclear division,cell wall organization or biogenesis,molecular adaptor activity;Ontology_id=GO:0061024,GO:0007010,GO:0065003,GO:0007163,GO:0005198,GO:0048856,GO:0000910,GO:0022414,GO:0008289,GO:0005856,GO:0003924,GO:0030154,GO:0140014,GO:0071554,GO:0060090;Enzyme_code=EC:3.6.1,EC:3.6,EC:3,EC:3.6.1.15;Enzyme_name=Acting on acid anhydrides,Acting on acid anhydrides,Hydrolases,nucleoside-triphosphate phosphatase
contig00009.g5462	1254	ID=contig00009.g5462;Description=WD40-repeat-containing domain protein [Fusarium oxysporum Fo47];Ontology_term=mRNA metabolic process,protein-containing complex assembly,DNA recombination,nuclear chromosome,meiotic nuclear division;Ontology_id=GO:0016071,GO:0065003,GO:0006310,GO:0000228,GO:0140013
contig00009.g5463	657	ID=contig00009.g5463;Description=DNA polymerase delta subunit 4 [Fusarium verticillioides 7600];Gene=FRV6_06444;Ontology_term=DNA repair,DNA replication,nuclear chromosome,mitotic cell cycle;Ontology_id=GO:0006281,GO:0006260,GO:0000228,GO:0000278
contig00009.g5464	1261	ID=contig00009.g5464;Description=cobyrinic acid a c-diamide synthase [Fusarium tjaetaba];Gene=FTJAE_4200;Ontology_term=tRNA metabolic process,hydrolase activity;Ontology_id=GO:0006399,GO:0016787;Enzyme_code=EC:3.2,EC:3,EC:3.2.2.1,EC:3.2.2;Enzyme_name=Glycosylases,Hydrolases,purine nucleosidase,Glycosylases

contig00009.g5465	2489	ID=contig00009.g5465;Description=ubiquitin-like 1-activating enzyme E1 B [Fusarium odoratissimum NRRL 54006];Gene=FOVG_07395;Ontology_term=nucleus,catalytic activity, acting on a protein,ATP-dependent activity,transferase activity,ligase activity;Ontology_id=GO:0005634,GO:0140096,GO:0140657,GO:0016740,GO:0016874;Enzyme_code=EC:6.2,EC:2.3,EC:6.2.1.45,EC:2,EC:6.2,EC:6,EC:2.3.2;Enzyme_name=Forming carbon-sulfur bonds,Acyltransferases,E1 ubiquitin-activating enzyme,Transferases,Forming carbon-sulfur bonds,Ligases,Acyltransferases
contig00009.g5466	1002	ID=contig00009.g5466;Description=related to 3-oxoacyl- [Fusarium mangiferae];Gene=FVEG_04659;Ontology_term=sulfur compound metabolic process,oxidoreductase activity,lipid metabolic process,transferase activity;Ontology_id=GO:0006790,GO:0016491,GO:0006629,GO:0016740;Enzyme_code=EC:1,EC:2.3.1.85,EC:1.1,EC:2.3,EC:1,EC:2,EC:1.1.1,EC:1.1.1.100,EC:2.3.1;Enzyme_name=Oxidoreductases,fatty-acid synthase system,Acting on the CH-OH group of donors,Acyltransferases,Oxidoreductases,Transferases,Acting on the CH-OH group of donors,3-oxoacyl-[acyl-carrier-protein] reductase,Acyltransferases
contig00009.g5467	3795	ID=contig00009.g5467;Description=Replication factor C subunit 3 [Fusarium pseudoanthophilum];Gene=FVEG_04658;Ontology_term=DNA binding,ATP-dependent activity,DNA replication,lipid metabolic process,signaling,endoplasmic reticulum,chromosome,hydrolase activity;Ontology_id=GO:0003677,GO:0140657,GO:0006260,GO:0006629,GO:0023052,GO:0005783,GO:0005694,GO:0016787;Enzyme_code=EC:3.6.1.15,EC:3.6.1,EC:3.1,EC:3.6,EC:3,EC:3.6.1.15,EC:3.1.3;Enzyme_name=nucleoside-triphosphate phosphatase,Acting on acid anhydrides,Acting on ester bonds,Acting on acid anhydrides,Hydrolases,nucleoside-triphosphate phosphatase,Acting on ester bonds
contig00009.g5468	1562	ID=contig00009.g5468;Description=Replication factor C subunit 3 [Fusarium pseudoanthophilum];Gene=FNYG_13321;Ontology_term=DNA binding,protein-containing complex assembly,ATP-dependent activity,DNA replication,lipid metabolic process,chromosome,hydrolase activity;Ontology_id=GO:0003677,GO:0065003,GO:0140657,GO:0006260,GO:0006629,GO:0005694,GO:0016787;Enzyme_code=EC:3.6.1.15;Enzyme_name=nucleoside-triphosphate phosphatase
contig00009.g5469	2022	ID=contig00009.g5469;Description=hypothetical protein FPANT_8429 [Fusarium pseudoanthophilum]

contig00009.g5470	862	ID=contig00009.g5470;Description=membrane steroid-binding 2 [Fusarium coicis];Gene=BFJ72_g5278;Ontology_term=membrane;Ontology_id=GO:0016020
contig00009.g5471	1128	ID=contig00009.g5471;Description=hypothetical protein FOC4_g10012636 [Fusarium odoratissimum]
contig00009.g5472	405	ID=contig00009.g5472;Description=Centromere X protein [Fusarium tjaetaba];Gene=FANTH_6220;Ontology_term=DNA binding,DNA repair,protein-containing complex assembly,DNA replication,DNA recombination,chromosome segregation,meiotic nuclear division,nucleoplasm;Ontology_id=GO:0003677,GO:0006281,GO:0065003,GO:0006260,GO:0006310,GO:0007059,GO:0140013,GO:0005654
contig00009.g5473	1337	ID=contig00009.g5473;Description=ADP,ATP carrier protein [Fusarium proliferatum];Gene=FGLOB1_11106;Ontology_term=transmembrane transport,mitochondrion,lyase activity,transporter activity;Ontology_id=GO:0055085,GO:0005739,GO:0016829,GO:0005215;Enzyme_code=EC:4.2.1.10;Enzyme_name=3-dehydroquinate dehydratase
contig00009.g5474	507	ID=contig00009.g5474;Description=40S ribosomal protein S25 [Fusarium oxysporum];Gene=FOC1_g10014477;Ontology_term=ribosome;Ontology_id=GO:0005840
contig00009.g5475	801	ID=contig00009.g5475;Description=40S ribosomal protein S5 [Fusarium proliferatum];Gene=F66182_8050;Ontology_term=nucleocytoplasmic transport,RNA binding,protein-containing complex assembly,cytoplasmic translation,structural molecule activity,cytosol,ribosome biogenesis,ribosome,intracellular protein transport;Ontology_id=GO:0006913,GO:0003723,GO:0065003,GO:0002181,GO:0005198,GO:0005829,GO:0042254,GO:0005840,GO:0006886
contig00009.g5476	834	ID=contig00009.g5476;Description=hypothetical protein FVEG_04648 [Fusarium verticillioides 7600];Gene=FPCIR_159;Ontology_term=nucleobase-containing small molecule metabolic process,carbohydrate derivative metabolic process,transferase activity;Ontology_id=GO:0055086,GO:1901135,GO:0016740;Enzyme_code=EC:2.3.1.4,EC:2.3,EC:2,EC:2.3.1.4,EC:2.3.1;Enzyme_name=glucosamine-phosphate N-acetyltransferase,Acyltransferases,Transferases,glucosamine-phosphate N-acetyltransferase,Acyltransferases
contig00009.g5477	756	ID=contig00009.g5477;Description=NPP1 domain protein [Fusarium tjaetaba]

contig00009.g5478	6143	ID=contig00009.g5478;Description=Dedicator of cytokinesis 1 [Fusarium tjaetaba];Gene=FVER53263_04646;Ontology_term=molecular function regulator activity,autophagy,signaling,mitochondrion,mitochondrion organization,plasma membrane;Ontology_id=GO:0098772,GO:0006914,GO:0023052,GO:0005739,GO:0007005,GO:0005886
contig00009.g5479	566	ID=contig00009.g5479;Description=hypothetical protein J7337_006684 [Fusarium musae]
contig00009.g5480	522	ID=contig00009.g5480;Description=hsp70 [Fusarium napiforme];Gene=FNYG_13332;Ontology_term=protein folding chaperone,ATP-dependent activity;Ontology_id=GO:0044183,GO:0140657
contig00009.g5481	1845	ID=contig00009.g5481;Description=related to RING finger protein Dorfin [Fusarium fujikuroi];Gene=3062;Ontology_term=nucleus,catalytic activity, acting on a protein,protein folding chaperone,ATP-dependent activity,lyase activity,regulation of DNA-templated transcription,chromosome,transferase activity;Ontology_id=GO:0005634,GO:0140096,GO:0044183,GO:0140657,GO:0016829,GO:0006355,GO:0005694,GO:0016740;Enzyme_code=EC:4.2.1.10,EC:2.3.2,EC:4.2.1,EC:2.3,EC:4.2.1.10,EC:4.2,EC:2,EC:4,EC:2.3.2;Enzyme_name=3-dehydroquinate dehydratase,Acyltransferases,Carbon-oxygen lyases,Acyltransferases,3-dehydroquinate dehydratase,Carbon-oxygen lyases,Transferases,Lyases,Acyltransferases
contig00009.g5482	2283	ID=contig00009.g5482;Description=ATP-binding protein cassette subfamily D (ALD) member 2 [Fusarium tjaetaba];Gene=FPANT_8441;Ontology_term=peroxisome,transmembrane transport,ATP-dependent activity,transporter activity,hydrolase activity;Ontology_id=GO:0005777,GO:0055085,GO:0140657,GO:0005215,GO:0016787;Enzyme_code=EC:7.2.2,EC:3.6.1.15,EC:3.6.1,EC:3.6,EC:3,EC:7.2.2,EC:7,EC:3.6.1.15;Enzyme_name=Catalysing the translocation of inorganic cations,nucleoside-triphosphate phosphatase,Acting on acid anhydrides,Acting on acid anhydrides,Hydrolases,Catalysing the translocation of inorganic cations,Translocases,nucleoside-triphosphate phosphatase
contig00009.g5483	475	ID=contig00009.g5483;Description=diphthamide biosynthesis protein 3 [Fusarium graminearum PH-1];Gene=F25303_3910;Ontology_term=nucleus,generation of precursor metabolites and energy,oxidoreductase activity,cytoplasmic translation,tRNA metabolic process,molecular carrier activity;Ontology_id=GO:0005634,GO:0006091,GO:0016491,GO:0002181,GO:0006399,GO:0140104;Enzyme_code=EC:1;Enzyme_name=Oxidoreductases
contig00009.g5484	304	ID=contig00009.g5484;Description=---NA---

contig00009.g5485	3522	ID=contig00009.g5485;Description=leucyl-tRNA synthetase, cytoplasmic [Fusarium verticillioides 7600];Gene=FVER53590_04639;Ontology_term=cytoplasmic translation,signaling,tRNA metabolic process,amino acid metabolic process,catalytic activity, acting on RNA,ligase activity,hydrolase activity;Ontology_id=GO:0002181,GO:0023052,GO:0006399,GO:0006520,GO:0140098,GO:0016874,GO:0016787;Enzyme_code=EC:6.1.1.4,EC:3.1.1,EC:3.1,EC:6.1,EC:6.1.1.4,EC:3,EC:6.1.1,EC:6,EC:6.1.1.9,EC:3.1.1;Enzyme_name=leucine--tRNA ligase,Acting on ester bonds,Acting on ester bonds,Forming carbon-oxygen bonds,leucine--tRNA ligase,Hydrolases,Forming carbon-oxygen bonds,Ligases,valine--tRNA ligase,Acting on ester bonds
contig00009.g5486	532	ID=contig00009.g5486;Description=hypothetical protein HG531_006501 [Fusarium graminearum];Gene=Forpe1208_v005074;Ontology_term=membrane;Ontology_id=GO:0016020
contig00009.g5487	623	ID=contig00009.g5487;Description=AP-3 complex subunit sigma [Fusarium sp. NRRL 52700];Gene=FVEG_04637;Ontology_term=intracellular protein transport;Ontology_id=GO:0006886
contig00009.g5488	666	ID=contig00009.g5488;Description=regulator of mitochondrial iron homeostasis [Fusarium pseudoanthophilum];Gene=FNAPI_2520;Ontology_term=transmembrane transport,sulfur compound metabolic process,oxidoreductase activity,mitochondrion,cellular modified amino acid metabolic process,molecular carrier activity,mitochondrion organization;Ontology_id=GO:0055085,GO:0006790,GO:0016491,GO:0005739,GO:0006575,GO:0140104,GO:0007005;Enzyme_code=EC:1.16.3.1,EC:1.16.3.1,EC:1.16,EC:1,EC:1.16.3;Enzyme_name=ferroxidase,ferroxidase,Oxidizing metal ions,Oxidoreductases,Oxidizing metal ions
contig00009.g5489	2514	ID=contig00009.g5489;Description=sugar transport STP1 [Fusarium tjaetaba];Gene=FVER53263_04634;Ontology_term=transmembrane transport,vacuole,transporter activity;Ontology_id=GO:0055085,GO:0005773,GO:0005215
contig00009.g5490	1101	ID=contig00009.g5490;Description=chitin synthase export chaperone [Fusarium tjaetaba];Gene=FOXG_07708;Ontology_term=membrane;Ontology_id=GO:0016020
contig00009.g5491	1460	ID=contig00009.g5491;Description=transcription initiation factor TFIIH subunit 2 [Fusarium verticillioides 7600];Gene=FPANT_8450;Ontology_term=DNA repair,catalytic activity, acting on a protein,translation regulator activity,regulation of DNA-templated transcription,transferase activity,transcription regulator activity,nucleoplasm;Ontology_id=GO:0006281,GO:0140096,GO:0045182,GO:0006355,GO:0016740,GO:0140110,GO:0005654;Enzyme_code=EC:2.3,EC:2,EC:2.3.2;Enzyme_name=Acyltransferases,Transferases,Acyltransferases

contig00009.g5492	1275	ID=contig00009.g5492;Description=4-hydroxyphenylpyruvate dioxygenase [Fusarium verticillioides 7600];Gene=FACUT_2528;Ontology_term=oxidoreductase activity,amino acid metabolic process;Ontology_id=GO:0016491,GO:0006520;Enzyme_code=EC:1.13.11.27,EC:1.13.11,EC:1.13,EC:1,EC:1.13.11.27;Enzyme_name=4-hydroxyphenylpyruvate dioxygenase,Acting on single donors with incorporation of molecular oxygen (oxygenases). The oxygen incorporated need not be derived from O2,Acting on single donors with incorporation of molecular oxygen (oxygenases). The oxygen incorporated need not be derived from O2,Oxidoreductases,4-hydroxyphenylpyruvate dioxygenase
contig00009.g5493	798	ID=contig00009.g5493;Description=tetratricopeptide repeat 36 [Fusarium tjaetaba]
contig00009.g5494	743	ID=contig00009.g5494;Description=probable 60S large subunit ribosomal protein [Fusarium fujikuroi];Gene=FMUND_7548;Ontology_term=structural molecule activity,ribosome;Ontology_id=GO:0005198,GO:0005840
contig00009.g5495	1402	ID=contig00009.g5495;Description=triose-phosphate transporter family-domain-containing protein [Fusarium redolens];Gene=FDENT_6827;Ontology_term=membrane;Ontology_id=GO:0016020
contig00009.g5496	1841	ID=contig00009.g5496;Description=UDP-galactopyranose mutase [Fusarium tjaetaba];Gene=FVEG_04626;Ontology_term=oxidoreductase activity;Ontology_id=GO:0016491;Enzyme_code=EC:1;Enzyme_name=Oxidoreductases
contig00009.g5497	7891	ID=contig00009.g5497;Description=serine threonine kinase [Fusarium tjaetaba];Gene=FVEG_15517;Ontology_term=catalytic activity, acting on a protein,vesicle-mediated transport,signaling,cell wall organization or biogenesis,cell adhesion,transferase activity,small molecule sensor activity;Ontology_id=GO:0140096,GO:0016192,GO:0023052,GO:0071554,GO:0007155,GO:0016740,GO:0140299;Enzyme_code=EC:2.7.3,EC:2.7.13.3,EC:2.7.1,EC:2.7.3,EC:2.7,EC:2,EC:2.7.13.3;Enzyme_name=Transferring phosphorus-containing groups,histidine kinase,Transferring phosphorus-containing groups,Transferring phosphorus-containing groups,Transferring phosphorus-containing groups,Transferases,histidine kinase

contig00009.g5498	1576	ID=contig00009.g5498;Description=hypothetical protein FVEG_04621 [Fusarium verticillioides 7600];Gene=PRKG1;Ontology_term=catalytic activity, acting on a protein,anatomical structure development,amino acid metabolic process,cell adhesion,Golgi apparatus,muscle system process,circulatory system process,plasma membrane,cell motility,wound healing,molecular function regulator activity,signaling,mitochondrion,cell differentiation,cytosol,transferase activity;Ontology_id=GO:0140096,GO:0048856,GO:0006520,GO:0007155,GO:0005794,GO:0003012,GO:0003013,GO:0005886,GO:0048870,GO:0042060,GO:0098772,GO:0023052,GO:0005739,GO:0030154,GO:0005829,GO:0016740;Enzyme_code=EC:2.7.11.12,EC:2.3.3.13,EC:2.7.1,EC:2.3.3,EC:2.7.11.12,EC:2.3,EC:2.7.11.1,EC:2.7,EC:2,EC:2.3.3.13;Enzyme_name=cGMP-dependent protein kinase,2-isopropylmalate synthase,Transferring phosphorus-containing groups,Acyltransferases,cGMP-dependent protein kinase,Acyltransferases,non-specific serine/threonine protein kinase,Transferring phosphorus-containing groups,Transferases,2-isopropylmalate synthase
contig00009.g5499	3364	ID=contig00009.g5499;Description=hypothetical protein FVEG_15515 [Fusarium verticillioides 7600];Ontology_term=membrane organization,vesicle-mediated transport,molecular function regulator activity,intracellular protein transport;Ontology_id=GO:0061024,GO:0016192,GO:0098772,GO:0006886
contig00009.g5500	2622	ID=contig00009.g5500;Description=allantoicase [Fusarium verticillioides 7600]
contig00009.g5501	1143	ID=contig00009.g5501;Description=Allantoicase [Fusarium musae];Gene=FNYG_13354;Ontology_term=nitrogen cycle metabolic process,hydrolase activity;Ontology_id=GO:0071941,GO:0016787;Enzyme_code=EC:3.5.3.4,EC:3.5.3,EC:4.3.2.3,EC:3.5,EC:3,EC:3.5.3.4;Enzyme_name=allantoicase,Acting on carbon-nitrogen bonds, other than peptide bonds,ureidoglycolate lyase,Acting on carbon-nitrogen bonds, other than peptide bonds,Hydrolases,allantoicase
contig00009.g5502	807	ID=contig00009.g5502;Description=cytochrome B-561 [Fusarium tjaetaba];Gene=FCIRC_10410;Ontology_term=oxidoreductase activity;Ontology_id=GO:0016491;Enzyme_code=EC:1,EC:1;Enzyme_name=Oxidoreductases,Oxidoreductases

contig00009.g5503	6294	ID=contig00009.g5503;Description=histidine kinase HHK2p [Fusarium subglutinans];Gene=FVEG_04615;Ontology_term=catalytic activity, acting on a protein,signaling,transferase activity,small molecule sensor activity;Ontology_id=GO:0140096,GO:0023052,GO:0016740,GO:0140299;Enzyme_code=EC:2.7.3,EC:2.7.13.3,EC:2.7.1,EC:2.7.3,EC:2.7,EC:2,EC:2.7.13.3;Enzyme_name=Transferring phosphorus-containing groups,histidine kinase,Transferring phosphorus-containing groups,Transferring phosphorus-containing groups,Transferring phosphorus-containing groups,Transferases,histidine kinase
contig00009.g5504	1566	ID=contig00009.g5504;Description=hypothetical protein FOWG_06278 [Fusarium oxysporum f. sp. lycopersici MN25];Gene=FOVG_07350;Ontology_term=catalytic activity;Ontology_id=GO:0003824
contig00009.g5505	639	ID=contig00009.g5505;Description=PRKR-interacting 1 protein [Fusarium tjaetaba];Gene=FVER53263_04613;Ontology_term=RNA binding;Ontology_id=GO:0003723
contig00009.g5506	1985	ID=contig00009.g5506;Description=T-complex protein 1 subunit zeta [Colletotrichum spaethianum];Gene=E0L32_010439;Ontology_term=cytoskeleton,protein folding chaperone,ATP-dependent activity,cytosol,hydrolase activity;Ontology_id=GO:0005856,GO:0044183,GO:0140657,GO:0005829,GO:0016787;Enzyme_code=EC:3.6.1.15,EC:3.6.1,EC:3.6,EC:3,EC:3.6.1.15;Enzyme_name=nucleoside-triphosphate phosphatase,Acting on acid anhydrides,Acting on acid anhydrides,Hydrolases,nucleoside-triphosphate phosphatase
contig00009.g5507	453	ID=contig00009.g5507;Description=impact like [Fusarium tjaetaba];Ontology_term=nucleus,molecular function regulator activity,cytoplasmic translation,cytoskeletal protein binding;Ontology_id=GO:0005634,GO:0098772,GO:0002181,GO:0008092
contig00009.g5508	1886	ID=contig00009.g5508;Description=hypothetical protein FCOIX_9918 [Fusarium coicis]
contig00009.g5509	1546	ID=contig00009.g5509;Description=cellobiose dehydrogenase [Fusarium tjaetaba];Gene=FTJAE_9870;Ontology_term=oxidoreductase activity;Ontology_id=GO:0016491;Enzyme_code=EC:1.1,EC:1.1,EC:1.1.99.18,EC:1;Enzyme_name=Acting on the CH-OH group of donors,Acting on the CH-OH group of donors,cellobiose dehydrogenase (acceptor),Oxidoreductases
contig00009.g5510	584	ID=contig00009.g5510;Description=hypothetical protein FVEG_04609 [Fusarium verticillioides 7600]
contig00009.g5511	940	ID=contig00009.g5511;Description=FPD1 [Fusarium oxysporum f. sp. cubense];Gene=FSARC_5088;Ontology_term=nucleus;Ontology_id=GO:0005634

contig00009.g5512	2607	ID=contig00009.g5512;Description=hypothetical protein FVEG_04607 [Fusarium verticillioides 7600];Gene=FVER53590_04607;Ontology_term=membrane;Ontology_id=GO:0016020
contig00009.g5513	1840	ID=contig00009.g5513;Description=glyoxal oxidase precursor [Fusarium tjaetaba]
contig00009.g5514	2747	ID=contig00009.g5514;Description=UBA domain-containing protein [Fusarium pseudocircinatum];Ontology_term=vesicle-mediated transport,cytosol,endoplasmic reticulum;Ontology_id=GO:0016192,GO:0005829,GO:0005783
contig00009.g5515	546	ID=contig00009.g5515;Description=ubiquitin-conjugating enzyme ubcP3 [Fusarium subglutinans];Gene=FACUT_2505;Ontology_term=catalytic activity, acting on a protein,protein catabolic process,regulation of DNA-templated transcription,endoplasmic reticulum,cell wall organization or biogenesis,transferase activity,chromatin organization;Ontology_id=GO:0140096,GO:0030163,GO:0006355,GO:0005783,GO:0071554,GO:0016740,GO:0006325;Enzyme_code=EC:2.3.2.23,EC:2.3,EC:2,EC:2.3.2.23,EC:2.3.2;Enzyme_name=E2 ubiquitin-conjugating enzyme,Acyltransferases,Transferases,E2 ubiquitin-conjugating enzyme,Acyltransferases
contig00009.g5516	592	ID=contig00009.g5516;Description=hypothetical protein FVER14953_04603 [Fusarium verticillioides]
contig00009.g5517	411	ID=contig00009.g5517;Description=hypothetical protein FGLOB1_14049, partial [Fusarium globosum]
contig00009.g5518	1167	ID=contig00009.g5518;Description=hypothetical protein FVEG_04601 [Fusarium verticillioides 7600];Gene=3022;Ontology_term=catalytic activity, acting on a protein,transferase activity;Ontology_id=GO:0140096,GO:0016740;Enzyme_code=EC:2.7.11.1,EC:2.7.1,EC:2.7.11.1,EC:2.7,EC:2;Enzyme_name=non-specific serine/threonine protein kinase,Transferring phosphorus-containing groups,non-specific serine/threonine protein kinase,Transferring phosphorus-containing groups,Transferases
contig00009.g5519	612	ID=contig00009.g5519;Description=hypothetical protein FVEG_04600 [Fusarium verticillioides 7600];Gene=FDENT_7262;Ontology_term=membrane;Ontology_id=GO:0016020

contig00009.g5520	1049	ID=contig00009.g5520;Description=nucleoside diphosphate kinase [Fusarium tricinctum];Gene=FIESC28_05991;Ontology_term=nucleobase-containing small molecule metabolic process,mitochondrion,carbohydrate derivative metabolic process,cytosol,transferase activity,extracellular region;Ontology_id=GO:0055086,GO:0005739,GO:1901135,GO:0005829,GO:0016740,GO:0005576;Enzyme_code=EC:2.7.4.6,EC:2.7.4.6,EC:2.7,EC:2,EC:2.7.4;Enzyme_name=nucleoside-diphosphate kinase,nucleoside-diphosphate kinase,Transferring phosphorus-containing groups,Transferases,Transferring phosphorus-containing groups
contig00009.g5521	2943	ID=contig00009.g5521;Description=alpha 1,3-glucosidase [Fusarium verticillioides 7600];Gene=FPANT_7679;Ontology_term=carbohydrate metabolic process,mitochondrion,protein glycosylation,endoplasmic reticulum,cell wall organization or biogenesis,hydrolase activity;Ontology_id=GO:0005975,GO:0005739,GO:0006486,GO:0005783,GO:0071554,GO:0016787;Enzyme_code=EC:3.2.1.106,EC:3.2.1.106,EC:3.2,EC:3.2.1.20,EC:3.2.1.84,EC:3,EC:3.2.1;Enzyme_name=mannosyl-oligosaccharide glucosidase,mannosyl-oligosaccharide glucosidase,Glycosylases,alpha-glucosidase,glucan 1,3-alpha-glucosidase,Hydrolases,Glycosylases
contig00009.g5522	2583	ID=contig00009.g5522;Description=Mechanosensitive ion channel family [Fusarium pseudoanthophilum];Gene=FOC1_g10014526;Ontology_term=transmembrane transport,endoplasmic reticulum,transporter activity;Ontology_id=GO:0055085,GO:0005783,GO:0005215
contig00009.g5523	4887	ID=contig00009.g5523;Description=general RNA polymerase II transcription factor TAF12 [Fusarium tjaetaba]
contig00009.g5524	558	ID=contig00009.g5524;Description=heterokaryon incompatibility (het-6OR allele) [Fusarium coicis]
contig00009.g5525	2252	ID=contig00009.g5525;Description=chitinase [Fusarium tjaetaba];Gene=F52700_6427;Ontology_term=carbohydrate metabolic process;Ontology_id=GO:0005975
contig00009.g5526	1077	ID=contig00009.g5526;Description=hypothetical protein FVEG_04592 [Fusarium verticillioides 7600]
contig00009.g5527	279	ID=contig00009.g5527;Description=hypothetical protein FVER14953_06658 [Fusarium verticillioides];Gene=fliP;Ontology_term=molecular function regulator activity,establishment or maintenance of cell polarity,signaling,cytokinesis,mitotic cell cycle;Ontology_id=GO:0098772,GO:0007163,GO:0023052,GO:0000910,GO:0000278

contig00009.g5528	1029	ID=contig00009.g5528;Description=putative DIM1-rRNA (adenine-N6,N6-)-dimethyltransferase [Fusarium fujikuroi];Gene=FGLOB1_8345;Ontology_term=RNA binding,mitochondrion,ribosome biogenesis,transferase activity,catalytic activity, acting on RNA;Ontology_id=GO:0003723,GO:0005739,GO:0042254,GO:0016740,GO:0140098;Enzyme_code=EC:2.1.1.183,EC:2.1,EC:2.1.1.183,EC:2,EC:2.1.1;Enzyme_name=18S rRNA (adenine(1779)-N(6)/adenine(1780)-N(6))-dimethyltransferase,Transferring one-carbon groups,18S rRNA (adenine(1779)-N(6)/adenine(1780)-N(6))-dimethyltransferase,Transferases,Transferring one-carbon groups
contig00009.g5529	2235	ID=contig00009.g5529;Description=hypothetical protein FVER14953_04590 [Fusarium verticillioides]
contig00009.g5530	480	ID=contig00009.g5530;Description=LSM domain-containing protein [Metarhizium robertsii ARSEF 23];Gene=MAJ_10696;Ontology_term=nucleus,mRNA metabolic process,RNA binding,protein-containing complex assembly,regulation of DNA-templated transcription,chromatin organization;Ontology_id=GO:0005634,GO:0016071,GO:0003723,GO:0065003,GO:0006355,GO:0006325
contig00009.g5531	2803	ID=contig00009.g5531;Description=tRNA (adenine57-N1/adenine58-N1)-methyltransferase [Fusarium verticillioides 7600];Gene=FMUND_7584;Ontology_term=nucleus,tRNA metabolic process,catalytic activity, acting on RNA,transferase activity;Ontology_id=GO:0005634,GO:0006399,GO:0140098,GO:0016740;Enzyme_code=EC:2.1.1,EC:2.1,EC:2.1.1.220,EC:2.1.1.219,EC:2,EC:2.1.1;Enzyme_name=Transferring one-carbon groups,Transferring one-carbon groups,tRNA (adenine(58)-N(1))-methyltransferase,tRNA (adenine(57)-N(1)/adenine(58)-N(1))-methyltransferase,Transferases,Transferring one-carbon groups
contig00009.g5532	2554	ID=contig00009.g5532;Description=cysteinyl-tRNA synthetase [Fusarium verticillioides 7600];Gene=FOZG_04071;Ontology_term=cytoplasmic translation,tRNA metabolic process,amino acid metabolic process,catalytic activity, acting on RNA,ligase activity;Ontology_id=GO:0002181,GO:0006399,GO:0006520,GO:0140098,GO:0016874;Enzyme_code=EC:6.1.1.16,EC:6.1,EC:6.1.1,EC:6,EC:6.1.1.16;Enzyme_name=cysteine--tRNA ligase,Forming carbon-oxygen bonds,Forming carbon-oxygen bonds,Ligases,cysteine--tRNA ligase
contig00009.g5533	1987	ID=contig00009.g5533;Description=nicotinamide mononucleotide permease [Fusarium fujikuroi];Gene=GE21DRAFT_3204;Ontology_term=cellular anatomical entity;Ontology_id=GO:0110165

contig00009.g5534	1227	ID=contig00009.g5534;Description=hypothetical protein FVEG_04585 [Fusarium verticillioides 7600]
contig00009.g5535	641	ID=contig00009.g5535;Description=DUF967 domain protein [Fusarium fujikuroi]
contig00009.g5536	2280	ID=contig00009.g5536;Description=putative transcriptional regulatory protein [Fusarium oxysporum f. sp. raphani];Gene=Forpi1262_v004003;Ontology_term=nucleus,DNA binding,regulation of DNA-templated transcription,transcription regulator activity;Ontology_id=GO:0005634,GO:0003677,GO:0006355,GO:0140110
contig00009.g5537	474	ID=contig00009.g5537;Description=Noranthrone monooxygenase [Fusarium oxysporum f. sp. raphani];Gene=FOIG_03707;Ontology_term=oxidoreductase activity;Ontology_id=GO:0016491;Enzyme_code=EC:1,EC:1;Enzyme_name=Oxidoreductases,Oxidoreductases
contig00009.g5538	1699	ID=contig00009.g5538;Description=related to cytochrome P450 7B1 [Fusarium fujikuroi];Gene=FVER53590_25839;Ontology_term=oxidoreductase activity;Ontology_id=GO:0016491;Enzyme_code=EC:1.14,EC:1,EC:1.14;Enzyme_name=Acting on paired donors, with incorporation or reduction of molecular oxygen. The oxygen incorporated need not be derived from O2,Oxidoreductases,Acting on paired donors, with incorporation or reduction of molecular oxygen. The oxygen incorporated need not be derived from O2
contig00009.g5539	918	ID=contig00009.g5539;Description=ubiquinol-cytochrome-c reductase cytochrome c1 [Fusarium tjaetaba]
contig00009.g5540	635	ID=contig00009.g5540;Description=guanylate kinase [Fusarium verticillioides 7600];Gene=FVER53263_04579;Ontology_term=nucleus,nucleobase-containing small molecule metabolic process,lipid metabolic process,carbohydrate derivative metabolic process,cytosol,transferase activity;Ontology_id=GO:0005634,GO:0055086,GO:0006629,GO:1901135,GO:0005829,GO:0016740;Enzyme_code=EC:2.7.4.4,EC:2.7.4.8,EC:2.4.1.80,EC:2.7.4.4,EC:2.7.4.8,EC:2.4.1.80,EC:2.4,EC:2.7,EC:2,EC:2.7.4,EC:2.4.1;Enzyme_name=nucleoside-phosphate kinase,guanylate kinase,ceramide glucosyltransferase,nucleoside-phosphate kinase,guanylate kinase,ceramide glucosyltransferase,Glycosyltransferases,Transferring phosphorus-containing groups,Transferases,Transferring phosphorus-containing groups,Glycosyltransferases

contig00009.g5541	1631	ID=contig00009.g5541;Description=ceramide glucosyltransferase [Fusarium verticillioides 7600];Gene=FNAPI_5942;Ontology_term=lipid metabolic process,transferase activity;Ontology_id=GO:0006629,GO:0016740;Enzyme_code=EC:2.4.1.80;Enzyme_name=ceramide glucosyltransferase
contig00009.g5542	595	ID=contig00009.g5542;Description=hypothetical protein FVER53263_04578 [Fusarium verticillioides];Gene=FANTH_1724;Ontology_term=lyase activity;Ontology_id=GO:0016829;Enzyme_code=EC:4.4,EC:4.4,EC:4;Enzyme_name=Carbon-sulfur lyases,Carbon-sulfur lyases,Lyases
contig00009.g5543	1815	ID=contig00009.g5543;Description=related to tRNA-splicing endonuclease beta chain [Fusarium mangiferae];Gene=FDENT_7283;Ontology_term=nucleus,mRNA metabolic process,mitochondrion,tRNA metabolic process,catalytic activity, acting on RNA,hydrolase activity;Ontology_id=GO:0005634,GO:0016071,GO:0005739,GO:0006399,GO:0140098,GO:0016787;Enzyme_code=EC:3.1.31.1,EC:3.1.27,EC:4.6.1.16,EC:3.1.27.9,EC:3.1.31.1,EC:3.1,EC:3,EC:3.1.27,EC:4.6.1.16;Enzyme_name=micrococcal nuclease,Acting on ester bonds,tRNA-intron lyase,Acting on ester bonds,micrococcal nuclease,Acting on ester bonds,Hydrolases,Acting on ester bonds,tRNA-intron lyase
contig00009.g5544	1527	ID=contig00009.g5544;Description=gamma-butyrobetaine 2-oxoglutarate dioxygenase [Fusarium tjaetaba];Gene=FVER53590_25838;Ontology_term=nucleus,oxidoreductase activity,tRNA metabolic process,catalytic activity, acting on RNA,hydrolase activity;Ontology_id=GO:0005634,GO:0016491,GO:0006399,GO:0140098,GO:0016787;Enzyme_code=EC:3.1.31.1,EC:1,EC:3.1.27,EC:4.6.1.16,EC:3.1.31.1,EC:3.1,EC:1,EC:3,EC:3.1.27,EC:4.6.1.16;Enzyme_name=micrococcal nuclease,Oxidoreductases,Acting on ester bonds,tRNA-intron lyase,micrococcal nuclease,Acting on ester bonds,Oxidoreductases,Hydrolases,Acting on ester bonds,tRNA-intron lyase
contig00009.g5545	1459	ID=contig00009.g5545;Description=SGT1 [Fusarium tjaetaba];Gene=FDENT_7285;Ontology_term=nucleus,protein-containing complex assembly,signaling,protein folding,molecular adaptor activity;Ontology_id=GO:0005634,GO:0065003,GO:0023052,GO:0006457,GO:0060090
contig00009.g5546	2489	ID=contig00009.g5546;Description=lanosterol synthase [Fusarium verticillioides 7600];Gene=FPCIR_225;Ontology_term=lipid droplet,lipid metabolic process,isomerase activity;Ontology_id=GO:0005811,GO:0006629,GO:0016853;Enzyme_code=EC:5.4,EC:5.4.99.7,EC:5.4,EC:5;Enzyme_name=Intramolecular transferases,lanosterol synthase,Intramolecular transferases,Isomerases

contig00009.g5547	1524	ID=contig00009.g5547;Description=related to meiotic recombination protein rec12 [Fusarium fujikuroi];Gene=FOXG_07650;Ontology_term=DNA repair,DNA binding,catalytic activity, acting on DNA,ATP-dependent activity,DNA recombination,nuclear chromosome,chromosome segregation,hydrolase activity,meiotic nuclear division,isomerase activity;Ontology_id=GO:0006281,GO:0003677,GO:0140097,GO:0140657,GO:0006310,GO:0000228,GO:0007059,GO:0016787,GO:0140013,GO:0016853;Enzyme_code=EC:5.6.2.2,EC:3.6.1,EC:3.1.31.1,EC:3.1,EC:3.1.22,EC:3.6,EC:3,EC:5.6.2.2,EC:5,EC:3.6.1.15;Enzyme_name=DNA topoisomerase (ATP-hydrolyzing),Acting on acid anhydrides,micrococcal nuclease,Acting on ester bonds,Acting on ester bonds,Acting on acid anhydrides,Hydrolases,DNA topoisomerase (ATP-hydrolyzing),Isomerases,nucleoside-triphosphate phosphatase
contig00009.g5548	656	ID=contig00009.g5548;Description=ankyrin repeat-containing protein [Fusarium tjaetaba];Ontology_term=nucleocytoplasmic transport,nucleus,ribosome biogenesis,intracellular protein transport;Ontology_id=GO:0006913,GO:0005634,GO:0042254,GO:0006886
contig00009.g5549	6832	ID=contig00009.g5549;Description=urea carboxylase [Fusarium tjaetaba];Gene=FNAPI_5951;Ontology_term=nucleus,DNA binding,DNA-templated transcription,lyase activity,ligase activity,hydrolase activity;Ontology_id=GO:0005634,GO:0003677,GO:0006351,GO:0016829,GO:0016874,GO:0016787;Enzyme_code=EC:3,EC:4,EC:6;Enzyme_name=Hydrolases,Lyases,Ligases
contig00009.g5550	1707	ID=contig00009.g5550;Description=related to HOL1, putative substrate-H+ antiporter [Fusarium proliferatum ET1];Gene=MAG1;Ontology_term=DNA repair,transmembrane transport,catalytic activity, acting on DNA,structural molecule activity,transferase activity,ribosome,transporter activity,hydrolase activity;Ontology_id=GO:0006281,GO:0055085,GO:0140097,GO:0005198,GO:0016740,GO:0005840,GO:0005215,GO:0016787;Enzyme_code=EC:3.2.2.21,EC:2.1.1,EC:2.1,EC:3.2,EC:3.2.2.21,EC:2,EC:3,EC:2.1.1,EC:3.2.2;Enzyme_name=DNA-3-methyladenine glycosylase II,Transferring one-carbon groups,Transferring one-carbon groups,Glycosylases,DNA-3-methyladenine glycosylase II,Transferases,Hydrolases,Transferring one-carbon groups,Glycosylases
contig00009.g5551	843	ID=contig00009.g5551;Description=related to Lactam utilization protein lamB [Fusarium fujikuroi IMI 58289];Gene=FOYG_01757;Ontology_term=carbohydrate metabolic process;Ontology_id=GO:0005975

contig00009.g5552	837	ID=contig00009.g5552;Description=S-adenosyl-L-methionine-dependent methyltransferase [Fusarium oxysporum Fo47];Gene=FPANT_12710;Ontology_term=mitochondrion,transferase activity;Ontology_id=GO:0005739,GO:0016740;Enzyme_code=EC:2.1.1,EC:2.1,EC:2,EC:2.1.1,EC:2.1.1.114;Enzyme_name=Transferring one-carbon groups,Transferring one-carbon groups,Transferases,Transferring one-carbon groups,polyprenyldihydroxybenzoate methyltransferase
contig00009.g5553	270	ID=contig00009.g5553;Description=GPI anchored glycoprotein [Fusarium coicis];Gene=FTJAE_9830;Ontology_term=transferase activity;Ontology_id=GO:0016740;Enzyme_code=EC:2.1.1,EC:2.1,EC:2,EC:2.1.1;Enzyme_name=Transferring one-carbon groups,Transferring one-carbon groups,Transferases,Transferring one-carbon groups
contig00009.g5554	1010	ID=contig00009.g5554;Description=phenylcoumaran benzylic ether reductase [Fusarium tjaetaba];Gene=FOVG_07296;Ontology_term=oxidoreductase activity;Ontology_id=GO:0016491;Enzyme_code=EC:1,EC:1;Enzyme_name=Oxidoreductases,Oxidoreductases
contig00009.g5555	636	ID=contig00009.g5555;Description=hypothetical protein FVER14953_04565 [Fusarium verticillioides]
contig00009.g5556	2106	ID=contig00009.g5556;Description=TOB3 (member of AAA-ATPase family) [Fusarium tjaetaba];Gene=FPANT_12714;Ontology_term=ATP-dependent activity,hydrolase activity;Ontology_id=GO:0140657,GO:0016787;Enzyme_code=EC:3.6.1.15,EC:3.6.1,EC:3.6,EC:3,EC:3.6.1.15;Enzyme_name=nucleoside-triphosphate phosphatase,Acting on acid anhydrides,Acting on acid anhydrides,Hydrolases,nucleoside-triphosphate phosphatase
contig00009.g5557	810	ID=contig00009.g5557;Description=carboxyphosphonoenolpyruvate phosphonomutase [Fusarium tjaetaba];Gene=FPHYL_581;Ontology_term=catalytic activity;Ontology_id=GO:0003824
contig00009.g5558	867	ID=contig00009.g5558;Description=acetyltransferase [Fusarium tjaetaba];Gene=FPCIR_237;Ontology_term=transferase activity;Ontology_id=GO:0016740;Enzyme_code=EC:2.3.1,EC:2.3,EC:2,EC:2.3.1;Enzyme_name=Acyltransferases,Acyltransferases,Transferases,Acyltransferases

contig00009.g5559	1717	ID=contig00009.g5559;Description=phospholipase A2 [Fusarium tjaetaba];Gene=FNYG_01760;Ontology_term=lipid metabolic process,hydrolase activity;Ontology_id=GO:0006629,GO:0016787;Enzyme_code=EC:3.1.1.47,EC:3.1.1.47,EC:3.1,EC:3,EC:3.1.1;Enzyme_name=1-alkyl-2-acetylglycerophosphocholine esterase,1-alkyl-2-acetylglycerophosphocholine esterase,Acting on ester bonds,Hydrolases,Acting on ester bonds
contig00009.g5560	1667	ID=contig00009.g5560;Description=cocaine esterase [Fusarium pseudoanthophilum];Gene=FVEG_04559;Ontology_term=hydrolase activity;Ontology_id=GO:0016787;Enzyme_code=EC:3,EC:3,EC:3.1.1.56,EC:3.1.1.84,EC:3.1.1.1;Enzyme_name=Hydrolases,Hydrolases,methylumbelliferyl-acetate deacetylase,cocaine esterase,carboxylesterase
contig00009.g5561	2089	ID=contig00009.g5561;Description=triacylglycerol lipase V [Fusarium coicis];Gene=FMEXI_8443;Ontology_term=oxidoreductase activity;Ontology_id=GO:0016491;Enzyme_code=EC:1,EC:1;Enzyme_name=Oxidoreductases,Oxidoreductases
contig00009.g5562	1318	ID=contig00009.g5562;Description=CYB2-lactate dehydrogenase cytochrome b2 [Fusarium pseudoanthophilum];Gene=FOYG_01769;Ontology_term=oxidoreductase activity;Ontology_id=GO:0016491;Enzyme_code=EC:1,EC:1.1.2.3,EC:1;Enzyme_name=Oxidoreductases,L-lactate dehydrogenase (cytochrome),Oxidoreductases
contig00009.g5563	1374	ID=contig00009.g5563;Description=integral membrane protein [Fusarium tjaetaba];Gene=FTJAE_9819;Ontology_term=Golgi apparatus,transferase activity;Ontology_id=GO:0005794,GO:0016740;Enzyme_code=EC:2.4,EC:2.4,EC:2;Enzyme_name=Glycosyltransferases,Glycosyltransferases,Transferases
contig00009.g5564	1693	ID=contig00009.g5564;Description=major facilitator superfamily transporter [Fusarium tjaetaba];Gene=FVER53263_04557;Ontology_term=transmembrane transport,transporter activity;Ontology_id=GO:0055085,GO:0005215
contig00009.g5565	3908	ID=contig00009.g5565;Description=related to ankyrin [Fusarium fujikuroi];Gene=KRS1;Ontology_term=RNA binding,cytosol,tRNA metabolic process,amino acid metabolic process,catalytic activity, acting on RNA,ligase activity;Ontology_id=GO:0003723,GO:0005829,GO:0006399,GO:0006520,GO:0140098,GO:0016874;Enzyme_code=EC:6.1.1.6;Enzyme_name=lysine--tRNA ligase
contig00009.g5566	1863	ID=contig00009.g5566;Description=beta-lactamase 2 [Fusarium coicis]

contig00009.g5567	4213	ID=contig00009.g5567;Description=Cullin-associated NEDD8-dissociated 1 [Fusarium coicis];Gene=FDENT_7310;Ontology_term=protein-containing complex assembly;Ontology_id=GO:0065003
contig00009.g5568	1850	ID=contig00009.g5568;Description=hypothetical protein FVEG_04553 [Fusarium verticillioides 7600]
contig00009.g5569	1557	ID=contig00009.g5569;Description=hypothetical protein FVER14953_04552 [Fusarium verticillioides]
contig00009.g5570	360	ID=contig00009.g5570;Description=Apc13p protein-domain-containing protein [Fusarium oxysporum Fo47];Gene=apc13;Ontology_term=nucleus,protein catabolic process,mitotic nuclear division,chromosome segregation;Ontology_id=GO:0005634,GO:0030163,GO:0140014,GO:0007059
contig00009.g5571	716	ID=contig00009.g5571;Description=mitochondrial chaperone BCS1 [Fusarium mundagurra];Gene=FocTR4_00002621;Ontology_term=vesicle-mediated transport,endosome,ATP-dependent activity,mitochondrion,cytosol,Golgi apparatus,endoplasmic reticulum,intracellular protein transport,hydrolase activity;Ontology_id=GO:0016192,GO:0005768,GO:0140657,GO:0005739,GO:0005829,GO:0005794,GO:0005783,GO:0006886,GO:0016787;Enzyme_code=EC:3.6.1.15,EC:3.6.1,EC:3.6,EC:3,EC:3.6.1.15;Enzyme_name=nucleoside-triphosphate phosphatase,Acting on acid anhydrides,Acting on acid anhydrides,Hydrolases,nucleoside-triphosphate phosphatase
contig00009.g5572	2006	ID=contig00009.g5572;Description=related to human BCS1 protein [Fusarium fujikuroi];Gene=FPRO_05806;Ontology_term=ATP-dependent activity,mitochondrion,hydrolase activity;Ontology_id=GO:0140657,GO:0005739,GO:0016787;Enzyme_code=EC:3.6.1.15,EC:3.6.1,EC:3.6,EC:3,EC:3.6.1.15;Enzyme_name=nucleoside-triphosphate phosphatase,Acting on acid anhydrides,Acting on acid anhydrides,Hydrolases,nucleoside-triphosphate phosphatase

contig00009.g5573	1099	ID=contig00009.g5573;Description=related to PDR16 protein [Fusarium fujikuroi];Gene=LSDV138;Ontology_term=membrane organization,anatomical structure development,reproductive process,cellular modified amino acid metabolic process,transporter activity,plasma membrane,lipid droplet,vesicle-mediated transport,endosome,lipid metabolic process,cell differentiation,cytosol,protein localization to plasma membrane;Ontology_id=GO:0061024,GO:0048856,GO:0022414,GO:0006575,GO:0005215,GO:0005886,GO:0005811,GO:0016192,GO:0005768,GO:0006629,GO:0030154,GO:0005829,GO:0072659
contig00009.g5574	3774	ID=contig00009.g5574;Description=inositol polyphosphate 5-phosphatase [Fusarium tjaetaba];Gene=FTJAE_9808;Ontology_term=lipid metabolic process,hydrolase activity;Ontology_id=GO:0006629,GO:0016787;Enzyme_code=EC:3.1.3;Enzyme_name=Acting on ester bonds
contig00009.g5575	519	ID=contig00009.g5575;Description=hypothetical protein FANTH_1689 [Fusarium anthophilum];Gene=FVER53263_04546;Ontology_term=membrane;Ontology_id=GO:0016020
contig00009.g5576	1664	ID=contig00009.g5576;Description=hypothetical protein FVER14953_20172 [Fusarium verticillioides];Gene=FPANT_499;Ontology_term=intracellular calcium ion homeostasis;Ontology_id=GO:0006874
contig00009.g5577	2184	ID=contig00009.g5577;Description=phosphatase 2A regulatory B subunit-domain-containing protein [Fusarium tricinctum];Gene=FALBO_10247;Ontology_term=molecular function regulator activity,microtubule organizing center,cytoskeleton organization,signaling,mitotic nuclear division,chromosome segregation,nuclear chromosome,cytokinesis,meiotic nuclear division;Ontology_id=GO:0098772,GO:0005815,GO:0007010,GO:0023052,GO:0140014,GO:0007059,GO:0000228,GO:0000910,GO:0140013

contig00009.g5578	1909	ID=contig00009.g5578;Description=transcription factor tau subunit sfc1 [Fusarium tjaetaba];Gene=FFC1_06530;Ontology_term=DNA repair,catalytic activity, acting on a protein,protein-containing complex assembly,regulation of DNA-templated transcription,nucleoplasm,DNA binding,translation regulator activity,mRNA metabolic process,GTPase activity,autophagy,cytosol,transferase activity;Ontology_id=GO:0006281,GO:0140096,GO:0065003,GO:0006355,GO:0005654,GO:0003677,GO:0045182,GO:0016071,GO:0003924,GO:0006914,GO:0005829,GO:0016740;Enzyme_code=EC:2.7.11.23,EC:2.7.11.1,EC:2.7.11.22,EC:3.6.1.15,EC:2.7.1,EC:2.7.11.23,EC:3.6.1,EC:2.7.11.1,EC:2.7,EC:2,EC:3.6,EC:3,EC:2.7.11.22,EC:3.6.1.15;Enzyme_name=[RNA-polymerase]-subunit kinase,non-specific serine/threonine protein kinase,cyclin-dependent kinase,nucleoside-triphosphate phosphatase,Transferring phosphorus-containing groups,[RNA-polymerase]-subunit kinase,Acting on acid anhydrides,non-specific serine/threonine protein kinase,Transferring phosphorus-containing groups,Transferases,Acting on acid anhydrides,Hydrolases,cyclin-dependent kinase,nucleoside-triphosphate phosphatase
contig00009.g5579	1073	ID=contig00009.g5579;Description=Palmitoyltransferase PFA3 [Fusarium tjaetaba];Gene=FPANT_10897;Ontology_term=transferase activity;Ontology_id=GO:0016740;Enzyme_code=EC:2;Enzyme_name=Transferases
contig00009.g5580	1449	ID=contig00009.g5580;Description=palmitoyltransferase PFA3 [Fusarium verticillioides 7600];Gene=FVER53590_04540;Ontology_term=catalytic activity, acting on a protein,transferase activity;Ontology_id=GO:0140096,GO:0016740;Enzyme_code=EC:2.3.1.225,EC:2.3,EC:2,EC:2.3.1.225,EC:2.3.1;Enzyme_name=protein S-acyltransferase,Acyltransferases,Transferases,protein S-acyltransferase,Acyltransferases
contig00009.g5581	672	ID=contig00009.g5581;Description=metal dependent phosphohydrolase [Fusarium pseudoanthophilum];Gene=FACUT_718;Ontology_term=hydrolase activity;Ontology_id=GO:0016787;Enzyme_code=EC:3,EC:3;Enzyme_name=Hydrolases,Hydrolases
contig00009.g5582	3282	ID=contig00009.g5582;Description=phosphatidylinositol phospholipase C, gamma-1 [Fusarium verticillioides 7600];Gene=FVER53590_04539;Ontology_term=lipid metabolic process,signaling,hydrolase activity;Ontology_id=GO:0006629,GO:0023052,GO:0016787;Enzyme_code=EC:3.1.4.11,EC:3.1.4,EC:3.1,EC:3,EC:3.1.4.11;Enzyme_name=phosphoinositide phospholipase C,Acting on ester bonds,Acting on ester bonds,Hydrolases,phosphoinositide phospholipase C

contig00009.g5583	2766	ID=contig00009.g5583;Description=Centromere kinetochore zw10 like [Fusarium tjaetaba];Gene=FTJAE_9799;Ontology_term=nucleus,chromosome,mitotic cell cycle;Ontology_id=GO:0005634,GO:0005694,GO:0000278
contig00009.g5584	3248	ID=contig00009.g5584;Description=metalloprotease 1 [Fusarium tjaetaba];Gene=FVER53263_04536;Ontology_term=catalytic activity, acting on a protein,protein catabolic process,mitochondrion organization,hydrolase activity;Ontology_id=GO:0140096,GO:0030163,GO:0007005,GO:0016787;Enzyme_code=EC:3.4,EC:3.4.24,EC:3.4,EC:3;Enzyme_name=Acting on peptide bonds (peptidases),Acting on peptide bonds (peptidases),Acting on peptide bonds (peptidases),Hydrolases
contig00009.g5585	1515	ID=contig00009.g5585;Description=STE24 endopeptidase [Fusarium verticillioides 7600];Gene=FVER53263_04535;Ontology_term=nuclear envelope,catalytic activity, acting on a protein,mitochondrion,reproductive process,endoplasmic reticulum,protein maturation,hydrolase activity;Ontology_id=GO:0005635,GO:0140096,GO:0005739,GO:0022414,GO:0005783,GO:0051604,GO:0016787;Enzyme_code=EC:3.4.24,EC:3.4.24,EC:3.4.24.84,EC:3.4,EC:3;Enzyme_name=Acting on peptide bonds (peptidases),Acting on peptide bonds (peptidases),Ste24 endopeptidase,Acting on peptide bonds (peptidases),Hydrolases
contig00009.g5586	1559	ID=contig00009.g5586;Description=muscle-derived [Fusarium agapanthi];Gene=FNYG_08641;Ontology_term=syntaxin binding;Ontology_id=GO:0019905
contig00009.g5587	3074	ID=contig00009.g5587;Description=mitochondrial presequence protease [Fusarium verticillioides 7600];Gene=FMUND_972;Ontology_term=catalytic activity, acting on a protein,protein catabolic process,mitochondrion,protein maturation,intracellular protein transport,mitochondrion organization,hydrolase activity;Ontology_id=GO:0140096,GO:0030163,GO:0005739,GO:0051604,GO:0006886,GO:0007005,GO:0016787;Enzyme_code=EC:3.4.24,EC:3.4.24,EC:3.4,EC:3;Enzyme_name=Acting on peptide bonds (peptidases),Acting on peptide bonds (peptidases),Acting on peptide bonds (peptidases),Hydrolases
contig00009.g5588	1398	ID=contig00009.g5588;Description=ornithine decarboxylase [Fusarium verticillioides 7600];Gene=FPANT_10888;Ontology_term=lyase activity,cellular modified amino acid metabolic process,amino acid metabolic process,vitamin metabolic process;Ontology_id=GO:0016829,GO:0006575,GO:0006520,GO:0006766;Enzyme_code=EC:4.1.1.17,EC:4.1.1,EC:4.1,EC:4;Enzyme_name=ornithine decarboxylase,Carbon-carbon lyases,Carbon-carbon lyases,Lyases

contig00009.g5589	3327	ID=contig00009.g5589;Description=adenosinetriphosphatase [Fusarium verticillioides 7600];Gene=FPANT_10887;Ontology_term=nucleus,DNA binding,catalytic activity, acting on DNA,ATP-dependent activity,chromatin organization,hydrolase activity;Ontology_id=GO:0005634,GO:0003677,GO:0140097,GO:0140657,GO:0006325,GO:0016787;Enzyme_code=EC:3;Enzyme_name=Hydrolases
contig00009.g5590	1968	ID=contig00009.g5590;Description=nucleotide exsicion repair RAD7 [Fusarium tjaetaba];Ontology_term=DNA repair,nucleus;Ontology_id=GO:0006281,GO:0005634
contig00009.g5591	1136	ID=contig00009.g5591;Description=triacylglycerol lipase precursor [Fusarium tjaetaba];Gene=FVER53590_04529;Ontology_term=lipid metabolic process;Ontology_id=GO:0006629
contig00009.g5592	827	ID=contig00009.g5592;Description=ribosomal L34 mitochondrial [Fusarium tjaetaba];Gene=FACUT_707;Ontology_term=structural molecule activity,ribosome;Ontology_id=GO:0005198,GO:0005840
contig00009.g5593	2655	ID=contig00009.g5593;Description=dynamin-related 1D [Fusarium napiforme];Gene=FMAN_05526;Ontology_term=GTPase activity;Ontology_id=GO:0003924;Enzyme_code=EC:3.6.1.15,EC:3.6.1,EC:3.6,EC:3,EC:3.6.1.15;Enzyme_name=nucleoside-triphosphate phosphatase,Acting on acid anhydrides,Acting on acid anhydrides,Hydrolases,nucleoside-triphosphate phosphatase
contig00009.g5594	1482	ID=contig00009.g5594;Description=acid phosphatase [Fusarium verticillioides 7600];Gene=FTJAE_13699;Ontology_term=membrane;Ontology_id=GO:0016020
contig00009.g5595	775	ID=contig00009.g5595;Description=METHIONYL-TRNA SYNTHETASE mitochondrial [Fusarium napiforme];Gene=FNAPI_11247;Ontology_term=catalytic activity, acting on RNA,transferase activity,ligase activity;Ontology_id=GO:0140098,GO:0016740,GO:0016874;Enzyme_code=EC:6.1.1,EC:2.3.1;Enzyme_name=Forming carbon-oxygen bonds,Acyltransferases
contig00009.g5596	1818	ID=contig00009.g5596;Description=methionyl-tRNA synthetase [Fusarium verticillioides 7600];Gene=FVER53263_04524;Ontology_term=mitochondrial gene expression,mitochondrion,tRNA metabolic process,amino acid metabolic process,catalytic activity, acting on RNA,ligase activity;Ontology_id=GO:0140053,GO:0005739,GO:0006399,GO:0006520,GO:0140098,GO:0016874;Enzyme_code=EC:6.1.1.10,EC:6.1,EC:6.1.1.10,EC:6.1.1,EC:6;Enzyme_name=methionine--tRNA ligase,Forming carbon-oxygen bonds,methionine--tRNA ligase,Forming carbon-oxygen bonds,Ligases

contig00009.g5597	559	ID=contig00009.g5597;Description=related to nuclear transport factor 2 domain protein [Fusarium proliferatum ET1];Gene=FPANT_12426;Ontology_term=nucleocytoplasmic transport;Ontology_id=GO:0006913
contig00009.g5598	1638	ID=contig00009.g5598;Description=protein farnesyltransferase/geranylgeranyltransferase type I alpha subunit [Fusarium verticillioides 7600];Gene=FNYG_08653;Ontology_term=catalytic activity, acting on a protein,protein maturation,transferase activity,protein localization to plasma membrane;Ontology_id=GO:0140096,GO:0051604,GO:0016740,GO:0072659;Enzyme_code=EC:2.5.1,EC:2.5.1,EC:2.5.1.59,EC:2.5.1.58,EC:2,EC:2.5.1.60;Enzyme_name=Transferring alkyl or aryl groups, other than methyl groups,Transferring alkyl or aryl groups, other than methyl groups,protein geranylgeranyltransferase type I,protein farnesyltransferase,Transferases,protein geranylgeranyltransferase type II
contig00009.g5599	1580	ID=contig00009.g5599;Description=mevalonate kinase [Fusarium verticillioides 7600];Gene=FPANT_12428;Ontology_term=nucleobase-containing small molecule metabolic process,sulfur compound metabolic process,lipid metabolic process,carbohydrate derivative metabolic process,transferase activity;Ontology_id=GO:0055086,GO:0006790,GO:0006629,GO:1901135,GO:0016740;Enzyme_code=EC:2.7.1.36;Enzyme_name=mevalonate kinase
contig00009.g5600	630	ID=contig00009.g5600;Description=hypothetical protein FVER14953_04520 [Fusarium verticillioides]
contig00009.g5601	420	ID=contig00009.g5601;Description=60S ribosomal protein L38 [Fusarium musae];Gene=FMUND_987
contig00009.g5602	344	ID=contig00009.g5602;Description=STE24 endopeptidase [Fusarium coicis]
contig00009.g5603	1275	ID=contig00009.g5603;Description=hypothetical protein FVER14953_04517 [Fusarium verticillioides]
contig00009.g5604	1099	ID=contig00009.g5604;Description=related to FMN-dependent 2-nitropropane dioxygenase [Fusarium fujikuroi];Gene=FVEG_04516;Ontology_term=oxidoreductase activity;Ontology_id=GO:0016491;Enzyme_code=EC:1.13.12.16,EC:1.13,EC:1.13.12.16,EC:1,EC:1.13.12;Enzyme_name=nitronate monooxygenase,Acting on single donors with incorporation of molecular oxygen (oxygenases). The oxygen incorporated need not be derived from O2,nitronate monooxygenase,Oxidoreductases,Acting on single donors with incorporation of molecular oxygen (oxygenases). The oxygen incorporated need not be derived from O2
contig00009.g5605	2595	ID=contig00009.g5605;Description=2-nitropropane dioxygenase [Fusarium coicis]

contig00009.g5606	1506	ID=contig00009.g5606;Description=24-dehydrocholesterol reductase precursor [Fusarium tjaetaba];Gene=FVER53263_04514;Ontology_term=oxidoreductase activity;Ontology_id=GO:0016491;Enzyme_code=EC:1.3.1.72,EC:1.3.1.72,EC:1.3,EC:1,EC:1.3.1;Enzyme_name=Delta(24)-sterol reductase,Delta(24)-sterol reductase,Acting on the CH-CH group of donors,Oxidoreductases,Acting on the CH-CH group of donors
contig00009.g5607	484	ID=contig00009.g5607;Description=hypothetical protein BFJ68_g7412 [Fusarium oxysporum]
contig00009.g5608	286	ID=contig00009.g5608;Description=General transcription and DNA repair factor IIH subunit tcf-29 [Fusarium oxysporum f. sp. albedinis];Gene=FMEXI_10294;Ontology_term=membrane;Ontology_id=GO:0016020
contig00009.g5609	4088	ID=contig00009.g5609;Description=Zonadhesin [Fusarium oxysporum f. sp. conglutinans];Gene=FMUND_996;Ontology_term=membrane;Ontology_id=GO:0016020
contig00009.g5610	663	ID=contig00009.g5610;Description=Dibenzothiophene desulfurization enzyme C [Fusarium oxysporum f. sp. albedinis]
contig00009.g5611	1967	ID=contig00009.g5611;Description=related to nitrate assimilation regulatory protein nirA [Fusarium proliferatum ET1];Gene=FMAN_05506;Ontology_term=nucleus,DNA binding,regulation of DNA-templated transcription,transcription regulator activity;Ontology_id=GO:0005634,GO:0003677,GO:0006355,GO:0140110
contig00009.g5612	2352	ID=contig00009.g5612;Description=nitrate assimilation regulatory nira [Fusarium tjaetaba];Gene=FVER53590_04507;Ontology_term=nucleus,DNA binding,regulation of DNA-templated transcription,transcription regulator activity;Ontology_id=GO:0005634,GO:0003677,GO:0006355,GO:0140110
contig00009.g5613	2300	ID=contig00009.g5613;Description=poly [ADP-ribose] polymerase [Fusarium oxysporum NRRL 32931];Gene=FOYG_01837;Ontology_term=nucleus,transferase activity;Ontology_id=GO:0005634,GO:0016740;Enzyme_code=EC:2.4.2.30,EC:2.7.7,EC:2.4.2,EC:2.4,EC:2.7,EC:2,EC:2.4.2.30,EC:2.7.7;Enzyme_name=NAD(+) ADP-ribosyltransferase,Transferring phosphorus-containing groups,Glycosyltransferases,Glycosyltransferases,Transferring phosphorus-containing groups,Transferases,NAD(+) ADP-ribosyltransferase,Transferring phosphorus-containing groups
contig00009.g5614	1515	ID=contig00009.g5614;Description=hypothetical protein FVEG_04505 [Fusarium verticillioides 7600]
contig00009.g5615	5028	ID=contig00009.g5615;Description=hypothetical protein FVEG_04504 [Fusarium verticillioides 7600]

contig00009.g5616	1806	ID=contig00009.g5616;Description=bzip transcription factor [Fusarium phyllophilum];Gene=BFJ72_g5124;Ontology_term=nuclear chromosome,regulation of DNA-templated transcription,chromatin organization,transcription regulator activity;Ontology_id=GO:0000228,GO:0006355,GO:0006325,GO:0140110
contig00009.g5617	870	ID=contig00009.g5617;Description=conserved oligomeric golgi complex subunit 2 [Fusarium napiforme];Gene=FVER53263_04502;Ontology_term=Golgi apparatus;Ontology_id=GO:0005794
contig00009.g5618	958	ID=contig00009.g5618;Description=ribosomal L1 [Fusarium sp. NRRL 25303];Gene=FNAPI_8510;Ontology_term=mitochondrial gene expression,mitochondrion,structural molecule activity,lyase activity,ribosome,hydrolase activity;Ontology_id=GO:0140053,GO:0005739,GO:0005198,GO:0016829,GO:0005840,GO:0016787;Enzyme_code=EC:4.1.3.1,EC:3,EC:4.1.3.1,EC:4.1,EC:3,EC:4,EC:4.1.3;Enzyme_name=isocitrate lyase,Hydrolases,isocitrate lyase,Carbon-carbon lyases,Hydrolases,Lyases,Carbon-carbon lyases
contig00009.g5619	940	ID=contig00009.g5619;Description=acid phosphatase precursor [Fusarium fujikuroi];Gene=FFB14_15416;Ontology_term=hydrolase activity;Ontology_id=GO:0016787;Enzyme_code=EC:3.1.3,EC:3.1,EC:3,EC:3.1.3;Enzyme_name=Acting on ester bonds,Acting on ester bonds,Hydrolases,Acting on ester bonds
contig00009.g5620	2654	ID=contig00009.g5620;Description=hypothetical protein FVEG_04499 [Fusarium verticillioides 7600]
contig00009.g5621	429	ID=contig00009.g5621;Description=hypothetical protein FVEG_04498 [Fusarium verticillioides 7600]
contig00009.g5622	1392	ID=contig00009.g5622;Description=hypothetical protein FVER53263_04497 [Fusarium verticillioides]
contig00009.g5623	2238	ID=contig00009.g5623;Description=hypothetical protein FVER14953_04496 [Fusarium verticillioides]
contig00009.g5624	2518	ID=contig00009.g5624;Description=Fusaridione A cluster transcription factor fsdR [Fusarium oxysporum f. sp. albedinis]
contig00009.g5625	699	ID=contig00009.g5625;Description=hypothetical protein FVER53263_04494 [Fusarium verticillioides];Gene=2917;Ontology_term=plasmid partitioning;Ontology_id=GO:0030541
contig00009.g5626	849	ID=contig00009.g5626;Description=hypothetical protein FVER14953_04493 [Fusarium verticillioides]
contig00009.g5627	884	ID=contig00009.g5627;Description=hypothetical protein FVER53263_04492 [Fusarium verticillioides]

contig00009.g5628	1629	ID=contig00009.g5628;Description=hypothetical protein FVEG_15489 [Fusarium verticillioides 7600]
contig00009.g5629	492	ID=contig00009.g5629;Description=hypothetical protein FVER53590_04491 [Fusarium verticillioides]
contig00009.g5630	431	ID=contig00009.g5630;Description=hypothetical protein FVER53590_04490 [Fusarium verticillioides];Gene=FNYG_08693;Ontology_term=membrane;Ontology_id=GO:0016020
contig00009.g5631	1206	ID=contig00009.g5631;Description=hypothetical protein FVER53590_04490 [Fusarium verticillioides]
contig00009.g5632	3276	ID=contig00009.g5632;Description=hypothetical protein FVER53263_04488 [Fusarium verticillioides]
contig00009.g5633	819	ID=contig00009.g5633;Description=ribosomal S21 [Fusarium pseudoanthophilum];Gene=FNYG_08697;Ontology_term=mitochondrial gene expression,mitochondrion,structural molecule activity,ribosome;Ontology_id=GO:0140053,GO:0005739,GO:0005198,GO:0005840
contig00009.g5634	2400	ID=contig00009.g5634;Description=hypothetical protein BFJ72_g6845 [Fusarium proliferatum];Gene=FOXB_04514;Ontology_term=nucleus,mRNA metabolic process;Ontology_id=GO:0005634,GO:0016071
contig00009.g5635	825	ID=contig00009.g5635;Description=enoyl-CoA hydratase [Fusarium verticillioides 7600];Gene=CEK26_006862;Ontology_term=carbohydrate metabolic process,transmembrane transport,mitochondrion,regulation of DNA-templated transcription;Ontology_id=GO:0005975,GO:0055085,GO:0005739,GO:0006355
contig00009.g5636	1927	ID=contig00009.g5636;Description=short chain dehydrogenase reductase family [Fusarium tjaetaba]
contig00009.g5637	1245	ID=contig00009.g5637;Description=alcohol dehydrogenase [Fusarium verticillioides 7600];Ontology_term=nuclear envelope,oxidoreductase activity,endoplasmic reticulum;Ontology_id=GO:0005635,GO:0016491,GO:0005783;Enzyme_code=EC:1.17.1,EC:1.17,EC:1;Enzyme_name=Acting on CH or CH2 groups,Acting on CH or CH2 groups,Oxidoreductases
contig00009.g5638	1994	ID=contig00009.g5638;Description=cercosporin resistance [Fusarium acutatum];Gene=BFJ65_g3964;Ontology_term=nucleus,regulation of DNA-templated transcription,transcription regulator activity;Ontology_id=GO:0005634,GO:0006355,GO:0140110
contig00009.g5639	1888	ID=contig00009.g5639;Description=hypothetical protein FVER53590_29576 [Fusarium verticillioides];Gene=FPCIR_10393;Ontology_term=membrane;Ontology_id=GO:0016020

contig00009.g5640	2785	ID=contig00009.g5640;Description=serine/threonine protein kinase [Fusarium verticillioides 7600];Gene=FNAPI_8402;Ontology_term=catalytic activity, acting on a protein,transferase activity;Ontology_id=GO:0140096,GO:0016740;Enzyme_code=EC:2.7.11.1,EC:2.7.1,EC:2.7.11.1,EC:2.7,EC:2;Enzyme_name=non-specific serine/threonine protein kinase,Transferring phosphorus-containing groups,non-specific serine/threonine protein kinase,Transferring phosphorus-containing groups,Transferases
contig00009.g5641	1124	ID=contig00009.g5641;Description=related to ras-related GTPase rag [Fusarium fujikuroi];Gene=FNAPI_8401;Ontology_term=GTPase activity,signaling,vacuole;Ontology_id=GO:0003924,GO:0023052,GO:0005773;Enzyme_code=EC:3.6.1.15;Enzyme_name=nucleoside-triphosphate phosphatase
contig00009.g5642	1323	ID=contig00009.g5642;Description=SEP domain-containing protein [Fusarium pseudoanthophilum];Ontology_term=membrane organization,nucleus,carbohydrate metabolic process,generation of precursor metabolites and energy,molecular function regulator activity,autophagy,protein catabolic process,cell differentiation,anatomical structure development,reproductive process,chromosome segregation,cytosol;Ontology_id=GO:0061024,GO:0005634,GO:0005975,GO:0006091,GO:0098772,GO:0006914,GO:0030163,GO:0030154,GO:0048856,GO:0022414,GO:0007059,GO:0005829
contig00009.g5643	2395	ID=contig00009.g5643;Description=preprotein translocase subunit YidC [Fusarium verticillioides 7600];Gene=FNAPI_8399;Ontology_term=membrane organization,transmembrane transport,protein-containing complex assembly,mitochondrion,molecular carrier activity,intracellular protein transport,mitochondrion organization,transporter activity;Ontology_id=GO:0061024,GO:0055085,GO:0065003,GO:0005739,GO:0140104,GO:0006886,GO:0007005,GO:0005215
contig00009.g5644	1782	ID=contig00009.g5644;Description=urg3 [Fusarium mundagurra]
contig00009.g5645	2483	ID=contig00009.g5645;Description=related to endocytosis ankyrin repeat protein Nuc-2 [Fusarium fujikuroi];Gene=RAG3;Ontology_term=nucleus,RNA binding,mRNA metabolic process,protein-containing complex assembly,ATP-dependent activity,cytoplasmic translation,catalytic activity, acting on RNA,hydrolase activity;Ontology_id=GO:0005634,GO:0003723,GO:0016071,GO:0065003,GO:0140657,GO:0002181,GO:0140098,GO:0016787;Enzyme_code=EC:3.6.4.13,EC:3.6.4.13,EC:3;Enzyme_name=RNA helicase,RNA helicase,Hydrolases

contig00009.g5646	1590	ID=contig00009.g5646;Description=HNM1-choline permease [Fusarium tjaetaba];Gene=FANTH_10369;Ontology_term=transmembrane transport,transporter activity;Ontology_id=GO:0055085,GO:0005215
contig00009.g5647	466	ID=contig00009.g5647;Description=hypothetical protein FVEG_15485 [Fusarium verticillioides 7600]
contig00009.g5648	1303	ID=contig00009.g5648;Description=DEAD/DEAH box helicase [Colletotrichum higginsianum IMI 349063];Gene=FOTG_02648;Ontology_term=translation regulator activity,ATP-dependent activity,catalytic activity, acting on RNA,hydrolase activity;Ontology_id=GO:0045182,GO:0140657,GO:0140098,GO:0016787;Enzyme_code=EC:3.6.4.13;Enzyme_name=RNA helicase
contig00009.g5649	1551	ID=contig00009.g5649;Description=RNA splicing factor PRP9 [Fusarium pseudocircinatum];Gene=FMUND_1513;Ontology_term=nucleus,mRNA metabolic process,RNA binding,protein-containing complex assembly;Ontology_id=GO:0005634,GO:0016071,GO:0003723,GO:0065003;Enzyme_code=EC:6.5.1.1;Enzyme_name=DNA ligase (ATP)
contig00009.g5650	1011	ID=contig00009.g5650;Description=Structure-specific endonuclease subunit SLX1 [Fusarium oxysporum f. sp. conglutinans];Gene=FPANT_4319;Ontology_term=DNA repair,catalytic activity, acting on DNA,DNA replication,DNA recombination,nuclear chromosome,nucleolus,hydrolase activity;Ontology_id=GO:0006281,GO:0140097,GO:0006260,GO:0006310,GO:0000228,GO:0005730,GO:0016787;Enzyme_code=EC:3.1.30,EC:3.1.21,EC:3.1.30,EC:3.1.21,EC:3.1,EC:3;Enzyme_name=Acting on ester bonds,Acting on ester bonds,Acting on ester bonds,Acting on ester bonds,Acting on ester bonds,Hydrolases

		ID=contig00009.g5651;Description=histone demethylase JARID1 [Fusarium tjaetaba];Gene=FPANT_4318;Ontology_term=DNA binding,catalytic activity, acting on a protein,oxidoreductase activity,regulation of DNA-templated transcription,nuclear chromosome,chromatin organization,transferase activity;Ontology_id=GO:0003677,GO:0140096,GO:0016491,GO:0006355,GO:0000228,GO:0006325,GO:0016740;Enzyme_code=EC:2.1.1,EC:2.1,EC:1.14.11,EC:2.3,EC:1,EC:2,EC:1.14,EC:2.1.1,EC:1.14.11.67,EC:2.3.2;Enzyme_name=Transferring one-carbon groups,Transferring one-carbon groups,Acting on paired donors, with incorporation or reduction of molecular oxygen. The oxygen incorporated need not be derived from O2,Acyltransferases,Oxidoreductases,Transferases,Acting on paired donors, with incorporation or reduction of molecular oxygen. The oxygen incorporated need not be derived from O2,Transferring one-carbon groups,[histone H3]-trimethyl-L-lysine(4) demethylase,Acyltransferases
contig00009.g5651	5555	
contig00009.g5652	1125	ID=contig00009.g5652;Description=GTP cyclohydrolase I [Fusarium verticillioides 7600];Gene=FNAPI_8390;Ontology_term=nucleus,cellular modified amino acid metabolic process,vitamin metabolic process,hydrolase activity;Ontology_id=GO:0005634,GO:0006575,GO:0006766,GO:0016787;Enzyme_code=EC:3.5.4.16,EC:3.5.4,EC:3.5.4.16,EC:3.5,EC:3;Enzyme_name=GTP cyclohydrolase I,Acting on carbon-nitrogen bonds, other than peptide bonds,GTP cyclohydrolase I,Acting on carbon-nitrogen bonds, other than peptide bonds,Hydrolases
contig00009.g5653	1189	ID=contig00009.g5653;Description=related to transcription initiation factor IID beta chain [Fusarium fujikuroi IMI 58289];Gene=BFJ69_g7124;Ontology_term=translation regulator activity,protein-containing complex assembly,DNA-templated transcription,nucleoplasm;Ontology_id=GO:0045182,GO:0065003,GO:0006351,GO:0005654
contig00009.g5654	962	ID=contig00009.g5654;Description=bzip transcription factor [Fusarium beomiforme]
contig00009.g5655	2208	ID=contig00009.g5655;Description=hypothetical protein FVEG_04464 [Fusarium verticillioides 7600];Gene=HEM3;Ontology_term=transferase activity;Ontology_id=GO:0016740;Enzyme_code=EC:2,EC:2;Enzyme_name=Transferases,Transferases
contig00009.g5656	621	ID=contig00009.g5656;Description=hypothetical protein FVEG_04463 [Fusarium verticillioides 7600]
contig00009.g5657	1140	ID=contig00009.g5657;Description=hypothetical protein FVEG_04462 [Fusarium verticillioides 7600]

contig00009.g5658	402	ID=contig00009.g5658;Description=hypothetical protein FVER53263_20352 [Fusarium verticillioides];Gene=FTJAE_10750;Ontology_term=membrane;Ontology_id=GO:0016020
contig00009.g5659	1026	ID=contig00009.g5659;Description=zinc-binding alcohol dehydrogenase domain-containing protein [Fusarium denticulatum];Gene=FMUND_1524;Ontology_term=oxidoreductase activity;Ontology_id=GO:0016491;Enzyme_code=EC:1.6,EC:1.6,EC:1;Enzyme_name=Acting on NADH or NADPH,Acting on NADH or NADPH,Oxidoreductases
contig00009.g5660	800	ID=contig00009.g5660;Description=related to endoglucanase I precursor [Fusarium proliferatum ET1];Gene=FPANT_4308;Ontology_term=carbohydrate metabolic process,generation of precursor metabolites and energy,nucleobase-containing small molecule metabolic process,carbohydrate derivative metabolic process,transferase activity,hydrolase activity;Ontology_id=GO:0005975,GO:0006091,GO:0055086,GO:1901135,GO:0016740,GO:0016787;Enzyme_code=EC:2.7.1.11,EC:3.2.1.4,EC:2.7.1,EC:3.2,EC:2.7,EC:2,EC:3,EC:3.2.1.151,EC:2.7.1.11,EC:3.2.1,EC:3.2.1.4;Enzyme_name=6-phosphofructokinase,cellulase,Transferring phosphorus-containing groups,Glycosylases,Transferring phosphorus-containing groups,Transferases,Hydrolases,xyloglucan-specific endo-beta-1,4-glucanase,6-phosphofructokinase,Glycosylases,cellulase
contig00009.g5661	1589	ID=contig00009.g5661;Description=heterokaryon incompatibility protein-domain-containing protein [Fusarium oxysporum]
contig00009.g5662	1761	ID=contig00009.g5662;Description=MFS transporter, SIT family, siderophore-iron:H+ symporter [Fusarium verticillioides 7600];Gene=FPCIR_10419;Ontology_term=nucleus,transmembrane transport,endosome,vacuole,transporter activity,plasma membrane;Ontology_id=GO:0005634,GO:0055085,GO:0005768,GO:0005773,GO:0005215,GO:0005886
contig00009.g5663	1581	ID=contig00009.g5663;Description=probable chitin binding protein [Fusarium fujikuroi];Gene=FPRO05_07214;Ontology_term=carbohydrate metabolic process,hydrolase activity;Ontology_id=GO:0005975,GO:0016787;Enzyme_code=EC:3.5,EC:3.5.1.41,EC:3.5,EC:3;Enzyme_name=Acting on carbon-nitrogen bonds, other than peptide bonds,chitin deacetylase,Acting on carbon-nitrogen bonds, other than peptide bonds,Hydrolases
contig00009.g5664	633	ID=contig00009.g5664;Description=infection structure specific [Fusarium pseudoanthophilum]
contig00009.g5665	508	ID=contig00009.g5665;Description=TM2 domain protein [Fusarium tjaetaba];Gene=FTJAE_14068;Ontology_term=membrane;Ontology_id=GO:0016020

contig00009.g5666	2788	ID=contig00009.g5666;Description=coatomer WD associated region-domain-containing protein [Fusarium solani];Gene=NECHADRAFT_101743;Ontology_term=vesicle-mediated transport,structural molecule activity,cytoplasmic vesicle,Golgi apparatus,intracellular protein transport;Ontology_id=GO:0016192,GO:0005198,GO:0031410,GO:0005794,GO:0006886
contig00009.g5667	2049	ID=contig00009.g5667;Description=Serine/threonine-protein kinase [Fusarium oxysporum];Gene=FGLOB1_11400;Ontology_term=catalytic activity, acting on a protein,signaling,regulation of DNA-templated transcription,chromosome,transferase activity;Ontology_id=GO:0140096,GO:0023052,GO:0006355,GO:0005694,GO:0016740;Enzyme_code=EC:2.7.11.1;Enzyme_name=non-specific serine/threonine protein kinase
contig00009.g5668	1795	ID=contig00009.g5668;Description=Phosphoglucose isomerase [Fusarium oxysporum];Gene=FNAPI_13669;Ontology_term=carbohydrate metabolic process,generation of precursor metabolites and energy,nucleobase-containing small molecule metabolic process,mitochondrion,carbohydrate derivative metabolic process,cytosol,extracellular matrix,extracellular region,plasma membrane,isomerase activity;Ontology_id=GO:0005975,GO:0006091,GO:0055086,GO:0005739,GO:1901135,GO:0005829,GO:0031012,GO:0005576,GO:0005886,GO:0016853;Enzyme_code=EC:5.3.1.9,EC:5.3,EC:5,EC:5.3.1.9,EC:5.3.1;Enzyme_name=glucose-6-phosphate isomerase,Intramolecular oxidoreductases,Isomerases,glucose-6-phosphate isomerase,Intramolecular oxidoreductases
contig00009.g5669	1225	ID=contig00009.g5669;Description=mannitol-1-phosphate dehydrogenase [Fusarium proliferatum];Gene=FMAN_05437;Ontology_term=carbohydrate metabolic process,oxidoreductase activity;Ontology_id=GO:0005975,GO:0016491;Enzyme_code=EC:1.1.1.17;Enzyme_name=mannitol-1-phosphate 5-dehydrogenase
contig00009.g5670	1398	ID=contig00009.g5670;Description=hypothetical protein FVER53590_04450 [Fusarium verticillioides]
contig00009.g5671	333	ID=contig00009.g5671;Description=hypothetical protein FVEG_04449 [Fusarium verticillioides 7600]
contig00009.g5672	1544	ID=contig00009.g5672;Description=hypothetical protein FNYG_08742 [Fusarium nygamai];Gene=HTA2;Ontology_term=nucleus,DNA binding,structural molecule activity,chromosome;Ontology_id=GO:0005634,GO:0003677,GO:0005198,GO:0005694

contig00009.g5673	1670	ID=contig00009.g5673;Description=sugar transporter stl1 [Fusarium tjaetaba];Gene=FOYG_01912;Ontology_term=transmembrane transport,transporter activity;Ontology_id=GO:0055085,GO:0005215
contig00009.g5674	1169	ID=contig00009.g5674;Description=DUF4419 domain-containing protein [Fusarium coicis]
contig00009.g5675	3414	ID=contig00009.g5675;Description=WD repeat-containing protein slp1 [Fusarium oxysporum f. sp. albedinis];Gene=FVEG_15480;Ontology_term=catalytic activity, acting on a protein,hydrolase activity;Ontology_id=GO:0140096,GO:0016787;Enzyme_code=EC:3.4.21,EC:3.4.21,EC:3.4,EC:3;Enzyme_name=Acting on peptide bonds (peptidases),Acting on peptide bonds (peptidases),Acting on peptide bonds (peptidases),Hydrolases
contig00009.g5676	973	ID=contig00009.g5676;Description=hypothetical protein FVER14953_04443 [Fusarium verticillioides];Gene=FVER53263_04442;Ontology_term=zinc ion binding;Ontology_id=GO:0008270
contig00009.g5677	4308	ID=contig00009.g5677;Description=hypothetical protein FVEG_15479 [Fusarium verticillioides 7600];Gene=FVER53590_04442;Ontology_term=zinc ion binding,cytoplasm;Ontology_id=GO:0008270,GO:0005737
contig00009.g5678	1248	ID=contig00009.g5678;Description=hypothetical protein FVER14953_04440 [Fusarium verticillioides]
contig00009.g5679	771	ID=contig00009.g5679;Description=hypothetical protein FVER53263_04439 [Fusarium verticillioides]
contig00009.g5680	1303	ID=contig00009.g5680;Description=hypothetical protein FPANT_6849 [Fusarium pseudoanthophilum];Gene=FVEG_04438;Ontology_term=transferase activity;Ontology_id=GO:0016740;Enzyme_code=EC:2.1.1,EC:2.1,EC:2,EC:2.1.1;Enzyme_name=Transferring one-carbon groups,Transferring one-carbon groups,Transferases,Transferring one-carbon groups
contig00009.g5681	704	ID=contig00009.g5681;Description=hypothetical protein FVEG_04437 [Fusarium verticillioides 7600]
contig00009.g5682	416	ID=contig00009.g5682;Description=hypothetical protein FVEG_04436 [Fusarium verticillioides 7600]
contig00009.g5683	1155	ID=contig00009.g5683;Description=SAM-dependent methyltransferase [Fusarium tjaetaba];Gene=FTJAE_13375;Ontology_term=transferase activity;Ontology_id=GO:0016740;Enzyme_code=EC:2.1.1,EC:2.1,EC:2,EC:2.1.1;Enzyme_name=Transferring one-carbon groups,Transferring one-carbon groups,Transferases,Transferring one-carbon groups

contig00009.g5684	1523	ID=contig00009.g5684;Description=hypothetical protein J7337_006471 [Fusarium musae]
contig00009.g5685	2049	ID=contig00009.g5685;Description=related to peptidase yuxL [Fusarium fujikuroi];Gene=FPCIR_10441;Ontology_term=catalytic activity, acting on a protein,hydrolase activity;Ontology_id=GO:0140096,GO:0016787;Enzyme_code=EC:3.4,EC:3.4,EC:3;Enzyme_name=Acting on peptide bonds (peptidases),Acting on peptide bonds (peptidases),Hydrolases
contig00009.g5686	914	ID=contig00009.g5686;Description=hypothetical protein FVER53590_04431 [Fusarium verticillioides]
contig00009.g5687	2493	ID=contig00009.g5687;Description=hypothetical protein FVEG_15478 [Fusarium verticillioides 7600];Gene=FFUJ_12977;Ontology_term=catalytic activity, acting on a protein,hydrolase activity;Ontology_id=GO:0140096,GO:0016787;Enzyme_code=EC:3.4.21,EC:3.4.21,EC:3.4,EC:3;Enzyme_name=Acting on peptide bonds (peptidases),Acting on peptide bonds (peptidases),Acting on peptide bonds (peptidases),Hydrolases
contig00009.g5688	4530	ID=contig00009.g5688;Description=Vault poly [Fusarium agapanthi]
contig00009.g5689	1650	ID=contig00009.g5689;Description=nicotinamide mononucleotide permease [Fusarium tjaetaba];Gene=FTJAE_13379;Ontology_term=transmembrane transport,transporter activity;Ontology_id=GO:0055085,GO:0005215
contig00009.g5690	1788	ID=contig00009.g5690;Description=hypothetical protein FVEG_04426 [Fusarium verticillioides 7600];Gene=FocTR4_00002481
contig00009.g5691	1063	ID=contig00009.g5691;Description=kelch repeat-containing protein [Fusarium tjaetaba]
contig00009.g5692	1855	ID=contig00009.g5692;Description=transcriptional regulatory [Fusarium tjaetaba];Gene=FNAPI_9825;Ontology_term=nucleus,DNA binding,regulation of DNA-templated transcription,transcription regulator activity;Ontology_id=GO:0005634,GO:0003677,GO:0006355,GO:0140110
contig00009.g5693	903	ID=contig00009.g5693;Description=L-fuculose-phosphate aldolase [Fusarium verticillioides 7600];Gene=FTJAE_13376;Ontology_term=nucleus,DNA binding,regulation of DNA-templated transcription,transcription regulator activity;Ontology_id=GO:0005634,GO:0003677,GO:0006355,GO:0140110
contig00009.g5694	1398	ID=contig00009.g5694;Description=capsule polysaccharide synthase Cps1 [Fusarium pseudoanthophilum];Gene=FNAPI_9824;Ontology_term=membrane;Ontology_id=GO:0016020

contig00009.g5695	1811	ID=contig00009.g5695;Description=family 43 glycoside hydrolase [Ilyonectria robusta];Gene=BKA56DRAFT_543381;Ontology_term=carbohydrate metabolic process,hydrolase activity;Ontology_id=GO:0005975,GO:0016787;Enzyme_code=EC:3.2.1,EC:3.2,EC:3,EC:3.2.1;Enzyme_name=Glycosylases,Glycosylases,Hydrolases,Glycosylases
contig00009.g5696	2138	ID=contig00009.g5696;Description=hypothetical protein FVER53590_29044 [Fusarium verticillioides];Gene=FVER53590_29044;Ontology_term=nucleus,DNA binding,regulation of DNA-templated transcription,transcription regulator activity;Ontology_id=GO:0005634,GO:0003677,GO:0006355,GO:0140110
contig00009.g5697	669	ID=contig00009.g5697;Description=glutathione S-transferase [Fusarium oxysporum Fo47];Gene=FVEG_04419;Ontology_term=transferase activity;Ontology_id=GO:0016740;Enzyme_code=EC:2,EC:2.5.1.18,EC:2;Enzyme_name=Transferases,glutathione transferase,Transferases
contig00009.g5698	816	ID=contig00009.g5698;Description=hypothetical protein FVER53590_04418 [Fusarium verticillioides]
contig00009.g5699	1655	ID=contig00009.g5699;Description=3-hydroxyphenylacetate 6-hydroxylase [Fusarium verticillioides 7600];Gene=FVEG_04417;Ontology_term=oxidoreductase activity;Ontology_id=GO:0016491;Enzyme_code=EC:1.14,EC:1,EC:1.14,EC:1.14.13.63;Enzyme_name=Acting on paired donors, with incorporation or reduction of molecular oxygen. The oxygen incorporated need not be derived from O2,Oxidoreductases,Acting on paired donors, with incorporation or reduction of molecular oxygen. The oxygen incorporated need not be derived from O2,3-hydroxyphenylacetate 6-hydroxylase
contig00009.g5700	2348	ID=contig00009.g5700;Description=activator of stress proteins 1 [Fusarium bulbicola];Gene=FVEG_04416;Ontology_term=nucleus,DNA binding,regulation of DNA-templated transcription,transcription regulator activity;Ontology_id=GO:0005634,GO:0003677,GO:0006355,GO:0140110
contig00009.g5701	372	ID=contig00009.g5701;Description=stress responsive A B barrel domain-containing protein [Fusarium phyllophilum]
contig00009.g5702	1339	ID=contig00009.g5702;Description=probable fumarylacetoacetate hydrolase [Fusarium fujikuroi];Gene=10;Ontology_term=amino acid metabolic process,hydrolase activity;Ontology_id=GO:0006520,GO:0016787;Enzyme_code=EC:3.7.1.2,EC:3.7.1,EC:3.7.1.2,EC:3,EC:3.7;Enzyme_name=fumarylacetoacetase,Acting on carbon-carbon bonds,fumarylacetoacetase,Hydrolases,Acting on carbon-carbon bonds

contig00009.g5703	3014	ID=contig00009.g5703;Description=homogentisate 1,2-dioxygenase [Fusarium proliferatum];Gene=F25303_2465;Ontology_term=oxidoreductase activity,amino acid metabolic process;Ontology_id=GO:0016491,GO:0006520;Enzyme_code=EC:1.13.11.5;Enzyme_name=homogentisate 1,2-dioxygenase
contig00009.g5704	1260	ID=contig00009.g5704;Description=hypothetical protein FOCG_15260 [Fusarium oxysporum f. sp. radicis-lycopersici 26381]
contig00009.g5705	1297	ID=contig00009.g5705;Description=CMGC protein kinase [Fusarium oxysporum NRRL 32931];Gene=FOYG_01947;Ontology_term=catalytic activity, acting on a protein,transferase activity;Ontology_id=GO:0140096,GO:0016740;Enzyme_code=EC:2.7.1,EC:2.7.1,EC:2.7,EC:2;Enzyme_name=Transferring phosphorus-containing groups,Transferring phosphorus-containing groups,Transferring phosphorus-containing groups,Transferases
contig00009.g5706	1599	ID=contig00009.g5706;Description=amidohydrolase ytcJ [Fusarium subglutinans];Gene=FSUBG_11379;Ontology_term=hydrolase activity;Ontology_id=GO:0016787;Enzyme_code=EC:3.5,EC:3.5,EC:3;Enzyme_name=Acting on carbon-nitrogen bonds, other than peptide bonds,Acting on carbon-nitrogen bonds, other than peptide bonds,Hydrolases
contig00009.g5707	2863	ID=contig00009.g5707;Description=amidohydrolase family-domain-containing protein [Fusarium sp. MPI-SDFR-AT-0072];Gene=FVER53590_29042;Ontology_term=nucleus,transmembrane transport,regulation of DNA-templated transcription,transcription regulator activity,transporter activity,hydrolase activity;Ontology_id=GO:0005634,GO:0055085,GO:0006355,GO:0140110,GO:0005215,GO:0016787;Enzyme_code=EC:3.5,EC:3.5,EC:3;Enzyme_name=Acting on carbon-nitrogen bonds, other than peptide bonds,Acting on carbon-nitrogen bonds, other than peptide bonds,Hydrolases
contig00009.g5708	2344	ID=contig00009.g5708;Description=major facilitator superfamily domain-containing protein [Fusarium oxysporum Fo47];Gene=FPCIR_6050;Ontology_term=transmembrane transport,transporter activity;Ontology_id=GO:0055085,GO:0005215
contig00009.g5709	1129	ID=contig00009.g5709;Description=related to 1-aminocyclopropane-1-carboxylate synthase, and related proteins [Fusarium mangiferae];Gene=FVEG_04406;Ontology_term=catalytic activity;Ontology_id=GO:0003824

contig00009.g5710	1551	ID=contig00009.g5710;Description=siderophore iron transporter-like protein mirB [Fusarium oxysporum Fo47];Gene=FVEG_04405;Ontology_term=transmembrane transport,transporter activity;Ontology_id=GO:0055085,GO:0005215
contig00009.g5711	2854	ID=contig00009.g5711;Description=Zn(2)-C6 fungal-type DNA-binding domain-containing protein [Fusarium denticulatum];Gene=FHETE_2322;Ontology_term=nucleus,DNA binding,regulation of DNA-templated transcription,transcription regulator activity;Ontology_id=GO:0005634,GO:0003677,GO:0006355,GO:0140110
contig00009.g5712	2447	ID=contig00009.g5712;Description=Mx2 (GTPase) [Fusarium phyllophilum];Gene=FVER53263_04403;Ontology_term=GTPase activity;Ontology_id=GO:0003924;Enzyme_code=EC:3.6.1.15,EC:3.6.1,EC:3.6,EC:3,EC:3.6.1.15;Enzyme_name=nucleoside-triphosphate phosphatase,Acting on acid anhydrides,Acting on acid anhydrides,Hydrolases,nucleoside-triphosphate phosphatase
contig00009.g5713	1464	ID=contig00009.g5713;Description=hypothetical protein FVEG_15473 [Fusarium verticillioides 7600];Gene=gB
contig00009.g5714	1869	ID=contig00009.g5714;Description=SERINE-TYPE CARBOXYPEPTIDASE F PRECURSOR [Fusarium tjaetaba];Gene=FPHYL_12921;Ontology_term=catalytic activity, acting on a protein,hydrolase activity;Ontology_id=GO:0140096,GO:0016787;Enzyme_code=EC:3.4.16,EC:3.4,EC:3.4.16,EC:3,EC:3.4.16.6;Enzyme_name=Acting on peptide bonds (peptidases),Acting on peptide bonds (peptidases),Acting on peptide bonds (peptidases),Hydrolases,carboxypeptidase D
contig00009.g5715	783	ID=contig00009.g5715;Description=ubiquitin carboxyl-terminal hydrolase family 1 [Fusarium phyllophilum];Gene=FPANT_13229;Ontology_term=catalytic activity, acting on a protein,protein catabolic process,hydrolase activity;Ontology_id=GO:0140096,GO:0030163,GO:0016787;Enzyme_code=EC:3.4.19.12,EC:3.4,EC:3,EC:3.4.19.12;Enzyme_name=ubiquitinyl hydrolase 1,Acting on peptide bonds (peptidases),Hydrolases,ubiquitinyl hydrolase 1
contig00009.g5716	1343	ID=contig00009.g5716;Description=O-methyltransferase [Fusarium oxysporum f. sp. cubense];Gene=Focb16_v006406;Ontology_term=transferase activity;Ontology_id=GO:0016740;Enzyme_code=EC:2.1.1,EC:2.1,EC:2,EC:2.1.1;Enzyme_name=Transferring one-carbon groups,Transferring one-carbon groups,Transferases,Transferring one-carbon groups
contig00009.g5717	1287	ID=contig00009.g5717;Description=hypothetical protein FGADI_698 [Fusarium gaditjirri];Gene=FACUT_10882;Ontology_term=membrane;Ontology_id=GO:0016020

contig00009.g5718	2249	ID=contig00009.g5718;Description=NADPH-ferrihemoprotein reductase [Fusarium verticillioides 7600];Gene=cprA;Ontology_term=generation of precursor metabolites and energy,oxidoreductase activity,lipid metabolic process,mitochondrion,endoplasmic reticulum,plasma membrane;Ontology_id=GO:0006091,GO:0016491,GO:0006629,GO:0005739,GO:0005783,GO:0005886;Enzyme_code=EC:1.6.2.4,EC:1.6.2.4,EC:1.6.2,EC:1.6,EC:1;Enzyme_name=NADPH--hemoprotein reductase,NADPH--hemoprotein reductase,Acting on NADH or NADPH,Acting on NADH or NADPH,Oxidoreductases
contig00009.g5719	822	ID=contig00009.g5719;Description=peroxin 11C [Fusarium acutatum]
contig00009.g5720	1584	ID=contig00009.g5720;Description=aldehyde dehydrogenase (NAD+) [Fusarium verticillioides 7600];Gene=F52700_6227;Ontology_term=oxidoreductase activity;Ontology_id=GO:0016491;Enzyme_code=EC:1.2.1,EC:1.2,EC:1,EC:1.2.1.3,EC:1.2.1;Enzyme_name=Acting on the aldehyde or oxo group of donors,Acting on the aldehyde or oxo group of donors,Oxidoreductases,aldehyde dehydrogenase (NAD(+)),Acting on the aldehyde or oxo group of donors
contig00009.g5721	2159	ID=contig00009.g5721;Description=copper amine oxidase 1 [Fusarium proliferatum];Gene=FMUND_10534;Ontology_term=oxidoreductase activity;Ontology_id=GO:0016491;Enzyme_code=EC:1.4.3.21,EC:1.4,EC:1.4.3,EC:1,EC:1.4.3.21;Enzyme_name=primary-amine oxidase,Acting on the CH-NH2 group of donors,Acting on the CH-NH2 group of donors,Oxidoreductases,primary-amine oxidase
contig00009.g5722	1789	ID=contig00009.g5722;Description=amino acid transporter [Fusarium tjaetaba];Gene=FVER53590_04393;Ontology_term=transmembrane transport,transporter activity;Ontology_id=GO:0055085,GO:0005215
contig00009.g5723	2323	ID=contig00009.g5723;Description=hypothetical protein FVEG_04392 [Fusarium verticillioides 7600];Gene=FVER53590_04392;Ontology_term=nucleus,DNA binding,regulation of DNA-templated transcription,transcription regulator activity;Ontology_id=GO:0005634,GO:0003677,GO:0006355,GO:0140110

contig00009.g5724	1458	ID=contig00009.g5724;Description=seryl-tRNA synthetase [Fusarium verticillioides 7600];Gene=FPANT_3690;Ontology_term=mitochondrial gene expression,mitochondrion,tRNA metabolic process,amino acid metabolic process,catalytic activity, acting on RNA,ligase activity;Ontology_id=GO:0140053,GO:0005739,GO:0006399,GO:0006520,GO:0140098,GO:0016874;Enzyme_code=EC:6.1.1.11,EC:6.1,EC:6.1.1.11,EC:6.1.1,EC:6;Enzyme_name=serine--tRNA ligase,Forming carbon-oxygen bonds,serine--tRNA ligase,Forming carbon-oxygen bonds,Ligases
contig00009.g5725	1998	ID=contig00009.g5725;Description=hypothetical protein FCOIX_7704 [Fusarium coicis]
contig00009.g5726	1409	ID=contig00009.g5726;Description=3-hydroxybenzoate 6-hydroxylase [Fusarium pseudoanthophilum];Gene=FVEG_04390;Ontology_term=oxidoreductase activity;Ontology_id=GO:0016491;Enzyme_code=EC:1;Enzyme_name=Oxidoreductases
contig00009.g5727	1496	ID=contig00009.g5727;Description=6-hydroxy-D-nicotine oxidase [Fusarium tjaetaba];Gene=FTJAE_1879;Ontology_term=oxidoreductase activity;Ontology_id=GO:0016491;Enzyme_code=EC:1,EC:1;Enzyme_name=Oxidoreductases,Oxidoreductases
contig00009.g5728	1908	ID=contig00009.g5728;Description=het-domain-containing protein [Fusarium pseudoanthophilum];Gene=FPANT_3694;Ontology_term=catalytic activity, acting on a protein,hydrolase activity;Ontology_id=GO:0140096,GO:0016787;Enzyme_code=EC:3.4,EC:3.4,EC:3;Enzyme_name=Acting on peptide bonds (peptidases),Acting on peptide bonds (peptidases),Hydrolases
contig00009.g5729	949	ID=contig00009.g5729;Description=Ammonia transport outward 2 [Fusarium coicis];Gene=LW93_8139;Ontology_term=transmembrane transport,mitochondrion,vacuole,transporter activity,plasma membrane;Ontology_id=GO:0055085,GO:0005739,GO:0005773,GO:0005215,GO:0005886
contig00009.g5730	1122	ID=contig00009.g5730;Description=zinc-binding alcohol dehydrogenase domain protein [Fusarium tjaetaba];Gene=FVER53263_20855;Ontology_term=oxidoreductase activity;Ontology_id=GO:0016491;Enzyme_code=EC:1.6,EC:1.6,EC:1;Enzyme_name=Acting on NADH or NADPH,Acting on NADH or NADPH,Oxidoreductases
contig00009.g5731	2511	ID=contig00009.g5731;Description=zeaxanthin epoxidase chloroplastic [Fusarium tjaetaba];Gene=FDENT_11866;Ontology_term=oxidoreductase activity;Ontology_id=GO:0016491;Enzyme_code=EC:1,EC:1;Enzyme_name=Oxidoreductases,Oxidoreductases

contig00009.g5732	1649	ID=contig00009.g5732;Description=tartrate transporter [Fusarium denticulatum];Gene=FDENT_11865;Ontology_term=transmembrane transport,transporter activity;Ontology_id=GO:0055085,GO:0005215
contig00009.g5733	1632	ID=contig00009.g5733;Description=hypothetical protein FVEG_04384 [Fusarium verticillioides 7600];Gene=FNAPI_39;Ontology_term=transmembrane transport,transporter activity;Ontology_id=GO:0055085,GO:0005215
contig00009.g5734	3241	ID=contig00009.g5734;Description=hypothetical protein J7337_006428 [Fusarium musae]
contig00009.g5735	304	ID=contig00009.g5735;Description=hypothetical protein H9Q71_006784 [Fusarium xylarioides]
contig00009.g5736	2484	ID=contig00009.g5736;Description=hypothetical protein FVER53263_04382 [Fusarium verticillioides];Gene=FVER53590_04382;Ontology_term=membrane;Ontology_id=GO:0016020
contig00009.g5737	3859	ID=contig00009.g5737;Description=hypothetical protein FVER53263_04381 [Fusarium verticillioides]
contig00009.g5738	1918	ID=contig00009.g5738;Description=C6 transcription factor [Fusarium pseudoanthophilum]
contig00009.g5739	1077	ID=contig00009.g5739;Description=asparaginase [Fusarium coicis]
contig00009.g5740	1725	ID=contig00009.g5740;Description=hypothetical protein J7337_006416 [Fusarium musae]
contig00009.g5741	1173	ID=contig00009.g5741;Description=hypothetical protein FVER53590_29036 [Fusarium verticillioides]
contig00009.g5742	1869	ID=contig00009.g5742;Description=related to allantoate transporter [Fusarium fujikuroi];Gene=FMUND_10555;Ontology_term=transmembrane transport,transporter activity;Ontology_id=GO:0055085,GO:0005215
contig00009.g5743	1662	ID=contig00009.g5743;Description=allantoate transporter [Fusarium pseudoanthophilum];Gene=FPANT_12965;Ontology_term=transmembrane transport,transporter activity;Ontology_id=GO:0055085,GO:0005215
contig00009.g5744	1209	ID=contig00009.g5744;Description=uncharacterized protein BKA55DRAFT_595044 [Fusarium redolens];Gene=FVEG_04374;Ontology_term=membrane;Ontology_id=GO:0016020
contig00009.g5745	723	ID=contig00009.g5745;Description=related to YSC84-protein involved in the organization of the actin cytoskeleton [Fusarium proliferatum]
contig00009.g5746	1374	ID=contig00009.g5746;Description=hypothetical protein FVER53590_04372 [Fusarium verticillioides]
contig00009.g5747	1824	ID=contig00009.g5747;Description=ankyrin protein [Fusarium mundagurra]
contig00009.g5748	1071	ID=contig00009.g5748;Description=Ff.00g062360.m01.CDS01 [Fusarium sp. VM40]

contig00009.g5749	1169	ID=contig00009.g5749;Description=cyanide hydratase [Fusarium verticillioides 7600];Gene=FOYG_01989;Ontology_term=lyase activity,hydrolase activity;Ontology_id=GO:0016829,GO:0016787;Enzyme_code=EC:4.2.1.66,EC:3.5.5.1;Enzyme_name=cyanide hydratase,nitrilase
contig00009.g5750	1948	ID=contig00009.g5750;Description=membrane primary amine oxidase [Fusarium pseudoanthophilum];Gene=FNAPI_2540;Ontology_term=oxidoreductase activity,transferase activity;Ontology_id=GO:0016491,GO:0016740;Enzyme_code=EC:2,EC:1.4.3.21,EC:1.4,EC:1.4.3,EC:1,EC:2,EC:1.4.3.21;Enzyme_name=Transferases,primary-amine oxidase,Acting on the CH-NH2 group of donors,Acting on the CH-NH2 group of donors,Oxidoreductases,Transferases,primary-amine oxidase
contig00009.g5751	2972	ID=contig00009.g5751;Description=membrane primary amine oxidase [Fusarium tjaetaba];Gene=FDENT_11884;Ontology_term=small molecule binding;Ontology_id=GO:0036094;Enzyme_code=EC:1.4.3.21;Enzyme_name=primary-amine oxidase
contig00009.g5752	2955	ID=contig00009.g5752;Description=multiple epidermal growth factor-like domain protein [Fusarium subglutinans]
contig00009.g5753	618	ID=contig00009.g5753;Description=hypothetical protein FVER14953_04366 [Fusarium verticillioides]
contig00009.g5754	349	ID=contig00009.g5754;Description=trypsin Inhibitor like cysteine rich domain protein [Fusarium tjaetaba]
contig00009.g5755	458	ID=contig00009.g5755;Description=hypothetical protein FOXB_14299 [Fusarium oxysporum f. sp. conglutinans Fo5176]
contig00009.g5756	1320	ID=contig00009.g5756;Description=RNA polymerase II mediator complex component [Fusarium tjaetaba];Gene=FACUT_10915;Ontology_term=nucleus,regulation of DNA-templated transcription,transcription regulator activity;Ontology_id=GO:0005634,GO:0006355,GO:0140110
contig00009.g5757	1349	ID=contig00009.g5757;Description=Sterol uptake control 2 [Fusarium acutatum];Gene=FMAN_05319;Ontology_term=nucleus,regulation of DNA-templated transcription,transcription regulator activity;Ontology_id=GO:0005634,GO:0006355,GO:0140110

contig00009.g5758	912	ID=contig00009.g5758;Description=NAD-dependent aldehyde dehydrogenase [Fusarium subglutinans];Gene=FMEXI_68;Ontology_term=oxidoreductase activity;Ontology_id=GO:0016491;Enzyme_code=EC:1,EC:1;Enzyme_name=Oxidoreductases,Oxidoreductases
contig00009.g5759	864	ID=contig00009.g5759;Description=hypothetical protein FVER14953_04360 [Fusarium verticillioides];Gene=FVER53590_04360;Ontology_term=DNA binding,regulation of DNA-templated transcription,transcription regulator activity;Ontology_id=GO:0003677,GO:0006355,GO:0140110
contig00009.g5760	1456	ID=contig00009.g5760;Description=glycosyltransferase family 4 [Fusarium coicis];Gene=FVER53263_04359;Ontology_term=transferase activity;Ontology_id=GO:0016740;Enzyme_code=EC:2.4,EC:2.4,EC:2;Enzyme_name=Glycosyltransferases,Glycosyltransferases,Transferases
contig00009.g5761	2108	ID=contig00009.g5761;Description=putative maltase MLT1 [Fusarium verticillioides];Gene=B0Z3Y4;Ontology_term=carbohydrate metabolic process,hydrolase activity;Ontology_id=GO:0005975,GO:0016787;Enzyme_code=EC:3.2.1.20;Enzyme_name=alpha-glucosidase
contig00009.g5762	621	ID=contig00009.g5762;Description=hypothetical protein FVER14953_04357 [Fusarium verticillioides]
contig00009.g5763	1125	ID=contig00009.g5763;Description=tyrosyl-tRNA synthetase [Fusarium verticillioides 7600];Gene=FPANT_8851;Ontology_term=nucleus,RNA binding,cytoplasmic translation,tRNA metabolic process,amino acid metabolic process,catalytic activity, acting on RNA,ligase activity;Ontology_id=GO:0005634,GO:0003723,GO:0002181,GO:0006399,GO:0006520,GO:0140098,GO:0016874;Enzyme_code=EC:6.1.1.1,EC:6.1.1.1,EC:6.1,EC:6.1.1,EC:6;Enzyme_name=tyrosine--tRNA ligase,tyrosine--tRNA ligase,Forming carbon-oxygen bonds,Forming carbon-oxygen bonds,Ligases
contig00009.g5764	2486	ID=contig00009.g5764;Description=homeobox 4 [Fusarium tjaetaba];Gene=FACUT_13000;Ontology_term=nucleus,DNA binding,regulation of DNA-templated transcription;Ontology_id=GO:0005634,GO:0003677,GO:0006355
contig00009.g5765	444	ID=contig00009.g5765;Description=dienelactone hydrolase family [Fusarium pseudoanthophilum];Gene=FMAN_05307;Ontology_term=hydrolase activity;Ontology_id=GO:0016787;Enzyme_code=EC:3,EC:3;Enzyme_name=Hydrolases,Hydrolases

contig00009.g5766	1164	ID=contig00009.g5766;Description=zinc cluster transcription factor [Fusarium tjaetaba];Gene=FPHYL_7186;Ontology_term=nucleus,regulation of DNA-templated transcription,transcription regulator activity,hydrolase activity;Ontology_id=GO:0005634,GO:0006355,GO:0140110,GO:0016787;Enzyme_code=EC:3;Enzyme_name=Hydrolases
contig00009.g5767	4386	ID=contig00009.g5767;Description=heterokaryon incompatibility protein-domain-containing protein [Fusarium oxysporum]
contig00009.g5768	2020	ID=contig00009.g5768;Description=hypothetical protein FVER53263_04351 [Fusarium verticillioides];Enzyme_code=EC:3.2.1.14;Enzyme_name=chitinase
contig00009.g5769	1524	ID=contig00009.g5769;Description=hypothetical protein FVER14953_04350 [Fusarium verticillioides]
contig00009.g5770	2372	ID=contig00009.g5770;Description=hypothetical protein FVER53263_04349 [Fusarium verticillioides];Gene=FVER53263_04349;Ontology_term=nucleus,DNA binding,regulation of DNA-templated transcription,transcription regulator activity;Ontology_id=GO:0005634,GO:0003677,GO:0006355,GO:0140110
contig00009.g5771	1411	ID=contig00009.g5771;Description=salicylate hydroxylase [Fusarium acutatum];Gene=FVER53263_04348;Ontology_term=oxidoreductase activity;Ontology_id=GO:0016491;Enzyme_code=EC:1,EC:1,EC:1.14.13.1;Enzyme_name=Oxidoreductases,Oxidoreductases,salicylate 1-monooxygenase
contig00009.g5772	1915	ID=contig00009.g5772;Description=benzoate 4-monooxygenase cytochrome P450 [Fusarium tjaetaba];Gene=BFJ65_g3337;Ontology_term=oxidoreductase activity;Ontology_id=GO:0016491;Enzyme_code=EC:1.14,EC:1,EC:1.14;Enzyme_name=Acting on paired donors, with incorporation or reduction of molecular oxygen. The oxygen incorporated need not be derived from O2,Oxidoreductases,Acting on paired donors, with incorporation or reduction of molecular oxygen. The oxygen incorporated need not be derived from O2
contig00009.g5773	1617	ID=contig00009.g5773;Description=aldehyde dehydrogenase n-terminal [Fusarium tjaetaba];Gene=FACUT_13827;Ontology_term=oxidoreductase activity;Ontology_id=GO:0016491;Enzyme_code=EC:1.2.1,EC:1.2,EC:1,EC:1.2.1.3,EC:1.2.1;Enzyme_name=Acting on the aldehyde or oxo group of donors,Acting on the aldehyde or oxo group of donors,Oxidoreductases,aldehyde dehydrogenase (NAD(+)),Acting on the aldehyde or oxo group of donors

contig00009.g5774	1689	ID=contig00009.g5774;Description=beta-carotene 15 15 monooxygenase [Fusarium subglutinans];Gene=FSUBG_9588;Ontology_term=oxidoreductase activity;Ontology_id=GO:0016491;Enzyme_code=EC:1.13.11,EC:1.13.11,EC:1.13,EC:1;Enzyme_name=Acting on single donors with incorporation of molecular oxygen (oxygenases). The oxygen incorporated need not be derived from O2,Acting on single donors with incorporation of molecular oxygen (oxygenases). The oxygen incorporated need not be derived from O2,Acting on single donors with incorporation of molecular oxygen (oxygenases). The oxygen incorporated need not be derived from O2,Oxidoreductases
contig00009.g5775	1179	ID=contig00009.g5775;Description=amidohydrolase 2 [Fusarium pseudocircinatum];Gene=FPHYL_7176;Ontology_term=oxidoreductase activity,lyase activity,hydrolase activity;Ontology_id=GO:0016491,GO:0016829,GO:0016787;Enzyme_code=EC:4.1.1,EC:1,EC:3,EC:4.1.1,EC:4.1,EC:4.1.1.46,EC:1,EC:3,EC:4;Enzyme_name=Carbon-carbon lyases,Oxidoreductases,Hydrolases,Carbon-carbon lyases,Carbon-carbon lyases,o-pyrocatechuate decarboxylase,Oxidoreductases,Hydrolases,Lyases
contig00009.g5776	1233	ID=contig00009.g5776;Description=alcohol dehydrogenase [Fusarium verticillioides 7600];Gene=FPANT_8860;Ontology_term=oxidoreductase activity,hydrolase activity;Ontology_id=GO:0016491,GO:0016787;Enzyme_code=EC:1,EC:3,EC:1,EC:3,EC:1.1.1.1;Enzyme_name=Oxidoreductases,Hydrolases,Oxidoreductases,Hydrolases,alcohol dehydrogenase
contig00009.g5777	877	ID=contig00009.g5777;Description=hypothetical protein FVEG_04342 [Fusarium verticillioides 7600];Gene=FNYG_13761;Ontology_term=membrane;Ontology_id=GO:0016020
contig00009.g5778	3384	ID=contig00009.g5778;Description=aldedh-domain-containing protein [Fusarium coicis];Gene=FVER53590_04341;Ontology_term=nucleus,DNA binding,regulation of DNA-templated transcription,transcription regulator activity;Ontology_id=GO:0005634,GO:0003677,GO:0006355,GO:0140110
contig00009.g5779	1761	ID=contig00009.g5779;Description=cytochrome P450 monooxygenase oxidoreductase [Fusarium tjaetaba];Gene=FTJAE_1826;Ontology_term=oxidoreductase activity;Ontology_id=GO:0016491;Enzyme_code=EC:1.14,EC:1,EC:1.14;Enzyme_name=Acting on paired donors, with incorporation or reduction of molecular oxygen. The oxygen incorporated need not be derived from O2,Oxidoreductases,Acting on paired donors, with incorporation or reduction of molecular oxygen. The oxygen incorporated need not be derived from O2

contig00009.g5780	870	ID=contig00009.g5780;Description=hypothetical protein FVER14953_04339 [Fusarium verticillioides]
contig00009.g5781	1633	ID=contig00009.g5781;Description=related to transporter (major facilitator superfamily) [Fusarium fujikuroi];Gene=FPCIR_5977;Ontology_term=transmembrane transport,transporter activity;Ontology_id=GO:0055085,GO:0005215
contig00009.g5782	1082	ID=contig00009.g5782;Description=retinol dehydrogenase 12 [Fusarium tjaetaba]
contig00009.g5783	2855	ID=contig00009.g5783;Description=transcriptional regulatory GAL4 [Fusarium tjaetaba];Gene=FLONG3_3573;Ontology_term=nucleus,DNA binding,regulation of DNA-templated transcription,transcription regulator activity;Ontology_id=GO:0005634,GO:0003677,GO:0006355,GO:0140110
contig00009.g5784	864	ID=contig00009.g5784;Description=dehydrogenase [Fusarium tjaetaba];Gene=FTJAE_1819;Ontology_term=oxidoreductase activity;Ontology_id=GO:0016491;Enzyme_code=EC:1,EC:1;Enzyme_name=Oxidoreductases,Oxidoreductases
contig00009.g5785	1595	ID=contig00009.g5785;Description=probable UGA2-succinate semialdehyde dehydrogenase [Fusarium fujikuroi];Gene=FVER53590_04334;Ontology_term=oxidoreductase activity;Ontology_id=GO:0016491;Enzyme_code=EC:1.2.1,EC:1.2.1.16,EC:1.2,EC:1.2.1.79,EC:1,EC:1.2.1.20,EC:1.2.1;Enzyme_name=Acting on the aldehyde or oxo group of donors,succinate-semialdehyde dehydrogenase [NAD(P)(+)],Acting on the aldehyde or oxo group of donors,succinate-semialdehyde dehydrogenase (NADP(+)),Oxidoreductases,glutarate-semialdehyde dehydrogenase,Acting on the aldehyde or oxo group of donors
contig00009.g5786	1879	ID=contig00009.g5786;Description=putative sterigmatocystin biosynthesis monooxygenase stcW [Phialophora attinorum];Gene=FFB14_06161;Ontology_term=oxidoreductase activity;Ontology_id=GO:0016491;Enzyme_code=EC:1.14.13.8,EC:1.14.13.8,EC:1,EC:1.14,EC:1.14.13;Enzyme_name=flavin-containing monooxygenase,flavin-containing monooxygenase,Oxidoreductases,Acting on paired donors, with incorporation or reduction of molecular oxygen. The oxygen incorporated need not be derived from O2,Acting on paired donors, with incorporation or reduction of molecular oxygen. The oxygen incorporated need not be derived from O2
contig00009.g5787	834	ID=contig00009.g5787;Description=glycoside hydrolase [Fusarium pseudocircinatum];Gene=Focb16_v004426;Ontology_term=hydrolase activity;Ontology_id=GO:0016787;Enzyme_code=EC:3.1.1.73;Enzyme_name=feruloyl esterase

contig00009.g5788	738	ID=contig00009.g5788;Description=hypothetical protein FVER14953_04331 [Fusarium verticillioides]
contig00009.g5789	1202	ID=contig00009.g5789;Description=related to PAF acetylhydrolase family protein [Fusarium fujikuroi];Gene=FVER53590_04330;Ontology_term=hydrolase activity;Ontology_id=GO:0016787;Enzyme_code=EC:3.1.1.47,EC:3.1.1.47,EC:3.1,EC:3,EC:3.1.1;Enzyme_name=1-alkyl-2-acetylglycerophosphocholine esterase,1-alkyl-2-acetylglycerophosphocholine esterase,Acting on ester bonds,Hydrolases,Acting on ester bonds
contig00009.g5790	3999	ID=contig00009.g5790;Description=ankyrin repeat [Fusarium mundagurra];Gene=FMUND_1534;Ontology_term=nucleobase-containing small molecule metabolic process,catalytic activity,carbohydrate derivative metabolic process;Ontology_id=GO:0055086,GO:0003824,GO:1901135
contig00009.g5791	3415	ID=contig00009.g5791;Description=ankyrin repeat domain-containing protein 52 [Metarhizium robertsii ARSEF 23]
contig00009.g5792	834	ID=contig00009.g5792;Description=unnamed protein product [Clonostachys chloroleuca]
contig00009.g5793	3032	ID=contig00009.g5793;Description=subtilisin-like protein [Hyaloscypha variabilis F];Gene=FMUND_1537;Ontology_term=catalytic activity, acting on a protein,hydrolase activity;Ontology_id=GO:0140096,GO:0016787;Enzyme_code=EC:3.4.21,EC:3.4.21,EC:3.4,EC:3;Enzyme_name=Acting on peptide bonds (peptidases),Acting on peptide bonds (peptidases),Acting on peptide bonds (peptidases),Hydrolases
contig00009.g5794	1569	ID=contig00009.g5794;Description=cytochrome p450 3a17 [Fusarium tjaetaba];Gene=FVEG_04329;Ontology_term=oxidoreductase activity;Ontology_id=GO:0016491;Enzyme_code=EC:1.14,EC:1,EC:1.14;Enzyme_name=Acting on paired donors, with incorporation or reduction of molecular oxygen. The oxygen incorporated need not be derived from O2,Oxidoreductases,Acting on paired donors, with incorporation or reduction of molecular oxygen. The oxygen incorporated need not be derived from O2
contig00009.g5795	6168	ID=contig00009.g5795;Description=related to transporter protein [Fusarium fujikuroi];Gene=FCIRC_6746;Ontology_term=catalytic activity;Ontology_id=GO:0003824
contig00009.g5796	2065	ID=contig00009.g5796;Description=transcriptional regulatory [Fusarium subglutinans];Gene=FOC1_g10001362;Ontology_term=nucleus,DNA binding,DNA-templated transcription;Ontology_id=GO:0005634,GO:0003677,GO:0006351
contig00009.g5797	915	ID=contig00009.g5797;Description=esterase lipase [Fusarium subglutinans]

contig00009.g5798	495	ID=contig00009.g5798;Description=hypothetical protein FVER53590_04326 [Fusarium verticillioides]
contig00009.g5799	1673	ID=contig00009.g5799;Description=hypothetical protein FVEG_04325 [Fusarium verticillioides 7600]
contig00009.g5800	1558	ID=contig00009.g5800;Description=l-sorbosone dehydrogenase [Fusarium tjaetaba]
contig00009.g5801	1563	ID=contig00009.g5801;Description=Purine-cytosine permease FCY21 [Fusarium napiforme];Gene=FPANT_13572;Ontology_term=transmembrane transport,transporter activity;Ontology_id=GO:0055085,GO:0005215
contig00009.g5802	1945	ID=contig00009.g5802;Description=fungal specific transcription factor factor [Fusarium subglutinans];Gene=FPRO_05542;Ontology_term=nucleus,DNA binding,DNA-templated transcription;Ontology_id=GO:0005634,GO:0003677,GO:0006351
contig00009.g5803	3838	ID=contig00009.g5803;Description=carboxylesterase 2 [Fusarium pseudoanthophilum];Gene=FOQG_13681;Ontology_term=hydrolase activity;Ontology_id=GO:0016787;Enzyme_code=EC:3,EC:3;Enzyme_name=Hydrolases,Hydrolases
contig00009.g5804	1086	ID=contig00009.g5804;Description=phytanoyl dioxygenase [Fusarium pseudocircinatum];Gene=FPCIR_5955;Ontology_term=oxidoreductase activity;Ontology_id=GO:0016491;Enzyme_code=EC:1,EC:1;Enzyme_name=Oxidoreductases,Oxidoreductases
contig00009.g5805	2033	ID=contig00009.g5805;Description=amino acid/polyamine transporter I [Fusarium oxysporum Fo47];Gene=FPCIR_5954;Ontology_term=transmembrane transport,transporter activity;Ontology_id=GO:0055085,GO:0005215
contig00009.g5806	1798	ID=contig00009.g5806;Description=major facilitator superfamily-domain-containing protein [Fusarium oxysporum Fo47];Gene=FVER53263_04315;Ontology_term=transmembrane transport,transporter activity;Ontology_id=GO:0055085,GO:0005215
contig00009.g5807	6615	ID=contig00009.g5807;Description=Aflatoxin B1 aldehyde reductase member 2 [Fusarium oxysporum f. sp. albedinis];Gene=FOC4_g10010258;Ontology_term=nucleus,regulation of DNA-templated transcription,transcription regulator activity;Ontology_id=GO:0005634,GO:0006355,GO:0140110;Enzyme_code=EC:1.14.13.12;Enzyme_name=Acting on paired donors, with incorporation or reduction of molecular oxygen. The oxygen incorporated need not be derived from O2
contig00009.g5808	1212	ID=contig00009.g5808;Description=transporter [Fusarium tjaetaba];Gene=FNAPI_1352;Ontology_term=transmembrane transport,transporter activity;Ontology_id=GO:0055085,GO:0005215

contig00009.g5809	2307	ID=contig00009.g5809;Description=hypothetical protein FVER14953_04312 [Fusarium verticillioides]
contig00009.g5810	795	ID=contig00009.g5810;Description=heterokaryon incompatibility 6 OR allele [Fusarium pseudocircinatum]
contig00009.g5811	1528	ID=contig00009.g5811;Description=acid phosphatase [Fusarium verticillioides 7600];Gene=FPCIR_5949;Ontology_term=hydrolase activity;Ontology_id=GO:0016787;Enzyme_code=EC:3.1.3.2,EC:3.1,EC:3,EC:3.1.3.2,EC:3.1.3;Enzyme_name=acid phosphatase,Acting on ester bonds,Hydrolases,acid phosphatase,Acting on ester bonds
contig00009.g5812	2409	ID=contig00009.g5812;Description=n-terminal binuclear Zn cluster-containing protein [Fusarium tjaetaba];Gene=FVER53590_04309;Ontology_term=nucleus,DNA binding,oxidoreductase activity,regulation of DNA-templated transcription,transcription regulator activity;Ontology_id=GO:0005634,GO:0003677,GO:0016491,GO:0006355,GO:0140110;Enzyme_code=EC:1;Enzyme_name=Oxidoreductases
contig00009.g5813	1983	ID=contig00009.g5813;Description=cytochrome p450 [Fusarium coicis];Gene=FVEG_04308;Ontology_term=oxidoreductase activity,regulation of DNA-templated transcription;Ontology_id=GO:0016491,GO:0006355;Enzyme_code=EC:1,EC:1;Enzyme_name=Oxidoreductases,Oxidoreductases
contig00009.g5814	951	ID=contig00009.g5814;Description=hypothetical protein FVER14953_04307 [Fusarium verticillioides]
contig00009.g5815	1613	ID=contig00009.g5815;Description=BAHD acyltransferase DCR [Fusarium pseudoanthophilum];Gene=FPCIR_5945;Ontology_term=transferase activity;Ontology_id=GO:0016740;Enzyme_code=EC:2.3,EC:2.3,EC:2;Enzyme_name=Acyltransferases,Acyltransferases,Transferases
contig00009.g5816	1883	ID=contig00009.g5816;Description=general substrate transporter [Fusarium sp. MPI-SDFR-AT-0072];Gene=FVER53263_04305;Ontology_term=transmembrane transport,transporter activity;Ontology_id=GO:0055085,GO:0005215
contig00009.g5817	1800	ID=contig00009.g5817;Description=beta-glucuronidase [Fusarium verticillioides 7600];Gene=FVER53263_04304;Ontology_term=carbohydrate metabolic process,hydrolase activity;Ontology_id=GO:0005975,GO:0016787;Enzyme_code=EC:3.2.1,EC:3.2,EC:3.2.1.31,EC:3,EC:3.2.1;Enzyme_name=Glycosylases,Glycosylases,beta-glucuronidase,Hydrolases,Glycosylases

contig00009.g5818	4512	ID=contig00009.g5818;Description=flavo protein subunit of succinate dehydrogenase/fumarate reductase [Fusarium oxysporum Fo47];Gene=BFJ71_g17088;Ontology_term=oxidoreductase activity;Ontology_id=GO:0016491;Enzyme_code=EC:1,EC:1;Enzyme_name=Oxidoreductases,Oxidoreductases
contig00010.g5819	2099	ID=contig00010.g5819;Description=metallo-beta-lactamase superfamily [Fusarium sp. NRRL 52700];Gene=Forpe1208_v012580;Ontology_term=cytoskeletal protein binding;Ontology_id=GO:0008092
contig00010.g5820	1780	ID=contig00010.g5820;Description=fungal specific transcription factor factor domain protein [Fusarium sp. NRRL 52700];Gene=FANTH_11205;Ontology_term=nucleus,DNA binding,regulation of DNA-templated transcription,transcription regulator activity;Ontology_id=GO:0005634,GO:0003677,GO:0006355,GO:0140110
contig00010.g5821	1289	ID=contig00010.g5821;Description=putative secondary metabolism biosynthetic enzyme [Fusarium musae]
contig00010.g5822	801	ID=contig00010.g5822;Description=putative secondary metabolism biosynthetic enzyme [Fusarium musae];Gene=F52700_5017;Ontology_term=biosynthetic process;Ontology_id=GO:0009058
contig00010.g5823	5778	ID=contig00010.g5823;Description=NRPS protein [Fusarium musae];Gene=FPHYL_9052;Ontology_term=ligase activity,isomerase activity;Ontology_id=GO:0016874,GO:0016853;Enzyme_code=EC:5,EC:6,EC:5,EC:6;Enzyme_name=Isomerases,Ligases,Isomerases,Ligases
contig00010.g5824	1539	ID=contig00010.g5824;Description=FAD dependent oxidoreductase [Fusarium tjaetaba];Gene=FPHYL_9053;Ontology_term=oxidoreductase activity;Ontology_id=GO:0016491;Enzyme_code=EC:1,EC:1;Enzyme_name=Oxidoreductases,Oxidoreductases
contig00010.g5825	2060	ID=contig00010.g5825;Description=dimethylaniline monooxygenase 2 [Fusarium globosum];Gene=FMAN_08292;Ontology_term=oxidoreductase activity;Ontology_id=GO:0016491;Enzyme_code=EC:1.14.13.8,EC:1.14.13.8,EC:1,EC:1.14,EC:1.14.13;Enzyme_name=flavin-containing monooxygenase,flavin-containing monooxygenase,Oxidoreductases,Acting on paired donors, with incorporation or reduction of molecular oxygen. The oxygen incorporated need not be derived from O2,Acting on paired donors, with incorporation or reduction of molecular oxygen. The oxygen incorporated need not be derived from O2

contig00010.g5826	1125	ID=contig00010.g5826;Description=putative secondary metabolism biosynthetic enzyme [Fusarium musae];Gene=FMAN_08293;Ontology_term=oxidoreductase activity,transferase activity;Ontology_id=GO:0016491,GO:0016740;Enzyme_code=EC:2.6.1,EC:1.14,EC:2.6.1,EC:1,EC:1.14,EC:2,EC:2.6;Enzyme_name=Transferring nitrogenous groups,Acting on paired donors, with incorporation or reduction of molecular oxygen. The oxygen incorporated need not be derived from O2,Transferring nitrogenous groups,Oxidoreductases,Acting on paired donors, with incorporation or reduction of molecular oxygen. The oxygen incorporated need not be derived from O2,Transferases,Transferring nitrogenous groups
contig00010.g5827	1554	ID=contig00010.g5827;Description=related to benzoate 4-monooxygenase cytochrome P450 [Fusarium proliferatum ET1];Gene=FVER53590_29991;Ontology_term=oxidoreductase activity;Ontology_id=GO:0016491;Enzyme_code=EC:1.14,EC:1,EC:1.14;Enzyme_name=Acting on paired donors, with incorporation or reduction of molecular oxygen. The oxygen incorporated need not be derived from O2,Oxidoreductases,Acting on paired donors, with incorporation or reduction of molecular oxygen. The oxygen incorporated need not be derived from O2
contig00010.g5828	3165	ID=contig00010.g5828;Description=putative NRPS-like protein biosynthetic cluster [Fusarium musae];Gene=FGLOB1_6227;Ontology_term=transferase activity,isomerase activity;Ontology_id=GO:0016740,GO:0016853;Enzyme_code=EC:5,EC:2.1.1,EC:2.1,EC:2,EC:5,EC:2.1.1;Enzyme_name=Isomerases,Transferring one-carbon groups,Transferring one-carbon groups,Transferases,Isomerases,Transferring one-carbon groups
contig00010.g5829	1461	ID=contig00010.g5829;Description=related to tetracycline resistance protein (probable transport protein) [Fusarium proliferatum ET1];Gene=FMAN_08296;Ontology_term=transmembrane transport,transporter activity;Ontology_id=GO:0055085,GO:0005215
contig00010.g5830	1638	ID=contig00010.g5830;Description=related to myo-inositol transporter [Fusarium proliferatum ET1];Gene=FGLOB1_6229;Ontology_term=transmembrane transport,transporter activity;Ontology_id=GO:0055085,GO:0005215

contig00010.g5831	1753	ID=contig00010.g5831;Description=cytochrome P450 monooxygenase [Fusarium subglutinans];Gene=FSUBG_13222;Ontology_term=nucleus,DNA binding,oxidoreductase activity,DNA-templated transcription;Ontology_id=GO:0005634,GO:0003677,GO:0016491,GO:0006351;Enzyme_code=EC:1.14,EC:1,EC:1.14;Enzyme_name=Acting on paired donors, with incorporation or reduction of molecular oxygen. The oxygen incorporated need not be derived from O2,Oxidoreductases,Acting on paired donors, with incorporation or reduction of molecular oxygen. The oxygen incorporated need not be derived from O2
contig00010.g5832	1115	ID=contig00010.g5832;Description=related to enoyl-CoA hydratase/isomerase [Fusarium oxysporum];Gene=FRV6_11987;Ontology_term=isomerase activity;Ontology_id=GO:0016853;Enzyme_code=EC:5,EC:5;Enzyme_name=Isomerases,Isomerases
contig00010.g5833	2185	ID=contig00010.g5833;Description=Dehydrocurvularin biosynthesis regulator [Fusarium oxysporum f. sp. cubense];Gene=FOXG_08999;Ontology_term=regulation of DNA-templated transcription;Ontology_id=GO:0006355
contig00010.g5834	1043	ID=contig00010.g5834;Description=Aldehyde/histidinol dehydrogenase [Fusarium oxysporum];Gene=BFJ69_g10256;Ontology_term=nucleus,DNA binding,DNA-templated transcription,oxidoreductase activity;Ontology_id=GO:0005634,GO:0003677,GO:0006351,GO:0016491;Enzyme_code=EC:1.2.1,EC:1.2,EC:1,EC:1.2.1.3,EC:1.2.1;Enzyme_name=Acting on the aldehyde or oxo group of donors,Acting on the aldehyde or oxo group of donors,Oxidoreductases,aldehyde dehydrogenase (NAD(+)),Acting on the aldehyde or oxo group of donors
contig00010.g5835	1677	ID=contig00010.g5835;Description=sugar transporter STL1 [Fusarium tjaetaba];Gene=FNYG_10452;Ontology_term=transmembrane transport,transporter activity;Ontology_id=GO:0055085,GO:0005215
contig00010.g5836	2148	ID=contig00010.g5836;Description=lactose regulatory [Fusarium coicis];Gene=FPHYL_9905;Ontology_term=nucleus,DNA binding,regulation of DNA-templated transcription,transcription regulator activity;Ontology_id=GO:0005634,GO:0003677,GO:0006355,GO:0140110
contig00010.g5837	1785	ID=contig00010.g5837;Description=heterokaryon incompatibility het-6 [Fusarium tjaetaba]
contig00010.g5838	2831	ID=contig00010.g5838;Description=beta-mannosidase [Fusarium avenaceum];Gene=MDC_LOCUS330829;Ontology_term=hydrolase activity;Ontology_id=GO:0016787;Enzyme_code=EC:3;Enzyme_name=Hydrolases

contig00010.g5839	1055	ID=contig00010.g5839;Description=hypothetical protein FVEG_06512 [Fusarium verticillioides 7600]
contig00010.g5840	1973	ID=contig00010.g5840;Description=putative Modin [Fusarium acutatum];Gene=FVER53263_06513;Ontology_term=membrane;Ontology_id=GO:0016020
contig00010.g5841	1550	ID=contig00010.g5841;Description=succinate-semialdehyde dehydrogenase [Fusarium verticillioides 7600];Gene=FPCIR_13073;Ontology_term=oxidoreductase activity;Ontology_id=GO:0016491;Enzyme_code=EC:1.2.1;Enzyme_name=Acting on the aldehyde or oxo group of donors
contig00010.g5842	1565	ID=contig00010.g5842;Description=related to ornithine aminotransferase [Fusarium oxysporum];Gene=FOXYS1_9889;Ontology_term=transferase activity;Ontology_id=GO:0016740;Enzyme_code=EC:2.6.1,EC:2.6.1,EC:2.6.1.13,EC:2,EC:2.6;Enzyme_name=Transferring nitrogenous groups,Transferring nitrogenous groups,ornithine aminotransferase,Transferases,Transferring nitrogenous groups
contig00010.g5843	2325	ID=contig00010.g5843;Description=transcriptional regulatory protein pro-1 [Fusarium denticulatum];Gene=FOTG_07142;Ontology_term=nucleus,regulation of DNA-templated transcription,transcription regulator activity;Ontology_id=GO:0005634,GO:0006355,GO:0140110
contig00010.g5844	539	ID=contig00010.g5844;Description=hypothetical protein FVEG_06517 [Fusarium verticillioides 7600];Gene=FVEG_06517;Ontology_term=membrane;Ontology_id=GO:0016020
contig00010.g5845	533	ID=contig00010.g5845;Description=hypothetical protein FVEG_06517 [Fusarium verticillioides 7600];Gene=FVEG_06517
contig00010.g5846	1012	ID=contig00010.g5846;Description=Chitinase II [Fusarium oxysporum f. sp. vasinfectum];Gene=AU210_000018
contig00010.g5847	1044	ID=contig00010.g5847;Description=F-box-like domain-containing [Fusarium acutatum];Gene=FOYG_16759;Ontology_term=chitin binding;Ontology_id=GO:0008061
contig00010.g5848	481	ID=contig00010.g5848;Description=hypothetical protein FVER14953_06519 [Fusarium verticillioides]
contig00010.g5849	942	ID=contig00010.g5849;Description=hypothetical protein FNAPI_5892 [Fusarium napiforme];Gene=FFB14_13548;Ontology_term=membrane,mycotoxin biosynthetic process;Ontology_id=GO:0016020,GO:0043386
contig00010.g5850	390	ID=contig00010.g5850;Description=hypothetical protein FVER53590_06521 [Fusarium verticillioides]

contig00010.g5851	2133	ID=contig00010.g5851;Description=galactose oxidase precursor [Fusarium tjaetaba];Enzyme_code=EC:1.1.3.9;Enzyme_name=galactose oxidase
contig00010.g5852	1022	ID=contig00010.g5852;Description=hypothetical protein FVER53590_06523 [Fusarium verticillioides]
contig00010.g5853	2604	ID=contig00010.g5853;Description=hypothetical protein FVEG_15879 [Fusarium verticillioides 7600];Gene=BFJ71_g5029;Ontology_term=protein heterodimerization activity;Ontology_id=GO:0046982
contig00010.g5854	1209	ID=contig00010.g5854;Description=protocatechuate 3 4-dioxygenase beta subunit [Fusarium phyllophilum];Gene=FVER53263_06525;Ontology_term=oxidoreductase activity;Ontology_id=GO:0016491;Enzyme_code=EC:1.13.11,EC:1.13.11,EC:1.13,EC:1;Enzyme_name=Acting on single donors with incorporation of molecular oxygen (oxygenases). The oxygen incorporated need not be derived from O2,Acting on single donors with incorporation of molecular oxygen (oxygenases). The oxygen incorporated need not be derived from O2,Acting on single donors with incorporation of molecular oxygen (oxygenases). The oxygen incorporated need not be derived from O2,Oxidoreductases
contig00010.g5855	3854	ID=contig00010.g5855;Description=general amidase [Fusarium mundagurra];Gene=FNYG_10476;Ontology_term=nucleus,regulation of DNA-templated transcription,transcription regulator activity,hydrolase activity;Ontology_id=GO:0005634,GO:0006355,GO:0140110,GO:0016787;Enzyme_code=EC:3.5.1.4,EC:3.5.1,EC:3.5.1.4,EC:3.5,EC:3;Enzyme_name=amidase,Acting on carbon-nitrogen bonds, other than peptide bonds,amidase,Acting on carbon-nitrogen bonds, other than peptide bonds,Hydrolases
contig00010.g5856	1951	ID=contig00010.g5856;Description=transporter HOL1 [Fusarium tjaetaba];Gene=FTJAE_136;Ontology_term=transmembrane transport,transporter activity;Ontology_id=GO:0055085,GO:0005215
contig00010.g5857	1119	ID=contig00010.g5857;Description=cytochrome P450 monooxygenase monooxygenase (lovA) [Fusarium tjaetaba];Gene=FVEG_06528;Ontology_term=oxidoreductase activity;Ontology_id=GO:0016491;Enzyme_code=EC:1.14,EC:1,EC:1.14;Enzyme_name=Acting on paired donors, with incorporation or reduction of molecular oxygen. The oxygen incorporated need not be derived from O2,Oxidoreductases,Acting on paired donors, with incorporation or reduction of molecular oxygen. The oxygen incorporated need not be derived from O2

contig00010.g5858	2057	ID=contig00010.g5858;Description=general substrate transporter [Fusarium oxysporum Fo47];Gene=MDC_LOCUS75834;Ontology_term=transmembrane transport,transporter activity;Ontology_id=GO:0055085,GO:0005215
contig00010.g5859	1996	ID=contig00010.g5859;Description=probable GAP1-General amino acid permease [Fusarium fujikuroi IMI 58289];Gene=FACUT_7914;Ontology_term=transmembrane transport,mitochondrion,lyase activity,anatomical structure development,amino acid metabolic process,transporter activity;Ontology_id=GO:0055085,GO:0005739,GO:0016829,GO:0048856,GO:0006520,GO:0005215;Enzyme_code=EC:4.3.1.19;Enzyme_name=threonine ammonia-lyase
contig00010.g5860	1835	ID=contig00010.g5860;Description=flavin-containing protein [Fusarium subglutinans]
contig00010.g5861	1859	ID=contig00010.g5861;Description=4-hydroxybenzoate transporter [Fusarium tjaetaba];Gene=FANTH_14428;Ontology_term=transmembrane transport,transporter activity;Ontology_id=GO:0055085,GO:0005215
contig00010.g5862	1056	ID=contig00010.g5862;Description=2 3-dihydroxybenzoate decarboxylase [Fusarium tjaetaba];Gene=FOZG_12162;Ontology_term=lyase activity,hydrolase activity;Ontology_id=GO:0016829,GO:0016787;Enzyme_code=EC:4.1.1,EC:3,EC:4.1.1,EC:4.1,EC:4.1.1.46,EC:3,EC:4;Enzyme_name=Carbon-carbon lyases,Hydrolases,Carbon-carbon lyases,Carbon-carbon lyases,o-pyrocatechuate decarboxylase,Hydrolases,Lyases
contig00010.g5863	537	ID=contig00010.g5863;Description=fungal specific transcription factor factor [Fusarium denticulatum]
contig00010.g5864	4210	ID=contig00010.g5864;Description=nitrate assimilation regulatory nirA [Fusarium tjaetaba];Gene=FVER53263_06536;Ontology_term=nucleus,DNA binding,regulation of DNA-templated transcription,transcription regulator activity;Ontology_id=GO:0005634,GO:0003677,GO:0006355,GO:0140110
contig00010.g5865	2028	ID=contig00010.g5865;Description=STB5-SIN3 binding protein [Fusarium fujikuroi];Gene=F25303_2245;Ontology_term=nucleus,DNA binding,regulation of DNA-templated transcription,transcription regulator activity;Ontology_id=GO:0005634,GO:0003677,GO:0006355,GO:0140110
contig00010.g5866	342	ID=contig00010.g5866;Description=hydrophobin 3 [Fusarium verticillioides];Ontology_term=cell wall;Ontology_id=GO:0005618
contig00010.g5867	2379	ID=contig00010.g5867;Description=hypothetical protein FVER53263_06539 [Fusarium verticillioides]

contig00010.g5868	348	ID=contig00010.g5868;Description=family 2 glycosyl transferase [Fusarium redolens];Gene=FACUT_7905;Ontology_term=transferase activity;Ontology_id=GO:0016740;Enzyme_code=EC:2,EC:2;Enzyme_name=Transferases,Transferases
contig00010.g5869	2657	ID=contig00010.g5869;Description=serine/threonine protein kinase [Fusarium verticillioides 7600];Gene=FMUND_1822;Ontology_term=catalytic activity, acting on a protein,oxidoreductase activity,transferase activity;Ontology_id=GO:0140096,GO:0016491,GO:0016740;Enzyme_code=EC:2.7.11.1,EC:1.14,EC:2.7.1,EC:2.7.11.1,EC:1,EC:1.14,EC:2.7,EC:2;Enzyme_name=non-specific serine/threonine protein kinase,Acting on paired donors, with incorporation or reduction of molecular oxygen. The oxygen incorporated need not be derived from O2,Transferring phosphorus-containing groups,non-specific serine/threonine protein kinase,Oxidoreductases,Acting on paired donors, with incorporation or reduction of molecular oxygen. The oxygen incorporated need not be derived from O2,Transferring phosphorus-containing groups,Transferases
contig00010.g5870	4008	ID=contig00010.g5870;Description=serine/threonine protein kinase [Fusarium verticillioides 7600];Gene=F52700_10576;Ontology_term=catalytic activity, acting on a protein,signaling,transferase activity;Ontology_id=GO:0140096,GO:0023052,GO:0016740;Enzyme_code=EC:2.7.11.1,EC:2.7.1,EC:2.7.11.1,EC:2.7,EC:2;Enzyme_name=non-specific serine/threonine protein kinase,Transferring phosphorus-containing groups,non-specific serine/threonine protein kinase,Transferring phosphorus-containing groups,Transferases
contig00010.g5871	414	ID=contig00010.g5871;Description=hypothetical protein FVEG_06545 [Fusarium verticillioides 7600];Gene=FNAPI_13988;Ontology_term=polyketide metabolic process;Ontology_id=GO:0030638
contig00010.g5872	2535	ID=contig00010.g5872;Description=hydroxylase [Fusarium tjaetaba];Gene=FVER53263_06546;Ontology_term=oxidoreductase activity;Ontology_id=GO:0016491;Enzyme_code=EC:1,EC:1;Enzyme_name=Oxidoreductases,Oxidoreductases
contig00010.g5873	1776	ID=contig00010.g5873;Description=sdr family [Fusarium tjaetaba]
contig00010.g5874	1086	ID=contig00010.g5874;Description=hypothetical protein FVER14953_06548 [Fusarium verticillioides]

contig00010.g5875	2244	ID=contig00010.g5875;Description=hypothetical protein FOTG_07181 [Fusarium oxysporum f. sp. vasinfectum 25433];Gene=FOTG_07181;Ontology_term=ATP-dependent activity,hydrolase activity;Ontology_id=GO:0140657,GO:0016787;Enzyme_code=EC:3.6.1.15,EC:3.6.1,EC:3.6,EC:3,EC:3.6.1.15;Enzyme_name=nucleoside-triphosphate phosphatase,Acting on acid anhydrides,Acting on acid anhydrides,Hydrolases,nucleoside-triphosphate phosphatase
contig00010.g5876	2969	ID=contig00010.g5876;Description=CHAT domain-containing protein [Fusarium oxysporum];Gene=Focb16_v014327;Ontology_term=ribosome;Ontology_id=GO:0005840
contig00010.g5877	2004	ID=contig00010.g5877;Description=hypothetical protein J7337_009199 [Fusarium musae]
contig00010.g5878	689	ID=contig00010.g5878;Description=acyl-CoA N-acyltransferase [Fusarium sp. MPI-SDFR-AT-0072];Gene=FPRO_10251;Ontology_term=transferase activity;Ontology_id=GO:0016740;Enzyme_code=EC:2.3.1,EC:2.3,EC:2,EC:2.3.1;Enzyme_name=Acyltransferases,Acyltransferases,Transferases,Acyltransferases
contig00010.g5879	1909	ID=contig00010.g5879;Description=glu asp-tRNA amidotransferase subunit A [Fusarium napiforme];Gene=FNAPI_10889;Ontology_term=transferase activity;Ontology_id=GO:0016740;Enzyme_code=EC:2.3.1;Enzyme_name=Acyltransferases
contig00010.g5880	1128	ID=contig00010.g5880;Description=7-alpha-cephem-methoxylase [Fusarium tjaetaba];Gene=FNYG_07069;Ontology_term=oxidoreductase activity;Ontology_id=GO:0016491;Enzyme_code=EC:1,EC:1;Enzyme_name=Oxidoreductases,Oxidoreductases
contig00010.g5881	4503	ID=contig00010.g5881;Description=serine/threonine protein kinase [Fusarium verticillioides 7600];Gene=FVER53590_26483;Ontology_term=catalytic activity, acting on a protein,transferase activity;Ontology_id=GO:0140096,GO:0016740;Enzyme_code=EC:2.7.11.1,EC:2.7.1,EC:2.7.11.1,EC:2.7,EC:2;Enzyme_name=non-specific serine/threonine protein kinase,Transferring phosphorus-containing groups,non-specific serine/threonine protein kinase,Transferring phosphorus-containing groups,Transferases
contig00010.g5882	1884	ID=contig00010.g5882;Description=het domain-containing [Fusarium sporotrichioides]
contig00010.g5883	2777	ID=contig00010.g5883;Description=ankyrin repeat [Fusarium coicis];Gene=EKO27_g2104;Ontology_term=catalytic activity, acting on a protein,transferase activity;Ontology_id=GO:0140096,GO:0016740;Enzyme_code=EC:2.7.1,EC:2.7.1,EC:2.7,EC:2;Enzyme_name=Transferring phosphorus-containing groups,Transferring phosphorus-containing groups,Transferring phosphorus-containing groups,Transferases

contig00010.g5884	1441	ID=contig00010.g5884;Description=hypothetical protein FVER14953_20015 [Fusarium verticillioides];Gene=FNAPI_10886;Ontology_term=membrane;Ontology_id=GO:0016020
contig00010.g5885	1964	ID=contig00010.g5885;Description=hypothetical protein FVER14953_20014 [Fusarium verticillioides]
contig00010.g5886	1246	ID=contig00010.g5886;Description=KES1-like protein [Fusarium tjaetaba];Gene=FOBC_12173;Ontology_term=lipid binding;Ontology_id=GO:0008289
contig00010.g5887	976	ID=contig00010.g5887;Description=hypothetical protein FVEG_15890 [Fusarium verticillioides 7600];Gene=F25303_2267;Ontology_term=nucleus,catalytic activity, acting on a protein,endoplasmic reticulum,catalytic activity, acting on RNA,transferase activity,hydrolase activity;Ontology_id=GO:0005634,GO:0140096,GO:0005783,GO:0140098,GO:0016740,GO:0016787;Enzyme_code=EC:2.7.11.1,EC:3.1,EC:2.7.1,EC:2.7.11.1,EC:3.1,EC:2.7,EC:2,EC:3;Enzyme_name=non-specific serine/threonine protein kinase,Acting on ester bonds,Transferring phosphorus-containing groups,non-specific serine/threonine protein kinase,Acting on ester bonds,Transferring phosphorus-containing groups,Transferases,Hydrolases
contig00010.g5888	2197	ID=contig00010.g5888;Description=cytosolic Fe-S cluster assembly factor cfd1 [Fusarium oxysporum];Gene=FPCIR_9109;Ontology_term=ATP-dependent activity,molecular carrier activity;Ontology_id=GO:0140657,GO:0140104
contig00010.g5889	1488	ID=contig00010.g5889;Description=adenosylmethionine decarboxylase [Fusarium verticillioides 7600];Gene=FACUT_6393;Ontology_term=nucleus,lyase activity,cellular modified amino acid metabolic process,cytosol,vitamin metabolic process;Ontology_id=GO:0005634,GO:0016829,GO:0006575,GO:0005829,GO:0006766;Enzyme_code=EC:4.1.1.50,EC:4.1.1,EC:4.1,EC:4,EC:4.1.1.50;Enzyme_name=adenosylmethionine decarboxylase,Carbon-carbon lyases,Carbon-carbon lyases,Lyases,adenosylmethionine decarboxylase
contig00010.g5890	1347	ID=contig00010.g5890;Description=UPF0271 protein [Fusarium verticillioides 7600];Gene=FNYG_07079;Ontology_term=RNA binding,protein-containing complex assembly,ribosome biogenesis,nucleolus,catalytic activity, acting on RNA,hydrolase activity;Ontology_id=GO:0003723,GO:0065003,GO:0042254,GO:0005730,GO:0140098,GO:0016787;Enzyme_code=EC:3.1,EC:3.1,EC:3;Enzyme_name=Acting on ester bonds,Acting on ester bonds,Hydrolases
contig00010.g5891	2199	ID=contig00010.g5891;Description=hypothetical protein FVER14953_06562 [Fusarium verticillioides]

contig00010.g5892	1287	ID=contig00010.g5892;Description=hypothetical protein FVEG_06563 [Fusarium verticillioides 7600]
contig00010.g5893	1509	ID=contig00010.g5893;Description=amino acid transporter [Fusarium pseudocircinatum];Gene=FDENT_2508;Ontology_term=membrane;Ontology_id=GO:0016020
contig00010.g5894	1530	ID=contig00010.g5894;Description=indoleamine 2,3-dioxygenase [Fusarium proliferatum];Gene=FVER53263_06565;Ontology_term=oxidoreductase activity,amino acid metabolic process;Ontology_id=GO:0016491,GO:0006520;Enzyme_code=EC:1,EC:1.13.11.52,EC:1;Enzyme_name=Oxidoreductases,indoleamine 2,3-dioxygenase,Oxidoreductases
contig00010.g5895	1100	ID=contig00010.g5895;Description=homoserine O-acetyltransferase [Fusarium verticillioides 7600];Gene=FPHYL_9968;Ontology_term=transferase activity;Ontology_id=GO:0016740;Enzyme_code=EC:2.3.1,EC:2.3,EC:2.3.1.31,EC:2,EC:2.3.1;Enzyme_name=Acyltransferases,Acyltransferases,homoserine O-acetyltransferase,Transferases,Acyltransferases
contig00010.g5896	1363	ID=contig00010.g5896;Description=formamidase [Fusarium proliferatum];Gene=BFJ72_g12509;Ontology_term=hydrolase activity;Ontology_id=GO:0016787;Enzyme_code=EC:3.5.1,EC:3.5.1,EC:3.5,EC:3,EC:3.5.1.49;Enzyme_name=Acting on carbon-nitrogen bonds, other than peptide bonds,Acting on carbon-nitrogen bonds, other than peptide bonds,Acting on carbon-nitrogen bonds, other than peptide bonds,Hydrolases,formamidase
contig00010.g5897	2220	ID=contig00010.g5897;Description=monocarboxylate transporter 4 [Fusarium fujikuroi];Gene=FMEXI_6796;Ontology_term=nucleus,DNA binding,regulation of DNA-templated transcription;Ontology_id=GO:0005634,GO:0003677,GO:0006355
contig00010.g5898	1962	ID=contig00010.g5898;Description=related to multidrug resistance protein [Fusarium fujikuroi];Gene=AU210_010118;Ontology_term=RNA binding,transmembrane transport,mRNA metabolic process,ribosome biogenesis,catalytic activity, acting on RNA,transporter activity,hydrolase activity;Ontology_id=GO:0003723,GO:0055085,GO:0016071,GO:0042254,GO:0140098,GO:0005215,GO:0016787;Enzyme_code=EC:3.1.13,EC:3.1.15,EC:3.1,EC:3.1.13,EC:3,EC:3.1.15;Enzyme_name=Acting on ester bonds,Acting on ester bonds,Acting on ester bonds,Acting on ester bonds,Hydrolases,Acting on ester bonds

contig00010.g5899	696	ID=contig00010.g5899;Description=multidrug resistance [Fusarium pseudocircinatum];Gene=FPCIR_9119;Ontology_term=transmembrane transport,transporter activity;Ontology_id=GO:0055085,GO:0005215
contig00010.g5900	621	ID=contig00010.g5900;Description=Vacuole-localized protein 4 like [Verticillium longisporum]
contig00010.g5901	1721	ID=contig00010.g5901;Description=heterokaryon incompatibility protein [Fusarium tjaetaba]
contig00010.g5902	1738	ID=contig00010.g5902;Description=hypothetical protein FVER53590_06574 [Fusarium verticillioides];Gene=F25303_12474;Ontology_term=membrane;Ontology_id=GO:0016020
contig00010.g5903	750	ID=contig00010.g5903;Description=acetyltransferase [Fusarium verticillioides 7600];Gene=BFJ63_vAg11546;Ontology_term=transferase activity;Ontology_id=GO:0016740;Enzyme_code=EC:2,EC:2,EC:2.3.1.47;Enzyme_name=Transferases,Transferases,8-amino-7-oxononanoate synthase
contig00010.g5904	3172	ID=contig00010.g5904;Description=adenosylmethionine-8-amino-7-oxononanoate aminotransferase [Fusarium verticillioides 7600];Gene=FVER53263_06577;Ontology_term=sulfur compound metabolic process,transferase activity,vitamin metabolic process,ligase activity;Ontology_id=GO:0006790,GO:0016740,GO:0006766,GO:0016874;Enzyme_code=EC:6.3.3.3,EC:2.6.1.62;Enzyme_name=dethiobiotin synthase,adenosylmethionine--8-amino-7-oxononanoate transaminase
contig00010.g5905	2572	ID=contig00010.g5905;Description=homeobox 4 [Fusarium sp. NRRL 52700];Gene=FNAPI_13449;Ontology_term=nucleus,DNA binding,regulation of DNA-templated transcription,transferase activity;Ontology_id=GO:0005634,GO:0003677,GO:0006355,GO:0016740;Enzyme_code=EC:2.6.1,EC:2.6.1,EC:2,EC:2.6;Enzyme_name=Transferring nitrogenous groups,Transferring nitrogenous groups,Transferases,Transferring nitrogenous groups
contig00010.g5906	2251	ID=contig00010.g5906;Description=glutamyl-tRNA(Gln) amidotransferase subunit A [Fusarium tjaetaba];Gene=FDENT_2519;Ontology_term=transferase activity;Ontology_id=GO:0016740;Enzyme_code=EC:2,EC:2;Enzyme_name=Transferases,Transferases
contig00010.g5907	1382	ID=contig00010.g5907;Description=glutamyl-tRNA(Gln) amidotransferase subunit A [Fusarium coicis];Gene=BFJ69_g10200;Ontology_term=membrane;Ontology_id=GO:0016020
contig00010.g5908	1356	ID=contig00010.g5908;Description=hypothetical protein FVEG_15896 [Fusarium verticillioides 7600]

contig00010.g5909	3074	ID=contig00010.g5909;Description=integral membrane protein [Fusarium pseudoanthophilum];Gene=FDENT_4817;Ontology_term=membrane;Ontology_id=GO:0016020
contig00010.g5910	1731	ID=contig00010.g5910;Description=6-hydroxy-d-nicotine oxidase [Fusarium tjaetaba];Gene=KTR1;Ontology_term=molecular transducer activity,anatomical structure development,regulation of DNA-templated transcription,reproductive process,immune system process,defense response to other organism,DNA binding,nucleus,lipid metabolic process,signaling,cell differentiation,transferase activity,transcription regulator activity;Ontology_id=GO:0060089,GO:0048856,GO:0006355,GO:0022414,GO:0002376,GO:0098542,GO:0003677,GO:0005634,GO:0006629,GO:0023052,GO:0030154,GO:0016740,GO:0140110;Enzyme_code=EC:2.4.1,EC:2.4,EC:2,EC:2.4.1;Enzyme_name=Glycosyltransferases,Glycosyltransferases,Transferases,Glycosyltransferases
contig00010.g5911	1545	ID=contig00010.g5911;Description=transcription activator [Fusarium tjaetaba];Gene=FTJAE_7195;Ontology_term=nucleus,regulation of DNA-templated transcription,transcription regulator activity;Ontology_id=GO:0005634,GO:0006355,GO:0140110
contig00010.g5912	1644	ID=contig00010.g5912;Description=MFS transporter, SP family, general alpha glucoside:H+ symporter [Fusarium verticillioides 7600];Gene=FPCIR_13030;Ontology_term=transmembrane transport,transporter activity;Ontology_id=GO:0055085,GO:0005215
contig00010.g5913	1779	ID=contig00010.g5913;Description=glucan 1,6-alpha-glucosidase [Fusarium verticillioides 7600];Gene=FTJAE_7197;Ontology_term=carbohydrate metabolic process,hydrolase activity;Ontology_id=GO:0005975,GO:0016787;Enzyme_code=EC:3.2.1.20,EC:3.2,EC:3.2.1.20,EC:3.2.1.10,EC:3,EC:3.2.1;Enzyme_name=alpha-glucosidase,Glycosylases,alpha-glucosidase,oligo-1,6-glucosidase,Hydrolases,Glycosylases
contig00010.g5914	1305	ID=contig00010.g5914;Description=hypothetical protein FVEG_06589 [Fusarium verticillioides 7600]
contig00010.g5915	459	ID=contig00010.g5915;Description=hypothetical protein FVEG_06590 [Fusarium verticillioides 7600]
contig00010.g5916	1214	ID=contig00010.g5916;Description=related to zinc-binding oxidoreductase [Fusarium proliferatum];Gene=FVEG_06591;Ontology_term=oxidoreductase activity;Ontology_id=GO:0016491;Enzyme_code=EC:1,EC:1;Enzyme_name=Oxidoreductases,Oxidoreductases

contig00010.g5917	1490	ID=contig00010.g5917;Description=neutral amino acid permease [Fusarium tjaetaba];Gene=FNAPI_5257;Ontology_term=membrane;Ontology_id=GO:0016020
contig00010.g5918	492	ID=contig00010.g5918;Description=acetyltransferase [Fusarium verticillioides 7600];Gene=FMAN_09913;Ontology_term=DNA binding,transferase activity;Ontology_id=GO:0003677,GO:0016740;Enzyme_code=EC:2.3.1,EC:2.3,EC:2,EC:2.3.1;Enzyme_name=Acyltransferases,Acyltransferases,Transferases,Acyltransferases
contig00010.g5919	2274	ID=contig00010.g5919;Description=glu leu phe val dehydrogenase family [Fusarium pseudoanthophilum];Gene=FVEG_06594;Ontology_term=oxidoreductase activity,amino acid metabolic process;Ontology_id=GO:0016491,GO:0006520;Enzyme_code=EC:1.4.1.3,EC:1.4,EC:1,EC:1.4.1.3,EC:1.4.1;Enzyme_name=glutamate dehydrogenase [NAD(P)(+)],Acting on the CH-NH2 group of donors,Oxidoreductases,glutamate dehydrogenase [NAD(P)(+)],Acting on the CH-NH2 group of donors
contig00010.g5920	1014	ID=contig00010.g5920;Description=methyltransferase [Fusarium pseudoanthophilum];Gene=F66182_2369;Ontology_term=transferase activity;Ontology_id=GO:0016740;Enzyme_code=EC:2.1.1,EC:2.1,EC:2,EC:2.1.1;Enzyme_name=Transferring one-carbon groups,Transferring one-carbon groups,Transferases,Transferring one-carbon groups
contig00010.g5921	1988	ID=contig00010.g5921;Description=Ff.00g117140.m01.CDS01 [Fusarium sp. VM40]
contig00010.g5922	623	ID=contig00010.g5922;Description=glu leu phe val dehydrogenase family [Fusarium napiforme];Gene=FNAPI_5254;Ontology_term=oxidoreductase activity;Ontology_id=GO:0016491;Enzyme_code=EC:1;Enzyme_name=Oxidoreductases
contig00010.g5923	1986	ID=contig00010.g5923;Description=serine threonine kinase [Fusarium tjaetaba];Gene=F52700_2456;Ontology_term=catalytic activity, acting on a protein,transferase activity;Ontology_id=GO:0140096,GO:0016740;Enzyme_code=EC:2.7.1,EC:2.7.1,EC:2.7,EC:2;Enzyme_name=Transferring phosphorus-containing groups,Transferring phosphorus-containing groups,Transferring phosphorus-containing groups,Transferases
contig00010.g5924	3222	ID=contig00010.g5924;Description=hypothetical protein FVER53590_06599 [Fusarium verticillioides];Gene=FDENT_13119;Ontology_term=catalytic activity,ATP-dependent activity;Ontology_id=GO:0003824,GO:0140657
contig00010.g5925	469	ID=contig00010.g5925;Description=hypothetical protein FPANT_2881 [Fusarium pseudoanthophilum]
contig00010.g5926	562	ID=contig00010.g5926;Description=---NA---

contig00010.g5927	1760	ID=contig00010.g5927;Description=global transactivator [Fusarium mundagurra];Gene=FVER53263_06600;Ontology_term=nucleus,catalytic activity, acting on DNA,ATP-dependent activity,chromatin organization,hydrolase activity;Ontology_id=GO:0005634,GO:0140097,GO:0140657,GO:0006325,GO:0016787;Enzyme_code=EC:3,EC:3;Enzyme_name=Hydrolases,Hydrolases
contig00010.g5928	1623	ID=contig00010.g5928;Description=uncharacterized protein FTJAE_3052 [Fusarium tjaetaba]
contig00010.g5929	623	ID=contig00010.g5929;Description=hypothetical protein FVEG_06603 [Fusarium verticillioides 7600]
contig00010.g5930	2496	ID=contig00010.g5930;Description=hypothetical protein FPHYL_515 [Fusarium phyllophilum]
contig00010.g5931	749	ID=contig00010.g5931;Description=uncharacterized protein FSUBG_12346 [Fusarium subglutinans]
contig00010.g5932	324	ID=contig00010.g5932;Description=hypothetical protein H9Q69_003695 [Fusarium xylarioides]
contig00010.g5933	1730	ID=contig00010.g5933;Description=speckle-type POZ [Fusarium pseudocircinatum];Gene=FPHYL_3690;Ontology_term=catalytic activity, acting on a protein,hydrolase activity;Ontology_id=GO:0140096,GO:0016787;Enzyme_code=EC:3.4;Enzyme_name=Acting on peptide bonds (peptidases)
contig00010.g5934	1155	ID=contig00010.g5934;Description=hypothetical protein FVER53263_06607 [Fusarium verticillioides]
contig00010.g5935	1530	ID=contig00010.g5935;Description=hypothetical protein FVEG_06609 [Fusarium verticillioides 7600]
contig00010.g5936	1533	ID=contig00010.g5936;Description=hypothetical protein FNAPI_11534 [Fusarium napiforme]
contig00010.g5937	2136	ID=contig00010.g5937;Description=hypothetical protein FVER53263_06611 [Fusarium verticillioides]
contig00010.g5938	1806	ID=contig00010.g5938;Description=hypothetical protein FVER53263_06612 [Fusarium verticillioides]
contig00010.g5939	378	ID=contig00010.g5939;Description=hypothetical protein FVER53263_06613 [Fusarium verticillioides];Gene=MCM3;Ontology_term=nucleus,DNA replication,anatomical structure development,structural molecule activity,cell differentiation,extracellular matrix organization,extracellular matrix,extracellular region;Ontology_id=GO:0005634,GO:0006260,GO:0048856,GO:0005198,GO:0030154,GO:0030198,GO:0031012,GO:0005576

contig00010.g5940	1241	ID=contig00010.g5940;Description=hypothetical protein FVEG_06614 [Fusarium verticillioides 7600]
contig00010.g5941	1284	ID=contig00010.g5941;Description=hypothetical protein FVEG_06615 [Fusarium verticillioides 7600];Gene=FPANT_13203;Ontology_term=nucleic acid binding,zinc ion binding;Ontology_id=GO:0003676,GO:0008270
contig00010.g5942	752	ID=contig00010.g5942;Description=hypothetical protein FVEG_06616 [Fusarium verticillioides 7600]
contig00010.g5943	435	ID=contig00010.g5943;Description=hypothetical protein FVEG_06617 [Fusarium verticillioides 7600];Gene=FANTH_12944;Ontology_term=catalytic activity, acting on a protein,extracellular region,hydrolase activity;Ontology_id=GO:0140096,GO:0005576,GO:0016787;Enzyme_code=EC:3.5.3,EC:3.4,EC:3.5.3,EC:3.4,EC:3.5,EC:3;Enzyme_name=Acting on carbon-nitrogen bonds, other than peptide bonds,Acting on peptide bonds (peptidases),Acting on carbon-nitrogen bonds, other than peptide bonds,Acting on peptide bonds (peptidases),Acting on carbon-nitrogen bonds, other than peptide bonds,Hydrolases
contig00010.g5944	1059	ID=contig00010.g5944;Description=Carbon-nitrogen hydrolase [Fusarium musae];Gene=FVER53590_06618;Ontology_term=hydrolase activity;Ontology_id=GO:0016787;Enzyme_code=EC:3.5.5.1,EC:3.5.5,EC:3.5,EC:3,EC:3.5.5.1;Enzyme_name=nitrilase,Acting on carbon-nitrogen bonds, other than peptide bonds,Acting on carbon-nitrogen bonds, other than peptide bonds,Hydrolases,nitrilase
contig00010.g5945	1574	ID=contig00010.g5945;Description=major facilitator superfamily transporter [Fusarium mundagurra];Gene=FNYG_07145;Ontology_term=transmembrane transport,transporter activity;Ontology_id=GO:0055085,GO:0005215
contig00010.g5946	2067	ID=contig00010.g5946;Description=Nudix hydrolase [Fusarium coicis];Gene=FVEG_15902;Ontology_term=nucleus,DNA binding,regulation of DNA-templated transcription,transcription regulator activity,hydrolase activity;Ontology_id=GO:0005634,GO:0003677,GO:0006355,GO:0140110,GO:0016787;Enzyme_code=EC:3,EC:3;Enzyme_name=Hydrolases,Hydrolases
contig00010.g5947	624	ID=contig00010.g5947;Description=YSA1-sugar-nucleotide hydrolase [Fusarium fujikuroi];Gene=LW93_5149;Ontology_term=hydrolase activity;Ontology_id=GO:0016787;Enzyme_code=EC:3,EC:3.6.1.13,EC:3;Enzyme_name=Hydrolases,ADP-ribose diphosphatase,Hydrolases
contig00010.g5948	3301	ID=contig00010.g5948;Description=hypothetical protein FVER14953_06623 [Fusarium verticillioides]

contig00010.g5949	1984	ID=contig00010.g5949;Description=beta-alanine synthase [Fusarium napiforme];Gene=FNAPI_9681;Ontology_term=catalytic activity, acting on a protein,extracellular region,hydrolase activity;Ontology_id=GO:0140096,GO:0005576,GO:0016787;Enzyme_code=EC:3.5.3,EC:3.4,EC:3.5.3,EC:3.4,EC:3.5,EC:3;Enzyme_name=Acting on carbon-nitrogen bonds, other than peptide bonds,Acting on peptide bonds (peptidases),Acting on carbon-nitrogen bonds, other than peptide bonds,Acting on peptide bonds (peptidases),Acting on carbon-nitrogen bonds, other than peptide bonds,Hydrolases
contig00010.g5950	1612	ID=contig00010.g5950;Description=signal recognition particle protein [Fusarium verticillioides 7600];Gene=BFJ63_vAg11188;Ontology_term=RNA binding,ATP-dependent activity,GTPase activity,endoplasmic reticulum;Ontology_id=GO:0003723,GO:0140657,GO:0003924,GO:0005783;Enzyme_code=EC:3.6.1.15;Enzyme_name=nucleoside-triphosphate phosphatase
contig00010.g5951	804	ID=contig00010.g5951;Description=flavin-nucleotide-binding protein [Fusarium tjaetaba]
contig00010.g5952	1677	ID=contig00010.g5952;Description=triacylglycerol lipase V precursor [Fusarium tjaetaba];Gene=FVER53263_20820;Ontology_term=hydrolase activity;Ontology_id=GO:0016787;Enzyme_code=EC:3,EC:3;Enzyme_name=Hydrolases,Hydrolases
contig00010.g5953	1083	ID=contig00010.g5953;Description=alcohol dehydrogenase (NADP+) [Fusarium verticillioides 7600];Gene=FNAPI_9676;Ontology_term=oxidoreductase activity;Ontology_id=GO:0016491;Enzyme_code=EC:1.1.1,EC:1.1,EC:1,EC:1.1.1,EC:1.1.1.2;Enzyme_name=Acting on the CH-OH group of donors,Acting on the CH-OH group of donors,Oxidoreductases,Acting on the CH-OH group of donors,alcohol dehydrogenase (NADP(+))
contig00010.g5954	727	ID=contig00010.g5954;Description=chaperone HSP31 [Fusarium tjaetaba]
contig00010.g5955	780	ID=contig00010.g5955;Description=filamentous hemagglutinin [Fusarium sp. NRRL 52700]
contig00010.g5956	1005	ID=contig00010.g5956;Description=Porphobilinogen deaminase [Fusarium oxysporum];Gene=BFJ65_g11394;Ontology_term=nucleus,transferase activity;Ontology_id=GO:0005634,GO:0016740;Enzyme_code=EC:2.5.1.61,EC:2.5.1,EC:2,EC:2.5.1.61;Enzyme_name=hydroxymethylbilane synthase,Transferring alkyl or aryl groups, other than methyl groups,Transferases,hydroxymethylbilane synthase
contig00010.g5957	550	ID=contig00010.g5957;Description=glycine cleavage system H [Fusarium tjaetaba];Gene=FIE12Z_12701;Ontology_term=mitochondrion,amino acid metabolic process;Ontology_id=GO:0005739,GO:0006520

contig00010.g5958	735	ID=contig00010.g5958;Description=rab family, other [Fusarium odoratissimum NRRL 54006];Gene=FOZG_12271;Ontology_term=cytoskeleton organization,microtubule organizing center,GTPase activity,signaling,mitotic nuclear division,chromosome segregation,cytokinesis;Ontology_id=GO:0007010,GO:0005815,GO:0003924,GO:0023052,GO:0140014,GO:0007059,GO:0000910;Enzyme_code=EC:3.6.1.15,EC:3.6.1,EC:3.6,EC:3,EC:3.6.1.15;Enzyme_name=nucleoside-triphosphate phosphatase,Acting on acid anhydrides,Acting on acid anhydrides,Hydrolases,nucleoside-triphosphate phosphatase
contig00010.g5959	396	ID=contig00010.g5959;Description=hypothetical protein FVEG_06635 [Fusarium verticillioides 7600];Gene=FVER53263_06635;Ontology_term=membrane;Ontology_id=GO:0016020
contig00010.g5960	237	ID=contig00010.g5960;Description=hypothetical protein FCOIX_402 [Fusarium coicis];Gene=FVER53263_06635;Ontology_term=membrane;Ontology_id=GO:0016020
contig00010.g5961	453	ID=contig00010.g5961;Description=hypothetical protein FBULB1_13480 [Fusarium bulbicola];Gene=Forpi1262_v006862;Ontology_term=membrane;Ontology_id=GO:0016020
contig00010.g5962	1145	ID=contig00010.g5962;Description=integral membrane protein [Fusarium napiforme]
contig00010.g5963	1141	ID=contig00010.g5963;Description=thymidylate kinase [Fusarium napiforme];Gene=FNAPI_13901;Ontology_term=transferase activity;Ontology_id=GO:0016740;Enzyme_code=EC:2.7;Enzyme_name=Transferring phosphorus-containing groups
contig00010.g5964	849	ID=contig00010.g5964;Description=thymidylate kinase [Fusarium tjaetaba];Gene=FVEG_06639;Ontology_term=nucleus,nucleobase-containing small molecule metabolic process,carbohydrate derivative metabolic process,transferase activity;Ontology_id=GO:0005634,GO:0055086,GO:1901135,GO:0016740;Enzyme_code=EC:2.7.4.4,EC:2.7.4.9,EC:2.7.4.4,EC:2.7,EC:2.7.4.6,EC:2,EC:2.7.4,EC:2.7.4.9;Enzyme_name=nucleoside-phosphate kinase,dTMP kinase,nucleoside-phosphate kinase,Transferring phosphorus-containing groups,nucleoside-diphosphate kinase,Transferases,Transferring phosphorus-containing groups,dTMP kinase

contig00010.g5965	1222	ID=contig00010.g5965;Description=formyltetrahydrofolate deformylase [Fusarium verticillioides 7600];Gene=FOC1_g10012307;Ontology_term=nucleobase-containing small molecule metabolic process,carbohydrate derivative metabolic process,amino acid metabolic process,hydrolase activity;Ontology_id=GO:0055086,GO:1901135,GO:0006520,GO:0016787;Enzyme_code=EC:3.5.1.10,EC:3.5.1,EC:3.5.1.10,EC:3.5,EC:3;Enzyme_name=formyltetrahydrofolate deformylase,Acting on carbon-nitrogen bonds, other than peptide bonds,formyltetrahydrofolate deformylase,Acting on carbon-nitrogen bonds, other than peptide bonds,Hydrolases
contig00010.g5966	1469	ID=contig00010.g5966;Description=Aspercryptin biosynthesis cluster-specific transcription regulator atnN [Fusarium oxysporum f. sp. cubense];Gene=FRV6_12027;Ontology_term=nucleus,regulation of DNA-templated transcription,transcription regulator activity;Ontology_id=GO:0005634,GO:0006355,GO:0140110
contig00010.g5967	3023	ID=contig00010.g5967;Description=hypothetical protein FVEG_15910 [Fusarium verticillioides 7600];Gene=FMAN_09974;Ontology_term=nucleus,regulation of DNA-templated transcription,transcription regulator activity;Ontology_id=GO:0005634,GO:0006355,GO:0140110
contig00010.g5968	1725	ID=contig00010.g5968;Description=clock-controlled protein 8 (ccg-8) [Fusarium fujikuroi];Gene=BFJ68_g317;Ontology_term=nuclear envelope,signaling,lipid metabolic process,regulation of DNA-templated transcription,lipid binding,endoplasmic reticulum,transcription regulator activity,molecular adaptor activity,nucleoplasm;Ontology_id=GO:0005635,GO:0023052,GO:0006629,GO:0006355,GO:0008289,GO:0005783,GO:0140110,GO:0060090,GO:0005654
contig00010.g5969	1473	ID=contig00010.g5969;Description=AP-1 complex subunit mu-1 [Fusarium oxysporum];Gene=FOMG_07829;Ontology_term=vesicle-mediated transport,intracellular protein transport;Ontology_id=GO:0016192,GO:0006886
contig00010.g5970	991	ID=contig00010.g5970;Description=hypothetical protein FVER53263_06645 [Fusarium verticillioides]

contig00010.g5971	1520	ID=contig00010.g5971;Description=ribosomal n-lysine methyltransferase 4 [Fusarium tjaetaba];Gene=FVER53263_06646;Ontology_term=nucleus,DNA repair,catalytic activity, acting on a protein,cytoplasmic translation,transferase activity,hydrolase activity;Ontology_id=GO:0005634,GO:0006281,GO:0140096,GO:0002181,GO:0016740,GO:0016787;Enzyme_code=EC:3.1,EC:2.1.1,EC:2.1,EC:3.1,EC:2,EC:3,EC:2.1.1;Enzyme_name=Acting on ester bonds,Transferring one-carbon groups,Transferring one-carbon groups,Acting on ester bonds,Transferases,Hydrolases,Transferring one-carbon groups
contig00010.g5972	1879	ID=contig00010.g5972;Description=UV-damage endonuclease [Fusarium musae];Gene=FPCIR_10097;Ontology_term=DNA repair,nucleus,DNA binding,catalytic activity, acting on DNA,mitochondrion,mitochondrion organization,hydrolase activity;Ontology_id=GO:0006281,GO:0005634,GO:0003677,GO:0140097,GO:0005739,GO:0007005,GO:0016787;Enzyme_code=EC:3.1,EC:3.1,EC:3.1.25,EC:3,EC:3.1.25.1;Enzyme_name=Acting on ester bonds,Acting on ester bonds,Acting on ester bonds,Hydrolases,deoxyribonuclease (pyrimidine dimer)
contig00010.g5973	3395	ID=contig00010.g5973;Description=putative ENA5-Plasma membrane P-type ATPase involved in Na+ and Li+ efflux [Fusarium fujikuroi];Gene=F52700_2520;Ontology_term=transmembrane transport,ATP-dependent activity,transporter activity,hydrolase activity;Ontology_id=GO:0055085,GO:0140657,GO:0005215,GO:0016787;Enzyme_code=EC:7.2.2,EC:3.6.1.15;Enzyme_name=Catalysing the translocation of inorganic cations,nucleoside-triphosphate phosphatase
contig00010.g5974	296	ID=contig00010.g5974;Description=hypothetical protein FVEG_06649 [Fusarium verticillioides 7600]
contig00010.g5975	827	ID=contig00010.g5975;Description=related to Glc8 protein [Fusarium fujikuroi];Gene=FPANT_9398;Ontology_term=molecular function regulator activity,signaling;Ontology_id=GO:0098772,GO:0023052
contig00010.g5976	402	ID=contig00010.g5976;Description=long chronological lifespan 2 [Fusarium subglutinans];Ontology_term=protein catabolic process;Ontology_id=GO:0030163
contig00010.g5977	836	ID=contig00010.g5977;Description=60s ribosomal L2 (mitochondrial) [Fusarium tjaetaba];Gene=FCIRC_1017;Ontology_term=structural molecule activity,ribosome;Ontology_id=GO:0005198,GO:0005840
contig00010.g5978	2098	ID=contig00010.g5978;Description=hypothetical protein BFJ70_g2363 [Fusarium oxysporum];Gene=FPANT_9395;Ontology_term=transmembrane transport,transporter activity;Ontology_id=GO:0055085,GO:0005215

contig00010.g5979	4139	ID=contig00010.g5979;Description=hypothetical protein FVER53263_06655 [Fusarium verticillioides];Gene=FVER53263_06655;Ontology_term=transmembrane transport,transporter activity;Ontology_id=GO:0055085,GO:0005215
contig00010.g5980	945	ID=contig00010.g5980;Description=FC receptor [Fusarium denticulatum];Gene=FVER53263_06655;Ontology_term=transmembrane transport,transporter activity;Ontology_id=GO:0055085,GO:0005215
contig00010.g5981	1460	ID=contig00010.g5981;Description=RNase III domain-containing protein [Fusarium mundagurra];Gene=F25303_10361;Ontology_term=gene expression,cellular nitrogen compound metabolic process,primary metabolic process;Ontology_id=GO:0010467,GO:0034641,GO:0044238
contig00010.g5982	847	ID=contig00010.g5982;Description=RNase III domain protein [Fusarium tjaetaba];Gene=FVER53263_06657;Ontology_term=mitochondrial gene expression,mitochondrion,structural molecule activity,catalytic activity, acting on RNA,ribosome,hydrolase activity;Ontology_id=GO:0140053,GO:0005739,GO:0005198,GO:0140098,GO:0005840,GO:0016787;Enzyme_code=EC:3.1.30,EC:3.1.26.3,EC:3.1.30,EC:3.1,EC:3,EC:3.1.26.3,EC:3.1.26;Enzyme_name=Acting on ester bonds,ribonuclease III,Acting on ester bonds,Acting on ester bonds,Hydrolases,ribonuclease III,Acting on ester bonds
contig00010.g5983	1062	ID=contig00010.g5983;Description=hypothetical protein FVER14953_20980 [Fusarium verticillioides];Gene=FPANT_13326;Ontology_term=membrane;Ontology_id=GO:0016020
contig00010.g5984	3378	ID=contig00010.g5984;Description=related to mitochondrial nicotinamide nucleotide transhydrogenase [Fusarium mangiferae];Gene=FNYG_07188;Ontology_term=transmembrane transport,oxidoreductase activity;Ontology_id=GO:0055085,GO:0016491;Enzyme_code=EC:1.6.1.1,EC:1.6.1.2,EC:1.6.1,EC:1.6.1.1,EC:1.6,EC:1;Enzyme_name=NAD(P)(+) transhydrogenase (Si-specific),NAD(P)(+) transhydrogenase (Re/Si-specific),Acting on NADH or NADPH,NAD(P)(+) transhydrogenase (Si-specific),Acting on NADH or NADPH,Oxidoreductases
contig00010.g5985	606	ID=contig00010.g5985;Description=NADH-ubiquinone oxidoreductase subunit [Fusarium tjaetaba];Gene=FMAN_09994;Ontology_term=protein-containing complex assembly,mitochondrion,mitochondrion organization;Ontology_id=GO:0065003,GO:0005739,GO:0007005

contig00010.g5986	1654	ID=contig00010.g5986;Description=phosphatidylserine synthase [Fusarium verticillioides 7600];Gene=FOIG_04815;Ontology_term=membrane organization,nucleobase-containing small molecule metabolic process,mitochondrion,lipid metabolic process,transferase activity,mitochondrion organization;Ontology_id=GO:0061024,GO:0055086,GO:0005739,GO:0006629,GO:0016740,GO:0007005;Enzyme_code=EC:2.7.8.5,EC:2.7,EC:2,EC:2.7.8,EC:2.7.8.5;Enzyme_name=CDP-diacylglycerol--glycerol-3-phosphate 1-phosphatidyltransferase,Transferring phosphorus-containing groups,Transferases,Transferring phosphorus-containing groups,CDP-diacylglycerol--glycerol-3-phosphate 1-phosphatidyltransferase
contig00010.g5987	1050	ID=contig00010.g5987;Description=ribosomal YML6 mitochondrial [Fusarium tjaetaba];Gene=FMUND_11280;Ontology_term=RNA binding,mitochondrial gene expression,mitochondrion,structural molecule activity,cytosol,ribosome;Ontology_id=GO:0003723,GO:0140053,GO:0005739,GO:0005198,GO:0005829,GO:0005840
contig00010.g5988	1359	ID=contig00010.g5988;Description=mitochondrial 54S ribosomal protein YmL35 [Fusarium oxysporum];Gene=FDENT_11147;Ontology_term=mitochondrial gene expression,mitochondrion,structural molecule activity,ribosome;Ontology_id=GO:0140053,GO:0005739,GO:0005198,GO:0005840
contig00010.g5989	1012	ID=contig00010.g5989;Description=transcription factor atf21 [Fusarium pseudocircinatum];Gene=FVER53263_06664;Ontology_term=DNA binding,reproductive process,regulation of DNA-templated transcription,transcription regulator activity;Ontology_id=GO:0003677,GO:0022414,GO:0006355,GO:0140110
contig00010.g5990	1420	ID=contig00010.g5990;Description=Replication factor A 3 [Fusarium tjaetaba];Gene=FMAN_09999;Ontology_term=nucleus,DNA binding,DNA repair,DNA replication,DNA recombination;Ontology_id=GO:0005634,GO:0003677,GO:0006281,GO:0006260,GO:0006310
contig00010.g5991	3963	ID=contig00010.g5991;Description=Rho-GTPase-activating 6 [Fusarium pseudocircinatum];Gene=FPHYL_5592;Ontology_term=molecular function regulator activity,signaling;Ontology_id=GO:0098772,GO:0023052

contig00010.g5992	1750	ID=contig00010.g5992;Description=nuclear SNF4 [Fusarium subglutinans];Ontology_term=nuclear envelope,carbohydrate metabolic process,molecular function regulator activity,signaling,peroxisome organization,regulation of DNA-templated transcription,cytosol,plasma membrane;Ontology_id=GO:0005635,GO:0005975,GO:0098772,GO:0023052,GO:0007031,GO:0006355,GO:0005829,GO:0005886
contig00010.g5993	1954	ID=contig00010.g5993;Description=vacuolar protein 8 [Fusarium verticillioides 7600];Gene=FCIRC_12779;Ontology_term=membrane organization,nucleus,autophagy,cytosol,intracellular protein transport,vacuole,molecular adaptor activity;Ontology_id=GO:0061024,GO:0005634,GO:0006914,GO:0005829,GO:0006886,GO:0005773,GO:0060090
contig00010.g5994	549	ID=contig00010.g5994;Description=Mitochondrial inner membrane protease subunit 1 [Fusarium odoratissimum];Gene=FOC4_g10008315;Ontology_term=catalytic activity, acting on a protein,protein-containing complex assembly,mitochondrion,protein maturation,intracellular protein transport,mitochondrion organization,hydrolase activity;Ontology_id=GO:0140096,GO:0065003,GO:0005739,GO:0051604,GO:0006886,GO:0007005,GO:0016787;Enzyme_code=EC:3.4.21,EC:3.4.21,EC:3.4.24,EC:3.4,EC:3;Enzyme_name=Acting on peptide bonds (peptidases),Acting on peptide bonds (peptidases),Acting on peptide bonds (peptidases),Acting on peptide bonds (peptidases),Hydrolases
contig00010.g5995	882	ID=contig00010.g5995;Description=translation initiation factor IF-3 [Fusarium verticillioides 7600];Gene=FPANT_4607;Ontology_term=translation regulator activity;Ontology_id=GO:0045182
contig00010.g5996	568	ID=contig00010.g5996;Description=microfibril-associated protein [Fusarium bulbicola];Gene=FGADI_8850;Ontology_term=nucleus;Ontology_id=GO:0005634
contig00010.g5997	2173	ID=contig00010.g5997;Description=related to D-mandelate dehydrogenase [Fusarium proliferatum]
contig00010.g5998	1201	ID=contig00010.g5998;Description=D-mandelate dehydrogenase [Fusarium globosum];Gene=FMUND_11291;Ontology_term=oxidoreductase activity;Ontology_id=GO:0016491;Enzyme_code=EC:1.1.1,EC:1.1,EC:1,EC:1.1.1;Enzyme_name=Acting on the CH-OH group of donors,Acting on the CH-OH group of donors,Oxidoreductases,Acting on the CH-OH group of donors

contig00010.g5999	1557	ID=contig00010.g5999;Description=regulator-Ty1 transposition protein 109 [Fusarium verticillioides 7600];Gene=FNYG_07205;Ontology_term=DNA repair,nucleus,catalytic activity, acting on a protein,DNA recombination,regulation of DNA-templated transcription,chromatin organization,transferase activity;Ontology_id=GO:0006281,GO:0005634,GO:0140096,GO:0006310,GO:0006355,GO:0006325,GO:0016740;Enzyme_code=EC:2.3.1.48,EC:2.3,EC:2,EC:2.3.1.48,EC:2.3.1;Enzyme_name=histone acetyltransferase,Acyltransferases,Transferases,histone acetyltransferase,Acyltransferases
contig00010.g6000	1035	ID=contig00010.g6000;Description=regulator-Ty1 transposition protein 109 [Fusarium oxysporum f. sp. lycopersici 4287]
contig00010.g6001	861	ID=contig00010.g6001;Description=peroxisomal short-chain alcohol dehydrogenase [Fusarium tjaetaba]
contig00010.g6002	936	ID=contig00010.g6002;Description=adenosine monophosphate transferase FICD like [Fusarium tjaetaba];Gene=FPCIR_6551;Ontology_term=transferase activity;Ontology_id=GO:0016740;Enzyme_code=EC:2,EC:2;Enzyme_name=Transferases,Transferases
contig00010.g6003	864	ID=contig00010.g6003;Description=hypothetical protein FVER53263_20570 [Fusarium verticillioides]
contig00010.g6004	1436	ID=contig00010.g6004;Description=transcription initiation factor TFIID subunit 6 [Fusarium proliferatum];Gene=FPHYL_5606;Ontology_term=translation regulator activity,protein-containing complex assembly,structural molecule activity,general transcription initiation factor activity,nuclear chromosome,regulation of DNA-templated transcription,cytosol,chromatin organization,nucleoplasm,molecular adaptor activity;Ontology_id=GO:0045182,GO:0065003,GO:0005198,GO:0140223,GO:0000228,GO:0006355,GO:0005829,GO:0006325,GO:0005654,GO:0060090
contig00010.g6005	660	ID=contig00010.g6005;Description=mitochondrial succinate dehydrogenase subunit C [Fusarium tjaetaba];Gene=FCIRC_8713;Ontology_term=generation of precursor metabolites and energy,oxidoreductase activity;Ontology_id=GO:0006091,GO:0016491;Enzyme_code=EC:1.3,EC:1.3,EC:1;Enzyme_name=Acting on the CH-CH group of donors,Acting on the CH-CH group of donors,Oxidoreductases
contig00010.g6006	1055	ID=contig00010.g6006;Description=Serine/threonine-protein kinase SRPK [Fusarium oxysporum f. sp. albedinis];Gene=FVER53590_06681;Ontology_term=membrane;Ontology_id=GO:0016020

contig00010.g6007	2271	ID=contig00010.g6007;Description=transcription factor [Fusarium coicis];Gene=FOIG_04837;Ontology_term=nucleus,DNA binding,regulation of DNA-templated transcription,transcription regulator activity;Ontology_id=GO:0005634,GO:0003677,GO:0006355,GO:0140110
contig00010.g6008	1400	ID=contig00010.g6008;Description=tRNA dimethylallyltransferase [Fusarium verticillioides 7600];Gene=FNAPI_7081;Ontology_term=tRNA metabolic process,catalytic activity, acting on RNA,transferase activity;Ontology_id=GO:0006399,GO:0140098,GO:0016740;Enzyme_code=EC:2.5.1.75;Enzyme_name=tRNA dimethylallyltransferase
contig00010.g6009	1616	ID=contig00010.g6009;Description=SecY subunit domain-containing protein [Fusarium oxysporum f. sp. albedinis];Gene=CEP51_006220;Ontology_term=transmembrane transport,ATP-dependent activity,protein catabolic process,endoplasmic reticulum,intracellular protein transport,vacuole,transporter activity,plasma membrane;Ontology_id=GO:0055085,GO:0140657,GO:0030163,GO:0005783,GO:0006886,GO:0005773,GO:0005215,GO:0005886;Enzyme_code=EC:7.2.2,EC:7.2.2,EC:7;Enzyme_name=Catalysing the translocation of inorganic cations,Catalysing the translocation of inorganic cations,Translocases
contig00010.g6010	5124	ID=contig00010.g6010;Description=ATPase [Fusarium verticillioides 7600];Gene=FVER53263_06686;Ontology_term=transmembrane transport,ATP-dependent activity,transporter activity,hydrolase activity;Ontology_id=GO:0055085,GO:0140657,GO:0005215,GO:0016787;Enzyme_code=EC:7.2.2,EC:3.6.1.15,EC:3.6.1,EC:3.6,EC:3,EC:7.2.2,EC:7,EC:3.6.1.15;Enzyme_name=Catalysing the translocation of inorganic cations,nucleoside-triphosphate phosphatase,Acting on acid anhydrides,Acting on acid anhydrides,Hydrolases,Catalysing the translocation of inorganic cations,Translocases,nucleoside-triphosphate phosphatase
contig00010.g6011	2831	ID=contig00010.g6011;Description=uncharacterized protein FSUBG_6792 [Fusarium subglutinans];Gene=FANTH_8311;Ontology_term=membrane;Ontology_id=GO:0016020
contig00010.g6012	1932	ID=contig00010.g6012;Description=Transcription elongation factor SPT4 [Fusarium musae];Gene=FVER53263_06689;Ontology_term=nucleus,DNA binding,regulation of DNA-templated transcription,transcription regulator activity;Ontology_id=GO:0005634,GO:0003677,GO:0006355,GO:0140110

contig00010.g6013	4719	ID=contig00010.g6013;Description=phospholipid-translocating ATPase [Fusarium verticillioides 7600];Gene=FVER53590_06690;Ontology_term=membrane organization,ATP-dependent activity,transporter activity,hydrolase activity;Ontology_id=GO:0061024,GO:0140657,GO:0005215,GO:0016787;Enzyme_code=EC:7.6.2.1,EC:3.6.1.15,EC:3.6.1,EC:3.6.3.1,EC:7.6.2.1,EC:3.6,EC:3,EC:3.6.1.15;Enzyme_name=P-type phospholipid transporter,nucleoside-triphosphate phosphatase,Acting on acid anhydrides,Acting on acid anhydrides,P-type phospholipid transporter,Acting on acid anhydrides,Hydrolases,nucleoside-triphosphate phosphatase
contig00010.g6014	762	ID=contig00010.g6014;Description=vesicle-associated membrane protein 7 [Fusarium proliferatum];Gene=FTJAE_13448;Ontology_term=vesicle-mediated transport;Ontology_id=GO:0016192
contig00010.g6015	2659	ID=contig00010.g6015;Description=ATP dependent RNA helicase [Fusarium coicis];Gene=FPCIR_13273;Ontology_term=ATP-dependent activity,mitochondrion,catalytic activity, acting on RNA,hydrolase activity;Ontology_id=GO:0140657,GO:0005739,GO:0140098,GO:0016787;Enzyme_code=EC:3.6.4.13;Enzyme_name=RNA helicase
contig00010.g6016	951	ID=contig00010.g6016;Description=fmi2 [Fusarium tjaetaba];Gene=FTJAE_13446;Ontology_term=catalytic activity, acting on a protein,hydrolase activity;Ontology_id=GO:0140096,GO:0016787;Enzyme_code=EC:3.1.3,EC:3.1,EC:3,EC:3.1.3;Enzyme_name=Acting on ester bonds,Acting on ester bonds,Hydrolases,Acting on ester bonds
contig00010.g6017	1644	ID=contig00010.g6017;Description=tyrosine phosphatase [Fusarium coicis]
contig00010.g6018	1017	ID=contig00010.g6018;Description=hypothetical protein FVER14953_06695 [Fusarium verticillioides]

contig00010.g6019	4630	ID=contig00010.g6019;Description=endoribonuclease Dicer [Fusarium tjaetaba];Gene=FVER53263_06696;Ontology_term=regulation of DNA-templated transcription,nuclear chromosome,chromatin organization,defense response to other organism,DNA binding,RNA binding,ATP-dependent activity,regulatory ncRNA-mediated gene silencing,cytosol,catalytic activity, acting on RNA,hydrolase activity;Ontology_id=GO:0006355,GO:0000228,GO:0006325,GO:0098542,GO:0003677,GO:0003723,GO:0140657,GO:0031047,GO:0005829,GO:0140098,GO:0016787;Enzyme_code=EC:3.1.30,EC:3.1.26.3,EC:3.1.30,EC:3.1,EC:3,EC:3.1.26.3,EC:3.1.26;Enzyme_name=Acting on ester bonds,ribonuclease III,Acting on ester bonds,Acting on ester bonds,Hydrolases,ribonuclease III,Acting on ester bonds
contig00010.g6020	2194	ID=contig00010.g6020;Description=ubiquitin thiolesterase L3 [Fusarium coicis];Gene=FVER53263_06697;Ontology_term=catalytic activity, acting on a protein,GTPase activity,protein catabolic process;Ontology_id=GO:0140096,GO:0003924,GO:0030163;Enzyme_code=EC:3.6.1.15,EC:3.4.19.12;Enzyme_name=nucleoside-triphosphate phosphatase,ubiquitinyl hydrolase 1
contig00010.g6021	793	ID=contig00010.g6021;Description=ubiquitin carboxyl-terminal hydrolase L3 [Fusarium verticillioides 7600];Gene=FFUJ_08934;Ontology_term=catalytic activity, acting on a protein,protein catabolic process,hydrolase activity;Ontology_id=GO:0140096,GO:0030163,GO:0016787;Enzyme_code=EC:3.4.19.12,EC:3.4,EC:3,EC:3.4.19.12;Enzyme_name=ubiquitinyl hydrolase 1,Acting on peptide bonds (peptidases),Hydrolases,ubiquitinyl hydrolase 1
contig00010.g6022	2295	ID=contig00010.g6022;Description=hypothetical protein FVEG_06699 [Fusarium verticillioides 7600];Gene=B52;Ontology_term=defense response to other organism,mRNA metabolic process,RNA binding,signaling,detoxification,regulation of DNA-templated transcription,cytosol,chromosome,transferase activity,mitotic cell cycle,immune system process,nucleoplasm;Ontology_id=GO:0098542,GO:0016071,GO:0003723,GO:0023052,GO:0098754,GO:0006355,GO:0005829,GO:0005694,GO:0016740,GO:0000278,GO:0002376,GO:0005654;Enzyme_code=EC:2.5.1.18,EC:2.5.1,EC:2.5.1.18,EC:2;Enzyme_name=glutathione transferase,Transferring alkyl or aryl groups, other than methyl groups,glutathione transferase,Transferases
contig00010.g6023	1123	ID=contig00010.g6023;Description=cysteine sulfinate desulfinase [Fusarium verticillioides 7600]

contig00010.g6024	1037	ID=contig00010.g6024;Description=oxidoreductase [Fusarium odoratissimum NRRL 54006];Gene=yhdF-0;Ontology_term=oxidoreductase activity;Ontology_id=GO:0016491;Enzyme_code=EC:1;Enzyme_name=Oxidoreductases
contig00010.g6025	2492	ID=contig00010.g6025;Description=amino-acid oxidase [Fusarium phyllophilum];Gene=FPHYL_13835;Ontology_term=oxidoreductase activity;Ontology_id=GO:0016491;Enzyme_code=EC:1,EC:1;Enzyme_name=Oxidoreductases,Oxidoreductases
contig00010.g6026	1356	ID=contig00010.g6026;Description=hypothetical protein FVEG_06703 [Fusarium verticillioides 7600];Gene=nbs;Ontology_term=DNA repair,nucleus,DNA binding,mRNA metabolic process,transmembrane transport,mitochondrion,structural molecule activity,regulation of DNA-templated transcription,chromosome,transporter activity;Ontology_id=GO:0006281,GO:0005634,GO:0003677,GO:0016071,GO:0055085,GO:0005739,GO:0005198,GO:0006355,GO:0005694,GO:0005215
contig00010.g6027	5219	ID=contig00010.g6027;Description=multiple ankyrin repeats single kh domain-containing protein [Fusarium globosum];Gene=FGLOB1_10456;Ontology_term=nucleobase-containing small molecule metabolic process,catalytic activity,carbohydrate derivative metabolic process;Ontology_id=GO:0055086,GO:0003824,GO:1901135
contig00010.g6028	1575	ID=contig00010.g6028;Description=aminopeptidase Y precursor vacuolar [Fusarium tjaetaba];Gene=FVEG_06704;Ontology_term=catalytic activity, acting on a protein,hydrolase activity;Ontology_id=GO:0140096,GO:0016787;Enzyme_code=EC:3.4.11,EC:3.4.11,EC:3.4,EC:3;Enzyme_name=Acting on peptide bonds (peptidases),Acting on peptide bonds (peptidases),Acting on peptide bonds (peptidases),Hydrolases
contig00010.g6029	5668	ID=contig00010.g6029;Description=hypothetical protein FVEG_15927 [Fusarium verticillioides 7600]
contig00010.g6030	1106	ID=contig00010.g6030;Description=bzip transcription factor [Fusarium bulbicola]
contig00010.g6031	3454	ID=contig00010.g6031;Description=5-methylthioadenosine s-adenosylhomocysteine deaminase [Fusarium tjaetaba];Gene=FTJAE_13512;Ontology_term=hydrolase activity;Ontology_id=GO:0016787;Enzyme_code=EC:3.5,EC:3.5,EC:3;Enzyme_name=Acting on carbon-nitrogen bonds, other than peptide bonds,Acting on carbon-nitrogen bonds, other than peptide bonds,Hydrolases

contig00010.g6032	1032	ID=contig00010.g6032;Description=glycosyltransferase family 17-domain-containing protein [Fusarium oxysporum Fo47];Gene=FNAPI_9358;Ontology_term=catalytic activity, acting on a protein,transferase activity;Ontology_id=GO:0140096,GO:0016740;Enzyme_code=EC:2.4.1.144,EC:2.4,EC:2,EC:2.4.1.144,EC:2.4.1;Enzyme_name=beta-1,4-mannosyl-glycoprotein 4-beta-N-acetylglucosaminyltransferase,Glycosyltransferases,Transferases,beta-1,4-mannosyl-glycoprotein 4-beta-N-acetylglucosaminyltransferase,Glycosyltransferases
contig00010.g6033	741	ID=contig00010.g6033;Description=serine threonine kinase fnkB [Fusarium pseudoanthophilum];Gene=FNAPI_9359;Ontology_term=transferase activity;Ontology_id=GO:0016740;Enzyme_code=EC:2.7,EC:2.7,EC:2;Enzyme_name=Transferring phosphorus-containing groups,Transferring phosphorus-containing groups,Transferases
contig00010.g6034	392	ID=contig00010.g6034;Description=serine threonine kinase fnkB [Fusarium pseudoanthophilum];Gene=FNAPI_9359;Ontology_term=transferase activity;Ontology_id=GO:0016740;Enzyme_code=EC:2.7;Enzyme_name=Transferring phosphorus-containing groups
contig00010.g6035	1134	ID=contig00010.g6035;Description=Opine oxidase subunit B [Fusarium napiforme];Gene=FVER53263_06712;Ontology_term=oxidoreductase activity;Ontology_id=GO:0016491;Enzyme_code=EC:1,EC:1;Enzyme_name=Oxidoreductases,Oxidoreductases
contig00010.g6036	1673	ID=contig00010.g6036;Description=pisatin demethylase [Fusarium pseudocircinatum];Gene=FGADI_9532;Ontology_term=oxidoreductase activity,transferase activity;Ontology_id=GO:0016491,GO:0016740;Enzyme_code=EC:1.14,EC:2.1.1,EC:2.1,EC:1,EC:1.14,EC:2,EC:2.1.1;Enzyme_name=Acting on paired donors, with incorporation or reduction of molecular oxygen. The oxygen incorporated need not be derived from O2,Transferring one-carbon groups,Transferring one-carbon groups,Oxidoreductases,Acting on paired donors, with incorporation or reduction of molecular oxygen. The oxygen incorporated need not be derived from O2,Transferases,Transferring one-carbon groups
contig00010.g6037	422	ID=contig00010.g6037;Description=thioredoxin [Fusarium verticillioides 7600];Gene=FTJAE_10574;Ontology_term=catalytic activity, acting on a protein,oxidoreductase activity;Ontology_id=GO:0140096,GO:0016491;Enzyme_code=EC:1.8;Enzyme_name=Acting on a sulfur group of donors
contig00010.g6038	312	ID=contig00010.g6038;Description=hypothetical protein FVER53263_06715 [Fusarium verticillioides]

contig00010.g6039	3486	ID=contig00010.g6039;Description=amino acid permease-domain-containing protein [Fusarium redolens];Gene=FVEG_06716;Ontology_term=transmembrane transport;Ontology_id=GO:0055085
contig00010.g6040	894	ID=contig00010.g6040;Description=hypothetical protein FVER14953_06717 [Fusarium verticillioides]
contig00010.g6041	2386	ID=contig00010.g6041;Description=mitotic spindle assembly checkpoint protein MAD1 [Fusarium verticillioides 7600];Gene=FPANT_6031;Ontology_term=nucleus,signaling,mitotic nuclear division,chromosome segregation;Ontology_id=GO:0005634,GO:0023052,GO:0140014,GO:0007059
contig00010.g6042	1536	ID=contig00010.g6042;Description=proline transport [Fusarium tjaetaba];Gene=FNYG_13694;Ontology_term=membrane organization,mitochondrion,lipid metabolic process,transferase activity,mitochondrion organization;Ontology_id=GO:0061024,GO:0005739,GO:0006629,GO:0016740,GO:0007005;Enzyme_code=EC:2.7.7.41,EC:2.7.7.41,EC:2.7,EC:2,EC:2.7.7;Enzyme_name=phosphatidate cytidylyltransferase,phosphatidate cytidylyltransferase,Transferring phosphorus-containing groups,Transferases,Transferring phosphorus-containing groups
contig00010.g6043	3020	ID=contig00010.g6043;Description=FCH domain protein [Fusarium tjaetaba]
contig00010.g6044	1922	ID=contig00010.g6044;Description=CMGC protein kinase [Fusarium verticillioides 7600];Gene=FVER53263_06721;Ontology_term=catalytic activity, acting on a protein,transferase activity;Ontology_id=GO:0140096,GO:0016740;Enzyme_code=EC:2.7.1;Enzyme_name=Transferring phosphorus-containing groups
contig00010.g6045	4905	ID=contig00010.g6045;Description=e3 ubiquitin ligase listerin [Fusarium tjaetaba];Gene=FVER53263_06722;Ontology_term=nucleus,catalytic activity, acting on a protein,protein catabolic process,regulation of DNA-templated transcription,cytosol,telomere organization,transferase activity,chromatin organization;Ontology_id=GO:0005634,GO:0140096,GO:0030163,GO:0006355,GO:0005829,GO:0032200,GO:0016740,GO:0006325;Enzyme_code=EC:2.3.2,EC:2.3,EC:1.14.19.1,EC:2,EC:2.3.2.27,EC:2.3.2;Enzyme_name=Acyltransferases,Acyltransferases,stearoyl-CoA 9-desaturase,Transferases,RING-type E3 ubiquitin transferase,Acyltransferases
contig00010.g6046	3153	ID=contig00010.g6046;Description=hypothetical protein FVER14953_06723 [Fusarium verticillioides];Gene=FVER53263_06723;Ontology_term=membrane;Ontology_id=GO:0016020

contig00010.g6047	294	ID=contig00010.g6047;Description=hypothetical protein FOXB_14357 [Fusarium oxysporum f. sp. conglutinans Fo5176]
contig00010.g6048	1620	ID=contig00010.g6048;Description=transcription factor PLM2 [Fusarium tjaetaba];Ontology_term=mitochondrion,regulation of DNA-templated transcription,nuclear chromosome,nucleoplasm;Ontology_id=GO:0005739,GO:0006355,GO:0000228,GO:0005654
contig00010.g6049	561	ID=contig00010.g6049;Description=hypothetical protein FVEG_06726 [Fusarium verticillioides 7600]
contig00010.g6050	3417	ID=contig00010.g6050;Description=beta-glucosidase G [Fusarium verticillioides 7600];Gene=FVER53590_06728;Ontology_term=carbohydrate metabolic process,extracellular space,hydrolase activity;Ontology_id=GO:0005975,GO:0005615,GO:0016787;Enzyme_code=EC:3.2.1,EC:3.2,EC:3.2.1.21,EC:3,EC:3.2.1;Enzyme_name=Glycosylases,Glycosylases,beta-glucosidase,Hydrolases,Glycosylases
contig00010.g6051	385	ID=contig00010.g6051;Description=putative transporter SEO1 [Fusarium oxysporum f. sp. albedinis]
contig00010.g6052	225	ID=contig00010.g6052;Description=hypothetical protein FVEG_15930 [Fusarium verticillioides 7600]
contig00010.g6053	429	ID=contig00010.g6053;Description=hypothetical protein FVER53590_06731 [Fusarium verticillioides]
contig00010.g6054	3924	ID=contig00010.g6054;Description=NACHT LRR and PYD domain protein [Fusarium tjaetaba]
contig00010.g6055	2630	ID=contig00010.g6055;Description=heterokaryon incompatibility (het-6OR allele) [Fusarium pseudocircinatum]
contig00010.g6056	1573	ID=contig00010.g6056;Description=aldehyde dehydrogenase family 3 member B1 [Fusarium tjaetaba];Gene=FTJAE_2606;Ontology_term=oxidoreductase activity;Ontology_id=GO:0016491;Enzyme_code=EC:1.2.1,EC:1.2,EC:1,EC:1.2.1;Enzyme_name=Acting on the aldehyde or oxo group of donors,Acting on the aldehyde or oxo group of donors,Oxidoreductases,Acting on the aldehyde or oxo group of donors
contig00010.g6057	412	ID=contig00010.g6057;Description=hypothetical protein FOMA001_g11428 [Fusarium oxysporum f. sp. matthiolae]
contig00010.g6058	552	ID=contig00010.g6058;Description=cupin domain-containing protein [Fusarium pseudoanthophilum]

contig00010.g6059	1335	ID=contig00010.g6059;Description=fumarylacetoacetate hydrolase [Fusarium tjaetaba];Gene=FPCIR_7813;Ontology_term=amino acid metabolic process,hydrolase activity;Ontology_id=GO:0006520,GO:0016787;Enzyme_code=EC:3.7.1.2,EC:3.7.1,EC:3.7.1.2,EC:3,EC:3.7;Enzyme_name=fumarylacetoacetase,Acting on carbon-carbon bonds,fumarylacetoacetase,Hydrolases,Acting on carbon-carbon bonds
contig00010.g6060	2533	ID=contig00010.g6060;Description=salicylate 1-monooxygenase [Fusarium fujikuroi];Gene=FPHYL_1102;Ontology_term=oxidoreductase activity,lyase activity;Ontology_id=GO:0016491,GO:0016829;Enzyme_code=EC:1,EC:4,EC:1,EC:4,EC:1.14.13.1;Enzyme_name=Oxidoreductases,Lyases,Oxidoreductases,Lyases,salicylate 1-monooxygenase
contig00010.g6061	1877	ID=contig00010.g6061;Description=probable hydrolase, CocE/NonD family [Fusarium fujikuroi IMI 58289];Gene=FVEG_06737;Ontology_term=catalytic activity, acting on a protein,hydrolase activity;Ontology_id=GO:0140096,GO:0016787;Enzyme_code=EC:3.4.14.1,EC:3.4.14.5,EC:3.4.14.4,EC:3.4.14.2,EC:3.4.14.1,EC:3.4,EC:3,EC:3.4.14.5,EC:3.4.14.4,EC:3.4.14.2;Enzyme_name=dipeptidyl-peptidase I,dipeptidyl-peptidase IV,dipeptidyl-peptidase III,dipeptidyl-peptidase II,dipeptidyl-peptidase I,Acting on peptide bonds (peptidases),Hydrolases,dipeptidyl-peptidase IV,dipeptidyl-peptidase III,dipeptidyl-peptidase II
contig00010.g6062	383	ID=contig00010.g6062;Description=hypothetical protein FVEG_06738 [Fusarium verticillioides 7600]
contig00010.g6063	1006	ID=contig00010.g6063;Description=stress response rds1p [Fusarium tjaetaba]
contig00010.g6064	973	ID=contig00010.g6064;Description=aromatic ring-opening dioxygenase family [Fusarium tjaetaba];Gene=FMUND_10125;Ontology_term=oxidoreductase activity;Ontology_id=GO:0016491;Enzyme_code=EC:1.13;Enzyme_name=Acting on single donors with incorporation of molecular oxygen (oxygenases). The oxygen incorporated need not be derived from O2
contig00010.g6065	2752	ID=contig00010.g6065;Description=zinc finger MSN2 [Fusarium tjaetaba];Gene=FVER53263_06741;Ontology_term=nucleus,DNA binding,regulation of DNA-templated transcription,transcription regulator activity;Ontology_id=GO:0005634,GO:0003677,GO:0006355,GO:0140110
contig00010.g6066	756	ID=contig00010.g6066;Description=Ff.00g080310.m01.CDS01 [Fusarium sp. VM40]
contig00010.g6067	1819	ID=contig00010.g6067;Description=HOL1 [Fusarium tjaetaba];Gene=FTJAE_2617;Ontology_term=transmembrane transport,transporter activity;Ontology_id=GO:0055085,GO:0005215

contig00010.g6068	2693	ID=contig00010.g6068;Description=ankyrin repeat [Fusarium tjaetaba]
contig00010.g6069	1137	ID=contig00010.g6069;Description=hypothetical protein FVER53590_06748 [Fusarium verticillioides]
contig00010.g6070	891	ID=contig00010.g6070;Description=phosphotransferase [Fusarium circinatum];Gene=FCIRC_2456;Ontology_term=transferase activity;Ontology_id=GO:0016740;Enzyme_code=EC:2,EC:2;Enzyme_name=Transferases,Transferases
contig00010.g6071	1514	ID=contig00010.g6071;Description=related to methyltransferase [Fusarium proliferatum ET1];Gene=BFJ71_g2214;Ontology_term=transferase activity;Ontology_id=GO:0016740;Enzyme_code=EC:2.1.1,EC:2.1,EC:2,EC:2.1.1;Enzyme_name=Transferring one-carbon groups,Transferring one-carbon groups,Transferases,Transferring one-carbon groups
contig00010.g6072	1178	ID=contig00010.g6072;Description=CIC1 adaptor [Fusarium coicis];Gene=FVER53590_06749;Ontology_term=ribonucleoprotein complex;Ontology_id=GO:1990904
contig00010.g6073	996	ID=contig00010.g6073;Description=palmitoyl-protein thioesterase [Fusarium verticillioides 7600];Gene=FNAPI_9401;Ontology_term=catalytic activity, acting on a protein,protein catabolic process,lipid metabolic process,protein glycosylation,endoplasmic reticulum,hydrolase activity;Ontology_id=GO:0140096,GO:0030163,GO:0006629,GO:0006486,GO:0005783,GO:0016787;Enzyme_code=EC:3.1.2.22,EC:3.6.1,EC:3.1,EC:3.1.2.22,EC:3.6.1.43,EC:3.6,EC:3,EC:3.1.2;Enzyme_name=palmitoyl-protein hydrolase,Acting on acid anhydrides,Acting on ester bonds,palmitoyl-protein hydrolase,dolichyldiphosphatase,Acting on acid anhydrides,Hydrolases,Acting on ester bonds
contig00010.g6074	1425	ID=contig00010.g6074;Description=CTK2 carboxy-terminal domain protein [Fusarium tjaetaba];Gene=FNAPI_9402;Ontology_term=molecular function regulator activity,signaling,regulation of DNA-templated transcription,cytosol,reproductive process,nucleolus,nucleoplasm;Ontology_id=GO:0098772,GO:0023052,GO:0006355,GO:0005829,GO:0022414,GO:0005730,GO:0005654;Enzyme_code=EC:3.2.1.21;Enzyme_name=beta-glucosidase

contig00010.g6075	1743	ID=contig00010.g6075;Description=hypothetical protein FVER53263_06752 [Fusarium verticillioides];Gene=FVER53263_06752;Ontology_term=catalytic activity, acting on a protein,endosome,lipid metabolic process,anatomical structure development,cell differentiation,reproductive process,cell wall organization or biogenesis,hydrolase activity;Ontology_id=GO:0140096,GO:0005768,GO:0006629,GO:0048856,GO:0030154,GO:0022414,GO:0071554,GO:0016787;Enzyme_code=EC:3.1.3,EC:3.1.3.16,EC:3.1,EC:3.1.3.48,EC:3.1.3.67,EC:3,EC:3.1.3;Enzyme_name=Acting on ester bonds,protein-serine/threonine phosphatase,Acting on ester bonds,protein-tyrosine-phosphatase,phosphatidylinositol-3,4,5-trisphosphate 3-phosphatase,Hydrolases,Acting on ester bonds
contig00010.g6076	1144	ID=contig00010.g6076;Description=Pore membrane of 33 kDa [Fusarium tjaetaba];Gene=FNYG_04672;Ontology_term=defense response to other organism,transmembrane transport,ATP-dependent activity,plasma membrane,transporter activity,immune system process,hydrolase activity;Ontology_id=GO:0098542,GO:0055085,GO:0140657,GO:0005886,GO:0005215,GO:0002376,GO:0016787;Enzyme_code=EC:7.6.2.2,EC:7.2.2,EC:3.6.1.15;Enzyme_name=ABC-type xenobiotic transporter,Catalysing the translocation of inorganic cations,nucleoside-triphosphate phosphatase
contig00010.g6077	645	ID=contig00010.g6077;Description=hypothetical protein FVEG_06754 [Fusarium verticillioides 7600]
contig00010.g6078	1077	ID=contig00010.g6078;Description=D-arabinitol 2-dehydrogenase [Fusarium tjaetaba];Gene=FVER53263_06755;Ontology_term=oxidoreductase activity;Ontology_id=GO:0016491;Enzyme_code=EC:1,EC:1;Enzyme_name=Oxidoreductases,Oxidoreductases
contig00010.g6079	1141	ID=contig00010.g6079;Description=hypothetical protein FVEG_06756 [Fusarium verticillioides 7600]
contig00010.g6080	1433	ID=contig00010.g6080;Description=phosphatidylinositol transporter [Fusarium denticulatum]
contig00010.g6081	954	ID=contig00010.g6081;Description=magnesium dependent phosphatase [Fusarium subglutinans];Gene=FSUBG_8763;Ontology_term=nucleus,catalytic activity, acting on a protein,hydrolase activity;Ontology_id=GO:0005634,GO:0140096,GO:0016787;Enzyme_code=EC:3.1.3,EC:3.1,EC:3.1.3.48,EC:3,EC:3.1.3.2,EC:3.1.3;Enzyme_name=Acting on ester bonds,Acting on ester bonds,protein-tyrosine-phosphatase,Hydrolases,acid phosphatase,Acting on ester bonds

contig00010.g6082	1847	ID=contig00010.g6082;Description=Transcriptional activator of proteases prtT [Fusarium oxysporum f. sp. conglutinans];Gene=FVEG_06759;Ontology_term=nucleus,DNA binding,catalytic activity, acting on a protein,regulation of DNA-templated transcription,transcription regulator activity,hydrolase activity;Ontology_id=GO:0005634,GO:0003677,GO:0140096,GO:0006355,GO:0140110,GO:0016787;Enzyme_code=EC:3.4;Enzyme_name=Acting on peptide bonds (peptidases)
contig00010.g6083	966	ID=contig00010.g6083;Description=alcohol dehydrogenase [Fusarium verticillioides 7600]
contig00010.g6084	1451	ID=contig00010.g6084;Description=L-lactate dehydrogenase (cytochrome) [Fusarium verticillioides 7600];Gene=FPANT_5958;Ontology_term=oxidoreductase activity;Ontology_id=GO:0016491;Enzyme_code=EC:1,EC:1.1.2.3,EC:1;Enzyme_name=Oxidoreductases,L-lactate dehydrogenase (cytochrome),Oxidoreductases
contig00010.g6085	2517	ID=contig00010.g6085;Description=putative glutamine amidotransferase-like protein C13C5.04 [Fusarium oxysporum f. sp. albedinis]
contig00010.g6086	537	ID=contig00010.g6086;Description=DNA-directed RNA polymerase [Fusarium oxysporum f. sp. albedinis]
contig00010.g6087	5799	ID=contig00010.g6087;Description=hypothetical protein FVER53590_06765 [Fusarium verticillioides];Ontology_term=signaling,anatomical structure development,cell differentiation,cytoskeletal protein binding,mitotic cell cycle,plasma membrane,nucleoplasm;Ontology_id=GO:0023052,GO:0048856,GO:0030154,GO:0008092,GO:0000278,GO:0005886,GO:0005654;Enzyme_code=EC:2.7.11.1;Enzyme_name=non-specific serine/threonine protein kinase
contig00010.g6088	774	ID=contig00010.g6088;Description=gnat family protein acetyltransferase [Fusarium tjaetaba];Gene=FVER53263_06767;Ontology_term=transferase activity;Ontology_id=GO:0016740;Enzyme_code=EC:2.3.1,EC:2.3,EC:2,EC:2.3.1;Enzyme_name=Acyltransferases,Acyltransferases,Transferases,Acyltransferases
contig00010.g6089	1074	ID=contig00010.g6089;Description=c-5 sterol desaturase [Fusarium falciforme];Gene=FLONG3_5785;Ontology_term=oxidoreductase activity,lipid metabolic process,endoplasmic reticulum,Golgi apparatus;Ontology_id=GO:0016491,GO:0006629,GO:0005783,GO:0005794;Enzyme_code=EC:1,EC:1.14.19.20,EC:1,EC:1.14,EC:1.14.19;Enzyme_name=Oxidoreductases,Delta(7)-sterol 5(6)-desaturase,Oxidoreductases,Acting on paired donors, with incorporation or reduction of molecular oxygen. The oxygen incorporated need not be derived from O2,Acting on paired donors, with incorporation or reduction of molecular oxygen. The oxygen incorporated need not be derived from O2

contig00010.g6090	3421	ID=contig00010.g6090;Description=potassium/sodium efflux P-type ATPase, fungal-type [Fusarium verticillioides 7600];Gene=AK830_g12628;Ontology_term=transmembrane transport,ATP-dependent activity,transporter activity,hydrolase activity;Ontology_id=GO:0055085,GO:0140657,GO:0005215,GO:0016787;Enzyme_code=EC:7.2.2,EC:3.6.1.15,EC:3.6.1,EC:3.6,EC:3,EC:3.6.3.7,EC:7.2.2,EC:7,EC:3.6.1.15;Enzyme_name=Catalysing the translocation of inorganic cations,nucleoside-triphosphate phosphatase,Acting on acid anhydrides,Acting on acid anhydrides,Hydrolases,Acting on acid anhydrides,Catalysing the translocation of inorganic cations,Translocases,nucleoside-triphosphate phosphatase
contig00010.g6091	1501	ID=contig00010.g6091;Description=cleavage polyadenylation factor IA subunit [Fusarium coicis];Gene=CLP1;Ontology_term=nucleus,mRNA metabolic process,transferase activity;Ontology_id=GO:0005634,GO:0016071,GO:0016740;Enzyme_code=EC:2.7.1;Enzyme_name=Transferring phosphorus-containing groups
contig00010.g6092	1409	ID=contig00010.g6092;Description=Argonaute-binding 1 [Fusarium tjaetaba];Gene=FDENT_12745;Ontology_term=nucleus,regulatory ncRNA-mediated gene silencing;Ontology_id=GO:0005634,GO:0031047
contig00010.g6093	1288	ID=contig00010.g6093;Description=transcriptional regulator ARG82 [Fusarium napiforme];Gene=FDENT_12744;Ontology_term=transferase activity;Ontology_id=GO:0016740;Enzyme_code=EC:2.7;Enzyme_name=Transferring phosphorus-containing groups
contig00010.g6094	556	ID=contig00010.g6094;Description=SNA2 integral membrane [Fusarium coicis];Gene=FVER53263_06773;Ontology_term=membrane;Ontology_id=GO:0016020
contig00010.g6095	3591	ID=contig00010.g6095;Description=adenosinetriphosphatase [Fusarium verticillioides 7600];Gene=FMEXI_13706;Ontology_term=nucleus,RNA binding,mRNA metabolic process,protein-containing complex assembly,ATP-dependent activity,catalytic activity, acting on RNA,hydrolase activity;Ontology_id=GO:0005634,GO:0003723,GO:0016071,GO:0065003,GO:0140657,GO:0140098,GO:0016787;Enzyme_code=EC:3.6.4.13,EC:3.6.1,EC:3.6.4.13,EC:3.6,EC:3,EC:3.6.1.15;Enzyme_name=RNA helicase,Acting on acid anhydrides,RNA helicase,Acting on acid anhydrides,Hydrolases,nucleoside-triphosphate phosphatase
contig00010.g6096	2404	ID=contig00010.g6096;Description=Phototropin-1 [Fusarium tjaetaba];Enzyme_code=EC:2.7.11.1;Enzyme_name=non-specific serine/threonine protein kinase
contig00010.g6097	1200	ID=contig00010.g6097;Description=hypothetical protein FCOIX_8778 [Fusarium coicis];Gene=FPHYL_12040;Ontology_term=membrane;Ontology_id=GO:0016020

contig00010.g6098	1368	ID=contig00010.g6098;Description=carbohydrate-binding module family 50 [Fusarium tjaetaba]
contig00010.g6099	858	ID=contig00010.g6099;Description=hypothetical protein FVEG_06778 [Fusarium verticillioides 7600];Gene=FNAPI_6489;Ontology_term=carbohydrate metabolic process,carbohydrate derivative metabolic process,extracellular region,hydrolase activity;Ontology_id=GO:0005975,GO:1901135,GO:0005576,GO:0016787;Enzyme_code=EC:3.2.1.14,EC:3.2,EC:3,EC:3.2.1.14,EC:3.2.1;Enzyme_name=chitinase,Glycosylases,Hydrolases,chitinase,Glycosylases
contig00010.g6100	4608	ID=contig00010.g6100;Description=killer toxin subunits alpha beta [Fusarium tjaetaba];Gene=FNAPI_6488;Ontology_term=carbohydrate metabolic process,carbohydrate derivative metabolic process,extracellular region,hydrolase activity;Ontology_id=GO:0005975,GO:1901135,GO:0005576,GO:0016787;Enzyme_code=EC:3.2.1.14,EC:3.2,EC:3,EC:3.2.1.14,EC:3.2.1;Enzyme_name=chitinase,Glycosylases,Hydrolases,chitinase,Glycosylases
contig00010.g6101	1829	ID=contig00010.g6101;Description=G coupled receptor [Fusarium pseudocircinatum];Gene=FPHYL_12042;Ontology_term=molecular transducer activity,signaling,plasma membrane;Ontology_id=GO:0060089,GO:0023052,GO:0005886
contig00010.g6102	3770	ID=contig00010.g6102;Description=related to protein RIS1 [Fusarium fujikuroi];Gene=FNYG_04694;Ontology_term=nucleus,catalytic activity, acting on DNA,ATP-dependent activity,chromatin organization,hydrolase activity;Ontology_id=GO:0005634,GO:0140097,GO:0140657,GO:0006325,GO:0016787;Enzyme_code=EC:3,EC:3;Enzyme_name=Hydrolases,Hydrolases
contig00010.g6103	801	ID=contig00010.g6103;Description=GMP synthase (glutamine-hydrolysing) [Fusarium verticillioides 7600];Gene=FPCIR_9803;Ontology_term=nucleus,amino acid metabolic process,hydrolase activity;Ontology_id=GO:0005634,GO:0006520,GO:0016787;Enzyme_code=EC:3.5.1,EC:3.4.19.16,EC:6.3.5.2,EC:3.5,EC:3;Enzyme_name=Acting on carbon-nitrogen bonds, other than peptide bonds,glucosinolate gamma-glutamyl hydrolase,GMP synthase (glutamine-hydrolyzing),Acting on carbon-nitrogen bonds, other than peptide bonds,Hydrolases

contig00010.g6104	1215	ID=contig00010.g6104;Description=exopolyphosphatase [Fusarium tjaetaba];Gene=FNAPI_6600;Ontology_term=generation of precursor metabolites and energy,mitochondrion,cytosol,plasma membrane,hydrolase activity;Ontology_id=GO:0006091,GO:0005739,GO:0005829,GO:0005886,GO:0016787;Enzyme_code=EC:3.6.1.11,EC:3.6.1.11,EC:3.6.1,EC:3.6,EC:3;Enzyme_name=exopolyphosphatase,exopolyphosphatase,Acting on acid anhydrides,Acting on acid anhydrides,Hydrolases
contig00010.g6105	1938	ID=contig00010.g6105;Description=ATPase synthesis protein 25 mitochondrial [Fusarium musae];Gene=FVER53263_06785;Ontology_term=mitochondrial gene expression,mRNA metabolic process,protein-containing complex assembly,mitochondrion,tRNA metabolic process,amino acid metabolic process,catalytic activity, acting on RNA,mitochondrion organization,ligase activity;Ontology_id=GO:0140053,GO:0016071,GO:0065003,GO:0005739,GO:0006399,GO:0006520,GO:0140098,GO:0007005,GO:0016874;Enzyme_code=EC:6.1,EC:6.1.1,EC:6.1.1.15,EC:6;Enzyme_name=Forming carbon-oxygen bonds,Forming carbon-oxygen bonds,proline--tRNA ligase,Ligases
contig00010.g6106	4341	ID=contig00010.g6106;Description=DNA-directed RNA polymerase, mitochondrial [Fusarium verticillioides 7600];Gene=FDENT_12734;Ontology_term=DNA binding,mitochondrial gene expression,DNA-templated transcription,DNA replication,mitochondrion,catalytic activity, acting on RNA,transferase activity,mitochondrion organization;Ontology_id=GO:0003677,GO:0140053,GO:0006351,GO:0006260,GO:0005739,GO:0140098,GO:0016740,GO:0007005;Enzyme_code=EC:2.7.7.6,EC:2.7,EC:2,EC:2.7.7.101,EC:2.7.7,EC:2.7.7.6;Enzyme_name=DNA-directed RNA polymerase,Transferring phosphorus-containing groups,Transferases,DNA primase DnaG,Transferring phosphorus-containing groups,DNA-directed RNA polymerase
contig00010.g6107	1173	ID=contig00010.g6107;Description=methionyl-tRNA synthetase [Fusarium tjaetaba];Gene=FTJAE_8671;Ontology_term=molecular function regulator activity,RNA binding,cytoplasmic translation,tRNA metabolic process,amino acid metabolic process,lipid binding,organelle,catalytic activity, acting on RNA,ligase activity;Ontology_id=GO:0098772,GO:0003723,GO:0002181,GO:0006399,GO:0006520,GO:0008289,GO:0043226,GO:0140098,GO:0016874;Enzyme_code=EC:6.1.1,EC:6.1,EC:6.1.1,EC:6;Enzyme_name=Forming carbon-oxygen bonds,Forming carbon-oxygen bonds,Forming carbon-oxygen bonds,Ligases

contig00010.g6108	2757	ID=contig00010.g6108;Description=proliferation associated SNF2 [Fusarium tjaetaba];Gene=FVER53263_06788;Ontology_term=nucleus,catalytic activity, acting on DNA,ATP-dependent activity,DNA recombination,chromatin organization,hydrolase activity;Ontology_id=GO:0005634,GO:0140097,GO:0140657,GO:0006310,GO:0006325,GO:0016787;Enzyme_code=EC:3,EC:3.6.1,EC:3.6,EC:3,EC:3.6.1.15;Enzyme_name=Hydrolases,Acting on acid anhydrides,Acting on acid anhydrides,Hydrolases,nucleoside-triphosphate phosphatase
contig00010.g6109	450	ID=contig00010.g6109;Description=hypothetical protein FVEG_15942 [Fusarium verticillioides 7600]
contig00010.g6110	5632	ID=contig00010.g6110;Description=ankyrin repeat domain protein [Fusarium tjaetaba]
contig00010.g6111	1039	ID=contig00010.g6111;Description=NADPH2:quinone reductase [Fusarium verticillioides 7600];Gene=FPHYL_12051;Ontology_term=oxidoreductase activity;Ontology_id=GO:0016491;Enzyme_code=EC:1.6.5.5,EC:1.6,EC:1,EC:1.6.5,EC:1.6.5.5;Enzyme_name=NADPH:quinone reductase,Acting on NADH or NADPH,Oxidoreductases,Acting on NADH or NADPH,NADPH:quinone reductase
contig00010.g6112	1364	ID=contig00010.g6112;Description=UDP-glucose 4-epimerase [Fusarium verticillioides 7600];Gene=HZS61_017224;Ontology_term=carbohydrate metabolic process,isomerase activity;Ontology_id=GO:0005975,GO:0016853;Enzyme_code=EC:5.1.3.2;Enzyme_name=UDP-glucose 4-epimerase
contig00010.g6113	3135	ID=contig00010.g6113;Description=spindle pole body-associated sad1 [Fusarium coicis];Gene=FVEG_06792;Ontology_term=membrane;Ontology_id=GO:0016020
contig00010.g6114	2439	ID=contig00010.g6114;Description=ATP-dependent RNA helicase DBP4 [Fusarium verticillioides 7600];Gene=F52700_1740;Ontology_term=RNA binding,ATP-dependent activity,ribosome biogenesis,catalytic activity, acting on RNA,nucleolus,hydrolase activity;Ontology_id=GO:0003723,GO:0140657,GO:0042254,GO:0140098,GO:0005730,GO:0016787;Enzyme_code=EC:3.6.4.13,EC:3.6.1.15,EC:3.6.1,EC:3.6.4.13,EC:3.6,EC:3,EC:3.6.1.15;Enzyme_name=RNA helicase,nucleoside-triphosphate phosphatase,Acting on acid anhydrides,RNA helicase,Acting on acid anhydrides,Hydrolases,nucleoside-triphosphate phosphatase
contig00010.g6115	2070	ID=contig00010.g6115;Description=pre-rRNA-processing esf1 [Fusarium tjaetaba];Gene=FPANT_12446;Ontology_term=RNA binding,ribosome biogenesis,nucleolus;Ontology_id=GO:0003723,GO:0042254,GO:0005730

contig00010.g6116	1240	ID=contig00010.g6116;Description=hypothetical protein FVEG_06795 [Fusarium verticillioides 7600];Gene=FPANT_12447;Ontology_term=transferase activity;Ontology_id=GO:0016740;Enzyme_code=EC:2.1.1,EC:2.1,EC:2,EC:2.1.1;Enzyme_name=Transferring one-carbon groups,Transferring one-carbon groups,Transferases,Transferring one-carbon groups
contig00010.g6117	660	ID=contig00010.g6117;Description=hypothetical protein FVEG_06796 [Fusarium verticillioides 7600]
contig00010.g6118	1555	ID=contig00010.g6118;Description=dimethylaniline monooxygenase (N-oxide forming) [Fusarium verticillioides 7600];Gene=FMUND_3357;Ontology_term=oxidoreductase activity;Ontology_id=GO:0016491;Enzyme_code=EC:1.14.13.8;Enzyme_name=flavin-containing monooxygenase
contig00010.g6119	2037	ID=contig00010.g6119;Description=Arginine metabolism regulation II [Fusarium tjaetaba];Gene=FPCIR_9787;Ontology_term=nucleus,regulation of DNA-templated transcription,transcription regulator activity;Ontology_id=GO:0005634,GO:0006355,GO:0140110
contig00010.g6120	354	ID=contig00010.g6120;Description=ethanolamine utilization [Fusarium tjaetaba]
contig00010.g6121	1200	ID=contig00010.g6121;Description=alpha-1,2-mannosyltransferase [Fusarium proliferatum];Gene=FDENT_12719;Ontology_term=transferase activity;Ontology_id=GO:0016740;Enzyme_code=EC:2.4,EC:2.4,EC:2;Enzyme_name=Glycosyltransferases,Glycosyltransferases,Transferases
contig00010.g6122	1554	ID=contig00010.g6122;Description=hypothetical protein FVER14953_06801 [Fusarium verticillioides];Gene=FPANT_12453;Ontology_term=membrane;Ontology_id=GO:0016020
contig00010.g6123	381	ID=contig00010.g6123;Description=integral membrane protein [Fusarium coicis];Gene=FMEXI_11054;Ontology_term=membrane;Ontology_id=GO:0016020
contig00010.g6124	1455	ID=contig00010.g6124;Description=FAD dependent oxidoreductase [Fusarium oxysporum Fo47];Gene=BFJ71_g2047;Ontology_term=oxidoreductase activity;Ontology_id=GO:0016491;Enzyme_code=EC:1,EC:1.5.3.7,EC:1,EC:1.5.3.1;Enzyme_name=Oxidoreductases,L-pipecolate oxidase,Oxidoreductases,sarcosine oxidasee (formaldehyde-forming)
contig00010.g6125	470	ID=contig00010.g6125;Description=hypothetical protein BFJ72_g2804 [Fusarium proliferatum]
contig00010.g6126	924	ID=contig00010.g6126;Description=hypothetical protein FVEG_06805 [Fusarium verticillioides 7600]

contig00010.g6127	784	ID=contig00010.g6127;Description=integral membrane protein Mpv17 PMP22 family [Fusarium coicis];Gene=BFJ68_g292;Ontology_term=membrane;Ontology_id=GO:0016020
contig00010.g6128	968	ID=contig00010.g6128;Description=acetylxylan esterase [Fusarium tjaetaba]
contig00010.g6129	339	ID=contig00010.g6129;Description=hypothetical protein FVEG_06808 [Fusarium verticillioides 7600]
contig00010.g6130	2124	ID=contig00010.g6130;Description=cercosporin resistance protein [Fusarium bulbicola];Gene=FMUND_3345;Ontology_term=nucleus,regulation of DNA-templated transcription,transcription regulator activity;Ontology_id=GO:0005634,GO:0006355,GO:0140110
contig00010.g6131	625	ID=contig00010.g6131;Description=hypothetical protein FOPG_03964 [Fusarium oxysporum f. sp. conglutinans race 2 54008];Gene=FOZG_12480;Ontology_term=endoplasmic reticulum;Ontology_id=GO:0005783
contig00010.g6132	937	ID=contig00010.g6132;Description=hypothetical protein FPANT_13348 [Fusarium pseudoanthophilum];Gene=FVER53263_06811;Ontology_term=membrane;Ontology_id=GO:0016020
contig00010.g6133	988	ID=contig00010.g6133;Description=coq5 family [Fusarium coicis];Gene=BFJ72_g2608;Ontology_term=transferase activity;Ontology_id=GO:0016740;Enzyme_code=EC:2.1.1,EC:2.1,EC:2,EC:2.1.1;Enzyme_name=Transferring one-carbon groups,Transferring one-carbon groups,Transferases,Transferring one-carbon groups
contig00010.g6134	4311	ID=contig00010.g6134;Description=nuclear pore protein [Fusarium denticulatum];Gene=FPCIR_11506;Ontology_term=nuclear envelope,nucleocytoplasmic transport,DNA binding,RNA binding,protein-containing complex assembly,structural molecule activity,ribosome biogenesis,chromosome segregation,regulation of DNA-templated transcription,chromatin organization,intracellular protein transport;Ontology_id=GO:0005635,GO:0006913,GO:0003677,GO:0003723,GO:0065003,GO:0005198,GO:0042254,GO:0007059,GO:0006355,GO:0006325,GO:0006886

contig00010.g6135	1057	ID=contig00010.g6135;Description=3-ketoacyl-CoA reductase [Fusarium verticillioides 7600];Gene=FPCIR_11507;Ontology_term=oxidoreductase activity,lipid metabolic process,endoplasmic reticulum;Ontology_id=GO:0016491,GO:0006629,GO:0005783;Enzyme_code=EC:1.1.1.330,EC:1.1,EC:1,EC:1.1.1,EC:1.1.1.330,EC:1.1.1.62;Enzyme_name=very-long-chain 3-oxoacyl-CoA reductase,Acting on the CH-OH group of donors,Oxidoreductases,Acting on the CH-OH group of donors,very-long-chain 3-oxoacyl-CoA reductase,17beta-estradiol 17-dehydrogenase
contig00010.g6136	571	ID=contig00010.g6136;Description=ERG28-like protein [Fusarium tjaetaba];Gene=FOPG_03959;Ontology_term=endoplasmic reticulum;Ontology_id=GO:0005783
contig00010.g6137	1244	ID=contig00010.g6137;Description=putative galactose-1-phosphate uridylyltransferase [Fusarium fujikuroi];Gene=FNAPI_6566;Ontology_term=peroxisome,carbohydrate metabolic process,peroxisome organization,transferase activity;Ontology_id=GO:0005777,GO:0005975,GO:0007031,GO:0016740;Enzyme_code=EC:2.7.7.12;Enzyme_name=UDP-glucose--hexose-1-phosphate uridylyltransferase
contig00010.g6138	1536	ID=contig00010.g6138;Description=transcriptional adapter 2-alpha [Fusarium tjaetaba];Gene=F25303_11111;Ontology_term=DNA repair,DNA recombination,regulation of DNA-templated transcription,nuclear chromosome,lipid binding,cell adhesion,chromatin organization,nucleoplasm,DNA binding,cell wall organization or biogenesis,telomere organization,transcription regulator activity,meiotic nuclear division,molecular adaptor activity;Ontology_id=GO:0006281,GO:0006310,GO:0006355,GO:0000228,GO:0008289,GO:0007155,GO:0006325,GO:0005654,GO:0003677,GO:0071554,GO:0032200,GO:0140110,GO:0140013,GO:0060090
contig00010.g6139	429	ID=contig00010.g6139;Description=hypothetical protein FCOIX_7542 [Fusarium coicis];Gene=FPANT_13427;Ontology_term=membrane;Ontology_id=GO:0016020
contig00010.g6140	720	ID=contig00010.g6140;Description=hypothetical protein FVER14953_06818 [Fusarium verticillioides];Gene=FDENT_3460;Ontology_term=membrane;Ontology_id=GO:0016020

contig00010.g6141	520	ID=contig00010.g6141;Description=CDC25-like phosphatase YCH1 [Fusarium odoratissimum];Gene=C2S_13919;Ontology_term=nucleus,catalytic activity, acting on a protein,transferase activity,hydrolase activity;Ontology_id=GO:0005634,GO:0140096,GO:0016740,GO:0016787;Enzyme_code=EC:2.8.1.1,EC:3.1.3,EC:2.8.1,EC:3.1,EC:3.1.3.48,EC:2,EC:3,EC:2.8,EC:2.8.1.1,EC:3.1.3;Enzyme_name=thiosulfate sulfurtransferase,Acting on ester bonds,Transferring sulfur-containing groups,Acting on ester bonds,protein-tyrosine-phosphatase,Transferases,Hydrolases,Transferring sulfur-containing groups,thiosulfate sulfurtransferase,Acting on ester bonds
contig00010.g6142	866	ID=contig00010.g6142;Description=hydrolase-HD superfamily protein [Fusarium verticillioides 7600];Gene=FVER53263_06820;Ontology_term=nucleus,hydrolase activity;Ontology_id=GO:0005634,GO:0016787;Enzyme_code=EC:3.1.3,EC:3.1,EC:3,EC:3.1.3;Enzyme_name=Acting on ester bonds,Acting on ester bonds,Hydrolases,Acting on ester bonds
contig00010.g6143	1383	ID=contig00010.g6143;Description=hypothetical protein FVEG_06821 [Fusarium verticillioides 7600]
contig00010.g6144	941	ID=contig00010.g6144;Description=polysaccharide deacetylase family [Fusarium subglutinans];Gene=Focb16_v014608;Ontology_term=carbohydrate metabolic process,cell wall organization or biogenesis,hydrolase activity;Ontology_id=GO:0005975,GO:0071554,GO:0016787;Enzyme_code=EC:3.2,EC:3.5,EC:3.5.1.104,EC:3.2,EC:3.5,EC:3;Enzyme_name=Glycosylases,Acting on carbon-nitrogen bonds, other than peptide bonds,peptidoglycan-N-acetylglucosamine deacetylase,Glycosylases,Acting on carbon-nitrogen bonds, other than peptide bonds,Hydrolases
contig00010.g6145	2744	ID=contig00010.g6145;Description=transcriptional activator acu-15 [Fusarium coicis];Gene=BFJ72_g2670;Ontology_term=nucleus,DNA binding,GTPase activity,regulation of DNA-templated transcription,transcription regulator activity;Ontology_id=GO:0005634,GO:0003677,GO:0003924,GO:0006355,GO:0140110;Enzyme_code=EC:3.6.1.15,EC:3.6.1,EC:3.6,EC:3,EC:3.6.1.15;Enzyme_name=nucleoside-triphosphate phosphatase,Acting on acid anhydrides,Acting on acid anhydrides,Hydrolases,nucleoside-triphosphate phosphatase

contig00010.g6146	2367	ID=contig00010.g6146;Description=Interferon-induced GTP-binding protein Mx [Fusarium oxysporum f. sp. conglutinans];Gene=FOVG_10424;Ontology_term=GTPase activity;Ontology_id=GO:0003924;Enzyme_code=EC:3.6.1.15,EC:3.6.1,EC:3.6,EC:3,EC:3.6.1.15;Enzyme_name=nucleoside-triphosphate phosphatase,Acting on acid anhydrides,Acting on acid anhydrides,Hydrolases,nucleoside-triphosphate phosphatase
contig00010.g6147	2466	ID=contig00010.g6147;Description=MFS transporter, PHS family, inorganic phosphate transporter [Fusarium verticillioides 7600];Gene=FVEG_06825;Ontology_term=transmembrane transport,transporter activity;Ontology_id=GO:0055085,GO:0005215
contig00010.g6148	1646	ID=contig00010.g6148;Description=homoserine O-acetyltransferase [Fusarium verticillioides 7600];Gene=FVER53590_06826;Ontology_term=sulfur compound metabolic process,amino acid metabolic process,transferase activity;Ontology_id=GO:0006790,GO:0006520,GO:0016740;Enzyme_code=EC:2.3.1,EC:2.3,EC:2.3.1.31,EC:2,EC:2.3.1;Enzyme_name=Acyltransferases,Acyltransferases,homoserine O-acetyltransferase,Transferases,Acyltransferases
contig00010.g6149	7387	ID=contig00010.g6149;Description=regulatory RLR1 [Fusarium pseudoanthophilum];Gene=FVER53590_06827;Ontology_term=DNA repair,nucleocytoplasmic transport,DNA binding,RNA binding,mRNA metabolic process,DNA recombination,regulation of DNA-templated transcription,chromosome,intracellular protein transport,nucleoplasm;Ontology_id=GO:0006281,GO:0006913,GO:0003677,GO:0003723,GO:0016071,GO:0006310,GO:0006355,GO:0005694,GO:0006886,GO:0005654
contig00010.g6150	1053	ID=contig00010.g6150;Description=1-phosphatidylinositol phosphodiesterase precursor [Fusarium subglutinans];Gene=FDENT_3470;Ontology_term=lipid metabolic process,hydrolase activity;Ontology_id=GO:0006629,GO:0016787;Enzyme_code=EC:3.1.4,EC:3.1.4,EC:4.6.1.13,EC:3.1,EC:3;Enzyme_name=Acting on ester bonds,Acting on ester bonds,phosphatidylinositol diacylglycerol-lyase,Acting on ester bonds,Hydrolases
contig00010.g6151	1410	ID=contig00010.g6151;Description=cell wall integrity and stress response component [Fusarium oxysporum f. sp. lycopersici 4287];Gene=FDENT_3471;Ontology_term=endoplasmic reticulum,intracellular protein transport,transporter activity;Ontology_id=GO:0005783,GO:0006886,GO:0005215
contig00010.g6152	3519	ID=contig00010.g6152;Description=hypothetical protein FVER14953_06830 [Fusarium verticillioides];Gene=FPCIR_9209;Ontology_term=membrane;Ontology_id=GO:0016020

contig00010.g6153	2834	ID=contig00010.g6153;Description=hypothetical protein FVEG_06831 [Fusarium verticillioides 7600];Gene=FPCIR_9208;Ontology_term=transmembrane transport,transporter activity;Ontology_id=GO:0055085,GO:0005215
contig00010.g6154	1404	ID=contig00010.g6154;Description=hypothetical protein FVEG_06832 [Fusarium verticillioides 7600]
contig00010.g6155	2177	ID=contig00010.g6155;Description=related to alcohol oxidase [Fusarium proliferatum ET1];Gene=FVER53263_06833;Ontology_term=oxidoreductase activity;Ontology_id=GO:0016491;Enzyme_code=EC:1.1,EC:1.1,EC:1;Enzyme_name=Acting on the CH-OH group of donors,Acting on the CH-OH group of donors,Oxidoreductases
contig00010.g6156	1223	ID=contig00010.g6156;Description=pyruvate formate lyase activating enzyme [Fusarium verticillioides 7600];Gene=FNAPI_13703;Ontology_term=lyase activity;Ontology_id=GO:0016829;Enzyme_code=EC:4,EC:1.97.1.4,EC:4;Enzyme_name=Lyases,[formate-C-acetyltransferase]-activating enzyme,Lyases
contig00010.g6157	930	ID=contig00010.g6157;Description=hypothetical protein FVER14953_20488 [Fusarium verticillioides]
contig00010.g6158	768	ID=contig00010.g6158;Description=adaptin ear-binding coat-associated protein 1 [Fusarium fujikuroi];Gene=FVEG_06836;Ontology_term=nucleus,DNA binding,vesicle-mediated transport,DNA recombination,hydrolase activity;Ontology_id=GO:0005634,GO:0003677,GO:0016192,GO:0006310,GO:0016787;Enzyme_code=EC:3.1,EC:3.1,EC:3;Enzyme_name=Acting on ester bonds,Acting on ester bonds,Hydrolases
contig00010.g6159	1151	ID=contig00010.g6159;Description=translation initiation factor eIF-2B alpha subunit [Fusarium fujikuroi];Gene=F25303_11133;Ontology_term=carbohydrate metabolic process,translation regulator activity,molecular function regulator activity,RNA binding,cytoplasmic translation,signaling,lyase activity,cytosol;Ontology_id=GO:0005975,GO:0045182,GO:0098772,GO:0003723,GO:0002181,GO:0023052,GO:0016829,GO:0005829;Enzyme_code=EC:4.1.1.49,EC:4.1.1.32,EC:4.1.1,EC:4.1,EC:4.1.1.49,EC:4,EC:4.1.1.32;Enzyme_name=phosphoenolpyruvate carboxykinase (ATP),phosphoenolpyruvate carboxykinase (GTP),Carbon-carbon lyases,Carbon-carbon lyases,phosphoenolpyruvate carboxykinase (ATP),Lyases,phosphoenolpyruvate carboxykinase (GTP)

		ID=contig00010.g6160;Description=Ergothioneine biosynthesis protein 1 [Fusarium musae];Gene=FVER53590_06838;Ontology_term=sulfur compound metabolic process,oxidoreductase activity,cellular modified amino acid metabolic process,amino acid metabolic process,transferase activity;Ontology_id=GO:0006790,GO:0016491,GO:0006575,GO:0006520,GO:0016740;Enzyme_code=EC:2.1.1,EC:2.1,EC:1.21,EC:1.21.3.10,EC:1,EC:2.1.1.44,EC:1.14,EC:2,EC:1.21.3,EC:1.14.99.51,EC:2.1.1;Enzyme_name=Transferring one-carbon groups,Transferring one-carbon groups,Catalyzing the reaction X-H + Y-H = 'X-Y',hercynylcysteine S-oxide synthase,Oxidoreductases,L-histidine N(alpha)-methyltransferase,Acting on paired donors, with incorporation or reduction of molecular oxygen. The oxygen incorporated need not be derived from O2,Transferases,Catalyzing the reaction X-H + Y-H = 'X-Y',Acting on paired donors, with incorporation or reduction of molecular oxygen. The oxygen incorporated need not be derived from O2,Transferring one-carbon groups
contig00010.g6160	3274	
		ID=contig00010.g6161;Description=transcriptional regulatory [Fusarium napiforme];Gene=FMUND_3314;Ontology_term=carbohydrate metabolic process,nucleus,DNA binding,regulation of DNA-templated transcription,transcription regulator activity,hydrolase activity;Ontology_id=GO:0005975,GO:0005634,GO:0003677,GO:0006355,GO:0140110,GO:0016787;Enzyme_code=EC:3.2.1.20;Enzyme_name=alpha-glucosidase
contig00010.g6161	3278	
contig00010.g6162	1662	ID=contig00010.g6162;Description=senescence-associated protein-domain-containing protein [Fusarium oxysporum]
contig00010.g6163	2404	ID=contig00010.g6163;Description=Spc97 Spc98 family [Fusarium tjaetaba];Gene=FMEXI_11094;Ontology_term=cytoskeleton organization,protein-containing complex assembly,microtubule organizing center,cytoskeletal protein binding;Ontology_id=GO:0007010,GO:0065003,GO:0005815,GO:0008092
		ID=contig00010.g6164;Description=hypothetical protein FVEG_06843 [Fusarium verticillioides 7600]
contig00010.g6164	1601	
contig00010.g6165	3790	ID=contig00010.g6165;Description=SH3 domain-containing protein [Fusarium pseudoanthophilum];Ontology_term=cytoskeleton,cytoskeleton organization,protein-containing complex assembly,cytoskeletal protein binding;Ontology_id=GO:0005856,GO:0007010,GO:0065003,GO:0008092

contig00010.g6166	804	ID=contig00010.g6166;Description=RNA polymerase II subunit B1 CTD phosphatase RPAP2 [Fusarium globosum];Gene=FNAPI_5530;Ontology_term=nucleus,catalytic activity, acting on a protein,cytoskeleton,cytoskeleton organization,protein-containing complex assembly,anatomical structure development,cell differentiation,reproductive process,regulation of DNA-templated transcription,cytoskeletal protein binding,hydrolase activity;Ontology_id=GO:0005634,GO:0140096,GO:0005856,GO:0007010,GO:0065003,GO:0048856,GO:0030154,GO:0022414,GO:0006355,GO:0008092,GO:0016787;Enzyme_code=EC:3.1.3.16,EC:3.1.3.16,EC:3.1,EC:3,EC:3.1.3;Enzyme_name=protein-serine/threonine phosphatase,protein-serine/threonine phosphatase,Acting on ester bonds,Hydrolases,Acting on ester bonds
contig00010.g6167	563	ID=contig00010.g6167;Description=ribosomal s17 domain-containing protein [Hirsutella rhossiliensis];Gene=PCL_11465;Ontology_term=protein-containing complex assembly,cytoplasmic translation,structural molecule activity,cytosol,ribosome biogenesis,ribosome;Ontology_id=GO:0065003,GO:0002181,GO:0005198,GO:0005829,GO:0042254,GO:0005840
contig00010.g6168	1308	ID=contig00010.g6168;Description=mitochondrial ribosomal subunit domain protein [Fusarium tjaetaba]
contig00010.g6169	909	ID=contig00010.g6169;Description=queuine tRNA-ribosyltransferase [Fusarium verticillioides 7600];Gene=FVER53590_06848;Ontology_term=tRNA metabolic process,catalytic activity, acting on RNA,transferase activity;Ontology_id=GO:0006399,GO:0140098,GO:0016740;Enzyme_code=EC:2.4.2.64,EC:2.4.2,EC:2.4,EC:2,EC:2.4.2.29,EC:2.4.2.64;Enzyme_name=tRNA-guanosine(34) queuine transglycosylase,Glycosyltransferases,Glycosyltransferases,Transferases,tRNA-guanosine(34) preQ1 transglycosylase,tRNA-guanosine(34) queuine transglycosylase
contig00010.g6170	1151	ID=contig00010.g6170;Description=queuine trna-ribosyltransferase [Fusarium denticulatum];Gene=FDENT_11758;Ontology_term=transferase activity;Ontology_id=GO:0016740;Enzyme_code=EC:2,EC:2;Enzyme_name=Transferases,Transferases

contig00010.g6171	3552	ID=contig00010.g6171;Description=histone deacetylase HOS3 [Fusarium verticillioides 7600];Gene=FPHYL_9360;Ontology_term=nucleus,catalytic activity, acting on a protein,regulation of DNA-templated transcription,chromatin organization,mitotic cell cycle,hydrolase activity;Ontology_id=GO:0005634,GO:0140096,GO:0006355,GO:0006325,GO:0000278,GO:0016787;Enzyme_code=EC:3.5.1,EC:3.5.1.98,EC:3.5,EC:3;Enzyme_name=Acting on carbon-nitrogen bonds, other than peptide bonds,histone deacetylase,Acting on carbon-nitrogen bonds, other than peptide bonds,Hydrolases
contig00010.g6172	1045	ID=contig00010.g6172;Description=histidinol-phosphatase (PHP family) [Fusarium verticillioides 7600];Gene=Focb16_v014637;Ontology_term=amino acid metabolic process,hydrolase activity;Ontology_id=GO:0006520,GO:0016787;Enzyme_code=EC:3.1.3.15,EC:3.1,EC:3.1.3.15,EC:3,EC:3.1.3;Enzyme_name=histidinol-phosphatase,Acting on ester bonds,histidinol-phosphatase,Hydrolases,Acting on ester bonds
contig00010.g6173	3165	ID=contig00010.g6173;Description=hypothetical protein FVEG_06852 [Fusarium verticillioides 7600];Gene=Cys;Ontology_term=molecular function regulator activity;Ontology_id=GO:0098772
contig00010.g6174	3180	ID=contig00010.g6174;Description=Msb2 [Fusarium coicis];Gene=FVER53263_06853;Ontology_term=molecular transducer activity,signaling,plasma membrane,small molecule sensor activity;Ontology_id=GO:0060089,GO:0023052,GO:0005886,GO:0140299
contig00010.g6175	680	ID=contig00010.g6175;Description=Erv1p [Fusarium tjaetaba];Gene=FGADI_9741;Ontology_term=catalytic activity, acting on a protein,oxidoreductase activity;Ontology_id=GO:0140096,GO:0016491;Enzyme_code=EC:1.8.3.2;Enzyme_name=thiol oxidase

contig00010.g6176	968	ID=contig00010.g6176;Description=proteasome component PRE3 [Fusarium proliferatum];Gene=FRV6_12258;Ontology_term=nucleus,catalytic activity, acting on a protein,oxidoreductase activity,protein catabolic process,mitotic nuclear division,chromosome segregation,cytosol,endoplasmic reticulum,hydrolase activity;Ontology_id=GO:0005634,GO:0140096,GO:0016491,GO:0030163,GO:0140014,GO:0007059,GO:0005829,GO:0005783,GO:0016787;Enzyme_code=EC:3.4.25,EC:3.5,EC:1.8.3.2,EC:3.4.25.1,EC:3.4,EC:3.4.25,EC:1,EC:1.8,EC:3.5,EC:1.8.3,EC:3,EC:1.8.3.2;Enzyme_name=Acting on peptide bonds (peptidases),Acting on carbon-nitrogen bonds, other than peptide bonds,thiol oxidase,proteasome endopeptidase complex,Acting on peptide bonds (peptidases),Acting on peptide bonds (peptidases),Oxidoreductases,Acting on a sulfur group of donors,Acting on carbon-nitrogen bonds, other than peptide bonds,Acting on a sulfur group of donors,Hydrolases,thiol oxidase
contig00010.g6177	1525	ID=contig00010.g6177;Description=PHO85 cyclin-6 [Fusarium phyllophilum];Gene=FNAPI_3159;Ontology_term=nucleus,carbohydrate metabolic process,generation of precursor metabolites and energy,molecular function regulator activity;Ontology_id=GO:0005634,GO:0005975,GO:0006091,GO:0098772
contig00010.g6178	922	ID=contig00010.g6178;Description=tyrosinase precursor (monophenol monooxygenase) [Fusarium beomiforme];Gene=BKA59DRAFT_388038;Ontology_term=oxidoreductase activity;Ontology_id=GO:0016491;Enzyme_code=EC:1,EC:1;Enzyme_name=Oxidoreductases,Oxidoreductases
contig00010.g6179	1922	ID=contig00010.g6179;Description=tyrosinase precursor (monophenol monooxygenase) [Fusarium napiforme];Gene=FNAPI_3157;Ontology_term=oxidoreductase activity;Ontology_id=GO:0016491;Enzyme_code=EC:1.14.18.1,EC:1,EC:1.14,EC:1.14.18.1,EC:1.14.18;Enzyme_name=tyrosinase,Oxidoreductases,Acting on paired donors, with incorporation or reduction of molecular oxygen. The oxygen incorporated need not be derived from O2,tyrosinase,Acting on paired donors, with incorporation or reduction of molecular oxygen. The oxygen incorporated need not be derived from O2
contig00010.g6180	3749	ID=contig00010.g6180;Description=DNA-directed RNA polymerase I subunit RPA2 [Fusarium verticillioides 7600];Gene=FTJAE_983;Ontology_term=DNA binding,DNA-templated transcription,nucleolus,catalytic activity, acting on RNA,transferase activity;Ontology_id=GO:0003677,GO:0006351,GO:0005730,GO:0140098,GO:0016740;Enzyme_code=EC:2.7.7.6,EC:2.7,EC:2,EC:2.7.7,EC:2.7.7.6;Enzyme_name=DNA-directed RNA polymerase,Transferring phosphorus-containing groups,Transferases,Transferring phosphorus-containing groups,DNA-directed RNA polymerase

contig00010.g6181	1861	ID=contig00010.g6181;Description=arf/Sar family, other [Fusarium verticillioides 7600];Gene=FOC1_g10012538;Ontology_term=GTPase activity;Ontology_id=GO:0003924;Enzyme_code=EC:3.6.1.15;Enzyme_name=nucleoside-triphosphate phosphatase
contig00010.g6182	1131	ID=contig00010.g6182;Description=beta-14-mannosyl-glyco 4-beta-N-acetylglucosaminyltransferase [Fusarium acutatum];Gene=FOPG_03903;Ontology_term=catalytic activity, acting on a protein,transferase activity;Ontology_id=GO:0140096,GO:0016740;Enzyme_code=EC:2.4.1.144,EC:2.4,EC:2,EC:2.4.1.144,EC:2.4.1;Enzyme_name=beta-1,4-mannosyl-glycoprotein 4-beta-N-acetylglucosaminyltransferase,Glycosyltransferases,Transferases,beta-1,4-mannosyl-glycoprotein 4-beta-N-acetylglucosaminyltransferase,Glycosyltransferases
contig00010.g6183	1262	ID=contig00010.g6183;Description=threonine aldolase [Fusarium proliferatum];Gene=FTJAE_980;Ontology_term=lyase activity,amino acid metabolic process;Ontology_id=GO:0016829,GO:0006520;Enzyme_code=EC:4,EC:4.1.2.48,EC:4;Enzyme_name=Lyases,low-specificity L-threonine aldolase,Lyases
contig00010.g6184	2223	ID=contig00010.g6184;Description=trehalase [Fusarium verticillioides 7600];Gene=FVER53263_06865;Ontology_term=carbohydrate metabolic process,hydrolase activity;Ontology_id=GO:0005975,GO:0016787;Enzyme_code=EC:3.2.1.28,EC:3.2,EC:3,EC:3.2.1.28,EC:3.2.1;Enzyme_name=alpha,alpha-trehalase,Glycosylases,Hydrolases,alpha,alpha-trehalase,Glycosylases
contig00010.g6185	572	ID=contig00010.g6185;Description=DUF74 domain protein [Fusarium subglutinans]
contig00010.g6186	2785	ID=contig00010.g6186;Description=dipeptidyl aminopeptidase [Fusarium tjaetaba];Gene=FACUT_9462;Ontology_term=catalytic activity, acting on a protein,protein maturation,vacuole,hydrolase activity;Ontology_id=GO:0140096,GO:0051604,GO:0005773,GO:0016787;Enzyme_code=EC:3.4.11,EC:3.4.14.1,EC:3.4.14.5,EC:3.4.14.4,EC:3.4.14.2,EC:3.4.11,EC:3.4.14.1,EC:3.4,EC:3,EC:3.4.14.5,EC:3.4.14.4,EC:3.4.14.2;Enzyme_name=Acting on peptide bonds (peptidases),dipeptidyl-peptidase I,dipeptidyl-peptidase IV,dipeptidyl-peptidase III,dipeptidyl-peptidase II,Acting on peptide bonds (peptidases),dipeptidyl-peptidase I,Acting on peptide bonds (peptidases),Hydrolases,dipeptidyl-peptidase IV,dipeptidyl-peptidase III,dipeptidyl-peptidase II

contig00010.g6187	5225	ID=contig00010.g6187;Description=clathrin heavy chain [Fusarium proliferatum];Gene=C2S_6790;Ontology_term=molecular transducer activity,vesicle-mediated transport,transmembrane transport,cytoskeleton,signaling,structural molecule activity,cytoplasmic vesicle,Golgi apparatus,intracellular protein transport,nervous system process,transporter activity,plasma membrane;Ontology_id=GO:0060089,GO:0016192,GO:0055085,GO:0005856,GO:0023052,GO:0005198,GO:0031410,GO:0005794,GO:0006886,GO:0050877,GO:0005215,GO:0005886
contig00010.g6188	570	ID=contig00010.g6188;Description=mfs multidrug [Fusarium acutatum]
contig00010.g6189	1975	ID=contig00010.g6189;Description=major facilitator superfamily transporter [Fusarium tjaetaba];Gene=FTJAE_13995;Ontology_term=nucleus,transmembrane transport,regulation of DNA-templated transcription,transcription regulator activity,transporter activity;Ontology_id=GO:0005634,GO:0055085,GO:0006355,GO:0140110,GO:0005215
contig00010.g6190	1867	ID=contig00010.g6190;Description=Zn(II)2Cys6 transcription factor [Fusarium tjaetaba];Gene=FVEG_06871;Ontology_term=nucleus,regulation of DNA-templated transcription,transcription regulator activity;Ontology_id=GO:0005634,GO:0006355,GO:0140110
contig00010.g6191	832	ID=contig00010.g6191;Description=DUF1640-domain protein [Fusarium tjaetaba];Gene=FOC1_g10012548;Ontology_term=transmembrane transport,protein-containing complex assembly,mitochondrion,mitochondrion organization;Ontology_id=GO:0055085,GO:0065003,GO:0005739,GO:0007005
contig00010.g6192	1170	ID=contig00010.g6192;Description=D-3-phosphoglycerate dehydrogenase [Fusarium verticillioides 7600];Gene=FPHYL_8409;Ontology_term=oxidoreductase activity;Ontology_id=GO:0016491;Enzyme_code=EC:1.1.1,EC:1.1,EC:1.1.1.26,EC:1,EC:1.1.1;Enzyme_name=Acting on the CH-OH group of donors,Acting on the CH-OH group of donors,glyoxylate reductase,Oxidoreductases,Acting on the CH-OH group of donors

contig00010.g6193	1406	ID=contig00010.g6193;Description=adenosylhomocysteinase [Fusarium subglutinans];Gene=AU210_010426;Ontology_term=sulfur compound metabolic process,nucleobase-containing small molecule metabolic process,lipid metabolic process,amino acid metabolic process,cytosol,carbohydrate derivative metabolic process,cellular modified amino acid metabolic process,extracellular matrix,extracellular region,hydrolase activity;Ontology_id=GO:0006790,GO:0055086,GO:0006629,GO:0006520,GO:0005829,GO:1901135,GO:0006575,GO:0031012,GO:0005576,GO:0016787;Enzyme_code=EC:3.13.2.1,EC:3.13,EC:3.3,EC:3,EC:3.13.2.1,EC:3.3.1.1,EC:3.3.1;Enzyme_name=adenosylhomocysteinase,Acting on carbon-sulfur bonds,Acting on ether bonds,Hydrolases,adenosylhomocysteinase,Acting on ether bonds,Acting on ether bonds
contig00010.g6194	1239	ID=contig00010.g6194;Description=translation initiation factor 3 subunit H [Fusarium proliferatum];Gene=FPCIR_11295;Ontology_term=nucleus,catalytic activity, acting on a protein,translation regulator activity,protein-containing complex assembly,cytoplasmic translation,hydrolase activity;Ontology_id=GO:0005634,GO:0140096,GO:0045182,GO:0065003,GO:0002181,GO:0016787;Enzyme_code=EC:3.4,EC:3.4,EC:3,EC:3.4.19.12;Enzyme_name=Acting on peptide bonds (peptidases),Acting on peptide bonds (peptidases),Hydrolases,ubiquitinyl hydrolase 1
contig00010.g6195	711	ID=contig00010.g6195;Description=hypothetical protein FVEG_06876 [Fusarium verticillioides 7600]
contig00010.g6196	1053	ID=contig00010.g6196;Description=PET8 protein, member of the mitochondrial carrier (MCF) family [Fusarium fujikuroi];Gene=FGLOB1_8545;Ontology_term=transmembrane transport,mitochondrion,cytoplasmic vesicle,extracellular region,nutrient reservoir activity,transporter activity;Ontology_id=GO:0055085,GO:0005739,GO:0031410,GO:0005576,GO:0045735,GO:0005215
contig00010.g6197	1083	ID=contig00010.g6197;Description=PTC7-2C phosphatase [Fusarium tjaetaba];Gene=FPCIR_11298;Ontology_term=nuclear envelope,catalytic activity, acting on a protein,signaling,mitochondrion,hydrolase activity;Ontology_id=GO:0005635,GO:0140096,GO:0023052,GO:0005739,GO:0016787;Enzyme_code=EC:3.1.3.16,EC:3.1.3.16,EC:3.1,EC:3,EC:3.1.3;Enzyme_name=protein-serine/threonine phosphatase,protein-serine/threonine phosphatase,Acting on ester bonds,Hydrolases,Acting on ester bonds

contig00010.g6198	959	ID=contig00010.g6198;Description=succinate dehydrogenase (ubiquinone) iron-sulfur subunit, mitochondrial [Fusarium proliferatum];Gene=FNAPI_914;Ontology_term=generation of precursor metabolites and energy,nucleobase-containing small molecule metabolic process,oxidoreductase activity,mitochondrion,carbohydrate derivative metabolic process;Ontology_id=GO:0006091,GO:0055086,GO:0016491,GO:0005739,GO:1901135;Enzyme_code=EC:1.3.5.1,EC:1.3.5,EC:1.3,EC:1,EC:1.3.5.1;Enzyme_name=succinate dehydrogenase,Acting on the CH-CH group of donors,Acting on the CH-CH group of donors,Oxidoreductases,succinate dehydrogenase
contig00010.g6199	340	ID=contig00010.g6199;Description=hypothetical protein FDENT_2026 [Fusarium denticulatum]
contig00010.g6200	1489	ID=contig00010.g6200;Description=BRO1 domain [Fusarium oxysporum f. sp. vasinfectum];Gene=FVEG_06880;Ontology_term=endosome,sulfur compound metabolic process,plasma membrane;Ontology_id=GO:0005768,GO:0006790,GO:0005886
contig00010.g6201	1864	ID=contig00010.g6201;Description=alpha-1,3-glucosyltransferase [Fusarium verticillioides 7600];Gene=FNYG_04793;Ontology_term=lipid metabolic process,carbohydrate derivative metabolic process,endoplasmic reticulum,transferase activity;Ontology_id=GO:0006629,GO:1901135,GO:0005783,GO:0016740;Enzyme_code=EC:2.4.1.267,EC:2.4.1.265;Enzyme_name=dolichyl-P-Glc:Man9GlcNAc2-PP-dolichol alpha-1,3-glucosyltransferase,dolichyl-P-Glc:Glc1Man9GlcNAc2-PP-dolichol alpha-1,3-glucosyltransferase
contig00010.g6202	1362	ID=contig00010.g6202;Description=putative GPI-anchored cupredoxin [Fusarium oxysporum f. sp. rapae];Gene=FTJAE_12458
contig00010.g6203	387	ID=contig00010.g6203;Description=60s acidic ribosomal protein-domain-containing protein [Fusarium redolens];Gene=FCIRC_3695;Ontology_term=molecular function regulator activity,cytoplasmic translation,structural molecule activity,cytosol,ribosome,vacuole;Ontology_id=GO:0098772,GO:0002181,GO:0005198,GO:0005829,GO:0005840,GO:0005773
contig00010.g6204	1191	ID=contig00010.g6204;Description=spt2 chromatin [Fusarium tjaetaba]
contig00010.g6205	1846	ID=contig00010.g6205;Description=nuclear fragile x mental retardation-interacting 1 [Fusarium acutatum];Gene=FVER53263_06885;Ontology_term=RNA binding;Ontology_id=GO:0003723

contig00010.g6206	795	ID=contig00010.g6206;Description=RNA polymerase III subunit Rpc25-domain-containing protein [Fusarium redolens];Gene=FCIRC_3698;Ontology_term=DNA binding,DNA-templated transcription,nuclear chromosome,tRNA metabolic process,catalytic activity, acting on RNA,transferase activity,nucleoplasm;Ontology_id=GO:0003677,GO:0006351,GO:0000228,GO:0006399,GO:0140098,GO:0016740,GO:0005654;Enzyme_code=EC:2.7.7.6,EC:2.7,EC:2,EC:2.7.7,EC:2.7.7.6;Enzyme_name=DNA-directed RNA polymerase,Transferring phosphorus-containing groups,Transferases,Transferring phosphorus-containing groups,DNA-directed RNA polymerase
contig00010.g6207	935	ID=contig00010.g6207;Description=DUF850 domain protein [Fusarium tjaetaba];Gene=FCIRC_3699;Ontology_term=endoplasmic reticulum,protein folding;Ontology_id=GO:0005783,GO:0006457
contig00010.g6208	795	ID=contig00010.g6208;Description=microsomal signal peptidase subunit [Fusarium tjaetaba];Gene=FGLOB1_11649;Ontology_term=protein maturation,endoplasmic reticulum;Ontology_id=GO:0051604,GO:0005783
contig00010.g6209	3751	ID=contig00010.g6209;Description=DNA repair transcription mms19 [Fusarium tjaetaba];Gene=FVER53590_06890;Ontology_term=DNA repair,nucleus,protein maturation;Ontology_id=GO:0006281,GO:0005634,GO:0051604
contig00010.g6210	1008	ID=contig00010.g6210;Description=ATP synthase mitochondrial F1 complex assembly factor 1 [Fusarium verticillioides 7600];Gene=BFJ65_g10782;Ontology_term=protein-containing complex assembly,mitochondrion,mitochondrion organization;Ontology_id=GO:0065003,GO:0005739,GO:0007005
contig00010.g6211	2172	ID=contig00010.g6211;Description=triacylglycerol lipase [Fusarium tjaetaba];Gene=FTJAE_12467;Ontology_term=lipid droplet,lipid metabolic process,carbohydrate derivative metabolic process,cell wall organization or biogenesis,plasma membrane,hydrolase activity;Ontology_id=GO:0005811,GO:0006629,GO:1901135,GO:0071554,GO:0005886,GO:0016787;Enzyme_code=EC:3.1,EC:3.1.1.13,EC:3,EC:3.1.1;Enzyme_name=Acting on ester bonds,sterol esterase,Hydrolases,Acting on ester bonds

contig00010.g6212	4275	ID=contig00010.g6212;Description=peroxisomal biogenesis factor 6 [Fusarium verticillioides 7600];Gene=FTJAE_12468;Ontology_term=peroxisome,transmembrane transport,ATP-dependent activity,peroxisome organization,signaling,lipid metabolic process,cytosol,intracellular protein transport,hydrolase activity;Ontology_id=GO:0005777,GO:0055085,GO:0140657,GO:0007031,GO:0023052,GO:0006629,GO:0005829,GO:0006886,GO:0016787;Enzyme_code=EC:3.6.1.15,EC:3.6.1,EC:3.6,EC:3,EC:3.6.1.15;Enzyme_name=nucleoside-triphosphate phosphatase,Acting on acid anhydrides,Acting on acid anhydrides,Hydrolases,nucleoside-triphosphate phosphatase
contig00010.g6213	1508	ID=contig00010.g6213;Description=cylicin I [Fusarium tjaetaba];Ontology_term=cellular bud,cytoplasm;Ontology_id=GO:0005933,GO:0005737
contig00010.g6214	570	ID=contig00010.g6214;Description=hypothetical protein FVEG_06895 [Fusarium verticillioides 7600]
contig00010.g6215	1701	ID=contig00010.g6215;Description=cyclopropane-fatty-acyl-phospholipid synthase [Fusarium verticillioides 7600];Gene=FMEXI_6163;Ontology_term=lipid metabolic process,carbohydrate derivative metabolic process,transferase activity,plasma membrane;Ontology_id=GO:0006629,GO:1901135,GO:0016740,GO:0005886;Enzyme_code=EC:2.1.1,EC:2.1,EC:2,EC:2.1.1.317,EC:2.1.1;Enzyme_name=Transferring one-carbon groups,Transferring one-carbon groups,Transferases,sphingolipid C(9)-methyltransferase,Transferring one-carbon groups
contig00010.g6216	907	ID=contig00010.g6216;Description=mesaconyl-C4 CoA hydratase [Fusarium verticillioides 7600]
contig00010.g6217	3195	ID=contig00010.g6217;Description=phosphatidylinositol-glycan biosynthesis class O (PIG-O) [Fusarium tjaetaba];Gene=FVER53263_06898;Ontology_term=lipid metabolic process,carbohydrate derivative metabolic process,endoplasmic reticulum,transferase activity;Ontology_id=GO:0006629,GO:1901135,GO:0005783,GO:0016740;Enzyme_code=EC:2.7.8,EC:2.7,EC:2,EC:2.7.8;Enzyme_name=Transferring phosphorus-containing groups,Transferring phosphorus-containing groups,Transferases,Transferring phosphorus-containing groups
contig00010.g6218	2690	ID=contig00010.g6218;Description=hypothetical protein FPANT_2341 [Fusarium pseudoanthophilum];Gene=FVER53263_06899;Ontology_term=membrane;Ontology_id=GO:0016020

contig00010.g6219	1331	ID=contig00010.g6219;Description=murein transglycosylase [Fusarium odoratissimum NRRL 54006];Gene=FNAPI_893;Ontology_term=carbohydrate metabolic process,extracellular region,hydrolase activity;Ontology_id=GO:0005975,GO:0005576,GO:0016787;Enzyme_code=EC:3.2.1,EC:3.2,EC:3.2.1.75,EC:3.2.1.21,EC:3,EC:3.2.1;Enzyme_name=Glycosylases,Glycosylases,glucan endo-1,6-beta-glucosidase,beta-glucosidase,Hydrolases,Glycosylases
contig00010.g6220	1949	ID=contig00010.g6220;Description=ATP-binding protein cassette [Fusarium tjaetaba];Gene=FPCIR_11321;Ontology_term=transmembrane transport,ATP-dependent activity,transporter activity,hydrolase activity;Ontology_id=GO:0055085,GO:0140657,GO:0005215,GO:0016787;Enzyme_code=EC:7.2.2,EC:3.6.1.15;Enzyme_name=Catalysing the translocation of inorganic cations,nucleoside-triphosphate phosphatase
contig00010.g6221	2132	ID=contig00010.g6221;Description=related to Zn(II)2Cys6 transcriptional activator [Fusarium proliferatum ET1];Gene=FVEG_06902;Ontology_term=nucleus,DNA binding,regulation of DNA-templated transcription,transcription regulator activity;Ontology_id=GO:0005634,GO:0003677,GO:0006355,GO:0140110
contig00010.g6222	1977	ID=contig00010.g6222;Description=hypothetical protein FVER53590_06904 [Fusarium verticillioides];Gene=FVER53590_06904;Ontology_term=catalytic activity, acting on a protein,transmembrane transport,ATP-dependent activity,transferase activity,transporter activity,plasma membrane,hydrolase activity;Ontology_id=GO:0140096,GO:0055085,GO:0140657,GO:0016740,GO:0005215,GO:0005886,GO:0016787;Enzyme_code=EC:2.7.1,EC:7.1.2.1,EC:7.2.2,EC:3.6.1.15,EC:2.7.1,EC:3.6.1,EC:7.1.2.1,EC:2.7,EC:3.6,EC:2,EC:3,EC:7.2.2,EC:7,EC:3.6.1.15;Enzyme_name=Transferring phosphorus-containing groups,P-type H(+)-exporting transporter,Catalysing the translocation of inorganic cations,nucleoside-triphosphate phosphatase,Transferring phosphorus-containing groups,Acting on acid anhydrides,P-type H(+)-exporting transporter,Transferring phosphorus-containing groups,Acting on acid anhydrides,Transferases,Hydrolases,Catalysing the translocation of inorganic cations,Translocases,nucleoside-triphosphate phosphatase
contig00010.g6223	387	ID=contig00010.g6223;Description=hypothetical protein FVER14953_21217 [Fusarium verticillioides]

contig00010.g6224	3135	ID=contig00010.g6224;Description=NAK BIKE kinase [Fusarium tjaetaba];Gene=FDENT_2749;Ontology_term=catalytic activity, acting on a protein,vesicle-mediated transport,cytoskeleton,cytoskeleton organization,signaling,transferase activity;Ontology_id=GO:0140096,GO:0016192,GO:0005856,GO:0007010,GO:0023052,GO:0016740;Enzyme_code=EC:2.7.1,EC:2.7.1,EC:2.7.11.1,EC:2.7,EC:2;Enzyme_name=Transferring phosphorus-containing groups,Transferring phosphorus-containing groups,non-specific serine/threonine protein kinase,Transferring phosphorus-containing groups,Transferases
contig00010.g6225	1470	ID=contig00010.g6225;Description=MAK11-like protein [Fusarium denticulatum];Gene=ENA1;Ontology_term=catalytic activity, acting on a protein,transmembrane transport,molecular function regulator activity,cytoskeleton organization,protein-containing complex assembly,ATP-dependent activity,ribosome biogenesis,transferase activity,nucleolus,transporter activity,plasma membrane;Ontology_id=GO:0140096,GO:0055085,GO:0098772,GO:0007010,GO:0065003,GO:0140657,GO:0042254,GO:0016740,GO:0005730,GO:0005215,GO:0005886;Enzyme_code=EC:2.7.1,EC:7.2.2.3,EC:7.2.2.6,EC:2.7.1,EC:2.7,EC:2,EC:7,EC:7.2.2,EC:7.2.2.3,EC:7.2.2.6;Enzyme_name=Transferring phosphorus-containing groups,P-type Na(+) transporter,P-type K(+) transporter,Transferring phosphorus-containing groups,Transferring phosphorus-containing groups,Transferases,Translocases,Catalysing the translocation of inorganic cations,P-type Na(+) transporter,P-type K(+) transporter
contig00010.g6226	1209	ID=contig00010.g6226;Description=insulin-induced protein-domain-containing protein [Fusarium oxysporum];Gene=FMAN_10249;Ontology_term=endoplasmic reticulum;Ontology_id=GO:0005783
contig00010.g6227	506	ID=contig00010.g6227;Description=hypothetical protein FPANT_11166 [Fusarium pseudoanthophilum]
contig00010.g6228	1779	ID=contig00010.g6228;Description=hypothetical protein FVEG_06909 [Fusarium verticillioides 7600];Gene=FPANT_9551;Ontology_term=catalytic activity, acting on a protein,protein catabolic process,cytosol,transferase activity;Ontology_id=GO:0140096,GO:0030163,GO:0005829,GO:0016740;Enzyme_code=EC:2.3,EC:2,EC:2.3.2;Enzyme_name=Acyltransferases,Transferases,Acyltransferases
contig00010.g6229	2486	ID=contig00010.g6229;Description=hypothetical protein FVER14953_06910 [Fusarium verticillioides]

contig00010.g6230	819	ID=contig00010.g6230;Description=dephospho-CoA kinase-domain-containing protein [Fusarium redolens];Gene=BFJ65_g11881;Ontology_term=nuclear envelope,lipid droplet,nucleobase-containing small molecule metabolic process,mitochondrion,carbohydrate derivative metabolic process,cytosol,endoplasmic reticulum,transferase activity;Ontology_id=GO:0005635,GO:0005811,GO:0055086,GO:0005739,GO:1901135,GO:0005829,GO:0005783,GO:0016740;Enzyme_code=EC:2.7.1.24,EC:2.7.1,EC:2.7,EC:2,EC:2.7.1.24;Enzyme_name=dephospho-CoA kinase,Transferring phosphorus-containing groups,Transferring phosphorus-containing groups,Transferases,dephospho-CoA kinase
contig00010.g6231	1557	ID=contig00010.g6231;Description=Uncharacterized protein HZ326_11043 [Fusarium oxysporum f. sp. albedinis]
contig00010.g6232	1476	ID=contig00010.g6232;Description=TIM23 translocase [Fusarium phyllophilum];Gene=FPHYL_9782;Ontology_term=transmembrane transport,transporter activity;Ontology_id=GO:0055085,GO:0005215
contig00010.g6233	776	ID=contig00010.g6233;Description=probable TIM23 translocase [Fusarium proliferatum ET1];Gene=FOQG_03838;Ontology_term=transmembrane transport,mitochondrion,intracellular protein transport,transporter activity,mitochondrion organization;Ontology_id=GO:0055085,GO:0005739,GO:0006886,GO:0005215,GO:0007005
contig00010.g6234	1273	ID=contig00010.g6234;Description=PQ loop repeat [Fusarium subglutinans];Gene=C2S_6837;Ontology_term=transmembrane transport,mitochondrion,endoplasmic reticulum,vacuole,transporter activity;Ontology_id=GO:0055085,GO:0005739,GO:0005783,GO:0005773,GO:0005215
contig00010.g6235	1297	ID=contig00010.g6235;Description=NPY1-NADH pyrophosphatase I of the Nudix family of hydrolase has a peroxisomal targetin [Fusarium phyllophilum];Gene=FVER53590_06917;Ontology_term=peroxisome,nucleobase-containing small molecule metabolic process,cytosol,hydrolase activity;Ontology_id=GO:0005777,GO:0055086,GO:0005829,GO:0016787;Enzyme_code=EC:3.6.1.22,EC:3.6.1.22,EC:3.6.1,EC:3.6,EC:3;Enzyme_name=NAD(+) diphosphatase,NAD(+) diphosphatase,Acting on acid anhydrides,Acting on acid anhydrides,Hydrolases
contig00010.g6236	1612	ID=contig00010.g6236;Description=hypothetical protein J7337_009574 [Fusarium musae]
contig00010.g6237	1662	ID=contig00010.g6237;Description=putative amino-acid permease P7G5.06 [Fusarium proliferatum];Gene=FMAN_10259;Ontology_term=transmembrane transport,Golgi apparatus,vacuole,transporter activity,plasma membrane;Ontology_id=GO:0055085,GO:0005794,GO:0005773,GO:0005215,GO:0005886

contig00010.g6238	3233	ID=contig00010.g6238;Description=hypothetical protein FVER14953_06922 [Fusarium verticillioides]
contig00010.g6239	999	ID=contig00010.g6239;Description=UPF0613 protein [Fusarium mundagurra]
contig00010.g6240	5268	ID=contig00010.g6240;Description=anion transporter [Fusarium sp. NRRL 25303];Gene=FVEG_06924;Ontology_term=membrane organization,transmembrane transport,cytoskeleton organization,sulfur compound metabolic process,anatomical structure development,reproductive process,cellular modified amino acid metabolic process,lipid binding,receptor ligand activity,vacuole,transporter activity,mitotic cell cycle,plasma membrane,cell motility,endosome,ATP-dependent activity,detoxification,cell differentiation,programmed cell death,hydrolase activity;Ontology_id=GO:0061024,GO:0055085,GO:0007010,GO:0006790,GO:0048856,GO:0022414,GO:0006575,GO:0008289,GO:0048018,GO:0005773,GO:0005215,GO:0000278,GO:0005886,GO:0048870,GO:0005768,GO:0140657,GO:0098754,GO:0030154,GO:0012501,GO:0016787;Enzyme_code=EC:7.2.2,EC:3.6.1.15,EC:7.6.2.3,EC:3.6.1,EC:3.6,EC:3,EC:7.2.2,EC:7,EC:7.4.2.5,EC:3.6.1.15;Enzyme_name=Catalysing the translocation of inorganic cations,nucleoside-triphosphate phosphatase,ABC-type glutathione-S-conjugate transporter,Acting on acid anhydrides,Acting on acid anhydrides,Hydrolases,Catalysing the translocation of inorganic cations,Translocases,bacterial ABC-type protein transporter,nucleoside-triphosphate phosphatase
contig00010.g6241	1737	ID=contig00010.g6241;Description=hypothetical protein FSARC_12758 [Fusarium sarcochroum]
contig00010.g6242	979	ID=contig00010.g6242;Description=putative CBF1-centromere binding factor 1 [Fusarium mangiferae];Gene=FACUT_5616;Ontology_term=sulfur compound metabolic process,chromosome segregation,regulation of DNA-templated transcription,amino acid metabolic process,nuclear chromosome,chromatin organization,nucleoplasm,DNA binding,carbohydrate metabolic process,wound healing,lipid metabolic process,mitochondrion,transcription regulator activity;Ontology_id=GO:0006790,GO:0007059,GO:0006355,GO:0006520,GO:0000228,GO:0006325,GO:0005654,GO:0003677,GO:0005975,GO:0042060,GO:0006629,GO:0005739,GO:0140110
contig00010.g6243	1376	ID=contig00010.g6243;Description=macrophage erythroblast attacher [Fusarium tjaetaba];Gene=FNAPI_11997;Ontology_term=carbohydrate metabolic process,catalytic activity, acting on a protein,protein catabolic process,transferase activity;Ontology_id=GO:0005975,GO:0140096,GO:0030163,GO:0016740;Enzyme_code=EC:2.3.2;Enzyme_name=Acyltransferases

		ID=contig00010.g6244;Description=CCR4-NOT core DEDD RNase subunit [Fusarium oxysporum];Gene=FNAPI_11998;Ontology_term=nucleus,mRNA metabolic process,RNA binding,regulation of DNA-templated transcription,catalytic activity, acting on RNA,hydrolase activity;Ontology_id=GO:0005634,GO:0016071,GO:0003723,GO:0006355,GO:0140098,GO:0016787;Enzyme_code=EC:3.1.15,EC:3.1.13.4,EC:3.1,EC:3.1.13,EC:3,EC:3.1.15,EC:3.1.13.4;Enzyme_name=Acting on ester bonds,poly(A)-specific ribonuclease,Acting on ester bonds,Acting on ester bonds,Hydrolases,Acting on ester bonds,poly(A)-specific ribonuclease
contig00010.g6244	1514	
contig00010.g6245	447	ID=contig00010.g6245;Description=NADH-ubiquinone oxidoreductase B12 subunit [Fusarium subglutinans];Gene=FACUT_5613;Ontology_term=generation of precursor metabolites and energy,mitochondrion;Ontology_id=GO:0006091,GO:0005739
		ID=contig00010.g6246;Description=hypothetical protein FVER14953_06931 [Fusarium verticillioides]
contig00010.g6246	1575	
		ID=contig00010.g6247;Description=hypothetical protein FVEG_06932 [Fusarium verticillioides 7600];Gene=FVER53263_06932;Ontology_term=membrane;Ontology_id=GO:0016020
contig00010.g6247	689	
		ID=contig00010.g6248;Description=hypothetical protein FVEG_06933 [Fusarium verticillioides 7600]
contig00010.g6248	1749	
		ID=contig00010.g6249;Description=ubiquitin-like 1-activating enzyme E1 A [Fusarium verticillioides 7600];Gene=FPANT_7691;Ontology_term=nucleus,catalytic activity, acting on a protein,ATP-dependent activity,protein catabolic process,anatomical structure development,chromosome segregation,cytosol,microtubule-based movement,ligase activity,meiotic nuclear division;Ontology_id=GO:0005634,GO:0140096,GO:0140657,GO:0030163,GO:0048856,GO:0007059,GO:0005829,GO:0007018,GO:0016874,GO:0140013;Enzyme_code=EC:6.2,EC:6.2.1.45,EC:6.2,EC:6;Enzyme_name=Forming carbon-sulfur bonds,E1 ubiquitin-activating enzyme,Forming carbon-sulfur bonds,Ligases
contig00010.g6249	1781	
contig00010.g6250	4232	ID=contig00010.g6250;Description=hypothetical protein J7337_009588 [Fusarium musae]

contig00010.g6251	1029	ID=contig00010.g6251;Description=hypothetical protein FVEG_06936 [Fusarium verticillioides 7600];Gene=FPCIR_9714;Ontology_term=ribosome biogenesis,transferase activity,catalytic activity, acting on RNA,nucleolus;Ontology_id=GO:0042254,GO:0016740,GO:0140098,GO:0005730;Enzyme_code=EC:2.1.1,EC:2.1,EC:2,EC:2.1.1,EC:2.1.1.313;Enzyme_name=Transferring one-carbon groups,Transferring one-carbon groups,Transferases,Transferring one-carbon groups,25S rRNA (uracil(2634)-N(3))-methyltransferase
contig00010.g6252	4928	ID=contig00010.g6252;Description=histone-lysine n-methyltransferase H3 lysine-36 specific [Fusarium tjaetaba];Gene=FPCIR_9715;Ontology_term=nucleus,catalytic activity, acting on a protein,regulation of DNA-templated transcription,chromosome,chromatin organization,transferase activity,ligase activity;Ontology_id=GO:0005634,GO:0140096,GO:0006355,GO:0005694,GO:0006325,GO:0016740,GO:0016874;Enzyme_code=EC:6.3.2.4,EC:2.1.1,EC:2.1,EC:6.3.2.4,EC:6.3,EC:2,EC:6.3.2,EC:6,EC:2.1.1;Enzyme_name=D-alanine--D-alanine ligase,Transferring one-carbon groups,Transferring one-carbon groups,D-alanine--D-alanine ligase,Forming carbon-nitrogen bonds,Transferases,Forming carbon-nitrogen bonds,Ligases,Transferring one-carbon groups
contig00010.g6253	868	ID=contig00010.g6253;Description=hypothetical protein FVER14953_06939 [Fusarium verticillioides]
contig00010.g6254	1562	ID=contig00010.g6254;Description=4-aminobutyrate aminotransferase [Fusarium verticillioides 7600];Gene=FACUT_5604;Ontology_term=amino acid metabolic process,transferase activity;Ontology_id=GO:0006520,GO:0016740;Enzyme_code=EC:2.6.1.19;Enzyme_name=4-aminobutyrate--2-oxoglutarate transaminase
contig00010.g6255	2945	ID=contig00010.g6255;Description=chloride channel, other eukaryote [Fusarium odoratissimum NRRL 54006];Gene=LLEC1_07796;Ontology_term=transmembrane transport;Ontology_id=GO:0055085
contig00010.g6256	1851	ID=contig00010.g6256;Description=SAS10-like protein [Fusarium tjaetaba];Ontology_term=RNA binding,ribosome biogenesis,nucleolus;Ontology_id=GO:0003723,GO:0042254,GO:0005730
contig00010.g6257	2457	ID=contig00010.g6257;Description=oligomeric golgi complex subunit 1 [Fusarium coicis];Gene=FVER53263_06943;Ontology_term=vesicle-mediated transport,Golgi apparatus;Ontology_id=GO:0016192,GO:0005794

contig00010.g6258	901	ID=contig00010.g6258;Description=delta3-cis-delta2-trans-enoyl-CoA isomerase [Fusarium fujikuroi];Gene=FDENT_2717;Ontology_term=nucleus,peroxisome,protein catabolic process,lipid metabolic process,isomerase activity;Ontology_id=GO:0005634,GO:0005777,GO:0030163,GO:0006629,GO:0016853;Enzyme_code=EC:5,EC:5.3,EC:5,EC:5.3.3.8,EC:5.3.3;Enzyme_name=Isomerases,Intramolecular oxidoreductases,Isomerases,Delta(3)-Delta(2)-enoyl-CoA isomerase,Intramolecular oxidoreductases
contig00010.g6259	1430	ID=contig00010.g6259;Description=DNA-directed RNA polymerase A (I) chain [Fusarium tjaetaba];Gene=FNAPI_8879;Ontology_term=DNA binding,DNA-templated transcription,nucleolus;Ontology_id=GO:0003677,GO:0006351,GO:0005730
contig00010.g6260	2282	ID=contig00010.g6260;Description=CAMK kinase [Fusarium tjaetaba];Gene=FPCIR_9723;Ontology_term=catalytic activity, acting on a protein,DNA replication,signaling,DNA recombination,nuclear chromosome,transferase activity,meiotic nuclear division;Ontology_id=GO:0140096,GO:0006260,GO:0023052,GO:0006310,GO:0000228,GO:0016740,GO:0140013;Enzyme_code=EC:2.7.1,EC:2.7.1,EC:2.7.11.1,EC:2.7,EC:2,EC:2.7.11.17;Enzyme_name=Transferring phosphorus-containing groups,Transferring phosphorus-containing groups,non-specific serine/threonine protein kinase,Transferring phosphorus-containing groups,Transferases,calcium/calmodulin-dependent protein kinase
contig00010.g6261	682	ID=contig00010.g6261;Description=hypothetical protein FNYG_04855 [Fusarium nygamai];Gene=FPRO_10704;Ontology_term=nucleus,regulation of DNA-templated transcription,transcription regulator activity;Ontology_id=GO:0005634,GO:0006355,GO:0140110
contig00010.g6262	3092	ID=contig00010.g6262;Description=ULK/ULK protein kinase [Fusarium verticillioides 7600];Gene=FVEG_06949;Ontology_term=catalytic activity, acting on a protein,autophagy,transferase activity;Ontology_id=GO:0140096,GO:0006914,GO:0016740;Enzyme_code=EC:2.7.11.1;Enzyme_name=non-specific serine/threonine protein kinase

contig00010.g6263	1367	ID=contig00010.g6263;Description=ornithine aminotransferase [Fusarium verticillioides 7600];Gene=FNAPI_8875;Ontology_term=nucleus,amino acid metabolic process,cytosol,transferase activity;Ontology_id=GO:0005634,GO:0006520,GO:0005829,GO:0016740;Enzyme_code=EC:2.6.1.13,EC:2.6.1,EC:2.6.1.13,EC:2,EC:2.6;Enzyme_name=ornithine aminotransferase,Transferring nitrogenous groups,ornithine aminotransferase,Transferases,Transferring nitrogenous groups
contig00010.g6264	2073	ID=contig00010.g6264;Description=GPI mannosyltransferase 3 [Fusarium verticillioides 7600];Gene=FNAPI_8874;Ontology_term=lipid metabolic process,carbohydrate derivative metabolic process,endoplasmic reticulum,transferase activity;Ontology_id=GO:0006629,GO:1901135,GO:0005783,GO:0016740;Enzyme_code=EC:2.4,EC:2.4,EC:2,EC:2.4.1;Enzyme_name=Glycosyltransferases,Glycosyltransferases,Transferases,Glycosyltransferases
contig00010.g6265	3184	ID=contig00010.g6265;Description=Rho-GTPase-activating 5 [Fusarium tjaetaba];Gene=FNAPI_8873;Ontology_term=molecular function regulator activity,signaling;Ontology_id=GO:0098772,GO:0023052
contig00010.g6266	385	ID=contig00010.g6266;Description=probable translocase of inner membrane [Fusarium fujikuroi];Gene=FCIRC_12278;Ontology_term=mitochondrion;Ontology_id=GO:0005739
contig00010.g6267	5639	ID=contig00010.g6267;Description=hypothetical protein FVEG_15967 [Fusarium verticillioides 7600];Gene=FOIG_05132;Ontology_term=nuclear envelope,nucleocytoplasmic transport,structural molecule activity,intracellular protein transport,mitotic cell cycle;Ontology_id=GO:0005635,GO:0006913,GO:0005198,GO:0006886,GO:0000278
contig00010.g6268	2336	ID=contig00010.g6268;Description=anthranilate synthase component 2 [Fusarium verticillioides 7600];Gene=FTJAE_37;Ontology_term=lyase activity,amino acid metabolic process,isomerase activity;Ontology_id=GO:0016829,GO:0006520,GO:0016853;Enzyme_code=EC:4.1.1.48,EC:4.1.3.27,EC:5.3.1.24,EC:4.1.1,EC:4.1.2,EC:4.1.1.48,EC:4.1,EC:4.1.3.27,EC:5.3,EC:4,EC:5,EC:4.1.2.8,EC:5.3.1,EC:5.3.1.24,EC:4.1.3;Enzyme_name=indole-3-glycerol-phosphate synthase,anthranilate synthase,phosphoribosylanthranilate isomerase,Carbon-carbon lyases,Carbon-carbon lyases,indole-3-glycerol-phosphate synthase,Carbon-carbon lyases,anthranilate synthase,Intramolecular oxidoreductases,Lyases,Isomerases,indole-3-glycerol-phosphate lyase,Intramolecular oxidoreductases,phosphoribosylanthranilate isomerase,Carbon-carbon lyases

contig00010.g6269	1568	ID=contig00010.g6269;Description=hypothetical protein FVER14953_06957 [Fusarium verticillioides]
contig00010.g6270	1148	ID=contig00010.g6270;Description=hypothetical protein FVER53590_06958 [Fusarium verticillioides];Gene=FVER53590_06958;Ontology_term=endoplasmic reticulum;Ontology_id=GO:0005783
contig00010.g6271	581	ID=contig00010.g6271;Description=pre-mRNA-splicing factor CWC21 [Fusarium verticillioides 7600];Gene=BFJ72_g14482;Ontology_term=nucleus;Ontology_id=GO:0005634
contig00010.g6272	1254	ID=contig00010.g6272;Description=D123-domain-containing protein [Fusarium oxysporum];Gene=Forpi1262_v006540;Ontology_term=protein-containing complex assembly,cytoplasmic translation;Ontology_id=GO:0065003,GO:0002181
contig00010.g6273	1002	ID=contig00010.g6273;Description=hypothetical protein FCOIX_6885 [Fusarium coicis]
contig00010.g6274	420	ID=contig00010.g6274;Description=hypothetical protein FVEG_15968 [Fusarium verticillioides 7600]
contig00010.g6275	1258	ID=contig00010.g6275;Description=Cell division control protein 48 [Fusarium oxysporum f. sp. albedinis];Gene=F52700_13182;Ontology_term=sulfur compound metabolic process,anatomical structure development,reproductive process,microtubule-based movement,intracellular protein transport,plasma membrane,nucleocytoplasmic transport,endosome,GTPase activity,signaling,cell differentiation,cytosol,nervous system process;Ontology_id=GO:0006790,GO:0048856,GO:0022414,GO:0007018,GO:0006886,GO:0005886,GO:0006913,GO:0005768,GO:0003924,GO:0023052,GO:0030154,GO:0005829,GO:0050877;Enzyme_code=EC:3.6.1.15,EC:3.6.1,EC:3.6,EC:3,EC:3.6.1.15;Enzyme_name=nucleoside-triphosphate phosphatase,Acting on acid anhydrides,Acting on acid anhydrides,Hydrolases,nucleoside-triphosphate phosphatase
contig00010.g6276	348	ID=contig00010.g6276;Description=uncharacterized protein FTJAE_29 [Fusarium tjaetaba]
contig00010.g6277	905	ID=contig00010.g6277;Description=nuclear WD PRL1 [Fusarium tjaetaba];Gene=FVEG_06963;Ontology_term=mRNA metabolic process;Ontology_id=GO:0016071
contig00010.g6278	789	ID=contig00010.g6278;Description=transmembrane [Fusarium acutatum];Gene=FNYG_04871;Ontology_term=membrane;Ontology_id=GO:0016020
contig00010.g6279	1070	ID=contig00010.g6279;Description=KTI12 (TOT4) [Fusarium tjaetaba];Gene=FVER53263_06965;Ontology_term=ATP binding;Ontology_id=GO:0005524

contig00010.g6280	2697	ID=contig00010.g6280;Description=cell division control protein 48 [Fusarium odoratissimum NRRL 54006];Gene=FPHYL_9131;Ontology_term=membrane organization,cytoskeleton organization,protein catabolic process,establishment or maintenance of cell polarity,regulation of DNA-templated transcription,chromosome segregation,nuclear chromosome,endoplasmic reticulum,chromatin organization,intracellular protein transport,cytoskeleton,molecular function regulator activity,ATP-dependent activity,autophagy,mitochondrion,signaling,mitotic nuclear division,cytosol,protein maturation,hydrolase activity,molecular adaptor activity;Ontology_id=GO:0061024,GO:0007010,GO:0030163,GO:0007163,GO:0006355,GO:0007059,GO:0000228,GO:0005783,GO:0006325,GO:0006886,GO:0005856,GO:0098772,GO:0140657,GO:0006914,GO:0005739,GO:0023052,GO:0140014,GO:0005829,GO:0051604,GO:0016787,GO:0060090;Enzyme_code=EC:3.6.1.15,EC:3.6.1,EC:3.6,EC:3,EC:3.6.1.15;Enzyme_name=nucleoside-triphosphate phosphatase,Acting on acid anhydrides,Acting on acid anhydrides,Hydrolases,nucleoside-triphosphate phosphatase
contig00010.g6281	544	ID=contig00010.g6281;Description=hypothetical protein FGLOB1_2694 [Fusarium globosum];Gene=PRS1;Ontology_term=nucleobase-containing small molecule metabolic process,carbohydrate derivative metabolic process,cell wall organization or biogenesis,transferase activity;Ontology_id=GO:0055086,GO:1901135,GO:0071554,GO:0016740;Enzyme_code=EC:2.7.6.1;Enzyme_name=ribose-phosphate diphosphokinase
contig00011.g6282	2628	ID=contig00011.g6282;Description=Ff.00g129820.m01.CDS01 [Fusarium sp. VM40]
contig00011.g6283	2460	ID=contig00011.g6283;Description=linalool dehydratase-isomerase precursor [Fusarium subglutinans];Gene=FVEG_10965;Ontology_term=isomerase activity;Ontology_id=GO:0016853;Enzyme_code=EC:5,EC:5;Enzyme_name=Isomerases,Isomerases
contig00011.g6284	3457	ID=contig00011.g6284;Description=related to zinc-containing long-chain alcohol dehydrogenase [Fusarium fujikuroi];Gene=FTJAE_2261;Ontology_term=oxidoreductase activity;Ontology_id=GO:0016491;Enzyme_code=EC:1,EC:1;Enzyme_name=Oxidoreductases,Oxidoreductases
contig00011.g6285	1648	ID=contig00011.g6285;Description=3-hydroxymethyl-3-methylglutaryl-coenzyme a lyase [Fusarium tjaetaba];Gene=FPCIR_953;Ontology_term=nucleus,DNA binding,lyase activity,regulation of DNA-templated transcription,transcription regulator activity;Ontology_id=GO:0005634,GO:0003677,GO:0016829,GO:0006355,GO:0140110;Enzyme_code=EC:4,EC:4;Enzyme_name=Lyases,Lyases

contig00011.g6286	4803	ID=contig00011.g6286;Description=hypothetical protein FVER53263_10970 [Fusarium verticillioides];Gene=FVER53263_10970;Ontology_term=transmembrane transport,ATP-dependent activity,transporter activity,hydrolase activity;Ontology_id=GO:0055085,GO:0140657,GO:0005215,GO:0016787;Enzyme_code=EC:7.2.2,EC:3.6.1.15,EC:3.6.1,EC:3.6,EC:3,EC:7.2.2,EC:7,EC:3.6.1.15;Enzyme_name=Catalysing the translocation of inorganic cations,nucleoside-triphosphate phosphatase,Acting on acid anhydrides,Acting on acid anhydrides,Hydrolases,Catalysing the translocation of inorganic cations,Translocases,nucleoside-triphosphate phosphatase
contig00011.g6287	1988	ID=contig00011.g6287;Description=4-coumarate ligase [Fusarium tjaetaba];Gene=FPCIR_951;Ontology_term=ATP-dependent activity,oxidoreductase activity,ligase activity,hydrolase activity;Ontology_id=GO:0140657,GO:0016491,GO:0016874,GO:0016787;Enzyme_code=EC:6,EC:3.6.1.15,EC:1.2.1,EC:1.2,EC:3.6.1,EC:1,EC:3.6,EC:3,EC:6,EC:3.6.1.15,EC:1.2.1;Enzyme_name=Ligases,nucleoside-triphosphate phosphatase,Acting on the aldehyde or oxo group of donors,Acting on the aldehyde or oxo group of donors,Acting on acid anhydrides,Oxidoreductases,Acting on acid anhydrides,Hydrolases,Ligases,nucleoside-triphosphate phosphatase,Acting on the aldehyde or oxo group of donors
contig00011.g6288	1632	ID=contig00011.g6288;Description=C6 zinc finger domain protein [Fusarium tjaetaba];Gene=FTJAE_2255;Ontology_term=nucleus,regulation of DNA-templated transcription,transcription regulator activity;Ontology_id=GO:0005634,GO:0006355,GO:0140110
contig00011.g6289	878	ID=contig00011.g6289;Description=antibiotic biosynthesis monooxygenase [Fusarium globosum];Gene=FGLOB1_3843;Ontology_term=oxidoreductase activity;Ontology_id=GO:0016491;Enzyme_code=EC:1,EC:1;Enzyme_name=Oxidoreductases,Oxidoreductases
contig00011.g6290	2838	ID=contig00011.g6290;Description=factor for adipocyte [Fusarium subglutinans]
contig00011.g6291	4624	ID=contig00011.g6291;Description=GAL4-transcription factor [Fusarium pseudocircinatum];Gene=FVER53263_10975;Ontology_term=nucleus,DNA binding,regulation of DNA-templated transcription,transcription regulator activity;Ontology_id=GO:0005634,GO:0003677,GO:0006355,GO:0140110
contig00011.g6292	817	ID=contig00011.g6292;Description=panthothenate kinase uridine kinase-related [Fusarium tjaetaba];Gene=FVEG_10976;Ontology_term=transferase activity;Ontology_id=GO:0016740;Enzyme_code=EC:2.7,EC:2.7,EC:2;Enzyme_name=Transferring phosphorus-containing groups,Transferring phosphorus-containing groups,Transferases

contig00011.g6293	1831	ID=contig00011.g6293;Description=MFS transporter, SP family, sugar:H+ symporter [Fusarium verticillioides 7600];Gene=FTJAE_2250;Ontology_term=transmembrane transport,transporter activity;Ontology_id=GO:0055085,GO:0005215
contig00011.g6294	1952	ID=contig00011.g6294;Description=related to protein-arginine deiminase type II [Fusarium fujikuroi];Gene=FMAN_12118;Ontology_term=hydrolase activity;Ontology_id=GO:0016787;Enzyme_code=EC:3.5.3.15;Enzyme_name=protein-arginine deiminase
contig00011.g6295	1452	ID=contig00011.g6295;Description=amine oxidase [Fusarium tjaetaba];Gene=FPCIR_943;Ontology_term=membrane;Ontology_id=GO:0016020;Enzyme_code=EC:1.21.99.1;Enzyme_name=beta-cyclopiazonate dehydrogenase
contig00011.g6296	1313	ID=contig00011.g6296;Description=integral membrane protein [Fusarium pseudoanthophilum];Gene=FPANT_10645;Ontology_term=membrane;Ontology_id=GO:0016020
contig00011.g6297	1583	ID=contig00011.g6297;Description=SCO2-Protein involved in stability of Cox1p and Cox2p [Fusarium fujikuroi]
contig00011.g6298	621	ID=contig00011.g6298;Description=isochorismatase hydrolase [Fusarium tjaetaba];Gene=F25303_4775;Ontology_term=hydrolase activity;Ontology_id=GO:0016787;Enzyme_code=EC:3,EC:3;Enzyme_name=Hydrolases,Hydrolases
contig00011.g6299	5365	ID=contig00011.g6299;Description=serine threonine kinase [Fusarium mundagurra];Gene=FFUJ_10168;Ontology_term=antioxidant activity,oxidoreductase activity,transferase activity;Ontology_id=GO:0016209,GO:0016491,GO:0016740;Enzyme_code=EC:2.7,EC:1.11.1;Enzyme_name=Transferring phosphorus-containing groups,Acting on a peroxide as acceptor
contig00011.g6300	1115	ID=contig00011.g6300;Description=acriflavine sensitivity control ACR-2 [Fusarium tjaetaba]
contig00011.g6301	1575	ID=contig00011.g6301;Description=related to fumarate reductase flavoprotein subunit precursor [Fusarium fujikuroi];Gene=FDENT_9770;Ontology_term=oxidoreductase activity;Ontology_id=GO:0016491;Enzyme_code=EC:1,EC:1;Enzyme_name=Oxidoreductases,Oxidoreductases
contig00011.g6302	2533	ID=contig00011.g6302;Description=Nit-4-like protein [Fusarium coicis];Gene=FVEG_10987;Ontology_term=nucleus,DNA binding,regulation of DNA-templated transcription,transcription regulator activity;Ontology_id=GO:0005634,GO:0003677,GO:0006355,GO:0140110

contig00011.g6303	1931	ID=contig00011.g6303;Description=related to maltose permease (MalP) [Fusarium fujikuroi];Gene=BFJ72_g6580;Ontology_term=transmembrane transport,transporter activity;Ontology_id=GO:0055085,GO:0005215
contig00011.g6304	1901	ID=contig00011.g6304;Description=monosaccharide transporter [Fusarium pseudoanthophilum];Gene=FVEG_10988;Ontology_term=transmembrane transport,transporter activity;Ontology_id=GO:0055085,GO:0005215
contig00011.g6305	3556	ID=contig00011.g6305;Description=related to ARCA protein [Fusarium oxysporum]
contig00011.g6306	1563	ID=contig00011.g6306;Description=trichothecene 3-o-acetyltransferase [Fusarium coicis];Gene=FMUND_6092;Ontology_term=transferase activity;Ontology_id=GO:0016740;Enzyme_code=EC:2,EC:2;Enzyme_name=Transferases,Transferases
contig00011.g6307	1591	ID=contig00011.g6307;Description=trichothecene 3-o-acetyltransferase [Fusarium tjaetaba];Gene=FMUND_6091;Ontology_term=transferase activity;Ontology_id=GO:0016740;Enzyme_code=EC:2,EC:2;Enzyme_name=Transferases,Transferases
contig00011.g6308	630	ID=contig00011.g6308;Description=hypothetical protein FVER53263_10994 [Fusarium verticillioides]
contig00011.g6309	1235	ID=contig00011.g6309;Description=penicillin-binding 4 [Fusarium pseudocircinatum]
contig00011.g6310	1818	ID=contig00011.g6310;Description=penicillin-binding 4 [Fusarium mexicanum]
contig00011.g6311	1155	ID=contig00011.g6311;Description=related to gibberellin 20-oxidase [Fusarium fujikuroi IMI 58289];Gene=FVER53263_10997;Ontology_term=transmembrane transport,structural molecule activity,anatomical structure development,regulation of DNA-templated transcription,reproductive process,chromosome,chromatin organization,DNA binding,nucleus,oxidoreductase activity,signaling,cell differentiation,external encapsulating structure,transcription regulator activity;Ontology_id=GO:0055085,GO:0005198,GO:0048856,GO:0006355,GO:0022414,GO:0005694,GO:0006325,GO:0003677,GO:0005634,GO:0016491,GO:0023052,GO:0030154,GO:0030312,GO:0140110;Enzyme_code=EC:1,EC:1;Enzyme_name=Oxidoreductases,Oxidoreductases
contig00011.g6312	1673	ID=contig00011.g6312;Description=major facilitator superfamily domain-containing protein [Fusarium redolens];Gene=FVEG_10998;Ontology_term=transmembrane transport,transporter activity;Ontology_id=GO:0055085,GO:0005215
contig00011.g6313	878	ID=contig00011.g6313;Description=D-arabinitol 2-dehydrogenase [Fusarium coicis];Gene=FVEG_10999;Ontology_term=oxidoreductase activity;Ontology_id=GO:0016491;Enzyme_code=EC:1;Enzyme_name=Oxidoreductases

contig00011.g6314	1097	ID=contig00011.g6314;Description=probable polysaccharide deacetylase family protein [Fusarium fujikuroi IMI 58289];Gene=FOBC_13283;Ontology_term=carbohydrate metabolic process,hydrolase activity;Ontology_id=GO:0005975,GO:0016787;Enzyme_code=EC:3.5,EC:3.5,EC:3;Enzyme_name=Acting on carbon-nitrogen bonds, other than peptide bonds,Acting on carbon-nitrogen bonds, other than peptide bonds,Hydrolases
contig00011.g6315	845	ID=contig00011.g6315;Description=hypothetical protein FVER14953_11001 [Fusarium verticillioides]
contig00011.g6316	1962	ID=contig00011.g6316;Description=general amidase [Fusarium tjaetaba];Enzyme_code=EC:3.5.1.4;Enzyme_name=amidase
contig00011.g6317	1991	ID=contig00011.g6317;Description=phenol 2-monooxygenase [Fusarium tjaetaba];Gene=FACUT_12988;Ontology_term=oxidoreductase activity;Ontology_id=GO:0016491;Enzyme_code=EC:1;Enzyme_name=Oxidoreductases
contig00011.g6318	1818	ID=contig00011.g6318;Description=major facilitator superfamily transporter [Fusarium pseudocircinatum];Gene=CEP51_004773;Ontology_term=transmembrane transport,transporter activity;Ontology_id=GO:0055085,GO:0005215
contig00011.g6319	1415	ID=contig00011.g6319;Description=OYE2-NADPH dehydrogenase (old yellow enzyme), isoform 1 [Fusarium fujikuroi];Gene=BFJ72_g6494;Ontology_term=transmembrane transport,oxidoreductase activity,transporter activity;Ontology_id=GO:0055085,GO:0016491,GO:0005215;Enzyme_code=EC:1;Enzyme_name=Oxidoreductases
contig00011.g6320	1466	ID=contig00011.g6320;Description=FAD binding domain protein [Fusarium tjaetaba];Gene=FPANT_13008;Ontology_term=oxidoreductase activity;Ontology_id=GO:0016491;Enzyme_code=EC:1;Enzyme_name=Oxidoreductases
contig00011.g6321	3082	ID=contig00011.g6321;Description=C2H2 zinc finger [Fusarium tjaetaba];Gene=FVER53263_11007;Ontology_term=nucleus,DNA binding,DNA-templated transcription,ligase activity;Ontology_id=GO:0005634,GO:0003677,GO:0006351,GO:0016874;Enzyme_code=EC:6,EC:6;Enzyme_name=Ligases,Ligases
contig00011.g6322	1410	ID=contig00011.g6322;Description=putative flavin-containing monoamine oxidase A [Fusarium oxysporum f. sp. rapae];Gene=BFJ63_vAg17226;Ontology_term=oxidoreductase activity;Ontology_id=GO:0016491;Enzyme_code=EC:1,EC:1,EC:1.4.3.4;Enzyme_name=Oxidoreductases,Oxidoreductases,monoamine oxidase

contig00011.g6323	366	ID=contig00011.g6323;Description=endoribonuclease L-PSP family protein [Talaromyces stipitatus ATCC 10500]
contig00011.g6324	945	ID=contig00011.g6324;Description=conidiospore surface protein [Fusarium napiforme];Gene=BFJ72_g6692;Ontology_term=extracellular region;Ontology_id=GO:0005576
contig00011.g6325	2245	ID=contig00011.g6325;Description=3-(3-hydroxyphenyl)propionate 2-hydroxylase [Fusarium tjaetaba];Gene=BFJ69_g10320;Ontology_term=transmembrane transport,oxidoreductase activity,transporter activity;Ontology_id=GO:0055085,GO:0016491,GO:0005215;Enzyme_code=EC:1,EC:1;Enzyme_name=Oxidoreductases,Oxidoreductases
contig00011.g6326	792	ID=contig00011.g6326;Description=hypothetical protein FVER53263_11013 [Fusarium verticillioides]
contig00011.g6327	417	ID=contig00011.g6327;Description=hypothetical protein FVEG_11014 [Fusarium verticillioides 7600]
contig00011.g6328	1460	ID=contig00011.g6328;Description=hypothetical protein FVEG_11015 [Fusarium verticillioides 7600];Gene=FPANT_3295;Ontology_term=membrane;Ontology_id=GO:0016020
contig00011.g6329	278	ID=contig00011.g6329;Description=---NA---
contig00011.g6330	1279	ID=contig00011.g6330;Description=hypothetical protein FVER53263_11017 [Fusarium verticillioides];Gene=FPCIR_962;Ontology_term=membrane;Ontology_id=GO:0016020
contig00011.g6331	430	ID=contig00011.g6331;Description=septin [Fusarium pseudoanthophilum]
contig00011.g6332	1239	ID=contig00011.g6332;Description=hypothetical protein FVER53263_11019 [Fusarium verticillioides];Enzyme_code=EC:3.5.1.23;Enzyme_name=ceramidase
contig00011.g6333	1850	ID=contig00011.g6333;Description=general substrate transporter [Fusarium oxysporum Fo47];Gene=FOBC_13350;Ontology_term=transmembrane transport,transporter activity;Ontology_id=GO:0055085,GO:0005215
contig00011.g6334	1426	ID=contig00011.g6334;Description=aspartate aminotransferase [Fusarium tjaetaba];Gene=FPCIR_966;Ontology_term=transferase activity;Ontology_id=GO:0016740;Enzyme_code=EC:2.6.1,EC:2.6.1,EC:2,EC:2.6;Enzyme_name=Transferring nitrogenous groups,Transferring nitrogenous groups,Transferases,Transferring nitrogenous groups

contig00011.g6335	1341	ID=contig00011.g6335;Description=transcription factor C6 [Fusarium coicis];Gene=FVEG_16948;Ontology_term=nucleus,regulation of DNA-templated transcription,transcription regulator activity;Ontology_id=GO:0005634,GO:0006355,GO:0140110
contig00011.g6336	2376	ID=contig00011.g6336;Description=N n-dimethylformamidase beta subunit [Fusarium coicis]
contig00011.g6337	2062	ID=contig00011.g6337;Description=putative MFS-type transporter [Fusarium oxysporum f. sp. conglutinans];Gene=BFJ69_g10306;Ontology_term=transmembrane transport,transporter activity;Ontology_id=GO:0055085,GO:0005215
contig00011.g6338	542	ID=contig00011.g6338;Description=hypothetical protein FVER53263_21111 [Fusarium verticillioides]
contig00011.g6339	351	ID=contig00011.g6339;Description=hypothetical protein H9Q73_005191 [Fusarium xylarioides];Gene=tra2;Ontology_term=nucleus,RNA binding,mRNA metabolic process,protein-containing complex assembly,anatomical structure development,cell differentiation,reproductive process;Ontology_id=GO:0005634,GO:0003723,GO:0016071,GO:0065003,GO:0048856,GO:0030154,GO:0022414
contig00011.g6340	1784	ID=contig00011.g6340;Description=related to HOL1 protein [Fusarium proliferatum ET1];Gene=BFJ72_g6482;Ontology_term=transmembrane transport,transporter activity;Ontology_id=GO:0055085,GO:0005215
contig00011.g6341	2262	ID=contig00011.g6341;Description=oleate-activated transcription factor 1 [Fusarium tjaetaba];Gene=FTJAE_2282;Ontology_term=nucleus,DNA binding,regulation of DNA-templated transcription,transcription regulator activity;Ontology_id=GO:0005634,GO:0003677,GO:0006355,GO:0140110
contig00011.g6342	5209	ID=contig00011.g6342;Description=related to pisatin demethylase / cytochrome P450 monooxygenase [Fusarium fujikuroi];Gene=FTJAE_2283;Ontology_term=nucleus,oxidoreductase activity,regulation of DNA-templated transcription,transferase activity,transcription regulator activity;Ontology_id=GO:0005634,GO:0016491,GO:0006355,GO:0016740,GO:0140110;Enzyme_code=EC:1.14,EC:2.1.1;Enzyme_name=Acting on paired donors, with incorporation or reduction of molecular oxygen. The oxygen incorporated need not be derived from O2,Transferring one-carbon groups

contig00011.g6343	1599	ID=contig00011.g6343;Description=Zn2-Cys6 binuclear cluster domain-containing protein [Fusarium denticulatum];Gene=FPCIR_973;Ontology_term=nucleus,oxidoreductase activity,regulation of DNA-templated transcription,transcription regulator activity;Ontology_id=GO:0005634,GO:0016491,GO:0006355,GO:0140110;Enzyme_code=EC:1.14,EC:1,EC:1.14;Enzyme_name=Acting on paired donors, with incorporation or reduction of molecular oxygen. The oxygen incorporated need not be derived from O2,Oxidoreductases,Acting on paired donors, with incorporation or reduction of molecular oxygen. The oxygen incorporated need not be derived from O2
contig00011.g6344	1320	ID=contig00011.g6344;Description=hydrolase or acyltransferase (alpha beta hydrolase superfamily) [Fusarium tjaetaba];Gene=FPCIR_974;Ontology_term=transferase activity,hydrolase activity;Ontology_id=GO:0016740,GO:0016787;Enzyme_code=EC:2.3,EC:3,EC:2.3,EC:2,EC:3;Enzyme_name=Acyltransferases,Hydrolases,Acyltransferases,Transferases,Hydrolases
contig00011.g6345	1126	ID=contig00011.g6345;Description=phytanoyl dioxygenase [Fusarium tjaetaba];Gene=FDENT_6123;Ontology_term=oxidoreductase activity;Ontology_id=GO:0016491;Enzyme_code=EC:1,EC:1;Enzyme_name=Oxidoreductases,Oxidoreductases
contig00011.g6346	1770	ID=contig00011.g6346;Description=cocaine esterase [Fusarium pseudoanthophilum];Gene=FOXYS1_13817;Ontology_term=catalytic activity, acting on a protein,hydrolase activity;Ontology_id=GO:0140096,GO:0016787;Enzyme_code=EC:3.4.14.1,EC:3.4.14.5,EC:3.4.14.4,EC:3.4.14.2,EC:3.4.14.1,EC:3.4,EC:3,EC:3.4.14.5,EC:3.4.14.4,EC:3.4.14.2;Enzyme_name=dipeptidyl-peptidase I,dipeptidyl-peptidase IV,dipeptidyl-peptidase III,dipeptidyl-peptidase II,dipeptidyl-peptidase I,Acting on peptide bonds (peptidases),Hydrolases,dipeptidyl-peptidase IV,dipeptidyl-peptidase III,dipeptidyl-peptidase II
contig00011.g6347	1275	ID=contig00011.g6347;Description=pyridine nucleotide-disulfide oxidoreductase family [Fusarium tjaetaba];Gene=FMUND_11499;Ontology_term=oxidoreductase activity;Ontology_id=GO:0016491;Enzyme_code=EC:1,EC:1;Enzyme_name=Oxidoreductases,Oxidoreductases
contig00011.g6348	2085	ID=contig00011.g6348;Description=sodium chloride dependent neurotransmitter transporter [Fusarium tjaetaba];Gene=FCIRC_10114;Ontology_term=membrane;Ontology_id=GO:0016020

contig00011.g6349	6505	ID=contig00011.g6349;Description=ankyrin protein [Fusarium globosum];Gene=F25303_12908;Ontology_term=carbohydrate derivative metabolic process;Ontology_id=GO:1901135;Enzyme_code=EC:2.4.1.232;Enzyme_name=initiation-specific alpha-1,6-mannosyltransferase
contig00011.g6350	879	ID=contig00011.g6350;Description=altered inheritance of mitochondria protein 6 [Fusarium bulbicola];Gene=FCIRC_150;Ontology_term=lipid metabolic process,hydrolase activity;Ontology_id=GO:0006629,GO:0016787;Enzyme_code=EC:3.1.4,EC:3.1.4,EC:3.1,EC:3;Enzyme_name=Acting on ester bonds,Acting on ester bonds,Acting on ester bonds,Hydrolases
contig00011.g6351	3482	ID=contig00011.g6351;Description=hypothetical protein FVEG_11037 [Fusarium verticillioides 7600]
contig00011.g6352	1024	ID=contig00011.g6352;Description=nitrogen metabolic regulation nmr [Fusarium tjaetaba]
contig00011.g6353	1773	ID=contig00011.g6353;Description=hypothetical protein FVER14953_11039 [Fusarium verticillioides]
contig00011.g6354	1496	ID=contig00011.g6354;Description=hypothetical protein AU210_011282 [Fusarium oxysporum f. sp. radicis-cucumerinum];Gene=BFJ71_g3889;Ontology_term=nucleus,regulation of DNA-templated transcription,transcription regulator activity;Ontology_id=GO:0005634,GO:0006355,GO:0140110
contig00011.g6355	1941	ID=contig00011.g6355;Description=hypothetical protein Ddc_24231 [Ditylenchus destructor];Gene=FPHYL_12602;Ontology_term=membrane;Ontology_id=GO:0016020
contig00011.g6356	3185	ID=contig00011.g6356;Description=heterokaryon incompatibility protein-domain-containing protein [Fusarium oxysporum];Gene=FOXB_13574;Ontology_term=membrane;Ontology_id=GO:0016020
contig00011.g6357	669	ID=contig00011.g6357;Description=yhhN family domain-containing protein [Ditylenchus destructor];Gene=FNYG_14872;Ontology_term=membrane;Ontology_id=GO:0016020
contig00011.g6358	3240	ID=contig00011.g6358;Description=related to promoter binding protein RUSH-1alpha [Fusarium proliferatum ET1];Gene=FPRO_12728;Ontology_term=nucleus,catalytic activity, acting on DNA,ATP-dependent activity,chromatin organization,hydrolase activity;Ontology_id=GO:0005634,GO:0140097,GO:0140657,GO:0006325,GO:0016787;Enzyme_code=EC:3,EC:3,EC:2.3.2.27;Enzyme_name=Hydrolases,Hydrolases,RING-type E3 ubiquitin transferase

contig00011.g6359	4279	ID=contig00011.g6359;Description=heterokaryon incompatibility protein het-E-1 [Fusarium napiforme];Gene=F52700_11588;Ontology_term=ATP-dependent activity,hydrolase activity;Ontology_id=GO:0140657,GO:0016787;Enzyme_code=EC:3.6.1.15,EC:3.6.1,EC:3.6,EC:3,EC:3.6.1.15;Enzyme_name=nucleoside-triphosphate phosphatase,Acting on acid anhydrides,Acting on acid anhydrides,Hydrolases,nucleoside-triphosphate phosphatase
contig00011.g6360	1834	ID=contig00011.g6360;Description=MFS transporter, SP family, general alpha glucoside:H+ symporter [Fusarium verticillioides 7600];Gene=FNAPI_1297;Ontology_term=transmembrane transport,transporter activity;Ontology_id=GO:0055085,GO:0005215
contig00011.g6361	1371	ID=contig00011.g6361;Description=DUF1295 domain protein [Fusarium tjaetaba];Gene=FNAPI_1298;Ontology_term=membrane;Ontology_id=GO:0016020
contig00011.g6362	1178	ID=contig00011.g6362;Description=ankyrin repeat-containing protein [Fusarium mundagurra]
contig00011.g6363	1807	ID=contig00011.g6363;Description=quinate permease [Fusarium coicis];Gene=FVER53590_11050;Ontology_term=transmembrane transport,transporter activity;Ontology_id=GO:0055085,GO:0005215
contig00011.g6364	1951	ID=contig00011.g6364;Description=putative amino acid permease NAAP1 [Fusarium fujikuroi];Gene=FPRO_12739;Ontology_term=transmembrane transport;Ontology_id=GO:0055085
contig00011.g6365	714	ID=contig00011.g6365;Description=hypothetical protein FVEG_11052 [Fusarium verticillioides 7600]
contig00011.g6366	354	ID=contig00011.g6366;Description=hypothetical protein FVEG_11053 [Fusarium verticillioides 7600]
contig00011.g6367	816	ID=contig00011.g6367;Description=hypothetical protein FVER53590_25715 [Fusarium verticillioides]
contig00011.g6368	603	ID=contig00011.g6368;Description=hypothetical protein FVER53263_11054 [Fusarium verticillioides]
contig00011.g6369	855	ID=contig00011.g6369;Description=ubiquinone menaquinone biosynthesis methyltransferase ubiE [Fusarium tjaetaba];Gene=FVER53263_11055;Ontology_term=transferase activity;Ontology_id=GO:0016740;Enzyme_code=EC:2.1.1,EC:2.1,EC:2,EC:2.1.1;Enzyme_name=Transferring one-carbon groups,Transferring one-carbon groups,Transferases,Transferring one-carbon groups

contig00011.g6370	1791	ID=contig00011.g6370;Description=related to phosphatase homologous to the C-terminal domain of histone macroH2A1 [Fusarium proliferatum ET1];Gene=FPRO_12746;Ontology_term=catalytic activity, acting on a protein,hydrolase activity;Ontology_id=GO:0140096,GO:0016787;Enzyme_code=EC:3.1.3,EC:3.1,EC:3,EC:3.1.3;Enzyme_name=Acting on ester bonds,Acting on ester bonds,Hydrolases,Acting on ester bonds
contig00011.g6371	2105	ID=contig00011.g6371;Description=hypothetical protein FVER14953_11058 [Fusarium verticillioides];Gene=FVER53263_11058;Ontology_term=catalytic activity, acting on a protein,hydrolase activity;Ontology_id=GO:0140096,GO:0016787;Enzyme_code=EC:3.4,EC:3.4,EC:3;Enzyme_name=Acting on peptide bonds (peptidases),Acting on peptide bonds (peptidases),Hydrolases
contig00011.g6372	1844	ID=contig00011.g6372;Description=major facilitator superfamily transporter [Fusarium tjaetaba];Gene=FNAPI_1310;Ontology_term=transmembrane transport,transporter activity;Ontology_id=GO:0055085,GO:0005215
contig00011.g6373	819	ID=contig00011.g6373;Description=oxidoreductase dltE [Fusarium coicis]
contig00011.g6374	1052	ID=contig00011.g6374;Description=carbonic anhydrase [Fusarium verticillioides 7600];Gene=FVER53590_11061;Ontology_term=lyase activity;Ontology_id=GO:0016829;Enzyme_code=EC:4.2.1.1,EC:4.2.1,EC:4.2.1.1,EC:4.2,EC:4;Enzyme_name=carbonic anhydrase,Carbon-oxygen lyases,carbonic anhydrase,Carbon-oxygen lyases,Lyases
contig00011.g6375	875	ID=contig00011.g6375;Description=Rtm1p [Fusarium coicis];Gene=FVEG_11062;Ontology_term=membrane;Ontology_id=GO:0016020
contig00011.g6376	880	ID=contig00011.g6376;Description=Pre-mRNA-splicing factor CWC24 [Fusarium keratoplasticum];Gene=FVER53590_11063;Ontology_term=DNA binding,nucleus,catalytic activity, acting on a protein,mRNA metabolic process,protein-containing complex assembly,transferase activity;Ontology_id=GO:0003677,GO:0005634,GO:0140096,GO:0016071,GO:0065003,GO:0016740;Enzyme_code=EC:2.3,EC:2,EC:2.3.1.258,EC:2.3.2,EC:2.3.1;Enzyme_name=Acyltransferases,Transferases,N-terminal methionine N(alpha)-acetyltransferase NatE,Acyltransferases,Acyltransferases

contig00011.g6377	1612	ID=contig00011.g6377;Description=mannan endo-1 6-alpha-mannosidase DCW1 [Fusarium tjaetaba];Gene=FDENT_3084;Ontology_term=carbohydrate metabolic process,hydrolase activity;Ontology_id=GO:0005975,GO:0016787;Enzyme_code=EC:3.2.1.101,EC:3.2.1.24,EC:3.2,EC:3.2.1.101,EC:3,EC:3.2.1.24,EC:3.2.1;Enzyme_name=mannan endo-1,6-alpha-mannosidase,alpha-mannosidase,Glycosylases,mannan endo-1,6-alpha-mannosidase,Hydrolases,alpha-mannosidase,Glycosylases
contig00011.g6378	444	ID=contig00011.g6378;Description=small secreted protein [Fusarium tjaetaba]
contig00011.g6379	1939	ID=contig00011.g6379;Description=glutamyl-tRNA synthetase [Fusarium subglutinans];Gene=HZS61_016115;Ontology_term=RNA binding,cytoplasmic translation,mitochondrion,tRNA metabolic process,amino acid metabolic process,catalytic activity, acting on RNA,ligase activity;Ontology_id=GO:0003723,GO:0002181,GO:0005739,GO:0006399,GO:0006520,GO:0140098,GO:0016874;Enzyme_code=EC:6.1.1.17,EC:6.1,EC:6.1.1,EC:6,EC:6.1.1.17;Enzyme_name=glutamate--tRNA ligase,Forming carbon-oxygen bonds,Forming carbon-oxygen bonds,Ligases,glutamate--tRNA ligase
contig00011.g6380	3434	ID=contig00011.g6380;Description=CMGC/SRPK protein kinase [Fusarium verticillioides 7600];Gene=F25303_2971;Ontology_term=catalytic activity, acting on a protein,chromosome,transferase activity;Ontology_id=GO:0140096,GO:0005694,GO:0016740;Enzyme_code=EC:2.7.11.1,EC:2.7.1,EC:2.7.11.1,EC:2.7,EC:2;Enzyme_name=non-specific serine/threonine protein kinase,Transferring phosphorus-containing groups,non-specific serine/threonine protein kinase,Transferring phosphorus-containing groups,Transferases
contig00011.g6381	2458	ID=contig00011.g6381;Description=Amidase 1 [Beauveria bassiana];Gene=FMUND_5678
contig00011.g6382	333	ID=contig00011.g6382;Description=hypothetical protein FMUND_5679 [Fusarium mundagurra]
contig00011.g6383	3150	ID=contig00011.g6383;Description=tetratricopeptide repeat domain-containing protein [Fusarium mundagurra];Gene=FMUND_5680;Ontology_term=ADP binding;Ontology_id=GO:0043531
contig00011.g6384	840	ID=contig00011.g6384;Description=N,N-dimethylformamidase beta subunit [Fusarium oxysporum f. sp. albedinis];Gene=ANO14919_101310;Ontology_term=membrane;Ontology_id=GO:0016020

contig00011.g6385	1446	ID=contig00011.g6385;Description=kinase-like domain-containing protein [Fusarium mundagurra];Gene=FMUND_5682;Ontology_term=catalytic activity, acting on a protein,transferase activity;Ontology_id=GO:0140096,GO:0016740;Enzyme_code=EC:2.7.1,EC:2.7.1,EC:2.7,EC:2;Enzyme_name=Transferring phosphorus-containing groups,Transferring phosphorus-containing groups,Transferring phosphorus-containing groups,Transferases
contig00011.g6386	963	ID=contig00011.g6386;Description=lipase/esterase [Fusarium fujikuroi];Gene=FVEG_11070;Ontology_term=hydrolase activity;Ontology_id=GO:0016787;Enzyme_code=EC:3,EC:3;Enzyme_name=Hydrolases,Hydrolases
contig00011.g6387	1555	ID=contig00011.g6387;Description=related to HOL1, putative substrate-H+ antiporter [Fusarium fujikuroi];Gene=FVEG_11071;Ontology_term=transmembrane transport,transferase activity,transporter activity;Ontology_id=GO:0055085,GO:0016740,GO:0005215;Enzyme_code=EC:2;Enzyme_name=Transferases
contig00011.g6388	693	ID=contig00011.g6388;Description=glutathione S-transferase [Fusarium verticillioides 7600];Gene=FVEG_11072;Ontology_term=transferase activity;Ontology_id=GO:0016740;Enzyme_code=EC:2,EC:2.5.1.18,EC:2;Enzyme_name=Transferases,glutathione transferase,Transferases
contig00011.g6389	684	ID=contig00011.g6389;Description=40S ribosomal s10 protein [Fusarium tjaetaba];Gene=FVER53590_11073;Ontology_term=membrane;Ontology_id=GO:0016020
contig00011.g6390	520	ID=contig00011.g6390;Description=histidine phosphatase superfamily [Fusarium oxysporum];Gene=RpL40;Ontology_term=nucleus,catalytic activity,structural molecule activity,ribosome;Ontology_id=GO:0005634,GO:0003824,GO:0005198,GO:0005840
contig00011.g6391	1297	ID=contig00011.g6391;Description=uncharacterized protein FTJAE_2336 [Fusarium tjaetaba]
contig00011.g6392	1248	ID=contig00011.g6392;Description=peptide chain release factor 1 [Fusarium denticulatum];Gene=FPCIR_10959;Ontology_term=translation regulator activity,RNA binding,transmembrane transport,mitochondrial gene expression,mitochondrion,cytosol,transporter activity;Ontology_id=GO:0045182,GO:0003723,GO:0055085,GO:0140053,GO:0005739,GO:0005829,GO:0005215
contig00011.g6393	2177	ID=contig00011.g6393;Description=peptide chain release factor 1 [Fusarium sp. NRRL 52700];Gene=F52700_1208;Ontology_term=translation regulator activity;Ontology_id=GO:0045182

contig00011.g6394	1116	ID=contig00011.g6394;Description=Methyltransferase fus9 [Fusarium musae];Gene=FACUT_11970;Ontology_term=transferase activity;Ontology_id=GO:0016740;Enzyme_code=EC:2.1.1,EC:2.1,EC:2,EC:2.1.1;Enzyme_name=Transferring one-carbon groups,Transferring one-carbon groups,Transferases,Transferring one-carbon groups
contig00011.g6395	1645	ID=contig00011.g6395;Description=fusarin C cluster-oxidoreductase [Fusarium tjaetaba];Gene=FDENT_3097;Ontology_term=oxidoreductase activity;Ontology_id=GO:0016491;Enzyme_code=EC:1.14,EC:1.2.1;Enzyme_name=Acting on paired donors, with incorporation or reduction of molecular oxygen. The oxygen incorporated need not be derived from O2,Acting on the aldehyde or oxo group of donors
contig00011.g6396	1749	ID=contig00011.g6396;Description=fusarin C cluster-oxidoreductase [Fusarium fujikuroi];Gene=FVER53263_11080;Ontology_term=oxidoreductase activity;Ontology_id=GO:0016491;Enzyme_code=EC:1.14,EC:1.2.1.5,EC:1.2.1.3,EC:1.2,EC:1,EC:1.14,EC:1.2.1.5,EC:1.2.1.3,EC:1.2.1;Enzyme_name=Acting on paired donors, with incorporation or reduction of molecular oxygen. The oxygen incorporated need not be derived from O2,aldehyde dehydrogenase [NAD(P)(+)],aldehyde dehydrogenase (NAD(+)),Acting on the aldehyde or oxo group of donors,Oxidoreductases,Acting on paired donors, with incorporation or reduction of molecular oxygen. The oxygen incorporated need not be derived from O2,aldehyde dehydrogenase [NAD(P)(+)],aldehyde dehydrogenase (NAD(+)),Acting on the aldehyde or oxo group of donors
contig00011.g6397	1674	ID=contig00011.g6397;Description=COP9 signalosome complex subunit 1 [Fusarium musae];Gene=FVER53263_11081;Ontology_term=transmembrane transport,transporter activity;Ontology_id=GO:0055085,GO:0005215
contig00011.g6398	702	ID=contig00011.g6398;Description=Esterase fus5 [Fusarium musae];Gene=FUS5;Ontology_term=hydrolase activity;Ontology_id=GO:0016787;Enzyme_code=EC:3,EC:3;Enzyme_name=Hydrolases,Hydrolases
contig00011.g6399	1320	ID=contig00011.g6399;Description=Secreted aspartic protease fus4 [Fusarium musae];Gene=FNYG_14907;Ontology_term=catalytic activity, acting on a protein,extracellular region,hydrolase activity;Ontology_id=GO:0140096,GO:0005576,GO:0016787;Enzyme_code=EC:3.4.23,EC:3.4.23,EC:3.4,EC:3;Enzyme_name=Acting on peptide bonds (peptidases),Acting on peptide bonds (peptidases),Acting on peptide bonds (peptidases),Hydrolases

contig00011.g6400	2731	ID=contig00011.g6400;Description=cell fusion- protein [Fusarium musae];Gene=FUS2;Ontology_term=hydrolase activity;Ontology_id=GO:0016787;Enzyme_code=EC:3,EC:3;Enzyme_name=Hydrolases,Hydrolases
contig00011.g6401	11860	ID=contig00011.g6401;Description=fus1 actin binding activity protein [Fusarium musae];Gene=FUS1;Ontology_term=oxidoreductase activity,lipid metabolic process,transferase activity,ligase activity,isomerase activity;Ontology_id=GO:0016491,GO:0006629,GO:0016740,GO:0016874,GO:0016853;Enzyme_code=EC:2.3.1.41,EC:1,EC:5,EC:6,EC:2.1.1,EC:2.3.1.41,EC:2.1,EC:2.3,EC:1,EC:2,EC:5,EC:6,EC:2.1.1,EC:2.3.1;Enzyme_name=beta-ketoacyl-[acyl-carrier-protein] synthase I,Oxidoreductases,Isomerases,Ligases,Transferring one-carbon groups,beta-ketoacyl-[acyl-carrier-protein] synthase I,Transferring one-carbon groups,Acyltransferases,Oxidoreductases,Transferases,Isomerases,Ligases,Transferring one-carbon groups,Acyltransferases
contig00011.g6402	852	ID=contig00011.g6402;Description=hypothetical protein FVEG_11087 [Fusarium verticillioides 7600]
contig00011.g6403	1403	ID=contig00011.g6403;Description=monocarboxylate transporter 4 [Fusarium tjaetaba];Gene=FMUND_5698;Ontology_term=translation regulator activity,transmembrane transport,transporter activity;Ontology_id=GO:0045182,GO:0055085,GO:0005215
contig00011.g6404	4563	ID=contig00011.g6404;Description=ZEB2-regulated ABC transporter 1 [Fusarium musae];Gene=FANTH_2790;Ontology_term=membrane organization,DNA repair,transmembrane transport,ATP-dependent activity,mitochondrion,DNA recombination,lipid binding,vacuole,transporter activity,plasma membrane,hydrolase activity;Ontology_id=GO:0061024,GO:0006281,GO:0055085,GO:0140657,GO:0005739,GO:0006310,GO:0008289,GO:0005773,GO:0005215,GO:0005886,GO:0016787;Enzyme_code=EC:7.2.2,EC:3.6.1.15,EC:3.6.1,EC:3.6,EC:7.6.2.1,EC:7.6.2.2,EC:3,EC:7.2.2,EC:7,EC:3.6.1.15;Enzyme_name=Catalysing the translocation of inorganic cations,nucleoside-triphosphate phosphatase,Acting on acid anhydrides,Acting on acid anhydrides,P-type phospholipid transporter,ABC-type xenobiotic transporter,Hydrolases,Catalysing the translocation of inorganic cations,Translocases,nucleoside-triphosphate phosphatase
contig00011.g6405	936	ID=contig00011.g6405;Description=hypothetical protein FVEG_11091 [Fusarium verticillioides 7600]
contig00011.g6406	10881	ID=contig00011.g6406;Description=hypothetical protein FVER53263_11092 [Fusarium verticillioides]

contig00011.g6407	1180	ID=contig00011.g6407;Description=PMU1-high copy suppressor of ts tps2 mutant phenotype [Fusarium tjaetaba];Ontology_term=nucleus;Ontology_id=GO:0005634
contig00011.g6408	1522	ID=contig00011.g6408;Description=ethanolamine-phosphate cytidylyltransferase [Fusarium verticillioides 7600];Gene=FVEG_11095;Ontology_term=nucleus,lipid metabolic process,transferase activity;Ontology_id=GO:0005634,GO:0006629,GO:0016740;Enzyme_code=EC:2.7.7.14,EC:2.7,EC:2,EC:2.7.7.14,EC:2.7.7;Enzyme_name=ethanolamine-phosphate cytidylyltransferase,Transferring phosphorus-containing groups,Transferases,ethanolamine-phosphate cytidylyltransferase,Transferring phosphorus-containing groups
contig00011.g6409	1285	ID=contig00011.g6409;Description=branched-chain amino acid aminotransferase [Fusarium verticillioides 7600];Gene=FPHYL_5062;Ontology_term=amino acid metabolic process,transferase activity;Ontology_id=GO:0006520,GO:0016740;Enzyme_code=EC:2.6.1.6,EC:2.6.1.42,EC:2.6.1,EC:2.6.1.6,EC:2,EC:2.6.1.42,EC:2.6;Enzyme_name=leucine transaminase,branched-chain-amino-acid transaminase,Transferring nitrogenous groups,leucine transaminase,Transferases,branched-chain-amino-acid transaminase,Transferring nitrogenous groups
contig00011.g6410	881	ID=contig00011.g6410;Description=hypothetical protein LB506_009179 [Fusarium annulatum]
contig00011.g6411	717	ID=contig00011.g6411;Description=phosphopantothenoylcysteine decarboxylase [Fusarium verticillioides 7600];Gene=FNYG_15411;Ontology_term=nucleobase-containing small molecule metabolic process,lyase activity,carbohydrate derivative metabolic process;Ontology_id=GO:0055086,GO:0016829,GO:1901135;Enzyme_code=EC:4.1.1,EC:4.1,EC:4.1.1.36,EC:4,EC:2.3.1.47;Enzyme_name=Carbon-carbon lyases,Carbon-carbon lyases,phosphopantothenoylcysteine decarboxylase,Lyases,8-amino-7-oxononanoate synthase
contig00011.g6412	529	ID=contig00011.g6412;Description=monothiol glutaredoxin grx5 [Fusarium oxysporum];Gene=BFJ68_g16144;Ontology_term=oxidoreductase activity;Ontology_id=GO:0016491;Enzyme_code=EC:1.8;Enzyme_name=Acting on a sulfur group of donors
contig00011.g6413	1120	ID=contig00011.g6413;Description=uncharacterized protein FTJAE_2357 [Fusarium tjaetaba]
contig00011.g6414	1161	ID=contig00011.g6414;Description=heterokaryon incompatibility 6 OR allele [Fusarium tjaetaba]

contig00011.g6415	1972	ID=contig00011.g6415;Description=related to lincomycin-condensing protein lmbA [Fusarium fujikuroi];Gene=FocTR4_00013777;Ontology_term=nucleus,DNA binding,catalytic activity, acting on a protein,cell differentiation,reproductive process,chromosome,transferase activity,hydrolase activity;Ontology_id=GO:0005634,GO:0003677,GO:0140096,GO:0030154,GO:0022414,GO:0005694,GO:0016740,GO:0016787;Enzyme_code=EC:2.3.2.2,EC:3.4.19.13,EC:2.3.2.2,EC:2.3,EC:3.4,EC:2,EC:3,EC:3.4.19,EC:2.3.2,EC:3.4.19.13;Enzyme_name=gamma-glutamyltransferase,glutathione gamma-glutamate hydrolase,gamma-glutamyltransferase,Acyltransferases,Acting on peptide bonds (peptidases),Transferases,Hydrolases,Acting on peptide bonds (peptidases),Acyltransferases,glutathione gamma-glutamate hydrolase
contig00011.g6416	628	ID=contig00011.g6416;Description=lincomycin-condensing lmbA [Fusarium mexicanum]
contig00011.g6417	822	ID=contig00011.g6417;Description=hypothetical protein FVER53263_11103 [Fusarium verticillioides]
contig00011.g6418	1491	ID=contig00011.g6418;Description=Protein arg-6, mitochondrial [Fusarium musae];Gene=FPANT_10217;Ontology_term=oxidoreductase activity,amino acid metabolic process;Ontology_id=GO:0016491,GO:0006520;Enzyme_code=EC:1.2.1.38,EC:1.2.1.38,EC:1.2,EC:1,EC:2.7.2.8,EC:1.2.1;Enzyme_name=N-acetyl-gamma-glutamyl-phosphate reductase,N-acetyl-gamma-glutamyl-phosphate reductase,Acting on the aldehyde or oxo group of donors,Oxidoreductases,acetylglutamate kinase,Acting on the aldehyde or oxo group of donors
contig00011.g6419	3723	ID=contig00011.g6419;Description=hypothetical protein FVEG_11105 [Fusarium verticillioides 7600]
contig00011.g6420	1494	ID=contig00011.g6420;Description=hypothetical protein FVER53590_11106 [Fusarium verticillioides];Gene=FVER53263_11106;Ontology_term=membrane;Ontology_id=GO:0016020
contig00011.g6421	1574	ID=contig00011.g6421;Description=hypothetical protein J7337_012011 [Fusarium musae]
contig00011.g6422	748	ID=contig00011.g6422;Description=hypothetical protein FVEG_11108 [Fusarium verticillioides 7600]
contig00011.g6423	672	ID=contig00011.g6423;Description=carbonic anhydrase [Fusarium verticillioides 7600];Gene=FOMG_10061;Ontology_term=nucleus,mitochondrion,detoxification,lyase activity;Ontology_id=GO:0005634,GO:0005739,GO:0098754,GO:0016829;Enzyme_code=EC:4.2.1.1,EC:4.2.1,EC:4.2.1.1,EC:4.2,EC:4;Enzyme_name=carbonic anhydrase,Carbon-oxygen lyases,carbonic anhydrase,Carbon-oxygen lyases,Lyases

contig00011.g6424	1435	ID=contig00011.g6424;Description=chromo domain protein [Fusarium tjaetaba];Gene=FDENT_11038;Ontology_term=chromatin organization;Ontology_id=GO:0006325
contig00011.g6425	1213	ID=contig00011.g6425;Description=hypothetical protein FVER53263_11111 [Fusarium verticillioides];Gene=FVEG_11111;Ontology_term=membrane;Ontology_id=GO:0016020
contig00011.g6426	1027	ID=contig00011.g6426;Description=meiotic chromosome segregation [Fusarium sp. NRRL 25303]
contig00011.g6427	1878	ID=contig00011.g6427;Description=hypothetical protein FVER14953_11114 [Fusarium verticillioides];Gene=FTJAE_2370;Ontology_term=membrane;Ontology_id=GO:0016020
contig00011.g6428	879	ID=contig00011.g6428;Description=hypothetical protein FVER14953_11115 [Fusarium verticillioides];Gene=FPCIR_7301;Ontology_term=hydrolase activity;Ontology_id=GO:0016787;Enzyme_code=EC:3.2,EC:3.2,EC:3;Enzyme_name=Glycosylases,Glycosylases,Hydrolases
contig00011.g6429	1257	ID=contig00011.g6429;Description=Protein RTA1 [Fusarium oxysporum f. sp. cubense race 1];Gene=FVEG_16967;Ontology_term=membrane;Ontology_id=GO:0016020
contig00011.g6430	2486	ID=contig00011.g6430;Description=conidial development fluffy [Fusarium sp. NRRL 25303];Gene=FMAN_12246;Ontology_term=nucleus,regulation of DNA-templated transcription,transcription regulator activity;Ontology_id=GO:0005634,GO:0006355,GO:0140110
contig00011.g6431	818	ID=contig00011.g6431;Description=hypothetical protein J7337_012001 [Fusarium musae]
contig00011.g6432	7512	ID=contig00011.g6432;Description=hypothetical protein FVER53590_11119 [Fusarium verticillioides]
contig00011.g6433	2355	ID=contig00011.g6433;Description=uncharacterized protein FFB14_07650 [Fusarium fujikuroi]
contig00011.g6434	6211	ID=contig00011.g6434;Description=transcriptional activator srcap [Fusarium mundagurra]
contig00011.g6435	3120	ID=contig00011.g6435;Description=hypothetical protein FVER14953_11122 [Fusarium verticillioides]
contig00011.g6436	4445	ID=contig00011.g6436;Description=hypothetical protein FVER14953_11123 [Fusarium verticillioides]
contig00011.g6437	1813	ID=contig00011.g6437;Description=hypothetical protein FVER14953_11124 [Fusarium verticillioides]

contig00011.g6438	1138	ID=contig00011.g6438;Description=related to PAF acetylhydrolase family protein [Fusarium fujikuroi];Gene=FFUJ_10008;Ontology_term=hydrolase activity;Ontology_id=GO:0016787;Enzyme_code=EC:3.1.1.47,EC:3.1.1.47,EC:3.1,EC:3,EC:3.1.1;Enzyme_name=1-alkyl-2-acetylglycerophosphocholine esterase,1-alkyl-2-acetylglycerophosphocholine esterase,Acting on ester bonds,Hydrolases,Acting on ester bonds
contig00011.g6439	723	ID=contig00011.g6439;Description=hypothetical protein FVEG_11126 [Fusarium verticillioides 7600]
contig00011.g6440	1697	ID=contig00011.g6440;Description=serine-type carboxypeptidase f precursor [Fusarium phyllophilum];Gene=FACUT_5529;Ontology_term=catalytic activity, acting on a protein,hydrolase activity;Ontology_id=GO:0140096,GO:0016787;Enzyme_code=EC:3.4.16;Enzyme_name=Acting on peptide bonds (peptidases)
contig00011.g6441	915	ID=contig00011.g6441;Description=hypothetical protein FVEG_11128 [Fusarium verticillioides 7600];Gene=FVER53590_11127;Ontology_term=catalytic activity, acting on a protein,hydrolase activity;Ontology_id=GO:0140096,GO:0016787;Enzyme_code=EC:3.4.16,EC:3.4,EC:3.4.16,EC:3;Enzyme_name=Acting on peptide bonds (peptidases),Acting on peptide bonds (peptidases),Acting on peptide bonds (peptidases),Hydrolases
contig00011.g6442	1327	ID=contig00011.g6442;Description=hypothetical protein FPANT_9250 [Fusarium pseudoanthophilum]
contig00011.g6443	2468	ID=contig00011.g6443;Description=TRI7-trichothecene biosynthesis [Fusarium tjaetaba];Gene=FPANT_9252;Ontology_term=lipid metabolic process,transferase activity;Ontology_id=GO:0006629,GO:0016740;Enzyme_code=EC:2.3.1,EC:2.3,EC:2,EC:2.3.1;Enzyme_name=Acyltransferases,Acyltransferases,Transferases,Acyltransferases
contig00011.g6444	994	ID=contig00011.g6444;Description=YSC84-like protein [Fusarium tjaetaba]
contig00011.g6445	2323	ID=contig00011.g6445;Description=dis1-suppressing protein kinase dsk1 [Fusarium fujikuroi];Gene=BFJ72_g6497;Ontology_term=catalytic activity, acting on a protein,transferase activity;Ontology_id=GO:0140096,GO:0016740;Enzyme_code=EC:2.7.1,EC:2.7.1,EC:2.7.11.1,EC:2.7,EC:2;Enzyme_name=Transferring phosphorus-containing groups,Transferring phosphorus-containing groups,non-specific serine/threonine protein kinase,Transferring phosphorus-containing groups,Transferases

contig00011.g6446	1605	ID=contig00011.g6446;Description=hypothetical protein FVEG_11133 [Fusarium verticillioides 7600];Gene=O3G_MSEX005174;Ontology_term=transmembrane transport,ATP-dependent activity,lipid binding,vacuole,transporter activity;Ontology_id=GO:0055085,GO:0140657,GO:0008289,GO:0005773,GO:0005215;Enzyme_code=EC:7.2.2,EC:7.2.2,EC:7;Enzyme_name=Catalysing the translocation of inorganic cations,Catalysing the translocation of inorganic cations,Translocases
contig00011.g6447	2711	ID=contig00011.g6447;Description=CMGC protein kinase [Fusarium verticillioides 7600];Gene=FFC1_08365;Ontology_term=catalytic activity, acting on a protein,transferase activity;Ontology_id=GO:0140096,GO:0016740;Enzyme_code=EC:2.7.1,EC:2.7.1,EC:2.7,EC:2;Enzyme_name=Transferring phosphorus-containing groups,Transferring phosphorus-containing groups,Transferring phosphorus-containing groups,Transferases
contig00011.g6448	1116	ID=contig00011.g6448;Description=hypothetical protein FMUND_6907 [Fusarium mundagurra];Gene=FACUT_5537;Ontology_term=membrane;Ontology_id=GO:0016020
contig00011.g6449	1873	ID=contig00011.g6449;Description=Amino-oxidase domain-containing protein [Fusarium keratoplasticum];Gene=FNYG_14590;Ontology_term=oxidoreductase activity;Ontology_id=GO:0016491;Enzyme_code=EC:1,EC:1;Enzyme_name=Oxidoreductases,Oxidoreductases
contig00011.g6450	1124	ID=contig00011.g6450;Description=monophenol monooxygenase (tyrosinase) [Fusarium tjaetaba];Gene=FDENT_11009;Ontology_term=oxidoreductase activity;Ontology_id=GO:0016491;Enzyme_code=EC:1,EC:1,EC:1.14.18.1;Enzyme_name=Oxidoreductases,Oxidoreductases,tyrosinase
contig00011.g6451	1178	ID=contig00011.g6451;Description=UPF0658 Golgi apparatus membrane protein [Fusarium oxysporum f. sp. vasinfectum];Gene=BFJ69_g8574;Ontology_term=membrane;Ontology_id=GO:0016020
contig00011.g6452	3300	ID=contig00011.g6452;Description=sodium/potassium-transporting ATPase subunit alpha [Fusarium verticillioides 7600];Gene=FVER53263_11139;Ontology_term=ATP-dependent activity,hydrolase activity;Ontology_id=GO:0140657,GO:0016787;Enzyme_code=EC:3.6.1.15,EC:3.6.1,EC:3.6,EC:3,EC:3.6.3.9,EC:3.6.1.15;Enzyme_name=nucleoside-triphosphate phosphatase,Acting on acid anhydrides,Acting on acid anhydrides,Hydrolases,Acting on acid anhydrides,nucleoside-triphosphate phosphatase

contig00011.g6453	1743	ID=contig00011.g6453;Description=alcohol oxidase [Fusarium tjaetaba];Gene=FPANT_9264;Ontology_term=oxidoreductase activity;Ontology_id=GO:0016491;Enzyme_code=EC:1.1,EC:1.1,EC:1;Enzyme_name=Acting on the CH-OH group of donors,Acting on the CH-OH group of donors,Oxidoreductases
contig00011.g6454	1709	ID=contig00011.g6454;Description=tachykinin family [Fusarium acutatum]
contig00011.g6455	622	ID=contig00011.g6455;Description=Adhesion defective 3 [Fusarium tjaetaba]
contig00011.g6456	324	ID=contig00011.g6456;Description=hypothetical protein FVER53590_25679 [Fusarium verticillioides];Gene=BFJ68_g9970;Ontology_term=catalytic activity, acting on a protein,transferase activity;Ontology_id=GO:0140096,GO:0016740;Enzyme_code=EC:2.7.1,EC:2.7.1,EC:2.7,EC:2;Enzyme_name=Transferring phosphorus-containing groups,Transferring phosphorus-containing groups,Transferring phosphorus-containing groups,Transferases
contig00011.g6457	1073	ID=contig00011.g6457;Description=phospholipid-translocating ATPase [Fusarium napiforme];Gene=FOXB_12670;Ontology_term=membrane;Ontology_id=GO:0016020
contig00011.g6458	1735	ID=contig00011.g6458;Description=hypothetical protein J7337_011977 [Fusarium musae]
contig00011.g6459	1509	ID=contig00011.g6459;Description=BAS1-transcription factor [Fusarium tjaetaba];Ontology_term=DNA binding,nucleus,cytoskeleton organization,cell differentiation,mitotic nuclear division,regulation of DNA-templated transcription,chromosome segregation,transcription regulator activity;Ontology_id=GO:0003677,GO:0005634,GO:0007010,GO:0030154,GO:0140014,GO:0006355,GO:0007059,GO:0140110
contig00011.g6460	1714	ID=contig00011.g6460;Description=hypothetical protein FVEG_16973 [Fusarium verticillioides 7600];Gene=FOPG_05395;Ontology_term=nucleus,DNA binding,DNA-templated transcription;Ontology_id=GO:0005634,GO:0003677,GO:0006351
contig00011.g6461	1289	ID=contig00011.g6461;Description=hypothetical protein FVEG_11148 [Fusarium verticillioides 7600];Gene=FMEXI_1546
contig00011.g6462	1325	ID=contig00011.g6462;Description=hypothetical protein FVEG_16974 [Fusarium verticillioides 7600]
contig00011.g6463	1413	ID=contig00011.g6463;Description=hypothetical protein FVER53590_11149 [Fusarium verticillioides]
contig00011.g6464	1362	ID=contig00011.g6464;Description=hypothetical protein FVER14953_11150 [Fusarium verticillioides]
contig00011.g6465	582	ID=contig00011.g6465;Description=uncharacterized protein FTJAE_2409 [Fusarium tjaetaba]

contig00011.g6466	1794	ID=contig00011.g6466;Description=hypothetical protein FVER53590_11152 [Fusarium verticillioides];Gene=FACUT_5554;Ontology_term=membrane;Ontology_id=GO:0016020
contig00011.g6467	1056	ID=contig00011.g6467;Description=hypothetical protein FVEG_11153 [Fusarium verticillioides 7600]
contig00011.g6468	3634	ID=contig00011.g6468;Description=uncharacterized protein FTJAE_2412 [Fusarium tjaetaba]
contig00011.g6469	834	ID=contig00011.g6469;Description=AB hydrolase superfamily yvaM [Fusarium tjaetaba];Gene=FOC4_g10006236;Ontology_term=hydrolase activity;Ontology_id=GO:0016787;Enzyme_code=EC:3,EC:3;Enzyme_name=Hydrolases,Hydrolases
contig00011.g6470	1566	ID=contig00011.g6470;Description=glucose transporter-3 [Fusarium pseudoanthophilum];Gene=FPANT_2915;Ontology_term=transmembrane transport,vacuole,transporter activity;Ontology_id=GO:0055085,GO:0005773,GO:0005215
contig00011.g6471	853	ID=contig00011.g6471;Description=DCG1 [Fusarium subglutinans];Gene=FPHYL_2879;Ontology_term=isomerase activity;Ontology_id=GO:0016853;Enzyme_code=EC:5.1.1,EC:5.1.1,EC:5.1,EC:5;Enzyme_name=Racemases and epimerases,Racemases and epimerases,Racemases and epimerases,Isomerases
contig00011.g6472	1323	ID=contig00011.g6472;Description=actin 2 [Fusarium subglutinans];Ontology_term=vesicle-mediated transport,cytoskeleton,cytoskeleton organization,protein-containing complex assembly,ATP-dependent activity,anatomical structure development,cell differentiation,reproductive process,cell wall organization or biogenesis,intracellular protein transport,mitochondrion organization,hydrolase activity;Ontology_id=GO:0016192,GO:0005856,GO:0007010,GO:0065003,GO:0140657,GO:0048856,GO:0030154,GO:0022414,GO:0071554,GO:0006886,GO:0007005,GO:0016787;Enzyme_code=EC:3.6.1,EC:3.6,EC:3,EC:3.6.1.15;Enzyme_name=Acting on acid anhydrides,Acting on acid anhydrides,Hydrolases,nucleoside-triphosphate phosphatase

contig00011.g6473	1359	ID=contig00011.g6473;Description=CMGC/CDK/CDK8 protein kinase [Fusarium proliferatum];Gene=FPHYL_4858;Ontology_term=catalytic activity, acting on a protein,mRNA metabolic process,nuclear chromosome,regulation of DNA-templated transcription,cell adhesion,transferase activity,mitotic cell cycle,nucleoplasm;Ontology_id=GO:0140096,GO:0016071,GO:0000228,GO:0006355,GO:0007155,GO:0016740,GO:0000278,GO:0005654;Enzyme_code=EC:2.7.1,EC:2.7.1,EC:2.7.11.23,EC:2.7.11.1,EC:2.7,EC:2,EC:2.7.11.22;Enzyme_name=Transferring phosphorus-containing groups,Transferring phosphorus-containing groups,[RNA-polymerase]-subunit kinase,non-specific serine/threonine protein kinase,Transferring phosphorus-containing groups,Transferases,cyclin-dependent kinase
contig00011.g6474	675	ID=contig00011.g6474;Description=ADP-ribosylation factor family protein [Colletotrichum tofieldiae];Gene=BN1708_008055;Ontology_term=cytoskeleton organization,establishment or maintenance of cell polarity,anatomical structure development,cytokinesis,Golgi apparatus,intracellular protein transport,mitotic cell cycle,RNA binding,vesicle-mediated transport,molecular function regulator activity,GTPase activity,lipid metabolic process,signaling,cell differentiation,cytosol;Ontology_id=GO:0007010,GO:0007163,GO:0048856,GO:0000910,GO:0005794,GO:0006886,GO:0000278,GO:0003723,GO:0016192,GO:0098772,GO:0003924,GO:0006629,GO:0023052,GO:0030154,GO:0005829;Enzyme_code=EC:3.6.1.15,EC:3.6.1,EC:3.6,EC:3,EC:3.6.1.15;Enzyme_name=nucleoside-triphosphate phosphatase,Acting on acid anhydrides,Acting on acid anhydrides,Hydrolases,nucleoside-triphosphate phosphatase
contig00011.g6475	1365	ID=contig00011.g6475;Description=SAGA-associated factor 29 [Fusarium proliferatum];Gene=FOBC_13492;Ontology_term=histone binding,regulation of DNA-templated transcription,chromosome,chromatin organization,transcription regulator activity,nucleoplasm;Ontology_id=GO:0042393,GO:0006355,GO:0005694,GO:0006325,GO:0140110,GO:0005654
contig00011.g6476	1395	ID=contig00011.g6476;Description=mannose-6-phosphate isomerase [Fusarium verticillioides 7600];Gene=FVER53590_11162;Ontology_term=nucleus,carbohydrate metabolic process,nucleobase-containing small molecule metabolic process,protein glycosylation,cell wall organization or biogenesis,isomerase activity;Ontology_id=GO:0005634,GO:0005975,GO:0055086,GO:0006486,GO:0071554,GO:0016853;Enzyme_code=EC:5.3.1.8,EC:5.3,EC:5,EC:5.3.1.8,EC:5.3.1;Enzyme_name=mannose-6-phosphate isomerase,Intramolecular oxidoreductases,Isomerases,mannose-6-phosphate isomerase,Intramolecular oxidoreductases

contig00011.g6477	1457	ID=contig00011.g6477;Description=transcriptional regulatory [Fusarium tjaetaba];Gene=FTJAE_10873;Ontology_term=nucleus,regulation of DNA-templated transcription,transcription regulator activity;Ontology_id=GO:0005634,GO:0006355,GO:0140110
contig00011.g6478	1848	ID=contig00011.g6478;Description=peptidyl-prolyl cis-trans isomerase-like 4 [Fusarium verticillioides 7600];Gene=C2S_4272;Ontology_term=nucleus,catalytic activity, acting on a protein,RNA binding,oxidoreductase activity,isomerase activity;Ontology_id=GO:0005634,GO:0140096,GO:0003723,GO:0016491,GO:0016853;Enzyme_code=EC:5.2.1.8,EC:1;Enzyme_name=peptidylprolyl isomerase,Oxidoreductases
contig00011.g6479	492	ID=contig00011.g6479;Description=hypothetical protein FVER14953_11167 [Fusarium verticillioides]
contig00011.g6480	1090	ID=contig00011.g6480;Description=FAM86A [Fusarium acutatum];Gene=Focb16_v015736;Ontology_term=catalytic activity, acting on a protein,cytoplasmic translation,transferase activity;Ontology_id=GO:0140096,GO:0002181,GO:0016740;Enzyme_code=EC:2.1.1,EC:2.1,EC:2,EC:2.1.1;Enzyme_name=Transferring one-carbon groups,Transferring one-carbon groups,Transferases,Transferring one-carbon groups
contig00011.g6481	766	ID=contig00011.g6481;Description=30S ribosomal protein S24e [Fusarium verticillioides 7600];Gene=FPANT_2904;Ontology_term=structural molecule activity,ribosome;Ontology_id=GO:0005198,GO:0005840
contig00011.g6482	429	ID=contig00011.g6482;Description=hypothetical protein FVEG_11170 [Fusarium verticillioides 7600]
contig00011.g6483	4584	ID=contig00011.g6483;Description=SRP40-suppressor of mutant AC40 of RNA polymerase I and III [Fusarium tjaetaba]
contig00011.g6484	1211	ID=contig00011.g6484;Description=GTP cyclohydrolase II-domain-containing protein [Fusarium redolens];Gene=FVER53590_11172;Ontology_term=nucleus,vitamin metabolic process,hydrolase activity;Ontology_id=GO:0005634,GO:0006766,GO:0016787;Enzyme_code=EC:3.5.4.25,EC:3.5.4.25,EC:3.5.4,EC:3.5,EC:3;Enzyme_name=GTP cyclohydrolase II,GTP cyclohydrolase II,Acting on carbon-nitrogen bonds, other than peptide bonds,Acting on carbon-nitrogen bonds, other than peptide bonds,Hydrolases
contig00011.g6485	663	ID=contig00011.g6485;Description=hypothetical protein FVEG_11173 [Fusarium verticillioides 7600]

contig00011.g6486	1389	ID=contig00011.g6486;Description=DNA-directed RNA polymerase I subunit RPA43 [Fusarium verticillioides 7600];Gene=FNYG_00371;Ontology_term=DNA binding,DNA-templated transcription,nucleolus;Ontology_id=GO:0003677,GO:0006351,GO:0005730
contig00011.g6487	729	ID=contig00011.g6487;Description=serine threonine kinase [Fusarium denticulatum];Gene=FDENT_13377;Ontology_term=transferase activity;Ontology_id=GO:0016740;Enzyme_code=EC:2.7,EC:2.7,EC:2;Enzyme_name=Transferring phosphorus-containing groups,Transferring phosphorus-containing groups,Transferases
contig00011.g6488	1456	ID=contig00011.g6488;Description=IBR finger domain protein [Fusarium tjaetaba];Gene=BFJ69_g8154;Ontology_term=catalytic activity, acting on a protein,transferase activity,hydrolase activity;Ontology_id=GO:0140096,GO:0016740,GO:0016787;Enzyme_code=EC:3.1.6,EC:2.3.2,EC:3.1.6,EC:2.3,EC:3.1,EC:2,EC:3,EC:2.3.2;Enzyme_name=Acting on ester bonds,Acyltransferases,Acting on ester bonds,Acyltransferases,Acting on ester bonds,Transferases,Hydrolases,Acyltransferases
contig00011.g6489	2006	ID=contig00011.g6489;Description=arylsulfatase [Fusarium odoratissimum NRRL 54006];Gene=FOIG_04583;Ontology_term=carbohydrate derivative metabolic process,hydrolase activity;Ontology_id=GO:1901135,GO:0016787;Enzyme_code=EC:3.1.6.14,EC:3.1.6,EC:3.1,EC:3,EC:3.1.6.14,EC:3.1.6.1;Enzyme_name=N-acetylglucosamine-6-sulfatase,Acting on ester bonds,Acting on ester bonds,Hydrolases,N-acetylglucosamine-6-sulfatase,arylsulfatase (type I)
contig00011.g6490	527	ID=contig00011.g6490;Description=NADH-ubiquinone oxidoreductase B18 subunit [Colletotrichum truncatum];Gene=CP532_2451;Ontology_term=mitochondrion;Ontology_id=GO:0005739
contig00011.g6491	1467	ID=contig00011.g6491;Description=neutral amino acid permease [Fusarium pseudocircinatum];Gene=FVER53263_11179;Ontology_term=membrane;Ontology_id=GO:0016020
contig00011.g6492	1124	ID=contig00011.g6492;Description=hypothetical protein F52700_1115 [Fusarium sp. NRRL 52700];Gene=F52700_1115;Ontology_term=lyase activity;Ontology_id=GO:0016829;Enzyme_code=EC:4,EC:4;Enzyme_name=Lyases,Lyases
contig00011.g6493	1550	ID=contig00011.g6493;Description=hypothetical protein FVER14953_11185 [Fusarium verticillioides];Gene=FDENT_12524;Ontology_term=nucleus,regulation of DNA-templated transcription,transcription regulator activity;Ontology_id=GO:0005634,GO:0006355,GO:0140110

contig00011.g6494	1248	ID=contig00011.g6494;Description=nitric oxide dioxygenase [Fusarium verticillioides 7600];Gene=FNAPI_994;Ontology_term=oxidoreductase activity;Ontology_id=GO:0016491;Enzyme_code=EC:1.14.12.17,EC:1.1.1.1,EC:1.1.1.71,EC:1.14.12,EC:1.14.12.17,EC:1.1,EC:1,EC:1.14,EC:1.1.1,EC:1.1.1.1,EC:1.1.1.71;Enzyme_name=nitric oxide dioxygenase,alcohol dehydrogenase,alcohol dehydrogenase [NAD(P)(+)],Acting on paired donors, with incorporation or reduction of molecular oxygen. The oxygen incorporated need not be derived from O2,nitric oxide dioxygenase,Acting on the CH-OH group of donors,Oxidoreductases,Acting on paired donors, with incorporation or reduction of molecular oxygen. The oxygen incorporated need not be derived from O2,Acting on the CH-OH group of donors,alcohol dehydrogenase,alcohol dehydrogenase [NAD(P)(+)]
contig00011.g6495	1704	ID=contig00011.g6495;Description=hypothetical protein J7337_011941 [Fusarium musae];Ontology_term=membrane;Ontology_id=GO:0016020
contig00011.g6496	2522	ID=contig00011.g6496;Description=trehalose synthase [Fusarium coicis];Gene=FNAPI_992;Ontology_term=transferase activity;Ontology_id=GO:0016740;Enzyme_code=EC:2.4,EC:2.4,EC:2,EC:2.4.1.231;Enzyme_name=Glycosyltransferases,Glycosyltransferases,Transferases,alpha,alpha-trehalose phosphorylase (configuration-retaining)
contig00011.g6497	1253	ID=contig00011.g6497;Description=phosphotransferase enzyme family [Fusarium napiforme];Gene=FNAPI_991;Ontology_term=transferase activity;Ontology_id=GO:0016740;Enzyme_code=EC:2.7,EC:2.7,EC:2;Enzyme_name=Transferring phosphorus-containing groups,Transferring phosphorus-containing groups,Transferases
contig00011.g6498	748	ID=contig00011.g6498;Description=Manganese/iron superoxide dismutase [Fusarium redolens];Gene=FVEG_11192;Ontology_term=antioxidant activity,oxidoreductase activity,mitochondrion;Ontology_id=GO:0016209,GO:0016491,GO:0005739;Enzyme_code=EC:1.15.1.1,EC:1,EC:1.15.1.1,EC:1.15;Enzyme_name=superoxide dismutase,Oxidoreductases,superoxide dismutase,Acting on superoxide as acceptor
contig00011.g6499	595	ID=contig00011.g6499;Description=DNA replication complex GINS protein PSF3 [Fusarium oxysporum f. sp. lycopersici 4287];Gene=FOMG_10152;Ontology_term=DNA repair,DNA replication,DNA recombination,nuclear chromosome,nucleoplasm;Ontology_id=GO:0006281,GO:0006260,GO:0006310,GO:0000228,GO:0005654
contig00011.g6500	2064	ID=contig00011.g6500;Description=key lime pathogenicity protein [Fusarium subglutinans]

contig00011.g6501	1595	ID=contig00011.g6501;Description=Protein transport protein yos1 [Fusarium musae];Gene=FPCIR_12408;Ontology_term=signaling,endoplasmic reticulum;Ontology_id=GO:0023052,GO:0005783
contig00011.g6502	2695	ID=contig00011.g6502;Description=hypothetical protein FVER53263_11196 [Fusarium verticillioides];Gene=FTJAE_90;Ontology_term=microtubule organizing center;Ontology_id=GO:0005815
contig00011.g6503	405	ID=contig00011.g6503;Description=related to rasp f 7 allergen [Fusarium fujikuroi IMI 58289]
contig00011.g6504	1592	ID=contig00011.g6504;Description=Putative FAD-linked oxidoreductase yvdP [Fusarium odoratissimum];Gene=FocTR4_00013878;Ontology_term=oxidoreductase activity;Ontology_id=GO:0016491;Enzyme_code=EC:1,EC:1;Enzyme_name=Oxidoreductases,Oxidoreductases
contig00011.g6505	3801	ID=contig00011.g6505;Description=histidine kinase [Fusarium tjaetaba];Gene=F52700_7169;Ontology_term=catalytic activity, acting on a protein,signaling,transferase activity,small molecule sensor activity;Ontology_id=GO:0140096,GO:0023052,GO:0016740,GO:0140299;Enzyme_code=EC:2.7.3,EC:2.7.13.3,EC:2.7.1,EC:2.7.3,EC:2.7,EC:2,EC:2.7.13.3;Enzyme_name=Transferring phosphorus-containing groups,histidine kinase,Transferring phosphorus-containing groups,Transferring phosphorus-containing groups,Transferring phosphorus-containing groups,Transferases,histidine kinase
contig00011.g6506	456	ID=contig00011.g6506;Description=hypothetical protein FVEG_11201 [Fusarium verticillioides 7600]
contig00011.g6507	576	ID=contig00011.g6507;Description=light induced alcohol dehydrogenase Bli-4 [Fusarium tjaetaba]
contig00011.g6508	384	ID=contig00011.g6508;Description=light induced alcohol dehydrogenase Bli-4 [Fusarium pseudoanthophilum]
contig00011.g6509	962	ID=contig00011.g6509;Description=Arginase [Fusarium mundagurra];Gene=F25303_12089;Ontology_term=metal ion binding;Ontology_id=GO:0046872
contig00011.g6510	411	ID=contig00011.g6510;Description=Virulence protein [Fusarium fujikuroi];Gene=FMUND_3245;Ontology_term=nucleus,DNA binding,oxidoreductase activity,DNA-templated transcription;Ontology_id=GO:0005634,GO:0003677,GO:0016491,GO:0006351;Enzyme_code=EC:1,EC:1;Enzyme_name=Oxidoreductases,Oxidoreductases

contig00011.g6511	1467	ID=contig00011.g6511;Description=hypothetical protein FVER53590_11205 [Fusarium verticillioides]
contig00011.g6512	1377	ID=contig00011.g6512;Description=O-methylsterigmatocystin oxidoreductase [Fusarium oxysporum];Gene=FIE12Z_3665;Ontology_term=oxidoreductase activity;Ontology_id=GO:0016491;Enzyme_code=EC:1.14,EC:1.14.13.175,EC:1,EC:1.14,EC:1.14.14.54;Enzyme_name=Acting on paired donors, with incorporation or reduction of molecular oxygen. The oxygen incorporated need not be derived from O2,Acting on paired donors, with incorporation or reduction of molecular oxygen. The oxygen incorporated need not be derived from O2,Oxidoreductases,Acting on paired donors, with incorporation or reduction of molecular oxygen. The oxygen incorporated need not be derived from O2,phenylacetate 2-hydroxylase
contig00011.g6513	1014	ID=contig00011.g6513;Description=integral membrane protein [Fusarium napiforme];Gene=FGADI_4100;Ontology_term=membrane;Ontology_id=GO:0016020
contig00011.g6514	1679	ID=contig00011.g6514;Description=hypothetical protein FVER53263_11208 [Fusarium verticillioides]
contig00011.g6515	1398	ID=contig00011.g6515;Description=integral membrane protein [Fusarium tjaetaba];Gene=FVER53590_11209;Ontology_term=membrane;Ontology_id=GO:0016020
contig00011.g6516	1974	ID=contig00011.g6516;Description=uncharacterized protein FTJAE_73 [Fusarium tjaetaba];Gene=FNAPI_11866;Ontology_term=membrane;Ontology_id=GO:0016020
contig00011.g6517	1224	ID=contig00011.g6517;Description=hypothetical protein FVEG_11211 [Fusarium verticillioides 7600]
contig00011.g6518	1829	ID=contig00011.g6518;Description=related to TEA1-TY1 enhancer activator [Fusarium fujikuroi];Gene=LY6E;Ontology_term=nucleus,DNA binding,oxidoreductase activity,regulation of DNA-templated transcription,transcription regulator activity;Ontology_id=GO:0005634,GO:0003677,GO:0016491,GO:0006355,GO:0140110;Enzyme_code=EC:1.1.1.1,EC:1.1.1.71;Enzyme_name=alcohol dehydrogenase,alcohol dehydrogenase [NAD(P)(+)]
contig00011.g6519	1119	ID=contig00011.g6519;Description=glutathione S-transferase [Fusarium verticillioides 7600];Gene=gto2-0;Ontology_term=transferase activity;Ontology_id=GO:0016740;Enzyme_code=EC:2.5.1.18,EC:2.5.1,EC:2.5.1.18,EC:2,EC:1.8.5.7;Enzyme_name=glutathione transferase,Transferring alkyl or aryl groups, other than methyl groups,glutathione transferase,Transferases,glutathionyl-hydroquinone reductase

contig00011.g6520	1260	ID=contig00011.g6520;Description=glutathione s-transferase omega 2 [Fusarium tjaetaba];Gene=FPCIR_2404;Ontology_term=transferase activity;Ontology_id=GO:0016740;Enzyme_code=EC:2,EC:2;Enzyme_name=Transferases,Transferases
contig00011.g6521	1353	ID=contig00011.g6521;Description=hypothetical protein FVEG_11214 [Fusarium verticillioides 7600];Gene=FVEG_11214;Ontology_term=transferase activity;Ontology_id=GO:0016740;Enzyme_code=EC:2.4,EC:2.4,EC:2;Enzyme_name=Glycosyltransferases,Glycosyltransferases,Transferases
contig00011.g6522	2030	ID=contig00011.g6522;Description=glutamine amidotransferase [Fusarium subglutinans];Gene=FSUBG_4350;Ontology_term=amino acid metabolic process,transferase activity;Ontology_id=GO:0006520,GO:0016740;Enzyme_code=EC:2;Enzyme_name=Transferases
contig00011.g6523	1536	ID=contig00011.g6523;Description=hypothetical protein FVER14953_11216 [Fusarium verticillioides];Ontology_term=hydrolase activity;Ontology_id=GO:0016787;Enzyme_code=EC:3.5.1,EC:3.4.19.16,EC:3.5,EC:3;Enzyme_name=Acting on carbon-nitrogen bonds, other than peptide bonds,glucosinolate gamma-glutamyl hydrolase,Acting on carbon-nitrogen bonds, other than peptide bonds,Hydrolases
contig00011.g6524	3222	ID=contig00011.g6524;Description=hypothetical protein FVER53263_11217 [Fusarium verticillioides];Gene=FVER53263_11217;Ontology_term=catalytic activity, acting on a protein,hydrolase activity;Ontology_id=GO:0140096,GO:0016787;Enzyme_code=EC:3.4.21,EC:3.4.21,EC:3.4,EC:3;Enzyme_name=Acting on peptide bonds (peptidases),Acting on peptide bonds (peptidases),Acting on peptide bonds (peptidases),Hydrolases
contig00011.g6525	7254	ID=contig00011.g6525;Description=ankyrin protein 1 [Fusarium denticulatum]
contig00011.g6526	1647	ID=contig00011.g6526;Description=peroxidase [Fusarium tjaetaba];Gene=FPHYL_1625;Ontology_term=antioxidant activity,oxidoreductase activity;Ontology_id=GO:0016209,GO:0016491;Enzyme_code=EC:1.11.1,EC:1.11,EC:1,EC:1.11.1;Enzyme_name=Acting on a peroxide as acceptor,Acting on a peroxide as acceptor,Oxidoreductases,Acting on a peroxide as acceptor

contig00011.g6527	1246	ID=contig00011.g6527;Description=related to D-amino acid oxidase [Fusarium fujikuroi];Gene=FMAN_12352;Ontology_term=oxidoreductase activity,amino acid metabolic process;Ontology_id=GO:0016491,GO:0006520;Enzyme_code=EC:1.4.3.3,EC:1.4.3.21,EC:1.4,EC:1.4.3,EC:1,EC:1.4.3.3,EC:1.4.3.21;Enzyme_name=D-amino-acid oxidase,primary-amine oxidase,Acting on the CH-NH2 group of donors,Acting on the CH-NH2 group of donors,Oxidoreductases,D-amino-acid oxidase,primary-amine oxidase
contig00011.g6528	522	ID=contig00011.g6528;Description=Endoribonuclease L-PSP/chorismate mutase-like protein [Fusarium flagelliforme];Enzyme_code=EC:3.5.99.10;Enzyme_name=2-iminobutanoate/2-iminopropanoate deaminase
contig00011.g6529	1084	ID=contig00011.g6529;Description=allantoate permease [Fusarium fujikuroi];Gene=FPCIR_2413;Ontology_term=transmembrane transport,transporter activity;Ontology_id=GO:0055085,GO:0005215
contig00011.g6530	1703	ID=contig00011.g6530;Description=major facilitator superfamily domain-containing protein [Fusarium oxysporum];Gene=FANTH_8469;Ontology_term=transmembrane transport,endoplasmic reticulum,transporter activity;Ontology_id=GO:0055085,GO:0005783,GO:0005215
contig00011.g6531	7917	ID=contig00011.g6531;Description=pectinesterase [Fusarium coicis];Gene=FVER53263_11228;Ontology_term=carbohydrate metabolic process,cell wall organization or biogenesis,hydrolase activity;Ontology_id=GO:0005975,GO:0071554,GO:0016787;Enzyme_code=EC:3.1.1.11,EC:3.1,EC:3.1.1.11,EC:3,EC:3.1.1;Enzyme_name=pectinesterase,Acting on ester bonds,pectinesterase,Hydrolases,Acting on ester bonds
contig00011.g6532	1720	ID=contig00011.g6532;Description=allantoate permease [Fusarium fujikuroi];Gene=FVEG_11229;Ontology_term=transmembrane transport,transporter activity;Ontology_id=GO:0055085,GO:0005215
contig00011.g6533	2561	ID=contig00011.g6533;Description=probable arginosuccinate synthetase [Fusarium fujikuroi];Gene=FTJAE_13732
contig00011.g6534	1593	ID=contig00011.g6534;Description=Argininosuccinate synthase [Fusarium tjaetaba]
contig00011.g6535	2571	ID=contig00011.g6535;Description=hypothetical protein FVER53590_11232 [Fusarium verticillioides]

contig00011.g6536	969	ID=contig00011.g6536;Description=pectate lyase 1 [Fusarium subglutinans];Gene=FANTH_8463;Ontology_term=carbohydrate metabolic process,lyase activity,extracellular region;Ontology_id=GO:0005975,GO:0016829,GO:0005576;Enzyme_code=EC:4.2.2.2,EC:4.2.2.2,EC:4.2,EC:4,EC:4.2.2;Enzyme_name=pectate lyase,pectate lyase,Carbon-oxygen lyases,Lyases,Carbon-oxygen lyases
contig00011.g6537	3231	ID=contig00011.g6537;Description=hypothetical protein FMEXI_11688 [Fusarium mexicanum];Gene=FANTH_8462;Ontology_term=nucleus;Ontology_id=GO:0005634
contig00011.g6538	2007	ID=contig00011.g6538;Description=hypothetical protein FVER53263_11235 [Fusarium verticillioides]
contig00011.g6539	1655	ID=contig00011.g6539;Description=multidrug resistance [Fusarium tjaetaba];Gene=FVER53263_11236;Ontology_term=transmembrane transport,transporter activity;Ontology_id=GO:0055085,GO:0005215
contig00011.g6540	4224	ID=contig00011.g6540;Description=hypothetical protein FVER53590_11237 [Fusarium verticillioides]
contig00011.g6541	1170	ID=contig00011.g6541;Description=Dip2/Utp12 family-domain-containing protein [Fusarium oxysporum Fo47];Ontology_term=RNA binding,ribosome biogenesis,regulation of DNA-templated transcription,chromosome,nucleolus;Ontology_id=GO:0003723,GO:0042254,GO:0006355,GO:0005694,GO:0005730
contig00011.g6542	2894	ID=contig00011.g6542;Description=bromodomain-containing protein [Fusarium denticulatum];Ontology_term=DNA repair,DNA binding,snRNA metabolic process,histone binding,nuclear chromosome,regulation of DNA-templated transcription,chromatin organization,nucleoplasm;Ontology_id=GO:0006281,GO:0003677,GO:0016073,GO:0042393,GO:0000228,GO:0006355,GO:0006325,GO:0005654
contig00011.g6543	982	ID=contig00011.g6543;Description=thiamine pyrophosphokinase [Fusarium napiforme];Gene=FPCIR_13904;Ontology_term=transferase activity;Ontology_id=GO:0016740;Enzyme_code=EC:2.7;Enzyme_name=Transferring phosphorus-containing groups
contig00011.g6544	1371	ID=contig00011.g6544;Description=hypothetical protein FVER14953_11241 [Fusarium verticillioides];Gene=FPANT_6509;Ontology_term=transferase activity;Ontology_id=GO:0016740;Enzyme_code=EC:2.1.1,EC:2.1,EC:2,EC:2.1.1;Enzyme_name=Transferring one-carbon groups,Transferring one-carbon groups,Transferases,Transferring one-carbon groups

contig00011.g6545	480	ID=contig00011.g6545;Description=hypothetical protein FVEG_11242 [Fusarium verticillioides 7600]
contig00011.g6546	2098	ID=contig00011.g6546;Description=hsp70 [Fusarium tjaetaba];Gene=FGADI_9664;Ontology_term=protein folding chaperone,ATP-dependent activity;Ontology_id=GO:0044183,GO:0140657
contig00011.g6547	2412	ID=contig00011.g6547;Description=CMGC/RCK/MAK protein kinase [Fusarium verticillioides 7600];Gene=FOYG_12160;Ontology_term=nucleus,catalytic activity, acting on a protein,signaling,cell differentiation,anatomical structure development,transferase activity,meiotic nuclear division;Ontology_id=GO:0005634,GO:0140096,GO:0023052,GO:0030154,GO:0048856,GO:0016740,GO:0140013;Enzyme_code=EC:2.7.1,EC:2.7.1,EC:2.7.11.1,EC:2.7,EC:2;Enzyme_name=Transferring phosphorus-containing groups,Transferring phosphorus-containing groups,non-specific serine/threonine protein kinase,Transferring phosphorus-containing groups,Transferases
contig00011.g6548	1551	ID=contig00011.g6548;Description=hypothetical protein FVEG_11245 [Fusarium verticillioides 7600]
contig00011.g6549	2192	ID=contig00011.g6549;Description=pyruvate dehydrogenase kinase [Fusarium verticillioides 7600];Gene=FOBC_13580;Ontology_term=catalytic activity, acting on a protein,nucleobase-containing small molecule metabolic process,sulfur compound metabolic process,ATP-dependent activity,mitochondrion,carbohydrate derivative metabolic process,transferase activity,hydrolase activity;Ontology_id=GO:0140096,GO:0055086,GO:0006790,GO:0140657,GO:0005739,GO:1901135,GO:0016740,GO:0016787;Enzyme_code=EC:2.7.1,EC:2.7.1,EC:2.7.11.2,EC:3.6.1,EC:2.7,EC:2,EC:3.6,EC:3,EC:3.6.1.15;Enzyme_name=Transferring phosphorus-containing groups,Transferring phosphorus-containing groups,[pyruvate dehydrogenase (acetyl-transferring)] kinase,Acting on acid anhydrides,Transferring phosphorus-containing groups,Transferases,Acting on acid anhydrides,Hydrolases,nucleoside-triphosphate phosphatase
contig00011.g6550	1293	ID=contig00011.g6550;Description=cytochrome P450 monooxygenase [Fusarium tjaetaba];Gene=FNAPI_1794;Ontology_term=oxidoreductase activity;Ontology_id=GO:0016491;Enzyme_code=EC:1,EC:1;Enzyme_name=Oxidoreductases,Oxidoreductases
contig00011.g6551	2054	ID=contig00011.g6551;Description=hypothetical protein J7337_011884 [Fusarium musae]

contig00011.g6552	1864	ID=contig00011.g6552;Description=sphinganine-1-phosphate aldolase [Fusarium verticillioides 7600];Gene=FFB14_07787;Ontology_term=lyase activity;Ontology_id=GO:0016829;Enzyme_code=EC:4.1;Enzyme_name=Carbon-carbon lyases
contig00011.g6553	1066	ID=contig00011.g6553;Description=alpha-1 2-galactosyltransferase [Fusarium pseudoanthophilum];Gene=FOTG_08099;Ontology_term=protein glycosylation,cell wall organization or biogenesis,endoplasmic reticulum,Golgi apparatus,transferase activity;Ontology_id=GO:0006486,GO:0071554,GO:0005783,GO:0005794,GO:0016740;Enzyme_code=EC:2.4,EC:2.4,EC:2,EC:2.4.1;Enzyme_name=Glycosyltransferases,Glycosyltransferases,Transferases,Glycosyltransferases
contig00011.g6554	1002	ID=contig00011.g6554;Description=RING finger domain protein [Fusarium tjaetaba];Gene=FDENT_12417;Ontology_term=zinc ion binding,membrane;Ontology_id=GO:0008270,GO:0016020
contig00011.g6555	1080	ID=contig00011.g6555;Description=Proteasome subunit alpha type-2 [Fusarium torreyae];Gene=BFJ65_g12863;Ontology_term=nucleus,protein catabolic process;Ontology_id=GO:0005634,GO:0030163
contig00011.g6556	1142	ID=contig00011.g6556;Description=e3 ubiquitin ligase HERC6 [Fusarium coicis]
contig00011.g6557	5081	ID=contig00011.g6557;Description=Dicer 2 [Fusarium tjaetaba];Gene=FTJAE_870;Ontology_term=defense response to other organism,nucleus,RNA binding,ATP-dependent activity,regulatory ncRNA-mediated gene silencing,catalytic activity, acting on RNA,chromatin organization,hydrolase activity;Ontology_id=GO:0098542,GO:0005634,GO:0003723,GO:0140657,GO:0031047,GO:0140098,GO:0006325,GO:0016787;Enzyme_code=EC:3.1.30,EC:3.1.26.3,EC:3.1.30,EC:3.1,EC:3,EC:3.1.26.3,EC:3.1.26;Enzyme_name=Acting on ester bonds,ribonuclease III,Acting on ester bonds,Acting on ester bonds,Hydrolases,ribonuclease III,Acting on ester bonds
contig00011.g6558	853	ID=contig00011.g6558;Description=uridine kinase udk [Fusarium tjaetaba];Gene=FPANT_6524;Ontology_term=transferase activity;Ontology_id=GO:0016740;Enzyme_code=EC:2.7;Enzyme_name=Transferring phosphorus-containing groups

contig00011.g6559	2276	ID=contig00011.g6559;Description=exostosin-like 3 [Fusarium tjaetaba];Gene=FDENT_12411;Ontology_term=transferase activity,catalytic activity, acting on RNA,hydrolase activity;Ontology_id=GO:0016740,GO:0140098,GO:0016787;Enzyme_code=EC:2.4,EC:3.1.1.29,EC:3.1,EC:2.4,EC:2,EC:3,EC:3.1.1.29,EC:3.1.1;Enzyme_name=Glycosyltransferases,aminoacyl-tRNA hydrolase,Acting on ester bonds,Glycosyltransferases,Transferases,Hydrolases,aminoacyl-tRNA hydrolase,Acting on ester bonds
contig00011.g6560	1706	ID=contig00011.g6560;Description=UTP7-u3 snoRNP [Fusarium tjaetaba];Gene=FNAPI_1783;Ontology_term=RNA binding,ribosome biogenesis,nucleolus;Ontology_id=GO:0003723,GO:0042254,GO:0005730
contig00011.g6561	2502	ID=contig00011.g6561;Description=Ribosome biogenesis protein [Fusarium oxysporum f. sp. matthiolae];Gene=FPRO05_09271;Ontology_term=RNA binding,GTPase activity,regulatory ncRNA-mediated gene silencing,ribosome biogenesis,nucleolus;Ontology_id=GO:0003723,GO:0003924,GO:0031047,GO:0042254,GO:0005730;Enzyme_code=EC:3.6.1,EC:3.6,EC:3,EC:3.6.1.15;Enzyme_name=Acting on acid anhydrides,Acting on acid anhydrides,Hydrolases,nucleoside-triphosphate phosphatase
contig00011.g6562	856	ID=contig00011.g6562;Description=urease accessory protein [Fusarium subglutinans];Gene=BFJ65_g12104;Ontology_term=GTPase activity,nitrogen cycle metabolic process;Ontology_id=GO:0003924,GO:0071941;Enzyme_code=EC:3.6.1.15,EC:3.6.1,EC:3.6,EC:3,EC:3.6.1.15;Enzyme_name=nucleoside-triphosphate phosphatase,Acting on acid anhydrides,Acting on acid anhydrides,Hydrolases,nucleoside-triphosphate phosphatase
contig00011.g6563	313	ID=contig00011.g6563;Description=hypothetical protein FVER53590_11262 [Fusarium verticillioides]
contig00011.g6564	1195	ID=contig00011.g6564;Description=KOW motif containing protein [Fusarium tjaetaba];Gene=FTJAE_861;Ontology_term=RNA binding,mitochondrial gene expression,mitochondrion,structural molecule activity,ribosome;Ontology_id=GO:0003723,GO:0140053,GO:0005739,GO:0005198,GO:0005840

contig00011.g6565	1210	ID=contig00011.g6565;Description=GTPase Rho [Fusarium tjaetaba];Gene=FANTH_13854;Ontology_term=cytoskeleton organization,establishment or maintenance of cell polarity,anatomical structure development,reproductive process,cytokinesis,Golgi apparatus,endoplasmic reticulum,plasma membrane,mitotic cell cycle,carbohydrate metabolic process,peroxisome,vesicle-mediated transport,GTPase activity,signaling,mitochondrion,cell wall organization or biogenesis;Ontology_id=GO:0007010,GO:0007163,GO:0048856,GO:0022414,GO:0000910,GO:0005794,GO:0005783,GO:0005886,GO:0000278,GO:0005975,GO:0005777,GO:0016192,GO:0003924,GO:0023052,GO:0005739,GO:0071554;Enzyme_code=EC:3.6.1.15,EC:3.6.1,EC:3.6,EC:3,EC:3.6.1.15;Enzyme_name=nucleoside-triphosphate phosphatase,Acting on acid anhydrides,Acting on acid anhydrides,Hydrolases,nucleoside-triphosphate phosphatase
contig00011.g6566	1836	ID=contig00011.g6566;Description=YBL089w-like protein [Fusarium denticulatum];Gene=FDENT_12404;Ontology_term=transmembrane transport,endoplasmic reticulum,transporter activity;Ontology_id=GO:0055085,GO:0005783,GO:0005215
contig00011.g6567	1689	ID=contig00011.g6567;Description=Exostosin-3 [Fusarium oxysporum f. sp. albedinis];Gene=FOPG_07186;Ontology_term=catalytic activity, acting on a protein,hydrolase activity;Ontology_id=GO:0140096,GO:0016787;Enzyme_code=EC:3.4.23,EC:3.4.23,EC:3.4,EC:3,EC:3.4.23.25,EC:3.4.23.5;Enzyme_name=Acting on peptide bonds (peptidases),Acting on peptide bonds (peptidases),Acting on peptide bonds (peptidases),Hydrolases,saccharopepsin,cathepsin D
contig00011.g6568	984	ID=contig00011.g6568;Description=prenyltransferase [Fusarium tjaetaba];Gene=FMUND_2551;Ontology_term=catalytic activity, acting on a protein,transferase activity;Ontology_id=GO:0140096,GO:0016740;Enzyme_code=EC:2.5.1,EC:2.5.1,EC:2;Enzyme_name=Transferring alkyl or aryl groups, other than methyl groups,Transferring alkyl or aryl groups, other than methyl groups,Transferases
contig00011.g6569	742	ID=contig00011.g6569;Description=GET complex subunit get1 [Fusarium irregulare];Gene=GET1;Ontology_term=membrane organization,endoplasmic reticulum;Ontology_id=GO:0061024,GO:0005783
contig00011.g6570	1172	ID=contig00011.g6570;Description=chorismate synthase [Fusarium tjaetaba];Gene=FGADI_9688;Ontology_term=membrane;Ontology_id=GO:0016020
contig00011.g6571	1940	ID=contig00011.g6571;Description=RUD3-suppressor of uso1-1 transport defect [Fusarium tjaetaba]

contig00011.g6572	1215	ID=contig00011.g6572;Description=RNA binding protein [Fusarium pseudocircinatum];Gene=FPCIR_3867;Ontology_term=RNA binding,cytoskeleton organization,cytokinesis,ribosome biogenesis,nucleolus;Ontology_id=GO:0003723,GO:0007010,GO:0000910,GO:0042254,GO:0005730
contig00011.g6573	666	ID=contig00011.g6573;Description=Survival motor neuron 1 [Fusarium pseudoanthophilum];Gene=FPCIR_3866;Ontology_term=nucleus,mRNA metabolic process;Ontology_id=GO:0005634,GO:0016071
contig00011.g6574	1055	ID=contig00011.g6574;Description=serine-threonine rich protein [Fusarium tjaetaba]
contig00011.g6575	1662	ID=contig00011.g6575;Description=ATPase [Fusarium verticillioides 7600];Gene=FNAPI_11338;Ontology_term=DNA repair,nucleus,DNA binding,molecular function regulator activity,catalytic activity, acting on DNA,ATP-dependent activity,DNA replication,mitotic cell cycle,hydrolase activity;Ontology_id=GO:0006281,GO:0005634,GO:0003677,GO:0098772,GO:0140097,GO:0140657,GO:0006260,GO:0000278,GO:0016787;Enzyme_code=EC:3.6.1.15,EC:3.6.1,EC:3.6,EC:3,EC:3.6.1.15;Enzyme_name=nucleoside-triphosphate phosphatase,Acting on acid anhydrides,Acting on acid anhydrides,Hydrolases,nucleoside-triphosphate phosphatase
contig00011.g6576	1948	ID=contig00011.g6576;Description=mRNA cleavage factor complex component Pcf11 [Fusarium napiforme];Gene=FPRO05_09286;Ontology_term=mRNA metabolic process,vesicle-mediated transport,RNA binding,DNA-templated transcription,cytosol,lipid binding,nucleoplasm;Ontology_id=GO:0016071,GO:0016192,GO:0003723,GO:0006351,GO:0005829,GO:0008289,GO:0005654
contig00011.g6577	852	ID=contig00011.g6577;Description=hypothetical protein FVEG_11276 [Fusarium verticillioides 7600];Gene=FNYG_00472;Ontology_term=membrane;Ontology_id=GO:0016020
contig00011.g6578	2482	ID=contig00011.g6578;Description=DNA repair endonuclease rad2 [Fusarium tjaetaba];Gene=FNYG_00473;Ontology_term=DNA repair,catalytic activity, acting on DNA,hydrolase activity;Ontology_id=GO:0006281,GO:0140097,GO:0016787;Enzyme_code=EC:3.1.31.1,EC:3.1.22,EC:3.1.21.10;Enzyme_name=micrococcal nuclease,Acting on ester bonds,crossover junction endodeoxyribonuclease

contig00011.g6579	1723	ID=contig00011.g6579;Description=seryl-tRNA synthetase [Fusarium phyllophilum];Gene=FGADI_9697;Ontology_term=tRNA metabolic process,amino acid metabolic process,catalytic activity, acting on RNA,ligase activity;Ontology_id=GO:0006399,GO:0006520,GO:0140098,GO:0016874;Enzyme_code=EC:6.1.1.11,EC:6.1,EC:6.1.1.11,EC:6.1.1,EC:6;Enzyme_name=serine--tRNA ligase,Forming carbon-oxygen bonds,serine--tRNA ligase,Forming carbon-oxygen bonds,Ligases
contig00011.g6580	1500	ID=contig00011.g6580;Description=related to mitochondrial serine--tRNA ligase [Fusarium fujikuroi];Gene=BFJ63_vAg13566;Ontology_term=mitochondrial gene expression,mitochondrion,tRNA metabolic process,amino acid metabolic process,catalytic activity, acting on RNA,ligase activity;Ontology_id=GO:0140053,GO:0005739,GO:0006399,GO:0006520,GO:0140098,GO:0016874;Enzyme_code=EC:6.1.1.11,EC:6.1,EC:6.1.1.11,EC:6.1.1,EC:6;Enzyme_name=serine--tRNA ligase,Forming carbon-oxygen bonds,serine--tRNA ligase,Forming carbon-oxygen bonds,Ligases
[truncated: 2,215,948 more chars]
