# Supplementary material for: Genomic Insights into Fusarium verticillioides Diversity: The Genome of Two Clinical Isolates and Their Demethylase Inhibitor Fungicides Susceptibility
Source: Pathogens. 2024 Dec 3;13(12):1062. doi: 10.3390/pathogens13121062 (PMC11728828; doi:10.3390/pathogens13121062)
Supplement: Supplementary file 1 [file pathogens-13-01062-s001.zip › Table S4.pdf]

Region	Type	From	To
Region 1.1	T1PKS	2,050,012	2,107,976
Region 1.2	NRPS-like	2,274,226	2,337,553
Region 1.3	T1PKS	2,686,362	2,754,565
Region 2.1	NRPS-like	181,882	246,428
Region 2.2	NRPS-like	415,535	479,732
Region 2.3	T1PKS	689,091	756,761
Region 2.4	betalactone	857,352	900,035
Region 3.1	terpene	361,886	393,736
Region 3.2	T1PKS	1,988,653	2,055,882
Region 3.3	NRPS-like	2,076,026	2,138,487
Region 4.1	terpene	1,749,180	1,782,310
Region 4.2	indole	1,821,830	1,849,925
Region 7.1	indole	475,298	506,538
Region 7.2	terpene	582,609	614,345
Region 7.3	NRPS,T1PKS	900,13	974,658
Region 7.4	NRPS-like	1,135,629	1,198,951
Region 8.1	phosphonate	1,500,340	1,527,360
Region 9.1	T1PKS	653,955	721,006
Region 9.2	terpene	878,722	911,826
Region 10.1	NRPS	1,090,630	1,172,963
Region 11.1	NRPS-like,NRPS	72,136	175,043
Region 11.2	NRPS-like	509,416	572,904
Region 11.3	T1PKS	818,726	887,444
Region 11.4	fungal-RiPP-like	1,140,158	1,230,489
Region 12.1	T3PKS	814,282	875,742
Region 13.1	CDPS	1,105,909	1,137,186
Region 15.1	terpene	1	17,799
Region 17.1	terpene	173,176	205,171
Region 17.2	NRPS,T1PKS	622,822	731,73
Region 17.3	T1PKS	785,775	853,385
Region 18.1	NRPS,T1PKS	615,623	687,482
Region 19.1	NRPS-like	86,293	149,283
Region 21.1	NRPS,T1PKS	549,119	621,395
Region 21.2	NRPS-like,T1PKS	767,759	868,759
Region 22.1	fungal-RiPP-like	377,89	469,233
Region 25.1	T1PKS	543,637	610,675
Region 26.1	T1PKS	4,057	71,847
Region 28.1	fungal-RiPP-like	339,821	430,94
Region 30.1	NRPS-like	526,833	561,742
Region 36.1	NRPS	80,141	147,934
Region 36.2	T1PKS	336,918	382,492

Region 44.1	NRPS	130,944	202,907
Region 45.1	NRPS,NRPS-like,fungal-RiPP-like	1	145,992

Most similar known cluster		Similarity
fumonisin B1	Polyketide	68%
choline	NRP	100%
bikaverin	Polyketide	71%
fusaridione A/(3R,5S)-5-(4-hydroxybenzyl)-3-methyl-3-((2E,4E,6E,8E,10E)-4,8,10-trimethyldodeca-2,4,6,8,10-pentaenoyl)pyrrolidine-2,4-dione	NRP+Polyketide	12%
ilicicolin H	Polyketide+NRP	25%
fosfonochlorin	Other	84%
oxyjavanicin	Polyketide	100%
trichoxide	Polyketide	41%
gibepyrone-A	Polyketide	60%
gregatin A	Polyketide	33%
lucilactaene	Polyketide	53%
equisetin	NRP+Polyketide	45%
fusaric acid	Polyketide	60%
depudecin	Polyketide:Iterative type I polyketide	66%
