# Supplementary material for: Genomic Insights into Fusarium verticillioides Diversity: The Genome of Two Clinical Isolates and Their Demethylase Inhibitor Fungicides Susceptibility
Source: Pathogens. 2024 Dec 3;13(12):1062. doi: 10.3390/pathogens13121062 (PMC11728828; doi:10.3390/pathogens13121062)
Supplement: Supplementary file 1 [file pathogens-13-01062-s001.zip › Table S5.pdf]

Region	Type	From	To	Most similar k
Region 1.1	NRPS-like	15,531	82,506	fusaridione A/(3R,5S)-5-(4-hydroxybenzyl)-3-methyl-3-((2E,4E,6E,8E,10E)-4,8,10-trimethyldodeca-2,4,6,8,10-pentaenoyl)pyrrolidine-2,4-dione
Region 1.2	T1PKS	102,465	168,741	bikaverin
Region 1.3	NRPS	500,11	565,86	
Region 1.4	terpene	1,788,356	1,820,205	
Region 2.1	T1PKS	1,851,048	1,919,210	fumonisin B1
Region 2.2	NRPS-like	2,087,638	2,150,965	
Region 3.1	CDPS	70,416	101,228	
Region 3.2	T1PKS	264,044	331,457	α-acorenol
Region 3.3	fungal-RiPP-like	461,801	550,255	
Region 3.4	NRPS-like,NRPS	1,242,189	1,345,085	
Region 3.5	NRPS-like	1,690,247	1,753,735	
Region 3.6	fungal-RiPP-like	2,329,841	2,420,172	
Region 4.1	betalactone	1,501,343	1,544,026	
Region 4.2	T1PKS	1,644,577	1,712,250	
Region 4.3	NRPS-like	1,920,962	1,982,101	choline
Region 4.4	NRPS-like	2,155,111	2,220,753	
Region 5.1	NRPS,T1PKS	1,341,661	1,413,937	equisetin
Region 5.2	NRPS-like,T1PKS	1,566,059	1,656,692	fusaric acid
Region 8.1	NRPS	6,624	75,894	
Region 8.2	indole	775,511	806,751	
Region 8.3	terpene	882,293	914,029	
Region 8.4	T1PKS,NRPS	1,187,747	1,262,271	ilicicolin H
Region 8.5	NRPS-like	1,429,026	1,492,348	
Region 10.1	NRPS-like,NRPS,fungal-RiPP-like	1	122,287	
Region 11.1	T1PKS,NRPS	327,342	399,201	lucilactaene
Region 12.1	T1PKS	437,404	505,194	
Region 13.1	T1PKS	323,849	390,9	oxyjavanicin
Region 14.1	NRPS	882,27	964,603	
Region 16.1	terpene	559,468	591,017	squalestatin S1
Region 18.1	terpene	91,448	123,093	koraiol
Region 18.2	fungal-RiPP-like	407,659	498,191	
Region 19.1	T1PKS	38,756	101,72	fujikurin A/fujikurin B/fujikurin C/fujikurin D
Region 19.2	terpene,T1PKS,NRPS	175,515	293,356	gibepyrone-A
Region 19.3	terpene	693,789	725,784	
Region 20.1	T3PKS	181,081	242,865	
Region 21.1	NRPS,indole	692,304	782,886	enniatin
Region 22.1	fungal-RiPP-like	696,556	782,552	lucilactaene

Region 24.1	fungal-RiPP-like	78,783	169,902	
Region 24.2	NRPS	409,31	477,103	
Region 24.3	T1PKS	659,363	728,462	
Region 25.1	phosphonate	9,819	36,839	fosfonochlorin
Region 28.1	NRPS-like	550,716	593,497	
Region 34.1	NRPS	246,735	321,703	
Region 39.1	NRPS-like	255,9	301,402	
Region 41.1	T1PKS	9,998	78,201	
Region 57.1	NRPS	1	43,363	acetylaranotin

known cluster	Similarity
NRP+Polyketide	12%
Polyketide	85%
Polyketide	62%
Terpene	100%
NRP	100%
NRP+Polyketide	45%
Polyketide	85%
Polyketide+NRP	25%
Polyketide	38%
Polyketide	100%
Terpene	40%
Terpene	100%
Polyketide	50%
Polyketide	60%
NRP	100%
Polyketide	15%

Other	69%
NRP	20%
