# Supplementary material for: Genomic Insights into Fusarium verticillioides Diversity: The Genome of Two Clinical Isolates and Their Demethylase Inhibitor Fungicides Susceptibility
Source: Pathogens. 2024 Dec 3;13(12):1062. doi: 10.3390/pathogens13121062 (PMC11728828; doi:10.3390/pathogens13121062)
Supplement: Supplementary file 1 [file pathogens-13-01062-s001.zip › Table S6.pdf]

Gene ID of IUM09-1037 of the effectors (effectorP- effectorp/SignalP) and gene id of the unique genes of the human strains compared to 7600.

list of effectors from IUM09-1037 (effector P threshold 90%)	list of effector with positive SignalP of IUM09-1037	IUM09-1037 codes of unique genes of human strains compared to 7600
Effectors (>90%)_IUM09-1037	SignalP (on Eff >90%)_IUM09-1037	118 unique_IUM09-1037
contig00001.g79	contig00001.g79	contig00001.g97
contig00001.g141	contig00001.g161	contig00001.g101
contig00001.g156	contig00001.g424	contig00001.g102
contig00001.g161	contig00001.g490	contig00001.g104
contig00001.g209	contig00002.g1445	contig00002.g1286
contig00001.g290	contig00002.g1479	contig00003.g1639
contig00001.g348	contig00003.g1878	contig00003.g1640
contig00001.g389	contig00003.g1946	contig00003.g1663
contig00001.g424	contig00003.g2118	contig00003.g1665
contig00001.g455	contig00004.g3241	contig00003.g1666
contig00001.g490	contig00005.g3649	contig00003.g1762
contig00001.g503	contig00005.g3659	contig00003.g1922
contig00001.g537	contig00007.g4673	contig00003.g2475
contig00001.g580	contig00008.g4776	contig00003.g2477
contig00001.g610	contig00008.g4827	contig00004.g3113
contig00001.g639	contig00008.g5085	contig00004.g3115
contig00001.g653	contig00010.g5848	contig00004.g3149
contig00001.g734	contig00011.g6439	contig00004.g3240
contig00002.g896	contig00011.g6465	contig00004.g3241
contig00002.g918	contig00011.g6485	contig00005.g3695
contig00002.g931	contig00013.g7169	contig00007.g4616
contig00002.g1023	contig00013.g7318	contig00007.g4617
contig00002.g1043	contig00013.g7360	contig00007.g4669
contig00002.g1065	contig00014.g7706	contig00008.g4849
contig00002.g1072	contig00016.g8232	contig00008.g5066
contig00002.g1075	contig00017.g8532	contig00008.g5236
contig00002.g1082	contig00017.g8591	contig00009.g5408

contig00002.g1139	contig00018.g8815	contig00010.g5885
contig00002.g1176	contig00018.g8852	contig00010.g5925
contig00002.g1203	contig00018.g8897	contig00011.g6382
contig00002.g1292	contig00021.g9501	contig00011.g6383
contig00002.g1380	contig00022.g10003	contig00011.g6384
contig00002.g1412	contig00025.g10725	contig00011.g6385
contig00002.g1445	contig00028.g11312	contig00011.g6414
contig00002.g1479	contig00031.g11770	contig00011.g6433
contig00002.g1525	contig00032.g11989	contig00013.g7336
contig00002.g1569	contig00041.g13055	contig00013.g7337
contig00003.g1679	contig00048.g13505	contig00013.g7338
contig00003.g1707		contig00013.g7339
contig00003.g1708		contig00013.g7340
contig00003.g1738		contig00013.g7341
contig00003.g1785		contig00013.g7342
contig00003.g1786		contig00013.g7343
contig00003.g1798		contig00013.g7344
contig00003.g1812		contig00013.g7349
contig00003.g1821		contig00013.g7351
contig00003.g1875		contig00013.g7352
contig00003.g1878		contig00013.g7353
contig00003.g1935		contig00013.g7354
contig00003.g1946		contig00013.g7356
contig00003.g1950		contig00017.g8590
contig00003.g2047		contig00018.g8853
contig00003.g2118		contig00019.g8991
contig00003.g2263		contig00019.g8992
contig00003.g2385		contig00019.g8993
contig00003.g2450		contig00019.g8994
contig00003.g2482		contig00019.g9033
contig00003.g2508		contig00019.g9040
contig00004.g2520		contig00021.g9701
contig00004.g2529		contig00023.g10162

contig00004.g2541
contig00004.g2558
contig00004.g2568
contig00004.g2668
contig00004.g2692
contig00004.g2724
contig00004.g2788
contig00004.g2791
contig00004.g2826
contig00004.g2853
contig00004.g2971
contig00004.g3005
contig00004.g3014
contig00004.g3071
contig00004.g3101
contig00004.g3129
contig00004.g3241
contig00005.g3442
contig00005.g3445
contig00005.g3512
contig00005.g3575
contig00005.g3649
contig00005.g3659
contig00005.g3660
contig00005.g3667
contig00005.g3696
contig00006.g3763
contig00006.g3841
contig00006.g3911
contig00006.g3925
contig00006.g3951
contig00006.g4054
contig00006.g4087
contig00023.g10199
contig00023.g10200
contig00023.g10201
contig00023.g10202
contig00023.g10230
contig00023.g10231
contig00023.g10232
contig00023.g10233
contig00023.g10235
contig00023.g10238
contig00023.g10240
contig00024.g10522
contig00024.g10526
contig00024.g10527
contig00024.g10528
contig00025.g10623
contig00025.g10740
contig00025.g10741
contig00028.g11204
contig00028.g11205
contig00028.g11206
contig00028.g11207
contig00028.g11208
contig00028.g11209
contig00028.g11210
contig00028.g11211
contig00028.g11213
contig00028.g11214
contig00028.g11215
contig00028.g11217
contig00028.g11220
contig00028.g11221
contig00028.g11224

contig00006.g4171
contig00006.g4178
contig00007.g4223
contig00007.g4229
contig00007.g4264
contig00007.g4268
contig00007.g4344
contig00007.g4389
contig00007.g4401
contig00007.g4471
contig00007.g4472
contig00007.g4481
contig00007.g4539
contig00007.g4660
contig00007.g4673
contig00007.g4722
contig00008.g4771
contig00008.g4776
contig00008.g4787
contig00008.g4827
contig00008.g4887
contig00008.g4935
contig00008.g5066
contig00008.g5085
contig00008.g5290
contig00009.g5423
contig00009.g5474
contig00009.g5494
contig00009.g5575
contig00009.g5597
contig00009.g5701
contig00009.g5753
contig00009.g5763
contig00028.g11225
contig00028.g11231
contig00028.g11232
contig00028.g11233
contig00028.g11235
contig00028.g11239
contig00028.g11240
contig00028.g11243
contig00028.g11245
contig00028.g11246
contig00028.g11247
contig00028.g11250
contig00028.g11251
contig00028.g11252
contig00028.g11253
contig00028.g11254
contig00028.g11277
contig00028.g11278
contig00028.g11279
contig00028.g11280
contig00028.g11281
contig00028.g11282
contig00028.g11290
contig00028.g11295
contig00028.g11758

contig00009.g5787
contig00010.g5845
contig00010.g5848
contig00010.g5961
contig00010.g5985
contig00010.g6052
contig00011.g6439
contig00011.g6465
contig00011.g6474
contig00011.g6485
contig00011.g6506
contig00011.g6610
contig00012.g6693
contig00012.g6721
contig00012.g6756
contig00012.g6761
contig00012.g6792
contig00012.g6836
contig00012.g6858
contig00013.g7037
contig00013.g7047
contig00013.g7169
contig00013.g7214
contig00013.g7244
contig00013.g7282
contig00013.g7318
contig00013.g7350
contig00013.g7360
contig00014.g7467
contig00014.g7495
contig00014.g7496
contig00014.g7512
contig00014.g7530

contig00014.g7556
contig00014.g7558
contig00014.g7600
contig00014.g7623
contig00014.g7656
contig00014.g7667
contig00014.g7706
contig00015.g7861
contig00015.g7924
contig00015.g7948
contig00015.g7950
contig00015.g7956
contig00015.g7957
contig00015.g7968
contig00015.g8034
contig00016.g8203
contig00016.g8208
contig00016.g8213
contig00016.g8232
contig00016.g8289
contig00016.g8290
contig00016.g8293
contig00017.g8373
contig00017.g8375
contig00017.g8393
contig00017.g8423
contig00017.g8428
contig00017.g8432
contig00017.g8532
contig00017.g8591
contig00018.g8665
contig00018.g8693
contig00018.g8746

contig00018.g8811
contig00018.g8815
contig00018.g8851
contig00018.g8852
contig00018.g8890
contig00018.g8892
contig00018.g8897
contig00018.g8902
contig00018.g8904
contig00018.g8916
contig00018.g8934
contig00019.g9015
contig00019.g9037
contig00019.g9095
contig00019.g9113
contig00019.g9128
contig00019.g9169
contig00020.g9266
contig00020.g9295
contig00020.g9341
contig00020.g9354
contig00020.g9370
contig00020.g9374
contig00020.g9384
contig00020.g9402
contig00020.g9431
contig00021.g9501
contig00021.g9599
contig00022.g9836
contig00022.g9893
contig00022.g9911
contig00022.g10003
contig00023.g10080

contig00023.g10162
contig00024.g10402
contig00024.g10449
contig00024.g10556
contig00025.g10725
contig00025.g10754
contig00026.g10923
contig00026.g10960
contig00027.g11201
contig00028.g11218
contig00028.g11232
contig00028.g11292
contig00028.g11312
contig00029.g11349
contig00029.g11351
contig00029.g11451
contig00029.g11479
contig00030.g11520
contig00030.g11576
contig00030.g11595
contig00030.g11651
contig00031.g11684
contig00031.g11770
contig00031.g11796
contig00031.g11844
contig00032.g11901
contig00032.g11977
contig00032.g11989
contig00033.g12059
contig00033.g12083
contig00033.g12088
contig00033.g12102
contig00034.g12201

contig00034.g12283
contig00034.g12298
contig00035.g12323
contig00035.g12360
contig00035.g12386
contig00035.g12411
contig00036.g12528
contig00037.g12583
contig00037.g12692
contig00038.g12721
contig00038.g12763
contig00038.g12793
contig00039.g12831
contig00040.g12904
contig00041.g13000
contig00041.g13055
contig00042.g13149
contig00042.g13154
contig00045.g13361
contig00046.g13399
contig00047.g13483
contig00048.g13505
contig00051.g13638
contig00051.g13644
contig00051.g13652
contig00053.g13692
contig00053.g13695
contig00054.g13708
contig00054.g13733
contig00068.g13796
contig00233.g13832
contig00257.g13836
contig00294.g13841

contig00345.g13847

Gene ID of IUM05-160 of the effectors (effectorP- effectorp/SignalP) and gene id of the unique genes of the human strains compared to 7600.

Effectors (>90%)_IUM06-0160	SignalP (on Eff >90%)_IUM06-0160	118 unique_IUM06-0160
Fv_160_1.g31	Fv_160_1.g705	Fv_160_1.g540
Fv_160_1.g134	Fv_160_1.g742	Fv_160_2.g1093
Fv_160_1.g156	Fv_160_1.g933	Fv_160_2.g961
Fv_160_1.g170	Fv_160_2.g961	Fv_160_2.g1057
Fv_160_1.g177	Fv_160_2.g1335	Fv_160_2.g962
Fv_160_1.g289	Fv_160_3.g1870	Fv_160_2.g1091
Fv_160_1.g311	Fv_160_3.g1936	Fv_160_3.g2271
Fv_160_1.g318	Fv_160_3.g2292	Fv_160_3.g2265
Fv_160_1.g321	Fv_160_4.g2504	Fv_160_3.g2270
Fv_160_1.g385	Fv_160_4.g2700	Fv_160_3.g2264
Fv_160_1.g387	Fv_160_7.g4198	Fv_160_4.g2905
Fv_160_1.g388	Fv_160_7.g4259	Fv_160_5.g3009
Fv_160_1.g426	Fv_160_7.g4396	Fv_160_6.g4135
Fv_160_1.g454	Fv_160_8.g5061	Fv_160_6.g4128
Fv_160_1.g635	Fv_160_9.g5405	Fv_160_6.g4127
Fv_160_1.g705	Fv_160_9.g5411	Fv_160_6.g4141
Fv_160_1.g727	Fv_160_9.g5566	Fv_160_6.g4125
Fv_160_1.g742	Fv_160_9.g5591	Fv_160_6.g4131
Fv_160_1.g790	Fv_160_10.g5839	Fv_160_6.g4134
Fv_160_1.g791	Fv_160_10.g6149	Fv_160_6.g4136
Fv_160_1.g792	Fv_160_11.g6336	Fv_160_6.g3687
Fv_160_1.g794	Fv_160_11.g6709	Fv_160_6.g4129
Fv_160_1.g795	Fv_160_13.g7195	Fv_160_6.g4138
Fv_160_1.g880	Fv_160_13.g7218	Fv_160_6.g4139
Fv_160_1.g933	Fv_160_13.g7292	Fv_160_6.g4126
Fv_160_2.g961	Fv_160_13.g7415	Fv_160_6.g4130
Fv_160_2.g1037	Fv_160_15.g7972	Fv_160_6.g4124
Fv_160_2.g1076	Fv_160_18.g9081	Fv_160_6.g4132
Fv_160_2.g1077	Fv_160_18.g9101	Fv_160_7.g4377

Fv_160_2.g1107	Fv_160_21.g10079	Fv_160_7.g4554
Fv_160_2.g1137	Fv_160_21.g10089	Fv_160_8.g5121
Fv_160_2.g1194	Fv_160_22.g10243	Fv_160_8.g5118
Fv_160_2.g1203	Fv_160_22.g10293	Fv_160_8.g5083
Fv_160_2.g1335	Fv_160_22.g10332	Fv_160_8.g5085
Fv_160_2.g1358	Fv_160_25.g11088	Fv_160_8.g5156
Fv_160_2.g1385	Fv_160_25.g11161	Fv_160_8.g5078
Fv_160_2.g1421	Fv_160_27.g11389	Fv_160_8.g5122
Fv_160_2.g1506	Fv_160_29.g11823	Fv_160_8.g5081
Fv_160_2.g1520	Fv_160_30.g12049	Fv_160_8.g5120
Fv_160_2.g1545	Fv_160_30.g12073	Fv_160_8.g5119
Fv_160_2.g1657	Fv_160_31.g12148	Fv_160_8.g5084
Fv_160_3.g1678	Fv_160_32.g12382	Fv_160_8.g5086
Fv_160_3.g1703	Fv_160_36.g12875	Fv_160_8.g5076
Fv_160_3.g1717	Fv_160_45.g13854	Fv_160_11.g6714
Fv_160_3.g1747	Fv_160_49.g14139	Fv_160_11.g6712
Fv_160_3.g1820		Fv_160_13.g7417
Fv_160_3.g1839		Fv_160_13.g7549
Fv_160_3.g1856		Fv_160_13.g7550
Fv_160_3.g1870		Fv_160_13.g7515
Fv_160_3.g1905		Fv_160_13.g7518
Fv_160_3.g1936		Fv_160_13.g7516
Fv_160_3.g1972		Fv_160_13.g7244
Fv_160_3.g2016		Fv_160_17.g8893
Fv_160_3.g2031		Fv_160_17.g8895
Fv_160_3.g2075		Fv_160_17.g8854
Fv_160_3.g2099		Fv_160_17.g8896
Fv_160_3.g2156		Fv_160_17.g8847
Fv_160_3.g2214		Fv_160_17.g8894
Fv_160_3.g2229		Fv_160_18.g9188
Fv_160_3.g2274		Fv_160_18.g9187
Fv_160_3.g2292		Fv_160_18.g9189
Fv_160_3.g2313		Fv_160_18.g9190

Fv_160_4.g2387
Fv_160_4.g2504
Fv_160_4.g2516
Fv_160_4.g2592
Fv_160_4.g2636
Fv_160_4.g2652
Fv_160_4.g2700
Fv_160_4.g2801
Fv_160_4.g2826
Fv_160_4.g2892
Fv_160_5.g3064
Fv_160_5.g3089
Fv_160_5.g3160
Fv_160_5.g3251
Fv_160_5.g3415
Fv_160_5.g3444
Fv_160_5.g3545
Fv_160_5.g3547
Fv_160_6.g3702
Fv_160_6.g3754
Fv_160_6.g3859
Fv_160_6.g3881
Fv_160_6.g3993
Fv_160_6.g4045
Fv_160_7.g4184
Fv_160_7.g4185
Fv_160_7.g4198
Fv_160_7.g4233
Fv_160_7.g4259
Fv_160_7.g4359
Fv_160_7.g4377
Fv_160_7.g4396
Fv_160_7.g4746
Fv_160_18.g9157
Fv_160_18.g9137
Fv_160_21.g9985
Fv_160_22.g10292
Fv_160_25.g11102
Fv_160_25.g11103
Fv_160_29.g11883
Fv_160_29.g11827
Fv_160_29.g11884
Fv_160_30.g11958
Fv_160_30.g12036
Fv_160_30.g12048
Fv_160_30.g11968
Fv_160_30.g12038
Fv_160_30.g12007
Fv_160_30.g11961
Fv_160_30.g11976
Fv_160_30.g12041
Fv_160_30.g11964
Fv_160_30.g11986
Fv_160_30.g12008
Fv_160_30.g12003
Fv_160_30.g12055
Fv_160_30.g12011
Fv_160_30.g11988
Fv_160_30.g11970
Fv_160_30.g11959
Fv_160_30.g11969
Fv_160_30.g11980
Fv_160_30.g12037
Fv_160_30.g11962
Fv_160_30.g12049
Fv_160_30.g11996

Fv_160_7.g4749
Fv_160_8.g4783
Fv_160_8.g4831
Fv_160_8.g4836
Fv_160_8.g4921
Fv_160_8.g4959
Fv_160_8.g4989
Fv_160_8.g5061
Fv_160_8.g5156
Fv_160_8.g5231
Fv_160_9.g5381
Fv_160_9.g5383
Fv_160_9.g5405
Fv_160_9.g5411
Fv_160_9.g5428
Fv_160_9.g5449
Fv_160_9.g5484
Fv_160_9.g5517
Fv_160_9.g5566
Fv_160_9.g5576
Fv_160_9.g5591
Fv_160_9.g5653
Fv_160_9.g5666
Fv_160_9.g5695
Fv_160_9.g5705
Fv_160_9.g5783
Fv_160_9.g5787
Fv_160_10.g5839
Fv_160_10.g5904
Fv_160_10.g5934
Fv_160_10.g5935
Fv_160_10.g5951
Fv_160_10.g5969
Fv_160_30.g11972
Fv_160_30.g12040
Fv_160_30.g12039
Fv_160_30.g11960
Fv_160_30.g11999
Fv_160_30.g11965
Fv_160_30.g11987
Fv_160_30.g11975
Fv_160_30.g12004
Fv_160_30.g11997
Fv_160_30.g11963
Fv_160_30.g12009
Fv_160_30.g11979
Fv_160_30.g12010
Fv_160_30.g11990
Fv_160_30.g12002
Fv_160_36.g12964
Fv_160_36.g12963
Fv_160_36.g12965
Fv_160_36.g12960
Fv_160_45.g13895
Fv_160_49.g14140
Fv_160_66.g14357

Fv_160_10.g5994
Fv_160_10.g5996
Fv_160_10.g6041
Fv_160_10.g6066
Fv_160_10.g6087
Fv_160_10.g6098
Fv_160_10.g6109
Fv_160_10.g6149
Fv_160_11.g6263
Fv_160_11.g6326
Fv_160_11.g6336
Fv_160_11.g6409
Fv_160_11.g6468
Fv_160_11.g6552
Fv_160_11.g6617
Fv_160_11.g6685
Fv_160_11.g6709
Fv_160_11.g6712
Fv_160_11.g6719
Fv_160_12.g6757
Fv_160_12.g6793
Fv_160_12.g6795
Fv_160_12.g6807
Fv_160_12.g6824
Fv_160_12.g6830
Fv_160_12.g6838
Fv_160_12.g6892
Fv_160_12.g6916
Fv_160_12.g6922
Fv_160_12.g6958
Fv_160_12.g6989
Fv_160_12.g7037
Fv_160_12.g7050

Fv_160_12.g7052
Fv_160_12.g7067
Fv_160_12.g7071
Fv_160_12.g7081
Fv_160_12.g7099
Fv_160_13.g7195
Fv_160_13.g7214
Fv_160_13.g7218
Fv_160_13.g7231
Fv_160_13.g7292
Fv_160_13.g7351
Fv_160_13.g7360
Fv_160_13.g7367
Fv_160_13.g7388
Fv_160_13.g7415
Fv_160_13.g7441
Fv_160_13.g7456
Fv_160_13.g7569
Fv_160_14.g7600
Fv_160_14.g7631
Fv_160_14.g7670
Fv_160_14.g7727
Fv_160_14.g7746
Fv_160_14.g7802
Fv_160_14.g7838
Fv_160_14.g7938
Fv_160_15.g7972
Fv_160_15.g7996
Fv_160_15.g8001
Fv_160_15.g8158
Fv_160_15.g8231
Fv_160_15.g8234
Fv_160_15.g8244

Fv_160_16.g8276
Fv_160_16.g8281
Fv_160_16.g8386
Fv_160_16.g8526
Fv_160_16.g8552
Fv_160_16.g8554
Fv_160_16.g8560
Fv_160_16.g8561
Fv_160_16.g8573
Fv_160_17.g8620
Fv_160_17.g8740
Fv_160_17.g8741
Fv_160_17.g8746
Fv_160_17.g8753
Fv_160_17.g8769
Fv_160_17.g8787
Fv_160_17.g8798
Fv_160_17.g8831
Fv_160_17.g8873
Fv_160_17.g8882
Fv_160_17.g8900
Fv_160_17.g8944
Fv_160_17.g8948
Fv_160_18.g9059
Fv_160_18.g9073
Fv_160_18.g9081
Fv_160_18.g9092
Fv_160_18.g9101
Fv_160_18.g9224
Fv_160_18.g9289
Fv_160_19.g9298
Fv_160_19.g9310
Fv_160_19.g9319

Fv_160_19.g9380
Fv_160_19.g9429
Fv_160_19.g9466
Fv_160_20.g9560
Fv_160_20.g9587
Fv_160_20.g9835
Fv_160_21.g9882
Fv_160_21.g9931
Fv_160_21.g9960
Fv_160_21.g10079
Fv_160_21.g10089
Fv_160_21.g10098
Fv_160_21.g10176
Fv_160_22.g10220
Fv_160_22.g10235
Fv_160_22.g10237
Fv_160_22.g10243
Fv_160_22.g10244
Fv_160_22.g10248
Fv_160_22.g10251
Fv_160_22.g10254
Fv_160_22.g10293
Fv_160_22.g10314
Fv_160_22.g10332
Fv_160_22.g10410
Fv_160_22.g10420
Fv_160_22.g10464
Fv_160_22.g10493
Fv_160_22.g10495
Fv_160_23.g10660
Fv_160_23.g10682
Fv_160_23.g10736
Fv_160_23.g10739

Fv_160_24.g10806
Fv_160_24.g10839
Fv_160_24.g10919
Fv_160_25.g10981
Fv_160_25.g11088
Fv_160_25.g11117
Fv_160_25.g11161
Fv_160_26.g11192
Fv_160_26.g11214
Fv_160_26.g11263
Fv_160_27.g11389
Fv_160_27.g11492
Fv_160_27.g11511
Fv_160_27.g11548
Fv_160_27.g11571
Fv_160_28.g11607
Fv_160_29.g11774
Fv_160_29.g11823
Fv_160_29.g11836
Fv_160_29.g11838
Fv_160_30.g11987
Fv_160_30.g12049
Fv_160_30.g12073
Fv_160_31.g12148
Fv_160_31.g12201
Fv_160_31.g12261
Fv_160_31.g12265
Fv_160_31.g12270
Fv_160_31.g12277
Fv_160_32.g12330
Fv_160_32.g12357
Fv_160_32.g12382
Fv_160_33.g12474

Fv_160_33.g12517
Fv_160_33.g12556
Fv_160_33.g12588
Fv_160_34.g12592
Fv_160_34.g12621
Fv_160_34.g12632
Fv_160_34.g12656
Fv_160_34.g12691
Fv_160_34.g12712
Fv_160_35.g12801
Fv_160_35.g12805
Fv_160_35.g12820
Fv_160_35.g12841
Fv_160_35.g12847
Fv_160_36.g12875
Fv_160_36.g12883
Fv_160_36.g12971
Fv_160_36.g12999
Fv_160_37.g13048
Fv_160_37.g13109
Fv_160_38.g13118
Fv_160_38.g13136
Fv_160_38.g13173
Fv_160_38.g13200
Fv_160_38.g13227
Fv_160_39.g13347
Fv_160_40.g13368
Fv_160_40.g13392
Fv_160_40.g13397
Fv_160_40.g13412
Fv_160_41.g13455
Fv_160_41.g13516
Fv_160_41.g13525

Fv_160_41.g13526
Fv_160_42.g13599
Fv_160_43.g13733
Fv_160_44.g13760
Fv_160_44.g13762
Fv_160_45.g13854
Fv_160_45.g13909
Fv_160_46.g13953
Fv_160_46.g13961
Fv_160_47.g13997
Fv_160_47.g14010
Fv_160_47.g14037
Fv_160_48.g14059
Fv_160_49.g14139
Fv_160_50.g14174
Fv_160_66.g14358
Fv_160_67.g14369
