# Supplementary material for: Genomic Insights into Fusarium verticillioides Diversity: The Genome of Two Clinical Isolates and Their Demethylase Inhibitor Fungicides Susceptibility
Source: Pathogens. 2024 Dec 3;13(12):1062. doi: 10.3390/pathogens13121062 (PMC11728828; doi:10.3390/pathogens13121062)
Supplement: Supplementary file 1 [file pathogens-13-01062-s001.zip › Table S7.pdf]

# BLASTP 2.2.30+											
# Query: contig00001.g79											
# Database: /data/phi-blast/fasta/Phi417/phi417.fas											
# Fields: query id	subject id	% identity	alignment length	mismatches	gap opens	q. start	q. end	s. start	s. end	evalue	bit score
# 4 hits found											
contig00001.g79	I1RNU2#PHI:1895#GzZC210#5518#Fusarium_graminearum#unaffected_pathogenicity	27.42	62	40	1	23	84	5	61	1.5	27.3
# BLASTP 2.2.30+											
# Query: contig00001.g141											
# Database: /data/phi-blast/fasta/Phi417/phi417.fas											
# Fields: query id	subject id	% identity	alignment length	mismatches	gap opens	q. start	q. end	s. start	s. end	evalue	bit score
# 9 hits found											
contig00001.g141	D4I1T1#PHI:7105#TssM-2#552#Erwinia_amylovora#unaffected_pathogenic	32.89	76	47	2	4	76	525	599	0.26	27.7
# BLASTP 2.2.30+											
# Query: contig00001.g156											
# Database: /data/phi-blast/fasta/Phi417/phi417.fas											
# Fields: query id	subject id	% identity	alignment length	mismatches	gap opens	q. start	q. end	s. start	s. end	evalue	bit score
# 5 hits found											
contig00001.g156	J4KLZ9#PHI:7155#Pmr1#176275#Beauveria_bassiana#reduced_virulence	43.48	46	21	2	1	45	190	231	0.056	32.0
# BLASTP 2.2.30+											
# Query: contig00001.g161											
# Database: /data/phi-blast/fasta/Phi417/phi417.fas											
# Fields: query id	subject id	% identity	alignment length	mismatches	gap opens	q. start	q. end	s. start	s. end	evalue	bit score
# 1 hits found											
contig00001.g161	Q9LBD5#PHI:10677#Hpa2#347#Xanthomonas_oryzae#reduced_virulence	32.65	49	32	1	19	67	32	79	0.96	26.6
# BLASTP 2.2.30+											
# Query: contig00001.g209											
# Database: /data/phi-blast/fasta/Phi417/phi417.fas											
# Fields: query id	subject id	% identity	alignment length	mismatches	gap opens	q. start	q. end	s. start	s. end	evalue	bit score
# 10 hits found											
contig00001.g209	I1S1A3#PHI:9095#Fgvps60_(FGSG_10503)#5518#Fusarium_graminearum#loss_of_pathogenicity	97.64	212	5	0	1	212	1	212	9,00E-151	420
# BLASTP 2.2.30+											
# Query: contig00001.g290											
# Database: /data/phi-blast/fasta/Phi417/phi417.fas											

# Fields: query id<br># 8 hits found	subject id	% identity	alignment length	mismatches	gap opens	q. start	q. end	s. start	s. end	evalue	bit score
contig00001.g290	Q5HJX6#PHI:9972#YycG#1280#Staphylococcus_aureus#reduced_virulence	27.71	83	48	4	7	87	394	466	0.14	29.3
# BLASTP 2.2.30+<br># Query: contig00001.g348<br># Database: /data/phi-blast/fasta/Phi417/phi417.fas											
# Fields: query id<br># 3 hits found	subject id	% identity	alignment length	mismatches	gap opens	q. start	q. end	s. start	s. end	evalue	bit score
contig00001.g348	A0A1C3YM49#PHI:1312#GzbHLH013#5518#Fusarium_graminearum#unaffected_pathogenicity	22.68	97	69	2	4	97	762	855	2.3	26.9
# BLASTP 2.2.30+<br># Query: contig00001.g389<br># Database: /data/phi-blast/fasta/Phi417/phi417.fas											
# Fields: query id<br># 10 hits found	subject id	% identity	alignment length	mismatches	gap opens	q. start	q. end	s. start	s. end	evalue	bit score
contig00001.g389	B4EDS9#PHI:5297#BCAL2200#95486#Burkholderia_cenocepacia#reduced_virulence	40.52	153	88	2	4	156	3	152	2,00E-34	120
# BLASTP 2.2.30+<br># Query: contig00001.g424<br># Database: /data/phi-blast/fasta/Phi417/phi417.fas											
# Fields: query id<br># 18 hits found	subject id	% identity	alignment length	mismatches	gap opens	q. start	q. end	s. start	s. end	evalue	bit score
contig00001.g424	Q0R411#PHI:6417#Sm1#29875#Trichoderma_virens#reduced_virulence	58.70	138	56	1	1	137	1	138	2,00E-54	169
# BLASTP 2.2.30+<br># Query: contig00001.g455<br># Database: /data/phi-blast/fasta/Phi417/phi417.fas											
# Fields: query id<br># 2 hits found	subject id	% identity	alignment length	mismatches	gap opens	q. start	q. end	s. start	s. end	evalue	bit score
contig00001.g455	O13359#PHI:306_PHI:9474#KEX2_Kex2#5476#Candida_albicans#reduced_virulence	35.09	57	35	1	21	77	410	464	0.42	28.1
# BLASTP 2.2.30+<br># Query: contig00001.g490<br># Database: /data/phi-blast/fasta/Phi417/phi417.fas											
# Fields: query id<br># 7 hits found	subject id	% identity	alignment length	mismatches	gap opens	q. start	q. end	s. start	s. end	evalue	bit score
contig00001.g490	P06996#PHI:7024#OmpC#562#Escherichia_coli#reduced_virulence	26.92	78	53	2	37	114	18	91	1.0	28.1

# BLASTP 2.2.30+											
# Query: contig00001.g503											
# Database: /data/phi-blast/fasta/Phi417/phi417.fas											
# Fields: query id	subject id	% identity	alignment length	mismatches	gap opens	q. start	q. end	s. start	s. end	evalue	bit score
# 24 hits found											
contig00001.g503	A0A0E0S4Y7#PHI:8646#FgLDHL2_(FGSG_16220)#5518#Fusarium_graminearum#reduced_virulence	40.32	62	36	1	8	69	6	66	8,00E-10	53.5
# BLASTP 2.2.30+											
# Query: contig00001.g537											
# Database: /data/phi-blast/fasta/Phi417/phi417.fas											
# Fields: query id	subject id	% identity	alignment length	mismatches	gap opens	q. start	q. end	s. start	s. end	evalue	bit score
# 14 hits found											
contig00001.g537	A0A125YP82#PHI:124379#MAPR_(TGME49_276990)#5811#Toxoplasma_gondii#reduced_virulence	36.90	84	46	3	38	114	131	214	8,00E-09	50.4
# BLASTP 2.2.30+											
# Query: contig00001.g580											
# Database: /data/phi-blast/fasta/Phi417/phi417.fas											
# Fields: query id	subject id	% identity	alignment length	mismatches	gap opens	q. start	q. end	s. start	s. end	evalue	bit score
# 9 hits found											
contig00001.g580	G4MVC5#PHI:6927#MoRAD6#318829#Magnaporthe_oryzae#reduced_virulence	28.70	115	81	1	54	167	13	127	3,00E-18	77.0
# BLASTP 2.2.30+											
# Query: contig00001.g610											
# Database: /data/phi-blast/fasta/Phi417/phi417.fas											
# Fields: query id	subject id	% identity	alignment length	mismatches	gap opens	q. start	q. end	s. start	s. end	evalue	bit score
# 3 hits found											
contig00001.g610	A0A0E0SG97#PHI:1690#GzZC005#5518#Fusarium_graminearum#unaffected_pathogenicity	29.09	55	35	1	76	130	331	381	0.58	28.5
# BLASTP 2.2.30+											
# Query: contig00001.g639											
# Database: /data/phi-blast/fasta/Phi417/phi417.fas											
# Fields: query id	subject id	% identity	alignment length	mismatches	gap opens	q. start	q. end	s. start	s. end	evalue	bit score
# 5 hits found											
contig00001.g639	G3XDB9#PHI:987#HopAA1-1#317#Pseudomonas_syringae#effector_(plant_avirulence_determinant)	33.96	53	33	1	7	57	145	197	2.2	27.3
# BLASTP 2.2.30+											
# Query: contig00001.g653											

# Database: /data/phi-blast/fasta/Phi417/phi417.fas											
# Fields: query id	subject id	% identity	alignment length	mismatches	gap opens	q. start	q. end	s. start	s. end	evalue	bit score
# 8 hits found											
contig00001.g653	A0A4E9DG31#PHI:123820#AP1sigma_(FGSG_10034)#5518#Fusarium_graminearum#loss_of_pathoge	94.70	151	8	0	1	151	1	151	1,00E-104	299
# BLASTP 2.2.30+											
# Query: contig00001.g734											
# Database: /data/phi-blast/fasta/Phi417/phi417.fas											
# Fields: query id	subject id	% identity	alignment length	mismatches	gap opens	q. start	q. end	s. start	s. end	evalue	bit score
# 4 hits found											
contig00001.g734	A1E140#PHI:4050#Rop18#5811#Toxoplasma_gondii#reduced_virulence_loss_of_pathogenicity	25.61	82	47	2	1	68	240	321	4.0	25.0
# BLASTP 2.2.30+											
# Query: contig00002.g896											
# Database: /data/phi-blast/fasta/Phi417/phi417.fas											
# Fields: query id	subject id	% identity	alignment length	mismatches	gap opens	q. start	q. end	s. start	s. end	evalue	bit score
# 4 hits found											
contig00002.g896	Q0E7C4#PHI:12092#VabF#55601#Vibrio_anguillarum#reduced_virulence	27.27	55	37	1	33	84	2655	2709	0.88	28.5
# BLASTP 2.2.30+											
# Query: contig00002.g918											
# Database: /data/phi-blast/fasta/Phi417/phi417.fas											
# Fields: query id	subject id	% identity	alignment length	mismatches	gap opens	q. start	q. end	s. start	s. end	evalue	bit score
# 5 hits found											
contig00002.g918	G2XDS1#PHI:8052#Vdmyo5#27337#Verticillium_dahliae#reduced_virulence	29.21	89	47	3	164	236	938	1026	1.3	28.9
# BLASTP 2.2.30+											
# Query: contig00002.g931											
# Database: /data/phi-blast/fasta/Phi417/phi417.fas											
# Fields: query id	subject id	% identity	alignment length	mismatches	gap opens	q. start	q. end	s. start	s. end	evalue	bit score
# 9 hits found											
contig00002.g931	E9EJ96#PHI:5431#Mero-Pbs2#568076#Metarhizium_robertsii#reduced_vir	32.67	101	53	5	7	92	226	326	0.12	29.3
# BLASTP 2.2.30+											
# Query: contig00002.g1023											
# Database: /data/phi-blast/fasta/Phi417/phi417.fas											
# 0 hits found											
# BLASTP 2.2.30+											

# Query: contig00002.g1043											
# Database: /data/phi-blast/fasta/Phi417/phi417.fas											
# Fields: query id	subject id	% identity	alignment length	mismatches	gap opens	q. start	q. end	s. start	s. end	evalue	bit score
# 9 hits found											
contig00002.g1043	Q63KU5#PHI:7014#GlbC#28450#Burkholderia_pseudomallei#increased_virulence_(hypervirulence)	39.29	28	17	0	40	67	2461	2488	0.51	26.9
# BLASTP 2.2.30+											
# Query: contig00002.g1065											
# Database: /data/phi-blast/fasta/Phi417/phi417.fas											
# Fields: query id	subject id	% identity	alignment length	mismatches	gap opens	q. start	q. end	s. start	s. end	evalue	bit score
# 5 hits found											
contig00002.g1065	N4X9G3#PHI:4233#Ftr1#5016#Bipolaris_maydis#reduced_virulence	40.00	40	23	1	38	76	315	354	0.46	27.7
# BLASTP 2.2.30+											
# Query: contig00002.g1072											
# Database: /data/phi-blast/fasta/Phi417/phi417.fas											
# Fields: query id	subject id	% identity	alignment length	mismatches	gap opens	q. start	q. end	s. start	s. end	evalue	bit score
# 4 hits found											
contig00002.g1072	G2WY50#PHI:4909#VdQase#27337#Verticillium_dahliae#reduced_virulence	21.05	57	37	1	8	64	597	645	0.64	26.6
# BLASTP 2.2.30+											
# Query: contig00002.g1075											
# Database: /data/phi-blast/fasta/Phi417/phi417.fas											
# Fields: query id	subject id	% identity	alignment length	mismatches	gap opens	q. start	q. end	s. start	s. end	evalue	bit score
# 12 hits found											
contig00002.g1075	I1SAJ7#PHI:9042#Nrps5_(FGSG_13878)#5518#Fusarium_graminearum#reduced_virulence	30.77	78	37	4	7	67	2344	2421	1.5	25.8
# BLASTP 2.2.30+											
# Query: contig00002.g1082											
# Database: /data/phi-blast/fasta/Phi417/phi417.fas											
# Fields: query id	subject id	% identity	alignment length	mismatches	gap opens	q. start	q. end	s. start	s. end	evalue	bit score
# 7 hits found											
contig00002.g1082	B8N3U5#PHI:3880_PHI:123103#MeaB_MeaB_(AFLA_031790)#5059#Aspergillus_flavus#reduced_virulence_unaffected_pathogenicity	35.00	40	26	0	3	42	15	54	1.6	25.8
# BLASTP 2.2.30+											
# Query: contig00002.g1139											
# Database: /data/phi-blast/fasta/Phi417/phi417.fas											

# Fields: query id	subject id	% identity	alignment length	mismatches	gap opens	q. start	q. end	s. start	s. end	evalue	bit score
# 6 hits found											
contig00002.g1139	A4VTH5#PHI:5301#AgaR1#1307#Streptococcus_suis#unaffected_pathogenicity	29.51	61	39	1	59	119	1	57	1.1	28.1
# BLASTP 2.2.30+											
# Query: contig00002.g1176											
# Database: /data/phi-blast/fasta/Phi417/phi417.fas											
# Fields: query id	subject id	% identity	alignment length	mismatches	gap opens	q. start	q. end	s. start	s. end	evalue	bit score
# 6 hits found											
contig00002.g1176	I1RNG8#PHI:1176#(Sc_Atg1)#5518#Fusarium_graminearum#reduced_virulence	27.78	36	26	0	11	46	687	722	2.4	25.0
# BLASTP 2.2.30+											
# Query: contig00002.g1203											
# Database: /data/phi-blast/fasta/Phi417/phi417.fas											
# Fields: query id	subject id	% identity	alignment length	mismatches	gap opens	q. start	q. end	s. start	s. end	evalue	bit score
# 371 hits found											
contig00002.g1203	I1RAY2#PHI:1218_PHI:9822#FGSG_00677_Fg00677#5518#Fusarium_graminearum#lethal_reduced_	99.12	340	3	0	1	340	1	340	0.0	701
# BLASTP 2.2.30+											
# Query: contig00002.g1292											
# Database: /data/phi-blast/fasta/Phi417/phi417.fas											
# Fields: query id	subject id	% identity	alignment length	mismatches	gap opens	q. start	q. end	s. start	s. end	evalue	bit score
# 163 hits found											
contig00002.g1292	I1RB57#PHI:1346#GzC2H006#5518#Fusarium_graminearum#unaffected_pathogenicity	68.63	153	45	2	1	150	1	153	2,00E-63	194
# BLASTP 2.2.30+											
# Query: contig00002.g1380											
# Database: /data/phi-blast/fasta/Phi417/phi417.fas											
# Fields: query id	subject id	% identity	alignment length	mismatches	gap opens	q. start	q. end	s. start	s. end	evalue	bit score
# 20 hits found											
contig00002.g1380	A0A2H3G405#PHI:124279#Gcn5#5518#Fusarium_graminearum#loss_of_pathogenicity_reduced_viru	97.98	396	8	0	1	396	1	396	0.0	815
# BLASTP 2.2.30+											
# Query: contig00002.g1412											
# Database: /data/phi-blast/fasta/Phi417/phi417.fas											
# Fields: query id	subject id	% identity	alignment length	mismatches	gap opens	q. start	q. end	s. start	s. end	evalue	bit score
# 7 hits found											
contig00002.g1412	Q2FWE8#PHI:10773#AtpA#1280#Staphylococcus_aureus#reduced_virulence	31.17	77	36	3	21	91	125	190	0.35	27.7

# BLASTP 2.2.30+											
# Query: contig00002.g1445											
# Database: /data/phi-blast/fasta/Phi417/phi417.fas											
# Fields: query id	subject id	% identity	alignment length	mismatches	gap opens	q. start	q. end	s. start	s. end	evalue	bit score
# 1 hits found											
contig00002.g1445	Q4W9V1#PHI:124370#Erg6#746128#Aspergillus_fumigatus#reduced_virulence	33.33	33	22	0	92	124	26	58	3.2	26.2
# BLASTP 2.2.30+											
# Query: contig00002.g1479											
# Database: /data/phi-blast/fasta/Phi417/phi417.fas											
# Fields: query id	subject id	% identity	alignment length	mismatches	gap opens	q. start	q. end	s. start	s. end	evalue	bit score
# 2 hits found											
contig00002.g1479	G4N6S9#PHI:124191#CEP3_(MGG_03671)#318829#Magnaporthe_oryzae#effector_(plant_avirulence_determinant)	35.48	93	45	6	1	78	1	93	6,00E-05	37.4
# BLASTP 2.2.30+											
# Query: contig00002.g1525											
# Database: /data/phi-blast/fasta/Phi417/phi417.fas											
# Fields: query id	subject id	% identity	alignment length	mismatches	gap opens	q. start	q. end	s. start	s. end	evalue	bit score
# 1 hits found											
contig00002.g1525	B1GVX4#PHI:2289#BcBOA2#40559#Botrytis_cinerea#reduced_virulence	43.48	23	13	0	7	29	134	156	5.2	24.6
# BLASTP 2.2.30+											
# Query: contig00002.g1569											
# Database: /data/phi-blast/fasta/Phi417/phi417.fas											
# Fields: query id	subject id	% identity	alignment length	mismatches	gap opens	q. start	q. end	s. start	s. end	evalue	bit score
# 1 hits found											
contig00002.g1569	A0A0H2WAC2#PHI:5327#BsaO#28450#Burkholderia_pseudomallei#effector_(plant_avirulence_deter	36.36	33	20	1	4	35	417	449	2.6	26.9
# BLASTP 2.2.30+											
# Query: contig00003.g1679											
# Database: /data/phi-blast/fasta/Phi417/phi417.fas											
# Fields: query id	subject id	% identity	alignment length	mismatches	gap opens	q. start	q. end	s. start	s. end	evalue	bit score
# 7 hits found											
contig00003.g1679	G4MQP9#PHI:2177#PAS1#318829#Magnaporthe_oryzae#unaffected_pathogenicity	29.41	34	24	0	38	71	1015	1048	1.5	26.6
# BLASTP 2.2.30+											
# Query: contig00003.g1707											

# Database: /data/phi-blast/fasta/Phi417/phi417.fas											
# Fields: query id	subject id	% identity	alignment length	mismatches	gap opens	q. start	q. end	s. start	s. end	evalue	bit score
# 1 hits found											
contig00003.g1707	Q8P712#PHI:10384#RpoN1_(XCC2802)#339#Xanthomonas_campestris#reduced_virulence	27.14	70	51	0	130	199	39	108	3.1	27.3
# BLASTP 2.2.30+											
# Query: contig00003.g1708											
# Database: /data/phi-blast/fasta/Phi417/phi417.fas											
# Fields: query id	subject id	% identity	alignment length	mismatches	gap opens	q. start	q. end	s. start	s. end	evalue	bit score
# 6 hits found											
contig00003.g1708	A0A1D8PF20#PHI:9413#RTG3#5476#Candida_albicans#reduced_virulence	31.91	47	31	1	30	75	315	361	2.2	26.6
# BLASTP 2.2.30+											
# Query: contig00003.g1738											
# Database: /data/phi-blast/fasta/Phi417/phi417.fas											
# Fields: query id	subject id	% identity	alignment length	mismatches	gap opens	q. start	q. end	s. start	s. end	evalue	bit score
# 5 hits found											
contig00003.g1738	A0A098DHQ5#PHI:1908#GzZC223#5518#Fusarium_graminearum#unaffected_pathogenicity	32.91	79	45	1	7	85	294	364	0.25	29.6
# BLASTP 2.2.30+											
# Query: contig00003.g1785											
# Database: /data/phi-blast/fasta/Phi417/phi417.fas											
# Fields: query id	subject id	% identity	alignment length	mismatches	gap opens	q. start	q. end	s. start	s. end	evalue	bit score
# 2 hits found											
contig00003.g1785	Q8Z111#PHI:5009#T4519#28901#Salmonella_enterica#reduced_virulence	30.61	49	34	0	80	128	73	121	6.4	25.8
# BLASTP 2.2.30+											
# Query: contig00003.g1786											
# Database: /data/phi-blast/fasta/Phi417/phi417.fas											
# Fields: query id	subject id	% identity	alignment length	mismatches	gap opens	q. start	q. end	s. start	s. end	evalue	bit score
# 2 hits found											
contig00003.g1786	Q4WW81#PHI:6256_PHI:7875#FleA#746128#Aspergillus_fumigatus#increased_virulence_(hypervirul	25.81	93	51	5	80	171	71	146	0.60	28.9
# BLASTP 2.2.30+											
# Query: contig00003.g1798											
# Database: /data/phi-blast/fasta/Phi417/phi417.fas											
# Fields: query id	subject id	% identity	alignment length	mismatches	gap opens	q. start	q. end	s. start	s. end	evalue	bit score
# 5 hits found											

contig00003.g1798	Q5A7M9#PHI:4992#Rhr2#5476#Candida_albicans#reduced_virulence	44.00	25	14	0	159	183	47	71	0.063	32.0
# BLASTP 2.2.30+											
# Query: contig00003.g1812											
# Database: /data/phi-blast/fasta/Phi417/phi417.fas											
# Fields: query id	subject id	% identity	alignment length	mismatches	gap opens	q. start	q. end	s. start	s. end	evalue	bit score
# 5 hits found											
contig00003.g1812	Q9HFW0#PHI:227#PKA1#5207#Cryptococcus_neoformans#reduced_virulence_loss_of_pathogenicity	26.76	71	50	2	49	117	423	493	0.16	30.8
# BLASTP 2.2.30+											
# Query: contig00003.g1821											
# Database: /data/phi-blast/fasta/Phi417/phi417.fas											
# Fields: query id	subject id	% identity	alignment length	mismatches	gap opens	q. start	q. end	s. start	s. end	evalue	bit score
# 3 hits found											
contig00003.g1821	I1RIV2#PHI:3657#NPS6#5518#Fusarium_graminearum#reduced_virulence	30.23	43	21	1	79	121	1291	1324	4.4	26.2
# BLASTP 2.2.30+											
# Query: contig00003.g1875											
# Database: /data/phi-blast/fasta/Phi417/phi417.fas											
# Fields: query id	subject id	% identity	alignment length	mismatches	gap opens	q. start	q. end	s. start	s. end	evalue	bit score
# 5 hits found											
contig00003.g1875	A0A0H5ASD5#PHI:6549#PblB#1313#Streptococcus_pneumoniae#unaffected_pathogenicity	36.59	41	26	0	75	115	559	599	4.0	26.6
# BLASTP 2.2.30+											
# Query: contig00003.g1878											
# Database: /data/phi-blast/fasta/Phi417/phi417.fas											
# Fields: query id	subject id	% identity	alignment length	mismatches	gap opens	q. start	q. end	s. start	s. end	evalue	bit score
# 10 hits found											
contig00003.g1878	A0A1D8PQ78#PHI:9146#CaCDC50_(orf19.5735)#5476#Candida_albicans#reduced_virulence	37.04	54	25	2	6	59	352	396	0.81	27.7
# BLASTP 2.2.30+											
# Query: contig00003.g1935											
# Database: /data/phi-blast/fasta/Phi417/phi417.fas											
# Fields: query id	subject id	% identity	alignment length	mismatches	gap opens	q. start	q. end	s. start	s. end	evalue	bit score
# 4 hits found											
contig00003.g1935	Q75T35#PHI:332#CAC1#5462#Colletotrichum_lagenaria#loss_of_pathogenicity	30.91	55	35	2	28	81	923	975	3.7	25.4
# BLASTP 2.2.30+											
# Query: contig00003.g1946											

# Database: /data/phi-blast/fasta/Phi417/phi417.fas											
# Fields: query id	subject id	% identity	alignment length	mismatches	gap opens	q. start	q. end	s. start	s. end	evalue	bit score
# 27 hits found											
contig00003.g1946	I1RII8#PHI:9746_PHI:11940#FGSG_03624#5518#Fusarium_graminearum#unaffected_pathogenicity_reduced_virulence	73.82	233	53	3	1	231	1	227	9,00E-121	345
# BLASTP 2.2.30+											
# Query: contig00003.g1950											
# Database: /data/phi-blast/fasta/Phi417/phi417.fas											
# Fields: query id	subject id	% identity	alignment length	mismatches	gap opens	q. start	q. end	s. start	s. end	evalue	bit score
# 2 hits found											
contig00003.g1950	I1RN14#PHI:3658#NPS2#5518#Fusarium_graminearum#unaffected_pathogenicity_reduced_virulence	28.95	38	26	1	77	113	1811	1848	2.8	26.2
# BLASTP 2.2.30+											
# Query: contig00003.g2047											
# Database: /data/phi-blast/fasta/Phi417/phi417.fas											
# Fields: query id	subject id	% identity	alignment length	mismatches	gap opens	q. start	q. end	s. start	s. end	evalue	bit score
# 5 hits found											
contig00003.g2047	A0A0H2WKF8#PHI:5345#WcbQ#28450#Burkholderia_pseudomallei#reduced_virulence	32.73	55	25	1	208	250	387	441	1.2	29.3
# BLASTP 2.2.30+											
# Query: contig00003.g2118											
# Database: /data/phi-blast/fasta/Phi417/phi417.fas											
# Fields: query id	subject id	% identity	alignment length	mismatches	gap opens	q. start	q. end	s. start	s. end	evalue	bit score
# 6 hits found											
contig00003.g2118	X0KJP1#PHI:11715#FocM35_1_(FOIG_01919)#5507#Fusarium_oxysporum#effector_(plant_avirulence	56.52	23	10	0	8	30	7	29	0.31	29.3
# BLASTP 2.2.30+											
# Query: contig00003.g2263											
# Database: /data/phi-blast/fasta/Phi417/phi417.fas											
# Fields: query id	subject id	% identity	alignment length	mismatches	gap opens	q. start	q. end	s. start	s. end	evalue	bit score
# 14 hits found											
contig00003.g2263	I1RYF8#PHI:1236#FGSG_09408#5518#Fusarium_graminearum#lethal	29.47	95	54	5	42	125	309	401	0.24	30.0
# BLASTP 2.2.30+											
# Query: contig00003.g2385											
# Database: /data/phi-blast/fasta/Phi417/phi417.fas											
# Fields: query id	subject id	% identity	alignment length	mismatches	gap opens	q. start	q. end	s. start	s. end	evalue	bit score

# 8 hits found											
contig00003.g2385	A0A098DGQ1#PHI:6198#SOD1#5518#Fusarium_graminearum#reduced_virulence	28.30	53	34	1	65	113	169	221	0.71	28.9
# BLASTP 2.2.30+											
# Query: contig00003.g2450											
# Database: /data/phi-blast/fasta/Phi417/phi417.fas											
# Fields: query id	subject id	% identity	alignment length	mismatches	gap opens	q. start	q. end	s. start	s. end	evalue	bit score
# 4 hits found											
contig00003.g2450	Q4WRQ8#PHI:3904#AGS3#746128#Aspergillus_fumigatus#reduced_virulence	33.33	39	26	0	5	43	709	747	7.2	25.8
# BLASTP 2.2.30+											
# Query: contig00003.g2482											
# Database: /data/phi-blast/fasta/Phi417/phi417.fas											
# Fields: query id	subject id	% identity	alignment length	mismatches	gap opens	q. start	q. end	s. start	s. end	evalue	bit score
# 8 hits found											
contig00003.g2482	Q00310#PHI:104#CaMNT1#5476#Candida_albicans#reduced_virulence	30.43	69	43	2	13	76	265	333	0.46	29.3
# BLASTP 2.2.30+											
# Query: contig00003.g2508											
# Database: /data/phi-blast/fasta/Phi417/phi417.fas											
# Fields: query id	subject id	% identity	alignment length	mismatches	gap opens	q. start	q. end	s. start	s. end	evalue	bit score
# 2 hits found											
contig00003.g2508	J4UFF8#PHI:11304#BbTFO1#176275#Beauveria_bassiana#reduced_virulence	39.06	64	39	0	21	84	775	838	1,00E-08	50.4
# BLASTP 2.2.30+											
# Query: contig00004.g2520											
# Database: /data/phi-blast/fasta/Phi417/phi417.fas											
# Fields: query id	subject id	% identity	alignment length	mismatches	gap opens	q. start	q. end	s. start	s. end	evalue	bit score
# 2 hits found											
contig00004.g2520	I1RS35#PHI:9743#Pex3_(FGSG_06942)#5518#Fusarium_graminearum#reduced_virulence	91.38	174	15	0	1	174	1	174	6,00E-111	328
# BLASTP 2.2.30+											
# Query: contig00004.g2529											
# Database: /data/phi-blast/fasta/Phi417/phi417.fas											
# Fields: query id	subject id	% identity	alignment length	mismatches	gap opens	q. start	q. end	s. start	s. end	evalue	bit score
# 6 hits found											
contig00004.g2529	G4MTV1#PHI:9551#Moarl8#318829#Magnaporthe_oryzae#unaffected_pathogenicity	34.04	47	31	0	71	117	3	49	0.076	30.8
# BLASTP 2.2.30+											

# Query: contig00004.g2541											
# Database: /data/phi-blast/fasta/Phi417/phi417.fas											
# Fields: query id	subject id	% identity	alignment length	mismatches	gap opens	q. start	q. end	s. start	s. end	evalue	bit score
# 13 hits found											
contig00004.g2541	I1RYH0#PHI:6409#FgCdc11#5518#Fusarium_graminearum#reduced_virulence	31.82	44	30	0	98	141	102	145	0.62	29.6
# BLASTP 2.2.30+											
# Query: contig00004.g2558											
# Database: /data/phi-blast/fasta/Phi417/phi417.fas											
# Fields: query id	subject id	% identity	alignment length	mismatches	gap opens	q. start	q. end	s. start	s. end	evalue	bit score
# 16 hits found											
contig00004.g2558	A0A0W0EMV0#PHI:123298#GAP1#5476#Candida_albicans#reduced_virulence	32.47	77	44	3	2	78	518	586	0.009	32.3
# BLASTP 2.2.30+											
# Query: contig00004.g2568											
# Database: /data/phi-blast/fasta/Phi417/phi417.fas											
# Fields: query id	subject id	% identity	alignment length	mismatches	gap opens	q. start	q. end	s. start	s. end	evalue	bit score
# 2 hits found											
contig00004.g2568	Q9UVT8#PHI:11240#MoMca1_(MGG_04626)#318829#Magnaporthe_oryzae#reduced_virulence	25.93	27	20	0	3	29	120	146	9.7	25.0
# BLASTP 2.2.30+											
# Query: contig00004.g2668											
# Database: /data/phi-blast/fasta/Phi417/phi417.fas											
# Fields: query id	subject id	% identity	alignment length	mismatches	gap opens	q. start	q. end	s. start	s. end	evalue	bit score
# 5 hits found											
contig00004.g2668	Q59RL7#PHI:2861#Hms1#5476#Candida_albicans#reduced_virulence	25.45	55	41	0	3	57	161	215	3.2	27.3
# BLASTP 2.2.30+											
# Query: contig00004.g2692											
# Database: /data/phi-blast/fasta/Phi417/phi417.fas											
# Fields: query id	subject id	% identity	alignment length	mismatches	gap opens	q. start	q. end	s. start	s. end	evalue	bit score
# 5 hits found											
contig00004.g2692	Q57DN5#PHI:11658#ExoR#235#Brucella_abortus#unaffected_pathogenicity	38.46	39	24	0	41	79	186	224	0.91	28.5
# BLASTP 2.2.30+											
# Query: contig00004.g2724											
# Database: /data/phi-blast/fasta/Phi417/phi417.fas											
# Fields: query id	subject id	% identity	alignment length	mismatches	gap opens	q. start	q. end	s. start	s. end	evalue	bit score

# 5 hits found											
contig00004.g2724	A0A075TRC0#PHI:3299_PHI:4505#PatK_PePatK#27334#Penicillium_expansum#unaffected_pathogenic	42.42	33	19	0	59	91	896	928	0.49	29.3
# BLAST processed 66 queries											
# BLASTP 2.2.30+											
# Query: contig00004.g2788											
# Database: /data/phi-blast/fasta/Phi417/phi417.fas											
# Fields: query id	subject id	% identity	alignment length	mismatches	gap opens	q. start	q. end	s. start	s. end	evalue	bit score
# 9 hits found											
contig00004.g2788	S7V464#PHI:10900#TGGT1_269950#5811#Toxoplasma_gondii#reduced_virulence	27.08	96	60	3	163	254	722	811	0.43	32.0
# BLASTP 2.2.30+											
# Query: contig00004.g2791											
# Database: /data/phi-blast/fasta/Phi417/phi417.fas											
# Fields: query id	subject id	% identity	alignment length	mismatches	gap opens	q. start	q. end	s. start	s. end	evalue	bit score
# 8 hits found											
contig00004.g2791	Q4IPZ1#PHI:1564#GzOB004#5518#Fusarium_graminearum#unaffected_pathogenicity	32.26	62	34	2	3	62	13	68	0.008	31.2
# BLASTP 2.2.30+											
# Query: contig00004.g2826											
# Database: /data/phi-blast/fasta/Phi417/phi417.fas											
# Fields: query id	subject id	% identity	alignment length	mismatches	gap opens	q. start	q. end	s. start	s. end	evalue	bit score
# 3 hits found											
contig00004.g2826	I1RQ27#PHI:1914#GzZC229#5518#Fusarium_graminearum#unaffected_pathogenicity	21.24	113	85	1	6	118	198	306	3.2	26.6
# BLASTP 2.2.30+											
# Query: contig00004.g2853											
# Database: /data/phi-blast/fasta/Phi417/phi417.fas											
# Fields: query id	subject id	% identity	alignment length	mismatches	gap opens	q. start	q. end	s. start	s. end	evalue	bit score
# 10 hits found											
contig00004.g2853	K9FEW0#PHI:6681#Pdac1#36651#Penicillium_digitatum#reduced_virulence	32.26	62	33	1	14	75	1040	1092	1.7	27.3
# BLASTP 2.2.30+											
# Query: contig00004.g2971											
# Database: /data/phi-blast/fasta/Phi417/phi417.fas											
# Fields: query id	subject id	% identity	alignment length	mismatches	gap opens	q. start	q. end	s. start	s. end	evalue	bit score
# 5 hits found											

contig00004.g2971	P0CL08#PHI:635_PHI:7270_PHI:9084_PHI:9231_PHI:9522_PHI:11209_PHI:12207#HilD#28901#Salmonella_enterica#reduced_virulence_increased_virulence_(hypervirulence)_unaffected_pathogenicity	29.31	58	38	1	41	95	218	275	2.2	26.2
# BLASTP 2.2.30+											
# Query: contig00004.g3005											
# Database: /data/phi-blast/fasta/Phi417/phi417.fas											
# Fields: query id	subject id	% identity	alignment length	mismatches	gap opens	q. start	q. end	s. start	s. end	evalue	bit score
# 6 hits found											
contig00004.g3005	P04046#PHI:502#ADE4#4932#Saccharomyces_cerevisiae#reduced_virulence	27.78	72	43	3	56	119	132	202	1.6	28.1
# BLASTP 2.2.30+											
# Query: contig00004.g3014											
# Database: /data/phi-blast/fasta/Phi417/phi417.fas											
# Fields: query id	subject id	% identity	alignment length	mismatches	gap opens	q. start	q. end	s. start	s. end	evalue	bit score
# 9 hits found											
contig00004.g3014	A0A1B2LQ61#PHI:6946#CgAP1#474922#Colletotrichum_gloeosporioides#loss_of_pathogenicity	34.55	55	20	2	93	143	381	423	0.17	30.4
# BLASTP 2.2.30+											
# Query: contig00004.g3071											
# Database: /data/phi-blast/fasta/Phi417/phi417.fas											
# Fields: query id	subject id	% identity	alignment length	mismatches	gap opens	q. start	q. end	s. start	s. end	evalue	bit score
# 15 hits found											
contig00004.g3071	A0A098DCZ4#PHI:1439#GzNot001#5518#Fusarium_graminearum#lethal	28.57	56	39	1	115	170	472	526	0.082	32.0
# BLASTP 2.2.30+											
# Query: contig00004.g3101											
# Database: /data/phi-blast/fasta/Phi417/phi417.fas											
# Fields: query id	subject id	% identity	alignment length	mismatches	gap opens	q. start	q. end	s. start	s. end	evalue	bit score
# 5 hits found											
contig00004.g3101	G4N7U7#PHI:8629#MoAbp1_(MGG_06358)#318829#Magnaporthe_oryzae#reduced_virulence	22.78	158	102	4	4	144	8	162	3,00E-08	50.4
# BLASTP 2.2.30+											
# Query: contig00004.g3129											
# Database: /data/phi-blast/fasta/Phi417/phi417.fas											
# Fields: query id	subject id	% identity	alignment length	mismatches	gap opens	q. start	q. end	s. start	s. end	evalue	bit score
# 4 hits found											
contig00004.g3129	I1RBU0#PHI:1632#GzWing004#5518#Fusarium_graminearum#unaffected_pathogenicity		56	35	1	44	96	23	78	0.42	28.1

# BLASTP 2.2.30+											
# Query: contig00004.g3241											
# Database: /data/phi-blast/fasta/Phi417/phi417.fas											
# Fields: query id	subject id	% identity	alignment length	mismatches	gap opens	q. start	q. end	s. start	s. end	evalue	bit score
# 4 hits found											
contig00004.g3241	Q2WCV5#PHI:123608#TcaA#630#Yersinia_enterocolitica#loss_of_pathogenicity	30.95	42	29	0	52	93	421	462	0.62	28.5
# BLASTP 2.2.30+											
# Query: contig00005.g3442											
# Database: /data/phi-blast/fasta/Phi417/phi417.fas											
# Fields: query id	subject id	% identity	alignment length	mismatches	gap opens	q. start	q. end	s. start	s. end	evalue	bit score
# 59 hits found											
contig00005.g3442	Q8J2P6#PHI:317#RHBA#746128#Aspergillus_fumigatus#reduced_virulence	71.66	187	52	1	1	186	1	187	5,00E-99	286
# BLASTP 2.2.30+											
# Query: contig00005.g3445											
# Database: /data/phi-blast/fasta/Phi417/phi417.fas											
# Fields: query id	subject id	% identity	alignment length	mismatches	gap opens	q. start	q. end	s. start	s. end	evalue	bit score
# 7 hits found											
contig00005.g3445	I1RH09#PHI:1897#GzZC212#5518#Fusarium_graminearum#unaffected_pathogenicity	39.47	38	16	1	22	52	182	219	0.20	29.6
# BLASTP 2.2.30+											
# Query: contig00005.g3512											
# Database: /data/phi-blast/fasta/Phi417/phi417.fas											
# Fields: query id	subject id	% identity	alignment length	mismatches	gap opens	q. start	q. end	s. start	s. end	evalue	bit score
# 17 hits found											
contig00005.g3512	G4MXC5#PHI:5190#MoPRX1#318829#Magnaporthe_oryzae#reduced_virulence	80.28	218	43	0	4	221	7	224	6,00E-133	376
# BLASTP 2.2.30+											
# Query: contig00005.g3575											
# Database: /data/phi-blast/fasta/Phi417/phi417.fas											
# Fields: query id	subject id	% identity	alignment length	mismatches	gap opens	q. start	q. end	s. start	s. end	evalue	bit score
# 55 hits found											
contig00005.g3575	A0A139Y2L7#PHI:10571#Rab11A#5811#Toxoplasma_gondii#reduced_virulence	55.42	166	72	2	3	166	5	170	1,00E-58	184
# BLASTP 2.2.30+											
# Query: contig00005.g3649											
# Database: /data/phi-blast/fasta/Phi417/phi417.fas											

# Fields: query id	subject id	% identity	alignment length	mismatches	gap opens	q. start	q. end	s. start	s. end	evalue	bit score
# 9 hits found											
contig00005.g3649	I1RWB8#PHI:1402#GzC2H065#5518#Fusarium_graminearum#unaffected_pathogenicity	29.69	64	44	1	23	85	630	693	0.20	28.9
# BLASTP 2.2.30+											
# Query: contig00005.g3659											
# Database: /data/phi-blast/fasta/Phi417/phi417.fas											
# Fields: query id	subject id	% identity	alignment length	mismatches	gap opens	q. start	q. end	s. start	s. end	evalue	bit score
# 6 hits found											
contig00005.g3659	Q8J230#PHI:6069#Kpp4#5270#Ustilago_maydis#loss_of_pathogenicity	25.64	78	48	3	25	99	464	534	1.0	27.7
# BLASTP 2.2.30+											
# Query: contig00005.g3660											
# Database: /data/phi-blast/fasta/Phi417/phi417.fas											
# Fields: query id	subject id	% identity	alignment length	mismatches	gap opens	q. start	q. end	s. start	s. end	evalue	bit score
# 2 hits found											
contig00005.g3660	Q5AG51#PHI:11355#Ccc2#5476#Candida_albicans#reduced_virulence	29.17	48	30	1	11	54	345	392	5.2	25.8
# BLASTP 2.2.30+											
# Query: contig00005.g3667											
# Database: /data/phi-blast/fasta/Phi417/phi417.fas											
# Fields: query id	subject id	% identity	alignment length	mismatches	gap opens	q. start	q. end	s. start	s. end	evalue	bit score
# 11 hits found											
contig00005.g3667	Q8ZMX2#PHI:5571_PHI:6366#Pat#28901#Salmonella_enterica#reduced_virulence_increased_virulence_(hypervirulence)	32.00	50	30	1	92	137	812	861	0.11	31.2
# BLASTP 2.2.30+											
# Query: contig00005.g3696											
# Database: /data/phi-blast/fasta/Phi417/phi417.fas											
# Fields: query id	subject id	% identity	alignment length	mismatches	gap opens	q. start	q. end	s. start	s. end	evalue	bit score
# 6 hits found											
contig00005.g3696	I1RK94#PHI:1367#GzC2H028#5518#Fusarium_graminearum#unaffected_pathogenicity	31.91	47	26	1	30	70	244	290	1.1	28.1
# BLASTP 2.2.30+											
# Query: contig00006.g3763											
# Database: /data/phi-blast/fasta/Phi417/phi417.fas											
# Fields: query id	subject id	% identity	alignment length	mismatches	gap opens	q. start	q. end	s. start	s. end	evalue	bit score
# 7 hits found											

contig00006.g3763	G4ZAL2#PHI:124292#ZFPK1_(345221)#67593#Phytophthora_sojae#reduced_virulence	25.64	117	80	3	42	153	555	669	0.53	30.0
# BLASTP 2.2.30+											
# Query: contig00006.g3841											
# Database: /data/phi-blast/fasta/Phi417/phi417.fas											
# Fields: query id	subject id	% identity	alignment length	mismatches	gap opens	q. start	q. end	s. start	s. end	evalue	bit score
# 7 hits found											
contig00006.g3841	Q9I074#PHI:10776#PA2769#287#Pseudomonas_aeruginosa#unaffected_pathogenicity	30.91	55	35	2	48	102	17	68	0.12	29.3
# BLASTP 2.2.30+											
# Query: contig00006.g3911											
# Database: /data/phi-blast/fasta/Phi417/phi417.fas											
# Fields: query id	subject id	% identity	alignment length	mismatches	gap opens	q. start	q. end	s. start	s. end	evalue	bit score
# 2 hits found											
contig00006.g3911	I1RCP1#PHI:1080_PHI:9405#Cch1_CCH1_(FGSG_01364)#5518#Fusarium_graminearum#unaffected_pathogenicity_reduced_virulence	30.77	52	36	0	87	138	713	764	4.6	27.3
# BLASTP 2.2.30+											
# Query: contig00006.g3925											
# Database: /data/phi-blast/fasta/Phi417/phi417.fas											
# Fields: query id	subject id	% identity	alignment length	mismatches	gap opens	q. start	q. end	s. start	s. end	evalue	bit score
# 13 hits found											
contig00006.g3925	Q01176#PHI:568#XYL2_(XYN33)#318829#Magnaporthe_oryzae#unaffected_pathogenicity	25.29	87	54	3	48	125	174	258	0.76	28.1
# BLASTP 2.2.30+											
# Query: contig00006.g3951											
# Database: /data/phi-blast/fasta/Phi417/phi417.fas											
# Fields: query id	subject id	% identity	alignment length	mismatches	gap opens	q. start	q. end	s. start	s. end	evalue	bit score
# 8 hits found											
contig00006.g3951	D4GHY9#PHI:124069#IucC_(PAGR_g3925)#553#Pantoea_ananatis#unaffected_pathogenicity	43.48	46	18	2	19	56	419	464	1.1	27.3
# BLASTP 2.2.30+											
# Query: contig00006.g4054											
# Database: /data/phi-blast/fasta/Phi417/phi417.fas											
# Fields: query id	subject id	% identity	alignment length	mismatches	gap opens	q. start	q. end	s. start	s. end	evalue	bit score
# 22 hits found											
contig00006.g4054	A0ST44#PHI:1047#CTB6#29003#Cercospora_nicotianae#reduced_virulence	28.90	353	221	13	4	340	6	344	3,00E-22	94.7
# BLASTP 2.2.30+											

# Query: contig00006.g4087											
# Database: /data/phi-blast/fasta/Phi417/phi417.fas											
# Fields: query id	subject id	% identity	alignment length	mismatches	gap opens	q. start	q. end	s. start	s. end	evalue	bit score
# 10 hits found											
contig00006.g4087	I1RD24#PHI:5091#FgRic8#5518#Fusarium_graminearum#reduced_virulence	64.26	470	159	2	1	470	1	461	0.0	604
# BLASTP 2.2.30+											
# Query: contig00006.g4171											
# Database: /data/phi-blast/fasta/Phi417/phi417.fas											
# Fields: query id	subject id	% identity	alignment length	mismatches	gap opens	q. start	q. end	s. start	s. end	evalue	bit score
# 2 hits found											
contig00006.g4171	I1RCE1#PHI:1568#GzOB008#5518#Fusarium_graminearum#unaffected_pathogenicity	30.77	39	21	1	61	93	3	41	7.8	24.6
# BLASTP 2.2.30+											
# Query: contig00006.g4178											
# Database: /data/phi-blast/fasta/Phi417/phi417.fas											
# Fields: query id	subject id	% identity	alignment length	mismatches	gap opens	q. start	q. end	s. start	s. end	evalue	bit score
# 5 hits found											
contig00006.g4178	A0A1D8PH27#PHI:11402#Ssy1#5476#Candida_albicans#reduced_virulence	41.18	34	13	1	93	126	736	762	2.1	26.9
# BLASTP 2.2.30+											
# Query: contig00007.g4223											
# Database: /data/phi-blast/fasta/Phi417/phi417.fas											
# Fields: query id	subject id	% identity	alignment length	mismatches	gap opens	q. start	q. end	s. start	s. end	evalue	bit score
# 5 hits found											
contig00007.g4223	A0A385EXL2#PHI:10561#PstS#470#Acinetobacter_baumannii#reduced_virulence_increased_virulence_(hypervirulence)	29.17	96	41	4	20	110	49	122	0.61	28.5
# BLASTP 2.2.30+											
# Query: contig00007.g4229											
# Database: /data/phi-blast/fasta/Phi417/phi417.fas											
# Fields: query id	subject id	% identity	alignment length	mismatches	gap opens	q. start	q. end	s. start	s. end	evalue	bit score
# 9 hits found											
contig00007.g4229	J5J977#PHI:11340#Dim5#176275#Beauveria_bassiana#loss_of_pathogenicity	36.71	79	47	1	23	101	333	408	7,00E-11	57.0
# BLASTP 2.2.30+											
# Query: contig00007.g4264											
# Database: /data/phi-blast/fasta/Phi417/phi417.fas											

# Fields: query id	subject id	% identity	alignment length	mismatches	gap opens	q. start	q. end	s. start	s. end	evalue	bit score
# 17 hits found											
contig00007.g4264	A3N3P9#PHI:11023#Hfq#715#Actinobacillus_pleuropneumoniae#reduced_virulence_unaffected_path	37.50	48	25	2	9	56	4	46	0.030	30.0
# BLASTP 2.2.30+											
# Query: contig00007.g4268											
# Database: /data/phi-blast/fasta/Phi417/phi417.fas											
# Fields: query id	subject id	% identity	alignment length	mismatches	gap opens	q. start	q. end	s. start	s. end	evalue	bit score
# 7 hits found											
contig00007.g4268	Q6XVV0#PHI:10400#Sfgtr4_(ORF186)#623#Shigella_flexneri#reduced_virulence	22.00	100	69	4	26	116	103	202	0.23	29.6
# BLASTP 2.2.30+											
# Query: contig00007.g4344											
# Database: /data/phi-blast/fasta/Phi417/phi417.fas											
# Fields: query id	subject id	% identity	alignment length	mismatches	gap opens	q. start	q. end	s. start	s. end	evalue	bit score
# 6 hits found											
contig00007.g4344	I1RN31#PHI:1223#FGSG_05393#5518#Fusarium_graminearum#lethal	50.00	24	12	0	62	85	72	95	2.2	25.8
# BLASTP 2.2.30+											
# Query: contig00007.g4389											
# Database: /data/phi-blast/fasta/Phi417/phi417.fas											
# Fields: query id	subject id	% identity	alignment length	mismatches	gap opens	q. start	q. end	s. start	s. end	evalue	bit score
# 6 hits found											
contig00007.g4389	G2XD68#PHI:8858#VdSSEP1_(VDAG-08100)#27337#Verticillium_dahliae#reduced_virul	29.06	117	65	5	4	120	692	790	0.032	32.0
# BLASTP 2.2.30+											
# Query: contig00007.g4401											
# Database: /data/phi-blast/fasta/Phi417/phi417.fas											
# Fields: query id	subject id	% identity	alignment length	mismatches	gap opens	q. start	q. end	s. start	s. end	evalue	bit score
# 65 hits found											
contig00007.g4401	G4MWX1#PHI:7134#MoRab5B#318829#Magnaporthe_oryzae#reduced_virulence	41.31	213	117	3	4	208	9	221	2,00E-52	170
# BLASTP 2.2.30+											
# Query: contig00007.g4471											
# Database: /data/phi-blast/fasta/Phi417/phi417.fas											
# Fields: query id	subject id	% identity	alignment length	mismatches	gap opens	q. start	q. end	s. start	s. end	evalue	bit score
# 2 hits found											
contig00007.g4471	Q4IPZ1#PHI:1564#GzOB004#5518#Fusarium_graminearum#unaffected_pathogenicity	36.36	66	39	3	39	102	7	71	7,00E-06	40.0

# BLASTP 2.2.30+											
# Query: contig00007.g4472											
# Database: /data/phi-blast/fasta/Phi417/phi417.fas											
# Fields: query id	subject id	% identity	alignment length	mismatches	gap opens	q. start	q. end	s. start	s. end	evalue	bit score
# 7 hits found											
contig00007.g4472	D0ZPH9#PHI:3771#SspH2#28901#Salmonella_enterica#effector_(plant_avirulence_determinant)	28.95	38	25	1	116	151	218	255	4.7	26.2
# BLASTP 2.2.30+											
# Query: contig00007.g4481											
# Database: /data/phi-blast/fasta/Phi417/phi417.fas											
# Fields: query id	subject id	% identity	alignment length	mismatches	gap opens	q. start	q. end	s. start	s. end	evalue	bit score
# 7 hits found											
contig00007.g4481	I1RX22#PHI:1595#GzOB035#5518#Fusarium_graminearum#unaffected_pathogenicity	28.95	76	53	1	66	140	36	111	0.008	34.7
# BLASTP 2.2.30+											
# Query: contig00007.g4539											
# Database: /data/phi-blast/fasta/Phi417/phi417.fas											
# Fields: query id	subject id	% identity	alignment length	mismatches	gap opens	q. start	q. end	s. start	s. end	evalue	bit score
# 6 hits found											
contig00007.g4539	P0CT09#PHI:7223#Moend3#318829#Magnaporthe_oryzae#reduced_virulence	35.71	42	27	0	109	150	341	382	1.2	28.1
# BLASTP 2.2.30+											
# Query: contig00007.g4660											
# Database: /data/phi-blast/fasta/Phi417/phi417.fas											
# Fields: query id	subject id	% identity	alignment length	mismatches	gap opens	q. start	q. end	s. start	s. end	evalue	bit score
# 5 hits found											
contig00007.g4660	I1S3I1#PHI:1857#GzZC172#5518#Fusarium_graminearum#unaffected_pathogenicity	35.56	45	18	2	42	75	59	103	0.97	26.2
# BLASTP 2.2.30+											
# Query: contig00007.g4673											
# Database: /data/phi-blast/fasta/Phi417/phi417.fas											
# Fields: query id	subject id	% identity	alignment length	mismatches	gap opens	q. start	q. end	s. start	s. end	evalue	bit score
# 11 hits found											
contig00007.g4673	Q04701#PHI:179#PELA#169388#Fusarium_solani#reduced_virulence	53.24	216	95	5	39	251	23	235	2,00E-67	211
# BLASTP 2.2.30+											
# Query: contig00007.g4722											
# Database: /data/phi-blast/fasta/Phi417/phi417.fas											

# Fields: query id	subject id	% identity	alignment length	mismatches	gap opens	q. start	q. end	s. start	s. end	evalue	bit score
# 3 hits found											
contig00007.g4722	A0A1C3YN74#PHI:1206#(Sp_Prp4)#5518#Fusarium_graminearum#reduced_virulence	41.67	24	14	0	5	28	525	548	2.7	27.7
# BLASTP 2.2.30+											
# Query: contig00008.g4771											
# Database: /data/phi-blast/fasta/Phi417/phi417.fas											
# Fields: query id	subject id	% identity	alignment length	mismatches	gap opens	q. start	q. end	s. start	s. end	evalue	bit score
# 5 hits found											
contig00008.g4771	Q4UUA3#PHI:2495#XC2317#339#Xanthomonas_campestris#unaffected_pathogenicity	34.88	43	27	1	61	103	156	197	1.5	27.3
# BLASTP 2.2.30+											
# Query: contig00008.g4776											
# Database: /data/phi-blast/fasta/Phi417/phi417.fas											
# Fields: query id	subject id	% identity	alignment length	mismatches	gap opens	q. start	q. end	s. start	s. end	evalue	bit score
# 6 hits found											
contig00008.g4776	G4N5V2#PHI:2978#MoCel12A#318829#Magnaporthe_oryzae#unaffected_pathogenicity	47.28	239	113	5	8	237	28	262	4,00E-67	210
# BLASTP 2.2.30+											
# Query: contig00008.g4787											
# Database: /data/phi-blast/fasta/Phi417/phi417.fas											
# Fields: query id	subject id	% identity	alignment length	mismatches	gap opens	q. start	q. end	s. start	s. end	evalue	bit score
# 9 hits found											
contig00008.g4787	B0YAP6#PHI:2297#TmpL#746128#Aspergillus_fumigatus#reduced_virulence	31.51	73	47	2	7	78	498	568	0.43	30.0
# BLASTP 2.2.30+											
# Query: contig00008.g4827											
# Database: /data/phi-blast/fasta/Phi417/phi417.fas											
# Fields: query id	subject id	% identity	alignment length	mismatches	gap opens	q. start	q. end	s. start	s. end	evalue	bit score
# 14 hits found											
contig00008.g4827	Q00845#PHI:180#PELD#169388#Fusarium_solani#reduced_virulence	84.98	233	35	0	1	233	1	233	1,00E-150	422
# BLASTP 2.2.30+											
# Query: contig00008.g4887											
# Database: /data/phi-blast/fasta/Phi417/phi417.fas											
# Fields: query id	subject id	% identity	alignment length	mismatches	gap opens	q. start	q. end	s. start	s. end	evalue	bit score
# 2 hits found											
contig00008.g4887	A0A0B5JJZ6#PHI:4608#BxCYP33C4#6326#Bursaphelenchus_xylophilus#reduced_virulence	30.61	49	34	0	95	143	296	344	0.28	30.4

# BLASTP 2.2.30+											
# Query: contig00008.g4935											
# Database: /data/phi-blast/fasta/Phi417/phi417.fas											
# Fields: query id	subject id	% identity	alignment length	mismatches	gap opens	q. start	q. end	s. start	s. end	evalue	bit score
# 9 hits found											
contig00008.g4935	A0A0K0GIM7#PHI:10490#PilC_(PXO_04887)#347#Xanthomonas_oryzae#reduced_virulence	44.44	36	12	2	51	78	39	74	4.2	26.6
# BLASTP 2.2.30+											
# Query: contig00008.g5066											
# Database: /data/phi-blast/fasta/Phi417/phi417.fas											
# Fields: query id	subject id	% identity	alignment length	mismatches	gap opens	q. start	q. end	s. start	s. end	evalue	bit score
# 6 hits found											
contig00008.g5066	J4UH12#PHI:123177#BbPlin1_(NCBI_EJP62332)#176275#Beauveria_bassiana#reduced_virulence	30.23	43	25	1	44	81	33	75	3.0	26.2
# BLASTP 2.2.30+											
# Query: contig00008.g5085											
# Database: /data/phi-blast/fasta/Phi417/phi417.fas											
# Fields: query id	subject id	% identity	alignment length	mismatches	gap opens	q. start	q. end	s. start	s. end	evalue	bit score
# 7 hits found											
contig00008.g5085	J5JFB6#PHI:7379#Blys6#176275#Beauveria_bassiana#unaffected_pathogenicity	34.09	44	23	1	1	38	1	44	0.53	26.6
# BLASTP 2.2.30+											
# Query: contig00008.g5290											
# Database: /data/phi-blast/fasta/Phi417/phi417.fas											
# Fields: query id	subject id	% identity	alignment length	mismatches	gap opens	q. start	q. end	s. start	s. end	evalue	bit score
# 12 hits found											
contig00008.g5290	Q8Y6G6#PHI:5256#LacR#1639#Listeria_monocytogenes#reduced_virulence_increased_virulence_(hyp	30.77	52	35	1	2	53	391	441	0.70	26.2
# BLASTP 2.2.30+											
# Query: contig00009.g5423											
# Database: /data/phi-blast/fasta/Phi417/phi417.fas											
# Fields: query id	subject id	% identity	alignment length	mismatches	gap opens	q. start	q. end	s. start	s. end	evalue	bit score
# 12 hits found											
contig00009.g5423	A0A380PMR4#PHI:9372#AmiC#632#Yersinia_pestis#unaffected_pathogenicity	24.81	129	88	1	8	127	22	150	1.2	28.1
# BLASTP 2.2.30+											
# Query: contig00009.g5474											
# Database: /data/phi-blast/fasta/Phi417/phi417.fas											

# Fields: query id	subject id	% identity	alignment length	mismatches	gap opens	q. start	q. end	s. start	s. end	evalue	bit score
# 3 hits found											
contig00009.g5474	M4BU64#PHI:9741#CesA3_(HpaG810051)#272952#Hyaloperonospora_arabidopsidis#reduced_virule	41.94	31	12	1	5	35	869	893	4.2	25.0
# BLASTP 2.2.30+											
# Query: contig00009.g5494											
# Database: /data/phi-blast/fasta/Phi417/phi417.fas											
# Fields: query id	subject id	% identity	alignment length	mismatches	gap opens	q. start	q. end	s. start	s. end	evalue	bit score
# 8 hits found											
contig00009.g5494	E9AS21#PHI:11740#FAZ7B_(LmxM.19.0690_)#5665#Leishmania_mexicana#reduced_virulence	38.46	26	16	0	99	124	329	354	2.1	26.9
# BLASTP 2.2.30+											
# Query: contig00009.g5575											
# Database: /data/phi-blast/fasta/Phi417/phi417.fas											
# Fields: query id	subject id	% identity	alignment length	mismatches	gap opens	q. start	q. end	s. start	s. end	evalue	bit score
# 11 hits found											
contig00009.g5575	A0A0F6B110#PHI:10148#STM14_RS07945_(ssaQ)#28901#Salmonella_enterica#reduced_virulence	24.27	103	66	4	9	111	194	284	0.19	29.3
# BLASTP 2.2.30+											
# Query: contig00009.g5597											
# Database: /data/phi-blast/fasta/Phi417/phi417.fas											
# Fields: query id	subject id	% identity	alignment length	mismatches	gap opens	q. start	q. end	s. start	s. end	evalue	bit score
# 3 hits found											
contig00009.g5597	Q4WIQ3#PHI:3277#GnoA#746128#Aspergillus_fumigatus#unaffected_pathogenicity	40.00	40	23	1	30	68	136	175	0.63	28.9
# BLASTP 2.2.30+											
# Query: contig00009.g5701											
# Database: /data/phi-blast/fasta/Phi417/phi417.fas											
# Fields: query id	subject id	% identity	alignment length	mismatches	gap opens	q. start	q. end	s. start	s. end	evalue	bit score
# 7 hits found											
contig00009.g5701	B5XY48#PHI:4160#OmpA2#573#Klebsiella_pneumoniae#reduced_virulence	39.29	28	17	0	15	42	235	262	2.2	25.8
# BLASTP 2.2.30+											
# Query: contig00009.g5753											
# Database: /data/phi-blast/fasta/Phi417/phi417.fas											
# Fields: query id	subject id	% identity	alignment length	mismatches	gap opens	q. start	q. end	s. start	s. end	evalue	bit score
# 4 hits found											
contig00009.g5753	Q5AG51#PHI:11355#Ccc2#5476#Candida_albicans#reduced_virulence	39.13	46	28	0	28	73	581	626	0.49	30.0

# BLASTP 2.2.30+											
# Query: contig00009.g5763											
# Database: /data/phi-blast/fasta/Phi417/phi417.fas											
# Fields: query id	subject id	% identity	alignment length	mismatches	gap opens	q. start	q. end	s. start	s. end	evalue	bit score
# 4 hits found											
contig00009.g5763	Q7A1N5#PHI:11420_PHI:11994#SarA#1280#Staphylococcus_aureus#reduced_virulence	31.34	67	40	1	270	330	20	86	0.89	28.9
# BLAST processed 60 queries											
# BLASTP 2.2.30+											
# Query: contig00009.g5787											
# Database: /data/phi-blast/fasta/Phi417/phi417.fas											
# Fields: query id	subject id	% identity	alignment length	mismatches	gap opens	q. start	q. end	s. start	s. end	evalue	bit score
# 9 hits found											
contig00009.g5787	A0A194VK59#PHI:10222#FAEC2_(VM1G_00022)#105487#Valsa_mali#unaffected_pathogenicity_reduced_virulence	30.86	81	46	3	21	97	263	337	0.021	33.9
# BLASTP 2.2.30+											
# Query: contig00010.g5845											
# Database: /data/phi-blast/fasta/Phi417/phi417.fas											
# Fields: query id	subject id	% identity	alignment length	mismatches	gap opens	q. start	q. end	s. start	s. end	evalue	bit score
# 4 hits found											
contig00010.g5845	P95129#PHI:4067#Rv2963#1773#Mycobacterium_tuberculosis#unaffected_pathogenicity	36.21	58	31	3	88	139	176	233	2.1	26.9
# BLASTP 2.2.30+											
# Query: contig00010.g5848											
# Database: /data/phi-blast/fasta/Phi417/phi417.fas											
# Fields: query id	subject id	% identity	alignment length	mismatches	gap opens	q. start	q. end	s. start	s. end	evalue	bit score
# 8 hits found											
contig00010.g5848	Q8P802#PHI:6663#GumG#339#Xanthomonas_campestris#reduced_virulence	47.62	21	11	0	11	31	327	347	1.3	27.3
# BLASTP 2.2.30+											
# Query: contig00010.g5961											
# Database: /data/phi-blast/fasta/Phi417/phi417.fas											
# Fields: query id	subject id	% identity	alignment length	mismatches	gap opens	q. start	q. end	s. start	s. end	evalue	bit score
# 6 hits found											
contig00010.g5961	Q2LD94#PHI:2036_PHI:2192#RGS1_MoRgs1#318829#Magnaporthe_oryzae#increased_virulence_(hypervirulence)_reduced_virulence_loss_of_pathogeni	35.21	71	44	2	10	79	139	208	0.41	29.3
# BLASTP 2.2.30+											

# Query: contig00010.g5985											
# Database: /data/phi-blast/fasta/Phi417/phi417.fas											
# Fields: query id	subject id	% identity	alignment length	mismatches	gap opens	q. start	q. end	s. start	s. end	evalue	bit score
# 16 hits found											
contig00010.g5985	Q9F8C5#PHI:11298#CzcD#1311#Streptococcus_agalactiae#reduced_virulence_unaffected_pathogenici	38.30	47	25	2	67	109	149	195	0.15	30.0
# BLASTP 2.2.30+											
# Query: contig00010.g6052											
# Database: /data/phi-blast/fasta/Phi417/phi417.fas											
# Fields: query id	subject id	% identity	alignment length	mismatches	gap opens	q. start	q. end	s. start	s. end	evalue	bit score
# 4 hits found											
contig00010.g6052	P62576#PHI:7006_PHI:7676_PHI:11278#NanA#1313#Streptococcus_pneumoniae#reduced_virulence	30.00	30	21	0	16	45	102	131	1.5	25.4
# BLASTP 2.2.30+											
# Query: contig00011.g6439											
# Database: /data/phi-blast/fasta/Phi417/phi417.fas											
# Fields: query id	subject id	% identity	alignment length	mismatches	gap opens	q. start	q. end	s. start	s. end	evalue	bit score
# 5 hits found											
contig00011.g6439	G4N8C1#PHI:11825#MoTPS2_(MGG_03441)#318829#Magnaporthe_oryzae#reduced_virulence	27.03	74	46	2	64	130	296	368	2.8	27.7
# BLASTP 2.2.30+											
# Query: contig00011.g6465											
# Database: /data/phi-blast/fasta/Phi417/phi417.fas											
# Fields: query id	subject id	% identity	alignment length	mismatches	gap opens	q. start	q. end	s. start	s. end	evalue	bit score
# 8 hits found											
contig00011.g6465	A0A656K4Z7#PHI:123950#HrcC#317#Pseudomonas_syringae#reduced_virulence	34.09	44	25	2	84	123	31	74	0.79	27.7
# BLASTP 2.2.30+											
# Query: contig00011.g6474											
# Database: /data/phi-blast/fasta/Phi417/phi417.fas											
# Fields: query id	subject id	% identity	alignment length	mismatches	gap opens	q. start	q. end	s. start	s. end	evalue	bit score
# 95 hits found											
contig00011.g6474	G4N9S6#PHI:9547#Moarf6#318829#Magnaporthe_oryzae#unaffected_pathogenicity	96.24	186	7	0	1	186	1	186	1,00E-132	372
# BLASTP 2.2.30+											
# Query: contig00011.g6485											
# Database: /data/phi-blast/fasta/Phi417/phi417.fas											
# Fields: query id	subject id	% identity	alignment length	mismatches	gap opens	q. start	q. end	s. start	s. end	evalue	bit score

# 6 hits found											
contig00011.g6485	R9UM22#PHI:3164#Sm1#29875#Trichoderma_virens#increased_virulence_(hypervirulence)_reduced	33.33	51	32	2	54	104	54	102	0.14	30.4
# BLASTP 2.2.30+											
# Query: contig00011.g6506											
# Database: /data/phi-blast/fasta/Phi417/phi417.fas											
# Fields: query id	subject id	% identity	alignment length	mismatches	gap opens	q. start	q. end	s. start	s. end	evalue	bit score
# 8 hits found											
contig00011.g6506	I6YE93#PHI:4963#TcdA#1496#Clostridioides_difficile#unaffected_pathogenicity_reduced_virulence	45.83	24	13	0	50	73	2608	2631	0.66	28.9
# BLASTP 2.2.30+											
# Query: contig00011.g6610											
# Database: /data/phi-blast/fasta/Phi417/phi417.fas											
# Fields: query id	subject id	% identity	alignment length	mismatches	gap opens	q. start	q. end	s. start	s. end	evalue	bit score
# 4 hits found											
contig00011.g6610	Q8XQE6#PHI:4025_PHI:10865#PopS_RSp1281#305#Ralstonia_solanacearum#effector_(plant_avirulence_determinant)	32.43	37	14	1	55	91	555	580	0.46	28.1
# BLASTP 2.2.30+											
# Query: contig00012.g6693											
# Database: /data/phi-blast/fasta/Phi417/phi417.fas											
# Fields: query id	subject id	% identity	alignment length	mismatches	gap opens	q. start	q. end	s. start	s. end	evalue	bit score
# 4 hits found											
contig00012.g6693	S7V2Q9#PHI:12162#PPM14_(TGGT1_232010)#5811#Toxoplasma_gondii#unaffected_pathogenicity	34.29	35	23	0	51	85	3053	3087	4.7	25.8
# BLASTP 2.2.30+											
# Query: contig00012.g6721											
# Database: /data/phi-blast/fasta/Phi417/phi417.fas											
# Fields: query id	subject id	% identity	alignment length	mismatches	gap opens	q. start	q. end	s. start	s. end	evalue	bit score
# 7 hits found											
contig00012.g6721	S7UQS3#PHI:6608#Gra39#5811#Toxoplasma_gondii#reduced_virulence	34.78	69	34	4	91	151	621	686	0.33	30.4
# BLASTP 2.2.30+											
# Query: contig00012.g6756											
# Database: /data/phi-blast/fasta/Phi417/phi417.fas											
# Fields: query id	subject id	% identity	alignment length	mismatches	gap opens	q. start	q. end	s. start	s. end	evalue	bit score
# 7 hits found											
contig00012.g6756	I1RGK4#PHI:1945#GzZC260#5518#Fusarium_graminearum#unaffected_pathogenicity	31.25	32	22	0	113	144	33	64	0.028	33.1

# BLASTP 2.2.30+											
# Query: contig00012.g6761											
# Database: /data/phi-blast/fasta/Phi417/phi417.fas											
# Fields: query id	subject id	% identity	alignment length	mismatches	gap opens	q. start	q. end	s. start	s. end	evalue	bit score
# 6 hits found											
contig00012.g6761	M2UDZ5#PHI:9195#Cla4#5016#Bipolaris_maydis#reduced_virulence	44.00	25	14	0	90	114	231	255	3.4	25.8
# BLASTP 2.2.30+											
# Query: contig00012.g6792											
# Database: /data/phi-blast/fasta/Phi417/phi417.fas											
# Fields: query id	subject id	% identity	alignment length	mismatches	gap opens	q. start	q. end	s. start	s. end	evalue	bit score
# 4 hits found											
contig00012.g6792	Q6ZX14#PHI:325#ACE1#318829#Magnaporthe_oryzae#effector_(plant_avirulence_determinant)	46.15	26	14	0	22	47	3444	3469	1.8	26.2
# BLASTP 2.2.30+											
# Query: contig00012.g6836											
# Database: /data/phi-blast/fasta/Phi417/phi417.fas											
# Fields: query id	subject id	% identity	alignment length	mismatches	gap opens	q. start	q. end	s. start	s. end	evalue	bit score
# 9 hits found											
contig00012.g6836	A0A2R8G1S3#PHI:11671#Mcr-4#562#Escherichia_coli#increased_virulence_(hypervirulence)	45.16	31	14	1	30	60	222	249	1.4	26.9
# BLASTP 2.2.30+											
# Query: contig00012.g6858											
# Database: /data/phi-blast/fasta/Phi417/phi417.fas											
# Fields: query id	subject id	% identity	alignment length	mismatches	gap opens	q. start	q. end	s. start	s. end	evalue	bit score
# 8 hits found											
contig00012.g6858	A0Q607#PHI:123791#TalA_(FTN_0781)#263#Francisella_tularensis#reduced_virulence	51.23	326	145	7	3	322	22	339	3,00E-94	286
# BLASTP 2.2.30+											
# Query: contig00013.g7037											
# Database: /data/phi-blast/fasta/Phi417/phi417.fas											
# Fields: query id	subject id	% identity	alignment length	mismatches	gap opens	q. start	q. end	s. start	s. end	evalue	bit score
# 4 hits found											
contig00013.g7037	A5IYN5#PHI:6514#MAG4460#2110#Mycoplasmopsis_agalactiae#reduced_virulence	29.55	44	31	0	86	129	404	447	4.6	27.7
# BLASTP 2.2.30+											
# Query: contig00013.g7047											

# Database: /data/phi-blast/fasta/Phi417/phi417.fas											
# Fields: query id	subject id	% identity	alignment length	mismatches	gap opens	q. start	q. end	s. start	s. end	evalue	bit score
# 4 hits found											
contig00013.g7047	Q57EK6#PHI:7603#RfbE#235#Brucella_abortus#unaffected_pathogenicity_reduced_virulence	26.92	52	38	0	103	154	166	217	2.6	26.9
# BLASTP 2.2.30+											
# Query: contig00013.g7169											
# Database: /data/phi-blast/fasta/Phi417/phi417.fas											
# Fields: query id	subject id	% identity	alignment length	mismatches	gap opens	q. start	q. end	s. start	s. end	evalue	bit score
# 8 hits found											
contig00013.g7169	A4L7H2#PHI:7940#Hyd1#176275#Beauveria_bassiana#reduced_virulence	41.41	128	65	3	5	123	9	135	9,00E-24	90.1
# BLASTP 2.2.30+											
# Query: contig00013.g7214											
# Database: /data/phi-blast/fasta/Phi417/phi417.fas											
# Fields: query id	subject id	% identity	alignment length	mismatches	gap opens	q. start	q. end	s. start	s. end	evalue	bit score
# 4 hits found											
contig00013.g7214	Q70ZY8#PHI:2524#NudC#746128#Aspergillus_fumigatus#lethal	60.62	193	68	2	3	187	7	199	5,00E-79	236
# BLASTP 2.2.30+											
# Query: contig00013.g7244											
# Database: /data/phi-blast/fasta/Phi417/phi417.fas											
# Fields: query id	subject id	% identity	alignment length	mismatches	gap opens	q. start	q. end	s. start	s. end	evalue	bit score
# 13 hits found											
contig00013.g7244	G8B9S9#PHI:7865#CPAR2_303700#5480#Candida_parapsilosis#reduced_virulence	27.18	195	88	5	4	155	5	188	1,00E-15	70.1
# BLASTP 2.2.30+											
# Query: contig00013.g7282											
# Database: /data/phi-blast/fasta/Phi417/phi417.fas											
# Fields: query id	subject id	% identity	alignment length	mismatches	gap opens	q. start	q. end	s. start	s. end	evalue	bit score
# 2 hits found											
contig00013.g7282	Q9A0L9#PHI:9798#AdcA#1314#Streptococcus_pyogenes#reduced_virulence_unaffected_pathogenicit	43.48	23	13	0	146	168	124	146	4.3	26.6
# BLASTP 2.2.30+											
# Query: contig00013.g7318											
# Database: /data/phi-blast/fasta/Phi417/phi417.fas											
# Fields: query id	subject id	% identity	alignment length	mismatches	gap opens	q. start	q. end	s. start	s. end	evalue	bit score
# 11 hits found											

contig00013.g7318	I1R9C7#PHI:8667#FgKP4L-2_(FGSG_00061)#5518#Fusarium_graminearum#reduced_virulence_increased_virulence_(hypervirul	73.39	124	33	0	1	124	1	124	1,00E-62	189
# BLASTP 2.2.30+											
# Query: contig00013.g7350											
# Database: /data/phi-blast/fasta/Phi417/phi417.fas											
# Fields: query id	subject id	% identity	alignment length	mismatches	gap opens	q. start	q. end	s. start	s. end	evalue	bit score
# 6 hits found											
contig00013.g7350	A0A0F2MLM2#PHI:11688#RmlD_(SPSK_0645)#29908#Sporothrix_schenckii#reduced_virulence	31.15	61	33	3	2	59	175	229	0.97	26.2
# BLASTP 2.2.30+											
# Query: contig00013.g7360											
# Database: /data/phi-blast/fasta/Phi417/phi417.fas											
# Fields: query id	subject id	% identity	alignment length	mismatches	gap opens	q. start	q. end	s. start	s. end	evalue	bit score
# 14 hits found											
contig00013.g7360	Q00845#PHI:180#PELD#169388#Fusarium_solani#reduced_virulence	73.82	233	52	2	1	224	1	233	3,00E-128	364
# BLASTP 2.2.30+											
# Query: contig00014.g7467											
# Database: /data/phi-blast/fasta/Phi417/phi417.fas											
# Fields: query id	subject id	% identity	alignment length	mismatches	gap opens	q. start	q. end	s. start	s. end	evalue	bit score
# 3 hits found											
contig00014.g7467	J9WUP1#PHI:3720#PdpacC#36651#Penicillium_digitatum#reduced_virulence	34.43	61	34	2	52	111	531	586	4.8	25.8
# BLASTP 2.2.30+											
# Query: contig00014.g7495											
# Database: /data/phi-blast/fasta/Phi417/phi417.fas											
# Fields: query id	subject id	% identity	alignment length	mismatches	gap opens	q. start	q. end	s. start	s. end	evalue	bit score
# 3 hits found											
contig00014.g7495	P09545#PHI:9438_PHI:12209#HlyA#666#Vibrio_cholerae#reduced_virulence_unaffected_pathogenici	40.00	30	18	0	47	76	573	602	1.9	25.4
# BLASTP 2.2.30+											
# Query: contig00014.g7496											
# Database: /data/phi-blast/fasta/Phi417/phi417.fas											
# Fields: query id	subject id	% identity	alignment length	mismatches	gap opens	q. start	q. end	s. start	s. end	evalue	bit score
# 12 hits found											
contig00014.g7496	G4NKK3#PHI:7793#Momyo2_(MGG_03060)#318829#Magnaporthe_oryzae#reduced_virulence	37.63	93	36	3	34	119	986	1063	0.005	34.7
# BLASTP 2.2.30+											

# Query: contig00014.g7512											
# Database: /data/phi-blast/fasta/Phi417/phi417.fas											
# Fields: query id	subject id	% identity	alignment length	mismatches	gap opens	q. start	q. end	s. start	s. end	evalue	bit score
# 44 hits found											
contig00014.g7512	G1XUA8#PHI:123037#AoRho2_(AOL_s00215g387)#2813651#Orbilia_oligospora#reduced_virulence	29.74	195	116	6	7	187	13	200	2,00E-19	82.0
# BLASTP 2.2.30+											
# Query: contig00014.g7530											
# Database: /data/phi-blast/fasta/Phi417/phi417.fas											
# Fields: query id	subject id	% identity	alignment length	mismatches	gap opens	q. start	q. end	s. start	s. end	evalue	bit score
# 18 hits found											
contig00014.g7530	A7EZX1#PHI:11004#SS1G_10888_(SsATX1)#5180#Sclerotinia_sclerotiorum#reduced_virulence_increased_virulence_(hypervirulence)	65.38	78	25	1	5	80	19	96	7,00E-31	105
# BLASTP 2.2.30+											
# Query: contig00014.g7556											
# Database: /data/phi-blast/fasta/Phi417/phi417.fas											
# Fields: query id	subject id	% identity	alignment length	mismatches	gap opens	q. start	q. end	s. start	s. end	evalue	bit score
# 2 hits found											
contig00014.g7556	B0XTA5#PHI:2293#CycA#746128#Aspergillus_fumigatus#reduced_virulence	77.32	97	22	0	5	101	66	162	2,00E-52	166
# BLASTP 2.2.30+											
# Query: contig00014.g7558											
# Database: /data/phi-blast/fasta/Phi417/phi417.fas											
# Fields: query id	subject id	% identity	alignment length	mismatches	gap opens	q. start	q. end	s. start	s. end	evalue	bit score
# 16 hits found											
contig00014.g7558	I1S297#PHI:8912#FgVPS20_(FGSG_10883)#5518#Fusarium_graminearum#reduced_virulence	83.33	204	17	3	1	201	1	190	6,00E-106	305
# BLASTP 2.2.30+											
# Query: contig00014.g7600											
# Database: /data/phi-blast/fasta/Phi417/phi417.fas											
# Fields: query id	subject id	% identity	alignment length	mismatches	gap opens	q. start	q. end	s. start	s. end	evalue	bit score
# 6 hits found											
contig00014.g7600	G4MZ47#PHI:2105#Calcium_permease#318829#Magnaporthe_oryzae#unaffected_pathogenicity_reduced_virulence	27.08	96	55	3	147	240	135	217	0.16	32.3
# BLASTP 2.2.30+											
# Query: contig00014.g7623											

# Database: /data/phi-blast/fasta/Phi417/phi417.fas											
# Fields: query id	subject id	% identity	alignment length	mismatches	gap opens	q. start	q. end	s. start	s. end	evalue	bit score
# 11 hits found											
contig00014.g7623	J9VLE6#PHI:11158#Ubi1_(CNAG_00370)#5207#Cryptococcus_neoformans#reduced_virulence	98.70	77	1	0	1	77	1	77	5,00E-49	156
# BLASTP 2.2.30+											
# Query: contig00014.g7656											
# Database: /data/phi-blast/fasta/Phi417/phi417.fas											
# Fields: query id	subject id	% identity	alignment length	mismatches	gap opens	q. start	q. end	s. start	s. end	evalue	bit score
# 4 hits found											
contig00014.g7656	G4MND3#PHI:7019#MoMyo5#318829#Magnaporthe_oryzae#loss_of_pathogenicity	26.87	67	45	1	55	117	1257	1323	6.0	25.4
# BLASTP 2.2.30+											
# Query: contig00014.g7667											
# Database: /data/phi-blast/fasta/Phi417/phi417.fas											
# Fields: query id	subject id	% identity	alignment length	mismatches	gap opens	q. start	q. end	s. start	s. end	evalue	bit score
# 7 hits found											
contig00014.g7667	A0A0K0GIX3#PHI:9026#PXO_RS06785#347#Xanthomonas_oryzae#unaffected_pathogenicity	35.48	31	20	0	31	61	110	140	1.5	25.8
# BLASTP 2.2.30+											
# Query: contig00014.g7706											
# Database: /data/phi-blast/fasta/Phi417/phi417.fas											
# Fields: query id	subject id	% identity	alignment length	mismatches	gap opens	q. start	q. end	s. start	s. end	evalue	bit score
# 9 hits found											
contig00014.g7706	F9XKL2#PHI:123546#Mycgr3G105826#1047171#Zymoseptoria_tritici#effector_(plant_avirulence_det	32.39	71	46	2	1	70	4	73	0.017	31.2
# BLASTP 2.2.30+											
# Query: contig00015.g7861											
# Database: /data/phi-blast/fasta/Phi417/phi417.fas											
# Fields: query id	subject id	% identity	alignment length	mismatches	gap opens	q. start	q. end	s. start	s. end	evalue	bit score
# 3 hits found											
contig00015.g7861	A0A2J9HLA0#PHI:9924#BcsQ_(yhjQ_BGLU_RS28225)#337#Burkholderia_glumae#reduced_virulence	29.03	62	44	0	118	179	162	223	0.90	28.5
# BLASTP 2.2.30+											
# Query: contig00015.g7924											
# Database: /data/phi-blast/fasta/Phi417/phi417.fas											
# Fields: query id	subject id	% identity	alignment length	mismatches	gap opens	q. start	q. end	s. start	s. end	evalue	bit score
# 6 hits found											

contig00015.g7924	J9VJS8#PHI:3098#URA4#5207#Cryptococcus_neoformans#reduced_virulence	28.05	82	55	3	187	266	153	232	1.4	29.3
# BLASTP 2.2.30+											
# Query: contig00015.g7948											
# Database: /data/phi-blast/fasta/Phi417/phi417.fas											
# Fields: query id	subject id	% identity	alignment length	mismatches	gap opens	q. start	q. end	s. start	s. end	evalue	bit score
# 66 hits found											
contig00015.g7948	Q96VL3#PHI:339#CLPT1#290576#Colletotrichum_lindemuthianum#reduced_virulence	94.58	203	10	1	1	203	1	202	3,00E-140	393
# BLASTP 2.2.30+											
# Query: contig00015.g7950											
# Database: /data/phi-blast/fasta/Phi417/phi417.fas											
# Fields: query id	subject id	% identity	alignment length	mismatches	gap opens	q. start	q. end	s. start	s. end	evalue	bit score
# 6 hits found											
contig00015.g7950	J9VS05#PHI:10004_PHI:10328#Atg7_(CNAG_04538)#5207#Cryptococcus_neoformans#reduced_virul	28.36	67	46	1	11	75	300	366	0.60	27.7
# BLASTP 2.2.30+											
# Query: contig00015.g7956											
# Database: /data/phi-blast/fasta/Phi417/phi417.fas											
# Fields: query id	subject id	% identity	alignment length	mismatches	gap opens	q. start	q. end	s. start	s. end	evalue	bit score
# 5 hits found											
contig00015.g7956	E9DTB1#PHI:12016#MaNmrA_(MAC_00749)#92637#Metarhizium_acridum#reduced_virulence	30.00	40	28	0	57	96	168	207	1.2	27.3
# BLASTP 2.2.30+											
# Query: contig00015.g7957											
# Database: /data/phi-blast/fasta/Phi417/phi417.fas											
# Fields: query id	subject id	% identity	alignment length	mismatches	gap opens	q. start	q. end	s. start	s. end	evalue	bit score
# 6 hits found											
contig00015.g7957	A0A1R3RGK0#PHI:10950#Pks#40993#Aspergillus_carbonarius#unaffected_pathogenicity	44.74	38	17	1	131	168	2483	2516	1.8	28.1
# BLASTP 2.2.30+											
# Query: contig00015.g7968											
# Database: /data/phi-blast/fasta/Phi417/phi417.fas											
# Fields: query id	subject id	% identity	alignment length	mismatches	gap opens	q. start	q. end	s. start	s. end	evalue	bit score
# 7 hits found											
contig00015.g7968	A0A290WHP2#PHI:7705#VEDA_05195#27337#Verticillium_dahliae#reduced_virulence_unaffected_p	41.18	51	27	3	1	49	191	240	0.016	30.8
# BLASTP 2.2.30+											
# Query: contig00015.g8034											

# Database: /data/phi-blast/fasta/Phi417/phi417.fas											
# Fields: query id	subject id	% identity	alignment length	mismatches	gap opens	q. start	q. end	s. start	s. end	evalue	bit score
# 4 hits found											
contig00015.g8034	A0A0D2XX76#PHI:123665#Nap-2sigma_(FOXG_08592)#5507#Fusarium_oxysporum#reduced_virulence	100.00	143	0	0	1	143	1	143	6,00E-103	293
# BLASTP 2.2.30+											
# Query: contig00016.g8203											
# Database: /data/phi-blast/fasta/Phi417/phi417.fas											
# Fields: query id	subject id	% identity	alignment length	mismatches	gap opens	q. start	q. end	s. start	s. end	evalue	bit score
# 8 hits found											
contig00016.g8203	Q9I3S1#PHI:3041#BdlA#287#Pseudomonas_aeruginosa#reduced_virulence_loss_of_pathogenicity	36.84	38	19	1	26	63	197	229	0.15	29.6
# BLASTP 2.2.30+											
# Query: contig00016.g8208											
# Database: /data/phi-blast/fasta/Phi417/phi417.fas											
# Fields: query id	subject id	% identity	alignment length	mismatches	gap opens	q. start	q. end	s. start	s. end	evalue	bit score
# 6 hits found											
contig00016.g8208	P45684#PHI:10649#RpoS#287#Pseudomonas_aeruginosa#unaffected_pathogenicity	35.94	64	32	3	347	409	72	127	2.3	29.3
# BLASTP 2.2.30+											
# Query: contig00016.g8213											
# Database: /data/phi-blast/fasta/Phi417/phi417.fas											
# Fields: query id	subject id	% identity	alignment length	mismatches	gap opens	q. start	q. end	s. start	s. end	evalue	bit score
# 9 hits found											
contig00016.g8213	W7MT31#PHI:3387#FVEG_12523#117187#Fusarium_verticillioides#unaffected_pathogenicity	40.48	42	23	1	114	153	1719	1760	0.26	30.4
# BLASTP 2.2.30+											
# Query: contig00016.g8232											
# Database: /data/phi-blast/fasta/Phi417/phi417.fas											
# Fields: query id	subject id	% identity	alignment length	mismatches	gap opens	q. start	q. end	s. start	s. end	evalue	bit score
# 4 hits found											
contig00016.g8232	I1S832#PHI:1723#GzZC038#5518#Fusarium_graminearum#unaffected_pathogenicity	23.68	76	49	2	105	180	105	171	0.98	28.9
# BLASTP 2.2.30+											
# Query: contig00016.g8289											
# Database: /data/phi-blast/fasta/Phi417/phi417.fas											
# Fields: query id	subject id	% identity	alignment length	mismatches	gap opens	q. start	q. end	s. start	s. end	evalue	bit score

# 19 hits found											
contig00016.g8289	G2WUQ0#PHI:10379#Isw2#27337#Verticillium_dahliae#reduced_virulence	38.10	63	37	2	1	63	308	368	0.006	35.0
# BLASTP 2.2.30+											
# Query: contig00016.g8290											
# Database: /data/phi-blast/fasta/Phi417/phi417.fas											
# Fields: query id	subject id	% identity	alignment length	mismatches	gap opens	q. start	q. end	s. start	s. end	evalue	bit score
# 7 hits found											
contig00016.g8290	P11557#PHI:6729#DamX#562#Escherichia_coli#reduced_virulence	37.04	27	17	0	74	100	13	39	2.4	25.8
# BLASTP 2.2.30+											
# Query: contig00016.g8293											
# Database: /data/phi-blast/fasta/Phi417/phi417.fas											
# Fields: query id	subject id	% identity	alignment length	mismatches	gap opens	q. start	q. end	s. start	s. end	evalue	bit score
# 6 hits found											
contig00016.g8293	B7UM99#PHI:10377#Tir#562#Escherichia_coli#effector_(plant_avirulence_determinant)	55.56	18	8	0	28	45	128	145	3.3	25.4
# BLASTP 2.2.30+											
# Query: contig00017.g8373											
# Database: /data/phi-blast/fasta/Phi417/phi417.fas											
# Fields: query id	subject id	% identity	alignment length	mismatches	gap opens	q. start	q. end	s. start	s. end	evalue	bit score
# 3 hits found											
contig00017.g8373	Q4WKJ9#PHI:2546#BRX1#746128#Aspergillus_fumigatus#lethal	32.14	28	19	0	96	123	150	177	5.5	25.8
# BLASTP 2.2.30+											
# Query: contig00017.g8375											
# Database: /data/phi-blast/fasta/Phi417/phi417.fas											
# Fields: query id	subject id	% identity	alignment length	mismatches	gap opens	q. start	q. end	s. start	s. end	evalue	bit score
# 7 hits found											
contig00017.g8375	Q5WV98#PHI:9873#RalF#446#Legionella_pneumophila#unaffected_pathogenicity	41.46	41	24	0	33	73	18	58	0.51	27.7
# BLASTP 2.2.30+											
# Query: contig00017.g8393											
# Database: /data/phi-blast/fasta/Phi417/phi417.fas											
# Fields: query id	subject id	% identity	alignment length	mismatches	gap opens	q. start	q. end	s. start	s. end	evalue	bit score
# 9 hits found											
contig00017.g8393	A0A290WHN4#PHI:7704#VEDA_05194#27337#Verticillium_dahliae#reduced_virulence_unaffected_	26.37	201	112	5	1	173	115	307	2,00E-11	60.1
# BLASTP 2.2.30+											

# Query: contig00017.g8423											
# Database: /data/phi-blast/fasta/Phi417/phi417.fas											
# Fields: query id	subject id	% identity	alignment length	mismatches	gap opens	q. start	q. end	s. start	s. end	evalue	bit score
# 12 hits found											
contig00017.g8423	A0A0E0S1S6#PHI:9097#Fgist1_(FGSG_12578)#5518#Fusarium_graminearum#reduced_virulence	98.06	206	4	0	1	206	1	206	4,00E-144	403
# BLASTP 2.2.30+											
# Query: contig00017.g8428											
# Database: /data/phi-blast/fasta/Phi417/phi417.fas											
# Fields: query id	subject id	% identity	alignment length	mismatches	gap opens	q. start	q. end	s. start	s. end	evalue	bit score
# 9 hits found											
contig00017.g8428	A0A194WBI8#PHI:10201#DCL2#105487#Valsa_mali#reduced_virulence	32.08	53	35	1	42	93	43	95	1.1	27.3
# BLASTP 2.2.30+											
# Query: contig00017.g8432											
# Database: /data/phi-blast/fasta/Phi417/phi417.fas											
# Fields: query id	subject id	% identity	alignment length	mismatches	gap opens	q. start	q. end	s. start	s. end	evalue	bit score
# 51 hits found											
contig00017.g8432	I1RGC9#PHI:1359#GzC2H019#5518#Fusarium_graminearum#unaffected_pathogenicity	83.91	174	20	2	1	166	1	174	2,00E-78	233
# BLAST processed 61 queries											
# BLASTP 2.2.30+											
# Query: contig00017.g8532											
# Database: /data/phi-blast/fasta/Phi417/phi417.fas											
# Fields: query id	subject id	% identity	alignment length	mismatches	gap opens	q. start	q. end	s. start	s. end	evalue	bit score
# 9 hits found											
contig00017.g8532	Q889C0#PHI:587#PSPTO0834#317#Pseudomonas_syringae#unaffected_pathogenicity	53.33	30	6	2	25	47	190	218	1.1	26.9
# BLASTP 2.2.30+											
# Query: contig00017.g8591											
# Database: /data/phi-blast/fasta/Phi417/phi417.fas											
# Fields: query id	subject id	% identity	alignment length	mismatches	gap opens	q. start	q. end	s. start	s. end	evalue	bit score
# 6 hits found											
contig00017.g8591	Q9P872#PHI:440#PMR1#5476#Candida_albicans#reduced_virulence	37.14	35	22	0	94	128	543	577	0.50	29.3
# BLASTP 2.2.30+											
# Query: contig00018.g8665											
# Database: /data/phi-blast/fasta/Phi417/phi417.fas											

# Fields: query id	subject id	% identity	alignment length	mismatches	gap opens	q. start	q. end	s. start	s. end	evalue	bit score
# 4 hits found											
contig00018.g8665	I1RPV7#PHI:1227#FGSG_06089#5518#Fusarium_graminearum#lethal	24.00	75	49	1	51	117	121	195	0.67	28.1
# BLASTP 2.2.30+											
# Query: contig00018.g8693											
# Database: /data/phi-blast/fasta/Phi417/phi417.fas											
# Fields: query id	subject id	% identity	alignment length	mismatches	gap opens	q. start	q. end	s. start	s. end	evalue	bit score
# 10 hits found											
contig00018.g8693	A0A0R6L508#PHI:11668#Mcr-1#562#Escherichia_coli#increased_virulence_(hypervirulence)	37.50	56	29	2	44	93	286	341	1.5	28.5
# BLASTP 2.2.30+											
# Query: contig00018.g8746											
# Database: /data/phi-blast/fasta/Phi417/phi417.fas											
# Fields: query id	subject id	% identity	alignment length	mismatches	gap opens	q. start	q. end	s. start	s. end	evalue	bit score
# 5 hits found											
contig00018.g8746	Q4UWE2#PHI:3966#XC_1565#339#Xanthomonas_campestris#reduced_virulence	41.38	29	17	0	12	40	197	225	1.3	26.9
# BLASTP 2.2.30+											
# Query: contig00018.g8811											
# Database: /data/phi-blast/fasta/Phi417/phi417.fas											
# Fields: query id	subject id	% identity	alignment length	mismatches	gap opens	q. start	q. end	s. start	s. end	evalue	bit score
# 5 hits found											
contig00018.g8811	G4NGB1#PHI:3311_PHI:5661#Pcf1_PCF1#318829#Magnaporthe_oryzae#reduced_virulence	24.10	83	58	2	119	201	400	477	2.3	28.5
# BLASTP 2.2.30+											
# Query: contig00018.g8815											
# Database: /data/phi-blast/fasta/Phi417/phi417.fas											
# Fields: query id	subject id	% identity	alignment length	mismatches	gap opens	q. start	q. end	s. start	s. end	evalue	bit score
# 3 hits found											
contig00018.g8815	D0CDA1#PHI:5094#PLD3#470#Acinetobacter_baumannii#reduced_virulence	21.24	113	75	3	30	140	185	285	0.23	29.6
# BLASTP 2.2.30+											
# Query: contig00018.g8851											
# Database: /data/phi-blast/fasta/Phi417/phi417.fas											
# Fields: query id	subject id	% identity	alignment length	mismatches	gap opens	q. start	q. end	s. start	s. end	evalue	bit score
# 7 hits found											

contig00018.g8851	A7H2F1#PHI:8906#HtrA#197#Campylobacter_jejuni#reduced_virulence	25.40	63	31	1	122	184	299	345	1.4	29.3
# BLASTP 2.2.30+											
# Query: contig00018.g8852											
# Database: /data/phi-blast/fasta/Phi417/phi417.fas											
# Fields: query id	subject id	% identity	alignment length	mismatches	gap opens	q. start	q. end	s. start	s. end	evalue	bit score
# 2 hits found											
contig00018.g8852	Q4WUA3#PHI:2532#HEM15#746128#Aspergillus_fumigatus#lethal	58.33	24	7	2	57	77	169	192	4.4	25.4
# BLASTP 2.2.30+											
# Query: contig00018.g8890											
# Database: /data/phi-blast/fasta/Phi417/phi417.fas											
# Fields: query id	subject id	% identity	alignment length	mismatches	gap opens	q. start	q. end	s. start	s. end	evalue	bit score
# 5 hits found											
contig00018.g8890	A4HTK3#PHI:2643#CFAS#5671#Leishmania_infantum#reduced_virulence	27.71	83	51	1	12	85	91	173	0.50	27.3
# BLASTP 2.2.30+											
# Query: contig00018.g8892											
# Database: /data/phi-blast/fasta/Phi417/phi417.fas											
# Fields: query id	subject id	% identity	alignment length	mismatches	gap opens	q. start	q. end	s. start	s. end	evalue	bit score
# 318 hits found											
contig00018.g8892	Q5AP71#PHI:427#TPK1#5476#Candida_albicans#unaffected_pathogenicity	28.57	84	60	0	100	183	168	251	3,00E-06	45.4
# BLASTP 2.2.30+											
# Query: contig00018.g8897											
# Database: /data/phi-blast/fasta/Phi417/phi417.fas											
# Fields: query id	subject id	% identity	alignment length	mismatches	gap opens	q. start	q. end	s. start	s. end	evalue	bit score
# 12 hits found											
contig00018.g8897	Q2VF46#PHI:2383#MfCUT1#38448#Monilinia_fructicola#increased_virulence_(hypervirulence)	26.21	206	114	6	22	221	27	200	3,00E-06	45.1
# BLASTP 2.2.30+											
# Query: contig00018.g8902											
# Database: /data/phi-blast/fasta/Phi417/phi417.fas											
# Fields: query id	subject id	% identity	alignment length	mismatches	gap opens	q. start	q. end	s. start	s. end	evalue	bit score
# 3 hits found											
contig00018.g8902	A0A1C3YMK9#PHI:1832#GzZC147#5518#Fusarium_graminearum#unaffected_pathogenicity	28.26	46	33	0	34	79	542	587	1.5	26.6
contig00018.g8902	G2WY50#PHI:4909#VdQase#27337#Verticillium_dahliae#reduced_virulence	37.50	32	17	1	8	36	417	448	4.2	25.0

contig00018.g8902	G2WUQ0#PHI:10379#Isw2#27337#Verticillium_dahliae#reduced_virulence	26.00	50	28	1	53	93	294	343	5.7	24.6
# BLASTP 2.2.30+											
# Query: contig00018.g8904											
# Database: /data/phi-blast/fasta/Phi417/phi417.fas											
# Fields: query id	subject id	% identity	alignment length	mismatches	gap opens	q. start	q. end	s. start	s. end	evalue	bit score
# 9 hits found											
contig00018.g8904	W2E906#PHI:3274#DhbF#1464#Paenibacillus_larvae#unaffected_pathogenicity	38.24	34	21	0	63	96	1243	1276	0.065	30.4
# BLASTP 2.2.30+											
# Query: contig00018.g8916											
# Database: /data/phi-blast/fasta/Phi417/phi417.fas											
# Fields: query id	subject id	% identity	alignment length	mismatches	gap opens	q. start	q. end	s. start	s. end	evalue	bit score
# 9 hits found											
contig00018.g8916	A0A0W0EMV0#PHI:123298#GAP1#5476#Candida_albicans#reduced_virulence	37.93	58	36	0	50	107	46	103	3,00E-05	40.8
# BLASTP 2.2.30+											
# Query: contig00018.g8934											
# Database: /data/phi-blast/fasta/Phi417/phi417.fas											
# Fields: query id	subject id	% identity	alignment length	mismatches	gap opens	q. start	q. end	s. start	s. end	evalue	bit score
# 4 hits found											
contig00018.g8934	C5AMV9#PHI:12130#Bglu_2g07410#337#Burkholderia_glumae#reduced_virulence	23.74	139	86	5	123	253	14	140	0.009	36.2
# BLASTP 2.2.30+											
# Query: contig00019.g9015											
# Database: /data/phi-blast/fasta/Phi417/phi417.fas											
# Fields: query id	subject id	% identity	alignment length	mismatches	gap opens	q. start	q. end	s. start	s. end	evalue	bit score
# 4 hits found											
contig00019.g9015	A0A0E0UWL3#PHI:9214#VirR#1639#Listeria_monocytogenes#reduced_virulence_unaffected_pathoge	27.68	177	105	5	3	169	49	212	0.094	31.6
# BLASTP 2.2.30+											
# Query: contig00019.g9037											
# Database: /data/phi-blast/fasta/Phi417/phi417.fas											
# Fields: query id	subject id	% identity	alignment length	mismatches	gap opens	q. start	q. end	s. start	s. end	evalue	bit score
# 9 hits found											
contig00019.g9037	W7N2B2#PHI:3388#FVEG_12522#117187#Fusarium_verticillioides#unaffected_pathogenicity	29.29	99	63	1	10	101	5	103	3,00E-08	47.0
# BLASTP 2.2.30+											
# Query: contig00019.g9095											

# Database: /data/phi-blast/fasta/Phi417/phi417.fas											
# Fields: query id	subject id	% identity	alignment length	mismatches	gap opens	q. start	q. end	s. start	s. end	evalue	bit score
# 3 hits found											
contig00019.g9095	Q7WYI8#PHI:11571#MtsA#1311#Streptococcus_agalactiae#reduced_virulence	34.69	49	27	2	23	69	112	157	2.4	25.0
# BLASTP 2.2.30+											
# Query: contig00019.g9113											
# Database: /data/phi-blast/fasta/Phi417/phi417.fas											
# Fields: query id	subject id	% identity	alignment length	mismatches	gap opens	q. start	q. end	s. start	s. end	evalue	bit score
# 12 hits found											
contig00019.g9113	A0A1B7Y872#PHI:11711#ChHxt3_(XP_018156754)#80884#Colletotrichum_higginsianum#unaffected_p	31.71	41	28	0	96	136	20	60	1.4	28.1
# BLASTP 2.2.30+											
# Query: contig00019.g9128											
# Database: /data/phi-blast/fasta/Phi417/phi417.fas											
# Fields: query id	subject id	% identity	alignment length	mismatches	gap opens	q. start	q. end	s. start	s. end	evalue	bit score
# 4 hits found											
contig00019.g9128	I1RH33#PHI:5901#FGSG_03072#5518#Fusarium_graminearum#unaffected_pathogenicity	30.91	55	27	1	20	63	389	443	0.28	28.1
# BLASTP 2.2.30+											
# Query: contig00019.g9169											
# Database: /data/phi-blast/fasta/Phi417/phi417.fas											
# Fields: query id	subject id	% identity	alignment length	mismatches	gap opens	q. start	q. end	s. start	s. end	evalue	bit score
# 2 hits found											
contig00019.g9169	Q5VDC5#PHI:8801#HexA#5059#Aspergillus_flavus#reduced_virulence	42.31	26	15	0	87	112	754	779	2.5	27.3
# BLASTP 2.2.30+											
# Query: contig00020.g9266											
# Database: /data/phi-blast/fasta/Phi417/phi417.fas											
# Fields: query id	subject id	% identity	alignment length	mismatches	gap opens	q. start	q. end	s. start	s. end	evalue	bit score
# 6 hits found											
contig00020.g9266	A0A8E5JM30#PHI:12047#AcrD#28901#Salmonella_enterica#reduced_virulence	35.90	39	24	1	68	106	616	653	1.9	26.9
# BLASTP 2.2.30+											
# Query: contig00020.g9295											
# Database: /data/phi-blast/fasta/Phi417/phi417.fas											
# Fields: query id	subject id	% identity	alignment length	mismatches	gap opens	q. start	q. end	s. start	s. end	evalue	bit score
# 9 hits found											

contig00020.g9295	Q2VLJ3#PHI:713#PKS13_(ZEA2)#5518#Fusarium_graminearum#unaffected_pathogenicity	31.91	94	51	4	75	161	1166	1253	0.23	30.4
# BLASTP 2.2.30+											
# Query: contig00020.g9341											
# Database: /data/phi-blast/fasta/Phi417/phi417.fas											
# Fields: query id	subject id	% identity	alignment length	mismatches	gap opens	q. start	q. end	s. start	s. end	evalue	bit score
# 9 hits found											
contig00020.g9341	O94072#PHI:9703#VMA4#5476#Candida_albicans#loss_of_pathogenicity	52.89	225	106	0	5	229	2	226	7,00E-71	218
# BLASTP 2.2.30+											
# Query: contig00020.g9354											
# Database: /data/phi-blast/fasta/Phi417/phi417.fas											
# Fields: query id	subject id	% identity	alignment length	mismatches	gap opens	q. start	q. end	s. start	s. end	evalue	bit score
# 4 hits found											
contig00020.g9354	G1XK81#PHI:123294#AoSW16_(AOL_s00109g110)#2813651#Orbilia_oligospora#reduced_virulence	35.71	28	18	0	120	147	306	333	3.7	26.2
# BLASTP 2.2.30+											
# Query: contig00020.g9370											
# Database: /data/phi-blast/fasta/Phi417/phi417.fas											
# Fields: query id	subject id	% identity	alignment length	mismatches	gap opens	q. start	q. end	s. start	s. end	evalue	bit score
# 11 hits found											
contig00020.g9370	B4EMX8#PHI:5294#BceF#95486#Burkholderia_cenocepacia#reduced_virulence	35.29	34	22	0	53	86	110	143	2.3	26.9
# BLASTP 2.2.30+											
# Query: contig00020.g9374											
# Database: /data/phi-blast/fasta/Phi417/phi417.fas											
# Fields: query id	subject id	% identity	alignment length	mismatches	gap opens	q. start	q. end	s. start	s. end	evalue	bit score
# 7 hits found											
contig00020.g9374	G4MKC3#PHI:123399#DHX35_(MGG_02518)#318829#Magnaporthe_oryzae#reduced_virulence	28.30	53	33	1	114	161	230	282	3.7	26.6
# BLASTP 2.2.30+											
# Query: contig00020.g9384											
# Database: /data/phi-blast/fasta/Phi417/phi417.fas											
# Fields: query id	subject id	% identity	alignment length	mismatches	gap opens	q. start	q. end	s. start	s. end	evalue	bit score
# 4 hits found											
contig00020.g9384	I1RW90#PHI:1627#GzTF2S002#5518#Fusarium_graminearum#unaffected_pathogenicity	90.00	110	11	0	1	110	1	110	1,00E-73	216
# BLASTP 2.2.30+											
# Query: contig00020.g9402											

# Database: /data/phi-blast/fasta/Phi417/phi417.fas											
# Fields: query id	subject id	% identity	alignment length	mismatches	gap opens	q. start	q. end	s. start	s. end	evalue	bit score
# 7 hits found											
contig00020.g9402	J4US35#PHI:8673#CryD_(EJP68422)#176275#Beauveria_bassiana#unaffected_pathogenicity	35.00	40	21	2	61	96	322	360	1.8	26.6
# BLASTP 2.2.30+											
# Query: contig00020.g9431											
# Database: /data/phi-blast/fasta/Phi417/phi417.fas											
# Fields: query id	subject id	% identity	alignment length	mismatches	gap opens	q. start	q. end	s. start	s. end	evalue	bit score
# 12 hits found											
contig00020.g9431	A0A384JPG6#PHI:10716_PHI:124333#PdeR_(BCIN_08g00160)#40559#Botrytis_cinerea#reduced_virul	31.71	41	28	0	67	107	341	381	0.83	27.7
# BLASTP 2.2.30+											
# Query: contig00021.g9501											
# Database: /data/phi-blast/fasta/Phi417/phi417.fas											
# Fields: query id	subject id	% identity	alignment length	mismatches	gap opens	q. start	q. end	s. start	s. end	evalue	bit score
# 12 hits found											
contig00021.g9501	Q9K5C7#PHI:123215#CelA#28447#Clavibacter_michiganensis#unaffected_pathogenicity	30.89	191	122	5	24	209	547	732	3,00E-27	108
# BLASTP 2.2.30+											
# Query: contig00021.g9599											
# Database: /data/phi-blast/fasta/Phi417/phi417.fas											
# Fields: query id	subject id	% identity	alignment length	mismatches	gap opens	q. start	q. end	s. start	s. end	evalue	bit score
# 9 hits found											
contig00021.g9599	Q5NGJ3#PHI:4730#FTT0846#263#Francisella_tularensis#reduced_virulence	30.65	62	43	0	29	90	51	112	0.30	29.6
# BLASTP 2.2.30+											
# Query: contig00022.g9836											
# Database: /data/phi-blast/fasta/Phi417/phi417.fas											
# Fields: query id	subject id	% identity	alignment length	mismatches	gap opens	q. start	q. end	s. start	s. end	evalue	bit score
# 9 hits found											
contig00022.g9836	Q8Z445#PHI:562#BarA#28901#Salmonella_enterica#unaffected_pathogenicity	38.89	36	15	2	32	60	754	789	1.8	25.4
# BLASTP 2.2.30+											
# Query: contig00022.g9893											
# Database: /data/phi-blast/fasta/Phi417/phi417.fas											
# Fields: query id	subject id	% identity	alignment length	mismatches	gap opens	q. start	q. end	s. start	s. end	evalue	bit score
# 9 hits found											

contig00022.g9893	Q8XZP7#PHI:123576#Cbl_(RSc1348)#305#Ralstonia_solanacearum#unaffected_pathogenicity	47.83	23	12	0	90	112	37	59	3.7	25.4
# BLASTP 2.2.30+											
# Query: contig00022.g9911											
# Database: /data/phi-blast/fasta/Phi417/phi417.fas											
# Fields: query id	subject id	% identity	alignment length	mismatches	gap opens	q. start	q. end	s. start	s. end	evalue	bit score
# 4 hits found											
contig00022.g9911	A0A098DWX2#PHI:1733#GzZC048#5518#Fusarium_graminearum#unaffected_pathogenicity	46.88	32	13	2	21	50	618	647	1.6	27.3
# BLASTP 2.2.30+											
# Query: contig00022.g10003											
# Database: /data/phi-blast/fasta/Phi417/phi417.fas											
# Fields: query id	subject id	% identity	alignment length	mismatches	gap opens	q. start	q. end	s. start	s. end	evalue	bit score
# 1 hits found											
contig00022.g10003	Q96VZ3#PHI:181#PGX1#5507#Fusarium_oxysporum#unaffected_pathogenicity	24.10	83	55	3	49	130	95	170	0.10	30.8
# BLASTP 2.2.30+											
# Query: contig00023.g10080											
# Database: /data/phi-blast/fasta/Phi417/phi417.fas											
# Fields: query id	subject id	% identity	alignment length	mismatches	gap opens	q. start	q. end	s. start	s. end	evalue	bit score
# 8 hits found											
contig00023.g10080	Q8Y0F9#PHI:11568#NagL#305#Ralstonia_solanacearum#reduced_virulence	37.78	45	28	0	1	45	33	77	0.003	35.8
# BLASTP 2.2.30+											
# Query: contig00023.g10162											
# Database: /data/phi-blast/fasta/Phi417/phi417.fas											
# Fields: query id	subject id	% identity	alignment length	mismatches	gap opens	q. start	q. end	s. start	s. end	evalue	bit score
# 1 hits found											
contig00023.g10162	Q83FB9#PHI:6350_PHI:6355_PHI:6415#CvpB_Cig2#777#Coxiella_burnetii#effector_(plant_avirulence_	32.56	43	29	0	126	168	385	427	8.3	25.8
# BLASTP 2.2.30+											
# Query: contig00024.g10402											
# Database: /data/phi-blast/fasta/Phi417/phi417.fas											
# Fields: query id	subject id	% identity	alignment length	mismatches	gap opens	q. start	q. end	s. start	s. end	evalue	bit score
# 4 hits found											
contig00024.g10402	Q9UVN5#PHI:160#AMT#5599#Alternaria_alternata#loss_of_pathogenicity	26.32	57	39	1	65	118	1405	1461	0.15	30.0
# BLASTP 2.2.30+											
# Query: contig00024.g10449											

# Database: /data/phi-blast/fasta/Phi417/phi417.fas											
# Fields: query id	subject id	% identity	alignment length	mismatches	gap opens	q. start	q. end	s. start	s. end	evalue	bit score
# 4 hits found											
contig00024.g10449	Q2T7Q2#PHI:10163#Ohr_(BTH_II0597)#57975#Burkholderia_thailandensis#increased_virulence_(hyp	37.78	135	77	4	41	173	3	132	2,00E-22	88.2
# BLASTP 2.2.30+											
# Query: contig00024.g10556											
# Database: /data/phi-blast/fasta/Phi417/phi417.fas											
# Fields: query id	subject id	% identity	alignment length	mismatches	gap opens	q. start	q. end	s. start	s. end	evalue	bit score
# 9 hits found											
contig00024.g10556	Q9UVB5#PHI:452#PMT6#5476#Candida_albicans#reduced_virulence	28.57	56	34	3	99	148	596	651	0.25	30.4
# BLASTP 2.2.30+											
# Query: contig00025.g10725											
# Database: /data/phi-blast/fasta/Phi417/phi417.fas											
# Fields: query id	subject id	% identity	alignment length	mismatches	gap opens	q. start	q. end	s. start	s. end	evalue	bit score
# 4 hits found											
contig00025.g10725	A0A0D2XG21#PHI:11461#Mt1_(FOXG_02862)#5507#Fusarium_oxysporum#unaffected_pathogenicity_reduced_virulence	35.56	45	21	2	89	132	35	72	1.8	26.6
# BLASTP 2.2.30+											
# Query: contig00025.g10754											
# Database: /data/phi-blast/fasta/Phi417/phi417.fas											
# Fields: query id	subject id	% identity	alignment length	mismatches	gap opens	q. start	q. end	s. start	s. end	evalue	bit score
# 20 hits found											
contig00025.g10754	Q9UUS7#PHI:166#CHIP2#474922#Colletotrichum_gloeosporioides#unaffected_pathogenicity	29.29	379	240	4	170	542	82	438	1,00E-38	148
# BLASTP 2.2.30+											
# Query: contig00026.g10923											
# Database: /data/phi-blast/fasta/Phi417/phi417.fas											
# Fields: query id	subject id	% identity	alignment length	mismatches	gap opens	q. start	q. end	s. start	s. end	evalue	bit score
# 18 hits found											
contig00026.g10923	Q4X1I3#PHI:2545#TIF35#746128#Aspergillus_fumigatus#lethal	38.03	71	39	2	16	83	212	280	2,00E-07	47.4
# BLASTP 2.2.30+											
# Query: contig00026.g10960											
# Database: /data/phi-blast/fasta/Phi417/phi417.fas											
# Fields: query id	subject id	% identity	alignment length	mismatches	gap opens	q. start	q. end	s. start	s. end	evalue	bit score

# 8 hits found											
contig00026.g10960	B4EZP9#PHI:123443#NikA#584#Proteus_mirabilis# reduced_virulence_unaffected_pathogenicity	34.48	29	19	0	58	86	93	121	1.8	25.8
# BLASTP 2.2.30+											
# Query: contig00027.g11201											
# Database: /data/phi-blast/fasta/Phi417/phi417.fas											
# Fields: query id	subject id	% identity	alignment length	mismatches	gap opens	q. start	q. end	s. start	s. end	evalue	bit score
# 4 hits found											
contig00027.g11201	A0A0F6B5U1#PHI:10094#STM14_RS15405_(prgI)# 28901#Salmonella_enterica#unaffected_pathogeni	29.69	64	38	1	32	95	24	80	0.84	26.9
# BLASTP 2.2.30+											
# Query: contig00028.g11218											
# Database: /data/phi-blast/fasta/Phi417/phi417.fas											
# Fields: query id	subject id	% identity	alignment length	mismatches	gap opens	q. start	q. end	s. start	s. end	evalue	bit score
# 8 hits found											
contig00028.g11218	F9XEG7#PHI:124178#BCK1#1047171#Zymoseptori a_tritici#reduced_virulence	27.17	92	62	3	42	129	1462	1552	0.092	31.2
# BLASTP 2.2.30+											
# Query: contig00028.g11232											
# Database: /data/phi-blast/fasta/Phi417/phi417.fas											
# Fields: query id	subject id	% identity	alignment length	mismatches	gap opens	q. start	q. end	s. start	s. end	evalue	bit score
# 3 hits found											
contig00028.g11232	Q9HV88#PHI:6944#PhuR#287#Pseudomonas_aeru ginosa#increased_virulence_(hypervirulence)	28.89	45	29	1	6	50	443	484	0.63	28.9
# BLASTP 2.2.30+											
# Query: contig00028.g11292											
# Database: /data/phi-blast/fasta/Phi417/phi417.fas											
# Fields: query id	subject id	% identity	alignment length	mismatches	gap opens	q. start	q. end	s. start	s. end	evalue	bit score
# 7 hits found											
contig00028.g11292	Q00LS5#PHI:1082_PHI:2271#CpkA#13684#Parasta gonospora_nodorum#reduced_virulence_unaffecte d_pathogenicity	32.00	75	44	2	50	121	304	374	0.54	28.9
# BLASTP 2.2.30+											
# Query: contig00028.g11312											
# Database: /data/phi-blast/fasta/Phi417/phi417.fas											
# Fields: query id	subject id	% identity	alignment length	mismatches	gap opens	q. start	q. end	s. start	s. end	evalue	bit score
# 2 hits found											
contig00028.g11312	Q2Q466#PHI:1063#MNH6#318829#Magnaporthe_ oryzae#reduced_virulence	30.56	36	25	0	87	122	50	85	0.87	27.7

# BLASTP 2.2.30+											
# Query: contig00029.g11349											
# Database: /data/phi-blast/fasta/Phi417/phi417.fas											
# Fields: query id	subject id	% identity	alignment length	mismatches	gap opens	q. start	q. end	s. start	s. end	evalue	bit score
# 6 hits found											
contig00029.g11349	Q4WUN7#PHI:6085#MpkC#746128#Aspergillus_fumigatus#reduced_virulence	27.78	72	40	3	46	106	279	349	3.5	26.2
# BLASTP 2.2.30+											
# Query: contig00029.g11351											
# Database: /data/phi-blast/fasta/Phi417/phi417.fas											
# Fields: query id	subject id	% identity	alignment length	mismatches	gap opens	q. start	q. end	s. start	s. end	evalue	bit score
# 8 hits found											
contig00029.g11351	G4N8G2#PHI:8522#MoYPEL1_(MGG_06263.7)#318829#Magnaporthe_oryzae#loss_of_pathogenicity	84.55	110	17	0	1	110	63	172	3,00E-69	207
# BLASTP 2.2.30+											
# Query: contig00029.g11451											
# Database: /data/phi-blast/fasta/Phi417/phi417.fas											
# Fields: query id	subject id	% identity	alignment length	mismatches	gap opens	q. start	q. end	s. start	s. end	evalue	bit score
# 6 hits found											
contig00029.g11451	B1GVX7#PHI:2290#BcBOA6#40559#Botrytis_cinerea#reduced_virulence	27.78	54	39	0	152	205	467	520	1.5	29.3
# BLASTP 2.2.30+											
# Query: contig00029.g11479											
# Database: /data/phi-blast/fasta/Phi417/phi417.fas											
# Fields: query id	subject id	% identity	alignment length	mismatches	gap opens	q. start	q. end	s. start	s. end	evalue	bit score
# 9 hits found											
contig00029.g11479	I1RV69#PHI:1234#FGSG_08133#5518#Fusarium_graminearum#lethal	26.67	60	42	1	80	137	1053	1112	0.36	30.4
# BLASTP 2.2.30+											
# Query: contig00030.g11520											
# Database: /data/phi-blast/fasta/Phi417/phi417.fas											
# Fields: query id	subject id	% identity	alignment length	mismatches	gap opens	q. start	q. end	s. start	s. end	evalue	bit score
# 10 hits found											
contig00030.g11520	E5AD52#PHI:4519#LmStuA#5022#Leptosphaeria_maculans#loss_of_pathogenicity	37.78	45	26	1	55	97	158	202	0.75	29.3
# BLASTP 2.2.30+											
# Query: contig00030.g11576											
# Database: /data/phi-blast/fasta/Phi417/phi417.fas											

# Fields: query id	subject id	% identity	alignment length	mismatches	gap opens	q. start	q. end	s. start	s. end	evalue	bit score
# 6 hits found											
contig00030.g11576	Q874K8#PHI:286#CLC-A#5207#Cryptococcus_neoformans#reduced_virule	28.70	108	70	4	7	114	604	704	0.001	37.7
# BLASTP 2.2.30+											
# Query: contig00030.g11595											
# Database: /data/phi-blast/fasta/Phi417/phi417.fas											
# Fields: query id	subject id	% identity	alignment length	mismatches	gap opens	q. start	q. end	s. start	s. end	evalue	bit score
# 73 hits found											
contig00030.g11595	G4MYS1#PHI:4736_PHI:11140#MoYpt7_MGG_08144_(MoYpt7)#318829#Magnaporthe_oryzae#loss_of_pathogenicity_reduced_virulence_unaffected_p	27.92	197	133	3	1	188	1	197	7,00E-27	103
# BLASTP 2.2.30+											
# Query: contig00030.g11651											
# Database: /data/phi-blast/fasta/Phi417/phi417.fas											
# Fields: query id	subject id	% identity	alignment length	mismatches	gap opens	q. start	q. end	s. start	s. end	evalue	bit score
# 7 hits found											
contig00030.g11651	G2WS86#PHI:123307#Vdchs5_(VDAG_00419)#27337#Verticillium_dahliae#reduced_virulence	37.74	53	26	3	136	186	987	1034	0.70	29.6
# BLAST processed 59 queries											
# BLASTP 2.2.30+											
# Query: contig00031.g11684											
# Database: /data/phi-blast/fasta/Phi417/phi417.fas											
# Fields: query id	subject id	% identity	alignment length	mismatches	gap opens	q. start	q. end	s. start	s. end	evalue	bit score
# 6 hits found											
contig00031.g11684	Q9HX66#PHI:11622_PHI:11623#RhlE1_(PA3950)_RhlE2_(PA0428)#287#Pseudomonas_aeruginosa#reduced_virulence	30.77	39	27	0	95	133	247	285	0.89	28.5
# BLASTP 2.2.30+											
# Query: contig00031.g11770											
# Database: /data/phi-blast/fasta/Phi417/phi417.fas											
# Fields: query id	subject id	% identity	alignment length	mismatches	gap opens	q. start	q. end	s. start	s. end	evalue	bit score
# 14 hits found											
contig00031.g11770	Q04701#PHI:179#PELA#169388#Fusarium_solani#reduced_virulence	87.60	242	28	1	1	240	1	242	3,00E-159	444
# BLASTP 2.2.30+											
# Query: contig00031.g11796											
# Database: /data/phi-blast/fasta/Phi417/phi417.fas											
# Fields: query id	subject id	% identity	alignment length	mismatches	gap opens	q. start	q. end	s. start	s. end	evalue	bit score

# 4 hits found											
contig00031.g11796	G4N6T3#PHI:9356#Leu4_(MGG_13485)#318829#Magnaporthe_oryzae#reduced_virulence	30.77	52	35	1	287	337	382	433	3.2	28.5
# BLASTP 2.2.30+											
# Query: contig00031.g11844											
# Database: /data/phi-blast/fasta/Phi417/phi417.fas											
# Fields: query id	subject id	% identity	alignment length	mismatches	gap opens	q. start	q. end	s. start	s. end	evalue	bit score
# 10 hits found											
contig00031.g11844	B8N4E0#PHI:9901#Aflste20_(AFLA_035530)#5059#Aspergillus_flavus#reduced_virulence	24.14	87	64	1	125	209	571	657	0.050	34.3
# BLASTP 2.2.30+											
# Query: contig00032.g11901											
# Database: /data/phi-blast/fasta/Phi417/phi417.fas											
# Fields: query id	subject id	% identity	alignment length	mismatches	gap opens	q. start	q. end	s. start	s. end	evalue	bit score
# 10 hits found											
contig00032.g11901	Q8Y0F9#PHI:11568#NagL#305#Ralstonia_solanacearum#reduced_virulence	22.82	206	140	7	32	232	15	206	3,00E-07	48.9
# BLASTP 2.2.30+											
# Query: contig00032.g11977											
# Database: /data/phi-blast/fasta/Phi417/phi417.fas											
# Fields: query id	subject id	% identity	alignment length	mismatches	gap opens	q. start	q. end	s. start	s. end	evalue	bit score
# 8 hits found											
contig00032.g11977	C1CHE9#PHI:4180#ArcD#1313#Streptococcus_pneumoniae#reduced_virulence	33.33	30	20	0	55	84	264	293	1.6	27.3
# BLASTP 2.2.30+											
# Query: contig00032.g11989											
# Database: /data/phi-blast/fasta/Phi417/phi417.fas											
# Fields: query id	subject id	% identity	alignment length	mismatches	gap opens	q. start	q. end	s. start	s. end	evalue	bit score
# 1 hits found											
contig00032.g11989	I1RF25#PHI:1657#GzCCCH001#5518#Fusarium_graminearum#unaffected_pathogenicity	20.87	115	76	4	30	134	1335	1444	1.7	28.5
# BLASTP 2.2.30+											
# Query: contig00033.g12059											
# Database: /data/phi-blast/fasta/Phi417/phi417.fas											
# Fields: query id	subject id	% identity	alignment length	mismatches	gap opens	q. start	q. end	s. start	s. end	evalue	bit score
# 6 hits found											
contig00033.g12059	Q4WIQ3#PHI:3277#GnoA#746128#Aspergillus_fumigatus#unaffected_pathogenicity	64.29	14	5	0	5	18	302	315	1.1	26.2
# BLASTP 2.2.30+											

# Query: contig00033.g12083											
# Database: /data/phi-blast/fasta/Phi417/phi417.fas											
# Fields: query id	subject id	% identity	alignment length	mismatches	gap opens	q. start	q. end	s. start	s. end	evalue	bit score
# 9 hits found											
contig00033.g12083	Q4WV27#PHI:7755_PHI:8601_PHI:9392#Cofilin_(AFUA_5G10570)#746128#Aspergillus_fumigatus#unaffected_pathogenicity_reduced_virulence_increased_virulence_(hypervirulence)	42.86	154	79	3	4	153	5	153	1,00E-36	125
# BLASTP 2.2.30+											
# Query: contig00033.g12088											
# Database: /data/phi-blast/fasta/Phi417/phi417.fas											
# Fields: query id	subject id	% identity	alignment length	mismatches	gap opens	q. start	q. end	s. start	s. end	evalue	bit score
# 8 hits found											
contig00033.g12088	J4VZT4#PHI:9444#Bbakr1_(XP_008600433.1)#176275#Beauveria_bassiana#unaffected_pathogenicity	38.04	276	148	8	13	281	38	297	9,00E-53	176
# BLASTP 2.2.30+											
# Query: contig00033.g12102											
# Database: /data/phi-blast/fasta/Phi417/phi417.fas											
# Fields: query id	subject id	% identity	alignment length	mismatches	gap opens	q. start	q. end	s. start	s. end	evalue	bit score
# 9 hits found											
contig00033.g12102	W7M5P0#PHI:2916#Ppr2#117187#Fusarium_verticillioides#unaffected_pathogenicity	23.27	159	105	4	8	159	488	636	0.32	29.6
# BLASTP 2.2.30+											
# Query: contig00034.g12201											
# Database: /data/phi-blast/fasta/Phi417/phi417.fas											
# Fields: query id	subject id	% identity	alignment length	mismatches	gap opens	q. start	q. end	s. start	s. end	evalue	bit score
# 10 hits found											
contig00034.g12201	I1RMV4#PHI:1464_PHI:8923_PHI:10389#GzCCAAT004_FgHLTF1_(FGSG_05304)_FCT2_(FGSG_05304)#5518#Fusarium_graminearum#reduced_virulence	83.52	182	25	2	1	177	1	182	5,00E-107	306
# BLASTP 2.2.30+											
# Query: contig00034.g12283											
# Database: /data/phi-blast/fasta/Phi417/phi417.fas											
# Fields: query id	subject id	% identity	alignment length	mismatches	gap opens	q. start	q. end	s. start	s. end	evalue	bit score
# 2 hits found											
contig00034.g12283	V5VGK1#PHI:11207#ClpB#470#Acinetobacter_baumannii#reduced_virulence	34.55	55	33	1	86	137	311	365	2.4	27.3
# BLASTP 2.2.30+											
# Query: contig00034.g12298											

# Database: /data/phi-blast/fasta/Phi417/phi417.fas											
# Fields: query id	subject id	% identity	alignment length	mismatches	gap opens	q. start	q. end	s. start	s. end	evalue	bit score
# 10 hits found											
contig00034.g12298	P15030#PHI:8008_PHI:11752#FecC#562#Escherichia_coli#reduced_virulence_unaffected_pathogenicit	56.25	16	7	0	28	43	5	20	2.5	26.2
# BLASTP 2.2.30+											
# Query: contig00035.g12323											
# Database: /data/phi-blast/fasta/Phi417/phi417.fas											
# Fields: query id	subject id	% identity	alignment length	mismatches	gap opens	q. start	q. end	s. start	s. end	evalue	bit score
# 8 hits found											
contig00035.g12323	H7C7L2#PHI:7821#OppF_(bb0335)#139#Borreliella_burgdorferi#reduced_virulence	34.62	52	33	1	101	151	190	241	0.75	28.9
# BLASTP 2.2.30+											
# Query: contig00035.g12360											
# Database: /data/phi-blast/fasta/Phi417/phi417.fas											
# Fields: query id	subject id	% identity	alignment length	mismatches	gap opens	q. start	q. end	s. start	s. end	evalue	bit score
# 5 hits found											
contig00035.g12360	B7VSJ8#PHI:3018#Vsp#212663#Vibrio_tasmaniensis#reduced_virulence	38.89	36	21	1	51	85	276	311	0.39	27.7
# BLASTP 2.2.30+											
# Query: contig00035.g12386											
# Database: /data/phi-blast/fasta/Phi417/phi417.fas											
# Fields: query id	subject id	% identity	alignment length	mismatches	gap opens	q. start	q. end	s. start	s. end	evalue	bit score
# 3 hits found											
contig00035.g12386	A0A125YUJ7#PHI:123717#PYK2#5811#Toxoplasma_gondii#unaffected_pathogenicity	37.04	27	16	1	59	84	939	965	4.3	26.6
# BLASTP 2.2.30+											
# Query: contig00035.g12411											
# Database: /data/phi-blast/fasta/Phi417/phi417.fas											
# Fields: query id	subject id	% identity	alignment length	mismatches	gap opens	q. start	q. end	s. start	s. end	evalue	bit score
# 31 hits found											
contig00035.g12411	W7MNK7#PHI:7147#FvPEX14#117187#Fusarium_verticillioides#unaffected_pathogenicity	20.00	135	97	3	49	178	192	320	0.092	32.7
# BLASTP 2.2.30+											
# Query: contig00036.g12528											
# Database: /data/phi-blast/fasta/Phi417/phi417.fas											
# Fields: query id	subject id	% identity	alignment length	mismatches	gap opens	q. start	q. end	s. start	s. end	evalue	bit score
# 8 hits found											

contig00036.g12528	I1RUR0#PHI:124331#HGG10_(FGSG_07962)#5518#Fusarium_graminearum#reduced_virulence	25.00	100	60	3	92	183	579	671	0.005	35.8
# BLASTP 2.2.30+											
# Query: contig00037.g12583											
# Database: /data/phi-blast/fasta/Phi417/phi417.fas											
# Fields: query id	subject id	% identity	alignment length	mismatches	gap opens	q. start	q. end	s. start	s. end	evalue	bit score
# 10 hits found											
contig00037.g12583	G2XE04#PHI:7873#VdSec22#27337#Verticillium_dahliae#reduced_virulence	27.34	128	86	3	75	196	69	195	3,00E-06	45.1
# BLASTP 2.2.30+											
# Query: contig00037.g12692											
# Database: /data/phi-blast/fasta/Phi417/phi417.fas											
# Fields: query id	subject id	% identity	alignment length	mismatches	gap opens	q. start	q. end	s. start	s. end	evalue	bit score
# 4 hits found											
contig00037.g12692	Q52EB3#PHI:2035_PHI:2069_PHI:8612_PHI:11533#ATG1_Moatg1_Atg_1_MoAtg1_(MGG_06393)#318829#Magnaporthe_oryzae#loss_of_pathogenicity_increased_virulence_(hypervirulence)_reduced_vi	29.31	58	40	1	5	62	431	487	1.4	27.7
# BLASTP 2.2.30+											
# Query: contig00038.g12721											
# Database: /data/phi-blast/fasta/Phi417/phi417.fas											
# Fields: query id	subject id	% identity	alignment length	mismatches	gap opens	q. start	q. end	s. start	s. end	evalue	bit score
# 6 hits found											
contig00038.g12721	J9VQN5#PHI:7881#Cer3_(CNAG_02087)#5207#Cryptococcus_neoformans#unaffected_pathogenicity	38.00	50	23	2	21	70	9	50	0.74	28.1
# BLASTP 2.2.30+											
# Query: contig00038.g12763											
# Database: /data/phi-blast/fasta/Phi417/phi417.fas											
# Fields: query id	subject id	% identity	alignment length	mismatches	gap opens	q. start	q. end	s. start	s. end	evalue	bit score
# 3 hits found											
contig00038.g12763	Q4WAU2#PHI:9148_PHI:11511#CpsA_Afcps1_(AFUA_7G02500)#746128#Aspergillus_fumigatus#reduc	34.38	32	21	0	86	117	43	74	2.6	26.2
# BLASTP 2.2.30+											
# Query: contig00038.g12793											
# Database: /data/phi-blast/fasta/Phi417/phi417.fas											
# Fields: query id	subject id	% identity	alignment length	mismatches	gap opens	q. start	q. end	s. start	s. end	evalue	bit score
# 51 hits found											
contig00038.g12793	Q5H5U8#PHI:7753#AnkB_(XOO0418)#347#Xanthomonas_oryzae#reduced_virulence	35.94	128	72	4	16	137	35	158	2,00E-13	63.9

# BLASTP 2.2.30+											
# Query: contig00039.g12831											
# Database: /data/phi-blast/fasta/Phi417/phi417.fas											
# Fields: query id	subject id	% identity	alignment length	mismatches	gap opens	q. start	q. end	s. start	s. end	evalue	bit score
# 15 hits found											
contig00039.g12831	P0AA28#PHI:2644#Thioredoxin_1#28901#Salmonella_enterica#reduced_virulence	20.69	87	69	0	22	108	19	105	0.22	28.9
# BLASTP 2.2.30+											
# Query: contig00040.g12904											
# Database: /data/phi-blast/fasta/Phi417/phi417.fas											
# Fields: query id	subject id	% identity	alignment length	mismatches	gap opens	q. start	q. end	s. start	s. end	evalue	bit score
# 5 hits found											
contig00040.g12904	A0A194VXQ8#PHI:11039#PKS16_(VM1G_03769)#105487#Valsa_mali#unaffected_pathogenicity	46.67	30	16	0	61	90	1635	1664	0.021	33.5
# BLASTP 2.2.30+											
# Query: contig00041.g13000											
# Database: /data/phi-blast/fasta/Phi417/phi417.fas											
# 0 hits found											
# BLASTP 2.2.30+											
# Query: contig00041.g13055											
# Database: /data/phi-blast/fasta/Phi417/phi417.fas											
# Fields: query id	subject id	% identity	alignment length	mismatches	gap opens	q. start	q. end	s. start	s. end	evalue	bit score
# 2 hits found											
contig00041.g13055	O51369#PHI:6524#FruA1#139#Borreliella_burgdorferi#unaffected_pathogenicity	27.59	58	39	1	33	87	153	210	7.3	25.0
# BLASTP 2.2.30+											
# Query: contig00042.g13149											
# Database: /data/phi-blast/fasta/Phi417/phi417.fas											
# Fields: query id	subject id	% identity	alignment length	mismatches	gap opens	q. start	q. end	s. start	s. end	evalue	bit score
# 8 hits found											
contig00042.g13149	Q4WV27#PHI:7755_PHI:8601_PHI:9392#Cofilin_(AFUA_5G10570)#746128#Aspergillus_fumigatus#unaffected_pathogenicity_reduced_virulence_increased_virulence_(hypervirulence)	52.63	152	70	2	1	150	1	152	2,00E-50	160
# BLASTP 2.2.30+											
# Query: contig00042.g13154											
# Database: /data/phi-blast/fasta/Phi417/phi417.fas											
# Fields: query id	subject id	% identity	alignment length	mismatches	gap opens	q. start	q. end	s. start	s. end	evalue	bit score

# 3 hits found											
contig00042.g13154	A0A2N6NZW9#PHI:124106#Atg16#176275#Beauveria_bassiana#reduced_virulence	34.62	26	17	0	61	86	157	182	3.7	24.6
# BLASTP 2.2.30+											
# Query: contig00045.g13361											
# Database: /data/phi-blast/fasta/Phi417/phi417.fas											
# Fields: query id	subject id	% identity	alignment length	mismatches	gap opens	q. start	q. end	s. start	s. end	evalue	bit score
# 8 hits found											
contig00045.g13361	A0A288Q8R8#PHI:7912#Ppxyn2#4792#Phytophthora_nicotianae#reduced_virulence	23.16	95	52	2	17	90	169	263	0.49	27.7
# BLASTP 2.2.30+											
# Query: contig00046.g13399											
# Database: /data/phi-blast/fasta/Phi417/phi417.fas											
# Fields: query id	subject id	% identity	alignment length	mismatches	gap opens	q. start	q. end	s. start	s. end	evalue	bit score
# 6 hits found											
contig00046.g13399	I1RJT0#PHI:8913#FgVPS2_(FGSG_04112)#5518#Fusarium_graminearum#lethal	95.61	228	9	1	1	227	1	228	8,00E-135	381
# BLASTP 2.2.30+											
# Query: contig00047.g13483											
# Database: /data/phi-blast/fasta/Phi417/phi417.fas											
# Fields: query id	subject id	% identity	alignment length	mismatches	gap opens	q. start	q. end	s. start	s. end	evalue	bit score
# 5 hits found											
contig00047.g13483	I1RAH2#PHI:1629#GzWing001#5518#Fusarium_graminearum#unaffected_pathogenicity	33.93	56	33	2	79	132	441	494	2.4	27.3
# BLASTP 2.2.30+											
# Query: contig00048.g13505											
# Database: /data/phi-blast/fasta/Phi417/phi417.fas											
# Fields: query id	subject id	% identity	alignment length	mismatches	gap opens	q. start	q. end	s. start	s. end	evalue	bit score
# 4 hits found											
contig00048.g13505	A0A142K489#PHI:9069#Cbu1524_(caeA)#777#Coxiella_burnetii#effector_(plant_avirulence_determin	31.11	45	30	1	53	96	73	117	4.9	24.6
# BLASTP 2.2.30+											
# Query: contig00051.g13638											
# Database: /data/phi-blast/fasta/Phi417/phi417.fas											
# Fields: query id	subject id	% identity	alignment length	mismatches	gap opens	q. start	q. end	s. start	s. end	evalue	bit score
# 6 hits found											
contig00051.g13638	B2SHS4#PHI:4098#Filp#347#Xanthomonas_oryzae#reduced_virulence	32.35	34	23	0	25	58	543	576	2.4	26.9
# BLASTP 2.2.30+											

# Query: contig00051.g13644											
# Database: /data/phi-blast/fasta/Phi417/phi417.fas											
# Fields: query id	subject id	% identity	alignment length	mismatches	gap opens	q. start	q. end	s. start	s. end	evalue	bit score
# 12 hits found											
contig00051.g13644	B2WBG9#PHI:124036#Pf2_(PTRG_06982)#45151#Pyrenophora_tritici-repentis#reduced_virulence	29.23	65	45	1	15	79	116	179	0.69	28.1
# BLASTP 2.2.30+											
# Query: contig00051.g13652											
# Database: /data/phi-blast/fasta/Phi417/phi417.fas											
# Fields: query id	subject id	% identity	alignment length	mismatches	gap opens	q. start	q. end	s. start	s. end	evalue	bit score
# 7 hits found											
contig00051.g13652	P71244#PHI:10990#WcaM#562#Escherichia_coli#unaffected_pathogenicity	34.21	38	25	0	96	133	386	423	1.5	27.3
# BLASTP 2.2.30+											
# Query: contig00053.g13692											
# Database: /data/phi-blast/fasta/Phi417/phi417.fas											
# Fields: query id	subject id	% identity	alignment length	mismatches	gap opens	q. start	q. end	s. start	s. end	evalue	bit score
# 13 hits found											
contig00053.g13692	I1RUQ2#PHI:1397#GzC2H060#5518#Fusarium_graminearum#unaffected_pathogenicity	92.44	119	9	0	1	119	1	119	6,00E-81	235
# BLASTP 2.2.30+											
# Query: contig00053.g13695											
# Database: /data/phi-blast/fasta/Phi417/phi417.fas											
# Fields: query id	subject id	% identity	alignment length	mismatches	gap opens	q. start	q. end	s. start	s. end	evalue	bit score
# 6 hits found											
contig00053.g13695	Q8DUX1#PHI:10238#MntH#1309#Streptococcus_mutans#unaffected_pathogenicity_reduced_virulenc	33.93	56	30	1	6	54	190	245	0.75	27.3
# BLASTP 2.2.30+											
# Query: contig00054.g13708											
# Database: /data/phi-blast/fasta/Phi417/phi417.fas											
# Fields: query id	subject id	% identity	alignment length	mismatches	gap opens	q. start	q. end	s. start	s. end	evalue	bit score
# 6 hits found											
contig00054.g13708	S7WL31#PHI:12153#EFPP_(TGGT1_269460)#5811#Toxoplasma_gondii#unaffected_pathogenicity	29.03	93	52	3	2	90	42	124	0.83	26.9
# BLASTP 2.2.30+											
# Query: contig00054.g13733											
# Database: /data/phi-blast/fasta/Phi417/phi417.fas											
# Fields: query id	subject id	% identity	alignment length	mismatches	gap opens	q. start	q. end	s. start	s. end	evalue	bit score

# 4 hits found											
contig00054.g13733	A0A3M7JTW8#PHI:123765#Ngg1#5059#Aspergillus_flavus#reduced_virulence	25.00	84	49	1	97	180	257	326	3.5	27.3
# BLASTP 2.2.30+											
# Query: contig00068.g13796											
# Database: /data/phi-blast/fasta/Phi417/phi417.fas											
# Fields: query id	subject id	% identity	alignment length	mismatches	gap opens	q. start	q. end	s. start	s. end	evalue	bit score
# 7 hits found											
contig00068.g13796	P0A2F4#PHI:11377#SodB#28901#Salmonella_enterica#increased_virulence_(hypervirulence)_reduce	25.81	62	45	1	68	129	7	67	2.7	26.6
# BLASTP 2.2.30+											
# Query: contig00233.g13832											
# Database: /data/phi-blast/fasta/Phi417/phi417.fas											
# Fields: query id	subject id	% identity	alignment length	mismatches	gap opens	q. start	q. end	s. start	s. end	evalue	bit score
# 8 hits found											
contig00233.g13832	B0YDQ1#PHI:123459#AreA_(AFUB_096370)#746128#Aspergillus_fumigatus#reduced_virulence	28.17	71	40	4	10	78	56	117	0.25	27.7
# BLASTP 2.2.30+											
# Query: contig00257.g13836											
# Database: /data/phi-blast/fasta/Phi417/phi417.fas											
# Fields: query id	subject id	% identity	alignment length	mismatches	gap opens	q. start	q. end	s. start	s. end	evalue	bit score
# 25 hits found											
contig00257.g13836	Q4WX28#PHI:11370#SltA_(Afu3g08010)#746128#Aspergillus_fumigatus#reduced_virulence	72.73	88	23	1	30	117	430	516	4,00E-38	136
# BLASTP 2.2.30+											
# Query: contig00294.g13841											
# Database: /data/phi-blast/fasta/Phi417/phi417.fas											
# Fields: query id	subject id	% identity	alignment length	mismatches	gap opens	q. start	q. end	s. start	s. end	evalue	bit score
# 7 hits found											
contig00294.g13841	S8EVM9#PHI:11890#G3PDH#5811#Toxoplasma_gondii#unaffected_pathogenicity	51.16	43	20	1	12	54	472	513	2,00E-08	49.7
# BLASTP 2.2.30+											
# Query: contig00345.g13847											
# Database: /data/phi-blast/fasta/Phi417/phi417.fas											
# Fields: query id	subject id	% identity	alignment length	mismatches	gap opens	q. start	q. end	s. start	s. end	evalue	bit score
# 6 hits found											
contig00345.g13847	A0A098DCZ4#PHI:1439#GzNot001#5518#Fusarium_graminearum#lethal	61.76	102	39	0	1	102	91	192	6,00E-39	139
