# Supplementary figures and images for: Genomic Insights into Fusarium verticillioides Diversity: The Genome of Two Clinical Isolates and Their Demethylase Inhibitor Fungicides Susceptibility
Source: Pathogens. 2024 Dec 3;13(12):1062. doi: 10.3390/pathogens13121062 (PMC11728828; doi:10.3390/pathogens13121062)

FV\_7600

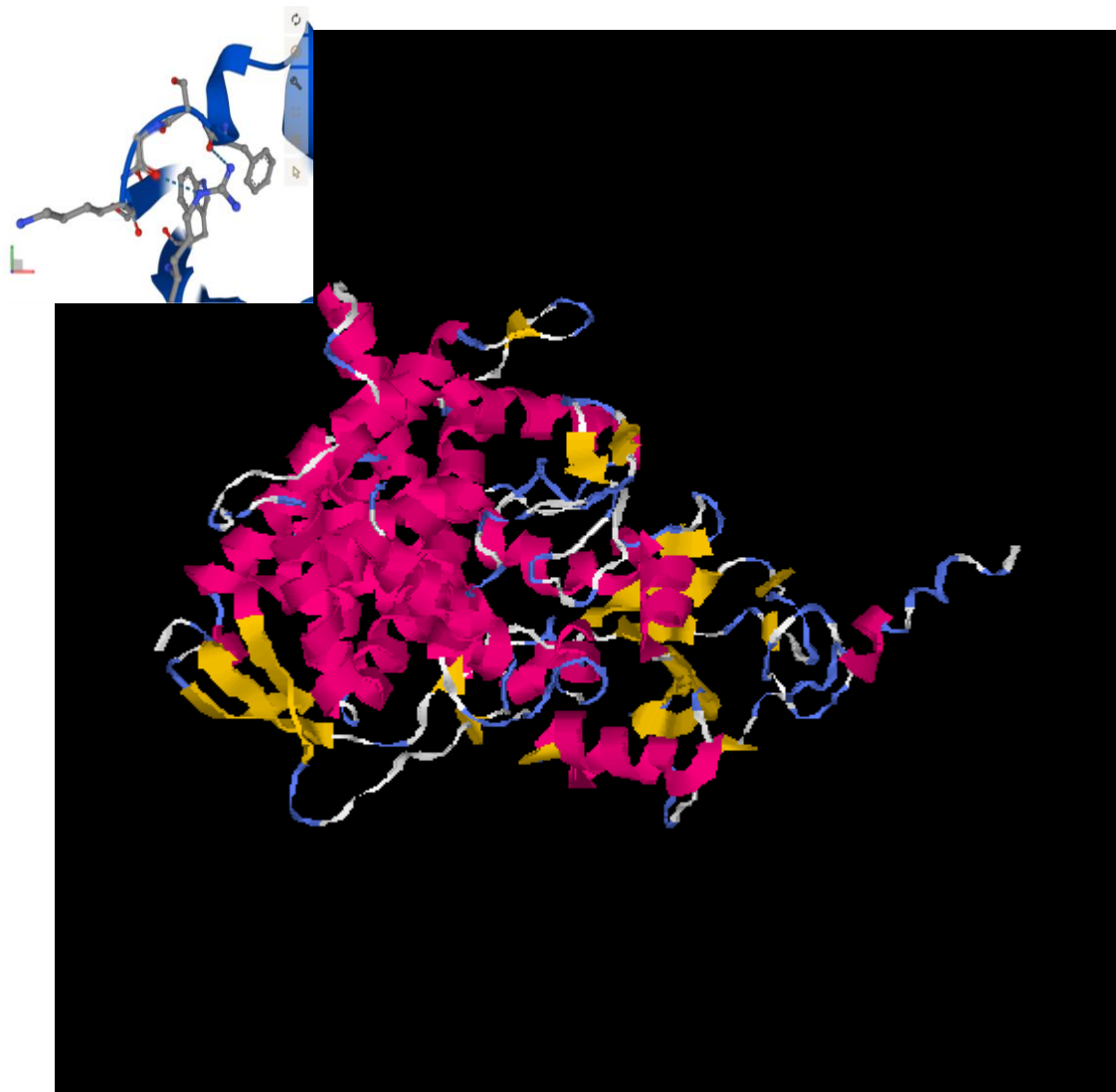

FV\_05-0160 & FV\_IUM\_09-1037

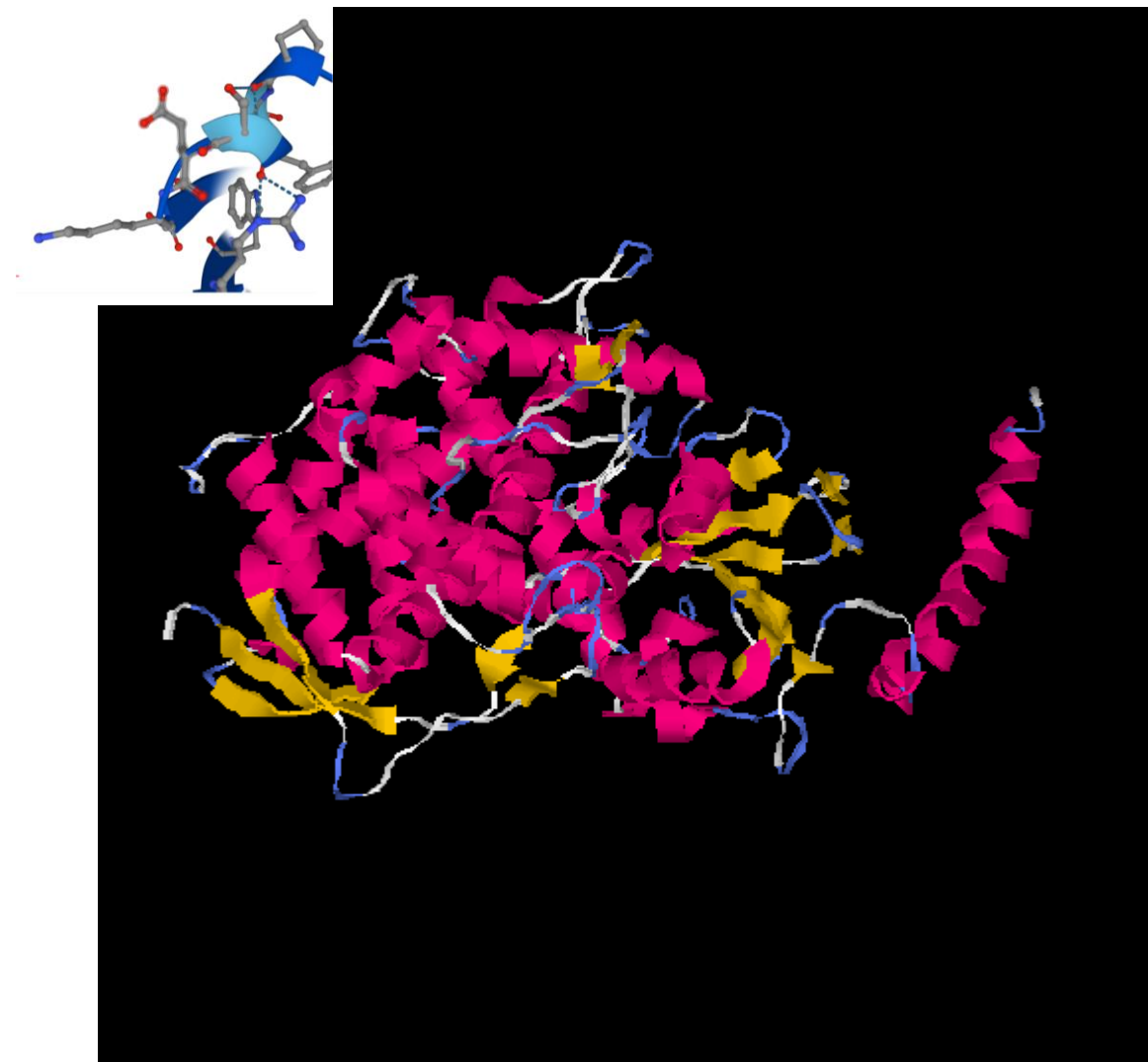

Supplement: Supplementary file 1 [file pathogens-13-01062-s001.zip › Figure S1.pdf]

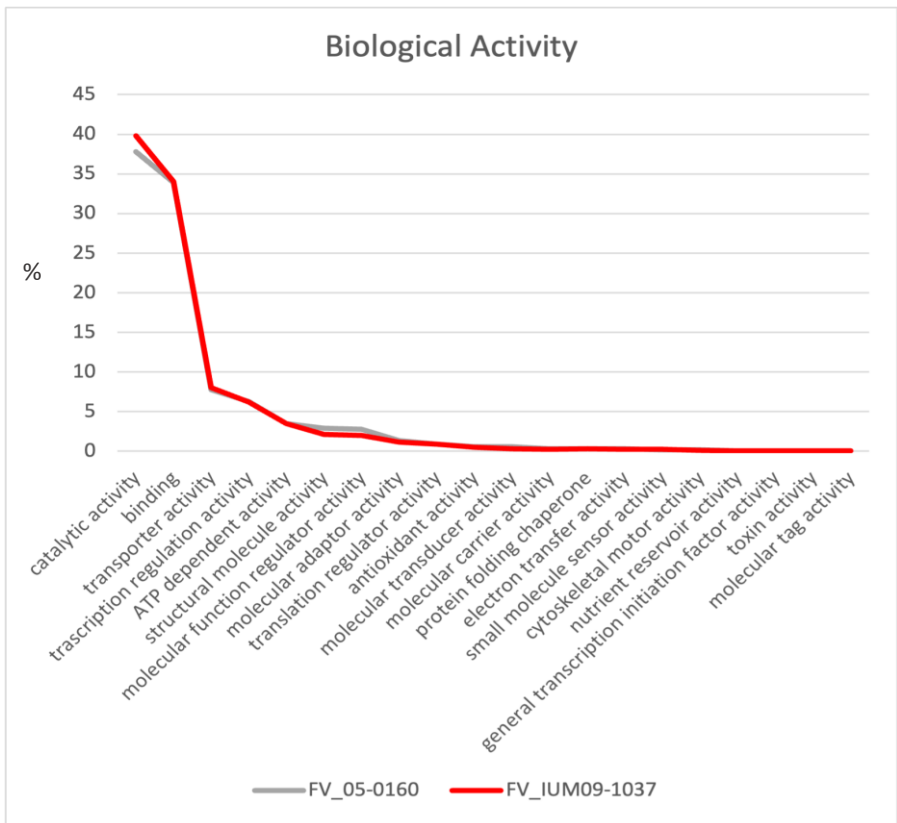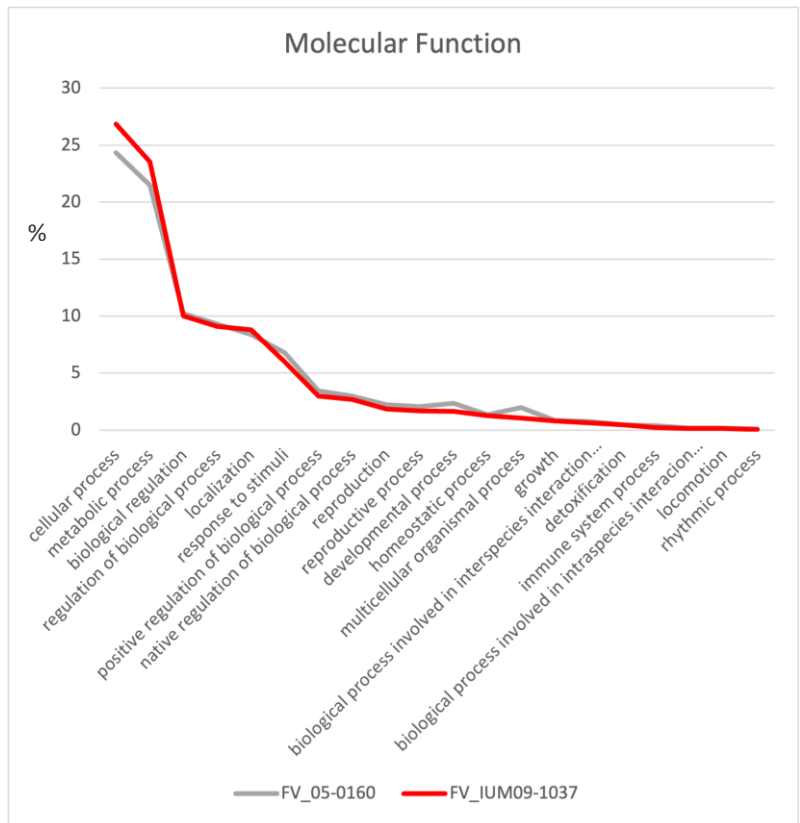

Supplement: Supplementary file 1 [file pathogens-13-01062-s001.zip › Figure S3.pdf]
