# Supplementary material for: Genomic Insights into Fusarium verticillioides Diversity: The Genome of Two Clinical Isolates and Their Demethylase Inhibitor Fungicides Susceptibility
Source: Pathogens. 2024 Dec 3;13(12):1062. doi: 10.3390/pathogens13121062 (PMC11728828; doi:10.3390/pathogens13121062)
Supplement: Supplementary file 1 [file Figure S2.pdf › Figure S2.pdf]

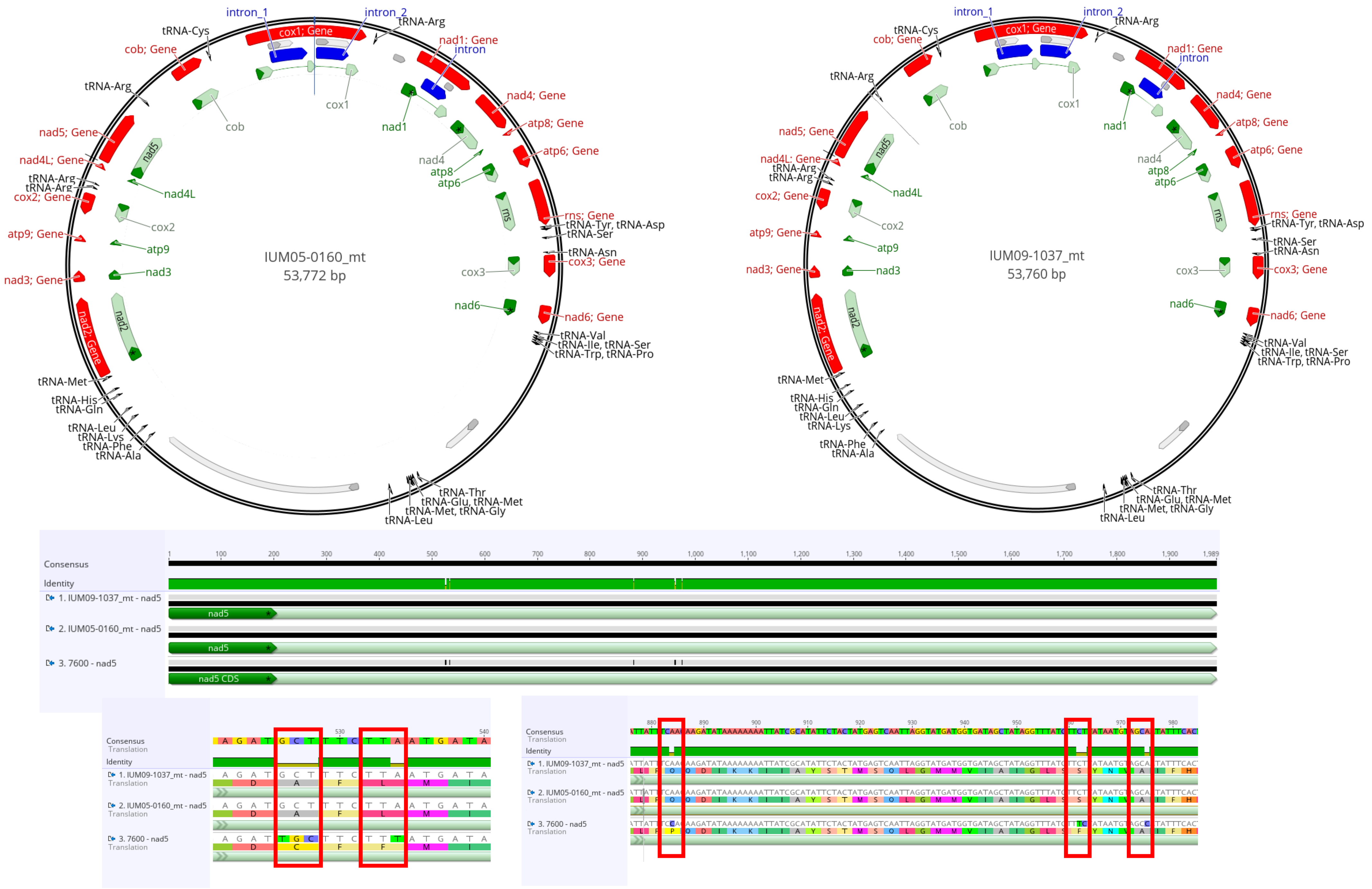

IUM05-0160_mt
53,772 bp
intron_1
intron_2
intron
cox1; Gene
cob; Gene
nad1: Gene
nad4; Gene
atp8; Gene
atp6; Gene
rns; Gene
cox3; Gene
nad6; Gene
nad5; Gene
nad4L; Gene
cox2; Gene
atp9; Gene
nad3; Gene
nad2; Gene
tRNA-Cys
tRNA-Arg
tRNA-Tyr, tRNA-Asp
tRNA-Ser
tRNA-Asn
tRNA-Val
tRNA-Ile, tRNA-Ser
tRNA-Trp, tRNA-Pro
tRNA-Thr
tRNA-Glu, tRNA-Met
tRNA-Met, tRNA-Gly
tRNA-Leu
tRNA-Met
tRNA-His
tRNA-Gln
tRNA-Lys
tRNA-Phe
tRNA-Ala
cob
cox1
nad1
nad4
atp8
atp6
rns
cox3
nad6
nad5
nad4L
cox2
atp9
nad3
nad2
IUM09-1037_mt
53,760 bp
Consensus
Identity
1. IUM09-1037_mt - nad5
2. IUM05-0160_mt - nad5
3. 7600 - nad5
nad5 CDS
Translation
